# Brain Short-Chain Fatty Acids Induce ACSS2 to Ameliorate Depressive-Like Behavior via PPARγ–TPH2 Axis

**DOI:** 10.34133/research.0400

**Published:** 2024-06-27

**Authors:** Nuo Chen, Xinyi Xu, Yaxin Guo, Ming Zhao, Yubin Li, Tian Zhou, Xinyue Zhang, Jie Gao, Faliang Zhu, Chun Guo, Yongyu Shi, Qun Wang, Wenxian Wu, Lining Zhang, Yan Li

**Affiliations:** ^1^ Department of Immunology, School of Basic Medical Science, Cheeloo College of Medicine, Shandong University, Jinan, China.; ^2^ Guangdong Key Laboratory of Age-Related Cardiac and Cerebral Diseases, Department of Neurology, Affiliated Hospital of Guangdong Medical University, Zhanjiang, China.; ^3^ Department of Pathogen Biology, School of Basic Medical Science, Cheeloo College of Medicine, Shandong University, Jinan, China.

## Abstract

Short-chain fatty acids (SCFAs) have been increasingly evidenced to be important bioactive metabolites of the gut microbiota and transducers in controlling diverse psychiatric or neurological disorders via the microbiota–gut–brain axis. However, the precise mechanism by which brain SCFAs extert multiple beneficial effects is not completely understood. Our previous research has demonstrated that the acetyl-coenzyme A synthetase short-chain family member 2 (ACSS2) is a novel target of the rapid and long-lasting antidepressant responses. Here, we show that micromolar SCFAs significantly augment both total cellular and nuclear ACSS2 to trigger tryptophan hydroxylase 2 (TPH2) promoter histone acetylation and its transcription in SH-SY5Y cells. In chronic-restraint-stress-induced depression mice, neuronal ACSS2 knockdown by stereotaxic injection of adeno-associated virus in the hippocampus abolished SCFA-mediated improvements in depressive-like behaviors of mice, supporting that ACSS2 is required for SCFA-mediated antidepressant responses. Mechanistically, the peroxisome-proliferator-activated receptor gamma (PPARγ) is identified as a novel partner of ACSS2 to activate TPH2 transcription. Importantly, PPARγ is also responsible for SCFA-mediated antidepressant-like effects via ACSS2–TPH2 axis. To further support brain SCFAs as a therapeutic target for antidepressant effects, d-mannose, which is a naturally present hexose, can significantly reverse the dysbiosis of gut microbiota in the chronic-restraint-stress-exposure mice and augment brain SCFAs to protect against the depressive-like behaviors via ACSS2–PPARγ–TPH2 axis. In summary, brain SCFAs can activate ACSS2–PPARγ–TPH2 axis to play the antidepressive-like effects, and d-mannose is suggested to be an inducer of brain SCFAs in resisting depression.

## Introduction

The major depressive disorder is a pervasive neuropsychiatric disorder with multiple impairments in neuron function and neuroinflammation [1,2]. In patients with major depression disorder or mouse model of depression, the balance of gut microbiota is often disrupted, and improving the dysbiosis of the gut microbiota is increasingly becoming a promising approach to preventing depression via microbiota–gut–brain axis (MGB) [3–5]. In this respect, d-mannose can alter the gut microbiome to combat diet-induced obesity in mice, suggesting that d-mannose may function like prebiotics [6]. Although reports from our laboratory and others all demonstrate that d-mannose, which is a naturally present hexose and shares the same transporter with glucose, can influence depressive-like behaviors of mice, its potential function in reversing the dysbiosis of gut microbiota needs to be explored.

The major products from the microbial fermentation of prebiotics in the gut are short-chain fatty acids (SCFAs)—in particular, acetate, propionate, and butyrate, which display multiple physiological roles in maintaining health and controlling development of disease [7–9]. Upon production in the colon, SCFAs are primarily transported into colonocyte cells via H^+^-linked monocarboxylate transporters (MCTs) and sodium-linked MCTs to generate adenosine 5′-triphosphate and energy for the cells in the mitochondria [8]. The residual SCFAs that are not metabolized in the colonocytes enter hepatocyte cells through the portal circulation, where the average concentrations of SCFAs are 260, 30, and 30 μM for acetate, propionate, and butyrate, respectively [10,11]. Therefore, only a minor fraction of the colon-derived acetate, propionate, and butyrate (36%, 9%, and 2%, respectively) can reach systemic circulation and peripheral tissues [12]. The plasma concentrations of acetate, propionate, and butyrate have been reported in ranges of 25 to 250, 1.4 to 13.4, and 0.5 to 14.2 μM, respectively [13]. Importantly, SCFAs can cross the blood–brain barrier and reach the brain, possibly owing to the abundant expression of MCTs on endothelial cells in the relative order of butyrate (highest), propionate, and acetate [8]. Therefore, microbiota-derived SCFAs can transduce information from the gut to brain via MGB and thus act as an important modulator in brain function through various pathways [8,14,15]. Increasing clinical and preclinical evidence supports SCFAs as a key role in MGB communication to regulate diverse psychiatric or neurological disorders, including anxiety and depression, Alzheimer’s disease (AD), autism spectrum disorder, multiple aclerosis, Parkinson’s disease, and stroke [16,17]. At present, 2 major SCFA signaling mechanisms have been identified, inhibition of histone deacetylases (HDACs) and activation of orphan G-protein-coupled receptors [18,19]. However, the physiological SCFA concentration, except in the gut lumen, presents much lower (micromolar or nanomolar) than millimolar that can act as HDAC inhibitor [19,20]. In the brain, physiological concentrations of acetate (within 171 μM), propionate (within 6 μM), and butyrate (within 2.8 μM) have been reported [21]. Therefore, it is important to explore the actual brain SCFA function, considering that the current studies of SCFAs mostly come from animal research regardless of physiological concentrations.

Since a major function of SCFAs has been well characterized to epigenetically modulate the specific gene expression via promoting the target gene histone acetylation, the nuclear acetyl-coenzyme A (CoA) is a crucial source for providing the acetyl-CoA group for histone in this process. The acetyl-CoA synthetase short-chain family member 2 (ACSS2) can largely generate nuclear acetyl-CoA in boosting local histone acetylation of lysosome- and memory-associated genes when it is allowed to translocate to the nucleus once being phosphorylated on serine-659 by adenosine 5′-monophosphate (AMP)-activated protein kinase (AMPK) [22,23]. In addition, ACSS2 can support tumor growth, the biosynthesis of cholesterol, glucose, and fatty acid, as well as ketogenesis and protein acetylation [24,25]. In the brain, ACSS2 is highly expressed and provides the acetyl-CoA group to histones in epigenetically regulating alcohol metabolism [23,26]. Recently, ACSS2 is discovered to be reduced in the brain of patients with AD and mouse model, and it-mediated histone acetylation of *N*-methyl-d-aspartate receptors (NMDARs) and AMPA receptors (AMPARs) can restore synaptic plasticity and improve cognition function in mouse model of AD [27]. More recently, our group also support ACSS2 as a novel target to trigger rapid and long-lasting antidepressant responses via brain-derived neurotrophic factor (BDNF) and tryptophan hydroxylase 2 (TPH2) [28]. However, at present, its upstream regulators are largely vague.

ACSS2-mediated gene histone acetylation is required for chromatin remodeling and the recruitment of transcriptionary elements. For activating gene transcription, the transcription activator can bind target gene promoter and is also indispensable. Peroxisome-proliferator-activated gamma (PPARγ) is a classical transcription activator and involved in multiple biological processes including adipocyte differentiation, glucose and lipid metabolism, and inflammation suppression [29–31]. In the brain, PPARγ can prevent microglial overactivation and neuroinflammation to improve various psychiatric disorders including anxiety- and depressive-like behaviors [31–36]. Interestingly, SCFAs can trigger PPARγ to play the protective role against inflammatory-related disorders [14,37,38]. However, neuronal PPARγ function in response to SCFAs remains to be further investigated.

We here reveal that micromolar SCFAs including acetate, propionate, and butyrate can induce both total cellular and nuclear ACSS2 to trigger TPH2 expression in vitro. Furthermore, the PPARγ is identified as a novel partner of ACSS2 to potentiate *TPH2* histone acetylation and transcription. Importantly, both neuronal ACSS2 and PPARγ are indispensable for SCFA-triggered antidepressive-like behavior via TPH2 in the chronic restraint stress (CRS)-exposure mice. To support brain SCFAs as a potent therapeutic agent, d-mannose remarkedly improves the gut microbiota dysbiosis in the CRS-exposure mice and enhances brain SCFA levels to resist depression by ACSS2–PPARγ–TPH2 axis. In summary, brain SCFAs act as inducers of ACSS2–PPARγ–TPH2 axis to prevent the depressive-like behavior in mice. In addition to directly activating ACSS2 upon entering the brain, d-mannose is also proved to significantly reverse the dysbiosis of gut microbiota and augment brain SCFA contents, thereby protecting against depressive-like behavior in mice.

## Results

### SCFAs augment ACSS2 levels to mediate the antidepressant responses in CRS-exposure mice

Recently, we have reported that ACSS2 is a novel protein for rapid and long-lasting antidepressant responses. To further support this finding, behaviors tests were carried out to examine the depressive-related and anxiety-like behaviors including tail suspension test (TST), forced swim test (FST), sucrose preference test (SPT), open field test (OFT), and elevated plus maze (EPM) in CRS-exposure mice with hippocampal ACSS2 overexpression by stereotaxically injecting the adeno-associated virus (AAV). The results showed that hippocampal ACSS2 overexpression showed antidepressive- and antianxiety-like effects in CRS-exposure mice (Fig. S1A to F). Correspondingly, the decrease in ACSS2 in the hippocampus of CRS mice was reversed by ACSS2 overexpression (Fig. S1G). In line with our previous finding, these data supported that the blockage of hippocampal ACSS2 reduction can exert an antidepressive- and antianxiety-like effects in mice.

**Fig. 1.**
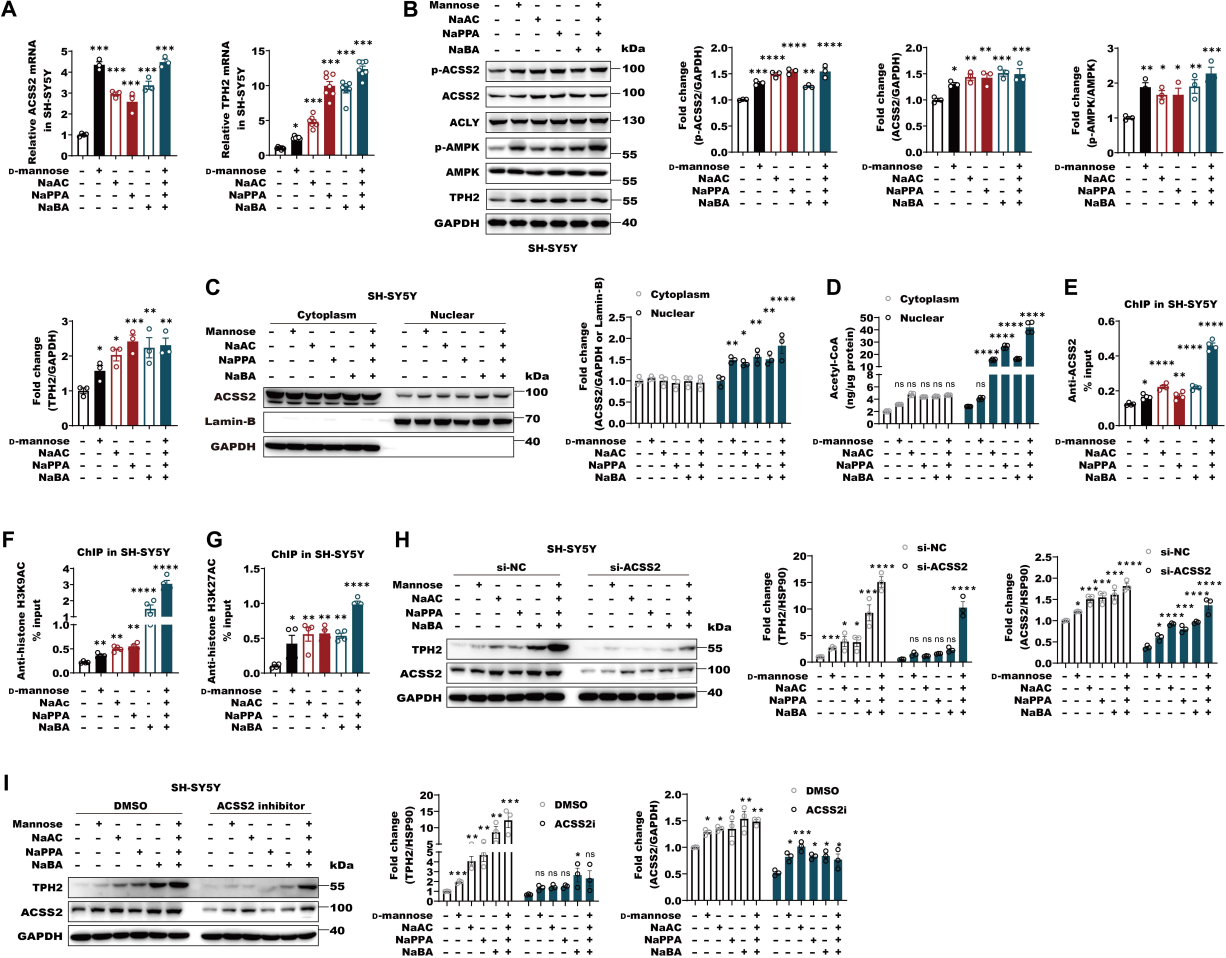
SCFAs augment ACSS2 levels to mediate the antidepressant responses in CRS-exposure mice. (A) The mRNA levels of *ACSS2* and *TPH2* in SH-SY5Y cells with or without 1 mM d-mannose, 10 μM NaAC, 1 μM NaPPA, and 0.1 μM NaBA alone or together for 12 h. Data were normalized with GAPDH mRNA levels. Data are shown as means ± SEM (*n* = 3 to 7 per group) and were analyzed using one-way ANOVA, ACSS2 (*F*_5,13_ = 73.43, *P*< 0.0001) and TPH2 (*F*_5,36_ = 138.4, *P* < 0.0001), and Tukey’s multiple comparison test, *P < 0.05 and ***P < 0.001. (B) The lysates of SH-SY5Y cells with or without 1 mM d-mannose, 10 μM NaAC, 1 μM NaPPA, and 0.1 μM NaBA alone or together for 24 h were subjected to Western blot with indicated antibodies. The levels of p-ACSS2, ACSS2, p-AMPK, and TPH2 were quantitatively analyzed. Data are shown as means ± SEM (*n* = 3 per group) and were analyzed using one-way ANOVA, ACSS2 (*F*_5,12_ = 8.214, *P* = 0.0014), p-ACSS2 (*F*_5,12_ = 35.67, *P* < 0.0001), p-AMPK (*F*_5,12_ = 7.579, *P* = 0.002), and TPH2 (*F*_5,12_ = 8.12, *P* = 0.0015), and Tukey’s multiple comparison test, *P < 0.05, **P < 0.01, ***P < 0.001, and ****P < 0.0001. (C) Subcellular fractions of cytoplasm and nuclear were extracted from SH-SY5Y with or without 1 mM d-mannose, 10 μM NaAC, 1 μM NaPPA, and 0.1 μM NaBA alone or together for 24 h. Immunoblot experiments were then carried out with indicated antibodies. The signals of GAPDH and Lamin-B were shown as controls for cytoplasm and nuclear fractions. The levels of ACSS2 were quantitatively analyzed. Data are shown as means ± SEM (*n* = 3 per group) and were analyzed using 2-way ANOVA with Tukey’s multiple comparison test, *P < 0.05, **P < 0.01, and ****P < 0.0001. (D) SH-SY5Y cells were pretreated with or without 1 mM d-mannose, 10 μM NaAC, 1 μM NaPPA, and 0.1 μM NaBA alone or together for 24 h. The levels of acetyl-CoA in cytoplasm and nuclear were measured by ELISA in the subcellular fraction of cytoplasm and nuclear. Data are shown as means ± SEM (*n* = 4 per group) and were analyzed using 2-way ANOVA with Tukey’s multiple comparison test, ****P < 0.0001. ns, not significant. (E to G) SH-SY5Y cells were incubated with or without 1 mM d-mannose, 10 μM NaAC, 1 μM NaPPA, and 0.1 μM NaBA alone or together for 24 h. ChIP analyses using an anti-ACSS2 (E), anti-H3K9Ac (F), and anti-H3K27Ac (G) antibodies were performed. The histogram shows the amount of immunoprecipitated DNA expressed as a percentage of the total input DNA. The data are presented as the means ± SEM of quadruplicate samples. Data are shown as means ± SEM (*n* = 4 per group) and were analyzed using one-way ANOVA, ACSS2 (*F*_5,18_ = 164.4, *P* < 0.0001), H3K9Ac (*F*_5,18_ = 65.25, *P* < 0.0001), and H3K27Ac (*F*_5,18_ = 15.61, *P* < 0.0001), and Tukey’s multiple comparison test, *P < 0.05, **P < 0.01, and ****P < 0.0001. (H) SH-SY5Y cells were transfected with small RNA interference against NC (si-NC) or ACSS2 (si-ACSS22) and treated with 1 mM d-mannose, 10 μM NaAC, 1 μM NaPPA, and 0.1 μM NaBA alone or together for 24 h. Immunoblots were performed with the indicated antibodies. Data are shown as means ± SEM (*n* = 3 per group) and were analyzed using 2-way ANOVA with Tukey’s multiple comparison test, *P < 0.05, ***P < 0.001, and ****P < 0.0001. (I) SH-SY5Y cells were stimulated with dimethyl sulfoxide (DMSO) or ACSS2 inhibitor (10 μM, 24 h) and treated with 1 mM d-mannose, 10 μM NaAC, 1 μM NaPPA, and 0.1 μM NaBA alone or together for 24 h. Immunoblots were performed with the indicated antibodies. Data are shown as means ± SEM (*n* = 3 per group) and were analyzed using 2-way ANOVA with Tukey’s multiple comparison test, *P < 0.05, **P < 0.01, and ***P < 0.001.

Next, we sought to explore whether SCFAs could induce ACSS2 to mediate the antidepressant response. To investigate the actual brain SCFA regulation on ACSS2, we selected increasing SCFAs, which contain sodium acetate (NaAC; 0 to 10,000 μM; acetate), sodium propionate (NaPPA; 0 to 1,000 μM; propionate), and sodium butyrate (NaBA; 0 to 1,000 μM; butyrate), to examine the changes of ACSS2 with d-mannose as a known ACSS2 inducer in human neuroblastoma SH-SY5Y cells [28]. Interestingly, no lower than 10 μM for acetate, 1 μM for propionate, and 0.1 μM for butyrate all triggered psychiatric disorder ACSS2 and its target TPH2 expressions at the mRNA and protein levels, indicating that physiological concentrations of acetate (within 171 μM), propionate (within 6 μM), and butyrate (within 2.8 μM) in the brain could induce expressions of *ACSS2* and *TPH2* (Fig. 1A and B and Fig. S1H to J). Importantly, the phosphorylations of AMPK and ACSS2 were both promoted as well in response to these physiological levels of SCFAs (acetate, 10 μM; propionate, 1 μM; butyrate, 0.1 μM). The data supported that physiological levels of SCFAs can enhance nuclear ACSS2 levels and TPH2 expression (Fig. 1A and B). Notably, another acetyl-CoA-producing enzyme from citrate, ACLY, was not altered by acetate, propionate, and butyrate, suggesting that ACSS2 is specially influenced by SCFAs (Fig. 1B and Fig. S1K). We thus selected 10 μM acetate, 1 μM propionate, and 0.1 μM butyrate to perform the SCFA stimulation experiments in vitro in our study. Importantly, the levels of ACSS2, rather than ACLY, could be significantly induced in mouse primary hippocampal neurons and neuronal HT22 cells (Fig. S1L and M).

To further examine whether SCFA-induced TPH2 expression is attributed to ACSS2-dependent acetyl-CoA generation and histone acetylation, we firstly examined the nuclear levels of ACSS2 and acetyl-CoA with SCFA treatment. Similar to d-mannose, SCFAs were able to significantly elevate nuclear levels of ACSS2 and acetyl-CoA as well in SH-SY5Y cells (Fig. 1C and D). In the chromatin immunoprecipitation (ChIP) assay, the occupancy of ACSS2 and acetylations of histone H3 at lysine 9 (H3K9) and H3K27 in the *Tph2* promoter were remarkedly augmented by SCFAs, suggesting that SCFAs can promote ACSS2 recruitment to *Tph2* promoters and *Tph2* histone acetylation (Fig. 1E to G). However, the ACSS2 knockdown by the small RNA interference technology or its inhibitor treatment all abolished SCFA-induced TPH2 expression, suggesting that SCFA-mediated TPH2 induction is dependent on ACSS2 (Fig. 1H and I). These data supported that SCFAs can induce nuclear ACSS2 to promote *Tph2* expression via epigenetically modifying histone acetylation.

**Fig. 2.**
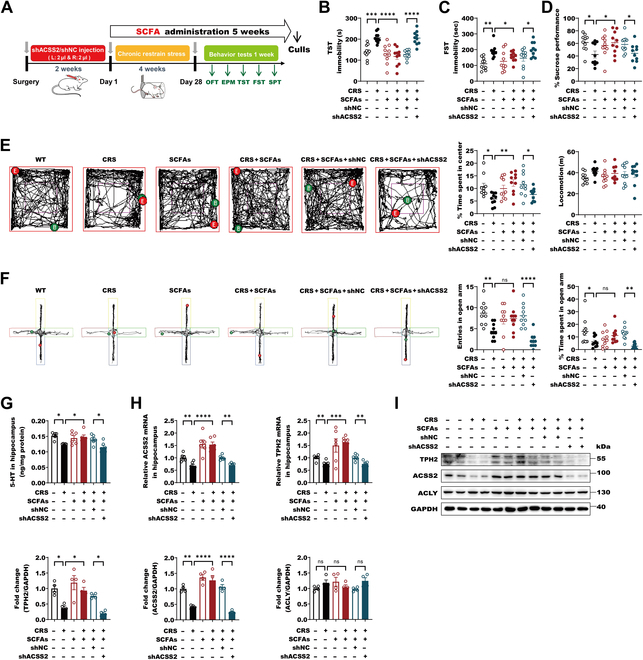
SCFAs augment ACSS2 levels to mediate the antidepressant responses in CRS-exposure mice. (A) Two weeks after injecting the shACSS2 virus into the hippocampus of mice using stereotaxic injection techniques, the CRS modeling experiment was initiated for 4 weeks, during which mice had free access to drinking SCFAs or water. Behavioral tests were then conducted for 1 week (*n* = 10 per group). (B) C57BL/6J male mice with or without CRS-exposure were free drinking with or without SCFAs (67.5 mM NaAC, 25 mM NaPPA, and 40 mM NaBA) supplementation for 4 weeks and injected with AAV-ACSS2 or control group with AAV-NC (2 μl was injected into the left and right hippocampus, respectively) in stereotactic location in the hippocampus. TST behavioral tests were performed 4 weeks after the injection of AAV. Immobility time in the TST in individual animals were detected. In the 4 groups that were not injected with AAV, data are shown as means ± SEM (*n* = 10 per group) and were analyzed using 2-way ANOVA and Tukey’s multiple comparison test, ***P < 0.001 and ****P < 0.0001; in the 2 groups that were injected with AAV, data are shown as means ± SEM (*n* = 10 per group) and were analyzed using unpaired 2-tailed Student’s *t* test, ****P < 0.0001. (C) FST behavioral tests were performed 4 weeks after the injection of AAV. Immobility time in the forced swimming test in individual animals was detected. In the 4 groups that were not injected with AAV, data are shown as means ± SEM (*n* = 10 per group) and were analyzed using 2-way ANOVA and Tukey’s multiple comparison test, *P < 0.05 and **P < 0.01; in the 2 groups that were injected with AAV, data are shown as means ± SEM (*n* = 10 per group) and were analyzed using unpaired 2-tailed Student’s *t* test, *P < 0.05. (D) SPTs were performed 4 weeks after the injection of AAV. In the 4 groups that were not injected with AAV, data are shown as means ± SEM (*n* = 10 per group) and were analyzed using 2-way ANOVA and Tukey’s multiple comparison test, *P < 0.05; in the 2 groups that were injected with AAV, data are shown as means ± SEM (*n* = 10 per group) and were analyzed using unpaired 2-tailed Student’s *t* test, *P < 0.05. (E) Raw traces of mice in the OFT were shown. Total distance traveled in the OFT and time spent exploring the center area in the OFT from mice in individual animals from wild-type (WT), CRS, SCFAs, CRS + SCFAs, CRS + SCFAs + shNC, and CRS + SCFAs + shACSS2 groups. In the 4 groups that were not injected with AAV, data are shown as means ± SEM (*n* = 10 per group) and were analyzed using 2-way ANOVA and Tukey’s multiple comparison test, *P < 0.05, **P < 0.01; in the 2 groups that were injected with AAV, data are shown as means ± SEM (*n* = 10 per group) and were analyzed using unpaired 2-tailed Student’s *t* test, *P < 0.05. (F) Raw traces of mice in the EPM were shown. Time spent in the open arms and probability of entering open arms in the EPM test from mice in individual animals from wild-type, CRS, SCFAs, CRS + SCFAs, CRS + SCFAs + shNC, and CRS + SCFAs + shACSS2 groups. In the 4 groups that were not injected with AAV, data are shown as means ± SEM (*n* = 10 per group) and were analyzed using 2-way ANOVA and Tukey’s multiple comparison test, **P < 0.01 and ****P < 0.0001; in the 2 groups that were injected with AAV, data are shown as means ± SEM (*n* = 10 per group) and were analyzed using unpaired 2-tailed Student’s *t* test, *P < 0.05, **P < 0.01 and ****P < 0.0001. (G) Analysis of 5-HT content of hippocampus by ELISA in male mice from control, CRS, SCFAs, CRS + SCFAs, CRS + SCFAs + AAV-shNC, and CRS + SCFAs + AAV-shACSS2 groups. In the 4 groups that were not injected with AAV, data are shown as means ± SEM (*n* = 6 per group) and were analyzed using 2-way ANOVA and Tukey’s multiple comparison test, *P < 0.05; in the 2 groups that were injected with AAV, data are shown as means ± SEM (*n* = 6 per group) and were analyzed using unpaired 2-tailed Student’s *t* test, *P < 0.05. (H) RT-PCR analysis of ACSS2 and TPH2 expression levels in hippocampus of male mice from control, CRS, SCFAs, CRS + SCFAs, CRS + SCFAs + AAV-shNC, and CRS + SCFAs + AAV-shACSS2 groups (*n* = 6 per group). Data were normalized with GAPDH mRNA levels and presented as fold changes compared with control group. Scale bars represent means values, and error bars represent SEM. In the 4 groups that were not injected with AAV, data are shown as means ± SEM (*n* = 6 per group) and were analyzed using 2-way ANOVA and Tukey’s multiple comparison test, **P < 0.01, ***P < 0.001, and ****P < 0.0001; in the 2 groups that were injected with AAV, data are shown as means ± SEM (*n* = 6 per group) and were analyzed using unpaired 2-tailed Student’s *t* test, **P < 0.01. (I) Representative immunoblots and quantification of ACSS2, TPH2, and ACLY protein levels normalized to loading controls in hippocampal male mice from control, CRS, SCFAs, CRS + SCFAs, CRS + SCFAs + AAV-shNC, and CRS + SCFAs + AAV-shACSS2 groups. In the 4 groups that were not injected with AAV, data are shown as means ± SEM (*n* = 4 per group) and were analyzed using 2-way ANOVA and Tukey’s multiple comparison test, *P < 0.05, **P < 0.01, and ****P < 0.0001; in the 2 groups that were injected with AAV, data are shown as means ± SEM (*n* = 4 per group) and were analyzed using unpaired 2-tailed Student’s *t* test, *P < 0.05 and ****P < 0.0001.

Then, we converted to investigate ACSS2 requirement in SCFA-mediated antidepressant responses in mice. Consistent with the previous reports with oral administration of SCFAs in mice [39], we observed that oral administration of SCFAs (67.5 mM acetate, 25 mM propionate, and 40 mM butyrate) in drinking water showed the protective effect against the depressive- and anxiety-like behaviors in CRS-exposure mice by behavioral tests of TST, FST, SPT, OFT, and EPM but did not cause obvious impairments in blood glucose levels and dysfunctions of the peripheral tissue, such as the heart, liver, spleen, lung, and kidney (Fig. 2A to F and Fig. S2A to G). The reductions of hippocampal serotonin (5-HT) in the CRS mice, which is mainly governed by TPH2, were significantly prevented by SCFAs as well (Fig. 2G). The decreases in ACSS2 and TPH2 in the hippocampus of CRS-exposure mice were remarkedly blocked as well by SCFA treatment (Fig. 2H and I). When we stereotaxically injected the AAV encoding Syn-short hairpin RNA (shRNA) to knockdown neuronal *Acss2* in the hippocampus, the SCFA-mediated antidepressive- and antianxiety-like effects were abrogated by the behavioral tests of TST, FST, SPT, OFT, and EPM (Fig. 2A to F). Similarly, the SCFA-induced recovery of hippocampal levels of 5-HT, ACSS2, and TPH2 in CRS mice was also abolished (Fig. 2G to I). These data supported that ACSS2 is required for SCFAs to exert the antidepressant- and antianxiety-like effects. In conclusion, SCFAs can obviously induce ACSS2 to mediate the antidepression responses in the CRS-exposure mice.

### PPARγ is identified as a novel transcription activator of *Tph2*

**Fig. 3.**
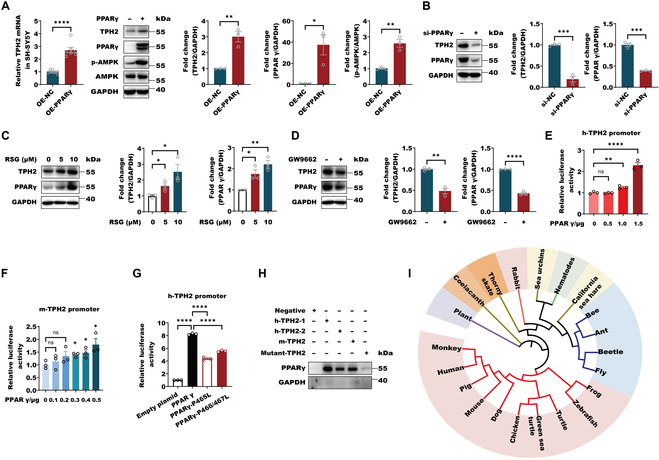
PPARγ is identified as a novel transcription activator of *Tph2*. (A) The mRNA and protein levels of TPH2 in SH-SY5Y cells that were transfected with PPARγ expression plasmid or empty plasmid. Immunoblotting analyses were conducted with the indicated antibodies. For the mRNA levels of TPH2, data are shown as means ± SEM (*n* = 7 to 8 per group) and were analyzed using unpaired 2-tailed Student’s *t* test, *t* = 8.542, df = 13, *P* < 0.0001; for the protein levels of TPH2, PPARγ, and p-AMPK, data are shown as means ± SEM (*n* = 3 per group) and were analyzed using unpaired 2-tailed Student’s *t* test, TPH2 (*t* = 6.095, df = 4, *P* = 0.0037), PPARγ (*t* = 3.828, df = 4, *P* = 0.0186), and p-AMPK (*t* = 6.428, df = 4, *P* = 0.0030). *P  < 0.05, **P  < 0.01, and ****P  < 0.0001. OE, overexpression. (B) The lysates of SH-SY5Y cells were transfected with small RNA interference against PPARγ to down-regulate PPARγ. Immunoblotting analyses were conducted with the indicated antibodies. Data are shown as means ± SEM (*n* = 3 per group) and were analyzed using unpaired 2-tailed Student’s *t* test, TPH2 (*t* = 12.04, df = 4, *P* = 0.0003) and PPARγ (*t* = 15.46, df = 4, *P* = 0.0001). ***P  < 0.001 (C) SH-SY5Y cells were treated with 10 μM PPARγ agonist rosiglitazone (RSG) for 24 h. Immunoblotting analyses were conducted with the indicated antibodies. Data are shown as means ± SEM (*n* = 3 per group) and were analyzed using one-way ANOVA, TPH2 (*F*_2,6_ = 6.806, *P* = 0.0286) and PPARγ (*F*_2,6_ = 15.67, *P* = 0.0042), and Tukey’s multiple comparison test, *P < 0.05 and **P < 0.01. (D) SH-SY5Y cells were treated with 10 μM PPARγ antagonist GW9662 for 24 h. Immunoblotting analyses were conducted with the indicated antibodies. Data are shown as means ± SEM (*n* = 3 per group) and were analyzed using unpaired 2-tailed Student’s *t* test, TPH2 (*t* = 7.702, df = 4, *P* = 0.0015) and PPARγ (*t* = 21.42, df = 4, *P* < 0.0001). (E and F) Analysis of luciferase activity was carried out in SH-SY5Y cells expressing luciferase with human *Tph2* promoter (E) or mouse *Tph2* promoter (F) that was transfected with increasing PPARγ. The luciferase activities were examined, and data of sample without exogenous PPARγ expression were normalized to 1. The data are presented as the means ± SEM from 3 independent experiments. For the human Tph2 promoter, data are shown as means ± SEM (*n* = 3 per group) and were analyzed using one-way ANOVA, *F*_3,8_ = 87.81, *P* < 0.0001, and Tukey’s multiple comparison test, **P < 0.01 and ****P < 0.0001; for the mouse Tph2 promoter, data are shown as means ± SEM (*n* = 3 per group) and were analyzed using one-way ANOVA, *F*_5,12_ = 3.288, *P* = 0.0423, and Tukey’s multiple comparison test, *P < 0.05. (G) Analysis of luciferase activity was carried out in SH-SY5Y cells expressing luciferase with human TPH2 promoter in the presence of wild-type PPARγ or its mutants P465L and L466/467A. The luciferase activity of samples without exogenous PPARγ was normalized to 1, and the relative luciferase activity is shown. The data are presented as the means ± SEM from 6 independent experiments. Data are shown as means ± SEM (*n* = 3 per group) and were analyzed using one-way ANOVA, *F*_3,8_ = 1324, *P* < 0.0001, and Tukey’s multiple comparison test, ****P < 0.0001. (H) The purified recombinant protein from BL21 (DE3) GST-PPARγ was incubated with 10 nM biotin-labeled DNA probes containing predicated PPARγ binding site within human (2 sites) and mouse (1 sites) TPH2 promoter or mutant sequences for 6 h. Streptavidin agarose (20 μl) was added for another 6 h. The precipitations were subject to immunoblot with anti-PPARγ antibody. GAPDH signals was presented as NC. (I) Phylogenetic trees of TPH2 in various species including animals from invertebrates to vertebrates and plant were generated from 21 TPH2 protein sequences. The colored pink is vertebrates including mammals (monkey, human, pig, mouse, dog, and rabbit), bony fishes (zebrafish), birds (chicken), amphibians (frog), and turtles (green sea turtle and turtle); the colored light orange is coelacanth, which is believed to be the ancestor of vertebrate; the colored orange is thorny skate, which belongs to vertebrate fish; the colored blue is arthropods of invertebrates (fly, beetle, ant, and bee); the colored yellow is mollusks (California sea hare and sea urchins); the colored green is nematodes; the light gray is plant.

Although ACSS2 can provide acetyl-CoA groups for histones to epigenetically regulate gene expression, the transcription activator, which can directly bind DNA, is also necessary for gene transcription via recruiting the necessary transcriptionary elements including RNA polymerase. Here, as for *Tph2* histone acetylation, we then tried to find the ACSS2 partner. By analyzing *Tph2* upstream 2,000-base-pair (bp) sequence from transcription start site (TSS) by bioinformatic method (http://jaspar.genereg.net/), which contains the core promoter region (−107 to +7 bp), we found that there were 3 putative PPARγ binding sites in human and one putative PPARγ binding site in mouse (Table S1). To further identify PPARγ role in *TPH2* expression, we examined TPH2 changes in response to PPARγ. The results showed that both PPARγ overexpression and its agonist rosiglitazone greatly triggered TPH2 transcription, while its down-regulation or antagonist GW9662 abolished TPH2 induction in SH-SY5Y cells (Fig. 3A to D). Besides, PPARγ overexpression activated AMPK signal, but AMPK activation or inactivation did not influence PPARγ expression, indicating that PPARγ may theoretically facilitate ACSS2 nuclear localization to induce target genes expression (Fig. 3A and Fig. S3A). To further explore PPARγ transactivation in TPH2 promoter, we examined the human and mouse TPH2-promoter-driven luciferase activities in response to increasing PPARγ in human embryonic kidney (HEK) 293T cells. The results showed that wild-type PPARγ, rather than the ligand-irresponsive mutants including P465L and P466/467L, could augment both human and mouse TPH2 promoter activities illustrated by the luciferase activities, suggesting that PPARγ triggers TPH2 promoter activity in a ligand-dependent manner (Fig. 3E to G). In vitro DNA probe pull-down experiment, the recombinant protein PPARγ could specifically bind the probes containing the putative PPARγ binding sites within human and mouse *Tph2* promoter but almost lost the binding ability when DNA sequence mutated (Fig. 3H). These data support that PPARγ can bind *Tph2* promoter to activate its activity. We next analyzed *Tph2* sequences or its homolog in various species including animals from invertebrate to vertebrate and plant and found that *Tph2* or its homolog promoter (−2,000 bp to TSS) in animals almost has PPARγ binding site, indicating that PPARγ regulation in *Tph2* or its homolog in different species is highly conserved and positively selected during the evolution (Fig. 3I and Table S2). In conclusion, PPARγ is a transcription activator of *Tph2* to activate its activity.

### PPARγ binds directly ACSS2 to allow *Tph2* histone acetylation

**Fig. 4.**
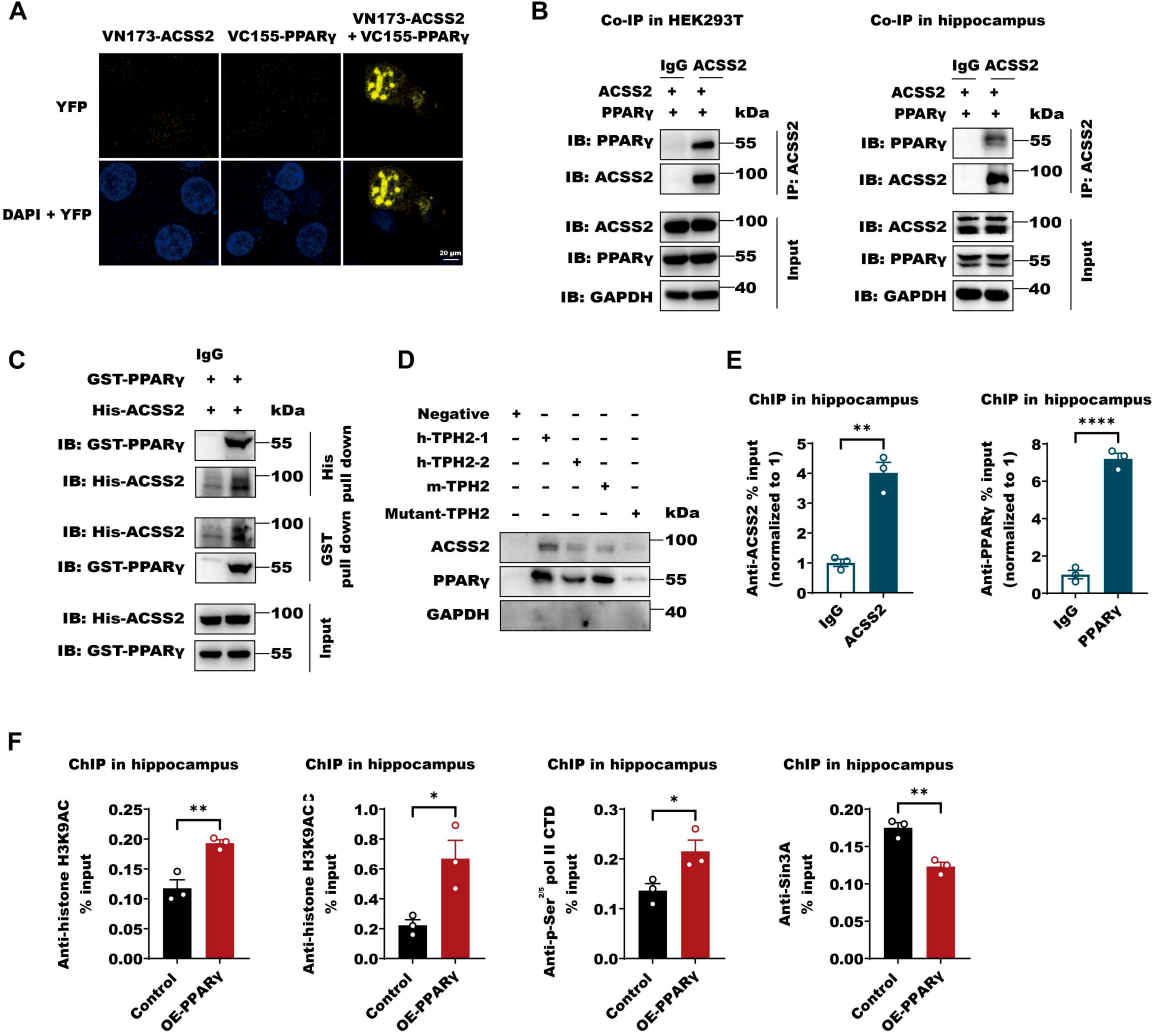
PPARγ binds directly ACSS2 to allow *Tph2* histone acetylation. (A) Representative BiFC fluorescent images of HEK293T cells transfected with 2 μg of plasmid encoding ACSS2 and PPARγ alone or together fused to the fluorescent protein fragments indicated in each panel. DAPI stain demonstrated nuclear locus. The intensity yellow fluorescent protein (YFP) signal indicates the amounts and localization of BiFC complex (ACSS2–PPARγ). (B) Co-IP of ACSS2 and PPARγ in HEK293T cells or hippocampus of mice. IB, immunoblot. (C) Pull-down assay of purified recombination proteins His-ACSS2 and GST-PPARγ from BL21 (DE3). (D) Pull-down assay of DNA probes and hippocampal ACSS2 was performed. The equal (10 mg) hippocampus lysate from 9 mice of individual groups were incubated with excess biotin-labelled DNA probes (20 nM) containing predicated PPARγ binding site within human TPH2 promoter for 6 h. Streptavidin agarose (20 μl) was added for another 6 h. The precipitations were subject to immunoblot with anti-PPARγ and anti-ACSS2 antibody. GAPDH signals was presented as NC. (E) ChIP analyses using an anti-ACSS2 and anti-PPARγ antibodies were performed in hippocampus of mice. The histogram shows the amount of immunoprecipitated DNA expressed as a percentage of the total input DNA. The data are presented as the means ± SEM of triplicate samples. Data are shown as means ± SEM (*n* = 3 per group) and were analyzed using unpaired 2-tailed Student’s *t* test, ACSS2 (*t* = 8.121, df = 4, *P* = 0.0013) and PPARγ (*t* = 16.69, df = 4, *P* < 0.0001). **P  < 0.01 and ****P  < 0.0001. (F) SH-SY5Y cells were transfected with PPARγ expression plasmid for 24 h. ChIP analyses using an anti-H3K9Ac, anti-H3K27Ac, anti-CTD, and anti-Sin3A antibodies were performed. The histogram shows the amount of immunoprecipitated DNA expressed as a percentage of the total input DNA. Data are shown as means ± SEM (*n* = 3 per group) and were analyzed using unpaired 2-tailed Student’s *t* test, H3K9Ac (*t* = 4.933, df = 4, *P* = 0.0079), H3K27Ac (*t* = 3.478, df = 4, *P* = 0.0254), p-Ser^2/5^ polymerase II (pol II) CTD (*t* = 2.928, df = 4, *P* = 0.0429), and Sin3a (*t* = 5.746, df = 4, *P* = 0.0045) *P  < 0.05 and **P  < 0.01.

Considering that both ACSS2 and PPARγ can decode *Tph2* transcription, how they cooperate with each other to control *Tph2* expression required further investigation. We primarily observed that there was a direct interaction between ACSS2 and PPARγ by bimolecular fluorescent complimentary (BiFC) with a positive pair of bfos–bJun in HEK293T cells (Fig. 4A and Fig. S4A). We noted that their interactions predominantly happened in the nuclear rather than cytoplasm, and it was important for facilitating target gene histone acetylation and transcription. Their physical interactions were further confirmed by coimmunoprecipitation (co-IP) in HEK293T cells containing the expressing plasmids of ACSS2 and PPARγ and the whole-cell lysates from the mouse hippocampus (Fig. 4B). Finally, the purified proteins of ACSS2 and PPARγ could pull down each other (Fig. 4C). These data supported that ACSS2 can directly bind PPARγ. Importantly, in the cocultures of the hippocampal lysates from mice with biotin-labeled DNA probes with PPARγ putative binding sites from human or mouse *Tph2* promoter, ACSS2 could be pulled down by the DNA probes along with PPARγ, while the mutated DNA probe lost the ability to pull down both ACSS2 and PPARγ (Fig. 4D). In the hippocampus of mice, both PPARγ and ACSS2 were able to occupy *Tph2* promoter in the ChIP assay (Fig. 4E). These observations suggest that a complex of PPARγ and ACSS2 can bind *Tph2* promoter. Since ACSS2 can potentiate *Tph2* histone acetylation, we further examined PPARγ function in *Tph2* histone acetylation. The results showed that PPARγ overexpression caused higher levels of acetylated H3K9 and H3K27 in *Tph2* promoter in the SH-SY5Y cells (Fig. 4F). Correspondingly, the recruitment of active RNA polymerase II with Ser^2^ and Ser^5^ phosphorylated at its C-terminal repeat domain (CTD) was promoted by PPARγ overexpression, while the repressor Sin3A binding was contrarily inhibited, suggesting that transcriptionary activities of *Tph2* is triggered by PPARγ via recruiting the necessary transcription activation elements (Fig. 4F). In conclusion, PPARγ acts a partner of ACSS2 to promote *Tph2* histone acetylation and transcription.

### SCFAs trigger PPARγ to mediate the antidepressant responses in CRS-exposure mice via TPH2

**Fig. 5.**
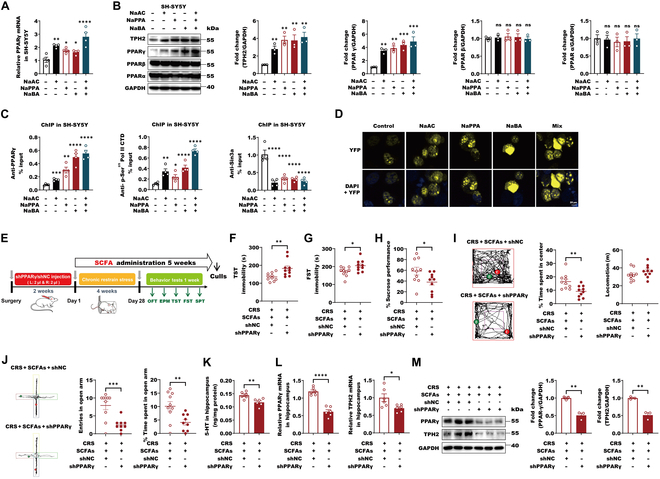
SCFAs trigger PPARγ to mediate the antidepressant responses in CRS-exposure mice via TPH2. (A) The mRNA levels of *PPARγ* in SH-SY5Y cells with or without 10 μM NaAC, 1 μM NaPPA, and 0.1 μM NaBA alone or together for 12 h. Data were normalized with GAPDH mRNA levels. Scale bars represent means values, and error bars represent SEM of triplicate samples. Data are shown as means ± SEM (*n* = 4 per group) and were analyzed using one-way ANOVA, *F*_4,15_ = 11.7, *P* = 0.0002, and Tukey’s multiple comparison test, *P < 0.05, **P < 0.01, and ****P < 0.0001. (B) The lysates of SH-SY5Y cells with or without 10 μM NaAC, 1 μM NaPPA, and 0.1 μM NaBA alone or together for 24 h were subjected to Western blot with indicated antibodies. The levels of TPH2, PPARα, PPARβ, and PPARγ were quantitatively analyzed (*n* = 3 biological replicates). Data are shown as means ± SEM (*n* = 3 per group) and were analyzed using one-way ANOVA, TPH2 (*F*_4,10_ = 8.281, *P* = 0.0032), PPARα (*F*_4,10_ = 0.1347, *P* = 0.9658), PPARβ (*F*_4,10_ = 0.1479, *P* = 0.9597), and PPARγ (*F*_4,10_ = 13.1, *P* = 0.0005), and Tukey’s multiple comparison test, **P < 0.01 and ***P < 0.001. (C) SH-SY5Y cells were incubated with or without 10 μM NaAC, 1 μM NaPPA, and 0.1 μM NaBA alone or together for 24 h. ChIP analyses using an anti-ACSS2, anti-CTD, and anti-Sin3A antibodies were performed. The histogram shows the amount of immunoprecipitated DNA expressed as a percentage of the total input DNA. The data are presented as the means ± SEM of quadruplicate samples. Data are shown as means ± SEM (*n* = 4 per group) and were analyzed using one-way ANOVA, PPARγ (*F*_4,15_ = 29.04, *P* < 0.0001), p-Ser^2/5^ pol II CTD (*F*_4,15_ = 33.26, *P* < 0.0001), and Sin3a (*F*_4,15_ = 23.71, *P* < 0.0001), and Tukey’s multiple comparison test, *P < 0.05, **P < 0.01, ***P < 0.001, and ****P < 0.0001. (D) Representative BiFC fluorescent images of SH-SY5Y cells transfected with 2 μg of plasmid encoding ACSS2 or PPARγ fused to the fluorescent protein fragments indicated in each panel in response to 10 μM NaAC, 1 μM NaPPA, and 0.1 μM NaBA alone or together for 24 h. DAPI stain demonstrated nuclear locus. The intensity YFP signal indicates the amounts and localization of BiFC complex (ACSS2–PPARγ). (E) Two weeks after injecting the shPPARγ virus into the hippocampus of mice using stereotaxic injection techniques, the CRS modeling experiment was initiated for 4 weeks, during which mice had free access to drinking SCFAs or water. Behavioral tests were then conducted for 1 week (*n* = 10 per group). (F) Immobility time in the TST in mice from CRS + SCFAs + shNC and CRS + SCFAs + shPPARγ groups were detected. Data are shown as means ± SEM (*n* = 10 per group) and were analyzed using unpaired 2-tailed Student’s *t* test, *t* = 2.946, df = 18, *P* = 0.0086. **P < 0.01. (G) Immobility time in the FST in mice from CRS + SCFAs + shNC and CRS + SCFAs + shPPARγ groups were detected. Data are shown as means ± SEM (*n* = 10 per group) and were analyzed using unpaired 2-tailed Student’s *t* test, *t* = 2.522, df = 18, *P* = 0.0213. *P < 0.05. (H) SPTs in mice from CRS + SCFAs + shNC and CRS + SCFAs + shPPARγ groups were detected. Data are shown as means ± SEM (*n* = 10 per group) and were analyzed using unpaired 2-tailed Student’s *t* test, *t* = 2.258, df = 18, *P* = 0.0366. *P < 0.05. (I) Raw traces of mice in the OFT were shown. Total distance traveled in the OFT and time spent exploring the center area in the OFT from mice in individual animals from CRS + SCFAs + shNC and CRS + SCFAs + shPPARγ groups. Data are shown as means ± SEM (*n* = 10 per group) and were analyzed using unpaired 2-tailed Student’s *t* test, time (*t* = 3.185, df = 18, *P* = 0.0051) and locomotion (*t* = 1.329, df = 18, *P* = 0.2004). **P < 0.01. (J) Raw traces of mice in the EPM were shown. Time spent in the open arms and probability of entering open arms in the EPM test from mice in individual animals from CRS + SCFAs + shNC and CRS + SCFAs + shPPARγ groups. Data are shown as means ± SEM (*n* = 10 per group) and were analyzed using unpaired 2-tailed Student’s *t* test, time (*t* = 2.923, df = 17, *P* = 0.0095) and entries (*t* = 4.064, df = 17, *P* = 0.0008). **P < 0.01 and ***P < 0.001. (K) Analysis of 5-HT content of hippocampus by ELISA in male mice from CRS + SCFAs + shNC and CRS + SCFAs + shPPARγ groups. Data are shown as means ± SEM (*n* = 6 per group) and were analyzed using unpaired 2-tailed Student’s *t* test, **P < 0.01. (L) RT-PCR analysis of *PPARγ* and *TPH2* expression levels in hippocampus of male mice from CRS + SCFAs + shNC and CRS + SCFAs + shPPARγ groups (*n* = 6 per group). Data were normalized with GAPDH mRNA levels and presented as fold changes compared with control group. Scale bars represent means values, and error bars represent SEM. Data are shown as means ± SEM (*n* = 6 per group) and were analyzed using unpaired 2-tailed Student’s *t* test, *P < 0.05 and ****P < 0.0001. (M) Representative immunoblots and quantification of PPARγ and TPH2 protein levels normalized to loading controls in hippocampal male mice from CRS + SCFAs + shNC and CRS + SCFAs + shPPARγ groups. Data are shown as means ± SEM (*n* = 3 per group) and were analyzed using unpaired 2-tailed Student’s *t* test, PPARγ (*t* = 6.951, df = 4, *P* = 0.0023) and TPH2 (*t* = 7.074, df = 4, *P* = 0.0021). **P < 0.01.

Butyrate has been reported to be a ligand of PPARγ to activate gene expression [40]. On this basis, SCFAs might trigger neuronal PPARγ to participate in SCFA-induced antidepressant actions via ACSS2–TPH2 axis. To explore this hypothesis, we started to evaluated the PPARγ changes in response to SCFAs. The results showed that as low as 1 μM acetate and 0.1 μM propionate and butyrate could specially promote PPARγ expression along with increased TPH2 in SH-SY5Y cells, while PPARα and PPARβ were not influenced (Fig. 5A to B). In primary mouse hippocampal neurons, physiological concentrations of SCFAs including as low as 10 μM acetate, 1 μM propionate, and 0.1 μM butyrate all augmented PPARγ expression as well (Fig. S5A to C). As for *Tph2* regulation, higher levels of PPARγ were recruited to the *Tph2* promoter by acetate, propionate, and butyrate alone or together along with higher levels of ACSS2, acetylated histone H3 including H3K9 and H3K27, phosphorylated RNA polymerase II at Ser^2^ and Ser^5^, and lower levels of Sin3A in SH-SY5Y cells (Figs. 1F and G and 5C). These findings indicate that SCFAs can trigger ACSS2 and PPARγ localization in *Tph2* promoter region, thereby activating its transcription by facilitating histone acetylation and transcription elements recruitment. To further support this finding, SCFAs indeed showed the ability to augment the complex of ACSS2–PPARγ formation by the BiFC assay in SH-SY5Y cells (Fig. 5D). We then further examined the requirement of neuronal PPARγ in SCFA-mediated antidepressant actions, given that SCFAs can trigger PPARγ–TPH2 axis. The behavioral tests of TST, FST, SPT, OFT, and EPM showed that AAV-mediated neuronal PPARγ knockdown remarkedly abolished SCFA-induced antidepressive- and antianxiety-like behaviors in CRS-exposure mice (Fig. 5E to J). The 5-HT levels in the hippocampus of SCFA-treated CRS mice were also decreased by PPARγ knockdown (Fig. 5K). Correspondingly, the levels of hippocampal TPH2 were reduced along with the decreased PPARγ (Fig. 5L and M). In conclusion, these data support that PPARγ is indispensable for SCFAs to exert the antidepressant action via TPH2 in CRS-exposure mice.

### D-Mannose significantly elevates brain SCFAs by altering the gut microbiota of CRS-exposure mice

**Fig. 6.**
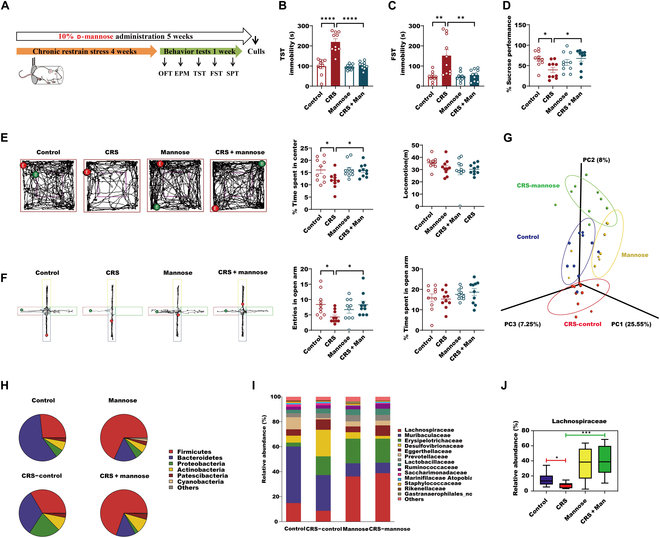
d-mannose significantly elevates brain SCFAs by altering the gut microbiota of the CRS-exposure mice. (A) CRS-induced male mice with depressive-like behaviors for 4 weeks and male mice with or without 10% d-mannose supplementation and behavioral testing started on day 29 and lasted for 1 week (*n* = 10 per group). (B) Immobility time in the TST in mice from control, CRS, mannose, and CRS + mannose groups. Data are shown as means ± SEM (*n* = 10 per group) and were analyzed using 2-way ANOVA and Tukey’s multiple comparison test, ****P < 0.0001. (C) Immobility time in the FST in mice from control, CRS, mannose, and CRS + mannose groups. Data are shown as means ± SEM (*n* = 10 per group) and were analyzed using 2-way ANOVA and Tukey’s multiple comparison test, **P < 0.01. (D) SPTs in mice from control, CRS, mannose, and CRS + mannose groups were detected. Data are shown as means ± SEM (*n* = 10 per group) and were analyzed using 2-way ANOVA and Tukey’s multiple comparison test, *P < 0.05. (E) Raw traces of mice in the OFT were shown. Total distance traveled in the OFT and time spent exploring the center area in the OFT from mice in individual animals from control, CRS, mannose, and CRS + mannose groups. Data are shown as means ± SEM (*n* = 10 per group) and were analyzed using 2-way ANOVA and Tukey’s multiple comparison test, *P < 0.05. (F) Raw traces of mice in the EPM were shown. Time spent in the open arms and probability of entering open arms in the EPM test from mice in individual animals from control, CRS, mannose, and CRS + mannose groups. Data are shown as means ± SEM (*n* = 10 per group) and were analyzed using 2-way ANOVA and Tukey’s multiple comparison test, *P < 0.05. (G) Principal coordinates analysis (PCoA) of cecum microbiota from the 4 mice groups of study is shown. Mice groups color coding: purple, control group (control); red, depression mouse group (CRS); yellow, mice with d-mannose administration (mannose); green, depression mice with d-mannose administration (CRS + mannose). (H) Microbial distribution at phylum level. Relative abundances of phylum-level distributions of cecum microbiota from control, CRS-control, mannose, and CRS-mannose groups are shown. (I) Microbial distribution at family level in control, CRS-control, mannose, and CRS-mannose groups. All families comprising less than 1% of the total abundance were combined into the “others” category. (J) Relative abundance of Lachnospiraceae with significant differences among the 4 mice groups of study. Data are shown as means ± SEM and were analyzed using 2-way ANOVA and Tukey’s multiple comparison test, *P < 0.05 and ***P < 0.001.

**Fig. 7.**
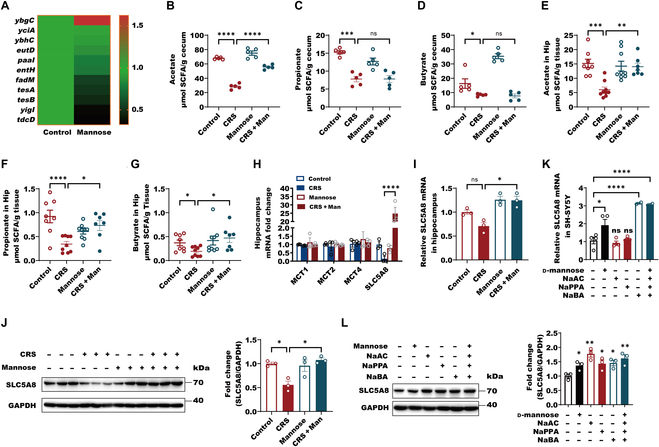
d-Mannose significantly elevates brain SCFAs by altering the gut microbiota of the CRS-exposure mice. (A) The mRNA analysis of *ybgC*, *yciA*, *eutD*, *paaI*, *entH*, *fadM*, *tesA*, *tesB*, *yigI*, and *tdcD* in the steady-phase *E. coli* MG1655 with 1 mM d-mannose treatment for 1 h by RNA sequencing. (B to D) Analysis of gut-microbiota-derived metabolites–SCFAs including acetate (B), propionate (C), and butyrate (D) in cecum content from control, CRS, mannose and CRS + mannose groups shown as individual mice by GC. Data are shown as means ± SEM (*n* = 5 per group) and were analyzed using 2-way ANOVA and Tukey’s multiple comparison test, *P < 0.05, **P < 0.01, ***P < 0.001, and ****P < 0.0001. (E to G) Analysis of gut-microbiota-derived SCFAs including acetate (E), propionate (F), and butyrate (G) in hippocampus (Hip) from control, CRS, mannose, and CRS + mannose groups shown as individual mice by GC. Data are shown as means ± SEM (*n* = 7 to 9 per group) and were analyzed using 2-way ANOVA and Tukey’s multiple comparison test, *P < 0.05, **P < 0.01, ***P < 0.001, and ****P < 0.0001. (H) The mRNA of *MCT1, MCT2, MCT4*, and *SLC5A8* analysis in hippocampus from mice in response to CRS and d-mannose administration (*n* = 10, 3 or 4 per group mice were combined as one sample for RNA sequencing) by RNA sequencing. Data are shown as means ± SEM (*n* = 3 per group) and were analyzed using 2-way ANOVA and Tukey’s multiple comparison test, ****P < 0.0001. (I) The mRNA levels of *SLC5A8* in hippocampus of mice exposure to CRS and d-mannose administration. Data are shown as means ± SEM (*n* = 3 per group) and were analyzed using 2-way ANOVA and Tukey’s multiple comparison test, *P < 0.05. (J) Representative immunoblots and quantification of hippocampal SLC5A8 protein levels normalized to loading controls from mice exposure to CRS and d-mannose. Data are shown as means ± SEM (*n* = 3 per group) and were analyzed using 2-way ANOVA and Tukey’s multiple comparison test, *P < 0.05. (K) The mRNA levels of *SLC5A8* in SH-SY5Y cells with or without 1 mM d-mannose, 10 μM NaAC, 1 μM NaPPA, and 0.1 μM NaBA alone or together for 12 h. Data were normalized with GAPDH mRNA levels and presented as fold changes without stimulation. Data are shown as means ± SEM (*n* = 2 to 4 per group) and were analyzed using one-way ANOVA, *F*_5,14_ = 19.93, *P* < 0.0001, and Tukey’s multiple comparison test, *P < 0.05 and ****P < 0.0001. (L) The lysates of SH-SY5Y cells with or without 1 mM d-mannose, 10 μM NaAC, 1 μM NaPPA, and 0.1 μM NaBA alone or together for 24 h were subjected to Western blot with indicated antibodies. The levels of SLC5A8 were quantitatively analyzed (*n* = 3 biological replicates). Data are shown as means ± SEM (*n* = 3 per group) and were analyzed using one-way ANOVA, *F*_5,12_ = 5.942, *P* = 0.0054, and Tukey’s multiple comparison test, *P < 0.05 and **P < 0.01.

As we observed that SCFAs can trigger ACSS2–PPARγ–TPH2 axis, the discovery of novel inducers of brain SCFAs is extremely important for treating depression. d-Mannose has been reported to be utilized by gut microbiota, thereby counteracting high-fat-diet-induced obesity in mice [6]. It is thus interesting to examine the alterations of the gut microbiota in the depressed mice in response to d-mannose. As we previously reported, oral supplement of 10% d-mannose produced significant antidepressive- and antianxiety-like behaviors in CRS-exposure mice by the behavioral tests of TST, FST, SPT, OFT, and EPM (Fig. 6A to F). Interestingly, when we performed microbial 16*S* ribosomal RNA (rRNA) gene sequencing of cecum content and β-diversity analysis, we found that d-mannose induced a major shift in the gut microbial composition in CRS-exposure mice (Fig. 6G and Fig. S6A to D). At the phylum level, d-mannose increased Firmicutes but decreased Bacteroidetes in CRS mice (Fig. 6H). Consistent with the phylum change, d-mannose significantly reversed CRS-induced Lachnospiraceae reduction at the family level, which belongs to Firmicutes and butyrate-producing bacteria (Fig. 6I and J). Besides, d-mannose decreased Muribaculaceae composition in both normal and depressed mice (Fig. S6E), which can degrade complex carbohydrate [41]. Interestingly, d-mannose treatment of *Escherichia coli*, which is rich in gut bacteria [42], significantly induced *ybgC* expression, which is proved to enhance butyrate production (Fig. 7A) [43]. Considering it, we speculated that d-mannose might enhance butyrate generation from the altered gut microbiota. Surprisingly, by examining the SCFA changes in the cecum and hippocampus, we observed that d-mannose significantly reversed the reductions of acetate, propionate, and butyrate in the hippocampus of the CRS-exposure mice, while reductions of propionate and butyrate in the cecum were not reversed by d-mannose (Fig. 7B to G). We then tried to investigate how brain SCFAs were enhanced by d-mannose. By the RNA sequencing of the hippocampus in normal or depressed mice with or without d-mannose administration, we found that the expression of SCFA transporter *SLC5A8*, which preferentially transports butyrate [44], but not *MCT1*, *MCT2*, and *MCT4*, was remarkedly decreased in CRS-exposed mice compared with normal mice (Fig. 7H). However, d-mannose-treated CRS-exposure mice displayed the resistance in the SLC5A8 reduction (Fig. 7H). We further confirmed that SLC5A8 levels were dramatically reduced in the hippocampus of CRS-exposure mice compared with normal mice, while d-mannose antagonized the CRS-induced reduction of SLC5A8 in the hippocampus (Fig. 7I and J). In SH-SY5Y cells, 1 mM d-mannose and 0.1 μM butyrate alone or together, rather than acetate and propionate alone, could enhance SLC5A8 expressions, suggesting that d-mannose and butyrate might specially promote SLC5A8 expression (Fig. 7K and L). Taken together, we speculate that d-mannose might facilitate hippocampal SCFA transport, especially butyrate, via up-regulating SLC5A8. In conclusion, d-mannose can reverse the dysbiosis of the gut microbiota of CRS-exposure mice and elevate brain SCFA content.

**Fig. 8.**
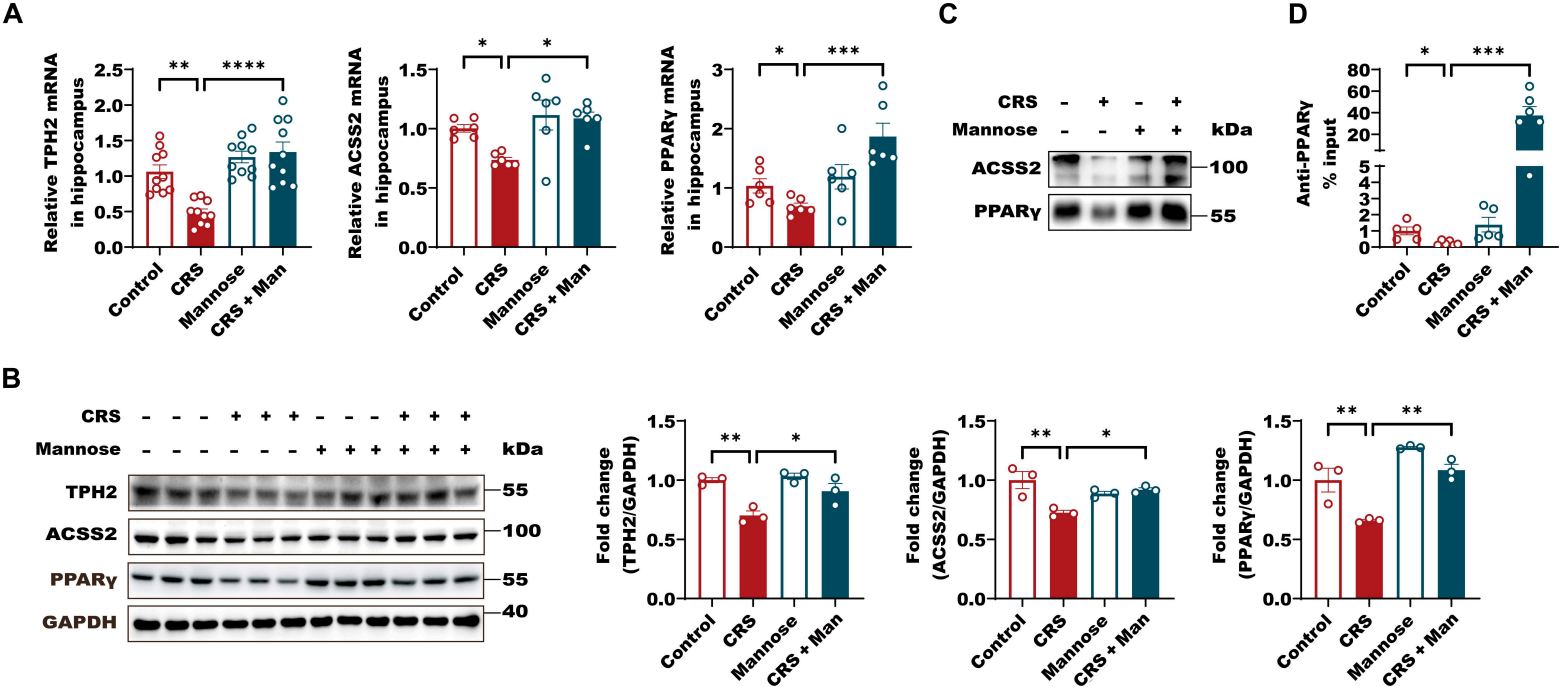
d-Mannose significantly elevates brain SCFAs by altering the gut microbiota of the CRS-exposure mice. (A) RT-PCR analysis of *ACSS2*, *TPH2* and *PPARγ* expression levels in hippocampus of male mice from control, CRS, mannose, and CRS + mannose groups. Data were normalized with GAPDH mRNA levels and presented as fold changes compared with control group. Data are shown as means ± SEM (*n* = 6 to 10 per group) and were analyzed using 2-way ANOVA and Tukey’s multiple comparison test, *P < 0.05, **P < 0.01, ***P < 0.001, and ****P < 0.0001. (B) Representative immunoblots and quantification of ACSS2, TPH2, and PPARγ protein levels normalized to loading controls in hippocampal male mice from control, CRS, mannose, and CRS + mannose groups. Data are shown as means ± SEM (*n* = 3 per group) and were analyzed using 2-way ANOVA and Tukey’s multiple comparison test, *P < 0.05 and **P < 0.01. (C) Pull-down assay of DNA probes and hippocampal ACSS2 was performed. The equal (10 mg) hippocampus lysates from 9 mice of individual groups were incubated with excess biotin-labeled DNA probes (20 nM) containing predicated PPARγ binding site within human TPH2 promoter for 6 h. Twenty microliters of streptavidin agarose was added for another 6 h. The precipitations were subject to immunoblot with anti-PPARγ and anti-ACSS2 antibody. GAPDH signals was presented as NC. (D) ChIP analyses using an anti-PPARγ antibody were performed in hippocampus of mice from control, CRS, mannose, and CRS + mannose groups (*n* = 5 per group). The histogram shows the amount of immunoprecipitated DNA expressed as a percentage of the total input DNA. Data are shown as means ± SEM (*n* = 5 to 6 per group) and were analyzed using 2-way ANOVA and Tukey’s multiple comparison test, *P < 0.05 and ***P < 0.001.

As the downstream effectors of SCFAs, the reductions of ACSS2, PPARγ, and their targets, TPH2, in the hippocampus of d-mannose-treated CRS mice were partially reversed compared to those of CRS mice (Fig. 8A and B). When we incubated DNA probe with the same amount of hippocampus lysate from depression mice fed with water or d-mannose, we found that CRS caused reduction in both PPARγ and ACSS2 bindings to DNA probe, while d-mannose administration reversed the reductions of ACSS2 and PPARγ binding to the DNA probe (Fig. 8C). In the ChIP assay, the reduction of PPARγ binding to the *tph2* promoter was partially prevented in the hippocampus of d-mannose-treated CRS mice compared with CRS mice without d-mannose treatment (Fig. 8D). Combined with our previous finding that d-mannose can rescue the defects of *Tph2* histone acetylation in CRS-exposure mice by directly entering the brain, we propose that d-mannose can also activate ACSS2–PPARγ–TPH2 axis via elevating brain SCFAs and reversing the dysbiosis of the gut microbiota in CRS mice.

## Discussion

Although increasing evidence has supported that SCFAs act as key metabolites in MGB communication to regulate multiple psychiatric and neurological disorders, its functions at the physiological concentrations are largely unknown. In human brain tissues, average concentrations of brain tissues for butyrate (17.0 pmol/mg) and for propionate (18.8 pmol/mg) have been reported [45]. By positron emission tomography (PET) imaging, only about 3% of acetate and 0.006% of butyrate were taken up in the rat brain, and no measurable brain uptake of ^11^C-acetate was detected in the human brain [46–48]. Therefore, despite being able to cross the blood–brain barrier, brain uptake of SCFAs seems to be minimal. Furthermore, the present SCFA studies of animal research mostly come from exogeneous supplements, and the regulation mechanism largely depends on their activities of inhibiting HDAC. However, millimolar concentrations of SCFAs have to be present for serving as HDAC inhibitors [19,20]. On the basis of these, it is extremely important to investigate micromolar or nanomolar SCFA function, particularly for the brain, where the low SCFAs are normally present. In this study, we show that micromolar or nanomolar SCFAs (acetate, propionate, and butyrate), much lower than millimolar that acts as HDAC inhibitors (butyrate and, to a lesser extent, propionate), can directly promote ACSS2-mediated target gene histone acetylation, for example, *Tph2*. In this respect, it provides a novel clue of SCFA-mediated gene regulation via ACSS2 and is generally meaningful for fully understanding SCFA function under the physiological condition.

Extensive studies have been carried out to explore the mechanism underlying the psychiatric disorders including the antidepressive effects for SCFAs, such as synapse plasticity involving BDNF, neuroinflammation suppression, and neurotransmitter [21,37,49]. The precise target of brain SCFAs remains vague. We reveal that neuronal ACSS2 and PPARγ are novel targets for brain SCFAs to exerting the antidepressive effects. Our group reports that ACSS2 is decreased in the CRS-exposure mice and, thus, a novel target for antidepression via BDNF and TPH2 [28]. However, how ACSS2 is regulated in the development of depression remains unclear. In this study, we revealed that the hippocampal SCFAs in CRS-treated mice were decreased, and it may provide an explanation for ACSS2 reduction in the stressed mice. Moreover, SCFAs, as brain ACSS2 inducers, are further supported to be a promising agent to prevent depression. Nevertheless, the alterations of brain SCFAs in different types of cells and their contributions to the pathogenesis of depression are worth further investigation, which will provide further evidence to link the gut microbiota and the brain function via SCFAs. The studies from our and other group identified multiple ACSS2 target including *NMDARs*, *AMPARs*, and *Tph2* [27,28]. However, it is still unclear which genes are governed by ACSS2 and what is the characteristics of ACSS2-targeted genes. In future, we should pay more attention to investigate other ACSS2 targets to advance our deeper understanding of its physiological role.

Among of the PPAR group including PPARα, PPARβ, and PPARγ, PPARγ presents the highest expression in central nervous system and can be expressed in neurons, astrocytes, and glial cells [50]. Microglial PPARγ has been evidenced to suppress microglial activation and neuroinflammation [32,33,35], whereas neuronal PPARγ function needs exploration. Here, we found that PPARγ is a transcription activator of *Tph2* together with ACSS2 and its expression can be induced by physiological brain levels of SCFAs. During the development of serotonergic neurons, PPARγ is hardly expressed [31,51], suggesting that ACSS2–PPARγ signal may not be involved in serotonergic neuron development but developed as novel *Tph2* regulators in adult mice to rapidly adapt to cell metabolic changes. In the view of human evolution, nutrient scarcity, which cause the insufficient cell energy and AMPK activation, is a selective pressure and driving force that has shaped the evolution of most cellular processes. We speculate that *Tph2* regulation governed by ACSS2 and PPARγ is perhaps evolutionarily positively selected for individuals who were adept at synchronizing 5-HT homeostasis with environmental nutrient levels via AMPK to make their brain function well in a fasted state. Besides, the animals from invertebrate to vertebrate *Tph2* or its homolog have putative PPRE (PPAR response elements) elements, suggesting that ACSS2–PPARγ function may be positively selected during animal evolution.

Since SCFAs are a proposing approach for preventing or treating diverse psychiatric and neurological disorders, how to elevate their levels in the brain via altering the gut microbiota is important. In this respect, d-mannose functions as prebiotics, such as fructo-oligosaccharides and galacto-oligosaccharides [5], and greatly improves gut microbiota dysbiosis in the CRS-treated mice. More importantly, d-mannose can enhance SCFA generation by enriching SCFA-producing Lachnospiraceae [52]. In addition to the productions of acetate and butyrate, Lachnospiraceae can also generate lantibiotics to prevent drug-resistant pathogen colonization [53,54]. On the basis of these findings, we suggest that d-mannose may abolish common clinical antibiotic treatment that caused Lachnospiraceae decrease and *Clostridium difficile* infection. Although we provide some clues about the special induction of SLC5A8 by butyrate in SH-SY5Y cells, it still needs further study to elucidate the precise mechanism of SLC5A8 regulation in reshaping SCFA influx into the brain, which would bring forward to better understand brain SCFA function. Combined with our previous finding, d-mannose not only enters brain directly to exert the antidepressive effects via ACSS2 in mice [28] but also can reverse the dysbiosis of the gut microbiota in CRS mice and elevate brain SCFAs, supporting it as a safe and promising agent to prevent depressive-like behaviors. Notably, increasing evidence demonstrates that the vagus nerve crucially controls the bidirectional communication between the gut and brain and is required for the pathogenesis of depression with abnormal gut microbiota [55–57]. Therefore, it is an interesting topic to further investigate whether the subdiaphragmatic vagotomy can block the antidepressive-like effects of SCFAs or d-mannose in CRS-exposure mice.

In summary, SCFAs significantly induce both ACSS2 and PPARγ to mediate the antidepressant responses via TPH2 in CRS mice. PPARγ is identified as a novel partner of ACSS2 to potentiate *Tph2* histone acetylation and transcription. To further support brain SCFAs as a therapeutic agent for depression, d-mannose can significantly reverse the dysbiosis of the gut microbiota in CRS mice and augment brain SCFAs to protect against depressive-like behaviors via ACSS2–PPARγ–TPH2 axis. We thus suggest d-mannose to be an inducer of brain SCFAs in resisting depression.

## Materials and Methods

### Table of key resources

**Table:** 

Reagent or resource	Source	Identifier
**Antibodies**		
Anti-TPH2	Abcam	Ab111828
Anti-ACSS2	Santa Cruz Biotechnology	398559
Anti-PPARγ	Cell Signaling Technology (CST)	2435S
Anti-glyceraldehyde-3-phosphate dehydrogenase (GAPDH)	ZSGB-BIO	TA-08
Anti-PPARα	Abcam	ab215270
Anti-PPARβ	Abcam	ab23673
Anti-p-ACSS2	Jiaxing Xinda Biological Technology	58003
Anti-AMPKα-p-T172	CST	2535S
Anti-AMPKα	CST	5831
Anti-ACLY	Santa Cruz Biotechnology	sc-517267
Anti-SLC5A8	Invitrogen	PA5-42514
Anti-Lamin-B	Proteintech	66095-1-Ig
Anti-Flag	Sigma-Aldrich	F7425
Anti-glutathione *S*-transferase (GST)	CST	2624S
Anti-hemagglutinin (HA)	Proteintech	51064-2-AP
**High-performance liquid chromatography (HPLC) standards**		
Acetate	Bnbio	Catalog no. BWJ4299-2016
Propionate	Bnbio	Catalog no. BWQ7048-2016
Butyrate	Bnbio	Catalog no. BWZ6668-2016
**Chemicals and inhibitors**		
Anti-HA magnetic beads	Bimake	B26201
Anti-Flag magnetic beads	Bimake	B26101
Glutathione agarose	Santa Cruz Biotechnology	sc-2009
Anti-His magnetic beads	Sigma-Aldrich	H9914
Streptavidin	Sigma-Aldrich	S1638
Ni–nitrilotriacetic acid His bind	Sigma-Aldrich	70666-4
GST-Sefinose resin 4FF (settled resin)	Sangon Biotech	C600031
Rosiglitazone	Selleck	S2556
GW9662	Selleck	S2915
Isopropyl-β-d-thiogalactopyranoside (IPTG)	Sangon Biotech	B300845-0005
Protease inhibitor cocktail	Bimake	B14002
Polyvinylidene difluoride membranes	Millipore	IPVH00010
Protein A/G-Sepharose	Santa Cruz Biotechnology	sc-2003
Lipofectamine 2000	Invitrogen	Catalog no. 11668019
ACSS2 inhibitor	Selleck	S8588
AICAR (acadesine)	Selleck	S1802
Compound C	Selleck	S7306
Phenylmethylsulfonyl fluoride (PMSF)	Sigma-Aldrich	Catalog no. 52332
4233-Diamidino-2-phenylindole (DAPI)	Invitrogen	Catalog no. S36964
**Critical commercial assays**		
Mouse 5-HT enzyme-linked immunosorbent assay (ELISA) assay kit	Jianglai Bio	Catalog no. JL12087
Human acetyl-CoA ELISA assay kit	Jianglai Bio	Catalog no. JL32777
QiaAmp DNA stool kit	QIAGEN	Catalog no. 51504
ECL (enhanced chemiluminescence)Western blot kit	Millipore	Catalog no. 69078
SimpleChIP plus sonication ChIP kit	CST	Catalog no. 56383
The nuclear/cytosol fractionation kit	Thermo Fisher Scientific	Catalog no. 78833
Dual luciferase assay system	Vazyme	Catalog no. DL101-01
**Experimental models: Cell lines**		
Cell line: HEK293T	Shanghai Cell Bank of Chinese Academy of Sciences	GNHu17
Cell line: SH-SY5Y	American Type Culture Collection	CRL-2266
Cell line: HT22	Fenghui Bio	CL0162
**Experimental models: Organism**		
Mouse: C57BL/6J	Charles River	N/A
Bacteria: *E. coli* DH5α	Zhuangmeng Bio	ZK206
Bacteria: *E. coli* MG1655	Our Lab	
Bacteria: *E. coli* BL21 (DE3)	Zhuangmeng Bio	ZK202
**Oligonucleotides**		
Human TPH2-1:AGAGTTATATGGAGGAAAATGTATTGCAAAGGAAAGGTAAGGGTTCAATTTAGCCACATG	This study	N/A
Anti-human TPH2-1:CATGTGGCTAAATTGAACCCTTACCTTTCCTTTGCAATACATTTTCCTCCATATAACTCT	This study	N/A
Human TPH2-2:GACAGTATGTTTAGTCATTAAAAGCTCAAATTGTCATAGTACTCTTAACCTCTGCTTTCTC	This study	N/A
Anti-human TPH2-2:GAGAAAGCAGAGGTTAAGAGTACTATGACATTTGAGCTTTTAATGACTAAACATACTGTC	This study	N/A
Mouse TPH2:GTTCGAAAATGGTGTAATCTAGATGTAGGGAAAGGATGACAAATTTAAAAGAGAAGCACC	This study	N/A
Anti-mouse TPH2:GGTGCTTCTCTTTTAAATTTGTCATCCTTTCCCTACATCTAGATTACACCATTTTCGAAC	This study	N/A
Mutant TPH2:AGAGTTATATGGAGGAAAATGTATTCATGCTAGCATTGCGGGGTTCAATTTAGCCACATG	This study	N/A
Anti-mutant TPH2:CATGTGGCTAAATTGAACCCCGCAATGCTAGCATGAATACATTTTCCTCCATATAACTCT	This study	N/A
Primers used for quantitative polymerase chain reaction (PCR), see Table S3	This study	N/A
Primers used for ChIP assay, see Table S3	This study	N/A
Primers used for plasmids construction, see Table S3	This study	N/A
**Recombinant DNA**		
PET28a	Miaoling Bio	P0023
PGEX-4T	Miaoling Bio	P0001
pGL3 basic	Promega	Catalog no. E1751
pBiFC-bFosVC155	Addgene	22013
pBiFC-bJunVN173	Addgene	22012
pBiFC-VC155	Addgene	22011
pBiFC-VN173	Addgene	22010
pBiFC-VC155-PPARγ	This study	N/A
pBiFC-VN173-ACSS2	This study	N/A
PGL3-TPH2 promoter-luciferase (human)	This study	N/A
PGL3-TPH2 promoter-luciferase (mouse)	This study	N/A
pCMV-ACSS2	This study	N/A
pCMV-PPARγ	This study	N/A
PET28a-ACSS2	This study	N/A
PGEX-4T-PPARγ	This study	N/A
PGL3-TPH2	This study	N/A
**Software**		
ImageJ		https://imagej.en.softonic.com
Zeiss Zen	Zeiss	https://www.zeiss.com
GraphPad Prism	GraphPad	https://www.graphpad.com/
MEGAX		https://www.megasoftware.net/
ITOL		https://itol.embl.de
Jaspar		http://jaspar.genereg.net/
**Other**		
Zebron ZB-FFAP column (30 m × 0.32 mm × 0.25 mm)	Phenomenex	UK

### Mice

Six- to 8-week-old male mice were used in all experiments. The mice were housed under specific-pathogen-free conditions on a 12-h light/dark cycle at 18 to 22 °C with food and water available ad libitum unless noted otherwise. All animal experiments were in accordance with the National Institutes of Health Guide for the Care and Use of Laboratory Animals and were approved by the Animal Care and Utilization Committee of Shandong University. Mice were divided randomly into stress groups and home cage controls with or without d-mannose or SCFAs. The mice were fed with water or 10% d-mannose or SCFAs (acetate, 67.5 mM; propionate, 25 mM; butyrate, 40 mM) in drinking water for 4 weeks.

### Cells

SH-SY5Y human neuroblastoma cells were grown in 1:1 mixture of minimum essential medium (MEM) and F12, supplemented with 10% heat-inactivated fetal bovine serum (FBS), 1 mM sodium pyruvate, 0.1 mM nonessential amino acid, sodium bicarbonate (1.5 g/l), penicillin (100 U/ml), and streptomycin (100 μg/ml). All media and supplements were purchased from Gibco (Gaithersburg, MD, USA). Cells were maintained at 37 °C in a humidified atmosphere of 5% CO_2_. Cells were treated with the indicated concentrations of d-mannose, acetate, propionate, and butyrate individually or together for 24 h before RNA or protein extraction.

HEK293T cell line was purchased from Shanghai Cell Bank of Chinese Academy of Sciences (GNHu17) and grown in Dulbecco’s modified Eagle’s medium (Gibco, 12100046) supplemented with 10% heat-inactivated FBS (Gibco, 10099-141). Cells were all cultured in a humidified cell incubator with an atmosphere of 5% CO_2_ at 37 °C.

HT22 cell line was derived from Fenghui Bio and grown in Dulbecco’s modified Eagle’s medium (Gibco, 12100046) supplemented with 10% heat-inactivated FBS (Gibco, 10099-141), penicillin (100 U/ml), streptomycin (100 mg/ml), and 2 mM glutamine.

### Bacteria

*E. coli* DH5α, MG1655, and Rossetta (DE3) were cultured in LB medium at 37 °C with 200 rpm/min and used for recombinant plasmid cloning and protein expression.

### Method Details

#### Chronic restraint stress

To generate the CRS-induced depression like behaviors in mice [58], we performed CRS with mice daily during 9:00 AM to 2:00 PM for 4 weeks using the well-ventilated polypropylene restrainers in deprivation of food and water. At the end of the stress session, mice were returned to the home cage. Then, mouse behavior was evaluated by one behavioral testing (TST and FST) per day after the last stressor during the light phase of the cycle between 9:00 AM and 4:00 PM. Before test, mice were allowed for 2-h habituation to the testing rooms.

#### Tail suspension test

Each mouse was taped with tail (1 cm from tip) and hung to a grid bar over 30-cm height from the ground. Then, we recorded the immobile time of the testing mice within 6 min. Immobility was defined as the absence of escape-orientated movement.

#### Forced swim test

To assess depressive-like behavior, mice were placed into a glass cylinder (25 cm in height and 10 cm in diameter) filled with water (22 °C) up to a height of 18 cm as previously described earlier. A testing period was defined as 6 min to determine the percentage of time spent immobile. Immobility was defined as being stationary with only enough motion of the tail or forepaws to keep the head above water. When mice used forepaws to move and swim in the center or along the sides of the cylinder, we stopped recording the immobility time. Eventually, we calculated the total immobility time of mice during the 6 min [59].

#### Sucrose preference test

Sucrose preference procedure was performed as described previously [28]. Mice were habituated to sucrose for 3 d by replacing water bottles with bottles containing sucrose solution (1%). Then, mice were deprived of water for 23 h before getting free access to 2 bottles with water or 1% sucrose solution. The weights of the 2 bottles were recorded before and after mouse uptake for 1 h, and, thus, the fluid consumption was calculated. Sucrose preference was determined as follows: sucrose preference (%) = sucrose intake / (sucrose intake + water intake) × 100. Sucrose preference was assessed for 2 consecutive days. The position of the sucrose and water bottles was alternated daily to avoid spurious effects from a side bias.

#### Open field test

The OFT apparatus consists of 40 × 40 × 40.5-cm arenas. The individual mouse was placed in the center of the arenas for 10 min. By using the Smart Video Tracking System (Smart 10.0, Panlab, DC, USA), we analyzed the total distance traveled and time spent in the central area of mice.

#### Elevated plus maze

The crossed maze was elevated 50 cm above the floor, with 2 open arms and 2 closed arms (30 × 5 × 10 cm, 0.5-cm-thick walls). The arms were interconnected by a central platform. Mouse was individually placed in the central platform and allowed to explore for 5 min. We used the video tracking system (Smart 10.0, Panlab, DC, USA) to record mice movement. The time spent in the open arms and the number of open arm entries were recorded and analyzed.

#### Enzyme-linked immunosorbent assay

The levels of 5-HT in the hippocampus were measured according to the manufacturer’s protocol using commercial ELISA kits (Jianglai, Shanghai, China). Samples were analyzed in duplicate in a single assay using 50 μl of protein lysate or 20 μl of plasma per sample. For acetyl-CoA level assay, SH-SY5Y cells were prepared for cytoplasm and nuclear isolation, and 50 μl of subcellular isolates were used to evaluate acetyl-CoA content by ELISA kit (Jianglai, Shanghai, China) according to the manufacturer’s instructions. Light absorbance was recorded with a multimode plate reader (Synergy HT, BioTek Instruments Inc.) at 405 nm.

#### Tissue collection

Mice were euthanized between 9:00 AM and 1:00 PM after the behavioral tests; brains were quickly excised and dissected, and the whole hippocampus of each mouse was snap-frozen on dry ice for further quantitative real-time PCR (RT-PCR), ELISA, HPLC, Western blotting, ChIPm and DNA probe pull-down experiments. For the gut microbiota composition sequencing and SCFA analysis for HPLC, the cecum contents from the indicated group mice were isolated, weighted, and stored at −80 °C before the gut microbiota and SCFA analysis.

#### Plasma collection

Mice were not restrained, and the end of the tail was held with 2 fingers. Using a single edge razor blade, a diagonal incision of 2 to 5 mm in length was made from the end of the tail. Approximately 100 μl of blood was collected in a collecting tube containing EDTA to avoid blood coagulation by increasing the pressure of the fingers on the tail above the incision. Blood was mixed with EDTA by gently inverting the tube and centrifuged at 3,500*g* at 4 °C temperature for 15 min. Plasma was carefully aspirated and stored at −80 °C.

#### Quantitative real-time PCR

Total RNAs were extracted with TRIzol reagent according to the manufacturer’s instructions (Tiangen, Beijing, China) and reversely transcribed into cDNA with PrimeScript RT reagent kit with gDNA Eraser (TAKARA, Japan). The expressions of genes were detected by quantitative RT-PCR using FastStart Universal SYBR Green Master (Roche Applied Science, Penzberg, Germany) on the Bio-Rad CFX 96 (Bio-Rad, CA, USA). Cycle threshold (*C*_t_) values were recorded. Data were normalized using β-actin and transformed using the 2^−ΔΔCt^ method. The primer sequences are shown in Table S3.

#### 16*S* rRNA sequencing of cecal contents

Mice cecum content was derived from killed mice cecum. Cecum content (200 mg) was prepared for gut microbiota genomic DNA extraction by QIAamp DNA stool kit (QIAGEN). 16*S* rRNA sequencing for microbiota analysis was performed by Sinotech Genomics (Shanghai, China). The diversity and composition alterations of gut microbiota were analyzed [60].

#### Bioinformatics analysis

Human and mouse TPH2 are located 2,000-bp upstream from their TSS separately and were analyzed online in searching of PPARγ binding sequence http://jaspardev.genereg.net/. (https://jaspar.elixir.no/).

#### SCFA concentration analysis by gas chromatography

Gas chromatography (GC) was utilized for measuring SCFAs in cecum content and hippocampus in GC system (GC2010 Plus, Shimadzu, Tokyo, Japan) with flame ionization system, fitted with a with a ZB-FFAP column (30 m × 0.32 mm × 0.25 mm; Phenomenex) [5]. Peaks for SCFAs were monitored by GC solution software. Briefly, 50 mg of fresh cecum content per mice was diluted in 500 μl of lysis buffer containing 50% ethanol with 0.5% hydrochloric acid and vibrated thoroughly. After incubation for 10 min at room temperature, supernatant was obtained by centrifugation with 10,000*g* for 10 min at 4 °C and filtered as described above. One microliter of 10-fold dilutions were prepared for injection onto ZB-FFAP column (30 m × 0.32 mm × 0.25 mm) for GC analysis. For mouse hippocampus sample, 10 mg of fresh hippocampus was collected and homogenized in 50 μl of lysis buffer containing 50% ethanol with 1% hydrochloric acid. After centrifugation with 10,000*g* for 10 min at 4 °C, the supernatant was collected and deproteinized by 50 μl of 6% perchloric acid. The mixture was centrifugated with 10,000*g* for 10 min at 4 °C, and 2 μl of the supernatant was prepared for GC analysis after filtration. Acetate, propionate, and *n*-butyrate standards were serially diluted and used for generation standard curve within linear range change. The retention time for acetate, propionate, *i*-butyrate, and *n*-butyrate was 6.51, 7.37, and 8.399 min separately. All SCFA data are represented as micromoles per gram.

#### Plasmid construction

The plasmids pCMV-ACSS2 and VN173-Flag-ACSS2 encode full-length human ACSS2 (NM_018677.4). Plasmids of pCMV-PPARγ, PGL3-PPARγ, and VN155-HA-PPARγ encodes full-length human PPARγ (NM_001354666.3). The mouse or human TPH2 promoter sequences were subcloned to pGL3 basic (Promega, Madison, MI, USA) to drive luciferase expression. For BiFC assay, full length of human ACSS2 was constructed to pVN173 plasmid. PPARγ was subcloned to pVC155. TPH2 and PPARγ expressing plasmid under their native promoters were generated by inserting their promoter and coding sequences into pGL3 basic, followed by deleting luciferase-coding region.

#### Protein purification and pull-down assay

The recombination protein of His-ACSS2 and GST-PPARγ was induced by IPTG in *E. coli* BL21 (DE3) harboring ACSS2-pET28a or PPARγ-pGEX-4t plasmid. These proteins were purified as described previously [22]. Briefly, BL21 (DE3) cells expressing (His)6-ACSS2 or GST-tagged PPARγ were cultured overnight, and 2.5 ml of the resulting cultures were transferred to 250 ml of fresh LB medium individually. IPTG (1 mM) was added to induce (His)6-ACSS2 or GST-tagged PPARγ expression for 20 h at 16 °C when optical density at 600 nm of the culture reached around 0.4. The bacterial cells were collected after centrifuged for 15 min at 4,000 rpm at 4 °C and resuspended with lysis buffer [25 mM tris and 50 mM NaCl (pH 8.0)] or phosphate-buffered saline [140 mM NaCl, 5 mM KH_2_PO_4_, and 1 mM NaHCO_3_ (pH 7.4)] before lysis via sonication. For His-ACSS2 affinity purification, cell lysates were loaded onto a Ni–nitrilotriacetic acid column and washed with 5 column volumes of 30 mM imidazole to remove contaminated proteins. Finally, the His-ACSS2 protein was eluted by elution buffer [imidazole (250 mM) and tris (25 mM) (pH 8.0)] and dialyzed to remove imidazole before use. For GST-PPARγ purification, cell lysates were loaded onto a GSTrap HP column (GE Healthcare Life Sciences) and washed with 5 column volumes of phosphate-buffered saline. The subsequent elution and dialysis were performed with 10 mM reduced glutathione to extract GST-PPARγ protein without glutathione. The purification efficiency was examined using SDS-polyacrylamide gel electrophoresis (PAGE) and Coomassie Brilliant Blue (G-250) staining.

For GST pull-down assay, 200 ng of purified His-ACSS2 was incubated with 100 ng of GST-PPARγ together with glutathione agarose beads in a binding buffer [50 mM tris-HCl (pH 7.5), 1% Triton X-100, 150 mM NaCl, 1 mM dithiothreitol, 0.5 mM EDTA, 100 μM PMSF, 100 μM leupeptin, 1 μM aprotinin, 100 μM sodium orthovanadate, 100 μM sodium pyrophosphate, and 1 mM sodium fluoride] at 4 °C. The glutathione beads were then washed 4 times with binding buffer, and the bound proteins were boiled with SDS buffer prior to electrophoresis on SDS-PAGE.

For biotin-labeled DNA probe binding assay, equal molar of purified His-ACSS2 and GST-PPARγ was incubated with biotin-labeled DNA probe containing putative PPARγ binding sites from human and mouse TPH2 promoter regions at 4 °C for 16 h. The streptavidin agarose was added to the mixture and pulled down the DNA–protein complex, followed by 3 washes with the co-IP buffer [120 mM NaCl, 1 mM EDTA, 40 mM Hepes (pH 7.4), 50 mM NaF, 10 mM β-glycerophosphate, 0.3% CHAPS, 1 mM Na_3_VO_4_, 1 mM PMSF, leupeptin (10 mg/ml), aprotinin (10 mg/ml), and 10 mM MgCl_2_]. The DNA binding ability of His-ACSS2 and GST-PPARγ was examined by Western blot. Endogenous ACSS2 and PPARγ from mouse hippocampus were also incubated with biotin–DNA probe to evaluate their bind ability to tph2 promoter region. Equal total lysates of mouse hippocampus were cultured with the overamount of DNA probe at 4 °C for 4 h, followed by incubation of streptavidin-coated beads for 2 h. After centrifugation at 4,000*g* for 5 min, the pellets were collected, extensively washed in IP buffer and subjected to analysis by SDS-PAGE and immunoblot.

#### Viral injection

For hippocampal ACSS2 and PPARγ RNA interference, we injected 2 μl of AAV liquid carrying small interfering RNA targeting *acss2* or *pparγ* with Syn promoter into each side of the hippocampus at a rate of 0.33 μl/min using a Hamilton microinjector plus a microinjection pump and diffused it for another 5 min before removing the microinjection pump. The coordinates were as follows: ventral hippocampus (vHIP): anteroposterior, −2.54 mm; lateral, ±2.75 mm; dorsoventral, −2.0 mm. Infection effects were evaluated by hippocampal ACSS2 and PPARγ changes by Western blot. Mice were anesthetized by isoflurane and prepared for virus liquid injection into the hippocampus by Hamilton microsyringe with a microinjection pump (KDS 200, KD Scientific). The shRNA sequence for ACSS2 and PPARγ were as listed: antisense, shACSS2 [titer: 1.85 × 10^13^ vector genomes (v.g.)/ml]: 5′-CAGGATTGATGACAT

GCTCAA-3′; shPPARγ (titer: 2.13 × 10^13^ v.g./ml): 5′-AATATGACCTGAAGCTCC

AAGAATAAG-3′. AAVs of shACSS2, shPPARγ, and shNC (negative control) were packaged and purified separately by Shanghai Genechem Co. Ltd. (Shanghai, China).

For hippocampal ACSS2 overexpression, we injected 2 μl of AAV liquid carrying ACSS2 with CMV promoter into both hippocampus at a rate of 0.33 μl/min using a Hamilton microinjector plus a microinjection pump and diffused it for another 5 min before removing the microinjection pump. The coordinates were as follows: vHIP: anteroposterior, −2.54 mm; lateral, ±2.75 mm; dorsoventral, −2.0 mm. The titer of AAV-ACSS2 is 2.94 × 10^13^ v.g./ml.

#### Primary hippocampus neuron culture

The hippocampus was dissected from mouse embryos of either sex at day 15.5. Hippocampal neurons were isolated by 0.25% trypsin digestion for 20 min at 37 °C, followed by trituration through a small-bore glass Pasteur pipette. The resulting cells were seeded on plates coated with poly-d-lysine (0.1 mg/ml; Sigma-Aldrich, MA, USA) in 1 × 10^6^ cells per well (6-well plate) and cultured in neurobasal medium supplemented with 2% B27 (Gibco, CA, USA) and l-glutamine for 7 d to use.

#### Western blotting and co-IP

Cells were lysed with radioimmunoprecipitation assay cell lysis buffer with 1% protease inhibitor cocktail. The supernatant was collected after high-speed (12,000*g*) centrifugation for 30 min at 4 °C, and protein concentration was determined using a bicinchoninic acid assay (BCA) method. Ten micrograms of denatured protein was separated by SDS-PAGE and then transferred to polyvinylidene difluoride membranes. The membranes were blocked with 5% bovine serum albumin in TBS-T (tris-buffered saline with Tween 20) for 2 to 3 h and incubated with primary antibodies overnight at 4 °C. Then, horseradish-peroxidase-conjugated secondary antibodies were added for 1 h. After 3 washes with TBS-T, signals were detected by the ECL detection system (Sage Creation Science, MiniChemi 500). The specific bands were analyzed using an eECL Western Blot Kit (Millipore, 69078). All protein signals were collected with different exposure times to make sure that the bands were not overexposed and within the linear range to perform quantitative analysis. The band intensity was quantified using the ImageJ software.

The Nuclear/Cytosol Fractionation Kit was used to extract the cytoplasmic and nuclear proteins from SH-SY5Y cells. The cytoplasmic and nuclear extractions were prepared for acetyl-CoA or Western blot analysis.

HEK-293 T cells were lysed by IP buffer. Cell extracts were clarified by centrifugation at 13,400*g*, and the supernatants were subjected to IP with the indicated antibodies. After overnight incubation at 4 °C, protein A or G agarose beads were added and left for an additional 3 h with rotation. Then, the protein complexes were washed 5 times with IP buffer and then subjected to immunoblot analyses with corresponding antibodies as indicated .

#### ChIP assay

ChIP was performed using a SimpleChIP plus sonication chromatin IP kit (CST, #56383). Chromatin was prepared from cells in a 10-cm dish that was used to determine total DNA input, and the cell lysates were incubated with specific antibodies or normal mouse immunoglobulin G for overnight. For mouse hippocampus ChIP, 2 mouse hippocampi were combined (about 30 mg) to extract chromatin. Primer sequences used for PCR were listed in Table S3.

#### BiFC assay

This assay allows for the rapid visualization of the compartment-specific interactions of a protein complex, and protein–protein interactions can be easily quantified in vivo. BiFC expression plasmids for ACSS2 and PPARγ were constructed by inserting the PCR fragment containing full-length ACSS2, PPARγ, or their derivatives (primers in Table S3) into pBiFC-VN173 and pBiFC-VC155 (Addgene, Cambridge, MA, USA) with ClonExpress II One Step Cloning Kit. The resulting plasmids were transfected into HEK293T cells, and cotransfection of pBiFC-bFosVC155 and pBiFC-bJunVN173 was defined as a positive. DAPI stain was indicated as cellular nuclear, and fluorescence could be detected at 561-nm excitation wavelength after 24 h by LSM780 with a 63× Plan-Apochromat objective and analyzed using ZEN lite 2012 software package.

#### Luciferase assay

Luciferase activity was measured with a dual luciferase assay system. The luciferase reporter vector PGL3 and its derivatives with or without human (−1,500 to +660, 2,160 bp) and mouse (−2000 to 0, 2,000 bp) TPH2 promoter region were transiently transfected into the HEK293T cells in the presence of PPARγ or its derivatives alone or together with ACSS2 or its derivatives. Twenty-four hours after transfection, luciferase activity was measured with a dual luciferase assay system (Vazyme, catalog no. DL101-01), and the readout was determined using a microplate luminometer (Centro LB 960, Berthold, Wildbad, Germany). Data were analyzed by GraphPad Prism 8. Three independent experiments have carried out for biological replicates.

#### Quantification analysis

The specific Western band signals were quantitively analyzed by ImageJ. All images of statistical analyses were performed using Image Pro-Plus software and Zeiss Auto-Measure software.

#### Statistical analysis

Mean values, SEM, and statistical significance were analyzed by GraphPad Prism 9.0. Differences between groups were analyzed with the Student’s *t* test (unpaired), 1-way or 2-way analysis of variance (ANOVA), followed by the Tukey’s test (for multiple comparisons). *P* < 0.05 was considered to be statistically significant; *P < 0.05, **P < 0.01, ***P < 0.001, and ****P < 0.0001. Results are presented followed by at least 3 independent experiments of biological replicates.

## Data Availability

This work did not generate new unique reagents. Gut microbiota genomic DNA was isolated from mouse cecum content by QIAamp DNA stool kit (QIAGEN), and 16*S* rRNA sequencing for microbiota analysis was performed by Sinotech Genomics (China). The related source data in the paper are available from the corresponding author on request. Further information and requests for resources and reagents should be directed to and will be fulfilled by lead contact, Y.L. (liyan2015@sdu.edu.cn).

## Supplementary Materials

Supplementary 1 Figs. S1 to S6 Tables S1 to S3 Control VS Mannose CRS-Control VS CRS-Mannose RNA sequencing for mannose-treated MG1655
