# Supplementary material for: Brain Short-Chain Fatty Acids Induce ACSS2 to Ameliorate Depressive-Like Behavior via PPARγ–TPH2 Axis
Source: Research (Wash D C). 2024 Jun 27;7:0400. doi: 10.34133/research.0400 (PMC11210491; doi:10.34133/research.0400)
Supplement: Supplementary 1 — Figs. S1 to S6 Tables S1 to S3 Control VS Mannose CRS-Control VS CRS-Mannose RNA sequencing for mannose-treated MG1655 [file research.0400.f1.zip › Control VS Mannose.pdf]

gene id	gene nam	descriptio	GO_id	GO_term	KO_ID	KO_Descr	kegg_id	kegg_tern	locus	A1_count	A2_count	A3_count	C1_count	C2_count	C3_count	A1_FPKM	A2_FPKM	A3_FPKM	C1_FPKM	C2_FPKM	C3_FPKM	A	C	Pvalue	Qvalue	updown
ENSMUS(	AL732309	-	-	-	-	-	-	-	2:2524481	24	28	30	262	198	185	0.745855	0.729371	0.642259	5.304273	5.395734	7.128558	0.705829	5.942855	7.80E-28	1.34E-23	DOWN
ENSMUS(	Tead1	TEA doma	GO:00081	metabolic	K09448	transcripti	mmu0439	Hippo sig	7:1126793	166	106	143	1160	865	520	0.387347	0.207325	0.229869	1.763325	1.769912	1.504466	0.274847	1.679234	2.96E-22	2.53E-18	DOWN
ENSMUS(	Irf7	interferon	GO:00081	metabolic	K09447	interferon	mmu0520	Viral carci	7:1412627	32	20	32	123	216	133	0.198718	0.104106	0.136896	0.497597	1.176214	1.024063	0.146573	0.899291	9.79E-12	5.59E-08	DOWN
ENSMUS(	Evi2b	ecotropic	GO:00325	developm	-	-	-	-	11:795150	20	29	18	2	0	0	0.684108	0.831449	0.424149	0.04456	0	0	0.646569	0.014853	4.87E-11	2.08E-07	UP
ENSMUS(	Rfng	RFNG O-f	GO:00325	developm	K05948	fringe[2.4.	mmu0516	Human pa	11:120780	360	500	516	1260	973	913	5.263195	6.127304	5.196937	12.00048	12.47389	16.55021	5.529145	13.67486	1.29E-10	4.43E-07	DOWN
ENSMUS(	Nfat5	nuclear fa	GO:00081	metabolic	-	-	-	-	8:1072934	1002	981	1284	3836	2377	1453	1.599762	1.312833	1.412226	3.989769	3.327817	2.876334	1.441607	3.397973	4.60E-09	1.31E-05	DOWN
ENSMUS(	Itgam	integrin al	GO:00081	metabolic	-	-	-	-	7:1280626	0	0	0	292	0	120	0	0	0	2.289639	0	1.790889	0	1.360176	1.43E-08	3.49E-05	DOWN
ENSMUS(	Ifi44	interferon	GO:00056	cell|intrac	-	-	-	-	3:1517309	14	17	22	61	100	49	0.118363	0.120475	0.128133	0.335973	0.741372	0.513654	0.122324	0.530333	6.70E-08	0.000143	DOWN
ENSMUS(	Bst2	bone mar	GO:00081	metabolic	-	-	-	-	8:7153425	28	35	42	89	165	115	1.57755	1.65292	1.630155	3.266638	8.151824	8.033617	1.620208	6.484026	8.53E-08	0.000162	DOWN
ENSMUS(	Car1	carbonic a	GO:00081	metabolic	K01672	carbonic a	mmu0091	Nitrogen r	3:1476621	16	49	53	10	0	4	0.554757	1.424031	1.265903	0.225862	0	0.171957	1.081564	0.132606	1.72E-07	0.000295	UP
ENSMUS(	Snx10	sorting ne	GO:00325	developm	-	-	-	-	6:5152390	901	1045	1205	2375	1907	1445	4.674387	4.544323	4.30664	8.026851	8.675477	9.295092	4.50845	8.665807	2.37E-07	0.000369	-
ENSMUS(	Gbp3	guanylate	GO:00023	immune s	K20899	guanylate	mmu0462	NOD-like	3:1425600	96	104	130	257	374	203	1.006117	0.913615	0.93858	1.754653	3.437088	2.637903	0.952771	2.609881	2.98E-07	0.000425	DOWN
ENSMUS(	Ifit1	interferon	GO:00023	immune s	K14217	interferon	mmu0516	Herpes sir	19:346408	37	50	57	114	259	127	0.885334	1.002831	0.939588	1.777033	5.434392	3.767887	0.942584	3.659771	3.24E-07	0.000427	DOWN
ENSMUS(	Muc2	mucin 2 [S	GO:00055	extracellul	-	-	-	-	7:1416903	18	93	51	12	8	2	0.144876	0.627426	0.28278	0.062923	0.056465	0.019965	0.351694	0.046451	6.22E-07	0.00076	UP
ENSMUS(	Oasl2	2'-5' oligo	GO:00081	metabolic	K14608	2'-5'-oligo	mmu0516	Human pa	5:1148969	96	98	113	228	381	189	0.569026	0.486901	0.461414	0.880395	1.980286	1.389022	0.50578	1.416568	1.70E-06	0.001945	DOWN
ENSMUS(	Tbl1xr1	transducir	GO:00325	developm	K04508	transducir	mmu0431	Wnt signa	3:2207665	1294	2752	3436	853	1256	748	3.793422	6.762359	6.939073	1.629023	3.228712	2.718844	5.831618	2.525527	2.49E-06	0.002244	UP
ENSMUS(	H2-Q4	histocomp	GO:00023	immune s	K06751	major hist	mmu0516	Human pa	17:353796	61	86	97	194	180	147	0.992504	1.172883	1.087247	2.056303	2.568146	2.965564	1.084211	2.530004	2.46E-06	0.002244	DOWN
ENSMUS(	Rnf213	ring finge	GO:00325	developm	-	-	-	-	11:119393	350	327	437	734	1261	614	1.110457	0.86963	0.955138	1.517089	3.508256	2.415391	0.978408	2.480246	2.31E-06	0.002244	DOWN
ENSMUS(	Ifit3	interferon	GO:00023	immune s	-	-	-	-	19:345835	158	173	234	429	949	297	5.754373	5.281277	5.870956	10.17841	30.30746	13.41169	5.635536	17.96585	2.18E-06	0.002244	DOWN
ENSMUS(	Zg16	zymogen	GO:00511	localizatio	-	-	-	-	7:1270501	8	9	18	3	0	0	0.308685	0.291083	0.478488	0.075403	0	0	0.359419	0.025134	3.43E-06	0.002939	UP
ENSMUS(	H2-Q6	histocomp	GO:00023	immune s	K06751|K0	major hist	mmu0516	Human pa	17:354248	2	14	10	40	33	27	0.056654	0.332357	0.195108	0.737994	0.819552	0.948127	0.194706	0.835224	3.71E-06	0.003021	DOWN
ENSMUS(	Yipf6	Yip1 dom	GO:00325	developm	-	-	-	-	X:9893631	262	278	360	1370	362	690	1.935699	1.721606	1.832274	6.593846	2.345246	6.3208	1.82986	5.086631	4.79E-06	0.003612	DOWN
ENSMUS(	H2-K1	histocomp	GO:00325	developm	K06751	major hist	mmu0516	Human pa	17:339960	321	358	424	818	606	577	2.805739	2.622876	2.553047	4.657759	4.644695	6.253219	2.660554	5.185224	4.85E-06	0.003612	-
ENSMUS(	Tgtp2	T cell spec	GO:00023	immune s	-	-	-	-	11:490571	23	30	37	99	141	45	0.459657	0.502547	0.509404	1.288922	2.470994	1.115075	0.490536	1.624997	5.20E-06	0.003709	DOWN
ENSMUS(	Usf3	upstream	GO:00325	developm	-	-	-	-	16:441732	318	893	914	2508	1730	888	0.789879	1.859252	1.563981	4.058289	3.768102	2.734843	1.40437	3.520411	5.91E-06	0.004044	DOWN
ENSMUS(	Usp18	ubiquitin s	GO:00081	metabolic	-	-	-	-	6:1212459	13	21	15	38	78	72	0.239679	0.324539	0.190523	0.456416	1.26105	1.645935	0.25158	1.121134	7.47E-06	0.004917	DOWN
ENSMUS(	Nlrc5	NLR famil	GO:00081	metabolic	-	-	-	-	8:9443435	15	23	41	73	82	50	0.072445	0.093109	0.136412	0.229676	0.347271	0.299415	0.100655	0.29212	9.99E-06	0.006335	DOWN
ENSMUS(	Ggnbp1	gametoge	GO:00325	developm	-	-	-	-	17:269732	52	61	64	71	373	321	0.398547	0.391886	0.337914	0.3545	2.506847	3.05048	0.376116	1.970609	1.21E-05	0.007373	DOWN
ENSMUS(	Tgtp1	T cell spec	GO:00023	immune s	-	-	-	-	11:489853	34	52	88	124	234	109	0.566993	0.726858	1.01095	1.347102	3.421811	2.253761	0.768267	2.340891	1.43E-05	0.008184	DOWN
ENSMUS(	Gm28635	predicted	GO:00325	developm	-	-	-	-	2:8460143	0	0	0	472	0	0	0	0	0	4.127921	0	0	0	1.375974	1.40E-05	0.008184	DOWN
ENSMUS(	Gm28551	predicted	GO:00081	metabolic	-	-	-	-	1:5332550	6	0	2	92	75	3	0.121367	0	0.027872	1.212271	1.330257	0.075245	0.049746	0.872591	1.52E-05	0.008376	DOWN
ENSMUS(	Prlr	prolactin r	GO:00325	developm	K05081	prolactin r	mmu0415	PI3K-Akt s	15:101772	514	215	719	238	66	132	1.441517	0.505415	1.389112	0.434826	0.162309	0.459004	1.112015	0.352047	1.63E-05	0.008435	UP
ENSMUS(	Ccdc137	coiled-co	GO:00056	cell|intrac	-	-	-	-	11:120458	147	149	246	329	406	349	1.58478	1.346453	1.827001	2.31063	3.838143	4.665132	1.586078	3.604635	1.58E-05	0.008435	DOWN
ENSMUS(	Gbp4	guanylate	GO:00081	metabolic	-	-	-	-	5:1051157	27	50	98	174	234	85	0.192777	0.299238	0.48203	0.809337	1.465063	0.752489	0.324682	1.008963	1.70E-05	0.008542	DOWN
ENSMUS(	Muc3	mucin 3, i	GO:00160	membran	-	-	-	-	5:1371349	2	15	16	0	1	0	0.051512	0.323775	0.283832	0	0.022579	0	0.219706	0.007526	2.02E-05	0.009879	UP
ENSMUS(	Ddx60	DEAD (As	GO:00023	immune s	-	-	-	-	8:6192808	13	10	14	39	39	30	0.078489	0.050606	0.058229	0.153398	0.206481	0.224588	0.062442	0.194822	2.93E-05	0.013936	DOWN
ENSMUS(	Isg15	ISG15 ubi	GO:00325	developm	K12159	ubiquitin c	mmu0516	Human pa	4:1561994	12	12	18	30	59	51	1.008738	0.845597	1.04242	1.642929	4.349161	5.315842	0.965585	3.769311	3.96E-05	0.018295	DOWN
ENSMUS(	Zc3h11a	zinc finge	GO:00081	metabolic	-	-	-	-	1:1336198	747	720	1231	684	499	264	4.553436	3.678777	5.169255	2.716167	2.667237	1.995297	4.467156	2.459567	4.54E-05	0.020449	-
ENSMUS(	Hc	hemolytic	GO:00325	developm	K03994	compleme	mmu0516	Herpes sir	2:3498333	0	0	0	9	4	5	0	0	0	0.053791	0.032178	0.056875	0	0.047615	5.24E-05	0.022673	DOWN
ENSMUS(	Dcaf10	DDB1 and	GO:00081	metabolic	-	-	-	-	4:4534210	132	167	333	483	413	284	0.723001	0.766717	1.256497	1.723435	1.983618	1.928721	0.915405	1.878592	5.56E-05	0.022673	DOWN
ENSMUS(	Pif1	PIF1 5'-to	GO:00081	metabolic	-	-	-	-	9:6558716	4	10	5	0	0	0	0.020202	0.042334	0.017397	0	0	0	0.026644	0	5.36E-05	0.022673	UP
ENSMUS(	Dmbt1	deleted in	GO:00511	localizatio	K13912	deleted in	mmu0497	Salivary se	7:1310320	40	137	80	24	4	24	0.282995	0.812437	0.389901	0.110613	0.024813	0.210529	0.495111	0.115318	5.56E-05	0.022673	UP
ENSMUS(	Hnf4a	hepatic nu	GO:00325	developm	K07292	hepatocyt	mmu0415	AMPK sign	2:1635068	3	9	7	0	0	0	0.023113	0.058125	0.037159	0	0	0	0.039465	0	6.74E-05	0.025773	UP
ENSMUS(	Gm16286	predicted	GO:00081	metabolic	-	-	-	-	18:802067	173	271	355	1216	1031	188	1.496486	1.964935	2.115463	6.852376	7.820369	2.016365	1.858962	5.563036	6.75E-05	0.025773	DOWN
ENSMUS(	Kcne2	potassium	GO:00325	developm	K04896	potassium	mmu0497	Gastric ac	16:922923	191	68	537	76	78	42	6.869624	2.050028	13.30531	1.78072	2.460012	1.873008	7.408322	2.037914	7.06E-05	0.025773	UP
ENSMUS(	Oas1a	2'-5' oligo	GO:00081	metabolic	K14216	2'-5'-oligo	mmu0516	Herpes sir	5:1208962	4	10	14	22	37	44	0.134855	0.28259	0.325158	0.483206	1.093884	1.839343	0.247535	1.138811	7.53E-05	0.025773	DOWN
ENSMUS(	Ifit3b	interferon	GO:00023	immune s	-	-	-	-	19:346079	69	74	87	135	279	139	2.192593	1.971031	1.904484	2.794603	7.774145	5.476549	2.022702	5.348432	7.22E-05	0.025773	DOWN
ENSMUS(	Ly6a	lymphocy	GO:00160	membran	-	-	-	-	15:749948	127	144	134	226	311	412	2.338125	2.222189	1.699505	2.710535	5.020753	9.404786	2.086606	5.712025	7.25E-05	0.025773	DOWN
ENSMUS(	Sprr2a1	small prol	GO:00325	developm	-	-	-	-	3:9221579	6	14	11	4	0	0	0.126984	0.248354	0.160368	0.055154	0	0	0.178569	0.018385	7.44E-05	0.025773	UP
ENSMUS(	Gm43302	predicted	GO:00023	immune s	-	-	-	-	5:1052149	26	13	43	58	165	67	0.383389	0.160682	0.436793	0.55715	2.13347	1.224956	0.326955	1.305192	7.37E-05	0.025773	DOWN
ENSMUS(	1810065E	RIKEN cDI	-	-	-	-	-	-	11:584211	4	6	7	0	0	0	0.274516	0.345151	0.330976	0	0	0	0.316881	0	7.93E-05	0.026605	UP
ENSMUS(	Irgm2	immunity-	GO:00081	metabolic	-	-	-	-	11:581996	59	93	120	189	238	124	0.358578	0.473765	0.502412	0.748293	1.268377	0.934407	0.444918	0.983692	8.38E-05	0.027581	DOWN
ENSMUS(	Muc13	mucin 13,	GO:00055	extracellul	-	-	-	-	16:337940	6	33	25	4	2	4	0.08998	0.414808	0.258261	0.039081	0.026298	0.074374	0.25435	0.046584	8.72E-05	0.028165	UP
ENSMUS(	Xdh	xanthine c	GO:00325	developm	K00106	xanthine c	mmu0110	Metabolic	17:738839	114	120	152	284	298	156	1.579472	1.393614	1.450787	2.563349	3.620485	2.679891	1.474624	2.954575	9.59E-05	0.0304	DOWN
ENSMUS(	Dhx58	DEXH (As	GO:00023	immune s	K12649	ATP-depe	mmu0462	RIG-I-like	11:100694	8	19	28	38	64	55	0.116207	0.231348	0.280204	0.359604	0.815233	0.990622	0.209253	0.72182	9.98E-05	0.030513	DOWN
ENSMUS(	Phactr1	phosphata	GO:00452	synapse|c	-	-	-	-	13:426806	2877	2638	3439	8368	3567	4376	11.58401	8.903211	9.538995	21.94939	12.59403	21.84652	10.00874	18.79665	9.98E-05	0.030513	-
ENSMUS(	Xaf1	XIAP asso	GO:00508	response	-	-	-	-	11:723016	41	56	55	101	160	84	0.451585	0.517016	0.417326	0.724708	1.545334	1.147162	0.461976	1.139068	0.000112	0.033697	DOWN
ENSMUS(	Ttpal	tocophero	GO:00511	localizatio	-	-	-	-	2:1636023	144	271	342	373	581	630	0.756397	1.193188	1.237553	1.276371	2.676118	4.103106	1.062379	2.685198	0.000115	0.033916	DOWN
ENSMUS(	Lypd8	LY6/PLAU	GO:00508	response	-	-	-	-	11:583790	11	52	26	7	0	6	0.400632	1.587424	0.652331	0.166083	0	0.270967	0.880129	0.145684	0.000125	0.03569	UP
ENSMUS(	Clca1	chloride c	GO:00081	metabolic	K05027	calcium-a	mmu0497	Pancreatic	3:1450038	17	47	56	10	0	11	0.263634	0.61096	0.598277	0.101026	0	0.211521	0.490957	0.104182	0.000125	0.03569	UP
ENSMUS(	Tmem80	transmem	GO:00160	membran	-	-	-	-	7:1413277	89	92	123	177	203	234	0.72127	0.624954	0.686701	0.934468	1.442604	2.351318	0.677641	1.57613	0.000137	0.038548	DOWN
ENSMUS(	Jmjd4	jumonji dc	-	-	-	-	-	-	11:594500	257	256	291	1028	301	478	1.339988	1.118821	1.045231	3.491747	1.376187	3.090165	1.168013	2.6527	0.000146	0.040201	DOWN
ENSMUS(	Csnk1g1	casein kin	GO:00325	developm	K08958	casein kin	mmu0434	Hedgehog	9:6590892	813	822	416	490	279	154	1.955219	1.657025	0.689206	0.767683	0.588371	0.459211	1.433817	0.605088	0.00015	0.040742	UP
ENSMUS(	Dlk1	delta-like	GO:00325	developm	-	-	-	-	12:109452	200	53	192	23	45	43	0.757079	0.168168	0.500685	0.056718	0.14937	0.201822	0.475311	0.13597	0.000169	0.045278	UP
ENSMUS(	Far1	fatty acyl	GO:00081	metabolic	K13356	alcohol-fo	mmu0414	Peroxisom	7:1135138	902	889	1032	2543	1341	1054	4.743076	3.918396	3.738389	8.711271	6.183366	6.871951	4.133287	7.255529	0.000207	0.054463	-
ENSMUS(	Igtp	interferon	GO:00508	response	K14140	interferon	mmu0514	Toxoplasm	11:581995	43	59	95	110	167	133	0.533642	0.613744	0.812188	0.889313	1.817361	2.046526	0.653191	1.5844	0.000219	0.056777	-
ENSMUS(	Gvin1	GTPase, v	GO:00971	organic cy	-	-	-	-	7:1061565	92	137	159	318	403	124	0.643152	0.802785	0.765725	1.448215	2.47043	1.074809	0.737221	1.664485	0.000231	0.059099	-
ENSMUS(	Meig1	meiosis ex	GO:00325	developm	-	-	-	-	2:3409043	36	58	60	22	24	18	1.015932	1.371988	1.166467	0.404459	0.593916	0.629852	1.184796	0.542742	0.000248	0.059741	-
ENSMUS(	Cfap206	cilia and f	GO:00990	supramole	-	-	-	-	4:3468855	54	44	59	29	14	21	0.996481	0.680577	0.750022	0.348623	0.226538	0.480476	0.809027	0.351879	0.00024	0.059741	-
ENSMUS(	Uba7	ubiquitin-	GO:00081	metabolic	K10698	ubiquitin-	mmu0501	Parkinson	9:1079755	21	57	50	81	108	111	0.108147	0.246046	0.177381	0.271738	0.487698	0.708753	0.177191	0.489396	0.000247	0.059741	-
ENSMUS(	Krt8	keratin 8 [	GO:00325	developm	-	-	-	-	15:101996	29	47	67	25	16	16	1.021076	1.387099	1.62511	0.573425	0.493987	0.698496	1.344428	0.588636	0.000247	0.059741	-
ENSMUS(	Agr2	anterior g	GO:00325	developm	-	-	-	-	12:359929	5	5	5	0	0	0	0.232797	0.195122	0.160374	0	0	0	0.196098	0	0.000251	0.05977	-
ENSMUS(	Pigr	polymeric	GO:00048	signal trar	K13073	polymeric	mmu0467	Intestinal i	1:1308266	13	52	35	10	0	8	0.184208	0.617626	0.341657	0.092309	0	0.140556	0.381164	0.077622	0.000259	0.060815	-
ENSMUS(	Pisd	phosphati	GO:00081	metabolic	K01613	phosphati	mmu0110	Metabolic	5:3273630	721	881	1133	873	420	301	3.175669	3.252584	3.437806	2.504931	1.622153	1.643814	3.288686	1.923633	0.000264	0.060957	-
ENSMUS(	Gm4070	predicted	GO:00056	cell|intrac	-	-	-	-	7:1058951	121	158	187	388	437	143	0.777311	0.850784	0.827564	1.623758	2.461682	1.139017	0.818553	1.741486	0.000283	0.064502	-
ENSMUS(	Oas2	2'-5' oligo	GO:00081	metabolic	K14216	2'-5'-oligo	mmu0516	Herpes sir	5:1207303	8	7	21	33	42	24	0.061955	0.04544	0.112041	0.166493	0.28523	0.230463	0.073145	0.227395	0.000289	0.065023	-
ENSMUS(	Stxbp6	syntaxin b	GO:00511	localizatio	-	-	-	-	12:448524	1594	714	837	3762	2837	1409	10.9981	4.129337	3.978381	16.90946	17.16451	12.05386	6.368606	15.37594	0.000344	0.075437	-
ENSMUS(	Gbp6	guanylate	GO:00023	immune s	-	-	-	-	5:1052707	29	39	55	82	128	59	0.20762	0.234039	0.27126	0.382444	0.803569	0.523732	0.23764	0.569915	0.000343	0.075437	-
ENSMUS(	Ifi47	interferon	GO:00508	response	K17072	interferon	mmu0466	TNF signa	11:490765	8	11	9	25	24	38	0.077253	0.089045	0.059871	0.157276	0.203236	0.455008	0.07539	0.27184	0.000369	0.079949	-
ENSMUS(	Cdh3	cadherin 3	GO:00325	developm	K06796	cadherin 3	mmu0451	Cell adhes	8:1065108	35	9	73	2	9	6	0.507385	0.109355	0.729024	0.018885	0.11441	0.107855	0.448588	0.080384	0.000377	0.08071	-
ENSMUS(	Ehf	ets homol	GO:00325	developm	-	-	-	-	2:1032634	8	6	15	4	1	0	0.052235	0.032838	0.067475	0.017017	0.005725	0	0.050849	0.007581	0.000393	0.081126	-
ENSMUS(	Zfpm1	zinc finge	GO:00325	developm	-	-	-	-	8:1222821	778	789	987	745	522	308	10.12994	8.611074	8.853107	6.319242	5.959924	4.972368	9.19804	5.750511	0.000391	0.081126	-
ENSMUS(	Gbp5	guanylate	GO:00511	localizatio	K20898	guanylate	mmu0462	NOD-like	3:1424939	44	62	74	124	138	78	0.27983	0.33051	0.324208	0.51374	0.769592	0.615068	0.311516	0.6328	0.00039	0.081126	-
ENSMUS(	Zmat1	zinc finge	GO:00971	organic cy	-	-	-	-	X:1349713	299	374	431	267	195	185	1.624131	1.702844	1.612794	0.944809	0.928812	1.245974	1.646589	1.039865	0.000406	0.082639	-
ENSMUS(	Eci3	enoyl-Coa	GO:00081	metabolic	-	-	mmu0414	Peroxisom	13:349466	3	2	12	0	0	0	0.120275	0.067215	0.331479	0	0	0	0.17299	0	0.000468	0.093092	-
ENSMUS(	AU019823	expressed	-	-	-	-	-	-	9:5060524	211	252	381	207	171	73	1.385442	1.386944	1.723383	0.885438	0.984565	0.594309	1.49859	0.821437	0.000463	0.093092	-
ENSMUS(	Trh	thyrotropi	GO:00081	metabolic	-	-	-	-	6:9224206	80	51	4	8	6	4	3.673574	1.962996	0.126551	0.239315	0.24158	0.227743	1.92104	0.236213	0.000497	0.096801	-
ENSMUS(	Apol9b	apolipopr	GO:00081	metabolic	-	-	-	-	15:777291	3	0	3	9	17	8	0.123389	0	0.085008	0.24117	0.613185	0.408021	0.069466	0.420792	0.000498	0.096801	-
ENSMUS(	Gm28042	predicted	GO:00081	metabolic	-	-	-	-	2:1200274	0	10	18	107	27	17	0	0.088692	0.131214	0.737593	0.250528	0.223038	0.073302	0.40372	0.000518	0.099544	-
ENSMUS(	Irgm1	immunity-	GO:00081	metabolic	K14139	immunity-	mmu0514	Toxoplasm	11:488619	109	179	188	258	349	228	0.84758	1.166706	1.007079	1.306939	2.379706	2.198238	1.007122	1.961628	0.000608	0.115581	-
ENSMUS(	Slc28a3	solute car	GO:00511	localizatio	-	-	-	-	13:585453	10	8	26	5	4	0	0.074358	0.049859	0.133183	0.024222	0.026079	0	0.0858	0.016767	0.000617	0.116004	-
ENSMUS(	Irak2	interleukin	GO:00081	metabolic	K04731	interleukin	mmu0515	Tuberculo	6:1136384	150	196	195	401	246	258	1.214547	1.33024	1.087698	2.115183	1.746627	2.590166	1.210828	2.150659	0.000637	0.11842	-
ENSMUS(	Ifitm3	interferon	GO:00511	localizatio	-	-	-	-	7:1410095	73	105	104	140	183	258	6.842755	8.249896	6.715676	8.549008	15.04182	29.98557	7.269442	17.8588	0.000675	0.121614	-
ENSMUS(	Gas7	growth ar	-	-	-	-	-	-	11:674554	10521	6446	14341	7789	6307	2500	44.33372	22.76776	41.63016	21.38162	23.30466	13.0618	36.24388	19.24936	0.000661	0.121614	-
ENSMUS(	Iigp1	interferon	GO:00081	metabolic	-	-	-	-	18:603760	41	50	82	128	175	63	0.834876	0.853418	1.15029	1.697991	3.124801	1.590633	0.946195	2.137808	0.000669	0.121614	-
ENSMUS(	Ackr1	atypical ch	GO:00508	response	K06574	Duffy bloo	mmu0514	Malaria -	1:1733318	17	44	63	83	79	112	0.442414	0.959831	1.129493	1.407186	1.802852	3.614046	0.843913	2.274695	0.00072	0.128345	-
ENSMUS(	Psmb8	proteasor	GO:00325	developm	K02740	20S prote	mmu0305	Proteason	17:341977	24	39	53	86	67	62	0.396381	0.539908	0.603015	0.925296	0.970327	1.269644	0.513101	1.055089	0.000741	0.129358	-
ENSMUS(	Tbr1	T-box bra	GO:00325	developm	-	-	-	-	2:6180293	1119	1389	1945	933	746	873	10.36221	10.78145	12.40775	5.628407	6.057644	10.02357	11.1838	7.236541	0.000741	0.129358	-
ENSMUS(	Smim20	small integ	GO:00099	cellular pr	-	-	-	-	5:5326708	123	128	112	241	206	211	1.701569	1.484252	1.067365	2.171918	2.498939	3.619207	1.417729	2.763355	0.000751	0.129827	-
ENSMUS(	Htr2c	5-hydroxy	GO:00325	developm	K04157	5-hydroxy	mmu0408	Neuroacti	X:1469625	1035	1131	1452	1025	824	404	4.520159	4.140272	4.36849	2.916212	3.155611	2.187667	4.342974	2.753163	0.000765	0.130905	-
ENSMUS(	Hirip3	HIRA inter	GO:00056	cell|intrac	-	-	-	-	7:1268619	313	416	552	360	253	167	2.878736	3.20704	3.497427	2.156963	2.040426	1.90441	3.194401	2.033933	0.000775	0.131246	-
ENSMUS(	Espnl	espin-like	GO:00990	supramole	-	-	-	-	1:9132207	4	5	4	0	0	0	0.034529	0.036178	0.023791	0	0	0	0.031499	0	0.000787	0.131982	-
ENSMUS(	Slc4a5	solute car	GO:00325	developm	K13857	solute car	mmu0497	Bile secret	6:8321982	204	87	389	91	112	54	1.689004	0.603771	2.218717	0.490823	0.813137	0.554351	1.503831	0.619437	0.000802	0.13233	-
ENSMUS(	CT010498	-	-	-	-	-	-	-	13:608426	2	3	10	0	0	0	0.076632	0.096326	0.263907	0	0	0	0.145621	0	0.000804	0.13233	-
ENSMUS(	Bend6	BEN doma	GO:00325	developm	-	-	-	-	1:3385205	1483	1987	2542	2089	747	637	21.07538	23.66928	24.88636	19.3399	9.308886	11.22431	23.21034	13.29103	0.000871	0.141971	-
ENSMUS(	Cdk2ap1	CDK2 (cyc	GO:00325	developm	-	-	-	-	5:1243454	45	73	78	129	102	124	0.78268	1.064242	0.93458	1.461638	1.555648	2.674081	0.927167	1.897122	0.000905	0.144828	-
ENSMUS(	Gm20547	predicted	GO:00081	metabolic	-	-	-	-	17:348564	7	8	9	23	20	19	0.109608	0.104988	0.097071	0.234599	0.2746	0.36886	0.103889	0.292687	0.000901	0.144828	-
ENSMUS(	Gm14399	predicted	GO:00081	metabolic	-	-	-	-	2:1751294	306	396	415	307	239	107	5.123084	5.557225	4.786405	3.348346	3.508732	2.221161	5.155571	3.026079	0.000923	0.14631	-
ENSMUS(	Chdh	choline de	GO:00081	metabolic	K00108	choline de	mmu0110	Metabolic	14:300090	166	128	252	86	107	75	1.309556	0.846407	1.369519	0.441973	0.740189	0.733609	1.175161	0.63859	0.000937	0.147138	-
ENSMUS(	Trim25	tripartite r	GO:00081	metabolic	K10652	tripartite r	mmu0516	Influenza	11:889993	132	114	174	238	252	222	1.442148	1.043982	1.309598	1.693932	2.414237	3.007297	1.265243	2.371822	0.000977	0.151949	-
ENSMUS(	Zfp697	zinc finge	GO:00971	organic cy	-	-	-	-	3:9838246	593	663	820	537	411	365	2.944523	2.759476	2.804951	1.737063	1.789552	2.247187	2.836317	1.924601	0.001002	0.154422	-
ENSMUS(	Helz2	helicase w	GO:00081	metabolic	-	-	-	-	2:1812276	106	123	174	235	266	167	0.333596	0.324468	0.377238	0.481795	0.73407	0.651653	0.3451	0.622506	0.001026	0.155058	-
ENSMUS(	Lgals3bp	lectin, gal	GO:00511	localizatio	-	-	-	-	11:118392	354	218	304	495	916	410	5.203001	2.685709	3.078048	4.739551	11.80562	7.471711	3.655586	8.005627	0.001033	0.155058	-
ENSMUS(	Pcdhga1	protocadh	GO:00160	membran	-	-	-	-	18:376617	198	180	238	207	167	3230	1.585027	1.207804	1.312498	1.0795	1.172277	32.05966	1.368443	11.43714	0.001026	0.155058	-
ENSMUS(	Pcdhga5	protocadh	GO:00160	membran	-	-	-	-	18:376943	2225	282	308	344	253	141	30.31849	3.220911	2.891215	3.053643	3.023015	2.382216	12.14354	2.819625	0.001052	0.156542	-
ENSMUS(	Ifi27l2a	interferon	GO:00325	developm	-	-	-	-	12:103442	9	8	7	21	18	37	1.235356	0.920402	0.661952	1.877854	2.166514	6.297075	0.939237	3.447147	0.001163	0.171552	-
ENSMUS(	H2-T23	histocomp	GO:00325	developm	K06751	major hist	mmu0516	Human pa	17:360297	76	79	126	134	201	181	0.748725	0.652364	0.85513	0.859992	1.73639	2.210929	0.752073	1.602437	0.001219	0.178321	-
ENSMUS(	Kcnc3	potassium	GO:00511	localizatio	-	-	-	-	7:4459066	884	968	1173	4475	1029	1206	6.675484	6.127149	6.102108	22.01431	6.813783	11.29181	6.30158	13.3733	0.001241	0.179991	-
ENSMUS(	Parp14	poly (ADP	GO:00081	metabolic	-	-	-	-	16:358328	81	118	160	222	341	123	0.4516	0.551449	0.614529	0.806315	1.667119	0.850277	0.539193	1.107904	0.001372	0.197252	-
ENSMUS(	Vil1	villin 1 [Sc	GO:00325	developm	-	-	-	-	1:7440937	11	29	14	5	6	4	0.170679	0.377156	0.14964	0.050543	0.081628	0.076952	0.232492	0.069708	0.001442	0.205273	-
ENSMUS(	C4b	compleme	GO:00081	metabolic	K03989	compleme	mmu0532	Systemic l	17:347283	584	625	1123	1271	1104	1106	5.894073	5.287317	7.807889	8.356597	9.770441	13.84023	6.32976	10.65576	0.001451	0.205273	-
ENSMUS(	Fsd1l	fibronecti	GO:00056	cell|intrac	-	-	-	-	4:5363147	280	771	985	446	358	105	1.656103	3.822408	4.013447	1.718488	1.856757	0.770022	3.163986	1.448422	0.001506	0.211275	-
ENSMUS(	Atf2	activating	GO:00325	developm	K04450	cyclic AMI	mmu0415	PI3K-Akt s	2:7381650	1504	1431	2307	1603	1140	656	5.473187	4.365005	5.783509	3.800209	3.63781	2.959935	5.207234	3.465985	0.001577	0.217686	-
ENSMUS(	Ugt2b34	UDP gluc	GO:00081	metabolic	K00699	glucuronc	mmu0110	Metabolic	5:8688976	3	8	2	0	0	0	0.030516	0.068216	0.014018	0	0	0	0.037584	0	0.001571	0.217686	-
ENSMUS(	Pagr1a	PAXIP1 as	GO:00325	developm	-	-	-	-	7:1270150	415	6	15	30	6	9	11.75334	0.142428	0.292661	0.5535	0.148996	0.31603	4.06281	0.339509	0.001652	0.225467	-
ENSMUS(	Slc6a14	solute car	GO:00081	metabolic	-	-	-	-	X:2171489	1	12	12	3	0	0	0.018511	0.186159	0.152998	0.036165	0	0	0.119223	0.012055	0.001673	0.225467	-
ENSMUS(	Wdr63	WD repea	GO:00038	catalytic a	-	-	-	-	3:1460405	35	7	57	11	6	7	0.601671	0.10086	0.675019	0.123181	0.090437	0.149206	0.459183	0.120942	0.001665	0.225467	-
ENSMUS(	Irf4	interferon	GO:00325	developm	K09445	interferon	mmu0465	Th17 cell c	13:307492	0	6	9	0	0	0	0	0.060693	0.074822	0	0	0	0.045172	0	0.001877	0.236489	-
ENSMUS(	Cd163l1	CD163 mo	GO:00511	localizatio	-	-	-	-	7:1402182	0	2	4	15	6	6	0	0.01727	0.028391	0.100669	0.054198	0.076647	0.01522	0.077171	0.001803	0.236489	-
ENSMUS(	Prr32	proline ric	-	-	-	-	-	-	X:4509090	77	36	112	37	36	22	4.40864	1.727714	4.417547	1.380063	1.80743	1.561823	3.517967	1.583105	0.001855	0.236489	-
ENSMUS(	Lrg1	leucine-ri	GO:00325	developm	-	-	-	-	17:561196	9	11	14	30	18	42	0.423368	0.433755	0.453681	0.919359	0.742484	2.449745	0.436935	1.37053	0.001792	0.236489	-
ENSMUS(	Tap1	transporte	GO:00511	localizatio	K05653	ATP-bind	mmu0516	Herpes sir	17:341875	43	54	55	84	92	111	0.232755	0.245008	0.205091	0.296205	0.436678	0.744973	0.227618	0.492619	0.001818	0.236489	-
ENSMUS(	Znfx1	zinc finge	GO:00081	metabolic	-	-	-	-	2:1670357	576	1094	749	1586	1326	964	2.981472	4.746555	2.670801	5.348023	6.01859	6.186873	3.466276	5.851162	0.00183	0.236489	-
ENSMUS(	Gbp7	guanylate	GO:00023	immune s	K20899	guanylate	mmu0462	NOD-like	3:1425303	125	131	214	292	375	156	1.214514	1.066879	1.432377	1.848233	3.194968	1.879325	1.237923	2.307508	0.001858	0.236489	-
ENSMUS(	Ugt1a6a	UDP gluc	GO:00081	metabolic	K00699	glucuronc	mmu0110	Metabolic	1:8813480	23	13	47	1	12	0	0.247663	0.11734	0.348649	0.007014	0.113309	0	0.237884	0.040108	0.001879	0.236489	-
ENSMUS(	Gapdh	glyceralde	GO:00325	developm	K00134|K0	glyceralde	mmu0110	Metabolic	6:1251617	644	932	1080	825	557	322	4.744741	5.755666	5.481533	3.959701	3.598523	2.941498	5.327313	3.499907	0.001773	0.236489	-
ENSMUS(	Igf2bp3	insulin-lik	GO:00081	metabolic	-	-	-	-	6:4908522	0	0	0	2	3	7	0	0	0	0.008988	0.018149	0.059887	0	0.029008	0.001983	0.246031	-
ENSMUS(	Cfap61	cilia and f	GO:00160	membran	-	-	-	-	2:1459347	38	37	78	37	20	15	0.172083	0.140447	0.243334	0.109154	0.079421	0.084225	0.185288	0.090933	0.001984	0.246031	-
ENSMUS(	Rprd2	regulation	GO:00329	macromo	-	-	-	-	3:9576034	1207	1453	1757	590	797	882	5.813891	5.866489	5.830192	1.851369	3.366362	5.267618	5.836857	3.495116	0.002008	0.24729	-
ENSMUS(	Nadk2	NAD kina	GO:00081	metabolic	K00858	NAD+ kin	mmu0110	Metabolic	15:907126	736	805	929	560	653	298	4.324214	3.964407	3.760072	2.143377	3.364228	2.170859	4.016231	2.559488	0.00208	0.253319	-
ENSMUS(	Zbp1	Z-DNA bi	GO:00023	immune s	K12965	Z-DNA bi	mmu0421	Necropto	2:1732066	1	14	12	16	57	27	0.013155	0.154348	0.108732	0.137091	0.657392	0.440314	0.092078	0.411599	0.002087	0.253319	-
ENSMUS(	Ly6c2	lymphocy	GO:00160	membran	-	-	-	-	15:751081	4	6	10	21	13	19	0.05829	0.073288	0.100395	0.199369	0.166126	0.343317	0.077324	0.23627	0.002184	0.263162	-
ENSMUS(	Slfn8	schlafen 8	GO:00056	cell|organ	-	-	-	-	11:830021	21	34	51	67	105	44	0.182502	0.247672	0.305323	0.379314	0.800152	0.474109	0.245166	0.551192	0.002214	0.263162	-
ENSMUS(	AC124712	-	-	-	-	-	-	-	12:739376	17	9	10	0	1	5	2.427807	1.077335	0.983868	0	0.125224	0.885335	1.496337	0.336853	0.002202	0.263162	-
ENSMUS(	Clic6	chloride ir	GO:00511	localizatio	-	-	-	-	16:924857	522	224	997	280	276	216	8.244208	2.965375	10.84737	2.880829	3.822345	4.229774	7.352319	3.644316	0.002294	0.266475	-
ENSMUS(	Cfap53	cilia and f	GO:00325	developm	-	-	-	-	18:742830	20	10	32	7	8	5	0.331613	0.138973	0.365508	0.075611	0.116319	0.102785	0.278698	0.098238	0.002304	0.266475	-
ENSMUS(	Ripor3	RIPOR far	-	-	-	-	-	-	2:1679801	3	4	4	0	0	0	0.029708	0.033204	0.027294	0	0	0	0.030069	0	0.002304	0.266475	-
ENSMUS(	Ugt1a10	UDP glyco	GO:00081	metabolic	K00699	glucuronc	mmu0110	Metabolic	1:8805538	0	3	2	11	25	2	0	0.054504	0.029868	0.155321	0.475178	0.053766	0.028124	0.228088	0.002293	0.266475	-
ENSMUS(	Prss32	protease,	GO:00081	metabolic	-	-	-	-	17:238438	3	3	5	0	0	0	0.079764	0.066864	0.091594	0	0	0	0.079407	0	0.002333	0.267922	-
ENSMUS(	Gsdmc2	gasdermin	GO:00325	developm	-	-	-	-	15:638243	2	4	5	0	0	0	0.038689	0.064843	0.066619	0	0	0	0.056717	0	0.002411	0.275034	-
ENSMUS(	Arhgap26	Rho GTPa	GO:00987	molecular	-	-	-	-	18:386015	686	2412	2574	1321	990	553	2.113401	6.228565	5.462833	2.6512	2.674457	2.112365	4.6016	2.479341	0.002541	0.288046	-
ENSMUS(	Pcdha2	protocadh	GO:00325	developm	-	-	-	-	18:369392	102	124	100	225	187	137	0.657581	0.670073	0.444119	0.944955	1.057136	1.095096	0.590591	1.032396	0.002584	0.290956	-
ENSMUS(	Sox17	SRY (sex c	GO:00325	developm	K04495	transcripti	mmu0431	Wnt signa	1:4490931	28	30	50	90	40	102	0.155047	0.139244	0.190735	0.324664	0.194227	0.700318	0.161675	0.406403	0.002612	0.292116	-
ENSMUS(	Kcnq5	potassium	GO:00511	localizatio	K04930	potassium	mmu0472	Cholinerg	1:2139840	1138	1596	1543	2847	2129	1342	5.063597	5.95254	4.729705	8.252504	8.306824	7.403811	5.248614	7.987713	0.002759	0.306059	-
ENSMUS(	4833427G	RIKEN cDI	-	-	-	-	-	-	9:5108111	5	8	16	2	3	1	0.326586	0.437971	0.719935	0.08509	0.171812	0.080964	0.494831	0.112622	0.002772	0.306059	-
ENSMUS(	Tmem72	transmem	GO:00160	membran	-	-	-	-	6:116679C	358	82	544	126	129	109	1.288699	0.24742	1.349021	0.295474	0.407194	0.486496	0.961714	0.396388	0.00279	0.306082	-
ENSMUS(	Gbp2	guanylate	GO:00023	immune s	K20897	guanylate	mmu0462	NOD-like	3:1426206	70	77	126	154	204	107	1.571975	1.449423	1.949274	2.252954	4.017201	2.979338	1.65689	3.083164	0.002848	0.308516	-
ENSMUS(	Lypd6	LY6/PLAU	GO:00452	synapse|m	-	-	-	-	2:5006642	237	312	532	105	273	78	1.466463	1.618195	2.267703	0.423249	1.481252	0.59842	1.784121	0.834307	0.002841	0.308516	-
ENSMUS(	H2-D1	histocomp	GO:00325	developm	K06751|K0	major hist	mmu0516	Human pa	17:352627	385	518	754	923	692	809	7.875084	8.881323	10.62472	12.29923	12.41206	20.51775	9.127042	15.07635	0.002874	0.309377	-
ENSMUS(	Lrch4	leucine-ri	GO:00081	metabolic	-	-	-	-	5:1376291	189	113	329	168	69	78	1.922762	0.963595	2.305745	1.113413	0.615538	0.983892	1.730701	0.904281	0.002963	0.316934	-
ENSMUS(	Map2k7	mitogen-a	GO:00048	signal trar	-	-	-	-	8:4238865	2	0	7	4	0	184	0.044158	0	0.106455	0.057529	0	5.036137	0.050204	1.697889	0.002986	0.317382	-
ENSMUS(	Cd200r2	Cd200 rec	GO:00160	membran	-	-	-	-	16:448670	5	4	2	0	0	0	0.181582	0.121756	0.050042	0	0	0	0.117793	0	0.003074	0.324746	-
ENSMUS(	Rsad2	radical S-	GO:00325	developm	K15045	radical S-	mmu0516	Influenza	12:264427	25	44	56	69	106	58	0.207312	0.305834	0.319903	0.372745	0.770777	0.596345	0.277683	0.579956	0.003162	0.329948	-
ENSMUS(	Duox2	dual oxida	GO:00325	developm	K13411	dual oxida	mmu0491	Thyroid ho	2:1222792	0	3	14	0	0	0	0	0.023905	0.091686	0	0	0	0.03853	0	0.00315	0.329948	-

ENSMUSC	Timd4	T cell imm	GO:00160	membran	-	-	-	-	11:468108	0	0	0	6	1	4	0	0	0	0.114321	0.025644	0.145051	0	0.095005	0.003271	0.339277	-
ENSMUSC	Cpne5	copine V	GO:00325	developm	-	-	-	-	17:291565	296	261	262	513	440	358	1.362475	1.007	0.830786	1.53828	1.775954	2.043176	1.066754	1.785803	0.003344	0.344751	-
ENSMUSC	Gbp9	guanylate	GO:00023	immune s	-	-	-	-	5:1050776	63	95	108	151	168	110	0.42044	0.531424	0.496522	0.656481	0.983139	0.910212	0.482795	0.849944	0.003452	0.351625	-
ENSMUSC	Msmp	microsem	GO:00055	extracellul	-	-	-	-	4:4358321	0	0	0	2	3	5	0	0	0	0.111431	0.224998	0.530248	0	0.288892	0.003439	0.351625	-
ENSMUSC	Dok2	docking p	GO:00048	signal trar	-	-	-	-	14:707660	3	5	2	10	12	12	0.04949	0.069144	0.022734	0.107478	0.173611	0.24548	0.047123	0.175523	0.003505	0.354942	-
ENSMUSC	Ankrd33b	ankyrin re	GO:00971	organic cy	-	-	-	-	15:312914	1511	1673	1549	1417	1043	788	4.512833	4.18826	3.187042	2.756997	2.731566	2.918078	3.962712	2.802214	0.003586	0.36098	-
ENSMUSC	Degs2	delta(4)-c	GO:00081	metabolic	K04712	sphingolip	mmu0110	Metabolic	12:108678	15	20	31	56	33	36	0.316712	0.353959	0.450907	0.77026	0.610988	0.942448	0.373859	0.774565	0.003641	0.362968	-
ENSMUSC	H2-T24	histocomp	GO:00023	immune s	K06751	major hist	mmu0516	Human pa	17:360056	50	33	67	97	142	59	1.147591	0.634871	1.059344	1.450336	2.857885	1.679007	0.947269	1.995742	0.003648	0.362968	-
ENSMUSC	Cyp2c55	cytochrom	GO:00081	metabolic	K07413	cytochrom	mmu0110	Metabolic	19:390070	0	5	8	0	0	0	0	0.133504	0.175563	0	0	0	0.103022	0	0.003741	0.368185	-
ENSMUSC	Trim5	tripartite r	GO:00048	signal trar	-	-	-	-	7:1042633	8	17	12	29	33	20	0.101742	0.181231	0.105142	0.240276	0.368036	0.315386	0.129372	0.307899	0.003743	0.368185	-
ENSMUSC	Phf11d	PHD finge	GO:00081	metabolic	-	-	-	-	14:593474	7	6	25	25	51	23	0.071562	0.051409	0.176051	0.166484	0.457161	0.291523	0.099674	0.305056	0.003772	0.368905	-
ENSMUSC	Mx1	MX dynar	GO:00038	catalytic a	-	-	-	-	16:974470	3	7	5	11	34	10	0.036249	0.070903	0.041626	0.086595	0.360296	0.149845	0.049593	0.198912	0.0039	0.372302	-
ENSMUSC	Rsph14	radial spo	-	-	-	-	-	-	10:749574	10	1	2	0	0	0	0.177178	0.01485	0.024414	0	0	0	0.072147	0	0.003908	0.372302	-
ENSMUSC	Thnsl1	threonine	-	-	-	-	-	-	2:2120572	209	281	402	185	169	165	1.710128	1.927264	2.266002	0.986135	1.212586	1.673995	1.967798	1.290905	0.003909	0.372302	-
ENSMUSC	Ly6c1	lymphocy	GO:00160	membran	-	-	-	-	15:750440	312	218	221	396	522	424	6.021428	3.526573	2.938251	4.978757	8.834008	10.14604	4.162084	7.986269	0.003916	0.372302	-
ENSMUSC	AC163623	-	-	-	-	-	-	-	9:2203638	0	0	0	6	0	7	0	0	0	0.341704	0	0.758756	0	0.36682	0.003837	0.372302	-
ENSMUSC	F5	coagulatic	GO:00508	response	K03902	coagulatic	mmu0461	Complem	1:1641518	592	140	967	250	274	150	5.063712	1.003758	5.698037	1.393055	2.055137	1.590836	3.921836	1.679676	0.003958	0.374277	-
ENSMUSC	Trim30a	tripartite r	GO:00081	metabolic	-	-	-	-	7:1044090	63	53	71	116	162	70	0.891528	0.628674	0.692156	1.069381	2.010249	1.22823	0.737452	1.435954	0.003996	0.37579	-
ENSMUSC	Tdrd12	tudor dor	GO:00325	developm	-	-	-	-	7:3546909	6	11	9	2	2	2	0.071612	0.110048	0.073993	0.015548	0.020929	0.029604	0.085218	0.022027	0.004032	0.377104	-
ENSMUSC	Gm21887	predicted	-	-	-	-	-	-	X:1700096	193	231	405	427	331	650	9.229314	9.259251	13.34189	13.30211	13.87975	38.53992	10.61015	21.90726	0.004098	0.381149	-
ENSMUSC	Fbxo34	F-box prc	-	-	-	-	-	-	14:474504	166	130	156	241	106	1045	0.59573	0.391055	0.385672	0.563431	0.333573	4.649908	0.457486	1.84897	0.004134	0.382435	-
ENSMUSC	Gm38394	predicted	GO:00081	metabolic	-	-	-	-	1:1336199	1080	1305	1296	2438	1381	1555	6.758297	6.84505	5.586885	9.938679	7.577916	12.06507	6.396744	9.860554	0.004194	0.385908	-
ENSMUSC	Areg	amphireg	GO:00325	developm	K09782	amphireg	mmu0439	Hippo sig	5:9113959	0	4	9	0	0	0	0	0.175369	0.324291	0	0	0	0.166553	0	0.004279	0.387485	-
ENSMUSC	Six3	sine oculis	GO:00325	developm	-	-	-	-	17:856136	13	22	20	11	2	7	0.092444	0.131135	0.097979	0.050957	0.012471	0.061723	0.107186	0.041717	0.004262	0.387485	-
ENSMUSC	Cav3	caveolin 3	GO:00325	developm	K12959	caveolin 3	mmu0414	Endocytos	6:1124595	6	11	5	3	0	1	0.329589	0.506485	0.189205	0.107337	0	0.068088	0.34176	0.058475	0.004267	0.387485	-
ENSMUSC	Ifih1	interferon	GO:00081	metabolic	K12647	interferon	mmu0516	Herpes sir	2:6259579	73	75	146	177	226	102	0.410347	0.353381	0.565367	0.64816	1.113981	0.71091	0.443032	0.824351	0.004372	0.393077	-
ENSMUSC	Apobec2	apolipopr	GO:00081	metabolic	-	-	-	-	17:484192	4	3	3	0	0	0	0.208704	0.131203	0.107831	0	0	0	0.149246	0	0.004387	0.393077	-
ENSMUSC	Synpr	synaptop	GO:00452	synapse|r	-	-	-	-	14:132847	830	1054	1780	898	647	707	4.02787	4.287368	5.95071	2.838934	2.753241	4.254053	4.755316	3.282076	0.004445	0.396218	-
ENSMUSC	Ipmk	inositol pc	GO:00325	developm	K00915	inositol-p	mmu0110	Metabolic	10:713477	887	1059	1213	1212	407	259	5.400604	5.404652	5.087812	4.807321	2.172981	1.955259	5.29769	2.97852	0.004564	0.404682	-
ENSMUSC	Awat2	acyl-CoA	GO:00081	metabolic	K11156	diacylglyc	mmu0083	Retinol m	X:1004022	0	0	0	3	5	2	0	0	0	0.043713	0.098072	0.055488	0	0.065758	0.004625	0.405938	-
ENSMUSC	Abi1	abl-intera	GO:00325	developm	-	-	-	-	2:2294007	3514	4139	4885	4243	2910	1710	13.66405	13.49044	13.0856	10.74812	9.922323	8.244414	13.41336	9.638285	0.004614	0.405938	-
ENSMUSC	Tmem168	transmem	GO:00160	membran	-	-	-	-	6:1358068	292	341	469	199	121	241	2.580655	2.526118	2.855425	1.145729	0.937722	2.640892	2.654066	1.574781	0.004718	0.411955	-
ENSMUSC	Slc5a8	solute car	GO:00511	localizatio	-	-	-	-	10:888859	4	7	5	4	3	4	0.047684	0.069945	0.041064	0.045816	0.027445	0.050637	0.052898	0.0413	0.004766	0.41408	-
ENSMUSC	Luzp1	leucine zi	GO:00325	developm	-	-	-	-	4:1364697	1807	1821	2567	3063	3405	2207	5.56181	4.698085	5.442963	6.141673	9.190051	8.422604	5.234286	7.918109	0.004935	0.420451	-
ENSMUSC	Elf5	E74-like f	GO:00325	developm	K17101	E74-like f	mmu0491	Prolactin s	2:1034116	4	8	12	2	3	0	0.028824	0.048321	0.059575	0.009388	0.018956	0	0.045574	0.009448	0.005025	0.420451	-
ENSMUSC	Fam198a	family wit	GO:00055	extracellul	-	-	-	-	9:1219509	32	69	66	94	86	116	0.326748	0.590563	0.464261	0.625281	0.770034	1.468621	0.460524	0.954645	0.005036	0.420451	-
ENSMUSC	Prokr2	prokinetic	GO:00048	signal trar	-	-	-	-	2:1323377	138	139	157	112	102	46	0.751076	0.634123	0.588647	0.397103	0.486798	0.310422	0.657949	0.398108	0.005025	0.420451	-
ENSMUSC	Olfr1507	olfactory	GO:00048	signal trar	K04257	olfactory r	mmu0474	Olfactory	14:524887	3	1	13	1	0	0	0.034374	0.009605	0.102625	0.007464	0	0	0.048868	0.002488	0.00496	0.420451	-
ENSMUSC	Ifi27	interferon	GO:00081	metabolic	-	-	-	-	12:103434	190	239	384	378	351	619	4.722354	4.979152	6.574878	6.120387	7.649896	19.07578	5.425461	10.94869	0.004916	0.420451	-
ENSMUSC	Gm8225	predicted	GO:00081	metabolic	-	-	-	-	17:265427	0	0	0	2	4	4	0	0	0	0.09419	0.253581	0.358585	0	0.235452	0.004994	0.420451	-
ENSMUSC	Gm28048	predicted	GO:00081	metabolic	-	-	-	-	11:228347	68	93	137	53	49	54	1.573787	1.804136	2.184279	0.799087	0.994436	1.549598	1.854067	1.114374	0.005036	0.420451	-
ENSMUSC	Wisp3	WNT1 ind	GO:00325	developm	-	-	-	-	10:391509	0	0	0	6	6	0	0	0	0	0.175066	0.235621	0	0	0.136896	0.005079	0.42196	-
ENSMUSC	Sp100	nuclear ar	GO:00325	developm	K15413	nuclear au	mmu0520	Viral carci	1:8564998	69	80	111	158	201	85	0.440678	0.428266	0.488364	0.657366	1.125661	0.673088	0.452436	0.818705	0.00516	0.423392	-
ENSMUSC	Rhof	ras homol	GO:00508	response	-	-	-	-	5:1231030	641	610	848	611	196	385	6.169544	4.921272	5.622665	3.831048	1.654222	4.594536	5.57116	3.359936	0.005171	0.423392	-
ENSMUSC	Cox16	cytochron	GO:00160	membran	-	-	-	-	12:813590	83	102	143	218	150	131	0.859388	0.885244	1.019991	1.470439	1.36189	1.68177	0.921541	1.5047	0.005126	0.423392	-
ENSMUSC	Nphs1	nephrosis	GO:00325	developm	-	-	-	-	7:3045831	25	27	36	57	42	65	0.212355	0.192234	0.210657	0.315408	0.31283	0.684566	0.205082	0.437601	0.005269	0.429423	-
ENSMUSC	Gm26920	predicted	GO:00081	metabolic	-	-	-	-	7:3005603	0	0	15	8	27	61	0	0	0.276635	0.139516	0.633813	2.024736	0.092212	0.932689	0.005351	0.434049	-
ENSMUSC	Drc7	dynein re	GO:00325	developm	-	-	-	-	8:9505510	231	118	386	135	128	107	4.088212	1.750479	4.706081	1.556448	1.986434	2.347961	3.514924	1.963614	0.005409	0.436684	-
ENSMUSC	Sostdc1	sclerostin	GO:00325	developm	-	-	-	-	12:363141	157	74	354	86	98	67	5.602367	2.213398	8.702158	1.999179	3.066472	2.964381	5.505974	2.676677	0.005443	0.437314	-
ENSMUSC	Pbrm1	polybrom	GO:00325	developm	K11757	protein pc	mmu0522	Hepatoce	14:310191	1329	1512	1895	1525	1095	746	5.440385	5.188109	5.343984	4.066829	3.930621	3.786426	5.324159	3.927959	0.005486	0.437345	-
ENSMUSC	B230104I2	RIKEN cDI	-	-	-	-	-	-	4:1543361	0	2	0	5	8	3	0	0.121617	0	0.236327	0.508962	0.269885	0.040539	0.338391	0.005494	0.437345	-
ENSMUSC	Aldh1a7	aldehyde	GO:00081	metabolic	K07249	retinal del	mmu0110	Metabolic	19:206929	8	13	11	1	5	2	0.246081	0.335208	0.233101	0.020037	0.134861	0.076303	0.271463	0.077067	0.005544	0.439247	-
ENSMUSC	Nhsl1	NHS-like	GO:00325	developm	-	-	-	-	10:183189	1043	1120	1156	1152	480	560	3.618021	3.256552	2.76246	2.603281	1.460062	2.40858	3.212344	2.157308	0.00587	0.462979	-
ENSMUSC	Sbno2	strawberry	GO:00325	developm	-	-	-	-	10:800569	268	329	393	556	465	370	1.112199	1.144449	1.123548	1.503156	1.692165	1.90386	1.126732	1.699727	0.00593	0.465555	-
ENSMUSC	Gh	growth hc	GO:00325	developm	K05438	growth hc	mmu0415	PI3K-Akt s	11:106300	0	32	22	3	0	3	0	1.977598	1.117369	0.144071	0	0.274268	1.031656	0.139446	0.005982	0.467493	-
ENSMUSC	Acox2	acyl-Coer	GO:00081	metabolic	K10214	3alpha,7a	mmu0110	Metabolic	14:822551	12	3	17	0	5	0	0.157303	0.032963	0.153519	0	0.057472	0	0.114595	0.019157	0.006065	0.470062	-
ENSMUSC	Rps17	ribosomal	GO:00081	metabolic	K02962	small subu	mmu0301	Ribosome	7:8134273	464	565	556	463	387	217	12.96203	13.22988	10.69992	8.425922	9.47999	7.516252	12.29728	8.474054	0.00607	0.470062	-
ENSMUSC	Lgals9	lectin, gal	GO:00325	developm	-	-	-	-	11:789629	82	88	137	169	191	118	1.594175	1.434022	1.834821	2.140375	3.256104	2.844398	1.621006	2.746959	0.006125	0.472143	-
ENSMUSC	4933405L	RIKEN cDI	-	-	-	-	-	-	8:1057076	3	7	3	0	0	1	0.076776	0.150172	0.052895	0	0	0.031727	0.093281	0.010576	0.006228	0.473964	-
ENSMUSC	Ncapg	non-SMC	GO:00511	localizatio	-	-	-	-	5:4566991	14	12	14	4	3	6	0.076801	0.055181	0.052907	0.014297	0.01443	0.040815	0.06163	0.02318	0.006212	0.473964	-
ENSMUSC	Ak9	adenylate	GO:00081	metabolic	K18533	adenylate	mmu0110	Metabolic	10:413039	24	18	43	18	11	0	0.218897	0.137612	0.270175	0.106948	0.087977	0	0.208895	0.064975	0.006231	0.473964	-
ENSMUSC	Per3	period cir	GO:00081	metabolic	K02633	period cir	mmu0516	Herpes sir	4:1510036	535	622	907	1102	890	827	2.675548	2.607368	3.124766	3.59023	3.902939	5.128028	2.802561	4.207066	0.006459	0.481597	-
ENSMUSC	Zc4h2	zinc finge	GO:00325	developm	-	-	-	-	X:9563919	282	387	506	207	204	248	3.106058	3.572931	3.839387	1.485295	1.970308	3.38686	3.506125	2.280821	0.0065	0.481597	-
ENSMUSC	Prickle1	prickle pla	GO:00325	developm	K04511	prickle[-]	mmu0431	Wnt signa	15:934991	608	1554	891	2336	1714	1077	8.531741	18.27837	8.613167	21.35444	21.09052	18.73853	11.80776	20.3945	0.006409	0.481597	-
ENSMUSC	Nlrc3	NLR famil	GO:00081	metabolic	-	-	-	-	16:394500	8	8	11	25	16	18	0.03886	0.032572	0.03681	0.079113	0.068153	0.108418	0.036081	0.085228	0.006495	0.481597	-
ENSMUSC	Gm5160	predicted	GO:00081	metabolic	-	-	-	-	18:144248	779	780	1064	891	579	307	111.5041	93.58375	104.9171	83.08303	72.67321	54.48502	103.335	70.08042	0.006486	0.481597	-
ENSMUSC	Lrrc61	leucine ric	-	-	-	-	-	-	6:4855479	197	247	244	418	279	333	1.78144	1.87221	1.520005	2.46242	2.212335	3.733661	1.724552	2.802805	0.006445	0.481597	-
ENSMUSC	Enpp2	ectonucle	GO:00325	developm	K01122	ectonucle	mmu0056	Ether lipid	15:548389	8753	3939	21018	5828	5732	4198	77.80133	29.34736	128.6983	33.74669	44.67648	46.26563	78.61567	41.56293	0.006627	0.488839	-
ENSMUSC	Aldoart1	aldolase 1	GO:00081	metabolic	K01623	fructose-l	mmu0110	Metabolic	4:7285058	100	149	167	93	86	10	1.549317	1.934992	1.782411	0.938653	1.168379	0.19211	1.755573	0.766381	0.006679	0.490555	-
ENSMUSC	Galnt5	polypeptic	GO:00081	metabolic	K00710	polypeptic	mmu0110	Metabolic	2:5799788	0	0	4	8	7	3	0	0	0.035898	0.067885	0.07996	0.048456	0.011966	0.065434	0.006785	0.496267	-
ENSMUSC	Neil1	nei endor	GO:00081	metabolic	K10567	endonucle	mmu0341	Base excis	9:5714280	51	61	81	118	85	105	1.210752	1.213867	1.324726	1.824948	1.769485	3.09073	1.249782	2.228388	0.00685	0.498872	-
ENSMUSC	Gbx1	gastrulatic	GO:00325	developm	-	-	-	-	5:2450275	4	6	5	3	0	0	0.073152	0.091974	0.062996	0.035738	0	0	0.07604	0.011913	0.007042	0.508474	-
ENSMUSC	Sprr2a3	small prol	GO:00325	developm	-	-	-	-	3:9228541	2	11	0	0	0	0	0.035691	0.164515	0	0	0	0	0.066735	0	0.00702	0.508474	-
ENSMUSC	Hmgn2	high mob	GO:00325	developm	-	-	-	-	4:1339647	219	223	269	368	508	244	1.641092	1.400707	1.388651	1.796466	3.338072	2.267066	1.476817	2.467201	0.007088	0.509395	-
ENSMUSC	Ddx58	DEAD (As	GO:00081	metabolic	K12646	ATP-depe	mmu0516	Epstein-B	4:4020377	136	133	188	262	332	145	0.791578	0.648868	0.753811	0.993428	1.694469	1.046419	0.731419	1.244772	0.007125	0.509395	-
ENSMUSC	Sp110	Sp110 nu	GO:00081	metabolic	-	-	-	-	1:8557689	21	13	25	37	38	39	0.144676	0.075072	0.118646	0.166056	0.229561	0.333137	0.112798	0.242918	0.007144	0.509395	-
ENSMUSC	Xkrx	X-linked	GO:00160	membran	-	-	-	-	X:1341490	6	6	12	26	14	15	0.116331	0.097501	0.160279	0.32838	0.23801	0.360588	0.124704	0.308993	0.007345	0.521621	-
ENSMUSC	Usmg5	upregulat	GO:00329	macromo	-	-	-	-	19:470834	717	1082	1213	876	741	251	133.6291	169.0293	155.7378	106.3573	121.0995	58.00176	152.7987	95.15286	0.007413	0.524258	-
ENSMUSC	Ccnd3	cyclin D3	GO:00081	metabolic	K10152	cyclin D3[	mmu0520	Pathways	17:475050	229	150	147	285	297	370	0.894288	0.491004	0.395468	0.725051	1.017048	1.791552	0.593587	1.177884	0.007665	0.534745	-
ENSMUSC	B3galt1	UDP-Gal:	GO:00081	metabolic	K07819	beta-1,3-	mmu0110	Metabolic	2:6756587	189	784	239	287	182	101	1.327971	4.617392	1.156847	1.313684	1.121352	0.879899	2.367403	1.104978	0.007718	0.534745	-
ENSMUSC	Tnfsf10	tumor nec	GO:00325	developm	K04721	tumor nec	mmu0406	Cytokine-	3:2731702	49	66	90	113	145	75	0.546719	0.617251	0.691767	0.821349	1.418655	1.037562	0.618579	1.092522	0.007694	0.534745	-
ENSMUSC	Synpo	synaptop	GO:00452	synapse|re	-	-	mmu0453	Tight junc	18:605939	1038	1089	3170	5825	1268	3291	2.035046	1.789606	4.281415	7.43968	2.17991	8.00001	2.702022	5.8732	0.007692	0.534745	-
ENSMUSC	Kcnj13	potassium	GO:00511	localizatio	K05006	potassium	mmu0497	Protein di	1:8738636	151	67	286	76	87	65	6.268114	2.331241	8.178583	2.055214	3.16683	3.345474	5.592646	2.855839	0.007707	0.534745	-
ENSMUSC	Haao	3-hydroxy	GO:00081	metabolic	K00452	3-hydroxy	mmu0110	Metabolic	17:838313	0	4	7	0	0	0	0	0.174364	0.250805	0	0	0	0.141723	0	0.008012	0.545781	-
ENSMUSC	Epsti1	epithelial	-	-	-	-	-	-	14:779042	6	15	23	37	27	22	0.122183	0.256032	0.322647	0.490827	0.482112	0.555471	0.233621	0.50947	0.007932	0.545781	-
ENSMUSC	Abcg3	ATP bindi	GO:00511	localizatio	K05682	ATP-bind	mmu0201	ABC trans	5:1049350	0	3	9	11	12	10	0	0.040202	0.099123	0.114566	0.168239	0.198248	0.046442	0.160351	0.008037	0.545781	-
ENSMUSC	Hist1h1e	histone cl	GO:00081	metabolic	-	-	-	-	13:236206	19	26	30	51	45	34	0.625651	0.717631	0.680529	1.094042	1.299361	1.388193	0.674603	1.260532	0.007975	0.545781	-
ENSMUSC	Ccl21a	chemokin	GO:00325	developm	-	-	-	-	JH584294	3	5	4	0	0	1	0.474219	0.66254	0.435687	0	0	0.195964	0.524149	0.065321	0.008011	0.545781	-
ENSMUSC	Gm20878	predicted	-	-	K16598	C-C motif	mmu0406	Cytokine-	4:4194157	10	8	10	5	0	3	0.178121	0.119436	0.122708	0.058022	0	0.066261	0.140088	0.041428	0.00807	0.54589	-
ENSMUSC	Armc4	armadillo	GO:00325	developm	-	-	-	-	18:708823	23	10	23	15	2	3	0.420393	0.153202	0.289612	0.17861	0.032053	0.067995	0.287736	0.092886	0.008206	0.552882	-
ENSMUSC	Rsph1	radial spo	GO:00325	developm	-	-	-	-	17:312550	105	93	178	108	74	43	5.732874	4.256149	6.695049	3.841392	3.542907	2.910982	5.561357	3.43176	0.008278	0.55337	-
ENSMUSC	4921507P	RIKEN cDI	GO:00056	cell|intrac	-	-	-	-	6:5057330	3	3	4	19	11	4	0.080167	0.067201	0.073653	0.330788	0.25779	0.132547	0.073673	0.240375	0.008256	0.55337	-
ENSMUSC	Cd274	CD274 an	GO:00400	locomotic	K06745	programn	mmu0451	Cell adhes	19:293674	53	53	51	87	109	71	0.92996	0.779507	0.616463	0.99446	1.677087	1.54466	0.77531	1.405402	0.008595	0.572339	-
ENSMUSC	Ltk	leukocyte	GO:00325	developm	-	-	-	-	2:1197513	122	178	231	251	238	317	1.038361	1.269884	1.354418	1.391696	1.776272	3.345304	1.220887	2.171091	0.008724	0.576426	-
ENSMUSC	Gm16486	predicted	-	-	-	-	-	-	8:7070345	0	2	0	4	4	6	0	0.023241	0	0.036131	0.048625	0.10315	0.007747	0.062635	0.008705	0.576426	-
ENSMUSC	Rtl5	retrotrans	-	-	-	-	-	-	X:1020665	188	252	116	114	130	24	1.266348	1.422812	0.538275	0.500244	0.767857	0.200443	1.075811	0.489515	0.008844	0.579896	-
ENSMUSC	H2-Q7	histocomp	GO:00325	developm	K06751	major hist	mmu0516	Human pa	17:354391	6	17	23	68	20	21	0.051587	0.122513	0.136226	0.380861	0.150785	0.223858	0.103442	0.251834	0.008833	0.579896	-
ENSMUSC	Trpv6	transient	GO:00511	localizatio	-	-	-	-	6:4162062	5	11	17	22	22	24	0.056133	0.103516	0.131472	0.160898	0.216578	0.334071	0.09704	0.237183	0.008897	0.581136	-
ENSMUSC	Srp14	signal rec	GO:00511	localizatio	K03104	signal rec	mmu0306	Protein ex	2:1184758	611	1114	1313	966	660	389	16.40511	25.07124	24.28589	16.89649	15.53905	12.95011	21.92075	15.12855	0.009091	0.587093	-
ENSMUSC	Thbs4	thrombos	GO:00325	developm	K04659	thrombos	mmu0415	PI3K-Akt s	13:927515	219	253	323	505	395	247	3.838417	3.716905	3.899977	5.766085	6.070829	5.367734	3.818433	5.734883	0.009034	0.587093	-
ENSMUSC	Zfp870	zinc finge	GO:00081	metabolic	-	-	-	-	17:328792	185	210	130	140	93	82	1.096149	1.042967	0.530631	0.54039	0.483196	0.602421	0.889915	0.542003	0.009064	0.587093	-
ENSMUSC	Tnpo1	transporti	GO:00511	localizatio	-	-	-	-	13:988390	1319	1596	2211	675	891	1145	7.088317	7.189255	8.185374	2.363107	4.198738	7.629388	7.487649	4.730411	0.009223	0.588959	-
ENSMUSC	Glod5	glyoxalase	-	-	-	-	-	-	X:8004200	5	4	0	0	0	0	0.472166	0.316602	0	0	0	0	0.262923	0	0.009218	0.588959	-
ENSMUSC	Reg3b	regenerat	GO:00508	response	-	-	-	-	6:7837065	2	25	21	0	0	5	0.075404	0.789893	0.545319	0	0	0.233674	0.470205	0.077891	0.009178	0.588959	-
ENSMUSC	Fgf14	fibroblast	GO:00325	developm	K04358	fibroblast	mmu0520	Pathways	14:123977	335	388	429	487	736	490	3.340174	3.242718	2.946677	3.163258	6.434941	6.057673	3.176523	5.218624	0.00927	0.589796	-
ENSMUSC	Rtbdn	retbindin	GO:00055	extracellul	-	-	-	-	8:8494695	10	6	11	24	15	22	0.145465	0.073154	0.110228	0.227429	0.19133	0.396801	0.109616	0.271853	0.009456	0.595211	-
ENSMUSC	Dnajb14	DnaJ heat	GO:00099	cellular pr	-	-	-	-	3:1378676	3475	4249	5044	3952	2756	2556	13.07869	13.40445	13.07783	9.689645	9.095598	11.92768	13.18699	10.23764	0.00946	0.595211	-
ENSMUSC	Fam228a	family wit	-	-	-	-	-	-	12:471367	45	39	24	89	79	47	0.503683	0.365898	0.185055	0.648955	0.775373	0.652268	0.351545	0.692199	0.009396	0.595211	-
ENSMUSC	Ccdc159	coiled-co	-	-	-	-	-	-	9:2192747	100	100	148	220	139	165	1.805482	1.513377	1.8408	2.587598	2.200656	3.693727	1.719887	2.827327	0.0097	0.601457	-
ENSMUSC	Stat1	signal trar	GO:00325	developm	K11220	signal trar	mmu0520	Pathways	1:5211944	540	530	705	782	1182	665	2.231381	1.835728	2.006876	2.105077	4.28292	3.407118	2.024661	3.265038	0.009645	0.601457	-
ENSMUSC	Pabpc1l	poly(A) bi	GO:00325	developm	K13126	polyadeny	mmu0301	RNA trans	2:1640254	7	2	13	3	2	0	0.128733	0.030827	0.164687	0.035934	0.032248	0	0.108082	0.022727	0.009642	0.601457	-
ENSMUSC	Gm27029	predicted	GO:00081	metabolic	-	-	-	-	11:101406	33	1	53	55	71	165	0.925936	0.023518	1.024459	1.005336	1.746894	5.740361	0.657971	2.830864	0.009697	0.601457	-
ENSMUSC	Rwdd3	RWD dom	GO:00081	metabolic	-	-	-	-	3:1211553	161	179	179	177	83	75	1.168305	1.088775	0.894821	0.836732	0.528142	0.674806	1.050634	0.679893	0.009818	0.606617	-
ENSMUSC	Fam109a	family wit	GO:00081	metabolic	-	-	-	-	5:1218489	70	90	28	122	126	155	0.883027	0.951638	0.243329	1.002583	1.393775	2.424352	0.692665	1.606903	0.00997	0.613744	-
ENSMUSC	Cxcl5	chemokin	GO:00400	locomotic	K05506	C-X-C mc	mmu0406	Cytokine-	5:9075936	12	5	25	4	2	5	0.336543	0.117538	0.483014	0.073092	0.049183	0.173863	0.312365	0.098713	0.010117	0.620612	-
ENSMUSC	Spata18	spermato	GO:00325	developm	-	-	-	-	5:7365137	29	22	46	27	14	8	0.567614	0.360934	0.620239	0.344264	0.240283	0.194146	0.516263	0.259564	0.010165	0.62127	-
ENSMUSC	Stxbp2	syntaxin b	GO:00511	localizatio	-	-	-	-	8:3630955	370	418	542	604	578	735	3.096061	2.931821	3.124345	3.292508	4.241104	7.625735	3.050742	5.053115	0.010302	0.62592	-
ENSMUSC	Mast4	microtubu	GO:00081	metabolic	-	-	-	-	13:102732	1048	1730	1483	2816	1826	1392	1.384746	1.916056	1.3499	2.423948	2.115693	2.280522	1.550234	2.273388	0.010314	0.62592	-
ENSMUSC	Pcdhga4	protocadh	GO:00325	developm	-	-	-	-	18:376852	19	5	121	452	276	8	0.218593	0.048215	0.959003	3.387677	2.784417	0.114119	0.408604	2.095404	0.0106	0.641021	-
ENSMUSC	Parp9	poly (ADP	GO:00081	metabolic	-	-	-	-	16:359384	58	82	105	133	153	90	0.597517	0.70809	0.745183	0.892594	1.382154	1.149613	0.683597	1.141454	0.010776	0.647339	-
ENSMUSC	Myh7	myosin, h	GO:00325	developm	K17751	myosin he	mmu0402	cGMP-PK	14:549706	310	486	372	303	275	191	1.853903	2.436208	1.532569	1.180455	1.442118	1.416265	1.940893	1.34628	0.01078	0.647339	-
ENSMUSC	Myadml2	myeloid-a	GO:00160	membran	-	-	-	-	11:120646	4	8	15	26	14	17	0.181314	0.303956	0.468425	0.767781	0.556489	0.955466	0.317898	0.759912	0.010919	0.653288	-
ENSMUSC	Nat8f7	N-acetylt	GO:00081	metabolic	-	-	mmu0048	Glutathior	6:8570672	7	4	15	33	11	19	0.177392	0.084957	0.261855	0.544754	0.244428	0.596972	0.174735	0.462051	0.010956	0.653288	-
ENSMUSC	Napepld	N-acyl ph	GO:00325	developm	K13985	N-acyl-ph	mmu0472	Retrograd	5:2166290	665	261	332	1063	744	649	6.397634	2.1047	2.200329	6.662123	6.27644	7.741552	3.567554	6.893371	0.011156	0.662905	-
ENSMUSC	Mslnl	mesotheli	GO:00160	membran	-	-	-	-	17:257360	2	9	2	0	0	1	0.048376	0.182426	0.033323	0	0	0.029976	0.088042	0.009992	0.011213	0.66402	-
ENSMUSC	Trhr	thyrotropi	GO:00048	signal trar	K04282	thyrotropi	mmu0408	Neuroacti	15:441961	170	265	153	125	149	67	1.691294	2.209889	1.048611	0.810144	1.299868	0.826481	1.649931	0.978831	0.011371	0.671071	-
ENSMUSC	Enpp1	ectonucle	GO:00325	developm	K01513	ectonucle	mmu0110	Metabolic	10:246379	95	96	139	78	85	34	0.490497	0.41547	0.494403	0.262355	0.384833	0.217663	0.46679	0.288284	0.011736	0.690203	-
ENSMUSC	Smyd1	SET and M	GO:00325	developm	-	-	-	-	6:7121394	37	36	46	32	21	15	0.288415	0.235222	0.247015	0.162498	0.143545	0.144978	0.256884	0.150341	0.011879	0.69621	-
ENSMUSC	Evi2	ecotropic	GO:00325	developm	-	-	-	-	11:795133	0	9	53	127	86	20	0	0.115941	0.561161	1.271582	1.159055	0.381132	0.225701	0.937257	0.012025	0.702382	-
ENSMUSC	Apol10b	apolipopr	GO:00081	metabolic	-	-	-	-	15:775841	6	5	14	20	20	14	0.192011	0.134109	0.308622	0.416944	0.561229	0.555504	0.211581	0.511225	0.012074	0.702813	-
ENSMUSC	Pnpla7	patatin-lik	GO:00325	developm	K14676	lysophosp	mmu0056	Glyceroph	2:2497603	228	289	348	516	372	304	1.340804	1.424564	1.409815	1.976799	1.918299	2.216612	1.391728	2.037237	0.012166	0.703927	-
ENSMUSC	AC149090	-	-	-	-	-	-	-	JH584304	2301	2707	3565	4672	3328	3149	25.75929	25.40144	27.49338	34.07232	32.66955	43.7095	26.21804	36.81712	0.012175	0.703927	-
ENSMUSC	Hist1h1d	histone cl	GO:00081	metabolic	-	-	-	-	13:235532	0	4	4	17	3	9	0	0.028697	0.023589	0.094791	0.022515	0.095511	0.017428	0.070939	0.012277	0.707452	-
ENSMUSC	Gm37240	predicted	GO:00511	localizatio	-	-	-	-	3:8449642	10	14	17	58	30	10	0.115426	0.135454	0.135173	0.436127	0.303641	0.143123	0.128684	0.294297	0.012369	0.710325	-
ENSMUSC	Lrrtm4	leucine ric	GO:00325	developm	-	-	-	-	6:8001887	161	722	167	214	131	128	0.585021	2.199058	0.418036	0.506573	0.417408	0.576689	1.067372	0.500223	0.012427	0.711308	-
ENSMUSC	Irf9	interferon	GO:00081	metabolic	K04693	interferon	mmu0516	Human pa	14:556035	184	193	266	305	333	270	1.262412	1.109926	1.25724	1.363218	2.003415	2.296859	1.209859	1.887831	0.012894	0.712679	-
ENSMUSC	Otx2	orthodent	GO:00325	developm	-	-	-	-	14:486576	136	44	223	73	51	58	0.818489	0.221961	0.924544	0.286203	0.269145	0.432802	0.654998	0.329383	0.012868	0.712679	-
ENSMUSC	Calml4	calmoduli	GO:00431	ion bindin	K02183	calmoduli	mmu0474	Olfactory	9:6285808	70	52	117	58	32	41	0.849311	0.528838	0.977928	0.458441	0.340453	0.616789	0.785359	0.471895	0.012567	0.712679	-
ENSMUSC	Pja1	praja ring	GO:00081	metabolic	-	-	-	-	X:9946573	966	1027	1193	3730	1046	976	5.176847	4.613296	4.404329	13.02203	4.915442	6.48521	4.731491	8.140893	0.012938	0.712679	-
ENSMUSC	Pkig	protein ki	GO:00081	metabolic	-	-	-	-	2:1636583	366	719	641	1033	1037	501	3.398157	5.595568	4.099892	6.248054	8.442754	5.767483	4.364539	6.81943	0.012705	0.712679	-
ENSMUSC	Vat1l	vesicle am	GO:00081	metabolic	-	-	-	-	8:1142056	684	678	1107	656	563	467	4.922464	4.089861	5.488142	3.075472	3.552855	4.167049	4.833489	3.598458	0.012811	0.712679	-
ENSMUSC	Taf13	TATA-box	GO:00081	metabolic	K03127	transcripti	mmu0516	Herpes sir	3:1085716	123	337	466	173	170	146	1.627526	3.737715	4.247766	1.49125	1.972492	2.395318	3.204335	1.95302	0.012771	0.712679	-
ENSMUSC	Gpr25	G protein	GO:00048	signal trar	-	-	-	-	1:1362597	14	18	24	30	24	65	0.826126	0.890326	0.975622	1.153254	1.241874	4.75573	0.897358	2.383619	0.012886	0.712679	-
ENSMUSC	Zfp457	zinc finge	GO:00081	metabolic	-	-	-	-	13:672881	2	8	6	10	20	11	0.018123	0.060748	0.037445	0.059017	0.158888	0.123565	0.038772	0.113824	0.012906	0.712679	-
ENSMUSC	Kl	klotho [Sc	GO:00325	developm	K14756	klotho[3.2	mmu0110	Metabolic	5:1509526	1440	804	2556	1189	1046	629	8.770131	4.104425	10.72398	4.717449	5.586222	4.749852	7.866178	5.017841	0.012951	0.712679	-
ENSMUSC	Ang4	angiogeni	GO:00325	developm	-	-	-	-	14:517638	2	7	5	2	0	0	0.179312	0.525921	0.30876	0.116774	0	0	0.337997	0.038925	0.012829	0.712679	-
ENSMUSC	Gm44503	predicted	-	-	-	-	-	-	9:7295885	680	233	966	250	249	351	9.85993	2.831869	9.649237	2.361488	3.16597	6.310406	7.447012	3.945955	0.012774	0.712679	-
ENSMUSC	Enkur	enkurin, T	GO:00056	cell|intrac	-	-	-	-	2:2118073	55	43	82	45	38	20	1.46865	0.962454	1.508427	0.782807	0.889788	0.662177	1.313177	0.778258	0.013165	0.717582	-
ENSMUSC	Phc3	polyhome	GO:00325	developm	-	-	-	-	3:3089937	465	569	2766	715	625	322	1.400378	1.436339	5.738461	1.402749	1.650498	1.202358	2.858393	1.418535	0.013146	0.717582	-
ENSMUSC	AC034099	-	GO:00081	metabolic	-	-	-	-	7:1423710	531	447	23	105	33	128	26.26199	18.53075	0.783642	3.383028	1.431186	7.84925	15.19213	4.221155	0.013166	0.717582	-
ENSMUSC	Slc17a7	solute car	GO:00325	developm	K12302	MFS trans	mmu0472	Retrograd	7:4516394	11790	13550	15697	18998	17108	17953	87.7286	84.51222	80.46286	92.09094	111.627	165.6343	84.23456	123.1174	0.013246	0.719674	-
ENSMUSC	Vti1b	vesicle tra	GO:00511	localizatio	K08493	vesicle tra	mmu0413	SNARE int	12:791560	1431	1877	2180	1644	1369	998	35.53902	39.07356	37.29699	26.59802	29.81343	30.7314	37.30319	29.04762	0.013484	0.725658	-
ENSMUSC	Spata1	spermato	-	-	-	-	-	-	3:1464571	36	28	41	20	24	11	0.39956	0.26049	0.313491	0.144612	0.233583	0.151382	0.324514	0.176526	0.013466	0.725658	-
ENSMUSC	Ucp3	uncouplin	GO:00325	developm	-	-	-	-	7:1004729	22	17	44	61	45	36	0.233764	0.151415	0.322085	0.422255	0.419289	0.474297	0.235754	0.438613	0.013425	0.725658	-
ENSMUSC	Rnf170	ring finge	GO:00081	metabolic	-	-	-	-	8:2611936	170	98	288	164	86	69	0.99274	0.479698	1.158594	0.623896	0.440383	0.499597	0.877011	0.521292	0.013565	0.727728	-
ENSMUSC	Slc16a11	solute car	GO:00081	metabolic	-	-	-	-	11:702127	70	98	128	165	116	166	0.502336	0.589493	0.632788	0.771371	0.729961	1.477043	0.574873	0.992792	0.013647	0.729876	-
ENSMUSC	Slfn3	schlafen 3	GO:00099	cellular pr	-	-	-	-	11:831913	8	6	18	23	27	15	0.113584	0.071405	0.176066	0.212743	0.336164	0.264077	0.120352	0.270995	0.013909	0.736735	-
ENSMUSC	Sar1a	secretion	GO:00511	localizatio	K07953	GTP-bind	mmu0414	Protein pr	10:616802	1162	599	871	1883	1591	925	14.54572	6.285052	7.511017	15.35539	17.46392	14.35677	9.447263	15.72536	0.013969	0.736735	-
ENSMUSC	Slc36a1	solute car	GO:00511	localizatio	K14209	solute car	mmu0497	Protein di	11:552043	1069	1275	1469	532	1204	259	9.255881	9.253437	8.762204	3.000775	9.14133	2.780517	9.090507	4.974207	0.013847	0.736735	-
ENSMUSC	Zfp764	zinc finge	GO:00081	metabolic	-	-	-	-	7:1274036	45	52	71	107	74	74	0.595939	0.577215	0.647733	0.923106	0.859334	1.215082	0.606962	0.999174	0.01393	0.736735	-
ENSMUSC	Pcdhgb5	protocadh	GO:00325	developm	-	-	-	-	18:377310	697	179	203	218	201	106	4.794507	1.032091	0.961964	0.976897	1.212409	0.904075	2.262854	1.031127	0.013991	0.736735	-
ENSMUSC	Mxd4	Max dime	GO:00055	protein bi	-	-	-	-	5:3417388	1224	1388	2052	1623	967	787	7.726352	7.344041	8.92322	6.674105	5.352569	6.159616	7.997871	6.062097	0.014125	0.741541	-
ENSMUSC	Apc	adenoma	GO:00081	metabolic	K02085	adenoma	mmu0520	Pathways	18:342209	3809	4385	11783	5517	4198	2186	8.925353	8.612652	19.02049	8.421686	8.625802	6.351123	12.18616	7.799537	0.014262	0.7464	-
ENSMUSC	Lrrc34	leucine ric	GO:00325	developm	-	-	-	-	3:3062426	10	5	19	7	3	2	0.336085	0.140847	0.439891	0.15326	0.088405	0.083364	0.305608	0.108343	0.014359	0.749202	-
ENSMUSC	Clca4a	chloride c	GO:00081	metabolic	K05030	calcium-a	mmu0497	Pancreatic	3:1449524	3	6	0	0	0	0	0.063461	0.106395	0	0	0	0	0.056618	0	0.014415	0.749841	-

ENSMUSC	Klk1	kallikrein	GO:00081	metabolic	K01325	tissue kalli	mmu0496	Endocrine	7:4422536	0	8	2	0	0	0	0	0.297796	0.061197	0	0	0	0.119664	0	0.014476	0.750724	-
ENSMUSC	Sh3rf2	SH3 dom	GO:00081	metabolic	-	-	-	-	18:420536	12	6	13	1	7	0	0.088758	0.037198	0.066243	0.004818	0.045406	0	0.064066	0.016741	0.014545	0.752058	-
ENSMUSC	Svbp	small vasc	GO:00081	metabolic	-	-	-	-	4:1191953	41	86	60	136	72	108	2.012083	3.537668	2.028482	4.347986	3.098408	6.571664	2.526078	4.672686	0.01466	0.754784	-
ENSMUSC	Rnh1	ribonucle	GO:00325	developm	-	-	-	-	7:1411603	206	271	350	404	311	488	4.434936	4.890373	5.190853	5.666077	5.871152	13.02644	4.83872	8.18789	0.014797	0.754784	-
ENSMUSC	Ggact	gamma-g	GO:00081	metabolic	-	-	-	-	14:122816	145	159	292	290	300	250	1.327457	1.22012	1.841557	1.729545	2.408328	2.837773	1.463045	2.325215	0.01481	0.754784	-
ENSMUSC	Arl15	ADP-ribo	GO:00508	response	-	-	-	-	13:113794	357	426	528	1512	425	402	4.077716	4.0786	4.154651	11.25078	4.256771	5.693258	4.103655	7.066935	0.014819	0.754784	-
ENSMUSC	Tgfbrap1	transform	GO:00081	metabolic	-	-	-	-	1:4304720	546	1350	671	1844	1311	978	1.905545	3.949235	1.613245	4.192459	4.012099	4.232052	2.489341	4.145537	0.014816	0.754784	-
ENSMUSC	Tmprss2	transmem	GO:00081	metabolic	K09633	transmem	mmu0520	Transcript	16:975646	1	2	5	0	0	0	0.020122	0.033725	0.069297	0	0	0	0.041048	0	0.015018	0.757073	-
ENSMUSC	Wdr37	WD repea	GO:00081	metabolic	-	-	-	-	13:880296	718	1505	1807	934	857	751	2.534356	4.452791	4.393925	2.14769	2.652567	3.286767	3.793691	2.695675	0.014968	0.757073	-
ENSMUSC	Trak1	trafficking	GO:00325	developm	-	-	-	-	9:1212975	3724	4330	3082	3210	2529	2067	12.11711	11.80946	6.908336	6.804184	7.215739	8.339036	10.2783	7.452986	0.015041	0.757073	-
ENSMUSC	Gm43720	predicted	GO:00055	extracellul	-	-	-	-	5:1382649	16	24	23	2	2	13	0.158613	0.199421	0.157071	0.012914	0.017384	0.159783	0.171702	0.06336	0.014933	0.757073	-
ENSMUSC	Cyp4f18	cytochron	GO:00081	metabolic	K17726	leukotrien	mmu0110	Metabolic	8:7198848	0	1	5	10	5	5	0	0.022429	0.092173	0.174318	0.117315	0.165884	0.038201	0.152505	0.015113	0.758484	-
ENSMUSC	Fam151a	family wit	GO:00055	extracellul	-	-	-	-	4:1067338	0	0	0	0	3	5	0	0	0	0	0.086934	0.204875	0	0.097269	0.0153	0.760881	-
ENSMUSC	Lin54	lin-54 hor	GO:00081	metabolic	-	-	mmu0421	Cellular se	5:1004419	356	431	358	369	265	162	1.301775	1.321043	0.901821	0.879012	0.849718	0.734494	1.174879	0.821074	0.01536	0.760881	-
ENSMUSC	Nfe2l1	nuclear fa	GO:00325	developm	-	-	-	-	11:968174	2273	2687	7482	3543	2514	1139	9.625908	9.538121	21.82787	9.774509	9.33577	5.980701	13.66397	8.36366	0.01535	0.760881	-
ENSMUSC	Gchfr	GTP cyclo	GO:00081	metabolic	-	-	-	-	2:1191677	4	5	2	0	0	1	0.394112	0.412932	0.135772	0	0	0.122136	0.314272	0.040712	0.015371	0.760881	-
ENSMUSC	Slfn2	schlafen 2	GO:00099	cellular pr	-	-	-	-	11:830651	18	36	70	74	84	50	0.687464	1.152501	1.841744	1.84119	2.813214	2.367788	1.227236	2.340731	0.015383	0.760881	-
ENSMUSC	Otogl	otogelin-l	GO:00081	metabolic	-	-	-	-	10:107760	43	40	46	40	21	14	0.311854	0.243163	0.229821	0.188983	0.133553	0.125896	0.261613	0.149478	0.015435	0.761273	-
ENSMUSC	Dlst	dihydrolip	GO:00081	metabolic	K00658	2-oxoglut	mmu0110	Metabolic	12:851108	2027	1110	1317	1290	937	701	9.886564	4.538031	4.425158	4.09886	4.007503	4.239313	6.283251	4.115225	0.015529	0.763668	-
ENSMUSC	Rtp4	receptor t	GO:00511	localizatio	-	-	-	-	16:235202	25	54	62	67	86	74	0.367787	0.66589	0.628337	0.642112	1.109419	1.34981	0.554004	1.03378	0.015865	0.777965	-
ENSMUSC	Cfap43	cilia and f	GO:00081	metabolic	-	-	-	-	19:477375	87	88	166	90	73	48	0.494599	0.419341	0.650119	0.333318	0.363916	0.338345	0.521353	0.345193	0.015925	0.778695	-
ENSMUSC	Cetn4	centrin 4	GO:00056	cell|intrac	-	-	-	-	3:3730774	185	197	148	167	117	54	2.212096	1.974472	1.219119	1.300864	1.226766	0.800601	1.801896	1.10941	0.016091	0.784543	-
ENSMUSC	Uchl4	ubiquitin	GO:00081	metabolic	-	-	-	-	9:6423520	122	158	159	129	102	62	6.672496	7.243378	5.990708	4.596236	4.891858	4.204465	6.635527	4.564186	0.016264	0.790725	-
ENSMUSC	Lsm2	LSM2 hon	GO:00081	metabolic	K12621	U6 snRNA	mmu0304	Spliceosoi	17:349818	51	70	79	106	95	97	0.500106	0.575364	0.533671	0.677147	0.816882	1.179369	0.53638	0.891133	0.016444	0.797228	-
ENSMUSC	Rp9	retinitis pi	GO:00081	metabolic	K19604	retinitis pi	mmu0304	Spliceosoi	9:2241104	115	163	206	207	316	189	1.61659	1.920625	1.994897	1.895632	3.895214	3.294204	1.844037	3.02835	0.016554	0.800304	-
ENSMUSC	Igsf5	immunog	GO:00160	membran	K06786	junctional	mmu0453	Tight junc	16:963616	127	174	216	164	113	70	2.055311	2.360351	2.408136	1.72902	1.603607	1.40463	2.274599	1.579086	0.017115	0.812359	-
ENSMUSC	Retn	resistin [S	GO:00508	response	-	-	-	-	8:3655770	0	0	3	5	4	4	0	0	0.076315	0.120292	0.129517	0.183147	0.025438	0.144319	0.017059	0.812359	-
ENSMUSC	Tmprss6	transmem	GO:00081	metabolic	-	-	-	-	15:784396	5	2	8	8	11	20	0.099583	0.033387	0.109761	0.103796	0.19211	0.493884	0.08091	0.263263	0.017742	0.812359	-
ENSMUSC	Krt19	keratin 19	GO:00325	developm	-	-	-	-	11:100140	8	21	30	5	0	10	0.217825	0.4793	0.562734	0.088699	0	0.337632	0.419953	0.14211	0.017645	0.812359	-
ENSMUSC	Tc2n	tandem C	GO:00081	metabolic	-	-	-	-	12:101645	16	10	33	15	7	3	0.140355	0.073524	0.199414	0.085718	0.053848	0.032632	0.137764	0.057399	0.017328	0.812359	-
ENSMUSC	Asnsd1	asparagin	GO:00081	metabolic	-	-	-	-	1:5334461	178	283	365	186	114	182	2.186404	2.913733	3.088545	1.488347	1.227887	2.771836	2.72956	1.829357	0.017218	0.812359	-
ENSMUSC	Nox1	NADPH o	GO:00325	developm	K08008	NADPH o	mmu0541	Fluid shea	X:1340864	0	0	0	1	1	6	0	0	0	0.010612	0.014285	0.121209	0	0.048702	0.017753	0.812359	-
ENSMUSC	Me1	malic enz	GO:00081	metabolic	K00029	malate de	mmu0110	Metabolic	9:8658137	527	615	700	786	1054	700	5.994713	5.863881	5.485384	5.824542	10.51334	9.872824	5.781326	8.736902	0.017547	0.812359	-
ENSMUSC	Trib3	tribbles ps	GO:00325	developm	K19518	tribbles hc	mmu0493	Insulin res	2:1523374	2	3	12	12	16	11	0.040117	0.050427	0.165792	0.156781	0.281365	0.273528	0.085446	0.237225	0.017177	0.812359	-
ENSMUSC	Tjp3	tight junct	GO:00056	cell|cell jur	K06097	tight junct	mmu0453	Tight junc	10:812732	40	30	44	30	16	18	0.505396	0.317714	0.38298	0.246929	0.177266	0.281993	0.40203	0.235396	0.017276	0.812359	-
ENSMUSC	Afg1l	AFG1 like	GO:00081	metabolic	-	-	-	-	10:423125	294	247	265	515	415	277	4.742274	3.339566	2.944671	5.411642	5.869906	5.539954	3.675504	5.607167	0.01706	0.812359	-
ENSMUSC	Cacna2d1	calcium cl	GO:00511	localizatio	K04858	voltage-d	mmu0401	MAPK sigi	5:1593469	2593	5817	6697	3858	3323	2682	8.565568	16.10666	15.24001	8.302281	9.625575	10.98496	13.30408	9.637605	0.01736	0.812359	-
ENSMUSC	Slc26a10	solute car	GO:00511	localizatio	-	-	-	-	10:127171	155	204	253	418	237	213	1.582958	1.746304	1.779954	2.780956	2.1224	2.697136	1.703072	2.533497	0.017708	0.812359	-
ENSMUSC	Chd3os	chromodc	-	-	-	-	-	-	11:693404	1457	609	744	749	527	432	26.48648	9.279717	9.317272	8.870093	8.400747	9.737215	15.02782	9.002685	0.017516	0.812359	-
ENSMUSC	Dupd1	dual speci	GO:00081	metabolic	-	-	-	-	14:216765	6	0	3	0	0	0	0.260296	0	0.08965	0	0	0	0.116649	0	0.017197	0.812359	-
ENSMUSC	Arfip1	ADP-ribo	GO:00511	localizatio	-	-	-	-	3:8449609	395	423	600	330	399	216	6.125774	5.498663	6.41013	3.333958	5.425995	4.153394	6.011523	4.304449	0.017552	0.812359	-
ENSMUSC	Gm20683	predicted	GO:00038	catalytic a	-	-	-	-	17:258344	222	317	398	412	369	579	5.056895	6.052625	6.245495	6.113789	7.370567	16.35297	5.785005	9.945776	0.017219	0.812359	-
ENSMUSC	Gm28539	predicted	-	-	-	-	-	-	16:188365	114	44	62	74	22	13	2.429589	0.786019	0.910266	1.027411	0.41115	0.343517	1.375292	0.594026	0.017696	0.812359	-
ENSMUSC	AC099934	-	-	-	-	-	-	-	12:691828	0	15	0	0	0	0	0	0.696666	0	0	0	0	0.232222	0	0.017436	0.812359	-
ENSMUSC	AC133183	-	-	-	-	-	-	-	12:840389	5	0	7	7	21	12	0.740718	0	0.714414	0.67556	2.728029	2.204171	0.485044	1.869254	0.017561	0.812359	-
ENSMUSC	D3Ertd751	DNA segr	-	-	-	-	-	-	3:4174261	275	414	289	294	243	105	1.049421	1.324253	0.759742	0.730881	0.813141	0.496811	1.044472	0.680277	0.017919	0.817788	-
ENSMUSC	Sulf1	sulfatase	GO:00325	developm	-	-	-	-	1:1269227	427	266	770	356	283	244	1.680216	0.877347	2.08727	0.912574	0.976487	1.190452	1.548278	1.026504	0.017985	0.818613	-
ENSMUSC	Sumo3	small ubic	-	-	-	-	-	-	10:776061	15	10	0	0	0	3	1.861922	1.040395	0	0	0.326511	0	0.967439	0.108837	0.018064	0.820038	-
ENSMUSC	Uhmk1	U2AF hon	GO:00325	developm	-	-	-	-	1:1701934	808	928	7832	8037	5782	5723	6.338828	6.102364	42.3274	41.07458	39.77574	55.66821	18.2562	45.50617	0.018208	0.824351	-
ENSMUSC	Cetn3	centrin 3	GO:00099	cellular pr	-	-	-	-	13:817830	415	685	892	613	461	302	5.888479	8.147017	8.719089	5.666266	5.735861	5.3131	7.584861	5.571742	0.018495	0.826868	-
ENSMUSC	Pfkfb3	6-phosph	GO:00325	developm	K01103	6-phosph	mmu0415	AMPK sigi	2:1147143	848	1001	1203	791	903	408	2.852987	2.822872	2.788182	1.733653	2.664003	1.701965	2.821347	2.033207	0.01847	0.826868	-
ENSMUSC	Trem3	triggering	GO:00081	metabolic	-	-	-	-	17:482477	0	0	1	0	4	7	0	0	0.044029	0	0.224243	0.554945	0.014676	0.259729	0.018509	0.826868	-
ENSMUSC	Tmem215	transmem	GO:00160	membran	-	-	-	-	4:4047218	185	245	220	219	140	86	1.677456	1.862085	1.374212	1.293615	1.113145	0.966866	1.637918	1.124542	0.018409	0.826868	-
ENSMUSC	Trim30b	tripartite	GO:00081	metabolic	-	-	-	-	7:1043553	4	4	4	10	9	12	0.019431	0.016287	0.013388	0.031647	0.038341	0.072282	0.016369	0.047423	0.018541	0.826868	-
ENSMUSC	Pld5	phospholi	GO:00038	catalytic a	-	-	-	-	1:1759623	53	55	78	46	43	25	0.228997	0.199192	0.232168	0.129477	0.162915	0.133929	0.220119	0.142107	0.018553	0.826868	-
ENSMUSC	Muc4	mucin 4 [	GO:00160	membran	-	-	-	-	16:327358	3	12	3	0	0	2	0.008873	0.029756	0.006113	0	0	0.007338	0.014914	0.002446	0.018657	0.829361	-
ENSMUSC	Paqr8	progestin	GO:00325	developm	-	-	-	-	1:2089060	2082	2842	3835	3731	819	759	10.73918	12.2876	13.62721	12.5371	3.704389	4.854203	12.218	7.031897	0.019069	0.84326	-
ENSMUSC	Klf10	Kruppel-li	GO:00325	developm	-	-	-	-	15:382914	595	683	731	324	528	381	5.86173	5.640048	4.961102	2.079389	4.561267	4.653934	5.487627	3.764864	0.019038	0.84326	-
ENSMUSC	Lgals12	lectin, gal	GO:00325	developm	-	-	-	-	19:759666	3	6	9	3	2	0	0.029796	0.049955	0.061584	0.019411	0.017419	0	0.047112	0.012277	0.019136	0.844055	-
ENSMUSC	Abhd17a	abhydrola	GO:00081	metabolic	-	-	-	-	10:805836	476	678	800	796	642	1721	9.465336	11.30086	10.959	10.31155	11.19456	42.43226	10.57507	21.31279	0.019193	0.844412	-
ENSMUSC	Diaph2	diaphano	GO:00325	developm	K05741	diaphano	mmu0481	Regulatio	X:1297497	416	504	663	812	898	465	1.387024	1.408555	1.522844	1.763714	2.625484	1.922336	1.439474	2.103844	0.019316	0.847607	-
ENSMUSC	H2-Oa	histocomp	GO:00325	developm	K06752	major hist	mmu0516	HTLV-I inf	17:340923	0	3	5	12	3	11	0	0.073204	0.10028	0.227594	0.07658	0.397074	0.057828	0.233749	0.019368	0.847719	-
ENSMUSC	Cfap73	cilia and f	GO:00099	cellular pr	-	-	-	-	5:1206283	11	5	25	7	4	3	0.759068	0.289187	1.188393	0.314675	0.242017	0.256698	0.745549	0.27113	0.019499	0.851272	-
ENSMUSC	Cldn7	claudin 7	GO:00400	locomotic	K06087	claudin[-]	mmu0453	Tight junc	11:699647	0	3	6	0	0	0	0	0.04887	0.080329	0	0	0	0.043066	0	0.019712	0.851913	-
ENSMUSC	Dkk2	dickkopf	GO:00325	developm	K02165	dickkopf[-	mmu0431	Wnt signa	3:1320852	14	27	30	13	13	9	0.240145	0.388204	0.354499	0.145272	0.195539	0.191409	0.327616	0.177407	0.019695	0.851913	-
ENSMUSC	Dusp22	dual speci	GO:00081	metabolic	-	-	-	-	13:306599	187	238	219	368	258	286	1.956284	2.086983	1.578286	2.507955	2.366752	3.709727	1.873851	2.861478	0.01965	0.851913	-
ENSMUSC	Phf11b	PHD finge	GO:00160	membran	-	-	-	-	14:593209	10	4	4	13	29	10	0.53677	0.17996	0.147927	0.45459	1.364967	0.665569	0.288219	0.828375	0.019564	0.851913	-
ENSMUSC	Rgl2	ral guanin	GO:00987	molecular	K17636	ral guanin	mmu0401	Ras signal	17:339295	461	569	698	937	634	718	3.545675	3.668287	3.698326	4.694826	4.275926	6.847137	3.637429	5.272629	0.019775	0.852482	-
ENSMUSC	Fopnl	Fgfr1op N	GO:00099	cellular pr	-	-	-	-	16:142992	179	164	315	377	193	465	4.668017	3.584905	5.659034	6.404774	4.413481	15.03556	4.637319	8.617937	0.02018	0.86774	-
ENSMUSC	Lrriq3	leucine-ri	-	-	-	-	-	-	3:1550934	10	5	9	6	0	2	0.115531	0.048417	0.071626	0.045159	0	0.028657	0.078525	0.024605	0.020333	0.87082	-
ENSMUSC	Nav2	neuron na	GO:00325	developm	-	-	-	-	7:4890871	1055	1082	1410	1639	1595	1384	1.605335	1.380046	1.478031	1.624702	2.12822	2.61117	1.487804	2.121364	0.020353	0.87082	-
ENSMUSC	Fmo4	flavin con	GO:00081	metabolic	K00485	dimethyla	mmu0098	Drug met	1:1627931	0	5	3	0	0	0	0	0.049886	0.0246	0	0	0	0.024829	0	0.020472	0.87153	-
ENSMUSC	Spx	spexin ho	GO:00081	metabolic	-	-	-	-	6:1424134	14	13	8	6	4	4	0.436788	0.33998	0.171943	0.121953	0.109425	0.154736	0.316237	0.128705	0.02046	0.87153	-
ENSMUSC	Fam83f	family wit	-	-	-	-	-	-	15:806718	0	0	0	3	5	0	0	0	0	0.041563	0.093247	0	0	0.044937	0.020555	0.872898	-
ENSMUSC	Sp6	trans-acti	GO:00325	developm	-	-	-	-	11:970133	3	13	11	17	15	25	0.026599	0.096629	0.067195	0.098203	0.116635	0.274865	0.063474	0.163234	0.020723	0.876567	-
ENSMUSC	Ube2n	ubiquitin-	GO:00081	metabolic	K10580	ubiquitin-	mmu0412	Ubiquitin	10:955151	1821	2215	2708	2860	655	302	17.16808	17.50404	17.58782	17.56546	5.414968	3.530244	17.41998	8.83689	0.020744	0.876567	-
ENSMUSC	1700007G	RIKEN cD	-	-	-	-	-	-	5:9832930	7	16	14	9	6	0	0.207316	0.397178	0.285612	0.173629	0.1558	0	0.296702	0.10981	0.020841	0.878231	-
ENSMUSC	Rbm47	RNA bind	GO:00325	developm	-	-	-	-	5:6601654	85	41	163	63	44	41	0.307491	0.124323	0.406213	0.148469	0.139574	0.183899	0.279342	0.157314	0.020886	0.878231	-
ENSMUSC	Mpped1	metalloph	GO:00038	catalytic a	-	-	-	-	15:837794	891	1028	1412	1253	1078	3125	6.235625	6.030431	6.807513	5.712617	6.615518	27.1168	6.357856	13.14831	0.021078	0.881987	-
ENSMUSC	Gm5136	predicted	GO:00081	metabolic	K11864	BRCA1/BR	mmu0462	NOD-like	10:108698	1	0	0	4	3	3	0.045502	0	0	0.118558	0.119666	0.169233	0.015167	0.135819	0.02103	0.881987	-
ENSMUSC	Tal2	T cell acut	GO:00325	developm	-	-	-	-	4:5377970	3	3	1	8	5	9	0.064166	0.053788	0.014731	0.111482	0.093781	0.238698	0.044228	0.147987	0.021202	0.882832	-
ENSMUSC	Jrk	jerky [Sou	GO:00329	macromo	-	-	-	-	15:747023	124	162	120	255	179	173	1.346416	1.474437	0.897612	1.803763	1.704332	2.329115	1.239488	1.945737	0.021187	0.882832	-
ENSMUSC	Hfe2	hemochrc	GO:00048	signal trar	-	-	-	-	3:9652517	0	0	0	2	0	6	0	0	0	0.040825	0	0.233155	0	0.091327	0.021334	0.88619	-
ENSMUSC	Tomm40l	translocas	GO:00511	localizatio	K11518	mitochon	mmu0501	Amyotrop	1:1712160	413	514	875	862	713	820	2.725017	2.842732	3.977215	3.705175	4.125264	6.708405	3.181655	4.846281	0.022035	0.905801	-
ENSMUSC	Slc2a8	solute car	GO:00081	metabolic	-	-	-	-	2:3297299	109	132	163	177	172	249	1.237454	1.256114	1.274803	1.309056	1.712277	3.50501	1.256124	2.175448	0.022005	0.905801	-
ENSMUSC	Cdc25a	cell divisic	GO:00081	metabolic	K06645	M-phase	mmu0421	Cellular se	9:1098755	122	149	139	116	59	84	0.751011	0.768824	0.589462	0.465187	0.318478	0.64114	0.703099	0.474935	0.021941	0.905801	-
ENSMUSC	Dnah6	dynein, ax	GO:00400	locomotic	K10408	dynein he	mmu0501	Huntingto	6:7301760	109	78	225	98	79	61	0.490426	0.294169	0.697399	0.287248	0.311686	0.340299	0.493998	0.313078	0.022071	0.905801	-
ENSMUSC	Kdm2a	lysine (K)-	GO:00081	metabolic	-	-	-	-	19:431441	1962	2170	2673	1471	2188	709	5.693913	5.278669	5.343947	2.781032	5.568032	2.551196	5.438843	3.63342	0.021901	0.905801	-
ENSMUSC	Sypl	synaptopl	GO:00452	synapsele	-	-	-	-	12:329538	599	699	993	447	562	469	3.445717	3.370406	3.935077	1.675104	2.83486	3.345117	3.583733	2.61836	0.022371	0.907502	-
ENSMUSC	Cox14	cytochron	GO:00099	cellular pr	-	-	-	-	15:997256	155	231	252	267	217	540	13.62482	17.02012	15.25981	15.28943	16.72635	58.85423	15.30158	30.29001	0.022536	0.907502	-
ENSMUSC	Nr4a3	nuclear re	GO:00325	developm	K08559	nuclear re	mmu0520	Transcript	4:4804515	2141	1903	2182	2211	1556	685	12.33832	9.192444	8.662547	8.300604	7.863059	4.894583	10.06444	7.019415	0.022351	0.907502	-
ENSMUSC	Ces2c	carboxyle	GO:00038	catalytic a	K03927	carboxyle	mmu0098	Drug met	8:1048470	0	6	3	0	0	0	0	0.119346	0.049043	0	0	0	0.05613	0	0.022198	0.907502	-
ENSMUSC	Parp10	poly (ADP	GO:00081	metabolic	-	-	-	-	15:762311	31	44	49	57	66	71	0.271224	0.322675	0.295333	0.324873	0.506345	0.770204	0.296411	0.533807	0.022255	0.907502	-
ENSMUSC	Ifi213	interferon	GO:00081	metabolic	-	-	-	-	1:1735662	2	8	8	11	26	8	0.012883	0.043185	0.035493	0.046149	0.146833	0.063883	0.03052	0.085622	0.022471	0.907502	-
ENSMUSC	Haus3	HAUS aug	GO:00990	supramole	-	-	-	-	5:3415388	129	174	226	148	135	70	1.195093	1.35118	1.442357	0.893213	1.096699	0.804079	1.329544	0.93133	0.022493	0.907502	-
ENSMUSC	Gm20425	predicted	GO:00508	response	-	-	-	-	9:1031902	0	0	0	15	0	0	0	0	0	0.205095	0	0	0	0.068365	0.022463	0.907502	-
ENSMUSC	Ube2l6	ubiquitin-	GO:00081	metabolic	K04553	ubiquitin-	mmu0501	Parkinson	2:8479882	62	89	87	116	138	97	1.188265	1.42976	1.148663	1.448309	2.319219	2.305034	1.255563	2.024187	0.022746	0.910461	-
ENSMUSC	Neil3	nei like 3	GO:00081	metabolic	K10569	endonucle	mmu0341	Base excis	8:5358686	4	9	12	2	3	3	0.038539	0.072683	0.079653	0.012552	0.025345	0.035843	0.063625	0.02458	0.022823	0.910461	-
ENSMUSC	Chmp3	charged r	GO:00400	locomotic	K12193	charged r	mmu0414	Endocytos	6:7154379	1385	1773	2197	2232	816	337	19.00279	20.39056	20.76582	19.95002	9.817488	5.733024	20.05306	11.83351	0.022819	0.910461	-
ENSMUSC	Uqcrh	ubiquinol-	GO:00081	metabolic	K00416	ubiquinol-	mmu0110	Metabolic	4:1160669	1659	1960	2565	1784	1697	1063	48.97093	48.49546	52.15919	34.30588	43.92549	38.90551	49.87519	39.04563	0.022737	0.910461	-
ENSMUSC	Trim12c	tripartite	GO:00048	signal trar	-	-	-	-	7:1043387	42	55	109	105	118	83	0.506204	0.555644	0.905014	0.824425	1.247105	1.240347	0.655621	1.103959	0.023012	0.915879	-
ENSMUSC	Zfp618	zinc finge	GO:00081	metabolic	-	-	-	-	4:6296557	72	90	96	66	69	27	0.394944	0.413806	0.362764	0.235847	0.331889	0.183635	0.390504	0.250457	0.023127	0.917435	-
ENSMUSC	Aurkaip1	aurora kir	GO:00081	metabolic	-	-	-	-	4:1558312	166	207	303	363	198	466	2.946045	3.079323	3.704472	4.196818	3.081339	10.25424	3.24328	5.844132	0.023158	0.917435	-
ENSMUSC	Fam109b	family wit	GO:00081	metabolic	-	-	-	-	15:823389	2	11	1	11	16	14	0.01993	0.091864	0.006861	0.071391	0.139778	0.172947	0.039552	0.128039	0.023325	0.919794	-
ENSMUSC	Gbp10	guanylate	GO:00023	immune s	-	-	-	-	5:1052156	7	8	4	11	35	8	0.105152	0.10072	0.041396	0.107634	0.461007	0.148994	0.082423	0.239212	0.023316	0.919794	-
ENSMUSC	Krt18	keratin 18	GO:00511	localizatio	-	-	-	-	15:102028	46	24	111	26	16	32	2.088154	0.913208	3.471223	0.768877	0.636891	1.801123	2.157528	1.068964	0.023419	0.921381	-
ENSMUSC	Sra1	steroid re	GO:00325	developm	-	-	-	-	18:366666	506	668	683	836	740	960	5.137032	5.684485	4.776763	5.529038	6.587733	12.08422	5.199427	8.066998	0.023565	0.923771	-
ENSMUSC	Rslcan18	regulator	GO:00081	metabolic	-	-	-	-	13:670966	26	0	2	0	2	0	0.200778	0	0.010641	0	0.013542	0	0.070473	0.004514	0.023588	0.923771	-
ENSMUSC	Elavl4	ELAV (em	GO:00325	developm	-	-	-	-	4:1102037	1392	1782	2202	1080	1676	774	7.363556	7.90149	8.024486	3.721808	7.774367	5.076631	7.763177	5.524269	0.023783	0.929278	-
ENSMUSC	Sim1	single-mi	GO:00325	developm	-	-	-	-	10:508947	2	6	0	0	0	0	0.01406	0.035347	0	0	0	0	0.016469	0	0.023956	0.933897	-
ENSMUSC	Tnfaip1	tumor nec	GO:00325	developm	-	-	-	-	11:785228	312	306	1129	1189	881	617	4.998377	4.109119	12.46003	12.40902	12.37635	12.25589	7.189177	12.34709	0.024046	0.935283	-
ENSMUSC	mt-Nd3	mitochon	GO:00081	metabolic	K03880	NADH-ub	mmu0110	Metabolic	MT:9459-	3927	5431	7212	5609	4151	2590	717.1628	831.3608	907.3274	667.3044	664.7408	586.4658	818.617	639.5036	0.024268	0.941643	-
ENSMUSC	Zfp82	zinc finge	GO:00081	metabolic	-	-	-	-	7:3005448	57	83	87	86	23	6	0.294346	0.359265	0.309496	0.28931	0.10415	0.03842	0.321036	0.14396	0.02432	0.941643	-
ENSMUSC	Tmem140	transmem	GO:00160	membran	-	-	-	-	6:3486314	8	30	31	37	35	53	0.069721	0.219158	0.186126	0.210075	0.267488	0.572728	0.158335	0.350097	0.024505	0.946666	-
ENSMUSC	Gm7173	predicted	-	-	-	-	-	-	X:7926655	23	29	32	23	18	3	0.13615	0.143893	0.130495	0.088696	0.093433	0.022021	0.136846	0.06805	0.024684	0.949294	-
ENSMUSC	Pcdhga9	protocadh	GO:00325	developm	-	-	-	-	18:377369	189	186	492	633	887	139	1.547673	1.276687	2.775459	3.376788	6.369194	1.4113	1.866607	3.719094	0.024631	0.949294	-
ENSMUSC	Spib	Spi-B trar	GO:00325	developm	-	-	-	-	7:4452599	0	0	0	2	2	3	0	0	0	0.025196	0.033916	0.071947	0	0.043686	0.025123	0.953326	-
ENSMUSC	Uts2	urotensin	GO:00325	developm	-	-	-	-	4:1509970	3	2	2	0	0	0	0.354342	0.198023	0.162775	0	0	0	0.23838	0	0.024915	0.953326	-
ENSMUSC	Fezf1	Fez family	GO:00325	developm	-	-	-	-	6:2324504	0	6	2	0	0	0	0	0.132673	0.036352	0	0	0	0.056342	0	0.025067	0.953326	-
ENSMUSC	Melk	maternal	GO:00325	developm	-	-	-	-	4:4430087	6	14	6	5	4	0	0.116616	0.228076	0.080329	0.06331	0.068164	0	0.141674	0.043825	0.025106	0.953326	-
ENSMUSC	Dlk2	delta-like	GO:00325	developm	-	-	-	-	17:462974	115	173	188	225	173	305	2.136393	2.693899	2.40598	2.722996	2.818197	7.025371	2.412091	4.188855	0.025108	0.953326	-
ENSMUSC	Gm42669	predicted	-	-	-	-	-	-	5:1075076	8	0	15	15	24	22	0.079055	0	0.10212	0.096568	0.207977	0.269573	0.060392	0.191372	0.025095	0.953326	-
ENSMUSC	Pde6a	phosphoc	GO:00081	metabolic	K08718	rod cGMP	mmu0023	Purine me	18:612204	0	0	0	0	2	5	0	0	0	0	0.024651	0.087143	0	0.037265	0.025279	0.954667	-
ENSMUSC	Hnrnpa3	heteroger	GO:00081	metabolic	K12741	heteroger	mmu0304	Spliceosoi	2:7565926	98	439	270	141	455	1158	0.84153	3.159825	1.597205	0.788762	3.426101	12.32935	1.866187	5.514737	0.025325	0.954667	-
ENSMUSC	Zfp788	zinc finge	GO:00081	metabolic	-	-	-	-	7:4163320	373	342	476	778	586	328	1.683016	1.293474	1.479577	2.286866	2.318568	1.835016	1.485356	2.146817	0.025283	0.954667	-
ENSMUSC	Ube2c	ubiquitin-	GO:00081	metabolic	K06688	ubiquitin-	mmu0412	Ubiquitin	2:1647698	7	10	15	10	3	0	0.160151	0.19175	0.236405	0.14903	0.060178	0	0.196102	0.069736	0.025843	0.972047	-
ENSMUSC	Hpn	hepsin [S	GO:00325	developm	K08665	hepsin[3.4	mmu0520	Viral carci	7:3109872	18	5	17	25	32	25	0.179413	0.041772	0.116729	0.162332	0.279688	0.308964	0.112638	0.250328	0.025931	0.972357	-
ENSMUSC	Chpf2	chondroit	GO:00038	catalytic a	K03419	chondroit	mmu0110	Metabolic	5:2458674	498	648	677	890	633	1007	3.152019	3.437851	2.951883	3.669701	3.513221	7.902681	3.180584	5.028534	0.025965	0.972357	-
ENSMUSC	Ftl1	ferritin ligl	GO:00511	localizatio	K13625	ferritin ligl	mmu0421	Necropto	7:4545794	898	1501	2235	1364	1207	524	17.81786	24.96392	30.54979	17.63093	21.00048	12.8913	24.44386	17.17424	0.026086	0.974746	-
ENSMUSC	Rag1	recombin	GO:00325	developm	K10628	V(D)J recc	mmu0406	FoxO sign	2:1016382	1	8	7	2	1	0	0.00962	0.064489	0.046381	0.012529	0.008433	0	0.040163	0.006987	0.026147	0.974892	-
ENSMUSC	Gm3512	predicted	-	-	-	-	-	-	14:715351	5	5	9	4	2	0	0.159923	0.134042	0.198295	0.083355	0.056089	0	0.164087	0.046481	0.026239	0.976205	-
ENSMUSC	Pkn3	protein ki	GO:00081	metabolic	K06071	protein ki	mmu0415	PI3K-Akt	2:3007768	14	14	23	37	21	29	0.139874	0.117247	0.158305	0.240822	0.183984	0.359244	0.138475	0.26135	0.026664	0.978122	-
ENSMUSC	Usp50	ubiquitin	GO:00081	metabolic	-	-	-	-	2:1267090	46	52	82	71	61	188	0.654301	0.619976	0.803504	0.657903	0.760848	3.31563	0.692594	1.578127	0.027008	0.978122	-
ENSMUSC	Slc52a3	solute car	GO:00511	localizatio	K14620	riboflavin	mmu0497	Vitamin d	2:1519965	22	25	34	32	47	59	0.205126	0.195387	0.218393	0.194373	0.384283	0.682096	0.206302	0.420251	0.026914	0.978122	-
ENSMUSC	Xlr4c	X-linked	GO:00099	cellular pr	-	-	-	-	X:7323368	3	8	4	4	0	0	0.055563	0.124205	0.051048	0.048273	0	0	0.076939	0.016091	0.026827	0.978122	-
ENSMUSC	Trim14	tripartite	GO:00081	metabolic	-	-	-	-	4:4649378	7	10	6	17	15	15	0.079136	0.09475	0.046723	0.12519	0.148688	0.210247	0.073536	0.161375	0.02687	0.978122	-
ENSMUSC	Fam159b	family wit	GO:00160	membran	-	-	-	-	13:104845	8	10	5	0	4	2	0.241867	0.253417	0.104144	0	0.106041	0.074996	0.199809	0.060346	0.026951	0.978122	-
ENSMUSC	Fam69c	family wit	GO:00160	membran	-	-	-	-	18:847200	41	34	57	63	64	81	0.740241	0.514553	0.708962	0.740994	1.013251	1.813269	0.654585	1.189171	0.027034	0.978122	-
ENSMUSC	Spata33	spermato	GO:00056	cell|intrac	-	-	-	-	8:1232128	44	47	66	114	42	109	0.234967	0.210378	0.2428	0.396588	0.196672	0.721711	0.229382	0.438324	0.026948	0.978122	-
ENSMUSC	Catsperg2	cation cha	GO:00325	developm	-	-	-	-	7:2969721	3	4	5	3	0	0	0.016706	0.018673	0.019184	0.010883	0	0	0.018188	0.003628	0.026418	0.978122	-
ENSMUSC	Tmc8	transmem	GO:00511	localizatio	-	-	-	-	11:117782	5	8	5	1	2	2	0.061133	0.081983	0.042114	0.007964	0.021441	0.030327	0.061743	0.019911	0.02688	0.978122	-
ENSMUSC	Ccdc146	coiled-co	GO:00056	cell|intrac	-	-	-	-	5:2129296	34	22	37	19	20	0	0.432768	0.234719	0.324435	0.157543	0.223233	0	0.330641	0.126925	0.02701	0.978122	-
ENSMUSC	Sap25	sin3 assoc	GO:00081	metabolic	-	-	-	-	5:1376413	18	14	29	34	29	42	0.282106	0.183923	0.313109	0.347137	0.39855	0.816179	0.259713	0.520622	0.026829	0.978122	-
ENSMUSC	Gm20695	predicted	GO:00038	catalytic a	-	-	-	-	16:388464	22	13	10	8	7	4	1.278009	0.633035	0.400202	0.302753	0.356606	0.288114	0.770415	0.315824	0.026735	0.978122	-
ENSMUSC	Foxg1	forkhead	GO:00325	developm	K09385	forkhead	mmu0406	FoxO sign	12:493826	823	976	3306	3282	2667	1699	6.401977	6.363805	17.71611	16.63161	18.19197	16.38677	10.16063	17.07012	0.02711	0.978837	-
ENSMUSC	Wdr90	WD repea	-	-	-	-	-	-	17:258447	188	210	282	338	248	356	0.83459	0.781424	0.862413	0.977492	0.965404	1.959526	0.826142	1.300807	0.027623	0.995244	-
ENSMUSC	Morn3	MORN re	GO:00056	cell|intrac	-	-	-	-	5:1230357	7	10	12	3	5	3	0.150253	0.1799	0.177438	0.041942	0.094097	0.079844	0.169197	0.071961	0.02777	0.995513	-
ENSMUSC	BC030499	cDNA seq	GO:00081	metabolic	-	-	-	-	11:782908	35	20	50	74	80	31	0.334644	0.160285	0.329331	0.460921	0.670722	0.367503	0.274753	0.499715	0.027805	0.995513	-
ENSMUSC	Tmem256	transmem	GO:00055	extracellul	-	-	-	-	11:698385	47	70	73	70	63	218	1.135735	1.417846	1.215226	1.101944	1.33495	6.531654	1.256269	2.989516	0.027782	0.995513	-
ENSMUSC	Etnppl	ethanolan	GO:00038	catalytic a	K14286	ethanolan	mmu0110	Metabolic	3:1306174	178	212	355	346	421	249	1.04532	1.043559	1.436177	1.323691	2.16797	1.81307	1.175019	1.768244	0.028211	0.997535	-
ENSMUSC	Arhgef6	Rac/Cdc4	GO:00987	molecular	K05729	Rho guan	mmu0481	Regulatio	X:5723148	599	742	836	564	641	337	3.839851	3.986982	3.691856	2.355309	3.603197	2.678572	3.839563	2.879026	0.028042	0.997535	-
ENSMUSC	Robo3	roundabo	GO:00325	developm	K06755	roundabo	mmu0436	Axon guic	9:3741566	356	466	639	840	580	479	2.192542	2.405675	2.711134	3.37023	3.132341	3.657796	2.436451	3.386789	0.02815	0.997535	-
ENSMUSC	Slc26a11	solute car	GO:00511	localizatio	-	-	-	-	11:119355	58	61	62	100	81	88	0.768094	0.677125	0.565621	0.862715	0.940616	1.444951	0.67028	1.082761	0.02817	0.997535	-
ENSMUSC	Pfdn4	prefoldin	GO:00329	macromo	-	-	-	-	2:1704964	187	206	236	180	144	117	3.36098	3.103446	2.922044	2.107547	2.269495	2.607318	3.128823	2.32812	0.028035	0.997535	-
ENSMUSC	Obscn	obscurin,	GO:00081	metabolic	-	-	-	-	11:589942	72	75	102	162	96	102	0.137495	0.120052	0.134186	0.201535	0.160755	0.241513	0.130577	0.201268	0.028142	0.997535	-
ENSMUSC	Gnai3	guanine n	GO:00048	signal trar	K04630	guanine n	mmu0520	Pathways	3:1081072	486	557	781	618	489	407	9.468655	9.09621	10.48225	7.843724	8.354174	9.831778	9.68237	8.676559	0.242728	1	-
ENSMUSC	Cdc45	cell divisic	GO:00081	metabolic	K06628	cell divisic	mmu0411	Cell cycle	16:187804	25	35	46	42	23	26	0.544683	0.639182	0.690407	0.59611	0.439415	0.702372	0.624757	0.579299	0.602926	1	-
ENSMUSC	Scml2	sex comb	GO:00081	metabolic	-	-	-	-	X:1611171	13	8	27	35	15	9	0.083952	0.043303	0.120118	0.147249	0.084943	0.072063	0.082458	0.101418	0.58386	1	-
ENSMUSC	Apoh	apolipopr	GO:00325	developm	K17305	beta-2-gl	mmu0497	Cholester	11:108343	11	5	9	5	0	5	0.317053	0.12079	0.178691	0.093888	0	0.178673	0.205511	0.090854	0.07967	1	-
ENSMUSC	Narf	nuclear pr	GO:00081	metabolic	-	-	-	-	11:121237	898	1101	1366	1450	1005	851	10.93307	11.23585	11.45692	11.50046	10.72937	12.84639	11.20861	11.69207	0.994255	1	-
ENSMUSC	Cav2	caveolin 2	GO:00325	developm	K12958	caveolin 2	mmu0414	Endocytos	6:1728118	187	320	386	352	236	250	2.700392	3.873354	3.839931	3.311384	2.988405	4.476215	3.471226	3.592002	0.940173	1	-
ENSMUSC	Klf6	Kruppel-li	GO:00081	metabolic	-	-	-	-	13:586148	654	784	1049	877	757	559	7.64319	7.680086	8.445471	6.676947	7.757749	8.100186	7.922916	7.511627	0.458855	1	-
ENSMUSC	Scmh1	sex comb	GO:00325	developm	-	-	-	-	4:1204052	664	793	940	854	775	379	3.940904	3.945062	3.843326	3.301927	4.033408	2.789026	3.909764	3.374787	0.217474	1	-
ENSMUSC	Cox5a	cytochron	GO:00081	metabolic	K02264	cytochron	mmu0110	Metabolic	9:5752127	522	585	794	667	598	826	12.36939	11.61946	12.96131	10.29638	12.4257	24.26853	12.31672	15.66354	0.305012	1	-
ENSMUSC	Tbx2	T-box 2 [	GO:00325	developm	-	-	-	-	11:858325	41	39	51	66	44	50	0.718601	0.572964	0.615783	0.753593	0.676239	1.086596	0.635783	0.83881	0.288481	1	-

ENSMUSC	Tbx4	T-box 4 [S	GO:00325	developm	-	-	-	-	11:858864	2	6	5	5	9	0	0.022829	0.057391	0.039309	0.037174	0.090066	0	0.039843	0.042413	1	1	-
ENSMUSC	Ngfr	nerve gro	GO:00325	developm	K02583	nerve gro	mmu0415	PI3K-Akt	11:955688	19	29	38	58	29	28	0.350408	0.448297	0.482783	0.696842	0.468984	0.640271	0.427163	0.602032	0.18217	1	-
ENSMUSC	Wnt3	wingless-	GO:00325	developm	K00312	wingless-t	mmu0520	Pathways	11:103774	30	32	49	51	23	19	0.619834	0.55419	0.697431	0.686444	0.416701	0.486738	0.623818	0.529961	0.4514	1	-
ENSMUSC	Wnt9a	wingless-	GO:00325	developm	K01064	wingless-t	mmu0520	Pathways	11:593069	115	111	144	165	124	129	1.145369	0.926663	0.988007	1.070563	1.082955	1.593021	1.020013	1.248846	0.381812	1	-
ENSMUSC	Fer	fer (fms/fp	GO:00325	developm	K08889	tyrosine-p	mmu0452	Adherens	17:638960	507	590	684	756	564	239	8.762956	8.547659	8.144232	8.512282	8.547997	5.121841	8.484949	7.39404	0.330809	1	-
ENSMUSC	Xpo6	exportin 6	GO:00511	localizatio	-	-	-	-	7:1261017	1275	1537	1320	2200	1508	970	3.238872	3.272732	2.309983	3.640725	3.359136	3.055211	2.940529	3.351691	0.493153	1	-
ENSMUSC	Tfe3	transcripti	GO:00325	developm	K09105	transcripti	mmu0520	Transcript	X:7762560	590	710	890	921	660	528	1.815272	1.831054	1.88639	1.845997	1.780641	2.014234	1.844238	1.880291	0.871465	1	-
ENSMUSC	Axin2	axin 2 [So	GO:00325	developm	K04385	axin 2[-]	mmu0520	Pathways	11:108920	408	346	558	504	456	374	3.016479	2.144219	2.842008	2.427458	2.956293	3.428445	2.667569	2.937399	0.731888	1	-
ENSMUSC	Brat1	BRCA1-as	GO:00081	metabolic	-	-	-	-	5:1407050	239	243	299	337	293	379	1.386253	1.18142	1.194722	1.273375	1.490234	2.725644	1.254132	1.829751	0.07373	1	-
ENSMUSC	Gna12	guanine n	GO:00325	developm	K04346	guanine n	mmu0520	Pathways	5:1407584	1510	1504	2276	2182	1603	1728	21.29717	17.78057	22.11407	20.04847	19.82536	30.21863	20.39727	23.36415	0.514074	1	-
ENSMUSC	Slc22a18	solute car	GO:00511	localizatio	-	-	-	-	7:1434737	2	3	8	2	4	3	0.055203	0.06939	0.152084	0.03595	0.096786	0.102657	0.092225	0.078464	0.711578	1	-
ENSMUSC	Itgb2l	integrin b	GO:00508	response	K06464	integrin b	mmu0516	HTLV-I inf	16:964222	0	0	0	5	0	0	0	0	0	0.045822	0	0	0	0.015274	0.185253	1	-
ENSMUSC	Pih1d2	PIH1 dom	GO:00055	protein bi	-	-	-	-	9:5061732	19	14	19	11	18	19	0.244089	0.15076	0.168149	0.092056	0.202769	0.302649	0.187666	0.199158	1	1	-
ENSMUSC	Dlat	dihydrolip	GO:00081	metabolic	K00627	pyruvate c	mmu0110	Metabolic	9:5063463	1815	2447	3097	2835	2156	1646	20.88894	23.60622	24.55457	21.25565	21.75861	23.48851	23.01658	22.16759	0.508811	1	-
ENSMUSC	Sdhd	succinate	GO:00081	metabolic	K00237	succinate	mmu0110	Metabolic	9:5059635	805	1030	1400	1157	923	1142	41.55983	44.57261	49.79178	38.91286	41.78519	73.10213	45.30807	51.26672	0.642775	1	-
ENSMUSC	Fgf23	fibroblast	GO:00325	developm	K04358	fibroblast	mmu0520	Pathways	6:1270729	2	2	6	5	10	3	0.070084	0.05873	0.144804	0.114125	0.307222	0.13033	0.091206	0.183892	0.209436	1	-
ENSMUSC	Ccnd2	cyclin D2	GO:00081	metabolic	K10151	cyclin D2[	mmu0520	Pathways	6:1271251	1972	2325	2757	2583	2075	1662	10.14461	10.02546	9.770498	8.656344	9.36029	10.60097	9.98019	9.539201	0.448028	1	-
ENSMUSC	Gpr107	G protein-	GO:00511	localizatio	-	-	-	-	2:3115231	788	1021	1272	1324	977	868	3.398695	3.691175	3.779414	3.720108	3.695073	4.641854	3.623095	4.019012	0.62201	1	-
ENSMUSC	Nalcn	sodium le	GO:00511	localizatio	-	-	-	-	14:123276	2190	2646	2982	3339	2359	1602	8.69067	8.801404	8.152076	8.631916	8.208801	7.882385	8.54805	8.241034	0.545526	1	-
ENSMUSC	Btbd17	BTB (POZ	GO:00055	extracellul	-	-	-	-	11:114791	86	147	185	172	131	186	0.570518	0.817411	0.845461	0.743327	0.762052	1.529925	0.744464	1.011768	0.220001	1	-
ENSMUSC	Slfn4	schlafen 4	GO:00971	organic cy	-	-	-	-	11:831751	2	6	13	6	5	9	0.015856	0.039861	0.070985	0.030983	0.03475	0.088447	0.042234	0.051393	0.907994	1	-
ENSMUSC	Th	tyrosine h	GO:00325	developm	K00501	tyrosine 3	mmu0110	Metabolic	7:1428927	15	23	25	21	14	17	0.239343	0.307613	0.274795	0.218289	0.195882	0.33632	0.273917	0.250164	0.63328	1	-
ENSMUSC	Ins2	insulin II [S	GO:00325	developm	K04526	insulin[-]	mmu0415	PI3K-Akt	7:1426786	2	0	0	0	0	0	0.038819	0	0	0	0	0	0.01294	0	0.53628	1	-
ENSMUSC	Scnn1g	sodium ch	GO:00511	localizatio	K04827	amiloride-	mmu0474	Taste tran	7:1217344	2	3	9	0	5	0	0.042505	0.053429	0.131733	0	0.093154	0	0.075889	0.031051	0.213496	1	-
ENSMUSC	Drp2	dystrophi	GO:00325	developm	-	-	-	-	X:1344045	674	759	1084	1116	859	682	3.605618	3.403409	3.994849	3.889245	4.029539	4.523664	3.667959	4.147483	0.512397	1	-
ENSMUSC	Tspan32	tetraspani	GO:00023	immune s	-	-	-	-	7:1430050	6	1	2	6	1	10	0.035143	0.004909	0.008071	0.022894	0.005135	0.072623	0.016041	0.033551	0.406274	1	-
ENSMUSC	Lhx2	LIM home	GO:00325	developm	-	-	-	-	2:3833928	1069	1325	1443	1558	1249	782	10.17191	10.56801	9.45894	9.657703	10.42149	9.226087	10.06629	9.768427	0.601459	1	-
ENSMUSC	Gmpr	guanosine	GO:00081	metabolic	K00364	GMP redu	mmu0023	Purine me	13:455074	576	742	878	949	716	709	13.58816	14.67221	14.2687	14.58433	14.81133	20.73817	14.17636	16.71128	0.372334	1	-
ENSMUSC	Glra1	glycine re	GO:00048	signal trar	K05193	glycine re	mmu0408	Neuroacti	11:555142	5	8	8	15	4	6	0.091313	0.122456	0.100646	0.178456	0.064051	0.135873	0.104805	0.126127	0.718652	1	-
ENSMUSC	Mid2	midline 2	GO:00081	metabolic	-	-	-	-	X:1406645	542	218	233	778	555	339	3.101819	1.045746	0.918596	2.900541	2.785179	2.405486	1.68872	2.697068	0.096723	1	-
ENSMUSC	Dgke	diacylglyc	GO:00081	metabolic	K00901	diacylglyc	mmu0110	Metabolic	11:890351	823	1049	1677	1825	1334	931	3.630972	3.87928	5.096913	5.245264	5.16085	5.092821	4.202388	5.166311	0.196165	1	-
ENSMUSC	Scpep1	serine car	GO:00081	metabolic	-	-	-	-	11:889240	191	232	309	316	216	228	5.788567	5.893569	6.451307	6.238879	5.740282	8.567572	6.044481	6.848911	0.609432	1	-
ENSMUSC	Mnt	max bindi	GO:00325	developm	-	-	-	-	11:748309	628	818	845	1067	814	718	6.973836	7.614098	6.464287	7.718953	7.926464	9.886055	7.017407	8.510491	0.2464	1	-
ENSMUSC	Itgb2	integrin b	GO:00325	developm	K06464	integrin b	mmu0516	HTLV-I inf	10:775302	111	156	218	285	163	144	1.513495	1.782925	2.047689	2.531534	1.948885	2.434472	1.78137	2.304963	0.180075	1	-
ENSMUSC	Hddc2	HD doma	GO:00055	extracellul	-	-	-	-	10:313133	98	152	163	146	127	117	1.167198	1.517456	1.337397	1.132805	1.32638	1.727788	1.340683	1.395657	0.964184	1	-
ENSMUSC	Tpd52l1	tumor pro	GO:00081	metabolic	-	-	-	-	10:313323	307	380	385	519	395	279	6.390696	6.630489	5.521052	7.038144	7.210229	7.201105	6.180746	7.149826	0.439261	1	-
ENSMUSC	Pemt	phosphati	GO:00081	metabolic	K00551	phosphati	mmu0110	Metabolic	11:599706	16	27	17	17	24	25	0.33166	0.469111	0.242747	0.229556	0.436236	0.642517	0.347839	0.436103	0.603475	1	-
ENSMUSC	Cdh1	cadherin 1	GO:00325	developm	K05689	cadherin 1	mmu0520	Pathways	8:1066033	24	48	56	47	24	40	0.277272	0.464821	0.44569	0.353731	0.243137	0.57298	0.395928	0.38995	0.781307	1	-
ENSMUSC	Cdh4	cadherin 4	GO:00325	developm	K06797	cadherin 4	mmu0451	Cell adhes	2:1794424	373	421	349	432	331	248	2.886312	2.730669	1.86042	2.177709	2.245974	2.37942	2.492467	2.267701	0.388085	1	-
ENSMUSC	Ckmt1	creatine k	GO:00081	metabolic	K00933	creatine k	mmu0110	Metabolic	2:1213577	1990	2761	3158	3078	2335	1914	20.88005	24.28275	22.82665	21.03918	21.48366	24.9004	22.66315	22.47441	0.669233	1	-
ENSMUSC	Bcl6b	B cell CLL	GO:00325	developm	-	-	-	-	11:702241	34	28	62	37	32	35	0.513135	0.354207	0.644601	0.363778	0.423485	0.654949	0.503981	0.480737	0.679025	1	-
ENSMUSC	Clec10a	C-type lec	GO:00508	response	-	-	-	-	11:701561	7	12	5	15	4	5	0.076496	0.109916	0.037639	0.106779	0.038325	0.06774	0.074684	0.070948	1	1	-
ENSMUSC	Alox12	arachidon	GO:00325	developm	K00458	arachidon	mmu0110	Metabolic	11:702414	12	14	8	9	20	5	0.169506	0.165772	0.07785	0.08282	0.247744	0.087569	0.137709	0.139378	1	1	-
ENSMUSC	Arvcf	armadillo	GO:00325	developm	-	-	-	-	16:183481	492	661	838	727	619	627	2.731314	3.075821	3.204807	2.629193	3.013282	4.315789	3.003981	3.319421	0.722322	1	-
ENSMUSC	Comt	catechol-	GO:00325	developm	-	-	-	-	16:184068	754	910	1025	1161	885	758	12.59037	12.73684	11.79078	12.62935	12.95845	15.6936	12.37267	13.76047	0.612887	1	-
ENSMUSC	Rtca	RNA 3'-te	GO:00081	metabolic	-	-	-	-	3:1164889	413	502	675	589	492	521	9.703277	9.886109	10.92506	9.014998	10.13623	15.17723	10.17148	11.44282	0.648521	1	-
ENSMUSC	Dbt	dihydrolip	GO:00081	metabolic	K09699	2-oxoisov	mmu0110	Metabolic	3:1165130	406	440	601	665	438	298	6.790138	6.168194	6.924336	7.24529	6.42346	6.179521	6.627556	6.61609	0.801246	1	-
ENSMUSC	Dazap2	DAZ asso	GO:00329	macromo	-	-	-	-	15:100615	1662	1961	2613	2306	1872	1293	57.84508	57.20919	62.65075	52.28489	57.13257	55.79814	59.23501	55.07187	0.348531	1	-
ENSMUSC	Mcts1	malignant	GO:00081	metabolic	-	-	-	-	X:3860057	362	517	598	551	460	233	11.07664	13.25999	12.60527	10.98329	12.34241	8.839797	12.31397	10.72184	0.265451	1	-
ENSMUSC	Rem1	rad and g	GO:00511	localizatio	-	-	-	-	2:1526269	8	12	16	6	10	8	0.147535	0.185511	0.203278	0.072089	0.161724	0.182935	0.178775	0.138916	0.364003	1	-
ENSMUSC	Rnf17	ring finge	GO:00325	developm	-	-	-	-	14:564025	5	5	21	17	15	5	0.045854	0.038433	0.132671	0.101561	0.120624	0.056851	0.072319	0.093012	0.622303	1	-
ENSMUSC	Trappc10	trafficking	-		-	-	-	-	10:781867	1765	1964	2431	2623	1930	1828	11.28254	10.52342	10.7053	10.92301	10.81838	14.48854	10.83709	12.07664	0.600534	1	-
ENSMUSC	Ccm2	cerebral c	GO:00325	developm	-	-	-	-	11:654688	649	724	919	867	710	561	7.140911	6.677295	6.96588	6.214548	6.850306	7.653457	6.928029	6.906104	0.720984	1	-
ENSMUSC	Tbrg4	transform	GO:00081	metabolic	-	-	-	-	11:661559	221	294	377	334	306	379	2.138108	2.384168	2.512639	2.105061	2.595975	4.546337	2.344972	3.082458	0.216913	1	-
ENSMUSC	Fap	fibroblast	GO:00325	developm	-	-	-	-	2:6250094	29	25	73	32	27	10	0.264046	0.190796	0.457886	0.189806	0.215569	0.112899	0.304243	0.172758	0.044347	1	-
ENSMUSC	Gcg	glucagon	GO:00081	metabolic	K05259	glucagon[	mmu0492	Glucagon	2:6247453	0	0	0	2	0	0	0	0	0	0.042611	0	0	0	0.014204	0.537449	1	-
ENSMUSC	Ndufa9	NADH de	GO:00081	metabolic	K03953	NADH de	mmu0110	Metabolic	6:1268217	988	1458	1716	1633	1325	1076	23.91103	29.57684	28.60949	25.74596	28.11901	32.2879	27.36579	28.71762	0.961976	1	-
ENSMUSC	Egfl6	EGF-like-	GO:00325	developm	-	-	-	-	X:1665230	33	59	82	106	65	37	0.775608	1.162346	1.327684	1.622998	1.339633	1.078234	1.088546	1.346955	0.396894	1	-
ENSMUSC	Lck	lymphocy	GO:00325	developm	K05856	lymphocy	mmu0516	HTLV-I inf	4:1295483	29	20	33	20	25	22	0.392137	0.226685	0.307395	0.176181	0.29643	0.368856	0.308739	0.280489	0.574167	1	-
ENSMUSC	Tssk3	testis-spe	GO:00325	developm	-	-	-	-	4:1294890	0	1	0	2	0	0	0	0.041616	0	0.064682	0	0	0.013872	0.021561	1	1	-
ENSMUSC	Cttnbp2	cortactin b	GO:00325	developm	-	-	-	-	6:1836647	2900	4088	5172	6053	3528	2184	11.38661	13.45428	13.98964	15.48278	12.14697	10.63249	12.94351	12.75408	0.746642	1	-
ENSMUSC	Galnt1	polypeptic	GO:00081	metabolic	K00710	polypeptic	mmu0110	Metabolic	18:242053	813	1009	1256	1304	982	614	5.7653	5.997567	6.135814	6.024077	6.106403	5.39865	5.966227	5.843043	0.666313	1	-
ENSMUSC	Myf5	myogenic	GO:00325	developm	K18484	myogenic	mmu0455	Signaling	10:107482	0	0	0	2	0	0	0	0	0	0.039747	0	0	0	0.013249	0.537905	1	-
ENSMUSC	Mkrn2	makorin, r	GO:00081	metabolic	-	-	-	-	6:1156019	518	630	748	761	662	480	4.418256	4.504171	4.395159	4.228512	4.951331	5.07632	4.439195	4.752055	0.833092	1	-
ENSMUSC	Pparg	peroxison	GO:00325	developm	K08530	peroxison	mmu0520	Pathways	6:1153609	17	28	37	39	22	9	0.260582	0.35976	0.390715	0.389451	0.295719	0.171049	0.337019	0.285406	0.572346	1	-
ENSMUSC	Raf1	v-raf-leuk	GO:00325	developm	K04366	RAF protc	mmu0520	Pathways	6:1156180	952	1093	1379	1483	1019	966	6.209205	5.975465	6.196046	6.301177	5.827945	7.811994	6.126905	6.647039	0.762839	1	-
ENSMUSC	1-Sep	septin 1 [S	GO:00511	localizatio	K13737	septin 1[-	mmu0510	Bacterial i	7:1272144	33	40	37	21	25	28	0.436108	0.443093	0.336848	0.180796	0.289711	0.458801	0.40535	0.309769	0.197209	1	-
ENSMUSC	Pdgfb	platelet de	GO:00325	developm	K17386	platelet-d	mmu0520	Pathways	15:799959	384	427	506	565	429	463	8.363394	7.795269	7.591934	8.016427	8.193155	12.50309	7.916866	9.57089	0.337915	1	-
ENSMUSC	Acvrl1	activin A r	GO:00325	developm	-	-	-	-	15:101128	97	182	219	281	197	126	0.986346	1.551247	1.534094	1.861416	1.756568	1.588597	1.357229	1.735527	0.223759	1	-
ENSMUSC	Grasp	GRP1 (ger	GO:00511	localizatio	-	-	-	-	15:101224	941	1050	1211	1385	996	627	29.70859	27.78654	26.33831	28.48549	27.57366	24.54404	27.94448	26.86773	0.570814	1	-
ENSMUSC	Acvr1b	activin A r	GO:00325	developm	K13567	activin rec	mmu0406	Cytokine-	15:101174	2198	2566	3043	3260	2325	1998	31.6541	30.97503	30.18947	30.5845	29.3608	35.67662	30.93953	31.87397	0.91465	1	-
ENSMUSC	Tom1l2	target of r	GO:00511	localizatio	-	-	-	-	11:602267	4358	5053	6321	7279	5367	4942	19.80436	19.24757	19.78841	21.54899	21.3869	27.84597	19.61345	23.59396	0.224536	1	-
ENSMUSC	Gpa33	glycoprot	GO:00055	extracellul	-	-	-	-	1:1661302	0	4	2	0	0	0	0	0.045773	0.018813	0	0	0	0.021529	0	0.054068	1	-
ENSMUSC	Zfp385a	zinc finge	GO:00325	developm	-	-	-	-	15:103313	516	602	719	801	663	507	14.31399	13.99782	13.74015	14.47523	16.1275	17.43835	14.01732	16.01369	0.471652	1	-
ENSMUSC	Itga5	integrin al	GO:00325	developm	-	-	-	-	15:103344	41	34	82	62	53	53	0.524064	0.364285	0.722055	0.516272	0.59405	0.83997	0.536801	0.650097	0.554579	1	-
ENSMUSC	Gabra2	gamma-a	GO:00048	signal trar	K05175	gamma-a	mmu0408	Neuroacti	5:7095759	1305	1923	1987	2015	1454	856	6.187921	7.643042	6.490579	6.224305	6.045626	5.032614	6.773847	5.767515	0.143694	1	-
ENSMUSC	Wdr77	WD repea	GO:00325	developm	-	-	-	-	3:1059593	427	515	599	613	451	377	3.001899	3.034786	2.90099	2.807434	2.780268	3.286209	2.979225	2.95797	0.711044	1	-
ENSMUSC	Adora3	adenosine	GO:00048	signal trar	K04268	adenosine	mmu0408	Neuroacti	3:1058708	10	10	14	25	13	13	0.121008	0.101425	0.116703	0.197074	0.137942	0.195043	0.113045	0.176686	0.172905	1	-
ENSMUSC	Atp5f1	ATP synth	GO:00325	developm	K02127	F-type H+	mmu0110	Metabolic	3:1059426	3166	3875	4418	4604	3360	2283	34.74509	35.64569	33.40102	32.91543	32.33438	31.06521	34.59727	32.10501	0.336805	1	-
ENSMUSC	Sox9	SRY (sex c	GO:00325	developm	K18435	transcripti	mmu0402	cAMP sigr	11:112782	612	630	948	916	741	697	9.406156	8.116222	10.03738	9.171448	9.986683	13.28248	9.186587	10.81354	0.387262	1	-
ENSMUSC	Hnrnpd	heteroger	GO:00325	developm	-	-	-	-	5:9995593	2555	3432	4061	3781	2616	2065	12.86978	14.49039	14.09173	12.40704	11.55475	12.89692	13.8173	12.28624	0.174689	1	-
ENSMUSC	Dynlt1c	dynein lig	GO:00325	developm	-	-	-	-	17:660167	33	36	54	61	31	7	0.310519	0.283945	0.350039	0.373927	0.255785	0.081672	0.314834	0.237128	0.358844	1	-
ENSMUSC	C1d	C1D nucle	GO:00081	metabolic	K12592	exosome	mmu0301	RNA degr	11:172575	356	257	517	508	333	320	4.956155	2.99903	4.958355	4.607241	4.065199	5.523706	4.304513	4.732048	0.758573	1	-
ENSMUSC	Gm2a	GM2 gang	GO:00081	metabolic	K12383	gangliosic	mmu0414	Lysosome	11:550981	1192	1468	1800	1749	1361	978	18.35599	18.94875	19.09526	17.54579	18.37818	18.67353	18.8	18.19917	0.54503	1	-
ENSMUSC	Krit1	KRIT1, ank	GO:00325	developm	K17705	Krev intera	mmu0401	Rap1 sign	5:3803184	824	1116	1295	1481	1092	552	1.806217	2.0505	1.955529	2.114851	2.09898	1.500265	1.937415	1.904699	0.7507	1	-
ENSMUSC	Clcn4	chloride c	GO:00511	localizatio	-	-	-	-	7:7282309	2563	3194	3924	3620	2986	2708	11.4138	11.92256	12.03822	10.502	11.66042	14.95259	11.79153	12.37167	0.984709	1	-
ENSMUSC	Sema4f	sema don	GO:00325	developm	K06521	semaphor	mmu0436	Axon guic	6:8291188	539	705	844	803	660	936	5.730189	6.282353	6.181216	5.561314	6.152713	12.33792	6.064586	8.017314	0.199321	1	-
ENSMUSC	Hk2	hexokinas	GO:00325	developm	K00844	hexokinas	mmu0110	Metabolic	6:8272502	169	190	226	237	190	122	1.566125	1.475859	1.442778	1.430768	1.543952	1.401799	1.494921	1.45884	0.705859	1	-
ENSMUSC	Myo18a	myosin XV	GO:00400	locomotic	-	-	-	-	11:777632	3976	4767	5686	6157	4786	3121	9.579814	9.627393	9.437761	9.664102	10.11174	9.323746	9.548323	9.699864	0.860321	1	-
ENSMUSC	Sez6	seizure re	GO:00325	developm	-	-	-	-	11:779308	3209	3208	3430	4202	3441	2565	20.96439	17.56709	15.43683	17.88341	19.71242	20.77714	17.98944	19.45766	0.767918	1	-
ENSMUSC	Cd52	CD52 anti	GO:00160	membran	-	-	-	-	4:1340824	17	19	44	50	32	25	0.771696	0.722964	1.375991	1.478617	1.273781	1.407112	0.956884	1.386503	0.181637	1	-
ENSMUSC	Abhd15	abhydrola	GO:00081	metabolic	-	-	-	-	11:775151	9	9	12	16	10	20	0.154796	0.129747	0.142191	0.179276	0.150825	0.426518	0.142244	0.252206	0.150922	1	-
ENSMUSC	Hoxb6	homeobo	GO:00325	developm	-	-	-	-	11:962924	2	0	6	3	4	6	0.038701	0	0.079962	0.037805	0.067853	0.143938	0.039554	0.083199	0.301517	1	-
ENSMUSC	Loxl3	lysyl oxida	GO:00325	developm	-	-	-	-	6:8303417	76	67	125	117	84	76	0.872791	0.644946	0.988911	0.875314	0.845896	1.082171	0.835549	0.93446	0.724457	1	-
ENSMUSC	Btn1a1	butyrophi	GO:00081	metabolic	-	-	-	-	13:234569	0	1	4	0	0	0	0	0.010601	0.034855	0	0	0	0.015152	0	0.161281	1	-
ENSMUSC	Kat2b	K(lysine) a	GO:00081	metabolic	K06062	histone ac	mmu0516	HTLV-I inf	17:535668	393	411	657	608	424	317	4.327907	3.793849	4.984276	4.361848	4.094429	4.328433	4.368678	4.26157	0.657567	1	-
ENSMUSC	Rab5b	RAB5B, m	GO:00452	synapse|c	K07888	Ras-relate	mmu0414	Endocytos	10:128677	2319	2863	3442	3524	2772	1964	33.5258	34.69386	34.27999	33.18914	35.14102	35.20512	34.16655	34.51176	0.804716	1	-
ENSMUSC	Cryba1	crystallin,	GO:00325	developm	-	-	-	-	11:777186	0	0	0	0	0	4	0	0	0	0	0	0.156658	0	0.052219	0.12758	1	-
ENSMUSC	Dnmt3l	DNA (cyto	GO:00325	developm	-	-	-	-	10:780419	4	2	5	6	1	0	0.057434	0.024071	0.04946	0.056127	0.01259	0	0.043655	0.022906	0.422847	1	-
ENSMUSC	Aire	autoimmu	GO:00325	developm	K10603	autoimmu	mmu0412	Ubiquitin	10:780300	0	5	7	6	12	6	0	0.041441	0.047687	0.038652	0.104053	0.073571	0.029709	0.072092	0.110279	1	-
ENSMUSC	Icosl	icos ligan	GO:00325	developm	K06710	inducible	mmu0451	Cell adhes	10:780693	192	213	237	233	216	190	2.135864	1.986113	1.816236	1.688532	2.107014	2.620668	1.979404	2.138738	0.864572	1	-
ENSMUSC	Spg7	SPG7, par	GO:00081	metabolic	-	-	-	-	8:1230629	522	628	797	855	662	639	3.910727	3.943657	4.113359	4.172867	4.348977	5.935723	3.989248	4.819189	0.280003	1	-
ENSMUSC	Sult5a1	sulfotrans	GO:00038	catalytic a	-	-	-	-	8:1231401	17	17	10	19	15	22	0.188514	0.15802	0.076395	0.137256	0.14586	0.302499	0.140976	0.195205	0.459657	1	-
ENSMUSC	Rpl13	ribosomal	GO:00081	metabolic	K02873	large subu	mmu0301	Ribosome	8:1231023	119	105	181	151	119	287	1.773225	1.311472	1.858001	1.465808	1.554915	5.302548	1.647566	2.774423	0.102049	1	-
ENSMUSC	Chmp1a	charged n	GO:00081	metabolic	K12197	charged n	mmu0414	Endocytos	8:1232042	789	1025	1241	1207	917	1515	13.05136	14.21201	14.14172	13.00671	13.3012	31.07258	13.8017	19.12683	0.147312	1	-
ENSMUSC	Rpa1	replicatior	GO:00325	developm	K07466	replicatior	mmu0346	Fanconi a	11:752981	840	1074	1038	1147	898	717	7.432066	7.965027	6.326728	6.611122	6.967053	7.865659	7.241273	7.147945	0.66794	1	-
ENSMUSC	Serpinf1	serine (or	GO:00325	developm	K19614	pigment e	mmu0431	Wnt signa	11:754097	263	309	395	359	239	162	4.197501	4.133772	4.342931	3.732591	3.344838	3.205801	4.224734	3.427743	0.095879	1	-
ENSMUSC	Tubgcp3	tubulin, ga	GO:00051	structural	-	-	-	-	8:1261427	755	904	1057	1132	871	614	3.842599	3.856552	3.705992	3.753236	3.887216	3.874646	3.801714	3.838366	0.821491	1	-
ENSMUSC	Oprm1	opioid rec	GO:00325	developm	K04215	mu-type c	mmu0408	Neuroacti	10:675850	35	25	43	50	44	15	0.152459	0.091279	0.129032	0.141882	0.168063	0.081015	0.124257	0.13032	0.939438	1	-
ENSMUSC	Polr3d	polymeras	GO:00081	metabolic	K03026	DNA-dire	mmu0110	Metabolic	14:704387	344	394	502	539	369	229	4.511399	4.331137	4.53532	4.604933	4.243475	3.723702	4.459285	4.190703	0.51216	1	-
ENSMUSC	Tcf7	transcripti	GO:00325	developm	K02620	transcripti	mmu0520	Pathways	11:522523	37	72	82	74	39	44	0.701505	1.144234	1.071019	0.914004	0.648395	1.034356	0.972253	0.865585	0.458962	1	-
ENSMUSC	Ddx3x	DEAD/H (	GO:00081	metabolic	K11594	ATP-depe	mmu0520	Viral carci	X:1328097	4405	5406	6835	7198	5112	2638	41.09071	42.26946	43.92256	43.74125	41.81492	30.51115	42.42758	38.68911	0.375642	1	-
ENSMUSC	Il12rb1	interleukir	GO:00048	signal trar	-	-	-	-	8:7080844	5	10	7	12	12	6	0.061051	0.102341	0.058883	0.095454	0.128482	0.090843	0.074091	0.104926	0.434951	1	-
ENSMUSC	Slc5a5	solute car	GO:00081	metabolic	K14385	solute car	mmu0491	Thyroid hc	8:7088288	300	495	426	466	402	393	6.511576	9.005822	6.369801	6.589194	7.651282	10.57654	7.295733	8.27234	0.628235	1	-
ENSMUSC	Kcnn3	potassium	GO:00511	localizatio	K04944	potassium	mmu0491	Insulin sec	3:8952016	698	802	897	962	734	436	3.961423	3.815247	3.507029	3.55674	3.652872	3.068093	3.761233	3.425902	0.347951	1	-
ENSMUSC	Usp32	ubiquitin s	GO:00081	metabolic	-	-	-	-	11:849844	3282	3773	4820	4714	3516	2191	19.98859	19.2612	20.22284	18.70316	18.77739	16.5452	19.82421	18.00858	0.273879	1	-
ENSMUSC	Car4	carbonic a	GO:00081	metabolic	K18246	carbonic a	mmu0496	Proximal t	11:849577	503	639	712	809	533	444	10.92522	11.63364	10.65352	11.44701	10.15154	11.95724	11.07079	11.18527	0.82374	1	-
ENSMUSC	Txnrd3	thioredox	GO:00081	metabolic	K00384	thioredox	mmu0520	Pathways	6:8964398	56	69	87	84	70	67	1.25492	1.296074	1.343077	1.226285	1.375525	1.861623	1.298024	1.487811	0.658157	1	-
ENSMUSC	Fasl	Fas liganc	GO:00325	developm	K04389	tumor nec	mmu0520	Pathways	1:1617806	0	0	4	0	3	0	0	0	0.074753	0	0.07135	0	0.024918	0.023783	1	1	-
ENSMUSC	Zfp512b	zinc finge	GO:00971	organic cy	-	-	-	-	2:1815821	1115	1348	1532	1944	1481	1443	5.323535	5.394713	5.0389	6.046491	6.200451	8.542357	5.252383	6.929766	0.062278	1	-
ENSMUSC	Dnajc5	DnaJ heat	GO:00452	synapse|c	K09525	DnaJ hom	mmu0414	Protein pr	2:1815204	1893	2549	3034	3120	2267	2042	8.322207	9.393137	9.18872	8.935611	8.739409	11.13089	8.968021	9.601969	0.832257	1	-
ENSMUSC	Tpd52l2	tumor pro	GO:00056	cell|organ	-	-	-	-	2:1814971	252	336	457	399	316	231	1.177599	1.316101	1.471177	1.214653	1.294872	1.338428	1.321626	1.282651	0.625393	1	-
ENSMUSC	Fmr1	fragile X r	GO:00325	developm	K15516	fragile X n	mmu0301	RNA trans	X:6867854	1541	1881	2202	2262	1591	746	6.963041	7.124232	6.854331	6.658412	6.303899	4.179472	6.980535	5.713927	0.129624	1	-
ENSMUSC	Bcl11a	B cell CLL	GO:00325	developm	-	-	-	-	11:240780	1918	2237	2919	3077	2532	1529	6.850324	6.697016	7.182043	7.159318	7.929923	6.771051	6.909794	7.286764	0.882086	1	-
ENSMUSC	Il4	interleukir	GO:00325	developm	K05430	interleukir	mmu0520	Pathways	11:536029	0	2	1	0	0	0	0	0.075079	0.030842	0	0	0	0.035307	0	0.285936	1	-
ENSMUSC	Pxmp4	peroxison	GO:00160	membran	K13350	peroxison	mmu0414	Peroxison	2:1545857	185	207	264	356	189	208	3.577995	3.355768	3.517419	4.485389	3.205326	4.987893	3.483727	4.226203	0.344391	1	-
ENSMUSC	Dlg3	discs, larg	GO:00325	developm	K12075	discs large	mmu0516	Human pa	X:1007677	5257	5824	7185	7911	5621	3299	26.35052	24.46951	24.81014	25.83236	24.70628	20.50309	25.21006	23.68058	0.452011	1	-
ENSMUSC	Gnb1l	guanine n	GO:00056	cell|behav	-	-	-	-	16:184987	46	47	56	60	51	59	0.33464	0.286597	0.280648	0.284353	0.32534	0.532185	0.300628	0.380626	0.416545	1	-
ENSMUSC	Dbh	dopamine	GO:00081	metabolic	K00503	dopamine	mmu0110	Metabolic	2:2716523	20	17	6	25	19	11	0.461034	0.328478	0.095276	0.37542	0.384048	0.314404	0.294929	0.357957	0.642709	1	-
ENSMUSC	Mmp11	matrix me	GO:00325	developm	-	-	-	-	10:759232	13	12	22	25	14	24	0.190494	0.1474	0.222083	0.238652	0.179893	0.436057	0.186659	0.284867	0.246131	1	-
ENSMUSC	Smarcb1	SWI/SNF r	GO:00325	developm	K11648	SWI/SNF-	mmu0522	Hepatoce	10:758967	666	961	1188	1125	827	765	3.144363	3.803076	3.863905	3.460136	3.423789	4.478225	3.603782	3.787383	0.968261	1	-
ENSMUSC	Vpreb3	pre-B lym	GO:00511	localizatio	-	-	-	-	10:759430	0	0	0	0	0	2	0	0	0	0	0	0.116426	0	0.038809	0.536484	1	-
ENSMUSC	Hip1r	huntingtir	GO:00325	developm	-	-	-	-	5:1239736	1156	1469	1852	1848	1507	1125	8.648298	9.211859	9.544765	9.006505	9.886186	10.43545	9.134974	9.776047	0.814413	1	-
ENSMUSC	Nsun5	NOL1/NO	GO:00081	metabolic	-	-	-	-	5:1353699	125	172	158	216	131	134	0.9299	1.072523	0.80972	1.046794	0.854555	1.235997	0.937381	1.045782	0.70483	1	-
ENSMUSC	Top1mt	DNA topo	GO:00081	metabolic	-	-	-	-	15:756570	306	408	403	516	358	273	10.21925	11.42118	9.27157	11.2261	10.4839	11.30437	10.304	11.00479	0.846983	1	-
ENSMUSC	Hoxa10	homeobo	GO:00325	developm	K17443	homrobox	mmu0520	Transcript	6:5223119	0	1	0	0	2	0	0	0.007885	0	0	0.016496	0	0.002628	0.005499	1	1	-
ENSMUSC	Hoxa4	homeobo	GO:00325	developm	-	-	-	-	6:5218967	0	0	0	2	0	0	0	0	0	0.05194	0	0	0	0.017313	0.536935	1	-
ENSMUSC	Gm38393	predicted	GO:00056	cell|intrace	-	-	-	-	7:5997414	1873	2291	2889	2901	1978	2018	20.75947	21.28416	22.05857	20.94632	19.22416	27.73228	21.3674	22.63425	0.924015	1	-
ENSMUSC	Mmp14	matrix me	GO:00325	developm	K07763	matrix me	mmu0466	TNF signa	14:544316	219	330	447	391	287	263	1.723604	2.177011	2.423552	2.004714	1.980699	2.566463	2.108056	2.183959	0.956732	1	-
ENSMUSC	Slc7a7	solute car	GO:00081	metabolic	K13867	solute car	mmu0497	Protein di	14:543694	22	27	36	37	21	32	0.254856	0.262176	0.287302	0.279233	0.213329	0.459638	0.268111	0.3174	0.699805	1	-
ENSMUSC	Oxa1l	oxidase as	GO:00081	metabolic	K03217	YidC/Oxa1	mmu0306	Protein ex	14:543608	524	636	764	808	628	579	5.740694	5.840405	5.766045	5.766688	6.033033	7.864988	5.782381	6.554903	0.53853	1	-
ENSMUSC	Heatr6	HEAT rep	GO:00971	organic cy	-	-	-	-	11:837536	353	434	530	591	445	375	2.756384	2.840586	2.850971	3.006311	3.046968	3.630629	2.81598	3.227969	0.479496	1	-
ENSMUSC	Ccl3	chemokin	GO:00325	developm	K05408	C-C motif	mmu0406	Cytokine-	11:836478	9	11	22	18	8	2	0.746697	0.765018	1.257405	0.972868	0.582051	0.205798	0.92304	0.586906	0.233279	1	-
ENSMUSC	Wfdc18	WAP four	GO:00081	metabolic	-	-	-	-	11:837090	4	2	5	0	6	0	0.138984	0.058248	0.119689	0	0.182803	0	0.10564	0.060934	0.472976	1	-
ENSMUSC	Icam4	intercellul	GO:00055	extracellul	-	-	-	-	9:2102937	10	8	8	8	9	5	0.317927	0.21318	0.175212	0.165689	0.250913	0.197086	0.235439	0.204563	0.726405	1	-
ENSMUSC	Ilf2	interleukir	GO:00081	metabolic	-	-	-	-	3:9047612	965	1200	1460	1641	1212	812	6.094471	6.35247	6.352034	6.751477	6.71203	6.358439	6.266325	6.607315	0.89016	1	-
ENSMUSC	Chtop	chromatir	GO:00081	metabolic	-	-	-	-	3:9049895	976	1135	1491	1493	1115	932	5.799155	5.652796	6.103006	5.779045	5.809414	6.866199	5.851652	6.151553	0.948318	1	-
ENSMUSC	Snapin	SNAP-ass	GO:00325	developm	-	-	-	-	3:9048802	225	293	423	357	483	224	1.931051	2.10781	2.500937	1.996001	3.634972	2.38366	2.179933	2.671544	0.339393	1	-
ENSMUSC	S100a4	S100 calci	GO:00508	response	-	-	-	-	3:9060377	9	20	23	41	17	17	0.685815	1.277479	1.20741	2.035319	1.135937	1.606191	1.056902	1.592482	0.184711	1	-
ENSMUSC	S100a3	S100 calci	GO:00056	cell|intrace	-	-	-	-	3:9056057	8	9	9	6	7	4	0.071435	0.067362	0.055362	0.034905	0.054816	0.044288	0.06472	0.04467	0.304675	1	-
ENSMUSC	S100a6	S100 calci	GO:00511	localizatio	-	-	-	-	3:9061288	44	66	80	85	47	74	1.448884	1.82169	1.814758	1.823389	1.357136	3.021336	1.695111	2.067287	0.532901	1	-
ENSMUSC	Icam2	intercellul	GO:00055	extracellul	K06523	intercellul	mmu0451	Cell adhes	11:106377	30	40	41	37	42	38	0.631746	0.706042	0.594781	0.507578	0.775545	0.992183	0.64419	0.758435	0.66546	1	-
ENSMUSC	Mapk7	mitogen-a	GO:00325	developm	K04464	mitogen-a	mmu0401	MAPK sigr	11:614888	401	438	489	458	418	342	1.82034	1.666614	1.529214	1.354425	1.6639	1.924953	1.672056	1.647759	0.674353	1	-
ENSMUSC	Epn2	epsin 2 [S	GO:00325	developm	K12471	epsin[-]	mmu0414	Endocytos	11:615172	2370	1090	1491	3841	1307	811	15.14231	5.837452	6.562556	15.98711	7.322546	6.42467	9.180774	9.911442	0.850933	1	-
ENSMUSC	B9d1	B9 proteir	GO:00325	developm	-	-	-	-	11:615051	76	120	129	122	127	137	2.421065	3.204264	2.830968	2.531835	3.54765	5.411257	2.818766	3.830247	0.205196	1	-
ENSMUSC	Sec24b	Sec24 rela	GO:00325	developm	K14007	protein tra	mmu0414	Protein pr	3:1299827	1207	1184	1672	1726	1156	762	8.581326	7.055891	8.189078	7.994099	7.206887	6.7172	7.942098	7.306062	0.369132	1	-
ENSMUSC	N4bp3	NEDD4 bi	GO:00325	developm	-	-	-	-	11:516430	252	299	378	406	269	220	4.958311	4.931252	5.123619	5.204053	4.641174	5.367122	5.004394	5.070783	0.858081	1	-
ENSMUSC	Rmnd5b	required f	GO:00081	metabolic	-	-	-	-	11:516236	422	424	575	581	496	735	7.876474	6.633419	7.393305	7.064422	8.117898	17.00953	7.301066	10.73062	0.080403	1	-
ENSMUSC	Nhp2	NHP2 ribo	GO:00081	metabolic	K11129	H/ACA rib	mmu0300	Ribosome	11:516197	470	523	627	622	429	302	16.96192	15.8209	15.58819	14.6234	13.57616	13.51356	16.12367	13.90438	0.172157	1	-
ENSMUSC	Vps9d1	VPS9 dor	-	-	-	-	-	-	8:1232423	582	574	702	652	789	505	3.665066	3.029865	3.045423	2.674779	4.35691	3.943085	3.246785	3.658258	0.610403	1	-
ENSMUSC	Zfp276	zinc finge	GO:00081	metabolic	-	-	-	-	8:1232541	159	190	212	244	165	165	0.922153	0.923658	0.847016	0.921886	0.839136	1.186523	0.897609	0.982515	0.780638	1	-
ENSMUSC	Mfsd10	major faci	GO:00511	localizatio	-	-	-	-	5:3463364	93	150	143	161	128	194	0.567768	0.767589	0.601413	0.640314	0.685234	1.468499	0.64559	0.931349	0.147345	1	-
ENSMUSC	Kctd10	potassium	GO:00325	developm	-	-	-	-	5:1143635	862	966	1123	1158	859	862	7.333691	6.888829	6.581831	6.418089	6.408423	9.093034	6.934784	7.306516	0.973359	1	-
ENSMUSC	Poldip2	polymeras	GO:00325	developm	-	-	-	-	11:785121	1111	1465	1808	1909	1289	1175	13.87724	15.33838	15.55747	15.53376	14.11837	18.19755	14.92436	15.94989	0.842297	1	-
ENSMUSC	Sebox	SEBOX hc	GO:00325	developm	-	-	-	-	11:785034	57	73	110	153	67	78	1.454826	1.561743	1.934115	2.543961	1.499525	2.468423	1.650228	2.170636	0.243266	1	-

ENSMUS(	Ift20	intraflagel	GO:00325	developm	-	-	-	-	11:785363	339	467	529	499	378	485	6.867858	7.930326	7.382927	6.585733	6.71516	12.18286	7.393704	8.494585	0.606996	1	-
ENSMUS(	Col6a1	collagen,	GO:00325	developm	K06238	collagen,	mmu0415	PI3K-Akt	10:767087	743	1071	684	684	651	658	10.42381	12.59448	6.610672	6.251381	8.008698	11.4459	9.87632	8.568661	0.274673	1	-
ENSMUS(	Pcbp3	poly(rC) b	GO:00081	metabolic	-	-	-	-	10:767618	691	871	899	937	770	544	8.375952	8.849677	7.507016	7.399074	8.184457	8.176007	8.244215	7.919846	0.536989	1	-
ENSMUS(	Araf	Araf protc	GO:00325	developm	K08845	A-Raf pro	mmu0520	Pathways	X:2079781	845	1174	1278	1400	1120	997	5.277348	6.145821	5.498465	5.695982	6.133659	7.720408	5.640545	6.516683	0.42634	1	-
ENSMUS(	Cfp	complem	GO:00081	metabolic	K15412	complem	mmu0516	Herpes sir	X:2092545	93	115	183	211	120	128	2.901551	3.007445	3.933209	4.288531	3.282982	4.95154	3.280735	4.174351	0.256689	1	-
ENSMUS(	Timp1	tissue inhi	GO:00325	developm	K16451	metallope	mmu0406	HIF-1 sigr	X:2087016	8	24	32	27	11	21	0.367344	0.923765	1.012278	0.807677	0.442937	1.195636	0.767796	0.815416	1	1	-
ENSMUS(	Uxt	ubiquitou	-	-	-	-	-	-	X:2094172	21	20	32	29	34	28	0.527942	0.421447	0.55419	0.474944	0.749525	0.872775	0.501193	0.699081	0.284722	1	-
ENSMUS(	Cnnm3	cyclin M3	GO:00511	localizatio	-	-	-	-	1:3651186	408	492	570	553	490	418	3.550549	3.588832	3.417129	3.135032	3.739154	4.510211	3.518837	3.794799	0.830663	1	-
ENSMUS(	Lman2l	lectin, ma	GO:00511	localizatio	-	-	-	-	1:3641987	900	1116	1385	1558	1224	982	6.010685	6.247389	6.372104	6.778458	7.168128	8.131658	6.21006	7.359414	0.281956	1	-
ENSMUS(	Mcm3ap	minichron	GO:00511	localizatio	-	-	-	-	10:764689	1111	1231	1612	1787	1330	1119	8.568857	7.958292	8.564961	8.978733	8.995041	10.70101	8.364036	9.558261	0.444474	1	-
ENSMUS(	Pcnt	pericentri	GO:00511	localizatio	-	-	-	-	10:763512	910	1020	1349	1501	1192	716	4.048244	3.803458	4.134174	4.349985	4.64991	3.949339	3.995292	4.316411	0.735671	1	-
ENSMUS(	Mxd1	MAX dim	GO:00081	metabolic	-	-	-	-	6:8664704	212	407	592	556	451	331	1.698168	2.732692	3.266759	2.901352	3.167837	3.287437	2.565873	3.118875	0.343474	1	-
ENSMUS(	Gmcl1	germ cell-	GO:00325	developm	-	-	-	-	6:8669176	429	496	647	588	495	412	3.829784	3.711521	3.978991	3.419606	3.874943	4.560377	3.840099	3.951642	0.910873	1	-
ENSMUS(	Snrnp27	small nucl	GO:00081	metabolic	K12846	U4/U6.U5	mmu0304	Spliceosor	6:8667515	294	361	469	429	334	220	8.071084	8.307012	8.869701	7.672281	8.040327	7.488469	8.415932	7.733693	0.387445	1	-
ENSMUS(	Oas1c	2'-5' oligc	GO:00023	immune s	-	-	-	-	5:1208001	10	14	18	10	11	15	0.197925	0.232267	0.245428	0.128933	0.190914	0.368112	0.225206	0.22932	0.871221	1	-
ENSMUS(	Ocrl	OCRL, ino	GO:00325	developm	K01099	inositol pc	mmu0110	Metabolic	X:4791238	1344	1679	1864	1944	1651	953	6.136151	6.425403	5.862653	5.781957	6.609776	5.394807	6.141402	5.928847	0.580883	1	-
ENSMUS(	Calm1	calmoduli	GO:00511	localizatio	K02183	calmoduli	mmu0474	Olfactory	12:100199	13603	16292	19703	21311	16143	9396	99.96665	100.357	99.748	102.0248	104.0273	85.61491	100.0239	97.22235	0.610293	1	-
ENSMUS(	Agpat3	1-acylglyc	GO:00081	metabolic	K13523	lysophosp	mmu0110	Metabolic	10:782691	3641	4265	5107	5430	4215	3761	19.42713	19.07479	18.77177	18.87424	19.72097	24.88154	19.09123	21.15891	0.612486	1	-
ENSMUS(	Slc26a3	solute car	GO:00325	developm	K14078	solute car	mmu0497	Pancreatic	12:313908	0	9	7	9	3	4	0	0.058537	0.037422	0.045496	0.020412	0.038486	0.031986	0.034798	1	1	-
ENSMUS(	Sema6b	sema don	GO:00325	developm	K06842	semaphor	mmu0436	Axon guic	17:561230	2385	2710	2965	3399	2683	2538	38.11262	36.29967	32.64049	35.38452	37.59619	50.28727	35.68426	41.08933	0.442839	1	-
ENSMUS(	Uhrf1	ubiquitin-	GO:00081	metabolic	-	-	-	-	17:563033	28	22	23	37	19	17	0.335371	0.220873	0.189781	0.288705	0.199552	0.252462	0.248675	0.246906	0.956794	1	-
ENSMUS(	Dpp9	dipeptidyl	GO:00081	metabolic	-	-	-	-	17:561868	1156	1359	1483	1691	1304	1270	11.16524	11.00228	9.867401	10.63985	11.0441	15.20897	10.67831	12.29764	0.457608	1	-
ENSMUS(	Ramp2	receptor (	GO:00325	developm	K08448	receptor a	mmu0427	Vascular s	11:101246	353	329	439	441	363	355	3.993273	3.119633	3.421135	3.249934	3.600843	4.9793	3.511347	3.943359	0.654787	1	-
ENSMUS(	Lsr	lipolysis st	GO:00325	developm	-	-	-	-	7:3095777	104	111	158	148	119	104	1.572193	1.406527	1.645442	1.457526	1.577476	1.94936	1.541387	1.661454	0.866377	1	-
ENSMUS(	Gramd1a	GRAM do	GO:00160	membran	-	-	-	-	7:3113012	1519	1763	2207	2431	1719	1687	12.94409	12.5927	12.9559	13.49523	12.84495	17.82439	12.8309	14.72152	0.450056	1	-
ENSMUS(	Gabrg1	gamma-a	GO:00048	signal trar	K05186	gamma-a	mmu0408	Neuroacti	5:7075104	243	322	382	306	306	138	1.415265	1.571954	1.532664	1.161008	1.562771	0.996547	1.506628	1.240109	0.174161	1	-
ENSMUS(	Ckb	creatine k	GO:00325	developm	K00933	creatine k	mmu0110	Metabolic	12:111669	7585	9029	11947	9482	9360	9765	84.52561	84.33838	91.71556	68.83579	91.46423	134.9245	86.85985	98.40819	0.601688	1	-
ENSMUS(	Sp1	trans-acti	GO:00325	developm	K04684	transcripti	mmu0520	Pathways	15:102406	488	488	615	751	113	31	2.579546	2.162199	2.239495	2.586097	0.523776	0.203177	2.32708	1.10435	0.07132	1	-
ENSMUS(	Itgb7	integrin b	GO:00325	developm	K06590	integrin b	mmu0415	PI3K-Akt	15:102215	10	16	15	12	13	11	0.227709	0.30539	0.235303	0.178014	0.259575	0.310574	0.256134	0.249388	0.860446	1	-
ENSMUS(	Myg1	melanocy	GO:00055	extracellul	-	-	-	-	15:102331	280	309	353	382	329	300	3.035105	2.807552	2.635985	2.697496	3.127194	4.03202	2.826214	3.28557	0.484495	1	-
ENSMUS(	Rarg	retinoic ac	GO:00325	developm	-	-	-	-	15:102234	139	156	168	216	158	165	1.567124	1.474227	1.304818	1.58644	1.562024	2.306532	1.448723	1.818332	0.277279	1	-
ENSMUS(	Pfdn5	prefoldin	GO:00325	developm	-	-	-	-	15:102326	1414	1758	2085	2091	1590	790	27.35585	28.50837	27.78806	26.35339	26.9737	18.95021	27.8841	24.09243	0.208003	1	-
ENSMUS(	Efnb2	ephrin B2	GO:00325	developm	K05463	ephrin-B[	mmu0436	Axon guic	8:8617434	926	1212	1337	1249	1057	677	7.221756	7.922957	7.183146	6.345648	7.228518	6.546455	7.442619	6.706874	0.258448	1	-
ENSMUS(	Rrp15	ribosomal	GO:00081	metabolic	-	-	-	-	1:1867209	130	186	202	238	128	122	1.960585	2.351298	2.098667	2.338302	1.692759	2.281332	2.13685	2.104131	0.743846	1	-
ENSMUS(	Rnd2	Rho famil	GO:00325	developm	-	-	-	-	11:101464	745	324	1139	1039	833	314	20.22512	7.372813	21.30149	18.37518	19.82998	10.5694	16.2998	16.25819	0.913257	1	-
ENSMUS(	Srr	serine rac	GO:00325	developm	K12235	serine rac	mmu0110	Metabolic	11:749063	880	1494	1418	1720	1450	661	3.747683	5.333144	4.160136	4.771887	5.414902	3.490336	4.413654	4.559041	0.981645	1	-
ENSMUS(	Sync	syncoilin [	GO:00452	synapse|st	-	-	-	-	4:1292876	13	12	23	26	29	8	0.256416	0.198408	0.312533	0.334087	0.501589	0.195653	0.255786	0.343776	0.385268	1	-
ENSMUS(	Fndc5	fibronecti	GO:00325	developm	-	-	-	-	4:1291369	1463	1783	2241	2408	1711	1384	33.65111	34.37632	35.50983	36.08223	34.51023	39.47092	34.51242	36.68779	0.858793	1	-
ENSMUS(	Acp5	acid phos	GO:00325	developm	K14379	tartrate-re	mmu0110	Metabolic	9:2212672	8	8	15	26	14	17	0.153088	0.128319	0.197751	0.324128	0.234929	0.403361	0.159719	0.320806	0.034114	1	-
ENSMUS(	Cnn1	calponin 1	GO:00099	cellular pr	-	-	-	-	9:2209921	6	21	22	28	11	19	0.098612	0.28929	0.249075	0.299783	0.158527	0.387175	0.212326	0.281828	0.504939	1	-
ENSMUS(	Fbxo9	f-box pro	GO:00325	developm	-	-	-	-	9:7808149	1192	1502	1444	1470	1356	1157	14.39391	15.20287	12.01216	11.56383	14.35837	17.32296	13.86965	14.41505	0.953658	1	-
ENSMUS(	Vps50	VPS50 EA	GO:00511	localizatio	-	-	-	-	6:3498382	1236	1508	1823	1982	1446	857	5.90391	6.037754	5.998731	6.167461	6.056646	5.07561	5.980131	5.766572	0.58857	1	-
ENSMUS(	Hars	histidyl-tR	GO:00081	metabolic	K01892	histidyl-tR	mmu0097	Aminoacy	18:367665	1259	1634	1879	2025	1539	1035	40.26836	43.80696	41.40154	42.19344	43.16379	41.04539	41.82562	42.1342	0.808528	1	-
ENSMUS(	Zmat2	zinc finge	GO:00081	metabolic	K12848	U4/U6.U5	mmu0304	Spliceosor	18:367939	2738	3643	4303	4354	3190	2227	77.09708	85.98378	83.46941	79.86832	78.76585	77.75184	82.18342	78.79534	0.49836	1	-
ENSMUS(	Smg5	Smg-5 hc	GO:00081	metabolic	K11125	protein SI	mmu0301	mRNA sur	3:8833626	1230	1462	1952	1944	1506	1402	8.25974	8.229276	9.030104	8.504316	8.868071	11.67335	8.506373	9.681912	0.4868	1	-
ENSMUS(	Cct3	chaperoni	GO:00081	metabolic	-	-	-	-	3:8829711	1370	1746	2033	2097	1530	1322	9.260543	9.89266	9.466831	9.234127	9.068801	11.07984	9.540011	9.794256	0.891051	1	-
ENSMUS(	Glmp	glycosylat	GO:00048	signal trar	-	-	-	-	3:8832502	352	360	398	451	346	357	1.655123	1.418871	1.289208	1.381484	1.426613	2.081337	1.454401	1.629811	0.670203	1	-
ENSMUS(	Mef2d	myocyte e	GO:00325	developm	K09262	MADS-bc	mmu0437	Apelin sig	3:8814237	4734	5524	5284	6087	4476	4179	33.32889	32.59862	25.62754	27.91757	27.63283	36.47968	30.51835	30.67669	0.746633	1	-
ENSMUS(	Tmem79	transmem	GO:00325	developm	-	-	-	-	3:8832865	9	11	22	16	6	7	0.093858	0.096161	0.158053	0.108701	0.054865	0.090518	0.116024	0.084695	0.314506	1	-
ENSMUS(	Snd1	staphyloc	GO:00325	developm	K15979	staphyloc	mmu0520	Viral carci	6:2847513	1374	1786	1914	2091	1705	1484	8.000167	8.716589	7.67725	7.931369	8.705215	10.71352	8.131336	9.116701	0.566493	1	-
ENSMUS(	Col18a1	collagen,	GO:00325	developm	K06823	collagen,	mmu0497	Protein di	10:770521	165	172	222	219	139	143	1.186492	1.036723	1.099731	1.025905	0.876478	1.274982	1.107649	1.059122	0.576024	1	-
ENSMUS(	Slc19a1	solute car	GO:00511	localizatio	K14609	solute car	mmu0152	Antifolate	10:770322	141	129	218	170	141	236	1.162401	0.891415	1.23807	0.912995	1.019297	2.412322	1.097295	1.448205	0.342002	1	-
ENSMUS(	Kpnb1	karyopher	GO:00511	localizatio	K14293	importin s	mmu0301	RNA trans	11:971597	4012	4743	5907	5922	4540	3364	36.37299	36.04326	36.89237	34.97584	36.09247	37.81464	36.43621	36.29432	0.701999	1	-
ENSMUS(	Npepps	aminopep	GO:00081	metabolic	-	-	-	-	11:972058	2249	3209	3271	3754	2791	1752	11.29441	13.50817	11.31634	12.28146	12.29069	10.90923	12.03964	11.82713	0.674234	1	-
ENSMUS(	Tbx21	T-box 21	GO:00325	developm	K10166	T-box prc	mmu0465	Th17 cell	11:970980	2	0	5	2	0	0	0.051098	0	0.087987	0.033277	0	0	0.046362	0.011092	0.256797	1	-
ENSMUS(	Mrpl10	mitochon	GO:00081	metabolic	-	-	mmu0301	Ribosome	11:970415	573	702	903	875	724	826	17.20164	17.66467	18.67474	17.11216	19.05884	30.74549	17.84702	22.3055	0.24578	1	-
ENSMUS(	Cyp51	cytochron	GO:00081	metabolic	K05917	sterol 14-	mmu0110	Metabolic	5:4081145	1138	1425	1593	1708	1241	931	13.36846	14.03158	12.89158	13.07099	12.7836	13.56046	13.43054	13.13835	0.612826	1	-
ENSMUS(	Tcf25	transcripti	GO:00081	metabolic	-	-	-	-	8:1233737	4790	6302	7469	8002	6138	6157	28.76761	31.72485	30.90169	31.30751	32.32495	45.84833	30.46472	36.4936	0.273322	1	-
ENSMUS(	Tubb6	tubulin, b	GO:00051	structural	K07375	tubulin be	mmu0414	Phagoson	18:673907	16	24	39	44	27	31	0.578754	0.727655	0.971814	1.036816	0.856387	1.390321	0.759408	1.094508	0.209142	1	-
ENSMUS(	Def8	differentia	GO:00511	localizatio	-	-	-	-	8:1234235	557	668	752	856	721	724	5.078767	5.105434	4.723598	5.084624	5.764762	8.185171	4.969266	6.344852	0.158945	1	-
ENSMUS(	Meox1	mesenchy	GO:00325	developm	-	-	-	-	11:101877	5	11	21	19	13	9	0.142625	0.263018	0.41266	0.353058	0.325168	0.318299	0.272768	0.332175	0.671481	1	-
ENSMUS(	Sost	sclerostin	GO:00325	developm	K16834	sclerostin[	mmu0431	Wnt signa	11:101962	0	0	10	2	0	0	0	0	0.211917	0.040074	0	0	0.070639	0.013358	0.295215	1	-
ENSMUS(	Nkx2-1	NK2 hom	GO:00325	developm	-	-	-	-	12:565319	8	3	16	0	4	10	0.157498	0.049506	0.217006	0	0.069051	0.244125	0.141337	0.104392	0.516246	1	-
ENSMUS(	Irx2	Iroquois r	GO:00325	developm	-	-	-	-	13:726288	0	0	1	5	2	0	0	0	0.008624	0.040793	0.02196	0	0.002875	0.020918	0.17364	1	-
ENSMUS(	Col1a1	collagen,	GO:00325	developm	K06236	collagen,	mmu0415	PI3K-Akt	11:949362	278	403	581	692	295	526	2.547988	3.096064	3.668428	4.131809	2.370917	5.977557	3.10416	4.160094	0.206616	1	-
ENSMUS(	Itga3	integrin al	GO:00325	developm	K06482	integrin al	mmu0520	Pathways	11:950444	260	326	369	364	285	274	2.448335	2.573165	2.393729	2.232953	2.35334	3.199141	2.471743	2.595145	0.99966	1	-
ENSMUS(	Sgca	sarcoglyca	GO:00325	developm	K12565	alpha-sar	mmu0541	Dilated ca	11:949627	3	7	6	6	6	0	0.072101	0.141028	0.099348	0.093956	0.126455	0	0.104159	0.07347	0.637465	1	-
ENSMUS(	Foxm1	forkhead	GO:00325	developm	K09406	forkhead	mmu0421	Cellular se	6:1283629	31	30	37	40	27	16	0.216075	0.175269	0.17766	0.181625	0.165022	0.138274	0.189668	0.161641	0.450868	1	-
ENSMUS(	Itfg2	integrin al	GO:00325	developm	-	-	-	-	6:1284094	176	212	289	285	228	313	1.011605	1.02138	1.14432	1.06715	1.149147	2.23064	1.059102	1.482312	0.125132	1	-
ENSMUS(	Nrip2	nuclear re	GO:00081	metabolic	-	-	-	-	6:1283992	136	277	184	306	256	311	1.264186	2.158255	1.178259	1.852995	2.086663	3.5844	1.533567	2.508019	0.028969	1	-
ENSMUS(	Tulp3	tubby-like	GO:00325	developm	-	-	-	-	6:1283211	334	406	481	489	428	332	1.915767	1.951977	1.900609	1.827202	2.152699	2.361136	1.922785	2.113679	0.703049	1	-
ENSMUS(	Gtf2h4	general tr	GO:00081	metabolic	K03144	transcripti	mmu0520	Viral carci	17:356677	175	212	262	247	167	211	1.74733	1.774296	1.802147	1.606632	1.462169	2.612197	1.774591	1.893666	0.954106	1	-
ENSMUS(	Tubb5	tubulin, b	GO:00051	structural	K07375	tubulin be	mmu0414	Phagoson	17:358339	4550	5868	6758	6340	5182	5061	57.94914	62.64387	59.29324	52.60251	57.87295	79.92044	59.96208	63.4653	0.940547	1	-
ENSMUS(	Ell2	elongatio	GO:00081	metabolic	-	-	-	-	13:757067	489	567	826	821	549	269	2.694187	2.618511	3.135092	2.946749	2.652368	1.837627	2.81593	2.478915	0.341553	1	-
ENSMUS(	Jup	junction p	GO:00325	developm	K10056	junction p	mmu0520	Pathways	11:100368	462	502	565	623	620	536	3.141609	2.861323	2.646735	2.759817	3.696959	4.519197	2.883222	3.658657	0.190201	1	-
ENSMUS(	Fkbp10	FK506 bin	GO:00081	metabolic	-	-	-	-	11:100415	102	135	198	172	143	156	1.447617	1.605969	1.935837	1.590236	1.779636	2.745123	1.663141	2.038332	0.399302	1	-
ENSMUS(	Klhl10	kelch-like	GO:00325	developm	-	-	-	-	11:100441	4	2	14	6	3	6	0.086199	0.036126	0.207841	0.084238	0.056688	0.160338	0.110056	0.100422	0.79881	1	-
ENSMUS(	Nom1	nucleolar	GO:00325	developm	-	-	-	-	5:2943466	495	606	795	794	628	361	3.325446	3.412483	3.679287	3.47494	3.699538	3.007034	3.472405	3.393837	0.689896	1	-
ENSMUS(	Ergic1	endoplasr	GO:00511	localizatio	-	-	-	-	17:265614	2023	2506	2853	3420	2562	1896	17.32018	17.98418	16.82712	19.075	19.23439	20.12709	17.37716	19.47883	0.51509	1	-
ENSMUS(	Tnk1	tyrosine k	GO:00325	developm	-	-	-	-	11:698510	2	1	4	2	0	0	0.02558	0.010718	0.035241	0.016659	0	0	0.023846	0.005553	0.230396	1	-
ENSMUS(	Acap1	ArfGAP w	GO:00048	signal trar	K12489	Arf-GAP v	mmu0414	Endocytos	11:698815	1	1	4	5	1	3	0.009938	0.008328	0.027384	0.032368	0.008712	0.036964	0.015217	0.026014	0.655817	1	-
ENSMUS(	Tcea3	transcripti	GO:00081	metabolic	-	-	-	-	4:1362477	6	6	24	13	9	10	0.216544	0.181493	0.596675	0.305641	0.284824	0.447492	0.331571	0.345986	1	1	-
ENSMUS(	Ifrd1	interferon	GO:00325	developm	-	-	-	-	12:402015	346	460	571	589	395	358	2.645815	2.948448	3.007948	2.934132	2.648638	3.394312	2.867404	2.99236	1	1	-
ENSMUS(	Stk38l	serine/thr	GO:00081	metabolic	-	-	-	-	6:1467249	495	553	698	772	524	354	5.280967	4.945226	5.129976	5.365467	4.902105	4.682714	5.118723	4.983429	0.650853	1	-
ENSMUS(	Brpf1	bromodo	GO:00081	metabolic	-	-	-	-	6:1133071	736	821	981	1165	829	572	3.151956	2.947122	2.89416	3.250198	3.113145	3.037276	2.997746	3.13354	0.951859	1	-
ENSMUS(	Akr1b3	aldo-ketc	GO:00325	developm	K00011	aldehyde	mmu0110	Metabolic	6:3430243	74	115	112	116	96	91	0.459986	0.599195	0.479605	0.469737	0.523272	0.701364	0.512928	0.564791	0.805316	1	-
ENSMUS(	Hoxc8	homeobo	GO:00325	developm	-	-	-	-	15:102990	10	10	18	25	7	11	0.15535	0.130208	0.192635	0.253002	0.095362	0.211883	0.159398	0.186749	0.810595	1	-
ENSMUS(	Hoxc6	homeobo	GO:00325	developm	-	-	-	-	15:102998	4	4	8	6	1	1	0.084312	0.070671	0.116169	0.082394	0.018482	0.026129	0.090384	0.042335	0.199339	1	-
ENSMUS(	Gstt1	glutathior	GO:00081	metabolic	K00799	glutathior	mmu0520	Pathways	10:757838	48	79	121	90	64	71	1.634806	2.255307	2.838978	1.996872	1.911384	2.998263	2.24303	2.302173	0.913583	1	-
ENSMUS(	Gstt3	glutathior	GO:00081	metabolic	K00799	glutathior	mmu0520	Pathways	10:757741	31	26	45	39	15	30	0.343415	0.24142	0.343412	0.281447	0.145707	0.412065	0.309416	0.27974	0.58899	1	-
ENSMUS(	Ddt	D-dopach	GO:00081	metabolic	-	-	-	-	10:757712	179	224	290	242	185	165	9.301683	9.756882	10.38149	8.192305	8.429929	10.63117	9.81335	9.084467	0.412021	1	-
ENSMUS(	Tat	tyrosine a	GO:00081	metabolic	K00815	tyrosine a	mmu0110	Metabolic	8:1099904	0	0	0	3	0	0	0	0	0	0.048625	0	0	0	0.016208	0.30305	1	-
ENSMUS(	Marveld3	MARVEL (	GO:00081	metabolic	-	-	mmu0453	Tight junc	8:1099479	12	4	10	3	9	6	0.181443	0.050695	0.104169	0.029548	0.119338	0.1125	0.112102	0.087128	0.538311	1	-
ENSMUS(	Ddx18	DEAD (As	GO:00081	metabolic	-	-	-	-	1:1215538	220	234	305	327	212	149	4.951015	4.414076	4.728486	4.794026	4.183596	4.157594	4.697859	4.378405	0.494141	1	-
ENSMUS(	Ubl3	ubiquitin-	GO:00055	extracellul	-	-	-	-	5:1485046	1394	2224	2417	2833	2171	2118	6.931609	9.269554	8.279408	9.176975	9.466158	13.0582	8.16019	10.56711	0.099531	1	-
ENSMUS(	Gramd3	GRAM do	GO:00990	supramol	-	-	-	-	18:564321	193	242	353	335	264	247	4.708544	4.948771	5.932737	5.3242	5.647742	7.471556	5.196684	6.147833	0.415188	1	-
ENSMUS(	Eef1e1	eukaryoti	GO:00325	developm	-	-	-	-	13:386442	217	271	290	276	184	203	3.720268	3.894357	3.425022	3.082511	2.766139	4.315145	3.679882	3.387932	0.396284	1	-
ENSMUS(	Akt1	thymoma	GO:00325	developm	K04456	RAC serin	mmu0520	Pathways	12:112653	2264	2680	3253	3465	2455	2367	26.32828	26.12363	26.06042	26.25009	25.03457	34.12951	26.17078	28.47139	0.74033	1	-
ENSMUS(	Cldn15	claudin 15	GO:00511	localizatio	K06087	claudin[-]	mmu0453	Tight junc	5:1369666	2	6	8	5	7	0	0.033368	0.083887	0.091929	0.054337	0.102395	0	0.069728	0.052244	0.65247	1	-
ENSMUS(	Il16	interleukir	GO:00081	metabolic	-	-	-	-	7:8364282	912	1064	1209	1368	1020	626	5.71432	5.588101	5.218519	5.583897	5.604188	4.863284	5.50698	5.350456	0.626722	1	-
ENSMUS(	Tcirg1	T cell, imn	GO:00081	metabolic	K02154	V-type H-	mmu0110	Metabolic	19:389605	163	221	342	359	267	239	1.419256	1.612942	2.051397	2.036331	2.038573	2.580218	1.694532	2.218374	0.142659	1	-
ENSMUS(	Naglu	alpha-N-	GO:00325	developm	K01205	alpha-N-	mmu0110	Metabolic	11:101070	99	98	138	152	94	152	1.653109	1.371664	1.58744	1.653459	1.376376	3.146996	1.537404	2.058944	0.259106	1	-
ENSMUS(	Coasy	Coenzym	GO:00081	metabolic	K02318	phosphop	mmu0110	Metabolic	11:101082	278	313	405	364	341	364	4.308157	4.065777	4.32367	3.674762	4.633866	6.994139	4.232535	5.100922	0.405673	1	-
ENSMUS(	Smo	smoothen	GO:00325	developm	K06226	smoothen	mmu0520	Pathways	6:2973550	317	365	405	401	335	378	3.207503	3.095655	2.823017	2.643221	2.972311	4.742256	3.042058	3.452596	0.644617	1	-
ENSMUS(	Tspan33	tetraspani	GO:00081	metabolic	-	-	-	-	6:2969422	532	615	737	658	605	419	4.967709	4.81362	4.740926	4.002687	4.953843	4.851139	4.840752	4.602556	0.493953	1	-
ENSMUS(	Crnkl1	Crn, crook	GO:00081	metabolic	K12869	crooked r	mmu0304	Spliceosor	2:1459174	451	504	613	681	503	313	6.534983	6.1214	6.118986	6.4283	6.391134	5.62339	6.258457	6.147608	0.710328	1	-
ENSMUS(	Rin2	Ras and R	GO:00511	localizatio	-	-	-	-	2:1457847	656	682	942	974	646	476	6.707014	5.844705	6.634791	6.487325	5.791622	6.03418	6.395503	6.104376	0.524182	1	-
ENSMUS(	Folh1	folate hyd	GO:00081	metabolic	K14592	glutamate	mmu0110	Metabolic	7:8671897	104	128	153	172	136	77	1.645792	1.697872	1.66796	1.773176	1.887222	1.510834	1.670541	1.723744	1	1	-
ENSMUS(	Chordc1	cysteine a	GO:00081	metabolic	-	-	-	-	9:1829212	1300	1728	2353	2155	1662	1082	9.157497	10.20305	11.41844	9.889224	10.26613	9.450318	10.25966	9.868558	0.550448	1	-
ENSMUS(	Rtcb	RNA 2',3'-	GO:00325	developm	-	-	-	-	10:859386	1479	1789	2159	2187	1632	1201	33.17857	33.63976	33.36519	31.96097	32.1035	33.40554	33.39451	32.49	0.57242	1	-
ENSMUS(	Pwp1	PWP1 hor	GO:00325	developm	-	-	-	-	10:858294	281	399	373	432	301	245	4.895391	5.826489	4.476529	4.902829	4.598229	5.292173	5.066136	4.931077	0.642929	1	-
ENSMUS(	Fbxo7	F-box prc	GO:00325	developm	-	-	-	-	10:860219	302	331	380	389	319	334	1.558635	1.431918	1.351053	1.30788	1.443677	2.137319	1.447202	1.629625	0.657879	1	-
ENSMUS(	Capns1	calpain, sr	GO:00055	extracellul	-	-	-	-	7:3018693	2017	1456	1763	1820	1415	1007	23.77785	14.38736	14.31761	13.97719	14.62736	14.71913	17.49427	14.44123	0.12309	1	-
ENSMUS(	Lrp3	low densit	GO:00160	membran	-	-	-	-	7:3520002	1131	827	1145	1472	1311	1075	10.13515	6.211922	7.068451	8.593235	10.30179	11.94433	7.805173	10.27978	0.121445	1	-
ENSMUS(	Folr1	folate rec	GO:00325	developm	K13649	folate rec	mmu0414	Endocytos	7:1018583	89	39	206	40	50	65	1.024302	0.376235	1.633272	0.299902	0.504608	0.927548	1.011269	0.577353	0.058068	1	-
ENSMUS(	Clpb	ClpB case	GO:00508	response	K03695	ATP-depe	mmu0421	Longevity	7:1016636	832	992	1325	1178	859	866	3.848895	3.846599	4.222599	3.55009	3.484563	4.967256	3.972698	4.000636	0.756629	1	-
ENSMUS(	7-Sep	septin 7 [S	GO:00329	macromo	-	-	-	-	9:2525243	5333	6496	8200	8352	6142	2323	20.47781	20.90792	21.69088	20.89219	20.68066	11.05979	21.02553	17.54421	0.226836	1	-
ENSMUS(	Zdhhc4	zinc finge	GO:00038	catalytic a	-	-	-	-	5:1433164	252	294	335	368	268	319	2.274584	2.224344	2.083043	2.163872	2.121185	3.570081	2.19399	2.61838	0.430791	1	-
ENSMUS(	Rac1	RAS-relat	GO:00325	developm	K04392	Ras-relate	mmu0520	Pathways	5:1435036	4613	5583	7120	6713	4980	867	45.8293	46.49222	48.72942	43.44683	43.38422	10.67984	47.01698	32.50363	0.150963	1	-
ENSMUS(	Nup214	nucleopo	GO:00081	metabolic	K14317	nuclear pc	mmu0516	Epstein-B	2:3197443	787	799	1055	1359	918	667	3.65776	3.11272	3.377881	4.114733	3.74133	3.843727	3.382787	3.89993	0.411196	1	-
ENSMUS(	Aif1l	allograft i	GO:00990	supramol	-	-	-	-	2:3195013	123	135	230	201	127	132	1.262844	1.161798	1.626765	1.344383	1.143386	1.680369	1.350469	1.38938	0.928304	1	-
ENSMUS(	Ltbp1	latent trar	GO:00325	developm	K19559	latent trar	mmu0435	TGF-beta	17:750055	124	147	242	220	171	135	0.529146	0.525804	0.711413	0.611586	0.639871	0.714289	0.588788	0.655249	0.691503	1	-
ENSMUS(	Ugp2	UDP-gluc	GO:00081	metabolic	-	-	mmu0110	Metabolic	11:213211	1066	1426	1792	1604	1175	798	15.85108	17.77354	18.35655	15.53774	15.32082	14.71264	17.32706	15.1904	0.174354	1	-
ENSMUS(	Kcnh6	potassium	GO:00511	localizatio	-	-	-	-	11:106008	16	27	18	31	23	23	0.207398	0.29335	0.160732	0.261769	0.261426	0.369651	0.220493	0.297615	0.355393	1	-
ENSMUS(	Trmt1	tRNA met	GO:00081	metabolic	-	-	-	-	8:8468630	502	678	690	809	616	682	2.227595	2.521824	2.109274	2.338637	2.396933	3.752348	2.286231	2.829306	0.257068	1	-
ENSMUS(	Nacc1	nucleus a	GO:00081	metabolic	-	-	-	-	8:8467048	2357	2778	3137	3485	2662	1946	34.83591	34.4154	31.93991	33.5546	34.49994	35.66125	33.73041	34.57193	0.900447	1	-
ENSMUS(	Nfix	nuclear fa	GO:00325	developm	-	-	-	-	8:8469987	5952	6619	7186	9743	7230	5069	45.23108	42.16181	37.61947	48.23345	48.17869	47.76197	41.67079	48.05804	0.379348	1	-
ENSMUS(	Slc1a5	solute car	GO:00511	localizatio	K05616	solute car	mmu0497	Protein di	7:1678134	28	24	27	47	25	46	0.25782	0.185235	0.171267	0.281929	0.201858	0.525181	0.204774	0.336323	0.116017	1	-
ENSMUS(	Uba1	ubiquitin-	GO:00081	metabolic	K03178	ubiquitin-	mmu0501	Parkinson	X:2065832	6839	7977	9507	10144	7526	5276	80.9987	79.19138	77.5676	78.26658	78.1614	77.4776	79.25256	77.96853	0.635373	1	-
ENSMUS(	Vwf	Von Wille	GO:00325	developm	K03900	von Willet	mmu0415	PI3K-Akt	6:1255467	338	420	614	688	502	549	1.083308	1.128333	1.355673	1.436499	1.410852	2.181692	1.189105	1.676348	0.04404	1	-
ENSMUS(	Siae	sialic acid	GO:00081	metabolic	-	-	-	-	9:3755569	272	317	417	482	342	267	1.221914	1.193668	1.290504	1.410588	1.347226	1.487198	1.235362	1.415004	0.479536	1	-
ENSMUS(	Vsig2	V-set and	GO:00160	membran	-	-	-	-	9:3753925	11	18	26	30	15	23	0.222078	0.3046	0.3616	0.394554	0.265541	0.575731	0.296093	0.411942	0.354493	1	-
ENSMUS(	Esam	endothelia	GO:00055	extracellul	K06787	endothelia	mmu0451	Cell adhes	9:3752807	121	137	198	188	135	231	2.248519	2.133953	2.534702	2.275877	2.199812	5.322403	2.305725	3.266031	0.175849	1	-
ENSMUS(	Spa17	sperm aut	GO:00099	cellular pr	-	-	-	-	9:3760329	20	31	44	28	20	21	0.944333	1.226871	1.431194	0.861264	0.828083	1.229391	1.200799	0.972913	0.285667	1	-
ENSMUS(	Fam50a	family wit	GO:00056	cell|intrac	-	-	-	-	X:7431303	396	515	578	622	485	233	8.068929	8.795917	8.113351	8.256432	8.665732	5.886585	8.326066	7.602916	0.446647	1	-
ENSMUS(	Emd	emerin [S	GO:00325	developm	K12569	emerin[-]	mmu0541	Dilated ca	X:7425468	409	550	739	653	508	352	5.574343	6.283274	6.938519	5.797835	6.07124	5.948397	6.265379	5.939157	0.502306	1	-
ENSMUS(	Taco1	translatior	GO:00081	metabolic	-	-	-	-	11:106066	218	193	295	317	242	158	10.01774	7.434017	9.338733	9.489751	9.751496	9.002388	8.930164	9.414545	0.918509	1	-
ENSMUS(	Grik3	glutamate	GO:00048	signal trar	K05203	glutamate	mmu0408	Neuroacti	4:1254907	1252	1586	1948	2229	1693	1222	8.867534	9.415741	9.504709	10.28466	10.51473	10.73138	9.262662	10.51026	0.452029	1	-
ENSMUS(	Gria3	glutamate	GO:00048	signal trar	K05199	glutamate	mmu0408	Neuroacti	X:4140085	2838	3592	5697	4601	4470	2709	9.83282	10.4317	13.59763	10.38483	13.58051	11.63753	11.28738	11.86762	0.916236	1	-
ENSMUS(	Npas1	neuronal	GO:00325	developm	-	-	-	-	7:1645572	133	162	176	217	156	182	1.831537	1.869963	1.669666	1.94673	1.883788	3.10758	1.790388	2.312699	0.233475	1	-
ENSMUS(	Sipa1l2	signal-inc	GO:00987	molecular	K17702	signal-ind	mmu0401	Rap1 sign	8:1254180	1561	2079	2473	3135	2107	1619	7.156189	7.988884	7.810062	9.362636	8.470051	9.202623	7.651712	9.01177	0.28656	1	-
ENSMUS(	Ap4e1	adaptor-r	GO:00511	localizatio	K12400	AP-4 com	mmu0414	Lysosome	2:1270087	223	290	334	369	270	178	1.213902	1.323215	1.252497	1.308542	1.288803	1.201395	1.263204	1.266246	0.829966	1	-
ENSMUS(	Blvra	biliverdin	GO:00081	metabolic	K00214	biliverdin	mmu0110	Metabolic	2:1270706	123	198	214	213	129	162	2.741847	3.69961	3.286277	3.09315	2.521575	4.477552	3.242578	3.364092	0.946063	1	-
ENSMUS(	Pdzd4	PDZ dom	GO:00056	cell|intrac	-	-	-	-	X:7379335	2019	2521	2942	2964	2343	1931	32.32079	33.82761	32.4444	30.91046	32.88975	38.3278	32.86427	34.04267	0.950149	1	-
ENSMUS(	Srpk3	serine/arg	GO:00325	developm	-	-	-	-	X:7377440	24	44	71	82	55	37	0.360926	0.55464	0.735559	0.803349	0.725288	0.689907	0.550375	0.739515	0.244049	1	-
ENSMUS(	Idh3g	isocitrate	GO:00081	metabolic	K00030	isocitrate	mmu0110	Metabolic	X:7377896	1227	1580	1790	1898	1367	907	14.28197	15.41535	14.35317	14.39201	13.95258	13.08991	14.6835	13.8115	0.42947	1	-
ENSMUS(	Pnck	pregnanc	GO:00081	metabolic	-	-	-	-	X:7365599	628	1011	864	995	744	824	13.5845	18.33109	12.87507	14.02133	14.11238	22.10026	14.93022	16.74465	0.66864	1	-
ENSMUS(	Ssr4	signal seq	GO:00329	macromo	K04571	translocor	mmu0414	Protein pr	X:7378702	174	208	249	274	183	355	1.491738	1.494715	1.470593	1.53029	1.375739	3.773601	1.485682	2.226543	0.111585	1	-
ENSMUS(	Bcap31	B cell rece	GO:00081	metabolic	K14009	B-cell rec	mmu0516	Human pa	X:7368617	536	728	904	718	623	653	27.94457	31.81389	32.46776	24.38589	28.4815	42.21156	30.74207	31.69298	0.889556	1	-
ENSMUS(	Fam98a	family wit	GO:00081	metabolic	-	-	-	-	17:755370	561	638	799	888	590	353	9.296795	8.862262	9.121553	9.586617	8.573645	7.253234	9.093537	8.471166	0.469958	1	-
ENSMUS(	Ltbp2	latent trar	GO:00099	cellular pr	-	-	-	-	12:847832	8	4	11	14	4	10	0.037341	0.01565	0.035372	0.042573	0.016371	0.05788	0.029454	0.038941	0.603564	1	-
ENSMUS(	Kmt2a	lysine (K)-	GO:00325	developm	K09186	histone-ly	mmu0520	Transcript	9:4480335	3343	3011	3584	4153	3144	2027	10.4964	7.924425	7.752175	8.494693	8.656241	7.891215	8.724335	8.347383	0.537557	1	-
ENSMUS(	Ift46	intraflagel	GO:00400	locomotic	-	-	-	-	9:4476790	412	554	638	549	544	422	2.85476	3.217622	3.045397	2.478141	3.305319	3.62552	3.03926	3.136327	0.928072	1	-
ENSMUS(	Tmem25	transmem	GO:00055	extracellul	-	-	-	-	9:4479376	389	570	554	414	497	696	2.924314	3.591711	2.869032	2.027479	3.27622	6.487375	3.128352	3.930358	0.435266	1	-
ENSMUS(	Cd3g	CD3 antig	GO:00048	signal trar	K06452	T-cell surf	mmu0516	HTLV-I in	9:4496957	0	0	1	0	0	2	0	0	0.012225	0	0	0.044034	0.004075	0.014678	1	1	-
ENSMUS(	Trappc6a	trafficking	GO:00511	localizatio	-	-	-	-	7:1950839	46	53	66	67	44	85	0.847367	0.81837	0.837554	0.804036	0.710737	1.941421	0.83443	1.152065	0.315257	1	-
ENSMUS(	Supt6	suppressc	GO:00325	developm	-	-	-	-	11:782067	6769	7244	8816	10112	7297	4005	54.35814	48.76086	48.77116	52.90038	51.3839	39.87755	50.63005	48.05394	0.523966	1	-
ENSMUS(	Spag5	sperm ass	GO:00511	localizatio	-	-	-	-	11:783015	109	132	172	181	134	112	0.841812	0.854505	0.9151	0.910643	0.907478	1.072488	0.870472	0.963536	0.707109	1	-
ENSMUS(	Unc119	unc-119 I	GO:00325	developm	-	-	-	-	11:783434	190	230	317	213	190	193	1.872683	1.900167	2.152394	1.367644	1.642125	2.358606	1.975081	1.789459	0.350038	1	-
ENSMUS(	Rab34	RAB34, m	GO:00038	catalytic a	-	-	-	-	11:781884	112	130	149	189	126	182	1.319853	1.284107	1.209601	1.450936	1.302028	2.659277	1.271187	1.80408	0.120764	1	-
ENSMUS(	Sdf2	stromal ce	GO:00160	membran	-	-	-	-	11:782457	341	371	535	487	438	429	8.268448	7.540431	8.936634	7.692716	9.312913	12.8977	8.248504	9.967778	0.348688	1	-
ENSMUS(	Ccne1	cyclin E1 [	GO:00081	metabolic	K06626	cyclin E[-]	mmu0520	Pathways	7:3809798	206	281	285	295	250	203	3.484677	3.984313	3.321173	3.25086	3.708323	4.257717	3.596721	3.738967	0.978241	1	-
ENSMUS(	Hsf2bp	heat shoc	GO:00056	cell|protei	-	-	-	-	17:319447	0	3	5	5	0	0	0	0.044763	0.06132	0.05799	0	0	0.035361	0.01933	0.643841	1	-
ENSMUS(	Bbc3	BCL2 binc	GO:00325	developm	K10132	Bcl-2-bin	mmu0520	Pathways	7:1630839	82	80	65	106	78	84	1.733075	1.41725	0.94639	1.459457	1.445574	2.201239	1.365572	1.70209	0.412747	1	-
ENSMUS(	Mybpc3	myosin bi	GO:00325	developm	K12568	myosin-b	mmu0541	Dilated ca	2:9111814	0	6	2	2	2	6	0	0.068191	0.018684	0.017664	0.023778	0.100883	0.028958	0.047442	0.744499	1	-

ENSMUSC	Psmc3	proteasor	GO:00325	developm	K03065	26S prote	mmu0516	Epstein-B	2:9105400	2725	2772	3252	3222	2415	1641	35.53908	30.30302	29.2174	27.37454	27.61847	26.53593	31.6865	27.17631	0.116413	1	-
ENSMUSC	Acp2	acid phos	GO:00325	developm	K14410	lysosomal	mmu0110	Metabolic	2:9120288	1430	1797	2098	2173	1604	1168	12.01332	12.65401	12.14183	11.89237	11.8161	12.16623	12.26972	11.95824	0.587168	1	-
ENSMUSC	Rapsn	receptor-	GO:00452	synapse|lc	-	-	-	-	2:9103562	0	2	8	0	2	0	0	0.036398	0.119661	0	0.038076	0	0.05202	0.012692	0.152856	1	-
ENSMUSC	Slc39a13	solute car	GO:00325	developm	-	-	-	-	2:9106179	262	336	471	430	353	364	2.440405	2.623329	3.022267	2.609223	2.883223	4.203864	2.695334	3.232103	0.380844	1	-
ENSMUSC	Celf2	CUGBP, El	GO:00081	metabolic	-	-	-	-	2:6539694	5575	6658	8463	9390	6571	5544	5.831559	5.837629	6.098395	6.39863	6.027177	7.190333	5.922528	6.538713	0.608226	1	-
ENSMUSC	Nr1h3	nuclear re	GO:00048	signal trar	K08536	liver X rec	mmu0493	Non-alco	2:9118406	51	60	82	69	53	33	0.62547	0.616799	0.692794	0.551277	0.569977	0.501804	0.645021	0.541019	0.280397	1	-
ENSMUSC	Ddb2	damage s	GO:00081	metabolic	K10140	DNA dam	mmu0520	Pathways	2:9121157	69	111	127	144	116	82	0.733554	0.989133	0.930112	0.997299	1.081388	1.080892	0.884266	1.053193	0.431714	1	-
ENSMUSC	Spi1	spleen foc	GO:00325	developm	K09438	transcripti	mmu0520	Pathways	2:9108239	41	50	53	73	66	50	0.788396	0.805906	0.702088	0.914464	1.112887	1.192133	0.765463	1.073161	0.126592	1	-
ENSMUSC	Sf3a1	splicing fa	GO:00081	metabolic	K12825	splicing fa	mmu0304	Spliceosor	11:416035	1398	1764	2238	2181	1752	1209	13.1762	13.93588	14.53097	13.39122	14.4797	14.12847	13.88101	13.9998	0.805909	1	-
ENSMUSC	Stat6	signal trar	GO:00325	developm	K11225	signal trar	mmu0520	Pathways	10:127642	156	185	310	262	221	191	1.715862	1.705625	2.348937	1.877334	2.131542	2.60482	1.923474	2.204566	0.571907	1	-
ENSMUSC	Clgn	calmegin	GO:00325	developm	-	-	-	-	8:8338986	224	266	355	390	239	149	3.212789	3.197933	3.50763	3.644015	3.005901	2.649751	3.306117	3.099889	0.554684	1	-
ENSMUSC	Napsa	napsin A a	GO:00081	metabolic	K08565	napsin-A	mmu0414	Lysosome	7:4457238	2	1	6	4	3	8	0.031887	0.01336	0.065883	0.041542	0.04193	0.158112	0.037043	0.080528	0.264157	1	-
ENSMUSC	Vrk3	vaccinia re	GO:00325	developm	-	-	-	-	7:4474841	255	317	429	437	307	263	1.866841	1.945263	2.163589	2.084154	1.97082	2.387304	1.991897	2.147426	0.813752	1	-
ENSMUSC	Smg9	smg-9 ho	GO:00325	developm	-	-	-	-	7:2439961	325	348	430	524	380	384	4.073109	3.655738	3.712468	4.278146	4.176081	5.967057	3.813771	4.807095	0.191192	1	-
ENSMUSC	Paxip1	PAX intera	GO:00325	developm	-	-	-	-	5:2774008	1006	1002	1312	1367	1083	825	5.489337	4.582924	4.93182	4.859283	5.181948	5.581645	5.00136	5.207625	0.999423	1	-
ENSMUSC	Rmnd5a	required f	GO:00081	metabolic	-	-	-	-	6:7138863	2031	2540	3208	3157	2393	1417	9.422991	9.877922	10.25333	9.541908	9.735636	8.151445	9.851413	9.142996	0.387211	1	-
ENSMUSC	Mov10	Moloney l	GO:00081	metabolic	-	-	-	-	3:1047948	118	207	218	250	194	201	0.940225	1.382529	1.196625	1.297693	1.355489	1.985793	1.173126	1.546325	0.179516	1	-
ENSMUSC	Ppm1j	protein pl	GO:00081	metabolic	-	-	-	-	3:1047810	2	0	7	2	2	4	0.059658	0	0.143822	0.038852	0.052299	0.14791	0.067827	0.079687	1	1	-
ENSMUSC	Rhoc	ras homol	GO:00325	developm	-	-	-	-	3:1047883	108	174	244	205	115	142	2.294018	3.09796	3.570386	2.836684	2.141983	3.739812	2.987455	2.90616	0.685743	1	-
ENSMUSC	Tead3	TEA doma	GO:00325	developm	K09448	transcripti	mmu0439	Hippo sig	17:283316	39	40	40	54	38	36	0.292527	0.251485	0.206685	0.263861	0.249935	0.334801	0.250232	0.282865	0.723207	1	-
ENSMUSC	Ppard	peroxison	GO:00325	developm	K04504	peroxison	mmu0520	Pathways	17:282327	375	409	517	540	421	370	3.692076	3.375332	3.506567	3.463501	3.634667	4.516765	3.524658	3.871644	0.718261	1	-
ENSMUSC	Def6	differentia	GO:00160	membran	-	-	-	-	17:282077	19	37	57	54	47	35	0.459827	0.750583	0.950322	0.851367	0.997438	1.050263	0.720244	0.966356	0.283718	1	-
ENSMUSC	Peg3	paternally	GO:00081	metabolic	-	-	-	-	7:6703892	7031	8446	10343	11104	8313	5274	27.69048	27.88157	28.06152	28.48882	28.70871	25.75369	27.87786	27.65041	0.71359	1	-
ENSMUSC	Zim1	zinc finge	GO:00081	metabolic	-	-	-	-	7:6671269	115	185	149	187	129	98	0.612367	0.825731	0.546576	0.648689	0.602347	0.647034	0.661558	0.63269	0.63784	1	-
ENSMUSC	Metrn	meteorin,	GO:00325	developm	-	-	-	-	17:257945	502	328	698	675	513	642	16.88916	9.249777	16.17748	14.79413	15.13436	26.78088	14.10547	18.90312	0.196617	1	-
ENSMUSC	Lmf1	lipase ma	GO:00081	metabolic	-	-	-	-	17:255791	182	207	292	280	229	254	0.747635	0.712756	0.826326	0.749302	0.824888	1.293712	0.762239	0.955967	0.288316	1	-
ENSMUSC	Narfl	nuclear pr	GO:00325	developm	-	-	-	-	17:257737	389	444	540	569	453	368	3.634542	3.477243	3.475717	3.463328	3.711418	4.263181	3.529167	3.812643	0.797432	1	-
ENSMUSC	Angptl4	angiopoie	GO:00325	developm	K08767	angiopoie	mmu0332	PPAR sign	17:337737	33	52	66	47	38	49	0.40231	0.531374	0.554299	0.373273	0.406234	0.740674	0.495994	0.506727	0.873319	1	-
ENSMUSC	Dbf4	DBF4 zinc	GO:00081	metabolic	K06629	activator c	mmu0411	Cell cycle	5:8396973	27	29	41	32	18	27	0.135313	0.121823	0.141554	0.104475	0.079102	0.167778	0.132897	0.117119	0.461512	1	-
ENSMUSC	Daxx	Fas death	GO:00081	metabolic	K02308	death-ass	mmu0401	MAPK sigi	17:339094	324	394	385	420	350	283	3.443339	3.509815	2.818691	2.907807	3.261716	3.729126	3.257282	3.299549	0.834626	1	-
ENSMUSC	Cd320	CD320 an	GO:00081	metabolic	-	-	-	-	17:338430	129	136	167	164	126	173	2.430578	2.147895	2.16764	2.013004	2.081778	4.041598	2.248705	2.712127	0.510062	1	-
ENSMUSC	Ipo4	importin 4	GO:00511	localizatio	-	-	-	-	14:556254	1082	969	1234	980	1033	1017	7.986566	5.99528	6.274796	4.712385	6.68615	9.307648	6.752214	6.902061	0.844406	1	-
ENSMUSC	Tm9sf1	transmem	GO:00081	metabolic	-	-	-	-	14:556359	272	207	243	283	235	355	1.374119	0.876554	0.845694	0.931374	1.041037	2.223665	1.032123	1.398692	0.254447	1	-
ENSMUSC	Rec8	REC8 mei	GO:00325	developm	K13054	meiotic re	mmu0411	Oocyte m	14:556180	23	41	36	27	52	27	0.370992	0.554341	0.400035	0.283712	0.735506	0.539999	0.441789	0.519739	0.690607	1	-
ENSMUSC	Gmpr2	guanosine	GO:00325	developm	K00364	GMP redu	mmu0023	Purine me	14:556719	252	315	406	456	324	251	2.517344	2.637581	2.793961	2.967488	2.838119	3.108865	2.649629	2.971491	0.585274	1	-
ENSMUSC	Mdp1	magnesiu	GO:00081	metabolic	-	-	-	-	14:556578	474	666	744	692	561	536	7.859156	9.256024	8.498091	7.474554	8.156486	11.01915	8.537757	8.883398	0.958079	1	-
ENSMUSC	Dhrs1	dehydrog	GO:00081	metabolic	-	-	-	-	14:557390	646	831	782	987	698	558	11.49364	12.39306	9.584797	11.43994	10.88987	12.30962	11.15717	11.54647	0.966281	1	-
ENSMUSC	Ncan	neurocan	GO:00071	cell adhes	-	-	-	-	8:7009308	5028	5753	6649	8071	5476	4428	44.41203	42.5944	40.45881	46.44236	42.41421	48.49522	42.48841	45.78393	0.762449	1	-
ENSMUSC	Tmem161	transmem	GO:00081	metabolic	-	-	-	-	8:7017235	325	383	445	485	249	392	1.91407	1.890713	1.80545	1.860793	1.285928	2.862509	1.870078	2.003077	0.937162	1	-
ENSMUSC	Armc6	armadillo	GO:00325	developm	-	-	-	-	8:7022017	306	347	346	397	354	336	3.175576	3.018448	2.473595	2.683939	3.221417	4.323414	2.889206	3.40959	0.450005	1	-
ENSMUSC	Borcs8	BLOC-1 re	GO:00325	developm	-	-	-	-	8:7013949	253	274	333	366	341	187	6.169958	5.600992	5.59445	5.814656	7.292204	5.654434	5.788467	6.253765	0.781256	1	-
ENSMUSC	Slc25a42	solute car	GO:00511	localizatio	-	-	-	-	8:7018434	461	612	515	581	455	501	3.275339	3.644684	2.520662	2.68914	2.83472	4.413462	3.146895	3.312441	1	1	-
ENSMUSC	Snx9	sorting ne	GO:00081	metabolic	-	-	-	-	17:584132	257	265	384	385	285	195	6.774415	5.855152	6.973025	6.611218	6.587596	6.373226	6.534197	6.524013	0.801747	1	-
ENSMUSC	Ranbp3	RAN bind	GO:00081	metabolic	K15304	Ran-bindi	mmu0516	HTLV-I inf	17:566732	1209	1400	1683	1712	1312	1140	30.5996	29.70095	29.34439	28.22766	29.1183	35.77505	29.88165	31.04034	0.9632	1	-
ENSMUSC	Ndufa11	NADH de	GO:00160	membran	K03956|K(	NADH de	mmu0110	Metabolic	17:567177	387	509	613	496	380	682	9.242773	10.1897	10.08563	7.717105	7.958251	20.19584	9.83937	11.95706	0.520044	1	-
ENSMUSC	Bmp8b	bone mor	GO:00325	developm	K16622	bone mor	mmu0439	Hippo sig	4:1231051	2	7	4	1	2	0	0.032766	0.096103	0.045141	0.010669	0.028724	0	0.058003	0.013131	0.047072	1	-
ENSMUSC	Nr2f6	nuclear re	GO:00325	developm	-	-	-	-	8:7137412	233	294	406	418	280	296	2.377624	2.514708	2.854075	2.778724	2.505462	3.745109	2.582136	3.009765	0.469002	1	-
ENSMUSC	Use1	unconven	GO:00511	localizatio	K08507	unconven	mmu0413	SNARE int	8:7136684	393	523	593	680	547	464	4.569402	5.097075	4.749768	5.150591	5.576951	6.689147	4.805415	5.805563	0.271544	1	-
ENSMUSC	Ocel1	occludin/E	-	-	-	-	-	-	8:7137129	257	304	340	419	369	217	1.739601	1.72482	1.585432	1.847618	2.19021	1.821224	1.683284	1.953017	0.437677	1	-
ENSMUSC	Dyrk1b	dual-spec	GO:00325	developm	-	-	-	-	7:2817946	371	397	502	573	460	264	6.914413	6.201905	6.445208	6.956934	7.517653	6.100577	6.520509	6.858388	0.906783	1	-
ENSMUSC	Braf	Braf trans	GO:00325	developm	K04365	B-Raf pro	mmu0520	Pathways	6:3960323	3696	4342	5414	6134	4231	2354	15.27852	15.04499	15.41768	16.51865	15.33681	12.0654	15.24706	14.64029	0.586787	1	-
ENSMUSC	Ndufb2	NADH de	GO:00081	metabolic	K03958	NADH de	mmu0110	Metabolic	6:3959257	380	537	674	614	463	548	3.393777	4.020014	4.146785	3.572316	3.625965	6.068297	3.853525	4.422193	0.575471	1	-
ENSMUSC	Hltf	helicase-l	GO:00081	metabolic	-	-	-	-	3:2005781	467	543	652	799	683	315	3.572799	3.482127	3.436301	3.982173	4.582002	2.988051	3.497076	3.850742	0.66406	1	-
ENSMUSC	Prpf6	pre-mRN.	GO:00081	metabolic	K12855	pre-mRN.	mmu0304	Spliceosor	2:1815918	1229	1453	1686	1788	1293	950	16.23839	16.09199	15.34617	15.39005	14.9807	15.56326	15.89218	15.31134	0.526562	1	-
ENSMUSC	Rgs19	regulator	GO:00987	molecular	-	-	-	-	2:1816884	221	256	291	280	188	164	2.173176	2.110058	1.971271	1.793665	1.62107	1.999544	2.084835	1.80476	0.205051	1	-
ENSMUSC	Rgs20	regulator	GO:00987	molecular	-	-	-	-	1:4909576	177	211	311	309	223	191	1.992717	1.991165	2.41204	2.266273	2.201506	2.666192	2.131974	2.37799	0.652261	1	-
ENSMUSC	Abhd3	abhydrola	GO:00081	metabolic	-	-	-	-	18:106444	488	801	1109	535	828	522	6.562396	9.028749	10.27367	4.686813	9.763722	8.703593	8.621605	7.718043	0.395801	1	-
ENSMUSC	Snrpd1	small nucl	GO:00081	metabolic	K11087	small nucl	mmu0532	Systemic l	18:106177	507	724	932	868	549	306	38.49628	46.07892	48.7504	42.93504	36.55324	28.80833	44.44187	36.09887	0.123543	1	-
ENSMUSC	Tchp	trichopleir	GO:00081	metabolic	-	-	-	-	5:1147077	161	220	310	266	237	151	1.098327	1.258007	1.456865	1.182141	1.417743	1.277231	1.271067	1.292372	0.897476	1	-
ENSMUSC	Tiam1	T cell lym	GO:00325	developm	K05731	T-lympho	mmu0401	Ras signal	16:897871	2037	2223	3096	3198	2759	2281	7.979872	7.299569	8.355208	8.161408	9.477621	11.0794	7.878216	9.572809	0.203103	1	-
ENSMUSC	Tsc2	tuberous	GO:00081	metabolic	K07207	tuberous	mmu0415	PI3K-Akt	17:245958	2344	2588	3025	3759	2640	2086	9.152071	8.469912	8.136516	9.561272	9.03875	10.09862	8.586166	9.566214	0.557202	1	-
ENSMUSC	Rpl3l	ribosomal	GO:00081	metabolic	K02925	large subu	mmu0301	Ribosome	17:247278	2	0	6	13	2	0	0.074828	0	0.154606	0.316795	0.065597	0	0.076478	0.127464	0.490418	1	-
ENSMUSC	Slc9a3r2	solute car	GO:00051	structural	K13358	Na(+)/H(+	mmu0496	Aldostero	17:246392	518	666	821	918	711	670	14.58592	15.71925	15.92573	16.83949	17.55564	23.39191	15.4103	19.26235	0.172756	1	-
ENSMUSC	Puf60	poly-U bi	GO:00081	metabolic	K12838	poly(U)-b	mmu0304	Spliceosor	15:760701	1906	2535	2834	2858	2221	1950	31.19548	34.77759	31.95361	30.47282	31.87572	39.57213	32.64223	33.97355	0.973875	1	-
ENSMUSC	Golga2	golgi autc	GO:00325	developm	-	-	-	-	2:3228738	2493	2897	3640	3974	2933	1718	14.1576	13.79014	14.24033	14.70201	14.60569	12.09696	14.06269	13.80155	0.680575	1	-
ENSMUSC	Uck1	uridine-cy	GO:00081	metabolic	K00876	uridine kir	mmu0110	Metabolic	2:3225500	498	543	729	648	632	492	7.070916	6.462473	7.130584	5.993807	7.86875	8.661584	6.887991	7.508047	0.762324	1	-
ENSMUSC	Scin	scinderin	GO:00325	developm	K05768	gelsolin[-]	mmu0520	Viral carci	12:400597	10	21	19	16	6	6	0.153957	0.270999	0.20151	0.160471	0.080995	0.114544	0.208822	0.11867	0.085262	1	-
ENSMUSC	Ikzf4	IKAROS fa	GO:00081	metabolic	-	-	-	-	10:128630	160	285	318	332	304	252	0.86261	1.287936	1.181066	1.166046	1.437183	1.684542	1.110537	1.429257	0.191583	1	-
ENSMUSC	Mien1	migration	GO:00400	locomotic	-	-	-	-	11:984377	311	375	480	407	371	459	26.45916	26.74236	28.13251	22.55755	27.67782	48.41876	27.11135	32.88471	0.423885	1	-
ENSMUSC	Pon1	paraoxon	GO:00081	metabolic	-	-	-	-	6:5168090	0	0	4	0	0	0	0	0	0.053808	0	0	0	0.017936	0	0.306145	1	-
ENSMUSC	Axl	AXL recep	GO:00325	developm	K05115	AXL recep	-	-	7:2575727	730	899	1138	1306	927	772	5.428067	5.603185	5.829298	6.326263	6.044287	7.117468	5.620184	6.496006	0.394331	1	-
ENSMUSC	Tgfb1	transform	GO:00325	developmental process|metabolic process|locomotion|localization|molecular function regulator|immune system process|response to stimulus|cellular process|reproductive process|extracellular region|cell|growth|reproduction|signaling|response to																						
ion|regula	K13375	transform	mmu0520	Pathways	7:2568700	103	108	111	147	84	81	1.146599	1.007748	0.851237	1.066044	0.819966	1.118008	1.001861	1.001339	0.000752	0.841839		1	-		
ENSMUSC	Ccdc97	coiled-co	GO:00329	macromo	-	-	-	-	7:2571110	695	809	970	1126	849	815	6.72595	6.56252	6.466842	7.09887	7.204763	9.77941	6.585104	8.027681	0.236919	1	-
ENSMUSC	Zfp40	zinc finge	GO:00081	metabolic	-	-	-	-	17:231738	326	432	509	535	387	219	2.501908	2.779011	2.691057	2.674783	2.604393	2.08393	2.657325	2.454369	0.45452	1	-
ENSMUSC	Akap8l	A kinase (	GO:00081	metabolic	K15978	A-kinase a	mmu0516	Epstein-B	17:323214	1552	2020	2313	2637	1910	1358	47.53459	51.85878	48.80284	52.61502	51.29724	51.5708	49.39874	51.82769	0.928333	1	-
ENSMUSC	Shh	sonic hed	GO:00325	developm	K11988	sonic hed	mmu0520	Pathways	5:2845681	19	29	44	47	18	38	0.424133	0.542617	0.676638	0.683483	0.352334	1.051777	0.547796	0.695865	0.53499	1	-
ENSMUSC	Pdcd2l	programn	GO:00099	cellular pr	-	-	-	-	7:3418449	69	110	108	122	94	126	1.923327	2.570075	2.073848	2.215356	2.297582	4.354707	2.189083	2.955882	0.233407	1	-
ENSMUSC	Gtf2f1	general tr	GO:00081	metabolic	K03138	transcripti	mmu0302	Basal tran	17:570034	1237	1517	1718	1709	1419	766	45.7863	47.06568	43.80669	41.20875	46.0565	35.15449	45.55289	40.80658	0.279844	1	-
ENSMUSC	Clpp	caseinolyt	GO:00081	metabolic	-	-	-	-	17:569902	437	525	650	636	496	492	23.00964	23.17079	23.57728	21.81561	22.90095	32.12034	23.25257	25.6123	0.723622	1	-
ENSMUSC	Alkbh7	alkB hom	GO:00081	metabolic	-	-	-	-	17:569973	97	125	149	125	91	106	3.004221	3.245052	3.179035	2.522027	2.471401	4.070541	3.14277	3.021323	0.604865	1	-
ENSMUSC	Dennd1c	DENN/MA	GO:00987	molecular	-	-	-	-	17:570660	14	20	29	31	21	10	0.353071	0.422785	0.503832	0.509306	0.464415	0.312712	0.426562	0.428811	1	1	-
ENSMUSC	Med6	mediator	GO:00325	developm	-	-	-	-	12:815735	119	162	153	193	150	101	2.663897	3.03976	2.359467	2.814555	2.944445	2.803342	2.687708	2.854114	0.88551	1	-
ENSMUSC	Prkd1	protein kir	GO:00325	developm	K06070	protein kir	mmu0401	Rap1 sign	12:503412	173	201	274	284	256	208	3.009769	2.931137	3.283893	3.218747	3.905425	4.486782	3.074933	3.870318	0.219186	1	-
ENSMUSC	Lcp2	lymphocy	GO:00081	metabolic	K07361	lymphocy	mmu0401	Rap1 sign	11:340469	76	84	105	110	91	45	0.915818	0.848453	0.871641	0.863514	0.961569	0.672343	0.878638	0.832475	0.679597	1	-
ENSMUSC	Cse1l	chromosc	GO:00511	localizatio	-	-	-	-	2:1669060	1039	1250	1399	1465	1214	600	8.272558	8.342329	7.673499	7.598778	8.475896	5.923272	8.096129	7.332649	0.358615	1	-
ENSMUSC	Naa20	N(alpha)-	GO:00081	metabolic	-	-	-	-	2:1459020	211	338	414	364	296	259	2.558614	3.435511	3.458391	2.875444	3.147437	3.894103	3.150839	3.305661	0.989696	1	-
ENSMUSC	Prkra	protein kir	GO:00325	developm	-	-	-	-	2:7662989	159	180	238	236	180	189	4.795894	4.550906	4.945382	4.637309	4.760883	7.06839	4.764061	5.488861	0.571708	1	-
ENSMUSC	Fkbp7	FK506 bin	GO:00081	metabolic	-	-	-	-	2:7666304	50	50	66	63	31	38	1.233571	1.033974	1.121723	1.012538	0.67064	1.162426	1.129756	0.948535	0.31474	1	-
ENSMUSC	Plekha3	pleckstrin	GO:00160	membran	-	-	-	-	2:7667528	881	1097	1370	1307	969	571	17.66252	18.43472	18.9212	17.07002	17.03505	14.19382	18.33948	16.09963	0.213193	1	-
ENSMUSC	Ykt6	YKT6 v-SN	GO:00511	localizatio	K08516	synaptobr	mmu0413	SNARE int	11:559569	1034	1368	1677	1721	1233	1334	21.02169	23.31234	23.48721	22.79343	21.98128	33.62707	22.60708	26.13393	0.456675	1	-
ENSMUSC	Baz1b	bromodo	GO:00081	metabolic	-	-	-	-	5:1351872	3281	4017	5171	5552	4275	2418	12.5093	12.83752	13.58165	13.78979	14.29241	11.43059	12.97616	13.17093	0.889004	1	-
ENSMUSC	Pex6	peroxison	GO:00511	localizatio	K13339	peroxin-6	mmu0414	Peroxisor	17:467114	805	837	1075	1251	863	1145	10.10272	8.804825	9.293995	10.22778	9.497211	17.81697	9.400512	12.51399	0.124406	1	-
ENSMUSC	Mrpl2	mitochon	GO:00081	metabolic	K02886	large subu	mmu0301	Ribosome	17:466462	207	262	309	409	315	240	4.833026	5.127467	4.970019	6.220897	6.449132	6.947754	4.976837	6.539261	0.093934	1	-
ENSMUSC	Mea1	male enha	GO:00325	developm	-	-	-	-	17:466811	595	762	903	814	640	617	45.01669	48.32417	47.0648	40.12016	42.45997	57.8799	46.80188	46.82001	0.724003	1	-
ENSMUSC	Gnmt	glycine N-	GO:00081	metabolic	K00552	glycine N-	mmu0026	Glycine, se	17:467256	4	7	11	3	0	7	0.160684	0.2357	0.304416	0.078501	0	0.348682	0.2336	0.142395	0.255991	1	-
ENSMUSC	Grin2d	glutamate	GO:00048	signal trar	K05212	glutamate	mmu0408	Neuroacti	7:4583188	230	277	314	308	234	258	1.54255	1.557196	1.450749	1.345686	1.376163	2.145443	1.516832	1.622431	0.92302	1	-
ENSMUSC	Kdelr1	KDEL (Lys	GO:00325	developm	-	-	-	-	7:4587277	408	328	451	705	386	390	6.116928	4.121922	4.658017	6.885624	5.074605	7.249752	4.965622	6.403327	0.175217	1	-
ENSMUSC	Tmem143	transmem	GO:00160	membran	-	-	-	-	7:4589694	184	289	300	309	268	234	0.833837	1.09778	0.936564	0.91223	1.064979	1.31482	0.95606	1.097343	0.548186	1	-
ENSMUSC	Ggct	gamma-c	GO:00508	response	K00682	gamma-c	mmu0048	Glutathior	6:5498258	237	298	345	368	301	193	1.657537	1.746966	1.662213	1.676662	1.845971	1.673632	1.688905	1.732088	0.947187	1	-
ENSMUSC	Jag2	jagged 2	GO:00325	developm	K06052	jagged[-]	mmu0520	Pathways	12:112907	533	657	752	874	715	667	3.67753	3.799682	3.574365	3.92846	4.325913	5.706125	3.683859	4.653499	0.157852	1	-
ENSMUSC	Btbd6	BTB (POZ	GO:00325	developm	-	-	-	-	12:112976	128	601	255	208	237	154	1.086375	4.275607	1.490943	1.150047	1.763848	1.620607	2.284308	1.511501	0.102296	1	-
ENSMUSC	Nudt14	nudix (nuc	GO:00038	catalytic a	-	-	-	-	12:112934	36	45	60	75	39	40	0.645382	0.676213	0.741011	0.87592	0.613095	0.88913	0.687536	0.792715	0.624292	1	-
ENSMUSC	Epdr1	ependymi	GO:00055	extracellul	-	-	-	-	13:195917	1024	1653	1441	1921	1410	1423	15.82258	21.40933	15.33885	19.33688	19.10465	27.26266	17.52358	21.9014	0.197245	1	-
ENSMUSC	Flii	flightless l	GO:00325	developm	-	-	-	-	11:607141	1128	1290	1654	1728	1293	962	12.16078	11.65722	12.28399	12.13606	12.22344	12.85918	12.034	12.40623	0.944931	1	-
ENSMUSC	Top3a	topoisom	GO:00081	metabolic	K03165	DNA topc	mmu0346	Fanconi a	11:607400	102	120	152	171	139	117	0.875886	0.863735	0.899171	0.956584	1.046657	1.245707	0.879597	1.082983	0.311351	1	-
ENSMUSC	Atg4d	autophag	GO:00081	metabolic	-	-	mmu0414	Autophag	9:2126529	219	226	363	334	227	381	1.456021	1.259463	1.662575	1.44661	1.323402	3.14076	1.459353	1.970257	0.222954	1	-
ENSMUSC	Qtrt1	queuine t	GO:00081	metabolic	-	-	-	-	9:2141183	99	123	131	159	126	103	1.401589	1.459637	1.277646	1.466447	1.564232	1.808049	1.379624	1.612909	0.494677	1	-
ENSMUSC	Plin4	perilipin 4	GO:00160	membran	K20254	perilipin-4	mmu0332	PPAR sign	17:561005	76	53	110	93	87	68	0.523296	0.305891	0.521771	0.417156	0.525289	0.580535	0.450319	0.50766	0.722393	1	-
ENSMUSC	Hdgfl2	HDGF like	GO:00099	cellular pr	-	-	-	-	17:560796	1405	1763	1994	2107	1740	1049	9.004841	9.471193	8.80392	8.797228	9.778932	8.336065	9.093318	8.970742	0.695006	1	-
ENSMUSC	Chaf1a	chromatir	GO:00081	metabolic	-	-	-	-	17:560404	80	86	103	134	99	74	1.536949	1.384909	1.363199	1.677088	1.667825	1.76275	1.428352	1.702555	0.41912	1	-
ENSMUSC	Adprh	ADP-ribo	GO:00081	metabolic	-	-	-	-	16:384453	853	1000	1112	1102	924	1060	34.24555	33.65179	30.7547	28.82163	32.52893	52.76515	32.88401	38.03857	0.5271	1	-
ENSMUSC	Tmem39a	transmem	GO:00160	membran	-	-	-	-	16:385586	112	136	161	151	134	102	0.89726	0.913255	0.888538	0.788059	0.941338	1.013179	0.899685	0.914192	0.872455	1	-
ENSMUSC	Timmdc1	translocas	GO:00160	membran	-	-	-	-	16:384983	258	304	364	427	296	293	7.864115	7.767069	7.643341	8.478896	7.911615	11.07348	7.758175	9.154664	0.399881	1	-
ENSMUSC	Pla1a	phospholi	GO:00081	metabolic	K13618	phospholi	mmu0401	Ras signal	16:383961	6	13	30	7	15	26	0.193374	0.351187	0.666035	0.146965	0.423896	1.038955	0.403532	0.536605	0.711344	1	-
ENSMUSC	Mcm2	minichron	GO:00325	developm	K02540	DNA repli	mmu0411	Cell cycle	6:8888347	101	118	173	160	134	100	0.717191	0.702343	0.846271	0.740143	0.834372	0.880437	0.755268	0.818317	0.820714	1	-
ENSMUSC	Tpra1	transmem	GO:00325	developm	-	-	-	-	6:8890225	142	185	202	197	158	146	0.821306	0.896897	0.804858	0.742274	0.801341	1.047025	0.84102	0.863547	0.909852	1	-
ENSMUSC	Nab1	Ngfi-A bi	GO:00325	developm	-	-	-	-	1:5245729	672	745	1023	1037	696	419	7.306691	6.789862	7.662644	7.34534	6.635957	5.648734	7.253066	6.543344	0.333353	1	-
ENSMUSC	Adgre5	adhesion	GO:00048	signal trar	-	-	-	-	8:8372325	135	139	216	208	171	127	1.388746	1.198552	1.530714	1.393906	1.542506	1.619862	1.372671	1.518758	0.71375	1	-
ENSMUSC	Il17ra	interleukir	GO:00048	signal trar	K05164	interleukir	mmu0406	Cytokine-	6:1204632	178	173	230	252	199	182	0.971026	0.791058	0.86435	0.895557	0.951931	1.231028	0.875478	1.026172	0.474168	1	-
ENSMUSC	Lamb1	laminin B1	GO:00325	developm	K05636	laminin, b	mmu0520	Pathways	12:312652	964	1087	1234	1305	1037	735	5.01146	4.736632	4.419304	4.419564	4.727252	4.737624	4.722465	4.628147	0.631497	1	-
ENSMUSC	Kcnn1	potassium	GO:00511	localizatio	-	-	-	-	8:7084204	252	256	339	355	334	247	1.843599	1.569852	1.708508	1.691906	2.142668	2.240521	1.70732	2.025032	0.379539	1	-
ENSMUSC	Arrdc2	arrestin do	GO:00160	membran	-	-	-	-	8:7083512	84	120	167	172	133	101	0.776386	0.929684	1.063329	1.035642	1.077939	1.157459	0.923133	1.090347	0.456882	1	-
ENSMUSC	Ppp1r17	protein pl	GO:00081	metabolic	K08067	protein pl	mmu0473	Long-terr	6:5601749	0	7	2	3	3	0	0	0.212224	0.049842	0.070683	0.095147	0	0.087355	0.055277	0.550728	1	-
ENSMUSC	Cd36	CD36 mol	GO:00325	developm	K06259	CD36 anti	mmu0414	Phagoson	5:1778169	104	141	228	270	115	171	0.791747	0.899759	1.195747	1.339057	0.767706	1.61412	0.962418	1.240294	0.298354	1	-
ENSMUSC	Map2k7	mitogen-	GO:00048	signal trar	K04431	mitogen-a	mmu0401	MAPK sigi	8:4238740	676	708	626	797	534	247	4.655593	4.087095	2.969986	3.57576	3.224874	2.109168	3.904225	2.969934	0.095318	1	-
ENSMUSC	Timm44	translocas	GO:00511	localizatio	-	-	-	-	8:4259731	555	734	827	923	665	537	5.400695	5.986945	5.543875	5.851128	5.674414	6.479129	5.643838	6.001557	0.870033	1	-
ENSMUSC	Ap2a2	adaptor-r	GO:00325	developm	K11824	AP-2 com	mmu0414	Endocytos	7:1415621	4997	5521	6407	7085	5288	4904	51.49576	47.6906	45.48501	47.56458	47.78551	62.66119	48.22379	52.67043	0.716892	1	-
ENSMUSC	Pnkp	polynucle	GO:00081	metabolic	-	-	-	-	7:4485713	217	243	272	390	219	204	1.625917	1.526151	1.403972	1.903635	1.438882	1.895192	1.51868	1.745903	0.51566	1	-
ENSMUSC	Med25	mediator	GO:00081	metabolic	-	-	-	-	7:4487676	939	1101	1342	1608	1201	1030	5.384972	5.292468	5.301784	6.007387	6.039535	7.323878	5.326408	6.456934	0.211554	1	-
ENSMUSC	Bcam	basal cell	GO:00055	extracellul	-	-	-	-	7:1975613	166	211	342	295	202	241	2.798352	2.981466	3.971658	3.239651	2.985988	5.037308	3.250492	3.754315	0.583295	1	-
ENSMUSC	Clptm1	cleft lip ar	GO:00325	developm	-	-	-	-	7:1963157	1813	2222	2618	2727	2232	1986	10.07006	10.34501	10.01741	9.86736	10.87104	13.67727	10.14416	11.47189	0.512939	1	-
ENSMUSC	Relb	avian retic	GO:00325	developm	K09253	transcripti	mmu0401	MAPK sigi	7:1960621	104	142	140	124	109	123	1.106007	1.265807	1.025664	0.85907	1.016468	1.621873	1.132493	1.165804	0.894943	1	-
ENSMUSC	Tomm40	translocas	GO:00511	localizatio	K11518	mitochon	mmu0501	Amyotrop	7:1970131	630	847	1052	1042	855	826	6.313213	7.114533	7.262348	6.802355	7.513091	10.26304	6.896698	8.192829	0.348354	1	-
ENSMUSC	Apoe	apolipopr	GO:00325	developm	K04524	apolipopr	mmu0501	Alzheimer	7:1969610	13370	17164	21976	25791	16432	13813	130.5028	140.43	147.7708	163.9979	140.6441	167.1716	139.5678	157.2712	0.475725	1	-
ENSMUSC	Apoc2	apolipopr	GO:00081	metabolic	-	-	mmu0497	Cholestero	7:1967157	3	3	6	8	2	4	0.156131	0.13088	0.215131	0.271263	0.091277	0.258146	0.167381	0.206895	0.732877	1	-
ENSMUSC	Hbp1	high mob	GO:00056	cell|intrace	-	-	-	-	12:319262	329	370	502	509	370	277	2.721455	2.56543	2.860622	2.742867	2.683796	2.841002	2.715836	2.755889	0.864533	1	-
ENSMUSC	Prkar2b	protein kir	GO:00081	metabolic	K04739	cAMP-de	mmu0491	Insulin sig	12:319584	409	723	470	799	658	469	3.976922	5.892715	3.148281	5.061186	5.610384	5.654353	4.339306	5.441974	0.254296	1	-
ENSMUSC	Cdkn1b	cyclin-dep	GO:00325	developm	K06624	cyclin-dep	mmu0520	Pathways	6:1349204	780	1046	1146	1410	623	727	6.539759	7.351085	6.619164	7.701362	4.580336	7.55766	6.836669	6.613119	0.614691	1	-
ENSMUSC	Klf4	Kruppel-li	GO:00325	developm	K17846	krueppel-	mmu0455	Signaling	4:5552714	68	116	137	98	95	61	0.979959	1.401232	1.360098	0.92004	1.200502	1.08996	1.247096	1.070167	0.294849	1	-
ENSMUSC	Ap1m1	adaptor-r	GO:00325	developm	K12393	AP-1 com	mmu0414	Lysosome	8:7224001	1066	1164	1482	1624	1289	849	13.14975	12.03556	12.59389	13.05052	13.94299	12.98536	12.59307	13.32629	0.873452	1	-
ENSMUSC	Rab8a	RAB8A, m	GO:00325	developm	K07901	Ras-relate	mmu0414	Endocytos	8:7216120	718	792	1047	989	743	500	9.097107	8.411175	9.138536	8.163126	8.254872	7.854793	8.882273	8.090931	0.310435	1	-
ENSMUSC	Fam32a	family wit	GO:00099	cellular pr	-	-	-	-	8:7221973	1239	1492	1611	1754	1258	1156	13.23175	13.35573	11.85204	12.20274	11.78066	15.307	12.81318	13.0968	0.853989	1	-
ENSMUSC	Elf3	E74-like f	GO:00325	developm	-	-	-	-	1:1352535	1	4	1	1	1	5	0.018361	0.061546	0.012641	0.011957	0.016096	0.1138	0.03085	0.047284	0.835205	1	-
ENSMUSC	Stard3nl	STARD3 N	GO:00511	localizatio	-	-	-	-	13:193576	205	238	318	334	247	491	1.685647	1.64037	1.80132	1.789125	1.780954	5.005883	1.709112	2.858654	0.042813	1	-
ENSMUSC	Stk11	serine/thr	GO:00325	developm	K07298	serine/thr	mmu0415	PI3K-Akt	10:801158	697	873	1022	1016	757	808	5.342071	5.608465	5.396091	5.072849	5.087631	7.678458	5.448876	5.946313	0.777775	1	-
ENSMUSC	Efna2	ephrin A2	GO:00325	developm	K05462	ephrin-A[	mmu0415	PI3K-Akt	10:801794	149	174	181	206	183	103	4.443647	4.349647	3.718618	4.002232	4.785713	3.808696	4.170637	4.19888	0.880906	1	-
ENSMUSC	Atp5d	ATP synth	GO:00081	metabolic	K02134	F-type H+	mmu0110	Metabolic	10:801386	1525	1711	1787	1910	1685	1983	26.93673	25.33249	21.74457	21.97809	26.09862	43.42938	24.67127	30.50203	0.301381	1	-
ENSMUSC	Ppp5c	protein pl	GO:00048	signal trar	K04460	serine/thr	mmu0401	MAPK sigi	7:1700464	1330	1842	2129	2130	1696	1324	14.59601	16.94435	16.09569	15.22803	16.32116	18.01592	15.87868	16.52171	0.993051	1	-
ENSMUSC	Cdk12	cyclin-dep	GO:00325	developm	-	-	-	-	11:982030	710	733	936	1081	824	437	2.677583	2.317082	2.431711	2.65578	2.724926	2.043394	2.475458	2.4747	0.830131	1	-
ENSMUSC	Lipe	lipase, ho	GO:00081	metabolic	K07188	hormone-	mmu0402	cAMP sigr	7:2537952	150	120	185	168	135	135	0.98634	0.661408	0.838026	0.719657	0.778413	1.100662	0.828591	0.866244	0.989709	1	-
ENSMUSC	Pafah1b2	platelet-a	GO:00325	developm	K16795	platelet-a	mmu0110	Metabolic	9:4596285	3674	4353	5402	5432	3974	3229	23.26562	23.10558	23.56576	22.40871	22.06714	25.353	23.31232	23.27628	0.703914	1	-
ENSMUSC	Tbc1d8	TBC1 dor	GO:00511	localizatio	-	-	-	-	1:3937149	193	197	201	331	224	188	1.739084	1.487923	1.247698	1.942997	1.769917	2.100416	1.491568	1.937776	0.159426	1	-
ENSMUSC	Cnot11	CCR4-NC	GO:00081	metabolic	-	-	-	-	1:3953499	490	525	751	751	518	368	4.017151	3.607733	4.241443	4.010918	3.723869	3.740733	3.955442	3.825174	0.604712	1	-
ENSMUSC	Slc2a3	solute car	GO:00511	localizatio	-	-	-	-	6:1227278	1698	2179	2690	2489	2129	2386	9.878524	10.62587	10.78098	9.433241	10.86107	17.21119	10.42846	12.50183	0.35088	1	-
ENSMUSC	Foxj2	forkhead	GO:00325	developm	-	-	-	-	6:1228199	852	1002	1129	1263	924	680	6.383777	6.29301	5.827512	6.164852	6.07089	6.317317	6.1681	6.184353	0.772873	1	-
ENSMUSC	Sri	sorcin [So	GO:00325	developm	-	-	-	-	5:8046078	389	478	629	596	481	458	3.405724	3.507843	3.793686	3.399281	3.692729	4.971764	3.569085	4.021258	0.602526	1	-
ENSMUSC	Dgcr2	DiGeorge	GO:00160	membran	-	-	-	-	16:178394	1569	944	2318	1789	1964	2065	6.539523	3.29798	6.655614	4.857528	7.178064	10.67159	5.497706	7.569061	0.172212	1	-
ENSMUSC	Irf3	interferon	GO:00081	metabolic	K05411	interferon	mmu0520	Viral carci	7:4499764	256	253	397	413	352	311	1.940547	1.607526	2.07313	2.039464	2.339755	2.923014	1.873734	2.434078	0.145589	1	-
ENSMUSC	Bcl2l12	BCL2-like	GO:00325	developm	-	-	-	-	7:4499122	18	13	33	20	21	13	0.321783	0.194807	0.406402	0.232925	0.329205	0.288148	0.307664	0.283426	0.704951	1	-
ENSMUSC	Zfp959	zinc finge	GO:00081	metabolic	-	-	-	-	17:558920	66	86	13	82	10	46	1.289818	1.408758	0.175018	1.043958	0.171371	1.114638	0.957865	0.776656	0.579267	1	-
ENSMUSC	Mpnd	MPN dom	GO:00081	metabolic	-	-	-	-	17:560057	461	811	850	961	599	763	7.007393	10.33308	8.900748	9.51614	7.984096	14.38025	8.747074	10.62683	0.357814	1	-
ENSMUSC	Sh3gl1	SH3-dom	GO:00452	synapse|lc	K11247	endophilir	mmu0414	Endocytos	17:560167	630	703	928	965	753	677	19.32355	18.07402	19.60856	19.28215	20.25274	25.74665	19.00204	21.76051	0.479365	1	-
ENSMUSC	Ebi3	Epstein-B	GO:00048	signal trar	-	-	-	-	17:559526	10	6	13	16	6	18	0.543193	0.273171	0.486461	0.566174	0.285767	1.212331	0.434275	0.688091	0.344044	1	-

ENSMUS(	Ccdc94	coiled-co	-	-	-	-	-	-	17:559591	111	162	182	201	141	86	5.38093	6.582668	6.077942	6.347623	5.993714	5.169159	6.013847	5.836832	0.723415	1	-
ENSMUS(	Ranbp2	RAN bind	GO:00081	metabolic	K12172	E3 SUMO	mmu0301	RNA trans	10:584469	4096	4550	5651	6236	4438	2178	20.79843	19.36577	19.7673	20.62806	19.76062	13.71243	19.97717	18.0337	0.356043	1	-
ENSMUS(	Edar	ectodyspl	GO:00325	developm	K05162	tumor nec	mmu0406	Cytokine-	10:586007	6	5	3	2	4	3	0.104848	0.073231	0.036112	0.022764	0.061286	0.065004	0.071397	0.049685	0.482405	1	-
ENSMUS(	Grk5	G protein-	GO:00325	developm	K08291	G protein-	mmu0414	Endocytos	19:608897	144	140	259	235	164	131	2.891515	2.356374	3.582726	3.074052	2.887671	3.261518	2.943538	3.074414	0.99066	1	-
ENSMUS(	Dvl3	dishevelle	GO:00325	developm	K02353	segment p	mmu0520	Pathways	16:205169	786	814	1056	1168	898	522	6.957193	6.039333	6.43913	6.734977	6.969964	5.728855	6.478552	6.477932	0.807422	1	-
ENSMUS(	Abcf3	ATP-bind	GO:00023	immune s	-	-	-	-	16:205485	1020	1317	1524	1433	1150	870	14.00693	15.15938	14.41715	12.81948	13.84787	14.81317	14.52782	13.82684	0.452614	1	-
ENSMUS(	Eif2b5	eukaryotic	GO:00325	developm	K03240	translation	mmu0301	RNA trans	16:204988	977	1106	1405	1446	1034	793	17.02997	16.15948	16.87125	16.41986	15.80457	17.13872	16.6869	16.45438	0.657787	1	-
ENSMUS(	Cyth2	cytohesin	GO:00987	molecular	K18441	cytohesin[	mmu0414	Endocytos	7:4580663	1164	1319	1612	1687	1314	834	12.56808	11.9375	11.99036	11.86622	12.44096	11.16524	12.16531	11.82414	0.607066	1	-
ENSMUS(	Sult2b1	sulfotrans	GO:00325	developm	K01015	alcohol su	mmu0014	Steroid hc	7:4572998	99	157	134	170	92	89	0.911053	1.211054	0.849507	1.019155	0.742404	1.015508	0.990538	0.925689	0.555997	1	-
ENSMUS(	Car11	carbonic a	GO:00055	extracellul	-	-	-	-	7:4569984	1616	1832	2120	2210	1927	1006	33.97342	32.28318	30.70331	30.26717	35.52405	26.22297	32.31997	30.6714	0.525	1	-
ENSMUS(	Dlgap1	discs, larg	GO:00452	synapse|c	K15008	discs, larg	mmu0472	Glutamate	17:699690	5903	10979	13031	9789	10923	6138	11.56382	18.02786	17.58562	12.49247	18.76347	14.90875	15.72577	15.38823	0.698822	1	-
ENSMUS(	Plag1	pleiomorp	GO:00325	developm	-	-	-	-	4:3900996	68	75	79	111	39	37	0.453	0.418798	0.362549	0.481716	0.227822	0.305613	0.411449	0.338384	0.341715	1	-
ENSMUS(	Hck	hemopoie	GO:00325	developm	K08893	hemopoie	mmu0516	Kaposi's s	2:1531084	23	37	40	56	41	30	0.51288	0.69159	0.614471	0.813503	0.801709	0.829479	0.606313	0.814897	0.237255	1	-
ENSMUS(	Mrpl4	mitochon	GO:00081	metabolic	K02926	large subu	mmu0301	Ribosome	9:2100273	369	460	520	545	436	420	5.031341	5.25736	4.884409	4.841005	5.212979	7.100561	5.057703	5.718182	0.592532	1	-
ENSMUS(	Keap1	kelch-like	GO:00325	developm	K10456	kelch-like	mmu0520	Pathways	9:2122973	1055	1315	1112	1613	879	995	7.235181	7.559196	5.253555	7.206311	5.286017	8.460691	6.682644	6.98434	0.991442	1	-
ENSMUS(	Ap1m2	adaptor p	GO:00511	localizatio	K12393	AP-1 com	mmu0414	Lysosome	9:2129427	4	5	14	9	5	16	0.04554	0.047714	0.109804	0.066752	0.049914	0.225866	0.067686	0.114178	0.383148	1	-
ENSMUS(	Glg1	golgi app	GO:00325	developm	K06816	golgi app	mmu0451	Cell adhes	8:1111544	6061	6743	7827	9599	6886	4496	42.69979	39.81874	37.9864	44.05438	42.53938	39.27297	40.16831	41.95557	0.947939	1	-
ENSMUS(	Atp8b3	ATPase, cl	GO:00511	localizatio	-	-	-	-	10:805195	2	5	13	10	5	2	0.022363	0.04685	0.100116	0.072824	0.04901	0.027729	0.056443	0.049854	0.896563	1	-
ENSMUS(	Btbd2	BTB (POZ	GO:00325	developm	-	-	-	-	10:806426	1000	1194	1399	1515	1225	1190	11.19482	11.20403	10.78913	11.04871	12.0253	16.51772	11.06266	13.19724	0.312675	1	-
ENSMUS(	Csnk1g2	casein kin	GO:00325	developm	K08958	casein kin	mmu0434	Hedgehog	10:806228	1753	2034	2577	2597	2090	1547	13.41301	13.04512	13.58345	12.94487	14.02275	14.67643	13.34719	13.88135	0.996345	1	-
ENSMUS(	Mob3a	MOB kina	GO:00056	cell|intrace	-	-	-	-	10:806852	163	184	216	198	120	136	2.553401	2.41603	2.330971	2.020593	1.648369	2.641528	2.433467	2.103497	0.237206	1	-
ENSMUS(	Cacnb3	calcium cl	GO:00511	localizatio	K04864	voltage-d	mmu0401	MAPK sigi	15:986322	3333	4097	4597	5047	3900	3628	67.89173	69.95203	64.50709	66.97249	69.66086	91.62939	67.45028	76.08758	0.522861	1	-
ENSMUS(	Ccdc65	coiled-co	GO:00511	localizatio	-	-	-	-	15:987082	99	111	160	159	138	100	3.328959	3.128592	3.706355	3.483006	4.069064	4.169265	3.387968	3.907112	0.540776	1	-
ENSMUS(	Fkbp11	FK506 bin	GO:00081	metabolic	-	-	-	-	15:987243	2	0	9	0	4	7	0.173915	0	0.539006	0	0.304922	0.754604	0.23764	0.353175	0.810221	1	-
ENSMUS(	Ddx23	DEAD (As	GO:00081	metabolic	K12858	ATP-depe	mmu0304	Spliceosor	15:986451	1224	1472	1752	1923	1490	1069	9.082184	9.155231	8.955592	9.295435	9.694772	9.834957	9.064336	9.608388	0.867787	1	-
ENSMUS(	Pld3	phospholi	GO:00081	metabolic	K16860	phospholi	mmu0110	Metabolic	7:2753200	5560	7033	7642	8568	6417	4599	76.89983	81.53501	72.81302	77.19901	77.82611	78.86785	77.08262	77.96432	0.81514	1	-
ENSMUS(	Grik5	glutamate	GO:00048	signal trar	K05205	glutamate	mmu0408	Neuroacti	7:2500984	3359	3953	4503	5091	3756	3375	31.05988	30.63864	28.68423	30.66723	30.45496	38.69452	30.12758	33.27224	0.632288	1	-
ENSMUS(	Cd79a	CD79A an	GO:00325	developm	K06506	CD79A an	mmu0466	B cell rece	7:2489738	0	3	1	3	5	3	0	0.109681	0.030038	0.085236	0.19123	0.162264	0.046573	0.146244	0.175159	1	-
ENSMUS(	Rabac1	Rab accep	GO:00452	synapse|m	-	-	-	-	7:2496975	404	532	734	580	421	1065	8.835304	9.752246	11.0583	8.263235	8.073563	28.8786	9.881948	15.0718	0.16155	1	-
ENSMUS(	Etv3	ets varian	GO:00325	developm	-	-	-	-	3:8752540	506	607	763	583	552	218	1.761493	1.771218	1.829813	1.322149	1.685048	0.940965	1.787508	1.316054	0.046323	1	-
ENSMUS(	Prkcsh	protein ki	GO:00325	developm	K08288	protein ki	mmu0414	Protein pr	9:2200280	1284	1546	1777	1820	1477	1343	15.65657	15.80135	14.92693	14.45722	15.79264	20.30457	15.46162	16.85148	0.741582	1	-
ENSMUS(	Elavl3	ELAV (em	GO:00325	developm	-	-	-	-	9:2201500	4277	5080	6419	6603	4864	4122	34.29858	34.14707	35.46138	34.49529	34.2037	40.98555	34.63568	36.56151	0.91574	1	-
ENSMUS(	Rab3b	RAB3B, m	GO:00081	metabolic	-	-	-	-	4:1088790	728	981	1154	1109	931	693	8.731187	9.861979	9.534536	8.664741	9.791171	10.30531	9.375901	9.587075	0.884678	1	-
ENSMUS(	St8sia6	ST8 alpha	GO:00081	metabolic	-	-	-	-	2:1365102	165	166	191	229	128	53	1.344215	1.133566	1.071939	1.215356	0.914407	0.535358	1.18324	0.888374	0.153005	1	-
ENSMUS(	Fcgrt	Fc recepto	GO:00048	signal trar	-	-	-	-	7:4509299	128	218	291	277	139	316	1.937312	2.76566	3.034132	2.73119	1.844799	5.930107	2.579035	3.502032	0.298616	1	-
ENSMUS(	Nosip	nitric oxid	GO:00325	developm	-	-	-	-	7:4506242	503	651	685	672	624	552	6.901379	7.486888	6.474548	6.006459	7.507486	9.390587	6.954272	7.634844	0.743057	1	-
ENSMUS(	Pih1d1	PIH1 dom	GO:00325	developm	-	-	-	-	7:4515430	196	263	312	328	206	261	3.456269	3.887412	3.790158	3.76796	3.185385	5.706613	3.71128	4.219986	0.635262	1	-
ENSMUS(	Rps11	ribosomal	GO:00325	developm	K02949	small subu	mmu0301	Ribosome	7:4512237	651	919	801	707	571	564	27.27293	32.27148	23.11716	19.29529	20.9763	29.2965	27.55386	23.18936	0.126189	1	-
ENSMUS(	Supt5	suppresso	GO:00081	metabolic	-	-	-	-	7:2831489	2556	3113	3477	3871	2972	2299	21.59839	22.04918	20.24028	21.30911	22.02176	24.08715	21.29595	22.47267	0.909192	1	-
ENSMUS(	Dll3	delta-like	GO:00325	developm	K06051	delta[-]	mmu0520	Pathways	7:2829355	19	30	38	25	20	18	0.314701	0.416497	0.433586	0.26975	0.290487	0.369671	0.388261	0.30997	0.243906	1	-
ENSMUS(	Paf1	Paf1, RNA	GO:00325	developm	-	-	-	-	7:2839295	1075	1343	1602	1638	1186	622	10.55616	11.05418	10.83706	10.47836	10.21233	7.573098	10.8158	9.421262	0.229477	1	-
ENSMUS(	Timm50	translocas	GO:00081	metabolic	-	-	-	-	7:2830551	235	310	358	388	388	383	4.119992	4.555572	4.323764	4.431403	5.964898	8.32554	4.333109	6.240614	0.04805	1	-
ENSMUS(	Med29	mediator	GO:00081	metabolic	-	-	-	-	7:2838614	151	145	199	220	150	89	7.491399	6.029871	6.801283	7.110341	6.525589	5.474707	6.774184	6.370212	0.599801	1	-
ENSMUS(	Bicd1	bicaudal [	GO:00511	localizatio	-	-	-	-	6:1494088	1177	1429	1715	1859	1385	596	2.203814	2.242764	2.212149	2.267565	2.274003	1.383665	2.219576	1.975078	0.364584	1	-
ENSMUS(	Ncstn	nicastrin [	GO:00081	metabolic	K06171	nicastrin[-	mmu0501	Alzheimer	1:1720660	887	1047	1298	1349	1032	859	11.63259	11.50939	11.72678	11.52515	11.86793	13.96792	11.62292	12.45366	0.825556	1	-
ENSMUS(	Pex19	peroxisom	GO:00511	localizatio	K13337	peroxin-1	mmu0414	Peroxisom	1:1721267	942	1220	1422	1427	1233	1258	9.910107	10.75821	10.30572	9.779868	11.37452	16.40944	10.32468	12.52128	0.288189	1	-
ENSMUS(	Phyhip	phytanoyl	GO:00055	protein bi	-	-	-	-	14:704574	10065	11826	13456	16051	11907	8753	212.4414	209.2262	195.6558	220.7034	220.3791	229.07	205.7745	223.3842	0.699163	1	-
ENSMUS(	Crhr2	corticotro	GO:00325	developm	K04579	corticotro	mmu0408	Neuroacti	6:5509004	42	36	36	41	23	32	0.317764	0.228305	0.187636	0.202078	0.152592	0.300187	0.244568	0.218286	0.528184	1	-
ENSMUS(	Inmt	indolethyl	GO:00081	metabolic	K00562	methyltrar	mmu0038	Tryptopha	6:5517062	17	8	28	20	6	24	0.54127	0.2135	0.614174	0.414855	0.167508	0.947484	0.456314	0.509949	1	1	-
ENSMUS(	Impdh1	inosine m	GO:00081	metabolic	K00088	IMP dehy(	mmu0110	Metabolic	6:2920043	474	653	749	686	604	406	4.038631	4.663601	4.396312	3.807688	4.512686	4.289112	4.366181	4.203162	0.564695	1	-
ENSMUS(	Dusp3	dual speci	GO:00325	developm	K17614	dual speci	mmu0401	MAPK sigi	11:101971	1547	1990	2493	2314	1766	1390	17.39192	18.75267	19.30771	16.94736	17.40967	19.37573	18.4841	17.91092	0.538494	1	-
ENSMUS(	Prodh	proline de	GO:00081	metabolic	-	-	-	-	16:180603	322	296	496	507	405	378	4.440993	3.421905	4.712562	4.555264	4.898031	6.463999	4.19182	5.305765	0.198597	1	-
ENSMUS(	Dgcr14	DiGeorge	GO:00329	macromo	-	-	-	-	16:179007	327	458	572	610	454	350	7.372048	8.654848	8.883583	8.958856	8.975115	9.783517	8.303493	9.239162	0.619776	1	-
ENSMUS(	Slc25a1	solute car	GO:00511	localizatio	-	-	-	-	16:179252	368	500	525	697	501	360	6.175736	7.033374	6.069485	7.620002	7.372608	7.490828	6.426199	7.494479	0.384828	1	-
ENSMUS(	Dgcr6	DiGeorge	-	-	-	-	-	-	16:180528	353	456	495	483	418	463	3.283696	3.555545	3.172084	2.926965	3.409631	5.340166	3.337108	3.892254	0.517921	1	-
ENSMUS(	Ddr1	discoidin	GO:00325	developm	-	-	-	-	17:356815	603	911	1240	1224	982	1152	5.672363	7.183199	8.035623	7.500831	8.100294	13.43646	6.963728	9.679196	0.06837	1	-
ENSMUS(	Ier3	immediat	GO:00508	response	-	-	-	-	17:358216	25	54	59	45	48	50	1.40481	2.543451	2.283887	1.647287	2.36516	3.483642	2.077383	2.498696	0.606187	1	-
ENSMUS(	Fosb	FBJ osteo	GO:00081	metabolic	K09029	protein Fo	mmu0503	Alcoholisr	7:1930269	77	167	232	189	190	176	0.296133	0.538349	0.614659	0.473519	0.640752	0.839252	0.483047	0.651174	0.222057	1	-
ENSMUS(	Klc4	kinesin lig	GO:00990	supramole	K10407	kinesin lig	mmu0513	Salmonell	17:466306	340	429	525	519	383	353	9.305787	9.842026	9.898854	9.253857	9.19211	11.97939	9.682223	10.14179	0.995791	1	-
ENSMUS(	Ercc1	excision re	GO:00325	developm	K10849	DNA excis	mmu0152	Platinum c	7:1934477	212	299	322	295	237	217	2.854505	3.374564	2.986769	2.587602	2.798241	3.62277	3.071946	3.002871	0.643767	1	-
ENSMUS(	As3mt	arsenic (+	GO:00081	metabolic	-	-	-	-	19:467074	141	105	200	196	132	90	5.117616	3.19442	5.000715	4.634329	4.201133	4.050236	4.437584	4.295233	0.72003	1	-
ENSMUS(	Homer3	homer sca	GO:00452	synapse|re	K15010	homer[-]	mmu0406	FoxO sign	8:7028282	960	1014	1016	1373	892	829	10.07945	8.923936	7.348698	9.391127	8.212456	10.79211	8.784027	9.46523	0.817528	1	-
ENSMUS(	Crtc1	CREB regu	GO:00325	developm	K15309	CREB-reg	mmu0516	HTLV-I inf	8:7038235	3941	4573	4931	6611	4933	4341	20.34791	19.79099	17.5388	22.23628	22.33403	27.79003	19.2259	24.12012	0.11906	1	-
ENSMUS(	Rnf215	ring finge	GO:00160	membran	-	-	-	-	11:413520	247	345	379	467	336	340	5.099928	5.970904	5.390856	6.281533	6.083451	8.704284	5.487229	7.023089	0.165181	1	-
ENSMUS(	Sec14l2	SEC14-lik	GO:00081	metabolic	-	-	-	-	11:409703	976	1146	1389	1568	1169	940	12.76817	12.56655	12.51793	13.36307	13.41022	15.24727	12.61755	14.00685	0.594465	1	-
ENSMUS(	Aven	apoptosis	GO:00099	cellular pr	-	-	-	-	2:1124929	71	81	126	111	92	92	0.878897	0.840463	1.074492	0.895125	0.998644	1.41206	0.931284	1.101943	0.526581	1	-
ENSMUS(	Cp	ceruloplas	GO:00081	metabolic	K13624	ceruloplas	mmu0421	Ferroptos	3:1995705	331	391	527	458	370	239	1.439348	1.425172	1.578698	1.297431	1.410852	1.288608	1.481072	1.332297	0.304684	1	-
ENSMUS(	Crot	carnitine (	GO:00081	metabolic	K05940	carnitine (	mmu0414	Peroxisom	5:8966033	419	514	619	654	529	404	4.59829	4.728233	4.67977	4.675652	5.090737	5.497311	4.668764	5.0879	0.731725	1	-
ENSMUS(	Rps6ka1	ribosomal	GO:00325	developm	K04373	ribosomal	mmu0401	MAPK sigi	4:1338472	200	227	264	271	187	188	1.913094	1.820059	1.739651	1.688719	1.568521	2.229716	1.824268	1.828985	0.778241	1	-
ENSMUS(	Calb2	calbindin	GO:00452	synapse|c	-	-	-	-	8:1101375	635	827	887	701	644	552	21.17322	23.11382	20.37457	15.22695	18.82963	22.82119	21.55387	18.95926	0.195361	1	-
ENSMUS(	Snrnp200	small nucl	GO:00325	developm	K12854	pre-mRN	mmu0304	Spliceosor	2:1272083	2507	3057	3719	4215	3089	2419	16.87612	17.2491	17.24629	18.48406	18.23386	20.19016	17.12383	18.96936	0.582044	1	-
ENSMUS(	Ciao1	cytosolic i	GO:00081	metabolic	-	-	-	-	2:1272409	763	973	1146	1209	1005	618	11.44734	12.23619	11.84449	11.81648	13.22174	11.4962	11.84267	12.17814	0.955351	1	-
ENSMUS(	Has1	hyalurona	GO:00081	metabolic	-	-	-	-	17:178433	8	12	8	5	3	7	0.243256	0.305872	0.167583	0.099054	0.079987	0.263931	0.238903	0.147657	0.191336	1	-
ENSMUS(	Taf6l	TATA-box	GO:00081	metabolic	K03131	transcripti	mmu0516	Herpes sir	19:877252	167	201	277	254	219	197	0.910783	0.918857	1.04071	0.902431	1.047336	1.332145	0.956784	1.093971	0.576967	1	-
ENSMUS(	Insig2	insulin ind	GO:00325	developm	-	-	-	-	1:1213043	613	678	914	896	671	672	5.149771	4.774297	5.289625	4.903621	4.94302	6.999748	5.071231	5.615463	0.682308	1	-
ENSMUS(	Kpna6	karyopher	GO:00081	metabolic	-	-	-	-	4:1296439	1804	2166	2796	2877	2090	1928	14.30438	14.39607	15.27289	14.86121	14.53187	18.95508	14.65778	16.11605	0.665725	1	-
ENSMUS(	Man1a	mannosid	GO:00081	metabolic	K01230	mannosyl	mmu0110	Metabolic	10:539047	1267	1278	1690	1848	1296	788	6.653036	5.625055	6.113376	6.321595	5.967479	5.130446	6.130489	5.806507	0.508858	1	-
ENSMUS(	Itpkc	inositol 1,4	GO:00081	metabolic	K00911	1D-myo-	mmu0110	Metabolic	7:2720717	137	140	181	197	169	132	1.179136	1.010011	1.073182	1.104565	1.275482	1.408652	1.087443	1.262899	0.507284	1	-
ENSMUS(	Coq8b	coenzyme	GO:00325	developm	-	-	-	-	7:2723302	349	447	496	504	423	302	2.536597	2.72324	2.483473	2.386371	2.695931	2.721566	2.581103	2.60129	0.81731	1	-
ENSMUS(	Brd8	bromodo	GO:00081	metabolic	-	-	-	-	18:345986	1006	1207	1479	1674	1228	829	5.124995	5.154135	5.190573	5.555623	5.485756	5.236442	5.156568	5.42594	0.904442	1	-
ENSMUS(	Kif20a	kinesin fa	GO:00511	localizatio	-	-	-	-	18:346246	18	17	28	28	16	22	0.301354	0.23857	0.322943	0.30539	0.234891	0.456697	0.287622	0.332326	0.759626	1	-
ENSMUS(	Farsa	phenylala	GO:00081	metabolic	K01889	phenylala	mmu0097	Aminoacy	8:8485698	397	537	620	708	456	473	3.798066	4.30625	4.086159	4.412523	3.825425	5.610725	4.063492	4.616224	0.552902	1	-
ENSMUS(	Gcdh	glutaryl-C	GO:00081	metabolic	K00252	glutaryl-C	mmu0110	Metabolic	8:8488639	336	439	514	572	424	392	5.31719	5.823174	5.603464	5.89685	5.883702	7.691569	5.581276	6.490707	0.434263	1	-
ENSMUS(	Mast2	microtubu	GO:00081	metabolic	-	-	-	-	4:1163067	1791	1916	2500	2623	2026	1460	7.184463	6.442392	6.908602	6.854544	7.126574	7.261685	6.845152	7.080934	0.967657	1	-
ENSMUS(	Dnase2a	deoxyribo	GO:00325	developm	K01158	deoxyribo	mmu0414	Lysosome	8:8490856	48	39	75	91	52	61	0.326856	0.222604	0.351824	0.403682	0.3105	0.515025	0.300428	0.409735	0.220652	1	-
ENSMUS(	Rad23a	RAD23 hc	GO:00081	metabolic	K10839	UV excisic	mmu0414	Protein pr	8:8483401	767	989	1028	1173	874	700	10.44017	11.28395	9.639551	10.4014	10.43196	11.81398	10.45456	10.88245	0.996338	1	-
ENSMUS(	Calr	calreticulir	GO:00325	developm	K08057	calreticulir	mmu0516	HTLV-I inf	8:8484185	7399	9539	12228	11270	9163	5754	110.6681	119.5928	125.9958	109.8133	120.1793	106.71	118.7523	112.2342	0.436303	1	-
ENSMUS(	Syce2	synaptone	GO:00099	cellular pr	-	-	-	-	8:8487211	75	62	71	84	42	50	1.602171	1.110181	1.044864	1.16899	0.786754	1.324369	1.252405	1.093371	0.404977	1	-
ENSMUS(	Nob1	NIN1/RPN	GO:00081	metabolic	K11883	RNA-binc	mmu0300	Ribosome	8:1074124	312	303	393	457	399	332	3.359065	2.734384	2.914793	3.205244	3.766852	4.431881	3.002747	3.801325	0.181635	1	-
ENSMUS(	Nqo1	NAD(P)H	GO:00325	developm	K00355	NAD(P)H	mmu0520	Pathways	8:1073882	58	66	103	61	59	56	2.373525	2.263917	2.903711	1.626211	2.11717	2.841436	2.513718	2.194939	0.348549	1	-
ENSMUS(	Ppfia3	protein ty	GO:00452	synapse|c	-	-	-	-	7:4533912	2882	3430	3995	4485	3467	2729	13.59054	13.55782	12.9781	13.77802	14.33638	15.9563	13.37549	14.69024	0.64049	1	-
ENSMUS(	Gys1	glycogen	GO:00325	developm	K00693	glycogen	mmu0110	Metabolic	7:4543484	268	258	449	431	354	307	2.411812	1.946168	2.783597	2.526781	2.793537	3.425566	2.380526	2.915295	0.290357	1	-
ENSMUS(	Ruvbl2	RuvB-like	GO:00081	metabolic	-	-	-	-	7:4542176	361	431	627	602	445	405	5.597125	5.601298	6.696949	6.080459	6.050079	7.78573	5.965124	6.638756	0.647787	1	-
ENSMUS(	Lin7b	lin-7 hom	GO:00452	synapse|c	-	-	-	-	7:4536789	329	426	434	481	377	502	7.597724	8.246122	6.904443	7.23626	7.63435	14.37398	7.582763	9.748198	0.253144	1	-
ENSMUS(	Bax	BCL2-asso	GO:00325	developm	K02159	apoptosis	mmu0520	Pathways	7:4546169	285	372	369	374	337	410	7.014946	7.674956	6.256884	5.996981	7.273659	12.51266	6.982262	8.594432	0.381266	1	-
ENSMUS(	Il7r	interleukir	GO:00325	developm	K05072	interleukir	mmu0520	Pathways	15:950578	11	16	39	30	17	10	0.133467	0.162723	0.325979	0.237124	0.180865	0.150446	0.20739	0.189478	0.729843	1	-
ENSMUS(	Tfam	transcripti	GO:00081	metabolic	K11830	transcripti	mmu0501	Huntingto	10:712254	454	631	704	790	569	446	5.430656	6.326717	5.801231	6.156089	5.968305	6.614803	5.852868	6.246399	0.846636	1	-
ENSMUS(	Zfp81	zinc finge	GO:00081	metabolic	-	-	-	-	17:333293	759	748	985	1050	732	335	7.265663	6.00189	6.49563	6.547936	6.144512	3.97616	6.587727	5.556203	0.22702	1	-
ENSMUS(	Efnb3	ephrin B3	GO:00325	developm	K05463	ephrin-B[	mmu0436	Axon guic	11:695540	1766	2286	2329	2652	2009	2332	35.26064	38.25855	32.03468	34.49488	35.17403	57.73166	35.18462	42.46686	0.3213	1	-
ENSMUS(	Mmd	monocyte	GO:00325	developm	-	-	-	-	11:902494	6817	8788	9504	10712	7504	5628	110.775	119.6992	106.3913	113.3967	106.9261	113.3936	112.2885	111.2388	0.676568	1	-
ENSMUS(	Hlf	hepatic le	GO:00325	developm	-	-	-	-	11:903365	1663	2262	2797	2637	2176	1580	9.118167	10.39586	10.56475	9.419046	10.46205	10.74133	10.02626	10.20748	0.856246	1	-
ENSMUS(	Fam162a	family wit	GO:00081	metabolic	-	-	-	-	16:360438	245	400	442	468	334	251	24.52048	33.55638	30.47445	30.51338	29.31237	31.1474	29.5171	30.32438	0.944639	1	-
ENSMUS(	Rpl8	ribosomal	GO:00081	metabolic	K02938	large subu	mmu0301	Ribosome	15:769040	1518	1793	2380	2208	1701	2624	111.9182	110.8057	120.8808	106.0497	109.9704	239.8715	114.5349	151.9639	0.192006	1	-
ENSMUS(	Grm3	glutamate	GO:00048	signal trar	K04605	metabotro	mmu0408	Neuroacti	5:9485541	816	937	1199	1458	1009	713	17.00861	16.37085	17.21668	19.79785	18.4422	18.42697	16.86538	18.889	0.534141	1	-
ENSMUS(	Ssbp2	single-stra	GO:00081	metabolic	-	-	-	-	13:914602	1120	1506	1703	1735	1441	874	7.459584	8.407648	7.813807	7.527971	8.415956	7.217623	7.89368	7.720517	0.647964	1	-
ENSMUS(	Fancl	Fanconi a	GO:00081	metabolic	K10606	E3 ubiquit	mmu0412	Ubiquitin	11:263861	54	101	113	104	83	69	0.839501	1.316134	1.210197	1.053267	1.131473	1.330019	1.121944	1.171586	1	1	-
ENSMUS(	Brinp2	bone mor	GO:00325	developm	-	-	-	-	1:1582452	1457	1796	2158	1051	1739	1318	8.900966	9.1968	9.081975	4.18275	9.31579	9.983413	9.059913	7.827318	0.262565	1	-
ENSMUS(	Gstm5	glutathior	GO:00081	metabolic	K00799	glutathior	mmu0520	Pathways	3:1078958	819	1152	1353	1242	980	1279	20.87004	24.60622	23.75138	20.61783	21.89821	40.41069	23.07588	27.64224	0.420484	1	-
ENSMUS(	Gstm7	glutathior	GO:00081	metabolic	K00799	glutathior	mmu0520	Pathways	3:1079263	266	284	362	477	350	275	7.340462	6.569223	6.881803	8.575164	8.469414	9.40937	6.930496	8.817983	0.145054	1	-
ENSMUS(	Gstm3	glutathior	GO:00081	metabolic	K00799	glutathior	mmu0520	Pathways	3:1079636	0	3	0	1	2	2	0	0.121895	0	0.031576	0.085011	0.120245	0.040632	0.078944	0.760287	1	-
ENSMUS(	Stat3	signal trar	GO:00325	developm	K04692	signal trar	mmu0520	Pathways	11:100885	867	942	1301	1246	909	804	3.500885	3.188326	3.618992	3.27762	3.218589	4.025324	3.436068	3.507177	0.855011	1	-
ENSMUS(	Stat5a	signal trar	GO:00325	developm	K11223	signal trar	mmu0520	Pathways	11:100859	22	40	86	80	55	64	0.14008	0.213489	0.377237	0.331842	0.30709	0.505274	0.243602	0.381402	0.118512	1	-
ENSMUS(	Cavin1	caveolae a	GO:00081	metabolic	-	-	-	-	11:100956	406	520	712	658	448	462	6.218975	6.676502	7.513181	6.565988	6.017461	8.77446	6.802886	7.119303	0.99839	1	-
ENSMUS(	Map3k11	mitogen-a	GO:00048	signal trar	K04419	mitogen-a	mmu0401	MAPK sigi	19:568913	331	411	498	519	419	374	5.534351	5.760143	5.736126	5.653106	6.143206	7.753463	5.676873	6.516591	0.499196	1	-
ENSMUS(	Akt2	thymoma	GO:00325	developm	K04456	RAC serin	mmu0520	Pathways	7:2759155	677	791	1135	1138	846	621	3.392366	3.322336	3.917971	3.714821	3.717295	3.85826	3.544224	3.763459	0.863556	1	-
ENSMUS(	Dnaja3	DnaJ heat	GO:00325	developm	K09504	DnaJ hom	mmu0520	Viral carci	16:463998	1212	1403	1662	1792	1410	1014	16.56835	16.07634	15.65161	15.95865	16.90201	17.18701	16.09876	16.68256	0.978752	1	-
ENSMUS(	Hmox2	heme oxy	GO:00081	metabolic	K00510	heme oxy	mmu0110	Metabolic	16:472636	842	991	1108	1181	833	745	13.3213	13.14197	12.07606	12.17211	11.55638	14.61424	12.84645	12.78091	0.698783	1	-
ENSMUS(	Cdip1	cell death	GO:00508	response	-	-	-	-	16:475034	2817	1875	2116	3881	3121	1388	17.31253	9.658902	8.958613	15.53813	16.81938	10.57667	11.97668	14.31139	0.435555	1	-
ENSMUS(	Map3k20	mitogen-a	GO:00325	developm	K04424	sterile alp	mmu0401	MAPK sigi	2:7228563	232	353	361	397	269	166	1.347001	1.717941	1.443909	1.501594	1.369541	1.195021	1.50295	1.355385	0.382394	1	-
ENSMUS(	Cwc15	CWC15 sp	GO:00081	metabolic	K12863	protein C\	mmu0304	Spliceosor	9:1450061	814	978	1276	1192	895	639	21.35044	21.50178	23.05605	20.36763	20.58488	20.78116	21.96943	20.57789	0.402271	1	-
ENSMUS(	Col5a3	collagen, t	GO:00051	structural	K19721	collagen, t	mmu0497	Protein di	9:2077005	45	56	70	92	60	90	0.413461	0.431282	0.443062	0.550666	0.48341	1.025295	0.429269	0.686457	0.057142	1	-
ENSMUS(	Dnmt1	DNA metl	GO:00325	developm	K00558	DNA (cyto	mmu0110	Metabolic	9:2090720	1189	1336	1660	1772	1342	815	8.161198	7.686548	7.849311	7.923505	8.077318	6.936104	7.899019	7.645642	0.600233	1	-
ENSMUS(	Ppan	peter pan	GO:00508	response	-	-	-	-	9:2088817	218	316	313	446	268	243	1.704125	2.070548	1.685547	2.271236	1.837056	2.355253	1.820073	2.154515	0.385195	1	-
ENSMUS(	Angptl2	angiopoie	GO:00055	extracellul	-	-	-	-	2:3321606	123	132	167	127	119	67	0.881683	0.793111	0.824662	0.593051	0.747994	0.595484	0.833152	0.64551	0.088278	1	-
ENSMUS(	Cacna1e	calcium cl	GO:00511	localizatio	K04852	voltage-d	mmu0401	MAPK sigi	1:1543907	11589	13118	15460	20036	13763	7413	21.5532	20.44967	19.80736	24.27495	22.4451	17.09408	20.60341	21.27138	0.99181	1	-
ENSMUS(	Cacna1b	calcium cl	GO:00452	synapse|c	K04849	voltage-d	mmu0401	MAPK sigi	2:2460388	2036	2325	2824	3079	2423	2011	7.354433	7.039589	7.027285	7.245407	7.674809	9.006781	7.140436	7.975666	0.55818	1	-
ENSMUS(	Trmt10a	tRNA met	GO:00081	metabolic	-	-	-	-	3:1381434	54	110	149	153	111	63	0.286731	0.489579	0.545023	0.529239	0.516823	0.414769	0.440444	0.486944	0.745636	1	-
ENSMUS(	Etv1	ets varian	GO:00325	developm	K09431	ets translo	mmu0520	Transcript	12:387793	1845	2510	2605	3162	2346	695	5.341196	6.090722	5.19519	5.963287	5.955426	2.49467	5.54237	4.804461	0.418965	1	-
ENSMUS(	Kifc2	kinesin fa	GO:00990	supramole	-	-	-	-	15:766606	8180	10328	11228	12913	10224	7417	165.4036	175.0495	156.4032	170.0985	181.2822	185.9542	165.6188	179.1117	0.730325	1	-
ENSMUS(	Psap	prosaposi	GO:00325	developm	K12382	saposin[-]	mmu0414	Lysosome	10:602776	22020	26558	32557	32324	24872	25216	350.8241	354.6666	357.3287	335.4894	347.4765	498.1203	354.2731	393.6954	0.62508	1	-
ENSMUS(	Ikbkg	inhibitor c	GO:00081	metabolic	K07210	inhibitor c	mmu0520	Pathways	X:7439329	470	766	610	793	551	465	1.331874	1.819478	1.190821	1.463929	1.369176	1.633819	1.447391	1.488975	0.931858	1	-
ENSMUS(	Pax2	paired bo	GO:00325	developm	-	-	-	-	19:447560	7	8	5	13	8	3	0.056238	0.053868	0.027672	0.068036	0.056358	0.029885	0.045926	0.051426	0.808753	1	-
ENSMUS(	Wars2	tryptopha	GO:00325	developm	K01867	tryptopha	mmu0097	Aminoacy	3:9914106	120	101	122	231	180	101	0.819952	0.578471	0.574271	1.028254	1.078506	0.855681	0.657565	0.98748	0.038142	1	-
ENSMUS(	Atn1	atrophin 1	GO:00325	developm	-	-	-	-	6:1247425	3313	3969	4549	5367	3772	2383	41.15542	41.32754	38.92897	43.4329	41.08847	36.70418	40.47064	40.40852	0.769118	1	-
ENSMUS(	Phb2	prohibitin	GO:00325	developm	-	-	-	-	6:1247123	874	1095	1260	1334	1064	915	11.45265	12.02711	11.37408	11.38759	12.22584	14.86625	11.61795	12.82656	0.651571	1	-
ENSMUS(	Ptpn6	protein ty	GO:00325	developm	K05697	tyrosine-p	mmu0520	Proteogly	6:1247207	103	87	151	187	101	94	1.307618	0.925799	1.320606	1.546565	1.124374	1.479656	1.184674	1.383532	0.524639	1	-
ENSMUS(	Eno2	enolase 2	GO:00081	metabolic	K01689	enolase[4	mmu0110	Metabolic	6:1247600	6603	8377	9847	9594	7686	9417	32.6061	34.6736	33.49754	30.86306	33.28133	57.65754	33.59242	40.60064	0.336008	1	-
ENSMUS(	Emg1	EMG1 N1	GO:00325	developm	K14568	rRNA sma	mmu0300	-	6:1247040	259	302	398	316	297	318	2.146335	2.097764	2.272126	1.70595	2.158227	3.267461	2.172075	2.377213	0.82583	1	-
ENSMUS(	Lpcat3	lysophosp	GO:00081	metabolic	K13515	lysophosp	mmu0056	Glyceroph	6:1246630	173	237	257	334	212	226	1.794172	2.060241	1.836122	2.256551	1.927949	2.90611	1.896845	2.363537	0.257045	1	-
ENSMUS(	Atp6v1f	ATPase, H	GO:00511	localizatio	K02151	V-type H-	mmu0110	Metabolic	6:2946771	509	718	783	640	545	737	30.43129	35.98156	32.24897	24.92669	28.57207	54.63313	32.88727	36.04396	0.846052	1	-
ENSMUS(	Clcn5	chloride c	GO:00511	localizatio	-	-	-	-	X:7153810	180	222	314	292	211	180	0.873713	0.903241	1.049973	0.92334	0.898094	1.083315	0.942309	0.96825	0.928453	1	-
ENSMUS(	Clcn3	chloride c	GO:00325	developm	-	-	-	-	8:6091038	3195	3787	4773	5179	3629	2622	7.925523	7.874176	8.156425	8.369211	7.893809	8.064468	7.985375	8.109163	0.849362	1	-
ENSMUS(	Hif3a	hypoxia ir	GO:00325	developm	-	-	-	-	7:1703150	69	84	122	149	85	88	0.59436	0.606498	0.723949	0.836112	0.642032	0.939865	0.641602	0.806003	0.305782	1	-
ENSMUS(	Pde1c	phosphoc	GO:00508	response	K13755	calcium/ca	mmu0474	Olfactory	6:5606980	176	97	219	259	216	119	0.38419	0.177484	0.329329	0.368312	0.413456	0.322083	0.297001	0.367951	0.372471	1	-
ENSMUS(	Utp20	UTP20 sm	GO:00081	metabolic	-	-	-	-	10:887466	532	708	853	996	709	450	2.898682	3.233514	3.201763	3.535327	3.387486	3.040093	3.11132	3.320969	0.814664	1	-
ENSMUS(	9330159F	RIKEN cDI	-	-	-	-	-	-	10:292116	1149	1843	2233	2454	1668	705	8.560655	11.50973	11.46113	11.91087	10.89747	6.512713	10.5105	9.773684	0.573377	1	-
ENSMUS(	Cul3	cullin 3 [S	GO:00325	developm	K03869	cullin 3[-]	mmu0412	Ubiquitin	1:8026492	2683	3290	4015	4071	2996	1519	27.21672	27.97459	28.0577	26.90282	26.65014	19.10551	27.74967	24.21949	0.227959	1	-
ENSMUS(	Sst	somatosta	GO:00400	locomotic	K05237	somatosta	mmu0497	Gastric ac	16:238895	2836	3680	3566	3553	2890	2936	300.8954	327.2728	260.6406	245.5761	268.8746	386.2348	296.2696	300.2285	0.784321	1	-
ENSMUS(	Il11	interleukir	GO:00081	metabolic	K05417	interleukir	mmu0406	Cytokine-	7:4772373	2	2	6	9	5	7	0.050509	0.042327	0.10436	0.148037	0.110696	0.219156	0.065732	0.159296	0.084253	1	-
ENSMUS(	Large1	LARGE xyl	GO:00325	developm	K09668	glycosyltra	mmu0110	Metabolic	8:7281459	2826	3355	3881	4225	3114	2390	19.12274	19.02933	18.09144	18.62458	18.47733	20.05219	18.74784	19.05137	0.836078	1	-
ENSMUS(	Ddx56	DEAD (As	GO:00081	metabolic	-	-	-	-	11:625891	421	537	596	638	459	414	8.032371	8.587937	7.833554	7.929834	7.679217	9.793719	8.151287	8.46759	0.969538	1	-
ENSMUS(	Tmed4	transmem	GO:00048	signal trar	-	-	-	-	11:627036	890	991	1208	1182	992	956	19.60562	18.29855	18.33195	16.96248	19.16216	26.11163	18.74537	20.74542	0.684672	1	-
ENSMUS(	Col26a1	collagen,	GO:00329	macromo	-	-	-	-	5:1367417	38	48	45	41	39	40	0.224318	0.237505	0.182998	0.157668	0.201878	0.29277	0.21494	0.217439	0.853907	1	-
ENSMUS(	Bid	BH3 inter	GO:00325	developm	K04726	BH3 intera	mmu0520	Pathways	6:1208919	174	174	235	251	197	150	2.023838	1.696394	1.882976	1.901866	2.009252	2.163234	1.867736	2.024784	0.791412	1	-
ENSMUS(	Ralb	v-ral sim	GO:00325	developm	K07835	Ras-relate	mmu0401	MAPK sigi	1:1194703	230	266	347	369	298	320	2.779993	2.694948	2.889323	2.90552	3.158457	4.795686	2.788088	3.619888	0.168887	1	-
ENSMUS(	Ppp1cc	protein p	GO:00325	developm	K06269|K0	serine/thr	mmu0481	Regulatio	5:1221582	171	198	1109	378	609	708	1.465815	1.422658	6.548875	2.110849	4.577657	7.524934	3.145783	4.737813	0.30993	1	-
ENSMUS(	Dnajb11	DnaJ heat	GO:00081	metabolic	K09517	DnaJ hom	mmu0414	Protein pr	16:228579	921	1292	1842	1686	1261	825	13.49293	15.85698	18.59035	16.0911	16.1996	14.98604	15.98303	15.75891	0.710905	1	-
ENSMUS(	Tbccd1	TBCC don	GO:00325	developm	-	-	-	-	16:228132	174	240	300	313	242	179	4.092617	4.731665	4.860984	4.795981	4.991236	5.220231	4.561755	5.002482	0.724973	1	-
ENSMUS(	Clec11a	C-type lec	GO:00508	response	-	-	-	-	7:4430266	47	73	64	69	72	56	1.003016	1.305823	0.940895	0.959274	1.34736	1.481783	1.083245	1.262806	0.60825	1	-
ENSMUS(	Zfp324	zinc finge	GO:00325	developm	-	-	-	-	7:1296583	216	316	316	323	244	200	1.031675	1.26511	1.039747	1.005016	1.021932	1.184415	1.112177	1.070454	0.582422	1	-
ENSMUS(	Gab2	growth fa	GO:00325	developm	K08091	growth fa	mmu0401	Ras signal	7:9708158	501	532	612	668	487	328	4.851448	4.318151	4.082601	4.213971	4.135284	3.938168	4.4174	4.095807	0.416737	1	-
ENSMUS(	Coro1c	coronin, a	GO:00325	developm	-	-	-	-	5:1138424	1016	2483	1332	2995	2260	1575	5.773413	11.82684	5.214283	11.08709	11.26134	11.097	7.604844	11.14847	0.062138	1	-
ENSMUS(	Tax1bp1	Tax1 (hun	GO:00081	metabolic	-		mmu0413	Mitophag	6:5271372	3510	4037	5125	5154	3624	1831	20.08383	19.36203	20.20155	19.21168	18.1832	12.99014	19.88247	16.79501	0.155141	1	-
ENSMUS(	Ctse	cathepsin	GO:00081	metabolic	K01382	cathepsin	mmu0414	Lysosome	1:1316383	2	4	7	5	0	2	0.030041	0.050348	0.072421	0.048918	0	0.03725	0.050936	0.028723	0.439942	1	-
ENSMUS(	Ndrg2	N-myc dc	GO:00325	developm	-	-	-	-	14:519052	9432	11420	15023	14051	11269	10292	106.0191	107.5968	116.3291	102.8891	111.073	143.4386	109.9817	119.1335	0.763074	1	-

ENSMUSC	Mettl17	methyltra	GO:00081	metabolic	-	-	-	-	14:518848	218	305	397	438	265	286	1.493267	1.751197	1.87337	1.95451	1.591732	2.429035	1.705945	1.991759	0.465749	1	-
ENSMUSC	Arhgef40	Rho guan	GO:00987	molecular	-	-	-	-	14:519847	281	406	520	596	462	361	0.805557	0.975592	1.02694	1.113058	1.16138	1.283168	0.93603	1.185869	0.152403	1	-
ENSMUSC	Pnpla6	patatin-lil	GO:00325	developm	K14676	lysophosp	mmu0056	Glyceroph	8:3515384	692	815	1076	1103	848	832	3.151916	3.111566	3.376231	3.272848	3.386934	4.698699	3.213238	3.78616	0.369377	1	-
ENSMUSC	Mcoln1	mucolipin	GO:00048	signal trar	K04992	mucolipin	mmu0414	Lysosome	8:3500457	729	842	1052	1097	826	834	4.493708	4.350531	4.467295	4.405201	4.464782	6.374257	4.437178	5.081413	0.502973	1	-
ENSMUSC	Arhgef18	rho/rac gu	GO:00325	developm	K16529	A-kinase a	mmu0453	Tight junc	8:3393006	1028	1160	1690	1617	1299	1109	5.485978	5.188859	6.212964	5.621503	6.078728	7.338011	5.629267	6.34608	0.536271	1	-
ENSMUSC	Pkn2	protein kii	GO:00081	metabolic	K06071	protein kii	mmu0415	PI3K-Akt s	3:1427909	547	583	928	827	607	392	2.142851	1.914372	2.504404	2.110533	2.085145	1.904046	2.187209	2.033241	0.434619	1	-
ENSMUSC	Cd33	CD33 anti	GO:00160	membran	K06473	CD33 anti	mmu0464	Hematopo	7:4352421	76	175	238	274	163	147	0.44739	0.863502	0.965165	1.050763	0.8414	1.072943	0.758686	0.988369	0.249434	1	-
ENSMUSC	Etfb	electron ti	GO:00081	metabolic	-	-	-	-	7:4344408	286	350	463	372	269	293	7.244382	7.431161	8.079188	6.138466	5.974887	9.202154	7.58491	7.105169	0.437289	1	-
ENSMUSC	Nkg7	natural kil	GO:00160	membran	-	-	-	-	7:4343707	2	0	1	0	0	0	0.079457	0	0.027353	0	0	0	0.035603	0	0.286761	1	-
ENSMUSC	Pcp2	Purkinje c	GO:00987	molecular	-	-	-	-	8:3623371	0	2	0	1	0	2	0	0.025959	0	0.010087	0	0.038412	0.008653	0.016166	1	1	-
ENSMUSC	Sgce	sarcoglyca	GO:00329	macromo	-	-	-	-	6:4674350	312	404	485	484	341	299	1.47853	1.604755	1.583316	1.494178	1.417007	1.756838	1.555534	1.556008	0.758241	1	-
ENSMUSC	Chn2	chimerin 2	GO:00452	synapse|n	-	-	-	-	6:5403955	360	376	626	529	400	310	2.514741	2.201562	3.012424	2.407283	2.450148	2.68496	2.576242	2.514131	0.650432	1	-
ENSMUSC	Wwox	WW dom	GO:00325	developm	-	-	-	-	8:1144396	200	264	343	301	237	167	1.11712	1.236026	1.319824	1.095263	1.16081	1.156572	1.224323	1.137548	0.442067	1	-
ENSMUSC	Slbp	stem-loop	GO:00081	metabolic	-	-	-	-	5:3363495	222	277	361	308	334	187	1.649759	1.725443	1.848109	1.49108	2.176492	1.723042	1.741104	1.796871	0.974993	1	-
ENSMUSC	Aqp1	aquaporir	GO:00325	developm	K09864	aquaporir	mmu0492	Renin secr	6:5533643	115	40	224	51	70	94	2.78211	0.811129	3.733155	0.803769	1.48496	2.819627	2.442131	1.702785	0.209191	1	-
ENSMUSC	Arid3b	AT rich int	GO:00325	developm	-	-	-	-	9:5779035	80	86	122	166	119	75	0.367942	0.331544	0.386546	0.49737	0.479933	0.4277	0.362011	0.468334	0.189247	1	-
ENSMUSC	Cnn2	calponin 2	GO:00325	developm	-	-	-	-	10:799885	93	138	170	197	104	109	2.029691	2.524515	2.555903	2.800861	1.990312	2.949547	2.370036	2.58024	0.81217	1	-
ENSMUSC	Polr2e	polymeras	GO:00081	metabolic	K03013	DNA-dire	mmu0110	Metabolic	10:800359	1070	1059	1232	1481	1210	561	14.14345	11.73331	11.21847	12.75289	14.0249	9.194336	12.36508	11.99071	0.692661	1	-
ENSMUSC	Abca13	ATP-bind	GO:00511	localizatio	K05647	ATP bindi	mmu0201	ABC trans	11:919194	2	0	3	2	1	1	0.006398	0	0.00661	0.004167	0.002804	0.003965	0.004336	0.003645	1	1	-
ENSMUSC	Myo9b	myosin IX	GO:00987	molecular	-	-	-	-	8:7127271	933	1000	1419	1581	1136	847	4.725453	4.24536	4.951029	5.216452	5.045247	5.319012	4.640614	5.19357	0.544294	1	-
ENSMUSC	Hdac9	histone de	GO:00325	developm	K11409	histone de	mmu0516	Human pa	12:340475	1007	1113	1225	1295	933	592	3.947075	3.656738	3.307755	3.306721	3.206788	2.877092	3.637189	3.1302	0.157771	1	-
ENSMUSC	Ly9	lymphocy	GO:00325	developm	-	-	-	-	1:1715886	14	8	21	34	14	12	0.171005	0.081904	0.176707	0.270544	0.149952	0.181739	0.143205	0.200745	0.350163	1	-
ENSMUSC	Cd244	CD244 na	GO:00081	metabolic	K06582	natural kil	mmu0465	Natural ki	1:1715591	4	4	4	7	3	3	0.048008	0.04024	0.033078	0.054734	0.031572	0.044649	0.040442	0.043652	1	1	-
ENSMUSC	Adgre1	adhesion	GO:00048	signal trar	-	-	-	-	17:573586	74	106	179	180	98	98	1.30274	1.564182	2.170871	2.064345	1.512849	2.139157	1.679264	1.905451	0.659368	1	-
ENSMUSC	Mtfp1	mitochon	GO:00508	response	-	-	-	-	11:409148	888	1224	1307	1205	992	1092	30.99125	35.80639	31.42339	27.39649	30.3585	47.2536	32.74035	35.00286	0.914782	1	-
ENSMUSC	Rab23	RAB23, m	GO:00325	developm	-	-	-	-	1:3371988	350	306	468	507	340	272	1.077693	0.789771	0.992713	1.016989	0.918012	1.038438	0.953392	0.991146	1	1	-
ENSMUSC	Rab11a	RAB11A, r	GO:00325	developm	K07904	Ras-relate	mmu0414	Endocytos	9:6471529	1145	1476	1801	1593	1329	1055	13.45317	14.53645	14.57754	12.19317	13.69263	15.36942	14.18905	13.75174	0.537673	1	-
ENSMUSC	Eif2b2	eukaryotic	GO:00325	developm	K03754	translatior	mmu0301	RNA trans	12:852194	338	381	413	496	380	264	4.906557	4.635941	4.13011	4.690547	4.837119	4.751705	4.557536	4.75979	0.970105	1	-
ENSMUSC	Pgf	placental	GO:00325	developm	K16859	placenta g	mmu0520	Pathways	12:851666	62	91	68	66	61	62	0.827443	1.01798	0.625185	0.573819	0.713874	1.025953	0.823536	0.771216	0.573828	1	-
ENSMUSC	Ulk2	unc-51 lik	GO:00325	developm	K08269	serine/thr	mmu0415	mTOR sig	11:617756	2104	2363	3238	3230	2257	1496	19.95456	18.78508	21.15557	19.95633	18.77028	17.59195	19.96507	18.77285	0.428383	1	-
ENSMUSC	Ccl24	chemokin	GO:00325	developm	K05514	C-C motif	mmu0406	Cytokine-	5:1355699	0	5	2	0	0	0	0	0.167721	0.055147	0	0	0	0.074289	0	0.035471	1	-
ENSMUSC	Dgkq	diacylglyc	GO:00048	signal trar	K00901	diacylglyc	mmu0110	Metabolic	5:1086466	345	458	491	585	410	508	1.679238	1.868578	1.646364	1.854942	1.749923	3.065791	1.731393	2.223552	0.200675	1	-
ENSMUSC	Grap	GRB2-rela	GO:00325	developm	-	-	-	-	11:616532	53	65	105	94	82	67	1.832598	1.883892	2.501108	2.117383	2.486256	2.87245	2.072533	2.492029	0.467966	1	-
ENSMUSC	Chmp2b	charged r	GO:00400	locomotic	K12192	charged m	mmu0414	Endocytos	16:655391	576	697	806	740	564	521	19.91649	20.20115	19.19893	16.66879	17.10064	22.33645	19.77219	18.70196	0.435118	1	-
ENSMUSC	Plod3	procollage	GO:00325	developm	K13646	lysyl hydro	mmu0031	Lysine deg	5:1369870	401	491	602	599	390	419	3.615885	3.711113	3.739541	3.518674	3.083741	4.68457	3.688846	3.762328	0.847262	1	-
ENSMUSC	Ap1s1	adaptor p	GO:00452	synapse|c	K12394	AP-1 com	mmu0414	Lysosome	5:1370349	2317	3170	3171	3302	2522	2600	44.71678	51.28101	42.15917	41.51481	42.68076	62.21623	46.05232	48.80393	0.940574	1	-
ENSMUSC	Mapk13	mitogen-a	GO:00048	signal trar	K04441	p38 MAP	mmu0401	MAPK sigi	17:287693	1	6	6	5	4	7	0.024957	0.125485	0.103131	0.081281	0.087514	0.216575	0.084524	0.128456	0.548816	1	-
ENSMUSC	Srpk1	serine/arg	GO:00325	developm	K15409	serine/thr	mmu0516	Herpes sir	17:285876	971	1231	1614	1603	1157	740	5.195756	5.521287	5.949554	5.587846	5.428819	4.909609	5.555532	5.308758	0.530721	1	-
ENSMUSC	Pax3	paired bo	GO:00325	developm	K09381	paired bo	mmu0520	Transcript	1:7810126	0	0	3	0	0	0	0	0	0.015231	0	0	0	0.005077	0	0.302416	1	-
ENSMUSC	Lbr	lamin B re	GO:00081	metabolic	-	-	-	-	1:1818153	129	134	155	185	126	167	1.054178	0.917868	0.872586	0.984867	0.902898	1.692102	0.948211	1.193289	0.343402	1	-
ENSMUSC	Crabp2	cellular re	GO:00325	developm	-	-	-	-	3:8794866	20	37	31	34	23	25	0.799919	1.240422	0.85414	0.885866	0.806654	1.239746	0.964827	0.977422	0.87992	1	-
ENSMUSC	Nes	nestin [So	GO:00325	developm	-	-	-	-	3:8797107	347	413	583	607	392	258	1.671179	1.667236	1.934257	1.904428	1.655475	1.540635	1.757557	1.700179	0.651333	1	-
ENSMUSC	Bcan	brevican [	GO:00325	developm	-	-	-	-	3:8798753	2365	3032	3417	3874	3488	3903	26.00396	27.94411	25.88241	27.74914	33.63004	53.20995	26.61016	38.19637	0.029046	1	-
ENSMUSC	Hapln2	hyalurona	GO:00325	developm	-	-	-	-	3:8802175	170	142	271	251	178	180	2.405698	1.68436	2.641889	2.313916	2.208802	3.158281	2.243982	2.560333	0.620305	1	-
ENSMUSC	Prcc	papillary r	GO:00099	cellular pr	K13105	proline-ric	mmu0520	Transcript	3:8785890	749	959	1171	1149	826	696	22.67804	24.33858	24.42485	22.66342	21.9304	26.12876	23.81382	23.57419	0.675564	1	-
ENSMUSC	Rrnad1	ribosomal	-	-	-	-	-	-	3:8792260	317	364	431	528	349	251	1.238783	1.19231	1.160283	1.344159	1.195925	1.216172	1.197126	1.252085	0.950819	1	-
ENSMUSC	Hdgf	hepatoma	GO:00081	metabolic	-	-	-	-	3:8790632	2240	2783	3491	3830	2613	2043	35.33352	36.79636	37.93501	39.35676	36.14273	39.95703	36.6883	38.48551	0.936688	1	-
ENSMUSC	Slc25a18	solute car	GO:00511	localizatio	-	-	-	-	6:1207735	635	895	959	962	756	714	21.11782	24.9489	21.97078	20.84164	22.0465	29.4415	22.67917	24.10988	0.912928	1	-
ENSMUSC	Thop1	thimet olig	GO:00081	metabolic	K01392	thimet olig	mmu0461	Renin-ang	10:810700	794	974	1108	1152	937	751	8.045458	8.272601	7.734303	7.604386	8.325536	9.435307	8.017454	8.455076	0.932789	1	-
ENSMUSC	Apba3	amyloid b	GO:00325	developm	-	-	-	-	10:812669	118	164	202	159	145	151	1.123313	1.30863	1.324713	0.986052	1.210404	1.782308	1.252219	1.326255	0.990098	1	-
ENSMUSC	Matk	megakary	GO:00081	metabolic	K08888	megakary	mmu0472	Neurotrop	10:812529	1544	2026	2198	2347	1819	1858	11.0977	12.20613	10.88341	10.98956	11.46465	16.55835	11.39574	13.00419	0.509292	1	-
ENSMUSC	Pias4	protein in	GO:00325	developm	K16065	E3 SUMO	mmu0463	Jak-STAT	10:811532	248	313	370	408	356	297	3.268591	3.45785	3.359403	3.503087	4.114352	4.853464	3.361948	4.156968	0.234535	1	-
ENSMUSC	Map2k1	mitogen-a	GO:00325	developm	K04368	mitogen-a	mmu0520	Pathways	9:6418577	3156	4178	4804	4974	3690	4873	49.34153	54.75161	51.74043	50.65975	50.58767	94.46218	51.94452	65.23653	0.234821	1	-
ENSMUSC	Sgta	small glut	GO:00081	metabolic	-	-	-	-	10:810440	2058	2427	2931	3284	2510	2031	27.86368	27.54328	27.33759	28.96529	29.79953	34.09486	27.58152	30.95323	0.52042	1	-
ENSMUSC	Nmrk2	nicotinam	GO:00325	developm	K10524	nicotinam	mmu0110	Metabolic	10:811981	0	2	3	0	0	0	0	0.057463	0.07084	0	0	0	0.042768	0	0.087965	1	-
ENSMUSC	Tmem242	transmem	GO:00160	membran	-	-	-	-	17:541087	314	385	505	456	346	538	9.029698	9.280192	10.0043	8.542605	8.724935	19.18279	9.438062	12.15011	0.298328	1	-
ENSMUSC	Dtx2	deltex 2, E	GO:00081	metabolic	K06058	deltex[2.3	mmu0433	Notch sigi	5:1359948	120	144	191	193	131	140	0.727013	0.731268	0.797159	0.761727	0.695945	1.051656	0.751813	0.836443	0.732992	1	-
ENSMUSC	Zp3	zona pellu	GO:00325	developm	-	-	-	-	5:1359800	0	0	0	0	5	0	0	0	0	0	0.170307	0	0	0.056769	0.124213	1	-
ENSMUSC	Hspb1	heat shoc	GO:00325	developm	K04455	heat shoc	mmu0401	MAPK sigi	5:1358879	18	44	77	59	47	83	0.624081	1.278728	1.839141	1.332631	1.428953	3.568111	1.247317	2.109899	0.134248	1	-
ENSMUSC	Rasa4	RAS p21 p	GO:00987	molecular	K17630	Ras GTPas	mmu0401	Ras signal	5:1360839	46	58	76	68	58	71	0.274164	0.289761	0.312049	0.264027	0.303127	0.524689	0.291991	0.363948	0.451407	1	-
ENSMUSC	Syt5	synaptota	GO:00452	synapse|c	-	-	-	-	7:4539765	2104	2839	3044	3033	2459	2139	51.60769	58.36963	51.43572	48.46438	52.88955	65.05272	53.80435	55.46888	0.906895	1	-
ENSMUSC	Hnrnpa2b	heteroger	GO:00081	metabolic	-	-	-	-	6:5146093	5506	7133	9025	8861	6485	3391	32.59341	35.39311	36.80377	34.17102	33.66249	24.88899	34.93009	30.9075	0.259283	1	-
ENSMUSC	Ccdc130	coiled-co	GO:00508	response	-	-	-	-	8:8425779	161	166	212	247	172	119	2.160021	1.866783	1.959384	2.158795	2.023502	1.979547	1.995396	2.053948	0.980473	1	-
ENSMUSC	Mri1	methylthic	GO:00081	metabolic	K08963	methylthic	mmu0110	Metabolic	8:8424990	197	225	249	301	185	237	2.46553	2.360365	2.146811	2.454096	2.030288	3.677719	2.324235	2.720701	0.523199	1	-
ENSMUSC	Prkacb	protein kii	GO:00325	developm	K04345	protein kii	mmu0474	Olfactory	3:1467295	4799	6518	7899	7459	3724	5060	19.83681	22.58334	22.49285	20.08553	13.49812	25.93328	21.63767	19.83898	0.342047	1	-
ENSMUSC	Sgsh	N-sulfogl	GO:00081	metabolic	K01565	N-sulfogl	mmu0110	Metabolic	11:119343	142	188	222	216	164	152	0.694781	0.77103	0.748281	0.688487	0.703632	0.922124	0.738031	0.771414	1	1	-
ENSMUSC	Chd5	chromodo	GO:00325	developm	-	-	-	-	4:1523386	6037	6863	8144	9250	7226	4697	28.72638	27.37326	26.69617	28.67366	30.15091	27.7119	27.5986	28.84549	0.948527	1	-
ENSMUSC	Cstb	cystatin B	GO:00081	metabolic	-	-	-	-	10:784256	137	177	240	213	151	162	14.43904	15.63665	17.42536	14.6245	13.95523	21.16998	15.83369	16.58324	1	1	-
ENSMUSC	Sh2b2	SH2B ada	GO:00325	developm	K07193	SH2B ada	mmu0491	Insulin sig	5:1362181	72	90	102	86	83	64	0.620282	0.649906	0.605354	0.482654	0.627011	0.683631	0.625181	0.597765	0.60844	1	-
ENSMUSC	Pex5	peroxison	GO:00325	developm	K13342	peroxin-5	mmu0414	Peroxisom	6:1243968	589	728	838	891	751	897	4.738922	4.909625	4.644719	4.670068	5.298422	8.948342	4.764422	6.305611	0.138363	1	-
ENSMUSC	Jkamp	JNK1/MAI	GO:00081	metabolic	-	-	-	-	12:720855	346	466	505	530	401	477	5.903187	6.664205	5.935436	5.89071	5.999241	10.09053	6.167609	7.326829	0.440305	1	-
ENSMUSC	Cd44	CD44 anti	GO:00325	developm	K06256	CD44 anti	mmu0516	Epstein-B	2:1028111	111	129	140	146	88	108	0.563811	0.549226	0.489879	0.483108	0.391955	0.680171	0.534305	0.518411	0.655802	1	-
ENSMUSC	Slc1a2	solute car	GO:00325	developm	K05613	solute car	mmu0472	Glutamate	2:1026586	69634	13467	83030	105828	84954	42373	294.1476	47.68345	241.6186	291.2233	314.6809	221.9316	194.4832	275.9453	0.290672	1	-
ENSMUSC	Eif2ak4	eukaryotic	GO:00325	developm	K16196	eukaryotic	mmu0516	Epstein-B	2:1183886	443	545	629	725	532	387	3.232373	3.333245	3.161696	3.446178	3.403862	3.50118	3.242438	3.450407	0.854919	1	-
ENSMUSC	Wdr1	WD repea	GO:00325	developm	-	-	-	-	5:3852681	2610	3023	4134	3791	2922	2640	13.69157	13.29241	14.93945	12.95532	13.44112	17.17128	13.97448	14.52257	0.960652	1	-
ENSMUSC	Slc2a9	solute car	GO:00081	metabolic	-	-	-	-	5:3834927	40	42	40	70	30	36	0.204057	0.179593	0.140572	0.232631	0.134199	0.227707	0.174741	0.198179	0.699548	1	-
ENSMUSC	Wisp1	WNT1 ind	GO:00508	response	-	-	-	-	15:668913	19	7	31	9	26	9	0.16573	0.051177	0.186279	0.05114	0.198867	0.097333	0.134395	0.11578	0.655691	1	-
ENSMUSC	Ndrg1	N-myc do	GO:00325	developm	-	-	-	-	15:669293	1560	1594	2259	1997	1623	1399	11.72176	10.03944	11.69327	9.775254	10.69374	13.03383	11.15149	11.16761	0.726482	1	-
ENSMUSC	4930550C	RIKEN cDI	-	-	-	-	-	-	9:5340232	4	15	11	16	14	5	0.058357	0.183443	0.110557	0.152072	0.179109	0.090444	0.117452	0.140541	0.718922	1	-
ENSMUSC	Man2b1	mannosid	GO:00081	metabolic	K12311	lysosomal	mmu0414	Lysosome	8:8508327	605	738	914	985	664	712	3.145673	3.21638	3.273829	3.336384	3.027392	4.590122	3.211961	3.651299	0.546315	1	-
ENSMUSC	Klf5	Kruppel-li	GO:00325	developm	-	-	-	-	14:992986	117	194	268	177	161	138	1.39506	1.938933	2.201377	1.374878	1.683359	2.04021	1.845124	1.699482	0.454227	1	-
ENSMUSC	Wdr83	WD repea	GO:00081	metabolic	-	-	-	-	8:8507503	233	277	362	425	287	127	1.908719	1.902035	2.042897	2.268072	2.061632	1.28996	1.951217	1.873221	0.719197	1	-
ENSMUSC	Prdx2	peroxired	GO:00325	developm	-	-	-	-	8:8496958	1085	1341	1664	1534	1245	1853	13.06957	13.53982	13.80817	12.03756	13.15054	27.67532	13.47252	17.62114	0.220562	1	-
ENSMUSC	Polr2a	polymeras	GO:00081	metabolic	K03006	DNA-dire	mmu0110	Metabolic	11:697339	2303	2855	3261	3330	2518	1925	15.79737	16.41533	15.40966	14.88047	15.14569	16.37221	15.87412	15.46612	0.561907	1	-
ENSMUSC	Senp3	SUMO/se	GO:00081	metabolic	-	-	-	-	11:696731	774	936	1117	1383	936	1257	7.793099	7.89947	7.747719	9.071371	8.263948	15.69243	7.813429	11.00925	0.052535	1	-
ENSMUSC	Corin	corin [Sou	GO:00325	developm	-	-	-	-	5:7230002	5	2	4	4	2	0	0.04062	0.013618	0.022389	0.021172	0.014246	0	0.025542	0.011806	0.281927	1	-
ENSMUSC	Plekha8	pleckstrin	GO:00511	localizatio	-	-	-	-	6:5459511	332	368	501	541	389	257	2.90828	2.70209	3.023349	3.087291	2.988075	2.791377	2.877906	2.955581	0.95901	1	-
ENSMUSC	Spc25	SPC25, NI	GO:00329	macromo	-	-	-	-	2:6919389	6	20	24	20	11	5	0.072538	0.202666	0.199875	0.157514	0.116611	0.074943	0.15836	0.116356	0.378792	1	-
ENSMUSC	Dnah2	dynein, ax	GO:00990	supramol	K10408	dynein he	mmu0501	Huntingto	11:694208	123	133	207	204	132	102	0.489419	0.443588	0.567411	0.528796	0.460567	0.503228	0.50014	0.49753	0.80822	1	-
ENSMUSC	Ripk4	receptor-	GO:00325	developm	-	-	-	-	16:977419	7	7	12	17	10	6	0.112547	0.094328	0.13291	0.178047	0.140982	0.119615	0.113262	0.146215	0.521182	1	-
ENSMUSC	Ufd1	ubiquitin r	GO:00081	metabolic	K14016	ubiquitin f	mmu0414	Protein pr	16:188117	448	569	689	662	516	509	4.221788	4.494522	4.472902	4.064044	4.263936	5.947333	4.396404	4.758438	0.820702	1	-
ENSMUSC	Zfp287	zinc finge	GO:00081	metabolic	-	-	-	-	11:627003	313	285	386	391	315	184	1.522313	1.161873	1.293299	1.238851	1.343425	1.109592	1.325828	1.230623	0.481211	1	-
ENSMUSC	Letm1	leucine zip	GO:00511	localizatio	-	-	-	-	5:3373967	1766	2176	2545	2555	2001	1369	9.72486	10.04395	9.654547	9.165692	9.662342	9.347209	9.807785	9.391748	0.489891	1	-
ENSMUSC	Ubqln1	ubiquilin 1	GO:00081	metabolic	K04523	ubiquilin[-	mmu0414	Protein pr	13:581761	3152	3784	4701	4712	3537	2158	26.62402	26.79115	27.35452	25.92831	26.19781	22.60085	26.92323	24.90899	0.352462	1	-
ENSMUSC	Fgfr4	fibroblast	GO:00048	signal trar	K05095	fibroblast	mmu0520	Pathways	13:551526	2	0	4	0	0	0	0.031727	0	0.04371	0	0	0	0.025146	0	0.051897	1	-
ENSMUSC	Cadm3	cell adhes	GO:00511	localizatio	K06780	cell adhes	mmu0451	Cell adhes	1:1733332	7153	9440	10911	12089	8272	5975	46.23623	51.14688	48.5859	50.90568	46.88646	47.88705	48.65634	48.55973	0.737805	1	-
ENSMUSC	Txn2	thioredox	GO:00081	metabolic	K03671	thioredox	mmu0462	NOD-like	15:779150	727	923	1072	1312	637	496	7.282953	7.750459	7.398085	8.562261	5.595704	6.160852	7.477166	6.772939	0.372982	1	-
ENSMUSC	Slc1a6	solute car	GO:00511	localizatio	K05617	solute car	mmu0472	Glutamate	10:787804	82	106	130	143	102	110	2.240481	2.427643	2.446925	2.545338	2.443826	3.726546	2.371683	2.905237	0.376948	1	-
ENSMUSC	Slc1a3	solute car	GO:00325	developm	K05614	solute car	mmu0472	Glutamate	15:863412	12295	12956	18273	18268	14292	8237	66.68808	58.90382	68.27803	64.54942	67.97596	55.39553	64.62331	62.6403	0.601321	1	-
ENSMUSC	Crbn	cereblon [	GO:00081	metabolic	-	-	-	-	6:1067802	814	1015	1174	1264	932	606	6.796127	7.103239	6.752379	6.874897	6.823324	6.273293	6.883915	6.657171	0.58909	1	-
ENSMUSC	Msh6	mutS hom	GO:00325	developm	K08737	DNA mism	mmu0520	Pathways	17:879750	513	599	637	721	467	311	7.664016	7.500989	6.555857	7.017063	6.117847	5.760824	7.240287	6.298578	0.220989	1	-
ENSMUSC	Fbxo11	F-box pro	GO:00081	metabolic	-	-	-	-	17:879908	3679	4183	6136	5668	3989	1705	44.15704	42.0834	50.73492	44.31815	41.98332	25.37351	45.65845	37.22499	0.150644	1	-
ENSMUSC	Mlxipl	MLX inter	GO:00081	metabolic	K09113	MAX-like	mmu0493	Non-alco	5:1350898	42	46	75	69	43	33	0.299474	0.274929	0.368403	0.320511	0.268857	0.291748	0.314269	0.293705	0.608225	1	-
ENSMUSC	Tbl2	transducir	GO:00508	response	-	-	-	-	5:1351496	608	804	953	930	806	587	4.16382	4.615267	4.496066	4.149087	4.840227	4.984395	4.425051	4.657903	0.934851	1	-
ENSMUSC	Bud23	BUD23, rR	GO:00081	metabolic	-	-	-	-	5:1350529	272	372	438	424	360	244	2.626317	3.010747	2.913426	2.667017	3.048056	2.921146	2.850163	2.87874	0.843645	1	-
ENSMUSC	Nid1	nidogen 1	GO:00325	developm	-	-	-	-	13:134375	232	312	464	416	273	227	1.865655	2.10306	2.570478	2.179311	1.925085	2.263371	2.179731	2.122589	0.657011	1	-
ENSMUSC	Mcm5	minichron	GO:00081	metabolic	K02209	DNA repli	mmu0411	Cell cycle	8:7510956	100	114	121	159	106	80	1.163761	1.112039	0.970067	1.205435	1.081713	1.154354	1.081955	1.147168	0.899513	1	-
ENSMUSC	Hmox1	heme oxy	GO:00325	developm	K00510	heme oxy	mmu0110	Metabolic	8:7509362	119	144	231	246	149	137	3.227829	3.274006	4.316448	4.346912	3.543984	4.607536	3.606094	4.166144	0.52794	1	-
ENSMUSC	Mprip	myosin ph	GO:00056	cell|cell ju	-	-	-	-	11:596613	7995	9440	11445	11708	9823	7051	22.93934	22.70321	22.62193	21.884	24.71435	25.08413	22.75482	23.89416	0.936021	1	-
ENSMUSC	Cic	capicua tr	GO:00325	developm	-	-	-	-	7:2526770	3330	3384	3667	4917	3594	2901	12.57765	10.71367	9.541519	12.09865	11.90353	13.58592	10.94428	12.52937	0.439833	1	-
ENSMUSC	Pafah1b3	platelet-a	GO:00325	developm	K16795	platelet-a	mmu0110	Metabolic	7:2529504	62	91	104	82	85	67	1.097267	1.349936	1.267956	0.945406	1.319107	1.470221	1.238386	1.244911	0.82086	1	-
ENSMUSC	Il27ra	interleukir	GO:00325	developm	K19598	interleukir	mmu0463	Jak-STAT	8:8403029	25	23	36	43	26	27	0.299555	0.231	0.297161	0.33565	0.27318	0.401131	0.275905	0.336654	0.544334	1	-
ENSMUSC	Prkaca	protein kii	GO:00325	developm	K04345	protein kii	mmu0474	Olfactory	8:8397299	2914	3841	4431	4568	3452	2941	66.35382	73.31173	69.50714	67.76165	68.92707	83.03425	69.72423	73.24099	0.953972	1	-
ENSMUSC	Asf1b	anti-silen	GO:00081	metabolic	-	-	-	-	8:8395550	9	6	10	12	11	5	0.20048	0.112026	0.15346	0.174146	0.21487	0.138091	0.155322	0.175702	0.911169	1	-
ENSMUSC	Ddx39	DEAD (As	GO:00081	metabolic	-	-	-	-	8:8371517	114	172	206	242	161	155	1.16125	1.468594	1.445573	1.605897	1.4381	1.957667	1.358472	1.667221	0.317653	1	-
ENSMUSC	Dnajb1	DnaJ heat	GO:00081	metabolic	K09507	DnaJ hom	mmu0516	Influenza	8:8360819	629	734	964	859	704	742	12.47654	12.20373	13.17262	11.09988	12.24501	18.24877	12.61763	13.86456	0.753899	1	-
ENSMUSC	Msh4	mutS hom	GO:00325	developm	-	-	-	-	3:1538571	0	4	0	0	0	0	0	0.025348	0	0	0	0	0.008449	0	0.187395	1	-
ENSMUSC	Usp40	ubiquitin s	GO:00081	metabolic	-	-	-	-	1:8794511	259	339	457	523	383	299	1.000623	1.097798	1.216292	1.316297	1.297512	1.432277	1.104904	1.348695	0.242547	1	-
ENSMUSC	Kbtbd4	kelch repe	GO:00081	metabolic	-	-	-	-	2:9090474	356	348	480	520	440	420	5.726359	4.692039	5.318903	5.448963	6.206174	8.376541	5.245767	6.677226	0.184146	1	-
ENSMUSC	Celf1	CUGBP, El	GO:00325	developm	-	-	-	-	2:9094038	2611	4313	5326	6625	4677	2837	5.905228	8.176399	8.298171	9.761067	9.275554	7.95564	7.459933	8.997421	0.248472	1	-
ENSMUSC	Ndufs3	NADH de	GO:00325	developm	-	-	-	-	2:9089463	639	826	990	990	770	786	11.60628	12.5755	12.38737	11.7141	12.26382	17.70112	12.18972	13.89302	0.537625	1	-
ENSMUSC	Por	P450 (cyto	GO:00325	developm	-	-	-	-	5:1356700	1440	1711	1832	2057	1613	1183	18.06481	17.99177	15.83244	16.81074	17.74386	18.40102	17.29634	17.65187	0.872364	1	-
ENSMUSC	Igf1r	insulin-lik	GO:00048	signal trar	K05087	insulin-lik	mmu0520	Pathways	7:6795282	1894	2137	2714	3057	2141	1562	6.355971	6.011174	6.274271	6.683118	6.300302	6.499336	6.213806	6.494252	0.955528	1	-
ENSMUSC	Insr	insulin rec	GO:00325	developm	K04527	insulin rec	mmu0415	PI3K-Akt s	8:3122061	1062	764	1578	1184	804	519	4.435973	2.674919	4.540698	3.221791	2.944847	2.68793	3.883863	2.951523	0.050917	1	-
ENSMUSC	Fcer2a	Fc recepto	GO:00081	metabolic	K06468	low affinit	mmu0516	Epstein-B	8:3681737	1	1	0	0	1	4	0.015459	0.012954	0	0	0.013552	0.076653	0.009471	0.030068	0.373169	1	-
ENSMUSC	Cyp2a5	cytochron	GO:00081	metabolic	K07411	cytochron	mmu0110	Metabolic	7:2683530	0	0	1	0	2	0	0	0	0.01388	0	0.035347	0	0.004627	0.011782	1	1	-
ENSMUSC	Atp4a	ATPase, H	GO:00511	localizatio	K01542	H+/K+-ex	mmu0019	Oxidative	7:3071220	7	9	11	10	4	6	0.087218	0.093984	0.09441	0.081161	0.043697	0.092695	0.091871	0.072518	0.541476	1	-
ENSMUSC	Trim28	tripartite r	GO:00325	developm	-	-	-	-	7:1299911	1373	1774	2040	2111	1559	1565	11.55585	12.51521	11.82805	11.57448	11.5059	16.33172	11.96637	13.13737	0.7092	1	-
ENSMUSC	Ube2m	ubiquitin-	GO:00081	metabolic	K10579	ubiquitin-	mmu0412	Ubiquitin	7:1303512	1109	1681	1667	1677	1283	740	12.60377	16.01362	13.05137	12.41606	12.78611	10.42765	13.88959	11.87661	0.161875	1	-
ENSMUSC	Adcy9	adenylate	GO:00081	metabolic	K08049	adenylate	mmu0520	Pathways	16:428752	1803	1836	2177	2687	1880	1564	11.43231	9.758071	9.509295	11.0991	10.45295	12.29592	10.23323	11.28266	0.642216	1	-
ENSMUSC	Mef2c	myocyte e	GO:00325	developm	K04454	MADS-bo	mmu0401	MAPK sigi	13:835040	1678	1710	2360	2995	3048	1105	2.53867	2.16852	2.459676	2.951841	4.043636	2.072825	2.388955	3.022768	0.212417	1	-
ENSMUSC	Ctr9	CTR9 hom	GO:00325	developm	-	-	-	-	7:1110289	1315	1540	1906	1985	1415	815	7.146591	7.015307	7.135877	7.027732	6.743296	5.491824	7.099258	6.420951	0.311813	1	-
ENSMUSC	Eif4g2	eukaryotic	GO:00325	developm	K03260	translatior	mmu0301	RNA trans	7:1110677	10161	11698	15226	15822	11502	6350	46.34435	44.72237	47.84071	47.01141	46.00196	35.91038	46.30247	42.97458	0.407664	1	-
ENSMUSC	Mrvi1	MRV integ	GO:00508	response	K12337	inositol 1,4	mmu0402	cGMP-PK	7:1108682	68	87	100	124	84	59	0.293628	0.31489	0.297467	0.348811	0.318059	0.315883	0.301995	0.327584	0.800354	1	-
ENSMUSC	Pcyt1a	phosphat	GO:00081	metabolic	K00968	choline-p	mmu0110	Metabolic	16:324309	480	654	817	868	614	523	2.118873	2.419884	2.484492	2.496119	2.376705	2.862544	2.341083	2.578456	0.668813	1	-
ENSMUSC	Zfp592	zinc finge	GO:00081	metabolic	-	-	-	-	7:8099368	688	809	905	1118	798	566	5.01485	4.942776	4.544324	5.308759	5.100528	5.115305	4.833983	5.174864	0.80873	1	-
ENSMUSC	Psmd4	proteasor	GO:00081	metabolic	K03029	26S prote	mmu0516	Epstein-B	3:9503269	971	1282	1604	1583	1264	911	9.119255	10.09208	10.37757	9.685065	10.40949	10.60826	9.862969	10.23427	0.989918	1	-
ENSMUSC	Tmod4	tropomod	GO:00325	developm	-	-	-	-	3:9512447	2	7	10	4	3	2	0.050751	0.148853	0.174779	0.066118	0.066736	0.062931	0.124795	0.065262	0.192261	1	-
ENSMUSC	Insrr	insulin rec	GO:00325	developm	K05086	insulin rec	mmu0481	Regulatior	3:8779695	1	4	0	2	4	2	0.008428	0.028252	0	0.010978	0.029554	0.020902	0.012227	0.020478	0.637927	1	-
ENSMUSC	Cabp5	calcium b	GO:00056	cell|intrace	-	-	-	-	7:1339813	0	0	2	0	0	0	0	0	0.031132	0	0	0	0.010377	0	0.537205	1	-
ENSMUSC	Snx6	sorting ne	GO:00081	metabolic	K17920	sorting ne	mmu0414	Endocytos	12:547463	506	695	827	786	615	444	7.616727	8.769107	8.575805	7.707654	8.117753	8.286806	8.320547	8.037404	0.568498	1	-
ENSMUSC	Mthfd2	methylene	GO:00081	metabolic	K13403	methylene	mmu0110	Metabolic	6:8330569	99	116	124	167	102	115	1.268494	1.245853	1.09453	1.39397	1.146036	1.827	1.202959	1.455669	0.414382	1	-
ENSMUSC	Kit	KIT proto-	GO:00325	developm	K05091	proto-onc	mmu0520	Pathways	5:7557491	1898	2234	2539	2495	1923	1679	9.308812	9.184049	8.578519	7.971689	8.270277	10.21021	9.023793	8.817392	0.569275	1	-
ENSMUSC	Nr1i3	nuclear re	GO:00048	signal trar	-	-	-	-	1:1712139	0	6	3	9	3	9	0	0.037082	0.015238	0.043231	0.019396	0.08228	0.01744	0.048302	0.09766	1	-
ENSMUSC	Apoa2	apolipopr	GO:00081	metabolic	K08758	apolipopr	mmu0332	PPAR sign	1:1712250	5	6	8	9	4	11	0.206477	0.207673	0.227582	0.242111	0.144833	0.563231	0.213911	0.316725	0.47782	1	-
ENSMUSC	Pan2	PAN2 pol	GO:00081	metabolic	K12571	PAB-depe	mmu0301	RNA degr	10:128303	396	442	539	706	508	307	1.839692	1.721175	1.725005	2.136664	2.069458	1.768374	1.761957	1.991498	0.52641	1	-
ENSMUSC	Cs	citrate syn	GO:00081	metabolic	K01647	citrate syn	mmu0110	Metabolic	10:128337	3864	5213	5937	6107	4685	3729	41.41825	46.83766	43.84026	42.64462	44.0359	49.56019	44.03206	45.41357	0.929928	1	-
ENSMUSC	Ampd3	adenosine	GO:00081	metabolic	K01490	AMP dear	mmu0110	Metabolic	7:1107682	272	383	422	533	372	265	2.183731	2.577399	2.333966	2.787656	2.61888	2.637927	2.365032	2.681488	0.521943	1	-
ENSMUSC	Bcas2	breast car	GO:00081	metabolic	K12861	pre-mRN	mmu0304	Spliceoso	3:1031716	662	815	865	1001	758	350	4.463407	4.605949	4.017687	4.396677	4.481473	2.925921	4.362348	3.93469	0.40318	1	-
ENSMUSC	Sh2d1a	SH2 dom	GO:00019	cell killing	K07990	SH2 dom	mmu0516	Measles -	X:4250245	0	0	2	0	0	7	0	0	0.011549	0	0	0.072744	0.00385	0.024248	0.255261	1	-
ENSMUSC	Ctcf	CCCTC-bi	GO:00081	metabolic	-	-	-	-	8:1056365	1785	1952	2420	2640	1807	806	18.18857	16.6722	16.98741	17.52451	16.14586	10.18314	17.28273	14.61784	0.221821	1	-
ENSMUSC	Pard6a	par-6 fan	GO:00081	metabolic	K06093	partitionin	mmu0516	Human pa	8:1057011	125	110	131	106	151	157	1.29152	0.952653	0.932426	0.713475	1.368071	2.011289	1.058866	1.364279	0.394755	1	-
ENSMUSC	Agrp	agouti rel	GO:00508	response	K05231	agouti rel	mmu0492	Adipocyto	8:1055666	7	5	6	7	6	3	0.385529	0.230798	0.227622	0.25114	0.289729	0.204869	0.281316	0.248579	0.884761	1	-
ENSMUSC	Pvalb	parvalbun	GO:00452	synapse|n	-	-	-	-	15:781911	232	265	289	208	199	340	8.739937	8.367973	7.500147	5.104652	6.5738	15.88132	8.202686	9.18659	0.841018	1	-
ENSMUSC	Tfap4	transcripti	GO:00081	metabolic	K09108	transcripti	mmu0520	Proteoglyc	16:454466	36	75	87	109	73	56	1.082764	1.890833	1.802635	2.13572	1.925328	2.088382	1.592077	2.04981	0.28428	1	-
ENSMUSC	Ranbp1	RAN bind	GO:00325	developm	-	-	-	-	16:182397	563	652	723	841	809	643	14.49184	14.06745	12.8205	14.10239	18.26024	20.52164	13.79326	17.62809	0.138163	1	-
ENSMUSC	Cd247	CD247 an	GO:00048	signal trar	K06453	CD3Z anti	mmu0465	Natural ki	1:1657886	0	0	4	2	3	3	0	0	0.021191	0.010017	0.020227	0.028605	0.007064	0.019616	0.297423	1	-
ENSMUSC	Rfx5	regulatory	GO:00081	metabolic	K08061	regulatory	mmu0515	Tuberculo	3:9495407	127	389	573	621	306	265	0.408896	1.049818	1.270917	1.302519	0.863922	1.057897	0.909877	1.074779	0.559336	1	-
ENSMUSC	Psmb4	proteasor	GO:00081	metabolic	K02736	20S prote	mmu0305	Proteason	3:9488409	715	978	1233	1046	829	1529	17.08928	19.59339	20.30169	16.28662	17.37461	45.3118	18.99478	26.32434	0.196669	1	-
ENSMUSC	Mmp8	matrix me	GO:00325	developm	-	-	-	-	9:7558456	6	3	7	9	5	2	0.157251	0.065899	0.126385	0.153653	0.114896	0.065007	0.116512	0.111185	1	1	-
ENSMUSC	Slc30a4	solute car	GO:00511	localizatio	-	-	-	-	2:1226812	783	1036	1400	1210	923	715	6.198556	6.874507	7.634987	6.240163	6.40727	7.018121	6.902683	6.555185	0.458803	1	-
ENSMUSC	Sqor	sulfide qu	GO:00081	metabolic	K17218	sulfide:qu	mmu0092	Sulfur me	2:1227652	90	96	123	109	77	59	1.027625	0.918795	0.9675	0.810776	0.770952	0.83528	0.971307	0.805669	0.184473	1	-
ENSMUSC	Bloc1s6	biogenesi	GO:00325	developm	-	-	-	-	2:1227385	289	380	209	461	159	317	2.644606	2.914742	1.317533	2.74819	1.275861	3.596744	2.292294	2.540265	0.843369	1	-
ENSMUSC	Metap1	methionyl	GO:00081	metabolic	-	-	-	-	3:1384589	777	1042	1188	1312	876	647	5.605071	6.300585	5.903749	6.165606	5.541243	5.786962	5.936468	5.83127	0.665436	1	-
ENSMUSC	Gpr108	G protein-	GO:00160	membran	-	-	-	-	17:572349	257	328	428	403	312	290	7.796236	8.340252	8.944321	7.964135	8.299458	10.90777	8.36027	9.057122	0.814629	1	-

ENSMUSC	Gata6	GATA bin	GO:00325	developm	-	-	-	-	18:110525	0	11	2	1	0	2	0	0.18347	0.027418	0.012961	0	0.049355	0.070296	0.020772	0.133896	1 -
ENSMUSC	Rsl1d1	ribosomal	GO:00325	developm	-	-	-	-	16:111929	815	1017	1179	1347	959	534	17.55191	18.35865	17.4917	18.89801	18.11041	14.25916	17.80076	17.08919	0.604892	1 -
ENSMUSC	Cnga2	cyclic nuc	GO:00511	localizatio	K04949	cyclic nucl	mmu0474	Olfactory	X:7199184	0	6	3	3	0	5	0	0.093126	0.038269	0.036186	0	0.114794	0.043798	0.050327	1	1 -
ENSMUSC	Reep5	receptor a	GO:00055	extracellul	-	-	-	-	18:343448	7141	8976	11084	10734	8052	6306	156.8183	165.2241	167.6817	153.561	155.0544	171.7028	163.2413	160.1061	0.59316	1 -
ENSMUSC	Ergic3	ERGIC anc	GO:00511	localizatio	-	-	-	-	2:156008C	965	1075	1259	1365	926	885	16.16039	15.08987	14.52451	14.89151	13.59809	18.37612	15.25825	15.6219	0.86772	1 -
ENSMUSC	Uqcc1	ubiquinol	GO:00081	metabolic	-	-	-	-	2:1558468	674	844	1273	1210	831	406	3.277584	3.440241	4.264555	3.833195	3.54354	2.447967	3.660793	3.274901	0.383573	1 -
ENSMUSC	Spo11	SPO11 me	GO:00325	developm	-	-	-	-	2:1729777	2	7	0	0	2	2	0.019265	0.056505	0	0	0.016889	0.023889	0.025257	0.013592	0.54804	1 -
ENSMUSC	Ncoa2	nuclear re	GO:00081	metabolic	K11255	nuclear re	mmu0491	Thyroid hc	1:1313910	2410	2539	3228	3498	2495	1625	7.159814	6.322668	6.606473	6.769962	6.49977	5.985826	6.696318	6.418519	0.523946	1 -
ENSMUSC	Nr2c2	nuclear re	GO:00325	developm	-	-	-	-	6:9209139	2470	2804	3567	4138	2887	1747	8.931776	8.499074	8.885776	9.74794	9.154409	7.832845	8.772209	8.911731	0.892521	1 -
ENSMUSC	Nr2c1	nuclear re	GO:00048	signal trar	-	-	-	-	10:94148C	216	185	337	257	197	150	2.561094	1.838638	2.752665	1.985115	2.048237	2.205207	2.384132	2.07952	0.273679	1 -
ENSMUSC	Smpd4	sphingom	GO:00081	metabolic	K12353	sphingom	mmu011C	Metabolic	16:176193	598	622	674	891	671	523	2.396564	2.089446	1.860797	2.326199	2.358049	2.598817	2.115602	2.427689	0.464953	1 -
ENSMUSC	Pex1	peroxison	GO:00511	localizatio	K13338	peroxin-1	mmu0414	Peroxisom	5:3596066	294	390	527	470	384	301	1.334135	1.483443	1.647459	1.389417	1.528012	1.693581	1.488346	1.537003	0.949218	1 -
ENSMUSC	Otx1	orthodent	GO:00325	developm	K09326	homeobo	mmu0455	Signaling	11:219947	66	61	92	109	55	42	0.498928	0.386526	0.47911	0.536788	0.364585	0.393673	0.454855	0.431682	0.692222	1 -
ENSMUSC	Kctd20	potassium	GO:00081	metabolic	-	-	-	-	17:289519	1000	1186	972	1462	1148	997	6.926755	6.88601	4.638183	6.59719	6.972917	8.56271	6.150316	7.377606	0.33203	1 -
ENSMUSC	Itgae	integrin al	GO:00508	response	K06524	integrin al	mmu0481	Regulatior	11:730905	0	5	3	1	0	6	0	0.056801	0.02801	0.008828	0	0.10084	0.02827	0.036556	1	1 -
ENSMUSC	Ctns	cystinosis,	GO:00325	developm	K12386	cystinosin	mmu0414	Lysosome	11:731835	183	249	281	335	198	250	2.111898	2.408648	2.233975	2.518529	2.003682	3.577233	2.251507	2.699815	0.417152	1 -
ENSMUSC	P2rx5	purinergic	GO:00048	signal trar	K05219	P2X purin	mmu0408	Neuroacti	11:731604	30	61	37	46	42	35	0.749453	1.277328	0.636755	0.748616	0.920045	1.084116	0.887845	0.917592	0.972926	1 -
ENSMUSC	Shpk	sedohept	GO:00081	metabolic	-	-	-	-	11:731994	66	68	91	93	79	67	0.879163	0.759254	0.83506	0.807033	0.922776	1.106594	0.824492	0.945468	0.635802	1 -
ENSMUSC	Trpv1	transient r	GO:00325	developm	K05222	transient r	mmu0408	Neuroacti	11:732342	0	0	3	5	1	4	0	0	0.022085	0.034811	0.00937	0.053001	0.007362	0.032394	0.076567	1 -
ENSMUSC	Ephb3	Eph recep	GO:00325	developm	K05112	Eph recep	mmu0436	Axon guic	16:212047	265	314	411	441	299	255	1.764249	1.752254	1.884983	1.912647	1.745536	2.104944	1.800495	1.921043	0.867206	1 -
ENSMUSC	Tuft1	tuftelin 1	GO:00325	developm	-	-	-	-	3:9461275	61	75	88	80	58	48	0.601975	0.620393	0.59825	0.514304	0.501901	0.587323	0.606873	0.534509	0.361894	1 -
ENSMUSC	Rcn1	reticuloca	GO:00325	developm	-	-	-	-	2:1053862	213	305	522	331	348	253	3.016214	3.620233	5.092192	3.053466	4.321202	4.442121	3.909546	3.93893	0.836509	1 -
ENSMUSC	Dnase1	deoxyribc	GO:00081	metabolic	-	-	-	-	16:403694	0	2	0	2	1	3	0	0.014154	0	0.010999	0.007403	0.031409	0.004718	0.016604	0.346991	1 -
ENSMUSC	Trap1	TNF recep	GO:00081	metabolic	-	-	-	-	16:403997	937	1248	1494	1459	1192	895	18.57429	20.73672	20.40209	18.84124	20.72009	21.99791	19.90437	20.51975	0.936311	1 -
ENSMUSC	Naa60	N(alpha)-	GO:00081	metabolic	-	-	-	-	16:387237	533	597	886	901	646	433	3.661234	3.437384	4.19262	4.031875	3.891129	3.68786	3.763746	3.870288	0.960614	1 -
ENSMUSC	1700037C	RIKEN cDI	-	-	-	-	-	-	16:389517	32	27	40	46	47	59	0.430135	0.304208	0.370398	0.402808	0.553991	0.983327	0.368247	0.646709	0.044467	1 -
ENSMUSC	Ankrd13d	ankyrin re	GO:00160	membran	-	-	-	-	19:427018	543	688	720	879	709	755	8.232173	8.742914	7.519666	8.681314	9.425487	14.19214	8.164917	10.76631	0.111124	1 -
ENSMUSC	Tyrp1	tyrosinase	GO:00325	developm	K00506	tyrosinase	mmu011C	Metabolic	4:8083412	8	5	4	5	4	0	0.105588	0.055315	0.036375	0.042995	0.046292	0	0.065759	0.029763	0.200765	1 -
ENSMUSC	Tpr	translocat	GO:00081	metabolic	K09291	nucleoprc	mmu052C	Pathways	1:1503928	3589	4028	5209	5465	4026	1770	15.79364	14.85768	15.79118	15.66681	15.5355	9.657572	15.48083	13.61996	0.306276	1 -
ENSMUSC	BC003331	cDNA seq	GO:00160	membran	-	-	-	-	1:1503613	1087	582	1646	1758	1159	594	8.508696	3.818647	8.875951	8.964663	7.955356	5.765084	7.067765	7.561701	0.846449	1 -
ENSMUSC	Prg4	proteogly	GO:00511	localizatio	-	-	-	-	1:1504494	32	110	64	87	36	52	0.280972	0.809584	0.387122	0.497642	0.277183	0.566117	0.492559	0.446981	0.625924	1 -
ENSMUSC	Dhx34	DEAH (As	GO:00081	metabolic	-	-	-	-	7:1619714	182	192	194	273	195	216	1.708264	1.510556	1.254399	1.669272	1.604948	2.513751	1.491073	1.929324	0.211368	1 -
ENSMUSC	Kptn	kaptin [Sc	GO:00452	synapse||c	-	-	-	-	7:1611989	132	204	192	253	151	220	1.494296	1.935741	1.497329	1.865805	1.498932	3.08796	1.642455	2.150899	0.246638	1 -
ENSMUSC	Napa	N-ethylm	GO:00325	developm	K15296	alpha-solı	mmu0472	Synaptic v	7:1609845	2783	3401	4164	4545	3410	3002	34.03272	34.86125	35.07886	36.20753	36.56625	45.51767	34.65761	39.43048	0.450909	1 -
ENSMUSC	Calcoco2	calcium b	GO:00081	metabolic	-	-	mmu0413	Mitophag	11:960993	2	0	0	0	0	0	0.061328	0	0	0	0	0	0.020443	0	0.536454	1 -
ENSMUSC	Atp5g1	ATP synth	GO:00081	metabolic	K02128	F-type H+	mmu011C	Metabolic	11:960688	455	707	897	784	571	472	2.951581	3.844283	4.008544	3.313145	3.248041	3.796394	3.60147	3.452526	0.537887	1 -
ENSMUSC	Snf8	SNF8, ESC	GO:00081	metabolic	K12188	ESCRT-II (	mmu0414	Endocytos	11:960348	527	645	737	814	581	595	8.091921	8.301438	7.795782	8.14229	7.822748	11.32776	8.063047	9.097598	0.58413	1 -
ENSMUSC	Tbcb	tubulin fo	GO:00325	developm	-	-	-	-	7:3022413	562	850	1054	1073	822	645	16.47453	20.88569	21.28477	20.49077	21.12961	23.44349	19.54833	21.68796	0.624203	1 -
ENSMUSC	Inpp5k	inositol pc	GO:00325	developm	K01106	inositol-1,	mmu011C	Metabolic	11:756309	370	454	527	630	420	267	2.407554	2.47619	2.362309	2.67053	2.396442	2.15413	2.415351	2.407034	0.804571	1 -
ENSMUSC	Crkl	v-crk avia	GO:00325	developm	K04438	proto-onc	mmu052C	Pathways	16:174519	716	800	976	1064	804	631	11.77943	11.032	11.06146	11.40341	11.59872	12.87143	11.29096	11.95785	0.89512	1 -
ENSMUSC	Eps8l1	EPS8-like	GO:00987	molecular	-	-	-	-	7:4460674	84	173	108	49	242	112	0.534163	0.922136	0.473121	0.202989	1.349442	0.88308	0.64314	0.811837	0.584572	1 -
ENSMUSC	Clint1	clathrin in	GO:00511	localizatio	-	-	-	-	11:45852C	1206	1374	1753	1693	1263	809	8.17197	7.804036	8.183006	7.473399	7.504566	6.796954	8.053004	7.258306	0.263221	1 -
ENSMUSC	Prss16	protease,	GO:00081	metabolic	-	-	-	-	13:220021	8	6	2	6	6	3	0.077277	0.04858	0.013311	0.037759	0.05082	0.035935	0.046389	0.041505	0.890352	1 -
ENSMUSC	Cdkal1	CDK5 reg	GO:00081	metabolic	-	-	-	-	13:291917	188	196	291	297	227	142	0.859135	0.750777	0.916109	0.884179	0.909642	0.804596	0.842007	0.866139	0.983987	1 -
ENSMUSC	5430419D	RIKEN cDI	GO:00511	localizatio	K13912	deleted in	mmu0497	Salivary se	7:1311744	0	2	6	1	0	1	0	0.012015	0.029624	0.004669	0	0.008884	0.01388	0.004518	0.252002	1 -
ENSMUSC	Htra1	HtrA serin	GO:00325	developm	-	-	-	-	7:1309361	1856	2114	2757	3049	2078	1639	28.02432	26.75562	28.67777	29.99137	27.51347	30.68471	27.81924	29.39652	0.889815	1 -
ENSMUSC	Zbtb17	zinc finge	GO:00325	developm	K10500	zinc finge	mmu052C	Pathways	4:1414446	440	471	670	685	497	368	4.026978	3.613271	4.224276	4.084123	3.988647	4.176002	3.954842	4.082924	0.966805	1 -
ENSMUSC	Fam131c	family witl	-	-	-	-	-	-	4:1413682	24	36	35	35	29	25	1.064392	1.338285	1.069317	1.011219	1.127788	1.374708	1.157331	1.171238	0.880707	1 -
ENSMUSC	Fblim1	filamin bir	GO:00325	developm	-	-	-	-	4:141576C	25	41	61	49	26	30	0.242645	0.333553	0.407857	0.309815	0.221281	0.361029	0.328018	0.297375	0.55081	1 -
ENSMUSC	Hspb7	heat shoc	GO:00056	cell||intrace	-	-	-	-	4:1414207	3	4	3	5	0	3	0.068847	0.076949	0.047431	0.074764	0	0.085381	0.064409	0.053382	0.842055	1 -
ENSMUSC	Epor	erythropo	GO:00325	developm	K05079	erythropo	mmu052C	Pathways	9:2195889	74	80	105	107	63	61	2.095763	1.89914	2.04859	1.974146	1.56458	2.142034	2.014498	1.893587	0.568278	1 -
ENSMUSC	Mob1b	MOB kina	GO:00081	metabolic	K06685	MOB kina	mmu0439	Hippo sig	5:8872085	609	747	1029	1011	743	496	2.170953	2.232066	2.52697	2.347828	2.322551	2.192307	2.309997	2.287562	0.730202	1 -
ENSMUSC	Atp6v1b1	ATPase, H	GO:00081	metabolic	K02147	V-type H-	mmu011C	Metabolic	6:8374299	1	3	6	0	1	0	0.011007	0.027672	0.045485	0	0.009649	0	0.028055	0.003216	0.039011	1 -
ENSMUSC	Vax1	ventral an	GO:00325	developm	-	-	-	-	19:591661	6	6	12	15	10	11	0.294694	0.246993	0.406023	0.479928	0.43068	0.66987	0.315904	0.526826	0.206222	1 -
ENSMUSC	Atp6v1b2	ATPase, H	GO:00081	metabolic	K02147	V-type H-	mmu011C	Metabolic	8:6908864	6196	8121	9808	8901	6983	9309	60.08155	66.00732	65.51814	56.22767	59.37647	111.9228	63.869	75.84233	0.438274	1 -
ENSMUSC	Eps15l1	epiderma	GO:00511	localizatio	K12472	epiderma	mmu0414	Endocytos	8:7234099	1034	1232	1515	1652	1188	783	8.034453	8.024158	8.109609	8.362337	8.094591	7.543687	8.056073	8.000205	0.735358	1 -
ENSMUSC	Tep1	telomeras	GO:00081	metabolic	-	-	-	-	14:508240	103	85	138	144	100	115	0.57903	0.400532	0.534436	0.527364	0.492958	0.801585	0.504666	0.607302	0.481098	1 -
ENSMUSC	Ttc5	tetratricop	GO:00081	metabolic	-	-	-	-	14:507654	454	571	628	693	546	574	4.293612	4.526427	4.091459	4.269548	4.527959	6.730764	4.303833	5.176091	0.342289	1 -
ENSMUSC	Osgep	O-sialogly	GO:00081	metabolic	-	-	-	-	14:509064	307	371	475	482	410	260	2.681527	2.716255	2.858174	2.742659	3.14029	2.815801	2.751985	2.899583	0.913102	1 -
ENSMUSC	Aamp	angio-ass	GO:00329	macromo	-	-	-	-	1:7427984	1761	2146	2647	2145	2211	1809	19.37283	19.78866	20.06037	15.37244	21.32874	24.67506	19.74062	20.45875	0.936593	1 -
ENSMUSC	Tmbim1	transmem	GO:00511	localizatio	-	-	-	-	1:7428824	384	342	554	520	435	497	1.418282	1.058789	1.409589	1.251168	1.408844	2.276005	1.295553	1.645339	0.254155	1 -
ENSMUSC	Arpc2	actin relat	GO:00325	developm	K05758	actin relat	mmu0414	Endocytos	1:7423608	4717	5077	6688	6429	4530	2568	104.8914	94.63111	102.4523	93.13201	88.33134	70.80352	100.6582	84.08896	0.104755	1 -
ENSMUSC	Kmt2b	lysine (K)-	GO:00325	developm	K14959	histone-ly	mmu0031	Lysine deg	7:3056885	1020	1373	1462	1760	1290	987	5.190076	5.855947	5.124747	5.834022	5.755804	6.226968	5.390257	5.938931	0.635083	1 -
ENSMUSC	Upk1a	uroplakin	GO:00325	developm	-	-	-	-	7:3060309	4	4	8	6	7	0	0.17889	0.149946	0.24648	0.17482	0.274544	0	0.191772	0.149788	0.762042	1 -
ENSMUSC	Tmem147	transmem	GO:00160	membran	-	-	-	-	7:307277C	309	308	365	374	305	446	6.688646	5.588355	5.442823	5.273912	5.78925	11.97018	5.906608	7.67778	0.28285	1 -
ENSMUSC	Rps9	ribosomal	GO:00451	translatior	K02997	small subı	mmu0301	Ribosome	7:3703993	1310	1840	1535	2000	1086	1337	13.42814	15.80939	10.83938	13.35538	9.761512	16.99267	13.35897	13.36985	0.749922	1 -
ENSMUSC	Tfpt	TCF3 (E2A	GO:00081	metabolic	-	-	-	-	7:3620324	150	201	282	258	196	179	2.774441	3.116253	3.593221	3.108735	3.178937	4.105082	3.161305	3.464251	0.774739	1 -
ENSMUSC	Susd2	sushi dom	GO:00511	localizatio	-	-	-	-	10:756367	29	62	81	71	70	40	0.298565	0.535038	0.574484	0.476188	0.631944	0.510605	0.469362	0.539579	0.646409	1 -
ENSMUSC	Ggt5	gamma-g	GO:00081	metabolic	K18592	gamma-g	mmu011C	Metabolic	10:755893	27	39	49	50	44	44	0.153783	0.186195	0.192265	0.185523	0.219756	0.310733	0.177415	0.238671	0.267904	1 -
ENSMUSC	Ggt1	gamma-g	GO:00081	metabolic	K18592	gamma-g	mmu011C	Metabolic	10:755616	17	28	31	48	24	26	0.151122	0.208639	0.189849	0.277979	0.187089	0.286588	0.183204	0.250552	0.261743	1 -
ENSMUSC	Crip2	cysteine ri	GO:00325	developm	-	-	-	-	12:11314C	262	527	713	391	347	330	5.43791	9.168414	10.19464	5.286762	6.315425	8.492407	8.266989	6.698198	0.138895	1 -
ENSMUSC	Crip1	cysteine-r	GO:00508	response	-	-	-	-	12:113146	18	30	40	30	31	40	0.250151	0.349464	0.382952	0.271606	0.377776	0.689255	0.327523	0.446212	0.39352	1 -
ENSMUSC	Cbfa2t3	core-binc	GO:00325	developm	-	-	-	-	8:1226251	840	875	994	1100	710	483	4.555771	3.977806	3.713816	3.886483	3.376631	3.247994	4.082464	3.503703	0.16952	1 -
ENSMUSC	Fbln1	fibulin 1 [S	GO:00325	developm	-	-	-	-	15:852059	259	331	347	321	279	302	3.496229	3.745249	3.226861	2.822844	3.302526	5.054668	3.489446	3.726679	0.941959	1 -
ENSMUSC	Pgrmc1	progester	GO:00055	extracellul	-	-	-	-	X:365982C	3737	5084	5511	5141	4219	3025	127.8932	145.842	129.929	114.6182	126.6125	128.3617	134.5547	123.1974	0.26794	1 -
ENSMUSC	Gcat	glycine C-	GO:00081	metabolic	K00639	glycine C-	mmu0026	Glycine, se	15:790308	77	92	134	136	90	127	1.648228	1.650687	1.975983	1.896478	1.68932	3.370667	1.758299	2.318821	0.266756	1 -
ENSMUSC	Tek	TEK recep	GO:00325	developm	K05121	endotheli	mmu0415	PI3K-Akt s	4:9473928	283	382	467	492	350	203	2.379667	2.692434	2.70519	2.695109	2.580717	2.116465	2.59243	2.464097	0.585504	1 -
ENSMUSC	Mpl	myelopro	GO:00325	developm	K05082	thrombop	mmu0406	Cytokine-	4:1184424	3	4	0	1	0	0	0.035408	0.039575	0	0.007689	0	0	0.024994	0.002563	0.115487	1 -
ENSMUSC	Elovl1	elongatior	GO:00081	metabolic	K10247	elongatior	mmu0006	Fatty acid	4:118428C	139	169	225	247	175	135	1.66363	1.695434	1.855134	1.92584	1.836629	2.003371	1.738066	1.921947	0.688738	1 -
ENSMUSC	Med8	mediator	GO:00081	metabolic	K15129	mediator	mmu0516	Herpes sir	4:1184093	240	280	352	390	336	282	2.667029	2.608112	2.694697	2.823331	3.27415	3.885538	2.656613	3.327673	0.201619	1 -
ENSMUSC	Cdc20	cell divisic	GO:00325	developm	K03363	cell divisio	mmu0516	HTLV-I inf	4:1184329	15	14	16	14	10	5	0.41466	0.324405	0.304696	0.252123	0.24241	0.171368	0.347921	0.221967	0.164656	1 -
ENSMUSC	Adamts4	a disinteg	GO:00081	metabolic	-	-	-	-	1:1712504	417	385	735	604	472	372	3.285597	2.542676	3.989483	3.100247	3.261089	3.634188	3.272585	3.331841	0.880065	1 -
ENSMUSC	Nectin4	nectin cell	GO:00099	cellular pr	K06593	poliovirus	mmu0452	Adherens	1:171370C	122	152	88	162	126	110	1.006287	1.050895	0.50003	0.870483	0.911335	1.124977	0.852404	0.968932	0.692458	1 -
ENSMUSC	Pfdn2	prefoldin	GO:00329	macromo	-	-	-	-	1:1713456	1045	1464	1710	1575	1201	730	24.08882	28.28742	27.15482	23.65166	24.27643	20.86452	26.51035	22.93087	0.16427	1 -
ENSMUSC	Rnf114	ring finge	GO:00325	developm	-	-	-	-	2:1674926	692	867	1074	1120	819	765	6.586593	6.917138	7.042232	6.944715	6.835671	9.028214	6.848654	7.602867	0.637225	1 -
ENSMUSC	C330007P	RIKEN cDI	-	-	-	-	-	-	X:3682373	649	792	977	131	701	547	3.552925	3.634288	3.684575	0.467191	3.36513	3.712914	3.62393	2.515078	0.189109	1 -
ENSMUSC	Neurl1a	neuralizec	GO:00325	developm	-	-	-	-	19:471788	2841	3244	3444	4447	3356	3522	40.93269	39.17714	34.18327	41.73955	42.39978	62.91792	38.0977	49.01909	0.111899	1 -
ENSMUSC	Srm	spermidin	GO:00081	metabolic	K00797	spermidin	mmu011C	Metabolic	4:1485915	400	449	513	574	407	415	13.09696	12.3228	11.57122	12.24345	11.68551	16.84784	12.33033	13.59227	0.724186	1 -
ENSMUSC	Epha2	Eph recep	GO:00325	developm	K05103	Eph recep	mmu0415	PI3K-Akt s	4:1413012	19	30	57	38	26	28	0.216321	0.286295	0.44707	0.281846	0.259575	0.395266	0.316562	0.312229	0.831885	1 -
ENSMUSC	Rbm14	RNA bind	GO:00081	metabolic	-	-	-	-	19:480056	409	331	634	505	594	432	3.690638	2.503569	3.941116	2.968596	4.700103	4.833353	3.378441	4.167351	0.322453	1 -
ENSMUSC	Actn3	actinin alp	GO:00325	developm	K05699	actinin alp	mmu0541	Arrhythmc	19:486122	18	17	32	28	24	16	0.377663	0.298981	0.462527	0.382721	0.441564	0.416238	0.379723	0.413508	0.90111	1 -
ENSMUSC	Zdhhc24	zinc finge	GO:00038	catalytic a	-	-	-	-	19:487866	219	189	181	190	214	344	1.649064	1.192912	0.938907	0.93203	1.413024	3.211728	1.260294	1.852261	0.202606	1 -
ENSMUSC	Bbs1	Bardet-Bi	GO:00325	developm	-	-	-	-	19:488689	652	822	995	1130	883	875	7.424572	7.846005	7.805458	8.382703	8.817133	12.3543	7.692012	9.851378	0.127722	1 -
ENSMUSC	Slc34a3	solute car	GO:00511	localizatio	-	-	-	-	2:2522889	0	0	1	2	2	0	0	0	0.012883	0.024372	0.032808	0	0.004294	0.01906	0.418103	1 -
ENSMUSC	Ndor1	NADPH d	GO:00081	metabolic	-	-	-	-	2:2524482	310	434	542	387	293	277	1.965034	2.305957	2.366787	1.598089	1.628614	2.177082	2.212593	1.801262	0.07405	1 -
ENSMUSC	Nsmf	NMDA rec	GO:00325	developm	-	-	-	-	2:2505435	6714	7894	9021	11087	8123	5363	36.49772	35.9695	33.78241	39.2627	38.72073	36.14749	35.41654	38.04364	0.762241	1 -
ENSMUSC	Pdk1	pyruvate c	GO:00081	metabolic	K12077	pyruvate c	mmu0436	Axon guic	2:7187322	847	715	1328	1484	1119	902	4.764931	3.371567	5.146623	5.438617	5.520084	6.291664	4.427707	5.750122	0.108201	1 -
ENSMUSC	Ptbp1	polypyrim	GO:00325	developm	-	-	-	-	10:798544	405	412	518	523	394	263	2.339055	1.994509	2.060947	1.967746	1.995375	1.883332	2.131503	1.948818	0.364884	1 -
ENSMUSC	Mvd	mevalona	GO:00081	metabolic	K01597	diphosphc	mmu011C	Metabolic	8:1224336	304	292	340	334	278	214	9.847143	7.928168	7.586962	7.047984	7.896313	8.594829	8.454091	7.846375	0.430004	1 -
ENSMUSC	Cyba	cytochron	GO:00081	metabolic	K08009	cytochron	mmu0414	Phagoson	8:1224247	63	59	94	106	41	87	3.089392	2.425143	3.175502	3.386256	1.763017	5.289772	2.896679	3.479681	0.624043	1 -
ENSMUSC	Itih3	inter-alph	GO:00081	metabolic	-	-	-	-	14:309085	254	330	325	346	262	240	3.213073	3.499077	2.832183	2.85131	2.906234	3.764289	3.181444	3.173944	0.744689	1 -
ENSMUSC	Tmem11C	transmem	GO:00511	localizatio	-	-	-	-	14:308255	185	228	349	324	169	252	1.857979	1.919365	2.414607	2.119807	1.488331	3.138018	2.063984	2.248719	0.854446	1 -
ENSMUSC	Sfmbt1	Scm-like	GO:00325	developm	-	-	-	-	14:307148	788	924	1084	1186	864	620	4.803816	4.721552	4.552403	4.710057	4.618667	4.686382	4.69259	4.671702	0.737787	1 -
ENSMUSC	Ihh	Indian hec	GO:00325	developm	K11989	indian hec	mmu052C	Proteoglyc	1:7494531	4	7	4	0	0	3	0.065975	0.096776	0.045457	0	0	0.06136	0.069403	0.020453	0.051716	1 -
ENSMUSC	Cryba2	crystallin,	GO:00325	developm	-	-	-	-	1:7488993	1	0	3	0	0	3	0.042377	0	0.087558	0	0	0.157613	0.043312	0.052538	1	1 -
ENSMUSC	Atp7b	ATPase, C	GO:00325	developm	K17686	Cu+-expc	mmu0152	Platinum c	8:2199278	55	69	64	80	74	58	0.452477	0.475815	0.362717	0.428753	0.533844	0.591636	0.430336	0.518078	0.449317	1 -
ENSMUSC	Slc4a1	solute car	GO:00511	localizatio	K06573	solute car	mmu0496	Collecting	11:102348	4	2	6	6	0	0	0.04789	0.020071	0.049487	0.046801	0	0	0.039149	0.0156	0.249844	1 -
ENSMUSC	Rundc3a	RUN dom	GO:00081	metabolic	-	-	-	-	11:102393	4273	5302	6282	6595	5120	4484	50.3079	52.32339	50.95099	50.58242	52.85862	65.4568	51.19409	56.29928	0.655942	1 -
ENSMUSC	Slc4a3	solute car	GO:00511	localizatio	-	-	-	-	1:7554626	1458	1790	1956	2164	1798	1916	6.51528	6.704732	6.021376	6.29962	7.045436	10.6159	6.413796	7.986986	0.210972	1 -
ENSMUSC	Cdt1	chromatir	GO:00081	metabolic	-	-	-	-	8:122568C	11	12	16	19	18	20	0.136012	0.124372	0.136283	0.153038	0.195155	0.306611	0.132222	0.218268	0.136473	1 -
ENSMUSC	Runx1t1	runt-relat	GO:00325	developm	K10053	runt-relat	mmu052C	Pathways	4:1374343	388	407	561	607	460	436	1.66567	1.464549	1.659095	1.69757	1.73164	2.320758	1.596438	1.916656	0.319995	1 -
ENSMUSC	Snai3	snail famil	GO:00081	metabolic	-	-	-	-	8:1224542	0	0	3	2	0	0	0	0	0.080674	0.050856	0	0	0.026891	0.016952	1	1 -
ENSMUSC	Aprt	adenine p	GO:00325	developm	K00759	adenine p	mmu011C	Metabolic	8:1225746	176	187	232	220	207	328	2.807561	2.500411	2.549509	2.286231	2.895545	6.487499	2.61916	3.889759	0.123634	1 -
ENSMUSC	Gtf2h1	general tr	GO:00081	metabolic	K03141	transcripti	mmu052C	Viral carci	7:467961C	832	1047	1213	1306	916	443	12.62563	13.31769	12.68066	12.91083	12.18899	8.335251	12.87466	11.14502	0.257833	1 -
ENSMUSC	Hfe	hemochrc	GO:00325	developm	-	-	-	-	13:23702C	77	90	173	127	86	99	1.299413	1.273062	2.011186	1.396175	1.272621	2.071464	1.527887	1.580087	0.93511	1 -
ENSMUSC	Slc5a6	solute car	GO:00511	localizatio	K14386	solute car	-	-	5:3103603	196	244	299	293	195	249	2.100218	2.19155	2.207144	2.045303	1.832255	3.308219	2.166304	2.395259	0.781399	1 -
ENSMUSC	Tcf23	transcripti	GO:00325	developm	-	-	-	-	5:3096866	6	3	4	5	3	0	0.092647	0.038825	0.042552	0.050297	0.040616	0	0.058008	0.030304	0.352016	1 -
ENSMUSC	Aplp1	amyloid b	GO:00081	metabolic	-	-	-	-	7:3043498	13727	16655	18856	18863	15235	17690	189.9809	193.2109	179.7775	170.0696	184.8925	303.5624	187.6564	219.5082	0.436882	1 -
ENSMUSC	Qrich1	glutamine	GO:00056	cell||intrace	-	-	-	-	9:1085168	1138	1465	1648	1726	1321	700	5.196016	5.606845	5.183664	5.133936	5.288998	3.96289	5.328842	4.795275	0.318829	1 -
ENSMUSC	P4htm	prolyl 4-h	GO:00325	developm	-	-	-	-	9:1085788	787	1006	1203	1150	975	1111	8.517747	9.126428	8.969476	8.108296	9.253314	14.90905	8.871217	10.75689	0.332126	1 -
ENSMUSC	Usp19	ubiquitin s	GO:00325	developm	-	-	-	-	9:1084906	1651	2082	2454	2604	1964	2025	8.181365	8.647935	8.377311	8.406232	8.5342	12.44198	8.402204	9.794139	0.400098	1 -
ENSMUSC	Pola1	polymeras	GO:00081	metabolic	K02320	DNA poly	mmu011C	Metabolic	X:9330476	142	149	177	202	118	93	0.902451	0.793734	0.774927	0.836313	0.657599	0.73283	0.823704	0.742247	0.408698	1 -
ENSMUSC	Cdc42	cell divisic	GO:00325	developm	K04393	cell divisio	mmu052C	Pathways	4:1373196	4004	5363	5169	5706	4617	3122	84.26034	94.59972	74.93542	78.22446	85.19845	81.46067	84.5985	81.62786	0.540427	1 -
ENSMUSC	Pknox1	Pbx/knott	GO:00325	developm	-	-	-	-	17:315647	153	145	286	355	224	204	0.845973	0.672026	1.089387	1.278719	1.086064	1.398557	0.869129	1.254447	0.048485	1 -
ENSMUSC	D130043k	RIKEN cDI	GO:00325	developm	-	-	-	-	13:248451	816	895	1085	1183	870	753	7.137249	6.561705	6.537656	6.740732	6.672715	8.166226	6.745537	7.193224	0.866317	1 -
ENSMUSC	Gmnn	geminin [	GO:00325	developm	-	-	-	-	13:247518	32	24	44	54	35	19	1.0318	0.648651	0.977366	1.134292	0.989611	0.759617	0.885939	0.961173	0.799891	1 -
ENSMUSC	Acot13	acyl-CoA	GO:00038	catalytic a	-	-	-	-	13:248179	637	885	1029	914	770	453	46.58607	54.25157	51.84215	43.54558	49.37987	41.07717	50.89326	44.66754	0.218375	1 -
ENSMUSC	Zfp184	zinc finge	GO:00081	metabolic	-	-	-	-	13:21945C	38	15	29	42	9	10	0.230925	0.076408	0.121405	0.16627	0.047961	0.075352	0.142913	0.096528	0.285978	1 -
ENSMUSC	Cyp27b1	cytochron	GO:00325	developm	K07438	calcidiol 1	mmu011C	Metabolic	10:127048	8	13	10	17	8	11	0.130662	0.177985	0.112522	0.180884	0.114585	0.222774	0.140389	0.172748	0.632719	1 -
ENSMUSC	Cdk4	cyclin-der	GO:00325	developm	K02089	cyclin-der	mmu052C	Pathways	10:127063	394	510	563	653	435	466	3.921669	4.254984	3.860424	4.23419	3.796707	5.751046	4.012359	4.593981	0.531694	1 -
ENSMUSC	B4galnt1	beta-1,4-	GO:00081	metabolic	K00725	(N-Acetyl	mmu011C	Metabolic	10:127165	1871	2093	2549	2639	2077	1636	20.98618	19.67803	19.69612	19.28327	20.42861	22.75249	20.12011	20.82145	0.951249	1 -
ENSMUSC	Mettl1	methyltrai	GO:00081	metabolic	-	-	-	-	10:127041	83	106	126	111	108	126	1.154503	1.235872	1.207364	1.005815	1.317287	2.173064	1.199246	1.498722	0.410084	1 -
ENSMUSC	Tspan31	tetraspani	GO:00508	response	-	-	-	-	10:127067	545	717	855	837	646	693	10.4927	11.57077	11.33987	10.49778	10.906	16.54282	11.13445	12.64887	0.575281	1 -
ENSMUSC	Kif5b	kinesin fai	GO:00325	developm	K10396	kinesin fai	mmu0414	Endocytos	18:62010C	5112	6029	8073	8169	5918	3170	34.67638	34.28002	37.725	36.09885	35.20145	26.66171	35.56047	32.654	0.384428	1 -
ENSMUSC	Saal1	serum am	GO:00329	macromo	-	-	-	-	7:466861C	180	252	288	260	190	176	1.074033	1.260373	1.183831	1.010651	0.994125	1.302098	1.172746	1.102291	0.475253	1 -
ENSMUSC	Tph2	tryptopha	GO:00081	metabolic	K00502	tryptopha	mmu011C	Metabolic	10:115078	9	10	18	23	17	9	0.195947	0.182488	0.269979	0.326221	0.324553	0.24295	0.216138	0.297908	0.315874	1 -
ENSMUSC	Krt23	keratin 23	GO:00051	structural	-	-	-	-	11:994779	8	2	4	0	0	5	0.324859	0.068074	0.111914	0	0	0.251741	0.168283	0.083914	0.258029	1 -
ENSMUSC	Cnp	2',3'-cyclic	GO:00325	developm	-	-	-	-	11:100574	3607	3432	5430	4595	3475	3926	41.38575	33.00691	42.91963	34.34567	34.96248	55.8523	39.1041	41.72015	0.916865	1 -
ENSMUSC	Ttc25	tetratricop	GO:00056	cell||intrace	-	-	-	-	11:100545	26	39	50	30	33	29	0.216851	0.272647	0.287279	0.162999	0.241348	0.299889	0.258925	0.234745	0.542208	1 -
ENSMUSC	Sulf2	sulfatase 2	GO:00325	developm	-	-	-	-	2:166073C	2841	3224	3848	3996	3079	1969	21.60513	20.55103	20.15915	19.7967	20.53232	18.56596	20.77177	19.63166	0.439373	1 -
ENSMUSC	Sod2	superoxid	GO:00325	developm	K04564	superoxid	mmu0501	Huntingto	17:130078	2479	2700	3609	3745	2710	2460	41.19977	37.61273	41.31964	40.54635	39.49391	50.69199	40.04405	43.57742	0.727382	1 -
ENSMUSC	Tmco6	transmem	GO:00511	localizatio	-	-	-	-	18:36735C	97	117	156	152	144	140	3.392769	3.430215	3.758869	3.463437	4.416584	6.071478	3.527284	4.6505	0.198394	1 -
ENSMUSC	Stambp	STAM bin	GO:00081	metabolic	K11866	STAM-bir	mmu0414	Endocytos	6:8354321	429	541	458	615	470	258	1.90406	2.012675	1.400361	1.778199	1.829213	1.419807	1.772365	1.67574	0.581728	1 -
ENSMUSC	Ezh1	enhancer	GO:00325	developm	K17451	histone-ly	mmu0031	Lysine deg	11:101191	1058	1739	1998	1538	1171	1366	6.030409	8.308328	7.845275	5.71083	5.852761	9.653794	7.394671	7.072462	0.510656	1 -
ENSMUSC	Hap1	huntingtir	GO:00325	developm	K04647	huntingtir	mmu0501	Huntingto	11:100347	1787	2622	2065	2273	1766	1652	21.40393	26.32415	17.03885	17.73576	18.54822	24.53381	21.58898	20.2726	0.427493	1 -
ENSMUSC	P3h4	prolyl 3-h	GO:00081	metabolic	-	-	-	-	11:100408	118	139	206	219	180	178	3.120781	3.081421	3.753195	3.773179	4.17444	5.836989	3.318466	4.59487	0.093296	1 -
ENSMUSC	Ctnnb1	catenin (c	GO:00325	developm	K02105	catenin be	mmu052C	Pathways	9:1209292	4244	6249	4863	6655	3268	6205	23.29582	28.75186	18.38901	23.79755	15.72995	42.2309	23.4789	27.2528	0.633337	1 -
ENSMUSC	Eif1b	eukaryotic	GO:00081	metabolic	K03113	translatior	mmu0301	RNA trans	9:1204922	1083	1382	1694	1614	1279	994	51.78924	55.39518	55.80537	50.28012	53.63203	58.93638	54.32993	54.28285	0.726621	1 -
ENSMUSC	Chrd	chordin [S	GO:00325	developm	K04657	chordin[-'	mmu0435	TGF-beta	16:207331	837	1092	1164	1377	1000	1525	6.173133	6.750803	5.91404	6.616001	6.467295	13.94556	6.279326	9.009618	0.07484	1 -
ENSMUSC	Psmd2	proteasor	GO:00081	metabolic	K03028	26S prote	mmu0516	Epstein-B	16:206516	1950	2444	2928	2900	2188	2396	21.93034	23.03907	22.68472	21.24665	21.57748	33.41055	22.55137	25.41156	0.589109	1 -
ENSMUSC	Syngr3	synaptogy	GO:00325	developm	-	-	-	-	17:24685C	1819	2344	2543	2588	2064	2366	55.04901	59.46032	53.01693	51.02262	54.77334	88.78048	55.84209	64.85881	0.47734	1 -
ENSMUSC	Vars	valyl-tRN/	GO:00081	metabolic	K01873	valyl-tRN/	mmu0097	Aminoacy	17:350009	497	618	687	791	618	745	3.257281	3.395004	3.101751	3.377199	3.551648	6.053976	3.251345	4.327607	0.12224	1 -
ENSMUSC	Vwa7	von Willet	GO:00055	extracellul	-	-	-	-	17:350165	8	10	5	4	5	2	0.086419	0.090546	0.037211	0.028153	0.047361	0.026796	0.071392	0.034103	0.12199	1 -
ENSMUSC	Hspa1l	heat shoc	GO:00325	developm	K03283	heat shoc	mmu0401	MAPK sigı	17:349727	21	39	48	30	33	14	0.539902	0.84044	0.850117	0.502449	0.743962	0.446291	0.743486	0.564234	0.23468	1 -
ENSMUSC	Slc44a4	solute car	GO:00325	developm	K15377	solute car	mmu0523	Choline m	17:349144	3	8	10	1	0	4	0.046159	0.103183	0.10601	0.010023	0	0.076319	0.085117	0.028781	0.038973	1 -
ENSMUSC	Msh5	mutS hon	GO:00081	metabolic	-	-	-	-	17:350286	0	6	1	7	0	2	0	0.035755	0.004896	0.032421	0	0.017635	0.01355	0.016686	1	1 -
ENSMUSC	Abhd16a	abhydrola	GO:00081	metabolic	-	-	-	-	17:350892	834	1032	1168	1253	1001	1084	12.3493	12.80881	11.91436	12.08674	12.9973	19.90177	12.35749	14.99527	0.293954	1 -
ENSMUSC	Neu1	neuramini	GO:00081	metabolic	K01186	sialidase-1	mmu0414	Lysosome	17:349312	777	861	1080	1096	893	829	8.064784	7.490792	7.722309	7.410783	8.127669	10.66873	7.759295	8.735727	0.577219	1 -
ENSMUSC	Ddah2	dimethyla	GO:00081	metabolic	-	-	-	-	17:35059C	106	147	142	158	133	137	2.898723	3.369536	2.675104	2.814735	3.189289	4.645205	2.981121	3.549743	0.488406	1 -
ENSMUSC	Clic1	chloride ir	GO:00325	developm	-	-	-	-	17:350499	130	144	241	256	133	151	3.979723	3.695085	5.082494	5.105405	3.570283	5.731545	4.252434	4.802411	0.657564	1 -
ENSMUSC	Pole	polymeras	GO:00325	developm	K02324	DNA poly	mmu011C	Metabolic	5:1102863	37	37	40	77	40	43	0.171438	0.143703	0.127679	0.232424	0.162522	0.247039	0.147607	0.213995	0.124244	1 -
ENSMUSC	Atp1a2	ATPase, N	GO:00081	metabolic	K01539	sodium/pı	mmu0402	cAMP sigı	1:1722717	17382	21449	28213	28878	22342	17914	139.7619	144.5603	156.2751	151.2648	157.5266	178.5943	146.8658	162.4619	0.58344	1 -
ENSMUSC	Casq1	calseques	GO:00325	developm	-	-	-	-	1:1722098	8	9	11	15	8	4	0.174529	0.164577	0.165323	0.213192	0.153056	0.108203	0.168143	0.15815	1	1 -
ENSMUSC	Stx1a	syntaxin 1	GO:00452	synapse||c	K04560	syntaxin 1	mmu0491	Insulin sec	5:1350234	1165	1594	1822	1949	1525	1484	16.2047	18.58472	17.45881	17.66072	18.60062	25.5938	17.41608	20.61838	0.326259	1 -
ENSMUSC	Ceacam9	carcinoen	-	-	-	-	-	-	7:1672192	0	0	0	0	2	0	0	0	0	0	0.059919	0	0	0.019973	0.534255	1 -

ENSMUSC	Zfp775	zinc finge	GO:00081	metabolic	-	-	-	-	6:4860206	92	85	121	140	103	148	1.207537	0.935153	1.09408	1.197073	1.185474	2.408564	1.078923	1.597037	0.104614	1	-
ENSMUSC	Scube2	signal pep	GO:00325	developm	-	-	-	-	7:1097986	9	24	19	43	18	12	0.091794	0.205182	0.133499	0.285715	0.160985	0.151756	0.143492	0.199485	0.338005	1	-
ENSMUSC	Mrpl49	mitochon	GO:00081	metabolic	-	-	-	-	19:605363	576	688	815	806	637	531	21.25814	21.28356	20.72105	19.37847	20.61507	24.29874	21.08758	21.43076	0.833426	1	-
ENSMUSC	Dennd2c	DENN/M/	GO:00987	molecular	-	-	-	-	3:1031026	8	8	11	13	10	6	0.040568	0.034005	0.038429	0.042949	0.04447	0.03773	0.037667	0.041716	0.910268	1	-
ENSMUSC	Mark3	MAP/micr	GO:00081	metabolic	-	-	-	-	12:111574	1159	1406	1684	1857	1346	984	4.812985	4.89406	4.817532	5.023711	4.901384	5.066551	4.841526	4.997216	0.959114	1	-
ENSMUSC	Gatad1	GATA zinc	GO:00081	metabolic	-	-	-	-	5:3632932	621	762	1052	1001	738	579	7.508833	7.723041	8.762901	7.884909	7.824917	8.680517	7.998258	8.130114	0.854867	1	-
ENSMUSC	Pcdha11	protocadł	GO:00325	developm	-	-	-	-	18:370052	47	56	55	58	36	54	0.557377	0.556667	0.449333	0.44809	0.37437	0.794028	0.521126	0.538829	0.9291	1	-
ENSMUSC	M6pr	mannose-	GO:00511	localizatio	-	-	-	-	6:1223087	919	1127	1405	1520	1071	892	11.50388	11.82513	12.11593	12.39522	11.75604	13.84457	11.81498	12.66528	0.812235	1	-
ENSMUSC	Lrrc8a	leucine ric	GO:00325	developm	-	-	-	-	2:3023771	829	736	1062	1024	891	470	8.323132	6.193882	7.345281	6.697523	7.844276	5.85081	7.287432	6.797536	0.483522	1	-
ENSMUSC	Mc5r	melanoco	GO:00048	signal trar	K04203	melanoco	mmu0408	Neuroacti	18:683376	3	7	5	10	9	2	0.164059	0.320893	0.188391	0.356286	0.431649	0.135664	0.224448	0.307866	0.586538	1	-
ENSMUSC	Ppp2r1a	protein pł	GO:00325	developm	K03456	serine/thr	mmu0415	PI3K-Akt	17:209453	6267	8007	9349	9734	7503	7935	79.21374	84.83275	81.40619	80.1519	83.16077	124.358	81.81756	95.89022	0.378542	1	-
ENSMUSC	Fance	Fanconi a	GO:00329	macromo	-	-	-	-	17:283135	159	168	225	242	194	170	1.248912	1.106104	1.217496	1.238313	1.336223	1.655654	1.190837	1.410063	0.417367	1	-
ENSMUSC	Tinf2	Terf1 (TRF	GO:00081	metabolic	-	-	-	-	14:556746	83	127	135	153	122	151	0.415643	0.533085	0.46572	0.49913	0.535725	0.937569	0.471483	0.657475	0.142583	1	-
ENSMUSC	Tssk4	testis-spe	GO:00325	developm	-	-	-	-	14:556501	4	3	5	6	2	0	0.047337	0.029759	0.040765	0.046261	0.020754	0	0.039287	0.022338	0.438563	1	-
ENSMUSC	Hapln4	hyalurona	GO:00325	developm	-	-	-	-	8:7008345	2200	2506	2908	3181	2334	1890	32.44005	30.97368	29.5396	30.55654	30.17883	34.55465	30.98444	31.76334	0.88792	1	-
ENSMUSC	Dus3l	dihydrour	GO:00081	metabolic	-	-	-	-	17:567647	854	1053	1222	1409	1097	852	23.56677	24.35697	23.23083	25.32997	26.54552	29.15195	23.71819	27.00915	0.456953	1	-
ENSMUSC	Gtpbp3	GTP bindi	GO:00081	metabolic	-	-	-	-	8:7148810	195	277	306	320	245	363	1.867515	2.223627	2.018845	1.996461	2.057497	4.310443	2.036662	2.788134	0.170893	1	-
ENSMUSC	Tgfbr1	transform	GO:00325	developm	K04674	TGF-beta	mmu0520	Pathways	4:4735322	620	740	942	1094	685	443	4.316229	4.318145	4.517676	4.961489	4.181635	3.823861	4.384017	4.322328	0.738581	1	-
ENSMUSC	Homer1	homer sca	GO:00325	developm	K15010	homer[-]	mmu0406	FoxO sign	13:932996	1961	2310	2925	3005	2151	1488	6.26363	6.184626	6.436146	6.252802	6.024648	5.893012	6.2948	6.05682	0.524901	1	-
ENSMUSC	Rad51c	RAD51 pa	GO:00081	metabolic	K10870	RAD51-lik	mmu0346	Fanconi a	11:873766	38	77	80	87	70	39	0.395969	0.672547	0.574272	0.590578	0.639611	0.503884	0.547596	0.578024	0.937562	1	-
ENSMUSC	Gabrb2	gamma-a	GO:00325	developm	K05181	gamma-a	mmu0408	Neuroacti	11:424197	2573	2873	3730	4230	2934	1683	21.2836	19.9202	21.25522	22.79435	21.28178	17.26137	20.81967	20.44584	0.703671	1	-
ENSMUSC	Cav1	caveolin 1	GO:00325	developm	K06278	caveolin 1	mmu0414	Endocytos	6:1730633	65	255	231	164	145	142	0.490084	1.611583	1.199838	0.805536	0.958673	1.327502	1.100502	1.030571	0.641132	1	-
ENSMUSC	Arpp19	cAMP-reg	GO:00081	metabolic	-	-	-	-	9:7503761	933	1187	751	1461	772	980	4.482197	4.779833	2.485423	4.572365	3.25214	5.837426	3.915818	4.553977	0.571863	1	-
ENSMUSC	Bcl2l1	BCL2-like	GO:00325	developm	K04570	Bcl-2-like	mmu0520	Pathways	2:1527806	644	720	1844	924	659	1471	3.437894	3.22175	6.781397	3.213368	3.084855	9.736555	4.480347	5.344926	0.66845	1	-
ENSMUSC	Khsrp	KH-type s	GO:00081	metabolic	-	-	-	-	17:570210	1722	2121	2552	2903	2092	1974	27.42814	28.31763	28.00241	30.12256	29.21915	38.9849	27.91606	32.77554	0.325762	1	-
ENSMUSC	Dio2	deiodinas	GO:00081	metabolic	K17904	type II thy	mmu0491	Thyroid hc	12:907245	1304	1696	2318	2161	1579	791	14.25161	15.53692	17.45224	15.3859	15.13252	10.71887	15.74693	13.74576	0.251118	1	-
ENSMUSC	Ccdc124	coiled-co	GO:00099	cellular pr	-	-	-	-	8:7086822	1056	1326	1444	1468	1137	880	37.63993	39.61695	35.45711	34.08729	35.53757	38.89137	37.57133	36.17208	0.513419	1	-
ENSMUSC	Cct4	chaperoni	GO:00081	metabolic	-	-	-	-	11:229905	1455	1751	2157	2135	1604	913	14.52098	14.64779	14.82978	13.88074	14.03719	11.2977	14.66618	13.07187	0.25345	1	-
ENSMUSC	0610009B	RIKEN cDI	GO:00511	localizatio	-	-	-	-	11:516853	50	105	124	137	73	116	3.184048	5.604639	5.439751	5.683405	4.076371	9.159015	4.742812	6.306264	0.305106	1	-
ENSMUSC	Cpt1c	carnitine p	GO:00081	metabolic	K19524	carnitine (	mmu0415	AMPK sigr	7:4495937	1694	2103	2496	2477	1989	1818	12.44323	12.94827	12.63035	11.85297	12.81141	16.55768	12.67395	13.74069	0.770048	1	-
ENSMUSC	Twist2	twist basic	GO:00325	developm	K09069	twist[-]	mmu0520	Proteoglyc	1:9180146	13	14	6	7	4	12	0.214367	0.193515	0.068157	0.075199	0.057835	0.245353	0.15868	0.126129	0.52153	1	-
ENSMUSC	Zfp655	zinc finge	GO:00081	metabolic	-	-	-	-	5:1452317	586	713	881	816	641	388	3.337101	3.40341	3.456202	3.02722	3.200905	2.739619	3.398904	2.989248	0.21939	1	-
ENSMUSC	Rhoa	ras homol	GO:00325	developm	K04513	Ras homo	mmu0520	Pathways	9:1083061	2048	2782	3783	3443	2434	1637	15.56524	17.72295	19.80677	17.04687	16.22143	15.42626	17.69832	16.23152	0.32422	1	-
ENSMUSC	Zmiz1	zinc finge	GO:00325	developm	-	-	-	-	14:254591	3180	3462	4312	4817	3707	2219	12.3585	11.27765	11.54433	12.19542	12.63293	10.69256	11.72683	11.84031	0.840312	1	-
ENSMUSC	Ankrd26	ankyrin re	-	-	-	-	-	-	6:1185013	471	549	729	673	530	291	3.832217	3.744161	4.086096	3.567189	3.781358	2.935676	3.887491	3.428074	0.270715	1	-
ENSMUSC	Aldh16a1	aldehyde	GO:00081	metabolic	-	-	-	-	7:4514068	69	88	98	75	53	96	0.430723	0.460448	0.421427	0.304993	0.290111	0.743023	0.437533	0.446042	0.844119	1	-
ENSMUSC	Hnrnpa0	heteroger	GO:00081	metabolic	-	-	-	-	13:581258	1108	1439	1689	1794	1287	1717	26.29453	28.62459	27.61256	27.73509	26.78223	50.52215	27.51056	35.01316	0.207987	1	-
ENSMUSC	Prrg2	proline-ric	GO:00081	metabolic	-	-	-	-	7:4505358	31	63	58	54	69	44	0.344255	0.586416	0.443703	0.39065	0.671897	0.605829	0.458125	0.556125	0.529656	1	-
ENSMUSC	Hnrnph1	heteroger	GO:00081	metabolic	-	-	-	-	11:503769	3725	4619	5312	5063	3853	2294	18.90855	19.65317	18.57553	16.74254	17.15037	14.43814	19.04575	16.11035	0.098618	1	-
ENSMUSC	Ift43	intraflagel	GO:00511	localizatio	-	-	-	-	12:860825	79	101	122	114	100	55	1.954325	2.094339	2.079135	1.837213	2.169284	1.687027	2.0426	1.897841	0.562726	1	-
ENSMUSC	Id3	inhibitor c	GO:00325	developm	K17694	DNA-binc	mmu0455	Signaling	4:1361434	166	271	341	328	226	486	4.600866	6.295848	6.510852	5.922253	5.492661	16.70144	5.802522	9.37212	0.078024	1	-
ENSMUSC	Tcap	titin-cap [	GO:00325	developm	-	-	-	-	11:983838	4	9	11	11	7	11	0.268146	0.505711	0.508002	0.480381	0.411526	0.914358	0.427286	0.602088	0.518682	1	-
ENSMUSC	Arid1a	AT rich int	GO:00325	developm	K11653	AT-rich in	mmu0522	Hepatoce	4:1336790	2066	1976	2432	2407	2084	1439	8.626273	6.915647	6.995325	6.547124	7.630152	7.449716	7.512415	7.208997	0.515092	1	-
ENSMUSC	Crlf1	cytokine r	GO:00325	developm	-	-	-	-	8:7049315	242	333	398	483	338	371	7.369339	8.499842	8.349272	9.581707	9.025544	14.0079	8.072818	10.87172	0.091809	1	-
ENSMUSC	Ctsd	cathepsin	GO:00081	metabolic	K01379	cathepsin	mmu0515	Tuberculo	7:1423759	4828	6317	9154	9257	5527	5429	85.08984	93.31987	111.1406	106.2826	85.41664	118.6359	96.51677	103.4451	0.82549	1	-
ENSMUSC	Rplp1	ribosomal	GO:00081	metabolic	K02942	large subu	mmu0301	Ribosome	9:6191328	1406	1722	2180	2040	1535	2637	179.0691	183.8322	191.2685	169.2573	171.4299	416.42	184.7233	252.3691	0.175603	1	-
ENSMUSC	Hmgcll1	3-hydroxy	GO:00081	metabolic	K01640	hydroxym	mmu0110	Metabolic	9:7601485	239	301	386	375	282	164	2.050651	2.164774	2.281563	2.096077	2.121704	1.744708	2.165663	1.987497	0.434215	1	-
ENSMUSC	Ttc9b	tetratricop	-	-	-	-	-	-	7:2765391	270	367	419	413	360	564	8.362225	9.527483	8.939736	8.332781	9.776952	21.65828	8.943148	13.256	0.091785	1	-
ENSMUSC	Phox2a	paired-lik	GO:00325	developm	-	-	-	-	7:1018183	0	0	3	0	2	0	0	0	0.059081	0	0.050134	0	0.019694	0.016711	1	1	-
ENSMUSC	Abhd8	abhydrola	GO:00038	catalytic a	-	-	-	-	8:7145670	2482	3290	3714	3677	3179	3198	30.41037	33.78847	31.34824	29.34912	34.15491	48.58299	31.84903	37.36234	0.394009	1	-
ENSMUSC	Ift22	intraflagel	GO:00511	localizatio	-	-	-	-	5:1369081	593	806	905	954	695	534	7.954189	9.062107	8.362591	8.336253	8.17464	8.881125	8.459629	8.464006	0.761283	1	-
ENSMUSC	Fzd3	frizzled cl	GO:00325	developm	K02329	frizzled 3[	mmu0520	Pathways	14:651924	2594	2949	3434	3764	2826	1762	12.67055	12.07405	11.55519	11.97724	12.10432	10.6713	12.09993	11.58428	0.519399	1	-
ENSMUSC	1700008C	RIKEN cDI	-	-	-	-	-	-	7:4436004	16	24	28	39	24	21	0.207609	0.261023	0.250284	0.329658	0.27307	0.337844	0.239639	0.313524	0.350995	1	-
ENSMUSC	Mid1ip1	Mid1 inter	GO:00081	metabolic	-	-	-	-	X:1071501	422	549	639	653	489	349	6.219711	6.782384	6.487985	6.269783	6.319878	6.377773	6.496693	6.322478	0.624992	1	-
ENSMUSC	Ap2s1	adaptor-r	GO:00511	localizatio	K11827	AP-2 com	mmu0414	Endocytos	7:1673841	826	1033	1402	1221	1089	1451	27.99722	29.34863	32.73664	26.96076	32.36722	60.98011	30.0275	40.1027	0.16466	1	-
ENSMUSC	Fgfrl1	fibroblast	GO:00325	developm	-	-	-	-	5:1086923	144	209	258	232	185	160	1.822311	2.216964	2.249216	1.912621	2.052928	2.510525	2.096163	2.158691	0.928071	1	-
ENSMUSC	4930432K	RIKEN cDI	-	-	-	-	-	-	8:8414802	51	41	42	54	34	30	1.069698	0.720826	0.60687	0.737852	0.625346	0.780203	0.799131	0.714467	0.517225	1	-
ENSMUSC	Fhl2	four and a	GO:00325	developm	K14380	four and a	mmu0438	Osteoclas	1:4312307	311	297	413	433	331	325	8.093774	6.478873	7.404431	7.34108	7.55372	10.48721	7.325692	8.460671	0.511978	1	-
ENSMUSC	Emc10	ER memb	GO:00329	macromo	-	-	-	-	7:4448993	2171	2679	2929	3257	2453	2703	27.53414	28.47982	25.59072	26.90984	27.28048	42.50536	27.20156	32.23189	0.357127	1	-
ENSMUSC	Clstn3	calsynteni	GO:00325	developm	-	-	-	-	6:1244307	5521	6755	8015	8198	5349	5211	56.6752	58.12373	56.68005	54.82326	48.14937	66.32578	57.15966	56.4328	0.62776	1	-
ENSMUSC	Fbxw9	F-box anc	GO:00081	metabolic	-	-	-	-	8:8506005	149	171	220	225	186	185	1.13815	1.094867	1.157674	1.119636	1.245857	1.752152	1.130231	1.372548	0.362878	1	-
ENSMUSC	Fnbp4	formin bir	GO:00056	cell|intrace	-	-	-	-	2:9074537	847	1003	1221	1391	1166	713	6.305427	6.258707	6.261782	6.745895	7.611534	6.581215	6.275305	6.979548	0.574674	1	-
ENSMUSC	Cers4	ceramide	GO:00081	metabolic	K04710	ceramide	mmu0110	Metabolic	8:4493026	916	1221	1385	1550	1137	978	3.003692	3.356049	3.12868	3.311108	3.269361	3.976351	3.162807	3.51894	0.595038	1	-
ENSMUSC	Scrn3	secernin 3	GO:00081	metabolic	-	-	-	-	2:7331260	665	736	952	729	727	407	6.760962	6.27216	6.667683	4.828315	6.481321	5.130581	6.566935	5.480072	0.116882	1	-
ENSMUSC	Phax	phosphor	GO:00511	localizatio	K14291	phosphor	mmu0301	RNA trans	18:565624	812	868	1092	1215	857	430	20.00971	17.92906	18.53783	19.50485	18.5186	13.13829	18.82553	17.05392	0.395768	1	-
ENSMUSC	Tle1	transducir	GO:00081	metabolic	-	-	-	-	4:7211714	922	964	1258	1502	1113	699	3.707662	3.249368	3.484987	3.934781	3.924705	3.485237	3.480672	3.781575	0.707097	1	-
ENSMUSC	1700109H	RIKEN cDI	GO:00431	ion bindin	-	-	-	-	5:3571566	34	66	71	103	68	40	0.572097	0.930861	0.823	1.129033	1.00332	0.834515	0.775319	0.988956	0.310872	1	-
ENSMUSC	Relt	RELT tum	GO:00325	developm	K05156	tumor nec	mmu0406	Cytokine-	7:1008458	138	152	199	201	169	201	1.964675	1.813876	1.951712	1.864188	2.109802	3.548093	1.910088	2.507361	0.21986	1	-
ENSMUSC	Snrpb2	U2 small r	GO:00081	metabolic	K11094	U2 small r	mmu0304	Spliceosor	2:1430630	258	340	388	406	274	167	4.971704	5.491828	5.150731	5.096753	4.629978	3.990138	5.204754	4.57229	0.285236	1	-
ENSMUSC	Ubc	ubiquitin (	GO:00055	extracellul	K08770	ubiquitin (	mmu0332	PPAR sign	5:1253859	6829	7515	9734	10652	8449	3602	73.65979	67.94465	72.32962	74.84903	79.91373	48.17293	71.31135	67.64523	0.596077	1	-
ENSMUSC	Prpf31	pre-mRN.	GO:00081	metabolic	K12844	U4/U6 sm	mmu0304	Spliceosor	7:3629985	614	758	853	870	650	497	4.40971	4.56314	4.220292	4.070456	4.093527	4.425723	4.397714	4.196569	0.494394	1	-
ENSMUSC	Sertad1	SERTA do	GO:00081	metabolic	-	-	-	-	7:2748691	49	83	120	102	87	86	1.947538	2.76515	3.285632	2.641001	3.032156	4.238128	2.666107	3.303762	0.41407	1	-
ENSMUSC	Carhsp1	calcium re	GO:00081	metabolic	-	-	-	-	16:865858	489	563	831	703	559	604	10.9082	10.52701	12.77015	10.21599	10.93449	16.70577	11.40178	12.61875	0.725541	1	-
ENSMUSC	Elk3	ELK3, mer	GO:00325	developm	-	-	-	-	10:932474	140	183	265	268	187	119	1.353635	1.483122	1.7651	1.688066	1.585463	1.426611	1.533952	1.566714	0.955423	1	-
ENSMUSC	Herpud2	HERPUD f	GO:00508	response	-	-	-	-	9:2510813	874	1089	1321	1425	964	676	8.246042	8.612212	8.58595	8.758514	7.975421	7.908	8.481401	8.213978	0.588107	1	-
ENSMUSC	Rdh13	retinol del	GO:00325	developm	-	-	-	-	7:4424770	288	303	418	396	314	241	2.128289	1.876869	2.127969	1.906401	2.034746	2.208215	2.044375	2.049787	0.787689	1	-
ENSMUSC	Adam21	a disinteg	GO:00081	metabolic	-	-	-	-	12:815585	0	4	10	1	6	9	0	0.073196	0.150402	0.014221	0.114856	0.243617	0.074533	0.124232	0.607175	1	-
ENSMUSC	Nutf2	nuclear tra	GO:00511	localizatio	-	-	-	-	8:1058605	231	322	362	377	231	51	5.305663	6.199193	5.727789	5.640923	4.652457	1.452395	5.744215	3.915258	0.129577	1	-
ENSMUSC	Fut1	fucosyltra	GO:00081	metabolic	K00718	galactosid	mmu0110	Metabolic	7:4561728	0	0	3	5	1	0	0	0	0.032429	0.051117	0.013759	0	0.01081	0.021625	0.773429	1	-
ENSMUSC	Arpc5	actin relat	GO:00325	developm	K05754	actin relat	mmu0414	Endocytos	1:1527665	1067	1259	1468	1520	1152	1760	27.28814	26.98909	25.86349	25.32416	25.83479	55.80954	26.71357	35.65616	0.176889	1	-
ENSMUSC	Rnf151	ring finge	GO:00325	developm	-	-	-	-	17:247158	0	2	0	2	0	0	0	0.089602	0	0.069632	0	0	0.029867	0.023211	1	1	-
ENSMUSC	Elavl2	ELAV (em	GO:00508	response	-	-	-	-	4:9125076	837	1725	1004	1182	1652	584	2.976215	5.141392	2.45937	2.738027	5.150999	2.574765	3.525659	3.48793	0.851446	1	-
ENSMUSC	Pou2f2	POU dom	GO:00325	developm	-	-	-	-	7:2508734	59	156	71	88	63	116	0.357042	0.791298	0.295988	0.346918	0.33431	0.870377	0.481443	0.517201	1	1	-
ENSMUSC	Mgst1	microsom	GO:00081	metabolic	K00799	glutathion	mmu0520	Pathways	6:1381403	82	96	93	132	127	96	1.072958	1.052916	0.838304	1.125183	1.457188	1.557487	0.98806	1.379953	0.100944	1	-
ENSMUSC	Nfib	nuclear fa	GO:00325	developm	-	-	-	-	4:8229017	4278	4889	5858	6733	4951	2891	9.348088	8.954779	8.818257	9.584561	9.486753	7.832781	9.040375	8.968032	0.743456	1	-
ENSMUSC	Rab25	RAB25, m	GO:00325	developm	-	-	-	-	3:8854202	3	0	0	0	0	0	0.071319	0	0	0	0	0	0.023773	0	0.308913	1	-
ENSMUSC	Ubqln4	ubiquilin 4	GO:00081	metabolic	K04523	ubiquilin[-	mmu0414	Protein pr	3:8855375	1945	2279	2810	3142	2347	1644	22.26416	21.86673	22.15871	23.43012	23.55821	23.33321	22.09653	23.44052	0.853049	1	-
ENSMUSC	Rbfox1	RNA bind	GO:00325	developm	-	-	-	-	16:680922	8028	9368	11635	12777	9083	4987	119.3738	116.7619	119.1844	123.7691	118.4332	91.94474	118.44	111.3823	0.479786	1	-
ENSMUSC	Rps18	ribosomal	GO:00081	metabolic	K02964	small subu	mmu0301	Ribosome	17:339519	218	305	335	371	253	464	3.350553	3.929288	3.546967	3.714635	3.409757	8.842291	3.608936	5.322228	0.111554	1	-
ENSMUSC	Rpl10	ribosomal	GO:00325	developm	K02866	large subu	mmu0301	Ribosome	X:7427081	58	59	81	76	63	159	0.714912	0.609578	0.687799	0.610266	0.680938	2.43	0.670763	1.240402	0.071027	1	-
ENSMUSC	Rps15a	ribosomal	GO:00081	metabolic	K02957	small subu	mmu0301	Ribosome	7:1181043	1048	1474	1741	1803	1366	1190	5.611553	6.615649	6.422029	6.289256	6.4138	7.900503	6.21641	6.867853	0.638895	1	-
ENSMUSC	Ncaph2	non-SMC	GO:00325	developm	-	-	-	-	15:893557	1578	1923	2317	2326	1892	1372	13.19908	13.48245	13.35102	12.67442	13.87716	14.22908	13.34418	13.59356	0.858366	1	-
ENSMUSC	Hipk1	homeodo	GO:00325	developm	K08826	homeodo	mmu0421	Cellular se	3:1037398	2884	3123	4051	4309	3139	2514	12.88394	11.6944	12.46715	12.5404	12.29666	13.9253	12.3485	12.92079	0.968413	1	-
ENSMUSC	Gprc5b	G protein-	GO:00325	developm	-	-	-	-	7:1189720	2714	3200	3309	4342	2470	2753	24.10999	23.82813	20.25048	25.12804	19.24096	30.32351	22.72953	24.8975	0.729982	1	-
ENSMUSC	Man1a2	mannosid	GO:00325	developm	K01230	mannosyl	mmu0110	Metabolic	3:1005622	2371	2753	3454	3151	2358	1464	15.52005	15.105	15.57526	13.43667	13.53468	11.88197	15.4001	12.95111	0.088597	1	-
ENSMUSC	Acyp1	acylphosp	GO:00038	catalytic a	K01512	acylphosp	mmu0062	Pyruvate r	12:852723	184	202	171	180	52	232	5.126579	4.717542	3.282155	3.267114	5.668161	5.561875	4.375425	4.832383	0.786833	1	-
ENSMUSC	Cd163	CD163 an	GO:00511	localizatio	-	-	-	-	6:1243046	21	21	46	52	35	23	0.191896	0.160846	0.289564	0.309547	0.280449	0.26059	0.214102	0.283528	0.301718	1	-
ENSMUSC	Hdac5	histone de	GO:00325	developm	K11406	histone de	mmu0516	Human pa	11:102194	2434	2663	3264	3290	2764	2462	9.629483	8.830929	8.89578	8.4793	9.588774	12.07692	9.118731	10.04833	0.670625	1	-
ENSMUSC	Rala	v-ral simia	GO:00325	developm	K07834	Ras-relate	mmu0520	Pathways	13:178805	1215	1496	2005	1904	1405	740	25.84233	26.67105	29.37795	26.38178	26.20442	19.5152	27.29711	24.0338	0.252773	1	-
ENSMUSC	Vdac3	voltage-d	GO:00511	localizatio	K15041	voltage-d	mmu0516	HTLV-I inf	8:2257707	516	780	888	845	578	608	5.057582	6.408264	5.995943	5.395495	4.967795	7.388949	5.820596	5.917413	0.827514	1	-
ENSMUSC	Slc1a7	solute car	GO:00511	localizatio	K05618	solute car	mmu0472	Glutamate	4:1079683	0	2	1	0	0	2	0	0.022237	0.009135	0	0	0.032904	0.010457	0.010968	1	1	-
ENSMUSC	Vps72	vacuolar p	GO:00325	developm	-	-	-	-	3:9511102	465	505	596	582	462	312	6.48643	5.904682	5.727297	5.288796	5.651151	5.396252	6.03947	5.4454	0.296443	1	-
ENSMUSC	Gabpa	GA repeat	GO:00325	developm	-	-	-	-	16:848349	755	914	1179	1301	925	448	6.631085	6.728786	7.133502	7.443829	7.123965	4.878675	6.831124	6.482156	0.599702	1	-
ENSMUSC	Bmp7	bone mor	GO:00325	developm	K16621	bone mor	mmu0406	Cytokine-	2:1728680	119	155	230	164	144	159	1.626409	1.775688	2.165521	1.460186	1.725792	2.694426	1.855873	1.960135	1	1	-
ENSMUSC	Dynll1	dynein lig	GO:00325	developm	K10418	dynein lig	mmu0496	Vasopress	5:1152971	1456	1872	2222	2040	1632	1342	14.44026	15.56225	15.18131	13.1803	14.19307	16.50259	15.06127	14.62532	0.541716	1	-
ENSMUSC	Pdcl	phosducir	GO:00081	metabolic	-	-	-	-	2:3735007	347	483	543	545	412	341	3.503793	4.087985	3.777116	3.584989	3.64795	4.269235	3.789631	3.834058	0.824539	1	-
ENSMUSC	Tmem184	transmem	GO:00511	localizatio	-	-	-	-	15:793606	907	1116	1410	1313	1014	744	6.218186	6.413178	6.659272	5.864119	6.095888	6.32434	6.430212	6.094782	0.441204	1	-
ENSMUSC	Nf2	neurofibro	GO:00325	developm	K16684	merlin[-]	mmu0453	Tight junc	11:476584	2112	2437	3173	3330	2484	1742	14.18858	13.72314	14.68475	14.57373	14.6332	14.51039	14.19882	14.57244	0.9234	1	-
ENSMUSC	Cabp7	calcium b	GO:00160	membran	-	-	-	-	11:473689	4335	5189	5781	7044	5069	4540	104.8335	105.1834	96.30862	110.9716	107.4919	136.1296	102.1085	118.1977	0.375284	1	-
ENSMUSC	Zmat5	zinc finge	GO:00081	metabolic	-	-	-	-	11:470467	114	156	159	190	125	159	4.674217	5.361429	4.491098	5.075053	4.494266	8.083278	4.842248	5.884199	0.423841	1	-
ENSMUSC	Ewsr1	Ewing sar	GO:00081	metabolic	K13209	RNA-binc	mmu0520	Transcript	11:506968	3698	4269	4987	5817	4201	2282	28.78371	27.85216	26.74058	29.49583	28.67314	22.02326	27.79215	26.73074	0.593985	1	-
ENSMUSC	Ap1b1	adaptor p	GO:00325	developm	K12392	AP-1 com	mmu0414	Lysosome	11:498682	2546	2824	3366	3818	2926	2642	22.796	21.19423	20.76183	22.26987	22.97297	29.33047	21.58402	24.85777	0.413386	1	-
ENSMUSC	Derl3	Der1-like	GO:00081	metabolic	K13989	Derlin-2/3	mmu0414	Protein pr	10:758934	7	4	11	12	12	10	0.106232	0.050877	0.114992	0.118634	0.159682	0.188165	0.0907	0.155494	0.195966	1	-
ENSMUSC	Tbx1	T-box 1 [S	GO:00325	developm	-	-	-	-	16:185817	15	22	26	20	22	16	0.536468	0.659513	0.640584	0.465982	0.689954	0.709502	0.612188	0.621813	0.947223	1	-
ENSMUSC	Gnat2	guanine n	GO:00325	developm	K04631	guanine n	mmu0474	Phototran	3:1080927	1	0	4	0	0	3	0.010438	0	0.02876	0	0	0.038821	0.013066	0.01294	1	1	-
ENSMUSC	Bcl2l13	BCL2-like	GO:00099	cellular pr	K15485	Bcl-2-like	mmu0413	Mitophag	6:1208362	891	1031	1292	1348	1031	783	5.86491	5.688467	5.858654	5.780365	5.950932	6.390452	5.80401	6.040583	1	1	-
ENSMUSC	2610028H	RIKEN cDI	-	-	-	-	-	-	10:764490	0	2	0	1	0	0	0	0.04604	0	0.01789	0	0	0.015347	0.005963	1	1	-
ENSMUSC	Dqx1	DEAQ RN	GO:00081	metabolic	-	-	-	-	6:8305784	11	9	19	5	10	4	0.170721	0.117073	0.203134	0.050555	0.136093	0.076971	0.163643	0.087873	0.066988	1	-
ENSMUSC	Lnpk	lunapark,	GO:00325	developm	-	-	-	-	2:7452029	726	831	569	1225	463	495	2.917267	2.798939	1.575081	3.206693	1.631409	2.466216	2.430429	2.434773	0.854182	1	-
ENSMUSC	Prr29	proline ric	-	-	-	-	-	-	11:106365	7	1	2	5	7	6	0.124692	0.014929	0.024544	0.058022	0.10934	0.132522	0.054722	0.099961	0.342104	1	-
ENSMUSC	Mymk	myomake	GO:00325	developm	-	-	-	-	2:2706163	0	7	10	4	7	0	0	0.168647	0.19802	0.07491	0.176448	0	0.122222	0.083786	0.619012	1	-
ENSMUSC	Fam163b	family witl	GO:00160	membran	-	-	-	-	2:2711038	4214	4946	6089	7416	5043	3794	90.50773	89.04266	90.09249	103.763	94.97796	101.0356	89.88096	99.92552	0.553891	1	-
ENSMUSC	Trpm5	transient r	GO:00511	localizatio	K04980	transient r	mmu0474	Taste tran	7:1430691	0	0	0	0	2	2	0	0	0	0	0.01121	0.015856	0	0.009022	0.156264	1	-
ENSMUSC	Ascl2	achaete-s	GO:00325	developm	-	-	-	-	7:1429668	0	5	5	9	3	0	0	0.110104	0.090496	0.154034	0.069108	0	0.066867	0.074381	1	1	-
ENSMUSC	Rarres2	retinoic ac	GO:00325	developm	-	-	-	-	6:4856969	60	70	103	82	71	110	1.056568	1.033232	1.249505	0.94069	1.096346	2.401758	1.113102	1.479598	0.343127	1	-
ENSMUSC	Pttg1ip	pituitary tı	GO:00081	metabolic	-	-	-	-	10:775817	708	829	1077	958	819	699	7.214294	7.080565	7.560096	6.359274	7.317905	8.831279	7.284985	7.50282	0.905796	1	-
ENSMUSC	Trpm2	transient r	GO:00325	developm	K04977	transient r	mmu0462	NOD-like	10:779077	443	609	691	781	563	564	1.576723	1.816862	1.694264	1.810859	1.757127	2.488955	1.69595	2.01898	0.345566	1	-
ENSMUSC	Ube2g2	ubiquitin-	GO:00081	metabolic	K04555	ubiquitin-	mmu0414	Protein pr	10:776222	582	1310	1594	805	1282	229	12.15506	22.93287	22.93369	10.95246	23.47821	5.930024	19.34054	13.45356	0.167248	1	-
ENSMUSC	Mpo	myelopero	GO:00325	developm	K10789	myelopero	mmu0520	Transcript	11:877935	4	0	0	4	3	0	0.052794	0	0	0.034398	0.034719	0	0.017598	0.023039	1	1	-
ENSMUSC	Lpo	lactopero:	GO:00081	metabolic	K12550	lactopero:	mmu0497	Salivary se	11:878064	7	9	20	11	6	11	0.134208	0.14462	0.264146	0.137376	0.100859	0.261482	0.180991	0.166572	0.711873	1	-
ENSMUSC	Met	met protc	GO:00325	developm	K05099	proto-onc	mmu0520	Pathways	6:1746380	96	105	103	91	92	72	0.527366	0.483484	0.389789	0.32566	0.44317	0.490409	0.46688	0.419746	0.4188	1	-
ENSMUSC	Slc16a12	solute car	GO:00511	localizatio	-	-	-	-	19:346684	63	87	86	71	63	37	0.988361	1.14405	0.92945	0.725626	0.866679	0.71971	1.020621	0.770672	0.096503	1	-
ENSMUSC	Syn2	synapsin I	GO:00452	synapse|c	-	-	-	-	6:1151349	10161	13278	14266	14967	11740	12185	68.91069	75.48066	66.65056	66.12514	69.81696	102.4617	70.34731	79.46794	0.555665	1	-
ENSMUSC	Elk1	ELK1, mer	GO:00325	developm	K04375	ETS doma	mmu0520	Pathways	X:2093339	1725	2129	2551	2978	2149	1498	19.48959	20.1624	19.85525	21.91896	21.29083	20.98512	19.83575	21.3983	0.740337	1	-
ENSMUSC	Nav1	neuron na	GO:00325	developm	-	-	-	-	1:1354345	3333	3319	3786	5152	3227	2518	9.231334	7.705295	7.223724	9.295797	7.837376	8.647105	8.053451	8.593426	0.831929	1	-
ENSMUSC	Myod1	myogenic	GO:00325	developm	-	-	-	-	7:4637647	1	3	2	2	0	0	0	0.08587	0.047057	0.044488	0	0	0.044309	0.014829	0.509013	1	-
ENSMUSC	Rnmt	RNA (gua	GO:00325	developm	K00565	mRNA (gu	mmu0301	mRNA sur	18:683003	975	1183	1596	1545	654	871	4.906889	4.990445	5.533322	5.065376	2.886171	5.435076	5.143552	4.462208	0.220737	1	-
ENSMUSC	Kcnq1	potassium	GO:00325	developm	K04926	potassium	mmu0426	Adrenergi	7:1431063	5	19	13	11	3	6	0.069993	0.22294	0.125365	0.100309	0.036822	0.104149	0.139433	0.080427	0.159223	1	-
ENSMUSC	6330409C	RIKEN cDI	-	-	-	-	-	-	2:3273262	14	16	13	26	11	11	0.48068	0.460478	0.307487	0.581539	0.331186	0.468294	0.416215	0.460339	0.87786	1	-
ENSMUSC	Cdk9	cyclin-dep	GO:00325	developm	K02211	cyclin-dep	mmu0520	Transcript	2:3270576	922	1204	1429	1466	1048	793	6.170586	6.754215	6.588388	6.391622	6.150345	6.58043	6.504396	6.374132	0.624541	1	-
ENSMUSC	Tor2a	torsin fam	GO:00099	cellular pr	-	-	-	-	2:3275723	147	177	191	191	183	277	1.452017	1.46548	1.299688	1.229053	1.585403	3.392512	1.405729	2.068879	0.129521	1	-
ENSMUSC	Fpgs	folylpolyg	GO:00081	metabolic	K01930	folylpolyg	mmu0110	Metabolic	2:3268260	122	130	131	163	115	115	1.093574	0.976754	0.808936	0.951829	0.903923	1.278127	0.959755	1.044626	0.83124	1	-
ENSMUSC	Mkl2	MKL/myo	GO:00325	developm	-	-	-	-	16:132564	6197	6804	8054	10187	7370	4002	32.00634	29.45586	28.65613	34.27539	33.37834	25.62817	30.03944	31.09397	0.981929	1	-
ENSMUSC	Cbx5	chromobc	GO:00081	metabolic	-	-	-	-	15:103191	4629	5340	6363	7101	4790	4349	22.55342	21.80818	21.35692	22.53858	20.46461	26.27246	21.90618	23.09188	0.93245	1	-
ENSMUSC	Apobec3	apolipopr	GO:00325	developm	-	-	-	-	15:798916	55	61	126	99	84	68	0.257677	0.239552	0.406667	0.302158	0.345093	0.395012	0.301299	0.347421	0.638882	1	-
ENSMUSC	Taf7l	TATA-bo	GO:00325	developm	K03132	transcripti	mmu0310	Basal tran	X:1344601	0	4	2	3	4	4	0	0.085366	0.035085	0.049755	0.089302	0.126281	0.04015	0.088446	0.409136	1	-
ENSMUSC	Sardh	sarcosine	GO:00081	metabolic	K00314	sarcosine	mmu0110	Metabolic	2:2718839	172	207	251	292	224	202	1.519689	1.533026	1.527746	1.680703	1.735468	2.21291	1.52682	1.87636	0.280455	1	-
ENSMUSC	Vav2	vav 2 onc	GO:00325	developm	K05730	guanine n	mmu0481	Regulatio	2:2726210	219	167	331	194	268	280	1.674056	1.070029	1.743031	0.966071	1.7964	2.653807	1.495705	1.805426	0.546798	1	-
ENSMUSC	Tex15	testis exp	GO:00325	developm	-	-	-	-	8:3351673	37	29	39	46	30	21	0.181257	0.119081	0.131618	0.146803	0.128871	0.127554	0.143985	0.134409	0.685651	1	-
ENSMUSC	Ppp2cb	protein pl	GO:00325	developm	K04382	serine/thr	mmu0516	Human pa	8:3359962	1280	1551	1953	2020	1411	1083	55.37635	56.24435	58.09363	56.93079	53.52836	58.09363	56.60891	56.18426	0.694602	1	-
ENSMUSC	G0s2	G0/G1 sw	GO:00508	response	-	-	-	-	1:1932721	59	94	111	87	65	64	4.304994	5.749054	5.579463	4.135401	4.158861	5.790061	5.21117	4.694774	0.40961	1	-
ENSMUSC	Dmap1	DNA metl	GO:00325	developm	-	-	-	-	4:1176746	271	250	349	283	284	252	3.639656	2.81439	3.229001	2.476053	3.344668	4.196403	3.227682	3.339041	0.92853	1	-
ENSMUSC	Mcu	mitochon	GO:00325	developm	K20858	calcium ui	mmu0421	Cellular se	10:594469	899	1200	1316	1468	1028	627	7.105357	7.94987	7.165284	7.558468	7.124621	6.144404	7.406837	6.942497	0.459347	1	-
ENSMUSC	Oit3	oncoprote	GO:00056	cell|intrac	-	-	-	-	10:594229	0	3	0	5	4	3	0	0.053662	0	0.069517	0.074848	0.079388	0.017887	0.074585	0.064212	1	-
ENSMUSC	Tex11	testis exp	GO:00325	developm	-	-	-	-	X:100838	0	4	3	5	5	8	0	0.054093	0.033343	0.052557	0.113189	0.106642	0.029145	0.075255	0.106737	1	-
ENSMUSC	Bcr	breakpoir	GO:00325	developm	K08878	breakpoir	mmu0520	Pathways	10:750605	3638	3982	4973	5657	4182	3125	25.29605	23.20834	23.82099	25.62464	25.49862	26.94176	24.10846	26.02167	0.742841	1	-
ENSMUSC	Fxyd5	FXYD don	GO:00511	localizatio	-	-	-	-	7:3103272	118	169	239	204	169	141	1.062365	1.275358	1.482318	1.196476	1.334205	1.57397	1.273347	1.368217	0.873025	1	-
ENSMUSC	Kcnd1	potassium	GO:00511	localizatio	-	-	-	-	X:7822289	201	209	234	275	179	213	2.115628	1.84392	1.696724	1.885636	1.652113	2.779774	1.885424	2.105841	0.722905	1	-
ENSMUSC	Tfcp2	transcripti	GO:00325	developm	-	-	-	-	15:100502	382	455	519	646	443	358	7.469924	7.457911	6.991514	8.229381	7.596253	8.680039	7.30645	8.168558	0.591858	1	-
ENSMUSC	Pou6f2	POU dom	GO:00325	developm	-	-	-	-	13:181210	22	36	19	25	30	23	0.161467	0.221476	0.096066	0.119533	0.193076	0.209308	0.15967	0.173972	0.925834	1	-
ENSMUSC	Pou6f1	POU dom	GO:00325	developm	-	-	-	-	15:100575	1061	2108	2694	2280	2245	1839	5.365621	8.935688	9.385415	7.511401	9.955511	11.53114	7.895575	9.666018	0.302722	1	-
ENSMUSC	Ubp1	upstream	GO:00325	developm	-	-	-	-	9:1139309	1101	1689	1906	2088	1112	769	6.679889	8.589436	7.966295	8.252664	5.916021	5.784877	7.745207	6.651187	0.205053	1	-
ENSMUSC	Nuak2	NUAK fan	GO:00325	developm	-	-	-	-	1:1323161	24	15	20	46	20	25	0.235636	0.123448	0.135276	0.294219	0.172192	0.304334	0.164787	0.256915	0.141573	1	-
ENSMUSC	Lck	intestinal	GO:00325	developm	-	-	-	-	9:7810919	505	440	648	717	506	432	1.964475	1.435078	1.736987	1.817486	1.726485	2.084204	1.712353	1.876058	0.72504	1	-
ENSMUSC	Sdhb	succinate	GO:00081	metabolic	K00235	succinate	mmu0110	Metabolic	4:1409612	1092	1532	1767	1623	1347	1346	26.61038	31.29249	29.66311	25.76491	28.78317	40.66864	29.18866	31.73891	0.787421	1	-

ENSMUSG	Cox4i2	cytochron	GO:00081	metabolic	K02263	cytochron	mmu0110	Metabolic	2:1527541	7	10	15	10	13	17	0.382545	0.458024	0.564688	0.35598	0.622935	1.151817	0.468419	0.710244	0.411304	1	-
ENSMUSG	Snap47	synaptosc	GO:00452	synapse|lc	-	-	-	-	11:594071	3192	4117	4870	4622	3846	4017	49.10704	53.09023	51.61327	46.32255	51.88394	76.62468	51.27018	58.27706	0.539877	1	-
ENSMUSG	Wnt3a	wingless-t	GO:00325	developm	K00312	wingless-t	mmu0520	Pathways	11:592480	0	0	0	0	3	0	0	0	0	0	0.060548	0	0	0.020183	0.30424	1	-
ENSMUSG	Kdsr	3-ketodih	GO:00081	metabolic	K04708	3-dehydro	mmu0110	Metabolic	1:1067204	539	692	901	851	621	593	3.986392	4.28993	4.59058	4.100175	4.027406	5.437908	4.288967	4.52183	0.956649	1	-
ENSMUSG	Vps4b	vacuolar p	GO:00081	metabolic	K12196	vacuolar p	mmu0414	Endocytos	1:1067640	852	891	1311	1248	888	403	5.323684	4.666628	5.643215	5.080056	4.865509	3.122217	5.211176	4.355927	0.201332	1	-
ENSMUSG	Rps25	ribosomal	GO:00329	macromo	K02975	small subu	mmu0301	Ribosome	9:4440713	425	728	954	802	624	314	6.110867	8.774011	9.449606	7.512249	7.867573	5.597956	8.111494	6.992593	0.274163	1	-
ENSMUSG	Nxf2	nuclear RI	GO:00081	metabolic	K14284	nuclear RI	mmu0516	Herpes sir	X:1349445	0	0	0	2	2	0	0	0	0	0.026794	0.036068	0	0	0.020954	0.155992	1	-
ENSMUSG	Taz	tafazzin [S	GO:00325	developm	K13511	monolyso	mmu0056	Glyceroph	X:7428191	181	322	359	479	267	460	0.775716	1.156731	1.059913	1.337339	1.003408	2.444378	0.997453	1.595042	0.034172	1	-
ENSMUSG	Kif19a	kinesin fai	GO:00990	supramole	-	-	-	-	11:114765	64	66	103	110	70	70	0.723225	0.625153	0.801825	0.809775	0.693633	0.980793	0.716734	0.828067	0.584815	1	-
ENSMUSG	Aldh3a2	aldehyde	GO:00325	developm	K00128	aldehyde	mmu0110	Metabolic	11:612234	697	797	982	1034	813	793	6.406776	6.140699	6.218274	6.191687	6.552998	9.03786	6.25525	7.260848	0.436751	1	-
ENSMUSG	Zmynd10	zinc finge	GO:00099	cellular pr	-	-	-	-	9:1075472	50	56	71	61	55	43	0.986245	0.925878	0.964764	0.783832	0.951293	1.05164	0.958962	0.928922	0.697019	1	-
ENSMUSG	Tmem115	transmem	GO:00081	metabolic	-	-	-	-	9:1075339	326	417	454	492	424	429	9.443163	10.12485	9.059558	9.284243	10.7698	15.40788	9.542522	11.82064	0.268956	1	-
ENSMUSG	Hyal2	hyalurono	GO:00325	developm	K01197	hyalurono	mmu0110	Metabolic	9:1075679	70	81	148	91	81	55	0.571517	0.554334	0.832428	0.484013	0.579909	0.55678	0.65276	0.540234	0.227283	1	-
ENSMUSG	Ifrd2	interferon	GO:00056	cell|intrace	-	-	-	-	9:1075876	84	80	115	128	79	114	1.148049	0.916488	1.082765	1.139662	0.946788	1.931859	1.049101	1.339436	0.349579	1	-
ENSMUSG	Hyal1	hyalurono	GO:00325	developm	K01197	hyalurono	mmu0110	Metabolic	9:1075769	38	63	74	151	60	64	0.303966	0.422411	0.407779	0.786868	0.420862	0.634757	0.378052	0.614162	0.036288	1	-
ENSMUSG	Tusc2	tumor sup	GO:00325	developm	-	-	-	-	9:1075632	1200	1391	1616	1695	1290	1085	32.08399	31.17361	29.76457	29.52284	30.244	35.96853	31.00739	31.91179	0.90736	1	-
ENSMUSG	Nprl2	nitrogen p	GO:00081	metabolic	K20405	nitrogen p	mmu0415	mTOR sig	9:1075422	289	363	485	403	305	291	5.014139	5.27908	5.796848	4.554972	4.640251	6.26005	5.363356	5.151758	0.540352	1	-
ENSMUSG	Slc38a3	solute car	GO:00325	developm	K13576	solute car	mmu0472	Glutamate	9:1076506	806	880	1074	1041	814	898	4.44998	4.072476	4.084874	3.74417	3.940853	6.147314	4.202443	4.610779	0.766741	1	-
ENSMUSG	Cacna2d2	calcium ch	GO:00325	developm	K04859	voltage-d	mmu0401	MAPK sigi	9:1073996	517	625	810	688	610	457	2.880165	2.918499	3.108585	2.496874	2.97988	3.156667	2.969083	2.877807	0.574459	1	-
ENSMUSG	Rassf1	Ras assoc	GO:00081	metabolic	K09850	Ras assoc	mmu0520	Pathways	9:1075515	120	144	141	178	122	176	0.864472	0.86953	0.699743	0.835354	0.770674	1.572052	0.811248	1.05936	0.284603	1	-
ENSMUSG	Epn3	epsin 3 [S	GO:00055	extracellul	K12471	epsin[-]	mmu0414	Endocytos	11:944895	66	40	139	67	65	54	0.813516	0.413273	1.180295	0.537999	0.702554	0.82529	0.802361	0.688614	0.4303	1	-
ENSMUSG	Rnf112	ring finge	GO:00325	developm	-	-	-	-	11:614484	5576	7261	7581	9825	6860	6166	59.54824	64.99732	55.77301	68.35343	64.2411	81.64614	60.10619	71.41356	0.267533	1	-
ENSMUSG	Slc3a2	solute car	GO:00081	metabolic	K06519	solute car	mmu0415	mTOR sig	19:870688	1597	2082	2229	2545	1875	1408	15.28296	16.70076	14.69481	15.86617	15.73427	16.70672	15.55951	16.10239	0.96877	1	-
ENSMUSG	Nxf1	nuclear RI	GO:00325	developm	K14284	nuclear RI	mmu0516	Herpes sir	19:875707	1180	1620	1947	2139	1492	1281	7.392071	8.506523	8.402365	8.729242	8.195877	9.949908	8.10032	8.958342	0.615531	1	-
ENSMUSG	Stx5a	syntaxin 5	GO:00081	metabolic	K08490	syntaxin 5	mmu0413	SNARE int	19:874141	451	573	751	754	581	393	1.812014	1.929708	2.078624	1.973503	2.046931	1.957777	1.940115	1.992737	0.944965	1	-
ENSMUSG	Slc47a1	solute car	GO:00511	localizatio	-	-	-	-	11:613434	23	55	63	38	42	59	0.306888	0.61514	0.579093	0.33031	0.491412	0.976101	0.500374	0.599274	0.698088	1	-
ENSMUSG	Pifo	primary ci	GO:00081	metabolic	-	-	-	-	3:1059969	16	12	31	8	7	22	0.747149	0.469707	0.997229	0.243359	0.286647	1.273787	0.738028	0.601264	0.46842	1	-
ENSMUSG	Tnfrsf13b	tumor nec	GO:00325	developm	K05150	tumor nec	mmu0406	Cytokine-	11:611267	19	27	34	15	19	21	0.255449	0.304272	0.314907	0.131379	0.223994	0.350066	0.291543	0.235146	0.313389	1	-
ENSMUSG	Spire2	spire hom	GO:00511	localizatio	-	-	-	-	8:1233327	273	321	358	347	286	250	6.912344	6.812708	6.24448	5.723657	6.349967	7.848535	6.656511	6.64072	0.744261	1	-
ENSMUSG	Prox1	prospero	GO:00325	developm	-	-	-	-	1:1901180	3602	4092	4796	6124	4144	2026	22.54905	21.47203	20.68308	24.97476	22.74819	15.7257	21.56805	21.14955	0.74434	1	-
ENSMUSG	Raver1	ribonucle	GO:00081	metabolic	-	-	-	-	9:2107415	537	276	322	466	601	496	9.83514	4.237096	4.062695	5.559989	9.652148	11.26353	6.044977	8.825222	0.157919	1	-
ENSMUSG	2610507B	RIKEN cDI	GO:00055	extracellul	-	-	-	-	11:782617	6221	7574	8748	9888	7155	5041	35.67617	36.40794	34.56035	36.94095	35.98075	35.84434	35.54815	36.25535	0.877756	1	-
ENSMUSG	AI597479	expressed	GO:00325	developm	-	-	-	-	1:4309871	486	594	767	691	530	447	8.93197	9.150612	9.710863	8.273143	8.541407	10.18601	9.264481	9.000187	0.580711	1	-
ENSMUSG	Tmem86a	transmem	GO:00160	membran	-	-	-	-	7:4705060	149	178	282	259	191	210	4.4374	4.443403	5.785524	5.024859	4.987912	7.754385	4.888776	5.922385	0.39416	1	-
ENSMUSG	Optc	opticin [S	-	-	-	-	-	-	1:1338971	12	22	9	17	0	0	0.144872	0.222636	0.07485	0.133704	0	0	0.147453	0.044568	0.093836	1	-
ENSMUSG	Tex14	testis expr	GO:00081	metabolic	-	-	-	-	11:874050	5	4	3	3	9	2	0.053245	0.035703	0.022007	0.020809	0.084044	0.026414	0.036985	0.043756	0.878401	1	-
ENSMUSG	Ifi35	interferon	GO:00056	cell|intrace	-	-	-	-	11:101448	25	41	50	59	47	59	0.52838	0.72634	0.727989	0.812342	0.871058	1.546115	0.660903	1.076505	0.059149	1	-
ENSMUSG	Rdm1	RAD52 m	GO:00081	metabolic	-	-	-	-	11:101627	40	51	60	56	43	58	0.955334	1.020964	0.987179	0.871283	0.900533	1.717547	0.987826	1.163121	0.671716	1	-
ENSMUSG	Nedd8	neural pre	GO:00081	metabolic	-	-	-	-	14:556622	529	706	792	807	685	461	12.18099	13.62651	12.5633	12.10548	13.83121	13.16176	12.79027	13.03282	0.883561	1	-
ENSMUSG	Gosr1	golgi SNA	GO:00511	localizatio	K08495	golgi SNA	mmu0413	SNARE int	11:767266	930	1133	1464	1536	1040	848	13.62792	13.91648	14.7788	14.66289	13.36358	15.40736	14.10773	14.47794	0.911798	1	-
ENSMUSG	Mrpl52	mitochon	GO:00081	metabolic	-	-	-	-	14:544269	81	100	116	136	118	148	2.102856	2.176098	2.074601	2.300099	2.686269	4.763999	2.117852	3.250122	0.059228	1	-
ENSMUSG	Kansl3	KAT8 regu	GO:00081	metabolic	-	-	-	-	1:3633573	1420	1774	2150	2266	1710	1307	10.15361	10.63258	10.59063	10.55537	10.72188	11.58761	10.45894	10.95495	0.955688	1	-
ENSMUSG	Eya4	EYA trans	GO:00325	developm	-	-	-	-	10:231029	85	74	178	156	127	45	0.428662	0.312812	0.618397	0.51251	0.561622	0.281379	0.45329	0.451837	0.935421	1	-
ENSMUSG	Ebf3	early B ce	GO:00325	developm	-	-	-	-	7:1371936	38	38	63	65	30	22	0.130739	0.109587	0.149319	0.145685	0.090507	0.093851	0.129882	0.110014	0.425402	1	-
ENSMUSG	Myt1	myelin tra	GO:00325	developm	-	-	-	-	2:1817633	287	364	398	547	383	271	1.343626	1.428401	1.283605	1.668266	1.572308	1.573082	1.351877	1.604552	0.328487	1	-
ENSMUSG	Faf1	Fas-assoc	GO:00081	metabolic	K20703	FAS-assoc	mmu0421	Necroptos	4:1096765	414	612	694	631	466	444	5.027886	6.23002	5.806261	4.992253	4.962649	6.685819	5.688056	5.546907	0.60554	1	-
ENSMUSG	Gm266	predicted	GO:00508	response	-	-	-	-	12:111484	29	20	39	36	19	39	1.516907	0.876887	1.405331	1.226727	0.871457	2.529372	1.266375	1.542519	0.669744	1	-
ENSMUSG	Tsacc	TSSK6 act	GO:00081	metabolic	-	-	-	-	3:8828275	11	8	17	8	17	4	0.470776	0.286967	0.501186	0.223038	0.637961	0.212254	0.419643	0.357751	0.673367	1	-
ENSMUSG	Mettl16	methyltrai	GO:00081	metabolic	-	-	-	-	11:747708	871	1030	1213	948	963	557	3.618191	3.586436	3.471245	2.565448	3.507857	2.868892	3.558624	2.980732	0.109788	1	-
ENSMUSG	Dazl	deleted in	GO:00325	developm	-	-	-	-	17:502793	62	78	103	86	73	27	1.339322	1.41235	1.532788	1.210244	1.382807	0.723181	1.428154	1.105411	0.194906	1	-
ENSMUSG	Apol7a	apolipopr	GO:00081	metabolic	-	-	-	-	15:773882	0	4	7	1	3	6	0	0.033153	0.047687	0.006441	0.026011	0.073571	0.026947	0.035341	1	1	-
ENSMUSG	Pigyl	phosphati	GO:00081	metabolic	K11001	phosphati	mmu0110	Metabolic	9:2215684	146	205	245	217	184	221	6.767844	7.965367	7.823782	6.552996	7.479269	12.70217	7.518997	8.911477	0.519339	1	-
ENSMUSG	Rbm25	RNA bind	GO:00081	metabolic	K12822	RNA-binc	mmu0304	Spliceosor	12:836312	2792	3456	3869	4371	3035	1576	8.247269	8.556996	7.873085	8.411186	7.861332	5.772139	8.225783	7.348219	0.306665	1	-
ENSMUSG	Psen2	presenilin	GO:00081	metabolic	K04522	presenilin	mmu0501	Alzheimer	1:1802270	172	192	211	258	174	209	1.496389	1.400134	1.264591	1.462233	1.327417	2.254494	1.387038	1.681381	0.399437	1	-
ENSMUSG	Acaa1b	acetyl-Co	GO:00081	metabolic	K07513	acetyl-Co	mmu0110	Metabolic	9:1191480	25	49	57	51	36	118	0.700238	1.150396	1.099844	0.930587	0.884199	4.09799	0.983493	1.970925	0.081383	1	-
ENSMUSG	Plcd1	phospholi	GO:00325	developm	K05857	phosphati	mmu0110	Metabolic	9:1190715	119	151	167	200	136	184	2.085715	2.218388	2.016396	2.283601	2.090205	3.998631	2.106833	2.790812	0.216924	1	-
ENSMUSG	Fads1	fatty acid	GO:00325	developm	K10224	acyl-CoA	mmu0121	Fatty acid	19:101828	4282	4850	6219	6137	4836	3288	78.74247	74.75788	78.78334	73.51909	77.98146	74.96873	77.4279	75.48976	0.58493	1	-
ENSMUSG	Lmbr1	limb regic	GO:00325	developm	-	-	-	-	5:2922980	410	428	647	711	551	398	2.104064	1.841074	2.287342	2.376985	2.479531	2.532469	2.077493	2.462995	0.327876	1	-
ENSMUSG	Tnfrsf22	tumor nec	GO:00325	developm	-	-	-	-	7:1436348	11	16	25	31	18	14	0.088888	0.108372	0.139162	0.163185	0.127539	0.140271	0.112141	0.143665	0.489629	1	-
ENSMUSG	Cars	cysteinyl-t	GO:00081	metabolic	K01883	cysteinyl-t	mmu0097	Aminoacy	7:1435572	628	741	933	928	795	547	4.607627	4.557102	4.715753	4.43555	5.114786	4.976128	4.626827	4.842155	0.961018	1	-
ENSMUSG	Wnt2	wingless-t	GO:00325	developm	K00182	wingless-t	mmu0520	Pathways	6:1798894	65	72	95	131	69	83	1.831894	1.700874	1.844441	2.405153	1.705217	2.900376	1.792403	2.336915	0.243979	1	-
ENSMUSG	Gabra1	gamma-a	GO:00048	signal trar	K05175	gamma-a	mmu0408	Neuroacti	11:421309	5470	6700	2547	2546	6678	3072	41.09646	42.19342	13.18249	12.46112	43.99529	28.61701	32.15746	28.35781	0.558111	1	-
ENSMUSG	Grid2ip	glutamate	GO:00452	synapse|re	-	-	-	-	5:1433573	15	19	37	28	34	20	0.115398	0.122521	0.196091	0.140329	0.229367	0.190774	0.14467	0.186823	0.432619	1	-
ENSMUSG	Kdelr3	KDEL (Lys	GO:00511	localizatio	-	-	-	-	15:795164	5	5	15	10	0	5	0.22473	0.18836	0.46445	0.29279	0	0.278624	0.292513	0.190471	0.374833	1	-
ENSMUSG	Apip	APAF1 int	GO:00081	metabolic	K08964	methylthic	mmu0110	Metabolic	2:1030736	109	127	154	135	169	86	3.789534	3.700976	3.688343	3.057552	5.152164	3.707203	3.726284	3.972306	0.89776	1	-
ENSMUSG	Pdhx	pyruvate c	GO:00081	metabolic	K13997	dihydrolip	mmu0110	Metabolic	2:1030210	831	949	1201	1267	958	639	12.33362	11.80615	12.27959	12.25033	12.46803	11.75916	12.13979	12.15917	0.783167	1	-
ENSMUSG	Vac14	Vac14 hoi	GO:00081	metabolic	K15305	vacuole m	mmu0516	HTLV-I int	8:1106185	392	489	495	586	402	383	2.161818	2.260446	1.880569	2.105292	1.944026	2.618889	2.100944	2.222736	0.937209	1	-
ENSMUSG	Mcoln2	mucolipin	GO:00400	locomotic	-	-	-	-	3:1461498	0	0	2	0	0	2	0	0	0.017656	0	0	0.031783	0.005885	0.010594	1	1	-
ENSMUSG	Slc5a1	solute car	GO:00511	localizatio	K14158	solute car	mmu0497	Bile secret	5:3310421	4	6	9	4	2	3	0.06194	0.077878	0.096007	0.040357	0.027156	0.057607	0.078608	0.041707	0.177433	1	-
ENSMUSG	Akt1s1	AKT1 subs	GO:00081	metabolic	K16184	proline-ric	mmu0415	mTOR sig	7:4484899	629	534	746	879	638	672	5.356402	3.811686	4.376357	4.876332	4.76416	7.095416	4.514815	5.578636	0.264787	1	-
ENSMUSG	Tbrg1	transform	GO:00081	metabolic	-	-	-	-	9:3764876	347	463	567	509	418	416	3.587583	4.012422	4.038385	3.42825	3.789589	5.332767	3.879464	4.183535	0.856545	1	-
ENSMUSG	Adssl1	adenylosu	GO:00081	metabolic	K01939	adenylosu	mmu0110	Metabolic	12:112620	164	156	291	248	179	175	3.171855	2.528976	3.877152	3.124652	3.035744	4.196551	3.192661	3.452316	0.853625	1	-
ENSMUSG	Cfap161	cilia and f	GO:00160	membran	-	-	-	-	7:8377410	26	24	60	29	20	12	0.360551	0.278964	0.57318	0.261981	0.243203	0.206325	0.404232	0.23717	0.035349	1	-
ENSMUSG	Brf1	BRF1, RNA	GO:00081	metabolic	-	-	-	-	12:112960	676	821	999	1130	845	623	4.409958	4.489354	4.489572	4.802286	4.833784	5.039207	4.462961	4.891759	0.668157	1	-
ENSMUSG	Vipr2	vasoactive	GO:00048	signal trar	K04590	vasoactive	mmu0408	Neuroacti	12:116077	6	17	9	11	7	6	0.073802	0.175269	0.076256	0.088136	0.075504	0.091511	0.108442	0.08505	0.540872	1	-
ENSMUSG	Odc1	ornithine c	GO:00325	developm	K01581	ornithine c	mmu0110	Metabolic	12:175447	770	993	1258	1114	858	503	7.091123	7.665251	7.980979	6.683298	6.928734	5.743524	7.579118	6.451852	0.145585	1	-
ENSMUSG	Thg1l	tRNA-hist	GO:00081	metabolic	-	-	-	-	11:459468	139	124	146	158	136	121	2.33886	1.748896	1.69236	1.731915	2.006641	2.524403	1.926705	2.087653	0.854281	1	-
ENSMUSG	Adam19	a disinteg	GO:00325	developm	-	-	-	-	11:460559	355	381	477	521	399	285	3.031216	2.726878	2.805805	2.898058	2.987468	3.017307	2.854633	2.967611	1	1	-
ENSMUSG	Pabpc4	poly(A) bi	GO:00325	developm	K13126	polyadeny	mmu0301	RNA trans	4:1232623	434	544	616	709	595	369	3.132538	3.291232	3.062943	3.333763	3.765878	3.302321	3.162238	3.467321	0.67767	1	-
ENSMUSG	Exoc3l2	exocyst cc	GO:00511	localizatio	-	-	-	-	7:1946333	8	3	8	5	5	4	0.083427	0.026223	0.057474	0.033972	0.045721	0.051722	0.055708	0.043805	0.566117	1	-
ENSMUSG	Zfp296	zinc finge	GO:00081	metabolic	-	-	-	-	7:1957728	14	19	23	16	10	10	0.410207	0.466643	0.46426	0.305405	0.256939	0.363317	0.447037	0.308554	0.162632	1	-
ENSMUSG	Plin5	perilipin 5	GO:00081	metabolic	K20255	perilipin-5	mmu0332	PPAR sign	17:561116	9	9	3	9	7	3	0.18233	0.152826	0.041867	0.118779	0.124363	0.075365	0.125674	0.106169	0.720814	1	-
ENSMUSG	Sugp1	SURP and	GO:00081	metabolic	-	-	-	-	8:7004281	951	1229	1410	1743	1303	686	8.928782	9.672003	9.11973	10.66082	10.7275	7.985865	9.240172	9.791397	0.84272	1	-
ENSMUSG	Dhdh	dihydrodi	GO:00081	metabolic	-	-	mmu0098	Metabolis	7:4547098	212	279	361	431	280	187	1.750911	1.931452	2.053935	2.318925	2.027812	1.914939	1.9121	2.087225	0.718242	1	-
ENSMUSG	Zfp790	zinc finge	GO:00081	metabolic	-	-	-	-	7:2981611	378	400	558	556	377	207	3.487161	3.093091	3.546218	3.341461	3.049751	2.367751	3.37549	2.919654	0.256452	1	-
ENSMUSG	Cpb1	carboxype	GO:00081	metabolic	K01291	carboxype	mmu0497	Pancreatic	3:2024826	0	0	0	1	0	1	0	0	0	0.011493	0	0.021871	0	0.011121	0.52785	1	-
ENSMUSG	Slc25a41	solute car	GO:00511	localizatio	-	-	-	-	17:570327	0	3	2	3	0	0	0	0.096037	0.052628	0.074633	0	0	0.049555	0.024878	0.513905	1	-
ENSMUSG	Fsd1	fibronecti	GO:00990	supramole	-	-	-	-	17:559865	504	650	687	719	620	553	18.32419	19.80892	17.20691	17.02963	19.76646	24.92905	18.44668	20.57505	0.644166	1	-
ENSMUSG	Fuz	fuzzy plar	GO:00325	developm	-	-	-	-	7:4489607	148	187	172	201	169	151	0.728458	0.771502	0.583206	0.644496	0.729411	0.921522	0.694389	0.765143	0.764552	1	-
ENSMUSG	Sptbn4	spectrin b	GO:00325	developm	-	-	-	-	7:2735638	3717	4951	5668	6801	5301	4425	19.48903	21.75923	20.47288	23.23015	24.37239	28.76715	20.57371	25.45657	0.136226	1	-
ENSMUSG	Pgam1	phosphog	GO:00081	metabolic	K01834	2,3-bisph	mmu0110	Metabolic	19:419118	5526	7018	7998	8107	6447	4978	191.8044	204.1804	191.2408	183.3114	196.2222	214.2341	195.7419	197.9226	0.792074	1	-
ENSMUSG	Evi5	ecotropic	GO:00511	localizatio	-	-	-	-	5:1077447	1260	1603	2113	1966	1459	941	7.645292	8.15286	8.832312	7.771209	7.762856	7.079446	8.210155	7.537837	0.328027	1	-
ENSMUSG	Evi5l	ecotropic	GO:00511	localizatio	-	-	-	-	8:4166567	1464	2161	2191	2862	1706	1542	5.651214	6.992111	5.826323	7.197008	5.774608	7.380251	6.156549	6.783956	0.651604	1	-
ENSMUSG	Snapc2	small nucl	GO:00081	metabolic	-	-	-	-	8:4253080	246	342	295	381	247	394	4.572685	5.328629	3.777557	4.613636	4.02603	9.080694	4.559624	5.906787	0.293373	1	-
ENSMUSG	Git1	G protein-	GO:00325	developm	K05737	G protein-	mmu0414	Endocytos	11:774935	7543	8342	10101	12238	9017	6539	79.3939	73.598	73.24177	83.91413	83.22384	85.33758	75.41122	84.15852	0.531828	1	-
ENSMUSG	Gltp	glycolipid	GO:00511	localizatio	-	-	-	-	5:1146693	615	482	900	604	624	358	12.07821	7.934644	12.17648	7.727625	10.74619	8.717592	10.72978	9.063803	0.195554	1	-
ENSMUSG	Bnip2	BCL2/ade	GO:00325	developm	-	-	-	-	9:6998946	363	426	554	555	361	258	3.960471	3.89585	4.163913	3.944709	3.453739	3.490166	4.006745	3.629538	0.330816	1	-
ENSMUSG	Ccnt1	cyclin T1 [	GO:00081	metabolic	K15188	cyclin T[-]	mmu0520	Transcript	15:985386	786	1036	1306	1299	949	646	3.861223	4.265946	4.419744	4.157127	4.088004	3.934788	4.182304	4.059973	0.601691	1	-
ENSMUSG	Scarf2	scavenger	GO:00511	localizatio	-	-	-	-	16:177972	33	64	46	82	48	78	0.635144	1.032503	0.609908	1.02815	0.810114	1.861409	0.759185	1.233225	0.082752	1	-
ENSMUSG	Brms1l	breast car	GO:00081	metabolic	-	-	-	-	12:558363	980	1515	1617	1749	1265	825	11.45942	14.84917	13.02561	13.32318	12.97089	11.96125	13.1114	12.75177	0.631167	1	-
ENSMUSG	Med15	mediator	GO:00081	metabolic	-	-	-	-	16:176512	1161	1200	1578	1349	1039	873	21.81054	18.89592	20.42173	16.50927	17.11561	20.33455	20.37606	17.98648	0.168825	1	-
ENSMUSG	Dhdds	dehydrod	GO:00081	metabolic	K11778	ditrans,po	mmu0090	Terpenoic	4:1339690	540	796	480	1186	446	823	2.229786	2.755086	1.365408	3.190329	1.614905	4.213617	2.11676	3.006283	0.162452	1	-
ENSMUSG	Crybg2	crystallin b	-	-	-	-	-	-	4:1340608	7	3	3	8	15	5	0.042187	0.015153	0.012454	0.031406	0.079264	0.037358	0.023265	0.049343	0.145608	1	-
ENSMUSG	Ubxn11	UBX dom	GO:00081	metabolic	-	-	-	-	4:1341025	156	189	218	211	160	131	1.409676	1.431562	1.357071	1.242107	1.26782	1.467753	1.399436	1.325893	0.533569	1	-
ENSMUSG	Mogat1	monoacyl	GO:00081	metabolic	K14458	2-acylglyc	mmu0056	Glycerolip	1:7851099	0	2	0	2	5	1	0	0.037355	0	0.02903	0.097694	0.027622	0.012452	0.051448	0.16638	1	-
ENSMUSG	Tjap1	tight junct	GO:00099	cellular pr	K06105	tight junct	mmu0453	Tight junc	17:462578	296	337	456	464	380	347	1.716082	1.63768	1.821223	1.752451	1.931847	2.494373	1.724995	2.059557	0.350068	1	-
ENSMUSG	Rpl15	ribosomal	GO:00081	metabolic	K02877	large subu	mmu0301	Ribosome	14:182678	559	778	1005	831	663	775	8.819796	10.28914	10.92356	8.54141	9.172814	15.16123	10.01083	10.95848	0.801237	1	-
ENSMUSG	Tmem167	transmem	GO:00511	localizatio	-	-	-	-	13:900891	725	923	1214	1132	832	554	4.126449	4.403449	4.760017	4.197265	4.152449	3.909614	4.429972	4.086443	0.365509	1	-
ENSMUSG	Steap4	STEAP far	GO:00325	developm	-	-	-	-	5:7960457	21	7	22	20	9	16	0.423023	0.118186	0.305284	0.262456	0.158978	0.399615	0.282165	0.273683	0.80742	1	-
ENSMUSG	Mplkip	M-phase	GO:00099	cellular pr	-	-	-	-	13:176951	130	132	162	144	123	87	1.091401	0.928894	0.936927	0.787563	0.905496	0.90562	0.985741	0.866227	0.302203	1	-
ENSMUSG	Kif11	kinesin fai	GO:00990	supramole	-	-	-	-	19:373764	22	41	55	39	26	27	0.290131	0.453228	0.499683	0.335062	0.300674	0.441502	0.414347	0.359079	0.411085	1	-
ENSMUSG	Rpa3	replicatior	GO:00081	metabolic	K10740	replicatior	mmu0346	Fanconi a	6:8255936	39	34	68	42	43	47	1.833268	1.339664	2.202021	1.286133	1.772425	2.739334	1.791651	1.932631	0.974723	1	-
ENSMUSG	Mlkl	mixed line	GO:00048	signal trar	K08849	mixed line	mmu0421	Necroptos	8:1113117	6	7	7	15	3	3	0.111209	0.108742	0.08938	0.18111	0.048753	0.068947	0.10311	0.099603	1	1	-
ENSMUSG	Tnpo3	transporti	GO:00511	localizatio	-	-	-	-	6:2954082	1363	1391	1982	2140	1569	1176	4.846027	4.145436	4.854507	4.956618	4.891652	5.18422	4.615323	5.01083	0.716844	1	-
ENSMUSG	Ttll5	tubulin tyr	GO:00325	developm	-	-	-	-	12:858246	766	790	1139	1023	834	444	2.375061	2.053175	2.432882	2.066345	2.267537	1.706928	2.287039	2.013603	0.260153	1	-
ENSMUSG	Zfp715	zinc finge	GO:00081	metabolic	-	-	-	-	7:4329619	572	143	737	879	536	370	5.480523	1.148459	4.864578	5.486517	4.50333	4.395555	3.831187	4.795134	0.493532	1	-
ENSMUSG	Acp4	acid phos	GO:00325	developm	-	-	-	-	7:4425214	0	0	0	2	0	0	0	0	0	0.042112	0	0	0	0.014037	0.538726	1	-
ENSMUSG	Cdh23	cadherin 2	GO:00325	developm	-	-	-	-	10:603027	12	59	55	39	35	30	0.035447	0.14609	0.111926	0.075052	0.090663	0.109883	0.097821	0.091866	0.714499	1	-
ENSMUSG	Rps5	ribosomal	GO:00081	metabolic	K02989	small subu	mmu0301	Ribosome	7:1292229	863	1188	1378	1343	988	1416	20.17889	23.28392	22.19664	20.45711	20.25752	41.05214	21.88648	27.25559	0.31534	1	-
ENSMUSG	Adgrl1	adhesion	GO:00325	developm	-	-	-	-	8:8390010	9493	10286	11357	12959	10721	9285	40.81098	37.06576	33.63478	36.29332	40.41583	49.49273	37.17051	42.06729	0.509859	1	-
ENSMUSG	Amotl1	angiomot	GO:00400	locomotic	K06104	angiomot	mmu0453	Tight junc	9:1454196	538	545	739	749	572	538	3.023389	2.567209	2.860935	2.74205	2.818714	3.748693	2.817178	3.103152	0.707535	1	-
ENSMUSG	Etv5	ets variar	GO:00325	developm	K15593	ets transl	mmu0520	Transcript	16:223813	1126	1325	1743	1889	1452	1220	10.07897	9.941373	10.74797	11.01517	11.3969	13.54015	10.2561	11.98408	0.33623	1	-
ENSMUSG	Tmem190	transmem	GO:00325	developm	-	-	-	-	7:4782937	0	2	0	0	0	2	0	0.106857	0	0	0	0.158116	0.035619	0.052705	1	1	-
ENSMUSG	Gfod2	glucose-f	GO:00081	metabolic	-	-	-	-	8:1057138	254	354	398	351	322	310	3.127175	3.653207	3.375616	2.815181	3.47629	4.732229	3.385333	3.674567	0.838653	1	-
ENSMUSG	Enkd1	enkurin d	GO:00990	supramole	-	-	-	-	8:1057036	72	133	139	130	103	130	0.759725	1.176323	1.010394	0.893612	0.953024	1.700802	0.982147	1.182479	0.535591	1	-
ENSMUSG	Atp6v0d1	ATPase, H	GO:00325	developm	K02146	V-type H-	mmu0110	Metabolic	8:1055244	2471	3105	3621	3691	2783	2626	73.79674	77.72827	74.49797	71.81087	72.88202	97.24007	75.341	80.64432	0.849382	1	-
ENSMUSG	Ptprs	protein ty	GO:00325	developm	-	-	-	-	17:564124	9132	10429	10924	13162	10433	9211	26.15204	25.03429	21.55127	24.55518	26.19941	32.70637	24.24586	27.82032	0.43615	1	-
ENSMUSG	Slc41a1	solute car	GO:00511	localizatio	-	-	-	-	1:1318274	1447	1773	2183	2338	1689	1423	17.22765	17.69372	17.90452	18.13359	17.63314	21.00624	17.60863	18.92433	0.795285	1	-
ENSMUSG	Iglon5	IgLON far	GO:00055	extracellul	-	-	-	-	7:4347290	429	521	667	725	527	398	9.124576	9.288512	9.773105	10.04559	9.828987	10.49601	9.395398	10.12353	0.783397	1	-
ENSMUSG	Igf2bp1	insulin-lik	GO:00325	developm	K17391	insulin-lik	mmu0520	MicroRNA	11:959571	0	0	3	0	1	0	0	0	0.012575	0	0.005336	0	0.004192	0.001779	0.665813	1	-
ENSMUSG	B4galnt2	beta-1,4-	GO:00081	metabolic	-	-	-	-	11:958659	7	10	4	1	2	3	0.258362	0.309339	0.101711	0.02404	0.064721	0.137293	0.223138	0.075351	0.045547	1	-
ENSMUSG	Zfp651	zinc finge	GO:00971	organic cy	-	-	-	-	9:1217593	1446	1560	2039	2189	1671	1402	10.05885	9.096149	9.771215	9.919902	10.19293	12.09244	9.642071	10.73509	0.584533	1	-
ENSMUSG	Nelfb	negative e	GO:00081	metabolic	-	-	-	-	2:2519971	525	692	825	784	569	495	5.286015	5.840204	5.72235	5.142409	5.023702	6.179605	5.61619	5.448572	0.568056	1	-
ENSMUSG	Card14	caspase re	GO:00081	metabolic	K20913	caspase re	mmu0406	NF-kappa	11:119307	11	21	7	23	17	15	0.103265	0.165242	0.045271	0.140656	0.139937	0.174595	0.104592	0.151729	0.315652	1	-
ENSMUSG	Tmem175	transmem	GO:00511	localizatio	-	-	-	-	5:1086297	258	333	413	439	387	409	1.810987	1.959263	1.997086	2.007437	2.382038	3.559631	1.922446	2.649702	0.079329	1	-
ENSMUSG	Bcas1	breast car	GO:00055	extracellul	-	-	-	-	2:1703469	2756	2443	4639	3681	2789	1675	25.63702	19.04867	29.72789	22.30672	22.7499	19.31919	24.80452	21.4586	0.202458	1	-
ENSMUSG	Tango2	transport	GO:00056	cell|intrace	-	-	-	-	16:183008	531	607	743	741	551	737	3.50469	3.358122	3.378276	3.186067	3.188961	6.031261	3.413696	4.13543	0.391707	1	-
ENSMUSG	Aldh1a2	aldehyde	GO:00325	developm	K07249	retinal del	mmu0110	Metabolic	9:7121578	103	121	172	195	115	117	2.891314	2.847073	3.326131	3.565952	2.830748	4.072206	3.021506	3.489635	0.544613	1	-
ENSMUSG	Ndufs2	NADH de	GO:00081	metabolic	K03935	NADH de	mmu0110	Metabolic	1:1712348	2073	2700	3075	2857	2408	2479	44.37366	48.44428	45.34427	39.83986	45.19861	65.79433	46.05407	50.2776	0.768684	1	-
ENSMUSG	Snx31	sorting ne	GO:00511	localizatio	-	-	-	-	15:365040	2	6	7	8	5	0	0.030414	0.076462	0.073321	0.079237	0.066656	0	0.060066	0.048631	0.878421	1	-
ENSMUSG	Atraid	all-trans r	GO:00325	developm	-	-	-	-	5:3104831	315	421	402	365	329	338	5.191701	5.816127	4.564324	3.918982	4.754859	6.907195	5.190717	5.193679	0.746516	1	-
ENSMUSG	Cad	carbamoy	GO:00325	developm	K11540	carbamoy	mmu0110	Metabolic	5:3105478	288	344	431	452	349	428	1.630873	1.632818	1.681341	1.667427	1.732989	3.005088	1.648344	2.135168	0.196182	1	-
ENSMUSG	Sh3bp5l	SH3 bindi	GO:00081	metabolic	-	-	-	-	11:583307	553	581	811	555	468	480	3.051559	2.68736	3.082969	1.995131	2.264569	3.284159	2.940629	2.51462	0.150894	1	-
ENSMUSG	Atad1	ATPase fa	GO:00081	metabolic	-	-	-	-	19:326725	1448	1782	2252	2043	1563	1283	32.71411	33.74636	35.04985	30.06881	30.96478	35.94008	33.83677	32.32456	0.4539	1	-
ENSMUSG	Pten	phosphata	GO:00325	developm	K01110	phosphati	mmu0520	Pathways	19:327574	3833	4741	5767	5931	4377	2224	18.79765	19.48891	19.48346	18.94847	18.82278	13.52338	19.25667	17.09821	0.274582	1	-
ENSMUSG	Pea15a	phosphop	GO:00511	localizatio	-	-	-	-	1:1721967	7228	9221	10633	10211	7706	7035	95.26362	101.8685	96.54197	87.67157	89.05957	114.9632	97.89135	97.23146	0.655677	1	-
ENSMUSG	Timm23	translocas	GO:00511	localizatio	-	-	-	-	14:321801	202	272	246	338	211	166	2.997368	3.383061	2.514632	3.267283	2.745447	3.054097	2.96502	3.022275	0.912163	1	-
ENSMUSG	Tnfaip8l2	tumor nec	GO:00023	immune s	-	-	-	-	3:9513952	31	29	49	30	35	20	1.755948	1.376856	1.912031	1.107	1.738434	1.404619	1.681611	1.416684	0.359118	1	-
ENSMUSG	Trnt1	tRNA nucl	GO:00081	metabolic	K00974	tRNA nucl	mmu0301	RNA trans	6:1067691	412	441	622	522	383	322	4.772851	4.282246	4.963887	3.939425	3.890645	4.625111	4.672995	4.151727	0.231854	1	-
ENSMUSG	Ly6g6e	lymphocy	GO:00048	signal trar	-	-	-	-	17:350769	30	47	51	59	31	46	0.717039	0.941599	0.839725	0.918658	0.649706	1.36322	0.832788	0.977195	0.676866	1	-
ENSMUSG	Ehmt2	euchroma	GO:00325	developm	K11420	euchroma	mmu0421	Longevity	17:348984	2523	3127	3391	3781	2865	2837	15.33807	15.93435	14.20146	14.97414	15.27288	21.38449	15.15796	17.2105	0.515039	1	-
ENSMUSG	Elof1	ELF1 hom	GO:00081	metabolic	-	-	-	-	9:2211298	244	292	360	342	273	380	3.96597	3.978278	4.031002	3.621319	3.891034	7.658248	3.99175	5.056867	0.316289	1	-
ENSMUSG	Med16	mediator	GO:00081	metabolic	K15159	mediator	mmu0491	Thyroid hc	10:798947	749	1006	925	1146	903	888	7.568961	8.521289	6.439434	7.54433	8.001752	11.12636	7.509895	8.890815	0.379005	1	-
ENSMUSG	St3gal1	ST3 beta-	GO:00081	metabolic	K00780	beta-gala	mmu0110	Metabolic	15:671028	425	528	658	837	594	559	5.418261	5.642317	5.778942	6.95149	6.640487	8.836271	5.613173	7.476083	0.064936	1	-
ENSMUSG	Tmem259	transmem	GO:00081	metabolic	-	-	-	-	10:799749	1287	1696	1898	1959	1462	1268	8.52629	9.418036	8.662222	8.454693	8.493206	10.41566	8.868849	9.121187	0.899815	1	-
ENSMUSG	Clip3	CAP-GLY	GO:00325	developm	-	-	-	-	7:3029172	8579	10086	11431	11727	9379	6073	88.09522	86.81354	80.86327	78.44842	84.45295	77.32232	85.25734	80.07457	0.390255	1	-
ENSMUSG	Thap8	THAP dor	-	-	-	-	-	-	7:3028009	2	3	3	2	2	1	0.039396	0.049521	0.0407	0.025656	0.034536	0.024412	0.043206	0.028202	0.61759	1	-
ENSMUSG	Dedd	death effe	GO:00325	developm	-	-	-	-	1:1713291	221	308	313	351	325	244	1.271983	1.485909	1.241034	1.316063	1.640268	1.741259	1.332975	1.565863	0.419418	1	-
ENSMUSG	Mcemp1	mast cell e	GO:00160	membran	-	-	-	-	8:3665754	4	0	2	2	0	0	0.170149	0	0.058616	0.055417	0	0	0.076255	0.018472	0.374354	1	-
ENSMUSG	Nit1	nitrilase 1	GO:00081	metabolic	-	-	-	-	1:1713380	327	458	499	553	467	257	2.190787	2.572003	2.303059	2.413571	2.743551	2.134876	2.355283	2.430666	0.998677	1	-
ENSMUSG	Pax5	paired bo	GO:00325	developm	K09383	paired bo	mmu0520	Transcript	4:4452475	4	12	8	9	12	5	0.025891	0.065112	0.035674	0.037952	0.068115	0.040128	0.042226	0.048731	0.82691	1	-
ENSMUSG	Prdm15	PR domai	GO:00056	cell|intrace	-	-	-	-	16:977914	382	445	550	613	425	275	1.196277	1.168103	1.18654	1.250577	1.167078	1.067792	1.18364	1.161816	0.710943	1	-
ENSMUSG	Rnf168	ring finge	GO:00325	developm	-	-	-	-	16:322774	748	890	908	978	748	519	5.963823	5.947937	4.987246	5.079769	5.22959	5.130705	5.633002	5.146688	0.325175	1	-
ENSMUSG	Tctex1d2	Tctex1 do	GO:00511	localizatio	-	-	-	-	16:324197	71	60	103	95	79	89	1.421631	1.007011	1.420751	1.239176	1.387072	2.209546	1.283131	1.611931	0.403202	1	-
ENSMUSG	Chp1	calcineuri	GO:00081	metabolic	-	-	-	-	2:1195476	884	986	1097	1022	834	660	8.858542	8.282083	7.572999	6.671789	7.328555	8.200489	8.237875	7.400278	0.235344	1	-

ENSMUSC	Trpv4	transient r	GO:00325	developm	K04973	transient r	mmu0421	Cellular se	5:1146221	100	40	212	67	84	55	0.923198	0.309534	1.348286	0.402952	0.68001	0.629572	0.86034	0.570845	0.12599	1	-
ENSMUSC	Klhl3	kelch-like	GO:00325	developm	-	-	-	-	13:580002	1335	2393	2756	1940	1653	1469	11.58744	17.41013	16.47924	10.96957	12.5812	15.80936	15.15894	13.12004	0.169746	1	-
ENSMUSC	Tvp23b	trans-gol	GO:00511	localizatio	-	-	-	-	11:628794	303	361	521	458	312	347	6.989688	6.980303	8.27949	6.88274	6.311203	9.924978	7.416494	7.706307	0.94681	1	-
ENSMUSC	Dnajc7	DnaJ heat	GO:00099	cellular pr	-	-	-	-	11:100582	2066	2662	3206	3442	2607	1456	21.86156	23.61085	23.37038	23.72702	24.18992	19.10284	22.9476	22.33993	0.649648	1	-
ENSMUSC	Zfp385c	zinc finge	GO:00508	response	-	-	-	-	11:100627	0	0	7	5	4	0	0	0	0.080611	0.054451	0.058626	0	0.02687	0.037692	0.858009	1	-
ENSMUSC	Cacybp	calcyclin b	GO:00325	developm	K04507	calcyclin b	mmu0431	Wnt signa	1:1602023	1326	1959	2103	1865	1459	658	26.88937	33.29848	29.37838	24.63755	25.94389	16.54434	29.85541	22.37526	0.037094	1	-
ENSMUSC	Cluap1	clusterin a	GO:00325	developm	-	-	-	-	16:390880	471	539	636	671	535	336	10.83368	10.39195	10.07776	10.05448	10.79076	9.582551	10.43446	10.1426	0.639737	1	-
ENSMUSC	Zswim7	zinc finge	GO:00081	metabolic	-	-	-	-	11:622672	39	61	55	70	37	61	3.386035	4.439237	3.289582	3.959172	2.816901	6.566563	3.704951	4.447545	0.601625	1	-
ENSMUSC	Pigl	phosphati	GO:00081	metabolic	K03434	N-acetylg	mmu0110	Metabolic	11:624584	122	131	142	194	140	84	3.156938	2.841391	2.531328	3.270325	3.176724	2.695084	2.843219	3.047378	0.835357	1	-
ENSMUSC	Ndufa2	NADH de	GO:00081	metabolic	K03946	NADH de	mmu0110	Metabolic	18:367423	223	306	362	293	215	430	24.77675	28.49803	27.70768	21.2075	20.94694	59.23717	26.99416	33.7972	0.475305	1	-
ENSMUSC	Pam16	presequer	GO:00081	metabolic	-	-	-	-	16:461646	20	31	30	35	33	51	1.10528	1.435973	1.142105	1.260067	1.599195	3.494588	1.227786	2.11795	0.091608	1	-
ENSMUSC	Glis2	GLIS famil	GO:00325	developm	-	-	-	-	16:459471	95	84	123	111	99	61	0.718582	0.532581	0.640931	0.546961	0.656649	0.572093	0.630698	0.591901	0.587457	1	-
ENSMUSC	Cox6c	cytochron	GO:00081	metabolic	K02268	cytochron	mmu0110	Metabolic	15:359258	802	992	1108	829	777	829	9.640536	9.995192	9.17525	6.491766	8.190123	12.35571	9.603659	9.012532	0.432484	1	-
ENSMUSC	Bicc1	BicC famil	GO:00325	developm	-	-	-	-	10:709228	142	185	283	240	211	130	1.086374	1.186361	1.491522	1.196147	1.415523	1.233168	1.254752	1.281613	0.939403	1	-
ENSMUSC	Ube2z	ubiquitin-	GO:00081	metabolic	K10585	ubiquitin-	mmu0412	Ubiquitin	11:960474	1647	1996	2505	2482	1830	1521	26.18759	26.60207	27.43855	25.70901	25.51501	29.98591	26.74274	27.06998	0.799382	1	-
ENSMUSC	Tmem87b	transmem	GO:00511	localizatio	-	-	-	-	2:1288181	345	344	803	511	602	562	2.209817	1.846918	3.543268	2.132256	3.381242	4.463329	2.533334	3.325609	0.249116	1	-
ENSMUSC	Anapc1	anaphase	GO:00081	metabolic	K03348	anaphase	mmu0516	HTLV-I in	2:1286101	1556	1795	2199	2501	1749	1432	4.914202	4.751828	4.784318	5.145633	4.843685	5.607539	4.816783	5.198952	0.760311	1	-
ENSMUSC	Mertk	c-mer prc	GO:00325	developm	-	-	-	-	2:1286989	766	944	1169	1204	976	638	7.972754	8.235768	8.381952	8.163709	8.907825	8.233521	8.196824	8.435018	0.951551	1	-
ENSMUSC	Tsg101	tumor sus	GO:00325	developm	K12183	ESCRT-I c	mmu0414	Endocytos	7:4688894	631	845	978	1100	794	488	8.460329	9.496578	9.033334	9.607977	9.335138	8.11267	8.996747	9.018595	0.820055	1	-
ENSMUSC	Hps5	HPS5, bio	GO:00508	response	-	-	-	-	7:4676046	200	265	355	328	288	250	0.932888	1.036093	1.14072	0.996677	1.177972	1.445858	1.036567	1.206836	0.475716	1	-
ENSMUSC	Map3k4	mitogen-	GO:00325	developm	K04428	mitogen-	mmu0401	MAPK sig	17:122276	1209	1390	1749	1835	1333	907	14.48362	13.95785	14.43418	14.32083	14.00308	13.47237	14.29188	13.93209	0.610978	1	-
ENSMUSC	Piezo1	piezo-typ	GO:00511	localizatio	-	-	-	-	8:1224816	124	144	161	198	151	156	0.582926	0.567423	0.521398	0.606372	0.622458	0.909288	0.557249	0.712706	0.235091	1	-
ENSMUSC	Blk	B lymphoi	GO:00325	developm	-	-	-	-	14:633728	0	0	0	2	0	1	0	0	0	0.028084	0	0.026722	0	0.018269	0.284257	1	-
ENSMUSC	Rnf166	ring finge	GO:00081	metabolic	-	-	-	-	8:1224661	353	437	502	563	403	397	8.877803	9.21224	8.697333	9.224037	8.887487	12.37963	8.929125	10.16372	0.546547	1	-
ENSMUSC	Ankrd28	ankyrin re	GO:00056	cell|intrac	-	-	-	-	14:316987	958	1163	1498	1540	1105	884	4.361614	4.438278	4.698347	4.567558	4.411505	4.990222	4.499413	4.656428	0.963683	1	-
ENSMUSC	Ankrd52	ankyrin re	-	-	-	-	-	-	10:128377	1711	1925	2300	2763	1919	1385	12.25645	11.55841	11.34995	12.89369	12.05403	12.30128	11.7216	12.41633	0.865187	1	-
ENSMUSC	Pkd2l2	polycystic	GO:00511	localizatio	-	-	-	-	18:344094	3	4	6	6	5	11	0.071506	0.079922	0.098528	0.09318	0.10451	0.325136	0.083319	0.174275	0.185737	1	-
ENSMUSC	Srp19	signal rec	GO:00511	localizatio	K03105	signal rec	mmu0306	Protein ex	18:343308	312	375	458	511	338	279	3.230986	3.255099	3.267357	3.447327	3.069303	3.582365	3.251147	3.366331	0.978204	1	-
ENSMUSC	Wdfy2	WD repea	GO:00325	developm	-	-	-	-	14:628376	564	710	915	935	666	526	3.739974	3.946395	4.17986	4.039081	3.872634	4.324747	3.95541	4.078821	0.947758	1	-
ENSMUSC	Rbsn	rabenosyr	GO:00511	localizatio	K12481	rabenosyr	mmu0414	Endocytos	6:9218671	942	1041	1304	1361	1017	933	5.706506	5.285948	5.44187	5.371047	5.40235	7.007885	5.478108	5.927094	0.783277	1	-
ENSMUSC	Mrps25	mitochon	GO:00081	metabolic	-	-	-	-	6:9216952	616	630	860	885	706	430	5.381998	4.613772	5.176217	5.037186	5.408918	4.658188	5.057329	5.034764	0.773178	1	-
ENSMUSC	Dguok	deoxygua	GO:00325	developm	K00904	deoxygua	mmu0110	Metabolic	6:8348021	140	157	182	225	170	150	1.421544	1.336242	1.273076	1.488316	1.513641	1.888466	1.34362	1.630141	0.339772	1	-
ENSMUSC	Camta1	calmoduli	GO:00081	metabolic	-	-	-	-	4:1509173	8494	9929	13167	13139	10324	5394	23.85309	23.37172	25.47245	24.03678	25.42278	18.78146	24.23242	22.74701	0.468932	1	-
ENSMUSC	Csf1	colony stir	GO:00325	developm	K05453	macropha	mmu0415	PI3K-Akt	3:1077410	443	460	525	615	561	464	3.539599	3.080784	2.889759	3.201161	3.930577	4.596791	3.170047	3.90951	0.243185	1	-
ENSMUSC	Strip1	striatin int	GO:00325	developm	-	-	-	-	3:1076125	1601	1868	2303	2432	1826	1351	12.47069	12.19632	12.35789	12.34083	12.47219	13.0479	12.34164	12.62031	0.888206	1	-
ENSMUSC	Kif1a	kinesin fa	GO:00325	developm	-	-	-	-	1:9301546	22940	25322	30296	35708	25788	18152	119.7262	110.7763	108.9262	121.4068	118.0201	117.4644	113.1429	118.9638	0.910716	1	-
ENSMUSC	Alx3	aristaless-	GO:00325	developm	-	-	-	-	3:1075950	2	5	6	4	8	11	0.06784	0.142124	0.140168	0.088381	0.237914	0.462546	0.116711	0.262947	0.142351	1	-
ENSMUSC	Slc25a11	solute car	GO:00511	localizatio	-	-	-	-	11:706441	644	902	1060	1142	810	1293	11.65712	13.68562	13.2179	13.46645	12.85681	29.01948	12.85355	18.44758	0.08501	1	-
ENSMUSC	Chrne	cholinergi	GO:00048	signal trar	K04817	nicotinic a	mmu0408	Neuroacti	11:706148	0	0	2	0	0	0	0	0	0.030836	0	0	0	0.010279	0	0.537272	1	-
ENSMUSC	Cmc2	COX asse	GO:00056	cell|intrac	-	-	-	-	8:1168886	66	80	119	95	101	114	0.972297	0.98787	1.207687	0.911717	1.304732	2.082323	1.055951	1.432924	0.243473	1	-
ENSMUSC	Chfr	checkpoir	GO:00081	metabolic	-	-	-	-	5:1101358	446	553	664	695	569	341	2.912822	3.027306	2.987432	2.956958	3.258617	2.761336	2.975853	2.992304	0.834712	1	-
ENSMUSC	Ceacam1	carcinoen	GO:00055	extracellul	-	-	-	-	7:1985209	0	0	13	0	0	0	0	0	0.268092	0	0	0	0.089364	0	0.029061	1	-
ENSMUSC	Hoxa2	homeobo	GO:00325	developm	-	-	-	-	6:5216241	0	1	3	0	0	0	0	0.03001	0.073993	0	0	0	0.034668	0	0.15986	1	-
ENSMUSC	Adam28	a disinteg	GO:00081	metabolic	-	-	-	-	14:686060	0	0	2	5	1	0	0	0	0.010865	0.025685	0.006914	0	0.003622	0.010866	0.52032	1	-
ENSMUSC	Tex261	testis expr	GO:00511	localizatio	-	-	-	-	6:8376888	433	575	685	753	470	687	4.804196	5.347542	5.235703	5.442638	4.572712	9.45096	5.129147	6.48877	0.270162	1	-
ENSMUSC	Fam120b	family wit	GO:00325	developm	-	-	-	-	17:153962	1218	1460	1658	1883	1342	945	17.13315	17.21455	16.06667	17.25528	16.55332	16.48192	16.80479	16.7635	0.744817	1	-
ENSMUSC	Tbp	TATA box	GO:00081	metabolic	K03120	transcripti	mmu0516	Human pa	17:154998	480	604	674	779	560	324	1.870351	1.97275	1.809229	1.977431	1.913433	1.565359	1.88411	1.818741	0.639119	1	-
ENSMUSC	Psmb1	proteasor	GO:00081	metabolic	K02732	20S prote	mmu0305	Proteason	17:154757	1109	1440	1799	1674	1282	1240	68.03119	74.04432	76.02546	66.89806	68.96158	94.31571	72.70032	76.72512	0.952393	1	-
ENSMUSC	Pdcd2	programn	GO:00325	developm	-	-	-	-	17:155192	102	138	153	162	104	110	1.851065	2.099197	1.912772	1.915211	1.654994	2.475142	1.954345	2.015116	0.936202	1	-
ENSMUSC	Dll1	delta-like	GO:00325	developm	K06051	delta[-]	mmu0520	Pathways	17:153673	137	121	159	155	130	127	1.44056	1.066475	1.151754	1.061756	1.198666	1.655776	1.219596	1.305399	0.932998	1	-
ENSMUSC	Nol3	nucleolar	GO:00081	metabolic	-	-	-	-	8:1052761	315	339	382	419	352	237	6.303274	5.686014	5.26588	5.461992	6.176483	5.880181	5.751722	5.839552	0.876488	1	-
ENSMUSC	Fhod1	formin ho	GO:00081	metabolic	-	-	-	-	8:1053291	42	54	86	67	60	60	0.425848	0.458941	0.600702	0.442553	0.533463	0.754299	0.495164	0.576772	0.659009	1	-
ENSMUSC	Plekhg4	pleckstrin	GO:00987	molecular	-	-	-	-	8:1053732	27	19	31	37	21	40	0.198348	0.116998	0.156887	0.177074	0.135282	0.364347	0.157411	0.225568	0.306026	1	-
ENSMUSC	Slc9a5	solute car	GO:00511	localizatio	-	-	-	-	8:1053488	230	299	424	432	329	298	2.195105	2.391942	2.787687	2.685917	2.753377	3.526382	2.458245	2.988559	0.293543	1	-
ENSMUSC	Elmo3	engulfme	GO:00400	locomotic	K19241	engulfme	mmu0510	Bacterial i	8:1053056	40	19	35	29	39	28	0.484495	0.192901	0.292039	0.228828	0.414222	0.420502	0.323145	0.354517	0.881396	1	-
ENSMUSC	Stc1	stanniocal	GO:00325	developm	-	-	-	-	14:690292	78	97	117	111	88	61	1.181119	1.231185	1.220488	1.094965	1.168487	1.145276	1.210931	1.136243	0.562967	1	-
ENSMUSC	4931428F	RIKEN cD	-	-	-	-	-	-	8:1052804	345	414	494	522	376	364	2.958948	2.976263	2.918746	2.916557	2.827801	3.870845	2.951319	3.205068	0.793936	1	-
ENSMUSC	Tppp3	tubulin pc	GO:00990	supramol	-	-	-	-	8:1054674	334	303	337	329	347	354	7.746972	5.890901	5.384775	4.971227	7.057603	10.18063	6.340883	7.403154	0.572238	1	-
ENSMUSC	Msh3	mutS hon	GO:00081	metabolic	K08736	DNA misn	mmu0520	Pathways	13:922118	480	604	671	683	571	261	1.683522	1.775693	1.621257	1.560558	1.756129	1.135024	1.693491	1.483904	0.304576	1	-
ENSMUSC	Adamts13	a disinteg	GO:00081	metabolic	-	-	-	-	2:2697341	17	25	9	20	15	9	0.198125	0.244225	0.072256	0.151852	0.153296	0.130051	0.171535	0.145066	0.609399	1	-
ENSMUSC	Tmem208	transmem	GO:00081	metabolic	-	-	-	-	8:1053263	155	163	197	212	158	220	3.228694	2.846	2.826914	2.876806	2.885982	5.682008	2.967203	3.814932	0.302549	1	-
ENSMUSC	E2f4	E2F transc	GO:00325	developm	K04682	transcripti	mmu0421	Cellular se	8:1052976	552	682	915	978	702	497	13.1934	13.66329	15.06573	15.22785	14.71287	14.72855	13.97414	14.88976	0.836307	1	-
ENSMUSC	Surf4	surfeit ger	GO:00099	cellular pr	-	-	-	-	2:2692004	842	1084	1350	1328	921	1201	14.4353	15.57743	15.94406	14.83178	13.84573	25.52951	15.31893	18.06901	0.447654	1	-
ENSMUSC	Surf2	surfeit ger	GO:00160	membran	-	-	-	-	2:2691636	263	294	351	400	321	245	2.189188	2.05129	2.012732	2.169047	2.34301	2.528598	2.084403	2.346885	0.576747	1	-
ENSMUSC	Dnajb9	DnaJ heat	GO:00081	metabolic	-	-	-	-	12:442055	737	980	1359	1147	918	476	16.46927	18.35632	20.92076	16.6975	17.98838	13.18863	18.58211	15.95817	0.204845	1	-
ENSMUSC	Naf1	nuclear as	GO:00081	metabolic	-	-	-	-	8:6686021	269	301	371	318	287	166	5.12003	4.80218	4.864572	3.943013	4.790099	3.917549	4.928928	4.216887	0.196494	1	-
ENSMUSC	Yes1	YES proto	GO:00325	developm	K05705	tyrosine-p	mmu0452	Adherens	5:3261117	258	346	419	429	328	188	1.56144	1.755234	1.746914	1.691394	1.740692	1.410745	1.687862	1.614277	0.608785	1	-
ENSMUSC	Ppp1cb	protein ph	GO:00081	metabolic	K06269	serine/thr	mmu0481	Regulation	5:3245884	4850	6225	7763	5539	4218	4464	24.49202	26.34967	27.00623	18.22199	18.67808	27.95072	25.94931	21.61693	0.06555	1	-
ENSMUSC	Gorasp2	golgi reas	GO:00099	cellular pr	-	-	-	-	2:7066157	1914	2420	2533	2612	2173	1400	18.13634	19.221	16.53463	16.12362	18.05553	16.44833	17.96399	16.87583	0.412221	1	-
ENSMUSC	Tsen15	tRNA splic	GO:00081	metabolic	-	-	-	-	1:1523707	169	240	286	261	205	169	1.822583	2.169516	2.124794	1.833674	1.93864	2.259819	2.038965	2.010711	0.707739	1	-
ENSMUSC	Efr3a	EFR3 hom	GO:00511	localizatio	-	-	-	-	15:657870	2665	3364	4471	4458	3185	2040	11.3018	11.95802	13.06191	12.31607	11.84411	10.72671	12.10724	11.62896	0.543452	1	-
ENSMUSC	Trappc2l	trafficking	GO:00511	localizatio	-	-	-	-	8:1226116	236	263	328	309	237	285	6.743947	6.299566	6.456936	5.752299	5.938713	10.09794	6.50015	7.262983	0.730328	1	-
ENSMUSC	Acsf3	acyl-CoA	GO:00081	metabolic	K18660	malonyl-C	mmu0028	Valine, leu	8:1227754	186	266	272	358	259	253	2.855969	3.423542	2.877141	3.581005	3.487254	4.816681	3.052217	3.961647	0.151091	1	-
ENSMUSC	Ddx19a	DEAD (As	GO:00081	metabolic	-	-	-	-	8:1109749	323	391	535	598	416	324	5.702112	5.785802	6.506366	6.877271	6.439765	7.091944	5.998093	6.802993	0.518458	1	-
ENSMUSC	Galns	galactosa	GO:00081	metabolic	K01132	N-acetylg	mmu0110	Metabolic	8:1225782	169	186	225	236	171	148	2.737126	2.525068	2.510388	2.490013	2.428543	2.972036	2.590861	2.630197	0.857798	1	-
ENSMUSC	Gata2	GATA bin	GO:00325	developm	-	-	-	-	6:8819389	51	92	92	106	92	77	0.381047	0.576169	0.473534	0.51594	0.602756	0.713321	0.476916	0.610672	0.285812	1	-
ENSMUSC	C8g	complem	GO:00081	metabolic	K03999	complem	mmu0532	Systemic l	2:2549865	12	11	12	8	11	11	0.242097	0.186033	0.166792	0.105147	0.194611	0.275178	0.198307	0.191645	0.780136	1	-
ENSMUSC	Entpd2	ectonucle	GO:00081	metabolic	K01509	adenosine	mmu0023	Purine me	2:2539587	76	155	168	179	125	171	1.451766	2.4818	2.210778	2.227499	2.093812	4.050096	2.048114	2.790469	0.212292	1	-
ENSMUSC	Rabl6	RAB, mem	GO:00056	cell|intrac	-	-	-	-	2:2558301	2155	2780	3157	3301	2564	1832	13.65607	14.76645	13.78175	13.62717	14.24752	14.39428	14.06809	14.08966	0.748237	1	-
ENSMUSC	Ptgds	prostagla	GO:00081	metabolic	K01830	prostagla	mmu0110	Metabolic	2:2546670	3494	5399	7289	4989	4214	5389	83.79404	108.5316	120.4231	77.94449	88.61911	160.245	104.2496	108.9362	0.948552	1	-
ENSMUSC	Edf1	endotheli	GO:00325	developm	-	-	-	-	2:2555784	630	687	809	856	630	577	15.11453	13.81541	13.3707	13.37857	13.25371	17.16389	14.10021	14.59872	0.940831	1	-
ENSMUSC	Clic3	chloride ir	GO:00511	localizatio	-	-	-	-	2:2545683	0	0	5	8	3	5	0	0	0.094931	0.14363	0.072495	0.170847	0.031644	0.128991	0.040087	1	-
ENSMUSC	Npdc1	neural prc	GO:00160	membran	-	-	-	-	2:2539935	1731	2082	2238	2683	1882	2229	13.92535	14.03921	12.40282	14.06084	13.27612	22.23336	13.45579	16.52344	0.255602	1	-
ENSMUSC	Fbxw5	F-box anc	GO:00081	metabolic	-	-	-	-	2:2550075	948	1253	1405	1609	1147	1171	10.2725	11.38078	10.48808	11.3581	10.89868	15.73299	10.71379	12.66326	0.344083	1	-
ENSMUSC	Slc25a13	solute car	GO:00081	metabolic	-	-	-	-	6:6041218	42	33	72	58	33	21	0.493659	0.325126	0.582992	0.444112	0.340128	0.30604	0.467259	0.363427	0.248928	1	-
ENSMUSC	Ube2i	ubiquitin-	GO:00325	developm	K10577	ubiquitin-	mmu0301	RNA trans	17:252619	700	935	1063	1297	960	707	4.606719	5.157728	4.819239	5.560522	5.539975	5.768984	4.861229	5.62316	0.385052	1	-
ENSMUSC	Tsr3	TSR3 20S	GO:00081	metabolic	-	-	-	-	17:252401	204	255	260	266	226	210	10.87651	11.396	9.549575	9.238947	10.566	13.88243	10.60736	11.22913	0.955829	1	-
ENSMUSC	Unkl	unkempt	GO:00081	metabolic	-	-	-	-	17:251883	906	1151	585	1069	1063	628	6.209319	6.61217	2.761995	4.77282	6.388396	5.336561	5.194495	5.499259	0.929886	1	-
ENSMUSC	Lrrk1	leucine-ri	GO:00325	developm	-	-	-	-	7:6622691	95	78	131	140	83	87	0.439445	0.302434	0.417451	0.421881	0.336667	0.498984	0.386443	0.419177	0.855678	1	-
ENSMUSC	Aldh1a3	aldehyde	GO:00325	developm	K00129	aldehyde	mmu0110	Metabolic	7:6639089	7	11	18	20	7	5	0.081469	0.107307	0.144309	0.15163	0.071439	0.072143	0.111028	0.098404	0.784008	1	-
ENSMUSC	Actn1	actinin, al	GO:00325	developm	K05699	actinin alp	mmu0520	Viral carci	12:801675	1474	1846	2179	2210	1675	1231	13.20698	13.86407	13.44977	12.89971	13.16023	13.67572	13.50694	13.24522	0.618658	1	-
ENSMUSC	Sirt2	sirtuin 2 [S	GO:00325	developm	-	-	-	-	7:2876673	1069	1163	1546	1538	1264	841	8.200141	7.477842	8.169671	7.685667	8.502239	7.998818	7.949218	8.062241	0.850498	1	-
ENSMUSC	Hnrnpl	heteroger	GO:00081	metabolic	-	-	-	-	7:2880854	2058	2707	3004	3256	2480	2051	11.78307	12.99135	11.84853	12.14449	12.4511	14.56011	12.20765	13.0519	0.830796	1	-
ENSMUSC	Nolc1	nucleolar	GO:00325	developm	-	-	-	-	19:460758	964	1078	1467	1634	1457	877	5.897112	5.527569	6.182212	6.511723	7.815642	6.651937	5.868964	6.993101	0.272599	1	-
ENSMUSC	Casd1	CAS1 don	GO:00081	metabolic	-	-	-	-	6:4600839	1011	1228	1597	1639	1147	801	12.51745	12.74431	13.62138	13.21982	12.45291	12.29654	12.96105	12.65642	0.627163	1	-
ENSMUSC	Cnksr3	Cnksr fam	GO:00081	metabolic	-	-	-	-	10:711906	111	94	127	111	75	57	1.806042	1.281983	1.423505	1.176541	1.070063	1.149905	1.503843	1.13217	0.074628	1	-
ENSMUSC	Mtmr1	myotubul	GO:00081	metabolic	K18081	myotubul	mmu0110	Metabolic	X:7136476	833	1046	1310	1284	974	690	5.800967	6.105766	6.284612	5.825085	5.947812	5.95787	6.063782	5.910256	0.603795	1	-
ENSMUSC	Hmgb3	high mob	GO:00325	developm	-	-	-	-	X:7155591	110	249	169	164	129	92	2.20392	4.181723	2.332605	2.140562	2.266397	2.285472	2.906083	2.23081	0.123267	1	-
ENSMUSC	Map2	microtubu	GO:00325	developm	-	-	-	-	1:6617527	30033	31517	34860	37846	28729	9933	58.90829	51.81738	47.10386	48.35922	49.41296	24.15706	52.60984	40.64308	0.118263	1	-
ENSMUSC	Cyp2j9	cytochron	GO:00081	metabolic	K07418	cytochron	mmu0110	Metabolic	4:9656842	456	536	760	835	674	338	8.330021	8.207287	9.564163	9.936874	10.79653	7.65569	8.70049	9.46303	0.717597	1	-
ENSMUSC	Abca1	ATP-bind	GO:00048	signal trar	K05641	ATP-bind	mmu0497	Cholester	4:5303078	958	976	1485	1578	1022	797	5.763871	4.922114	6.154982	6.184967	5.391909	5.945561	5.613655	5.840812	0.988822	1	-
ENSMUSC	Nipsnap3	nipsnap h	GO:00056	cell|intrac	-	-	-	-	4:5301188	100	131	143	137	114	118	2.662462	2.923526	2.622834	2.376221	2.661537	3.895408	2.736274	2.977722	0.862031	1	-
ENSMUSC	Lage3	L antigen	GO:00081	metabolic	-	-	-	-	X:7435127	82	105	119	138	107	130	1.48726	1.596298	1.486861	1.630546	1.701758	2.923495	1.523473	2.085267	0.161166	1	-
ENSMUSC	Ubl4a	ubiquitin-	GO:00511	localizatio	-	-	-	-	X:7436571	605	824	989	923	145	695	5.188874	5.923764	5.843399	5.157046	1.090507	7.390755	5.652012	4.546103	0.345097	1	-
ENSMUSC	Gdi1	guanosine	GO:00325	developm	-	-	-	-	X:7430499	6975	9993	10357	10559	8428	6760	43.90672	52.7273	44.91301	43.30033	46.52153	52.76177	47.18234	47.52788	0.766389	1	-
ENSMUSC	Sash1	SAM and	GO:00325	developm	-	-	-	-	10:872221	1964	2329	2846	3255	2302	1572	12.92916	12.85142	12.9067	13.95923	13.28853	12.8312	12.89576	13.35965	0.997056	1	-
ENSMUSC	Gadd45b	growth ar	GO:00325	developm	K04402	growth ar	mmu0520	Pathways	10:809300	136	155	189	152	143	204	3.513512	3.356483	3.363685	2.558157	3.239516	6.534561	3.411227	4.110744	0.554938	1	-
ENSMUSC	Slamf6	SLAM fan	GO:00325	developm	-	-	-	-	1:1719175	3	1	6	6	3	1	0.015743	0.004399	0.021693	0.020515	0.013806	0.006506	0.013945	0.013609	1	1	-
ENSMUSC	Slamf1	signaling	GO:00325	developm	K06536	signaling l	mmu0516	Measles -	1:1717671	0	0	0	0	3	0	0	0	0	0	0.026211	0	0	0.008737	0.304378	1	-
ENSMUSC	Zdhhc12	zinc finge	GO:00081	metabolic	-	-	-	-	2:3009094	57	65	72	85	53	85	1.126405	1.076677	0.980173	1.09426	0.918414	2.082681	1.061085	1.365118	0.399892	1	-
ENSMUSC	Endog	endonucle	GO:00325	developm	K01173	endonucle	mmu0421	Apoptosis	2:3017149	102	117	97	118	102	166	6.098241	5.863313	3.995097	4.595847	5.34745	12.30545	5.318883	7.41625	0.262413	1	-
ENSMUSC	Cybb	cytochron	GO:00325	developm	K08008	NADPH o	mmu0421	Necropto	X:9435252	32	33	65	77	35	48	0.323371	0.279529	0.452504	0.506906	0.310148	0.601424	0.351801	0.472826	0.30209	1	-
ENSMUSC	Golga7	golgi autc	GO:00081	metabolic	-	-	-	-	8:2324134	728	824	1138	1082	850	643	5.215484	4.948156	5.616392	5.04978	5.339804	5.711638	5.260011	5.367074	0.875224	1	-
ENSMUSC	Xk	X-linked I	GO:00325	developm	-	-	-	-	X:9272756	464	593	682	734	511	311	5.809419	6.223315	5.882342	5.986765	5.610198	4.827925	5.971692	5.474963	0.395183	1	-
ENSMUSC	Pcolce2	procollag	GO:00081	metabolic	-	-	-	-	9:9563760	96	65	188	97	89	52	1.302262	0.739081	1.756853	0.857199	1.058669	0.874611	1.266065	0.93016	0.124035	1	-
ENSMUSC	Cd48	CD48 anti	GO:00023	immune s	K06479	CD48 anti	mmu0465	Natural ki	1:1716820	12	13	16	33	7	14	0.422496	0.383679	0.388087	0.756922	0.216137	0.611208	0.398087	0.528089	0.463631	1	-
ENSMUSC	Clpx	caseinolyt	GO:00081	metabolic	-	-	-	-	9:6529426	421	551	672	676	513	457	5.274163	5.78596	5.799517	5.516953	5.635485	7.098612	5.61988	6.083683	0.791416	1	-
ENSMUSC	Trabd	TraB dom	-	-	-	-	-	-	15:890751	236	358	418	429	337	334	1.386186	1.762563	1.691361	1.641528	1.735729	2.432435	1.61337	1.936564	0.36359	1	-
ENSMUSC	Dennd6b	DENN/M	GO:00987	molecular	-	-	-	-	15:891822	600	801	963	1119	758	934	8.276924	9.261964	9.151567	10.0561	9.169169	15.97536	8.896818	11.73355	0.117642	1	-
ENSMUSC	Cd83	CD83 anti	GO:00325	developm	-	-	-	-	13:437847	219	224	299	319	290	224	3.212858	2.75454	3.021831	3.048735	3.730682	4.074562	2.99641	3.617993	0.325249	1	-
ENSMUSC	Tmem27	transmem	GO:00081	metabolic	-	-	-	-	X:1640888	4	2	20	0	1	4	0.107213	0.044933	0.369314	0	0.023503	0.132938	0.17382	0.052147	0.061003	1	-
ENSMUSC	Ace2	angiotens	GO:00081	metabolic	K09708	angiotens	mmu0497	Protein di	X:1641393	35	39	45	40	39	17	0.511351	0.477602	0.452909	0.380703	0.499637	0.307946	0.48062	0.396095	0.34778	1	-
ENSMUSC	Gzmb	granzyme	GO:00081	metabolic	K01353	granzyme	mmu0520	Transcript	14:562588	1	0	2	2	2	0	0.044266	0	0.060984	0.057655	0.077611	0	0.035083	0.045089	1	1	-
ENSMUSC	Ager	advanced	GO:00325	developm	K19722	receptor f	-	-	17:345978	0	7	8	4	6	17	0	0.061786	0.058036	0.027444	0.055401	0.221966	0.039941	0.101604	0.174767	1	-
ENSMUSC	Atf6b	activating	GO:00081	metabolic	K09049	cyclic AMI	mmu0415	PI3K-Akt	17:346471	1098	1222	1469	1689	1287	1198	18.44112	17.20319	16.99645	18.47976	18.95423	24.94753	17.54692	20.79384	0.314786	1	-
ENSMUSC	Egfl8	EGF-like c	GO:00055	extracellul	-	-	-	-	17:346133	5	11	19	11	16	25	0.089336	0.164747	0.233858	0.12803	0.250674	0.553825	0.162647	0.310843	0.14625	1	-
ENSMUSC	Notch4	notch 4 [S	GO:00325	developm	K02599	Notch[-]	mmu0520	Pathways	17:345642	100	114	135	176	114	172	0.521225	0.49806	0.484742	0.597613	0.521044	1.111576	0.501342	0.743411	0.084107	1	-
ENSMUSC	Ppt2	palmitoyl-	GO:00081	metabolic	K01074	palmitoyl-	mmu0110	Metabolic	17:346166	378	350	441	403	374	402	4.114945	3.193694	3.307213	2.857976	3.570144	5.426051	3.538617	3.95139	0.732367	1	-
ENSMUSC	Prrt1	proline-ri	GO:00452	synapse|re	-	-	-	-	17:346295	3467	3890	4277	5089	3863	2485	78.52399	73.85007	66.73276	75.08662	76.72128	69.78479	73.03561	73.86423	0.83695	1	-
ENSMUSC	Rnf5	ring finge	GO:00081	metabolic	K10666	E3 ubiquit	mmu0414	Protein pr	17:346010	634	873	987	969	865	756	14.92318	17.22422	16.00445	14.85859	17.85382	22.06378	16.05062	18.25873	0.530224	1	-
ENSMUSC	Fam163a	family wit	GO:00160	membran	-	-	-	-	1:1560759	244	332	409	449	366	289	3.994575	4.555874	4.612704	4.788599	5.254175	5.866308	4.387718	5.303027	0.284892	1	-
ENSMUSC	Cacfd1	calcium cl	GO:00511	localizatio	-	-	-	-	2:2700992	411	570	646	650	511	756	3.186179	3.70387	3.449943	3.282639	3.473697	7.266677	3.446664	4.674337	0.167182	1	-
ENSMUSC	Hivep2	human im	GO:00325	developm	-	-	-	-	10:139660	5120	4162	6055	7126	5260	3790	16.6594	11.35127	13.57235	15.10486	15.00783	15.29025	13.861	15.13431	0.705425	1	-
ENSMUSC	Arnt	aryl hydro	GO:00325	developm	K09097	aryl hydro	mmu0520	Pathways	3:9543438	456	447	669	714	513	365	2.200299	1.807912	2.223795	2.244383	2.170588	2.183717	2.077336	2.199563	0.884886	1	-
ENSMUSC	Itga2	integrin al	GO:00325	developm	K06481	integrin al	mmu0520	Pathways	13:114833	4	9	10	11	10	2	0.035538	0.067023	0.061208	0.063666	0.077911	0.022038	0.05459	0.054539	1	1	-
ENSMUSC	Mocs2	molybden	GO:00081	metabolic	K03635	molybdop	mmu0110	Metabolic	13:114818	317	446	645	562	436	446	1.272587	1.500774	1.78377	1.469758	1.534818	2.219977	1.519044	1.741517	0.553767	1	-
ENSMUSC	Nat9	N-acetylti	GO:00038	catalytic a	-	-	-	-	11:115182	131	147	183	200	175	139	1.889576	1.777304	1.818417	1.879332	2.213459	2.485951	1.828432	2.192914	0.383083	1	-
ENSMUSC	Lpl	lipoprotei	GO:00325	developm	K01059	lipoprotei	mmu0501	Alzheimer	8:6888049	778	936	1124	1168	743	581	4.25693	4.29285	4.236767	4.163335	3.564911	3.941657	4.262182	3.889968	0.309558	1	-
ENSMUSC	Atp6v0e	ATPase, H	GO:00511	localizatio	K02153	V-type H-	mmu0110	Metabolic	17:266763	177	185	270	316	180	327	3.161576	2.769843	3.322348	3.677046	2.819331	7.242083	3.084589	4.579487	0.092624	1	-
ENSMUSC	Zfp318	zinc finge	GO:00056	cell|intrac	-	-	-	-	17:463837	1153	1459	1721	2112	1395	792	6.928573	7.348909	7.124378	8.267804	7.350748	5.901001	7.133953	7.173184	0.857499	1	-
ENSMUSC	Ttbk1	tau tubuli	GO:00325	developm	-	-	-	-	17:464424	3252	3631	4528	5113	3890	3174	14.31858	13.40073	13.73432	14.66584	15.01902	17.32775	13.81788	15.67087	0.456089	1	-
ENSMUSC	Srf	serum res	GO:00325	developm	K04378	serum res	mmu0401	MAPK sig	17:465468	1230	1416	1691	1790	1335	1123	19.09851	18.42937	18.08795	18.10628	18.17684	21.62021	18.53861	19.30111	0.987298	1	-
ENSMUSC	Gata3	GATA bin	GO:00325	developm	K17895	GATA-bin	mmu0465	Th17 cell	2:9857078	0	0	5	0	0	1	0	0	0.040979	0	0	0.014747	0.01366	0.004916	0.45929	1	-
ENSMUSC	Lama5	laminin, a	GO:00325	developm	K06240	laminin, a	mmu0110	Metabolic	2:1801763	222	249	370	337	285	331	1.141946	1.073608	1.311132	1.12929	1.285528	2.111091	1.175562	1.508636	0.234979	1	-
ENSMUSC	Steap1	six transm	GO:00081	metabolic	K14737	metallore	mmu0497	Mineral al	5:5736317	27	9	31	7	19	21	0.965079	0.269637	0.763346	0.163001	0.595512	0.930686	0.66602	0.563066	0.58774	1	-
ENSMUSC	Steap2	six transm	GO:00081	metabolic	K14738	metallore	mmu0497	Mineral al	5:5664829	215	186	486	341	215	133	0.40195	0.291474	0.625924	0.415307	0.352464	0.308297	0.439783	0.358689	0.2344	1	-
ENSMUSC	Hspa8	heat shoc	GO:00081	metabolic	K03283	heat shoc	mmu0401	MAPK sig	9:4080098	13477	15720	21134	18434	14561	9112	92.72534	90.65892	100.1701	82.62403	87.84945	77.73289	94.51814	82.73546	0.145991	1	-
ENSMUSC	Serac1	serine act	GO:00081	metabolic	-	-	-	-	17:604219	379	414	511	554	452	286	4.286637	3.924917	3.981522	4.08196	4.482887	4.010783	4.064359	4.191877	0.975942	1	-
ENSMUSC	Pdzd11	PDZ dom	GO:00452	synapse|c	-	-	-	-	X:1006228	456	548	610	608	488	293	7.480677	7.53546	6.893788	6.497734	7.020029	5.959785	7.303308	6.492516	0.272697	1	-
ENSMUSC	Psma2	proteasor	GO:00081	metabolic	K02726	20S prote	mmu0305	Proteason	13:146132	791	1111	1359	1224	953	845	12.80123	15.071	15.15117	12.90441	13.52417	16.95579	14.34113	14.46146	0.776691	1	-
ENSMUSC	Mrpl32	mitochon	GO:00081	metabolic	K02911	large sub	mmu0301	Ribosome	13:146081	188	264	276	264	217	201	1.618092	1.904591	1.636456	1.480232	1.637751	2.145001	1.719713	1.754328	0.864689	1	-
ENSMUSC	Setdb1	SET doma	GO:00081	metabolic	K11421	histone-ly	mmu0455	Signaling	3:9532352	836	1011	1335	1330	971	694	6.190902	6.275545	6.81052	6.416244	6.305355	6.372258	6.425656	6.364619	0.703699	1	-
ENSMUSC	Anxa9	annexin A	GO:00048	signal trar	-	-	-	-	3:9529609	11	16	23	34	13	15	0.260276	0.317329	0.3749	0.524066	0.269722	0.440063	0.317501	0.411283	0.461745	1	-
ENSMUSC	Arnt2	aryl hydro	GO:00325	developm	K15589	aryl hydro	mmu0520	Pathways	7:8424627	4011	4705	5672	6131	4862	3928	18.41444	18.10583	17.93881	18.3366	19.57328	22.35956	18.15303	20.08981	0.60068	1	-
ENSMUSC	Prune1	prune exc	GO:00325	developm	K01514	exopolypl	mmu0023	Purine me	3:9525367	416	470	595	692	515	451	6.919147	6.552535	6.817535	7.498028	7.511192	9.300817	6.763072	8.103346	0.295991	1	-
ENSMUSC	Cers2	ceramide	GO:00325	developm	K04710	ceramide	mmu0110	Metabolic	3:9531479	523	640	892	843	638	618	5.133312	5.265376	6.03132	5.390208	5.491108	7.520911	5.47667	6.134076	0.615566	1	-
ENSMUSC	Nlrp5	NLR famil	GO:00325	developm	-	-	-	-	7:2338588	0	2	0	0	0	0	0	0.016456	0	0	0	0	0.005485	0	0.536429	1	-
ENSMUSC	Capza2	capping p	GO:00329	macromo	K10364	capping p	mmu0414	Endocytos	6:1763623	4341	5277	6412	6628	4577	3026	35.13546	35.80105	35.75204	34.94781	32.48474	30.36761	35.56285	32.60006	0.308148	1	-
ENSMUSC	Plekho1	pleckstrin	GO:00325	developm	-	-	-	-	3:9598842	219	291	341	386	248	249	3.61697	4.028534	3.879778	4.153078	3.591664	5.099024	3.841761	4.281255	0.665245	1	-
ENSMUSC	Vps45	vacuolar p	GO:00511	localizatio	K12479	vacuolar p	mmu0414	Endocytos	3:9599983	588	710	868	938	714	548	8.446922	8.549333	8.589976	8.778197	8.994182	9.76085	8.528743	9.177743	0.791386	1	-
ENSMUSC	Prpf3	pre-mRN	GO:00081	metabolic	K12843	U4/U6 sm	mmu0304	Spliceoso	3:9583012	502	645	699	697	475	301	3.77513	4.06575	3.621232	3.414619	3.132306	2.806603	3.820704	3.117843	0.088982	1	-
ENSMUSC	Anp32e	acidic (leu	GO:00081	metabolic	-	-	-	-	3:9592924	1265	1491	2151	1824	1691	1410	8.32759	8.227336	9.754852	7.822315	9.761469	11.5089	8.769926	9.697563	0.660125	1	-
ENSMUSC	Aph1a	aph1 hom	GO:00325	developm	K06172	gamma-s	mmu0501	Alzheimer	3:9589396	306	366	433	467	360	427	2.857785	2.865118	2.785784	2.841228	2.948171	4.944488	2.836229	3.577963	0.246586	1	-
ENSMUSC	Tab2	TGF-beta	GO:00325	developm	K04404	TAK1-bin	mmu0401	MAPK sig	10:790565	1588	1088	2465	2548	1766	863	12.27465	7.049209	13.12584	12.83039	11.96994	8.270949	10.81657	11.02376	0.961637	1	-
ENSMUSC	Ppil4	peptidylp	GO:00081	metabolic	-	-	-	-	10:779289	908	1153	1484	1416	1125	670	7.974868	8.488279	8.978892	8.101816	8.664289	7.296232	8.48068	8.020779	0.483566	1	-

ENSMUSC	Cnih1	cornichon	GO:00511	localizatio	-	-	-	-	14:467755	596	846	1050	1009	772	539	14.35845	17.08379	17.42617	15.83559	16.30878	16.10039	16.28947	16.08159	0.699718	1	-
ENSMUSC	Eps8	epiderma	GO:00325	developm	-	-	-	-	6:1374772	961	1121	1370	1479	1085	538	3.830561	3.745393	3.76193	3.840507	3.792376	2.658929	3.779295	3.430604	0.385933	1	-
ENSMUSC	Med22	mediator	GO:00081	metabolic	-	-	-	-	2:2690526	313	368	497	503	408	609	3.990384	3.93253	4.364945	4.177539	4.561144	9.626625	4.095953	6.121769	0.063066	1	-
ENSMUSC	Surf1	surfeit ger	GO:00081	metabolic	-	-	-	-	2:2691338	389	509	639	602	495	498	6.536793	7.169435	7.397195	6.590112	7.29395	10.37599	7.034474	8.086685	0.525299	1	-
ENSMUSC	Med28	mediator	GO:00325	developm	-	-	-	-	5:4552022	306	473	611	446	337	373	2.834873	3.673046	3.89946	2.691713	2.737695	4.284568	3.469126	3.237992	0.441611	1	-
ENSMUSC	Qdpr	quinoid d	GO:00325	developm	K00357	dihydropt	mmu0110	Metabolic	5:4543402	1303	1413	1335	1801	1303	1403	17.97473	16.33853	12.68676	16.18502	15.76177	23.99725	15.66667	18.64801	0.37604	1	-
ENSMUSC	Gnrh1	gonadotr	GO:00325	developm	K05252	gonadotr	mmu0491	GnRH sigr	14:677451	2	2	8	1	4	0	0.112208	0.09403	0.309134	0.036537	0.196732	0	0.171791	0.077756	0.31147	1	-
ENSMUSC	Tnr	tenascin R	GO:00325	developm	K06252	tenascin[-	mmu0415	PI3K-Akt s	1:1595237	6938	7947	9567	11864	8432	4723	30.90781	29.67495	29.36034	34.43076	32.93875	26.08782	29.98103	31.15245	0.95919	1	-
ENSMUSC	Sqstm1	sequestos	GO:00325	developm	K14381	sequestos	mmu0421	Cellular se	11:501993	5445	4877	8128	8186	4945	6129	49.34352	37.0457	50.74198	48.32653	39.29536	68.86648	45.7104	52.16279	0.560219	1	-
ENSMUSC	Nfe2l2	nuclear fa	GO:00325	developm	K05638	nuclear fa	mmu0520	Pathways	2:7567551	352	364	587	591	394	233	5.703887	4.944043	6.552665	6.238746	5.598445	4.681351	5.733532	5.506181	0.645511	1	-
ENSMUSC	Rxrg	retinoid X	GO:00048	signal trar	K08526	retinoid X	mmu0520	Pathways	1:1675983	67	107	78	73	71	81	1.044921	1.398765	0.838026	0.741669	0.970973	1.566308	1.093904	1.092984	0.804474	1	-
ENSMUSC	Rxra	retinoid X	GO:00325	developm	K08524	retinoid X	mmu0520	Pathways	2:2767644	617	788	717	916	647	700	4.731205	5.064839	3.787536	4.575765	4.35044	6.655341	4.52786	5.193849	0.541162	1	-
ENSMUSC	Adamtsl4	ADAMTS-	GO:00325	developm	-	-	-	-	3:9567620	106	70	151	146	75	110	1.184359	0.655579	1.162263	1.062702	0.734822	1.5239	1.000734	1.107141	0.830914	1	-
ENSMUSC	Fcrls	Fc recepto	GO:00511	localizatio	-	-	-	-	3:8725075	330	472	552	576	379	398	7.840187	9.399561	9.034476	8.914906	7.895769	11.72415	8.758075	9.511607	0.802033	1	-
ENSMUSC	Cd5l	CD5 antig	GO:00511	localizatio	-	-	-	-	3:8735788	11	15	40	28	14	13	0.34337	0.392474	0.860138	0.569382	0.383201	0.503127	0.531994	0.485237	0.685927	1	-
ENSMUSC	Prpsap1	phosphor	GO:00081	metabolic	-	-	-	-	11:116470	407	466	580	473	403	369	4.427609	4.249258	4.34664	3.352101	3.844348	4.977223	4.341169	4.057891	0.40983	1	-
ENSMUSC	Fam184b	family witl	-	-	-	-	-	-	5:4552970	143	173	162	203	174	105	1.007436	1.021597	0.786225	0.931662	1.074912	0.917181	0.938419	0.974585	1	1	-
ENSMUSC	Lcorl	ligand dep	GO:00081	metabolic	-	-	-	-	5:4569718	394	509	653	606	416	252	0.7976	0.863694	0.910655	0.799178	0.738458	0.632521	0.857316	0.723386	0.162363	1	-
ENSMUSC	Lta4h	leukotrien	GO:00081	metabolic	K01254	leukotrien	mmu0110	Metabolic	10:934534	371	446	558	584	435	387	3.064087	3.087556	3.174776	3.142117	3.150354	3.962999	3.108806	3.41849	0.704778	1	-
ENSMUSC	Amdhd1	amidohyc	GO:00081	metabolic	K01468	imidazolo	mmu0110	Metabolic	10:935233	0	0	3	0	0	0	0	0	0.054249	0	0	0	0.018083	0	0.302542	1	-
ENSMUSC	Dstn	destrin [Sc	GO:00099	cellular pr	-	-	-	-	2:1439153	3444	4195	5501	4663	3671	1855	114.5351	116.9392	126.0284	101.0235	107.0539	76.49013	119.1676	94.85584	0.054033	1	-
ENSMUSC	H2afy	H2A histo	GO:00325	developm	K11251	histone H2	mmu0503	Alcoholisr	13:560736	1086	1333	1586	1585	1283	949	7.89144	8.119134	7.939282	7.503044	8.175157	8.550253	7.983286	8.076151	0.812062	1	-
ENSMUSC	Gtf2ird2	GTF2I rep	GO:00325	developm	-	-	-	-	5:1341840	210	249	236	301	216	192	1.700361	1.689951	1.31639	1.587706	1.533619	1.927561	1.568901	1.682962	0.865221	1	-
ENSMUSC	Bola1	bolA-like	GO:00056	cell|intrace	-	-	-	-	3:9619658	58	61	85	82	75	85	2.399794	2.115574	2.422783	2.210255	2.721138	4.360614	2.312717	3.097336	0.247997	1	-
ENSMUSC	Gatsl2	GATS pro	GO:00508	response	-	-	-	-	5:1340997	2819	3535	4794	4485	3616	3094	21.98502	23.10862	25.75617	22.78641	24.72879	29.91838	23.6166	25.81119	0.694694	1	-
ENSMUSC	Fcgr1	Fc recepto	GO:00048	signal trar	K06498	high affini	mmu0520	Transcript	3:9628290	61	80	118	128	122	87	1.328099	1.459976	1.769851	1.815495	2.329183	2.348594	1.519309	2.164424	0.079228	1	-
ENSMUSC	Ncf1	neutrophi	GO:00081	metabolic	K08011	neutrophi	mmu0406	Chemokin	5:1342200	104	160	192	218	131	123	0.98122	1.265342	1.247922	1.339898	1.083797	1.438883	1.164828	1.287526	0.72276	1	-
ENSMUSC	Wnt11	wingless-t	GO:00325	developm	K01384	wingless-t	mmu0520	Pathways	7:9883511	12	5	18	23	8	14	0.103742	0.036232	0.107206	0.129539	0.060652	0.150079	0.082393	0.113423	0.504093	1	-
ENSMUSC	Adss	adenylosu	GO:00081	metabolic	K01939	adenylosu	mmu0110	Metabolic	1:1777629	1292	1640	2020	1977	1468	981	13.29724	14.14802	14.32194	13.25524	13.24854	12.51856	13.9224	13.00744	0.386758	1	-
ENSMUSC	Il17rb	interleukir	GO:00048	signal trar	K05165	interleukir	mmu0406	Cytokine-	14:299961	11	14	36	18	13	15	0.083415	0.088988	0.188061	0.088917	0.086442	0.141034	0.120155	0.105465	0.566261	1	-
ENSMUSC	Cacna1d	calcium cl	GO:00511	localizatio	K04851	voltage-d	mmu0401	MAPK sigı	14:300399	1015	1301	1279	1761	1294	797	3.009812	3.233728	2.612735	3.401844	3.36473	2.930336	2.952092	3.232303	0.669374	1	-
ENSMUSC	Actr8	ARP8 acti	GO:00081	metabolic	-	-	-	-	14:299783	484	723	795	842	680	482	2.85208	3.571144	3.227265	3.232286	3.513725	3.52167	3.21683	3.42256	0.859815	1	-
ENSMUSC	Lrrc27	leucine ric	GO:00508	response	-	-	-	-	7:1392129	65	78	92	105	74	64	0.749034	0.753424	0.73035	0.788249	0.747768	0.914441	0.744269	0.816819	0.782504	1	-
ENSMUSC	Stk32c	serine/thr	GO:00081	metabolic	-	-	-	-	7:1391036	1232	1550	1768	1914	1510	1208	34.01273	35.86867	33.62517	34.42345	36.55531	41.35078	34.50219	37.44318	0.73296	1	-
ENSMUSC	Fnta	farnesyltra	GO:00081	metabolic	K05955	protein fa	mmu0090	Terpenoic	8:2599872	502	614	803	763	572	472	7.091269	7.270112	7.814248	7.021444	7.085316	8.267008	7.391876	7.457922	0.799466	1	-
ENSMUSC	Skiv2l2	superkille	GO:00081	metabolic	K12598	ATP-depe	-	-	13:112867	1345	1505	1895	2039	1495	791	10.7345	10.06813	10.41886	10.60128	10.46269	7.827478	10.40716	9.630482	0.429476	1	-
ENSMUSC	Lbp	lipopolysa	GO:00081	metabolic	K05399	lipopolysa	mmu0515	Tuberculo	2:1583064	191	129	381	181	141	144	2.237122	1.26648	3.074203	1.38107	1.448168	2.091241	2.192602	1.640159	0.106583	1	-
ENSMUSC	Celsr1	cadherin,	GO:00325	developm	-	-	-	-	15:858989	175	207	298	337	236	179	0.816815	0.80986	0.958197	1.024704	0.96592	1.035918	0.861624	1.008848	0.415142	1	-
ENSMUSC	Fli1	Friend leu	GO:00325	developm	K09436	friend leul	mmu0520	Transcript	9:3242220	119	174	207	165	166	93	2.165131	2.653621	2.59453	1.9557	2.648435	2.097995	2.471094	2.234043	0.424152	1	-
ENSMUSC	Stard13	StAR-rela	GO:00325	developm	-	-	-	-	5:1510375	391	416	602	653	448	421	1.971065	1.757805	2.090607	2.144468	1.980364	2.631434	1.939826	2.252089	0.437909	1	-
ENSMUSC	Tenm1	teneurin t	GO:00325	developm	-	-	-	-	X:4252786	1768	1934	1965	2354	1726	941	5.488021	5.032026	4.201915	4.76016	4.698041	3.621672	4.907321	4.359958	0.286968	1	-
ENSMUSC	Camk1g	calcium/ca	GO:00081	metabolic	K08794	calcium/ca	mmu0492	Oxytocin s	1:1933463	363	419	446	542	397	496	7.379957	7.140259	6.246456	7.178405	7.077504	12.50302	6.922224	8.919644	0.211287	1	-
ENSMUSC	Diexf	digestive	GO:00056	cell|intrace	-	-	-	-	1:1930911	231	349	396	407	293	280	1.251024	1.584279	1.477406	1.435917	1.391435	1.880166	1.43757	1.569173	0.774974	1	-
ENSMUSC	Hsd11b1	hydroxyst	GO:00325	developm	K15680	corticoste	mmu0110	Metabolic	1:1932216	230	357	421	500	356	369	2.318351	3.016282	2.923373	3.283242	3.146615	4.611727	2.752669	3.680528	0.099709	1	-
ENSMUSC	Syt14	synaptota	GO:00511	localizatio	-	-	-	-	1:1928912	509	570	812	881	618	165	2.177321	2.043772	2.392833	2.455062	2.318121	0.875138	2.204642	1.882774	0.435792	1	-
ENSMUSC	H2-M3	histocomp	GO:00325	developm	K06751	major hist	mmu0516	Human pa	17:372702	33	24	33	46	26	37	1.458445	0.889076	1.0047	1.324394	1.007613	2.0275	1.117407	1.453169	0.408784	1	-
ENSMUSC	Lonrf3	LON pept	GO:00081	metabolic	-	-	-	-	X:3632835	163	181	272	240	208	124	2.36943	2.205404	2.723808	2.272735	2.651321	2.234925	2.432881	2.386327	0.739631	1	-
ENSMUSC	Atp5e	ATP synth	GO:00081	metabolic	K02135	F-type H+	mmu0110	Metabolic	2:1744610	226	299	411	289	263	745	5.699604	6.320613	7.140495	4.748041	5.816128	23.2958	6.386904	11.28666	0.092716	1	-
ENSMUSC	Nelfcd	negative e	GO:00081	metabolic	-	-	-	-	2:1744158	445	530	583	573	513	436	6.004481	5.994371	5.419204	5.036761	6.069812	7.294374	5.806019	6.133649	0.951892	1	-
ENSMUSC	Ctsz	cathepsin	GO:00325	developm	K08568	cathepsin	mmu0421	Apoptosis	2:1744274	656	667	1064	1180	747	596	28.93185	24.6576	32.327	33.90282	28.8892	32.5915	28.63882	31.79451	0.617241	1	-
ENSMUSC	Prelid3b	PRELI don	GO:00511	localizatio	-	-	-	-	2:1744650	733	885	1057	1023	896	553	14.70001	14.87681	14.60294	13.36506	15.75667	13.7507	14.72659	14.29081	0.606483	1	-
ENSMUSC	Sertad4	SERTA do	GO:00056	cell|intrace	-	-	-	-	1:1928444	354	385	534	681	453	294	3.321195	3.027639	3.451307	4.162168	3.726759	3.419986	3.266714	3.769638	0.439734	1	-
ENSMUSC	Ube2a	ubiquitin-	GO:00325	developm	K10573	ubiquitin-	mmu0412	Ubiquitin	X:3687390	384	488	327	432	509	311	4.116104	4.38457	2.41465	3.016617	4.784266	4.133335	3.638442	3.978073	0.794857	1	-
ENSMUSC	Slc25a5	solute car	GO:00511	localizatio	K05863	solute car	mmu0516	HTLV-I inf	X:3679565	1653	2261	2892	2511	2059	1861	35.08789	40.22886	42.28964	34.72264	38.32511	48.97976	39.20213	40.67583	0.947911	1	-
ENSMUSC	Ppdpf	pancreatic	GO:00325	developm	-	-	-	-	2:1811872	265	323	367	406	277	363	4.6808	4.782222	4.465732	4.671788	4.290395	7.95	4.642918	5.637394	0.390008	1	-
ENSMUSC	Kcnq2	potassium	GO:00511	localizatio	K04927	potassium	mmu0472	Cholinerg	2:1810755	2366	2423	2889	3707	2816	2220	3.989663	3.424741	3.355989	4.072169	4.163866	4.641521	3.590131	4.292519	0.243574	1	-
ENSMUSC	Eef1a2	eukaryotic	GO:00081	metabolic	K03231	elongatio	mmu0301	RNA trans	2:1811476	14815	17767	20170	21271	16118	11741	451.7936	454.1563	423.7364	422.579	431.0148	443.9453	443.2287	432.513	0.564774	1	-
ENSMUSC	Col20a1	collagen,	GO:00329	macromo	-	-	-	-	2:1809865	98	92	129	198	93	81	1.030812	0.811137	0.934754	1.35676	0.857789	1.05639	0.925568	1.090313	0.486021	1	-
ENSMUSC	Pls3	plastin 3 (	GO:00325	developm	-	-	-	-	X:7578565	3858	4578	4981	5758	5301	2120	35.3806	35.19099	31.46813	34.39982	42.62882	24.10597	34.01324	33.71154	0.812091	1	-
ENSMUSC	Mpped2	metalloph	GO:00038	catalytic a	-	-	-	-	2:1066932	661	739	873	1016	741	374	10.20368	9.562079	9.283693	10.21716	10.03035	7.158346	9.683152	9.135286	0.558758	1	-
ENSMUSC	Nkap	NFKB acti	GO:00325	developm	-	-	-	-	X:3712679	239	300	433	411	303	257	3.428707	3.607497	4.279292	3.8411	3.81169	4.57143	3.771832	4.07474	0.808863	1	-
ENSMUSC	Ndufa1	NADH de	GO:00081	metabolic	K03945	NADH de	mmu0110	Metabolic	X:3718758	480	603	678	592	442	402	72.80529	76.66416	70.84407	58.49594	58.78783	75.60212	73.43784	64.29529	0.179771	1	-
ENSMUSC	Wt1	Wilms tun	GO:00081	metabolic	K09234	Wilms tun	mmu0520	Transcript	2:1051265	0	2	6	1	4	0	0	0.00981	0.024187	0.003812	0.020525	0	0.011332	0.008112	0.825111	1	-
ENSMUSC	E2f3	E2F transc	GO:00081	metabolic	K06620	transcripti	mmu0520	Pathways	13:299065	595	784	887	238	701	583	6.475003	7.151423	6.649646	1.687261	6.689348	7.866437	6.758691	5.414348	0.277966	1	-
ENSMUSC	Cr1l	complem	GO:00325	developm	-	-	-	-	1:1950973	325	384	544	470	360	268	1.611134	1.595628	1.857798	1.517846	1.564922	1.647283	1.688187	1.576684	0.429255	1	-
ENSMUSC	Ppfibp1	PTPRF int	GO:00300	cell junctic	-	-	-	-	6:1468884	597	682	866	845	577	404	2.450346	2.346337	2.448626	2.25939	2.076689	2.055989	2.415103	2.130689	0.216155	1	-
ENSMUSC	Cd46	CD46 anti	GO:00325	developm	K04007	membran	mmu0516	Measles -	1:1950368	67	101	109	150	106	65	0.509702	0.644046	0.57124	0.743384	0.707115	0.61311	0.574996	0.68787	0.408576	1	-
ENSMUSC	Cd34	CD34 anti	GO:00325	developm	K06474	CD34 anti	mmu0451	Cell adhes	1:1949388	432	620	836	733	746	625	2.792115	3.358883	3.722268	3.086283	4.227968	5.008595	3.291089	4.107615	0.225028	1	-
ENSMUSC	Plgrkt	plasminog	GO:00081	metabolic	-	-	-	-	19:293485	79	96	127	126	110	98	0.782888	0.797445	0.867021	0.813441	0.955892	1.204172	0.815785	0.991168	0.395418	1	-
ENSMUSC	Gtf3a	general tr	GO:00971	organic cy	-	-	-	-	5:1469486	268	307	360	370	260	257	3.972065	3.813933	3.675657	3.572439	3.379074	4.722816	3.820552	3.891443	0.851477	1	-
ENSMUSC	Mtif3	mitochon	GO:00081	metabolic	-	-	-	-	5:1469515	284	394	434	495	269	123	2.322621	2.7009	2.44512	2.637218	1.929094	1.247241	2.489547	1.937851	0.152576	1	-
ENSMUSC	Lnx2	ligand of	GO:00099	cellular pr	-	-	-	-	5:1470166	138	150	194	175	157	111	1.671172	1.5226	1.618432	1.380584	1.667189	1.666677	1.604068	1.571483	0.705016	1	-
ENSMUSC	Dyrk3	dual-spec	GO:00325	developm	-	-	-	-	1:1311274	221	228	265	271	222	141	3.780682	3.269378	3.123016	3.020147	3.330222	2.990755	3.391025	3.113708	0.433072	1	-
ENSMUSC	Mapkapk2	MAP kina	GO:00325	developm	K04443	mitogen-a	mmu0401	MAPK sigı	1:1310537	364	454	527	440	349	477	7.60714	7.952958	7.587198	5.990376	6.395704	12.36016	7.715765	8.248746	0.962526	1	-
ENSMUSC	Lamp2	lysosomal	GO:00081	metabolic	K06528	lysosomal	mmu0515	Tuberculo	X:3840135	1333	1519	2161	2045	1487	1135	8.508203	8.126777	9.501975	8.503205	8.322642	8.982343	8.712318	8.60273	0.662969	1	-
ENSMUSC	Atxn10	ataxin 10	GO:00325	developm	-	-	-	-	15:853363	3603	4503	5297	5105	3969	3198	94.42535	98.91889	95.63256	87.1569	91.2112	103.9175	96.3256	94.0952	0.561294	1	-
ENSMUSC	Foxred2	FAD-dep	GO:00081	metabolic	-	-	-	-	15:779405	376	443	329	590	444	317	1.922748	1.898853	1.158995	1.965478	1.990952	2.009928	1.660199	1.988786	0.381115	1	-
ENSMUSC	Eif3d	eukaryotic	GO:00081	metabolic	K03251	translatior	mmu0301	RNA trans	15:779589	1152	1353	1642	1609	1133	965	38.33146	37.73579	37.63806	34.8771	33.0579	39.8122	37.90177	35.91573	0.423261	1	-
ENSMUSC	H3f3b	H3 histon	GO:00325	developm	K11253|K1	histone H3	mmu0503	Alcoholisr	11:116021	1860	2111	2767	2977	2232	1148	20.70928	19.70123	21.22329	21.59298	21.7916	15.84821	20.5446	19.74426	0.610892	1	-
ENSMUSC	Nup50	nucleopor	GO:00325	developm	K14295	nuclear pc	mmu0301	RNA trans	15:849234	839	932	1147	1129	843	637	11.24678	10.47213	10.59207	9.859201	9.909149	10.58745	10.77033	10.1186	0.409983	1	-
ENSMUSC	Phf21b	PHD finge	GO:00081	metabolic	-	-	-	-	15:847853	119	118	158	152	127	113	0.909866	0.756252	0.832223	0.757106	0.851487	1.071256	0.83278	0.893283	0.900484	1	-
ENSMUSC	Ift27	intraflagel	GO:00325	developm	-	-	-	-	15:781594	72	88	131	90	92	151	4.521563	4.63223	5.667347	3.68198	5.066244	11.7576	4.94038	6.835274	0.301326	1	-
ENSMUSC	Pacsin2	protein kii	GO:00325	developm	-	-	-	-	15:833756	754	775	1070	1085	819	576	8.895297	7.663791	8.696098	8.338739	8.472578	8.425539	8.418395	8.412285	0.765017	1	-
ENSMUSC	Cmah	cytidine m	GO:00081	metabolic	K08080	CMP-N-a	mmu0052	Amino suç	13:243274	13	7	6	13	6	5	0.029661	0.013387	0.00943	0.019323	0.012004	0.014144	0.017493	0.015157	0.756922	1	-
ENSMUSC	Ttll12	tubulin tyı	GO:00081	metabolic	-	-	-	-	15:835751	314	369	463	527	364	308	3.522618	3.469891	3.578232	3.851486	3.580805	4.284224	3.52358	3.905505	0.646488	1	-
ENSMUSC	Scube1	signal pep	GO:00325	developm	-	-	-	-	15:836025	746	748	926	1183	852	548	3.212315	2.699813	2.746891	3.318528	3.217078	2.925808	2.88634	3.153805	0.688976	1	-
ENSMUSC	Tox4	TOX high	GO:00329	macromo	-	-	-	-	14:522791	1442	1518	1917	2071	1372	943	9.727571	8.583489	8.908664	9.101246	8.115883	7.887451	9.073241	8.368193	0.352529	1	-
ENSMUSC	Mrps18c	mitochon	GO:00081	metabolic	K02963	small subu	mmu0301	Ribosome	5:1007986	77	125	134	101	105	152	2.2666	3.084228	2.717319	1.936806	2.710288	5.547691	2.689382	3.398262	0.468788	1	-
ENSMUSC	Srsf6	serine/arg	GO:00325	developm	K12893	splicing fa	mmu0516	Herpes sir	2:1629315	1428	2034	2661	2522	1906	1501	12.35417	14.74989	15.85921	14.21385	14.45942	16.10097	14.32109	14.92475	0.991464	1	-
ENSMUSC	Plcg1	phospholi	GO:00325	developm	K01116	phosphati	mmu0110	Metabolic	2:1607313	1212	1653	2082	1987	1535	1468	6.566602	7.506955	7.770882	7.013222	7.292715	9.86166	7.28148	8.055866	0.657189	1	-
ENSMUSC	Kctd2	potassium	GO:00099	cellular pr	-	-	-	-	11:115420	557	692	837	903	665	926	6.149932	6.404338	6.366378	6.495084	6.438429	12.67688	6.306882	8.536797	0.127782	1	-
ENSMUSC	Kctd5	potassium	GO:00099	cellular pr	-	-	-	-	17:240477	292	355	427	374	317	339	6.627675	6.753973	6.676638	5.530073	6.309281	9.540329	6.686095	7.126561	0.943724	1	-
ENSMUSC	Pom121l2	POM121	GO:00511	localizatio	K14316	nuclear pc	mmu0301	RNA trans	13:219811	0	1	0	1	0	0	0	0.010864	0	0.008443	0	0	0.003621	0.002814	1	1	-
ENSMUSC	Etaa1	Ewing tun	GO:00081	metabolic	-	-	-	-	11:179387	150	182	212	238	192	70	2.277892	2.316678	2.217826	2.354505	2.556733	1.318036	2.270799	2.076425	0.574776	1	-
ENSMUSC	Matn4	matrilin 4	GO:00431	ion bindin	-	-	-	-	2:1643893	122	146	215	218	161	196	1.399033	1.40338	1.698474	1.628573	1.618965	2.786833	1.500296	2.011457	0.175617	1	-
ENSMUSC	Slpi	secretory	GO:00081	metabolic	-	-	-	-	2:1643540	2	4	4	8	6	0	0.07691	0.128901	0.105956	0.20037	0.202267	0	0.103922	0.134212	0.741272	1	-
ENSMUSC	Rbpjl	recombina	GO:00081	metabolic	K06053	recombini	mmu0516	Human pa	2:1644031	8	13	12	13	10	10	0.069397	0.094532	0.071716	0.073469	0.076072	0.107567	0.078548	0.085702	1	1	-
ENSMUSC	Sdc4	syndecan	GO:00325	developm	K16338	syndecan	mmu0520	Proteoglyc	2:1644242	1273	1406	1949	2057	1437	1229	28.04262	25.96142	29.57696	29.51931	27.75809	33.5682	27.86033	30.28187	0.723527	1	-
ENSMUSC	Il13ra1	interleukir	GO:00048	signal trar	K05076	interleukir	mmu0520	Pathways	X:3611211	125	162	189	177	134	88	2.140123	2.324859	2.229167	1.974164	2.011755	1.868082	2.231383	1.951334	0.288803	1	-
ENSMUSC	Nbr1	neighbor	GO:00325	developm	K17987	next to BR	mmu0413	Mitophag	11:101552	2027	3486	2744	3011	2291	2474	11.76241	16.95598	10.96928	11.38243	11.65763	17.80034	13.22922	13.61347	0.887001	1	-
ENSMUSC	Cyth1	cytohesin	GO:00987	molecular	K18441	cytohesin[	mmu0414	Endocytos	11:118132	1171	1380	1739	2032	1458	1627	11.76238	11.61904	12.03343	13.29669	12.84217	20.26337	11.80495	15.46741	0.088886	1	-
ENSMUSC	Rnd3	Rho famil	GO:00508	response	-	-	-	-	2:5113043	221	313	378	316	265	219	2.286008	2.713827	2.693569	2.129383	2.403661	2.808761	2.564468	2.447268	0.531985	1	-
ENSMUSC	Brca1	breast car	GO:00325	developm	K10605	breast car	mmu0415	PI3K-Akt s	11:101488	33	36	32	39	37	31	0.179175	0.163841	0.119692	0.137946	0.176162	0.208697	0.154236	0.174268	0.795718	1	-
ENSMUSC	Gast	gastrin [Sc	GO:00508	response	K13768	gastrin[-]	mmu0497	Gastric ac	11:100334	0	0	2	0	0	0	0	0	0.190376	0	0	0	0.063459	0	0.536229	1	-
ENSMUSC	Cntnap1	contactin	GO:00325	developm	K07379	contactin	mmu0451	Cell adhes	11:101170	3939	4676	5621	6865	4768	3901	42.03078	41.82239	41.31868	47.72035	44.61289	51.61112	41.72395	47.98145	0.379801	1	-
ENSMUSC	Nt5c3b	5'-nucleo	GO:00081	metabolic	K01081	5'-nucleo	mmu0110	Metabolic	11:100422	344	430	447	514	422	382	3.932765	4.120607	3.520451	3.828112	4.230527	5.414878	3.857941	4.491172	0.452751	1	-
ENSMUSC	Coa3	cytochron	GO:00081	metabolic	-	-	-	-	11:101277	704	916	1031	992	770	643	38.01304	41.45797	38.35038	34.89416	36.45804	43.04838	39.2738	38.13353	0.56321	1	-
ENSMUSC	Gsdma	gasdermi	GO:00099	cellular pr	-	-	-	-	11:986643	0	6	0	1	0	3	0	0.117245	0	0.015186	0	0.086727	0.039082	0.033971	1	1	-
ENSMUSC	Med24	mediator	GO:00325	developm	K15167	mediator	mmu0491	Thyroid hc	11:987045	1699	2151	2454	2868	2123	1681	12.17735	12.92268	12.11673	13.39122	13.34295	14.93871	12.40559	13.89096	0.528658	1	-
ENSMUSC	Gsdma2	gasdermi	GO:00099	cellular pr	-	-	-	-	11:986467	3	1	0	1	1	0	0.038929	0.010878	0	0.008453	0.011379	0	0.016602	0.006611	0.720278	1	-
ENSMUSC	Psmd3	proteasor	GO:00081	metabolic	K03033	26S prote	mmu0516	Epstein-B	11:986825	1702	2368	2365	2823	1950	1506	14.00585	16.3337	13.40703	15.13361	14.07108	15.36601	14.5822	14.8569	0.86827	1	-
ENSMUSC	Exosc10	exosome	GO:00081	metabolic	K12591	exosome	mmu0301	RNA degr	4:1485584	748	1054	1075	1190	900	653	9.063419	10.70493	8.973268	9.393322	9.562591	9.810465	9.58054	9.588793	0.76808	1	-
ENSMUSC	Glod4	glyoxalase	GO:00055	extracellul	-	-	-	-	11:761209	563	696	842	826	579	491	4.755495	4.927756	4.899492	4.545159	4.288531	5.142274	4.860914	4.658655	0.506051	1	-
ENSMUSC	Vps53	VPS53 GA	GO:00511	localizatio	-	-	-	-	11:760462	1232	1463	1904	1912	1484	964	9.483686	9.439818	10.09682	9.588173	10.01712	9.200876	9.673442	9.602058	0.721488	1	-
ENSMUSC	Taok1	TAO kinas	GO:00325	developm	K04429	thousand	mmu0401	MAPK sigı	11:775291	2502	5949	7766	8773	6155	3267	12.57689	25.06587	26.89272	28.72868	27.13043	20.36203	21.51183	25.40705	0.443521	1	-
ENSMUSC	Dnttip1	deoxynuc	GO:00081	metabolic	-	-	-	-	2:1647459	356	475	523	526	419	244	9.010293	10.07711	9.118904	8.672748	9.299214	7.657102	9.402103	8.543021	0.36511	1	-
ENSMUSC	Tnnc2	troponin C	GO:00990	supramol	K12042	troponin C	mmu0402	Calcium si	2:1647771	0	0	2	0	5	6	0	0	0.109466	0	0.348278	0.591047	0.036489	0.313108	0.0605	1	-
ENSMUSC	Acot8	acyl-CoA	GO:00081	metabolic	K11992	acyl-CoA	mmu0110	Metabolic	2:1647927	123	122	170	163	182	145	2.7888	2.318602	2.655294	2.407587	3.618491	4.076293	2.587565	3.367457	0.217584	1	-
ENSMUSC	Cd300lg	CD300 mo	GO:00511	localizatio	-	-	-	-	11:102041	4	3	15	5	3	3	0.088541	0.055662	0.228747	0.072108	0.058228	0.082347	0.124317	0.070895	0.26203	1	-
ENSMUSC	Pyy	peptide Y	GO:00508	response	-	-	-	-	11:102106	0	0	0	0	2	0	0	0	0	0	0.088311	0	0	0.029437	0.535389	1	-
ENSMUSC	Mpp2	membran	GO:00452	synapse	-	-	-	-	11:102057	3219	3838	4287	4852	3620	2711	27.43424	27.41763	25.16963	26.93856	27.05348	28.64755	26.67383	27.54653	0.949645	1	-
ENSMUSC	Vtn	vitronecti	GO:00325	developm	K06251	vitronecti	mmu0415	PI3K-Akt s	11:784990	811	1113	1336	1459	1208	1026	15.20846	17.49494	17.25926	17.82385	19.86434	23.85601	16.65422	20.51473	0.182705	1	-
ENSMUSC	Nlk	nemo like	GO:00048	signal trar	K04468	nemo like	mmu0401	MAPK sigı	11:785671	1032	1265	1664	1653	1328	996	8.94527	9.190882	9.936159	9.334008	10.09379	10.70432	9.357437	10.04404	0.797723	1	-
ENSMUSC	Traf4	TNF recep	GO:00325	developm	K09848	TNF recep	mmu0401	MAPK sigı	11:781584	124	180	205	207	160	154	1.550378	1.886437	1.765722	1.686042	1.754196	2.387391	1.734179	1.942543	0.690672	1	-
ENSMUSC	Aldoc	aldolase C	GO:00325	developm	K01623	fructose-b	mmu0110	Metabolic	11:783229	10722	13575	16872	15231	13776	10109	122.8661	130.3914	133.1908	113.7015	138.4274	143.6317	128.8161	131.9202	0.879413	1	-
ENSMUSC	Stac2	SH3 and C	GO:00508	response	-	-	-	-	11:980366	1397	1465	2138	2191	1748	1422	27.72752	24.37279	29.23304	28.3295	30.42278	34.99455	27.11112	31.24894	0.40717	1	-
ENSMUSC	Rpl19	ribosomal	GO:00081	metabolic	K02885	large subu	mmu0301	Ribosome	11:980267	496	641	736	676	528	480	27.10429	29.36077	27.70674	24.06492	25.30072	32.52249	28.05727	27.29604	0.581324	1	-
ENSMUSC	Nek8	NIMA (ne	GO:00325	developm	-	-	-	-	11:786161	43	51	63	72	72	50	0.509249	0.529536	0.296998	0.320976	0.432049	0.424249	0.294598	0.392425	0.205199	1	-
ENSMUSC	Cacnb4	calcium cl	GO:00325	developm	K04865	voltage-d	mmu0401	MAPK sigı	2:5242832	2590	1315	4594	1636	1437	1949	10.1381	4.314552	12.38795	4.171787	4.932379	9.459209	8.946869	6.187791	0.06329	1	-
ENSMUSC	Plxdc1	plexin dor	GO:00325	developm	-	-	-	-	11:979232	134	177	347	252	257	200	1.24833	1.382132	2.226922	1.529351	2.099428	2.310147	1.619128	1.979642	0.393547	1	-
ENSMUSC	Arl5b	ADP-ribo	GO:00511	localizatio	-	-	-	-	2:1504939	680	745	289	140	696	461	2.2644	2.079473	0.662968	0.303706	2.032338	1.903404	1.668947	1.413149	0.540642	1	-
ENSMUSC	Zfp207	zinc finge	GO:00990	supramol	-	-	-	-	11:803832	1899	2143	2914	2943	2292	1139	13.49063	12.76093	14.26094	13.62006	14.2779	10.03268	13.50417	12.64355	0.491322	1	-
ENSMUSC	Psmd11	proteasor	GO:00081	metabolic	K03036	26S prote	mmu0516	Epstein-B	11:804286	1079	1339	1541	1742	1230	779	6.636379	6.903083	6.529257	6.979742	6.633727	5.940639	6.689573	6.518036	0.6384	1	-
ENSMUSC	C1qtnf1	C1q and t	GO:00081	metabolic	-	-	-	-	11:118426	35	52	58	60	40	49	0.663399	0.826142	0.757325	0.740862	0.664821	1.151546	0.748955	0.85241	0.73686	1	-
ENSMUSC	Pipox	pipecolic	GO:00081	metabolic	K00306	sarcosine	mmu0110	Metabolic	11:778806	18	24	23	21	20	19	0.37642	0.420695	0.331353	0.286096	0.366765	0.492664	0.376156	0.381842	0.898392	1	-
ENSMUSC	Timp2	tissue inhi	GO:00325	developm	-	-	-	-	11:118301	5397	7063	7176	6795	5581	4702	83.82101	91.94806	76.77757	68.74982	76.00727	90.54596	84.18221	78.43435	0.332377	1	-
ENSMUSC	Zc3h18	zinc finge	GO:00329	macromo	-	-	-	-	8:1223766	866	960	1237	1235	994	663	4.505315	4.186308	4.433311	4.185581	4.534572	4.27668	4.374978	4.332278	0.706017	1	-
ENSMUSC	Top2b	topoisom	GO:00325	developm	K03164	DNA topo	mmu0152	Platinum	14:163651	2530	3168	3785	3998	2835	1490	9.8354	10.32309	10.13652	10.12502	9.66423	7.181973	10.09833	8.990408	0.283353	1	-
ENSMUSC	Rarb	retinoic a	GO:00325	developm	K08528	retinoic a	mmu0520	Pathways	14:164308	91	114	132	110	111	67	0.666129	0.679945	0.665644	0.524553	0.712804	0.677083	0.61505	[illegible]	0.447389	1	-
ENSMUSC	Igfbp4	insulin-lik	GO:00081	metabolic	-	-	-	-	11:990412	1197	1518	952	938	1548	1345	12.4526	13.23704	6.822669	6.356972	14.12146	17.34899	10.83743	12.60914	0.655372	1	-
ENSMUSC	Cdc6	cell divisi	GO:00325	developm	K02213	cell divisio	mmu0411	Cell cycle	11:989078	8	18	16	13	10	6	0.072664	0.044293	0.072809	0.055944	0.057925	0.049146	0.063256	0.054339	0.612547	1	-
ENSMUSC	Suz12	suppressc	GO:00325	developm	-	-	-	-	11:799931	688	503	1022	1044	784	543	5.406093	3.312957	5.532195	5.344131	5.401992	5.29031	4.750415	5.345477	0.597816	1	-
ENSMUSC	Atad5	ATPase fa	GO:00325	developm	-	-	-	-	11:800894	90	127	142	160	159	84	0.615756	0.728922	0.66928	0.713131	0.953908	0.712577	0.671119	0.793206	0.446301	1	-
ENSMUSC	Crlf3	cytokine r	GO:00081	metabolic	-	-	-	-	11:800464	173	176	220	195	176	188	1.905161	1.624612	1.669009	1.398945	1.699571	2.567013	1.732927	1.88851	0.86006	1	-
ENSMUSC	Tns4	tensin 4 [	GO:00511	localizatio	-	-	-	-	11:990656	5	5	49	11	0	4	0.051889	0.043491	0.35031	0.074363	0	0.051469	0.148563	0.041944	0.055692	1	-
ENSMUSC	Abr	active BCF	GO:00325	developm	-	-	-	-	11:764167	12898	13987	18609	21416	15234	10540	46.87253	42.60626	46.58761	50.70093	48.54591	47.49223	45.35547	48.91302	0.732277	1	-
ENSMUSC	Rab11fip4	RAB11 far	GO:00325	developm	K12485	Rab11 far	mmu0414	Endocytos	11:795912	6216	7252	9155	9649	7289	5463	51.91819	50.77141	52.67667	52.50159	53.38499	56.57514	51.78876	54.15391	0.962063	1	-
ENSMUSC	Cd40	CD40 anti	GO:00325	developm	K03160	tumor nec	mmu0516	HTLV-I inf	2:1650556	5	15	6	14	11	10	0.057452	0.144472	0.047491	0.104797	0.110834	0.142476	0.083139	0.119369	0.454575	1	-
ENSMUSC	Slc35c2	solute car	GO:00081	metabolic	-	-	-	-	2:1652765	499	612	786	940	614	720	4.890196	5.027247	5.306399	6.001168	5.276396	8.74872	5.074614	6.675428	0.109696	1	-
ENSMUSC	Zfp334	zinc finge	GO:00081	metabolic	-	-	-	-	2:1653742	594	685	877	843	612	440	4.600916	4.447348	4.679599	4.249656	4.156731	4.225679	4.575954	4.212036	0.343327	1	-
ENSMUSC	Elmo2	engulfmer	GO:00400	locomotic	K18985	engulfmer	mmu0510	Bacterial i	2:1652880	3308	3797	4552	5141	3883	3117	21.06547	20.26745	19.96913	21.32725	21.68281	24.61095	20.43402	22.54034	0.613858	1	-
ENSMUSC	Wsb1	WD repea	GO:00081	metabolic	-	-	-	-	11:792393	1029	1503	1677	1851	1276	1001	9.466708	11.59033	10.62841	11.09359	10.29383	11.41836	10.56181	10.93526	0.975899	1	-
ENSMUSC	Rhot1	ras homo	GO:00511	localizatio	K07870	Ras homo	mmu0413	Mitophag	11:802090	1139	1368	1720	1720	1257	841	4.832878	4.865429	5.027618	4.754365	4.676925	4.424503	4.908642	4.618597	0.430857	1	-
ENSMUSC	Rhbdl3	rhomboid	GO:00081	metabolic	-	-	-	-	11:803009	469	654	683	708	509	474	5.607973	6.554858	5.626065	5.515031	5.336955	7.027436	5.929632	5.959807	0.770088	1	-
ENSMUSC	Ada	adenosine	GO:00081	metabolic	K01488	adenosine	mmu0110	Metabolic	2:1637265	20	7	10	15	7	5	0.595071	0.174568	0.204973	0.290743	0.182644	0.184445	0.324871	0.219277	0.378611	1	-
ENSMUSC	Serinc3	serine inc	GO:00081	metabolic	-	-	-	-	2:1636232	3130	4230	5305	5101	3614	3304	48.02533	54.40254	56.07417	50.98741	48.62469	62.85676	52.83401	54.15629	0.814798	1	-
ENSMUSC	Tha1	threonine	GO:00081	metabolic	K01620	threonine	mmu0110	Metabolic	11:117867	33	32	72	57	49	69	0.562716	0.457389	0.845783	0.633185	0.732686	1.458846	0.621963	0.941572	0.17943	1	-
ENSMUSC	Pgs1	phosphati	GO:00081	metabolic	K00995	CDP-diac	mmu0110	Metabolic	11:117798	822	1107	1296	1364	964	1077	8.415043	9.499153	9.139885	9.096631	8.653742	13.67055	9.018027	10.47364	0.451322	1	-
ENSMUSC	Birc5	baculovira	GO:00081	metabolic	K08731	baculovira	mmu0520	Pathways	11:117849	18	14	22	17	11	10	0.27426	0.178808	0.230921	0.168741	0.146973	0.188932	0.227996	0.168215	0.264921	1	-
ENSMUSC	Afmid	arylforma	GO:00081	metabolic	K01432	arylforma	mmu0110	Metabolic	11:117825	14	16	15	13	39	18	0.171169	0.163976	0.126343	0.103546	0.418126	0.272879	0.153829	0.264851	0.178209	1	-
ENSMUSC	Pigt	phosphati	GO:00081	metabolic	K05292	phosphati	mmu0110	Metabolic	2:1644975	1204	1414	1704	1960	1444	1056	12.86663	12.66603	12.54467	13.64508	13.53157	13.99227	12.69244	13.72297	0.736438	1	-
ENSMUSC	Wfdc2	WAP four	GO:00081	metabolic	-	-	-	-	2:1645624	11	6	25	3	4	14	0.411963	0.188338	0.649967	0.073182	0.131348	0.650106	0.41509	0.284879	0.35881	1	-
ENSMUSC	Etv4	ets varian	GO:00325	developm	K15592	ets transc	mmu0520	Transcript	11:101769	117	141	145	170	133	85	1.221365	1.23377	1.042753	1.156091	1.217462	1.100181	1.165963	1.157911	0.825506	1	-
ENSMUSC	Dbndd2	dysbindin	GO:00081	metabolic	-	-	-	-	2:1644861	1563	1439	2705	2380	1673	1448	13.7829	10.63642	16.43237	13.67226	12.9366	15.83201	13.61723	14.14696	0.981635	1	-
ENSMUSC	Mmp9	matrix me	GO:00325	developm	K01403	matrix me	mmu0520	Pathways	2:1649407	19	27	32	42	17	25	0.306163	0.364679	0.355221	0.440885	0.240206	0.499482	0.342021	0.393524	0.719131	1	-

ENSMUSG	Slc12a5	solute car	GO:00325	developm	K14427	solute car	mmu0472	GABAergi	2:1649608	5179	5939	6946	8049	6057	5091	26.54149	25.51206	24.52255	26.87219	27.21947	32.34957	25.52537	28.81374	0.489153	1	-
ENSMUSG	Ghdc	GH3 dom	GO:00160	membran	-	-	-	-	11:100766	61	58	102	104	55	93	1.122061	0.894274	1.292532	1.246239	0.88714	2.12107	1.102956	1.418149	0.391949	1	-
ENSMUSG	Pltp	phospholi	GO:00081	metabolic	K08761	phospholi	mmu0332	PPAR sign	2:1648395	582	712	1061	956	733	948	9.263172	9.498819	11.63331	9.912346	10.23019	18.70815	10.13177	12.95023	0.227578	1	-
ENSMUSG	Slc12a7	solute car	GO:00511	localizatio	K13627	solute car	mmu0496	Collecting	13:737330	71	79	151	128	87	82	0.249931	0.233101	0.366177	0.293532	0.268551	0.357902	0.28307	0.306662	0.866427	1	-
ENSMUSG	Ctsa	cathepsin	GO:00081	metabolic	K13289	cathepsin	mmu0414	Lysosome	2:1648328	1068	1263	1592	1712	1145	933	7.119962	7.05769	7.311406	7.435189	6.693521	7.712129	7.16302	7.28028	0.847854	1	-
ENSMUSG	Zswim1	zinc finge	GO:00056	cell|intrace	-	-	-	-	2:1648226	431	425	552	584	433	295	7.702859	6.366721	6.796182	6.799378	6.785862	6.537053	6.955254	6.707431	0.601785	1	-
ENSMUSG	Slc12a4	solute car	GO:00511	localizatio	-	-	-	-	8:1059435	396	369	637	528	401	402	4.677879	3.653705	5.183758	4.063209	4.15375	5.887972	4.505114	4.701643	0.977083	1	-
ENSMUSG	Spata25	spermato	GO:00325	developm	-	-	-	-	2:1648263	0	0	2	0	0	0	0	0	0.069392	0	0	0	0.023131	0	0.536401	1	-
ENSMUSG	Myo1c	myosin IC	GO:00400	locomotic	-	-	-	-	11:756505	255	350	521	458	318	302	1.792506	2.062248	2.52295	2.097329	1.960149	2.632162	2.125901	2.22988	0.986033	1	-
ENSMUSG	Crk	v-crk avia	GO:00325	developm	K04438	proto-onc	mmu0520	Pathways	11:756792	2364	2991	2163	2165	2641	1099	16.44296	17.4382	10.36431	9.810068	16.10807	9.477983	14.74849	11.79871	0.15156	1	-
ENSMUSG	Cox7c	cytochron	GO:00081	metabolic	K02272	cytochron	mmu0110	Metabolic	13:860448	148	229	232	260	144	207	5.341196	6.927319	5.767885	6.112689	4.557032	9.2626	6.012133	6.644107	0.794608	1	-
ENSMUSG	Pitpna	phosphati	GO:00325	developm	-	-	-	-	11:755880	4110	5307	6410	4841	4819	2951	29.59806	32.0349	31.80027	22.7111	30.43134	26.34975	31.14441	26.49739	0.104267	1	-
ENSMUSG	Mlx	MAX-like	GO:00081	metabolic	K09113	MAX-like	mmu0493	Non-alco	11:101087	186	281	396	374	221	312	3.689406	4.671992	5.411161	4.832773	3.843961	7.673322	4.590853	5.450019	0.502315	1	-
ENSMUSG	Retreg3	reticuloph	GO:00325	developm	-	-	-	-	11:101096	860	426	1474	1226	1022	930	7.939512	3.296532	9.374417	7.373395	8.273494	10.64545	6.870154	8.764114	0.334012	1	-
ENSMUSG	Jph2	junctophil	GO:00325	developm	-	-	-	-	2:1633362	15	19	19	35	15	21	0.192315	0.204186	0.16781	0.292329	0.168635	0.333823	0.188104	0.264929	0.286402	1	-
ENSMUSG	Rab5a	RAB5A, m	GO:00081	metabolic	K07887	Ras-relate	mmu0414	Endocytos	17:534792	1923	2210	2739	2988	2049	974	51.85084	49.94842	50.87682	52.48544	48.44636	32.56278	50.89202	44.49819	0.279817	1	-
ENSMUSG	Nkiras2	NFKB inhi	GO:00508	response	-	-	-	-	11:100619	118	90	150	93	136	117	1.264201	0.808219	1.107078	0.649082	1.27766	1.554193	1.059833	1.160312	0.876538	1	-
ENSMUSG	Ppp2r5c	protein pl	GO:00081	metabolic	K11584	serine/thr	mmu0415	PI3K-Akt s	12:110447	3061	3767	4701	4630	3643	2278	18.99949	19.59869	20.10112	18.72152	19.82807	17.53147	19.56643	18.69369	0.492209	1	-
ENSMUSG	Ift52	intraflagel	GO:00325	developm	-	-	-	-	2:1630173	376	432	503	569	452	239	7.765979	7.479035	7.156947	7.656015	8.186332	6.120575	7.46732	7.320974	0.736803	1	-
ENSMUSG	Mybl2	myeloblas	GO:00325	developm	K09421	myb-relat	mmu0516	HTLV-I int	2:1630546	12	12	19	22	14	11	0.136717	0.114606	0.149128	0.163292	0.139871	0.155398	0.133483	0.152854	0.804304	1	-
ENSMUSG	Sgk2	serum/glu	GO:00081	metabolic	K13303	serum/glu	mmu0415	PI3K-Akt s	2:1629873	25	24	34	23	17	20	0.331008	0.266352	0.310118	0.198383	0.197369	0.32833	0.302493	0.241361	0.266337	1	-
ENSMUSG	Eya2	EYA trans	GO:00325	developm	-	-	-	-	2:1655950	56	64	48	73	34	58	0.757869	0.726006	0.447506	0.643591	0.403492	0.973252	0.643794	0.673445	0.980267	1	-
ENSMUSG	B4galt5	UDP-Gal:l	GO:00081	metabolic	K09905	beta-1,4-	mmu0051	Mucin typ	2:1672984	911	1003	1309	1461	1004	814	12.32371	11.37305	12.19872	12.87523	11.90966	13.65317	11.96516	12.81269	0.814809	1	-
ENSMUSG	Gdap1l1	gangliosic	GO:00081	metabolic	-	-	-	-	2:1634384	1115	1409	1492	1442	1301	921	13.91352	14.73758	12.82574	11.72221	14.23582	14.24978	13.82562	13.4026	0.563078	1	-
ENSMUSG	Ptgis	prostaglai	GO:00325	developm	K01831	prostacycl	mmu0110	Metabolic	2:1671918	8	12	14	13	16	28	0.162222	0.203979	0.195572	0.17174	0.284508	0.704013	0.187258	0.386754	0.086706	1	-
ENSMUSG	Cadps2	Ca2+-dep	GO:00452	synapse|c	-	-	-	-	6:2326277	1662	1900	2128	2700	1859	1279	5.865129	5.620216	5.173322	6.207145	5.752657	5.596327	5.552889	5.852043	0.895241	1	-
ENSMUSG	Ddx27	DEAD (As	GO:00081	metabolic	-	-	-	-	2:1670151	533	667	818	824	633	407	5.4224	5.687787	5.73283	5.461019	5.64691	5.133866	5.614339	5.413932	0.584012	1	-
ENSMUSG	Cyth3	cytohesin	GO:00511	localizatio	K18441	cytohesin[	mmu0414	Endocytos	5:1436224	1051	1156	1398	1568	1057	644	6.45979	5.955609	5.919352	6.278318	5.696828	4.907806	6.111584	5.627651	0.386043	1	-
ENSMUSG	Cyth4	cytohesin	GO:00987	molecular	K18441	cytohesin[	mmu0414	Endocytos	15:785970	254	327	482	487	357	276	5.783761	6.241333	7.560916	7.224152	7.128317	7.792388	6.52867	7.381619	0.554985	1	-
ENSMUSG	Rac3	RAS-relat	GO:00325	developm	K07861	Ras-relate	mmu0520	Pathways	11:120721	84	141	173	167	102	169	3.279133	4.613754	4.652411	4.246986	3.491609	8.180046	4.181766	5.306214	0.395919	1	-
ENSMUSG	Rrp7a	ribosomal	GO:00325	developm	K14545	ribosomal	mmu0300	Ribosome	15:831134	1627	1909	2153	2296	1841	1433	15.61237	15.35466	14.23236	14.35274	15.49094	17.04957	15.06646	15.63108	0.968988	1	-
ENSMUSG	Cyb5r3	cytochron	GO:00081	metabolic	K00326	cytochron	mmu0052	Amino su	15:831534	1065	1274	1450	1523	1186	930	15.26132	15.30258	14.31403	14.21753	14.90287	16.52386	14.95931	15.21475	0.840799	1	-
ENSMUSG	Ints2	integrator	GO:00081	metabolic	-	-	-	-	11:862106	380	480	557	660	510	337	1.678374	1.77705	1.694776	1.899026	1.975234	1.845532	1.716733	1.906597	0.609615	1	-
ENSMUSG	Med13l	mediator	GO:00081	metabolic	K15164	mediator	mmu0491	Thyroid hc	5:1185606	2396	2800	3475	3934	2953	2265	7.634646	7.478474	7.627959	8.166163	8.251032	8.948625	7.580359	8.455273	0.540595	1	-
ENSMUSG	Hist1h2bc	histone cl	GO:00023	immune s	K11252	histone H:	mmu0520	Viral carci	13:236841	287	308	473	378	273	314	15.77831	14.19326	17.91393	13.53792	13.16085	21.40395	15.96183	16.03424	0.766474	1	-
ENSMUSG	Baiap2l2	BAI1-assc	GO:00508	response	-	-	-	-	15:792581	20	13	15	12	9	19	0.26547	0.144641	0.137162	0.103767	0.104757	0.312699	0.182424	0.173741	0.799296	1	-
ENSMUSG	Mafk	v-maf mu	GO:00325	developm	-	-	-	-	5:1397915	189	235	284	228	194	173	1.874745	1.953897	1.940665	1.473326	1.687432	2.127721	1.923102	1.762826	0.376656	1	-
ENSMUSG	Med1	mediator	GO:00325	developm	K15144	mediator	mmu0491	Thyroid hc	11:981521	1189	1278	1678	1873	1319	852	5.008918	4.512796	4.869736	5.140216	4.872472	4.450281	4.79715	4.820989	0.816966	1	-
ENSMUSG	Erbb3	erb-b2 re	GO:00325	developm	K05084	receptor t	mmu0520	Proteogly	10:128567	267	203	373	346	266	200	2.820587	1.797537	2.714491	2.381148	2.464065	2.619651	2.444205	2.488288	0.895303	1	-
ENSMUSG	Stard3	START do	GO:00081	metabolic	-	-	mmu0497	Cholester	11:983583	318	330	542	449	376	437	3.780365	3.288321	4.438721	3.477243	3.919567	6.441316	3.835802	4.612709	0.422546	1	-
ENSMUSG	Ikzf3	IKAROS fa	GO:00325	developm	-	-	-	-	11:984649	2	4	3	5	0	0	0.013368	0.022405	0.01381	0.021769	0	0	0.016528	0.007256	0.350323	1	-
ENSMUSG	Mfng	MFNG O-	GO:00325	developm	K05948	fringe[2.4.	mmu0516	Human pa	15:787558	47	60	61	56	52	56	0.948549	1.015006	0.848094	0.73626	0.920259	1.401318	0.937216	1.019279	0.929798	1	-
ENSMUSG	Vmp1	vacuole m	GO:00325	developm	-	-	mmu0414	Autophag	11:865838	866	1001	1396	1326	940	893	9.169766	8.88438	10.18303	9.146721	8.727913	11.72405	9.412391	9.866229	0.985903	1	-
ENSMUSG	Uchl5	ubiquitin c	GO:00325	developm	-	-	-	-	1:1437772	435	582	666	669	575	313	5.187759	5.817901	5.471622	5.197547	6.013137	4.628296	5.492427	5.27966	0.613833	1	-
ENSMUSG	Glrx2	glutaredo	GO:00325	developm	-	-	-	-	1:1437163	432	546	1204	763	582	788	1.512998	1.602879	2.904914	1.740851	1.787396	3.4219	2.00693	2.316715	0.648023	1	-
ENSMUSG	Trove2	TROVE dc	GO:00325	developm	K11089	60 kDa SS	mmu0532	Systemic l	1:1437507	1247	1605	2142	1723	1410	952	6.704223	7.232856	7.933279	6.034605	6.647274	6.346066	7.290119	6.342648	0.143171	1	-
ENSMUSG	Stk4	serine/thr	GO:00325	developm	K04411	serine/thr	mmu0520	Pathways	2:1640703	1225	1518	1944	1937	1431	918	12.27956	12.75474	13.42439	12.64906	12.57851	11.40973	12.81956	12.21243	0.497973	1	-
ENSMUSG	Pmp22	periphera	GO:00325	developm	-	-	-	-	11:631289	229	254	388	338	231	235	3.231987	3.004834	3.772398	3.107655	2.858839	4.112328	3.336406	3.359607	0.799488	1	-
ENSMUSG	Gdf9	growth di	GO:00325	developm	K05498	growth di	mmu0491	Ovarian st	11:534310	14	12	20	4	19	15	0.341681	0.245496	0.336268	0.063605	0.406613	0.45392	0.307815	0.308046	0.906752	1	-
ENSMUSG	Zcchc10	zinc finge	GO:00971	organic cy	-	-	-	-	11:533246	89	130	149	149	117	100	3.541763	4.336373	4.084772	3.862765	4.082819	4.934196	3.987636	4.29326	0.87772	1	-
ENSMUSG	Tbx5	T-box 5 [S	GO:00325	developm	-	-	-	-	5:1198326	0	2	0	0	0	0	0	0.021282	0	0	0	0	0.007094	0	0.536096	1	-
ENSMUSG	Psmb6	proteasor	GO:00081	metabolic	K02738	20S prote	mmu0305	Proteason	11:705253	948	1217	1376	1324	1009	863	19.39113	20.86595	19.38942	17.64267	18.09793	21.88727	19.88217	19.20929	0.518925	1	-
ENSMUSG	Spag7	sperm ass	GO:00056	cell|intrace	-	-	-	-	11:706637	652	734	894	970	689	449	16.09186	15.18475	15.20014	15.59597	14.91147	13.74014	15.49225	14.7492	0.525513	1	-
ENSMUSG	Pfn1	profilin 1		GO:00325	developm	K05759	profilin[-]	mmu0481	Regulatior	11:706518	868	953	1216	1439	926	1576	13.16563	12.11624	12.70595	14.21885	12.31617	29.63904	12.66261	18.72469	0.060901	1	-
ENSMUSG	Tomm34	translocas	GO:00511	localizatio	-	-	-	-	2:1640535	1777	2102	2455	2745	2039	1669	33.38271	33.09937	31.77145	33.59372	33.58876	38.87554	32.75118	35.35267	0.763553	1	-
ENSMUSG	Ywhab	tyrosine 3	GO:00081	metabolic	K16197	14-3-3 pr	mmu0415	PI3K-Akt s	2:1639949	9131	11460	12989	13957	9822	10322	168.9381	177.7243	165.5528	168.2221	159.35	236.7877	170.7384	188.1199	0.67997	1	-
ENSMUSG	Ksr1	kinase sur	GO:00048	signal trar	K14958	kinase sur	mmu0401	Ras signal	11:790134	3144	3552	4478	3867	1808	2728	18.19935	17.23452	17.85701	14.5824	9.177292	19.57959	17.76363	14.44643	0.090652	1	-
ENSMUSG	Gpx3	glutathior	GO:00081	metabolic	K00432	glutathior	mmu0059	Arachidor	11:549024	58	117	112	107	72	73	1.553998	2.627614	2.067233	1.867615	1.691585	2.42509	2.082949	1.994763	0.646264	1	-
ENSMUSG	Anxa6	annexin A	GO:00511	localizatio	-	-	-	-	11:549791	1881	2317	2826	3062	2283	1661	39.31049	40.58809	40.68584	41.68756	41.83775	43.0403	40.19481	42.18854	0.930468	1	-
ENSMUSG	Il12rb2	interleukir	GO:00048	signal trar	K05064	interleukir	mmu0520	Pathways	6:6729131	2	3	8	7	0	6	0.02817	0.03541	0.077609	0.064218	0	0.104772	0.047063	0.05633	1	1	-
ENSMUSG	Zkscan6	zinc finge	GO:00081	metabolic	-	-	-	-	11:658071	279	276	377	428	349	292	4.944595	4.100034	4.602767	4.94141	5.423685	6.416437	4.549132	5.593844	0.250518	1	-
ENSMUSG	Kpna2	karyopher	GO:00511	localizatio	K15043	importin s	mmu0516	Influenza	11:106988	34	37	27	40	30	21	0.544971	0.497107	0.298129	0.417669	0.421651	0.417342	0.446736	0.418887	0.752878	1	-
ENSMUSG	Smurf2	SMAD spe	GO:00325	developm	K04678	E3 ubiquit	mmu0414	Endocytos	11:106820	635	799	1110	1034	757	481	5.04074	5.316429	6.070088	5.347155	5.269378	4.734254	5.475752	5.116929	0.44465	1	-
ENSMUSG	Cep95	centroson	GO:00056	cell|intrace	-	-	-	-	11:106789	191	249	326	377	235	125	2.046296	2.23608	2.406044	2.631226	2.207729	1.660463	2.229473	2.166473	0.748856	1	-
ENSMUSG	Vezf1	vascular e	GO:00325	developm	-	-	-	-	11:880682	602	677	793	801	628	341	7.730636	7.287187	7.015252	6.700898	7.071656	5.429493	7.344359	6.400682	0.231693	1	-
ENSMUSG	Cuedc1	CUE dom	-	-	-	-	-	-	11:880980	458	430	525	515	451	293	4.215391	3.317372	3.328768	3.087888	3.639916	3.343694	3.62051	3.357166	0.429582	1	-
ENSMUSG	Srsf1	serine/arg	GO:00325	developm	-	-	-	-	11:880473	1191	1759	1925	2068	1597	1066	6.648362	8.230415	7.402615	7.520303	7.817188	7.378128	7.427131	7.571873	0.891084	1	-
ENSMUSG	Abi3	ABI gene	GO:00400	locomotic	-	-	-	-	11:958300	67	61	92	88	54	76	0.744806	0.568396	0.704544	0.63728	0.526385	1.047531	0.672582	0.737065	0.891845	1	-
ENSMUSG	Shroom1	shroom fa	GO:00325	developm	-	-	-	-	11:534572	96	87	115	116	96	93	0.587491	0.446273	0.48482	0.462455	0.51516	0.705662	0.506195	0.561092	0.764852	1	-
ENSMUSG	Kif3a	kinesin fa	GO:00325	developm	K10394	kinesin fa	mmu0434	Hedgehoc	11:535673	2511	2928	3261	3754	2722	1550	32.91021	32.1668	29.44328	32.05235	31.28346	25.18846	31.50676	29.50809	0.450569	1	-
ENSMUSG	8-Sep	septin 8 [S	GO:00056	cell|intrace	K16939	septin 6/8	mmu0510	Bacterial i	11:535192	5226	7040	7563	8429	6219	3949	29.97004	33.84102	29.87882	31.49022	31.27384	28.07961	31.22996	30.28122	0.587129	1	-
ENSMUSG	Mtmr4	myotubul	GO:00081	metabolic	K18082	myotubul	mmu0110	Metabolic	11:875921	1745	2071	2765	2174	1667	1602	14.7395	14.66291	16.08918	11.96268	12.34712	16.77783	15.16386	13.69588	0.223728	1	-
ENSMUSG	Mrm1	mitochon	GO:00081	metabolic	-	-	-	-	11:848130	98	136	151	158	101	125	1.94691	2.264724	2.066569	2.04484	1.759492	3.079046	2.092734	2.294459	0.833724	1	-
ENSMUSG	Mapt	microtubu	GO:00325	developm	K04380	microtubu	mmu0401	MAPK sign	11:104231	8192	10159	11999	12399	9904	6593	48.35388	50.26263	48.7908	47.67709	51.2619	48.25145	49.13577	49.06348	0.733954	1	-
ENSMUSG	Kansl1	KAT8 regu	GO:00081	metabolic	-	-	-	-	11:104333	1406	1753	1666	1902	1325	1232	8.126183	8.492512	6.633271	7.161327	6.715216	8.828727	7.750655	7.568423	0.588117	1	-
ENSMUSG	Gid4	GID comp	GO:00081	metabolic	-	-	-	-	11:604171	1369	1546	2184	2336	1746	1116	15.53921	14.70916	17.07771	17.27349	17.37851	15.70637	15.77536	16.78613	0.825982	1	-
ENSMUSG	Myo1b	myosin IB	GO:00511	localizatio	-	-	-	-	1:5174976	290	329	403	398	268	245	1.106862	1.052555	1.059624	0.989603	0.896959	1.159441	1.073014	1.015334	0.481423	1	-
ENSMUSG	Dhx40	DEAH (As	GO:00081	metabolic	-	-	-	-	11:867688	597	785	911	915	683	406	8.176972	9.012409	8.595836	8.164332	8.203167	6.894932	8.595072	7.754144	0.316668	1	-
ENSMUSG	Ypel2	yippee-lik	GO:00056	cell|intrace	-	-	-	-	11:869364	625	757	922	763	644	592	8.194889	8.319777	8.328092	6.517326	7.40443	9.624337	8.28092	7.848698	0.444624	1	-
ENSMUSG	Akap1	A kinase (	GO:00508	response	-	-	-	-	11:888307	733	1032	1285	1357	901	732	9.458747	11.16252	11.42311	11.40753	10.19524	11.71189	10.68146	11.10489	1	1	-
ENSMUSG	Nol11	nucleolar	GO:00081	metabolic	-	-	-	-	11:107166	307	393	406	408	356	276	2.853289	3.06163	2.599471	2.470296	2.901346	3.180543	2.83813	2.850729	0.78776	1	-
ENSMUSG	Derl2	Der1-like	GO:00081	metabolic	K13989	Derlin-2/3	mmu0414	Protein pr	11:710071	376	413	508	520	413	352	2.356135	2.16928	2.192944	2.122742	2.269367	2.734902	2.239453	2.37567	0.91557	1	-
ENSMUSG	C1qbp	compleme	GO:00325	developm	K15414	compleme	mmu0516	Herpes sir	11:709778	513	710	745	814	753	547	8.68711	10.07787	8.690912	8.979733	11.18137	11.485	9.151964	10.5487	0.448607	1	-
ENSMUSG	Rpain	RPA intera	GO:00081	metabolic	-	-	-	-	11:709702	129	148	212	176	151	129	1.719811	1.653885	1.947053	1.528572	1.765262	2.132382	1.773583	1.808739	0.881367	1	-
ENSMUSG	Slc13a3	solute car	GO:00511	localizatio	-	-	-	-	2:1654050	167	302	292	319	233	273	1.389	2.105451	1.673091	1.728454	1.699359	2.815367	1.722514	2.08106	0.421142	1	-
ENSMUSG	Kcnab3	potassium	GO:00511	localizatio	-	-	-	-	11:693262	326	500	400	439	449	347	6.73985	8.664733	5.696962	5.912601	8.139938	8.895027	7.033848	7.649189	0.810353	1	-
ENSMUSG	Chd3	chromodc	GO:00329	macromo	-	-	-	-	11:693432	16384	19417	22023	24870	18900	12644	99.63185	98.97221	92.25845	98.52256	100.7819	95.3342	96.95417	98.2129	0.831925	1	-
ENSMUSG	Kdm6b	KDM1 lysi	GO:00325	developm	-	-	-	-	11:693985	881	985	1116	1319	893	690	7.701537	7.217566	6.720742	7.511527	6.845353	7.478888	7.213282	7.27859	0.814663	1	-
ENSMUSG	1700125H	RIKEN cDl	-	-	-	-	-	-	11:851704	0	0	2	0	0	0	0	0	0.088013	0	0	0	0.029338	0	0.537003	1	-
ENSMUSG	Appbp2	amyloid b	GO:00511	localizatio	-	-	-	-	11:851872	2009	2492	3087	3204	2409	1618	19.75522	20.54011	20.91173	20.52467	20.77215	19.72724	20.40236	20.34135	0.739176	1	-
ENSMUSG	Wnt9b	wingless-t	GO:00325	developm	K01064	wingless-t	mmu0520	Pathways	11:103727	12	5	11	24	13	8	0.168793	0.058951	0.106593	0.219928	0.160353	0.139529	0.111446	0.17327	0.229948	1	-
ENSMUSG	Adora2b	adenosine	GO:00048	signal trar	K04267	adenosine	mmu0408	Neuroacti	11:622489	31	29	56	40	33	35	1.067845	0.837307	1.328855	0.897593	0.996788	1.494846	1.078003	1.129742	1	1	-
ENSMUSG	Ncor1	nuclear re	GO:00081	metabolic	-	-	mmu0520	Transcript	11:623164	5823	6280	7973	9514	6517	3113	17.67452	15.97767	16.67148	18.81246	17.34568	11.71564	16.77456	15.95793	0.600048	1	-
ENSMUSG	Trpv2	transient r	GO:00325	developm	K04971	transient r	mmu0462	NOD-like	11:625744	194	217	268	346	194	237	2.201261	2.06387	2.094865	2.557576	1.930259	3.334307	2.119999	2.607381	0.321705	1	-
ENSMUSG	Cenpv	centrome	GO:00081	metabolic	-	-	-	-	11:625249	198	228	263	336	245	215	4.379925	4.227546	4.007823	4.841956	4.752366	5.896913	4.205098	5.163745	0.259466	1	-
ENSMUSG	Pcgf2	polycomb	GO:00325	developm	K11460	polycomb	mmu0455	Signaling	11:976888	196	232	247	262	252	198	2.367236	2.348691	2.0551	2.061429	2.668875	2.965083	2.257009	2.565129	0.595882	1	-
ENSMUSG	Cwc25	CWC25 sp	GO:00056	cell|intrace	-	-	-	-	11:977454	214	440	246	609	438	245	1.776892	3.062338	1.40713	3.294175	3.189071	2.522321	2.08212	3.001856	0.088102	1	-
ENSMUSG	1700001P	RIKEN cDl	-	-	-	-	-	-	11:977714	3	2	0	2	4	3	0.305506	0.170732	0	0.132681	0.357208	0.378876	0.158746	0.289588	0.490859	1	-
ENSMUSG	Pip4k2b	phosphati	GO:00081	metabolic	K00920	1-phosph	mmu0481	Regulatior	11:977151	3613	3949	4847	5279	3989	2675	43.0882	39.47575	39.82125	41.0132	41.71546	39.55489	40.79507	40.76118	0.747385	1	-
ENSMUSG	Trim37	tripartite r	GO:00081	metabolic	K10608	tripartite r	mmu0412	Ubiquitin	11:871270	6998	8863	11231	11147	8646	5228	58.31943	61.91173	64.47764	60.51722	63.18256	54.02078	61.5696	59.24019	0.546251	1	-
ENSMUSG	Ybx2	Y box pro	GO:00325	developm	-	-	-	-	11:699357	39	65	53	90	72	66	0.696621	0.973186	0.652164	1.047264	1.127723	1.461716	0.77399	1.212234	0.043157	1	-
ENSMUSG	Ctdnep1	CTD nucle	GO:00325	developm	-	-	-	-	11:699811	704	884	1052	1126	815	773	13.50071	14.20982	13.89795	14.06707	13.70515	18.38014	13.86949	15.38412	0.645702	1	-
ENSMUSG	Elp5	elongator	GO:00081	metabolic	-	-	-	-	11:699682	363	468	529	534	449	522	4.570076	4.938728	4.588004	4.379652	4.956848	8.148411	4.698936	5.828304	0.299664	1	-
ENSMUSG	Slc2a4	solute car	GO:00325	developm	K07191	MFS trans	mmu0491	Insulin sig	11:699425	10	11	19	21	15	28	0.100703	0.092854	0.131807	0.137767	0.132455	0.349608	0.108455	0.20661	0.08111	1	-
ENSMUSG	Gabarap	gamma-a	GO:00081	metabolic	K08341	GABA(A) r	mmu0516	Kaposi's sa	11:699911	1138	1519	1840	1883	1325	1401	21.72525	24.30709	24.19867	23.41825	22.18099	33.16242	23.41034	26.25389	0.596095	1	-
ENSMUSG	2810408A	RIKEN cDl	GO:00081	metabolic	-	-	-	-	11:698973	4	3	4	8	4	7	0.033108	0.020813	0.022811	0.043138	0.029031	0.071844	0.025578	0.048004	0.276026	1	-
ENSMUSG	Phf23	PHD finge	GO:00081	metabolic	-	-	-	-	11:699957	320	603	631	478	374	425	2.787413	4.402725	3.78645	2.712445	2.856703	4.590144	3.658863	3.38643	0.434592	1	-
ENSMUSG	Acadvl	acyl-Coer	GO:00325	developm	K09479	very long	mmu0110	Metabolic	11:700101	512	603	784	785	531	553	6.699435	6.613605	7.06701	6.691439	6.092631	8.971788	6.79335	7.251953	0.892436	1	-
ENSMUSG	Dnah11	dynein, ax	GO:00325	developm	K10408	dynein he	mmu0501	Huntingto	12:117877	62	15	69	41	27	38	0.263882	0.053514	0.20231	0.113679	0.100768	0.200536	0.173236	0.138328	0.416273	1	-
ENSMUSG	G3bp1	GTPase ac	GO:00081	metabolic	-	-	-	-	11:554696	1161	1470	1821	1740	1447	1171	10.27361	10.90337	11.10074	10.03048	11.22799	12.84795	10.75924	11.3688	0.917833	1	-
ENSMUSG	Atox1	antioxidar	GO:00511	localizatio	K07213	copper ch	mmu0497	Mineral ak	11:554466	93	125	170	138	129	289	7.462702	8.407635	9.39746	7.213933	9.077034	28.7538	8.422599	15.01492	0.06439	1	-
ENSMUSG	Glra2	glycine re	GO:00048	signal trar	K05194	glycine re	mmu0408	Neuroacti	X:1651290	150	203	196	208	139	105	3.026342	3.43301	2.724161	2.733825	2.459146	2.62663	3.061171	2.606534	0.215035	1	-
ENSMUSG	Sparc	secreted a	GO:00325	developm	-	-	-	-	11:553945	3539	4402	5348	5693	3965	4400	43.01284	44.84566	44.77754	45.07548	42.25746	66.3065	44.21201	51.21315	0.432526	1	-
ENSMUSG	Glra4	glycine re	GO:00048	signal trar	-	-	-	-	X:1367576	2	0	0	0	0	0	0.057138	0	0	0	0	0	0.019046	0	0.535777	1	-
ENSMUSG	Mief2	mitochon	GO:00325	developm	-	-	-	-	11:607283	148	185	162	242	138	304	2.732669	2.86319	2.060587	2.910863	2.23432	6.959596	2.552149	4.034926	0.094154	1	-
ENSMUSG	Tbx3	T-box 3 [S	GO:00325	developm	K10177	T-box prc	mmu0455	Signaling	5:1196706	59	86	127	85	76	66	0.270401	0.330373	0.400967	0.253778	0.30543	0.375048	0.333914	0.311418	0.534094	1	-
ENSMUSG	Crhr1	corticotro	GO:00325	developm	K04578	corticotro	mmu0408	Neuroacti	11:104132	95	143	200	175	129	135	2.45428	3.096638	3.559452	2.945246	2.922362	4.32435	3.03679	3.397319	0.733953	1	-
ENSMUSG	Dusp14	dual speci	GO:00081	metabolic	-	-	-	-	11:840480	388	331	482	318	374	256	5.4338	3.885552	4.65018	2.901217	4.592883	4.445256	4.656511	3.979785	0.214523	1	-
ENSMUSG	Tada2a	transcripti	GO:00081	metabolic	-	-	-	-	11:840789	444	499	673	649	514	323	6.919455	6.518421	7.225296	6.588935	7.024163	6.241328	6.887724	6.618142	0.582628	1	-
ENSMUSG	Ikzf1	IKAROS fa	GO:00325	developm	-	-	-	-	11:116850	68	66	100	116	83	52	0.398416	0.324132	0.403626	0.442758	0.426428	0.377759	0.375391	0.415648	0.705069	1	-
ENSMUSG	Pnpo	pyridoxine	GO:00081	metabolic	K00275	pyridoxan	mmu0110	Metabolic	11:969378	573	689	789	870	620	544	10.44932	10.53187	9.912017	10.33557	9.914426	12.30037	10.29773	10.85012	0.943741	1	-
ENSMUSG	Cog1	compone	GO:00511	localizatio	-	-	-	-	11:113649	725	915	1140	1207	979	719	6.209693	6.569106	6.726488	6.734743	7.352878	7.63567	6.501762	7.241097	0.580246	1	-
ENSMUSG	Cbx1	chromobc	GO:00081	metabolic	-	-	-	-	11:967891	598	691	842	906	569	270	6.315174	6.116666	6.125587	6.23295	5.269127	3.535361	6.185809	5.012479	0.153872	1	-
ENSMUSG	Cdk5rap3	CDK5 regi	GO:00081	metabolic	-	-	-	-	11:969074	420	414	465	513	378	349	4.173274	3.44811	3.182967	3.320675	3.293536	4.299712	3.60145	3.637974	0.80937	1	-
ENSMUSG	Copz2	coatomer	GO:00511	localizatio	-	-	-	-	11:968498	76	103	135	119	81	79	1.698918	1.929966	2.078951	1.732952	1.587765	2.189642	1.902612	1.836786	0.655257	1	-
ENSMUSG	Slc25a39	solute car	GO:00081	metabolic	-	-	-	-	11:102402	486	563	626	529	488	532	4.904216	4.762059	4.351704	3.477526	4.318131	6.656274	4.67266	4.817311	0.882834	1	-
ENSMUSG	Sp2	Sp2 trans	GO:00325	developm	-	-	-	-	11:969533	293	350	344	347	340	236	3.768682	3.773484	3.048112	2.907585	3.834803	3.76373	3.530092	3.502039	0.742475	1	-
ENSMUSG	Aatf	apoptosis	GO:00325	developm	-	-	-	-	11:844228	301	426	454	521	333	197	5.922433	7.025798	6.153768	6.678095	5.745397	4.806034	6.367333	5.743176	0.390244	1	-
ENSMUSG	Lhx1	LIM home	GO:00325	developm	-	-	-	-	11:845182	10	4	7	5	8	1	0.11505	0.038572	0.055482	0.037477	0.080712	0.014261	0.069701	0.04415	0.45422	1	-
ENSMUSG	Dync1h1	dynein cy	GO:00511	localizatio	K10413	dynein he	mmu0414	Phagoson	12:110601	16965	18164	21841	25675	18949	13000	61.09689	54.83143	54.18626	60.23617	59.84036	58.04897	56.70486	59.37517	0.939348	1	-
ENSMUSG	Cpsf4l	cleavage a	GO:00081	metabolic	-	-	-	-	11:113698	0	0	0	0	0	2	0	0	0	0	0	0.055566	0	0.018522	0.536392	1	-
ENSMUSG	Pex12	peroxison	GO:00081	metabolic	K13345	peroxin-1	mmu0414	Peroxison	11:832946	103	68	188	172	130	131	0.866552	0.479534	1.089603	0.942689	0.959059	1.366522	0.811896	1.089424	0.242843	1	-
ENSMUSG	Ndel1	nudE neu	GO:00325	developm	-	-	-	-	11:688214	1306	1571	2000	1897	1596	1033	16.28732	16.42236	17.18257	15.41188	17.45348	15.97324	16.63075	16.27953	0.626758	1	-
ENSMUSG	Slc25a35	solute car	GO:00511	localizatio	-	-	-	-	11:689681	92	112	156	177	126	110	1.024333	1.045254	1.196542	1.283829	1.230175	1.518561	1.08871	1.344188	0.295921	1	-
ENSMUSG	Zbtb4	zinc finge	GO:00081	metabolic	-	-	-	-	11:697659	2065	2162	3070	3691	2631	1382	8.529079	7.48498	8.735184	9.931341	9.528963	7.077441	8.249747	8.845915	0.774	1	-
ENSMUSG	Tnfsfm13	tumor nec	GO:00023	immune s	-	-	-	-	11:696825	54	78	131	99	83	83	1.05955	1.282845	1.770718	1.265444	1.428053	2.019249	1.371037	1.570915	0.677066	1	-
ENSMUSG	Mpdu1	mannose-	GO:00081	metabolic	-	-	-	-	11:696566	233	294	312	333	301	388	2.752898	2.91162	2.539455	2.563069	3.118487	5.683983	2.734658	3.788513	0.135994	1	-
ENSMUSG	Fxr2	fragile X r	GO:00055	extracellul	K15516	fragile X n	mmu0301	RNA trans	11:696329	1870	2352	2810	2805	2290	1696	22.94288	24.18781	23.75003	22.41924	24.63681	25.79991	23.62691	24.28532	0.913732	1	-
ENSMUSG	Atp5g3	ATP synth	GO:00081	metabolic	K02128	F-type H+	mmu0110	Metabolic	2:7390844	919	1158	1415	1246	964	2206	19.92671	21.04658	21.13626	17.60029	18.32905	59.3078	20.70318	31.74571	0.123806	1	-
ENSMUSG	Cd68	CD68 anti	GO:00508	response	K06501	CD68 anti	mmu0414	Lysosome	11:696642	191	249	340	388	212	198	5.872581	6.417236	7.201558	7.77157	5.715759	7.548267	6.497125	7.011865	0.809394	1	-
ENSMUSG	Acsl1	acyl-CoA	GO:00081	metabolic	K01897	long-chai	mmu0110	Metabolic	8:4647103	878	963	601	1246	585	406	3.943709	3.62568	1.85967	3.645944	2.304138	2.261113	3.14302	2.737065	0.400243	1	-
ENSMUSG	Abca5	ATP-bind	GO:00325	developm	K05648	ATP-bind	mmu0201	ABC trans	11:110269	870	1030	1431	1459	1088	689	3.99878	3.968242	4.531052	4.368629	4.385103	3.926568	4.166025	4.226767	0.877722	1	-
ENSMUSG	Smyd4	SET and M	GO:00081	metabolic	-	-	-	-	11:753484	139	204	234	275	195	218	1.581431	1.945444	1.834017	2.038216	1.945418	3.075233	1.786964	2.352955	0.164046	1	-
ENSMUSG	Lsp1	lymphocy	GO:00048	signal trar	K14957	lymphocy	mmu0515	Tuberculo	7:1424608	53	75	101	102	72	48	0.378462	0.448913	0.496842	0.47449	0.450836	0.424984	0.441406	0.450103	0.959338	1	-
ENSMUSG	Zfyve27	zinc finge	GO:00325	developm	K19368	protrudin[	mmu0414	Endocytos	19:421639	908	1106	1387	1516	1227	1205	5.051308	5.157342	5.315522	5.494127	5.985563	8.31172	5.174724	6.597137	0.127554	1	-
ENSMUSG	Avpi1	arginine v	GO:00081	metabolic	-	-	-	-	19:421232	136	164	212	203	159	175	5.391915	5.45004	5.790145	5.242998	5.52767	8.602537	5.544033	6.457735	0.552231	1	-
ENSMUSG	Sfrp5	secreted f	GO:00325	developm	K02222	secreted f	mmu0431	Wnt signa	19:421979	20	9	15	11	16	7	0.668985	0.252323	0.345648	0.239685	0.469288	0.290324	0.422319	0.333099	0.501574	1	-
ENSMUSG	Myh11	myosin, h	GO:00325	developm	K10352	myosin he	mmu0453	Tight junc	16:141945	353	575	583	621	502	324	3.382722	4.618625	3.848681	3.876721	4.218304	3.849668	3.950009	3.981564	0.846671	1	-
ENSMUSG	Rad51d	RAD51 pa	GO:00081	metabolic	K10871	RAD51-lik	mmu0344	Homologo	11:828764	193	238	302	297	255	195	1.83153	1.89315	1.974303	1.836087	2.121964	2.294438	1.899661	2.084163	0.729737	1	-
ENSMUSG	Fndc8	fibronecti	GO:00056	cell|intrace	-	-	-	-	11:828921	0	1	0	0	0	3	0	0.024593	0	0	0	0.10915	0.008198	0.036383	0.454892	1	-
ENSMUSG	Unc45b	unc-45 m	GO:00325	developm	-	-	-	-	11:829105	17	19	20	27	23	21	0.219588	0.205721	0.177976	0.2272	0.260523	0.336333	0.201095	0.274686	0.30233	1	-
ENSMUSG	Pank3	pantother	GO:00081	metabolic	K09680	type II par	mmu0110	Metabolic	11:357694	1463	1592	2104	2244	1602	917	12.60548	11.4977	12.48856	12.59562	12.10376	9.796494	12.19725	11.49863	0.491519	1	-
ENSMUSG	Rars	arginyl-tR	GO:00081	metabolic	K01887	arginyl-tR	mmu0097	Aminoacy	11:358083	726	1020	1210	1140	884	587	11.65433	13.72473	13.38097	11.92168	12.44358	11.68355	12.92001	12.01627	0.395923	1	-
ENSMUSG	Wwc1	WW, C2 a	GO:00325	developm	K16685	protein Kl	mmu0439	Hippo sig	11:358384	1890	2326	2936	3004	2324	1680	22.12881	22.82752	23.6812	22.91278	23.86026	24.38887	22.87918	23.72064	0.984326	1	-
ENSMUSG	Mrpl58	mitochon	GO:00081	metabolic	-	-	-	-	11:115403	207	221	436	360	370	343	1.619736	1.449503	2.350234	1.835092	2.538735	3.327766	1.806491	2.567198	0.083625	1	-
ENSMUSG	Fdxr	ferredoxir	GO:00081	metabolic	-	-	-	-	11:115268	126	164	211	208	158	175	2.265904	2.472104	2.613989	2.436771	2.491548	3.902056	2.450666	2.943458	0.436951	1	-
ENSMUSG	Otop3	otopetrin	GO:00160	membran	-	-	-	-	11:115334	0	2	0	0	3	0	0	0.026228	0	0	0.041155	0	0.008743	0.013718	1	1	-
ENSMUSG	Sult4a1	sulfotrans	GO:00081	metabolic	-	-	-	-	15:840760	5747	7376	8617	8704	6833	6060	153.2686	164.8868	158.3141	151.2212	159.7961	200.3875	158.8232	170.4683	0.818416	1	-
ENSMUSG	Mrpl45	mitochon	GO:00329	macromo	-	-	-	-	11:973157	415	519	653	634	470	385	9.604696	10.0683	10.41118	9.558868	9.538418	11.04797	10.02806	10.04842	0.766336	1	-
ENSMUSG	Mb	myoglobi	GO:00325	developm	-	-	-	-	15:770154	4	0	4	5	3	0	0.180157	0	0.124129	0.14672	0.118479	0	0.101429	0.0884	1	1	-
ENSMUSG	Irf1	interferon	GO:00325	developm	K09444	interferon	mmu0516	Human pa	11:537700	129	113	181	191	175	145	1.421595	1.043797	1.37409	1.371203	1.691088	1.981247	1.279827	1.681179	0.178782	1	-
ENSMUSG	Slc22a5	solute car	GO:00325	developm	K08202	MFS trans	mmu0523	Choline m	11:538645	117	156	213	180	145	172	1.783562	1.993335	2.236846	1.787548	1.938272	3.251007	2.004581	2.325609	0.608888	1	-
ENSMUSG	P4ha2	procollag	GO:00081	metabolic	K00472	prolyl 4-h	mmu0110	Metabolic	11:541000	220	257	310	310	248	240	1.644902	1.610656	1.596724	1.509942	1.625967	2.224916	1.617427	1.786942	0.735566	1	-
ENSMUSG	Alox12e	arachidon	GO:00081	metabolic	K00458	arachidon	mmu0110	Metabolic	11:703156	2	0	2	0	2	0	0.031129	0	0.021443	0	0.027289	0	0.017524	0.009096	0.720891	1	-
ENSMUSG	Arrb1	arrestin, b	GO:00325	developm	K04439	beta-arre	mmu0474	Olfactory	7:9953546	1796	1850	1349	1708	1248	924	7.609415	6.570063	3.937387	4.714269	4.636627	4.854031	6.038955	4.734976	0.073628	1	-

ENSMUS(	Tm4sf5	transmem	GO:00160	membran	-	-	-	-	11:705052	0	0	0	4	0	0	0	0	0	0.115904	0	0	0	0.038635	0.30509	1	-
ENSMUS(	Cxcl16	chemokin	GO:00400	locomotic	K10035	C-X-C mc	mmu0406	Cytokine-	11:704539	20	27	33	46	31	22	0.276922	0.313354	0.314762	0.414919	0.376377	0.377696	0.301679	0.389664	0.319338	1	-
ENSMUS(	Pelp1	proline, gl	GO:00081	metabolic	-	-	-	-	11:703928	517	683	725	801	566	634	8.587801	9.509658	8.296223	8.667732	8.244232	13.05768	8.797894	9.989881	0.583475	1	-
ENSMUS(	Med11	mediator	GO:00081	metabolic	-	-	-	-	11:704519	78	82	110	126	97	82	2.376393	2.094059	2.3087	2.500765	2.5914	3.097589	2.259717	2.729918	0.39495	1	-
ENSMUS(	Ccl6	chemokin	GO:00081	metabolic	K05510	C-C motif	mmu0406	Cytokine-	11:835878	25	33	41	46	28	25	0.624053	0.690478	0.705047	0.748028	0.612881	0.773746	0.673193	0.711552	1	1	-
ENSMUS(	Ccl4	chemokin	GO:00081	metabolic	K12964	C-C motif	mmu0406	Cytokine-	11:836625	2	2	9	9	3	3	0.192624	0.161419	0.596989	0.564558	0.253293	0.35821	0.317011	0.392021	0.874348	1	-
ENSMUS(	Natd1	N-acetylt	-	-	-	-	-	-	11:609022	233	263	334	401	251	205	3.353984	3.173326	3.312095	3.760378	3.168266	3.658864	3.279802	3.529169	0.81093	1	-
ENSMUS(	Map2k3	mitogen-	GO:00048	signal trar	K04432	mitogen-a	mmu0401	MAPK sigı	11:609320	100	100	170	132	98	142	1.254253	1.05133	1.468875	1.078551	1.077834	2.208312	1.258152	1.454899	0.688812	1	-
ENSMUS(	Ywhah	tyrosine 3	GO:00325	developm	K16198	14-3-3 pr	mmu0415	PI3K-Akt s	5:3301881	10221	28423	32512	34875	11059	4905	121.8715	284.0741	267.057	270.8974	115.6292	72.51601	224.3342	153.0142	0.160609	1	-
ENSMUS(	Sart3	squamous	GO:00325	developm	-	-	-	-	5:1137424	866	885	1166	1335	965	715	6.591249	5.646061	6.113636	6.619308	6.440498	6.747482	6.116982	6.602429	0.76191	1	-
ENSMUS(	E2f2	E2F transc	GO:00081	metabolic	K09389	transcripti	mmu0520	Pathways	4:1361723	12	28	30	32	16	38	0.15592	0.304961	0.268538	0.270875	0.182304	0.612228	0.24314	0.355136	0.357991	1	-
ENSMUS(	Nars2	asparagin	GO:00081	metabolic	K01893	asparagin	mmu0097	Aminoacy	7:9695150	133	162	266	182	164	203	0.942838	0.962619	1.299031	0.840504	1.019462	1.784299	1.068163	1.214755	0.717162	1	-
ENSMUS(	Slc35b4	solute car	GO:00081	metabolic	-	-	-	-	6:3415338	750	969	1160	1166	915	971	3.861058	4.181398	4.113907	3.910437	4.13056	6.197979	4.052121	4.746325	0.417784	1	-
ENSMUS(	Dnah1	dynein, ax	GO:00400	locomotic	K10408	dynein he	mmu0501	Huntingto	14:312603	187	222	190	257	159	167	0.893163	0.888782	0.625164	0.799656	0.665929	0.988988	0.802369	0.818191	0.886711	1	-
ENSMUS(	Dalrd3	DALR anti	GO:00081	metabolic	-	-	-	-	9:1085698	675	830	914	1041	757	815	6.815746	7.024905	6.357803	6.847655	6.702675	10.20359	6.732818	7.917973	0.404366	1	-
ENSMUS(	Fis1	fission, mi	GO:00325	developm	K17969	mitochon	mmu0413	Mitophag	5:1369532	719	869	1209	1035	874	916	14.84072	15.03486	17.19111	13.91709	15.81905	23.4427	15.6889	17.72628	0.607255	1	-
ENSMUS(	Plod1	procollag	GO:00325	developm	K00473	procollag	mmu0031	Lysine deg	4:1479097	344	360	622	559	456	367	2.709747	2.376979	3.375296	2.868557	3.149764	3.584444	2.820674	3.200922	0.557779	1	-
ENSMUS(	Rab3d	RAB3D, m	GO:00081	metabolic	K07884	Ras-relate	mmu0497	Pancreatic	9:2190749	100	113	81	148	128	118	0.743484	0.704209	0.414868	0.716827	0.834498	1.087779	0.620853	0.879701	0.127043	1	-
ENSMUS(	Mfsd3	major faci	GO:00511	localizatio	-	-	-	-	15:767015	39	49	61	61	33	80	1.142728	1.203435	1.231281	1.164364	0.84789	2.906376	1.192481	1.639543	0.386908	1	-
ENSMUS(	Slc25a22	solute car	GO:00511	localizatio	-	-	-	-	7:1414297	1084	1301	3799	1626	3455	1237	4.045301	4.069598	9.766572	3.95297	11.30608	5.723703	5.96049	6.994252	0.61557	1	-
ENSMUS(	Atp6ap1	ATPase, H	GO:00325	developm	K03662	V-type H-	mmu0110	Metabolic	X:7429709	2226	3242	3543	3643	2779	2056	21.37963	26.10002	23.44215	22.79375	23.40487	24.48409	23.6406	23.5609	0.719199	1	-
ENSMUS(	Dnase1l1	deoxyribo	GO:00081	metabolic	-	-	-	-	X:7427321	19	24	27	27	8	29	0.310094	0.328323	0.303565	0.287063	0.114497	0.586839	0.313994	0.329467	0.963008	1	-
ENSMUS(	Aldh3a1	aldehyde	GO:00325	developm	K00129	aldehyde	mmu0110	Metabolic	11:612075	5	8	22	11	12	19	0.141167	0.189314	0.427886	0.202311	0.29709	0.665129	0.252789	0.388176	0.410295	1	-
ENSMUS(	Ccl9	chemokin	GO:00325	developm	K05510	C-C motif	mmu0406	Cytokine-	11:835729	26	24	36	38	30	30	0.512692	0.396677	0.489029	0.488137	0.518723	0.733483	0.466133	0.580115	0.491537	1	-
ENSMUS(	Scrn1	secernin 1	GO:00081	metabolic	-	-	-	-	6:5450117	3569	4403	5421	5495	4164	2933	22.73208	23.50684	23.78611	22.80037	23.25659	23.16278	23.34168	23.07325	0.664039	1	-
ENSMUS(	BC005537	cDNA seq	GO:00056	cell|intrace	-	-	-	-	13:248016	3491	4188	5334	5736	4011	3233	36.89118	37.09644	38.83087	39.48785	37.1679	42.36082	37.60616	39.67219	0.903992	1	-
ENSMUS(	Isyna1	myo-inos	GO:00081	metabolic	K01858	myo-inos	mmu0110	Metabolic	8:7059437	187	298	335	290	271	319	2.429856	3.245691	2.998714	2.454815	3.087813	5.139427	2.89142	3.560685	0.392988	1	-
ENSMUS(	Hars2	histidyl-tR	GO:00081	metabolic	K01892	histidyl-tR	mmu0097	Aminoacy	18:367830	216	276	383	387	245	295	2.093878	2.242636	2.557688	2.443935	2.082603	3.545723	2.298067	2.690754	0.494643	1	-
ENSMUS(	Cacng2	calcium cl	GO:00081	metabolic	K04867	voltage-d	mmu0401	MAPK sigı	15:779917	1028	1196	1494	1693	1217	998	11.85708	11.56295	11.87098	12.72105	12.30886	14.27253	11.76367	13.10082	0.575289	1	-
ENSMUS(	Tmem160	transmem	GO:00160	membran	-	-	-	-	7:1645277	127	156	205	209	131	271	5.331049	5.488914	5.928113	5.715287	4.821968	14.10474	5.582692	8.214	0.156088	1	-
ENSMUS(	Rab5c	RAB5C, m	GO:00452	synapse|c	K07889	Ras-relate	mmu0414	Endocytos	11:100715	1422	1547	1611	2097	2414	1348	23.69491	21.6072	18.49279	22.76331	35.27243	27.85039	21.26497	28.62871	0.069201	1	-
ENSMUS(	Styxl1	serine/thr	GO:00081	metabolic	-	-	-	-	5:1357472	2	0	7	3	0	0	0.029092	0	0.070134	0.028419	0	0	0.033075	0.009473	0.204387	1	-
ENSMUS(	Mdh2	malate de	GO:00081	metabolic	K00026	malate de	mmu0110	Metabolic	5:1357784	2880	3528	4044	4265	3376	3271	35.45084	36.40118	34.2923	34.20067	36.44006	49.92298	35.38144	40.18791	0.503361	1	-
ENSMUS(	H13	histocomp	GO:00081	metabolic	-	-	-	-	2:1526694	875	1105	1233	1290	1030	891	5.279493	5.588551	5.125053	5.070554	5.449589	6.665729	5.331032	5.728624	0.823025	1	-
ENSMUS(	Rnf145	ring finge	GO:00081	metabolic	-	-	-	-	11:445189	1643	2034	2412	2617	1995	1329	11.44553	11.87687	11.57517	11.87637	12.18664	11.47914	11.63253	11.84738	0.879791	1	-
ENSMUS(	Scn1b	sodium cl	GO:00325	developm	K04845	voltage-g	mmu0426	Adrenergi	7:3111652	5000	6068	7404	7641	5917	4924	109.9533	111.8502	112.1647	109.4637	114.0992	134.2587	111.3227	119.2738	0.813807	1	-
ENSMUS(	Atp6v1e1	ATPase, H	GO:00511	localizatio	K02150	V-type H-	mmu0110	Metabolic	6:1207943	2925	3657	4868	4071	3226	3652	32.44198	33.99847	37.19491	29.41469	31.37534	50.22249	34.54512	37.00417	0.893755	1	-
ENSMUS(	Chtf18	CTF18, ch	GO:00081	metabolic	-	-	-	-	17:257189	28	27	29	34	26	22	0.249506	0.201668	0.178022	0.19737	0.203161	0.243077	0.209732	0.214536	1	1	-
ENSMUS(	Lhx9	LIM home	GO:00325	developm	-	-	-	-	1:1388251	215	245	221	267	390	206	1.138753	1.087703	0.806372	0.921265	1.811335	1.352834	1.010943	1.361811	0.16799	1	-
ENSMUS(	Rps6kl1	ribosomal	GO:00081	metabolic	-	-	-	-	12:851352	593	697	816	723	708	640	5.363163	5.283863	5.084028	4.259772	5.614902	7.176821	5.243685	5.683832	0.823792	1	-
ENSMUS(	Ppp1r12c	protein pl	GO:00987	molecular	K17457	protein pl	mmu0481	Regulatior	7:4481520	1089	1337	1608	1588	1370	1195	7.944127	8.175278	8.080824	7.546581	8.763577	10.80867	8.066743	9.03961	0.580377	1	-
ENSMUS(	Ahr	aryl-hydrc	GO:00048	signal trar	K09093	aryl hydro	mmu0465	Th17 cell c	12:354979	158	191	229	245	192	128	1.534207	1.554577	1.531842	1.549797	1.634825	1.541067	1.540209	1.57523	0.94305	1	-
ENSMUS(	Map1s	microtubu	GO:00325	developm	-	-	-	-	8:7090593	961	327	1170	1389	1078	1068	4.589649	1.309052	3.849403	4.321554	4.51458	6.324313	3.249368	5.053482	0.089528	1	-
ENSMUS(	Dpep1	dipeptida	GO:00081	metabolic	-	-	-	-	8:1231862	5	11	13	1	1	7	0.118526	0.218576	0.212294	0.015441	0.020785	0.20575	0.183132	0.080658	0.072261	1	-
ENSMUS(	Tmem129	transmem	GO:00081	metabolic	-	-	-	-	5:3365321	342	325	348	399	167	279	3.596146	2.864498	2.520825	2.73317	1.539826	3.637488	2.993823	2.636828	0.347513	1	-
ENSMUS(	Nop9	NOP9 nuc	GO:00971	organic cy	-	-	-	-	14:557456	139	189	218	231	177	213	2.246091	2.559922	2.426717	2.431678	2.507997	4.26755	2.41091	3.069075	0.275962	1	-
ENSMUS(	Hsd17b1	hydroxyst	GO:00325	developm	K00044	17beta-es	mmu0110	Metabolic	11:101078	6	3	7	9	4	11	0.28479	0.119346	0.22889	0.278274	0.166466	0.647358	0.211009	0.364032	0.346482	1	-
ENSMUS(	Atp6v0a1	ATPase, H	GO:00081	metabolic	K02154	V-type H-	mmu0110	Metabolic	11:101009	4923	5956	6626	7863	6058	4486	32.31476	32.77016	29.96221	33.62335	34.86928	36.51037	31.68238	35.001	0.598237	1	-
ENSMUS(	Psmc3ip	proteasor	GO:00081	metabolic	-	-	-	-	11:101091	35	39	61	57	35	44	0.730022	0.68184	0.876485	0.774493	0.640145	1.137893	0.762782	0.850844	0.829906	1	-
ENSMUS(	Grb7	growth fa	GO:00081	metabolic	-	-	-	-	11:984463	1	20	39	26	30	20	0.012883	0.215934	0.346063	0.218165	0.338842	0.319413	0.191627	0.29214	0.404031	1	-
ENSMUS(	Noxo1	NADPH o	GO:00081	metabolic	-	-	-	-	17:246962	13	26	37	27	23	29	0.12198	0.204492	0.239171	0.165041	0.189248	0.337392	0.188548	0.23056	0.658897	1	-
ENSMUS(	Aoc3	amine oxi	GO:00081	metabolic	K00276	primary-a	mmu0110	Metabolic	11:101330	32	21	29	36	23	30	0.365113	0.200841	0.227945	0.26759	0.230121	0.424419	0.264633	0.307377	0.748697	1	-
ENSMUS(	Zfp687	zinc finge	GO:00081	metabolic	-	-	-	-	3:9500659	529	584	604	578	537	431	1.92651	1.782713	1.515321	1.371278	1.714877	1.94616	1.741515	1.677438	0.557166	1	-
ENSMUS(	Gdpd2	glyceroph	GO:00325	developm	-	-	-	-	X:1007298	103	155	197	183	153	138	0.970775	1.224521	1.279092	1.123612	1.264498	1.612683	1.158129	1.333598	0.573983	1	-
ENSMUS(	D8Ertd738	DNA segr	GO:00056	cell|intrace	-	-	-	-	8:8424623	459	564	677	631	532	515	47.35526	48.77389	48.11665	42.40984	48.12928	65.8793	48.08193	52.13947	0.817605	1	-
ENSMUS(	Sec14l4	SEC14-lik	GO:00511	localizatio	-	-	-	-	11:403146	10	8	16	14	12	6	0.144703	0.097028	0.159494	0.131974	0.152265	0.107659	0.133742	0.130633	0.921452	1	-
ENSMUS(	Calm3	calmoduli	GO:00511	localizatio	K02183	calmoduli	mmu0474	Olfactory	7:1691537	32081	39416	44961	48245	37354	28367	561.9747	578.7543	542.5706	550.5576	573.7842	616.1241	561.0999	580.1553	0.950181	1	-
ENSMUS(	Cops3	COP9 sigr	GO:00325	developm	-	-	-	-	11:598177	926	1099	1306	1338	975	519	12.59364	12.52827	12.23587	11.85436	11.62753	8.751733	12.45259	10.74454	0.202108	1	-
ENSMUS(	Fkbp8	FK506 bin	GO:00325	developm	-	-	-	-	8:7052772	3041	3831	4108	4508	3622	3578	21.91458	23.14098	20.39382	21.16325	22.888	31.97003	21.81646	25.34043	0.410117	1	-
ENSMUS(	Ddx39b	DEAD (As	GO:00325	developm	K12812	ATP-depe	mmu0516	Influenza	17:352417	1962	2448	2611	2790	2153	2018	20.91078	21.86933	19.17033	19.37123	20.12139	26.66727	20.65015	22.0533	0.871662	1	-
ENSMUS(	Gipc1	GIPC PDZ	GO:00081	metabolic	-	-	-	-	8:8365267	550	629	772	748	637	545	16.01933	15.35624	15.48995	14.19269	16.26909	19.68175	15.62184	16.71451	0.865575	1	-
ENSMUS(	Tlcd1	TLC doma	GO:00160	membran	-	-	-	-	11:781767	143	138	179	155	179	114	1.034617	0.836907	0.892173	0.730562	1.135639	1.022671	0.921232	0.962957	1	1	-
ENSMUS(	Plscr3	phospholi	GO:00508	response	-	-	-	-	11:698463	143	171	196	229	171	213	1.923403	1.927892	1.816107	2.00656	2.016848	3.552228	1.889134	2.525212	0.17749	1	-
ENSMUS(	Ptger1	prostaglar	GO:00048	signal trar	K04258	prostaglar	mmu0520	Pathways	8:8366667	5	32	21	7	29	6	0.035105	0.188322	0.10157	0.032017	0.178537	0.052236	0.108332	0.087597	0.63579	1	-
ENSMUS(	Arhgef25	Rho guan	GO:00987	molecular	-	-	-	-	10:127182	3899	4795	5215	7113	4671	3492	30.35937	31.29543	27.9734	36.08062	31.89276	33.71318	29.87607	33.89552	0.451751	1	-
ENSMUS(	Xab2	XPA bindi	GO:00325	developm	K12867	pre-mRN	mmu0304	Spliceoso	8:3608421	631	719	905	985	697	581	6.081583	5.808563	6.00879	6.184504	5.890642	6.943032	5.966312	6.339392	0.878253	1	-
ENSMUS(	Cdc37	cell divisic	GO:00081	metabolic	K09554	cell divisio	mmu0415	PI3K-Akt s	9:2113322	2501	2946	3552	3826	2939	1988	17.93771	17.71083	17.55003	17.8764	18.48399	17.67892	17.73286	18.0131	0.85477	1	-
ENSMUS(	Rab4a	RAB4A, m	GO:00452	synapse|c	K07879	Ras-relate	mmu0414	Endocytos	8:1238059	298	340	392	446	334	298	8.103904	7.750127	7.343694	7.90122	7.964638	10.048	7.732575	8.637953	0.634194	1	-
ENSMUS(	Trip10	thyroid hc	GO:00511	localizatio	K07196	thyroid hc	mmu0491	Insulin sig	17:572494	155	153	196	194	138	86	1.868857	1.546274	1.627984	1.523794	1.459027	1.28567	1.681039	1.42283	0.230337	1	-
ENSMUS(	Cops6	COP9 sigr	GO:00081	metabolic	-	-	-	-	5:1381610	1086	1460	1541	1810	1168	1080	16.02846	18.06208	15.66809	17.40293	15.11638	19.76387	16.58621	17.42772	0.956286	1	-
ENSMUS(	Ubb	ubiquitin I	GO:00325	developm	K04551	ubiquitin I	mmu0414	Endocytos	11:625511	11396	14216	16474	16205	12752	9705	303.1603	316.9934	301.9051	280.8346	297.4685	320.1116	307.353	299.4716	0.546929	1	-
ENSMUS(	Ap4m1	adaptor-r	GO:00511	localizatio	K12402	AP-4 com	mmu0414	Lysosome	5:1381720	360	454	533	607	394	382	2.872816	3.036784	2.930109	3.155553	2.757045	3.779672	2.94657	3.230756	0.726762	1	-
ENSMUS(	Gyg	glycogeni	GO:00081	metabolic	K00750	glycogeni	mmu0110	Metabolic	3:2012208	357	385	511	502	335	267	5.190668	4.692113	5.118319	4.754892	4.271124	4.813403	5.000367	4.61314	0.380602	1	-
ENSMUS(	Rcn3	reticuloca	GO:00056	cell|intrace	-	-	-	-	7:4508291	122	106	201	167	138	98	1.914902	1.394591	2.17338	1.707605	1.899366	1.907226	1.827624	1.838065	0.85242	1	-
ENSMUS(	Slc6a8	solute car	GO:00325	developm	-	-	-	-	X:7367315	575	1514	1732	931	740	885	3.703558	8.173905	7.685113	3.906448	4.179508	7.067738	6.520859	5.051231	0.111313	1	-
ENSMUS(	Arid3a	AT rich int	GO:00081	metabolic	-	-	-	-	10:799270	143	131	142	156	124	122	0.678468	0.520975	0.464124	0.482169	0.515889	0.717696	0.554522	0.571918	0.922622	1	-
ENSMUS(	Pdk4	pyruvate c	GO:00081	metabolic	-	-	-	-	6:5483351	163	149	208	226	182	155	2.600841	1.992804	2.286342	2.349175	2.54648	3.066504	2.293329	2.654053	0.521499	1	-
ENSMUS(	Ubxn6	UBX dom	GO:00081	metabolic	K14011	UBX dom	mmu0414	Protein pr	17:560670	1005	1105	1347	1249	1056	862	6.720407	6.193624	6.205101	5.440939	6.192074	7.146987	6.373044	6.26	0.615663	1	-
ENSMUS(	Mydgf	myeloid d	GO:00325	developm	-	-	-	-	17:561757	286	390	439	476	373	387	7.403729	8.462574	7.828895	8.027375	8.46712	12.4217	7.898399	9.638732	0.302344	1	-
ENSMUS(	Cyb561	cytochron	GO:00081	metabolic	-	-	-	-	11:105933	341	461	759	486	445	779	3.459155	3.919844	5.304057	3.211686	3.958384	9.798024	4.227685	5.656031	0.307284	1	-
ENSMUS(	Sema6a	sema don	GO:00325	developm	K06842	semaphor	mmu0436	Axon guic	18:472355	392	497	627	664	467	460	1.143523	1.215257	1.26002	1.261852	1.194589	1.663806	1.206267	1.373416	0.535978	1	-
ENSMUS(	Ccdc12	coiled-co	GO:00081	metabolic	K12871	coiled-co	mmu0304	Spliceoso	9:1106565	195	283	226	278	232	302	2.400315	2.91994	1.916438	2.229259	2.504179	4.609215	2.412231	3.114217	0.295471	1	-
ENSMUS(	Fmc1	formation	GO:00081	metabolic	-	-	-	-	6:3853350	39	50	70	55	53	119	2.531745	2.720651	3.1304	2.325931	3.016975	9.57826	2.794266	4.973722	0.100337	1	-
ENSMUS(	Akt3	thymoma	GO:00325	developm	K04456	RAC serin	mmu0520	Pathways	1:1770200	5508	6273	7497	8338	6079	3303	39.84633	38.03842	37.36227	39.29505	38.56285	29.62708	38.41567	35.82833	0.442131	1	-
ENSMUS(	Mrpl24	mitochon	GO:00081	metabolic	K02895	large subu	mmu0301	Ribosome	3:8791950	434	568	730	722	547	393	7.300689	8.008943	8.459571	7.912118	8.068717	8.19696	7.923068	8.059265	0.876798	1	-
ENSMUS(	Gle1	GLE1 RNA	GO:00081	metabolic	-	-	-	-	2:2993542	767	1013	1207	1212	945	811	6.581844	7.286425	7.135283	6.775428	7.110939	8.628975	7.001184	7.505114	0.832706	1	-
ENSMUS(	L3hypdh	L-3-hydro	GO:00081	metabolic	K18384	trans-L-3	mmu0033	Arginine a	12:720734	27	27	42	39	32	28	0.515598	0.43218	0.552528	0.485175	0.535846	0.662973	0.500102	0.561331	0.809373	1	-
ENSMUS(	Lyst	lysosomal	GO:00511	localizatio	-	-	-	-	13:135903	2713	3145	3849	4397	3042	1771	8.228076	7.995066	8.041698	8.687335	8.090033	6.659672	8.08828	7.812346	0.605791	1	-
ENSMUS(	Slc35e1	solute car	GO:00511	localizatio	-	-	-	-	8:7248064	1299	1439	1786	1993	1419	1086	17.31446	16.07732	16.39958	17.30569	16.58536	17.94798	16.59712	17.27968	0.993171	1	-
ENSMUS(	Calr3	calreticuli	GO:00325	developm	-	-	-	-	8:7242417	13	26	18	30	12	14	0.325906	0.546362	0.310876	0.489962	0.263806	0.435199	0.394381	0.396323	0.949344	1	-
ENSMUS(	Tmc4	transmem	GO:00160	membran	-	-	-	-	7:3665790	67	88	111	132	76	57	0.624257	0.687262	0.712463	0.801204	0.620934	0.658485	0.674661	0.693541	1	1	-
ENSMUS(	Syne4	spectrin re	GO:00325	developm	-	-	-	-	7:3031480	33	65	85	74	23	63	0.254984	0.420984	0.452449	0.372491	0.155839	0.60357	0.376139	0.3773	0.848475	1	-
ENSMUS(	Polr2i	polymeras	GO:00081	metabolic	K03017	DNA-dire	mmu0110	Metabolic	7:3023194	73	99	118	105	110	75	1.514655	1.721783	1.686646	1.419259	2.00135	1.929466	1.641028	1.783358	0.841016	1	-
ENSMUS(	Krt10	keratin 10	GO:00325	developm	K07604	type I kera	-	-	11:993852	100	67	92	105	60	70	2.397321	1.346334	1.519381	1.639831	1.261315	2.080717	1.754345	1.660621	0.642006	1	-
ENSMUS(	Iyd	iodotyrosi	GO:00081	metabolic	K17231	iodotyrosi	mmu0491	Thyroid ho	10:354024	114	95	119	162	99	56	3.707797	2.589942	2.666305	3.432482	2.823524	2.258316	2.988015	2.838108	0.708103	1	-
ENSMUS(	Rmnd1	required f	GO:00081	metabolic	-	-	-	-	10:440191	230	281	385	412	248	206	1.731281	1.772955	1.99642	2.020309	1.636944	1.922618	1.833552	1.859957	0.877307	1	-
ENSMUS(	Ccdc170	coiled-co	-	-	-	-	-	-	10:448250	40	23	44	37	44	20	0.631589	0.304403	0.478606	0.380589	0.276919	0.391548	0.471532	0.349686	0.201561	1	-
ENSMUS(	Esr1	estrogen i	GO:00325	developm	K08550	estrogen i	mmu0520	Pathways	10:461159	63	113	44	59	43	26	0.210685	0.316753	0.101367	0.128537	0.126095	0.10781	0.209602	0.120814	0.034378	1	-
ENSMUS(	Vip	vasoactive	GO:00081	metabolic	-	-	-	-	10:563921	249	341	372	352	312	220	4.226685	4.851847	4.350057	3.892469	4.644061	4.63029	4.476196	4.38894	0.678501	1	-
ENSMUS(	Fbxo5	F-box pro	GO:00325	developm	K10292	F-box pro	mmu0411	Oocyte m	10:579916	15	20	35	27	19	8	0.408092	0.456086	0.655964	0.478521	0.453262	0.26986	0.506714	0.400548	0.419131	1	-
ENSMUS(	Mtrf1l	mitochon	GO:00081	metabolic	-	-	-	-	10:581188	114	169	188	174	146	86	2.015309	2.504244	2.289529	2.003869	2.26325	1.885053	2.269694	2.050724	0.413461	1	-
ENSMUS(	Rgs17	regulator	GO:00452	synapse|m	-	-	-	-	10:582566	1087	1512	1802	1686	1372	853	7.184826	8.377055	8.205274	7.259822	7.95213	6.990722	7.922385	7.400892	0.41089	1	-
ENSMUS(	Hdac2	histone de	GO:00325	developm	K06067	histone de	mmu0520	Pathways	10:369745	1181	1630	2006	2003	1345	852	13.16312	15.22823	15.4025	14.54358	13.1454	11.77428	14.59795	13.15442	0.28963	1	-
ENSMUS(	Frk	fyn-relate	GO:00325	developm	-	-	-	-	10:344833	14	16	18	16	12	7	0.099814	0.095619	0.088408	0.074313	0.075022	0.061882	0.094613	0.070406	0.294753	1	-
ENSMUS(	Rwdd1	RWD dom	GO:00325	developm	-	-	-	-	10:339965	355	360	538	550	401	212	10.38258	8.825341	10.83953	10.47902	10.28402	7.687718	10.01582	9.483585	0.589627	1	-
ENSMUS(	Clvs2	clavesin 2	GO:00099	cellular pr	-	-	-	-	10:335122	651	858	1019	1149	746	480	4.635634	5.121156	4.998661	5.33003	4.658108	4.237945	4.918484	4.742028	0.611059	1	-
ENSMUS(	Trdn	triadin [Sc	GO:00511	localizatio	-	-	-	-	10:330805	0	8	0	2	0	0	0	0.037762	0	0.007337	0	0	0.012587	0.002446	0.293846	1	-
ENSMUS(	Hey2	hairy/enh	GO:00325	developm	K09091	hairy and	mmu0520	Pathways	10:308323	80	103	169	130	104	79	1.069013	1.153678	1.555724	1.13167	1.218627	1.308905	1.259472	1.219734	0.680538	1	-
ENSMUS(	Stxbp5	syntaxin b	GO:00325	developm	-	-	-	-	10:975554	2315	2839	3281	3882	2559	1977	6.901131	7.093941	6.737936	7.538873	6.689325	7.307382	6.911003	7.178527	1	1	-
ENSMUS(	Hint3	histidine t	GO:00038	catalytic a	-	-	-	-	10:306081	91	94	134	153	119	76	0.892627	0.77287	0.905494	0.977691	1.02357	0.92433	0.856997	0.975197	0.580823	1	-
ENSMUS(	Trmt11	tRNA met	GO:00081	metabolic	-	-	-	-	10:305342	73	88	83	119	87	65	0.69162	0.69884	0.541715	0.734464	0.722781	0.763554	0.644058	0.740266	0.573369	1	-
ENSMUS(	Katna1	katanin p6	GO:00990	supramole	-	-	-	-	10:772600	186	208	248	261	200	102	3.254641	3.05075	2.989466	2.975177	3.068757	2.212983	3.098286	2.752305	0.377969	1	-
ENSMUS(	Pcmt1	protein-L	GO:00081	metabolic	-	-	-	-	10:762937	992	1384	1642	1478	1200	1257	8.030133	9.390744	9.15663	7.794121	8.517947	12.61631	8.859169	9.642792	0.793476	1	-
ENSMUS(	Lrp11	low densit	GO:00511	localizatio	-	-	-	-	10:758980	1748	1693	1877	3573	1879	1231	17.941	14.56515	13.27153	23.8902	16.91121	15.66568	15.25923	18.82236	0.243675	1	-
ENSMUS(	1700021F	RIKEN cDI	GO:00056	cell|intrace	-	-	-	-	10:435251	322	304	401	363	274	196	4.59764	3.638359	3.944343	3.3765	3.430615	3.469923	4.060114	3.425679	0.150447	1	-
ENSMUS(	Sec63	SEC63-lik	GO:00325	developm	K09540	translocat	mmu0414	Protein pr	10:427614	1959	2308	3171	3152	2239	1200	9.161175	9.047014	10.21561	9.602505	9.181494	6.957992	9.4746	8.580664	0.34328	1	-
ENSMUS(	Nr2e1	nuclear re	GO:00325	developm	-	-	-	-	10:425619	251	355	393	414	369	228	3.866161	4.583404	4.170146	4.154218	4.983975	4.354386	4.20657	4.497527	0.837574	1	-
ENSMUS(	Snx3	sorting ne	GO:00081	metabolic	K17918	sorting ne	mmu0414	Endocytos	10:425020	1772	2206	1618	2653	1993	1377	39.30749	41.01757	24.72529	38.33808	38.76694	37.87313	35.01678	38.32605	0.724363	1	-
ENSMUS(	Aig1	androgen	GO:00081	metabolic	-	-	-	-	10:136470	589	723	835	802	615	524	3.442408	3.541915	3.3619	3.053535	3.151844	3.797206	3.448741	3.334195	0.544913	1	-
ENSMUS(	Adat2	adenosine	GO:00081	metabolic	-	-	-	-	10:135528	69	87	103	107	92	82	2.991259	3.161356	3.076033	3.021817	3.497298	4.40762	3.076216	3.642245	0.498028	1	-
ENSMUS(	Pex3	peroxison	GO:00511	localizatio	K13336	peroxin-3	mmu0414	Peroxisom	10:135238	228	236	333	447	253	236	2.203813	1.912074	2.217359	2.814684	2.144387	2.828379	2.111082	2.595817	0.259897	1	-
ENSMUS(	Fuca2	fucosidas	GO:00081	metabolic	K01206	alpha-L-f	mmu0051	Other glyc	10:134995	465	582	717	743	531	369	3.559645	3.734473	3.781149	3.705303	3.564431	3.502402	3.691756	3.590712	0.628787	1	-
ENSMUS(	Cep57l1	centroson	GO:00055	protein bi	-	-	-	-	10:417188	79	99	129	120	115	78	0.512993	0.53886	0.57707	0.507634	0.654829	0.628014	0.542974	0.596826	0.770344	1	-
ENSMUS(	Ltv1	LTV1 ribo	GO:00511	localizatio	-	-	-	-	10:131781	328	435	443	421	335	188	8.564244	9.520458	7.968399	7.161084	7.670124	6.086374	8.684367	6.972527	0.090875	1	-
ENSMUS(	Plagl1	pleiomorp	GO:00325	developm	-	-	-	-	10:130605	381	810	558	474	580	379	1.498557	2.670463	1.511938	1.21453	2.00041	1.848303	1.893653	1.687748	0.391288	1	-
ENSMUS(	Cd164	CD164 an	GO:00325	developm	K06546	CD164 an	mmu0414	Lysosome	10:415194	929	1087	1404	1323	891	766	11.54718	11.32512	12.02206	10.71277	9.711377	11.80525	11.63145	10.74313	0.319824	1	-
ENSMUS(	Utrn	utrophin [	GO:00325	developm	-	-	-	-	10:123821	1268	1474	2083	2320	1635	945	3.712748	3.617652	4.201622	4.425332	4.197944	3.430791	3.844007	4.018022	0.923026	1	-
ENSMUS(	Smpd2	sphingom	GO:00081	metabolic	K12351	sphingom	mmu0110	Metabolic	10:414856	137	168	174	189	123	162	0.764554	0.78587	0.668944	0.687118	0.601915	1.120961	0.739789	0.803331	0.886781	1	-
ENSMUS(	Mical1	microtubu	GO:00081	metabolic	-	-	-	-	10:414763	172	176	232	260	205	155	1.082718	0.928648	1.006066	1.066209	1.131577	1.209775	1.005811	1.135854	0.591754	1	-
ENSMUS(	Zbtb24	zinc finge	GO:00325	developm	-	-	-	-	10:414503	167	226	258	307	234	162	1.564468	1.774647	1.66503	1.873573	1.922247	1.881707	1.668048	1.892509	0.549381	1	-
ENSMUS(	Grm1	glutamate	GO:00048	signal trar	K04603	metabotro	mmu0408	Neuroacti	10:106860	1172	1476	1947	2176	1568	880	6.210113	6.555584	7.107053	7.511258	7.285523	5.7815	6.62425	6.859427	0.977517	1	-
ENSMUS(	Wasf1	WAS prot	GO:00325	developm	K05753	WAS prot	mmu0481	Regulatior	10:408834	6367	6879	7634	9176	6184	5245	107.1048	96.99566	88.46627	100.5563	91.21921	109.397	97.52225	100.3908	0.91557	1	-
ENSMUS(	Rab32	RAB32, m	GO:00325	developm	-	-	-	-	10:105450	42	55	61	70	41	49	0.910063	0.998947	0.910552	0.988106	0.779021	1.316446	0.939854	1.027858	0.871175	1	-
ENSMUS(	Gtf3c6	general tr	GO:00081	metabolic	-	-	-	-	10:402477	471	610	743	746	496	298	7.400111	8.033414	8.041883	7.635515	6.833484	5.805236	7.825136	6.758078	0.213383	1	-
ENSMUS(	Slc16a10	solute car	GO:00511	localizatio	K08187	MFS trans	mmu0491	Thyroid ho	10:400335	60	91	118	151	97	75	0.420739	0.53488	0.570032	0.689801	0.596454	0.652097	0.50855	0.646117	0.250977	1	-
ENSMUS(	Rev3l	REV3 like,	GO:00081	metabolic	K02350	DNA poly	mmu0110	Metabolic	10:397321	1133	1322	1638	1854	1440	723	3.840912	3.756545	3.825337	4.094451	4.280649	3.038986	3.807598	3.804695	0.828064	1	-
ENSMUS(	Traf3ip2	TRAF3 int	GO:00511	localizatio	-	-	mmu0421	Cellular se	10:396129	20	35	47	32	19	25	0.258453	0.379116	0.418407	0.269387	0.215295	0.40056	0.351992	0.295081	0.362361	1	-
ENSMUS(	Fyn	Fyn proto	GO:00325	developm	K05703	tyrosine-p	mmu0451	Focal adh	10:393688	2036	2669	3181	3297	2350	1872	14.50767	15.94122	15.61475	15.30455	14.68353	16.5391	15.35455	15.50906	0.796793	1	-
ENSMUS(	Tube1	epsilon-tu	GO:00990	supramole	-	-	-	-	10:391339	23	40	37	51	35	19	0.126621	0.184584	0.140324	0.182909	0.168964	0.129695	0.15051	0.160523	0.894653	1	-
ENSMUS(	Lama4	laminin, a	GO:00325	developm	K06241	laminin, a	mmu0520	Pathways	10:389655	117	158	183	231	124	103	0.504011	0.570515	0.543072	0.648261	0.468402	0.550148	0.539199	0.555603	0.989078	1	-
ENSMUS(	Popdc3	popeye de	GO:00160	membran	-	-	-	-	10:451780	87	116	103	85	75	79	1.682119	1.879961	1.371909	1.070624	1.271575	1.89387	1.644663	1.412023	0.287272	1	-
ENSMUS(	Prep	prolyl end	GO:00081	metabolic	K01322	prolyl olig	mmu0461	Renin-ang	10:450672	639	817	909	1014	750	513	3.714476	3.980812	3.64009	3.839868	3.822968	3.697431	3.778459	3.786756	0.791029	1	-
ENSMUS(	Tnfaip3	tumor nec	GO:00325	developm	K11859	tumor nec	mmu0516	Epstein-B	10:190009	147	151	179	122	116	92	0.669314	0.576291	0.561459	0.361872	0.463142	0.519381	0.602355	0.448132	0.041109	1	-
ENSMUS(	Perp	PERP, TP5	GO:00325	developm	K10136	TP53 apop	mmu0411	p53 signa	10:188450	83	82	149	106	61	84	2.702318	2.237811	3.341896	2.248252	1.741532	3.390946	2.760675	2.460243	0.401142	1	-
ENSMUS(	Arfgef3	ARFGEF fa	GO:00081	metabolic	-	-	-	-	10:185818	4247	4701	5867	6577	4749	2851	17.80172	16.51668	16.94132	17.95929	17.45518	14.81708	17.08657	16.74385	0.666063	1	-
ENSMUS(	Hebp2	heme bin	GO:00511	localizatio	-	-	-	-	10:185394	40	28	49	46	47	37	0.478386	0.280686	0.403707	0.358389	0.4929	0.548654	0.387593	0.466648	0.55699	1	-
ENSMUS(	Reps1	RalBP1 as	GO:00511	localizatio	-	-	-	-	10:180558	686	816	988	1090	781	523	5.259029	5.24354	5.217831	5.443644	5.250191	4.971296	5.240133	5.22171	0.763372	1	-
ENSMUS(	Fam184a	family wit	GO:00055	extracellul	-	-	-	-	10:536331	480	546	734	743	626	275	3.69315	3.521287	3.890485	3.724144	4.223508	2.62346	3.701641	3.523704	0.630329	1	-
ENSMUS(	Asf1a	anti-silenc	GO:00325	developm	-	-	-	-	10:535967	139	186	248	209	151	129	1.681042	1.885513	2.066174	1.646612	1.601333	1.93436	1.877576	1.727435	0.418862	1	-
ENSMUS(	Gopc	golgi asso	GO:00325	developm	-	-	-	-	10:523358	1332	1459	828	867	1480	723	4.413128	4.051823	1.889835	1.871298	4.299785	2.970069	3.451595	3.047051	0.457532	1	-
ENSMUS(	Qrsl1	glutaminy	GO:00081	metabolic	K02433	aspartyl-t	mmu0110	Metabolic	10:438741	137	197	242	229	163	191	1.725474	2.07973	2.099696	1.878913	1.800189	2.98269	1.9683	2.220597	0.688659	1	-
ENSMUS(	Rtn4ip1	reticulon 4	GO:00081	metabolic	-	-	-	-	10:439018	213	299	342	415	291	214	2.930671	3.448353	3.241643	3.719788	3.510942	3.650802	3.206889	3.627177	0.549237	1	-
ENSMUS(	Nmbr	neuromec	GO:00048	signal trar	K04168	neuromec	mmu0408	Neuroacti	10:147055	40	41	74	70	44	29	0.535639	0.460199	0.68264	0.610643	0.516655	0.48149	0.559493	0.536263	0.736561	1	-
ENSMUS(	Crybg1	crystallin b	-	-	-	-	-	-	10:439506	22	40	44	38	41	29	0.142857	0.217721	0.196831	0.160751	0.23346	0.233489	0.185803	0.209233	0.797726	1	-
ENSMUS(	Vta1	vesicle (m	GO:00511	localizatio	K12199	vacuolar p	mmu0414	Endocytos	10:146547	394	434	563	503	452	308	6.982671	6.447163	6.873622	5.807315	7.024357	6.768022	6.767819	6.533231	0.585055	1	-
ENSMUS(	Smpdl3a	sphingom	GO:00081	metabolic	-	-	-	-	10:577943	369	417	586	537	381	286	8.590139	8.136965	9.397725	8.143836	7.77752	8.255161	8.708276	8.058839	0.394456	1	-
ENSMUS(	Reep3	receptor a	GO:00990	supramole	-	-	-	-	10:670091	827	899	1400	1340	927	528	4.948532	4.509031	5.770989	5.223445	4.863997	3.917335	5.076184	4.668259	0.421883	1	-
ENSMUS(	Fabp7	fatty acid	GO:00325	developm	K08756	fatty acid-	mmu0332	PPAR sign	10:577848	180	262	327	295	192	211	7.57083	9.236907	9.474825	8.083046	7.081356	11.00373	8.760854	8.72271	0.740649	1	-
ENSMUS(	Pkib	protein kii	GO:00081	metabolic	-	-	-	-	10:576319	100	164	69	154	126	56	0.345772	0.475322	0.164358	0.346891	0.382037	0.240084	0.328484	0.323004	0.86667	1	-
ENSMUS(	Serinc1	serine inc	GO:00081	metabolic	-	-	-	-	10:575157	18413	22717	27394	28585	20360	10049	182.3596	188.5853	186.9007	184.4267	176.8172	123.3994	185.9485	161.5478	0.22101	1	-
ENSMUS(	Hsf2	heat shoc	GO:00081	metabolic	-	-	-	-	10:574863	463	614	778	715	541	339	2.274839	2.528661	2.633301	2.288535	2.330824	2.065166	2.478934	2.228175	0.30043	1	-
ENSMUS(	Rspo3	R-spondir	GO:00325	developm	-	-	-	-	10:294524	232	261	300	281	211	152	3.917182	3.693848	3.489473	3.090821	3.124004	3.182111	3.700168	3.132312	0.160591	1	-
ENSMUS(	Echdc1	enoyl Coe	GO:00081	metabolic	K18426	ethylmalo	mmu0064	Propanoa	10:293131	328	414	811	409	581	419	3.833984	4.056301	6.530541	3.114453	5.955188	6.072623	4.806942	5.047421	0.989363	1	-
ENSMUS(	Mgat4c	MGAT4 fa	GO:00081	metabolic	K13748	alpha-1,3	mmu0110	Metabolic	10:101681	191	181	47	57	94	96	0.658813	0.523312	0.111681	0.12808	0.284314	0.410568	0.431269	0.274321	0.151606	1	-
ENSMUS(	Ptprk	protein ty	GO:00325	developm	-	-	-	-	10:280748	1222	1382	1558	1760	1371	729	4.743573	4.496714	4.166325	4.450704	4.666749	3.508711	4.468871	4.208721	0.504788	1	-

ENSMUSC	Nts	neurotens	GO:00452	synapse|re	-	-	-	-	10:102481	65	141	136	212	117	53	3.310034	6.018583	4.771011	7.03296	5.224569	3.346419	4.699876	5.201316	0.729198	1	-
ENSMUSC	Dcbld1	discoidin,	GO:00160	membran	-	-	-	-	10:522336	177	218	291	297	217	142	1.151135	1.188396	1.303758	1.258317	1.237531	1.145059	1.214429	1.213636	0.820998	1	-
ENSMUSC	Lrriq1	leucine-ri	-	-	-	-	-	-	10:103046	88	66	138	89	66	25	0.602596	0.378824	0.650985	0.397023	0.396303	0.212257	0.544135	0.335194	0.030443	1	-
ENSMUSC	Slc6a15	solute car	GO:00511	localizatio	-	-	-	-	10:103367	876	1167	1299	1254	922	933	5.121657	5.719135	5.231991	4.776235	4.72694	6.763544	5.357594	5.42224	0.784445	1	-
ENSMUSC	Ccdc59	coiled-co	GO:00081	metabolic	-	-	-	-	10:105841	383	497	571	604	489	256	4.652297	5.060318	4.778107	4.779548	5.208585	3.855614	4.830241	4.614582	0.607524	1	-
ENSMUSC	Lama2	laminin, a	GO:00325	developm	K05637	laminin, a	mmu0520	Pathways	10:269800	323	377	494	585	382	242	1.116541	1.092362	1.176389	1.317377	1.15792	1.037228	1.128431	1.170842	0.976125	1	-
ENSMUSC	Lin7a	lin-7 hom	GO:00325	developm	-	-	-	-	10:107271	402	503	531	617	442	256	5.45089	5.716918	4.960063	5.450145	5.255408	4.303941	5.375957	5.003165	0.481893	1	-
ENSMUSC	Ppp1r12a	protein pl	GO:00081	metabolic	K06270	protein pl	mmu0481	Regulatioı	10:108162	2208	3488	4675	4868	2538	1838	14.11721	18.693	20.59125	20.27598	14.22932	14.57072	17.80049	16.35867	0.43017	1	-
ENSMUSC	P4ha1	procollag	GO:00081	metabolic	K00472	prolyl 4-h	mmu0110	Metabolic	10:593232	1299	1501	2222	1985	1474	890	12.62894	12.23182	14.88172	12.57187	12.56602	10.72837	13.24749	11.95542	0.291984	1	-
ENSMUSC	10-Sep	septin 10	GO:00971	organic cy	-	-	-	-	10:591416	99	112	128	137	94	59	1.907742	1.809068	1.699204	1.719842	1.588383	1.409694	1.805338	1.57264	0.333322	1	-
ENSMUSC	Lims1	LIM and s	GO:00325	developm	-	-	-	-	10:583234	283	509	514	474	364	331	2.916886	4.39747	3.649612	3.182675	3.289857	4.230057	3.654656	3.567529	0.645362	1	-
ENSMUSC	Zwint	ZW10 inte	GO:00511	localizatio	-	-	-	-	10:726548	7312	10043	11113	11304	8734	7696	83.57906	96.22273	87.50726	84.17345	87.54214	109.0718	89.10302	93.59579	0.962624	1	-
ENSMUSC	Ube2d1	ubiquitin-	GO:00081	metabolic	K06689	ubiquitin-	mmu0414	Protein pr	10:712549	630	768	1035	1078	760	446	7.983728	8.157919	9.035603	8.899503	8.445426	7.007866	8.392417	8.117598	0.640489	1	-
ENSMUSC	Dcn	decorin [S	GO:00325	developm	K04660	decorin[-]	mmu0520	Proteoglyı	10:974796	859	1153	1244	1082	892	509	7.835802	8.816017	7.817398	6.429826	7.135065	5.756971	8.156406	6.44062	0.043164	1	-
ENSMUSC	Kera	keratocan	GO:00325	developm	-	-	-	-	10:976068	3	3	3	9	0	0	0.083612	0.07009	0.057604	0.163425	0	0	0.070435	0.054475	0.858449	1	-
ENSMUSC	Mrln	myoreguli	GO:00511	localizatio	-	-	-	-	10:702046	1	1	0	0	1	0	0.0351	0.029414	0	0	0.03077	0	0.021505	0.010257	1	1	-
ENSMUSC	Slc17a8	solute car	GO:00325	developm	K12302	MFS trans	mmu0472	Retrograd	10:895740	153	152	164	229	120	69	1.811741	1.508691	1.337826	1.766535	1.246029	1.013065	1.552753	1.341876	0.386518	1	-
ENSMUSC	Epyc	epiphycar	GO:00325	developm	-	-	-	-	10:976440	0	0	8	0	0	0	0	0	0.101462	0	0	0	0.033821	0	0.065045	1	-
ENSMUSC	Cdk1	cyclin-dep	GO:00325	developm	K02087	cyclin-dep	mmu0520	Viral carci	10:693351	8	11	19	14	7	5	0.078216	0.090154	0.127975	0.089174	0.060019	0.060611	0.098782	0.069935	0.312012	1	-
ENSMUSC	Atp2b1	ATPase, C	GO:00325	developm	K05850	Ca2+ tran	mmu0402	cAMP sigr	10:989144	20729	23295	28863	34680	17108	9291	67.25497	63.35218	64.51176	73.30047	48.67298	37.37614	65.03964	53.11653	0.164926	1	-
ENSMUSC	Rhobtb1	Rho-relat	GO:00508	response	K07868	Rho-relat	mmu0412	Ubiquitin	10:691514	69	38	128	147	56	33	0.411368	0.189897	0.525701	0.570923	0.292757	0.243935	0.375655	0.369205	0.91215	1	-
ENSMUSC	Cabcoco1	ciliary ass	GO:00056	cell|intrace	-	-	-	-	10:684309	30	43	55	50	32	30	1.172576	1.408758	1.480919	1.273102	1.096737	1.453885	1.354084	1.274574	0.644672	1	-
ENSMUSC	Arid5b	AT rich int	GO:00325	developm	-	-	-	-	10:680925	635	728	999	928	778	524	2.656234	2.552566	2.878787	2.528845	2.853742	2.717753	2.695862	2.700113	0.778229	1	-
ENSMUSC	Actr6	ARP6 actiı	GO:00099	cellular pr	-	-	-	-	10:897119	170	225	291	234	208	178	3.953161	4.385633	4.661662	3.544814	4.241338	5.132194	4.333485	4.306115	0.734933	1	-
ENSMUSC	Uhrf1bp1l	UHRF1 (IC	-	-	-	-	-	-	10:897449	1259	2043	2432	2540	1334	1081	6.643409	9.036217	8.840572	8.731337	6.172537	7.072567	8.173399	7.32548	0.30574	1	-
ENSMUSC	Poc1b	POC1 cen	GO:00099	cellular pr	-	-	-	-	10:991070	130	169	157	183	113	76	1.125754	1.226701	0.936589	1.032364	0.858068	0.816015	1.096348	0.902149	0.19908	1	-
ENSMUSC	Dusp6	dual speci	GO:00325	developm	K04459	dual speci	mmu0401	MAPK sigı	10:992632	1114	794	1553	1450	1024	929	11.943	7.13512	11.46967	10.12693	9.626548	12.34894	10.1826	10.70081	0.969277	1	-
ENSMUSC	Tmpo	thymopoi	GO:00081	metabolic	-	-	-	-	10:911475	428	561	758	534	547	318	1.338023	1.470063	1.632454	1.087534	1.499514	1.232631	1.48018	1.273226	0.193061	1	-
ENSMUSC	Kitl	kit ligand	GO:00325	developm	K05461	KIT ligand	mmu0520	Pathways	10:100015	296	514	640	510	474	363	2.201743	3.204728	3.279489	2.471307	3.091689	3.347858	2.89532	2.970285	0.913964	1	-
ENSMUSC	Psen1	presenilin	GO:00325	developm	K04505	presenilin	mmu0516	Human pa	12:836881	993	1272	1538	1653	1222	868	9.686591	10.40068	10.33545	10.50452	10.45287	10.4985	10.14091	10.4853	0.975876	1	-
ENSMUSC	Sgk1	serum/glu	GO:00325	developm	K13302	serum/glu	mmu0415	PI3K-Akt s	10:218821	851	893	1145	1105	830	901	5.284701	4.648311	4.898324	4.470279	4.519719	6.937478	4.943779	5.309159	0.877213	1	-
ENSMUSC	Cep290	centroson	GO:00325	developm	-	-	-	-	10:100487	521	573	736	778	583	234	2.29016	2.111228	2.228727	2.227867	2.247185	1.275352	2.210038	1.916801	0.33494	1	-
ENSMUSC	Ikbip	IKBKB inte	GO:00160	membran	-	-	-	-	10:910829	221	239	350	292	230	127	1.913259	1.734327	2.087372	1.646818	1.746025	1.363235	1.911652	1.58536	0.164971	1	-
ENSMUSC	Hbs1l	Hbs1-like	GO:00081	metabolic	K14416	elongatioı	mmu0301	mRNA sur	10:212959	530	685	822	731	695	501	3.270521	3.54311	3.494333	2.938599	3.760699	3.833229	3.435988	3.510842	0.879861	1	-
ENSMUSC	Epb41l2	erythrocyt	GO:00325	developm	-	-	-	-	10:253597	1240	1439	1641	1669	1509	977	3.422913	3.329568	3.120572	3.001319	3.652634	3.343912	3.291018	3.332622	0.834618	1	-
ENSMUSC	Apaf1	apoptotic	GO:00325	developm	K02084	apoptotic	mmu0520	Pathways	10:909893	196	453	516	294	223	324	0.817563	1.583859	1.482744	0.798903	0.815667	1.675702	1.294722	1.096757	0.277574	1	-
ENSMUSC	Myb	myeloblas	GO:00325	developm	K09420	myb protc	mmu0415	PI3K-Akt s	10:211249	15	19	24	32	19	18	0.084289	0.089492	0.092904	0.117139	0.093618	0.125414	0.088895	0.112057	0.466428	1	-
ENSMUSC	Med23	mediator	GO:00081	metabolic	-	-	-	-	10:248699	495	663	763	823	590	476	3.307962	3.713831	3.512625	3.582922	3.457406	3.944108	3.511473	3.661478	0.993459	1	-
ENSMUSC	Ahi1	Abelson h	GO:00056	cell|cell ju	-	-	-	-	10:209525	4350	5465	5812	6354	5088	3129	28.02672	29.51387	25.79646	26.66932	28.7457	24.99623	27.77902	26.80375	0.556914	1	-
ENSMUSC	Arg1	arginase,	GO:00325	developm	K01476	arginase[3	mmu0110	Metabolic	10:249152	19	21	39	33	20	24	0.460529	0.426654	0.651212	0.521069	0.425095	0.721273	0.512798	0.555813	0.956858	1	-
ENSMUSC	Nedd1	neural pre	GO:00511	localizatio	-	-	-	-	10:926847	50	53	76	86	39	20	0.670255	0.595523	0.701831	0.75101	0.458431	0.332416	0.65587	0.513952	0.285755	1	-
ENSMUSC	Enpp3	ectonucle	GO:00081	metabolic	K01513	ectonucle	mmu0110	Metabolic	10:247724	3	10	6	2	5	3	0.024481	0.068407	0.033733	0.010632	0.03578	0.030361	0.042207	0.025591	0.28057	1	-
ENSMUSC	Pde7b	phosphoc	GO:00081	metabolic	K18436	high affini	mmu0023	Purine me	10:203980	411	438	602	586	490	294	1.678898	1.499717	1.694066	1.559415	1.755177	1.489072	1.624227	1.601221	0.728328	1	-
ENSMUSC	Mtfr2	mitochon	GO:00081	metabolic	-	-	-	-	10:203477	2	2	10	9	5	4	0.047034	0.039414	0.161976	0.13785	0.103079	0.11661	0.082808	0.11918	0.563952	1	-
ENSMUSC	Map7	microtubu	GO:00099	cellular pr	-	-	-	-	10:201484	1311	1516	2114	2055	1625	1170	6.77437	6.566266	7.525282	6.917669	7.36313	7.496145	6.955306	7.258982	0.97373	1	-
ENSMUSC	Ctgf	connectiv	GO:00325	developm	K06827	connectiv	mmu0439	Hippo sig	10:245954	148	237	316	268	283	173	1.97768	2.654574	2.908931	2.332981	3.316073	2.86634	2.513728	2.838465	0.624598	1	-
ENSMUSC	Stx7	syntaxin 7	GO:00511	localizatio	K08488	syntaxin 7	mmu0414	Phagoson	10:241493	1585	1968	2419	2366	1757	1495	15.89577	16.54361	16.71243	15.45784	15.45137	18.59001	16.38394	16.49974	0.762903	1	-
ENSMUSC	Moxd1	monooxy	GO:00081	metabolic	-	-	-	-	10:242235	176	215	266	270	191	111	3.176746	3.252826	3.30753	3.174794	3.023063	2.484159	3.2457	2.894005	0.362969	1	-
ENSMUSC	Pex7	peroxison	GO:00325	developm	K13341	peroxin-7	mmu0414	Peroxisom	10:198539	313	373	464	463	353	269	1.512475	1.510797	1.544593	1.457495	1.49576	1.611696	1.522622	1.52165	0.767172	1	-
ENSMUSC	Il20ra	interleukiı	GO:00048	signal trar	K05136	interleukiı	mmu0406	Cytokine-	10:197125	0	0	0	3	0	0	0	0	0	0.048436	0	0	0	0.016145	0.30226	1	-
ENSMUSC	Ifngr1	interferon	GO:00048	signal trar	K05132	interferon	mmu0520	Pathways	10:195919	247	269	359	334	273	200	3.769831	3.441365	3.774605	3.32088	3.653685	3.78478	3.661934	3.586448	0.672099	1	-
ENSMUSC	Vnn3	vanin 3 [S	GO:00081	metabolic	K08069	pantetheiı	mmu0077	Pantother	10:238514	0	0	0	1	3	0	0	0	0	0.022858	0.09231	0	0	0.038389	0.163382	1	-
ENSMUSC	Cfap54	cilia and f	GO:00325	developm	-	-	-	-	10:927756	151	130	213	180	116	111	0.599368	0.432527	0.582438	0.465448	0.403756	0.546295	0.538111	0.471833	0.300964	1	-
ENSMUSC	Cdk17	cyclin-dep	GO:00081	metabolic	-	-	-	-	10:931608	3108	3700	4447	4542	3479	2621	28.04526	27.9855	27.64375	26.69975	27.52806	29.32455	27.8915	27.85079	0.712472	1	-
ENSMUSC	Hal	histidine a	GO:00081	metabolic	K01745	histidine a	mmu0110	Metabolic	10:934887	4	0	3	8	6	4	0.03616	0	0.018682	0.047114	0.04756	0.044836	0.018281	0.046503	0.087123	1	-
ENSMUSC	Snrpf	small nucl	GO:00081	metabolic	K11098	small nucl	mmu0304	Spliceosoı	10:935830	97	132	150	176	133	107	7.159886	8.166928	7.627397	8.463068	8.608488	9.792714	7.651404	8.954757	0.472982	1	-
ENSMUSC	Ntn4	netrin 4 [S	GO:00325	developm	K06845	netrin 4[-]	mmu0436	Axon guic	10:936406	102	130	215	194	142	106	1.533214	1.637935	2.226334	1.899697	1.871685	1.975579	1.799161	1.915654	0.888435	1	-
ENSMUSC	Fgd6	FYVE, Rhc	GO:00987	molecular	-	-	-	-	10:940360	543	469	707	714	558	287	4.070929	2.947265	3.651443	3.487173	3.668347	2.66785	3.556546	3.274457	0.470227	1	-
ENSMUSC	Ndufa12	NADH de	GO:00081	metabolic	K11352	NADH de	mmu0110	Metabolic	10:941989	815	1128	1245	1324	670	395	25.6923	29.80626	27.03749	27.19037	18.52094	15.43932	27.51202	20.38354	0.046799	1	-
ENSMUSC	Tmcc3	transmem	GO:00160	membran	-	-	-	-	10:943119	906	723	1123	1663	754	877	4.037233	2.700518	3.447362	4.82758	2.946249	4.845532	3.395038	4.206454	0.27743	1	-
ENSMUSC	Cep83	centroson	GO:00511	localizatio	-	-	-	-	10:946886	674	834	1147	1091	854	446	4.84282	5.022931	5.677448	5.10675	5.380701	3.973369	5.181066	4.820273	0.47371	1	-
ENSMUSC	Socs2	suppressc	GO:00325	developm	K04695	suppresso	mmu0463	Jak-STAT	10:953853	266	327	395	329	287	296	1.234759	1.272337	1.263133	0.994899	1.168223	1.70364	1.256743	1.288921	0.867162	1	-
ENSMUSC	Nudt4	nudix (nuc	GO:00081	metabolic	-	-	-	-	10:955470	1665	2135	2991	2861	2032	1260	32.96441	35.43087	40.79425	36.90035	35.27748	30.93055	36.39651	34.36946	0.480894	1	-
ENSMUSC	Nuak1	NUAK fan	GO:00325	developm	-	-	-	-	10:843709	1602	1995	2472	2360	1774	1225	17.60538	18.37717	18.7147	16.89571	17.0954	16.69187	18.23241	16.89433	0.340394	1	-
ENSMUSC	Tcp11l2	t-complex	-	-	-	-	-	-	10:845766	308	401	464	511	331	320	4.762603	5.197462	4.942699	5.147504	4.488123	6.135211	4.967588	5.256946	0.930531	1	-
ENSMUSC	Rfx4	regulatory	GO:00325	developm	-	-	-	-	10:847560	212	239	289	274	223	166	1.769073	1.671703	1.661338	1.489501	1.631762	1.717528	1.700705	1.61293	0.521292	1	-
ENSMUSC	Cry1	cryptochrı	GO:00048	signal trar	K02295	cryptochrı	mmu0471	Circadian	10:851317	232	300	361	299	262	218	3.583056	3.883648	3.840833	3.008289	3.548219	4.174544	3.769179	3.577017	0.497283	1	-
ENSMUSC	Btbd11	BTB (POZ	GO:00081	metabolic	-	-	-	-	10:853868	243	418	505	475	441	418	1.055452	1.521814	1.511037	1.344025	1.679632	2.251098	1.362768	1.758252	0.192719	1	-
ENSMUSC	Timp3	tissue inhi	GO:00081	metabolic	K16866	metallopr	mmu0520	Proteoglyı	10:863003	2195	2644	3785	3281	2471	1713	15.44325	15.5926	18.34516	15.03807	15.24472	14.94333	16.46033	15.07537	0.292871	1	-
ENSMUSC	Hsp90b1	heat shoc	GO:00081	metabolic	K09487	heat shoc	mmu0520	Pathways	10:866902	6789	8115	11578	10946	8337	4843	79.90026	80.05413	93.87011	83.92269	86.03889	70.67125	84.60816	80.21095	0.493393	1	-
ENSMUSC	Pah	phenylala	GO:00081	metabolic	K00500	phenylala	mmu0110	Metabolic	10:875217	0	0	0	0	0	4	0	0	0	0	0	0.087847	0	0.029282	0.127651	1	-
ENSMUSC	Ascl1	achaete-s	GO:00325	developm	-	-	-	-	10:874908	145	150	234	216	176	88	3.714313	3.22072	4.12931	3.604498	3.953336	2.794971	3.688114	3.450935	0.592185	1	-
ENSMUSC	Igf1	insulin-lik	GO:00325	developm	K05459	insulin-lik	mmu0520	Pathways	10:878582	215	290	402	455	234	188	1.039554	1.175329	1.339017	1.433183	0.992127	1.127072	1.184633	1.184128	0.835948	1	-
ENSMUSC	Washc3	WASH co	GO:00511	localizatio	K18463	WASH co	mmu0414	Endocytos	10:882010	86	140	141	170	128	121	0.886978	1.210309	1.001807	1.142207	1.157625	1.547334	1.033031	1.282389	0.323714	1	-
ENSMUSC	Dram1	DNA-dan	GO:00081	metabolic	-	-	-	-	10:883228	22	32	26	37	37	21	0.417356	0.508862	0.339796	0.457275	0.615514	0.493958	0.422005	0.522249	0.4866	1	-
ENSMUSC	Sycp3	synaptone	GO:00325	developm	K19528	synaptone	mmu0344	Homologı	10:884595	7	5	8	10	13	2	0.213791	0.127987	0.168308	0.198945	0.348137	0.075753	0.170029	0.207612	0.736037	1	-
ENSMUSC	Mybpc1	myosin bi	GO:00325	developm	-	-	-	-	10:885182	33	42	60	44	45	22	0.374843	0.399888	0.469506	0.325592	0.448216	0.309852	0.414745	0.36122	0.458005	1	-
ENSMUSC	Sirt1	sirtuin 1 [S	GO:00325	developm	K11411	NAD-dep	mmu0421	Cellular se	10:633190	232	175	416	393	150	142	1.399551	0.884895	1.728805	1.544457	0.793477	1.062128	1.33775	1.133354	0.34639	1	-
ENSMUSC	Herc4	hect dom	GO:00325	developm	K10615	E3 ubiquit	mmu0412	Ubiquitin	10:632438	434	552	613	628	493	311	2.305802	2.458238	2.243594	2.173569	2.296791	2.0487	2.335878	2.17302	0.430757	1	-
ENSMUSC	Mypn	myopallac	GO:00325	developm	-	-	-	-	10:631157	19	22	35	30	33	16	0.178124	0.17288	0.226041	0.183221	0.27129	0.185984	0.192348	0.213498	0.827263	1	-
ENSMUSC	Hnrnph3	heteroger	GO:00325	developm	-	-	-	-	10:630146	905	1060	1308	1306	1070	393	11.4527	11.24393	11.40299	10.76674	11.87369	6.166491	11.36654	9.602307	0.263247	1	-
ENSMUSC	Rufy2	RUN and	GO:00056	cell|intrace	-	-	-	-	10:629802	1188	1536	1871	1935	1447	838	6.190634	6.709079	6.716514	6.568727	6.611955	5.414381	6.538742	6.198354	0.505503	1	-
ENSMUSC	Pbld2	phenazine	GO:00081	metabolic	-	-	-	-	10:630243	4	13	15	13	9	13	0.07924	0.215879	0.204717	0.167779	0.156352	0.319316	0.166612	0.214482	0.654273	1	-
ENSMUSC	Ccar1	cell divisic	GO:00081	metabolic	-	-	-	-	10:627439	1026	1130	1603	1602	1198	607	4.084527	3.770732	4.396218	4.154688	4.182095	2.99619	4.083826	3.777658	0.45361	1	-
ENSMUSC	Ddx21	DEAD (As	GO:00325	developm	-	-	-	-	10:625802	562	619	855	938	607	421	6.366627	5.87783	6.67253	6.922414	6.029822	5.913457	6.305662	6.288564	0.784589	1	-
ENSMUSC	Ddx50	DEAD (As	GO:00081	metabolic	-	-	-	-	10:626158	1181	1351	1757	1860	1315	702	5.772216	5.534786	5.915832	5.922256	5.63587	4.25418	5.740945	5.270769	0.408109	1	-
ENSMUSC	Srgn	serglycin [	GO:00325	developm	-	-	-	-	10:624938	75	37	198	192	128	109	1.225311	0.506692	2.228452	2.043477	1.83375	2.20799	1.320151	2.028406	0.157025	1	-
ENSMUSC	Vps26a	VPS26 ret	GO:00511	localizatio	K18466	vacuolar p	mmu0414	Endocytos	10:624552	946	1229	1810	1298	1128	740	9.502317	10.34768	12.52473	8.493655	9.935509	9.216284	10.79158	9.21515	0.130076	1	-
ENSMUSC	Supv3l1	suppressc	GO:00325	developm	-	-	-	-	10:624292	304	363	537	616	466	260	3.669534	3.672796	4.46543	4.843957	4.932487	3.891314	3.93592	4.555919	0.439464	1	-
ENSMUSC	Hkdc1	hexokinas	GO:00081	metabolic	K00844	hexokinas	mmu0110	Metabolic	10:623831	81	118	120	160	87	60	0.846259	1.033367	0.863673	1.088982	0.797044	0.777239	0.914433	0.887755	0.759602	1	-
ENSMUSC	Tacr2	tachykinin	GO:00048	signal trar	K04223	tachykinin	mmu0408	Neuroacti	10:622524	0	2	7	12	5	0	0	0.047182	0.135732	0.220035	0.123393	0	0.060971	0.114476	0.424908	1	-
ENSMUSC	Fam241b	family witl	GO:00160	membran	-	-	-	-	10:621076	236	244	299	309	238	250	2.129869	1.845795	1.858928	1.816688	1.883474	2.797475	1.944864	2.165879	0.714551	1	-
ENSMUSC	Aifm2	apoptosis	GO:00081	metabolic	-	-	-	-	10:617152	70	57	120	110	90	94	0.88058	0.601039	1.03993	0.901459	0.992793	1.466184	0.840516	1.120145	0.24157	1	-
ENSMUSC	H2afy2	H2A histo	GO:00325	developm	K11251	histone H2	mmu0503	Alcoholisr	10:617386	213	271	339	285	260	159	6.223801	6.637416	6.823821	5.425047	6.661825	5.760497	6.56168	5.949123	0.367095	1	-
ENSMUSC	Tysnd1	trypsin do	GO:00081	metabolic	-	-	-	-	10:616955	207	237	282	289	236	264	4.0466	3.883468	3.797696	3.680426	4.045516	6.398944	3.909254	4.708295	0.408988	1	-
ENSMUSC	Ppa1	pyrophos	GO:00081	metabolic	K01507	inorganic	mmu0019	Oxidative	10:616485	817	1139	1227	1315	940	687	15.12903	17.67932	15.65254	15.8634	15.26368	15.77363	16.15363	15.63357	0.571682	1	-
ENSMUSC	Npffr1	neuropep	GO:00048	signal trar	K04240	neuropep	mmu0408	Neuroacti	10:615954	42	56	62	56	61	67	0.767236	0.857482	0.780228	0.666422	0.97714	1.517552	0.801649	1.053705	0.350836	1	-
ENSMUSC	Eif4ebp2	eukaryotic	GO:00451	translatior	K18644	eukaryotic	mmu0301	RNA trans	10:614284	1162	1277	1790	1840	1352	1412	10.51975	9.690423	11.16358	10.85172	10.73293	15.84965	10.45792	12.4781	0.313947	1	-
ENSMUSC	Pald1	phosphata	GO:00056	cell|intrace	-	-	-	-	10:613196	218	276	303	412	259	303	2.819401	2.992007	2.699576	3.471199	2.93727	4.858802	2.836995	3.755757	0.130836	1	-
ENSMUSC	Tbata	thymus, b	GO:00325	developm	-	-	-	-	10:611719	116	118	146	145	98	89	1.548775	1.320582	1.342866	1.261187	1.14735	1.47334	1.404074	1.293959	0.464051	1	-
ENSMUSC	Sgpl1	sphingosiı	GO:00325	developm	K01634	sphingani	mmu0110	Metabolic	10:610986	408	489	633	695	480	435	2.603641	2.615663	2.782756	2.889262	2.685992	3.441875	2.667353	3.00571	0.563761	1	-
ENSMUSC	Pcbd1	pterin 4 al	GO:00081	metabolic	K01724	4a-hydrox	mmu0079	Folate bio	10:610893	23	28	26	19	20	48	1.36736	1.395289	1.064844	0.73584	1.042657	3.53822	1.275831	1.772239	0.502863	1	-
ENSMUSC	Unc5b	unc-5 net	GO:00325	developm	K07521	netrin rec	mmu0436	Axon guic	10:607625	456	430	530	562	438	557	2.865066	2.264602	2.294024	2.300321	2.413163	4.339216	2.474564	3.017567	0.39083	1	-
ENSMUSC	Slc29a3	solute car	GO:00511	localizatio	-	-	-	-	10:607120	399	478	589	645	342	299	2.430987	2.441131	2.472162	2.560066	1.82717	2.258744	2.448093	2.215327	0.340156	1	-
ENSMUSC	Vsir	V-set imn	GO:00325	developm	-	-	-	-	10:603468	450	504	684	697	558	428	4.153794	3.899559	4.349505	4.191278	4.51657	4.898491	4.134286	4.535446	0.690756	1	-
ENSMUSC	Slc16a7	solute car	GO:00511	localizatio	-	-	-	-	10:125219	955	1035	1255	1371	1171	664	4.347334	3.949235	3.935635	4.06573	4.674327	3.747776	4.077401	4.162611	0.919484	1	-
ENSMUSC	Lrig3	leucine-ri	GO:00325	developm	-	-	-	-	10:125966	47	30	68	51	38	33	0.238044	0.127359	0.23726	0.168274	0.168768	0.207232	0.200888	0.181425	0.539352	1	-
ENSMUSC	Anapc16	anaphase	GO:00081	metabolic	-	-	-	-	10:599874	479	556	736	781	589	578	3.842706	3.738774	4.067525	4.081633	4.143424	5.749297	3.883002	4.658118	0.310542	1	-
ENSMUSC	Ddit4	DNA-dan	GO:00325	developm	K08270	DNA-dan	mmu0415	PI3K-Akt s	10:599496	570	692	801	789	659	757	20.64116	21.00477	19.98218	18.61305	20.92604	33.98917	20.5427	24.50942	0.407539	1	-
ENSMUSC	Dnajb12	DnaJ heat	GO:00081	metabolic	K09518	DnaJ hom	mmu0414	Protein pr	10:598795	603	793	801	862	634	807	4.798108	5.28906	4.390726	4.468292	4.423685	7.961812	4.825965	5.61793	0.527378	1	-
ENSMUSC	Micu1	mitochon	GO:00511	localizatio	-	-	-	-	10:597024	945	1131	1080	1378	897	913	4.531622	4.546083	3.567773	4.304797	3.771872	5.428487	4.21516	4.501719	0.883906	1	-
ENSMUSC	Cand1	cullin assc	GO:00325	developm	-	-	-	-	10:119199	2790	3471	3994	4282	3114	2185	18.91943	19.72928	18.65792	18.91612	18.51677	18.37136	19.10221	18.60142	0.584328	1	-
ENSMUSC	Tbk1	TANK-bin	GO:00081	metabolic	K05410	TANK-bin	mmu0516	Human pa	10:121546	1068	1176	1523	1634	1120	727	11.55312	10.66322	11.34957	11.51494	10.62402	9.75099	11.18864	10.62998	0.504258	1	-
ENSMUSC	Pno1	partner of	GO:00056	cell|intrace	-	-	-	-	11:172031	296	378	471	445	340	240	9.752043	10.43875	10.68996	9.550936	9.822582	9.803933	10.29358	9.725817	0.491864	1	-
ENSMUSC	Plek	pleckstrin	GO:00325	developm	-	-	-	-	11:169712	302	367	576	637	365	266	2.67052	2.720244	3.508831	3.66952	2.830247	2.916461	2.966532	3.138743	0.879845	1	-
ENSMUSC	Srgap1	SLIT-ROB	GO:00400	locomotic	K07526	SLIT-ROB	mmu0436	Axon guic	10:121780	390	397	515	466	327	341	2.440975	2.082775	2.220536	1.900055	1.79469	2.646303	2.248095	2.113683	0.443557	1	-
ENSMUSC	Egfr	epiderma	GO:00325	developm	K04361	epiderma	mmu0520	Pathways	11:167522	413	474	623	636	419	229	2.138452	2.057224	2.222233	2.145303	1.902422	1.470186	2.139303	1.839303	0.242569	1	-
ENSMUSC	Avpr1a	arginine v	GO:00325	developm	K04226	arginine v	mmu0408	Neuroacti	10:122448	5	0	7	5	0	5	0.119014	0	0.114788	0.077537	0	0.147556	0.077934	0.075031	0.889333	1	-
ENSMUSC	Usp15	ubiquitin s	GO:00081	metabolic	K11835	ubiquitin c	mmu0413	Mitophag	10:123105	1947	2241	2853	2941	2030	1074	5.761942	5.559012	5.816424	5.669956	5.267949	3.940879	5.712459	4.959595	0.217436	1	-
ENSMUSC	Vps54	VPS54 GA	GO:00511	localizatio	-	-	-	-	11:212392	581	716	986	916	700	397	3.272849	3.380771	3.826294	3.361455	3.457726	2.772852	3.493305	3.197344	0.386262	1	-
ENSMUSC	Tbc1d15	TBC1 dom	GO:00511	localizatio	K20168	TBC1 dom	mmu0413	Mitophag	10:115197	702	807	1010	1104	735	465	8.804858	8.484218	8.726859	9.020611	8.083795	7.231429	8.671978	8.111945	0.46091	1	-
ENSMUSC	Pcsk4	proprotei	GO:00325	developm	-	-	-	-	10:803212	15	32	36	32	24	37	0.162265	0.29016	0.268279	0.225506	0.227659	0.496263	0.240234	0.316476	0.469038	1	-
ENSMUSC	Rab21	RAB21, m	GO:00325	developm	-	-	-	-	10:115287	1591	1893	2539	2506	1830	1594	18.56985	18.52003	20.41514	19.05465	18.72975	23.06814	19.16834	20.28418	0.905362	1	-
ENSMUSC	2310011J(	RIKEN cDI	-	-	-	-	-	-	10:803182	283	276	327	362	310	286	10.90693	8.916144	8.681905	9.088785	10.4766	13.66682	9.501658	11.0774	0.488553	1	-
ENSMUSC	Peli1	pellino 1 [	GO:00081	metabolic	-	-	-	-	11:210912	733	906	1121	1129	854	547	6.063299	6.28183	6.387961	6.083883	6.194494	5.610207	6.244364	5.962861	0.527048	1	-
ENSMUSC	Apc2	adenoma	GO:00511	localizatio	K02085	adenomat	mmu0520	Pathways	10:802959	3168	3457	4105	4731	3539	2998	10.07637	9.216607	8.994634	9.802875	9.870567	11.82324	9.429203	10.49889	0.572119	1	-
ENSMUSC	Thap2	THAP dor	GO:00056	cell|intrace	-	-	-	-	10:115368	380	432	673	594	453	241	2.631305	2.507408	3.210368	2.679515	2.750607	2.069149	2.783027	2.499757	0.369345	1	-
ENSMUSC	Lgr5	leucine ric	GO:00325	developm	-	-	-	-	10:115450	21	55	33	50	36	25	0.100545	0.220726	0.108841	0.155949	0.151141	0.148407	0.143371	0.151832	0.941214	1	-
ENSMUSC	Slc1a4	solute car	GO:00511	localizatio	-	-	-	-	11:203021	1112	1330	1539	1561	1338	1031	10.15241	10.17816	9.679534	9.284297	10.71182	11.67102	10.00337	10.55571	0.925276	1	-
ENSMUSC	Dock2	dedicator	GO:00325	developm	K12367	dedicator	mmu0406	Chemokin	11:342268	103	139	200	220	161	93	0.540361	0.611248	0.722821	0.751886	0.740656	0.604944	0.62481	0.699162	0.638453	1	-
ENSMUSC	Rab1a	RAB1A, m	GO:00325	developm	K07874	Ras-relate	mmu0513	Legionellc	11:202014	2974	2831	3441	3352	3361	2607	25.07385	20.00657	19.98555	18.4105	24.84799	27.25254	21.68866	23.50368	0.78903	1	-
ENSMUSC	Gamt	guanidinc	GO:00325	developm	K00542	guanidino	mmu0110	Metabolic	10:802581	106	132	166	174	118	149	2.465824	2.573838	2.660207	2.636862	2.407023	4.297601	2.566623	3.113829	0.436591	1	-
ENSMUSC	Ptprr	protein ty	GO:00325	developm	K04458	receptor-	mmu0401	MAPK sigı	10:116018	340	374	428	453	341	236	2.513147	2.317199	2.179385	2.181317	2.210222	2.162897	2.336577	2.184812	0.456711	1	-
ENSMUSC	Actr2	ARP2 actiı	GO:00325	developm	K17260	actin-rela	mmu0453	Tight junc	11:200623	5926	7394	9385	9990	6758	5295	66.85871	69.92439	72.94274	73.42492	66.85859	74.07101	69.90861	71.45151	0.88307	1	-
ENSMUSC	Ndufs7	NADH de	GO:00081	metabolic	K03940	NADH de	mmu0110	Metabolic	10:802491	430	523	613	677	517	833	9.449447	9.633683	9.280039	9.691888	9.962556	22.69703	9.454389	14.11716	0.066673	1	-
ENSMUSC	Ptprb	protein ty	GO:00325	developm	K05694	receptor-	mmu0452	Adherens	10:116275	1045	1287	1592	1792	1234	842	4.665465	4.816263	4.896357	5.211927	4.830994	4.660975	4.792695	4.901299	0.919263	1	-
ENSMUSC	Kcnmb1	potassium	GO:00325	developm	K04937	potassium	mmu0402	cGMP-PK	11:339630	26	28	21	42	18	17	0.394275	0.355897	0.219377	0.414901	0.239345	0.319629	0.323183	0.324625	1	1	-
ENSMUSC	Mum1	melanoma	GO:00081	metabolic	-	-	-	-	10:802264	392	561	572	699	522	527	1.297609	1.556587	1.304382	1.507357	1.515203	2.16299	1.386192	1.728517	0.215792	1	-
ENSMUSC	Meis1	Meis hom	GO:00325	developm	K15613	homeobo	mmu0520	Transcript	11:188798	35	38	60	40	33	29	0.099064	0.090153	0.11699	0.073753	0.081904	0.10177	0.102069	0.085809	0.312813	1	-
ENSMUSC	Uqcr11	ubiquinol-	GO:00081	metabolic	K00420	ubiquinol-	mmu0110	Metabolic	10:804029	620	753	865	968	688	465	35.53008	36.17032	34.14853	36.13778	34.57287	33.04026	35.28298	34.58364	0.669592	1	-
ENSMUSC	Cnot2	CCR4-NC	GO:00325	developm	K12605	CCR4-NO	mmu0301	RNA degr	10:116485	872	991	1366	1487	1099	552	5.636514	5.369336	6.082701	6.26162	6.229236	4.424044	5.696183	5.6383	0.788133	1	-
ENSMUSC	Tcf3	transcripti	GO:00325	developm	K09063	transcripti	mmu0516	HTLV-I inf	10:804095	326	464	442	530	414	372	2.194503	2.618115	2.049706	2.324213	2.443772	3.1049	2.287441	2.624295	0.516679	1	-
ENSMUSC	Best3	bestrophi	GO:00511	localizatio	-	-	-	-	10:116986	41	39	32	51	37	28	0.958311	0.764092	0.515261	0.776573	0.758357	0.811466	0.745888	0.782132	1	1	-
ENSMUSC	Frs2	fibroblast	GO:00325	developm	K12461	fibroblast	mmu0520	Proteoglyı	10:117069	1248	1309	1677	1880	1338	808	8.579144	7.542613	7.941702	8.419157	8.06544	6.886941	8.021153	7.790513	0.634939	1	-
ENSMUSC	Yeats4	YEATS do	GO:00081	metabolic	-	-	-	-	10:117215	323	332	409	447	312	230	7.192572	6.196882	6.274183	6.484425	6.092279	6.350329	6.554546	6.309011	0.594986	1	-
ENSMUSC	Cobl	cordon-b	GO:00325	developm	-	-	-	-	11:122366	423	476	797	634	610	358	2.024467	1.909549	2.62773	1.976704	2.560018	2.124416	2.187249	2.220379	0.89033	1	-
ENSMUSC	Rab36	RAB36, m	GO:00511	localizatio	-	-	-	-	10:750370	383	502	597	644	513	453	2.352452	2.58451	2.526082	2.576849	2.763005	3.449895	2.487681	2.929916	0.373719	1	-
ENSMUSC	Grb10	growth fa	GO:00081	metabolic	K20064	growth fa	mmu0415	mTOR sig	11:119305	593	756	846	812	561	545	3.731751	3.98779	3.667579	3.328859	3.095733	4.252459	3.795707	3.559017	0.401171	1	-
ENSMUSC	Adora2a	adenosine	GO:00325	developm	K04266	adenosine	mmu0408	Neuroacti	10:753168	25	47	35	55	29	25	0.381108	0.600555	0.367554	0.546195	0.387652	0.472525	0.449739	0.468791	1	1	-
ENSMUSC	Snrpd3	small nucl	GO:00081	metabolic	K11088	small nucl	mmu0532	Systemic l	10:755175	1067	1252	1599	1605	1194	646	16.96973	16.69044	17.51905	16.62905	16.65167	12.73883	17.05974	15.33985	0.31327	1	-
ENSMUSC	Nav3	neuron na	GO:00400	locomotic	-	-	-	-	10:109681	2119	2162	2873	3252	2443	1295	7.552935	6.459416	7.054591	7.551217	7.635737	5.723222	7.022314	6.970059	0.777084	1	-
ENSMUSC	Ddc	dopa dec	GO:00325	developm	K01593	aromatic-	mmu0110	Metabolic	11:118141	103	96	145	145	118	93	1.222397	0.954998	1.185486	1.121055	1.228009	1.368496	1.12096	1.239187	0.738968	1	-
ENSMUSC	Cpm	carboxype	GO:00081	metabolic	-	-	-	-	10:117629	259	218	386	405	259	161	2.039936	1.439212	2.094378	2.078034	1.78879	1.572269	1.857842	1.813031	0.743728	1	-
ENSMUSC	Mdm2	transform	GO:00325	developm	K06643	E3 ubiquit	mmu0520	Pathways	10:117688	629	899	1008	982	790	547	4.079897	4.887775	4.504126	4.149462	4.493331	4.399186	4.490599	4.347326	0.583293	1	-
ENSMUSC	E2f7	E2F transc	GO:00325	developm	-	-	-	-	10:110745	2	4	8	10	3	3	0.009903	0.016597	0.027282	0.032248	0.013022	0.018416	0.017927	0.021229	0.875777	1	-
ENSMUSC	Csrp2	cysteine a	GO:00325	developm	-	-	-	-	10:110919	73	62	120	74	61	93	1.381593	0.983559	1.56454	0.91237	1.01235	2.18234	1.309897	1.36902	0.943344	1	-
ENSMUSC	Osbpl8	oxysterol	GO:00325	developm	-	-	-	-	10:111164	1707	1985	2439	2490	1844	1199	10.2354	9.976647	10.07476	9.726401	9.695601	8.91408	10.0956	9.445361	0.400138	1	-
ENSMUSC	Mknk2	MAP kina	GO:00325	developm	K04372	MAP kina	mmu0401	MAPK sigı	10:806653	568	654	851	503	492	693	5.746277	5.545856	5.930881	3.315032	4.364611	8.692749	5.741004	5.457464	0.545783	1	-
ENSMUSC	Spata48	spermato	-	-	-	-	-	-	11:114620	0	0	4	9	2	2	0	0	0.083008	0.176592	0.052819	0.074712	0.027669	0.101374	0.086409	1	-
ENSMUSC	Zpbp	zona pellu	GO:00099	cellular pr	-	-	-	-	11:112796	6	11	6	17	9	10	0.089768	0.137948	0.061835	0.165683	0.118073	0.185507	0.096517	0.156421	0.219833	1	-
ENSMUSC	Cabin1	calcineuri	GO:00987	molecular	-	-	-	-	10:756461	1736	1879	2238	2631	1955	1557	7.204388	6.536227	6.398218	7.112952	7.114379	8.011653	6.712945	7.412995	0.620532	1	-
ENSMUSC	Ap3d1	adaptor-r	GO:00325	developm	K12396	AP-3 com	mmu0414	Lysosome	10:807069	3364	4017	4746	5211	3959	3146	19.75534	19.77349	19.20029	19.93569	20.38716	22.90726	19.57637	21.0767	0.769399	1	-
ENSMUSC	Phlda1	pleckstrin	GO:00081	metabolic	-	-	-	-	10:111506	183	205	251	228	200	204	5.032549	4.725451	4.755119	4.084638	4.822901	6.955872	4.837707	5.287804	0.828125	1	-
ENSMUSC	Sf3a2	splicing fa	GO:00325	developm	K12826	splicing fa	mmu0304	Spliceosoı	10:807981	392	422	449	546	463	276	8.312575	7.500919	6.559145	7.542649	8.609399	7.256775	7.457547	7.802941	0.94874	1	-
ENSMUSC	Mdm1	transform	GO:00325	developm	-	-	-	-	10:118141	114	140	160	164	151	107	1.088661	1.12065	1.052594	1.020265	1.264463	1.266946	1.087301	1.183892	0.794822	1	-

ENSMUSC	Jsrp1	junctional	GO:00511	localizatio	-	-	-	-	10:808084	25	34	41	45	33	44	0.581354	0.662724	0.656806	0.681698	0.672914	1.268628	0.633628	0.874414	0.279519	1	-
ENSMUSC	Wif1	Wnt inhib	GO:00325	developm	K01691	WNT inhit	mmu0431	Wnt signa	10:121033	13	3	13	16	20	12	0.17169	0.03321	0.118279	0.137661	0.231629	0.196507	0.107726	0.188599	0.178982	1	-
ENSMUSC	Timm13	translocas	GO:00511	localizatio	-	-	-	-	10:808994	333	279	539	430	259	461	8.348378	5.86293	9.308901	7.022772	5.693785	14.32996	7.840069	9.015507	0.697625	1	-
ENSMUSC	Vps13d	vacuolar p	GO:00055	extracellul	-	-	-	-	4:1449726	2866	3488	4000	4503	3348	2495	5.924504	6.04373	5.696225	6.064002	6.068815	6.394879	5.888153	6.175899	0.940172	1	-
ENSMUSC	Llph	LLP homo	GO:00325	developm	-	-	-	-	10:120227	382	558	581	563	455	219	7.069671	8.656117	7.407361	6.787752	7.383976	5.025343	7.71105	6.399024	0.17114	1	-
ENSMUSC	Tmbim4	transmem	GO:00508	response	-	-	-	-	10:120201	174	217	369	279	223	330	2.523563	2.638005	3.686736	2.636024	2.836033	5.93422	2.949434	3.802093	0.327919	1	-
ENSMUSC	Irak3	interleukir	GO:00325	developm	K04732	interleukir	mmu0472	Neurotrop	10:120141	22	19	37	25	29	19	0.199279	0.144263	0.230887	0.147525	0.230348	0.213399	0.191476	0.19709	1	1	-
ENSMUSC	Helb	helicase (I	GO:00081	metabolic	-	-	-	-	10:120083	87	93	153	169	149	83	0.952312	0.853282	1.153725	1.205113	1.430168	1.126481	0.98644	1.253921	0.240525	1	-
ENSMUSC	Slc5a4a	solute car	GO:00511	localizatio	-	-	-	-	10:761474	0	0	0	3	0	0	0	0	0	0	0.060433	0	0	0.020144	0.302755	1	-
ENSMUSC	Prmt2	protein ar	GO:00081	metabolic	-	-	-	-	10:762072	919	1291	1329	1341	938	998	9.16735	10.79463	9.132822	8.714432	8.204911	12.34369	9.698267	9.754344	0.751309	1	-
ENSMUSC	Dip2a	disco inte	GO:00325	developm	-	-	-	-	10:762594	1198	1334	1507	1596	1236	1021	5.617686	5.243357	4.868165	4.875457	5.082318	5.936254	5.24307	5.298009	0.791429	1	-
ENSMUSC	Hmg20b	high mob	GO:00325	developm	-	-	-	-	10:813460	269	325	407	373	272	266	2.330078	2.359687	2.428642	2.104785	2.065997	2.856836	2.372802	2.342539	0.67764	1	-
ENSMUSC	4930404N	RIKEN cDI	-	-	-	-	-	-	10:813630	0	6	4	0	5	3	0	0.097028	0.053171	0	0.084585	0.071773	0.050066	0.052119	1	1	-
ENSMUSC	Fzr1	fizzy/cell c	GO:00325	developm	K03364	cell divisio	mmu0412	Ubiquitin	10:813662	927	1114	1224	1326	1080	1046	8.640899	8.703963	7.859808	8.052012	8.827658	12.08918	8.401557	9.656282	0.482039	1	-
ENSMUSC	Ncln	nicalin [Sc	GO:00508	response	-	-	-	-	10:814862	668	786	889	967	756	958	5.60441	5.527504	5.138147	5.285201	5.561829	9.965634	5.423354	6.937555	0.214202	1	-
ENSMUSC	Col6a2	collagen,	GO:00508	response	K06238	collagen,	mmu0415	PI3K-Akt	10:765957	228	418	328	335	267	278	1.716833	2.638292	1.701449	1.643311	1.762979	2.595521	2.018858	2.000603	0.728705	1	-
ENSMUSC	Hcfc2	host cell f	GO:00160	membran	K14966	host cell f	mmu0516	Herpes sir	10:826961	471	810	669	719	486	377	4.758138	6.858899	4.655796	4.731806	4.305221	4.722195	5.424278	4.586407	0.161746	1	-
ENSMUSC	Nfyb	nuclear tr	GO:00081	metabolic	K08065	nuclear tr	mmu0516	HTLV-I inf	10:827487	557	714	878	884	670	542	4.246013	4.562231	4.610756	4.389955	4.478616	5.122846	4.473	4.663805	0.999888	1	-
ENSMUSC	Txnrd1	thioredox	GO:00325	developm	K00384	thioredox	mmu0520	Pathways	10:828339	957	1149	1447	1477	1101	992	5.423117	5.457701	5.648806	5.452541	5.471003	6.970031	5.509875	5.964525	0.772452	1	-
ENSMUSC	Glt8d2	glycosyltra	GO:00038	catalytic a	-	-	-	-	10:826504	260	335	401	431	321	189	2.3837	2.574395	2.532641	2.574167	2.580624	2.148456	2.496912	2.434416	0.699665	1	-
ENSMUSC	Ppm1m	protein pl	GO:00081	metabolic	-	-	-	-	9:1061949	191	248	335	303	247	259	3.350435	3.646469	4.048222	3.462516	3.799337	5.633175	3.681709	4.298342	0.504946	1	-
ENSMUSC	D10Wsu1	DNA segr	-	-	-	-	-	-	10:833602	844	1007	1242	1642	1158	682	0.435454	0.435494	0.441441	0.551892	0.523903	0.436285	0.437463	0.504027	0.407517	1	-
ENSMUSC	Aldh1l2	aldehyde	GO:00081	metabolic	K00289	formyltetr	mmu0067	One carbo	10:834874	117	126	165	165	125	75	0.66988	0.604695	0.6508	0.615429	0.627578	0.532428	0.641792	0.591812	0.509435	1	-
ENSMUSC	Wdr82	WD repea	GO:00081	metabolic	K14962	COMPASS	mmu0301	mRNA sur	9:1061709	2207	2666	3318	3422	2537	2391	19.25347	19.49484	19.94042	19.4477	19.4075	25.86259	19.56291	21.5726	0.653764	1	-
ENSMUSC	Glyctk	glycerate	GO:00081	metabolic	K11529	glycerate	mmu0110	Metabolic	9:1061528	68	82	93	104	81	93	0.467202	0.472239	0.440178	0.465487	0.488002	0.792248	0.459873	0.581912	0.348307	1	-
ENSMUSC	Pofut2	protein O	GO:00325	developm	K03691	peptide-(	mmu0051	Other typ	10:772592	345	437	489	518	186	363	1.986033	2.108635	1.939221	1.942578	0.938906	2.590951	2.011296	1.824145	0.44	1	-
ENSMUSC	Adarb1	adenosine	GO:00325	developm	-	-	-	-	10:772907	2648	3246	3727	2703	3184	2207	7.042238	7.235925	6.828158	4.68296	7.425205	7.277479	7.03544	6.461881	0.335881	1	-
ENSMUSC	Appl2	adaptor p	GO:00329	macromo	-	-	-	-	10:836000	985	1239	1889	2025	1337	1008	6.173544	6.509119	8.156083	8.268083	7.348054	7.833302	6.946249	7.81648	0.523162	1	-
ENSMUSC	Slc36a2	solute car	GO:00511	localizatio	-	-	-	-	11:551584	4	6	10	6	6	2	0.075791	0.095292	0.130537	0.074066	0.099686	0.047001	0.10054	0.073584	0.556681	1	-
ENSMUSC	Sumo3	small ubic	GO:00081	metabolic	K12160	small ubic	mmu0301	RNA trans	10:776060	1263	1588	1686	1907	1399	415	10.63426	11.20747	9.779424	10.46012	10.32915	4.332493	10.54039	8.37392	0.218277	1	-
ENSMUSC	Hint1	histidine t	GO:00081	metabolic	-	-	-	-	11:548663	594	886	1049	801	644	1029	31.04483	38.81405	37.76848	27.27199	29.51421	66.68128	35.87579	41.15583	0.685835	1	-
ENSMUSC	Lyrm7	LYR motif	GO:00081	metabolic	-	-	-	-	11:548268	100	234	285	152	246	100	1.07353	2.105633	2.107711	1.063018	2.315755	1.331066	1.762291	1.569946	0.525116	1	-
ENSMUSC	Fbxw11	F-box anc	GO:00081	metabolic	K03362	F-box anc	mmu0421	Cellular se	11:326427	2696	3320	4028	4206	2978	3002	18.94294	19.55323	19.49703	19.25211	18.34826	26.15316	19.33107	21.25118	0.686233	1	-
ENSMUSC	Stk10	serine/thr	GO:00081	metabolic	K08837	serine/thr	mmu0491	Progester	11:325333	156	165	233	265	181	123	1.811815	1.606302	1.86422	2.005012	1.843367	1.771256	1.760779	1.873212	0.859653	1	-
ENSMUSC	Papolg	poly(A) pc	GO:00081	metabolic	K14376	poly(A) pc	mmu0301	mRNA sur	11:238626	476	548	765	799	567	288	4.955154	4.781715	5.486092	5.418495	5.175783	3.717305	5.07432	4.770528	0.558518	1	-
ENSMUSC	Rel	reticuloen	GO:00081	metabolic	K09254	c-Rel prot	mmu0401	Ras signal	11:237368	103	110	177	174	112	88	0.876766	0.784861	1.037942	0.964895	0.836008	0.928787	0.899856	0.909897	0.896417	1	-
ENSMUSC	Pfkl	phosphof	GO:00081	metabolic	-	-	mmu0110	Metabolic	10:779869	4531	5483	5968	6866	4952	4040	36.65462	37.17969	33.25944	36.18429	35.12837	40.52303	35.69791	37.27856	0.985807	1	-
ENSMUSC	Pus10	pseudouri	GO:00081	metabolic	-	-	-	-	11:236656	112	134	183	191	136	87	1.256703	1.260286	1.414539	1.396139	1.338115	1.210372	1.310509	1.314875	0.868018	1	-
ENSMUSC	Rhbdf1	rhomboid	GO:00081	metabolic	-	-	-	-	11:322095	182	182	225	253	184	220	1.133541	0.950147	0.965382	1.026522	1.004908	1.698923	1.016357	1.243451	0.375939	1	-
ENSMUSC	Pex13	peroxison	GO:00325	developm	K13344	peroxin-1	mmu0414	Peroxisom	11:236464	386	524	697	602	496	414	4.607711	5.243028	5.731687	4.681406	5.191856	6.127512	5.194142	5.333591	0.903505	1	-
ENSMUSC	1810043G	RIKEN cDI	GO:00325	developm	-	-	-	-	10:779785	349	385	373	384	313	417	1.538569	1.422671	1.132795	1.10282	1.209981	2.279363	1.364678	1.530721	0.755131	1	-
ENSMUSC	1700093K	RIKEN cDI	GO:00160	membran	-	-	-	-	11:235162	0	8	5	9	3	3	0	0.172111	0.088413	0.150488	0.067518	0.095484	0.086841	0.104497	0.883854	1	-
ENSMUSC	Mpg	N-methyl	GO:00081	metabolic	K03652	DNA-3-m	mmu0341	Base excis	11:322265	121	176	176	226	169	166	1.436834	1.7518	1.439743	1.748275	1.759745	2.444076	1.542792	1.984032	0.207627	1	-
ENSMUSC	Ahsa2	AHA1, act	GO:00987	molecular	-	-	-	-	11:234878	390	504	575	561	410	311	2.388518	2.587305	2.425962	2.238253	2.201865	2.361621	2.467262	2.267246	0.353935	1	-
ENSMUSC	Nprl3	nitrogen p	GO:00325	developm	K20406	nitrogen p	mmu0415	mTOR sig	11:322256	284	336	417	452	347	299	1.408544	1.39683	1.424751	1.460397	1.509117	1.838689	1.410042	1.602734	0.537349	1	-
ENSMUSC	Xpo1	exportin 1	GO:00081	metabolic	K14290	exportin-1	mmu0516	HTLV-I inf	11:232560	1472	1954	2294	2156	1772	1085	8.634858	9.607819	9.270265	8.23906	9.114938	7.891563	9.170981	8.415187	0.326804	1	-
ENSMUSC	Hbq1a	hemoglob	GO:00511	localizatio	-	-	-	-	11:323000	2	1	0	5	0	0	0.210483	0.088192	0	0.342753	0	0	0.099559	0.114251	1	1	-
ENSMUSC	Nsg2	neuron sp	GO:00511	localizatio	-	-	-	-	11:320004	14837	19764	21333	21543	16401	11413	217.4668	242.8145	215.4022	205.7002	210.7948	207.4114	225.2278	207.9688	0.296249	1	-
ENSMUSC	4930524B	RIKEN cDI	-	-	-	-	-	-	11:319655	0	1	2	1	0	1	0	0.026634	0.043786	0.020698	0	0.039389	0.023474	0.020029	1	1	-
ENSMUSC	Cpeb4	cytoplasm	GO:00451	translatior	K02602	cytoplasm	mmu0411	Oocyte m	11:318722	1388	1613	2012	2919	1511	1193	11.07212	10.78521	11.05658	15.169	10.56935	11.79961	10.9713	12.51266	0.47086	1	-
ENSMUSC	Stc2	stanniocal	GO:00325	developm	-	-	-	-	11:313573	136	147	185	171	139	103	0.919591	0.833153	0.861743	0.753237	0.824163	0.863531	0.871496	0.813644	0.482574	1	-
ENSMUSC	Asb3	ankyrin re	GO:00081	metabolic	-	-	-	-	11:308854	163	250	291	334	206	158	1.522061	1.956758	1.871925	2.031758	1.686766	1.829313	1.783581	1.849279	1	1	-
ENSMUSC	Cdc34	cell divisic	GO:00081	metabolic	K02207	ubiquitin-	mmu0516	Herpes sir	10:796821	238	273	322	389	293	175	2.657345	2.554975	2.476731	2.82945	2.868673	2.422675	2.563017	2.706933	0.888778	1	-
ENSMUSC	Tpgs1	tubulin pc	GO:00325	developm	-	-	-	-	10:796693	329	431	448	454	419	484	11.6875	12.83383	10.96364	10.50663	13.05217	21.31853	11.82832	14.95911	0.277495	1	-
ENSMUSC	Chac2	ChaC, cati	GO:00081	metabolic	-	-	-	-	11:309767	81	142	173	154	119	87	2.206512	3.242375	3.246518	2.732901	2.842568	2.93851	2.898468	2.837993	0.738093	1	-
ENSMUSC	Erlec1	endoplasr	GO:00081	metabolic	K14008	endoplasr	mmu0414	Protein pr	11:309307	844	1023	1116	1248	897	482	13.35293	13.56634	12.16326	12.86265	12.44427	9.45512	13.02751	11.58734	0.300348	1	-
ENSMUSC	Shc2	SHC (Src I	GO:00081	metabolic	K17447	SHC-trans	mmu0401	Ras signal	10:796180	669	899	949	1185	851	815	5.452292	6.141372	5.328077	6.291479	6.081704	8.235623	5.64058	6.869602	0.240325	1	-
ENSMUSC	Sptbn1	spectrin b	GO:00081	metabolic	-	-	-	-	11:300993	29291	33334	40022	40672	29399	18268	86.12618	82.15637	81.06832	77.9073	75.80122	66.60057	83.11696	73.43636	0.181578	1	-
ENSMUSC	Theg	testicular	GO:00325	developm	-	-	-	-	10:795763	0	0	2	2	6	0	0	0	0.036935	0.034919	0.141015	0	0.012312	0.058645	0.205939	1	-
ENSMUSC	Wdpcp	WD repea	GO:00325	developm	-	-	-	-	11:215722	100	132	176	155	110	109	0.893225	0.988296	1.082995	0.901933	0.861579	1.207179	0.988172	0.990231	0.786439	1	-
ENSMUSC	Mdh1	malate de	GO:00081	metabolic	K00025	malate de	mmu0110	Metabolic	11:215567	3337	4666	5877	4974	3739	6477	62.01064	72.67877	75.23452	60.214	60.92676	149.2347	69.97465	90.12516	0.303511	1	-
ENSMUSC	Prss57	protease,	GO:00081	metabolic	-	-	-	-	10:797814	10	10	18	11	10	13	0.103038	0.086362	0.127767	0.073833	0.090353	0.166078	0.105722	0.110088	0.92906	1	-
ENSMUSC	Fstl3	follistatin-	GO:00325	developm	-	-	-	-	10:797772	31	34	25	34	23	17	0.503494	0.462874	0.279711	0.359734	0.327568	0.342337	0.41536	0.343213	0.421843	1	-
ENSMUSC	Ccng1	cyclin G1	GO:00099	cellular pr	K10145	cyclin G1[	mmu0520	MicroRNA	11:407485	1430	1740	2459	2378	1814	1048	21.05671	21.47616	24.94391	22.8112	23.42258	19.13384	22.49226	21.78921	0.622987	1	-
ENSMUSC	Fgf22	fibroblast	GO:00508	response	K04358	fibroblast	mmu0520	Pathways	10:797550	6	19	19	6	21	7	0.106906	0.283754	0.233203	0.069644	0.328098	0.154644	0.207954	0.184128	0.697665	1	-
ENSMUSC	Nudcd2	NudC dor	GO:00325	developm	-	-	-	-	11:407336	148	153	200	198	152	99	2.422938	2.099539	2.255603	2.111681	2.182067	2.009566	2.25936	2.101104	0.504388	1	-
ENSMUSC	Polrmt	polymeras	GO:00081	metabolic	-	-	-	-	10:797361	349	394	500	549	415	437	2.384178	2.256121	2.353067	2.443245	2.486018	3.701524	2.331122	2.876929	0.260117	1	-
ENSMUSC	Hmmr	hyalurona	GO:00160	membran	K06267	hyalurona	mmu0451	ECM-rece	11:407013	19	11	18	23	11	4	0.202942	0.098488	0.132449	0.16004	0.103029	0.052974	0.144626	0.105348	0.390797	1	-
ENSMUSC	Hcn2	hyperpola	GO:00511	localizatio	K04955	hyperpola	mmu0402	cAMP sigr	10:797166	1557	1713	2000	2257	1857	1714	22.80001	21.026	20.17567	21.53077	23.8452	31.12025	21.33389	25.49874	0.291532	1	-
ENSMUSC	Acsl6	acyl-CoA	GO:00325	developm	K01897	long-chai	mmu0110	Metabolic	11:543037	3104	3599	7104	4541	3245	3161	11.29896	10.98124	17.8144	10.76837	10.35796	14.26682	13.36487	11.79772	0.237329	1	-
ENSMUSC	Slc22a4	solute car	GO:00081	metabolic	K08202	MFS trans	mmu0523	Choline m	11:539831	57	104	110	112	80	122	1.186544	1.814661	1.577448	1.518823	1.460298	3.148881	1.526218	2.042667	0.295626	1	-
ENSMUSC	Zfp354b	zinc finge	GO:00081	metabolic	-	-	-	-	11:509218	75	89	101	97	93	44	1.066083	1.060408	0.989016	0.898228	1.159196	0.775478	1.038502	0.944301	0.524863	1	-
ENSMUSC	Cyfip2	cytoplasm	GO:00325	developm	K05749	cytoplasm	mmu0481	Regulatior	11:461938	20737	23796	29710	33667	24514	15302	125.0023	120.2344	123.3747	132.2081	129.5772	114.3684	122.8704	125.3846	0.90392	1	-
ENSMUSC	Mgat1	mannosid	GO:00325	developm	K00726	alpha-1,3	mmu0110	Metabolic	11:492441	170	218	291	563	192	148	0.741981	0.797542	0.874962	1.600793	0.734835	0.800925	0.804828	1.045517	0.256365	1	-
ENSMUSC	Ppp2ca	protein pl	GO:00325	developm	K04382	serine/thr	mmu0415	PI3K-Akt	11:520986	5925	7258	9022	8629	6350	4407	49.10689	50.42243	51.51198	46.59037	46.14986	45.28799	50.3471	46.00941	0.269262	1	-
ENSMUSC	Sgcd	sarcoglyca	GO:00325	developm	K12563	delta-sarc	mmu0541	Dilated ca	11:468962	25	37	59	47	37	36	0.186527	0.231396	0.303249	0.228443	0.242072	0.333032	0.240391	0.267849	0.844402	1	-
ENSMUSC	Flt4	FMS-like	GO:00325	developm	K05097	FMS-like	mmu0520	Pathways	11:496092	85	77	114	99	87	66	0.623859	0.473711	0.5764	0.473354	0.559928	0.600622	0.55799	0.544635	0.709632	1	-
ENSMUSC	Hnrnpab	heteroger	GO:00325	developm	-	-	-	-	11:516001	2894	3891	4677	4511	3463	1938	47.92142	54.0064	53.35191	48.66152	50.28359	39.78973	51.75991	46.24494	0.255175	1	-
ENSMUSC	Phykpl	5-phosph	GO:00038	catalytic a	K18202	5-phosph	mmu0110	Metabolic	11:515847	55	76	97	95	76	57	0.282501	0.327212	0.34323	0.317881	0.342309	0.36301	0.317648	0.341067	0.896968	1	-
ENSMUSC	Hspa4	heat shoc	GO:00511	localizatio	K09489	heat shoc	mmu0453	Tight junc	11:532598	5991	7105	8964	9159	6709	4431	62.00067	61.63309	63.90727	61.74854	60.8832	56.85711	62.51368	59.82962	0.497558	1	-
ENSMUSC	Cnot6	CCR4-NC	GO:00081	metabolic	K12603	CCR4-NO	mmu0301	RNA degr	11:496715	891	937	1457	1456	1081	638	6.042009	5.325941	6.806357	6.43201	6.427944	5.364264	6.058103	6.074739	0.827025	1	-
ENSMUSC	Gfpt2	glutamine	GO:00081	metabolic	K00820	glucosam	mmu0110	Metabolic	11:497941	94	88	138	141	90	75	1.027867	0.806575	1.039538	1.004409	0.862971	1.016854	0.957994	0.961411	0.848482	1	-
ENSMUSC	Zfp354a	zinc finge	GO:00325	developm	-	-	-	-	11:510592	124	204	257	239	171	103	0.756947	1.043824	1.080756	0.950437	0.91534	0.779592	0.960509	0.88179	0.491857	1	-
ENSMUSC	Mapk9	mitogen-	GO:00325	developm	K04440	c-Jun N-t	mmu0520	Pathways	11:498467	1181	1320	1700	1766	1287	764	5.053945	4.73486	5.01165	4.923261	4.829494	4.053775	4.933485	4.602177	0.431074	1	-
ENSMUSC	Canx	calnexin [	GO:00325	developm	K08054	calnexin[-	mmu0516	HTLV-I inf	11:502939	6591	8200	10048	9977	7629	5195	55.46582	57.84167	58.25124	54.69602	56.29689	54.20579	57.18624	55.06623	0.511686	1	-
ENSMUSC	Rack1	receptor f	GO:00325	developm	K14753	guanine n	mmu0516	Measles -	11:488003	1684	1668	2561	2669	2041	1974	13.24545	10.99699	13.87669	13.67587	14.07701	19.25121	12.70638	15.66803	0.197685	1	-
ENSMUSC	Rasgef1c	RasGEF dc	GO:00987	molecular	-	-	-	-	11:499018	36	35	69	48	41	50	0.446416	0.363804	0.589443	0.38776	0.445828	0.76878	0.466554	0.534123	0.776761	1	-
ENSMUSC	Rufy1	RUN and	GO:00511	localizatio	K12482	RUN and	mmu0414	Endocytos	11:503892	721	814	1030	1195	883	543	13.88116	13.13614	13.6609	14.98788	14.90712	12.96214	13.5594	14.28572	0.890057	1	-
ENSMUSC	Rnf130	ring finge	GO:00081	metabolic	-	-	-	-	11:500253	696	688	955	862	718	485	20.47819	16.96773	19.35695	16.52234	18.52464	17.69335	18.93429	17.58011	0.390804	1	-
ENSMUSC	Ltc4s	leukotrien	GO:00081	metabolic	K00807	leukotrien	mmu0110	Metabolic	11:502364	36	36	59	36	30	44	0.834688	0.699658	0.942385	0.54377	0.60994	1.264926	0.825577	0.806212	0.705418	1	-
ENSMUSC	Rad50	RAD50 dc	GO:00081	metabolic	K10866	DNA repa	mmu0421	Cellular se	11:536495	538	617	709	851	602	245	5.128469	4.929967	4.655895	5.284657	5.03205	2.895732	4.904777	4.404147	0.452886	1	-
ENSMUSC	Mrnip	MRN com	GO:00081	metabolic	-	-	-	-	11:501744	130	153	184	208	160	129	3.816123	3.764624	3.720903	3.977621	4.118513	4.69518	3.767217	4.263771	0.598908	1	-
ENSMUSC	Clk4	CDC like k	GO:00081	metabolic	-	-	-	-	11:512617	945	1153	1338	1372	1078	615	6.038366	6.175466	5.889727	5.71115	6.040169	4.872468	6.03452	5.541262	0.366437	1	-
ENSMUSC	Sar1b	secretion	GO:00511	localizatio	K07953	GTP-bind	mmu0414	Protein pr	11:517636	305	441	566	527	402	501	14.01566	16.98655	17.91768	15.77633	16.19881	28.5455	16.30663	20.17355	0.326675	1	-
ENSMUSC	Jade2	jade famil	GO:00325	developm	-	-	-	-	11:518134	1047	1224	1503	1699	1214	1078	3.432552	3.363603	3.394539	3.628652	3.490049	4.382026	3.396898	3.833576	0.515958	1	-
ENSMUSC	Pdlim4	PDZ and I	GO:00452	synapse|s	-	-	-	-	11:540549	69	84	122	91	88	105	1.339397	1.366751	1.631429	1.150751	1.497905	2.527149	1.445859	1.725268	0.583568	1	-
ENSMUSC	Cdkl3	cyclin-dep	GO:00325	developm	-	-	-	-	11:520042	149	194	219	206	175	106	0.983631	1.073493	0.995958	0.88592	1.013037	0.867638	1.017694	0.922198	0.402417	1	-
ENSMUSC	Ube2b	ubiquitin-	GO:00325	developm	K10574	ubiquitin-	mmu0412	Ubiquitin	11:519854	1660	2155	2682	2268	1976	1716	36.88741	40.13934	41.05635	32.8318	38.50345	47.27956	39.36103	39.53827	0.738674	1	-
ENSMUSC	Cdkn2aipl	CDKN2A i	GO:00508	response	-	-	-	-	11:519676	578	738	906	996	749	457	15.50597	16.59511	16.74368	17.40652	17.61958	15.20104	16.28158	16.74238	0.969146	1	-
ENSMUSC	Kremen1	kringle co	GO:00325	developm	-	-	-	-	11:519155	250	380	464	524	377	362	2.047458	2.608619	2.617847	2.79568	2.70744	3.675944	2.424641	3.059688	0.188894	1	-
ENSMUSC	Itk	IL2 inducil	GO:00325	developm	K07363	IL2-induci	mmu0406	Chemokir	11:463251	0	6	4	3	10	2	0	0.051885	0.028433	0.020161	0.090471	0.025591	0.026772	0.045408	0.477425	1	-
ENSMUSC	Nefh	neurofilar	GO:00325	developm	K04574	neurofilar	mmu0501	Amyotrop	11:493875	1274	1508	2107	2074	1536	1123	20.27204	20.11325	23.09638	21.49901	21.43196	22.15611	21.16056	21.69569	0.914009	1	-
ENSMUSC	Med7	mediator	GO:00325	developm	-	-	-	-	11:464369	110	268	382	225	365	134	0.97651	1.994214	2.336141	1.301211	2.841316	1.474944	1.768955	1.87249	0.912256	1	-
ENSMUSC	Havcr2	hepatitis A	GO:00325	developm	-	-	-	-	11:464549	76	69	122	173	90	63	0.973207	0.740614	1.076224	1.443174	1.010596	1.000275	0.930015	1.151348	0.356207	1	-
ENSMUSC	Tnip1	TNFAIP3 i	GO:00081	metabolic	-	-	-	-	11:549107	476	580	647	717	538	361	3.920583	4.004285	3.67113	3.8472	3.885697	3.686696	3.865332	3.806531	0.700327	1	-
ENSMUSC	Vdac1	voltage-d	GO:00325	developm	K05862	voltage-d	mmu0516	HTLV-I inf	11:523608	5409	6452	7013	6998	5297	6039	99.87166	99.85561	89.20321	84.17456	85.76259	138.2535	96.31016	102.7302	0.920488	1	-
ENSMUSC	Upp1	uridine ph	GO:00081	metabolic	K00757	uridine ph	mmu0110	Metabolic	11:911810	28	36	51	47	48	45	0.544182	0.586472	0.682822	0.595069	0.818042	1.084385	0.604492	0.832498	0.236078	1	-
ENSMUSC	Slu7	SLU7 splic	GO:00081	metabolic	K12819	pre-mRN.	mmu0304	Spliceosor	11:434337	1296	1495	2013	2062	1398	775	17.29623	16.72403	18.50726	17.92739	16.3605	12.82432	17.50917	15.70407	0.311646	1	-
ENSMUSC	Nipal4	NIPA-like	GO:00511	localizatio	-	-	-	-	11:461481	60	44	97	71	51	45	1.107188	0.680577	1.233098	0.853516	0.825252	1.029599	1.006954	0.902789	0.465739	1	-
ENSMUSC	Ascc2	activating	GO:00081	metabolic	-	-	-	-	11:463774	580	762	932	948	694	443	6.772132	7.457708	7.496617	7.210874	7.105589	6.41338	7.242152	6.909948	0.540534	1	-
ENSMUSC	Hus1	HUS1 che	GO:00325	developm	K10903	HUS1 che	mmu0421	Cellular se	11:899313	197	203	263	257	180	199	1.013024	0.874988	0.931667	0.860929	0.81165	1.268796	0.939893	0.980458	0.968562	1	-
ENSMUSC	Pttg1	pituitary t	GO:00081	metabolic	K06635	securin[-]	mmu0516	HTLV-I inf	11:434202	198	235	286	262	230	248	2.654192	2.640502	2.641091	2.287964	2.703558	4.121953	2.645262	3.037825	0.603515	1	-
ENSMUSC	Zfp607a	zinc finge	GO:00081	metabolic	-	-	-	-	7:2785752	46	33	77	52	45	36	0.321325	0.193225	0.370537	0.236634	0.275639	0.311801	0.295029	0.274692	0.630367	1	-
ENSMUSC	Tns3	tensin 3 [S	GO:00325	developm	-	-	-	-	11:843165	1369	1433	1999	2142	1472	1279	7.353906	6.452289	7.397395	7.495761	6.933702	8.51866	7.067863	7.649374	0.755512	1	-
ENSMUSC	Btg2	B cell tran	GO:00325	developm	K14443	protein Tc	mmu0301	RNA degr	1:1340751	148	233	303	310	198	259	3.429036	4.525002	4.836207	4.679005	4.022704	7.440391	4.263415	5.3807	0.299561	1	-
ENSMUSC	Gatsl3	GATS pro	GO:00508	response	-	-	-	-	11:421822	29	30	31	43	36	42	1.170929	1.015311	0.86228	1.13106	1.274617	2.102672	1.016173	1.502783	0.160281	1	-
ENSMUSC	Igfbp3	insulin-lik	GO:00325	developm	K10138	insulin-lik	mmu0520	Transcript	11:720608	328	455	447	359	336	284	5.510275	6.40714	5.173192	3.928939	4.949739	5.915662	5.696869	4.931447	0.192364	1	-
ENSMUSC	Igfbp1	insulin-lik	GO:00325	developm	-	-	-	-	11:719778	1	0	1	1	0	0	0.041683	0	0.028698	0.027145	0	0	0.02346	0.009048	1	1	-
ENSMUSC	Pes1	pescadillo	GO:00081	metabolic	-	-	-	-	11:396397	596	713	835	839	634	493	11.13719	11.16791	10.74898	10.21347	10.3887	11.42253	11.01803	10.6749	0.568329	1	-
ENSMUSC	Adcy1	adenylate	GO:00325	developm	K08041	adenylate	mmu0520	Pathways	11:706348	22255	23981	33709	40456	28636	20871	87.81648	79.31742	91.6317	103.9951	99.08398	102.1122	86.2552	101.7304	0.257483	1	-
ENSMUSC	Tcn2	transcoba	GO:00511	localizatio	K14619	transcoba	mmu0497	Vitamin d	11:391719	418	364	657	585	426	349	10.40546	7.595197	11.26685	9.486872	9.29902	10.77202	9.755834	9.852636	0.834831	1	-
ENSMUSC	4921536K	RIKEN cDI	-	-	-	-	-	-	11:388608	13	17	21	12	11	10	0.318985	0.349657	0.354984	0.191829	0.236689	0.30426	0.341209	0.24426	0.19302	1	-
ENSMUSC	Osbp2	oxysterol	GO:00325	developm	-	-	-	-	11:370373	1712	1947	2448	2659	1933	1311	13.40265	12.77632	13.20231	13.56085	13.26971	12.72553	13.12709	13.18537	0.790533	1	-
ENSMUSC	Gabrg2	gamma-a	GO:00325	developm	K05186	gamma-a	mmu0408	Neuroacti	11:419102	1866	2856	3092	3207	2437	1879	13.90595	17.84021	15.87376	15.56932	15.92528	17.36208	15.87331	16.28556	0.907396	1	-
ENSMUSC	Myo1g	myosin IG	GO:00400	locomotic	-	-	-	-	11:650654	9	14	22	14	21	17	0.062259	0.081181	0.104841	0.063093	0.127389	0.145811	0.08276	0.112097	0.487096	1	-
ENSMUSC	Smtn	smootheli	GO:00056	cell|intrac	-	-	-	-	11:351752	149	169	210	229	187	138	1.658972	1.577217	1.610727	1.661001	1.825727	1.905102	1.615639	1.797277	0.660841	1	-
ENSMUSC	Arf5	ADP-ribo	GO:00511	localizatio	K07940	ADP-ribo	mmu0414	Endocytos	6:2842356	1345	1561	1883	1874	1629	1751	35.4096	34.44726	34.15077	32.14032	37.60648	57.15718	34.66921	42.30133	0.283114	1	-
ENSMUSC	2310033P	RIKEN cDI	GO:00971	organic c	-	-	-	-	11:592083	222	266	293	308	228	156	10.97956	11.02727	9.982797	9.923515	9.888032	9.566274	10.66321	9.792607	0.407447	1	-
ENSMUSC	Guk1	guanylate	GO:00081	metabolic	-	-	mmu0110	Metabolic	11:591838	730	745	980	992	753	937	18.47618	15.80515	17.08705	16.35621	16.71194	29.40459	17.12279	20.82424	0.349856	1	-
ENSMUSC	Npc1l1	NPC1 like	GO:00081	metabolic	K14461	Niemann-	mmu0497	Fat digest	11:621101	10	12	7	14	9	6	0.142274	0.14311	0.068611	0.129758	0.112285	0.105851	0.117998	0.115965	1	1	-
ENSMUSC	Rnf185	ring finge	GO:00081	metabolic	K10666	E3 ubiquit	mmu0414	Protein pr	11:341598	535	664	834	762	550	437	10.48115	10.90376	11.25571	9.725056	9.448454	10.61507	10.88021	9.929526	0.299988	1	-
ENSMUSC	Limk2	LIM motif	GO:00048	signal trar	K05744	LIM doma	mmu0481	Regulatior	11:334425	1268	1662	1860	1968	1515	1794	10.88254	11.95625	10.99703	11.00317	11.40161	19.09059	11.27861	13.83179	0.277746	1	-
ENSMUSC	Patz1	POZ (BTB	GO:00325	developm	-	-	-	-	11:328887	462	392	610	485	487	408	2.495663	1.774939	2.269999	1.706742	2.306831	2.732692	2.1802	2.248755	0.918131	1	-
ENSMUSC	Eif4enif1	eukaryotic	GO:00325	developm	-	-	-	-	11:320239	1127	1149	1614	1565	1188	707	6.212529	5.309057	6.12914	5.620055	5.742532	4.832256	5.883575	5.398281	0.36126	1	-
ENSMUSC	Trim11	tripartite r	GO:00325	developm	-	-	-	-	11:589780	401	400	533	579	438	440	3.504023	2.929781	3.208496	3.295966	3.356132	4.76718	3.2141	3.806426	0.395225	1	-
ENSMUSC	Ogdh	oxoglutar	GO:00325	developm	K00164	2-oxoglut	mmu0110	Metabolic	11:629163	4758	5761	7230	7249	5652	3844	36.99807	37.54961	38.72972	36.72099	38.53891	37.06158	37.75913	37.44049	0.690782	1	-
ENSMUSC	Drg1	developm	GO:00329	macromo	-	-	-	-	11:318736	644	833	892	836	698	855	9.123525	9.891787	8.705484	7.715511	8.671114	15.01858	9.240265	10.4684	0.655087	1	-
ENSMUSC	Rtn4	reticulon 4	GO:00325	developm	K20720	reticulon-	mmu0501	Alzheimer	11:296929	9149	10120	12694	13795	8225	4848	57.0661	52.91002	54.54494	56.05417	44.9866	37.49326	54.84035	46.17801	0.140166	1	-
ENSMUSC	Mtif2	mitochon	GO:00081	metabolic	-	-	-	-	11:295264	474	534	698	807	598	333	4.661016	4.401457	4.728341	5.1696	5.156387	4.06006	4.596938	4.795349	0.940591	1	-
ENSMUSC	Rps27a	ribosomal	GO:00081	metabolic	K02977	small subu	mmu0301	Ribosome	11:295458	50	80	76	68	56	84	1.149667	1.541849	1.203828	1.018568	1.129085	2.394763	1.298448	1.514139	0.721044	1	-
ENSMUSC	Clhc1	clathrin he	-	-	-	-	-	-	11:295479	0	5	1	2	2	0	0	0.078244	0.012857	0.024322	0.032741	0	0.030367	0.019021	0.799738	1	-
ENSMUSC	Cfap36	cilia and f	GO:00056	cell|intrac	-	-	-	-	11:292215	1456	1821	2283	2063	1552	913	25.92692	27.18018	28.00573	23.93153	24.23394	20.15796	27.03761	22.77447	0.106972	1	-
ENSMUSC	Ppp4r3b	protein pl	GO:00081	metabolic	K17491	protein pl	mmu0492	Glucagon	11:291728	807	715	1061	969	720	474	6.970275	5.176487	6.313104	5.452323	5.453203	5.07622	6.153289	5.327248	0.188313	1	-
ENSMUSC	Pnpt1	polyribon	GO:00325	developm	K00962	polyribon	mmu0023	Purine me	11:291307	452	618	707	762	602	261	6.481485	7.428104	6.984043	7.118249	7.569648	4.640486	6.964544	6.442794	0.523096	1	-
ENSMUSC	Efemp1	epiderma	GO:00325	developm	-	-	-	-	11:288532	181	255	347	270	228	131	5.153714	6.086037	6.806478	5.008266	5.692707	4.624868	6.01541	5.108614	0.213716	1	-
ENSMUSC	Myl7	myosin, lic	GO:00452	synapse|s	K12754	myosin re	mmu0481	Regulatior	11:589663	0	0	0	0	0	2	0	0	0	0	0	0.267189	0	0.089063	0.536262	1	-
ENSMUSC	Pold2	polymeras	GO:00081	metabolic	K02328	DNA poly	mmu0110	Metabolic	11:587218	186	218	261	238	190	169	4.381341	4.304301	4.235323	3.652188	3.924546	4.935921	4.306988	4.170885	0.610171	1	-
ENSMUSC	Zkscan17	zinc finge	GO:00081	metabolic	-	-	-	-	11:594855	169	161	210	231	185	154	1.970008	1.573113	1.686359	1.75418	1.891013	2.225808	1.74316	1.957	0.647005	1	-
ENSMUSC	Aebp1	AE bindin	GO:00081	metabolic	-	-	-	-	11:586194	298	313	431	462	334	366	4.251139	3.742705	4.235621	4.293507	4.178083	6.473742	4.076488	4.981777	0.309055	1	-
ENSMUSC	Polm	polymeras	GO:00325	developm	K03513	DNA poly	mmu0345	Non-hom	11:582786	90	124	139	186	115	124	0.8361	0.965587	0.889578	1.12567	0.936824	1.428313	0.897088	1.163602	0.20833	1	-
ENSMUSC	Pgam2	phosphoc	GO:00081	metabolic	K01834	2,3-bisph	mmu0110	Metabolic	11:580164	141	154	146	158	108	74	10.66779	9.766285	7.609575	7.787434	7.165102	6.941878	9.347884	7.298138	0.107538	1	-
ENSMUSC	Dbnl	drebrin-lil	GO:00325	developm	-	-	-	-	11:578848	1297	1492	1727	2048	1521	1028	21.24439	20.48453	19.48714	21.85325	21.84622	20.87775	20.40535	21.52574	0.890014	1	-
ENSMUSC	Mrps24	mitochon	GO:00081	metabolic	-	-	-	-	11:570398	94	113	111	156	105	160	1.967061	1.98208	1.600169	2.126655	1.926741	4.151419	1.84977	2.734938	0.103371	1	-
ENSMUSC	Ccdc117	coiled-co	-	-	-	-	-	-	11:552888	263	293	371	400	256	226	4.956835	4.628806	4.816965	4.911232	4.230877	5.281329	4.800869	4.807813	0.789641	1	-
ENSMUSC	Dynll2	dynein lig	GO:00511	localizatio	K10418	dynein lig	mmu0496	Vasopress	11:879795	2736	5385	6513	6337	4935	4421	49.76561	82.10163	81.61044	75.08939	78.71237	99.70563	71.15923	84.50246	0.363003	1	-
ENSMUSC	Xbp1	X-box bin	GO:00325	developm	K09027	X box-bin	mmu0516	HTLV-I inf	11:552065	830	1127	1546	1416	1090	1061	11.71679	13.33544	15.03459	13.02193	13.49273	18.57086	13.36227	15.02851	0.584176	1	-
ENSMUSC	Supt4a	suppressc	GO:00081	metabolic	-	-	-	-	11:877375	288	385	410	434	349	353	12.31715	13.80163	12.0796	12.09173	13.08838	18.71881	12.73279	14.63297	0.541652	1	-
ENSMUSC	4-Sep	septin 4 [S	GO:00325	developm	K16943	septin 4[-	mmu0421	Apoptosis	11:875689	1704	1554	2595	2452	1752	1338	12.58944	9.623664	13.20765	11.80154	11.35046	12.25685	11.80692	11.80295	0.759861	1	-
ENSMUSC	Btnl10	butyrophi	GO:00160	membran	-	-	-	-	11:589179	0	2	0	2	0	0	0	0.0143	0	0.011113	0	0	0.004767	0.003704	1	1	-
ENSMUSC	2810021J	RIKEN cDI	GO:00081	metabolic	-	-	-	-	11:588672	200	210	277	252	232	151	3.200855	2.817139	3.053991	2.627357	3.255874	2.996396	3.023995	2.959875	0.697506	1	-
ENSMUSC	Ska2	spindle ar	GO:00990	supramol	-	-	-	-	11:871092	168	234	346	342	223	164	2.531875	2.955976	3.592197	3.357685	2.947	3.064514	3.026683	3.123066	0.985538	1	-
ENSMUSC	Prr11	proline ric	GO:00099	cellular pr	-	-	-	-	11:870891	21	17	32	33	20	9	0.349016	0.236823	0.366369	0.357281	0.291475	0.185453	0.317403	0.27807	0.641936	1	-
ENSMUSC	Smg8	smg-8 ho	GO:00081	metabolic	-	-	-	-	11:870777	299	349	381	397	343	246	4.486972	4.389968	3.938755	3.881097	4.513562	4.577243	4.271898	4.323967	0.845211	1	-
ENSMUSC	Rnf187	ring finge	GO:00081	metabolic	-	-	-	-	11:589322	4735	5817	6608	6929	5296	5659	128.49	132.3126	123.5295	122.4902	126.0201	190.4035	128.1107	146.3046	0.508235	1	-
ENSMUSC	Tubd1	tubulin, de	GO:00325	developm	-	-	-	-	11:865449	65	113	155	168	105	88	1.505987	2.194523	2.47397	2.535723	2.133253	2.528007	2.05816	2.398994	0.538975	1	-

ENSMUSC	Mrpl22	mitochon	GO:00081	metabolic	K02890	large subu	mmu0301	Ribosome	11:581716	223	264	325	303	223	113	11.02903	10.94436	11.07306	9.762408	9.671205	6.9294	11.01548	8.787671	0.12121	1	-
ENSMUSC	Cnot8	CCR4-NC	GO:00081	metabolic	K12581	CCR4-NC	mmu0301	RNA degr	11:581041	777	822	1244	1272	957	645	17.54821	15.56096	19.35459	18.71462	18.95251	18.06165	17.48792	18.57626	0.852354	1	-
ENSMUSC	Rps6kb1	ribosomal	GO:00325	developm	K04688	ribosomal	mmu0520	Pathways	11:864988	1218	1388	1753	1851	1250	588	7.148834	6.828575	7.087958	7.077437	6.4334	4.279088	7.021789	5.929975	0.205729	1	-
ENSMUSC	Sap30l	SAP30-lik	GO:00081	metabolic	-	-	-	-	11:578016	275	336	380	463	333	268	15.18423	15.5508	14.45427	16.65419	16.12305	18.34767	15.0631	17.04164	0.545091	1	-
ENSMUSC	Galnt10	polypeptic	GO:00081	metabolic	K00710	polypeptic	mmu0110	Metabolic	11:576454	148	218	235	246	171	209	1.479604	1.826806	1.618465	1.602142	1.499073	2.5907	1.641625	1.897305	0.587615	1	-
ENSMUSC	Rnft1	ring finge	GO:00081	metabolic	-	-	-	-	11:864846	239	344	406	331	227	157	3.06914	3.702793	3.591671	2.769035	2.556152	2.499786	3.454535	2.608324	0.03145	1	-
ENSMUSC	Mfap3	microfibri	GO:00160	membran	-	-	-	-	11:575186	616	671	870	935	617	434	3.662518	3.344062	3.563442	3.621532	3.216821	3.199444	3.523341	3.345932	0.511122	1	-
ENSMUSC	Fam114a2	family witl	-	-	-	-	-	-	11:574829	576	722	935	1075	807	504	8.939319	9.392312	9.99644	10.86857	10.98242	9.698374	9.442691	10.51645	0.575703	1	-
ENSMUSC	Gria1	glutamate	GO:00048	signal trar	K05197	glutamate	mmu0408	Neuroacti	11:570113	24821	28765	32245	38679	27450	17095	131.6186	127.8543	117.7909	133.6149	127.6389	112.3966	125.7546	124.5501	0.714262	1	-
ENSMUSC	Ppm1d	protein pł	GO:00081	metabolic	K10147	protein pł	mmu0411	p53 signa	11:853112	311	321	422	400	309	175	5.967692	5.163013	5.578405	5.000203	5.199333	4.163607	5.569703	4.787714	0.219563	1	-
ENSMUSC	Znhit3	zinc finge	GO:00056	cell|intrac	-	-	-	-	11:849109	225	280	301	224	239	217	4.442198	4.633673	4.093862	2.88101	4.137666	5.312045	4.389911	4.11024	0.472226	1	-
ENSMUSC	Myo19	myosin XI	GO:00325	developm	-	-	-	-	11:848801	104	147	195	198	173	116	0.643699	0.762641	0.831451	0.798358	0.938942	0.890212	0.74593	0.875837	0.458536	1	-
ENSMUSC	Prpsap2	phosphor	GO:00325	developm	-	-	-	-	11:617296	349	452	558	566	447	305	7.739012	8.401398	8.52404	8.176319	8.691808	8.385832	8.221483	8.417986	0.924181	1	-
ENSMUSC	Ggnbp2	gametoge	GO:00325	developm	-	-	-	-	11:848323	1035	1328	1575	1538	1035	748	8.389969	9.023419	8.795329	8.121907	7.357041	7.51809	8.736239	7.665679	0.174219	1	-
ENSMUSC	Acaca	acetyl-Co	GO:00081	metabolic	K11262	acetyl-Co	mmu0110	Metabolic	11:841296	1149	1315	1580	1724	1406	865	3.602308	3.455728	3.412475	3.521112	3.865351	3.362507	3.490171	3.58299	0.942463	1	-
ENSMUSC	Shmt1	serine hyc	GO:00451	translatior	K00600	glycine hy	mmu0110	Metabolic	11:607881	57	65	71	77	65	61	0.634305	0.606303	0.544295	0.558209	0.634279	0.841661	0.594968	0.678049	0.68023	1	-
ENSMUSC	Llgl1	lethal giar	GO:00325	developm	K06094	lethal(2) g	mmu0516	Human pa	11:606997	797	964	1290	1300	1027	1021	8.010714	8.121622	8.93211	8.512122	9.051613	12.72403	8.354815	10.09592	0.272937	1	-
ENSMUSC	Drg2	developm	GO:00160	membran	-	-	-	-	11:604545	549	785	871	948	706	627	10.44629	12.52023	11.4172	11.75113	11.77978	14.79256	11.46124	12.77449	0.615537	1	-
ENSMUSC	Srebf1	sterol regi	GO:00325	developm	K07197	sterol regi	mmu0493	Non-alco	11:601990	541	642	843	914	742	734	3.735977	3.716163	4.010382	4.111823	4.493172	6.284764	3.820841	4.963253	0.112489	1	-
ENSMUSC	Tom1l1	target of r	GO:00081	metabolic	-	-	-	-	11:906434	278	312	434	373	308	196	1.574806	1.481459	1.693647	1.376487	1.529941	1.376649	1.583304	1.427692	0.328459	1	-
ENSMUSC	Myocd	myocardi	GO:00325	developm	-	-	-	-	11:651765	9	19	10	15	15	6	0.046509	0.082302	0.035601	0.050498	0.067972	0.038448	0.054804	0.052306	0.868225	1	-
ENSMUSC	Cox11	cytochron	GO:00081	metabolic	K02258	cytochron	mmu0110	Metabolic	11:906381	113	124	184	123	115	97	3.838306	3.530507	4.305588	2.721757	3.425341	4.085245	3.891467	3.410781	0.288779	1	-
ENSMUSC	Stxbp4	syntaxin b	GO:00511	localizatio	-	-	-	-	11:904764	475	568	733	729	592	337	2.343762	2.349207	2.491584	2.343307	2.561442	2.061752	2.394851	2.322167	0.647824	1	-
ENSMUSC	Bzw2	basic leuc	GO:00325	developm	-	-	-	-	12:360918	494	675	735	756	537	425	4.669823	5.348469	4.78643	4.655609	4.451333	4.981361	4.934907	4.696101	0.49436	1	-
ENSMUSC	1700086D	RIKEN cDI	-	-	-	-	-	-	11:651519	3	1	1	2	5	0	0.170974	0.047774	0.039251	0.074254	0.249886	0	0.086	0.108046	0.815491	1	-
ENSMUSC	Elac2	elaC ribor	GO:00081	metabolic	K00784	ribonuclea	mmu0301	RNA trans	11:649790	483	607	720	751	566	376	4.579459	4.824012	4.702736	4.638615	4.705719	4.420193	4.702069	4.588176	0.649731	1	-
ENSMUSC	Pctp	phosphati	GO:00082	lipid bindi	-	-	-	-	11:899826	28	26	40	53	35	14	0.653498	0.50864	0.64313	0.805837	0.716314	0.405153	0.601756	0.642434	0.898013	1	-
ENSMUSC	Twistnb	twist basic	GO:00081	metabolic	K03004	DNA-dire	mmu0110	Metabolic	12:334296	218	288	298	324	234	135	2.489584	2.756872	2.344439	2.410448	2.343311	1.911573	2.530299	2.221777	0.30482	1	-
ENSMUSC	Efcab10	EF-hand c	GO:00431	ion bindin	-	-	-	-	12:333948	6	18	25	17	8	9	0.566618	1.424786	1.626315	1.045795	0.662483	1.053745	1.205906	0.920674	0.3481	1	-
ENSMUSC	Atxn7l1	ataxin 7-li	-	-	-	-	-	-	12:331476	554	664	782	918	591	385	2.358862	2.369811	2.293776	2.546344	2.206596	2.032542	2.340816	2.261827	0.624866	1	-
ENSMUSC	Atp6v1c2	ATPase, H	GO:00511	localizatio	K02148	V-type H-	mmu0110	Metabolic	12:172847	12	6	11	18	13	5	0.205112	0.085963	0.129528	0.200435	0.194856	0.105964	0.140201	0.167085	0.710662	1	-
ENSMUSC	Pdia6	protein di	GO:00081	metabolic	K09584	protein di	mmu0414	Protein pr	12:172665	1405	1800	2674	2461	1852	1597	19.97137	21.44652	26.1845	22.78897	23.08422	28.14637	22.53413	24.67318	0.694873	1	-
ENSMUSC	Nampt	nicotinam	GO:00081	metabolic	K03462	nicotinam	mmu0110	Metabolic	12:328195	1358	1698	2057	2024	1512	846	16.25635	17.03781	16.96325	15.78394	15.8715	12.55682	16.75247	14.73742	0.21971	1	-
ENSMUSC	Pik3cg	phosphati	GO:00325	developm	K00922	phosphati	mmu0415	PI3K-Akt s	12:321734	54	53	75	113	65	35	0.211167	0.173725	0.202041	0.287866	0.222887	0.169702	0.195644	0.226818	0.55958	1	-
ENSMUSC	Nbas	neuroblas	GO:00081	metabolic	-	-	-	-	12:132691	783	765	988	1181	788	628	4.678639	3.831531	4.066926	4.597158	4.128827	4.652682	4.192365	4.459556	0.863204	1	-
ENSMUSC	Tspan13	tetraspani	GO:00511	localizatio	-	-	-	-	12:360145	2961	3235	4004	3765	3247	3174	66.82545	61.19707	62.25138	55.35413	64.25818	88.81704	63.42463	69.47645	0.738783	1	-
ENSMUSC	Rock2	Rho-asso	GO:00325	developm	K17388	Rho-asso	mmu0520	Pathways	12:168948	7220	8574	10612	11977	8129	3370	31.38313	31.23887	31.77662	33.91476	30.9841	18.16247	31.46621	27.68711	0.350722	1	-
ENSMUSC	Matn3	matrilin 3	GO:00055	extracellul	-	-	-	-	12:894792	0	2	0	0	0	0	0	0.035347	0	0	0	0	0.011782	0	0.53623	1	-
ENSMUSC	Laptm4a	lysosomal	GO:00160	membran	K12387	lysosomal	mmu0414	Lysosome	12:892166	1928	2532	3304	3072	2177	2558	69.10897	76.07534	81.5866	71.73487	68.42717	113.6878	75.5903	84.61661	0.638964	1	-
ENSMUSC	Fam49a	family witl	GO:00056	cell|intrac	-	-	-	-	12:122621	6555	7659	9661	10276	7334	3573	29.42851	28.82176	29.87921	30.05393	28.87215	19.88907	29.37649	26.27172	0.322215	1	-
ENSMUSC	Snx13	sorting ne	GO:00511	localizatio	-	-	-	-	12:350471	1658	2010	2633	2806	1969	1145	10.69321	10.86607	11.69838	11.78943	11.13556	9.156194	11.08589	10.69373	0.603554	1	-
ENSMUSC	Ntsr2	neurotens	GO:00048	signal trar	K04212	neurotens	mmu0408	Neuroacti	12:166533	815	979	1158	1381	1095	885	7.46766	7.519044	7.309488	8.243325	8.797995	10.05441	7.432064	9.031909	0.204963	1	-
ENSMUSC	Sdc1	syndecan	GO:00325	developm	K06257	syndecan	mmu0520	Proteogly	12:877132	17	26	44	26	16	25	0.167786	0.2151	0.299175	0.167173	0.138476	0.305941	0.227354	0.203864	0.548208	1	-
ENSMUSC	Lpin1	lipin 1 [So	GO:00325	developm	K15728	phosphati	mmu0110	Metabolic	12:165356	346	411	755	717	445	410	1.387692	1.381693	2.086004	1.873343	1.565018	2.038854	1.618463	1.825738	0.601195	1	-
ENSMUSC	Pum2	pumilio Rl	GO:00081	metabolic	-	-	-	-	12:867413	2577	3005	4301	5369	4210	2432	8.808955	8.610084	10.12817	11.95598	12.61929	10.30765	9.182402	11.62764	0.104109	1	-
ENSMUSC	Nrcam	neuronal c	GO:00325	developm	K06756	neuronal c	mmu0451	Cell adhes	12:443288	8405	9874	10421	13435	9778	5782	22.69461	22.34761	19.38411	23.63221	23.15142	19.35749	21.47544	22.04704	0.953609	1	-
ENSMUSC	Rgs9	regulator	GO:00325	developm	K13765	regulator	mmu0503	Cocaine a	11:109225	232	218	314	295	261	181	1.993277	1.569961	1.858497	1.65114	1.966364	1.928174	1.807245	1.848559	0.912932	1	-
ENSMUSC	Trib2	tribbles ps	GO:00325	developm	-	-	-	-	12:157917	682	922	1186	1217	873	712	6.883142	7.799848	8.245911	8.001556	7.726079	8.909807	7.642967	8.212481	0.80109	1	-
ENSMUSC	Arsg	arylsulfata	GO:00081	metabolic	K12381	arylsulfata	mmu0414	Lysosome	11:109473	331	213	309	332	232	259	3.807433	2.053695	2.448578	2.487847	2.340104	3.693935	2.769902	2.840629	0.891435	1	-
ENSMUSC	Hs1bp3	HCLS1 bir	GO:00325	developm	-	-	-	-	12:831343	223	271	333	337	239	221	4.298545	4.378641	4.42194	4.23184	4.039777	5.281974	4.366376	4.517864	0.950655	1	-
ENSMUSC	Fam84a	family witl	-	-	-	-	-	-	12:141468	1815	2077	2916	3224	2249	1544	25.43522	24.39766	28.15125	29.43306	27.637	26.82823	25.99471	27.9661	0.7556	1	-
ENSMUSC	Smc6	structural	GO:00325	developm	-	-	-	-	12:112658	1112	1286	1713	1877	1182	502	4.148817	4.021735	4.402793	4.562106	3.867049	2.32225	4.191115	3.583802	0.294953	1	-
ENSMUSC	Apob	apolipopr	GO:00325	developm	K14462	apolipopr	mmu0497	Cholestere	12:797764	1	3	2	0	0	0	0.003599	0.009048	0.004958	0	0	0	0.005868	0	0.045131	1	-
ENSMUSC	Amz2	archaelysi	GO:00081	metabolic	-	-	-	-	11:109425	654	787	1047	1147	869	714	5.158703	5.20344	5.689326	5.893966	6.010701	6.983083	5.350489	6.295917	0.327611	1	-
ENSMUSC	Gna13	guanine n	GO:00325	developm	K04639	guanine n	mmu0520	Pathways	11:109362	797	1009	1264	1307	964	1088	7.888451	8.371	8.618504	8.427342	8.366677	13.35206	8.292652	10.04869	0.295419	1	-
ENSMUSC	Prkar1a	protein kii	GO:00325	developm	K04739	cAMP-de	mmu0491	Insulin sig	11:109649	11342	14224	16465	16840	13178	11666	158.1435	166.2403	158.1521	152.9627	161.1217	201.6832	160.8453	171.9225	0.842986	1	-
ENSMUSC	Fam20a	family witl	GO:00325	developm	-	-	-	-	11:109669	190	217	280	331	264	163	2.104769	2.014945	2.136785	2.388697	2.564471	2.238849	2.0855	2.397339	0.483631	1	-
ENSMUSC	Abca8b	ATP-bind	GO:00511	localizatio	K05650	ATP-bind	mmu0201	ABC trans	11:109932	599	744	920	972	772	451	4.822433	5.020709	5.102444	5.097849	5.450033	4.501954	4.981862	5.016612	0.845986	1	-
ENSMUSC	Rdh14	retinol del	GO:00325	developm	-	-	-	-	12:103907	299	417	494	411	372	319	12.83941	15.0094	14.61345	11.49735	14.00747	16.98442	14.15409	14.16308	0.751247	1	-
ENSMUSC	Map2k6	mitogen-a	GO:00048	signal trar	K04433	mitogen-a	mmu0401	MAPK sigi	11:110399	316	332	414	513	324	207	5.438062	4.789034	4.90805	5.751161	4.889281	4.416853	5.045049	5.019098	0.811294	1	-
ENSMUSC	Klhl29	kelch-like	GO:00081	metabolic	-	-	-	-	12:507747	1231	1511	1911	1932	1475	914	10.49976	10.80286	11.22881	10.7352	11.03205	9.666155	10.84381	10.4778	0.578296	1	-
ENSMUSC	Trappc12	trafficking	GO:00081	metabolic	-	-	-	-	12:286906	963	1199	1317	1512	1108	1046	5.066771	5.287825	4.773558	5.182492	5.11196	6.823744	5.042718	5.706065	0.528241	1	-
ENSMUSC	Adi1	acireducto	GO:00081	metabolic	K08967	1,2-dihyd	mmu0110	Metabolic	12:286752	186	230	395	298	235	259	4.042701	4.190245	5.914347	4.219464	4.478881	6.97982	4.715764	5.226055	0.76776	1	-
ENSMUSC	Rnaseh1	ribonuclea	GO:00081	metabolic	K03469	ribonuclea	mmu0303	DNA repli	12:286496	102	162	178	206	153	117	1.540868	2.051314	1.852398	2.027277	2.026743	2.191461	1.81486	2.081827	0.554371	1	-
ENSMUSC	Dcdc2c	doublecor	GO:00508	response	-	-	-	-	12:284377	0	0	0	1	6	0	0	0	0	0.007959	0.064285	0	0	0.024081	0.046718	1	-
ENSMUSC	Ubxn2a	UBX dom	GO:00081	metabolic	-	-	-	-	12:487903	662	849	1036	1057	789	446	8.921135	9.590098	9.617751	9.279382	9.323565	7.452173	9.376328	8.68504	0.430336	1	-
ENSMUSC	Fkbp1b	FK506 bin	GO:00325	developm	-	-	-	-	12:483317	390	475	528	553	399	301	23.36066	23.84885	21.78744	21.57883	20.95733	22.35495	22.99898	21.63037	0.456776	1	-
ENSMUSC	Allc	allantoica	GO:00081	metabolic	K01477	allantoica	mmu0110	Metabolic	12:285537	0	0	4	2	0	0	0	0	0.106407	0.050299	0	0	0.035469	0.016766	1	1	-
ENSMUSC	Cmpk2	cytidine m	GO:00081	metabolic	K13809	UMP-CM	mmu0110	Metabolic	12:264692	265	378	459	432	397	271	3.366284	4.024854	4.016706	3.574959	4.422202	4.268355	3.802615	4.088506	0.820801	1	-
ENSMUSC	Pfn4	profilin fa	GO:00511	localizatio	K05759	profilin[-]	mmu0481	Regulatior	12:476902	115	134	74	183	148	59	1.006556	0.983096	0.446193	1.043454	1.135908	0.640293	0.811948	0.939885	0.628793	1	-
ENSMUSC	Itsn2	intersectir	GO:00325	developm	-	-	-	-	12:459263	1778	2119	2735	2988	2064	1192	6.164273	6.157915	6.532189	6.748577	6.274835	5.124037	6.284792	6.04915	0.595947	1	-
ENSMUSC	Rnf144a	ring finge	GO:00081	metabolic	-	-	-	-	12:263009	116	477	590	581	133	344	0.595106	2.051192	2.085154	1.941747	0.598314	2.188164	1.577151	1.576075	0.878185	1	-
ENSMUSC	Id2	inhibitor c	GO:00325	developm	K17693	DNA-binc	mmu0520	Transcript	12:250937	657	908	1212	990	911	754	10.10266	11.70332	12.83881	9.917164	12.28377	14.37567	11.54827	12.1922	0.944115	1	-
ENSMUSC	Mboat2	membran	GO:00081	metabolic	K13517	lysophosp	mmu0110	Metabolic	12:248308	1068	1244	1604	1777	1268	815	4.866609	4.751478	5.035135	5.275023	5.066605	4.604674	4.884407	4.982101	0.909602	1	-
ENSMUSC	Ncoa1	nuclear re	GO:00325	developm	K09101	nuclear re	mmu0520	Pathways	12:424736	3510	4126	5003	5741	4003	2556	18.7565	18.48105	18.41733	19.98545	18.75741	16.93525	18.55163	18.55937	0.779237	1	-
ENSMUSC	Dus4l	dihydrour	GO:00081	metabolic	-	-	-	-	12:316400	61	122	110	132	86	57	1.681145	2.818319	2.088442	2.369911	2.078357	1.947757	2.195968	2.132009	0.761054	1	-
ENSMUSC	Rrm2	ribonuclec	GO:00081	metabolic	K10808	ribonuclec	mmu0110	Metabolic	12:247082	23	26	34	32	23	20	0.393038	0.372419	0.40026	0.356238	0.344655	0.423765	0.388572	0.374886	0.774888	1	-
ENSMUSC	Bcap29	B cell rece	GO:00325	developm	-	-	-	-	12:315909	449	602	773	746	360	424	3.179421	3.573149	3.77079	3.441299	2.235354	3.722664	3.507787	3.133106	0.29591	1	-
ENSMUSC	Slc26a4	solute car	GO:00325	developm	K14702	solute car	mmu0491	Thyroid hc	12:315198	224	266	272	309	254	234	4.578922	4.557748	3.830327	4.114864	4.552933	5.930857	4.322333	4.866218	0.642741	1	-
ENSMUSC	Cenpo	centrome	GO:00329	macromo	-	-	-	-	12:419600	35	53	76	79	57	55	0.217224	0.27572	0.32494	0.319406	0.310207	0.42324	0.272628	0.350951	0.308005	1	-
ENSMUSC	Klf11	Kruppel-li	GO:00081	metabolic	-	-	-	-	12:246512	58	71	111	66	91	106	0.729918	0.748951	0.962319	0.541095	1.004221	1.65401	0.813729	1.066442	0.400985	1	-
ENSMUSC	Adcy3	adenylate	GO:00081	metabolic	K08043	adenylate	mmu0474	Olfactory	12:413310	957	1059	1289	1413	988	1073	6.034953	5.597714	5.599719	5.804778	5.463379	8.38972	5.744129	6.552626	0.528162	1	-
ENSMUSC	Grhl1	grainyhea	GO:00325	developm	-	-	-	-	12:245722	614	841	840	1103	793	406	5.335183	6.125336	5.028193	6.243648	6.042224	4.374143	5.496237	5.553339	0.903081	1	-
ENSMUSC	Dnajc27	DnaJ heat	GO:00081	metabolic	-	-	-	-	12:408258	1901	2527	2904	3058	2316	2122	15.93856	17.75927	16.77316	16.70268	17.02739	22.05963	16.82366	18.59657	0.639475	1	-
ENSMUSC	Efr3b	EFR3 hom	GO:00081	metabolic	-	-	-	-	12:396255	3430	4094	4779	1455	4066	3552	14.21499	14.22175	13.64397	3.92823	14.77619	18.25204	14.0269	12.31882	0.447485	1	-
ENSMUSC	Cbll1	Casitas B-	GO:00081	metabolic	-	-	-	-	12:314848	564	87	157	322	114	268	3.784596	0.489341	0.725755	1.407597	0.670794	2.229771	1.666564	1.436054	0.598568	1	-
ENSMUSC	Pomc	pro-opior	GO:00081	metabolic	K05228	proopiom	mmu0492	Aldostero	12:395495	17	14	25	20	11	7	0.343959	0.237443	0.348459	0.263626	0.195169	0.175618	0.309954	0.211471	0.182185	1	-
ENSMUSC	Dnmt3a	DNA metl	GO:00325	developm	K17398	DNA (cytc	mmu0110	Metabolic	12:380600	1141	1738	2076	2413	1834	1496	2.481911	3.16886	3.110851	3.419317	3.498182	4.034762	2.920541	3.650754	0.137379	1	-
ENSMUSC	Dld	dihydrolip	GO:00325	developm	K00382	dihydrolip	mmu0110	Metabolic	12:313312	1861	2153	2789	2716	2012	1263	28.14663	27.29461	29.05895	26.76033	26.68399	23.68479	28.16673	25.7097	0.303973	1	-
ENSMUSC	Kif3c	kinesin fai	GO:00511	localizatio	-	-	-	-	12:336513	3802	4418	5342	4236	4421	3280	17.87193	17.40756	17.29875	12.9717	18.22311	19.11698	17.52608	16.7706	0.478378	1	-
ENSMUSC	Sh3yl1	Sh3 doma	GO:00081	metabolic	-	-	-	-	12:309116	155	222	242	258	175	109	2.296211	2.756674	2.469713	2.489886	2.273311	2.002117	2.507533	2.255105	0.39626	1	-
ENSMUSC	Rab10	RAB10, m	GO:00325	developm	K07903	Ras-relate	mmu0414	Endocytos	12:324742	3754	4665	5658	5747	4063	2805	67.9129	70.73958	70.51352	67.72996	64.45364	62.91828	69.722	65.03396	0.361481	1	-
ENSMUSC	Sntg2	syntrophir	GO:00051	structural	-	-	-	-	12:301744	7	34	43	36	33	29	0.073757	0.30027	0.312099	0.247094	0.304886	0.378838	0.228708	0.310273	0.429089	1	-
ENSMUSC	Tpo	thyroid pe	GO:00325	developm	K00431	thyroid pe	mmu0110	Metabolic	12:300546	0	4	0	1	0	0	0	0.046553	0	0.009044	0	0	0.015518	0.003015	0.455459	1	-
ENSMUSC	Pxdn	peroxidas	GO:00081	metabolic	-	-	-	-	12:299376	1450	1836	2129	2358	1812	1439	5.075559	5.386932	5.133847	5.377016	5.561819	6.245435	5.198779	5.72809	0.633019	1	-
ENSMUSC	Ccl11	chemokin	GO:00325	developm	K16597	C-C motif	mmu0406	Cytokine-	11:820578	2	0	0	3	3	0	0.117497	0	0	0.114778	0.154504	0	0.039166	0.089761	0.359614	1	-
ENSMUSC	Ddx52	DEAD (As	GO:00081	metabolic	-	-	-	-	11:839420	336	405	545	529	339	228	6.116825	6.180075	6.83493	6.273692	5.41164	5.146433	6.377276	5.610589	0.259541	1	-
ENSMUSC	Hnf1b	HNF1 hor	GO:00325	developm	K08034	transcripti	-	-	11:838500	7	7	14	13	19	0	0.083361	0.069867	0.114844	0.10085	0.198392	0	0.089357	0.099747	0.888882	1	-
ENSMUSC	Taf15	TATA-box	GO:00081	metabolic	K14651	transcripti	mmu0520	Transcript	11:834730	1200	1401	1618	1810	1299	701	20.36412	19.92853	18.91534	20.00988	19.33019	14.74986	19.736	18.02998	0.381253	1	-
ENSMUSC	Ace	angiotens	GO:00325	developm	K01283	peptidyl-c	mmu0514	Chagas di	11:105967	611	352	1067	484	519	366	5.728115	2.766089	6.891067	2.955955	4.266582	4.254391	5.128424	3.825643	0.092876	1	-
ENSMUSC	Mmp28	matrix me	GO:00081	metabolic	-	-	-	-	11:834407	35	42	57	60	52	46	0.371224	0.373393	0.41648	0.414571	0.483627	0.60494	0.387032	0.501046	0.301707	1	-
ENSMUSC	Rasl10b	RAS-like,	GO:00511	localizatio	-	-	-	-	11:834091	1126	1137	1456	1384	1158	1008	7.742987	6.553651	6.897362	6.199945	6.982667	8.594425	7.064666	7.259013	0.886162	1	-
ENSMUSC	Gas2l2	growth ar	GO:00990	supramole	-	-	-	-	11:834219	8	7	19	6	5	7	0.181249	0.132933	0.296554	0.088563	0.099331	0.196654	0.203579	0.128183	0.195685	1	-
ENSMUSC	Cdc27	cell divisic	GO:00081	metabolic	K03350	anaphase	mmu0516	HTLV-I inf	11:104502	1340	1650	2092	2176	1487	900	11.10313	11.45981	11.94136	11.74577	10.80424	9.246322	11.50143	10.59878	0.376187	1	-
ENSMUSC	Itgb3	integrin b	GO:00325	developm	K06493	integrin b	mmu0415	PI3K-Akt s	11:104608	40	46	52	92	58	42	0.2944	0.283781	0.263652	0.441107	0.374319	0.383278	0.280611	0.399568	0.093663	1	-
ENSMUSC	Mettl2	methyltrai	GO:00081	metabolic	-	-	-	-	11:105126	186	259	307	327	233	166	2.922338	3.410904	3.322823	3.34693	3.210086	3.233794	3.218688	3.263603	0.886121	1	-
ENSMUSC	Nle1	notchless	GO:00325	developm	-	-	-	-	11:829007	85	132	140	127	116	111	1.04832	1.364589	1.189476	1.020375	1.254519	1.697404	1.200795	1.324099	0.808779	1	-
ENSMUSC	Tlk2	tousled-lil	GO:00325	developm	-	-	-	-	11:105178	946	1167	1526	1481	1127	618	4.97939	5.148832	5.533384	5.07834	5.201776	4.033286	5.220535	4.771134	0.365953	1	-
ENSMUSC	Mrc2	mannose	GO:00325	developm	K06560	mannose	mmu0515	Tuberculo	11:105292	118	159	207	241	139	152	0.970147	1.09574	1.172405	1.290788	1.00211	1.549478	1.079431	1.280792	0.44882	1	-
ENSMUSC	Rffl	ring finge	GO:00081	metabolic	-	-	-	-	11:828024	125	100	212	174	134	158	0.839672	0.563058	0.981038	0.761432	0.789307	1.315961	0.794589	0.955567	0.509099	1	-
ENSMUSC	Lig3	ligase III, [	GO:00081	metabolic	K10776	DNA ligas	mmu0341	Base excis	11:827811	288	349	682	778	337	404	1.113948	1.13149	1.817222	1.96035	1.142997	1.937492	1.35422	1.68028	0.331868	1	-
ENSMUSC	Cct6b	chaperoni	GO:00511	localizatio	-	-	-	-	11:827192	2	0	4	1	2	0	0.060916	0	0.083922	0.019835	0.053402	0	0.04828	0.024412	0.566035	1	-
ENSMUSC	Map3k3	mitogen-a	GO:00325	developm	K04421	mitogen-a	mmu0401	MAPK sigi	11:106084	543	534	697	853	561	444	7.392731	6.093962	6.537168	7.565467	6.697474	7.49505	6.67462	7.252664	0.742411	1	-
ENSMUSC	Tmem132	transmem	GO:00160	membran	-	-	-	-	11:823889	167	175	150	189	167	190	1.277025	1.12169	0.79018	0.941511	1.119806	1.801453	1.062965	1.28759	0.481954	1	-
ENSMUSC	5530401A	RIKEN cDI	-	-	-	-	-	-	11:818606	0	0	2	0	0	0	0	0	0.015951	0	0	0	0.005317	0	0.537335	1	-
ENSMUSC	Asic2	acid-sens	GO:00325	developm	K04828	acid-sens	mmu0475	Inflammat	11:808801	1191	1290	1597	1837	1380	1075	17.75131	16.11616	16.39741	17.83651	18.03602	19.86612	16.75496	18.57955	0.596829	1	-
ENSMUSC	Ddx42	DEAD (As	GO:00081	metabolic	K12835	ATP-depe	mmu0304	Spliceosor	11:106216	2300	2793	3187	3693	2563	1456	32.80339	33.38987	31.31298	34.31246	32.054	25.74768	32.50208	30.70472	0.500537	1	-
ENSMUSC	Ftsj3	FtsJ RNA r	GO:00081	metabolic	-	-	-	-	11:106249	581	773	937	932	734	477	6.418265	7.15771	7.130714	6.707172	7.11018	6.533503	6.90223	6.783618	0.682932	1	-
ENSMUSC	Rnf135	ring finge	GO:00081	metabolic	-	-	-	-	11:801838	41	41	58	68	51	50	1.199102	1.00511	1.168574	1.295592	1.307947	1.813161	1.124262	1.472233	0.261418	1	-
ENSMUSC	Psmc5	protease (	GO:00081	metabolic	K03066	26S prote	mmu0516	Epstein-B	11:106256	2178	2557	3266	3439	2511	1649	86.29581	84.92103	89.1455	88.76578	87.2411	81.00996	86.78745	85.67228	0.686927	1	-
ENSMUSC	Adap2	ArfGAP w	GO:00325	developm	-	-	-	-	11:801541	65	66	131	148	77	61	1.98316	1.687884	2.753411	2.941642	2.060066	2.307597	2.141485	2.436435	0.629909	1	-
ENSMUSC	Tcam1	testicular	GO:00160	membran	-	-	-	-	11:106276	2	4	15	9	5	3	0.023613	0.039575	0.121978	0.069207	0.05175	0.043911	0.061722	0.054956	0.803089	1	-
ENSMUSC	Ern1	endoplasr	GO:00081	metabolic	K08852	serine/thr	mmu0501	Alzheimer	11:106394	226	331	358	430	300	245	1.350668	1.658137	1.473918	1.674133	1.572185	1.815483	1.494241	1.687267	0.567725	1	-
ENSMUSC	Nf1	neurofibrc	GO:00325	developm	K08052	neurofibrc	mmu0401	MAPK sigi	11:793396	2813	3015	3657	4496	3153	1850	8.91911	8.012939	7.987818	9.286663	8.766342	7.272928	8.306622	8.441978	0.899357	1	-
ENSMUSC	Pecam1	platelet/ei	GO:00325	developm	K06471	platelet/ei	mmu0451	Cell adhes	11:106654	320	347	525	518	382	355	2.267473	2.060985	2.562732	2.391132	2.373548	3.118933	2.297063	2.627871	0.521483	1	-
ENSMUSC	Polg2	polymeras	GO:00325	developm	K02333	DNA poly	mmu0110	Metabolic	11:106768	32	28	46	34	42	41	0.325388	0.23865	0.322227	0.225222	0.374495	0.516919	0.295422	0.372212	0.502545	1	-
ENSMUSC	Ddx5	DEAD (As	GO:00325	developm	K12823	ATP-depe	mmu0520	Proteogly	11:106780	15775	18430	24090	24072	18903	9237	105.5426	103.3562	111.0316	104.9185	110.9	76.62582	106.6434	97.48145	0.37495	1	-
ENSMUSC	Psmd12	proteasor	GO:00081	metabolic	K03035	26S prote	mmu0516	Epstein-B	11:107479	1072	1358	1573	1773	1177	776	21.80819	23.15676	22.04475	23.49716	20.99638	19.57369	22.33657	21.35574	0.537849	1	-
ENSMUSC	Helz	helicase w	GO:00038	catalytic a	-	-	-	-	11:107547	1747	2017	2434	2824	2018	1229	6.387476	6.181519	6.130676	6.726399	6.469938	5.571526	6.233224	6.255954	0.813742	1	-
ENSMUSC	Cacng1	calcium cł	GO:00511	localizatio	K04866	voltage-d	mmu0401	MAPK sigi	11:107703	0	0	0	3	0	0	0	0	0	0.099431	0	0	0	0.033144	0.303001	1	-
ENSMUSC	Cacng4	calcium cł	GO:00511	localizatio	K04869	voltage-d	mmu0401	MAPK sigi	11:107732	422	508	595	694	461	339	7.093202	7.157243	6.889659	7.599249	6.794742	7.065041	7.046701	7.153011	0.876797	1	-
ENSMUSC	Cep112	centroson	GO:00452	synapse|lc	-	-	-	-	11:108425	74	93	106	128	74	47	0.524233	0.552241	0.517311	0.590729	0.459697	0.412839	0.531262	0.487755	0.561862	1	-
ENSMUSC	Rab37	RAB37, m	GO:00038	catalytic a	-	-	-	-	11:115091	51	57	64	88	67	30	0.574576	0.538282	0.496718	0.645866	0.661907	0.419078	0.536525	0.575617	0.84469	1	-
ENSMUSC	Slc9a3r1	solute car	GO:00325	developm	K13365	Na(+)/H(+	mmu0516	Human pa	11:115163	763	1002	1146	1221	1037	765	25.1901	27.72847	26.06403	26.26048	30.02112	31.31501	26.32753	29.19887	0.609202	1	-
ENSMUSC	Grin2c	glutamate	GO:00048	signal trar	K05211	glutamate	mmu0408	Neuroacti	11:115249	676	724	1001	1140	776	702	7.881455	7.075409	8.039806	8.658579	7.933491	10.14807	7.665557	8.913378	0.394766	1	-
ENSMUSC	Nt5c	5',3'-nucle	GO:00081	metabolic	K01081	5'-nucleot	mmu0110	Metabolic	11:115490	206	324	389	349	281	260	4.650774	6.131352	6.050044	5.132921	5.562963	7.278071	5.610723	5.991318	0.903654	1	-
ENSMUSC	Jpt1	Jupiter mi	GO:00160	membran	-	-	-	-	11:115497	488	722	686	749	573	686	9.886479	12.26061	9.574088	9.885192	10.17933	17.23185	10.57372	12.43212	0.479931	1	-
ENSMUSC	Sumo2	small ubic	GO:00081	metabolic	K12160	small ubic	mmu0301	RNA trans	11:115523	272	379	98	432	339	437	7.041311	8.223876	1.747679	7.285345	7.69533	14.02659	5.670955	9.669088	0.10085	1	-
ENSMUSC	Nup85	nucleopoi	GO:00081	metabolic	K14304	nuclear pc	mmu0301	RNA trans	11:115564	291	404	448	444	365	356	3.762751	4.378713	3.990631	3.740044	4.138552	5.707542	4.044032	4.528713	0.656472	1	-
ENSMUSC	Gga3	golgi assc	GO:00081	metabolic	K12404	ADP-ribos	mmu0414	Lysosome	11:115584	638	781	1377	1054	771	1067	3.876367	3.977481	5.763547	4.171829	4.107728	8.038123	4.539132	5.439227	0.463171	1	-
ENSMUSC	Cluh	clustered	GO:00511	localizatio	-	-	-	-	11:746494	1782	2055	2429	2858	2071	1862	12.92235	12.49103	12.13424	13.50136	13.16912	16.74169	12.51588	14.47072	0.388238	1	-
ENSMUSC	Mif4gd	MIF4G do	GO:00081	metabolic	-	-	-	-	11:115607	139	190	260	181	206	123	1.853909	2.124122	2.388897	1.572651	2.409261	2.034064	2.122309	2.005325	0.572209	1	-
ENSMUSC	Slc25a19	solute car	GO:00511	localizatio	-	-	-	-	11:115614	432	552	656	565	471	511	3.254489	3.485709	3.404511	2.77287	3.111453	4.773169	3.38157	3.552498	0.989939	1	-
ENSMUSC	Pafah1b1	platelet-a	GO:00325	developm	K16794	platelet-a	mmu0110	Metabolic	11:746739	8618	10107	13143	13138	9414	7776	22.76675	22.3805	23.91887	22.61026	21.80778	25.47046	23.02204	23.29617	0.7931	1	-
ENSMUSC	Tmem94	transmem	GO:00160	membran	-	-	-	-	11:115765	984	1029	1292	1385	1131	967	8.25669	7.23734	7.468353	7.570807	8.321777	10.06058	7.654127	8.651055	0.531326	1	-
ENSMUSC	Recql5	RecQ pro	GO:00081	metabolic	-	-	-	-	11:115892	383	537	590	721	499	537	2.652083	3.116847	2.814436	3.252406	3.029921	4.610502	2.861122	3.630943	0.175026	1	-
ENSMUSC	Sap30bp	SAP30 bir	GO:00081	metabolic	-	-	-	-	11:115933	765	957	1215	1141	990	691	8.722294	9.14606	9.543265	8.474939	9.897987	9.768621	9.137206	9.380516	0.918427	1	-
ENSMUSC	Itgb4	integrin b	GO:00325	developm	K06525	integrin b	mmu0415	PI3K-Akt s	11:115974	137	128	239	244	160	153	0.933599	0.731144	1.121991	1.083211	0.956099	1.292756	0.928911	1.110689	0.424015	1	-
ENSMUSC	Galk1	galactokir	GO:00081	metabolic	K00849	galactokir	mmu0110	Metabolic	11:116008	102	107	119	134	95	118	4.610548	4.05403	3.70552	3.94581	3.765443	6.613328	4.123366	4.77486	0.630139	1	-
ENSMUSC	Unk	unkempt	GO:00325	developm	-	-	-	-	11:116030	357	391	510	590	466	422	3.875049	3.557453	3.813562	4.171984	4.435447	5.679465	3.748688	4.762299	0.155525	1	-
ENSMUSC	Trim47	tripartite r	GO:00056	cell|intrac	-	-	-	-	11:116105	29	45	53	46	27	53	0.337677	0.439204	0.425138	0.348933	0.275682	0.765176	0.400673	0.463264	0.779805	1	-
ENSMUSC	Aspa	aspartoac	GO:00325	developm	K01437	aspartoac	mmu0110	Metabolic	11:733049	121	159	191	218	131	102	1.106943	1.219242	1.203713	1.299202	1.050879	1.156982	1.176633	1.169021	0.800337	1	-
ENSMUSC	Mrpl38	mitochon	GO:00329	macromo	-	-	-	-	11:116131	477	630	665	717	563	505	7.352603	8.139844	7.061486	7.199854	7.609813	9.651617	7.517978	8.153761	0.789539	1	-
ENSMUSC	Fbf1	Fas (TNFR	GO:00990	supramole	-	-	-	-	11:116142	1382	1740	2039	2134	1818	1183	6.35346	6.705087	6.457597	6.391141	7.328901	6.743312	6.505381	6.821118	0.934872	1	-
ENSMUSC	Acox1	acyl-Coer	GO:00081	metabolic	K00232	acyl-CoA	mmu0110	Metabolic	11:116171	3589	4343	5398	5439	4133	3267	36.4073	36.92817	37.7224	35.94312	36.76403	41.09133	37.01929	37.93282	0.88407	1	-
ENSMUSC	Ten1	TEN1 telo	GO:00081	metabolic	-	-	-	-	11:116198	109	157	175	193	113	224	1.95575	2.361247	2.1631	2.255935	1.777912	4.983343	2.160032	3.00573	0.22606	1	-
ENSMUSC	Srp68	signal rec	GO:00511	localizatio	K03107	signal rec	mmu0306	Protein ex	11:116245	1135	1328	1643	1666	1284	937	23.99622	23.53413	23.92964	22.94584	23.80427	24.56252	23.82	23.77088	0.731691	1	-
ENSMUSC	Tsen54	tRNA splic	GO:00081	metabolic	-	-	-	-	11:115814	67	83	91	86	88	92	0.930112	0.965809	0.870264	0.77775	1.071241	1.583556	0.922062	1.144182	0.433104	1	-
ENSMUSC	Llgl2	lethal giar	GO:00325	developm	K06094	lethal(2) g	mmu0516	Human pa	11:115824	34	25	40	25	24	22	0.305847	0.188501	0.247876	0.146501	0.189313	0.245382	0.247408	0.193732	0.234271	1	-
ENSMUSC	Ncbp3	nuclear ca	GO:00081	metabolic	-	-	-	-	11:730477	545	698	813	781	617	349	1.934778	2.077029	1.988276	1.806206	1.920713	1.536195	2.000028	1.754371	0.23531	1	-
ENSMUSC	Camkk1	calcium/ca	GO:00081	metabolic	K07359	calcium/ca	mmu0503	Alcoholisr	11:730190	2316	1600	3374	2569	1605	1804	24.98961	14.47084	25.07941	18.05787	15.18583	24.13478	21.51329	19.12616	0.297991	1	-
ENSMUSC	P2rx1	purinergic	GO:00048	signal trar	K05215	P2X purin	mmu0408	Neuroacti	11:729991	5	6	6	8	0	16	0.077372	0.077821	0.063958	0.080646	0	0.306985	0.07305	0.12921	0.52507	1	-
ENSMUSC	Atp2a3	ATPase, C	GO:00511	localizatio	K05853	Ca2+ tran	mmu0402	Calcium si	11:729611	171	168	253	255	182	174	1.46978	1.210372	1.49806	1.427837	1.371738	1.854353	1.392737	1.551309	0.692669	1	-
ENSMUSC	Ankfy1	ankyrin re	GO:00511	localizatio	-	-	-	-	11:726900	579	665	807	936	660	410	2.332037	2.245077	2.239141	2.455919	2.331005	2.047511	2.272085	2.278145	0.822865	1	-
ENSMUSC	Exoc7	exocyst cc	GO:00511	localizatio	K07195	exocyst cc	mmu0491	Insulin sig	11:116288	911	1130	1330	1348	1112	777	10.78353	11.21176	10.8454	10.39475	11.54225	11.4038	10.9469	11.1136	0.845925	1	-

ENSMUS(	Galr2	galanin re	GO:00325	developm	K04231	galanin re	mmu0408	Neuroacti	11:11628(	0	0	1	4	0	0	0	0	0.012252	0.046368	0	0	0.004084	0.015456	0.667729	1	-
ENSMUS(	Ube2g1	ubiquitin-	GO:00081	metabolic	K10575	ubiquitin-	mmu0414	Protein pr	11:726072	790	1025	1266	1233	866	591	10.00136	10.877	11.04123	10.16898	9.613754	9.276969	10.63987	9.686567	0.307936	1	-
ENSMUS(	Spns3	spinster h	GO:00400	locomotic	-	-	-	-	11:724949	2	3	16	9	0	3	0.01972	0.024787	0.108655	0.057796	0	0.036671	0.051054	0.031489	0.435622	1	-
ENSMUS(	Tekt1	tektin 1 [S	GO:00400	locomotic	-	-	-	-	11:723447	60	37	84	50	49	36	2.044587	1.056853	1.971919	1.109954	1.464193	1.521055	1.69112	1.365068	0.262809	1	-
ENSMUS(	Med31	mediator	GO:00325	developm	-	-	-	-	11:722117	89	105	149	141	99	97	2.945935	2.913245	3.397594	3.040429	2.873524	3.980986	3.085591	3.298313	0.91382	1	-
ENSMUS(	Ube2o	ubiquitin-	GO:00081	metabolic	K10581	ubiquitin-	mmu0412	Ubiquitin	11:116537	2546	3057	3492	3682	2956	2638	28.07182	28.25273	26.52392	26.44707	28.57981	36.06392	27.61616	30.3636	0.673768	1	-
ENSMUS(	Txndc17	thioredox	GO:00081	metabolic	-	-	-	-	11:722075	230	302	381	313	269	242	9.370002	10.31266	10.69271	8.306884	9.609609	12.22399	10.12512	10.04683	0.711249	1	-
ENSMUS(	Aanat	arylalkylar	GO:00081	metabolic	K00669	arylalkylar	mmu011C	Metabolic	11:11658€	2	3	3	3	6	4	0.037702	0.047392	0.038949	0.03683	0.099154	0.093475	0.041348	0.076486	0.449586	1	-
ENSMUS(	Slc13a5	solute car	GO:00511	localizatio	-	-	-	-	11:72241‌9	51	112	145	146	133	96	0.401087	0.738313	0.785581	0.748007	0.917202	0.936111	0.64166	0.867107	0.19371	1	-
ENSMUS(	Rhbdf2	rhomboid	GO:00081	metabolic	-	-	-	-	11:116598	29	23	45	34	32	21	0.255269	0.169698	0.272874	0.194964	0.246994	0.229191	0.232614	0.223716	0.773391	1	-
ENSMUS(	4933427D	RIKEN cDI	GO:00511	localizatio	-	-	-	-	11:721539	520	659	752	839	582	404	3.339824	3.547798	3.327284	3.510461	3.277825	3.217274	3.404969	3.335187	0.66926	1	-
ENSMUS(	Pimreg	PICALM ir	GO:00099	cellular pr	-	-	-	-	11:72042C	10	9	10	13	4	5	0.174742	0.131816	0.12038	0.147989	0.061286	0.108324	0.142312	0.105866	0.431781	1	-
ENSMUS(	Cygb	cytoglobir	GO:00081	metabolic	-	-	-	-	11:116645	1047	1298	1592	1703	1298	983	28.5457	29.66343	29.90117	30.24754	31.03211	33.23022	29.3701	31.50329	0.797978	1	-
ENSMUS(	Wscd1	WSC dom	GO:00038	catalytic a	-	-	-	-	11:717499	1971	2200	2755	2839	2191	2056	22.17436	20.74627	21.35195	20.8071	21.61472	28.67963	21.42419	23.70048	0.646327	1	-
ENSMUS(	1810032C	RIKEN cDI	-	-	-	-	-	-	11:116671	18	22	27	28	16	36	0.23689	0.242691	0.244788	0.240062	0.184644	0.587444	0.241456	0.337384	0.404337	1	-
ENSMUS(	Mxra7	matrix-rei	GO:00055	extracellul	-	-	-	-	11:116803	202	238	281	321	235	123	4.158642	4.107037	3.985261	4.305135	4.242387	3.139719	4.083647	3.895747	0.648732	1	-
ENSMUS(	Rabep1	rabaptin,	GO:00511	localizatio	K12480	Rab GTPa	mmu0414	Endocytos	11:708447	2123	2611	2914	3231	2181	1520	12.10181	12.47556	11.44303	11.99827	10.90181	10.7431	12.0068	11.21439	0.387619	1	-
ENSMUS(	Mfsd11	major faci	GO:00160	membran	-	-	-	-	11:116852	366	510	600	651	469	481	1.676907	1.958624	1.893787	1.943081	1.884274	2.732495	1.843106	2.186617	0.37567	1	-
ENSMUS(	Kif1c	kinesin fa	GO:00511	localizatio	-	-	-	-	11:707005	2518	2772	3899	3789	3000	2143	12.82983	11.8389	13.68579	12.57684	13.40384	13.53859	12.78484	13.17309	0.94117	1	-
ENSMUS(	Sec14l1	SEC14-lik	GO:00511	localizatio	-	-	-	-	11:117115	4229	5156	4405	5002	5236	2957	29.90605	30.56238	21.45946	23.04341	32.46861	25.92738	27.3093	27.14647	0.756953	1	-
ENSMUS(	Nos2	nitric oxid	GO:00081	metabolic	K13241	nitric-oxic	mmu011C	Metabolic	11:789207	7	16	2	16	9	4	0.097974	0.1877	0.019285	0.145876	0.110455	0.069411	0.101653	0.108581	1	1	-
ENSMUS(	Mink1	misshapeı	GO:00325	developm	-	-	-	-	11:705628	6070	6581	7589	9164	6925	4930	34.34533	31.21216	29.58115	33.77892	34.35908	34.58692	31.71288	34.24164	0.74463	1	-
ENSMUS(	Pld2	phospholi	GO:00081	metabolic	K01115	phospholi	mmu011C	Metabolic	11:705400	285	326	495	465	365	281	2.316188	2.220753	2.771314	2.461863	2.601149	2.831537	2.436085	2.631516	0.795781	1	-
ENSMUS(	Slc46a1	solute car	GO:00511	localizatio	K14613	MFS trans	mmu0497	Mineral al	11:784656	123	136	178	188	140	115	1.809492	1.677049	1.803944	1.801736	1.806027	2.097667	1.763495	1.90181	0.844876	1	-
ENSMUS(	Vmo1	vitelline m	GO:00055	extracellul	-	-	-	-	11:705135	0	4	0	2	0	0	0	0.317073	0	0.123204	0	0	0.105691	0.041068	0.743884	1	-
ENSMUS(	0610010K	RIKEN cDI	GO:00081	metabolic	-	-	-	-	11:702352	104	100	105	124	96	104	1.47369	1.187757	1.02498	1.144661	1.192851	1.827226	1.228809	1.388246	0.721458	1	-
ENSMUS(	Eral1	Era (G-prı	GO:00099	cellular pr	-	-	-	-	11:780733	218	261	292	336	232	246	5.114262	5.13239	4.719116	5.135082	4.772637	7.155639	4.98859	5.687786	0.584544	1	-
ENSMUS(	Dhrs13	dehydrog	GO:00081	metabolic	-	-	-	-	11:780322	49	51	61	60	45	65	0.698859	0.609692	0.599338	0.557475	0.562784	1.149444	0.635963	0.756568	0.624329	1	-
ENSMUS(	Coro6	coronin 6	GO:00099	cellular pr	-	-	-	-	11:774624	152	310	498	543	393	320	2.271883	3.883807	5.127708	5.28718	5.150842	5.930319	3.761133	5.456114	0.0658	1	-
ENSMUS(	Slc6a4	solute car	GO:00325	developm	K05037	solute car	mmu0472	Serotoner	11:769986	0	0	1	1	0	0	0	0	0.007543	0.007135	0	0	0.002514	0.002378	1	1	-
ENSMUS(	Tmigd1	transmem	GO:00400	locomotic	-	-	-	-	11:769021	1	1	0	0	0	0	0.02485	0.020824	0	0	0	0	0.015225	0	0.52541	1	-
ENSMUS(	Blmh	bleomycir	GO:00081	metabolic	-	-	-	-	11:769248	759	960	1226	1108	925	693	5.989909	6.350421	6.66531	5.696389	6.401219	6.781057	6.335213	6.292888	0.702911	1	-
ENSMUS(	Cpd	carboxype	GO:00081	metabolic	-	-	-	-	11:767784	2784	2901	3789	4026	2862	1715	20.29265	17.72434	19.02591	19.11722	18.29287	15.49956	19.0143	17.63655	0.39459	1	-
ENSMUS(	Timm22	translocas	GO:00511	localizatio	-	-	-	-	11:764069	190	306	321	308	231	273	2.981496	4.024907	3.47006	3.148572	3.178605	5.31166	3.492154	3.879612	0.758022	1	-
ENSMUS(	Nxn	nucleored	GO:00325	developm	-	-	-	-	11:762571	147	170	289	244	164	192	2.590042	2.51068	3.507832	2.800672	2.533817	4.194464	2.869518	3.176317	0.772667	1	-
ENSMUS(	Rflnb	refilin B [S	GO:00325	developm	-	-	-	-	11:760191	241	274	412	388	286	184	4.361135	4.156089	5.136063	4.573985	4.538274	4.128427	4.551096	4.413562	0.663507	1	-
ENSMUS(	Rph3al	rabphilin :	GO:00511	localizatio	-	-	-	-	11:758309	49	88	83	88	76	47	0.730327	1.099394	0.852209	0.85444	0.993291	0.868569	0.893977	0.905433	0.919867	1	-
ENSMUS(	Doc2b	double C2	GO:00452	synapse|lc	-	-	-	-	11:757689	2459	2991	3686	4142	3022	1743	35.8351	36.53586	37.0047	39.32256	38.61777	31.49439	36.45855	36.47824	0.80308	1	-
ENSMUS(	Ywhae	tyrosine 3	GO:00325	developm	K06630	14-3-3 pr	mmu0415	PI3K-Akt s	11:757328	13047	17431	20450	20759	15359	10653	240.4803	269.3048	259.6651	249.2625	248.2415	243.4595	256.4834	246.9878	0.507809	1	-
ENSMUS(	Prpf8	pre-mRN.	GO:00081	metabolic	K12856	pre-mRN.	mmu0304	Spliceosoı	11:754868	4789	5440	6679	7271	4572	3754	35.66386	33.95746	34.26465	35.27437	29.85605	34.6628	34.62866	33.26441	0.497573	1	-
ENSMUS(	Nme2	NME/NM	GO:00325	developm	K00940	nucleosid	mmu011C	Metabolic	11:939498	249	317	400	213	317	307	10.33619	11.02993	11.43858	5.760007	11.53885	15.80101	10.9349	11.03329	0.813233	1	-
ENSMUS(	Spag9	sperm ass	GO:00325	developm	-	-	-	-	11:939960	7746	8518	10987	11771	8364	5004	23.35967	21.53177	22.82547	23.12513	22.118	18.7108	22.5723	21.31798	0.468865	1	-
ENSMUS(	Luc7l3	LUC7-like	GO:00081	metabolic	-	-	-	-	11:942878	2193	2702	3472	3674	2717	1498	10.98627	11.34618	11.98237	11.99036	11.93559	9.304847	11.43827	11.07693	0.626864	1	-
ENSMUS(	Ankrd40	ankyrin re	-	-	-	-	-	-	11:943280	1589	1852	2987	2545	1955	1273	16.45255	16.07323	21.3057	17.16638	17.75002	16.34269	17.94383	17.08636	0.51084	1	-
ENSMUS(	Abcc3	ATP-bind	GO:00511	localizatio	K05667	ATP-bind	mmu0497	Bile secret	11:943432	39	62	73	94	50	66	0.338604	0.451201	0.436618	0.53166	0.380662	0.71049	0.408807	0.540937	0.266168	1	-
ENSMUS(	Cacna1g	calcium cl	GO:00511	localizatio	K04854	voltage-d	mmu0401	MAPK sigı	11:944083	1117	1457	1715	1875	1469	1422	4.581099	5.008746	4.845427	5.009559	5.283005	7.231063	4.811758	5.841209	0.234854	1	-
ENSMUS(	Spata20	spermato	GO:00325	developm	-	-	-	-	11:944789	4	2	0	0	2	0	0.069492	0.029124	0	0	0.030467	0	0.032872	0.010156	0.37524	1	-
ENSMUS(	Xylt2	xylosyltrar	GO:00081	metabolic	K00771	protein xy	mmu011C	Metabolic	11:946638	434	478	552	655	579	406	5.065565	4.67648	4.438425	4.980367	5.92597	5.875571	4.726823	5.593969	0.341424	1	-
ENSMUS(	Lrrc59	leucine ric	GO:00160	membran	-	-	-	-	11:946297	1433	1785	2255	2219	1640	1198	26.21516	27.37142	28.4187	26.4451	26.30831	27.17371	27.33509	26.64237	0.588061	1	-
ENSMUS(	Cdc34b	cell divisic	GO:00081	metabolic	-	-	-	-	11:947417	4	2	7	3	4	11	0.102295	0.042872	0.123333	0.049976	0.089697	0.348818	0.0895	0.16283	0.390193	1	-
ENSMUS(	Dlx4	distal-less	GO:00325	developm	-	-	-	-	11:951404	0	2	1	2	2	0	0	0.027493	0.011294	0.021366	0.028761	0	0.012929	0.016709	1	1	-
ENSMUS(	Tac4	tachykinin	GO:00048	signal trar	-	-	-	-	11:952615	0	0	0	0	2	0	0	0	0	0	0.089088	0	0	0.029696	0.53523	1	-
ENSMUS(	Slc35b1	solute car	GO:00511	localizatio	-	-	-	-	11:953846	312	460	570	481	383	418	5.722529	7.072021	7.202105	5.747241	6.159907	9.505937	6.665551	7.137695	0.912123	1	-
ENSMUS(	Hoxb9	homeobo	GO:00325	developm	-	-	-	-	11:962714	7	3	12	17	2	6	0.154963	0.055662	0.183	0.245148	0.038819	0.164694	0.131209	0.149554	0.921024	1	-
ENSMUS(	Snx11	sorting ne	GO:00511	localizatio	-	-	-	-	11:967675	340	465	607	694	486	502	3.406059	3.904618	4.189028	4.529124	4.269253	6.235372	3.833235	5.01125	0.114858	1	-
ENSMUS(	Scrn2	secernin 2	GO:00081	metabolic	-	-	-	-	11:970299	34	45	51	54	45	63	0.631816	0.700929	0.652874	0.653711	0.733265	1.451569	0.661873	0.946182	0.208099	1	-
ENSMUS(	Lrrc46	leucine ric	GO:00511	localizatio	-	-	-	-	11:970346	2	15	23	10	10	15	0.095159	0.598111	0.753728	0.309884	0.417141	0.884737	0.482333	0.537254	1	1	-
ENSMUS(	Cacnb1	calcium cl	GO:00511	localizatio	K04862	voltage-d	mmu0401	MAPK sigı	11:980015	2338	2849	3309	3561	2836	2137	23.59272	24.09787	23.00285	23.40923	25.09472	26.7377	23.56448	25.08055	0.847529	1	-
ENSMUS(	Fbxl20	F-box anc	GO:00081	metabolic	-	-	-	-	11:980825	1199	1329	1682	1735	1319	973	5.737957	5.33109	5.545176	5.409029	5.535099	5.773471	5.538074	5.572533	0.783717	1	-
ENSMUS(	Asgr1	asialoglyc	GO:00511	localizatio	K10063	asialoglyc	mmu0491	Thyroid hı	11:700540	14	15	13	14	12	11	0.427348	0.383802	0.273371	0.278401	0.321205	0.416336	0.361507	0.338647	0.729795	1	-
ENSMUS(	Dlg4	discs, larg	GO:00325	developm	K11828	discs large	mmu0501	Huntingto	11:700170	12457	14325	16025	18451	13890	8602	116.4406	112.2377	103.1907	112.3551	113.8508	99.69571	110.623	108.6338	0.656264	1	-
ENSMUS(	Dvl2	dishevelle	GO:00325	developm	K02353	segment ļ	mmu052C	Pathways	11:700005	297	332	417	429	376	274	2.143211	2.008164	2.072983	2.016729	2.379245	2.451573	2.074786	2.282515	0.696422	1	-
ENSMUS(	Nr1d1	nuclear re	GO:00325	developm	K03728	nuclear re	mmu0471	Circadian	11:987679	1248	1462	1774	1970	1515	1221	22.64825	22.23925	22.17813	23.28991	24.10878	27.47398	22.35521	24.95756	0.556176	1	-
ENSMUS(	Gucy2e	guanylate	GO:00081	metabolic	K12321	guanylate	mmu0474	Olfactory	11:692181	59	89	139	170	116	70	0.349748	0.442224	0.567634	0.656495	0.602979	0.514502	0.453202	0.591325	0.219067	1	-
ENSMUS(	Alox8	arachidon	GO:00325	developm	K08022	arachidon	mmu011C	Metabolic	11:691839	116	134	194	188	169	63	1.620613	1.569194	1.867121	1.711035	2.070379	1.091309	1.685642	1.624241	0.767166	1	-
ENSMUS(	Aloxe3	arachidon	GO:00325	developm	-	-	-	-	11:691258	147	194	190	202	139	162	1.139719	1.260768	1.014814	1.020267	0.945018	1.557338	1.138434	1.174208	0.917125	1	-
ENSMUS(	Per1	period cir	GO:00081	metabolic	K02633	period cir	mmu0516	Herpes sir	11:690952	1050	1209	1472	1676	1284	893	8.073885	7.792422	7.797449	8.395546	8.657671	8.513946	7.887919	8.522387	0.743123	1	-
ENSMUS(	Vamp2	vesicle-as	GO:00452	synapse|lc	K13504	vesicle-as	mmu0491	Insulin sec	11:690884	15856	20365	22583	24775	17529	16331	203.2466	218.8099	199.4172	206.883	197.0287	259.5544	207.1579	221.1554	0.850737	1	-
ENSMUS(	Tmem107	transmem	GO:00325	developm	-	-	-	-	11:690708	60	69	111	98	81	69	0.91839	0.885276	1.170451	0.97721	1.08719	1.309518	0.991372	1.124639	0.67255	1	-
ENSMUS(	Aurkb	aurora kir	GO:00325	developm	-	-	-	-	11:690456	0	9	7	5	5	5	0	0.082515	0.052751	0.035632	0.047956	0.06781	0.045089	0.050466	1	1	-
ENSMUS(	Ctc1	CTS telom	GO:00325	developm	-	-	-	-	11:690159	497	632	700	762	570	489	3.235261	3.448439	3.13908	3.231392	3.253644	3.946823	3.27426	3.477286	0.897419	1	-
ENSMUS(	Pfas	phosphor	GO:00081	metabolic	K01952	phosphor	mmu011C	Metabolic	11:689856	259	437	459	570	466	434	1.161217	1.642281	1.417678	1.664829	1.832066	2.412618	1.407058	1.969838	0.049334	1	-
ENSMUS(	Myh10	myosin, hı	GO:00325	developm	K10352	myosin he	mmu0481	Regulatioı	11:686915	6315	7567	8914	10278	6271	4401	23.90905	24.01405	23.24947	25.35007	20.81941	20.6598	23.72419	22.27643	0.428956	1	-
ENSMUS(	Pik3r5	phosphoi	GO:00081	metabolic	K02649	phosphoi	mmu0415	PI3K-Akt s	11:684321	74	69	82	86	50	52	0.670596	0.524122	0.511915	0.507705	0.397326	0.58428	0.568878	0.496437	0.348595	1	-
ENSMUS(	Ntn1	netrin 1 [S	GO:00325	developm	K06843	netrin 1[-]	mmu0436	Axon guic	11:682093	149	201	58	244	214	155	1.346043	1.522025	0.360954	1.435966	1.695227	1.73616	1.076341	1.622451	0.178865	1	-
ENSMUS(	Stx8	syntaxin 8	GO:00511	localizatio	K08501	syntaxin 8	mmu0413	SNARE int	11:679661	184	277	312	313	246	217	1.39029	1.754368	1.624031	1.54069	1.629922	2.032989	1.589563	1.734534	0.781724	1	-
ENSMUS(	Cfap52	cilia and f	GO:00056	cell|cell pr	-	-	-	-	11:679248	41	23	70	21	25	30	0.712705	0.335126	0.838255	0.237814	0.381078	0.646613	0.628696	0.421835	0.121015	1	-
ENSMUS(	Usp43	ubiquitin s	GO:00081	metabolic	-	-	-	-	11:678545	8	14	24	23	11	30	0.07769	0.113968	0.160566	0.145513	0.093677	0.361249	0.117408	0.200147	0.218499	1	-
ENSMUS(	Rcvrn	recoverin	GO:00511	localizatio	K13764	recoverin[	mmu0474	Phototran	11:676953	3	4	3	4	2	6	0.183304	0.204878	0.126286	0.159256	0.107162	0.454652	0.171489	0.240357	0.727819	1	-
ENSMUS(	Myh3	myosin, hı	GO:00081	metabolic	K10352	myosin he	mmu0453	Tight junc	11:670783	23	24	30	45	17	18	0.219608	0.192081	0.197329	0.27991	0.142333	0.213105	0.203006	0.211783	0.958259	1	-
ENSMUS(	Adprm	ADP-ribo:	GO:00038	catalytic a	K01517	manganes	mmu0023	Purine me	11:670379	108	147	248	170	201	92	1.489195	1.699024	2.35577	1.527074	2.430343	1.572905	1.847996	1.84344	0.859329	1	-
ENSMUS(	Krt12	keratin 12	GO:00051	structural	-	-	-	-	11:994156	13	19	26	29	20	32	0.446097	0.546517	0.614643	0.648304	0.601836	1.36154	0.535752	0.87056	0.149764	1	-
ENSMUS(	Top2a	topoisom	GO:00325	developm	K03164	DNA topo	mmu0152	Platinum (	11:989929	51	70	102	70	43	49	0.301955	0.347395	0.416033	0.269994	0.223246	0.359711	0.355128	0.284317	0.163608	1	-
ENSMUS(	Acly	ATP citrat	GO:00081	metabolic	K01648	ATP citrat	mmu011C	Metabolic	11:100476	2630	3183	3755	3990	2915	2296	20.40835	20.7034	20.07306	20.17004	19.83506	22.09073	20.39494	20.69861	0.823388	1	-
ENSMUS(	Kat2a	K(lysine) a	GO:00325	developm	K06062	histone ac	mmu0516	HTLV-I inf	11:100704	1188	1408	1658	1833	1287	1037	16.53185	16.4233	15.89428	16.61687	15.70458	17.89245	16.28315	16.73797	0.916267	1	-
ENSMUS(	Stat5b	signal trar	GO:00325	developm	K11224	signal trar	mmu052C	Pathways	11:100780	347	389	495	432	360	324	3.761364	3.534428	3.696346	3.050575	3.42185	4.354597	3.664046	3.609007	0.65797	1	-
ENSMUS(	Tmem101	transmem	GO:00048	signal trar	-	-	-	-	11:102152	272	315	391	416	312	343	6.789638	6.590844	6.723664	6.764767	6.829287	10.61591	6.701382	8.069989	0.366142	1	-
ENSMUS(	Lsm12	LSM12 hc	-	-	-	-	-	-	11:102162	677	915	988	1032	820	423	13.45805	15.24641	13.53013	13.36457	14.29387	10.42604	14.07819	12.69483	0.349938	1	-
ENSMUS(	Ubtf	upstream	GO:00056	cell|intrace	-	-	-	-	11:102304	2256	3024	3593	3370	2667	1897	11.05525	12.42122	12.12937	10.75824	11.46028	11.52611	11.86861	11.24821	0.424881	1	-
ENSMUS(	Ccdc43	coiled-co	-	-	-	-	-	-	11:102684	344	428	549	535	379	421	3.581038	3.734621	3.937082	3.628154	3.459653	5.433987	3.750914	4.173931	0.695176	1	-
ENSMUS(	Adam11	a disinteg	GO:00081	metabolic	-	-	-	-	11:102761	2102	4998	5001	5966	4271	4148	13.97661	27.85596	22.90748	25.84247	24.90241	34.19745	21.58002	28.31411	0.145798	1	-
ENSMUS(	Higd1b	HIG1 dom	GO:00160	membran	-	-	-	-	11:102835	11	24	22	22	24	18	0.649723	1.188188	0.895141	0.846508	1.243028	1.318241	0.911018	1.135926	0.569658	1	-
ENSMUS(	Eftud2	elongatior	GO:00081	metabolic	K12852	116 kDa U	mmu0304	Spliceosoı	11:102838	1537	2006	2286	2544	1971	1529	8.922267	9.760793	9.141743	9.620553	10.033	11.00512	9.274934	10.21956	0.626431	1	-
ENSMUS(	Ccdc103	coiled-co	GO:00325	developm	-	-	-	-	11:102881	34	34	49	42	42	39	0.820976	0.688156	0.815083	0.660658	0.889283	1.167624	0.774738	0.905855	0.666499	1	-
ENSMUS(	Gfap	glial fibrill	GO:00325	developm	K05640	glial fibrill	mmu0463	Jak-STAT	11:102887	7758	8280	13279	13826	8656	7217	104.0837	93.1142	122.7297	120.8401	101.834	120.0536	106.6425	114.2426	0.803056	1	-
ENSMUS(	Dcakd	dephosph	GO:00081	metabolic	-	-	-	-	11:102994	868	1096	1192	1392	1024	634	12.64939	13.38792	11.96679	13.21511	13.08557	11.45579	12.66804	12.58549	0.754959	1	-
ENSMUS(	Nmt1	N-myristo	GO:00325	developm	-	-	-	-	11:103028	3787	4384	5603	5980	4206	3452	43.38055	42.09428	44.21521	44.62544	42.24853	49.02938	43.23001	45.30111	0.956345	1	-
ENSMUS(	Plcd3	phospholi	GO:00325	developm	K05857	phosphati	mmu011C	Metabolic	11:103070	122	99	169	173	100	179	1.695853	1.153504	1.618334	1.566595	1.218917	3.085097	1.48923	1.95687	0.329057	1	-
ENSMUS(	1700023F	RIKEN cDI	GO:00431	ion bindin	-	-	-	-	11:103198	9	20	8	20	21	11	0.272496	0.507583	0.166864	0.394497	0.557563	0.412964	0.315648	0.455008	0.325885	1	-
ENSMUS(	Map3k14	mitogen-.	GO:00048	signal trar	K04466	mitogen-.	mmu0401	MAPK sigı	11:103219	72	79	107	91	92	94	0.545911	0.502076	0.558884	0.449482	0.611673	0.883698	0.535624	0.648284	0.492697	1	-
ENSMUS(	Gosr2	golgi SNA	GO:00511	localizatio	K08496	golgi SNA	mmu0413	SNARE int	11:103676	1087	1320	1595	1716	1263	1082	15.22638	15.49866	15.39144	15.65908	15.51363	18.79232	15.37216	16.65501	0.752608	1	-
ENSMUS(	Klhl28	kelch-like	GO:00081	metabolic	-	-	-	-	12:649388	500	658	817	893	627	359	3.66807	4.04619	4.128965	4.267771	4.033464	3.265486	3.947742	3.855574	0.703415	1	-
ENSMUS(	Fkbp3	FK506 bin	GO:00081	metabolic	-	-	-	-	12:650624	1195	1522	1991	1774	1349	950	6.034154	6.441929	6.925817	5.835576	5.973146	5.947822	6.4673	5.918848	0.290516	1	-
ENSMUS(	Scfd1	Sec1 fami	GO:00325	developm	-	-	-	-	12:513775	419	512	650	651	467	299	1.674445	1.715064	1.78946	1.694804	1.636504	1.481542	1.726323	1.604283	0.43375	1	-
ENSMUS(	Coch	cochlin [S	GO:00325	developm	-	-	-	-	12:515933	90	146	152	91	108	111	1.216194	1.653739	1.415004	0.801097	1.279755	1.859818	1.428312	1.313557	0.496359	1	-
ENSMUS(	Strn3	striatin, ca	GO:00081	metabolic	-	-	-	-	12:516096	1450	1702	2250	2099	1561	784	11.73759	11.54844	12.54715	11.06892	11.08043	7.868882	11.9444	10.00608	0.140403	1	-
ENSMUS(	Ap4s1	adaptor-ı	GO:00511	localizatio	K12403	AP-4 com	mmu0414	Lysosome	12:516910	261	298	384	323	260	254	11.62391	11.12451	11.78133	9.371229	10.1538	14.02594	11.50992	11.18366	0.595076	1	-
ENSMUS(	Dtd2	D-tyrosyl-	GO:00081	metabolic	-	-	-	-	12:519883	158	173	195	238	187	155	2.971702	2.727383	2.526588	2.91613	3.084123	3.614645	2.741891	3.204966	0.471669	1	-
ENSMUS(	Ston2	stonin 2 [S	GO:00325	developm	-	-	-	-	12:916330	797	812	1157	1269	916	582	3.76817	3.217964	3.768401	3.908548	3.797611	3.411779	3.584845	3.705979	0.996472	1	-
ENSMUS(	Gtf2a1	general tr	GO:00081	metabolic	K03122	transcripti	mmu052C	Viral carci	12:915552	741	939	1147	1230	845	661	6.186652	6.571368	6.597087	6.689968	6.18638	6.842654	6.451702	6.573	0.873389	1	-
ENSMUS(	Tshr	thyroid sti	GO:00325	developm	K04249	thyroid sti	mmu0408	Neuroacti	12:913845	6	13	5	10	2	3	0.0527	0.095708	0.030253	0.057215	0.015402	0.032673	0.059553	0.035096	0.282554	1	-
ENSMUS(	Sel1l	sel-1 supı	GO:00081	metabolic	K14026	SEL1 prot	mmu0414	Protein pr	12:918060	4099	4620	3765	4435	3133	3374	16.55674	15.64198	10.47643	11.67004	11.09686	16.89772	14.22505	13.22154	0.399164	1	-
ENSMUS(	Dnaaf2	dynein, ax	GO:00400	locomotic	-	-	-	-	12:691890	71	87	134	125	79	79	1.331049	1.367123	1.730588	1.526608	1.298692	1.836328	1.476253	1.553876	1	1	-
ENSMUS(	Pole2	polymeras	GO:00081	metabolic	K02325	DNA poly	mmu011C	Metabolic	12:692017	3	0	17	14	12	2	0.054143	0	0.211378	0.164621	0.189932	0.044772	0.088507	0.133108	0.595094	1	-
ENSMUS(	Klhdc2	kelch don	GO:00160	membran	-	-	-	-	12:692966	1481	1927	2374	2275	1773	1307	11.62431	12.67788	12.83643	11.63255	12.20291	12.7196	12.37954	12.18502	0.640578	1	-
ENSMUS(	Nemf	nuclear ex	GO:00511	localizatio	-	-	-	-	12:693105	1319	1550	1893	2207	1451	526	5.526897	5.444035	5.464349	6.024493	5.331463	2.7328	5.478427	4.696252	0.34167	1	-
ENSMUS(	Sec23a	SEC23 ho	GO:00511	localizatio	K14006	protein tra	mmu0414	Protein pr	12:589583	2552	3377	3586	3809	2797	1776	27.8242	30.86214	26.93418	27.0542	26.74097	24.00883	28.54017	25.93467	0.285819	1	-
ENSMUS(	L2hgdh	L-2-hydrc	GO:00081	metabolic	K00109	2-hydroxy	-	-	12:696904	205	297	349	338	293	278	3.275928	3.978227	3.842	3.518675	4.10573	5.508222	3.698718	4.377542	0.430222	1	-
ENSMUS(	Cdkl1	cyclin-dep	GO:00325	developm	-	-	-	-	12:697468	103	160	175	210	149	123	3.038967	3.956983	3.556963	4.03637	3.854947	4.499681	3.517638	4.130333	0.45426	1	-
ENSMUS(	Trappc6b	trafficking	GO:00511	localizatio	-	-	-	-	12:590430	1066	1172	1387	1508	1025	561	10.31322	9.504238	9.244104	9.504292	8.695677	6.729543	9.687187	8.309838	0.193208	1	-
ENSMUS(	Pnn	pinin [Sou	GO:00329	macromo	K13114	pinin[-]	mmu0301	RNA trans	12:590668	1726	2126	2564	2578	2168	975	15.74006	16.25106	16.10776	15.31547	17.33675	11.02443	16.03296	14.55888	0.388414	1	-
ENSMUS(	Ctage5	melanom.	GO:00511	localizatio	-	-	-	-	12:590957	880	1245	1559	1638	1213	629	3.140536	3.724289	3.832821	3.808172	3.795984	2.783283	3.565882	3.46248	0.681897	1	-
ENSMUS(	Galc	galactosyl	GO:00081	metabolic	K01202	galactosyl	mmu011C	Metabolic	12:982022	492	662	850	944	612	474	1.861639	2.099623	2.215648	2.326933	2.030601	2.223793	2.05897	2.193776	0.842288	1	-
ENSMUS(	Spata7	spermato	GO:00511	localizatio	-	-	-	-	12:986281	300	339	376	442	333	147	1.440457	1.364368	1.243708	1.382557	1.402058	0.87515	1.349511	1.219922	0.459295	1	-
ENSMUS(	Ptpn21	protein ty	GO:00081	metabolic	-	-	-	-	12:986767	174	173	313	302	202	164	0.959168	0.799361	1.188611	1.084508	0.976422	1.120917	0.98238	1.060616	0.821083	1	-
ENSMUS(	Npas3	neuronal	GO:00081	metabolic	-	-	-	-	12:532486	157	310	344	334	353	227	1.04754	1.733748	1.581178	1.451774	2.065326	1.877944	1.454155	1.798348	0.305142	1	-
ENSMUS(	Zc3h14	zinc finge	GO:00081	metabolic	-	-	-	-	12:987469	1218	1553	1663	1922	1336	711	6.263762	6.694408	5.891577	6.439068	6.024724	4.533604	6.283249	5.665799	0.346001	1	-
ENSMUS(	Ttc8	tetratricoŗ	GO:00325	developm	-	-	-	-	12:989205	298	433	491	540	434	264	3.281154	3.996233	3.724285	3.873337	4.190272	3.604118	3.667224	3.889242	0.866172	1	-
ENSMUS(	Polr2h	polymeras	GO:00081	metabolic	K03016	DNA-dire	mmu011C	Metabolic	16:207176	140	196	229	206	147	135	5.755137	6.753591	6.485063	5.51666	5.298905	6.88092	6.331264	5.898828	0.456362	1	-
ENSMUS(	Ppp2r3c	protein pl	GO:00325	developm	K11583	serine/thr	mmu0415	PI3K-Akt s	12:552789	349	425	572	495	353	209	3.474312	3.546375	3.922748	3.210185	3.08149	2.579743	3.647812	2.957139	0.095255	1	-
ENSMUS(	1110008L	RIKEN cDI	GO:00081	metabolic	-	-	-	-	12:552995	90	119	159	138	130	93	0.651528	0.722091	0.792938	0.650807	0.825235	0.834753	0.722186	0.770265	0.905617	1	-
ENSMUS(	Psma6	proteasor	GO:00325	developm	K02730	20S prote	mmu0305	Proteason	12:553842	868	1014	1253	1281	956	766	28.94229	28.34029	28.78165	27.82557	27.95207	31.66855	28.68808	29.14873	0.836727	1	-
ENSMUS(	Nfkbia	nuclear fa	GO:00325	developm	K04734	NF-kappa	mmu052C	Pathways	12:554894	289	349	409	393	262	272	9.280845	9.394398	9.048265	8.221758	7.377928	10.8304	9.24117	8.810028	0.513515	1	-
ENSMUS(	Ralgapa1	Ral GTPas	GO:00081	metabolic	-	-	-	-	12:556028	2875	3245	4729	4584	3255	1860	9.635846	9.116319	10.91874	10.00871	9.566337	7.729491	9.890303	9.101514	0.384762	1	-
ENSMUS(	Mbip	MAP3K12	GO:00081	metabolic	-	-	-	-	12:563283	146	157	224	183	158	139	3.663132	3.30183	3.871693	2.991124	3.476167	4.32416	3.612218	3.59715	0.7597	1	-
ENSMUS(	Ngb	neurogloł	GO:00511	localizatio	-	-	-	-	12:870975	34	58	36	17	24	36	0.746394	1.06726	0.544435	0.243116	0.462003	0.979885	0.786029	0.561668	0.193194	1	-
ENSMUS(	Gstz1	glutathior	GO:00081	metabolic	K01800	maleylace	mmu011C	Metabolic	12:871471	428	592	630	750	506	416	4.186006	4.853228	4.244711	4.778585	4.339595	5.044709	4.427982	4.720963	0.856701	1	-
ENSMUS(	Sptlc2	serine pal	GO:00081	metabolic	K00654	serine pal	mmu011C	Metabolic	12:873050	545	690	870	770	634	509	3.139346	3.331535	3.452336	2.889449	3.20239	3.63535	3.307739	3.242396	0.623648	1	-
ENSMUS(	Ahsa1	AHA1, act	GO:00987	molecular	-	-	-	-	12:872664	1969	2302	2941	2720	2191	1270	19.92609	19.52696	20.50326	17.93193	19.44292	15.93551	19.98544	17.77012	0.226074	1	-
ENSMUS(	Vipas39	VPS33B in	GO:00325	developm	-	-	-	-	12:872388	964	741	874	1374	1051	662	9.652607	6.219256	6.028797	8.962642	9.228116	8.218866	7.30022	8.803208	0.327968	1	-
ENSMUS(	Snw1	SNW dom	GO:00325	developm	K06063	SNW dom	mmu052C	Viral carci	12:874490	1067	1472	1725	1716	1429	951	8.700415	10.06089	9.689834	9.115377	10.21764	9.61484	9.483712	9.649287	0.870732	1	-
ENSMUS(	Slirp	SRA stem	GO:00325	developm	-	-	-	-	12:874438	119	94	111	102	76	98	1.903071	1.260047	1.222877	1.062648	1.065776	1.94322	1.461998	1.357214	0.545216	1	-
ENSMUS(	Adck1	aarF dom	GO:00081	metabolic	-	-	-	-	12:883605	413	471	589	647	505	554	3.324976	3.178434	3.266674	3.393322	3.565108	5.530118	3.256695	4.162849	0.179073	1	-
ENSMUS(	Nova1	neuro-on	GO:00081	metabolic	-	-	-	-	12:466945	712	915	1309	1281	901	501	4.716955	5.081076	5.974102	5.528566	5.234188	4.115336	5.257378	4.959364	0.54018	1	-
ENSMUS(	Mthfd1	methylene	GO:00325	developm	-	-	mmu011C	Metabolic	12:762552	708	779	1027	1015	738	549	8.349518	7.700485	8.343533	7.797861	7.631798	8.027608	8.131179	7.819089	0.536982	1	-
ENSMUS(	Ppp2r5e	protein pl	GO:00987	molecular	K11584	serine/thr	mmu0415	PI3K-Akt s	12:754508	2215	2585	3241	3303	2452	1194	17.51087	17.12961	17.65081	17.01078	16.99797	11.70373	17.43043	15.23749	0.248793	1	-
ENSMUS(	Sgpp1	sphingosi	GO:00081	metabolic	K04716	sphingosi	mmu0407	Sphingolip	12:757142	812	998	1366	1074	881	648	7.911245	8.150275	9.168345	6.816708	7.526747	7.82798	8.409955	7.390478	0.172838	1	-
ENSMUS(	Esr2	estrogen	GO:00325	developm	K08551	estrogen	mmu052C	Pathways	12:761204	3	2	2	4	4	5	0.035976	0.020105	0.016526	0.031256	0.042064	0.074349	0.024202	0.049223	0.32917	1	-
ENSMUS(	Akap5	A kinase (	GO:00081	metabolic	-	-	-	-	12:763248	2633	2895	3613	3466	2144	1495	10.46891	9.64833	9.896236	8.977613	7.475119	7.370175	10.00449	7.940969	0.032986	1	-
ENSMUS(	Sptb	spectrin b	GO:00051	structural	-	-	-	-	12:765804	6942	7764	9697	11403	8350	5548	33.63973	31.53597	32.37104	35.99719	35.48109	33.33422	32.51558	34.9375	0.758927	1	-
ENSMUS(	Rab15	RAB15, m	GO:00511	localizatio	-	-	-	-	12:767979	3746	4824	5414	5924	4425	3525	39.89101	43.05935	39.71705	41.09642	41.32028	46.54279	40.88914	42.9865	0.9353	1	-
ENSMUS(	Fut8	fucosyltra	GO:00081	metabolic	K00717	glycoprot	mmu011C	Metabolic	12:772381	1358	1709	2039	2053	1742	938	7.415789	7.822629	7.670547	7.303456	8.341583	6.351065	7.636322	7.332035	0.57451	1	-
ENSMUS(	Atl1	atlastin G	GO:00325	developm	-	-	-	-	12:698926	1851	2347	2671	2711	1956	1449	9.512138	10.10969	9.455775	9.075759	8.814209	9.232641	9.692533	9.040869	0.354808	1	-
ENSMUS(	Sav1	salvador f	GO:00325	developm	K16686	scaffold p	mmu0439	Hippo sig	12:699650	425	349	674	637	467	249	3.923026	2.700293	4.285914	3.830478	3.780003	2.849825	3.636411	3.486769	0.669764	1	-
ENSMUS(	Nin	ninein [So	GO:00325	developm	-	-	-	-	12:700114	1338	1500	1417	2113	1449	1024	4.119662	3.871235	3.005568	4.238249	3.912158	3.909232	3.665489	4.01988	0.676928	1	-
ENSMUS(	Pygl	liver glycc	GO:00081	metabolic	K00688	glycogen	mmu011C	Metabolic	12:701908	79	89	111	98	72	56	0.629629	0.594568	0.609445	0.508826	0.50319	0.553392	0.611214	0.521803	0.261036	1	-
ENSMUS(	Bdkrb2	bradykinir	GO:00048	signal trar	K03916	bradykinir	mmu052C	Pathways	12:105563	16	20	19	19	14	5	0.268162	0.280965	0.219366	0.20744	0.205748	0.103896	0.256164	0.172362	0.189249	1	-
ENSMUS(	Trim9	tripartite ı	GO:00081	metabolic	-	-	-	-	12:702445	3706	4422	5263	5779	5464	3415	8.83217	8.833516	8.640656	8.972145	11.41867	10.0911	8.768781	10.16064	0.361717	1	-
ENSMUS(	Tmx1	thioredox	GO:00081	metabolic	-	-	-	-	12:704530	731	908	1143	1032	797	492	12.19671	12.69883	13.1378	11.21726	11.66075	10.17833	12.67778	11.01878	0.178546	1	-
ENSMUS(	Actr10	ARP10 act	GO:00329	macromo	-	-	-	-	12:709378	1440	1917	2280	2337	1731	1408	8.043976	8.976011	8.773941	8.504497	8.479069	9.752072	8.597976	8.911879	0.966736	1	-
ENSMUS(	Timm9	translocas	GO:00511	localizatio	-	-	-	-	12:711231	175	180	345	341	243	296	1.214033	1.046693	1.648786	1.541094	1.478228	2.546069	1.303171	1.85513	0.087564	1	-
ENSMUS(	Ccdc175	coiled-co	-	-	-	-	-	-	12:721013	0	0	3	0	0	0	0	0	0.050091	0	0	0	0.016697	0	0.303405	1	-
ENSMUS(	Rtn1	reticulon	GO:00160	membran	-	-	-	-	12:722117	8029	8720	9591	12084	9140	11214	77.79648	70.82194	64.01973	76.27652	77.6582	134.724	70.87938	96.21958	0.074228	1	-
ENSMUS(	Lrrc9	leucine ric	-	-	-	-	-	-	12:724418	24	21	74	27	38	22	0.148214	0.108705	0.31482	0.108622	0.205781	0.16846	0.19058	0.160954	0.501178	1	-
ENSMUS(	Serpina3n	serine (or	GO:00055	extracellul	-	-	-	-	12:104406	1228	1394	1851	2149	1331	1020	37.81159	35.97842	39.26307	43.10667	35.93743	38.94151	37.68436	39.32854	0.961196	1	-

ENSMUSC	Dhrs7	dehydrog	GO:00081	metabolic	-	-	-	-	12:726503	344	415	484	500	424	263	5.016576	5.072826	4.862343	4.750078	5.421971	4.755436	4.983915	4.975828	0.789286	1	-
ENSMUSC	Ppm1a	protein pl	GO:00048	signal trar	K04457	protein pl	mmu0401	MAPK sigi	12:727574	2645	3073	4052	3714	3251	3030	10.61491	10.33727	11.20241	9.709888	11.44064	15.07715	10.7182	12.07589	0.562413	1	-
ENSMUSC	Clmn	calmin [Sc	GO:00325	developm	-	-	-	-	12:104763	2804	3306	3982	4158	3168	2325	13.08678	12.93333	12.80287	12.64215	12.9653	13.45438	12.94099	13.02061	0.773729	1	-
ENSMUSC	4930447C	RIKEN cDI	GO:00325	developm	-	-	-	-	12:728811	41	112	66	121	61	66	0.362097	0.829114	0.401551	0.696167	0.472411	0.722733	0.530921	0.630437	0.593824	1	-
ENSMUSC	Six6	sine oculis	GO:00325	developm	-	-	-	-	12:729398	0	0	2	0	0	0	0	0	0.021844	0	0	0	0.007281	0	0.536657	1	-
ENSMUSC	Glrx5	glutaredo	GO:00325	developm	-	-	-	-	12:105032	249	337	392	368	312	430	3.564124	4.043298	3.865363	3.431492	3.916073	7.631455	3.824262	4.993007	0.248687	1	-
ENSMUSC	Mnat1	menage a	GO:00325	developm	K10842	CDK-activ	mmu0342	Nucleotid	12:731237	199	255	340	312	278	204	2.17191	2.332824	2.556349	2.218324	2.660582	2.760608	2.353694	2.546505	0.803291	1	-
ENSMUSC	Prkch	protein kii	GO:00325	developm	K18051	novel prot	mmu0427	Vascular s	12:735847	61	64	83	104	60	46	0.377443	0.331937	0.353794	0.419215	0.325552	0.352916	0.354392	0.365894	1	1	-
ENSMUSC	Hif1a	hypoxia ir	GO:00325	developm	K08268	hypoxia-ii	mmu0520	Pathways	12:739013	1491	1797	2326	2396	1803	1095	13.63222	13.77177	14.65042	14.27109	14.4553	12.41334	14.01814	13.71325	0.65693	1	-
ENSMUSC	Papola	poly (A) p	GO:00081	metabolic	K14376	poly(A) pc	mmu0301	mRNA sur	12:105784	1091	1133	1588	1465	983	762	4.902171	4.267235	4.915479	4.288281	3.873113	4.245267	4.694962	4.135554	0.181253	1	-
ENSMUSC	Mpp5	membran	GO:00325	developm	K06091	MAGUK p	mmu0516	Human pa	12:787489	947	1017	1510	955	1003	605	4.564982	4.109253	5.01438	2.998984	4.239673	3.616014	4.562872	3.618224	0.045839	1	-
ENSMUSC	Snapc1	small nucl	GO:00081	metabolic	-	-	-	-	12:739644	671	901	1075	1025	772	681	4.56086	5.133364	5.033667	4.538686	4.601343	5.739279	4.909297	4.95977	0.792158	1	-
ENSMUSC	Atp6v1d	ATPase, H	GO:00511	localizatio	K02149	V-type H-	mmu0110	Metabolic	12:788407	3187	3846	4998	4712	3684	2741	37.11626	37.54432	40.09866	35.74943	37.62222	39.58012	38.25308	37.65059	0.623106	1	-
ENSMUSC	Vrk1	vaccinia re	GO:00325	developm	-	-	-	-	12:106010	202	268	251	280	222	217	2.033861	2.261815	1.740982	1.836574	1.96004	2.709042	2.012219	2.168552	0.872001	1	-
ENSMUSC	Eif2s1	eukaryotic	GO:00325	developm	K03237	translatior	mmu0516	Herpes sir	12:788618	644	819	981	985	686	464	7.961123	8.486433	8.354277	7.932429	7.436271	7.112016	8.267278	7.493572	0.303596	1	-
ENSMUSC	Plek2	pleckstrin	GO:00508	response	-	-	-	-	12:788886	6	9	7	11	0	3	0.240893	0.302852	0.193609	0.287682	0	0.149349	0.245785	0.145677	0.332405	1	-
ENSMUSC	Pigh	phosphati	GO:00081	metabolic	K03858	phosphati	mmu0110	Metabolic	12:790806	178	227	273	289	207	134	2.022971	2.162459	2.137384	2.139675	2.062921	1.888255	2.107605	2.030284	0.643234	1	-
ENSMUSC	Rdh12	retinol del	GO:00081	metabolic	K11153	retinol del	mmu0110	Metabolic	12:792089	45	40	62	63	46	26	0.627036	0.467186	0.595136	0.57187	0.562046	0.449204	0.563119	0.527707	0.696587	1	-
ENSMUSC	Arg2	arginase t	GO:00325	developm	K01476	arginase[3	mmu0110	Metabolic	12:791307	20	29	41	39	5	14	0.890106	1.081815	1.257038	1.130716	0.195114	0.772571	1.07632	0.699467	0.183931	1	-
ENSMUSC	Zfp36l1	zinc finge	GO:00325	developm	K18753	butyrate r	mmu0421	Cellular se	12:801077	493	559	327	724	571	427	5.438579	5.168959	2.485065	5.203057	5.52353	5.840521	4.364201	5.52237	0.296795	1	-
ENSMUSC	Galnt16	polypeptic	GO:00081	metabolic	K00710	polypeptic	mmu0110	Metabolic	12:805182	2218	2548	2620	3265	2383	1598	12.65923	12.18985	10.30146	12.13978	11.92649	11.30859	11.71684	11.79162	0.807687	1	-
ENSMUSC	Erh	enhancer	GO:00325	developm	-	-	-	-	12:806340	210	277	345	257	241	234	3.649475	4.034999	4.130306	2.909548	3.672572	5.042121	3.93826	3.874747	0.668946	1	-
ENSMUSC	Susd6	sushi dom	GO:00508	response	-	-	-	-	12:807905	1727	1926	2578	2727	1954	1694	12.49215	11.67761	12.84633	12.85025	12.39402	15.19302	12.3387	13.4791	0.6933	1	-
ENSMUSC	Srsf5	serine/arg	GO:00325	developm	K12893	splicing fa	mmu0516	Herpes sir	12:809455	1603	2056	2410	2696	1936	1493	9.513958	10.2283	9.85363	10.42388	10.07571	10.98686	9.865298	10.49548	0.84493	1	-
ENSMUSC	Smoc1	SPARC rel	GO:00325	developm	-	-	-	-	12:810268	214	324	340	322	249	264	2.366922	3.003786	2.590606	2.320109	2.414975	3.620426	2.653771	2.78517	0.993899	1	-
ENSMUSC	Gm20498	predicted	GO:00325	developm	-	-	-	-	12:813588	162	82	77	146	95	119	2.368979	1.00511	0.775689	1.390853	1.218181	2.157643	1.383259	1.588892	0.75324	1	-
ENSMUSC	Pcnx	pecanex h	GO:00160	membran	-	-	-	-	12:818600	2791	4466	5719	5926	3103	2195	8.469081	11.35921	11.95496	11.71439	8.256594	8.258419	10.59442	9.409802	0.303919	1	-
ENSMUSC	Pacs2	phosphof	GO:00081	metabolic	-	-	-	-	12:113014	1450	1347	3181	2215	1648	2006	7.754931	6.038517	11.71993	7.717301	7.72876	13.30231	8.504461	9.58279	0.691558	1	-
ENSMUSC	Mta1	metastasis	GO:00081	metabolic	-	-	-	-	12:113098	1241	1264	1424	1526	1240	970	13.08599	11.1721	10.34419	10.48265	11.46566	12.68214	11.53409	11.54348	0.742593	1	-
ENSMUSC	Gtpbp4	GTP bindi	GO:00325	developm	K06943	nucleolar	mmu0300	Ribosome	13:896633	831	991	1233	1254	892	346	4.719621	4.717722	4.824145	4.639649	4.442359	2.436509	4.753829	3.839506	0.176404	1	-
ENSMUSC	Zmynd11	zinc finge	GO:00081	metabolic	-	-	-	-	13:968483	4462	5525	6324	6991	4016	2691	11.21198	11.63692	10.94703	11.4439	8.8489	8.384026	11.26531	9.558941	0.134681	1	-
ENSMUSC	Esyt2	extended	GO:00511	localizatio	-	-	-	-	12:116281	750	994	1118	1232	895	655	3.995035	4.438117	4.102537	4.275151	4.180468	4.326002	4.178563	4.26054	0.883639	1	-
ENSMUSC	Cdca7l	cell divisic	GO:00081	metabolic	-	-	-	-	12:117804	31	28	30	41	27	19	0.537858	0.407197	0.358564	0.463406	0.410776	0.408737	0.43454	0.42764	0.880735	1	-
ENSMUSC	Efcab11	EF-hand c	GO:00431	ion bindin	-	-	-	-	12:996930	0	5	1	7	2	2	0	0.035765	0.005877	0.038917	0.014966	0.021168	0.013881	0.025017	0.547139	1	-
ENSMUSC	Tdp1	tyrosyl-DI	GO:00081	metabolic	-	-	-	-	12:998845	246	324	358	379	301	237	1.483021	1.637237	1.486781	1.488449	1.591191	1.771529	1.53568	1.617056	0.948096	1	-
ENSMUSC	Psmc1	protease (	GO:00081	metabolic	K03062	26S prote	mmu0516	Human pa	12:100110	1651	2031	2374	2087	1809	1198	42.68756	44.01665	42.285	35.15267	41.01433	38.4058	42.9964	38.19093	0.19142	1	-
ENSMUSC	Nrde2	nrde-2 ne	GO:00081	metabolic	-	-	-	-	12:100125	291	362	439	548	350	302	3.898382	4.064924	4.05142	4.782486	4.111522	5.01631	4.004909	4.636773	0.435764	1	-
ENSMUSC	Rps6ka5	ribosomal	GO:00081	metabolic	K04445	ribosomal	mmu0520	Pathways	12:100548	472	557	508	589	475	289	1.732029	1.713253	1.284187	1.408024	1.528444	1.314915	1.57649	1.417128	0.338168	1	-
ENSMUSC	Ccdc88c	coiled-co	GO:00081	metabolic	-	-	-	-	12:100911	1056	1208	1468	1801	1299	885	4.707962	4.514283	4.508647	5.230753	5.078328	4.892134	4.576964	5.067072	0.593519	1	-
ENSMUSC	Dglucy	D-glutam	GO:00081	metabolic	-	-	-	-	12:100779	103	138	158	166	90	104	0.803182	0.902011	0.848765	0.843271	0.61541	1.005535	0.851319	0.821405	0.636467	1	-
ENSMUSC	Fbln5	fibulin 5 [S	GO:00081	metabolic	-	-	-	-	12:101746	69	103	137	91	85	77	0.533218	0.667181	0.729332	0.458118	0.575986	0.737781	0.643244	0.590628	0.459621	1	-
ENSMUSC	Trip11	thyroid hc	GO:00325	developm	-	-	-	-	12:101834	1603	1165	1551	1660	1290	841	8.07318	4.918017	5.381142	5.446292	5.696965	5.251621	6.124113	5.464959	0.310555	1	-
ENSMUSC	Atxn3	ataxin 3 [S	GO:00081	metabolic	K11863	Ataxin-3 [	mmu0414	Protein pr	12:101918	515	682	864	942	669	445	5.225897	5.800844	6.03975	6.227106	5.952817	5.598862	5.68883	5.926262	0.964748	1	-
ENSMUSC	Lgmn	legumain	GO:00325	developm	K01369	legumain[	mmu0414	Lysosome	12:102394	1618	1993	2849	2892	2113	1495	32.03388	33.07434	38.85751	37.30018	36.68371	36.69934	34.65524	36.89441	0.830125	1	-
ENSMUSC	Golga5	golgi autc	GO:00511	localizatio	-	-	-	-	12:102469	416	517	641	690	469	317	6.102969	6.357572	6.478246	6.594444	6.033419	5.766247	6.312929	6.13137	0.642956	1	-
ENSMUSC	Pitrm1	pitrilysin n	GO:00081	metabolic	-	-	-	-	13:654814	700	730	904	981	755	428	6.851545	5.989168	6.095517	6.255203	6.480076	5.194226	6.312077	5.976501	0.530291	1	-
ENSMUSC	Chga	chromogr	GO:00081	metabolic	-	-	-	-	12:102554	2418	3083	3371	3522	2655	1804	47.82793	51.11541	45.93413	45.38333	46.05031	44.24334	48.29249	45.22566	0.382899	1	-
ENSMUSC	Pfkp	phosphof	GO:00081	metabolic	-	-	mmu0110	Metabolic	13:657976	4039	5190	5789	6060	4841	3153	16.13797	17.38182	15.93419	15.77355	16.96106	15.62014	16.48466	16.11825	0.613822	1	-
ENSMUSC	Unc79	unc-79 hc	GO:00325	developm	-	-	-	-	12:102948	1601	1846	2348	2629	1960	1092	5.94985	5.750414	6.01124	6.36483	6.387241	5.0318	5.903835	5.927957	0.834133	1	-
ENSMUSC	Asb2	ankyrin re	GO:00325	developm	-	-	-	-	12:103321	37	38	49	78	56	26	0.526286	0.453066	0.480147	0.722768	0.698476	0.458554	0.4865	0.626599	0.293441	1	-
ENSMUSC	Otub2	OTU dom	GO:00081	metabolic	-	-	-	-	12:103388	616	732	927	988	762	664	4.467495	4.449869	4.631425	4.667904	4.845973	5.970866	4.516263	5.161581	0.476494	1	-
ENSMUSC	Ppp4r4	protein pl	GO:00081	metabolic	-	-	-	-	12:103532	934	1163	1298	1499	1055	766	11.94817	12.4706	11.4388	12.49217	11.83449	12.14981	11.95252	12.15882	0.869509	1	-
ENSMUSC	Akr1c12	aldo-keto	GO:00081	metabolic	-	-	-	-	13:426817	0	0	0	2	0	0	0	0	0	0.065657	0	0	0	0.021886	0.536837	1	-
ENSMUSC	Akr1c13	aldo-keto	GO:00081	metabolic	-	-	-	-	13:419115	3	8	11	9	5	2	0.109561	0.244911	0.276774	0.214143	0.160128	0.090598	0.210415	0.154956	0.498455	1	-
ENSMUSC	Akr1c18	aldo-keto	GO:00081	metabolic	K05295	20alpha-h	mmu0014	Steroid hc	13:413261	30	35	39	69	26	23	1.241281	1.213863	1.111639	1.859842	0.943325	1.179949	1.188928	1.327705	0.755637	1	-
ENSMUSC	Net1	neuroepit	GO:00325	developm	-	-	-	-	13:388201	624	890	1189	1068	725	490	5.077079	6.069774	6.664427	5.660858	5.172614	4.943235	5.937093	5.258902	0.250592	1	-
ENSMUSC	Tshz3	teashirt zi	GO:00081	metabolic	-	-	-	-	7:3669811	222	328	533	455	408	252	2.55177	3.160215	4.220544	3.407083	4.112371	3.591495	3.310843	3.70365	0.635425	1	-
ENSMUSC	Gdi2	guanosine	GO:00511	localizatio	-	-	-	-	13:353806	1821	2277	2894	3678	2096	1895	8.098104	8.487688	8.865913	10.65533	8.173487	10.44886	8.483901	9.759224	0.410522	1	-
ENSMUSC	Rgs6	regulator	GO:00048	signal trar	-	-	-	-	12:825882	88	126	340	402	368	86	0.35752	0.429081	0.951583	1.063956	1.311012	0.433214	0.579395	0.936061	0.105506	1	-
ENSMUSC	Dpf3	D4, zinc a	GO:00325	developm	-	-	mmu0522	Hepatoce	12:832137	189	212	303	380	230	162	1.650157	1.551504	1.822464	2.161372	1.760897	1.753744	1.674708	1.892004	0.56511	1	-
ENSMUSC	Dcaf4	DDB1 anc	GO:00081	metabolic	-	-	-	-	12:835204	265	269	348	335	301	271	3.488301	2.96807	3.155731	2.87273	3.474387	4.42307	3.204034	3.590062	0.66051	1	-
ENSMUSC	Papln	papilin, pr	GO:00081	metabolic	-	-	-	-	12:837636	15	19	22	32	21	17	0.139842	0.148474	0.14129	0.194345	0.171677	0.196503	0.143202	0.187508	0.385951	1	-
ENSMUSC	Numb	numb hor	GO:00325	developm	K06057	numb[-]	mmu0433	Notch sigi	12:837940	435	201	491	464	413	286	2.754366	1.066797	2.141733	1.913955	2.293108	2.245351	1.987632	2.150805	0.851255	1	-
ENSMUSC	Acot2	acyl-CoA	GO:00081	metabolic	K01068	acyl-coen	mmu0110	Metabolic	12:839878	93	146	140	174	137	154	2.691466	3.541682	2.791151	3.280468	3.476688	5.526005	3.0081	4.094387	0.154224	1	-
ENSMUSC	Acot3	acyl-CoA	GO:00081	metabolic	K01068	acyl-coen	mmu0110	Metabolic	12:840521	5	110	11	4	12	13	0.049466	0.912169	0.074967	0.025782	0.104102	0.159459	0.345534	0.096448	0.039071	1	-
ENSMUSC	Fam161b	family witl	-	-	-	-	-	-	12:843453	267	302	386	375	291	214	2.181341	2.0681	2.172456	1.99584	2.08472	2.167766	2.140632	2.082776	0.638321	1	-
ENSMUSC	Coq6	coenzyme	GO:00081	metabolic	K06126	ubiquinon	mmu0110	Metabolic	12:843616	91	149	134	203	139	165	0.78333	1.075083	0.794622	1.138362	1.049209	1.761062	0.884345	1.316211	0.055295	1	-
ENSMUSC	Entpd5	ectonucle	GO:00081	metabolic	K01511	ectonucle	mmu0023	Purine me	12:843738	463	544	807	795	608	443	2.044542	2.01357	2.454934	2.286988	2.354296	2.425516	2.171015	2.3556	0.745618	1	-
ENSMUSC	Aldh6a1	aldehyde	GO:00325	developm	K00140	malonate-	mmu0110	Metabolic	12:844307	1007	800	1151	1597	882	867	10.08317	6.714448	7.939519	10.41728	7.744241	10.76397	8.245712	9.641831	0.430398	1	-
ENSMUSC	Vsx2	visual syst	GO:00325	developm	-	-	-	-	12:845697	10	5	4	7	6	4	0.103693	0.043456	0.028577	0.047286	0.054552	0.051427	0.058575	0.051088	0.798069	1	-
ENSMUSC	Abcd4	ATP-bind	GO:00081	metabolic	K05678	ATP-bind	mmu0414	Peroxisom	12:846014	134	183	217	256	175	225	0.624944	0.715386	0.697183	0.777781	0.715675	1.301084	0.679171	0.931513	0.127135	1	-
ENSMUSC	Isca2	iron-sulfu	GO:00081	metabolic	-	-	-	-	12:847732	150	194	209	243	207	161	1.488133	1.613255	1.42839	1.570494	1.800789	1.98043	1.509926	1.783904	0.409473	1	-
ENSMUSC	Npc2	NPC intra	GO:00081	metabolic	K13443	Niemann-	mmu0414	Lysosome	12:847545	443	929	1335	1081	798	773	4.285884	7.533646	8.897531	6.813092	6.769905	9.272611	6.905687	7.618536	0.749234	1	-
ENSMUSC	Fcf1	FCF1 rRN/	GO:00081	metabolic	K14566	U3 small r	mmu0300	Ribosome	12:849708	35	55	80	49	52	93	1.526682	2.010921	2.403901	1.392359	1.988946	5.029716	1.980501	2.803674	0.334747	1	-
ENSMUSC	Ylpm1	YLP motif	GO:00081	metabolic	-	-	-	-	12:849963	3444	4040	4694	5662	4163	2057	15.19765	14.94333	14.26947	16.27664	16.10877	11.25469	14.80348	14.5467	0.737195	1	-
ENSMUSC	Mlh3	mutL hom	GO:00081	metabolic	K08739	DNA misn	mmu0343	Mismatch	12:852345	573	744	833	905	608	410	3.471816	3.778575	3.476949	3.572171	3.230339	3.080149	3.57578	3.294219	0.380357	1	-
ENSMUSC	Tmed10	transmem	GO:00325	developm	-	-	-	-	12:853406	1327	2110	2774	2704	2070	1455	11.70829	15.60479	16.8609	15.54215	16.01532	15.91738	14.72466	15.82495	0.783613	1	-
ENSMUSC	Fos	FBJ osteos	GO:00325	developm	K04379	proto-onc	mmu0520	Pathways	12:854738	106	98	237	117	103	194	1.776538	1.376728	2.736328	1.277423	1.51373	4.031401	1.963198	2.274185	0.776461	1	-
ENSMUSC	Erg28	ergostero	GO:00081	metabolic	-	-	-	-	12:858154	516	645	733	765	579	465	5.835117	6.113817	5.710249	5.635635	5.741442	6.519867	5.886394	5.965648	0.829376	1	-
ENSMUSC	Tgfb3	transform	GO:00325	developm	K13377	transform	mmu0520	Pathways	12:860567	227	285	427	348	267	217	4.264412	4.487788	5.526038	4.258876	4.398327	5.054529	4.759413	4.570577	0.577372	1	-
ENSMUSC	Gpatch2l	G patch d	-	-	-	-	-	-	12:862418	619	737	944	967	701	571	3.39923	3.392426	3.571195	3.45938	3.375597	3.887877	3.454284	3.574285	0.960416	1	-
ENSMUSC	Esrrb	estrogen r	GO:00325	developm	K08553	estrogen-	mmu0455	Signaling	12:863611	36	30	38	52	12	33	0.211079	0.14744	0.153489	0.198625	0.061698	0.239907	0.170669	0.166743	0.838005	1	-
ENSMUSC	Vash1	vasohibin	GO:00325	developm	-	-	-	-	12:866787	522	498	652	839	519	326	12.76441	10.20733	10.9832	13.36514	11.12857	9.884015	11.31831	11.45924	0.900686	1	-
ENSMUSC	Angel1	angel hon	GO:00056	cell|intrace	-	-	-	-	12:867005	125	151	199	193	119	147	1.541055	1.560402	1.690096	1.550053	1.286462	2.24704	1.597184	1.694518	0.969374	1	-
ENSMUSC	Ccnk	cyclin K [S	GO:00081	metabolic	-	-	-	-	12:108179	449	524	657	666	487	219	5.871462	5.743597	5.918572	5.673559	5.584335	3.550829	5.844544	4.936241	0.231807	1	-
ENSMUSC	Cyp46a1	cytochron	GO:00081	metabolic	K07440	cholesterc	mmu0012	Primary b	12:108334	1980	2496	2775	2989	2231	2194	30.35825	32.07812	29.31069	29.85514	29.99535	41.70942	30.58235	33.8533	0.652171	1	-
ENSMUSC	Hhipl1	hedgehog	GO:00511	localizatio	-	-	-	-	12:108306	178	196	210	244	211	144	1.971841	1.81995	1.602585	1.760854	2.049634	1.977876	1.798125	1.929455	0.840797	1	-
ENSMUSC	Evl	Ena-vaso	GO:00325	developm	-	-	-	-	12:108554	1139	1385	1714	1679	1259	919	15.69533	15.9974	16.2708	15.07228	15.21302	15.70175	15.98784	15.32902	0.499088	1	-
ENSMUSC	Yy1	YY1 trans	GO:00325	developm	-	-	-	-	12:108792	1520	1893	2366	2336	1772	1199	14.47634	15.11188	15.5232	14.49337	14.79863	14.1586	15.03714	14.48353	0.535955	1	-
ENSMUSC	Slc25a29	solute car	GO:00511	localizatio	-	-	-	-	12:108825	189	248	294	260	227	292	3.839992	4.223516	4.114981	3.441319	4.044244	7.355928	4.059496	4.947164	0.436602	1	-
ENSMUSC	Wars	tryptopha	GO:00325	developm	K01867	tryptopha	mmu0097	Aminoacy	12:108860	1514	1719	1201	1232	1797	879	19.94595	18.98268	10.89992	10.57355	20.75963	14.35832	16.60951	15.2305	0.5066	1	-
ENSMUSC	Hsp90aa1	heat shoc	GO:00325	developm	K04079	molecular	mmu0520	Pathways	12:110690	8043	10337	11467	12135	8891	4497	77.78981	83.80155	76.4022	76.45855	75.40461	53.9279	79.33119	68.59702	0.203069	1	-
ENSMUSC	Zfp839	zinc finge	GO:00971	organic cy	-	-	-	-	12:110850	213	238	208	262	171	173	2.579906	2.416318	1.735563	2.067322	1.816201	2.598115	2.243929	2.160546	0.617094	1	-
ENSMUSC	Fdft1	farnesyl d	GO:00081	metabolic	K00801	farnesyl-c	mmu0110	Metabolic	14:631451	842	1066	1080	961	777	968	8.091888	8.587122	7.150113	6.01648	6.547888	11.5345	7.943041	8.032955	0.776806	1	-
ENSMUSC	Tecpr2	tectonin b	-	-	-	-	-	-	12:110889	1684	1946	2016	2382	1942	1505	6.378025	6.177885	5.260005	5.87716	6.44965	7.067515	5.938638	6.464775	0.72518	1	-
ENSMUSC	Cinp	cyclin-dep	GO:00081	metabolic	-	-	-	-	12:110872	460	593	668	649	452	419	3.923555	4.239639	3.92509	3.606184	3.380666	4.431197	4.029428	3.806016	0.445511	1	-
ENSMUSC	Traf3	TNF recep	GO:00081	metabolic	K03174	TNF recep	mmu0520	Pathways	12:111166	927	1768	1346	1900	1099	954	2.919408	4.667133	2.920192	3.898075	3.034978	3.725197	3.502244	3.55275	0.873008	1	-
ENSMUSC	Amn	amnionles	GO:00325	developm	-	-	-	-	12:111271	28	35	64	62	36	49	0.721604	0.75608	1.136248	1.040917	0.813563	1.565748	0.871311	1.140076	0.354982	1	-
ENSMUSC	Cdc42bpb	CDC42 bii	GO:00081	metabolic	-	-	-	-	12:111292	3240	3536	4549	5007	3541	3010	22.00147	20.12666	21.28012	22.1496	21.08508	25.34306	21.13608	22.85925	0.747207	1	-
ENSMUSC	Exoc3l4	exocyst cc	GO:00511	localizatio	-	-	-	-	12:111417	28	25	33	30	28	32	0.175128	0.131066	0.142187	0.122237	0.153567	0.248162	0.14946	0.174655	0.743176	1	-
ENSMUSC	Tnfaip2	tumor nec	GO:00325	developm	-	-	-	-	12:111442	9	24	23	25	22	15	0.064673	0.14456	0.11386	0.117032	0.138631	0.133651	0.107698	0.129771	0.605633	1	-
ENSMUSC	Eif5	eukaryotic	GO:00081	metabolic	K03262	translatior	mmu0301	RNA trans	12:111538	3227	2466	2626	4377	1718	1255	10.79114	6.912166	6.04943	9.535125	5.037721	5.20353	7.917578	6.592125	0.278128	1	-
ENSMUSC	Ppp1r13b	protein pl	GO:00508	response	-	-	-	-	12:111828	2445	2753	3124	3756	2593	1681	26.72178	25.22002	23.52063	26.74198	24.85029	22.77929	25.15414	24.79052	0.690181	1	-
ENSMUSC	Zfyve21	zinc finge	GO:00056	cell|cell jui	-	-	-	-	12:111814	93	91	131	113	120	104	1.60654	1.317646	1.558944	1.271651	1.817732	2.227537	1.494377	1.772306	0.519105	1	-
ENSMUSC	Xrcc3	X-ray rep	GO:00081	metabolic	K10880	DNA-repa	mmu0344	Homologo	12:111803	73	79	72	100	60	89	0.848617	0.769783	0.576593	0.757302	0.611623	1.282806	0.731664	0.88391	0.538317	1	-
ENSMUSC	Klc1	kinesin lig	GO:00511	localizatio	K10407	kinesin lig	mmu0513	Salmonell	12:111758	10802	13592	15982	15713	12822	8356	99.21948	104.6475	101.1289	94.02287	103.2741	95.16498	101.6653	97.4873	0.487742	1	-
ENSMUSC	2010107E	RIKEN cDI	GO:00329	macromo	-	-	-	-	12:111961	179	264	347	241	177	501	6.21637	7.684974	8.301672	5.452346	5.390137	21.57291	7.401006	10.80513	0.276577	1	-
ENSMUSC	Kif26a	kinesin fai	GO:00325	developm	-	-	-	-	12:112146	132	119	151	189	148	141	1.165783	0.880939	0.918698	1.087395	1.146169	1.544004	0.988474	1.259189	0.249421	1	-
ENSMUSC	Gpr132	G protein-	GO:00048	signal trar	-	-	-	-	12:112850	0	0	0	2	0	0	0	0	0	0.030161	0	0	0	0.010054	0.537288	1	-
ENSMUSC	Hecw1	HECT, C2	GO:00081	metabolic	-	-	-	-	13:142264	1187	893	1819	1405	990	708	3.489564	2.200519	3.683873	2.690783	2.552104	2.580713	3.124652	2.607867	0.141937	1	-
ENSMUSC	Ggps1	geranylge	GO:00081	metabolic	K00804	geranylge	mmu0110	Metabolic	13:140506	956	1184	1433	1525	1023	333	5.284569	5.486006	5.45694	5.491652	4.958724	2.282349	5.409172	4.244242	0.184369	1	-
ENSMUSC	Gng4	guanine n	GO:00048	signal trar	K04541	guanine n	mmu0520	Pathways	13:137840	521	865	1000	864	690	487	9.184779	12.78202	12.14457	9.922619	10.66651	10.64501	11.37046	10.41138	0.354404	1	-
ENSMUSC	Gpr137b	G protein-	GO:00160	membran	-	-	-	-	13:133576	160	232	295	298	195	231	0.944584	1.148055	1.199765	1.146095	1.009485	1.690907	1.097468	1.282162	0.521162	1	-
ENSMUSC	Mtr	5-methylt	GO:00325	developm	K00548	5-methylt	mmu0110	Metabolic	13:121827	514	652	823	890	702	465	3.327855	3.538359	3.670732	3.753817	3.985484	3.73285	3.512315	3.82405	0.706236	1	-
ENSMUSC	Ryr2	ryanodine	GO:00325	developm	K04962	ryanodine	mmu0402	cAMP sigr	13:115531	7853	8995	10440	13978	9313	6318	15.02851	14.42894	13.76361	17.42637	15.62833	14.99153	14.40702	16.01541	0.558166	1	-
ENSMUSC	Amph	amphiphy	GO:00452	synapse|lc	K12562	amphiphy	mmu0414	Endocytos	13:189482	4520	5365	5841	6286	4900	3566	46.81545	46.5772	41.67632	42.41376	44.50303	45.795	45.02299	44.23726	0.621158	1	-
ENSMUSC	Gli3	GLI-Krupp	GO:00325	developm	K06230	zinc finge	mmu0520	Pathways	13:154632	217	223	289	345	237	165	1.330667	1.146218	1.220836	1.378192	1.274382	1.254527	1.232574	1.302367	0.90303	1	-
ENSMUSC	Sfrp4	secreted f	GO:00325	developm	K02185	secreted f	mmu0431	Wnt signa	13:196231	47	43	98	87	39	40	0.430525	0.330159	0.618414	0.51916	0.313263	0.454304	0.459699	0.428909	0.65665	1	-
ENSMUSC	Aoah	acyloxyac	GO:00081	metabolic	-	-	-	-	13:207941	4	0	0	7	0	2	0.037986	0	0	0.043308	0	0.023557	0.012662	0.022288	0.708203	1	-
ENSMUSC	Trim27	tripartite r	GO:00081	metabolic	-	-	-	-	13:211794	399	508	554	462	433	336	1.92863	2.058224	1.844752	1.45479	1.8353	2.013735	1.943869	1.767942	0.315532	1	-
ENSMUSC	Zkscan3	zinc finge	GO:00081	metabolic	-	-	-	-	13:213870	523	622	937	847	664	339	2.235553	2.228574	2.759147	2.35857	2.488823	1.796672	2.407758	2.214688	0.448071	1	-
ENSMUSC	Scgn	secretago	GO:00452	synapse|c	-	-	-	-	13:239534	16	12	17	13	11	19	0.536324	0.337169	0.392543	0.283879	0.323325	0.789665	0.422012	0.465623	1	1	-
ENSMUSC	Carmil1	capping p	GO:00325	developm	-	-	-	-	13:240123	500	651	858	880	705	421	2.536235	2.767919	2.998186	2.907931	3.135824	2.647816	2.767447	2.897191	0.93115	1	-
ENSMUSC	Mrs2	MRS2 ma	GO:00081	metabolic	-	-	-	-	13:249874	1685	2079	2638	2769	2021	1357	13.26153	13.71516	14.30276	14.19705	13.9477	13.24217	13.75981	13.79564	0.781806	1	-
ENSMUSC	Gpld1	glycosylpl	GO:00325	developm	-	-	mmu0056	Glycosylpl	13:249431	425	590	679	741	568	607	4.665751	5.429218	5.135158	5.29947	5.467936	8.262424	5.076709	6.343277	0.221742	1	-
ENSMUSC	Exoc2	exocyst cc	GO:00511	localizatio	K17637	exocyst cc	mmu0401	Ras signal	13:308139	928	1072	1150	1092	1036	710	7.504418	7.266351	6.406459	5.752717	7.346345	7.118908	7.059076	6.739323	0.498947	1	-
ENSMUSC	Tfap2a	transcripti	GO:00325	developm	-	-	-	-	13:407153	7	0	4	3	0	3	0.039858	0	0.015691	0.011126	0	0.021181	0.018517	0.010769	0.495902	1	-
ENSMUSC	Gcnt2	glucosam	GO:00325	developm	K00742	N-acetylla	mmu0110	Metabolic	13:408597	293	324	325	552	313	190	1.314861	1.218737	1.004723	1.613734	1.231683	1.057186	1.17944	1.300868	0.682162	1	-
ENSMUSC	Tmem14c	transmem	GO:00325	developm	-	-	-	-	13:410162	246	349	370	340	276	354	17.10505	20.34083	17.72324	15.40106	16.82836	30.51962	18.3897	20.91634	0.67711	1	-
ENSMUSC	Mak	male gerr	GO:00081	metabolic	-	-	-	-	13:410250	35	44	63	43	60	21	0.163569	0.172359	0.202825	0.130913	0.245882	0.121682	0.179584	0.166159	0.676914	1	-
ENSMUSC	Elovl2	elongatior	GO:00081	metabolic	K10205	elongatior	mmu0121	Fatty acid	13:411823	281	347	434	105	347	235	2.597584	2.688724	2.763786	0.632319	2.812776	2.6935	2.683364	2.046198	0.223582	1	-
ENSMUSC	Nedd9	neural pre	GO:00099	cellular pr	-	-	-	-	13:413095	257	343	366	395	278	150	1.711708	1.914887	1.679301	1.71386	1.623618	1.238725	1.768632	1.525401	0.262491	1	-
ENSMUSC	Hivep1	human im	GO:00081	metabolic	-	-	-	-	13:420520	1118	1119	1823	1947	1542	890	6.418453	5.384825	7.209857	7.281756	7.762747	6.335261	6.337712	7.126588	0.532194	1	-
ENSMUSC	Edn1	endothelir	GO:00325	developm	K16366	endothelir	mmu0541	Fluid shea	13:423014	26	38	32	24	23	28	0.772513	0.946376	0.654976	0.464533	0.59924	1.031498	0.791288	0.698424	0.484296	1	-
ENSMUSC	Tbc1d7	TBC1 dom	GO:00081	metabolic	K20396	TBC1 dom	mmu0415	mTOR sig	13:431517	74	107	85	97	62	116	0.762714	0.924419	0.603535	0.65131	0.560362	1.482435	0.763556	0.898036	0.702047	1	-
ENSMUSC	Mcur1	mitochon	GO:00511	localizatio	-	-	-	-	13:435383	561	626	838	824	605	444	4.675218	4.37287	4.810993	4.473509	4.421171	4.587843	4.619693	4.494175	0.607558	1	-
ENSMUSC	Cap2	CAP, ader	GO:00325	developm	-	-	-	-	13:465018	4568	5723	6318	7271	5473	3369	46.05174	48.36114	43.87842	47.75245	48.38247	42.11209	46.0971	46.08234	0.774617	1	-
ENSMUSC	Nup153	nucleopoi	GO:00511	localizatio	K14296	nuclear pc	mmu0301	RNA trans	13:466799	928	1031	1465	1376	1025	671	5.916062	5.509293	6.433888	5.714583	5.729953	5.303873	5.953081	5.582803	0.429774	1	-
ENSMUSC	Kif13a	kinesin fai	GO:00511	localizatio	-	-	-	-	13:467490	597	622	970	949	760	519	3.332263	2.910103	3.729822	3.450745	3.719815	3.591854	3.324063	3.587471	0.770159	1	-
ENSMUSC	Tpmt	thiopurine	GO:00081	metabolic	K00569	thiopurine	mmu0098	Drug meta	13:470224	154	195	203	224	182	132	2.073994	2.201268	1.883363	1.965241	2.149317	2.204171	2.052875	2.106243	0.941378	1	-
ENSMUSC	Dek	DEK onco	GO:00081	metabolic	-	-	-	-	13:470847	991	1320	1599	1448	1122	593	10.90203	12.17196	12.11808	10.37729	10.82354	8.088617	11.73069	9.763147	0.118624	1	-
ENSMUSC	Id4	inhibitor c	GO:00325	developm	K17695	DNA-binc	mmu0455	Signaling	13:482612	1523	1661	2317	2139	1701	1036	25.14711	22.9885	26.35517	23.0081	24.62834	21.20968	24.83026	22.9487	0.3641	1	-
ENSMUSC	Susd3	sushi dom	GO:00160	membran	-	-	-	-	13:492306	23	14	20	33	24	24	0.361719	0.18456	0.21669	0.338095	0.330982	0.467998	0.254323	0.379025	0.193571	1	-
ENSMUSC	Ippk	inositol 1,:	GO:00081	metabolic	K10572	inositol-p	mmu0110	Metabolic	13:494212	615	766	820	1024	678	458	4.982166	5.201457	4.576235	5.40412	4.816325	4.600387	4.919953	4.940277	0.823738	1	-
ENSMUSC	Aspn	asporin [S	GO:00325	developm	-	-	-	-	13:495444	31	52	52	61	37	14	0.496516	0.698098	0.573749	0.636468	0.51965	0.278032	0.589454	0.47805	0.393557	1	-
ENSMUSC	Ogn	osteoglyci	GO:00325	developm	-	-	-	-	13:496080	86	190	228	196	136	72	2.129992	3.944443	3.89014	3.162397	2.953656	2.211043	3.321525	2.775699	0.307504	1	-
ENSMUSC	Cenpp	centrome	GO:00099	cellular pr	-	-	-	-	13:494640	17	21	10	21	13	14	0.393006	0.406943	0.159266	0.316277	0.263541	0.401321	0.319738	0.327046	1	1	-
ENSMUSC	Nol8	nucleolar	GO:00081	metabolic	-	-	-	-	13:496530	680	812	1020	1084	754	376	3.20594	3.208893	3.31282	3.329331	3.117167	2.197964	3.242551	2.881487	0.335134	1	-
ENSMUSC	Spin1	spindlin 1	GO:00325	developm	-	-	-	-	13:511008	3271	4042	4934	5144	3732	3274	25.88493	26.81116	26.89782	26.51851	25.89708	32.12412	26.53131	28.1799	0.876811	1	-
ENSMUSC	Nxnl2	nucleored	GO:00162	antioxidar	-	-	-	-	13:511710	1	5	0	5	1	4	0.015614	0.065424	0	0.050853	0.013688	0.077425	0.027013	0.047322	0.552084	1	-
ENSMUSC	Wrnip1	Werner he	GO:00081	metabolic	-	-	-	-	13:328020	978	1268	1436	1397	1137	819	10.21779	11.10428	10.33532	9.508158	10.41649	10.60933	10.55247	10.17799	0.531796	1	-
ENSMUSC	Serpinb9b	serine (or	GO:00081	metabolic	K13963	serpin B[-	mmu0514	Amoebias	13:330274	0	0	2	0	0	0	0	0	0.02471	0	0	0	0.008237	0	0.53714	1	-
ENSMUSC	Ripk1	receptor (	GO:00325	developm	K02861	receptor-	mmu0516	Epstein-B	13:340023	94	138	241	178	152	53	0.565074	0.695364	0.998038	0.697077	0.801247	0.395037	0.752825	0.63112	0.37428	1	-
ENSMUSC	Pxdc1	PX domai	GO:00431	ion bindin	-	-	-	-	13:346278	94	132	106	126	127	153	1.062794	1.250974	0.825618	0.928055	1.259129	2.144857	1.046462	1.444014	0.215537	1	-
ENSMUSC	Prpf4b	pre-mRN.	GO:00081	metabolic	-	-	-	-	13:348753	1945	2381	2995	3287	2429	1207	8.746861	8.975213	9.278563	9.629723	9.578615	6.730167	9.000212	8.646168	0.621899	1	-
ENSMUSC	Fam217a	family witl	-	-	-	-	-	-	13:349099	0	13	17	12	13	9	0	0.111592	0.119926	0.080058	0.116738	0.114272	0.077173	0.103689	0.666183	1	-
ENSMUSC	Eci2	enoyl-Coe	GO:00081	metabolic	K13239	peroxisom	mmu0414	Peroxisom	13:349777	378	398	584	534	441	287	2.735799	2.414505	2.911766	2.517764	2.798813	2.575489	2.687356	2.630688	0.678535	1	-
ENSMUSC	Rpp40	ribonuclea	GO:00081	metabolic	K14530	ribonuclea	mmu0301	RNA trans	13:358934	67	94	109	121	82	37	0.546044	0.642142	0.61197	0.642423	0.586014	0.373884	0.600052	0.534107	0.513584	1	-
ENSMUSC	Fars2	phenylala	GO:00081	metabolic	K01889	phenylala	mmu0097	Aminoacy	13:361174	212	231	289	288	212	220	1.558864	1.423759	1.46393	1.379575	1.366941	2.005762	1.482184	1.584093	0.919608	1	-
ENSMUSC	Ly86	lymphocy	GO:00023	immune s	-	-	-	-	13:373452	108	115	149	171	96	91	1.797722	1.604548	1.708586	1.85429	1.401241	1.878141	1.703619	1.711224	0.853245	1	-
ENSMUSC	Ssr1	signal seq	GO:00081	metabolic	K13249	translocor	mmu0414	Protein pr	13:379666	2290	1739	2387	2398	1779	2196	12.47954	7.943565	8.961215	8.51322	8.501227	14.8382	9.794773	10.61755	0.869091	1	-
ENSMUSC	Riok1	RIO kinas	GO:00081	metabolic	K07178	RIO kinas	mmu0300	Ribosome	13:380369	206	243	346	375	236	129	0.842955	0.833842	0.975359	0.999655	0.846821	0.654502	0.883932	0.833659	0.610749	1	-
ENSMUSC	Snrnp48	small nucl	GO:00081	metabolic	-	-	-	-	13:382049	292	360	615	600	426	283	1.39551	1.442132	2.024774	1.868026	1.785261	1.676957	1.620805	1.776748	0.703905	1	-
ENSMUSC	Slc35b3	solute car	GO:00511	localizatio	-	-	-	-	13:389321	68	126	107	131	79	137	0.459502	0.713675	0.498093	0.576674	0.468109	1.147842	0.55709	0.730875	0.357224	1	-
ENSMUSC	Shc3	src homol	GO:00325	developm	K17448	SHC-trans	mmu0401	Ras signal	13:514310	971	962	1208	1580	1114	713	13.12979	10.90351	11.2527	13.918	13.20889	11.95401	11.762	13.02696	0.607532	1	-

ENSMUSC	Sema4d	sema don	GO:00325	developm	K06521	semaphor	mmu0436	Axon guic	13:517012	825	816	1109	1166	588	902	4.795685	3.975939	4.440991	4.415471	2.997209	6.501137	4.404205	4.637939	0.996336	1	-
ENSMUSC	Gadd45g	growth ar	GO:00081	metabolic	K04402	growth ar	mmu0520	Pathways	13:518466	221	227	267	300	231	228	7.262272	6.252571	6.044248	6.422185	6.656334	9.289658	6.519697	7.456059	0.571041	1	-
ENSMUSC	Fbp2	fructose b	GO:00081	metabolic	K03841	fructose-1	mmu0110	Metabolic	13:628368	0	0	3	1	2	0	0	0	0.089406	0.02818	0.075867	0	0.029802	0.034682	1	1	-
ENSMUSC	Syk	spleen tyr	GO:00325	developm	K05855	spleen tyr	mmu0415	PI3K-Akt s	13:525831	85	122	160	139	131	76	0.39575	0.47612	0.513188	0.421599	0.534832	0.438735	0.461686	0.465056	0.879751	1	-
ENSMUSC	2010111I(	RIKEN cDI	GO:00081	metabolic	-	-	-	-	13:629648	328	259	400	382	344	345	0.887301	0.587286	0.745433	0.673197	0.816016	1.157186	0.740007	0.882133	0.452322	1	-
ENSMUSC	Auh	AU RNA b	GO:00081	metabolic	K05607	methylglu	mmu0110	Metabolic	13:528351	810	964	1200	1218	887	595	4.077136	4.067243	4.161051	3.99392	3.915042	3.713415	4.10181	3.874126	0.461206	1	-
ENSMUSC	Fancc	Fanconi a	GO:00325	developm	K10890	fanconi ar	mmu0346	Fanconi a	13:632850	72	61	112	74	51	102	0.330695	0.234843	0.354374	0.221416	0.205404	0.580874	0.306637	0.335898	0.947172	1	-
ENSMUSC	Ror2	receptor t	GO:00325	developm	-	-	-	-	13:531093	20	25	32	36	13	13	0.190508	0.199604	0.209981	0.223392	0.108584	0.153532	0.200031	0.161836	0.389044	1	-
ENSMUSC	Ptch1	patched 1	GO:00325	developm	K06225	patched 1	mmu0520	Pathways	13:635083	1026	1080	1545	1353	1000	854	2.525872	2.228644	2.620258	2.169918	2.158771	2.606801	2.458258	2.31183	0.397413	1	-
ENSMUSC	Sptlc1	serine pal	GO:00081	metabolic	K00654	serine pal	mmu0110	Metabolic	13:533327	385	520	657	658	544	478	6.639861	7.51718	7.805769	7.392751	8.22697	10.22144	7.320937	8.613721	0.383106	1	-
ENSMUSC	Msx2	msh hom	GO:00325	developm	K09341	homeobo	mmu0516	HTLV-I inf	13:534668	5	6	20	13	6	8	0.079007	0.079465	0.217713	0.133823	0.08313	0.156736	0.125395	0.124563	0.923983	1	-
ENSMUSC	Ercc6l2	excision r	GO:00081	metabolic	-	-	-	-	13:638152	429	466	586	634	485	307	1.37928	1.255839	1.29791	1.327903	1.367351	1.223825	1.31101	1.306359	0.782676	1	-
ENSMUSC	Sfxn1	sideroflex	GO:00325	developm	-	-	-	-	13:540718	618	867	967	976	752	713	3.840777	4.5165	4.140076	3.951497	4.098177	5.494214	4.165784	4.514629	0.794829	1	-
ENSMUSC	Habp4	hyaluronic	GO:00081	metabolic	-	-	-	-	13:641618	2078	2550	3179	3051	2456	1921	17.02284	17.50973	17.94024	16.28209	17.64239	19.5119	17.49094	17.81213	0.841568	1	-
ENSMUSC	Ctsl	cathepsin	GO:00325	developm	K01365	cathepsin	mmu0520	Proteogly	13:643593	959	1223	1643	1611	1129	875	7.109219	7.599461	8.390588	7.780013	7.339052	8.042614	7.699756	7.72056	0.769437	1	-
ENSMUSC	Drd1	dopamine	GO:00325	developm	K04144	dopamine	mmu0408	Neuroacti	13:540511	82	112	122	135	68	63	0.6942	0.794766	0.711509	0.744532	0.504801	0.661298	0.733492	0.636877	0.330726	1	-
ENSMUSC	Zfp346	zinc finge	GO:00508	response	-	-	-	-	13:551053	293	354	425	450	407	249	2.101937	2.128666	2.100352	2.103029	2.560286	2.214815	2.110318	2.29271	0.745685	1	-
ENSMUSC	Aaed1	AhpC/TSA	GO:00081	metabolic	-	-	-	-	13:642752	169	206	291	272	205	151	1.389814	1.420003	1.648592	1.4572	1.478313	1.539685	1.486137	1.491733	0.820677	1	-
ENSMUSC	Cdk20	cyclin-dep	GO:00325	developm	-	-	-	-	13:644323	92	130	140	166	152	125	0.403318	0.477698	0.422802	0.474074	0.584313	0.679442	0.434606	0.579276	0.14555	1	-
ENSMUSC	Lman2	lectin, ma	GO:00511	localizatio	K10082	lectin, ma	mmu0414	Protein pr	13:553438	1410	1692	2326	2292	1623	1499	9.871091	9.928855	11.21778	10.45303	9.963387	13.01168	10.33924	11.1427	0.795945	1	-
ENSMUSC	Mxd3	Max dime	GO:00081	metabolic	-	-	-	-	13:553251	20	15	18	19	19	13	0.302204	0.189985	0.187368	0.187022	0.25174	0.243549	0.226519	0.227437	0.943798	1	-
ENSMUSC	Prelid1	PRELI don	GO:00325	developm	-	-	-	-	13:553205	1109	1301	1547	1666	1315	1101	19.45895	19.13453	18.69947	19.0434	20.2328	23.95306	19.09765	21.07642	0.643962	1	-
ENSMUSC	Nsd1	nuclear re	GO:00081	metabolic	K15588	histone-ly	mmu0031	Lysine deg	13:552097	3724	4101	5301	5977	4333	2847	9.710393	8.963337	9.52218	10.15294	9.907373	9.204507	9.398637	9.754941	0.989016	1	-
ENSMUSC	Slc34a1	solute car	GO:00325	developm	-	-	-	-	13:553981	0	0	2	0	0	0	0	0	0.011287	0	0	0	0.003762	0	0.536201	1	-
ENSMUSC	F12	coagulatic	GO:00081	metabolic	K01328	coagulatic	mmu0461	Complem	13:554179	6	1	3	6	5	6	0.170925	0.023876	0.058869	0.111349	0.124887	0.21194	0.084557	0.149392	0.348851	1	-
ENSMUSC	Pdlim7	PDZ and l	GO:00325	developm	-	-	-	-	13:554957	511	574	683	720	596	620	3.813032	3.590161	3.510923	3.499963	3.899763	5.736238	3.638039	4.378655	0.34093	1	-
ENSMUSC	Ddx41	DEAD (As	GO:00325	developm	-	-	-	-	13:555304	588	716	877	976	702	510	9.450982	9.646398	9.710701	10.21953	9.894161	10.16378	9.602693	10.09249	0.924891	1	-
ENSMUSC	Fam193b	family witl	GO:00056	cell|intrace	-	-	-	-	13:555393	751	1217	1213	1582	1125	937	3.073893	4.175341	3.420273	4.218293	4.037792	4.75526	3.556502	4.337115	0.216616	1	-
ENSMUSC	Pcbd2	pterin 4 a	GO:00081	metabolic	K01724	4a-hydro:	mmu0079	Folate bio	13:557273	42	53	70	46	38	90	3.466512	3.666721	3.980081	2.473348	2.750255	9.210342	3.704438	4.811315	0.543162	1	-
ENSMUSC	Txndc15	thioredox	GO:00099	cellular pr	-	-	-	-	13:557146	816	972	1152	1232	894	791	10.77035	10.75373	10.47475	10.59332	10.34713	12.94501	10.66628	11.29515	0.911155	1	-
ENSMUSC	Catsper3	cation cha	GO:00325	developm	-	-	-	-	13:557845	0	0	3	5	3	0	0	0	0.069564	0.109652	0.088545	0	0.023188	0.066066	0.429632	1	-
ENSMUSC	Ddx46	DEAD (As	GO:00081	metabolic	K12811	ATP-depe	mmu0304	Spliceoso	13:556350	1833	2347	2802	3073	2193	1194	14.52889	15.59323	15.29994	15.86771	15.24233	11.73438	15.14068	14.28148	0.507593	1	-
ENSMUSC	Caml	calcium m	GO:00081	metabolic	-	-	-	-	13:556230	441	585	693	712	526	367	11.97219	13.312	12.96043	12.59204	12.52169	12.35339	12.74821	12.48904	0.664974	1	-
ENSMUSC	B4galt7	xylosylpro	GO:00081	metabolic	K00733	xylosylpro	mmu0110	Metabolic	13:555998	306	388	442	473	425	369	4.105389	4.363327	4.085133	4.134046	4.999931	6.13826	4.184616	5.090746	0.286435	1	-
ENSMUSC	Cxcl14	chemokin	GO:00325	developm	K10033	C-X-C mo	mmu0406	Cytokine-	13:562886	1131	1332	1736	1844	1457	1029	35.53062	35.07498	37.57002	37.73835	40.13678	40.08129	36.05854	39.3188	0.683554	1	-
ENSMUSC	Slc25a48	solute car	GO:00511	localizatio	-	-	-	-	13:564383	0	0	0	4	0	0	0	0	0	0.049089	0	0	0	0.016363	0.305167	1	-
ENSMUSC	Zfp729a	zinc finge	GO:00081	metabolic	-	-	-	-	13:676127	430	508	545	635	472	211	2.38213	2.35892	2.079911	2.291671	2.292882	1.449323	2.273654	2.011292	0.372129	1	-
ENSMUSC	Zfp369	zinc finge	GO:00081	metabolic	K12458	zinc finge	mmu0472	Neurotrop	13:652788	500	658	792	846	590	704	3.933708	4.33921	4.292488	4.335948	4.070306	6.867376	4.188469	5.09121	0.317694	1	-
ENSMUSC	Ptdss1	phosphati	GO:00081	metabolic	K08729	phosphati	mmu0110	Metabolic	13:669328	681	878	1049	1056	751	732	6.289722	6.797229	6.674389	6.353752	6.08229	8.382653	6.587114	6.939565	0.961399	1	-
ENSMUSC	Mterf3	mitochon	GO:00325	developm	-	-	-	-	13:669069	247	263	349	424	271	168	2.065743	1.843694	2.010743	2.310081	1.987432	1.742104	1.973393	2.013206	0.950002	1	-
ENSMUSC	Uqcrb	ubiquinol	GO:00081	metabolic	K00417	ubiquinol	mmu0110	Metabolic	13:669006	892	1141	1358	1237	924	445	51.53571	55.25634	54.04988	46.55805	46.81208	31.87788	53.61397	41.74934	0.059817	1	-
ENSMUSC	Fastkd3	FAST kina	GO:00081	metabolic	-	-	-	-	13:685822	114	142	115	185	127	88	0.786649	0.821331	0.546673	0.83163	0.768465	0.752912	0.718218	0.784336	0.765637	1	-
ENSMUSC	1700001L	RIKEN cDI	-	-	-	-	-	-	13:685974	82	133	156	127	103	97	2.093756	2.846522	2.744019	2.112492	2.306173	3.070909	2.561432	2.496525	0.684446	1	-
ENSMUSC	Adcy2	adenylate	GO:00081	metabolic	K08042	adenylate	mmu0520	Pathways	13:686200	2782	3144	4132	4392	3216	2145	41.98634	39.7728	42.95983	43.18121	42.56079	40.13879	41.57299	41.96026	0.822949	1	-
ENSMUSC	Smad5	SMAD far	GO:00325	developm	K16790	mothers a	mmu0455	Signaling	13:567030	591	665	790	914	469	546	2.669876	2.518131	2.458569	2.689871	1.857888	3.058314	2.548859	2.535358	0.727078	1	-
ENSMUSC	Trpc7	transient r	GO:00511	localizatio	-	-	-	-	13:567731	35	67	70	51	38	42	0.421044	0.675588	0.580099	0.399678	0.400851	0.626463	0.55891	0.475664	0.321535	1	-
ENSMUSC	Hnrnpk	heteroger	GO:00081	metabolic	K12886	heteroger	mmu0520	Viral carci	13:583911	2067	2655	3175	2822	2263	1875	7.2629	7.819643	7.685369	6.459638	6.972633	8.168774	7.589304	7.200348	0.410596	1	-
ENSMUSC	Ccnh	cyclin H [S	GO:00081	metabolic	K06634	cyclin H[-]	mmu0411	Cell cycle	13:851894	620	785	994	977	819	355	8.214063	8.717444	9.072024	8.432229	9.51465	5.8315	8.667843	7.926127	0.457865	1	-
ENSMUSC	Rasa1	RAS p21 p	GO:00325	developm	K04352	Ras GTPas	mmu0401	MAPK sign	13:852147	1285	1503	1793	1857	1314	677	8.458377	8.292695	8.130467	7.963011	7.584412	5.52533	8.293846	7.024251	0.167104	1	-
ENSMUSC	2210016F	RIKEN cDI	GO:00081	metabolic	-	-	-	-	13:583798	151	156	189	221	151	121	3.145356	2.723768	2.712116	2.998946	2.758114	3.125097	2.860413	2.960719	0.989407	1	-
ENSMUSC	Gkap1	G kinase a	GO:00508	response	-	-	-	-	13:582333	183	174	279	275	232	94	1.686517	1.344127	1.771312	1.651026	1.874866	1.074124	1.600652	1.533339	0.717209	1	-
ENSMUSC	Naa35	N(alpha)-	GO:00081	metabolic	-	-	-	-	13:595852	671	768	879	941	710	533	3.433774	3.294304	3.098775	3.137046	3.186035	3.381914	3.275618	3.234998	0.681775	1	-
ENSMUSC	Golm1	golgi mer	GO:00081	metabolic	-	-	-	-	13:596346	877	967	2508	2839	992	1321	7.641345	7.062355	15.05391	16.11452	7.57922	14.27115	9.919204	12.65496	0.326076	1	-
ENSMUSC	Agtpbp1	ATP/GTP l	GO:00325	developm	-	-	-	-	13:594457	5094	5643	7014	7957	5633	2938	12.32165	11.44123	11.68765	12.53837	11.94792	8.811456	11.81684	11.09925	0.498962	1	-
ENSMUSC	Dapk1	death ass	GO:00081	metabolic	K08803	death-ass	mmu0520	Pathways	13:606019	3176	3965	4919	4671	3446	2496	14.70633	15.38933	15.69105	14.09014	13.99206	14.33027	15.26224	14.13749	0.311076	1	-
ENSMUSC	Slc6a19	solute car	GO:00511	localizatio	K05334	solute car	mmu0497	Protein di	13:736797	0	0	2	5	2	2	0	0	0.010445	0.024693	0.013293	0.018803	0.003482	0.018929	0.097952	1	-
ENSMUSC	Nkd2	naked cut	GO:00081	metabolic	K03213	naked cut	mmu0431	Wnt signa	13:738185	293	359	359	353	282	185	2.432853	2.498589	2.053495	1.909433	2.053235	1.904612	2.328312	1.95576	0.146699	1	-
ENSMUSC	Trip13	thyroid hc	GO:00325	developm	-	-	-	-	13:739113	8	14	19	22	18	7	0.110044	0.16143	0.180051	0.197152	0.21712	0.119397	0.150508	0.17789	0.689838	1	-
ENSMUSC	Cep72	centroson	GO:00511	localizatio	-	-	-	-	13:740365	29	19	43	42	21	29	0.139751	0.076748	0.142749	0.131851	0.088742	0.173275	0.11975	0.131289	0.895705	1	-
ENSMUSC	Tppp	tubulin pc	GO:00990	supramole	-	-	-	-	13:740094	13594	14913	19792	21349	15277	8518	161.6653	148.6577	162.1477	165.3975	159.3127	125.6011	157.4903	150.1037	0.533754	1	-
ENSMUSC	Ahrr	aryl-hydrc	GO:00081	metabolic	-	-	-	-	13:742111	17	21	32	32	26	13	0.094878	0.098242	0.123034	0.116347	0.127246	0.089959	0.105385	0.111184	0.952232	1	-
ENSMUSC	Pdcd6	programn	GO:00325	developm	-	-	-	-	13:743035	380	461	604	636	454	322	5.203646	5.291494	5.69788	5.673657	5.451594	5.467218	5.397673	5.530823	0.932282	1	-
ENSMUSC	Sdha	succinate	GO:00325	developm	K00234	succinate	mmu0110	Metabolic	13:743222	7327	9098	10413	10890	8023	5608	78.85739	82.07577	77.20466	76.35293	75.7174	74.83594	79.37928	75.63542	0.453348	1	-
ENSMUSC	Ccdc127	coiled-co	GO:00160	membran	-	-	-	-	13:743503	1196	1383	2238	1560	1190	1103	5.39916	5.233234	6.95996	4.587768	4.710695	6.173855	5.864118	5.157439	0.179403	1	-
ENSMUSC	Lrrc14b	leucine ric	-	-	-	-	-	-	13:743595	3	3	13	3	8	8	0.069196	0.058005	0.206591	0.045078	0.161833	0.228818	0.111264	0.145243	0.739815	1	-
ENSMUSC	Erap1	endoplasr	GO:00325	developm	-	-	-	-	13:746395	147	209	279	274	204	120	1.166036	1.389614	1.524584	1.415881	1.418956	1.180216	1.360078	1.338351	0.767885	1	-
ENSMUSC	Cast	calpastati	GO:00081	metabolic	-	-	-	-	13:746923	163	188	348	281	189	164	0.715411	0.691637	1.052201	0.803443	0.727396	0.892476	0.81975	0.807772	0.738432	1	-
ENSMUSC	Pcsk1	proprotei	GO:00081	metabolic	-	-	-	-	13:750898	553	710	913	748	624	417	5.658478	6.089559	6.435712	4.986065	5.598885	5.2905	6.061249	5.291817	0.180351	1	-
ENSMUSC	Rhobtb3	Rho-relat	GO:00325	developm	-	-	-	-	13:758695	383	448	602	525	457	401	3.347657	3.282256	3.62485	2.989393	3.502685	4.345827	3.418254	3.612635	0.951419	1	-
ENSMUSC	Spata9	spermato	GO:00325	developm	-	-	-	-	13:759671	0	0	2	2	0	0	0	0	0.040922	0.038688	0	0	0.013641	0.012896	1	1	-
ENSMUSC	Glrx	glutaredo	GO:00081	metabolic	-	-	-	-	13:758398	277	314	393	381	283	189	2.421484	2.300828	2.366715	2.169741	2.16936	2.048569	2.363009	2.129223	0.322975	1	-
ENSMUSC	Arsk	arylsulfata	GO:00081	metabolic	-	-	-	-	13:760604	125	143	205	188	125	100	1.161084	1.113377	1.311775	1.137606	1.01814	1.151697	1.195412	1.102481	0.458458	1	-
ENSMUSC	Srd5a1	steroid 5 a	GO:00325	developm	K12343	3-oxo-5-	mmu0014	Steroid hc	13:695734	140	198	240	231	179	178	2.308625	2.73679	2.726387	2.481523	2.588338	3.63941	2.590601	2.90309	0.688831	1	-
ENSMUSC	Nsun2	NOL1/NC	GO:00325	developm	-	-	-	-	13:695337	1249	1510	1815	2033	1332	991	9.88145	10.01355	9.892054	10.47797	9.240706	9.721154	9.929018	9.813278	0.687966	1	-
ENSMUSC	Mctp1	multiple C	GO:00452	synapse|m	-	-	-	-	13:763845	824	977	1156	1190	968	637	4.777635	4.748249	4.617374	4.494843	4.921575	4.579427	4.714419	4.665282	0.704844	1	-
ENSMUSC	Slf1	SMC5-SM	GO:00081	metabolic	-	-	-	-	13:770430	155	271	337	224	239	167	1.437226	2.106271	2.152653	1.353074	1.943266	1.91997	1.898716	1.73877	0.441535	1	-
ENSMUSC	Med10	mediator	GO:00325	developm	-	-	-	-	13:698023	373	439	515	440	415	249	3.417716	3.371665	3.250767	2.626403	3.334404	2.82887	3.346716	2.929893	0.227575	1	-
ENSMUSC	8030423J2	RIKEN cDI	-	-	-	-	-	-	13:708829	2	0	3	0	0	0	0.146297	0	0.151137	0	0	0	0.099144	0	0.088837	1	-
ENSMUSC	Ndufs6	NADH de	GO:00325	developm	K03939	NADH de	mmu0110	Metabolic	13:733198	386	579	576	498	428	323	12.49692	15.71258	12.84665	10.50332	12.15074	12.96594	13.68538	11.87333	0.187059	1	-
ENSMUSC	Mrpl36	mitochon	GO:00081	metabolic	K02919	large subu	mmu0301	Ribosome	13:733285	138	148	204	128	142	169	3.780306	3.398306	3.849722	2.284237	3.410985	5.740136	3.676111	3.811786	0.91484	1	-
ENSMUSC	Lpcat1	lysophosp	GO:00325	developm	K13510	lysophosp	mmu0110	Metabolic	13:734671	224	343	335	327	249	618	2.009864	2.579677	2.070692	1.911384	1.95912	6.875321	2.220078	3.581941	0.116445	1	-
ENSMUSC	Slc6a3	solute car	GO:00325	developm	K05036	solute car	mmu0503	Alcoholisr	13:735367	4	0	0	4	6	2	0.073554	0	0	0.047924	0.096744	0.045614	0.024518	0.063427	0.246635	1	-
ENSMUSC	Clptm1l	CLPTM1-l	GO:00099	cellular pr	-	-	-	-	13:736040	957	1387	1604	1626	1219	1251	15.78517	19.17637	18.22607	17.47186	17.63127	25.58471	17.7292	20.22928	0.51761	1	-
ENSMUSC	Tert	telomeras	GO:00325	developm	K11126	telomeras	mmu0520	Pathways	13:736269	29	23	48	40	26	32	0.181903	0.120926	0.207411	0.163448	0.143007	0.248872	0.17008	0.185109	0.931081	1	-
ENSMUSC	Hapln1	hyalurona	GO:00325	developm	-	-	-	-	13:895397	754	1001	1377	1336	1093	435	7.755133	8.629883	9.756708	8.951711	9.857816	5.547441	8.713908	8.118989	0.567158	1	-
ENSMUSC	Vcan	versican [S	GO:00325	developm	K06793	versican c	mmu0451	Cell adhes	13:896553	458	488	648	848	550	315	1.455145	1.299611	1.418297	1.755168	1.532309	1.240901	1.391018	1.509459	0.729717	1	-
ENSMUSC	Xrcc4	X-ray rep	GO:00081	metabolic	K10886	DNA-repa	mmu0345	Non-hom	13:897740	191	193	270	278	185	100	2.676654	2.267088	2.606596	2.537963	2.273385	1.737577	2.51678	2.182975	0.316335	1	-
ENSMUSC	Atg10	autophag	GO:00081	metabolic	K17888	ubiquitin-	mmu0414	Autophag	13:909353	106	119	152	173	133	91	2.859351	2.690678	2.824602	3.040098	3.145971	3.043608	2.791544	3.076559	0.702326	1	-
ENSMUSC	Acot12	acyl-CoA	GO:00081	metabolic	K01067	acetyl-Co	mmu0062	Pyruvate r	13:917415	12	11	13	9	9	6	0.199062	0.152964	0.148567	0.097261	0.130926	0.123424	0.166864	0.117204	0.248402	1	-
ENSMUSC	Zcchc9	zinc finge	GO:00081	metabolic	-	-	-	-	13:917965	126	175	258	203	169	159	1.471734	1.713355	2.076009	1.544666	1.73096	2.302717	1.753699	1.859448	0.963606	1	-
ENSMUSC	Cd180	CD180 an	GO:00023	immune s	-	-	-	-	13:102693	60	61	92	134	72	60	0.629233	0.536225	0.664666	0.91548	0.662119	0.780189	0.610041	0.785929	0.249852	1	-
ENSMUSC	Slc30a5	solute car	GO:00511	localizatio	-	-	-	-	13:100802	706	915	1081	1019	814	442	4.779846	5.192582	5.041809	4.494332	4.832551	3.710371	5.004746	4.345751	0.209202	1	-
ENSMUSC	Rad17	RAD17 ch	GO:00325	developm	-	-	-	-	13:100617	253	344	377	358	297	172	1.645575	1.87546	1.689236	1.516915	1.693935	1.38711	1.736757	1.532653	0.283412	1	-
ENSMUSC	Marveld2	MARVEL (	GO:00325	developm	K17291	MARVEL (	mmu0453	Tight junc	13:100595	17	27	31	22	13	22	0.209051	0.278308	0.262622	0.176247	0.140185	0.335453	0.249993	0.217295	0.488618	1	-
ENSMUSC	Ocln	occludin [	GO:00508	response	K06088	occludin[-	mmu0453	Tight junc	13:100496	297	308	456	399	278	235	2.496727	2.170292	2.640773	2.18509	2.049281	2.449446	2.435931	2.227939	0.353775	1	-
ENSMUSC	Gtf2h2	general tr	GO:00081	metabolic	K03142	transcripti	mmu0520	Viral carci	13:100460	244	375	421	456	333	215	2.630524	3.388726	3.126695	3.202571	3.148029	2.873932	3.048649	3.074844	0.866546	1	-
ENSMUSC	Naip1	NLR famil	GO:00081	metabolic	K12807	baculovira	mmu0462	NOD-like	13:100407	8	7	4	6	3	9	0.04767	0.034963	0.016423	0.023293	0.015675	0.066495	0.033019	0.035154	1	1	-
ENSMUSC	Serf1	small EDR	-	-	-	-	-	-	13:100108	71	85	110	96	65	61	2.273177	2.281114	2.42617	2.002288	1.82487	2.421524	2.32682	2.082894	0.379974	1	-
ENSMUSC	Smn1	survival m	GO:00325	developm	K13129	survival m	mmu0301	RNA trans	13:100124	209	262	279	331	224	118	3.260325	3.425863	2.998275	3.363759	3.06412	2.282361	3.228154	2.903413	0.426067	1	-
ENSMUSC	Mccc2	methylcro	GO:00081	metabolic	K01969	3-methylc	mmu0110	Metabolic	13:999485	339	405	474	508	367	292	5.316997	5.324443	5.121488	5.190536	5.047491	5.678515	5.254309	5.305514	0.82317	1	-
ENSMUSC	Cartpt	CART pre	GO:00325	developm	-	-	-	-	13:998984	29	45	22	10	21	31	1.206969	1.56986	0.630761	0.271123	0.766421	1.599734	1.135863	0.879093	0.378511	1	-
ENSMUSC	Ptcd2	pentatrico	GO:00325	developm	-	-	-	-	13:993196	328	439	448	466	372	296	12.2332	13.7241	11.51053	11.32228	12.16611	13.68811	12.48928	12.39217	0.718645	1	-
ENSMUSC	Btf3	basic tran	GO:00325	developm	-	-	-	-	13:983098	278	377	462	449	355	338	5.575182	6.337355	6.382745	5.865996	6.242879	8.404589	6.098427	6.837821	0.636467	1	-
ENSMUSC	Ankra2	ankyrin re	GO:00081	metabolic	-	-	-	-	13:982630	399	537	507	633	457	346	3.260173	3.677863	2.853828	3.369408	3.274366	3.50534	3.263955	3.383038	0.986597	1	-
ENSMUSC	Arhgef28	Rho guan	GO:00987	molecular	-	-	-	-	13:978994	1834	1986	2402	2554	1963	1226	14.26812	12.95088	12.87335	12.94404	13.39153	11.82615	13.36412	12.72057	0.49254	1	-
ENSMUSC	Hexb	hexosami	GO:00325	developm	K12373	hexosami	mmu0110	Metabolic	13:971763	904	1126	1395	1586	1022	1287	24.91411	26.01166	26.48515	28.47486	24.6985	43.9786	25.80364	32.38398	0.209324	1	-
ENSMUSC	Gfm2	G elongat	GO:00081	metabolic	-	-	-	-	13:971379	383	518	588	703	526	419	1.776572	2.014033	1.878937	2.124324	2.1395	2.409813	1.889848	2.224546	0.35083	1	-
ENSMUSC	Polk	polymeras	GO:00081	metabolic	K03511	DNA poly	mmu0520	Pathways	13:964806	193	217	269	304	245	132	1.683007	1.586133	1.615965	1.726961	1.873429	1.427207	1.628368	1.675865	1	1	-
ENSMUSC	Col4a3bp	collagen,	GO:00325	developm	-	-	-	-	13:965426	727	1529	1093	1208	948	838	4.039435	7.121087	4.183674	4.372547	4.618883	5.773194	5.114732	4.921541	0.602033	1	-
ENSMUSC	Hmgcr	3-hydrox	GO:00325	developm	K00021	hydroxym	mmu0110	Metabolic	13:966489	1682	1862	2181	2408	1790	1462	10.59532	9.831516	9.464443	9.881575	9.887435	11.41882	9.96376	10.39595	0.996359	1	-
ENSMUSC	Poc5	POC5 cen	GO:00099	cellular pr	-	-	-	-	13:963882	802	831	1102	1176	888	531	6.772457	5.882012	6.410693	6.469352	6.575485	5.559718	6.355054	6.201518	0.666054	1	-
ENSMUSC	F2rl2	coagulatic	GO:00048	signal trar	K04235	coagulatic	mmu0408	Neuroacti	13:956968	2	6	6	6	4	3	0.050812	0.127742	0.104986	0.099288	0.089088	0.094492	0.094513	0.094289	1	1	-
ENSMUSC	Iqgap2	IQ motif c	GO:00508	response	K05767	Ras GTPas	mmu0481	Regulatior	13:956271	3143	3547	4312	5482	3560	1674	34.61222	32.74154	32.71261	39.32835	34.37777	22.85738	33.35546	32.18783	0.684563	1	-
ENSMUSC	F2rl1	coagulatic	GO:00325	developm	K04234	coagulatic	mmu0408	Neuroacti	13:955117	2	2	10	2	4	6	0.046382	0.038868	0.159731	0.030205	0.08132	0.172506	0.08166	0.094677	1	1	-
ENSMUSC	Crhbp	corticotro	GO:00081	metabolic	-	-	-	-	13:954313	413	476	537	609	425	298	9.229031	8.915928	8.266699	8.865548	8.327944	8.256744	8.803886	8.483412	0.595832	1	-
ENSMUSC	Aggf1	angiogeni	GO:00325	developm	-	-	-	-	13:953506	1303	1548	1912	2022	1517	973	19.19554	19.11524	19.40418	19.40523	19.59675	17.77276	19.23832	18.92492	0.672133	1	-
ENSMUSC	Pde8b	phosphoc	GO:00081	metabolic	K18437	high affini	mmu0023	Purine me	13:950244	1190	1347	1327	1733	1248	810	6.222486	5.903876	4.780125	5.903329	5.722347	5.251556	5.635496	5.625744	0.77959	1	-
ENSMUSC	Otp	orthopedi	GO:00325	developm	-	-	-	-	13:948756	0	9	5	3	0	6	0	0.143366	0.065464	0.037138	0	0.141399	0.06961	0.059512	0.804802	1	-
ENSMUSC	Ap3b1	adaptor-r	GO:00325	developm	K12397	AP-3 com	mmu0414	Lysosome	13:943589	1322	1606	2004	2243	1515	826	16.94918	17.259	17.69972	18.73389	17.03227	13.13056	17.30263	16.29891	0.516443	1	-
ENSMUSC	Scamp1	secretory	GO:00452	synapse|c	-	-	-	-	13:942013	3318	4135	5300	5107	3655	3264	34.65389	36.19961	38.13316	34.74746	33.47384	42.26799	36.32889	36.82977	0.797922	1	-
ENSMUSC	Jmy	junction-r	GO:00081	metabolic	-	-	-	-	13:934301	1446	1694	2152	2260	1582	1023	8.927302	8.766338	9.152624	9.089541	8.56448	7.830931	8.948755	8.494984	0.485764	1	-
ENSMUSC	Dimt1	DIM1 dim	GO:00081	metabolic	-	-	-	-	13:106947	208	302	341	359	284	136	2.306176	2.806655	2.604568	2.593018	2.76116	1.869623	2.572466	2.407934	0.572081	1	-
ENSMUSC	Kif2a	kinesin fa	GO:00325	developm	-	-	-	-	13:106958	1787	2296	2619	2549	2041	1053	13.20266	14.21875	13.3298	12.26841	13.22277	9.646084	13.58374	11.71242	0.182105	1	-
ENSMUSC	Ercc8	excision r	GO:00081	metabolic	K10570	DNA excis	mmu0412	Ubiquitin	13:108158	120	153	138	183	112	124	0.710355	0.759166	0.56276	0.705711	0.581375	0.910124	0.677427	0.732403	0.865696	1	-
ENSMUSC	Elovl7	ELOVL far	GO:00081	metabolic	K10250	elongatio	-	-	13:108214	184	178	457	233	217	214	1.998586	1.620616	3.419588	1.648706	2.066852	2.882081	2.346263	2.199213	0.567627	1	-
ENSMUSC	Depdc1b	DEP dom	GO:00400	locomotic	-	-	-	-	13:108316	5	5	7	4	4	2	0.06964	0.05837	0.067167	0.036298	0.048849	0.034548	0.065059	0.039898	0.311508	1	-
ENSMUSC	Pde4d	phosphoc	GO:00325	developm	K13293	cAMP-spe	mmu0402	cAMP sign	13:108449	599	674	866	838	664	344	2.19439	2.069661	2.185527	1.999918	2.133031	1.562542	2.14986	1.898497	0.280616	1	-
ENSMUSC	Rab3c	RAB3C, m	GO:00452	synapse|c	-	-	-	-	13:110054	11722	14185	15783	17438	12670	7037	52.14309	52.89045	48.36554	50.5328	49.42132	38.81223	51.13303	46.25545	0.302971	1	-
ENSMUSC	Plk2	polo like l	GO:00325	developm	K08861	polo-like	mmu0406	FoxO sign	13:110395	3579	4110	4777	5208	3745	2652	30.85826	29.70328	28.37372	29.25246	28.31419	28.35105	29.64509	28.63923	0.533494	1	-
ENSMUSC	Serinc5	serine inc	GO:00325	developm	-	-	-	-	13:926110	1265	1306	1792	1939	1299	1331	10.96339	9.487475	10.69902	10.94747	9.872027	14.30272	10.38329	11.70741	0.556008	1	-
ENSMUSC	Mtx3	metaxin 3	GO:00511	localizatio	-	-	-	-	13:928447	1073	1278	1551	1709	1238	846	8.349745	8.335991	8.314513	8.663577	8.447668	8.162628	8.333416	8.424624	0.837109	1	-
ENSMUSC	Zfyve16	zinc finge	GO:00511	localizatio	K04679	MAD, mo	mmu0414	Endocytos	13:924871	365	374	533	514	384	246	2.545471	2.186247	2.56067	2.335178	2.348272	2.12714	2.430796	2.270196	0.465058	1	-
ENSMUSC	Dhfr	dihydrofo	GO:00325	developm	K00287	dihydrofo	mmu0110	Metabolic	13:923547	87	69	145	137	90	56	1.034061	0.687426	1.18726	1.060789	0.938019	0.825276	0.969582	0.941361	0.772437	1	-
ENSMUSC	Rasgrf2	RAS prote	GO:00987	molecular	-	-	-	-	13:918804	2189	2629	3269	3480	2651	1580	13.58702	13.67798	13.978	14.07147	14.42883	12.15966	13.74767	13.55332	0.697046	1	-
ENSMUSC	Erbin	Erbb2 inte	GO:00081	metabolic	K12796	erbb2-int	mmu0462	NOD-like	13:103818	1674	1617	2519	2513	1882	999	9.112438	7.378057	9.446237	8.911551	8.983415	6.742649	8.645577	8.212539	0.560661	1	-
ENSMUSC	Nln	neurolysir	GO:00325	developm	K01393	neurolysir	mmu0461	Renin-an	13:104023	458	606	713	733	559	378	2.443731	2.710275	2.620771	2.547848	2.615428	2.500723	2.591592	2.554666	0.703192	1	-
ENSMUSC	Trappc13	trafficking	-	-	-	-	-	-	13:104142	697	909	1170	1130	837	606	4.282747	4.681732	4.952529	4.52324	4.509806	4.616872	4.639003	4.549973	0.650519	1	-
ENSMUSC	Trim23	tripartite r	GO:00081	metabolic	-	-	-	-	13:104178	1115	1550	1857	1752	1416	938	8.203471	9.558889	9.412096	8.397282	9.135429	8.556807	9.058152	8.696506	0.524353	1	-
ENSMUSC	Ppwd1	peptidylp	GO:00081	metabolic	-	-	-	-	13:104205	183	238	295	275	212	114	1.698586	1.851676	1.886296	1.662841	1.725496	1.311982	1.812186	1.566773	0.27466	1	-
ENSMUSC	Cenpk	centrome	GO:00081	metabolic	-	-	-	-	13:104228	11	7	9	14	12	9	0.112036	0.059756	0.063143	0.09289	0.107171	0.113649	0.078312	0.10457	0.481778	1	-
ENSMUSC	Cwc27	CWC27 sp	GO:00081	metabolic	-	-	-	-	13:104631	275	389	427	481	365	180	5.437789	6.447526	5.816611	6.196076	6.328868	4.413143	5.900642	5.646029	0.654146	1	-
ENSMUSC	Srek1ip1	splicing re	GO:00081	metabolic	-	-	-	-	13:104792	166	243	299	282	228	179	1.339315	1.643369	1.661873	1.482195	1.613066	1.790664	1.548186	1.628642	0.9606	1	-
ENSMUSC	Rgs7bp	regulator	GO:00508	response	-	-	-	-	13:104945	7712	9597	11657	12819	9040	4290	69.25545	72.23959	72.11492	74.99334	71.18657	47.76724	71.20332	64.64905	0.392651	1	-
ENSMUSC	Rnf180	ring finge	GO:00081	metabolic	-	-	-	-	13:105130	302	313	310	385	292	167	2.501046	2.172759	1.76859	2.077098	2.120506	1.714813	2.147465	1.970805	0.465288	1	-
ENSMUSC	Htr1a	5-hydroxy	GO:00048	signal trar	K04153	5-hydroxy	mmu0408	Neuroacti	13:105443	617	815	1055	1090	760	629	8.74492	9.682354	10.30088	10.06419	9.445542	11.05368	9.576052	10.1878	0.86551	1	-
ENSMUSC	Parp8	poly (ADP	GO:00038	catalytic a	-	-	-	-	13:116854	658	769	849	986	690	474	3.978486	3.897368	3.536316	3.883746	3.658336	3.553499	3.804057	3.698527	0.626294	1	-
ENSMUSC	Emb	embigin [	GO:00452	synapse|c	-	-	-	-	13:117208	466	579	1161	684	552	270	3.351707	3.490696	5.752597	3.204929	3.481466	2.407855	4.198333	3.031417	0.044835	1	-
ENSMUSC	Hcn1	hyperpola	GO:00325	developm	-	-	-	-	13:117602	2657	2996	3425	3964	2921	1522	8.813169	8.329812	7.826231	8.565563	8.496006	6.259523	8.323071	7.773697	0.468742	1	-
ENSMUSC	Mrps30	mitochon	GO:00081	metabolic	-	-	-	-	13:118378	276	388	473	429	347	290	2.462878	2.902146	2.907681	2.493869	2.715225	3.208619	2.757568	2.805904	0.854682	1	-
ENSMUSC	Fgf10	fibroblast	GO:00325	developm	K04358	fibroblast	mmu0520	Pathways	13:118669	262	394	421	466	309	194	3.316248	4.180181	3.670956	3.842494	3.429634	3.044627	3.722462	3.438918	0.464898	1	-
ENSMUSC	Slc4a7	solute car	GO:00452	synapse|c	-	-	-	-	14:147022	688	788	1020	1150	777	606	2.179359	2.092278	2.225829	2.37312	2.158261	2.380118	2.165822	2.303833	0.853996	1	-
ENSMUSC	Psmd6	proteasor	GO:00081	metabolic	K03037	26S prote	mmu0516	Epstein-B	14:141121	506	706	818	782	562	771	7.045974	8.240398	7.846843	7.093797	6.862299	13.31162	7.711071	9.089238	0.499083	1	-
ENSMUSC	Atxn7	ataxin 7 [S	GO:00081	metabolic	-	-	-	-	14:139614	327	232	293	417	384	255	2.027889	1.205973	1.251746	1.68467	2.088193	1.960753	1.495203	1.911205	0.228868	1	-
ENSMUSC	Fezf2	Fez family	GO:00325	developm	-	-	-	-	14:123420	735	962	1055	1265	938	601	8.9127	9.777996	8.813041	9.992962	9.973953	9.036125	9.167912	9.66768	0.889672	1	-
ENSMUSC	Ptprg	protein ty	GO:00325	developm	-	-	-	-	14:115535	1386	2586	2551	1941	2186	1012	5.722002	8.948826	7.255152	5.220256	7.91366	5.180257	7.30866	6.104724	0.1842	1	-
ENSMUSC	4930452B	RIKEN cDI	-	-	-	-	-	-	14:843119	161	187	224	252	158	106	2.889584	2.813227	2.769556	2.946409	2.486623	2.358867	2.824122	2.5973	0.474821	1	-
ENSMUSC	Pdhb	pyruvate c	GO:00081	metabolic	-	-	mmu0110	Metabolic	14:816599	1522	2017	2368	2295	1720	1305	13.09254	14.54347	14.03274	12.86097	12.97419	13.91892	13.88958	13.25136	0.461833	1	-
ENSMUSC	Oit1	oncoprote	GO:00511	localizatio	-	-	-	-	14:834893	0	4	3	0	0	0	0	0.11759	0.072482	0	0	0	0.063357	0	0.030877	1	-
ENSMUSC	Fam107a	family witl	GO:00099	cellular pr	-	-	-	-	14:829627	7142	7876	11004	10812	9764	6366	79.9957	73.94441	84.90803	78.89227	95.8997	88.40958	79.61605	87.73385	0.61754	1	-
ENSMUSC	Kctd6	potassium	GO:00081	metabolic	-	-	-	-	14:821414	695	894	1100	1091	809	701	9.53569	10.28154	10.39709	9.751555	9.733275	11.92535	10.07144	10.47006	0.976755	1	-
ENSMUSC	Map3k1	mitogen-	GO:00081	metabolic	K04416	mitogen-	mmu0401	MAPK sigi	13:111746	231	223	372	358	265	187	1.773682	1.43523	1.967695	1.790719	1.784233	1.780289	1.725536	1.78508	0.99329	1	-
ENSMUSC	Il6st	interleukir	GO:00325	developm	K05060	interleukir	mmu0520	Pathways	13:112464	1533	1448	1971	2040	1251	1038	5.556132	4.398977	4.921171	4.816617	3.975853	4.664591	4.95876	4.485687	0.270241	1	-
ENSMUSC	Ddx4	DEAD (As	GO:00325	developm	-	-	-	-	13:112598	69	90	94	92	96	37	1.173451	1.282942	1.101271	1.019255	1.431613	0.780183	1.185888	1.077017	0.557517	1	-
ENSMUSC	Plpp1	phospholi	GO:00081	metabolic	K01080	phosphati	mmu0110	Metabolic	13:112800	148	265	256	234	178	244	3.995686	5.996929	4.761253	4.115538	4.213989	8.167793	4.917956	5.499107	0.785545	1	-
ENSMUSC	Gpx8	glutathior	GO:00081	metabolic	K00432	glutathior	mmu0059	Arachidor	13:113042	64	85	127	77	60	61	3.995499	4.447949	5.461895	3.131561	3.284622	4.721733	4.635114	3.712639	0.145487	1	-

ENSMUSG	BC067074	cDNA seq	GO:00160	membran	-	-	-	-	13:113293	95	58	124	101	72	33	0.511222	0.261619	0.459684	0.35407	0.339749	0.220182	0.410842	0.304667	0.161379	1	-
ENSMUSG	Ndufs4	NADH de	GO:00325	developm	K03937	NADH de	mmu0110	Metabolic	13:114287	328	554	618	562	394	558	6.927673	9.80792	8.991952	7.732716	7.297143	14.61281	8.575848	9.88089	0.623997	1	-
ENSMUSG	Fst	follistatin	GO:00325	developm	K04661	follistatin[	mmu0435	TGF-beta	13:114452	29	51	47	56	37	38	0.302287	0.445594	0.337498	0.380266	0.338194	0.491128	0.361793	0.403196	0.791118	1	-
ENSMUSG	Kat6b	K(lysine) a	GO:00081	metabolic	-	-	-	-	14:214814	1464	1361	1925	2318	1608	953	2.862114	2.230267	2.592555	2.952167	2.756605	2.310069	2.561645	2.672947	0.941953	1	-
ENSMUSG	Dusp13	dual speci	GO:00081	metabolic	-	-	-	-	14:217333	8	5	4	7	2	0	0.088005	0.046104	0.030318	0.050167	0.019292	0	0.054809	0.023153	0.217271	1	-
ENSMUSG	Samd8	sterile alpl	GO:00081	metabolic	-	-	-	-	14:217505	1817	2208	2764	2823	2022	1558	13.70145	13.95608	14.35824	13.86769	13.37012	14.56683	14.00526	13.93488	0.702557	1	-
ENSMUSG	Vdac2	voltage-d	GO:00452	synapse|lc	K15040	voltage-d	mmu0516	HTLV-I inf	14:218252	1723	2193	2627	2537	1956	1137	16.6568	17.77043	17.49516	15.97749	16.58128	13.62865	17.30746	15.3958	0.23338	1	-
ENSMUSG	Nkiras1	NFKB inhi	GO:00508	response	-	-	-	-	14:182711	1016	1328	1767	1778	1262	940	8.804179	9.645978	10.54832	10.03711	9.589528	10.09971	9.666159	9.908781	0.917906	1	-
ENSMUSG	Comtd1	catechol-	GO:00081	metabolic	-	-	-	-	14:218458	76	89	115	112	112	107	1.317159	1.292906	1.373018	1.264512	1.702111	2.299298	1.327694	1.755307	0.21967	1	-
ENSMUSG	Ube2e1	ubiquitin-	GO:00081	metabolic	K20217	ubiquitin-	mmu0412	Ubiquitin	14:182827	975	1229	1403	1431	1089	567	12.03656	12.71752	11.93182	11.5085	11.78876	8.678944	12.22863	10.65874	0.225583	1	-
ENSMUSG	Nr1d2	nuclear re	GO:00325	developm	-	-	-	-	14:182040	1439	1859	2443	2349	1692	1070	13.31385	14.417	15.57103	14.15817	13.72732	12.27474	14.43396	13.38674	0.385321	1	-
ENSMUSG	Thrb	thyroid hc	GO:00325	developm	K08362	thyroid hc	mmu0408	Neuroacti	14:176602	744	1040	1174	1266	955	567	4.062848	4.760407	4.41649	4.503738	4.573029	3.839079	4.413248	4.305282	0.665873	1	-
ENSMUSG	Dlg5	discs, larg	GO:00325	developm	-	-	-	-	14:241339	1360	1201	1563	1513	1241	1099	7.742729	5.73127	6.130071	5.611459	6.195402	7.757801	6.53469	6.521554	0.718751	1	-
ENSMUSG	Ngly1	N-glycana	GO:00081	metabolic	K01456	peptide-N	mmu0414	Protein pr	14:162492	402	408	513	504	444	275	2.328078	1.980545	2.046633	1.901442	2.254743	1.974647	2.118418	2.043611	0.609856	1	-
ENSMUSG	Oxsm	3-oxoacyl	GO:00081	metabolic	K09458	3-oxoacyl	mmu0110	Metabolic	14:162386	147	200	172	241	175	126	1.235261	1.408724	0.995684	1.31929	1.289502	1.312803	1.213223	1.307198	0.831279	1	-
ENSMUSG	Dydc1	DPY30 do	GO:00081	metabolic	-	-	-	-	14:410729	0	0	2	0	0	0	0	0	0.058499	0	0	0	0.0195	0	0.536886	1	-
ENSMUSG	Dydc2	DPY30 do	GO:00081	metabolic	-	-	-	-	14:410491	9	4	14	7	7	8	0.69923	0.26048	0.749295	0.354298	0.476927	0.770651	0.569669	0.533959	0.828559	1	-
ENSMUSG	Fam213a	family witl	GO:00325	developm	-	-	-	-	14:409937	1698	2210	2612	2641	2156	1563	24.80758	27.064	26.28884	25.13604	27.62093	28.31338	26.05348	27.02345	0.981279	1	-
ENSMUSG	Glud1	glutamate	GO:00325	developm	K00261	glutamate	mmu0110	Metabolic	14:343107	2928	3955	4642	4562	3491	3201	39.75288	45.00873	43.41642	40.34915	41.56139	53.88515	42.72601	45.26523	0.911079	1	-
ENSMUSG	Bmpr1a	bone mor	GO:00325	developm	K04673	bone mor	mmu0406	Cytokine-	14:344107	1217	1421	1802	1816	1355	849	11.04441	10.80933	11.2657	10.73617	10.78286	9.553134	11.03982	10.35739	0.429092	1	-
ENSMUSG	Ldb3	LIM doma	GO:00325	developm	-	-	-	-	14:345266	68	120	162	80	146	99	0.395427	0.584912	0.648965	0.303058	0.744476	0.713801	0.543101	0.587112	0.917809	1	-
ENSMUSG	Opn4	opsin 4 (n	GO:00048	signal trar	-	-	-	-	14:345906	0	0	0	2	0	0	0	0	0	0.02111	0	0	0	0.007037	0.538335	1	-
ENSMUSG	Cdhr1	cadherin-	GO:00325	developm	-	-	-	-	14:370778	128	177	187	200	126	96	1.877833	2.17657	1.889911	1.911435	1.620923	1.746241	1.981438	1.759533	0.33697	1	-
ENSMUSG	Nid2	nidogen 2	GO:00055	extracellul	-	-	-	-	14:197512	134	104	202	148	129	114	1.287004	0.837262	1.336524	0.926015	1.086446	1.357585	1.153597	1.123349	0.694215	1	-
ENSMUSG	Rtraf	RNA trans	GO:00081	metabolic	-	-	-	-	14:198113	893	1095	1443	1334	951	469	27.5232	28.28878	30.63834	26.78459	25.70221	17.92282	28.81678	23.46987	0.114787	1	-
ENSMUSG	Nudt13	nudix (nuc	GO:00038	catalytic a	-	-	-	-	14:202946	151	216	207	255	166	208	0.739613	0.886818	0.698473	0.813674	0.712985	1.263215	0.774968	0.929958	0.453699	1	-
ENSMUSG	Ecd	ecdysonel	GO:00081	metabolic	-	-	-	-	14:203198	688	823	944	1097	810	519	6.040127	6.056337	5.709274	6.274019	6.235703	5.649516	5.935246	6.053079	0.909479	1	-
ENSMUSG	Dnajc9	DnaJ heat	GO:00055	extracellul	-	-	-	-	14:203846	294	437	516	555	371	298	4.600975	5.732413	5.562936	5.658195	5.091192	5.782364	5.298775	5.510583	1	1	-
ENSMUSG	Anxa7	annexin A	GO:00325	developm	-	-	-	-	14:204552	1139	1288	1498	2062	1189	1126	6.510195	6.170768	5.898393	7.677869	5.959293	7.979848	6.193119	7.20567	0.383413	1	-
ENSMUSG	Mss51	MSS51 mi	GO:00076	behavior|i	-	-	-	-	14:204828	5	4	5	4	5	0	0.174024	0.116688	0.119885	0.090704	0.152586	0	0.136866	0.081097	0.371225	1	-
ENSMUSG	Ppp3cb	protein pl	GO:00325	developm	K04348	serine/thre	mmu0401	MAPK sigi	14:204993	4389	5495	5891	10383	4788	2971	19.6101	20.57951	18.13238	30.2217	18.75906	16.45898	19.44066	21.81325	0.592046	1	-
ENSMUSG	Zswim8	zinc finge	GO:00056	cell|intrace	-	-	-	-	14:207075	2536	3041	3564	4354	3235	2126	9.775015	9.825111	9.463618	10.93297	10.93415	10.16055	9.687915	10.67589	0.604951	1	-
ENSMUSG	Camk2g	calcium/ca	GO:00325	developm	K04515	calcium/ca	mmu0474	Olfactory	14:207348	1511	1821	2269	2225	2971	1498	8.258391	8.342455	8.543128	7.922144	14.2389	10.15147	8.381325	10.77084	0.149874	1	-
ENSMUSG	Plau	plasminog	GO:00081	metabolic	K01348	urokinase	mmu0520	Proteogly	14:208366	33	48	101	90	51	45	0.789922	0.963081	1.665496	1.403451	1.070497	1.33557	1.1395	1.26984	0.780083	1	-
ENSMUSG	Vcl	vinculin [S	GO:00325	developm	K05700	vinculin[-]	mmu0481	Regulation	14:209293	1059	1120	1590	1562	1263	808	8.070827	7.154729	8.347777	7.755053	8.440499	7.635181	7.857778	7.943578	0.841762	1	-
ENSMUSG	Ap3m1	adaptor-r	GO:00511	localizatio	K12398	AP-3 com	mmu0414	Lysosome	14:210314	823	916	1093	1239	859	467	5.953125	5.55384	5.44649	5.838452	5.448546	4.188392	5.651151	5.158463	0.398593	1	-
ENSMUSG	Txndc16	thioredox	GO:00099	cellular pr	-	-	-	-	14:451334	1410	1668	1937	2243	1674	737	4.442065	4.404682	4.203845	4.603376	4.624488	2.878849	4.350197	4.035571	0.506228	1	-
ENSMUSG	Ero1l	ERO1-like	GO:00325	developm	K10950	ERO1-like	mmu0414	Protein pr	14:452830	573	733	899	949	661	464	6.287272	6.741621	6.795456	6.783526	6.359918	6.312651	6.608116	6.485365	0.67285	1	-
ENSMUSG	Psmc6	proteasor	GO:00081	metabolic	K03064	26S prote	mmu0516	Epstein-B	14:453297	1122	1429	1853	1739	1307	661	13.36078	14.26343	15.20078	13.49025	13.64762	9.759468	14.27499	12.29912	0.208373	1	-
ENSMUSG	Bmp4	bone mor	GO:00325	developm	K04662	bone mor	mmu0520	Pathways	14:463835	43	52	57	58	53	41	0.667835	0.676944	0.60986	0.586832	0.721803	0.789526	0.651546	0.699387	0.916986	1	-
ENSMUSG	Samd4	sterile alpl	GO:00451	translation	-	-	-	-	14:468828	578	613	964	867	654	377	2.501781	2.223998	2.874418	2.44468	2.482229	2.023247	2.533399	2.316719	0.391532	1	-
ENSMUSG	Mapk1ip1	mitogen-	-	-	-	-	-	-	14:472982	973	1038	1355	1357	1037	591	4.800642	4.292763	4.605506	4.361623	4.486504	3.615432	4.566304	4.15452	0.339519	1	-
ENSMUSG	Ktn1	kinectin 1	GO:00511	localizatio	-	-	-	-	14:476484	2132	2510	3321	3439	2364	1237	7.501245	7.402404	8.049455	7.882429	7.293507	5.396371	7.651035	6.857436	0.320403	1	-
ENSMUSG	Peli2	pellino 2 [	GO:00081	metabolic	-	-	-	-	14:481208	452	589	757	846	618	400	2.058027	2.24792	2.374433	2.50937	2.467426	2.258184	2.226794	2.41166	0.737926	1	-
ENSMUSG	Slc35f4	solute car	GO:00511	localizatio	-	-	-	-	14:492985	103	146	150	200	138	153	0.744618	0.884715	0.747036	0.941911	0.874819	1.37143	0.792123	1.06272	0.14878	1	-
ENSMUSG	Anxa11	annexin A	GO:00511	localizatio	-	-	-	-	14:258421	358	363	486	558	373	419	4.499106	3.823862	4.207566	4.568344	4.110496	6.52894	4.176845	5.06926	0.326055	1	-
ENSMUSG	Tmem254	transmem	GO:00160	membran	-	-	-	-	14:262033	1	2	3	4	0	5	0.018033	0.030223	0.037259	0.046986	0	0.111766	0.028505	0.052917	0.673899	1	-
ENSMUSG	Ppif	peptidylpr	GO:00081	metabolic	K09565	peptidyl-p	mmu0501	Huntingto	14:256941	371	429	455	583	403	374	3.457714	3.351398	2.921313	3.539691	3.293544	4.321882	3.243475	3.718372	0.504647	1	-
ENSMUSG	Slmap	sarcolemr	GO:00511	localizatio	-	-	-	-	14:264131	2289	2734	3320	3424	2404	1139	7.257312	7.265767	7.251362	7.072052	6.68355	4.477545	7.258147	6.077716	0.164969	1	-
ENSMUSG	Rnase4	ribonuclea	GO:00081	metabolic	-	-	-	-	14:510910	185	225	351	321	201	221	3.94805	4.024827	5.160244	4.46271	3.7614	5.847773	4.377707	4.690628	0.896209	1	-
ENSMUSG	Arf4	ADP-ribos	GO:00325	developm	-	-	-	-	14:266380	949	1199	1543	1572	1151	858	8.073868	8.550419	9.043425	8.712636	8.586846	9.050839	8.555904	8.783441	0.921718	1	-
ENSMUSG	Dnah12	dynein, ax	GO:00038	catalytic a	K10408	dynein he	mmu0501	Huntingto	14:266932	40	13	55	36	21	12	0.207912	0.05664	0.196939	0.1219	0.095716	0.077336	0.153831	0.098318	0.163152	1	-
ENSMUSG	Rnase6	ribonuclea	GO:00081	metabolic	-	-	-	-	14:511239	6	5	5	9	8	2	0.087382	0.061031	0.050163	0.085382	0.102166	0.036123	0.066192	0.074557	0.88789	1	-
ENSMUSG	Hacl1	2-hydroxy	GO:00081	metabolic	K12261	2-hydroxy	mmu0414	Peroxisom	14:315987	85	74	111	104	109	95	0.800058	0.583836	0.719744	0.637701	0.899646	1.108699	0.701213	0.882015	0.346745	1	-
ENSMUSG	Gpr65	G-protein	GO:00048	signal trar	-	-	-	-	12:982686	6	8	12	14	6	6	0.184308	0.205967	0.253936	0.28015	0.161598	0.228534	0.214737	0.223428	1	1	-
ENSMUSG	Eaf1	ELL associ	GO:00081	metabolic	-	-	-	-	14:314943	295	116	445	199	336	337	2.753029	0.907406	2.860883	1.20983	2.749613	3.899466	2.173772	2.619636	0.651317	1	-
ENSMUSG	Mettl6	methyltrai	GO:00081	metabolic	-	-	-	-	14:314735	281	361	494	418	331	236	1.686185	1.815762	2.042103	1.634019	1.741685	1.755886	1.848017	1.71053	0.405875	1	-
ENSMUSG	Sh3bp5	SH3-dom	GO:00081	metabolic	-	-	-	-	14:313598	2695	3221	3731	4509	3088	1536	46.69448	46.77894	44.53316	50.89424	46.91663	32.99771	46.00219	43.60286	0.569309	1	-
ENSMUSG	Capn7	calpain 7	GO:00081	metabolic	-	-	-	-	14:313366	937	1265	1405	1450	1041	598	7.373599	8.344174	7.616715	7.433435	7.183451	5.834809	7.778163	6.817232	0.222461	1	-
ENSMUSG	Arhgef3	Rho guan	GO:00987	molecular	-	-	-	-	14:271148	1853	2279	2763	2979	1955	1329	13.7929	14.21926	14.16812	14.44549	12.76055	12.26566	14.06009	13.15723	0.4108	1	-
ENSMUSG	Asb14	ankyrin re	GO:00081	metabolic	-	-	-	-	14:268945	2	2	2	0	1	2	0.033403	0.027992	0.023009	0	0.014641	0.041419	0.028135	0.018687	0.549225	1	-
ENSMUSG	Btd	biotinidas	GO:00081	metabolic	K01435	biotinidas	mmu0110	Metabolic	14:316410	136	151	161	191	126	128	3.018945	2.809596	2.462024	2.762043	2.452615	3.522978	2.763522	2.912545	0.977819	1	-
ENSMUSG	Bap1	Brca1 ass	GO:00081	metabolic	-	-	-	-	14:312514	1873	2222	2856	2868	2080	1974	13.34321	13.26843	14.01626	13.31016	12.99357	17.43636	13.54263	14.58003	0.808154	1	-
ENSMUSG	Phf7	PHD finge	GO:00160	membran	-	-	-	-	14:312376	51	63	66	88	64	56	0.357037	0.369691	0.318304	0.401336	0.392889	0.486093	0.348344	0.426773	0.397351	1	-
ENSMUSG	Galnt15	polypepti	GO:00081	metabolic	K00710	polypepti	mmu0110	Metabolic	14:320289	57	67	97	101	97	65	0.401165	0.395253	0.470298	0.463075	0.598634	0.567212	0.422238	0.542973	0.243103	1	-
ENSMUSG	Sema3g	sema don	GO:00325	developm	K06840	semaphor	mmu0436	Axon guic	14:312178	87	112	152	171	110	90	1.217869	1.314165	1.465807	1.559401	1.35025	1.562106	1.332613	1.490586	0.661625	1	-
ENSMUSG	Dph3	diphtham	GO:00081	metabolic	-	-	-	-	14:320805	349	394	511	540	392	380	3.521754	3.332596	3.552266	3.549841	3.468665	4.754486	3.468872	3.924331	0.576663	1	-
ENSMUSG	Oxnad1	oxidoredu	GO:00081	metabolic	-	-	-	-	14:320853	167	136	151	150	231	95	1.065277	0.727176	0.663551	0.62333	1.292116	0.751374	0.818668	0.88894	0.836047	1	-
ENSMUSG	Nisch	nischarin |	GO:00081	metabolic	-	-	-	-	14:311709	5112	5215	6133	6447	4611	4445	13.92555	11.90772	11.50922	11.44091	11.01436	15.0134	12.44749	12.48956	0.72794	1	-
ENSMUSG	Parg	poly (ADP	GO:00081	metabolic	-	-	-	-	14:322019	667	834	1009	1071	797	490	3.97616	4.167323	4.143629	4.159197	4.166193	3.621762	4.095704	3.982384	0.639889	1	-
ENSMUSG	Ogdhl	oxoglutar	GO:00081	metabolic	K00164	2-oxoglut	mmu0110	Metabolic	14:323220	1259	1550	2018	2130	1598	1565	17.08954	17.63556	18.87023	18.83502	19.0206	26.33935	17.86511	21.39832	0.275632	1	-
ENSMUSG	Glt8d1	glycosyltra	GO:00081	metabolic	-	-	-	-	14:310014	366	501	589	640	461	342	3.231955	3.708303	3.583038	3.681683	3.569671	3.744524	3.507766	3.665292	0.967638	1	-
ENSMUSG	Spcs1	signal pep	GO:00081	metabolic	K12946	signal pep	mmu0306	Protein ex	14:309998	384	489	375	542	337	505	5.228017	5.580417	3.517127	4.807132	4.023262	8.524768	4.775187	5.785054	0.469163	1	-
ENSMUSG	Nek4	NIMA (ne	GO:00325	developm	-	-	-	-	14:309513	340	406	468	555	422	254	1.53608	1.537493	1.456572	1.633463	1.671821	1.422835	1.510048	1.57604	0.950226	1	-
ENSMUSG	Chat	choline ac	GO:00038	catalytic a	K00623	choline O	mmu0472	Cholinerg	14:324082	2	0	9	7	0	2	0.023344	0	0.072349	0.053216	0	0.028946	0.031898	0.027387	0.877269	1	-
ENSMUSG	Itih4	inter alpha	GO:00081	metabolic	-	-	-	-	14:308864	0	2	0	0	3	0	0	0.021001	0	0	0.032954	0	0.007	0.010985	1	1	-
ENSMUSG	Ebpl	emopami	GO:00081	metabolic	-	-	-	-	14:613317	156	169	246	219	185	232	0.60324	0.547779	0.655319	0.551685	0.627308	1.112345	0.602113	0.763779	0.316687	1	-
ENSMUSG	Kpna3	karyopher	GO:00511	localizatio	-	-	-	-	14:613652	1888	2519	2746	2633	2019	1131	25.73746	28.78359	25.78789	23.38279	24.13476	19.11669	26.76965	22.21141	0.092232	1	-
ENSMUSG	Spryd7	SPRY dom	GO:00160	membran	-	-	-	-	14:615319	683	919	1068	1082	812	639	8.38453	9.456411	9.031931	8.653003	8.740913	9.72624	8.957624	9.040052	0.803049	1	-
ENSMUSG	Rnaseh2b	ribonuclea	GO:00325	developm	K10744	ribonuclea	mmu0303	DNA repli	14:622925	69	96	126	119	81	73	0.647834	0.755505	0.814957	0.727845	0.666866	0.849803	0.739432	0.748171	0.863848	1	-
ENSMUSG	Gucy1b2	guanylate	GO:00081	metabolic	K12319	guanylate	mmu0023	Purine me	14:623926	0	5	2	4	13	0	0	0.079434	0.026118	0.049396	0.216067	0	0.035184	0.088488	0.332081	1	-
ENSMUSG	Mapk8	mitogen-	GO:00325	developm	K04440	c-Jun N-t	mmu0520	Pathways	14:333778	1709	2298	2755	2799	2205	1096	6.755324	7.613897	7.502	7.207565	7.642851	5.371564	7.290407	6.74066	0.435515	1	-
ENSMUSG	Pspc1	paraspeck	GO:00081	metabolic	-	-	-	-	14:567207	675	1028	1352	1103	1040	469	6.841835	8.734026	9.44054	7.28326	9.243672	5.894234	8.3388	7.473722	0.375208	1	-
ENSMUSG	Ctsb	cathepsin	GO:00325	developm	K01363	cathepsin	mmu0462	NOD-like	14:631224	8432	10285	13965	13805	9485	9205	84.43027	86.32265	96.32965	90.0504	83.28132	114.2819	89.02753	95.8712	0.797909	1	-
ENSMUSG	Ptpn20	protein ty	GO:00081	metabolic	-	-	-	-	14:335892	5	9	12	15	8	9	0.077391	0.116759	0.127958	0.151249	0.108585	0.172716	0.107369	0.144183	0.520468	1	-
ENSMUSG	Gdf10	growth di	GO:00325	developm	-	-	-	-	14:339235	241	259	295	321	250	173	2.93979	2.648197	2.478977	2.550854	2.674121	2.616566	2.688988	2.613847	0.656436	1	-
ENSMUSG	Zmym2	zinc finge	GO:00325	developm	-	-	-	-	14:568866	1927	2201	2647	2928	2011	1034	11.59393	11.09997	10.9712	11.47629	10.60971	7.713568	11.2217	9.93319	0.286733	1	-
ENSMUSG	Cryl1	crystallin,	GO:00081	metabolic	K13247	L-gulonat	mmu0110	Metabolic	14:572749	173	176	241	209	184	207	4.113238	3.507532	3.947347	3.237166	3.836168	6.102296	3.856039	4.391877	0.666142	1	-
ENSMUSG	Prkcd	protein kii	GO:00325	developm	K06068	novel prot	mmu0406	Chemokin	14:305953	292	342	537	522	352	252	1.815979	1.782815	2.300665	2.114853	1.919611	1.943182	1.966486	1.992549	0.881628	1	-
ENSMUSG	Anxa8	annexin A	GO:00508	response	-	-	-	-	14:340859	11	9	10	22	18	7	0.306758	0.210362	0.192111	0.399668	0.440147	0.242043	0.23641	0.360619	0.228368	1	-
ENSMUSG	Eef1akmt1	EEF1A alp	GO:00081	metabolic	-	-	-	-	14:575495	198	191	254	246	186	222	11.53394	9.32604	10.19287	9.335302	9.500941	16.03432	10.35095	11.62352	0.720575	1	-
ENSMUSG	Xpo4	exportin 4	GO:00511	localizatio	-	-	-	-	14:575775	390	456	526	556	426	311	1.638938	1.606261	1.522774	1.52214	1.569821	1.620482	1.589324	1.570814	0.708292	1	-
ENSMUSG	Tkt	transketol	GO:00081	metabolic	K00615	transketol	mmu0110	Metabolic	14:305483	2164	2182	2515	2719	1868	2060	14.32143	12.10422	11.46618	11.72249	10.84048	16.90372	12.63061	13.15557	0.949559	1	-
ENSMUSG	Pinx1	PIN2/TERI	GO:00081	metabolic	-	-	-	-	14:638603	102	110	120	134	104	46	2.165864	1.957825	1.755337	1.853595	1.936448	1.211093	1.959675	1.667045	0.342185	1	-
ENSMUSG	Lats2	large tum	GO:00325	developm	K08791	serine/thre	mmu0439	Hippo sig	14:576896	1070	1358	1422	1488	1323	1067	6.870944	7.309458	6.290477	6.224683	7.449638	8.495379	6.823626	7.3899	0.776735	1	-
ENSMUSG	Dcp1a	decappin	GO:00081	metabolic	K12610	mRNA-de	mmu0301	RNA degr	14:304795	435	463	432	586	485	291	1.771697	1.580643	1.21209	1.554818	1.732147	1.469531	1.521477	1.585499	0.973236	1	-
ENSMUSG	Sap18	Sin3-asso	GO:00329	macromo	K14324	histone de	mmu0301	RNA trans	14:577981	167	379	268	452	75	13	1.434043	2.727958	1.585372	2.528514	0.564744	0.138409	1.915791	1.077222	0.199662	1	-
ENSMUSG	Ska3	spindle ar	GO:00990	supramole	-	-	-	-	14:578065	8	17	12	17	7	6	0.131472	0.23419	0.135866	0.182007	0.100886	0.122275	0.167176	0.135056	0.576843	1	-
ENSMUSG	Mrpl57	mitochon	GO:00081	metabolic	-	-	-	-	14:578262	199	252	316	283	226	328	3.724096	3.952954	4.073874	3.450143	3.708678	7.610776	3.916974	4.923199	0.364468	1	-
ENSMUSG	Zdhhc20	zinc finge	GO:00081	metabolic	-	-	-	-	14:578327	646	838	1105	1034	768	558	4.445135	4.833359	5.238005	4.63505	4.633998	4.760713	4.838833	4.676587	0.570559	1	-
ENSMUSG	Hmbox1	homeobo	GO:00081	metabolic	-	-	-	-	14:648116	920	1077	1399	1622	1091	820	4.827341	4.736843	5.056958	5.544377	5.019815	5.334822	4.873714	5.299671	0.704475	1	-
ENSMUSG	Micu2	mitochon	GO:00511	localizatio	-	-	-	-	14:579162	254	382	499	472	328	285	4.326582	5.454146	5.855487	5.237625	4.899226	6.019247	5.212072	5.385366	0.955085	1	-
ENSMUSG	Fgf9	fibroblast	GO:00325	developm	K04358	fibroblast	mmu0520	Pathways	14:580705	210	312	356	306	262	199	2.404275	2.994142	2.807803	2.282278	2.630322	2.824905	2.735407	2.579168	0.492183	1	-
ENSMUSG	Ints9	integrator	GO:00081	metabolic	-	-	-	-	14:649500	256	306	387	466	343	186	2.247796	2.252124	2.340882	2.665538	2.640914	2.024962	2.280267	2.443805	0.799608	1	-
ENSMUSG	Extl3	exostoses	GO:00081	metabolic	K02370	alpha-1,4	mmu0110	Metabolic	14:650520	2010	2487	3116	3272	2339	2304	13.49193	13.99286	14.40876	14.3078	13.76736	19.17549	13.96452	15.75022	0.530254	1	-
ENSMUSG	Cab39l	calcium b	GO:00055	extracellul	K08272	calcium b	mmu0415	mTOR sig	14:594409	620	592	993	707	548	375	5.197581	4.15991	5.734696	3.861094	4.028405	3.897862	5.030729	3.92912	0.038717	1	-
ENSMUSG	Cdadc1	cytidine a	GO:00038	catalytic a	-	-	-	-	14:595593	716	967	1232	1157	893	541	5.628195	6.371406	6.671419	5.924766	6.155315	5.272775	6.223673	5.784285	0.420303	1	-
ENSMUSG	Atp8a2	ATPase, a	GO:00325	developm	-	-	-	-	14:596385	1294	1532	1819	2026	1455	1085	5.070761	5.032113	4.910468	5.172016	4.999707	5.271747	5.004447	5.147823	0.93211	1	-
ENSMUSG	Amer2	APC mem	GO:00508	response	-	-	-	-	14:603732	1853	1772	2233	2406	1819	857	8.87176	7.111332	7.365029	7.504323	7.636768	5.087464	7.782707	6.742851	0.253621	1	-
ENSMUSG	Mtmr6	myotubula	GO:00081	metabolic	K18083	myotubula	mmu0110	Metabolic	14:602652	2801	3402	2212	4457	1746	1940	34.97288	35.60455	19.02632	36.25289	19.11636	30.03351	29.86792	28.46759	0.634821	1	-
ENSMUSG	Spata13	spermato	GO:00987	molecular	K05769	Rho guan	mmu0481	Regulation	14:606340	477	540	792	818	628	429	1.749165	1.659813	2.000734	1.954103	2.019366	1.950544	1.803237	1.974671	0.684927	1	-
ENSMUSG	Cacna2d3	calcium ch	GO:00511	localizatio	K04860	voltage-d	mmu0401	MAPK sigi	14:289049	1632	1904	2397	2575	1820	1355	10.21656	9.990892	10.33723	10.50131	9.990759	10.51743	10.18156	10.3365	0.845844	1	-
ENSMUSG	Mipep	mitochon	GO:00081	metabolic	-	-	-	-	14:607845	597	632	784	797	654	423	5.062195	4.491943	4.579649	4.402551	4.862785	4.447238	4.711262	4.570858	0.611493	1	-
ENSMUSG	Wnt5a	wingless-	GO:00325	developm	K00444	wingless-t	mmu0520	Pathways	14:285047	200	230	250	157	226	212	1.013847	0.977289	0.873041	0.518469	1.0046	1.332489	0.954726	0.951853	0.765156	1	-
ENSMUSG	Esd	esterase D	GO:00081	metabolic	-	-	mmu0120	Carbon m	14:747322	326	407	365	461	330	309	4.246423	4.443775	3.275288	3.911904	3.769309	4.99057	3.988495	4.223928	0.938619	1	-
ENSMUSG	Lcp1	lymphocy	GO:00325	developm	-	-	-	-	14:751311	234	256	438	467	270	231	1.12996	1.036191	1.457045	1.46908	1.143285	1.383072	1.207732	1.331812	0.699102	1	-
ENSMUSG	Zc3h13	zinc finge	GO:00056	cell|intrace	-	-	-	-	14:752843	2303	2888	3478	3708	2803	1394	18.74041	19.69859	19.49692	19.65651	20.00096	14.06481	19.31197	17.90743	0.446919	1	-
ENSMUSG	Slc25a30	solute car	GO:00511	localizatio	-	-	-	-	14:757601	139	159	256	221	162	206	1.7369	1.66537	2.203695	1.799014	1.775075	3.191636	1.868655	2.255241	0.462223	1	-
ENSMUSG	Gpalpp1	GPALPP n	-	-	-	-	-	-	14:760862	1237	1440	1715	1922	1296	719	14.58806	14.23454	13.93296	14.76601	13.40219	10.51339	14.25186	12.89386	0.347477	1	-
ENSMUSG	Nufip1	nuclear fr	GO:00081	metabolic	-	-	-	-	14:761108	169	241	303	284	211	198	2.702688	3.230569	3.338127	2.958752	2.95892	3.926087	3.090461	3.281253	0.930754	1	-
ENSMUSG	Tsc22d1	TSC22 do	GO:00081	metabolic	-	-	-	-	14:764149	3678	5950	6135	5989	5391	5338	11.219	15.21292	12.89165	11.90087	14.41965	20.18864	13.10786	15.50305	0.384039	1	-
ENSMUSG	Enox1	ecto-NOX	GO:00081	metabolic	-	-	-	-	14:771567	841	973	1193	1355	999	573	5.554203	5.386312	5.427719	5.829703	5.785405	4.692087	5.456078	5.435732	0.790512	1	-
ENSMUSG	Dnajc15	DnaJ heat	GO:00081	metabolic	-	-	-	-	14:778206	1127	1231	1602	1790	1329	732	10.13933	9.283171	9.928859	10.49108	10.48463	8.165492	9.783788	9.713733	0.77626	1	-
ENSMUSG	Akap11	A kinase (	GO:00081	metabolic	-	-	-	-	14:784922	4580	6706	6409	9233	5627	3941	22.02427	27.0304	21.23136	28.92415	23.7277	23.49787	23.42868	25.38324	0.725809	1	-
ENSMUSG	Rgcc	regulator	GO:00325	developm	-	-	-	-	14:792887	129	151	159	170	163	207	8.930653	8.76239	7.583009	7.666974	9.895156	17.76845	8.425351	11.77686	0.162901	1	-
ENSMUSG	Tdrd3	tudor don	GO:00081	metabolic	-	-	-	-	14:874166	375	492	647	653	474	288	3.290852	3.619061	3.911411	3.733127	3.647527	3.133685	3.607108	3.50478	0.667609	1	-
ENSMUSG	Naa16	N(alpha)-	GO:00081	metabolic	-	-	-	-	14:793252	144	142	234	235	169	124	0.500582	0.413765	0.560377	0.532183	0.51516	0.534465	0.491575	0.527269	0.842995	1	-
ENSMUSG	Diaph3	diaphano	GO:00325	developm	K05745	diaphano	mmu0481	Regulation	14:866553	41	30	62	51	43	33	0.342623	0.210138	0.356925	0.277646	0.315098	0.341926	0.303229	0.311557	0.970927	1	-
ENSMUSG	Mtrf1	mitochon	GO:00048	signal trar	-	-	-	-	14:793977	110	138	173	174	169	93	2.727598	2.868274	2.95518	2.810733	3.674661	2.859265	2.850351	3.114886	0.751241	1	-
ENSMUSG	Wbp4	WW dom	GO:00081	metabolic	-	-	-	-	14:794599	688	846	934	1050	765	429	4.510934	4.649442	4.218675	4.48486	4.39827	3.487565	4.459684	4.123562	0.433846	1	-
ENSMUSG	Sugt1	SGT1, sup	GO:00329	macromo	K12795	suppresso	mmu0462	NOD-like	14:795876	2203	2601	3375	3560	2673	1525	16.31786	16.14886	17.22162	17.17831	17.36163	14.0057	16.56278	16.18188	0.661143	1	-
ENSMUSG	Cnmd	chondron	GO:00325	developm	-	-	-	-	14:796376	20	18	61	31	28	18	0.357444	0.269652	0.751026	0.360925	0.438807	0.398883	0.459374	0.399538	0.531838	1	-
ENSMUSG	Olfm4	olfactome	GO:00325	developm	-	-	-	-	14:799840	88	96	121	116	102	55	1.398872	1.279143	1.325046	1.201249	1.421796	1.084035	1.334354	1.235693	0.558897	1	-
ENSMUSG	Elp3	elongator	GO:00325	developm	-	-	-	-	14:655304	1504	1875	2170	2295	1675	1356	21.87271	22.85645	21.74032	21.74298	21.36053	24.45121	22.15649	22.51824	0.833787	1	-
ENSMUSG	Scara5	scavenger	GO:00081	metabolic	-	-	-	-	14:656664	33	13	60	25	19	16	0.26043	0.085998	0.326199	0.128528	0.131481	0.156561	0.224209	0.138857	0.106196	1	-
ENSMUSG	Pbk	PDZ bindi	GO:00081	metabolic	-	-	-	-	14:658058	5	7	30	11	9	9	0.154481	0.181273	0.638513	0.221391	0.243838	0.34476	0.324755	0.269996	0.556291	1	-
ENSMUSG	Esco2	establishn	GO:00325	developm	-	-	-	-	14:658190	5	9	2	5	8	3	0.036941	0.055733	0.010181	0.024067	0.051831	0.027484	0.034285	0.034461	1	1	-
ENSMUSG	Ccdc25	coiled-co	GO:00055	extracellul	-	-	-	-	14:658373	620	820	948	998	707	418	6.85148	7.595552	7.216933	7.184638	6.851016	5.727364	7.221321	6.587673	0.367739	1	-
ENSMUSG	Clu	clusterin [	GO:00325	developm	K17252	clusterin[-	mmu0461	Complem	14:659684	11001	13171	16900	16529	13802	10971	123.9402	124.3803	131.1651	121.3132	136.353	153.2541	126.4952	136.9735	0.737189	1	-
ENSMUSG	Ephx2	epoxide h	GO:00081	metabolic	K08726	soluble ep	mmu0110	Metabolic	14:660843	220	290	294	391	286	287	4.875058	5.386517	4.488026	5.644374	5.557328	7.885414	4.916534	6.362372	0.154678	1	-
ENSMUSG	Chrna2	cholinergi	GO:00048	signal trar	K04804	nicotinic a	mmu0408	Neuroacti	14:661350	7	6	12	16	13	4	0.111114	0.079823	0.131217	0.144763	0.180937	0.07872	0.107385	0.134807	0.596083	1	-
ENSMUSG	Trim35	tripartite r	GO:00400	locomotic	-	-	-	-	14:662970	2473	2957	2980	3829	2744	2957	27.66531	27.72781	22.96566	27.90476	26.91771	41.01556	26.11959	31.94601	0.241184	1	-
ENSMUSG	Stmn4	stathmin-	GO:00325	developm	-	-	-	-	14:663442	2703	3189	4568	3820	2871	1612	44.41153	43.91947	51.70436	40.88788	41.36428	32.8398	46.67845	38.36399	0.080061	1	-
ENSMUSG	Dpysl2	dihydropy	GO:00325	developm	K07528	dihydropy	mmu0436	Axon guic	14:668028	4190	5050	6071	5876	4667	4355	58.91307	59.51713	58.80434	53.82215	57.5411	75.92272	59.07818	62.42866	0.938624	1	-
ENSMUSG	Bnip3l	BCL2/ade	GO:00325	developm	K15465	BCL2/ade	mmu0413	Mitophag	14:669852	1214	1528	2003	1993	1416	1165	15.05432	15.88248	17.11097	16.1002	15.39743	17.91241	16.01592	16.47001	0.916497	1	-
ENSMUSG	Ppp2r2a	protein pl	GO:00325	developm	K04354	serine/thre	mmu0415	PI3K-Akt	14:670140	1802	1441	1700	2713	1957	683	15.02328	10.06999	9.763612	14.73471	14.30684	7.0602	11.61895	12.03392	0.955058	1	-
ENSMUSG	Ebf2	early B ce	GO:00325	developm	-	-	-	-	14:672332	0	3	12	5	7	2	0	0.014209	0.046714	0.018407	0.034687	0.014016	0.020308	0.02237	1	1	-
ENSMUSG	Nefm	neurofilan	GO:00325	developm	K04573	neurofilan	mmu0501	Amyotrop	14:680825	3793	4668	6109	5820	4534	2270	41.69086	43.00725	46.25722	41.67376	43.70008	30.9364	43.65178	38.77008	0.273605	1	-
ENSMUSG	Nefl	neurofilan	GO:00325	developm	K04572	neurofilan	mmu0501	Amyotrop	14:680839	8887	10782	12885	13437	9945	7926	167.0993	169.9306	166.8996	164.59	163.9709	184.7819	167.9765	171.1143	0.840845	1	-
ENSMUSG	Adamdec	ADAM-lik	GO:00081	metabolic	-	-	-	-	14:685633	2	1	1	5	2	0	0.02603	[illegible]	0.109067	0.071722	0.033904	0	0.06894	0.011301	0.069424	1	-
ENSMUSG	Nkx3-1	NK-3 tran	GO:00325	developm	K09348	homeobo	mmu0520	Pathways	14:691906	14	17	27	18	14	15	0.278478	0.283447	0.369979	0.233245	0.204193	0.369956	0.310635	0.282465	0.611268	1	-
ENSMUSG	Pibf1	progester	GO:00081	metabolic	-	-	-	-	14:990994	247	352	365	377	282	118	1.364176	1.629558	1.388731	1.35643	1.365731	0.808059	1.460822	1.17674	0.177221	1	-
ENSMUSG	Entpd4b	ectonucle	GO:00038	catalytic a	K12305	ectonucle	mmu0023	Purine me	14:695554	1818	2251	2773	2857	2110	2059	11.26334	11.68966	11.83518	11.53096	11.463	15.81669	11.59606	12.93689	0.598164	1	-
ENSMUSG	Bora	bora, aurc	GO:00081	metabolic	-	-	-	-	14:990462	26	34	30	66	23	27	0.229214	0.251245	0.182192	0.379044	0.177802	0.295131	0.220884	0.283992	0.401178	1	-
ENSMUSG	Tnfrsf10b	tumor nec	GO:00081	metabolic	K04722	tumor nec	mmu0421	Necropto	14:697674	6	16	16	12	16	8	0.059667	0.133366	0.109607	0.07774	0.139516	0.098638	0.10088	0.105298	1	1	-
ENSMUSG	Rhobtb2	Rho-relat	GO:00508	response	K07868	Rho-relat	mmu0412	Ubiquitin	14:697849	1235	1779	2226	2180	1738	1311	6.289099	7.593645	7.809048	7.23202	7.760933	8.277711	7.230597	7.756888	0.801099	1	-
ENSMUSG	Klhl1	kelch-like	GO:00325	developm	-	-	-	-	14:961027	47	51	52	69	48	24	0.449508	0.408847	0.342609	0.429905	0.402558	0.284602	0.400321	0.372355	0.690702	1	-
ENSMUSG	Pebp4	phosphati	-	-	-	-	-	-	14:698404	0	0	0	0	2	0	0	0	0	0	0.103289	0	0	0.03443	0.534652	1	-
ENSMUSG	Bin3	bridging i	GO:00325	developm	-	-	-	-	14:701001	139	211	237	274	191	134	3.076933	3.915057	3.614126	3.951249	3.70749	3.677856	3.535372	3.778865	0.84785	1	-
ENSMUSG	Pdlim2	PDZ and l	GO:00055	extracellul	-	-	-	-	14:701642	110	108	168	125	100	123	0.92692	0.762826	0.97524	0.686184	0.738914	1.285112	0.888329	0.903403	0.837535	1	-
ENSMUSG	Sorbs3	sorbin anc	GO:00081	metabolic	-	-	-	-	14:701804	434	449	714	607	481	452	6.644664	5.762118	7.530658	6.054162	6.457605	8.580411	6.645813	7.030726	0.947678	1	-
ENSMUSG	Ppp3cc	protein pl	GO:00325	developm	K04348	serine/thre	mmu0401	MAPK sigi	14:702178	365	430	466	524	416	253	6.215669	6.137849	5.466779	5.813097	6.211992	5.341972	5.940099	5.78902	0.672855	1	-
ENSMUSG	Slc39a14	solute car	GO:00511	localizatio	K14720	solute car	mmu0421	Ferroptos	14:703034	650	801	1036	1142	735	568	2.822263	2.915206	3.098812	3.230218	2.798428	3.057866	2.945427	3.028837	0.945135	1	-
ENSMUSG	Fam160b	family witl	GO:00325	developm	-	-	-	-	14:705832	757	981	1102	1137	882	772	11.92899	12.95776	11.96302	11.67213	12.18763	15.08381	12.28326	12.98119	0.93045	1	-
ENSMUSG	Hr	hairless [S	GO:00081	metabolic	-	-	-	-	14:705522	186	250	411	428	357	247	1.112133	1.252958	1.69292	1.667127	1.871778	1.83116	1.35267	1.790022	0.114541	1	-

ENSMUSC	Sftpc	surfactant	GO:00055	extracellul	-	-	-	-	14:705209	9	5	10	4	4	5	0.42973	0.200106	0.328942	0.124437	0.167466	0.295999	0.319592	0.195967	0.231889	1	-
ENSMUSC	Bmp1	bone mor	GO:00325	developm	-	-	-	-	14:704745	761	1148	1198	1471	994	982	3.899683	4.931047	4.229145	4.910646	4.466565	6.239386	4.353292	5.205532	0.298768	1	-
ENSMUSC	Dmtn	dematin a	GO:00325	developm	-	-	-	-	14:706012	3955	4957	5793	6276	4853	3534	14.27488	14.99677	14.40393	14.75675	15.35957	15.81534	14.55853	15.31055	0.919902	1	-
ENSMUSC	Xpo7	exportin 7	GO:00511	localizatio	-	-	-	-	14:706542	1603	1582	1888	2381	1799	1122	4.542942	3.758055	3.686014	4.395868	4.470721	3.942597	3.99567	4.269729	0.811373	1	-
ENSMUSC	Fgf17	fibroblast	GO:00325	developm	K04358	fibroblast	mmu0520	Pathways	14:706362	5	2	5	5	8	2	0.110298	0.036979	0.075984	0.071858	0.154756	0.054718	0.07442	0.093777	0.75336	1	-
ENSMUSC	Gfra2	glial cell li	GO:00325	developm	-	-	-	-	14:708901	541	650	883	867	609	743	6.926309	6.975422	7.78783	7.23111	6.836976	11.79449	7.229854	8.620859	0.395643	1	-
ENSMUSC	Rb1	RB transcr	GO:00325	developm	K06618	retinoblas	mmu0520	Pathways	14:731836	800	959	1226	1387	953	475	7.481222	7.517168	7.898138	8.449688	7.814804	5.507593	7.632176	7.257362	0.604037	1	-
ENSMUSC	Rcbtb2	regulator	GO:00056	cell|intrace	-	-	-	-	14:731230	312	310	366	446	335	256	1.086794	0.905122	0.878262	1.012066	1.023244	1.105649	0.956726	1.046987	0.725959	1	-
ENSMUSC	Itm2b	integral m	GO:00325	developm	-	-	-	-	14:733622	8903	11598	14043	15453	11607	7442	162.7301	177.6918	176.8246	184.0034	186.0348	168.658	172.4155	179.5654	0.962063	1	-
ENSMUSC	Med4	mediator	GO:00081	metabolic	K15146	mediator	mmu0491	Thyroid h	14:735100	372	366	572	560	335	237	5.150701	4.24774	5.455952	5.051179	4.067338	4.068727	4.951464	4.395748	0.30638	1	-
ENSMUSC	Sucla2	succinate-	GO:00081	metabolic	K01900	succinyl-C	mmu0110	Metabolic	14:735253	2437	3015	3548	3653	2565	1302	27.61253	28.63458	27.694	26.96386	25.48477	18.29144	27.98037	23.58002	0.152793	1	-
ENSMUSC	Uchl3	ubiquitin c	GO:00325	developm	-	-	-	-	14:101653	240	325	343	341	307	120	2.672169	3.033116	2.630862	2.473364	2.997317	1.656604	2.778715	2.375762	0.310867	1	-
ENSMUSC	Gpc5	glypican 5	GO:00055	extracellul	-	-	-	-	14:115092	701	745	927	1010	732	487	7.435022	6.623288	6.773227	6.978586	6.80798	6.404419	6.943845	6.730328	0.60967	1	-
ENSMUSC	Spry2	sprouty h	GO:00325	developm	K17383	protein sp	mmu0520	MicroRNA	14:105891	538	718	943	831	681	565	16.38309	18.32697	19.7823	16.48529	18.18459	21.33281	18.16412	18.66756	0.905776	1	-
ENSMUSC	Rbm26	RNA bind	GO:00081	metabolic	-	-	-	-	14:105106	1347	1633	2107	2330	1568	765	8.770199	8.912116	9.450564	9.882787	8.952214	6.175739	9.044293	8.336913	0.465263	1	-
ENSMUSC	Rnf219	ring finge	-	-	-	-	-	-	14:104477	257	288	384	395	263	208	3.112848	2.923955	3.20411	3.116762	2.793338	3.123731	3.080304	3.011277	0.670094	1	-
ENSMUSC	Ednrb	endotheli	GO:00325	developm	K04198	endotheli	mmu0520	Pathways	14:103814	305	1059	521	875	337	484	2.074672	6.038078	2.441399	3.877389	2.010124	4.082078	3.51805	3.323197	0.684181	1	-
ENSMUSC	Scel	sciellin [Sc	GO:00325	developm	-	-	-	-	14:103513	42	30	54	55	46	9	1.04634	0.62646	0.92676	0.892625	1.004897	0.277997	0.86652	0.725173	0.572191	1	-
ENSMUSC	Fbxl3	F-box anc	GO:00081	metabolic	K10269	F-box anc	mmu0471	Circadian	14:103080	1366	1380	1124	1940	1255	738	7.629266	6.460468	4.324637	7.058548	6.146364	5.110631	6.138124	6.105181	0.817675	1	-
ENSMUSC	Cln5	ceroid-lip	GO:00325	developm	K12390	ceroid-lip	mmu0414	Lysosome	14:103070	200	236	289	259	195	150	4.63383	4.583261	4.612741	3.909242	3.961758	4.309118	4.609944	4.060039	0.253248	1	-
ENSMUSC	Acod1	aconitate	GO:00325	developm	-	-	-	-	14:103046	0	0	0	2	0	0	0	0	0	0.031564	0	0	0	0.010521	0.537856	1	-
ENSMUSC	Dct	dopachro	GO:00325	developm	K01827	dopachro	mmu0110	Metabolic	14:118012	8	7	11	11	8	9	0.071516	0.052452	0.067743	0.06406	0.062716	0.099757	0.063903	0.075511	0.821477	1	-
ENSMUSC	Tgds	TDP-gluc	GO:00038	catalytic a	-	-	-	-	14:118111	48	58	92	95	58	59	0.767048	0.776896	1.012793	0.988973	0.812733	1.169014	0.852246	0.99024	0.609986	1	-
ENSMUSC	Gpr180	G protein-	GO:00508	response	-	-	-	-	14:118137	167	247	326	290	208	285	5.660454	7.017541	7.612081	6.403467	6.182174	11.9775	6.763359	8.187714	0.452066	1	-
ENSMUSC	Cldn10	claudin 10	GO:00511	localizatio	K06087	claudin[-]	mmu0453	Tight junc	14:118787	367	385	534	504	397	315	6.285084	5.526603	6.29996	5.622858	5.96181	6.688694	6.037216	6.091121	0.811289	1	-
ENSMUSC	Dnajc3	DnaJ heat	GO:00081	metabolic	K09523	DnaJ hom	mmu0516	Influenza	14:118937	1529	1764	2763	2518	1730	897	18.90149	18.27847	23.52994	20.27804	18.75329	13.74887	20.23663	17.5934	0.253055	1	-
ENSMUSC	Mbnl2	muscleblir	GO:00081	metabolic	-	-	-	-	14:120275	2652	3101	3918	4198	3159	2195	8.745916	8.572084	8.901182	9.018949	9.135328	8.975369	8.739728	9.043215	0.979018	1	-
ENSMUSC	Nipbl	Nipped-B	GO:00325	developm	-	-	-	-	15:829122	1654	1990	2584	2669	1810	845	11.92475	12.02596	12.83387	12.53557	11.44287	7.553647	12.26153	10.5107	0.239284	1	-
ENSMUSC	Nup155	nucleopor	GO:00081	metabolic	K14312	nuclear pc	mmu0301	RNA trans	15:810931	434	576	680	817	565	402	4.308341	4.792875	4.650296	5.283531	4.918261	4.94803	4.583838	5.049941	0.648275	1	-
ENSMUSC	Gdnf	glial cell li	GO:00325	developm	-	-	-	-	15:781101	0	2	6	5	0	0	0	0.030361	0.074858	0.058998	0	0	0.035073	0.019666	0.655341	1	-
ENSMUSC	Osmr	oncostatir	GO:00048	signal trar	K05057	oncostatir	mmu0415	PI3K-Akt s	15:681357	150	115	187	195	169	85	0.664226	0.426851	0.57045	0.562522	0.656226	0.466688	0.553842	0.561812	0.940716	1	-
ENSMUSC	Fyb	FYN bindi	GO:00511	localizatio	K17698	FYN bindi	mmu0401	Rap1 sign	15:652285	208	282	374	347	227	277	1.668013	1.895562	2.066143	1.812792	1.596265	2.754249	1.876573	2.054435	0.810752	1	-
ENSMUSC	Dab2	disabled 2	GO:00325	developm	K12475	disabled h	mmu0414	Endocytos	15:629978	239	342	368	451	236	207	1.181393	1.417017	1.253128	1.452293	1.022943	1.268682	1.283846	1.247972	0.673509	1	-
ENSMUSC	Ttc33	tetratricop	-	-	-	-	-	-	15:518552	624	682	839	930	692	567	17.10831	15.67329	15.84659	16.61067	16.63687	19.27489	16.2094	17.50748	0.780734	1	-
ENSMUSC	Mroh2b	maestro h	GO:00325	developm	-	-	-	-	15:489873	0	0	2	0	0	0	0	0	0.014274	0	0	0	0.004758	0	0.536281	1	-
ENSMUSC	Rab2b	RAB2B, m	GO:00452	synapse|c	-	-	-	-	14:522617	291	654	443	428	319	384	1.852727	3.490189	1.943006	1.775185	1.780949	3.031351	2.428641	2.195828	0.421589	1	-
ENSMUSC	Mettl3	methyltrai	GO:00081	metabolic	-	-	-	-	14:522948	389	389	473	507	370	242	2.490646	2.087684	2.086289	2.114715	2.077333	1.921159	2.22154	2.037736	0.394652	1	-
ENSMUSC	Dad1	defender	GO:00325	developm	K12668	oligosacch	mmu0110	Metabolic	14:542354	451	630	810	626	582	632	6.560411	7.681524	8.116902	5.932121	7.423685	11.39875	7.452946	8.251518	0.753507	1	-
ENSMUSC	Lrp10	low-densi	GO:00325	developm	-	-	-	-	14:544641	372	523	710	665	480	542	4.026863	4.745463	5.294611	4.689508	4.556259	7.274598	4.688979	5.506788	0.45425	1	-
ENSMUSC	Rem2	rad and g	GO:00511	localizatio	-	-	-	-	14:544761	221	267	281	311	213	149	4.15171	4.204347	3.636565	3.806062	3.508779	3.470602	3.997541	3.595147	0.345305	1	-
ENSMUSC	Haus4	HAUS aug	GO:00990	supramole	-	-	-	-	14:545417	39	43	62	68	57	39	0.672613	0.621612	0.736612	0.763995	0.862013	0.833972	0.676945	0.819993	0.440162	1	-
ENSMUSC	Ajuba	ajuba LIM	GO:00325	developm	K16682	LIM doma	mmu0439	Hippo sig	14:545674	23	32	50	37	30	39	0.276002	0.321882	0.413343	0.28925	0.315681	0.580285	0.337076	0.395072	0.718337	1	-
ENSMUSC	4931414P	RIKEN cDI	GO:00055	extracellul	-	-	-	-	14:545836	54	75	101	117	88	43	0.933591	1.086869	1.202908	1.317734	1.334097	0.921758	1.074456	1.191196	0.702531	1	-
ENSMUSC	Slc7a8	solute car	GO:00511	localizatio	K13781	solute car	mmu0497	Protein di	14:547222	1606	1786	1899	2326	1792	1380	17.35229	16.17504	14.13473	16.37203	16.9782	18.48741	15.88735	17.27921	0.724787	1	-
ENSMUSC	C6	complem	GO:00325	developm	K03995	complem	mmu0532	Systemic l	15:472717	0	0	2	2	2	3	0	0	0.020635	0.019508	0.02626	0.055707	0.006878	0.033825	0.228674	1	-
ENSMUSC	Fbxo4	F-box prc	GO:00325	developm	K10291	F-box prc	mmu0412	Ubiquitin	15:396544	64	66	112	105	60	63	1.735247	1.499941	2.091927	1.854604	1.426512	2.117903	1.775705	1.799673	0.887706	1	-
ENSMUSC	Acin1	apoptotic	GO:00325	developm	K12875	apoptotic	mmu0301	RNA trans	14:546421	1792	2011	2590	2757	2129	1289	6.338678	5.962459	6.311197	6.353002	6.603572	5.653265	6.204111	6.203279	0.781087	1	-
ENSMUSC	Oxct1	3-oxoacic	GO:00325	developm	K01027	3-oxoacic	mmu0028	Valine, leu	15:402638	4178	4830	5506	6086	4353	4230	56.11253	54.37401	50.94235	53.24821	51.26521	70.43973	53.80963	58.31772	0.77101	1	-
ENSMUSC	Drosha	drosha, ril	GO:00325	developm	K03685	ribonuclea	mmu0520	Proteogly	15:128248	2180	2811	3319	3542	2728	1679	17.40959	18.81681	18.25958	18.42733	19.10377	16.62527	18.162	18.05212	0.737843	1	-
ENSMUSC	Psmb5	proteasor	GO:00081	metabolic	K02737	20S prote	mmu0305	Proteason	14:546141	657	876	1042	990	771	1066	27.45188	30.6806	29.99342	26.94782	28.24907	55.22681	29.3753	36.8079	0.301707	1	-
ENSMUSC	Pabpn1	poly(A) bi	GO:00081	metabolic	K14396	polyadeny	mmu0516	Influenza	14:548925	1180	1647	1436	1562	1088	1285	11.6611	13.64282	9.776049	10.05588	9.42821	15.74514	11.69332	11.74308	0.746707	1	-
ENSMUSC	6030458C	RIKEN cDI	-	-	-	-	-	-	15:128081	255	313	509	594	237	680	1.443617	1.48528	1.985092	2.190682	1.176527	4.773159	1.637996	2.713456	0.071416	1	-
ENSMUSC	Pdzd2	PDZ dom	GO:00056	cell|intrace	-	-	-	-	15:123597	1567	1769	2402	3218	2359	1487	4.878396	4.616244	5.151485	6.526429	6.439891	5.739908	4.882042	6.235409	0.087373	1	-
ENSMUSC	Slc22a17	solute car	GO:00048	signal trar	-	-	-	-	14:549063	4063	5268	6191	6314	5098	6707	18.57535	20.18779	19.49855	18.80513	20.43773	38.01932	19.42056	25.75406	0.133928	1	-
ENSMUSC	Golph3	golgi pho	GO:00081	metabolic	-	-	-	-	15:123214	997	1363	1800	1609	1258	873	9.542521	10.93495	11.86839	10.03242	10.55824	10.36021	10.78195	10.31695	0.511213	1	-
ENSMUSC	Zfr	zinc finge	GO:00325	developm	-	-	-	-	15:121178	6590	8250	9246	9972	7333	3762	52.26685	54.84636	50.51802	51.52344	50.99943	36.99523	52.54374	46.50603	0.261255	1	-
ENSMUSC	Efs	embryona	GO:00071	cell adhes	-	-	-	-	14:549165	206	155	277	180	163	228	2.560018	1.614577	2.371411	1.457232	1.776252	3.51314	2.182002	2.248875	0.880843	1	-
ENSMUSC	Ngdn	neuroguic	GO:00081	metabolic	-	-	-	-	14:550154	185	227	266	225	219	186	5.50435	5.66127	5.452159	4.361128	5.713764	6.861741	5.53926	5.645544	0.857684	1	-
ENSMUSC	Sub1	SUB1 hon	GO:00081	metabolic	-	-	-	-	15:119813	2892	3597	4378	3972	3029	2273	41.12672	42.87647	42.88969	36.79736	37.7718	40.07848	42.29763	38.21588	0.215247	1	-
ENSMUSC	Npr3	natriuretic	GO:00325	developm	-	-	-	-	15:118398	604	689	770	759	610	387	2.630799	2.515493	2.310431	2.153646	2.329825	2.090006	2.485574	2.191159	0.221917	1	-
ENSMUSC	Jph4	junctophil	GO:00511	localizatio	-	-	-	-	14:551068	9015	11014	11767	14003	10671	8610	55.13717	56.46466	49.57879	55.79323	57.23037	65.29319	53.72687	59.43893	0.600249	1	-
ENSMUSC	Dhrs4	dehydrog	GO:00081	metabolic	K11147	dehydrog	mmu0110	Metabolic	14:554787	130	131	215	199	138	124	1.930352	1.630481	2.199285	1.924986	1.796853	2.282965	1.920039	2.001601	1	1	-
ENSMUSC	Carmil3	capping p	GO:00160	membran	-	-	-	-	14:554906	629	850	843	1071	770	778	3.728647	4.223503	3.442547	4.13592	4.002529	5.718295	3.798232	4.618914	0.269351	1	-
ENSMUSC	Cpne6	copine VI	GO:00325	developm	-	-	-	-	14:555104	7664	8950	9024	11981	7825	5615	62.38891	61.06999	50.60605	63.53699	55.85717	56.67449	58.02165	58.68955	0.836797	1	-
ENSMUSC	Dcaf11	DDB1 anc	GO:00081	metabolic	-	-	-	-	14:555600	744	928	1132	1164	720	763	2.374143	2.482188	2.488466	2.41974	2.014695	3.018871	2.448266	2.484435	0.816087	1	-
ENSMUSC	Psme1	proteasor	GO:00081	metabolic	K06696	proteasor	mmu0461	Antigen p	14:555781	183	229	308	313	215	240	1.775877	1.862728	2.059036	1.978736	1.829538	2.887737	1.899214	2.232004	0.472068	1	-
ENSMUSC	Emc9	ER memb	GO:00329	macromo	-	-	-	-	14:555815	174	146	227	255	174	180	3.260098	2.292905	2.929934	3.112448	2.85872	4.181557	2.827645	3.384242	0.42371	1	-
ENSMUSC	Tgm1	transgluta	GO:00325	developm	-	-	-	-	14:557000	5	8	16	11	6	4	0.069594	0.09333	0.153416	0.099737	0.073225	0.069031	0.105447	0.080665	0.485371	1	-
ENSMUSC	Cideb	cell death	GO:00081	metabolic	-	-	-	-	14:557540	24	37	45	41	38	47	0.780591	1.008716	1.008266	0.868708	1.083775	1.895384	0.932524	1.282622	0.301395	1	-
ENSMUSC	Adcy4	adenylate	GO:00081	metabolic	K08044	adenylate	mmu0520	Pathways	14:557690	44	69	76	89	50	58	0.37646	0.494839	0.447951	0.496065	0.375127	0.615292	0.43975	0.495495	0.741139	1	-
ENSMUSC	Ripk3	receptor-	GO:00325	developm	K08847	receptor-	mmu0421	Necroptos	14:557849	2	4	6	6	3	8	0.024787	0.041543	0.051214	0.048434	0.032594	0.122908	0.039181	0.067979	0.363018	1	-
ENSMUSC	Sdr39u1	short chai	GO:00081	metabolic	-	-	-	-	14:558972	353	429	495	514	393	424	2.921121	2.975675	2.821833	2.770895	2.851742	4.350379	2.90621	3.324339	0.565165	1	-
ENSMUSC	Zscan26	zinc finge	GO:00081	metabolic	-	-	-	-	13:214421	608	1805	1094	1051	876	472	8.685155	21.61243	10.76571	9.780433	10.97288	8.359916	13.68776	9.70441	0.063044	1	-
ENSMUSC	Sema5a	sema don	GO:00055	extracellul	K06841	semaphor	mmu0436	Axon guic	15:322448	3275	3840	4741	6018	4247	2535	8.613123	8.465126	8.589556	10.31058	9.794319	8.266341	8.555935	9.45708	0.589405	1	-
ENSMUSC	Cct5	chaperoni	GO:00081	metabolic	-	-	-	-	15:315908	2311	2970	3483	3494	2619	1813	35.67428	38.42948	37.03908	35.1366	35.4514	34.70076	37.04761	35.09625	0.431389	1	-
ENSMUSC	Cmbl	carboxym	GO:00038	catalytic a	K01061	carboxym	mmu0110	Metabolic	15:315653	55	106	121	82	57	70	1.8553	2.997186	2.811854	1.801991	1.686049	2.927803	2.55478	2.138615	0.256964	1	-
ENSMUSC	Ropn1l	ropporin	-	-	-	-	-	-	15:314412	7	7	11	6	9	7	0.46489	0.389632	0.503225	0.259581	0.524112	0.576401	0.452582	0.453365	1	1	-
ENSMUSC	Ctnnd2	catenin (c	GO:00325	developm	-	-	-	-	15:301725	4659	5035	6608	6922	5487	3486	20.13967	18.24366	19.67801	19.49274	20.79875	18.68414	19.35378	19.65854	0.864283	1	-
ENSMUSC	Tars	threonyl-t	GO:00081	metabolic	K01868	threonyl-t	mmu0097	Aminoacy	15:113823	634	645	939	816	621	423	5.207107	4.440379	5.31281	4.365956	4.472409	4.307579	4.986766	4.381981	0.204429	1	-
ENSMUSC	Slc45a2	solute car	GO:00081	metabolic	-	-	-	-	15:110007	0	0	2	0	0	0	0	0	0.029357	0	0	0	0.009786	0	0.536713	1	-
ENSMUSC	Amacr	alpha-me	GO:00081	metabolic	K01796	alpha-me	mmu0110	Metabolic	15:109817	133	154	186	236	161	139	2.796077	2.713754	2.693776	3.232157	2.968013	3.623255	2.734536	3.274475	0.366052	1	-
ENSMUSC	Skor1	SKI family	GO:00325	developm	-	-	-	-	9:6313817	3	4	5	3	3	0	0.040769	0.045567	0.046816	0.026559	0.035751	0	0.044384	0.02077	0.196125	1	-
ENSMUSC	Rai14	retinoic ac	GO:00325	developm	-	-	-	-	15:105689	133	193	250	280	204	140	0.912506	1.109926	1.181617	1.25148	1.227321	1.190961	1.068016	1.223254	0.524126	1	-
ENSMUSC	Brix1	BRX1, bio	GO:00099	cellular pr	-	-	-	-	15:104747	385	530	660	612	482	257	7.547158	8.708664	8.912892	7.815487	8.285393	6.246588	8.389572	7.449156	0.312194	1	-
ENSMUSC	Rad1	RAD1 che	GO:00325	developm	K02830	cell cycle c	mmu0421	Cellular se	15:104860	74	61	81	80	74	68	0.433406	0.299467	0.326817	0.305236	0.380052	0.493811	0.35323	0.393033	0.795344	1	-
ENSMUSC	Mtdh	metadher	GO:00325	developm	-	-	-	-	15:340826	795	1605	1094	1413	1419	502	2.481563	4.199391	2.352486	2.873308	3.884038	1.942888	3.011147	2.900078	0.748664	1	-
ENSMUSC	Laptm4b	lysosomal	GO:00081	metabolic	K12387	lysosomal	mmu0414	Lysosome	15:342380	998	792	1015	1356	1100	549	16.36798	10.88786	11.46785	14.4879	15.81976	11.16407	12.90789	13.82391	0.818097	1	-
ENSMUSC	Sdc2	syndecan	GO:00325	developm	K16336	syndecan	mmu0520	Proteogly	15:329207	671	837	1193	1028	755	478	12.09074	12.6418	14.80889	12.06717	11.92944	10.67934	13.18048	11.55865	0.215131	1	-
ENSMUSC	Dnah5	dynein, ax	GO:00325	developm	K10408	dynein he	mmu0501	Huntingto	15:282037	193	214	275	285	205	158	0.784406	0.729036	0.769957	0.754587	0.730601	0.796208	0.761133	0.760465	0.791136	1	-
ENSMUSC	Trio	triple func	GO:00325	developm	-	-	-	-	15:277306	5769	5911	7687	9009	6391	4101	13.15716	11.29992	12.0773	13.38504	12.78124	11.59678	12.17812	12.58769	0.988256	1	-
ENSMUSC	Ank	progressiv	GO:00325	developm	-	-	-	-	15:274666	3022	3733	4513	4811	3757	2677	37.99351	39.33923	39.08694	39.40324	41.41893	41.73008	38.80656	40.85075	0.909329	1	-
ENSMUSC	11-Mar	membran	GO:00081	metabolic	-	-	-	-	15:263090	33	47	40	45	47	33	0.534194	0.637726	0.446063	0.47455	0.66716	0.662341	0.539327	0.60135	0.787028	1	-
ENSMUSC	Retreg1	reticuloph	GO:00081	metabolic	-	-	-	-	15:258431	692	779	1064	1070	794	560	5.079543	4.793014	5.380365	5.116627	5.110713	5.096742	5.084307	5.108027	0.798866	1	-
ENSMUSC	Myo10	myosin X	GO:00325	developm	K12559	myosin X[	mmu0466	Fc gamma	15:256225	1161	1192	1665	1714	1234	622	4.854919	4.178099	4.7964	4.669197	4.524885	3.22497	4.609806	4.139684	0.349399	1	-
ENSMUSC	Rnf19a	ring finge	GO:00081	metabolic	-	-	-	-	15:362399	940	1224	1447	1437	1034	641	8.166757	8.913663	8.660473	8.133177	7.877441	6.90503	8.580298	7.638549	0.241297	1	-
ENSMUSC	Pabpc1	poly(A) bi	GO:00081	metabolic	K13126	polyadeny	mmu0301	RNA trans	15:365956	4078	4293	5596	6360	4344	1712	28.91223	25.5122	27.3315	29.37467	27.0064	15.04957	27.25197	23.81021	0.350817	1	-
ENSMUSC	Ywhaz	tyrosine 3	GO:00511	localizatio	K16197	14-3-3 pr	mmu0415	PI3K-Akt s	15:367707	12527	16048	18972	19831	14550	10644	98.763	106.0525	103.0414	101.8529	100.5896	104.049	102.619	102.1638	0.699128	1	-
ENSMUSC	Grhl2	grainyhea	GO:00325	developm	-	-	-	-	15:372330	2	3	6	1	0	9	0.010225	0.012852	0.021126	0.003329	0	0.057035	0.014734	0.020121	0.897658	1	-
ENSMUSC	Rrm2b	ribonucle	GO:00325	developm	K10808	ribonucle	mmu0110	Metabolic	15:379239	998	1258	1453	1614	1195	660	6.28664	6.642345	6.305291	6.623281	6.600831	5.154872	6.411425	6.126328	0.569522	1	-
ENSMUSC	Atp6v1c1	ATPase, H	GO:00511	localizatio	K02148	V-type H-	mmu0110	Metabolic	15:386619	2938	4087	4665	4542	3323	2755	24.26494	28.29339	26.54181	24.43747	24.06581	28.21208	26.36672	25.57179	0.528221	1	-
ENSMUSC	Baalc	brain and	GO:00452	synapse|r	-	-	-	-	15:389331	3832	4509	5690	6008	4618	2524	37.72814	37.21114	38.59256	38.53465	39.8691	30.81167	37.84395	36.40514	0.58107	1	-
ENSMUSC	Fzd6	frizzled cl	GO:00325	developm	-	-	-	-	15:390060	158	213	290	287	209	175	1.313286	1.484	1.660549	1.554051	1.523319	1.80354	1.485945	1.62697	0.747402	1	-
ENSMUSC	Slc25a32	solute car	GO:00081	metabolic	-	-	-	-	15:390912	460	523	653	637	462	268	3.612308	3.442557	3.532573	3.258722	3.181347	2.609436	3.529146	3.016502	0.186917	1	-
ENSMUSC	Dcaf13	DDB1 anc	GO:00081	metabolic	-	-	-	-	15:391128	485	564	667	777	547	306	2.700708	2.632499	2.558664	2.818637	2.670955	2.112729	2.630624	2.534107	0.641445	1	-
ENSMUSC	Dpys	dihydropy	GO:00081	metabolic	K01464	dihydropy	mmu0110	Metabolic	15:397684	0	3	0	2	4	4	0	0.036286	0	0.018799	0.050613	0.07157	0.012095	0.046994	0.110715	1	-
ENSMUSC	Lrp12	low densit	GO:00325	developm	-	-	-	-	15:398705	512	1034	641	794	974	833	4.165279	7.050941	3.592392	4.208004	6.948251	8.402434	4.936204	6.519563	0.233369	1	-
ENSMUSC	Zfpm2	zinc finge	GO:00325	developm	K17442	zinc finge	mmu0520	MicroRNA	15:406550	593	685	874	942	694	444	7.576789	7.336246	7.692958	7.840846	7.775577	7.033955	7.535331	7.550126	0.806323	1	-
ENSMUSC	Oxr1	oxidation	GO:00081	metabolic	-	-	-	-	15:414474	6117	7165	8605	9375	6959	3445	54.37876	53.38999	52.69788	54.29299	54.24757	37.97223	53.48888	48.83759	0.385692	1	-
ENSMUSC	Angpt1	angiopoie	GO:00325	developm	K05465	angiopoie	mmu0415	PI3K-Akt s	15:424247	143	173	182	231	193	89	1.569616	1.59168	1.376197	1.651777	1.857626	1.211243	1.512498	1.573549	0.953909	1	-
ENSMUSC	Csmd3	CUB and S	GO:00325	developm	-	-	-	-	15:475806	599	771	853	926	699	414	1.577371	1.701822	1.547417	1.588545	1.614086	1.351739	1.60887	1.518123	0.502641	1	-
ENSMUSC	Eif3h	eukaryotic	GO:00081	metabolic	K03247	translatior	mmu0301	RNA trans	15:517865	1798	1996	2748	2386	2045	1025	53.82397	50.08407	56.67015	46.53056	53.6812	38.04486	53.52606	46.08554	0.183112	1	-
ENSMUSC	Utp23	UTP23 sm	GO:00081	metabolic	-	-	-	-	15:518774	99	110	118	134	110	68	1.298067	1.208948	1.065854	1.144586	1.264728	1.105496	1.190956	1.171603	0.793563	1	-
ENSMUSC	Rad21	RAD21 co	GO:00081	metabolic	K06670	cohesin cc	mmu0411	Cell cycle	15:519622	2440	2892	3695	3677	2801	1646	36.6421	36.40337	38.22578	35.9721	36.8847	30.64829	37.09041	34.50169	0.394337	1	-
ENSMUSC	Cdh10	cadherin 1	GO:00160	membran	-	-	-	-	15:188189	576	725	935	929	659	417	3.145971	3.319124	3.517998	3.305443	3.15617	2.823934	3.327697	3.095182	0.428806	1	-
ENSMUSC	Shcbp1	Shc SH2-c	GO:00508	response	-	-	-	-	8:4735976	15	20	9	17	12	4	0.110515	0.123512	0.045677	0.081593	0.077527	0.03654	0.093235	0.06522	0.32848	1	-
ENSMUSC	Rida	reactive ir	GO:00325	developm	-	-	-	-	15:344840	486	643	945	717	662	364	6.412031	7.110882	8.589006	6.162549	7.658785	5.954529	7.37064	6.591954	0.31592	1	-
ENSMUSC	Matn2	matrilin 2	GO:00325	developm	-	-	-	-	15:343066	938	1092	1449	1605	1041	848	6.53863	6.380571	6.958312	7.288546	6.363228	7.329356	6.625838	6.99371	0.902309	1	-
ENSMUSC	Pop1	processin	GO:00081	metabolic	K01164	ribonuclea	mmu0301	RNA trans	15:344953	173	174	250	262	196	137	1.900222	1.601987	1.891689	1.874739	1.887803	1.865789	1.797966	1.87611	0.972823	1	-
ENSMUSC	Stk3	serine/thr	GO:00325	developm	K04412	serine/thr	mmu0401	MAPK sigi	15:348754	461	633	692	735	587	234	3.421055	3.937457	3.537668	3.553271	3.819799	2.153087	3.63206	3.175386	0.353302	1	-
ENSMUSC	Osr2	odd-skipp	GO:00325	developm	-	-	-	-	15:352960	0	3	1	0	0	0	0	0.02615	0.007162	0	0	0	0.011104	0	0.162968	1	-
ENSMUSC	Khdrbs3	KH domai	GO:00081	metabolic	-	-	-	-	15:689284	3516	4429	4845	4856	3984	2106	118.4167	125.0325	112.4112	106.5429	117.6593	87.94448	118.6201	104.0489	0.208442	1	-
ENSMUSC	Zfat	zinc finge	GO:00325	developm	-	-	-	-	15:680837	105	154	141	184	155	110	0.653581	0.803495	0.604616	0.746123	0.846029	0.848966	0.687231	0.813706	0.4367	1	-
ENSMUSC	Eif3e	eukaryotic	GO:00081	metabolic	K03250	translatior	mmu0301	RNA trans	15:432500	1112	1363	1680	1749	1271	617	9.577306	9.839822	9.967802	9.813185	9.599021	6.588849	9.794977	8.667018	0.304374	1	-
ENSMUSC	Emc2	ER memb	GO:00329	macromo	-	-	-	-	15:434772	995	1213	1493	1434	1110	488	15.60979	15.95099	16.1356	14.65564	15.27001	9.492484	15.89879	13.13938	0.155946	1	-
ENSMUSC	Eny2	enhancer	GO:00081	metabolic	-	-	-	-	15:444280	739	908	1061	1063	833	661	4.670412	4.810047	4.619314	4.37649	4.616346	5.179622	4.699924	4.724153	0.769729	1	-
ENSMUSC	Ebag9	estrogen r	GO:00099	cellular pr	-	-	-	-	15:446191	475	564	753	781	582	276	6.428375	6.397938	7.020281	6.885582	6.906746	4.631294	6.615531	6.141207	0.515623	1	-
ENSMUSC	Sybu	syntabulin	GO:00325	developm	-	-	-	-	15:446718	1204	1415	2037	2180	1616	1377	6.730393	6.630148	7.844339	7.938747	7.921326	9.54407	7.068294	8.468048	0.246252	1	-
ENSMUSC	Kcnv1	potassium	GO:00511	localizatio	-	-	-	-	15:451062	740	916	931	985	761	638	8.145	8.450993	7.059279	7.062802	7.344914	8.706941	7.885091	7.704886	0.60649	1	-
ENSMUSC	Myc	myelocytc	GO:00325	developm	K04377	Myc protc	mmu0520	Pathways	15:619853	60	74	89	103	98	46	0.458587	0.47409	0.468616	0.512851	0.656811	0.435935	0.467098	0.535199	0.579965	1	-
ENSMUSC	Washc5	WASH co	GO:00325	developm	K18464	WASH co	mmu0414	Endocytos	15:593319	741	814	1116	1201	861	582	11.02101	10.148	11.43454	11.63664	11.2292	10.73277	10.86785	11.19954	0.96832	1	-
ENSMUSC	Sqle	squalene	GO:00081	metabolic	K00511	squalene	mmu0110	Metabolic	15:593150	710	791	1061	1019	877	787	5.275032	4.926021	5.430421	4.931996	5.713593	7.249826	5.210491	5.965138	0.496416	1	-
ENSMUSC	Mtss1	metastasi	GO:00099	cellular pr	-	-	-	-	15:589412	575	857	1041	1014	834	561	2.067263	2.582624	2.578278	2.374909	2.629275	2.500785	2.409389	2.501656	0.999957	1	-
ENSMUSC	Ndufb9	NADH de	GO:00081	metabolic	K03965	NADH de	mmu0110	Metabolic	15:589337	1704	2041	2401	2244	1801	1634	93.1965	93.56769	90.46352	79.95293	86.37466	110.8072	92.40924	92.37827	0.70209	1	-
ENSMUSC	Klhl38	kelch-like	GO:00081	metabolic	-	-	-	-	15:583145	8	7	14	10	9	6	0.229839	0.16857	0.27709	0.187163	0.226752	0.21376	0.225167	0.209225	0.822091	1	-
ENSMUSC	Fbxo32	F-box prc	GO:00081	metabolic	K10305	F-box prc	mmu0406	FoxO sign	15:581758	334	330	477	541	328	264	3.042819	2.519978	2.993636	3.210765	2.620271	2.982077	2.852144	2.937704	0.963327	1	-
ENSMUSC	Wdyhv1	WDYHV n	GO:00081	metabolic	-	-	-	-	15:581413	70	102	91	111	82	69	0.833713	1.018296	0.74664	0.861239	0.856398	1.01895	0.866216	0.912196	0.964954	1	-
ENSMUSC	Atad2	ATPase fa	GO:00081	metabolic	-	-	-	-	15:580940	196	192	267	245	208	117	0.630416	0.517636	0.591608	0.513357	0.586648	0.466596	0.579887	0.5222	0.392192	1	-
ENSMUSC	Zhx1	zinc finge	GO:00081	metabolic	-	-	-	-	15:580470	1395	1842	2117	2146	1606	753	6.445868	7.13427	6.738751	6.459792	6.507217	4.314076	6.772963	5.760361	0.195159	1	-
ENSMUSC	Gm29394	predicted	-	-	-	-	-	-	15:580282	25	8	23	36	24	18	0.748746	0.200823	0.474543	0.702391	0.630301	0.668439	0.474704	0.667043	0.333288	1	-
ENSMUSC	Tbc1d31	TBC1 don	GO:00056	cell|intrace	-	-	-	-	15:579121	155	141	212	216	167	75	1.131619	0.862859	1.066238	1.027313	1.069122	0.678915	1.020239	0.925117	0.516206	1	-
ENSMUSC	Derl1	Der1-like	GO:00081	metabolic	K11519	Derlin-1[-	mmu0414	Protein pr	15:578695	746	965	1230	1109	846	1053	13.27283	14.39146	15.07582	12.85399	13.19889	23.22946	14.24671	16.42745	0.548221	1	-
ENSMUSC	Has2	hyalurona	GO:00325	developm	-	-	-	-	15:566656	2	9	10	12	4	4	0.030005	0.113149	0.103333	0.117262	0.052607	0.074391	0.082163	0.08142	1	1	-
ENSMUSC	Mtbp	Mdm2, tra	GO:00511	localizatio	-	-	-	-	15:555574	31	27	39	46	39	33	0.292051	0.213207	0.25311	0.282312	0.322179	0.385465	0.252789	0.329986	0.309246	1	-
ENSMUSC	Mrpl13	mitochon	GO:00081	metabolic	K02871	large subu	mmu0301	Ribosome	15:555340	245	331	385	366	278	269	1.853191	2.098623	2.006162	1.803498	1.843914	2.522855	1.985992	2.056756	0.938313	1	-
ENSMUSC	Col14a1	collagen,	GO:00325	developm	K08133	collagen,	mmu0497	Protein di	15:553077	32	46	95	70	28	16	0.231864	0.27938	0.474201	0.33042	0.177904	0.143745	0.328482	0.217356	0.132235	1	-
ENSMUSC	Sla	src-like ac	GO:00325	developm	-	-	-	-	15:667808	128	125	147	181	129	111	0.645156	0.528103	0.510417	0.594312	0.570149	0.693689	0.561225	0.619383	0.738551	1	-
ENSMUSC	Lrrc6	leucine ric	GO:00325	developm	-	-	-	-	15:663798	60	108	107	101	91	59	1.829728	2.760669	2.247872	2.006509	2.433452	2.230887	2.279423	2.223616	0.733393	1	-
ENSMUSC	Adcy8	adenylate	GO:00081	metabolic	K08048	adenylate	mmu0520	Pathways	15:646970	271	320	413	411	376	294	1.633582	1.616867	1.715036	1.613969	1.987481	2.197381	1.655161	1.932943	0.427813	1	-
ENSMUSC	Asap1	ArfGAP w	GO:00325	developm	K12488	Arf-GAP v	mmu0414	Endocytos	15:640868	1205	1491	2087	2191	1711	1100	5.769279	5.98363	6.883483	6.833739	7.183349	6.529998	6.212131	6.849029	0.612173	1	-
ENSMUSC	Fam49b	family wit	GO:00019	cell killing	-	-	-	-	15:639290	1558	1972	2542	2692	1859	1150	7.071029	7.50196	7.947711	7.959241	7.398386	6.471407	7.5069	7.276345	0.616629	1	-
ENSMUSC	Wnt7b	wingless-t	GO:00325	developm	K00572	wingless-t	mmu0520	Pathways	15:855354	812	946	1016	1102	863	865	11.69387	11.41947	10.07969	10.33869	10.89823	15.44559	11.06434	12.2275	0.701428	1	-
ENSMUSC	Ppara	peroxisor	GO:00325	developm	K07294	peroxisor	mmu0402	cAMP sigr	15:857349	27	29	34	44	35	28	0.283434	0.255175	0.245881	0.300906	0.322187	0.364449	0.261497	0.329181	0.382673	1	-
ENSMUSC	Gtse1	G two S p	GO:00990	supramole	K10129	G-2 and S	mmu0411	p53 signa	15:858597	8	10	11	16	6	4	0.186845	0.195767	0.176989	0.243449	0.122876	0.115838	0.186533	0.160721	0.742351	1	-
ENSMUSC	Trmu	tRNA 5-m	GO:00081	metabolic	-	-	-	-	15:858793	147	156	189	229	168	136	1.49071	1.326028	1.320355	1.512844	1.493926	1.710017	1.379031	1.572262	0.567216	1	-
ENSMUSC	Brd1	bromodo	GO:00081	metabolic	-	-	-	-	15:886873	1330	1442	1955	2031	1527	878	7.971094	7.244101	8.071694	7.929723	8.025056	6.524496	7.762296	7.493091	0.601423	1	-
ENSMUSC	Ttll8	tubulin tyi	GO:00081	metabolic	-	-	-	-	15:888906	24	33	44	49	28	17	0.278793	0.321323	0.352109	0.370804	0.285212	0.244848	0.317408	0.300288	0.778998	1	-
ENSMUSC	Tef	thyrotropl	GO:00325	developm	-	-	-	-	15:818024	4361	5056	6019	6906	5777	6261	20.7918	20.20533	19.76886	21.44935	24.15188	37.01141	20.25533	27.53755	0.053072	1	-
ENSMUSC	Zc3h7b	zinc finge	GO:00081	metabolic	-	-	-	-	15:817448	3913	4755	5947	6456	4845	3532	41.73933	42.51469	43.70036	44.86222	45.31813	46.71348	42.65146	45.63128	0.796934	1	-
ENSMUSC	Rangap1	RAN GTPa	GO:00511	localizatio	K14319	Ran GTPa	mmu0301	RNA trans	15:817042	2585	3100	3700	3753	3522	3678	40.99938	41.21272	40.42685	38.77725	48.98339	72.32933	40.87965	53.36332	0.117053	1	-
ENSMUSC	L3mbtl2	l(3)mbt-lil	GO:00325	developm	-	-	-	-	15:816638	482	592	736	891	569	356	2.362347	2.432041	2.484995	2.844827	2.445408	2.163382	2.426461	2.484539	0.959316	1	-
ENSMUSC	Rbx1	ring-box	GO:00081	metabolic	K03868|K(	RING-box	mmu0520	Pathways	15:814663	1053	1359	1743	1630	1172	932	40.43583	43.74319	46.10912	40.77626	39.46466	44.37514	43.42938	41.53869	0.481674	1	-
ENSMUSC	Xpnpep3	X-prolyl a	GO:00081	metabolic	-	-	-	-	15:814001	72	358	229	122	352	231	0.461971	1.925384	1.012207	0.509946	1.980465	1.83772	1.133187	1.44271	0.558794	1	-
ENSMUSC	St13	suppressi	GO:00329	macromo	-	-	-	-	15:813636	2882	3394	4380	4212	3099	1989	39.44009	38.93216	41.29233	37.55034	37.18846	33.74928	39.88819	36.1627	0.267364	1	-
ENSMUSC	Slc25a17	solute car	GO:00081	metabolic	K13354	solute car	mmu0414	Peroxisom	15:813189	365	530	609	629	462	461	13.91534	16.93671	15.99444	15.62185	15.4449	21.79152	15.6155	17.61942	0.598192	1	-
ENSMUSC	Adsl	adenylosu	GO:00081	metabolic	K01756	adenylosu	mmu0110	Metabolic	15:809484	209	236	308	347	276	204	1.491753	1.411937	1.514446	1.613475	1.727439	1.805371	1.472712	1.715428	0.431299	1	-

ENSMUSC	Mief1	mitochon	GO:00325	developm	-	-	-	-	15:802339	370	477	559	603	433	446	2.087773	2.256074	2.172926	2.216567	2.14246	3.120345	2.172258	2.493124	0.517377	1	-
ENSMUSC	Tab1	TGF-beta	GO:00325	developm	K04403	TAK1-bin	mmu0401	MAPK sig	15:801331	313	382	388	509	384	382	6.786785	6.942825	5.79567	7.18985	7.301195	10.26998	6.508427	8.253674	0.185586	1	-
ENSMUSC	Syngr1	synaptog	GO:00452	synapse|c	-	-	-	-	15:800913	7953	9542	11320	11726	9555	7990	93.04807	93.5769	91.23768	89.37331	98.02791	115.9068	92.62088	101.1027	0.694961	1	-
ENSMUSC	Cacna1i	calcium cl	GO:00400	locomotic	K04856	voltage-d	mmu0401	MAPK sig	15:802872	1602	1836	2108	2370	1916	1672	8.678136	8.336613	7.866584	8.363616	9.101276	11.23017	8.293778	9.565022	0.417225	1	-
ENSMUSC	Deptor	DEP dom	GO:00081	metabolic	K20402	DEP dom	mmu0415	mTOR sig	15:551123	273	304	551	474	351	290	1.495559	1.395943	2.079425	1.69161	1.686129	1.969812	1.656976	1.782517	0.835332	1	-
ENSMUSC	Dnal4	dynein, ax	GO:00990	supramole	K10412	dynein lig	mmu0501	Huntingto	15:797614	427	739	836	853	690	574	7.847647	11.38435	10.58446	10.21272	11.11994	13.08003	9.938819	11.4709	0.469764	1	-
ENSMUSC	Nptxr	neuronal	-	-	-	-	-	-	15:797863	23159	26772	25727	30588	23562	18205	145.3941	140.8836	111.2673	125.1007	129.7125	141.711	132.515	132.1747	0.710086	1	-
ENSMUSC	Dscc1	DNA repli	GO:00081	metabolic	-	-	-	-	15:550760	2	0	2	7	3	6	0.024387	0	0.016799	0.055595	0.032069	0.090704	0.013729	0.059456	0.030586	1	-
ENSMUSC	Josd1	Josephin	GO:00081	metabolic	-	-	-	-	15:796742	955	1146	1486	1376	1090	1075	17.88248	17.98713	19.16878	16.78512	17.89755	24.9585	18.34613	19.88039	0.798817	1	-
ENSMUSC	Tomm22	translocas	GO:00511	localizatio	-	-	-	-	15:796708	513	643	874	772	552	766	19.2233	20.19641	22.56178	18.84557	18.13811	35.58977	20.6605	24.19115	0.529314	1	-
ENSMUSC	Cby1	chibby far	GO:00325	developm	-	-	-	-	15:796592	470	597	725	723	562	481	26.69343	28.42062	28.36582	26.75015	27.98886	33.87171	27.82662	29.53691	0.903418	1	-
ENSMUSC	Dmc1	DNA meic	GO:00325	developm	-	-	-	-	15:795615	12	14	9	13	12	6	0.347904	0.340241	0.17975	0.245545	0.305086	0.215711	0.289299	0.255447	0.704481	1	-
ENSMUSC	Ribc2	RIB43A dc	-	-	-	-	-	-	15:851320	5	1	7	10	3	6	0.192354	0.032245	0.185523	0.250608	0.101195	0.286221	0.136707	0.212675	0.48722	1	-
ENSMUSC	Smc1b	structural	GO:00329	macromo	K06636	structural	mmu0411	Cell cycle	15:850646	0	0	2	0	0	0	0	0	0.021204	0	0	0	0.007068	0	0.537213	1	-
ENSMUSC	Csnk1e	casein kin	GO:00325	developm	K08960	casein kin	mmu0439	Hippo sig	15:794178	1146	1425	1752	2023	1544	1425	8.319826	8.67156	8.76224	9.567691	9.829228	12.82715	8.584542	10.74136	0.133892	1	-
ENSMUSC	Fam118a	family witl	GO:00160	membran	-	-	-	-	15:850370	378	473	554	609	455	313	9.446736	9.908413	9.537867	9.914911	9.971126	9.698855	9.631005	9.86163	0.927944	1	-
ENSMUSC	Upk3a	uroplakin	GO:00325	developm	K19520	uroplakin-	mmu0521	Bladder ca	15:850171	0	2	3	0	0	0	0	0.096326	0.11875	0	0	0	0.071692	0	0.087969	1	-
ENSMUSC	Sh3bp1	SH3-dom	GO:00081	metabolic	K07758	pyridoxal	mmu0110	Metabolic	15:788996	1800	2268	2616	2701	2144	2737	18.44492	19.48051	18.46688	18.03062	19.26512	34.77483	18.79744	24.02352	0.197259	1	-
ENSMUSC	Samm50	SAMM50	GO:00511	localizatio	-	-	-	-	15:841922	796	889	1204	1298	927	791	30.238	28.30708	31.50781	32.12155	30.87897	37.25655	30.01763	33.41902	0.59322	1	-
ENSMUSC	Parvb	parvin, be	GO:00325	developm	K06275	parvin[-]	mmu0451	Focal adh	15:842320	442	485	575	700	486	386	5.88404	5.411879	5.273189	6.070628	5.67326	6.371282	5.523036	6.03839	0.709999	1	-
ENSMUSC	Parvg	parvin, ga	GO:00099	cellular pr	K06275	parvin[-]	mmu0451	Focal adh	15:843240	178	217	281	386	229	197	2.044912	2.089613	2.223879	2.888836	2.306914	2.806126	2.119468	2.667292	0.196945	1	-
ENSMUSC	C1qtnf6	C1q and t	GO:00329	macromo	-	-	-	-	15:785233	33	37	43	63	46	44	0.475673	0.447047	0.426986	0.591585	0.581423	0.786383	0.449902	0.65313	0.112744	1	-
ENSMUSC	Efcab6	EF-hand	GO:00081	metabolic	-	-	-	-	15:838667	107	156	201	158	138	123	0.846322	1.034252	1.095211	0.814119	0.957129	1.206262	0.991928	0.992503	0.789403	1	-
ENSMUSC	Ttll1	tubulin tyr	GO:00325	developm	-	-	-	-	15:834837	464	560	671	660	644	422	6.800879	6.879991	6.775181	6.301909	8.277046	7.669112	6.818683	7.416023	0.745723	1	-
ENSMUSC	Myh9	myosin, h	GO:00325	developm	K10352	myosin he	mmu0481	Regulatio	15:777605	3302	3722	4668	5455	3820	2399	14.7337	13.92079	14.34884	15.85664	14.94654	13.27244	14.33444	14.69187	0.939797	1	-
ENSMUSC	Cyp2d26	cytochron	GO:00081	metabolic	K07414	cytochron	mmu0472	Serotoner	15:827901	2	4	1	1	1	0	0.077284	0.129528	0.026605	0.025165	0.033875	0	0.077805	0.01968	0.230673	1	-
ENSMUSC	Adamts20	a disinteg	GO:00325	developm	-	-	-	-	15:942701	202	343	420	520	307	255	1.335316	1.900546	1.912638	2.239323	1.77956	2.090056	1.716167	2.036313	0.385177	1	-
ENSMUSC	Ndufa6	NADH de	GO:00081	metabolic	K03950	NADH de	mmu0110	Metabolic	15:823501	689	787	1051	876	717	600	78.19294	74.86449	82.16794	64.764	71.35266	84.42778	78.40845	73.51481	0.393405	1	-
ENSMUSC	Twf1	twinfilin a	GO:00081	metabolic	-	-	-	-	15:945779	1126	1340	1708	1745	1237	696	18.42923	18.38342	19.25786	18.60569	17.75338	14.1242	18.69017	16.82776	0.312692	1	-
ENSMUSC	Smdt1	single-pa	GO:00511	localizatio	-	-	-	-	15:823389	652	794	816	796	707	460	21.34804	21.79134	18.4057	16.97873	20.29891	18.67474	20.51502	18.6508	0.316349	1	-
ENSMUSC	Naga	N-acetyl	GO:00081	metabolic	K01204	alpha-N-	mmu0414	Lysosome	15:823295	279	313	450	407	330	246	9.268854	8.716023	10.29876	8.80841	9.613414	10.1331	9.427878	9.518309	0.829916	1	-
ENSMUSC	Nell2	NEL-like	GO:00099	cellular pr	-	-	-	-	15:952194	15966	18653	21306	24125	17435	13564	281.0768	275.252	258.3939	276.6801	269.1495	296.0756	271.5742	280.6351	0.947962	1	-
ENSMUSC	3-Sep	septin 3 [	GO:00452	synapse|c	K16938	septin 3/9	mmu0510	Bacterial i	15:822749	10348	12823	15414	17939	12623	8908	104.5377	108.5823	107.2712	118.0582	111.8206	111.579	106.7971	113.8193	0.811346	1	-
ENSMUSC	Slc38a2	solute car	GO:00325	developm	K14207	solute car	mmu0472	Glutamate	15:966873	1377	1725	2619	2066	1423	1462	18.91754	19.86427	24.78663	18.49023	17.14265	24.9037	21.18948	20.17886	0.468727	1	-
ENSMUSC	Srebf2	sterol regi	GO:00081	metabolic	-	-	-	-	15:821472	3519	3727	4602	5242	4212	3921	44.82723	39.79558	40.38505	43.5012	47.04935	61.93061	41.66929	50.82705	0.204582	1	-
ENSMUSC	Slc38a4	solute car	GO:00511	localizatio	-	-	-	-	15:969948	16	22	22	29	24	13	0.237642	0.273885	0.225093	0.280593	0.312573	0.239394	0.24554	0.27752	0.745295	1	-
ENSMUSC	Rpap3	RNA poly	GO:00329	macromo	-	-	-	-	15:976751	384	425	581	567	436	283	10.7984	10.01773	11.25526	10.38705	10.75119	9.867332	10.69046	10.33519	0.616366	1	-
ENSMUSC	Endou	endonucl	GO:00081	metabolic	-	-	-	-	15:977110	9	17	18	9	17	7	0.210438	0.333191	0.289945	0.137089	0.348555	0.202949	0.277858	0.229531	0.568049	1	-
ENSMUSC	Rapgef3	Rap guan	GO:00325	developm	K08014	Rap guan	mmu0401	Rap1 sign	15:977447	381	443	558	587	451	424	1.810098	1.764143	1.826261	1.816758	1.878868	2.497639	1.800168	2.064422	0.504447	1	-
ENSMUSC	Xrcc6	X-ray rep	GO:00325	developm	K10884	ATP-depe	-	-	15:819878	280	311	401	413	348	205	2.717596	2.530118	2.681165	2.611311	2.961753	2.466995	2.64296	2.68002	0.893377	1	-
ENSMUSC	Desi1	desumoyl	GO:00081	metabolic	-	-	-	-	15:819925	1518	1586	2049	1854	1594	1648	15.74563	13.78937	14.64137	12.52794	14.49837	21.19491	14.72545	16.07374	0.783857	1	-
ENSMUSC	Pmm1	phosphor	GO:00081	metabolic	K17497	phosphon	mmu0110	Metabolic	15:819511	1065	1275	1567	1438	1268	1223	16.15369	16.21008	16.37354	14.20897	16.8649	23.00036	16.24577	18.02474	0.674365	1	-
ENSMUSC	Hdac7	histone de	GO:00325	developm	K11408	histone de	mmu0516	Human pa	15:977926	312	369	456	595	403	519	1.276709	1.265658	1.285445	1.586117	1.446055	2.63324	1.275937	1.888471	0.029688	1	-
ENSMUSC	Polr3h	polymeras	GO:00081	metabolic	K03022	DNA-dire	mmu0110	Metabolic	15:819150	362	420	574	601	459	541	9.151225	8.899649	9.996179	9.897536	10.17481	16.95719	9.349018	12.34318	0.137431	1	-
ENSMUSC	Aco2	aconitase	GO:00325	developm	K01681	aconitate	mmu0110	Metabolic	15:818723	9170	10701	12680	13466	10444	6680	113.6247	111.1426	108.2366	108.6985	113.4783	102.628	111.0013	108.2683	0.59988	1	-
ENSMUSC	Vdr	vitamin D	GO:00325	developm	K08539	vitamin D	mmu0515	Tuberculo	15:978544	8	0	13	6	3	9	0.083331	0	0.093289	0.040718	0.027401	0.116238	0.058874	0.061452	1	1	-
ENSMUSC	Col2a1	collagen,	GO:00325	developm	K19719	collagen,	mmu0415	PI3K-Akt	15:979756	10	14	17	19	16	15	0.087745	0.10297	0.102756	0.108603	0.123105	0.163193	0.097823	0.131634	0.463851	1	-
ENSMUSC	Hoxc10	homeobo	GO:00325	developm	-	-	-	-	15:102966	0	0	7	0	0	0	0	0	0.160547	0	0	0	0.053516	0	0.087566	1	-
ENSMUSC	Hoxc5	homeobo	GO:00325	developm	-	-	-	-	15:102967	1	2	2	0	0	1	0.017967	0.030112	0.024752	0	0	0.022266	0.024277	0.007422	0.255283	1	-
ENSMUSC	Nckap1l	NCK asso	GO:00325	developm	K05750	NCK-asso	mmu0481	Regulatio	15:103453	188	242	344	372	279	183	2.532964	2.732997	3.19286	3.265086	3.296224	3.057083	2.819607	3.206131	0.53109	1	-
ENSMUSC	Pde1b	phosphoc	GO:00325	developm	K13755	calcium/ca	mmu0474	Olfactory	15:103503	1684	2103	2437	2683	2084	2017	14.17151	14.83428	14.12801	14.70877	15.37852	21.04582	14.37793	17.04437	0.311844	1	-
ENSMUSC	Ppp1r1a	protein pl	GO:00081	metabolic	K08050	protein pl	mmu0426	Adrenergi	15:103530	1101	1305	1544	1707	1236	1224	39.7116	39.45422	38.36444	40.10933	39.09231	54.7391	39.17675	44.64691	0.49899	1	-
ENSMUSC	Glycam1	glycosylat	GO:00055	extracellul	K06815	glycosylat	mmu0451	Cell adhes	15:103562	0	4	5	6	6	4	0	0.060224	0.061874	0.070215	0.094502	0.089089	0.040699	0.084602	0.224107	1	-
ENSMUSC	Shisa9	shisa fami	GO:00452	synapse|c	-	-	-	-	16:119841	1234	1761	1949	2180	1653	733	9.062212	10.84005	9.860125	10.42935	10.64473	6.674349	9.920796	9.249478	0.528571	1	-
ENSMUSC	Tnfrsf17	tumor nec	GO:00023	immune s	K05153	tumor nec	mmu0406	Cytokine-	16:113138	1	2	0	6	3	2	0.077995	0.13072	0	0.304809	0.205121	0.193425	0.069572	0.234451	0.090253	1	-
ENSMUSC	Txndc11	thioredox	GO:00508	response	-	-	-	-	16:110749	361	422	573	544	391	409	1.642277	1.609181	1.79575	1.612204	1.559766	2.307008	1.682403	1.826326	0.813029	1	-
ENSMUSC	Litaf	LPS-induc	GO:00325	developm	K19363	lipopolysa	mmu0414	Lysosome	16:109592	177	156	246	234	151	273	3.524088	2.603451	3.374113	3.035081	2.63628	6.739418	3.167217	4.136926	0.336135	1	-
ENSMUSC	Nubp1	nucleotide	GO:00081	metabolic	-	-	-	-	16:104119	154	170	234	260	179	188	1.956653	1.810481	2.048144	2.152028	1.994293	2.961662	1.938426	2.369328	0.331672	1	-
ENSMUSC	Ciita	class II tra	GO:00325	developm	K08060	class II, ma	mmu0515	Tuberculo	16:104881	3	9	0	2	1	1	0.036284	0.091247	0	0.015758	0.010606	0.014994	0.04251	0.013786	0.181222	1	-
ENSMUSC	Emp2	epithelial	GO:00325	developm	-	-	-	-	16:102817	305	402	508	479	296	327	5.634785	6.225243	6.465359	5.764941	4.795267	7.490503	6.108463	6.016904	0.671344	1	-
ENSMUSC	1810013L	RIKEN cD	-	-	-	-	-	-	16:883010	919	1020	1352	1294	1017	783	14.36429	13.36355	14.55784	13.17603	13.93902	15.17457	14.09523	14.09654	0.737914	1	-
ENSMUSC	Bcl6	B cell leuk	GO:00325	developm	K15618	B-cell lym	mmu0520	Transcript	16:239650	1094	1236	1620	1875	1317	728	9.322473	8.828461	9.509993	10.40871	9.841066	7.691853	9.220309	9.313875	0.882734	1	-
ENSMUSC	Trp63	transform	GO:00325	developm	K10149	tumor pro	mmu0520	MicroRNA	16:256837	24	33	44	42	64	24	0.121294	0.139798	0.153192	0.138279	0.28363	0.150392	0.138095	0.190767	0.258793	1	-
ENSMUSC	Cldn1	claudin 1	GO:00325	developm	K06087	claudin[-]	mmu0453	Tight junc	16:263566	72	60	150	73	58	50	1.409246	0.984371	2.022541	0.930799	0.995455	1.213427	1.472053	1.04656	0.073394	1	-
ENSMUSC	Il1rap	interleukir	GO:00325	developm	K04723	interleukir	mmu0401	MAPK sig	16:265817	1112	1168	1683	1938	1446	788	4.689512	4.12875	4.889433	5.324249	5.347291	4.120358	4.569232	4.930632	0.743363	1	-
ENSMUSC	Anks3	ankyrin re	GO:00055	protein bi	-	-	-	-	16:494141	400	543	603	685	553	472	10.2381	11.64963	10.63232	11.42173	12.41158	14.97917	10.84001	12.93749	0.314872	1	-
ENSMUSC	Nudt16l1	nudix (nu	GO:00081	metabolic	K16867	NUDT16-	mmu0520	Proteogly	16:493911	263	352	444	433	354	581	13.17135	14.77645	15.31824	14.12681	15.54607	36.0775	14.42202	21.91679	0.072308	1	-
ENSMUSC	Mgrn1	mahogun	GO:00081	metabolic	K10604	E3 ubiquit	mmu0412	Ubiquitin	16:488610	3121	3604	4303	4341	3504	3290	58.13275	56.26841	55.21408	52.67427	57.23132	75.9817	56.53842	61.96243	0.705547	1	-
ENSMUSC	Srl	sarcalume	GO:00511	localizatio	-	-	-	-	16:448021	249	194	337	440	255	229	1.822074	1.189927	1.698824	2.097494	1.636246	2.077727	1.570275	1.937156	0.290839	1	-
ENSMUSC	Crebbp	CREB binc	GO:00081	metabolic	K04498	E1A/CREB	mmu0520	Pathways	16:408132	2464	2707	3374	3945	2756	1581	8.755151	8.062395	8.258848	9.131699	8.587067	6.965316	8.358798	8.228027	0.715121	1	-
ENSMUSC	Fgf12	fibroblast	GO:00081	metabolic	K04358	fibroblast	mmu0520	Pathways	16:281601	1886	2469	2844	2735	2129	1067	39.10636	42.91208	40.62437	36.94406	38.71007	27.4319	40.88094	34.36201	0.139738	1	-
ENSMUSC	Hrasls	HRAS-like	GO:00160	membran	-	-	-	-	16:292096	171	257	359	338	233	171	2.732601	3.442437	3.952088	3.518675	3.264973	3.388149	3.375709	3.390599	0.838028	1	-
ENSMUSC	Zfp251	zinc finge	GO:00081	metabolic	-	-	-	-	15:768521	1171	1386	1686	1614	1260	855	21.28127	21.11332	21.10812	19.10846	20.07954	19.26604	21.16757	19.48468	0.32025	1	-
ENSMUSC	Hes1	hairy and	GO:00325	developm	K06054	hairy and	mmu0520	Pathways	16:300643	154	194	249	173	120	117	3.422091	3.613467	3.811716	2.504361	2.338264	3.223593	3.615758	2.688739	0.029078	1	-
ENSMUSC	Zfp263	zinc finge	GO:00081	metabolic	-	-	-	-	16:374409	224	422	272	296	414	185	1.00614	1.588823	0.841649	0.86613	1.63062	1.030313	1.145537	1.175688	0.969685	1	-
ENSMUSC	Atp13a3	ATPase ty	GO:00511	localizatio	-	-	-	-	16:303124	1487	1743	2191	2263	1609	1116	9.137816	8.978044	9.275245	9.059366	8.670222	8.503189	9.130368	8.744259	0.50733	1	-
ENSMUSC	Mefv	Mediterra	GO:00081	metabolic	K12803	pyrin[-]	mmu0462	NOD-like	16:370721	0	0	0	4	0	0	0	0	0	0.033494	0	0	0	0.011165	0.304988	1	-
ENSMUSC	Glyr1	glyoxylate	GO:00081	metabolic	-	-	-	-	16:501390	3879	4812	5528	5896	4343	2782	71.64258	74.49548	70.3349	70.9399	70.33701	63.70809	72.15765	68.32833	0.450472	1	-
ENSMUSC	Tmem44	transmem	GO:00160	membran	-	-	-	-	16:305118	658	756	1020	873	790	575	3.027867	2.915987	3.233424	2.617019	3.187724	3.280689	3.059093	3.028477	0.690825	1	-
ENSMUSC	Lsg1	large 60S	GO:00511	localizatio	K14539	large subu	mmu0300	Ribosome	16:305604	251	310	392	384	284	190	1.932378	2.000478	2.079008	1.925893	1.917258	1.813674	2.003955	1.885608	0.502202	1	-
ENSMUSC	Rogdi	rogdi hon	GO:00325	developm	-	-	-	-	16:500873	1639	2276	2339	2959	2308	1807	23.66812	27.54926	23.26839	27.83629	29.22559	32.35407	24.82859	29.80532	0.231028	1	-
ENSMUSC	4930451G	RIKEN cD	-	-	-	-	-	-	16:496893	37	36	44	68	52	31	1.065932	0.869339	0.873249	1.276211	1.313642	1.107341	0.936173	1.232398	0.2412	1	-
ENSMUSC	Eef2kmt	eukaryotic	GO:00081	metabolic	-	-	-	-	16:524415	70	54	70	65	51	57	1.046012	0.67638	0.720588	0.632751	0.668274	1.056085	0.814327	0.785703	0.697094	1	-
ENSMUSC	Ercc4	excision r	GO:00081	metabolic	K10848	DNA excis	mmu0346	Fanconi a	16:131096	459	554	663	731	559	361	2.964215	2.998886	2.949592	3.075361	3.165569	2.890615	2.970898	3.043848	0.937299	1	-
ENSMUSC	Gpt	glutamic	GO:00081	metabolic	K00814	alanine tr	mmu0110	Metabolic	15:766967	144	151	246	233	176	171	5.232501	4.599128	6.157901	5.515483	5.607906	7.704214	5.329843	6.275868	0.474966	1	-
ENSMUSC	Apod	apolipopr	GO:00325	developm	-	-	-	-	16:312961	1467	1676	2409	2469	1824	1375	21.30537	20.40261	24.10166	23.3594	23.22878	24.75982	21.93654	23.78267	0.724455	1	-
ENSMUSC	Adck5	aarF dom	GO:00160	membran	-	-	-	-	15:765763	102	162	162	163	131	186	0.77867	1.036622	0.851957	0.810627	0.876932	1.760562	0.889083	1.149374	0.327118	1	-
ENSMUSC	Cyc1	cytochron	GO:00081	metabolic	K00413	ubiquinol-	mmu0110	Metabolic	15:763435	1524	1884	2060	2060	1721	2003	81.11793	84.05538	75.53537	71.43001	80.32585	132.19	80.23622	94.64864	0.435594	1	-
ENSMUSC	Sharpin	SHANK-a	GO:00325	developm	K20894	shank-ass	mmu0421	Necropto	15:763470	254	380	415	431	379	381	1.697421	2.128589	1.910536	1.876354	2.220944	3.156943	1.912182	2.41808	0.226423	1	-
ENSMUSC	Maf1	MAF1 hor	GO:00081	metabolic	-	-	-	-	15:763512	356	468	582	554	420	704	2.694717	2.969354	3.034855	2.73184	2.787762	6.607273	2.899642	4.042292	0.160812	1	-
ENSMUSC	Hgh1	HGH1 hor	-	-	-	-	-	-	15:763688	65	67	106	104	76	107	2.108689	1.821915	2.368964	2.197935	2.162017	4.303995	2.099856	2.887982	0.218816	1	-
ENSMUSC	Dgat1	diacylglyc	GO:00081	metabolic	K11155	diacylglyc	mmu0110	Metabolic	15:765020	130	202	250	244	156	180	1.504627	1.9597	1.993319	1.839742	1.58326	2.583107	1.819215	2.002036	0.790817	1	-
ENSMUSC	Hsf1	heat shoc	GO:00325	developm	K09414	heat shoc	mmu0513	Legionella	15:764774	370	473	615	528	443	341	1.789815	1.917879	2.049433	1.663881	1.879116	2.045255	1.919042	1.862751	0.596771	1	-
ENSMUSC	Bop1	block of p	GO:00081	metabolic	-	-	-	-	15:764529	817	1015	1145	1363	919	877	20.97044	21.83758	20.24614	22.79097	20.68444	27.91073	21.01806	23.79538	0.520362	1	-
ENSMUSC	Mroh1	maestro h	-	-	-	-	-	-	15:763802	758	957	1196	1271	999	1089	2.244684	2.375478	2.439881	2.451959	2.594145	3.998527	2.353348	3.014877	0.145626	1	-
ENSMUSC	Fbxl6	F-box and	-	-	-	-	-	-	15:765357	149	204	231	275	193	373	5.531198	6.347693	5.907382	6.650393	6.282508	17.1683	5.928758	10.03373	0.03491	1	-
ENSMUSC	Slc52a2	solute car	GO:00511	localizatio	-	-	-	-	15:765389	118	145	161	173	114	225	2.777495	2.860839	2.610657	2.652767	2.352996	6.566617	2.749664	3.85746	0.228895	1	-
ENSMUSC	Gpaa1	GPI ancho	GO:00081	metabolic	K05289	glycosylpl	mmu0110	Metabolic	15:763312	259	297	287	344	283	406	2.704608	2.59964	2.06461	2.340156	2.591397	5.256734	2.456286	3.396096	0.164497	1	-
ENSMUSC	Oplah	5-oxopro	GO:00081	metabolic	K01469	5-oxopro	mmu0048	Glutathior	15:762965	336	332	462	519	444	460	2.630431	2.178603	2.491613	2.64689	3.047986	4.4651	2.433549	3.386659	0.064324	1	-
ENSMUSC	Grina	glutamate	GO:00508	response	-	-	-	-	15:762468	6234	7884	8728	9834	7513	5848	234.0162	248.0725	225.7072	240.4867	247.3063	272.1902	235.932	253.3277	0.785988	1	-
ENSMUSC	Plec	plectin [S	GO:00051	structural	-	-	-	-	15:761709	4056	4336	5361	6664	4748	3833	6.886196	6.170547	6.270161	7.370519	7.068624	8.06875	6.442301	7.502631	0.329849	1	-
ENSMUSC	Scrib	scribbled	GO:00325	developm	K16175	protein sc	mmu0516	Human pa	15:760471	749	871	963	1181	865	763	7.361769	7.175821	6.520456	7.561919	7.4552	9.298458	7.019349	8.105192	0.427624	1	-
ENSMUSC	Tsta3	tissue spe	GO:00081	metabolic	K02377	GDP-L-fu	mmu0110	Metabolic	15:759246	181	285	312	341	220	217	8.774287	11.58062	10.41932	10.76885	9.351888	13.04308	10.25807	11.0546	0.848007	1	-
ENSMUSC	Pycrl	pyrroline-	GO:00081	metabolic	K00286	pyrroline-	mmu0110	Metabolic	15:759164	147	186	235	192	185	190	6.904863	7.323258	7.604274	5.875185	7.619958	11.06569	7.277465	8.186943	0.715041	1	-
ENSMUSC	Naprt	nicotinate	GO:00081	metabolic	K00763	nicotinate	mmu0110	Metabolic	15:758909	51	67	94	92	80	80	1.714912	1.888429	2.177487	2.015321	2.358884	3.335412	1.926943	2.569872	0.221679	1	-
ENSMUSC	Gsdmd	gasdermir	GO:00511	localizatio	K20917	gasdermir	mmu0462	NOD-like	15:758623	28	28	40	48	39	35	1.001957	0.839844	0.98606	1.118959	1.223773	1.552923	0.94262	1.298551	0.196149	1	-
ENSMUSC	Ly6h	lymphocy	GO:00987	molecular	-	-	-	-	15:755512	1395	1841	1690	1779	1389	1588	13.33782	14.75425	11.13137	11.08072	11.64543	18.82551	13.07448	13.85055	0.970636	1	-
ENSMUSC	Rhpn1	rhophilin,	GO:00987	molecular	-	-	-	-	15:757042	102	164	150	140	137	139	1.081847	1.458013	1.095993	0.967328	1.274167	1.827954	1.211951	1.356483	0.763993	1	-
ENSMUSC	Ly6g	lymphocy	GO:00160	membran	-	-	-	-	15:751552	3	0	1	0	0	0	0.149284	0	0.034271	0	0	0	0.061185	0	0.166117	1	-
ENSMUSC	Ly6e	lymphocy	GO:00160	membran	-	-	-	-	15:749550	1605	2239	1627	2398	1886	2099	8.170012	9.553324	5.705408	7.952036	8.418444	13.24787	7.809581	9.872782	0.243521	1	-
ENSMUSC	Gm9747	predicted	-	-	-	-	-	-	1:8223311	0	5	5	0	2	9	0	0.09438	0.077573	0	0.039493	0.251301	0.057318	0.096931	0.711817	1	-
ENSMUSC	Lynx1	Ly6/neurc	GO:00987	molecular	-	-	-	-	15:747478	7075	8275	10281	11043	7917	6643	90.25242	88.48166	90.34814	91.77008	88.55961	105.0709	89.69407	95.13354	0.876785	1	-
ENSMUSC	Lypd2	Ly6/Plaur	GO:00099	cellular pr	-	-	-	-	15:747322	2	2	0	2	0	0	0.143328	0.120109	0	0.09334	0	0	0.087812	0.031113	0.719015	1	-
ENSMUSC	Zbtb11	zinc finge	GO:00056	cell|intrace	-	-	-	-	16:559738	1415	1661	1949	2067	1479	755	10.44212	10.27435	9.908212	9.936973	9.57068	6.908202	10.20823	8.805285	0.208491	1	-
ENSMUSC	Arc	activity re	GO:00325	developm	K15867	activity-re	mmu0503	Amphetar	15:746690	2231	2192	3821	3027	2258	2042	27.27718	22.46432	32.18314	24.10982	24.20842	30.95577	27.30821	26.42467	0.551285	1	-
ENSMUSC	Cep97	centroson	GO:00329	macromo	-	-	-	-	16:558998	479	451	714	512	523	456	2.520026	1.988836	2.587729	1.754771	2.412758	2.974543	2.36553	2.380691	0.783829	1	-
ENSMUSC	Ptk2	PTK2 prot	GO:00325	developm	K05725	focal adhe	mmu0520	Pathways	15:732053	1717	2151	2672	2773	2042	1433	6.928726	7.275727	7.427985	7.289784	7.225726	7.169933	7.210813	7.228481	0.769028	1	-
ENSMUSC	Mapk12	mitogen-	GO:00325	developm	K04441	p38 MAP	mmu0401	MAPK sig	15:891305	47	66	69	96	51	56	1.58125	1.861229	1.599205	2.104046	1.50459	2.336025	1.680561	1.981553	0.537665	1	-
ENSMUSC	Lmf2	lipase ma	GO:00081	metabolic	-	-	-	-	15:893510	216	271	343	340	255	273	4.723835	4.967784	5.16757	4.843966	4.890156	7.402692	4.953063	5.712271	0.540106	1	-
ENSMUSC	Tymp	thymidine	GO:00325	developm	K00758	thymidine	mmu0110	Metabolic	15:893719	25	14	16	15	24	13	0.77999	0.366131	0.343886	0.304873	0.656602	0.50288	0.496669	0.488119	0.908494	1	-
ENSMUSC	Chkb	choline ki	GO:00325	developm	K14156	choline/et	mmu0110	Metabolic	15:894164	203	297	392	360	284	322	1.239073	1.519536	1.648311	1.431484	1.52007	2.436934	1.468973	1.796163	0.355661	1	-
ENSMUSC	Mapk8ip2	mitogen-	GO:00325	developm	K04435	mitogen-	mmu0401	MAPK sig	15:894539	6902	8136	9273	10407	7916	6991	73.8456	72.96481	68.3473	72.53655	74.26738	92.74168	71.71924	79.84854	0.580346	1	-
ENSMUSC	Arsa	arylsulfata	GO:00325	developm	K01134	arylsulfata	mmu0414	Lysosome	15:894724	336	521	457	624	511	488	3.16119	4.108671	2.961955	3.824522	4.215746	5.69268	3.410605	4.577649	0.097669	1	-
ENSMUSC	Rabl2	RAB, mem	GO:00325	developm	-	-	-	-	15:895825	276	295	373	425	320	231	2.902154	2.600078	2.701916	2.911268	2.950556	3.011684	2.734716	2.957836	0.775107	1	-
ENSMUSC	Acr	acrosin pr	GO:00081	metabolic	-	-	-	-	15:895683	11	9	9	24	14	11	0.464828	0.318759	0.261977	0.660662	0.518749	0.576338	0.348522	0.585249	0.114539	1	-
ENSMUSC	Shank3	SH3 and r	GO:00325	developm	K15009	SH3 and r	mmu0472	Glutamate	15:894996	4292	4546	5164	8361	5338	4497	22.47051	19.94966	18.62477	28.51629	24.50611	29.19188	20.34831	27.40476	0.032342	1	-
ENSMUSC	Kif21a	kinesin fa	GO:00990	supramole	-	-	-	-	15:909332	4520	5432	6876	7452	5042	3120	44.36442	44.68986	46.49258	47.64867	43.39521	37.96968	45.18228	43.00452	0.510892	1	-
ENSMUSC	Yaf2	YY1 assoc	GO:00081	metabolic	-	-	-	-	15:932842	1366	1701	2011	2043	1501	1151	26.57283	27.73601	26.94949	25.89029	25.60415	27.76185	27.08611	26.41876	0.581128	1	-
ENSMUSC	Zcrb1	zinc finge	GO:00081	metabolic	-	-	-	-	15:933860	527	668	963	908	656	369	7.359363	7.819133	9.264177	8.260322	8.032965	6.389132	8.147558	7.560806	0.464858	1	-
ENSMUSC	Alcam	activated	GO:00325	developm	K06547	activated	mmu0451	Cell adhes	16:522489	2209	2906	3150	3179	2411	2052	15.99688	17.63957	15.71453	14.99725	15.31015	18.4248	16.45033	16.24406	0.632165	1	-
ENSMUSC	Cblb	Casitas B-	GO:00325	developm	K04707	E3 ubiquit	mmu0520	Pathways	16:520312	976	1070	1400	1624	1103	627	4.324602	3.974044	4.273421	4.687742	4.285627	3.444687	4.190689	4.139352	0.754936	1	-
ENSMUSC	Bbx	bobby so	GO:00325	developm	-	-	-	-	16:501918	813	801	984	994	520	600	2.566619	2.11961	2.140017	2.044273	1.439518	2.348594	2.275415	1.944129	0.160636	1	-
ENSMUSC	Retnlb	resistin lik	GO:00508	response	-	-	-	-	16:488168	0	10	0	0	0	0	0	0.76645	0	0	0	0	0.255483	0	0.035521	1	-
ENSMUSC	Retnlg	resistin lik	GO:00400	locomotic	-	-	-	-	16:488726	2	1	3	4	0	0	0.209099	0.087612	0.216016	0.272411	0	0	0.170909	0.090804	0.564362	1	-
ENSMUSC	Morc1	microrchi	GO:00325	developm	-	-	-	-	16:484312	0	0	7	8	0	0	0	0	0	0.100486	0.108594	0	0	0.033495	0.036198	1	1 -
ENSMUSC	Nectin3	nectin cell	GO:00325	developm	K06592	poliovirus	mmu0451	Cell adhes	16:463877	768	944	1145	1147	864	270	3.142461	3.237677	3.227495	3.05741	3.100026	1.369805	3.202544	2.50908	0.178674	1	-
ENSMUSC	Cd96	CD96 anti	GO:00023	immune s	-	-	-	-	16:460356	0	0	2	0	0	0	0	0	0.040098	0	0	0	0.013366	0	0.535705	1	-
ENSMUSC	Tagln3	transgelin	GO:00081	metabolic	-	-	-	-	16:457112	2467	3279	3845	3796	3051	2654	126.4397	140.8668	135.757	126.7425	137.1195	168.6558	134.3545	144.1726	0.821509	1	-
ENSMUSC	Cd200	CD200 an	GO:00023	immune s	-	-	-	-	16:453821	831	1250	1573	438	514	1026	7.920299	9.986274	10.3281	2.719547	4.295822	12.12478	9.411559	6.380049	0.06966	1	-
ENSMUSC	Atg3	autophag	GO:00081	metabolic	K08343	ubiquitin-	mmu0516	Kaposi's s	16:451587	896	1070	1335	1320	1021	507	16.55815	16.5745	16.9956	15.89131	16.54522	11.61712	16.70942	14.68455	0.266613	1	-
ENSMUSC	Slc35a5	solute car	GO:00511	localizatio	-	-	-	-	16:451395	724	941	1192	1159	861	455	4.060033	4.423171	4.604886	4.234053	4.233863	3.163648	4.362697	3.877188	0.298663	1	-
ENSMUSC	Ccdc80	coiled-co	GO:00099	cellular pr	-	-	-	-	16:450934	79	111	122	163	95	73	0.771816	0.908999	0.821107	1.037428	0.813868	0.884291	0.833974	0.911862	0.751229	1	-
ENSMUSC	Cd200r1	CD200 rec	GO:00329	macromo	-	-	mmu0516	Kaposi's s	16:447657	0	3	5	7	7	0	0	0.065872	0.090235	0.119462	0.160811	0	0.052036	0.093424	0.414697	1	-
ENSMUSC	Gtpbp8	GTP-bind	GO:00056	cell|intrace	-	-	-	-	16:447367	374	402	506	532	413	289	4.978812	4.485722	4.640398	4.613676	4.821098	4.770215	4.701644	4.734997	0.823894	1	-
ENSMUSC	Mzt2	mitotic sp	GO:00329	macromo	-	-	-	-	16:158484	183	254	241	233	209	225	5.148394	5.989716	4.670765	4.270288	5.155954	7.848546	5.269625	5.758263	0.84082	1	-
ENSMUSC	Prkdc	protein ki	GO:00325	developm	K06642	DNA-dep	mmu0411	Cell cycle	16:156378	672	792	949	1077	760	564	3.155346	3.117137	3.069697	3.29439	3.129207	3.283545	3.11406	3.235714	0.999739	1	-
ENSMUSC	Mcm4	minichron	GO:00081	metabolic	K02212	DNA repli	mmu0411	Cell cycle	16:156238	231	223	283	318	229	138	4.09049	3.309947	3.452236	3.668354	3.555822	3.029903	3.617558	3.418026	0.581306	1	-
ENSMUSC	Ube2v2	ubiquitin-	GO:00325	developm	-	-	-	-	16:155526	553	653	867	798	591	478	5.575001	5.518052	6.021302	5.240881	5.224565	5.97494	5.704785	5.480128	0.521324	1	-
ENSMUSC	Snai2	snail famil	GO:00325	developm	K05706	snail 2[-]	mmu0439	Hippo sig	16:147058	11	20	17	10	13	8	0.344216	0.524578	0.366451	0.203843	0.356708	0.310385	0.411748	0.290312	0.199209	1	-
ENSMUSC	Nde1	nudE neu	GO:00325	developm	-	-	-	-	16:141632	291	353	537	544	455	275	2.538979	2.581629	3.22769	3.092049	3.481133	2.974983	2.782766	3.182722	0.490183	1	-
ENSMUSC	Mpv17l	Mpv17 tra	GO:00081	metabolic	K13349	Mpv17-lik	mmu0414	Peroxisom	16:139031	1154	1350	1617	1836	604	215	6.240122	6.118911	6.0235	6.46758	2.863961	1.44149	6.127511	3.59101	0.043504	1	-
ENSMUSC	Pdxdc1	pyridoxal-	GO:00081	metabolic	-	-	-	-	16:138331	1527	1621	2308	2065	1775	1370	8.036224	7.150707	8.367586	7.079697	8.191323	8.939633	7.851506	8.070217	0.905616	1	-
ENSMUSC	Ntan1	N-termina	GO:00081	metabolic	-	-	-	-	16:138192	304	345	494	574	335	256	5.010398	4.766193	5.608898	6.163003	4.841567	5.231476	5.128497	5.412016	0.898963	1	-
ENSMUSC	Rrn3	RRN3 RN	GO:00325	developm	-	-	-	-	16:137806	1216	1441	1909	1849	1391	821	21.48469	21.34089	23.23555	21.28208	21.55091	17.98561	22.02038	20.27287	0.363665	1	-
ENSMUSC	Bfar	bifunction	GO:00081	metabolic	-	-	-	-	16:136718	525	671	798	835	592	481	4.310766	4.618176	4.513875	4.466458	4.262452	4.896956	4.480939	4.541955	0.834723	1	-
ENSMUSC	Parn	poly(A)-sp	GO:00081	metabolic	K01148	poly(A)-sp	mmu0301	RNA degr	16:135379	463	567	695	780	500	443	10.13957	10.40817	10.48516	11.12795	9.601771	12.02896	10.3443	10.91956	0.923267	1	-
ENSMUSC	B3gnt5	UDP-GlcN	GO:00325	developm	K03766	beta-1,3-	mmu0110	Metabolic	16:197602	18	6	29	22	15	8	0.074003	0.020676	0.082136	0.058924	0.054077	0.040781	0.058938	0.051261	0.665108	1	-
ENSMUSC	Boc	biregional	GO:00325	developm	K20020	brother of	mmu0436	Axon guic	16:444850	239	293	402	431	324	250	3.412531	3.506703	3.954175	4.009015	4.056642	4.425932	3.62447	4.163863	0.476078	1	-
ENSMUSC	Sidt1	SID1 trans	GO:00511	localizatio	-	-	-	-	16:442401	1012	1229	1273	1576	1069	722	8.139161	8.285217	7.053084	8.257279	7.539097	7.199834	7.825821	7.665403	0.666323	1	-
ENSMUSC	Naa50	N(alpha)-	GO:00081	metabolic	-	-	-	-	16:441398	1099	1256	1602	1601	1192	950	5.282866	5.060743	5.305004	5.013543	5.024477	5.66215	5.216204	5.23339	0.753553	1	-
ENSMUSC	Ccdc191	coiled-co	-	-	-	-	-	-	16:438898	59	94	133	109	90	71	0.706415	0.943373	1.097002	0.850184	0.944911	1.054024	0.915597	0.949706	0.983576	1	-
ENSMUSC	Hira	histone ce	GO:00325	developm	-	-	-	-	16:188770	675	1062	1225	1316	981	968	5.69245	7.507108	7.116764	7.229906	7.254493	10.12177	6.772108	8.202055	0.273892	1	-
ENSMUSC	Qtrt2	queuine tl	GO:00081	metabolic	-	-	-	-	16:438614	218	295	381	396	256	251	1.962955	2.226533	2.363364	2.322903	2.021326	2.802295	2.184284	2.382175	0.769477	1	-
ENSMUSC	Drd3	dopamine	GO:00325	developm	K04146	dopamine	mmu0408	Neuroacti	16:437622	0	0	0	0	5	0	0	0	0	0	0.191493	0	0	0.063831	0.124269	1	-
ENSMUSC	Mrpl40	mitochon	GO:00329	macromo	-	-	-	-	16:188720	272	319	362	357	290	224	9.582271	9.419824	8.785361	8.193118	8.9586	9.784369	9.262485	8.978696	0.601115	1	-

ENSMUSG	Gbe1	glucan (1,	GO:00081	metabolic	K00700	1,4-alpha	mmu0110	Metabolic	16:703139	154	155	257	247	173	82	1.011072	0.85299	1.162371	1.056427	0.995977	0.667518	1.008811	0.90664	0.474902	1	-
ENSMUSG	Zbtb20	zinc finge	GO:00081	metabolic	-	-	-	-	16:428758	10664	11166	10344	19752	8657	7059	18.40605	16.15441	12.2993	22.20922	13.10239	15.1067	15.61992	16.8061	0.808382	1	-
ENSMUSG	Usp7	ubiquitin s	GO:00081	metabolic	K11838	ubiquitin c	mmu0520	Viral carci	16:868959	3051	3790	4748	4940	3560	1593	16.10467	16.76879	17.2652	16.98706	16.47792	10.42585	16.71289	14.63028	0.289072	1	-
ENSMUSG	Pmm2	phosphor	GO:00081	metabolic	K17497	phosphon	mmu0110	Metabolic	16:863767	247	306	395	438	345	234	2.926469	3.03894	3.224003	3.380675	3.584337	3.437553	3.063137	3.467522	0.532859	1	-
ENSMUSG	Tmem114	transmem	GO:00160	membran	-	-	-	-	16:840927	31	35	46	62	25	39	1.401262	1.326098	1.432373	1.825677	0.990912	2.185766	1.386578	1.667452	0.57674	1	-
ENSMUSG	Dgcr8	DGCR8, m	GO:00081	metabolic	-	-	-	-	16:182539	504	756	907	992	762	469	2.533472	3.185375	3.140831	3.248472	3.358796	2.923108	2.953226	3.176792	0.77506	1	-
ENSMUSG	Trmt2a	TRM2 tRN	GO:00081	metabolic	-	-	-	-	16:182486	429	510	605	566	441	385	2.915025	2.904744	2.831992	2.505435	2.627646	3.243636	2.883921	2.792239	0.563512	1	-
ENSMUSG	Arl6	ADP-ribo	GO:00325	developm	-	-	-	-	16:596129	258	383	438	397	316	229	4.762323	5.925846	5.569612	4.773873	5.114802	5.241087	5.41926	5.043254	0.432403	1	-
ENSMUSG	Crybg3	beta-gam	GO:00329	macromo	-	-	-	-	16:594907	112	135	246	214	118	93	0.759898	0.767752	1.149799	0.94587	0.702038	0.782353	0.892483	0.810087	0.477631	1	-
ENSMUSG	Riox2	ribosomal	GO:00081	metabolic	-	-	-	-	16:594717	225	249	310	323	202	171	1.986861	1.843049	1.885807	1.8581	1.564148	1.872262	1.905239	1.764837	0.435293	1	-
ENSMUSG	Cpox	copropor	GO:00081	metabolic	K00228	copropor	mmu0110	Metabolic	16:586702	978	982	1370	1461	889	631	19.50874	16.4193	18.82618	18.98551	15.55016	15.60651	18.25141	16.71406	0.35744	1	-
ENSMUSG	Cldnd1	claudin dc	GO:00160	membran	-	-	-	-	16:587279	543	568	833	741	497	524	7.802231	6.84101	8.245472	6.93616	6.262077	9.335479	7.629571	7.511238	0.650756	1	-
ENSMUSG	St3gal6	ST3 beta-	GO:00081	metabolic	K03792	beta-gala	mmu0110	Metabolic	16:584681	211	258	328	314	246	147	1.388024	1.422611	1.486412	1.34563	1.419032	1.198995	1.432349	1.321219	0.447929	1	-
ENSMUSG	Cmss1	cms small	GO:00329	macromo	-	-	-	-	16:573021	74	104	92	108	76	95	5.056873	5.957163	4.331051	4.807936	4.554185	8.049395	5.115029	5.803839	0.734085	1	-
ENSMUSG	Tbc1d23	TBC1 dom	GO:00325	developm	-	-	-	-	16:571688	621	795	969	1013	782	340	3.249335	3.486766	3.492836	3.452979	3.587996	2.205811	3.409646	3.082262	0.426736	1	-
ENSMUSG	Klhl22	kelch-like	GO:00081	metabolic	-	-	-	-	16:177596	1164	1440	1688	1873	1440	1499	8.339988	8.648243	8.331754	8.742423	9.047275	13.31681	8.439995	10.36883	0.21758	1	-
ENSMUSG	Nit2	nitrilase fa	GO:00081	metabolic	K13566	omega-am	mmu0025	Alanine, a	16:571566	56	101	100	98	80	89	2.780431	4.203404	3.420411	3.169825	3.483039	5.478984	3.468082	4.043949	0.621268	1	-
ENSMUSG	Tomm70a	translocas	GO:00055	extracellul	-	-	-	-	16:571217	4250	5299	6406	6625	4726	2539	71.49294	74.71727	74.23566	72.60084	69.71248	52.95694	73.48195	65.09009	0.254689	1	-
ENSMUSG	Tmem30c	transmem	GO:00160	membran	-	-	-	-	16:572661	2	8	5	8	3	0	0.027536	0.092299	0.047414	0.071737	0.036208	0	0.05575	0.035982	0.640984	1	-
ENSMUSG	Tmem45a	transmem	GO:00160	membran	-	-	-	-	16:568051	6	10	15	13	8	10	0.109327	0.152719	0.188283	0.15431	0.127824	0.225927	0.15011	0.169354	0.918031	1	-
ENSMUSG	Slc7a4	solute car	GO:00511	localizatio	-	-	-	-	16:175720	661	844	629	957	761	901	4.469948	4.784051	2.930237	4.215939	4.512613	7.554602	4.061412	5.427718	0.163369	1	-
ENSMUSG	Tfg	Trk-fused	GO:00048	signal trar	K09292	protein TF	mmu0520	Pathways	16:566903	779	1061	1250	1037	882	528	13.87551	15.84089	15.33814	12.03294	13.77598	11.66088	15.01818	12.48993	0.094229	1	-
ENSMUSG	P2rx6	purinergic	GO:00048	signal trar	K05221	P2X purin	mmu0408	Neuroacti	16:175618	35	36	40	48	34	35	0.440994	0.380207	0.347193	0.393987	0.375654	0.546786	0.389465	0.438809	0.736054	1	-
ENSMUSG	Lrrc74b	leucine ric	-	-	-	-	-	-	16:175444	21	15	27	33	19	31	0.308944	0.184971	0.27363	0.31626	0.245097	0.565462	0.255849	0.375606	0.26333	1	-
ENSMUSG	Thap7	THAP dor	GO:00081	metabolic	-	-	-	-	16:175279	129	210	186	186	205	222	1.6148	2.203439	1.603956	1.516783	2.250227	3.445627	1.807398	2.404212	0.24118	1	-
ENSMUSG	Lztr1	leucine-zi	GO:00056	cell|intrace	-	-	-	-	16:175086	2029	2365	2892	3101	2357	2383	13.77661	13.45997	13.52726	13.71651	14.03339	20.06181	13.58795	15.93724	0.365074	1	-
ENSMUSG	Ncam2	neural cel	GO:00325	developm	K06491	neural cel	mmu0451	Cell adhes	16:812006	1895	2083	2222	2815	1977	742	19.54446	18.00763	15.78738	18.91361	17.87984	9.488652	17.77982	15.42737	0.355842	1	-
ENSMUSG	Aifm3	apoptosis	GO:00081	metabolic	-	-	-	-	16:174896	960	1169	1512	1655	1185	1150	10.19397	10.40493	11.06051	11.44858	11.034	15.14104	10.55314	12.5412	0.303568	1	-
ENSMUSG	Snap29	synaptosc	GO:00081	metabolic	K08509	synaptosc	mmu0414	Autophag	16:174060	564	681	805	836	648	407	10.45317	10.57959	10.27818	10.09384	10.5314	9.352949	10.43698	9.99273	0.551711	1	-
ENSMUSG	Serpind1	serine (or	GO:00081	metabolic	K03912	heparin cc	mmu0461	Complem	16:173313	30	41	43	49	29	21	0.49872	0.571306	0.492435	0.530648	0.422736	0.432843	0.520821	0.462076	0.526827	1	-
ENSMUSG	Ccdc116	coiled-co	GO:00056	cell|intrace	-	-	-	-	16:171390	32	38	31	44	49	24	0.28978	0.288444	0.193393	0.259573	0.389102	0.269475	0.257206	0.30605	0.578272	1	-
ENSMUSG	Sdf2l1	stromal ce	GO:00081	metabolic	-	-	-	-	16:171301	126	165	291	243	202	181	7.227169	7.932926	11.49849	9.079951	10.1599	12.87248	8.886194	10.70411	0.414911	1	-
ENSMUSG	Dlg1	discs, larg	GO:00325	developm	K12076	disks large	mmu0516	Human pa	16:316634	1709	2109	2749	2606	2102	1179	5.856994	6.058459	6.490212	5.818201	6.31696	5.009941	6.135221	5.715034	0.416936	1	-
ENSMUSG	Ppil2	peptidylpr	GO:00081	metabolic	K10598	peptidyl-p	mmu0412	Ubiquitin	16:170865	924	1070	1382	1457	1108	827	6.841775	6.641001	7.049471	7.028106	7.194147	7.592564	6.844082	7.271606	0.858658	1	-
ENSMUSG	Senp5	SUMO/se	GO:00081	metabolic	-	-	-	-	16:319625	636	534	633	990	749	472	6.197444	4.36164	4.249229	6.28452	6.399998	5.70274	4.936104	6.129086	0.223124	1	-
ENSMUSG	Ypel1	yippee-lik	GO:00056	cell|intrace	-	-	-	-	16:170696	291	348	438	178	372	361	2.348435	2.354063	2.43507	0.935809	2.632515	3.61226	2.379189	2.393528	0.820168	1	-
ENSMUSG	Ncbp2	nuclear ca	GO:00081	metabolic	K12883	nuclear ca	mmu0301	RNA trans	16:319485	763	1098	1224	1275	898	600	14.78833	17.83816	16.34284	16.09854	15.26207	14.41889	16.32311	15.25984	0.429869	1	-
ENSMUSG	Top3b	topoisom	GO:00081	metabolic	K03165	DNA topo	mmu0346	Fanconi a	16:168707	500	657	705	788	680	502	2.08836	2.300136	2.028506	2.144092	2.490505	2.599711	2.139001	2.411436	0.542902	1	-
ENSMUSG	Meltf	melanotra	GO:00325	developm	-	-	-	-	16:318788	10	8	13	3	7	5	0.153771	0.103108	0.137711	0.030048	0.094393	0.095324	0.13153	0.073255	0.123436	1	-
ENSMUSG	Pak2	p21 prote	GO:00325	developm	K04410	p21-activa	mmu0401	MAPK sign	16:320162	836	972	1259	1293	920	854	7.782382	7.584475	8.073898	7.841267	7.509944	9.857122	7.813585	8.402778	0.817946	1	-
ENSMUSG	Spag6l	sperm ass	GO:00325	developm	-	-	-	-	16:167530	103	156	183	180	160	87	1.123187	1.425912	1.374734	1.278704	1.529955	1.176312	1.307944	1.328324	0.932892	1	-
ENSMUSG	Wdr53	WD repea	GO:00056	cell|intrace	-	-	-	-	16:322472	139	130	223	201	140	117	3.033611	2.378155	3.352743	2.857738	2.67927	3.166028	2.921503	2.901012	0.78095	1	-
ENSMUSG	Fgd4	FYVE, Rhc	GO:00081	metabolic	-	-	-	-	16:164169	1344	1654	1913	2069	1418	684	3.949654	4.074255	3.872812	3.960975	3.654087	2.492309	3.965574	3.369124	0.201578	1	-
ENSMUSG	Dnm1l	dynamin 1	GO:00325	developm	K17065	dynamin 1	mmu0421	Necroptos	16:163122	2475	2983	3563	3769	2809	2501	36.96678	37.34588	36.66096	36.67282	36.7901	46.31655	36.99121	39.92649	0.777735	1	-
ENSMUSG	Igsf11	immunog	GO:00055	extracellul	-	-	-	-	16:388926	659	722	1045	1162	777	487	8.135477	7.471158	8.887204	9.345127	8.411263	7.454405	8.164613	8.403598	0.982229	1	-
ENSMUSG	Tnk2	tyrosine k	GO:00081	metabolic	-	-	-	-	16:326438	2222	2662	2401	3485	3047	2799	13.92101	13.97937	10.36264	14.22366	16.73948	21.74281	12.75434	17.56865	0.042806	1	-
ENSMUSG	Yars2	tyrosyl-tR	GO:00081	metabolic	K01866	tyrosyl-tR	mmu0097	Aminoacy	16:163029	100	126	146	174	118	131	0.950396	1.003756	0.955891	1.0773	0.983399	1.543698	0.970014	1.201466	0.328514	1	-
ENSMUSG	B4galt4	UDP-Gal:l	GO:00081	metabolic	K07969	beta-1,4-	mmu0110	Metabolic	16:387422	512	638	823	780	590	375	6.024654	6.292682	6.671339	5.979133	6.087749	5.471152	6.329558	5.846011	0.392741	1	-
ENSMUSG	Tfrc	transferrin	GO:00325	developm	K06503	transferrin	mmu0414	Endocytos	16:326089	1494	1922	2247	2374	1795	1487	9.846334	10.61769	10.20184	10.19263	10.37364	12.15125	10.22196	10.90584	0.84612	1	-
ENSMUSG	Arhgap31	Rho GTPa	GO:00987	molecular	-	-	-	-	16:385983	759	867	1117	1103	898	659	4.007039	3.836662	4.062436	3.793491	4.157197	4.313734	3.968712	4.088141	0.93649	1	-
ENSMUSG	Fyttd1	forty-two	GO:00081	metabolic	-	-	-	-	16:328775	522	473	659	543	393	424	2.837384	2.155071	2.467652	1.922775	1.873194	2.857587	2.486702	2.217852	0.268169	1	-
ENSMUSG	Lrch3	leucine-ri	GO:00508	response	-	-	-	-	16:329141	670	772	1027	1118	751	594	4.324214	4.176403	4.566193	4.700626	4.250252	4.753399	4.355603	4.568093	0.944946	1	-
ENSMUSG	Lmln	leishmanc	GO:00081	metabolic	-	-	-	-	16:330625	440	492	604	591	478	290	3.554059	3.331119	3.360938	3.109858	3.38565	2.904396	3.415372	3.133301	0.381704	1	-
ENSMUSG	Popdc2	popeye d	GO:00325	developm	-	-	-	-	16:383622	0	4	6	5	9	5	0	0.046381	0.057178	0.045064	0.109181	0.085758	0.03452	0.080001	0.174301	1	-
ENSMUSG	Maats1	MYCBP-a	GO:00329	macromo	-	-	-	-	16:382977	51	61	99	62	58	28	0.640424	0.642071	0.856415	0.507193	0.638657	0.435956	0.71297	0.527269	0.108277	1	-
ENSMUSG	Osbpl11	oxysterol	GO:00325	developm	-	-	-	-	16:331850	488	542	672	620	497	384	6.861479	6.387779	6.509059	5.678987	6.127692	6.694457	6.586106	6.167045	0.413062	1	-
ENSMUSG	Snx4	sorting ne	GO:00511	localizatio	K17919	sorting ne	mmu0414	Endocytos	16:332514	601	748	861	897	681	363	15.64101	16.31718	15.43637	15.20773	15.54105	11.71339	15.79819	14.15406	0.324485	1	-
ENSMUSG	Zfp148	zinc finge	GO:00325	developm	-	-	-	-	16:333807	2513	2941	3769	3720	2786	1515	12.83523	12.59096	13.26138	12.37757	12.47771	9.594218	12.89586	11.48317	0.25912	1	-
ENSMUSG	Gsk3b	glycogen	GO:00325	developm	K03083	glycogen	mmu0520	Pathways	16:380890	4999	5639	7436	8054	5804	3782	29.43316	27.82971	30.16091	30.89207	29.96565	27.60967	29.14126	29.48913	0.845349	1	-
ENSMUSG	Umps	uridine mo	GO:00325	developm	K13421	uridine mo	mmu0110	Metabolic	16:339547	303	323	403	442	298	274	3.317814	2.964582	3.039938	3.152916	2.861331	3.720021	3.107445	3.244756	1	1	-
ENSMUSG	Fstl1	follistatin-	GO:00508	response	-	-	-	-	16:377768	697	911	1034	1165	652	707	7.221462	7.91159	7.380134	7.863212	5.923561	9.082341	7.504395	7.623038	0.83357	1	-
ENSMUSG	Itgb5	integrin b	GO:00325	developm	K06588	integrin b	mmu0415	PI3K-Akt s	16:338296	559	594	869	982	674	617	7.713013	6.869912	8.260066	8.82684	8.154824	10.55561	7.61433	9.17909	0.269514	1	-
ENSMUSG	Ndufb4	NADH de	GO:00081	metabolic	K03960|K0	NADH de	mmu0110	Metabolic	16:376471	39	59	82	65	44	66	1.726029	2.188708	2.500039	1.874019	1.707551	3.621716	2.138259	2.401095	0.837537	1	-
ENSMUSG	Abcc5	ATP-bind	GO:00511	localizatio	K05668	ATP-bind	mmu0201	ABC trans	16:203313	2279	2966	3797	4000	2745	2497	9.097823	9.924693	10.44203	10.40243	9.608998	12.3594	9.821517	10.79028	0.65881	1	-
ENSMUSG	Rabl3	RAB, mem	GO:00971	organic cy	-	-	-	-	16:375398	115	162	191	187	150	128	0.879707	1.038743	1.006523	0.931885	1.006172	1.214045	0.974991	1.050701	0.867509	1	-
ENSMUSG	Gtf2e1	general tr	GO:00081	metabolic	K03136	transcripti	mmu0520	Viral carci	16:375097	349	473	469	527	414	280	3.222901	3.661304	2.983632	3.170396	3.352463	3.206009	3.289279	3.242956	0.714563	1	-
ENSMUSG	Stxbp5l	syntaxin b	GO:00511	localizatio	-	-	-	-	16:371149	1606	1705	2283	2715	1851	954	10.05479	8.94756	9.846563	11.07334	10.16194	7.405624	9.616304	9.546967	0.803029	1	-
ENSMUSG	Hcls1	hematopo	GO:00325	developm	K06106	cortactin[-	mmu0520	Proteogly	16:369349	88	98	144	148	109	83	2.000247	1.86715	2.254818	2.191502	2.172536	2.33918	2.040738	2.234406	0.764666	1	-
ENSMUSG	Ccdc14	coiled-co	GO:00325	developm	-	-	-	-	16:346906	50	39	72	66	67	50	0.773723	0.50586	0.767527	0.665334	0.909135	0.959337	0.68237	0.844602	0.435962	1	-
ENSMUSG	Mylk	myosin, lig	GO:00325	developm	K00907	myosin-lig	mmu0481	Regulation	16:347452	528	693	744	880	641	431	3.249972	3.575461	3.154788	3.528665	3.459768	3.28934	3.32674	3.425924	0.964868	1	-
ENSMUSG	Iqcb1	IQ calmoc	GO:00325	developm	-	-	-	-	16:368283	356	470	477	486	396	285	3.075281	3.403186	2.838604	2.734975	2.999667	3.052577	3.10569	2.929073	0.474411	1	-
ENSMUSG	Eaf2	ELL associ	GO:00325	developm	-	-	-	-	16:367928	5	9	19	19	7	4	0.051957	0.078387	0.13601	0.128616	0.063788	0.051536	0.088784	0.081313	0.856614	1	-
ENSMUSG	Adcy5	adenylate	GO:00081	metabolic	K08045	adenylate	mmu0520	Pathways	16:351548	2401	2761	3039	3440	2736	2577	21.77691	20.99052	18.9883	20.32563	21.76019	28.9804	20.58524	23.68874	0.443353	1	-
ENSMUSG	Ap2m1	adaptor-r	GO:00081	metabolic	K11826	AP-2 com	mmu0414	Endocytos	16:205354	1457	2526	2576	2952	2703	1045	12.18539	17.70785	14.84147	16.08339	19.82296	10.83632	14.91157	15.58089	0.915829	1	-
ENSMUSG	Ece2	endotheli	GO:00081	metabolic	-	-	-	-	16:206298	437	563	504	689	560	378	3.987457	4.306022	3.168088	4.095583	4.480701	4.276541	3.820522	4.284275	0.590187	1	-
ENSMUSG	Clcn2	chloride c	GO:00325	developm	K05011	chloride c	mmu0497	Mineral al	16:207029	671	854	866	1073	820	995	3.913016	4.174458	3.479035	4.076343	4.193201	7.19446	3.855503	5.154668	0.114687	1	-
ENSMUSG	Pdia5	protein di	GO:00081	metabolic	-	-	-	-	16:353973	29	38	49	73	42	46	1.019382	1.119634	1.186559	1.671628	1.294576	2.004869	1.108525	1.657024	0.082965	1	-
ENSMUSG	Thpo	thrombop	GO:00325	developm	K06854	thrombop	mmu0406	Cytokine-	16:207251	99	129	150	220	79	135	3.425004	3.740838	3.574953	4.958272	2.396606	5.79091	3.580265	4.38193	0.460471	1	-
ENSMUSG	Dirc2	disrupted	GO:00511	localizatio	-	-	-	-	16:356940	1067	1354	1534	1588	1291	1063	10.76366	11.44899	10.66037	10.43584	11.41996	13.2958	10.95767	11.7172	0.843807	1	-
ENSMUSG	Hspbap1	Hspb asso	GO:00056	cell|intrace	-	-	-	-	16:357703	96	97	130	121	96	39	1.329215	1.12577	1.239988	1.091419	1.165564	0.66954	1.231658	0.975508	0.207564	1	-
ENSMUSG	Ehhadh	enoyl-Co	GO:00081	metabolic	K07514	enoyl-Co/	mmu0110	Metabolic	16:217612	35	49	48	66	56	33	0.737036	0.864894	0.696319	0.90541	1.03406	0.861613	0.766083	0.933694	0.430498	1	-
ENSMUSG	Senp2	SUMO/se	GO:00325	developm	K03345	sentrin-sp	mmu0301	RNA trans	16:220094	1744	2299	2671	2826	2094	1483	24.6358	27.22149	25.99235	26.00602	25.93817	25.9745	25.94988	25.9729	0.756196	1	-
ENSMUSG	Tmem41a	transmem	GO:00160	membran	-	-	-	-	16:219343	111	114	176	174	150	99	5.190876	4.468612	5.669983	5.300889	6.151052	5.740349	5.109824	5.730763	0.647557	1	-
ENSMUSG	Tra2b	transform	GO:00325	developm	K12897	transform	mmu0304	Spliceoso	16:222445	779	914	1324	1137	920	367	6.64356	6.533749	7.778623	6.316919	6.880085	3.880743	6.98531	5.692582	0.17005	1	-
ENSMUSG	Chodl	chondrole	GO:00325	developm	-	-	-	-	16:789309	3	12	11	4	2	5	0.048594	0.162955	0.12276	0.042219	0.028409	0.100427	0.111436	0.057018	0.124588	1	-
ENSMUSG	Dgkg	diacylglyc	GO:00325	developm	K00901	diacylglyc	mmu0110	Metabolic	16:224684	3592	3975	4540	5314	3693	2737	35.64128	33.06031	31.03304	34.34948	32.13209	33.67268	33.24488	33.38475	0.770203	1	-
ENSMUSG	Btg3	B cell tran	GO:00099	cellular pr	K14443	protein Tc	mmu0301	RNA degr	16:783598	62	52	85	71	55	58	2.56029	1.799905	2.41806	1.910013	1.991597	2.969715	2.259418	2.290442	0.871692	1	-
ENSMUSG	D16Ertd47	DNA segr	GO:00325	developm	-	-	-	-	16:785440	124	125	158	188	158	61	2.034221	1.718856	1.785606	2.009163	2.272878	1.240765	1.846228	1.840936	0.896929	1	-
ENSMUSG	Cxadr	coxsackie	GO:00325	developm	K06788	coxsackie	mmu0541	Viral myoc	16:783014	318	458	553	527	388	319	3.056539	3.68996	3.661674	3.299859	3.270229	3.801715	3.469391	3.457268	0.745869	1	-
ENSMUSG	Usp25	ubiquitin s	GO:00081	metabolic	K11849	ubiquitin c	mmu0465	IL-17 sign	16:770140	967	1020	1395	1474	1031	566	13.92922	12.31555	13.84288	13.83184	13.02272	10.10887	13.36255	12.32114	0.426816	1	-
ENSMUSG	Ahsg	alpha-2-H	GO:00325	developm	-	-	-	-	16:228920	0	0	0	0	0	3	0	0	0	0	0	0.160393	0	0.053464	0.188116	1	-
ENSMUSG	Fetub	fetuin bet	GO:00081	metabolic	-	-	-	-	16:229183	2	1	3	0	0	0	0.053082	0.022241	0.054838	0	0	0	0.043387	0	0.04493	1	-
ENSMUSG	Kng1	kininogen	GO:00081	metabolic	K03898	kininogen	mmu0461	Complem	16:230578	0	2	4	5	2	0	0	0.020792	0.034181	0.040403	0.02175	0	0.018324	0.020718	1	1	-
ENSMUSG	Samsn1	SAM dom	GO:00081	metabolic	-	-	-	-	16:758587	26	39	45	40	34	15	0.275723	0.346666	0.328742	0.276332	0.316169	0.197231	0.317044	0.263244	0.385731	1	-
ENSMUSG	Adipoq	adiponect	GO:00325	developm	K07296	adiponect	mmu0493	Non-alco	16:231465	0	0	0	4	0	0	0	0	0	0.103516	0	0	0	0.034505	0.30484	1	-
ENSMUSG	Rfc4	replicatior	GO:00081	metabolic	K10755	replicatior	mmu0342	Nucleotid	16:231139	23	23	26	14	11	29	0.604261	0.5065	0.470574	0.239616	0.253421	0.944668	0.527112	0.479235	0.613445	1	-
ENSMUSG	Robo1	roundabo	GO:00325	developm	K06753	roundabo	mmu0436	Axon guic	16:726631	1623	1877	2191	2649	1938	1114	13.63831	13.22084	12.6834	14.50126	14.28033	11.60682	13.18085	13.4628	0.932028	1	-
ENSMUSG	Eif4a2	eukaryotic	GO:00081	metabolic	K03257	translatior	mmu0301	RNA trans	16:231074	3644	9472	6206	5896	8175	5008	14.39324	31.3599	16.88664	15.17117	28.31461	24.52621	20.87992	22.67066	0.83409	1	-
ENSMUSG	St6gal1	beta galac	GO:00081	metabolic	K00778	beta-gala	mmu0110	Metabolic	16:232247	331	432	542	480	405	358	2.234077	2.44403	2.520115	2.110534	2.396997	2.995978	2.399407	2.50117	0.979055	1	-
ENSMUSG	Masp1	mannan-l	GO:00081	metabolic	K03992	mannan-l	mmu0461	Complem	16:234517	102	145	172	218	152	94	2.355534	2.806782	2.736323	3.279634	3.078047	2.691555	2.63288	3.016412	0.530577	1	-
ENSMUSG	Mrpl39	mitochon	GO:00081	metabolic	-	-	-	-	16:847175	508	649	836	846	583	296	11.46076	12.27289	12.99292	12.43372	11.53348	8.279921	12.24219	10.74904	0.306082	1	-
ENSMUSG	Atp5j	ATP synth	GO:00325	developm	K02131	F-type H+	mmu0110	Metabolic	16:848278	713	980	1139	891	849	844	8.55614	9.857508	9.415932	6.96542	8.933851	12.55788	9.276527	9.485718	0.844893	1	-
ENSMUSG	App	amyloid b	GO:00325	developm	K04520	amyloid b	mmu0501	Alzheimer	16:849496	27313	30677	33962	36645	28706	19537	86.94332	81.85261	74.47517	75.99118	80.1276	77.10999	81.09037	77.74292	0.482236	1	-
ENSMUSG	Adamts1	a disinteg	GO:00325	developm	-	-	-	-	16:857938	365	409	600	605	432	369	3.672135	3.449069	4.158416	3.965172	3.811113	4.602966	3.759873	4.126417	0.707692	1	-
ENSMUSG	Adamts5	a disinteg	GO:00325	developm	-	-	-	-	16:858581	36	41	76	68	35	29	0.282769	0.269942	0.411245	0.347957	0.241073	0.282431	0.321319	0.290487	0.542398	1	-
ENSMUSG	Ets2	E26 avian	GO:00325	developm	K02678	C-ets[-]	mmu0516	HTLV-I inf	16:957020	1302	1699	2092	2233	1606	1144	13.94674	15.25485	15.43742	15.58232	15.08516	15.19406	14.87967	15.28718	0.935259	1	-
ENSMUSG	Dyrk1a	dual-spec	GO:00081	metabolic	-	-	-	-	16:945700	1840	2225	2572	2656	1881	1343	9.079004	9.202441	8.742641	8.537486	8.138637	8.216413	9.008028	8.297512	0.311036	1	-
ENSMUSG	Dscr3	DSCR3 ar	GO:00511	localizatio	-	-	-	-	16:944977	304	389	531	510	368	426	4.200934	4.505838	5.054972	4.591182	4.459264	7.299079	4.587248	5.449842	0.423493	1	-
ENSMUSG	Slc15a2	solute car	GO:00511	localizatio	-	-	-	-	16:367501	352	549	562	493	424	217	2.114028	2.763716	2.325179	1.928844	2.232938	1.615902	2.400974	1.925895	0.103039	1	-
ENSMUSG	Ildr1	immunog	GO:00511	localizatio	-	-	-	-	16:366939	0	3	3	3	8	4	0	0.018114	0.014888	0.014077	0.050539	0.035728	0.011001	0.033448	0.069379	1	-
ENSMUSG	Cd86	CD86 anti	GO:00023	immune s	K05413	CD86 anti	mmu0516	Kaposi's sa	16:366038	25	26	55	67	32	31	0.340514	0.296834	0.516068	0.594497	0.382189	0.523531	0.384472	0.500072	0.341837	1	-
ENSMUSG	Kpna1	karyopher	GO:00511	localizatio	K15042	importin s	mmu0516	Influenza	16:359787	860	3257	4764	1464	3267	699	4.620866	14.66883	17.63388	5.124451	15.39276	4.656805	12.30786	8.391339	0.207833	1	-
ENSMUSG	Arl13b	ADP-ribo	GO:00325	developm	-	-	-	-	16:627936	167	200	286	283	193	117	1.864281	1.871452	2.19944	2.058086	1.889277	1.619441	1.978391	1.855601	0.556116	1	-
ENSMUSG	Pros1	protein S	GO:00508	response	K03908	protein S[	mmu0461	Complem	16:628543	194	295	325	329	300	206	2.690804	3.429697	3.105388	2.972755	3.648759	3.542702	3.075296	3.388072	0.705272	1	-
ENSMUSG	Psmg1	proteasor	GO:00325	developm	-	-	-	-	16:959799	87	123	102	128	113	89	3.090624	3.662555	2.496198	2.96223	3.520047	3.920123	3.083126	3.467467	0.683284	1	-
ENSMUSG	Brwd1	bromodo	GO:00325	developm	-	-	-	-	16:959920	2226	2600	3128	3252	2695	2275	5.242506	5.132628	5.07495	4.989372	5.56564	6.643256	5.150028	5.732756	0.587633	1	-
ENSMUSG	Grik1	glutamate	GO:00048	signal trar	K05201	glutamate	mmu0472	Glutamate	16:878959	210	287	348	358	248	154	0.951867	1.090416	1.086646	1.057113	0.985716	0.865495	1.042977	0.969441	0.493258	1	-
ENSMUSG	Fam3b	family witl	GO:00511	localizatio	-	-	-	-	16:974710	2	0	0	0	0	0	0.079457	0	0	0	0	0	0.026486	0	0.536422	1	-
ENSMUSG	Pigp	phosphati	GO:00081	metabolic	K03861	phosphati	mmu0110	Metabolic	16:943587	32	45	79	66	43	103	0.659215	0.777043	1.121141	0.885747	0.776764	2.630901	0.852466	1.431137	0.133291	1	-
ENSMUSG	Ripply3	ripply trar	GO:00325	developm	-	-	-	-	16:943284	1	2	0	0	0	4	0.041519	0.069586	0	0	0	0.205876	0.037035	0.068625	0.785421	1	-
ENSMUSG	Chaf1b	chromatir	GO:00081	metabolic	-	-	-	-	16:938839	4	9	11	1	7	9	0.037224	0.070203	0.070521	0.006062	0.057128	0.103847	0.059316	0.055679	0.759511	1	-
ENSMUSG	Dopey2	dopey far	GO:00511	localizatio	-	-	-	-	16:937119	2027	2174	2695	3032	2357	1800	9.723877	8.741746	8.906274	9.475379	9.914892	10.70642	9.123966	10.03223	0.640329	1	-
ENSMUSG	Cbr3	carbonyl r	GO:00081	metabolic	-	-	mmu0110	Metabolic	16:936832	62	84	128	89	89	120	3.367766	3.824567	4.789721	3.149374	4.239201	8.08196	3.994018	5.156845	0.398441	1	-
ENSMUSG	Setd4	SET doma	-	-	-	-	-	-	16:935834	37	87	113	116	74	89	0.31597	0.622756	0.664779	0.645336	0.554144	0.942368	0.534501	0.713949	0.280152	1	-
ENSMUSG	Rcan1	regulator	GO:00325	developm	K17901	calcipressi	mmu0516	Kaposi's sa	16:923919	603	809	1018	893	757	667	15.29229	17.19715	17.78501	14.75327	16.83425	20.97331	16.75815	17.52028	0.996834	1	-
ENSMUSG	Runx1	runt relate	GO:00325	developm	K08367	runt-relat	mmu0520	Pathways	16:926014	25	28	36	27	33	32	0.087184	0.081846	0.086487	0.061338	0.100915	0.138365	0.085172	0.100206	0.71609	1	-
ENSMUSG	Atp5o	ATP synth	GO:00081	metabolic	K02137	F-type H+	mmu0110	Metabolic	16:916843	1038	1423	1708	1241	1369	1126	21.17075	24.32744	23.99816	16.48892	24.48413	28.47495	23.16545	23.14933	0.732418	1	-
ENSMUSG	Itsn1	intersectin	GO:00081	metabolic	-	-	-	-	16:917292	3474	4076	4898	5466	4186	2917	6.494386	6.386977	6.307808	6.656708	6.861991	6.761308	6.39639	6.760002	0.877975	1	-
ENSMUSG	Donson	downstrea	GO:00325	developm	-	-	-	-	16:916772	144	150	222	279	169	195	1.92221	1.678346	2.041467	2.426183	1.978189	3.227441	1.880675	2.543938	0.120862	1	-
ENSMUSG	Son	Son DNA	GO:00081	metabolic	-	-	-	-	16:916475	6156	7470	9241	10809	7581	4789	18.02499	18.33368	18.64003	20.61786	19.4646	17.3863	18.3329	19.15625	0.933945	1	-
ENSMUSG	Gart	phosphor	GO:00325	developm	K11787	phosphor	mmu0110	Metabolic	16:916211	444	581	759	819	591	526	5.980826	6.560053	7.043221	7.186935	6.980851	8.785158	6.528033	7.650982	0.373151	1	-
ENSMUSG	Tmem50b	transmem	GO:00160	membran	-	-	-	-	16:915745	1456	1817	2139	2204	1790	1249	31.58129	33.03515	31.96176	31.14309	34.04585	33.59053	32.19273	32.92649	0.893519	1	-
ENSMUSG	Ifngr2	interferon	GO:00048	signal trar	K05133	interferon	mmu0520	Pathways	16:915470	1122	1308	1507	1749	1263	851	20.35002	19.88533	18.82941	20.66538	20.08714	19.1376	19.68825	19.96337	0.8592	1	-
ENSMUSG	Ifnar1	interferon	GO:00048	signal trar	K05130	interferon	mmu0520	Pathways	16:914852	680	747	997	1131	765	628	4.449761	4.097331	4.494423	4.821382	4.389666	5.095339	4.347172	4.768796	0.676014	1	-
ENSMUSG	Il10rb	interleukir	GO:00160	membran	K05135	interleukir	mmu0406	Cytokine-	16:914061	131	151	201	212	144	133	1.575898	1.522596	1.665723	1.661392	1.519011	1.983771	1.588073	1.721391	0.827872	1	-
ENSMUSG	Ifnar2	interferon	GO:00048	signal trar	K05131	interferon	mmu0520	Pathways	16:913727	269	296	383	397	279	180	2.79617	2.579022	2.742591	2.688329	2.543064	2.319895	2.705927	2.517096	0.471762	1	-
ENSMUSG	1110004E	RIKEN cDI	GO:00325	developm	-	-	-	-	16:909258	261	380	424	441	333	278	6.280695	7.664855	7.028855	6.913339	7.026745	8.29466	6.991468	7.411581	0.912703	1	-
ENSMUSG	Synj1	synaptoja	GO:00325	developm	K20279	synaptoja	mmu0110	Metabolic	16:909360	9032	9999	11652	13758	9764	6968	34.69391	32.19429	30.83339	34.42756	32.88818	33.18665	32.57386	33.5008	0.933684	1	-
ENSMUSG	Paxbp1	PAX3 and	GO:00325	developm	-	-	-	-	16:910140	764	1064	1382	1455	1030	644	6.337881	7.398525	7.897873	7.863125	7.492563	6.624033	7.211426	7.326574	0.899117	1	-
ENSMUSG	Mis18a	MIS18 kin	GO:00081	metabolic	-	-	-	-	16:907193	72	104	125	111	93	73	1.061676	1.28542	1.269753	1.066257	1.202497	1.334644	1.205616	1.201133	0.80094	1	-
ENSMUSG	Sod1	superoxid	GO:00325	developm	K04565	superoxid	mmu0501	Huntingto	16:902207	1712	2174	2657	2418	1903	1511	168.4253	179.2735	180.0721	154.9678	164.1667	184.3122	175.9236	167.8156	0.442854	1	-
ENSMUSG	Scaf4	SR-relate	GO:00056	cell|intrace	-	-	-	-	16:902289	825	946	1184	1300	906	574	8.798661	8.456811	8.698931	9.032077	8.47293	7.590323	8.651468	8.36511	0.607458	1	-
ENSMUSG	Krt75	keratin 75	GO:00325	developm	-	-	-	-	15:101563	3	6	0	3	5	1	0.05837	0.09786	0	0.038025	0.08531	0.024121	0.052076	0.049152	1	1	-
ENSMUSG	Zfp641	zinc finge	GO:00081	metabolic	-	-	-	-	15:982855	166	212	254	307	223	205	1.412287	1.511833	1.488677	1.701515	1.663654	2.162501	1.470933	1.842557	0.222449	1	-
ENSMUSG	Kansl2	KAT8 regu	GO:00081	metabolic	-	-	-	-	15:985188	304	411	470	562	387	293	5.274391	5.977151	5.617572	6.3521	5.88779	6.303086	5.623038	6.180992	0.67589	1	-
ENSMUSG	4930415C	RIKEN cDI	-	-	-	-	-	-	15:985710	0	2	0	0	0	0	0	0.190926	0	0	0	0	0.063642	0	0.535988	1	-
ENSMUSG	Adcy6	adenylate	GO:00081	metabolic	K08046	adenylate	mmu0520	Pathways	15:985899	815	1055	1073	1160	909	1015	3.780431	4.101937	3.428744	3.505286	3.697348	5.837618	3.770371	4.346751	0.519122	1	-
ENSMUSG	Enah	enabled h	GO:00325	developm	K05746	enabled[-	mmu0481	Regulation	1:1818963	3746	4249	5503	5632	3571	898	8.22461	7.819646	8.323357	8.055488	6.875118	2.444612	8.122538	5.791739	0.124587	1	-
ENSMUSG	Wnt10b	wingless-	GO:00325	developm	K01357	wingless-t	mmu0520	Pathways	15:987707	5	26	10	8	5	6	0.031876	0.138933	0.043918	0.033224	0.027949	0.047431	0.071576	0.036201	0.100404	1	-
ENSMUSG	Lmbr1l	limb regic	GO:00511	localizatio	-	-	-	-	15:989039	77	71	95	103	82	138	0.850763	0.657546	0.72309	0.74137	0.794461	1.890529	0.7438	1.14212	0.122114	1	-
ENSMUSG	Dhh	desert hec	GO:00325	developm	K11990	desert hec	mmu0434	Hedgehog	15:988930	4	3	7	4	1	6	0.107576	0.067628	0.129701	0.070091	0.023582	0.200101	0.101635	0.097925	1	1	-
ENSMUSG	Tuba1b	tubulin, al	GO:00508	response	K07374	tubulin alp	mmu0414	Phagoson	15:989314	4617	5759	6534	6649	5309	4775	129.3188	135.2078	126.0759	121.322	130.3938	165.8292	130.2008	139.1817	0.854085	1	-
ENSMUSG	Prpf40b	pre-mRN.	-	-	K12821	pre-mRN/	mmu0304	Spliceoso	15:992950	915	1115	1270	1541	1096	846	6.098048	6.228701	5.830756	6.69043	6.405056	6.990791	6.052501	6.695426	0.606128	1	-
ENSMUSG	Fmnl3	formin-lik	GO:00325	developm	-	-	-	-	15:993172	171	185	262	299	234	170	1.937175	1.756702	2.044682	2.206615	2.324514	2.38786	1.912853	2.306329	0.320044	1	-
ENSMUSG	Nckap5l	NCK-asso	GO:00990	supramole	-	-	-	-	15:994220	346	446	491	514	449	384	4.196445	4.534112	4.1024	4.061159	4.775223	5.774608	4.277652	4.87033	0.544809	1	-
ENSMUSG	Tmbim6	transmem	GO:00081	metabolic	-	-	-	-	15:993928	3753	4589	5737	5913	4530	4503	47.2774	48.45581	49.78646	48.52488	50.03983	70.33361	48.50656	56.29944	0.397653	1	-
ENSMUSG	Faim2	Fas apopt	GO:00325	developm	-	-	-	-	15:994970	5917	7808	8490	9530	6988	6433	83.19538	92.02172	82.23502	87.29155	86.15753	112.1495	85.81738	95.19951	0.608096	1	-
ENSMUSG	Racgap1	Rac GTPa:	GO:00325	developm	-	-	-	-	15:996204	112	142	171	192	119	67	0.60718	0.645267	0.638625	0.678079	0.565701	0.450359	0.630357	0.564713	0.446458	1	-
ENSMUSG	Asic1	acid-sens	GO:00452	synapse|c	K04829	acid-sens	mmu0475	Inflammat	15:996703	538	682	782	828	661	541	3.903585	4.147812	3.908768	3.913754	4.205577	4.867038	3.986721	4.328789	0.755735	1	-
ENSMUSG	Smarcd1	SWI/SNF r	GO:00325	developm	K11650	SWI/SNF-	mmu0522	Hepatoce	15:997022	1941	2253	2634	2970	2202	1726	39.12349	38.06503	36.57459	38.9987	38.91993	43.1359	37.92104	40.35151	0.851315	1	-
ENSMUSG	Gpd1	glycerol-3	GO:00081	metabolic	K00006	glycerol-3	mmu0056	Glyceroph	15:997175	2393	2933	3435	3777	2664	1887	38.50187	39.55523	38.07301	39.58837	37.58512	37.6441	38.71004	38.27253	0.679304	1	-
ENSMUSG	Cers5	ceramide	GO:00081	metabolic	K04710	ceramide	mmu0110	Metabolic	15:997348	251	375	358	299	353	435	2.588314	3.241366	2.543184	2.008617	3.19199	5.561843	2.790955	3.587483	0.339397	1	-
ENSMUSG	Lima1	LIM doma	GO:00099	cellular pr	-	-	-	-	15:997784	311	392	522	555	401	234	2.454974	2.593734	2.838624	2.854047	2.775702	2.290279	2.629111	2.640009	0.855812	1	-
ENSMUSG	Larp4	La ribonu	GO:00325	developm	-	-	-	-	15:999700	1251	1467	1790	1539	1241	684	11.57781	11.38025	11.41231	9.278746	10.07125	7.848941	11.45679	9.066312	0.042722	1	-
ENSMUSG	Dip2b	disco inte	GO:00081	metabolic	-	-	-	-	15:100038	1346	1507	1943	2125	1555	1343	8.23235	7.725825	8.186589	8.466801	8.339726	10.18453	8.048255	8.997017	0.559061	1	-

ENSMUSG	Atf1	activating	GO:00325	developm	K09053	activating	mmu0516	HTLV-I inf	15:100227	172	198	211	247	202	141	2.315425	2.234185	1.956755	2.166111	2.384489	2.353453	2.168788	2.301351	0.899704	1	-
ENSMUSG	Slc11a2	solute car	GO:00325	developm	K12347	natural re	mmu0414	Lysosome	15:100387	523	608	735	777	530	744	4.594084	4.476658	4.447708	4.446318	4.082403	8.103197	4.50615	5.543972	0.342839	1	-
ENSMUSG	Cela1	chymotryp	GO:00325	developm	-	-	-	-	15:100674	14	4	18	16	6	22	0.806653	0.193176	0.714478	0.600565	0.303125	1.571735	0.571436	0.825142	0.528261	1	-
ENSMUSG	Slc4a8	solute car	GO:00511	localizatio	-	-	-	-	15:100761	1614	986	1487	3745	912	770	7.029025	3.599326	4.461224	10.6249	3.482803	4.157846	5.029859	6.088518	0.519835	1	-
ENSMUSG	Scn8a	sodium ch	GO:00511	localizatio	-	-	-	-	15:100869	6225	7321	8943	10026	7174	5821	12.87482	12.69185	12.74197	13.50862	13.01086	14.92744	12.76955	13.81564	0.732488	1	-
ENSMUSG	Nr4a1	nuclear re	GO:00325	developm	K04465	nuclear re	mmu0415	PI3K-Akt s	15:101266	806	759	1166	1286	860	881	20.70482	16.34297	20.63414	21.52082	19.37213	28.0607	19.22731	22.98455	0.324369	1	-
ENSMUSG	Pcdhgc4	protocadh	GO:00099	cellular pr	-	-	-	-	18:378150	180	217	280	278	193	190	1.382086	1.39661	1.48106	1.39056	1.299463	1.808853	1.419918	1.499625	0.967434	1	-
ENSMUSG	Krt7	keratin 7 [	GO:00051	structural	-	-	-	-	15:101411	0	0	2	0	0	0	0	0	0.01019	0	0	0	0.003397	0	0.536196	1	-
ENSMUSG	Csad	cysteine s	GO:00081	metabolic	K01594	sulfinoala	mmu0110	Metabolic	15:102176	191	260	273	293	230	241	5.359217	6.114957	5.276932	5.355713	5.658971	8.384393	5.583702	6.466359	0.539347	1	-
ENSMUSG	Soat2	sterol O-a	GO:00081	metabolic	K00637	sterol O-a	mmu0497	Cholester	15:102150	0	0	2	0	0	0	0	0	0.033046	0	0	0	0.011015	0	0.536475	1	-
ENSMUSG	Igfbp6	insulin-lik	GO:00508	response	-	-	-	-	15:102144	97	154	234	165	143	206	5.543761	7.377409	9.212966	6.143219	7.166555	14.59772	7.378045	9.302497	0.427188	1	-
ENSMUSG	Amhr2	anti-Mulle	GO:00325	developm	K04672	anti-mulle	mmu0406	Cytokine-	15:102445	2	2	14	3	3	7	0.046399	0.038882	0.223695	0.045325	0.061012	0.201319	0.102992	0.102552	0.903968	1	-
ENSMUSG	Prr13	proline ric	GO:00081	metabolic	-	-	-	-	15:102459	476	561	753	744	540	385	7.861542	7.766349	8.56737	8.004892	7.820552	7.884024	8.065087	7.903156	0.664751	1	-
ENSMUSG	Map3k12	mitogen-	GO:00048	signal trar	K04423	mitogen-a	mmu0401	MAPK sigi	15:102497	1876	2190	2970	3080	2339	1955	9.128355	8.932164	9.955604	9.763198	9.980047	11.79485	9.338708	10.5127	0.508861	1	-
ENSMUSG	Tarbp2	TARBP2, F	GO:00325	developm	-	-	-	-	15:102518	100	124	163	143	143	150	0.616361	0.640634	0.69211	0.574188	0.772884	1.14634	0.649702	0.831137	0.295503	1	-
ENSMUSG	Npff	neuropep	GO:00325	developm	-	-	-	-	15:102523	8	12	12	9	10	5	0.331208	0.416464	0.342276	0.242742	0.363062	0.256661	0.363316	0.287488	0.50848	1	-
ENSMUSG	Calcoco1	calcium b	GO:00081	metabolic	-	-	-	-	15:102706	1848	2155	2677	2838	1957	1331	23.41894	22.89103	23.37034	23.42929	21.74697	20.91362	23.22677	22.02996	0.463894	1	-
ENSMUSG	Sncg	synuclein,	GO:00511	localizatio	-	-	-	-	14:343702	58	52	87	57	35	49	4.32639	3.251237	4.470619	2.769812	2.289346	4.531849	4.016082	3.197002	0.192118	1	-
ENSMUSG	Rttn	rotatin [Sc	GO:00325	developm	-	-	-	-	18:889717	42	52	88	94	79	69	0.367256	0.38113	0.530095	0.535464	0.605746	0.74809	0.42616	0.629767	0.071544	1	-
ENSMUSG	Cdkn1a	cyclin-dep	GO:00325	developm	K06625	cyclin-dep	mmu0520	Pathways	17:290909	116	130	217	147	145	111	2.398233	2.252826	3.090603	1.979845	2.628711	2.845388	2.580554	2.484648	0.636262	1	-
ENSMUSG	Nus1	NUS1 deh	GO:00325	developm	K19177	dehydrod	mmu0090	Terpenoic	10:524175	1402	1690	2147	2082	1600	1276	17.74573	17.93021	18.72101	17.16754	17.7586	20.02546	18.13232	18.3172	0.791138	1	-
ENSMUSG	Rgn	regucalcir	GO:00325	developm	-	-	mmu0110	Metabolic	X:2054978	2	0	5	1	2	3	0.079111	0	0.136223	0.02576	0.069352	0.147118	0.071778	0.080743	1	1	-
ENSMUSG	Cep89	centroson	GO:00329	macromo	-	-	-	-	7:3539703	246	316	330	371	306	186	2.085647	2.245672	1.927401	2.049095	2.274942	1.955267	2.08624	2.093101	0.835802	1	-
ENSMUSG	Slc10a2	solute car	GO:00511	localizatio	-	-	-	-	8:5083219	0	0	0	3	0	0	0	0	0	0.029911	0	0	0	0.00997	0.303613	1	-
ENSMUSG	Mospd1	motile spe	GO:00325	developm	-	-	-	-	X:5334459	129	144	260	150	117	87	2.073429	1.94006	2.87888	1.57062	1.649029	1.733825	2.297457	1.651158	0.029603	1	-
ENSMUSG	Akirin1	akirin 1 [S	GO:00160	membran	-	-	-	-	4:1237345	1113	1296	1683	1505	1218	717	17.61317	17.19097	18.34755	15.51532	16.90178	14.06848	17.71723	15.49519	0.187488	1	-
ENSMUSG	Gtf2ird1	general tr	GO:00325	developm	K03121	transcripti	mmu0516	Herpes sir	5:1343576	254	320	358	335	292	279	1.158246	1.22312	1.124607	0.99516	1.167594	1.577454	1.168658	1.246736	0.923265	1	-
ENSMUSG	Lrrc71	leucine ric	-	-	-	-	-	-	3:8773692	19	4	32	13	11	12	0.161496	0.028497	0.187374	0.071985	0.081988	0.126466	0.125789	0.09348	0.396514	1	-
ENSMUSG	Noct	nocturnin	GO:00325	developm	-	-	-	-	3:5122444	968	299	1630	428	1207	892	3.817992	0.988515	4.42893	1.099727	4.174551	4.362248	3.078479	3.212175	0.976237	1	-
ENSMUSG	Abcc1	ATP-bind	GO:00511	localizatio	K05665	ATP-bind	mmu0520	MicroRNA	16:143615	329	350	460	494	351	337	1.371078	1.222609	1.320613	1.341142	1.282674	1.741335	1.304766	1.455051	0.649072	1	-
ENSMUSG	Ndufa5	NADH de	GO:00081	metabolic	K03949	NADH de	mmu0110	Metabolic	6:2451866	586	749	927	923	784	450	27.52554	29.48989	29.99643	28.24369	32.29217	26.20816	29.00395	28.91468	0.785552	1	-
ENSMUSG	Fhl1	four and a	GO:00325	developm	K14365	four and a	mmu0463	Jak-STAT	X:5673178	484	663	689	656	541	606	3.551516	4.077882	3.482883	3.135838	3.481031	5.513486	3.704094	4.043452	0.816226	1	-
ENSMUSG	Msrb2	methionir	GO:00081	metabolic	-	-	-	-	2:1937144	307	349	472	419	306	281	11.75349	11.19971	12.44864	10.45017	10.27287	13.33893	11.80061	11.35399	0.549196	1	-
ENSMUSG	Rfc2	replicatior	GO:00081	metabolic	K10755	replicatior	mmu0342	Nucleotid	5:1345813	339	407	508	510	460	420	2.828843	2.846793	2.920278	2.772433	3.365966	4.345551	2.865305	3.49465	0.288452	1	-
ENSMUSG	Denr	density-re	GO:00081	metabolic	-	-	-	-	5:1239071	920	1220	1409	1534	1085	572	7.957098	8.844627	8.395168	8.643191	8.228858	6.134075	8.398964	7.668708	0.39407	1	-
ENSMUSG	Prmt5	protein ar	GO:00325	developm	K02516	type II prc	mmu0301	RNA trans	14:545071	1003	1211	1450	1481	1085	700	11.58134	11.72073	11.53393	11.14024	10.98576	10.02171	11.612	10.7159	0.357262	1	-
ENSMUSG	Sympk	symplekin	GO:00081	metabolic	K06100	symplekin	mmu0453	Tight junc	7:1902437	1962	2218	2725	3214	2292	1803	10.04439	9.517848	9.610419	10.71894	10.2892	11.44475	9.724217	10.81763	0.562362	1	-
ENSMUSG	Gm853	predicted	GO:00081	metabolic	K01581	ornithine (	mmu0110	Metabolic	4:1302091	0	1	7	0	2	0	0	0.020109	0.115698	0	0.042072	0	0.045269	0.014024	0.29562	1	-
ENSMUSG	Sult1c2	sulfotrans	GO:00081	metabolic	-	-	-	-	17:538296	10	5	19	5	21	4	0.344277	0.14428	0.450614	0.112147	0.633979	0.170746	0.313057	0.305624	1	1	-
ENSMUSG	Gzma	granzyme	GO:00081	metabolic	K01352	granzyme	mmu0408	Neuroacti	13:113093	0	3	2	8	0	0	0	0.121064	0.066343	0.250918	0	0	0.062469	0.083639	0.844397	1	-
ENSMUSG	Nagpa	N-acetylg	GO:00081	metabolic	K01125	N-acetylg	mmu0414	Lysosome	16:519528	81	143	178	187	97	172	1.347589	1.99417	2.040062	2.026728	1.415095	3.548023	1.79394	2.329949	0.344587	1	-
ENSMUSG	Wrb	tryptopha	GO:00511	localizatio	-	-	-	-	16:961454	626	706	764	844	671	718	8.497265	8.032716	7.144132	7.463262	7.986746	12.08413	7.891371	9.178046	0.482433	1	-
ENSMUSG	Ivns1abp	influenza	GO:00508	response	K15046	influenza	mmu0516	Influenza	1:1513444	1413	2032	2557	2680	2017	1287	9.93807	11.97944	12.38916	12.27938	12.43966	11.2234	11.43556	11.98081	0.923918	1	-
ENSMUSG	Lrrc69	leucine ric	-	-	-	-	-	-	4:1462362	0	2	4	2	0	0	0	0.060021	0.098674	0.046644	0	0	0.052898	0.015548	0.507916	1	-
ENSMUSG	Tmem52	transmem	GO:00160	membran	-	-	-	-	4:1554691	10	7	6	7	7	12	0.231778	0.135988	0.095797	0.105695	0.142278	0.344854	0.154521	0.197609	0.690616	1	-
ENSMUSG	Rpp14	ribonucle	GO:00081	metabolic	K14529	ribonucle	mmu0301	RNA trans	14:808036	387	474	582	543	414	372	8.545872	8.773579	8.853594	7.811359	8.016556	10.18532	8.724348	8.671079	0.707314	1	-
ENSMUSG	Psg29	pregnanc	GO:00224	reproduct	-	-	-	-	7:1720347	0	3	3	0	0	0	0	0.029349	0.024121	0	0	0	0.017823	0	0.050274	1	-
ENSMUSG	Slc38a1	solute car	GO:00511	localizatio	K14990	solute car	mmu0472	Glutamate	15:965714	969	3997	5261	5236	4046	2707	7.339157	25.37519	27.44998	25.83472	26.87144	25.42123	20.05478	26.04246	0.330083	1	-
ENSMUSG	Gps2	G protein	GO:00325	developm	K15307	G protein	mmu0516	HTLV-I inf	11:699138	289	362	457	504	409	192	7.160543	7.518129	7.800393	8.13506	8.88617	5.898413	7.493022	7.639881	0.961676	1	-
ENSMUSG	Bsg	basigin [S	GO:00325	developm	-	-	-	-	10:797044	4151	4458	6035	5420	4133	3577	68.86153	61.98932	68.9688	58.574	60.1218	73.57477	66.60655	64.09019	0.477208	1	-
ENSMUSG	Vwa5a	von Willet	-	-	-	-	-	-	9:3871826	262	301	433	417	292	231	2.236218	2.153435	2.545966	2.318627	2.185437	2.444617	2.311873	2.316227	0.793794	1	-
ENSMUSG	P3h3	prolyl 3-h	GO:00081	metabolic	-	-	-	-	6:1248410	213	370	432	453	356	383	1.790341	2.606825	2.501449	2.480488	2.623913	3.991546	2.299538	3.031982	0.15598	1	-
ENSMUSG	Grm2	glutamate	GO:00048	signal trar	K04605	metabotro	mmu0408	Neuroacti	9:1066430	678	859	1178	1369	997	885	5.757475	6.114322	6.891269	7.573339	7.424039	9.318194	6.254355	8.105191	0.081272	1	-
ENSMUSG	Il15ra	interleukir	GO:00325	developm	K05074	interleukir	mmu0520	Pathways	2:1170529	57	53	83	87	71	53	0.296587	0.231159	0.297513	0.294902	0.323949	0.34193	0.275086	0.32026	0.560041	1	-
ENSMUSG	Serping1	serine (or	GO:00325	developm	K04001	C1 inhibit	mmu0461	Complem	2:8476538	142	167	248	224	134	153	3.186621	3.14132	3.833949	3.274708	2.636876	4.257148	3.387297	3.389577	0.779645	1	-
ENSMUSG	Serinc2	serine inc	GO:00081	metabolic	-	-	-	-	4:1302534	123	134	190	199	131	139	2.929912	2.67551	3.117853	3.088059	2.736304	4.105359	2.907758	3.309907	0.620448	1	-
ENSMUSG	Ccl25	chemokin	GO:00081	metabolic	K13072	C-C motif	mmu0406	Cytokine-	8:4325210	126	159	176	232	176	103	0.900349	0.952336	0.866371	1.079962	1.102791	0.912562	0.906352	1.031772	0.547516	1	-
ENSMUSG	Scg5	secretogr	GO:00081	metabolic	-	-	-	-	2:1137763	2009	2699	3119	2954	2252	1701	70.89283	79.8322	75.82095	67.90699	69.68405	74.42395	75.51533	70.67166	0.360246	1	-
ENSMUSG	Kcnk5	potassium	GO:00511	localizatio	K04916	potassium	mmu0497	Protein di	14:201400	11	21	12	14	4	10	0.188437	0.301533	0.141616	0.156235	0.06008	0.212408	0.210529	0.142908	0.226417	1	-
ENSMUSG	Guca2a	guanylate	GO:00081	metabolic	-	-	-	-	4:1196377	2	0	5	0	0	0	0.214027	0	0.368536	0	0	0	0.194188	0	0.03258	1	-
ENSMUSG	Parp3	poly (ADP	GO:00081	metabolic	K10798	poly [ADP	mmu0421	Necroptos	9:1064703	66	60	92	120	80	50	0.567129	0.43216	0.544602	0.671744	0.602797	0.53272	0.51463	0.602421	0.506947	1	-
ENSMUSG	Slc26a6	solute car	GO:00511	localizatio	-	-	-	-	9:1088532	70	93	118	90	102	123	0.463405	0.516058	0.538145	0.388142	0.592119	1.009616	0.50587	0.663292	0.337032	1	-
ENSMUSG	Acy1	aminoacy	GO:00081	metabolic	K14677	aminoacy	mmu0110	Metabolic	9:1064329	71	92	113	95	73	75	0.859805	0.933859	0.942699	0.749456	0.775194	1.126135	0.912121	0.883595	0.640592	1	-
ENSMUSG	Frs3	fibroblast	GO:00508	response	-	-	-	-	17:476890	183	278	359	382	282	279	2.481379	3.159645	3.353415	3.374315	3.35299	4.690628	2.998146	3.805978	0.206686	1	-
ENSMUSG	Gabrr2	gamma-a	GO:00048	signal trar	K05190	gamma-a	mmu0408	Neuroacti	4:3306299	10	5	17	19	14	13	0.242201	0.101502	0.283636	0.299776	0.297332	0.390384	0.209113	0.329164	0.239657	1	-
ENSMUSG	Creld2	cysteine-r	GO:00055	extracellul	-	-	-	-	15:888196	198	296	441	349	303	255	9.225459	11.56022	14.15507	10.59324	12.37961	14.73152	11.64691	12.56812	0.845689	1	-
ENSMUSG	Cd4	CD4 antig	GO:00325	developm	K06454	T-cell surf	mmu0451	Cell adhes	6:1248646	8	8	4	4	5	5	0.102521	0.085934	0.035319	0.033399	0.056185	0.079446	0.074591	0.056344	0.588686	1	-
ENSMUSG	Twf2	twinfilin a	GO:00325	developm	-	-	-	-	9:1062031	312	376	480	562	418	428	3.666524	3.703737	3.885907	4.302467	4.307434	6.236336	3.752056	4.948746	0.104228	1	-
ENSMUSG	Bmp15	bone mor	GO:00325	developm	K05498	growth di	mmu0491	Ovarian st	X:6314107	0	5	5	0	0	4	0	0.08704	0.071539	0	0	0.103005	0.05286	0.034335	0.580145	1	-
ENSMUSG	Zfp605	zinc finge	GO:00081	metabolic	-	-	-	-	5:1101100	203	223	266	292	183	189	2.169733	1.997881	1.958596	2.033181	1.715162	2.504725	2.04207	2.084356	0.882299	1	-
ENSMUSG	Ube2j2	ubiquitin-	GO:00081	metabolic	K04554	ubiquitin-	mmu0414	Protein pr	4:1559438	356	400	531	514	412	333	2.665196	2.510107	2.738581	2.506829	2.7047	3.091079	2.637961	2.767536	0.971857	1	-
ENSMUSG	5-Mar	membran	GO:00325	developm	-	-	-	-	19:372075	842	982	1175	1438	1016	670	12.71363	12.42858	12.22212	14.14483	13.4522	12.54349	12.45478	13.38017	0.772387	1	-
ENSMUSG	Ache	acetylchol	GO:00325	developm	K01049	acetylchol	mmu0472	Cholinerg	5:1372875	702	718	825	1091	727	773	4.85782	4.164677	3.93287	4.91825	4.411448	6.63238	4.318455	5.320693	0.2409	1	-
ENSMUSG	Dtwd1	DTW dom	-	-	-	-	-	-	2:1261521	94	103	133	122	99	96	2.697049	2.477153	2.628842	2.280366	2.49082	3.415221	2.601014	2.728802	1	1	-
ENSMUSG	Wfdc1	WAP four	GO:00081	metabolic	-	-	-	-	8:1196663	68	79	91	105	53	75	2.733472	2.661861	2.519968	2.749646	1.868196	3.738112	2.638434	2.785318	1	1	-
ENSMUSG	Poc1a	POC1 cen	GO:00325	developm	-	-	-	-	9:1062810	102	134	163	166	108	151	1.216216	1.339261	1.338902	1.289429	1.129209	2.232399	1.298126	1.550346	0.524299	1	-
ENSMUSG	Trip6	thyroid hc	GO:00081	metabolic	K12792	thyroid re	mmu0462	NOD-like	5:1373096	83	117	119	131	82	120	1.466071	1.732268	1.448016	1.507398	1.270078	2.628097	1.548785	1.801858	0.636634	1	-
ENSMUSG	Clec4n	C-type lec	GO:00511	localizatio	-	-	-	-	6:1232298	0	12	15	6	3	9	0	0.242884	0.249518	0.094384	0.063516	0.269442	0.164134	0.142447	0.737908	1	-
ENSMUSG	4921501E	RIKEN cDI	GO:00056	cell|intrace	-	-	-	-	17:330641	1	0	1	1	0	0	0.016232	0	0.011176	0.010571	0	0	0.009136	0.003524	1	1	-
ENSMUSG	Agap3	ArfGAP w	GO:00987	molecular	K12491	Arf-GAP v	mmu0414	Endocytos	5:2445217	3722	4372	4871	5083	4347	3889	31.43446	30.95014	28.33996	27.96607	32.19304	40.72429	30.24152	33.6278	0.619448	1	-
ENSMUSG	Tmem176	transmem	GO:00325	developm	-	-	-	-	6:4884091	101	125	154	143	132	178	1.11053	1.15205	1.166486	1.024301	1.272696	2.426693	1.143022	1.574563	0.198849	1	-
ENSMUSG	Dlx2	distal-less	GO:00325	developm	-	-	-	-	2:7154340	86	93	94	99	70	57	1.763653	1.598631	1.327993	1.322611	1.25879	1.449348	1.563426	1.343583	0.299953	1	-
ENSMUSG	Slc17a9	solute car	GO:00081	metabolic	-	-	-	-	2:1807252	2	10	5	1	8	5	0.030198	0.126528	0.051998	0.009833	0.105903	0.093581	0.069574	0.069772	1	1	-
ENSMUSG	Stk31	serine thr	GO:00081	metabolic	-	-	-	-	6:4939560	4	0	0	0	0	0	0.062596	0	0	0	0	0	0.020865	0	0.194637	1	-
ENSMUSG	Nfatc4	nuclear fa	GO:00325	developm	K17334	nuclear fa	mmu0516	HTLV-I inf	14:558231	19	22	36	43	18	33	0.11575	0.112342	0.151087	0.170657	0.096156	0.249267	0.126393	0.172026	0.361726	1	-
ENSMUSG	Gnb3	guanine n	GO:00048	signal trar	K07825	guanine n	mmu0520	Pathways	6:1248342	4	2	11	3	9	0	0.063774	0.026728	0.120819	0.031156	0.125834	0	0.07044	0.05233	0.718287	1	-
ENSMUSG	Tpi1	triosephos	GO:00325	developm	K01803	triosephos	mmu0110	Metabolic	6:1248105	4077	6089	5646	6538	4612	4614	65.62959	82.1595	62.61109	68.56232	65.10149	92.09214	70.13339	75.25199	0.849259	1	-
ENSMUSG	Rab12	RAB12, m	GO:00081	metabolic	-	-	-	-	17:664945	1441	1873	654	2268	1619	839	22.90642	24.95652	7.1618	23.48648	22.56747	16.53641	18.34158	20.86345	0.694051	1	-
ENSMUSG	Celsr3	cadherin,	GO:00325	developm	-	-	-	-	9:1088263	1995	2552	2866	3562	2543	2258	8.989524	9.638897	8.896545	10.45609	10.04807	12.61548	9.174989	11.03988	0.218879	1	-
ENSMUSG	Prph	peripherin	GO:00051	structural	K07607	peripherin	mmu0501	Amyotrop	15:990551	11	33	41	40	31	23	0.323808	0.814246	0.831427	0.767049	0.800171	0.839463	0.656494	0.802228	0.59077	1	-
ENSMUSG	Pcbp4	poly(rC) b	GO:00081	metabolic	-	-	-	-	9:1064532	937	801	1338	1137	997	770	6.170261	4.42129	6.069757	4.877596	5.757073	6.28696	5.553769	5.640543	0.843208	1	-
ENSMUSG	Cdca3	cell divisic	GO:00081	metabolic	-	-	-	-	6:1248295	17	8	21	19	15	19	0.217554	0.085813	0.18514	0.1584	0.168329	0.301491	0.162836	0.209407	0.595702	1	-
ENSMUSG	C1qtnf12	C1q and t	GO:00081	metabolic	-	-	-	-	4:1559623	60	76	95	82	98	96	2.03912	2.165025	2.224179	1.815482	2.920527	4.045302	2.142775	2.927104	0.222585	1	-
ENSMUSG	Ccndbp1	cyclin D-t	GO:00099	cellular pr	-	-	-	-	2:1210084	598	732	944	784	679	646	7.236237	7.424639	7.869276	6.18029	7.204835	9.692376	7.51005	7.6925	0.86016	1	-
ENSMUSG	Ogfod2	2-oxoglut	GO:00081	metabolic	-	-	-	-	5:1241122	132	170	192	168	142	138	1.303648	1.407308	1.306294	1.080884	1.229757	1.689871	1.339083	1.333504	0.751518	1	-
ENSMUSG	Mrps23	mitochon	GO:00081	metabolic	-	-	-	-	11:882043	470	600	460	662	396	416	13.34075	14.27534	8.994795	12.24114	9.856432	14.6407	12.20363	12.24609	0.793987	1	-
ENSMUSG	Rhebl1	Ras homo	GO:00081	metabolic	-	-	-	-	15:988777	25	42	55	64	40	34	0.672098	0.946435	1.018601	1.120852	0.942955	1.133339	0.879045	1.065715	0.46454	1	-
ENSMUSG	Sfi1	Sfi1 homo	GO:00081	metabolic	-	-	-	-	11:313185	239	286	354	389	293	270	1.046591	1.04978	1.067908	1.109711	1.125094	1.465983	1.05476	1.233596	0.427369	1	-
ENSMUSG	Hes7	hairy and	GO:00325	developm	K09087	hairy and	mmu0516	Human pa	11:691204	11	10	11	13	17	11	0.386458	0.294462	0.266217	0.297531	0.523699	0.479166	0.315712	0.433466	0.423533	1	-
ENSMUSG	Pigx	phosphati	GO:00081	metabolic	K07541	phosphati	mmu0110	Metabolic	16:320844	110	121	124	113	113	88	1.208859	1.114607	0.938764	0.808992	1.088944	1.199087	1.08741	1.032341	0.582745	1	-
ENSMUSG	Tiam2	T cell lymp	GO:00048	signal trar	-	-	-	-	17:332657	568	682	884	963	750	420	2.17524	2.189255	2.332181	2.40252	2.518621	1.994318	2.232225	2.305153	1	1	-
ENSMUSG	Synj2	synaptoja	GO:00081	metabolic	K20279	synaptoja	mmu0110	Metabolic	17:594128	1091	1235	1757	1584	1343	1049	4.121519	3.910682	4.572523	3.898248	4.448886	4.913536	4.201574	4.420224	0.944045	1	-
ENSMUSG	Rsph3b	radial spo	GO:00056	cell|intrace	-	-	-	-	17:690471	192	237	246	259	235	198	7.560218	7.822277	6.672967	6.643773	8.114156	9.666833	7.351821	8.141587	0.721742	1	-
ENSMUSG	Rps6ka2	ribosomal	GO:00325	developm	K04373	ribosomal	mmu0401	MAPK sigi	17:717011	898	1154	1361	1444	1162	985	10.56081	11.37573	11.02631	11.06292	11.98311	14.36293	10.98762	12.46965	0.493928	1	-
ENSMUSG	Park2	Parkinson	GO:00325	developm	K04556	parkin[2.3	mmu0414	Protein pr	17:108403	106	129	141	174	144	120	0.531029	0.541693	0.48661	0.567863	0.632582	0.74538	0.519777	0.648608	0.274004	1	-
ENSMUSG	Agpat4	1-acylglyc	GO:00081	metabolic	K13523	lysophosp	mmu0110	Metabolic	17:121187	755	891	1023	1069	762	710	13.22197	13.07916	12.34173	12.19575	11.70164	15.41678	12.88096	13.10472	0.830458	1	-
ENSMUSG	Slc22a3	solute car	GO:00511	localizatio	K08200	MFS trans	mmu0523	Choline m	17:124199	5	8	11	12	12	9	0.090765	0.121721	0.137557	0.141913	0.191017	0.202562	0.116681	0.178497	0.324809	1	-
ENSMUSG	Slc22a1	solute car	GO:00511	localizatio	K08198	MFS trans	mmu0523	Choline m	17:126488	0	0	0	0	2	0	0	0	0	0	0.055892	0	0	0.018631	0.5355	1	-
ENSMUSG	Igf2r	insulin-lik	GO:00325	developm	K06564	insulin-lik	mmu0414	Endocytos	17:126824	1122	1234	1560	1708	1102	930	7.315736	6.744242	7.007145	7.254954	6.30071	7.51855	7.022374	7.024738	0.745671	1	-
ENSMUSG	Acat2	acetyl-Co	GO:00081	metabolic	K00626	acetyl-Co	mmu0110	Metabolic	17:129428	490	606	715	724	582	525	8.887258	9.212924	8.933667	8.554456	9.256301	11.80639	9.011283	9.872383	0.731269	1	-
ENSMUSG	Lnpep	leucyl/cys	GO:00081	metabolic	K01257	cystinyl ar	mmu0461	Renin-anc	17:175277	1800	1747	2014	2758	1752	829	22.0798	17.96257	17.01898	22.03933	18.84513	12.6085	19.02045	17.83099	0.57655	1	-
ENSMUSG	Chd1	chromodo	GO:00081	metabolic	K07179	RIO kinase	mmu0300	Ribosome	17:157049	1188	1202	1653	1760	1312	702	5.564635	4.719302	5.333895	5.370499	5.388859	4.077027	5.205944	4.945462	0.554899	1	-
ENSMUSG	Mpc1	mitochon	GO:00081	metabolic	-	-	-	-	17:828290	344	186	298	460	243	164	5.333554	2.417265	3.182924	4.646202	3.303743	3.152734	3.644581	3.700893	0.939385	1	-
ENSMUSG	Pde10a	phosphoc	GO:00081	metabolic	-	-	mmu0023	Purine me	17:852537	1636	1956	2460	2650	1986	1307	4.086174	4.095015	4.232727	4.311826	4.349663	4.047579	4.137972	4.236356	0.919404	1	-
ENSMUSG	1700010I1	RIKEN cDI	-	-	-	-	-	-	17:898833	21	15	37	31	17	5	0.513915	0.307692	0.62376	0.494205	0.364795	0.151704	0.481789	0.336901	0.289165	1	-
ENSMUSG	Zfp54	zinc finge	GO:00081	metabolic	-	-	-	-	17:214232	5	17	8	11	19	17	0.057608	0.164179	0.063497	0.08256	0.191954	0.242851	0.095095	0.172455	0.167593	1	-
ENSMUSG	Phf10	PHD finge	GO:00056	cell|intrace	-	-	mmu0522	Hepatoce	17:149450	1269	1512	1851	1863	1432	828	14.05765	14.03963	14.12567	13.44453	13.91032	11.3728	14.07432	12.90922	0.349345	1	-
ENSMUSG	Thbs2	thrombos	GO:00325	developm	K04659	thrombos	mmu0415	PI3K-Akt s	17:146655	150	172	264	254	155	166	1.609756	1.547207	1.951746	1.775756	1.458622	2.208831	1.702903	1.814403	0.920874	1	-
ENSMUSG	Smoc2	SPARC rel	GO:00508	response	-	-	-	-	17:142795	473	545	804	797	563	590	10.61461	10.25161	12.4294	11.65152	11.07883	16.4165	11.09854	13.04895	0.40694	1	-
ENSMUSG	Zfp51	zinc finge	GO:00081	metabolic	-	-	-	-	17:214503	67	88	124	97	64	50	1.637713	1.803006	2.088027	1.544607	1.371786	1.515384	1.842916	1.477259	0.147997	1	-
ENSMUSG	Zscan10	zinc finge	GO:00325	developm	-	-	-	-	17:236008	3	4	7	2	1	0	0.019196	0.021455	0.030861	0.008337	0.005611	0	0.023838	0.004649	0.039303	1	-
ENSMUSG	Mmp25	matrix me	GO:00325	developm	-	-	-	-	17:236294	2	3	4	15	3	3	0.037691	0.047378	0.051926	0.184117	0.049562	0.070092	0.045665	0.101257	0.177923	1	-
ENSMUSG	Hcfc1r1	host cell f	GO:00056	cell|intrace	-	-	-	-	17:236735	474	613	587	499	492	552	18.246	19.7789	15.56605	12.51329	16.6072	26.34597	17.86365	18.48882	0.900518	1	-
ENSMUSG	Tnfrsf12a	tumor nec	GO:00325	developm	K05149	tumor nec	mmu0406	Cytokine-	17:236754	16	33	44	28	29	42	1.05594	1.825501	2.000403	1.203802	1.678214	3.436766	1.627281	2.106261	0.556242	1	-
ENSMUSG	Cldn6	claudin 6	GO:00051	structural	K06087	claudin[-]	mmu0453	Tight junc	17:236793	0	0	3	0	0	0	0	0	0.08721	0	0	0	0.02907	0	0.302792	1	-
ENSMUSG	Pkmyt1	protein ki	GO:00081	metabolic	K06633	membran	mmu0411	Cell cycle	17:237263	23	37	42	41	46	35	0.716875	0.966665	0.901822	0.832494	1.25723	1.352619	0.861787	1.147448	0.300627	1	-
ENSMUSG	Paqr4	progestin	GO:00160	membran	-	-	-	-	17:237361	640	895	1091	1089	827	833	16.22414	19.01769	19.05277	17.98421	18.38358	26.18261	18.0982	20.85013	0.48042	1	-
ENSMUSG	Flywch2	FLYWCH t	GO:00971	organic cy	-	-	-	-	17:237769	42	39	31	79	49	55	1.678751	1.306646	0.853603	2.057058	1.717434	2.725769	1.279667	2.166754	0.035034	1	-
ENSMUSG	Slc25a27	solute car	GO:00325	developm	-	-	-	-	17:436419	389	515	621	662	494	523	9.200652	10.21007	10.11839	10.20019	10.24562	15.33757	9.843039	11.92779	0.30879	1	-
ENSMUSG	Pla2g7	phospholi	GO:00081	metabolic	K01062	platelet-a	mmu0110	Metabolic	17:435680	2054	2501	3187	2992	2361	2357	34.0919	34.79504	36.4405	32.35146	34.36287	48.50609	35.10915	38.40681	0.733901	1	-
ENSMUSG	Mep1a	meprin 1	GO:00081	metabolic	K01395	meprin A[	mmu0497	Protein di	17:434743	0	5	0	0	0	0	0	0.059069	0	0	0	0	0.01969	0	0.124515	1	-
ENSMUSG	Tnfrsf21	tumor nec	GO:00325	developm	K05157	tumor nec	mmu0406	Cytokine-	17:430165	2193	2691	2891	3274	2286	2152	38.43678	39.53438	34.90663	37.38253	35.13397	46.76667	37.62593	39.76106	0.929861	1	-
ENSMUSG	Adgrf4	adhesion	GO:00048	signal trar	-	-	-	-	17:426568	11	40	29	37	25	21	0.146839	0.44756	0.266678	0.321754	0.292632	0.347566	0.287026	0.320651	0.819144	1	-
ENSMUSG	Cenpq	centrome	GO:00511	localizatio	-	-	-	-	17:409230	39	57	87	90	65	34	0.928306	1.137247	1.426579	1.395567	1.356692	1.003451	1.164044	1.251903	0.848253	1	-
ENSMUSG	Mut	methylma	GO:00325	developm	K01847	methylma	mmu0110	Metabolic	17:409346	764	849	1072	1110	943	559	13.21211	12.30664	12.77101	12.505	14.29991	11.98607	12.76325	12.93033	0.86856	1	-
ENSMUSG	Tbc1d5	TBC1 dom	GO:00081	metabolic	-	-	-	-	17:507331	608	892	818	1316	976	613	1.868484	2.297754	1.731769	2.63465	2.630135	2.33578	1.966002	2.533522	0.110789	1	-
ENSMUSG	Satb1	special AT	GO:00325	developm	-	-	-	-	17:517361	1261	1555	2834	2854	1876	1119	2.418747	2.500106	3.744782	3.566243	3.155375	2.661284	2.887879	3.127634	0.742761	1	-
ENSMUSG	Efhb	EF hand d	GO:00431	ion bindin	-	-	-	-	17:533988	10	8	19	9	4	4	0.238834	0.160145	0.312602	0.140026	0.083765	0.11845	0.237194	0.11408	0.048011	1	-
ENSMUSG	Cdc5l	cell divisic	GO:00325	developm	K12860	pre-mRNA	mmu0304	Spliceoso	17:453918	1000	1261	1514	1629	1289	589	21.26229	22.47388	22.17622	22.56384	24.03288	15.52785	21.9708	20.70819	0.5585	1	-
ENSMUSG	Spats1	spermato	-	-	-	-	-	-	17:454489	7	10	14	16	17	12	0.383534	0.459209	0.528382	0.571055	0.816699	0.815163	0.457042	0.734306	0.203195	1	-
ENSMUSG	Aars2	alanyl-tR	GO:00081	metabolic	K01872	alanyl-tR	mmu0097	Aminoacy	17:455068	497	566	731	812	565	594	9.434245	9.00575	9.55916	10.04126	9.404632	13.9805	9.333052	11.14213	0.337135	1	-
ENSMUSG	Mrpl14	mitochon	GO:00081	metabolic	K02874	large subu	mmu0301	Ribosome	17:456863	97	91	85	134	120	111	3.864992	3.039261	2.333162	3.47825	4.192749	5.483839	3.079138	4.384946	0.111702	1	-
ENSMUSG	Sgo1	shugoshir	GO:00329	macromo	K11580	shugoshir	mmu0411	Oocyte m	17:536747	4	4	8	12	3	4	0.059756	0.050088	0.082334	0.116793	0.039298	0.074094	0.064059	0.076728	0.776152	1	-
ENSMUSG	Slc29a1	solute car	GO:00325	developm	K15014	solute car	mmu0503	Alcoholisr	17:455852	193	206	230	260	224	244	1.208687	1.081374	0.992281	1.060746	1.230117	1.894657	1.094114	1.395173	0.256018	1	-
ENSMUSG	Sult1c1	sulfotrans	GO:00081	metabolic	-	-	-	-	17:539616	1	2	0	0	0	6	0.040852	0.068468	0	0	0	0.30388	0.03644	0.101293	0.518471	1	-
ENSMUSG	Hsp90ab1	heat shoc	GO:00325	developm	K04079	molecular	mmu0520	Pathways	17:455677	25326	34463	44596	41660	33226	20183	278.2271	317.3501	337.5049	298.1492	320.0763	274.9188	311.0274	297.7148	0.513046	1	-
ENSMUSG	Slc5a7	solute car	GO:00081	metabolic	K14387	solute car	mmu0472	Cholinerg	17:542735	29	18	31	33	18	20	0.356075	0.185255	0.262216	0.263957	0.193798	0.304479	0.267849	0.254078	0.749055	1	-
ENSMUSG	Nfkbie	nuclear fa	GO:00081	metabolic	K05872	NF-kappa	mmu0516	Epstein-B	17:455557	48	37	51	74	29	44	1.259516	0.813803	0.921894	1.264963	0.667267	1.431528	0.998404	1.121253	0.785111	1	-
ENSMUSG	Tcte1	t-complex	-	-	-	-	-	-	17:455234	50	50	96	68	46	64	1.033728	0.866466	1.367267	0.915849	0.833927	1.64058	1.089154	1.130119	0.940543	1	-
ENSMUSG	Vegfa	vascular e	GO:00325	developm	K05448	vascular e	mmu0520	Pathways	17:460169	779	993	1274	1228	981	1013	6.177665	6.600686	6.959974	6.344053	6.821794	9.960526	6.579442	7.708791	0.409429	1	-
ENSMUSG	Gtpbp2	GTP bindi	GO:00038	catalytic a	-	-	-	-	17:461610	713	819	1184	1340	1060	1024	3.486444	3.356832	3.988376	4.268539	4.545082	6.208392	3.610551	5.007338	0.030521	1	-
ENSMUSG	Polh	polymeras	GO:00081	metabolic	K03509	DNA poly	mmu0152	Platinum (	17:461720	79	73	102	90	79	57	1.954325	1.513717	1.738302	1.450433	1.713727	1.748357	1.735448	1.637506	0.599046	1	-
ENSMUSG	Clic5	chloride ir	GO:00325	developm	-	-	-	-	17:441885	119	174	230	294	172	108	1.288382	1.579062	1.715447	2.073609	1.632935	1.449796	1.52763	1.71878	0.611838	1	-
ENSMUSG	Enpp5	ectonucle	GO:00081	metabolic	-	-	-	-	17:440788	3102	4066	4662	4699	3455	2363	23.11696	25.39857	23.93385	22.81266	22.5777	21.83426	24.14979	22.40821	0.335222	1	-
ENSMUSG	Enpp4	ectonucle	GO:00081	metabolic	K18424	ectonucle	mmu0023	Purine me	17:440963	201	474	658	246	412	442	1.601575	3.165793	3.611839	1.276933	2.878661	4.366757	2.793069	2.840783	0.871355	1	-
ENSMUSG	Cyp39a1	cytochron	GO:00081	metabolic	K07439	24-hydrox	mmu0012	Primary b	17:436674	26	24	45	35	21	13	0.54124	0.418765	0.645317	0.47464	0.383336	0.335528	0.535107	0.397835	0.204607	1	-
ENSMUSG	Calcr	calcitonin	GO:00325	developm	K04576	calcitonin	mmu0408	Neuroacti	6:3685680	0	5	0	0	0	0	0	0.030201	0	0	0	0	0.010067	0	0.124302	1	-
ENSMUSG	Fbxl17	F-box anc	-	-	-	-	-	-	17:630574	435	570	621	750	850	481	2.849177	3.129375	2.802032	3.200169	4.881933	3.90626	2.926862	3.996121	0.060034	1	-
ENSMUSG	Rsph9	radial spo	GO:00400	locomotic	-	-	-	-	17:461220	120	148	163	153	108	183	4.030851	4.167056	3.771854	3.348024	3.181124	7.621702	3.98992	4.71695	0.633438	1	-
ENSMUSG	Mrps18a	mitochon	GO:00081	metabolic	K02963	small subu	mmu0301	Ribosome	17:461109	341	471	430	526	407	464	4.030427	4.666274	3.501195	4.050078	4.218259	6.799865	4.065965	5.022734	0.311068	1	-
ENSMUSG	Crip3	cysteine-r	GO:00023	immune s	-	-	-	-	17:464289	6	13	16	12	13	17	0.102043	0.18532	0.187449	0.132951	0.193865	0.35846	0.158271	0.228425	0.423921	1	-
ENSMUSG	Rrp36	ribosomal	GO:00081	metabolic	-	-	-	-	17:466674	155	213	304	254	221	180	8.243302	9.495116	11.13764	8.800022	10.30632	11.86932	9.625353	10.32522	0.880589	1	-
ENSMUSG	Ptk7	PTK7 prot	GO:00325	developm	-	-	-	-	17:465644	184	187	213	240	177	203	2.748232	2.34116	2.191636	2.335228	2.318202	3.759399	2.427009	2.804276	0.58261	1	-
ENSMUSG	Cnpy3	canopy F(	GO:00023	immune s	-	-	-	-	17:467357	716	867	1093	1081	872	845	9.168639	9.306011	9.641922	9.017771	9.791536	13.41636	9.372191	10.74189	0.495404	1	-
ENSMUSG	Ubr2	ubiquitin	GO:00081	metabolic	-	-	-	-	17:469282	1714	1908	2392	2788	1924	1439	8.688671	8.107252	8.353243	9.206974	8.552442	9.044598	8.383055	8.934671	0.827447	1	-

ENSMUSC	Prph2	peripherir	GO:00325	developm	-	-	-	-	17:469104	0	0	3	4	1	0	0	0	0.032437	0.040905	0.013763	0	0.010812	0.018223	1	1	-
ENSMUSC	Guca1b	guanylate	GO:00081	metabolic	K08328	guanylate	mmu0474	Phototran	17:473853	4	5	10	11	8	9	0.082694	0.086643	0.142427	0.148146	0.145039	0.2307	0.103922	0.174628	0.273819	1	-
ENSMUSC	Taf8	TATA-box	GO:00325	developm	K14649	transcripti	mmu0302	Basal tran	17:474880	262	351	406	415	289	253	5.991681	6.728338	6.39625	6.182692	5.795463	7.17389	6.37209	6.384015	0.774649	1	-
ENSMUSC	Guca1a	guanylate	GO:00081	metabolic	K08328	guanylate	mmu0474	Phototran	17:473945	0	3	1	0	3	0	0	0.183473	0.050246	0	0.191933	0	0.077906	0.063978	1	1	-
ENSMUSC	Gm20517	predicted	GO:00081	metabolic	-	-	-	-	17:476115	24	43	52	45	35	21	0.217585	0.326767	0.32477	0.265775	0.278249	0.236057	0.289708	0.260027	0.547783	1	-
ENSMUSC	Pgc	progastric	GO:00081	metabolic	-	-	-	-	17:477268	0	0	0	3	0	0	0	0	0	0.081811	0	0	0	0.02727	0.302552	1	-
ENSMUSC	Bysl	bystin-like	GO:00325	developm	-	-	-	-	17:475993	473	548	635	630	532	454	7.799839	7.574564	7.213556	6.767791	7.692692	9.282522	7.52932	7.914335	0.974811	1	-
ENSMUSC	Tfeb	transcripti	GO:00325	developm	K15590	transcripti	mmu0413	Mitophag	17:477370	108	93	178	131	93	80	0.744517	0.537386	0.845323	0.588309	0.562183	0.683798	0.709075	0.61143	0.293057	1	-
ENSMUSC	Foxp4	forkhead	GO:00325	developm	-	-	-	-	17:478671	608	535	628	957	766	416	4.28812	3.162788	3.051227	4.397003	4.737339	3.637834	3.500712	4.257392	0.286427	1	-
ENSMUSC	Trem2	triggering	GO:00325	developm	K14378	triggering	mmu0438	Osteoclas	17:483464	138	182	247	245	168	186	1.433291	1.584458	1.767271	1.657692	1.530059	2.395273	1.595007	1.861008	0.520608	1	-
ENSMUSC	Treml1	triggering	GO:00508	response	-	-	-	-	17:483599	0	0	3	0	0	0	0	0	0.039393	0	0	0	0.013131	0	0.303266	1	-
ENSMUSC	Nfya	nuclear tr	GO:00325	developm	K08064	nuclear tr	mmu0515	Tuberculo	17:483868	388	397	582	548	467	296	1.606632	1.377932	1.660193	1.478246	1.695682	1.519712	1.548252	1.564546	0.85589	1	-
ENSMUSC	Tspo2	translocat	GO:00511	localizatio	-	-	-	-	17:484479	0	0	0	3	0	0	0	0	0	0.023624	0	0	0	0.007875	0.303442	1	-
ENSMUSC	Kif6	kinesin fa	GO:00990	supramole	-	-	-	-	17:496151	73	76	77	105	72	57	0.652975	0.569824	0.474475	0.61185	0.564735	0.632164	0.565758	0.602916	0.902554	1	-
ENSMUSC	Brd4	bromodo	GO:00325	developm	-	-	-	-	17:321962	1934	1984	2999	3303	2517	1784	11.17479	9.608984	11.93743	12.43292	12.75289	12.78097	10.90707	12.65559	0.355795	1	-
ENSMUSC	Stk38	serine/thr	GO:00081	metabolic	-	-	-	-	17:289708	497	457	935	722	516	456	2.524243	1.945557	3.271431	2.388874	2.298089	2.871612	2.58041	2.519525	0.663624	1	-
ENSMUSC	Ppil1	peptidylpr	GO:00081	metabolic	K12733	peptidyl-p	mmu0304	Spliceosor	17:292508	128	185	224	212	165	172	2.400346	2.907967	2.893773	2.589889	2.713266	3.999247	2.734029	3.100801	0.653847	1	-
ENSMUSC	Pi16	peptidase	GO:00055	extracellul	-	-	-	-	17:293188	6	17	11	23	12	8	0.051083	0.121315	0.064513	0.127561	0.089585	0.084446	0.07897	0.100531	0.559116	1	-
ENSMUSC	Mtch1	mitochon	GO:00081	metabolic	-	-	-	-	17:293320	3499	4417	4474	4584	3345	2839	50.37879	53.30697	44.37631	42.99619	42.23209	50.68216	49.35403	45.30348	0.271677	1	-
ENSMUSC	Fgd2	FYVE, Rhc	GO:00081	metabolic	-	-	-	-	17:293609	49	68	111	84	70	93	0.437806	0.509265	0.683216	0.488928	0.548431	1.030268	0.543429	0.689209	0.432804	1	-
ENSMUSC	Pim1	proviral in	GO:00325	developm	K04702	proto-onc	mmu0520	Pathways	17:294908	30	36	60	45	60	49	0.722748	0.726976	0.995786	0.706248	1.26753	1.46366	0.81517	1.145813	0.219635	1	-
ENSMUSC	Ccdc167	coiled-co	GO:00160	membran	-	-	-	-	17:296893	163	194	221	300	185	156	1.35662	1.353394	1.267111	1.626572	1.350157	1.60983	1.325708	1.528853	0.494851	1	-
ENSMUSC	Cmtr1	cap meth	GO:00081	metabolic	-	-	-	-	17:296605	1710	2154	2515	3000	2083	1569	10.51327	11.10044	10.652	12.01557	11.22984	11.96053	10.75524	11.73531	0.674152	1	-
ENSMUSC	Glo1	glyoxalase	GO:00325	developm	K01759	lactoylglut	mmu0062	Pyruvate r	17:305928	895	1108	1560	1493	1137	1030	25.21273	26.16313	30.27422	27.39923	28.08665	35.9766	27.21669	30.4875	0.578068	1	-
ENSMUSC	Glp1r	glucagon-	GO:00048	signal trar	K04581	glucagon-	mmu0408	Neuroacti	17:309018	17	15	16	19	5	21	0.729982	0.539916	0.473309	0.531495	0.188258	1.118081	0.581069	0.612612	1	1	-
ENSMUSC	Abcg1	ATP bindi	GO:00325	developm	K05679	ATP-bind	mmu0201	ABC trans	17:310576	1072	1233	1516	1684	1259	1030	16.22114	15.63878	15.80293	16.60009	16.70534	19.32458	15.88762	17.54334	0.628257	1	-
ENSMUSC	Tmprss3	transmem	GO:00081	metabolic	-	-	-	-	17:311792	0	0	0	4	0	0	0	0	0	0.054825	0	0	0	0.018275	0.304634	1	-
ENSMUSC	Slc37a1	solute car	GO:00511	localizatio	-	-	-	-	17:312954	67	71	61	108	75	67	1.174312	1.043079	0.736528	1.233144	1.152694	1.456029	0.984639	1.280622	0.25571	1	-
ENSMUSC	Wdr4	WD repea	GO:00081	metabolic	-	-	-	-	17:314943	178	263	307	353	279	311	1.05714	1.309245	1.256034	1.365741	1.452975	2.290117	1.207473	1.702945	0.066643	1	-
ENSMUSC	Ndufv3	NADH de	GO:00081	metabolic	K03944	NADH de	mmu0110	Metabolic	17:315201	598	580	801	576	633	571	11.79172	9.586429	10.88077	7.399123	10.94516	13.96041	10.75297	10.76823	0.749994	1	-
ENSMUSC	Cbs	cystathion	GO:00325	developm	K01697	cystathion	mmu0110	Metabolic	17:316126	315	393	520	519	431	401	3.635888	3.802293	4.134804	3.902553	4.362346	5.738923	3.857661	4.66794	0.306336	1	-
ENSMUSC	Cryaa	crystallin,	GO:00325	developm	K09541	crystallin,	mmu0414	Protein pr	17:316779	4	5	3	2	2	1	0.047981	0.050272	0.02479	0.015627	0.021036	0.014869	0.041014	0.017178	0.194393	1	-
ENSMUSC	Sik1	salt induci	GO:00325	developm	K19008	serine/thr	mmu0492	Glucagon	17:318442	215	219	262	265	207	184	3.029018	2.586183	2.542821	2.43215	2.557271	3.214151	2.719341	2.734524	0.80388	1	-
ENSMUSC	Arhgap28	Rho GTPa	GO:00987	molecular	-	-	-	-	17:678427	13	9	14	11	9	13	0.120048	0.069662	0.089062	0.066173	0.072882	0.148847	0.092924	0.095967	0.929354	1	-
ENSMUSC	Epb41l3	erythrocyt	GO:00325	developm	-	-	-	-	17:690756	2764	3093	4104	4214	3001	1886	11.14244	10.45141	11.39727	11.0667	10.60842	9.426917	10.99704	10.36735	0.448641	1	-
ENSMUSC	Akap8	A kinase (	GO:00081	metabolic	-	-	-	-	17:323036	1232	1575	1882	2086	1550	1197	21.19007	22.70675	22.29938	23.37317	23.37739	25.52712	22.0654	24.09256	0.677954	1	-
ENSMUSC	Myl12a	myosin, lig	GO:00325	developm	K12757	myosin re	mmu0481	Regulation	17:709936	320	453	556	466	238	277	3.823457	4.536882	4.576485	3.627216	2.493593	4.10367	4.312275	3.40816	0.066655	1	-
ENSMUSC	Myom1	myomesir	GO:00325	developm	-	-	-	-	17:710195	54	60	72	94	69	32	0.525074	0.489023	0.482288	0.595433	0.588321	0.385797	0.498795	0.523184	0.914528	1	-
ENSMUSC	Wiz	widely-int	GO:00081	metabolic	-	-	-	-	17:323540	668	844	846	1010	957	582	2.49169	2.638837	2.173898	2.45426	3.130201	2.691697	2.434808	2.758719	0.519575	1	-
ENSMUSC	Lpin2	lipin 2 [So	GO:00081	metabolic	K15728	phosphati	mmu0110	Metabolic	17:711825	1047	1263	1596	1690	1204	800	3.975624	4.019894	4.174869	4.180487	4.008924	3.766468	4.056796	3.985293	0.670946	1	-
ENSMUSC	Emilin2	elastin mi	GO:00329	macromo	-	-	-	-	17:712521	59	65	123	92	56	60	0.95899	0.885574	1.377265	0.974158	0.798157	1.209194	1.073943	0.993836	0.529373	1	-
ENSMUSC	Smchd1	SMC hing	GO:00325	developm	-	-	-	-	17:713444	1069	1206	1456	1658	1233	580	3.255303	3.078321	3.054404	3.289122	3.292452	2.189917	3.129343	2.92383	0.520856	1	-
ENSMUSC	Cyp4f13	cytochron	GO:00081	metabolic	K17726	leukotrien	mmu0110	Metabolic	17:329246	74	74	142	142	135	106	0.973684	0.816162	1.287151	1.217189	1.557631	1.729348	1.025666	1.501389	0.068021	1	-
ENSMUSC	Ndc80	NDC80 ki	GO:00511	localizatio	-	-	-	-	17:714961	0	6	11	4	10	3	0	0.145211	0.218803	0.07525	0.253203	0.107414	0.121338	0.145289	0.908746	1	-
ENSMUSC	Clip4	CAP-GLY	GO:00056	cell|intrace	-	-	-	-	17:717819	902	1087	1219	1392	1032	697	14.13682	14.27998	13.16135	14.21236	14.18298	13.54452	13.85938	13.97995	0.824887	1	-
ENSMUSC	Lbh	limb-bud	GO:00325	developm	-	-	-	-	17:729183	652	745	994	961	754	844	12.56795	12.03723	13.19942	12.06763	12.74474	20.17183	12.60153	14.99474	0.386222	1	-
ENSMUSC	Galnt14	polypeptic	GO:00081	metabolic	K00710	polypeptic	mmu0110	Metabolic	17:734932	319	339	426	476	368	312	3.107511	2.768053	2.858795	3.020724	3.143494	3.768443	2.911453	3.310887	0.535229	1	-
ENSMUSC	Ehd3	EH-doma	GO:00081	metabolic	K12476	EH domai	mmu0414	Endocytos	17:738048	3715	4477	5040	5824	4172	2733	64.54332	65.19771	60.32181	65.9167	63.55934	58.87324	63.35428	62.78309	0.708431	1	-
ENSMUSC	Dpy30	dpy-30, h	GO:00081	metabolic	-	-	-	-	17:742994	179	147	288	153	217	199	11.67963	8.039837	12.94557	6.503554	12.41597	16.09964	10.88834	11.67305	0.94828	1	-
ENSMUSC	Spast	spastin [S	GO:00325	developm	-	-	-	-	17:743389	1297	1501	1926	1866	1344	939	10.98311	10.65416	11.23552	10.29387	9.979925	9.859091	10.9576	10.04429	0.300712	1	-
ENSMUSC	Slc30a6	solute car	GO:00511	localizatio	-	-	-	-	17:743956	168	184	242	225	165	181	4.772873	4.381686	4.736284	4.164224	4.110531	6.375818	4.630281	4.883524	1	1	-
ENSMUSC	Prkd3	protein ki	GO:00081	metabolic	K06070	protein ki	mmu0401	Rap1 sign	17:789494	257	453	527	613	343	281	1.053613	1.556681	1.488367	1.637157	1.233062	1.428372	1.366221	1.432864	0.941609	1	-
ENSMUSC	Yipf4	Yip1 dom	GO:00160	membran	-	-	-	-	17:744894	451	588	785	698	511	591	13.43762	14.68505	16.11263	13.54822	13.35085	21.83328	14.7451	16.24412	0.756624	1	-
ENSMUSC	Birc6	baculovira	GO:00325	developm	K10586	baculovira	mmu0412	Ubiquitin	17:745282	3560	3936	5015	5720	4196	2442	5.344247	4.952728	5.186315	5.593894	5.523504	4.545358	5.161097	5.220919	0.86517	1	-
ENSMUSC	Crim1	cysteine ri	GO:00081	metabolic	-	-	-	-	17:782002	2942	3302	3965	4431	3103	2196	31.18815	29.34113	28.9562	30.60062	28.84506	28.86457	29.82849	29.43675	0.67022	1	-
ENSMUSC	Vit	vitrin [Sou	GO:00099	cellular pr	-	-	-	-	17:785080	127	124	176	146	124	76	2.967356	2.428522	2.832907	2.222297	2.540564	2.20174	2.742928	2.321533	0.230514	1	-
ENSMUSC	Strn	striatin, ca	GO:00452	synapse|re	-	-	-	-	17:786499	2215	2420	3090	3439	2336	1361	13.92658	12.75379	13.38384	14.0859	12.87913	10.60999	13.35474	12.52501	0.466448	1	-
ENSMUSC	Ttc27	tetratricop	-	-	-	-	-	-	17:747177	217	324	334	372	276	268	4.890437	6.120481	5.185426	5.461494	5.454297	7.48871	5.398781	6.134834	0.588568	1	-
ENSMUSC	Eif2ak2	eukaryotic	GO:00325	developm	K16195	eukaryotic	mmu0516	Human pa	17:788525	158	177	262	277	337	168	1.712378	1.60793	1.956116	1.955707	3.202692	2.257546	1.758808	2.471982	0.078071	1	-
ENSMUSC	Cebpz	CCAAT/er	GO:00081	metabolic	-	-	-	-	17:789190	739	993	1131	1269	832	458	11.52815	12.98427	12.15428	12.8961	11.38101	8.858625	12.22223	11.04525	0.37176	1	-
ENSMUSC	Ndufaf7	NADH de	GO:00081	metabolic	-	-	-	-	17:789371	388	518	667	574	493	430	10.70716	11.98188	12.68	10.31895	11.92975	14.71284	11.78968	12.32051	0.994771	1	-
ENSMUSC	Pja2	praja ring	GO:00081	metabolic	-	-	-	-	17:642810	13058	15558	18251	19814	14125	10651	83.88509	83.77506	80.76928	82.9205	79.56814	84.83683	82.80981	82.44182	0.696346	1	-
ENSMUSC	Qpct	glutaminy	GO:00081	metabolic	-	-	-	-	17:790519	119	132	189	160	136	122	3.930771	3.654743	4.300762	3.442972	3.939232	4.99664	3.962092	4.126281	0.976618	1	-
ENSMUSC	Man2a1	mannosid	GO:00325	developm	K01231	alpha-ma	mmu0110	Metabolic	17:646007	322	408	580	559	380	273	2.622594	2.785403	3.25427	2.965976	2.713944	2.756917	2.887422	2.812279	0.657479	1	-
ENSMUSC	Cyp1b1	cytochron	GO:00325	developm	K07410	cytochron	mmu0520	MicroRNA	17:797069	125	127	226	206	145	111	1.549169	1.319303	1.929518	1.663174	1.575791	1.705679	1.59933	1.648215	0.978797	1	-
ENSMUSC	Vapa	vesicle-as	GO:00325	developm	K06096	vesicle-as	mmu0497	Cholester	17:655800	1450	1819	2349	2303	1659	1270	56.19015	59.08508	62.70855	58.13904	56.37431	61.02134	59.32793	58.51157	0.654769	1	-
ENSMUSC	Hnrnpll	heteroger	GO:00081	metabolic	-	-	-	-	17:800294	770	922	1068	1145	851	803	3.117528	3.128983	2.978805	3.019997	3.021286	4.031076	3.075105	3.357453	0.739602	1	-
ENSMUSC	Ralbp1	ralA bindi	GO:00511	localizatio	K08773	RalA-binc	mmu0520	Pathways	17:658484	1234	1422	1930	1922	1416	1224	18.09098	17.47429	19.49197	18.35617	18.20342	22.2492	18.35241	19.60293	0.844366	1	-
ENSMUSC	Srsf7	serine/arg	GO:00081	metabolic	K12896	splicing fa	mmu0516	Herpes sir	17:802000	1068	1569	1897	1746	1335	956	29.78262	36.67478	36.44271	31.71887	32.64494	33.05487	34.30004	32.47289	0.449838	1	-
ENSMUSC	Twsg1	twisted ga	GO:00325	developm	-	-	-	-	17:659230	607	861	1012	1033	709	530	9.534525	11.33616	10.95071	10.57042	9.765606	10.32219	10.60713	10.21941	0.563764	1	-
ENSMUSC	Ndufv2	NADH de	GO:00325	developm	-	-	mmu0110	Metabolic	17:660787	1014	1643	1876	1538	1437	1121	26.22824	35.62226	33.42838	25.91606	32.59345	35.95196	31.75962	31.48715	0.694297	1	-
ENSMUSC	Washc1	WASH co	GO:00325	developm	K18461	WAS prot	mmu0414	Endocytos	17:661115	456	529	638	700	502	453	9.689115	9.421672	9.33881	9.689461	9.353323	11.93449	9.483199	10.32576	0.753722	1	-
ENSMUSC	Washc2	WASH co	GO:00511	localizatio	K18462	WASH co	mmu0414	Endocytos	6:1162080	1888	2307	2639	3182	2121	1360	10.87045	11.13387	10.46734	11.93514	10.70851	9.708912	10.82389	10.78419	0.767378	1	-
ENSMUSC	Lhcgr	luteinizing	GO:00325	developm	K04248	luteinizing	mmu0408	Neuroacti	17:887415	3	4	2	0	2	0	0.074671	0.08346	0.034302	0	0.043654	0	0.064144	0.014551	0.101492	1	-
ENSMUSC	Nrxn1	neurexin I	GO:00325	developm	K07377	neurexin[-	mmu0451	Cell adhes	17:900336	5022	5529	6669	7002	5610	3688	6.885938	6.354565	6.29939	6.254464	6.745155	6.269939	6.513298	6.423186	0.662657	1	-
ENSMUSC	Cacna1h	calcium cl	GO:00081	metabolic	K04855	voltage-d	mmu0401	MAPK sigr	17:253742	1909	2225	3634	4567	2294	2542	7.555751	7.381668	9.908504	11.77564	7.961735	12.47479	8.281974	10.73739	0.117875	1	-
ENSMUSC	Prss41	protease,	GO:00081	metabolic	-	-	-	-	17:238367	0	0	2	0	0	0	0	0	0.024985	0	0	0	0.008328	0	0.536968	1	-
ENSMUSC	Tedc2	tubulin ep	-	-	-	-	-	-	17:242150	97	128	139	137	103	89	1.172881	1.297308	1.157839	1.079155	1.092097	1.334304	1.209343	1.168519	0.636882	1	-
ENSMUSC	Lrpprc	leucine-ri	GO:00081	metabolic	-	-	-	-	17:847052	1419	1734	2012	2200	1552	1147	8.375753	8.579132	8.181271	8.459521	8.032963	8.394425	8.378719	8.295637	0.685826	1	-
ENSMUSC	Atp6v0c	ATPase, H	GO:00511	localizatio	K02155	V-type H-	mmu0110	Metabolic	17:241638	2279	3915	3248	2967	2717	3294	31.81141	45.80607	31.23244	26.97973	33.25606	57.00961	36.28331	39.0818	0.910402	1	-
ENSMUSC	Pdpk1	3-phosph	GO:00325	developm	K06276	3-phosph	mmu0415	PI3K-Akt s	17:240736	2542	3138	3905	4049	3020	2829	10.01685	10.36481	10.60054	10.39406	10.43531	13.82212	10.3274	11.55049	0.568712	1	-
ENSMUSC	Prss30	protease,	GO:00081	metabolic	-	-	-	-	17:239721	5	8	3	2	2	0	0.110914	0.148742	0.045842	0.028898	0.0389	0	0.101833	0.022599	0.034588	1	-
ENSMUSC	Sbpl	spermine	-	-	-	-	-	-	17:239530	0	0	4	2	0	0	0	0	0.201317	0.095164	0	0	0.067106	0.031721	1	1	-
ENSMUSC	Prepl	prolyl end	GO:00081	metabolic	-	-	-	-	17:850634	6446	7730	9398	10433	7521	5199	106.9615	107.5152	107.4297	112.779	109.4348	106.9654	107.3021	109.7264	0.90095	1	-
ENSMUSC	Abca3	ATP-bind	GO:00511	localizatio	K05643	ATP-bind	mmu0201	ABC trans	17:243519	1671	1979	2330	2659	1919	1672	9.656914	9.586512	9.276183	10.01064	9.724775	11.98076	9.506536	10.57206	0.585824	1	-
ENSMUSC	Slc3a1	solute car	GO:00081	metabolic	K14210	solute car	mmu0497	Protein di	17:850283	16	11	15	13	9	19	0.440776	0.254011	0.284669	0.233305	0.217415	0.648983	0.326485	0.366568	1	1	-
ENSMUSC	Eci1	enoyl-Co	GO:00081	metabolic	K13238	3,2-trans-	mmu0007	Fatty acid	17:244266	194	220	271	293	197	214	11.30088	10.74207	10.87509	11.11887	10.06283	15.45651	10.97268	12.21274	0.710797	1	-
ENSMUSC	Six2	sine oculis	GO:00325	developm	-	-	-	-	17:856842	4	5	8	5	4	6	0.064666	0.067754	0.089099	0.052664	0.056703	0.120284	0.07384	0.07655	1	1	-
ENSMUSC	Srbd1	S1 RNA b	GO:00081	metabolic	-	-	-	-	17:859846	186	248	325	307	251	129	3.348685	3.742538	4.030846	3.600657	3.962576	2.879622	3.707356	3.480952	0.567457	1	-
ENSMUSC	Dnase1l2	deoxyribo	GO:00325	developm	-	-	-	-	17:244400	48	57	87	76	48	57	0.596743	0.593984	0.745103	0.615519	0.523278	0.878624	0.645277	0.672474	0.95509	1	-
ENSMUSC	E4f1	E4F transc	GO:00325	developm	-	-	-	-	17:244437	298	355	418	465	363	329	2.200653	2.197435	2.126485	2.237017	2.350631	3.01243	2.174858	2.533359	0.437885	1	-
ENSMUSC	Epas1	endothelia	GO:00325	developm	K09095	hypoxia-ir	mmu0520	Pathways	17:867539	3338	3755	4891	5208	3974	2967	39.95855	37.67786	40.33411	40.61402	41.71516	44.03792	39.32351	42.12237	0.794749	1	-
ENSMUSC	Mlst8	MTOR ass	GO:00081	metabolic	K08266	target of r	mmu0415	PI3K-Akt s	17:244735	579	705	869	953	762	665	10.43893	10.65416	10.79315	11.19313	12.04688	14.86567	10.62875	12.70189	0.291394	1	-
ENSMUSC	Rhog	ras homol	GO:00325	developm	K07194	Ras homo	mmu0491	Insulin sig	17:869630	620	663	874	874	592	437	8.812986	7.899471	8.558437	8.093278	7.378971	7.701919	8.423631	7.724723	0.338804	1	-
ENSMUSC	Pigf	phosphati	GO:00081	metabolic	K05287	phosphati	mmu0110	Metabolic	17:869972	58	85	111	113	72	80	3.730854	4.583008	4.918737	4.735225	4.061173	6.380495	4.410866	5.058965	0.648934	1	-
ENSMUSC	Cript	cysteine-r	GO:00452	synapse|c	-	-	-	-	17:870255	269	278	439	374	318	305	7.17707	6.217153	8.068824	6.500506	7.439845	10.08975	7.154349	8.010035	0.674251	1	-
ENSMUSC	Mcfd2	multiple c	GO:00081	metabolic	-	-	-	-	17:872546	742	958	1129	1144	857	769	9.528462	10.31186	9.987665	9.570317	9.650328	12.24421	9.942663	10.48828	0.942204	1	-
ENSMUSC	Msh2	mutS hon	GO:00325	developm	K08735	DNA misn	mmu0520	Pathways	17:876723	456	554	648	757	591	367	4.103093	4.178399	4.016736	4.437358	4.663125	4.094478	4.099409	4.39832	0.792604	1	-
ENSMUSC	Meiob	meiosis sp	GO:00081	metabolic	-	-	-	-	17:248043	0	2	0	0	0	0	0	0.062558	0	0	0	0	0.020853	0	0.536329	1	-
ENSMUSC	Hagh	hydroxyac	GO:00081	metabolic	K01069	hydroxyac	mmu0062	Pyruvate r	17:248401	837	948	1252	1179	1166	626	20.12633	19.10733	20.73933	18.46862	24.58557	18.66375	19.991	20.57265	0.967309	1	-
ENSMUSC	Spsb3	splA/ryan	GO:00081	metabolic	-	-	-	-	17:248866	471	497	568	635	508	532	3.782341	3.345411	3.142244	3.321967	3.577229	5.297096	3.423332	4.065431	0.403089	1	-
ENSMUSC	Mapk8ip3	mitogen-a	GO:00325	developm	K04436	mitogen-a	mmu0401	MAPK sigr	17:248921	7062	7799	9325	10996	8516	5876	17.78316	16.46163	16.17638	18.03838	18.8044	18.34632	16.80706	18.39637	0.64862	1	-
ENSMUSC	C3	complem	GO:00325	developm	K03990	complem	mmu0520	Viral carci	17:572039	78	57	191	175	68	88	0.872739	0.534586	1.472218	1.275582	0.667173	1.220831	0.959847	1.054529	0.838927	1	-
ENSMUSC	Jpt2	Jupiter mi	GO:00160	membran	-	-	-	-	17:249421	83	113	149	126	106	87	1.798474	2.052361	2.224133	1.778587	2.01406	2.337383	2.024989	2.043343	0.838466	1	-
ENSMUSC	Tmem204	transmem	GO:00325	developm	-	-	-	-	17:250577	92	102	141	139	108	105	2.255746	2.096306	2.38161	2.220228	2.322024	3.192085	2.244554	2.578112	0.607877	1	-
ENSMUSC	Ift140	intraflagel	GO:00325	developm	-	-	-	-	17:250160	641	721	846	935	716	561	4.222815	3.981367	3.83942	4.012704	4.136178	4.582395	4.014534	4.243759	0.910182	1	-
ENSMUSC	Telo2	telomere	GO:00081	metabolic	K11137	telomere	mmu0415	mTOR sig	17:250995	308	373	345	524	354	354	1.41015	1.431453	1.088144	1.56289	1.42122	2.009576	1.309916	1.664562	0.190584	1	-
ENSMUSC	St6gal2	beta galac	GO:00081	metabolic	-	-	-	-	17:554453	276	386	432	473	359	218	1.811112	2.123128	1.952861	2.02199	2.065729	1.773696	1.962367	1.953805	0.798995	1	-
ENSMUSC	Pot1b	protectior	GO:00325	developm	-	-	-	-	17:556520	83	131	149	162	128	86	1.507552	1.994415	1.864356	1.916853	2.038664	1.936772	1.788774	1.964097	0.750287	1	-
ENSMUSC	Tekt4	tektin 4 [S	-	-	-	-	-	-	17:254715	7	4	19	9	8	9	0.290405	0.139082	0.542973	0.243217	0.291026	0.462905	0.324153	0.332382	1	1	-
ENSMUSC	Sox8	SRY (sex c	GO:00325	developm	-	-	-	-	17:255658	611	624	807	873	644	571	10.10432	8.649744	9.193704	9.405061	9.338869	11.70815	9.315922	10.15069	0.74794	1	-
ENSMUSC	Nme4	NME/NM	GO:00081	metabolic	K00940	nucleoside	mmu0110	Metabolic	17:260917	34	33	39	56	45	41	1.151818	0.937078	0.910169	1.235865	1.336763	1.722144	0.999688	1.431591	0.141168	1	-
ENSMUSC	Tmem8	transmem	GO:00038	catalytic a	-	-	-	-	17:261133	195	213	240	287	255	206	3.483088	3.189057	2.953202	3.339593	3.994039	4.562299	3.208449	3.96531	0.268346	1	-
ENSMUSC	Mrpl28	mitochon	GO:00051	structural	K02902	large subu	mmu0301	Ribosome	17:261235	403	535	584	551	444	493	13.05397	14.52593	13.03171	11.62707	12.61139	19.80022	13.5372	14.67956	0.848804	1	-
ENSMUSC	Axin1	axin 1 [So	GO:00048	signal trar	K02157	axin 1[-]	mmu0520	Pathways	17:261386	561	615	830	788	574	417	6.929681	6.367639	7.062854	6.341012	6.217353	6.386646	6.786725	6.315004	0.39454	1	-
ENSMUSC	Pdia2	protein di	GO:00081	metabolic	-	-	-	-	17:261959	1	4	0	0	0	5	0.022948	0.076922	0	0	0	0.142229	0.03329	0.04741	1	1	-
ENSMUSC	Rgs11	regulator	GO:00048	signal trar	-	-	-	-	17:262029	317	422	516	691	406	388	2.164178	2.414895	2.4268	3.073212	2.430535	3.284364	2.335291	2.92937	0.181981	1	-
ENSMUSC	Fam234a	family witl	GO:00055	extracellul	-	-	-	-	17:262118	207	193	352	279	257	234	2.335019	1.824857	2.735352	2.050241	2.542119	3.272814	2.298409	2.621725	0.6145	1	-
ENSMUSC	Luc7l	Luc7-like	GO:00325	developm	-	-	-	-	17:262529	630	850	936	1090	800	634	3.102549	3.508731	3.17545	3.496924	3.454704	3.871266	3.262243	3.607631	0.6307	1	-
ENSMUSC	Dusp1	dual speci	GO:00325	developm	K04459	dual speci	mmu0401	MAPK sigr	17:265055	158	173	316	258	205	232	1.952816	1.792265	2.690563	2.077326	2.221782	3.555308	2.145215	2.618139	0.405952	1	-
ENSMUSC	Bnip1	BCL2/ade	GO:00081	metabolic	K08497	protein tra	mmu0413	SNARE int	17:267810	163	218	207	259	178	159	0.988563	1.108217	0.864843	1.023289	0.94663	1.195637	0.987208	1.055185	0.884785	1	-
ENSMUSC	Phf1	PHD finge	GO:00081	metabolic	-	-	-	-	17:269331	470	554	759	784	587	548	12.29722	12.14987	13.68049	13.36306	13.46758	17.77769	12.70919	14.86944	0.39536	1	-
ENSMUSC	Cuta	cutA dival	GO:00511	localizatio	-	-	-	-	17:269379	169	262	291	245	216	381	16.10267	20.92499	19.10093	15.20752	18.04703	45.01129	18.70953	26.08861	0.232807	1	-
ENSMUSC	Plin3	perilipin 3	GO:00511	localizatio	-	-	-	-	17:562789	215	242	382	331	228	242	6.328809	5.971074	7.74638	6.347363	5.885188	8.832528	6.682088	7.021693	1	1	-
ENSMUSC	Kdm4b	lysine (K)-	GO:00081	metabolic	-	-	-	-	17:563260	1366	1581	1958	2376	1678	1321	10.10516	9.803411	9.9783	11.45039	10.88497	12.11661	9.96229	11.48399	0.380339	1	-
ENSMUSC	Rfx2	regulatory	GO:00325	developm	-	-	-	-	17:567758	55	84	109	67	72	59	1.042465	1.334549	1.423245	0.827296	1.196671	1.38657	1.266753	1.136846	0.417962	1	-
ENSMUSC	Uqcc2	ubiquinol-	GO:00325	developm	-	-	-	-	17:271226	250	350	418	328	298	479	10.62051	12.46309	12.23297	9.077357	11.10105	25.23048	11.77219	15.13629	0.362704	1	-
ENSMUSC	1700061G	RIKEN cDI	GO:00081	metabolic	K15013	long-chai	mmu0110	Metabolic	17:568754	2	2	6	6	3	0	0.038397	0.032177	0.079334	0.075028	0.05049	0	0.049969	0.04184	1	1	-
ENSMUSC	Ip6k3	inositol he	GO:00081	metabolic	K07756	inositol-h	mmu0407	Phosphati	17:271439	5	7	6	2	2	8	0.137741	0.161629	0.11386	0.035888	0.048309	0.273252	0.137743	0.11915	0.593363	1	-
ENSMUSC	Grm8	glutamate	GO:00048	signal trar	K04608	metabotro	mmu0408	Neuroacti	6:2727511	130	158	253	258	169	88	0.656854	0.669169	0.880637	0.84923	0.74878	0.551306	0.735553	0.716438	0.783729	1	-
ENSMUSC	Mllt1	myeloid/ly	GO:00081	metabolic	K15187	YEATS do	mmu0520	Transcript	17:568926	1079	1490	1685	1709	1444	1335	19.00601	21.99929	20.4466	19.61071	22.30383	29.1566	20.48397	23.69038	0.432206	1	-
ENSMUSC	Nudt3	nudix (nu	GO:00081	metabolic	-	-	-	-	17:275793	4858	5278	6902	7275	5381	3705	66.94287	60.96339	65.51989	65.30725	65.02082	63.30252	64.47538	64.54353	0.757421	1	-
ENSMUSC	Spdef	SAM poin	GO:00325	developm	-	-	-	-	17:277143	4	5	5	4	1	6	0.043357	0.045427	0.037338	0.028249	0.009504	0.080648	0.042041	0.039467	0.873076	1	-
ENSMUSC	Snrpc	U1 small r	GO:00081	metabolic	K11095	U1 small r	mmu0304	Spliceosor	17:278399	212	247	313	276	220	161	1.97064	1.924512	2.004314	1.671328	1.793232	1.855585	1.966489	1.773382	0.322873	1	-
ENSMUSC	Taf11	TATA-box	GO:00081	metabolic	K03135	transcripti	mmu0302	Basal tran	17:279011	352	481	591	652	414	321	6.595119	7.554021	7.628154	7.958098	6.801783	7.457129	7.259098	7.40567	0.90712	1	-
ENSMUSC	Anks1	ankyrin re	GO:00325	developm	-	-	-	-	17:279093	512	475	552	652	470	363	2.67284	2.078498	1.985151	2.217338	2.151511	2.349606	2.245496	2.239485	0.766562	1	-
ENSMUSC	Zfp523	zinc finge	GO:00081	metabolic	-	-	-	-	17:281772	1292	1231	1539	1818	1422	961	12.40902	9.91028	10.18275	11.37497	11.97615	11.44417	10.83401	11.59843	0.812002	1	-
ENSMUSC	Fkbp5	FK506 bin	GO:00081	metabolic	K09571	FK506-bir	mmu0491	Estrogen s	17:283990	1164	1482	1711	1979	1306	1078	6.641143	7.087466	6.724984	7.355587	6.533948	7.625961	6.817864	7.171832	0.927573	1	-
ENSMUSC	Pdzph1	PDZ and p	-	-	-	-	-	-	17:588788	7	5	17	8	13	5	0.092131	0.055155	0.154123	0.068588	0.150026	0.081585	0.10047	0.100066	1	1	-
ENSMUSC	Nudt12	nudix (nu	GO:00081	metabolic	K03426	NAD+ dip	mmu0110	Metabolic	17:589996	149	163	248	220	175	96	1.363094	1.249913	1.56294	1.311119	1.403844	1.088918	1.391982	1.26796	0.459276	1	-
ENSMUSC	Cul2	cullin 2 [S	GO:00081	metabolic	K03870	cullin 2[-]	mmu0520	Pathways	18:338298	1106	1346	1615	1602	1210	630	9.919505	10.11889	9.978354	9.360069	9.516188	7.005855	10.00558	8.62737	0.197058	1	-
ENSMUSC	Bambi	BMP and	GO:00325	developm	K10162	BMP and	mmu0431	Wnt signa	18:350795	77	80	107	113	91	88	0.97095	0.84557	0.929477	0.928255	1.006213	1.375858	0.915332	1.103442	0.450868	1	-
ENSMUSC	Mtpap	mitochon	GO:00081	metabolic	-	-	-	-	18:437559	242	300	485	491	335	284	5.801512	6.028371	8.009753	7.668138	7.042279	8.441701	6.613212	7.717373	0.436913	1	-
ENSMUSC	Map3k8	mitogen-a	GO:00048	signal trar	K04415	mitogen-a	mmu0401	MAPK sigr	18:433132	16	22	38	40	29	26	0.133168	0.153477	0.217872	0.216875	0.211645	0.268311	0.168172	0.232277	0.253782	1	-
ENSMUSC	Svil	supervillin	GO:00325	developm	-	-	-	-	18:492054	251	290	400	426	282	187	0.988828	0.957633	1.085573	1.093299	0.97418	0.91343	1.010678	0.993636	0.739377	1	-
ENSMUSC	Zeb1	zinc finge	GO:00325	developm	K09299	zinc finge	mmu0520	Transcript	18:559186	1004	1235	1883	1594	1757	762	3.835722	3.954878	4.955814	3.967192	5.886103	3.609561	4.248805	4.487619	0.876677	1	-
ENSMUSC	Epc1	enhancer	GO:00325	developm	-	-	-	-	18:643595	763	956	1237	1325	928	521	8.317485	8.735312	9.289422	9.409465	8.87071	7.041922	8.78074	8.440699	0.615951	1	-
ENSMUSC	Sos1	son of sev	GO:00325	developm	K03099	son of sev	mmu0520	Pathways	17:803937	1888	2008	2494	2729	1821	893	13.45308	11.99324	12.24244	12.66791	11.37817	7.889647	12.56292	10.64524	0.198323	1	-
ENSMUSC	Map4k3	mitogen-a	GO:00048	signal trar	K04406	mitogen-a	mmu0401	MAPK sigr	17:805805	2091	1847	3037	2434	2272	1094	30.18155	22.34638	30.19838	22.88704	28.75666	19.57901	27.57543	23.7409	0.217476	1	-
ENSMUSC	Tmem178	transmem	GO:00325	developm	-	-	-	-	17:809446	743	962	1180	1235	917	1021	27.8419	30.21608	30.46096	30.14803	30.13161	47.43753	29.50631	35.90572	0.279803	1	-
ENSMUSC	Thumpd2	THUMP d	GO:00081	metabolic	-	-	-	-	17:810263	58	79	98	93	66	47	1.986037	2.267458	2.31172	2.074544	1.981731	1.995463	2.188405	2.017246	0.521895	1	-
ENSMUSC	Pkdcc	protein ki	GO:00325	developm	-	-	-	-	17:832152	159	157	212	224	203	177	2.742184	2.269616	2.518752	2.516682	3.069986	3.784941	2.510184	3.12387	0.286163	1	-
ENSMUSC	Cox7a2l	cytochron	GO:00081	metabolic	K02270	cytochron	mmu0501	Huntingto	17:835019	1047	1323	1578	1694	1185	1093	58.01223	61.44482	60.23253	61.14595	57.57496	75.08944	59.89653	64.60345	0.792538	1	-
ENSMUSC	Thada	thyroid ad	GO:00099	cellular pr	-	-	-	-	17:841900	255	294	368	406	302	277	2.055561	1.986505	2.043565	2.132051	2.134711	2.768572	2.028544	2.345111	0.479428	1	-
ENSMUSC	Dync2li1	dynein cy	GO:00325	developm	K10417	dynein lig	mmu0496	Vasopress	17:846264	220	268	304	305	263	165	10.56017	10.78291	10.05248	9.537377	11.06997	9.82018	10.46519	10.14251	0.653312	1	-
ENSMUSC	Adcyap1	adenylate	GO:00325	developm	K05262	adenylate	mmu0491	Insulin sec	17:931994	92	141	147	139	106	104	2.800237	3.597314	3.082304	2.756145	2.829137	3.92486	3.159952	3.170047	0.792123	1	-
ENSMUSC	Polr2d	polymeras	GO:00081	metabolic	K03012	DNA-dire	mmu0110	Metabolic	18:317891	167	220	268	236	176	181	11.10183	12.25899	12.27337	10.22051	10.25965	14.91915	11.87806	11.79977	0.7266	1	-
ENSMUSC	Slc25a46	solute car	GO:00511	localizatio	-	-	-	-	18:315801	1991	2405	2962	3034	2238	1053	28.94854	29.3105	29.66824	28.73774	28.5337	18.98321	29.30909	25.41821	0.240424	1	-
ENSMUSC	Sap130	Sin3A ass	GO:00081	metabolic	-	-	-	-	18:316343	974	1063	1291	1439	1126	558	13.6285	12.46738	12.44419	13.1169	13.81561	9.680746	12.84669	12.20442	0.571946	1	-
ENSMUSC	Syt4	synaptota	GO:00325	developm	-	-	-	-	18:314378	2606	3431	4045	3892	2974	2289	42.45556	46.8526	45.39731	41.30615	42.4858	46.23725	44.90182	43.34306	0.509203	1	-
ENSMUSC	Adad2	adenosine	GO:00081	metabolic	-	-	-	-	8:1196127	0	0	2	1	0	0	0	0	0.045469	0.021493	0	0	0.015156	0.007164	1	1	-
ENSMUSC	Celf4	CUGBP, El	GO:00325	developm	-	-	-	-	18:254776	6607	8371	9875	10190	7930	5937	29.15126	30.95874	30.01522	29.28929	30.68095	32.47925	30.04174	30.8165	0.899071	1	-
ENSMUSC	Tpgs2	tubulin pc	-	-	-	-	-	-	18:251272	827	1085	1336	1306	924	611	7.499765	8.247544	8.346411	7.715549	7.347787	6.870201	8.03124	7.311179	0.309762	1	-
ENSMUSC	Slc39a6	solute car	GO:00511	localizatio	-	-	-	-	18:245798	1230	1481	1698	1494	1258	817	15.61218	15.75674	14.8473	12.35352	14.00174	12.8578	15.40541	13.07102	0.100415	1	-
ENSMUSC	Elp2	elongator	GO:00081	metabolic	-	-	-	-	18:246039	1127	1579	1818	1770	1390	1294	25.68098	30.1594	28.53862	26.27499	27.77443	36.56015	28.12633	30.20319	0.836272	1	-

ENSMUSC	2700062C	RIKEN cDI	-	-	-	-	-	-	18:244708	143	142	207	213	161	110	8.738536	7.273519	8.714127	8.479395	8.627216	8.334563	8.242061	8.480391	0.968105	1	-
ENSMUSC	Zfp397	zinc finge	GO:00081	metabolic	-	-	-	-	18:239546	736	817	1081	1009	830	440	4.661189	4.337042	4.71624	4.162859	4.609347	3.455077	4.57149	4.075761	0.292793	1	-
ENSMUSC	Mapre2	microtubu	GO:00990	supramole	-	-	-	-	18:237523	1343	1735	1924	1920	1985	2252	5.423633	5.873088	5.352673	5.051228	7.029385	11.27635	5.549798	7.785653	0.073436	1	-
ENSMUSC	Wac	WW dom	GO:00081	metabolic	-	-	-	-	18:786883	1934	1635	2720	2051	1701	990	9.241468	6.548699	8.953747	6.384571	7.127404	5.865508	8.247971	6.459161	0.043629	1	-
ENSMUSC	Ccny	cyclin Y [S	GO:00081	metabolic	-	-	-	-	18:931404	1999	2437	3070	3070	2240	1860	31.6262	32.31787	33.45986	31.6413	31.07598	36.48658	32.46798	33.06796	0.841436	1	-
ENSMUSC	Thoc1	THO com	GO:00325	developm	K12878	THO com	mmu0301	RNA trans	18:995818	490	645	798	932	587	290	8.078068	8.91301	9.062877	10.00943	8.485799	5.92783	8.684652	8.141021	0.580453	1	-
ENSMUSC	Rock1	Rho-asso	GO:00325	developm	K04514	Rho-asso	mmu0520	Pathways	18:100644	755	927	1191	1318	949	406	7.755379	7.981572	8.427899	8.819687	8.548006	5.17092	8.05495	7.512871	0.564291	1	-
ENSMUSC	Cyp4f14	cytochron	GO:00081	metabolic	K17726	leukotrien	mmu0110	Metabolic	17:329050	97	128	175	165	146	110	2.364657	2.615517	2.938895	2.620353	3.120975	3.324874	2.63969	3.022067	0.578995	1	-
ENSMUSC	Esco1	establishn	GO:00081	metabolic	-	-	-	-	18:105666	259	311	359	478	403	214	1.971051	1.98386	1.882104	2.369776	2.68934	2.019286	1.945672	2.359467	0.284767	1	-
ENSMUSC	Mib1	mindbom	GO:00325	developm	-	-	-	-	18:107255	2460	3738	3747	5152	2845	2861	10.39359	13.23801	10.90601	14.18039	10.54036	14.98768	11.51254	13.23614	0.449317	1	-
ENSMUSC	Zfp871	zinc finge	GO:00081	metabolic	-	-	-	-	17:327712	1226	1253	1759	2011	1418	858	8.582946	7.352751	8.483271	9.171484	8.704922	7.44765	8.139656	8.441352	0.979803	1	-
ENSMUSC	Adamts10	a disinteg	GO:00081	metabolic	-	-	-	-	17:335242	271	354	398	471	318	347	1.645443	1.801649	1.664749	1.863016	1.693103	2.612335	1.703947	2.056151	0.338912	1	-
ENSMUSC	Myo1f	myosin IF	GO:00038	catalytic a	-	-	-	-	17:335557	75	62	98	129	53	48	0.750623	0.520124	0.675678	0.841075	0.465137	0.595647	0.648808	0.633953	0.816307	1	-
ENSMUSC	Kifc5b	kinesin fai	GO:00990	supramole	-	-	-	-	17:269170	36	40	50	43	44	38	0.87091	0.811129	0.833294	0.677689	0.9334	1.139859	0.838444	0.916983	0.877149	1	-
ENSMUSC	Dtna	dystrobre	GO:00452	synapse|r	-	-	-	-	18:234151	2493	2779	4257	4851	3132	2016	7.937361	7.416436	9.337034	10.06159	8.744162	7.958483	8.230277	8.921413	0.712446	1	-
ENSMUSC	Cdh2	cadherin 2	GO:00325	developm	K06736	cadherin 2	mmu0451	Cell adhes	18:165888	2332	2671	3018	3526	2674	1611	15.37881	14.76458	13.71087	15.1481	15.46316	13.17273	14.61809	14.59466	0.772714	1	-
ENSMUSC	Tapbp	TAP bindi	GO:00023	immune s	K08058	TAP bindi	mmu0461	Antigen p	17:339158	422	448	619	676	552	611	5.695348	5.068015	5.755054	5.943407	6.532647	10.22433	5.506139	7.566794	0.073549	1	-
ENSMUSC	Pfdn6	prefoldin	GO:00329	macromo	-	-	-	-	17:339388	271	365	446	480	312	469	6.1975	6.996692	7.02642	7.151059	6.256698	13.29861	6.740204	8.902124	0.200219	1	-
ENSMUSC	Wdr46	WD repea	GO:00081	metabolic	-	-	-	-	17:339406	182	226	275	301	211	148	3.617972	3.765782	3.765976	3.897999	3.678057	3.647879	3.716576	3.741312	0.851708	1	-
ENSMUSC	Mep1b	meprin 1	GO:00081	metabolic	K08606	meprin B[	mmu0497	Protein di	18:210723	0	0	4	0	0	0	0	0	0.076617	0	0	0	0.025539	0	0.306439	1	-
ENSMUSC	Rnf138	ring finge	GO:00081	metabolic	-	-	-	-	18:210013	228	304	390	296	289	134	4.659187	5.207183	5.490252	3.940476	5.178647	3.395217	5.118874	4.171446	0.152382	1	-
ENSMUSC	Vps52	VPS52 GA	GO:00325	developm	-	-	-	-	17:339558	1341	1570	1724	1960	1587	1119	15.65477	15.3628	13.86458	14.90582	16.24567	16.19697	14.96072	15.78282	0.905438	1	-
ENSMUSC	Ring1	ring finge	GO:00325	developm	-	-	-	-	17:340207	385	452	538	613	496	463	4.936027	4.857456	4.751725	5.119872	5.576244	7.360099	4.848403	6.018738	0.216264	1	-
ENSMUSC	Slc39a7	solute car	GO:00511	localizatio	-	-	-	-	17:340282	727	949	1226	1172	931	862	7.096154	7.764389	8.243856	7.452428	7.968579	10.43234	7.701466	8.617782	0.600877	1	-
ENSMUSC	Col11a2	collagen,	GO:00325	developm	K19721	collagen,	mmu0497	Protein di	17:340394	411	506	653	861	518	457	3.336351	3.442968	3.651694	4.553176	3.687244	4.599727	3.477005	4.280049	0.205952	1	-
ENSMUSC	Dsc2	desmocol	GO:00511	localizatio	K07601	desmocol	mmu0541	Arrhythmo	18:200306	11	7	21	8	13	8	0.084453	0.045044	0.111066	0.040011	0.087518	0.076153	0.080188	0.067894	0.601492	1	-
ENSMUSC	Brd2	bromodo	GO:00081	metabolic	-	-	-	-	17:341120	3236	3112	4014	4243	3602	2486	17.05569	13.74844	14.57437	14.56852	16.64743	16.24605	15.12616	15.82067	0.9654	1	-
ENSMUSC	Tap2	transporte	GO:00511	localizatio	K05654	ATP-bind	mmu0516	Herpes sir	17:342035	146	160	195	225	209	202	1.731103	1.590176	1.592788	1.737942	2.173004	2.969676	1.638022	2.293541	0.081472	1	-
ENSMUSC	Btnl2	butyrophi	GO:00511	localizatio	-	-	-	-	17:343548	0	0	0	3	0	0	0	0	0	0.036154	0	0	0	0.012051	0.302393	1	-
ENSMUSC	Pfdn1	prefoldin	GO:00329	macromo	-	-	-	-	18:364036	465	619	646	682	544	413	27.43935	30.61711	26.26057	26.2172	28.14892	30.21736	28.10567	28.19449	0.778305	1	-
ENSMUSC	Psd2	pleckstrin	GO:00987	molecular	K12494	PH and SE	mmu0414	Endocytos	18:359648	741	756	1145	1127	901	741	3.853748	3.295638	4.102246	3.818304	4.108966	4.778249	3.750544	4.235173	0.539776	1	-
ENSMUSC	Tmem173	transmem	GO:00081	metabolic	K12654	transmem	mmu0462	NOD-like	18:357336	49	47	64	64	56	46	1.352786	1.087625	1.217197	1.15104	1.355687	1.574632	1.219203	1.360453	0.769033	1	-
ENSMUSC	Dnajc18	DnaJ heat	GO:00160	membran	-	-	-	-	18:356711	2077	2301	2898	3172	2334	1651	25.53678	23.71368	24.54592	25.40647	25.16362	25.1688	24.59879	25.2463	0.921812	1	-
ENSMUSC	Spata24	spermato	GO:00325	developm	-	-	-	-	18:356566	19	31	39	46	32	40	1.163301	1.590914	1.644968	1.834757	1.71801	3.036575	1.466394	2.196447	0.143733	1	-
ENSMUSC	Mzb1	marginal z	GO:00081	metabolic	-	-	-	-	18:356472	0	0	2	2	0	0	0	0	0.096766	0.091484	0	0	0.032255	0.030495	1	1	-
ENSMUSC	Slc23a1	solute car	GO:00325	developm	K14611	solute car	mmu0497	Vitamin di	18:356146	2	0	0	5	0	3	0.030335	0	0	0.049397	0	0.056411	0.010112	0.035269	0.185612	1	-
ENSMUSC	Sil1	endoplasr	GO:00511	localizatio	K14001	nucleotide	mmu0414	Protein pr	18:352664	238	249	357	329	263	236	8.923662	7.82562	9.221169	8.036085	8.646987	10.97144	8.656817	9.218171	0.913194	1	-
ENSMUSC	Hspa9	heat shoc	GO:00325	developm	K04043	molecular	mmu0515	Tuberculo	18:349374	4529	5068	6646	6662	4800	2824	57.47435	53.909	58.10099	55.07545	53.41407	44.43471	56.49478	50.97475	0.281526	1	-
ENSMUSC	Etf1	eukaryotic	GO:00081	metabolic	K03265	peptide cl	mmu0301	mRNA sur	18:349027	2034	2458	2993	2892	2086	1291	33.86608	34.30432	34.32986	31.36851	30.45587	26.65178	34.16675	29.49205	0.141659	1	-
ENSMUSC	Cyp21a1	cytochron	GO:00081	metabolic	K00513	steroid 21	mmu0110	Metabolic	17:348013	0	0	0	2	0	0	0	0	0	0.028027	0	0	0	0.009342	0.537797	1	-
ENSMUSC	Gfra3	glial cell li	GO:00325	developm	-	-	-	-	18:346899	0	0	4	0	3	0	0	0	0.087925	0	0.083922	0	0.029308	0.027974	1	1	-
ENSMUSC	Nelfe	negative e	GO:00081	metabolic	-	-	-	-	17:348503	219	290	263	279	247	193	3.434025	3.811628	2.840976	2.850002	3.396249	3.752355	3.36221	3.332869	0.74181	1	-
ENSMUSC	Cdc23	CDC23 ce	GO:00081	metabolic	K03355	anaphase	mmu0516	HTLV-I inf	18:346309	808	1006	1150	1228	868	793	4.452128	4.646297	4.365213	4.407945	4.193905	5.417701	4.487879	4.673184	0.98095	1	-
ENSMUSC	C2	complem	GO:00081	metabolic	K01332	complem	mmu0532	Systemic l	17:348626	6	14	21	12	8	21	0.067576	0.132165	0.162929	0.088045	0.07901	0.293241	0.12089	0.153432	0.745563	1	-
ENSMUSC	Epb41l4a	erythrocyt	GO:00051	structural	-	-	-	-	18:337963	140	128	116	101	107	94	2.309224	1.769699	1.318096	1.085275	1.547615	1.92244	1.799007	1.518443	0.280655	1	-
ENSMUSC	Stard4	StAR-rela	GO:00081	metabolic	-	-	-	-	18:331993	307	393	496	453	390	392	2.873883	3.083727	3.198628	2.762552	3.201385	4.54991	3.052079	3.504616	0.548787	1	-
ENSMUSC	Tslp	thymic str	GO:00508	response	K05436	thymic str	mmu0406	Cytokine-	18:328153	3	0	4	5	2	0	0.168258	0	0.154586	0.182721	0.098366	0	0.107615	0.093696	1	1	-
ENSMUSC	Bin1	bridging i	GO:00325	developm	K12562	amphiphy	mmu0414	Endocytos	18:323772	3643	4430	5569	5948	4533	3263	94.46088	96.28295	99.47672	100.4721	103.0674	104.9049	96.74018	102.8148	0.841379	1	-
ENSMUSC	Ercc3	excision re	GO:00325	developm	K10843	DNA excis	mmu0342	Nucleotid	18:322403	522	608	721	847	591	487	9.051758	8.837278	8.612891	9.568137	8.986536	10.47074	8.833976	9.675139	0.694212	1	-
ENSMUSC	Map3k2	mitogen-a	GO:00048	signal trar	K04420	mitogen-a	mmu0401	MAPK sigr	18:321630	925	929	1214	1325	976	605	5.447736	4.586092	4.925433	5.083605	5.040421	4.417897	4.986421	4.847308	0.63601	1	-
ENSMUSC	Iws1	IWS1, SUF	GO:00081	metabolic	-	-	-	-	18:320677	791	638	1166	1224	743	635	3.355163	2.268356	3.407119	3.382208	2.763561	3.339621	3.010213	3.161797	0.946978	1	-
ENSMUSC	Proc	protein C	GO:00325	developm	K01344	protein C	mmu0461	Complem	18:321231	0	0	0	0	2	0	0	0	0	0	0.071168	0	0	0.023723	0.535276	1	-
ENSMUSC	Csnk2b	casein kin	GO:00325	developm	K03115	casein kin	mmu0516	Epstein-B	17:351161	1112	1183	1521	1537	1004	742	18.18136	16.21285	17.13176	16.37107	14.39454	15.04221	17.17532	15.26927	0.215668	1	-
ENSMUSC	Myo7b	myosin VI	GO:00325	developm	-	-	-	-	18:319592	0	4	0	0	0	0	0	0.032181	0	0	0	0	0.010727	0	0.187505	1	-
ENSMUSC	Bag6	BCL2-ass	GO:00325	developm	-	-	-	-	17:351351	2610	3102	3498	3881	3008	2960	14.72336	14.66766	13.5937	14.26237	14.87945	20.70352	14.32824	16.61511	0.411189	1	-
ENSMUSC	Prrc2a	proline-ri	GO:00325	developm	-	-	-	-	17:351490	9237	9575	11376	14222	10705	7071	63.4157	55.10075	53.80305	63.60747	64.44588	60.19115	57.43983	62.74817	0.664352	1	-
ENSMUSC	Lims2	LIM and s	GO:00325	developm	-	-	-	-	18:319313	123	158	200	162	64	140	1.916403	2.063448	2.146666	1.644293	0.874392	2.704554	2.042172	1.741079	0.324629	1	-
ENSMUSC	Aif1	allograft i	GO:00081	metabolic	-	-	-	-	17:351709	39	73	62	71	61	55	0.773347	1.213335	0.846931	0.917164	1.060677	1.352254	0.944538	1.110031	0.595327	1	-
ENSMUSC	Ltb	lymphoto	GO:00325	developm	K03157	lymphoto	mmu0406	Cytokine-	17:351944	2	0	5	5	2	1	0.036511	0	0.062869	0.059455	0.032007	0.022624	0.033127	0.038029	1	1	-
ENSMUSC	Wdr33	WD repea	GO:00081	metabolic	K15542	polyaden	mmu0301	mRNA sur	18:318040	926	1124	1398	1560	1116	889	9.176686	9.336716	9.544067	10.0712	9.697991	10.92352	9.35249	10.2309	0.67666	1	-
ENSMUSC	Tnf	tumor nec	GO:00325	developm	K03156	tumor nec	mmu0516	Human pa	17:351993	0	2	2	0	2	0	0	0.034976	0.02875	0	0.036589	0	0.021242	0.012196	0.719445	1	-
ENSMUSC	Lta	lymphoto	GO:00325	developm	K05468	lymphoto	mmu0516	HTLV-I inf	17:352031	2	3	0	0	0	0	0.091925	0.115549	0	0	0	0	0.069158	0	0.088807	1	-
ENSMUSC	Atp6v1g2	ATPase, H	GO:00452	synapse|c	K02152	V-type H-	mmu0110	Metabolic	17:352336	3068	4400	4026	4511	3677	2801	30.94439	37.19908	27.97382	29.6402	32.52093	35.02884	32.0391	32.39666	0.804413	1	-
ENSMUSC	Riok3	RIO kinas	GO:00081	metabolic	-	-	-	-	18:121288	802	964	1215	1201	887	755	13.92229	14.02705	14.52995	13.58191	13.50214	16.2506	14.15976	14.44488	0.855757	1	-
ENSMUSC	Psors1c2	psoriasis s	-	-	-	-	-	-	17:355332	1	0	2	0	0	3	0.075404	0	0.103883	0	0	0.280449	0.059762	0.093483	1	1	-
ENSMUSC	3110002H	RIKEN cDI	GO:00081	metabolic	-	-	-	-	18:121687	437	570	664	796	568	357	3.556946	3.888874	3.723194	4.220763	4.054032	3.602888	3.723005	3.959228	0.839062	1	-
ENSMUSC	Aqp4	aquaporir	GO:00511	localizatio	K09866	aquaporir	mmu0497	Bile secret	18:153893	3282	3770	5206	5726	3741	2168	41.04308	39.51806	44.84945	46.64815	41.02342	33.61604	41.80353	40.42921	0.629421	1	-
ENSMUSC	Npc1	NPC intra	GO:00081	metabolic	K12385	Niemann-	mmu0414	Lysosome	18:121896	1303	1348	1991	2047	1409	852	5.285942	4.58375	5.564173	5.409754	5.012237	4.285518	5.144622	4.902503	0.543641	1	-
ENSMUSC	Mrpl27	mitochon	GO:00081	metabolic	K02899	large subu	mmu0301	Ribosome	11:946537	149	180	286	222	198	274	5.426597	5.494991	7.175594	5.267152	6.323367	12.37307	6.032394	7.987862	0.266969	1	-
ENSMUSC	Zfp521	zinc finge	GO:00325	developm	-	-	-	-	18:136870	223	185	330	303	229	134	2.30369	1.601934	2.348472	2.039125	2.074422	1.716373	2.084699	1.943306	0.543427	1	-
ENSMUSC	Lama3	laminin, a	GO:00325	developm	K06240	laminin, a	mmu0110	Metabolic	18:123338	78	102	137	151	88	68	0.461345	0.505689	0.558216	0.581821	0.456411	0.498682	0.508417	0.512305	0.892944	1	-
ENSMUSC	Dhx16	DEAH (As	GO:00081	metabolic	K12813	pre-mRN	mmu0304	Spliceoso	17:358798	424	479	667	672	517	392	4.144335	3.924439	4.491239	4.278978	4.431208	4.750744	4.186671	4.486977	0.82489	1	-
ENSMUSC	Impact	impact, R\	GO:00325	developm	-	-	-	-	18:129722	3053	4041	4982	4578	3626	2603	56.53476	62.72353	63.55416	55.22633	58.87885	59.76529	60.93749	57.95682	0.435145	1	-
ENSMUSC	Ttc39c	tetratricop	-	-	-	-	-	-	18:125999	477	542	568	695	467	419	5.082961	4.841174	4.169638	4.824641	4.363736	5.536025	4.697924	4.908134	0.997055	1	-
ENSMUSC	Ndfip1	Nedd4 fai	GO:00325	developm	-	-	-	-	18:384189	3258	3945	4953	4769	3561	4416	222.6405	225.9712	233.1701	212.3059	213.3869	374.1693	227.2606	266.6207	0.439458	1	-
ENSMUSC	Atat1	alpha tub	GO:00325	developm	-	-	-	-	17:358975	574	794	866	883	807	656	4.135522	4.795034	4.298212	4.144389	5.098405	5.860141	4.409589	5.034312	0.505544	1	-
ENSMUSC	Spry4	sprouty h	GO:00325	developm	-	-	-	-	18:385862	304	360	467	467	401	321	4.211924	4.18083	4.457342	4.215077	4.87185	5.514406	4.283365	4.867111	0.536987	1	-
ENSMUSC	Gnl1	guanine n	GO:00508	response	-	-	-	-	17:359798	2431	3072	3610	3745	2792	2419	46.46538	49.21738	47.53386	46.63135	46.79533	57.32792	47.73888	50.25153	0.942499	1	-
ENSMUSC	Cabyr	calcium-b	GO:00325	developm	-	-	-	-	18:127413	10	18	13	10	10	15	0.216463	0.326594	0.193855	0.14101	0.189816	0.402591	0.245638	0.244472	0.874374	1	-
ENSMUSC	Nr3c1	nuclear re	GO:00048	signal trar	K05771	glucocorti	mmu0408	Neuroacti	18:394105	1504	1877	2525	1603	1827	1186	5.709559	5.972716	6.603398	3.96433	6.081853	5.582455	6.095225	5.209546	0.139621	1	-
ENSMUSC	Mrps18b	mitochon	GO:00081	metabolic	K16174	small subu	mmu0520	Viral carci	17:359103	142	206	215	231	222	197	2.563054	3.116667	2.673371	2.716214	3.51371	4.40883	2.784364	3.546251	0.233693	1	-
ENSMUSC	Pcdh12	protocadh	GO:00325	developm	-	-	-	-	18:382670	16	17	22	13	19	7	0.10086	0.089825	0.095534	0.053386	0.105021	0.054713	0.095406	0.07104	0.297435	1	-
ENSMUSC	0610009C	RIKEN cDI	GO:00081	metabolic	-	-	-	-	18:382502	484	463	684	676	484	445	12.79521	10.25972	12.45688	11.64206	11.21992	14.58639	11.83727	12.48279	0.951894	1	-
ENSMUSC	Rpp21	ribonuclea	GO:00081	metabolic	K03540	ribonuclea	mmu0301	RNA trans	17:362556	123	160	196	178	107	136	3.443615	3.754776	3.780223	3.246473	2.626855	4.721004	3.659538	3.531444	0.616308	1	-
ENSMUSC	Arap3	ArfGAP w	GO:00325	developm	K12490	Arf-GAP w	mmu0414	Endocytos	18:379726	180	217	271	326	214	196	2.189802	2.212813	2.271195	2.58364	2.28291	2.956475	2.224603	2.607675	0.430098	1	-
ENSMUSC	Hdac3	histone de	GO:00081	metabolic	K11404	histone de	mmu0516	Human pa	18:379358	793	1003	1127	1319	1248	1020	7.274472	7.712267	7.122024	7.882333	10.03889	11.60151	7.369588	9.84091	0.055551	1	-
ENSMUSC	Diaph1	diaphano	GO:00325	developm	K05740	diaphano	mmu0481	Regulation	18:378436	1399	1814	2175	2295	1710	1235	7.076621	7.691276	7.579122	7.562624	7.584849	7.74571	7.449006	7.631061	0.908272	1	-
ENSMUSC	Trim26	tripartite r	GO:00081	metabolic	-	-	-	-	17:368371	346	414	645	618	444	315	1.955132	1.960888	2.510791	2.274938	2.200013	2.206966	2.14227	2.227306	0.989343	1	-
ENSMUSC	H2-M5	histocomp	GO:00023	immune s	K06751	major hist	mmu0516	Human pa	17:369840	16	18	11	31	17	11	0.271455	0.255975	0.12856	0.342619	0.252902	0.231397	0.218663	0.275639	0.461095	1	-
ENSMUSC	Gabbr1	gamma-a	GO:00048	signal trar	K04615	gamma-a	mmu0408	Neuroacti	17:370459	13737	16318	18210	21829	14285	9625	66.24385	65.95891	60.49438	68.57552	60.40551	57.54934	64.23238	62.17679	0.572506	1	-
ENSMUSC	Myot	myotilin [S	GO:00990	supramole	-	-	-	-	18:443340	0	0	2	0	0	0	0	0	0	0.033285	0	0	0	0.011095	0	0.535767	1 -
ENSMUSC	Dcp2	decapping	GO:00081	metabolic	K12613	mRNA-de	mmu0301	RNA degr	18:443805	1584	1858	2425	2611	1844	1352	11.16796	10.98037	11.77827	11.99243	11.40045	11.81901	11.30887	11.7373	0.999016	1	-
ENSMUSC	Ik	IK cytokin	GO:00081	metabolic	-	-	-	-	18:367446	2431	2888	3605	4133	2812	1577	64.96946	64.69553	66.3714	71.95671	65.89952	52.2567	65.34546	63.37098	0.644363	1	-
ENSMUSC	Pggt1b	protein ge	GO:00081	metabolic	-	-	-	-	18:462399	206	182	255	256	196	146	4.785062	3.543602	4.080476	3.873838	3.992261	4.204939	4.13638	4.023679	0.677305	1	-
ENSMUSC	Mal2	mal, T cell	GO:00325	developm	-	-	-	-	15:545711	4046	4958	6234	6433	4459	2222	87.16456	89.531	92.5193	90.28368	84.23532	59.3532	89.73829	77.9574	0.233087	1	-
ENSMUSC	Ap3s1	adaptor-r	GO:00511	localizatio	K12399	AP-3 com	mmu0414	Lysosome	18:467418	286	387	425	487	283	262	5.648277	6.406406	5.782167	6.265571	4.900946	6.415603	5.945617	5.860707	0.705455	1	-
ENSMUSC	Ankhd1	ankyrin re	GO:00023	immune s	-	-	-	-	18:365599	1559	1774	2316	2571	1819	786	6.539876	6.237779	6.692886	7.025985	6.691119	4.0882	6.49018	5.935102	0.470073	1	-
ENSMUSC	Hbegf	heparin-b	GO:00325	developm	K08523	heparin-b	mmu0520	Proteogly	18:365049	170	252	266	282	198	147	4.552881	5.657072	4.907632	4.920037	4.649927	4.88137	5.039195	4.817111	0.587107	1	-
ENSMUSC	Yipf5	Yip1 dom	GO:00511	localizatio	-	-	-	-	18:402048	614	790	962	910	667	504	16.75463	18.06952	18.08394	16.17669	15.96009	17.05231	17.63603	16.39637	0.374303	1	-
ENSMUSC	Rbm27	RNA bind	GO:00081	metabolic	-	-	-	-	18:422753	1345	1432	1905	2035	1387	647	13.29168	11.86188	12.96893	13.10097	12.01923	7.927715	12.7075	11.01597	0.271149	1	-
ENSMUSC	Lars	leucyl-tRN	GO:00081	metabolic	K01869	leucyl-tRN	mmu0097	Aminoacy	18:422023	607	682	894	899	676	634	10.00433	9.421858	10.15051	9.65251	9.769857	12.95609	9.858899	10.79282	0.729893	1	-
ENSMUSC	Pou4f3	POU dom	GO:00325	developm	-	-	-	-	18:423945	0	0	0	2	0	0	0	0	0	0.035082	0	0	0	0.011694	0.53904	1	-
ENSMUSC	Tcerg1	transcripti	GO:00081	metabolic	K12824	transcripti	mmu0304	Spliceoso	18:425115	1757	2158	2370	2923	1969	911	24.08599	24.79687	22.38167	26.10376	23.66907	15.48449	23.75484	21.75244	0.458724	1	-
ENSMUSC	Ppp2r2b	protein pl	GO:00325	developm	K04354	serine/thr	mmu0415	PI3K-Akt s	18:426374	1602	2002	2116	2156	1729	1194	17.89942	18.74966	16.2871	15.69303	16.94003	16.54119	17.64539	16.39142	0.35256	1	-
ENSMUSC	Dpysl3	dihydropy	GO:00325	developm	-	-	-	-	18:433209	1023	1211	1500	1693	1164	997	8.670945	8.603748	8.758576	9.348227	8.651402	10.47784	8.677757	9.492491	0.686663	1	-
ENSMUSC	Jakmip2	janus kina	GO:00056	cell|intrac	-	-	-	-	18:435314	1383	1693	2015	2180	1509	854	25.03384	25.6871	25.12648	25.70653	23.95175	19.16678	25.28247	22.94169	0.335042	1	-
ENSMUSC	Dtwd2	DTW dom	GO:00511	localizatio	-	-	-	-	18:496961	51	56	88	90	29	32	0.519837	0.478457	0.61792	0.59762	0.259201	0.404422	0.538738	0.420415	0.241238	1	-
ENSMUSC	Hsd17b4	hydroxyst	GO:00325	developm	K12405	3-hydroxy	mmu0110	Metabolic	18:501282	1135	1145	1666	1655	1194	851	18.40588	15.56393	18.61175	17.48399	16.97884	17.11104	17.52719	17.19129	0.650401	1	-
ENSMUSC	Rab27b	RAB27B, r	GO:00452	synapse|c	K07886	Ras-relate	mmu0497	Pancreatic	18:699791	903	1120	1266	1391	980	592	4.580809	4.762395	4.424249	4.59688	4.359366	3.723592	4.589151	4.226613	0.382722	1	-
ENSMUSC	Dynap	dynactin a	GO:00160	membran	-	-	-	-	18:702404	3	0	1	2	2	0	0.155368	0	0.035668	0.067476	0.09083	0	0.063679	0.052769	1	1	-
ENSMUSC	Mbd2	methyl-Cp	GO:00325	developm	-	-	-	-	18:705681	698	815	1130	1176	893	505	10.21885	10.00131	11.39663	11.21593	11.46411	9.166924	10.53893	10.61566	0.859392	1	-
ENSMUSC	Smad4	SMAD far	GO:00325	developm	K04501	mothers a	mmu0520	Pathways	18:736390	575	702	982	1155	712	687	6.397586	6.546937	7.526812	8.371656	6.946571	9.477433	6.823778	8.26522	0.253906	1	-
ENSMUSC	Sec11c	SEC11 ho	GO:00081	metabolic	K13280	signal pep	mmu0306	Protein ex	18:658005	610	768	985	864	690	585	8.234351	8.689882	9.159831	7.597927	8.167537	9.791323	8.694688	8.518929	0.615039	1	-
ENSMUSC	Grp	gastrin rel	GO:00511	localizatio	-	-	-	-	18:658728	226	323	294	320	275	231	6.561444	7.860409	5.880155	6.052314	7.001093	8.315494	6.767336	7.122967	0.974342	1	-
ENSMUSC	Rax	retina anc	GO:00325	developm	-	-	-	-	18:659346	0	2	0	0	0	0	0	0.063833	0	0	0	0	0.021278	0	0.535832	1	-
ENSMUSC	Cplx4	complexir	GO:00452	synapse|c	K15295	complexir	mmu0472	Synaptic v	18:659557	5	0	0	0	0	0	0.181478	0	0	0	0	0	0.060493	0	0.090479	1	-
ENSMUSC	Pmaip1	phorbol-1	GO:00081	metabolic	K10131	phorbol-1	mmu0520	Pathways	18:664586	28	22	40	24	25	12	0.672515	0.442915	0.661845	0.375524	0.526539	0.357366	0.592425	0.419809	0.14023	1	-
ENSMUSC	Gnal	guanine n	GO:00048	signal trar	K04633	guanine n	mmu0474	Olfactory	18:670883	2239	2878	3670	3864	2909	1732	24.69974	26.61227	27.89047	27.76879	28.14003	23.69039	26.40083	26.53307	0.821407	1	-
ENSMUSC	Impa2	inositol (m	GO:00081	metabolic	K01092	myo-inos	mmu0110	Metabolic	18:672892	44	41	54	55	56	45	1.683532	1.314933	1.423339	1.370913	1.878863	2.134822	1.473934	1.794866	0.499449	1	-
ENSMUSC	Cidea	cell death	GO:00081	metabolic	-	-	-	-	18:673435	95	114	175	178	123	77	5.186881	5.21722	6.582215	6.33118	5.888839	5.212638	5.662106	5.810886	0.987258	1	-
ENSMUSC	Afg3l2	AFG3-like	GO:00325	developm	-	-	-	-	18:674047	1192	1456	1883	1856	1462	1141	24.55597	25.14174	26.72284	24.9081	26.41015	29.14423	25.47352	26.82083	0.928149	1	-
ENSMUSC	Srfbp1	serum res	GO:00081	metabolic	-	-	-	-	18:524656	270	373	427	401	325	109	11.72883	13.58168	12.77825	11.34796	12.37993	5.870869	12.69626	9.866254	0.160392	1	-
ENSMUSC	Lox	lysyl oxida	GO:00325	developm	-	-	-	-	18:525160	26	48	60	44	26	24	0.221621	0.342943	0.352318	0.244325	0.194333	0.253645	0.305627	0.230768	0.158572	1	-
ENSMUSC	Prelid3a	PRELI dor	GO:00511	localizatio	-	-	-	-	18:674648	285	365	458	503	340	532	8.358371	8.972684	9.25324	9.610074	8.743775	19.34528	8.861432	12.56638	0.104526	1	-
ENSMUSC	Spire1	spire hom	GO:00325	developm	-	-	-	-	18:674882	3405	4040	5522	5340	4100	2260	13.30207	13.22928	14.86106	13.59017	14.04522	10.94703	13.79747	12.86081	0.42697	1	-
ENSMUSC	Sncaip	synuclein,	GO:00081	metabolic	K04558	synuclein,	mmu0501	Parkinson	18:527677	119	128	207	184	145	87	0.859117	0.774583	1.0295	0.865377	0.917944	0.778774	0.887733	0.854031	0.668907	1	-
ENSMUSC	Snx24	sorting ne	GO:00511	localizatio	-	-	-	-	18:532456	137	149	196	197	154	170	3.493878	3.185128	3.443462	3.272916	3.443914	5.375559	3.374156	4.030796	0.461943	1	-
ENSMUSC	Psmg2	proteasor	GO:00329	macromo	-	-	-	-	18:676415	113	144	142	147	127	116	6.781369	7.243623	5.870577	5.746972	6.68325	8.631442	6.631856	7.020555	0.963575	1	-
ENSMUSC	Ppic	peptidylpi	GO:00081	metabolic	-	-	-	-	18:534063	30	37	46	56	46	40	1.482588	1.532683	1.566032	1.802864	1.993384	2.450983	1.527101	2.08241	0.198735	1	-
ENSMUSC	Ptpn2	protein ty	GO:00325	developm	K18026	tyrosine-p	mmu0463	Jak-STAT	18:676655	371	512	592	549	488	219	5.824642	6.737811	6.402783	5.614997	6.718278	4.2631	6.321745	5.532125	0.310136	1	-
ENSMUSC	Cep192	centroson	GO:00081	metabolic	-	-	-	-	18:678001	185	219	293	319	224	123	0.759025	0.753149	0.82814	0.852622	0.805889	0.625714	0.780105	0.761409	0.747869	1	-
ENSMUSC	Ldlrad4	low densi	GO:00325	developm	-	-	-	-	18:679332	459	429	603	726	514	257	11.7011	9.166941	10.58967	12.0568	11.48999	8.12332	10.4859	10.5567	0.896749	1	-
ENSMUSC	Setbp1	SET bindir	GO:00056	cell|intrac	-	-	-	-	18:787503	1449	1585	2102	2368	1694	864	9.252314	8.483289	9.246262	9.850207	9.485019	6.840402	8.993955	8.72521	0.674386	1	-
ENSMUSC	Slc14a2	solute car	GO:00511	localizatio	-	-	-	-	18:781469	4	11	7	13	6	9	0.075297	0.173579	0.090783	0.15943	0.099036	0.210062	0.11322	0.156176	0.503202	1	-
ENSMUSC	Galr1	galanin re	GO:00048	signal trar	K04230	galanin re	mmu0408	Neuroacti	18:823924	10	18	14	12	4	6	0.221364	0.333989	0.213488	0.173054	0.077638	0.164694	0.25628	0.138462	0.060158	1	-
ENSMUSC	Me2	malic enz	GO:00081	metabolic	-	-	mmu0120	Carbon m	18:737700	359	415	500	536	424	294	9.2935	9.005042	8.916736	9.039216	9.624826	9.436653	9.071759	9.366898	0.970571	1	-
ENSMUSC	Mapk4	mitogen-a	GO:00048	signal trar	K06855	mitogen-a	mmu0465	IL-17 sign	18:739284	974	486	1335	702	1097	773	8.284346	3.46488	7.822254	3.889718	8.181791	8.152005	6.523827	6.741171	0.948113	1	-
ENSMUSC	Cxxc1	CXXC fing	GO:00081	metabolic	-	-	-	-	18:742161	763	891	1085	1144	949	700	18.52213	18.12998	18.14462	18.09148	20.20111	21.06931	18.26557	19.7873	0.749244	1	-
ENSMUSC	Mbd1	methyl-Cp	GO:00056	cell|intrac	-	-	-	-	18:742676	868	1071	1261	1299	1079	776	6.587533	6.81311	6.592796	6.422349	7.180708	7.302149	6.66448	6.968402	0.969532	1	-
ENSMUSC	Smad2	SMAD far	GO:00325	developm	K04500	mothers a	mmu0520	Pathways	18:762415	260	290	336	351	246	174	3.147992	2.943138	2.802529	2.768521	2.611787	2.612134	2.964553	2.664147	0.326574	1	-
ENSMUSC	Sall3	sal-like 3	GO:00325	developm	-	-	-	-	18:809663	109	135	150	161	138	104	1.005993	1.04437	0.953702	0.968004	1.116836	1.190112	1.001355	1.09165	0.784726	1	-
ENSMUSC	Atp9b	ATPase, cl	GO:00511	localizatio	-	-	-	-	18:807341	700	905	1401	1442	918	982	2.857965	3.097137	3.940476	3.83536	3.286582	4.971146	3.298526	4.031029	0.258642	1	-
ENSMUSC	Rbfa	ribosome	GO:00081	metabolic	-	-	-	-	18:801922	381	472	520	572	454	373	17.58438	18.25986	16.53321	17.19807	18.37389	21.34505	17.45915	18.97234	0.766352	1	-
ENSMUSC	Csnk1a1	casein kin	GO:00325	developm	K08957	casein kin	mmu0516	Human pa	18:615552	4768	6144	7355	7590	6023	3965	18.58795	20.07702	19.75283	19.27608	20.58973	19.16568	19.4726	19.67717	0.822027	1	-
ENSMUSC	Il17b	interleukir	GO:00400	locomotic	K05490	interleukir	mmu0406	Cytokine-	18:616879	6	5	4	4	1	0	0.551061	0.384886	0.253101	0.239344	0.080527	0	0.396349	0.106624	0.044836	1	-
ENSMUSC	Pcyox1l	prenylcyst	GO:00081	metabolic	-	-	-	-	18:616968	355	403	470	490	362	416	7.394737	7.036416	6.744401	6.649229	6.612196	10.74419	7.058518	8.001871	0.626669	1	-
ENSMUSC	Grpel2	GrpE-like	GO:00511	localizatio	-	-	-	-	18:617124	289	341	468	465	350	295	4.557517	4.507523	5.084266	4.777113	4.839965	5.768179	4.716435	5.128419	0.765989	1	-
ENSMUSC	Napg	N-ethylm	GO:00511	localizatio	-	-	-	-	18:629778	3052	3996	4586	4874	3654	2518	30.53106	33.507	31.60405	31.76321	32.05299	31.23193	31.8807	31.68271	0.707338	1	-
ENSMUSC	Txnl1	thioredox	GO:00081	metabolic	-	-	-	-	18:636628	829	1101	1335	1397	949	822	20.59633	22.92851	22.84906	22.61068	20.67495	25.32174	22.12463	22.86912	0.948861	1	-
ENSMUSC	Nars	asparagin	GO:00081	metabolic	K01893	asparagin	mmu0097	Aminoacy	18:644996	3902	4749	5939	5827	4458	2908	94.2188	96.11811	98.79046	91.65937	94.39152	87.06241	96.37579	91.03777	0.425302	1	-
ENSMUSC	Fech	ferrochela	GO:00081	metabolic	K01772	ferrochela	mmu0110	Metabolic	18:644565	1092	1333	1572	1720	1226	756	23.92274	24.47775	23.72425	24.54701	23.55166	20.53506	24.04158	22.87791	0.517781	1	-
ENSMUSC	Nedd4l	neural pre	GO:00325	developm	K13305	E3 ubiquit	mmu0414	Endocytos	18:648877	3027	2598	3327	5797	2582	3241	9.090156	6.539595	6.882768	11.3408	6.799199	12.06768	7.504173	10.06923	0.104746	1	-
ENSMUSC	Lmnb1	lamin B1 [	GO:00081	metabolic	K07611	lamin B[-]	mmu0421	Apoptosis	18:567078	185	255	273	294	226	177	3.668422	4.238389	3.729252	3.797843	3.929695	4.351797	3.878687	4.026445	0.984154	1	-
ENSMUSC	C330018D	RIKEN cDI	GO:00081	metabolic	-	-	-	-	18:569558	81	122	173	148	145	90	1.445197	1.824549	2.126369	1.720224	2.268573	1.991006	1.798705	1.993268	0.721212	1	-
ENSMUSC	Megf10	multiple E	GO:00325	developm	-	-	-	-	18:571330	549	582	933	813	618	462	4.428866	3.935465	5.185053	4.2726	4.371709	4.621126	4.516461	4.421812	0.651316	1	-
ENSMUSC	Prrc1	proline-ri	GO:00056	cell|intrac	-	-	-	-	18:573547	333	366	512	490	377	293	4.408069	4.061055	4.669023	4.225536	4.376116	4.809041	4.379382	4.470231	0.886105	1	-
ENSMUSC	Slc12a2	solute car	GO:00325	developm	K10951	solute car	mmu0497	Pancreatic	18:578786	1456	1419	2607	2014	1453	1235	14.19221	11.59375	17.50576	12.7888	12.4193	14.92594	14.43057	13.37801	0.375097	1	-
ENSMUSC	Fbn2	fibrillin 2 [	GO:00325	developm	-	-	-	-	18:580086	127	143	233	252	166	134	0.770153	0.726882	0.973379	0.995538	0.882727	1.00755	0.823472	0.961938	0.468186	1	-
ENSMUSC	Slc27a6	solute car	GO:00081	metabolic	K08749	solute car	mmu0493	Insulin res	18:585562	2	0	0	3	2	4	0.05116	0	0	0.049976	0.044849	0.12684	0.017053	0.073888	0.10459	1	-
ENSMUSC	Isoc1	isochorism	GO:00081	metabolic	-	-	-	-	18:586594	190	205	302	276	220	286	4.711299	4.260834	5.158762	4.458396	4.783584	8.793051	4.710298	6.011677	0.285674	1	-
ENSMUSC	Dctn4	dynactin 4	GO:00511	localizatio	K10426	dynactin 4	mmu0501	Huntingto	18:605261	1723	2033	2511	2655	1995	1302	7.724452	7.639635	7.754978	7.754051	7.842744	7.237349	7.706355	7.611381	0.688396	1	-
ENSMUSC	Rbm22	RNA bind	GO:00081	metabolic	K12872	pre-mRN	mmu0304	Spliceoso	18:605607	665	791	895	1088	737	557	6.357216	6.338325	5.894131	6.775724	6.178104	6.602162	6.196557	6.518663	0.921553	1	-
ENSMUSC	Rps14	ribosomal	GO:00081	metabolic	K02955	small subu	mmu0301	Ribosome	18:607745	470	627	860	639	606	1590	14.75788	16.50236	18.60267	13.07098	16.68556	61.90257	16.62097	30.55304	0.055372	1	-
ENSMUSC	Cd74	CD74 anti	GO:00325	developm	K06505	CD74 anti	mmu0516	Herpes sir	18:608035	55	81	121	115	63	70	1.745075	2.154238	2.6448	2.377032	1.75283	2.275386	2.181371	2.294574	0.98284	1	-
ENSMUSC	Tcof1	treacle rib	GO:00325	developm	K14562	Treacher (	mmu0300	Ribosome	18:608137	475	510	669	675	551	406	4.288018	3.859101	4.160456	3.969617	4.36172	4.544387	4.102525	4.291908	0.972935	1	-

ENSMUS(	Tmx3	thioredox	GO:00081	metabolic	-	-	-	-	18:905101	1053	1472	1763	1577	1281	992	14.5134	17.00597	16.73959	14.15971	15.4822	16.9527	16.08632	15.53154	0.530379		1	-
ENSMUS(	Camk2a	calcium/ca	GO:00325	developm	K04515	calcium/ca	mmu0474	Olfactory	18:609256	43383	47122	58774	67349	52465	47918	246.1934	224.1473	229.7701	248.983	261.0774	337.1642	233.3703	282.4082	0.201978		1	-
ENSMUS(	Cdx1	caudal typ	GO:00325	developm	-	-	-	-	18:610188	0	0	0	2	0	0	0	0	0	0.04731	0	0	0	0.01577	0.537734		1	-
ENSMUS(	Pdgfrb	platelet de	GO:00325	developm	K05089	platelet-d	mmu0520	Pathways	18:610451	355	536	685	642	598	442	2.935	3.714473	3.901406	3.457768	4.335336	4.530929	3.516959	4.108011	0.417582		1	-
ENSMUS(	Csf1r	colony stir	GO:00325	developm	K05090	macropha	mmu0520	Pathways	18:611055	1216	1499	1824	2172	1491	1484	13.18104	13.6198	13.62048	15.33761	14.17217	19.94509	13.47377	16.48496	0.199357		1	-
ENSMUS(	Hmgxb3	HMG box	GO:00056	cell|intrace	-	-	-	-	18:611312	854	971	1170	1197	882	891	10.63993	10.14034	10.04195	9.715309	9.635894	13.76399	10.27407	11.0384	0.851567		1	-
ENSMUS(	Gnaq	guanine n	GO:00325	developm	K04634	guanine n	mmu0520	Pathways	19:161328	5544	6681	8212	8676	6473	4235	53.00706	53.54326	54.08923	54.03954	54.26986	50.20532	53.54652	52.83824	0.675433		1	-
ENSMUS(	Psat1	phosphos	GO:00081	metabolic	K00831	phosphos	mmu0110	Metabolic	19:159046	1438	1505	2310	2169	1478	901	13.13441	11.52235	14.535	12.90604	11.83774	10.20382	13.06392	11.6492	0.267615		1	-
ENSMUS(	Tle4	transducir	GO:00081	metabolic	-	-	-	-	19:144480	919	1056	1325	1429	1131	846	8.406043	8.096425	8.349174	8.515109	9.071552	9.594729	8.283881	9.060463	0.678128		1	-
ENSMUS(	Cndp2	CNDP dip	GO:00081	metabolic	K08660	cytosolic r	mmu0110	Metabolic	18:846674	674	722	882	906	727	811	19.39101	17.41128	17.48077	16.98049	18.34076	28.92992	18.09435	21.41705	0.417799		1	-
ENSMUS(	Timm21	translocas	GO:00511	localizatio	-	-	-	-	18:849472	100	145	149	127	122	101	4.832926	5.873966	4.960746	3.998474	5.170254	6.052235	5.222546	5.073654	0.653097		1	-
ENSMUS(	Cyb5a	cytochron	GO:00081	metabolic	-	-	-	-	18:848513	296	476	472	463	354	368	5.449505	7.345567	5.986309	5.553005	5.714936	8.400394	6.260461	6.556111	0.988667		1	-
ENSMUS(	Cbln2	cerebellin	GO:00325	developm	-	-	-	-	18:867111	66	120	160	151	70	138	0.69595	1.060645	1.162272	1.037277	0.647252	1.804268	0.972955	1.162932	0.616638		1	-
ENSMUS(	Slc22a6	solute car	GO:00511	localizatio	-	-	-	-	19:861799	73	115	130	117	105	91	1.152357	1.52165	1.413698	1.203176	1.453431	1.781108	1.362568	1.479238	0.867888		1	-
ENSMUS(	Asrgl1	asparagin	GO:00081	metabolic	-	-	-	-	19:910986	3876	4748	5933	5966	4681	3243	54.55846	56.01985	57.5313	54.70704	57.77762	56.59933	56.03654	56.36133	0.779778		1	-
ENSMUS(	Anxa1	annexin A	GO:00325	developm	-	-	-	-	19:203734	27	43	62	60	22	45	0.455874	0.608565	0.721151	0.659965	0.32573	0.942067	0.595197	0.642587	0.950636		1	-
ENSMUS(	Incenp	inner cent	GO:00990	supramole	-	-	-	-	19:987229	216	289	356	379	274	175	4.267164	4.785596	4.844923	4.877596	4.746555	4.286561	4.632561	4.636904	0.82766		1	-
ENSMUS(	Fth1	ferritin he	GO:00081	metabolic	K00522	ferritin he	mmu0421	Necropto	19:998270	25075	27727	38484	35977	26664	20436	1840.175	1705.587	1945.583	1719.986	1715.877	1859.517	1830.449	1765.126	0.490868		1	-
ENSMUS(	Rab3il1	RAB3A int	GO:00511	localizatio	-	-	-	-	19:100016	136	154	210	211	135	147	1.115689	1.058949	1.186786	1.127628	0.971132	1.495223	1.120475	1.197994	0.918751		1	-
ENSMUS(	Fads3	fatty acid	GO:00081	metabolic	-	-	-	-	19:100415	407	491	641	569	456	426	7.912539	8.0012	8.584816	7.20635	7.773722	10.26873	8.166185	8.416266	0.907019		1	-
ENSMUS(	Fads2	fatty acid	GO:00081	metabolic	K10226	acyl-CoA	mmu0332	PPAR sign	19:100641	2330	2589	3000	3250	2339	1319	98.19531	91.45758	87.09785	89.22773	86.43859	68.92323	92.25025	81.52985	0.238053		1	-
ENSMUS(	Tmem138	transmem	GO:00099	cellular pr	-	-	-	-	19:105704	37	66	85	78	61	78	1.478897	2.211234	2.340499	2.031022	2.138031	3.865644	2.01021	2.678233	0.306119		1	-
ENSMUS(	Tmem216	transmem	GO:00329	macromo	-	-	-	-	19:105338	49	43	52	66	50	54	0.904214	0.66511	0.661044	0.793417	0.809072	1.235539	0.743456	0.946009	0.381488		1	-
ENSMUS(	Sdhaf2	succinate	GO:00325	developm	-	-	-	-	19:105005	489	622	758	728	543	497	10.03794	10.70234	10.71904	9.735271	9.774115	12.64962	10.48644	10.71967	0.86486		1	-
ENSMUS(	Cd5	CD5 antig	GO:00511	localizatio	K06455	T-cell surf	mmu0464	Hematop	19:107181	1	1	0	0	0	2	0.030723	0.025746	0	0	0	0.076192	0.018823	0.025397	1		1	-
ENSMUS(	Cd6	CD6 antig	GO:00511	localizatio	K06456	T-cell surf	mmu0451	Cell adhes	19:107893	8	7	11	3	5	4	0.083495	0.061238	0.079091	0.020396	0.045758	0.051765	0.074608	0.039306	0.13248		1	-
ENSMUS(	Ms4a7	membran	GO:00160	membran	-	-	-	-	19:113210	11	13	24	27	10	22	0.12576	0.12458	0.189017	0.201084	0.100252	0.311859	0.146452	0.204398	0.419135		1	-
ENSMUS(	Ms4a1	membran	GO:00023	immune s	K06466	B-lympho	mmu0464	Hematop	19:112496	2	0	2	0	0	0	0.01516	0	0.010443	0	0	0	0.008534	0	0.156899		1	-
ENSMUS(	Ms4a4c	membran	GO:00160	membran	-	-	-	-	19:114047	0	0	0	3	0	2	0	0	0	0.053808	0	0.068302	0	0.040703	0.088802		1	-
ENSMUS(	Ms4a6b	membran	GO:00160	membran	-	-	-	-	19:115165	17	14	25	22	28	16	0.131512	0.090786	0.133233	0.110876	0.189945	0.153474	0.118511	0.151431	0.452067		1	-
ENSMUS(	Ms4a4d	membran	GO:00160	membran	-	-	-	-	19:115368	0	0	0	0	2	2	0	0	0	0	0.073661	0.104191	0	0.059284	0.156325		1	-
ENSMUS(	Ms4a6d	membran	GO:00160	membran	-	-	-	-	19:115866	15	13	36	43	27	21	0.410553	0.298251	0.678785	0.766705	0.648007	0.712644	0.46253	0.709119	0.143931		1	-
ENSMUS(	Mrpl16	mitochon	GO:00081	metabolic	K02878	large subu	mmu0301	Ribosome	19:117704	195	239	269	212	211	189	11.0453	11.34732	10.49657	7.822758	10.48017	13.27371	10.96306	10.52554	0.578373		1	-
ENSMUS(	Osbp	oxysterol	GO:00511	localizatio	-	-	-	-	19:119658	1292	1637	1868	1950	1505	1157	17.93588	19.04854	17.86441	17.63504	18.32059	19.91497	18.28295	18.62353	0.851879		1	-
ENSMUS(	Fam111a	family wit	GO:00081	metabolic	-	-	-	-	19:125457	47	71	71	103	50	38	0.293446	0.371569	0.305381	0.418937	0.273744	0.294175	0.323465	0.328952	0.978571		1	-
ENSMUS(	Zfp91	zinc finge	GO:00081	metabolic	-	-	-	-	19:127636	2935	3784	4890	4850	3507	2056	17.44394	18.85127	20.02151	18.77846	18.27743	15.15115	18.77224	17.40234	0.390395		1	-
ENSMUS(	Lpxn	leupaxin [	GO:00081	metabolic	-	-	-	-	19:127986	19	18	29	29	12	18	0.720899	0.572466	0.758003	0.716811	0.399253	0.846822	0.68379	0.654295	0.755829		1	-
ENSMUS(	Gna14	guanine n	GO:00048	signal trar	K04636	guanine n	mmu0402	Calcium si	19:164356	17	17	11	13	15	16	0.319354	0.267695	0.142355	0.1591	0.247095	0.372683	0.243135	0.259626	1		1	-
ENSMUS(	Rfk	riboflavin	GO:00081	metabolic	K00861	riboflavin	mmu0110	Metabolic	19:173940	968	1236	1487	1374	1021	933	17.68813	18.93119	18.71841	16.35592	16.35969	21.13851	18.44591	17.95137	0.557243		1	-
ENSMUS(	Pcsk5	proprotei	GO:00325	developm	-	-	-	-	19:174328	270	304	408	468	348	249	1.836003	1.732756	1.911268	2.073186	2.075065	2.099406	1.826676	2.082552	0.498304		1	-
ENSMUS(	Ostf1	osteoclast	GO:00055	extracellul	-	-	-	-	19:185793	345	398	544	541	343	252	3.925135	3.795518	4.26369	4.00972	3.421936	3.554847	3.994781	3.662168	0.380475		1	-
ENSMUS(	Carnmt1	carnosine	GO:00081	metabolic	K19787	carnosine	mmu0034	Histidine r	19:186707	66	59	101	87	71	29	2.006932	1.50382	2.115737	1.723413	1.893165	1.093375	1.875497	1.569984	0.355813		1	-
ENSMUS(	Trpm6	transient r	GO:00081	metabolic	K04981	transient r	mmu0497	Mineral ab	19:187499	10	11	10	20	19	7	0.097655	0.090044	0.067274	0.127236	0.162695	0.08476	0.084991	0.124897	0.265543		1	-
ENSMUS(	Ms4a8a	membran	GO:00160	membran	-	-	-	-	19:110674	0	3	0	0	0	1	0	0.083318	0	0	0	0.041073	0.027773	0.013691	0.668406		1	-
ENSMUS(	Ccdc86	coiled-co	GO:00056	cell|intrace	-	-	-	-	19:109414	134	208	209	251	189	163	1.197428	1.557976	1.286595	1.461164	1.480976	1.806001	1.347333	1.582713	0.450355		1	-
ENSMUS(	Prpf19	pre-mRN	GO:00325	developm	K10599	pre-mRN	mmu0412	Ubiquitin	19:108952	2111	2556	3071	3034	2524	2128	13.58174	13.78417	13.61126	12.71641	14.23964	16.97558	13.65906	14.64388	0.827256		1	-
ENSMUS(	Tmem132	transmem	GO:00055	extracellul	-	-	-	-	19:108578	2103	2796	3247	3202	2710	2973	22.9682	25.59634	24.42988	22.78194	25.95373	40.25953	24.33147	29.66506	0.277683		1	-
ENSMUS(	Slc15a3	solute car	GO:00511	localizatio	-	-	-	-	19:108397	32	37	79	90	57	44	0.27939	0.270783	0.475164	0.511906	0.436395	0.476324	0.341779	0.474875	0.180531		1	-
ENSMUS(	Ddb1	damage s	GO:00081	metabolic	K10610	DNA dam	mmu0520	Viral carci	19:106056	3438	4047	5144	5286	3971	3472	52.5861	51.88612	54.20227	52.67128	53.26079	65.8462	52.8915	57.25942	0.749942		1	-
ENSMUS(	Fen1	flap struct	GO:00081	metabolic	K04799	flap endo	mmu0303	DNA repli	19:101991	152	192	198	229	189	167	1.378825	1.459888	1.237322	1.353268	1.503383	1.878315	1.358679	1.578322	0.509458		1	-
ENSMUS(	Syt7	synaptota	GO:00452	synapse|lc	-	-	-	-	19:103890	2090	6070	7329	6948	2783	4211	10.11312	24.61958	24.43066	21.90184	11.80851	25.26447	19.72112	19.65827	0.823629		1	-
ENSMUS(	Zfand5	zinc finge	GO:00325	developm	-	-	-	-	19:212722	1835	2180	2783	2873	2033	1816	13.46027	13.40377	14.06314	13.72888	13.07669	16.51656	13.64239	14.44071	0.904633		1	-
ENSMUS(	Tmem2	transmem	GO:00325	developm	-	-	-	-	19:217783	309	363	457	494	361	285	2.945532	2.900445	3.001051	3.06771	3.017554	3.368503	2.94901	3.151256	0.848719		1	-
ENSMUS(	Rtn3	reticulon 3	GO:00511	localizatio	K20723	reticulon-	mmu0501	Alzheimer	19:742590	13642	17691	21062	21383	15939	12023	133.3421	144.9421	141.8209	136.1568	136.6133	145.7095	140.035	139.4932	0.694846		1	-
ENSMUS(	Atl3	atlastin G	GO:00038	catalytic a	-	-	-	-	19:749404	654	697	937	979	682	601	6.205385	5.543402	6.124663	6.051394	5.674376	7.070518	5.957817	6.265429	0.953079		1	-
ENSMUS(	Naa40	N(alpha)-	GO:00081	metabolic	-	-	-	-	19:722566	372	434	487	582	424	465	7.218844	7.059397	6.51036	7.357482	7.214946	11.18829	6.929534	8.586906	0.257713		1	-
ENSMUS(	Lipo3	lipase, me	GO:00081	metabolic	-	-	-	-	19:335177	88	64	96	108	64	62	1.026559	0.625794	0.771472	0.820738	0.65467	0.896767	0.807942	0.790725	0.76164		1	-
ENSMUS(	Otub1	OTU dom	GO:00081	metabolic	-	-	-	-	19:719820	2644	3703	3948	4066	3165	3126	42.57263	49.97762	43.79228	42.6499	44.68743	62.40852	45.44751	49.91528	0.706829		1	-
ENSMUS(	Cdc42bpg	CDC42 bi	GO:00081	metabolic	-	-	-	-	19:630645	117	165	205	198	153	144	1.179329	1.394081	1.423499	1.300166	1.352341	1.799694	1.332303	1.484067	0.704589		1	-
ENSMUS(	Lipn	lipase, fan	GO:00081	metabolic	-	-	-	-	19:340673	0	0	0	2	0	0	0	0	0	0.037446	0	0	0	0.012482	0.537174		1	-
ENSMUS(	Ehd1	EH-doma	GO:00325	developm	-	-	-	-	19:627672	1334	1831	2051	2407	1697	1261	16.55208	19.04311	17.53128	19.45603	18.4638	19.39981	17.70882	19.10655	0.750143		1	-
ENSMUS(	Atg2a	autophag	GO:00081	metabolic	-	-	-	-	19:624166	909	979	1214	1465	1041	929	6.08743	5.495479	5.600669	6.391301	6.113124	7.71386	5.727859	6.739428	0.328044		1	-
ENSMUS(	Ankrd22	ankyrin re	-	-	-	-	-	-	19:341225	0	0	2	0	0	0	0	0	0.033864	0	0	0	0.011288	0	0.53597		1	-
ENSMUS(	Stambpl1	STAM bin	GO:00081	metabolic	-	-	-	-	19:341922	299	341	412	434	319	215	2.935629	2.806321	2.78663	2.775887	2.746398	2.617307	2.84286	2.713197	0.560024		1	-
ENSMUS(	Ppp2r5b	protein pl	GO:00325	developm	-	-	-	-	19:622776	1645	2019	2344	2467	1873	2001	29.05633	29.89264	28.52227	28.38741	29.01051	43.82359	29.15708	33.7405	0.449942		1	-
ENSMUS(	Fas	Fas (TNF r	GO:00325	developm	K04390	tumor nec	mmu0520	Pathways	19:342906	17	22	39	25	20	15	0.599875	0.650724	0.948072	0.574699	0.618878	0.656307	0.73289	0.616628	0.431234		1	-
ENSMUS(	Cdc37l1	cell divisic	GO:00099	cellular pr	-	-	-	-	19:289903	2312	2641	3762	3676	2334	1635	8.485971	8.125214	9.512262	8.789634	7.512029	7.440759	8.707816	7.914141	0.293008		1	-
ENSMUS(	Lipa	lysosomal	GO:00325	developm	K01052	lysosomal	mmu0414	Lysosome	19:344923	377	421	677	567	394	380	7.794237	7.295702	9.642114	7.636541	7.142844	9.740973	8.244017	8.173453	0.70963		1	-
ENSMUS(	Ak3	adenylate	GO:00081	metabolic	K00944	nucleoside	mmu0023	Purine me	19:290208	969	1141	1661	1404	1079	915	18.58825	18.3465	21.95006	17.54541	18.15009	21.76314	19.62827	19.15288	0.586427		1	-
ENSMUS(	Rcl1	RNA term	GO:00081	metabolic	K11108	RNA 3'-te	mmu0300	Ribosome	19:291013	182	204	230	283	218	230	6.415228	6.027315	5.584954	6.498439	6.738145	10.05205	6.009166	7.762877	0.193355		1	-
ENSMUS(	Majin	membran	GO:00511	localizatio	-	-	-	-	19:618438	0	0	2	1	0	0	0	0	0.035657	0.016855	0	0	0.011886	0.005618	1		1	-
ENSMUS(	Snx15	sorting ne	GO:00511	localizatio	-	-	-	-	19:611939	345	396	473	505	423	384	6.899247	6.637894	6.516206	6.578925	7.417629	9.52138	6.684449	7.839311	0.419575		1	-
ENSMUS(	Jak2	Janus kina	GO:00325	developm	K04447	Janus kina	mmu0520	Pathways	19:292518	888	1039	1474	1436	1055	509	11.2197	11.00365	12.82972	11.81964	11.68862	7.973919	11.68435	10.49406	0.365293		1	-
ENSMUS(	Sac3d1	SAC3 don	-	-	-	-	-	-	19:611600	74	83	113	113	116	140	3.422774	3.217959	3.600644	3.404951	4.704903	8.029022	3.413792	5.379625	0.057		1	-
ENSMUS(	Cdca5	cell divisic	GO:00081	metabolic	K17390	sororin[-]	mmu0520	MicroRNA	19:608498	1	3	6	0	3	2	0.02969	0.07464	0.122688	0	0.078082	0.07363	0.075673	0.050571	0.661945		1	-
ENSMUS(	Zfpl1	zinc finge	GO:00511	localizatio	-	-	-	-	19:608076	261	392	317	393	342	272	3.89465	4.903065	3.258662	3.820347	4.475043	5.032486	4.018792	4.442626	0.717409		1	-
ENSMUS(	Tnfrsf25	tumor nec	GO:00325	developm	K05160	tumor nec	mmu0406	Cytokine-	4:1521159	226	276	276	367	238	227	3.90087	3.993135	3.281801	4.126667	3.602233	4.858072	3.725268	4.195657	0.614429		1	-
ENSMUS(	Kif20b	kinesin fai	GO:00325	developm	-	-	-	-	19:349223	26	28	32	48	22	11	0.182164	0.164432	0.154448	0.21908	0.135161	0.095559	0.167014	0.149933	0.671907		1	-
ENSMUS(	Vps51	VPS51 GA	GO:00081	metabolic	-	-	-	-	19:606784	586	763	842	910	815	779	4.063064	4.43439	4.021796	4.110356	4.955147	6.696991	4.173084	5.254165	0.186506		1	-
ENSMUS(	Htr7	5-hydroxy	GO:00048	signal trar	K04163	5-hydroxy	mmu0408	Neuroacti	19:359587	84	210	86	57	142	111	0.550127	1.152809	0.388005	0.243186	0.815487	0.901352	0.69698	0.653342	0.706028		1	-
ENSMUS(	Tm7sf2	transmem	GO:00081	metabolic	K00222	Delta14-s	mmu0110	Metabolic	19:606282	113	126	150	166	151	186	1.006231	0.940467	0.920161	0.962961	1.179067	2.053619	0.95562	1.398549	0.090149		1	-
ENSMUS(	Rpp30	ribonuclea	GO:00081	metabolic	K03539	ribonuclea	mmu0301	RNA trans	19:360837	95	157	205	167	110	113	5.227299	7.241157	7.770704	5.986228	5.307476	7.709332	6.746386	6.334345	0.525633		1	-
ENSMUS(	Pcgf5	polycomb	GO:00081	metabolic	K11489	polycomb	mmu0455	Signaling	19:363483	166	303	322	90	323	184	0.710663	1.087307	0.94965	0.251005	1.212556	0.976695	0.915873	0.813418	0.557392		1	-
ENSMUS(	Syvn1	synovial a	GO:00081	metabolic	K10601	E3 ubiquit	mmu0414	Protein pr	19:604657	840	979	1278	1146	939	788	4.883773	4.771027	5.118688	4.340526	4.78723	5.680522	4.924496	4.936093	0.73745		1	-
ENSMUS(	Il33	interleukir	GO:00325	developm	K12967	interleukir	mmu0421	Necropto	19:299251	606	214	865	844	620	446	7.327458	2.168937	7.205235	6.64821	6.573778	6.686539	5.56721	6.636176	0.558741		1	-
ENSMUS(	Tnks2	tankyrase	GO:00325	developm	-	-	-	-	19:368342	3538	4204	5410	5601	3998	2613	18.64742	18.57276	19.64308	19.23127	18.47763	17.076	18.95442	18.26164	0.541509		1	-
ENSMUS(	Tjp2	tight junct	GO:00325	developm	K06098	tight junct	mmu0453	Tight junc	19:240945	696	629	1078	953	707	505	9.634695	7.298477	10.28014	8.594156	8.58205	8.667745	9.071104	8.61465	0.516445		1	-
ENSMUS(	Trpd52l3	tumor prc	-	-	-	-	-	-	19:300037	2	0	0	4	0	0	0.056984	0	0	0.074239	0	0	0.018995	0.024746	1		1	-
ENSMUS(	Frmd8	FERM dor	GO:00056	cell|intrace	-	-	-	-	19:584970	177	171	297	328	200	330	1.911126	1.54762	2.20914	2.307123	1.893599	4.417899	1.889295	2.872874	0.064931		1	-
ENSMUS(	Uhrf2	ubiquitin-	GO:00325	developm	-	-	-	-	19:300305	1005	1318	1690	1697	1297	737	5.487649	6.03238	6.357092	6.036484	6.210166	4.989691	5.95904	5.745447	0.605252		1	-
ENSMUS(	Slc25a45	solute car	GO:00511	localizatio	-	-	-	-	19:587780	55	49	57	83	65	48	0.66013	0.492965	0.471302	0.64898	0.684111	0.714326	0.541466	0.682472	0.32528		1	-
ENSMUS(	Rad9a	RAD9 che	GO:00081	metabolic	-	-	-	-	19:419519	104	109	152	165	102	105	3.23045	2.837978	3.252572	3.338837	2.778269	4.043932	3.107	3.387013	0.815656		1	-
ENSMUS(	Dpf2	D4, zinc a	GO:00081	metabolic	-	-	-	-	19:589651	763	949	1173	1179	783	706	5.567911	5.804793	5.896813	5.604843	5.010401	6.38791	5.756505	5.667718	0.644005		1	-
ENSMUS(	Gldc	glycine de	GO:00081	metabolic	-	-	mmu0110	Metabolic	19:300984	815	922	1246	1294	1069	774	13.79747	13.08355	14.53152	14.27111	15.86946	16.24684	13.80418	15.46247	0.545491		1	-
ENSMUS(	Mrpl21	mitochon	GO:00081	metabolic	K02888	large subu	mmu0301	Ribosome	19:328290	258	284	303	306	272	239	5.429364	5.009576	4.392621	4.195008	5.019278	6.236096	4.943854	5.150127	0.974368		1	-
ENSMUS(	Rps6kb2	ribosomal	GO:00081	metabolic	-	-	-	-	19:415671	319	446	473	533	394	397	3.997121	4.684295	4.082907	4.350766	4.329082	6.167846	4.254775	4.949231	0.467441		1	-
ENSMUS(	Ighmbp2	immunog	GO:00038	catalytic a	-	-	-	-	19:326092	180	190	211	273	180	199	1.729069	1.529843	1.396288	1.70838	1.5162	2.37017	1.551733	1.864917	0.406771		1	-
ENSMUS(	Pola2	polymera	GO:00081	metabolic	K02321	DNA poly	mmu0110	Metabolic	19:594054	77	108	93	110	79	79	1.703896	2.003215	1.417703	1.585715	1.532927	2.167532	1.708271	1.762058	0.93481		1	-
ENSMUS(	Coro1b	coronin, a	GO:00325	developm	-	-	-	-	19:414861	1041	1250	1444	1808	1283	861	15.65886	15.76059	14.96332	17.71697	16.92306	16.05827	15.46092	16.89943	0.667298		1	-
ENSMUS(	Eif1ad	eukaryoti	GO:00081	metabolic	-	-	-	-	19:536681	313	390	472	423	349	305	8.05673	8.414588	8.36968	7.09312	7.877414	9.734222	8.280333	8.234919	0.714547		1	-
ENSMUS(	Chka	choline ki	GO:00081	metabolic	K14156	choline/et	mmu0110	Metabolic	19:385177	566	759	936	1015	768	647	1.855609	2.085762	2.113964	2.167793	2.207872	2.63003	2.018445	2.335232	0.412162		1	-
ENSMUS(	Banf1	barrier to	GO:00081	metabolic	-	-	-	-	19:536464	303	229	289	467	327	373	23.48365	14.87684	15.43018	23.57873	22.22352	35.84407	17.93022	27.21544	0.033135		1	-
ENSMUS(	Cst6	cystatin E/	GO:00325	developm	-	-	-	-	19:534470	16	29	31	25	25	30	0.25737	0.390998	0.343515	0.261967	0.352625	0.598334	0.330628	0.404309	0.654226		1	-
ENSMUS(	Aip	aryl-hydr	GO:00081	metabolic	-	-	-	-	19:411444	963	1128	1337	1348	1069	921	21.22842	20.84267	20.30366	19.35811	20.66387	25.17313	20.79158	21.7317	1		1	-
ENSMUS(	Pitpnm1	phosphati	GO:00511	localizatio	-	-	-	-	19:409999	2226	2739	2851	2974	2781	2312	13.78034	14.2128	12.1586	11.99382	15.09658	17.74634	13.38392	14.94558	0.603876		1	-
ENSMUS(	Sf3b2	splicing fa	GO:00081	metabolic	K12829	splicing fa	mmu0304	Spliceoso	19:527392	5275	6399	7454	7981	6158	3491	103.758	105.5028	101.0043	102.2676	106.2139	85.14025	103.4217	97.87393	0.482596		1	-
ENSMUS(	Pold4	polymera	GO:00081	metabolic	-	-	-	-	19:423189	91	136	133	169	109	137	6.355305	7.961374	6.398774	7.688885	6.675176	11.86314	6.905151	8.742401	0.318676		1	-
ENSMUS(	Pacs1	phosphof	GO:00511	localizatio	-	-	-	-	19:513368	1521	1865	2028	2384	1769	1248	22.27797	22.89697	20.46283	22.74754	22.72046	22.66455	21.87926	22.71085	0.998574		1	-
ENSMUS(	Cdk2ap2	CDK2-ass	GO:00990	supramole	-	-	-	-	19:409718	163	233	289	273	222	223	3.045018	3.648466	3.719209	3.322352	3.636618	5.165263	3.470897	4.041411	0.517549		1	-
ENSMUS(	Grk2	G protein-	GO:00325	developm	K00910	beta-adre	mmu0474	Olfactory	19:428600	3311	3019	3868	5634	4236	3004	25.41046	19.42089	20.4499	28.16773	28.50703	28.58507	21.76042	28.41994	0.063357		1	-
ENSMUS(	Klc2	kinesin lig	GO:00038	catalytic a	K10407	kinesin lig	mmu0513	Salmonell	19:510774	4141	4765	4650	5108	4705	2838	33.98411	32.77831	26.28906	27.30884	33.85892	28.87813	31.01716	30.0153	0.583107		1	-
ENSMUS(	Acy3	aspartoac	GO:00081	metabolic	-	-	-	-	19:398666	11	14	20	25	22	17	0.46452	0.495554	0.581822	0.687729	0.814651	0.890075	0.513965	0.797485	0.143306		1	-
ENSMUS(	Pip5k1b	phosphati	GO:00081	metabolic	K00889	1-phosph	mmu0110	Metabolic	19:242947	996	1033	1374	1457	1107	633	14.06327	12.22588	13.36489	13.40196	13.70621	11.08196	13.21801	12.73004	0.599169		1	-
ENSMUS(	Nudt8	nudix (nu	GO:00038	catalytic a	-	-	-	-	19:400062	81	76	121	137	93	83	1.318594	1.037038	1.35695	1.452879	1.327552	1.675294	1.237528	1.485242	0.432656		1	-
ENSMUS(	Rab1b	RAB1B, m	GO:00081	metabolic	K07875	Ras-relate	mmu0513	Legionellc	19:509920	1890	2186	2503	2794	2050	2040	65.45787	63.46045	59.71897	63.03883	62.25821	87.60243	62.8791	70.96649	0.544722		1	-
ENSMUS(	Doc2g	double C2	GO:00160	membran	-	-	-	-	19:400332	25	26	25	28	29	36	0.296647	0.258594	0.204352	0.216442	0.30174	0.529643	0.253197	0.349275	0.373822		1	-
ENSMUS(	Cnih2	cornichon	GO:00452	synapse|lc	-	-	-	-	19:509287	2763	3322	3678	4634	3187	4090	141.9539	143.0602	130.1756	155.0973	143.5791	260.5408	138.3966	186.4057	0.079019		1	-
ENSMUS(	Yif1a	Yip1 inter	GO:00511	localizatio	-	-	-	-	19:508853	107	126	168	183	95	142	4.554721	4.495724	4.926496	5.074693	3.546023	7.494653	4.65898	5.37179	0.652289		1	-
ENSMUS(	Cbwd1	COBW do	GO:00971	organic cy	-	-	-	-	19:249199	237	288	375	351	260	175	7.747964	7.891972	8.445448	7.475291	7.453431	7.093553	8.028461	7.340758	0.385535		1	-
ENSMUS(	Rin1	Ras and R	GO:00511	localizatio	K17638	Ras and R	mmu0401	Ras signal	19:505048	1496	1814	1847	2263	1735	1488	13.65829	13.88208	11.6167	13.45956	13.89014	16.84431	13.05236	14.73134	0.52745		1	-
ENSMUS(	Aldh3b1	aldehyde	GO:00081	metabolic	K00129	aldehyde	mmu0110	Metabolic	19:391349	48	66	90	116	69	80	1.626959	1.875127	2.101496	2.561382	2.050808	3.362096	1.86786	2.658095	0.109022		1	-
ENSMUS(	Asah2	N-acylsph	GO:00081	metabolic	K12349	neutral ce	mmu0110	Metabolic	19:319846	221	246	371	306	259	113	2.999836	2.798937	3.469202	2.705879	3.082816	1.901811	3.089325	2.563502	0.219046		1	-
ENSMUS(	Rce1	Ras conve	GO:00081	metabolic	K08658	prenyl prc	mmu0090	Terpenoic	19:462259	115	172	118	172	172	183	1.628114	2.041114	1.150857	1.586339	2.135293	3.212355	1.606695	2.311329	0.128976		1	-
ENSMUS(	Slc29a2	solute car	GO:00511	localizatio	-	-	-	-	19:502400	141	179	266	265	208	193	3.624978	3.857399	4.711089	4.43828	4.689149	6.152222	4.064489	5.093217	0.250005		1	-
ENSMUS(	Pcx	pyruvate c	GO:00081	metabolic	K01958	pyruvate c	mmu0110	Metabolic	19:451047	1048	1215	1537	1619	1308	1054	10.8705	10.56373	10.98282	10.93999	11.89703	13.55548	10.80568	12.13083	0.538981		1	-
ENSMUS(	Minpp1	multiple ir	GO:00081	metabolic	K03103	multiple ir	mmu0110	Metabolic	19:324857	695	842	1056	1051	819	749	16.86495	17.12637	17.6529	16.61441	17.42718	22.53555	17.21474	18.85905	0.718283		1	-
ENSMUS(	Apba1	amyloid b	GO:00325	developm	-	-	-	-	19:237588	4620	5251	6666	7040	5564	3450	44.27245	42.17805	44.00564	43.94869	46.75428	40.99177	43.48538	43.89825	0.828831		1	-
ENSMUS(	Papss2	3'-phosph	GO:00325	developm	K13811	3'-phosph	mmu0110	Metabolic	19:326200	382	477	513	568	487	274	6.678749	6.990417	6.178747	6.469371	7.466263	5.939753	6.615971	6.625129	0.825563		1	-
ENSMUS(	Cpt1a	carnitine p	GO:00325	developm	K08765	carnitine C	mmu0415	AMPK sig	19:332330	621	555	946	865	618	432	9.178238	6.875651	9.631862	8.328474	8.009378	7.916582	8.561917	8.084812	0.502669		1	-
ENSMUS(	Peli3	pellino 3 [	GO:00508	response	-	-	-	-	19:493065	409	463	565	682	449	341	4.351048	4.128617	4.140673	4.726468	4.188512	4.497918	4.206779	4.470966	0.86266		1	-
ENSMUS(	Mrpl11	mitochon	GO:00081	metabolic	K02867	large subu	mmu0301	Ribosome	19:496230	435	556	664	618	480	421	9.592494	10.27709	10.08699	8.877941	9.281678	11.51093	9.985523	9.890182	0.685228		1	-
ENSMUS(	Lao1	L-amino a	GO:00081	metabolic	K03334	L-amino-	mmu0110	Metabolic	4:1189615	5	0	8	3	0	0	0.100975	0	0.111296	0.039463	0	0	0.070757	0.013154	0.06138		1	-
ENSMUS(	Tesmin	testis expr	GO:00325	developm	-	-	-	-	19:338885	1	3	0	0	0	0	0.011166	0.02807	0	0	0	0	0.013079	0	0.160189		1	-
ENSMUS(	Mus81	MUS81 str	GO:00081	metabolic	K08991	crossover	mmu0346	Fanconi a	19:548234	195	232	292	337	248	243	1.682893	1.678274	1.736025	1.894673	1.876795	2.600245	1.699064	2.123905	0.231907		1	-
ENSMUS(	Gal	galanin [S	GO:00325	developm	-	-	-	-	19:340991	1	5	10	9	0	7	0.089278	0.374075	0.614918	0.523326	0	0.774741	0.359423	0.432689	0.908118		1	-
ENSMUS(	Ppp6r3	protein pl	GO:00081	metabolic	-	-	-	-	19:345492	1450	1677	2221	2264	1648	1155	6.315231	6.122195	6.663789	6.423613	6.293918	6.237196	6.367072	6.318242	0.709733		1	-
ENSMUS(	Efemp2	epiderma	GO:00099	cellular pr	-	-	-	-	19:547397	88	129	178	163	103	137	1.137882	1.398153	1.585562	1.373035	1.167866	2.196431	1.373866	1.579111	0.659651		1	-
ENSMUS(	Ctsw	cathepsin	GO:00081	metabolic	K08569	cathepsin	mmu0421	Apoptosis	19:546524	0	0	5	2	2	0	0	0	0.177687	0.067202	0.090462	0	0.059229	0.052555	1		1	-
ENSMUS(	Fibp	fibroblast	GO:00508	response	-	-	-	-	19:546061	193	291	378	368	256	311	1.798762	2.273324	2.426938	2.23432	2.092171	3.593862	2.166341	2.640118	0.372567		1	-
ENSMUS(	Fosl1	fos-like an	GO:00325	developm	K04502	fos-like an	mmu0516	HTLV-I int	19:544770	2	8	4	5	2	4	0.074477	0.249646	0.102605	0.121279	0.065289	0.184649	0.142243	0.123739	0.742065		1	-
ENSMUS(	Lrp5	low densit	GO:00325	developm	K03068	low densit	mmu0520	Pathways	19:358482	269	301	448	413	326	236	1.431807	1.342921	1.642709	1.432066	1.521571	1.557503	1.472479	1.503714	0.904302		1	-
ENSMUS(	Drap1	Dr1 assoc	GO:00081	metabolic	-	-	-	-	19:542280	1179	1454	1575	1742	1296	995	14.39835	14.88388	13.25046	13.85889	13.87863	15.06634	14.17756	14.26795	0.780341		1	-
ENSMUS(	Smarca2	SWI/SNF r	GO:00325	developm	K11647	SWI/SNF-	mmu0522	Hepatoce	19:266050	4308	5158	6067	6757	5236	3435	11.05984	11.0996	10.72997	11.30079	11.78733	10.93417	10.96314	11.34076	0.982568		1	-
ENSMUS(	Vldlr	very low c	GO:00325	developm	-	-	-	-	19:272164	1709	1842	2398	2413	1771	1393	7.604824	6.870519	7.351021	6.994972	6.910483	7.685711	7.275455	7.197055	0.664476		1	-
ENSMUS(	Rnaseh2c	ribonuclea	GO:00081	metabolic	K10745	ribonuclea	mmu0303	DNA repli	19:560187	85	119	150	126	94	97	1.72698	2.026611	2.099485	1.66771	1.674708	2.443572	1.951025	1.928663	0.736281		1	-
ENSMUS(	Kat5	K(lysine) a	GO:00081	metabolic	K11304	histone ac	mmu0516	HTLV-I int	19:560301	735	865	1002	1036	903	531	23.26268	22.94781	21.84699	21.36062	25.06126	20.83787	22.68582	22.41992	0.722646		1	-
ENSMUS(	Rela	v-rel retic	GO:00325	developm	K04735	transcripti	mmu0520	Pathways	19:563748	393	362	483	514	371	315	9.196006	7.100148	7.785834	7.835226	7.612419	9.13908	8.027329	8.195575	0.881907		1	-
ENSMUS(	Slc1a1	solute car	GO:00511	localizatio	K05612	solute car	mmu0472	Glutamate	19:288350	1974	2428	2973	3248	2300	1922	15.06046	15.52718	15.62563	16.14317	15.38727	18.18153	15.40442	16.57066	0.781287		1	-
ENSMUS(	Kcnk7	potassium	GO:00511	localizatio	-	-	-	-	19:570436	4	0	1	0	4	0	0.207343	0	0.035697	0	0.181809	0	0.081013	0.060603	1		1	-
ENSMUS(	Ehbp1l1	EH domai	GO:00160	membran	-	-	-	-	19:570737	253	309	415	372	311	273	2.480931	2.539836	2.803455	2.376394	2.674223	3.319275	2.608074	2.789964	0.883893		1	-
ENSMUS(	Fam89b	family wit	GO:00400	locomotic	-	-	-	-	19:572808	74	109	97	111	69	54	1.326253	1.63748	1.197628	1.295987	1.084395	1.199999	1.38712	1.19346	0.309955		1	-
ENSMUS(	Ltbp3	latent trar	GO:00325	developm	-	-	-	-	19:574090	376	462	626	481	409	502	4.604223	4.742021	5.280738	3.837031	4.39172	7.621811	4.875661	5.28352	0.882084		1	-
ENSMUS(	Scyl1	SCY1-like	GO:00081	metabolic	-	-	-	-	19:575842	949	1251	1383	1337	1234	852	22.87137	25.2718	22.96146	20.99129	26.07858	25.45959	23.70154	24.17648	0.87178		1	-
ENSMUS(	Capn1	calpain 1	GO:00325	developm	K01367	calpain-1	mmu0421	Cellular se	19:598854	449	488	602	688	498	414	8.945237	8.149253	8.262159	8.92926	8.699966	10.22661	8.452216	9.285278	0.691717		1	-
ENSMUS(	Smc5	structural	GO:00325	developm	-	-	-	-	19:232064	567	801	971	985	718	387	4.518438	5.350454	5.330598	5.113557	5.017331	3.823859	5.066497	4.651583	0.425573		1	-
ENSMUS(	Arl2	ADP-ribo	GO:00511	localizatio	-	-	-	-	19:613437	421	492	591	624	499	490	6.79082	6.652092	6.567183	6.557017	7.058024	9.799901	6.670031	7.804981	0.431794		1	-
ENSMUS(	Men1	multiple e	GO:00325	developm	-	-	-	-	19:633497	933	1030	1292	1368	1132	672	5.335642	4.93736	5.090012	5.096506	5.676673	4.764968	5.121004	5.179382	0.856713		1	-
ENSMUS(	Map4k2	mitogen-	GO:00048	signal trar	-	-	-	-	19:634113	728	824	1054	1130	855	881	2.599245	2.466016	2.592437	2.628307	2.676855	3.900122	2.552566	3.068428	0.304141		1	-
ENSMUS(	Sf1	splicing fa	GO:00325	developm	-	-	-	-	19:636369	2051	2098	2419	2631	2304	1540	9.895028	8.484176	8.039679	8.269016	9.747125	9.212086	8.806294	9.076076	0.942397		1	-
ENSMUS(	Rps6ka4	ribosomal	GO:00081	metabolic	K16510	ribosomal	mmu0401	MAPK sig	19:682900	677	729	846	924	682	720	10.66304	9.624396	9.179401	9.48083	9.419323	14.06083	9.822276	10.98699	0.648135		1	-
ENSMUS(	Prdx5	peroxired	GO:00081	metabolic	K11187	peroxired	mmu0414	Peroxisom	19:690669	1754	1801	2850	2392	1777	2274	28.76697	24.75888	32.20035	25.55685	25.55609	46.24245	28.5754	32.4518	0.621583		1	-
ENSMUS(	Esrra	estrogen	GO:00325	developm	-	-	-	-	19:691097	506	704	750	807	557	502	7.883752	9.194077	8.049988	8.191008	7.609918	9.697763	8.375939	8.499565	0.830898		1	-
ENSMUS(	Kcnk4	potassium	GO:00325	developm	-	-	-	-	19:692571	124	93	130	134	124	115	4.595086	2.888722	3.318686	3.234874	4.029349	5.283918	3.600831	4.182714	0.60574		1	-
ENSMUS(	Gpr137	G protein	GO:00160	membran	-	-	-	-	19:693806	938	1176	1248	1383	1099	1321	14.69632	15.69692	13.69053	14.3469	15.34599	26.08213	14.77474	18.59167	0.228853		1	-
ENSMUS(	Bad	BCL2-ass	GO:00160	membran	K02158	Bcl-2-ant	mmu0520	Pathways	19:694186	272	351	408	363	336	300	4.829957	5.224379	4.990978	4.199163	5.23187	6.605123	5.015105	5.345385	0.719652		1	-

ENSMUS(	Plcb3	phospholi	GO:00048	signal trar	K05858	phosphati	mmu011C	Metabolic	19:695371	313	442	503	533	427	363	4.735079	5.604778	5.242067	5.252817	5.664395	6.808879	5.193975	5.908697	0.535107	1	-
ENSMUS(	Vegfb	vascular e	GO:00325	developm	K16858	vascular e	mmu052C	Pathways	19:698247	564	738	753	859	623	604	16.64063	18.25154	15.30513	16.51069	16.11836	22.09598	16.73243	18.24168	0.764556	1	-
ENSMUS(	Dnajc4	DnaJ heat	GO:00160	membran	-	-	-	-	19:698791	143	164	209	212	198	143	6.731909	6.471418	6.777974	6.501581	8.173538	8.346885	6.660434	7.674001	0.521346	1	-
ENSMUS(	Fermt3	fermitin fa	GO:00325	developm	K17084	kindlin 3[-	mmu0461	Platelet ac	19:699895	48	82	85	111	52	82	1.179184	1.688522	1.438498	1.776409	1.120176	2.497708	1.435401	1.798097	0.440925	1	-
ENSMUS(	Stip1	stress-ind	GO:00329	macromo	K09553	stress-ind	mmu0502	Prion dise	19:702069	3883	4562	5835	5742	4366	2390	113.5648	111.8367	117.5625	109.401	111.9703	86.66829	114.3213	102.6799	0.277817	1	-
ENSMUS(	Rcor2	REST core	GO:00081	metabolic	-	-	-	-	19:726732	220	256	292	377	273	226	2.976723	2.903407	2.721756	3.323065	3.239077	3.791493	2.867295	3.451211	0.306728	1	-
ENSMUS(	Mark2	MAP/micr	GO:00325	developm	-	-	-	-	19:727539	1184	1386	1787	1787	1439	1715	4.720033	4.631368	4.907601	4.64087	5.030332	8.477021	4.753001	6.049408	0.18502	1	-
ENSMUS(	AI846148	expressed	GO:00081	metabolic	-	-	-	-	19:735646	359	425	480	460	369	359	7.167937	7.112805	6.602258	5.983281	6.460534	8.887497	6.961	7.110437	0.851305	1	-
ENSMUS(	Hrasls5	HRAS-like	GO:00081	metabolic	-	-	-	-	19:761254	0	0	0	0	2	0	0	0	0	0	0.0552	0	0	0.0184	0.534765	1	-
ENSMUS(	Smc3	structural	GO:00325	developm	K06669	structural	mmu0411	Cell cycle	19:536003	1882	2172	2622	2804	2050	864	19.87814	19.22954	19.07832	19.29371	18.98684	11.31503	19.39533	16.53186	0.242655	1	-
ENSMUS(	Pdcd4	programn	GO:00325	developm	K16865	programn	mmu052C	Proteoglyc	19:538922	1661	2199	2633	2629	1876	1289	37.55302	41.67286	41.00884	38.72108	37.19211	36.13383	40.07824	37.34901	0.373599	1	-
ENSMUS(	Shoc2	soc-2 (sup	GO:00987	molecular	K19613	leucine-ri	mmu0401	Ras signal	19:539443	1295	1659	2030	1989	1472	878	19.58153	21.02693	21.1458	19.59264	19.51764	16.46104	20.58475	18.52377	0.274125	1	-
ENSMUS(	Gpam	glycerol-3	GO:00325	developm	K00629	glycerol-3	mmu011C	Metabolic	19:550697	800	939	1252	1352	987	704	13.26785	13.05356	14.3043	14.60727	14.35392	14.47669	13.5419	14.47929	0.809555	1	-
ENSMUS(	Tectb	tectorin b	GO:00051	structural	-	-	-	-	19:551807	0	0	2	0	0	0	0	0	0.013603	0	0	0	0.004534	0	0.536056	1	-
ENSMUS(	Acsl5	acyl-CoA	GO:00081	metabolic	K01897	long-chai	mmu011C	Metabolic	19:552519	975	1273	1537	1586	1085	795	11.0237	12.06435	11.97148	11.68173	10.7571	11.14489	11.68651	11.19457	0.513625	1	-
ENSMUS(	Zdhhc6	zinc finge	GO:00081	metabolic	-	-	-	-	19:552712	222	419	368	432	298	237	1.127166	1.783211	1.287167	1.428898	1.326769	1.492007	1.399181	1.415892	0.866446	1	-
ENSMUS(	Vti1a	vesicle tra	GO:00081	metabolic	K08493	vesicle tra	mmu0413	SNARE int	19:553162	1114	1313	1563	1721	1283	857	4.386498	4.333614	4.239776	4.414639	4.429983	4.184076	4.319963	4.342899	0.801846	1	-
ENSMUS(	Tcf7l2	transcripti	GO:00325	developm	K04491	transcripti	mmu052C	Pathways	19:557418	108	95	136	131	99	76	0.31254	0.230442	0.271127	0.246966	0.251226	0.272699	0.27137	0.256963	0.590423	1	-
ENSMUS(	Hhex	hematopo	GO:00325	developm	-	-	-	-	19:374348	27	44	46	68	46	19	0.570639	0.779484	0.669743	0.936256	0.852508	0.497906	0.673288	0.762223	0.670464	1	-
ENSMUS(	Cyp26a1	cytochron	GO:00081	metabolic	K07437	cytochron	mmu011C	Metabolic	19:376978	12	16	5	11	2	6	0.430122	0.480736	0.123469	0.256853	0.062859	0.266688	0.344776	0.195467	0.22036	1	-
ENSMUS(	Cep55	centroson	GO:00325	developm	-	-	-	-	19:380550	6	3	10	9	1	5	0.1247	0.052258	0.143172	0.121847	0.018223	0.128834	0.10671	0.089634	0.791049	1	-
ENSMUS(	Rbp4	retinol bir	GO:00325	developm	-	-	-	-	19:381166	56	98	124	104	101	92	2.743988	4.025098	4.185714	3.31978	4.339718	5.589451	3.6516	4.416316	0.480962	1	-
ENSMUS(	Eif3a	eukaryotic	GO:00081	metabolic	K03254	translatior	mmu0301	RNA trans	19:607611	5381	6250	7731	8176	6041	2916	66.07009	64.32425	65.39265	65.39798	65.04192	44.39309	65.26233	58.27766	0.314879	1	-
ENSMUS(	Pde6c	phosphoc	GO:00325	developm	K13757	cone cGM	mmu0023	Purine me	19:381327	4	1	4	2	2	0	0.072381	0.015167	0.049871	0.023574	0.031734	0	0.045806	0.018436	0.33496	1	-
ENSMUS(	Fam45a	family witl	-	-	-	-	-	-	19:608115	537	388	782	754	603	301	4.499991	2.725354	4.514359	4.116148	4.43096	3.127452	3.913235	3.89152	0.839054	1	-
ENSMUS(	Prdx3	peroxired	GO:00325	developm	-	-	-	-	19:608640	436	567	666	682	483	613	18.74772	20.43608	19.7282	19.10414	18.21175	32.68195	19.63733	23.33261	0.438117	1	-
ENSMUS(	Plce1	phospholi	GO:00325	developm	K05860	phosphati	mmu011C	Metabolic	19:384811	335	387	525	514	422	241	0.977964	0.946983	1.055817	0.977514	1.080271	0.872331	0.993588	0.976705	0.728707	1	-
ENSMUS(	Noc3l	NOC3 like	GO:00325	developm	-	-	-	-	19:387881	341	427	466	509	385	211	5.449234	5.719536	5.130003	5.298831	5.394903	4.180698	5.432924	4.958144	0.4132	1	-
ENSMUS(	Hells	helicase, l	GO:00325	developm	-	-	-	-	19:389309	64	74	109	100	75	51	0.484678	0.46974	0.568653	0.493347	0.498054	0.478882	0.507691	0.490094	0.708957	1	-
ENSMUS(	Sorbs1	sorbin anc	GO:00329	macromo	K06086	sorbin anc	mmu0491	Insulin sig	19:402947	1919	2102	2559	3095	2397	1351	6.992617	6.420227	6.423717	7.34695	7.659058	6.103882	6.612187	7.03663	0.821029	1	-
ENSMUS(	Aldh18a1	aldehyde	GO:00081	metabolic	K12657	delta-1-p	mmu011C	Metabolic	19:405502	443	473	545	596	511	369	5.117043	4.579624	4.336739	4.484803	5.175821	5.284794	4.677802	4.981806	0.871813	1	-
ENSMUS(	Tctn3	tectonic fa	GO:00508	response	-	-	-	-	19:405964	297	333	418	424	380	314	4.202907	3.949938	4.074934	3.908775	4.71541	5.509456	4.075926	4.711213	0.47954	1	-
ENSMUS(	Ccnj	cyclin J [S	GO:00056	cell|intrace	-	-	-	-	19:408312	194	238	314	290	199	134	1.75256	1.802192	1.95413	1.706681	1.576404	1.500942	1.836294	1.594675	0.245728	1	-
ENSMUS(	Tll2	tolloid-lik	GO:00325	developm	-	-	-	-	19:410839	16	8	15	27	12	4	0.295344	0.123772	0.190744	0.324666	0.194234	0.091547	0.203287	0.203482	1	1	-
ENSMUS(	Dntt	deoxynuc	GO:00081	metabolic	K00977	DNA nucl	mmu0464	Hematopc	19:410292	6	3	0	0	3	0	0.179198	0.075096	0	0	0.078559	0	0.084765	0.026186	0.226556	1	-
ENSMUS(	Tm9sf3	transmem	GO:00160	membran	-	-	-	-	19:412108	3896	4558	5864	5559	4075	2664	40.29989	39.51956	41.78595	37.4596	36.96198	34.16686	40.53513	36.19615	0.205635	1	-
ENSMUS(	Pik3ap1	phosphoir	GO:00023	immune s	K12230	phosphoir	mmu0415	PI3K-Akt s	19:412742	37	36	71	82	41	69	0.899215	0.73337	1.188708	1.298261	0.873755	2.079204	0.940431	1.417073	0.140571	1	-
ENSMUS(	Lcor	ligand dep	GO:00081	metabolic	-	-	-	-	19:414826	279	273	347	477	309	166	1.890534	1.550583	1.619795	2.105619	1.836037	1.394679	1.686971	1.778778	0.880963	1	-
ENSMUS(	Slit1	slit homol	GO:00325	developm	K06838	slit 1[-]	mmu0436	Axon guic	19:416002	5112	6111	6931	8461	6316	4915	42.1051	42.18995	39.32704	45.3991	45.6172	50.19418	41.20736	47.07016	0.412514	1	-
ENSMUS(	Smndc1	survival m	GO:00081	metabolic	K12839	survival of	mmu0304	Spliceosor	19:533792	496	627	683	633	558	281	14.83402	15.71801	14.07178	12.33283	14.63369	10.42005	14.8746	12.46219	0.157189	1	-
ENSMUS(	Mxi1	MAX inter	GO:00081	metabolic	-	-	-	-	19:533105	1137	1322	1935	1883	1366	894	12.8599	12.53318	15.07681	13.87424	13.54785	12.5372	13.48996	13.31976	0.707461	1	-
ENSMUS(	Add3	adducin 3	GO:00452	synapse|st	-	-	-	-	19:531404	3356	3774	4766	5055	3787	2317	43.19237	40.71363	42.2562	42.38262	42.73887	36.97405	42.05407	40.69851	0.57908	1	-
ENSMUS(	Xpnpep1	X-prolyl a	GO:00081	metabolic	-	-	-	-	19:529434	801	967	1083	1227	944	811	5.682736	5.750482	5.293038	5.670891	5.872727	7.13398	5.575419	6.225866	0.589143	1	-
ENSMUS(	Trim8	tripartite r	GO:00325	developm	-	-	-	-	19:465016	1550	1722	1901	2045	1681	1416	30.49757	28.40008	25.76719	26.21251	29.00307	34.5448	28.22161	29.92013	0.90947	1	-
ENSMUS(	Arl3	ADP-ribo	GO:00325	developm	-	-	-	-	19:465311	1326	1866	2161	1884	1547	891	102.5198	120.9285	115.0987	94.89141	104.8811	85.41377	112.849	95.0621	0.107887	1	-
ENSMUS(	Sfxn2	sideroflex	GO:00511	localizatio	-	-	-	-	19:465733	68	74	91	123	96	66	1.346299	1.228056	1.241143	1.58642	1.666647	1.620182	1.271833	1.624416	0.227705	1	-
ENSMUS(	Maoa	monoami	GO:00081	metabolic	K00274	monoami	mmu011C	Metabolic	X:1661969	568	721	934	928	678	362	8.662853	9.217243	9.813202	9.220239	9.067452	6.84553	9.231099	8.37774	0.380642	1	-
ENSMUS(	Efhc2	EF-hand c	GO:00508	response	-	-	-	-	X:1713204	35	39	52	40	40	22	0.870938	0.813456	0.8914	0.648418	0.872805	0.678788	0.858598	0.733337	0.370258	1	-
ENSMUS(	Fundc1	FUN14 dc	GO:00081	metabolic	K17986	FUN14 dc	mmu0413	Mitophag	X:1755656	388	458	619	570	393	326	6.891723	6.818898	7.574217	6.595572	6.121123	7.179585	7.094946	6.632093	0.420544	1	-
ENSMUS(	Nt5c2	5'-nucleo	GO:00081	metabolic	K01081	5'-nucleo	mmu011C	Metabolic	19:468868	810	970	1449	1209	967	938	8.957355	8.991235	11.03862	8.70969	9.377008	12.86127	9.662402	10.31599	0.889698	1	-
ENSMUS(	Msr1	macropha	GO:00325	developm	K06558	macropha	mmu0414	Phagoson	8:3958168	26	39	83	104	27	62	0.238408	0.29975	0.524285	0.621231	0.217093	0.704889	0.354148	0.514405	0.281981	1	-
ENSMUS(	Pdcd11	programn	GO:00081	metabolic	-	-	-	-	19:470907	526	676	820	1012	744	511	5.533669	5.961103	5.942818	6.935686	6.86346	6.665528	5.81253	6.821558	0.335254	1	-
ENSMUS(	Taf5	TATA-box	GO:00081	metabolic	K03130	transcripti	mmu0516	Herpes sir	19:470677	103	152	180	185	127	95	2.009188	2.485311	2.418849	2.350924	2.172364	2.297709	2.304449	2.273666	0.760222	1	-
ENSMUS(	Pcgf6	polycomb	GO:00081	metabolic	K11470	polycomb	mmu0455	Signaling	19:470336	133	187	194	208	137	169	4.028857	4.748159	4.048395	4.104647	3.639088	6.347529	4.275137	4.697088	0.817844	1	-
ENSMUS(	Nr0b1	nuclear re	GO:00325	developm	-	-	-	-	X:8619176	0	0	0	0	2	0	0	0	0	0	0.061642	0	0	0.020547	0.534879	1	-
ENSMUS(	5430427C	RIKEN cDI	-	-	-	-	-	-	X:8587035	11	4	7	9	13	5	0.186229	0.056759	0.081642	0.099256	0.192987	0.104948	0.10821	0.132397	0.680856	1	-
ENSMUS(	Gk	glycerol k	GO:00081	metabolic	K00864	glycerol k	mmu011C	Metabolic	X:8570193	431	352	731	723	323	362	3.019996	2.0674	3.528563	3.300267	1.984607	3.145019	2.871986	2.809964	0.724714	1	-
ENSMUS(	Slk	STE20-like	GO:00081	metabolic	K08836	STE20-like	mmu0411	Oocyte m	19:475796	2683	3022	3782	4435	3028	1654	16.77449	15.83712	16.28927	18.06359	16.60073	12.82183	16.30029	15.82872	0.65937	1	-
ENSMUS(	Col17a1	collagen,	GO:00329	macromo	K07603	collagen,	mmu0497	Protein dig	19:476463	11	7	11	13	8	5	0.111004	0.059206	0.076467	0.085461	0.070792	0.062555	0.082225	0.072936	0.744039	1	-
ENSMUS(	Sfr1	SWI5 dep	GO:00081	metabolic	-	-	-	-	19:477317	1905	2370	2679	2602	1945	878	76.48041	79.75471	74.09338	68.05251	68.47271	43.70547	76.77617	60.0769	0.069207	1	-
ENSMUS(	Gsto1	glutathion	GO:00081	metabolic	K00799	glutathion	mmu052C	Pathways	19:478549	425	494	656	638	491	472	10.8387	10.5601	11.52506	10.59963	10.98024	14.92507	10.97462	12.16831	0.679084	1	-
ENSMUS(	Gsto2	glutathion	GO:00081	metabolic	K00799	glutathion	mmu052C	Pathways	19:478655	4	9	10	7	12	10	0.152949	0.288456	0.26343	0.174378	0.402376	0.474148	0.234945	0.350301	0.458005	1	-
ENSMUS(	Casp7	caspase 7	GO:00325	developm	K04397	caspase 7	mmu052C	Pathways	19:563971	25	32	29	33	36	37	0.676102	0.7254	0.540279	0.58138	0.853722	1.240658	0.64726	0.89192	0.303363	1	-
ENSMUS(	Dclre1a	DNA cros	GO:00081	metabolic	-	-	-	-	19:565291	117	106	135	164	148	107	0.681984	0.517903	0.542095	0.622752	0.756474	0.773322	0.580661	0.717516	0.321119	1	-
ENSMUS(	Nhlrc2	NHL repe	GO:00099	cellular pr	-	-	-	-	19:565482	264	270	384	383	279	164	4.264874	3.656104	4.273504	4.03072	3.952285	3.284966	4.064827	3.75599	0.470551	1	-
ENSMUS(	Tdrd1	tudor don	GO:00325	developm	-	-	-	-	19:568262	9	29	18	28	16	3	0.098718	0.266626	0.136015	0.20008	0.153891	0.040804	0.16712	0.131592	0.588223	1	-
ENSMUS(	Vwa2	von Willet	GO:00508	response	-	-	-	-	19:568742	0	0	3	0	0	0	0	0	0.032437	0	0	0	0.010812	0	0.302599	1	-
ENSMUS(	Afap1l2	actin filar	GO:00081	metabolic	-	-	-	-	19:569123	267	259	424	384	300	204	2.587473	2.103859	2.83062	2.424253	2.549347	2.451208	2.507317	2.474936	0.736811	1	-
ENSMUS(	Ablim1	actin-binc	GO:00325	developm	K07520	actin-binc	mmu0436	Axon guic	19:570327	2218	2572	2493	3366	2221	1539	4.309271	4.188573	3.336691	4.26028	3.783847	3.707378	3.944845	3.917168	0.7389	1	-
ENSMUS(	Trub1	TruB pseu	GO:00081	metabolic	-	-	-	-	19:574529	383	579	631	774	442	349	3.309875	4.194152	3.756587	4.357476	3.349483	3.739591	3.753538	3.815517	0.898323	1	-
ENSMUS(	Gfra1	glial cell li	GO:00325	developm	-	-	-	-	19:582356	1008	1310	1404	1658	1222	597	5.279934	5.751646	5.066247	5.657625	5.612829	3.877288	5.365943	5.049248	0.542996	1	-
ENSMUS(	Ccdc172	coiled-co	GO:00056	cell|cilium	-	-	-	-	19:585120	0	0	3	0	0	0	0	0	0.120937	0	0	0	0.040312	0	0.303485	1	-
ENSMUS(	Pnliprp2	pancreatic	GO:00081	metabolic	K14075	pancreatic	mmu011C	Metabolic	19:587597	2	0	1	2	2	0	0.081131	0	0.027929	0.052835	0.071122	0	0.036353	0.041319	1	1	-
ENSMUS(	Hspa12a	heat shoc	GO:00055	extracellul	-	-	-	-	19:587957	6163	7524	8844	9316	7041	4574	68.31976	69.91266	67.53895	67.2768	68.44333	62.86897	68.59046	66.19637	0.542076	1	-
ENSMUS(	Slc18a2	solute car	GO:00325	developm	K08155	MFS trans	mmu0503	Alcoholisr	19:592608	28	28	47	42	28	14	0.467914	0.392207	0.54108	0.457232	0.410307	0.290095	0.467067	0.385878	0.39228	1	-
ENSMUS(	3110040N	RIKEN cDI	-	-	-	-	-	-	7:8178218	74	113	131	92	90	80	2.876386	3.681698	3.507862	2.329637	3.067639	3.855614	3.355315	3.084297	0.461674	1	-
ENSMUS(	Btbd1	BTB (POZ	GO:00325	developm	-	-	-	-	7:8179207	2138	2604	3253	3209	2324	1384	33.51661	34.21734	35.13079	32.77206	31.94708	26.90137	34.28825	30.54017	0.236922	1	-
ENSMUS(	Hdgfl3	HDGF like	GO:00325	developm	-	-	-	-	7:8188125	2127	2718	3297	3341	2648	1309	12.89367	13.81056	13.76827	13.19372	14.07569	9.83863	13.49083	12.36935	0.397224	1	-
ENSMUS(	Bnc1	basonucli	GO:00056	cell|intrace	-	-	-	-	7:8196667	0	0	0	2	0	0	0	0	0	0.01782	0	0	0	0.00594	0.537142	1	-
ENSMUS(	Gcgr	glucagon	GO:00048	signal trar	K04583	glucagon	mmu0408	Neuroacti	11:120530	0	2	2	1	0	0	0	0.020755	0.017061	0.008065	0	0	0.012605	0.002688	0.418141	1	-
ENSMUS(	Bhlhe22	basic helix	GO:00325	developm	-	-	-	-	3:1805417	2213	2624	2938	3117	2180	1271	42.05825	41.80099	38.46566	38.5912	36.33031	29.95032	40.77497	34.95727	0.152298	1	-
ENSMUS(	P4hb	prolyl 4-h	GO:00081	metabolic	K09580	protein di	mmu0414	Protein pr	11:120560	1599	2003	2504	2425	1814	1607	26.91241	28.25778	29.03286	26.58873	26.7722	33.53559	28.06768	28.9655	0.919485	1	-
ENSMUS(	Arhgdia	Rho GDP	GO:00325	developm	K12462	Rho GDP-	mmu0472	Neurotrop	11:120578	3682	4489	5186	5635	4134	4132	65.36374	66.79679	63.42158	65.16714	64.35261	90.94929	65.19404	73.48968	0.541822	1	-
ENSMUS(	Ints4	integrator	GO:00081	metabolic	-	-	-	-	7:9748095	479	561	696	696	519	351	3.378681	3.31686	3.381986	3.198175	3.210118	3.069757	3.359176	3.15935	0.459659	1	-
ENSMUS(	Alyref	Aly/REF e	GO:00081	metabolic	K12881	THO com	mmu0516	Herpes sir	11:120592	371	442	613	640	460	330	5.222181	5.214988	5.944158	5.868675	5.677784	5.759413	5.460442	5.768624	0.896889	1	-
ENSMUS(	Anapc11	anaphase	GO:00081	metabolic	K03358	anaphase	mmu0516	HTLV-I inf	11:120598	412	515	606	635	561	645	4.271429	4.475443	4.328122	4.288752	5.100132	8.291271	4.358331	5.893385	0.11236	1	-
ENSMUS(	Pcyt2	phosphate	GO:00081	metabolic	-	-	mmu011C	Metabolic	11:120610	737	949	1134	1111	898	929	5.862152	6.327151	6.21375	5.756853	6.263372	9.162019	6.134351	7.060748	0.496611	1	-
ENSMUS(	Sirt7	sirtuin 7 [S	GO:00081	metabolic	-	-	-	-	11:120618	311	316	433	481	446	286	1.702118	1.449669	1.632558	1.714966	2.140458	1.940802	1.594782	1.932075	0.279364	1	-
ENSMUS(	Tollip	toll interac	GO:00325	developm	K05402	toll-intera	mmu0462	Toll-like r	7:1418748	2533	3036	3787	3589	2860	2495	20.95818	21.05586	21.58565	19.34521	20.75043	25.59617	21.19989	21.89727	0.919758	1	-
ENSMUS(	Pycr1	pyrroline-	GO:00081	metabolic	K00286	pyrroline-	mmu011C	Metabolic	11:120635	15	26	29	38	20	20	0.202744	0.294561	0.270025	0.334595	0.237048	0.335173	0.255777	0.302272	0.642808	1	-
ENSMUS(	Aspscr1	alveolar s	GO:00081	metabolic	K15627	tether cor	mmu052C	Transcript	11:120672	251	290	298	331	256	337	1.567588	1.518134	1.282115	1.346694	1.401977	2.609608	1.455946	1.786093	0.397697	1	-
ENSMUS(	Cenpx	centrome	GO:00081	metabolic	K15360	centrome	mmu0346	Fanconi a	11:120710	107	106	167	179	126	95	3.45363	2.8678	3.713281	3.763784	3.566195	3.801898	3.344904	3.710626	0.699084	1	-
ENSMUS(	Lrrc45	leucine ric	GO:00160	membran	-	-	-	-	11:120713	607	774	880	1009	774	727	7.245809	7.744473	7.236561	7.846411	8.101805	10.76015	7.408947	8.90279	0.284809	1	-
ENSMUS(	Mob2	MOB kina	GO:00325	developm	-	-	-	-	7:1420085	184	210	293	246	235	352	1.159747	1.109477	1.27223	1.010098	1.298844	2.750905	1.180485	1.686616	0.152921	1	-
ENSMUS(	Cbr2	carbonyl r	GO:00081	metabolic	K00081	carbonyl r	mmu011C	Metabolic	11:120729	8	10	17	10	12	14	0.261525	0.274014	0.38285	0.212965	0.344007	0.567506	0.30613	0.374826	0.726123	1	-
ENSMUS(	Maged1	melanom	GO:00325	developm	K12464	melanom	mmu0472	Neurotrop	X:9453547	7537	8706	10245	11341	8492	6550	64.18317	62.14329	60.1016	62.91521	63.41257	69.1592	62.14269	65.16233	0.948998	1	-
ENSMUS(	Fasn	fatty acid	GO:00325	developm	K00665	fatty acid	mmu011C	Metabolic	11:120805	5305	5069	6768	7562	5678	4935	24.21698	19.39589	21.28366	22.4881	22.72859	27.93236	21.63217	24.38302	0.524099	1	-
ENSMUS(	Arhgap19	Rho GTPa	GO:00987	molecular	-	-	-	-	19:417665	42	64	125	125	73	88	0.273822	0.349746	0.561411	0.530899	0.417339	0.711358	0.394993	0.553199	0.187029	1	-
ENSMUS(	Dus1l	dihydrour	GO:00081	metabolic	-	-	-	-	11:120789	312	400	516	546	419	403	2.872456	3.086817	3.272647	3.27471	3.382634	4.600328	3.077307	3.752557	0.274987	1	-
ENSMUS(	Gps1	G protein	GO:00081	metabolic	-	-	-	-	11:120784	329	404	473	514	414	391	4.371511	4.499555	4.329606	4.449189	4.823677	6.441663	4.400224	5.238177	0.361777	1	-
ENSMUS(	Zdhhc16	zinc finge	GO:00325	developm	-	-	-	-	19:419334	302	371	418	491	341	272	2.963716	3.051802	2.8259	3.139011	2.934448	3.30966	2.947139	3.127706	0.887482	1	-
ENSMUS(	Mms19	MMS19 (N	GO:00081	metabolic	-	-	-	-	19:419410	475	539	653	764	530	572	1.902183	1.80926	1.801456	1.993124	1.861133	2.84015	1.837633	2.231469	0.292565	1	-
ENSMUS(	Slc16a3	solute car	GO:00511	localizatio	K08180	MFS trans	mmu0523	Central ca	11:120948	18	42	47	39	27	49	0.130736	0.2557	0.235169	0.184533	0.171962	0.441273	0.207201	0.265923	0.535048	1	-
ENSMUS(	Csnk1d	casein kin	GO:00325	developm	K08959	casein kin	mmu0439	Hippo sig	11:120961	1938	2224	2908	2806	2027	2362	20.14487	19.37752	20.82363	19.00115	18.47596	30.44223	20.11534	22.63978	0.611782	1	-
ENSMUS(	Cd7	CD7 antig	GO:00023	immune s	K06457	T-cell surf	mmu0464	Hematopc	11:121036	0	2	8	2	4	0	0	0.073575	0.241884	0.057177	0.153935	0	0.105153	0.070371	0.707052	1	-
ENSMUS(	Ogfod3	2-oxoglut	GO:00081	metabolic	-	-	-	-	11:121177	96	132	166	148	109	59	4.664446	5.375937	5.556335	4.684587	4.644045	3.554411	5.198906	4.294347	0.214989	1	-
ENSMUS(	Rab40b	Rab40B, n	GO:00081	metabolic	-	-	-	-	11:121356	396	453	481	463	409	495	9.118474	8.743361	7.629983	6.94528	8.258343	14.13249	8.497273	9.778704	0.617136	1	-
ENSMUS(	Ubtd1	ubiquitin c	GO:00055	protein bi	-	-	-	-	19:419817	134	141	190	180	126	132	5.577019	4.918921	5.447564	4.880345	4.598453	6.811711	5.314501	5.43017	0.878652	1	-
ENSMUS(	Ankrd2	ankyrin re	GO:00325	developm	-	-	-	-	19:420360	3	2	1	3	1	0	0.173305	0.096851	0.039786	0.112899	0.050659	0	0.103314	0.054519	0.55491	1	-
ENSMUS(	Wdr45b	WD repea	GO:00081	metabolic	-	-	-	-	11:121327	715	900	992	1185	860	636	8.233446	8.687025	7.869347	8.889464	8.683936	9.080689	8.263273	8.884697	0.785845	1	-
ENSMUS(	Fn3k	fructosam	GO:00325	developm	-	-	-	-	11:121434	219	245	352	307	224	315	2.2742	2.132574	2.518132	2.07685	2.039742	4.05584	2.308302	2.724144	0.54255	1	-
ENSMUS(	Hoga1	4-hydroxy	GO:00081	metabolic	K18123	4-hydroxy	mmu011C	Metabolic	19:420456	22	19	30	28	28	23	0.408455	0.295691	0.383704	0.338668	0.455856	0.529477	0.362617	0.441334	0.572208	1	-
ENSMUS(	Pi4k2a	phosphati	GO:00081	metabolic	K13711	phosphati	mmu011C	Metabolic	19:420904	927	1235	1542	1590	1108	1256	15.62282	17.44614	17.90257	17.45655	16.37427	26.24552	16.99051	20.02545	0.390068	1	-
ENSMUS(	R3hcc1l	R3H dom	GO:00329	macromo	-	-	-	-	19:425187	303	346	456	445	334	201	2.12007	2.029254	2.197979	2.028378	2.049252	1.743773	2.115767	1.940468	0.409481	1	-
ENSMUS(	Loxl4	lysyl oxida	GO:00081	metabolic	-	-	-	-	19:425939	4	2	4	2	2	2	0.047417	0.019873	0.03267	0.015444	0.020789	0.029405	0.03332	0.021879	0.506006	1	-
ENSMUS(	Hps1	HPS1, bio	GO:00987	molecular	-	-	-	-	19:427551	156	277	228	291	237	273	1.061142	1.579363	1.068404	1.289511	1.413646	2.302496	1.236303	1.668551	0.166806	1	-
ENSMUS(	Cnnm1	cyclin M1	GO:00511	localizatio	-	-	-	-	19:434404	2347	2802	3176	3735	2806	2155	25.17875	25.19661	23.47216	26.10317	26.3968	28.66515	24.61584	27.05504	0.634555	1	-
ENSMUS(	Got1	glutamic-	GO:00081	metabolic	K14454	aspartate	mmu011C	Metabolic	19:434997	2627	4613	3721	3898	2976	3439	43.52288	64.06102	42.46867	42.07082	43.23476	70.64406	50.01752	51.98321	0.932482	1	-
ENSMUS(	Entpd7	ectonucle	GO:00081	metabolic	-	-	-	-	19:436896	273	314	427	417	303	263	1.76142	1.698175	1.897928	1.75274	1.714293	2.103979	1.785841	1.857004	0.984343	1	-
ENSMUS(	Cutc	cutC copp	GO:00099	cellular pr	-	-	-	-	19:437529	64	69	95	81	73	92	1.73229	1.565446	1.771386	1.428243	1.732631	3.087529	1.689708	2.082801	0.488021	1	-
ENSMUS(	Abcc2	ATP-bind	GO:00081	metabolic	K05666	ATP-bind	mmu0152	Platinum c	19:437821	0	5	2	4	2	0	0	0.023652	0.007777	0.014708	0.009897	0	0.010476	0.008202	0.816503	1	-
ENSMUS(	Dnmbp	dynamin l	GO:00987	molecular	-	-	-	-	19:438468	224	271	315	352	275	186	1.310854	1.329317	1.269896	1.341934	1.411178	1.349602	1.303355	1.367571	0.943381	1	-
ENSMUS(	Cpn1	carboxype	GO:00081	metabolic	-	-	-	-	19:439563	2	11	3	8	0	0	0.032598	0.150257	0.033676	0.084926	0	0	0.072177	0.028309	0.238641	1	-
ENSMUS(	Erlin1	ER lipid ra	GO:00081	metabolic	-	-	-	-	19:440349	344	413	513	565	352	236	3.342339	3.363529	3.433687	3.576201	2.999008	2.843081	3.379851	3.13943	0.459695	1	-
ENSMUS(	Chuk	conservec	GO:00325	developm	K04467	inhibitor c	mmu052C	Pathways	19:440733	417	502	689	668	457	386	3.194122	3.223084	3.635682	3.333288	3.069546	3.665964	3.350962	3.356266	0.770583	1	-
ENSMUS(	Cwf19l1	CWF19-lik	-	-	-	-	-	-	19:441086	200	266	271	292	252	171	3.556406	3.964756	3.319732	3.382567	3.929384	3.770193	3.613632	3.694048	0.916986	1	-
ENSMUS(	Scd3	stearoyl-c	GO:00081	metabolic	K00507	stearoyl-C	mmu0415	AMPK sigı	19:442032	46	37	76	66	40	33	0.842483	0.56802	0.958899	0.787472	0.642405	0.74938	0.789801	0.726419	0.598884	1	-
ENSMUS(	Scd2	stearoyl-C	GO:00081	metabolic	K00507	stearoyl-C	mmu0415	AMPK sigı	19:442936	24210	25825	35812	36070	26838	24314	282.16	252.2864	287.5283	273.8599	274.2796	351.3524	273.9916	299.8306	0.683667	1	-
ENSMUS(	Ndufb8	NADH de	GO:00081	metabolic	K03964	NADH de	mmu011C	Metabolic	19:445485	773	1005	1112	1093	911	1060	9.916527	10.80685	9.82735	9.134436	10.24805	16.86056	10.18357	12.08102	0.421936	1	-
ENSMUS(	Sema4g	sema don	GO:00325	developm	K06521	semaphor	mmu0436	Axon guic	19:449891	400	514	489	590	445	416	2.602231	2.802866	2.191524	2.500457	2.538568	3.355565	2.532207	2.798197	0.704121	1	-
ENSMUS(	Mrpl43	mitochon	GO:00329	macromo	-	-	-	-	19:450050	465	531	628	655	472	379	24.36288	23.31967	22.66661	22.35622	21.68503	24.62068	23.44972	22.88731	0.622948	1	-
ENSMUS(	Twnk	twinkle m	GO:00081	metabolic	-	-	-	-	19:450065	292	342	433	397	321	247	5.320381	5.223218	5.434995	4.712283	5.128694	5.580104	5.326198	5.14036	0.576984	1	-
ENSMUS(	Sfxn3	sideroflex	GO:00511	localizatio	-	-	-	-	19:450475	1256	1539	1850	1722	1504	1535	24.67468	25.34273	25.03716	22.03823	25.90909	37.39006	25.01819	28.44579	0.553644	1	-
ENSMUS(	Kazald1	Kazal-typ	GO:00325	developm	-	-	-	-	19:450752	23	23	41	28	14	33	0.643074	0.539033	0.789727	0.510014	0.343246	1.144016	0.657278	0.665759	0.830034	1	-
ENSMUS(	Btrc	beta-tran	GO:00325	developm	K03362	F-box anc	mmu0421	Cellular se	19:453637	1432	1785	1879	2200	1469	1176	4.829287	5.045811	4.365341	4.833307	4.344147	4.917375	4.746813	4.698276	0.680708	1	-
ENSMUS(	Poll	polymera	GO:00081	metabolic	K03512	DNA poly	mmu0341	Base excis	19:455522	128	123	140	169	145	148	3.500333	2.819411	2.637422	3.010708	3.477047	5.018188	2.985722	3.835314	0.261609	1	-
ENSMUS(	Mgea5	meningioi	GO:00081	metabolic	K15719	protein O	mmu0493	Insulin res	19:457502	3996	5018	6016	6305	4665	3529	50.54893	53.20715	52.42588	51.95809	51.74648	55.35083	52.06065	53.01847	0.853033	1	-
ENSMUS(	Kcnip2	Kv channe	GO:00325	developm	-	-	-	-	19:457918	1701	2259	2595	2813	2367	2434	19.10972	21.27252	20.08347	20.58739	23.31798	33.90443	20.15524	25.9366	0.119069	1	-
ENSMUS(	Ldb1	LIM doma	GO:00325	developm	K15617	LIM doma	mmu052C	Transcript	19:460325	1474	1550	1959	1897	1524	1187	11.42265	10.06825	10.45816	9.57675	10.35612	11.40529	10.64968	10.44605	0.606221	1	-
ENSMUS(	Gbf1	golgi-spe	GO:00081	metabolic	K18443	golgi-spe	mmu0414	Endocytos	19:461525	1379	1597	1978	2092	1622	1244	6.259971	6.076671	6.185655	6.186592	6.456565	7.001876	6.174099	6.548344	0.874877	1	-
ENSMUS(	Nfkb2	nuclear fa	GO:00325	developm	K04469	nuclear fa	mmu052C	Pathways	19:463047	48	49	51	74	40	42	0.901462	0.77135	0.659819	0.905361	0.658731	0.978016	0.777544	0.847369	0.843684	1	-
ENSMUS(	Fbxl15	F-box anc	GO:00325	developm	-	-	-	-	19:463281	118	155	164	194	146	292	3.883603	4.275996	3.718339	4.15946	4.213559	11.91576	3.959313	6.762928	0.046611	1	-
ENSMUS(	Mfsd13a	major faci	GO:00160	membran	-	-	-	-	19:463411	270	297	382	433	300	329	5.274911	4.863636	5.141234	5.510888	5.139445	7.969558	5.09326	6.20663	0.314841	1	-
ENSMUS(	Actr1a	ARP1 acti	GO:00329	macromo	-	-	-	-	19:463768	2655	3407	4118	4146	3013	2996	61.53656	66.19019	65.75162	62.60077	61.23651	86.09868	64.49279	69.97865	0.76758	1	-
ENSMUS(	Sufu	suppressc	GO:00325	developm	K06229	suppressc	mmu052C	Pathways	19:463968	286	297	357	415	311	264	2.087773	1.817299	1.7953	1.973547	1.99077	2.389498	1.900124	2.117938	0.642344	1	-
ENSMUS(	Hexa	hexosami	GO:00325	developm	K12373	hexosami	mmu011C	Metabolic	9:5953954	912	986	1348	1342	1004	935	29.03825	26.31511	29.56766	27.83619	28.03185	36.91246	28.30701	30.92684	0.727642	1	-
ENSMUS(	Arih1	ariadne R	GO:00081	metabolic	-	-	-	-	9:5938825	1819	2308	2890	2939	2112	1502	12.12025	12.89043	13.26563	12.75734	12.34001	12.40894	12.75877	12.5021	0.624641	1	-
ENSMUS(	Bbs4	Bardet-Bi	GO:00325	developm	-	-	-	-	9:5932199	792	1113	1278	1295	1007	579	7.792844	9.179494	8.6627	8.300846	8.688467	7.063755	8.545013	8.017689	0.469507	1	-
ENSMUS(	Adpgk	ADP-depe	GO:00081	metabolic	K08074	ADP-depe	mmu011C	Metabolic	9:5929155	247	292	367	375	311	263	1.029349	1.020004	1.05362	1.018075	1.136501	1.358963	1.034325	1.17118	0.571757	1	-
ENSMUS(	Parp6	poly (ADP	GO:00038	catalytic a	-	-	-	-	9:5961728	1371	1953	2158	2241	1728	1502	7.703907	9.198744	8.35365	8.203459	8.514513	10.46476	8.418767	9.060912	0.804206	1	-
ENSMUS(	Limd1	LIM doma	GO:00325	developm	K16682	LIM doma	mmu0439	Hippo sig	9:1234787	125	133	131	196	120	174	0.9299	0.829333	0.671351	0.949867	0.782797	1.60495	0.810195	1.112538	0.177898	1	-
ENSMUS(	Sacm1l	SAC1 sup	GO:00081	metabolic	-	-	mmu011C	Metabolic	9:1235297	1347	1657	2283	2020	1506	993	18.98978	19.58067	22.17224	18.55176	18.61744	17.35751	20.24756	18.17557	0.240404	1	-
ENSMUS(	Fyco1	FYVE and	GO:00081	metabolic	-	-	-	-	9:1237895	962	985	1964	1723	1164	911	4.143261	3.555957	5.827206	4.834309	4.396057	4.864875	4.508808	4.698414	0.980846	1	-
ENSMUS(	Slc6a20b	solute car	GO:00511	localizatio	-	-	-	-	9:1235908	1	2	10	6	5	18	0.005294	0.008873	0.036464	0.02069	0.023205	0.118133	0.016877	0.054009	0.080444	1	-
ENSMUS(	Lztfl1	leucine zip	GO:00511	localizatio	-	-	-	-	9:1236944	726	896	1121	1209	846	487	2.910458	3.010825	3.095862	3.157421	2.973976	2.420695	3.005715	2.850697	0.541707	1	-
ENSMUS(	Tbl1x	transducir	GO:00325	developm	K04508	transducir	mmu0431	Wnt signa	X:7751101	1099	1224	1589	1535	1226	766	9.161172	8.552393	9.124908	8.335724	8.961615	7.917137	8.946158	8.404825	0.433025	1	-
ENSMUS(	Zfhx4	zinc finge	GO:00081	metabolic	-	-	-	-	3:5218526	296	330	605	649	485	171	0.725956	0.678399	1.022175	1.036919	1.043045	0.519997	0.808843	0.866653	0.81243	1	-
ENSMUS(	Ribc1	RIB43A dc	-	-	-	-	-	-	X:1520045	8	0	7	3	3	0	0.296792	0	0.178916	0.072498	0.097591	0	0.158569	0.056696	0.157051	1	-
ENSMUS(	Hsd17b1C	hydroxyst	GO:00081	metabolic	K08683	3-hydroxy	mmu011C	Metabolic	X:1520018	230	330	397	286	219	251	7.349021	8.838293	8.738624	5.95318	6.136049	9.943992	8.308646	7.344407	0.266736	1	-
ENSMUS(	Huwe1	HECT, UB	GO:00325	developm	K10592	E3 ubiquit	mmu0412	Ubiquitin	X:1518008	9424	10205	12481	14871	10782	6463	19.66327	17.84787	17.93995	20.21354	19.72706	16.72017	18.4837	18.88692	0.921998	1	-
ENSMUS(	Fam120c	family witl	GO:00056	cell|intrace	-	-	-	-	X:1513441	1231	1430	1685	1877	1389	894	12.99564	12.65402	12.25437	12.90879	12.85833	11.70208	12.63468	12.48974	0.705762	1	-
ENSMUS(	Tsr2	TSR2 20S	-	-	-	-	-	-	X:1510870	903	1429	1248	1307	1224	825	6.338456	8.407766	6.034782	5.976581	7.533897	7.180188	6.927001	6.896889	0.746822	1	-
ENSMUS(	Fgd1	FYVE, Rhc	GO:00325	developm	K05720	FYVE, Rhc	mmu0481	Regulatior	X:1510461	337	359	384	467	313	266	3.980178	3.554023	3.12432	3.593116	3.241607	3.8953	3.55284	3.576674	0.815018	1	-
ENSMUS(	Gnl3l	guanine n	GO:00081	metabolic	K14538	nuclear G	mmu030C	Ribosome	X:1509831	4073	3306	3646	3884	4545	3349	39.80492	27.08183	24.54655	24.72767	38.94924	40.58106	30.47777	34.75266	0.591374	1	-
ENSMUS(	Maged2	melanom	GO:00511	localizatio	-	-	-	-	X:1508063	467	674	729	806	591	520	4.679793	5.661382	5.03256	5.261704	5.19326	6.460994	5.124578	5.638653	0.689755	1	-

ENSMUSC	Apex2	apurinic/a	GO:00081	metabolic	K10772	AP endon	mmu0341	Base excis	X:1505195	84	128	86	128	81	89	0.370415	0.473123	0.261255	0.36771	0.313212	0.486615	0.368264	0.389179	0.972933	1	-
ENSMUSC	Alas2	aminolevu	GO:00325	developm	K00643	5-aminole	mmu011C	Metabolic	X:1505473	19	69	73	63	50	64	0.377818	1.150084	1.000014	0.816113	0.871854	1.577955	0.842638	1.088641	0.490283	1	-
ENSMUSC	Pfkfb1	6-phosph	GO:00325	developm	K19028	6-phosph	mmu0415	AMPK sig	X:1505882	5	9	6	19	6	4	0.062198	0.093837	0.051414	0.153966	0.065443	0.061694	0.06915	0.093701	0.516065	1	-
ENSMUSC	Tro	trophinin	GO:00099	cellular pr	-	-	-	-	X:1506453	3446	3787	2737	3008	2243	2677	23.23153	21.39985	12.71127	13.21058	13.25973	22.37674	19.11422	16.28235	0.20589	1	-
ENSMUSC	Abhd6	abhydrola	GO:00081	metabolic	K13700	abhydrola	mmu0472	Retrograd	14:800290	543	651	807	788	600	496	8.635953	8.678494	8.841693	8.164292	8.367675	9.780893	8.718713	8.770953	0.776586	1	-
ENSMUSC	Flnb	filamin, be	GO:00325	developm	K04437	filamin[-]	mmu0401	MAPK sig	14:781795	1322	1802	2257	2591	1967	1575	9.121385	10.42166	10.72784	11.64604	11.90081	13.47398	10.0903	12.34028	0.175071	1	-
ENSMUSC	Dnase1l3	deoxyribc	GO:00325	developm	-	-	-	-	14:796453	2	3	0	3	0	3	0.024216	0.030439	0	0.023655	0	0.045032	0.018218	0.022896	1	1	-
ENSMUSC	Polr3a	polymera	GO:00081	metabolic	K03018	DNA-dire	mmu011C	Metabolic	14:244486	467	539	669	816	573	429	3.872048	3.745983	3.821215	4.407537	4.166022	4.410292	3.813082	4.32795	0.495574	1	-
ENSMUSC	Sat1	spermidin	GO:00325	developm	K00657	diamine N	mmu011C	Metabolic	X:1552131	365	335	602	578	395	356	4.738892	3.645701	5.384328	4.888696	4.497003	5.730862	4.58964	5.038854	0.732081	1	-
ENSMUSC	Acot9	acyl-CoA	GO:00081	metabolic	-	-	-	-	X:1552624	137	183	206	234	135	121	2.938489	3.290099	3.04385	3.269651	2.539101	3.217911	3.090813	3.008888	0.681417	1	-
ENSMUSC	Prdx4	peroxired	GO:00325	developm	-	-	-	-	X:1553239	79	94	150	100	90	127	2.040089	2.034712	2.668504	1.682311	2.038029	4.06644	2.247769	2.595594	0.716984	1	-
ENSMUSC	Rps24	ribosomal	GO:00081	metabolic	K02974	small subu	mmu0301	Ribosome	14:244871	942	1170	1364	1425	1019	554	5.411458	5.633807	5.397952	5.332853	5.133101	3.946014	5.481072	4.803989	0.240387	1	-
ENSMUSC	Ptprj	protein ty	GO:00081	metabolic	K05698	receptor-t	mmu0452	Adherens	2:9042975	2794	2628	3147	4583	2488	1883	19.45728	15.3403	15.09746	20.79156	15.19318	16.25893	16.63168	17.41456	0.938901	1	-
ENSMUSC	Banp	BTG3 assc	GO:00325	developm	-	-	-	-	8:1219497	388	511	575	624	402	348	2.266414	2.501961	2.313805	2.374508	2.059095	2.520421	2.360727	2.318008	0.665157	1	-
ENSMUSC	Car5a	carbonic a	GO:00081	metabolic	K01672	carbonic a	mmu0091	Nitrogen r	8:1219161	7	6	5	12	3	6	0.311337	0.22366	0.153192	0.347682	0.116986	0.330887	0.229396	0.265185	0.897501	1	-
ENSMUSC	Jph3	junctophil	GO:00048	signal trar	-	-	-	-	8:1217296	3930	4441	5648	6435	4964	4275	43.69548	41.3883	43.26035	46.60946	48.39699	58.934	42.78138	51.31349	0.225826	1	-
ENSMUSC	Itgb8	integrin b	GO:00325	developm	K06591	integrin b	mmu0415	PI3K-Akt s	12:119158	1259	1443	1927	1998	1338	794	5.873392	5.642642	6.192935	6.072123	5.473463	4.59272	5.90299	5.379436	0.348262	1	-
ENSMUSC	Sp4	trans-acti	GO:00081	metabolic	-	-	-	-	12:118234	531	652	804	858	707	323	4.922927	5.066744	5.134948	5.182002	5.747657	3.71293	5.04154	4.880863	0.694796	1	-
ENSMUSC	Atp10a	ATPase, cl	GO:00511	localizatio	-	-	-	-	7:5865616	252	284	351	365	310	227	2.622457	2.477308	2.516327	2.474471	2.828871	2.929003	2.538697	2.744115	0.793643	1	-
ENSMUSC	Ube3a	ubiquitin p	GO:00325	developm	K10587	ubiquitin-	mmu0516	Human pa	7:5922875	1411	1375	1496	1194	913	1524	3.086541	2.521162	2.254385	1.701497	1.751291	4.133481	2.620696	2.528757	0.60292	1	-
ENSMUSC	Padi4	peptidyl a	GO:00325	developm	-	-	-	-	4:1407458	0	0	3	2	0	0	0	0	0.022509	0.014189	0	0	0.007503	0.00473	1	1	-
ENSMUSC	Kdm5c	lysine (K)-	GO:00081	metabolic	-	-	-	-	X:1522330	1232	1207	1807	1685	1408	1023	6.367706	5.229167	6.434013	5.673536	6.381423	6.555921	6.010295	6.203627	0.947461	1	-
ENSMUSC	Sbds	SBDS ribo	GO:00325	developm	K14574	ribosome	mmu0300	Ribosome	5:1302457	813	1009	1694	1670	1080	810	16.80827	17.48542	24.12665	22.49212	19.57937	20.76363	19.47345	20.94504	0.786605	1	-
ENSMUSC	Rabgef1	RAB guan	GO:00081	metabolic	-	-	-	-	5:1301717	351	483	492	688	522	479	2.771066	3.196245	2.675818	3.538427	3.613713	4.688795	2.881043	3.946978	0.048552	1	-
ENSMUSC	Mettl7b	methyltrai	GO:00081	metabolic	-	-	-	-	10:128958	3	0	0	0	0	0	0.154989	0	0	0	0	0	0.051663	0	0.309014	1	-
ENSMUSC	Itga7	integrin al	GO:00325	developm	K06583	integrin al	mmu0415	PI3K-Akt s	10:128933	516	589	806	963	567	514	3.29714	3.154683	3.547923	4.008628	3.176971	4.072269	3.333249	3.752623	0.553307	1	-
ENSMUSC	Rdh5	retinol del	GO:00081	metabolic	K00061	11-cis-ret	mmu0083	Retinol m	10:128913	77	38	179	52	51	86	1.040304	0.430336	1.665996	0.457674	0.604203	1.440639	1.045545	0.834172	0.36742	1	-
ENSMUSC	Cd63	CD63 anti	GO:00325	developm	K06497	CD63 anti	mmu052C	Proteoglyc	10:128900	580	634	1038	754	529	477	7.319443	6.706444	9.024004	6.198743	5.853944	7.46371	7.683297	6.505465	0.120354	1	-
ENSMUSC	Gdf11	growth di	GO:00325	developm	-	-	-	-	10:128882	521	639	786	821	565	340	8.151435	8.380119	8.471688	8.367984	7.751528	6.595713	8.334414	7.571742	0.361783	1	-
ENSMUSC	Ormdl2	ORM1-lik	GO:00081	metabolic	-	-	-	-	10:128817	39	40	38	47	43	32	0.696621	0.598886	0.467585	0.546901	0.673502	0.708705	0.587697	0.643036	0.842469	1	-
ENSMUSC	Dnajc14	DnaJ heat	GO:00511	localizatio	-	-	-	-	10:128804	625	1052	907	947	1225	507	3.790136	5.347408	3.789077	3.741163	6.514087	3.812143	4.308874	4.689131	0.765989	1	-
ENSMUSC	Mmp19	matrix me	GO:00325	developm	-	-	-	-	10:128790	22	18	25	29	30	33	0.285977	0.19613	0.223872	0.245584	0.341961	0.531878	0.235326	0.373141	0.134765	1	-
ENSMUSC	Dgka	diacylglyc	GO:00081	metabolic	K00901	diacylglyc	mmu011C	Metabolic	10:128720	223	263	329	367	284	228	2.489864	2.46139	2.530568	2.669429	2.780564	3.156395	2.493941	2.868796	0.482149	1	-
ENSMUSC	Cdk2	cyclin-dep	GO:00325	developm	K02206	cyclin-dep	mmu052C	Pathways	10:128697	50	44	76	76	41	28	0.266316	0.196439	0.278862	0.263705	0.191491	0.184912	0.247206	0.21337	0.435261	1	-
ENSMUSC	Pmel	premelan	GO:00099	cellular pr	-	-	-	-	10:128704	2	4	12	15	2	4	0.048358	0.081047	0.199846	0.236222	0.042392	0.119892	0.10975	0.132835	0.81796	1	-
ENSMUSC	Rps26	ribosomal	GO:00081	metabolic	K02976	small subu	mmu0301	Ribosome	10:128624	346	446	570	420	390	254	10.73176	11.59529	12.17925	8.486424	10.60723	9.768188	11.5021	9.620613	0.115943	1	-
ENSMUSC	Pa2g4	proliferati	GO:00325	developm	-	-	-	-	10:128557	1300	1756	2082	2153	1462	928	18.08646	20.47799	19.95456	19.51353	17.8361	16.00825	19.50634	17.78596	0.322206	1	-
ENSMUSC	Esyt1	extended	GO:00511	localizatio	-	-	-	-	10:128509	111	195	251	230	190	206	0.670698	0.98762	1.044788	0.905342	1.006696	1.543322	0.901035	1.151787	0.277095	1	-
ENSMUSC	Smarcc2	SWI/SNF r	GO:00325	developm	K11649	SWI/SNF r	mmu0522	Hepatoce	10:128459	5985	7062	8423	8903	6876	4141	47.65878	47.13672	46.20586	46.18456	48.01283	40.88557	47.00045	45.02765	0.524107	1	-
ENSMUSC	Cdh9	cadherin 9	GO:00325	developm	-	-	-	-	15:167287	757	951	1070	1217	1000	400	10.07109	10.60509	9.806548	10.54758	11.66604	6.598227	10.16091	9.60395	0.619458	1	-
ENSMUSC	Chmp6	charged n	GO:00081	metabolic	K12195	charged n	mmu0414	Endocytos	11:119913	250	352	359	417	310	289	4.580074	5.405396	4.530831	4.976793	4.980085	6.564719	4.838767	5.507199	0.561779	1	-
ENSMUSC	Baiap2	brain-spe	GO:00325	developm	K05627	BAI1-assc	mmu0481	Regulatior	11:119942	3965	4616	5264	6111	4856	2732	34.86271	34.02018	31.88492	35.00354	37.4403	29.78411	33.58927	34.07598	0.880531	1	-
ENSMUSC	Rnf41	ring finge	GO:00325	developm	K11981	E3 ubiquit	mmu0414	Endocytos	10:128411	1028	1071	1330	1551	1070	1130	8.237614	7.193668	7.341946	8.096581	7.518556	11.22723	7.591076	8.947455	0.370552	1	-
ENSMUSC	Nabp2	nucleic ac	GO:00081	metabolic	-	-	-	-	10:128401	836	1162	1475	1450	1081	561	12.00685	13.98885	14.59373	13.56665	13.61417	9.99014	13.52981	12.39032	0.410313	1	-
ENSMUSC	Aatk	apoptosis	GO:00081	metabolic	-	-	-	-	11:120007	4212	4432	5899	5850	4856	5470	16.59344	14.63526	16.0095	15.01362	16.77526	26.71906	15.74607	19.50265	0.237094	1	-
ENSMUSC	Tepsin	TEPSIN, a	GO:00329	macromo	-	-	-	-	11:120090	259	297	354	359	278	226	3.417116	3.284494	3.217468	3.085567	3.216227	3.69704	3.306359	3.332945	0.809841	1	-
ENSMUSC	Fscn2	fascin acti	GO:00325	developm	-	-	-	-	11:120361	9	0	2	2	3	0	0.213183	0	0.03264	0.030858	0.062308	0	0.081941	0.031056	0.353304	1	-
ENSMUSC	Cnpy2	canopy FC	GO:00325	developm	-	-	-	-	10:128322	349	398	455	501	378	435	8.230058	7.867074	7.391627	7.696563	7.816496	12.71897	7.829586	9.410678	0.385802	1	-
ENSMUSC	Il23a	interleukir	GO:00325	developm	K05426	interleukir	mmu052C	Pathways	10:128296	0	5	8	4	3	0	0	0.090685	0.119254	0.056393	0.05692	0	0.06998	0.037771	0.369518	1	-
ENSMUSC	Faap100	Fanconi a	GO:00081	metabolic	K10993	fanconi ar	mmu0346	Fanconi a	11:120369	177	240	266	288	232	227	1.114966	1.267218	1.154308	1.181848	1.281502	1.772963	1.17883	1.412104	0.386913	1	-
ENSMUSC	Pde6g	phosphoc	GO:00081	metabolic	K13759	retinal roc	mmu0023	Purine me	11:120447	0	0	5	4	6	2	0	0	0.057008	0.043132	0.087069	0.041052	0.019003	0.057085	0.149814	1	-
ENSMUSC	Atp5b	ATP synth	GO:00325	developm	K02133	F-type H+	mmu011C	Metabolic	10:128083	14507	18690	21246	20323	15992	13913	133.2702	143.9189	134.4572	121.6256	128.8253	158.4756	137.2154	136.3088	0.650648	1	-
ENSMUSC	Prim1	DNA prim	GO:00081	metabolic	K02684	DNA prim	mmu011C	Metabolic	10:128015	63	76	96	87	85	49	0.688184	0.695874	0.72241	0.619102	0.814182	0.663653	0.702156	0.698979	0.841783	1	-
ENSMUSC	Tac2	tachykinin	GO:00508	response	-	-	-	-	10:127724	38	46	38	43	36	40	1.250654	1.269003	0.861558	0.921952	1.038968	1.632297	1.127072	1.197739	1	1	-
ENSMUSC	Myo1a	myosin IA	GO:00511	localizatio	-	-	-	-	10:127705	13	37	45	28	20	11	0.195775	0.467069	0.466861	0.274707	0.264123	0.205406	0.376568	0.248078	0.122211	1	-
ENSMUSC	Nab2	Ngfi-A bir	GO:00325	developm	-	-	-	-	10:127660	648	723	802	910	715	569	9.180191	8.585552	7.827128	8.398466	8.882298	9.99482	8.530957	9.091861	0.867787	1	-
ENSMUSC	Shmt2	serine hyc	GO:00081	metabolic	K00600	glycine hy	mmu011C	Metabolic	10:127517	330	372	441	503	431	301	3.574046	3.377085	3.290305	3.548915	4.09323	4.042024	3.413812	3.894723	0.496788	1	-
ENSMUSC	R3hdm2	R3H dom	GO:00056	cell|intrace	-	-	-	-	10:127380	2752	3312	4554	4562	3299	2322	12.34283	12.45115	14.07054	13.32917	12.97452	12.91262	12.95484	13.0721	0.813846	1	-
ENSMUSC	Gli1	GLI-Krupp	GO:00325	developm	K16797	zinc finge	mmu052C	Pathways	10:127329	20	29	28	43	30	16	0.213375	0.259331	0.20579	0.298857	0.280652	0.211648	0.226165	0.263719	0.598225	1	-
ENSMUSC	Ddit3	DNA-dan	GO:00325	developm	K04452	DNA dam	mmu0401	MAPK sig	10:127290	209	282	445	310	334	232	5.772519	6.52861	8.46702	5.577797	8.089247	7.944978	6.922716	7.204007	0.988717	1	-
ENSMUSC	Mbd6	methyl-C	GO:00056	cell|intrace	-	-	-	-	10:127281	459	471	608	649	498	426	4.225209	3.634206	3.855585	3.8919	4.019828	4.862176	3.905	4.257968	0.74987	1	-
ENSMUSC	Dctn2	dynactin 2	GO:00511	localizatio	K10424	dynactin 2	mmu0501	Huntingto	10:127266	3009	3875	4361	4563	3489	3045	31.11471	33.58676	31.0657	30.738	31.63641	39.04063	31.92239	33.80501	0.904972	1	-
ENSMUSC	Ttc4	tetratricop	-	-	-	-	-	-	4:1066622	638	807	656	988	767	369	5.92443	6.281346	4.196442	5.976745	6.245453	4.24853	5.467406	5.490243	0.879603	1	-
ENSMUSC	Pip4k2c	phosphati	GO:00081	metabolic	K00920	1-phosph	mmu0481	Regulatior	10:127197	2513	2846	3335	3438	2527	2271	27.00063	25.63121	24.68475	24.06406	23.80835	30.25411	25.7722	26.04217	0.773005	1	-
ENSMUSC	Katnal2	katanin p6	GO:00990	supramole	-	-	-	-	18:769771	94	90	140	121	69	73	0.858945	0.689339	0.881289	0.72029	0.552878	0.827076	0.809858	0.700081	0.288258	1	-
ENSMUSC	Hdhd2	haloacid c	GO:00081	metabolic	-	-	-	-	18:769301	966	1159	1669	1496	1059	863	7.499662	7.542243	8.926311	7.566198	7.209468	8.307323	7.989405	7.69433	0.5258	1	-
ENSMUSC	Agap2	ArfGAP w	GO:00325	developm	K17848	Arf-GAP w	mmu0414	Endocytos	10:127075	21512	23645	24480	28212	21710	19068	228.2391	210.2817	178.9255	194.9956	201.9816	250.8421	205.8154	215.9398	0.978127	1	-
ENSMUSC	Pias2	protein in	GO:00325	developm	K16063	E3 SUMO	mmu0463	Jak-STAT	18:770652	1370	1532	1995	1942	1522	782	16.0257	15.02131	16.07647	14.79882	15.61183	11.34199	15.70783	13.91755	0.266149	1	-
ENSMUSC	St8sia5	ST8 alpha	GO:00081	metabolic	K03369	alpha-N-	mmu011C	Metabolic	18:771858	607	758	932	1022	823	583	11.69345	12.23984	12.36862	12.82585	13.90262	13.92543	12.10064	13.5513	0.552417	1	-
ENSMUSC	Rnf165	ring finge	GO:00325	developm	-	-	-	-	18:774561	1790	2107	2356	2772	1961	1576	7.751951	7.648485	7.028856	7.820471	7.446947	8.462527	7.476431	7.909981	0.892552	1	-
ENSMUSC	Atp5a1	ATP synth	GO:00081	metabolic	K02132	F-type H+	mmu011C	Metabolic	18:777737	8918	10447	13064	13319	10247	8501	107.5253	105.5814	108.5102	104.6156	108.3384	127.0864	107.2057	113.3468	0.903736	1	-
ENSMUSC	Pstpip2	proline-se	GO:00400	locomotic	-	-	-	-	18:777945	125	132	163	149	114	58	5.694722	5.040663	5.115662	4.422105	4.554185	3.276266	5.283682	4.084185	0.11043	1	-
ENSMUSC	Avil	advillin [S	GO:00325	developm	-	-	-	-	10:127000	0	10	15	12	9	5	0	0.129073	0.159131	0.120387	0.121535	0.095463	0.096068	0.112462	0.855697	1	-
ENSMUSC	Crisp3	cysteine-r	GO:00055	extracellul	-	-	-	-	17:402217	0	0	0	2	0	0	0	0	0	0.058428	0	0	0	0.019476	0.537018	1	-
ENSMUSC	Atp23	ATP23 me	GO:00081	metabolic	-	-	-	-	10:126868	93	109	102	118	87	72	1.255674	1.233589	0.948736	1.037898	1.030044	1.20534	1.146	1.091094	0.615831	1	-
ENSMUSC	Usp33	ubiquitin s	GO:00325	developm	-	-	-	-	3:1523464	2609	3440	3794	4034	3013	1687	11.3444	12.53773	11.36466	11.42681	11.48813	9.095122	11.74893	10.67002	0.321143	1	-
ENSMUSC	Clns1a	chloride c	GO:00081	metabolic	K05019	chloride c	mmu0301	RNA trans	7:9769663	903	1198	1328	1313	999	830	11.95591	13.29549	12.11279	11.32508	11.59853	13.62571	12.45473	12.18311	0.594701	1	-
ENSMUSC	Paip1	polyadeny	GO:00451	translatior	K14322	polyadeny	mmu0301	RNA trans	13:119428	867	1142	1306	1306	1033	584	10.06033	11.1074	10.4397	9.872296	10.51082	8.402185	10.53581	9.5951	0.340008	1	-
ENSMUSC	Nnt	nicotinam	GO:00081	metabolic	K00323	NAD(P) tr	mmu011C	Metabolic	13:119335	268	331	435	414	299	281	2.03881	2.110682	2.279727	2.051748	1.9946	2.650541	2.143073	2.232296	0.980787	1	-
ENSMUSC	Paox	polyamine	GO:00081	metabolic	K00308	N1-acetyl	mmu0414	Peroxisom	7:1401157	67	110	139	146	84	102	0.692817	0.953428	0.990173	0.983509	0.761664	1.30777	0.878806	1.017648	0.609416	1	-
ENSMUSC	Echs1	enoyl Coe	GO:00081	metabolic	K07511	enoyl-Co/	mmu011C	Metabolic	7:1401057	330	410	573	567	361	420	1.884491	1.962534	2.254168	2.109329	1.807712	2.973826	2.033731	2.296956	0.62435	1	-
ENSMUSC	Fuom	fucose mu	GO:00325	developm	-	-	-	-	7:1400967	145	181	237	200	160	161	1.519909	1.590306	1.71139	1.365716	1.470655	2.092468	1.607202	1.642946	0.869085	1	-
ENSMUSC	Caly	calcyon ne	GO:00511	localizatio	K15493	neuron-sp	mmu0472	Dopamine	7:1400698	2097	2764	3222	3052	2506	1797	35.38786	39.09729	37.45698	33.55228	37.08341	37.60017	37.31404	36.07862	0.530386	1	-
ENSMUSC	Msx3	msh hom	GO:00325	developm	K09341	homeobo	mmu0516	HTLV-I inf	7:1400461	0	2	2	3	3	0	0	0.024071	0.019786	0.028059	0.037771	0	0.014619	0.021943	0.784458	1	-
ENSMUSC	Zfp511	zinc finge	GO:00081	metabolic	-	-	-	-	7:1400363	169	198	253	264	194	179	2.706775	2.658162	2.791495	2.754544	2.724642	3.554703	2.718811	3.011296	0.709715	1	-
ENSMUSC	Adam8	a disinteg	GO:00325	developm	-	-	-	-	7:1399789	103	142	238	258	139	132	1.020729	1.179551	1.624812	1.665619	1.207907	1.621937	1.275031	1.498488	0.497706	1	-
ENSMUSC	Tubgcp2	tubulin, ga	GO:00051	structural	-	-	-	-	7:1399959	725	927	957	1095	847	578	7.661444	8.211171	6.966843	7.538216	7.848715	7.573317	7.613153	7.653416	0.804143	1	-
ENSMUSC	Adgra1	adhesion	GO:00048	signal trar	-	-	-	-	7:1398341	1320	1619	1712	1872	1514	1089	13.12421	13.4927	11.72613	12.12514	13.19982	13.42497	12.78101	12.91664	0.809535	1	-
ENSMUSC	Inpp5a	inositol pc	GO:00081	metabolic	K01106	inositol-1,	mmu011C	Metabolic	7:1393891	557	757	976	907	669	518	4.793366	5.460523	5.786111	5.084806	5.048408	5.527148	5.346667	5.220121	0.620292	1	-
ENSMUSC	Dpysl4	dihydropy	GO:00325	developm	-	-	-	-	7:1390860	664	877	870	1032	900	834	4.378874	4.847823	3.952436	4.433593	5.204502	6.819403	4.393044	5.485833	0.200071	1	-
ENSMUSC	Cyp2e1	cytochron	GO:00081	metabolic	K07415	cytochron	mmu011C	Metabolic	7:1407637	4	6	5	0	6	0	0.074481	0.093645	0.06414	0	0.097963	0	0.077422	0.032654	0.182164	1	-
ENSMUSC	Syce1	synaptone	GO:00099	cellular pr	-	-	-	-	7:1407772	1	2	0	0	2	4	0.03958	0.066337	0	0	0.069395	0.196262	0.035306	0.088552	0.430735	1	-
ENSMUSC	Urah	urate (5-h	GO:00081	metabolic	K07127	5-hydroxy	mmu011C	Metabolic	7:1408350	8	15	8	9	9	0	0.159775	0.251124	0.110072	0.117099	0.157629	0	0.173657	0.091576	0.179541	1	-
ENSMUSC	Odf3	outer den	GO:00325	developm	-	-	-	-	7:1408478	0	1	6	1	1	0	0	0.015498	0.076426	0.012044	0.016213	0	0.030641	0.009419	0.371978	1	-
ENSMUSC	Bet1l	Bet1 golg	GO:00511	localizatio	K08504	blocked e	mmu0413	SNARE int	7:1408533	268	341	385	369	295	404	2.46237	2.626184	2.436862	2.208649	2.376744	4.602418	2.508472	3.062604	0.420216	1	-
ENSMUSC	Ric8a	RIC8 guar	GO:00325	developm	-	-	-	-	7:1408569	573	748	723	885	600	702	2.113884	2.313029	1.837454	2.126922	1.940978	3.21107	2.088122	2.426324	0.496252	1	-
ENSMUSC	Sirt3	sirtuin 3 [S	GO:00081	metabolic	K11413	NAD-dep	mmu0523	Central ca	7:1408636	454	645	678	660	614	664	3.391287	4.038511	3.488912	3.211692	4.021795	6.149823	3.63957	4.461103	0.329214	1	-
ENSMUSC	Psmd13	proteasor	GO:00081	metabolic	K03039	26S prote	mmu0516	Epstein-B	7:1408819	616	739	963	893	730	546	5.796366	5.828706	6.242415	5.474045	6.023388	6.370204	5.955829	5.955879	0.749589	1	-
ENSMUSC	Cox8b	cytochron	GO:00081	metabolic	K02273	cytochron	mmu011C	Metabolic	7:1408989	0	0	12	0	0	6	0	0	1.459429	0	0	1.313438	0.486476	0.437813	0.920457	1	-
ENSMUSC	Ifitm1	interferon	GO:00325	developm	K19831	interferon	mmu0466	B cell rece	7:1409672	14	19	24	27	18	14	0.681792	0.775593	0.805169	0.856571	0.768656	0.845388	0.754185	0.823538	0.887662	1	-
ENSMUSC	Sigirr	single imr	GO:00081	metabolic	-	-	-	-	7:1410911	11	27	22	23	12	11	0.130747	0.268991	0.180133	0.178088	0.12507	0.162112	0.19329	0.15509	0.410501	1	-
ENSMUSC	Ptdss2	phosphati	GO:00081	metabolic	K08730	phosphati	mmu011C	Metabolic	7:1411223	652	760	861	936	696	662	5.139097	5.021179	4.675135	4.806145	4.810513	6.469694	4.945137	5.362117	0.790108	1	-
ENSMUSC	Drd4	dopamine	GO:00048	signal trar	K04147	dopamine	mmu0408	Neuroacti	7:1412920	6	4	0	0	5	0	0.103764	0.057979	0	0	0.075816	0	0.053914	0.025272	0.433175	1	-
ENSMUSC	Cdhr5	cadherin-	GO:00325	developm	-	-	-	-	7:1412690	5	1	3	1	0	1	0.075803	0.012707	0.031331	0.009875	0	0.018792	0.039947	0.009556	0.109557	1	-
ENSMUSC	Hras	Harvey ra	GO:00325	developm	K02833	GTPase H	mmu052C	Pathways	7:1411891	1052	1388	1558	1660	1212	902	7.908421	8.746133	8.068497	8.129496	7.989491	8.407491	8.241017	8.175493	0.699907	1	-
ENSMUSC	Lmntd2	lamin tail	GO:00081	metabolic	-	-	-	-	7:1412099	2	0	2	4	0	0	0.049238	0	0.033917	0.064146	0	0	0.027718	0.021382	1	1	-
ENSMUSC	Taldo1	transaldol	GO:00081	metabolic	K00616	transaldol	mmu011C	Metabolic	7:1413921	800	795	1013	882	870	849	17.34643	14.44908	15.13147	12.45863	16.54178	22.82518	15.64233	17.2752	0.746286	1	-
ENSMUSC	Eps8l2	EPS8-like	GO:00987	molecular	-	-	-	-	7:1413388	86	85	180	113	102	73	0.684907	0.56742	0.987545	0.586266	0.712323	0.720841	0.746624	0.673144	0.452381	1	-
ENSMUSC	Pidd1	p53 induc	GO:00081	metabolic	K10130	leucine-ri	mmu0421	Apoptosis	7:1414381	21	14	35	33	20	31	0.108419	0.060586	0.124479	0.110987	0.090544	0.19844	0.097828	0.133324	0.390052	1	-
ENSMUSC	Rplp2	ribosomal	GO:00081	metabolic	K02943	large subu	mmu0301	Ribosome	7:1414473	593	804	949	889	721	518	13.35943	15.18244	14.72823	13.04717	14.24331	14.46933	14.42337	13.91994	0.557202	1	-
ENSMUSC	Pnpla2	patatin-lik	GO:00081	metabolic	K16816	patatin-lik	mmu011C	Metabolic	7:1414551	256	259	403	340	275	210	2.328212	1.9744	2.524871	2.014391	2.193101	2.368039	2.275828	2.191844	0.589711	1	-
ENSMUSC	Cd151	CD151 an	GO:00400	locomotic	-	-	-	-	7:1414673	264	341	304	407	300	332	2.508674	2.716106	1.990054	2.519511	2.499793	3.911691	2.404945	2.976998	0.308906	1	-
ENSMUSC	Tspan4	tetraspani	GO:00508	response	-	-	-	-	7:1414752	53	65	83	91	62	146	1.0425	1.071679	1.124674	1.166066	1.069388	3.560729	1.079617	1.932061	0.062158	1	-
ENSMUSC	Chid1	chitinase c	GO:00081	metabolic	-	-	-	-	7:1414931	764	856	1047	1121	820	997	2.507721	2.355117	2.367467	2.397028	2.360164	4.05758	2.410102	2.938258	0.313882	1	-
ENSMUSC	Tktl2	transketol	GO:00081	metabolic	K00615	transketol	mmu011C	Metabolic	8:6651175	0	4	7	2	5	0	0	0.028387	0.040832	0.01103	0.03712	0	0.023073	0.01605	0.724129	1	-
ENSMUSC	Tmem192	transmem	GO:00055	extracellul	-	-	-	-	8:6494718	184	241	270	183	196	156	3.875945	4.255313	3.918102	2.511275	3.620427	4.074465	4.016453	3.402056	0.183141	1	-
ENSMUSC	Apool	apolipopr	GO:00329	macromo	-	-	-	-	X:1123113	34	43	73	65	37	28	0.539798	0.572231	0.798412	0.672269	0.515107	0.55118	0.636814	0.579519	0.572642	1	-
ENSMUSC	Zfp711	zinc finge	GO:00081	metabolic	-	-	-	-	X:1126005	242	244	352	345	293	98	1.772684	1.498166	1.776276	1.646333	1.88203	0.890083	1.682375	1.472815	0.434841	1	-
ENSMUSC	Chm	choroider	GO:00081	metabolic	-	-	-	-	X:1130405	1155	1494	1672	1758	1260	561	6.891721	7.47221	6.872797	6.833546	6.592635	4.150443	7.078909	5.858874	0.169545	1	-
ENSMUSC	Crcp	calcitonin	GO:00048	signal trar	-	-	-	-	5:1300292	387	449	597	433	466	322	6.124258	5.955814	6.508311	4.463874	6.466519	6.318067	6.196128	5.749487	0.415711	1	-
ENSMUSC	Asl	argininosu	GO:00325	developm	K01755	argininosu	mmu011C	Metabolic	5:1300112	138	183	183	187	168	216	1.319637	1.466832	1.205529	1.164927	1.408732	2.561031	1.330666	1.711563	0.31733	1	-
ENSMUSC	Gusb	glucuronic	GO:00081	metabolic	K01195	beta-gluc	mmu011C	Metabolic	5:1299890	210	220	304	320	214	220	1.667641	1.4644	1.663061	1.655444	1.490182	2.166163	1.598368	1.770596	0.714061	1	-
ENSMUSC	Phkg1	phosphor	GO:00081	metabolic	K00871	phosphor	mmu0402	Calcium si	5:1298634	321	414	496	562	449	312	2.757201	2.980688	2.934922	3.144715	3.381831	3.32279	2.890937	3.283112	0.508079	1	-
ENSMUSC	Sumf2	sulfatase r	GO:00056	cell|intrace	-	-	-	-	5:1298469	149	190	211	217	133	91	2.402794	2.568242	2.344034	2.279664	1.880718	1.819523	2.438357	1.993302	0.152804	1	-
ENSMUSC	Tm9sf2	transmem	GO:00055	extracellul	-	-	-	-	14:122107	1943	2364	2972	2871	2164	1567	32.94651	33.59981	34.71656	31.71403	32.17633	32.94515	33.75429	32.2785	0.471627	1	-
ENSMUSC	Clybl	citrate lya	GO:00038	catalytic a	-	-	-	-	14:122181	207	269	283	258	224	182	7.626374	8.307156	7.182651	6.192239	7.236651	8.313903	7.705394	7.247598	0.476254	1	-
ENSMUSC	Farp1	FERM, Rh	GO:00325	developm	-	-	-	-	14:121035	1041	1218	1556	1604	1373	1001	11.17357	10.95825	11.50541	11.21572	12.92273	13.32175	11.21241	12.48673	0.575923	1	-
ENSMUSC	Slc15a1	solute car	GO:00511	localizatio	K14206	solute car	mmu0497	Protein di	14:121459	0	0	3	3	0	0	0	0	0.02609	0.02467	0	0	0.008697	0.008223	1	1	-
ENSMUSC	Dock9	dedicator	GO:00987	molecular	-	-	-	-	14:121542	7089	7443	9097	11235	7737	4559	23.15145	20.37484	20.46647	23.90276	22.15688	18.46073	21.33092	21.50679	0.849656	1	-
ENSMUSC	Tnrc6c	trinucleoti	GO:00081	metabolic	-	-	-	-	11:117654	2566	2920	3322	4248	3223	2317	6.340474	6.047848	5.654787	6.838035	6.983419	7.098673	6.01437	6.973376	0.347445	1	-
ENSMUSC	Tmc6	transmem	GO:00511	localizatio	-	-	-	-	11:117765	30	52	75	70	48	82	0.3365	0.488891	0.579522	0.511492	0.472114	1.140411	0.468304	0.708006	0.169315	1	-
ENSMUSC	6030468B	RIKEN cDI	GO:00055	extracellul	-	-	-	-	11:117797	0	0	4	1	0	0	0	0	0.163535	0.038652	0	0	0.054512	0.012884	0.669028	1	-
ENSMUSC	Tk1	thymidine	GO:00081	metabolic	K00857	thymidine	mmu011C	Metabolic	11:117815	3	2	9	13	4	14	0.025814	0.014426	0.053353	0.072882	0.030183	0.149388	0.031198	0.084151	0.064062	1	-
ENSMUSC	Cant1	calcium ac	GO:00048	signal trar	K12304	soluble ca	mmu0023	Purine me	11:118406	651	843	949	993	669	796	5.725579	6.214675	5.74984	5.689437	5.159493	8.680346	5.896698	6.509759	0.733585	1	-
ENSMUSC	Rbfox3	RNA bind	GO:00325	developm	-	-	-	-	11:118489	3038	3869	4481	4959	3811	2663	21.79412	23.265	22.14511	23.1754	23.97359	23.68692	22.40141	23.61197	0.896305	1	-
ENSMUSC	Cbx2	chromobc	GO:00325	developm	-	-	-	-	11:119022	40	37	49	37	40	33	0.569604	0.441638	0.480685	0.343238	0.499472	0.582646	0.497309	0.475119	0.663841	1	-
ENSMUSC	Cbx8	chromobc	GO:00081	metabolic	-	-	-	-	11:119036	148	163	192	211	134	167	1.963232	1.812387	1.754541	1.823364	1.558679	2.746707	1.843387	2.042917	0.769804	1	-
ENSMUSC	Gaa	glucosidas	GO:00325	developm	K12316	lysosomal	mmu011C	Metabolic	11:119267	5617	6649	6982	7587	6068	5735	58.59771	58.14139	50.17733	51.56179	55.50913	74.1815	55.63881	60.41747	0.773239	1	-
ENSMUSC	Eif4a3	eukaryotic	GO:00325	developm	K13025	ATP-depe	mmu0301	RNA trans	11:119288	622	715	903	932	717	544	11.64359	11.21902	11.6449	11.36564	11.76951	12.62645	11.5025	11.92054	0.976162	1	-
ENSMUSC	Nptx1	neuronal p	GO:00325	developm	-	-	-	-	11:119538	13798	16252	18461	21072	15739	13002	168.086	165.9491	154.9252	167.2256	168.1261	196.3864	162.9867	177.246	0.704557	1	-
ENSMUSC	Rptor	regulatory	GO:00081	metabolic	K07204	regulatory	mmu0415	PI3K-Akt s	11:119602	1596	1856	2175	2357	1951	1578	4.96795	4.842561	4.663961	4.779532	5.3253	6.090279	4.824824	5.39837	0.558052	1	-
ENSMUSC	Pde8a	phosphoc	GO:00081	metabolic	K18437	high affini	mmu0023	Purine me	7:8121359	356	273	546	451	327	228	5.246949	3.372662	5.543726	4.330272	4.22618	4.166558	4.721112	4.241003	0.371935	1	-
ENSMUSC	Cpeb1	cytoplasm	GO:00325	developm	K02602	cytoplasm	mmu0411	Oocyte m	7:8134702	991	1219	953	1728	1192	1240	8.741292	9.012776	5.790908	9.929505	9.219793	13.56156	7.848325	10.90362	0.05807	1	-
ENSMUSC	Nat1	N-acetyl t	GO:00325	developm	K00622	arylamine	mmu011C	Metabolic	8:6748092	7	22	28	16	25	8	0.174129	0.45869	0.479801	0.259266	0.545293	0.246729	0.370873	0.350429	0.86103	1	-
ENSMUSC	Tma16	translatior	GO:00329	macromo	-	-	-	-	8:6647311	247	286	383	409	315	128	1.457528	1.41462	1.556936	1.572264	1.629948	0.936517	1.476361	1.379576	0.608217	1	-
ENSMUSC	Dach2	dachshun	GO:00325	developm	-	-	-	-	X:1132975	23	24	20	25	13	9	0.112129	0.098074	0.067171	0.079398	0.055575	0.054401	0.092458	0.063125	0.168653	1	-
ENSMUSC	Klhl4	kelch-like	GO:00081	metabolic	-	-	-	-	X:1144743	183	203	284	241	178	128	2.234433	2.077614	2.388826	1.916963	1.905808	1.937807	2.233624	1.920193	0.206026	1	-
ENSMUSC	Zfp202	zinc finge	GO:00081	metabolic	-	-	-	-	9:4019231	61	83	89	103	65	54	0.562414	0.641444	0.565289	0.61865	0.525514	0.617321	0.589716	0.587162	0.837188	1	-
ENSMUSC	Copg2	coatomer	GO:00511	localizatio	-	-	-	-	6:3074755	990	1350	1650	1660	1170	1014	9.172985	10.48485	10.53199	10.01994	9.506132	11.64928	10.06328	10.39178	0.938181	1	-
ENSMUSC	Podxl	podocaly	GO:00325	developm	-	-	-	-	6:3151948	664	823	982	1009	840	615	7.373616	7.660637	7.512344	7.29937	8.179644	8.46786	7.515532	7.982291	0.874088	1	-
ENSMUSC	Mkln1	muskelin 1	GO:00325	developm	-	-	-	-	6:3139873	2077	2469	3279	3206	2283	1522	4.135585	4.120732	4.49773	4.158585	3.986108	3.757518	4.251349	3.967404	0.385641	1	-
ENSMUSC	Map3k7cl	Map3k7 C	GO:00048	signal trar	-	-	-	-	16:875533	22	14	12	26	18	8	0.846333	0.451458	0.318035	0.651591	0.607201	0.381594	0.538609	0.546795	1	1	-
ENSMUSC	Bach1	BTB and C	GO:00081	metabolic	-	-	-	-	16:876989	208	266	365	357	207	165	1.892755	2.028929	2.288106	2.116321	1.651754	1.861672	2.06993	1.876582	0.37994	1	-
ENSMUSC	Cct8	chaperoni	GO:00081	metabolic	-	-	-	-	16:874833	2499	3042	3511	3204	2721	1838	29.02393	29.61435	28.09133	24.24176	27.71158	26.46802	28.90987	26.14045	0.23179	1	-
ENSMUSC	Usp16	ubiquitin s	GO:00081	metabolic	-	-	-	-	16:874547	367	482	605	649	489	269	1.899654	2.091261	2.157324	2.188441	2.219524	1.726423	2.049413	2.044796	0.825433	1	-
ENSMUSC	Phf6	PHD finge	GO:00081	metabolic	-	-	-	-	X:5291226	545	679	897	858	642	333	3.618512	3.778816	4.10277	3.711097	3.737759	2.741347	3.833366	3.396734	0.303076	1	-
ENSMUSC	Hprt	hypoxanth	GO:00325	developm	K00760	hypoxanth	mmu011C	Metabolic	X:5298813	1577	2036	2556	2212	1730	1297	77.75261	84.14218	86.81514	71.04768	74.79492	79.28829	82.90331	75.04363	0.235602	1	-
ENSMUSC	Ccdc51	coiled-co	GO:00160	membran	-	-	-	-	9:1090824	57	78	84	79	59	85	2.373865	2.722893	2.40998	2.14333	2.15463	4.38919	2.502246	2.895717	0.698659	1	-
ENSMUSC	Atrip	ATR intera	GO:00081	metabolic	K10905	ATR intera	mmu0346	Fanconi a	9:1090597	42	69	49	66	65	110	0.367508	0.506081	0.295374	0.376226	0.498743	1.193439	0.389654	0.689469	0.069014	1	-
ENSMUSC	Shisa5	shisa fami	GO:00048	signal trar	K10135	scotin[-]	mmu0411	p53 signa	9:1090385	447	595	731	651	660	549	3.144584	3.508533	3.542624	2.983448	4.071388	4.788663	3.39858	3.947833	0.465877	1	-
ENSMUSC	Pfkfb4	6-phosph	GO:00081	metabolic	K19030	6-phosph	mmu0415	AMPK sig	9:1089917	136	156	121	188	202	175	1.001882	0.96328	0.614064	0.902225	1.304877	1.598454	0.859742	1.268519	0.075313	1	-
ENSMUSC	Col7a1	collagen, t	GO:00325	developm	K16628	collagen, t	mmu0497	Protein di	9:1089535	64	67	96	100	43	62	0.363195	0.318701	0.3753	0.369691	0.213976	0.436252	0.352398	0.339973	0.708833	1	-
ENSMUSC	Uqcrc1	ubiquinol-	GO:00081	metabolic	-	-	-	-	9:1089366	2313	3310	3316	3225	2972	3066	23.78995	28.53637	23.49546	21.60873	26.8046	39.09993	25.27393	29.17109	0.506687	1	-
ENSMUSC	Tmem89	transmem	GO:00160	membran	-	-	-	-	9:1089146	0	0	0	0	2	3	0	0	0	0	0.075151	0.159419	0	0.07819	0.05383	1	-
ENSMUSC	Arhgef9	CDC42 gu	GO:00987	molecular	-	-	-	-	X:9504893	4681	11182	12739	14569	4944	5203	9.432799	18.88749	17.68434	19.12552	8.736211	12.99995	15.33488	13.62056	0.452541	1	-

ENSMUSC	Cnksr2	connector	GO:00452	synapse|re	-	-	-	-	X:1578214	9939	10478	12477	15160	9955	4239	67.13289	59.32311	58.05696	66.70732	58.96259	35.50111	61.50432	53.72368	0.329997	1	-
ENSMUSC	Rps6ka6	ribosomal	GO:00081	metabolic	K04373	ribosomal	mmu0401	MAPK sig	X:1113881	95	103	132	101	97	69	0.70988	0.645137	0.679497	0.49166	0.635586	0.639285	0.678171	0.588844	0.286546	1	-
ENSMUSC	Tmem47	transmem	GO:00160	membran	-	-	-	-	X:8107069	2239	2843	3745	3937	2670	1517	23.28128	24.77893	26.82601	26.66857	24.34483	19.558	24.96207	23.5238	0.49969	1	-
ENSMUSC	Alox5	arachidon	GO:00081	metabolic	K00461	arachidon	mmu0110	Metabolic	6:1164100	5	12	22	15	15	16	0.042901	0.086306	0.130035	0.083843	0.112856	0.170215	0.086414	0.122305	0.454571	1	-
ENSMUSC	8-Mar	membran	GO:00081	metabolic	-	-	-	-	6:1163380	289	417	1496	477	944	324	1.079955	1.306163	3.851159	1.161204	3.093308	1.501205	2.079092	1.918573	0.720918	1	-
ENSMUSC	Myo3a	myosin III,	GO:00081	metabolic	-	-	-	-	2:2222750	0	0	2	0	0	0	0	0	0.009921	0	0	0	0.003307	0	0.53611	1	-
ENSMUSC	Wdr73	WD repea	GO:00099	cellular pr	-	-	-	-	7:8089072	326	260	286	299	291	452	1.871065	1.250824	1.130806	1.117954	1.464557	3.216589	1.417565	1.933033	0.274417	1	-
ENSMUSC	Nmb	neuromed	GO:00508	response	-	-	-	-	7:8090222	14	17	18	12	14	22	0.447554	0.45554	0.396413	0.249918	0.392454	0.872044	0.433169	0.504805	0.898323	1	-
ENSMUSC	Sec11a	SEC11 hor	GO:00081	metabolic	K13280	signal pep	-	-	7:8090488	307	374	479	459	355	258	2.961565	3.024176	3.183239	2.884539	3.002988	3.085946	3.056327	2.991158	0.659029	1	-
ENSMUSC	Slc28a1	solute car	GO:00511	localizatio	-	-	-	-	7:8111479	0	0	0	2	0	0	0	0	0	0.027802	0	0	0	0.009267	0.537207	1	-
ENSMUSC	A930017K	RIKEN cDI	-	-	-	-	-	-	17:259463	9	7	17	13	9	15	0.225274	0.14686	0.293132	0.211987	0.197548	0.465541	0.221755	0.291692	0.666882	1	-
ENSMUSC	Pigq	phosphati	GO:00081	metabolic	K03860	phosphati	mmu0110	Metabolic	17:259264	941	1243	1234	1482	1114	1138	3.804287	4.212185	3.436768	3.903134	3.949222	5.704422	3.817746	4.518926	0.361251	1	-
ENSMUSC	Rab40c	Rab40C, r	GO:00081	metabolic	-	-	-	-	17:258821	446	488	554	548	440	561	4.204817	3.856419	3.598101	3.36569	3.637531	6.557835	3.886445	4.520352	0.564193	1	-
ENSMUSC	Mettl26	methyltrai	-	-	-	-	-	-	17:258754	172	152	158	230	85	228	4.194597	3.107115	2.654427	3.654026	1.817695	6.894167	3.318713	4.121962	0.551106	1	-
ENSMUSC	Mcrip2	MAPK reg	GO:00329	macromo	-	-	-	-	17:258636	75	97	106	75	60	96	5.841247	6.332455	5.687257	3.805314	4.097727	9.270484	5.953653	5.724508	0.647161	1	-
ENSMUSC	Rhot2	ras homol	GO:00511	localizatio	K07871	Ras homo	mmu0413	Mitophag	17:258384	754	948	1264	1257	932	926	4.516822	4.760173	5.21627	4.905448	4.895762	6.877944	4.831088	5.559718	0.46352	1	-
ENSMUSC	Rhbdl1	rhomboid	GO:00081	metabolic	-	-	-	-	17:258344	305	352	420	457	412	376	6.690943	6.472668	6.347291	6.531085	7.925509	10.22732	6.503634	8.227972	0.191763	1	-
ENSMUSC	Jmjd8	jumonji do	GO:00055	extracellul	-	-	-	-	17:258288	205	255	271	327	281	293	2.099317	2.188855	1.911815	2.18149	2.523323	3.720304	2.066662	2.808372	0.10689	1	-
ENSMUSC	Wdr24	WD repea	GO:00081	metabolic	K20408	WD repea	mmu0415	mTOR sig	17:258236	361	458	580	561	373	440	3.972747	4.224765	4.397068	4.021878	3.599442	6.003757	4.198193	4.541693	0.850722	1	-
ENSMUSC	Fbxl16	F-box and	GO:00081	metabolic	-	-	-	-	17:258090	17962	20388	22741	27402	20901	17389	328.2171	312.2727	286.2645	326.1898	334.9009	393.9736	308.9181	351.6881	0.436959	1	-
ENSMUSC	Gng13	guanine n	GO:00048	signal trar	K04547	guanine n	mmu0474	Olfactory	17:257171	72	120	162	139	112	212	4.899167	6.844225	7.593726	6.16149	6.682697	17.88588	6.445706	10.24336	0.108021	1	-
ENSMUSC	Prps2	phosphor	GO:00325	developm	-	-	mmu0110	Metabolic	X:1673463	515	515	785	647	491	393	7.955711	6.668562	8.35397	6.511153	6.651134	7.527496	7.659414	6.896594	0.272354	1	-
ENSMUSC	Sdc3	syndecan	GO:00400	locomotic	K16337	syndecan	mmu0451	Cell adhes	4:1307925	3947	4733	5258	6112	4545	3524	35.97357	36.15812	33.01326	36.28954	36.32395	39.82339	35.04832	37.47896	0.814501	1	-
ENSMUSC	Hadha	hydroxyac	GO:00081	metabolic	K07515	enoyl-Co/	mmu0110	Metabolic	5:3011830	1187	1386	1826	1876	1352	931	7.0555	6.905469	7.477035	7.264265	7.046875	6.861399	7.146001	7.057513	0.687457	1	-
ENSMUSC	Il6	interleukir	GO:00325	developm	K05405	interleukir	mmu0520	Pathways	5:3001311	2	0	0	0	3	2	0.031114	0	0	0	0.040914	0.038581	0.010371	0.026498	0.364886	1	-
ENSMUSC	Tyms	thymidylat	GO:00325	developm	K00560	thymidylat	mmu0110	Metabolic	5:3005820	31	44	39	57	25	34	0.188804	0.22462	0.16363	0.22615	0.133514	0.256753	0.192351	0.205472	0.969019	1	-
ENSMUSC	Agbl1	ATP/GTP	GO:00081	metabolic	-	-	-	-	7:7622988	3	3	8	1	4	0	0.03582	0.030027	0.065811	0.007778	0.041882	0	0.043886	0.016554	0.164266	1	-
ENSMUSC	Hspa4l	heat shoc	GO:00508	response	K09485	heat shoc	mmu0414	Protein pr	3:4074449	3934	4651	6245	6114	4237	2139	17.952	17.79007	19.6319	18.17547	16.95429	12.1025	18.45799	15.74409	0.183368	1	-
ENSMUSC	Plk4	polo like k	GO:00325	developm	K08863	polo-like	mmu0406	FoxO sign	3:4080001	36	58	59	80	42	32	0.193462	0.261265	0.218423	0.280071	0.197919	0.213223	0.224383	0.230404	1	1	-
ENSMUSC	Mfsd8	major faci	GO:00325	developm	K12307	MFS trans	mmu0414	Lysosome	3:4081810	197	295	326	361	266	252	1.111108	1.394646	1.266654	1.326412	1.315568	1.762281	1.257469	1.468087	0.460286	1	-
ENSMUSC	Larp1b	La ribonu	GO:00329	macromo	-	-	-	-	3:4095035	177	212	297	262	204	143	0.723541	0.726404	0.836367	0.697706	0.731246	0.724789	0.762104	0.717914	0.517809	1	-
ENSMUSC	Jade1	jade famil	GO:00081	metabolic	-	-	-	-	3:4155573	406	487	701	517	540	489	1.358171	1.365557	1.615465	1.12668	1.584034	2.028257	1.446397	1.579657	0.801857	1	-
ENSMUSC	Crispld1	cysteine-r	GO:00325	developm	-	-	-	-	1:1772704	132	165	214	298	156	111	1.301222	1.363376	1.453261	1.913714	1.348485	1.356712	1.37262	1.539637	0.623941	1	-
ENSMUSC	Gdap1	gangliosid	GO:00081	metabolic	-	-	-	-	1:1714536	2288	2538	3362	3591	2637	1319	14.15589	13.16211	14.32947	14.47365	14.30652	10.1184	13.88249	12.96619	0.487259	1	-
ENSMUSC	Ly96	lymphocy	GO:00048	signal trar	K05400	lymphocy	mmu0514	Toxoplasr	1:1668805	10	21	26	19	19	13	0.401222	0.706241	0.718635	0.496599	0.668447	0.646696	0.608699	0.603914	0.888463	1	-
ENSMUSC	Itih5	inter-alph	GO:00081	metabolic	-	-	-	-	2:1015357	757	948	1282	1429	945	666	6.089823	6.39249	7.104745	7.488984	6.66628	6.643081	6.529019	6.932782	0.846634	1	-
ENSMUSC	Atp5c1	ATP synth	GO:00081	metabolic	K02136	F-type H+	mmu0110	Metabolic	2:1005601	1998	2464	3533	3009	2099	1419	35.43927	36.63388	43.17024	34.76907	32.64709	31.20741	38.41446	32.87452	0.121427	1	-
ENSMUSC	Taf3	TATA-box	GO:00081	metabolic	K14650	transcripti	mmu0516	Herpes sir	2:9914552	432	573	639	610	435	282	2.235739	2.485678	2.278191	2.056597	1.974102	1.80956	2.333203	1.946753	0.115997	1	-
ENSMUSC	Clec3b	C-type lec	GO:00325	developm	-	-	-	-	9:1231509	25	38	63	31	26	29	1.601653	2.040624	2.780466	1.293801	1.460632	2.303581	2.140914	1.686005	0.216997	1	-
ENSMUSC	Exosc7	exosome	GO:00081	metabolic	K12589	exosome	mmu0301	RNA degr	9:1231132	150	186	241	282	185	236	1.326051	1.378268	1.467695	1.624043	1.434108	2.586812	1.390671	1.881654	0.1371	1	-
ENSMUSC	Zdhhc3	zinc finge	GO:00081	metabolic	-	-	-	-	9:1230661	674	812	1041	1072	846	692	2.25446	2.276625	2.398749	2.335926	2.481392	2.869951	2.309945	2.562423	0.620189	1	-
ENSMUSC	Tgm4	transgluta	GO:00081	metabolic	-	-	-	-	9:1230347	0	2	13	22	14	5	0	0.018457	0.098607	0.157804	0.135169	0.068256	0.039021	0.12041	0.050342	1	-
ENSMUSC	St8sia2	ST8 alpha	GO:00081	metabolic	-	-	-	-	7:7393911	82	123	158	145	120	35	0.898975	1.130293	1.193279	1.035579	1.153603	0.475762	1.074182	0.888314	0.375254	1	-
ENSMUSC	Slco3a1	solute car	GO:00511	localizatio	-	-	-	-	7:7427541	447	647	747	793	531	480	2.684575	3.257058	3.090584	3.102583	2.796438	3.57434	3.010739	3.157787	0.970062	1	-
ENSMUSC	Pgm2	phosphoc	GO:00081	metabolic	K01835	phosphoc	mmu0110	Metabolic	4:9992941	945	1242	1432	1498	1158	1066	20.56063	22.65056	21.46343	21.23235	22.09305	28.75727	21.5582	24.02756	0.608845	1	-
ENSMUSC	Slc25a10	solute car	GO:00511	localizatio	K13577	solute car	mmu0496	Proximal t	11:120491	159	152	161	231	147	190	1.968624	1.577468	1.373227	1.863197	1.595969	2.916788	1.639773	2.125318	0.257428	1	-
ENSMUSC	Hgs	HGF-regu	GO:00081	metabolic	K12182	hepatocyt	mmu0414	Endocytos	11:120467	1170	1476	1625	1800	1508	1194	15.95644	16.87289	15.26705	15.99206	18.03409	20.19019	16.03213	18.07211	0.517363	1	-
ENSMUSC	Rpl14	ribosomal	GO:00081	metabolic	K02875	large subu	mmu0301	Ribosome	9:1205714	372	570	683	665	450	753	7.710931	9.903563	9.752946	8.979811	8.179353	19.35284	9.12248	12.17067	0.223076	1	-
ENSMUSC	Rassf3	Ras assoc	GO:00508	response	-	-	-	-	10:121410	218	314	427	390	337	288	3.54608	4.281295	4.784896	4.132743	4.806907	5.808586	4.20409	4.916079	0.45323	1	-
ENSMUSC	Ccr1	chemokin	GO:00325	developm	K04176	C-C chem	mmu0406	Cytokine-	9:1239621	5	8	18	11	9	8	0.112167	0.150422	0.278175	0.160749	0.177048	0.222518	0.180255	0.186771	1	1	-
ENSMUSC	Itgb1	integrin b	GO:00325	developm	K05719	integrin b	mmu0520	Pathways	8:1286856	1400	1596	2262	2175	1444	1042	15.73373	15.03451	17.51248	15.92371	14.23028	14.51968	16.09357	14.89122	0.354379	1	-
ENSMUSC	Nrp1	neuropilin	GO:00325	developm	K06724	neuropilin	mmu0516	HTLV-I inf	8:1283586	1572	1908	2171	2466	1692	1351	8.723834	8.875359	8.299768	8.915167	8.233754	9.296	8.632987	8.814974	0.874749	1	-
ENSMUSC	Pard3	par-3 fam	GO:00325	developm	K04237	partitionir	mmu0516	Human pa	8:1270638	413	522	661	777	639	308	1.081741	1.146033	1.192687	1.325796	1.467633	1.000253	1.140154	1.26456	0.624193	1	-
ENSMUSC	Homer2	homer sca	GO:00452	synapse|re	K15010	homer[-]	mmu0406	FoxO sign	7:8160048	1965	2125	3035	3449	2280	1762	8.517948	7.721192	9.063222	9.739739	8.666624	9.470313	8.434121	9.292225	0.620495	1	-
ENSMUSC	Dhtkd1	dehydrog	GO:00325	developm	-	-	-	-	2:5896115	69	115	116	134	121	94	0.533802	0.745729	0.618213	0.675327	0.820835	0.901663	0.632582	0.799275	0.277161	1	-
ENSMUSC	Sec61a2	Sec61, alp	GO:00511	localizatio	K10956	protein tra	mmu0414	Phagoson	2:5870987	768	944	1165	1263	910	732	6.345385	6.537649	6.630928	6.798008	6.592965	7.498835	6.504654	6.96327	0.820413	1	-
ENSMUSC	Nudt5	nudix (nu	GO:00081	metabolic	K13987	ADP-suga	mmu0023	Purine me	2:5845019	207	282	371	333	270	292	1.627149	1.85805	2.009005	1.705226	1.861072	2.84593	1.831401	2.137409	0.510775	1	-
ENSMUSC	Zfp282	zinc finge	GO:00081	metabolic	-	-	-	-	6:4787720	361	421	503	569	455	309	2.88079	2.816046	2.765184	2.958006	3.183899	3.057382	2.820673	3.066429	0.734821	1	-
ENSMUSC	Pdia4	protein di	GO:00081	metabolic	K09582	protein di	mmu0414	Protein pr	6:4779614	990	1233	1967	1612	1295	759	22.91242	23.91947	31.3611	24.30426	26.28136	21.78028	26.06433	24.12196	0.419712	1	-
ENSMUSC	Iscu	iron-sulfu	GO:00081	metabolic	-	-	-	-	5:1137727	289	350	391	560	300	354	4.580247	4.649572	4.26894	5.781773	4.169231	6.956342	4.499586	5.635782	0.235353	1	-
ENSMUSC	Fam20c	family wit	GO:00325	developm	-	-	-	-	5:1387545	495	680	708	708	567	470	3.161045	3.639886	3.114659	2.945377	3.175056	3.721426	3.305197	3.280619	0.702842	1	-
ENSMUSC	Prkar1b	protein ki	GO:00325	developm	K04739	cAMP-de	mmu0491	Insulin sig	5:1390173	6889	8737	10487	10015	7867	7553	56.34707	59.90048	59.09052	53.36392	56.42439	76.59855	58.44602	62.12895	0.901382	1	-
ENSMUSC	Pdgfa	platelet de	GO:00325	developm	K04359	platelet-d	mmu0520	Pathways	5:1389760	283	360	415	465	305	433	3.444842	3.67314	3.480022	3.687371	3.255552	6.535162	3.532668	4.492695	0.269037	1	-
ENSMUSC	Dnaaf5	dynein, ax	GO:00099	cellular pr	-	-	-	-	5:1391502	83	164	161	165	114	129	1.204871	1.995526	1.610045	1.560361	1.451139	2.321851	1.603481	1.777784	0.787413	1	-
ENSMUSC	Get4	golgi to E	GO:00081	metabolic	-	-	-	-	5:1392523	435	632	754	710	613	612	6.096046	7.42384	7.279163	6.481849	7.532901	10.63399	6.933016	8.216246	0.399042	1	-
ENSMUSC	Xiap	X-linked i	GO:00081	metabolic	K04725	E3 ubiquit	mmu0520	Pathways	X:4205967	1958	469	2401	1694	1011	757	10.21146	2.050219	8.626179	5.755326	4.623478	4.895043	6.962618	5.091282	0.2035	1	-
ENSMUSC	Stag2	stromal an	GO:00325	developm	K06671	cohesin co	mmu0411	Cell cycle	X:4214931	1645	1829	2700	2721	1899	911	9.298646	8.666038	10.51403	10.01992	9.412857	6.38496	9.492904	8.605911	0.406399	1	-
ENSMUSC	Cplx2	complexir	GO:00325	developm	K15294	complexir	mmu0472	Synaptic v	13:543713	28533	34101	38961	46146	33329	25616	367.9704	368.6255	346.1361	387.6869	376.9037	409.6026	360.9107	391.3977	0.704948	1	-
ENSMUSC	Higd2a	HIG1 dom	GO:00081	metabolic	-	-	-	-	13:545902	748	966	1042	1035	788	732	73.36054	79.41289	70.40122	66.12766	67.76884	89.01395	74.39155	74.30348	0.716619	1	-
ENSMUSC	Nop16	NOP16 nu	GO:00056	cell|intrace	-	-	-	-	13:545841	285	348	445	443	318	252	6.562532	6.716756	7.058918	6.645262	6.42091	7.194702	6.779402	6.753624	0.752581	1	-
ENSMUSC	Arl10	ADP-ribos	GO:00508	response	-	-	-	-	13:545750	220	191	291	292	215	212	5.801522	4.221871	5.28644	5.016304	4.971638	6.931706	5.103278	5.639883	0.744503	1	-
ENSMUSC	4833439L	RIKEN cDI	GO:00081	metabolic	-	-	-	-	13:545512	1933	2069	2798	2924	2151	1680	16.82849	15.09825	16.78079	16.58335	16.42088	18.13463	16.23584	17.04628	0.941124	1	-
ENSMUSC	Thoc3	THO com	GO:00081	metabolic	K12880	THO com	mmu0301	RNA trans	13:544588	425	493	681	694	451	433	8.125762	7.900876	8.969616	8.644031	7.561257	10.26477	8.332085	8.823351	0.921907	1	-
ENSMUSC	Faf2	Fas associ	GO:00081	metabolic	-	-	-	-	13:546217	1008	1148	1352	1482	1070	820	6.275612	5.990875	5.798608	6.010704	5.841465	6.329875	6.021698	6.060681	0.786628	1	-
ENSMUSC	Tspan17	tetraspani	GO:00160	membran	-	-	-	-	13:547893	135	177	198	183	205	353	2.588918	2.845172	2.615775	2.286215	3.447315	8.393528	2.683289	4.709019	0.042705	1	-
ENSMUSC	Unc5a	unc-5 net	GO:00325	developm	K07521	netrin rec	mmu0436	Axon guic	13:549494	2855	3263	3473	3986	3119	2784	35.30723	33.82419	29.58787	32.11267	33.82326	42.68867	32.90643	36.2082	0.676654	1	-
ENSMUSC	Hk3	hexokinas	GO:00081	metabolic	K00844	hexokinas	mmu0110	Metabolic	13:550059	29	44	49	71	45	32	0.301891	0.383933	0.3514	0.481492	0.410773	0.413034	0.345741	0.4351	0.341187	1	-
ENSMUSC	Uimc1	ubiquitin i	GO:00081	metabolic	K20775	BRCA1-A	mmu0344	Homologo	13:550278	248	240	338	417	307	183	1.8606	1.509262	1.746907	2.03807	2.019677	1.702309	1.70559	1.920019	0.571875	1	-
ENSMUSC	Smad7	SMAD far	GO:00325	developm	K19631	mothers a	mmu0439	Hippo sig	18:753675	275	320	409	357	303	369	2.261816	2.206112	2.317396	1.912823	2.185297	3.763031	2.261775	2.620384	0.583472	1	-
ENSMUSC	Myo5b	myosin VE	GO:00038	catalytic a	-	-	-	-	18:744409	2389	2773	2874	3622	2469	1539	11.72599	11.4087	9.717881	11.58147	10.62667	9.366082	10.95086	10.52474	0.57155	1	-
ENSMUSC	Casp12	caspase 1	GO:00081	metabolic	K04741	caspase 1	mmu0501	Alzheimer	9:5345430	8	27	25	32	16	17	0.086126	0.243655	0.185416	0.224436	0.15105	0.226929	0.171732	0.200805	0.7304	1	-
ENSMUSC	Casp1	caspase 1	GO:00081	metabolic	K01370	caspase 1	mmu0421	Necropto	9:5298517	31	21	33	27	41	35	1.316081	0.747285	0.965102	0.74671	1.526299	1.842345	1.00949	1.371785	0.370201	1	-
ENSMUSC	Snca	synuclein,	GO:00325	developm	K04528	alpha-syn	mmu0501	Alzheimer	6:6073157	5182	6835	6876	7380	5502	4254	90.12908	99.64579	82.38627	83.61912	83.9133	91.73837	90.72038	86.42359	0.445574	1	-
ENSMUSC	Gria4	glutamate	GO:00048	signal trar	K05200	glutamate	mmu0408	Neuroacti	9:4417896	1242	1530	1829	1845	1571	782	8.581523	8.861086	8.705783	8.304639	9.518345	6.699395	8.716131	8.174126	0.486496	1	-
ENSMUSC	Kbtbd3	kelch repe	GO:00081	metabolic	-	-	-	-	9:4309833	72	100	142	142	109	79	1.064392	1.239146	1.446136	1.367532	1.412982	1.448046	1.249892	1.40952	0.635393	1	-
ENSMUSC	Aasdhppt	aminoadi	GO:00081	metabolic	K06133	4'-phosph	mmu0077	Pantother	9:4294793	210	494	654	406	438	293	1.871828	3.690853	4.015831	2.357516	3.423443	3.238177	3.192837	3.006379	0.595815	1	-
ENSMUSC	Cwf19l2	CWF19-lik	-	-	-	-	-	-	9:3403592	258	348	423	430	369	170	1.394513	1.57665	1.57505	1.514095	1.748927	1.1393	1.515404	1.467441	0.711689	1	-
ENSMUSC	Alkbh8	alkB hom	GO:00081	metabolic	-	-	-	-	9:3335140	501	781	1100	933	323	574	1.837068	2.400441	2.778635	2.228696	1.038564	2.609666	2.338715	1.958975	0.244212	1	-
ENSMUSC	Rp1	retinitis pi	GO:00325	developm	-	-	-	-	1:3999557	0	2	4	3	1	0	0	0.008559	0.01407	0.009977	0.004477	0	0.007543	0.004818	0.757491	1	-
ENSMUSC	Lypla1	lysophosp	GO:00081	metabolic	K06128	lysophosp	mmu0523	Choline m	1:4807788	391	452	607	552	420	281	2.693975	2.610408	2.881092	2.477634	2.537516	2.400544	2.728492	2.471898	0.31991	1	-
ENSMUSC	Oprk1	opioid rec	GO:00048	signal trar	K04214	kappa-typ	mmu0408	Neuroacti	1:5588466	10	23	21	21	18	17	0.045399	0.087522	0.065677	0.062108	0.071655	0.09569	0.066199	0.076484	0.786008	1	-
ENSMUSC	Rb1cc1	RB1-induc	GO:00325	developm	K17589	RB1-induc	mmu0414	Autophag	1:6206197	1135	1321	1670	1764	1437	626	3.201911	3.123698	3.245499	3.241855	3.554781	2.189644	3.190369	2.995427	0.551951	1	-
ENSMUSC	Sntg1	syntrophir	GO:00051	structural	-	-	-	-	1:8361475	419	497	560	635	367	348	1.451867	1.443519	1.336758	1.433403	1.115121	1.49513	1.410715	1.347885	0.534496	1	-
ENSMUSC	Adhfe1	alcohol de	GO:00081	metabolic	-	-	-	-	1:9547948	238	319	397	438	317	206	2.965224	3.331378	3.407395	3.554977	3.46324	3.182251	3.234666	3.400156	0.917441	1	-
ENSMUSC	Mybl1	myeloblas	GO:00081	metabolic	-	-	-	-	1:9667415	35	74	107	73	51	26	0.341949	0.606005	0.720149	0.464612	0.436921	0.314961	0.556034	0.405498	0.153337	1	-
ENSMUSC	Sgk3	serum/glu	GO:00081	metabolic	K13304	serum/glu	mmu0415	PI3K-Akt s	1:9798107	127	177	190	186	180	166	0.338302	0.395209	0.348664	0.322772	0.420454	0.548273	0.360725	0.4305	0.458907	1	-
ENSMUSC	Ppp1r42	protein pł	GO:00081	metabolic	-	-	-	-	1:9968624	1	7	5	0	7	1	0.029745	0.174487	0.102438	0	0.182558	0.036864	0.102223	0.073141	0.762638	1	-
ENSMUSC	Cops5	COP9 sigr	GO:00081	metabolic	-	-	-	-	1:1002460	433	655	745	696	544	511	4.84309	6.140855	5.740407	5.071375	5.335513	7.086669	5.574784	5.831186	0.997116	1	-
ENSMUSC	Stau2	staufen (R	GO:00511	localizatio	-	-	-	-	1:1622867	2122	3118	3532	2389	2669	1863	5.773093	7.11037	6.619655	4.234099	6.367288	6.284376	6.50104	5.628588	0.15837	1	-
ENSMUSC	Rdh10	retinol del	GO:00325	developm	K11151	retinol del	mmu0110	Metabolic	1:1610577	129	180	217	238	150	145	1.478244	1.72895	1.713039	1.776705	1.507263	2.060197	1.640078	1.781388	0.803944	1	-
ENSMUSC	Terf1	telomeric	GO:00081	metabolic	-	-	-	-	1:1580564	186	202	247	275	184	116	1.981703	1.803975	1.812898	1.908713	1.719041	1.532388	1.866192	1.720047	0.481268	1	-
ENSMUSC	Tfap2b	transcripti	GO:00325	developm	-	-	-	-	1:1920891	2	9	10	9	9	0	0.01275	0.048081	0.043909	0.037369	0.050303	0	0.034914	0.029224	0.74199	1	-
ENSMUSC	Msc	musculin		GO:00325	developm	-	-	-	-	1:1475334	13	25	19	12	19	15	0.186117	0.300013	0.187392	0.111926	0.238527	0.266278	0.224507	0.205577	0.649303	1	-
ENSMUSC	Eya1	EYA trans	GO:00325	developm	K15616	eyes abse	mmu0520	Transcript	1:1416895	318	339	466	435	256	239	1.420926	1.269688	1.434439	1.266239	1.00306	1.324124	1.375018	1.197807	0.211498	1	-
ENSMUSC	Tmem14a	transmem	GO:00511	localizatio	-	-	-	-	1:2121857	159	251	331	282	238	297	4.693427	6.210387	6.73087	5.422781	6.160437	10.87014	5.878228	7.484451	0.307841	1	-
ENSMUSC	Gsta3	glutathior	GO:00325	developm	K00799	glutathior	mmu0520	Pathways	1:2124058	13	8	15	28	12	13	0.173894	0.089696	0.13823	0.244004	0.14076	0.215611	0.13394	0.200125	0.241044	1	-
ENSMUSC	Tram1	translocat	GO:00511	localizatio	K14010	translocat	mmu0414	Protein pr	1:1356469	452	581	855	735	514	410	7.820839	8.426444	10.19137	8.284839	7.798676	8.795992	8.812884	8.293169	0.459623	1	-
ENSMUSC	Lactb2	lactamase	GO:00081	metabolic	-	-	-	-	1:1362333	307	351	481	529	371	180	3.986676	3.820607	4.302974	4.475165	4.224628	2.89821	4.036752	3.866001	0.6721	1	-
ENSMUSC	Slco5a1	solute car	GO:00511	localizatio	-	-	-	-	1:1286654	35	66	55	87	67	44	0.083819	0.132484	0.090737	0.135727	0.140697	0.130649	0.102346	0.135691	0.232464	1	-
ENSMUSC	Ube2w	ubiquitin-	-	-	K10688	ubiquitin-	mmu0412	Ubiquitin	1:1654079	931	1180	1363	1356	1021	818	10.5808	11.24097	10.67128	10.03945	10.17507	11.52679	10.83102	10.58044	0.588944	1	-
ENSMUSC	Tmem70	transmem	GO:00099	cellular pr	-	-	-	-	1:1666520	261	401	494	401	299	428	4.015327	5.171041	5.235515	4.018899	4.033622	8.164151	4.807294	5.405557	0.741678	1	-
ENSMUSC	Pth2r	parathyro	GO:00048	signal trar	K04586	parathyro	mmu0408	Neuroacti	1:6528205	5	10	7	9	7	14	0.121425	0.203547	0.117113	0.14238	0.149074	0.421563	0.147361	0.237672	0.377129	1	-
ENSMUSC	Pikfyve	phosphoi	GO:00325	developm	K00921	1-phosph	mmu0481	Regulatior	1:6518668	1154	1301	1564	1759	1246	1009	3.271485	3.0915	3.054411	3.24853	3.09742	3.546635	3.139132	3.297528	0.943575	1	-
ENSMUSC	Idh1	isocitrate	GO:00325	developm	K00031	isocitrate	mmu0110	Metabolic	1:6515861	664	790	675	942	666	529	9.336107	9.310601	6.538116	8.6284	8.211355	9.2223	8.394942	8.687351	0.966883	1	-
ENSMUSC	Mettl21a	methyltrai	GO:00081	metabolic	-	-	-	-	1:6460647	94	116	158	190	131	122	1.183666	1.224377	1.370604	1.558615	1.446495	1.904802	1.259549	1.636637	0.179936	1	-
ENSMUSC	Creb1	cAMP res	GO:00325	developm	K05870	cyclic AMI	mmu0415	PI3K-Akt s	1:6453264	1342	389	1621	1415	1429	730	4.120798	1.001225	3.428965	2.830523	3.847722	2.779315	2.850329	3.15252	0.797921	1	-
ENSMUSC	Klf7	Kruppel-li	GO:00325	developm	-	-	-	-	1:6402944	972	1215	1492	1720	1118	765	7.000624	7.334993	7.40271	8.070135	7.060819	6.831526	7.246109	7.320827	0.851045	1	-
ENSMUSC	4933402D	RIKEN cDI	-	-	-	-	-	-	1:6375490	0	0	0	0	3	2	0	0	0	0	0.095093	0.089671	0	0.061588	0.088975	1	-
ENSMUSC	Fastkd2	FAST kina	GO:00081	metabolic	-	-	-	-	1:6373061	239	305	399	416	292	173	1.935666	2.07055	2.226162	2.194864	2.073756	1.737264	2.07746	2.001961	0.654065	1	-
ENSMUSC	Mdh1b	malate de	GO:00081	metabolic	-	-	-	-	1:6369881	16	14	29	19	11	15	0.591547	0.433862	0.738601	0.457599	0.356617	0.687614	0.588003	0.50061	0.438929	1	-
ENSMUSC	Adam23	a disinteg	GO:00081	metabolic	-	-	-	-	1:6344589	1795	2115	4266	3111	1972	983	5.459828	5.392346	8.938956	6.164477	5.259738	3.707267	6.597043	5.043827	0.092027	1	-
ENSMUSC	Eef1b2	eukaryotic	GO:00081	metabolic	-	-	-	-	1:6317682	867	1134	1414	830	833	820	10.08242	11.0538	11.32783	6.287898	8.494414	11.82349	10.82135	8.8686	0.082708	1	-
ENSMUSC	Ndufs1	NADH de	GO:00081	metabolic	K03934	NADH de	mmu0110	Metabolic	1:6314359	2099	2776	3200	3339	2317	1800	11.98112	13.28181	12.58306	12.41604	11.59721	12.73924	12.61533	12.25083	0.557991	1	-
ENSMUSC	Nrp2	neuropilin	GO:00325	developm	-	-	-	-	1:6270328	2213	2600	2627	2798	2307	1570	10.12256	9.968618	8.277903	8.337544	9.25335	8.904191	9.456359	8.831695	0.380748	1	-
ENSMUSC	Maip1	matrix AA	GO:00511	localizatio	-	-	-	-	1:5740632	271	321	470	433	303	191	2.046928	2.032315	2.445587	2.130606	2.006868	1.788763	2.174943	1.975412	0.385655	1	-
ENSMUSC	Rftn2	raftlin fam	GO:00511	localizatio	-	-	-	-	1:5517015	586	619	851	886	646	433	5.668501	5.018969	5.670897	5.58324	5.479562	5.193315	5.452789	5.418706	0.751475	1	-
ENSMUSC	Mob4	MOB fam	GO:00452	synapse|c	-	-	-	-	1:5513123	258	320	383	323	249	262	1.619745	1.68395	1.656446	1.321026	1.370784	2.039456	1.65338	1.577089	0.512583	1	-
ENSMUSC	Hspd1	heat shoc	GO:00325	developm	K04077	chaperoni	mmu0515	Tuberculo	1:5507783	2373	2764	3586	3564	2594	1383	54.30727	53.02139	56.5356	53.13486	52.05624	39.24356	54.62142	48.14489	0.240793	1	-
ENSMUSC	Coq10b	coenzyme	-	-	-	-	-	-	1:5505277	119	203	277	247	189	194	1.401817	2.004445	2.247896	1.895498	1.952308	2.833563	1.884719	2.227123	0.493767	1	-
ENSMUSC	Sf3b1	splicing fa	GO:00081	metabolic	K12828	splicing fa	mmu0304	Spliceosor	1:5498516	4222	5060	6691	6955	5164	3696	16.02489	16.09831	17.49522	17.19712	17.18723	17.39382	16.53947	17.25939	0.965535	1	-
ENSMUSC	Ccdc150	coiled-co	-	-	-	-	-	-	1:5425068	1	1	5	2	4	0	0.009392	0.007871	0.032345	0.012233	0.032934	0	0.016536	0.015056	1	1	-
ENSMUSC	Slc39a10	solute car	GO:00081	metabolic	-	-	-	-	1:4680754	2477	3279	3911	3916	3067	1963	19.98994	22.18093	21.74327	20.5878	21.70411	19.64228	21.30472	20.64473	0.573429	1	-
ENSMUSC	Cps1	carbamoy	GO:00325	developm	K01948	carbamoy	mmu0110	Metabolic	1:6712302	6	3	8	9	6	1	0.068647	0.028768	0.063051	0.067076	0.060188	0.014181	0.053489	0.047149	0.887743	1	-
ENSMUSC	Slc40a1	solute car	GO:00325	developm	K14685	solute car	mmu0497	Mineral al	1:4590806	113	140	169	201	116	159	0.667361	0.693051	0.687578	0.773323	0.600738	1.164305	0.682663	0.846122	0.366117	1	-
ENSMUSC	Wdr75	WD repea	GO:00081	metabolic	K14552	NET1-ass	mmu0300	Ribosome	1:4579516	413	494	606	585	397	326	3.544546	3.553785	3.582902	3.270761	2.987748	3.469082	3.560411	3.24253	0.313987	1	-
ENSMUSC	Ikzf2	IKAROS fa	GO:00081	metabolic	-	-	-	-	1:6953121	99	91	129	44	99	95	0.363873	0.280355	0.326631	0.105355	0.319077	0.432938	0.32362	0.28579	0.485289	1	-
ENSMUSC	Lancl1	LanC (bac	GO:00508	response	-	-	-	-	1:6700051	1968	2425	2841	3016	2444	2063	16.27698	16.81177	16.18719	16.25031	17.72526	21.15602	16.42531	18.3772	0.555955	1	-
ENSMUSC	Acadl	acyl-Coer	GO:00081	metabolic	-	-	mmu0110	Metabolic	1:6683083	612	677	947	873	717	506	19.07526	17.68724	20.33388	17.72616	19.59661	19.55488	19.03213	18.95922	0.740811	1	-
ENSMUSC	Kansl1l	KAT8 regu	GO:00329	macromo	-	-	-	-	1:6671924	330	365	296	369	345	158	2.075252	1.923986	1.282331	1.511698	1.902472	1.231971	1.760523	1.548714	0.381198	1	-
ENSMUSC	Rpe	ribulose-5	GO:00081	metabolic	K01783	ribulose-p	mmu0110	Metabolic	1:6670083	189	568	269	286	569	164	3.02024	7.608194	2.961315	2.977338	7.973248	3.249448	4.529916	4.733345	0.958268	1	-
ENSMUSC	Icos	inducible	GO:00325	developm	K06713	inducible	mmu0451	Cell adhes	1:6097792	0	5	5	4	7	0	0	0.075773	0.062279	0.04712	0.110989	0	0.046017	0.052703	1	1	-
ENSMUSC	Ctla4	cytotoxic	GO:00325	developm	-	-	-	-	1:6088700	0	0	2	1	0	0	0	0	0.030302	0.014324	0	0	0.010101	0.004775	1	1	-
ENSMUSC	Cd28	CD28 anti	GO:00325	developm	-	-	-	-	1:6071680	3	3	4	7	4	9	0.028072	0.023532	0.025791	0.042676	0.032823	0.104428	0.025798	0.059976	0.137837	1	-
ENSMUSC	Raph1	Ras assoc	GO:00325	developm	-	-	-	-	1:6048229	978	1169	1305	1396	1070	696	2.798255	2.803599	2.572234	2.60205	2.68457	2.469128	2.724696	2.585249	0.476978	1	-
ENSMUSC	Carf	calcium re	GO:00081	metabolic	-	-	-	-	1:6009824	268	395	261	457	233	268	1.010454	1.248336	0.677913	1.122482	0.770337	1.252857	0.978901	1.048559	0.899541	1	-
ENSMUSC	Ica1l	islet cell a	GO:00325	developm	-	-	-	-	1:5998249	366	450	542	673	452	483	2.839055	2.925897	2.896302	3.400868	3.074496	4.645428	2.887085	3.706931	0.146234	1	-
ENSMUSC	Wdr12	WD repea	GO:00081	metabolic	-	-	-	-	1:6006978	464	672	768	728	569	440	2.039605	2.475996	2.325631	2.084687	2.193222	2.398098	2.280411	2.225336	0.622198	1	-
ENSMUSC	Nop58	NOP58 rib	GO:00081	metabolic	K14565	nucleolar	mmu0300	Ribosome	1:5968497	841	969	1035	1390	892	600	3.65482	3.52977	3.098574	3.935196	3.399206	3.233011	3.427721	3.522471	0.961017	1	-
ENSMUSC	Sumo1	small ubic	GO:00325	developm	K12160	small ubic	mmu0301	RNA trans	1:5958655	812	1064	1444	1157	974	679	19.45892	21.37259	23.83862	18.06249	20.46747	20.17523	21.55671	19.5684	0.278882	1	-
ENSMUSC	Cdk15	cyclin-dep	GO:00081	metabolic	-	-	-	-	1:5925690	5	3	9	9	2	7	0.104495	0.05255	0.129567	0.122528	0.036649	0.181393	0.095537	0.113523	0.893344	1	-
ENSMUSC	Als2	amyotrop	GO:00325	developm	K04575	amyotrop	mmu0501	Amyotrop	1:5916292	1394	1757	2096	2303	1428	859	8.506279	8.986725	8.810885	9.154875	7.640958	6.49914	8.767963	7.764991	0.260723	1	-
ENSMUSC	Stradb	STE20-rel	GO:00325	developm	K17532	STE20-rel	mmu0415	mTOR sig	1:5897352	341	417	528	563	416	384	2.293777	2.351176	2.446706	2.467098	2.453763	3.202683	2.363886	2.707848	0.50199	1	-
ENSMUSC	Trak2	trafficking	GO:00325	developm	K15374	trafficking	mmu0110	Metabolic	1:5890044	3985	4691	6332	6383	4524	3152	28.01535	27.64305	30.66621	29.23305	27.889	27.47514	28.77487	28.19906	0.628858	1	-
ENSMUSC	Casp8	caspase 8	GO:00325	developm	K04398	caspase 8	mmu0520	Pathways	1:5879537	49	56	83	74	62	58	0.838029	0.802793	0.977885	0.824473	0.929815	1.229922	0.872902	0.994737	0.688018	1	-
ENSMUSC	Cflar	CASP8 an	GO:00325	developm	K04724	CASP8 an	mmu0421	Necropto	1:5871150	536	643	870	805	617	371	1.897528	1.908039	2.121748	1.856525	1.915363	1.628481	1.975772	1.800123	0.349813	1	-
ENSMUSC	Ndufb3	NADH de	GO:00081	metabolic	K03959	NADH de	mmu0110	Metabolic	1:5858638	676	757	912	945	758	407	56.30644	52.85185	52.33085	51.27727	55.36351	42.03311	53.82971	49.55796	0.414552	1	-
ENSMUSC	Clk1	CDC-like	GO:00081	metabolic	K08823	CDC-like	mmu0513	Legionellc	1:5841018	993	1415	1637	1621	1226	641	5.53289	6.608641	6.283517	5.88393	5.990124	4.428397	6.141682	5.43415	0.277315	1	-
ENSMUSC	Ppil3	peptidylpı	GO:00081	metabolic	-	-	-	-	1:5843099	128	167	208	222	163	113	1.341044	1.466571	1.501236	1.515196	1.497487	1.4679	1.436284	1.493527	1	1	-
ENSMUSC	Nif3l1	Ngg1 inte	GO:00325	developm	-	-	-	-	1:5844515	260	338	470	447	359	229	1.511923	1.647498	1.882804	1.693343	1.830596	1.651117	1.680742	1.725019	0.9529	1	-
ENSMUSC	Orc2	origin rec	GO:00081	metabolic	K02604	origin rec	mmu0411	Cell cycle	1:5846277	485	692	794	823	543	319	6.562313	7.848279	7.400947	7.25433	6.44255	5.351701	7.270513	6.349527	0.257438	1	-
ENSMUSC	Sgo2a	shugoshir	GO:00511	localizatio	-	-	-	-	1:5799597	22	33	31	23	26	15	0.172143	0.216444	0.167105	0.117242	0.178398	0.145532	0.185231	0.147057	0.283344	1	-
ENSMUSC	Col5a2	collagen,	GO:00325	developm	K19721	collagen,	mmu0497	Protein di	1:4537432	205	294	341	371	219	211	1.289554	1.550193	1.477718	1.520342	1.208017	1.645713	1.439155	1.458024	0.852003	1	-
ENSMUSC	Col3a1	collagen,	GO:00325	developm	K19720	collagen,	mmu0492	Relaxin sig	1:4531153	458	579	1238	1371	532	732	2.71169	2.873467	5.049478	5.288025	2.76203	5.373676	3.544878	4.474577	0.324952	1	-
ENSMUSC	Kdelc1	KDEL (Lys	GO:00081	metabolic	-	-	-	-	1:4410654	156	198	235	218	170	140	1.662628	1.768839	1.725404	1.513593	1.588777	1.850055	1.718957	1.650808	0.582793	1	-
ENSMUSC	Ercc5	excision re	GO:00081	metabolic	K10846	DNA excis	mmu0342	Nucleotid	1:4414774	298	473	576	516	432	302	2.381044	3.167852	3.170476	2.685852	3.026754	2.991875	2.906457	2.901494	0.780434	1	-

ENSMUSC	Tex30	testis expr	GO:00038	catalytic a	-	-	-	-	1:4408661	34	35	57	47	44	32	0.41779	0.360498	0.482511	0.376233	0.4741	0.487543	0.420266	0.445958	1	1	-
ENSMUSC	1500015C	RIKEN cDI	GO:00325	developm	-	-	-	-	1:4373060	136	65	250	57	56	117	9.582306	3.838796	12.1345	2.616274	3.459858	10.22115	8.518533	5.432427	0.10385	1	-
ENSMUSC	Khdrbs2	KH domai	GO:00081	metabolic	-	-	-	-	1:3217271	132	169	191	214	165	115	0.744826	0.799321	0.742448	0.786644	0.816413	0.804574	0.762199	0.802544	0.935436	1	-
ENSMUSC	Slc9a2	solute car	GO:00511	localizatio	-	-	-	-	1:4068057	574	661	751	875	542	472	4.607154	4.447082	4.152522	4.575201	3.814718	4.697306	4.402253	4.362408	0.708265	1	-
ENSMUSC	Ptp4a1	protein ty	GO:00325	developm	-	-	-	-	1:3094030	426	328	514	653	480	423	2.470219	1.594236	2.05324	2.466725	2.440675	3.041245	2.039232	2.649548	0.144831	1	-
ENSMUSC	Slc9a4	solute car	GO:00325	developm	K13961	solute car	mmu0497	Gastric ac	1:4058008	407	524	494	667	391	280	4.066356	4.388285	3.40008	4.341282	3.425551	3.468598	3.951574	3.745144	0.561637	1	-
ENSMUSC	Il18rap	interleukir	GO:00048	signal trar	K05174	interleukir	mmu0406	Cytokine-	1:4051536	0	4	5	6	0	0	0	0.0286	0.029384	0.033345	0	0	0.019328	0.011115	0.692188	1	-
ENSMUSC	Il1rl1	interleukir	GO:00048	signal trar	-	-	-	-	1:4042957	4	4	2	5	0	3	0.02298	0.019262	0.007917	0.018715	0	0.021372	0.016719	0.013362	0.8458	1	-
ENSMUSC	Il18r1	interleukir	GO:00325	developm	K05173	interleukir	mmu0406	Cytokine-	1:4046555	2	0	3	0	0	0	0.01484	0	0.015331	0	0	0	0.010057	0	0.088828	1	-
ENSMUSC	Il1r1	interleukir	GO:00048	signal trar	K04386	interleukir	mmu0401	MAPK sigı	1:4022508	244	343	442	520	337	231	1.579923	1.861625	1.971602	2.193464	1.913458	1.854571	1.804383	1.987164	0.6697	1	-
ENSMUSC	Il1r2	interleukir	GO:00048	signal trar	K04387	interleukir	mmu0516	HTLV-I inf	1:4007407	3	0	3	0	6	3	0.081329	0	0.056031	0	0.142639	0.100861	0.045787	0.081167	0.574182	1	-
ENSMUSC	Map4k4	mitogen-a	GO:00325	developm	K04407	mitogen-a	mmu0401	MAPK sigı	1:3990091	3009	3347	4680	4707	3393	2025	14.70669	13.71201	15.75759	14.98713	14.54183	12.27166	14.72543	13.93354	0.484296	1	-
ENSMUSC	Npas2	neuronal	GO:00081	metabolic	K09026	neuronal	mmu0471	Circadian	1:3919373	486	615	684	657	589	367	6.253645	6.633226	6.06324	5.507368	6.645921	5.855294	6.316703	6.002861	0.516037	1	-
ENSMUSC	Pdcl3	phosducir	GO:00325	developm	-	-	-	-	1:3898560	346	448	446	491	365	212	4.288935	4.654824	3.808547	3.964937	3.967424	3.258321	4.250769	3.730227	0.27318	1	-
ENSMUSC	Chst10	carbohydı	GO:00081	metabolic	K09674	carbohydı	mmu0110	Metabolic	1:3886386	212	265	338	288	283	275	0.590751	0.618967	0.648838	0.522807	0.691509	0.950137	0.619519	0.721484	0.526425	1	-
ENSMUSC	Rev1	REV1, DN	GO:00081	metabolic	K03515	DNA repa	mmu0346	Fanconi a	1:3805278	483	595	752	775	543	421	3.288987	3.39613	3.527641	3.437938	3.242329	3.554537	3.404253	3.411601	0.779365	1	-
ENSMUSC	Eif5b	eukaryotic	GO:00081	metabolic	K03243	translatior	mmu0301	RNA trans	1:3799801	2115	2773	3431	3450	2516	1178	11.99702	13.18456	13.40711	12.74864	12.51458	8.285031	12.8629	11.18275	0.255868	1	-
ENSMUSC	Mrpl30	mitochon	GO:00081	metabolic	K02907	large subı	mmu0301	Ribosome	1:3789047	235	338	413	346	297	338	3.277369	3.951176	3.967878	3.143512	3.632092	5.844666	3.732141	4.206757	0.689673	1	-
ENSMUSC	Mitd1	MIT, micro	GO:00511	localizatio	-	-	-	-	1:3787480	94	115	140	140	111	64	1.491626	1.529629	1.530429	1.447248	1.544534	1.259212	1.517228	1.416998	0.569552	1	-
ENSMUSC	2010300C	RIKEN cDI	-	-	-	-	-	-	1:3761167	9948	10602	12553	16045	11151	7386	83.77174	74.83459	72.82168	88.02035	82.3414	77.11823	77.14267	82.49333	0.793016	1	-
ENSMUSC	Stk17b	serine/thr	GO:00081	metabolic	-	-	-	-	1:5375550	59	91	115	108	83	60	0.680394	0.879626	0.913602	0.811356	0.839315	0.857911	0.824541	0.836194	0.90926	1	-
ENSMUSC	Osgepl1	O-sialogly	GO:00081	metabolic	-	-	-	-	1:5331362	134	147	210	174	190	127	0.939138	0.863564	1.013898	0.794432	1.167669	1.103607	0.938867	1.021903	0.8121	1	-
ENSMUSC	Ormdl1	ORM1-lik	GO:00081	metabolic	-	-	-	-	1:5329709	104	124	153	179	108	87	1.351083	1.350282	1.369282	1.5149	1.230312	1.401379	1.356882	1.382197	0.919875	1	-
ENSMUSC	Pms1	PMS1 hor	GO:00081	metabolic	-	-	-	-	1:5318918	76	98	122	132	96	61	0.513286	0.554788	0.567619	0.580767	0.568537	0.51081	0.545231	0.553371	0.934016	1	-
ENSMUSC	Mstn	myostatin	GO:00325	developm	-	-	-	-	1:5306164	0	3	8	14	3	2	0	0.030934	0.067799	0.112201	0.03236	0.030515	0.032911	0.058359	0.431852	1	-
ENSMUSC	Inpp1	inositol pc	GO:00081	metabolic	K01107	inositol pc	mmu0110	Metabolic	1:5278542	472	551	651	731	560	394	3.852191	3.769382	3.660144	3.886558	4.007716	3.987016	3.760572	3.96043	0.917245	1	-
ENSMUSC	Gls	glutamina	GO:00081	metabolic	K01425	glutamina	mmu0110	Metabolic	1:5216344	4595	5736	6523	7425	5500	4897	11.89855	12.45003	11.6361	12.52525	12.4886	15.72261	11.99489	13.57882	0.483234	1	-
ENSMUSC	Nabp1	nucleic ac	GO:00081	metabolic	-	-	-	-	1:5146586	187	212	321	331	262	166	0.593035	0.563543	0.701285	0.68383	0.728589	0.652729	0.619287	0.688383	0.642748	1	-
ENSMUSC	Tmeff2	transmem	GO:00325	developm	-	-	-	-	1:5090064	1482	1756	1951	1818	1519	1065	13.14707	13.05744	11.9231	10.50645	11.81629	11.71429	12.7092	11.34568	0.198066	1	-
ENSMUSC	Mgat4a	mannosid	GO:00081	metabolic	K00738	alpha-1,3	mmu0110	Metabolic	1:3743934	654	774	932	949	680	624	2.663825	2.642541	2.615144	2.518117	2.428733	3.151364	2.640503	2.699405	0.863837	1	-
ENSMUSC	Unc50	unc-50 hc	GO:00511	localizatio	-	-	-	-	1:3743013	152	249	277	271	195	351	2.383431	3.272743	2.992203	2.768282	2.681249	6.824207	2.882792	4.091246	0.177667	1	-
ENSMUSC	Coa5	cytochron	GO:00099	cellular pr	-	-	-	-	1:3741708	1350	1639	2055	2096	1531	1401	16.03075	16.31367	16.8106	16.2141	15.94181	20.62737	16.38501	17.59443	0.820263	1	-
ENSMUSC	Inpp4a	inositol pc	GO:00160	membran	K01109	inositol pc	mmu0110	Metabolic	1:3729986	1428	1666	2004	3235	2404	1268	5.734831	5.608159	5.544233	8.463468	8.46583	6.313899	5.629075	7.747732	0.033469	1	-
ENSMUSC	Cnga3	cyclic nucl	GO:00325	developm	K04950	cyclic nucl	mmu0474	Olfactory	1:3721443	0	5	6	0	2	0	0	0.0474	0.046748	0	0.019834	0	0.031383	0.006611	0.092692	1	-
ENSMUSC	Tmem131	transmem	GO:00160	membran	-	-	-	-	1:3679219	2441	2776	3419	3862	2656	1298	11.69667	11.14979	11.28613	12.05558	11.16002	7.71179	11.37753	10.30913	0.383542	1	-
ENSMUSC	Zap70	zeta-chair	GO:00325	developm	K07360	tyrosine-p	mmu0401	Ras signal	1:3676179	69	71	54	92	69	73	1.046334	0.902459	0.564105	0.908842	0.917505	1.37254	0.837633	1.066296	0.375954	1	-
ENSMUSC	Sema4c	sema don	GO:00325	developm	K06521	semaphor	mmu0436	Axon guic	1:3654863	133	175	250	233	168	211	1.116584	1.231488	1.44588	1.274317	1.236781	2.196385	1.264651	1.569161	0.358735	1	-
ENSMUSC	Plekhb2	pleckstrin	GO:00325	developm	-	-	-	-	1:3484997	3439	3978	4967	5077	3806	3674	25.79778	25.01311	25.66822	24.8107	25.03581	34.17238	25.49304	28.00629	0.683301	1	-
ENSMUSC	Ptpn18	protein ty	GO:00081	metabolic	-	-	-	-	1:3445976	18	19	24	14	23	11	0.151636	0.134166	0.139282	0.076833	0.169906	0.114901	0.141695	0.120547	0.508998	1	-
ENSMUSC	Imp4	IMP4, U3	GO:00081	metabolic	K14561	U3 small r	mmu0300	Ribosome	1:3443985	472	515	694	661	504	378	2.938579	2.687546	2.976505	2.680889	2.751493	2.917918	2.867543	2.783433	0.598409	1	-
ENSMUSC	Dst	dystonin [	GO:00325	developm	-	-	-	-	1:3390822	10870	12266	16301	17876	13702	8082	12.77382	12.08223	13.19645	13.68496	14.11946	11.77596	12.68417	13.19346	0.962069	1	-
ENSMUSC	Prim2	DNA prim	GO:00081	metabolic	K02685	DNA prim	mmu0110	Metabolic	1:3345381	111	134	136	146	119	96	1.156459	1.170207	0.976102	0.990926	1.087166	1.240117	1.100923	1.10607	0.827724	1	-
ENSMUSC	Zfp142	zinc finge	GO:00971	organic cy	-	-	-	-	1:7456512	719	659	999	1107	827	636	2.173242	1.669622	2.080158	2.17976	2.191935	2.383541	1.974341	2.251745	0.48586	1	-
ENSMUSC	Col19a1	collagen,	GO:00325	developm	-	-	-	-	1:2426189	254	382	392	579	462	290	2.058989	2.595589	2.189058	3.057597	3.284011	2.914769	2.281212	3.085459	0.061176	1	-
ENSMUSC	Rhbdd1	rhomboid	GO:00325	developm	-	-	-	-	1:8231645	158	169	224	232	161	150	1.440038	1.291088	1.406424	1.377482	1.286721	1.695097	1.379183	1.4531	0.961159	1	-
ENSMUSC	Col9a1	collagen,	GO:00329	macromo	K08131	collagen,	mmu0415	PI3K-Akt s	1:2417761	1	5	14	13	8	6	0.012057	0.05052	0.11626	0.102093	0.08457	0.089689	0.059612	0.092117	0.445283	1	-
ENSMUSC	Mff	mitochon	GO:00325	developm	-	-	-	-	1:8272489	1468	1922	2344	2073	1778	1666	6.044039	6.632963	6.648297	5.560095	6.419123	8.504764	6.441767	6.827994	0.930833	1	-
ENSMUSC	Fam135a	family wit	GO:00081	metabolic	-	-	-	-	1:2401109	533	693	774	908	724	362	1.972614	2.149816	1.973366	2.189187	2.349614	1.661153	2.031932	2.066651	0.932996	1	-
ENSMUSC	Sdhaf4	succinate	GO:00325	developm	-	-	-	-	1:2399593	44	76	85	78	59	142	2.521502	3.650674	3.35563	2.911926	2.964802	10.08974	3.175936	5.322155	0.128582	1	-
ENSMUSC	Smap1	small ArfC	GO:00325	developm	K12486	stromal m	mmu0414	Endocytos	1:2384484	1136	1427	1957	1731	1193	1014	13.83865	14.57109	16.42319	13.73707	12.74379	15.31578	14.94431	13.93221	0.357218	1	-
ENSMUSC	B3gat2	beta-1,3-	GO:00081	metabolic	K10157	galactosyl	mmu0110	Metabolic	1:2376175	415	450	531	585	362	242	2.970101	2.699535	2.617997	2.727476	2.271825	2.147458	2.762544	2.382253	0.216275	1	-
ENSMUSC	Ogfrl1	opioid grc	GO:00160	membran	-	-	-	-	1:2336642	4786	5950	6695	7082	5152	2571	32.80818	34.18845	31.61634	31.6262	30.96906	21.85232	32.87099	28.1492	0.187024	1	-
ENSMUSC	Agfg1	ArfGAP w	GO:00325	developm	K15044	Arf-GAP c	mmu0516	Influenza	1:8283948	1722	1762	2236	2293	1675	1269	7.875523	6.754683	7.044814	6.831751	6.717438	7.196044	7.225007	6.915078	0.487841	1	-
ENSMUSC	Nhej1	nonhomo	GO:00081	metabolic	-	-	-	-	1:7496713	11	21	27	24	22	12	0.625315	1.000614	1.057319	0.888762	1.096645	0.845787	0.894416	0.943731	1	1	-
ENSMUSC	Sphkap	SPHK1 int	GO:00055	extracellul	-	-	-	-	1:8325413	4885	6631	6319	9386	6548	4027	19.90233	22.64494	17.73533	24.9116	23.39326	20.34262	20.0942	22.88249	0.456785	1	-
ENSMUSC	Wnt10a	wingless-t	GO:00325	developm	K01357	wingless-t	mmu0520	Pathways	1:7479151	25	26	71	52	28	54	0.559254	0.487514	1.094147	0.757796	0.549242	1.497781	0.713638	0.934939	0.485756	1	-
ENSMUSC	Cyp27a1	cytochron	GO:00081	metabolic	K00488	cholestan	mmu0110	Metabolic	1:7471357	33	48	55	69	38	34	0.674789	0.82271	0.774766	0.91915	0.681369	0.862038	0.757422	0.820852	0.853274	1	-
ENSMUSC	Rnf25	ring finge	GO:00081	metabolic	-	-	-	-	1:7459374	436	534	712	567	474	427	6.880838	7.063972	7.740797	5.829334	6.559571	8.355412	7.228536	6.914772	0.506364	1	-
ENSMUSC	Bcs1l	BCS1-like	GO:00099	cellular pr	-	-	-	-	1:7458828	243	320	353	354	296	181	4.639043	5.120646	4.642469	4.402582	4.955157	4.284381	4.800719	4.547373	0.542961	1	-
ENSMUSC	Plcd4	phospholi	GO:00048	signal trar	K05857	phosphati	mmu0110	Metabolic	1:7454288	220	269	387	357	322	288	1.56342	1.602353	1.894588	1.652732	2.006554	2.537643	1.686787	2.065643	0.299272	1	-
ENSMUSC	Cnot9	CCR4-NC	GO:00081	metabolic	K12606	CCR4-NO	mmu0301	RNA degr	1:7450605	443	543	625	614	495	425	6.615125	6.796529	6.429329	5.972886	6.481607	7.868818	6.613661	6.774437	0.881038	1	-
ENSMUSC	Ctdsp1	CTD (carb	GO:00325	developm	-	-	-	-	1:7439150	306	361	531	400	427	207	3.450537	3.412124	4.124879	2.938374	4.222165	2.894152	3.662513	3.351563	0.446824	1	-
ENSMUSC	Slc11a1	solute car	GO:00081	metabolic	K12347	natural re:	-	-	1:7437519	27	43	70	67	31	51	0.22363	0.298533	0.399409	0.361518	0.225149	0.523756	0.30719	0.370141	0.616739	1	-
ENSMUSC	Pnkd	paroxysm	GO:00081	metabolic	-	-	-	-	1:7428493	3362	4050	4787	5437	4201	2866	16.54012	16.70124	16.22392	17.42536	18.12326	17.48248	16.48842	17.67703	0.777568	1	-
ENSMUSC	Cxcr2	chemokin	GO:00325	developm	K05050	C-X-C ch	mmu0406	Cytokine-	1:7415398	4	2	6	3	4	3	0.048272	0.020231	0.049881	0.023583	0.042328	0.044895	0.039462	0.036935	0.853926	1	-
ENSMUSC	Ppm1f	protein pl	GO:00081	metabolic	-	-	-	-	16:168964	1191	1480	1825	1715	1336	1479	15.11414	15.74296	15.9546	14.17808	14.86692	23.27158	15.6039	17.43886	0.649385	1	-
ENSMUSC	Igfbp5	insulin-lik	GO:00325	developm	-	-	-	-	1:7285793	3823	4723	6377	6884	5040	3225	24.45035	25.3193	28.09627	28.6816	28.26533	25.57387	25.95531	27.50693	0.844691	1	-
ENSMUSC	Xrcc5	X-ray rep	GO:00325	developm	K10885	ATP-depe	mmu0345	Non-hom	1:7230742	255	328	332	351	302	225	6.342874	6.83868	5.688991	5.687658	6.587109	6.939282	6.290182	6.404683	0.87361	1	-
ENSMUSC	Tmem169	transmem	GO:00160	membran	-	-	-	-	1:7228436	192	448	485	482	357	28	3.794214	7.420797	6.602567	6.205095	6.186297	0.686062	5.939192	4.359151	0.399308	1	-
ENSMUSC	Pecr	peroxisor	GO:00081	metabolic	K07753	peroxisor	mmu0414	Peroxisor	1:7225916	60	48	68	86	58	32	1.687983	1.131909	1.317899	1.576157	1.430828	1.116234	1.379263	1.374406	0.910432	1	-
ENSMUSC	Atic	5-aminoir	GO:00325	developm	K00602	phosphor	mmu0110	Metabolic	1:7155715	410	478	564	653	478	381	2.960992	2.893572	2.805974	3.072194	3.027079	3.411651	2.886846	3.170308	0.688013	1	-
ENSMUSC	Fn1	fibronecti	GO:00325	developm	K05717	fibronecti	mmu0520	Pathways	1:7158552	1354	1766	2212	2523	1923	1406	4.760234	5.204193	5.357305	5.778413	5.92832	6.12888	5.107244	5.945204	0.335395	1	-
ENSMUSC	Bard1	BRCA1 as	GO:00081	metabolic	K10683	BRCA1-as	mmu0344	Homologı	1:7102749	6	9	18	16	18	9	0.067385	0.084717	0.13926	0.117057	0.177257	0.125317	0.097121	0.139877	0.340227	1	-
ENSMUSC	Zfand2b	zinc finge	GO:00511	localizatio	-	-	-	-	1:7516864	172	248	283	312	228	232	2.601409	3.144017	2.948613	3.074084	3.023827	4.350641	2.898013	3.482851	0.371643	1	-
ENSMUSC	Abcb6	ATP-bind	GO:00325	developm	K05661	ATP-bind	mmu0201	ABC trans	1:7517171	254	334	363	410	270	298	1.578726	1.740092	1.554284	1.660115	1.471567	2.296546	1.624367	1.809409	0.691083	1	-
ENSMUSC	Ankzf1	ankyrin re	GO:00508	response	-	-	-	-	1:7519215	314	423	506	585	392	370	1.968788	2.223118	2.185602	2.389501	2.155254	2.876452	2.125836	2.473736	0.431103	1	-
ENSMUSC	Glb1l	galactosic	GO:00081	metabolic	-	-	-	-	1:7519823	104	137	179	177	139	107	0.666012	0.735401	0.789686	0.738422	0.780563	0.84961	0.730366	0.789532	0.827923	1	-
ENSMUSC	Stk16	serine/thr	GO:00081	metabolic	-	-	-	-	1:7521083	404	492	615	642	510	373	2.324827	2.373166	2.438016	2.406724	2.573492	2.661367	2.37867	2.547194	0.826664	1	-
ENSMUSC	Tuba4a	tubulin, al	GO:00051	structural	K07374	tubulin alp	mmu0414	Phagoson	1:7521422	2285	2908	3550	3515	2584	3128	18.88654	20.14714	20.21371	18.92663	18.72844	32.05671	19.74913	23.23726	0.417076	1	-
ENSMUSC	Dnajb2	DnaJ heat	GO:00081	metabolic	K09508	DnaJ hom	mmu0414	Protein pr	1:7523640	734	842	1182	924	740	913	5.528312	5.315711	6.132894	4.533675	4.88732	8.526161	5.658972	5.982385	0.995014	1	-
ENSMUSC	Ptprn	protein ty	GO:00325	developm	K07817	receptor-	mmu0494	Type I dia	1:7524702	9984	11857	12703	14173	10941	8735	127.157	126.5795	111.4534	117.5923	122.1899	137.9382	121.7299	125.9068	0.942994	1	-
ENSMUSC	Slc23a3	solute car	GO:00511	localizatio	-	-	-	-	1:7512554	4	0	4	1	2	8	0.102749	0	0.070795	0.016733	0.045048	0.254807	0.057848	0.105529	0.576097	1	-
ENSMUSC	Speg	SPEG com	GO:00325	developm	-	-	-	-	1:7537529	2412	2676	3307	4183	3064	2205	7.867071	7.316016	7.430556	8.888023	8.763289	8.917237	7.537881	8.856183	0.286982	1	-
ENSMUSC	Des	desmin [S	GO:00325	developm	K07610	desmin[-]	mmu0541	Dilated ca	1:7536032	37	45	78	61	56	44	0.539446	0.549937	0.783426	0.579377	0.715942	0.795403	0.62427	0.696907	0.773751	1	-
ENSMUSC	Dnpep	aspartyl a	GO:00081	metabolic	-	-	-	-	1:7530789	288	315	361	382	296	331	2.311021	2.118723	1.995584	1.996896	2.082793	3.293249	2.141776	2.457646	0.583376	1	-
ENSMUSC	Obsl1	obscurin-	GO:00325	developm	-	-	-	-	1:7547931	210	250	255	330	249	231	0.894573	0.892667	0.74832	0.915783	0.930119	1.220096	0.845186	1.021999	0.331093	1	-
ENSMUSC	Stk11ip	serine/thr	GO:00511	localizatio	-	-	-	-	1:7552152	348	431	545	589	454	516	2.456291	2.549947	2.650015	2.708304	2.809957	4.515818	2.552084	3.344693	0.139488	1	-
ENSMUSC	Trip12	thyroid hc	GO:00325	developm	K10590	E3 ubiquit	mmu0412	Ubiquitin	1:8472118	3381	4292	5236	5638	4295	2499	9.817817	10.44678	10.4742	10.66538	10.93643	8.997503	10.24626	10.19977	0.761039	1	-
ENSMUSC	Slc16a14	solute car	GO:00511	localizatio	-	-	-	-	1:8490589	135	152	182	203	168	142	2.39455	2.259878	2.223886	2.345667	2.613015	3.122947	2.292771	2.693876	0.463615	1	-
ENSMUSC	Itm2c	integral m	GO:00325	developm	-	-	-	-	1:8589428	8176	10270	11368	11551	9061	9419	135.6328	142.8063	129.915	124.8318	131.8083	193.738	136.1181	150.126	0.69303	1	-
ENSMUSC	4933407L	RIKEN cDI	-	-	-	-	-	-	1:8592848	22	24	29	33	16	14	0.314048	0.287172	0.285188	0.306883	0.20028	0.247806	0.295469	0.251656	0.502448	1	-
ENSMUSC	Spata3	spermato	GO:00160	membran	-	-	-	-	1:8601681	3	4	7	9	6	2	0.030202	0.033757	0.048556	0.059032	0.05297	0.024975	0.037505	0.045659	0.752203	1	-
ENSMUSC	2810459N	RIKEN cDI	GO:00055	protein bi	-	-	-	-	1:8604586	242	289	396	431	355	294	3.99891	4.002928	4.507893	4.639658	5.143963	6.023656	4.169911	5.269093	0.173325	1	-
ENSMUSC	Htr2b	5-hydroxy	GO:00325	developm	K04157	5-hydroxy	mmu0408	Neuroacti	1:8609902	2	0	9	14	9	4	0.048841	0	0.151369	0.222678	0.192691	0.12109	0.066736	0.17882	0.066577	1	-
ENSMUSC	Psmd1	proteasor	GO:00081	metabolic	K03032	26S prote	mmu0516	Epstein-B	1:8606438	2313	2725	3387	3451	2488	1418	17.28982	17.07396	17.44141	16.80512	16.30827	13.14248	17.2684	15.41863	0.260454	1	-
ENSMUSC	Ncl	nucleolin	GO:00325	developm	-	-	-	-	1:8634471	6721	7996	9761	9821	7186	3437	35.23383	35.13595	35.25103	33.54002	33.0336	22.34043	35.20694	29.63802	0.157612	1	-
ENSMUSC	Epha4	Eph recep	GO:00325	developm	K05105	Eph recep	mmu0436	Axon guic	1:7736718	8655	9407	11422	13301	8990	5447	44.02171	40.10549	40.02151	44.07231	40.09611	34.3513	41.3829	39.50657	0.529729	1	-
ENSMUSC	Nmur1	neuromec	GO:00048	signal trar	K05052	neuromec	mmu0408	Neuroacti	1:8638630	0	0	1	2	0	0	0	0	0.013367	0.025288	0	0	0.004456	0.008429	1	1	-
ENSMUSC	Ptma	prothymo	GO:00099	cellular pr	K13784	prothymo	mmu0516	Epstein-B	1:8652672	673	1228	903	1353	828	566	14.52332	22.21274	13.42425	19.02086	15.66837	15.14443	16.7201	16.61122	0.802482	1	-
ENSMUSC	Pde6d	phosphoc	GO:00987	molecular	K13758	retinal roc	mmu0023	Purine me	1:8654299	166	425	574	238	196	386	2.837489	6.089311	6.759116	2.650243	2.937823	8.180877	5.228639	4.589648	0.452667	1	-
ENSMUSC	Cops7b	COP9 sigr	GO:00081	metabolic	-	-	-	-	1:8658290	214	277	356	405	263	256	1.231134	1.335746	1.410891	1.517847	1.326753	1.826068	1.325924	1.556889	0.418014	1	-
ENSMUSC	Nppc	natriuretic	GO:00325	developm	K12336	natriuretic	mmu0541	Fluid shea	1:8666629	34	38	40	31	21	31	2.073714	1.942705	1.680654	1.231719	1.123152	2.344332	1.899024	1.566401	0.29669	1	-
ENSMUSC	Farsb	phenylala	GO:00081	metabolic	K01890	phenylala	mmu0097	Aminoacy	1:7841797	593	748	880	819	657	552	10.60409	11.21175	10.84059	9.540788	10.30212	12.23894	10.88548	10.69395	0.631118	1	-
ENSMUSC	Ecel1	endotheli	GO:00081	metabolic	-	-	-	-	1:8714765	58	170	92	83	36	66	0.664758	1.633187	0.726398	0.61972	0.361812	0.937923	1.008115	0.639818	0.066377	1	-
ENSMUSC	Mrpl44	mitochon	GO:00081	metabolic	-	-	-	-	1:7977601	205	297	397	311	267	266	5.642427	6.85206	7.527554	5.576399	6.44415	9.077786	6.674014	7.032778	0.994042	1	-
ENSMUSC	Serpine2	serine (or	GO:00325	developm	-	-	-	-	1:7979419	2015	2729	3302	3196	2458	2032	14.54228	16.50874	16.4167	15.02609	15.55543	18.18306	15.82257	16.25486	0.898056	1	-
ENSMUSC	Eif4e2	eukaryotic	GO:00325	developm	K03259	translatior	mmu0415	PI3K-Akt s	1:8721391	857	955	1275	1225	933	883	6.114156	5.711001	6.26639	5.693428	5.836873	7.810926	6.030516	6.447075	0.869687	1	-
ENSMUSC	Efhd1	EF hand d	GO:00325	developm	-	-	-	-	1:8726436	333	280	448	383	280	243	6.495745	4.578206	6.020243	4.867051	4.789446	5.877289	5.698065	5.177929	0.352739	1	-
ENSMUSC	3110079C	RIKEN cDI	GO:00325	developm	-	-	-	-	1:8747027	0	0	3	1	1	0	0	0	0.154152	0.048587	0.065404	0	0.051384	0.037997	1	1	-
ENSMUSC	Ngef	neuronal	GO:00325	developm	K07525	neuronal	mmu0436	Axon guic	1:8747683	958	1276	1540	1641	1278	1706	14.58643	16.28498	16.1531	16.27698	17.06307	32.20688	15.67484	21.84897	0.073037	1	-
ENSMUSC	Ndufa10	NADH de	GO:00081	metabolic	K03954	NADH de	mmu0110	Metabolic	1:9243901	1746	2155	2584	2552	1945	1506	22.75242	23.53875	23.19676	21.66435	22.2252	24.33293	23.16264	22.74082	0.609754	1	-
ENSMUSC	Rnpepl1	arginyl an	GO:00081	metabolic	-	-	-	-	1:9291078	375	447	491	580	418	396	1.37624	1.375066	1.241357	1.38667	1.345187	1.80196	1.330888	1.511272	0.553435	1	-
ENSMUSC	Capn10	calpain 1C	GO:00081	metabolic	-	-	-	-	1:9293437	281	392	412	389	319	356	2.840074	3.320953	2.868616	2.561265	2.8272	4.461287	3.009881	3.283251	0.830721	1	-
ENSMUSC	Gpr35	G protein-	GO:00048	signal trar	K04276	G protein-	mmu0408	Neuroacti	1:9295086	4	13	17	7	6	7	0.017747	0.048348	0.051959	0.020233	0.023342	0.03851	0.039351	0.027361	0.297849	1	-
ENSMUSC	Mterf4	mitochon	GO:00325	developm	-	-	-	-	1:9329921	230	283	225	259	236	171	2.104406	2.170408	1.418192	1.543773	1.89346	1.939918	1.897669	1.792384	0.550202	1	-
ENSMUSC	Pask	PAS dom	GO:00081	metabolic	-	-	-	-	1:9330877	61	70	85	77	64	43	0.32263	0.310331	0.309703	0.265307	0.296824	0.281989	0.314221	0.281374	0.421327	1	-
ENSMUSC	Ppp1r7	protein pl	GO:00081	metabolic	-	-	-	-	1:9334285	1843	2163	2665	2864	2122	1495	10.76051	10.58564	10.71905	10.89337	10.86415	10.82268	10.6884	10.86007	0.854682	1	-
ENSMUSC	2-Sep	septin 2 [S	GO:00325	developm	K16942	septin 2[-	mmu0510	Bacterial i	1:9347896	877	1056	1485	1518	1045	822	4.738648	4.782686	5.527552	5.343284	4.951237	5.506972	5.016295	5.267164	0.935122	1	-
ENSMUSC	Stk25	serine/thr	GO:00325	developm	-	-	-	-	1:9361975	2803	3365	4299	4360	3440	3404	23.97888	24.12924	25.33521	24.29819	25.80518	36.10615	24.48111	28.73651	0.349699	1	-
ENSMUSC	Bok	BCL2-rela	GO:00325	developm	K02561	Bcl-2-rela	mmu0421	Apoptosis	1:9368566	559	778	459	920	739	570	9.805724	11.43932	5.546669	10.51326	11.36723	12.39733	8.93057	11.42594	0.234418	1	-
ENSMUSC	Thap4	THAP dor	GO:00971	organic cy	-	-	-	-	1:9370193	222	98	155	125	99	123	1.510248	0.558826	0.726408	0.553971	0.590577	1.037499	0.931827	0.727349	0.221689	1	-
ENSMUSC	Atg4b	autophag	GO:00081	metabolic	-	-	mmu0414	Autophag	1:9375150	756	1003	1140	1273	944	854	4.579308	5.092507	4.757009	5.023287	5.014095	6.413894	4.809608	5.483759	0.480879	1	-
ENSMUSC	Dtymk	deoxythyr	GO:00325	developm	K00943	dTMP kin	mmu0110	Metabolic	1:9379257	249	387	384	431	373	357	4.539504	5.913888	4.822709	5.118796	5.962931	8.069807	5.092034	6.383845	0.240521	1	-
ENSMUSC	Ing5	inhibitor c	GO:00081	metabolic	-	-	-	-	1:9380396	248	316	290	386	313	244	1.776905	1.897808	1.431404	1.801696	1.966526	2.167642	1.702039	1.978621	0.463622	1	-
ENSMUSC	Pdcd1	programn	GO:00325	developm	K06744	programn	mmu0451	Cell adhes	1:9403830	0	0	1	0	0	1	0	0	0.022676	0	0	0.040817	0.007559	0.013606	1	1	-
ENSMUSC	Inpp5d	inositol pc	GO:00325	developm	K03084	phosphati	mmu0407	Phosphati	1:8762031	194	230	334	337	252	232	0.993014	0.986814	1.177747	1.123742	1.13109	1.472406	1.052525	1.242413	0.415544	1	-
ENSMUSC	Atg16l1	autophag	GO:00081	metabolic	K17890	autophag	mmu0462	NOD-like	1:8775587	753	1051	1247	1265	940	927	2.929624	3.42746	3.342219	3.20619	3.206914	4.471802	3.233101	3.628302	0.591784	1	-
ENSMUSC	Spp2	secreted p	GO:00055	extracellul	-	-	-	-	1:8840696	0	2	0	0	0	0	0	0.078336	0	0	0	0	0.026112	0	0.536637	1	-
ENSMUSC	Iqca	IQ motif c	GO:00971	organic cy	-	-	-	-	1:9004213	26	22	26	36	19	8	0.261167	0.185231	0.179914	0.235574	0.16735	0.099635	0.208771	0.167519	0.452134	1	-
ENSMUSC	Mlph	melanopł	GO:00511	localizatio	-	-	-	-	1:9091508	36	28	54	45	41	24	0.38144	0.248677	0.394159	0.310617	0.380939	0.315302	0.341425	0.335619	0.870942	1	-
ENSMUSC	Rab17	RAB17, m	GO:00325	developm	-	-	-	-	1:9095813	0	2	0	0	0	0	0	0.026641	0	0	0	0	0.00888	0	0.535908	1	-
ENSMUSC	Lrrfip1	leucine ric	GO:00081	metabolic	-	-	-	-	1:9099873	1081	1406	1546	1959	1428	857	5.536371	6.035836	5.454567	6.536052	6.413134	5.442098	5.675591	6.130428	0.735293	1	-
ENSMUSC	Scly	selenocys	GO:00081	metabolic	K01763	selenocys	mmu0110	Metabolic	1:9129833	181	234	277	335	249	198	1.900709	2.059707	2.003866	2.291729	2.292869	2.578035	1.988094	2.387544	0.319452	1	-
ENSMUSC	Klhl30	kelch-like	GO:00081	metabolic	-	-	-	-	1:9135101	2	2	3	2	0	0	0.041156	0.034489	0.042518	0.026802	0	0	0.039388	0.008934	0.22333	1	-
ENSMUSC	Ilkap	integrin-li	GO:00081	metabolic	-	-	-	-	1:9137386	357	466	630	605	384	327	2.709064	2.964079	3.293389	2.990805	2.5552	3.076696	2.988844	2.874234	0.569515	1	-
ENSMUSC	Asb1	ankyrin re	GO:00325	developm	-	-	-	-	1:9154054	578	649	752	768	683	732	3.973782	3.740018	3.561605	3.439686	4.117557	6.239835	3.758468	4.599026	0.312579	1	-
ENSMUSC	Cdh7	cadherin 7	GO:00160	membran	-	-	-	-	1:1099824	139	68	109	102	123	42	0.4469	0.183255	0.241419	0.213637	0.34677	0.167431	0.290525	0.242613	0.435594	1	-
ENSMUSC	Hdac4	histone de	GO:00325	developm	K11406	histone de	mmu0516	Human pa	1:9192877	1171	1208	1443	1518	1126	1213	4.025999	3.48126	3.417704	3.39993	3.394672	5.170867	3.641655	3.98849	0.759481	1	-
ENSMUSC	Serpinb8	serine (or	GO:00081	metabolic	-	-	-	-	1:1075900	85	68	105	119	77	61	0.698835	0.468616	0.594701	0.637359	0.555125	0.621827	0.587384	0.60477	1	1	-
ENSMUSC	Cln8	ceroid-lip	GO:00325	developm	-	-	-	-	8:1488133	461	497	711	735	481	460	3.285624	2.969113	3.490908	3.412607	3.006114	4.065008	3.248548	3.494576	0.828688	1	-
ENSMUSC	2310035C	RIKEN cDI	-	-	-	-	-	-	1:1056638	668	848	951	992	647	622	2.580911	2.746285	2.531213	2.496835	2.192015	2.9797	2.619469	2.556183	0.596591	1	-
ENSMUSC	Tnfrsf11a	tumor nec	GO:00325	developm	K05147	tumor nec	mmu0406	Cytokine-	1:1057807	59	86	85	114	51	66	0.598794	0.7316	0.594282	0.753721	0.453876	0.830531	0.641559	0.679376	0.964215	1	-
ENSMUSC	Htr4	5 hydroxy	GO:00048	signal trar	K04160	5-hydroxy	mmu0408	Neuroacti	18:623242	294	297	318	370	233	225	4.012145	3.397339	2.989565	3.289375	2.788232	3.807143	3.46635	3.294917	0.537044	1	-
ENSMUSC	Serpinb11	serine (or	GO:00081	metabolic	-	-	-	-	1:1073611	0	0	2	0	0	0	0	0	0.036113	0	0	0	0.012038	0	0.537149	1	-
ENSMUSC	Gin1	gypsy retr	GO:00081	metabolic	-	-	-	-	1:9777017	133	165	175	208	164	64	1.021826	1.062583	0.926221	1.041048	1.104869	0.609664	1.003543	0.918527	0.571369	1	-
ENSMUSC	Pam	peptidylgl	GO:00325	developm	-	-	-	-	1:9779511	2379	2817	3312	3627	2775	2081	8.840641	8.774633	8.478733	8.780482	9.042632	9.588422	8.698002	9.137179	0.933806	1	-
ENSMUSC	Ccdc93	coiled-co	GO:00511	localizatio	-	-	-	-	1:1214310	843	1088	1321	1313	994	654	4.171221	4.512503	4.502878	4.232356	4.312857	4.01236	4.395534	4.185858	0.496845	1	-
ENSMUSC	Actr3	ARP3 actii	GO:00511	localizatio	K18584	actin-rela	mmu0453	Tight junc	1:1253929	3742	4470	3912	4340	4249	2838	27.78541	27.82105	20.01075	20.99348	27.66575	26.12832	25.20574	24.92918	0.696094	1	-
ENSMUSC	Slc35f5	solute car	GO:00511	localizatio	-	-	-	-	1:1255605	281	324	395	365	246	293	5.266407	5.089872	5.099838	4.456383	4.042841	6.808672	5.152039	5.102632	0.69326	1	-
ENSMUSC	Gpr39	G protein-	GO:00048	signal trar	-	-	-	-	1:1256769	22	21	14	8	22	38	0.366102	0.292926	0.160493	0.086727	0.321039	0.784082	0.273174	0.397283	0.511856	1	-
ENSMUSC	Lypd1	Ly6/Plaur	GO:00511	localizatio	-	-	-	-	1:1258676	2068	3168	2563	2663	2238	1368	12.83222	16.47743	10.95599	10.76476	12.17739	10.52503	13.42188	11.15572	0.106435	1	-
ENSMUSC	Tmem163	transmem	GO:00452	synapse|m	-	-	-	-	1:1274865	95	113	173	133	113	156	0.864976	0.862403	1.085118	0.788884	0.902201	1.761126	0.937499	1.150737	0.486681	1	-
ENSMUSC	Acmsd	amino car	GO:00081	metabolic	K03392	aminocarl	mmu0110	Metabolic	1:1277294	0	0	0	3	0	0	0	0	0	0.043868	0	0	0	0.014623	0.302813	1	-
ENSMUSC	Ccnt2	cyclin T2 [	GO:00325	developm	K15188	cyclin T[-]	mmu0520	Transcript	1:1277741	1235	1556	1638	2311	1739	1108	4.497619	4.749831	4.109426	5.482736	5.553388	5.003128	4.452292	5.346417	0.232681	1	-
ENSMUSC	Ubxn4	UBX dom	GO:00081	metabolic	-	-	-	-	1:1282439	2003	2362	3064	3020	2272	1030	24.16872	23.88942	25.46906	23.73894	24.03941	15.40976	24.50907	21.06271	0.227487	1	-
ENSMUSC	Lct	lactase [S	GO:00081	metabolic	K01229	lactase-pl	mmu0110	Metabolic	1:1282847	969	1100	1425	1792	1136	718	9.248065	8.7998	9.369002	11.14158	9.507098	8.496455	9.138956	9.715044	0.829014	1	-
ENSMUSC	Mcm6	minichron	GO:00081	metabolic	K02542	DNA repli	mmu0411	Cell cycle	1:1283315	317	379	437	531	386	255	3.463949	3.471392	3.289609	3.779968	3.698633	3.454917	3.408317	3.644506	0.820573	1	-
ENSMUSC	Dars	aspartyl-t	GO:00081	metabolic	K01876	aspartyl-t	mmu0097	Aminoacy	1:1283637	952	1288	1439	1527	1057	690	12.57064	14.25572	13.08978	13.13533	12.23877	11.2968	13.30538	12.22364	0.352568	1	-
ENSMUSC	Rgs18	regulator	GO:00987	molecular	-	-	-	-	1:1447526	0	4	1	0	2	1	0	0.10491	0.021548	0	0.054874	0.038788	0.042153	0.031221	0.766369	1	-
ENSMUSC	Rgs1	regulator	GO:00987	molecular	-	-	-	-	1:1442422	4	10	14	22	5	5	0.014057	0.029456	0.033893	0.050367	0.015407	0.021786	0.025802	0.029187	0.854642	1	-
ENSMUSC	Rgs2	regulator	GO:00325	developm	K18154	regulator	mmu0474	Olfactory	1:1439993	401	541	654	552	427	419	5.432692	6.143571	6.10379	4.871818	5.072718	7.038339	5.893351	5.660959	0.527308	1	-
ENSMUSC	Cdc73	cell divisic	GO:00325	developm	-	-	-	-	1:1435988	1225	1489	1777	1904	1424	840	3.823417	3.8955	3.820801	3.871366	3.89734	3.250729	3.846573	3.673145	0.533546	1	-
ENSMUSC	Cfh	complem	GO:00081	metabolic	K04004	complem	mmu0461	Complem	1:1400847	471	622	875	720	609	427	2.292521	2.537676	2.933946	2.283004	2.599276	2.576951	2.588048	2.48641	0.555483	1	-
ENSMUSC	Tsn	translin [S	GO:00081	metabolic	-	-	-	-	1:1182947	945	1357	1534	1568	1130	1266	5.018601	6.040648	5.612127	5.424735	5.262256	8.336242	5.557125	6.341078	0.543156	1	-
ENSMUSC	Nifk	nucleolar	GO:00081	metabolic	-	-	-	-	1:1183218	561	657	801	834	555	301	15.30838	15.02742	15.05742	14.82567	13.28012	10.18403	15.13107	12.76327	0.182988	1	-

ENSMUS(	Tfcp2l1	transcripti	GO:00325	developm	-	-	-	-	1:1186279	88	93	132	142	95	115	0.582876	0.516327	0.602305	0.612717	0.551768	0.944442	0.567169	0.702976	0.376669	1	-
ENSMUS(	Epb41l5	erythrocyt	GO:00325	developm	-	-	-	-	1:1195450	135	161	216	224	138	121	0.463591	0.463425	0.510982	0.501106	0.415547	0.515193	0.479333	0.477282	0.780183	1	-
ENSMUS(	Ptpn4	protein ty	GO:00081	metabolic	-	-	-	-	1:1196524	1769	1972	2660	2803	1839	1098	7.548865	7.053645	7.819643	7.792175	6.881425	5.809545	7.474051	6.827715	0.357184	1	-
ENSMUS(	Dbi	diazepam	GO:00325	developm	K08762	diazepam	mmu0332	PPAR sign	1:1201132	865	1042	1434	1149	919	745	18.64136	18.8227	21.28933	16.13107	17.36679	19.90689	19.58446	17.80158	0.261079	1	-
ENSMUS(	Sctr	secretin re	GO:00048	signal trar	K04588	secretin re	mmu0408	Neuroacti	1:1200068	3	8	9	8	8	5	0.069907	0.156269	0.144486	0.121456	0.163495	0.144472	0.123554	0.143141	0.895379	1	-
ENSMUS(	3110009E	RIKEN cDI	-	-	-	-	-	-	1:1201211	7	13	16	17	16	13	0.162254	0.252567	0.255469	0.256681	0.325181	0.373584	0.22343	0.318482	0.341043	1	-
ENSMUS(	Steap3	STEAP far	GO:00511	localizatio	K10142	metallore	mmu0411	p53 signa	1:1201907	95	122	154	196	135	64	0.590179	0.635293	0.659071	0.793227	0.735421	0.492976	0.628181	0.673875	0.814802	1	-
ENSMUS(	Nek7	NIMA (ne	GO:00081	metabolic	K20876	NIMA (ne	mmu0462	NOD-like	1:1384828	250	638	970	371	634	738	1.132208	2.421918	3.026278	1.094564	2.517778	4.14408	2.193468	2.585474	0.69366	1	-
ENSMUS(	Ptprc	protein ty	GO:00081	metabolic	K06478	receptor-	mmu0451	Cell adhes	1:1380628	98	113	144	179	100	86	0.231101	0.223361	0.233932	0.274987	0.206786	0.251457	0.229465	0.24441	0.877368	1	-
ENSMUS(	Nr5a2	nuclear re	GO:00325	developm	K08027	nuclear re	mmu0495	Maturity c	1:1368425	0	3	2	3	2	0	0	0.018065	0.0099	0.014039	0.012599	0	0.009322	0.008879	1	1	-
ENSMUS(	Cd55	CD55 mol	GO:00081	metabolic	K04006	decay acc	mmu0464	Hematopo	1:1304390	87	129	162	161	136	56	0.88807	1.103746	1.139181	1.070619	1.217328	0.708762	1.043666	0.998903	0.734849	1	-
ENSMUS(	Cd55b	CD55 mol	GO:00160	membran	K04006	decay acc	mmu0464	Hematopo	1:1303885	0	5	14	0	9	0	0	0.15901	0.365925	0	0.299448	0	0.174978	0.099816	0.574588	1	-
ENSMUS(	Ddx59	DEAD (As	GO:00081	metabolic	-	-	-	-	1:1364152	123	157	172	204	173	92	1.862522	1.992742	1.794225	2.012371	2.29713	1.72731	1.883163	2.01227	0.851006	1	-
ENSMUS(	C4bp	complem	GO:00081	metabolic	K04002	complem	mmu0461	Complem	1:1306347	10	6	7	12	2	6	0.08642	0.043461	0.041676	0.06756	0.015155	0.064297	0.057186	0.049004	0.731401	1	-
ENSMUS(	Cacna1s	calcium cl	GO:00511	localizatio	K04857	voltage-d	mmu0401	MAPK sigi	1:1360527	9	7	12	13	6	5	0.058382	0.03806	0.053628	0.054939	0.034127	0.040214	0.050024	0.043093	0.730284	1	-
ENSMUS(	Pfkfb2	6-phosph	GO:00081	metabolic	K19029	6-phosph	mmu0415	AMPK sigi	1:1306891	517	543	596	676	567	411	2.140516	1.884432	1.699907	1.82329	2.058512	2.109867	1.908285	1.997223	0.973527	1	-
ENSMUS(	Tmem9	transmem	GO:00511	localizatio	-	-	-	-	1:1360081	593	461	919	485	395	843	8.705694	5.672862	9.294277	4.638442	5.084968	15.34483	7.890944	8.356079	0.959014	1	-
ENSMUS(	Tnnt2	troponin T	GO:00325	developm	K12045	troponin T	mmu0426	Adrenergi	1:1358363	24	50	84	50	62	51	0.174377	0.304506	0.420445	0.23666	0.395015	0.459446	0.299776	0.363707	0.593091	1	-
ENSMUS(	Tnni1	troponin I	GO:00325	developm	-	-	-	-	1:1357794	33	35	55	50	26	45	0.486374	0.432397	0.558435	0.480071	0.336027	0.822342	0.492402	0.546146	0.889315	1	-
ENSMUS(	Csrp1	cysteine a	GO:00508	response	-	-	-	-	1:1357200	3149	3277	5114	4964	3592	2752	102.3151	89.24761	114.4668	105.0705	102.3402	110.8668	102.0098	106.0925	0.998018	1	-
ENSMUS(	Gpr37l1	G protein-	GO:00325	developm	-	-	-	-	1:1351602	2068	2528	3160	3325	2581	2802	57.92315	59.35148	60.97337	60.67012	63.39168	97.30961	59.416	73.79047	0.194458	1	-
ENSMUS(	Srgap2	SLIT-ROB	GO:00325	developm	K07526	SLIT-ROB	mmu0436	Axon guic	1:1312852	1778	2020	2220	2692	2043	1144	7.60822	7.245285	6.544185	7.504259	7.665882	6.069644	7.132563	7.079928	0.762842	1	-
ENSMUS(	Arl8a	ADP-ribo	GO:00508	response	-	-	-	-	1:1351468	1787	1970	2109	2925	2012	866	41.1036	37.98169	33.41822	43.82913	40.58129	24.69785	37.50117	36.36942	0.732185	1	-
ENSMUS(	Eif2d	eukaryotic	GO:00081	metabolic	-	-	-	-	1:1311531	398	489	520	612	479	396	2.665907	2.745509	2.399476	2.670511	2.81345	3.288842	2.60363	2.924268	0.585187	1	-
ENSMUS(	Ube2t	ubiquitin-	GO:00081	metabolic	K13960	ubiquitin-	mmu0346	Fanconi a	1:1349625	9	29	18	26	13	21	0.091078	0.245992	0.125489	0.171406	0.115362	0.263497	0.154186	0.183422	0.738692	1	-
ENSMUS(	Rassf5	Ras assoc	GO:00081	metabolic	K08015	Ras assoc	mmu0520	Pathways	1:1311764	344	392	537	490	363	337	5.024647	4.799392	5.403468	4.66256	4.649399	6.103279	5.075836	5.138412	0.812679	1	-
ENSMUS(	Avpr1b	arginine v	GO:00048	signal trar	K04227	arginine v	mmu0408	Neuroacti	1:1315992	19	15	18	19	13	7	0.259902	0.171992	0.169623	0.16931	0.155935	0.118729	0.200506	0.147991	0.311866	1	-
ENSMUS(	Rab29	RAB29, m	GO:00325	developm	-	-	-	-	1:1318672	39	52	47	63	50	41	0.875204	0.978127	0.726598	0.921009	0.983914	1.140799	0.859976	1.015241	0.548313	1	-
ENSMUS(	Nucks1	nuclear ca	GO:00081	metabolic	-	-	-	-	1:1319105	3324	4209	4950	5727	4177	3206	23.69073	25.14485	24.30379	26.59048	26.10505	28.33132	24.37979	27.00895	0.57374	1	-
ENSMUS(	Slc45a3	solute car	GO:00325	developm	K15379	solute car	mmu0520	Transcript	1:1319629	13	6	14	17	20	14	0.121711	0.047084	0.090295	0.103686	0.164202	0.162527	0.086363	0.143471	0.164503	1	-
ENSMUS(	Elk4	ELK4, mer	GO:00325	developm	K04376	ETS doma	mmu0401	MAPK sigi	1:1320076	544	527	699	834	579	250	4.048815	3.287702	3.583913	4.04368	3.778769	2.307041	3.640143	3.376497	0.562503	1	-
ENSMUS(	Cdk18	cyclin-dep	GO:00081	metabolic	-	-	-	-	1:1321122	429	504	577	547	464	390	4.506485	4.437762	4.175493	3.743259	4.274065	5.079619	4.373247	4.365648	0.731455	1	-
ENSMUS(	Rbbp5	retinoblas	GO:00081	metabolic	-	-	-	-	1:1324773	1074	1349	1708	1797	1340	903	7.794442	8.206268	8.539256	8.495923	8.527628	8.12558	8.179989	8.383044	0.926321	1	-
ENSMUS(	Nfasc	neurofasc	GO:00325	developm	K06757	neurofasc	mmu0451	Cell adhes	1:1325646	5748	5823	8409	9130	6657	4870	20.08592	17.0559	20.24281	20.78392	20.39841	21.10036	19.12821	20.7609	0.709533	1	-
ENSMUS(	Lrrn2	leucine ric	GO:00325	developm	-	-	-	-	1:1328802	3822	4308	4638	5764	4023	3711	59.44679	56.16507	49.69583	58.4041	54.86943	71.56729	55.10256	61.61361	0.57044	1	-
ENSMUS(	Pik3c2b	phosphati	GO:00081	metabolic	K00923	phosphati	mmu0110	Metabolic	1:1330456	447	455	707	706	459	392	2.737346	2.335538	2.982586	2.816492	2.464778	2.976425	2.685157	2.752565	0.914526	1	-
ENSMUS(	Chit1	chitinase 1	GO:00081	metabolic	K01183	chitinase[	mmu0110	Metabolic	1:1341112	0	0	0	0	0	3	0	0	0	0	0	0.087336	0	0.029112	0.188367	1	-
ENSMUS(	Syt2	synaptota	GO:00325	developm	-	-	-	-	1:1346466	484	594	1098	910	711	616	3.12281	3.212469	4.880378	3.824926	4.022649	4.927953	3.738552	4.258509	0.553203	1	-
ENSMUS(	Klhl12	kelch-like	GO:00325	developm	-	-	-	-	1:1344555	678	898	1108	1039	867	724	3.084391	3.424277	3.472409	3.079192	3.458609	4.083797	3.327026	3.540533	0.885324	1	-
ENSMUS(	Cyb5r1	cytochron	GO:00081	metabolic	K00326	cytochron	mmu0052	Amino suc	1:1344055	281	225	256	271	208	213	2.50644	1.682237	1.573051	1.574715	1.626888	2.355688	1.920576	1.85243	0.622226	1	-
ENSMUS(	Adipor1	adiponect	GO:00325	developm	K07297	adiponect	mmu0493	Non-alco	1:1344153	916	1097	1549	1464	1097	1014	7.430064	7.458591	8.655655	7.736058	7.802724	10.19814	7.848103	8.578974	0.721801	1	-
ENSMUS(	Ppfia4	protein ty	GO:00452	synapse|c	-	-	-	-	1:1342967	2070	2464	3008	3413	2595	2229	8.85771	8.837812	8.867076	9.514131	9.737134	11.82626	8.854199	10.35917	0.319314	1	-
ENSMUS(	Myog	myogenin	GO:00325	developm	-	-	-	-	1:1342899	0	0	3	4	4	0	0	0	0.087851	0.110787	0.149096	0	0.029284	0.086627	0.430449	1	-
ENSMUS(	Atp2b4	ATPase, C	GO:00325	developm	K05850	Ca2+ tran	mmu0402	cAMP sigr	1:1336994	2679	3661	3799	3468	3108	2189	8.587639	9.836794	8.389222	7.242054	8.736242	8.700272	8.937885	8.226189	0.30341	1	-
ENSMUS(	Tor1aip1	torsin A ir	GO:00511	localizatio	-	-	-	-	1:1560045	462	544	706	726	534	443	2.212954	2.18415	2.329629	2.265421	2.242926	2.630994	2.242244	2.37978	0.892605	1	-
ENSMUS(	Lhx4	LIM home	GO:00325	developm	-	-	-	-	1:1556980	4	0	4	2	2	3	0.042998	0	0.029626	0.014004	0.018851	0.03999	0.024208	0.024282	1	1	-
ENSMUS(	Xpr1	xenotropi	GO:00511	localizatio	-	-	-	-	1:1552757	3343	3936	5070	5621	3980	2006	9.502112	9.377599	9.927574	10.40828	9.919951	7.069696	9.602428	9.132642	0.570071	1	-
ENSMUS(	Stx6	syntaxin 6	GO:00452	synapse|c	K08498	syntaxin 6	mmu0413	SNARE int	1:1551587	1029	910	1497	2409	1045	450	5.123875	3.7982	5.135189	7.814511	4.562912	2.778316	4.685755	5.051913	0.797104	1	-
ENSMUS(	Mr1	major hist	GO:00023	immune s	-	-	-	-	1:1551272	133	160	221	166	150	44	0.78475	0.791323	0.898307	0.63807	0.776091	0.321898	0.824793	0.578687	0.087163	1	-
ENSMUS(	Glul	glutamate	GO:00081	metabolic	K01915	glutamine	mmu0110	Metabolic	1:1538999	24934	30503	39012	39154	31737	19656	427.5852	438.4561	460.872	437.4098	477.2426	417.9379	442.3044	444.1968	0.787013	1	-
ENSMUS(	Rgs16	regulator	GO:00987	molecular	-	-	-	-	1:1537403	134	175	192	207	136	95	3.640918	3.98562	3.593845	3.664024	3.240309	3.200486	3.740128	3.368273	0.394174	1	-
ENSMUS(	Lamc1	laminin, g	GO:00325	developm	K05635	laminin, g	mmu0520	Pathways	1:1532189	885	1078	1298	1516	1090	742	5.507681	5.623376	5.564824	6.146192	5.948323	5.725526	5.565293	5.940013	0.813073	1	-
ENSMUS(	Lamc2	laminin, g	GO:00325	developm	K06246	laminin, g	mmu0520	Pathways	1:1531227	47	75	156	74	97	69	0.337245	0.451093	0.771126	0.345912	0.610326	0.613878	0.519821	0.523372	0.879781	1	-
ENSMUS(	Ncf2	neutrophi	GO:00081	metabolic	K08010	neutrophi	mmu0414	Phagoson	1:1528001	36	49	85	83	48	30	0.214925	0.245209	0.349591	0.322813	0.251291	0.222078	0.269908	0.265394	0.860342	1	-
ENSMUS(	Rgl1	ral guanin	GO:00987	molecular	K17635	ral guanin	mmu0401	Ras signal	1:1525167	2007	2350	3034	3207	2346	1771	12.37637	12.14693	12.88882	12.88328	12.68576	13.54099	12.47071	13.03668	0.962737	1	-
ENSMUS(	Fam129a	family witl	GO:00081	metabolic	-	-	-	-	1:1515711	88	72	139	106	93	67	0.559381	0.383624	0.608682	0.438947	0.51838	0.528062	0.517229	0.495129	0.666151	1	-
ENSMUS(	Rnf2	ring finge	GO:00325	developm	-	-	-	-	1:1514580	740	750	856	1141	651	475	6.347581	5.392507	5.058267	6.375936	4.896666	5.05191	5.599452	5.441504	0.660493	1	-
ENSMUS(	Coq8a	coenzyme	GO:00081	metabolic	-	-	-	-	1:1801652	685	830	606	797	778	784	5.148271	5.228802	3.137581	3.902214	5.127354	7.305888	4.504885	5.445152	0.429366	1	-
ENSMUS(	Cdc42bpa	CDC42 bi	GO:00081	metabolic	-	-	-	-	1:1799604	6709	7564	10800	11095	6741	4265	13.98279	13.21418	15.50641	15.06416	12.31978	11.02151	14.23446	12.80182	0.295175	1	-
ENSMUS(	Ahctf1	AT hook c	GO:00325	developm	-	-	-	-	1:1797448	1426	1253	1683	2224	1221	691	4.463484	3.287445	3.629026	4.534934	3.351297	2.681752	3.793318	3.522661	0.527093	1	-
ENSMUS(	Tfb2m	transcripti	GO:00081	metabolic	-	-	-	-	1:1795280	192	264	273	301	235	167	3.004726	3.463064	2.943179	3.068678	3.224882	3.240452	3.13699	3.178004	0.868606	1	-
ENSMUS(	Kif26b	kinesin far	GO:00325	developm	-	-	-	-	1:1785291	772	960	1190	1412	1083	698	3.137215	3.270026	3.33139	3.738037	3.859207	3.516971	3.24621	3.704738	0.438725	1	-
ENSMUS(	Efcab2	EF-hand c	GO:00431	ion bindin	-	-	-	-	1:1784060	402	455	555	531	398	215	5.819664	5.521233	5.53498	5.007812	5.052392	3.859187	5.625292	4.639797	0.133737	1	-
ENSMUS(	Parp1	poly (ADP	GO:00325	developm	K10798	poly [ADP	mmu0421	Necropto	1:1805689	2256	2522	3072	3429	2477	1774	19.45658	18.23163	18.25157	19.26534	18.73252	18.96999	18.64659	18.98928	0.868568	1	-
ENSMUS(	Acbd3	acyl-Coer	GO:00160	membran	-	-	-	-	1:1807260	802	996	1144	1237	827	477	7.239988	7.536606	7.114444	7.274695	6.546538	5.339097	7.297013	6.386776	0.242908	1	-
ENSMUS(	Cox20	COX20 Cc	GO:00081	metabolic	-	-	-	-	1:1783191	157	169	251	218	135	129	9.395307	8.477171	10.34753	8.498638	7.084141	9.571624	9.40667	8.384801	0.322765	1	-
ENSMUS(	Desi2	desumoyl	GO:00081	metabolic	-	-	-	-	1:1781874	380	485	624	602	435	370	1.908045	2.041267	2.158447	1.969171	1.915302	2.303525	2.035919	2.062666	0.830545	1	-
ENSMUS(	Sdccag8	serologica	GO:00325	developm	-	-	-	-	1:1768146	338	492	587	638	487	268	4.82717	5.889717	5.775181	5.935793	6.098846	4.745655	5.497356	5.593431	0.931601	1	-
ENSMUS(	Capn2	calpain 2	GO:00325	developm	K03853	calpain-2	mmu0451	Focal adh	1:1824672	1449	1640	2152	2164	1770	1115	11.06963	10.50175	11.32553	10.76971	11.85717	10.5615	10.96564	11.06279	0.824557	1	-
ENSMUS(	Trp53bp2	transform	GO:00508	response	K16823	apoptosis	mmu0439	Hippo sig	1:1824091	747	814	1061	852	797	502	9.047861	8.264229	8.853025	6.722738	8.464986	7.539013	8.721705	7.575579	0.171586	1	-
ENSMUS(	Srp9	signal rec	GO:00081	metabolic	K03109	signal rec	mmu0306	Protein ex	1:1821247	633	931	1155	980	811	543	5.733898	7.068858	7.207422	5.783015	6.441844	6.098636	6.67006	6.107832	0.338671	1	-
ENSMUS(	Cnih3	cornichon	GO:00452	synapse|c	-	-	-	-	1:1813526	526	697	777	833	634	690	6.757408	7.5055	6.876488	6.971415	7.142101	10.99081	7.046465	8.368107	0.384652	1	-
ENSMUS(	Nvl	nuclear V(	GO:00081	metabolic	K14571	ribosome	mmu0300	Ribosome	1:1810871	1137	1461	1586	1794	1364	1053	4.806105	5.176495	4.618355	4.940111	5.055799	5.518831	4.866985	5.17158	0.864874	1	-
ENSMUS(	Tmem63a	transmem	GO:00511	localizatio	-	-	-	-	1:1809423	360	286	608	504	364	416	3.751289	2.498025	4.36448	3.421287	3.325997	5.374736	3.537931	4.040674	0.633066	1	-
ENSMUS(	Pycr2	pyrroline-	GO:00081	metabolic	K00286	pyrroline-	mmu0110	Metabolic	1:1809042	262	358	409	303	329	403	2.878285	3.296617	3.095336	2.168487	3.169358	5.48938	3.090079	3.609075	0.61	1	-
ENSMUS(	Opn3	opsin 3 [S	GO:00048	signal trar	-	-	-	-	1:1756624	11	14	18	16	11	13	0.365448	0.389863	0.411955	0.346275	0.320452	0.535477	0.389089	0.400734	0.931691	1	-
ENSMUS(	Fh1	fumarate	GO:00081	metabolic	K01679	fumarate	mmu0110	Metabolic	1:1756003	1005	1093	1407	1407	1077	861	13.57508	12.37512	13.09248	12.3809	12.7566	14.42002	13.01423	13.18584	0.815814	1	-
ENSMUS(	Rgs7	regulator	GO:00048	signal trar	-	-	-	-	1:1750590	1324	1683	1760	2165	1426	831	10.56955	11.26174	9.679047	11.25921	9.982298	8.22537	10.50345	9.822294	0.471856	1	-
ENSMUS(	Spta1	spectrin a	GO:00325	developm	K06114	spectrin a	mmu0421	Apoptosis	1:1741727	0	0	5	2	0	1	0	0	0.021223	0.008026	0	0.007637	0.007074	0.005221	1	1	-
ENSMUS(	Ifi202b	interferon	GO:00081	metabolic	K12971|K1	interferon	mmu0462	Cytosolic I	1:1739625	5	2	7	7	4	3	0.187253	0.062779	0.180603	0.170781	0.131348	0.139316	0.143545	0.147148	1	1	-
ENSMUS(	Ifi211	interferon	GO:00081	metabolic	-	-	-	-	1:1738963	4	7	19	15	13	6	0.14241	0.208895	0.466014	0.347914	0.405868	0.264895	0.27244	0.339559	0.713761	1	-
ENSMUS(	Dusp23	dual speci	GO:00081	metabolic	-	-	-	-	1:1726307	46	52	63	84	61	63	2.073345	1.964578	1.956186	2.466486	2.410971	3.520827	1.998036	2.799428	0.130651	1	-
ENSMUS(	Cfap45	cilia and f	GO:00056	cell|intrace	-	-	-	-	1:1725208	78	88	135	84	73	70	0.743313	0.702926	0.886258	0.521479	0.610019	0.827108	0.777499	0.652869	0.213317	1	-
ENSMUS(	Tagln2	transgelin	GO:00325	developm	-	-	-	-	1:1725000	145	182	215	265	158	161	3.071737	3.231765	3.137647	3.657143	2.935044	4.228879	3.14705	3.607022	0.550857	1	-
ENSMUS(	Slamf9	SLAM fan	GO:00160	membran	-	-	-	-	1:1724753	11	5	9	12	14	6	0.532446	0.202849	0.300086	0.378399	0.59421	0.36012	0.345127	0.444243	0.551084	1	-
ENSMUS(	Copa	coatomer	GO:00511	localizatio	-	-	-	-	1:1720825	3191	3917	4796	4958	3828	3106	13.74154	14.13888	14.22785	13.90903	14.45517	16.58426	14.03609	14.98282	0.833406	1	-
ENSMUS(	Dcaf8	DDB1 anc	GO:00081	metabolic	-	-	-	-	1:1721480	2952	3457	4317	4532	3453	2624	11.25629	11.0492	11.33998	11.25771	11.54564	12.40591	11.21515	11.73642	0.960639	1	-
ENSMUS(	Vangl2	vang-like	GO:00325	developm	K04510	vang-like[	mmu0431	Wnt signa	1:1720009	258	342	459	451	314	260	1.922688	2.136326	2.356424	2.189509	2.051925	2.402412	2.138479	2.214615	0.974574	1	-
ENSMUS(	Uck2	uridine-cy	GO:00081	metabolic	K00876	uridine kir	mmu0110	Metabolic	1:1672228	567	639	731	852	561	466	5.146321	4.861474	4.570704	5.037733	4.464983	5.244277	4.8595	4.915664	0.829062	1	-
ENSMUS(	Tada1	transcripti	GO:00081	metabolic	-	-	-	-	1:1663790	372	478	621	610	446	412	4.814033	5.18498	5.536168	5.14254	5.061087	6.610735	5.178393	5.604787	0.799277	1	-
ENSMUS(	Dusp27	dual speci	GO:00081	metabolic	-	-	-	-	1:1660981	0	14	19	9	4	2	0	0.099203	0.110646	0.049562	0.029649	0.020969	0.06995	0.033393	0.207492	1	-
ENSMUS(	Pou2f1	POU dom	GO:00325	developm	-	-	-	-	1:1658651	442	570	697	762	586	408	1.279803	1.383404	1.390291	1.437336	1.48786	1.464765	1.351166	1.46332	0.747448	1	-
ENSMUS(	Mpzl1	myelin prc	GO:00508	response	K06770	myelin prc	mmu0451	Cell adhes	1:1655922	275	285	451	423	352	366	2.695412	2.341485	3.045239	2.700943	3.025372	4.447952	2.694046	3.391422	0.243816	1	-
ENSMUS(	Adcy10	adenylate	GO:00081	metabolic	K11265	adenylate	mmu0402	cAMP sigr	1:1654851	0	3	4	4	3	1	0	0.014055	0.015404	0.014567	0.014703	0.006929	0.00982	0.012066	0.816993	1	-
ENSMUS(	Mpc2	mitochon	GO:00081	metabolic	-	-	-	-	1:1654606	448	574	611	607	537	932	3.192615	3.428728	2.999585	2.817989	3.35572	8.235127	3.206976	4.802945	0.102728	1	-
ENSMUS(	Dcaf6	DDB1 anc	GO:00081	metabolic	-	-	-	-	1:1653286	1823	2265	2414	2787	2086	1154	14.26636	14.85756	13.01413	14.2084	14.31475	11.19743	14.04602	13.24019	0.491391	1	-
ENSMUS(	Dpt	dermatop	GO:00099	cellular pr	-	-	-	-	1:1647966	0	5	6	4	2	2	0	0.15449	0.152364	0.096071	0.064646	0.091439	0.102285	0.084052	0.84478	1	-
ENSMUS(	Nme7	NME/NM	GO:00081	metabolic	K00940	nucleoside	mmu0110	Metabolic	1:1643041	216	265	304	347	270	241	1.676535	1.72408	1.625486	1.754565	1.837661	2.319327	1.675367	1.970518	0.414655	1	-
ENSMUS(	Atp1b1	ATPase, N	GO:00081	metabolic	K01540	sodium/p	mmu0402	cAMP sigr	1:1644371	30691	40030	48154	48433	36709	23296	515.4613	563.5383	557.1457	529.9172	540.6301	485.1235	545.3818	518.5569	0.458581	1	-
ENSMUS(	Blzf1	basic leuc	GO:00511	localizatio	-	-	-	-	1:1642898	389	516	591	745	502	268	3.214844	3.574478	3.36472	4.010952	3.637944	2.746185	3.384681	3.465027	0.977758	1	-
ENSMUS(	Ccdc181	coiled-co	GO:00990	supramole	-	-	-	-	1:1642755	440	632	637	678	546	307	14.64816	17.63599	14.60902	14.70421	15.93918	12.67226	15.63106	14.43855	0.438706	1	-
ENSMUS(	Selp	selectin, p	GO:00400	locomotic	K06496	selectin, p	mmu0451	Cell adhes	1:1641152	1	0	1	0	0	7	0.01039	0	0.007153	0	0	0.090163	0.005848	0.030054	0.22731	1	-
ENSMUS(	Sell	selectin, ly	GO:00400	locomotic	K06495	selectin, ly	mmu0451	Cell adhes	1:1640619	2	8	0	4	2	4	0.01696	0.05685	0	0.022095	0.014868	0.042049	0.024603	0.026337	1	1	-
ENSMUS(	Sele	selectin, e	GO:00048	signal trar	K06494	selectin, e	mmu0451	Cell adhes	1:1640482	0	2	4	0	2	3	0	0.036585	0.060146	0	0.038272	0.081188	0.032244	0.03982	1	1	-
ENSMUS(	Scyl3	SCY1-like	GO:00081	metabolic	-	-	-	-	1:1639291	284	347	430	456	366	334	1.335485	1.367738	1.392965	1.396904	1.50919	1.947388	1.365396	1.617827	0.373888	1	-
ENSMUS(	Kifap3	kinesin-as	GO:00511	localizatio	-	-	-	-	1:1637795	5425	6488	7636	7957	5826	3327	85.93595	86.14676	83.3283	82.11192	80.92605	65.34524	85.137	76.12774	0.255337	1	-
ENSMUS(	Prrx1	paired rel	GO:00325	developm	-	-	-	-	1:1632451	178	199	290	272	170	95	0.708401	0.663843	0.795075	0.705194	0.593267	0.468779	0.72244	0.58908	0.17724	1	-
ENSMUS(	Astn1	astrotactir	GO:00325	developm	-	-	-	-	1:1583622	6898	8302	9060	10495	3563	4949	26.93963	27.17716	24.3752	26.70131	12.20189	23.9647	26.164	20.95596	0.115622	1	-
ENSMUS(	Sec16b	SEC16 ho	GO:00081	metabolic	-	-	-	-	1:1575067	8	11	18	25	20	7	0.073099	0.084256	0.11331	0.148818	0.160259	0.079312	0.090222	0.129463	0.276714	1	-
ENSMUS(	Ralgps2	Ral GEF w	GO:00987	molecular	-	-	-	-	1:1568041	1206	1321	1826	1922	1374	921	3.537566	3.247977	3.689858	3.672759	3.534163	3.349679	3.4918	3.518867	0.823169	1	-
ENSMUS(	Abl2	v-abl Abe	GO:00325	developm	K08887	abelson ty	mmu0401	Ras signal	1:1565587	2173	2330	2845	3181	2276	1710	10.77397	9.68333	9.717387	10.27451	9.895341	10.5123	10.05823	10.22739	0.85026	1	-
ENSMUS(	Soat1	sterol O-a	GO:00325	developm	K00637	sterol O-a	mmu0497	Cholester	1:1564245	671	766	1121	1154	807	656	3.245855	3.105909	3.73563	3.636589	3.423126	3.934564	3.362465	3.66476	0.715533	1	-
ENSMUS(	Axdnd1	axonemal	-	-	-	-	-	-	1:1563235	9	4	3	12	6	1	0.074543	0.027769	0.017117	0.064751	0.043574	0.010267	0.03981	0.039531	1	1	-
ENSMUS(	Nphs2	nephrosis	GO:00325	developm	-	-	-	-	1:1563107	4	2	2	1	3	4	0.067914	0.028463	0.023396	0.01106	0.044663	0.08421	0.039924	0.046644	1	1	-
ENSMUS(	Smyd2	SET and N	GO:00325	developm	-	-	-	-	1:1898804	756	903	1171	1060	801	675	12.66037	12.67549	13.50929	11.5641	11.7625	14.01568	12.94838	12.44743	0.509265	1	-
ENSMUS(	Ptpn14	protein ty	GO:00325	developm	-	-	-	-	1:1897282	352	419	466	475	344	336	1.208244	1.205533	1.101918	1.062153	1.035412	1.430007	1.171899	1.175858	0.758944	1	-
ENSMUS(	Cenpf	centrome	GO:00325	developm	-	-	-	-	1:1896406	65	63	74	101	51	47	0.230559	0.187311	0.180823	0.233385	0.15863	0.206708	0.199564	0.199574	0.895033	1	-
ENSMUS(	Kctd3	potassium	GO:00099	cellular pr	-	-	-	-	1:1889710	1280	1416	2014	2010	1616	1025	7.086668	6.571254	7.681446	7.249528	7.845418	7.036261	7.113122	7.377069	0.996463	1	-
ENSMUS(	Ush2a	usherin [S	GO:00325	developm	-	-	-	-	1:1882620	3	0	2	3	4	1	0.010251	0	0.004709	0.006678	0.011986	0.004236	0.004987	0.007634	0.633906	1	-
ENSMUS(	Esrrg	estrogen-	GO:00325	developm	-	-	-	-	1:1876087	315	282	520	440	405	218	1.408216	1.05672	1.601451	1.281425	1.587657	1.208374	1.355462	1.359152	0.853684	1	-
ENSMUS(	Spata17	spermato	GO:00056	cell|protei	-	-	-	-	1:1870446	10	6	15	8	6	2	0.133713	0.067244	0.138172	0.069685	0.070344	0.033167	0.113043	0.057732	0.094829	1	-
ENSMUS(	Slc30a10	solute car	GO:00325	developm	-	-	-	-	1:1854548	267	398	452	530	329	307	2.907582	3.632927	3.390866	3.759921	3.141661	4.145194	3.310459	3.682259	0.650544	1	-
ENSMUS(	Eprs	glutamyl-	GO:00081	metabolic	K14163	bifunction	mmu0110	Metabolic	1:1853630	1858	2204	2586	2948	2101	1421	5.595484	5.563605	5.365026	5.783636	5.54831	5.306056	5.508038	5.546	0.808876	1	-
ENSMUS(	Cr2	complem	GO:00325	developm	K04012	complem	mmu0516	Epstein-B	1:1951368	7	7	13	6	3	4	0.039119	0.032786	0.050045	0.021843	0.014699	0.027714	0.04065	0.021419	0.10304	1	-
ENSMUS(	Bpnt1	bisphospl	GO:00081	metabolic	K01082	3'(2'), 5'-b	mmu0110	Metabolic	1:1853321	973	1268	1604	1633	1178	899	19.95388	21.7965	22.66055	21.81636	21.18374	22.85915	21.47031	21.95308	0.893017	1	-
ENSMUS(	Iars2	isoleucine	GO:00081	metabolic	K01870	isoleucyl-	mmu0097	Aminoacy	1:1852847	885	1222	1264	1315	1083	820	4.979587	5.763334	4.899461	4.820113	5.343439	5.720705	5.214127	5.294752	0.836045	1	-
ENSMUS(	Mark1	MAP/micr	GO:00325	developm	-	-	-	-	1:1848967	1319	1567	1878	1963	1525	1015	18.03883	17.96327	17.69337	17.48902	18.28841	17.21136	17.89849	17.66293	0.676044	1	-
ENSMUS(	1-Mar	mitochon	GO:00081	metabolic	-	-	-	-	1:1847867	0	0	3	0	2	3	0	0	0.030061	0	0.025509	0.054113	0.01002	0.026541	0.585402	1	-
ENSMUS(	Nek2	NIMA (ne	GO:00325	developm	-	-	-	-	1:1918214	14	19	26	39	21	28	0.163135	0.185579	0.208713	0.296051	0.214581	0.404543	0.185809	0.305058	0.089204	1	-
ENSMUS(	Lpgat1	lysophosp	GO:00081	metabolic	K13514	lysophosp	mmu0056	Glyceroph	1:1917178	3738	4366	5557	5708	4184	2861	22.82486	22.34629	23.37548	22.70568	22.40284	21.66071	22.84888	22.25641	0.58588	1	-
ENSMUS(	Ppp2r5a	protein pl	GO:00081	metabolic	K11584	serine/thr	mmu0415	PI3K-Akt s	1:1913519	1072	1303	1767	1618	1252	977	6.457094	6.578693	7.332137	6.348959	6.612861	7.296644	6.789308	6.752821	0.703165	1	-
ENSMUS(	Tmem206	transmem	GO:00160	membran	-	-	-	-	1:1913259	165	211	250	238	177	134	4.668842	5.004509	4.873248	4.387179	4.391786	4.701302	4.848866	4.493422	0.441386	1	-
ENSMUS(	Atf3	activating	GO:00325	developm	K09032	activating	mmu0516	HTLV-I inf	1:1911702	26	22	30	25	18	30	0.644716	0.457259	0.512454	0.403836	0.391376	0.922363	0.538143	0.572525	1	1	-
ENSMUS(	Batf3	basic leuc	GO:00325	developm	-	-	-	-	1:1910978	6	6	8	10	12	6	0.119428	0.100097	0.109693	0.12966	0.209442	0.148086	0.109739	0.162396	0.406666	1	-
ENSMUS(	Tatdn3	TatD DNa	GO:00081	metabolic	-	-	-	-	1:1910458	115	193	206	269	184	206	1.342259	1.888199	1.656369	2.045373	1.88321	2.981205	1.628942	2.303263	0.075592	1	-
ENSMUS(	Angel2	angel hor	GO:00081	metabolic	-	-	-	-	1:1909251	631	757	1030	985	765	631	5.134037	5.162714	5.773222	5.220921	5.457999	6.365683	5.356658	5.681535	0.896159	1	-
ENSMUS(	Traf5	TNF recep	GO:00081	metabolic	-	-	-	-	1:1919972	10	19	11	16	24	28	0.098487	0.156849	0.07463	0.102654	0.207268	0.341915	0.109989	0.217279	0.080959	1	-
ENSMUS(	Irf6	interferon	GO:00325	developm	-	-	-	-	1:1931531	10	19	23	15	12	13	0.079373	0.126408	0.125763	0.077561	0.083521	0.127934	0.110514	0.096338	0.532644	1	-
ENSMUS(	Lamb3	laminin, b	GO:00325	developm	K06244	laminin, b	mmu0520	Pathways	1:1932076	11	28	23	47	19	11	0.086651	0.184874	0.124812	0.241184	0.131237	0.107438	0.132112	0.159953	0.616486	1	-
ENSMUS(	Plxna2	plexin A2	GO:00325	developm	K06820	plexin A[-	mmu0436	Axon guic	1:1946182	3464	3910	4781	5317	3847	2166	10.9119	10.3241	10.37511	10.91116	10.62643	8.459935	10.53704	9.999175	0.511791	1	-
ENSMUS(	Usf1	upstream	GO:00081	metabolic	-	-	-	-	1:1714113	790	889	988	1124	883	595	8.021544	7.566343	6.91098	7.434965	7.862026	7.490906	7.499622	7.595966	0.849391	1	-
ENSMUS(	Nmt2	N-myristo	GO:00081	metabolic	-	-	-	-	2:3284212	1593	2065	2578	2613	1910	1185	12.69625	13.79535	14.15449	13.56691	13.3486	11.7102	13.5487	12.87524	0.497586	1	-
ENSMUS(	Acbd7	acyl-Coer	GO:00480	cofactor b	-	-	-	-	2:3336168	0	2	2	0	2	0	0	0.1634	0.134314	0	0.170934	0	0.099238	0.056978	0.719473	1	-
ENSMUS(	Suv39h2	suppressc	GO:00081	metabolic	K11419	histone-ly	mmu0031	Lysine deg	2:3455815	86	116	154	97	107	57	0.653697	0.739078	0.806398	0.480323	0.713188	0.5372	0.733058	0.576903	0.131337	1	-
ENSMUS(	Dclre1c	DNA cros	GO:00325	developm	K10887	DNA cros	mmu0534	Primary in	2:3424131	174	160	199	282	211	142	0.68164	0.525386	0.537043	0.719673	0.72482	0.689732	0.581356	0.711408	0.297814	1	-
ENSMUS(	Cfap126	cilia and f	GO:00099	cellular pr	-	-	-	-	1:1711139	46	26	50	25	28	19	0.835262	0.395722	0.625447	0.295723	0.445827	0.427773	0.61881	0.389775	0.052891	1	-
ENSMUS(	Fam107b	family witl	GO:00508	neurologi	-	-	-	-	2:3570488	300	109	468	100	332	199	3.308328	1.007549	3.555369	0.718405	3.210465	2.720987	2.623749	2.216619	0.512293	1	-
ENSMUS(	Fcgr2b	Fc recepto	GO:00160	membran	K12560	low affinit	mmu0515	Tuberculo	1:1709586	45	47	104	123	66	63	0.380004	0.332675	0.605	0.676642	0.488718	0.659629	0.439226	0.60833	0.177632	1	-
ENSMUS(	Frmd4a	FERM dor	GO:00099	cellular pr	-	-	-	-	2:4017717	1432	1657	1890	2285	1664	903	2.585452	2.507661	2.350751	2.68758	2.634448	2.021469	2.481288	2.447832	0.742338	1	-
ENSMUS(	Dusp12	dual speci	GO:00081	metabolic	-	-	-	-	1:1708734	120	172	205	212	149	157	1.371156	1.64735	1.613657	1.578054	1.492908	2.224279	1.544054	1.76508	0.610227	1	-
ENSMUS(	Sephs1	selenopho	GO:00081	metabolic	K01008	selenide, v	mmu0110	Metabolic	2:4881564	185	481	576	562	433	289	1.456192	3.17355	3.123355	2.881811	2.98867	2.820536	2.584365	2.897006	0.670644	1	-
ENSMUS(	Atf6	activating	GO:00325	developm	K09054	cyclic AMI	mmu0501	Alzheimer	1:1707046	2181	2428	3081	3729	2532	1478	14.85469	13.86147	14.4561	16.54558	15.12218	12.48155	14.39075	14.71643	0.94423	1	-
ENSMUS(	Phyh	phytanoyl	GO:00081	metabolic	K00477	phytanoyl	mmu0414	Peroxisom	2:4919019	1058	1265	1636	1518	1168	795	30.39741	30.46453	32.38066	28.41216	29.42636	28.32071	31.08087	28.71974	0.339454	1	-
ENSMUS(	Ucma	upper zor	GO:00325	developm	-	-	-	-	2:4976122	26	27	29	31	22	37	0.599349	0.521689	0.460521	0.465524	0.444704	1.057507	0.527186	0.655912	0.600828	1	-
ENSMUS(	Mcm10	minichron	GO:00081	metabolic	-	-	-	-	2:4989714	11	28	12	14	21	16	0.065039	0.138763	0.048878	0.053924	0.108878	0.117293	0.084227	0.093365	0.954324	1	-
ENSMUS(	Uap1	UDP-N-a	GO:00081	metabolic	K00972	UDP-N-a	mmu0110	Metabolic	1:1701419	704	1027	1215	1302	947	576	6.440384	7.875208	7.657141	7.759456	7.596813	6.533522	7.324244	7.296597	0.787286	1	-
ENSMUS(	Optn	optineurir	GO:00081	metabolic	K19946	optineurir	mmu0413	Mitophag	2:5020642	336	413	472	481	383	268	3.816593	3.932232	3.69343	3.559283	3.814849	3.774471	3.814085	3.716201	0.636775	1	-
ENSMUS(	Ddr2	discoidin	GO:00325	developm	-	-	-	-	1:1699723	306	370	511	483	382	216	1.239782	1.256547	1.426251	1.274832	1.357156	1.085083	1.307527	1.239024	0.559412	1	-
ENSMUS(	Hsd17b7	hydroxyst	GO:00081	metabolic	K13373	17beta-es	mmu0110	Metabolic	1:1699495	267	368	436	460	336	400	3.070693	3.547532	3.454325	3.446396	3.388502	5.703896	3.357517	4.179598	0.280982	1	-
ENSMUS(	Ccdc3	coiled-co	GO:00325	developm	-	-	-	-	2:5137776	279	287	338	426	269	176	6.640943	5.726114	5.54234	6.605656	5.614603	5.194249	5.969799	5.804836	0.698788	1	-
ENSMUS(	Rgs5	regulator	GO:00987	molecular	-	-	-	-	1:1696555	2326	3022	3398	3842	2578	1468	31.81755	34.65008	32.02077	34.23701	30.92306	24.89825	32.82947	30.01944	0.366041	1	-
ENSMUS(	Nuf2	NUF2, ND	GO:00329	macromo	-	-	-	-	1:1694979	58	70	92	107	84	46	0.480585	0.486172	0.525147	0.577573	0.610324	0.472595	0.497301	0.553497	0.691653	1	-
ENSMUS(	Lmx1a	LIM home	GO:00325	developm	-	-	-	-	1:1676892	27	10	42	12	15	8	0.491102	0.152456	0.526278	0.142197	0.239245	0.180422	0.389945	0.187288	0.029238	1	-
ENSMUS(	Aldh9a1	aldehyde	GO:00081	metabolic	K00149	aldehyde	mmu0110	Metabolic	1:1673499	482	553	690	617	507	499	7.205963	6.929831	7.106332	6.009134	6.646546	9.249792	7.080709	7.301824	0.899494	1	-
ENSMUS(	Mgst3	microsom	GO:00081	metabolic	K00799	glutathior	mmu0520	Pathways	1:1673719	413	651	784	727	514	654	23.92649	31.61282	31.2894	27.43756	26.1117	46.97789	28.9429	33.50905	0.55359	1	-

ENSMUSC	Mettl13	methyltrai	GO:00081	metabolic	-	-	-	-	1:1625321	213	193	296	347	250	252	0.878783	0.66744	0.84129	0.93264	0.904451	1.289105	0.795838	1.042065	0.151965	1	-
ENSMUSC	Vamp4	vesicle-as	GO:00452	synapse|lc	K08513	vesicle-as	mmu0413	SNARE int	1:1625705	1103	892	1088	1683	1067	978	9.080178	6.155122	6.170201	9.025777	7.702396	9.982599	7.135167	8.903591	0.212361	1	-
ENSMUSC	Myoc	myocilin [	GO:00325	developm	-	-	-	-	1:1626391	393	532	790	657	618	316	12.0426	13.66443	16.67654	13.11518	16.60575	12.00604	14.12786	13.90899	0.758132	1	-
ENSMUSC	Pigc	phosphati	GO:00081	metabolic	K03859	phosphati	mmu0110	Metabolic	1:1619691	174	208	234	271	189	183	1.017787	1.019818	0.942919	1.03266	0.969416	1.327221	0.993508	1.109766	0.674844	1	-
ENSMUSC	Prdx6	peroxired	GO:00081	metabolic	K11188	peroxired	mmu0110	Metabolic	1:1612401	1187	1384	1791	1570	1588	1744	8.258063	8.070797	8.583703	7.115555	9.687702	15.04389	8.304188	10.61572	0.204464	1	-
ENSMUSC	Klhl20	kelch-like	GO:00081	metabolic	-	-	-	-	1:1610883	476	304	455	823	300	409	1.737876	0.930335	1.144392	1.957465	0.960454	1.851491	1.270868	1.589803	0.372493	1	-
ENSMUSC	Nsun6	NOL1/NC	GO:00081	metabolic	-	-	-	-	2:1499513	126	123	119	141	128	77	1.132471	0.926646	0.736807	0.825573	1.008809	0.858084	0.931974	0.897488	0.696474	1	-
ENSMUSC	Cenpl	centrome	GO:00056	cell|intrace	-	-	-	-	1:1610707	15	27	17	19	24	22	0.063847	0.096329	0.049847	0.052683	0.089579	0.116109	0.070008	0.086124	0.652457	1	-
ENSMUSC	Dars2	aspartyl-t	GO:00081	metabolic	K01876	aspartyl-t	mmu0097	Aminoacy	1:1610406	286	340	379	442	407	293	1.852253	1.84572	1.690926	1.864825	2.31138	2.352818	1.796299	2.176341	0.283144	1	-
ENSMUSC	Mrc1	mannose	GO:00048	signal trar	K06560	mannose	mmu0515	Tuberculo	2:1422939	42	94	106	114	75	40	0.358621	0.67277	0.623512	0.634126	0.561557	0.42348	0.551634	0.539721	0.837273	1	-
ENSMUSC	Serpinc1	serine (or	GO:00325	developm	K03911	antithrom	mmu0461	Complem	1:1609785	1	0	0	2	0	3	0.005912	0	0	0.0077	0	0.021988	0.001971	0.009896	0.275771	1	-
ENSMUSC	Stam	signal trar	GO:00511	localizatio	K04705	signal trar	mmu0414	Endocytos	2:1407409	1152	1451	1742	1765	1306	755	6.446517	6.806011	6.715403	6.434262	6.408527	5.238482	6.655977	6.02709	0.313736	1	-
ENSMUSC	Rabgap1l	RAB GTPa	GO:00325	developm	-	-	-	-	1:1602191	4365	5112	6594	7005	4865	2237	10.72402	10.5273	11.16024	11.21148	10.4809	6.814363	10.80385	9.50225	0.30226	1	-
ENSMUSC	Trdmt1	tRNA aspa	GO:00081	metabolic	-	-	-	-	2:1350901	106	99	116	161	109	47	1.65682	1.297054	1.249046	1.639369	1.493952	0.910866	1.400973	1.348062	0.789518	1	-
ENSMUSC	Tnn	tenascin N	GO:00325	developm	K06252	tenascin[-	mmu0415	PI3K-Akt s	1:1600850	0	4	2	0	0	2	0	0.035406	0.014552	0	0	0.026195	0.016653	0.008732	0.373808	1	-
ENSMUSC	Cubn	cubilin (in	GO:00081	metabolic	K14616	cubilin[-]	mmu0497	Vitamin d	2:1327633	7	11	17	8	10	1	0.027995	0.036874	0.046832	0.020841	0.035068	0.004957	0.037234	0.020289	0.144204	1	-
ENSMUSC	Rsu1	Ras suppr	GO:00055	extracellul	-	-	-	-	2:1307682	358	442	567	450	384	273	1.911288	1.977963	2.085346	1.565082	1.797699	1.807142	1.991532	1.723308	0.169264	1	-
ENSMUSC	Vim	vimentin [	GO:00325	developm	K07606	vimentin[-	mmu0516	Epstein-B	2:1357392	429	544	1046	1599	617	679	3.491386	3.711013	5.864404	8.477559	4.403202	6.851672	4.355601	6.577478	0.039943	1	-
ENSMUSC	Pter	phosphot	GO:00325	developm	-	-	-	-	2:1292404	15	31	83	67	21	25	0.115092	0.199368	0.438708	0.334894	0.141292	0.237831	0.251056	0.238006	0.827709	1	-
ENSMUSC	Ptf1a	pancreas	GO:00325	developm	-	-	-	-	2:1944566	0	0	0	0	2	0	0	0	0	0	0.073905	0	0	0.024635	0.534535	1	-
ENSMUSC	4930426L	RIKEN cDl	-	-	-	-	-	-	2:1899834	7	16	5	12	9	2	0.304935	0.584198	0.150042	0.340533	0.34378	0.108048	0.346391	0.26412	0.639222	1	-
ENSMUSC	Pip4k2a	phosphati	GO:00325	developm	K00920	1-phosph	mmu0481	Regulation	2:1884225	1471	1598	2284	2222	1613	873	24.5823	22.38408	26.29404	24.18998	23.6367	18.08882	24.42014	21.97184	0.318673	1	-
ENSMUSC	Bmi1	Bmi1 poly	GO:00325	developm	K11459	polycomb	mmu0520	Transcript	2:1867701	1025	1144	1557	1520	1097	598	10.09951	9.448337	10.56857	9.756672	9.478193	7.305723	10.03881	8.846863	0.255819	1	-
ENSMUSC	Dnajc1	DnaJ heat	GO:00081	metabolic	K09521	DnaJ hom	mmu0414	Protein pr	2:1819565	618	767	889	608	764	391	2.513168	2.614455	2.490495	1.610713	2.724386	1.971492	2.539373	2.102197	0.139626	1	-
ENSMUSC	Mllt10	myeloid/ly	GO:00081	metabolic	-	-	-	-	2:1805523	490	542	651	747	527	333	2.942267	2.727963	2.692889	2.922057	2.77485	2.47923	2.787706	2.725379	0.68574	1	-
ENSMUSC	Plxdc2	plexin dor	GO:00055	extracellul	-	-	-	-	2:1635630	706	818	1149	1090	884	478	4.898299	4.757149	5.491768	4.926616	5.378182	4.112011	5.049072	4.805603	0.559115	1	-
ENSMUSC	Nek6	NIMA (ne	GO:00048	signal trar	-	-	-	-	2:3851164	637	722	687	889	639	586	2.949388	2.802092	2.191292	2.681486	2.594394	3.36415	2.647591	2.88001	0.777065	1	-
ENSMUSC	Psmb7	proteasor	GO:00081	metabolic	K02739	20S prote	-	-	2:3858803	1329	1631	2112	1980	1487	1191	23.72527	24.40579	25.97356	23.02676	23.27768	26.36234	24.70154	24.22226	0.60509	1	-
ENSMUSC	Nr5a1	nuclear re	GO:00325	developm	-	-	-	-	2:3869265	0	0	3	0	0	0	0	0	0.021591	0	0	0	0.007197	0	0.303563	1	-
ENSMUSC	Ppp6c	protein pł	GO:00081	metabolic	-	-	-	-	2:3919435	848	1122	1402	1256	928	718	8.28867	9.192528	9.440358	7.997617	7.953898	8.701614	8.973852	8.217709	0.29241	1	-
ENSMUSC	Golga1	golgi autc	GO:00160	membran	-	-	-	-	2:3901615	556	550	632	761	553	385	2.687928	2.228732	2.1048	2.396672	2.344284	2.307753	2.340487	2.34957	0.813168	1	-
ENSMUSC	Arpc5l	actin relat	GO:00400	locomotic	K05754	actin relat	mmu0414	Endocytos	2:3900534	914	1212	1529	1610	1192	854	10.37462	11.53138	11.95596	11.9051	11.86437	12.01903	11.28732	11.9295	0.883864	1	-
ENSMUSC	Orc4	origin rec	GO:00081	metabolic	K02606	origin rec	mmu0411	Cell cycle	2:4890282	646	791	924	990	708	360	4.036105	4.142468	3.97698	4.029457	3.878875	2.788809	4.051851	3.565714	0.291206	1	-
ENSMUSC	Kif5c	kinesin fa	GO:00325	developm	K10396	kinesin fa	mmu0414	Endocytos	2:4961929	19056	21890	27851	30364	21619	13917	151.4022	145.7804	152.4378	157.1596	150.6186	137.0983	149.8735	148.2922	0.696192	1	-
ENSMUSC	Lypd6b	LY6/PLAU	GO:00987	molecular	-	-	-	-	2:4978768	88	110	142	156	85	71	2.001679	2.097269	2.225104	2.311615	1.695387	2.002419	2.108018	2.00314	0.631439	1	-
ENSMUSC	Mmadhc	methylma	GO:00081	metabolic	-	-	-	-	2:5027988	245	350	448	403	213	349	4.52631	5.419995	5.70173	4.850251	3.450639	7.994464	5.216012	5.431785	0.937241	1	-
ENSMUSC	Mindy3	MINDY ly	GO:00081	metabolic	-	-	-	-	2:1234726	698	650	821	852	595	428	3.675534	2.869003	2.978239	2.922711	2.747415	2.794441	3.174259	2.821522	0.251652	1	-
ENSMUSC	Itga8	integrin al	GO:00325	developm	K06584	integrin al	mmu0415	PI3K-Akt s	2:1210663	379	404	532	613	435	249	2.469662	2.206641	2.388145	2.602195	2.485591	2.011796	2.354816	2.366527	0.864174	1	-
ENSMUSC	Il2ra	interleukir	GO:00325	developm	K05068	interleukir	mmu0520	Pathways	2:1164280	6	4	5	7	10	4	0.064338	0.03595	0.036934	0.048897	0.094027	0.05318	0.045741	0.065368	0.493984	1	-
ENSMUSC	Spopl	speckle-ty	GO:00081	metabolic	K10523	speckle-ty	mmu0434	Hedgehog	2:2350622	433	498	653	560	476	225	2.228213	2.148085	2.314908	1.877321	2.147922	1.435617	2.230402	1.820287	0.131921	1	-
ENSMUSC	4931423N	RIKEN cDl	-	-	-	-	-	-	2:2320747	1	3	3	3	2	2	0.023692	0.059562	0.048952	0.046287	0.041539	0.058756	0.044069	0.048861	1	1	-
ENSMUSC	Yme1l1	YME1-like	GO:00081	metabolic	-	-	-	-	2:2315636	1840	2325	2855	2717	1900	1237	12.8207	13.57905	13.70412	12.33289	11.60887	10.68684	13.36796	11.54287	0.138748	1	-
ENSMUSC	Prkcq	protein ki	GO:00081	metabolic	K18052	novel prot	mmu0516	Measles -	2:1117210	159	210	252	212	180	133	1.105695	1.224079	1.207227	0.960404	1.097623	1.146762	1.179	1.068263	0.359407	1	-
ENSMUSC	Mastl	microtubu	GO:00081	metabolic	-	-	-	-	2:2311560	25	30	32	17	30	9	0.257552	0.259053	0.227102	0.11409	0.271007	0.114957	0.247903	0.166685	0.156319	1	-
ENSMUSC	Acbd5	acyl-Coer	GO:00081	metabolic	-	-	-	-	2:2306816	1652	2019	2510	2611	1906	928	3.791196	3.883787	3.96818	3.903504	3.835586	2.640583	3.881054	3.459891	0.319956	1	-
ENSMUSC	Abi2	abl-intera	GO:00325	developm	K05751	abl intera	mmu0481	Regulation	1:6040961	1940	1744	2391	2567	1322	789	6.385915	4.811944	5.421911	5.504638	3.815888	3.220209	5.539923	4.180245	0.054928	1	-
ENSMUSC	Pdss1	prenyl (so	GO:00081	metabolic	K12504	decapreny	mmu0090	Terpenoic	2:2289552	93	129	139	187	141	102	1.016941	1.182368	1.047076	1.332089	1.351989	1.382924	1.082128	1.355667	0.241181	1	-
ENSMUSC	Apbb1ip	amyloid b	GO:00023	immune s	K17704	amyloid b	mmu0401	Rap1 sign	2:2277409	85	140	175	251	105	67	0.678046	0.936102	0.961679	1.304355	0.734469	0.662679	0.858609	0.900501	0.913624	1	-
ENSMUSC	Gad2	glutamic a	GO:00081	metabolic	K01580	glutamate	mmu0110	Metabolic	2:2262220	3663	4518	5381	5482	4068	2120	38.86389	40.17985	39.32998	37.89046	37.84712	27.88888	39.45791	34.54216	0.219542	1	-
ENSMUSC	Zbtb43	zinc finge	GO:00081	metabolic	-	-	-	-	2:3345028	265	298	383	344	317	260	2.101512	1.980868	2.092363	1.777164	2.204391	2.556497	2.058248	2.179351	0.941219	1	-
ENSMUSC	Odf2	outer den	GO:00325	developm	-	-	-	-	2:2988922	1330	1659	2005	2250	1607	1138	5.170703	5.406263	5.369872	5.698521	5.478435	5.485626	5.315613	5.554194	0.953539	1	-
ENSMUSC	Lrsam1	leucine ric	GO:00081	metabolic	-	-	-	-	2:3292521	617	705	830	991	728	494	4.573387	4.380208	4.238207	4.785285	4.731801	4.540099	4.397268	4.685728	0.834032	1	-
ENSMUSC	Fam129b	family witl	GO:00081	metabolic	-	-	-	-	2:3287611	534	516	763	631	510	402	6.516378	5.277983	6.414182	5.016218	5.457304	6.082428	6.069514	5.51865	0.30646	1	-
ENSMUSC	Stxbp1	syntaxin b	GO:00325	developm	K15292	syntaxin-b	mmu0472	Synaptic v	2:3278760	13797	16212	19265	20826	15928	10661	91.92167	90.5363	88.42065	90.39008	93.05446	88.06781	90.29287	90.50411	0.759267	1	-
ENSMUSC	Coq4	coenzyme	GO:00081	metabolic	-	-	-	-	2:2978749	165	192	254	190	155	147	1.608069	1.568468	1.705324	1.206308	1.324637	1.776337	1.627287	1.43576	0.266203	1	-
ENSMUSC	Med27	mediator	GO:00325	developm	K15170	mediator	mmu0491	Thyroid hc	2:2934681	272	325	367	388	304	285	3.780104	3.785923	3.513602	3.512757	3.704683	4.910958	3.69321	4.042799	0.762846	1	-
ENSMUSC	Ttf1	transcripti	GO:00325	developm	K15225	transcripti	mmu0491	Thyroid hc	2:2906026	188	214	307	343	240	143	1.476519	1.40879	1.661001	1.754913	1.652851	1.392523	1.515437	1.600096	0.889634	1	-
ENSMUSC	Ddx31	DEAD/H (	GO:00081	metabolic	-	-	-	-	2:2884040	104	130	149	158	109	85	1.569952	1.644938	1.549497	1.553787	1.442853	1.590951	1.588129	1.529197	0.649769	1	-
ENSMUSC	Ak8	adenylate	GO:00325	developm	K00939	adenylate	mmu0110	Metabolic	2:2870016	15	23	30	26	15	14	0.425772	0.547219	0.58661	0.480763	0.373346	0.492734	0.519867	0.448948	0.502544	1	-
ENSMUSC	Spaca9	sperm acr	GO:00990	supramole	-	-	-	-	2:2869208	11	2	6	3	1	4	0.568838	0.086686	0.213731	0.101049	0.045341	0.256466	0.289752	0.134285	0.134225	1	-
ENSMUSC	Dpm2	dolichol-p	GO:00081	metabolic	K09658	dolichyl-p	mmu0110	Metabolic	2:3257085	198	235	281	281	198	239	3.555673	3.537332	3.476265	3.287327	3.117908	5.321563	3.52309	3.908933	0.759957	1	-
ENSMUSC	St6galnac	ST6 (alpha	GO:00081	metabolic	K03376	N-acetylg	mmu0110	Metabolic	2:3259970	1282	1673	1875	1760	1569	1455	9.278548	10.14941	9.348561	8.29825	9.957668	13.05693	9.592174	10.43762	0.774753	1	-
ENSMUSC	Tsc1	tuberous	GO:00325	developm	K07206	tuberous	mmu0415	PI3K-Akt s	2:2864122	1316	1638	1911	2148	1646	1290	4.820504	5.029251	4.822239	5.125693	5.286997	5.858847	4.890665	5.423845	0.589867	1	-
ENSMUSC	Eng	endoglin		GO:00325	developm	-	-	-	-	2:3264659	314	367	465	453	445	352	2.729909	2.674473	2.784987	2.565659	3.392513	3.794442	2.729789	3.250872	0.359406	1	-
ENSMUSC	Gtf3c5	general tr	GO:00325	developm	-	-	-	-	2:2856631	334	380	450	456	356	273	3.151233	3.005179	2.924811	2.802725	2.94528	3.19362	3.027074	2.980542	0.681655	1	-
ENSMUSC	Ak1	adenylate	GO:00081	metabolic	K00939	adenylate	mmu0110	Metabolic	2:3262175	1453	1763	1996	1943	1595	1400	15.13068	15.38855	14.31874	13.18096	14.56451	18.07618	14.94599	15.27389	0.847407	1	-
ENSMUSC	Slc25a25	solute car	GO:00325	developm	-	-	-	-	2:3241448	891	826	1224	978	845	623	3.904683	3.03418	3.695226	2.792087	3.247188	3.385185	3.544696	3.141487	0.218426	1	-
ENSMUSC	Ptges2	prostaglai	GO:00081	metabolic	K05309	microsom	mmu0110	Metabolic	2:3239589	280	391	484	479	395	434	3.07178	3.595527	3.657878	3.423338	3.799901	5.90348	3.441728	4.375573	0.217897	1	-
ENSMUSC	Ralgds	ral guanin	GO:00987	molecular	K08732	ral guanin	mmu0520	Pathways	2:2851312	1304	1413	1724	1609	1659	1092	7.405338	6.726085	6.744595	5.952575	8.261449	7.689108	6.958672	7.301044	0.955847	1	-
ENSMUSC	Lcn2	lipocalin 2	GO:00081	metabolic	-	-	mmu0465	IL-17 sign	2:3238463	20	15	23	37	14	38	0.261161	0.164183	0.2069	0.314746	0.160304	0.615247	0.210748	0.363432	0.137009	1	-
ENSMUSC	Kcnj3	potassium	GO:00511	localizatio	K04997	potassium	mmu0492	Oxytocin s	2:5543597	1082	1078	1291	1661	1033	603	6.399659	5.344431	5.26026	6.400015	5.357632	4.422147	5.668117	5.393265	0.580498	1	-
ENSMUSC	Dnm1	dynamin 1	GO:00081	metabolic	K01528	dynamin (	mmu0414	Endocytos	2:3230847	22609	26220	29906	34799	24584	17959	106.8543	103.8715	97.36895	107.1418	101.884	105.2395	102.6982	104.7551	0.870073	1	-
ENSMUSC	Nr4a2	nuclear re	GO:00325	developm	K08558	nuclear re	mmu0492	Aldostero	2:5710683	968	1180	1645	1727	815	763	9.668285	9.878907	11.31857	11.23694	7.137966	9.448976	10.28859	9.274627	0.321672	1	-
ENSMUSC	Gpd2	glycerol p	GO:00325	developm	K00111	glycerol-3	mmu0056	Glyceroph	2:5723763	3126	3972	4766	5078	3553	2958	25.25638	26.8995	26.52697	26.72739	25.17213	29.63236	26.22762	27.17729	0.963876	1	-
ENSMUSC	Gbgt1	globoside	GO:00081	metabolic	K00722	globoside	mmu0110	Metabolic	2:2849689	14	10	16	19	8	10	0.56925	0.340808	0.448174	0.503271	0.285253	0.504181	0.452744	0.430902	0.861571	1	-
ENSMUSC	Ermn	ermin, ERI	GO:00325	developm	-	-	-	-	2:5804511	1156	1012	2137	1642	1146	631	20.21108	14.83081	25.73877	18.70193	17.56948	13.67878	20.26022	16.65006	0.160438	1	-
ENSMUSC	1700007K	RIKEN cDl	-	-	-	-	-	-	2:2846200	24	30	45	26	21	13	1.145961	1.200676	1.480205	0.808737	0.879282	0.769622	1.275614	0.819214	0.046744	1	-
ENSMUSC	Cytip	cytohesin	GO:00056	cell|intrace	-	-	-	-	2:5812913	4	5	6	8	3	5	0.028364	0.029719	0.02931	0.036957	0.018654	0.043961	0.029131	0.03319	1	1	-
ENSMUSC	Olfm1	olfactome	GO:00325	developm	-	-	-	-	2:2819299	15583	19418	22178	25027	17701	15078	185.7364	194.0007	182.1041	194.3285	185.0063	222.831	187.2804	200.7219	0.801739	1	-
ENSMUSC	Acvr1c	activin A r	GO:00325	developm	K13568	activin rec	mmu0455	Signaling	2:5826745	587	802	863	882	675	423	2.869883	3.286648	2.906623	2.809159	2.893826	2.564201	3.021051	2.755729	0.342126	1	-
ENSMUSC	Acvr1	activin A r	GO:00325	developm	K04675	activin rec	mmu0406	Cytokine-	2:5838864	494	639	945	845	582	477	4.499816	4.878894	5.929947	5.014245	4.648714	5.387307	5.102886	5.016756	0.671744	1	-
ENSMUSC	Col5a1	collagen,	GO:00325	developm	K19721	collagen,	mmu0497	Protein di	2:2788642	247	424	404	470	361	447	1.787268	2.571649	2.013846	2.215506	2.290565	4.010387	2.124254	2.838819	0.161068	1	-
ENSMUSC	Upp2	uridine ph	GO:00081	metabolic	K00757	uridine ph	mmu0110	Metabolic	2:5856729	374	432	454	624	453	301	2.75485	2.667245	2.30374	2.99428	2.925947	2.749026	2.575278	2.889751	0.571375	1	-
ENSMUSC	Lamc3	laminin ga	GO:00325	developm	K06247	laminin, g	mmu0520	Pathways	2:3188729	116	101	158	222	121	101	1.083664	0.79088	1.016822	1.351048	0.991204	1.169879	0.963789	1.17071	0.363318	1	-
ENSMUSC	Fibcd1	fibrinoger	GO:00160	membran	-	-	-	-	2:3181329	2423	2719	2878	3109	2388	2693	32.4119	30.48689	26.52121	27.09277	28.01097	44.66562	29.80667	33.25645	0.670139	1	-
ENSMUSC	Abl1	c-abl onc	GO:00325	developm	K06619	abelson ty	mmu0520	Pathways	2:3168837	1118	1216	819	1655	710	1065	3.426188	3.123601	1.729038	3.304067	1.907964	4.046736	2.759609	3.086256	0.753406	1	-
ENSMUSC	Fubp3	far upstre	GO:00081	metabolic	-	-	-	-	2:3157265	575	566	1010	1043	669	465	3.096078	2.554541	3.74642	3.658556	3.15873	3.104436	3.132346	3.307241	0.890485	1	-
ENSMUSC	Tor1b	torsin fam	GO:00038	catalytic a	-	-	-	-	2:3095295	304	320	391	457	318	371	1.552064	1.369427	1.375197	1.519967	1.423659	2.348529	1.432229	1.764052	0.30373	1	-
ENSMUSC	Tor1a	torsin fam	GO:00325	developm	-	-	-	-	2:3096062	431	524	663	609	480	387	6.446544	6.569517	6.831474	5.934012	6.295551	7.177048	6.615845	6.468871	0.623314	1	-
ENSMUSC	BC005624	cDNA seq	GO:00056	cell|intrace	-	-	-	-	2:3097217	1232	1583	1901	1851	1459	858	20.86259	22.4694	22.1764	20.41953	21.66484	18.01486	21.83613	20.03308	0.342304	1	-
ENSMUSC	Crat	carnitine a	GO:00081	metabolic	K00624	carnitine (	mmu0414	Peroxisom	2:3040047	581	727	884	893	770	573	2.935624	3.079016	3.077004	2.939394	3.4116	3.589757	3.030548	3.313584	0.704915	1	-
ENSMUSC	Usp20	ubiquitin s	GO:00081	metabolic	-	-	-	-	2:3098227	1286	1530	1784	1416	1528	1617	6.541476	6.523473	6.251452	4.692232	6.815545	10.19837	6.4388	7.235381	0.685185	1	-
ENSMUSC	Dolpp1	dolichyl p	GO:00081	metabolic	K07252	dolichyldi	mmu0051	N-Glycan	2:3039225	186	209	272	311	231	220	1.354826	1.276056	1.36487	1.475749	1.475456	1.986912	1.331917	1.646039	0.262484	1	-
ENSMUSC	Ntmt1	N-termina	GO:00081	metabolic	-	-	-	-	2:3080782	138	205	270	252	220	293	1.658844	2.065541	2.235845	1.973375	2.318957	4.366977	1.986744	2.886436	0.098797	1	-
ENSMUSC	Miga2	mitoguarc	GO:00325	developm	-	-	-	-	2:3036423	880	1089	1066	1176	838	1076	4.97126	5.15661	4.14851	4.327853	4.151169	7.536713	4.758793	5.338578	0.689207	1	-
ENSMUSC	Sh3glb2	SH3-dom	GO:00056	cell|intrace	K11248	endophilir	mmu0414	Endocytos	2:3034480	1480	1935	2301	2402	1948	1877	10.79519	11.83048	11.56209	11.41361	12.45948	16.97534	11.39592	13.61615	0.290524	1	-
ENSMUSC	Hspa5	heat shoc	GO:00325	developm	K09490	heat shoc	mmu0414	Protein pr	2:3477197	2309	4770	7691	6009	4764	1954	21.10815	36.55086	48.43514	35.78576	38.18922	22.14813	35.36472	32.04103	0.537521	1	-
ENSMUSC	Kynu	kynurenin	GO:00081	metabolic	K01556	kynurenin	mmu0110	Metabolic	2:4355532	0	3	1	3	0	1	0	0.023356	0.006396	0.018151	0	0.011514	0.009918	0.009888	1	1	-
ENSMUSC	Gapvd1	GTPase ac	GO:00511	localizatio	-	-	-	-	2:3467459	1538	1826	2062	2416	1930	1399	4.513925	4.492122	4.169059	4.619305	4.967044	5.090983	4.391702	4.892444	0.563246	1	-
ENSMUSC	Psmd5	proteasor	GO:00329	macromo	-	-	-	-	2:3484973	494	613	767	871	602	519	3.39959	3.536	3.636184	3.904803	3.632775	4.428462	3.523925	3.98868	0.516303	1	-
ENSMUSC	Cutal	cutA dival	GO:00508	response	-	-	-	-	2:3487439	6	10	14	9	9	2	0.063961	0.089346	0.102805	0.062497	0.084129	0.026441	0.085371	0.057689	0.303597	1	-
ENSMUSC	Zeb2	zinc finge	GO:00325	developm	K09299	zinc finge	mmu0520	MicroRNA	2:4498363	5698	7644	9592	11325	8230	2610	10.59372	11.91241	12.28532	13.71658	13.4174	6.016619	11.59715	11.0502	0.722015	1	-
ENSMUSC	Phf19	PHD finge	GO:00325	developm	-	-	-	-	2:3489375	13	18	19	17	27	22	0.099157	0.115084	0.099836	0.084472	0.180589	0.208068	0.104692	0.15771	0.250985	1	-
ENSMUSC	Traf1	TNF recep	GO:00081	metabolic	K03172	TNF recep	mmu0520	Pathways	2:3494175	8	15	23	16	14	14	0.03806	0.05982	0.075384	0.04959	0.058407	0.08259	0.057755	0.063529	0.937622	1	-
ENSMUSC	Rab14	RAB14, m	GO:00325	developm	K07881	Ras-relate	mmu0415	AMPK sigr	2:3518020	3928	4785	6108	5697	4558	2740	13.5923	13.87894	14.56036	12.84249	13.83052	11.75596	14.01053	12.80966	0.302224	1	-
ENSMUSC	Gsn	gelsolin [S	GO:00325	developm	K05768	gelsolin[-	mmu0520	Viral carci	2:3525638	1085	1076	1790	1667	1200	1128	15.65736	13.01529	17.7948	15.67134	15.18492	20.18292	15.48915	17.01306	0.705094	1	-
ENSMUSC	Stom	stomatin [	GO:00511	localizatio	-	-	-	-	2:3531398	230	284	382	357	275	185	3.975307	4.114474	4.548391	4.019686	4.167907	3.964618	4.212724	4.050737	0.605732	1	-
ENSMUSC	Dab2ip	disabled 2	GO:00325	developm	K19901	disabled h	mmu0421	Apoptosis	2:3555826	1174	1183	1853	2236	1588	1479	3.844552	3.247248	4.180272	4.770139	4.560064	6.00526	3.757357	5.111821	0.033898	1	-
ENSMUSC	Ttll11	tubulin ty	GO:00081	metabolic	-	-	-	-	2:3575124	489	598	699	720	549	416	3.642885	3.734139	3.587274	3.49422	3.586339	3.842518	3.654766	3.641026	0.738171	1	-
ENSMUSC	Mrrf	mitochon	GO:00081	metabolic	-	-	-	-	2:3613638	327	394	441	509	309	245	3.83216	3.87031	3.560301	3.88594	3.175399	3.559994	3.754257	3.540444	0.501292	1	-
ENSMUSC	Grb14	growth fa	GO:00508	response	-	-	-	-	2:6491247	338	548	648	637	567	330	2.491984	3.386586	3.291207	3.059494	3.665679	3.016676	3.056592	3.247283	0.862124	1	-
ENSMUSC	Rbm18	RNA bind	GO:00971	organic cy	-	-	-	-	2:3611607	506	679	885	811	694	500	4.237984	4.766846	5.106268	4.42498	5.096958	5.192362	4.703699	4.904767	0.990394	1	-
ENSMUSC	Lhx6	LIM home	GO:00325	developm	-	-	-	-	2:3608195	378	507	511	534	389	330	2.821925	3.172591	2.628001	2.597026	2.546515	3.054593	2.874172	2.732711	0.497736	1	-
ENSMUSC	Gca	grancalcir	GO:00081	metabolic	-	-	-	-	2:6266428	163	172	288	236	215	194	2.879141	2.54657	3.504442	2.715623	3.330085	4.248769	2.976718	3.431492	0.565939	1	-
ENSMUSC	Morn5	MORN re	-	-	-	-	-	-	2:3604945	7	15	14	8	10	7	0.623109	1.119155	0.858437	0.463882	0.780532	0.772571	0.8669	0.672328	0.359276	1	-
ENSMUSC	Ndufa8	NADH de	GO:00081	metabolic	K03952	NADH de	mmu0110	Metabolic	2:3603632	735	924	1137	1049	844	929	54.25257	57.16862	57.81557	50.44176	54.62834	85.02268	56.41225	63.36426	0.641103	1	-
ENSMUSC	Slc4a10	solute car	GO:00325	developm	-	-	-	-	2:6204646	4577	4248	6873	6558	4966	3330	37.57196	29.22943	38.86697	35.07002	35.7464	33.89325	35.22279	34.90322	0.715547	1	-
ENSMUSC	Psmd14	proteasor	GO:00081	metabolic	K03030	26S prote	mmu0516	Epstein-B	2:6171169	698	986	1224	1054	808	817	11.44479	13.55133	13.82564	11.25834	11.61732	16.60966	12.94059	13.16177	0.820352	1	-
ENSMUSC	Strbp	spermatid	GO:00325	developm	-	-	-	-	2:3748322	2308	2830	4122	3660	3410	2040	7.022229	7.217362	8.639701	7.254409	9.097797	7.695828	7.626431	8.016011	0.912228	1	-
ENSMUSC	Wdr5	WD repea	GO:00325	developm	-	-	-	-	2:2751515	479	592	679	688	480	496	7.28973	7.551791	7.118638	6.820963	6.405602	9.359302	7.320053	7.528623	0.891018	1	-
ENSMUSC	Brd3	bromodo	GO:00081	metabolic	-	-	-	-	2:2744557	1013	1146	1395	1508	1132	879	5.144164	4.878011	4.880125	4.988709	5.04074	5.534521	4.967433	5.18799	0.980794	1	-
ENSMUSC	Egfl7	EGF-like c	GO:00508	response	-	-	-	-	2:2658001	307	317	353	426	288	332	2.532882	2.19224	2.006332	2.289641	2.083586	3.39626	2.243818	2.589829	0.551003	1	-
ENSMUSC	Agpat2	1-acylglyc	GO:00325	developm	K13509	lysophosp	mmu0110	Metabolic	2:2659305	25	11	22	16	25	23	0.581141	0.214339	0.352294	0.242291	0.509591	0.662912	0.382591	0.471598	0.656798	1	-
ENSMUSC	Notch1	notch 1 [S	GO:00325	developm	K02599	Notch[-]	mmu0520	Pathways	2:2645790	744	759	998	1124	920	784	2.987328	2.554492	2.760528	2.940071	3.239221	3.903121	2.76745	3.360804	0.242664	1	-
ENSMUSC	Sec16a	SEC16 ho	GO:00511	localizatio	-	-	-	-	2:2640943	990	1112	1400	1589	1108	1216	4.673017	4.399665	4.552412	4.88616	4.586109	7.116748	4.541698	5.529672	0.258518	1	-
ENSMUSC	Inpp5e	inositol pc	GO:00081	metabolic	K20278	inositol pc	mmu0110	Metabolic	2:2639624	394	481	590	644	490	432	2.10207	2.151044	2.168476	2.238302	2.292398	2.857732	2.14053	2.462811	0.467094	1	-
ENSMUSC	Pmpca	peptidase	GO:00081	metabolic	-	-	-	-	2:2638933	1658	2073	2464	2692	1930	1297	19.81774	20.76929	20.28904	20.96168	20.22877	19.22185	20.29202	20.13744	0.718556	1	-
ENSMUSC	Sdccag3	serologica	GO:00511	localizatio	-	-	-	-	2:2638280	840	1133	1300	1469	1049	944	6.015836	6.801417	6.413743	6.853621	6.587717	8.382516	6.410332	7.274618	0.492993	1	-
ENSMUSC	Card9	caspase re	GO:00081	metabolic	K12794	caspase re	mmu0515	Tuberculo	2:2635217	27	27	25	35	34	33	0.614801	0.515333	0.392157	0.519196	0.678896	0.931691	0.50743	0.709928	0.268088	1	-
ENSMUSC	Gpsm1	G-protein	GO:00325	developm	K15839	G-protein	mmu0503	Cocaine a	2:2631551	538	638	505	732	611	585	4.283039	4.257394	2.769573	3.79632	4.265339	5.774464	3.770002	4.612041	0.329972	1	-
ENSMUSC	1700019N	RIKEN cDl	-	-	-	-	-	-	19:587858	4	0	1	3	3	0	0.183143	0	0.031531	0.089474	0.120442	0	0.071558	0.069972	1	1	-
ENSMUSC	Nacc2	nucleus ac	GO:00081	metabolic	-	-	-	-	2:2605553	209	1754	2441	596	307	1960	1.341538	9.437121	10.79385	2.492211	1.727976	15.59908	7.190836	6.606423	0.719969	1	-
ENSMUSC	Camsap1	calmoduli	GO:00325	developm	-	-	-	-	2:2592683	2129	2585	2943	3705	2635	1645	9.657699	9.829048	9.196861	10.94883	10.48145	9.252304	9.561202	10.22753	0.788396	1	-
ENSMUSC	Lhx3	LIM home	GO:00325	developm	-	-	-	-	2:2620021	0	0	4	0	5	2	0	0	0.037091	0	0.059005	0.033384	0.012364	0.030797	0.359444	1	-
ENSMUSC	Fcna	ficolin A [S	GO:00048	signal trar	-	-	-	-	2:2562466	0	6	7	4	2	2	0	0.204747	0.196338	0.106103	0.071396	0.100987	0.133695	0.092829	0.58953	1	-
ENSMUSC	Tmem141	transmem	GO:00099	cellular pr	-	-	-	-	2:2562006	105	122	118	109	81	82	2.433649	2.370183	1.884098	1.645797	1.646249	2.356517	2.22931	1.882855	0.229331	1	-
ENSMUSC	Ccdc183	coiled-co	GO:00099	cellular pr	-	-	-	-	2:2560863	0	0	0	0	3	0	0	0	0	0	0	0.045072	0	0	0.015024	0.304305	1	-
ENSMUSC	Mamdc4	MAM dor	GO:00511	localizatio	-	-	-	-	2:2556311	17	16	39	35	17	22	0.173554	0.136923	0.274293	0.232783	0.152188	0.278494	0.194923	0.221155	0.832497	1	-
ENSMUSC	Traf2	TNF recep	GO:00081	metabolic	K03173	TNF recep	mmu0520	Pathways	2:2551798	209	186	243	229	202	184	2.016176	1.504002	1.61488	1.439131	1.708739	2.200825	1.711686	1.782899	0.968376	1	-
ENSMUSC	Abca2	ATP-bind	GO:00081	metabolic	K05642	ATP-bind	mmu0414	Lysosome	2:2542870	4749	4732	6602	7095	5403	4511	30.08505	25.12732	28.81214	29.28078	30.01416	35.43296	28.00817	31.57597	0.507638	1	-
ENSMUSC	Nmi	N-myc (a	GO:00081	metabolic	-	-	-	-	2:5194848	58	59	67	87	86	34	1.045994	0.89188	0.832385	1.022115	1.360015	0.760276	0.92342	1.047469	0.656444	1	-
ENSMUSC	Neb	nebulin [S	GO:00325	developm	-	-	-	-	2:5213664	109	146	124	157	105	60	0.239574	0.268979	0.187753	0.224798	0.202369	0.163512	0.232102	0.196893	0.302515	1	-
ENSMUSC	Sapcd2	suppressc	GO:00325	developm	-	-	-	-	2:2537232	17	7	34	47	22	40	0.126448	0.043642	0.174224	0.227748	0.143498	0.36891	0.114771	0.246718	0.035888	1	-
ENSMUSC	Uap1l1	UDP-N-a	GO:00081	metabolic	K00972	UDP-N-a	mmu0110	Metabolic	2:2535988	134	146	192	189	135	161	1.413931	1.291306	1.395651	1.299169	1.249105	2.106365	1.366962	1.551546	0.673957	1	-
ENSMUSC	Dpp7	dipeptidyl	GO:00081	metabolic	-	-	-	-	2:2535227	253	298	401	362	264	314	2.056127	2.03001	2.245047	1.916548	1.881377	3.164062	2.110395	2.320662	0.790809	1	-
ENSMUSC	Grin1	glutamate	GO:00325	developm	K05208	glutamate	mmu0408	Neuroacti	2:2529118	6675	7517	8988	10135	7606	8810	29.15977	27.52519	27.04876	28.84285	29.13612	47.71937	27.91124	35.23278	0.169959	1	-
ENSMUSC	Arl6ip6	ADP-ribo	GO:00160	membran	-	-	-	-	2:5319172	120	147	216	158	124	107	2.591359	2.660823	3.213302	2.222713	2.348057	2.864943	2.821828	2.478571	0.281152	1	-
ENSMUSC	Lrrc26	leucine ric	GO:00511	localizatio	-	-	-	-	2:2528991	0	2	0	0	0	0	0	0.089526	0	0	0	0	0.029842	0	0.53666	1	-
ENSMUSC	Tmem210	transmem	GO:00160	membran	-	-	-	-	2:2528814	1	0	0	2	0	0	0.078283	0	0	0.101962	0	0	0.026094	0.033987	1	1	-
ENSMUSC	Anapc2	anaphase	GO:00325	developm	K03349	anaphase	mmu0516	HTLV-I inf	2:2527247	1170	1387	1709	1790	1341	1388	15.49428	15.39624	15.59118	15.44259	15.57245	22.79086	15.4939	17.9353	0.436903	1	-
ENSMUSC	Ssna1	SS nuclea	GO:00511	localizatio	-	-	-	-	2:2527103	187	226	294	263	191	376	4.753769	4.815682	5.148664	4.355454	4.257682	11.85142	4.906038	6.821519	0.232976	1	-
ENSMUSC	Fam166a	family witl	GO:00056	cell|intrace	-	-	-	-	2:2521874	0	0	0	0	2	0	0	0	0	0	0	0.094368	0	0	0.031456	0.535011	1	-
ENSMUSC	Rbms1	RNA bind	GO:00081	metabolic	-	-	-	-	2:6075019	275	349	486	436	320	253	2.392478	2.54504	2.912753	2.471062	2.441227	2.729123	2.616757	2.547137	0.630869	1	-
ENSMUSC	Itgb6	integrin b	GO:00511	localizatio	K06589	integrin b	mmu0415	PI3K-Akt s	2:6059829	0	3	2	0	0	0	0	0.017837	0.009775	0	0	0	0.009204	0	0.089508	1	-
ENSMUSC	Arrdc1	arrestin d	GO:00081	metabolic	-	-	-	-	2:2492535	61	65	65	92	48	89	0.548411	0.489828	0.402574	0.538825	0.378412	0.992092	0.480271	0.636443	0.357545	1	-
ENSMUSC	Zmynd19	zinc finge	GO:00452	synapse|m	-	-	-	-	2:2494979	415	608	636	669	473	401	5.838941	7.170384	6.164457	6.131886	5.835658	6.995464	6.39126	6.321003	0.69749	1	-
ENSMUSC	Dph7	diphtham	GO:00081	metabolic	-	-	-	-	2:2496240	252	271	277	339	262	211	3.158222	2.846851	2.39152	2.767732	2.879298	3.278768	2.798864	2.975266	0.905522	1	-
ENSMUSC	Pax8	paired bo	GO:00325	developm	K09293	paired bo	mmu0520	Pathways	2:2442056	0	2	0	2	3	0	0	0.014046	0	0.010915	0.02204	0	0.004682	0.010985	0.511299	1	-
ENSMUSC	7-Mar	membran	GO:00325	developm	-	-	-	-	2:6020988	622	825	1079	1140	856	504	2.698109	2.999683	3.224346	3.221481	3.256006	2.710726	2.974046	3.062737	0.984741	1	-
ENSMUSC	Psd4	pleckstrin	GO:00987	molecular	K12494	PH and SE	mmu0414	Endocytos	2:2436758	15	12	18	31	12	13	0.054885	0.036805	0.045372	0.073893	0.038502	0.058977	0.045688	0.057124	0.52076	1	-
ENSMUSC	Ly75	lymphocy	GO:00048	signal trar	-	-	-	-	2:6029210	35	40	59	56	50	19	0.303669	0.290899	0.352638	0.316516	0.380402	0.204397	0.315735	0.300438	0.776197	1	-
ENSMUSC	Il1rn	interleukir	GO:00325	developm	-	-	-	-	2:2433685	2	2	6	7	4	3	0.015691	0.013149	0.032421	0.035771	0.027511	0.02918	0.020421	0.030821	0.468352	1	-
ENSMUSC	Hnmt	histamine	GO:00325	developm	K00546	histamine	mmu0034	Histidine r	2:2400291	314	340	461	451	333	159	5.944484	5.395308	6.012246	5.562149	5.528041	3.732225	5.784012	4.940805	0.258342	1	-
ENSMUSC	Baz2b	bromodo	GO:00056	cell|intrace	-	-	-	-	2:5989936	710	921	1225	1298	966	498	1.971195	2.143304	2.342927	2.347619	2.351749	1.714298	2.152475	2.137889	0.805566	1	-
ENSMUSC	Wdsub1	WD repea	GO:00081	metabolic	-	-	-	-	2:5985236	116	128	157	151	123	112	1.120901	1.036742	1.045101	0.950535	1.042209	1.341873	1.067581	1.111539	0.974655	1	-

ENSMUS(	Dapl1	death ass	GO:00325	developm	-	-	-	-	2:5948465	4	8	0	4	4	0	0.327158	0.54845	0	0.213161	0.28687	0	0.29187	0.166677	0.438191	1	-
ENSMUS(	Pkp4	plakophili	GO:00452	synapse|c	-	-	-	-	2:5916085	5408	6009	7859	8024	5496	4074	30.35904	28.27525	30.39275	29.34431	27.05458	28.35687	29.67568	28.25192	0.451986	1	-
ENSMUS(	Galnt3	polypepti	GO:00081	metabolic	K00710	polypepti	mmu011C	Metabolic	2:6608276	75	97	85	106	82	41	0.913815	0.99066	0.713457	0.84137	0.8761	0.619391	0.872644	0.778954	0.489681	1	-
ENSMUS(	Nup35	nucleopor	GO:00081	metabolic	K14313	nuclear pc	mmu0301	RNA trans	2:8061723	96	117	130	142	110	107	1.305885	1.334054	1.218225	1.258352	1.312101	1.804693	1.286055	1.458382	0.664643	1	-
ENSMUS(	Dusp19	dual speci	GO:00081	metabolic	-	-	-	-	2:8061704	270	279	335	367	279	190	2.965144	2.568261	2.534424	2.625607	2.686762	2.587157	2.689276	2.633175	0.691926	1	-
ENSMUS(	Nckap1	NCK-asso	GO:00325	developm	K05750	NCK-asso	mmu0481	Regulatio	2:8050051	12720	15194	17939	20271	13531	8872	117.1756	117.3208	113.8412	121.6485	109.3006	101.3345	116.1125	110.7612	0.494795	1	-
ENSMUS(	Frzb	frizzled-re	GO:00325	developm	-	-	-	-	2:8041197	472	590	582	637	463	466	8.231892	8.625066	6.992493	7.237333	7.080782	10.07696	7.949817	8.13169	0.862084	1	-
ENSMUS(	Dnajc10	DnaJ heat	GO:00081	metabolic	K09530	DnaJ hom	mmu0414	Protein pr	2:8031546	1029	1264	1543	1470	1140	1021	11.30441	11.63945	11.67751	10.52039	10.98197	13.90735	11.54046	11.80324	0.85738	1	-
ENSMUS(	Ssfa2	sperm spe	GO:00160	membran	-	-	-	-	2:7963535	973	1009	1554	1360	1122	867	9.386319	8.158803	10.32726	8.546791	9.49113	10.37021	9.290793	9.469377	0.860601	1	-
ENSMUS(	Itga4	integrin al	GO:00325	developm	K06483	integrin al	mmu0415	PI3K-Akt :	2:7925542	943	1135	1407	1559	1106	538	3.840711	3.874795	3.947713	4.136452	3.950008	2.716865	3.88774	3.601108	0.481883	1	-
ENSMUS(	Slc25a12	solute car	GO:00081	metabolic	-	-	-	-	2:7127106	2796	3335	4140	4427	3169	2068	20.57837	20.57418	20.99063	21.22584	20.45214	18.87167	20.71439	20.18321	0.610901	1	-
ENSMUS(	Ube2e3	ubiquitin-	GO:00081	metabolic	K20217	ubiquitin-	mmu0412	Ubiquitin	2:7886812	604	704	915	854	667	617	6.644623	6.491715	6.934357	6.120311	6.434317	8.41598	6.690232	6.990203	0.990964	1	-
ENSMUS(	Dync1i2	dynein cyt	GO:00511	localizatio	K10415	dynein int	mmu0414	Phagoson	2:7121170	1365	1722	2135	2260	1655	1222	6.553589	6.929988	7.061483	7.068651	6.967665	7.274507	6.848353	7.103608	0.990534	1	-
ENSMUS(	Cwc22	CWC22 sp	GO:00081	metabolic	-	-	-	-	2:7788115	314	443	362	637	379	278	1.95337	2.309998	1.55137	2.581523	2.067461	2.144303	1.938246	2.264429	0.438376	1	-
ENSMUS(	Cybrd1	cytochron	GO:00081	metabolic	K08370	cytochron	mmu0497	Mineral al	2:7111792	114	121	134	139	109	89	1.357002	1.2073	1.098837	1.077886	1.137743	1.313561	1.221047	1.176396	0.637393	1	-
ENSMUS(	Zfp385b	zinc finge	GO:00508	response	-	-	-	-	2:7741063	530	876	739	879	858	324	3.069355	4.252342	2.94827	3.316208	4.357135	2.326499	3.423322	3.333281	0.768661	1	-
ENSMUS(	Hat1	histone ar	GO:00081	metabolic	K11303	histone ac	mmu0503	Alcoholisr	2:7138895	280	354	413	428	337	145	4.947129	5.242662	5.026861	4.926298	5.221181	3.176494	5.072217	4.441324	0.351747	1	-
ENSMUS(	Stk39	serine/thr	GO:00081	metabolic	-	-	-	-	2:6821044	1310	1420	2130	1808	1468	803	10.64908	9.675684	11.92811	9.574597	10.46428	8.093604	10.75096	9.377493	0.206128	1	-
ENSMUS(	Cers6	ceramide	GO:00081	metabolic	K04710	ceramide	mmu011C	Metabolic	2:6886144	1491	1734	2095	2261	1671	1311	8.652868	8.434985	8.375637	8.548002	8.503573	9.433461	8.48783	8.828345	0.994758	1	-
ENSMUS(	Ssrp1	structure :	GO:00081	metabolic	-	-	-	-	2:8503723	1733	2128	2434	2712	2010	1302	18.05531	18.58364	17.46941	18.40677	18.36306	16.81913	18.03612	17.86299	0.710151	1	-
ENSMUS(	Dhrs9	dehydrog	GO:00081	metabolic	K11149	dehydrog	mmu011C	Metabolic	2:6938044	3	7	3	8	5	5	0.044531	0.087101	0.030679	0.077368	0.065083	0.092029	0.054104	0.07816	0.544681	1	-
ENSMUS(	Lrp2	low densit	GO:00325	developm	K06233	low densit	mmu0491	Thyroid h	2:6942434	31	45	64	65	23	50	0.113072	0.137579	0.160812	0.154447	0.073564	0.226125	0.137154	0.151379	0.908389	1	-
ENSMUS(	P2rx3	purinergic	GO:00048	signal trar	K05217	P2X purin	mmu0408	Neuroacti	2:8499858	16	31	36	34	22	21	0.241824	0.392715	0.374825	0.334754	0.291569	0.393522	0.336455	0.339948	0.913079	1	-
ENSMUS(	Prg2	proteogly	GO:00081	metabolic	K10786	bone mar	mmu0531	Asthma -	2:8498046	0	0	3	2	0	0	0	0	0.156728	0.098798	0	0	0.052243	0.032933	1	1	-
ENSMUS(	Slc43a3	solute car	GO:00511	localizatio	-	-	-	-	2:8493657	22	27	51	34	22	25	0.222563	0.228955	0.355433	0.224075	0.195169	0.313587	0.268984	0.244277	0.534412	1	-
ENSMUS(	Slc43a1	solute car	GO:00511	localizatio	-	-	-	-	2:8483885	4	0	5	7	2	0	0.028701	0	0.024716	0.032722	0.012583	0	0.017806	0.015102	1	1	-
ENSMUS(	Timm10	translocas	GO:00511	localizatio	-	-	-	-	2:8482699	174	235	307	267	220	247	9.708737	10.99088	11.80054	9.705234	10.76411	17.08815	10.83339	12.51917	0.580517	1	-
ENSMUS(	Clp1	CLP1, clea	GO:00325	developm	K14399	polyribon	mmu0301	mRNA sur	2:8472210	65	69	108	91	82	100	0.817034	0.726991	0.935201	0.745166	0.903825	1.558527	0.826408	1.069173	0.337656	1	-
ENSMUS(	Med19	mediator	GO:00081	metabolic	-	-	-	-	2:8467840	380	525	636	589	519	382	8.512555	9.858001	9.814893	8.595548	10.19498	10.61026	9.39515	9.800263	0.99727	1	-
ENSMUS(	Tfpi	tissue fact	GO:00081	metabolic	K03909	tissue fact	mmu0461	Complem	2:8443285	81	91	107	129	103	58	0.437589	0.412073	0.398212	0.453997	0.487935	0.388507	0.415958	0.44348	0.852927	1	-
ENSMUS(	Fastkd1	FAST kina	GO:00081	metabolic	-	-	-	-	2:6968681	149	249	281	249	196	161	1.073143	1.503221	1.394209	1.168292	1.237854	1.437742	1.323524	1.281296	0.627307	1	-
ENSMUS(	Itgav	integrin al	GO:00325	developm	K06487	integrin al	mmu052C	Pathways	2:8372439	1159	633	1663	932	776	929	5.337144	2.443329	5.275568	2.795911	3.133502	5.304292	4.352014	3.744568	0.301535	1	-
ENSMUS(	Phospho2	phosphat	GO:00081	metabolic	K13248	pyridoxal	mmu011C	Metabolic	2:6978962	337	426	539	411	386	240	2.769245	2.934229	3.051211	2.200165	2.781388	2.445279	2.918228	2.47561	0.14969	1	-
ENSMUS(	Zc3h15	zinc finge	GO:00508	response	-	-	-	-	2:8364443	1714	2205	2515	2667	1803	1042	26.32428	28.38619	26.6094	26.68396	24.28197	19.84263	27.10663	23.60285	0.203439	1	-
ENSMUS(	Mtx2	metaxin 2	GO:00511	localizatio	-	-	-	-	2:7482580	348	418	517	510	391	308	5.131425	5.166399	5.251715	4.899036	5.055668	5.63112	5.18318	5.195275	0.773394	1	-
ENSMUS(	Hoxd8	homeobo	GO:00325	developm	-	-	-	-	2:7470461	6	8	6	6	0	6	0.077053	0.086107	0.053076	0.050196	0	0.095542	0.072079	0.048579	0.346639	1	-
ENSMUS(	Chrna1	cholinergi	GO:00325	developm	K04803	nicotinic a	mmu0408	Neuroacti	2:7356321	37	33	23	58	40	17	0.536125	0.400811	0.22959	0.547496	0.508241	0.305418	0.388842	0.453718	0.641943	1	-
ENSMUS(	Ola1	Obg-like	GO:00081	metabolic	-	-	-	-	2:7309280	2595	3377	2950	4119	2078	1302	32.06689	34.97869	25.11268	33.15836	22.51686	19.9488	30.71942	25.208	0.155557	1	-
ENSMUS(	Sp3	trans-acti	GO:00325	developm	-	-	-	-	2:7293642	902	900	1085	1426	871	422	6.025945	5.039808	4.993442	6.206116	5.102457	3.495561	5.353065	4.934711	0.519533	1	-
ENSMUS(	Itga6	integrin al	GO:00325	developm	K06485	integrin al	mmu052C	Pathways	2:7174561	445	518	638	672	514	309	2.297782	2.241978	2.269449	2.260474	2.327311	1.978306	2.269737	2.188697	0.61555	1	-
ENSMUS(	Kif18a	kinesin fa	GO:00325	developm	-	-	-	-	2:1092807	11	17	27	17	12	11	0.058842	0.076224	0.099494	0.059239	0.056287	0.072958	0.078186	0.062828	0.388839	1	-
ENSMUS(	Arl14ep	ADP-ribo	GO:00160	membran	-	-	-	-	2:1069625	655	763	997	1003	747	554	6.122553	5.978177	6.420038	6.107644	6.122862	6.420761	6.173589	6.217089	0.802552	1	-
ENSMUS(	Slc12a6	solute car	GO:00511	localizatio	-	-	-	-	2:1122658	2239	2673	3490	3688	2584	1928	9.414171	9.420627	10.10892	10.10184	9.527155	10.05127	9.647906	9.893422	0.913118	1	-
ENSMUS(	Emc4	ER memb	GO:00329	macromo	-	-	-	-	2:1123630	992	1161	1460	1414	1113	807	41.23255	40.44957	41.80544	38.28774	40.56641	41.58992	41.16252	40.14802	0.594235	1	-
ENSMUS(	Katnbl1	katanin p	GO:00099	cellular pr	-	-	-	-	2:1123792	137	183	241	259	157	110	1.950872	2.184307	2.364163	2.402655	1.960432	1.942179	2.166448	2.101755	0.704548	1	-
ENSMUS(	Nop10	NOP10 rib	GO:00081	metabolic	K11130	H/ACA rib	mmu030C	Ribosome	2:1122619	156	218	271	196	110	226	8.93981	10.4716	10.69858	7.317142	5.527624	16.05829	10.03666	9.634352	0.624184	1	-
ENSMUS(	Lpcat4	lysophosp	GO:00081	metabolic	K13512	lysophosp	mmu011C	Metabolic	2:1122394	751	1001	1070	1227	918	837	12.16939	13.59614	11.94439	12.95254	13.0441	16.81668	12.56997	14.2711	0.513394	1	-
ENSMUS(	Ccdc34	coiled-co	-	-	-	-	-	-	2:1100178	139	182	231	209	176	124	0.839004	0.920818	0.960532	0.82182	0.931544	0.928017	0.906785	0.893794	0.74187	1	-
ENSMUS(	Lin7c	lin-7 hom	GO:00325	developm	-	-	-	-	2:1098908	2512	2980	3942	3973	2836	1722	33.37762	33.18982	36.08309	34.3903	33.0434	28.3697	34.21685	31.93447	0.413103	1	-
ENSMUS(	Commd9	COMM dc	GO:00081	metabolic	-	-	-	-	2:1018862	269	356	431	381	283	277	8.331278	9.241907	9.195755	7.687144	7.685791	10.63714	8.92298	8.670027	0.597967	1	-
ENSMUS(	Traf6	TNF recep	GO:00325	developm	K03175	TNF recep	mmu052C	Pathways	2:1016784	405	471	568	611	439	330	2.948334	2.874062	2.848536	2.897642	2.802391	2.978666	2.890311	2.892899	0.780403	1	-
ENSMUS(	B230118H	RIKEN cDI	GO:00056	cell|cilium	-	-	-	-	2:1015607	289	332	591	406	349	126	2.530558	2.436746	3.56499	2.31594	2.67971	1.367971	2.844098	2.121207	0.102797	1	-
ENSMUS(	Dnajc24	DnaJ heat	GO:00431	ion bindin	-	-	-	-	2:1059667	58	76	101	102	73	74	1.576597	1.73165	1.891313	1.806228	1.740042	2.494088	1.733187	2.013453	0.601138	1	-
ENSMUS(	Elp4	elongator	GO:00081	metabolic	-	-	-	-	2:1057010	173	214	253	267	203	178	2.946057	3.054644	2.968021	2.962021	3.031323	3.758383	2.989574	3.250576	0.795042	1	-
ENSMUS(	Pax6	paired bo	GO:00325	developm	K08031	paired bo	mmu0455	Signaling	2:1056689	116	149	154	177	166	110	0.325698	0.350667	0.29787	0.323751	0.408703	0.382944	0.324745	0.371799	0.562778	1	-
ENSMUS(	Eif3m	eukaryotic	GO:00081	metabolic	-	-	-	-	2:1049996	453	614	720	820	553	500	8.788006	9.984198	9.622234	10.36305	9.407191	12.02675	9.464813	10.599	0.589656	1	-
ENSMUS(	Prrg4	proline ric	GO:00055	extracellul	-	-	-	-	2:1048307	37	32	45	22	26	19	0.689574	0.499909	0.577757	0.267109	0.424911	0.439063	0.58908	0.377028	0.037315	1	-
ENSMUS(	Depdc7	DEP dom	GO:00508	response	-	-	-	-	2:1047217	31	16	39	32	41	29	0.859587	0.371878	0.744973	0.578031	0.996889	0.997013	0.658813	0.857311	0.44587	1	-
ENSMUS(	Tcp11l1	t-complex	GO:00990	supramol	-	-	-	-	2:1046572	554	618	746	989	662	512	3.517671	3.289176	3.263139	4.090942	3.68592	4.030895	3.356662	3.935919	0.344749	1	-
ENSMUS(	Cstf3	cleavage :	GO:00081	metabolic	K14408	cleavage :	mmu0301	mRNA sur	2:1045905	402	577	759	705	546	420	3.523907	4.239621	4.583437	4.025953	4.19695	4.564926	4.115655	4.26261	0.974895	1	-
ENSMUS(	Hipk3	homeodo	GO:00081	metabolic	K08826	homeodo	mmu0421	Cellular se	2:1044264	1743	1933	2603	2507	1803	1479	11.90978	11.07111	12.25271	11.15944	10.80301	12.53027	11.74454	11.49758	0.593235	1	-
ENSMUS(	Fbxo3	F-box pro	GO:00056	cell|intrace	-	-	-	-	2:1040277	940	1156	1471	1497	1012	870	3.910186	4.030697	4.215352	4.056702	3.691414	4.487195	4.052078	4.078437	0.776053	1	-
ENSMUS(	Caprin1	cell cycle	GO:00325	developm	-	-	-	-	2:1037629	4942	4458	5587	5908	4079	2818	19.73602	14.92278	15.37046	15.37018	14.28411	13.95352	16.67642	14.53593	0.172259	1	-
ENSMUS(	Nat10	N-acetyltr	GO:00081	metabolic	K14521	N-acetyltr	mmu030C	Ribosome	2:1037212	494	619	694	801	597	440	2.919942	3.066837	2.825908	3.084333	3.094308	3.224674	2.937562	3.134438	0.841287	1	-
ENSMUS(	Cat	catalase [	GO:00325	developm	K03781	catalase[1	mmu0406	FoxO sign	2:1034538	914	1134	1523	1521	1135	703	18.52279	19.26309	21.26235	20.08034	20.16965	17.6645	19.68274	19.30483	0.679432	1	-
ENSMUS(	Pamr1	peptidase	GO:00055	extracellul	-	-	-	-	2:1025500	133	169	259	269	216	169	1.250746	1.332163	1.677912	1.647981	1.781203	1.97057	1.420274	1.799918	0.204722	1	-
ENSMUS(	Trim44	tripartite r	GO:00081	metabolic	-	-	-	-	2:1023001	7648	9083	11118	11604	8631	4962	79.48543	79.12648	79.60086	78.56498	78.65809	63.94143	79.40426	73.7215	0.391331	1	-
ENSMUS(	Api5	apoptosis	GO:00329	macromo	-	-	-	-	2:9441168	1553	1783	2526	2638	1822	1141	18.42753	17.73371	20.64811	20.39163	18.95775	16.78677	18.93645	18.71205	0.726573	1	-
ENSMUS(	Ttc17	tetratricop	GO:00099	cellular pr	-	-	-	-	2:9430076	961	1051	1272	1353	989	553	6.200453	5.684017	5.653773	5.686948	5.595498	4.423957	5.846081	5.235468	0.298209	1	-
ENSMUS(	Hsd17b12	hydroxyst	GO:00081	metabolic	K10251	17beta-es	mmu011C	Metabolic	2:9403268	898	521	802	1259	973	804	14.00506	6.810816	8.616563	12.79134	13.30651	15.54713	9.810812	13.88166	0.076251	1	-
ENSMUS(	Ext2	exostoses	GO:00325	developm	K02367	glucurony	mmu011C	Metabolic	2:9366102	458	559	707	676	564	545	2.879628	2.94602	3.062256	2.768847	3.109516	4.248673	2.962634	3.375678	0.550092	1	-
ENSMUS(	Gatm	glycine ar	GO:00325	developm	-	-	-	-	2:1225944	1608	1634	2583	2244	1616	1073	30.07452	25.61637	33.2804	27.34117	26.50311	24.88275	29.6571	26.24235	0.21664	1	-
ENSMUS(	Sema6d	sema don	GO:00325	developm	K06842	semaphor	mmu0436	Axon guic	2:1240899	933	1022	1439	1567	2014	784	3.077222	2.825405	3.269558	3.36688	5.824773	3.206113	3.057395	4.132589	0.105931	1	-
ENSMUS(	Myef2	myelin ba	GO:00081	metabolic	-	-	-	-	2:1250846	1041	1347	1577	1799	1325	719	6.128642	6.647124	6.395827	6.899638	6.840252	5.248418	6.390531	6.329436	0.768159	1	-
ENSMUS(	Slc12a1	solute car	GO:00511	localizatio	-	-	-	-	2:1251525	1	1	1	0	0	1	0.008689	0.007281	0.005982	0	0	0.010768	0.007317	0.003589	0.648685	1	-
ENSMUS(	Dut	deoxyurid	GO:00081	metabolic	K01520	dUTP pyrc	mmu011C	Metabolic	2:1252471	316	408	510	452	399	372	6.842509	7.405281	7.607635	6.37599	7.576056	9.987515	7.285141	7.979854	0.767337	1	-
ENSMUS(	Fbn1	fibrillin 1 [	GO:00325	developm	-	-	-	-	2:1253005	212	244	378	377	242	253	0.916985	0.884645	1.126338	1.062304	0.917874	1.356853	0.975989	1.112344	0.577055	1	-
ENSMUS(	Cops2	COP9 sigr	GO:00325	developm	-	-	-	-	2:1258303	1565	1985	2264	2152	1813	938	17.93373	19.06644	17.87245	16.06498	18.21783	13.32739	18.29087	15.87007	0.194766	1	-
ENSMUS(	Galk2	galactokir	GO:00081	metabolic	-	-	-	-	2:1258591	216	293	337	341	275	257	1.637146	1.861459	1.759604	1.683716	1.827715	2.415198	1.752736	1.975543	0.619815	1	-
ENSMUS(	Fgf7	fibroblast	GO:00325	developm	K04358	fibroblast	mmu052C	Pathways	2:1260346	19	18	27	28	16	9	0.218949	0.173868	0.21434	0.210202	0.161677	0.128589	0.202386	0.166823	0.502233	1	-
ENSMUS(	Fam227b	family witl	-	-	-	-	-	-	2:1259834	0	0	0	2	0	0	0	0	0	0.026068	0	0	0	0.008689	0.538279	1	-
ENSMUS(	Meis2	Meis hom	GO:00325	developm	-	-	-	-	2:1158630	959	1233	1644	1326	1217	594	3.322286	3.580423	3.923482	2.992566	3.69702	2.55147	3.60873	3.080352	0.181289	1	-
ENSMUS(	Cd82	CD82 anti	GO:00508	response	K06509	CD82 anti	mmu0411	p53 signa	2:9341911	249	244	446	382	236	283	3.12186	2.564231	3.852125	3.120027	2.594589	4.399324	3.179405	3.371313	0.962244	1	-
ENSMUS(	Tspan18	tetraspani	GO:00508	response	-	-	-	-	2:9320176	728	833	925	1073	804	694	5.400556	5.179708	4.727162	5.185478	5.230052	6.383415	5.102475	5.599648	0.696075	1	-
ENSMUS(	Slc28a2	solute car	GO:00511	localizatio	-	-	-	-	2:1224264	0	2	3	5	0	0	0	0.013998	0.017256	0.0272	0	0	0.010418	0.009067	1	1	-
ENSMUS(	Syt13	synaptota	GO:00452	synapse|c	-	-	-	-	2:9291509	3506	4356	5148	5240	3887	3968	59.24404	61.69834	59.92703	57.68272	57.5957	83.13624	60.2898	66.13822	0.708614	1	-
ENSMUS(	Chst1	carbohydi	GO:00081	metabolic	K01022	keratan su	mmu0053	Glycosam	2:9259970	4311	5094	5949	6635	4783	4930	102.2302	101.2542	97.18444	102.5	99.45907	144.9553	100.223	115.6381	0.432461	1	-
ENSMUS(	Pex16	peroxison	GO:00511	localizatio	K13335	peroxin-1	mmu0414	Peroxisor	2:9237467	183	225	264	290	210	271	4.691497	4.834988	4.66246	4.843284	4.720865	8.614192	4.729649	6.059447	0.2541	1	-
ENSMUS(	Mapk8ip1	mitogen-	GO:00081	metabolic	K04434	mitogen-	mmu0401	MAPK sigr	2:9238367	5202	5856	6769	7327	6030	4851	54.49196	51.4181	48.84702	49.99999	55.38882	63.00563	51.58569	56.13148	0.719012	1	-
ENSMUS(	Duoxa1	dual oxid	GO:00325	developm	-	-	-	-	2:1223021	0	0	0	0	3	0	0	0	0	0	0.025668	0	0	0.008556	0.304699	1	-
ENSMUS(	Sord	sorbitol d	GO:00081	metabolic	K00008	L-iditol 2-	mmu011C	Metabolic	2:1222347	213	249	354	342	270	243	5.755428	5.639637	6.589517	6.020135	6.397436	8.141244	5.994861	6.852938	0.548059	1	-
ENSMUS(	Creb3l1	cAMP resp	GO:00081	metabolic	K09048	cyclic AMI	mmu0415	PI3K-Akt :	2:9198232	95	100	130	123	111	60	2.319451	2.046519	2.186533	1.956361	2.376435	1.816345	2.184168	2.049714	0.597334	1	-
ENSMUS(	Patl2	protein as	GO:00451	translatior	-	-	-	-	2:1221201	0	0	1	0	3	0	0	0	0.017981	0	0.068683	0	0.005994	0.022894	0.668036	1	-
ENSMUS(	Eif3j1	eukaryotic	GO:00081	metabolic	K03245	translatior	mmu0301	RNA trans	2:1220285	850	1038	1297	1227	856	441	8.992852	9.205107	9.453007	8.456775	7.94136	5.784997	9.216989	7.394377	0.087889	1	-
ENSMUS(	Frmd5	FERM dor	GO:00400	locomotic	-	-	-	-	2:1215455	465	596	761	762	527	460	2.108159	2.264903	2.376765	2.250541	2.095093	2.585795	2.249942	2.310476	0.909648	1	-
ENSMUS(	Mdk	midkine [	GO:00325	developm	-	-	-	-	2:9192980	122	164	148	216	147	132	4.236852	4.773997	3.540774	4.886754	4.476561	5.683871	4.183874	5.015729	0.405438	1	-
ENSMUS(	Wdr76	WD repea	GO:00508	response	-	-	-	-	2:1215067	43	41	82	80	84	49	0.284843	0.227653	0.374198	0.345228	0.487929	0.402453	0.295565	0.41187	0.167676	1	-
ENSMUS(	Harbi1	harbinger	GO:00081	metabolic	-	-	-	-	2:9171085	60	69	103	76	63	54	0.446453	0.430356	0.527978	0.368403	0.411066	0.498208	0.468262	0.425893	0.436616	1	-
ENSMUS(	Atg13	autophag	GO:00081	metabolic	K08331	autophag	mmu0414	Autophag	2:9167461	1012	1239	1563	1658	1241	1233	8.868677	9.101278	9.436015	9.465523	9.53658	13.39761	9.135323	10.7999	0.336502	1	-
ENSMUS(	Hypk	huntingtir	GO:00329	macromo	-	-	-	-	2:1214532	249	357	394	379	231	303	2.133573	2.564064	2.325709	2.115576	1.735655	3.219112	2.341115	2.356781	0.781288	1	-
ENSMUS(	Ell3	elongatior	GO:00325	developm	-	-	-	-	2:1214390	0	0	0	0	0	2	0	0	0	0	0	0.04849	0	0.016163	0.536046	1	-
ENSMUS(	Arhgap1	Rho GTPa	GO:00511	localizatio	-	-	-	-	2:9164986	845	984	1220	1145	922	517	6.712787	6.552304	6.676634	5.925612	6.42273	5.092401	6.647241	5.813581	0.210929	1	-
ENSMUS(	Pdia3	protein di	GO:00081	metabolic	K08056	protein di:	mmu0414	Protein pr	2:1214137	4057	5180	7073	6208	4888	2610	65.27457	69.85888	78.39604	65.06873	68.96249	52.06736	71.1765	62.03286	0.200351	1	-
ENSMUS(	F2	coagulatic	GO:00325	developm	K01313	coagulatic	mmu0408	Neuroacti	2:9162532	0	0	0	0	0	2	0	0	0	0	0	0.028569	0	0.009523	0.536093	1	-
ENSMUS(	Lrp4	low densit	GO:00325	developm	-	-	-	-	2:9145751	960	1105	1398	1646	1207	967	4.490718	4.332711	4.505086	5.015982	4.951018	5.60863	4.442838	5.191877	0.332893	1	-
ENSMUS(	Map1a	microtubu	GO:00452	synapse|s	-	-	-	-	2:1212896	34877	37020	43904	55207	39756	25827	157.5925	140.2122	136.6633	162.507	157.5222	144.6959	144.8226	154.9084	0.7894	1	-
ENSMUS(	Arfgap2	ADP-ribo	GO:00511	localizatio	K12493	ADP-ribo:	mmu0414	Endocytos	2:9126497	702	822	1081	1055	747	992	8.987554	8.821224	9.534139	8.799108	8.386248	15.74716	9.114305	10.9775	0.389158	1	-
ENSMUS(	Pacsin3	protein ki	GO:00081	metabolic	-	-	-	-	2:9125595	270	345	486	441	368	265	2.272753	2.434231	2.818234	2.418295	2.716312	2.765805	2.508406	2.633471	0.953185	1	-
ENSMUS(	Adal	adenosine	GO:00081	metabolic	-	-	-	-	2:1211404	214	215	306	399	251	144	1.010201	0.850717	0.995102	1.227013	1.038988	0.842834	0.952006	1.036278	0.732967	1	-
ENSMUS(	Tubgcp4	tubulin, g	GO:00051	structural	-	-	-	-	2:1211706	252	344	402	418	518	272	1.241981	1.421103	1.364873	1.342062	2.238653	1.662145	1.342652	1.74762	0.158222	1	-
ENSMUS(	Lamp5	lysosomal	GO:00452	synapse|n	-	-	-	-	2:1360522	546	601	816	866	701	472	6.642413	6.128588	6.838718	6.863291	7.478137	7.119691	6.536573	7.153707	0.68287	1	-
ENSMUS(	Ubr1	ubiquitin	GO:00081	metabolic	-	-	-	-	2:1208602	1369	1584	2063	2256	1626	1098	7.698112	7.466016	7.99156	8.264214	8.017595	7.65542	7.718563	7.979076	0.984504	1	-
ENSMUS(	Snap25	synaptosc	GO:00325	developm	K18211	synaptosc	mmu0491	Insulin sec	2:1367134	37592	48845	57248	55746	44592	27410	323.7242	352.576	339.6186	312.734	336.7281	292.6677	338.6396	314.0433	0.340442	1	-
ENSMUS(	Mkks	McKusick-	GO:00325	developm	-	-	-	-	2:1368737	212	220	241	276	252	163	3.486869	3.033021	2.730657	2.957264	3.634477	3.324088	3.083516	3.305277	0.84815	1	-
ENSMUS(	Jag1	jagged 1	GO:00325	developm	K06052	jagged[-]	mmu052C	Pathways	2:1370814	200	221	319	262	196	144	1.865365	1.727746	2.049635	1.591905	1.602998	1.665259	1.880915	1.620054	0.20759	1	-
ENSMUS(	Slx4ip	SLX4 inter	-	-	-	-	-	-	2:1368912	218	177	323	299	263	107	1.264215	0.86038	1.290384	1.129584	1.337407	0.76937	1.138326	1.078787	0.682814	1	-
ENSMUS(	Mtch2	mitochon	GO:00081	metabolic	-	-	-	-	2:9084715	1010	1302	1600	1534	1163	939	5.147848	5.562477	5.617923	5.093445	5.197885	5.934115	5.442749	5.408482	0.691734	1	-
ENSMUS(	Cdan1	congenita	GO:00081	metabolic	-	-	-	-	2:1207161	297	417	552	479	347	431	1.039272	1.223098	1.330648	1.091917	1.064741	1.869972	1.197673	1.34221	0.705498	1	-
ENSMUS(	Haus2	HAUS aug	GO:00990	supramol	-	-	-	-	2:1206093	255	332	269	378	237	295	3.905071	4.261663	2.837867	3.771044	3.182578	5.601388	3.6682	4.185003	0.640536	1	-
ENSMUS(	Lrrc57	leucine ric	GO:00055	extracellul	-	-	-	-	2:1206042	404	201	412	292	356	379	6.648212	2.772513	4.670599	3.130319	5.137081	7.73301	4.697108	5.33347	0.761378	1	-
ENSMUS(	Snap23	synaptosc	GO:00452	synapse|c	K08508	synaptosc	mmu0461	Platelet ac	2:1205676	199	270	401	342	221	179	2.377708	2.704101	3.300671	2.662034	2.315482	2.651826	2.79416	2.543114	0.383278	1	-
ENSMUS(	Zfp106	zinc finge	GO:00971	organic cy	-	-	-	-	2:1205068	7292	7789	10133	11365	8660	4671	30.64801	27.44038	29.33899	31.11764	31.91657	24.34173	29.14246	29.12531	0.803816	1	-
ENSMUS(	Vps39	VPS39 HC	GO:00081	metabolic	-	-	-	-	2:1203164	1706	1977	2455	2548	1954	1210	8.682747	8.434078	8.607578	8.448101	8.720579	7.635706	8.574801	8.268129	0.562747	1	-
ENSMUS(	Ehd4	EH-doma	GO:00081	metabolic	K12477	EH domai	mmu0414	Endocytos	2:1200891	188	252	302	287	248	168	2.672331	3.002512	2.95726	2.657632	3.091192	2.960915	2.877367	2.903246	0.84627	1	-
ENSMUS(	Itpka	inositol 1,	GO:00325	developm	K00911	1D-myo-	mmu011C	Metabolic	2:1197423	2496	3170	3657	4121	2994	3689	87.25429	92.88688	88.06811	93.84831	91.77761	159.8957	89.4031	115.1739	0.144128	1	-
ENSMUS(	Tyro3	TYRO3 pr	GO:00325	developm	-	-	-	-	2:1197977	1460	1742	2330	2239	1823	1871	10.03541	10.03652	11.0329	10.02578	10.98782	15.94564	10.36828	12.31975	0.339797	1	-
ENSMUS(	Ubox5	U box dor	GO:00081	metabolic	K10600	U-box do	mmu0412	Ubiquitin	2:1305900	226	256	311	336	271	219	2.168328	2.058772	2.05555	2.100081	2.279966	2.605228	2.094216	2.328425	0.656775	1	-
ENSMUS(	Oxt	oxytocin [	GO:00325	developm	K05243	oxytocin-	mmu0492	Oxytocin :	2:1305761	0	0	0	0	1	3	0	0	0	0	0.10377	0.440259	0	0.181343	0.096986	1	-
ENSMUS(	Ptpra	protein ty	GO:00081	metabolic	-	-	-	-	2:1304502	5183	6219	6996	7920	5739	3371	82.76262	83.23893	76.95809	82.38722	80.35851	66.74178	80.98655	76.49584	0.474231	1	-
ENSMUS(	Rtf1	RTF1, Paf	GO:00325	developm	-	-	-	-	2:1196750	2251	2693	3301	3344	2574	1293	21.95485	22.0163	22.17951	21.24726	22.01437	15.6365	22.05022	19.63271	0.286597	1	-
ENSMUS(	Ndufaf1	NADH de	GO:00056	cell|intrace	-	-	-	-	2:1196554	235	256	387	428	325	214	5.03709	4.599428	5.714436	5.976356	6.108546	5.687371	5.116985	5.924091	0.446264	1	-
ENSMUS(	Nusap1	nucleolar	GO:00511	localizatio	-	-	-	-	2:1196182	15	18	26	37	15	9	0.156768	0.157685	0.187193	0.251911	0.137465	0.116621	0.167215	0.168665	1	1	-
ENSMUS(	4930402H	RIKEN cDI	-	-	-	-	-	-	2:1307062	1876	2067	2933	2961	2208	1809	9.043192	8.351842	9.739854	9.298415	9.333202	10.81218	9.044963	9.814601	0.729256	1	-
ENSMUS(	Atrn	attractin [	GO:00325	developm	-	-	-	-	2:1309064	4631	5135	6429	7036	5039	3496	19.38189	18.01419	18.53601	19.18354	18.49304	18.14173	18.64403	18.6061	0.739782	1	-
ENSMUS(	Chac1	ChaC, cati	GO:00325	developm	-	-	-	-	2:1193512	69	84	118	114	122	127	2.678794	2.733503	3.155892	2.883201	4.153258	6.113323	2.856063	4.38326	0.059443	1	-
ENSMUS(	Dll4	delta-like	GO:00325	developm	K06051	delta[-]	mmu052C	Pathways	2:1193257	30	59	67	88	57	50	0.486628	0.802191	0.748679	0.929895	0.810749	1.005615	0.679166	0.91542	0.212333	1	-
ENSMUS(	Spint1	serine pro	GO:00325	developm	K15619	Kunitz-typ	mmu052C	Transcript	2:1192373	20	18	25	35	16	28	0.246475	0.185938	0.212238	0.280992	0.1729	0.427841	0.214884	0.293911	0.363825	1	-
ENSMUS(	Gfra4	glial cell li	GO:00048	signal trar	-	-	-	-	2:1310396	485	379	372	646	332	436	6.250899	4.094426	3.302903	5.423948	3.752162	6.967451	4.549409	5.381187	0.530803	1	-
ENSMUS(	Adam33	a disinteg	GO:00081	metabolic	-	-	-	-	2:1310505	21	14	8	19	12	20	0.113374	0.063354	0.029753	0.066821	0.056808	0.133875	0.068827	0.085835	0.688365	1	-
ENSMUS(	Siglec1	sialic acid	GO:00160	membran	K06548	sialoadhe:	mmu0451	Cell adhes	2:1310692	17	13	32	42	28	23	0.140839	0.09028	0.182635	0.226679	0.203415	0.236267	0.137918	0.22212	0.081765	1	-
ENSMUS(	Rad51	RAD51 re	GO:00081	metabolic	K04482	DNA repa	mmu052C	Pathways	2:1191127	19	30	32	22	25	26	0.114684	0.151781	0.13306	0.086508	0.132322	0.194588	0.133175	0.137806	0.958679	1	-
ENSMUS(	Rpusd2	RNA pseu	GO:00081	metabolic	-	-	-	-	2:1190347	86	125	133	152	123	102	2.019793	2.460771	2.151842	2.325597	2.533115	2.970271	2.210802	2.609661	0.467395	1	-
ENSMUS(	Knl1	kinetocho	GO:00081	metabolic	-	-	-	-	2:1190471	11	11	21	19	8	2	0.048181	0.040386	0.063364	0.054212	0.030727	0.010864	0.050644	0.031934	0.240717	1	-
ENSMUS(	1700037H	RIKEN cDI	-	-	-	-	-	-	2:1311463	287	427	396	458	342	384	4.546292	5.66964	4.321375	4.726308	4.750551	7.54211	4.845769	5.67299	0.49545	1	-
ENSMUS(	Spef1	sperm flag	GO:00400	locomotic	-	-	-	-	2:1311702	69	79	125	113	67	106	0.921839	0.884676	1.150438	0.983478	0.78491	1.755886	0.985651	1.174758	0.589202	1	-
ENSMUS(	Cdc25b	cell divisic	GO:00325	developm	K05866	M-phase	mmu0401	MAPK sigr	2:1311869	219	253	337	333	260	293	2.050096	1.985197	2.173263	2.030748	2.134257	3.400826	2.069518	2.521944	0.355858	1	-
ENSMUS(	Knstrn	kinetocho	GO:00990	supramol	-	-	-	-	2:1188140	16	40	67	53	22	13	0.13472	0.282305	0.388623	0.290712	0.162434	0.135714	0.268549	0.196286	0.27736	1	-
ENSMUS(	Ivd	isovaleryl	GO:00081	metabolic	K00253	isovaleryl-	mmu011C	Metabolic	2:1188619	606	687	1382	974	708	889	5.692996	5.40976	8.943918	5.96086	5.83236	10.35515	6.682225	7.382789	0.780027	1	-
ENSMUS(	Smox	spermine	GO:00081	metabolic	K12259	spermine	mmu0033	Arginine a	2:1314914	411	412	592	627	555	456	2.937505	2.468238	2.914807	2.919347	3.478341	4.04099	2.773517	3.479559	0.185643	1	-
ENSMUS(	Adra1d	adrenergi	GO:00048	signal trar	K04137	adrenergi	mmu0408	Neuroacti	2:1315458	680	765	809	978	748	868	22.75728	21.4598	18.65138	21.32219	21.95106	36.01776	20.95615	26.43034	0.223037	1	-
ENSMUS(	Rassf2	Ras assoc	GO:00325	developm	K09851	Ras assoc	mmu0439	Hippo sig	2:1319894	1027	1139	1862	1670	1280	989	4.033928	3.750027	5.038353	4.273224	4.408696	4.816593	4.274103	4.499504	0.927756	1	-
ENSMUS(	Slc23a2	solute car	GO:00081	metabolic	-	-	-	-	2:1320524	2219	2544	3194	3351	2549	2428	15.53814	14.93178	15.40735	15.28614	15.65143	21.08028	15.29242	17.33928	0.50288	1	-
ENSMUS(	Tmem230	transmem	GO:00452	synapse|c	-	-	-	-	2:1322394	106	141	179	146	242	179	1.242692	1.385573	1.445646	1.115043	2.487792	2.60193	1.35797	2.068255	0.070378	1	-
ENSMUS(	Pcna	proliferati	GO:00081	metabolic	K04802	proliferati	mmu0516	HTLV-I inf	2:1322491	274	327	455	445	288	291	4.961125	4.962838	5.675337	5.248933	4.572611	6.532924	5.199767	5.45149	0.993484	1	-
ENSMUS(	Fsip1	fibrous sh	GO:00056	cell|cilium	-	-	-	-	2:1182048	9	0	15	14	0	0	0.268279	0	0.308036	0.271872	0	0	0.192105	0.090624	0.463119	1	-
ENSMUS(	Gpcpd1	glyceroph	GO:00325	developm	K18695	glyceroph	mmu0523	Choline m	2:1325290	899	1130	1412	1410	1115	1125	5.445499	5.73732	5.892016	5.563889	5.922369	8.449214	5.691612	6.645157	0.408322	1	-
ENSMUS(	Rasgrp1	RAS guan	GO:00325	developm	K04350	RAS guan	mmu052C	Pathways	2:1172799	8734	6045	15575	10067	12342	8163	60.15084	34.89616	73.8938	45.16586	74.5344	69.70505	56.3136	63.1351	0.680898	1	-
ENSMUS(	Fam98b	family witl	GO:00081	metabolic	-	-	-	-	2:1172497	775	1009	1265	1342	905	426	11.24255	12.26893	12.64168	12.68227	11.51209	7.662284	12.05106	10.61888	0.3335	1	-
ENSMUS(	Chgb	chromogr	GO:00055	extracellul	-	-	-	-	2:1327812	14561	17181	21144	22953	16297	8344	337.7355	334.0311	337.8502	346.822	331.4636	239.9638	336.5389	306.0832	0.357511	1	-
ENSMUS(	Spred1	sprouty p	GO:00325	developm	-	-	-	-	2:1171213	2853	3397	4785	4400	3304	2050	25.67498	25.62459	29.66482	25.79542	26.073	22.87433	26.98813	24.91425	0.352738	1	-
ENSMUS(	Mcm8	minichron	GO:00081	metabolic	-	-	-	-	2:1328161	60	76	89	113	80	74	0.85401	0.906741	0.872685	1.047795	0.998497	1.305975	0.877812	1.117422	0.265828	1	-
ENSMUS(	Tmco5	transmem	GO:00160	membran	-	-	-	-	2:1168786	8	20	28	15	18	16	0.329277	0.690037	0.793971	0.40222	0.649674	0.816571	0.604428	0.622821	0.948602	1	-
ENSMUS(	Fermt1	fermitin fa	GO:00325	developm	-	-	-	-	2:1329043	14	22	18	17	19	18	0.130384	0.17174	0.115485	0.103139	0.155161	0.207859	0.139203	0.155386	0.893211	1	-
ENSMUS(	Crls1	cardiolipir	GO:00081	metabolic	K08744	cardiolipir	mmu011C	Metabolic	2:1328466	617	808	400	1031	723	340	10.27843	11.28252	4.590422	11.18875	10.56143	7.022748	8.717124	9.590976	0.759202	1	-
ENSMUS(	Bmp2	bone mor	GO:00325	developm	K04662	bone mor	mmu052C	Pathways	2:1335521	54	60	70	66	57	52	0.965368	0.899087	0.862071	0.768644	0.893535	1.152617	0.908842	0.938265	0.951929	1	-

ENSMUSG	Slc27a2	solute car	GO:00081	metabolic	K08746	solute car	mmu0493	Insulin res	2:1265524	88	118	130	135	90	85	1.314072	1.476967	1.337299	1.313253	1.178475	1.573755	1.376113	1.355161	0.731419	1	-
ENSMUSG	Hdc	histidine c	GO:00081	metabolic	K01590	histidine c	mmu011C	Metabolic	2:1265936	29	21	37	38	26	20	0.245641	0.149098	0.215901	0.209685	0.193116	0.210047	0.203547	0.204283	0.959194	1	-
ENSMUSG	Gabpb1	GA repeat	GO:00081	metabolic	-	-	-	-	2:1266274	256	403	496	245	367	200	1.302502	1.718686	1.73849	0.812058	1.637373	1.261694	1.586559	1.237042	0.10799	1	-
ENSMUSG	Usp8	ubiquitin s	GO:00081	metabolic	K11839	ubiquitin c	mmu0414	Endocytos	2:1267073	1672	1867	2412	2629	2018	873	15.73999	14.73212	15.64216	16.12279	16.65834	10.18986	15.37142	14.32367	0.52799	1	-
ENSMUSG	Trpm7	transient r	GO:00081	metabolic	K04982	transient r	mmu0421	Cellular se	2:1267915	1066	1347	1598	1719	1260	642	3.960917	4.195259	4.090409	4.160982	4.105364	2.957734	4.082195	3.74136	0.411825	1	-
ENSMUSG	Sppl2a	signal pep	GO:00081	metabolic	-	-	-	-	2:1268903	749	951	1189	1317	976	684	5.206869	5.541515	5.694141	5.964337	5.949593	5.895721	5.480842	5.93655	0.729059	1	-
ENSMUSG	Stard7	START do	GO:00511	localizatio	-	-	-	-	2:1272702	1184	1401	1104	1491	1283	916	9.570945	9.492794	6.147856	7.851676	9.094368	9.18088	8.403865	8.708975	0.970181	1	-
ENSMUSG	Dusp2	dual speci	GO:00325	developm	K04459	dual speci	mmu0401	MAPK sigı	2:1273361	0	5	2	9	3	6	0	0.112238	0.036904	0.15702	0.070448	0.199257	0.049714	0.142242	0.106424	1	-
ENSMUSG	Fahd2a	fumarylac	GO:00081	metabolic	-	-	-	-	2:1274362	101	109	136	153	128	146	2.047484	1.852154	1.899273	2.020562	2.27537	3.669765	1.932971	2.655233	0.148004	1	-
ENSMUSG	Mrps5	mitochon	GO:00081	metabolic	K02988	small subu	mmu0301	Ribosome	2:1275872	628	818	912	899	758	544	4.638148	5.063972	4.640144	4.325401	4.909048	4.981616	4.780755	4.738689	0.702065	1	-
ENSMUSG	Mal	myelin an	GO:00325	developm	-	-	-	-	2:1276332	4190	3366	7068	5334	4465	3062	84.85886	57.1413	98.61231	70.3749	79.29528	76.89078	80.20416	75.52032	0.488079	1	-
ENSMUSG	Prom2	prominin	GO:00081	metabolic	-	-	-	-	2:1275264	5	0	13	0	0	5	0.041045	0	0.073516	0	0	0.050888	0.038187	0.016963	0.28082	1	-
ENSMUSG	Mall	mal, T cell	GO:00325	developm	-	-	-	-	2:1277043	7	24	27	22	14	16	0.221785	0.637333	0.589274	0.454059	0.388932	0.628507	0.482797	0.4905	0.949659	1	-
ENSMUSG	Nphp1	nephrono	GO:00099	cellular pr	-	-	-	-	2:1277407	288	363	473	417	308	270	4.631399	4.893034	5.240006	4.368546	4.343221	5.383554	4.92148	4.69844	0.515325	1	-
ENSMUSG	Bub1	BUB1, mit	GO:00081	metabolic	K02178	checkpoir	mmu0411	Cell cycle	2:1278011	2	12	8	10	6	0	0.02443	0.122842	0.067304	0.079555	0.064248	0	0.071525	0.047934	0.50821	1	-
ENSMUSG	Bcl2l11	BCL2-like	GO:00325	developm	K16341	Bcl-2-like	mmu052C	Pathways	2:1281260	75	78	104	54	67	83	0.307018	0.267641	0.293284	0.144006	0.240503	0.421278	0.289314	0.268596	0.552059	1	-
ENSMUSG	Ndufaf5	NADH de	GO:00081	metabolic	-	-	-	-	2:1401706	233	341	437	371	266	317	6.880966	8.441132	8.890504	7.137554	6.888391	11.60752	8.070867	8.544488	0.980536	1	-
ENSMUSG	Fbln7	fibulin 7 [S	GO:00055	extracellul	-	-	-	-	2:1288629	17	29	55	20	12	20	0.214275	0.306393	0.477583	0.16423	0.132636	0.312571	0.33275	0.203146	0.061407	1	-
ENSMUSG	Zc3h8	zinc finge	GO:00325	developm	-	-	-	-	2:1289262	102	126	124	152	108	61	3.991644	4.133076	3.342901	3.87504	3.706087	2.959806	3.82254	3.513645	0.537271	1	-
ENSMUSG	Ttl	tubulin tyr	GO:00325	developm	-	-	-	-	2:1290659	1142	1247	1592	1776	1252	889	11.24885	10.29583	10.8028	11.39637	10.81406	10.8575	10.78249	11.02264	0.904811	1	-
ENSMUSG	Polr1b	polymeras	GO:00325	developm	K03002	DNA-dire	mmu011C	Metabolic	2:1291009	251	261	313	343	211	204	2.392288	2.08513	2.055113	2.129687	1.76346	2.410771	2.17751	2.101306	0.602143	1	-
ENSMUSG	Slc20a1	solute car	GO:00048	signal trar	-	-	-	-	2:1291987	1323	1613	2039	2336	1764	1476	8.417323	8.602036	8.936813	9.682077	9.841364	11.64357	8.652058	10.38901	0.228212	1	-
ENSMUSG	Il1b	interleukir	GO:00325	developm	-	-	mmu0401	MAPK sigı	2:1293645	0	4	2	2	0	4	0	0.090592	0.037233	0.035201	0	0.134012	0.042609	0.056404	1	1	-
ENSMUSG	Il1a	interleukir	GO:00325	developm	K04383	interleukir	mmu0401	MAPK sigı	2:1292996	9	8	22	11	13	6	0.289752	0.215879	0.487929	0.2307	0.367008	0.239533	0.331187	0.27908	0.590631	1	-
ENSMUSG	Pdyn	prodynorp	GO:00508	response	K15840	proenkep	mmu0503	Alcoholisr	2:1296865	153	200	237	306	193	217	3.045291	3.336729	3.249657	3.967718	3.36851	5.3553	3.210559	4.230509	0.147103	1	-
ENSMUSG	Tgm3	transgluta	GO:00325	developm	-	-	-	-	2:1300123	2	16	10	12	3	8	0.030745	0.206129	0.105882	0.120154	0.040429	0.152453	0.114252	0.104345	0.856235	1	-
ENSMUSG	Snrpb	small nucl	GO:00081	metabolic	K11086	small nucl	mmu0532	Systemic l	2:1301714	586	607	760	715	622	872	11.81537	10.25868	10.55638	9.391563	10.99722	21.79978	10.87681	14.06285	0.265194	1	-
ENSMUSG	Nop56	NOP56 rit	GO:00081	metabolic	K14564	nucleolar	mmu0300	Ribosome	2:1302744	747	1016	1048	1197	848	578	3.110406	3.546034	3.006139	3.246923	3.096239	2.984075	3.22086	3.109079	0.585877	1	-
ENSMUSG	Idh3b	isocitrate	GO:00081	metabolic	K00030	isocitrate	mmu011C	Metabolic	2:1302793	2983	3709	3991	4556	3303	2813	41.38368	43.13061	38.14249	41.17572	40.18158	48.38732	40.88559	43.24821	0.904641	1	-
ENSMUSG	Cpxm1	carboxype	GO:00038	catalytic a	-	-	-	-	2:1303907	81	100	126	133	110	113	1.751545	1.812551	1.87698	1.873567	2.085789	3.029697	1.813692	2.329684	0.260345	1	-
ENSMUSG	Vps16	VSP16 CC	GO:00081	metabolic	-	-	-	-	2:1304243	838	930	1177	1270	873	788	9.083642	8.449913	8.789092	8.968121	8.297991	10.59079	8.774216	9.285633	0.913587	1	-
ENSMUSG	Lpin3	lipin 3 [So	GO:00081	metabolic	K15728	phosphati	mmu011C	Metabolic	2:1608806	13	27	19	35	25	19	0.153936	0.267989	0.154991	0.269998	0.259591	0.278965	0.192305	0.269518	0.265795	1	-
ENSMUSG	Pcsk2	proprotei	GO:00325	developm	-	-	-	-	2:1435461	4777	5654	6995	7437	5654	4148	36.10331	35.81793	36.41925	36.61602	37.47056	38.87017	36.11349	37.65225	0.980268	1	-
ENSMUSG	Bfsp1	beaded fil	GO:00325	developm	-	-	-	-	2:1438265	12	13	11	19	9	8	0.266087	0.24164	0.168034	0.274464	0.175009	0.219956	0.225254	0.223143	1	1	-
ENSMUSG	Rrbp1	ribosome	GO:00325	developm	K14000	ribosome-	mmu0414	Protein pr	2:1439473	1286	1280	1694	1844	1327	816	8.647677	7.214747	7.847353	8.077947	7.824777	6.80353	7.903259	7.568751	0.556477	1	-
ENSMUSG	Snx5	sorting ne	GO:00511	localizatio	K17920	sorting ne	mmu0414	Endocytos	2:1442501	919	1110	1557	1381	1022	896	10.62685	10.75883	12.40308	10.40315	10.36294	12.84645	11.26292	11.20418	0.696387	1	-
ENSMUSG	Mgme1	mitochon	GO:00081	metabolic	-	-	-	-	2:1442706	131	158	214	182	147	106	1.79777	1.817492	2.023143	1.627104	1.768971	1.80366	1.879468	1.733245	0.445812	1	-
ENSMUSG	Kat14	lysine ace	GO:00081	metabolic	-	-	-	-	2:1443689	441	471	560	573	455	345	3.315224	2.967892	2.900103	2.806144	2.999358	3.215727	3.061073	3.007077	0.665026	1	-
ENSMUSG	Polr3f	polymeras	GO:00081	metabolic	K03025	DNA-dire	mmu011C	Metabolic	2:1445277	560	686	806	820	620	362	3.683091	3.781823	3.651831	3.513335	3.575678	2.952015	3.705582	3.347009	0.324791	1	-
ENSMUSG	Rbbp9	retinoblas	GO:00038	catalytic a	-	-	-	-	2:1445422	499	584	733	717	512	378	11.91319	11.68675	12.05545	11.15139	10.71867	11.18937	11.88513	11.01981	0.382954	1	-
ENSMUSG	Sec23b	SEC23 ho	GO:00511	localizatio	K14006	protein tra	mmu0414	Protein pr	2:1445562	571	668	820	875	714	553	4.810917	4.717603	4.759454	4.802657	5.275124	5.777008	4.762658	5.28493	0.620838	1	-
ENSMUSG	Dtd1	D-tyrosyl-	GO:00081	metabolic	-	-	-	-	2:1445998	869	1021	1082	1315	952	658	18.17294	17.89716	15.58777	17.91483	17.45761	17.06152	17.21929	17.47799	0.865229	1	-
ENSMUSG	Xrn2	5'-3' exor	GO:00325	developm	K12619	5'-3' exor	mmu0301	RNA degr	2:1470129	1088	1267	1559	1785	1179	666	15.22027	14.85672	15.02419	16.26722	14.46272	11.55191	15.03372	14.09395	0.495463	1	-
ENSMUSG	Nkx2-2	NK2 hom	GO:00325	developm	K08029	homeobo	mmu0495	Maturity c	2:1471775	86	72	97	113	96	71	1.404667	0.985729	1.091439	1.202365	1.374952	1.437872	1.160612	1.338396	0.58228	1	-
ENSMUSG	Cd93	CD93 anti	GO:00055	extracellul	-	-	-	-	2:1484366	197	226	339	290	249	188	1.875088	1.803086	2.222826	1.798188	2.078246	2.218696	1.967	2.03171	0.955222	1	-
ENSMUSG	Napb	N-ethylm	GO:00452	synapse|c	-	-	-	-	2:1486939	6512	8448	13469	9826	7196	7699	54.69238	59.4729	77.92901	53.76143	52.99638	80.17384	64.03143	62.31055	0.581266	1	-
ENSMUSG	Gzf1	GDNF-inc	GO:00325	developm	-	-	-	-	2:1486810	824	1050	1206	1274	856	542	9.398359	10.03846	9.475969	9.46621	8.561333	7.664961	9.637596	8.564168	0.254475	1	-
ENSMUSG	9230104L	RIKEN cD	GO:00081	metabolic	-	-	-	-	2:1488467	0	0	0	0	3	0	0	0	0	0	0.151288	0	0	0.050429	0.304448	1	-
ENSMUSG	Cst3	cystatin C	GO:00325	developm	K13899	cystatin-C	mmu0497	Salivary se	2:1488717	8375	10566	14036	11333	10905	8830	267.0629	282.418	308.3355	235.4265	304.9279	349.1206	285.9388	296.4917	0.947178	1	-
ENSMUSG	Acss1	acyl-CoA	GO:00081	metabolic	K01895	acetyl-Co	mmu011C	Metabolic	2:1506181	498	569	746	773	640	520	6.395114	6.124683	6.599466	6.466656	7.206784	8.279577	6.373088	7.317672	0.45962	1	-
ENSMUSG	Gins1	GINS com	GO:00325	developm	-	-	-	-	2:1509054	7	6	9	15	14	8	0.239322	0.171925	0.211949	0.334065	0.419687	0.339103	0.207732	0.364285	0.106018	1	-
ENSMUSG	Nsfl1c	NSFL1 (p9	GO:00081	metabolic	K14012	UBX doma	mmu0414	Protein pr	2:1514941	1016	1243	1513	1725	1254	629	9.213734	9.448565	9.452186	10.1909	9.971996	7.07259	9.371495	9.078496	0.673768	1	-
ENSMUSG	Sdcbp2	syndecan	GO:00055	extracellul	-	-	-	-	2:1515726	13	13	6	18	11	5	0.590545	0.495025	0.187759	0.532678	0.438188	0.281611	0.424443	0.417492	1	1	-
ENSMUSG	Snph	syntaphilir	GO:00325	developm	-	-	-	-	2:1515905	3159	4183	9331	6043	4825	3170	11.77363	13.06777	23.95744	14.67219	15.76888	14.64893	16.26628	15.03	0.495458	1	-
ENSMUSG	Fam110a	family witl	GO:00056	cell|intrace	-	-	-	-	2:1519693	71	90	87	116	103	81	1.316294	1.398586	1.111134	1.40099	1.674464	1.861933	1.275338	1.645796	0.233604	1	-
ENSMUSG	Angpt4	angiopoie	GO:00325	developm	K05467	angiopoie	mmu0415	PI3K-Akt s	2:1519112	2	2	0	2	0	0	0.054446	0.045626	0	0.035457	0	0	0.033357	0.011819	0.719047	1	-
ENSMUSG	Tbc1d20	TBC1 dom	GO:00325	developm	-	-	-	-	2:1522938	879	1022	1345	1441	1046	967	10.36998	10.10632	10.93108	11.07477	10.82091	14.14495	10.46913	12.01354	0.443668	1	-
ENSMUSG	Rbck1	RanBP-typ	GO:00081	metabolic	K10630	RanBP-typ	mmu0421	Necroptos	2:1523163	961	1231	1493	1643	1197	1051	9.559315	10.26394	10.23092	10.64688	10.44095	12.96259	10.01806	11.35014	0.495354	1	-
ENSMUSG	Tpx2	TPX2, mic	GO:00081	metabolic	-	-	-	-	2:1528479	52	54	80	79	52	37	0.456836	0.397656	0.48417	0.452132	0.400594	0.403034	0.44622	0.418586	0.640846	1	-
ENSMUSG	Mylk2	myosin, lig	GO:00325	developm	K00907	myosin-lig	mmu0481	Regulatior	2:1529113	0	0	0	1	1	0	0	0	0	0.014028	0.018883	0	0	0.01097	0.527838	1	-
ENSMUSG	Pdrg1	p53 and D	GO:00329	macromo	-	-	-	-	2:1530088	178	251	308	317	230	289	2.762504	3.265192	3.292949	3.204963	3.130054	5.561174	3.106882	3.965397	0.255465	1	-
ENSMUSG	Ccm2l	cerebral c	GO:00325	developm	-	-	-	-	2:1530659	60	64	80	97	58	40	1.25474	1.121858	1.152512	1.32148	1.063587	1.037172	1.17637	1.140746	0.760932	1	-
ENSMUSG	Kif3b	kinesin fa	GO:00511	localizatio	-	-	-	-	2:1532914	1660	1919	2464	2425	1897	1466	18.72191	18.14137	19.14407	17.81704	18.76083	20.50039	18.66912	19.02609	0.852719	1	-
ENSMUSG	Dnmt3b	DNA metl	GO:00325	developm	K17399	DNA (cytc	mmu011C	Metabolic	2:1536494	4	17	17	24	15	5	0.028264	0.100691	0.082751	0.110478	0.092942	0.043804	0.070568	0.082408	0.772401	1	-
ENSMUSG	Mapre1	microtubu	GO:00511	localizatio	-	-	-	-	2:1537412	1473	1831	2157	2147	1548	1116	11.53302	12.01661	11.63435	10.95101	10.62807	10.83405	11.72799	10.80438	0.311491	1	-
ENSMUSG	Bpifb1	BPI fold c	GO:00023	immune s	-	-	-	-	2:1541908	0	5	0	3	0	0	0	0.063415	0	0.029569	0	0	0.021138	0.009856	0.575303	1	-
ENSMUSG	Cdk5rap1	CDK5 regu	GO:00081	metabolic	-	-	-	-	2:1543353	116	129	134	166	122	112	1.618834	1.508986	1.28825	1.509147	1.492948	1.937967	1.472023	1.646687	0.692991	1	-
ENSMUSG	Snta1	syntrophir	GO:00081	metabolic	-	-	-	-	2:1543763	330	523	620	605	504	643	5.251002	6.975616	6.796283	6.271405	7.032358	12.68601	6.340967	8.663259	0.129018	1	-
ENSMUSG	Necab3	N-termin	GO:00081	metabolic	-	-	-	-	2:1545443	182	277	401	341	297	318	3.182024	4.059419	4.829785	3.883896	4.553354	6.89358	4.023743	5.110276	0.267617	1	-
ENSMUSG	E2f1	E2F transc	GO:00325	developm	K17454	transcripti	mmu052C	Pathways	2:1545594	103	124	173	175	136	108	1.396317	1.40904	1.615641	1.5455	1.616698	1.815338	1.473666	1.659179	0.634931	1	-
ENSMUSG	Fam210b	family witl	GO:00325	developm	-	-	-	-	2:1723455	830	957	1171	1165	902	505	14.58363	14.09459	14.17411	13.33507	13.89751	11.00183	14.28411	12.7448	0.281088	1	-
ENSMUSG	Aurka	aurora kir	GO:00325	developm	K11481	aurora kir	mmu0411	Oocyte m	2:1723561	23	33	41	43	25	20	0.376826	0.453199	0.462761	0.458956	0.359171	0.406286	0.430929	0.408137	0.724738	1	-
ENSMUSG	Cstf1	cleavage s	GO:00081	metabolic	K14406	cleavage s	mmu0301	mRNA sur	2:1723706	410	470	569	670	486	355	7.146661	6.867042	6.832552	7.608098	7.428454	7.672438	6.948752	7.569663	0.718526	1	-
ENSMUSG	Pkia	protein ki	GO:00081	metabolic	K15985	cAMP-de	mmu0503	Alcoholisr	3:7366669	2150	3018	3563	3422	2503	1946	32.47896	38.21516	37.07924	33.67637	33.15638	36.44958	35.92445	34.42744	0.478845	1	-
ENSMUSG	Stmn2	stathmin-	GO:00325	developm	-	-	-	-	3:8509360	4266	5493	6384	6241	4775	3631	42.76295	46.154	44.08503	40.75517	41.97231	45.12934	44.33399	42.61894	0.482913	1	-
ENSMUSG	Rtfdc1	replicatior	GO:00325	developm	-	-	-	-	2:1724405	773	1371	1775	1781	1344	1077	17.06966	25.37674	27.00196	25.62069	26.02479	29.48812	23.14945	27.04453	0.40131	1	-
ENSMUSG	Tpd52	tumor prc	GO:00325	developm	-	-	-	-	3:8925593	675	868	962	963	698	613	3.21456	3.464897	3.156051	2.987622	2.914846	3.619627	3.278503	3.174032	0.549702	1	-
ENSMUSG	Pag1	phosphop	GO:00048	signal trar	-	-	-	-	3:9687479	606	653	1002	935	747	497	2.775921	2.507265	3.161939	2.790151	3.000529	2.822778	2.815042	2.871153	0.900661	1	-
ENSMUSG	Rae1	ribonuclei	GO:00511	localizatio	K14298	mRNA exp	mmu0516	Influenza	2:1730001	597	734	904	890	637	456	11.023	11.35989	11.4986	10.70525	10.31352	10.43942	11.29383	10.48606	0.38292	1	-
ENSMUSG	Rbm38	RNA bind	GO:00325	developm	-	-	-	-	2:1730204	40	48	46	58	39	14	0.263982	0.265522	0.209129	0.249358	0.225693	0.114562	0.246211	0.196537	0.348325	1	-
ENSMUSG	Rab22a	RAB22A, r	GO:00511	localizatio	K07891	Ras-relate	mmu0414	Endocytos	2:1736597	715	1233	1621	1708	1290	904	3.239725	4.682936	5.059838	5.041639	5.125481	5.078746	4.3275	5.081955	0.367633	1	-
ENSMUSG	Zdbf2	zinc finge	-	-	-	-	-	-	1:6327326	235	724	1108	988	595	438	0.59604	1.539213	1.935968	1.632472	1.323325	1.377422	1.357074	1.444406	0.89159	1	-
ENSMUSG	Stx16	syntaxin 1	GO:00511	localizatio	K08489	syntaxin 1	mmu0413	SNARE int	2:1740763	712	1175	1218	1370	595	727	6.00687	8.309192	7.078919	7.529572	4.401773	7.604814	7.13166	6.512053	0.397005	1	-
ENSMUSG	Gnas	GNAS (gu	GO:00325	developm	K04632	guanine n	mmu052C	Pathways	2:1742843	10152	12616	13738	13607	13060	11551	41.29481	43.01485	38.49629	36.05686	46.58324	58.25718	40.93532	46.96576	0.471054	1	-
ENSMUSG	Edn3	endothelir	GO:00325	developm	-	-	-	-	2:1747606	79	130	152	120	117	100	0.791528	1.091784	1.049151	0.78326	1.027946	1.2423	0.977488	1.017835	0.960751	1	-
ENSMUSG	Phactr3	phosphata	GO:00987	molecular	-	-	-	-	2:1781189	3082	3328	4147	4471	3490	1911	22.60216	20.45755	20.95091	21.36012	22.44324	17.37657	21.33687	20.39331	0.549391	1	-
ENSMUSG	Impa1	inositol (m	GO:00081	metabolic	K01092	myo-inos	mmu011C	Metabolic	3:1031195	580	736	981	868	669	591	4.731192	5.03238	5.512686	4.612582	4.785329	5.977459	5.092086	5.125123	0.772365	1	-
ENSMUSG	Fabp5	fatty acid	GO:00081	metabolic	K08754	fatty acid-	mmu0332	PPAR sign	3:1001254	962	1167	1366	1469	1037	784	14.47052	14.71408	14.15506	14.39504	13.67826	14.62215	14.44655	14.23182	0.656132	1	-
ENSMUSG	Snx16	sorting ne	GO:00511	localizatio	-	-	-	-	3:1041781	404	558	682	672	495	305	5.894259	6.823945	6.85463	6.387033	6.332809	5.5174	6.524278	6.079081	0.458131	1	-
ENSMUSG	Chmp4c	charged r	GO:00325	developm	K12194	charged r	mmu0414	Endocytos	3:1036690	7	13	10	8	3	8	0.042981	0.066906	0.042297	0.031998	0.01615	0.060902	0.050728	0.03635	0.312455	1	-
ENSMUSG	Ptpn1	protein ty	GO:00081	metabolic	K05696	tyrosine-p	mmu0491	Insulin sig	2:1679320	1000	1099	1374	1457	1012	959	10.46656	9.641732	9.907009	9.934492	9.288114	12.4454	10.0051	10.556	0.943564	1	-
ENSMUSG	Nfatc2	nuclear fa	GO:00325	developm	K17332	nuclear fa	mmu0516	HTLV-I inf	2:1684764	243	218	281	355	244	223	0.85649	0.644058	0.682297	0.815129	0.754136	0.974556	0.727615	0.84794	0.472755	1	-
ENSMUSG	Atp9a	ATPase, cl	GO:00511	localizatio	-	-	-	-	2:1686344	5548	6476	7783	8341	6379	5354	45.32032	44.3421	43.79807	44.38705	45.69322	54.22766	44.48683	48.10264	0.754001	1	-
ENSMUSG	Sall4	sal-like 4	GO:00325	developm	-	-	-	-	2:1687483	11	11	9	5	11	6	0.085853	0.071964	0.048387	0.025423	0.075282	0.058067	0.068735	0.052924	0.472875	1	-
ENSMUSG	Lrrcc1	leucine ric	GO:00056	cell|intrace	-	-	-	-	3:1453378	290	377	487	532	373	210	1.821002	1.984298	2.106652	2.176236	2.053827	1.635002	1.970651	1.955022	0.805679	1	-
ENSMUSG	Zfp64	zinc finge	GO:00325	developm	-	-	-	-	2:1688933	245	269	311	374	296	214	2.298909	2.115731	2.01033	2.286166	2.435511	2.489747	2.141656	2.403808	0.588647	1	-
ENSMUSG	E2f5	E2F transc	GO:00325	developm	K04682	transcripti	mmu0421	Cellular se	3:1457864	84	134	181	166	112	71	1.853621	2.478563	2.751519	2.386339	2.167236	1.942625	2.361234	2.1654	0.519293	1	-
ENSMUSG	Car13	carbonic a	GO:00081	metabolic	K01672	carbonic a	mmu0091	Nitrogen r	3:1464172	19	22	50	29	29	27	0.525231	0.509767	0.952181	0.522252	0.702967	0.925445	0.662393	0.716888	0.963832	1	-
ENSMUSG	Car3	carbonic a	GO:00081	metabolic	K01672	carbonic a	mmu0091	Nitrogen r	3:1486351	3	4	14	6	7	5	0.051219	0.057247	0.164676	0.066743	0.104816	0.10585	0.091047	0.09247	1	1	-
ENSMUSG	Dok5	docking p	GO:00325	developm	-	-	-	-	2:1707318	210	312	385	364	276	275	7.548717	9.400726	9.533808	8.523869	8.699727	12.25666	8.82775	9.826752	0.695423	1	-
ENSMUSG	Car2	carbonic a	GO:00325	developm	K18245	carbonic a	mmu0497	Pancreatic	3:1488627	1937	2214	3285	2806	2087	1704	40.25578	38.56818	47.03113	37.98988	38.03328	43.90907	41.9517	39.97741	0.451424	1	-
ENSMUSG	Psma7	proteasor	GO:00081	metabolic	K02731	20S prote	mmu0305	Proteason	2:1800363	649	988	1048	1104	827	714	9.632392	12.29134	10.71525	10.67432	10.76311	13.13935	10.87966	11.52559	0.910307	1	-
ENSMUSG	Ntsr1	neurotens	GO:00048	signal trar	K04211	neurotens	mmu0408	Neuroacti	2:1804999	84	97	108	108	90	92	0.86062	0.83303	0.762272	0.720842	0.808575	1.168712	0.818641	0.899376	0.830135	1	-
ENSMUSG	Mrgbp	MRG/MO	GO:00081	metabolic	-	-	-	-	2:1805813	255	305	333	267	272	223	1.942472	1.947454	1.747469	1.324974	1.816879	2.106224	1.879132	1.749359	0.439152	1	-
ENSMUSG	Col9a3	collagen, t	GO:00325	developm	K08131	collagen, t	mmu0415	PI3K-Akt s	2:1805977	320	208	564	279	248	288	3.51061	1.912709	4.262485	1.993968	2.385762	3.917512	3.228601	2.765747	0.30957	1	-
ENSMUSG	Gid8	GID comp	GO:00056	cell|cell jur	-	-	-	-	2:1807101	637	828	904	926	738	556	11.44566	12.47052	11.18976	10.83911	11.62787	12.38688	11.70198	11.61795	0.707512	1	-
ENSMUSG	Nkain4	Na+/K+ t	GO:00511	localizatio	-	-	-	-	2:1809347	156	236	342	280	215	232	5.901339	7.483266	8.912589	6.900271	7.131933	10.88178	7.432398	8.304662	0.718002	1	-
ENSMUSG	Arfgap1	ADP-ribo	GO:00452	synapse|c	K12492	ADP-ribo	mmu0414	Endocytos	2:1809672	1041	965	1352	1598	1103	893	4.347397	3.377992	3.889617	4.347474	4.039213	4.623982	3.871669	4.33689	0.563623	1	-
ENSMUSG	Chrna4	cholinergi	GO:00048	signal trar	K04806	nicotinic a	mmu0408	Neuroacti	2:1810183	363	388	662	676	513	432	3.820127	3.4226	4.799325	4.63446	4.734031	5.636911	4.014017	5.001801	0.20612	1	-
ENSMUSG	Srms	src-relate	GO:00081	metabolic	-	-	-	-	2:1812055	4	0	8	6	0	1	0.09922	0	0.136709	0.096963	0	0.030748	0.078643	0.04257	0.498242	1	-
ENSMUSG	Stmn3	stathmin-	GO:00325	developm	-	-	-	-	2:1813064	5106	6304	7430	7286	5908	5590	285.6529	295.6153	286.3506	265.5392	289.8284	387.7533	289.2062	314.3736	0.758716	1	-
ENSMUSG	Zgpat	zinc finge	GO:00081	metabolic	-	-	-	-	2:1813649	475	421	483	564	632	734	2.552435	1.896253	1.78797	1.974341	2.977976	4.890393	2.078886	3.280904	0.035065	1	-
ENSMUSG	Zbtb46	zinc finge	GO:00325	developm	-	-	-	-	2:1813877	128	165	158	170	147	137	0.765194	0.826797	0.650685	0.662053	0.770587	1.01547	0.747559	0.816036	0.830609	1	-
ENSMUSG	Oprl1	opioid rec	GO:00048	signal trar	K04216	nociceptir	mmu0408	Neuroacti	2:1817150	161	547	576	327	407	611	0.705949	2.010425	1.73989	0.934064	1.564894	3.321814	1.485421	1.940257	0.457367	1	-
ENSMUSG	Pcmtd2	protein-L	GO:00081	metabolic	-	-	-	-	2:1818378	703	1416	1806	1015	1301	1077	4.262334	7.196282	7.543295	4.009032	6.916907	8.096432	6.33397	6.34079	0.795319	1	-
ENSMUSG	Raly	hnRNP-as	GO:00081	metabolic	-	-	-	-	2:1547910	462	526	736	611	513	789	4.570591	4.361833	5.016023	3.937798	4.450313	9.67817	4.649483	6.022094	0.289822	1	-
ENSMUSG	Ahcy	S-adenos	GO:00081	metabolic	K01251	adenosylh	mmu011C	Metabolic	2:1550593	144	190	211	234	156	164	2.908978	3.217241	2.936376	3.079459	2.763408	4.107773	3.020865	3.31688	0.778042	1	-
ENSMUSG	Itch	itchy, E3 u	GO:00325	developm	K05632	atrophin-	mmu0414	Endocytos	2:1551335	1314	1596	2171	2156	1550	949	6.395701	6.51146	7.279539	6.836331	6.615568	5.727235	6.7289	6.393045	0.505819	1	-
ENSMUSG	Armc1	armadillo	GO:00511	localizatio	-	-	-	-	3:1913140	905	1263	1638	1468	1102	1063	15.65898	18.31771	19.52453	16.54714	16.72013	22.80522	17.83374	18.69083	0.993228	1	-
ENSMUSG	Mtfr1	mitochon	GO:00081	metabolic	-	-	-	-	3:1918732	71	110	165	99	88	58	0.677414	0.879713	1.084503	0.615339	0.736247	0.686142	0.880544	0.679243	0.106248	1	-
ENSMUSG	Map1lc3a	microtubu	GO:00081	metabolic	K10435	microtubu	-	-	2:1552762	790	1019	1278	1088	925	1499	19.83676	21.44723	22.10683	17.79732	20.36705	46.66933	21.13027	28.2779	0.225561	1	-
ENSMUSG	Ggt7	gamma-g	GO:00081	metabolic	K00681	gamma-g	mmu011C	Metabolic	2:1554903	1007	1288	1443	1597	1224	1782	15.84498	16.98756	15.64156	16.37001	16.88831	34.76609	16.15803	22.6748	0.087697	1	-
ENSMUSG	Acss2	acyl-CoA	GO:00081	metabolic	K01895	acetyl-Co	mmu011C	Metabolic	2:1555179	815	924	1156	1226	853	845	4.046222	3.845182	3.953678	3.965194	3.713505	5.201578	3.948361	4.293426	0.765766	1	-
ENSMUSG	Gss	glutathior	GO:00325	developm	K01920	glutathior	mmu011C	Metabolic	2:1555631	167	233	289	396	225	205	1.31712	1.540343	1.57021	2.03463	1.556087	2.004701	1.475891	1.865139	0.196159	1	-
ENSMUSG	Procr	protein C	GO:00508	response	K06557	protein C	mmu0461	Complem	2:1557511	12	16	21	31	15	14	0.202229	0.226026	0.24381	0.340348	0.221671	0.292557	0.224022	0.284858	0.471734	1	-
ENSMUSG	Mmp24	matrix me	GO:00325	developm	-	-	-	-	2:1557753	852	932	1186	1290	1018	886	10.9322	10.0239	10.48344	10.78299	11.45402	14.09573	10.47985	12.11091	0.415385	1	-
ENSMUSG	Eif6	eukaryotic	GO:00325	developm	K03264	translatior	mmu0300	Ribosome	2:1558198	269	292	391	463	298	471	4.198375	3.820006	4.203941	4.707508	4.078381	9.114539	4.074107	5.966809	0.071955	1	-
ENSMUSG	Hps3	HPS3, bio	GO:00329	macromo	-	-	-	-	3:1999594	171	210	263	298	197	202	0.637505	0.656236	0.675455	0.723747	0.644017	0.93374	0.656399	0.767168	0.473017	1	-
ENSMUSG	Nfs1	nitrogen f	GO:00081	metabolic	-	-	mmu011C	Metabolic	2:1561236	472	549	679	700	629	584	6.495672	6.332966	6.437296	6.275699	7.590591	9.965086	6.421978	7.943792	0.235144	1	-
ENSMUSG	Rbm39	RNA bind	GO:00081	metabolic	-	-	-	-	2:1561472	2423	3328	3637	3846	3046	1818	9.673278	11.1367	10.00265	10.00256	10.66333	8.999121	10.27088	9.888339	0.560314	1	-
ENSMUSG	Epb41l1	erythrocyt	GO:00051	structural	-	-	-	-	2:1564209	10147	11490	14295	16086	12229	9071	46.54774	44.18083	45.17483	48.07183	49.19201	51.59434	45.30113	49.61939	0.63995	1	-
ENSMUSG	Aar2	AAR2 splic	GO:00081	metabolic	-	-	-	-	2:1565475	509	589	742	682	447	579	4.61067	4.472131	4.630224	4.024504	3.550559	6.502964	4.571008	4.692676	0.863269	1	-
ENSMUSG	Ndrg3	N-myc dc	GO:00508	response	-	-	-	-	2:1569273	7400	8750	10882	11680	8515	6195	48.02331	47.59719	48.64974	49.37929	48.45602	49.84798	48.09008	49.22776	0.896731	1	-
ENSMUSG	Dsn1	DSN1 hor	GO:00329	macromo	-	-	-	-	2:1569952	31	46	72	60	39	39	0.360178	0.447979	0.576277	0.454133	0.397335	0.561826	0.461478	0.471098	0.944648	1	-
ENSMUSG	Sla2	Src-like-a	GO:00325	developm	-	-	-	-	2:1568724	13	12	21	16	11	17	0.164151	0.127016	0.182676	0.131616	0.121801	0.266156	0.157948	0.173191	1	1	-
ENSMUSG	1110008F	RIKEN cD	GO:00056	cell|intrace	-	-	-	-	2:1568631	279	343	512	446	332	382	4.184876	4.31245	5.290531	4.358072	4.366747	7.104391	4.595952	5.276404	0.578856	1	-
ENSMUSG	Samhd1	SAM dom	GO:00081	metabolic	-	-	-	-	2:1570975	292	356	430	481	433	339	1.6031	1.638251	1.626289	1.7203	2.08453	2.307612	1.622547	2.037481	0.178001	1	-
ENSMUSG	Rbl1	retinoblas	GO:00081	metabolic	K04681	retinoblas	mmu0516	Human pa	2:1571458	54	40	50	56	52	34	0.486792	0.302246	0.310505	0.328863	0.411049	0.38003	0.366514	0.373314	0.947751	1	-
ENSMUSG	Rpn2	ribophorir	GO:00325	developm	K12667	oligosacch	mmu011C	Metabolic	2:1572790	1445	1739	2168	2276	1747	1513	14.24447	14.36914	14.72276	14.61613	15.10129	18.49283	14.44545	16.07008	0.592494	1	-
ENSMUSG	Ghrh	growth hc	GO:00325	developm	-	-	-	-	2:1573294	0	0	0	2	2	0	0	0	0	0.096271	0.129592	0	0	0.075288	0.155941	1	-
ENSMUSG	Src	Rous sarc	GO:00325	developm	K05704	tyrosine-p	mmu0414	Endocytos	2:1574184	1402	1503	1893	2027	1642	1458	8.720033	7.835777	8.110959	8.21307	8.955422	11.24382	8.222257	9.47077	0.429704	1	-
ENSMUSG	Ctnnbl1	catenin, b	GO:00325	developm	K12864	beta-cate	mmu0304	Spliceosor	2:1577374	553	607	800	729	595	420	11.72666	10.78924	11.68668	10.07067	11.06391	11.04295	11.40086	10.72584	0.439396	1	-
ENSMUSG	Tti1	TELO2 int	GO:00508	response	K20403	TELO2-int	mmu0415	mTOR sig	2:1579818	338	418	446	523	384	281	2.67109	2.768861	2.428055	2.6925	2.661005	2.753372	2.622669	2.702292	0.95726	1	-
ENSMUSG	Rprd1b	regulatior	GO:00081	metabolic	-	-	-	-	2:1580284	174	163	188	223	123	111	1.248673	0.980482	0.92941	1.042522	0.774007	0.987661	1.052855	0.93473	0.356043	1	-
ENSMUSG	Ralgapb	Ral GTPas	GO:00987	molecular	-	-	-	-	2:1584098	2120	2568	3018	3398	2685	2142	7.706921	7.825154	7.558153	8.047297	8.559175	9.65496	7.696743	8.753811	0.444871	1	-
ENSMUSG	Fam83d	family witl	GO:00325	developm	-	-	-	-	2:1587680	27	33	31	37	23	24	0.416888	0.427103	0.329745	0.372174	0.311412	0.45947	0.391245	0.381019	0.798533	1	-
ENSMUSG	Dhx35	DEAH (As	GO:00081	metabolic	-	-	-	-	2:1587948	263	273	272	362	286	228	2.603902	2.265605	1.855194	2.334851	2.483006	2.798909	2.241567	2.538922	0.587849	1	-
ENSMUSG	Wisp2	WNT1 ind	GO:00099	cellular pr	-	-	-	-	2:1638208	2	9	11	16	5	9	0.054988	0.207359	0.208298	0.286515	0.120511	0.306735	0.156882	0.23792	0.401063	1	-
ENSMUSG	Skil	SKI-like [S	GO:00325	developm	K18499	Ski-like pr	mmu0455	Signaling	3:3109505	812	943	1030	1342	870	409	2.478748	2.412908	2.166033	2.668766	2.328832	1.548052	2.352563	2.181883	0.534289	1	-
ENSMUSG	Slc2a10	solute car	GO:00511	localizatio	-	-	-	-	2:1655037	17	19	38	31	18	31	0.275465	0.25807	0.424189	0.327244	0.255761	0.622844	0.319241	0.40195	0.59545	1	-
ENSMUSG	Zmat3	zinc finge	GO:00099	cellular pr	K10137	zinc finge	mmu0411	p53 signa	3:3233479	1594	1675	891	2408	1680	1583	5.330641	4.69525	2.052675	5.246012	4.926552	6.563839	4.026189	5.578801	0.151943	1	-
ENSMUSG	Pik3ca	phosphati	GO:00325	developm	K00922	phosphati	mmu052C	Pathways	3:3239767	955	1008	1440	1356	1002	601	4.495121	3.976961	4.669302	4.157949	4.135693	3.507505	4.380461	3.933716	0.28596	1	-
ENSMUSG	Zfp639	zinc finge	GO:00081	metabolic	-	-	-	-	3:3251025	356	274	496	373	244	237	2.301612	1.484861	2.209103	1.570988	1.383298	1.899843	1.998525	1.618043	0.108092	1	-
ENSMUSG	Mfn1	mitofusin	GO:00325	developm	K06030	mitofusin	mmu0462	NOD-like	3:3252946	915	1109	1574	1484	1077	828	7.295319	7.411527	8.645291	7.707949	7.52977	8.185391	7.784045	7.807703	0.770764	1	-
ENSMUSG	Gnb4	guanine n	GO:00325	developm	K04538	guanine n	mmu052C	Pathways	3:3258033	179	183	230	235	183	266	0.745378	0.638747	0.659787	0.63749	0.668219	1.373385	0.681304	0.893031	0.271716	1	-
ENSMUSG	Actl6a	actin-like	GO:00325	developm	K11340	actin-like	mmu0522	Hepatoce	3:3270629	151	151	211	189	135	123	1.670115	1.399909	1.607699	1.361799	1.309321	1.686793	1.559241	1.452638	0.473971	1	-
ENSMUSG	Ndufb5	NADH de	GO:00081	metabolic	K03961	NADH de	mmu011C	Metabolic	3:3273699	538	420	546	492	543	515	5.288709	3.460745	3.697532	3.150755	4.68069	6.277131	4.148995	4.702858	0.680174	1	-
ENSMUSG	Pex5l	peroxisom	GO:00511	localizatio	K13342	peroxin-5	mmu0414	Peroxisom	3:3294940	3889	3246	4149	5225	3559	3066	17.92426	12.54021	13.17341	15.68813	14.38381	17.5211	14.54596	15.86435	0.734207	1	-
ENSMUSG	Ccdc39	coiled-co	GO:00325	developm	-	-	-	-	3:3381236	459	560	680	713	508	261	3.376646	3.453136	3.446139	3.41699	3.277022	2.380673	3.425307	3.024895	0.314826	1	-
ENSMUSG	Ttc14	tetratricop	GO:00971	organic cy	-	-	-	-	3:3379983	1564	2338	2783	3195	2326	1367	4.477942	5.610986	5.489167	5.959287	5.83975	4.852844	5.192699	5.550627	0.792947	1	-
ENSMUSG	Ncoa3	nuclear re	GO:00325	developm	K11256	nuclear re	mmu052C	Pathways	2:1659926	853	941	1423	1392	999	722	5.309049	4.909195	6.101324	5.644022	5.452253	5.571745	5.439856	5.556007	0.90075	1	-
ENSMUSG	Dnajc19	DnaJ heat	GO:00325	developm	-	-	-	-	3:3405602	148	242	301	284	240	152	1.894812	2.597008	2.654743	2.368671	2.694373	2.412866	2.382188	2.49197	0.961875	1	-
ENSMUSG	Fxr1	fragile X r	GO:00325	developm	K15516	fragile X n	mmu0301	RNA trans	3:3401994	1498	1899	2480	2313	1688	839	10.39441	11.045	11.85472	10.45551	10.27077	7.218317	11.09804	9.314867	0.154247	1	-
ENSMUSG	Mecom	MDS1 and	GO:00325	developm	K04462	ecotropic	mmu052C	Pathways	3:2995129	35	52	61	85	47	29	0.099627	0.124067	0.119615	0.157618	0.117314	0.102349	0.114437	0.125761	0.772454	1	-
ENSMUSG	Slc2a2	solute car	GO:00325	developm	K07593	MFS trans	mmu0493	Insulin res	3:2869790	0	0	0	0	2	0	0	0	0	0	0.015635	0	0	0.005212	0.534369	1	-

ENSMUSC	Tnik	TRAF2 an	GO:00325	developm	-	-	-	-	3:2826321	4627	4225	6586	5259	5326	2477	11.64916	8.916095	11.42269	8.625415	11.75815	7.732272	10.66265	9.371945	0.270383	1	-
ENSMUSC	Pld1	phospholi	GO:00081	metabolic	K01115	phospholi	mmu011C	Metabolic	3:2793869	168	159	202	223	137	123	0.596509	0.473215	0.494094	0.515815	0.426549	0.5415	0.521273	0.494622	0.571527	1	-
ENSMUSC	Nceh1	neutral ch	GO:00081	metabolic	K14349	neutral ch	mmu0497	Bile secret	3:2718296	2217	2651	3323	3336	2628	1972	24.96404	25.02141	25.77691	24.47129	25.94879	27.53227	25.25412	25.98412	0.920691	1	-
ENSMUSC	Ect2	ect2 onco	GO:00325	developm	-	-	-	-	3:2709722	14	13	31	8	23	13	0.087255	0.067916	0.133101	0.032481	0.125701	0.100458	0.096091	0.086213	0.691495	1	-
ENSMUSC	Sec62	SEC62 ho	GO:00511	localizatio	K12275	translocat	mmu0414	Protein pr	3:3079287	2727	3413	4494	4316	3069	1739	34.28468	35.96699	38.92238	35.34908	33.83409	27.10818	36.39135	32.09711	0.230038	1	-
ENSMUSC	Dcun1d1	DCN1, de	GO:00081	metabolic	-	-	-	-	3:358921C	714	1027	1214	1087	795	439	3.439723	4.14714	4.028983	3.411433	3.35842	2.622258	3.871949	3.130704	0.084936	1	-
ENSMUSC	Mccc1	methylcro	GO:00081	metabolic	K01968	3-methylc	mmu011C	Metabolic	3:3595931	321	374	521	563	407	336	1.533413	1.49754	1.714522	1.75204	1.704869	1.990122	1.581825	1.815677	0.470521	1	-
ENSMUSC	Acad9	acyl-Coer	GO:00081	metabolic	-	-	-	-	3:3606597	475	639	717	718	561	511	2.643633	2.981	2.749024	2.60324	2.737876	3.526268	2.791219	2.955795	0.93161	1	-
ENSMUSC	Anxa5	annexin A	GO:00508	response	-	-	-	-	3:3644892	608	720	1082	1031	631	562	16.94005	16.81496	20.76775	18.71333	15.4164	19.41479	18.17425	17.84817	0.663395	1	-
ENSMUSC	1810062G	RIKEN cDI	-	-	-	-	-	-	3:3647593	0	0	0	0	0	5	0	0	0	0	0	0.432463	0	0.144154	0.093197	1	-
ENSMUSC	Exosc9	exosome	GO:00081	metabolic	K03678	exosome	mmu0301	RNA degr	3:365526C	420	490	650	656	465	307	3.982731	3.894756	4.246162	4.052446	3.86658	3.609572	4.041216	3.842866	0.534101	1	-
ENSMUSC	Ccna2	cyclin A2 |	GO:00325	developm	K06627	cyclin A[-]	mmu0516	Human pa	3:3656486	32	39	63	54	45	30	0.324092	0.331086	0.439557	0.356285	0.399644	0.376736	0.364912	0.377555	1	1	-
ENSMUSC	Trpc3	transient r	GO:00511	localizatio	K04966	transient r	mmu0436	Axon guic	3:3662048	66	88	129	127	100	73	0.610019	0.681765	0.821376	0.764688	0.810484	0.836579	0.704387	0.803917	0.605625	1	-
ENSMUSC	Il21	interleukir	GO:00325	developm	K05434	interleukir	mmu0406	Cytokine-	3:3722275	0	0	0	3	0	0	0	0	0	0.021621	0	0	0	0.007207	0.303299	1	-
ENSMUSC	Spata5	spermato	GO:00325	developm	K14575	AAA famil	mmu030C	Ribosome	3:3741989	256	253	369	339	237	165	2.630059	2.17871	2.611587	2.26886	2.135095	2.10183	2.473452	2.168595	0.26063	1	-
ENSMUSC	Slc7a11	solute car	GO:00325	developm	K13869	solute car	mmu0421	Ferroptos	3:4989252	804	1245	1656	1593	1064	625	3.255393	4.225416	4.619118	4.201888	3.777735	3.137711	4.033309	3.705778	0.425388	1	-
ENSMUSC	Rab33b	RAB33B, r	GO:00325	developm	K07920	Ras-relate	mmu0414	Autophag	3:5148392	278	374	530	459	379	313	3.663988	4.131745	4.812122	3.940968	4.38017	5.114929	4.202618	4.478689	0.889847	1	-
ENSMUSC	Cog6	compone	GO:00081	metabolic	-	-	-	-	3:5298187	671	795	924	960	727	336	5.955872	5.914841	5.649977	5.551059	5.658483	3.697848	5.84023	4.96913	0.221162	1	-
ENSMUSC	Stoml3	stomatin (	GO:00508	response	-	-	-	-	3:5348865	11	17	27	7	20	8	0.300173	0.388842	0.507549	0.124438	0.478575	0.270671	0.398855	0.291228	0.315622	1	-
ENSMUSC	Ufm1	ubiquitin-	GO:00081	metabolic	-	-	-	-	3:5385337	1079	1417	1738	1550	1245	682	10.67794	11.75408	11.8486	9.992618	10.80383	8.368285	11.42687	9.721579	0.145105	1	-
ENSMUSC	Trpc4	transient r	GO:00325	developm	K04967	transient r	mmu0436	Axon guic	3:5415603	856	1009	1139	1150	912	496	4.703155	4.646857	4.311124	4.116185	4.393926	3.378961	4.553712	3.963024	0.214034	1	-
ENSMUSC	Postn	periostin,	GO:00325	developm	-	-	-	-	3:543611C	98	99	201	139	71	96	0.515235	0.436286	0.727996	0.476078	0.327326	0.625804	0.559839	0.476403	0.297374	1	-
ENSMUSC	Supt20	suppressc	GO:00325	developm	-	-	mmu0414	Autophag	3:546928C	402	485	773	743	500	351	2.785469	2.816868	3.689799	3.353841	3.037979	3.015538	3.097379	3.135786	0.877469	1	-
ENSMUSC	Exosc8	exosome	GO:00081	metabolic	K12586	exosome	mmu0301	RNA degr	3:5472867	122	151	209	193	142	116	0.842399	0.873954	0.994162	0.868157	0.859788	0.993122	0.903505	0.907022	0.820842	1	-
ENSMUSC	Mbnl1	musclebli	GO:00325	developm	-	-	-	-	3:6047283	1172	1558	2085	1828	1274	838	1.654614	1.843697	2.027808	1.68123	1.57718	1.466896	1.84204	1.575102	0.130913	1	-
ENSMUSC	P2ry1	purinergic	GO:00325	developm	K04270	purinergic	mmu0408	Neuroacti	3:6100279	45	56	79	83	50	39	0.362106	0.377713	0.437923	0.435091	0.352804	0.38911	0.392581	0.392335	0.875955	1	-
ENSMUSC	Dhx36	DEAH (As	GO:00081	metabolic	K14442	ATP-depe	mmu0301	RNA degr	3:6246801	1509	1826	2080	2227	1733	1080	8.644447	8.768019	8.20848	8.310933	8.70541	7.671103	8.540315	8.229149	0.557723	1	-
ENSMUSC	Gfm1	G elongat	GO:00081	metabolic	-	-	-	-	3:6743009	949	1138	1430	1560	1147	735	12.43542	12.4994	12.90868	13.31682	13.17954	11.94174	12.6145	12.8127	0.880708	1	-
ENSMUSC	Mfsd1	major faci	GO:00511	localizatio	-	-	-	-	3:6758274	400	472	510	610	378	462	8.248278	8.158282	7.244779	8.194359	6.834996	11.81221	7.883779	8.947189	0.613846	1	-
ENSMUSC	Il12a	interleukir	GO:00325	developm	K05406	interleukir	mmu052C	Pathways	3:6869064	8	6	7	18	8	6	0.121164	0.07617	0.073042	0.177602	0.106256	0.112688	0.090125	0.132182	0.323359	1	-
ENSMUSC	Schip1	schwannc	-	-	-	-	-	-	3:680648C	3559	4347	5848	4982	4059	2797	34.40078	35.21947	38.94029	31.37081	34.40346	33.52115	36.18685	33.09847	0.267823	1	-
ENSMUSC	Ift80	intraflagel	GO:00325	developm	-	-	-	-	3:6889249	543	636	835	933	646	375	2.052169	2.014759	2.173959	2.297084	2.140861	1.757237	2.080296	2.06506	0.787129	1	-
ENSMUSC	Kpna4	karyopher	GO:00511	localizatio	-	-	-	-	3:6906714	991	2625	1608	1568	2391	1407	5.577489	12.38362	6.234514	5.749009	11.80015	9.818504	8.065206	9.122554	0.696376	1	-
ENSMUSC	Ppm1l	protein pl	GO:00081	metabolic	-	-	-	-	3:6931686	3103	3526	4472	4671	3404	2155	17.61388	16.77677	17.48745	17.27288	16.94359	15.16724	17.2927	16.46123	0.483605	1	-
ENSMUSC	Nmd3	NMD3 rib	GO:00081	metabolic	K07562	nonsense-	mmu0301	RNA trans	3:6972198	435	547	722	737	537	256	2.466372	2.599617	2.820056	2.722188	2.669847	1.799676	2.628682	2.397237	0.453156	1	-
ENSMUSC	Sis	sucrase-is	GO:00081	metabolic	K01203	sucrase-is	mmu011C	Metabolic	3:7288855	2	2	2	6	0	0	0.015399	0.012904	0.010607	0.03009	0	0	0.01297	0.01003	1	1	-
ENSMUSC	Bche	butyrylchc	GO:00325	developm	-	-	-	-	3:736358C	86	121	149	128	75	51	0.711569	0.839184	0.849288	0.68994	0.544157	0.52321	0.800014	0.585769	0.058671	1	-
ENSMUSC	Ccna1	cyclin A1 |	GO:00325	developm	K06627	cyclin A[-]	mmu0516	Human pa	3:5504546	15	13	14	20	12	10	0.279561	0.203091	0.179743	0.24283	0.196114	0.231095	0.220798	0.223346	1	1	-
ENSMUSC	Smad9	SMAD far	GO:00325	developm	K16791	mothers a	mmu0455	Signaling	3:5475558	322	295	405	426	318	208	3.812239	2.927509	3.303162	3.2856	3.301362	3.053319	3.347637	3.213427	0.608333	1	-
ENSMUSC	Dclk1	doublecoı	GO:00325	developm	-	-	-	-	3:5524236	10242	11206	14606	16062	12115	8870	17.75386	16.28214	17.44178	18.138	18.4151	19.06416	17.15926	18.53909	0.729706	1	-
ENSMUSC	Nbea	neurobea	GO:00452	synapse|c	-	-	-	-	3:5562519	6178	8822	8312	12077	7112	5380	10.48721	12.55254	9.720058	13.35527	10.58635	11.32348	10.91994	11.75503	0.768969	1	-
ENSMUSC	Tm4sf1	transmem	GO:00160	membran	-	-	-	-	3:5728561	101	112	144	121	101	147	1.069276	0.993888	1.05022	0.834519	0.937634	1.929617	1.037795	1.233923	0.603043	1	-
ENSMUSC	Wwtr1	WW dom	GO:00325	developm	K16820	WW dom	mmu0439	Hippo sig	3:5745564	393	511	364	637	466	256	3.037377	3.31039	1.938023	3.207204	3.158159	2.453189	2.76193	2.939517	0.862039	1	-
ENSMUSC	Ppid	peptidylpı	GO:00081	metabolic	K05864	peptidyl-p	mmu0421	Cellular se	3:7959134	1289	1829	2316	2235	1682	822	13.42948	15.9725	16.6225	15.1693	15.3665	10.61852	15.34149	13.71811	0.334704	1	-
ENSMUSC	Pfn2	profilin 2 |	GO:00081	metabolic	K05759	profilin[-]	mmu0481	Regulatio	3:5784189	6849	8664	10435	10951	7941	5920	78.42785	83.15999	82.31652	81.69181	79.7372	84.05253	81.30145	81.82718	0.775747	1	-
ENSMUSC	Tsc22d2	TSC22 do	GO:00081	metabolic	-	-	-	-	3:5841471	936	1154	1422	1378	1002	587	3.301272	3.411646	3.455067	3.166184	3.098958	2.567023	3.389328	2.944055	0.187938	1	-
ENSMUSC	Serp1	stress-ass	GO:00325	developm	-	-	-	-	3:5851981	753	988	1366	1216	925	563	10.37853	11.41434	12.9701	10.91833	11.17957	9.621342	11.58765	10.57308	0.347136	1	-
ENSMUSC	Etfdh	electron tı	GO:00081	metabolic	-	-	-	-	3:7960378	668	757	857	902	676	448	4.430537	4.208512	3.915724	3.89734	3.931597	3.684209	4.184924	3.837715	0.349796	1	-
ENSMUSC	Eif2a	eukaryotic	GO:00081	metabolic	-	-	-	-	3:5852582	678	849	981	996	798	411	5.309135	5.572561	5.291934	5.080835	5.479486	3.990446	5.39121	4.850256	0.338527	1	-
ENSMUSC	4930579G	RIKEN cDI	GO:00056	cell|intrace	-	-	-	-	3:7962907	21	16	33	27	28	28	0.677481	0.432662	0.73338	0.567424	0.792079	1.119987	0.614508	0.826497	0.385308	1	-
ENSMUSC	Mme	membran	GO:00325	developm	K01389	neprilysin[	mmu0501	Alzheimer	3:6324153	135	88	100	162	105	86	0.588415	0.321502	0.300262	0.459987	0.401311	0.464767	0.403393	0.442022	0.806101	1	-
ENSMUSC	Slc33a1	solute car	GO:00081	metabolic	K03372	MFS trans	mmu011C	Metabolic	3:639335C	279	523	613	510	505	394	2.922584	4.592156	4.423582	3.480278	4.638695	5.117339	3.979441	4.412104	0.709365	1	-
ENSMUSC	Gmps	guanine n	GO:00081	metabolic	K01951	GMP synt	mmu011C	Metabolic	3:639761C	1673	2090	2454	2470	1872	1185	13.11834	13.73669	13.25589	12.61716	12.87157	11.52093	13.37031	12.33655	0.34412	1	-
ENSMUSC	Vmn2r1	vomerona	GO:00048	signal trar	-	-	-	-	3:6408164	13	3	7	11	11	5	0.301633	0.058344	0.111896	0.166265	0.223813	0.143839	0.157291	0.177972	0.848958	1	-
ENSMUSC	Kcnab1	potassiur	GO:00325	developm	-	-	-	-	3:6510938	1903	2317	2887	3177	2242	1788	10.6971	10.91707	11.17958	11.63392	11.0511	12.4618	10.93125	11.7156	0.796985	1	-
ENSMUSC	Ssr3	signal seq	GO:00511	localizatio	K13251	translocor	mmu0414	Protein pr	3:6537965	2488	3319	4444	4012	3022	1863	31.97571	35.75444	39.34552	33.59018	34.05705	29.68716	35.69189	32.4448	0.299153	1	-
ENSMUSC	Ccnl1	cyclin L1 [	GO:00081	metabolic	-	-	-	-	3:6594615	356	563	638	813	501	360	1.629797	2.160456	2.012129	2.42469	2.011242	2.043493	1.934127	2.159808	0.597845	1	-
ENSMUSC	Veph1	ventricula	GO:00160	membran	-	-	-	-	3:6605355	6	5	0	4	3	2	0.047009	0.032833	0	0.020417	0.020608	0.019433	0.026614	0.020153	0.732118	1	-
ENSMUSC	Ptx3	pentraxin	GO:00081	metabolic	-	-	-	-	3:6621991	11	12	19	17	10	12	0.226687	0.207287	0.269726	0.228217	0.180707	0.306611	0.234566	0.238512	0.931002	1	-
ENSMUSC	Shox2	short statı	GO:00325	developm	-	-	-	-	3:6697172	2	0	0	0	0	0	0.016859	0	0	0	0	0	0.00562	0	0.536591	1	-
ENSMUSC	Serpini1	serine (or	GO:00325	developm	-	-	-	-	3:7555754	3210	4146	5365	4963	4020	2096	25.26692	27.35454	29.09166	25.44915	27.74701	20.45618	27.23771	24.55078	0.303882	1	-
ENSMUSC	Pdcd10	programn	GO:00325	developm	-	-	-	-	3:7551649	488	503	678	680	533	210	7.678604	6.634116	7.349261	6.970322	7.354151	4.097018	7.220661	6.140497	0.286456	1	-
ENSMUSC	Wnt2b	wingless-t	GO:00325	developm	K00182	wingless-t	mmu052C	Pathways	3:1049452	6	10	5	10	6	4	0.082433	0.11515	0.047322	0.089495	0.072276	0.068136	0.081635	0.076636	0.897444	1	-
ENSMUSC	Ptpn22	protein ty	GO:00325	developm	-	-	-	-	3:1038597	11	20	17	26	22	6	0.081357	0.123987	0.086613	0.125268	0.14268	0.055026	0.097319	0.107658	0.841789	1	-
ENSMUSC	Dclre1b	DNA cros	GO:00081	metabolic	-	-	-	-	3:1038006	113	130	73	187	154	79	0.386933	0.373124	0.172201	0.417139	0.462405	0.335407	0.310753	0.404984	0.299048	1	-
ENSMUSC	Olfml3	olfactome	GO:00055	extracellul	-	-	-	-	3:1037222	137	157	615	691	166	99	0.807152	0.775335	2.496101	2.65213	0.857605	0.723198	1.359529	1.410978	0.943935	1	-
ENSMUSC	Syt6	synaptota	GO:00452	synapse|c	-	-	-	-	3:1035752	35	98	115	116	63	91	0.203269	0.477068	0.460099	0.438873	0.320837	0.655281	0.380145	0.471664	0.507021	1	-
ENSMUSC	Nras	neuroblas	GO:00508	response	K07828	GTPase N	mmu052C	Pathways	3:1030582	290	250	293	363	705	178	2.119894	1.531826	1.475488	1.728641	4.519064	1.613329	1.709069	2.620345	0.105458	1	-
ENSMUSC	Sike1	suppressc	GO:00056	cell|intrace	K12656	suppresso	mmu0462	RIG-I-like	3:1029957	825	981	1397	1306	1023	676	3.836057	3.823429	4.474857	3.955997	4.17109	3.897299	4.044781	4.008129	0.720391	1	-
ENSMUSC	Tshb	thyroid sti	GO:00508	response	K05251	thyroid sti	mmu0408	Neuroacti	3:1027754	0	2	0	0	0	1	0	0.017534	0	0	0	0.012965	0.005845	0.004322	1	1	-
ENSMUSC	Tspan2	tetraspani	GO:00325	developm	-	-	-	-	3:1027345	371	298	3180	3393	2575	444	4.417038	2.9739	26.08171	26.31619	26.88298	6.554307	11.15755	19.91783	0.196658	1	-
ENSMUSC	Ngf	nerve gro	GO:00325	developm	K02582	nerve gro	mmu0415	PI3K-Akt	3:1024699	65	93	120	129	80	51	1.270271	1.523419	1.615537	1.642321	1.370938	1.235786	1.469742	1.416348	0.734429	1	-
ENSMUSC	Vangl1	vang-like	GO:00325	developm	K04510	vang-like	mmu0431	Wnt signa	3:1021535	84	86	111	120	93	65	0.486461	0.417467	0.442839	0.452726	0.472277	0.466732	0.448922	0.463912	1	1	-
ENSMUSC	Casq2	calseques	GO:00325	developm	-	-	-	-	3:1020864	48	24	30	56	32	25	0.753594	0.315833	0.324462	0.572748	0.440537	0.486649	0.464629	0.499978	0.917838	1	-
ENSMUSC	Ptgfrn	prostaglaı	GO:00099	cellular pr	-	-	-	-	3:1010402	267	276	512	420	317	280	1.547246	1.340633	2.043948	1.585546	1.610831	2.011831	1.643943	1.736069	0.948207	1	-
ENSMUSC	Gdap2	gangliosic	GO:00508	response	-	-	-	-	3:1001623	620	791	1036	1107	690	339	5.787729	6.189364	6.662355	6.732025	5.648174	3.923755	6.213149	5.434651	0.324946	1	-
ENSMUSC	Spag17	sperm ass	GO:00990	supramole	-	-	-	-	3:998854C	32	16	29	31	5	7	0.214954	0.09009	0.134199	0.135657	0.02945	0.058304	0.146414	0.07447	0.079701	1	-
ENSMUSC	Tbx15	T-box 15	GO:00325	developm	-	-	-	-	3:9924038	15	21	36	37	13	24	0.247611	0.290568	0.409387	0.397888	0.188175	0.491215	0.315855	0.359093	0.838338	1	-
ENSMUSC	Hmgcs2	3-hydroxy	GO:00081	metabolic	K01641	hydroxym	mmu011C	Metabolic	3:9828043	126	179	179	151	137	127	1.891733	2.252655	1.851369	1.476889	1.80364	2.364165	1.998586	1.881564	0.510111	1	-
ENSMUSC	Notch2	notch 2 [S	GO:00325	developm	K02599	Notch[-]	mmu052C	Pathways	3:9801352	800	846	1092	1146	888	739	4.537473	4.022048	4.26676	4.234386	4.416519	5.197024	4.275427	4.615976	0.787319	1	-
ENSMUSC	Sec22b	SEC22 ho	GO:00081	metabolic	K08517	vesicle tra	mmu0414	Phagoson	3:9790119	724	886	1101	1169	867	787	7.525737	7.71964	7.884054	7.916017	7.902645	10.14312	7.709811	8.653926	0.567104	1	-
ENSMUSC	Prpf38b	PRP38 pre	GO:00081	metabolic	K12850	pre-mRN	mmu0304	Spliceoso	3:1089028	902	844	1219	1128	876	546	3.861817	3.028868	3.595342	3.146125	3.28876	2.898434	3.495342	3.111106	0.260693	1	-
ENSMUSC	Stxbp3	syntaxin b	GO:00325	developm	-	-	-	-	3:1087931	324	331	451	454	334	160	3.059154	2.619616	2.933492	2.792507	2.765318	1.873103	2.870754	2.476976	0.287813	1	-
ENSMUSC	Gpsm2	G-protein	GO:00325	developm	-	-	-	-	3:1086786	328	381	410	447	389	210	4.42953	4.312824	3.81434	3.932543	4.606562	3.516332	4.185565	4.018479	0.626685	1	-
ENSMUSC	Clcc1	chloride c	GO:00511	localizatio	-	-	-	-	3:1086539	299	371	506	482	376	284	2.967723	3.086595	3.459825	3.116599	3.27253	3.495072	3.171381	3.294734	0.995461	1	-
ENSMUSC	1700013F	RIKEN cDI	-	-	-	-	-	-	3:1085341	16	20	23	30	20	8	0.539454	0.565208	0.534207	0.658915	0.5913	0.334426	0.54629	0.528214	0.896133	1	-
ENSMUSC	Sypl2	synaptopl	GO:00325	developm	-	-	-	-	3:1082114	58	73	74	99	86	57	0.75861	0.800317	0.666764	0.843542	0.986354	0.924373	0.741897	0.91809	0.329384	1	-
ENSMUSC	Ampd2	adenosine	GO:00325	developm	K01490	AMP dear	mmu011C	Metabolic	3:108074C	673	957	1181	1306	822	715	3.937326	4.693007	4.759789	4.977499	4.216975	5.186548	4.463374	4.793674	0.806429	1	-
ENSMUSC	Gstm4	glutathior	GO:00081	metabolic	K00799	glutathior	mmu052C	Pathways	3:1080404	120	124	149	168	135	120	2.644372	2.290423	2.261921	2.411751	2.608654	3.278743	2.398905	2.766382	0.551417	1	-
ENSMUSC	Ahcyl1	S-adenos	GO:00081	metabolic	K01251	adenosylh	mmu011C	Metabolic	3:1076631	5422	6341	8071	8466	6332	4125	30.24262	29.6463	31.01264	30.76237	30.97016	28.5279	30.30052	30.08681	0.716077	1	-
ENSMUSC	Slc6a17	solute car	GO:00325	developm	-	-	-	-	3:1074675	2361	4184	5256	5566	2569	2785	7.943284	11.79909	12.18179	12.19913	7.578982	11.61757	10.64139	10.46523	0.710495	1	-
ENSMUSC	Kcnc4	potassiur	GO:00452	synapse|c	-	-	-	-	3:1074383	1014	1412	1567	1618	1380	1243	17.57371	20.51223	18.70879	18.26776	20.97234	26.71052	18.93157	21.98354	0.412185	1	-
ENSMUSC	Slc16a4	solute car	GO:00511	localizatio	-	-	-	-	3:1072912	49	48	76	54	52	30	0.673905	0.553339	0.720056	0.483811	0.627114	0.511581	0.6491	0.540835	0.285058	1	-
ENSMUSC	Dram2	DNA-dan	GO:00081	metabolic	-	-	-	-	3:1065477	282	301	482	470	357	179	2.793322	2.49914	3.289046	3.032853	3.100862	2.19842	2.860502	2.777379	0.724876	1	-
ENSMUSC	Dennd2d	DENN/M/	GO:00987	molecular	-	-	-	-	3:1064824	15	4	22	12	7	6	0.117099	0.026173	0.118311	0.061029	0.047921	0.058081	0.087194	0.055677	0.216309	1	-
ENSMUSC	Ddx20	DEAD (As	GO:00325	developm	K13131	ATP-depe	mmu0301	RNA trans	3:1056782	245	248	361	303	254	153	3.07596	2.609869	3.122289	2.478209	2.796339	2.381715	2.936039	2.552088	0.247647	1	-
ENSMUSC	S100a11	S100 calci	GO:00099	cellular pr	-	-	-	-	3:9352048	36	37	39	52	35	32	4.47726	3.857203	3.341442	4.213106	3.81707	4.934583	3.891968	4.321586	0.800472	1	-
ENSMUSC	Crct1	cysteine-r	-	-	-	-	-	-	3:930142C	0	0	0	0	0	2	0	0	0	0	0	0.133821	0	0.044607	0.536039	1	-
ENSMUSC	Lelp1	late cornif	GO:00325	developm	-	-	-	-	3:9213499	0	0	0	0	0	2	0	0	0	0	0	0.183946	0	0.061315	0.536796	1	-
ENSMUSC	Npr1	natriuretic	GO:00048	signal trar	K12323	atrial natri	mmu0402	cAMP sigr	3:9045059	89	81	102	84	80	66	0.909064	0.693497	0.717727	0.558943	0.716536	0.83587	0.773429	0.703783	0.44449	1	-
ENSMUSC	Slc27a3	solute car	GO:00081	metabolic	K08772	solute car	mmu0493	Insulin res	3:9038523	127	141	131	189	116	129	1.432081	1.332717	1.017629	1.388377	1.147008	1.803595	1.260809	1.446327	0.619328	1	-
ENSMUSC	Ints3	integrator	GO:00081	metabolic	-	-	-	-	3:9039138	1271	1473	1870	1983	1509	1213	8.930447	8.67527	9.051503	9.076784	9.297357	10.56755	8.88574	9.64723	0.724933	1	-
ENSMUSC	Rab13	RAB13, m	GO:00325	developm	K06109	Ras-relate	mmu0453	Tight junc	3:9021369	51	72	98	93	88	66	1.127761	1.334541	1.492886	1.339719	1.706382	1.809598	1.318396	1.618566	0.394792	1	-
ENSMUSC	Crtc2	CREB regı	GO:00081	metabolic	K16333	CREB-reg	mmu0415	PI3K-Akt	3:9025416	416	455	557	600	418	367	3.85226	3.531723	3.553276	3.619556	3.39423	4.213798	3.645753	3.742528	0.907136	1	-
ENSMUSC	Jtb	jumping t	GO:00081	metabolic	-	-	-	-	3:9023159	155	224	324	240	197	306	3.280302	3.973582	4.723651	3.308823	3.655861	8.029495	3.992512	4.99806	0.427021	1	-
ENSMUSC	Creb3l4	cAMP res	GO:00325	developm	K09048	cyclic AMI	mmu0415	PI3K-Akt	3:902375C	0	3	6	4	0	0	0	0.078683	0.129333	0.081549	0	0	0.069339	0.027183	0.340976	1	-
ENSMUSC	Nup210l	nucleopoı	GO:00325	developm	K14314	nuclear pc	mmu0301	RNA trans	3:9010413	6	3	20	11	1	2	0.057612	0.024143	0.132292	0.068802	0.008419	0.023816	0.071349	0.033679	0.165514	1	-
ENSMUSC	Tpm3	tropomyo	GO:00051	structural	K09290	tropomyo	mmu052C	Pathways	3:9007264	2033	2451	3053	3037	2277	1693	10.97174	11.08752	11.35054	10.67738	10.77566	11.32873	11.1366	10.92726	0.613792	1	-
ENSMUSC	4933434E	RIKEN cDI	GO:00160	membran	-	-	-	-	3:9005163	409	521	659	637	497	469	2.24796	2.400249	2.495182	2.280796	2.395327	3.19613	2.38113	2.624084	0.709044	1	-
ENSMUSC	Hax1	HCLS1 ass	GO:00325	developm	-	-	-	-	3:8999544	267	415	316	381	278	186	2.764524	3.601723	2.253971	2.569896	2.524041	2.387859	2.873406	2.493932	0.270555	1	-
ENSMUSC	Il6ra	interleukir	GO:00325	developm	K05055	interleukir	mmu052C	Pathways	3:8986405	137	164	215	248	182	153	0.794628	0.797336	0.85908	0.937082	0.925676	1.100323	0.817015	0.987694	0.326588	1	-
ENSMUSC	Chrnb2	cholinergi	GO:00325	developm	K04813	nicotinic a	mmu0408	Neuroacti	3:8974619	462	1392	449	1763	1241	249	3.339567	8.434137	2.235869	8.301993	7.86616	2.231693	4.669858	6.133282	0.435681	1	-
ENSMUSC	Adar	adenosine	GO:00325	developm	K12968	double-st	mmu0516	Influenza	3:8971502	2326	2882	2567	3740	2383	2343	14.91969	15.49519	11.34299	15.628	13.40346	18.63409	13.91929	15.88852	0.50203	1	-
ENSMUSC	Pmvk	phosphor	GO:00081	metabolic	K13273	phosphon	mmu011C	Metabolic	3:8945454	507	583	728	824	578	484	10.28452	9.91282	10.17322	10.88892	10.28126	12.17327	10.12352	11.11448	0.683685	1	-
ENSMUSC	Slc50a1	solute car	GO:00081	metabolic	-	-	-	-	3:8926824	215	282	297	341	255	296	3.748669	4.121353	3.567352	3.87324	3.898709	6.39905	3.812458	4.723666	0.308132	1	-
ENSMUSC	Efna1	ephrin A1	GO:00325	developm	K05462	ephrin-A[	mmu0415	PI3K-Akt	3:8927173	83	88	129	114	87	89	1.404773	1.24842	1.50407	1.256937	1.29119	1.86767	1.385754	1.471932	0.968624	1	-
ENSMUSC	Fam198b	family witl	GO:00160	membran	-	-	-	-	3:7988453	88	126	103	145	96	74	0.323707	0.3885	0.26101	0.347471	0.309657	0.337511	0.324406	0.331546	0.956627	1	-
ENSMUSC	Tmem144	transmem	GO:00511	localizatio	-	-	-	-	3:7981256	190	216	359	344	227	157	2.492787	2.375406	3.244726	2.940171	2.61156	2.553972	2.704307	2.701901	0.83098	1	-
ENSMUSC	Slc35a3	solute car	GO:00511	localizatio	-	-	-	-	3:1166694	337	399	532	478	380	284	1.716821	1.70381	1.867061	1.586373	1.697548	1.793904	1.762564	1.692608	0.548714	1	-
ENSMUSC	Sass6	SAS-6 cer	GO:00099	cellular pr	-	-	-	-	3:1165949	90	149	193	183	98	38	0.662086	0.91878	0.978096	0.877013	0.632178	0.346615	0.852987	0.618602	0.150302	1	-
ENSMUSC	Lrrc39	leucine ric	GO:00990	supramole	-	-	-	-	3:1165629	2	3	2	12	0	0	0.02306	0.028987	0.015885	0.090121	0	0	0.022644	0.03004	0.759146	1	-
ENSMUSC	Vcam1	vascular c	GO:00325	developm	K06527	vascular c	mmu0516	HTLV-I inf	3:1161099	537	623	824	805	632	382	3.535474	3.438071	3.737254	3.45264	3.648661	3.118337	3.570266	3.406546	0.547298	1	-
ENSMUSC	Extl2	exostoses	GO:00081	metabolic	K02369	alpha-1,4	mmu011C	Metabolic	3:1160074	1069	1470	1786	1863	1493	1234	7.686182	8.859375	8.846387	8.726246	9.413163	11.00104	8.463981	9.713485	0.425896	1	-
ENSMUSC	Olfm3	olfactome	GO:00325	developm	-	-	-	-	3:114904C	296	449	571	495	391	225	2.323578	2.954366	3.087828	2.531351	2.691444	2.189954	2.788591	2.470916	0.303172	1	-
ENSMUSC	Col11a1	collagen,	GO:00325	developm	K19721	collagen, t	mmu0497	Protein di	3:1140305	275	339	462	670	330	212	1.222428	1.263117	1.414767	1.940206	1.286316	1.168456	1.300104	1.464993	0.589067	1	-
ENSMUSC	Neurog2	neurogen	GO:00325	developm	-	-	-	-	3:1276331	19	20	34	23	22	23	0.542212	0.478409	0.668418	0.427588	0.550538	0.813831	0.563013	0.597319	1	1	-
ENSMUSC	Larp7	La ribonu	GO:00081	metabolic	-	-	-	-	3:1275367	262	347	427	420	326	203	4.392214	4.876016	4.931308	4.586833	4.792288	4.219531	4.733179	4.532884	0.590379	1	-
ENSMUSC	Ndst4	N-deacet	GO:00081	metabolic	K02579	heparan s	mmu011C	Metabolic	3:125404C	341	833	442	562	819	160	1.300036	2.661941	1.160844	1.395785	2.737959	0.75632	1.707607	1.630022	0.817753	1	-
ENSMUSC	Ndst3	N-deacet	GO:00081	metabolic	K02578	heparan s	mmu011C	Metabolic	3:1235261	643	810	1012	1069	770	394	2.167422	2.288604	2.349982	2.347426	2.275969	1.646701	2.268669	2.090032	0.452049	1	-
ENSMUSC	Prss12	protease,	GO:00081	metabolic	-	-	-	-	3:1234469	342	434	487	575	426	255	8.353231	8.885291	8.194246	9.149092	9.123904	7.722443	8.477589	8.665146	0.944137	1	-
ENSMUSC	Rnpc3	RNA-binc	GO:00325	developm	-	-	-	-	3:113605C	256	312	421	463	311	185	1.478512	1.510404	1.675015	1.742	1.575031	1.324782	1.554644	1.547271	0.820898	1	-
ENSMUSC	Cyp2u1	cytochron	GO:00081	metabolic	K07422	cytochron	mmu011C	Metabolic	3:1312884	167	155	248	220	162	174	1.514678	1.178386	1.549556	1.299892	1.28843	1.95677	1.414207	1.515031	0.922166	1	-
ENSMUSC	Hadh	hydroxyac	GO:00081	metabolic	K00022	3-hydroxy	mmu011C	Metabolic	3:1312334	262	289	413	376	325	343	3.077228	2.845179	3.341641	2.876924	3.347226	4.995032	3.088016	3.739727	0.369008	1	-
ENSMUSC	Lef1	lymphoid	GO:00325	developm	K04492	lymphoid	mmu052C	Pathways	3:1311104	168	177	199	271	226	164	1.154761	1.019782	0.942293	1.213481	1.362175	1.397691	1.038945	1.324449	0.185413	1	-
ENSMUSC	Trim2	tripartite r	GO:00081	metabolic	-	-	-	-	3:8416043	4054	9398	11597	5896	8772	3265	5.958463	11.57814	11.74215	5.645333	11.30555	5.950035	9.759583	7.633639	0.182434	1	-
ENSMUSC	Mcub	mitochon	GO:00511	localizatio	-	-	-	-	3:1299149	44	74	88	94	70	44	1.436233	2.024685	1.978804	1.998844	2.00359	1.780771	1.813241	1.927735	0.894157	1	-
ENSMUSC	Tlr2	toll-like re	GO:00325	developm	K10159	toll-like re	mmu0415	PI3K-Akt	3:8383627	16	23	48	45	26	29	0.337382	0.40651	0.697243	0.618143	0.480739	0.758179	0.480379	0.61902	0.450517	1	-
ENSMUSC	Sfrp2	secreted f	GO:00325	developm	K02176	secreted f	mmu0431	Wnt signa	3:8376632	25	20	17	16	22	22	0.56282	0.377406	0.263642	0.234652	0.434307	0.614107	0.401289	0.427689	1	1	-
ENSMUSC	Casp6	caspase 6	GO:00325	developm	K04396	caspase 6	mmu0421	Apoptosis	3:1299014	55	57	94	82	42	53	0.574427	0.499005	0.676327	0.557921	0.38465	0.686332	0.583253	0.542968	0.572001	1	-
ENSMUSC	Plrg1	pleiotropi	GO:00081	metabolic	K12862	pleiotropi	mmu0304	Spliceoso	3:8305552	411	493	715	630	548	433	6.644694	6.680873	7.963259	6.635224	7.768847	8.679746	7.096275	7.694605	0.780299	1	-
ENSMUSC	Pla2g12a	phospholi	GO:00081	metabolic	-	-	mmu011C	Metabolic	3:1298786	221	275	356	238	273	271	2.717731	2.834647	3.015889	1.906657	2.943875	4.132078	2.856089	2.994203	0.966216	1	-
ENSMUSC	Lrat	lecithin-re	GO:00081	metabolic	K00678	phosphati	mmu0083	Retinol m	3:8289257	3	0	4	5	4	0	0.035626	0	0.032731	0.038689	0.041655	0	0.022786	0.026781	1	1	-
ENSMUSC	Npy2r	neuropep	GO:00325	developm	K04205	neuropep	mmu0408	Neuroacti	3:8253838	887	1003	678	1072	890	595	5.881222	5.574396	3.096888	4.630422	5.1746	4.891558	4.850835	4.89886	0.859874	1	-
ENSMUSC	Gucy1b3	guanylate	GO:00081	metabolic	K12319	guanylate	mmu0023	Purine me	3:820320C	2436	3049	3705	3369	2758	1807	26.62341	27.93166	27.89498	23.98662	26.43159	24.48672	27.48335	24.96831	0.255037	1	-
ENSMUSC	Snx7	sorting ne	GO:00511	localizatio	-	-	-	-	3:1177816	189	257	288	294	225	163	1.6188	1.845091	1.699324	1.640443	1.689885	1.731035	1.721071	1.687121	0.697885	1	-
ENSMUSC	Gar1	GAR1 ribc	GO:00081	metabolic	K11128	H/ACA rib	mmu030C	Ribosome	3:1298249	205	269	320	322	243	113	3.798357	4.177797	4.084541	3.88669	3.948118	2.596	4.020232	3.476936	0.315201	1	-
ENSMUSC	Tdo2	tryptopha	GO:00081	metabolic	K00453	tryptopha	mmu011C	Metabolic	3:8195709	107	120	148	168	113	74	1.362219	1.28055	1.298	1.393327	1.261491	1.168104	1.31359	1.274308	0.721603	1	-
ENSMUSC	Rrh	retinal pig	GO:00048	signal trar	-	-	-	-	3:1298044	11	8	4	4	9	6	0.087728	0.053475	0.021978	0.020784	0.062941	0.059335	0.054394	0.047686	0.752269	1	-
ENSMUSC	Ppa2	pyrophos	GO:00081	metabolic	K01507	inorganic	mmu0019	Oxidative	3:1333101	560	676	759	779	573	328	3.255553	3.294093	3.039692	2.950229	2.921017	2.36427	3.196446	2.745172	0.192232	1	-
ENSMUSC	Ctso	cathepsin	GO:00081	metabolic	K01374	cathepsin	mmu0421	Apoptosis	3:819326C	383	425	634	561	431	357	4.205393	3.911551	4.79566	4.012845	4.149806	4.860288	4.304202	4.34098	0.806833	1	-
ENSMUSC	Ints12	integrator	GO:00081	metabolic	-	-	-	-	3:1330918	149	203	243	259	205	149	1.063023	1.213963	1.194301	1.203755	1.282488	1.318034	1.157096	1.268092	0.730938	1	-
ENSMUSC	Egf	epiderma	GO:00325	developm	K04357	epiderma	mmu052C	Pathways	3:1296775	15	20	22	45	25	20	0.064653	0.072256	0.065322	0.126353	0.094488	0.106882	0.06741	0.109241	0.064837	1	-
ENSMUSC	Gstcd	glutathior	GO:00055	extracellul	-	-	-	-	3:1329817	75	90	131	131	88	65	0.58947	0.59292	0.709294	0.670742	0.606498	0.633431	0.630562	0.63689	0.899983	1	-
ENSMUSC	Pdgfc	platelet-d	GO:00325	developm	K05450	platelet de	mmu0415	PI3K-Akt	3:8103641	94	114	134	117	92	89	0.807948	0.821321	0.79344	0.655124	0.693405	0.948485	0.807569	0.765672	0.546662	1	-
ENSMUSC	Glrb	glycine re	GO:00325	developm	K05196	glycine re	mmu0408	Neuroacti	3:8084359	1838	2351	3079	3385	2145	1794	20.04641	21.49298	23.13408	24.0509	20.5145	24.26048	21.55782	22.94196	0.84485	1	-
ENSMUSC	Pitx2	paired-lik	GO:00325	developm	K04686	paired-lik	mmu0435	TGF-beta	3:1291998	0	1	1	0	0	0	0	0.004305	0.003537	0	0	0	0.002614	0	0.527817	1	-
ENSMUSC	Enpep	glutamyl a	GO:00325	developm	K11141	glutamyl a	mmu0461	Renin-an	3:1292691	60	79	114	120	68	27	0.630273	0.695604	0.824967	0.821193	0.62637	0.351669	0.716948	0.599744	0.420187	1	-
ENSMUSC	Alpk1	alpha-kin	GO:00081	metabolic	-	-	-	-	3:1276703	113	164	199	244	168	93	0.795907	0.968238	0.965582	1.119586	1.037618	0.81218	0.909909	0.989795	0.756686	1	-
ENSMUSC	Aimp1	aminoacy	GO:00325	developm	-	-	-	-	3:1326604	350	544	747	623	523	525	3.35145	4.366325	4.927613	3.886281	4.391459	6.233178	4.215129	4.836973	0.55711	1	-
ENSMUSC	Tbck	TBC1 dor	GO:00081	metabolic	-	-	-	-	3:1326841	908	1154	1338	1403	1002	683	4.382302	4.668473	4.448604	4.41119	4.240593	4.087174	4.499793	4.246319	0.448635	1	-
ENSMUSC	Papss1	3'-phospl	GO:00081	metabolic	K13811	3'-phospl	mmu011C	Metabolic	3:1315647	1170	1477	1781	1784	1405	1125	17.28429	18.28939	18.12513	17.16889	18.20056	20.6065	17.8996	18.65865	1	1	-
ENSMUSC	Fubp1	far upstre	GO:00325	developm	-	-	-	-	3:1522104	2111	2187	2674	3144	2270	1072	4.383181	3.806299	3.824847	4.252706	4.133041	2.759828	4.004776	3.715192	0.489045	1	-
ENSMUSC	Dnajb4	DnaJ heat	GO:00987	molecular	-	-	-	-	3:1521785	986	718	889	871	1267	747	5.233713	3.194554	3.250769	3.011848	5.897287	4.916316	3.893012	4.608484	0.495259	1	-
ENSMUSC	Ptgfr	prostaglaı	GO:00048	signal trar	K04262	prostaglaı	mmu0408	Neuroacti	3:1517965	4	14	29	19	9	12	0.030126	0.088387	0.150469	0.093223	0.059442	0.112063	0.08966	0.088243	0.893885	1	-
ENSMUSC	Efna3	ephrin A3	GO:00325	developm	K05462	ephrin-A[	mmu0415	PI3K-Akt	3:8931389	959	1188	1254	1349	1038	842	21.40756	22.2289	19.28402	19.61744	20.31836	23.30488	20.97349	21.08023	0.762238	1	-

ENSMUSC	Efna4	ephrin A4	GO:00325	developm	K05462	ephrin-A[	mmu0415	PI3K-Akt s	3:8933339	0	8	15	8	6	5	0	0.266508	0.410714	0.207137	0.209097	0.246388	0.225741	0.220874	1	1	-
ENSMUSC	Adam15	a disinteg	GO:00325	developm	-	-	-	-	3:8933854	1188	1498	1537	1846	1290	1608	6.947729	7.343299	6.192299	7.032987	6.615441	11.65999	6.827775	8.43614	0.264605	1	-
ENSMUSC	Zbtb7b	zinc finge	GO:00325	developm	-	-	-	-	3:8937764	274	220	393	349	344	201	2.459541	1.65531	2.430229	2.040844	2.70772	2.237103	2.181693	2.328555	0.865827	1	-
ENSMUSC	Cks1b	CDC28 pr	GO:00081	metabolic	K02219	cyclin-der	mmu0520	Pathways	3:8941547	9	14	22	29	8	18	0.332928	0.434114	0.560636	0.69887	0.259518	0.825627	0.442559	0.594671	0.514678	1	-
ENSMUSC	Thbs3	thrombos	GO:00325	developm	K04659	thrombos	mmu0415	PI3K-Akt s	3:8921518	309	411	411	487	351	268	2.207742	2.461417	2.022939	2.266735	2.199076	2.374161	2.230699	2.279991	0.907037	1	-
ENSMUSC	Gba	glucosida	GO:00325	developm	K01201	glucosylce	mmu0110	Metabolic	3:8920292	431	530	682	673	487	385	6.708637	6.914888	7.312949	6.824226	6.647041	7.430253	6.978825	6.967173	0.752555	1	-
ENSMUSC	Scamp3	secretory	GO:00511	localizatio	-	-	-	-	3:8917747	392	487	591	604	432	581	5.117664	5.329262	5.315263	5.136934	4.945522	9.404756	5.254063	6.495737	0.329893	1	-
ENSMUSC	Hcn3	hyperpola	GO:00452	synapse|c	-	-	-	-	3:8914607	226	299	276	331	262	232	2.594473	2.877158	2.182729	2.475426	2.637449	3.302275	2.551453	2.80505	0.746178	1	-
ENSMUSC	Ash1l	ash1 (abs	GO:00325	developm	K06101	histone-ly	mmu0031	Lysine deg	3:8895062	4371	4866	6428	7373	4950	2813	15.69436	14.64495	15.89974	17.24601	15.58514	12.52329	15.41302	15.11815	0.705119	1	-
ENSMUSC	Rit1	Ras-like w	GO:00508	response	-	-	-	-	3:8871683	371	346	529	480	358	244	2.938453	2.297072	2.886371	2.476669	2.486398	2.396184	2.707299	2.453084	0.34637	1	-
ENSMUSC	Arhgef2	rho/rac gu	GO:00325	developm	K12791	Rho guan	mmu0453	Tight junc	3:8860596	2475	2860	3716	3957	2285	2232	8.028464	7.776358	8.303945	8.361901	6.499594	8.977128	8.036256	7.946208	0.661139	1	-
ENSMUSC	2810403A	RIKEN cDI	GO:00081	metabolic	-	-	-	-	3:8868580	764	833	1221	1106	876	516	4.086048	3.734289	4.498597	3.853423	4.108251	3.421731	4.106311	3.794469	0.402461	1	-
ENSMUSC	Lamtor2	late endos	GO:00081	metabolic	K20398	ragulator	mmu0415	mTOR sig	3:8854981	462	557	671	552	490	354	12.78253	12.91765	12.78937	9.949368	11.88813	12.14407	12.82985	11.32719	0.211806	1	-
ENSMUSC	Lmna	lamin A [S	GO:00325	developm	K12641	lamin A/C	mmu0421	Apoptosis	3:8848014	535	588	809	885	627	503	3.475505	3.2018	3.620462	3.745322	3.571696	4.051524	3.432589	3.789514	0.644554	1	-
ENSMUSC	Sema4a	sema don	GO:00325	developm	K06521	semaphor	mmu0436	Axon guic	3:8843595	379	491	526	580	412	497	2.580248	2.80192	2.466942	2.572358	2.459582	4.195311	2.61637	3.07575	0.469851	1	-
ENSMUSC	Pmf1	polyamine	GO:00081	metabolic	-	-	-	-	3:8839414	41	51	85	62	48	67	0.459388	0.478982	0.656099	0.452557	0.471614	0.930811	0.53149	0.618327	0.721127	1	-
ENSMUSC	Iqgap3	IQ motif c	GO:00325	developm	K05767	Ras GTPas	mmu0481	Regulation	3:8808197	8	13	25	28	9	13	0.036088	0.049158	0.077691	0.082287	0.035603	0.072712	0.054312	0.063534	0.781362	1	-
ENSMUSC	Gpatch4	G patch d	GO:00325	developm	-	-	-	-	3:8804310	138	326	195	369	291	101	1.703634	3.373406	1.65838	2.967603	3.150163	1.545977	2.24514	2.554581	0.652992	1	-
ENSMUSC	Naxe	NAD(P)H	GO:00081	metabolic	-	-	-	-	3:8805652	431	622	754	725	519	487	30.1667	36.49166	36.35573	33.0575	31.8537	42.26352	34.33803	35.72491	0.974634	1	-
ENSMUSC	Ntrk1	neurotrop	GO:00325	developm	K03176	neurotrop	mmu0520	Pathways	3:8777824	0	5	1	1	2	0	0	0.102914	0.016911	0.015996	0.043064	0	0.039942	0.019686	0.5912	1	-
ENSMUSC	Pear1	platelet er	GO:00511	localizatio	-	-	-	-	3:8774789	67	75	101	147	99	84	0.304082	0.28532	0.315782	0.434624	0.393999	0.472693	0.301728	0.433772	0.054954	1	-
ENSMUSC	Cd1d1	CD1d1 an	GO:00160	membran	K06448	T-cell surf	mmu0453	Tight junc	3:8699583	8	6	14	10	12	14	0.125377	0.078819	0.151153	0.102097	0.16492	0.272067	0.118449	0.179695	0.361755	1	-
ENSMUSC	Dclk2	doublecor	GO:00325	developm	-	-	-	-	3:8678615	1281	1622	1652	2098	1499	1359	4.605761	4.888278	4.091792	4.914045	4.726029	6.058395	4.528611	5.232823	0.406874	1	-
ENSMUSC	Lrba	LPS-respc	GO:00160	membran	-	-	-	-	3:8622468	1008	1162	1457	1504	1080	656	2.952412	2.852828	2.939865	2.869763	2.773849	2.382353	2.915035	2.675322	0.35502	1	-
ENSMUSC	Rps3a1	ribosomal	GO:00325	developm	K02984	small subu	mmu0301	Ribosome	3:8613794	2291	2664	3527	3814	2646	1427	149.0276	145.2542	158.0514	161.6233	150.9295	115.0937	150.7777	142.5488	0.525212	1	-
ENSMUSC	Sh3d19	SH3 dom	GO:00325	developm	-	-	-	-	3:8597110	647	591	977	778	655	366	2.45764	1.881717	2.556586	1.925199	2.181716	1.723777	2.298648	1.943564	0.166091	1	-
ENSMUSC	Gatb	glutamyl-	GO:00081	metabolic	K02434	aspartyl-t	mmu0110	Metabolic	3:8557411	281	370	395	364	342	285	3.159658	3.487297	3.059722	2.666348	3.372118	3.973428	3.235559	3.337298	0.920541	1	-
ENSMUSC	Fbxw7	F-box anc	GO:00325	developm	K10260	F-box anc	mmu0412	Ubiquitin	3:8481526	2156	2494	3015	3251	2544	1376	13.21187	12.81046	12.72783	12.97819	13.67021	10.4549	12.91672	12.36777	0.563474	1	-
ENSMUSC	Fmo5	flavin con	GO:00081	metabolic	K00485	dimethyla	mmu0098	Drug met	3:9762880	72	100	137	122	89	60	0.543253	0.632445	0.712101	0.599669	0.588848	0.561314	0.629267	0.583277	0.533664	1	-
ENSMUSC	Chd1l	chromodc	GO:00081	metabolic	-	-	-	-	3:9756074	193	213	294	327	242	157	2.57684	2.383753	2.70413	2.844187	2.83326	2.599042	2.554908	2.75883	0.778615	1	-
ENSMUSC	Acp6	acid phos	GO:00325	developm	-	-	-	-	3:9715877	179	238	317	358	241	207	1.04568	1.165403	1.275728	1.362421	1.234543	1.499351	1.16227	1.365438	0.406121	1	-
ENSMUSC	Gpr89	G protein	GO:00325	developm	-	-	-	-	3:9686828	504	640	708	826	636	436	3.522953	3.749814	3.40927	3.761304	3.898309	3.778762	3.560679	3.812791	0.811597	1	-
ENSMUSC	Rnf115	ring finge	GO:00081	metabolic	-	-	-	-	3:9672766	1011	1267	1486	1582	1198	673	14.10893	14.82084	14.28609	14.3824	14.66029	11.64512	14.40528	13.5626	0.486518	1	-
ENSMUSC	Polr3c	polymeras	GO:00081	metabolic	K03023	DNA-dire	mmu0110	Metabolic	3:9671149	380	432	449	497	456	244	4.119072	3.925122	3.35285	3.509571	4.334351	3.279376	3.799015	3.707766	0.702263	1	-
ENSMUSC	Nudt17	nudix (nuc	GO:00038	catalytic a	-	-	-	-	3:9670606	79	97	123	117	73	128	1.226645	1.262468	1.315687	1.183481	0.993945	2.464277	1.268267	1.547235	0.537341	1	-
ENSMUSC	Pias3	protein in	GO:00081	metabolic	K16064	E3 SUMO	mmu0463	Jak-STAT	3:9669638	231	323	380	414	309	293	1.561286	1.829888	1.769314	1.822855	1.831351	2.455408	1.720163	2.036538	0.389631	1	-
ENSMUSC	Pex11b	peroxison	GO:00508	response	K13352	peroxin-1	mmu0414	Peroxison	3:9663537	196	208	326	303	420	249	2.019192	1.796129	2.313609	2.033505	3.79414	3.180587	2.042977	3.002744	0.048319	1	-
ENSMUSC	Polr3gl	polymeras	GO:00081	metabolic	K03024	DNA-dire	mmu0110	Metabolic	3:9657787	302	314	391	541	382	232	7.22629	6.297824	6.445199	8.433094	8.015194	6.883055	6.656437	7.777114	0.403144	1	-
ENSMUSC	Tars2	threonyl-t	GO:00081	metabolic	K01868	threonyl-t	mmu0097	Aminoacy	3:9573997	385	488	565	581	449	435	2.354946	2.502032	2.380787	2.315145	2.408294	3.299099	2.412589	2.674179	0.685684	1	-
ENSMUSC	Ecm1	extracellul	GO:00325	developm	-	-	-	-	3:9573414	85	121	148	181	93	123	1.124947	1.34231	1.349358	1.560534	1.079293	2.018391	1.272205	1.552739	0.417367	1	-
ENSMUSC	Ctsk	cathepsin	GO:00081	metabolic	K01371	cathepsin	mmu0421	Apoptosis	3:9549925	31	42	57	60	24	24	1.303025	1.479743	1.650494	1.642929	0.884589	1.25078	1.477754	1.259433	0.416638	1	-
ENSMUSC	Mettl14	methyltra	GO:00325	developm	-	-	-	-	3:1233682	311	300	477	799	384	262	1.453095	1.174916	1.535334	2.431989	1.573284	1.517822	1.387782	1.841031	0.136845	1	-
ENSMUSC	Bnipl	BCL2/ade	GO:00099	cellular pr	-	-	-	-	3:9524127	0	2	0	3	2	0	0	0.029977	0	0.034944	0.031359	0	0.009992	0.022101	0.507403	1	-
ENSMUSC	Bcar3	breast car	GO:00325	developm	-	-	-	-	3:1222941	186	221	307	266	220	162	1.220155	1.215201	1.387368	1.136749	1.265517	1.317661	1.274241	1.239976	0.651176	1	-
ENSMUSC	Gclm	glutamate	GO:00325	developm	K11205	glutamate	mmu0110	Metabolic	3:1222455	924	919	1413	1361	1015	678	7.093001	5.913255	7.47226	6.806094	6.832299	6.453186	6.826172	6.697193	0.670535	1	-
ENSMUSC	Abca4	ATP-bind	GO:00511	localizatio	K05644	ATP-bind	mmu0201	ABC trans	3:1220444	176	64	392	107	129	97	0.783231	0.238732	1.201751	0.310201	0.503397	0.535221	0.741238	0.449606	0.067966	1	-
ENSMUSC	Pip5k1a	phosphati	GO:00081	metabolic	K00889	1-phosph	mmu0110	Metabolic	3:9505853	724	1388	1094	1151	1048	1028	4.137058	6.648067	4.306476	4.284599	5.251185	7.283372	5.030534	5.606385	0.711395	1	-
ENSMUSC	Abcd3	ATP-bind	GO:00081	metabolic	K05677	ATP-bind	mmu0414	Peroxison	3:1217587	1127	1444	1716	1811	977	1008	7.85612	8.437313	8.240486	8.22402	5.97202	8.712261	8.177973	7.6361	0.394587	1	-
ENSMUSC	F3	coagulatic	GO:00325	developm	K03901	coagulatic	mmu0493	AGE-RAG	3:1217235	611	633	852	891	742	454	11.54307	10.02389	11.08845	10.96577	12.29211	10.63461	10.88514	11.2975	0.992525	1	-
ENSMUSC	Tmem56	transmem	GO:00160	membran	-	-	-	-	3:1212017	876	197	1284	1338	525	437	5.509937	1.03863	5.563644	5.482523	2.895642	3.408084	4.037404	3.92875	0.850844	1	-
ENSMUSC	Ptbp2	polypyrim	GO:00325	developm	-	-	-	-	3:1197187	1516	2054	2124	2256	1768	1028	6.959932	7.904222	6.717566	6.747247	7.117553	5.851742	7.193907	6.572181	0.338358	1	-
ENSMUSC	Snx27	sorting ne	GO:00511	localizatio	-	-	-	-	3:9449754	2264	2634	3070	3396	2541	2034	12.47045	12.16114	11.64918	12.18582	12.27306	13.89129	12.09359	12.78339	0.900871	1	-
ENSMUSC	Celf3	CUGBP, El	GO:00081	metabolic	-	-	-	-	3:9447829	2548	2911	3033	3940	2797	2089	12.128	11.61408	9.945204	12.21709	11.67414	12.32861	11.22909	12.07328	0.780177	1	-
ENSMUSC	Adh5	alcohol de	GO:00081	metabolic	K00121	S-(hydrox	mmu0110	Metabolic	3:1384430	504	685	809	746	612	662	4.390171	5.001438	4.854575	4.233231	4.674613	7.149828	4.748728	5.352558	0.632767	1	-
ENSMUSC	Riiad1	regulatory	-	-	-	-	-	-	3:9446498	18	21	26	21	12	24	1.23803	1.210699	1.231946	0.940959	0.723754	2.04673	1.226892	1.237148	0.861318	1	-
ENSMUSC	Mrpl9	mitochon	GO:00081	metabolic	K02939	large subu	mmu0301	Ribosome	3:9444331	432	592	751	756	530	502	8.763125	10.06584	10.49463	9.990317	9.427445	12.62598	9.774531	10.68125	0.741821	1	-
ENSMUSC	Oaz3	ornithine	GO:00081	metabolic	-	-	-	-	3:9443241	5	0	3	0	3	15	0.053496	0	0.022111	0	0.028144	0.198991	0.025202	0.075712	0.261454	1	-
ENSMUSC	Them4	thioestera	GO:00081	metabolic	K16339	thioestera	mmu0415	PI3K-Akt s	3:9431008	304	462	476	548	363	244	4.171005	5.313275	4.499102	4.898125	4.367343	4.150903	4.661127	4.472124	0.607268	1	-
ENSMUSC	Rap1gds1	RAP1, GTI	GO:00508	response	-	-	-	-	3:1389259	4712	5904	7270	7272	5527	3683	21.9724	23.07658	23.35382	22.09061	22.59978	21.29412	22.80093	21.99484	0.533505	1	-
ENSMUSC	Rorc	RAR-relat	GO:00325	developm	K08534	RAR-relat	mmu0465	Th17 cell	3:9437279	34	27	69	67	41	26	0.393949	0.262223	0.550756	0.505729	0.416567	0.373531	0.402309	0.431943	0.894067	1	-
ENSMUSC	Tspan5	tetraspani	GO:00081	metabolic	-	-	-	-	3:1387421	777	2633	1373	3688	1065	3038	4.03998	11.47525	4.917905	12.49197	4.855686	19.58538	6.811044	12.31101	0.061397	1	-
ENSMUSC	Eif4e	eukaryotic	GO:00325	developm	K03259	translatior	mmu0415	PI3K-Akt s	3:1385261	1228	1632	2097	1790	1606	1316	11.18898	12.46421	13.1626	10.62494	12.83158	14.86736	12.27193	12.77463	0.977111	1	-
ENSMUSC	Mttp	microsom	GO:00081	metabolic	K14463	microsom	mmu0497	Fat digest	3:1380898	179	224	253	239	205	165	1.724152	1.808528	1.678789	1.499697	1.731495	1.970585	1.737157	1.733925	0.769544	1	-
ENSMUSC	Dapp1	dual adap	GO:00160	membran	K12229	dual adap	mmu0466	B cell rece	3:1379310	80	102	142	61	153	76	0.638401	0.682273	0.78063	0.317115	1.070631	0.751975	0.700435	0.713241	0.92814	1	-
ENSMUSC	Ppp3ca	protein pl	GO:00325	developm	K04348	serine/thr	mmu0401	MAPK sigr	3:1366701	34455	43265	53476	53837	38938	20608	190.9916	201.0257	204.2078	194.4127	189.2685	141.6393	198.7417	175.1068	0.230542	1	-
ENSMUSC	Nfkb1	nuclear fa	GO:00325	developm	K02580	nuclear fa	mmu0520	Pathways	3:1355846	281	373	481	482	398	319	1.100535	1.224503	1.29776	1.229777	1.366858	1.549081	1.207599	1.381905	0.500518	1	-
ENSMUSC	Manba	mannosid	GO:00081	metabolic	K01192	beta-mar	mmu0414	Lysosome	3:1354856	189	211	343	315	215	157	1.262773	1.181679	1.578738	1.371061	1.259635	1.300614	1.341063	1.310437	0.705506	1	-
ENSMUSC	Cisd2	CDGSH irc	GO:00325	developm	-	-	-	-	3:1354064	809	1004	1363	1227	927	610	5.830621	6.065304	6.767269	5.760926	5.85853	5.451066	6.221064	5.690174	0.324428	1	-
ENSMUSC	Bdh2	3-hydroxy	GO:00325	developm	K00019	3-hydroxy	mmu0110	Metabolic	3:1352812	17	23	46	32	31	34	0.22602	0.256323	0.421322	0.277165	0.361416	0.560505	0.301221	0.399695	0.437559	1	-
ENSMUSC	Tacr3	tachykinin	GO:00325	developm	K04224	tachykinin	mmu0408	Neuroacti	3:1348290	54	105	83	27	63	49	0.912249	1.486823	0.965928	0.297136	0.933259	1.026352	1.121667	0.752249	0.09974	1	-
ENSMUSC	Wls	wntless hc	GO:00325	developm	-	-	-	-	3:1598396	354	541	484	414	444	295	1.949716	2.497573	1.836389	1.485424	2.144341	2.014539	2.094559	1.881434	0.33205	1	-
ENSMUSC	Rpe65	retinal pig	GO:00325	developm	K11158	retinoid is	mmu0083	Retinol me	3:1595991	5	4	5	4	3	0	0.145632	0.09765	0.100326	0.075906	0.076615	0	0.114536	0.05084	0.172655	1	-
ENSMUSC	Depdc1a	DEP dom	GO:00081	metabolic	-	-	-	-	3:1594954	2	3	3	3	0	0	0.027752	0.034884	0.02867	0.02711	0	0	0.030435	0.009037	0.149655	1	-
ENSMUSC	Lrrc7	leucine ric	-	-	-	-	-	-	3:1580828	2570	2636	3006	4346	2880	1369	11.37563	9.780035	9.166057	12.5318	11.17834	7.513312	10.10724	10.40782	0.992251	1	-
ENSMUSC	Cth	cystathior	GO:00325	developm	K01758	cystathior	mmu0110	Metabolic	3:1578942	34	57	51	57	49	50	0.606969	0.852935	0.627199	0.662887	0.767056	1.106741	0.695701	0.845562	0.516006	1	-
ENSMUSC	Zranb2	zinc finge	GO:00081	metabolic	-	-	-	-	3:1575341	1346	1305	1692	1816	1421	769	7.20478	5.855161	6.239178	6.33247	6.669793	5.10373	6.43304	6.035331	0.485812	1	-
ENSMUSC	Adgrl2	adhesion	GO:00325	developm	-	-	-	-	3:1488155	924	1032	1434	1556	1174	704	1.913922	1.791781	2.046224	2.099634	2.132373	1.808052	1.917309	2.013353	0.9054	1	-
ENSMUSC	Uox	urate oxic	GO:00081	metabolic	K00365	urate oxid	mmu0110	Metabolic	3:1465704	6	11	8	7	7	5	0.058685	0.090182	0.053901	0.044601	0.060038	0.06063	0.067589	0.05509	0.61212	1	-
ENSMUSC	Rpf1	ribosome	GO:00081	metabolic	-	-	-	-	3:1465059	165	207	271	287	212	103	2.023195	2.127543	2.289161	2.292547	2.279466	1.565952	2.146633	2.045988	0.664331	1	-
ENSMUSC	Ctbs	chitobiase	GO:00081	metabolic	-	-	-	-	3:1464497	127	146	163	217	140	109	0.947103	0.912642	0.837403	1.054231	0.915517	1.007872	0.899049	0.99254	0.69772	1	-
ENSMUSC	Bcl10	B cell leuk	GO:00325	developm	K07368	B-cell CLL	mmu0515	Tuberculo	3:1459228	125	158	225	202	161	150	1.59617	1.69114	1.979257	1.680356	1.802754	2.374907	1.755522	1.952672	0.715518	1	-
ENSMUSC	Ddah1	dimethyla	GO:00325	developm	-	-	-	-	3:1457586	1666	2069	2607	2840	2126	1391	17.78886	18.51768	19.17633	19.75483	19.90578	18.41562	18.49429	19.35874	0.932742	1	-
ENSMUSC	Cyr61	cysteine ri	GO:00325	developm	-	-	-	-	3:1456469	41	40	81	83	50	47	1.290564	1.055391	1.756459	1.702005	1.380111	1.834364	1.367472	1.638827	0.532952	1	-
ENSMUSC	Col24a1	collagen,	GO:00325	developm	K19721	collagen, t	mmu0497	Protein di	3:1452924	10	22	46	19	20	16	0.065927	0.121572	0.208913	0.081599	0.115622	0.130787	0.132137	0.109336	0.438708	1	-
ENSMUSC	Cryz	crystallin,	GO:00081	metabolic	-	-	-	-	3:1545967	122	195	239	232	177	219	0.868733	1.163897	1.172402	1.076211	1.105205	1.933561	1.068344	1.371659	0.276514	1	-
ENSMUSC	Asph	aspartate	GO:00325	developm	-	-	-	-	4:9448069	1876	2284	2854	3007	2054	1643	6.116635	6.24207	6.4104	6.386968	5.872497	6.642065	6.256368	6.30051	0.780027	1	-
ENSMUSC	Trp53inp1	transform	GO:00325	developm	K15310	tumor prc	mmu0516	HTLV-I inf	4:1115643	259	309	413	439	303	225	2.28837	2.28843	2.51378	2.526809	2.347531	2.464878	2.363527	2.446406	0.991601	1	-
ENSMUSC	Ccne2	cyclin E2 [	GO:00081	metabolic	K06626	cyclin E[-]	mmu0520	Pathways	4:1119135	59	72	110	102	61	40	0.481712	0.492738	0.618698	0.542518	0.436725	0.404929	0.53105	0.461391	0.384508	1	-
ENSMUSC	Gem	GTP bindi	GO:00511	localizatio	-	-	-	-	4:1170445	20	34	41	41	18	15	0.520718	0.742002	0.735375	0.695394	0.410936	0.484231	0.666031	0.530187	0.340221	1	-
ENSMUSC	Cdh17	cadherin 1	GO:00325	developm	K06811	cadherin 1	mmu0522	Gastric ca	4:1175814	3	3	5	2	0	1	0.039691	0.033272	0.045578	0.017238	0	0.016402	0.039514	0.011213	0.08781	1	-
ENSMUSC	Fam92a	family witl	GO:00099	cellular pr	-	-	-	-	4:1215340	488	728	755	722	553	342	4.614475	5.770143	4.918139	4.44755	4.585323	4.009722	5.100919	4.347532	0.162625	1	-
ENSMUSC	Tmem55a	transmem	GO:00081	metabolic	K13084	phosphati	mmu0407	Phosphati	4:1486407	563	721	882	810	670	458	15.29728	16.4208	16.5092	14.33747	15.96333	15.42969	16.07576	15.2435	0.474905	1	-
ENSMUSC	Calb1	calbindin	GO:00325	developm	K14757	calbindin	mmu0496	Endocrine	4:1588126	1984	2605	2789	2935	2118	1387	23.25081	25.58923	22.51627	22.40713	21.76532	20.15389	23.78544	21.44212	0.253874	1	-
ENSMUSC	Decr1	2,4-dieno	GO:00081	metabolic	-	-	-	-	4:1591724	263	303	399	382	327	230	4.152658	4.010204	4.340049	3.929296	4.527525	4.502817	4.167637	4.319879	0.985226	1	-
ENSMUSC	Nbn	nibrin [So	GO:00325	developm	K10867	nijmegen	mmu0421	Cellular se	4:1595792	283	340	475	444	323	161	6.148896	6.192153	7.10977	6.284553	6.153979	4.337311	6.483606	5.591948	0.278745	1	-
ENSMUSC	Mmp16	matrix me	GO:00325	developm	K07996	matrix me	mmu0520	MicroRNA	4:1785289	535	574	790	865	627	440	2.909289	2.61636	2.959454	3.064297	2.989809	2.966689	2.828368	3.006932	0.852618	1	-
ENSMUSC	Cpne3	copine III	GO:00081	metabolic	-	-	-	-	4:1951925	359	393	566	542	372	317	3.058384	2.806357	3.321735	3.008002	2.778962	3.348443	3.062159	3.045135	0.734571	1	-
ENSMUSC	Rmdn1	regulator	GO:00990	supramole	-	-	-	-	4:1957516	84	120	116	144	114	70	2.561626	3.067421	2.436959	2.860774	3.048507	2.646824	2.688669	2.852035	0.897637	1	-
ENSMUSC	Tmem68	transmem	GO:00081	metabolic	-	-	-	-	4:3549041	462	641	821	695	552	449	6.004392	6.982941	7.350587	5.884286	6.290845	7.235351	6.779307	6.470161	0.502212	1	-
ENSMUSC	Tgs1	trimethylg	GO:00081	metabolic	K14292	trimethylg	mmu0301	RNA trans	4:3574875	664	808	1030	1021	744	538	6.530363	6.66091	6.978434	6.541487	6.416305	6.560502	6.723235	6.506098	0.575231	1	-
ENSMUSC	Rps20	ribosomal	GO:00081	metabolic	K02969	small subu	mmu0301	Ribosome	4:3831334	579	747	762	850	554	728	7.786122	8.420076	7.059089	7.446342	6.53273	12.13835	7.755095	8.705807	0.685292	1	-
ENSMUSC	Atp6v0d2	ATPase, H	GO:00511	localizatio	K02146	V-type H-	mmu0110	Metabolic	4:1987684	20	19	56	49	24	30	0.463726	0.369263	0.894469	0.74012	0.48796	0.862465	0.575819	0.696848	0.643829	1	-
ENSMUSC	Ubxn2b	UBX dom	GO:00081	metabolic	-	-	-	-	4:6191098	1007	1239	1721	1771	1311	755	9.635332	9.937138	11.34409	11.03919	10.99975	8.957157	10.30552	10.33203	0.827277	1	-
ENSMUSC	Nsmaf	neutral sp	GO:00987	molecular	K18953	factor ass	mmu0407	Sphingolip	4:6396207	456	652	810	866	731	432	2.492916	2.987745	3.050559	3.084201	3.504317	2.928284	2.84374	3.172267	0.584004	1	-
ENSMUSC	Faxc	failed axo	GO:00160	membran	-	-	-	-	4:2193132	1205	1407	1645	1895	1335	718	6.105509	5.9756	5.741851	6.254974	5.931421	4.510715	5.940987	5.565703	0.492477	1	-
ENSMUSC	Coq3	coenzyme	GO:00081	metabolic	K00591	polypreny	mmu0110	Metabolic	4:2187967	251	281	347	355	284	242	4.864831	4.56513	4.633138	4.48234	4.826767	5.815623	4.6877	5.041577	0.843446	1	-
ENSMUSC	Pnisr	PNN inter	GO:00056	cell|intrace	-	-	-	-	4:2184758	2531	2642	3305	4907	2528	1880	8.835632	7.730917	7.948191	11.15946	7.738649	8.137467	8.17158	9.011858	0.639586	1	-
ENSMUSC	Sdcbp	syndecan	GO:00325	developm	-	-	-	-	4:6365650	1956	2522	2970	2877	2278	2304	14.22633	15.37525	14.881	13.63156	14.52849	20.77747	14.82752	16.3125	0.706091	1	-
ENSMUSC	Tstd3	thiosulfate	GO:00038	catalytic a	-	-	-	-	4:2175738	323	342	480	467	378	269	12.9267	11.47266	13.23362	12.17541	13.2654	13.34825	12.54433	12.92969	0.954994	1	-
ENSMUSC	Ccnc	cyclin C [S	GO:00081	metabolic	-	-	-	-	4:2172770	481	632	821	816	622	216	4.500071	4.956148	5.291384	4.973321	5.102786	2.50561	4.915868	4.193906	0.338705	1	-
ENSMUSC	Odf2l	outer den	GO:00056	cell|intrace	-	-	-	-	3:1451185	174	278	303	304	227	155	0.610852	0.818055	0.732791	0.695249	0.698801	0.674688	0.720566	0.689579	0.60423	1	-
ENSMUSC	Fhl5	four and a	GO:00081	metabolic	-	-	-	-	4:2519990	2	2	0	2	0	3	0.108567	0.090979	0	0.070703	0	0.201895	0.066515	0.090866	1	1	-
ENSMUSC	Ndufaf4	NADH de	GO:00099	cellular pr	-	-	-	-	4:2489808	120	151	1005	173	694	348	1.162734	1.226387	6.708353	1.092007	5.896588	4.180847	3.032491	3.723147	0.690407	1	-
ENSMUSC	Clca3a2	chloride c	GO:00081	metabolic	K05030	calcium-a	mmu0497	Pancreatic	3:1447965	0	0	3	0	0	0	0	0	0.027592	0	0	0	0.009197	0	0.302445	1	-
ENSMUSC	Spaca1	sperm acr	GO:00325	developm	-	-	-	-	4:3402487	0	0	2	5	0	0	0	0	0.037633	0.088965	0	0	0.012544	0.029655	0.743789	1	-
ENSMUSC	Lmo4	LIM dom	GO:00325	developm	-	-	-	-	3:1441885	1187	1389	1779	1719	1662	985	11.36448	11.14689	11.73346	10.72152	13.95316	11.69287	11.41494	12.12251	0.854121	1	-
ENSMUSC	Gtf2b	general tr	GO:00081	metabolic	K03124	transcripti	mmu0520	Viral carci	3:1427650	616	669	849	796	594	288	8.410023	7.655885	7.98501	7.079639	7.111241	4.875219	8.016973	6.355367	0.087789	1	-
ENSMUSC	Pdlim5	PDZ and l	GO:00325	developm	-	-	-	-	3:1422395	483	606	848	827	644	543	2.423505	2.54872	2.93119	2.703235	2.833513	3.37818	2.634472	2.971643	0.549828	1	-
ENSMUSC	Rngtt	RNA guar	GO:00081	metabolic	K13917	mRNA-ca	mmu0301	mRNA sur	4:3331031	565	740	893	878	647	412	4.299781	4.720434	4.681668	4.352853	4.31762	3.887597	4.567294	4.186023	0.35697	1	-
ENSMUSC	Ube2j1	ubiquitin-	GO:00325	developm	K10578	ubiquitin-	mmu0414	Protein pr	4:3303141	1883	2382	2848	2820	2111	1549	32.57222	34.53755	33.93817	31.77808	32.02049	33.22266	33.68265	32.34041	0.491041	1	-
ENSMUSC	Rragd	Ras-relate	GO:00508	response	K16186	Ras-relate	mmu0415	mTOR sig	4:3298303	1794	2195	969	2798	2068	1612	18.74307	19.22231	6.974188	19.04354	18.94573	20.88186	14.97986	19.62371	0.266803	1	-
ENSMUSC	Gabrr1	gamma-a	GO:00048	signal trar	K05190	gamma-a	mmu0408	Neuroacti	4:3313252	2	0	2	1	1	3	0.055613	0	0.038308	0.018109	0.024376	0.10342	0.031307	0.048635	1	1	-
ENSMUSC	Casp8ap2	caspase 8	GO:00081	metabolic	-	-	-	-	4:3261545	215	490	716	690	455	161	1.912372	3.653276	4.387311	3.998199	3.548856	1.775596	3.317653	3.10755	0.722234	1	-
ENSMUSC	Map3k7	mitogen-	GO:00325	developm	K04427	mitogen-a	mmu0401	MAPK sigr	4:3196409	1153	1318	1625	1738	1241	902	9.232278	8.846021	8.963644	9.065905	8.713528	8.955138	9.013981	8.911524	0.684535	1	-
ENSMUSC	Epha7	Eph recep	GO:00325	developm	K05108	EphA7[2.7	mmu0436	Axon guic	4:2881313	2423	3190	4122	3760	2934	1557	12.7127	14.02904	14.89855	12.8515	13.49855	10.12883	13.88009	12.15963	0.217991	1	-
ENSMUSC	Akirin2	akirin 2 [S	GO:00325	developm	-	-	-	-	4:3455093	793	1053	1265	1178	922	610	23.13937	25.75487	25.42848	22.39263	23.59129	22.06956	24.77424	22.68449	0.32076	1	-
ENSMUSC	Rars2	arginyl-tR	GO:00081	metabolic	K01887	arginyl-tR	mmu0097	Aminoacy	4:3461495	335	413	493	526	358	151	5.788545	5.98174	5.868447	5.920954	5.424376	3.235097	5.879577	4.860143	0.227705	1	-
ENSMUSC	Slc35a1	solute car	GO:00511	localizatio	-	-	-	-	4:3466325	185	252	342	383	220	212	3.539221	4.041009	4.50727	4.773285	3.690646	5.028721	4.029166	4.497551	0.656114	1	-
ENSMUSC	Smim8	small inte	GO:00160	membran	-	-	-	-	4:3476866	42	124	161	168	119	76	0.910063	2.252158	2.403265	2.371459	2.261067	2.041845	1.855162	2.224791	0.520097	1	-
ENSMUSC	3110043C	RIKEN cDI	GO:00325	developm	-	-	-	-	4:3519128	286	207	453	456	206	284	2.353205	1.427635	2.567693	2.44422	1.486293	2.897326	2.116178	2.275946	0.896901	1	-
ENSMUSC	Aldob	aldolase E	GO:00081	metabolic	K01623	fructose-b	mmu0110	Metabolic	4:4953599	14	26	34	20	14	24	0.317764	0.494653	0.531631	0.295732	0.278642	0.675421	0.448016	0.416598	0.665308	1	-
ENSMUSC	Rnf20	ring finge	GO:00081	metabolic	-	-	-	-	4:4963200	1326	1621	2092	2187	1662	1185	7.747658	7.938948	8.420541	8.324486	8.515312	8.584821	8.035716	8.474873	0.897066	1	-
ENSMUSC	Ppp3r2	protein pl	GO:00081	metabolic	K06268	serine/thr	mmu0401	MAPK sigr	4:4967874	6	0	3	8	5	4	0.126019	0	0.043403	0.109455	0.092076	0.104163	0.056474	0.101898	0.276125	1	-
ENSMUSC	Smc2	structural	GO:00329	macromo	-	-	-	-	4:5243924	339	345	480	555	309	186	3.588353	3.061033	3.500165	3.827103	2.86812	2.441152	3.383183	3.045458	0.417623	1	-
ENSMUSC	Polr1e	polymeras	GO:00081	metabolic	K03005	DNA-dire	mmu0110	Metabolic	4:4501858	76	93	99	108	74	68	0.837528	0.859053	0.751572	0.775339	0.715093	0.929138	0.816051	0.806524	0.766013	1	-
ENSMUSC	Exosc3	exosome	GO:00325	developm	K03681	exosome	mmu0301	RNA degr	4:4531661	72	92	147	145	123	80	2.09324	2.241944	2.944122	2.746226	3.135685	2.883774	2.426435	2.921895	0.407193	1	-
ENSMUSC	Stra6l	STRA6-lik	GO:00511	localizatio	-	-	-	-	4:4584866	0	0	11	9	0	6	0	0	0.04138	0.032017	0	0.040629	0.013793	0.024215	0.631753	1	-
ENSMUSC	Tmod1	tropomoc	GO:00325	developm	-	-	-	-	4:4603893	631	692	897	999	754	399	9.411397	8.651342	9.216548	9.7067	9.861396	7.378753	9.093095	8.982283	0.768412	1	-
ENSMUSC	Xpa	xeroderm	GO:00325	developm	K10847	DNA-repa	mmu0152	Platinum	4:4615534	104	114	140	172	106	70	2.515988	2.311703	2.33322	2.710725	2.248663	2.099726	2.38697	2.353038	0.811307	1	-
ENSMUSC	Ncbp1	nuclear ca	GO:00081	metabolic	K12882	nuclear ca	mmu0301	RNA trans	4:4613861	1107	1521	1831	1897	1320	840	18.96312	21.83958	21.60738	21.16952	19.82799	17.84133	20.80336	19.61295	0.469428	1	-
ENSMUSC	Trmo	tRNA met	GO:00081	metabolic	-	-	-	-	4:4637650	34	30	57	68	37	40	0.657181	0.48604	0.758986	0.856241	0.62712	0.958627	0.634069	0.813996	0.365551	1	-
ENSMUSC	Hemgn	hemogen	GO:00325	developm	-	-	-	-	4:4639398	0	0	0	0	2	2	0	0	0	0	0.042168	0.059645	0	0.033937	0.156276	1	-
ENSMUSC	Anp32b	acidic (leu	GO:00325	developm	-	-	-	-	4:4645090	965	1320	1452	1395	1079	494	35.73931	40.97751	37.04561	33.65696	35.04152	22.68462	37.92081	30.46103	0.101765	1	-
ENSMUSC	Nans	N-acetyln	GO:00081	metabolic	K05304	sialic acid	mmu0110	Metabolic	4:4648924	208	234	297	289	230	189	2.562318	2.416234	2.520449	2.31926	2.484507	2.886819	2.499667	2.563529	0.914333	1	-
ENSMUSC	Coro2a	coronin, a	GO:00329	macromo	-	-	-	-	4:4653693	406	510	590	619	513	409	2.674668	2.81622	2.677609	2.656538	2.963493	3.340821	2.722832	2.986951	0.707154	1	-
ENSMUSC	Col15a1	collagen,	GO:00051	structural	K08135	collagen, t	mmu0497	Protein di	4:4720816	88	119	147	147	105	87	0.597957	0.677776	0.688106	0.650705	0.625629	0.732978	0.654613	0.669771	0.926949	1	-
ENSMUSC	Erp44	endoplasr	GO:00081	metabolic	-	-	-	-	4:4819332	767	952	1296	1180	898	389	12.66108	13.17241	14.73777	12.68934	12.99853	7.961797	13.52375	11.21656	0.184648	1	-
ENSMUSC	Invs	inversin [S	GO:00508	response	K19626	inversin[-]	mmu0431	Wnt signa	4:4827976	232	309	346	414	334	182	1.167031	1.302887	1.19901	1.356681	1.473275	1.13515	1.222976	1.321702	0.76329	1	-
ENSMUSC	Tex10	testis expr	GO:00329	macromo	-	-	-	-	4:4843085	220	321	313	445	239	215	2.153013	2.633184	2.110178	2.837043	2.050993	2.608844	2.298792	2.49896	0.77103	1	-
ENSMUSC	Tmeff1	transmem	GO:00325	developm	-	-	-	-	4:4858517	1661	1971	2474	2543	1927	872	29.12846	28.97259	29.88815	29.05199	29.63278	18.96052	29.32973	25.88176	0.307832	1	-
ENSMUSC	Cavin4	caveolae a	GO:00325	developm	-	-	-	-	4:4866351	10	8	17	17	13	8	0.309414	0.207471	0.362347	0.342658	0.352713	0.30691	0.293077	0.334094	0.852564	1	-
ENSMUSC	Brinp1	bone mor	GO:00325	developm	-	-	-	-	4:6876151	4604	5131	6357	6865	4827	3143	44.35319	41.43282	42.18842	43.08358	40.77647	37.54223	42.65815	40.46742	0.466665	1	-
ENSMUSC	Fmn2	formin 2 [	GO:00325	developm	-	-	-	-	1:1745018	1958	2300	2709	3024	2256	1160	14.50651	14.28337	13.82644	14.59531	14.65656	10.656	14.20544	13.30262	0.484626	1	-
ENSMUSC	Kif12	kinesin fa	GO:00990	supramole	-	-	-	-	4:6316563	0	0	0	0	0	3	0	0	0	0	0	0.044989	0	0.014996	0.188327	1	-
ENSMUSC	Slc44a5	solute car	GO:00160	membran	K15377	solute car	mmu0523	Choline m	3:1539734	876	1140	1320	853	1094	479	6.205819	6.769446	6.441997	3.93664	6.796028	4.207424	6.472421	4.980031	0.064691	1	-
ENSMUSC	Tnfsf8	tumor nec	GO:00325	developm	K05471	tumor nec	mmu0406	Cytokine-	4:6383130	0	3	6	6	4	2	0	0.041977	0.068998	0.065253	0.058549	0.041408	0.036992	0.05507	0.574816	1	-
ENSMUSC	Tnc	tenascin C	GO:00325	developm	K06252	tenascin[-	mmu0415	PI3K-Akt s	4:6395978	215	264	291	379	207	171	1.723282	1.773679	1.606801	1.978967	1.454894	1.699416	1.701254	1.711092	0.85241	1	-
ENSMUSC	Txn1	thioredox	GO:00081	metabolic	K03671	thioredox	mmu0462	NOD-like	4:5794337	225	340	388	353	276	457	13.60555	17.23316	16.16281	13.90561	14.63475	34.26381	15.66717	20.93472	0.256277	1	-
ENSMUSC	Svep1	sushi, von	GO:00055	extracellul	-	-	-	-	4:5804244	18	29	31	26	20	7	0.058364	0.078818	0.069246	0.05492	0.056867	0.028144	0.06881	0.046644	0.166612	1	-
ENSMUSC	Pappa	pregnanc	GO:00081	metabolic	-	-	-	-	4:6512417	25	45	46	68	41	23	0.144086	0.217392	0.182635	0.255312	0.207207	0.16436	0.181371	0.20896	0.627564	1	-
ENSMUSC	Astn2	astrotacti	GO:00325	developm	-	-	-	-	4:6538080	536	624	943	964	627	494	6.126696	5.978591	7.42548	7.178275	6.284515	7.001224	6.510256	6.821338	0.947926	1	-
ENSMUSC	Ptgr1	prostaglai	GO:00081	metabolic	-	-	-	-	4:5896543	25	40	58	49	47	35	0.692003	0.928064	1.105972	0.883566	1.140798	1.201215	0.90868	1.075193	0.593762	1	-
ENSMUSC	Ugcg	UDP-gluc	GO:00081	metabolic	K00720	ceramide	mmu0110	Metabolic	4:5918925	1822	2146	2602	2768	1992	1215	19.75325	19.50172	19.43341	19.54961	18.93748	16.3325	19.56279	18.2732	0.415829	1	-
ENSMUSC	Ptbp3	polypyrim	GO:00325	developm	-	-	-	-	4:5947186	714	758	1090	1247	815	569	2.403815	2.13907	2.528022	2.734961	2.406043	2.375206	2.356969	2.505403	0.843773	1	-
ENSMUSC	Hsdl2	hydroxyst	GO:00081	metabolic	-	-	-	-	4:5958156	567	658	872	793	618	328	8.581696	8.347735	9.091974	7.818893	8.202031	6.155322	8.673802	7.392082	0.181714	1	-

ENSMUS(	Snx30	sorting ne	GO:00511	localizatio	-	-	-	-	4:5980584	1106	1246	1739	1828	1285	936	7.033885	6.642189	7.618882	7.573527	7.166154	7.380772	7.098319	7.373484	0.99575	1	-
ENSMUS(	Zfp37	zinc finge	GO:00325	developm	-	-	-	-	4:6218954	236	291	372	431	309	142	1.295882	1.339364	1.407172	1.541742	1.487834	0.96678	1.347473	1.332119	0.835301	1	-
ENSMUS(	Wdr31	WD repea	-	-	-	-	-	-	4:6244865	86	111	119	131	79	65	0.897317	0.970783	0.855354	0.890432	0.722798	0.8409	0.907818	0.818043	0.426756	1	-
ENSMUS(	Bspry	B-box and	GO:00511	localizatio	-	-	-	-	4:6248005	5	15	3	11	8	0	0.088293	0.222027	0.036493	0.126535	0.123882	0	0.115605	0.083472	0.660645	1	-
ENSMUS(	Alad	aminolevu	GO:00081	metabolic	K01698	porphobil	mmu011C	Metabolic	4:6250916	160	180	274	265	176	287	4.905187	4.625547	5.786811	5.292536	4.731417	10.90949	5.105848	6.977814	0.197933	1	-
ENSMUS(	Pole3	polymera	GO:00081	metabolic	K02326	DNA poly	mmu011C	Metabolic	4:6252264	321	405	566	483	346	246	8.423011	8.907779	10.23127	8.256409	7.961233	8.003561	9.187355	8.073734	0.230307	1	-
ENSMUS(	Kdm4c	lysine (K)-	GO:00325	developm	-	-	-	-	4:7424249	706	807	1041	1039	751	411	7.136698	6.837845	7.24928	6.842104	6.656948	5.151337	7.074608	6.216797	0.258174	1	-
ENSMUS(	Dmac1	distal mer	GO:00329	macromo	-	-	-	-	4:7527735	129	137	186	168	123	151	11.06389	9.849013	10.98959	9.386625	9.250484	16.05761	10.63416	11.56491	0.877265	1	-
ENSMUS(	Ptprd	protein ty	GO:00325	developm	-	-	-	-	4:7594123	3815	4189	5925	6067	4526	2467	7.438859	6.8466	7.958873	7.706681	7.738717	5.964403	7.414778	7.1366	0.596052	1	-
ENSMUS(	Mpdz	multiple P	GO:00452	synapse|n	K06095	multiple P	mmu0453	Tight junc	4:8127850	1823	2232	2615	2952	2156	1201	6.871716	7.052216	6.790498	7.248974	7.126396	5.613161	6.90481	6.662844	0.608684	1	-
ENSMUS(	Zdhhc21	zinc finge	GO:00325	developm	-	-	-	-	4:8279873	970	1135	1515	1494	1141	715	3.814991	3.741711	4.10474	3.82784	3.935048	3.486693	3.887147	3.74986	0.576231	1	-
ENSMUS(	Aco1	aconitase	GO:00325	developm	K01681	aconitate	mmu011C	Metabolic	4:4014308	503	511	727	772	556	459	6.435914	5.480439	6.408078	6.434887	6.238199	7.281823	6.108144	6.651636	0.739603	1	-
ENSMUS(	Smim27	small inte	-	-	-	-	-	-	4:4026957	4	7	17	12	7	28	0.189845	0.278475	0.555834	0.371052	0.291357	1.647778	0.341384	0.770062	0.147469	1	-
ENSMUS(	Smu1	smu-1 sup	GO:00081	metabolic	-	-	-	-	4:4073654	1097	1356	1602	1697	1272	717	18.84261	19.52302	18.95607	18.98882	19.15862	15.27001	19.10723	17.80582	0.438456	1	-
ENSMUS(	Dnaja1	DnaJ heat	GO:00081	metabolic	K09502	DnaJ hom	mmu0414	Protein pr	4:4072215	1716	2017	2638	2393	1851	1420	7.899242	7.782643	8.365549	7.176169	7.471665	8.104803	8.015811	7.584212	0.408028	1	-
ENSMUS(	Aptx	aprataxin	GO:00081	metabolic	-	-	-	-	4:4068238	452	520	675	623	519	357	3.349183	3.229661	3.445527	3.007258	3.372177	3.279859	3.341457	3.219764	0.568809	1	-
ENSMUS(	Slc44a1	solute car	GO:00511	localizatio	K06515	solute car	mmu0523	Choline m	4:5344041	1143	1228	2041	1869	1256	1146	9.163759	8.252368	11.27254	9.761538	8.82998	11.39194	9.562888	9.994486	0.988629	1	-
ENSMUS(	B4galt1	UDP-Gal:I	GO:00325	developm	K07966	beta-1,4-	mmu011C	Metabolic	4:4080460	147	160	204	237	169	167	2.20649	2.013071	2.109437	2.317479	2.224411	3.108048	2.109666	2.549979	0.365086	1	-
ENSMUS(	Fktn	fukutin [S	GO:00325	developm	K19872	fukutin [E	mmu011C	Metabolic	4:5371399	646	803	891	918	699	777	3.289685	3.427597	3.125724	3.045411	3.121341	4.906008	3.281002	3.69092	0.634465	1	-
ENSMUS(	Spink4	serine pep	GO:00081	metabolic	-	-	-	-	4:4092005	0	0	3	0	0	2	0	0	0.211835	0	0	0.25426	0.070612	0.084753	1	1	-
ENSMUS(	Bag1	BCL2-asso	GO:00325	developm	K09555	BCL2-asso	mmu0414	Protein pr	4:4093639	1209	1543	1842	2039	1307	996	21.60731	23.11496	22.67856	23.73959	20.48295	22.07089	22.46694	22.09781	0.659038	1	-
ENSMUS(	Chmp5	charged r	GO:00081	metabolic	K12198	charged n	mmu0414	Endocytos	4:4094840	944	1298	1581	1362	1119	919	39.49574	45.52038	45.56817	37.12248	41.05358	47.67379	43.5281	41.94995	0.515505	1	-
ENSMUS(	Tmem38b	transmem	GO:00511	localizatio	-	-	-	-	4:5382604	193	246	249	277	180	192	2.842586	3.036992	2.526421	2.657767	2.324723	3.506241	2.802	2.829577	0.822628	1	-
ENSMUS(	Nfx1	nuclear tr	GO:00081	metabolic	K12236	transcripti	mmu0516	Human pa	4:4097090	1515	1890	2288	2757	1994	1354	9.322505	9.748425	9.69901	11.05194	10.75939	10.33058	9.58998	10.71397	0.531739	1	-
ENSMUS(	Rad23b	RAD23 ho	GO:00325	developm	K10839	UV excisio	mmu0414	Protein pr	4:5535004	4240	5134	5898	6192	4605	2313	39.56898	40.16037	37.91798	37.6445	37.68437	26.76398	39.21578	34.03095	0.211768	1	-
ENSMUS(	Nol6	nucleolar	GO:00081	metabolic	K14544	U3 small r	mmu030C	Ribosome	4:4111442	2383	2886	3234	3527	2736	2562	25.66461	26.05312	23.99396	24.74557	25.83862	34.21177	25.23723	28.26532	0.573041	1	-
ENSMUS(	Ikbkap	inhibitor c	GO:00081	metabolic	-	-	-	-	4:5674968	1048	1201	1467	1497	1133	900	6.943654	6.669951	6.695893	6.461457	6.58263	7.393587	6.769832	6.812558	0.771991	1	-
ENSMUS(	Ubap2	ubiquitin-	GO:00081	metabolic	-	-	-	-	4:4119431	1137	1198	1404	1539	1166	736	10.61863	9.378158	9.032885	9.363284	9.548812	8.522591	9.676558	9.144896	0.474186	1	-
ENSMUS(	Epb41l4b	erythrocyt	GO:00081	metabolic	-	-	mmu0453	Tight junc	4:5699197	827	980	1049	1096	888	575	5.59429	5.556717	4.888395	4.829827	5.267387	4.822735	5.346468	4.973316	0.399041	1	-
ENSMUS(	Dcaf12	DDB1 and	GO:00081	metabolic	-	-	-	-	4:4129130	649	821	993	1081	836	906	11.98312	12.70636	12.63066	13.00267	13.5355	20.74147	12.44005	15.75988	0.174365	1	-
ENSMUS(	Ubap1	ubiquitin-	GO:00081	metabolic	-	-	-	-	4:4134899	658	761	989	1038	721	559	7.782965	7.544953	8.058736	7.998308	7.478204	8.198178	7.795551	7.891564	0.835338	1	-
ENSMUS(	Kif24	kinesin fa	GO:00990	supramol	-	-	-	-	4:4139074	18	14	19	26	20	12	0.109259	0.071233	0.079449	0.102811	0.106457	0.090314	0.086647	0.09986	0.671432	1	-
ENSMUS(	Fam219a	family wit	-	-	-	-	-	-	4:4151769	2475	3103	3808	3656	2932	3251	20.84464	21.90554	22.09366	20.0589	21.65339	33.94863	21.61462	25.22031	0.434838	1	-
ENSMUS(	1110017D	RIKEN cDI	GO:00325	developm	-	-	-	-	4:4150500	34	24	37	21	17	26	0.733472	0.433975	0.549866	0.295128	0.321579	0.695458	0.572438	0.437388	0.22816	1	-
ENSMUS(	Nudt2	nudix (nu	GO:00038	catalytic a	K01518	bis(5'-nuc	mmu0023	Purine me	4:4146515	190	228	246	284	191	121	14.29001	14.37366	12.74576	13.91489	12.59669	11.28363	13.80314	12.59841	0.431435	1	-
ENSMUS(	Cntfr	ciliary neu	GO:00325	developm	K05059	ciliary neu	mmu0406	Cytokine-	4:4165749	1019	1001	1467	1088	859	910	19.67808	16.20299	19.51595	13.68733	14.54601	21.78892	18.46567	16.67409	0.268066	1	-
ENSMUS(	Enho	energy ho	GO:00508	response	-	-	-	-	4:4163814	376	449	522	496	468	586	18.83051	18.84835	18.00927	16.1822	20.55244	36.38796	18.56271	24.3742	0.213032	1	-
ENSMUS(	Dctn3	dynactin 3	GO:00990	supramol	-	-	-	-	4:4171479	581	708	803	794	611	816	20.49072	20.92992	19.5096	18.24247	18.89578	35.68267	20.31008	24.27364	0.447354	1	-
ENSMUS(	1700022I1	RIKEN cDI	-	-	-	-	-	-	4:4296960	1	0	2	0	0	14	0.010047	0	0.013841	0	0	0.174369	0.007963	0.058123	0.128539	1	-
ENSMUS(	Vcp	valosin co	GO:00081	metabolic	K13525	transition	mmu0414	Protein pr	4:4297996	1749	2350	2953	2509	2126	2225	19.37835	21.82467	22.53937	18.10962	20.65537	30.56631	21.24746	23.11043	0.796716	1	-
ENSMUS(	Fancg	Fanconi a	GO:00325	developm	K10894	fanconi ar	mmu0346	Fanconi a	4:4300234	102	147	208	187	146	167	0.982038	1.186305	1.379564	1.172868	1.232598	1.993558	1.182636	1.466341	0.368169	1	-
ENSMUS(	Pigo	phosphati	GO:00081	metabolic	K05288	phosphati	mmu011C	Metabolic	4:4301763	223	251	329	345	258	415	2.116849	1.997156	2.151455	2.133471	2.147576	4.884489	2.088487	3.055179	0.097651	1	-
ENSMUS(	Stoml2	stomatin (	GO:00081	metabolic	-	-	-	-	4:4302769	349	331	485	524	393	426	3.709652	2.949094	3.551406	3.628446	3.66305	5.614395	3.403384	4.301964	0.22045	1	-
ENSMUS(	Unc13b	unc-13 ho	GO:00325	developm	K15293	protein ur	mmu0472	Synaptic v	4:4305895	908	1016	1467	1680	1213	726	2.003129	1.878753	2.229485	2.414426	2.346531	1.985847	2.037122	2.248935	0.616206	1	-
ENSMUS(	Atp8b5	ATPase, cl	GO:00511	localizatio	-	-	-	-	4:4326715	7	0	1	8	7	0	0.056416	0	0.00555	0.042	0.049471	0	0.020655	0.03049	0.640944	1	-
ENSMUS(	Tesk1	testis spec	GO:00048	signal trar	-	-	-	-	4:4344193	1315	1578	1732	1902	1511	1230	12.05253	12.12306	10.93582	11.3565	12.14394	13.97794	11.7038	12.49279	0.849629	1	-
ENSMUS(	Cd72	CD72 anti	GO:00160	membran	K06504	CD72 anti	mmu0466	B cell rece	4:4344646	16	11	33	24	16	23	0.158268	0.091207	0.224865	0.154651	0.138778	0.282086	0.158113	0.191838	0.696759	1	-
ENSMUS(	Ccdc107	coiled-co	GO:00160	membran	-	-	-	-	4:4349290	132	198	209	223	167	173	2.394798	3.011016	2.612123	2.63562	2.656782	3.89161	2.672645	3.061337	0.600651	1	-
ENSMUS(	Car9	carbonic a	GO:00325	developm	K01672	carbonic a	mmu0091	Nitrogen r	4:4350696	2	9	8	4	2	5	0.034065	0.12846	0.09385	0.04438	0.029863	0.105566	0.085459	0.059937	0.369254	1	-
ENSMUS(	Tpm2	tropomyo	GO:00051	structural	K10374	tropomyo	mmu0426	Adrenergi	4:4351471	96	148	211	201	164	126	1.096137	1.41647	1.659691	1.495101	1.642019	1.783818	1.390766	1.640313	0.470099	1	-
ENSMUS(	Tln1	talin 1 [So	GO:00511	localizatio	K06271	talin[-]	mmu0516	HTLV-I inf	4:4353151	991	1035	1430	1442	1028	1046	5.527559	4.838974	5.494743	5.239706	5.028004	7.233983	5.287092	5.833898	0.689115	1	-
ENSMUS(	Creb3	cAMP resp	GO:00081	metabolic	K09048	cyclic AMI	mmu0415	PI3K-Akt s	4:4356233	369	504	606	608	476	455	5.664512	6.485136	6.408548	6.080247	6.40745	8.660294	6.186065	7.04933	0.543442	1	-
ENSMUS(	Gba2	glucosida	GO:00325	developm	K17108	non-lysos	mmu011C	Metabolic	4:4356692	988	1137	1290	1822	1128	1308	6.9651	6.718674	6.264862	8.367591	6.973041	11.4331	6.649545	8.924578	0.066503	1	-
ENSMUS(	Rgp1	RAB6A GE	GO:00081	metabolic	-	-	-	-	4:4357871	616	762	1067	1141	898	729	4.905232	5.086117	5.853219	5.918971	6.270435	7.197672	5.281523	6.462359	0.203323	1	-
ENSMUS(	Npr2	natriuretic	GO:00325	developm	K12324	atrial natri	mmu0023	Purine me	4:4363193	671	926	1132	1307	987	742	5.822507	6.735215	6.766834	7.388307	7.510132	7.983216	6.441519	7.627218	0.293544	1	-
ENSMUS(	Hint2	histidine t	GO:00081	metabolic	-	-	-	-	4:4365422	98	120	147	150	107	94	1.98729	2.039727	2.053558	1.981567	1.902668	2.363484	2.026858	2.082573	0.928856	1	-
ENSMUS(	Spaar	small regu	GO:00325	developm	-	-	-	-	4:4373003	18	11	20	26	19	10	0.21645	0.11088	0.165685	0.203676	0.200344	0.149108	0.164338	0.184376	0.778747	1	-
ENSMUS(	Reck	reversion-	GO:00325	developm	K17461	reversion-	mmu052C	MicroRNA	4:4387553	147	231	286	257	234	183	1.439488	1.896078	1.929339	1.639479	2.009326	2.22192	1.754968	1.956908	0.68096	1	-
ENSMUS(	Clta	clathrin, li	GO:00452	synapse|c	K04644	clathrin li	mmu0414	Endocytos	4:4400445	2759	3229	4052	4101	3172	2576	48.30379	47.38604	48.8709	46.77361	48.69735	55.91925	48.18691	50.4634	0.970087	1	-
ENSMUS(	Gne	glucosam	GO:00081	metabolic	K12409	bifunction	mmu011C	Metabolic	4:4403407	636	720	870	943	665	494	3.320987	3.151338	3.12954	3.20777	3.04491	3.198324	3.200621	3.150335	0.674939	1	-
ENSMUS(	Glipr2	GLI patho	GO:00325	developm	-	-	-	-	4:4395740	38	37	75	64	37	67	1.005417	0.820579	1.367016	1.10312	0.858439	2.197986	1.064337	1.386515	0.444421	1	-
ENSMUS(	Snapc3	small nucl	GO:00081	metabolic	-	-	-	-	4:8341772	397	457	602	626	485	296	2.370847	2.28761	2.476629	2.435392	2.539787	2.191744	2.378362	2.388974	0.83508	1	-
ENSMUS(	Psip1	PC4 and S	GO:00081	metabolic	-	-	-	-	4:8345568	3395	4272	4677	5320	3917	2001	29.63363	31.25569	28.12315	30.2509	29.98067	21.65599	29.67082	27.29585	0.406498	1	-
ENSMUS(	Bnc2	basonucli	GO:00325	developm	-	-	-	-	4:8427509	0	10	7	13	0	0	0	0.031705	0.018242	0.032036	0	0	0.016649	0.010679	0.652735	1	-
ENSMUS(	Sh3gl2	SH3-dom	GO:00325	developm	K11247	endophilir	mmu0414	Endocytos	4:8520512	3785	4977	5133	6182	4118	3431	33.12879	36.51406	30.95012	35.24929	31.60594	37.23457	33.53099	34.6966	0.95797	1	-
ENSMUS(	Plin2	perilipin 2	GO:00511	localizatio	K17284	perilipin-2	mmu0332	PPAR sign	4:8664838	125	135	248	269	129	163	1.516346	1.372691	2.072484	2.125798	1.37221	2.451666	1.65384	1.983225	0.4671	1	-
ENSMUS(	Rps6	ribosomal	GO:00325	developm	K02991|K(	small subu	mmu0415	PI3K-Akt s	4:8685466	434	567	703	631	457	829	11.25337	12.32335	12.5574	10.65869	10.39087	26.65223	12.0447	15.9006	0.279152	1	-
ENSMUS(	Mllt3	myeloid/ly	GO:00325	developm	K15187	YEATS do	mmu052C	Transcript	4:8776992	820	1085	1268	1315	980	528	3.40544	3.776958	3.627691	3.55768	3.568852	2.718815	3.603363	3.281782	0.370804	1	-
ENSMUS(	Hacd4	3-hydroxy	GO:00081	metabolic	K10703	very-long	mmu0121	Fatty acid	4:8839614	57	75	87	118	54	57	0.433834	0.478483	0.456164	0.585076	0.3604	0.537908	0.45616	0.494461	0.846559	1	-
ENSMUS(	Usp24	ubiquitin s	GO:00081	metabolic	-	-	-	-	4:1063162	2137	2436	3032	3340	2392	1465	8.144205	7.781699	7.960216	8.292248	7.993704	6.92258	7.96204	7.736178	0.617266	1	-
ENSMUS(	Plpp3	phospholi	GO:00325	developm	K01080	phosphati	mmu011C	Metabolic	4:1051573	5122	5949	7299	7603	6137	4321	103.0448	100.319	101.1583	99.64445	108.2643	107.7846	101.5074	105.2311	0.980848	1	-
ENSMUS(	Prkaa2	protein ki	GO:00081	metabolic	K07198	5'-AMP-a	mmu0415	PI3K-Akt s	4:1050298	631	689	914	1006	737	495	5.032245	4.605793	5.02146	5.226511	5.153976	4.894676	4.886499	5.091721	0.969394	1	-
ENSMUS(	Dab1	disabled 1	GO:00325	developm	-	-	-	-	4:1036193	2465	2748	3470	3783	2859	1799	17.84262	16.6729	17.30304	17.83855	18.14673	16.14577	17.27286	17.37702	0.810136	1	-
ENSMUS(	Slc35d1	solute car	GO:00325	developm	-	-	-	-	4:1031706	65	93	348	222	110	262	0.336504	0.403565	1.24111	0.748711	0.499359	1.68177	0.660393	0.976614	0.304662	1	-
ENSMUS(	Mier1	MEIR1 tre	GO:00048	signal trar	-	-	-	-	4:1031143	778	915	1157	1207	756	477	2.241861	2.210055	2.296751	2.265781	1.910263	1.70425	2.249556	1.960098	0.21785	1	-
ENSMUS(	Tctex1d1	Tctex1 do	-	-	-	-	-	-	4:1029786	4	2	3	6	2	0	0.046421	0.019455	0.023984	0.045365	0.020352	0	0.029954	0.021906	0.834756	1	-
ENSMUS(	Sgip1	SH3-dom	GO:00511	localizatio	-	-	-	-	4:1027412	4050	4163	5282	5813	4138	2049	14.08965	12.1396	12.65887	13.17429	12.62346	8.838385	12.96271	11.54538	0.309413	1	-
ENSMUS(	Pde4b	phosphoc	GO:00081	metabolic	K13293	cAMP-spe	mmu0402	cAMP sign	4:1020875	1414	1553	2136	1950	1605	1038	5.389144	4.961289	5.608193	4.841573	5.36399	4.905164	5.319542	5.036909	0.449395	1	-
ENSMUS(	Ak4	adenylate	GO:00325	developm	K00939	adenylate	mmu011C	Metabolic	4:1014192	925	452	1002	964	656	998	9.95201	4.07625	7.42657	6.756596	6.188932	13.3133	7.15161	8.752942	0.506393	1	-
ENSMUS(	Dnajc6	DnaJ heat	GO:00081	metabolic	K09526	DnaJ hom	mmu0414	Endocytos	4:1014966	6194	7923	8885	9578	7408	5294	33.83885	36.28164	33.439	34.08795	35.48853	35.86033	34.51983	35.1456	0.855749	1	-
ENSMUS(	Jak1	Janus kina	GO:00325	developm	K11217	Janus kina	mmu052C	Pathways	4:1011523	4106	4665	5593	6283	4559	2781	28.4723	27.11491	26.71775	28.38261	27.72145	23.91061	27.43499	26.67156	0.613044	1	-
ENSMUS(	Cachd1	cache dor	GO:00511	localizatio	-	-	-	-	4:1007766	1093	1206	1702	1861	1328	791	10.38007	9.600204	11.13502	11.51352	11.05913	9.314137	10.37177	10.62893	0.953936	1	-
ENSMUS(	St3gal3	ST3 beta-	GO:00081	metabolic	K00781	N-acetylla	mmu011C	Metabolic	4:1179321	705	857	1021	991	790	470	8.811181	8.977984	8.790675	8.06864	8.657946	7.283309	8.859946	8.003298	0.302635	1	-
ENSMUS(	Artn	artemin [S	GO:00325	developm	-	-	-	-	4:1179261	5	6	7	2	5	6	0.07798	0.078432	0.075211	0.020317	0.068374	0.116034	0.077207	0.068242	0.664468	1	-
ENSMUS(	Dph2	DPH2 hor	GO:00081	metabolic	-	-	-	-	4:1178886	154	169	201	189	162	188	1.462738	1.345502	1.315202	1.169466	1.349279	2.214054	1.374481	1.5776	0.644111	1	-
ENSMUS(	B4galt2	UDP-Gal:I	GO:00325	developm	K07967	beta-1,4-	mmu011C	Metabolic	4:1178692	529	647	697	654	527	624	3.673864	3.766385	3.334663	2.958879	3.209377	5.373263	3.591637	3.847173	0.925379	1	-
ENSMUS(	Slc6a9	solute car	GO:00452	synapse|c	-	-	-	-	4:1178345	725	595	791	1049	736	571	4.714614	3.24323	3.543529	4.443912	4.196901	4.60395	3.833791	4.414921	0.471468	1	-
ENSMUS(	Bend5	BEN dom	GO:00081	metabolic	-	-	-	-	4:1114150	68	107	114	119	125	80	0.864497	1.140221	0.99841	0.985553	1.393501	1.261038	1.001043	1.213364	0.411993	1	-
ENSMUS(	Itgb3bp	integrin b	GO:00081	metabolic	-	-	-	-	4:9976540	66	66	73	99	73	42	0.632367	0.530056	0.48184	0.617936	0.61333	0.498961	0.548088	0.576742	0.918084	1	-
ENSMUS(	Atg4c	autophag	GO:00081	metabolic	-	-	mmu0414	Autophag	4:9919393	483	687	919	860	612	504	5.776461	6.886907	7.571495	6.700304	6.418137	7.473624	6.744955	6.864022	0.864574	1	-
ENSMUS(	Cdkn2c	cyclin-dep	GO:00325	developm	K06622	cyclin-dep	mmu0516	HTLV-I inf	4:1096608	49	36	36	58	40	35	1.101951	0.678614	0.557728	0.849723	0.788799	0.975934	0.779431	0.871485	0.783484	1	-
ENSMUS(	Eps15	epiderma	GO:00081	metabolic	K12472	epiderma	mmu0414	Endocytos	4:1092802	3909	4675	6228	6005	4739	2842	22.79372	22.84988	25.01782	22.81099	24.23142	20.54752	23.55381	22.52998	0.517522	1	-
ENSMUS(	Angptl3	angiopoie	GO:00325	developm	-	-	mmu0497	Cholester	4:9903095	0	6	2	4	2	0	0	0.080163	0.021965	0.041542	0.027953	0	0.034043	0.023165	0.667761	1	-
ENSMUS(	Ttc39a	tetratricop	-	-	-	-	-	-	4:1094066	30	33	59	50	26	35	0.325082	0.299737	0.440422	0.352952	0.24705	0.470246	0.355081	0.356749	0.846075	1	-
ENSMUS(	Dock7	dedicator	GO:00325	developm	-	-	-	-	4:9893667	624	736	896	880	627	489	1.881979	1.860634	1.861615	1.728999	1.658215	1.828627	1.868076	1.738614	0.3778	1	-
ENSMUS(	Rnf11	ring finge	GO:00081	metabolic	-	-	-	-	4:1094510	3473	4534	5211	5227	3913	2853	35.67473	39.03825	36.8747	34.97761	35.2459	36.33656	37.1959	35.52002	0.454729	1	-
ENSMUS(	Calr4	calreticuli	GO:00099	cellular pr	-	-	-	-	4:1092344	9	21	15	6	18	10	0.138794	0.27146	0.159362	0.060281	0.243411	0.191224	0.189872	0.164972	0.629744	1	-
ENSMUS(	Osbpl9	oxysterol	GO:00511	localizatio	-	-	-	-	4:1090611	455	609	825	716	602	463	2.492384	2.796233	3.113211	2.555042	2.891629	3.144631	2.800609	2.863768	0.88867	1	-
ENSMUS(	Usp1	ubiquitin s	GO:00325	developm	K11832	ubiquitin c	mmu0346	Fanconi a	4:9892381	429	716	685	899	531	254	5.706202	7.982808	6.276707	7.789887	6.193367	4.188995	6.655239	6.057416	0.511026	1	-
ENSMUS(	Tm2d1	TM2 dom	GO:00048	signal trar	-	-	-	-	4:9835537	213	328	359	336	262	194	8.284436	10.69329	9.619005	8.513426	8.935691	9.355613	9.532242	8.93491	0.474492	1	-
ENSMUS(	Nfia	nuclear fa	GO:00325	developm	-	-	-	-	4:9777273	1104	1464	2384	3054	2092	580	3.571158	3.969482	5.312483	6.435622	5.933954	2.326237	4.284374	4.898604	0.595844	1	-
ENSMUS(	Txndc12	thioredox	GO:00325	developm	K05360	protein-d	mmu0048	Glutathior	4:1088346	339	486	583	540	426	307	8.241955	9.904201	9.764519	8.552755	9.082019	9.254533	9.303558	8.963102	0.578364	1	-
ENSMUS(	Btf3l4	basic tran	-	-	-	-	-	-	4:1088142	740	898	1159	1105	916	606	8.707504	8.857092	9.394996	8.470438	9.451489	8.841382	8.986531	8.921103	0.725731	1	-
ENSMUS(	Cyp2j13	cytochron	GO:00081	metabolic	K07418	cytochron	mmu011C	Metabolic	4:9602753	0	0	1	0	3	0	0	0	0.007589	0	0.028988	0	0.00253	0.009663	0.668088	1	-
ENSMUS(	Hook1	hook micr	GO:00325	developm	-	-	-	-	4:9596724	846	1558	1454	1313	1117	574	4.010582	6.190951	4.748459	4.054929	4.643364	3.373917	4.983331	4.02407	0.105062	1	-
ENSMUS(	Fggy	FGGY carb	GO:00081	metabolic	-	-	-	-	4:9555750	128	169	147	194	141	118	0.943928	1.044646	0.746792	0.931993	0.911785	1.078944	0.911789	0.974241	0.89047	1	-
ENSMUS(	Eqtn	equatorin	GO:00325	developm	-	-	-	-	4:9490726	0	5	9	15	3	6	0	0.1592	0.235513	0.371206	0.099924	0.282629	0.131571	0.251253	0.24956	1	-
ENSMUS(	Ift74	intraflagel	GO:00325	developm	-	-	-	-	4:9461449	516	592	789	748	576	269	11.78769	11.33583	12.41674	11.13172	11.53837	7.619348	11.84675	10.09648	0.230625	1	-
ENSMUS(	Plaa	phospholi	GO:00325	developm	K14018	phospholi	mmu0414	Protein pr	4:9456751	1128	1286	1494	1619	1123	629	8.536296	8.157446	7.78865	7.98158	7.452166	5.901966	8.160798	7.111904	0.22061	1	-
ENSMUS(	Caap1	caspase a	GO:00099	cellular pr	-	-	-	-	4:9450008	135	198	251	291	164	92	2.051572	2.52214	2.627709	2.880894	2.185432	1.733505	2.400474	2.26661	0.652835	1	-
ENSMUS(	Pum1	pumilio RI	GO:00325	developm	-	-	-	-	4:1306633	1961	2174	2766	2948	2391	1420	13.08287	12.15732	12.71244	12.81252	13.98775	11.74626	12.65088	12.84884	0.877706	1	-
ENSMUS(	Laptm5	lysosomal	GO:00511	localizatio	K12387	lysosomal	mmu0414	Lysosome	4:1309131	486	556	711	948	549	454	5.590362	5.360827	5.634104	7.103853	5.537568	6.475084	5.528431	6.372168	0.438311	1	-
ENSMUS(	Cc2d1b	coiled-co	GO:00081	metabolic	-	-	-	-	4:1086199	320	445	493	617	468	388	2.666793	3.108507	2.830337	3.349702	3.420017	4.009198	2.868546	3.592972	0.172808	1	-
ENSMUS(	Pdpn	podoplan	GO:00325	developm	-	-	-	-	4:1432674	236	288	373	346	256	177	6.902226	7.060282	7.51513	6.592261	6.565355	6.418549	7.159213	6.525389	0.366449	1	-
ENSMUS(	Lrrc38	leucine ric	GO:00325	developm	-	-	-	-	4:1433497	13	15	26	25	11	18	0.355496	0.343837	0.48981	0.445367	0.26378	0.610339	0.396381	0.439828	0.946266	1	-
ENSMUS(	Orc1	origin rec	GO:00081	metabolic	K02603	origin rec	mmu0411	Cell cycle	4:1085794	0	0	0	2	2	0	0	0	0	0.018787	0.025289	0	0	0.014692	0.156106	1	-
ENSMUS(	1700012P	RIKEN cDI	-	-	-	-	-	-	4:1444181	1	0	1	0	0	0	0.065263	0	0.044933	0	0	0	0.036732	0	0.527877	1	-
ENSMUS(	Gpx7	glutathior	GO:00081	metabolic	K00432	glutathior	mmu0059	Arachidor	4:1084003	38	54	47	61	51	47	1.490748	1.775706	1.270201	1.558954	1.754426	2.28616	1.512218	1.866513	0.439035	1	-
ENSMUS(	Tnfrsf1b	tumor nec	GO:00325	developm	K05141	tumor nec	mmu0406	Cytokine-	4:1452134	87	70	114	159	90	95	1.411572	0.951988	1.274202	1.680593	1.280467	1.911141	1.212587	1.624067	0.180518	1	-
ENSMUS(	Podn	podocan	GO:00081	metabolic	-	-	-	-	4:1080147	50	50	79	85	59	58	0.343125	0.287606	0.373471	0.379996	0.355034	0.49351	0.334734	0.409513	0.419111	1	-
ENSMUS(	Echdc2	enoyl Coe	GO:00081	metabolic	-	-	-	-	4:1081654	181	217	203	281	244	266	1.961646	1.971306	1.515619	1.983945	2.318865	3.574456	1.81619	2.625755	0.065299	1	-
ENSMUS(	Tnfrsf8	tumor nec	GO:00325	developm	K05145	tumor nec	mmu0406	Cytokine-	4:1452671	22	42	24	23	12	26	0.246065	0.393765	0.184925	0.167589	0.117696	0.360581	0.274918	0.215289	0.338158	1	-
ENSMUS(	Scp2	sterol carr	GO:00081	metabolic	K08764	sterol carr	mmu011C	Metabolic	4:1080438	991	1266	1538	1533	995	684	10.72017	11.47929	11.46136	10.80319	9.438308	9.174243	11.22027	9.805246	0.183311	1	-
ENSMUS(	Cpt2	carnitine p	GO:00081	metabolic	K08766	carnitine C	mmu0332	PPAR sign	4:1079039	84	84	150	112	83	104	1.008202	0.845089	1.240262	0.875725	0.873553	1.547707	1.031184	1.098995	0.986603	1	-
ENSMUS(	0610037L	RIKEN cDI	GO:00055	extracellul	-	-	-	-	4:1078898	551	747	882	1001	722	440	3.206459	3.643739	3.53585	3.794806	3.684288	3.174773	3.462016	3.551289	0.959817	1	-
ENSMUS(	Magoh	mago hor	GO:00081	metabolic	K12877	protein m	mmu0301	RNA trans	4:1078797	164	194	235	207	145	98	9.544611	9.463841	9.421779	7.848125	7.39987	7.071755	9.476744	7.439916	0.077034	1	-
ENSMUS(	Dmrtb1	DMRT-like	GO:00081	metabolic	-	-	-	-	4:1076762	9	5	8	7	9	0	0.176698	0.08228	0.108202	0.089532	0.154951	0	0.122393	0.081494	0.491916	1	-
ENSMUS(	Lrp8	low densit	GO:00325	developm	-	-	-	-	4:1078022	851	1017	1195	1322	1068	677	3.803884	3.810409	3.679738	3.849556	4.186118	3.752079	3.764677	3.929251	0.958485	1	-
ENSMUS(	Ndc1	NDC1 trar	GO:00511	localizatio	K14315	nucleopor	mmu0301	RNA trans	4:1073677	348	481	569	645	453	271	2.629779	3.046759	2.962127	3.175274	3.001792	2.539189	2.879555	2.905418	0.879222	1	-
ENSMUS(	Lrrc42	leucine ric	-	-	-	-	-	-	4:1072335	245	294	409	395	302	233	4.441103	4.467086	5.107392	4.664475	4.800361	5.236811	4.67186	4.900549	0.962117	1	-
ENSMUS(	Tmem59	transmem	GO:00081	metabolic	-	-	-	-	4:1071783	560	766	1014	951	696	1208	8.145961	9.339764	10.16117	9.011897	8.877814	21.78748	9.215631	13.22573	0.118551	1	-
ENSMUS(	Tceanc2	transcripti	GO:00081	metabolic	-	-	-	-	4:1071341	340	407	564	577	330	292	2.318957	2.326806	2.649986	2.563723	1.973649	2.469343	2.431916	2.335572	0.576722	1	-
ENSMUS(	Cyb5rl	cytochron	GO:00081	metabolic	K00326	cytochron	mmu0052	Amino sug	4:1070669	67	63	99	53	56	52	0.259748	0.204725	0.264401	0.133856	0.190373	0.249958	0.242958	0.191396	0.144712	1	-
ENSMUS(	Mrpl37	mitochon	GO:00081	metabolic	-	-	-	-	4:1070558	441	594	586	722	492	441	4.675046	5.278201	4.279531	4.986152	4.573559	5.796576	4.744259	5.118763	0.807213	1	-
ENSMUS(	Col9a2	collagen,	GO:00329	macromo	K08131	collagen,	mmu0415	PI3K-Akt s	4:1210393	70	74	121	119	90	76	1.219487	1.080609	1.452175	1.350543	1.374888	1.641648	1.250757	1.455693	0.545931	1	-
ENSMUS(	Exo5	exonuclea	GO:00081	metabolic	-	-	-	-	4:1209211	154	144	203	213	142	124	1.923959	1.507961	1.747118	1.73355	1.555629	1.920796	1.726346	1.736659	0.833839	1	-
ENSMUS(	Dyrk2	dual-spec	GO:00081	metabolic	-	-	-	-	10:118855	1439	1687	1345	1803	1032	608	13.16436	12.9362	8.476422	10.74524	8.278678	6.896495	11.52566	8.640137	0.0558	1	-
ENSMUS(	Kcnq4	potassium	GO:00325	developm	K04929	potassium	mmu0472	Cholinerg	4:1206961	105	108	138	137	126	77	1.658317	1.42973	1.50144	1.409552	1.744989	1.507831	1.529829	1.554124	0.916195	1	-
ENSMUS(	Ctps	cytidine 5'	GO:00081	metabolic	K01937	CTP synth	mmu011C	Metabolic	4:1205398	520	636	854	772	565	501	8.406909	8.61872	9.511353	8.130777	8.009847	10.04284	8.845661	8.727823	0.659955	1	-
ENSMUS(	Hivep3	human im	GO:00325	developm	-	-	-	-	4:1197337	1137	1292	1607	1841	1360	898	2.923485	2.784555	2.846477	3.083726	3.066351	2.862877	2.851506	3.004318	0.894649	1	-
ENSMUS(	Ppcs	phosphop	GO:00081	metabolic	K01922	phosphop	mmu011C	Metabolic	4:1194185	30	56	58	55	37	56	0.826087	1.292539	1.100222	0.986606	0.893402	1.911937	1.072949	1.263982	0.678826	1	-
ENSMUS(	Ccdc30	coiled-co	-	-	-	-	-	-	4:1193228	267	308	369	431	304	204	1.745387	1.687658	1.661723	1.835438	1.742595	1.653468	1.698256	1.743834	0.960813	1	-
ENSMUS(	Ybx1	Y box pro	GO:00325	developm	-	-	-	-	4:1192779	1823	2037	2586	2895	1959	1213	23.52429	22.033	22.98845	24.33661	22.167	19.40781	22.84858	21.97048	0.571423	1	-
ENSMUS(	Tfap2c	transcripti	GO:00325	developm	-	-	-	-	2:1725495	6	2	6	7	3	0	0.047288	0.013211	0.032574	0.035939	0.020731	0	0.031025	0.01889	0.514084	1	-
ENSMUS(	P3h1	prolyl 3-h	GO:00325	developm	-	-	-	-	4:1192329	101	131	185	207	128	165	0.571378	0.621191	0.720983	0.762876	0.634973	1.157372	0.637851	0.85174	0.181532	1	-
ENSMUS(	Tmem269	transmem	GO:00081	metabolic	-	-	-	-	4:1192050	8	18	14	18	16	11	0.23183	0.437246	0.279491	0.339816	0.406588	0.395263	0.316189	0.380556	0.682208	1	-
ENSMUS(	Ermap	erythrobla	GO:00160	membran	-	-	-	-	4:1191754	8	17	12	10	9	15	0.043666	0.077782	0.045125	0.035558	0.04308	0.101521	0.055524	0.060053	1	1	-
ENSMUS(	Slc2a1	solute car	GO:00325	developm	K07299	MFS trans	mmu052C	Pathways	4:1191087	1206	1522	1876	1966	1522	1535	13.95066	14.75758	14.94968	14.81537	15.43849	22.01617	14.55264	17.42334	0.29245	1	-
ENSMUS(	Rragc	Ras-relate	GO:00081	metabolic	K16186	Ras-relate	mmu0415	mTOR sig	4:1239174	1075	1291	1631	1489	1156	975	12.0175	12.0972	12.56062	10.84381	11.33199	13.51438	12.22511	11.89673	0.55834	1	-
ENSMUS(	Mycbp	MYC bind	GO:00081	metabolic	-	-	-	-	4:1239048	52	69	96	77	82	52	0.929083	1.033362	1.181608	0.896241	1.284717	1.151969	1.048018	1.110976	0.938689	1	-
ENSMUS(	Ndufs5	NADH de	GO:00081	metabolic	K03938	NADH de	mmu011C	Metabolic	4:1237127	179	259	330	268	212	319	8.85978	10.74541	11.25218	8.641478	9.201304	19.57706	10.28579	12.47328	0.497799	1	-
ENSMUS(	Macf1	microtubu	GO:00325	developm	-	-	-	-	4:1233496	9125	11521	14689	15719	10839	8148	13.06129	13.8228	14.48428	14.65749	13.60458	14.46073	13.78946	14.24093	0.961384	1	-
ENSMUS(	Ppie	peptidylpr	GO:00081	metabolic	K09564	peptidyl-p	mmu0304	Spliceoso	4:1231271	103	154	168	186	134	116	3.0041	3.764883	3.37552	3.534055	3.427092	4.194905	3.381501	3.718684	0.753409	1	-
ENSMUS(	Trit1	tRNA isop	GO:00081	metabolic	-	-	mmu011C	Metabolic	4:1230165	147	261	291	320	236	185	4.097491	6.09809	5.58786	5.810752	5.768409	6.393816	5.261147	5.990992	0.568698	1	-
ENSMUS(	Mycl	v-myc avi	GO:00325	developm	-	-	-	-	4:1229956	589	603	792	611	608	605	6.241912	5.356392	5.78201	4.218178	5.649996	7.949563	5.793438	5.939246	0.859013	1	-
ENSMUS(	Mfsd2a	major faci	GO:00325	developm	-	-	-	-	4:1229468	186	318	321	381	331	304	4.396005	6.299774	5.226382	5.866129	6.859868	8.90849	5.307387	7.211496	0.095521	1	-
ENSMUS(	Cap1	CAP, ader	GO:00325	developm	-	-	-	-	4:1228590	4857	5428	6666	7433	5216	3219	63.59228	59.5702	60.12472	63.39896	59.8848	52.25686	61.09573	58.51354	0.5353	1	-
ENSMUS(	Ppt1	palmitoyl-	GO:00325	developm	K01074	palmitoyl-	mmu011C	Metabolic	4:1228362	1405	1742	2163	2316	1524	968	15.56695	16.17811	16.50954	16.71657	14.80658	13.29805	16.08487	14.9404	0.404663	1	-
ENSMUS(	Epha8	Eph recep	GO:00325	developm	K05109	Eph recep	mmu0436	Axon guic	4:1369294	53	68	84	99	98	79	0.714686	0.768597	0.780316	0.869674	1.158792	1.320847	0.754533	1.116437	0.060005	1	-
ENSMUS(	Ephb2	Eph recep	GO:00325	developm	K05111	Eph recep	mmu0436	Axon guic	4:1366475	744	818	908	958	871	470	2.75384	2.537885	2.315279	2.310005	2.827005	2.156996	2.535668	2.431335	0.588082	1	-
ENSMUS(	Eloa	elongin A	GO:00081	metabolic	-	-	-	-	4:1360033	666	877	1059	1103	799	552	8.990288	9.923209	9.847981	9.699669	9.457781	9.238997	9.587159	9.465482	0.701716	1	-
ENSMUS(	Pithd1	PITH (C-te	GO:00508	response	-	-	-	-	4:1359754	629	869	952	959	725	866	10.56697	12.23693	11.01763	10.49542	10.68022	18.03864	11.27384	13.07143	0.51552	1	-
ENSMUS(	Lypla2	lysophosp	GO:00081	metabolic	K06130	lysophosp	mmu0056	Glyceroph	4:1359682	481	629	803	838	655	721	9.11147	9.987275	10.47878	10.34115	10.87997	16.9342	9.859175	12.71844	0.151529	1	-
ENSMUS(	Gale	galactose	GO:00081	metabolic	K01784	UDP-gluc	mmu011C	Metabolic	4:1359637	111	164	161	217	135	195	1.877169	2.324751	1.875672	2.390675	2.001958	4.088837	2.025864	2.827157	0.138023	1	-
ENSMUS(	Hmgcl	3-hydroxy	GO:00325	developm	K01640	hydroxym	mmu011C	Metabolic	4:1359464	171	186	227	264	241	303	3.921889	3.575737	3.586552	3.944436	4.84684	8.616439	3.694726	5.802572	0.031192	1	-

ENSMUSC	Fuca1	fucosidas	GO:00081	metabolic	K01206	alpha-L-f	mmu0414	Lysosome	4:1359207	437	470	639	587	486	429	6.663301	6.007014	6.712138	5.8308	6.498115	8.110577	6.460818	6.813164	0.959751	1	-
ENSMUSC	Pnrc2	proline-ri	GO:00081	metabolic	-	-	-	-	4:1358709	717	988	1245	1083	855	511	18.1183	20.92704	21.67299	17.82823	18.94554	16.01052	20.23944	17.59476	0.192043	1	-
ENSMUSC	Srsf10	serine/arg	GO:00081	metabolic	K12900	FUS-intera	mmu0304	Spliceoso	4:1358557	748	885	1291	1264	861	445	4.567413	4.529652	5.430582	5.028028	4.610143	3.369107	4.842549	4.33576	0.358763	1	-
ENSMUSC	Rnf220	ring finge	GO:00081	metabolic	-	-	-	-	4:1172714	2144	3020	3221	3677	2005	2489	10.45723	12.34672	10.82266	11.68334	8.575287	15.05229	11.20887	11.7703	0.992932	1	-
ENSMUSC	Kif2c	kinesin fa	GO:00511	localizatio	-	-	-	-	4:1171596	9	7	10	4	9	5	0.091927	0.059929	0.070367	0.026619	0.080613	0.06332	0.074074	0.056851	0.477451	1	-
ENSMUSC	Plk3	polo like k	GO:00081	metabolic	K08862	polo-like	mmu0515	Tuberculo	4:1171286	131	137	199	181	135	142	2.174888	1.90651	2.275978	1.957598	1.965349	2.923071	2.119125	2.282006	0.894397	1	-
ENSMUSC	Ptch2	patched 2	GO:00325	developm	K11101	patched 2	mmu0520	Pathways	4:1170960	19	5	23	34	24	29	0.148873	0.032837	0.12415	0.173547	0.164899	0.281735	0.101953	0.206727	0.047216	1	-
ENSMUSC	Eif2b3	eukaryotic	GO:00325	developm	K03241	translation	mmu0301	RNA trans	4:1170194	186	231	269	315	231	158	1.552312	1.615961	1.546572	1.712611	1.690525	1.634971	1.571615	1.679369	0.84855	1	-
ENSMUSC	Urod	uroporphy	GO:00081	metabolic	K01599	uroporphy	mmu0110	Metabolic	4:1169899	352	382	480	424	445	371	4.455416	4.052862	4.185419	3.496178	4.93911	5.822444	4.231232	4.752578	0.648128	1	-
ENSMUSC	Mutyh	mutY DNA	GO:00081	metabolic	K03575	A/G-spec	mmu0341	Base excis	4:1168077	23	18	34	26	19	30	0.305413	0.200351	0.311025	0.224912	0.221233	0.493944	0.272263	0.313363	0.84447	1	-
ENSMUSC	Toe1	target of E	GO:00081	metabolic	-	-	-	-	4:1167943	131	176	176	202	130	118	1.421457	1.600757	1.315606	1.427886	1.236936	1.58756	1.44594	1.417461	0.711194	1	-
ENSMUSC	Ccdc163	coiled-co	-	-	-	-	-	-	4:1167085	11	19	28	29	34	33	0.101407	0.146816	0.17782	0.17416	0.274847	0.37719	0.142014	0.275399	0.030268	1	-
ENSMUSC	Mmachc	methylma	GO:00081	metabolic	K14618	methylma	mmu0497	Vitamin d	4:1167022	298	357	430	418	370	160	7.329266	7.359776	7.285575	6.697326	7.979723	4.879202	7.324872	6.51875	0.384535	1	-
ENSMUSC	Prdx1	peroxired	GO:00081	metabolic	K13279	peroxired	mmu0414	Peroxisom	4:1166855	715	1079	1299	1210	788	934	11.11284	14.05703	13.90847	12.25141	10.7396	17.99914	13.02611	13.66338	0.994111	1	-
ENSMUSC	Akr1a1	aldo-keto	GO:00081	metabolic	-	-	mmu0110	Metabolic	4:1166365	2314	2805	3466	3184	2553	4092	43.27888	43.97425	44.65732	38.79426	41.87031	94.89302	43.97015	58.5192	0.207367	1	-
ENSMUSC	Nasp	nuclear au	GO:00325	developm	-	-	-	-	4:1166010	379	479	597	639	432	269	2.600304	2.754691	2.821697	2.856059	2.599028	2.288354	2.725564	2.581147	0.546404	1	-
ENSMUSC	Ipp	IAP prom	GO:00081	metabolic	-	-	-	-	4:1165075	154	151	217	237	175	162	3.26784	2.685768	3.172125	3.276196	3.256261	4.26227	3.041911	3.598243	0.438856	1	-
ENSMUSC	Pik3r3	phosphoir	GO:00081	metabolic	K02649	phosphoir	mmu0520	Pathways	4:1162216	957	1158	1474	1440	1055	759	8.969217	9.097114	9.51681	8.791974	8.670365	8.820032	9.19438	8.76079	0.474203	1	-
ENSMUSC	Tspan1	tetraspani	GO:00400	locomotic	-	-	-	-	4:1161618	0	8	8	4	2	0	0	0.151492	0.124511	0.058879	0.039619	0	0.092001	0.032833	0.137733	1	-
ENSMUSC	Pomgnt1	protein O	GO:00081	metabolic	K09666	beta-1,2-	mmu0110	Metabolic	4:1161238	805	916	1161	1093	916	953	5.663694	5.401974	5.627139	5.009631	5.651222	8.313485	5.564269	6.324779	0.568425	1	-
ENSMUSC	Lurap1	leucine ric	GO:00400	locomotic	-	-	-	-	4:1161324	366	449	555	558	416	354	3.751069	3.857209	3.918491	3.725547	3.738615	4.498457	3.842256	3.98754	0.971146	1	-
ENSMUSC	Rad54l	RAD54 lik	GO:00325	developm	K10875	DNA repa	mmu0344	Homologo	4:1160942	16	11	13	22	13	14	0.104348	0.060134	0.058405	0.093468	0.074343	0.11321	0.074296	0.093674	0.534413	1	-
ENSMUSC	Lrrc41	leucine ric	GO:00081	metabolic	-	-	-	-	4:1160752	475	601	704	775	621	818	6.370046	6.755802	6.503891	6.770685	7.302701	13.60156	6.543247	9.224983	0.075794	1	-
ENSMUSC	Nsun4	NOL1/NC	GO:00081	metabolic	-	-	-	-	4:1160328	118	149	171	190	123	146	0.908338	0.961402	0.906807	0.952802	0.830258	1.393497	0.925516	1.058852	0.62822	1	-
ENSMUSC	Dmbx1	diencepha	GO:00325	developm	-	-	-	-	4:1159151	3	2	0	2	3	0	0.020094	0.01123	0	0.008727	0.017621	0	0.010441	0.008783	1	1	-
ENSMUSC	Mknk1	MAP kina	GO:00081	metabolic	K04372	MAP kina	mmu0401	MAPK sig	4:1158391	335	450	430	567	404	435	3.210234	3.614576	2.83865	3.539615	3.394814	5.168522	3.221153	4.034317	0.234027	1	-
ENSMUSC	Mob3c	MOB kina	GO:00081	metabolic	-	-	-	-	4:1158280	68	77	114	119	82	59	1.106405	1.050145	1.277791	1.261337	1.169928	1.190264	1.14478	1.207176	0.945065	1	-
ENSMUSC	Atpaf1	ATP synth	GO:00099	cellular pr	-	-	-	-	4:1157848	394	372	565	530	285	283	3.687755	2.918518	3.643061	3.231649	2.33913	3.284267	3.416445	2.951682	0.207671	1	-
ENSMUSC	Cyp4b1	cytochron	GO:00081	metabolic	-	-	-	-	4:1156247	3	6	2	5	4	0	0.065646	0.110058	0.030156	0.071289	0.076755	0	0.06862	0.049348	0.709902	1	-
ENSMUSC	Pdzk1ip1	PDZK1 int	GO:00055	extracellul	-	-	-	-	4:1150887	5	8	11	2	4	3	0.078988	0.105927	0.119708	0.02058	0.055406	0.058767	0.101541	0.044918	0.046754	1	-
ENSMUSC	Tal1	T cell acut	GO:00325	developm	-	-	-	-	4:1150564	17	31	49	40	28	25	0.10683	0.163292	0.212133	0.163755	0.154296	0.194794	0.160752	0.170949	1	1	-
ENSMUSC	Stil	Scl/Tal1 ir	GO:00511	localizatio	-	-	-	-	4:1150001	3	11	3	10	10	3	0.028436	0.087411	0.019591	0.061755	0.083129	0.035265	0.045146	0.06005	0.644467	1	-
ENSMUSC	Cmpk1	cytidine m	GO:00081	metabolic	K13800	UMP-CM	mmu0110	Metabolic	4:1149593	920	1134	1359	1281	949	740	15.18669	15.69066	15.45419	13.77546	13.73676	15.14583	15.44385	14.21935	0.305574	1	-
ENSMUSC	Ebna1bp2	EBNA1 bi	GO:00081	metabolic	-	-	-	-	4:1186207	653	810	1191	1118	756	674	8.378781	8.711758	10.52764	9.345259	8.506129	10.72294	9.20606	9.524777	0.955342	1	-
ENSMUSC	Cfap57	cilia and f	-	-	-	-	-	-	4:1185545	24	23	49	16	21	17	0.347285	0.278967	0.488456	0.150825	0.266467	0.305001	0.371569	0.240764	0.068906	1	-
ENSMUSC	Aldh4a1	aldehyde	GO:00081	metabolic	K00294	1-pyrrolin	mmu0110	Metabolic	4:1396228	377	407	505	486	417	442	3.50029	3.167448	3.230022	2.939549	3.395015	5.088276	3.299253	3.807613	0.545083	1	-
ENSMUSC	Mrto4	mRNA tur	GO:00081	metabolic	-	-	-	-	4:1393474	140	186	226	241	205	198	2.296707	2.557658	2.554097	2.575579	2.948994	4.027431	2.469487	3.184001	0.194298	1	-
ENSMUSC	Akr7a5	aldo-keto	GO:00081	metabolic	K15303	aflatoxin E	mmu0098	Metabolis	4:1393107	213	264	272	292	249	307	6.802396	7.067087	5.984164	6.075022	6.973088	12.15646	6.617882	8.401522	0.297085	1	-
ENSMUSC	Pqlc2	PQ loop r	GO:00511	localizatio	-	-	-	-	4:1392940	141	140	215	185	138	157	1.612841	1.342319	1.694194	1.378565	1.384185	2.226681	1.549785	1.663144	0.922827	1	-
ENSMUSC	Capzb	capping p	GO:00325	developm	K10365	capping p	mmu0414	Endocytos	4:1391928	3905	4086	5184	6205	4224	2709	34.50223	30.26059	31.55313	35.7149	32.72598	29.67706	32.10531	32.70598	0.899137	1	-
ENSMUSC	Htr6	5-hydroxy	GO:00325	developm	K04162	5-hydroxy	mmu0408	Neuroacti	4:1390611	43	69	58	82	59	46	0.7711	1.037152	0.71651	0.957944	0.927755	1.0228	0.841588	0.9695	0.613054	1	-
ENSMUSC	Pla2g2f	phospholi	GO:00081	metabolic	-	-	mmu0110	Metabolic	4:1387505	41	55	75	127	78	50	1.057058	1.188605	1.332078	2.13306	1.763424	1.598377	1.19258	1.83162	0.037606	1	-
ENSMUSC	Pla2g2c	phospholi	GO:00081	metabolic	-	-	mmu0110	Metabolic	4:1387247	1	0	5	4	0	0	0.013568	0	0.046726	0.035352	0	0	0.020098	0.011784	0.779559	1	-
ENSMUSC	Vwa5b1	von Willeb	GO:00055	extracellul	-	-	-	-	4:1385653	76	80	113	88	57	45	0.536968	0.473782	0.550004	0.40504	0.353142	0.394212	0.520251	0.384131	0.057037	1	-
ENSMUSC	Cda	cytidine d	GO:00081	metabolic	K01489	cytidine d	mmu0110	Metabolic	4:1383384	5	9	16	2	9	10	0.202917	0.306139	0.447316	0.052869	0.320291	0.503215	0.318791	0.292125	0.7198	1	-
ENSMUSC	Pink1	PTEN indu	GO:00325	developm	K05688	PTEN indu	mmu0501	Parkinson	4:1383134	3906	4588	6490	5210	4530	4843	58.88	57.97114	67.39561	51.16287	59.87926	90.5182	61.41558	67.18678	0.771904	1	-
ENSMUSC	Ddost	dolichyl-c	GO:00081	metabolic	K12670	oligosacch	mmu0110	Metabolic	4:1383047	1189	1491	1896	1790	1307	1359	35.64365	37.46542	39.15524	34.95707	34.35725	50.5133	37.42144	39.94254	0.890049	1	-
ENSMUSC	Kif17	kinesin fa	GO:00325	developm	-	-	-	-	4:1382504	324	402	507	620	461	282	3.50667	3.646941	3.780155	4.371427	4.375157	3.784297	3.644589	4.17696	0.463703	1	-
ENSMUSC	Hp1bp3	heterochr	GO:00081	metabolic	-	-	-	-	4:1382162	3629	4372	5318	5854	4397	3445	15.70862	15.86295	15.85808	16.50765	16.68976	18.48954	15.80988	17.22898	0.68376	1	-
ENSMUSC	Eif4g3	eukaryotic	GO:00971	organic cy	K03260	translation	mmu0301	RNA trans	4:1379930	5208	6315	7306	8624	6297	3739	22.67325	23.04459	21.91159	24.45869	24.03915	20.18293	22.54314	22.89359	0.885925	1	-
ENSMUSC	Hspg2	perlecan (	GO:00055	extracellul	K06255	heparan s	mmu0520	Proteoglyc	4:1374687	281	338	441	465	339	338	1.068149	1.076949	1.154824	1.15149	1.129974	1.593046	1.099974	1.291504	0.421791	1	-
ENSMUSC	Alpl	alkaline pl	GO:00325	developm	K01077	alkaline pl	mmu0110	Metabolic	4:1377417	117	169	199	202	138	132	1.625639	1.968246	1.904776	1.828406	1.681358	2.274045	1.832887	1.927936	0.978466	1	-
ENSMUSC	Ptpn12	protein ty	GO:00325	developm	-	-	-	-	5:2098664	603	684	757	884	593	384	6.235354	5.928607	5.392506	5.954938	5.37701	4.923356	5.852156	5.418435	0.420055	1	-
ENSMUSC	Zcchc17	zinc finge	GO:00329	macromo	-	-	-	-	4:1303153	805	862	1170	1125	1010	658	9.373424	8.413227	9.385124	8.533691	10.31257	9.499807	9.057258	9.448688	0.976577	1	-
ENSMUSC	Fabp3	fatty acid	GO:00081	metabolic	K08752	fatty acid-	mmu0332	PPAR sign	4:1303085	545	660	797	719	557	359	42.08554	42.72021	42.39809	36.16986	37.71672	34.37291	42.40128	36.08649	0.136448	1	-
ENSMUSC	Tinagl1	tubulointe	GO:00081	metabolic	-	-	-	-	4:1301644	117	166	221	167	132	152	1.115128	1.326177	1.451059	1.036907	1.103207	1.796261	1.297455	1.312125	0.817426	1	-
ENSMUSC	Hcrtr1	hypocretin	GO:00048	signal tran	K04238	hypocretin	mmu0408	Neuroacti	4:1301302	22	32	38	42	32	31	0.42062	0.512843	0.500502	0.52312	0.53649	0.734896	0.477988	0.598169	0.445173	1	-
ENSMUSC	Pef1	penta-EF	GO:00325	developm	-	-	-	-	4:1301025	575	704	866	812	682	679	12.6359	12.96772	13.11014	11.62453	13.14211	18.50094	12.90458	14.42252	0.650513	1	-
ENSMUSC	Sema3c	sema don	GO:00325	developm	K06840	semaphor	mmu0436	Axon guic	5:1757428	510	593	772	895	622	372	2.913702	2.839765	3.038392	3.33104	3.116076	2.635145	2.93062	3.02742	0.998762	1	-
ENSMUSC	Adgrb2	adhesion	GO:00325	developm	-	-	-	-	4:1299848	7110	7893	8972	10420	8550	7661	40.90358	38.06157	35.5576	39.0518	43.13209	54.6466	38.17425	45.61017	0.26733	1	-
ENSMUSC	Spocd1	SPOC dor	GO:00081	metabolic	-	-	-	-	4:1299292	0	7	2	4	3	0	0	0.112346	0.026385	0.049902	0.050369	0	0.046244	0.033424	0.705839	1	-
ENSMUSC	Hpca	hippocalc	GO:00325	developm	-	-	-	-	4:1291115	18742	21751	25466	28203	19957	16659	209.0401	203.3507	195.6707	204.923	195.1873	230.382	202.6872	210.1641	0.96201	1	-
ENSMUSC	Tmem54	transmem	GO:00160	membran	-	-	-	-	4:1291055	0	0	2	0	0	8	0	0	0.041523	0	0	0.298906	0.013841	0.099635	0.222059	1	-
ENSMUSC	Ptp4a2	protein ty	GO:00081	metabolic	-	-	-	-	4:1298112	1932	3162	4046	3816	2889	1141	14.35068	19.68702	20.70345	18.46526	18.81723	10.50841	18.24705	15.9303	0.355673	1	-
ENSMUSC	Azin2	antizyme	GO:00081	metabolic	K01583	arginine d	mmu0110	Metabolic	4:1289302	241	287	352	359	303	322	3.023353	3.017909	3.042039	2.933908	3.33316	5.008559	3.027767	3.758542	0.283868	1	-
ENSMUSC	Khdrbs1	KH domai	GO:00081	metabolic	-	-	-	-	4:1297031	1990	2585	3300	3121	2553	1533	15.67363	17.06593	17.90531	16.0137	17.63234	14.97079	16.88162	16.20561	0.540575	1	-
ENSMUSC	Ak2	adenylate	GO:00081	metabolic	K00939	adenylate	mmu0110	Metabolic	4:1289919	274	380	418	457	299	226	6.890997	8.010638	7.241999	7.487358	6.593942	7.047346	7.381211	7.042882	0.556631	1	-
ENSMUSC	Rnf19b	ring finge	GO:00081	metabolic	-	-	-	-	4:1290582	734	703	839	1085	748	538	9.84964	7.907384	7.755996	9.484963	8.801744	8.951429	8.50434	9.079379	0.836491	1	-
ENSMUSC	A3galt2	alpha 1,3-	GO:00081	metabolic	K20736	alpha-1,3	mmu0060	Glycosphi	4:1287553	2	3	6	9	8	8	0.024486	0.030779	0.050592	0.071766	0.085873	0.121416	0.035286	0.093018	0.041633	1	-
ENSMUSC	Ccdc28b	coiled coil	GO:00099	cellular pr	-	-	-	-	4:1296192	218	229	247	283	221	216	3.252238	2.863617	2.538482	2.750396	2.891091	3.995448	2.884779	3.212312	0.708223	1	-
ENSMUSC	Phc2	polyhome	GO:00325	developm	-	-	-	-	4:1286547	1185	1336	1552	1630	1334	1133	7.418275	7.010417	6.693104	6.64743	7.322896	8.794282	7.040599	7.588202	0.80301	1	-
ENSMUSC	Tmem234	transmem	GO:00160	membran	-	-	-	-	4:1296006	469	609	729	659	571	600	2.813249	3.062	3.012411	2.575147	3.003403	4.46244	2.962554	3.346997	0.626558	1	-
ENSMUSC	Eif3i	eukaryotic	GO:00081	metabolic	K03246	translation	mmu0301	RNA trans	4:1295919	708	817	782	839	678	901	10.57475	10.22849	8.046274	8.163575	8.879923	16.68582	9.616503	11.2431	0.558941	1	-
ENSMUSC	Zfp362	zinc finge	GO:00971	organic cy	-	-	-	-	4:1287730	468	590	696	693	580	379	6.128747	6.476353	6.278939	5.912088	6.66034	6.153902	6.29468	6.24211	0.732982	1	-
ENSMUSC	Hdac1	histone de	GO:00325	developm	K06067	histone de	mmu0520	Pathways	4:1295161	269	350	433	524	365	314	2.90992	3.17358	3.226768	3.692677	3.462295	4.211569	3.103423	3.788847	0.243717	1	-
ENSMUSC	Stpg1	sperm tail	GO:00511	localizatio	-	-	-	-	4:1354947	61	67	111	125	74	43	1.133544	1.043605	1.420968	1.513216	1.205831	0.990755	1.199373	1.236601	0.982461	1	-
ENSMUSC	Nipal3	NIPA-like	GO:00511	localizatio	-	-	-	-	4:1354454	1486	1757	2123	2366	1779	1634	9.544193	9.458993	9.393376	9.899578	10.01933	13.01243	9.465521	10.97711	0.387832	1	-
ENSMUSC	Csmd2	CUB and S	GO:00160	membran	-	-	-	-	4:1279878	1370	1632	1806	2173	1756	1244	4.832258	4.825058	4.388324	4.993107	5.431213	5.440463	4.68188	5.288261	0.487569	1	-
ENSMUSC	Zbtb8a	zinc finge	GO:00081	metabolic	-	-	-	-	4:1293536	64	85	123	109	101	83	1.752442	1.950888	2.320166	1.944324	2.425082	2.817901	2.007832	2.395769	0.485569	1	-
ENSMUSC	Srrm1	serine/arg	GO:00081	metabolic	K13171	serine/arg	mmu0301	RNA trans	4:1353204	1916	2545	2704	3007	2393	1230	9.826307	10.94046	9.553287	10.04638	10.76168	7.82142	10.10668	9.543161	0.514603	1	-
ENSMUSC	Yars	tyrosyl-tR	GO:00081	metabolic	K01866	tyrosyl-tR	mmu0097	Aminoacy	4:1291897	1358	1601	2007	2114	1664	1626	11.00407	10.87422	11.20347	11.15939	11.82361	16.33655	11.02726	13.10652	0.312055	1	-
ENSMUSC	CK137956	cDNA seq	-	-	-	-	-	-	4:1279275	16	24	13	29	6	9	0.438495	0.551311	0.245433	0.517748	0.144177	0.305809	0.411746	0.322578	0.510205	1	-
ENSMUSC	Sfpq	splicing fa	GO:00081	metabolic	-	-	-	-	4:1270213	3267	4116	5298	5362	4267	2641	34.96005	36.91909	39.05585	37.37931	40.03945	35.04105	36.97833	37.4866	0.85833	1	-
ENSMUSC	Syf2	SYF2 hom	GO:00325	developm	K12868	pre-mRN	mmu0304	Spliceoso	4:1349308	709	831	1093	1069	758	550	16.57802	16.28698	17.60591	16.28343	15.54171	15.94538	16.82364	15.92351	0.464398	1	-
ENSMUSC	Tmem50a	transmem	GO:00160	membran	-	-	-	-	4:1348978	821	1008	1337	1188	974	890	32.85707	33.81418	36.86112	30.97304	34.18119	44.16331	34.51079	36.43918	0.94829	1	-
ENSMUSC	Rhd	Rh blood	GO:00511	localizatio	-	-	-	-	4:1348645	0	0	2	0	0	0	0	0	0.050042	0	0	0	0.016681	0	0.535873	1	-
ENSMUSC	Tmem57	transmem	GO:00325	developm	-	-	-	-	4:1348027	1010	1196	1498	1570	1209	742	6.543839	6.495242	6.686128	6.626629	6.868792	5.960758	6.57507	6.485393	0.70359	1	-
ENSMUSC	AU040320	expressed	GO:00055	extracellul	-	-	-	-	4:1267535	1614	1653	2011	1898	1659	1172	12.59356	10.81112	10.80957	9.647682	11.351	11.33858	11.40475	10.77909	0.429312	1	-
ENSMUSC	Stmn1	stathmin 1	GO:00325	developm	K04381	stathmin[-	mmu0401	MAPK sig	4:1344683	1667	2291	3283	2699	2030	2372	36.04725	41.52547	48.90563	38.02078	38.49241	63.59709	42.15945	46.70343	0.713624	1	-
ENSMUSC	Ncdn	neurocho	GO:00325	developm	-	-	-	-	4:1267437	37191	43246	48787	58342	43797	39339	524.196	510.9221	473.7088	535.6965	541.3061	687.4881	502.9423	588.1636	0.319309	1	-
ENSMUSC	Trim63	tripartite r	GO:00081	metabolic	-	-	-	-	4:1343151	0	3	2	4	0	0	0	0.032316	0.017709	0.033494	0	0	0.016675	0.011165	0.77025	1	-
ENSMUSC	Slc30a2	solute car	GO:00511	localizatio	-	-	-	-	4:1343431	3	0	0	2	0	2	0.026858	0	0	0.011664	0	0.022209	0.008953	0.011291	1	1	-
ENSMUSC	Psmb2	proteasor	GO:00081	metabolic	K02734	20S prote	mmu0305	Proteason	4:1266776	537	697	804	761	596	525	20.88613	22.7232	21.54227	19.28191	20.32698	25.31799	21.7172	21.64229	0.711491	1	-
ENSMUSC	Extl1	exostoses	GO:00081	metabolic	K02368	alpha-1,4	mmu0110	Metabolic	4:1343563	850	999	1224	1364	1030	1047	12.22727	12.04561	12.12951	12.78223	12.99242	18.67424	12.13413	14.8163	0.23417	1	-
ENSMUSC	Zfp593	zinc finge	GO:00081	metabolic	-	-	-	-	4:1342432	83	103	118	117	114	133	1.314132	1.366939	1.287044	1.206773	1.582732	2.610927	1.322705	1.800144	0.209428	1	-
ENSMUSC	Cnksr1	connector	GO:00508	response	-	-	-	-	4:1342280	0	0	3	2	0	4	0	0	0.03255	0.020519	0	0.078116	0.01085	0.032878	0.598765	1	-
ENSMUSC	Ago3	argonaute	GO:00081	metabolic	-	-	-	-	4:1263317	1364	1607	2067	2406	1787	1054	4.986843	4.924704	5.205989	5.730448	5.728997	4.777909	5.039179	5.412452	0.762371	1	-
ENSMUSC	Sh3bgrl3	SH3 doma	GO:00081	metabolic	-	-	-	-	4:1341274	1291	1417	1743	2030	1486	1154	50.36646	46.33805	46.84505	51.59324	50.8366	55.82217	47.84986	52.75067	0.625316	1	-
ENSMUSC	Tekt2	tektin 2 [S	GO:00400	locomotic	-	-	-	-	4:1263221	9	33	28	18	21	9	0.194614	0.598148	0.417112	0.253561	0.398205	0.241297	0.403291	0.297688	0.308085	1	-
ENSMUSC	Trappc3	trafficking	GO:00511	localizatio	-	-	-	-	4:1262623	559	807	926	909	735	631	17.98792	21.76685	20.52727	19.05525	20.73954	25.17587	20.09401	21.65689	0.813013	1	-
ENSMUSC	Gpn2	GPN-loop	GO:00511	localizatio	-	-	-	-	4:1335843	55	78	74	72	72	99	1.330056	1.581101	1.232804	1.134287	1.526803	2.968468	1.38132	1.876519	0.302981	1	-
ENSMUSC	Map7d1	MAP7 dor	GO:00051	structural	-	-	-	-	4:1262321	4139	4613	4957	4641	4522	3707	51.72978	48.32607	42.67912	37.78665	49.55853	57.44519	47.57832	48.26345	0.802116	1	-
ENSMUSC	Gpatch3	G patch d	GO:00081	metabolic	-	-	-	-	4:1335747	63	83	117	105	75	95	1.748407	1.930775	2.23685	1.898337	1.825182	3.268968	1.972011	2.330829	0.568555	1	-
ENSMUSC	Nudc	nudC nuc	GO:00325	developm	-	-	-	-	4:1335325	1485	1745	2029	1987	1540	859	53.02035	52.22333	49.90564	46.21634	48.2147	38.02727	51.71644	44.15277	0.141227	1	-
ENSMUSC	Slc9a1	solute car	GO:00325	developm	K05742	solute car	mmu0481	Regulation	4:1333697	1154	1393	1450	1685	1318	1086	10.61209	10.7374	9.185753	10.09431	10.62804	12.38257	10.17842	11.03498	0.757732	1	-
ENSMUSC	Tmem222	transmem	GO:00160	membran	-	-	-	-	4:1332660	715	872	1023	1102	845	969	15.17715	15.51507	14.9593	15.23869	15.72836	25.50317	15.21718	18.82341	0.252843	1	-
ENSMUSC	Csf3r	colony stir	GO:00325	developm	K05061	granulocy	mmu0520	Pathways	4:1260245	100	101	154	180	102	105	1.151531	0.974879	1.221651	1.350297	1.029958	1.499164	1.11602	1.29314	0.547897	1	-
ENSMUSC	Sytl1	synaptota	GO:00511	localizatio	-	-	-	-	4:1332530	3	7	9	7	6	8	0.055369	0.1083	0.114439	0.084175	0.097109	0.183094	0.092703	0.12146	0.698569	1	-
ENSMUSC	Mrps15	mitochon	GO:00081	metabolic	K02956	small subu	mmu0301	Ribosome	4:1260469	318	399	394	501	368	223	9.858455	10.36831	8.414529	10.11817	10.00398	8.571817	9.547098	9.564653	0.837756	1	-
ENSMUSC	Map3k6	mitogen-a	GO:00048	signal tran	K04425	mitogen-a	mmu0401	MAPK sig	4:1332408	51	47	69	84	60	60	0.56447	0.436035	0.526105	0.605668	0.582331	0.823397	0.50887	0.670465	0.232786	1	-
ENSMUSC	Meaf6	MYST/Esa	GO:00081	metabolic	-	-	-	-	4:1250851	1361	1828	2040	2148	1602	1374	12.32834	13.87955	12.72999	12.67542	12.72482	15.4319	12.9793	13.61071	0.968691	1	-
ENSMUSC	Hgf	hepatocyt	GO:00325	developm	K05460	hepatocyt	mmu0520	Pathways	5:1655349	86	90	108	107	53	22	0.486173	0.426468	0.420599	0.394057	0.262731	0.15421	0.444413	0.270332	0.031022	1	-
ENSMUSC	Cd164l2	CD164 sia	GO:00160	membran	-	-	-	-	4:1332121	14	22	37	25	32	25	0.34486	0.454246	0.62787	0.401175	0.691199	0.763549	0.475658	0.618641	0.479893	1	-
ENSMUSC	Wasf2	WAS prot	GO:00325	developm	K05748	WAS prot	mmu0481	Regulation	4:1331305	438	442	596	638	514	451	3.480396	2.943945	3.262511	3.30261	3.581463	4.443414	3.228951	3.775829	0.412826	1	-
ENSMUSC	Gnl2	guanine n	GO:00038	catalytic a	K14537	nuclear G	mmu0300	Ribosome	4:1250165	825	1007	1117	1295	962	579	10.7353	10.98355	10.01302	10.97772	10.97686	9.341686	10.57729	10.43209	0.720994	1	-
ENSMUSC	Rspo1	R-spondin	GO:00081	metabolic	-	-	-	-	4:1249864	15	17	24	31	27	21	0.418116	0.397199	0.460853	0.562917	0.659944	0.725772	0.425389	0.649544	0.127701	1	-
ENSMUSC	Cdca8	cell divisic	GO:00511	localizatio	-	-	-	-	4:1249184	21	18	33	34	27	18	0.203855	0.146461	0.220675	0.215005	0.229826	0.216653	0.19033	0.220495	0.68848	1	-
ENSMUSC	Fgr	FGR proto	GO:00325	developm	K08891	tyrosine-p	mmu0516	Epstein-B	4:1329740	7	8	13	10	9	10	0.094099	0.090132	0.120381	0.087564	0.106086	0.166674	0.101537	0.120108	0.831194	1	-
ENSMUSC	Epha10	Eph recep	GO:00048	signal tran	-	-	-	-	4:1248808	540	740	921	1099	823	852	4.339187	4.984239	5.0983	5.752992	5.799055	8.488675	4.807242	6.680241	0.036987	1	-
ENSMUSC	Fam76a	family witl	GO:00056	cell|intrace	-	-	-	-	4:1328992	781	987	1268	1276	942	793	11.3633	12.03714	12.70938	12.09444	12.01842	14.30581	12.03661	12.80622	0.869746	1	-
ENSMUSC	Stx12	syntaxin 1	GO:00081	metabolic	K13813	syntaxin 1	mmu0414	Phagoson	4:1328535	2112	2700	3230	3329	2376	1999	39.79365	42.64188	41.92506	40.86159	39.25626	46.70018	41.45353	42.27268	0.849031	1	-
ENSMUSC	Ppp1r8	protein pl	GO:00325	developm	-	-	-	-	4:1328269	476	632	778	761	561	469	7.764686	8.641456	8.742749	8.086921	8.024592	9.485844	8.382964	8.532452	0.854972	1	-
ENSMUSC	Sema3a	sema don	GO:00325	developm	K06840	semaphor	mmu0436	Axon guic	5:1312541	102	112	204	197	137	66	0.370848	0.341322	0.510947	0.466597	0.436774	0.297528	0.407706	0.400299	0.832322	1	-
ENSMUSC	Rpa2	replication	GO:00081	metabolic	K10739	replication	mmu0346	Fanconi a	4:1327683	97	130	190	140	106	139	1.49664	1.681279	2.019527	1.407193	1.434143	2.659164	1.732482	1.8335	1	1	-
ENSMUSC	Smpdl3b	sphingom	GO:00081	metabolic	-	-	-	-	4:1327329	30	53	32	30	26	26	0.950453	1.407465	0.698401	0.61917	0.722307	1.021346	1.018773	0.787607	0.23843	1	-
ENSMUSC	Eya3	EYA trans	GO:00325	developm	-	-	-	-	4:1326389	291	542	608	717	553	504	1.1731	1.831447	1.688485	1.882969	1.954834	2.519178	1.564344	2.118993	0.082536	1	-
ENSMUSC	Yrdc	yrdC dom	GO:00081	metabolic	-	-	-	-	4:1248506	129	200	261	223	202	258	3.327248	4.323935	4.637541	3.746987	4.568658	8.250899	4.096242	5.522181	0.209466	1	-
ENSMUSC	Mtf1	metal resp	GO:00081	metabolic	-	-	-	-	4:1248021	604	705	892	898	659	515	2.57296	2.517321	2.617655	2.492036	2.461639	2.720132	2.569312	2.557936	0.725694	1	-
ENSMUSC	Sesn2	sestrin 2 [	GO:00081	metabolic	K20394	sestrin 2[-	mmu0415	mTOR sig	4:1324920	319	381	398	476	333	365	5.834072	5.840619	5.014369	5.671139	5.340326	8.27676	5.56302	6.429408	0.521391	1	-
ENSMUSC	Inpp5b	inositol pc	GO:00325	developm	K01099	inositol pc	mmu0110	Metabolic	4:1247418	667	766	997	1010	782	573	5.88666	5.666634	6.061635	5.806928	6.05191	6.270234	5.871643	6.043024	0.937263	1	-
ENSMUSC	Rcc1	regulator	GO:00987	molecular	-	-	-	-	4:1323319	130	132	142	142	123	131	1.432121	1.218881	1.077646	1.019071	1.18818	1.789341	1.242883	1.332197	0.933847	1	-
ENSMUSC	Trnau1ap	tRNA sele	GO:00081	metabolic	-	-	-	-	4:1323117	108	140	238	164	165	150	1.319943	1.434216	2.003831	1.305743	1.768319	2.27306	1.585997	1.782374	0.726954	1	-
ENSMUSC	Taf12	TATA-box	GO:00081	metabolic	K03126	transcripti	mmu0302	Basal tran	4:1322743	284	354	439	436	379	212	4.225962	4.415326	4.500102	4.226436	4.945254	3.911359	4.380464	4.361016	0.799447	1	-
ENSMUSC	Gmeb1	glucocorti	GO:00081	metabolic	-	-	-	-	4:1322210	332	299	366	518	430	259	1.489346	1.124299	1.131072	1.513804	1.691491	1.440601	1.248239	1.548632	0.232572	1	-
ENSMUSC	Sf3a3	splicing fa	GO:00081	metabolic	K12827	splicing fa	mmu0304	Spliceoso	4:1247147	545	608	880	837	571	371	10.80025	10.09935	12.01355	10.80548	9.922374	9.115838	10.97105	9.947898	0.336565	1	-
ENSMUSC	Epb41	erythrocyt	GO:00325	developm	-	-	-	-	4:1319234	534	628	987	1037	708	480	1.423127	1.402862	1.812052	1.800374	1.654544	1.586097	1.546014	1.680338	0.722668	1	-
ENSMUSC	Utp11	UTP11 sm	GO:00325	developm	-	-	-	-	4:1246781	293	366	459	479	354	179	3.419217	3.580082	3.689971	3.641461	3.622468	2.589986	3.56309	3.284638	0.486128	1	-
ENSMUSC	Ptpru	protein ty	GO:00325	developm	-	-	-	-	4:1317684	1105	1457	1460	1877	1343	1116	8.120495	8.974968	7.391364	8.985999	8.654442	10.16882	8.162276	9.269755	0.481351	1	-
ENSMUSC	Mecr	mitochon	GO:00081	metabolic	K07512	mitochon	mmu0110	Metabolic	4:1318434	125	163	223	249	175	170	1.608125	1.757722	1.976354	2.086843	1.974191	2.711719	1.780734	2.257584	0.225825	1	-
ENSMUSC	Srsf4	serine/arg	GO:00081	metabolic	K12893	splicing fa	mmu0516	Herpes sir	4:1318736	1263	1560	1817	1813	1436	1029	17.44186	18.05789	17.28605	16.31057	17.38948	17.61939	17.59527	17.10648	0.568657	1	-
ENSMUSC	Casp9	caspase 9	GO:00325	developm	K04399	caspase 9	mmu0520	Pathways	4:1417936	374	454	583	573	441	468	3.134905	3.189784	3.366454	3.128877	3.241411	4.863898	3.230381	3.744728	0.493287	1	-
ENSMUSC	Plekhm2	pleckstrin	GO:00511	localizatio	K15348	pleckstrin	mmu0513	Salmonell	4:1416257	1424	1715	1946	2369	1181	1472	14.46603	14.60348	13.61864	15.67784	10.52039	18.541	14.22938	14.91308	0.997784	1	-
ENSMUSC	Arhgef19	Rho guan	GO:00987	molecular	-	-	-	-	4:1412394	231	320	403	370	301	334	2.337702	2.714432	2.809522	2.439271	2.671073	4.190915	2.620552	3.10042	0.462947	1	-
ENSMUSC	Fbxo42	F-box pro	-	-	-	-	-	-	4:1411479	489	683	763	797	617	602	4.993163	5.845751	5.367137	5.301593	5.524518	7.621644	5.402017	6.149252	0.539318	1	-
ENSMUSC	Necap2	NECAP er	GO:00511	localizatio	-	-	-	-	4:1410665	261	323	386	397	284	328	3.106239	3.222179	3.164707	3.077986	2.963852	4.840112	3.164375	3.627316	0.578781	1	-
ENSMUSC	Cdk14	cyclin-dep	GO:00081	metabolic	K08821	cyclin-dep	mmu0520	Transcript	5:4803391	5038	6426	7170	8357	5512	3687	14.54108	15.54648	14.25638	15.71341	13.95052	13.19464	14.78131	14.28619	0.573385	1	-
ENSMUSC	Padi2	peptidyl a	GO:00325	developm	-	-	-	-	4:1409063	702	701	992	1185	679	474	8.644491	7.235581	8.415215	9.506094	7.331873	7.237129	8.098429	8.025032	0.770542	1	-
ENSMUSC	Kcnab2	potassium	GO:00325	developm	-	-	-	-	4:1523907	10596	12288	16934	16779	13689	12087	56.81806	55.23044	62.55405	58.61276	64.36635	80.36167	58.20085	67.78026	0.349001	1	-
ENSMUSC	Psmc2	proteasor	GO:00325	developm	K03061	26S prote	mmu0516	Epstein-B	5:2178528	1322	1578	1988	1942	1504	1085	16.0002	16.00863	16.57532	15.31175	15.9619	16.28208	16.19472	15.85191	0.607152	1	-
ENSMUSC	Xrcc2	X-ray rep	GO:00325	developm	K10879	DNA-repa	mmu0344	Homologo	5:2568981	91	114	128	139	120	80	0.924002	0.970258	0.895346	0.919449	1.068451	1.007178	0.929869	0.998359	0.853247	1	-
ENSMUSC	Rpl22	ribosomal	GO:00325	developm	K02891	large subu	mmu0301	Ribosome	4:1523257	948	1102	1420	1476	950	1228	9.484923	9.241877	9.787355	9.620411	8.334735	15.23386	9.504718	11.063	0.484424	1	-
ENSMUSC	Acot7	acyl-CoA	GO:00081	metabolic	K17360	acyl-coen	mmu0104	Biosynthe	4:1521781	3684	4160	4777	5230	3969	3174	69.9102	66.17092	62.4493	64.65533	66.04573	74.68163	66.17681	68.4609	0.947028	1	-
ENSMUSC	Espn	espin [Sou	GO:00452	synapse|s	-	-	-	-	4:1521203	20	40	43	34	29	30	0.089123	0.149406	0.132	0.098699	0.113317	0.165756	0.12351	0.125924	0.894219	1	-
ENSMUSC	Prkag2	protein ki	GO:00081	metabolic	K07200	5'-AMP-a	mmu0453	Tight junc	5:2486274	1895	2447	2767	2996	2234	1551	13.64989	14.77429	13.73031	14.05864	14.11061	13.85216	14.0515	14.00714	0.731887	1	-
ENSMUSC	Rheb	Ras homo	GO:00325	developm	K07208	Ras homo	mmu0415	PI3K-Akt s	5:2480282	1148	1470	1746	1787	1329	1045	8.46783	9.088674	8.872095	8.586903	8.596033	9.557245	8.809533	8.913394	0.808115	1	-
ENSMUSC	Hes3	hairy and	GO:00325	developm	K09088	hairy and	mmu0516	Human pa	4:1522859	0	0	3	0	0	2	0	0	0.056489	0	0	0.067803	0.01883	0.022601	1	1	-
ENSMUSC	Nol9	nucleolar	GO:00081	metabolic	-	-	-	-	4:1520393	395	439	547	587	436	387	2.840075	2.645759	2.709392	2.749494	2.748919	3.450082	2.731742	2.982832	0.745052	1	-
ENSMUSC	Smarcd3	SWI/SNF	GO:00325	developm	K11650	SWI/SNF-	mmu0522	Hepatoce	5:2459081	715	831	839	922	650	729	6.30591	6.143217	5.097474	5.297296	5.026869	7.971776	5.848867	6.098647	0.955119	1	-
ENSMUSC	Tas1r1	taste rece	GO:00048	signal tran	K04624	taste rece	mmu0474	Taste tran	4:1520279	0	0	5	0	3	5	0	0	0.076462	0	0.058391	0.137609	0.025487	0.065333	0.443891	1	-
ENSMUSC	Zbtb48	zinc finge	GO:00081	metabolic	-	-	-	-	4:1520197	87	116	129	129	121	140	0.818402	0.914659	0.835965	0.790531	0.998099	1.632901	0.856342	1.14051	0.231135	1	-
ENSMUSC	Abcf2	ATP-bind	GO:00038	catalytic a	-	-	-	-	5:2456534	1653	1958	2427	2524	1878	1397	40.89262	40.60108	41.3612	40.67645	40.73899	42.85034	40.95164	41.42193	0.814335	1	-
ENSMUSC	Nub1	negative r	GO:00081	metabolic	-	-	-	-	5:2468553	1589	2120	2546	2778	2029	1469	16.30643	18.23575	17.99885	18.57158	18.25827	18.69145	17.51368	18.5071	0.881396	1	-
ENSMUSC	Vamp3	vesicle-as	GO:00325	developm	K13505	vesicle-as	mmu0414	Phagoson	4:1510473	312	380	514	447	341	355	6.039769	6.165971	6.854557	5.637084	5.788451	8.520776	6.353432	6.64877	0.98372	1	-
ENSMUSC	Tmub1	transmem	GO:00081	metabolic	-	-	-	-	5:2444545	229	275	344	362	260	211	4.110036	4.137095	4.25324	4.232535	4.091915	4.695463	4.16679	4.339971	1	1	-
ENSMUSC	Fastk	Fas-activa	GO:00081	metabolic	-	-	-	-	5:2443844	499	605	677	772	646	502	2.71445	2.758608	2.537007	2.735777	3.081462	3.385877	2.670022	3.067705	0.453706	1	-

ENSMUSC	Ube4b	ubiquitina	GO:00325	developm	K10597	ubiquitin (	mmu0414	Protein pr	4:1493284	2215	2543	3102	3418	2509	1747	12.94436	12.45679	12.48821	13.01249	12.85731	12.6586	12.62979	12.8428	0.86255	1 -
ENSMUSC	Pgd	phosphog	GO:00081	metabolic	K00033	6-phosph	mmu011C	Metabolic	4:1491499	427	631	783	728	578	446	3.706756	4.591428	4.682511	4.116979	4.399834	4.800498	4.326898	4.439103	0.91424	1 -
ENSMUSC	Slc4a2	solute car	GO:00325	developm	K13855	solute car	mmu0497	Pancreatic	5:2442383	603	448	901	618	524	570	3.222814	2.007004	3.317371	2.15173	2.455793	3.777272	2.849063	2.794932	0.675417	1 -
ENSMUSC	Park7	Parkinson	GO:00081	metabolic	K05687	protein D.	mmu0501	Parkinson	4:1508971	1414	1570	1837	1796	1384	1039	22.38206	20.83066	20.03139	18.51989	19.21008	20.39163	21.08137	19.37387	0.29247	1 -
ENSMUSC	Tnfrsf9	tumor nec	GO:00325	developm	K05146	tumor nec	mmu0406	Cytokine-	4:1509145	8	7	13	4	13	5	0.118897	0.087202	0.133105	0.038734	0.169428	0.092136	0.113068	0.100099	0.759849	1 -
ENSMUSC	Errfi1	ERBB rece	GO:00325	developm	-	-	-	-	4:1508539	839	887	1159	1120	780	518	8.664439	7.67812	8.24543	7.534914	7.063431	6.632757	8.195996	7.077034	0.163292	1 -
ENSMUSC	Cdk5	cyclin-dep	GO:00325	developm	K02090	cyclin-dep	mmu0501	Alzheimer	5:2441824	1131	1432	1714	1633	1361	1309	28.63683	30.39194	29.89679	26.93582	30.21783	41.0949	29.64185	32.74952	0.68047	1 -
ENSMUSC	Abcb1b	ATP-bind	GO:00511	localizatio	K05658	ATP-bind	mmu0522	Gastric ca	5:8798147	103	86	111	128	110	52	0.661272	0.462802	0.49093	0.53535	0.619268	0.413936	0.538335	0.522851	0.776736	1 -
ENSMUSC	Cort	cortistatin	GO:00508	response	-	-	-	-	4:1491250	42	32	60	46	37	58	3.942709	2.518005	3.880184	2.813114	3.045748	6.750948	3.446966	4.20327	0.626748	1 -
ENSMUSC	Abcb8	ATP-bind	GO:00511	localizatio	K05655	ATP-bind	mmu0201	ABC trans	5:2439366	330	469	514	562	473	529	2.152129	2.563773	2.309237	2.38766	2.704943	4.277557	2.341713	3.123387	0.124889	1 -
ENSMUSC	Dffa	DNA fragi	GO:00081	metabolic	K02310	DNA fragi	mmu0421	Apoptosis	4:1491041	693	776	929	1011	796	593	11.9291	11.19667	11.01644	11.33724	12.01518	12.65654	11.38074	12.00299	0.917814	1 -
ENSMUSC	Pex14	peroxisom	GO:00081	metabolic	K13343	peroxin-1	mmu0414	Peroxisom	4:1489605	598	798	952	894	793	593	5.486461	6.136865	6.016989	5.343308	6.379793	6.745769	5.880105	6.15629	0.975814	1 -
ENSMUSC	Slc2a5	solute car	GO:00511	localizatio	K08143	MFS trans	mmu0497	Carbohyd	4:1501192	66	75	91	102	70	75	1.05125	1.001336	0.998514	1.058386	0.977692	1.481192	1.017033	1.172423	0.617869	1 -
ENSMUSC	Casz1	castor zin	GO:00325	developm	-	-	-	-	4:1488044	31	16	33	24	24	22	0.138413	0.059881	0.1015	0.069807	0.093965	0.121795	0.099932	0.095189	0.768028	1 -
ENSMUSC	Nos3	nitric oxid	GO:00325	developm	K13242	nitric-oxic	mmu011C	Metabolic	5:2436481	116	191	236	227	175	175	1.620613	2.236692	2.271345	2.065984	2.143878	3.031406	2.042883	2.413756	0.480948	1 -
ENSMUSC	Masp2	mannan-l	GO:00081	metabolic	K03993	mannan-l	mmu0461	Complem	4:1486025	8	12	8	5	10	25	0.127547	0.160379	0.08787	0.051937	0.139814	0.494219	0.125265	0.228657	0.321126	1 -
ENSMUSC	H6pd	hexose-6	GO:00081	metabolic	K13937	hexose-6	mmu011C	Metabolic	4:1499794	103	132	205	207	142	138	1.112119	1.194653	1.524832	1.456023	1.344459	1.847489	1.277201	1.549324	0.375551	1 -
ENSMUSC	Slc25a33	solute car	GO:00081	metabolic	-	-	-	-	4:1497440	249	325	427	379	305	483	8.386174	9.174893	9.907041	8.315436	9.007545	20.16958	9.156036	12.49752	0.197615	1 -
ENSMUSC	Klhl7	kelch-like	GO:00081	metabolic	-	-	-	-	5:2410060	1064	1519	1789	1619	1311	1049	8.944497	10.70348	10.36039	8.86631	9.664064	10.93392	10.00279	9.821431	0.620254	1 -
ENSMUSC	Ctnnbip1	catenin be	GO:00325	developm	K04493	Beta-cate	mmu0431	Wnt signa	4:1495182	505	638	828	861	625	618	10.36636	10.97764	11.70892	11.51383	11.25015	15.72929	11.01764	12.83109	0.420948	1 -
ENSMUSC	Angptl7	angiopoie	GO:00055	extracellul	-	-	-	-	4:1484951	1	2	3	1	3	7	0.02947	0.049391	0.060889	0.019192	0.077503	0.255733	0.046583	0.117476	0.35702	1 -
ENSMUSC	Lzic	leucine zip	GO:00508	response	-	-	-	-	4:1494852	142	140	209	244	152	163	2.669974	2.206486	2.707179	2.988764	2.506149	3.800082	2.52788	3.098332	0.337898	1 -
ENSMUSC	Mtor	mechanist	GO:00325	developm	K07203	serine/thr	mmu052C	Pathways	4:1484486	2525	2680	3154	3806	2632	2083	13.92014	12.38425	11.9783	13.66887	12.72362	14.23828	12.7609	13.54359	0.868193	1 -
ENSMUSC	Nmnat1	nicotinam	GO:00081	metabolic	K06210	nicotinam	mmu011C	Metabolic	4:1494675	106	116	150	173	128	98	1.397931	1.282306	1.36277	1.486298	1.480242	1.602481	1.347669	1.523007	0.614579	1 -
ENSMUSC	Fam126a	family witl	GO:00325	developm	-	-	-	-	5:2391527	596	599	679	773	571	386	2.955723	2.489989	2.319737	2.497345	2.48311	2.373508	2.588483	2.451321	0.510746	1 -
ENSMUSC	Tomm7	translocas	GO:00081	metabolic	K17771	mitochon	mmu0413	Mitophag	5:2383894	232	343	424	322	288	318	13.10602	16.24165	16.50063	11.85009	14.26655	22.27392	15.28276	16.13019	1	1 -
ENSMUSC	Rint1	RAD50 int	GO:00511	localizatio	-	-	-	-	5:2378771	267	332	413	432	347	201	1.335168	1.391604	1.422741	1.407311	1.521586	1.246258	1.383171	1.391718	0.858829	1 -
ENSMUSC	Fbxo44	F-box prc	-	-	-	-	-	-	4:1481528	937	1264	1361	1326	1198	1022	10.82122	12.23591	10.82795	9.976125	12.1321	14.63435	11.29503	12.24753	0.783544	1 -
ENSMUSC	Mad2l2	MAD2 mi	GO:00325	developm	K13728	mitotic sp	mmu0411	Cell cycle	4:1481303	67	73	91	87	87	76	1.009257	0.921721	0.944316	0.853741	1.149186	1.419467	0.958431	1.140798	0.522871	1 -
ENSMUSC	Kmt2e	lysine (K)-	GO:00325	developm	K09189	histone-ly	mmu0031	Lysine deg	5:2343444	3011	3232	4101	4251	2984	1383	15.07469	13.56319	14.14422	13.8647	13.10024	8.585109	14.2607	11.85001	0.157761	1 -
ENSMUSC	Draxin	dorsal inh	GO:00325	developm	-	-	-	-	4:1480984	23	30	28	35	49	21	0.281098	0.307327	0.235748	0.278669	0.525139	0.318223	0.274724	0.37401	0.29813	1 -
ENSMUSC	Agtrap	angiotens	GO:00048	signal trar	-	-	-	-	4:1480770	169	191	262	283	226	221	2.463979	2.334184	2.63149	2.68793	2.889349	3.995106	2.476551	3.190795	0.181539	1 -
ENSMUSC	Mthfr	5,10-meth	GO:00081	metabolic	K00297	methylene	mmu011C	Metabolic	4:1480390	425	477	406	445	513	368	2.060105	1.93808	1.355746	1.405214	2.180521	2.211743	1.784643	1.932493	0.839609	1 -
ENSMUSC	Orc5	origin rec	GO:00081	metabolic	K02607	origin rec	mmu0411	Cell cycle	5:2248648	229	269	361	353	278	217	1.923812	1.894229	2.08923	1.931897	2.047927	2.26034	1.96909	2.080055	0.924673	1 -
ENSMUSC	Dnajc2	DnaJ heat	GO:00081	metabolic	-	-	-	-	5:2175726	592	706	843	874	619	267	5.153888	5.151942	5.055828	4.956856	4.725486	2.882108	5.120553	4.18815	0.1736	1 -
ENSMUSC	Clcn6	chloride c	GO:00511	localizatio	-	-	-	-	4:1480042	1503	1791	2184	2489	1798	1591	12.75643	12.74145	12.76951	13.76185	13.38144	16.74273	12.7558	14.62867	0.42517	1 -
ENSMUSC	Pmpcb	peptidase	GO:00081	metabolic	-	-	-	-	5:2173714	750	956	1298	1126	869	521	7.427889	7.936243	8.855848	7.264809	7.546863	6.397756	8.073327	7.06981	0.212143	1 -
ENSMUSC	Mfn2	mitofusin	GO:00325	developm	K06030	mitofusin	mmu0462	NOD-like	4:1478735	3781	4189	5367	5991	4135	4035	32.03919	29.7535	31.32982	33.07164	30.72508	42.394	31.04084	35.39691	0.463649	1 -
ENSMUSC	Miip	migration	GO:00400	locomotic	-	-	-	-	4:1478607	158	189	212	284	220	181	1.585064	1.589293	1.465131	1.856051	1.935332	2.251413	1.546496	2.014265	0.132124	1 -
ENSMUSC	Trp73	transform	GO:00325	developm	K10148	tumor prc	mmu0439	Hippo sig	4:1540562	67	47	107	93	77	69	0.605009	0.355742	0.66561	0.547081	0.609707	0.772537	0.542121	0.643108	0.557775	1 -
ENSMUSC	Dffb	DNA fragi	GO:00081	metabolic	K02311	DNA fragi	mmu0421	Apoptosis	4:1539644	93	116	142	158	91	103	1.321068	1.381188	1.389571	1.462102	1.133508	1.81411	1.363942	1.469907	0.878098	1 -
ENSMUSC	Lrrc47	leucine ric	GO:00038	catalytic a	-	-	-	-	4:1540117	857	511	542	1306	541	501	7.318584	3.657799	3.188579	7.265596	4.051224	5.304813	4.721654	5.540545	0.568562	1 -
ENSMUSC	Wrap73	WD repea	GO:00099	cellular pr	-	-	-	-	4:1541423	197	236	291	315	223	283	2.432005	2.442091	2.47481	2.53332	2.414044	4.331813	2.449635	3.093059	0.281114	1 -
ENSMUSC	Tprgl	transform	GO:00452	synapse|e	-	-	-	-	4:1541574	1342	1668	1884	1885	1592	1401	22.42065	23.35848	21.68343	20.51581	23.32283	29.02148	22.48752	24.28671	0.79771	1 -
ENSMUSC	Arhgef16	Rho guan	GO:00400	locomotic	-	-	-	-	4:1542784	9	12	10	12	12	5	0.068197	0.076222	0.052202	0.059239	0.079736	0.046974	0.06554	0.061983	0.836087	1 -
ENSMUSC	Acap3	ArfGAP w	GO:00987	molecular	K12489	Arf-GAP v	mmu0414	Endocytos	4:1558918	1342	1565	1738	2048	1568	1250	8.160764	7.977108	7.280808	8.113156	8.361168	9.424842	7.806227	8.633055	0.615388	1 -
ENSMUSC	Ints11	integrator	GO:00081	metabolic	-	-	-	-	4:1558695	291	425	427	456	326	293	3.70917	4.540726	3.749416	3.786433	3.643715	4.630607	3.999771	4.020252	0.788243	1 -
ENSMUSC	Atad3a	ATPase fa	GO:00099	cellular pr	-	-	-	-	4:1557406	540	611	781	806	596	531	5.521904	5.237076	5.501703	5.369224	5.344212	6.732492	5.420228	5.815309	0.838014	1 -
ENSMUSC	Ssu72	Ssu72 RN.	GO:00081	metabolic	K15544	RNA polyi	mmu0301	mRNA sur	4:1557048	576	658	746	982	549	608	10.12068	9.690943	9.029792	11.24037	8.458684	13.24577	9.613806	10.98161	0.528874	1 -
ENSMUSC	Pex10	peroxisom	GO:00511	localizatio	K13346	peroxin-1	mmu0414	Peroxisom	4:1550670	171	233	219	275	192	161	1.877277	2.14408	1.65626	1.966743	1.848321	2.191507	1.892539	2.00219	0.923135	1 -
ENSMUSC	Rer1	retention	GO:00511	localizatio	-	-	-	-	4:1550741	754	486	744	1046	820	709	20.43453	11.04033	13.89048	18.46743	19.48721	23.82458	15.12178	20.59307	0.10105	1 -
ENSMUSC	Morn1	MORN re	-	-	-	-	-	-	4:1550865	87	86	95	145	85	73	0.954944	0.79124	0.718345	1.036831	0.81812	0.993491	0.82151	0.949481	0.545668	1 -
ENSMUSC	Ski	ski sarcon	GO:00325	developm	-	-	-	-	4:1551540	3599	4028	5097	2001	4205	3201	35.79457	33.57976	34.92218	12.96475	36.67279	39.47361	34.7655	29.70372	0.299609	1 -
ENSMUSC	Prkcz	protein kii	GO:00325	developm	K18952	atypical p	mmu0516	Human pa	4:1552601	2586	3107	3515	3776	3115	2282	18.45571	18.58645	17.2814	17.55561	19.49409	20.19314	18.10785	19.08094	0.917379	1 -
ENSMUSC	Gabrd	gamma-a	GO:00048	signal trar	K05184	gamma-a	mmu0408	Neuroacti	4:1553849	272	369	455	500	367	380	4.617099	5.250249	5.320629	5.529071	5.462722	7.997796	5.062659	6.329863	0.220476	1 -
ENSMUSC	Plch2	phospholi	GO:00048	signal trar	K19006	phosphati	mmu011C	Metabolic	4:1549831	2527	2833	2876	3361	2519	2277	7.50355	7.051166	5.883039	6.501471	6.558921	8.383213	6.812585	7.147868	0.98169	1 -
ENSMUSC	Pank4	pantother	GO:00081	metabolic	K09680	type II par	mmu011C	Metabolic	4:1549641	404	573	616	752	530	502	3.943993	4.688806	4.142736	4.782494	4.537048	6.076375	4.258512	5.131972	0.291747	1 -
ENSMUSC	Fam213b	family witl	GO:00081	metabolic	K15717	prostamic	mmu011C	Metabolic	4:1548955	340	457	545	500	416	644	15.22765	17.15627	16.81518	14.58833	16.33767	35.76231	16.3997	22.22944	0.199525	1 -
ENSMUSC	Mib2	mindbom	GO:00048	signal trar	-	-	-	-	4:1556546	1282	1571	1920	2087	1630	1273	9.326343	9.579713	9.622237	9.890712	10.39809	11.48253	9.509431	10.59045	0.565515	1 -
ENSMUSC	Mmp23	matrix me	GO:00081	metabolic	-	-	-	-	4:1556506	15	14	33	32	26	28	0.268159	0.209791	0.406402	0.372672	0.40758	0.62064	0.294784	0.466964	0.147818	1 -
ENSMUSC	Cdk11b	cyclin-dep	GO:00325	developm	-	-	-	-	4:1556248	1156	1625	1804	2044	1471	902	13.54236	15.95668	14.55876	15.59908	15.11095	13.10174	14.68593	14.60392	0.762861	1 -
ENSMUSC	Nadk	NAD kina	GO:00081	metabolic	K00858	NAD+ kin	mmu011C	Metabolic	4:1555623	512	539	770	736	509	668	4.88943	4.314494	5.06559	4.578755	4.262346	7.909526	4.756505	5.583542	0.494556	1 -
ENSMUSC	Gnb1	guanine n	GO:00325	developm	K04536	guanine n	mmu0474	Olfactory	4:1554913	8010	9784	12449	12573	9614	8140	54.40415	55.70174	58.24859	55.63141	57.25933	68.55038	56.11816	60.48037	0.771663	1 -
ENSMUSC	Mrpl20	mitochon	GO:00081	metabolic	K02887	large subu	mmu0301	Ribosome	4:1558028	311	496	572	526	331	349	6.495233	8.682983	8.229649	7.156513	6.061842	9.037448	7.802622	7.418601	0.515529	1 -
ENSMUSC	Ccnl2	cyclin L2 [	GO:00081	metabolic	-	-	-	-	4:1558124	904	1225	1573	1469	1168	974	6.173645	7.012327	7.400366	6.535465	6.994532	8.247417	6.862113	7.259138	0.912517	1 -
ENSMUSC	Mxra8	matrix-rei	GO:00325	developm	-	-	-	-	4:1558396	89	98	161	139	124	148	0.947119	0.874171	1.180304	0.963636	1.157122	1.952825	1.000532	1.357861	0.206619	1 -
ENSMUSC	Dvl1	dishevelle	GO:00325	developm	K02353	segment p	mmu052C	Pathways	4:1558474	1239	1489	1722	1929	1440	1407	6.929088	6.979951	6.634216	7.027791	7.061715	9.756296	6.847751	7.948601	0.402549	1 -
ENSMUSC	Tas1r3	taste rece	GO:00048	signal trar	K04626	taste rece	mmu0474	Taste tran	4:1558592	1	2	6	7	5	4	0.018043	0.03024	0.07456	0.082264	0.079087	0.089468	0.040948	0.083606	0.214947	1 -
ENSMUSC	Cptp	ceramide-	GO:00511	localizatio	-	-	-	-	4:1558647	251	291	378	341	281	263	4.321804	4.199891	4.483687	3.824969	4.242682	5.61479	4.335127	4.560814	0.987549	1 -
ENSMUSC	Ttll10	tubulin tyi	GO:00081	metabolic	-	-	-	-	4:1560348	0	0	3	1	0	0	0	0	0.021897	0.006902	0	0	0.007299	0.002301	0.665759	1 -
ENSMUSC	Tnfrsf4	tumor nec	GO:00023	immune s	K05142	tumor nec	mmu0406	Cytokine-	4:1560138	9	10	20	23	3	8	0.28162	0.262276	0.431139	0.468852	0.082311	0.310385	0.325012	0.287183	0.761762	1 -
ENSMUSC	Sdf4	stromal ce	GO:00325	developm	-	-	-	-	4:1559928	4546	5321	6578	6974	5178	3778	36.18637	35.50273	36.07123	36.16422	36.14268	37.28752	35.92011	36.53147	0.849846	1 -
ENSMUSC	Bst1	bone mar	GO:00081	metabolic	K18152	ADP-ribos	mmu011C	Metabolic	5:4381888	3	4	10	11	0	4	0.033837	0.037819	0.077711	0.080831	0	0.055945	0.049789	0.045592	0.899001	1 -
ENSMUSC	Cd38	CD38 anti	GO:00081	metabolic	K01242	ADP-ribos	mmu011C	Metabolic	5:4386855	166	166	220	242	203	171	2.541505	2.130318	2.320369	2.413681	2.725343	3.246126	2.330731	2.79505	0.369545	1 -
ENSMUSC	Prom1	prominin	GO:00325	developm	K06532	prominin	mmu052C	Transcript	5:4399362	172	212	284	301	191	261	0.950202	0.981693	1.080827	1.083267	0.925261	1.787781	1.004241	1.265436	0.299618	1 -
ENSMUSC	Kcnip4	Kv channe	GO:00511	localizatio	-	-	-	-	5:4838950	947	453	1605	1685	497	492	7.304858	2.928957	8.528802	8.467265	3.361715	4.705577	6.254205	5.511519	0.546039	1 -
ENSMUSC	5730480H	RIKEN cDI	-	-	-	-	-	-	5:4832795	113	166	187	153	175	129	0.550093	0.677361	0.627123	0.485213	0.747032	0.778634	0.618193	0.670293	0.848692	1 -
ENSMUSC	Adgra3	adhesion	GO:00048	signal trar	-	-	-	-	5:4995995	432	498	632	549	491	348	3.094204	2.98984	3.118416	2.561649	3.083823	3.090519	3.067487	2.911997	0.488293	1 -
ENSMUSC	Sorcs2	sortilin-re	GO:00160	membran	-	-	-	-	5:3601718	1084	1317	1572	1712	1377	1270	6.629279	6.751117	6.622791	6.820599	7.384374	9.630004	6.667729	7.944992	0.291946	1 -
ENSMUSC	Afap1	actin filan	GO:00508	response	-	-	-	-	5:3589331	564	597	771	860	601	440	2.696044	2.392078	2.538948	2.678106	2.519214	2.607875	2.542356	2.601732	0.915538	1 -
ENSMUSC	Ablim2	actin-binc	GO:00081	metabolic	K07520	actin-binc	mmu0436	Axon guic	5:3575788	1224	1492	1870	2059	1490	1118	6.394485	6.533495	6.730032	7.00748	6.825789	7.241886	6.552671	7.025052	0.790661	1 -
ENSMUSC	Htra3	HtrA serin	GO:00081	metabolic	-	-	-	-	5:3565202	41	61	70	74	77	57	0.397081	0.495203	0.467032	0.466891	0.653934	0.684475	0.453105	0.601767	0.224094	1 -
ENSMUSC	Trmt44	tRNA met	GO:00081	metabolic	-	-	-	-	5:3555620	92	125	148	120	118	128	0.936702	1.066781	1.038067	0.795934	1.053506	1.615874	1.01385	1.155105	0.712912	1 -
ENSMUSC	Acox3	acyl-Coer	GO:00081	metabolic	K00232	acyl-CoA	mmu011C	Metabolic	5:3558304	467	532	637	703	564	431	2.046139	1.953811	1.92269	2.006575	2.166907	2.34143	1.974213	2.171637	0.675661	1 -
ENSMUSC	Rgs12	regulator	GO:00048	signal trar	-	-	-	-	5:3494944	530	663	702	737	446	520	1.999472	2.096553	1.824434	1.811291	1.475424	2.432365	1.973486	1.90636	0.564729	1 -
ENSMUSC	Hgfac	hepatocyt	GO:00081	metabolic	-	-	-	-	5:3504150	0	0	2	2	2	0	0	0	0.014669	0.013868	0.018668	0	0.00489	0.010845	0.718238	1 -
ENSMUSC	Lrpap1	low densit	GO:00081	metabolic	-	-	mmu0497	Cholestero	5:3509150	3045	3532	4142	4350	3427	2436	34.87456	33.90744	32.68006	32.4558	34.41741	34.59272	33.82069	33.82198	0.734883	1 -
ENSMUSC	Htt	huntingtin	GO:00081	metabolic	K04533	huntingtin	mmu0501	Huntingto	5:3476174	3651	4176	5275	6002	4507	3606	8.465542	8.116266	8.42591	9.066101	9.163749	10.36704	8.335906	9.532295	0.406404	1 -
ENSMUSC	Add1	adducin 1	GO:00325	developm	-	-	-	-	5:3457366	3897	4791	5183	5932	4442	2899	15.82228	16.30488	14.49677	15.68994	15.81466	14.59394	15.54131	15.36618	0.690208	1 -
ENSMUSC	Pcdh7	protocadh	GO:00160	membran	-	-	-	-	5:5771796	1628	1505	1066	2051	1659	394	4.339218	3.362377	1.957337	3.561264	3.877448	1.302084	3.219644	2.913599	0.639777	1 -
ENSMUSC	Rnf4	ring finge	GO:00081	metabolic	-	-	-	-	5:3433628	1185	1503	1788	1863	1366	948	4.747547	5.047341	4.934801	4.862339	4.798928	4.709175	4.909896	4.790147	0.607715	1 -
ENSMUSC	Nelfa	negative e	GO:00081	metabolic	-	-	-	-	5:3389791	375	449	561	531	403	311	5.498927	5.518816	5.667106	5.072518	5.181975	5.654487	5.561616	5.302993	0.508994	1 -
ENSMUSC	Man2b2	mannosid	GO:00081	metabolic	K12312	epididymi	mmu0051	Other glyc	5:3680692	414	443	567	646	427	358	6.326263	5.674186	5.968732	6.43075	5.721597	6.782911	5.989727	6.311753	0.930545	1 -
ENSMUSC	Ppp2r2c	protein pl	GO:00325	developm	K04354	serine/thr	mmu0415	PI3K-Akt s	5:3686851	9645	11506	14314	15123	11779	9202	127.9417	127.9344	130.8044	130.6861	137.0128	151.3485	128.8935	139.6825	0.7182	1 -
ENSMUSC	Crmp1	collapsin r	GO:00325	developm	-	-	-	-	5:3724194	4172	4721	5174	6070	4322	3745	26.83364	25.45201	22.9252	25.43346	24.37601	29.86572	25.07028	26.5584	0.8994	1 -
ENSMUSC	Evc	EvC ciliary	GO:00325	developm	K19605	ellis van ci	mmu0434	Hedgehog	5:3728909	221	239	340	389	332	232	1.361632	1.234289	1.443104	1.561341	1.79369	1.772312	1.346342	1.709114	0.161498	1 -
ENSMUSC	Stk32b	serine/thr	GO:00081	metabolic	-	-	-	-	5:3744682	137	182	181	200	110	103	2.517855	2.803729	2.291606	2.394545	1.77274	2.347117	2.53773	2.171467	0.245625	1 -
ENSMUSC	Stx18	syntaxin 1	GO:00511	localizatio	K08492	syntaxin 1	mmu0414	Phagoson	5:3803874	259	296	409	354	306	186	2.330489	2.232499	2.535257	2.075067	2.414408	2.075135	2.366082	2.188203	0.430731	1 -
ENSMUSC	Nsg1	neuron sp	GO:00081	metabolic	-	-	-	-	5:3813719	2455	2827	3578	3090	2860	2334	27.60974	26.64952	27.7206	22.63867	28.20458	32.546	27.32662	27.79642	0.819107	1 -
ENSMUSC	Zbtb49	zinc finge	GO:00081	metabolic	-	-	-	-	5:3818973	111	99	136	201	128	129	0.490809	0.366925	0.414264	0.578984	0.496298	0.707233	0.424	0.594172	0.0845	1 -
ENSMUSC	Rab28	RAB28, m	GO:00511	localizatio	-	-	-	-	5:4162497	535	678	955	838	599	395	5.813107	6.174997	7.148405	5.93171	5.707216	5.321537	6.378837	5.653488	0.247448	1 -
ENSMUSC	Rnf32	ring finge	GO:00056	cell|intrace	-	-	-	-	5:2919599	191	228	314	271	217	122	1.14117	1.141839	1.292404	1.054794	1.136895	0.903783	1.191804	1.031824	0.26252	1 -
ENSMUSC	Dnajb6	DnaJ heat	GO:00325	developm	-	-	-	-	5:2973568	993	1338	1659	1557	1315	643	7.142162	8.066579	8.220123	7.295424	8.29371	5.734262	7.809621	7.107798	0.380545	1 -
ENSMUSC	Plb1	phospholi	GO:00081	metabolic	K14621	phospholi	mmu011C	Metabolic	5:3223270	13	21	26	35	11	14	0.060517	0.081943	0.083381	0.106144	0.044904	0.080811	0.07528	0.077286	1	1 -
ENSMUSC	Fosl2	fos-like ar	GO:00325	developm	K09030	fos-like ar	mmu0438	Osteoclas	5:3213580	1123	1276	1526	1448	1203	1142	6.14835	5.855746	5.75552	5.164506	5.775463	7.752302	5.919872	6.230757	0.979338	1 -
ENSMUSC	Rbks	ribokinase	GO:00081	metabolic	K00852	ribokinase	mmu0003	Pentose p	5:3162443	10	17	40	27	17	22	0.419219	0.59737	1.155173	0.737351	0.624915	1.143551	0.723921	0.835273	0.8344	1 -
ENSMUSC	Slc4a1ap	solute car	GO:00160	membran	-	-	-	-	5:3152699	620	647	883	932	662	399	4.047962	3.540804	3.971521	3.964081	3.790052	3.230005	3.853429	3.661379	0.545537	1 -
ENSMUSC	Eif2b4	eukaryotic	GO:00325	developm	K03680	translatior	mmu0301	RNA trans	5:3118755	262	324	333	333	334	277	2.891782	2.997526	2.531979	2.394364	3.232608	3.790799	2.807096	3.139257	0.673799	1 -
ENSMUSC	Snx17	sorting ne	GO:00325	developm	-	-	-	-	5:3119322	686	910	1042	1064	878	820	6.333142	7.041898	6.626959	6.399096	7.107757	9.38631	6.667333	7.631054	0.500164	1 -
ENSMUSC	Ppm1g	protein pl	GO:00081	metabolic	-	-	-	-	5:3120266	1183	1574	2201	1947	1070	854	10.6191	11.84296	13.61051	11.38546	8.42227	9.504883	12.02419	9.77087	0.069366	1 -
ENSMUSC	Nrbp1	nuclear re	GO:00081	metabolic	-	-	-	-	5:3124086	912	1173	1322	1225	960	856	7.282366	7.851062	7.27211	6.37229	6.721892	8.47495	7.468513	7.18971	0.501492	1 -
ENSMUSC	Krtcap3	keratinocy	GO:00160	membran	-	-	-	-	5:3125169	6	5	8	13	6	6	0.065975	0.04608	0.060597	0.09312	0.057845	0.081806	0.05755	0.07759	0.610673	1 -
ENSMUSC	Slc30a3	solute car	GO:00452	synapse|c	-	-	-	-	5:3108610	939	1120	1309	1440	1188	1245	16.66935	16.66572	16.00826	16.65318	18.4932	27.40364	16.44778	20.85001	0.163239	1 -
ENSMUSC	Ociad1	OCIA don	GO:00325	developm	-	-	-	-	5:7329278	4680	5757	6169	7166	5254	2770	33.31408	34.35034	30.25159	33.23077	32.79553	24.44825	32.63867	30.15818	0.417061	1 -
ENSMUSC	Ociad2	OCIA don	GO:00160	membran	-	-	-	-	5:7332219	1425	1758	1970	1976	1447	1389	15.60355	16.13543	14.86026	14.0954	13.89376	18.85805	15.53308	15.61574	0.740333	1 -
ENSMUSC	Cwh43	cell wall b	GO:00081	metabolic	-	-	-	-	5:7340607	15	13	36	29	17	12	0.252063	0.183115	0.416748	0.317466	0.250495	0.250024	0.283975	0.272662	0.855457	1 -
ENSMUSC	Sgcb	sarcoglyca	GO:00325	developm	K12566	beta-sarc	mmu0541	Dilated ca	5:7363274	1100	1428	1790	1891	1321	719	16.45289	17.90319	18.44394	18.42564	17.32586	13.33412	17.60001	16.36187	0.45866	1 -
ENSMUSC	Cgref1	cell growt	GO:00431	ion bindin	-	-	-	-	5:3093314	178	204	215	244	175	192	5.063766	4.864481	4.213492	4.521944	4.365489	6.77235	4.713913	5.219928	0.755814	1 -
ENSMUSC	Khk	ketohexol	GO:00081	metabolic	K00846	ketohexol	mmu011C	Metabolic	5:3092143	68	92	80	120	90	90	1.272562	1.443136	1.031356	1.462962	1.476911	2.088328	1.249018	1.676067	0.181157	1 -
ENSMUSC	Emilin1	elastin mi	GO:00400	locomotic	-	-	-	-	5:3091340	63	72	92	95	72	82	0.490909	0.470264	0.493855	0.48224	0.491962	0.792247	0.485009	0.588816	0.462883	1 -
ENSMUSC	Agbl5	ATP/GTP	GO:00081	metabolic	-	-	-	-	5:3088869	145	212	211	242	199	201	0.690433	0.846137	0.692128	0.75067	0.830898	1.186685	0.7429	0.922751	0.30566	1 -
ENSMUSC	Mapre3	microtubu	GO:00081	metabolic	-	-	-	-	5:3081464	2128	2295	3564	3025	2301	1161	22.57023	20.40328	26.04074	20.9012	21.40048	15.26801	23.00475	19.1899	0.136598	1 -
ENSMUSC	Ppargc1a	peroxisom	GO:00325	developm	K07202	alpha per	mmu0501	Huntingto	5:5145425	1060	1169	1522	1669	1237	786	8.00549	7.400301	7.918595	8.211452	8.192083	7.360218	7.774795	7.921251	0.89977	1 -
ENSMUSC	Dpysl5	dihydropy	GO:00325	developm	K07529	dihydropy	mmu0436	Axon guic	5:3071156	749	867	954	956	782	693	4.020711	3.901151	3.527938	3.343188	3.681038	4.612539	3.8166	3.878921	0.821711	1 -
ENSMUSC	Dhx15	DEAH (As	GO:00081	metabolic	K12820	pre-mRN.	mmu0304	Spliceosoi	5:5215020	1680	2061	2620	2721	1961	1125	10.09064	10.37625	10.84082	10.64681	10.32832	8.378145	10.4359	9.784425	0.457604	1 -
ENSMUSC	Pgm1	phosphog	GO:00081	metabolic	K15779	phosphog	mmu011C	Metabolic	5:6409295	129	133	232	237	163	103	2.293242	1.981816	2.841188	2.744673	2.54091	2.270302	2.372082	2.518628	0.882527	1 -
ENSMUSC	Sepsecs	Sep (O-pl	GO:00081	metabolic	K03341	O-phospl	mmu0097	Aminoacy	5:5264008	241	276	243	419	311	298	1.470883	1.411958	1.021687	1.665926	1.664423	2.255083	1.30151	1.861811	0.045284	1 -
ENSMUSC	Tbc1d1	TBC1 dor	GO:00511	localizatio	K18341	TBC1 dor	mmu0415	AMPK sigi	5:6415630	618	723	873	993	682	597	3.711566	3.639651	3.611885	3.885075	3.591662	4.445581	3.654367	3.974106	0.741115	1 -
ENSMUSC	Slc35f6	solute car	GO:00511	localizatio	-	-	-	-	5:3064793	279	253	343	392	363	272	1.463584	1.112464	1.239534	1.339617	1.669792	1.769159	1.271861	1.592856	0.219191	1 -
ENSMUSC	Anapc4	anaphase	GO:00081	metabolic	K03351	anaphase	mmu0516	HTLV-I inf	5:5283401	774	1114	1248	1348	925	631	5.684081	6.857363	6.313718	6.448974	5.956669	5.745592	6.285054	6.050411	0.570642	1 -
ENSMUSC	Cenpa	centrome	GO:00511	localizatio	-	-	-	-	5:3066677	25	23	41	37	22	26	0.163832	0.126338	0.185095	0.157957	0.126423	0.211262	0.158421	0.165214	1	1 -
ENSMUSC	Klf3	Kruppel-li	GO:00081	metabolic	K15605	krueppel-	mmu052C	Transcript	5:6480338	1055	1292	1728	1782	1202	701	4.606556	4.728674	5.197795	5.068895	4.602257	3.795139	4.844342	4.488764	0.430299	1 -
ENSMUSC	Zcchc4	zinc finge	GO:00081	metabolic	-	-	-	-	5:5277540	122	187	205	219	211	162	0.631851	0.811803	0.731413	0.738892	0.958255	1.040298	0.725022	0.912482	0.250147	1 -
ENSMUSC	1700001C	RIKEN cDI	GO:00056	cell|intrace	-	-	-	-	5:3046604	12	7	19	14	2	11	0.335211	0.163902	0.365641	0.254783	0.048989	0.381016	0.288252	0.228263	0.479649	1 -
ENSMUSC	Fam114a1	family witl	GO:00056	cell|intrace	-	-	-	-	5:6497007	112	132	217	175	146	94	2.318556	2.290464	3.09463	2.360034	2.650287	2.412756	2.567883	2.474359	0.659546	1 -
ENSMUSC	Pi4k2b	phosphati	GO:00081	metabolic	K13711	phosphati	mmu011C	Metabolic	5:5274157	38	47	67	61	48	34	0.509281	0.527987	0.618584	0.532582	0.564107	0.564996	0.551951	0.553895	0.884548	1 -
ENSMUSC	Slc34a2	solute car	GO:00325	developm	K14683	solute car	mmu0497	Mineral al	5:5303808	0	3	0	0	0	0	0	0.030643	0	0	0	0	0.010214	0	0.304972	1 -
ENSMUSC	Sel1l3	sel-1 supp	GO:00160	membran	-	-	-	-	5:5310708	910	1052	1314	1339	1060	830	5.122971	4.964198	5.095976	4.910693	5.232742	5.793551	5.061048	5.312329	0.954021	1 -
ENSMUSC	D5Ertd579	DNA segr	GO:00971	organic cy	-	-	-	-	5:3660048	3091	2807	4418	4830	3574	2296	9.329518	7.101588	9.186233	9.497056	9.459267	8.592473	8.539113	9.182932	0.769564	1 -
ENSMUSC	Rfc1	replicatior	GO:00081	metabolic	K10754	replicatior	mmu0342	Nucleotid	5:6526185	847	942	1277	1305	1002	537	5.943406	5.540583	6.172968	5.96546	6.165411	4.672098	5.885652	5.60099	0.561098	1 -
ENSMUSC	Tbc1d14	TBC1 dor	GO:00081	metabolic	-	-	-	-	5:3649060	1483	1590	2086	2215	1592	1427	4.412411	3.96538	4.275634	4.293291	4.153561	5.264351	4.217808	4.570401	0.758834	1 -
ENSMUSC	Klb	klotho be	GO:00081	metabolic	-	-	-	-	5:6534831	1	5	8	8	6	6	0.008999	0.037704	0.049582	0.046887	0.047331	0.066936	0.032095	0.053718	0.38623	1 -
ENSMUSC	Tada2b	transcripti	GO:00081	metabolic	-	-	-	-	5:3647367	627	819	979	973	725	521	7.794945	8.534576	8.38454	7.880246	7.903617	8.030987	8.23802	7.938283	0.55398	1 -
ENSMUSC	Grpel1	GrpE-like	GO:00511	localizatio	-	-	-	-	5:3646499	530	710	815	727	666	544	9.547363	10.72059	10.11385	8.531464	10.52022	12.15045	10.12727	10.40071	0.893212	1 -
ENSMUSC	Lias	lipoic acid	GO:00325	developm	K03644	lipoyl synt	mmu011C	Metabolic	5:6539149	323	427	441	523	381	317	3.170286	3.512988	2.981855	3.344105	3.279171	3.85782	3.22171	3.493699	0.774317	1 -
ENSMUSC	Ugdh	UDP-gluc	GO:00325	developm	K00012	UDPgluco	mmu011C	Metabolic	5:6541322	285	415	541	431	362	337	2.102204	2.565852	2.749029	2.071045	2.341436	3.082099	2.472362	2.498193	0.803004	1 -
ENSMUSC	Pds5a	PDS5 coh	GO:00081	metabolic	-	-	-	-	5:6560572	1251	1449	1916	2224	1455	928	2.622969	2.546578	2.767469	3.037752	2.67511	2.412512	2.645672	2.708458	0.943545	1 -
ENSMUSC	Ube2k	ubiquitin-	GO:00081	metabolic	K04649	ubiquitin-	mmu0412	Ubiquitin	5:6553723	1214	1412	1628	1515	1114	543	4.902674	4.779713	4.529185	3.985735	3.944956	2.718944	4.737191	3.549878	0.030226	1 -
ENSMUSC	Rhoh	ras homol	GO:00325	developm	K07873	Ras homo	mmu0467	Leukocyte	5:6586121	10	15	12	19	15	12	0.050961	0.064075	0.042129	0.063075	0.067029	0.075823	0.052388	0.068643	0.439637	1 -
ENSMUSC	Nsun7	NOL1/NC	GO:00081	metabolic	-	-	-	-	5:6625989	199	200	225	420	265	148	1.82024	1.533416	1.417784	2.502689	2.125517	1.678507	1.59048	2.102238	0.143251	1 -
ENSMUSC	Apbb2	amyloid b	GO:00325	developm	-	-	-	-	5:6629870	717	1012	1357	1386	989	529	1.447875	1.712951	1.887743	1.823293	1.75126	1.324504	1.682856	1.633019	0.685101	1 -
ENSMUSC	Guf1	GUF1 hon	GO:00081	metabolic	-	-	-	-	5:6955692	325	565	659	598	422	325	1.281073	1.866773	1.78948	1.535581	1.45863	1.5884	1.645775	1.527537	0.43725	1 -
ENSMUSC	Gnpda2	glucosam	GO:00081	metabolic	K02564	glucosam	mmu011C	Metabolic	5:6957310	269	377	392	437	344	165	1.615706	1.898032	1.621984	1.709909	1.8118	1.228799	1.711907	1.583503	0.515054	1 -
ENSMUSC	Gabra4	gamma-a	GO:00325	developm	K05175	gamma-a	mmu0408	Neuroacti	5:7156974	1522	2062	2494	2485	1875	1140	10.97308	12.46107	12.38687	11.67137	11.85381	10.19072	11.94034	11.23863	0.455046	1 -
ENSMUSC	Gabrb1	gamma-a	GO:00325	developm	K05181	gamma-a	mmu0408	Neuroacti	5:7165811	6476	7449	7873	9993	6937	3542	29.53705	28.47811	24.73728	29.69189	27.74436	20.03064	27.58415	25.8223	0.503088	1 -
ENSMUSC	Commd8	COMM dc	GO:00081	metabolic	-	-	-	-	5:7215657	377	612	819	697	711	348	2.943787	4.005614	4.405553	3.545515	4.8683	3.369232	3.784985	3.927682	0.97656	1 -
ENSMUSC	Tec	tec protei	GO:00325	developm	K07364	tyrosine-p	mmu0438	Osteoclas	5:7275571	32	38	50	62	34	33	0.305265	0.303858	0.328589	0.385305	0.284419	0.390326	0.312571	0.35335	0.686164	1 -
ENSMUSC	Slc10a4	solute car	GO:00511	localizatio	-	-	-	-	5:7300688	14	7	11	12	8	8	0.405717	0.170031	0.219601	0.226556	0.203306	0.287457	0.265117	0.239107	0.768105	1 -
ENSMUSC	Slc30a9	solute car	GO:00081	metabolic	-	-	-	-	5:6730695	2938	3286	4458	4272	3143	2073	9.662529	9.058566	10.10021	9.152758	9.06413	8.45326	9.607103	8.89005	0.342973	1 -
ENSMUSC	Uchl1	ubiquitin (	GO:00325	developm	K05611	ubiquitin (	mmu0501	Parkinson	5:6667609	3093	4613	5086	5052	3941	5474	81.69969	102.1355	92.54826	86.93317	91.28294	179.2798	92.12783	119.1653	0.205551	1 -
ENSMUSC	Fip1l1	FIP1 like 1	GO:00081	metabolic	K14405	pre-mRN.	mmu0301	mRNA sur	5:7453544	1372	1497	1662	1702	1314	548	4.373076	3.999517	3.649348	3.53406	3.672576	2.165705	4.007313	3.124114	0.089538	1 -
ENSMUSC	Lnx1	ligand of	GO:00081	metabolic	-	-	-	-	5:7459244	194	281	290	283	191	321	1.878029	2.280131	1.933977	1.78472	1.621356	3.852944	2.030712	2.419674	0.56598	1 -
ENSMUSC	Chic2	cysteine-r	GO:00511	localizatio	-	-	-	-	5:7499798	201	261	291	276	208	218	1.283577	1.39707	1.280177	1.148198	1.164748	1.72611	1.320275	1.346352	0.848325	1 -
ENSMUSC	Pdgfra	platelet de	GO:00325	developm	K04363	platelet-d	mmu052C	Pathways	5:7515229	1165	1389	1630	1885	1319	850	4.946503	4.943418	4.767728	5.213932	4.910884	4.474838	4.885883	4.866551	0.762654	1 -
ENSMUSC	Srd5a3	steroid 5 a	GO:00081	metabolic	K12345	3-oxo-5-	mmu0014	Steroid hc	5:7614027	127	143	196	222	165	204	1.003008	0.946654	1.066373	1.142187	1.142694	1.997641	1.005345	1.427507	0.089845	1 -

ENSMUS(	Tmem165	transmem	GO:00081	metabolic	-	-	-	-	5:7618388	226	381	444	386	301	258	3.876654	5.478043	5.246653	4.313372	4.527487	5.487237	4.867117	4.776032	0.678679	1	-
ENSMUS(	Nmu	neuromed	GO:00452	synapse|lc	-	-	-	-	5:7633349	0	0	2	0	0	0	0	0	0.105764	0	0	0	0.035255	0	0.536164	1	-
ENSMUS(	Clock	circadian	GO:00325	developm	K02223	circadian	mmu0516	Herpes sir	5:7620986	1892	2320	2974	3070	2177	1268	5.406336	5.556773	5.854289	5.71481	5.454852	4.492489	5.605799	5.220717	0.42257	1	-
ENSMUS(	Epha5	Eph recep	GO:00325	developm	K05106	Eph recep	mmu0436	Axon guic	5:8405476	2205	3138	3683	3878	2699	1816	6.470329	7.718334	7.445106	7.413226	6.944856	6.607233	7.211256	6.988438	0.586572	1	-
ENSMUS(	Ppat	phosphor	GO:00325	developm	K00764	amidophc	mmu011C	Metabolic	5:7691324	318	364	493	474	375	222	2.520558	2.418376	2.691961	2.44754	2.606421	2.181772	2.543632	2.411911	0.547298	1	-
ENSMUS(	Paics	phosphor	GO:00081	metabolic	K01587	phosphor	mmu011C	Metabolic	5:7695130	794	940	1354	1370	929	788	8.382244	8.318027	9.847129	9.42197	8.599994	10.31458	8.849134	9.445516	0.843021	1	-
ENSMUS(	Thegl	theg sperı	-	-	-	-	-	-	5:7701602	4	5	16	18	6	10	0.097321	0.101968	0.268184	0.285305	0.128004	0.301698	0.155824	0.238336	0.394454	1	-
ENSMUS(	Rest	RE1-silenc	GO:00325	developm	K09222	RE1-silenc	mmu0501	Huntingto	5:7726549	68	149	224	246	148	127	0.598397	1.09905	1.357934	1.410249	1.142043	1.385698	1.01846	1.312663	0.284359	1	-
ENSMUS(	Polr2b	polymeras	GO:00081	metabolic	K03010	DNA-dire	mmu011C	Metabolic	5:7731014	1589	1837	2248	2466	1773	875	18.24494	17.67993	17.78142	18.44562	17.8513	12.45697	17.90209	16.2513	0.383444	1	-
ENSMUS(	Cenpc1	centrome	GO:00511	localizatio	-	-	-	-	5:8601202	404	532	626	729	488	240	3.417917	3.772634	3.648433	4.017811	3.620286	2.517544	3.612995	3.385214	0.569335	1	-
ENSMUS(	Stap1	signal trar	GO:00325	developm	-	-	-	-	5:8607174	3	0	0	3	0	0	0.021369	0	0	0.013921	0	0	0.007123	0.00464	1	1	-
ENSMUS(	Pigg	phosphati	GO:00081	metabolic	K05310	ethanolan	mmu0056	Glycosylpl	5:1083126	336	396	551	524	427	303	3.553642	3.510605	4.014547	3.610331	3.96008	3.973399	3.692932	3.847937	0.990898	1	-
ENSMUS(	Dr1	down-rec	GO:00081	metabolic	-	-	-	-	5:1082688	1048	1268	1445	1497	1149	635	18.63036	18.89436	17.69618	17.33659	17.91111	13.99652	18.40697	16.41474	0.273403	1	-
ENSMUS(	Mtf2	metal resp	GO:00325	developm	-	-	-	-	5:1080656	403	611	563	490	512	368	1.546145	1.964892	1.488005	1.224678	1.722493	1.750568	1.666347	1.565913	0.479824	1	-
ENSMUS(	Sult1b1	sulfotrans	GO:00325	developm	-	-	-	-	5:8751333	2	0	0	0	0	0	0.025381	0	0	0	0	0	0.00846	0	0.536233	1	-
ENSMUS(	Fam69a	family witl	GO:00160	membran	-	-	-	-	5:1079080	417	520	595	616	442	345	7.471561	7.809644	7.344179	7.190142	6.944488	7.664437	7.541795	7.266356	0.560002	1	-
ENSMUS(	Sult1d1	sulfotrans	GO:00081	metabolic	-	-	-	-	5:8755464	0	2	0	0	0	4	0	0.04147	0	0	0	0.122692	0.013823	0.040897	0.573733	1	-
ENSMUS(	Glmn	glomulin,	GO:00325	developm	-	-	-	-	5:1075489	107	160	158	201	140	101	1.743193	2.184912	1.773247	2.133231	2.000009	2.040166	1.900451	2.057802	0.791927	1	-
ENSMUS(	Brdt	bromodoı	GO:00325	developm	-	-	-	-	5:1073311	252	311	402	422	313	223	2.28856	2.367421	2.515009	2.496644	2.492582	2.511035	2.39033	2.500087	0.961296	1	-
ENSMUS(	Cdc7	cell divisic	GO:00325	developm	K02214	cell divisio	mmu0411	Cell cycle	5:1069643	226	355	388	484	260	220	1.835997	2.417375	2.171432	2.561475	1.852163	2.216005	2.141601	2.209881	0.982936	1	-
ENSMUS(	Tgfbr3	transform	GO:00325	developm	-	-	-	-	5:1071065	453	427	639	617	459	346	3.686237	2.912496	3.582099	3.270785	3.27522	3.490978	3.39361	3.345661	0.698044	1	-
ENSMUS(	Zfp326	zinc finge	GO:00081	metabolic	-	-	-	-	5:1058765	418	553	646	632	420	276	2.285959	2.534955	2.433752	2.251601	2.014117	1.871487	2.418222	2.045735	0.151014	1	-
ENSMUS(	Rufy3	RUN and	GO:00325	developm	-	-	-	-	5:8856504	3465	4685	5494	5888	4373	2619	13.68026	15.50435	14.94278	15.144	15.13956	12.82073	14.70913	14.3681	0.645804	1	-
ENSMUS(	Spp1	secreted p	GO:00325	developm	K06250	secreted p	mmu0415	PI3K-Akt s	5:1044351	1265	1413	2902	3239	1389	1826	14.96548	14.01184	23.65097	24.96276	14.40937	26.7847	17.54276	22.05228	0.280424	1	-
ENSMUS(	Ibsp	integrin b	GO:00325	developm	K06253	integrin b	mmu0415	PI3K-Akt s	5:1042991	9	17	4	6	9	3	0.276581	0.437917	0.084693	0.120125	0.242541	0.114322	0.266397	0.158996	0.263984	1	-
ENSMUS(	Dmp1	dentin ma	GO:00325	developm	-	-	-	-	5:1042026	121	152	181	226	119	78	2.782176	2.929502	2.866995	3.385226	2.399316	2.223724	2.859558	2.669422	0.592779	1	-
ENSMUS(	Sparcl1	SPARC-lik	GO:00325	developm	-	-	-	-	5:1040791	34443	41540	52192	51930	41581	20605	619.3991	626.1652	646.584	608.3724	655.703	459.4393	630.7161	574.5049	0.350277	1	-
ENSMUS(	Nudt9	nudix (nuc	GO:00081	metabolic	K13988	ADP-ribos	mmu0023	Purine me	5:1040463	775	987	1331	1358	980	707	15.54722	16.59667	18.39419	17.74731	17.23931	17.58557	16.84603	17.52406	0.986462	1	-
ENSMUS(	Hsd17b11	hydroxyst	GO:00081	metabolic	-	-	-	-	5:1039897	575	746	896	911	722	474	9.844561	10.70582	10.56787	10.16081	10.83947	10.06218	10.37275	10.35415	0.76978	1	-
ENSMUS(	Klhl8	kelch-like	GO:00081	metabolic	-	-	-	-	5:1038619	378	631	797	795	530	338	3.787332	5.299361	5.501124	5.189078	4.656501	4.198982	4.862606	4.68152	0.643921	1	-
ENSMUS(	Aff1	AF4/FMR2	GO:00329	macromo	K15184	AF4/FMR2	mmu052C	Transcript	5:1036923	562	613	817	844	584	474	3.609578	3.300148	3.614879	3.531379	3.289088	3.774717	3.508202	3.531728	0.796681	1	-
ENSMUS(	Gpat3	glycerol-3	GO:00081	metabolic	K13506	glycerol-3	mmu011C	Metabolic	5:1008457	48	71	89	66	65	60	0.516954	0.640943	0.660319	0.463062	0.613857	0.801211	0.606072	0.626043	0.93723	1	-
ENSMUS(	Coq2	coenzyme	GO:00081	metabolic	K06125	4-hydroxy	mmu011C	Metabolic	5:1006547	628	873	1007	1008	763	951	10.60905	12.36188	11.71921	11.09326	11.30276	19.91974	11.56338	14.10525	0.335545	1	-
ENSMUS(	Plac8	placenta-	GO:00325	developm	-	-	-	-	5:1005537	3	7	13	3	8	6	0.077526	0.151638	0.23146	0.050504	0.181314	0.192289	0.153541	0.141369	0.809997	1	-
ENSMUS(	Enoph1	enolase-p	GO:00081	metabolic	K09880	enolase-p	mmu011C	Metabolic	5:1000399	595	889	1050	1051	736	613	9.309212	11.65872	11.31714	10.71225	10.09757	11.89166	10.76169	10.90049	0.832053	1	-
ENSMUS(	Hnrnpdl	heteroger	GO:00055	extracellul	-	-	-	-	5:1000335	2530	3046	3527	4040	2787	2040	19.45422	19.63252	18.68315	20.23747	18.792	19.44954	19.25663	19.493	0.828681	1	-
ENSMUS(	Cds1	CDP-diac	GO:00081	metabolic	K00981	phosphati	mmu011C	Metabolic	5:1017651	1178	1467	1986	1833	1505	1289	18.66038	19.47862	21.67234	18.91556	20.9052	25.3171	19.93711	21.71262	0.731338	1	-
ENSMUS(	Prkg2	protein kiı	GO:00081	metabolic	K19477	cGMP-de	mmu0474	Olfactory	5:9892977	65	95	127	93	85	49	0.549325	0.672972	0.739389	0.512015	0.629909	0.513449	0.653895	0.551791	0.26776	1	-
ENSMUS(	Bmp3	bone mor	GO:00325	developm	-	-	-	-	5:9885441	153	175	209	223	160	119	0.865321	0.829613	0.814298	0.821623	0.793502	0.834486	0.836411	0.816537	0.697146	1	-
ENSMUS(	Fgf5	fibroblast	GO:00325	developm	K04358	fibroblast	mmu052C	Pathways	5:9825418	55	62	76	89	67	38	1.12718	1.065072	1.073003	1.188251	1.204069	0.965627	1.088418	1.119316	1	1	-
ENSMUS(	Antxr2	anthrax tc	GO:00511	localizatio	K20909	anthrax tc	mmu0462	NOD-like	5:9788278	57	63	80	83	69	57	0.501734	0.464828	0.485109	0.475949	0.532585	0.622095	0.48389	0.543543	0.694671	1	-
ENSMUS(	Crybb1	crystallin,	GO:00051	structural	-	-	-	-	5:1122558	18	24	32	25	27	75	0.852414	0.952676	1.043959	0.771261	1.121216	4.403841	0.949683	2.098773	0.059606	1	-
ENSMUS(	Tpst2	protein-ty	GO:00081	metabolic	-	-	-	-	5:1122766	243	167	321	359	198	195	3.01393	1.736186	2.742729	2.900698	2.15345	2.9988	2.497615	2.684316	0.862632	1	-
ENSMUS(	Tfip11	tuftelin int	GO:00325	developm	-	-	-	-	5:1123263	885	1082	1223	1352	1046	757	11.92883	12.2246	11.35618	11.8717	12.36314	12.65132	11.83654	12.29539	0.996883	1	-
ENSMUS(	Srrd	SRR1 dom	GO:00485	rhythmic p	-	-	-	-	5:1123373	139	183	205	197	129	151	3.13147	3.455712	3.181554	2.891211	2.548391	4.217897	3.256245	3.219166	0.713437	1	-
ENSMUS(	Asphd2	aspartate	GO:00081	metabolic	-	-	-	-	5:1123843	1307	1592	1770	2138	1508	1397	10.79451	11.02106	10.07053	11.50315	10.92123	14.30572	10.6287	12.24337	0.41792	1	-
ENSMUS(	Crybb3	crystallin,	GO:00051	structural	-	-	-	-	5:1130758	22	21	28	27	16	16	0.299709	0.239804	0.262785	0.239619	0.191135	0.270265	0.267433	0.233673	0.480438	1	-
ENSMUS(	Tesc	tescalcin [	GO:00325	developm	-	-	-	-	5:1180277	517	682	672	811	552	466	10.474	11.58135	9.378689	10.70346	9.806259	11.70559	10.47801	10.73844	0.904285	1	-
ENSMUS(	Nos1	nitric oxid	GO:00325	developm	K13240	nitric-oxic	mmu011C	Metabolic	5:1177810	2637	3003	3073	3574	2672	1693	11.26802	10.75588	9.045896	9.948868	10.0119	8.969753	10.3566	9.643508	0.394564	1	-
ENSMUS(	Rfc5	replicatior	GO:00081	metabolic	K10756	replicatior	mmu0342	Nucleotid	5:1173781	89	144	180	171	117	109	0.693586	0.940649	0.966353	0.86814	0.799542	1.053229	0.866863	0.90697	1	1	-
ENSMUS(	Wsb2	WD repea	GO:00081	metabolic	-	-	-	-	5:1173573	3187	4021	4603	4813	3624	3869	26.95189	28.50323	26.81634	26.51579	26.87435	40.56877	27.42382	31.31964	0.501362	1	-
ENSMUS(	Dck	deoxycyti	GO:00081	metabolic	K00893	deoxycitic	mmu011C	Metabolic	5:8876499	314	404	522	455	377	311	5.120768	5.522549	5.864449	4.833913	5.391258	6.288567	5.502589	5.504579	0.753047	1	-
ENSMUS(	Afm	afamin [S	GO:00511	localizatio	-	-	-	-	5:9051893	0	6	0	1	0	0	0	0.094003	0	0.012175	0	0	0.031334	0.004058	0.241628	1	-
ENSMUS(	Rassf6	Ras assoc	GO:00508	response	K09854	Ras assoc	mmu0439	Hippo sig	5:9060307	0	5	16	15	8	5	0	0.047148	0.124004	0.109936	0.078926	0.069742	0.057051	0.086201	0.472619	1	-
ENSMUS(	Ppbp	pro-plate	GO:00400	locomotic	K10029	platelet ba	mmu0406	Cytokine-	5:9076851	1	0	3	4	0	2	0.058694	0	0.121272	0.152933	0	0.14556	0.059989	0.099498	0.788213	1	-
ENSMUS(	Pf4	platelet fa	GO:00325	developm	K05407	platelet fa	mmu0406	Cytokine-	5:9077243	9	9	11	10	10	16	0.420876	0.35277	0.354368	0.304639	0.410081	0.927731	0.376004	0.547484	0.422978	1	-
ENSMUS(	Mthfd2l	methylene	GO:00081	metabolic	K13403	methylene	mmu011C	Metabolic	5:9093111	108	119	140	139	107	70	0.950388	0.877768	0.848708	0.796847	0.825666	0.763775	0.892288	0.795429	0.373175	1	-
ENSMUS(	Cxcl1	chemokin	GO:00325	developm	K05505	C-X-C mc	mmu0406	Cytokine-	5:9089124	2	1	5	2	2	2	0.045018	0.018863	0.077518	0.029318	0.039465	0.055822	0.047133	0.041535	0.814357	1	-
ENSMUS(	Shroom3	shroom fa	GO:00325	developm	-	-	-	-	5:9268343	73	62	111	111	74	65	0.204857	0.145838	0.214585	0.202922	0.182097	0.226163	0.188427	0.203727	0.856431	1	-
ENSMUS(	2010109A	RIKEN cDI	-	-	-	-	-	-	5:9320651	3	3	3	0	3	3	0.130038	0.109007	0.089589	0	0.114034	0.161268	0.109545	0.091767	0.830221	1	-
ENSMUS(	Ccng2	cyclin G2	GO:00099	cellular pr	K10146	cyclin G2[	mmu0406	FoxO sign	5:9326725	536	678	951	861	734	540	3.298899	3.49773	4.032144	3.452141	3.961346	4.120822	3.609591	3.84477	0.862786	1	-
ENSMUS(	Gtf2h3	general tr	GO:00081	metabolic	K03143	transcripti	mmu052C	Viral carci	5:1245791	211	258	306	377	238	247	1.79298	1.837657	1.791289	2.086968	1.773422	2.602407	1.807309	2.154266	0.37655	1	-
ENSMUS(	Eif2b1	eukaryotic	GO:00325	developm	K03239	translatior	mmu0301	RNA trans	5:1245702	331	373	453	471	325	305	5.735024	5.417122	5.406996	5.316299	4.937797	6.552289	5.519714	5.602128	0.83441	1	-
ENSMUS(	Ddx55	DEAD (As	GO:00081	metabolic	-	-	-	-	5:1245528	379	430	579	604	461	320	3.030525	2.88204	3.189394	3.146275	3.232375	3.172586	3.033987	3.183746	0.938205	1	-
ENSMUS(	Tmed2	transmem	GO:00325	developm	-	-	-	-	5:1245406	628	822	432	938	773	191	8.659419	9.500663	4.103592	8.42585	9.346558	3.265492	7.421225	7.012633	0.770703	1	-
ENSMUS(	Rilpl1	Rab intera	GO:00325	developm	-	-	-	-	5:1244930	1289	1582	1801	1955	1386	872	24.92995	25.64649	23.99571	24.63185	23.50576	20.91081	24.85739	23.01614	0.377429	1	-
ENSMUS(	Rchy1	ring finge	GO:00081	metabolic	K10144	RING fing	mmu0412	Ubiquitin	5:9194890	296	395	423	449	367	387	3.730258	4.172508	3.672306	3.686167	4.055608	6.047051	3.858358	4.596275	0.405509	1	-
ENSMUS(	Rilpl2	Rab intera	GO:00325	developm	-	-	-	-	5:1244632	139	173	184	172	181	195	1.982468	2.068184	1.807845	1.598087	2.263668	3.44835	1.952832	2.436702	0.374124	1	-
ENSMUS(	Snrnp35	small nucl	GO:00081	metabolic	-	-	-	-	5:1244831	77	210	248	250	85	59	1.782729	4.075359	3.955462	3.770638	1.725651	1.693686	3.271183	2.396658	0.208919	1	-
ENSMUS(	Cdkl2	cyclin-dep	GO:00081	metabolic	-	-	-	-	5:9200607	718	896	1029	1077	738	469	5.40909	5.657968	5.340307	5.28563	4.875268	4.38085	5.469122	4.847249	0.246114	1	-
ENSMUS(	Arl6ip4	ADP-ribo	GO:00081	metabolic	-	-	-	-	5:1241160	240	517	394	396	268	234	5.037232	9.095443	5.696759	5.414481	4.932386	6.08951	6.609811	5.478792	0.177189	1	-
ENSMUS(	G3bp2	GTPase ac	GO:00511	localizatio	-	-	-	-	5:9205214	4706	5293	6678	6676	4796	2765	20.28627	19.12516	19.83116	18.74771	18.12893	14.77854	19.74753	17.21839	0.18387	1	-
ENSMUS(	Pitpnm2	phosphati	GO:00511	localizatio	-	-	-	-	5:1241186	5022	5528	6741	8465	6603	4547	15.5152	14.31534	14.34685	17.03685	17.88812	17.41773	14.7258	17.44757	0.25446	1	-
ENSMUS(	Uso1	USO1 ves	GO:00511	localizatio	-	-	-	-	5:9213793	1754	2337	2818	2794	2018	1209	12.03541	13.44135	13.3206	12.48934	12.14214	10.28593	12.93245	11.63913	0.276459	1	-
ENSMUS(	Abcb9	ATP-bind	GO:00511	localizatio	K05656	ATP-bind	mmu0414	Lysosome	5:1240615	386	442	545	615	469	499	3.456108	3.317226	3.361614	3.587213	3.682273	5.539711	3.378316	4.269732	0.195037	1	-
ENSMUS(	Ppef2	protein pł	GO:00081	metabolic	-	-	-	-	5:9222667	3	18	20	2	10	7	0.049761	0.250295	0.228568	0.021611	0.145471	0.143987	0.176208	0.10369	0.23783	1	-
ENSMUS(	Naaa	N-acyleth	GO:00081	metabolic	-	-	-	-	5:9225765	462	579	804	728	603	500	4.632609	4.866486	5.553813	4.755506	5.302052	6.216416	5.017636	5.424658	0.795398	1	-
ENSMUS(	Kntc1	kinetocho	GO:00990	supramole	-	-	-	-	5:1237497	16	26	34	40	21	7	0.111291	0.151584	0.162916	0.181248	0.128086	0.060372	0.141931	0.123235	0.651117	1	-
ENSMUS(	Sdad1	SDA1 dor	GO:00511	localizatio	-	-	-	-	5:9228401	396	437	570	615	405	329	3.164067	2.926745	3.137452	3.201161	2.837582	3.259363	3.076088	3.099369	0.818797	1	-
ENSMUS(	Slc15a4	solute car	GO:00511	localizatio	-	-	-	-	5:1275956	208	228	309	295	256	303	1.439351	1.322489	1.473039	1.329865	1.55341	2.599755	1.411626	1.827677	0.228962	1	-
ENSMUS(	Cxcl9	chemokin	GO:00325	developm	K05416	C-X-C mc	mmu0406	Cytokine-	5:9232134	0	0	1	4	3	0	0	0	0.013429	0.050821	0.051296	0	0.004476	0.034039	0.169258	1	-
ENSMUS(	Gm996	predicted	-	-	-	-	-	-	2:2557541	2179	2334	2290	3024	2383	1894	10.66312	9.573727	7.719943	9.640302	10.22572	11.49193	9.318931	10.45265	0.568419	1	-
ENSMUS(	Rimbp2	RIMS binc	GO:00081	metabolic	-	-	-	-	5:1287577	3035	3779	4535	5322	3881	2453	14.36962	14.99741	14.7916	16.41508	16.11287	14.40025	14.71954	15.64273	0.825221	1	-
ENSMUS(	Rsrc2	arginine/s	GO:00971	organic cy	-	-	-	-	5:1237284	1569	1468	2248	2276	1842	753	5.113572	4.010332	5.047186	4.832318	5.264217	3.042861	4.723697	4.379799	0.532104	1	-
ENSMUS(	Piwil1	piwi-like F	GO:00325	developm	-	-	-	-	5:1287025	3	4	4	2	2	3	0.038257	0.04276	0.035149	0.016615	0.022366	0.047445	0.038722	0.028809	0.531066	1	-
ENSMUS(	Scarb2	scavenger	GO:00325	developm	K12384	lysosome	mmu0414	Lysosome	5:9244131	2821	3093	4242	4425	3164	2516	26.63542	24.47875	27.59171	27.2177	26.19604	29.4546	26.2353	27.62278	0.920357	1	-
ENSMUS(	Zcchc8	zinc finge	GO:00081	metabolic	-	-	-	-	5:1236982	424	557	724	608	541	305	2.461089	2.710004	2.895021	2.299043	2.753604	2.195068	2.688705	2.415905	0.319374	1	-
ENSMUS(	Stx2	syntaxin 2	GO:00325	developm	K08486	syntaxin 1	mmu0472	Synaptic v	5:1289845	241	311	369	468	338	216	1.922468	2.07948	2.027774	2.432031	2.364292	2.136389	2.009907	2.310904	0.460858	1	-
ENSMUS(	Ran	RAN, mer	GO:00325	developm	K07936	GTP-bind	mmu0516	HTLV-I inf	5:1290200	1395	1935	2251	2015	1626	1804	30.9771	36.01641	34.43448	29.14897	31.66138	49.66941	33.80933	36.82659	0.80126	1	-
ENSMUS(	B3gnt4	UDP-GlcN	GO:00081	metabolic	K07971	beta-1,3-	mmu011C	Metabolic	5:1235104	6	4	7	5	5	7	0.267979	0.149735	0.215379	0.145483	0.195799	0.387643	0.211031	0.242975	1	1	-
ENSMUS(	Nipsnap2	nipsnap h	GO:00081	metabolic	-	-	-	-	5:1297250	763	807	1100	1048	789	654	10.58523	9.384312	10.51284	9.471503	9.598326	11.24967	10.16079	10.1065	0.705565	1	-
ENSMUS(	Diablo	diablo, IAI	GO:00081	metabolic	K10522	diablo[-]	mmu0421	Apoptosis	5:1235097	347	436	306	313	254	360	2.047621	2.156552	1.243923	1.203226	1.314305	2.633962	1.816032	1.717164	0.565268	1	-
ENSMUS(	Vps33a	VPS33A C	GO:00325	developm	-	-	-	-	5:1235286	2036	2322	3012	3247	2411	1434	23.78127	22.73382	24.23616	24.70707	24.69433	20.7679	23.58375	23.38977	0.740533	1	-
ENSMUS(	Mmp17	matrix me	GO:00325	developm	-	-	-	-	5:1295841	4934	5628	6367	7339	5409	4100	45.54401	43.54508	40.48727	44.13171	43.78162	46.92475	43.19212	44.94602	0.999882	1	-
ENSMUS(	Bcl7a	B cell CLL/	-	-	-	-	-	-	5:1233438	1999	1515	1700	3030	1599	1826	9.7951	6.222455	5.738474	9.672091	6.870485	11.09386	7.25201	9.212146	0.2341	1	-
ENSMUS(	Sfswap	splicing fa	GO:00081	metabolic	-	-	-	-	5:1295012	1212	1760	1805	2122	1696	1081	4.796453	5.838255	4.92092	5.470723	5.885538	5.304315	5.185209	5.553525	0.793408	1	-
ENSMUS(	Psmd9	proteasor	GO:00081	metabolic	-	-	-	-	5:1231694	371	475	526	507	482	386	2.953175	3.169292	2.884382	2.62909	3.364384	3.809682	3.002283	3.267719	0.779902	1	-
ENSMUS(	Wdr66	WD repea	GO:00056	cell|intrace	-	-	-	-	5:1232521	104	153	125	163	150	80	0.581771	0.717403	0.481704	0.594003	0.735787	0.554874	0.593626	0.628222	0.907892	1	-
ENSMUS(	Hpd	4-hydroxy	GO:00081	metabolic	K00457	4-hydroxy	mmu011C	Metabolic	5:1231718	0	0	0	5	0	0	0	0	0	0.072589	0	0	0	0.024196	0.185324	1	-
ENSMUS(	Psph	phosphos	GO:00081	metabolic	K01079	phosphos	mmu011C	Metabolic	5:1297655	202	271	234	357	196	231	1.99809	2.246907	1.594524	2.300449	1.700051	2.833092	1.946507	2.277864	0.51105	1	-
ENSMUS(	Tmem116	transmem	GO:00160	membran	-	-	-	-	5:1214518	35	24	38	36	36	29	0.435128	0.250096	0.325444	0.291564	0.392458	0.447017	0.336889	0.377013	0.828188	1	-
ENSMUS(	Mapkapk5	MAP kina	GO:00325	developm	K04442	mitogen-a	mmu0401	MAPK sigı	5:1215250	127	142	162	185	277	141	1.505261	1.410753	1.322741	1.428443	2.878934	2.07211	1.412919	2.126495	0.052626	1	-
ENSMUS(	Aldh2	aldehyde	GO:00081	metabolic	K00128	aldehyde	mmu011C	Metabolic	5:1215660	1567	1644	2005	2032	1454	1080	14.72971	12.95329	12.98348	12.44316	11.98484	12.58737	13.55549	12.33846	0.269301	1	-
ENSMUS(	Acad10	acyl-Coer	GO:00081	metabolic	-	-	-	-	5:1216210	37	37	51	68	51	49	0.294815	0.24712	0.279943	0.352974	0.35634	0.484094	0.27396	0.397803	0.101971	1	-
ENSMUS(	Brap	BRCA1 as	GO:00081	metabolic	K10632	BRCA1-as	mmu0401	Ras signal	5:1216605	1509	1728	2138	2289	1733	1093	4.998254	4.797619	4.878521	4.939198	5.033503	4.488851	4.891465	4.820517	0.686745	1	-
ENSMUS(	Fam168a	family witl	GO:00081	metabolic	-	-	-	-	7:1007066	4193	1275	2206	2097	2069	2956	14.68521	3.742986	5.322458	4.784485	6.354162	12.83647	7.916884	7.991706	0.84469	1	-
ENSMUS(	Vps29	VPS29 ret	GO:00511	localizatio	K18467	vacuolar p	mmu0414	Endocytos	5:1223543	1650	2009	2506	2623	1813	903	11.22965	11.4608	11.74937	11.62953	10.81989	7.620016	11.47994	10.02314	0.25886	1	-
ENSMUS(	Fam216a	family witl	-	-	-	-	-	-	5:1223645	759	995	1165	1184	951	726	34.75269	38.18763	36.74719	35.31668	38.18297	41.21634	36.5625	38.23867	0.974853	1	-
ENSMUS(	Gpn3	GPN-loop	GO:00511	localizatio	-	-	-	-	5:1223718	205	254	267	355	209	187	2.923124	3.035846	2.622745	3.297642	2.61326	3.306138	2.860572	3.072347	0.831249	1	-
ENSMUS(	Arpc3	actin relat	GO:00508	response	K05756	actin relat	mmu0414	Endocytos	5:1223918	1414	2200	2592	2658	2022	1126	22.21606	28.97297	28.0546	27.20534	27.85746	21.93521	26.41455	25.666	0.659931	1	-
ENSMUS(	Anapc7	anaphase	GO:00081	metabolic	K03354	anaphase	mmu0516	HTLV-I inf	5:1224216	418	522	600	646	491	387	3.804803	3.982719	3.762348	3.830634	3.919044	4.3677	3.849956	4.039126	0.959799	1	-
ENSMUS(	Atp2a2	ATPase, C	GO:00325	developm	K05853	Ca2+ tran	mmu0402	cAMP sigr	5:1224535	11538	13613	18188	17890	13313	12089	77.80928	76.94981	84.49621	78.59462	78.72623	101.0828	79.75176	86.13454	0.769372	1	-
ENSMUS(	P2rx7	purinergic	GO:00325	developm	K05220	P2X purin	mmu0408	Neuroacti	5:1226439	184	214	246	243	196	180	1.095739	1.06821	1.009199	0.942709	1.023503	1.329071	1.057716	1.098427	0.957579	1	-
ENSMUS(	Ift81	intraflagel	GO:00325	developm	-	-	-	-	5:1225502	627	788	925	969	723	440	5.166981	5.443125	5.251245	5.202048	5.224564	4.495811	5.287117	4.974141	0.478371	1	-
ENSMUS(	P2rx4	purinergic	GO:00048	signal trar	K05218	P2X purin	mmu0408	Neuroacti	5:1227075	118	130	172	244	155	123	0.789976	0.729505	0.793254	1.064155	0.909929	1.020996	0.770911	0.99836	0.164115	1	-
ENSMUS(	Camkk2	calcium/ca	GO:00081	metabolic	K07359	calcium/ca	mmu0503	Alcoholisr	5:1227311	1762	2430	2552	2874	2133	2045	7.365679	8.514632	7.349182	7.826638	7.818804	10.59951	7.743164	8.748319	0.532358	1	-
ENSMUS(	Anapc5	anaphase	GO:00081	metabolic	K03352	anaphase	mmu0516	HTLV-I inf	5:1227874	1755	2198	2441	2690	1822	1760	8.612781	9.041633	8.252502	8.600036	7.840748	10.7094	8.635638	9.05006	0.985948	1	-
ENSMUS(	Rnf34	ring finge	GO:00081	metabolic	-	-	-	-	5:1228500	1095	1344	1524	1538	1196	751	14.22829	14.63829	13.6419	13.01896	13.62738	12.09942	14.1695	12.91525	0.30665	1	-
ENSMUS(	Kdm2b	lysine (K)-	GO:00325	developm	-	-	-	-	5:1228706	423	556	593	624	524	352	1.430931	1.576546	1.381925	1.375134	1.554364	1.476413	1.463134	1.468637	0.802125	1	-
ENSMUS(	Ncor2	nuclear re	GO:00325	developm	K06065	nuclear re	mmu0516	Epstein-B	5:1250171	4799	4826	5944	7363	5362	3340	17.00061	14.33025	14.50587	16.99222	16.65648	14.67054	15.27891	16.10641	0.886907	1	-
ENSMUS(	Dhx37	DEAH (As	GO:00081	metabolic	-	-	-	-	5:1254138	395	450	527	556	403	339	4.352189	4.156011	4.000116	3.990868	3.893668	4.631228	4.169439	4.171921	0.759668	1	-
ENSMUS(	Aacs	acetoacet	GO:00325	developm	K01907	acetoacet	mmu0028	Valine, leu	5:1254758	599	732	773	799	645	775	8.084154	8.280782	7.186842	7.024827	7.633255	12.96866	7.850593	9.208914	0.49142	1	-
ENSMUS(	Anxa3	annexin A	GO:00325	developm	-	-	-	-	5:9679333	105	143	211	220	118	156	1.316706	1.503103	1.822778	1.797227	1.297551	2.425545	1.547529	1.840107	0.503709	1	-
ENSMUS(	Mrpl1	mitochon	GO:00081	metabolic	K02863	large subu	mmu0301	Ribosome	5:9620949	306	386	458	451	360	94	2.76239	2.920809	2.848263	2.652292	2.849759	1.052153	2.843821	2.184735	0.20625	1	-
ENSMUS(	Mfsd7a	major faci	GO:00511	localizatio	-	-	-	-	5:1084410	5	9	21	13	11	11	0.126702	0.191154	0.36659	0.214607	0.244428	0.345619	0.228149	0.268218	0.859155	1	-
ENSMUS(	Pde6b	phosphoc	GO:00325	developm	K13756	rod cGMP	mmu0023	Purine me	5:1083883	2	0	0	8	6	0	0.018902	0	0	0.049244	0.04971	0	0.006301	0.032984	0.038152	1	-
ENSMUS(	Pxmp2	peroxison	GO:00329	macromo	K13347	peroxison	mmu0414	Peroxison	5:1102742	30	52	26	66	43	57	0.893025	1.297449	0.53317	1.279873	1.122397	2.10376	0.907881	1.50201	0.085703	1	-
ENSMUS(	Pgam5	phosphog	GO:00048	signal trar	K15637	serine/thr	mmu0421	Necropto	5:1102591	657	837	968	483	386	768	9.408361	10.04678	9.549387	4.505863	4.84706	13.63628	9.668178	7.663067	0.164719	1	-
ENSMUS(	Ankle2	ankyrin re	GO:00325	developm	-	-	-	-	5:1102310	663	621	1147	1065	863	506	3.688021	2.895507	4.395363	3.859324	4.209529	3.489925	3.65963	3.852926	0.904106	1	-
ENSMUS(	Golga3	golgi autc	GO:00329	macromo	-	-	-	-	5:1101767	1897	2199	2637	2905	2085	1295	7.738131	7.518768	7.410206	7.719629	7.457916	6.549746	7.555702	7.24243	0.539664	1	-
ENSMUS(	P2rx2	purinergic	GO:00325	developm	K05216	P2X purin	mmu0408	Neuroacti	5:1103398	0	0	2	0	0	0	0	0	0.013189	0	0	0	0.004396	0	0.535548	1	-
ENSMUS(	Ddx51	DEAD (As	GO:00081	metabolic	-	-	-	-	5:1106534	367	475	489	603	432	397	3.636979	3.945678	3.338375	3.892905	3.754062	4.878109	3.640344	4.175025	0.499812	1	-
ENSMUS(	Ep400	E1A bindi	GO:00081	metabolic	-	-	-	-	5:1106643	1760	2202	2439	3248	2467	1635	5.676967	5.953521	5.419597	6.82498	6.977754	6.538941	5.683362	6.780558	0.239142	1	-
ENSMUS(	Pus1	pseudouri	GO:00081	metabolic	-	-	-	-	5:1107736	153	233	242	289	186	169	3.033889	3.872724	3.305793	3.733249	3.234174	4.155106	3.404135	3.70751	0.783649	1	-
ENSMUS(	Gpc2	glypican 2	GO:00055	extracellul	-	-	-	-	5:1382736	16	19	42	53	64	17	0.093188	0.092756	0.168513	0.20109	0.326855	0.122761	0.118152	0.216902	0.04992	1	-
ENSMUS(	Ulk1	unc-51 lik	GO:00325	developm	K08269	serine/thr	mmu0415	mTOR sig	5:1107844	1554	1855	2248	2715	2041	1459	10.16586	10.17162	10.13074	11.5703	11.7079	11.83408	10.15607	11.70409	0.374748	1	-
ENSMUS(	Prkab1	protein kiı	GO:00325	developm	K07199	5'-AMP-a	mmu0453	Tight junc	5:1160135	188	223	295	282	205	144	1.728586	1.718663	1.868557	1.689127	1.652836	1.641648	1.771936	1.661204	0.501732	1	-
ENSMUS(	Cit	citron [So	GO:00081	metabolic	-	-	-	-	5:1158452	1255	1491	2209	1509	1696	795	3.983569	3.966971	4.830319	3.120322	4.7206	3.128828	4.260287	3.656584	0.20913	1	-
ENSMUS(	Rab35	RAB35, m	GO:00325	developm	K07876	Ras-relate	mmu0414	Endocytos	5:1156319	1337	1684	1953	1952	1544	1218	24.38174	25.74118	24.53505	23.18969	24.69012	27.54016	24.88599	25.13999	0.790028	1	-
ENSMUS(	Chek2	checkpoir	GO:00325	developm	K06641	serine/thr	mmu0516	HTLV-I inf	5:1108399	35	49	36	34	25	26	0.497401	0.583687	0.352446	0.314768	0.311544	0.458144	0.477845	0.361485	0.171453	1	-
ENSMUS(	Sirt4	sirtuin 4 [S	GO:00081	metabolic	-	-	-	-	5:1154780	146	206	184	242	216	208	2.007945	2.374764	1.743293	2.168186	2.604928	3.546898	2.042001	2.773337	0.123373	1	-
ENSMUS(	Pxn	paxillin [S	GO:00325	developm	K05760	paxillin[-]	mmu0516	Human pa	5:1155066	630	815	954	913	791	567	2.679946	2.906003	2.795666	2.530101	2.950561	2.990576	2.793872	2.823746	0.817869	1	-
ENSMUS(	Ccr9	chemokin	GO:00325	developm	K04184	C-C chem	mmu0406	Cytokine-	9:1236784	2	8	8	6	5	2	0.005608	0.018799	0.015451	0.010959	0.012291	0.006954	0.013286	0.010068	0.648568	1	-
ENSMUS(	St7	suppressic	GO:00160	membran	-	-	-	-	6:1769293	301	422	521	518	393	307	1.343172	1.578448	1.6016	1.505832	1.537802	1.698592	1.50774	1.580742	0.963085	1	-
ENSMUS(	Triap1	TP53 regu	GO:00081	metabolic	-	-	-	-	5:1153412	116	145	186	185	129	183	6.77589	7.099508	7.484637	7.039807	6.607547	13.25391	7.120011	8.967089	0.360026	1	-
ENSMUS(	Gatc	glutamyl-	GO:00081	metabolic	K02435	aspartyl-t	mmu011C	Metabolic	5:1153332	448	615	724	771	536	341	15.84404	18.23122	17.63917	17.76332	16.62247	14.95301	17.23814	16.44627	0.562867	1	-
ENSMUS(	Srsf9	serine/arg	GO:00081	metabolic	K12890	splicing fa	mmu0516	Herpes sir	5:1153271	395	553	647	723	490	692	10.49474	12.31551	11.84216	12.51397	11.41599	22.79644	11.5508	15.57546	0.137042	1	-
ENSMUS(	Cabp1	calcium b	GO:00452	synapse|lc	-	-	-	-	5:1151686	740	940	1061	1296	891	903	6.117225	6.513336	6.042121	6.979262	6.458672	9.255425	6.224227	7.564453	0.244824	1	-
ENSMUS(	Acads	acyl-Coer	GO:00081	metabolic	K00248	butyryl-Cc	mmu011C	Metabolic	5:1151102	78	93	94	113	84	101	1.616288	1.615311	1.341849	1.5254	1.526308	2.594943	1.524483	1.882217	0.415549	1	-
ENSMUS(	Uncx	UNC hom	GO:00325	developm	-	-	-	-	5:1395434	0	0	1	0	0	4	0	0	0.011848	0	0	0.085327	0.003949	0.028442	0.32612	1	-
ENSMUS(	Ints1	integrator	GO:00081	metabolic	-	-	-	-	5:1397512	1481	1684	1959	2318	1848	1702	7.606435	7.249717	6.931254	7.755701	8.322825	10.83856	7.262469	8.972361	0.177545	1	-
ENSMUS(	Sppl3	signal pep	GO:00081	metabolic	-	-	-	-	5:1150111	687	923	1143	1036	811	1197	4.939017	5.562094	5.660846	4.852049	5.112667	10.66999	5.387319	6.878235	0.27816	1	-
ENSMUS(	Psmg3	proteasor	GO:00329	macromo	-	-	-	-	5:1398235	69	101	114	113	86	140	1.160102	1.423375	1.320384	1.237673	1.267908	2.918486	1.301287	1.808022	0.238147	1	-
ENSMUS(	Tes	testis deri	GO:00099	cellular pr	-	-	-	-	6:1706514	23	35	60	54	26	33	0.167897	0.214162	0.301733	0.256799	0.166431	0.298685	0.22793	0.240638	1	1	-
ENSMUS(	Tfec	transcripti	GO:00081	metabolic	-	-	-	-	6:1683337	4	4	0	2	1	0	0.036497	0.030592	0	0.011887	0.008001	0	0.022363	0.006629	0.226594	1	-
ENSMUS(	Mad1l1	MAD1 mit	GO:00325	developm	K06638	mitotic sp	mmu052C	Viral carci	5:1400086	307	349	391	506	379	226	4.143299	3.948081	3.635262	4.448762	4.485279	3.781831	3.908881	4.238624	0.744074	1	-
ENSMUS(	Mrm2	mitochon	GO:00081	metabolic	-	-	-	-	5:1403276	71	101	135	119	99	125	3.126996	3.728591	4.095951	3.414263	3.8234	6.825965	3.650513	4.687876	0.334203	1	-
ENSMUS(	2210016L	RIKEN cDI	-	-	-	-	-	-	5:1149421	1184	1588	1814	1868	1561	1228	15.99634	17.98341	16.8833	16.44097	18.4933	20.57089	16.95435	18.50172	0.702759	1	-
ENSMUS(	Snx8	sorting ne	GO:00511	localizatio	-	-	-	-	5:1403402	175	166	235	175	182	190	1.481715	1.178118	1.370715	0.965268	1.35127	1.994654	1.343516	1.437064	0.952962	1	-
ENSMUS(	Foxp2	forkhead	GO:00325	developm	-	-	-	-	6:1490134	37	49	69	33	41	25	0.094477	0.104876	0.121375	0.054893	0.091802	0.07915	0.106909	0.075282	0.068728	1	-
ENSMUS(	Lfng	LFNG O-f	GO:00325	developm	K05948	fringe[2.4.	mmu0516	Human pa	5:1406073	197	211	314	347	194	228	4.19008	3.761756	4.600837	4.808025	3.618266	6.012784	4.184225	4.813025	0.560907	1	-
ENSMUS(	Tmem106	transmem	GO:00325	developm	-	-	-	-	6:1306975	3813	4720	5805	5822	4229	2627	30.96839	32.13266	32.47921	30.80389	30.11842	26.45439	31.86008	29.12557	0.305336	1	-
ENSMUS(	Mmab	methylma	GO:00081	metabolic	-	-	mmu011C	Metabolic	5:1144310	538	630	714	868	623	481	8.234947	8.082991	7.528845	8.655257	8.361982	9.128718	7.948928	8.715319	0.680393	1	-
ENSMUS(	Radil	Ras assoc	GO:00325	developm	-	-	-	-	5:1424848	134	136	167	207	147	179	1.087487	0.92515	0.933655	1.094388	1.046112	1.801183	0.982097	1.313894	0.178133	1	-
ENSMUS(	Ube3b	ubiquitin p	GO:00081	metabolic	K10588	ubiquitin-	mmu0412	Ubiquitin	5:1143806	1687	1988	2341	2926	2066	1889	13.57482	13.40875	12.97693	15.33822	14.5778	18.84677	13.32017	16.25426	0.186034	1	-

ENSMUSC	Wipi2	WD repea	GO:00081	metabolic	K17908	autophag	mmu0414	Autophag	5:1426276	1706	2126	2567	2692	2009	1946	8.485674	8.86387	8.795985	8.72296	8.762537	12.00152	8.715177	9.829004	0.53818	1	-
ENSMUSC	Actb	actin, beta	GO:00325	developm	K05692	actin beta	mmu0481	Regulation	5:1429031	10748	12750	14417	16614	12192	9551	115.735	115.08	106.9457	116.5448	115.121	127.5181	112.5869	119.7279	0.849219	1	-
ENSMUSC	Fscn1	fascin acti	GO:00325	developm	-	-	-	-	5:1429603	1282	2183	2477	1462	1300	2292	14.71198	20.99857	19.58216	10.9298	13.08185	32.61248	18.4309	18.87471	0.844819	1	-
ENSMUSC	Zfp12	zinc finge	GO:00081	metabolic	-	-	-	-	5:1432351	171	713	917	214	166	569	0.736035	2.572438	2.719089	0.600066	0.626548	3.036692	2.009187	1.421102	0.244531	1	-
ENSMUSC	Ung	uracil DN/	GO:00325	developm	K03648	uracil-DN	mmu0341	Base excis	5:1141303	20	22	30	32	22	24	0.1461	0.134707	0.150968	0.152281	0.140925	0.217377	0.143925	0.170194	0.684503	1	-
ENSMUSC	Usp30	ubiquitin s	GO:00081	metabolic	K11851	ubiquitin c	mmu0413	Mitophag	5:1140654	675	888	961	1072	782	613	6.041164	6.661656	5.925044	6.250189	6.137136	6.802411	6.209288	6.396579	0.937357	1	-
ENSMUSC	Rbm19	RNA bind	GO:00325	developm	-	-	-	-	5:1201164	241	273	348	354	259	250	1.509144	1.432941	1.501219	1.444102	1.422184	1.94106	1.481101	1.602449	0.830496	1	-
ENSMUSC	Lhx5	LIM home	GO:00325	developm	K18493	LIM home	mmu0455	Signaling	5:1204316	2	0	7	1	5	3	0.047279	0	0.113977	0.015395	0.103616	0.087921	0.053752	0.068977	0.860464	1	-
ENSMUSC	Sdsl	serine del	GO:00038	catalytic a	K17989	L-serine/L	mmu011C	Metabolic	5:1204581	27	30	43	39	38	40	0.880405	0.819959	0.965921	0.828457	1.086555	1.617221	0.888762	1.177411	0.343414	1	-
ENSMUSC	Sds	serine del	GO:00081	metabolic	K17989	L-serine/L	mmu011C	Metabolic	5:1204765	0	1	0	1	0	0	0	0.023675	0	0.018398	0	0	0.007892	0.006133	1	1	-
ENSMUSC	Plbd2	phospholi	GO:00081	metabolic	-	-	-	-	5:1204832	1236	1483	1936	1980	1435	1224	8.935447	8.986526	9.64172	9.324909	9.096878	10.97148	9.187898	9.797756	0.849418	1	-
ENSMUSC	Ddx54	DEAD (As	GO:00081	metabolic	-	-	-	-	5:1206127	792	988	1171	1246	912	761	6.187336	6.469761	6.302123	6.341298	6.247639	7.371382	6.31974	6.653439	0.937756	1	-
ENSMUSC	Rita1	RBPJ inter	GO:00325	developm	-	-	-	-	5:1205943	156	183	215	204	169	172	2.526567	2.484343	2.398813	2.152376	2.400142	3.453988	2.469908	2.668835	0.873353	1	-
ENSMUSC	Iqcd	IQ motif c	-	-	-	-	-	-	5:1205890	16	16	35	18	27	19	0.429603	0.360095	0.647373	0.314836	0.635687	0.632533	0.479023	0.527685	0.956086	1	-
ENSMUSC	Rasal1	RAS prote	GO:00325	developm	K17632	RAS prote	mmu0401	Ras signal	5:1206488	1194	1515	1649	1811	1376	1688	16.22457	17.25577	15.43622	16.0313	16.3957	28.43985	16.30552	20.28895	0.246529	1	-
ENSMUSC	Dtx1	deltex 1, E	GO:00325	developm	K06058	deltex[2.3	mmu0433	Notch sigı	5:1206802	1429	1749	1812	1486	1127	1123	9.515641	9.762216	8.312189	6.446234	6.580704	9.271952	9.196682	7.432963	0.040799	1	-
ENSMUSC	Ankrd61	ankyrin re	GO:00971	organic cy	-	-	-	-	5:1438907	0	0	2	1	0	2	0	0	0.02372	0.011212	0	0.042698	0.007907	0.01797	1	1	-
ENSMUSC	Rph3a	rabphilin :	GO:00452	synapse|lc	-	-	-	-	5:1209404	7003	7986	10104	11649	8224	4818	79.05182	75.56312	78.57285	85.66393	81.40545	67.43424	77.72926	78.16787	0.831649	1	-
ENSMUSC	Aimp2	aminoacy	GO:00325	developm	-	-	-	-	5:1439027	273	359	405	393	311	235	6.485975	7.149233	6.62855	6.082564	6.479118	6.922547	6.754586	6.494743	0.565822	1	-
ENSMUSC	Eif2ak1	eukaryotic	GO:00325	developm	K16194	eukaryotic	mmu0516	Epstein-B	5:1438177	1370	1615	1952	2188	1583	1035	14.21512	14.04609	13.9528	14.78969	14.40302	13.31546	14.07134	14.16939	0.817235	1	-
ENSMUSC	Rpl6	ribosomal	GO:00081	metabolic	K02934	large subu	mmu0301	Ribosome	5:1212044	944	1253	1469	1436	1136	893	10.99396	12.23168	11.7857	10.89478	11.6012	12.89495	11.67045	11.79698	0.801556	1	-
ENSMUSC	Erp29	endoplasr	GO:00081	metabolic	K09586	endoplasr	mmu0414	Protein pr	5:1214285	888	815	1411	1376	647	970	5.144492	3.957673	5.631291	5.193134	3.286823	6.967664	4.911152	5.149207	0.988123	1	-
ENSMUSC	Ccz1	CCZ1 vacı	GO:00511	localizatio	-	-	-	-	5:1439879	362	403	499	501	368	291	3.919949	3.657881	3.72241	3.534203	3.494319	3.907065	3.766747	3.645196	0.585213	1	-
ENSMUSC	Ocm	oncomod	GO:00325	developm	-	-	-	-	5:1440198	0	4	0	0	0	0	0	0.201392	0	0	0	0	0.067131	0	0.187581	1	-
ENSMUSC	Arpc1a	actin relat	GO:00329	macromo	K05757	actin relat	mmu0414	Endocytos	5:1450838	1782	2226	2646	2835	2188	2419	30.1843	31.60468	30.87554	31.28297	32.49849	50.80366	30.88817	38.19504	0.215437	1	-
ENSMUSC	Arpc1b	actin relat	GO:00508	response	K05757	actin relat	mmu0414	Endocytos	5:1451142	228	247	415	349	255	267	1.800904	1.635333	2.258165	1.795821	1.766193	2.614888	1.898134	2.058967	0.840175	1	-
ENSMUSC	Pdap1	PDGFA as	GO:00508	response	-	-	-	-	5:1451287	1398	1665	2049	2048	1552	847	13.79185	13.76837	13.92543	13.16215	13.4261	10.3606	13.82855	12.31628	0.262995	1	-
ENSMUSC	Ptcd1	pentatricc	GO:00081	metabolic	-	-	-	-	5:1451475	443	605	759	770	632	353	8.379158	9.59191	9.889856	9.487885	10.4823	8.278617	9.286975	9.416267	0.898248	1	-
ENSMUSC	Cpsf4	cleavage a	GO:00081	metabolic	K14404	cleavage a	mmu0516	Influenza	5:1451672	269	370	324	477	330	295	2.463369	2.840087	2.043966	2.84562	2.649922	3.349533	2.44914	2.948358	0.333153	1	-
ENSMUSC	Zkscan14	zinc finge	GO:00081	metabolic	-	-	-	-	5:1451949	139	154	209	179	147	134	1.802096	1.673537	1.866639	1.51181	1.671176	2.154044	1.780758	1.77901	0.769013	1	-
ENSMUSC	Phf14	PHD finge	GO:00325	developm	-	-	-	-	6:1190780	864	1044	1268	1344	1041	606	2.904819	2.942109	2.936812	2.943651	3.069013	2.526176	2.927913	2.84628	0.638099	1	-
ENSMUSC	Ndufa4	NADH de	GO:00081	metabolic	K03948	NADH de	mmu011C	Metabolic	6:1190029	674	685	1138	983	696	1164	15.98311	13.61585	18.59068	15.18575	14.47282	34.22475	16.06321	21.29444	0.237865	1	-
ENSMUSC	Rnf6	ring finge	GO:00325	developm	-	-	-	-	5:1462091	1056	689	1078	847	1627	1013	6.219256	3.401312	4.373657	3.249673	8.402426	7.397243	4.664742	6.349781	0.213905	1	-
ENSMUSC	Cdk8	cyclin-dep	GO:00081	metabolic	-	-	-	-	5:1462312	370	412	468	521	385	291	1.663219	1.552382	1.449257	1.525693	1.517579	1.62191	1.554952	1.555061	0.775834	1	-
ENSMUSC	Wasf3	WAS prot	GO:00325	developm	K06083	WAS prot	mmu0523	Choline m	5:1463849	3068	3733	4688	4723	3714	2427	37.52514	38.27171	39.50081	37.6328	39.83378	36.80635	38.43255	38.09098	0.695854	1	-
ENSMUSC	Glcci1	glucocorti	GO:00056	cell|protei	-	-	-	-	6:8509600	166	176	334	292	197	513	0.971883	0.863716	1.347112	1.113707	1.011382	3.723999	1.060903	1.949696	0.043733	1	-
ENSMUSC	Usp12	ubiquitin s	GO:00081	metabolic	-	-	-	-	5:1467348	546	1756	2091	863	1581	1016	4.491905	12.10919	11.85067	4.625197	11.40544	10.36376	9.483922	8.798134	0.625332	1	-
ENSMUSC	Rasl11a	RAS-like,	GO:00081	metabolic	-	-	-	-	5:1468450	96	103	110	131	100	89	3.684237	3.313341	2.908181	3.275132	3.365272	4.234964	3.30192	3.625123	0.776689	1	-
ENSMUSC	Polr1d	polymeras	GO:00081	metabolic	K03020	DNA-dire	mmu011C	Metabolic	5:1470770	272	340	441	339	319	355	2.873407	3.010646	3.20936	2.332973	2.955026	4.649885	3.031138	3.312628	0.839514	1	-
ENSMUSC	Cdx2	caudal typ	GO:00325	developm	K09312	homeobo	mmu0522	Gastric ca	5:1473008	0	2	0	0	0	0	0	0.047119	0	0	0	0	0.015706	0	0.536678	1	-
ENSMUSC	Pan3	PAN3 pol	GO:00081	metabolic	K12572	PAB-depe	mmu0301	RNA degr	5:1474301	645	892	1090	1148	821	591	1.56738	1.816906	1.824704	1.817349	1.749445	1.78069	1.73633	1.782494	0.93554	1	-
ENSMUSC	Flt1	FMS-like	GO:00325	developm	K05096	FMS-like t	mmu0415	PI3K-Akt s	5:1475616	1708	1880	2536	2931	2053	1216	7.540187	6.956728	7.712495	8.429304	7.947415	6.656008	7.403136	7.677575	0.975245	1	-
ENSMUSC	Pomp	proteasor	GO:00099	cellular pr	K11599	proteason	mmu0305	Proteason	5:1478604	535	884	891	886	611	474	5.406402	7.487891	6.20274	5.832705	5.414247	5.939072	6.365678	5.728675	0.290147	1	-
ENSMUSC	Slc46a3	solute car	GO:00511	localizatio	-	-	-	-	5:1478784	56	73	101	74	83	55	0.802831	0.877225	0.997494	0.691121	1.043416	0.977661	0.892517	0.904066	0.882167	1	-
ENSMUSC	Mtus2	microtubu	GO:00990	supramole	-	-	-	-	5:1479573	911	1096	1223	1333	1043	673	3.245517	3.272871	3.001536	3.093693	3.25831	2.972805	3.173308	3.10827	0.651263	1	-
ENSMUSC	N4bp2l2	NEDD4 bi	GO:00325	developm	-	-	-	-	5:1506080	560	675	923	984	960	427	1.601697	1.618261	1.818633	1.833448	2.40772	1.514277	1.67953	1.918482	0.501213	1	-
ENSMUSC	Hsph1	heat shoc	GO:00081	metabolic	K09485	heat shoc	mmu0414	Protein pr	5:1496142	5361	7628	9986	8424	6795	4529	30.19388	36.01112	38.74503	30.90815	33.5587	31.62724	34.98334	32.03136	0.301108	1	-
ENSMUSC	Wdr95	WD40 rep	-	-	-	-	-	-	5:1495286	5	12	19	18	17	10	0.0725	0.145853	0.189787	0.170024	0.216147	0.179793	0.136046	0.188655	0.408234	1	-
ENSMUSC	Medag	mesenteri	GO:00325	developm	-	-	-	-	5:1494117	108	85	164	180	136	77	1.061507	0.70028	1.110441	1.152534	1.172144	0.938372	0.957409	1.087683	0.614485	1	-
ENSMUSC	Tex26	testis expr	-	-	-	-	-	-	5:1494396	2	0	5	4	7	0	0.041985	0	0.072295	0.054698	0.12884	0	0.038094	0.061179	0.724696	1	-
ENSMUSC	Col1a2	collagen, t	GO:00325	developm	K06236	collagen, t	mmu0415	PI3K-Akt s	6:4504814	340	476	827	780	417	570	1.333089	1.564372	2.233762	1.992307	1.433702	2.771025	1.710407	2.065678	0.421237	1	-
ENSMUSC	Tfpi2	tissue fact	GO:00081	metabolic	-	-	-	-	6:3962595	2	1	5	6	1	3	0.068424	0.02867	0.117821	0.133702	0.029992	0.127244	0.071638	0.096979	0.842209	1	-
ENSMUSC	Tspan12	tetraspani	GO:00325	developm	-	-	-	-	6:2177139	245	281	357	322	230	223	3.996537	3.842162	4.011777	3.421804	3.289931	4.510324	3.950158	3.740686	0.488925	1	-
ENSMUSC	Ing3	inhibitor c	GO:00081	metabolic	-	-	-	-	6:2194957	207	458	546	482	400	192	1.395809	2.588649	2.536292	2.117303	2.365141	1.605245	2.173583	2.02923	0.605081	1	-
ENSMUSC	Wnt16	wingless-t	GO:00325	developm	K01558	wingless-t	mmu052C	Pathways	6:2228822	11	23	14	21	9	20	0.125693	0.220285	0.110198	0.156319	0.090179	0.283348	0.152058	0.176615	0.850723	1	-
ENSMUSC	Fam3c	family witl	GO:00325	developm	-	-	-	-	6:2230652	866	1134	1310	1375	1040	778	11.45648	12.57474	11.93867	11.84998	12.06449	12.76142	11.98996	12.22529	0.871723	1	-
ENSMUSC	Auts2	autism sus	GO:00325	developm	-	-	-	-	5:1314373	1919	2458	2546	2905	2247	1455	2.776005	2.980438	2.537202	2.737619	2.850303	2.609722	2.764548	2.732548	0.694349	1	-
ENSMUSC	Limk1	LIM-dom	GO:00325	developm	K05743	LIM doma	mmu0481	Regulation	5:1346560	620	566	950	988	773	456	3.555573	2.720741	3.753123	3.691107	3.88723	3.242419	3.343146	3.606918	0.769253	1	-
ENSMUSC	Eln	elastin [Sc	GO:00325	developm	K14211	elastin[-]	mmu0497	Protein di	5:1347025	193	254	330	305	247	284	1.267779	1.398529	1.493313	1.305167	1.422739	2.31307	1.38654	1.680325	0.395165	1	-
ENSMUSC	Pot1a	protectior	GO:00081	metabolic	-	-	-	-	6:2574373	252	285	365	374	269	231	1.587721	1.505124	1.58423	1.535065	1.486169	1.804562	1.559025	1.608599	0.940496	1	-
ENSMUSC	Bcl7b	B cell CLL/	GO:00325	developm	-	-	-	-	5:1351682	425	513	666	630	526	354	6.172311	6.244956	6.663225	5.960481	6.698649	6.374527	6.360164	6.344552	0.764593	1	-
ENSMUSC	Wasl	Wiskott-A	GO:00325	developm	K05747	Wiskott-A	mmu0414	Endocytos	6:2461380	3768	4569	5611	5907	4303	2086	33.30567	33.85176	34.16638	34.01385	33.35195	22.86165	33.7746	30.07582	0.305429	1	-
ENSMUSC	Cul1	cullin 1 [S	GO:00325	developm	K03347	cullin 1[-]	mmu052C	Pathways	6:4745339	1906	2274	2964	2960	2184	1133	20.19541	20.19636	21.63512	20.43163	20.292	14.88489	20.67563	18.53617	0.308917	1	-
ENSMUSC	Ezh2	enhancer	GO:00325	developm	K11430	histone-ly	mmu052C	MicroRNA	6:4753013	89	110	145	148	90	53	0.62458	0.647059	0.701002	0.676617	0.553842	0.461167	0.657547	0.563875	0.329392	1	-
ENSMUSC	Aass	aminoadip	GO:00081	metabolic	K14157	alpha-am	mmu011C	Metabolic	6:2307217	74	106	141	102	90	71	1.085116	1.302884	1.42435	0.97438	1.157266	1.2909	1.270783	1.140848	0.380881	1	-
ENSMUSC	Ssc4d	scavenger	GO:00511	localizatio	-	-	-	-	5:1359602	11	7	11	12	8	16	0.101717	0.054253	0.070069	0.072288	0.06487	0.18344	0.075346	0.106866	0.493705	1	-
ENSMUSC	Slc13a1	solute car	GO:00511	localizatio	-	-	-	-	6:2408828	6	0	1	1	0	0	0.043167	0	0.004954	0.004686	0	0	0.01604	0.001562	0.116425	1	-
ENSMUSC	Rbm28	RNA bind	GO:00081	metabolic	K14573	nucleolar	mmu030C	Ribosome	6:2912357	893	1134	1325	1500	1120	719	8.060338	8.579611	8.238895	8.820123	8.864666	8.04668	8.292948	8.577156	0.993998	1	-
ENSMUSC	Lrwd1	leucine-ri	GO:00081	metabolic	-	-	-	-	5:1361227	185	211	294	308	223	182	0.989671	0.946139	1.083473	1.073377	1.046086	1.207195	1.006428	1.108886	0.709767	1	-
ENSMUSC	Cux1	cut-like h	GO:00081	metabolic	-	-	-	-	5:1362481	837	913	1292	1267	1157	751	1.543597	1.411341	1.64143	1.522182	1.871045	1.717253	1.532123	1.703493	0.593658	1	-
ENSMUSC	Gcc1	golgi coile	GO:00160	membran	-	-	-	-	6:2841609	521	514	675	641	554	382	3.879907	3.208483	3.462888	3.109734	3.617727	3.527221	3.517093	3.418227	0.615945	1	-
ENSMUSC	Ephb4	Eph recep	GO:00325	developm	K05113	Eph recep	mmu0436	Axon guic	5:1373501	102	121	183	164	119	132	0.704	0.700019	0.870107	0.737387	0.720212	1.129614	0.758042	0.862404	0.665402	1	-
ENSMUSC	Epo	erythropo	GO:00325	developm	K05437	erythropo	mmu052C	Pathways	5:1374830	4	1	20	4	14	4	0.220853	0.04628	0.760767	0.143898	0.677844	0.273846	0.342633	0.365196	1	1	-
ENSMUSC	Actl6b	actin-like	GO:00325	developm	K11652	actin-like	mmu0522	Hepatoce	5:1375535	1241	1492	1619	1862	1411	1064	12.19943	12.29389	10.96392	11.92419	12.1629	12.96867	11.81908	12.35192	0.972769	1	-
ENSMUSC	Gnb2	guanine n	GO:00048	signal trar	K04537	guanine n	mmu052C	Pathways	5:1375281	1660	2196	2750	2870	1721	2190	11.63281	12.89917	13.2758	13.10208	10.57548	19.0286	12.6026	14.23539	0.598417	1	-
ENSMUSC	Gigyf1	GRB10 int	GO:00508	response	-	-	-	-	5:1375185	1155	1347	1601	2044	1325	1264	13.33401	13.03464	12.73273	15.37239	13.41335	18.09305	13.03379	15.62627	0.253991	1	-
ENSMUSC	Pop7	processin	GO:00081	metabolic	K14527	ribonuclea	mmu0301	RNA trans	5:1375014	88	136	136	153	126	128	2.992344	3.876325	3.185779	3.389225	3.757002	5.396622	3.351483	4.18095	0.332142	1	-
ENSMUSC	Tfr2	transferrir	GO:00081	metabolic	-	-	-	-	5:1375698	76	111	100	83	84	83	0.54694	0.669578	0.495768	0.389123	0.530086	0.740612	0.570762	0.553274	0.658818	1	-
ENSMUSC	Pcolce	procollag	GO:00081	metabolic	-	-	-	-	5:1376051	125	121	276	156	130	112	1.166198	0.946238	1.773868	0.948129	1.063525	1.295581	1.295435	1.102412	0.273717	1	-
ENSMUSC	Gm20605	predicted	GO:00081	metabolic	-	-	-	-	5:1376291	333	368	328	595	455	363	4.740846	4.391502	3.216896	5.518366	5.680236	6.407729	4.116415	5.868777	0.034183	1	-
ENSMUSC	Agfg2	ArfGAP w	GO:00987	molecular	-	-	-	-	5:1376504	612	1063	1213	1308	742	597	5.25885	7.656414	7.180447	7.32198	5.590949	6.360619	6.69857	6.424516	0.593873	1	-
ENSMUSC	Tsc22d4	TSC22 do	GO:00081	metabolic	-	-	-	-	5:1377457	638	765	952	919	738	1137	3.700538	3.719281	3.803932	3.472495	3.75356	8.176941	3.74125	5.134332	0.153953	1	-
ENSMUSC	Ppp1r35	protein pł	GO:00081	metabolic	-	-	-	-	5:1377788	73	71	101	113	78	157	2.925213	2.384744	2.788077	2.949813	2.740757	7.800403	2.699345	4.496991	0.073655	1	-
ENSMUSC	Mepce	methylph	GO:00081	metabolic	-	-	-	-	5:1377819	675	808	970	1022	760	477	9.875301	9.908563	9.776186	9.740443	9.749956	8.65268	9.85335	9.381027	0.524865	1	-
ENSMUSC	Cyp3a13	cytochron	GO:00081	metabolic	K07424	cytochron	mmu011C	Metabolic	5:1378929	4	5	8	8	1	5	0.085995	0.090102	0.118487	0.112047	0.018851	0.133279	0.098195	0.088059	0.881266	1	-
ENSMUSC	Zkscan1	zinc finge	GO:00081	metabolic	-	-	-	-	5:1380850	1564	1730	2112	1676	1687	923	5.686971	5.272825	5.290415	3.970087	5.379006	4.161328	5.416737	4.503474	0.094149	1	-
ENSMUSC	Mcm7	minichron	GO:00081	metabolic	K02210	DNA repli	mmu0411	Cell cycle	5:1381645	120	162	204	237	171	141	1.124667	1.272656	1.317115	1.447015	1.405339	1.638498	1.238146	1.496951	0.335054	1	-
ENSMUSC	Tpk1	thiamine p	GO:00081	metabolic	K00949	thiamine p	mmu011C	Metabolic	6:4334500	22	37	41	32	25	32	0.166445	0.234646	0.213696	0.15772	0.16586	0.300187	0.204929	0.207922	0.892632	1	-
ENSMUSC	Asns	asparagin	GO:00325	developm	K01953	asparagin	mmu011C	Metabolic	6:7675169	1294	1497	1881	1988	1470	985	21.92418	21.26003	21.95479	21.94255	21.83982	20.69242	21.713	21.4916	0.700816	1	-
ENSMUSC	Dlx6	distal-less	GO:00325	developm	-	-	-	-	6:6863334	65	83	91	68	66	42	1.330838	1.424444	1.283528	0.906998	1.184952	1.066239	1.34627	1.05273	0.127009	1	-
ENSMUSC	Dlx5	distal-less	GO:00325	developm	K18489	homeobo	mmu0455	Signaling	6:6877805	103	80	93	81	45	58	1.895175	1.233836	1.178819	0.970908	0.726047	1.323217	1.435944	1.006724	0.057305	1	-
ENSMUSC	Dync1i1	dynein cyt	GO:00511	localizatio	K10415	dynein int	mmu0414	Phagoson	6:5725639	1974	2345	2752	2973	2362	1647	22.17278	22.07846	21.29477	21.75453	23.26461	22.93785	21.84867	22.65233	0.985685	1	-
ENSMUSC	Pon3	paraoxon	GO:00081	metabolic	-	-	-	-	6:5220852	76	43	129	76	59	39	1.170631	0.555172	1.368827	0.762611	0.796889	0.744834	1.031544	0.768112	0.163969	1	-
ENSMUSC	Cald1	caldesmo	GO:00160	membran	K12327	caldesmo	mmu0427	Vascular s	6:3459850	721	742	1111	1156	830	518	1.92893	1.663942	2.047606	2.014748	1.947161	1.71829	1.880159	1.8934	0.846865	1	-
ENSMUSC	Akr1b8	aldo-keto	GO:00081	metabolic	K00011	aldehyde	mmu011C	Metabolic	6:3435411	14	9	19	27	9	12	0.523375	0.282008	0.489314	0.657544	0.295044	0.556229	0.431566	0.502939	0.767434	1	-
ENSMUSC	Exoc4	exocyst cc	GO:00325	developm	-	-	-	-	6:3324908	1408	1759	2099	2183	1659	1176	6.426042	6.72914	6.599405	6.490471	6.639417	6.654789	6.584862	6.594892	0.758273	1	-
ENSMUSC	Plxna4	plexin A4	GO:00325	developm	K06820	plexin A[-	mmu0436	Axon guic	6:3214426	5641	6626	8401	10045	7228	5363	25.0631	24.67646	25.71349	29.07436	28.16044	29.54421	25.15102	28.92634	0.369766	1	-
ENSMUSC	Calu	calumenir	GO:00055	extracellul	-	-	-	-	6:2934806	1140	1214	1555	1689	1085	795	6.678689	5.961533	6.275787	6.446112	5.573898	5.774834	6.305336	5.931615	0.439361	1	-
ENSMUSC	Ccdc136	coiled-co	GO:00160	membran	-	-	-	-	6:2939630	919	1263	1544	1682	1214	738	8.404833	9.682107	9.727752	10.02124	9.735876	8.368668	9.271564	9.375262	0.869671	1	-
ENSMUSC	Irf5	interferon	GO:00081	metabolic	K09446	interferon	mmu0462	Toll-like re	6:2952662	47	46	92	101	38	47	0.841403	0.690266	1.134613	1.177906	0.596534	1.043262	0.888761	0.939234	0.960543	1	-
ENSMUSC	Ahcyl2	S-adenos	GO:00081	metabolic	K01251	adenosylh	mmu011C	Metabolic	6:2976801	1514	3587	5724	5784	1622	1369	2.920869	5.800567	7.607417	7.269358	2.743971	3.274729	5.442951	4.429353	0.366174	1	-
ENSMUSC	Klhdc10	kelch dom	GO:00056	cell|intrace	-	-	-	-	6:3040186	1843	2066	2781	1500	1982	1690	8.891536	8.354772	9.242806	4.714377	8.384898	10.10937	8.829705	7.736215	0.240985	1	-
ENSMUSC	Hibadh	3-hydroxy	GO:00081	metabolic	-	-	mmu011C	Metabolic	6:5254622	447	592	712	682	550	481	2.571341	2.854478	2.821523	2.55575	2.774329	3.430706	2.749114	2.920261	0.908396	1	-
ENSMUSC	Gars	glycyl-tRN	GO:00081	metabolic	K01880	glycyl-tRN	mmu0097	Aminoacy	6:5503800	1940	2457	2932	3039	2129	1845	33.72343	35.80041	35.11117	34.41456	32.45251	39.76603	34.87834	35.54437	0.842643	1	-
ENSMUSC	Adcyap1r	adenylate	GO:00325	developm	K04587	pituitary a	mmu0408	Neuroacti	6:5545197	1728	2012	4012	2717	3203	3292	10.64247	10.38674	17.02201	10.90109	17.29809	25.13878	12.68374	17.77932	0.111358	1	-
ENSMUSC	Nt5c3	5'-nucleo	GO:00081	metabolic	K01081	5'-nucleo	mmu011C	Metabolic	6:5688240	249	328	441	384	305	213	2.789478	3.079998	3.403404	2.802441	2.996164	2.958624	3.09096	2.919077	0.500369	1	-
ENSMUSC	Fkbp9	FK506 bin	GO:00081	metabolic	-	-	-	-	6:5683205	689	741	1036	981	753	586	12.30693	11.09433	12.74798	11.41513	11.79418	12.97817	12.04974	12.06249	0.755158	1	-
ENSMUSC	Tmem209	transmem	GO:00160	membran	-	-	-	-	6:3047905	364	492	575	619	499	335	1.778801	2.01532	1.935733	1.970598	2.138302	2.029814	1.909951	2.046238	0.815709	1	-
ENSMUSC	Ssmem1	serine-ricl	GO:00160	membran	-	-	-	-	6:3050984	0	0	0	2	2	0	0	0	0	0.02976	0.040061	0	0	0.023274	0.155963	1	-
ENSMUSC	Avl9	AVL9 cell	GO:00400	locomotic	-	-	-	-	6:5671489	1304	1516	1906	2135	1471	870	7.980849	7.777206	8.036107	8.512378	7.89454	6.602018	7.931388	7.669645	0.616154	1	-
ENSMUSC	Cep41	centroson	GO:00081	metabolic	-	-	-	-	6:3065345	116	139	174	174	135	99	0.651539	0.654411	0.67326	0.636669	0.664901	0.689451	0.659737	0.663674	0.85029	1	-
ENSMUSC	Sspo	SCO-spor	GO:00325	developm	-	-	-	-	6:4844822	23	13	46	60	28	32	0.081125	0.038436	0.111773	0.137868	0.086602	0.139947	0.077111	0.121473	0.152519	1	-
ENSMUSC	Herc6	hect dom	GO:00325	developm	-	-	-	-	6:5758100	107	89	162	165	236	88	0.639718	0.44601	0.667219	0.64264	1.23725	0.652335	0.584316	0.844075	0.121789	1	-
ENSMUSC	Abcg2	ATP bindi	GO:00325	developm	K05681	ATP-bind	mmu0497	Bile secret	6:5858452	268	356	398	425	346	246	1.586458	1.76643	1.623038	1.638944	1.796023	1.805572	1.658642	1.746847	0.93075	1	-
ENSMUSC	Herc3	hect dom	GO:00081	metabolic	K10614	E3 ubiquit	mmu0412	Ubiquitin	6:5883146	2717	3371	3980	4494	3142	2766	14.89207	15.48734	15.02796	16.04649	15.1013	18.79765	15.13579	16.64848	0.643889	1	-
ENSMUSC	Tmem176	transmem	GO:00325	developm	-	-	-	-	6:4883381	273	283	427	459	303	330	3.488836	3.031501	3.759217	3.821304	3.395493	5.228987	3.426518	4.148595	0.335732	1	-
ENSMUSC	Aoc1	amine oxi	GO:00081	metabolic	K11182	diamine o	mmu011C	Metabolic	6:4887289	0	0	2	0	0	0	0	0	0.013207	0	0	0	0.004402	0	0.537187	1	-
ENSMUSC	Malsu1	mitochon	GO:00081	metabolic	-	-	-	-	6:4907379	143	93	182	197	97	158	1.969248	1.07349	1.726583	1.767304	1.171324	2.697791	1.589774	1.878806	0.613102	1	-
ENSMUSC	Gpnmb	glycoprot	GO:00325	developm	-	-	-	-	6:4903654	798	858	1801	1981	735	978	6.787381	6.117013	10.55272	10.97654	5.481877	10.31393	7.819037	8.924113	0.619835	1	-
ENSMUSC	Tra2a	transform	GO:00081	metabolic	K12897	transform	mmu0304	Spliceosor	6:4924392	1281	1479	2499	2530	1844	1200	9.186575	8.890485	12.34588	11.8197	11.59599	10.67018	10.14098	11.36196	0.550278	1	-
ENSMUSC	Npy	neuropep	GO:00325	developm	K05232	neuropep	mmu0503	Alcoholisr	6:4982271	671	800	866	989	604	639	75.34283	75.29435	66.98679	72.34318	59.47019	88.96244	72.54132	73.59194	0.818565	1	-
ENSMUSC	Gsdme	gasdermir	GO:00325	developm	-	-	-	-	6:5018888	106	125	141	141	107	126	0.425938	0.42102	0.390309	0.369096	0.377021	0.627765	0.412422	0.457961	0.784136	1	-
ENSMUSC	Osbpl3	oxysterol	GO:00511	localizatio	-	-	-	-	6:5029333	344	417	635	584	462	245	1.482688	1.506539	1.885456	1.639782	1.746127	1.309315	1.624894	1.565075	0.658972	1	-
ENSMUSC	Luc7l2	LUC7-like	GO:00081	metabolic	-	-	-	-	6:3855133	2521	3285	3981	4631	3487	1678	5.811075	6.347048	6.321603	6.954085	7.048199	4.795805	6.159909	6.26603	0.936498	1	-
ENSMUSC	Zc3hav1	zinc finge	GO:00081	metabolic	-	-	-	-	6:3830528	152	183	222	290	211	144	1.236407	1.247736	1.244007	1.536734	1.505024	1.452332	1.242717	1.49803	0.313281	1	-
ENSMUSC	Svopl	SV2 relate	GO:00511	localizatio	-	-	-	-	6:3798373	1	0	6	3	0	2	0.00771	0	0.031859	0.015062	0	0.01912	0.013189	0.011394	1	1	-
ENSMUSC	Nfe2l3	nuclear fa	GO:00081	metabolic	-	-	-	-	6:5143267	37	34	66	58	45	32	0.631769	0.486627	0.776346	0.645168	0.673769	0.677478	0.631581	0.665472	1	1	-
ENSMUSC	Trim24	tripartite r	GO:00081	metabolic	-	-	-	-	6:3787081	543	670	776	854	653	357	3.40261	3.519169	3.349854	3.4862	3.588133	2.773748	3.423878	3.282694	0.603985	1	-
ENSMUSC	Cbx3	chromobc	GO:00056	cell|intrace	-	-	-	-	6:5147036	881	1160	1084	1218	859	606	7.761322	8.565861	6.578708	6.990191	6.635836	6.619394	7.635297	6.748474	0.223156	1	-
ENSMUSC	Ptn	pleiotroph	GO:00325	developm	-	-	-	-	6:3671492	4264	5304	6637	6517	4536	4275	69.8248	72.80293	74.87136	69.52193	65.13407	86.79896	72.4997	73.81832	0.823759	1	-
ENSMUSC	Mtpn	myotroph	GO:00325	developm	-	-	-	-	6:3550890	6537	7879	9729	9964	7338	3983	97.63713	98.64169	100.1051	96.95091	96.10742	73.76202	98.79464	88.94012	0.292997	1	-
ENSMUSC	Slc13a4	solute car	GO:00511	localizatio	-	-	-	-	6:3526795	323	271	514	312	291	203	3.603863	2.534477	3.950757	2.267785	2.847093	2.808329	3.363032	2.641069	0.074777	1	-
ENSMUSC	Slc23a4	solute car	GO:00511	localizatio	-	-	-	-	6:3494527	0	12	6	7	10	3	0	0.140253	0.05763	0.063584	0.122269	0.051869	0.065961	0.07924	0.839087	1	-
ENSMUSC	Tcaf2	TRPM8 ch	GO:00325	developm	-	-	-	-	6:4262301	9	10	17	23	11	12	0.115293	0.107374	0.150022	0.191945	0.123569	0.190605	0.12423	0.168706	0.441085	1	-
ENSMUSC	Epha1	Eph recep	GO:00325	developm	K05102	Eph recep	mmu0436	Axon guic	6:4235848	1	0	3	5	0	2	0.012909	0	0.026673	0.042044	0	0.032015	0.013194	0.024686	0.614699	1	-
ENSMUSC	Zyx	zyxin [Sou	GO:00508	response	K06273	zyxin[-]	mmu0451	Focal adh	6:4234963	425	472	625	718	596	476	3.010481	2.802477	3.049846	3.313241	3.701993	4.180614	2.954268	3.731949	0.141931	1	-
ENSMUSC	Fam131b	family witl	GO:00056	cell|intrace	-	-	-	-	6:4231531	525	802	868	1050	713	664	4.325856	5.539108	4.927013	5.636166	5.151632	6.783703	4.930659	5.857167	0.32855	1	-
ENSMUSC	Clcn1	chloride c	GO:00511	localizatio	-	-	-	-	6:4228668	12	31	32	35	20	20	0.096386	0.208717	0.177072	0.183149	0.140875	0.199189	0.160725	0.174404	0.914612	1	-
ENSMUSC	Casp2	caspase 2	GO:00325	developm	K02186	caspase 2	mmu0421	Apoptosis	6:4226498	70	140	216	140	111	61	0.431702	0.723719	0.917685	0.562468	0.600277	0.466446	0.691035	0.543064	0.191068	1	-
ENSMUSC	Gstk1	glutathior	GO:00325	developm	K13299	glutathior	mmu052C	Chemical	6:4224593	56	56	94	89	70	95	1.565062	1.311865	1.809785	1.62039	1.715475	3.291969	1.562237	2.209278	0.186274	1	-
ENSMUSC	Ephb6	Eph recep	GO:00048	signal trar	K05114	Eph recep	-	-	6:4160548	1207	1374	1496	1714	1324	1374	14.97043	14.28455	12.78233	13.84903	14.39984	21.13002	14.01244	16.45963	0.392938	1	-
ENSMUSC	Ccdc184	coiled-co	GO:00056	cell|intrace	-	-	-	-	15:981678	154	206	264	218	208	268	4.20231	4.711802	4.962747	3.875291	4.977061	9.067481	4.62562	5.973278	0.302707	1	-
ENSMUSC	Mad2l1	MAD2 mi	GO:00081	metabolic	K02537	mitotic sp	mmu0516	HTLV-I inf	6:6653539	122	195	199	188	173	92	0.744521	0.997482	0.836609	0.747407	0.925778	0.696132	0.859537	0.789772	0.501478	1	-
ENSMUSC	Ssbp1	single-stra	GO:00325	developm	K03111	single-stra	mmu0344	Homolog	6:4047135	163	202	226	254	176	134	0.993017	1.03151	0.948483	1.008055	0.940206	1.012186	0.991003	0.986816	0.785535	1	-
ENSMUSC	Prdm5	PR domai	GO:00081	metabolic	-	-	-	-	6:6577898	125	186	213	218	173	76	0.705706	0.880196	0.82841	0.801773	0.85645	0.532001	0.804771	0.730074	0.511782	1	-
ENSMUSC	Clec5a	C-type lec	GO:00325	developm	-	-	-	-	6:4057489	42	47	72	87	40	26	0.43686	0.409773	0.515914	0.589515	0.364836	0.335322	0.454182	0.429891	0.764852	1	-
ENSMUSC	Agk	acylglycer	GO:00081	metabolic	K09881	acylglycer	mmu011C	Metabolic	6:4032517	424	470	624	611	423	317	2.042637	1.897911	2.070911	1.917557	1.786933	1.893525	2.003819	1.866005	0.415972	1	-
ENSMUSC	C130060K	RIKEN cDI	GO:00048	signal trar	-	-	-	-	6:6538110	4	4	15	2	3	2	0.066026	0.055344	0.17058	0.021505	0.043422	0.040946	0.097317	0.035291	0.045115	1	-
ENSMUSC	Mrps33	mitochon	GO:00329	macromo	-	-	-	-	6:3980180	398	564	649	608	504	418	9.285654	11.02963	10.43099	9.240904	10.31103	12.0918	10.24876	10.54791	0.915518	1	-
ENSMUSC	Hpgds	hematopc	GO:00081	metabolic	K04097	prostaglar	mmu011C	Metabolic	6:6511729	119	134	212	270	170	84	0.982948	0.92777	1.206343	1.452879	1.231334	0.860297	1.03902	1.181503	0.586949	1	-
ENSMUSC	Smarcad1	SWI/SNF-	GO:00081	metabolic	K14439	SWI/SNF-	mmu0455	Signaling	6:6504258	567	566	786	720	619	370	2.022935	1.692654	1.931847	1.673451	1.936566	1.636766	1.882479	1.748928	0.431141	1	-
ENSMUSC	Mkrn1	makorin, r	GO:00081	metabolic	-	-	-	-	6:3939780	1293	1725	2234	2018	1592	1672	11.75931	13.14999	13.99645	11.956	12.69606	18.85409	12.96858	14.50205	0.632355	1	-
ENSMUSC	Rab19	RAB19, m	GO:00038	catalytic a	-	-	-	-	6:3938117	0	5	0	0	3	5	0	0.169106	0	0	0.106142	0.250142	0.056369	0.118761	0.602746	1	-
ENSMUSC	Slc37a3	solute car	GO:00511	localizatio	-	-	-	-	6:3933477	559	880	792	1177	737	778	3.005596	3.966013	2.933563	4.122652	3.474792	5.186619	3.301724	4.261354	0.13957	1	-
ENSMUSC	Tbxas1	thrombox	GO:00081	metabolic	K01832	thrombox	mmu011C	Metabolic	6:3887540	37	60	58	87	43	35	0.219188	0.297936	0.236699	0.335751	0.22337	0.257083	0.251274	0.272068	0.851728	1	-
ENSMUSC	Gfpt1	glutamine	GO:00081	metabolic	K00820	glucosam	mmu011C	Metabolic	6:8704284	1847	1893	2659	2673	1906	1155	10.60078	9.10698	10.51334	9.994278	9.592601	8.219378	10.0737	9.268752	0.365119	1	-
ENSMUSC	Nfu1	NFU1 iror	GO:00081	metabolic	-	-	-	-	6:8700923	309	450	527	567	406	315	3.146589	3.84103	3.696951	3.761373	3.625357	3.977206	3.561523	3.787978	0.871739	1	-
ENSMUSC	Anxa4	annexin A	GO:00325	developm	-	-	-	-	6:8673684	69	97	139	136	69	92	0.580435	0.683955	0.805507	0.745287	0.50897	0.959566	0.689966	0.737941	0.943135	1	-
ENSMUSC	Pcyox1	prenylcyst	GO:00081	metabolic	K05906	prenylcyst	mmu009C	Terpenoic	6:8638600	1749	2137	2628	2744	1967	1734	16.3366	16.73128	16.91019	16.69697	16.11087	20.08199	16.65936	17.62994	0.905714	1	-
ENSMUSC	Tgfa	transform	GO:00325	developm	K08774	transform	mmu052C	Pathways	6:8619522	589	620	898	980	598	519	5.131288	4.52747	5.389379	5.561842	4.568297	5.606148	5.016046	5.245429	0.970959	1	-
ENSMUSC	Add2	adducin 2	GO:00325	developm	-	-	-	-	6:8602868	7636	5418	10359	12532	3900	2296	32.42188	19.28254	30.29993	34.66366	14.52044	12.08733	27.33478	20.42381	0.191127	1	-
ENSMUSC	Figla	folliculoge	GO:00325	developm	-	-	-	-	6:8601617	0	0	0	0	0	2	0	0	0	0	0	0.163359	0	0.054453	0.536611	1	-
ENSMUSC	Dusp11	dual speci	GO:00081	metabolic	-	-	-	-	6:8594226	983	1208	1635	1751	1208	941	6.267316	6.455768	7.181206	7.272705	6.753637	7.438816	6.634763	7.155053	0.760591	1	-
ENSMUSC	Nat8	N-acetyltı	GO:00325	developm	K20838	N-acetyltı	mmu0048	Glutathior	6:8583038	8	23	11	14	12	16	0.45272	1.091026	0.428839	0.516145	0.595502	1.122694	0.657528	0.744781	0.890321	1	-
ENSMUSC	Cct7	chaperoni	GO:00081	metabolic	-	-	-	-	6:8545151	2573	3123	3598	3653	2856	2313	20.8947	21.25793	20.12838	19.32536	20.33748	23.28938	20.76034	20.98407	0.784035	1	-

ENSMUSG	Pradc1	protease-	GO:00055	extracellul	-	-	-	-	6:8544681	72	75	83	116	75	78	0.918471	0.801953	0.72939	0.963989	0.838954	1.233716	0.816605	1.01222	0.354135	1	-
ENSMUSG	Zfp638	zinc finge	GO:00081	metabolic	-	-	-	-	6:8386710	1091	1362	1708	1611	1118	847	2.959296	3.096662	3.19156	2.846695	2.659183	2.848611	3.082506	2.78483	0.245465	1	-
ENSMUSG	Reg3g	regenerat	GO:00325	developm	-	-	-	-	6:7846626	3	6	2	3	0	1	0.074005	0.124072	0.033996	0.04821	0	0.030581	0.077357	0.026264	0.182517	1	-
ENSMUSG	Fbxl14	F-box anc	GO:00081	metabolic	-	-	-	-	6:1194796	470	481	649	632	504	474	10.08098	8.647735	9.589609	8.830867	9.479324	12.60575	9.439441	10.30531	0.76833	1	-
ENSMUSG	Prickle2	prickle pla	GO:00325	developm	K04511	prickle[-]	mmu0431	Wnt signa	6:9237090	6822	7692	9239	11234	7676	5058	27.13303	25.64358	25.31413	29.10734	26.77097	24.94315	26.03024	26.94049	0.996411	1	-
ENSMUSG	Adamts9	a disinteg	GO:00325	developm	-	-	-	-	6:9277269	249	279	347	335	253	186	0.903082	0.848172	0.866978	0.791505	0.804619	0.836425	0.872744	0.81085	0.431665	1	-
ENSMUSG	Lrig1	leucine-ri	GO:00325	developm	-	-	-	-	6:9460452	357	657	451	772	497	336	2.493506	3.846453	2.170055	3.512703	3.043979	2.909835	2.836671	3.155506	0.669136	1	-
ENSMUSG	1700003E	RIKEN cDI	-	-	-	-	-	-	6:8315640	397	502	539	552	508	326	6.16884	6.538368	5.769698	5.587701	6.921807	6.28082	6.158969	6.263443	0.880414	1	-
ENSMUSG	Kbtbd8	kelch repe	GO:00325	developm	-	-	-	-	6:9511724	121	145	188	160	141	85	1.424326	1.430684	1.524515	1.226945	1.45541	1.240582	1.459842	1.307646	0.378542	1	-
ENSMUSG	Wdr54	WD repea	-	-	-	-	-	-	6:8314936	97	121	121	149	131	150	0.707525	0.739788	0.608003	0.708004	0.837881	1.356583	0.685105	0.967489	0.119266	1	-
ENSMUSG	Ino80b	INO80 co	GO:00081	metabolic	-	-	-	-	6:8312176	115	223	144	196	213	128	3.102123	5.04219	2.675921	3.444274	5.038285	4.281106	3.606745	4.254555	0.5235	1	-
ENSMUSG	Wbp1	WW dom	GO:00160	membran	-	-	-	-	6:8311904	258	347	450	409	317	309	2.717381	3.06347	3.265085	2.806311	2.927741	4.035298	3.015312	3.25645	0.842528	1	-
ENSMUSG	Mogs	mannosyl	GO:00081	metabolic	K01228	mannosyl	mmu0110	Metabolic	6:8311549	306	317	426	475	332	399	5.614096	4.87495	5.384174	5.677185	5.341198	9.076463	5.291073	6.698282	0.23321	1	-
ENSMUSG	Mrpl53	mitochon	GO:00329	macromo	-	-	-	-	6:8310904	25	20	21	18	39	15	1.130847	0.758304	0.654382	0.530403	1.546919	0.841287	0.847845	0.97287	0.748183	1	-
ENSMUSG	M1ap	meiosis 1	GO:00325	developm	-	-	-	-	6:8294690	0	0	0	0	4	0	0	0	0	0	0.077181	0	0	0.025727	0.187287	1	-
ENSMUSG	Pole4	polymera	GO:00081	metabolic	K03506	DNA poly	mmu0110	Metabolic	6:8261899	188	222	276	278	221	342	3.219615	3.186777	3.256155	3.101499	3.318795	7.262016	3.220849	4.56077	0.141557	1	-
ENSMUSG	Tacr1	tachykinin	GO:00048	signal trar	K04222	tachykinin	mmu0408	Neuroacti	6:8240233	34	65	36	45	33	31	0.179201	0.287162	0.130713	0.15451	0.152519	0.202587	0.199025	0.169872	0.413912	1	-
ENSMUSG	Mrpl19	mitochon	GO:00081	metabolic	K02884	large subu	mmu0301	Ribosome	6:8195785	493	568	669	675	575	424	5.313151	5.131048	4.966866	4.739038	5.433951	5.665747	5.137022	5.279579	0.920924	1	-
ENSMUSG	Arhgap25	Rho GTPa	GO:00511	localizatio	-	-	-	-	6:8745854	53	69	104	108	93	61	0.539189	0.588389	0.728866	0.715764	0.82964	0.769445	0.618815	0.771617	0.320742	1	-
ENSMUSG	Gkn3	gastrokine	GO:00099	cellular pr	-	-	-	-	6:8738325	20	25	30	26	38	29	1.484897	1.555793	1.53437	1.25751	2.473944	2.669571	1.52502	2.133675	0.294278	1	-
ENSMUSG	Gkn1	gastrokine	-	-	-	-	-	-	6:8734565	0	0	0	2	0	0	0	0	0	0.066234	0	0	0	0.022078	0.537406	1	-
ENSMUSG	Aplf	aprataxin	GO:00325	developm	-	-	-	-	6:8762842	123	112	159	198	112	63	1.037839	0.792128	0.924217	1.088362	0.828683	0.659103	0.918061	0.858716	0.649417	1	-
ENSMUSG	Gp9	glycoprot	GO:00508	response	K06263	platelet gl	mmu0461	Platelet ac	6:8777807	3	2	0	3	3	5	0.216386	0.120927	0	0.140964	0.189754	0.44719	0.112438	0.259303	0.217486	1	-
ENSMUSG	Rab43	RAB43, m	GO:00511	localizatio	-	-	-	-	6:8778885	347	230	507	519	384	341	2.675346	1.486386	2.692839	2.606752	2.596118	3.259804	2.284857	2.820891	0.323852	1	-
ENSMUSG	Isy1	ISY1 splici	GO:00081	metabolic	K12870	pre-mRN.	mmu0304	Spliceosoı	6:8781426	470	584	697	732	564	395	3.778614	3.935501	3.860278	3.833779	3.976089	3.937466	3.858131	3.915778	0.864279	1	-
ENSMUSG	Cnbp	cellular nu	GO:00081	metabolic	-	-	-	-	6:8784261	1824	2519	2836	2615	2121	2147	15.82535	18.31933	16.95064	14.78025	16.13659	23.09652	17.03177	18.00445	0.956513	1	-
ENSMUSG	Copg1	coatomer	GO:00511	localizatio	-	-	-	-	6:8788781	4729	6214	7041	7463	5685	4660	19.3424	21.30419	19.83932	19.88548	20.38985	23.63267	20.16197	21.30267	0.906828	1	-
ENSMUSG	Tmf1	TATA eler	GO:00325	developm	-	-	-	-	6:9715299	919	1126	1411	1585	1044	597	8.889678	9.129816	9.402628	9.988071	8.855513	7.160295	9.140707	8.66796	0.548135	1	-
ENSMUSG	Hmces	5-hydrox	GO:00081	metabolic	-	-	-	-	6:8791393	185	242	287	291	227	187	2.880277	3.158145	3.078202	2.951469	3.099068	3.609862	3.038875	3.220133	0.920607	1	-
ENSMUSG	Uba3	ubiquitin-	GO:00081	metabolic	K10686	ubiquitin-	mmu0412	Ubiquitin	6:9718367	1100	1589	2019	1710	1111	711	7.865475	9.523768	9.945345	7.96544	6.966082	6.303587	9.111529	7.07837	0.034311	1	-
ENSMUSG	Rpn1	ribophorir	GO:00081	metabolic	K12666	oligosacch	mmu0110	Metabolic	6:8808448	1951	2429	3007	2855	2163	1597	21.29713	22.22513	22.61251	20.3026	20.70443	21.61498	22.04492	20.874	0.416439	1	-
ENSMUSG	Frmd4b	FERM dor	GO:00099	cellular pr	-	-	-	-	6:9728686	647	748	1027	1122	680	405	2.001498	1.939571	2.188634	2.26113	1.844603	1.553432	2.043234	1.886388	0.456052	1	-
ENSMUSG	Foxp1	forkhead	GO:00325	developm	-	-	-	-	6:9892533	846	760	917	2686	875	394	2.210858	1.664779	1.650864	4.572757	2.005125	1.276652	1.842167	2.618178	0.18847	1	-
ENSMUSG	Gm20696	predicted	GO:00081	metabolic	-	-	-	-	6:9925748	9	10	21	17	13	28	0.116991	0.108955	0.188056	0.14396	0.148184	0.451294	0.138001	0.247813	0.17582	1	-
ENSMUSG	Prok2	prokinetic	GO:00325	developm	-	-	-	-	6:9971129	2	5	9	3	0	2	0.032842	0.068804	0.101786	0.032082	0	0.040724	0.067811	0.024269	0.084315	1	-
ENSMUSG	Gxylt2	glucoside	GO:00081	metabolic	K13676	UDP-xylo:	mmu0051	Other type	6:1007047	54	79	81	91	56	63	0.386473	0.473919	0.399357	0.424275	0.351441	0.55905	0.419917	0.444922	0.979409	1	-
ENSMUSG	Cntn3	contactin	GO:00325	developm	-	-	-	-	6:1021626	320	406	523	481	336	227	1.833315	1.94969	2.064146	1.795203	1.687989	1.612496	1.94905	1.698563	0.217087	1	-
ENSMUSG	Chl1	cell adhes	GO:00325	developm	-	-	-	-	6:1035105	5980	7220	7658	8427	6692	2402	27.25133	27.57887	24.04104	25.01734	26.74146	13.57205	26.29041	21.77695	0.219431	1	-
ENSMUSG	Ruvbl1	RuvB-like	GO:00081	metabolic	K04499	RuvB-like	mmu0431	Wnt signa	6:8846540	493	607	778	859	593	424	15.91247	16.42222	17.29901	18.06197	16.7837	16.96847	16.54457	17.27138	0.961167	1	-
ENSMUSG	Sec61a1	Sec61 alp	GO:00325	developm	K10956	protein tra	mmu0414	Phagoson	6:8850357	998	1165	1447	1532	1125	1092	6.129291	5.997339	6.122087	6.129429	6.058639	8.315501	6.082906	6.834523	0.570829	1	-
ENSMUSG	Abtb1	ankyrin re	GO:00160	membran	-	-	-	-	6:8883591	214	292	420	430	320	326	1.423373	1.62795	1.924448	1.863185	1.866369	2.688493	1.658591	2.139349	0.170009	1	-
ENSMUSG	Plxna1	plexin A1	GO:00325	developm	K06820	plexin A[-	mmu0436	Axon guic	6:8931631	3952	4365	5061	6335	4481	3597	22.05493	20.41859	19.45703	23.03122	21.92837	24.88944	20.64351	23.28301	0.487604	1	-
ENSMUSG	Chchd6	coiled-co	GO:00508	response	-	-	-	-	6:8938314	645	715	790	754	611	528	12.23268	11.36634	10.32144	9.315687	10.16121	12.416	11.30682	10.63096	0.417554	1	-
ENSMUSG	Klf15	Kruppel-li	GO:00325	developm	-	-	-	-	6:9046257	310	350	478	529	417	333	2.998239	2.837435	3.184814	3.333048	3.536581	3.99333	3.006829	3.620986	0.280951	1	-
ENSMUSG	Aldh1l1	aldehyde	GO:00081	metabolic	K00289	formyltetr	mmu0067	One carbo	6:9048642	821	984	1188	1302	1110	825	8.069443	8.106779	8.043927	8.336675	9.566782	10.05404	8.073383	9.319166	0.398468	1	-
ENSMUSG	Slc41a3	solute car	GO:00511	localizatio	-	-	-	-	6:9060472	33	47	64	76	42	57	0.720455	0.860087	0.962551	1.080911	0.804052	1.54295	0.847698	1.142638	0.257528	1	-
ENSMUSG	Nup210	nucleopor	GO:00511	localizatio	K14314	nuclear pc	mmu0301	RNA trans	6:9101306	401	507	535	697	456	492	1.839789	1.949774	1.690941	2.083233	1.834558	2.79882	1.826835	2.23887	0.269116	1	-
ENSMUSG	Cntn6	contactin	GO:00325	developm	-	-	-	-	6:1044927	113	159	185	175	132	92	0.867013	1.022585	0.977849	0.87472	0.888106	0.875229	0.955816	0.879352	0.45472	1	-
ENSMUSG	Wnt7a	wingless-t	GO:00325	developm	K00572	wingless-t	mmu0520	Pathways	6:9136398	291	348	423	376	246	278	4.915982	4.927763	4.922765	4.137969	3.644145	5.823019	4.92217	4.535044	0.376075	1	-
ENSMUSG	Xpc	xeroderm	GO:00081	metabolic	K10838	xeroderm	mmu0342	Nucleotid	6:9148930	339	389	436	498	358	293	3.507729	3.373877	3.10788	3.356888	3.248267	3.75907	3.329829	3.454742	0.981016	1	-
ENSMUSG	Tmem43	transmem	GO:00099	cellular pr	-	-	-	-	6:9147370	495	662	806	773	592	494	6.650892	7.455659	7.460389	6.766064	6.974922	8.229769	7.18898	7.323585	0.854998	1	-
ENSMUSG	Slc6a6	solute car	GO:00511	localizatio	-	-	-	-	6:9168405	1997	2446	3226	3519	2477	2313	11.73513	12.04811	13.05947	13.47133	12.76375	16.85277	12.2809	14.36261	0.334865	1	-
ENSMUSG	Grip2	glutamate	GO:00452	synapse|c	-	-	-	-	6:9176150	211	261	299	360	249	234	1.319982	1.368604	1.288569	1.467134	1.365926	1.815044	1.325718	1.549368	0.437152	1	-
ENSMUSG	Sumf1	sulfatase r	GO:00081	metabolic	K13444	sulfatase r	mmu0414	Lysosome	6:1081070	359	468	599	502	392	327	5.234123	5.719362	6.016276	4.767982	5.011621	5.911294	5.656587	5.230299	0.367242	1	-
ENSMUSG	Itpr1	inositol 1,4	GO:00325	developm	K04958	inositol 1,4	mmu0516	Kaposi's sa	6:1082130	9173	9551	13303	14855	10445	6249	27.87871	24.33117	27.85238	29.41139	27.83636	23.5482	26.68742	26.93199	0.850416	1	-
ENSMUSG	Bhlhe40	basic helix	GO:00081	metabolic	K03729	class B ba	mmu0471	Circadian	6:1086606	2406	2604	3367	3781	2679	1636	23.89569	21.67793	23.03661	24.46314	23.33133	20.14621	22.87008	22.64689	0.730227	1	-
ENSMUSG	Edem1	ER degrac	GO:00081	metabolic	K10084	ER degrac	mmu0414	Protein pr	6:1088286	198	339	512	347	391	334	1.078187	1.547321	1.920656	1.230947	1.867015	2.255072	1.515388	1.784345	0.521335	1	-
ENSMUSG	Arl8b	ADP-ribos	GO:00511	localizatio	-	-	-	-	6:1087830	4888	5996	7273	7383	5436	2689	65.39939	67.24453	67.03592	64.35125	63.77707	44.60866	66.55995	57.579	0.21071	1	-
ENSMUSG	Slc6a13	solute car	GO:00511	localizatio	K05039	solute car	mmu0472	GABAergi	6:1213002	71	119	162	136	119	150	1.349762	1.896272	2.121609	1.684308	1.98376	3.535719	1.789215	2.401263	0.253434	1	-
ENSMUSG	Slc6a12	solute car	GO:00511	localizatio	-	-	-	-	6:1213430	11	17	15	23	17	20	0.15867	0.205541	0.149054	0.216123	0.215018	0.35769	0.171088	0.262944	0.194268	1	-
ENSMUSG	Ret	ret proto-	GO:00325	developm	K05126	proto-onc	mmu0520	Pathways	6:1181517	82	103	143	118	80	66	0.746185	0.785638	0.896435	0.699511	0.638358	0.744671	0.809419	0.69418	0.252246	1	-
ENSMUSG	A2m	alpha-2-r	GO:00325	developm	K03910	alpha-2-r	mmu0461	Complem	6:1216353	168	123	376	324	167	179	1.401722	0.860222	2.161181	1.761079	1.221834	1.851787	1.474375	1.611567	0.829876	1	-
ENSMUSG	Klrg1	killer cell l	GO:00023	immune s	-	-	-	-	6:1222705	2	0	5	5	1	4	0.089909	0	0.154817	0.146409	0.039409	0.222911	0.081575	0.136243	0.692681	1	-
ENSMUSG	Mfap5	microfibril	GO:00325	developm	-	-	-	-	6:1225058	8	5	17	16	9	9	0.101356	0.053098	0.148377	0.132062	0.099995	0.141382	0.100944	0.12448	0.697164	1	-
ENSMUSG	Gdf3	growth di	GO:00325	developm	-	-	-	-	6:1226054	0	0	0	2	0	1	0	0	0	0.032442	0	0.030869	0	0.021104	0.284445	1	-
ENSMUSG	Mlf2	myeloid le	GO:00508	response	-	-	-	-	6:1249313	5282	6602	7495	7436	6175	4630	44.42654	46.54495	43.42774	40.74414	45.54323	48.28488	44.79974	44.85742	0.727865	1	-
ENSMUSG	Ptms	parathym	GO:00023	immune s	-	-	-	-	6:1249136	4648	4522	5090	5008	4052	1603	98.39919	80.2433	74.2326	69.06713	75.22072	42.07701	84.2917	62.12162	0.045218	1	-
ENSMUSG	Plxnd1	plexin D1	GO:00325	developm	-	-	-	-	6:1159548	689	800	970	932	732	537	5.328309	5.185763	5.167651	4.695347	4.96391	5.149086	5.227241	4.936114	0.44028	1	-
ENSMUSG	Lag3	lymphocy	GO:00048	signal trar	-	-	-	-	6:1249043	80	79	115	124	89	90	2.350544	1.945632	2.32772	2.373465	2.293049	3.278763	2.207965	2.648425	0.454827	1	-
ENSMUSG	Lrrc23	leucine ric	-	-	-	-	-	-	6:1247698	46	52	115	54	63	30	1.017194	0.963832	1.751864	0.777902	1.221614	0.822553	1.244297	0.94069	0.183918	1	-
ENSMUSG	Tmcc1	transmem	GO:00099	cellular pr	-	-	-	-	6:1160186	1377	1730	2209	2062	1713	1111	3.030842	3.191745	3.349472	2.956647	3.3062	3.031998	3.190686	3.098282	0.583946	1	-
ENSMUSG	Cops7a	COP9 sigr	GO:00081	metabolic	-	-	-	-	6:1249584	1982	2350	2770	2911	2232	1782	32.79408	32.59211	31.57352	31.37731	32.38387	36.55825	32.3199	33.43981	0.950042	1	-
ENSMUSG	Rasgef1a	RasGEF dc	GO:00400	locomotic	-	-	-	-	6:1180114	4812	5490	6645	7389	5454	3977	60.94402	58.2815	57.97648	60.9639	60.5708	62.45209	59.06733	61.32893	0.993895	1	-
ENSMUSG	Tuba8	tubulin, al	GO:00051	structural	K07374	tubulin alp	mmu0414	Phagoson	6:1212106	199	261	201	245	168	108	5.588612	6.1439	3.888651	4.482288	4.137177	3.760629	5.207054	4.126698	0.128268	1	-
ENSMUSG	Bms1	BMS1, rib	GO:00081	metabolic	K14569	ribosome	mmu0300	Ribosome	6:1183833	806	1056	1217	1314	876	538	9.289756	10.20202	9.662983	9.866119	8.853527	7.688423	9.718254	8.80269	0.331843	1	-
ENSMUSG	Clec4e	C-type lec	GO:00325	developm	K10059	C-type lec	mmu0515	Tuberculo	6:1232817	3	0	1	0	0	0	0.014447	0	0.003317	0	0	0	0.005921	0	0.166079	1	-
ENSMUSG	Clec4d	C-type lec	GO:00325	developm	-	-	-	-	6:1232621	5	4	18	12	5	10	0.132791	0.08904	0.329323	0.207621	0.116432	0.329308	0.183718	0.217787	0.924518	1	-
ENSMUSG	Zfp248	zinc finge	GO:00081	metabolic	-	-	-	-	6:1184273	156	193	216	215	169	108	1.105761	1.146693	1.054734	0.992791	1.05043	0.949175	1.102396	0.997465	0.393615	1	-
ENSMUSG	Clec4a2	C-type lec	GO:00023	immune s	-	-	-	-	6:1231064	4	9	8	6	4	7	0.059407	0.112039	0.081853	0.058055	0.052091	0.128912	0.084433	0.079686	0.894687	1	-
ENSMUSG	Clec2e	C-type lec	GO:00160	membran	-	-	-	-	6:1290919	0	0	0	0	2	0	0	0	0	0	0.053096	0	0	0.017699	0.534826	1	-
ENSMUSG	Cd69	CD69 anti	GO:00508	response	-	-	-	-	6:1292673	3	3	3	1	0	0	0.025147	0.02108	0.017325	0.005461	0	0	0.021184	0.00182	0.035089	1	-
ENSMUSG	Clec2d	C-type lec	GO:00325	developm	-	-	-	-	6:1291806	44	60	59	93	74	42	2.305314	2.634998	2.129494	3.17424	3.399788	2.728447	2.356602	3.100825	0.212267	1	-
ENSMUSG	Clec12b	C-type lec	GO:00019	cell killing	-	-	-	-	6:1293755	0	0	2	2	0	0	0	0	0.038578	0.036473	0	0	0.012859	0.012158	1	1	-
ENSMUSG	Clec1b	C-type lec	GO:00325	developm	-	-	-	-	6:1293972	0	1	3	2	0	0	0	0.009047	0.022306	0.014061	0	0	0.010451	0.004687	0.720721	1	-
ENSMUSG	Gabarapl1	gamma-a	GO:00081	metabolic	K08341	GABA(A) r	mmu0516	Kaposi's sa	6:1295331	7593	8904	10444	10187	7822	5184	151.0826	148.5044	143.1593	132.0472	136.4777	127.8946	147.5821	132.1398	0.202514	1	-
ENSMUSG	Olr1	oxidized l	GO:00511	localizatio	K08763	oxidised l	mmu0414	Phagoson	6:1294852	2	2	0	0	2	0	0.03048	0.025542	0	0	0.02672	0	0.018674	0.008907	0.720673	1	-
ENSMUSG	Klrd1	killer cell l	GO:00023	immune s	K06516	killer cell l	mmu0465	Natural ki	6:1295917	0	0	2	0	2	0	0	0	0.03149	0	0.040075	0	0.010497	0.013358	1	1	-
ENSMUSG	Rad52	RAD52 hc	GO:00081	metabolic	K10873	DNA repa	mmu0344	Homolog	6:1199026	113	160	191	209	198	171	0.66409	0.788177	0.773275	0.800161	1.02037	1.246039	0.741847	1.02219	0.09428	1	-
ENSMUSG	Adipor2	adiponect	GO:00325	developm	K07297	adiponect	mmu0493	Non-alco	6:1193531	918	1014	1473	1319	1048	860	6.220459	5.759312	6.875965	5.822455	6.227069	7.225432	6.285245	6.424985	0.869333	1	-
ENSMUSG	Wnt5b	wingless-t	GO:00325	developm	K00444	wingless-t	mmu0520	Pathways	6:1194325	39	43	48	60	29	31	0.439542	0.406214	0.372671	0.440523	0.286597	0.433196	0.406142	0.386772	0.698057	1	-
ENSMUSG	Erc1	ELKS/RAB	GO:00081	metabolic	K16072	ELKS/RAB	mmu0406	NF-kappa	6:1195707	1854	2029	2564	2766	1896	833	3.538783	3.246227	3.371425	3.439362	3.173401	1.971402	3.385478	2.861389	0.224161	1	-
ENSMUSG	Ccdc77	coiled-co	GO:00160	membran	-	-	-	-	6:1203243	122	112	151	190	128	70	0.885096	0.681085	0.754675	0.897983	0.814302	0.629678	0.773619	0.780654	0.92939	1	-
ENSMUSG	Kdm5a	lysine (K)-	GO:00325	developm	-	-	-	-	6:1203641	1052	1121	1533	1625	1151	861	3.689927	3.295797	3.704209	3.713101	3.540137	3.744478	3.563311	3.665905	0.937531	1	-
ENSMUSG	Klra2	killer cell l	GO:00160	membran	-	-	-	-	6:1312192	1	0	6	12	2	2	0.0328	0	0.135539	0.256366	0.057507	0.081342	0.056113	0.131739	0.252715	1	-
ENSMUSG	Magohb	mago hor	GO:00081	metabolic	K12877	protein m	mmu0301	RNA trans	6:1312843	32	33	62	31	39	53	0.716084	0.618999	0.955779	0.451919	0.765289	1.470539	0.763621	0.895916	0.7707	1	-
ENSMUSG	Ybx3	Y box pro	GO:00325	developm	K06099	cold shocl	mmu0453	Tight junc	6:1313648	269	379	585	497	346	279	3.826272	4.518714	5.732314	4.60533	4.315601	4.92054	4.692433	4.613824	0.70592	1	-
ENSMUSG	Etv6	ets varian	GO:00325	developm	K03211	ETS transl	mmu0520	Transcript	6:1340357	269	348	484	420	314	224	1.601778	1.736929	1.985392	1.629222	1.63954	1.653799	1.7747	1.640854	0.411423	1	-
ENSMUSG	Lrp6	low densit	GO:00325	developm	K03068	low densit	mmu0520	Pathways	6:1344464	1615	1668	2297	2366	1697	943	6.578524	5.695145	6.445673	6.278446	6.061503	4.762702	6.239781	5.700884	0.369298	1	-
ENSMUSG	Dusp16	dual speci	GO:00081	metabolic	K04459	dual speci	mmu0401	MAPK sigı	6:1347154	166	265	378	332	209	189	0.812835	1.087662	1.275082	1.059046	0.897396	1.147476	1.058526	1.034639	0.696076	1	-
ENSMUSG	Ddx47	DEAD (As	GO:00081	metabolic	-	-	-	-	6:1350116	472	619	779	845	573	499	2.712698	2.98197	3.084243	3.163718	2.887734	3.555875	2.926304	3.202442	0.706217	1	-
ENSMUSG	Gprc5d	G protein-	GO:00325	developm	-	-	-	-	6:1351059	0	0	6	5	0	0	0	0	0.180906	0.142578	0	0	0.060302	0.047526	1	1	-
ENSMUSG	Gsg1	germ cell	GO:00160	membran	-	-	-	-	6:1352373	0	0	8	2	2	0	0	0	0.130059	0.030744	0.041385	0	0.043353	0.024043	0.67558	1	-
ENSMUSG	Fam234b	family witl	GO:00160	membran	-	-	-	-	6:1351979	3243	3849	4971	5232	3895	4244	20.53631	20.43037	21.68556	21.58365	21.62846	33.32244	20.88408	25.51152	0.230239	1	-
ENSMUSG	Emp1	epithelial	GO:00099	cellular pr	-	-	-	-	6:1353625	98	120	234	205	107	103	0.976508	1.002274	1.606274	1.330724	0.934927	1.272551	1.195019	1.179401	0.787389	1	-
ENSMUSG	Grin2b	glutamate	GO:00048	signal trar	K05210	glutamate	mmu0408	Neuroacti	6:1357132	25195	26033	30512	42416	28906	14861	53.17012	46.05007	44.35835	58.31289	53.49139	38.88546	47.85951	50.22992	0.895631	1	-
ENSMUSG	Atf7ip	activating	GO:00081	metabolic	-	-	-	-	6:1365061	1417	1669	2090	2338	1756	1094	3.584263	3.538664	3.641898	3.852621	3.894909	3.431099	3.588275	3.72621	0.979969	1	-
ENSMUSG	Plbd1	phospholi	GO:00055	extracellul	-	-	-	-	6:1366120	2	8	9	5	8	5	0.034314	0.115019	0.106346	0.055877	0.120338	0.106336	0.085226	0.094183	1	1	-
ENSMUSG	Wbp11	WW dom	GO:00081	metabolic	K12866	WW dom	mmu0304	Spliceosoı	6:1368136	1932	2194	2461	2677	1955	1383	18.8841	17.9754	16.57113	17.04587	16.75632	16.7609	17.81021	16.85436	0.436459	1	-
ENSMUSG	Art4	ADP-ribos	GO:00081	metabolic	-	-	-	-	6:1368484	0	2	0	0	0	0	0	0.042327	0	0	0	0	0.014109	0	0.536438	1	-
ENSMUSG	Mgp	matrix Gla	GO:00325	developm	-	-	-	-	6:1368724	174	258	367	255	148	303	8.301991	10.3182	12.06284	7.925993	6.192065	17.92504	10.22768	10.68103	0.926747	1	-
ENSMUSG	Erp27	endoplasr	GO:00056	cell|intrace	-	-	-	-	6:1369073	0	4	7	1	0	0	0	0.062302	0.089615	0.012104	0	0	0.050639	0.004035	0.034551	1	-
ENSMUSG	Arhgdib	Rho, GDP	GO:00325	developm	K12462	Rho GDP-	mmu0472	Neurotrop	6:1369236	144	104	194	225	104	117	3.866347	2.340584	3.588311	3.935517	2.44858	3.895004	3.265081	3.426367	0.980781	1	-
ENSMUSG	Rerg	RAS-like,	GO:00508	response	-	-	-	-	6:1370548	415	472	552	556	418	292	4.821663	4.596677	4.418139	4.208286	4.258611	4.206461	4.61216	4.224453	0.353225	1	-
ENSMUSG	Ptpro	protein ty	GO:00325	developm	-	-	-	-	6:1372523	936	1085	1110	1378	908	671	5.930179	5.762017	4.844694	5.687516	5.044526	5.271091	5.512296	5.334378	0.589457	1	-
ENSMUSG	Strap	serine/thr	GO:00325	developm	K13137	serine-thr	mmu0301	RNA trans	6:1377350	1801	2114	2624	2808	1894	1656	34.17706	33.62628	34.30333	34.7136	31.51691	38.96435	34.03556	35.06495	0.920014	1	-
ENSMUSG	Dera	deoxyribo	GO:00081	metabolic	K01619	deoxyribo	mmu0003	Pentose p	6:1377545	31	20	38	45	28	22	0.172701	0.093392	0.145834	0.163314	0.136781	0.151966	0.137309	0.150687	0.814236	1	-
ENSMUSG	Lmo3	LIM doma	GO:00081	metabolic	-	-	-	-	6:1383629	2376	3228	3527	3910	2671	2048	15.10624	17.20268	15.4478	16.19453	14.89109	16.14454	15.91891	15.74339	0.673159	1	-
ENSMUSG	Pik3c2g	phosphati	GO:00081	metabolic	K00923	phosphati	mmu0110	Metabolic	6:1395872	2	3	6	4	10	0	0.008002	0.010059	0.016534	0.010425	0.035079	0	0.011532	0.015168	0.768159	1	-
ENSMUSG	Plcz1	phospholi	GO:00325	developm	K05861	phosphati	mmu0110	Metabolic	6:1399896	28	47	51	54	30	17	0.524301	0.737688	0.657875	0.658718	0.492588	0.394686	0.639955	0.515331	0.315907	1	-
ENSMUSG	Plekha5	pleckstrin	GO:00325	developm	-	-	-	-	6:1404240	1019	1235	1609	1815	1378	821	5.20625	5.288962	5.663152	6.041002	6.173658	5.200918	5.386121	5.805193	0.7455	1	-
ENSMUSG	Aebp2	AE bindin	GO:00081	metabolic	-	-	-	-	6:1406226	796	980	1245	1114	878	552	3.118877	3.21858	3.360517	2.843492	3.016634	2.681701	3.232658	2.847276	0.205135	1	-
ENSMUSG	Slco1c1	solute car	GO:00511	localizatio	K08747	solute car	mmu0491	Thyroid hc	6:1415243	686	888	1211	1093	839	674	5.100895	5.534622	6.203231	5.294489	5.470501	6.213962	5.612916	5.659651	0.796811	1	-
ENSMUSG	Slco1b2	solute car	GO:00325	developm	K05043	solute car	mmu0497	Bile secret	6:1416295	0	0	6	1	0	1	0	0	0.044043	0.006941	0	0.013209	0.014681	0.006717	0.549378	1	-
ENSMUSG	Slco1a4	solute car	GO:00511	localizatio	K03460	solute car	mmu0497	Bile secret	6:1418054	530	700	878	873	635	524	4.691893	5.194256	5.354489	5.034636	4.929335	5.751612	5.080213	5.238528	0.937911	1	-
ENSMUSG	Recql	RecQ pro	GO:00081	metabolic	-	-	-	-	6:1423503	279	410	414	530	356	265	1.630616	2.008556	1.666859	2.017923	1.824481	1.920345	1.768677	1.920916	0.749193	1	-
ENSMUSG	Gys2	glycogen	GO:00081	metabolic	K00693	glycogen	mmu0110	Metabolic	6:1424226	0	5	0	0	0	0	0	0.089647	0	0	0	0	0.029882	0	0.124345	1	-
ENSMUSG	Golt1b	golgi tran	GO:00048	signal trar	-	-	-	-	6:1423872	506	604	662	775	556	472	10.36346	10.36918	9.340357	10.3404	9.985541	11.98623	10.02433	10.77072	0.82211	1	-
ENSMUSG	Ldhb	lactate de	GO:00081	metabolic	K00016	L-lactate c	mmu0110	Metabolic	6:1424902	3796	4323	5613	5983	4676	3326	122.585	117.017	124.8698	125.8669	132.4123	133.1739	121.4906	130.4844	0.771775	1	-
ENSMUSG	Kcnj8	potassium	GO:00325	developm	K05001	potassium	mmu0402	cGMP-PK	6:1425648	57	60	71	107	61	30	0.829141	0.731575	0.711483	1.013958	0.778089	0.541089	0.7574	0.777712	1	1	-
ENSMUSG	Abcc9	ATP-bind	GO:00048	signal trar	K05033	ATP-bind	mmu0201	ABC trans	6:1425878	178	205	286	250	206	143	1.100219	1.062101	1.217799	1.006652	1.116527	1.095924	1.126706	1.073035	0.561966	1	-
ENSMUSG	Rad18	RAD18 E3	GO:00081	metabolic	-	-	-	-	6:1126198	105	138	163	175	136	86	0.702428	0.77383	0.751195	0.762664	0.797798	0.713344	0.742484	0.757935	0.949293	1	-
ENSMUSG	Sspn	sarcospan	GO:00329	macromo	-	-	-	-	6:1459316	224	95	31	339	107	197	2.849453	1.01296	0.271663	2.809281	1.193547	3.107184	1.378025	2.370004	0.221749	1	-
ENSMUSG	Bhlhe41	basic helix	GO:00325	developm	K03730	class B ba	mmu0471	Circadian	6:1458582	602	767	1038	1017	724	503	5.253181	5.610146	6.239858	5.781341	5.539965	5.442271	5.701062	5.587859	0.667853	1	-
ENSMUSG	Srgap3	SLIT-ROB	GO:00987	molecular	K07526	SLIT-ROB	mmu0436	Axon guic	6:1127179	3739	3727	6258	5718	3925	7233	15.10647	12.62175	17.41784	15.04989	13.9056	36.23363	15.04869	21.7297	0.113511	1	-
ENSMUSG	Rassf8	Ras assoc	GO:00508	response	-	-	-	-	6:1457467	372	413	514	629	351	267	3.142591	2.924477	2.991295	3.461601	2.600131	2.796675	3.019454	2.952802	0.700897	1	-
ENSMUSG	Lrmp	lymphoid-	GO:00160	membran	-	-	-	-	6:1451156	35	42	57	80	35	33	0.341529	0.343524	0.383164	0.50854	0.299481	0.399255	0.356072	0.402425	0.704428	1	-
ENSMUSG	Thumpd3	THUMP d	GO:00081	metabolic	-	-	-	-	6:1130462	541	645	781	886	634	328	3.781172	3.778693	3.760375	4.034079	3.885623	2.842428	3.773413	3.587377	0.593134	1	-
ENSMUSG	Kras	Kirsten rat	GO:00325	developm	K07827	GTPase Kf	mmu0520	Pathways	6:1452166	2080	2326	3110	2972	2215	1300	16.00172	14.99913	16.48221	14.89478	14.94238	12.40032	15.82769	14.07916	0.235118	1	-
ENSMUSG	Bcat1	branched	GO:00081	metabolic	K00826	branched	mmu0110	Metabolic	6:1449938	1553	1970	2439	2612	1793	1839	8.791898	9.348245	9.512055	9.633096	8.900901	12.90859	9.217399	10.48086	0.501668	1	-
ENSMUSG	Mtmr14	myotubul	GO:00081	metabolic	K18086	myotubul	mmu0110	Metabolic	6:1132378	140	214	206	277	214	223	1.333549	1.708623	1.351758	1.718865	1.787458	2.633727	1.464643	2.046683	0.079384	1	-
ENSMUSG	Cpne9	copine fa	GO:00325	developm	-	-	-	-	6:1132823	186	199	324	365	298	162	0.879659	0.788875	1.055598	1.124542	1.235836	0.949955	0.908044	1.103445	0.308239	1	-
ENSMUSG	Ogg1	8-oxogua	GO:00325	developm	K03660	N-glycosy	mmu0341	Base excis	6:1133269	56	59	79	77	71	96	0.605882	0.535067	0.588818	0.542719	0.673598	1.28783	0.576589	0.834716	0.165306	1	-
ENSMUSG	Camk1	calcium/c	GO:00325	developm	K08794	calcium/c	mmu0492	Oxytocin s	6:1133341	581	796	876	878	711	559	4.486547	5.152312	4.660061	4.41685	4.81447	5.352225	4.766307	4.861182	0.86528	1	-
ENSMUSG	Etnk1	ethanolan	GO:00081	metabolic	K00894	ethanolan	mmu0110	Metabolic	6:1431672	7388	8962	10941	11542	8648	6212	31.12766	31.65027	31.75616	31.67979	31.95052	32.45167	31.51136	32.02733	0.846236	1	-
ENSMUSG	Ttll3	tubulin tyr	GO:00081	metabolic	-	-	-	-	6:1133892	159	183	267	275	220	207	1.01384	0.978084	1.172828	1.142317	1.230092	1.636544	1.054917	1.336318	0.21808	1	-
ENSMUSG	C2cd5	C2 calciur	GO:00511	localizatio	-	-	-	-	6:1430109	863	1013	1238	1537	1144	852	2.858361	2.812344	2.824739	3.316362	3.322577	3.498902	2.831815	3.37928	0.247256	1	-
ENSMUSG	Il17rc	interleukir	GO:00048	signal trar	K05166	interleukir	mmu0406	Cytokine-	6:1134714	18	26	48	22	29	67	0.336948	0.407961	0.618996	0.268289	0.47603	1.555099	0.454635	0.766473	0.242656	1	-
ENSMUSG	Cmas	cytidine m	GO:00160	membran	K00983	N-acylneu	mmu0110	Metabolic	6:1427566	963	1314	1473	1407	1041	1039	10.04456	11.48823	10.58422	9.560501	9.521344	13.43712	10.70567	10.83966	0.788016	1	-
ENSMUSG	St8sia1	ST8 alpha	GO:00081	metabolic	K03371	alpha-N-	mmu0110	Metabolic	6:1428215	1335	1517	1926	2286	1517	995	7.445002	7.091241	7.399314	8.305037	7.418427	6.880066	7.311852	7.53451	0.976772	1	-
ENSMUSG	Creld1	cysteine-r	GO:00160	membran	-	-	-	-	6:1134832	928	1139	1454	1484	1134	1113	8.175384	8.410789	8.824209	8.516781	8.760232	12.15741	8.470127	9.811475	0.426301	1	-
ENSMUSG	Emc3	ER memb	GO:00329	macromo	-	-	-	-	6:1135148	691	896	1148	1110	836	918	11.43921	12.43309	13.09216	11.97079	12.13577	18.84285	12.32148	14.31647	0.464446	1	-
ENSMUSG	Itpr2	inositol 1,4	GO:00048	signal trar	K04959	inositol 1,4	mmu0516	Kaposi's sa	6:1461082	614	694	949	1119	814	437	2.359364	2.235317	2.512144	2.801165	2.742799	2.082065	2.368942	2.54201	0.778852	1	-
ENSMUSG	Med21	mediator	GO:00325	developm	-	-	-	-	6:1466425	182	179	245	220	190	138	2.696819	2.223243	2.50091	2.123653	2.46874	2.535398	2.473657	2.37593	0.603691	1	-
ENSMUSG	Sec13	SEC13 hoı	GO:00511	localizatio	K14004	protein tra	mmu0301	RNA trans	6:1137280	507	628	797	766	598	626	8.578645	8.906827	9.290103	8.443468	8.872664	13.1332	8.925191	10.14978	0.564916	1	-
ENSMUSG	Ccdc91	coiled-co	GO:00511	localizatio	-	-	-	-	6:1474758	761	955	1212	1135	887	550	15.75884	16.57661	17.28994	15.31147	16.10665	14.12172	16.54179	15.17995	0.351366	1	-
ENSMUSG	Atp2b2	ATPase, C	GO:00325	developm	K05850	Ca2+ tran	mmu0402	cAMP sigr	6:1137438	8681	9913	14152	14863	11269	5062	24.70905	23.65071	27.74952	27.55969	28.12641	17.86464	25.36976	24.51692	0.678305	1	-
ENSMUSG	Far2	fatty acyl	GO:00081	metabolic	K13356	alcohol-fc	mmu0414	Peroxisor	6:1480472	348	406	460	579	410	228	2.905852	2.841665	2.646084	3.149595	3.002069	2.360557	2.797867	2.837407	0.918429	1	-
ENSMUSG	Ergic2	ERGIC anc	GO:00511	localizatio	-	-	-	-	6:1481790	776	937	1073	1105	870	461	4.84975	4.908518	4.61965	4.498853	4.767818	3.572274	4.792639	4.279648	0.300496	1	-
ENSMUSG	Tmtc1	transmem	GO:00081	metabolic	-	-	-	-	6:1482324	1502	1576	2229	1001	1705	1058	10.91432	9.599212	11.15804	4.738518	10.8641	9.532311	10.55719	8.37831	0.110699	1	-
ENSMUSG	Slc6a11	solute car	GO:00325	developm	-	-	-	-	6:1141312	1962	2780	3006	3155	2473	2440	24.21655	28.76146	25.55957	25.36846	26.76579	37.34127	26.17919	29.82517	0.504134	1	-
ENSMUSG	Caprin2	caprin fan	GO:00325	developm	-	-	-	-	6:1488424	150	171	174	215	184	136	0.503937	0.48154	0.402703	0.470548	0.542056	0.566511	0.462727	0.526372	0.582937	1	-
ENSMUSG	Slc6a1	solute car	GO:00511	localizatio	K05034	solute car	mmu0472	GABAergi	6:1142826	6274	7925	8491	9362	7559	6424	38.91963	41.2075	36.28561	37.83332	41.11792	49.41009	38.80425	42.78711	0.641238	1	-
ENSMUSG	Dennd5b	DENN/MA	GO:00511	localizatio	-	-	-	-	6:1489880	1741	1998	2519	2706	1951	1011	5.519869	5.309804	5.501861	5.589069	5.424128	3.974358	5.443845	4.995852	0.404597	1	-
ENSMUSG	Atg7	autophag	GO:00325	developm	K08337	ubiquitin-	mmu0414	Autophag	6:1146430	348	368	410	417	438	242	2.031641	1.800814	1.648933	1.585935	2.242251	1.751739	1.827129	1.859975	0.907555	1	-
ENSMUSG	Vgll4	vestigial li	GO:00081	metabolic	-	-	-	-	6:1148606	324	430	570	589	454	363	2.787487	3.100914	3.378272	3.301146	3.425051	3.872221	3.088891	3.532806	0.489018	1	-

ENSMUS(	Tamm41	TAM41 m	GO:00081	metabolic	-	-	-	-	6:1150043	103	141	131	169	114	76	0.973663	1.117238	0.853095	1.040739	0.944978	0.890783	0.981332	0.958833	0.76854	1	-
ENSMUS(	Timp4	tissue inhi	GO:00325	developm	-	-	-	-	6:1152418	356	466	504	581	519	522	3.632763	3.985896	3.542989	3.862292	4.644053	6.604557	3.720549	5.036967	0.087133	1	-
ENSMUS(	Cand2	cullin-ass	GO:00081	metabolic	-	-	-	-	6:1157745	107	91	92	143	110	69	1.242272	0.885572	0.73582	1.08156	1.119864	0.993262	0.954555	1.064895	0.703791	1	-
ENSMUS(	Efcab12	EF-hand (	GO:00431	ion bindin	-	-	-	-	6:1158107	58	70	87	119	79	59	1.149029	1.162386	1.187334	1.53578	1.372371	1.449242	1.16625	1.452464	0.300823	1	-
ENSMUS(	Mbd4	methyl-C	GO:00081	metabolic	K10801	methyl-C	mmu0341	Base excis	6:1158406	98	160	218	182	177	114	0.636762	0.871418	0.975798	0.770383	1.008481	0.918428	0.827993	0.899097	0.820692	1	-
ENSMUS(	Ift122	intraflagel	GO:00325	developm	-	-	-	-	6:1158534	554	638	824	957	689	599	2.438253	2.353655	2.498318	2.743864	2.659075	3.268752	2.430075	2.890564	0.300134	1	-
ENSMUS(	Rho	rhodopsir	GO:00325	developm	K04250	rhodopsin	mmu0474	Phototran	6:1159317	2	9	18	15	15	2	0.01433	0.054037	0.088827	0.069999	0.09422	0.017768	0.052398	0.060662	0.812972	1	-
ENSMUS(	Necap1	NECAP er	GO:00511	localizatio	-	-	-	-	6:1228744	3583	4282	5141	5488	4103	2673	54.79076	54.88581	54.15762	54.67091	55.01799	50.68103	54.6114	53.45664	0.624878	1	-
ENSMUS(	Pianp	PILR alpha	GO:00160	membran	-	-	-	-	6:1249966	1814	1104	2580	3028	1251	1852	21.82191	11.13211	21.38093	23.72974	13.1964	27.62374	18.11165	21.51663	0.518562	1	-
ENSMUS(	Ing4	inhibitor c	GO:00081	metabolic	-	-	-	-	6:1250397	408	498	643	699	465	450	2.282138	2.334875	2.47767	2.547064	2.280744	3.120898	2.364895	2.649569	0.605867	1	-
ENSMUS(	Mrpl51	mitochon	GO:00081	metabolic	-	-	-	-	6:1251918	544	731	883	905	713	551	7.27696	8.196366	8.136979	7.886444	8.363405	9.138796	7.870101	8.462882	0.802082	1	-
ENSMUS(	Vamp1	vesicle-as	GO:00452	synapse|lc	K08510	vesicle-as	mmu0413	SNARE int	6:1252155	1331	1798	2285	2274	1717	1942	6.27981	7.110679	7.426877	6.989411	7.103651	11.36067	6.939122	8.484576	0.265369	1	-
ENSMUS(	Ltbr	lymphoto:	GO:00325	developm	K03159	lymphoto:	mmu0516	HTLV-I int	6:1253065	103	141	179	160	152	113	2.361448	2.709665	2.827145	2.38971	3.055838	3.212222	2.632753	2.885924	0.777585	1	-
ENSMUS(	Scnn1a	sodium ch	GO:00511	localizatio	K04824	amiloride-	mmu0474	Taste tran	6:1253206	103	114	160	154	134	75	0.590682	0.547992	0.632107	0.575335	0.673852	0.533293	0.59026	0.59416	0.895126	1	-
ENSMUS(	Tnfrsf1a	tumor nec	GO:00325	developm	K03158	tumor nec	mmu0516	Human pa	6:1253493	96	148	225	220	122	161	1.620908	2.094599	2.617099	2.419858	1.806292	3.37052	2.110869	2.532223	0.490159	1	-
ENSMUS(	Cd9	CD9 antig	GO:00325	developm	K06460	CD9 antig	mmu0464	Hematop	6:1254602	804	798	1279	1287	791	569	21.56887	17.94431	23.63704	22.49217	18.60759	18.92645	21.05007	20.00874	0.536996	1	-
ENSMUS(	Akap3	A kinase (	GO:00511	localizatio	-	-	-	-	6:1268530	2	7	8	6	4	7	0.035158	0.103119	0.096861	0.0687	0.061642	0.152548	0.078379	0.094297	0.889562	1	-
ENSMUS(	Dyrk4	dual-spec	GO:00081	metabolic	-	-	-	-	6:1268760	0	0	2	8	3	0	0	0	0.025186	0.095258	0.04808	0	0.008395	0.047779	0.096108	1	-
ENSMUS(	Rad51ap1	RAD51 as	GO:00081	metabolic	-	-	-	-	6:1269230	28	35	30	39	24	26	0.234234	0.245425	0.172887	0.21254	0.176056	0.269688	0.217515	0.219428	0.922591	1	-
ENSMUS(	D6Wsu16	DNA segr	GO:00511	localizatio	-	-	-	-	6:1269399	414	626	735	542	473	383	3.146488	3.987982	3.848262	2.683532	3.152316	3.609194	3.660911	3.148347	0.158267	1	-
ENSMUS(	Prmt8	protein ar	GO:00081	metabolic	-	-	-	-	6:1276890	1362	1710	1939	1962	1463	1235	31.77651	33.44091	31.16437	29.82014	29.93063	35.72581	32.12726	31.82553	0.65874	1	-
ENSMUS(	Tspan11	tetraspani	GO:00508	response	-	-	-	-	6:1278875	19	43	40	32	24	50	0.214668	0.407225	0.311332	0.235528	0.237777	0.700444	0.311075	0.39125	0.634547	1	-
ENSMUS(	Tspan9	tetraspani	GO:00508	response	-	-	-	-	6:1279613	729	831	1060	1025	753	781	5.803598	5.545287	5.813361	5.315886	5.256631	7.709154	5.720749	6.09389	0.911076	1	-
ENSMUS(	Tead4	TEA doma	GO:00081	metabolic	K09448	transcripti	mmu0439	Hippo sig	6:1282242	10	10	17	14	13	6	0.129966	0.108933	0.152201	0.118534	0.148154	0.096695	0.130367	0.121128	0.774287	1	-
ENSMUS(	Fkbp4	FK506 bin	GO:00325	developm	K09571	FK506-bir	mmu0491	Estrogen s	6:1284297	1953	1840	3087	2863	1932	1823	32.1802	25.41307	35.04084	30.7319	27.91491	37.24425	30.87804	31.96369	0.945207	1	-
ENSMUS(	Clec2i	C-type lec	GO:00325	developm	-	-	-	-	6:1288875	0	2	2	3	0	0	0	0.019459	0.015995	0.022683	0	0	0.011818	0.007561	0.720914	1	-
ENSMUS(	Strn4	striatin, ca	GO:00452	synapse|m	-	-	-	-	7:1681588	2406	3252	3578	3903	3026	2957	11.47876	13.00477	11.75956	12.13052	12.65933	17.49188	12.08103	14.09391	0.381224	1	-
ENSMUS(	Slc8a2	solute car	GO:00511	localizatio	-	-	-	-	7:1612982	8482	10054	11752	12971	10242	6837	76.5162	76.02332	73.03286	76.22725	81.01819	76.47274	75.1908	77.90606	0.987624	1	-
ENSMUS(	Mzf1	myeloid z	GO:00081	metabolic	-	-	-	-	7:1304230	179	248	246	324	250	223	0.651992	0.757173	0.617272	0.768807	0.798497	1.00712	0.675479	0.858141	0.189371	1	-
ENSMUS(	Slc27a5	solute car	GO:00081	metabolic	K08748	solute car	mmu0110	Metabolic	7:1298834	0	0	8	6	4	2	0	0	0.09027	0.064026	0.057448	0.040629	0.03009	0.054034	0.510366	1	-
ENSMUS(	Zfp606	zinc finge	GO:00081	metabolic	-	-	-	-	7:1247829	197	259	367	306	262	180	0.732161	0.806848	0.939632	0.740873	0.853855	0.829464	0.826214	0.808064	0.689167	1	-
ENSMUS(	Zik1	zinc finge	GO:00081	metabolic	-	-	-	-	7:1048722	247	252	349	367	261	173	3.738409	3.197007	3.638874	3.618573	3.463966	3.246561	3.524763	3.443034	0.701992	1	-
ENSMUS(	Mark4	MAP/micr	GO:00325	developm	-	-	-	-	7:1942477	1845	2259	2093	2586	1801	1578	13.12314	13.46823	10.25563	11.98262	11.23306	13.91664	12.28233	12.37744	0.779859	1	-
ENSMUS(	Ercc2	excision re	GO:00325	developm	K10844	DNA excis	mmu0342	Nucleotid	7:1938201	266	403	481	477	564	314	2.319896	2.946083	2.889907	2.710114	4.313295	3.395494	2.718629	3.472968	0.1866	1	-
ENSMUS(	Rtn2	reticulon 2	GO:00511	localizatio	-	-	-	-	7:1928262	740	1031	1186	1223	992	1040	9.257722	10.81145	10.22136	9.967366	10.88245	16.13214	10.09684	12.32732	0.268482	1	-
ENSMUS(	Vasp	vasodilato	GO:00325	developm	K06274	vasodilato	mmu0401	Rap1 sign	7:1925692	133	193	209	214	199	203	2.551326	3.103304	2.761927	2.674304	3.347419	4.828325	2.805519	3.616683	0.233159	1	-
ENSMUS(	Gipr	gastric inh	GO:00325	developm	K04580	gastric inh	mmu0408	Neuroacti	7:1915606	30	25	32	42	22	30	0.318884	0.222739	0.234319	0.290827	0.205059	0.395387	0.258648	0.297091	0.744611	1	-
ENSMUS(	Qpctl	glutaminy	GO:00081	metabolic	-	-	-	-	7:1913970	285	289	378	438	308	275	3.738409	3.177557	3.415746	3.742815	3.542706	4.472606	3.443904	3.919376	0.542546	1	-
ENSMUS(	Dmpk	dystrophia	GO:00325	developm	-	-	-	-	7:1908384	178	190	294	251	216	202	1.047062	0.936824	1.191379	0.961847	1.114159	1.473294	1.058421	1.1831	0.698163	1	-
ENSMUS(	Dmwd	dystrophia	GO:00081	metabolic	-	-	-	-	7:1907622	1560	2029	1978	2612	1787	1617	24.05208	26.22183	21.00902	26.23511	24.15991	30.91179	23.76098	27.10227	0.474126	1	-
ENSMUS(	Nova2	neuro-on	GO:00081	metabolic	-	-	-	-	7:1892588	2510	2857	3140	3809	2800	2285	19.05376	18.179	16.42056	18.83647	18.63836	21.50696	17.88444	19.6606	0.649231	1	-
ENSMUS(	Pglyrp1	peptidogl	GO:00325	developm	-	-	-	-	7:1887133	26	30	36	35	23	52	0.644464	0.623284	0.614719	0.565163	0.499912	1.59811	0.627489	0.887728	0.379039	1	-
ENSMUS(	Pdcd5	programn	GO:00081	metabolic	-	-	-	-	7:3564198	60	74	106	88	76	71	0.9392	0.970951	1.143055	0.897372	1.0432	1.378019	1.017735	1.106197	0.886438	1	-
ENSMUS(	Uri1	URI1, pref	GO:00081	metabolic	-	-	-	-	7:3795999	900	1154	815	1429	664	529	7.381298	7.933212	4.604678	7.634917	4.775302	5.379377	6.639729	5.929866	0.404857	1	-
ENSMUS(	Pop4	processin	GO:00081	metabolic	K03538	ribonuclea	mmu0301	RNA trans	7:3826199	228	294	368	352	299	270	3.245257	3.507638	3.608391	3.26392	3.73189	4.765018	3.453762	3.920276	0.579702	1	-
ENSMUS(	Lilra6	leukocyte	-	-	K06512	leukocyte	mmu0438	Osteoclas	7:3908280	0	2	0	0	0	0	0	0.021614	0	0	0	0	0.007205	0	0.537037	1	-
ENSMUS(	Ttyh1	tweety far	GO:00511	localizatio	-	-	-	-	7:4119408	2989	3527	4951	4733	3955	4362	11.71291	11.58503	13.36544	12.0825	13.59027	21.19388	12.22113	15.62222	0.147995	1	-
ENSMUS(	Tmem238	transmem	GO:00160	membran	-	-	-	-	7:4784557	25	21	33	21	25	28	0.983193	0.692256	0.894033	0.538024	0.862143	1.365331	0.856494	0.921833	1	1	-
ENSMUS(	Rpl28	ribosomal	GO:00081	metabolic	K02903	large subu	mmu0301	Ribosome	7:4792874	163	189	200	218	184	95	3.961434	3.850162	3.348471	3.451454	3.921254	2.862691	3.720022	3.4118	0.498888	1	-
ENSMUS(	Sbk2	SH3-bind	GO:00081	metabolic	-	-	-	-	7:4940512	0	0	5	4	3	4	0	0	0.050186	0.03797	0.038325	0.07226	0.016729	0.049518	0.19228	1	-
ENSMUS(	U2af2	U2 small r	GO:00081	metabolic	K12837	splicing fa	mmu0304	Spliceosor	7:5062143	1822	2019	2503	2724	2253	1553	15.81231	14.68711	14.9644	15.40054	17.14553	16.71108	15.15461	16.41905	0.725557	1	-
ENSMUS(	Zfp583	zinc finge	GO:00081	metabolic	-	-	-	-	7:6315660	110	123	176	99	136	79	1.033841	0.96899	1.139531	0.606148	1.120834	0.920608	1.047454	0.88253	0.261802	1	-
ENSMUS(	Zfp273	zinc finge	GO:00081	metabolic	-	-	-	-	13:678137	129	119	169	212	153	54	1.351746	1.045216	1.219954	1.447178	1.405855	0.701599	1.205639	1.184877	0.869945	1	-
ENSMUS(	Cyfip1	cytoplasm	GO:00325	developm	K05749	cytoplasm	mmu0481	Regulatio	7:5584174	2064	2395	2950	3246	2469	1694	8.519415	8.286258	8.38829	8.72831	8.936425	8.669605	8.397988	8.778113	0.952565	1	-
ENSMUS(	Oca2	oculocuta	GO:00325	developm	-	-	-	-	7:5623976	22	9	41	13	9	22	0.240437	0.082444	0.308693	0.09256	0.086255	0.29813	0.210525	0.158982	0.394858	1	-
ENSMUS(	Herc2	HECT and	GO:00081	metabolic	K10595	E3 ubiquit	mmu0412	Ubiquitin	7:5605019	7406	8067	10087	12152	8415	5589	25.65118	23.42009	24.06784	27.4191	25.55763	24.0018	24.37971	25.65951	0.894767	1	-
ENSMUS(	Nipa2	non impri	GO:00511	localizatio	-	-	-	-	7:5593128	436	504	674	545	423	468	2.659992	2.57737	2.832727	2.166067	2.262957	3.540182	2.69003	2.656402	0.66417	1	-
ENSMUS(	Psd3	pleckstrin	GO:00452	synapse|m	K12494	PH and SE	mmu0414	Endocytos	8:6768908	15971	17217	21969	24640	16317	10286	32.93226	29.75773	31.20693	33.09876	29.50342	26.29795	31.29897	29.63338	0.472239	1	-
ENSMUS(	Siglecg	sialic acid	GO:00325	developm	-	-	-	-	7:4340820	1	0	0	4	1	0	0.005327	0	0	0.013881	0.00467	0	0.001776	0.006184	0.42367	1	-
ENSMUS(	Zfp719	zinc finge	GO:00081	metabolic	-	-	-	-	7:4357961	326	439	490	481	391	267	3.745173	4.22738	3.87794	3.599817	3.938888	3.803215	3.950165	3.78064	0.549274	1	-
ENSMUS(	Zdhhc13	zinc finge	GO:00048	signal trar	-	-	-	-	7:4878900	292	351	432	396	316	206	6.490902	6.540067	6.615409	5.734539	6.159588	5.677741	6.548793	5.857289	0.293207	1	-
ENSMUS(	Siglece	sialic acid	GO:00048	signal trar	-	-	-	-	7:4365107	9	12	15	30	15	14	0.267026	0.298446	0.306598	0.579857	0.390253	0.515047	0.29069	0.495053	0.095886	1	-
ENSMUS(	Zfp108	zinc finge	GO:00081	metabolic	-	-	-	-	7:2425479	140	154	174	224	151	77	1.316969	1.214281	1.127581	1.372702	1.24556	0.898092	1.21961	1.172118	0.724063	1	-
ENSMUS(	Faap24	Fanconi a	GO:00081	metabolic	K10898	fanconi ar	mmu0346	Fanconi a	7:3539215	76	82	104	90	76	85	1.18238	1.069329	1.114621	0.912158	1.036816	1.639634	1.12211	1.196202	0.983326	1	-
ENSMUS(	Rhpn2	rhophilin,	GO:00508	response	-	-	-	-	7:3533417	114	109	164	152	128	90	0.88809	0.711756	0.880132	0.771398	0.874392	0.869326	0.826659	0.838372	0.88239	1	-
ENSMUS(	Slc7a10	solute car	GO:00511	localizatio	-	-	-	-	7:3518638	362	396	476	492	386	410	7.113846	6.522952	6.443983	6.298587	6.651593	9.990006	6.693594	7.646729	0.575428	1	-
ENSMUS(	Gas2	growth ar	GO:00325	developm	-	-	-	-	7:5186201	71	107	105	114	80	80	0.474126	0.598925	0.483034	0.495934	0.468455	0.662386	0.518695	0.542258	1	1	-
ENSMUS(	Kctd15	potassium	GO:00325	developm	-	-	-	-	7:3463901	138	142	173	239	187	197	1.111996	0.959105	0.96033	1.254596	1.32132	1.968238	1.010477	1.514718	0.031169	1	-
ENSMUS(	Slc17a6	solute car	GO:00452	synapse|lc	K12302	MFS trans	mmu0472	Retrograd	7:5162200	224	389	743	461	447	212	2.161863	3.146902	4.939927	2.898433	3.782949	2.536889	3.416231	3.072757	0.496579	1	-
ENSMUS(	Prmt3	protein ar	GO:00081	metabolic	-	-	-	-	7:4977834	358	445	536	524	415	257	3.023519	3.15023	3.118502	2.882987	3.073412	2.691221	3.097417	2.88254	0.440581	1	-
ENSMUS(	Asb7	ankyrin re	GO:00081	metabolic	-	-	-	-	7:6664456	458	530	651	544	457	149	3.618508	3.509882	3.543205	2.799911	3.166089	1.459603	3.557198	2.475201	0.044862	1	-
ENSMUS(	Cers3	ceramide	GO:00325	developm	K04710	ceramide	mmu0110	Metabolic	7:6674350	0	7	0	3	2	2	0	0.104069	0	0.034661	0.031105	0.043997	0.03469	0.036588	1	1	-
ENSMUS(	Snrpa1	small nucl	GO:00081	metabolic	K11092	U2 small r	mmu0304	Spliceosor	7:6605900	149	216	245	247	184	146	1.940056	2.3574	2.197583	2.095106	2.100815	2.35704	2.165013	2.18432	0.844371	1	-
ENSMUS(	Pcsk6	proprotei	GO:00325	developm	-	-	-	-	7:6586173	387	433	598	584	456	374	2.814397	2.639462	2.995897	2.766745	2.907918	3.372344	2.816585	3.015669	0.848797	1	-
ENSMUS(	Tarsl2	threonyl-t	GO:00081	metabolic	K01868	threonyl-t	mmu0097	Aminoacy	7:6564489	637	705	883	997	681	453	5.745571	5.330103	5.486629	5.858282	5.386211	5.066146	5.520768	5.43688	0.709577	1	-
ENSMUS(	Tjp1	tight junct	GO:00325	developm	K05701	tight junct	mmu0453	Tight junc	7:6529616	2862	2845	3762	4173	3174	1527	17.08196	14.23322	15.46815	16.2255	16.61184	11.30038	15.59444	14.71257	0.543382	1	-
ENSMUS(	Fam189a1	family witl	GO:00160	membran	-	-	-	-	7:6475609	1976	2250	2546	2992	2131	1614	13.90398	13.27051	12.34133	13.71499	13.14856	14.08127	13.17194	13.64827	0.976762	1	-
ENSMUS(	Apba2	amyloid b	GO:00325	developm	-	-	-	-	7:6450170	4009	4739	5053	5105	4602	2403	41.49577	41.11567	36.03035	34.42272	41.76928	30.8395	39.54726	35.67717	0.297978	1	-
ENSMUS(	Mphosph	M-phase	GO:00081	metabolic	K14559	U3 small r	mmu0300	Ribosome	7:6437652	548	655	820	909	567	227	7.163109	7.17654	7.383909	7.740461	6.499004	3.679025	7.241186	5.97283	0.246316	1	-
ENSMUS(	Mtmr10	myotubul	GO:00056	cell|intrace	-	-	-	-	7:6428765	301	348	578	547	322	232	2.136415	2.070387	2.826172	2.529229	2.004089	2.041701	2.344325	2.191673	0.515697	1	-
ENSMUS(	Trpm1	transient r	GO:00325	developm	-	-	-	-	7:6415383	1	2	1	3	2	1	0.002842	0.004764	0.001957	0.005553	0.004983	0.003522	0.003188	0.004686	0.775289	1	-
ENSMUS(	Chrna7	cholinergi	GO:00325	developm	K04809	nicotinic a	mmu0408	Neuroacti	7:6309869	380	524	537	644	441	365	5.253452	6.072195	5.114312	5.800012	5.346177	6.256633	5.479986	5.800941	0.90544	1	-
ENSMUS(	Crtc3	CREB regu	GO:00081	metabolic	K16334	CREB-reg	mmu0516	HTLV-I int	7:8058662	396	409	474	593	452	351	2.354035	2.037954	1.941097	2.296436	2.356126	2.587084	2.111029	2.413215	0.495676	1	-
ENSMUS(	Blm	Bloom syr	GO:00325	developm	K10901	bloom syr	mmu0346	Fanconi a	7:8045473	79	96	109	128	109	51	0.441144	0.449347	0.419308	0.465638	0.533733	0.353113	0.436599	0.450828	1	1	-
ENSMUS(	Furin	furin (pair	GO:00081	metabolic	K01349	furin[3.4.2	mmu0516	Influenza	7:8038858	830	882	1063	1186	867	754	3.335799	2.971277	2.94311	3.105189	3.05551	3.75733	3.083395	3.306009	0.834218	1	-
ENSMUS(	Hddc3	HD doma	GO:00038	catalytic a	K01139	guanosine	mmu0023	Purine me	7:8034310	103	119	142	144	137	104	0.973084	0.942357	0.924177	0.886257	1.13495	1.218241	0.946539	1.079816	0.608659	1	-
ENSMUS(	Unc45a	unc-45 m	GO:00325	developm	-	-	-	-	7:8032529	371	443	539	606	477	501	2.70051	2.702891	2.70279	2.873599	3.044619	4.521638	2.702064	3.479952	0.155256	1	-
ENSMUS(	Vps33b	vacuolar p	GO:00325	developm	-	-	-	-	7:8026964	227	288	362	394	280	239	2.366941	2.517144	2.600294	2.676334	2.560134	3.089911	2.494793	2.77546	0.643282	1	-
ENSMUS(	Iqgap1	IQ motif c	GO:00325	developm	K16848	Ras GTPas	mmu0481	Regulatio	7:8071158	612	663	1006	1100	721	621	2.200037	1.997768	2.491311	2.57604	2.272775	2.767935	2.229705	2.538917	0.491948	1	-
ENSMUS(	Cib1	calcium ar	GO:00325	developm	-	-	-	-	7:8022714	91	81	117	106	84	154	1.205865	0.899695	1.068054	0.915052	0.976061	2.530256	1.057871	1.473789	0.278186	1	-
ENSMUS(	Sema4b	sema don	GO:00325	developm	K06521	semaphor	mmu0436	Axon guic	7:8018684	288	399	409	422	287	356	2.207343	2.563318	2.159493	2.107033	1.928864	3.383089	2.310051	2.472995	0.925944	1	-
ENSMUS(	Idh2	isocitrate	GO:00325	developm	K00031	isocitrate	mmu0110	Metabolic	7:8009484	380	463	655	571	480	456	4.67482	4.774363	5.551043	4.576143	5.178048	6.955572	5.000075	5.569921	0.671773	1	-
ENSMUS(	Mesp2	mesoderm	GO:00055	extracellul	-	-	-	-	7:7981072	11	12	18	11	17	8	0.35962	0.328843	0.405386	0.23426	0.487331	0.324276	0.364616	0.348622	0.796956	1	-
ENSMUS(	Pex11a	peroxison	GO:00325	developm	K13351	peroxin-1	mmu0414	Peroxisom	7:7973595	157	183	215	238	170	128	1.959893	1.914862	1.848937	1.935495	1.860908	1.9812	1.907898	1.925867	0.851606	1	-
ENSMUS(	Plin1	perilipin 1	GO:00081	metabolic	K08768	perilipin-1	mmu0437	Apelin sig	7:7972021	4	7	4	11	0	2	0.039956	0.05861	0.02753	0.071581	0	0.024779	0.042032	0.03212	0.788333	1	-
ENSMUS(	Rhcg	Rhesus bl	GO:00511	localizatio	-	-	-	-	7:7959336	7	3	6	2	4	8	0.148251	0.05325	0.087529	0.027588	0.074275	0.210061	0.096343	0.103975	1	1	-
ENSMUS(	Nr2f2	nuclear re	GO:00325	developm	-	-	-	-	7:7035194	664	1199	840	811	710	573	3.631601	5.496698	3.164906	2.889565	3.405109	3.885711	4.097735	3.393462	0.137959	1	-
ENSMUS(	Pgpep1l	pyrogluta	GO:00081	metabolic	-	-	-	-	7:6823661	17	12	18	15	14	4	0.907877	0.537203	0.662243	0.521872	0.655629	0.264871	0.702441	0.48079	0.290326	1	-
ENSMUS(	Synm	synemin, i	GO:00051	structural	-	-	-	-	7:6773016	1354	1433	1034	1078	1367	631	4.794982	4.253708	2.522553	2.486961	4.245019	2.770665	3.857081	3.167548	0.210461	1	-
ENSMUS(	Ttc23	tetratricor	-	-	-	-	-	-	7:6764741	51	68	93	91	52	49	0.264716	0.295851	0.332542	0.307707	0.236679	0.315349	0.297703	0.286578	0.677414	1	-
ENSMUS(	Lrrc28	leucine ric	GO:00508	response	-	-	-	-	7:6751341	156	346	392	486	309	273	0.792126	1.472652	1.371222	1.607639	1.375854	1.718778	1.212	1.567424	0.203313	1	-
ENSMUS(	Mef2a	myocyte e	GO:00325	developm	K09260	MADS-bo	mmu0402	cGMP-PK	7:6723116	1619	1898	6001	2836	1805	1129	4.173793	4.101409	10.65759	4.762904	4.08041	3.608809	6.31093	4.150707	0.037338	1	-
ENSMUS(	Rab38	RAB38, m	GO:00325	developm	-	-	-	-	7:8843027	17	24	39	20	30	15	0.501101	0.592992	0.791965	0.384068	0.775438	0.548241	0.628686	0.569249	0.632487	1	-
ENSMUS(	Ctsc	cathepsin	GO:00325	developm	K01275	cathepsin	mmu0421	Apoptosis	7:8827808	186	229	381	362	214	195	1.75046	1.806458	2.470106	2.21937	1.766019	2.275411	2.009008	2.086933	0.993273	1	-
ENSMUS(	Nox4	NADPH o	GO:00325	developm	-	-	mmu0493	AGE-RAG	7:8724609	3	4	1	7	0	2	0.017902	0.020009	0.00411	0.027215	0	0.014803	0.014007	0.014006	1	1	-
ENSMUS(	Cd22	CD22 anti	GO:00055	extracellul	K06467	CD22 anti	mmu0451	Cell adhes	7:3086540	12	9	16	17	12	20	0.071439	0.04491	0.06562	0.065932	0.062646	0.147633	0.060656	0.09207	0.297227	1	-
ENSMUS(	Tyrobp	TYRO pro	GO:00325	developm	K07992	TYRO pro	mmu0438	Osteoclas	7:3041376	84	100	133	160	112	185	4.106483	4.09776	4.479142	5.095598	4.801261	11.21377	4.227795	7.036876	0.03456	1	-
ENSMUS(	Sipa1l3	signal-inc	GO:00325	developm	K17703	signal-ind	mmu0401	Rap1 sign	7:2932037	3955	4041	4882	6484	4475	3362	10.37102	8.882124	8.819102	11.07643	10.28989	10.93097	9.357417	10.76577	0.39056	1	-
ENSMUS(	Dpf1	D4, zinc a	GO:00325	developm	-	-	mmu0522	Hepatoce	7:2930395	601	683	879	942	635	508	3.378027	3.217825	3.403527	3.449223	3.129716	3.540288	3.333127	3.373076	0.832875	1	-
ENSMUS(	2200002D	RIKEN cD	GO:00056	cell|intrace	-	-	-	-	7:2924656	0	0	3	0	0	8	0	0	0.071379	0	0	0.342605	0.023793	0.114202	0.236032	1	-
ENSMUS(	Yif1b	Yip1 inter	GO:00511	localizatio	-	-	-	-	7:2923832	241	277	352	335	276	337	6.792153	6.543676	6.834133	6.150573	6.820895	11.77622	6.723321	8.249228	0.368213	1	-
ENSMUS(	Rasgrp4	RAS guan	GO:00325	developm	K12363	RAS guan	mmu0520	Pathways	7:2913485	12	10	12	16	11	10	0.060171	0.04203	0.041455	0.052266	0.048369	0.062177	0.047885	0.054271	0.85085	1	-
ENSMUS(	Fam98c	family witl	GO:00329	macromo	-	-	-	-	7:2915251	250	352	422	415	314	252	2.990453	3.529328	3.477447	3.233898	3.293584	3.737507	3.332409	3.421663	0.92132	1	-
ENSMUS(	Psmd8	proteasor	GO:00081	metabolic	K03031	26S prote	mmu0516	Epstein-B	7:2917418	1046	1290	1616	1432	1085	895	12.59024	13.01502	13.39969	11.22863	11.45183	13.35707	13.00165	12.01251	0.30962	1	-
ENSMUS(	Ryr1	ryanodine	GO:00325	developm	K04961	ryanodine	mmu0402	Calcium si	7:2900334	658	731	818	1239	846	498	1.778727	1.656356	1.523309	2.181907	2.005378	1.669162	1.652797	1.952149	0.33951	1	-
ENSMUS(	Nfkbib	nuclear fa	GO:00508	response	K02581	NF-kappa	mmu0516	Epstein-B	7:2875825	246	284	338	362	284	247	5.262205	5.092198	4.98083	5.044567	5.327152	6.551129	5.111745	5.640949	0.699088	1	-
ENSMUS(	Fbxo17	F-box pro	GO:00081	metabolic	-	-	-	-	7:2871680	26	17	27	27	17	20	0.341054	0.186917	0.24398	0.230717	0.195536	0.32528	0.257317	0.250511	0.817009	1	-
ENSMUS(	Lrfn1	leucine ric	GO:00325	developm	-	-	-	-	7:2845198	862	974	983	1126	943	1066	7.214891	6.833366	5.667972	6.139639	6.921133	11.06282	6.572077	8.041197	0.318269	1	-
ENSMUS(	Pak4	p21 prote	GO:00325	developm	K05734	p21-activ	mmu0401	Ras signal	7:2855881	293	227	255	492	278	247	3.705683	2.406462	2.221728	4.053658	3.083098	3.873314	2.777958	3.670023	0.164904	1	-
ENSMUS(	Psmc4	proteasor	GO:00325	developm	K03063	26S prote	mmu0516	Epstein-B	7:2804170	864	1257	1650	1607	1147	1115	14.2957	17.43331	18.80733	17.32165	16.6417	22.87454	16.84545	18.94597	0.574765	1	-
ENSMUS(	Zfp626	zinc finge	GO:00081	metabolic	-	-	-	-	7:2780719	209	251	276	341	254	191	2.161524	2.175909	1.96641	2.297471	2.30351	2.449252	2.101281	2.350078	0.612042	1	-
ENSMUS(	Mfge8	milk fat gl	GO:00325	developm	-	-	-	-	7:7913376	1530	1586	2254	1822	1350	1560	12.15147	10.55829	12.33227	9.426872	9.401867	15.36201	11.68068	11.39692	0.581079	1	-
ENSMUS(	Hapln3	hyalurona	GO:00325	developm	-	-	-	-	7:7911510	23	23	24	22	15	25	0.299715	0.251225	0.215449	0.186763	0.171401	0.403928	0.255463	0.254031	0.825484	1	-
ENSMUS(	Acan	aggrecan	GO:00325	developm	-	-	-	-	7:7905348	124	131	170	215	139	94	0.840057	0.743893	0.79339	0.948872	0.825745	0.789592	0.792446	0.854736	0.794226	1	-
ENSMUS(	Aen	apoptosis	GO:00081	metabolic	-	-	-	-	7:7889585	341	472	504	608	422	258	2.04141	2.368484	2.078535	2.371159	2.215288	1.915054	2.16281	2.167167	0.839528	1	-
ENSMUS(	Det1	de-etiolat	GO:00081	metabolic	K10571	de-etiolat	mmu0412	Ubiquitin	7:7882154	67	52	80	101	76	37	0.831651	0.541026	0.684079	0.816712	0.827225	0.569443	0.685585	0.737793	0.828569	1	-
ENSMUS(	Mrps11	mitochon	GO:00081	metabolic	K02948	small subu	mmu0301	Ribosome	7:7878311	174	231	261	228	170	223	2.923916	3.253714	3.021391	2.495928	2.504992	4.646278	3.06634	3.215733	0.96422	1	-
ENSMUS(	Mrpl46	mitochon	GO:00051	structural	-	-	-	-	7:7877523	313	463	482	505	381	309	12.90854	16.00542	13.69404	13.5677	13.77847	15.80077	14.20267	14.38232	0.836741	1	-
ENSMUS(	Ccdc90b	coiled-co	GO:00160	membran	-	-	-	-	7:9256114	171	229	275	308	189	154	6.034178	6.773464	6.685078	7.080354	5.848251	6.737983	6.497573	6.555529	0.859701	1	-
ENSMUS(	Tmem126	transmem	GO:00160	membran	-	-	-	-	7:9046743	530	672	862	784	602	440	6.531529	6.941623	7.318088	6.294141	6.505464	6.72323	6.930413	6.507612	0.423941	1	-
ENSMUS(	Tmem126	transmem	GO:00325	developm	-	-	-	-	7:9045070	168	175	222	213	160	87	3.059289	2.671165	2.784935	2.526811	2.554894	1.964339	2.838463	2.348682	0.194195	1	-
ENSMUS(	Sytl2	synaptota	GO:00081	metabolic	-	-	-	-	7:9030225	385	407	697	613	529	411	1.100769	0.975399	1.372841	1.141767	1.326276	1.457012	1.14967	1.308352	0.540912	1	-
ENSMUS(	Eed	embryoni	GO:00325	developm	-	-	-	-	7:8995465	187	230	317	284	223	129	1.830344	1.886998	2.137477	1.810886	1.913984	1.565542	1.951606	1.763471	0.394743	1	-
ENSMUS(	Me3	malic enz	GO:00081	metabolic	K00029	malate de	mmu0110	Metabolic	7:8963239	676	842	954	1104	863	704	2.752022	2.873229	2.675502	2.927899	3.080768	3.553562	2.766917	3.18741	0.423478	1	-
ENSMUS(	Zfand6	zinc finge	GO:00511	localizatio	-	-	-	-	7:8461376	410	708	717	700	512	396	3.763248	5.447099	4.533666	4.185612	4.120897	4.506721	4.581337	4.271077	0.437442	1	-
ENSMUS(	Fah	fumarylac	GO:00081	metabolic	K01555	fumarylac	mmu0110	Metabolic	7:8458515	230	252	304	296	251	190	5.056106	4.643456	4.603764	4.238976	4.838428	5.178788	4.767775	4.752064	0.75959	1	-
ENSMUS(	Sh3gl3	SH3-dom	GO:00511	localizatio	-	-	-	-	7:8217384	592	799	742	925	667	420	6.248693	7.069147	5.395392	6.360496	6.173567	5.496714	6.237744	6.010259	0.611285	1	-
ENSMUS(	Ddias	DNA dam	-	-	-	-	-	-	7:9285752	3	7	23	10	9	3	0.045346	0.088696	0.239529	0.098479	0.119309	0.056237	0.124524	0.091342	0.467493	1	-
ENSMUS(	Rab30	RAB30, m	GO:00038	catalytic a	-	-	-	-	7:9274160	994	1224	1650	1685	1178	652	5.135494	5.300662	5.872608	5.67123	5.336835	4.176661	5.436255	5.061575	0.462782	1	-
ENSMUS(	Ndufc2	NADH de	GO:00081	metabolic	K03968	NADH de	mmu0110	Metabolic	7:9740000	411	598	678	580	505	842	22.6542	27.62874	25.74472	20.82651	24.40847	57.54465	25.34256	34.25987	0.238017	1	-
ENSMUS(	Anapc15	anaphase	GO:00329	macromo	-	-	-	-	7:1018637	181	209	265	292	203	209	0.963327	0.932383	0.971611	1.012419	0.9474	1.379204	0.955774	1.113008	0.494425	1	-
ENSMUS(	Tmc5	transmem	GO:00511	localizatio	-	-	-	-	7:1185972	6	3	2	5	6	0	0.062279	0.026099	0.014302	0.033811	0.054605	0	0.034227	0.029472	1	1	-
ENSMUS(	Art2b	ADP-ribo	GO:00081	metabolic	K19980	ADP-ribo	mmu0110	Metabolic	7:1015769	2	2	0	0	0	0	0.034462	0.02888	0	0	0	0	0.021114	0	0.156187	1	-
ENSMUS(	Coq7	demethyl	GO:00325	developm	K06134	ubiquinor	mmu0110	Metabolic	7:1185096	294	340	401	412	281	254	2.418713	2.344602	2.272656	2.208087	2.027151	2.590941	2.345324	2.275393	0.602782	1	-
ENSMUS(	Gm45837	predicted	GO:00508	response	-	-	-	-	7:1014108	657	181	183	487	387	124	10.08802	2.329555	1.935721	4.871372	5.210673	2.360737	4.784431	4.147594	0.628911	1	-
ENSMUS(	Arl6ip1	ADP-ribo	GO:00081	metabolic	-	-	-	-	7:1181188	3330	4121	5568	4855	3743	3585	60.12258	62.36615	69.25388	57.10371	59.25923	80.25435	63.91421	65.5391	0.86297	1	-
ENSMUS(	Smg1	SMG1 hor	GO:00081	metabolic	K08873	serine/thr	mmu0301	mRNA sur	7:1181313	3868	5197	6396	7113	5350	3295	8.671619	9.766058	9.878104	10.38839	10.51744	9.159148	9.438594	10.02166	0.83125	1	-
ENSMUS(	Xylt1	xylosyltrar	GO:00081	metabolic	K00771	protein xy	mmu0110	Metabolic	7:1173809	1742	1853	2109	2179	1765	1277	10.66358	9.507875	8.893713	8.689484	9.474197	9.692412	9.688391	9.285364	0.497494	1	-
ENSMUS(	Nucb2	nucleobin	GO:00099	cellular pr	-	-	-	-	7:1165043	312	439	484	570	391	181	6.692056	7.892632	7.151566	7.964548	7.354009	4.813603	7.245418	6.71072	0.547624	1	-
ENSMUS(	Pik3c2a	phosphati	GO:00081	metabolic	K00923	phosphati	mmu0110	Metabolic	7:1163372	630	669	872	961	661	378	2.481001	2.208337	2.365671	2.465422	2.282603	1.845713	2.35167	2.197912	0.490943	1	-
ENSMUS(	Ipo5	importin 5	GO:00511	localizatio	-	-	-	-	14:120911	3018	3636	4352	4398	3309	2493	27.18296	27.45075	27.00337	25.80563	26.13467	27.84106	27.21236	26.59379	0.578018	1	-
ENSMUS(	1110004F	RIKEN cD	-	-	-	-	-	-	7:1160393	1072	1436	1605	1645	1181	1176	14.52949	16.31411	14.98588	14.52458	14.03617	19.76283	15.27649	16.10786	0.958864	1	-
ENSMUS(	Calcb	calcitonin	GO:00508	response	-	-	-	-	7:1147186	4	4	5	0	2	3	0.155952	0.13072	0.134301	0	0.068374	0.145042	0.140324	0.071139	0.231192	1	-
ENSMUS(	Calca	calcitonin	GO:00325	developm	-	-	-	-	7:1146314	5	13	20	21	13	11	0.090999	0.198322	0.250757	0.248982	0.207467	0.248228	0.180026	0.234892	0.509309	1	-
ENSMUS(	Cyp2r1	cytochron	GO:00081	metabolic	K07419	vitamin D	mmu0110	Metabolic	7:1145496	11	10	15	17	7	6	0.151485	0.115424	0.142304	0.152507	0.084534	0.102457	0.136404	0.113166	0.62459	1	-
ENSMUS(	Pde3b	phosphoc	GO:00325	developm	K13296	cGMP-inh	mmu0402	cAMP sigr	7:1144152	346	381	562	583	394	187	2.172648	2.00535	2.431088	2.384858	2.16946	1.455929	2.203029	2.003416	0.472208	1	-
ENSMUS(	Mylpf	myosin lig	GO:00325	developm	K12758	fast skelet	mmu0481	Regulatio	7:1272088	15	5	11	4	10	8	0.542266	0.151502	0.27394	0.094213	0.317008	0.358585	0.322569	0.256602	0.60257	1	-
ENSMUS(	Qprt	quinolinat	GO:00081	metabolic	K00767	nicotinate	mmu0110	Metabolic	7:1271071	3	11	8	11	14	5	0.063334	0.194686	0.116362	0.151296	0.259202	0.130888	0.124794	0.180462	0.401837	1	-
ENSMUS(	Kif22	kinesin far	GO:00081	metabolic	K10403	kinesin far	mmu0491	Progester	7:1270277	15	15	19	21	14	21	0.136109	0.114089	0.118766	0.124136	0.111393	0.236259	0.122988	0.157263	0.58195	1	-
ENSMUS(	Maz	MYC-assc	GO:00081	metabolic	-	-	-	-	7:1270221	1891	2338	2638	3018	1713	1914	18.53466	19.20837	17.81227	19.27055	14.72289	23.2606	18.51843	19.08468	0.904463	1	-
ENSMUS(	Mvp	major vau	GO:00081	metabolic	-	-	-	-	7:1269868	148	191	263	248	188	178	1.209133	1.307971	1.4802	1.319914	1.346828	1.803096	1.332435	1.489946	0.676489	1	-
ENSMUS(	Cdipt	CDP-diac	GO:00081	metabolic	K00999	CDP-diac	mmu0110	Metabolic	7:1269759	1115	1423	1160	1689	1378	734	9.813264	10.49776	7.033123	9.683897	10.63483	8.009788	9.114715	9.44284	0.988365	1	-
ENSMUS(	Sez6l2	seizure re	GO:00325	developm	-	-	-	-	7:1269505	5390	6096	7079	8167	6289	5388	48.27378	45.76362	43.6763	47.65042	49.39088	59.83234	45.90457	52.29121	0.451413	1	-
ENSMUS(	Kctd13	potassium	GO:00081	metabolic	-	-	-	-	7:1269288	1978	2357	2956	3073	2222	1958	42.26896	42.21897	43.5162	42.77986	41.63723	51.87928	42.66804	45.43212	0.864177	1	-
ENSMUS(	Stard10	START do	GO:00511	localizatio	-	-	-	-	7:1013170	195	246	298	292	251	197	2.052472	2.170358	2.160783	2.002202	2.316644	2.570966	2.127871	2.296604	0.82569	1	-

ENSMUSC	Ino80e	INO80 co	GO:00329	macromo	-	-	-	-	7:1268509	331	369	432	393	397	272	1.576617	1.472319	1.416632	1.2187	1.657125	1.605375	1.488189	1.493733	0.795564	1	-
ENSMUSC	Fchsd2	FCH and	GO:00452	synapse|c	-	-	-	-	7:1010928	1016	1136	1280	1306	1010	545	3.11255	2.917116	2.701369	2.606439	2.713231	2.070165	2.910345	2.463278	0.14943	1	-
ENSMUSC	Klk10	kallikrein	GO:00081	metabolic	-	-	-	-	7:4378103	11	9	11	16	8	8	0.509178	0.349173	0.350755	0.482464	0.324728	0.459135	0.403035	0.422109	1	1	-
ENSMUSC	Aldoa	aldolase A	GO:00325	developm	K01623	fructose-b	mmu0110	Metabolic	7:1267952	18204	23626	24197	25206	24479	16487	198.0686	215.4726	181.3685	178.6629	233.5529	222.4214	198.3032	211.5457	0.838938	1	-
ENSMUSC	Ppp4c	protein ph	GO:00048	signal trar	K15423	serine/thr	mmu0492	Glucagon	7:1267858	259	330	401	349	327	331	2.028123	2.166013	2.163168	1.780332	2.245353	3.213717	2.119101	2.413134	0.619306	1	-
ENSMUSC	Tbx6	T-box 6 [	GO:00325	developm	-	-	-	-	7:1267814	10	17	19	26	17	11	0.278744	0.397199	0.36484	0.472118	0.415514	0.380181	0.346927	0.422604	0.590281	1	-
ENSMUSC	Plekhb1	pleckstrin	GO:00325	developm	-	-	-	-	7:1006428	2368	1702	3704	2827	1904	1739	16.08009	9.687662	17.32723	12.50588	11.33747	14.64172	14.36499	12.82836	0.297937	1	-
ENSMUSC	Gdpd3	glyceroph	GO:00081	metabolic	-	-	-	-	7:1267663	15	15	29	25	13	9	0.285335	0.239173	0.380023	0.309797	0.216843	0.212263	0.30151	0.246301	0.435419	1	-
ENSMUSC	Rab6a	RAB6A, m	GO:00081	metabolic	-	-	-	-	7:1006074	5511	6811	8351	7714	6668	2780	77.23056	80.006	80.62111	70.4239	81.94021	48.30472	79.28589	66.88961	0.188082	1	-
ENSMUSC	Mrpl48	mitochon	GO:00329	macromo	-	-	-	-	7:1005457	650	785	917	986	646	626	3.539497	3.583033	3.439924	3.497734	3.08463	4.226569	3.520818	3.602978	0.872184	1	-
ENSMUSC	Coro1a	coronin, a	GO:00325	developm	K13882	coronin-1	mmu0515	Tuberculo	7:1266997	554	718	763	787	625	1135	6.800922	7.388139	6.45259	6.293821	6.727922	17.27587	6.88055	10.09921	0.117701	1	-
ENSMUSC	Dnajb13	DnaJ heat	GO:00099	cellular pr	-	-	-	-	7:1005017	16	6	16	7	10	15	0.275425	0.086568	0.189734	0.078498	0.150948	0.320154	0.183909	0.1832	0.888141	1	-
ENSMUSC	Sult1a1	sulfotrans	GO:00038	catalytic a	K01014	aryl sulfot	mmu0520	Chemical	7:1266728	104	105	144	140	117	67	2.16138	1.829114	2.061632	1.895431	2.132198	1.726476	2.017375	1.918035	0.632431	1	-
ENSMUSC	Klk7	kallikrein	GO:00081	metabolic	-	-	-	-	7:4381129	3	13	7	17	6	6	0.100599	0.365456	0.161733	0.371409	0.176436	0.249519	0.209263	0.265788	0.613183	1	-
ENSMUSC	Sgf29	SAGA con	GO:00081	metabolic	-	-	-	-	7:1266493	113	131	189	199	153	137	1.915059	1.860922	2.206578	2.197051	2.273736	2.878789	1.994186	2.449858	0.320239	1	-
ENSMUSC	Nupr1	nuclear pr	GO:00325	developm	K15626	nuclear pr	mmu0520	Transcript	7:1266232	54	92	82	109	45	84	0.673182	0.961336	0.704209	0.885205	0.491912	1.298377	0.779576	0.891831	0.747291	1	-
ENSMUSC	Ppme1	protein ph	GO:00081	metabolic	-	-	-	-	7:1003267	2285	2698	3111	3537	2338	2296	31.53499	31.21054	29.57726	31.79971	28.29396	39.28841	30.77426	33.12736	0.808181	1	-
ENSMUSC	Cln3	ceroid lipo	GO:00081	metabolic	K12389	battenin[-	mmu0414	Lysosome	7:1265712	112	124	153	130	94	128	0.622526	0.577714	0.585843	0.470721	0.45815	0.882131	0.595361	0.603667	0.829133	1	-
ENSMUSC	Nfatc2ip	nuclear fa	GO:00081	metabolic	-	-	-	-	7:1263828	81	122	150	165	95	120	1.346532	1.699985	1.71781	1.786885	1.384832	2.473422	1.588109	1.881713	0.516122	1	-
ENSMUSC	Cd19	CD19 anti	GO:00048	signal trar	K06465	B-lympho	mmu0415	PI3K-Akt	7:1264084	0	3	0	0	0	0	0	0.031236	0	0	0	0	0.010412	0	0.304895	1	-
ENSMUSC	Lipt2	lipoyl(octa	GO:00081	metabolic	K03801	lipoyl(octa	mmu0110	Metabolic	7:1001592	47	40	61	58	42	60	1.296433	0.924842	1.159136	1.042238	1.015883	2.05206	1.126804	1.37006	0.584079	1	-
ENSMUSC	Pold3	polymeras	GO:00081	metabolic	K03504	DNA poly	mmu0110	Metabolic	7:1000821	420	519	544	637	483	269	3.542907	3.669702	3.161266	3.50051	3.572733	2.813516	3.457958	3.295586	0.588997	1	-
ENSMUSC	Rabep2	rabaptin,	GO:00511	localizatio	-	-	-	-	7:1264287	222	238	312	295	232	168	1.824013	1.639098	1.765964	1.578989	1.671503	1.711474	1.743025	1.653989	0.535832	1	-
ENSMUSC	Pgm2l1	phosphog	GO:00081	metabolic	K11809	glucose-1	mmu0050	Starch and	7:1002273	8209	10865	12444	14109	10121	5215	40.03274	44.41275	41.80579	44.82321	43.28039	31.533	42.08376	39.87887	0.539497	1	-
ENSMUSC	Atp2a1	ATPase, C	GO:00511	localizatio	K05853	Ca2+ tran	mmu0402	Calcium si	7:1264458	1	4	5	5	0	1	0.016781	0.056249	0.057791	0.054652	0	0.020797	0.043607	0.02515	0.496154	1	-
ENSMUSC	Syt3	synaptota	GO:00325	developm	-	-	-	-	7:4438410	772	833	1008	984	814	984	10.73117	9.705721	9.652542	8.91059	9.92195	16.95943	10.02981	11.93066	0.435332	1	-
ENSMUSC	Chrdl2	chordin-li	GO:00055	extracellul	-	-	-	-	7:1000061	5	6	7	7	7	2	0.078056	0.078509	0.075285	0.07119	0.095831	0.038723	0.077283	0.068581	0.768163	1	-
ENSMUSC	Sh2b1	SH2B ada	GO:00048	signal trar	K12459	SH2B ada	mmu0472	Neurotrop	7:1264669	1418	1719	1749	2175	1672	1217	9.374615	9.525896	7.9656	9.367377	9.692952	9.975936	8.95537	9.678755	0.752528	1	-
ENSMUSC	Slco2b1	solute car	GO:00325	developm	-	-	-	-	7:9965780	337	317	534	578	427	299	2.377063	1.874231	2.594801	2.655956	2.641079	2.614979	2.282032	2.637338	0.452826	1	-
ENSMUSC	Eif3c	eukaryotic	GO:00081	metabolic	K03252	translation	mmu0301	RNA trans	7:1265464	3529	4285	5110	5541	4029	2503	57.64035	58.66493	57.49727	58.95826	57.70518	50.68994	57.93418	55.78446	0.549969	1	-
ENSMUSC	Myh14	myosin, h	GO:00325	developm	K10352	myosin he	mmu0481	Regulation	7:4460580	810	763	1236	1248	998	744	4.679811	3.695057	4.919415	4.697205	5.056112	5.329693	4.431427	5.02767	0.507427	1	-
ENSMUSC	Spns1	spinster h	GO:00400	locomotic	-	-	-	-	7:1263700	453	585	695	757	563	823	3.53636	3.827957	3.737616	3.84978	3.853981	7.96607	3.700644	5.223277	0.091158	1	-
ENSMUSC	Lat	linker for	GO:00048	signal trar	K07362	linker for	mmu0401	Ras signal	7:1263638	6	7	4	12	6	8	0.074189	0.072544	0.034075	0.096661	0.065048	0.122645	0.06027	0.094785	0.318979	1	-
ENSMUSC	Rps3	ribosomal	GO:00081	metabolic	K02985	small subu	mmu0301	Ribosome	7:9947789	2102	2600	3314	3196	2516	2911	20.53943	21.2952	22.30794	20.34436	21.55802	35.26823	21.38086	25.72354	0.325686	1	-
ENSMUSC	Il21r	interleukin	GO:00048	signal trar	K05075	interleukin	mmu0406	Cytokine-	7:1256034	29	29	27	36	37	34	0.362233	0.303623	0.232328	0.292939	0.405262	0.526575	0.299394	0.408259	0.292789	1	-
ENSMUSC	Dgat2	diacylglyc	GO:00325	developm	K11160	diacylglyc	mmu0110	Metabolic	7:9915365	935	1138	1378	1636	1152	1318	15.13552	15.44119	15.3669	17.25245	16.35239	26.45376	15.31454	20.01953	0.103655	1	-
ENSMUSC	Il4ra	interleukin	GO:00325	developm	K05071	interleukin	mmu0520	Pathways	7:1255521	59	80	120	92	93	124	0.40944	0.465349	0.573676	0.415916	0.565927	1.066946	0.482822	0.68293	0.212472	1	-
ENSMUSC	Nsmce1	NSE1 hon	GO:00081	metabolic	-	-	-	-	7:1254676	83	138	150	162	123	147	3.70951	5.169752	4.618276	4.716646	4.8204	8.145915	4.49918	5.89432	0.243181	1	-
ENSMUSC	Psma1	proteason	GO:00081	metabolic	K02725	20S prote	mmu0305	Proteason	7:1142646	759	936	1173	1039	885	716	13.09358	13.5346	13.9401	11.67652	13.38759	15.31494	13.52276	13.45968	0.702202	1	-
ENSMUSC	Kdm8	lysine (K)-	GO:00081	metabolic	-	-	-	-	7:1254446	36	72	95	92	58	76	0.864983	1.450084	1.572482	1.440059	1.222019	2.264171	1.29585	1.642083	0.414208	1	-
ENSMUSC	Thap12	THAP dor	GO:00081	metabolic	-	-	-	-	7:9870310	1377	1711	2205	2291	1697	1003	16.78092	17.47772	18.51151	18.18815	18.13453	15.15544	17.59005	17.15937	0.652666	1	-
ENSMUSC	Copb1	coatomer	GO:00511	localizatio	-	-	-	-	7:1142155	1194	1443	1798	1678	1193	744	12.31456	12.4748	12.77484	11.27426	10.78941	9.514221	12.5214	10.52596	0.103219	1	-
ENSMUSC	Zkscan2	zinc finge	GO:00081	metabolic	-	-	-	-	7:1234753	375	437	458	673	453	449	1.850338	1.807401	1.556815	2.163301	1.960021	2.746962	1.738185	2.290094	0.098707	1	-
ENSMUSC	Acer3	alkaline ce	GO:00081	metabolic	K04711	dihydroce	mmu0060	Sphingolip	7:9820638	379	528	620	647	492	277	1.231926	1.438572	1.388316	1.370033	1.402337	1.116378	1.352938	1.296249	0.609838	1	-
ENSMUSC	Myo7a	myosin VI	GO:00325	developm	-	-	-	-	7:9805106	489	431	719	655	478	394	2.588924	1.912673	2.622349	2.259088	2.219118	2.586379	2.374649	2.354862	0.726871	1	-
ENSMUSC	Lcmt1	leucine ca	GO:00081	metabolic	-	-	-	-	7:1233697	600	890	940	957	666	626	8.032818	9.987573	8.669546	8.346618	7.81869	10.39147	8.896645	8.852258	0.710261	1	-
ENSMUSC	Arhgap17	Rho GTPa	GO:00511	localizatio	K20638	Rho GTPa	mmu0453	Tight junc	7:1232792	309	381	403	463	352	272	1.368683	1.414563	1.229704	1.336003	1.367195	1.493822	1.33765	1.399007	0.978389	1	-
ENSMUSC	Disp1	dispatche	GO:00325	developm	-	-	-	-	1:1830862	95	116	141	175	108	67	0.981074	1.004133	1.003109	1.177333	0.978012	0.857907	0.996105	1.004417	0.932458	1	-
ENSMUSC	Slc5a11	solute car	GO:00511	localizatio	-	-	-	-	7:1232147	3	3	0	6	9	4	0.047387	0.039723	0	0.06175	0.124677	0.078349	0.029036	0.088258	0.041807	1	-
ENSMUSC	Parva	parvin, alp	GO:00325	developm	K06275	parvin[-]	mmu0451	Focal adh	7:1124275	1418	1573	2067	2097	599	882	7.398257	6.879148	7.429252	7.127433	2.740457	5.705685	7.235552	5.191192	0.047988	1	-
ENSMUSC	Micalcl	MICAL C-	GO:00325	developm	-	-	-	-	7:1123683	12	42	18	9	3	20	0.142757	0.418827	0.147523	0.069751	0.031294	0.295017	0.236369	0.132021	0.158256	1	-
ENSMUSC	Dkk3	dickkopf \	GO:00325	developm	-	-	-	-	7:1121160	5474	6840	8875	9215	6878	5963	103.5693	108.4763	115.6767	113.5803	114.1119	139.8868	109.2408	122.5264	0.526002	1	-
ENSMUSC	Pak1	p21 prote	GO:00325	developm	K04409	p21-activ	mmu0401	MAPK sig	7:9778854	4450	5822	7109	7631	5357	3535	51.82535	56.83387	57.03509	57.89556	54.70745	51.04551	55.23144	54.5495	0.69365	1	-
ENSMUSC	Trat1	T cell rece	GO:00081	metabolic	-	-	-	-	16:487346	3	0	2	1	0	2	0.278708	0	0.128031	0.060521	0	0.23047	0.135579	0.096997	0.762889	1	-
ENSMUSC	Rbbp6	retinoblas	GO:00325	developm	-	-	-	-	7:1229656	2012	2572	3166	3320	2590	1361	7.256193	7.775078	7.865807	7.800109	8.190744	6.085901	7.632359	7.358918	0.612515	1	-
ENSMUSC	BC017158	cDNA seq	GO:00160	membran	-	-	-	-	7:1282713	250	308	400	411	353	275	1.721933	1.778196	1.897958	1.844161	2.132029	2.348518	1.799362	2.108236	0.406718	1	-
ENSMUSC	Slc5a2	solute car	GO:00511	localizatio	-	-	-	-	7:1282656	0	1	0	3	1	4	0	0.008878	0	0.020698	0.009287	0.052533	0.002959	0.027506	0.066219	1	-
ENSMUSC	Tgfb1i1	transform	GO:00325	developm	-	-	-	-	7:1282468	123	184	199	229	170	143	0.762486	0.95609	0.849829	0.924795	0.924101	1.099131	0.856135	0.982676	0.54413	1	-
ENSMUSC	Cox6a2	cytochron	GO:00081	metabolic	K02266	cytochron	mmu0110	Metabolic	7:1282054	40	66	85	65	57	62	3.738447	5.170386	5.472638	3.957487	4.671348	7.184689	4.793824	5.271174	0.874082	1	-
ENSMUSC	Itgam	integrin al	GO:00508	response	K06461	integrin al	mmu0481	Regulation	7:1280626	439	454	566	341	544	159	3.871204	3.355757	3.438344	1.958923	4.206519	1.73846	3.555102	2.634634	0.122835	1	-
ENSMUSC	Lyve1	lymphatic	GO:00048	signal trar	-	-	-	-	7:1108506	25	22	30	30	15	9	0.469238	0.346118	0.387897	0.366821	0.246876	0.209442	0.401084	0.27438	0.146484	1	-
ENSMUSC	Rnf141	ring finge	GO:00081	metabolic	-	-	-	-	7:1108004	1129	1366	1773	1395	1337	920	4.832064	4.900522	5.227561	3.88951	5.017797	4.882165	4.986716	4.596491	0.327792	1	-
ENSMUSC	Itgax	integrin al	GO:00511	localizatio	K06462	integrin al	mmu0481	Regulation	7:1281295	15	22	44	54	23	19	0.179699	0.220915	0.363127	0.421431	0.241616	0.282225	0.25458	0.315091	0.533808	1	-
ENSMUSC	Adm	adrenome	GO:00325	developm	-	-	-	-	7:1106276	20	24	21	39	21	17	0.719746	0.723947	0.520616	0.914305	0.662698	0.75853	0.65477	0.778511	0.55872	1	-
ENSMUSC	Dkkl1	dickkopf-	GO:00325	developm	-	-	-	-	7:4520751	15	20	32	23	39	26	0.330206	0.36904	0.485277	0.329837	0.75283	0.709671	0.394841	0.597446	0.212122	1	-
ENSMUSC	Pycard	PYD and	GO:00081	metabolic	K12799	apoptosis	mmu0421	Necroptos	7:1279897	34	56	62	76	63	42	0.510106	0.704245	0.640796	0.742808	0.828829	0.781305	0.618382	0.784314	0.300366	1	-
ENSMUSC	Fus	fused in s	GO:00081	metabolic	K13098	RNA-binc	mmu0520	Transcript	7:1279674	3136	3804	4273	5338	3839	1538	9.541471	9.70136	8.956197	10.58034	10.24236	5.802052	9.399676	8.874916	0.616677	1	-
ENSMUSC	Tead2	TEA doma	GO:00325	developm	K09448	transcripti	mmu0439	Hippo sig	7:4521575	40	43	58	62	54	39	0.281928	0.254036	0.281614	0.284674	0.333741	0.340821	0.272526	0.319745	0.540871	1	-
ENSMUSC	Cd37	CD37 anti	GO:00023	immune s	K06475	CD37 anti	mmu0464	Hematopo	7:4523363	52	64	69	92	62	72	0.485279	0.500635	0.443597	0.559318	0.507371	0.833118	0.476504	0.633269	0.229143	1	-
ENSMUSC	Prss8	protease,	GO:00081	metabolic	-	-	-	-	7:1279257	1	4	7	10	6	9	0.025215	0.084519	0.121572	0.164222	0.132625	0.281305	0.077102	0.192717	0.074932	1	-
ENSMUSC	Kat8	K(lysine) a	GO:00325	developm	-	-	-	-	7:1279125	234	320	423	356	301	241	4.41419	5.059849	5.497014	4.374885	4.979026	5.636881	4.990351	4.996931	0.773812	1	-
ENSMUSC	Bckdk	branched	GO:00081	metabolic	-	-	-	-	7:1279040	410	551	476	545	522	407	4.203376	4.734985	3.361807	3.63993	4.692755	5.173619	4.100056	4.502101	0.743444	1	-
ENSMUSC	Stx4a	syntaxin 4	GO:00452	synapse|c	K13502	syntaxin 4	mmu0496	Vasopress	7:1278242	524	616	803	741	620	449	7.460072	7.350979	7.875516	6.872454	7.740093	7.925821	7.562189	7.512789	0.718649	1	-
ENSMUSC	Stx1b	syntaxin 1	GO:00325	developm	K08486	syntaxin 1	mmu0472	Synaptic v	7:1278039	10732	13224	16042	17371	12816	9547	137.6213	142.1414	141.7147	145.1147	144.112	151.7951	140.4924	147.0072	0.952598	1	-
ENSMUSC	Fbxl19	F-box and	GO:00081	metabolic	-	-	-	-	7:1277445	764	888	1049	994	826	774	4.656613	4.536724	4.404572	3.946798	4.414683	5.849299	4.532636	4.736927	0.98238	1	-
ENSMUSC	Bcl7c	B cell CLL	GO:00099	cellular pr	-	-	-	-	7:1276614	204	214	267	291	180	120	1.464122	1.287401	1.32011	1.360576	1.132827	1.067859	1.357211	1.187087	0.300047	1	-
ENSMUSC	Phkg2	phosphor	GO:00081	metabolic	K00871	phosphor	mmu0402	Calcium si	7:1275733	636	737	925	882	761	543	4.43296	4.305828	4.4415	4.004854	4.651188	4.692699	4.39343	4.44958	0.830816	1	-
ENSMUSC	Rnf40	ring finge	GO:00081	metabolic	-	-	-	-	7:1275887	1196	1357	1741	1808	1310	1031	8.199501	7.798106	8.222553	8.074887	7.875361	8.763969	8.073386	8.238072	0.86808	1	-
ENSMUSC	Prr14	proline ric	GO:00325	developm	-	-	-	-	7:1274596	318	388	475	494	435	270	2.374276	2.428229	2.443148	2.402775	2.847976	2.499507	2.415217	2.583419	0.82645	1	-
ENSMUSC	9130019C	RIKEN cD	GO:00081	metabolic	-	-	-	-	7:1273822	60	65	67	102	65	65	0.586729	0.532789	0.45135	0.649786	0.557373	0.788109	0.523623	0.665089	0.2981	1	-
ENSMUSC	Nucb1	nucleobin	GO:00511	localizatio	-	-	-	-	7:4549045	1346	1621	1958	1833	1428	1099	8.033654	8.109686	8.050674	7.127088	7.473759	8.133014	8.064672	7.577954	0.375319	1	-
ENSMUSC	Hsd17b14	hydroxyst	GO:00081	metabolic	-	-	-	-	7:4555489	0	0	2	2	0	3	0	0	0.026369	0.02493	0	0.071189	0.00879	0.03204	0.515833	1	-
ENSMUSC	Bcat2	branched	GO:00081	metabolic	K00826	branched	mmu0110	Metabolic	7:4557015	117	183	212	221	152	222	1.351203	1.7715	1.686644	1.66269	1.539303	3.178893	1.603116	2.126962	0.240676	1	-
ENSMUSC	Itgal	integrin al	GO:00048	signal trar	K05718	integrin al	mmu0516	HTLV-I inf	7:1272962	8	3	16	18	21	7	0.053931	0.016952	0.074307	0.079051	0.124146	0.058514	0.048397	0.087237	0.160298	1	-
ENSMUSC	Abcc6	ATP-bind	GO:00511	localizatio	K05669	ATP-bind	mmu0201	ABC trans	7:4596755	14	29	15	29	26	18	0.13123	0.227851	0.096863	0.177088	0.213709	0.209208	0.151981	0.200002	0.398083	1	-
ENSMUSC	Nomo1	nodal mo	GO:00160	membran	-	-	-	-	7:4603369	2359	2831	3319	3450	2612	2389	21.55594	21.68361	20.89285	20.53713	20.92931	27.06706	21.37747	22.8445	0.854449	1	-
ENSMUSC	Ush1c	USH1 pro	GO:00511	localizatio	-	-	-	-	7:4619535	0	0	3	2	0	0	0	0	0.024604	0.01551	0	0	0.008201	0.00517	1	1	-
ENSMUSC	Sergef	secretion	GO:00511	localizatio	-	-	-	-	7:4644315	204	320	275	282	242	234	2.688676	3.535178	2.496853	2.421246	2.796831	3.823939	2.906903	3.014005	0.942844	1	-
ENSMUSC	Lamtor1	late endos	GO:00081	metabolic	K20397	ragulator	mmu0415	mTOR sig	7:1019059	704	778	927	1048	785	797	13.29609	12.31639	12.06097	12.89419	13.0006	18.66358	12.55782	14.85279	0.366089	1	-
ENSMUSC	Rgs10	regulator	GO:00452	synapse|n	-	-	-	-	7:1283736	281	287	361	421	273	262	7.33404	6.278737	6.490771	7.158143	6.248026	8.478608	6.701183	7.294926	0.77892	1	-
ENSMUSC	Tial1	Tia1 cytot	GO:00325	developm	-	-	-	-	7:1284397	1382	1495	2192	2203	1518	998	5.132969	4.654306	5.608576	5.330364	4.943961	4.59597	5.13195	4.956765	0.583289	1	-
ENSMUSC	Bag3	BCL2-ass	GO:00325	developm	-	-	-	-	7:1285236	205	224	333	278	220	133	5.085232	4.657559	5.690529	4.492452	4.785451	4.090656	5.14444	4.456186	0.253525	1	-
ENSMUSC	Fgfr2	fibroblast	GO:00325	developm	K05093	fibroblast	mmu0520	Pathways	7:1301624	620	602	1037	989	662	512	2.274992	1.85156	2.621309	2.364102	2.130044	2.329401	2.249287	2.274516	0.848649	1	-
ENSMUSC	Ate1	arginyltra	GO:00081	metabolic	-	-	-	-	7:1303914	1413	1615	1935	2089	1520	1201	12.57885	12.05103	11.86673	12.11487	11.86547	13.25646	12.16554	12.41227	0.86372	1	-
ENSMUSC	Tacc2	transform	GO:00325	developm	-	-	-	-	7:1305774	1390	1159	1320	1853	1148	1004	3.900163	2.725863	2.551486	3.387077	2.82457	3.492913	3.059171	3.234853	0.922342	1	-
ENSMUSC	Ptpn5	protein ty	GO:00081	metabolic	K18018	tyrosine-p	mmu0401	MAPK sig	7:4707779	1805	2425	2507	2721	2169	1860	10.78943	12.15026	10.3235	10.59574	11.36903	13.78542	11.08773	11.91673	0.81402	1	-
ENSMUSC	1700007K	RIKEN cD	GO:00055	extracellul	-	-	-	-	7:1313259	1	2	1	2	1	8	0.061121	0.102439	0.042081	0.079609	0.053581	0.606147	0.068547	0.246446	0.185378	1	-
ENSMUSC	Fam24a	family wit	GO:00055	extracellul	-	-	-	-	7:1313338	1	1	0	0	0	0	0.04439	0.037198	0	0	0	0	0.027196	0	0.525398	1	-
ENSMUSC	Acadsb	acyl-Coer	GO:00081	metabolic	K09478	short/brar	mmu0110	Metabolic	7:1314106	536	1318	859	817	609	512	3.141896	6.475823	3.468736	3.119827	3.130304	3.721198	4.362152	3.323776	0.076355	1	-
ENSMUSC	Cpxm2	carboxype	GO:00081	metabolic	-	-	-	-	7:1320326	49	44	69	72	44	50	0.527995	0.397407	0.51219	0.50541	0.415741	0.668023	0.479197	0.529725	0.827175	1	-
ENSMUSC	Chp2	calcineuri	GO:00081	metabolic	-	-	-	-	7:1222194	8	4	25	33	14	7	0.305348	0.127972	0.657363	0.820567	0.468587	0.331301	0.363561	0.540152	0.368514	1	-
ENSMUSC	Ern2	endoplasr	GO:00081	metabolic	-	-	-	-	7:1221698	1	2	3	0	3	0	0.017458	0.02926	0.036072	0	0.045914	0	0.027597	0.015305	0.561248	1	-
ENSMUSC	Plk1	polo like k	GO:00081	metabolic	K06631	polo-like	mmu0411	Cell cycle	7:1221594	12	24	28	26	21	15	0.235372	0.394596	0.378361	0.332231	0.361211	0.364817	0.33611	0.352753	1	1	-
ENSMUSC	Dctn5	dynactin 5	GO:00325	developm	K10427	dynactin 5	mmu0496	Vasopress	7:1221330	1151	1089	1651	1624	1411	822	19.26508	15.27834	19.03681	17.70774	20.70929	17.05899	17.86008	18.49201	1	1	-
ENSMUSC	Ndufab1	NADH de	GO:00081	metabolic	K03955	NADH de	mmu0110	Metabolic	7:1220854	158	185	284	258	167	203	1.170324	1.148613	1.449165	1.244943	1.084698	1.864364	1.256034	1.398002	0.746983	1	-
ENSMUSC	Ubfd1	ubiquitin	GO:00971	organic cy	-	-	-	-	7:1220671	932	1154	1383	1434	1065	1001	5.105277	5.298609	5.218874	5.117221	5.115588	6.798661	5.207586	5.677157	0.739359	1	-
ENSMUSC	Ears2	glutamyl-	GO:00081	metabolic	K01885	glutamyl-	mmu0110	Metabolic	7:1220372	87	87	147	177	96	113	0.817193	0.684976	0.951205	1.083077	0.790709	1.316033	0.817791	1.063273	0.234634	1	-
ENSMUSC	Gga2	golgi asso	GO:00511	localizatio	K12404	ADP-ribo	mmu0414	Lysosome	7:1219867	359	417	534	638	457	485	1.566894	1.525576	1.605601	1.814043	1.749057	2.624657	1.566024	2.062586	0.105362	1	-
ENSMUSC	Scnn1b	sodium ch	GO:00511	localizatio	K04825	amiloride-	mmu0474	Taste tran	7:1218650	1	0	0	1	0	3	0.016158	0	0	0.010523	0	0.060096	0.005386	0.02354	0.433613	1	-
ENSMUSC	Mettl9	methyltra	-	-	-	-	-	-	7:1210344	592	882	1014	932	725	643	7.118899	8.890219	8.400027	7.301109	7.644898	9.587122	8.136382	8.17771	0.766022	1	-
ENSMUSC	Mfsd13b	major faci	GO:00099	cellular pr	-	-	-	-	7:1209825	9	16	21	22	20	9	0.176425	0.262907	0.283593	0.280951	0.3438	0.218745	0.240975	0.281165	0.706598	1	-
ENSMUSC	Cdr2	cerebellar	GO:00056	cell|intrac	-	-	-	-	7:1209570	189	139	269	176	146	159	1.88091	1.159514	1.844214	1.141043	1.274097	1.961961	1.628213	1.459034	0.386213	1	-
ENSMUSC	Mrpl17	mitochon	GO:00081	metabolic	K02879	large subu	mmu0301	Ribosome	7:1058032	255	386	446	395	387	358	1.62597	2.063057	1.959107	1.64078	2.163838	2.830353	1.882712	2.211657	0.471578	1	-
ENSMUSC	Polr3e	polymeras	GO:00081	metabolic	K14721	DNA-dire	mmu0516	Epstein-B	7:1209177	508	632	817	805	668	506	4.941071	5.152604	5.474323	5.100754	5.697394	6.102295	5.189332	5.633481	0.747847	1	-
ENSMUSC	Arfip2	ADP-ribo	GO:00099	cellular pr	-	-	-	-	7:1056342	1299	1395	1629	2131	1330	952	5.686023	5.11831	4.912152	6.076654	5.104976	5.166805	5.238828	5.449478	0.976722	1	-
ENSMUSC	Dnhd1	dynein he	GO:00038	catalytic a	-	-	-	-	7:1056508	109	108	143	182	114	116	0.441115	0.366355	0.398669	0.479822	0.404552	0.582062	0.402046	0.488812	0.374544	1	-
ENSMUSC	Uqcrc2	ubiquinol	GO:00081	metabolic	K00415	ubiquinol-	mmu0110	Metabolic	7:1206351	2551	3269	4004	3798	2553	1993	47.08793	50.5786	50.91487	45.67049	41.32307	45.61338	49.52713	44.20231	0.187963	1	-
ENSMUSC	Pdzd9	PDZ dom	-	-	-	-	-	-	7:1206587	0	0	0	3	0	0	0	0	0	0.028321	0	0	0	0.00944	0.303775	1	-
ENSMUSC	Rrp8	ribosomal	GO:00081	metabolic	-	-	-	-	7:1057317	168	108	268	238	167	107	1.857178	1.000737	2.040936	1.713968	1.618839	1.466608	1.63295	1.599805	0.793934	1	-
ENSMUSC	Vwa3a	von Willet	-	-	-	-	-	-	7:1207393	193	238	291	286	217	108	1.136666	1.174907	1.180643	1.097292	1.12067	0.788648	1.164072	1.002203	0.284557	1	-
ENSMUSC	Ilk	integrin lin	GO:00325	developm	K06272	integrin-li	mmu0451	Focal adh	7:1057365	546	687	887	877	633	611	4.28236	4.516479	4.792538	4.480962	4.35348	5.941795	4.530459	4.925412	0.772314	1	-
ENSMUSC	Tpp1	tripeptidy	GO:00325	developm	K01279	tripeptidyl	mmu0414	Lysosome	7:1057448	1081	1275	1743	1829	1230	931	8.579025	8.481547	9.529295	9.456	8.559729	9.161099	8.863289	9.058943	0.900193	1	-
ENSMUSC	Hpx	hemopexi	GO:00081	metabolic	-	-	-	-	7:1055916	0	3	3	7	0	0	0	0.091474	0.075179	0.165893	0	0	0.055551	0.055298	1	1	-
ENSMUSC	Cnga4	cyclic nuc	GO:00511	localizatio	K04951	cyclic nuc	mmu0474	Olfactory	7:1054045	15	28	20	15	19	11	0.275124	0.430466	0.252711	0.179229	0.305576	0.250162	0.319433	0.244989	0.28597	1	-
ENSMUSC	Cckbr	cholecyst	GO:00325	developm	K04195	cholecyst	mmu0408	Neuroacti	7:1054257	133	243	286	189	177	151	2.624198	4.018881	3.88743	2.429337	3.062388	3.694097	3.51017	3.061941	0.282961	1	-
ENSMUSC	Crym	crystallin,	GO:00081	metabolic	-	-	-	-	7:1201863	1442	2002	1756	1926	1451	2910	25.7715	29.99103	21.61973	22.42396	22.73968	64.4842	25.79408	36.54928	0.188035	1	-
ENSMUSC	Anks4b	ankyrin re	GO:00325	developm	-	-	-	-	7:1201738	8	12	6	7	5	8	0.13036	0.163916	0.067353	0.074312	0.071442	0.161639	0.120543	0.102464	0.654885	1	-
ENSMUSC	Zp2	zona pellu	GO:00099	cellular pr	-	-	-	-	7:1201267	1	2	10	5	3	5	0.016671	0.02794	0.114823	0.054294	0.043843	0.103324	0.053145	0.067154	1	1	-
ENSMUSC	Tmem159	transmem	GO:00160	membran	-	-	-	-	7:1201023	118	173	177	117	107	163	2.340586	2.876351	2.418624	1.511857	1.861099	4.008826	2.545187	2.460594	0.650862	1	-
ENSMUSC	Lyrm1	LYR motif	GO:00056	cell|intrac	-	-	-	-	7:1198958	106	109	136	124	135	72	0.588763	0.507473	0.520383	0.448681	0.657521	0.495852	0.538873	0.534018	0.815001	1	-
ENSMUSC	Rexo5	RNA exon	GO:00055	extracellul	K14570	RNA exon	mmu0300	Ribosome	7:1197940	8	20	23	17	20	8	0.065949	0.138204	0.130624	0.091299	0.144584	0.081774	0.111593	0.105885	0.830109	1	-
ENSMUSC	Eri2	exoribonu	GO:00081	metabolic	-	-	-	-	7:1197686	157	169	246	284	116	117	0.612625	0.552757	0.661274	0.72193	0.396914	0.566063	0.608885	0.561636	0.532963	1	-
ENSMUSC	Chst15	carbohydr	GO:00081	metabolic	K08106	N-acetylg	mmu0053	Glycosam	7:1322357	486	1178	1278	1752	1088	957	4.452465	9.046122	8.065789	10.45635	8.740496	10.87082	7.188125	10.02256	0.074162	1	-
ENSMUSC	Oat	ornithine	GO:00081	metabolic	K00819	ornithine-	mmu0110	Metabolic	7:1325574	868	1184	1501	1436	1025	1110	22.18094	25.36092	26.42363	23.90542	22.9682	35.16974	24.65516	27.34779	0.676139	1	-
ENSMUSC	Acsm3	acyl-CoA	GO:00081	metabolic	K01896	medium-c	mmu0110	Metabolic	7:1197609	1	2	1	1	0	0	0.008187	0.013722	0.005637	0.005332	0	0	0.009182	0.001777	0.411476	1	-
ENSMUSC	Thumpd1	THUMP d	GO:00081	metabolic	-	-	-	-	7:1197150	1177	1512	1668	1867	1227	905	21.78907	23.4621	21.27207	22.51585	19.91817	20.7729	22.17441	21.06897	0.475189	1	-
ENSMUSC	Lhpp	phospholy	GO:00081	metabolic	K11725	phospholy	mmu0019	Oxidative	7:1326106	149	228	223	247	227	157	2.133231	2.736143	2.199412	2.30372	2.84983	2.786988	2.356262	2.646846	0.631694	1	-
ENSMUSC	Fam53b	family wit	GO:00511	localizatio	-	-	-	-	7:1327120	515	393	875	707	700	139	1.489817	0.952953	1.743754	1.332375	1.775687	0.498571	1.395508	1.202211	0.526713	1	-
ENSMUSC	Eef1akmt2	EEF1A lysi	GO:00081	metabolic	-	-	-	-	7:1328274	193	248	367	363	261	133	4.026841	4.337215	5.275017	4.933947	4.775179	3.440663	4.546358	4.383263	0.706888	1	-
ENSMUSC	Abraxas2	BRISC con	GO:00081	metabolic	-	-	-	-	7:1328592	446	605	803	784	596	335	2.069256	2.352812	2.566528	2.369607	2.424754	1.927123	2.329532	2.240495	0.627266	1	-
ENSMUSC	Trim21	tripartite r	GO:00081	metabolic	K10651	tripartite r	mmu0532	Systemic l	7:1025579	58	32	52	49	66	52	0.572551	0.264786	0.353625	0.315108	0.57131	0.636464	0.396987	0.507627	0.464331	1	-
ENSMUSC	Zranb1	zinc finge	GO:00325	developm	-	-	-	-	7:1329311	1026	1279	1740	1671	1275	872	9.30044	9.718058	10.86568	9.86766	10.13466	9.800729	9.961391	9.934348	0.749331	1	-
ENSMUSC	Ctbp2	C-termina	GO:00325	developm	K04496	C-termina	mmu0520	Pathways	7:1329875	310	536	414	594	349	436	1.308109	1.895834	1.203468	1.632866	1.291371	2.281157	1.469137	1.735131	0.491769	1	-
ENSMUSC	Acsm5	acyl-CoA	GO:00081	metabolic	K01896	medium-c	mmu0110	Metabolic	7:1195194	0	0	2	0	2	2	0	0	0.012496	0	0.015903	0.022494	0.004165	0.012799	0.719704	1	-
ENSMUSC	Rrm1	ribonucle	GO:00325	developm	K10807	ribonucle	mmu0110	Metabolic	7:1024416	310	367	505	463	359	253	1.566839	1.554827	1.75835	1.524493	1.591107	1.585513	1.626672	1.567038	0.584841	1	-
ENSMUSC	Uros	uroporphy	GO:00081	metabolic	K01719	uroporphy	mmu0110	Metabolic	7:1336862	207	204	246	233	213	300	2.029856	1.676786	1.661807	1.48844	1.831538	3.647544	1.789483	2.322508	0.316384	1	-
ENSMUSC	Knop1	lysine rich	GO:00056	cell|intrac	-	-	-	-	7:1188422	348	510	561	376	484	205	1.423469	1.748605	1.580822	1.001933	1.73603	1.039704	1.584299	1.259222	0.130771	1	-
ENSMUSC	Mmp21	matrix me	GO:00325	developm	-	-	-	-	7:1336742	0	2	4	4	3	2	0	0.028608	0.047032	0.044475	0.044891	0.042331	0.025213	0.043899	0.479456	1	-
ENSMUSC	9030624J	RIKEN cD	GO:00511	localizatio	-	-	-	-	7:1187402	819	954	1180	1247	945	774	2.956876	2.887027	2.934828	2.932901	2.991741	3.464787	2.926244	3.12981	0.8352	1	-
ENSMUSC	Bccip	BRCA2 an	GO:00325	developm	-	-	-	-	7:1337093	504	649	837	791	622	470	10.54682	11.38384	12.06614	10.78324	11.41364	12.19481	11.33227	11.4639	0.825913	1	-
ENSMUSC	Dhx32	DEAH (As	GO:00081	metabolic	-	-	-	-	7:1337209	344	456	533	503	378	331	3.155635	3.506276	3.368272	3.005923	3.040626	3.764799	3.343394	3.270449	0.628359	1	-
ENSMUSC	Stim1	stromal in	GO:00325	developm	K16059	stromal in	mmu0402	Calcium si	7:1022678	947	1158	1424	1486	1108	1338	3.641159	3.732079	3.771821	3.722116	3.735699	6.378694	3.71502	4.61217	0.24626	1	-
ENSMUSC	Pgap2	post-GPI	GO:00081	metabolic	-	-	-	-	7:1022102	99	174	199	161	137	231	0.521573	0.768391	0.722245	0.552572	0.632911	1.508963	0.670736	0.898149	0.322136	1	-
ENSMUSC	D7Ertd443	DNA segr	GO:00508	response	-	-	-	-	7:1342657	60	60	68	106	66	56	0.499039	0.418303	0.389626	0.574347	0.481363	0.57751	0.435656	0.544406	0.32051	1	-
ENSMUSC	Art1	ADP-ribo	GO:00081	metabolic	-	-	-	-	7:1021017	3	0	0	3	0	0	0.055725	0	0	0.036302	0	0	0.018575	0.012101	1	1	-
ENSMUSC	Mki67	antigen id	GO:00325	developm	-	-	-	-	7:1356897	91	116	159	195	84	85	0.558938	0.59722	0.672775	0.780257	0.45242	0.647328	0.609644	0.626668	0.987697	1	-
ENSMUSC	Atp6ap2	ATPase, H	GO:00325	developm	K19514	renin rece	mmu0461	Renin-ang	X:1258780	2028	2828	3296	2854	2284	2645	46.86745	54.78172	52.47374	42.96739	46.28521	75.79054	51.3743	55.01438	0.904793	1	-
ENSMUSC	Usp9x	ubiquitin s	GO:00325	developm	-	-	-	-	X:1307149	5065	5458	6837	7609	5378	3109	18.15237	16.39609	16.87994	17.76492	16.90119	13.81531	17.1428	16.16047	0.47814	1	-
ENSMUSC	Cask	calcium/ca	GO:00081	metabolic	-	-	-	-	X:1351708	1255	1391	1813	1848	1406	1012	6.292129	5.845666	6.261857	6.035836	6.18133	6.291013	6.133217	6.169393	0.78446	1	-
ENSMUSC	Swap70	SWA-70 p	GO:00325	developm	-	-	-	-	7:1102217	228	249	316	343	228	167	1.614133	1.477601	1.541147	1.581904	1.415408	1.46591	1.544294	1.487741	0.617834	1	-
ENSMUSC	Wee1	WEE 1 ho	GO:00325	developm	K06632	wee1-like	mmu0411	Cell cycle	7:1101220	220	296	348	318	246	283	4.089403	4.611916	4.456235	3.850752	4.009732	6.522425	4.385851	4.794303	0.82827	1	-
ENSMUSC	Tmem9b	TMEM9 d	GO:00048	signal trar	-	-	-	-	7:1097358	544	1566	690	1773	1308	398	9.903419	23.89631	8.653399	21.02696	20.88029	8.983698	14.15104	16.96365	0.544743	1	-
ENSMUSC	BC051019	cDNA seq	-	-	-	-	-	-	7:1097121	4	3	4	0	4	2	0.090367	0.056809	0.062263	0	0.079239	0.05604	0.069813	0.045093	0.54732	1	-
ENSMUSC	Akip1	A kinase (	GO:00325	developm	-	-	-	-	7:1097036	34	53	55	38	49	33	1.098531	1.435371	1.224186	0.79983	1.388267	1.321987	1.252696	1.170028	0.618665	1	-
ENSMUSC	St5	suppressi	GO:00081	metabolic	-	-	-	-	7:1095239	469	575	712	810	560	492	3.616401	3.716418	3.782115	4.068837	3.78647	4.703864	3.704978	4.18639	0.536595	1	-

ENSMUSC	Trim66	tripartite r	GO:00081	metabolic	-	-	-	-	7:1094490	235	253	515	476	327	225	1.35563	1.223337	2.046593	1.7888	1.654106	1.609319	1.541853	1.684075	0.74757	1 -
ENSMUSC	Stk33	serine/thr	GO:00081	metabolic	-	-	-	-	7:1092792	28	29	47	46	23	20	0.563482	0.48918	0.651591	0.603065	0.405881	0.499044	0.568085	0.502664	0.537325	1 -
ENSMUSC	Tub	tubby bip	GO:00325	developm	-	-	-	-	7:1089503	4606	5481	6876	7716	5699	5152	32.1464	32.06425	33.05946	35.08184	34.87785	44.5831	32.42337	38.18093	0.295928	1 -
ENSMUSC	Eif3f	eukaryotic	GO:00081	metabolic	K03249	translation	mmu0301	RNA trans	7:1089344	1039	1356	1611	1647	1198	945	18.64771	20.39965	19.91853	19.25686	18.85427	21.02946	19.6553	19.71353	0.757305	1 -
ENSMUSC	Ndufb11	NADH de	GO:00081	metabolic	K11351	NADH de	mmu0110	Metabolic	X:2061532	612	722	900	736	629	887	42.8353	42.3585	43.39547	33.55904	38.60501	76.97679	42.86309	49.71361	0.609282	1 -
ENSMUSC	Rbm10	RNA bind	GO:00081	metabolic	-	-	-	-	X:2061750	835	1121	1341	1475	1124	667	9.893132	11.13284	10.94529	11.38469	11.67767	9.798493	10.65709	10.95362	0.96724	1 -
ENSMUSC	Cdk16	cyclin-dep	GO:00325	developm	-	-	-	-	X:2068795	3544	3632	4526	5842	4348	3319	32.06148	27.54156	28.20692	34.42968	34.49229	37.22914	29.26999	35.3837	0.194332	1 -
ENSMUSC	Usp11	ubiquitin s	GO:00081	metabolic	-	-	-	-	X:2070390	2474	2889	3190	3685	2687	2254	21.29624	20.84508	18.9167	20.66438	20.28213	24.05709	20.35268	21.66787	0.860628	1 -
ENSMUSC	Glrx3	glutaredo	GO:00081	metabolic	-	-	-	-	7:1374376	786	1009	1215	1111	850	553	8.972992	9.655145	9.555265	8.262485	8.508964	7.827551	9.394467	8.199666	0.177601	1 -
ENSMUSC	Mrgprf	MAS-rela	GO:00048	signal trar	-	-	-	-	7:1453008	10	20	22	21	13	17	0.162666	0.272695	0.246525	0.222536	0.18543	0.342862	0.227295	0.250276	0.943432	1 -
ENSMUSC	Oraov1	oral cance	-	-	-	-	-	-	7:1449151	141	160	201	244	162	121	0.863206	0.821052	0.847704	0.973126	0.869669	0.918473	0.843988	0.920423	0.745312	1 -
ENSMUSC	Fgf3	fibroblast	GO:00325	developm	K04358	fibroblast	mmu0520	Pathways	7:1448380	3	0	0	0	0	2	0.06823	0	0	0	0	0.056421	0.022743	0.018807	1	1 -
ENSMUSC	Ano1	anoctamir	GO:00325	developm	-	-	-	-	7:1445885	52	40	74	88	58	60	0.361135	0.232853	0.354039	0.398135	0.353212	0.516659	0.316009	0.422668	0.236651	1 -
ENSMUSC	Fadd	Fas (TNFR	GO:00325	developm	K02373	FAS-assoc	mmu0520	Pathways	7:1445773	48	39	89	81	43	39	0.781591	0.5323	0.998345	0.859215	0.613968	0.787391	0.770745	0.753525	0.82184	1 -
ENSMUSC	Cttn	cortactin [	GO:00325	developm	K06106	cortactin[-	mmu0520	Proteogly	7:1444357	3742	3891	4728	5586	4320	2641	37.54584	32.72443	32.68037	36.51242	38.00883	32.85582	34.31688	35.79236	0.955231	1 -
ENSMUSC	Zfp300	zinc finge	GO:00081	metabolic	-	-	-	-	X:2107917	69	65	94	111	78	36	0.743252	0.58688	0.697534	0.77891	0.736753	0.480808	0.675889	0.66549	0.874377	1 -
ENSMUSC	Nadsyn1	NAD synt	GO:00081	metabolic	K01950	NAD+ syr	mmu0110	Metabolic	7:1437955	50	59	75	74	74	51	0.619914	0.613146	0.640572	0.597686	0.804515	0.783999	0.624544	0.728733	0.563005	1 -
ENSMUSC	Dock11	dedicator	GO:00325	developm	-	-	-	-	X:3588883	454	512	609	686	506	233	2.751843	2.601303	2.54294	2.708779	2.689431	1.751098	2.632029	2.383103	0.43432	1 -
ENSMUSC	Cul4b	cullin 4B [	GO:00325	developm	K10609	cullin 4[-]	mmu0412	Ubiquitin	X:3853327	1107	1133	1485	1492	1073	505	12.24385	10.50396	11.31484	10.75031	10.4067	6.925447	11.35422	9.360819	0.147607	1 -
ENSMUSC	Tnni2	troponin I	GO:00081	metabolic	-	-	-	-	7:1424418	4	4	3	8	7	0	0.078216	0.065561	0.040412	0.101911	0.120039	0	0.061396	0.073983	0.758578	1 -
ENSMUSC	Smarca1	SWI/SNF r	GO:00325	developm	-	-	-	-	X:4780936	787	1048	1159	1390	885	462	5.587779	6.237042	5.668906	6.429261	5.509984	4.067169	5.831242	5.335471	0.444062	1 -
ENSMUSC	Sash3	SAM and	GO:00325	developm	-	-	-	-	X:4814643	20	35	38	47	20	38	0.474812	0.696486	0.621468	0.726886	0.416362	1.118569	0.597589	0.753939	0.522875	1 -
ENSMUSC	Elf4	E74-like f	GO:00325	developm	-	-	-	-	X:4841104	29	39	50	55	41	33	0.168885	0.190375	0.200592	0.208658	0.209371	0.23828	0.186618	0.21877	0.588529	1 -
ENSMUSC	Rab33a	RAB33A, r	GO:00081	metabolic	-	-	-	-	X:4851928	93	94	140	108	106	167	5.104024	4.324202	5.293064	3.861296	5.101241	11.36397	4.907097	6.775501	0.274189	1 -
ENSMUSC	Slc25a14	solute car	GO:00511	localizatio	-	-	-	-	X:4862341	585	752	914	788	592	603	10.8932	11.73736	11.72459	9.558895	9.666387	13.92206	11.45172	11.04911	0.532798	1 -
ENSMUSC	Rbmx2	RNA bind	GO:00081	metabolic	-	-	-	-	X:4869500	83	98	123	129	94	57	2.4208	2.395848	2.471366	2.451045	2.404079	2.061279	2.429338	2.305468	0.636523	1 -
ENSMUSC	Enox2	ecto-NOX	GO:00081	metabolic	-	-	-	-	X:4900970	265	307	305	388	277	202	2.436562	2.366051	1.931893	2.32405	2.233339	2.302874	2.244835	2.286755	0.904445	1 -
ENSMUSC	Igsf1	immunog	GO:00048	signal trar	-	-	-	-	X:4978253	130	157	181	162	97	122	1.306643	1.322717	1.253262	1.060742	0.85492	1.520407	1.294207	1.145356	0.315315	1 -
ENSMUSC	Stk26	serine/thr	GO:00325	developm	-	-	-	-	X:5084104	149	143	182	211	151	103	1.522413	1.224715	1.281058	1.404459	1.352895	1.304876	1.342729	1.354077	0.882059	1 -
ENSMUSC	Gpc4	glypican 4	GO:00055	extracellul	K08110	glypican 4	mmu0431	Wnt signa	X:5205302	607	749	977	1000	814	591	14.55175	15.05085	16.13513	15.61737	17.11171	17.5671	15.24591	16.76539	0.658369	1 -
ENSMUSC	3830403N	RIKEN cDI	GO:00325	developm	-	-	-	-	X:5613657	0	0	3	6	0	0	0	0	0.12851	0.243071	0	0	0.042837	0.081024	0.572339	1 -
ENSMUSC	Slc9a9	solute car	GO:00511	localizatio	-	-	-	-	9:9466990	215	258	310	390	277	164	2.263734	2.276978	2.248535	2.675054	2.557462	2.140996	2.263082	2.457838	0.740708	1 -
ENSMUSC	Brs3	bombesin	GO:00048	signal trar	K04170	bombesin	mmu0408	Neuroacti	X:5704307	2	5	0	0	0	0	0.045863	0.096083	0	0	0	0	0.047315	0	0.034414	1 -
ENSMUSC	Rbmx	RNA bind	GO:00325	developm	K12885	heteroger	mmu0304	Spliceosor	X:5738353	1285	1628	1555	2034	1541	920	10.59217	11.24836	8.830056	10.9223	11.13851	9.402759	10.22353	10.48786	0.949535	1 -
ENSMUSC	Fgf13	fibroblast	GO:00325	developm	K04358	fibroblast	mmu0520	Pathways	X:5906214	1957	2127	2438	2843	1920	2517	23.89495	21.76886	20.50692	22.61382	20.55698	38.10522	22.05691	27.09201	0.29227	1 -
ENSMUSC	F9	coagulatic	GO:00081	metabolic	K01321	coagulatic	mmu0461	Complem	X:5999946	7	0	4	3	0	0	0.162729	0	0.064062	0.045424	0	0	0.075597	0.015141	0.09446	1 -
ENSMUSC	Mcf2	mcf.2 trar	GO:00325	developm	-	-	-	-	X:6005595	80	113	154	131	95	64	0.883292	1.045791	1.17135	0.942255	0.919773	0.876154	1.033478	0.912727	0.361825	1 -
ENSMUSC	Cacna1f	calcium cl	GO:00511	localizatio	K04853	voltage-d	mmu0401	MAPK sigr	X:7607083	0	1	1	0	3	0	0	0.004311	0.003542	0	0.01353	0	0.002618	0.00451	1	1 -
ENSMUSC	Ccdc22	coiled-co	GO:00081	metabolic	-	-	-	-	X:7593809	338	455	561	607	443	395	5.254625	5.929113	6.00813	6.147462	6.039096	7.613912	5.730622	6.600157	0.468891	1 -
ENSMUSC	Syp	synaptopl	GO:00452	synapse|lc	-	-	-	-	X:7638471	23378	30906	34658	35980	27353	21534	235.421	260.8757	240.4323	236.0374	241.5381	268.8739	245.5764	248.8164	0.801209	1 -
ENSMUSC	Prickle3	prickle pla	GO:00325	developm	-	-	-	-	X:7657260	8	7	33	27	20	14	0.069359	0.05087	0.197102	0.1525	0.15206	0.150509	0.105777	0.15169	0.391609	1 -
ENSMUSC	Plp2	proteolipi	GO:00055	extracellul	-	-	-	-	X:7667941	37	40	47	61	40	27	0.817042	0.740388	0.714984	0.877521	0.774547	0.739263	0.757471	0.79711	0.968041	1 -
ENSMUSC	Magix	MAGI fam	-	-	-	-	-	-	X:7673164	11	10	18	9	6	8	0.201123	0.153246	0.226717	0.107195	0.096187	0.181356	0.193695	0.128246	0.150624	1 -
ENSMUSC	Gpkow	G patch d	GO:00056	cell|intrace	-	-	-	-	X:7697131	815	976	1237	1236	1014	691	12.30886	12.35558	12.87009	12.16075	13.42892	12.93971	12.51151	12.84313	0.925744	1 -
ENSMUSC	Praf2	PRA1 don	GO:00511	localizatio	-	-	-	-	X:7728439	471	539	688	653	490	322	22.35508	21.44358	22.49548	20.19067	20.39361	18.94948	22.09805	19.84459	0.286097	1 -
ENSMUSC	Ccdc120	coiled-co	GO:00511	localizatio	-	-	-	-	X:7731714	121	151	173	174	181	156	1.236722	1.293643	1.218097	1.158557	1.62221	1.976948	1.249487	1.585905	0.265119	1 -
ENSMUSC	Gripap1	GRIP1 ass	GO:00452	synapse|lc	-	-	-	-	X:7789765	1888	2240	2678	3124	2233	1485	15.63566	15.54945	15.27834	16.85415	16.21608	15.24849	15.48782	16.10624	0.976486	1 -
ENSMUSC	Otud5	OTU dom	GO:00325	developm	K12655	OTU dom	mmu0462	RIG-I-like	X:7841364	925	1218	1351	1560	1223	990	3.905303	4.310356	3.929342	4.290613	4.527747	5.182439	4.048333	4.666933	0.40528	1 -
ENSMUSC	Pim2	proviral in	GO:00081	metabolic	K08806	proto-onc	mmu0520	Pathways	X:7878262	439	522	613	660	504	478	7.404397	7.379868	7.122577	7.251879	7.45415	9.99629	7.302281	8.234106	0.583984	1 -
ENSMUSC	Slc35a2	solute car	GO:00511	localizatio	-	-	-	-	X:7884028	233	84	151	130	137	176	1.166339	0.352453	0.520712	0.423931	0.601355	1.092363	0.679835	0.705883	0.934399	1 -
ENSMUSC	Pqbp1	polyglutar	GO:00325	developm	K12865	polyglutar	mmu0304	Spliceosor	X:7894519	791	1080	1373	1222	920	686	19.05626	21.80912	22.78679	19.17848	19.43533	20.4914	21.21739	19.70174	0.359239	1 -
ENSMUSC	Timm17b	translocas	GO:00511	localizatio	-	-	-	-	X:7899357	174	212	217	254	217	192	3.364238	3.435776	2.89033	3.199278	3.679085	4.602808	3.230115	3.827057	0.430292	1 -
ENSMUSC	Hdac6	histone de	GO:00325	developm	K11407	histone de	mmu0516	Human pa	X:7930120	625	721	753	918	677	630	4.086484	3.951466	3.391691	3.910158	3.881511	5.107354	3.809881	4.299675	0.569048	1 -
ENSMUSC	Gata1	GATA bin	GO:00325	developm	-	-	-	-	X:7959260	0	2	2	0	0	0	0	0.0386	0.031729	0	0	0	0.023443	0	0.155922	1 -
ENSMUSC	Was	Wiskott-A	GO:00400	locomotic	K05747	Wiskott-A	mmu0414	Endocytos	X:8081453	21	30	39	44	20	16	0.512731	0.613946	0.655965	0.699841	0.428198	0.484359	0.594214	0.537466	0.610604	1 -
ENSMUSC	Wdr13	WD repea	GO:00099	cellular pr	-	-	-	-	X:8123301	3170	2942	4669	5229	3632	2273	14.72037	11.45131	14.93602	15.8183	14.78932	13.08715	13.70257	14.56492	0.832993	1 -
ENSMUSC	Rbm3	RNA bind	GO:00081	metabolic	-	-	-	-	X:8138975	409	582	760	809	541	498	2.384475	2.844106	3.052355	3.072558	2.765729	3.599853	2.760312	3.146047	0.513321	1 -
ENSMUSC	Ebp	phenylalk	GO:00325	developm	K01824	cholesten	mmu0110	Metabolic	X:8185329	178	224	282	227	231	178	3.170531	3.344357	3.460288	2.634014	3.608002	3.931134	3.325059	3.39105	0.873782	1 -
ENSMUSC	Porcn	porcupine	GO:00081	metabolic	K00181	O-palmitc	mmu0431	Wnt signa	X:8193848	641	829	839	903	703	863	7.907123	8.571717	7.129736	7.256542	7.604278	13.19948	7.869525	9.353435	0.438458	1 -
ENSMUSC	Slc38a5	solute car	GO:00511	localizatio	K14992	solute car	mmu0472	GABAergi	X:8271133	50	57	66	98	68	54	1.130445	1.080202	1.027947	1.443392	1.348111	1.513766	1.079531	1.43509	0.16776	1 -
ENSMUSC	Ftsj1	FtsJ RNA r	GO:00081	metabolic	-	-	-	-	X:8238668	185	284	291	299	274	246	1.680333	2.162201	1.820826	1.769202	2.182316	2.77042	1.887787	2.240646	0.432212	1 -
ENSMUSC	Rpgr	retinitis pi	-	-	-	-	-	-	X:1007362	283	413	469	487	319	188	2.065639	2.526799	2.358259	2.315678	2.041741	1.701417	2.316899	2.019612	0.277629	1 -
ENSMUSC	Dynlt3	dynein lig	GO:00511	localizatio	-	-	-	-	X:9654267	2694	3282	3772	3933	2927	1224	57.97895	59.20578	55.92368	55.14146	55.23802	32.6618	57.7028	47.68043	0.168783	1 -
ENSMUSC	Aff2	AF4/FMR2	GO:00081	metabolic	-	-	-	-	X:6936029	302	321	363	431	328	164	2.292526	2.042511	1.898299	2.131405	2.183351	1.543604	2.077778	1.952787	0.577422	1 -
ENSMUSC	F8	coagulatic	GO:00081	metabolic	K03899	coagulatic	mmu0461	Complem	X:7517271	55	72	64	86	84	26	0.314871	0.345507	0.252408	0.32074	0.421691	0.184562	0.304262	0.308998	1	1 -
ENSMUSC	Vbp1	von Hippe	GO:00329	macromo	-	-	-	-	X:7551429	529	691	912	773	591	500	14.54761	15.92821	17.27756	13.84833	14.25169	17.04874	15.91779	15.04959	0.441647	1 -
ENSMUSC	Fundc2	FUN14 dc	GO:00081	metabolic	-	-	-	-	X:7538239	322	454	554	460	366	408	6.855644	8.102159	8.12556	6.380162	6.833068	10.77056	7.694454	7.994597	0.93304	1 -
ENSMUSC	Mtcp1	mature T	GO:00081	metabolic	K16837	mature T-	mmu0415	PI3K-Akt s	X:7541044	67	68	83	93	74	59	0.691916	0.588625	0.590481	0.625667	0.670124	0.755471	0.623674	0.683754	0.79384	1 -
ENSMUSC	Brcc3	BRCA1/BF	GO:00081	metabolic	K11864	BRCA1/BF	mmu0462	NOD-like	X:7541662	292	349	416	536	338	192	1.548522	1.551361	1.519773	1.851742	1.571788	1.262469	1.539885	1.562	0.932393	1 -
ENSMUSC	Rab39b	RAB39B, r	GO:00081	metabolic	-	-	-	-	X:7557204	955	1094	1392	1500	1050	479	17.84567	17.13561	17.91927	18.26007	17.20527	11.09815	17.63352	15.52116	0.324759	1 -
ENSMUSC	Msn	moesin [S	GO:00325	developm	K05763	moesin[-]	mmu0481	Regulatior	X:9609604	799	908	1326	1315	874	600	7.221115	6.878532	8.25567	7.742204	6.926457	6.723486	7.451772	7.130715	0.548754	1 -
ENSMUSC	Heph	hephaesti	GO:00081	metabolic	K14735	hephaesti	mmu0497	Mineral al	X:9645535	171	156	226	223	173	115	1.143111	0.874115	1.040767	0.971135	1.014101	0.953184	1.019331	0.979473	0.646064	1 -
ENSMUSC	Gpr165	G protein-	GO:00048	signal trar	-	-	-	-	X:9671344	112	93	125	152	90	107	0.80785	0.56227	0.621114	0.714228	0.569242	0.956932	0.663745	0.746801	0.724027	1 -
ENSMUSC	Pgr15l	G protein-	GO:00048	signal trar	-	-	-	-	X:9707259	4	7	4	2	3	0	0.047258	0.069321	0.032561	0.015392	0.031079	0	0.049713	0.01549	0.081256	1 -
ENSMUSC	Ophn1	oligophre	GO:00325	developm	-	-	-	-	X:9855427	678	906	1019	1102	814	535	2.471263	2.768026	2.558675	2.616692	2.601695	2.41785	2.599321	2.545412	0.664151	1 -
ENSMUSC	Stard8	START do	GO:00987	molecular	-	-	-	-	X:9900324	244	252	309	311	221	171	2.5334	2.193143	2.21016	2.103563	2.012098	2.201384	2.312235	2.105681	0.362354	1 -
ENSMUSC	Efnb1	ephrin B1	GO:00325	developm	K05463	ephrin-B[	mmu0436	Axon guic	X:9913613	284	321	305	338	226	199	5.0586	4.792572	3.742501	3.922021	3.529897	4.394912	4.531225	3.948943	0.235909	1 -
ENSMUSC	Igbp1	immunog	GO:00081	metabolic	K17606	immunog	mmu0414	Autophag	X:1004942	494	617	721	812	604	417	5.053149	5.290212	5.080677	5.410936	5.417693	5.288797	5.141346	5.372475	0.958257	1 -
ENSMUSC	Magee2	melanom	-	-	-	-	-	-	X:1048549	189	226	220	261	196	122	5.18631	5.198275	4.15883	4.665735	4.716247	4.150922	4.847805	4.510968	0.515873	1 -
ENSMUSC	Pbdc1	polysacch	GO:00056	cell|intrace	-	-	-	-	X:1050797	166	206	253	237	197	190	1.557394	1.619986	1.635172	1.44851	1.62069	2.210197	1.604184	1.759799	0.784015	1 -
ENSMUSC	Magee1	melanom	GO:00452	synapse|n	-	-	-	-	X:1051203	3199	3908	4288	4802	3569	2481	57.26932	58.64286	52.88274	56.00303	56.02694	55.07068	56.26497	55.70022	0.680911	1 -
ENSMUSC	Atrx	ATRX, chr	GO:00325	developm	-	-	-	-	X:1057976	4500	6562	7842	7468	4943	2554	7.835087	9.5768	9.406101	8.470661	7.546831	5.513636	8.93933	7.177043	0.087483	1 -
ENSMUSC	Fgf16	fibroblast	GO:00400	locomotic	K04358	fibroblast	mmu0520	Pathways	X:1057642	19	19	51	49	24	16	0.734046	0.615288	1.357342	1.233233	0.813069	0.766435	0.902226	0.937579	1	1 -
ENSMUSC	Cox7b	cytochron	GO:00325	developm	K02271	cytochron	mmu0110	Metabolic	X:1060157	1126	1571	1941	1509	1308	1014	41.9219	49.02658	49.78282	36.59935	42.70246	46.80869	46.91043	42.03683	0.213341	1 -
ENSMUSC	Magt1	magnesiu	GO:00160	membran	-	-	-	-	X:1059680	222	277	479	356	284	224	1.62356	1.698043	2.413254	1.696086	1.821285	2.031185	1.911619	1.849519	0.627822	1 -
ENSMUSC	Itm2a	integral m	GO:00325	developm	-	-	-	-	X:1073970	609	768	906	885	566	551	22.19252	23.4587	22.74413	21.00945	18.08626	24.89588	22.79845	21.33053	0.396476	1 -
ENSMUSC	2610002N	RIKEN cDI	GO:00511	localizatio	K12197	charged n	mmu0414	Endocytos	X:1077827	1464	1728	2101	2145	1534	813	14.31189	14.15965	14.14925	13.66045	13.14995	9.854458	14.20693	12.22162	0.185809	1 -
ENSMUSC	Hmgn5	high-mob	GO:00081	metabolic	-	-	-	-	X:1090045	294	392	456	465	303	145	9.661096	10.79739	10.32277	9.954377	8.731018	5.907885	10.26042	8.19776	0.141964	1 -
ENSMUSC	Sh3bgrl	SH3-bind	GO:00055	extracellul	-	-	-	-	X:1090953	1652	2308	2629	2562	1793	1241	30.34381	35.53437	33.26612	30.65638	28.87905	28.26296	33.0481	29.26613	0.194034	1 -
ENSMUSC	Tnmd	tenomodu	GO:00325	developm	-	-	-	-	X:1338509	2	2	0	3	1	0	0.093963	0.078741	0	0.091788	0.041186	0	0.057568	0.044325	1	1 -
ENSMUSC	Srpx2	sushi-rep	GO:00325	developm	-	-	-	-	X:1339084	13	23	27	28	20	12	0.218448	0.323962	0.312555	0.306521	0.294712	0.250024	0.284988	0.283752	0.947016	1 -
ENSMUSC	Sytl4	synaptota	GO:00452	synapse|lc	-	-	-	-	X:1339363	29	43	28	39	29	26	0.318865	0.396305	0.212092	0.279353	0.279606	0.354467	0.309087	0.304475	0.826269	1 -
ENSMUSC	Cstf2	cleavage s	GO:00081	metabolic	K14407	cleavage s	mmu0301	mRNA sur	X:1340591	1732	2392	2924	3134	2274	1249	8.610279	9.967433	10.01378	10.14963	9.91294	7.698705	9.530498	9.253757	0.653432	1 -
ENSMUSC	Cenpi	centrome	GO:00329	macromo	-	-	-	-	X:1343080	7	6	8	12	3	2	0.057288	0.041155	0.0451	0.063976	0.021526	0.020299	0.047848	0.035267	0.604513	1 -
ENSMUSC	Btk	Bruton ag	GO:00325	developm	K07370	Bruton ag	mmu0438	Osteoclas	X:1345423	29	18	43	42	27	17	0.462379	0.240562	0.472298	0.43624	0.377489	0.336067	0.391746	0.383265	0.888757	1 -
ENSMUSC	Gla	galactosic	GO:00081	metabolic	K01189	alpha-gal	mmu0414	Lysosome	X:1345881	167	170	299	242	210	168	3.183369	2.716264	3.926386	3.005156	3.510205	3.970681	3.275339	3.495347	0.901776	1 -
ENSMUSC	Col4a6	collagen,	GO:00508	response	K06237	collagen, t	mmu0520	Pathways	X:1411654	34	37	43	40	28	37	0.262744	0.239668	0.228913	0.201369	0.189737	0.354517	0.243775	0.248541	0.86945	1 -
ENSMUSC	Col4a5	collagen,	GO:00452	synapse|re	K06237	collagen, t	mmu0520	Pathways	X:1414753	89	78	141	130	112	102	0.694864	0.510459	0.75837	0.661207	0.766786	0.987414	0.654564	0.805136	0.388293	1 -
ENSMUSC	Acsl4	acyl-CoA	GO:00325	developm	K01897	long-chai	mmu0110	Metabolic	X:1423179	2134	1335	1481	3363	1219	1250	21.23741	11.13631	10.15345	21.80297	10.63785	15.42421	14.17572	15.95501	0.701083	1 -
ENSMUSC	Chrdl1	chordin-li	GO:00325	developm	-	-	-	-	X:1432856	64	41	131	122	68	45	0.345399	0.185471	0.487038	0.428924	0.321802	0.301116	0.339302	0.350614	1	1 -
ENSMUSC	Pak3	p21 prote	GO:00325	developm	K05733	p21-activ	mmu0401	Ras signal	X:1435185	2751	3659	3874	4233	3236	704	10.19918	11.37077	9.894314	10.22362	10.52024	3.236184	10.48809	7.993348	0.224772	1 -
ENSMUSC	Dcx	doublecoi	GO:00325	developm	-	-	-	-	X:1438558	408	496	542	611	527	304	1.388395	1.414774	1.270583	1.354488	1.572554	1.282659	1.357917	1.403233	0.995194	1 -
ENSMUSC	Glt28d2	glycosyltra	GO:00081	metabolic	K07432	beta-1,4-	mmu0110	Metabolic	3:8586984	7	7	11	5	3	10	0.162848	0.136485	0.176276	0.075777	0.061191	0.288446	0.158536	0.141805	0.649734	1 -
ENSMUSC	Il13ra2	interleukir	GO:00048	signal trar	K05077	interleukir	mmu0463	Jak-STAT	X:1473834	3	6	2	9	10	0	0.073861	0.123832	0.03393	0.144366	0.215924	0	0.077208	0.120097	0.522353	1 -
ENSMUSC	Lrch2	leucine-ri	GO:00508	response	-	-	-	-	X:1474703	583	748	876	963	654	260	2.898264	3.116908	3.000024	3.118728	2.850952	1.60261	3.005065	2.524097	0.266133	1 -
ENSMUSC	Cdkl5	cyclin-dep	GO:00325	developm	-	-	-	-	X:1607719	3777	3675	4358	4842	3971	2470	16.22987	13.23665	12.9005	13.55422	14.96272	13.15985	14.12234	13.89226	0.67128	1 -
ENSMUSC	Rs1	retinoschi	GO:00325	developm	-	-	-	-	X:1607680	14	3	22	11	6	6	0.128001	0.02299	0.138566	0.065516	0.048101	0.068024	0.096519	0.060547	0.255695	1 -
ENSMUSC	Phka2	phosphor	GO:00081	metabolic	K07190	phosphor	mmu0402	Calcium si	X:1605021	485	541	723	841	585	413	3.772728	3.527478	3.874396	4.261787	3.990361	3.983366	3.724867	4.078505	0.680615	1 -
ENSMUSC	Slc7a3	solute car	GO:00511	localizatio	-	-	-	-	X:1010792	45	105	104	87	76	151	0.471621	0.922399	0.750862	0.593986	0.698449	1.962185	0.714961	1.084873	0.218525	1 -
ENSMUSC	Adgrg2	adhesion	GO:00048	signal trar	-	-	-	-	X:1603906	27	24	38	56	18	11	0.291871	0.217467	0.282985	0.394367	0.170624	0.147442	0.264108	0.237478	0.710642	1 -
ENSMUSC	Pdha1	pyruvate c	GO:00081	metabolic	K00161	pyruvate c	mmu0110	Metabolic	X:1601222	4624	5800	7142	6959	5277	3173	72.13282	75.83959	76.75141	70.72022	72.18465	61.37212	74.90794	68.09233	0.290058	1 -
ENSMUSC	Nlgn3	neuroligin	GO:00325	developm	K07378	neuroligin	mmu0451	Cell adhes	X:1012991	3319	4286	4720	5595	4135	3092	19.23338	20.81868	18.84264	21.12175	21.01195	22.2164	19.63157	21.45004	0.661957	1 -
ENSMUSC	Map3k15	mitogen-	GO:00048	signal trar	-	-	-	-	X:1599884	64	55	79	61	39	35	0.935469	0.673853	0.795473	0.580843	0.499867	0.634312	0.801598	0.571674	0.056233	1 -
ENSMUSC	Il2rg	interleukir	GO:00325	developm	K05070	interleukir	mmu0520	Pathways	X:1012643	11	27	37	36	24	14	0.192431	0.395897	0.445886	0.41026	0.368153	0.303669	0.344738	0.360694	1	1 -
ENSMUSC	Rps6ka3	ribosomal	GO:00081	metabolic	K04373	ribosomal	mmu0401	MAPK sigr	X:1592103	354	437	505	597	430	489	2.030852	2.101402	1.995802	2.231159	2.163148	3.47832	2.042685	2.624209	0.171988	1 -
ENSMUSC	Zmym3	zinc finge	GO:00325	developm	-	-	-	-	X:1014043	2109	2741	2764	2704	2575	2381	8.957049	9.757763	8.086823	7.481295	9.589773	12.53816	8.933879	9.869742	0.702698	1 -
ENSMUSC	Nono	non-POU	GO:00081	metabolic	-	-	-	-	X:1014293	2255	2700	3249	3582	2544	1385	23.05535	23.13885	22.8837	23.85791	22.80787	17.55742	23.02597	21.40773	0.439142	1 -
ENSMUSC	Itgb1bp2	integrin b	GO:00990	supramole	-	-	-	-	X:1014490	6	5	7	5	6	4	0.182544	0.127497	0.146713	0.099101	0.160051	0.150884	0.152251	0.136679	0.765558	1 -
ENSMUSC	Taf1	TATA-box	GO:00325	developm	K03125	transcripti	mmu0302	Basal tran	X:1015327	1811	1812	2433	2818	1775	966	9.355757	7.846419	8.658726	9.483815	8.04084	6.187618	8.620301	7.904091	0.428377	1 -
ENSMUSC	Rps4x	ribosomal	GO:00325	developm	K02987	small subu	mmu0301	Ribosome	X:1021849	733	1028	1294	1223	930	1067	21.57681	25.36468	26.24033	23.45263	24.00541	38.94338	24.39394	28.80047	0.415001	1 -
ENSMUSC	Dmrtc1a	DMRT-lik	GO:00081	metabolic	-	-	-	-	X:1029032	25	45	60	60	45	37	0.57776	0.871701	0.955231	0.903312	0.911915	1.060198	0.801564	0.958475	0.557797	1 -
ENSMUSC	Chic1	cysteine-r	GO:00511	localizatio	-	-	-	-	X:1033564	828	936	1276	1115	849	573	7.135176	6.760875	7.574889	6.259368	6.415414	6.122299	7.15698	6.265694	0.178038	1 -
ENSMUSC	Flna	filamin, al	GO:00325	developm	K04437	filamin[-]	mmu0401	MAPK sigr	X:7422346	1413	1780	2276	2459	1818	1947	5.694743	6.013188	6.319106	6.456127	6.424931	9.729352	6.009012	7.536803	0.16499	1 -
ENSMUSC	Tsx	testis spec	GO:00325	developm	-	-	-	-	X:1034144	0	1	2	0	0	0	0	0.065601	0.107849	0	0	0	0.057817	0	0.284757	1 -
ENSMUSC	Zcchc13	zinc finge	GO:00971	organic cy	-	-	-	-	X:1036305	0	0	1	0	0	1	0	0	0.040336	0	0	0.072606	0.013445	0.024202	1	1 -
ENSMUSC	Abcb7	ATP-bind	GO:00511	localizatio	K05662	ATP-bind	mmu0201	ABC trans	X:1042806	956	1246	1684	1665	1195	609	9.828	10.73688	11.92616	11.15072	10.77253	7.762654	10.83035	9.895301	0.413187	1 -
ENSMUSC	Mtm1	X-linked r	GO:00325	developm	K01108	myotubul	mmu0110	Metabolic	X:7121076	77	130	163	140	104	62	0.225324	0.318869	0.328591	0.266886	0.266866	0.224956	0.290928	0.252902	0.346107	1 -
ENSMUSC	Gabre	gamma-a	GO:00048	signal trar	K05185	gamma-a	mmu0408	Neuroacti	X:7225599	2	13	12	6	5	6	0.025386	0.138287	0.104911	0.049604	0.055635	0.094416	0.089528	0.066552	0.421577	1 -
ENSMUSC	Gpm6b	glycoprot	GO:00325	developm	-	-	-	-	X:1662389	7200	9816	11640	12546	9198	7973	54.9978	62.84927	61.25157	62.43092	61.60967	75.51279	59.69954	66.51779	0.560022	1 -
ENSMUSC	Gabra3	gamma-a	GO:00048	signal trar	K05175	gamma-a	mmu0408	Neuroacti	X:7243268	1455	1928	1827	1885	1457	851	19.28459	21.4194	16.68159	16.27573	16.93362	13.98502	19.12853	15.73146	0.082976	1 -
ENSMUSC	Gabrq	gamma-a	GO:00048	signal trar	K05192	gamma-a	mmu0408	Neuroacti	X:7282517	17	55	30	20	23	12	0.181973	0.4935	0.221226	0.139473	0.215896	0.159271	0.2989	0.171546	0.061267	1 -
ENSMUSC	Cetn2	centrin 2 [	GO:00081	metabolic	K10840	centrin-2[	mmu0342	Nucleotid	X:7291353	418	306	644	545	399	292	10.77693	6.612931	11.43817	9.153722	9.020578	9.334418	9.609345	9.169573	0.60183	1 -
ENSMUSC	Nsdhl	NAD(P) de	GO:00325	developm	K07748	sterol-4al	mmu0110	Metabolic	X:7291855	354	441	464	511	381	255	7.466902	7.797014	6.74228	7.021654	7.047006	6.669033	7.335399	6.912564	0.492796	1 -
ENSMUSC	Zfp185	zinc finge	GO:00056	cell|intrace	-	-	-	-	X:7298733	61	46	125	60	45	57	0.992504	0.627354	1.401084	0.635973	0.64203	1.149905	1.006981	0.809302	0.256356	1 -
ENSMUSC	Hccs	holocytoc	GO:00081	metabolic	K01764	cytochron	mmu0086	Porphyrin	X:1692501	143	326	400	375	245	264	1.671524	3.194095	3.220979	2.855553	2.511228	3.826185	2.695532	3.064322	0.656149	1 -
ENSMUSC	Rbbp7	retinoblas	GO:00081	metabolic	-	-	-	-	X:1627604	1700	2263	2317	2591	1917	1247	24.87684	27.75771	23.35736	24.69991	24.59864	22.62553	25.33064	23.97469	0.463524	1 -
ENSMUSC	Arhgap6	Rho GTPa	GO:00987	molecular	-	-	-	-	X:1687950	110	190	117	119	92	77	0.549033	0.794898	0.402292	0.38693	0.402658	0.476519	0.582074	0.422035	0.068394	1 -
ENSMUSC	Syap1	synapse a	GO:00055	extracellul	-	-	-	-	X:1628570	479	570	838	751	517	324	11.70843	11.67859	14.11101	11.95871	11.08143	9.819615	12.49934	10.95325	0.245308	1 -
ENSMUSC	Msl3	male-spe	GO:00081	metabolic	-	-	-	-	X:1686541	222	243	390	413	280	158	3.588184	3.292171	4.342493	4.348644	3.968468	3.166405	3.740949	3.827839	0.975804	1 -
ENSMUSC	Ctps2	cytidine 5	GO:00081	metabolic	K01937	CTP synth	mmu0110	Metabolic	X:1629012	354	501	617	575	526	393	2.661514	3.1573	3.195667	2.816271	3.467797	3.663565	3.004827	3.315878	0.683653	1 -
ENSMUSC	Grpr	gastrin rel	GO:00048	signal trar	K04169	gastrin-re	mmu0408	Neuroacti	X:1635139	22	21	25	44	27	16	0.554159	0.443395	0.433813	0.722031	0.596382	0.499717	0.477122	0.606043	0.378951	1 -
ENSMUSC	Zfp275	zinc finge	GO:00081	metabolic	-	-	-	-	X:7334262	238	431	595	526	456	370	2.205542	3.347874	3.798455	3.175455	3.705497	4.251344	3.11729	3.710765	0.408185	1 -
ENSMUSC	Ap1s2	adaptor-r	GO:00325	developm	K12394	AP-1 com	mmu0414	Lysosome	X:1639090	831	1009	1219	1136	888	410	6.784758	6.905215	6.856277	6.042174	6.357541	4.150524	6.84875	5.516746	0.10673	1 -
ENSMUSC	Zrsr2	zinc finge	GO:00081	metabolic	-	-	-	-	X:1639354	735	986	1117	1154	766	547	5.441689	6.118939	5.697065	5.565879	4.972991	5.021345	5.752564	5.186738	0.279279	1 -
ENSMUSC	Haus7	HAUS aug	GO:00990	supramole	-	-	-	-	X:7343731	66	82	106	83	94	57	2.373791	2.472104	2.626373	1.944736	2.964623	2.541895	2.490756	2.483751	0.820196	1 -
ENSMUSC	Car5b	carbonic a	GO:00081	metabolic	K01672	carbonic a	mmu0091	Nitrogen r	X:1639768	58	78	93	119	101	42	0.559091	0.630234	0.617571	0.747275	0.853726	0.501988	0.602298	0.700996	0.540055	1 -
ENSMUSC	Zfp92	zinc finge	GO:00081	metabolic	-	-	-	-	X:7341109	72	74	83	92	61	63	0.608325	0.524071	0.483092	0.506376	0.451937	0.659979	0.538496	0.539431	0.822393	1 -
ENSMUSC	Bgn	biglycan [	GO:00081	metabolic	-	-	-	-	X:7348360	380	578	778	775	441	462	2.497427	3.184129	3.522415	3.318126	2.541507	3.76476	3.06799	3.208131	0.993733	1 -
ENSMUSC	Atp2b3	ATPase, C	GO:00511	localizatio	K05850	Ca2+ tran	mmu0402	cAMP sigr	X:7350308	4638	4472	5609	6443	4687	2724	61.15329	49.42466	50.94786	55.34251	54.19094	44.53302	53.84194	51.35549	0.53982	1 -
ENSMUSC	Bmx	BMX non-	GO:00325	developm	-	-	-	-	X:1641928	23	31	34	28	23	16	0.411981	0.465436	0.419551	0.326737	0.361267	0.355351	0.432323	0.347785	0.269786	1 -
ENSMUSC	Abcd1	ATP-bind	GO:00081	metabolic	K05675	ATP-bind	mmu0414	Peroxisom	X:7371659	193	243	278	290	260	223	2.239094	2.363055	2.221826	2.191767	2.645029	3.207781	2.274658	2.681526	0.427806	1 -
ENSMUSC	Pir	pirin [Sou	GO:00325	developm	-	-	-	-	X:1642693	74	57	91	88	65	59	0.761602	0.491733	0.645193	0.590013	0.586618	0.752902	0.632843	0.643178	0.901514	1 -
ENSMUSC	Vegfd	vascular e	GO:00325	developm	K05449	vascular e	mmu0520	Pathways	X:1643733	82	90	62	70	65	46	1.918056	1.764576	0.999048	1.066659	1.333223	1.334119	1.56056	1.244667	0.222879	1 -
ENSMUSC	Piga	phosphati	GO:00081	metabolic	K03857	phosphati	mmu0110	Metabolic	X:1644197	101	132	219	161	148	112	0.452192	0.495366	0.675455	0.469579	0.581038	0.621733	0.541004	0.55745	0.945035	1 -
ENSMUSC	Asb11	ankyrin re	GO:00081	metabolic	-	-	-	-	X:1644369	16	23	44	28	25	26	0.264397	0.318571	0.500881	0.30142	0.362252	0.532715	0.361283	0.398796	0.921226	1 -
ENSMUSC	Dusp9	dual speci	GO:00081	metabolic	K18498	dual speci	mmu0401	MAPK sigr	X:7363941	7	7	9	4	9	8	0.163446	0.136987	0.144751	0.060847	0.184267	0.231592	0.148395	0.158902	1	1 -
ENSMUSC	Plxnb3	plexin B3	GO:00325	developm	K06821	plexin B[-	mmu0436	Axon guic	X:7375709	309	332	578	457	362	345	2.180047	1.963355	2.80923	2.100417	2.239544	3.017957	2.317544	2.452639	0.951893	1 -
ENSMUSC	Hcfc1	host cell fa	GO:00081	metabolic	K14966	host cell fa	mmu0516	Herpes sir	X:7394279	2549	2088	3053	3777	1747	1903	10.92873	7.503837	9.017336	10.54942	6.568037	10.11637	9.149969	9.077944	0.739536	1 -
ENSMUSC	Renbp	renin binc	GO:00081	metabolic	K01787	N-acylglu	mmu0052	Amino suc	X:7392212	48	43	62	62	34	45	0.506988	0.380695	0.451124	0.426608	0.314907	0.58932	0.446269	0.443612	0.819403	1 -
ENSMUSC	Naa10	N(alpha)-	GO:00081	metabolic	-	-	-	-	X:7391687	301	356	444	467	380	229	2.357296	2.336955	2.395422	2.38257	2.6096	2.223668	2.363224	2.405279	0.911367	1 -
ENSMUSC	Arhgap4	Rho GTPa	GO:00325	developm	-	-	-	-	X:7389144	63	96	117	119	94	93	0.274839	0.351044	0.351619	0.338192	0.359589	0.503041	0.325834	0.400274	0.383627	1 -
ENSMUSC	L1cam	L1 cell adl	GO:00325	developm	K06550	L1 cell adl	mmu0436	Axon guic	X:7385377	2303	2710	2910	3432	2490	1777	12.02058	11.85642	10.46347	11.66972	11.39655	11.50019	11.44683	11.52215	0.791219	1 -
ENSMUSC	Irak1	interleukir	GO:00325	developm	K04730	interleukir	mmu0401	MAPK sigr	X:7401391	611	700	751	878	774	505	2.964869	2.847184	2.510473	2.775496	3.293427	3.038377	2.774175	3.035766	0.698301	1 -
ENSMUSC	Mecp2	methyl Cp	GO:00325	developm	-	-	-	-	X:7402659	3353	3695	4845	5185	3943	2879	7.569652	6.992132	7.535074	7.625577	7.805698	8.058791	7.365619	7.830022	0.844196	1 -
ENSMUSC	Opn1mw	opsin 1 (c	GO:00048	signal trar	-	-	-	-	X:7412746	0	2	5	2	3	0	0	0.036448	0.074892	0.028325	0.057192	0	0.037113	0.028506	1	1 -

ENSMUSC	Tktl1	transketol	GO:00081	metabolic	K00615	transketol	mmu011C	Metabolic	X:7417725	4	0	0	0	0	0	0.093251	0	0	0	0	0	0.031084	0	0.194793	1	-
ENSMUSC	Plxna3	plexin A3	GO:00325	developm	K06820	plexin A[-	mmu0436	Axon guic	X:7432906	442	512	580	624	442	472	2.284702	2.218354	2.065317	2.101232	2.003424	3.025073	2.189457	2.376576	0.816243	1	-
ENSMUSC	Fam3a	family witl	GO:00023	immune s	-	-	-	-	X:7438471	195	163	356	351	269	224	2.183373	1.5298	2.745968	2.560248	2.641119	3.109761	2.153047	2.770376	0.212546	1	-
ENSMUSC	G6pdx	glucose-6	GO:00325	developm	K00036	glucose-6	mmu011C	Metabolic	X:7440948	424	533	711	703	493	408	6.984569	7.359602	8.068539	7.54416	7.121362	8.333366	7.470903	7.666296	0.914123	1	-
ENSMUSC	Mpp1	membran	GO:00400	locomotic	-	-	-	-	X:7510973	498	591	659	769	532	351	4.226121	4.2039	3.852556	4.251288	3.958824	3.693218	4.094192	3.967777	0.63023	1	-
ENSMUSC	Dkc1	dyskerato	GO:00081	metabolic	K11131	H/ACA rib	mmu030C	Ribosome	X:7509585	467	493	599	634	472	251	5.078584	4.493927	4.4875	4.491556	4.501017	3.384428	4.68667	4.125667	0.293733	1	-
ENSMUSC	Tceal6	transcripti	GO:00081	metabolic	-	-	-	-	X:1352086	745	1159	1269	1202	1031	557	42.50179	55.42262	49.87282	44.67218	51.57653	39.39962	49.26574	45.21611	0.397616	1	-
ENSMUSC	Morf4l2	mortality	GO:00325	developm	-	-	-	-	X:1367329	4111	5092	5703	6461	4783	2815	30.33749	31.4973	28.99258	31.06086	30.95103	25.75707	30.27579	29.25632	0.58842	1	-
ENSMUSC	Plp1	proteolipi	GO:00325	developm	-	-	-	-	X:1368226	16820	14619	28933	22498	15772	14919	130.4248	95.01774	154.5536	113.6475	107.2417	143.4366	126.6654	121.4419	0.522901	1	-
ENSMUSC	Zcchc18	zinc finge	GO:00081	metabolic	-	-	-	-	X:1369931	4092	5437	5949	6027	4888	3923	29.41178	32.75652	29.45649	28.22074	30.80772	34.96149	30.5416	31.32998	0.884867	1	-
ENSMUSC	Psmd10	proteasor	GO:00081	metabolic	-	-	-	-	X:1409484	203	310	323	326	224	185	7.620338	9.754251	8.352814	7.972199	7.373434	8.610692	8.575801	7.985442	0.454152	1	-
ENSMUSC	Vsig1	V-set and	GO:00325	developm	-	-	-	-	X:1409076	0	0	2	3	0	0	0	0	0.026251	0.037227	0	0	0.00875	0.012409	1	1	-
ENSMUSC	Tsc22d3	TSC22 do	GO:00325	developm	-	-	-	-	X:1405395	616	873	1123	1220	879	1209	4.862583	5.77635	6.106854	6.273759	6.084402	11.8331	5.581929	8.063753	0.049077	1	-
ENSMUSC	Prps1	phosphor	GO:00325	developm	-	-	mmu011C	Metabolic	X:1404566	952	1272	1397	1401	1135	761	27.79168	31.1256	28.09478	26.64387	29.05468	27.54533	29.00402	27.74796	0.508169	1	-
ENSMUSC	Rbm41	RNA bind	GO:00325	developm	-	-	-	-	X:1398895	173	137	171	194	157	77	0.897525	0.595765	0.611151	0.655667	0.714237	0.495307	0.70148	0.621737	0.438817	1	-
ENSMUSC	Morc4	microrchi	GO:00056	cell|intrace	-	-	-	-	X:1398216	119	131	181	140	113	70	0.596718	0.550612	0.625247	0.457332	0.496873	0.43522	0.590859	0.463142	0.093884	1	-
ENSMUSC	Rnf128	ring finge	GO:00081	metabolic	-	-	-	-	X:1395633	431	592	645	665	475	317	5.347787	6.157024	5.51325	5.375264	5.168121	4.876881	5.672687	5.140089	0.32805	1	-
ENSMUSC	Atp11a	ATPase, cl	GO:00511	localizatio	-	-	-	-	8:1275701	747	773	1102	1451	1146	624	2.720269	2.359518	2.764547	3.442231	3.65947	2.817484	2.614778	3.306395	0.141867	1	-
ENSMUSC	Mcf2l	mcf.2 tran	GO:00987	molecular	-	-	-	-	8:1287380	2473	3237	4034	3933	3288	2852	6.00262	6.585857	6.745343	6.219025	6.998278	8.583257	6.444606	7.266853	0.523603	1	-
ENSMUSC	F7	coagulatic	GO:00325	developm	K01320	coagulatic	mmu0461	Complem	8:1302603	3	7	13	17	4	5	0.102548	0.20058	0.306164	0.378601	0.119902	0.211928	0.203097	0.23681	0.829419	1	-
ENSMUSC	F10	coagulatic	GO:00081	metabolic	K01314	coagulatic	mmu0461	Complem	8:1303730	0	0	1	5	0	0	0	0	0.007985	0.037771	0	0	0.002662	0.01259	0.452765	1	-
ENSMUSC	Proz	protein Z,	GO:00081	metabolic	-	-	-	-	8:1306091	2	0	0	0	0	4	0.017608	0	0	0	0	0.043656	0.005869	0.014552	0.580058	1	-
ENSMUSC	Cul4a	cullin 4A [	GO:00325	developm	K10609	cullin 4[-]	mmu0412	Ubiquitin	8:1310562	1222	1624	2068	2242	1521	1233	9.376045	10.44449	10.93076	11.20639	10.23341	11.72998	10.25043	11.05659	0.75973	1	-
ENSMUSC	Lamp1	lysosomal	GO:00452	synapse|lc	K06528	lysosomal	mmu0515	Tuberculo	8:1315916	4357	5071	6763	6396	4961	3535	97.26043	94.88447	104.0015	93.01195	97.10923	97.8417	98.71546	95.98763	0.561803	1	-
ENSMUSC	Adprhl1	ADP-ribo	GO:00081	metabolic	-	-	-	-	8:1322166	0	5	1	1	1	0	0	0.039788	0.006538	0.006184	0.008325	0	0.015442	0.004836	0.377597	1	-
ENSMUSC	Grk1	G protein	GO:00081	metabolic	K00909	rhodopsin	mmu0414	Endocytos	8:1340508	2	0	0	2	0	0	0.007173	0	0	0.004671	0	0	0.002391	0.001557	1	1	-
ENSMUSC	Gas6	growth ar	GO:00325	developm	K05464	growth ar	mmu0152	EGFR tyro	8:1346537	1464	1605	2179	1942	1649	1429	36.51554	33.55557	37.44081	31.55495	36.06616	44.19313	35.83731	37.27141	0.963319	1	-
ENSMUSC	Rasa3	RAS p21 p	GO:00048	signal trar	K12380	Ras GTPas	mmu0401	Ras signal	8:1356694	1381	1494	2059	1469	1239	1289	11.5437	10.46778	11.85657	7.999347	9.081655	13.35949	11.28935	10.14683	0.239602	1	-
ENSMUSC	Coprs	coordinat	GO:00325	developm	-	-	-	-	8:1388479	150	143	202	165	119	186	2.611059	2.086477	2.42229	1.871065	1.816409	4.014428	2.373276	2.567301	0.942656	1	-
ENSMUSC	Myom2	myomesir	GO:00325	developm	-	-	-	-	8:1505765	2	4	10	3	6	2	0.01358	0.022759	0.046766	0.013265	0.035713	0.016838	0.027702	0.021939	0.633932	1	-
ENSMUSC	Angpt2	angiopoie	GO:00325	developm	K05466	angiopoie	mmu0415	PI3K-Akt s	8:1869026	38	46	99	60	54	39	0.633529	0.642824	1.137019	0.651655	0.789439	0.806188	0.804457	0.749094	0.609058	1	-
ENSMUSC	Agpat5	1-acylglyc	GO:00325	developm	K19007	lysophosp	mmu011C	Metabolic	8:1884627	835	673	1354	1196	894	824	4.053683	2.738616	4.52828	3.782476	3.805778	4.959939	3.773526	4.182731	0.692359	1	-
ENSMUSC	Nek3	NIMA (ne	GO:00325	developm	-	-	-	-	8:2212828	42	32	55	42	33	28	0.863265	0.551323	0.778775	0.562371	0.594785	0.713575	0.731121	0.623577	0.369091	1	-
ENSMUSC	Vps36	vacuolar p	GO:00081	metabolic	K12190	ESCRT-II c	mmu0414	Endocytos	8:2219280	575	714	826	945	645	411	9.079008	9.449769	8.984667	9.720387	8.930434	8.046325	9.171148	8.899049	0.638946	1	-
ENSMUSC	Thsd1	thrombos	GO:00160	membran	-	-	-	-	8:2222731	57	74	86	79	89	72	0.414049	0.450573	0.430358	0.37384	0.566907	0.648478	0.43166	0.529741	0.427482	1	-
ENSMUSC	Slc25a15	solute car	GO:00511	localizatio	-	-	-	-	8:2237555	138	184	239	233	187	195	1.196985	1.337769	1.4281	1.31658	1.422308	2.097154	1.320951	1.612014	0.361675	1	-
ENSMUSC	Erlin2	ER lipid ra	GO:00081	metabolic	-	-	-	-	8:2702326	1050	1206	1515	1614	1224	933	9.426566	9.075373	9.369755	9.439509	9.635811	10.38561	9.290564	9.82031	0.897053	1	-
ENSMUSC	Plpbp	pyridoxal	GO:00055	extracellul	-	-	-	-	8:2704255	451	846	1089	813	833	421	1.389492	2.184751	2.311311	1.63174	2.250435	1.608226	1.961851	1.830134	0.537708	1	-
ENSMUSC	Adgra2	adhesion	GO:00325	developm	-	-	-	-	8:2708558	230	215	305	408	273	285	1.409023	1.104029	1.287184	1.62829	1.466546	2.164817	1.266745	1.753218	0.066232	1	-
ENSMUSC	Brf2	BRF2, RN/	GO:00081	metabolic	-	-	-	-	8:2712336	168	204	246	236	187	218	1.830433	1.863064	1.84642	1.675092	1.786602	2.945011	1.846639	2.135568	0.58837	1	-
ENSMUSC	Rab11fip1	RAB11 far	GO:00511	localizatio	K12484	Rab11 far	mmu0414	Endocytos	8:2713877	89	87	178	129	109	113	0.401348	0.328855	0.552972	0.37897	0.431023	0.631823	0.427725	0.480605	0.752237	1	-
ENSMUSC	Adrb3	adrenergi	GO:00325	developm	K04143	adrenergi	mmu0408	Neuroacti	8:2722577	3	8	2	7	5	9	0.027056	0.060481	0.012429	0.041132	0.039543	0.100649	0.033322	0.060441	0.305974	1	-
ENSMUSC	Eif4ebp1	eukaryotic	GO:00325	developm	K07205	eukaryotic	mmu0415	PI3K-Akt s	8:2726032	20	22	35	36	20	27	0.48607	0.448166	0.585977	0.569971	0.426233	0.813613	0.506738	0.603272	0.67084	1	-
ENSMUSC	Chrna6	cholinergi	GO:00048	signal trar	K04808	nicotinic a	mmu0408	Neuroacti	8:2740321	0	0	4	0	0	3	0	0	0.06027	0	0	0.081355	0.02009	0.027118	1	1	-
ENSMUSC	Chrnb3	cholinergi	GO:00048	signal trar	K04814	nicotinic a	mmu0408	Neuroacti	8:2736871	2	12	17	16	11	8	0.014798	0.074412	0.086633	0.077107	0.071357	0.073378	0.058614	0.073947	0.655135	1	-
ENSMUSC	Ggn	gametoge	GO:00325	developm	-	-	-	-	7:2917021	8	15	24	18	9	11	0.076314	0.119945	0.157722	0.111861	0.075289	0.130113	0.117994	0.105754	0.685685	1	-
ENSMUSC	Cd209a	CD209a a	GO:00081	metabolic	K06563	CD209 an	mmu0414	Phagoson	8:3743397	3	4	3	8	4	0	0.068011	0.076016	0.046856	0.118163	0.079521	0	0.063628	0.065895	1	1	-
ENSMUSC	Tnfsf13b	tumor nec	GO:00325	developm	K05476	tumor nec	mmu0406	Cytokine-	8:1000646	9	18	26	30	14	8	0.052716	0.088376	0.104914	0.114475	0.071908	0.058101	0.082002	0.081494	1	1	-
ENSMUSC	Col4a1	collagen,	GO:00325	developm	K06237	collagen,	mmu052C	Pathways	8:1119842	997	1266	1400	1497	1178	1126	6.279717	6.683926	6.074696	6.142547	6.506285	8.79365	6.346113	7.147494	0.568332	1	-
ENSMUSC	Col4a2	collagen,	GO:00325	developm	K06237	collagen,	mmu052C	Pathways	8:1131280	1401	1739	1834	2440	1662	1276	12.96414	13.48833	11.69113	14.7088	13.48588	14.64005	12.71454	14.27824	0.521691	1	-
ENSMUSC	Rab20	RAB20, m	GO:00511	localizatio	-	-	-	-	8:1145351	15	8	27	16	12	22	0.684845	0.306139	0.849197	0.475879	0.480423	1.245419	0.613394	0.733907	0.816438	1	-
ENSMUSC	Naxd	NAD(P)H)	GO:00081	metabolic	-	-	-	-	8:1149750	478	613	672	748	555	501	4.516555	4.855035	4.374212	4.604289	4.598485	5.869524	4.581934	5.024099	0.718282	1	-
ENSMUSC	Ptpn7	protein ty	GO:00081	metabolic	K18019	tyrosine-p	mmu0401	MAPK sigr	1:1351327	0	4	5	7	0	5	0	0.022048	0.022652	0.029989	0	0.040767	0.0149	0.023585	0.633967	1	-
ENSMUSC	Ankrd10	ankyrin re	GO:00508	response	-	-	-	-	8:1161158	547	716	839	926	656	779	2.574881	2.825114	2.720717	2.839637	2.707801	4.546672	2.706904	3.364703	0.245693	1	-
ENSMUSC	Arhgef7	Rho guan	GO:00325	developm	K13710	Rho guan	mmu0481	Regulatior	8:1172772	2235	2761	3335	3547	2766	2333	7.858857	8.137689	8.07847	8.125024	8.528578	10.17144	8.025006	8.941682	0.568281	1	-
ENSMUSC	Leprotl1	leptin rece	GO:00511	localizatio	-	-	-	-	8:3413557	700	861	1208	1113	799	848	15.45227	15.93127	18.37015	16.00557	15.46621	23.21005	16.58456	18.22728	0.729862	1	-
ENSMUSC	Dctn6	dynactin 6	GO:00081	metabolic	K10428	dynactin 6	mmu0496	Vasopress	8:3409042	607	665	873	787	652	402	6.959532	6.390956	6.895357	5.878245	6.555137	5.714827	6.748615	6.049403	0.271358	1	-
ENSMUSC	Gpm6a	glycoprot	GO:00325	developm	-	-	-	-	8:5495484	20215	26141	30755	31537	22954	19184	399.6031	433.1419	418.8151	406.1226	397.8837	470.1974	417.1867	424.7346	0.829051	1	-
ENSMUSC	Asb5	ankyrin re	GO:00081	metabolic	-	-	-	-	8:5452958	0	7	0	10	4	5	0	0.193201	0	0.21451	0.115491	0.204132	0.0644	0.178044	0.156464	1	-
ENSMUSC	Vegfc	vascular e	GO:00325	developm	K05449	vascular e	mmu052C	Pathways	8:5407760	24	41	44	66	29	28	0.524149	0.750551	0.661989	0.939013	0.555368	0.758205	0.645563	0.750862	0.63484	1	-
ENSMUSC	Aga	aspartylgl	GO:00081	metabolic	K01444	N4-(beta-	mmu0414	Lysosome	8:5351172	78	77	107	118	94	54	1.691285	1.399477	1.598283	1.666798	1.78727	1.45179	1.563015	1.635286	0.947829	1	-
ENSMUSC	Dlc1	deleted in	GO:00325	developm	-	-	-	-	8:3656775	1306	1539	1801	1892	1561	1048	4.527119	4.471677	4.300747	4.272496	4.744872	4.50429	4.433181	4.507219	0.859175	1	-
ENSMUSC	Eri1	exoribonu	GO:00081	metabolic	-	-	-	-	8:3546525	317	418	548	517	380	227	2.182464	2.412218	2.599077	2.31878	2.294107	1.937755	2.39792	2.183548	0.382787	1	-
ENSMUSC	Tnks	tankyrase	GO:00081	metabolic	-	-	-	-	8:3482646	2450	2903	3622	3929	2846	2142	9.361203	9.297484	9.533785	9.779781	9.53549	10.14778	9.397491	9.821017	0.963323	1	-
ENSMUSC	Dusp4	dual speci	GO:00325	developm	K04459	dual speci	mmu0401	MAPK sigr	8:3480729	314	372	456	570	350	291	7.283077	7.232386	7.28622	8.612758	7.118635	8.368819	7.267228	8.033404	0.653611	1	-
ENSMUSC	Saraf	store-ope	GO:00511	localizatio	-	-	-	-	8:3415457	2324	2942	3582	3707	2787	2802	33.14569	35.17114	35.19394	34.44254	34.85544	49.55016	34.50359	39.61605	0.45398	1	-
ENSMUSC	Mrps31	mitochon	GO:00051	structural	-	-	-	-	8:2241136	221	300	349	358	244	182	3.954176	4.499215	4.301698	4.172799	3.828216	4.037579	4.251697	4.012865	0.512828	1	-
ENSMUSC	Smim19	small inte	GO:00160	membran	-	-	-	-	8:2246261	238	279	144	274	247	199	5.878588	5.776341	2.450241	4.408875	5.349785	6.09446	4.701724	5.284373	0.738798	1	-
ENSMUSC	Polb	polymeras	GO:00325	developm	K02330	DNA poly	mmu0516	HTLV-I inf	8:2262812	704	885	1124	1369	946	467	10.8701	11.45399	11.95579	13.77038	12.80839	8.940527	11.42663	11.83976	0.95928	1	-
ENSMUSC	Ikbkb	inhibitor c	GO:00325	developm	K07209	inhibitor c	mmu052C	Pathways	8:2265921	690	868	1056	1177	921	589	1.79609	1.893879	1.893629	1.995894	2.102237	1.900993	1.861199	1.999708	0.777184	1	-
ENSMUSC	Plat	plasminog	GO:00081	metabolic	K01343	tissue plas	mmu052C	Transcript	8:2275772	722	877	988	1061	741	563	6.748829	6.871373	6.362086	6.460825	6.073678	6.525079	6.660763	6.353194	0.496	1	-
ENSMUSC	Ap3m2	adaptor-r	GO:00511	localizatio	K12398	AP-3 com	mmu0414	Lysosome	8:2278735	2416	2937	3653	3712	2896	2397	31.639	32.23908	32.95543	31.66762	33.25577	38.92063	32.27784	34.61467	0.809347	1	-
ENSMUSC	Kat6a	K(lysine) a	GO:00325	developm	K11305	histone ac	mmu0455	Signaling	8:2285953	2067	2135	2864	3342	2313	1137	13.98234	12.1057	13.34639	14.72744	13.72011	9.536428	13.14481	12.66133	0.654211	1	-
ENSMUSC	Ank1	ankyrin 1,	GO:00325	developm	K10380	ankyrin[-]	mmu052C	Proteoglyc	8:2297484	807	942	1430	1414	1137	766	1.962549	1.920218	2.39571	2.240154	2.424654	2.309733	2.092826	2.324847	0.590521	1	-
ENSMUSC	Gpat4	glycerol-3	GO:00325	developm	K13506	glycerol-3	mmu011C	Metabolic	8:2317126	1393	1926	2202	2197	1728	1478	11.25182	13.0401	12.25295	11.56068	12.23934	14.8024	12.18162	12.86748	0.924584	1	-
ENSMUSC	Gins4	GINS com	GO:00325	developm	-	-	-	-	8:2322661	225	212	322	290	218	194	10.60003	8.371679	10.45037	8.900297	9.005839	11.33215	9.807359	9.746095	0.74524	1	-
ENSMUSC	Sfrp1	secreted f	GO:00325	developm	K02166	secreted f	mmu0431	Wnt signa	8:2341150	116	134	163	132	112	76	1.686223	1.632722	1.632285	1.250004	1.427639	1.369792	1.65041	1.349145	0.13952	1	-
ENSMUSC	Ido2	indoleami	GO:00081	metabolic	K00463	indoleami	mmu011C	Metabolic	8:2453189	24	22	21	30	22	13	0.244592	0.187934	0.147435	0.199175	0.196608	0.164267	0.19332	0.186683	0.856073	1	-
ENSMUSC	Ido1	indoleami	GO:00325	developm	K00463	indoleami	mmu011C	Metabolic	8:2458413	0	0	0	2	0	1	0	0	0	0.045366	0	0.043166	0	0.029511	0.284399	1	-
ENSMUSC	Adam18	a disinteg	GO:00325	developm	-	-	-	-	8:2460224	6	11	13	7	16	6	0.133942	0.205832	0.199915	0.101797	0.313188	0.166083	0.179897	0.193689	1	1	-
ENSMUSC	Adam5	a disinteg	GO:00081	metabolic	-	-	-	-	8:2472709	0	4	9	5	0	2	0	0.05363	0.099173	0.052107	0	0.039678	0.050934	0.030595	0.512535	1	-
ENSMUSC	Adam9	a disinteg	GO:00325	developm	-	-	-	-	8:2494961	1142	1330	1878	1431	1247	596	11.14863	10.88328	12.62997	9.100732	10.67491	7.214187	11.55396	8.996611	0.050618	1	-
ENSMUSC	Tm2d2	TM2 dom	GO:00160	membran	-	-	-	-	8:2501721	332	614	539	404	316	778	5.980604	9.271041	6.688794	4.741003	4.991571	17.37694	7.31348	9.036504	0.59745	1	-
ENSMUSC	Plekha2	pleckstrin	GO:00511	localizatio	-	-	-	-	8:2503914	797	952	1229	1480	1028	669	6.159016	6.166561	6.54269	7.45069	6.966084	6.410101	6.289422	6.942292	0.612653	1	-
ENSMUSC	Slit2	slit homol	GO:00325	developm	K06839	slit 2[-]	mmu0436	Axon guic	5:4798313	1202	1435	1394	1388	1203	669	2.123852	2.125322	1.696813	1.597685	1.863923	1.465655	1.981995	1.642421	0.104502	1	-
ENSMUSC	Tenm3	teneurin t	GO:00325	developm	-	-	-	-	8:4822768	2536	2550	3705	3930	2946	1653	11.92265	10.04886	11.99951	12.03644	12.14504	9.635684	11.32367	11.27239	0.780757	1	-
ENSMUSC	Dctd	dCMP dea	GO:00081	metabolic	K01493	dCMP dea	mmu011C	Metabolic	8:4809909	63	90	141	170	86	28	0.649765	0.778051	1.001807	1.142207	0.777782	0.358062	0.809874	0.75935	0.7767	1	-
ENSMUSC	Wwc2	WW, C2 a	GO:00325	developm	-	-	-	-	8:4782395	1119	1216	1571	1655	1190	772	4.983591	4.539401	4.819918	4.801664	4.647313	4.262999	4.78097	4.570659	0.526318	1	-
ENSMUSC	Fgfr1	fibroblast	GO:00325	developm	K04362	fibroblast	mmu052C	Pathways	8:2551365	1777	2236	2431	2983	1814	2008	4.552127	4.801219	4.290059	4.978079	4.074805	6.377882	4.547802	5.143589	0.548406	1	-
ENSMUSC	Rwdd4a	RWD dom	-	-	-	-	-	-	8:4753366	254	283	266	386	233	220	2.329026	2.175105	1.68025	2.305734	1.873438	2.501198	2.06146	2.22679	0.823525	1	-
ENSMUSC	Plpp5	phospholi	GO:00081	metabolic	-	-	mmu0056	Glyceroph	8:2572003	179	221	295	187	246	119	1.146192	1.186183	1.301307	0.780061	1.381287	0.944798	1.211227	1.035382	0.275142	1	-
ENSMUSC	Star	steroidog	GO:00325	developm	K16931	steroidog	mmu0492	Aldostero	8:2580655	15	36	47	66	37	29	0.222683	0.447971	0.480665	0.638292	0.481657	0.533789	0.383773	0.551246	0.180749	1	-
ENSMUSC	Ash2l	ash2 (abs	GO:00081	metabolic	-	-	-	-	8:2581599	645	839	956	1026	789	491	2.783061	3.034433	2.841661	2.883978	2.985262	2.626823	2.886385	2.832021	0.684138	1	-
ENSMUSC	Kcnu1	potassium	GO:00511	localizatio	K05274	potassium	mmu0402	cGMP-PK	8:2584962	38	48	48	50	41	30	0.541361	0.573186	0.47108	0.464036	0.512188	0.52993	0.528542	0.502051	0.660223	1	-
ENSMUSC	Tti2	TELO2 int	GO:00056	cell|intrace	-	-	-	-	8:3115031	341	415	521	540	398	262	1.606494	1.638797	1.690882	1.657294	1.644181	1.530423	1.645391	1.610633	0.682921	1	-
ENSMUSC	Mak16	MAK16 hc	GO:00081	metabolic	-	-	-	-	8:3115946	420	607	747	707	468	332	6.831908	8.276262	8.370761	7.491926	6.675466	6.696031	7.82631	6.954474	0.266827	1	-
ENSMUSC	Wrn	Werner sy	GO:00325	developm	-	-	-	-	8:3323438	160	251	296	315	265	148	1.015018	1.334694	1.293595	1.30181	1.474156	1.164131	1.214436	1.313366	0.785719	1	-
ENSMUSC	Gsr	glutathior	GO:00081	metabolic	K00383	glutathior	mmu0491	Thyroid hc	8:3365252	372	495	494	540	413	367	7.944125	8.860567	7.267443	7.5124	7.733845	9.717506	8.024045	8.32125	0.956653	1	-
ENSMUSC	Gtf2e2	general tr	GO:00081	metabolic	K03137	transcripti	mmu052C	Viral carci	8:3373183	320	357	470	474	316	162	5.222641	4.883837	5.284326	5.039644	4.522409	3.278248	5.130268	4.280101	0.197819	1	-
ENSMUSC	Rbpms	RNA bind	GO:00081	metabolic	-	-	-	-	8:3378264	52	31	69	68	56	43	0.328178	0.163989	0.29999	0.279575	0.30991	0.336483	0.264052	0.308656	0.618563	1	-
ENSMUSC	Frg1	FSHD reg	GO:00325	developm	-	-	-	-	8:4139745	275	319	459	406	306	218	9.791066	9.520091	11.25803	9.416859	9.55348	9.62367	10.18973	9.531336	0.457502	1	-
ENSMUSC	Asah1	N-acylsph	GO:00081	metabolic	K12348	acid cerar	mmu011C	Metabolic	8:4134019	689	868	954	808	776	558	7.827681	8.26583	7.466438	5.980078	7.730686	7.860198	7.853316	7.190321	0.320429	1	-
ENSMUSC	Pcm1	pericentri	GO:00325	developm	-	-	-	-	8:4123975	2625	2938	3674	4151	2869	1167	19.45727	18.25397	18.76045	20.04412	18.64772	10.7253	18.8239	16.47238	0.341011	1	-
ENSMUSC	Fgl1	fibrinoger	GO:00055	extracellul	-	-	-	-	8:4119143	0	3	9	6	4	3	0	0.031789	0.078379	0.049417	0.04434	0.04703	0.036723	0.046929	0.873562	1	-
ENSMUSC	Pdgfrl	platelet-d	-	-	-	-	-	-	8:4092621	23	27	55	41	37	31	0.404122	0.397649	0.665738	0.469299	0.570078	0.675365	0.489169	0.571581	0.675777	1	-
ENSMUSC	Slc7a2	solute car	GO:00081	metabolic	-	-	-	-	8:4086239	227	276	403	407	240	246	0.77729	0.792172	0.950636	0.907893	0.720629	1.04443	0.840033	0.890984	0.926232	1	-
ENSMUSC	Vps37a	vacuolar p	GO:00081	metabolic	K12185	ESCRT-I c	mmu0414	Endocytos	8:4051178	1061	1275	1694	1619	1116	664	10.57058	10.64747	11.62648	10.50781	9.749681	8.202336	10.94818	9.486608	0.187329	1	-
ENSMUSC	Cnot7	CCR4-NC	GO:00081	metabolic	K12581	CCR4-NO	mmu0301	RNA degr	8:4049254	1228	1114	1798	1950	1103	839	5.451842	4.145555	5.499027	5.639764	4.294009	4.618412	5.032141	4.850728	0.60739	1	-
ENSMUSC	Fgf20	fibroblast	GO:00325	developm	K04358	fibroblast	mmu052C	Pathways	8:4027916	3	0	2	3	0	3	0.097313	0	0.044703	0.063394	0	0.120683	0.047339	0.061359	1	1	-
ENSMUSC	Msmo1	methylste	GO:00081	metabolic	K07750	methylste	mmu011C	Metabolic	8:6471813	716	884	944	940	807	594	4.613602	4.774546	4.190351	3.945815	4.559772	4.745688	4.526166	4.417091	0.608325	1	-
ENSMUSC	Klhl2	kelch-like	GO:00081	metabolic	-	-	-	-	8:6473967	5894	7622	9085	9618	6997	4864	32.99405	35.7641	35.03498	35.07453	34.3463	33.7602	34.59771	34.39368	0.711764	1	-
ENSMUSC	Galnt7	polypeptic	GO:00081	metabolic	K00710	polypeptic	mmu011C	Metabolic	8:5752382	235	271	398	359	237	126	1.932329	1.86782	2.254487	1.92304	1.708849	1.284608	2.018212	1.638832	0.157213	1	-
ENSMUSC	Sap30	sin3 assoc	GO:00325	developm	-	-	-	-	8:5748270	78	118	135	115	97	78	4.208112	5.336137	5.017366	4.041762	4.588847	5.217635	4.853872	4.616081	0.586359	1	-
ENSMUSC	Scrg1	scrapie re	GO:00055	extracellul	-	-	-	-	8:5745592	145	223	230	222	176	150	13.16459	16.97057	14.38525	13.13026	14.01175	16.88559	14.84013	14.67587	0.730372	1	-
ENSMUSC	Hpgd	hydroxypr	GO:00325	developm	K00069	15-hydrox	mmu052C	Transcript	8:5629458	130	125	194	219	141	73	4.909035	3.956534	5.046663	5.387374	4.668901	3.417893	4.637411	4.491389	0.767291	1	-
ENSMUSC	Ednra	endothelir	GO:00325	developm	K04197	endothelir	mmu052C	Pathways	8:7766303	120	149	175	185	134	115	1.277449	1.329537	1.283367	1.282966	1.25086	1.51791	1.296784	1.350579	1	1	-
ENSMUSC	Tmem184	transmem	GO:00511	localizatio	-	-	-	-	8:7759598	1291	1539	1887	1989	1430	710	25.32314	25.30362	25.49849	25.41601	24.59631	17.26775	25.37508	22.42669	0.287747	1	-
ENSMUSC	Nr3c2	nuclear re	GO:00081	metabolic	K08555	mineraloc	mmu0496	Aldostero	8:7689944	2044	3458	2807	3223	2268	2278	9.30666	13.19747	8.804542	9.559933	9.05521	12.86034	10.43622	10.49183	0.76714	1	-
ENSMUSC	Iqcm	IQ motif c	-	-	-	-	-	-	8:7544869	2	0	2	1	0	6	0.046912	0	0.032315	0.015275	0	0.174479	0.026409	0.063251	0.541094	1	-
ENSMUSC	Sin3b	transcripti	GO:00081	metabolic	-	-	-	-	8:7272328	1919	2288	2838	3141	2276	1873	13.57958	13.57126	13.83488	14.47975	14.12298	16.43369	13.66191	15.01214	0.641946	1	-
ENSMUSC	Sorbs2	sorbin anc	GO:00508	response	-	-	-	-	8:4550778	1618	2546	2288	3342	2431	975	2.143114	2.82669	2.08773	2.883732	2.823543	1.601243	2.352511	2.436173	0.953444	1	-
ENSMUSC	Irf2	interferon	GO:00081	metabolic	-	-	-	-	8:4673973	430	540	599	635	497	281	2.413264	2.540288	2.315873	2.321622	2.445882	1.955371	2.423142	2.240958	0.43766	1	-
ENSMUSC	Casp3	caspase 3	GO:00325	developm	K02187	caspase 3	mmu052C	Pathways	8:4661729	136	179	193	210	139	90	1.774064	1.957203	1.734353	1.784553	1.589965	1.455658	1.821873	1.610059	0.34899	1	-
ENSMUSC	Cenpu	centrome	GO:00325	developm	-	-	-	-	8:4655202	28	15	20	41	23	26	0.350636	0.157453	0.17254	0.334474	0.252566	0.403708	0.226877	0.33025	0.244542	1	-
ENSMUSC	Cfap97	cilia and f	-	-	-	-	-	-	8:4615177	634	734	1159	989	804	437	3.227022	3.131566	4.063942	3.279372	3.588487	2.757911	3.474177	3.20859	0.440487	1	-
ENSMUSC	Slc25a4	solute car	GO:00511	localizatio	K05863	solute car	mmu0516	HTLV-I inf	8:4620679	7787	9510	11711	11511	8667	7854	182.4133	186.7324	188.9869	175.6633	178.0319	228.1199	186.0442	193.9384	0.981587	1	-
ENSMUSC	Ufsp2	UFM1-spe	GO:00081	metabolic	-	-	-	-	8:4597552	512	588	841	782	700	348	5.34568	5.145918	6.048952	5.318891	6.408751	4.50504	5.513517	5.410894	0.736886	1	-
ENSMUSC	Anxa10	annexin A	GO:00056	cell|intrace	-	-	-	-	8:6205704	0	2	4	0	0	0	0	0.035787	0.058833	0	0	0	0.03154	0	0.088047	1	-
ENSMUSC	Pdlim3	PDZ and l	GO:00325	developm	-	-	-	-	8:4588546	20	31	25	20	22	11	0.191715	0.249075	0.165084	0.124894	0.184924	0.130741	0.201958	0.146853	0.200929	1	-
ENSMUSC	Lrp2bp	Lrp2 bindi	GO:00056	cell|protei	-	-	-	-	8:4599930	6	5	14	3	4	10	0.046538	0.032504	0.074802	0.015156	0.027203	0.096172	0.051281	0.046177	0.677854	1	-
ENSMUSC	Tlr3	toll-like re	GO:00325	developm	K05401	toll-like re	mmu0516	Human pa	8:4539566	253	206	257	317	328	147	1.35859	0.927232	0.950719	1.108942	1.544488	0.978749	1.078847	1.210726	0.65078	1	-
ENSMUSC	Cbr4	carbonyl r	GO:00081	metabolic	-	-	-	-	8:6148773	147	252	271	285	200	175	1.089987	1.566239	1.384287	1.376675	1.3004	1.608898	1.346838	1.428657	0.920968	1	-
ENSMUSC	Sh3rf1	SH3 doma	GO:00081	metabolic	-	-	-	-	8:6122387	1391	1515	1827	1979	1496	1030	10.95986	10.00561	9.916715	10.15793	10.33599	10.06239	10.29406	10.18544	0.689828	1	-
ENSMUSC	Nek1	NIMA (ne	GO:00325	developm	-	-	-	-	8:6099319	740	966	1131	1237	895	520	3.248548	3.554573	3.420358	3.537601	3.44527	2.830395	3.407826	3.271088	0.592872	1	-
ENSMUSC	F11	coagulatic	GO:00081	metabolic	K01323	coagulatic	mmu0461	Complem	8:4524117	0	0	0	2	0	0	0	0	0	0.035473	0	0	0	0.011824	0.538397	1	-
ENSMUSC	Mfap3l	microfibril	GO:00160	membran	-	-	-	-	8:6063282	1637	1716	2379	2518	1718	829	7.5784	6.658846	7.587081	7.593924	6.974206	4.758495	7.274775	6.442208	0.323969	1	-
ENSMUSC	N4bp1	NEDD4 bi	GO:00081	metabolic	-	-	-	-	8:8680816	1216	1376	1541	1770	1283	869	5.8014	5.502634	5.064696	5.501159	5.367459	5.140499	5.456243	5.336372	0.63856	1	-
ENSMUSC	Cbln1	cerebellin	GO:00325	developm	-	-	-	-	8:8746840	58	98	142	110	101	105	0.594434	0.841889	1.002571	0.734426	0.907695	1.334282	0.812964	0.992134	0.483711	1	-
ENSMUSC	Heatr3	HEAT rep	GO:00511	localizatio	-	-	-	-	8:8813785	392	447	496	522	415	433	3.957552	3.782687	3.44964	3.433151	3.673936	5.420188	3.72996	4.175758	0.670538	1	-
ENSMUSC	Adcy7	adenylate	GO:00081	metabolic	K08047	adenylate	mmu052C	Pathways	8:8827240	55	92	115	118	64	76	0.309847	0.434438	0.446313	0.433063	0.316164	0.530869	0.396866	0.426699	0.901154	1	-
ENSMUSC	Brd7	bromodoi	GO:00081	metabolic	K11723	bromodoi	mmu0522	Hepatoce	8:8833103	940	1103	1485	1522	1103	612	8.488182	8.348632	9.23772	8.9533	8.733837	6.852109	8.691511	8.179749	0.499971	1	-
ENSMUSC	Nkd1	naked cut	GO:00325	developm	K03213	naked cut	mmu0439	Hippo sig	8:8852135	224	263	345	349	255	185	1.925067	1.894555	2.042533	1.953911	1.921676	1.971319	1.954052	1.948968	0.787492	1	-
ENSMUSC	Snx20	sorting ne	GO:00056	cell|intrace	-	-	-	-	8:8862656	15	18	20	16	17	13	0.142412	0.143245	0.13081	0.098958	0.141525	0.153028	0.138822	0.13117	0.761626	1	-
ENSMUSC	Sall1	sal-like 1	GO:00325	developm	-	-	-	-	8:8902723	476	503	646	669	549	310	5.74572	5.089306	5.37182	5.260727	5.811028	4.639652	5.402282	5.237136	0.652869	1	-
ENSMUSC	Rbl2	RB transcr	GO:00081	metabolic	K16332	retinoblas	mmu0415	PI3K-Akt s	8:9107005	845	1063	1365	1352	1087	733	4.128723	4.353566	4.594549	4.303456	4.657269	4.440672	4.358946	4.467132	0.919789	1	-
ENSMUSC	Aktip	thymoma	GO:00081	metabolic	-	-	-	-	8:9111178	1004	1293	1723	1749	1190	935	6.161974	6.651773	7.284886	6.992901	6.404361	7.11514	6.699544	6.837468	0.88385	1	-
ENSMUSC	Eif2ak3	eukaryotic	GO:00325	developm	K08860	eukaryotic	mmu0516	Epstein-B	6:7084451	532	547	774	775	526	434	4.256607	3.668526	4.266228	4.039566	3.690455	4.305534	4.063787	4.011852	0.689488	1	-
ENSMUSC	Gins3	GINS com	GO:00081	metabolic	-	-	-	-	8:9563345	35	46	59	63	57	43	0.701032	0.77228	0.81408	0.822029	1.001108	1.06788	0.762464	0.963672	0.341039	1	-
ENSMUSC	Setd6	SET doma	GO:00325	developm	-	-	-	-	8:9571588	110	96	139	137	118	80	2.051301	1.500591	1.785682	1.664331	1.929573	1.849744	1.779191	1.814549	0.916421	1	-
ENSMUSC	Got2	glutamati	GO:00081	metabolic	K14455	aspartate	mmu011C	Metabolic	8:9586413	3619	4310	5242	5193	4226	2733	41.75714	41.68428	41.66684	39.03392	42.7577	39.09918	41.70276	40.29693	0.539292	1	-
ENSMUSC	Cdh11	cadherin 1	GO:00325	developm	-	-	-	-	8:1026320	2072	2367	3102	3314	2466	1227	13.78723	13.20196	14.21938	14.36552	14.38875	10.1232	13.73619	12.95916	0.531449	1	-
ENSMUSC	Smad1	SMAD far	GO:00325	developm	K04676	mothers a	mmu052C	Transcript	8:7933839	350	358	430	481	316	261	3.037495	2.604251	2.570794	2.719403	2.404788	2.808498	2.737513	2.64423	0.599867	1	-
ENSMUSC	Lsm6	LSM6 hon	GO:00081	metabolic	K12625	U6 snRNA	mmu0304	Spliceosor	8:7880486	276	411	487	463	369	252	2.703965	3.375105	3.286793	2.954983	3.170014	3.061103	3.121954	3.062033	0.689742	1	-
ENSMUSC	Slc10a7	solute car	GO:00511	localizatio	-	-	-	-	8:7850934	83	101	117	133	105	86	0.351217	0.358237	0.34106	0.36663	0.389607	0.451213	0.350171	0.402483	0.578049	1	-
ENSMUSC	Tnpo2	transporti	GO:00325	developm	-	-	-	-	8:8503691	1792	2709	2608	2741	2644	1655	12.04516	15.26288	12.07629	12.00232	15.58399	13.79299	13.12811	13.7931	0.931389	1	-
ENSMUSC	Vps35	VPS35 ret	GO:00325	developm	K18468	vacuolar p	mmu0414	Endocytos	8:8526039	2006	2709	3240	3101	2386	1806	13.69212	15.49893	15.23478	13.78869	14.28081	15.28422	14.80861	14.45124	0.581077	1	-
ENSMUSC	Orc6	origin rec	GO:00081	metabolic	K02608	origin rec	mmu0411	Cell cycle	8:8529963	245	345	446	405	339	184	3.273866	3.864264	4.105639	3.525579	3.972253	3.048578	3.747923	3.51547	0.531085	1	-
ENSMUSC	Mylk3	myosin lig	GO:00325	developm	K00907	myosin-lig	mmu0481	Regulatior	8:8532430	49	38	71	81	32	23	0.500099	0.325084	0.499192	0.538544	0.286381	0.291055	0.441458	0.371993	0.452576	1	-
ENSMUSC	Gpt2	glutamic p	GO:00081	metabolic	K00814	alanine tra	mmu011C	Metabolic	8:8549257	1476	1664	2280	2113	1578	1171	14.01318	13.24209	14.91203	13.06867	13.13712	13.78458	14.05577	13.33012	0.436716	1	-
ENSMUSC	Dnaja2	DnaJ heat	GO:00987	molecular	K09503	DnaJ hom	mmu0414	Protein pr	8:8553763	3769	4766	5741	5726	4399	2456	74.59709	79.06835	78.27711	73.82931	76.34709	60.27125	77.31419	70.14921	0.305138	1	-
ENSMUSC	Itfg1	integrin al	GO:00055	extracellul	-	-	-	-	8:8571757	3563	4522	5365	5200	3863	2427	17.7141	18.84459	18.37488	16.84179	16.84111	14.96095	18.31119	16.21462	0.189255	1	-
ENSMUSC	Rfx1	regulatory	GO:00081	metabolic	-	-	-	-	8:8406683	363	438	572	643	500	425	2.962217	2.995968	3.215565	3.418241	3.577856	4.300165	3.057917	3.765421	0.209592	1	-
ENSMUSC	Tecr	trans-2,3-	GO:00081	metabolic	K10258	very-long	mmu0121	Fatty acid	8:8357169	2393	3280	3884	3866	3121	1930	29.63991	34.05343	33.14093	31.19447	33.89772	29.63994	32.27809	31.57737	0.63795	1	-
ENSMUSC	Tbc1d9	TBC1 dom	GO:00081	metabolic	-	-	-	-	8:8316535	1775	1769	2562	2300	1721	1308	14.30285	11.94827	14.22181	12.07352	12.1604	13.06827	13.49098	12.43406	0.11494	1	-
ENSMUSC	Zfp330	zinc finge	GO:00056	cell|intrace	-	-	-	-	8:8276360	228	340	341	319	226	240	1.700114	2.125079	1.751658	1.549586	1.477728	2.218912	1.85895	1.748742	0.474395	1	-

ENSMUSC	Il15	interleukir	GO:00325	developm	K05433	interleukir	mmu052C	Pathways	8:8233163	4	8	13	12	12	2	0.055165	0.09248	0.123515	0.107821	0.145128	0.03421	0.090387	0.09572	0.915873	1 -
ENSMUSC	Gab1	growth fa	GO:00325	developm	K09593	GRB2-ass	mmu0401	Ras signal	8:8076443	670	646	1076	925	487	448	5.225888	4.223481	5.78161	4.700116	3.330862	4.332608	5.076993	4.121196	0.089182	1 -
ENSMUSC	Smarca5	SWI/SNF r	GO:00081	metabolic	-	-	-	-	8:8069850	1295	1591	1908	1965	1463	797	8.794739	9.056827	8.926535	8.693551	8.712444	6.711152	8.926034	8.039049	0.307586	1 -
ENSMUSC	Hp	haptoglot	GO:00081	metabolic	-	-	-	-	8:1095751	6	3	10	5	8	7	0.160562	0.067286	0.184346	0.087167	0.187727	0.232259	0.137398	0.169051	0.800796	1 -
ENSMUSC	Txnl4b	thioredox	GO:00081	metabolic	-	-	-	-	8:1095658	295	356	382	446	348	195	7.300672	7.384888	6.51263	7.19048	7.552008	5.983586	7.066063	6.908691	0.720877	1 -
ENSMUSC	Ces1f	carboxyle	GO:00081	metabolic	-	-	mmu011C	Metabolic	8:9325623	1	4	0	0	2	0	0.015354	0.051467	0	0	0.02692	0	0.022274	0.008973	0.524475	1 -
ENSMUSC	Pmfbp1	polyamine	GO:00056	cell|intrace	-	-	-	-	8:1094940	4	2	3	5	8	1	0.070749	0.029651	0.036554	0.057618	0.124089	0.021925	0.045651	0.067877	0.500178	1 -
ENSMUSC	Zfp821	zinc finge	GO:00081	metabolic	-	-	-	-	8:1097055	548	705	823	871	660	500	4.203625	4.533	4.349053	4.352548	4.439459	4.755539	4.361893	4.515849	0.972595	1 -
ENSMUSC	Ist1	increased	GO:00325	developm	K19476	vacuolar p	mmu0414	Endocytos	8:1096713	942	1194	1445	1499	1121	1125	4.731444	5.026894	4.999908	4.904852	4.937314	7.006177	4.919415	5.616114	0.500381	1 -
ENSMUSC	Dhodh	dihydroor	GO:00325	developm	K00254	dihydroor	mmu011C	Metabolic	8:1095913	172	255	312	242	214	281	1.122061	1.394379	1.402148	1.028453	1.224176	2.272902	1.306196	1.50851	0.648506	1 -
ENSMUSC	Ap1g1	adaptor p	GO:00325	developm	K12391	AP-1 com	mmu0414	Lysosome	8:1097785	2252	2352	3056	3049	2200	1805	13.48675	11.80672	12.60793	11.89537	11.55325	13.403	12.6338	12.28387	0.552029	1 -
ENSMUSC	Phlpp2	PH domai	GO:00325	developm	K16340	PH domai	mmu0415	PI3K-Akt s	8:1098685	707	1046	1154	1219	889	799	3.260903	4.043924	3.666704	3.662721	3.595531	4.569316	3.657177	3.942523	0.804336	1 -
ENSMUSC	Irx3	Iroquois r	GO:00325	developm	-	-	-	-	8:9179852	7	12	12	6	7	3	0.09194	0.132108	0.108575	0.051337	0.080621	0.048857	0.110875	0.060272	0.11107	1 -
ENSMUSC	Irx5	Iroquois r	GO:00325	developm	-	-	-	-	8:9235762	0	0	4	0	2	0	0	0	0.038125	0	0.02426	0	0.012708	0.008087	1	1 -
ENSMUSC	Irx6	Iroquois r	GO:00325	developm	-	-	-	-	8:9267428	0	4	0	6	2	1	0	0.043202	0	0.050369	0.022597	0.015973	0.014401	0.029646	0.480485	1 -
ENSMUSC	Mmp2	matrix me	GO:00325	developm	K01398	matrix me	mmu052C	Pathways	8:9282729	45	63	105	118	63	47	0.610962	0.716953	0.982063	1.043663	0.750036	0.791194	0.769993	0.861631	0.707118	1 -
ENSMUSC	Gnao1	guanine n	GO:00325	developm	K04534	guanine n	mmu0401	Rap1 sign	8:9380996	13618	15801	12736	21170	11563	9715	49.75366	48.38927	32.05503	50.3865	37.0446	44.00889	43.39932	43.81333	0.83856	1 -
ENSMUSC	St3gal2	ST3 beta-	GO:00081	metabolic	K03368	beta-gala	mmu011C	Metabolic	8:1109199	1882	2252	2036	1992	1489	2026	19.22012	19.27781	14.32404	13.25279	13.3344	25.6544	17.60732	17.41386	0.674648	1 -
ENSMUSC	Il34	interleukir	GO:00325	developm	-	-	-	-	8:1107418	311	487	504	493	352	462	5.704183	7.487114	6.368177	5.890628	5.66133	10.50656	6.519825	7.35284	0.694054	1 -
ENSMUSC	Amfr	autocrine	GO:00325	developm	K10636	autocrine	mmu0414	Protein pr	8:9397158	2157	2666	3351	3220	2521	3088	27.29666	28.27955	29.21361	26.54587	27.97531	48.45323	28.26327	34.3248	0.317167	1 -
ENSMUSC	Cog4	compone	GO:00511	localizatio	-	-	-	-	8:1108466	503	668	862	835	691	556	4.716316	5.250055	5.567923	5.100381	5.681402	6.463912	5.178098	5.748565	0.631261	1 -
ENSMUSC	Nudt21	nudix (nuc	GO:00081	metabolic	K14397	cleavage a	mmu0301	mRNA sur	8:9401549	808	1068	1273	1332	1018	725	7.929408	8.785234	8.606156	8.515595	8.760311	8.821714	8.440266	8.699207	0.952017	1 -
ENSMUSC	Bbs2	Bardet-Bi	GO:00325	developm	-	-	-	-	8:9406795	293	436	571	518	391	293	4.567343	5.696851	6.131729	5.260255	5.344599	5.663038	5.465307	5.42263	0.741462	1 -
ENSMUSC	Cenpn	centrome	GO:00329	macromo	-	-	-	-	8:1169217	6	20	14	28	16	14	0.073291	0.20477	0.117802	0.222806	0.171372	0.212037	0.131954	0.202072	0.201807	1 -
ENSMUSC	Cdyl2	chromodc	GO:00081	metabolic	-	-	-	-	8:1165744	73	83	122	130	78	93	1.924259	1.833876	2.215387	2.232361	1.80292	3.039526	1.991174	2.358269	0.518979	1 -
ENSMUSC	Mt3	metallothi	GO:00325	developm	-	-	-	-	8:9415260	2781	3220	3943	3883	3162	2894	135.7457	131.7449	132.5877	123.4736	135.3411	175.1498	133.3594	144.6548	0.767592	1 -
ENSMUSC	Mt2	metallothi	GO:00325	developm	K14739	metallothi	mmu0497	Mineral al	8:9417266	311	362	454	400	370	854	23.72749	23.15006	23.86155	19.88076	24.75343	80.7858	23.5797	41.80666	0.052676	1 -
ENSMUSC	Mt1	metallothi	GO:00508	response	K14739	metallothi	mmu0497	Mineral al	8:9417908	1450	1514	2086	1849	1659	1944	40.64925	35.5765	40.28564	33.76782	40.78248	67.57201	38.83713	47.3741	0.335973	1 -
ENSMUSC	Slc12a3	solute car	GO:00511	localizatio	-	-	-	-	8:9432920	6	5	8	9	3	4	0.028155	0.019665	0.02586	0.027511	0.012343	0.023272	0.02456	0.021042	0.66433	1 -
ENSMUSC	Nudt7	nudix (nuc	GO:00325	developm	K17879	peroxisom	mmu0414	Peroxisom	8:1141335	33	60	93	55	43	60	0.256826	0.39141	0.498609	0.27885	0.293451	0.57898	0.382282	0.38376	0.804953	1 -
ENSMUSC	Herpud1	homocyst	GO:00081	metabolic	K14027	homocyst	mmu0414	Protein pr	8:9438643	638	747	1242	848	845	673	8.151749	8.000257	10.93209	7.058427	9.467375	10.66182	9.028034	9.062539	0.778012	1 -
ENSMUSC	Cntnap4	contactin	GO:00452	synapse|c	-	-	-	-	8:1125700	498	636	648	756	604	401	3.848422	4.119673	3.449684	3.805893	4.092914	3.842231	3.805926	3.913679	0.949227	1 -
ENSMUSC	Fam192a	family wit	GO:00056	cell|intrace	-	-	-	-	8:9457413	612	763	909	901	785	512	5.186619	5.420139	5.306988	4.974385	5.833716	5.380082	5.304582	5.396061	0.874858	1 -
ENSMUSC	Pllp	plasma m	GO:00325	developm	-	-	-	-	8:9467489	488	287	673	634	513	348	10.07599	4.967097	9.572695	8.527827	9.288114	8.90909	8.205259	8.908343	0.804328	1 -
ENSMUSC	Arl2bp	ADP-ribo	GO:00081	metabolic	-	-	-	-	8:9466660	1501	2043	2408	2202	1850	1480	23.04181	26.28797	25.465	22.02088	24.90292	28.16977	24.93159	25.03119	0.748715	1 -
ENSMUSC	Cx3cl1	chemokin	GO:00325	developm	K05508	C-X3-C m	mmu0406	Cytokine-	8:9477200	1530	1942	1363	2201	1697	1167	19.14096	20.36454	11.74681	17.938	18.61645	18.10212	17.0841	18.21886	0.866357	1 -
ENSMUSC	Ccl22	chemokin	GO:00081	metabolic	K05514	C-C motif	mmu0406	Cytokine-	8:9474559	2	10	8	11	5	4	0.050832	0.212988	0.140043	0.182088	0.111404	0.126028	0.134621	0.13984	1	1 -
ENSMUSC	Ccl17	chemokin	GO:00325	developm	K05514	C-C motif	mmu0406	Cytokine-	8:9481045	4	6	7	8	0	7	0.494556	0.621808	0.596272	0.644381	0	1.073182	0.570879	0.572521	1	1 -
ENSMUSC	Ciapin1	cytokine i	GO:00325	developm	-	-	-	-	8:9481980	595	877	933	1048	781	776	3.033129	3.74737	3.276478	3.480304	3.491143	4.904805	3.352326	3.958751	0.371915	1 -
ENSMUSC	Coq9	coenzyme	GO:00081	metabolic	-	-	-	-	8:9483832	765	992	1024	1152	990	721	10.06169	10.93638	9.278129	9.870594	11.41792	11.75791	10.09206	11.01547	0.712662	1 -
ENSMUSC	Polr2c	polymeras	GO:00081	metabolic	K03011	DNA-dire	mmu011C	Metabolic	8:9485745	314	385	392	460	407	358	2.940273	3.021839	2.528689	2.806068	3.341915	4.156494	2.830267	3.434826	0.307517	1 -
ENSMUSC	Adgrg1	adhesion	GO:00325	developm	-	-	-	-	8:9497475	846	1066	1218	1231	967	857	4.943078	5.2208	4.902592	4.685615	4.95446	6.208605	5.022157	5.282893	0.963342	1 -
ENSMUSC	Katnb1	katanin p8	GO:00325	developm	-	-	-	-	8:9508118	526	681	745	791	672	653	4.114832	4.465462	4.014893	4.031105	4.609757	6.333812	4.198396	4.991558	0.365383	1 -
ENSMUSC	Kifc3	kinesin fai	GO:00990	supramole	-	-	-	-	8:9509982	821	912	1136	1256	867	812	6.706558	6.244597	6.39273	6.683869	6.210382	8.224289	6.447961	7.039513	0.727556	1 -
ENSMUSC	Cngb1	cyclic nucl	GO:00325	developm	K04952	cyclic nucl	mmu0474	Olfactory	8:9523904	4	5	8	20	12	0	0.019628	0.020565	0.027044	0.063937	0.051637	0	0.022412	0.038525	0.324809	1 -
ENSMUSC	Mmp15	matrix me	GO:00325	developm	-	-	-	-	8:9535226	412	407	530	518	338	391	4.70848	3.898791	4.17264	3.856511	3.387217	5.540456	4.259971	4.261395	0.74343	1 -
ENSMUSC	Tmem38a	transmem	GO:00511	localizatio	-	-	-	-	8:7257205	1418	1837	2092	2361	1644	1626	28.87477	31.35483	29.34644	31.31988	29.35532	41.05337	29.85868	33.90952	0.505143	1 -
ENSMUSC	Usb1	U6 snRNA	GO:00081	metabolic	-	-	-	-	8:9533228	81	86	121	123	101	98	1.348649	1.200231	1.387879	1.334139	1.474617	2.023148	1.312253	1.610635	0.378016	1 -
ENSMUSC	Cfap20	cilia and f	GO:00081	metabolic	-	-	-	-	8:9542024	366	527	474	542	435	378	2.338902	2.822894	2.086704	2.256379	2.437604	2.995084	2.416167	2.563022	0.92464	1 -
ENSMUSC	Tpm4	tropomyo	GO:00325	developm	K10375	tropomyo	mmu0426	Adrenergi	8:7213497	459	626	835	839	599	351	13.05183	14.92058	16.35673	15.54184	14.9358	12.37517	14.77638	14.28427	0.651125	1 -
ENSMUSC	B3gnt3	UDP-GlcN	GO:00081	metabolic	K07970	beta-1,3-	mmu011C	Metabolic	8:7169075	23	23	39	52	30	43	0.478464	0.401055	0.558912	0.704713	0.547249	1.109127	0.479477	0.78703	0.068922	1 -
ENSMUSC	Jak3	Janus kina	GO:00081	metabolic	K11218	Janus kina	mmu052C	Pathways	8:7167629	83	130	143	151	111	107	0.673079	0.883653	0.798865	0.797712	0.789315	1.075862	0.785199	0.887629	0.664114	1 -
ENSMUSC	Pgls	6-phosph	GO:00081	metabolic	K01057	6-phosph	mmu011C	Metabolic	8:7159217	202	197	246	212	199	251	2.355114	1.92521	1.975815	1.610188	2.034489	3.628435	2.08538	2.424371	0.62895	1 -
ENSMUSC	Slc27a1	solute car	GO:00081	metabolic	K08745	solute car	mmu0493	Insulin res	8:7156888	1190	1394	1751	2010	1443	1686	6.525852	6.407755	6.614966	7.180717	6.939037	11.46393	6.516191	8.527896	0.102667	1 -
ENSMUSC	Map1lc3b	microtubu	GO:00081	metabolic	K10435	microtubu	mmu0421	Ferroptos	8:1215903	1005	1123	1537	1335	1108	742	6.040358	5.657565	6.363884	5.227088	5.839547	5.529513	6.020602	5.532049	0.316526	1 -
ENSMUSC	Mvb12a	multivesic	GO:00511	localizatio	K12186	ESCRT-I c	mmu0414	Endocytos	8:7154292	159	173	276	214	191	232	2.099948	1.915176	2.511137	1.841224	2.212013	3.799126	2.17542	2.617454	0.488784	1 -
ENSMUSC	Mthfsd	methenylt	GO:00511	localizatio	-	-	-	-	8:1210916	371	502	576	597	502	444	1.338679	1.518306	1.431782	1.403326	1.58836	1.986424	1.429589	1.65937	0.449281	1 -
ENSMUSC	Cox4i1	cytochron	GO:00081	metabolic	K02263	cytochron	mmu011C	Metabolic	8:1206682	2431	2817	4103	3595	3062	3117	58.0599	56.39375	67.50628	55.93347	64.12673	92.30261	60.65331	70.7876	0.421351	1 -
ENSMUSC	Emc8	ER memb	GO:00329	macromo	-	-	-	-	8:1206539	931	1061	1570	1513	1096	838	4.991383	4.768036	5.798588	5.284353	5.152574	5.570591	5.186002	5.33584	0.935478	1 -
ENSMUSC	Babam1	BRISC anc	GO:00325	developm	K20776	BRISC anc	mmu0344	Homologe	8:7139686	738	896	1013	1072	831	743	16.3081	16.59618	15.42084	15.43206	16.10241	20.35739	16.10837	17.29728	0.836351	1 -
ENSMUSC	Gins2	GINS com	GO:00081	metabolic	-	-	-	-	8:1205786	47	49	53	39	53	61	0.622547	0.544029	0.483618	0.336528	0.615594	1.001818	0.550065	0.651313	0.696996	1 -
ENSMUSC	Gse1	genetic su	-	-	-	-	-	-	8:1202284	2784	3268	2709	3361	3387	2296	13.51655	13.29938	9.060599	10.63033	14.41964	13.82148	11.95884	12.95715	0.787994	1 -
ENSMUSC	Zdhhc7	zinc finge	GO:00081	metabolic	-	-	-	-	8:1200808	464	450	606	652	426	492	5.989967	4.869362	5.389272	5.483215	4.822344	7.875127	5.416201	6.060229	0.672244	1 -
ENSMUSC	6430548M	RIKEN cDI	-	-	-	-	-	-	8:1201141	2564	4892	5891	4147	4840	3353	11.90458	19.03862	18.84242	12.5433	19.70535	19.30259	16.59521	17.18375	0.965131	1 -
ENSMUSC	Crispld2	cysteine-r	GO:00325	developm	-	-	-	-	8:1199924	45	52	23	21	36	64	0.434241	0.420597	0.152897	0.132014	0.304623	0.765737	0.335912	0.400791	0.822284	1 -
ENSMUSC	Usp10	ubiquitin s	GO:00081	metabolic	-	-	-	-	8:1199103	561	707	830	994	629	432	2.54593	2.689406	2.594858	2.938679	2.503096	2.430827	2.610065	2.624201	0.835358	1 -
ENSMUSC	Cotl1	coactosin	GO:00508	response	-	-	-	-	8:1198092	784	830	1023	1084	820	832	12.92492	11.46946	11.6182	11.64186	11.85409	17.00673	12.00419	13.50089	0.604377	1 -
ENSMUSC	Klhl36	kelch-like	GO:00081	metabolic	-	-	-	-	8:1198622	85	101	114	158	106	100	1.399118	1.393514	1.292683	1.694236	1.529976	2.040899	1.361772	1.755037	0.212249	1 -
ENSMUSC	Dnaaf1	dynein, ax	GO:00325	developm	-	-	-	-	8:1195752	6	6	20	5	7	2	0.135177	0.113297	0.310402	0.073386	0.138294	0.055881	0.186292	0.089187	0.092619	1 -
ENSMUSC	Taf1c	TATA-box	GO:00081	metabolic	-	-	-	-	8:1195978	136	189	182	213	201	180	1.412525	1.645395	1.302203	1.441178	1.830601	2.318	1.453374	1.86326	0.230877	1 -
ENSMUSC	Mast3	microtubu	GO:00081	metabolic	-	-	-	-	8:7077811	6192	6876	8139	10418	7321	6169	46.23123	43.03217	41.86267	50.67228	47.93112	57.10909	43.70869	51.90417	0.25085	1 -
ENSMUSC	Pik3r2	phosphoir	GO:00081	metabolic	-	-	-	-	8:7076817	2703	3052	3638	4208	3016	2383	28.93932	27.38922	26.8322	29.34943	28.31497	31.63386	27.72025	29.76609	0.787073	1 -
ENSMUSC	Mbtps1	membran	GO:00081	metabolic	K08653	membran	mmu0414	Protein pr	8:1195081	1364	1669	2008	1961	1630	1344	6.987449	7.166607	7.0863	6.544306	7.322083	8.536691	7.080119	7.467693	0.934389	1 -
ENSMUSC	Necab2	N-termina	GO:00081	metabolic	-	-	-	-	8:1194467	1164	1505	1549	1508	1238	1291	34.66527	37.56912	31.77932	29.25665	32.32994	47.67097	34.67124	36.41919	0.995772	1 -
ENSMUSC	Hsbp1	heat shoc	GO:00325	developm	-	-	-	-	8:1193444	2085	2742	3214	3068	2220	2403	37.7086	41.56757	40.04347	36.14692	35.20708	53.88576	39.77321	41.74659	1	1 -
ENSMUSC	Rab3a	RAB3A, m	GO:00325	developm	K07882	Ras-relate	mmu0491	Insulin sec	8:7075467	4613	5948	6365	6408	5391	5369	69.20917	74.80042	65.78547	62.63027	70.92383	99.87564	69.93169	77.80991	0.647737	1 -
ENSMUSC	Cdh13	cadherin 1	GO:00325	developm	-	-	-	-	8:1182837	980	1159	1327	1444	1034	678	4.953628	4.910586	4.620825	4.754951	4.583113	4.249259	4.828346	4.529108	0.429731	1 -
ENSMUSC	Pde4c	phosphoc	GO:00081	metabolic	-	-	-	-	8:7072372	22	23	36	51	30	27	0.113513	0.099474	0.127965	0.17143	0.135735	0.172737	0.113651	0.159967	0.180411	1 -
ENSMUSC	Mphosph	M phase p	GO:00081	metabolic	K12593	M-phase	mmu0301	RNA degr	8:1177916	198	229	307	334	220	143	3.684781	3.572184	3.935818	4.049237	3.590139	3.299647	3.730928	3.646341	0.726186	1 -
ENSMUSC	Hsd17b2	hydroxyst	GO:00325	developm	K13368	17beta-es	mmu011C	Metabolic	8:1177019	1	0	2	2	0	0	0.028015	0	0.038595	0.036489	0	0	0.022203	0.012163	1	1 -
ENSMUSC	Bco1	beta-carc	GO:00081	metabolic	K00515	beta-carc	mmu011C	Metabolic	8:1170958	2	1	1	0	0	2	0.037613	0.01576	0.012948	0	0	0.046639	0.022107	0.015546	0.717657	1 -
ENSMUSC	1700030J2	RIKEN cDI	GO:00056	cell|intrace	-	-	-	-	8:1169695	151	162	188	205	136	118	2.279449	2.049852	1.955073	2.016	1.800257	2.208631	2.094792	2.008296	0.606711	1 -
ENSMUSC	Lsm4	LSM4 hon	GO:00081	metabolic	-	-	-	-	8:7067324	322	392	521	535	423	364	9.197347	9.385238	10.25168	9.955002	10.59471	12.89122	9.611423	11.14698	0.44175	1 -
ENSMUSC	Comp	cartilage c	GO:00325	developm	K04659	thrombos	mmu0415	PI3K-Akt s	8:7037355	2	10	8	21	8	2	0.037271	0.156166	0.102682	0.254895	0.13071	0.046216	0.098706	0.14394	0.453254	1 -
ENSMUSC	Ntpcr	nucleosid	GO:00081	metabolic	K06928	nucleosid	mmu011C	Metabolic	8:1257299	90	128	205	208	104	161	1.312474	1.564629	2.059468	1.976032	1.329919	2.911114	1.645524	2.072355	0.382644	1 -
ENSMUSC	Map3k21	mitogen-	GO:00048	signal trar	-	-	-	-	8:1259104	29	48	67	64	28	44	0.323738	0.449145	0.515251	0.46543	0.27409	0.609022	0.429378	0.449514	1	1 -
ENSMUSC	Mau2	MAU2 sist	GO:00329	macromo	-	-	-	-	8:7001612	1101	1304	1567	1812	1409	1174	4.868284	4.833025	4.773195	5.219494	5.46314	6.436394	4.824834	5.706343	0.289548	1 -
ENSMUSC	Pbx4	pre B cell	GO:00081	metabolic	-	-	-	-	8:6983249	79	136	109	122	88	93	1.203418	1.736541	1.143849	1.210693	1.175489	1.756543	1.36127	1.380908	0.863336	1 -
ENSMUSC	Lpar2	lysophosp	GO:00048	signal trar	K04291	lysophosp	mmu052C	Pathways	8:6982242	23	21	40	26	20	22	0.135859	0.103977	0.16277	0.100049	0.103597	0.161132	0.134202	0.121593	0.546219	1 -
ENSMUSC	Atp13a1	ATPase ty	GO:00511	localizatio	-	-	-	-	8:6979116	851	974	1232	1279	1014	932	13.81446	13.25307	13.77737	13.5256	14.43394	18.75884	13.61497	15.5728	0.477773	1 -
ENSMUSC	Ints10	integrator	GO:00081	metabolic	-	-	-	-	8:6879392	803	998	1256	1350	1043	773	4.238628	4.415635	4.567206	4.642214	4.827658	5.059118	4.407156	4.842996	0.647539	1 -
ENSMUSC	Dctn1	dynactin 1	GO:00511	localizatio	K04648	dynactin 1	mmu0501	Huntingto	6:8316592	8489	9585	10932	12216	9858	6963	57.36937	54.29608	50.89493	53.78165	58.41912	58.3453	54.18679	56.84869	0.939545	1 -
ENSMUSC	Pgr	progester	GO:00325	developm	K08556	progester	mmu0522	Breast car	9:8899833	365	427	499	535	434	270	1.844535	1.808731	1.737184	1.761282	1.923207	1.691778	1.796817	1.792089	0.786972	1 -
ENSMUSC	Cdh5	cadherin 5	GO:00325	developm	K06533	cadherin 5	mmu0451	Cell adhes	8:1041016	329	434	584	615	401	246	4.816617	5.325853	5.889937	5.865477	5.147932	4.465475	5.344136	5.159628	0.659848	1 -
ENSMUSC	Bean1	brain expi	GO:00160	membran	-	-	-	-	8:1041704	117	155	191	129	153	94	0.753972	0.837249	0.847921	0.541556	0.864582	0.751081	0.813047	0.719073	0.345118	1 -
ENSMUSC	Cmtm3	CKLF-like	GO:00400	locomotic	-	-	-	-	8:1043394	81	117	142	145	115	112	1.069338	1.294703	1.291429	1.24704	1.331286	1.833299	1.21849	1.470542	0.426255	1 -
ENSMUSC	Ces2g	carboxyle	GO:00038	catalytic a	K03927	carboxyles	mmu0098	Drug meta	8:1049617	2	3	8	5	0	4	0.030708	0.0386	0.084601	0.050005	0	0.076134	0.051303	0.042047	0.728382	1 -
ENSMUSC	Nae1	NEDD8 ac	GO:00081	metabolic	K04532	amyloid b	mmu0501	Alzheimer	8:1045110	477	638	857	744	603	393	2.266017	2.540498	2.80464	2.302498	2.511915	2.314854	2.537052	2.376423	0.449655	1 -
ENSMUSC	Fam96b	family wit	GO:00081	metabolic	-	-	-	-	8:1046398	123	167	191	179	192	170	3.783643	4.306011	4.047529	3.587072	5.179056	6.483975	4.045728	5.083368	0.305403	1 -
ENSMUSC	Rrad	Ras-relate	GO:00511	localizatio	-	-	-	-	8:1046280	24	30	35	21	17	17	0.758088	0.794284	0.761596	0.43213	0.470861	0.665787	0.771323	0.522926	0.071365	1 -
ENSMUSC	Car7	carbonic a	GO:00081	metabolic	K01672	carbonic a	mmu0091	Nitrogen r	8:1045346	183	183	225	274	205	230	2.251301	1.887062	1.906845	2.195903	2.21146	3.508293	2.01507	2.638552	0.184324	1 -
ENSMUSC	Cbfb	core bindi	GO:00325	developm	-	-	-	-	8:1051706	350	467	550	560	398	283	3.233548	3.616431	3.500458	3.37039	3.224306	3.241775	3.450146	3.278824	0.521508	1 -
ENSMUSC	Ces2e	carboxyle	GO:00038	catalytic a	K03927	carboxyles	mmu0098	Drug meta	8:1049262	0	4	3	0	0	0	0	0.064941	0.04003	0	0	0	0.03499	0	0.030856	1 -
ENSMUSC	Tradd	TNFRSF1A	GO:00325	developm	K03171	tumor nec	mmu0516	Human pa	8:1052582	38	51	59	75	51	65	0.89744	1.009582	0.959894	1.153897	1.056176	1.903352	0.955639	1.371142	0.148936	1 -
ENSMUSC	D230025D	RIKEN cDI	-	-	-	-	-	-	8:1052251	330	390	433	597	337	407	3.004656	2.97645	2.71594	3.541086	2.690628	4.594737	2.899015	3.608817	0.253871	1 -
ENSMUSC	Tsnaxip1	translin-a	GO:00325	developm	-	-	-	-	8:1058277	4	7	24	16	11	4	0.093388	0.136987	0.386019	0.243359	0.225211	0.115796	0.205465	0.194789	0.871039	1 -
ENSMUSC	Ctrl	chymotryp	GO:00081	metabolic	K09632	chymotryp	mmu0497	Pancreatic	8:1059320	4	5	6	4	4	4	0.165496	0.173399	0.171013	0.10783	0.145116	0.205206	0.169969	0.152717	0.737224	1 -
ENSMUSC	Psmb10	proteasor	GO:00325	developm	K02733	20S prote	mmu0305	Proteason	8:1059357	145	189	225	193	144	245	2.276485	2.487197	2.433489	1.973943	1.982444	4.769231	2.399057	2.908539	0.544916	1 -
ENSMUSC	Dus2	dihydrour	GO:00081	metabolic	-	-	-	-	8:1059913	120	166	198	229	178	149	1.744765	2.023096	1.983226	2.169068	2.269442	2.686124	1.917029	2.374878	0.268427	1 -
ENSMUSC	Nfatc3	nuclear fa	GO:00081	metabolic	K17333	nuclear fa	mmu0401	MAPK sigr	8:1060588	297	222	396	412	309	190	1.457887	0.913426	1.339102	1.317484	1.330053	1.156397	1.236805	1.267978	0.961533	1 -
ENSMUSC	Pla2g15	phospholi	GO:00081	metabolic	K06129	lysophosp	mmu0414	Lysosome	8:1061503	309	436	516	486	395	392	3.714368	4.393051	4.272949	3.80579	4.163576	5.842508	4.12679	4.603958	0.676704	1 -
ENSMUSC	Slc7a6	solute car	GO:00511	localizatio	-	-	-	-	8:1061688	267	334	478	519	336	303	2.164369	2.269444	2.669313	2.740747	2.388375	3.045428	2.367708	2.72485	0.48179	1 -
ENSMUSC	Smpd3	sphingom	GO:00325	developm	K12352	sphingom	mmu011C	Metabolic	8:1062525	2185	2531	2764	3210	2350	1869	24.34917	23.64165	21.21887	23.3034	22.96378	25.82426	23.0699	24.03048	1	1 -
ENSMUSC	Zfp90	zinc finge	GO:00081	metabolic	-	-	-	-	8:1064153	148	249	327	286	240	176	0.494862	0.697871	0.75322	0.622974	0.703681	0.72966	0.648651	0.685438	0.934638	1 -
ENSMUSC	Has3	hyalurona	GO:00081	metabolic	-	-	-	-	8:1068702	36	38	65	64	56	49	0.23504	0.207962	0.292356	0.27221	0.320607	0.396665	0.245119	0.329827	0.217318	1 -
ENSMUSC	Vps4a	vacuolar p	GO:00325	developm	K12196	vacuolar p	mmu0414	Endocytos	8:1070312	1351	1892	2111	2071	1594	1414	27.88572	32.73411	30.01692	27.84763	28.85077	36.18778	30.21225	30.96206	0.87304	1 -
ENSMUSC	Cog8	compone	GO:00511	localizatio	-	-	-	-	8:1070462	398	460	574	616	495	370	3.451709	3.343962	3.429366	3.480272	3.764427	3.978673	3.408346	3.741124	0.688606	1 -
ENSMUSC	Nip7	NIP7, nucl	GO:00329	macromo	-	-	-	-	8:1070568	374	503	582	625	405	483	5.999204	6.763047	6.431258	6.531056	5.69665	9.606258	6.397836	7.277988	0.593204	1 -
ENSMUSC	Mtmr2	myotubul	GO:00325	developm	K18081	myotubul	mmu011C	Metabolic	9:1374841	890	1039	1433	1323	1010	430	7.215488	7.060638	8.003372	6.98742	7.18025	4.322447	7.426499	6.163372	0.184052	1 -
ENSMUSC	Terf2	telomeric	GO:00325	developm	-	-	-	-	8:1070694	576	438	831	1008	666	458	3.906361	2.489873	3.882419	4.453405	3.960658	3.851247	3.426218	4.088437	0.354894	1 -
ENSMUSC	Cep57	centroson	GO:00325	developm	-	-	-	-	9:1380779	216	283	371	412	278	145	1.305513	1.433728	1.544727	1.622204	1.473378	1.086624	1.427989	1.394068	0.758793	1 -
ENSMUSC	Cyb5b	cytochron	GO:00081	metabolic	-	-	-	-	8:1071506	4320	5066	6428	6548	4797	2872	40.45219	39.76274	41.46537	39.94372	39.38861	33.34484	40.5601	37.55906	0.371677	1 -
ENSMUSC	Maml2	mastermir	GO:00081	metabolic	K06061	mastermir	mmu0516	Human pa	9:1329795	548	521	806	978	521	333	3.436653	2.738709	3.482104	3.995544	2.865074	2.58932	3.219155	3.149979	0.752923	1 -
ENSMUSC	1700012B	RIKEN cDI	-	-	-	-	-	-	9:1475658	16	11	16	6	5	9	0.587107	0.338339	0.404444	0.143429	0.160867	0.409452	0.443297	0.237916	0.04976	1 -
ENSMUSC	Mre11a	MRE11A h	GO:00081	metabolic	K10865	double-st	mmu0421	Cellular se	9:1478465	217	247	353	367	262	196	1.876586	1.790433	2.102977	2.067545	1.98679	2.101596	1.923332	2.051977	0.860385	1 -
ENSMUSC	Wwp2	WW dom	GO:00081	metabolic	K05630	atrophin-	mmu0412	Ubiquitin	8:1074363	611	677	830	865	681	595	3.302789	3.067474	3.090787	3.046052	3.22797	3.987886	3.153683	3.420636	0.776531	1 -
ENSMUSC	Ankrd49	ankyrin re	GO:00325	developm	-	-	-	-	9:1477961	118	175	102	123	148	104	0.722679	0.898368	0.430346	0.49074	0.794818	0.789738	0.683798	0.691765	0.875159	1 -
ENSMUSC	Gpr83	G protein-	GO:00048	signal trar	K04210	G protein-	mmu0408	Neuroacti	9:1486021	253	348	424	460	306	269	2.001857	2.308049	2.311156	2.371113	2.123128	2.639072	2.20702	2.377771	0.815996	1 -
ENSMUSC	Panx1	pannexin	GO:00511	localizatio	K03443	pannexin	mmu0462	NOD-like	9:1500212	232	273	347	389	271	243	2.119344	2.090401	2.183708	2.314968	2.170834	2.752365	2.131151	2.412723	0.56481	1 -
ENSMUSC	Med17	mediator	GO:00325	developm	K15133	mediator	mmu0491	Thyroid h	9:1526035	327	432	549	549	427	301	3.831453	4.242808	4.431395	4.190544	4.387191	4.372898	4.168552	4.316877	0.988369	1 -
ENSMUSC	Vstm5	V-set and	GO:00325	developm	-	-	-	-	9:1523904	84	130	149	130	124	111	2.68128	3.478246	3.276434	2.703276	3.470785	4.393131	3.14532	3.522397	0.725837	1 -
ENSMUSC	4931406C	RIKEN cDI	GO:00038	catalytic a	-	-	-	-	9:1528333	416	487	632	674	428	255	1.836869	1.802463	1.922442	1.93877	1.657188	1.396081	1.853925	1.664013	0.353287	1 -
ENSMUSC	Taf1d	TATA-box	GO:00081	metabolic	-	-	-	-	9:1530621	212	297	351	395	262	172	0.779793	0.915697	0.889409	0.946501	0.845058	0.784434	0.861633	0.858665	0.811392	1 -
ENSMUSC	Kars	lysyl-tRNA	GO:00081	metabolic	K04567	lysyl-tRNA	mmu0097	Aminoacy	8:1119934	1275	1621	1768	1770	1357	822	15.96967	17.01851	15.25525	14.44244	14.90418	12.76567	16.08114	14.03743	0.176271	1 -
ENSMUSC	Adat1	adenosine	GO:00081	metabolic	-	-	-	-	8:1119669	124	151	199	207	162	105	1.546423	1.578468	1.709663	1.681741	1.771591	1.623604	1.611518	1.692312	0.944904	1 -
ENSMUSC	Gabarapl2	gamma-a	GO:00081	metabolic	K08341	GABA(A) r	mmu0516	Kaposi's sa	8:1119407	948	1231	1519	1332	993	1381	20.35413	22.15416	22.46745	18.63074	18.69548	36.7641	21.65858	24.69677	0.6356	1 -
ENSMUSC	Tmem231	transmem	GO:00325	developm	-	-	-	-	8:1119120	242	302	179	362	313	228	2.007546	2.099954	1.022953	1.956325	2.276868	2.345151	1.710151	2.192781	0.270967	1 -
ENSMUSC	Chst5	carbohydi	GO:00081	metabolic	K09671	carbohydi	mmu0053	Glycosam	8:1118891	0	13	5	7	9	15	0	0.317388	0.100326	0.132821	0.22987	0.541709	0.139238	0.301466	0.245643	1 -
ENSMUSC	Tmem170	transmem	GO:00099	cellular pr	-	-	-	-	8:1118630	40	57	105	72	67	67	0.65553	0.782993	1.185414	0.768671	0.96282	1.361414	0.874646	1.030968	0.624725	1 -
ENSMUSC	Cfdp1	craniofaci	GO:00325	developm	-	-	-	-	8:1117684	1196	1609	1938	1909	1431	801	64.52408	72.76118	72.02711	67.09318	67.69759	53.58078	69.77079	62.79051	0.298706	1 -
ENSMUSC	Bcar1	breast car	GO:00048	signal trar	K05726	breast car	mmu0481	Regulatior	8:1117104	1104	1127	1096	1494	1157	1016	11.38079	9.738226	7.783325	10.0331	10.45873	12.98622	9.634112	11.15935	0.454259	1 -
ENSMUSC	Ldhd	lactate de	GO:00081	metabolic	K00102	D-lactate	mmu0062	Pyruvate r	8:1116237	132	165	199	231	205	142	0.948657	0.993971	0.985236	1.081509	1.291915	1.265353	0.975955	1.212926	0.246062	1 -
ENSMUSC	Wdr59	WD repea	GO:00508	response	K20409	WD repea	mmu0415	mTOR sig	8:1114487	560	634	850	835	633	493	2.880358	2.733381	3.011818	2.797862	2.854989	3.144062	2.875186	2.932304	0.872656	1 -
ENSMUSC	Aars	alanyl-tRN	GO:00325	developm	K01872	alanyl-tRN	mmu0097	Aminoacy	8:1110331	3224	3803	4760	5049	3814	2952	15.72969	15.55264	15.99865	16.04767	16.3173	17.85778	15.76033	16.74092	0.856727	1 -
ENSMUSC	Cdh15	cadherin 1	GO:00452	synapse|e	K06809	cadherin 1	mmu0451	Cell adhes	8:1228479	27	36	35	20	15	23	0.52781	0.589899	0.471341	0.254706	0.257128	0.55749	0.529684	0.356441	0.07242	1 -
ENSMUSC	Bmper	BMP-binc	GO:00325	developm	-	-	-	-	9:2322307	70	134	171	150	96	91	0.834338	1.338759	1.404088	1.16471	1.003364	1.34485	1.192395	1.170975	0.751409	1 -
ENSMUSC	Afg3l1	AFG3-like	GO:00081	metabolic	-	-	-	-	8:1234779	310	384	491	565	412	352	3.471006	3.603939	3.787275	4.1212	4.045137	4.886782	3.62074	4.35104	0.287864	1 -
ENSMUSC	Acad8	acyl-Coer	GO:00081	metabolic	K11538	isobutyryl	mmu011C	Metabolic	9:2697413	351	340	464	525	311	298	2.903055	2.357112	2.643739	2.828719	2.255549	3.055984	2.634635	2.713417	0.935799	1 -
ENSMUSC	Dbndd1	dysbindin	GO:00056	cell|intrace	-	-	-	-	8:1235047	100	107	123	132	111	131	2.100231	1.883664	1.779612	1.80602	2.044236	3.411337	1.921169	2.420531	0.360159	1 -
ENSMUSC	Ccsap	centriole,	GO:00325	developm	-	-	-	-	8:1238408	980	1120	1318	1346	1164	838	12.98352	12.43762	12.02911	11.61697	13.52266	13.76564	12.48342	12.96843	0.985998	1 -
ENSMUSC	Acta1	actin, alpl	GO:00325	developm	-	-	-	-	8:1238917	48	62	67	86	52	66	1.986034	2.150254	1.909716	2.318058	1.88665	3.385928	2.015335	2.530212	0.383161	1 -
ENSMUSC	Abcb10	ATP-bind	GO:00511	localizatio	K05657	ATP-bind	mmu0201	ABC trans	8:1239524	391	449	652	582	504	607	5.770836	5.554703	6.629203	5.595855	6.522814	11.10803	5.984914	7.742233	0.210574	1 -
ENSMUSC	Urb2	URB2 ribc	GO:00056	cell|intrace	-	-	-	-	8:1240215	239	295	360	391	286	279	1.938384	2.005471	2.011386	2.065858	2.033999	2.805637	1.98508	2.301832	0.476203	1 -
ENSMUSC	Cog2	compone	GO:00511	localizatio	-	-	-	-	8:1245207	292	343	454	419	358	396	3.112628	3.064719	3.333893	2.909642	3.346336	5.233904	3.170413	3.829961	0.381191	1 -
ENSMUSC	Agt	angiotens	GO:00325	developm	K09821	angiotens	mmu0492	Renin secr	8:1245565	319	469	493	483	366	335	10.72099	13.21204	11.41414	10.57484	10.7862	13.95966	11.78239	11.77357	0.744589	1 -
ENSMUSC	Capn9	calpain 9	GO:00081	metabolic	-	-	-	-	8:1245761	0	0	0	1	0	3	0	0	0	0.015395	0	0.087921	0	0.034438	0.167962	1 -
ENSMUSC	Arv1	ARV1 hon	GO:00081	metabolic	-	-	-	-	8:1247221	68	65	90	83	69	64	1.726576	1.383378	1.574233	1.372892	1.536263	2.01484	1.561396	1.641331	1	1 -
ENSMUSC	2310022B	RIKEN cDI	GO:00056	cell|intrace	-	-	-	-	8:1246357	892	1010	1261	1181	1037	1254	17.14221	16.26957	16.69431	14.78541	17.47525	29.88032	16.70203	20.71366	0.295856	1 -
ENSMUSC	2810004N	RIKEN cDI	GO:00056	cell|intrace	-	-	-	-	8:1248369	354	405	512	553	381	258	2.783	2.668808	2.772882	2.832154	2.6265	2.514871	2.741564	2.657842	0.636681	1 -
ENSMUSC	Gnpat	glycerone	GO:00325	developm	K00649	glycerone	mmu0056	Glyceroph	8:1248630	690	769	996	921	819	758	7.837632	7.321757	7.793757	6.815178	8.157601	10.67558	7.651049	8.549452	0.63888	1 -
ENSMUSC	Sprtn	SprT-like	GO:00081	metabolic	-	-	-	-	8:1248978	305	362	468	483	357	326	3.753614	3.734315	3.967776	3.87239	3.852659	4.974546	3.818568	4.233198	0.671147	1 -
ENSMUSC	Egln1	egl-9 fam	GO:00325	developm	K09592	hypoxia-i	mmu052C	Pathways	8:1249085	1331	1557	1961	2103	1547	1376	23.53618	23.07808	23.8884	24.22584	23.98784	30.1691	23.50089	26.1276	0.596491	1 -
ENSMUSC	Vps26b	VPS26 ret	GO:00511	localizatio	K18466	vacuolar p	mmu0414	Endocytos	9:2700777	2289	2844	3460	3630	2702	2071	24.62719	25.64789	25.64464	25.44236	25.4916	27.62709	25.30657	26.18702	0.963105	1 -
ENSMUSC	Jam3	junction a	GO:00325	developm	K06785	junctional	mmu0453	Tight junc	9:2709738	369	375	613	541	422	405	6.019271	5.127449	6.888571	5.74905	6.036319	8.191403	6.011764	6.658924	0.70489	1 -
ENSMUSC	Spata19	spermato	GO:00325	developm	-	-	-	-	9:2739680	0	0	4	0	0	0	0	0	0.072255	0	0	0	0.024085	0	0.306173	1 -

ENSMUSC	Snx19	sorting ne	GO:00325	developm	-	-	-	-	9:3042710	1020	1113	1394	1642	1211	1004	6.553179	5.993768	6.169729	6.872372	6.822425	7.997825	6.238892	7.230874	0.379042	1	-
ENSMUSC	Adamts8	a disinteg	GO:00081	metabolic	-	-	-	-	9:3094256	68	111	86	140	92	72	0.724375	0.991123	0.63111	0.971543	0.859376	0.950978	0.782203	0.927299	0.482207	1	-
ENSMUSC	St14	suppressi	GO:00325	developm	K08670	suppresso	mmu0520	MicroRNA	9:3108940	7	14	21	20	6	7	0.046089	0.077262	0.095246	0.085782	0.034637	0.057144	0.072866	0.059188	0.498967	1	-
ENSMUSC	Aplp2	amyloid b	GO:00325	developm	-	-	-	-	9:3114955	9011	10796	10362	11908	8633	7562	63.27913	63.5482	50.12825	54.47635	53.16086	65.84308	58.9852	57.82676	0.597181	1	-
ENSMUSC	Trpc6	transient r	GO:00325	developm	K04969	transient r	mmu0436	Axon guic	9:8544142	424	514	598	752	517	295	4.260311	4.329043	4.139311	4.922377	4.555211	3.675219	4.242888	4.384269	0.99078	1	-
ENSMUSC	Birc3	baculovira	GO:00081	metabolic	K16060	baculovira	mmu0520	Pathways	9:7848699	17	25	33	41	40	23	0.15678	0.193261	0.209659	0.246329	0.323486	0.26301	0.186567	0.277609	0.127711	1	-
ENSMUSC	Dcun1d5	DCN1, de	GO:00081	metabolic	-	-	-	-	9:7184520	391	537	669	678	450	363	6.365067	7.327464	7.502462	7.190148	6.423641	7.32687	7.064998	6.98022	0.707358	1	-
ENSMUSC	Pdgfd	platelet-d	GO:00325	developm	K05450	platelet de	mmu0415	PI3K-Akt s	9:6168584	73	81	98	98	74	60	0.715734	0.66568	0.661918	0.625945	0.636215	0.729396	0.681111	0.663852	0.711804	1	-
ENSMUSC	Sesn3	sestrin 3 [	GO:00081	metabolic	K10141	sestrin 1/3	mmu0411	p53 signa	9:1427506	748	480	639	688	572	363	2.776082	1.493224	1.633742	1.663416	1.86153	1.67041	1.967683	1.731785	0.33699	1	-
ENSMUSC	Usp2	ubiquitin s	GO:00325	developm	-	-	-	-	9:4406702	687	738	1020	1088	881	740	5.357825	4.824373	5.480036	5.527674	6.024907	7.155655	5.220744	6.236079	0.28776	1	-
ENSMUSC	Thy1	thymus ce	GO:00325	developm	K06514	Thy-1 cell	mmu0467	Leukocyte	9:4404338	8877	11080	12701	13162	10015	8093	115.0173	120.3344	113.3669	111.0966	113.7865	130.0147	116.2396	118.2993	0.829231	1	-
ENSMUSC	Nectin1	nectin cell	GO:00325	developm	K06081	poliovirus	mmu0516	Herpes sir	9:4374398	1159	1424	1340	1580	1165	1014	12.20108	12.56544	9.717872	10.83561	10.75434	13.23545	11.4948	11.60847	0.792564	1	-
ENSMUSC	Trim29	tripartite r	GO:00081	metabolic	-	-	-	-	9:4331084	3	2	10	1	6	4	0.050313	0.028117	0.115551	0.010925	0.088241	0.083187	0.06466	0.060785	0.884228	1	-
ENSMUSC	Oaf	out at first	GO:00055	extracellul	-	-	-	-	9:4322123	123	113	158	211	154	173	2.329957	1.79421	2.061829	2.603797	2.558043	4.063282	2.061999	3.07504	0.043647	1	-
ENSMUSC	Pou2f3	POU dom	GO:00081	metabolic	K09364	POU dom	mmu0516	Herpes sir	9:4312393	2	9	5	3	7	5	0.031313	0.118082	0.053919	0.030588	0.09609	0.097038	0.067772	0.074572	1	1	-
ENSMUSC	Grik4	glutamate	GO:00048	signal trar	K05204	glutamate	mmu0408	Neuroacti	9:4251813	1161	1480	1548	1707	1283	1157	10.74331	11.47943	9.867981	10.29013	10.41059	13.27471	10.69691	11.32515	0.919919	1	-
ENSMUSC	Sc5d	sterol-C5-	GO:00081	metabolic	K00227	Delta7-ste	mmu0110	Metabolic	9:4225159	1521	1681	2246	2269	1630	978	15.63638	14.4853	15.90627	15.19579	14.69391	12.46614	15.34265	14.11861	0.36513	1	-
ENSMUSC	Ubash3b	ubiquitin a	GO:00325	developm	-	-	-	-	9:4101109	708	872	1093	1226	890	660	5.333122	5.505763	5.671786	6.016173	5.878695	6.164217	5.503557	6.019695	0.678776	1	-
ENSMUSC	Crtam	cytotoxic a	GO:00511	localizatio	-	-	-	-	9:4096969	2	0	0	1	1	0	0.020097	0	0	0.006544	0.008809	0	0.006699	0.005118	1	1	-
ENSMUSC	Jhy	junctional	GO:00325	developm	-	-	-	-	9:4089484	5	11	18	10	10	8	0.067827	0.125081	0.168211	0.088368	0.118954	0.134556	0.120373	0.113959	0.764939	1	-
ENSMUSC	Clmp	CXADR-lil	GO:00325	developm	-	-	-	-	9:4068596	615	718	960	1088	722	371	6.800955	6.655367	7.313369	7.838005	7.001238	5.086914	6.92323	6.642052	0.651658	1	-
ENSMUSC	Rexo2	RNA exon	GO:00081	metabolic	K13288	oligoribor	mmu0300	Ribosome	9:4846851	709	778	1013	1028	771	517	7.325484	6.737871	7.210264	6.919347	6.985341	6.623184	7.091207	6.842624	0.57496	1	-
ENSMUSC	Nxpe2	neurexopl	GO:00160	membran	-	-	-	-	9:4831800	6	11	9	10	13	4	0.061734	0.094869	0.063787	0.067024	0.117286	0.051027	0.073463	0.078446	0.914412	1	-
ENSMUSC	Cul5	cullin 5 [S	GO:00081	metabolic	K10612	cullin 5[-]	mmu0412	Ubiquitin	9:5361458	1464	1881	2365	2454	1808	1113	7.219242	7.774854	8.034025	7.883278	7.81793	6.805056	7.67604	7.502088	0.653011	1	-
ENSMUSC	Barx2	BarH-like	GO:00325	developm	-	-	-	-	9:3184604	14	25	20	17	25	15	0.452103	0.676707	0.44494	0.357632	0.707938	0.600614	0.524583	0.555395	1	1	-
ENSMUSC	Kcnj5	potassium	GO:00511	localizatio	K04999	potassium	mmu0492	Oxytocin s	9:3231470	2	14	17	14	25	16	0.014151	0.083015	0.082843	0.064518	0.155078	0.140337	0.060003	0.119978	0.103187	1	-
ENSMUSC	Ets1	E26 avian	GO:00325	developm	K02678	C-ets[-]	mmu0520	Pathways	9:3263622	271	337	320	480	238	156	1.469401	1.531631	1.195289	1.695486	1.131595	1.048772	1.398773	1.291951	0.542991	1	-
ENSMUSC	Kirrel3	kin of IRR	GO:00325	developm	-	-	-	-	9:3448589	721	681	965	1162	979	895	4.039292	3.197939	3.724334	4.240895	4.809446	6.216971	3.653855	5.089104	0.034894	1	-
ENSMUSC	St3gal4	ST3 beta-	GO:00081	metabolic	K03494	beta-gala	mmu0110	Metabolic	9:3504657	194	307	314	314	251	327	3.338549	4.428417	3.72253	3.52021	3.787678	6.977344	3.829832	4.761744	0.362385	1	-
ENSMUSC	Dcps	decapping	GO:00081	metabolic	K12584	m7GpppX	mmu0301	RNA degr	9:3512440	201	209	294	274	199	175	5.134309	4.474916	5.173496	4.5595	4.457396	5.542551	4.927574	4.853149	0.697417	1	-
ENSMUSC	Tirap	toll-interle	GO:00081	metabolic	K05403	toll-interle	mmu0515	Tuberculo	9:3518455	42	42	32	72	50	38	0.329207	0.275946	0.172791	0.36765	0.343666	0.369315	0.259315	0.360211	0.190208	1	-
ENSMUSC	Srpr	signal rec	GO:00511	localizatio	K13431	signal rec	mmu0306	Protein ex	9:3520017	1355	1588	1918	2166	1558	880	23.19265	22.78319	22.61576	24.15189	23.38413	18.67582	22.86387	22.07061	0.611157	1	-
ENSMUSC	Rpusd4	RNA pseu	GO:00081	metabolic	-	-	-	-	9:3526786	107	151	177	148	108	143	1.92804	2.280656	2.197128	1.737295	1.70646	3.194867	2.135275	2.212874	0.916312	1	-
ENSMUSC	Abhd12	abhydrola	GO:00081	metabolic	-	-	-	-	2:1508324	3032	3606	4585	4469	3318	3273	41.27951	41.15133	43.00273	39.63668	39.61177	55.25062	41.81119	44.83302	0.843669	1	-
ENSMUSC	Acat1	acetyl-Co	GO:00325	developm	K00626	acetyl-Co	mmu0110	Metabolic	9:5358052	1213	1553	1789	1911	1514	989	18.09195	19.41551	18.38173	18.56807	19.80127	18.28971	18.62973	18.88635	0.851714	1	-
ENSMUSC	Rdx	radixin [Sc	GO:00325	developm	K05762	radixin[-]	mmu0481	Regulatior	9:5204717	1999	2276	3014	2994	2281	1178	17.60566	16.80214	18.28665	17.17801	17.61601	12.86384	17.56482	15.88595	0.333471	1	-
ENSMUSC	Fdx1	ferredoxir	GO:00081	metabolic	-	-	-	-	9:5194330	38	77	117	106	95	150	0.60755	1.031916	1.288651	1.104047	1.331877	2.973562	0.976039	1.803162	0.033559	1	-
ENSMUSC	Pou2af1	POU dom	GO:00081	metabolic	-	-	-	-	9:5121370	0	6	3	1	3	2	0	0.082014	0.033702	0.010623	0.042898	0.040452	0.038572	0.031324	0.829046	1	-
ENSMUSC	Ppp2r1b	protein pł	GO:00325	developm	K03456	serine/thre	mmu0415	PI3K-Akt s	9:5084530	124	106	134	169	134	84	0.822006	0.588995	0.611942	0.729831	0.778933	0.690427	0.674315	0.733064	0.791068	1	-
ENSMUSC	Alg9	asparagin	GO:00081	metabolic	K03846	alpha-1,2	mmu0110	Metabolic	9:5077501	286	247	371	338	296	272	2.806695	2.031793	2.508154	2.160867	2.547208	3.30966	2.44888	2.672578	0.801299	1	-
ENSMUSC	Cryab	crystallin,	GO:00325	developm	K09542	crystallin,	mmu0414	Protein pr	9:5075132	365	450	618	457	384	566	7.439666	7.68822	8.677608	6.06817	6.863311	14.30415	7.935165	9.078544	0.688675	1	-
ENSMUSC	2310030G	RIKEN cDl	GO:00055	extracellul	-	-	-	-	9:5073969	17	17	29	33	13	24	0.527269	0.441978	0.619647	0.666786	0.353574	0.922976	0.529631	0.647779	0.654657	1	-
ENSMUSC	Dixdc1	DIX doma	GO:00325	developm	-	-	-	-	9:5066275	1721	2035	2753	2852	2025	1534	6.713811	6.654349	7.398541	7.248022	6.927174	7.419929	6.922234	7.198375	0.995548	1	-
ENSMUSC	Bco2	beta-carc	GO:00081	metabolic	-	-	-	-	9:5053308	68	69	85	94	61	38	2.040425	1.735443	1.757032	1.837464	1.605038	1.413791	1.8443	1.618764	0.411331	1	-
ENSMUSC	Pts	6-pyruvoy	GO:00081	metabolic	K01737	6-pyruvoy	mmu0110	Metabolic	9:5052161	206	208	305	333	236	268	1.128128	0.954789	1.150646	1.188001	1.133302	1.819747	1.077854	1.38035	0.216502	1	-
ENSMUSC	Plet1	placenta e	GO:00325	developm	-	-	-	-	9:5049452	3	19	4	18	0	4	0.087972	0.467074	0.080824	0.343893	0	0.145453	0.211957	0.163115	0.689479	1	-
ENSMUSC	Cadm1	cell adhes	GO:00325	developm	K06781	cell adhes	mmu0451	Cell adhes	9:4753017	2166	2395	2353	2906	2486	1221	8.325117	7.715967	6.230247	7.276276	8.378682	5.818801	7.423777	7.157919	0.636005	1	-
ENSMUSC	Bud13	BUD13 hc	GO:00056	cell|intrace	-	-	-	-	9:4628300	174	225	298	325	174	128	1.766212	1.91438	2.083821	2.149104	1.548759	1.610971	1.921471	1.769611	0.489555	1	-
ENSMUSC	Zpr1	ZPR1 zinc	GO:00325	developm	-	-	-	-	9:4627306	445	541	695	680	540	445	5.658484	5.766205	6.088021	5.632879	6.021105	7.015941	5.83757	6.223308	0.872436	1	-
ENSMUSC	Apoa1	apolipopr	GO:00325	developm	K08757	apolipopr	mmu0332	PPAR sign	9:4622858	0	2	0	3	2	0	0	0.071839	0	0.083742	0.075151	0	0.023946	0.052964	0.507458	1	-
ENSMUSC	Tagln	transgelin	GO:00325	developm	-	-	-	-	9:4592961	161	237	294	228	181	191	4.071635	5.023937	5.122029	3.756307	4.01389	5.989118	4.7392	4.586438	0.610265	1	-
ENSMUSC	Bace1	beta-site	GO:00081	metabolic	K04521	beta-site	mmu0501	Alzheimer	9:4583858	2008	2342	1476	2824	2112	1810	10.97758	10.73206	5.558797	10.05749	10.12465	12.26896	9.089479	10.81703	0.433893	1	-
ENSMUSC	Dscaml1	DS cell ad	GO:00325	developm	-	-	-	-	9:4542662	2383	2666	3084	3718	2970	1706	14.03065	13.1573	12.50891	14.26083	15.33391	12.45428	13.23229	14.01634	0.851722	1	-
ENSMUSC	Il10ra	interleukir	GO:00048	signal trar	K05134	interleukir	mmu0406	Cytokine-	9:4525383	62	80	133	61	96	73	0.476398	0.515255	0.704012	0.305345	0.646831	0.69548	0.565221	0.549218	0.73371	1	-
ENSMUSC	Tmprss4	transmem	GO:00081	metabolic	K09635	transmem	mmu0516	Influenza	9:4517272	0	4	0	0	0	0	0	0.047678	0	0	0	0	0.015893	0	0.18743	1	-
ENSMUSC	Mpzl2	myelin prc	GO:00160	membran	-	-	-	-	9:4503979	33	50	59	63	36	33	0.248136	0.315135	0.305617	0.308601	0.23737	0.307661	0.289629	0.284544	0.792563	1	-
ENSMUSC	Cd3e	CD3 antig	GO:00325	developm	K06451	T-cell surf	mmu0516	HTLV-I inf	9:4499874	0	0	0	2	0	0	0	0	0	0.018456	0	0	0	0.006152	0.537554	1	-
ENSMUSC	Cd3d	CD3 antig	GO:00325	developm	K06450	T-cell surf	mmu0516	HTLV-I inf	9:4498178	0	0	0	2	0	0	0	0	0	0.038366	0	0	0	0.012789	0.537593	1	-
ENSMUSC	Arcn1	archain 1	GO:00325	developm	-	-	-	-	9:4474156	2310	2661	3434	3593	2662	1764	9.238984	8.920925	9.461585	9.361621	9.336043	8.747759	9.207165	9.148475	0.720553	1	-
ENSMUSC	Ddx6	DEAD (As	GO:00325	developm	K12614	ATP-depe	mmu0301	RNA degr	9:4460489	3943	4840	5940	6345	4718	2501	30.39659	31.27493	31.5454	31.86481	31.89325	23.9055	31.07231	29.22119	0.485334	1	-
ENSMUSC	Treh	trehalase	GO:00325	developm	K01194	alpha,alph	mmu0110	Metabolic	9:4467323	3	0	0	0	1	0	0.063694	0	0	0	0.018618	0	0.021231	0.006206	0.671457	1	-
ENSMUSC	Ddx25	DEAD (As	GO:00325	developm	-	-	-	-	9:3553664	606	796	943	950	713	599	5.554243	6.115305	5.954091	5.672279	5.730392	6.80715	5.874547	6.06994	0.942277	1	-
ENSMUSC	Pus3	pseudouri	GO:00081	metabolic	-	-	-	-	9:3555859	145	194	268	232	193	92	1.598475	1.79263	2.035272	1.66612	1.865681	1.257505	1.808792	1.596436	0.378733	1	-
ENSMUSC	Pdzd3	PDZ dom	GO:00081	metabolic	-	-	-	-	9:4424730	16	15	15	15	10	16	0.387232	0.304294	0.250088	0.236492	0.212224	0.480116	0.313871	0.309611	0.820075	1	-
ENSMUSC	Nlrx1	NLR famil	GO:00023	immune s	K12653	NLR famil	mmu0462	NOD-like	9:4425271	88	89	140	116	115	184	0.666431	0.564953	0.730382	0.572282	0.763681	1.727721	0.653922	1.021228	0.11149	1	-
ENSMUSC	Trappc4	trafficking	GO:00325	developm	-	-	-	-	9:4440369	237	326	419	269	272	390	2.814283	3.244823	3.427567	2.080914	2.832253	5.742096	3.162224	3.551755	0.793922	1	-
ENSMUSC	Chek1	checkpoir	GO:00325	developm	K02216	serine/thre	mmu0516	HTLV-I inf	9:3670848	20	25	49	28	19	13	0.187723	0.196686	0.316836	0.17121	0.156376	0.151287	0.233748	0.159624	0.113464	1	-
ENSMUSC	Slc37a4	solute car	GO:00325	developm	K08171	MFS trans	mmu0497	Carbohyd	9:4439685	163	193	274	245	190	345	1.633162	1.620881	1.891228	1.599152	1.669309	4.285947	1.71509	2.518136	0.143574	1	-
ENSMUSC	Hyou1	hypoxia u	GO:00511	localizatio	K09486	hypoxia u	mmu0414	Protein pr	9:4437949	1614	1946	2629	2929	2003	1323	8.506764	8.59719	9.545593	10.05685	9.257301	8.64583	8.883182	9.319993	0.911634	1	-
ENSMUSC	Stt3a	STT3, sub	GO:00081	metabolic	K07151	dolichyl-d	mmu0110	Metabolic	9:3672934	764	958	1295	1247	812	731	4.531029	4.762356	5.29084	4.817829	4.222817	5.375351	4.861408	4.805332	0.673664	1	-
ENSMUSC	Fez1	fasciculati	GO:00325	developm	-	-	-	-	9:3682186	1869	3160	3971	4501	3163	1657	18.51606	26.24094	27.10134	29.04892	27.47776	20.35392	23.95278	25.62687	0.793555	1	-
ENSMUSC	Hinfp	histone H	GO:00325	developm	-	-	-	-	9:4429244	398	449	647	665	442	404	2.425364	2.293465	2.716122	2.639956	2.361885	3.052539	2.478317	2.684793	0.783221	1	-
ENSMUSC	C2cd2l	C2 calciur	GO:00511	localizatio	-	-	-	-	9:4430923	2442	2275	3032	3236	2052	2424	15.00497	11.7172	12.83424	12.95328	11.05629	18.4675	13.18547	14.15903	0.873925	1	-
ENSMUSC	Tmem218	transmem	GO:00160	membran	-	-	-	-	9:3720822	165	161	267	259	189	141	4.003902	3.274765	4.463374	4.09433	4.02165	4.242327	3.914014	4.119435	0.946395	1	-
ENSMUSC	Slc37a2	solute car	GO:00081	metabolic	-	-	-	-	9:3722758	83	53	161	103	56	67	0.56332	0.301514	0.752753	0.455399	0.333276	0.563815	0.539195	0.45083	0.343564	1	-
ENSMUSC	Dpagt1	dolichyl-p	GO:00081	metabolic	-	-	mmu0110	Metabolic	9:4432601	193	222	238	292	167	159	2.071919	1.997657	1.760122	2.04211	1.572079	2.116399	1.943233	1.910196	0.716256	1	-
ENSMUSC	Robo4	roundabo	GO:00325	developm	-	-	-	-	9:3740189	98	97	134	164	97	120	0.688728	0.571413	0.648755	0.750842	0.597773	1.045659	0.636298	0.798091	0.327526	1	-
ENSMUSC	Hmbs	hydroxym	GO:00081	metabolic	K01749	hydroxym	mmu0110	Metabolic	9:4433633	142	183	250	195	143	139	1.335184	1.442309	1.619369	1.194454	1.179051	1.620521	1.465621	1.331342	0.365919	1	-
ENSMUSC	Vps11	VPS11, CC	GO:00081	metabolic	-	-	-	-	9:4434774	907	1015	1290	1386	947	904	7.383448	6.925817	7.234242	7.350158	6.759967	9.124438	7.181169	7.744854	0.802282	1	-
ENSMUSC	Abcg4	ATP bindi	GO:00511	localizatio	K05680	ATP-bind	mmu0201	ABC trans	9:4427318	1087	1292	1511	1630	1306	1171	9.116139	9.082323	8.729663	8.905346	9.604334	12.17656	8.976042	10.22875	0.486584	1	-
ENSMUSC	Mcam	melanom	GO:00325	developm	-	-	-	-	9:4413446	267	284	406	400	345	299	3.046983	2.716631	3.191808	2.973725	3.452394	4.230734	2.985141	3.552284	0.373261	1	-
ENSMUSC	Pin1	protein (p	GO:00325	developm	K09578	peptidyl-p	mmu0462	RIG-I-like	9:2065209	883	1039	1206	1111	927	910	14.60247	14.40238	13.73929	11.9691	13.44276	18.6592	14.24805	14.69035	0.891535	1	-
ENSMUSC	Olfm2	olfactome	GO:00325	developm	-	-	-	-	9:2066769	411	657	884	688	709	679	5.207395	6.977447	7.71582	5.678696	7.877121	10.66681	6.633554	8.074208	0.357959	1	-
ENSMUSC	Icam5	intercellul	GO:00511	localizatio	-	-	-	-	9:2103207	5300	6316	6782	7797	6200	5907	114.2962	114.1697	100.7547	109.5381	117.2439	157.9461	109.7402	128.2427	0.372273	1	-
ENSMUSC	Tyk2	tyrosine k	GO:00081	metabolic	K11219	non-recep	mmu0516	Human pa	9:2110406	363	403	482	616	443	413	2.181536	2.030081	1.99551	2.411672	2.334546	3.077456	2.069042	2.607891	0.170127	1	-
ENSMUSC	Pde4a	phosphoc	GO:00081	metabolic	K13293	cAMP-spe	mmu0402	cAMP sigr	9:2116571	2132	2611	2999	3485	2427	1856	12.97472	13.31897	12.573	13.81641	12.9516	14.00473	12.95556	13.59091	0.93768	1	-
ENSMUSC	Ilf3	interleukir	GO:00081	metabolic	-	-	-	-	9:2136787	1404	1610	1946	2088	1577	1175	4.788474	4.602658	4.57219	4.63919	4.716332	4.968829	4.654441	4.774784	0.907566	1	-
ENSMUSC	Bmp5	bone mor	GO:00325	developm	K04663	bone mor	mmu0439	Hippo sig	9:7577536	44	47	57	69	58	35	0.663585	0.594142	0.592204	0.677911	0.767024	0.654483	0.616643	0.699806	0.65584	1	-
ENSMUSC	Tmed1	transmem	GO:00511	localizatio	-	-	-	-	9:2150755	63	99	70	119	62	76	0.802697	1.057302	0.614407	0.987727	0.692701	1.200628	0.824802	0.960352	0.630753	1	-
ENSMUSC	Scg3	secretogr	GO:00055	extracellul	-	-	-	-	9:7564318	3986	4752	6052	6394	5739	2733	46.78159	46.74842	48.93144	48.88683	59.06314	39.77069	47.48715	49.24022	0.967858	1	-
ENSMUSC	Yipf2	Yip1 dom	GO:00160	membran	-	-	-	-	9:2158868	154	259	243	228	146	228	1.813784	2.556914	1.971614	1.74937	1.507854	3.32954	2.114104	2.195588	0.921101	1	-
ENSMUSC	Lysmd2	LysM, put	-	-	-	-	-	-	9:7562574	448	648	625	695	516	366	11.56917	14.0266	11.11874	11.69206	11.68468	11.71902	12.23817	11.69859	0.545153	1	-
ENSMUSC	Carm1	coactivatc	GO:00325	developm	K05931	type I pro	mmu0152	Endocrine	9:2154689	1137	1438	1499	1678	1400	1007	6.256258	6.632327	5.682078	6.01489	6.75499	6.870201	6.190221	6.546693	0.897428	1	-
ENSMUSC	Tmod2	tropomoc	GO:00325	developm	-	-	-	-	9:7556562	13196	14630	18055	20188	14878	8980	37.45291	34.8049	35.30142	37.32658	37.02802	31.60134	35.85308	35.31865	0.685262	1	-
ENSMUSC	Smarca4	SWI/SNF r	GO:00325	developm	K11647	SWI/SNF-	mmu0522	Hepatoce	9:2161616	3791	4233	5220	5716	4275	2806	26.5487	24.84796	25.18323	26.07734	26.25235	24.36482	25.52663	25.56484	0.768278	1	-
ENSMUSC	Gnb5	guanine n	GO:00048	signal trar	K04539	guanine n	mmu0520	Pathways	9:7530628	842	1411	1159	914	1120	1403	10.34842	14.53589	9.812875	7.317947	12.07041	21.37988	11.56573	13.58941	0.614466	1	-
ENSMUSC	Ldlr	low densit	GO:00081	metabolic	K12473	low-densi	mmu0414	Endocytos	9:2172348	344	348	429	489	364	415	1.264006	1.071823	1.085923	1.170525	1.172829	1.890706	1.140584	1.411354	0.289801	1	-
ENSMUSC	Kank2	KN motif a	GO:00081	metabolic	-	-	-	-	9:2176678	249	290	366	363	323	261	2.516252	2.456437	2.547924	2.389697	2.862202	3.270255	2.506871	2.840718	0.570597	1	-
ENSMUSC	Dock6	dedicator	GO:00987	molecular	-	-	-	-	9:2179986	315	349	461	521	399	348	1.934222	1.796282	1.950059	2.084082	2.148379	2.649486	1.893521	2.293982	0.272734	1	-
ENSMUSC	Polr2m	polymeras	GO:00081	metabolic	-	-	-	-	9:7147843	2591	2726	3820	3265	2976	2151	39.0759	34.46039	39.68774	32.07794	39.35656	40.2224	37.74134	37.21897	0.645189	1	-
ENSMUSC	Rab27a	RAB27A, r	GO:00325	developm	-	-	-	-	9:7304485	125	228	249	293	279	125	1.037229	1.585813	1.423359	1.583848	2.030068	1.286051	1.3488	1.633322	0.372796	1	-
ENSMUSC	Aqp9	aquaporir	GO:00511	localizatio	K09877	aquaporir	mmu0497	Bile secret	9:7111065	15	25	29	24	29	8	0.093571	0.130718	0.124623	0.09753	0.15863	0.061876	0.116304	0.106012	0.736397	1	-
ENSMUSC	Lipc	lipase, hep	GO:00081	metabolic	K01046	triacylglyc	mmu0110	Metabolic	9:7079812	5	0	8	9	0	2	0.11049	0	0.121783	0.129558	0	0.054813	0.077424	0.061457	0.895435	1	-
ENSMUSC	Sltm	SAFB-like	GO:00081	metabolic	-	-	-	-	9:7054275	1623	1868	2235	2400	1673	833	7.052755	6.804087	6.690665	6.79412	6.374979	4.488192	6.849169	5.885764	0.21411	1	-
ENSMUSC	Rsl24d1	ribosomal	GO:00081	metabolic	K02896	large subu	mmu0301	Ribosome	9:7311342	125	166	392	343	221	393	2.819069	3.138024	6.09021	5.039302	4.370493	10.9894	4.015768	6.79973	0.04828	1	-
ENSMUSC	Nedd4	neural pre	GO:00325	developm	K10591	E3 ubiquit	mmu0414	Endocytos	9:7266234	8434	5961	7623	8003	5846	3609	30.00314	17.77481	18.68144	18.54674	18.23621	15.91864	22.15313	17.5672	0.081241	1	-
ENSMUSC	Rnf111	ring finge	GO:00325	developm	-	-	-	-	9:7042542	952	1021	1182	1333	1059	750	4.261039	3.830514	3.644578	3.88678	4.156398	4.162227	3.912043	4.068469	1	1	-
ENSMUSC	Ccnb2	cyclin B2 [	GO:00325	developm	K05868	cyclin B[-]	mmu0516	HTLV-I inf	9:7040769	13	11	12	22	9	13	0.539629	0.382759	0.343171	0.594933	0.327613	0.669084	0.421853	0.530543	0.618648	1	-
ENSMUSC	Myo1e	myosin IE	GO:00325	developm	-	-	-	-	9:7020735	270	291	444	384	329	190	2.60938	2.357329	2.956029	2.417617	2.788129	2.276745	2.640913	2.494164	0.546347	1	-
ENSMUSC	Mns1	meiosis-s	GO:00325	developm	-	-	-	-	9:7243801	111	84	148	111	106	62	1.182236	0.749915	1.085909	0.770164	0.989984	0.818768	1.00602	0.859639	0.320437	1	-
ENSMUSC	Fam81a	family witl	-	-	-	-	-	-	9:7008851	2793	3627	4472	4083	3532	2169	35.77258	38.93855	39.45777	34.06751	39.66822	34.4449	38.0563	36.06021	0.453823	1	-
ENSMUSC	Gcnt3	glucosam	GO:00325	developm	K09662	N-acetylg	mmu0110	Metabolic	9:7003149	2	0	5	0	0	0	0.029559	0	0.050898	0	0	0	0.026819	0	0.032557	1	-
ENSMUSC	Tcf12	transcripti	GO:00325	developm	-	-	-	-	9:7184268	679	734	951	881	724	679	1.384291	1.254314	1.33564	1.170079	1.294311	1.71638	1.324749	1.39359	0.981621	1	-
ENSMUSC	Anxa2	annexin A	GO:00325	developm	-	-	-	-	9:6945362	157	213	352	289	196	198	2.441354	2.776282	3.770729	2.927591	2.672578	3.817536	2.996122	3.139235	1	1	-
ENSMUSC	Cgnl1	cingulin-li	GO:00508	response	-	-	mmu0453	Tight junc	9:7162650	674	578	1117	759	630	400	4.665077	3.353357	5.32603	3.422332	3.823685	3.432774	4.448155	3.559597	0.081022	1	-
ENSMUSC	Ice2	interactor	GO:00081	metabolic	-	-	-	-	9:6939790	153	194	240	256	153	100	0.904186	0.960993	0.977078	0.98557	0.792869	0.732742	0.947419	0.83706	0.352712	1	-
ENSMUSC	Rora	RAR-relat	GO:00325	developm	K08532	RAR-relat	mmu0465	Th17 cell c	9:6865378	745	865	1231	1214	920	606	3.182991	3.097761	3.62317	3.378932	3.446749	3.210239	3.301307	3.345307	0.864077	1	-
ENSMUSC	Itga11	integrin al	GO:00325	developm	K06587	integrin al	mmu0415	PI3K-Akt s	9:6267782	53	63	94	123	64	68	0.627949	0.625663	0.767235	0.949368	0.664922	0.998945	0.673616	0.871078	0.260839	1	-
ENSMUSC	Fem1b	feminizati	GO:00325	developm	-	-	-	-	9:6279182	3680	4308	5245	5193	3850	2577	34.46943	33.82321	33.84411	31.68737	31.62203	29.92861	34.04558	31.07934	0.270275	1	-
ENSMUSC	Cln6	ceroid-lip	GO:00081	metabolic	-	-	-	-	9:6283876	145	151	168	166	166	165	2.523332	2.202594	2.014036	1.881888	2.533128	3.560219	2.246654	2.658412	0.521874	1	-
ENSMUSC	Anp32a	acidic (leu	GO:00325	developm	-	-	-	-	9:6234129	3888	4746	5548	5748	4286	2066	55.24124	56.52204	54.30316	53.20294	53.39887	36.39599	55.35548	47.66593	0.204467	1	-
ENSMUSC	Irak1bp1	interleukir	GO:00081	metabolic	-	-	-	-	9:8282954	229	287	367	345	273	216	4.567997	4.798721	5.043221	4.483235	4.775249	5.342313	4.803313	4.866932	0.843667	1	-
ENSMUSC	Glce	glucurony	GO:00081	metabolic	K01793	heparosar	mmu0110	Metabolic	9:6205724	553	593	669	746	491	420	3.722572	3.345996	3.102384	3.271439	2.898291	3.505531	3.390317	3.225087	0.499128	1	-
ENSMUSC	Phip	pleckstrin	GO:00325	developm	-	-	-	-	9:8286615	1488	1508	2353	2248	1695	938	2.471561	2.099532	2.692414	2.432464	2.468771	1.931778	2.421169	2.277671	0.497448	1	-
ENSMUSC	Kif23	kinesin far	GO:00081	metabolic	K17387	kinesin far	mmu0520	MicroRNA	9:6191590	26	18	32	26	26	12	0.152563	0.088531	0.12935	0.099384	0.133778	0.087304	0.123481	0.106822	0.537333	1	-
ENSMUSC	Lca5	Leber con	GO:00056	cell|macr	-	-	-	-	9:8339029	153	181	225	207	172	49	1.095125	1.085936	1.109445	0.965216	1.079551	0.434861	1.096835	0.826543	0.173782	1	-
ENSMUSC	Drd2	dopamine	GO:00325	developm	K04145	dopamine	mmu0401	Neuroacti	9:4934062	57	49	54	60	50	33	1.422269	1.024833	0.928216	0.975308	1.094011	1.020946	1.125106	1.030088	0.557874	1	-
ENSMUSC	Sh3bgrl2	SH3 doma	GO:00325	developm	-	-	-	-	9:8354832	469	572	812	779	600	413	2.903378	2.800888	3.267793	2.964602	3.075557	2.991461	2.990686	3.009873	0.81907	1	-
ENSMUSC	Elovl4	elongation	GO:00081	metabolic	K10249	elongation	mmu0006	Fatty acid	9:8377869	969	1227	1584	1423	1096	753	16.84894	17.88324	18.97384	16.11889	16.71098	16.23414	17.90201	16.35467	0.295905	1	-
ENSMUSC	Bckdhb	branched	GO:00081	metabolic	K00167	2-oxoisov	mmu0110	Metabolic	9:8392514	266	329	374	407	313	255	6.719032	6.965857	6.508023	6.697333	6.932865	7.986405	6.730971	7.205534	0.854203	1	-
ENSMUSC	Zw10	zw10 kine	GO:00511	localizatio	-	-	-	-	9:4905561	229	292	365	382	249	255	2.944888	3.147525	3.233535	3.200206	2.80786	4.065924	3.108649	3.357997	0.829121	1	-
ENSMUSC	Fam46a	family witl	GO:00081	metabolic	-	-	-	-	9:8532043	184	186	293	243	198	108	2.021387	1.712769	2.217441	1.739081	1.907394	1.471098	1.983866	1.705858	0.266017	1	-
ENSMUSC	Usp28	ubiquitin s	GO:00081	metabolic	-	-	-	-	9:4898537	373	463	603	602	494	348	2.691947	2.800858	2.997962	2.830321	3.126277	3.114033	2.830256	3.023544	0.840273	1	-
ENSMUSC	Tmprss5	transmem	GO:00081	metabolic	-	-	-	-	9:4908126	15	12	20	23	8	17	0.206477	0.13846	0.189656	0.206246	0.096567	0.290137	0.178198	0.19765	0.944316	1	-
ENSMUSC	Htr3a	5-hydrox	GO:00048	signal trar	K04819	5-hydrox	mmu0472	Serotoner	9:4889921	203	194	223	239	206	134	4.324918	3.464447	3.272943	3.31713	3.848517	3.566176	3.687444	3.577274	0.676344	1	-
ENSMUSC	Nnmt	nicotinam	GO:00081	metabolic	K00541	nicotinam	mmu0110	Metabolic	9:4859187	4	21	17	13	[illegible]	4	0.083702	0.368352	0.245065	0.177226	0.073394	0.103785	0.232373	0.118135	0.106001	1	-
ENSMUSC	Cyp19a1	cytochron	GO:00325	developm	K07434	aromatase	mmu0110	Metabolic	9:5416593	0	0	1	3	3	0	0	0	0.007034	0.01996	0.026868	0	0.002345	0.015609	0.169963	1	-
ENSMUSC	Pagr5	progestin	GO:00325	developm	-	-	-	-	9:6195348	63	56	97	90	35	50	0.583567	0.434803	0.618975	0.543093	0.284291	0.57426	0.545781	0.467215	0.368124	1	-
ENSMUSC	Idh3a	isocitrate	GO:00081	metabolic	K00030	isocitrate	mmu0110	Metabolic	9:5458633	3191	3500	4286	3931	3675	2733	29.84952	27.443	27.6194	23.95495	30.14467	31.69831	28.30397	28.59931	0.786428	1	-
ENSMUSC	Tle3	transducin	GO:00325	developm	-	-	-	-	9:6137236	745	791	745	969	714	525	1.681954	1.496881	1.158685	1.425158	1.413508	1.469613	1.44584	1.436093	0.743342	1	-
ENSMUSC	Acsbg1	acyl-CoA	GO:00081	metabolic	K15013	long-chai	mmu0110	Metabolic	9:5460487	2157	2514	2850	3623	2717	1957	32.20955	31.4668	29.31776	35.24394	35.5768	36.23358	30.99803	35.68477	0.376972	1	-
ENSMUSC	Dnaja4	DnaJ heat	GO:00508	response	-	-	-	-	9:5469887	1091	1324	1666	1620	1189	998	16.06867	16.34542	16.9037	15.54362	15.35606	18.22518	16.43926	16.37495	0.701815	1	-
ENSMUSC	Imp3	IMP3, U3	GO:00081	metabolic	K14560	U3 small r	mmu0300	Ribosome	9:5693747	132	192	219	196	171	286	9.078978	11.06924	10.37672	8.782154	10.31342	24.39025	10.17498	14.49528	0.184319	1	-
ENSMUSC	Thsd4	thrombos	GO:00081	metabolic	-	-	-	-	9:5996693	1305	1458	1516	1895	1309	799	4.73032	4.429863	3.785564	4.47477	4.160661	3.590977	4.315249	4.075469	0.502948	1	-
ENSMUSC	Ptpn9	protein ty	GO:00081	metabolic	-	-	-	-	9:5699492	622	744	903	907	680	578	6.710234	6.727801	6.710988	6.374349	6.432782	7.731443	6.716341	6.846191	0.853289	1	-
ENSMUSC	Crabp1	cellular re	GO:00081	metabolic	-	-	-	-	9:5476474	6	12	11	19	11	8	0.47579	0.797097	0.600481	0.980814	0.76437	0.786026	0.624352	0.843737	0.431511	1	-
ENSMUSC	Ireb2	iron respc	GO:00325	developm	-	-	-	-	9:5486378	1052	1345	1615	1611	1291	853	6.672434	7.150615	7.056549	6.656493	7.180212	6.708163	6.959866	6.848289	0.664866	1	-
ENSMUSC	Pkm	pyruvate	GO:00081	metabolic	K00873	pyruvate	mmu0110	Metabolic	9:5965636	14924	17955	17424	21475	14282	14653	128.5706	129.6566	103.4084	120.5233	107.8917	156.5196	120.5452	128.3116	0.897692	1	-
ENSMUSC	Man2c1	mannosid	GO:00081	metabolic	K01191	alpha-ma	mmu0051	Other glyc	9:5713069	832	889	1034	1302	986	914	4.208877	3.76962	3.603418	4.29077	4.373834	5.732908	3.860638	4.799171	0.175536	1	-
ENSMUSC	Celf6	CUGBP, El	GO:00081	metabolic	-	-	-	-	9:5957791	289	317	356	361	291	258	2.759019	2.536702	2.341312	2.245157	2.436088	3.05396	2.545678	2.578402	0.823347	1	-
ENSMUSC	Commd4	COMM dc	GO:00081	metabolic	-	-	-	-	9:5715503	287	375	405	294	248	428	3.192111	3.49607	3.103145	2.130222	2.418747	5.902364	3.263775	3.483778	1	1	-
ENSMUSC	1700017B	RIKEN cDl	GO:00056	cell|intrac	-	-	-	-	9:5725311	521	441	544	663	399	327	3.12487	2.217101	2.247733	2.590535	2.098502	2.431798	2.529901	2.373612	0.499854	1	-
ENSMUSC	Psma4	proteasor	GO:00325	developm	K02728	20S prote	mmu0305	Proteason	9:5495079	760	928	1186	1127	832	526	13.76469	14.08813	14.79752	13.29714	13.21352	11.81202	14.21678	12.77423	0.275717	1	-
ENSMUSC	Chrna3	cholinergi	GO:00325	developm	K04805	nicotinic a	mmu0408	Neuroacti	9:5501011	2	12	15	12	11	6	0.019628	0.098699	0.101394	0.076708	0.094646	0.073003	0.07324	0.081452	0.923515	1	-
ENSMUSC	Fam219b	family witl	-	-	-	-	-	-	9:5753752	207	310	347	374	279	310	2.124252	2.666551	2.453102	2.500275	2.510623	3.944416	2.414635	2.985105	0.29874	1	-
ENSMUSC	Mpi	mannose	GO:00081	metabolic	K01809	mannose-	mmu0110	Metabolic	9:5754425	517	743	852	856	673	471	8.006073	9.6443	9.089073	8.635436	9.138741	9.043471	8.913149	8.939216	0.789871	1	-
ENSMUSC	Ube2q2	ubiquitin-	GO:00081	metabolic	K10582	ubiquitin-	mmu0412	Ubiquitin	9:5514885	925	688	1118	1028	1042	689	9.153927	5.706991	7.621826	6.62736	9.042241	8.454166	7.494248	8.041256	0.845938	1	-
ENSMUSC	Ulk3	unc-51-lil	GO:00081	metabolic	-	-	-	-	9:5758945	234	276	297	410	291	322	2.536484	2.507713	2.217806	2.895221	2.765997	4.327709	2.420668	3.329642	0.0767	1	-
ENSMUSC	Fbxo22	F-box prc	GO:00325	developm	-	-	-	-	9:5520892	446	644	717	733	555	400	5.35007	6.475346	5.925085	5.72809	5.83794	5.949364	5.916834	5.838465	0.700254	1	-
ENSMUSC	Nrg4	neuregulir	GO:00325	developm	K05458	neuregulir	mmu0401	ErbB signa	9:5522022	7	10	3	20	0	2	0.048676	0.05828	0.01437	0.090596	0	0.017247	0.040442	0.035948	0.939362	1	-
ENSMUSC	Csk	c-src tyro	GO:00325	developm	-	-	-	-	9:5762664	324	406	505	468	343	320	3.170311	3.329932	3.40408	2.983217	2.943023	3.882327	3.301441	3.269522	0.688872	1	-
ENSMUSC	Tmem266	transmem	GO:00511	localizatio	-	-	-	-	9:5532691	100	139	140	145	102	83	1.009258	1.175902	0.973379	0.953351	0.902706	1.038645	1.052846	0.964901	0.447032	1	-
ENSMUSC	Etfa	electron t	GO:00081	metabolic	-	-	-	-	9:5545450	258	348	489	420	313	278	7.466612	8.441824	9.74909	7.918344	7.943119	9.9755	8.552509	8.612321	0.799846	1	-
ENSMUSC	Cyp1a1	cytochron	GO:00325	developm	K07408	cytochron	mmu0110	Metabolic	9:5768792	0	2	0	0	[illegible]	3	0	0.026377	0	0	0.04139	[illegible]	0.008792	0.026807	0.36089	1	-
ENSMUSC	Clk3	CDC-like	GO:00081	metabolic	-	-	-	-	9:5775062	686	895	1148	1173	870	703	3.16129	3.457135	3.644463	3.521438	3.515622	4.016814	3.420963	3.684625	0.788618	1	-

ENSMUSC	Rcn2	reticuloca	GO:00056	cellintrace	-	-	-	-	9:5604184	1563	2027	2420	2248	1710	1138	14.01233	15.23203	14.94576	13.12892	13.44281	12.64967	14.73004	13.0738	0.196619	1	-
ENSMUSC	Pstpip1	proline-se	GO:00160	membran	K12804	proline-se	mmu0462	NOD-like	9:5608996	29	49	42	56	32	32	0.319751	0.452854	0.319017	0.402235	0.309385	0.437469	0.363874	0.38303	1	1	-
ENSMUSC	Cyp11a1	cytochron	GO:00081	metabolic	K00498	cholesterc	mmu011C	Metabolic	9:5800641	45	35	64	52	42	27	0.487374	0.317739	0.477503	0.366888	0.398874	0.362576	0.427539	0.376113	0.460889	1	-
ENSMUSC	Tspan3	tetraspani	GO:00508	response	-	-	-	-	9:5613172	2178	2684	3112	3124	2460	3075	28.2602	29.19125	27.81687	26.40645	27.9895	49.47075	28.42277	34.62223	0.319603	1	-
ENSMUSC	Stra6	stimulatec	GO:00325	developm	-	-	-	-	9:5806378	162	196	271	278	228	230	1.173286	1.189863	1.352105	1.311642	1.447991	2.065395	1.238418	1.608343	0.177818	1	-
ENSMUSC	Tmem30a	transmem	GO:00325	developm	-	-	-	-	9:7976894	4067	5282	6278	6150	4592	4547	40.25387	43.82125	42.80615	39.65429	39.85456	55.80131	42.29375	45.10339	0.881969	1	-
ENSMUSC	Hmg20a	high mob	GO:00325	developm	-	-	-	-	9:5641860	1462	1830	2161	2307	1825	1454	8.273777	8.680813	8.424859	8.505222	9.056532	10.2025	8.459816	9.254753	0.680578	1	-
ENSMUSC	Cox7a2	cytochron	GO:00081	metabolic	K02270	cytochron	mmu0501	Huntingto	9:7975536	724	1095	1197	899	771	1206	35.25851	44.69845	40.15792	28.52113	32.92478	72.82135	40.03829	44.75575	0.792469	1	-
ENSMUSC	Col12a1	collagen,	GO:00325	developm	K08132	collagen,	mmu0497	Protein di	9:7959899	215	320	345	385	209	175	0.772364	0.963576	0.853797	0.901003	0.658374	0.779485	0.863246	0.779621	0.377852	1	-
ENSMUSC	Stoml1	stomatin-	GO:00160	membran	-	-	-	-	9:5825316	556	798	915	904	725	917	6.920388	8.325512	7.845629	7.330024	7.912904	14.15177	7.697176	9.798233	0.235441	1	-
ENSMUSC	Loxl1	lysyl oxida	GO:00325	developm	-	-	-	-	9:5828773	124	118	150	136	97	86	2.390225	1.906572	1.991868	1.707808	1.63957	2.055434	2.096222	1.800937	0.248405	1	-
ENSMUSC	Nptn	neuroplas	GO:00325	developm	-	-	-	-	9:5858224	7601	6523	7830	11539	8865	3838	47.60682	34.24512	33.78404	47.08128	48.68777	29.80501	38.54533	41.85802	0.741679	1	-
ENSMUSC	Hcn4	hyperpola	GO:00511	localizatio	K04957	hyperpola	mmu0402	cAMP sigr	9:5882341	52	97	56	65	52	42	0.540168	0.844603	0.400742	0.439865	0.473667	0.540962	0.595171	0.484831	0.28344	1	-
ENSMUSC	Neo1	neogenin	GO:00325	developm	K06766	neogenin|	mmu0451	Cell adhes	9:5887468	2116	2327	2928	3015	2249	1624	11.33498	10.4485	10.80507	10.52141	10.56421	10.78641	10.86285	10.62401	0.602284	1	-
ENSMUSC	Mto1	mitochon	GO:00081	metabolic	-	-	-	-	9:7844820	293	411	458	488	371	244	3.543493	4.166376	3.815751	3.84472	3.934411	3.658798	3.841873	3.812643	0.761573	1	-
ENSMUSC	Mb21d1	Mab-21 c	GO:00325	developm	K17834	cyclic GM	mmu0462	Cytosolic	9:7843052	21	33	47	61	49	19	0.149926	0.197479	0.231153	0.283701	0.306754	0.168187	0.192853	0.252881	0.324399	1	-
ENSMUSC	Ooep	oocyte ex	GO:00325	developm	-	-	-	-	9:7837610	3	0	7	0	0	4	0.089166	0	0.143351	0	0	0.147426	0.077505	0.049142	0.596474	1	-
ENSMUSC	Gsta4	glutathior	GO:00081	metabolic	K00799	glutathior	mmu052C	Pathways	9:7818277	586	767	878	794	585	518	26.07987	28.61253	26.91868	23.02026	22.83001	28.58402	27.20369	24.81143	0.287879	1	-
ENSMUSC	Elovl5	ELOVL far	GO:00081	metabolic	K10244	elongatioi	mmu0121	Fatty acid	9:7791736	1287	1399	2158	1929	1376	1060	21.07516	19.20273	24.34419	20.57815	19.75849	21.52209	21.54069	20.61958	0.499496	1	-
ENSMUSC	Gclc	glutamate	GO:00325	developm	K11204	glutamate	mmu011C	Metabolic	9:7775453	1157	1389	1672	1884	1322	825	18.60599	18.72294	18.52282	19.73703	18.64204	16.44975	18.61725	18.27627	0.684805	1	-
ENSMUSC	Lrrc1	leucine ric	GO:00508	response	-	-	-	-	9:7743082	115	142	190	173	131	113	0.597792	0.618719	0.680389	0.585839	0.597126	0.728307	0.6323	0.637091	0.832	1	-
ENSMUSC	Tmed3	transmem	GO:00511	localizatio	-	-	-	-	9:8969920	273	293	414	371	315	306	5.931618	5.336183	6.196728	5.251287	6.001551	8.243614	5.82151	6.498818	0.685931	1	-
ENSMUSC	Mlip	muscular	GO:00081	metabolic	-	-	-	-	9:7710208	314	369	535	527	413	308	1.490116	1.467812	1.749023	1.629231	1.718632	1.812285	1.568984	1.72005	0.701202	1	-
ENSMUSC	Rasgrf1	RAS prote	GO:00325	developm	K04349	Ras-speci	mmu0401	MAPK sigi	9:8990990	11189	13723	14939	18342	12806	7578	57.40189	59.01153	52.79683	61.30039	57.6091	48.20315	56.40342	55.70422	0.718564	1	-
ENSMUSC	Fam83b	family witl	GO:00508	response	-	-	-	-	9:7649005	0	1	3	7	4	0	0	0.007921	0.01953	0.043095	0.033145	0	0.00915	0.025413	0.166533	1	-
ENSMUSC	Ctsh	cathepsin	GO:00325	developm	K01366	cathepsin	mmu0421	Apoptosis	9:9005415	122	154	242	224	153	130	1.131724	1.197443	1.546499	1.353667	1.244564	1.495246	1.291889	1.364492	0.949785	1	-
ENSMUSC	Hcrtr2	hypocretir	GO:00048	signal trar	K04239	hypocretir	mmu0408	Neuroacti	9:7622588	42	77	86	62	50	31	0.395088	0.607144	0.557312	0.379944	0.41244	0.361574	0.519848	0.384653	0.098779	1	-
ENSMUSC	Adamts7	a disinteg	GO:00325	developm	-	-	-	-	9:9016306	5	7	8	9	0	4	0.02742	0.032175	0.030222	0.032152	0	0.027198	0.029939	0.019783	0.399657	1	-
ENSMUSC	Tpm1	tropomyo	GO:00325	developm	K10373	tropomyo	mmu0426	Adrenergi	9:6702259	3420	3755	4653	4949	3724	2364	13.14254	12.09528	12.31793	12.38945	12.54889	11.26384	12.51858	12.0674	0.545053	1	-
ENSMUSC	Zic1	zinc finge	GO:00325	developm	-	-	-	-	9:9135805	242	232	603	275	224	197	1.812802	1.45672	3.111746	1.341994	1.471384	1.829737	2.127089	1.547705	0.05449	1	-
ENSMUSC	Plscr1	phospholi	GO:00325	developm	-	-	-	-	9:9224975	21	35	53	34	26	37	0.370939	0.518203	0.644914	0.391229	0.402709	0.810324	0.511352	0.534754	0.968799	1	-
ENSMUSC	Lactb	lactamase	GO:00081	metabolic	-	-	-	-	9:6695538	181	288	303	359	284	132	3.567956	4.758683	4.114676	4.610167	4.909109	3.226266	4.147105	4.248514	0.993748	1	-
ENSMUSC	Plscr2	phospholi	GO:00508	response	-	-	-	-	9:9227560	28	41	52	50	21	22	0.381371	0.468089	0.48792	0.443649	0.25082	0.371544	0.445793	0.355337	0.284668	1	-
ENSMUSC	Car12	carbonic a	GO:00081	metabolic	K01672	carbonic a	mmu0091	Nitrogen	9:6671368	888	852	1114	968	751	526	4.904412	3.944269	4.238483	3.482819	3.637111	3.602014	4.362388	3.573981	0.066983	1	-
ENSMUSC	Plod2	procollag	GO:00081	metabolic	K13645	procollag	mmu0031	Lysine deg	9:9254222	149	158	216	221	155	100	1.278096	1.136024	1.276387	1.234955	1.165874	1.063559	1.230169	1.154796	0.559479	1	-
ENSMUSC	Aph1b	aph1 hor	GO:00081	metabolic	K06172	gamma-s	mmu0501	Alzheimer	9:6677520	675	969	1083	1111	922	524	4.363575	5.250672	4.823013	4.678797	5.226512	4.200063	4.81242	4.701791	0.677902	1	-
ENSMUSC	Usp3	ubiquitin	GO:00081	metabolic	-	-	-	-	9:6651463	289	302	451	541	283	241	1.282508	1.123368	1.378768	1.564018	1.101267	1.32607	1.261548	1.330452	0.912305	1	-
ENSMUSC	Plscr4	phospholi	GO:00508	response	-	-	-	-	9:9245737	75	101	129	124	83	69	1.095989	1.237148	1.298639	1.180456	1.063569	1.250199	1.210592	1.164742	0.656076	1	-
ENSMUSC	Dapk2	death-ass	GO:00081	metabolic	K08803	death-ass	mmu052C	Pathways	9:6615822	6	11	11	11	10	21	0.094671	0.145482	0.11956	0.113059	0.138357	0.410814	0.119904	0.220743	0.193474	1	-
ENSMUSC	Fam96a	family witl	GO:00099	cellular pr	-	-	-	-	9:6612661	137	197	257	226	180	222	7.021568	8.463161	9.073996	7.545778	8.089661	14.10762	8.186241	9.914353	0.444684	1	-
ENSMUSC	Snx1	sorting ne	GO:00325	developm	K17917	sorting ne	mmu0414	Endocytos	9:6608813	868	994	1238	1251	1009	825	10.07194	9.667915	9.896128	9.456542	10.26662	11.86953	9.87866	10.5309	0.863563	1	-
ENSMUSC	Ppib	peptidylpı	GO:00325	developm	-	-	-	-	9:6606022	1448	1812	2299	2490	1803	1570	24.51379	25.71301	26.81219	27.46141	26.7658	32.95547	25.67967	29.06089	0.489687	1	-
ENSMUSC	Trip4	thyroid hc	GO:00325	developm	-	-	-	-	9:6582893	798	952	1163	1231	941	586	3.362193	3.362092	3.375598	3.378774	3.476579	3.061278	3.366628	3.305544	0.681111	1	-
ENSMUSC	Rbpms2	RNA bind	GO:00325	developm	-	-	-	-	9:6562964	60	46	66	46	38	43	0.770957	0.49544	0.584222	0.385054	0.428163	0.685076	0.616873	0.499431	0.228199	1	-
ENSMUSC	Spg21	SPG21, m	GO:00056	cellintrace	K19367	maspardir	mmu0414	Endocytos	9:6546094	237	285	376	376	257	278	3.554045	3.582395	3.884312	3.673204	3.379494	5.168994	3.673584	4.073897	0.713835	1	-
ENSMUSC	Parp16	poly (ADP	GO:00081	metabolic	-	-	-	-	9:6519535	64	51	87	64	55	55	0.560482	0.374368	0.524868	0.365123	0.42236	0.597213	0.486573	0.461565	0.61104	1	-
ENSMUSC	Dpp8	dipeptidyl	GO:00081	metabolic	-	-	-	-	9:6503241	4844	5347	6864	7510	5965	3042	18.56312	17.17552	18.12072	18.74858	20.04472	14.45413	17.95312	17.74914	0.751455	1	-
ENSMUSC	Igdcc3	immunog	GO:00160	membran	-	-	-	-	9:6514118	25	50	56	71	40	52	0.362005	0.606862	0.55861	0.669745	0.507894	0.933596	0.509159	0.703745	0.23087	1	-
ENSMUSC	Dis3l	DIS3 like e	GO:00081	metabolic	K18681	DIS3-like	mmu0301	RNA degr	9:6430675	586	643	775	928	659	468	4.986884	4.586652	4.54344	5.144716	4.917675	4.938146	4.705659	5.000179	0.855632	1	-
ENSMUSC	Tipin	timeless ir	GO:00081	metabolic	-	-	-	-	9:6428158	109	134	203	177	175	126	2.505341	2.58165	3.214318	2.650307	3.527126	3.590861	2.767103	3.256098	0.479062	1	-
ENSMUSC	Snapc5	small nucl	GO:00081	metabolic	-	-	-	-	9:6417927	221	265	310	322	245	134	11.43749	11.49574	11.05224	10.85615	11.11852	8.598636	11.32849	10.1911	0.384085	1	-
ENSMUSC	Rpl4	ribosomal	GO:00081	metabolic	K02930	large subı	mmu0301	Ribosome	9:6417337	5608	6785	8526	8602	6481	5251	136.397	138.3246	142.8544	136.2943	138.2233	158.3524	139.192	144.29	0.963304	1	-
ENSMUSC	Zwilch	zwilch kin	GO:00329	macromo	-	-	-	-	9:6413714	30	55	43	76	56	34	0.278662	0.428224	0.27515	0.459885	0.456123	0.391583	0.327346	0.435864	0.234602	1	-
ENSMUSC	Lctl	lactase-lik	GO:00081	metabolic	-	-	-	-	9:6411714	1	0	4	0	0	4	0.01658	0	0.045682	0	0	0.082211	0.020754	0.027404	1	1	-
ENSMUSC	Smad3	SMAD far	GO:00325	developm	K04500	mothers a	mmu052C	Pathways	9:6364676	1532	1824	2231	2384	1933	1278	16.99183	16.95739	17.04639	17.22541	18.79992	17.57511	16.99854	17.86681	0.916291	1	-
ENSMUSC	2300009A	RIKEN cDI	GO:00055	extracellul	-	-	-	-	9:6339444	75	88	106	133	38	73	1.492316	1.467695	1.452975	1.723986	0.663023	1.800975	1.470995	1.395995	0.692149	1	-
ENSMUSC	Pias1	protein in	GO:00325	developm	K04706	E3 SUMO	mmu0463	Jak-STAT	9:6287836	1292	1524	1813	2018	1363	799	8.130554	8.038881	7.859719	8.272952	7.52136	6.23434	8.009718	7.342884	0.37181	1	-
ENSMUSC	U2surp	U2 snRNP	GO:00081	metabolic	K12842	U2-associ	mmu0304	Spliceosoı	9:9545689	1811	2228	2814	2899	2223	1198	6.868854	7.083271	7.352607	7.163011	7.393463	5.63389	7.101578	6.730121	0.518853	1	-
ENSMUSC	Atr	ataxia tela	GO:00325	developm	K06640	serine/thr	mmu0516	Human pa	9:9585759	206	299	345	373	271	200	0.468271	0.56971	0.540258	0.552357	0.540185	0.563696	0.52608	0.55208	0.95245	1	-
ENSMUSC	Xrn1	5'-3' exor	GO:00081	metabolic	K12618	5'-3' exor	mmu0301	RNA degr	9:9595476	925	1078	1329	1560	1127	779	2.721976	2.658976	2.694134	2.990535	2.9081	2.842275	2.691695	2.913637	0.72576	1	-
ENSMUSC	Tfdp2	transcripti	GO:00325	developm	K09392	transcripti	mmu0411	Cell cycle	9:9619627	248	365	429	650	252	147	0.721862	0.890529	0.860223	1.232531	0.6432	0.530525	0.824205	0.802085	0.820291	1	-
ENSMUSC	Atp1b3	ATPase, N	GO:00511	localizatio	K01540	sodium/p	mmu0402	cAMP sigr	9:9633265	1166	1453	1933	1703	1256	1006	28.19742	29.45299	32.20282	26.82915	26.63442	30.1644	29.95108	27.87599	0.341965	1	-
ENSMUSC	Rasa2	RAS p21 p	GO:00987	molecular	K08053	Ras GTPas	mmu0401	MAPK sigi	9:9653930	446	583	781	691	556	356	1.624988	1.780477	1.960276	1.640115	1.776365	1.608242	1.78858	1.674907	0.452587	1	-
ENSMUSC	Ube2cbp	ubiquitin-	GO:00081	metabolic	-	-	-	-	9:8630730	27	24	27	18	17	13	0.891382	0.664151	0.614069	0.387125	0.492141	0.532139	0.723201	0.470468	0.073426	1	-
ENSMUSC	Rwdd2a	RWD dor	-	-	-	-	-	-	9:8657199	205	320	292	303	239	222	4.223132	5.52565	4.14395	4.066352	4.317389	5.670485	4.630911	4.684742	0.828439	1	-
ENSMUSC	Tbx18	T-box18 [	GO:00325	developm	-	-	-	-	9:8770280	34	64	53	70	40	41	0.450638	0.711017	0.483921	0.604403	0.464889	0.673773	0.548525	0.581022	0.974311	1	-
ENSMUSC	Nt5e	5' nucleot	GO:00081	metabolic	K19970	5'-nucleo	mmu011C	Metabolic	9:8832719	44	62	88	66	60	61	0.28657	0.33847	0.394828	0.28003	0.342667	0.492593	0.339956	0.371763	0.893114	1	-
ENSMUSC	Snx14	sorting ne	GO:00081	metabolic	-	-	-	-	9:8837675	827	1074	1282	1225	976	619	6.353766	6.916439	6.785237	6.131172	6.575336	5.89661	6.685148	6.201039	0.378954	1	-
ENSMUSC	Syncrip	synaptota	GO:00325	developm	-	-	-	-	9:8844700	1552	1762	2183	2302	1715	641	3.845241	3.659237	3.72595	3.715513	3.725963	1.969136	3.743476	3.13687	0.244519	1	-
ENSMUSC	Zfp949	zinc finge	GO:00081	metabolic	-	-	-	-	9:8854802	374	424	521	524	425	247	4.008237	3.808902	3.846542	3.658428	3.994043	3.282193	3.887894	3.644888	0.489559	1	-
ENSMUSC	Crtap	cartilage a	GO:00081	metabolic	-	-	-	-	9:1143751	77	71	108	79	66	70	2.137872	1.652339	2.065694	1.428887	1.606855	2.409778	1.951969	1.815173	0.511054	1	-
ENSMUSC	Cmtm6	CKLF-like	GO:00400	locomotic	-	-	-	-	9:1147311	294	351	494	492	317	456	5.254376	5.25817	6.08209	5.728242	4.967938	10.10475	5.531545	6.933643	0.310226	1	-
ENSMUSC	Dync1li1	dynein cy	GO:00511	localizatio	K10416	dynein lig	mmu0414	Phagoson	9:1146887	1650	2223	2513	2546	1868	1403	24.85481	28.06848	26.07778	24.98426	24.67437	26.20416	26.33369	25.2876	0.497378	1	-
ENSMUSC	Cmtm7	CKLF-like	GO:00325	developm	-	-	-	-	9:1147568	18	24	32	42	20	35	0.238122	0.26613	0.291631	0.361959	0.232015	0.574103	0.265294	0.389359	0.223386	1	-
ENSMUSC	Stt3b	STT3, sub	GO:00081	metabolic	K07151	dolichyl-d	mmu011C	Metabolic	9:1152425	1489	1785	2369	2424	1727	1439	22.41896	22.52743	24.57181	23.7758	22.80109	26.8638	23.17274	24.48023	0.906385	1	-
ENSMUSC	Tgfbr2	transform	GO:00325	developm	K04388	TGF-beta	mmu052C	Pathways	9:1160842	346	413	576	588	420	338	2.169005	2.170136	2.48747	2.40128	2.308744	2.62716	2.275537	2.445728	0.814111	1	-
ENSMUSC	Eomes	eomesode	GO:00325	developm	-	-	-	-	9:1184782	12	9	23	22	16	8	0.106778	0.067126	0.140994	0.127533	0.124846	0.088266	0.104966	0.113548	0.938208	1	-
ENSMUSC	Slc25a36	solute car	GO:00511	localizatio	-	-	-	-	9:9707496	939	1257	1560	1493	1242	742	3.91012	4.387456	4.475078	4.050103	4.53512	3.831023	4.257551	4.138749	0.623335	1	-
ENSMUSC	Clstn2	calsynteni	GO:00325	developm	-	-	-	-	9:9744439	3593	3868	4819	5908	4107	2834	38.21689	34.48558	35.31072	40.93741	38.30589	37.37524	36.0044	38.87285	0.732308	1	-
ENSMUSC	Rbp2	retinol bir	GO:00081	metabolic	K14622	retinol bin	mmu0497	Vitamin d	9:9848611	0	0	4	0	0	0	0	0	0.096499	0	0	0	0.032166	0	0.306733	1	-
ENSMUSC	Nmnat3	nicotinam	GO:00081	metabolic	K06210	nicotinam	mmu011C	Metabolic	9:9828743	18	22	35	42	22	22	0.150202	0.153881	0.201199	0.228316	0.160983	0.227629	0.168427	0.205643	0.547606	1	-
[truncated: 1,958,945 more chars]
