# Supplementary material for: Brain Short-Chain Fatty Acids Induce ACSS2 to Ameliorate Depressive-Like Behavior via PPARγ–TPH2 Axis
Source: Research (Wash D C). 2024 Jun 27;7:0400. doi: 10.34133/research.0400 (PMC11210491; doi:10.34133/research.0400)
Supplement: Supplementary 1 — Figs. S1 to S6 Tables S1 to S3 Control VS Mannose CRS-Control VS CRS-Mannose RNA sequencing for mannose-treated MG1655 [file research.0400.f1.zip › CRS-Control VS CRS-Mannose.pdf]

gene id	gene nam	descriptio	GO_id	GO_term	KO_ID	KO_Descr	kegg_id	kegg_tern	locus	B1_count	B2_count	B3_count	D1_count	D2_count	D3_count	B1_FPKM	B2_FPKM	B3_FPKM	D1_FPKM	D2_FPKM	D3_FPKM	B	D	Pvalue	Qvalue	updown
ENSMUSC	Car1	carbonic a	GO:00081	metabolic	K01672	carbonic a	mmu0091	Nitrogen i	3:1476621	15	28	14	3443	6223	3469	0.499645	0.744884	0.515412	88.48064	133.4859	90.90674	0.586647	104.2911	######	######	DOWN
ENSMUSC	Zg16	zymogen	GO:00511	localizatio	-	-	-	-	7:1270501	0	9	3	690	1153	672	0	0.266474	0.122927	19.73465	27.5254	19.59881	0.1298	22.28629	######	3.69E-98	DOWN
ENSMUSC	Lypd8	LY6/PLAU	GO:00508	response	-	-	-	-	11:58379C	12	23	10	2407	6051	2510	0.419874	0.642733	0.386706	64.9762	136.342	69.09277	0.483104	90.137	######	9.78E-97	DOWN
ENSMUSC	Pigr	polymeric	GO:00048	signal trar	K13073	polymeric	mmu0467	Intestinal i	1:1308266	14	24	4	3269	8024	3297	0.190598	0.260934	0.060189	34.33414	70.34391	35.31106	0.170574	46.66303	2.03E-98	8.84E-95	DOWN
ENSMUSC	Muc13	mucin 13,	GO:00055	extracellul	-	-	-	-	16:33794C	3	14	3	1671	4057	1706	0.043221	0.161087	0.047773	18.57331	37.63935	19.33625	0.084027	25.18297	6.52E-94	2.27E-90	DOWN
ENSMUSC	Dmbt1	deleted in	GO:00511	localizatio	K13912	deleted in	mmu0497	Salivary se	7:131032C	23	59	15	5804	13851	5746	0.156333	0.320272	0.112684	30.43545	60.62584	30.72547	0.19643	40.59558	3.05E-90	8.84E-87	DOWN
ENSMUSC	Cyp2c55	cytochron	GO:00081	metabolic	K07413	cytochron	mmu011C	Metabolic	19:39007C	3	4	2	831	1938	839	0.091814	0.097761	0.067656	19.62147	38.19521	20.20103	0.085744	26.0059	7.76E-87	1.93E-83	DOWN
ENSMUSC	Muc3	mucin 3, i	GO:00160	membran	-	-	-	-	5:1371349	2	8	0	615	1267	593	0.049471	0.158069	0	11.73826	20.18503	11.54155	0.06918	14.48828	3.41E-79	7.42E-76	DOWN
ENSMUSC	Gsdmc2	gasdermi	GO:00325	developm	-	-	-	-	15:638243	1	3	0	604	1533	685	0.018587	0.044525	0	8.658505	18.34309	10.01331	0.021037	12.3383	1.58E-78	3.05E-75	DOWN
ENSMUSC	Krt20	keratin 20	GO:00511	localizatio	-	-	-	-	11:994284	0	10	1	711	1611	703	0	0.253038	0.035022	17.38048	32.87092	17.52378	0.09602	22.59173	3.07E-72	5.34E-69	DOWN
ENSMUSC	Ceacam1	carcinoen	GO:00325	developm	-	-	-	-	7:254617C	79	120	78	3099	7136	2735	0.487184	0.591006	0.531627	14.74403	28.3383	13.26883	0.536606	18.78372	3.88E-71	6.14E-68	DOWN
ENSMUSC	1810065E	RIKEN cDI	-	-	-	-	-	-	11:584211	7	2	0	631	1528	655	0.461573	0.105335	0	32.09906	64.87991	33.97699	0.188969	43.65199	2.56E-69	3.71E-66	DOWN
ENSMUSC	Gsdmc4	gasdermi	GO:00099	cellular pr	-	-	-	-	15:638912	0	0	0	469	1198	463	0	0	0	7.087764	15.11187	7.135075	0	9.778235	1.21E-68	1.61E-65	DOWN
ENSMUSC	Adamdec	ADAM-lik	GO:00081	metabolic	-	-	-	-	14:685633	2	0	0	407	993	430	0.049997	0	0	7.850956	15.98828	8.458176	0.016666	10.7658	3.92E-67	4.87E-64	DOWN
ENSMUSC	Sprr2a1	small prol	GO:00325	developm	-	-	-	-	3:9221579	3	13	1	786	1900	788	0.060996	0.211094	0.022473	12.32938	24.8769	12.60449	0.098188	16.60359	5.07E-67	5.88E-64	DOWN
ENSMUSC	Cdh17	cadherin 1	GO:00325	developm	K06811	cadherin 1	mmu0522	Gastric ca	4:1175814	1	3	0	447	1059	433	0.012716	0.030462	0	4.383976	8.669243	4.330418	0.014393	5.794546	7.63E-67	8.30E-64	DOWN
ENSMUSC	Sprr2a3	small prol	GO:00325	developm	-	-	-	-	3:9228541	5	6	0	278	470	351	0.0857	0.082137	0	3.676411	5.188022	4.733341	0.055946	4.532591	1.32E-66	1.36E-63	DOWN
ENSMUSC	Slc6a14	solute car	GO:00081	metabolic	-	-	-	-	X:2171489	3	4	0	483	1171	492	0.05334	0.056794	0	6.625534	13.40771	6.882066	0.036712	8.97177	4.04E-66	3.90E-63	DOWN
ENSMUSC	Epcam	epithelial	GO:00325	developm	-	-	-	-	17:876359	16	15	12	773	1916	821	0.478885	0.358557	0.396949	17.84937	36.92864	19.33157	0.411464	24.70319	5.75E-65	5.27E-62	DOWN
ENSMUSC	Itln1	intelectin	GO:00081	metabolic	-	-	-	-	1:1715181	5	3	0	490	1189	497	0.211694	0.101464	0	16.00681	32.42012	16.55561	0.104386	21.66085	1.56E-64	1.36E-61	DOWN
ENSMUSC	Reg3b	regenerat	GO:00508	response	-	-	-	-	6:7837065	5	13	2	632	1499	633	0.181058	0.375988	0.080056	17.65765	34.95763	18.03435	0.212367	23.54988	1.70E-64	1.40E-61	DOWN
ENSMUSC	Hnf4a	hepatic nu	GO:00325	developm	K07292	hepatocyt	mmu0415	AMPK sigi	2:1635068	2	8	0	514	1181	481	0.014803	0.047298	0	2.935539	5.629881	2.80124	0.0207	3.788886	8.73E-63	6.90E-60	DOWN
ENSMUSC	Myo15b	myosin XV	GO:00038	catalytic a	-	-	-	-	11:115858	8	10	6	491	949	414	0.040182	0.040109	0.033308	1.902516	3.069288	1.635791	0.037866	2.202532	1.20E-62	9.07E-60	DOWN
ENSMUSC	Agr2	anterior g	GO:00325	developm	-	-	-	-	12:359929	0	6	0	394	896	377	0	0.214339	0	13.59683	25.80913	13.26671	0.071446	17.55756	2.45E-60	1.78E-57	DOWN
ENSMUSC	Tspan1	tetraspani	GO:00400	locomotic	-	-	-	-	4:1161618	3	10	0	414	975	462	0.065116	0.17334	0	6.932731	13.62803	7.889081	0.079485	9.48328	3.11E-59	2.16E-56	DOWN
ENSMUSC	Fcgbp	Fc fragme	GO:00055	extracellul	-	-	-	-	7:2807123	24	38	18	823	1978	877	0.106406	0.134547	0.0882	2.814989	5.647122	3.058839	0.109718	3.840317	1.24E-58	8.33E-56	DOWN
ENSMUSC	B4galnt2	beta-1,4-	GO:00081	metabolic	-	-	-	-	11:958659	3	1	2	372	946	381	0.10637	0.028322	0.078382	10.17616	21.60012	10.62787	0.071025	14.13472	1.03E-57	6.62E-55	DOWN
ENSMUSC	Gpa33	glycoprot	GO:00055	extracellul	-	-	-	-	1:1661302	0	2	0	282	645	279	0	0.020954	0	2.853668	5.448011	2.878984	0.006985	3.726888	3.54E-57	2.20E-54	DOWN
ENSMUSC	Clca1	chloride c	GO:00081	metabolic	K05027	calcium-a	mmu0497	Pancreatic	3:1450038	6	40	6	2805	7247	3000	0.089395	0.475968	0.098809	32.24294	69.53193	35.16443	0.221391	45.64643	3.62E-56	2.17E-53	DOWN
ENSMUSC	Muc2	mucin 2 [S	GO:00055	extracellul	-	-	-	-	7:1416903	17	50	5	3454	8786	3536	0.131459	0.308779	0.042735	20.60577	43.75041	21.51094	0.160991	28.62237	2.79E-55	1.62E-52	DOWN
ENSMUSC	Cdhr5	cadherin-	GO:00325	developm	-	-	-	-	7:141269C	0	2	0	258	590	249	0	0.023268	0	2.899159	5.533872	2.853206	0.007756	3.762079	1.55E-53	8.68E-51	DOWN
ENSMUSC	Lgals4	lectin, gal	GO:00055	extracellul	-	-	-	-	7:2883385	31	40	29	743	1732	759	0.485825	0.500646	0.502306	8.983453	17.47941	9.357868	0.496259	11.94024	8.08E-53	4.39E-50	DOWN
ENSMUSC	Vil1	villin 1 [Sc	GO:00325	developm	-	-	-	-	1:7440937	10	19	6	529	1337	548	0.149069	0.226193	0.098858	6.083718	12.83422	6.426501	0.15804	8.448146	5.03E-52	2.65E-49	DOWN
ENSMUSC	Krt8	keratin 8 [	GO:00325	developm	-	-	-	-	15:101996	23	66	18	1457	3564	1473	0.778029	1.783019	0.672962	38.02385	77.63517	39.19942	1.078003	51.61948	1.05E-50	5.39E-48	DOWN
ENSMUSC	Retnlb	resistin lik	GO:00508	response	-	-	-	-	16:488168	0	8	2	388	979	370	0	0.561314	0.194207	26.29784	55.3855	25.57238	0.25184	35.75191	1.74E-50	8.65E-48	DOWN
ENSMUSC	Myo1a	myosin IA	GO:00511	localizatio	-	-	-	-	10:127705	13	19	7	404	905	415	0.188094	0.219547	0.111945	4.509644	8.432069	4.72378	0.173195	5.888498	1.10E-49	5.32E-47	DOWN
ENSMUSC	Gcnt3	glucosam	GO:00325	developm	K09662	N-acetylg	mmu011C	Metabolic	9:7003149	0	0	0	172	412	207	0	0	0	1.883794	3.766395	2.311829	0	2.654006	1.25E-49	5.90E-47	DOWN
ENSMUSC	Ugt2b34	UDP gluc	GO:00081	metabolic	K00699	glucuronc	mmu011C	Metabolic	5:8688976	0	4	0	215	540	255	0	0.03122	0	1.621216	3.398759	1.96075	0.010407	2.326908	3.91E-48	1.79E-45	DOWN
ENSMUSC	Cdhr2	cadherin-	GO:00325	developm	-	-	-	-	13:547014	11	3	7	423	1060	426	0.16267	0.035435	0.114412	4.825802	10.0939	4.955858	0.104172	6.625188	5.88E-47	2.62E-44	DOWN
ENSMUSC	Slc44a4	solute car	GO:00325	developm	K15377	solute car	mmu0523	Choline m	17:349144	0	4	3	223	522	243	0	0.047223	0.049022	2.543483	4.969565	2.826253	0.032082	3.446434	8.36E-47	3.63E-44	DOWN
ENSMUSC	Ang4	angiogeni	GO:00325	developm	-	-	-	-	14:517638	1	5	0	265	678	268	0.086145	0.343868	0	17.6065	37.59944	18.15696	0.143338	24.4543	1.25E-46	5.32E-44	DOWN
ENSMUSC	Krt19	keratin 19	GO:00325	developm	-	-	-	-	11:10014C	19	19	0	1044	2467	1049	0.497042	0.396953	0	21.07043	41.55912	21.58882	0.297998	28.07279	7.84E-46	3.25E-43	DOWN
ENSMUSC	Adgrg7	adhesion	GO:00048	signal trar	-	-	-	-	16:567246	0	1	0	188	412	170	0	0.018076	0	3.282394	6.004175	3.026645	0.006025	4.104404	1.03E-45	4.15E-43	DOWN
ENSMUSC	Oit1	oncoprote	GO:00511	localizatio	-	-	-	-	14:834893	1	0	0	176	368	154	0.033707	0	0	4.575386	7.985241	4.082411	0.011236	5.547679	1.20E-44	4.74E-42	DOWN
ENSMUSC	Reg3g	regenerat	GO:00325	developm	-	-	-	-	6:7846626	3	8	0	205	400	209	0.071106	0.151441	0	3.748704	6.105366	3.897212	0.074183	4.583761	1.89E-44	7.29E-42	DOWN
ENSMUSC	Clca3b	chloride c	GO:00081	metabolic	K05030	calcium-a	mmu0497	Pancreatic	3:1448226	0	1	0	190	480	188	0	0.014511	0	2.66292	5.615268	2.686845	0.004837	3.655011	4.60E-44	1.74E-41	DOWN
ENSMUSC	Elf3	E74-like f	GO:00325	developm	-	-	-	-	1:1352535	0	5	0	201	450	189	0	0.070422	0	2.734911	5.110737	2.622337	0.023474	3.489328	3.24E-43	1.20E-40	DOWN
ENSMUSC	Guca2a	guanylate	GO:00081	metabolic	-	-	-	-	4:1196377	0	0	0	167	396	157	0	0	0	13.2435	26.21243	12.69598	0	17.38397	6.09E-43	2.21E-40	DOWN
ENSMUSC	Ceacam2	carcinoen	GO:00329	macromo	-	-	-	-	7:2551604	32	33	17	781	3150	1403	0.363507	0.299381	0.213434	6.844569	23.04253	12.53815	0.292108	14.14175	7.52E-43	2.67E-40	DOWN
ENSMUSC	Prss32	protease,	GO:00081	metabolic	-	-	-	-	17:238438	0	2	0	174	440	177	0	0.040812	0	3.429446	7.238573	3.557384	0.013604	4.741801	1.48E-42	5.13E-40	DOWN
ENSMUSC	Ces2c	carboxyle	GO:00038	catalytic a	K03927	carboxyle	mmu0098	Drug met	8:104847C	0	0	0	150	370	160	0	0	0	2.638489	5.432367	2.869882	0	3.646913	1.85E-42	6.32E-40	DOWN
ENSMUSC	Mgam	maltase-g	GO:00081	metabolic	K12047	maltase-g	mmu011C	Metabolic	6:4062883	2	1	0	182	472	196	0.01292	0.005161	0	0.90722	1.963851	0.996274	0.006027	1.289115	2.08E-42	6.94E-40	DOWN
ENSMUSC	Pla2g4c	phospholi	GO:00081	metabolic	K16342	cytosolic p	mmu011C	Metabolic	7:1332465	4	5	2	282	739	256	0.043445	0.043366	0.024008	2.362829	5.168347	2.187273	0.03694	3.239483	2.90E-42	9.51E-40	DOWN
ENSMUSC	Duox2	dual oxida	GO:00325	developm	K13411	dual oxida	mmu0491	Thyroid hc	2:1222792	4	1	0	191	473	209	0.036536	0.007295	0	1.345877	2.782005	1.501753	0.014611	1.876545	4.10E-42	1.32E-39	DOWN
ENSMUSC	Clec2h	C-type lec	GO:00048	signal trar	K10071	C-type lec	mmu0516	Kaposi's s	6:1286623	0	0	0	159	302	121	0	0	0	2.008525	3.184293	1.558639	0	2.250485	5.98E-42	1.89E-39	DOWN
ENSMUSC	Saa1	serum am	GO:00081	metabolic	-	-	-	-	7:4674049	1	2	1	159	372	169	0.019992	0.031928	0.022091	2.451649	4.787738	2.657232	0.02467	3.298873	8.10E-42	2.52E-39	DOWN
ENSMUSC	Mep1a	meprin 1	GO:00081	metabolic	K01395	meprin A[	mmu0497	Protein di	17:434743	0	0	0	152	396	164	0	0	0	1.587946	3.453134	1.747099	0	2.262727	1.51E-41	4.60E-39	DOWN
ENSMUSC	Gucy2c	guanylate	GO:00081	metabolic	K12320	heat-stab	mmu0023	Purine me	6:1366972	0	6	0	187	432	191	0	0.057741	0	1.738462	3.352206	1.810661	0.019247	2.300443	5.05E-41	1.51E-38	DOWN
ENSMUSC	Klk1	kallikrein 1	GO:00081	metabolic	K01325	tissue kalli	mmu0496	Endocrine	7:4422536	1	0	1	170	449	177	0.042681	0	0.047161	5.596069	12.33687	5.941403	0.029947	7.958114	1.27E-40	3.74E-38	DOWN
ENSMUSC	Clca4a	chloride c	GO:00081	metabolic	K05030	calcium-a	mmu0497	Pancreatic	3:1449524	0	11	0	307	770	313	0	0.178554	0	4.81406	10.07833	5.004936	0.059518	6.632442	3.87E-40	1.12E-37	DOWN
ENSMUSC	Spp1	secreted p	GO:00325	developm	K06250	secreted p	mmu0415	PI3K-Akt s	5:1044351	1078	2550	1163	122	119	201	12.25254	23.14693	14.60945	1.069783	0.870981	1.797275	16.66964	1.246013	8.31E-40	2.37E-37	UP
ENSMUSC	Ms4a8a	membran	GO:00160	membran	-	-	-	-	19:110674	0	3	0	144	339	153	0	0.076283	0	3.536601	6.949438	3.831744	0.025428	4.772594	1.55E-39	4.34E-37	DOWN
ENSMUSC	Ehf	ets homol	GO:00325	developm	-	-	-	-	2:1032634	0	10	0	247	612	265	0	0.050098	0	1.195432	2.472308	1.307834	0.016699	1.658525	1.17E-38	3.23E-36	DOWN
ENSMUSC	Tspan8	tetraspani	GO:00081	metabolic	-	-	-	-	10:115816	6	8	0	234	542	229	0.134684	0.143424	0	4.052472	7.834803	4.044082	0.092703	5.310453	1.23E-38	3.34E-36	DOWN
ENSMUSC	Esrp1	epithelial	GO:00325	developm	-	-	-	-	4:1133193	3	0	0	129	304	150	0.019524	0	0	0.647694	1.274019	0.767981	0.006508	0.896565	1.65E-38	4.41E-36	DOWN
ENSMUSC	Cyp2c65	cytochron	GO:00081	metabolic	K07413	cytochron	mmu011C	Metabolic	19:39061C	0	3	1	144	332	143	0	0.075887	0.035004	3.518258	6.770629	3.562729	0.036964	4.617205	6.45E-38	1.70E-35	DOWN
ENSMUSC	Sult1d1	sulfotrans	GO:00081	metabolic	-	-	-	-	5:8755464	0	0	0	116	286	120	0	0	0	2.126995	4.37723	2.243741	0	2.915989	1.34E-37	3.48E-35	DOWN
ENSMUSC	Ces2e	carboxyle	GO:00038	catalytic a	K03927	carboxyle	mmu0098	Drug met	8:1049262	0	0	1	133	315	128	0	0	0.020569	1.909498	3.774867	1.873949	0.006856	2.519438	1.75E-37	4.46E-35	DOWN
ENSMUSC	Sectm1b	secreted a	GO:00023	immune s	-	-	-	-	11:121053	0	2	1	140	417	183	0	0.017613	0.012186	1.190827	2.96061	1.587272	0.009933	1.912903	3.16E-37	7.96E-35	DOWN
ENSMUSC	Cdh1	cadherin 1	GO:00325	developm	K05689	cadherin 1	mmu052C	Pathways	8:1066033	32	45	12	531	1278	587	0.355181	0.398895	0.147205	4.547016	9.134554	5.125673	0.300427	6.269081	5.57E-37	1.38E-34	DOWN
ENSMUSC	Cldn7	claudin 7	GO:00400	locomotic	K06087	claudin[-]	mmu0453	Tight junc	11:699647	2	2	0	158	386	141	0.037337	0.029829	0	2.276062	4.641272	2.071224	0.022389	2.996186	1.24E-35	3.03E-33	DOWN
ENSMUSC	Gpnmb	glycoprot	GO:00325	developm	-	-	-	-	6:4903654	680	1628	752	179	206	164	5.556679	10.62446	6.791585	1.128471	1.083995	1.054292	7.657574	1.08892	2.15E-35	5.12E-33	UP
ENSMUSC	Pof1b	premature	GO:00325	developm	-	-	-	-	X:1126384	0	0	0	120	297	109	0	0	0	1.454632	3.005053	1.347345	0	1.935677	2.15E-35	5.12E-33	DOWN
ENSMUSC	Hao2	hydroxyac	GO:00081	metabolic	K11517	(S)-2-hyd	mmu011C	Metabolic	3:9887452	0	2	0	112	221	97	0	0.047604	0	2.574841	4.240812	2.273963	0.015868	3.029872	2.20E-35	5.16E-33	DOWN
ENSMUSC	Fut2	fucosyltra	GO:00081	metabolic	K00718	galactosic	mmu011C	Metabolic	7:4564859	7	3	5	173	420	195	0.065066	0.022273	0.051368	1.240571	2.513903	1.425901	0.046235	1.726792	9.91E-35	2.30E-32	DOWN
ENSMUSC	Fabp2	fatty acid	GO:00511	localizatio	K08751	fatty acid-	mmu0497	Fat digest	3:122895C	0	0	0	104	241	96	0	0	0	5.076683	9.819469	4.778556	0	6.558236	1.96E-34	4.47E-32	DOWN
ENSMUSC	Eps8l3	EPS8-like	GO:00056	cell|intrace	-	-	-	-	3:1078772	2	4	2	168	343	127	0.046725	0.074639	0.051655	3.028607	5.161219	2.334627	0.057673	3.508151	1.98E-34	4.47E-32	DOWN
ENSMUSC	Slc5a8	solute car	GO:00511	localizatio	-	-	-	-	10:888859	3	0	0	121	232	128	0.023296	0	0	1.069182	1.711108	1.153334	0.007765	1.311208	9.20E-34	2.05E-31	UP
ENSMUSC	Plac8	placenta-	GO:00325	developm	-	-	-	-	5:1005537	5	5	6	196	476	182	0.124141	0.099147	0.164669	3.754656	7.611076	3.555233	0.129319	4.973655	9.63E-34	2.12E-31	DOWN
ENSMUSC	Gsdmc3	gasdermi	GO:00099	cellular pr	-	-	-	-	15:638577	4	2	0	140	298	120	0.067138	0.026812	0	1.812754	3.220711	1.584434	0.031316	2.205966	2.65E-33	5.76E-31	DOWN
ENSMUSC	Fam83e	family wit	GO:00055	protein bi	-	-	-	-	7:4572121	2	2	1	141	381	137	0.019927	0.01592	0.011015	1.08404	2.444976	1.074053	0.015621	1.534356	5.16E-33	1.09E-30	DOWN
ENSMUSC	Jchain	immunog	GO:00081	metabolic	-	-	-	-	5:885198C	10	9	8	202	538	239	0.167147	0.120143	0.147777	2.604811	5.790699	3.14269	0.145022	3.846067	5.16E-33	1.09E-30	DOWN
ENSMUSC	Sprr2a2	small prol	GO:00325	developm	-	-	-	-	3:9221592	0	0	0	82	183	85	0	0	0	1.076254	2.004825	1.13762	0	1.406233	6.35E-33	1.33E-30	DOWN
ENSMUSC	Tmprss2	transmem	GO:00081	metabolic	K09633	transmem	mmu052C	Transcript	16:975646	0	0	0	94	243	99	0	0	0	1.401686	3.024504	1.505362	0	1.977184	7.08E-33	1.47E-30	DOWN
ENSMUSC	Plet1	placenta e	GO:00325	developm	-	-	-	-	9:5049452	12	10	0	277	750	305	0.338108	0.225014	0	6.021349	13.60819	6.760763	0.187707	8.796766	4.17E-32	8.53E-30	DOWN
ENSMUSC	Clca4b	chloride c	GO:00081	metabolic	K05030	calcium-a	mmu0492	Renin sec	3:1449109	0	0	0	106	268	92	0	0	0	1.666074	3.515983	1.474534	0	2.218864	7.92E-32	1.60E-29	DOWN
ENSMUSC	Myo7b	myosin VI	GO:00325	developm	-	-	-	-	18:319592	1	1	3	154	416	140	0.009225	0.007366	0.030579	1.095646	2.470404	1.015686	0.015723	1.527245	8.39E-32	1.68E-29	DOWN
ENSMUSC	Akr1c19	aldo-keto	GO:00081	metabolic	-	-	-	-	13:42287C	0	0	0	72	159	79	0	0	0	0.492035	0.90696	0.550523	0	0.649839	1.58E-31	3.12E-29	DOWN
ENSMUSC	Cdx2	caudal typ	GO:00325	developm	K09312	homeobo	mmu0522	Gastric ca	5:1473008	2	0	0	118	293	106	0.054	0	0	2.458414	5.095248	2.251946	0.018	3.268536	3.25E-31	6.34E-29	DOWN
ENSMUSC	Mep1b	meprin 1	GO:00081	metabolic	K08606	meprin B[	mmu0497	Protein di	18:210723	0	2	0	101	241	95	0	0.042669	0	2.081217	4.145139	1.996199	0.014223	2.740852	3.56E-31	6.88E-29	DOWN
ENSMUSC	Prr15l	proline ric	-	-	-	-	-	-	11:969281	0	2	0	88	189	83	0	0.013037	0	0.554036	0.993213	0.532863	0.004346	0.693371	1.01E-30	1.94E-28	DOWN
ENSMUSC	Naip1	NLR famil	GO:00081	metabolic	K12807	baculovira	mmu0462	NOD-like	13:100407	9	4	4	158	383	169	0.051525	0.018287	0.025312	0.697864	1.412005	0.761167	0.031708	0.957012	1.59E-30	2.98E-28	DOWN
ENSMUSC	Apol10a	apolipopr	GO:00081	metabolic	-	-	-	-	15:77477C	0	0	0	79	189	80	0	0	0	0.539324	1.076988	0.55692	0	0.724411	1.59E-30	2.98E-28	DOWN
ENSMUSC	Cdx1	caudal typ	GO:00325	developm	-	-	-	-	18:610188	0	0	0	81	237	96	0	0	0	2.180321	5.324861	2.635033	0	3.380072	5.20E-30	9.62E-28	DOWN
ENSMUSC	Tmc5	transmem	GO:00511	localizatio	-	-	-	-	7:1185972	0	4	2	116	287	115	0	0.031852	0.022044	0.892414	1.842962	0.902172	0.017965	1.212516	8.43E-30	1.54E-27	DOWN
ENSMUSC	Ush1c	USH1 pro	GO:00511	localizatio	-	-	-	-	7:4619535	1	0	1	93	289	121	0.011442	0	0.012643	0.820684	2.128714	1.088828	0.008028	1.346076	1.48E-29	2.67E-27	DOWN
ENSMUSC	Tmem45b	transmem	GO:00160	membran	-	-	-	-	9:3142619	0	2	0	86	201	85	0	0.019235	0	0.798871	1.558474	0.805151	0.006412	1.054165	1.50E-29	2.70E-27	DOWN
ENSMUSC	Btnl1	butyrophi	GO:00160	membran	-	-	-	-	17:343771	0	0	0	57	122	72	0	0	0	1.44278	2.577581	1.858405	0	1.959589	1.63E-29	2.90E-27	DOWN
ENSMUSC	Slfn4	schlafen 4	GO:00971	organic cy	-	-	-	-	11:831751	2	15	0	237	548	225	0.015227	0.091227	0	1.392375	2.687279	1.347939	0.035485	1.809198	1.69E-29	2.96E-27	DOWN
ENSMUSC	Actg2	actin, gan	GO:00325	developm	K12315	actin, gan	mmu0427	Vascular s	6:835129C	0	10	0	195	497	182	0	0.18512	0	3.487333	7.418887	3.319028	0.061707	4.741749	1.84E-29	3.21E-27	DOWN
ENSMUSC	Dsc2	desmocol	GO:00511	localizatio	K07601	desmocol	mmu0541	Arrhythmi	18:200306	7	24	8	244	580	236	0.051633	0.141374	0.065212	1.388475	2.754865	1.369433	0.086073	1.837591	7.04E-29	1.21E-26	DOWN
ENSMUSC	Fam3b	family wit	GO:00511	localizatio	-	-	-	-	16:97471C	0	0	0	87	177	63	0	0	0	2.561367	4.349611	1.891371	0	2.934116	1.77E-28	3.02E-26	DOWN
ENSMUSC	Chmp4c	charged n	GO:00325	developm	K12194	charged n	mmu0414	Endocytos	3:103669C	7	8	6	129	318	154	0.041292	0.037688	0.039119	0.58706	1.207933	0.714649	0.039367	0.836547	2.03E-28	3.43E-26	DOWN
ENSMUSC	Nr1h4	nuclear re	GO:00325	developm	K08537	farnesoid	mmu0497	Bile secret	10:894542	0	5	3	123	238	94	0	0.077104	0.064029	1.832381	2.959462	1.427978	0.047044	2.073274	2.30E-28	3.84E-26	DOWN
ENSMUSC	Atp8b1	ATPase, cl	GO:00325	developm	-	-	-	-	18:645289	37	58	39	347	745	366	0.350804	0.439167	0.408663	2.53815	4.548501	2.729919	0.399544	3.27219	3.12E-28	5.18E-26	DOWN
ENSMUSC	Cd177	CD177 an	GO:00081	metabolic	-	-	-	-	7:2474398	5	11	3	142	347	150	0.098855	0.173697	0.065564	2.166135	4.418246	2.33329	0.112705	2.972557	3.76E-28	6.16E-26	DOWN
ENSMUSC	Ugt1a6b	UDP gluc	GO:00081	metabolic	K00699	glucuronc	mmu011C	Metabolic	1:8810325	3	3	1	119	293	104	0.044545	0.035581	0.016412	1.363226	2.801643	1.214886	0.03218	1.793252	4.42E-28	7.19E-26	DOWN
ENSMUSC	Vdr	vitamin D	GO:00325	developm	K08539	vitamin D:	mmu0515	Tuberculo	15:978544	2	17	4	209	584	240	0.020012	0.135871	0.044246	1.613688	3.763654	1.889574	0.06671	2.422305	5.23E-28	8.42E-26	DOWN
ENSMUSC	Ifit1bl1	interferon	GO:00023	immune s	K14217	interferon	mmu0516	Herpes sir	19:345928	42	55	30	302	610	342	0.581769	0.608433	0.459272	3.227297	5.441097	3.726818	0.549825	4.131737	8.70E-28	1.39E-25	DOWN
ENSMUSC	Reg4	regenerat	GO:00055	extracellul	-	-	mmu0522	Gastric ca	3:9822215	0	2	0	84	210	82	0	0.095534	0	3.875513	8.087065	3.857822	0.031845	5.273466	9.07E-28	1.43E-25	DOWN
ENSMUSC	Cldn3	claudin 3	GO:00325	developm	K06087	claudin[-]	mmu0453	Tight junc	5:1349862	5	8	3	116	270	126	0.242465	0.30986	0.160811	4.340151	8.432107	4.807258	0.237712	5.859838	2.33E-27	3.65E-25	DOWN
ENSMUSC	Hsd3b3	hydroxy-c	GO:00081	metabolic	-	-	-	-	3:9874152	0	0	0	56	130	63	0	0	0	0.317284	0.614794	0.363988	0	0.432022	9.47E-27	1.47E-24	DOWN
ENSMUSC	Ces1f	carboxyle	GO:00081	metabolic	-	-	mmu011C	Metabolic	8:9325623	0	0	0	66	174	69	0	0	0	0.75096	1.652523	0.800582	0	1.068022	1.43E-26	2.21E-24	DOWN
ENSMUSC	Ildr1	immunog	GO:00511	localizatio	-	-	-	-	16:366939	9	9	8	138	350	159	0.062289	0.049749	0.061191	0.73686	1.559909	0.865733	0.057743	1.054167	2.70E-26	4.12E-24	DOWN
ENSMUSC	Gipc2	GIPC PDZ	GO:00055	extracellul	-	-	-	-	3:1520935	6	10	3	110	191	100	0.05742	0.076427	0.031734	0.812167	1.177091	0.752897	0.055194	0.914052	3.49E-26	5.28E-24	DOWN
ENSMUSC	Tmprss4	transmem	GO:00081	metabolic	K09635	transmem	mmu0516	Influenza	9:4517272	0	2	0	63	170	84	0	0.021826	0	0.66405	1.495674	0.90287	0.007275	1.020864	3.56E-26	5.34E-24	DOWN
ENSMUSC	Fbp2	fructose b	GO:00081	metabolic	K03841	fructose-1	mmu011C	Metabolic	13:628368	0	0	2	65	144	70	0	0	0.091881	2.084318	3.854229	2.288907	0.030627	2.742485	8.45E-26	1.26E-23	DOWN
ENSMUSC	Slc13a2	solute car	GO:00511	localizatio	-	-	-	-	11:78397C	0	2	0	70	201	84	0	0.04044	0	1.367091	3.276543	1.672854	0.01348	2.105496	8.97E-26	1.32E-23	DOWN
ENSMUSC	Osr2	odd-skipp	GO:00325	developm	-	-	-	-	15:35296C	0	0	0	55	122	55	0	0	0	0.423963	0.784958	0.432317	0	0.547079	2.15E-25	3.14E-23	DOWN
ENSMUSC	2200002D	RIKEN cDI	GO:00056	cell|intrace	-	-	-	-	7:2924656	0	7	2	80	159	88	0	0.185512	0.073355	2.048071	3.397649	2.297311	0.086289	2.58101	4.36E-25	6.31E-23	DOWN
ENSMUSC	Slc26a3	solute car	GO:00325	developm	K14078	solute car	mmu0497	Pancreatic	12:313908	0	9	4	116	287	125	0	0.053588	0.032961	0.667186	1.377833	0.733131	0.028849	0.92605	4.65E-25	6.68E-23	DOWN
ENSMUSC	Lpo	lactopero:	GO:00081	metabolic	K12550	lactopero:	mmu0497	Salivary se	11:878064	15	15	18	205	502	207	0.276274	0.22065	0.366424	2.91302	5.954129	2.99945	0.287783	3.955533	4.90E-25	6.99E-23	DOWN
ENSMUSC	Prss30	protease,	GO:00081	metabolic	-	-	-	-	17:239721	0	2	0	59	140	65	0	0.034046	0	0.970065	1.921334	1.089784	0.011349	1.327061	1.20E-24	1.70E-22	DOWN
ENSMUSC	Btnl4	butyrophi	GO:00160	membran	-	-	-	-	17:34469C	0	0	0	51	140	65	0	0	0	1.364218	3.12585	1.772988	0	2.087685	1.46E-24	2.04E-22	DOWN
ENSMUSC	Misp	mitotic sp	GO:00099	cellular pr	-	-	-	-	10:798209	3	7	7	265	663	134	0.033322	0.062096	0.085939	2.270877	4.742266	1.170937	0.060452	2.728027	1.78E-24	2.47E-22	DOWN
ENSMUSC	Dpep1	dipeptida	GO:00081	metabolic	-	-	-	-	8:1231862	7	15	6	136	314	137	0.159424	0.27283	0.151034	2.389555	4.605009	2.454578	0.194429	3.149714	1.91E-24	2.63E-22	DOWN
ENSMUSC	Alpi	alkaline pl	GO:00081	metabolic	K01077	alkaline pl	mmu011C	Metabolic	1:870980C	0	0	0	53	115	50	0	0	0	1.068298	1.9348	1.027692	0	1.343597	2.72E-24	3.72E-22	DOWN
ENSMUSC	Aqp8	aquaporir	GO:00511	localizatio	K09869	aquaporir	mmu0497	Bile secret	7:1234622	0	4	0	78	194	76	0	0.062053	0	1.169019	2.42692	1.161508	0.020684	1.585816	3.58E-24	4.87E-22	DOWN
ENSMUSC	St14	suppressi	GO:00325	developm	K08670	suppresso	mmu052C	MicroRNA	9:310894C	0	21	4	215	458	242	0	0.106084	0.027965	1.04918	1.865527	1.204225	0.044683	1.372977	4.48E-24	6.05E-22	DOWN
ENSMUSC	Aoc1	amine oxi	GO:00081	metabolic	K11182	diamine o	mmu011C	Metabolic	6:4887289	1	0	0	46	61	51	0.009211	0	0	0.326779	0.361701	0.369441	0.00307	0.35264	4.62E-24	6.18E-22	DOWN
ENSMUSC	Slc10a2	solute car	GO:00511	localizatio	-	-	-	-	8:5083219	0	0	0	63	202	69	0	0	0	0.714749	1.912903	0.798268	0	1.141973	6.18E-24	8.20E-22	DOWN
ENSMUSC	Hmgcs2	3-hydrox	GO:00081	metabolic	K01641	hydroxym	mmu011C	Metabolic	3:9828043	113	162	122	745	1615	724	1.629945	1.866185	1.944924	8.290517	15.00107	8.215698	1.813685	10.50243	7.29E-24	9.61E-22	DOWN
ENSMUSC	Oas3	2'-5' oligo	GO:00081	metabolic	K14216	2'-5'-olig	mmu0516	Herpes sir	5:120753C	2	4	1	94	212	75	0.014551	0.023243	0.008043	0.527699	0.993392	0.429338	0.015279	0.650143	8.79E-24	1.15E-21	DOWN
ENSMUSC	Krt7	keratin 7 [	GO:00051	structural	-	-	-	-	15:101411	3	0	0	70	143	61	0.021314	0	0	0.383689	0.654242	0.340948	0.007105	0.459626	1.39E-23	1.81E-21	DOWN
ENSMUSC	Akr1c13	aldo-keto	GO:00081	metabolic	-	-	-	-	13:419115	2	9	4	94	227	108	0.070169	0.252232	0.155142	2.544785	5.129497	2.981459	0.159181	3.551914	1.66E-23	2.14E-21	DOWN
ENSMUSC	Cftr	cystic fibr	GO:00325	developm	K05031	cystic fibr	mmu0402	cAMP sigr	6:1817068	9	7	2	92	205	117	0.051573	0.032036	0.012668	0.406734	0.756481	0.527455	0.032092	0.563557	1.85E-23	2.37E-21	DOWN
ENSMUSC	Gpx2	glutathior	GO:00081	metabolic	K00432	glutathior	mmu0059	Arachidor	12:767923	2	5	0	83	184	75	0.023291	0.046509	0	0.745858	1.380128	0.687252	0.023267	0.937746	3.59E-23	4.56E-21	DOWN
ENSMUSC	Mettl7b	methyltra	GO:00081	metabolic	-	-	-	-	10:128958	0	3	0	85	183	59	0	0.118952	0	3.255255	5.849852	2.304118	0.039651	3.803075	4.01E-23	5.05E-21	DOWN
ENSMUSC	Pck1	phosphoe	GO:00325	developm	K01596	phosphoe	mmu011C	Metabolic	2:173153C	0	2	0	53	104	50	0	0.017384	0	0.44495	0.728771	0.428037	0.005795	0.533919	4.18E-23	5.23E-21	DOWN
ENSMUSC	Rab25	RAB25, m	GO:00325	developm	-	-	-	-	3:8854202	0	0	2	51	80	47	0	0	0.050495	0.898761	1.176765	0.844607	0.016832	0.973377	4.72E-23	5.87E-21	DOWN
ENSMUSC	Tmem30b	transmem	GO:00511	localizatio	-	-	-	-	12:735434	4	5	5	81	165	85	0.08166	0.081512	0.112817	1.275681	2.169032	1.365068	0.091996	1.603261	5.72E-23	7.01E-21	DOWN
ENSMUSC	Cdcp1	CUB dom	GO:00160	membran	-	-	-	-	9:1231708	0	3	0	60	155	68	0	0.019443	0	0.375595	0.809886	0.434068	0.006481	0.53985	5.71E-23	7.01E-21	DOWN
ENSMUSC	Cd74	CD74 anti	GO:00325	developm	K06505	CD74 anti	mmu0516	Herpes sir	18:608038	62	130	91	624	1563	680	1.88997	3.164831	3.065854	14.67496	30.68147	16.30731	2.706885	20.55458	8.93E-23	1.09E-20	DOWN
ENSMUSC	Duoxa2	dual oxida	GO:00081	metabolic	K17232	dual oxida	mmu0491	Thyroid hc	2:1222989	0	0	0	44	101	48	0	0	0	1.301916	2.494451	1.44829	0	1.748219	1.10E-22	1.33E-20	DOWN
ENSMUSC	Trim31	tripartite r	GO:00081	metabolic	-	-	-	-	17:368981	0	1	0	59	144	53	0	0.021608	0	1.231386	2.508579	1.127979	0.007203	1.622648	1.14E-22	1.37E-20	DOWN
ENSMUSC	Tns4	tensin 4 [S	GO:00511	localizatio	-	-	-	-	11:990656	4	13	6	130	254	101	0.039883	0.103513	0.06612	0.999955	1.630787	0.792207	0.069839	1.140983	1.77E-22	2.11E-20	DOWN
ENSMUSC	Tmigd1	transmem	GO:00400	locomotic	-	-	-	-	11:769021	0	0	0	52	153	57	0	0	0	0.957584	2.351733	1.070359	0	1.459892	1.98E-22	2.35E-20	DOWN
ENSMUSC	Ugt1a1	UDP gluc	GO:00325	developm	K00699	glucuronc	mmu011C	Metabolic	1:8821195	0	0	0	99	259	52	0	0	0	2.096891	4.578926	1.123114	0	2.599644	2.04E-22	2.39E-20	DOWN
ENSMUSC	Mall	mal, T cell	GO:00325	developm	-	-	-	-	2:1277043	10	18	16	140	270	139	0.30438	0.437546	0.538247	3.287537	5.292135	3.328421	0.426724	3.969364	2.07E-22	2.42E-20	DOWN
ENSMUSC	2610528A	RIKEN cDI	GO:00048	signal trar	-	-	-	-	14:371021	0	7	0	109	306	100	0	0.247709	0	3.726061	8.731138	3.485837	0.08257	5.314345	2.53E-22	2.94E-20	DOWN
ENSMUSC	Slc5a1	solute car	GO:00511	localizatio	K14158	solute car	mmu0497	Bile secret	5:3310421	6	11	9	106	177	92	0.089264	0.130696	0.147981	1.216672	1.695755	1.076798	0.122647	1.329742	3.42E-22	3.94E-20	DOWN
ENSMUSC	Slc17a4	solute car	GO:00511	localizatio	-	-	-	-	13:238958	0	0	0	37	99	59	0	0	0	0.187329	0.418373	0.304607	0	0.303437	4.38E-22	5.01E-20	DOWN
ENSMUSC	Lad1	ladinin [Sc	GO:00051	structural	-	-	-	-	1:1358185	40	50	24	293	591	255	0.618785	0.617715	0.410325	3.496823	5.887333	3.103321	0.548942	4.162492	5.56E-22	6.32E-20	DOWN
ENSMUSC	Mmp12	matrix me	GO:00325	developm	-	-	-	-	9:7344381	31	62	24	0	0	1	0.190152	0.303729	0.162704	0	0	0.004827	0.218862	0.001609	6.32E-22	7.14E-20	UP
ENSMUSC	Spink4	serine per	GO:00081	metabolic	-	-	-	-	4:4092005	3	0	0	70	169	63	0.295435	0	0	5.318425	10.71751	4.880956	0.098478	6.972297	6.72E-22	7.54E-20	DOWN
ENSMUSC	Tm4sf20	transmem	GO:00081	metabolic	-	-	-	-	1:8275664	0	1	0	54	134	51	0	0.026826	0	1.399188	2.898064	1.347504	0.008942	1.881585	7.27E-22	8.11E-20	DOWN
ENSMUSC	Tff3	trefoil fact	GO:00081	metabolic	-	-	-	-	17:311253	0	0	0	46	79	35	0	0	0	4.395282	6.300572	3.410146	0	4.702	1.87E-21	2.08E-19	DOWN
ENSMUSC	Nr5a2	nuclear re	GO:00325	developm	K08027	nuclear re	mmu0495	Maturity c	1:1368425	0	0	0	37	77	41	0	0	0	0.197028	0.342248	0.222634	0	0.25397	2.51E-21	2.77E-19	DOWN
ENSMUSC	Abca12	ATP-bind	GO:00325	developm	K05646	ATP-bind	mmu0201	ABC trans	1:7124227	12	2	7	141	320	130	0.04639	0.006176	0.029911	0.420534	0.79663	0.395373	0.027492	0.537512	2.73E-21	2.99E-19	DOWN
ENSMUSC	Anks4b	ankyrin re	GO:00325	developm	-	-	-	-	7:1201738	6	13	7	123	306	115	0.093933	0.162542	0.12113	1.485638	3.085002	1.41641	0.125869	1.995683	3.92E-21	4.26E-19	DOWN
ENSMUSC	Trim40	tripartite r	GO:00081	metabolic	-	-	-	-	17:368815	0	0	2	62	132	49	0	0	0.031186	0.674809	1.199182	0.543825	0.010395	0.805939	6.82E-21	7.37E-19	DOWN
ENSMUSC	Ceacam2(	carcinoen	GO:00023	immune s	-	-	-	-	7:1996541	0	0	0	44	131	51	0	0	0	0.642881	1.597627	0.759852	0	1.00012	8.43E-21	9.05E-19	DOWN
ENSMUSC	Rasef	RAS and E	GO:00038	catalytic a	-	-	-	-	4:7371457	0	0	0	34	88	50	0	0	0	0.270857	0.58516	0.406176	0	0.420731	8.63E-21	9.21E-19	DOWN
ENSMUSC	Hsd17b2	hydroxyst	GO:00325	developm	K13368	17beta-es	mmu011C	Metabolic	8:1177019	0	6	2	59	140	78	0	0.128943	0.059486	1.224873	2.426012	1.65126	0.06281	1.767382	9.80E-21	1.04E-18	DOWN

ENSMUS(	Ifi27l2b	interferon	GO:00160	membran	-	-	-	-	12:103450	0	0	0	47	150	53	0	0	0	2.025605	5.395977	2.329231	0	3.250271	1.18E-20	1.24E-18	DOWN
ENSMUS(	Tmem54	transmem	GO:00160	membran	-	-	-	-	4:1291055	0	0	0	45	120	45	0	0	0	1.005103	2.237183	1.024915	0	1.4224	1.40E-20	1.47E-18	DOWN
ENSMUS(	Scin	scinderin	GO:00325	developm	K05768	gelsolin[-]	mmu0520	Viral carci	12:400597	11	7	14	119	250	128	0.162709	0.08269	0.228864	1.357942	2.381216	1.489444	0.158087	1.742867	2.22E-20	2.30E-18	DOWN
ENSMUS(	Myh11	myosin, h	GO:00325	developm	K10352	myosin he	mmu0453	Tight junc	16:141945	397	610	314	1522	3118	1868	3.655012	4.485127	3.195035	10.81045	18.48543	13.52966	3.778392	14.27518	2.20E-20	2.30E-18	DOWN
ENSMUS(	S100g	S100 calci	GO:00160	membran	K14734	S100 calci	mmu0497	Mineral al	X:1629619	0	0	0	33	71	40	0	0	0	3.238565	5.815877	4.002862	0	4.352435	3.09E-20	3.18E-18	DOWN
ENSMUS(	Muc4	mucin 4 [S	GO:00160	membran	-	-	-	-	16:327358	1	1	0	85	602	119	0.002843	0.00227	0	0.18637	1.101741	0.266064	0.001704	0.518058	3.15E-20	3.22E-18	DOWN
ENSMUS(	Apob	apolipopr	GO:00325	developm	K14462	apolipopr	mmu0497	Cholestero	12:797764	0	0	0	43	157	59	0	0	0	0.114683	0.349508	0.160461	0	0.208218	3.21E-20	3.27E-18	DOWN
ENSMUS(	Gm1123	predicted	-	-	-	-	-	-	9:9900679	0	0	1	41	73	39	0	0	0.043908	1.256548	1.867431	1.218822	0.014636	1.4476	3.81E-20	3.86E-18	DOWN
ENSMUS(	Tnk1	tyrosine k	GO:00325	developm	-	-	-	-	11:698510	0	0	0	39	104	44	0	0	0	0.369645	0.822764	0.425252	0	0.53922	4.72E-20	4.74E-18	DOWN
ENSMUS(	Hepacam	HEPACAM	GO:00099	cellular pr	-	-	-	-	6:3457096	2	3	3	56	129	66	0.016019	0.019196	0.026563	0.34609	0.665454	0.415938	0.020592	0.475827	5.48E-20	5.48E-18	DOWN
ENSMUS(	Isx	intestine s	GO:00081	metabolic	-	-	-	-	8:7487317	0	0	0	45	132	46	0	0	0	0.794811	1.946029	0.828493	0	1.189778	5.92E-20	5.89E-18	DOWN
ENSMUS(	Sult1b1	sulfotrans	GO:00325	developm	-	-	-	-	5:8751333	0	0	0	32	79	44	0	0	0	0.300938	0.62012	0.421941	0	0.447666	6.76E-20	6.68E-18	DOWN
ENSMUS(	Sdcbp2	syndecan	GO:00055	extracellul	-	-	-	-	2:1515726	6	10	8	93	215	100	0.261858	0.348538	0.385867	3.131389	6.042519	3.433512	0.332088	4.202473	6.87E-20	6.75E-18	DOWN
ENSMUS(	Cldn15	claudin 15	GO:00511	localizatio	K06087	claudin[-]	mmu0453	Tight junc	5:1369666	3	5	0	61	144	67	0.048076	0.06399	0	0.754189	1.486053	0.844704	0.037355	1.028315	1.07E-19	1.04E-17	DOWN
ENSMUS(	Ano9	anoctamir	GO:00511	localizatio	-	-	-	-	7:1411012	2	0	0	49	108	46	0.013914	0	0	0.263039	0.483922	0.251805	0.004638	0.332922	1.31E-19	1.28E-17	DOWN
ENSMUS(	Cnn1	calponin 1	GO:00099	cellular pr	-	-	-	-	9:2209921	15	31	7	136	254	166	0.236837	0.390906	0.122164	1.656684	2.582607	2.062001	0.249969	2.100431	1.33E-19	1.28E-17	DOWN
ENSMUS(	Slc51a	solute car	GO:00511	localizatio	K14360	organic sc	mmu0497	Bile secret	16:324755	0	0	0	43	86	33	0	0	0	1.265961	2.113371	0.990727	0	1.456686	1.63E-19	1.57E-17	DOWN
ENSMUS(	Ddx60	DEAD (As	GO:00023	immune s	-	-	-	-	8:6192808	28	25	23	165	260	149	0.162417	0.115814	0.147451	0.738406	0.971199	0.67995	0.141894	0.796518	2.21E-19	2.11E-17	DOWN
ENSMUS(	Slc9a3	solute car	GO:00511	localizatio	K12040	solute car	mmu0497	Protein di	13:741214	26	25	19	206	577	203	0.251866	0.19341	0.203424	1.539549	3.599372	1.547041	0.216233	2.228654	3.17E-19	3.02E-17	DOWN
ENSMUS(	Cfb	complem	GO:00081	metabolic	K01335	compone	mmu0461	Complem	17:348563	0	0	8	96	181	80	0	0	0.112464	0.942114	1.482637	0.800572	0.037488	1.075108	4.04E-19	3.82E-17	DOWN
ENSMUS(	Clrn3	clarin 3 [S	GO:00055	extracellul	-	-	-	-	7:1355114	0	0	2	55	118	43	0	0	0.040839	0.783914	1.403812	0.624964	0.013613	0.937563	4.12E-19	3.87E-17	DOWN
ENSMUS(	Casp1	caspase 1	GO:00081	metabolic	K01370	caspase 1	mmu0421	Necroptos	9:5298517	17	22	16	116	172	115	0.693388	0.716633	0.721258	3.650134	4.51756	3.690046	0.710426	3.95258	7.09E-19	6.63E-17	DOWN
ENSMUS(	Tmem236	transmem	GO:00160	membran	-	-	-	-	2:1417452	4	5	2	60	155	72	0.066752	0.066631	0.036888	0.772438	1.66559	0.945202	0.056757	1.127743	1.25E-18	1.17E-16	DOWN
ENSMUS(	Abcc3	ATP-bind	GO:00511	localizatio	K05667	ATP-bind	mmu0497	Bile secret	11:943432	54	62	45	277	574	271	0.450426	0.413021	0.414851	1.78255	3.083179	1.778329	0.426099	2.214686	2.19E-18	2.02E-16	DOWN
ENSMUS(	Igf2bp2	insulin-lik	GO:00081	metabolic	-	-	-	-	16:220590	10	8	10	82	156	95	0.044409	0.028374	0.04908	0.280942	0.44612	0.331901	0.040621	0.352988	2.71E-18	2.50E-16	DOWN
ENSMUS(	Nlrp6	NLR famil	GO:00048	signal trar	K20863	NACHT, L	mmu0462	NOD-like	7:1409209	14	12	16	119	322	148	0.119107	0.081531	0.150442	0.781049	1.764051	0.990541	0.117027	1.178547	4.28E-18	3.92E-16	DOWN
ENSMUS(	Slc25a24	solute car	GO:00511	localizatio	-	-	-	-	3:1091231	80	121	59	388	708	369	1.32017	1.594682	1.07607	4.93973	7.523663	4.790476	1.330307	5.751289	7.08E-18	6.45E-16	DOWN
ENSMUS(	Lyz2	lysozyme	GO:00081	metabolic	K13915	lysozyme	mmu0497	Salivary se	10:117277	935	2545	998	288	557	339	43.38093	94.30205	51.1758	10.30885	16.6417	12.37366	62.95293	13.10807	7.27E-18	6.58E-16	UP
ENSMUS(	Nr1i2	nuclear re	GO:00048	signal trar	-	-	-	-	16:382483	0	0	0	29	78	39	0	0	0	0.535081	1.201266	0.733776	0	0.823374	7.67E-18	6.91E-16	DOWN
ENSMUS(	Dsg2	desmogle	GO:00325	developm	K07597	desmogle	mmu0541	Arrhythmo	18:205580	94	135	102	472	1089	509	0.646263	0.741246	0.775047	2.503535	4.821299	2.753025	0.720852	3.359287	1.12E-17	1.00E-15	DOWN
ENSMUS(	Cyp2d34	cytochron	GO:00081	metabolic	K07414	cytochron	mmu0472	Serotoner	15:826159	0	0	0	30	83	38	0	0	0	0.871254	2.011999	1.12536	0	1.336205	1.23E-17	1.10E-15	DOWN
ENSMUS(	Sqor	sulfide qu	GO:00081	metabolic	K17218	sulfide:qu	mmu0092	Sulfur met	2:1227652	69	114	76	396	794	373	0.756922	0.998739	0.921428	3.351392	5.608873	3.218988	0.892363	4.059751	1.29E-17	1.14E-15	DOWN
ENSMUS(	Ptprh	protein ty	GO:00048	signal trar	-	-	-	-	7:4548612	13	19	5	130	265	104	0.155516	0.181522	0.066112	1.199789	2.041425	0.978764	0.134383	1.406659	1.66E-17	1.46E-15	DOWN
ENSMUS(	Mapk13	mitogen-	GO:00048	signal trar	K04441	p38 MAP	mmu0401	MAPK sigi	17:287693	2	12	0	90	220	91	0.047936	0.229739	0	1.664517	3.396182	1.71619	0.092558	2.258963	1.73E-17	1.52E-15	DOWN
ENSMUS(	Ppp1r14d	protein pl	GO:00081	metabolic	-	-	-	-	2:1192181	0	0	0	31	73	32	0	0	0	1.000196	1.965919	1.052821	0	1.339645	1.88E-17	1.65E-15	DOWN
ENSMUS(	Ces1g	carboxyle	GO:00081	metabolic	-	-	-	-	8:9330236	0	0	0	36	73	28	0	0	0	0.710134	1.201944	0.563229	0	0.825102	1.96E-17	1.71E-15	DOWN
ENSMUS(	Cblc	Casitas B-	GO:00048	signal trar	K04707	E3 ubiquit	mmu0520	Pathways	7:1977888	0	0	0	28	54	29	0	0	0	0.315387	0.507697	0.333091	0	0.385392	3.00E-17	2.60E-15	DOWN
ENSMUS(	Akr1c12	aldo-keto	GO:00081	metabolic	-	-	-	-	13:426817	0	0	0	59	154	31	0	0	0	2.203995	4.80178	1.180868	0	2.728881	4.34E-17	3.73E-15	DOWN
ENSMUS(	Ceacam1(	carcinoem	-	-	-	-	-	-	7:2477720	0	0	0	30	63	29	0	0	0	0.593521	1.040344	0.585043	0	0.739636	4.56E-17	3.91E-15	DOWN
ENSMUS(	Selenbp1	selenium	GO:00325	developm	-	-	-	-	3:9493305	104	137	130	466	908	538	1.919604	2.019498	2.651963	6.635814	10.79241	7.812154	2.197021	8.41346	5.35E-17	4.56E-15	DOWN
ENSMUS(	Oas1g	2'-5' oligo	GO:00023	immune s	K14216	2'-5'-oligo	mmu0516	Herpes sir	5:1208761	1	8	0	58	127	59	0.018226	0.116417	0	0.815316	1.490143	0.845737	0.044881	1.050399	6.01E-17	5.10E-15	DOWN
ENSMUS(	Foxa1	forkhead	GO:00325	developm	-	-	-	-	12:575406	0	0	0	31	96	37	0	0	0	0.405073	1.047039	0.493007	0	0.648373	8.36E-17	7.06E-15	DOWN
ENSMUS(	Tmem238	transmem	GO:00160	membran	-	-	-	-	11:669566	0	0	0	54	145	30	0	0	0	0.911402	2.042713	0.516313	0	1.15681	1.18E-16	9.90E-15	DOWN
ENSMUS(	Hgfac	hepatocyt	GO:00081	metabolic	-	-	-	-	5:3504150	1	0	0	30	75	35	0.010231	0	0	0.236713	0.493953	0.281608	0.00341	0.337425	1.37E-16	1.14E-14	DOWN
ENSMUS(	Zbp1	Z-DNA bi	GO:00023	immune s	K12965	Z-DNA bi	mmu0421	Necroptos	2:1732066	15	13	14	102	212	106	0.189538	0.131191	0.195519	0.994366	1.725073	1.053736	0.172083	1.257725	2.14E-16	1.78E-14	DOWN
ENSMUS(	Slc22a18	solute car	GO:00511	localizatio	-	-	-	-	7:1434737	0	0	0	34	56	23	0	0	0	0.69543	0.956078	0.47973	0	0.710413	2.64E-16	2.19E-14	DOWN
ENSMUS(	Atp6v0d2	ATPase, H	GO:00511	localizatio	K02146	V-type H-	mmu0110	Metabolic	4:1987684	20	42	16	0	0	0	0.445521	0.747194	0.393916	0	0	0	0.528877	0	3.24E-16	2.67E-14	UP
ENSMUS(	H2-T3	histocomp	GO:00160	membran	K06751|K0	major hist	mmu0516	Human pa	17:361855	0	0	0	30	101	37	0	0	0	0.165071	0.463867	0.207603	0	0.278847	3.29E-16	2.70E-14	DOWN
ENSMUS(	AA467197	expressed	GO:00081	metabolic	-	-	-	-	2:1226369	0	5	4	51	159	71	0	0.125091	0.138506	1.232626	3.207632	1.749845	0.087866	2.063368	4.04E-16	3.30E-14	DOWN
ENSMUS(	Atoh1	atonal bH	GO:00325	developm	-	-	-	-	6:6472912	0	0	0	33	78	27	0	0	0	0.732914	1.445937	0.611479	0	0.93011	4.89E-16	3.97E-14	DOWN
ENSMUS(	Esrp2	epithelial	GO:00325	developm	-	-	-	-	8:1061303	3	2	0	44	98	41	0.024703	0.013154	0	0.279521	0.519654	0.265599	0.012619	0.354925	5.56E-16	4.50E-14	DOWN
ENSMUS(	Gprc5a	G protein-	GO:00048	signal trar	-	-	-	-	6:1350656	0	0	1	31	75	31	0	0	0.032399	0.70105	1.415699	0.71487	0.0108	0.943873	7.11E-16	5.73E-14	DOWN
ENSMUS(	Nox1	NADPH o	GO:00325	developm	K08008	NADPH o	mmu0541	Fluid shea	X:1340864	1	0	0	27	53	29	0.015657	0	0	0.326029	0.534191	0.357084	0.005219	0.405768	7.44E-16	5.96E-14	DOWN
ENSMUS(	Ugt2b5	UDP gluc	GO:00081	metabolic	K00699	glucuronc	mmu0110	Metabolic	5:8712496	0	0	0	30	58	24	0	0	0	0.760589	1.227378	0.62046	0	0.869476	7.91E-16	6.31E-14	DOWN
ENSMUS(	Pkp3	plakophili	GO:00081	metabolic	-	-	-	-	7:1410782	3	0	0	36	66	34	0.027474	0	0	0.254357	0.389235	0.244961	0.009158	0.296185	8.12E-16	6.45E-14	DOWN
ENSMUS(	Ido1	indoleami	GO:00325	developm	K00463	indoleami	mmu0110	Metabolic	8:2458413	1	0	0	27	64	32	0.033467	0	0	0.696898	1.378855	0.842257	0.011156	0.97267	9.01E-16	7.13E-14	DOWN
ENSMUS(	Pklr	pyruvate k	GO:00081	metabolic	K12406	pyruvate k	mmu0110	Metabolic	3:8913614	3	4	0	44	74	38	0.038603	0.041103	0	0.436807	0.613191	0.384686	0.026568	0.478228	9.98E-16	7.85E-14	DOWN
ENSMUS(	Atp2a3	ATPase, C	GO:00511	localizatio	K05853	Ca2+ tran	mmu0402	Calcium si	11:729611	166	234	138	604	1082	603	1.370795	1.543215	1.259479	3.847964	5.75368	3.91735	1.391163	4.506331	1.07E-15	8.36E-14	DOWN
ENSMUS(	Apol7a	apolipopr	GO:00081	metabolic	-	-	-	-	15:773882	0	2	1	34	67	31	0	0.015177	0.010501	0.249195	0.409887	0.23169	0.008559	0.296924	1.08E-15	8.45E-14	DOWN
ENSMUS(	Hsd11b2	hydroxyst	GO:00081	metabolic	K00071	corticoste	mmu0014	Steroid hc	8:1055187	0	0	0	31	102	33	0	0	0	0.793634	2.179628	0.861501	0	1.278255	1.24E-15	9.66E-14	DOWN
ENSMUS(	BC030870	cDNA seq	GO:00160	membran	-	-	-	-	8:6508561	0	0	0	26	44	23	0	0	0	0.812692	1.147981	0.733125	0	0.897932	1.73E-15	1.33E-13	DOWN
ENSMUS(	Slc10a5	solute car	GO:00511	localizatio	-	-	-	-	3:1033173	0	1	0	29	70	30	0	0.012432	0	0.348219	0.701583	0.367329	0.004144	0.472377	1.75E-15	1.35E-13	DOWN
ENSMUS(	Arhgef16	Rho guan	GO:00400	locomotic	-	-	-	-	4:1542784	3	12	5	57	135	80	0.02184	0.069768	0.040233	0.32014	0.632883	0.458179	0.043947	0.470401	1.76E-15	1.35E-13	DOWN
ENSMUS(	Frk	fyn-relate	GO:00325	developm	-	-	-	-	10:344833	13	8	7	90	195	81	0.089046	0.043764	0.052996	0.475603	0.860121	0.436481	0.061935	0.590735	1.82E-15	1.39E-13	DOWN
ENSMUS(	Dhrs9	dehydrog	GO:00081	metabolic	K11149	dehydrog	mmu0110	Metabolic	2:6938044	3	3	3	48	107	46	0.042787	0.034177	0.047293	0.52816	0.982739	0.516139	0.041419	0.675679	1.86E-15	1.42E-13	DOWN
ENSMUS(	Rnf186	ring finge	GO:00160	membran	-	-	-	-	4:1389671	0	0	0	27	76	30	0	0	0	1.018285	2.392484	1.153747	0	1.521505	2.10E-15	1.59E-13	DOWN
ENSMUS(	Ern2	endoplasr	GO:00081	metabolic	-	-	-	-	7:1221698	2	0	2	51	125	38	0.033533	0	0.037071	0.659815	1.349852	0.501328	0.023534	0.836998	2.17E-15	1.63E-13	DOWN
ENSMUS(	Cyp2d26	cytochron	GO:00081	metabolic	K07414	cytochron	mmu0472	Serotoner	15:827901	0	2	0	37	90	31	0	0.059295	0	1.059521	2.15117	0.905212	0.019765	1.371968	3.39E-15	2.54E-13	DOWN
ENSMUS(	Selenbp2	selenium	GO:00084	selenium	-	-	-	-	3:9469355	12	12	6	69	144	85	0.208919	0.166851	0.115461	0.926807	1.614446	1.16422	0.163744	1.235158	3.41E-15	2.55E-13	DOWN
ENSMUS(	Dpt	dermatop	GO:00099	cellular pr	-	-	-	-	1:1647966	2	4	3	49	121	48	0.07082	0.113128	0.117437	1.338842	2.759611	1.3374	0.100461	1.811951	3.71E-15	2.76E-13	DOWN
ENSMUS(	Irf7	interferon	GO:00081	metabolic	K09447	interferon	mmu0520	Viral carci	7:1412627	66	69	52	231	312	270	0.393771	0.328773	0.342882	1.063263	1.198692	1.267278	0.355142	1.176411	4.37E-15	3.24E-13	DOWN
ENSMUS(	Oas2	2'-5' oligo	GO:00081	metabolic	K14216	2'-5'-oligo	mmu0516	Herpes sir	5:1207303	12	9	16	96	162	87	0.089286	0.053483	0.131577	0.551078	0.776213	0.509262	0.091449	0.612185	6.02E-15	4.43E-13	DOWN
ENSMUS(	Fgfbp1	fibroblast	GO:00508	response	-	-	-	-	5:4397885	7	7	11	68	176	93	0.093322	0.074529	0.162072	0.699388	1.510932	0.975376	0.109974	1.061899	6.51E-15	4.78E-13	DOWN
ENSMUS(	Xpnpep2	X-prolyl a	GO:00081	metabolic	K14208	Xaa-Pro a	mmu0497	Protein di	X:4810872	4	4	1	42	88	42	0.036206	0.028911	0.010004	0.293274	0.512904	0.29906	0.02504	0.368413	9.34E-15	6.83E-13	DOWN
ENSMUS(	Tmem150	transmem	GO:00081	metabolic	-	-	-	-	7:4706832	0	0	0	23	47	23	0	0	0	0.277307	0.472991	0.282779	0	0.344359	1.86E-14	1.35E-12	DOWN
ENSMUS(	Il22ra1	interleukir	GO:00048	signal trar	K05138	interleukir	mmu0406	Cytokine-	4:1357281	0	0	0	22	51	25	0	0	0	0.403396	0.78056	0.467442	0	0.550466	1.97E-14	1.43E-12	DOWN
ENSMUS(	Entpd8	ectonucle	GO:00081	metabolic	K01510	apyrase[3	mmu0516	Epstein-B	2:2508030	0	0	0	28	50	20	0	0	0	0.184882	0.275574	0.134663	0	0.198373	2.04E-14	1.47E-12	DOWN
ENSMUS(	Mmrn1	multimeri	GO:00508	response	-	-	-	-	6:6092497	1	0	0	26	44	23	0.008319	0	0	0.166811	0.235632	0.150479	0.002773	0.184307	2.74E-14	1.97E-12	DOWN
ENSMUS(	2210407C	RIKEN cDI	-	-	-	-	-	-	11:586082	0	0	2	32	56	26	0	0	0.06649	0.742561	1.084655	0.615223	0.022163	0.814146	4.41E-14	3.16E-12	DOWN
ENSMUS(	Fer1l6	fer-1-like	GO:00160	membran	-	-	-	-	15:585100	0	2	0	42	120	29	0	0.011327	0	0.229756	0.547929	0.161768	0.003776	0.313151	6.02E-14	4.29E-12	DOWN
ENSMUS(	Des	desmin [S	GO:00325	developm	K07610	desmin[-]	mmu0541	Dilated ca	1:7536032	27	61	49	233	561	240	0.378203	0.682392	0.758581	2.51792	5.06027	2.64471	0.606392	3.407633	6.32E-14	4.49E-12	DOWN
ENSMUS(	Sh2d4a	SH2 doma	GO:00081	metabolic	-	-	-	-	8:6827656	2	2	0	35	61	26	0.036709	0.029327	0	0.495697	0.721118	0.375497	0.022012	0.530771	6.66E-14	4.71E-12	DOWN
ENSMUS(	Slc51b	solute car	GO:00081	metabolic	K14361	organic sc	mmu0497	Bile secret	9:6541271	0	0	0	19	68	35	0	0	0	1.262325	3.771017	2.37123	0	2.468191	6.90E-14	4.84E-12	DOWN
ENSMUS(	Cyp4f40	cytochron	GO:00081	metabolic	-	-	-	-	17:326594	0	0	0	28	80	24	0	0	0	0.709876	1.692945	0.62046	0	1.007761	6.87E-14	4.84E-12	DOWN
ENSMUS(	Slc16a5	solute car	GO:00511	localizatio	-	-	-	-	11:115462	0	0	0	22	51	23	0	0	0	0.137371	0.265808	0.14645	0	0.18321	7.09E-14	4.95E-12	DOWN
ENSMUS(	Acsm3	acyl-CoA	GO:00081	metabolic	K01896	medium-c	mmu0110	Metabolic	7:1197609	0	0	0	25	77	26	0	0	0	0.151682	0.389944	0.160858	0	0.234161	7.23E-14	5.03E-12	DOWN
ENSMUS(	Oasl2	2'-5' oligo	GO:00081	metabolic	K14608	2'-5'-oligo	mmu0516	Human pa	5:1148969	115	194	96	452	791	453	0.654888	0.882299	0.604204	1.985805	2.900673	2.029443	0.713797	2.305307	7.85E-14	5.44E-12	DOWN
ENSMUS(	Adh1	alcohol de	GO:00081	metabolic	K13951	alcohol de	mmu0110	Metabolic	3:1382609	7	11	2	67	163	63	0.156105	0.1959	0.049296	1.152698	2.340729	1.105257	0.133767	1.532895	7.91E-14	5.46E-12	DOWN
ENSMUS(	Lrrc66	leucine ric	GO:00081	metabolic	-	-	-	-	5:7360664	0	0	0	23	79	27	0	0	0	0.303229	0.869349	0.362985	0	0.511854	2.14E-13	1.47E-11	DOWN
ENSMUS(	A4gnt	alpha-1,4	GO:00081	metabolic	-	-	-	-	9:9961250	1	5	2	33	75	41	0.022153	0.08843	0.048957	0.563842	1.069605	0.714334	0.05318	0.782593	2.35E-13	1.61E-11	DOWN
ENSMUS(	Nxpe2	neurexopl	GO:00160	membran	-	-	-	-	9:4831800	2	14	7	71	167	70	0.019766	0.110521	0.076479	0.541442	1.063007	0.544343	0.068922	0.716264	3.76E-13	2.57E-11	DOWN
ENSMUS(	EPPK1	epiplakin	GO:00056	cell|intrace	-	-	-	-	15:761014	16	22	26	108	225	123	0.079264	0.087043	0.142355	0.412771	0.717782	0.479371	0.102887	0.536642	4.02E-13	2.73E-11	DOWN
ENSMUS(	Casp4	caspase 4	GO:00325	developm	K12808	caspase 1	mmu0462	NOD-like	9:5308828	0	3	4	43	53	29	0	0.030192	0.055705	0.417981	0.43002	0.287451	0.028632	0.378484	4.79E-13	3.24E-11	DOWN
ENSMUS(	Atp10b	ATPase, cl	GO:00511	localizatio	-	-	-	-	11:431498	46	74	67	260	544	252	0.266656	0.342586	0.42925	1.162774	2.030696	1.149219	0.346164	1.447563	4.99E-13	3.36E-11	DOWN
ENSMUS(	Bspry	B-box and	GO:00511	localizatio	-	-	-	-	4:6248005	4	11	5	50	175	80	0.067865	0.149034	0.093758	0.654425	1.911853	1.067727	0.103552	1.211335	5.37E-13	3.60E-11	DOWN
ENSMUS(	F2rl1	coagulatic	GO:00325	developm	K04234	coagulatic	mmu0408	Neuroacti	13:955117	4	3	4	38	103	49	0.089108	0.053379	0.098485	0.653055	1.477499	0.858691	0.080324	0.996415	5.48E-13	3.66E-11	DOWN
ENSMUS(	2010109I(	RIKEN cDI	GO:00056	cell|cell su	-	-	-	-	15:748769	0	0	4	41	96	33	0	0	0.054033	0.386572	0.755519	0.317286	0.018011	0.486459	5.67E-13	3.78E-11	DOWN
ENSMUS(	H2-Q2	histocomp	GO:00325	developm	K06751	major hist	mmu0516	Human pa	17:353422	10	3	2	65	202	68	0.269932	0.064682	0.05967	1.353609	3.511216	1.444016	0.131428	2.102947	7.45E-13	4.95E-11	DOWN
ENSMUS(	Ripk3	receptor-	GO:00325	developm	K08847	receptor-	mmu0421	Necroptos	14:557849	0	0	2	25	54	27	0	0	0.026316	0.229608	0.413958	0.252865	0.008772	0.29881	8.95E-13	5.92E-11	DOWN
ENSMUS(	Lrrc19	leucine ric	GO:00081	metabolic	-	-	-	-	4:9463665	0	0	0	20	46	20	0	0	0	0.322086	0.618348	0.32844	0	0.422958	9.03E-13	5.95E-11	DOWN
ENSMUS(	Oasl1	2'-5' oligo	GO:00023	immune s	K14608	2'-5'-oligo	mmu0516	Human pa	5:1149232	0	11	3	60	136	55	0	0.095815	0.036167	0.504886	0.955224	0.471934	0.043994	0.644015	1.41E-12	9.24E-11	DOWN
ENSMUS(	Cyp3a13	cytochron	GO:00081	metabolic	K07424	cytochron	mmu0110	Metabolic	5:1378929	5	5	5	39	59	47	0.103269	0.082477	0.114152	0.621494	0.78477	0.763746	0.099966	0.723337	1.56E-12	1.02E-10	DOWN
ENSMUS(	Mlkl	mixed line	GO:00048	signal trar	K08849	mixed line	mmu0421	Necroptos	8:1113117	6	8	3	53	77	40	0.106836	0.113769	0.059044	0.728087	0.882917	0.560331	0.093216	0.723778	1.82E-12	1.19E-10	DOWN
ENSMUS(	Chst4	carbohydi	GO:00081	metabolic	K04746	carbohydi	mmu0053	Glycosam	8:1100291	0	0	0	17	42	22	0	0	0	0.264207	0.544832	0.348656	0	0.385898	1.88E-12	1.22E-10	DOWN
ENSMUS(	Nipal1	NIPA-like	GO:00511	localizatio	-	-	-	-	5:7264779	2	0	0	23	46	23	0.024871	0	0	0.220704	0.368439	0.22506	0.00829	0.271401	1.95E-12	1.26E-10	DOWN
ENSMUS(	Itga2	integrin al	GO:00325	developm	K06481	integrin al	mmu0520	Pathways	13:114833	3	18	4	69	147	71	0.025607	0.122706	0.037739	0.454398	0.808029	0.476786	0.062018	0.579738	2.19E-12	1.41E-10	DOWN
ENSMUS(	Gm3336	predicted	GO:00160	membran	-	-	-	-	8:7071854	0	0	0	19	54	21	0	0	0	0.514361	1.220223	0.57973	0	0.771438	3.25E-12	2.09E-10	DOWN
ENSMUS(	B3gnt3	UDP-GlcN	GO:00081	metabolic	K07970	beta-1,3-	mmu0110	Metabolic	8:7169075	14	32	23	131	246	102	0.279814	0.510771	0.508041	2.019914	3.166081	1.603772	0.432875	2.263256	3.46E-12	2.21E-10	DOWN
ENSMUS(	Acer1	alkaline ce	GO:00325	developm	-	-	mmu0110	Metabolic	17:569534	0	0	0	32	51	14	0	0	0	0.620584	0.825549	0.276851	0	0.574328	3.48E-12	2.22E-10	DOWN
ENSMUS(	Clec2e	C-type lec	GO:00160	membran	-	-	-	-	6:1290919	1	0	2	30	76	27	0.029098	0	0.064304	0.673261	1.423636	0.617888	0.031134	0.904928	3.74E-12	2.38E-10	DOWN
ENSMUS(	Cxcl9	chemokin	GO:00325	developm	K05416	C-X-C mc	mmu0406	Cytokine-	5:9232134	1	1	0	28	32	19	0.018741	0.014965	0	0.40471	0.386071	0.280038	0.011235	0.35694	3.80E-12	2.41E-10	DOWN
ENSMUS(	Irf6	interferon	GO:00325	developm	-	-	-	-	1:1931531	22	19	9	101	222	92	0.167761	0.11571	0.075848	0.594188	1.090138	0.551914	0.119773	0.745413	3.88E-12	2.44E-10	DOWN
ENSMUS(	Hsd17b13	hydroxyst	GO:00081	metabolic	-	-	-	-	5:1039554	0	0	0	23	75	21	0	0	0	0.41086	1.118279	0.38253	0	0.637223	3.88E-12	2.44E-10	DOWN
ENSMUS(	Spdef	SAM poin	GO:00325	developm	-	-	-	-	17:277143	4	2	6	51	92	37	0.041659	0.016637	0.069064	0.409754	0.61697	0.303137	0.042453	0.443287	4.41E-12	2.76E-10	DOWN
ENSMUS(	Mkrn2os	makorin, r	-	-	-	-	-	-	6:1155835	0	3	0	24	66	29	0	0.044798	0	0.346157	0.794559	0.426511	0.014933	0.522409	4.90E-12	3.05E-10	DOWN
ENSMUS(	A1cf	APOBEC1	GO:00325	developm	-	-	-	-	19:318687	0	0	0	19	57	21	0	0	0	0.158041	0.395755	0.178127	0	0.243974	4.99E-12	3.10E-10	DOWN
ENSMUS(	Apol9b	apolipopr	GO:00081	metabolic	-	-	-	-	15:777291	8	4	0	39	73	49	0.316177	0.126234	0	1.189085	1.857762	1.523413	0.14747	1.52342	6.68E-12	4.14E-10	DOWN
ENSMUS(	Tat	tyrosine a	GO:00081	metabolic	K00815	tyrosine a	mmu0110	Metabolic	8:1099904	0	0	0	18	34	17	0	0	0	0.331998	0.523424	0.319735	0	0.391719	6.81E-12	4.20E-10	DOWN
ENSMUS(	Slc17a9	solute car	GO:00081	metabolic	-	-	-	-	2:1807252	5	3	2	36	78	36	0.072509	0.034753	0.03206	0.402803	0.728464	0.410747	0.046441	0.514005	7.97E-12	4.90E-10	DOWN
ENSMUS(	Sdc1	syndecan	GO:00325	developm	K06257	syndecan	mmu0520	Proteogly	12:877132	18	24	15	114	226	81	0.170682	0.18175	0.157204	0.833992	1.38003	0.604254	0.169879	0.939425	1.24E-11	7.57E-10	DOWN
ENSMUS(	Ihh	Indian hec	GO:00325	developm	K11989	indian hec	mmu0520	Proteogly	1:7494531	0	3	2	47	86	20	0	0.037973	0.035031	0.574614	0.673509	0.249335	0.024335	0.499152	1.41E-11	8.62E-10	DOWN
ENSMUS(	Slc44a3	solute car	GO:00160	membran	K15282	solute car	mmu0523	Choline m	3:1214595	0	7	0	34	58	31	0	0.066917	0	0.313973	0.447063	0.291918	0.022306	0.350985	1.54E-11	9.36E-10	DOWN
ENSMUS(	Helz2	helicase w	GO:00081	metabolic	-	-	-	-	2:1812276	99	151	101	351	666	352	0.299333	0.364622	0.337511	0.818762	1.296732	0.837289	0.333822	0.984261	1.59E-11	9.58E-10	DOWN
ENSMUS(	Ces2a	carboxyle	GO:00081	metabolic	K03927	carboxyle	mmu0098	Drug met	8:1047340	0	4	0	26	71	30	0	0.058533	0	0.367565	0.837821	0.432482	0.019511	0.545956	1.59E-11	9.58E-10	DOWN
ENSMUS(	Btnl2	butyrophi	GO:00511	localizatio	-	-	-	-	17:343548	0	0	1	19	28	19	0	0	0.019647	0.26055	0.320502	0.265689	0.006549	0.282247	1.73E-11	1.04E-09	DOWN
ENSMUS(	Prelid2	PRELI dor	GO:00511	localizatio	-	-	-	-	18:418756	0	6	0	34	44	26	0	0.404106	0	2.212121	2.389513	1.725004	0.134702	2.108879	1.76E-11	1.06E-09	DOWN
ENSMUS(	Akr1c14	aldo-keto	GO:00325	developm	-	-	-	-	13:404901	26	33	28	113	166	109	0.325377	0.329816	0.387272	1.090994	1.337751	1.073126	0.347488	1.16729	1.91E-11	1.14E-09	DOWN
ENSMUS(	Bcl2l14	BCL2-like	GO:00325	developm	-	-	-	-	6:1343963	0	0	0	18	53	19	0	0	0	0.128124	0.314488	0.137903	0	0.193636	2.12E-11	1.26E-09	DOWN
ENSMUS(	Klf4	Kruppel-li	GO:00325	developm	K17846	krueppel-	mmu0455	Signaling	4:5552714	77	114	92	320	653	297	1.066091	1.260539	1.407799	3.418106	5.822007	3.234982	1.24481	4.158365	2.16E-11	1.28E-09	DOWN
ENSMUS(	Tmc4	transmem	GO:00160	membran	-	-	-	-	7:3665790	77	64	35	234	387	222	0.689263	0.457529	0.34627	1.615998	2.230803	1.563362	0.497687	1.803388	2.44E-11	1.44E-09	DOWN
ENSMUS(	Samd9l	sterile alp	-	-	-	-	-	-	6:3372257	99	166	89	356	609	346	1.049614	1.405564	1.042877	2.911903	4.157848	2.885916	1.166018	3.318556	2.62E-11	1.55E-09	DOWN
ENSMUS(	Cldn4	claudin 4	GO:00325	developm	K06087	claudin[-]	mmu0453	Tight junc	5:1349451	0	1	0	21	46	17	0	0.028656	0	0.544721	0.995962	0.449671	0.008952	0.663451	4.25E-11	2.50E-09	DOWN
ENSMUS(	Grhl2	grainyhea	GO:00325	developm	-	-	-	-	15:372330	2	2	3	27	55	31	0.009819	0.007845	0.016283	0.102287	0.173921	0.119758	0.011316	0.131989	4.62E-11	2.70E-09	DOWN
ENSMUS(	Gal3st2c	galactose	GO:00081	metabolic	-	-	-	-	1:939905C	0	0	0	15	30	17	0	0	0	0.073093	0.122018	0.084473	0	0.093195	4.83E-11	2.82E-09	DOWN
ENSMUS(	Foxa3	forkhead	GO:00325	developm	K08038	forkhead	mmu0495	Maturity c	7:1901328	0	0	0	16	35	16	0	0	0	0.370005	0.67557	0.3773	0	0.474291	5.49E-11	3.19E-09	DOWN
ENSMUS(	Hnf4g	hepatocyt	GO:00048	signal trar	K08037	hepatocyt	mmu0495	Maturity c	3:3508030	0	3	0	18	55	33	0	0.042781	0	0.247931	0.632321	0.463498	0.01426	0.447917	5.60E-11	3.24E-09	DOWN
ENSMUS(	Themis3	thymocyte	-	-	-	-	-	-	17:665552	0	0	0	20	33	13	0	0	0	0.524263	0.722045	0.347483	0	0.531264	7.35E-11	4.25E-09	DOWN
ENSMUS(	Gcg	glucagon	GO:00081	metabolic	K05259	glucagon[	mmu0492	Glucagon	2:6247453	0	0	0	19	47	15	0	0	0	0.460622	0.951095	0.370826	0	0.594181	8.64E-11	4.97E-09	DOWN
ENSMUS(	Ap1m2	adaptor p	GO:00511	localizatio	K12393	AP-1 com	mmu0414	Lysosome	9:2129427	7	2	4	35	50	42	0.07657	0.017474	0.04836	0.295358	0.352194	0.361423	0.047468	0.336325	8.88E-11	5.10E-09	DOWN
ENSMUS(	Gm5485	predicted	-	-	-	-	-	-	16:483994	0	0	0	17	63	15	0	0	0	1.275178	3.944363	1.147317	0	2.122286	8.95E-11	5.12E-09	DOWN
ENSMUS(	Galnt5	polypeptic	GO:00081	metabolic	K00710	polypeptic	mmu0051	Metabolic	2:5799788	3	4	4	34	97	38	0.037543	0.039974	0.055328	0.328259	0.781694	0.374121	0.044282	0.494691	9.27E-11	5.29E-09	DOWN
ENSMUS(	Tpm2	tropomyo	GO:00051	structural	K10374	tropomyo	mmu0426	Adrenergi	4:4351471	143	202	115	386	610	459	1.568688	1.769687	1.394261	3.266766	4.309083	3.961175	1.577545	3.845675	9.47E-11	5.38E-09	DOWN
ENSMUS(	Phf11b	PHD finge	GO:00160	membran	-	-	-	-	14:593209	6	6	3	45	49	34	0.309409	0.247105	0.170997	1.79034	1.627194	1.379356	0.242504	1.598963	1.17E-10	6.65E-09	DOWN
ENSMUS(	Fam83f	family witl	-	-	-	-	-	-	15:806718	1	0	1	17	52	24	0.020441	0	0.022586	0.268009	0.68426	0.385815	0.014342	0.446028	1.23E-10	6.93E-09	DOWN
ENSMUS(	Anxa4	annexin A	GO:00325	developm	-	-	-	-	6:8673684	65	124	64	245	428	283	0.525318	0.800342	0.57166	1.527586	2.227445	1.799311	0.63244	1.851447	1.37E-10	7.72E-09	DOWN
ENSMUS(	Zan	zonadhes	GO:00160	membran	-	-	-	-	5:1373786	10	5	2	47	82	41	0.028708	0.011463	0.006346	0.104095	0.151587	0.092596	0.015506	0.116093	1.47E-10	8.26E-09	DOWN
ENSMUS(	Klf5	Kruppel-li	GO:00325	developm	-	-	-	-	14:992986	169	191	130	404	756	499	1.935991	1.747411	1.645908	3.570488	5.5769	4.49705	1.776437	4.548146	1.55E-10	8.69E-09	DOWN
ENSMUS(	Slc34a2	solute car	GO:00325	developm	K14683	solute car	mmu0497	Mineral al	5:5303808	1	0	0	18	40	17	0.011712	0	0	0.162593	0.301577	0.156587	0.003904	0.206919	1.58E-10	8.82E-09	DOWN
ENSMUS(	Cfd	complem	GO:00081	metabolic	K01334	compone	mmu0461	Complem	10:798990	0	0	0	20	49	14	0	0	0	0.748293	1.53026	0.534131	0	0.937561	1.61E-10	8.95E-09	DOWN
ENSMUS(	Fam83b	family witl	GO:00508	response	-	-	-	-	9:7649005	4	0	0	36	93	33	0.036319	0	0.040141	0.252164	0.543739	0.235712	0.025487	0.343871	2.47E-10	1.37E-08	DOWN
ENSMUS(	Slc26a2	solute car	GO:00511	localizatio	-	-	-	-	18:611929	207	337	170	645	1202	635	0.802528	1.043437	0.728425	1.929215	3.000887	1.936755	0.85813	2.288952	2.69E-10	1.49E-08	DOWN
ENSMUS(	Slc39a4	solute car	GO:00511	localizatio	K14710	solute car	mmu0497	Mineral al	15:766123	12	41	16	95	220	128	0.329442	0.89896	0.485487	2.012157	3.889422	2.76458	0.571296	2.88872	2.73E-10	1.51E-08	DOWN
ENSMUS(	Spint1	serine prc	GO:00325	developm	K15619	Kunitz-typ	mmu0520	Transcript	2:1192373	27	40	15	107	299	132	0.31968	0.378227	0.196284	0.977377	2.27967	1.229504	0.298064	1.495517	2.89E-10	1.59E-08	DOWN
ENSMUS(	Ovol1	ovo like zi	GO:00325	developm	-	-	-	-	19:554913	0	0	0	16	42	15	0	0	0	0.259896	0.569444	0.248454	0	0.359265	2.93E-10	1.60E-08	DOWN
ENSMUS(	Dnajc22	DnaJ heat	GO:00160	membran	-	-	-	-	15:990331	0	0	0	15	55	14	0	0	0	0.363284	1.111845	0.345744	0	0.606957	2.97E-10	1.62E-08	DOWN
ENSMUS(	Parp14	poly (ADP	GO:00081	metabolic	-	-	-	-	16:358328	133	215	109	413	590	416	0.712407	0.919729	0.645285	1.706699	2.03509	1.752995	0.75914	1.831595	2.98E-10	1.62E-08	DOWN
ENSMUS(	Sfn	stratifin [S	GO:00325	developm	K06644	stratifin[-]	mmu0411	Cell cycle	4:1336005	19	28	14	83	170	86	0.719216	0.84648	0.585709	2.423922	4.143935	2.561044	0.717135	3.042967	3.24E-10	1.75E-08	DOWN
ENSMUS(	S100a14	S100 calci	GO:00400	locomotic	-	-	-	-	3:9052685	0	0	0	14	37	16	0	0	0	0.211982	0.46763	0.247046	0	0.308886	3.65E-10	1.97E-08	DOWN
ENSMUS(	H2-Ab1	histocomp	GO:00325	developm	K06752	major hist	mmu0516	HTLV-I inf	17:342632	31	53	44	153	305	153	0.804068	1.09789	1.261352	3.061666	5.094375	3.122041	1.054436	3.759361	3.80E-10	2.04E-08	DOWN
ENSMUS(	Arl14	ADP-ribo	GO:00508	response	-	-	-	-	3:6922241	0	0	0	16	30	13	0	0	0	0.628083	0.982954	0.520356	0	0.710464	4.09E-10	2.19E-08	DOWN
ENSMUS(	Hoxb6	homeobo	GO:00325	developm	-	-	-	-	11:962924	6	4	0	34	85	35	0.111519	0.059371	0	0.487542	1.017377	0.511781	0.056963	0.672234	4.34E-10	2.32E-08	DOWN
ENSMUS(	Serpinb5	serine (or	GO:00325	developm	K10139	serpin B5[	mmu0520	MicroRNA	1:1068611	0	1	0	17	36	13	0	0.0068	0	0.166454	0.294219	0.129792	0.002267	0.196821	4.39E-10	2.34E-08	DOWN
ENSMUS(	Mvp	major vau	GO:00081	metabolic	-	-	-	-	7:1269868	156	206	101	424	748	429	1.22446	1.291317	0.876167	2.567534	3.780732	2.649038	1.130648	2.999102	5.68E-10	3.02E-08	DOWN
ENSMUS(	Naip6	NLR famil	GO:00081	metabolic	K12807|K1	baculovira	mmu0462	NOD-like	13:100281	17	23	18	82	149	75	0.139142	0.150344	0.162828	0.517785	0.785316	0.482949	0.150772	0.59534	6.96E-10	3.67E-08	DOWN
ENSMUS(	Gja1	gap juncti	GO:00325	developm	K07372	gap juncti	mmu0454	Gap junct	10:563773	2884	3798	2920	6922	8382	6403	15.16985	15.95463	16.97523	28.08975	28.3915	26.496	16.03323	27.65908	8.34E-10	4.41E-08	-

ENSMUSC	Plscr1	phospholi	GO:00325	developm	-	-	-	-	9:9224975	18	34	20	92	138	86	0.305453	0.460796	0.375117	1.204488	1.508049	1.148128	0.380455	1.286888	9.44E-10	4.98E-08	DOWN
ENSMUSC	Dio1	deiodinas	GO:00081	metabolic	K01562	type I thyr	mmu0491	Thyroid ho	4:1072914	0	0	0	10	35	15	0	0	0	0.105433	0.307998	0.161261	0	0.191564	9.50E-10	4.99E-08	DOWN
ENSMUSC	Tmem171	transmem	GO:00160	membran	-	-	-	-	13:986862	5	6	10	37	95	58	0.15363	0.147243	0.339608	0.877158	1.879852	1.402124	0.213494	1.386378	1.17E-09	6.13E-08	DOWN
ENSMUSC	Car3	carbonic a	GO:00081	metabolic	K01672	carbonic a	mmu0091	Nitrogen r	3:1486351	0	10	4	52	89	33	0	0.131006	0.072528	0.658114	0.940184	0.42589	0.067845	0.674729	1.18E-09	6.17E-08	DOWN
ENSMUSC	Ethe1	ethylmalo	GO:00081	metabolic	K17725	sulfur diox	mmu0092	Sulfur met	7:2458754	81	105	63	240	417	233	3.332686	3.450199	2.864829	7.61816	11.04842	7.541833	3.215905	8.736138	1.21E-09	6.28E-08	DOWN
ENSMUSC	Adtrp	androgen	GO:00081	metabolic	-	-	-	-	13:417631	0	3	0	17	46	23	0	0.02666	0	0.14592	0.329568	0.201315	0.008887	0.225601	1.23E-09	6.39E-08	DOWN
ENSMUSC	Ptk6	PTK6 prot	GO:00325	developm	-	-	-	-	2:1811937	0	0	2	20	29	18	0	0	0.036588	0.255381	0.30909	0.234372	0.012196	0.266281	1.25E-09	6.45E-08	DOWN
ENSMUSC	Mboat1	membran	GO:00081	metabolic	K13517	lysophosp	mmu0110	Metabolic	13:301364	31	61	26	121	291	171	0.434322	0.682548	0.402599	1.307896	2.625447	1.884794	0.50649	1.939379	1.34E-09	6.93E-08	DOWN
ENSMUSC	Galnt12	polypeptid	GO:00081	metabolic	K00710	polypeptid	mmu0110	Metabolic	4:4709190	21	18	21	86	188	87	0.388443	0.265895	0.429312	1.227227	2.239294	1.265982	0.361216	1.577501	1.35E-09	6.97E-08	DOWN
ENSMUSC	Cnksr1	connector	GO:00508	response	-	-	-	-	4:1342280	0	2	0	20	40	15	0	0.024174	0	0.233482	0.389771	0.178566	0.008058	0.267273	1.65E-09	8.45E-08	DOWN
ENSMUSC	Tstd1	thiosulfate	GO:00329	macromo	-	-	-	-	1:1714188	2	2	2	30	57	19	0.100077	0.079951	0.110634	1.15834	1.837016	0.748068	0.096887	1.247808	1.70E-09	8.68E-08	DOWN
ENSMUSC	Slc15a1	solute car	GO:00511	localizatio	K14206	solute car	mmu0497	Protein di	14:121459	0	0	0	12	37	16	0	0	0	0.112287	0.288994	0.152674	0	0.184651	1.85E-09	9.41E-08	DOWN
ENSMUSC	Pitx2	paired-lik	GO:00325	developm	K04686	paired-lik	mmu0435	TGF-beta	3:1291998	0	0	0	13	46	12	0	0	0	0.049492	0.146179	0.046588	0	0.080753	1.92E-09	9.74E-08	DOWN
ENSMUSC	Klk6	kallikrein r	GO:00325	developm	-	-	-	-	7:4382449	20	65	18	3	4	5	0.542502	1.408076	0.539625	0.062788	0.069871	0.106696	0.830068	0.079785	2.19E-09	1.11E-07	UP
ENSMUSC	H2-D1	histocomp	GO:00325	developm	K06751|K0	major hist	mmu0516	Human pa	17:352627	643	869	634	1496	2654	1644	12.63611	13.63853	13.77013	22.68109	33.58595	25.41644	13.34826	27.22783	2.19E-09	1.11E-07	DOWN
ENSMUSC	Crybg1	crystallin b	-	-	-	-	-	-	10:439506	10	43	34	138	260	134	0.062387	0.214241	0.234435	0.664205	1.044533	0.657673	0.170355	0.788803	2.45E-09	1.23E-07	DOWN
ENSMUSC	1700066B	RIKEN cDI	GO:00160	membran	-	-	-	-	18:357032	0	0	0	8	25	17	0	0	0	0.091159	0.237773	0.197533	0	0.175488	2.55E-09	1.28E-07	DOWN
ENSMUSC	Tcim	transcripti	GO:00325	developm	-	-	-	-	8:2443718	20	33	10	88	226	95	0.67655	0.891509	0.373851	2.296565	4.923	2.52815	0.647304	3.249238	2.83E-09	1.42E-07	DOWN
ENSMUSC	Gbp2b	guanylate	GO:00038	catalytic a	K20899	guanylate	mmu0462	NOD-like	3:1425948	0	0	0	13	37	10	0	0	0	0.216919	0.515337	0.170154	0	0.300803	2.94E-09	1.47E-07	DOWN
ENSMUSC	Phgr1	proline/hi	-	-	-	-	-	-	2:1187727	0	0	0	12	23	10	0	0	0	0.54091	0.865391	0.459659	0	0.621987	2.97E-09	1.48E-07	DOWN
ENSMUSC	Ugt1a6a	UDP gluc	GO:00081	metabolic	K00699	glucuronc	mmu0110	Metabolic	1:8813480	17	10	7	75	106	48	0.175873	0.082617	0.080042	0.5986	0.70616	0.39066	0.112844	0.56514	3.10E-09	1.54E-07	DOWN
ENSMUSC	Arhgef38	Rho guan	GO:00987	molecular	-	-	-	-	3:1331122	2	0	2	24	68	21	0.013829	0	0.015288	0.12805	0.302826	0.114252	0.009705	0.181709	3.13E-09	1.55E-07	DOWN
ENSMUSC	Mgat4c	MGAT4 fa	GO:00081	metabolic	K13748	alpha-1,3	mmu0110	Metabolic	10:101681	83	79	191	579	855	590	0.27505	0.209078	0.699545	1.480284	1.824553	1.53815	0.394558	1.614329	3.14E-09	1.55E-07	DOWN
ENSMUSC	Pparg	peroxisom	GO:00325	developm	K08530	peroxisom	mmu0520	Pathways	6:1153609	18	28	19	85	159	77	0.265079	0.329323	0.309258	0.965741	1.507881	0.892104	0.30122	1.121909	3.22E-09	1.59E-07	DOWN
ENSMUSC	Fam110c	family wit	GO:00400	locomotic	-	-	-	-	12:310738	4	3	0	25	40	23	0.079095	0.04738	0	0.381367	0.509303	0.357778	0.042159	0.416149	3.40E-09	1.67E-07	DOWN
ENSMUSC	Tjp3	tight junct	GO:00056	cell|cell ju	K06097	tight junct	mmu0453	Tight junc	10:812732	31	57	22	114	310	163	0.376297	0.552578	0.295158	1.067609	2.423211	1.556586	0.408011	1.682468	3.47E-09	1.70E-07	DOWN
ENSMUSC	Slc39a5	solute car	GO:00325	developm	-	-	-	-	10:128395	0	3	0	13	37	23	0	0.042532	0	0.178013	0.422906	0.321168	0.014177	0.307362	3.81E-09	1.86E-07	DOWN
ENSMUSC	Trim15	tripartite r	GO:00081	metabolic	-	-	-	-	17:368606	0	0	4	22	60	25	0	0	0.072489	0.278283	0.633493	0.322465	0.024163	0.411414	4.35E-09	2.12E-07	DOWN
ENSMUSC	Zc3h12d	zinc finge	GO:00329	macromo	-	-	-	-	10:783247	2	0	0	17	31	15	0.029837	0	0	0.195702	0.297873	0.176079	0.009946	0.223218	4.58E-09	2.23E-07	DOWN
ENSMUSC	Cmah	cytidine m	GO:00081	metabolic	K08080	CMP-N-a	mmu0052	Amino sug	13:243274	6	5	4	31	105	43	0.013152	0.008753	0.009692	0.052426	0.148217	0.074155	0.010532	0.0916	4.62E-09	2.24E-07	DOWN
ENSMUSC	Ces2b	carboxyes	GO:00038	catalytic a	K03927	carboxyles	mmu0098	Drug meta	8:1048315	0	2	0	16	38	16	0	0.023555	0	0.182009	0.360808	0.185597	0.007852	0.242805	5.30E-09	2.56E-07	DOWN
ENSMUSC	Cutal	cutA dival	GO:00508	response	-	-	-	-	2:3487439	7	16	5	52	97	43	0.07169	0.130867	0.056598	0.410852	0.639699	0.346445	0.086385	0.465665	5.37E-09	2.59E-07	DOWN
ENSMUSC	Marveld3	MARVEL (	GO:00081	metabolic	-	-	mmu0453	Tight junc	8:1099479	4	13	7	41	83	44	0.058112	0.150825	0.112398	0.459508	0.77646	0.502855	0.107112	0.579608	5.51E-09	2.65E-07	DOWN
ENSMUSC	Slfn9	schlafen 9	GO:00056	cell|intrace	-	-	-	-	11:829783	20	36	22	92	118	89	0.154559	0.222185	0.187906	0.548506	0.587217	0.541083	0.188217	0.558935	5.89E-09	2.82E-07	DOWN
ENSMUSC	Apol9a	apolipopr	GO:00081	metabolic	-	-	-	-	15:774037	3	0	0	24	57	16	0.115784	0	0	0.714633	1.416662	0.485815	0.038595	0.87237	6.19E-09	2.96E-07	DOWN
ENSMUSC	Lamb3	laminin, b	GO:00325	developm	K06244	laminin, b	mmu0520	Pathways	1:1932076	24	27	40	129	211	110	0.181633	0.163188	0.334565	0.753181	1.02829	0.654912	0.226462	0.812127	6.39E-09	3.05E-07	DOWN
ENSMUSC	H2-Aa	histocomp	GO:00325	developm	K06752	major hist	mmu0516	HTLV-I inf	17:342827	14	30	13	69	161	84	0.333658	0.570985	0.342413	1.268662	2.470841	1.574912	0.415685	1.771472	7.38E-09	3.50E-07	DOWN
ENSMUSC	Ubd	ubiquitin I	GO:00325	developm	-	-	-	-	17:371938	0	0	0	9	22	12	0	0	0	0.455383	0.929133	0.619153	0	0.66789	8.36E-09	3.96E-07	DOWN
ENSMUSC	Ifit1	interferon	GO:00023	immune s	K14217	interferon	mmu0516	Herpes sir	19:346408	69	110	51	236	335	203	1.58623	2.01954	1.295794	4.185598	4.95924	3.671311	1.633855	4.27205	9.50E-09	4.49E-07	DOWN
ENSMUSC	Capn13	calpain 13	GO:00081	metabolic	-	-	-	-	17:733064	0	0	0	11	21	9	0	0	0	0.198235	0.315866	0.165379	0	0.226493	1.01E-08	4.77E-07	DOWN
ENSMUSC	Ms4a12	membran	GO:00160	membran	-	-	-	-	19:112150	0	0	0	8	20	13	0	0	0	0.47582	0.992883	0.788418	0	0.752374	1.04E-08	4.90E-07	DOWN
ENSMUSC	Nupr1	nuclear pr	GO:00325	developm	K15626	nuclear pr	mmu0520	Transcript	7:1266232	52	61	52	149	249	176	0.622798	0.583473	0.688319	1.376768	1.920429	1.658318	0.63153	1.651838	1.08E-08	5.06E-07	DOWN
ENSMUSC	Ltf	lactotrans	GO:00325	developm	-	-	-	-	9:1110192	11	26	7	0	2	0	0.120911	0.228232	0.085042	0	0.014153	0	0.144729	0.004718	1.13E-08	5.29E-07	UP
ENSMUSC	Rnf213	ring finge	GO:00325	developm	-	-	-	-	11:119393	441	629	418	1106	1474	1027	1.344248	1.531219	1.408199	2.600915	2.893296	2.46276	1.427889	2.652323	1.15E-08	5.38E-07	-
ENSMUSC	Slc13a1	solute car	GO:00511	localizatio	-	-	-	-	6:2408828	0	4	0	14	40	24	0	0.022077	0	0.074652	0.178031	0.130497	0.007359	0.127727	1.16E-08	5.40E-07	DOWN
ENSMUSC	Casp7	caspase 7	GO:00325	developm	K04397	caspase 7	mmu0520	Pathways	19:563971	31	37	30	117	214	98	0.805436	0.767747	0.861478	2.345261	3.580499	2.003147	0.811554	2.642969	1.21E-08	5.58E-07	DOWN
ENSMUSC	Mptx1	mucosal p	GO:00055	extracellul	-	-	-	-	1:1743305	0	0	0	10	15	12	0	0	0	0.589579	0.738138	0.721441	0	0.683053	1.21E-08	5.58E-07	DOWN
ENSMUSC	Lgals9	lectin, gal	GO:00325	developm	-	-	-	-	11:789629	81	158	95	303	536	294	1.512911	2.356846	1.961105	4.366178	6.446854	4.320032	1.943621	5.044355	1.24E-08	5.72E-07	DOWN
ENSMUSC	Clca3a2	chloride c	GO:00081	metabolic	K05030	calcium-a	mmu0497	Pancreatic	3:1447965	0	0	0	7	21	16	0	0	0	0.069273	0.173461	0.161462	0	0.134732	1.28E-08	5.90E-07	DOWN
ENSMUSC	Muc16	mucin 16	GO:00081	metabolic	-	-	-	-	9:1849545	0	0	0	12	25	8	0	0	0	0.021193	0.036853	0.014407	0	0.024151	1.33E-08	6.10E-07	DOWN
ENSMUSC	Dnase1l3	deoxyribo	GO:00325	developm	-	-	-	-	14:796453	0	0	0	15	21	10	0	0	0	0.134588	0.157271	0.091494	0	0.127784	1.40E-08	6.40E-07	DOWN
ENSMUSC	Gm21885	predicted	-	-	-	-	-	-	11:942818	0	0	0	12	24	8	0	0	0	0.512932	0.856301	0.348689	0	0.572641	1.46E-08	6.67E-07	DOWN
ENSMUSC	Erbb2	erb-b2 re	GO:00325	developm	K05083	receptor t	mmu0520	Pathways	11:984124	39	58	40	139	319	146	0.352728	0.418936	0.399833	0.969884	1.857891	1.03882	0.390499	1.288865	1.56E-08	7.12E-07	DOWN
ENSMUSC	Noxa1	NADPH o	GO:00081	metabolic	-	-	-	-	2:2508566	0	3	0	14	59	21	0	0.071476	0	0.322167	1.133258	0.492785	0.023825	0.649403	1.64E-08	7.47E-07	DOWN
ENSMUSC	Ccl6	chemokin	GO:00081	metabolic	K05510	C-C motif	mmu0406	Cytokine-	11:835878	22	40	27	105	211	96	0.527599	0.76611	0.715637	1.942699	3.25854	1.811197	0.669782	2.337479	1.69E-08	7.66E-07	DOWN
ENSMUSC	Ccdc68	coiled-co	GO:00511	localizatio	-	-	-	-	18:699254	6	7	3	30	85	36	0.12854	0.119769	0.071039	0.495851	1.172661	0.606753	0.10645	0.758422	1.76E-08	7.97E-07	DOWN
ENSMUSC	Epha1	Eph recep	GO:00325	developm	K05102	Eph recep	mmu0436	Axon guic	6:4235848	0	3	3	25	57	20	0	0.029714	0.041117	0.239168	0.45515	0.195103	0.02361	0.296474	1.79E-08	8.06E-07	DOWN
ENSMUSC	Gata6	GATA bin	GO:00325	developm	-	-	-	-	18:110525	2	0	2	24	50	16	0.038226	0	0.042259	0.35396	0.615513	0.240626	0.026828	0.403366	1.85E-08	8.30E-07	DOWN
ENSMUSC	Pyy	peptide Y	GO:00508	response	-	-	-	-	11:102106	0	0	0	8	26	12	0	0	0	0.298613	0.810035	0.45676	0	0.521803	1.85E-08	8.30E-07	DOWN
ENSMUSC	Ugt2b35	UDP gluc	GO:00081	metabolic	K00699	glucuronc	mmu0110	Metabolic	5:8700086	0	0	0	11	17	15	0	0	0	0.154268	0.198997	0.214503	0	0.189256	1.86E-08	8.33E-07	DOWN
ENSMUSC	Arhgef5	Rho guan	GO:00081	metabolic	-	-	-	-	6:4326558	66	111	60	219	416	215	0.267445	0.359216	0.268712	0.684639	1.085515	0.685388	0.298457	0.818514	1.93E-08	8.60E-07	DOWN
ENSMUSC	2610528J1	RIKEN cDI	GO:00160	membran	-	-	-	-	4:1185269	0	0	0	12	19	8	0	0	0	0.357528	0.472519	0.243046	0	0.357698	2.02E-08	9.01E-07	DOWN
ENSMUSC	Mcpt1	mast cell p	GO:00081	metabolic	-	-	-	-	14:560179	0	0	0	11	18	9	0	0	0	0.511032	0.697971	0.426331	0	0.545111	2.10E-08	9.34E-07	DOWN
ENSMUSC	Ereg	epiregulin	GO:00325	developm	K09784	epiregulin	mmu0401	ErbB signa	5:9107462	0	0	0	7	33	14	0	0	0	0.079878	0.314321	0.162905	0	0.185701	2.14E-08	9.49E-07	DOWN
ENSMUSC	Plbd1	phospholi	GO:00055	extracellul	-	-	-	-	6:1366120	3	8	10	43	92	39	0.049439	0.105294	0.182133	0.546704	0.976328	0.505622	0.112288	0.676218	2.31E-08	1.02E-06	DOWN
ENSMUSC	Mgst2	microsom	GO:00081	metabolic	K00799	glutathion	mmu0522	Hepatoce	3:5166036	4	9	0	30	76	34	0.04647	0.083501	0	0.268869	0.568533	0.310723	0.043324	0.382708	2.34E-08	1.03E-06	DOWN
ENSMUSC	Cracr2a	calcium re	GO:00511	localizatio	-	-	-	-	6:1275613	32	48	40	121	209	122	0.231719	0.277589	0.320123	0.675974	0.974571	0.694996	0.276477	0.781847	2.42E-08	1.06E-06	DOWN
ENSMUSC	Hoxd4	homeobo	GO:00325	developm	-	-	-	-	2:7471192	0	0	0	10	22	9	0	0	0	0.089252	0.163892	0.081906	0	0.111684	2.47E-08	1.08E-06	DOWN
ENSMUSC	Nxpe5	neurexopl	GO:00160	membran	-	-	-	-	5:1382258	1	3	0	18	45	17	0.007062	0.016916	0	0.098037	0.204569	0.094416	0.007993	0.132341	2.72E-08	1.19E-06	DOWN
ENSMUSC	Nlrc4	NLR famil	GO:00081	metabolic	K12805	NLR famil	mmu0462	NOD-like	17:744262	2	6	0	23	72	27	0.031812	0.076231	0	0.282292	0.737606	0.337923	0.036014	0.452607	3.02E-08	1.32E-06	DOWN
ENSMUSC	Slc22a1	solute car	GO:00511	localizatio	K08198	MFS trans	mmu0523	Choline m	17:126488	0	0	0	11	26	8	0	0	0	0.259873	0.51267	0.192706	0	0.32175	3.09E-08	1.34E-06	DOWN
ENSMUSC	Ciita	class II tra	GO:00325	developm	K08060	class II, m	mmu0515	Tuberculo	16:104881	0	7	6	29	40	39	0	0.064968	0.077069	0.260004	0.299339	0.356553	0.047346	0.305299	3.26E-08	1.42E-06	DOWN
ENSMUSC	Rnasel	ribonucle	GO:00325	developm	K01165	ribonucle	mmu0516	Herpes sir	1:1537494	199	226	113	400	676	614	1.262268	1.144859	0.792176	1.957438	2.761208	3.063918	1.066434	2.594188	3.32E-08	1.44E-06	DOWN
ENSMUSC	Tubal3	tubulin, al	GO:00051	structural	K07374	tubulin alp	mmu0414	Phagoson	13:391827	0	0	0	10	12	13	0	0	0	0.112348	0.112531	0.148921	0	0.1246	3.38E-08	1.46E-06	DOWN
ENSMUSC	Tpsg1	tryptase g	GO:00081	metabolic	-	-	-	-	17:253692	0	0	0	8	19	11	0	0	0	0.137637	0.272846	0.192983	0	0.201155	3.55E-08	1.53E-06	DOWN
ENSMUSC	Oas1a	2'-5' oligo	GO:00081	metabolic	K14216	2'-5'-oligo	mmu0516	Herpes sir	5:1208962	7	28	7	83	151	56	0.226746	0.724335	0.250614	2.074157	3.149662	1.42702	0.400565	2.216947	3.64E-08	1.56E-06	DOWN
ENSMUSC	Pcsk9	proprotei	GO:00325	developm	K13050	proprotei	mmu0497	Cholester	4:1064423	0	4	0	16	22	18	0	0.055391	0	0.214058	0.245675	0.245555	0.018464	0.235096	4.13E-08	1.77E-06	DOWN
ENSMUSC	Golph3l	golgi pho	GO:00511	localizatio	-	-	-	-	3:9558893	403	433	313	278	192	208	1.871354	1.605772	1.606357	0.995922	0.574127	0.759847	1.694495	0.776632	4.66E-08	1.99E-06	UP
ENSMUSC	Afm	afamin [S	GO:00511	localizatio	-	-	-	-	5:9051893	0	0	0	9	29	9	0	0	0	0.124695	0.335363	0.127147	0	0.195735	4.74E-08	2.02E-06	DOWN
ENSMUSC	Papss2	3'-phospl	GO:00325	developm	K13811	3'-phospl	mmu0110	Metabolic	19:326200	436	454	355	928	1669	903	7.323606	6.090324	6.590436	12.02589	18.05302	11.93267	6.668122	14.00386	5.05E-08	2.15E-06	DOWN
ENSMUSC	Glod5	glyoxalase	-	-	-	-	-	-	X:8004200	0	1	0	13	14	13	0	0.072467	0	0.909901	0.817923	0.927826	0.024156	0.885217	5.24E-08	2.22E-06	DOWN
ENSMUSC	Fam83g	family wit	GO:00081	metabolic	-	-	-	-	11:616840	0	3	0	13	37	16	0	0.020147	0	0.084324	0.20033	0.105833	0.006716	0.130162	5.59E-08	2.37E-06	DOWN
ENSMUSC	Gzmb	granzyme	GO:00081	metabolic	K01353	granzyme	mmu0520	Transcript	14:562588	0	4	4	28	56	20	0	0.135816	0.187986	0.918489	1.533321	0.669002	0.107934	1.040271	5.65E-08	2.39E-06	DOWN
ENSMUSC	Nos2	nitric oxid	GO:00081	metabolic	K13241	nitric-oxid	mmu0110	Metabolic	11:789207	9	20	10	46	105	60	0.121011	0.214767	0.148602	0.47718	0.909149	0.634675	0.16146	0.673668	5.76E-08	2.43E-06	DOWN
ENSMUSC	Hoxb3	homeobo	GO:00325	developm	-	-	-	-	11:963233	2	0	3	16	34	23	0.020302	0	0.033665	0.125324	0.222286	0.183708	0.017989	0.177106	5.95E-08	2.50E-06	DOWN
ENSMUSC	Degs2	delta(4)-c	GO:00081	metabolic	K04712	sphingolip	mmu0110	Metabolic	12:108678	18	24	18	73	140	66	0.365123	0.388801	0.403554	1.142429	1.828778	1.053248	0.385826	1.341485	6.46E-08	2.71E-06	DOWN
ENSMUSC	H2-Q1	histocomp	GO:00023	immune s	K06751	major hist	mmu0516	Human pa	17:353204	30	31	20	78	124	101	0.390817	0.322516	0.28796	0.783918	1.040218	1.035093	0.333765	0.953076	6.52E-08	2.72E-06	DOWN
ENSMUSC	Tmem139	transmem	GO:00160	membran	-	-	-	-	6:4226197	0	3	0	14	34	14	0	0.127559	0	0.574957	1.165497	0.586287	0.04252	0.77558	6.56E-08	2.74E-06	DOWN
ENSMUSC	Malrd1	MAM anc	GO:00081	metabolic	-	-	-	-	2:1552647	0	0	0	7	29	11	0	0	0	0.048937	0.169224	0.078419	0	0.09886	7.03E-08	2.93E-06	DOWN
ENSMUSC	Gm11127	predicted	GO:00023	immune s	K06751	major hist	mmu0516	Human pa	17:360558	0	0	0	12	17	7	0	0	0	0.420574	0.497346	0.250193	0	0.389371	8.00E-08	3.32E-06	DOWN
ENSMUSC	Smim24	small inte	GO:00055	extracellul	-	-	-	-	10:813930	12	21	11	54	108	55	0.399063	0.55775	0.404297	1.385471	2.312863	1.438937	0.453703	1.712424	8.09E-08	3.35E-06	DOWN
ENSMUSC	Arg1	arginase,	GO:00325	developm	K01476	arginase[3	mmu0110	Metabolic	10:249152	16	37	12	6	1	2	0.372588	0.688103	0.308839	0.10779	0.014992	0.036647	0.45651	0.053143	8.53E-08	3.52E-06	UP
ENSMUSC	Otc	ornithine	GO:00325	developm	K00611	ornithine c	mmu0110	Metabolic	X:1025230	0	0	0	12	15	12	0	0	0	0.128817	0.134406	0.131365	0	0.13153	8.56E-08	3.53E-06	DOWN
ENSMUSC	Prap1	proline-ri	GO:00055	extracellul	-	-	mmu0414	Autophag	7:1400933	0	0	0	8	23	9	0	0	0	0.607822	1.458602	0.697257	0	0.921227	9.48E-08	3.90E-06	DOWN
ENSMUSC	Ces1d	carboxyle	GO:00325	developm	K01044	carboxyles	mmu0110	Metabolic	8:9316606	2	0	0	21	41	10	0.038748	0	0	0.313936	0.511608	0.152442	0.012916	0.325995	9.74E-08	4.00E-06	DOWN
ENSMUSC	Ces1e	carboxyle	GO:00038	catalytic a	K01044	carboxyles	mmu0110	Metabolic	8:9320121	0	0	0	9	19	8	0	0	0	0.140664	0.247861	0.12749	0	0.172005	1.04E-07	4.26E-06	DOWN
ENSMUSC	Hoxb5	homeobo	GO:00325	developm	-	-	-	-	11:963033	0	4	2	21	41	16	0	0.041665	0.028834	0.211325	0.344387	0.164187	0.0235	0.239966	1.06E-07	4.34E-06	DOWN
ENSMUSC	Krt18	keratin 18	GO:00511	localizatio	-	-	-	-	15:102028	31	65	35	131	316	135	1.351983	2.263984	1.687076	4.407741	8.874778	4.631918	1.767681	5.971479	1.22E-07	4.96E-06	DOWN
ENSMUSC	Stap2	signal trar	GO:00160	membran	-	-	-	-	17:559970	17	27	14	69	140	64	0.696179	0.883033	0.633635	2.179954	3.691899	2.061862	0.737615	2.644572	1.22E-07	4.96E-06	DOWN
ENSMUSC	Foxd2	forkhead	GO:00325	developm	-	-	-	-	4:1149062	4	3	2	21	40	23	0.094231	0.056447	0.052073	0.381641	0.606762	0.426242	0.067584	0.471548	1.23E-07	4.97E-06	DOWN
ENSMUSC	Tfcp2l1	transcripti	GO:00325	developm	-	-	-	-	1:1186279	89	117	101	248	518	272	0.566357	0.594606	0.710339	1.21753	2.12267	1.361689	0.623767	1.567297	1.25E-07	5.04E-06	DOWN
ENSMUSC	Cldn8	claudin 8	GO:00051	structural	K06087	claudin[-]	mmu0453	Tight junc	16:885608	0	0	0	10	22	7	0	0	0	0.199947	0.367158	0.142725	0	0.23661	1.27E-07	5.13E-06	DOWN
ENSMUSC	Tagln	transgelin	GO:00325	developm	-	-	-	-	9:4592961	156	250	136	365	654	514	3.79032	4.851058	3.652055	6.841866	10.23254	9.824827	4.097811	8.96641	1.55E-07	6.25E-06	DOWN
ENSMUSC	Hnf1a	HNF1 hor	GO:00325	developm	K08036	transcripti	mmu0455	Signaling	5:1149489	0	0	1	16	38	9	0	0	0.011188	0.12495	0.247697	0.071668	0.003729	0.148105	1.61E-07	6.47E-06	DOWN
ENSMUSC	Plek2	pleckstrin	GO:00508	response	-	-	-	-	12:788886	10	16	8	48	84	41	0.385715	0.492878	0.341016	1.428333	2.086401	1.244105	0.406536	1.58628	1.71E-07	6.87E-06	DOWN
ENSMUSC	Slc6a5	solute car	GO:00511	localizatio	-	-	-	-	7:4991014	16	148	21	1	8	0	0.118015	0.87182	0.171196	0.005691	0.038	0	0.38701	0.014564	1.75E-07	6.98E-06	UP
ENSMUSC	Gstm3	glutathion	GO:00081	metabolic	K00799	glutathion	mmu0520	Pathways	3:1079636	0	2	0	11	25	12	0	0.074402	0	0.39526	0.749774	0.439688	0.024801	0.528241	1.85E-07	7.39E-06	DOWN
ENSMUSC	Pkp1	plakophili	GO:00081	metabolic	-	-	-	-	1:1358713	2	0	0	13	34	13	0.017467	0	0	0.087606	0.191248	0.089331	0.005822	0.122728	1.86E-07	7.41E-06	DOWN
ENSMUSC	1600014C	RIKEN cDI	GO:00081	metabolic	-	-	-	-	7:3818321	533	604	408	384	394	345	3.072218	2.780401	2.599148	1.707599	1.46243	1.564424	2.817256	1.578151	1.89E-07	7.50E-06	-
ENSMUSC	Ano7	anoctamir	GO:00511	localizatio	-	-	-	-	1:9337393	3	5	0	22	91	24	0.036774	0.048946	0	0.208056	0.718327	0.231442	0.028573	0.385942	2.11E-07	8.33E-06	DOWN
ENSMUSC	Smim6	small inte	GO:00160	membran	-	-	-	-	11:115912	0	0	0	9	23	7	0	0	0	0.433499	0.924676	0.343824	0	0.567333	2.10E-07	8.33E-06	DOWN
ENSMUSC	Tes	testis deri	GO:00099	cellular pr	-	-	-	-	6:1706514	30	40	23	83	158	102	0.210402	0.224043	0.178275	0.449091	0.713568	0.562775	0.20424	0.575145	2.13E-07	8.40E-06	DOWN
ENSMUSC	Mki67	antigen id	GO:00325	developm	-	-	-	-	7:1356897	88	184	80	277	662	341	0.519293	0.867146	0.521757	1.261067	2.515601	1.58305	0.636065	1.786573	2.15E-07	8.43E-06	DOWN
ENSMUSC	Lgi2	leucine-ri	GO:00055	extracellul	-	-	-	-	5:5253351	647	635	460	1371	1425	1377	5.580531	4.374124	4.385067	9.12303	7.91482	9.343644	4.779907	8.793831	2.14E-07	8.43E-06	-
ENSMUSC	Msr1	macropha	GO:00325	developm	K06558	macropha	mmu0414	Phagoson	8:3958168	34	108	31	9	23	10	0.299525	0.759834	0.301826	0.061169	0.130477	0.069304	0.453728	0.086983	2.20E-07	8.60E-06	UP
ENSMUSC	Marveld2	MARVEL (	GO:00325	developm	K17291	MARVEL c	mmu0453	Tight junc	13:100595	10	19	6	39	85	58	0.118147	0.179274	0.078352	0.355483	0.646688	0.539091	0.125258	0.513754	2.20E-07	8.61E-06	DOWN
ENSMUSC	Chp2	calcineuri	GO:00081	metabolic	-	-	-	-	7:1222194	8	13	6	36	64	39	0.293389	0.38073	0.243196	1.018499	1.511357	1.125123	0.305772	1.218326	2.26E-07	8.81E-06	DOWN
ENSMUSC	Dennd2d	DENN/MA	GO:00987	molecular	-	-	-	-	3:1064824	12	12	10	40	128	57	0.089999	0.071876	0.082889	0.231452	0.618202	0.33632	0.081588	0.395324	2.27E-07	8.85E-06	DOWN
ENSMUSC	Ms4a4d	membran	GO:00160	membran	-	-	-	-	19:115368	0	2	0	14	24	9	0	0.064468	0	0.435873	0.623691	0.285723	0.021489	0.448429	2.32E-07	9.00E-06	DOWN
ENSMUSC	Hoxb9	homeobo	GO:00325	developm	-	-	-	-	11:962714	2	10	4	26	88	36	0.042526	0.169838	0.094026	0.426585	1.205174	0.602315	0.10213	0.744691	2.35E-07	9.11E-06	DOWN
ENSMUSC	Muc3a	mucin 3A,	GO:00160	membran	-	-	-	-	5:1372088	91	104	79	224	308	229	2.845004	2.596685	2.729713	5.402815	6.200783	5.632311	2.7238	5.745303	2.45E-07	9.47E-06	DOWN
ENSMUSC	C3ar1	complem	GO:00325	developm	K04009	C3a anapl	mmu0408	Neuroacti	6:1228471	70	126	64	49	44	35	0.992809	1.427207	1.003227	0.536159	0.40186	0.390523	1.141081	0.442847	2.51E-07	9.68E-06	UP
ENSMUSC	H2-DMb2	histocomp	GO:00023	immune s	K06752	major hist	mmu0516	HTLV-I inf	17:341433	0	3	0	18	23	10	0	0.058014	0	0.336211	0.358578	0.190461	0.019338	0.295083	2.65E-07	1.02E-05	DOWN
ENSMUSC	Cgn	cingulin [S	GO:00038	catalytic a	K06102	cingulin[-]	mmu0453	Tight junc	3:9476006	128	162	79	286	532	314	1.176497	1.189156	0.802518	2.028033	3.148801	2.270491	1.056057	2.482442	2.65E-07	1.02E-05	DOWN
ENSMUSC	Sult1a1	sulfotrans	GO:00038	catalytic a	K01014	aryl sulfot	mmu0520	Chemical	7:1266728	85	139	96	248	431	265	1.697172	2.216494	2.118467	3.820209	5.541634	4.162574	2.010711	4.508139	2.66E-07	1.02E-05	DOWN
ENSMUSC	Gmds	GDP-mar	GO:00081	metabolic	K01711	GDPmanr	mmu0110	Metabolic	13:318195	122	179	119	347	542	297	1.860407	2.179959	2.005595	4.082339	5.322344	3.563004	2.01532	4.322562	2.68E-07	1.02E-05	DOWN
ENSMUSC	Erbb3	erb-b2 re	GO:00325	developm	K05084	receptor t	mmu0520	Proteogly	10:128567	181	292	210	519	810	494	1.837019	2.366816	2.355603	4.063811	5.293894	3.944332	2.18648	4.434012	2.70E-07	1.03E-05	DOWN
ENSMUSC	Rassf6	Ras assoc	GO:00508	response	K09854	Ras assoc	mmu0439	Hippo sig	5:9060307	3	13	7	41	71	32	0.032425	0.112217	0.083627	0.341886	0.49418	0.272105	0.07609	0.36939	2.79E-07	1.06E-05	DOWN
ENSMUSC	Psmb9	proteasor	GO:00081	metabolic	K02741	20S prote	mmu0305	Proteason	17:341819	15	25	17	65	88	61	0.145304	0.19341	0.18201	0.485779	0.548953	0.464875	0.173575	0.499869	2.82E-07	1.07E-05	DOWN
ENSMUSC	Galnt4	polypeptid	GO:00081	metabolic	K00710	polypeptid	mmu0110	Metabolic	10:991081	94	149	110	291	501	271	1.127817	1.427716	1.458654	2.693611	3.870825	2.557942	1.338062	3.040793	2.98E-07	1.13E-05	DOWN
ENSMUSC	Cdkn2b	cyclin-dep	GO:00325	developm	K04685	cyclin-dep	mmu0520	Pathways	4:8930628	6	12	5	40	53	30	0.262986	0.420061	0.242236	1.352655	1.495964	1.03448	0.308428	1.294366	3.01E-07	1.14E-05	DOWN
ENSMUSC	Myb	myeloblas	GO:00325	developm	K09420	myb prote	mmu0415	PI3K-Akt s	10:211249	18	25	23	84	132	62	0.097172	0.107786	0.137229	0.349858	0.458891	0.26332	0.114063	0.357357	3.03E-07	1.14E-05	DOWN
ENSMUSC	Tnip3	TNFAIP3 i	GO:00081	metabolic	-	-	-	-	6:6552531	0	2	0	9	25	14	0	0.006851	0	0.029777	0.069037	0.047231	0.002284	0.048682	3.05E-07	1.15E-05	DOWN
ENSMUSC	AI661453	expressed	-	-	-	-	-	-	17:474366	5	8	10	38	71	33	0.050101	0.064026	0.11075	0.293784	0.458169	0.260161	0.074959	0.337371	3.16E-07	1.19E-05	DOWN
ENSMUSC	Mlst8	MTOR ass	GO:00081	metabolic	K08266	target of r	mmu0415	PI3K-Akt s	17:244735	358	368	344	833	927	834	6.201087	5.0907	6.585517	11.13165	10.33996	11.36479	5.959101	10.94546	3.24E-07	1.22E-05	-
ENSMUSC	Slc5a9	solute car	GO:00511	localizatio	-	-	-	-	4:1118753	0	0	2	11	26	14	0	0	0.01283	0.049257	0.097173	0.063923	0.004277	0.070118	3.30E-07	1.23E-05	DOWN
ENSMUSC	Tlr1	toll-like re	GO:00048	signal trar	K05398	toll-like re	mmu0515	Tuberculo	5:6492467	11	9	6	33	88	44	0.109072	0.07127	0.065755	0.252438	0.561879	0.343212	0.082032	0.385843	3.33E-07	1.24E-05	DOWN
ENSMUSC	Hk2	hexokinas	GO:00325	developm	K00844	hexokinas	mmu0110	Metabolic	6:8272502	150	214	134	371	468	393	1.335482	1.52162	1.318553	2.548299	2.68316	2.752643	1.391885	2.661367	3.37E-07	1.26E-05	-
ENSMUSC	Rep15	RAB15 eff	GO:00081	metabolic	-	-	-	-	6:1470325	15	18	3	47	73	60	0.551053	0.52811	0.121817	1.332122	1.726981	1.734092	0.400327	1.597731	3.55E-07	1.32E-05	DOWN
ENSMUSC	Sptssb	serine pal	GO:00081	metabolic	-	-	-	-	3:6981954	15	33	19	87	183	65	0.370489	0.65096	0.518683	1.657843	2.910728	1.263039	0.513378	1.94387	3.97E-07	1.47E-05	DOWN
ENSMUSC	Aoc3	amine oxi	GO:00081	metabolic	K00276	primary-a	mmu0110	Metabolic	11:101330	17	24	16	59	78	72	0.186356	0.210106	0.193846	0.498965	0.550598	0.620914	0.196769	0.556826	4.01E-07	1.48E-05	DOWN
ENSMUSC	Kcne3	potassium	GO:00511	localizatio	K04897	potassium	mmu0497	Protein di	7:1001765	5	10	7	31	43	38	0.053302	0.085141	0.082488	0.254983	0.295215	0.318724	0.073644	0.289641	4.03E-07	1.49E-05	DOWN
ENSMUSC	Apoa4	apolipopr	GO:00325	developm	K08760	apolipopr	mmu0497	Cholester	9:4624069	0	0	0	5	21	11	0	0	0	0.129759	0.454917	0.291123	0	0.291933	4.11E-07	1.51E-05	DOWN
ENSMUSC	Cd8a	CD8 antig	GO:00325	developm	K06458	T-cell surf	mmu0451	Cell adhes	6:7137342	0	0	0	8	17	7	0	0	0	0.048973	0.086866	0.043698	0	0.059846	4.26E-07	1.57E-05	DOWN
ENSMUSC	BC016579	cDNA seq	GO:00160	membran	-	-	-	-	16:456268	0	0	0	6	25	9	0	0	0	0.128935	0.448428	0.197217	0	0.258193	4.42E-07	1.62E-05	DOWN
ENSMUSC	Llgl2	lethal giar	GO:00325	developm	K06094	lethal(2) g	mmu0516	Human pa	11:115824	27	44	22	86	187	96	0.233346	0.303687	0.210141	0.573401	1.040702	0.652697	0.249058	0.7556	4.60E-07	1.68E-05	DOWN
ENSMUSC	Fzd5	frizzled cl	GO:00325	developm	K02375	frizzled 5/	mmu0520	Pathways	1:6473055	17	74	22	125	245	156	0.105811	0.367835	0.15134	0.600225	0.981964	0.763855	0.208329	0.782015	4.71E-07	1.72E-05	DOWN
ENSMUSC	Nlrp9b	NLR famil	GO:00023	immune s	-	-	-	-	7:1999146	0	0	0	6	41	10	0	0	0	0.053895	0.307413	0.091599	0	0.150969	4.96E-07	1.81E-05	DOWN
ENSMUSC	Cd36	CD36 mol	GO:00325	developm	K06259	CD36 anti	mmu0414	Phagoson	5:1778169	110	212	103	35	99	29	0.804551	1.238348	0.832616	0.197495	0.466285	0.166865	0.958505	0.276881	5.41E-07	1.97E-05	UP
ENSMUSC	Cd14	CD14 anti	GO:00511	localizatio	K04391	monocyte	mmu0401	MAPK sigr	18:367250	19	38	27	80	159	89	0.737975	1.178738	1.159041	2.397233	3.976892	2.719526	1.025251	3.031217	5.81E-07	2.11E-05	DOWN
ENSMUSC	Cd24a	CD24a an	GO:00325	developm	K06469	CD24 anti	mmu0464	Hematopo	10:435782	222	283	187	499	895	487	4.326483	4.404686	4.027825	7.502629	11.23209	7.466595	4.252998	8.733773	5.88E-07	2.13E-05	DOWN
ENSMUSC	Ttll10	tubulin tyr	GO:00081	metabolic	-	-	-	-	4:1560348	0	0	0	14	18	4	0	0	0	0.109949	0.117996	0.032032	0	0.086659	6.44E-07	2.33E-05	DOWN
ENSMUSC	Capn9	calpain 9	GO:00081	metabolic	-	-	-	-	8:1245761	0	9	2	22	42	27	0	0.163214	0.050195	0.385394	0.614127	0.482316	0.071136	0.493945	6.69E-07	2.41E-05	DOWN
ENSMUSC	Baiap2l1	BAI1-assc	GO:00508	response	-	-	-	-	5:1442645	17	58	16	102	214	111	0.308379	0.840236	0.320773	1.427444	2.499755	1.584023	0.489796	1.837074	6.71E-07	2.41E-05	DOWN
ENSMUSC	Crybg2	crystallin b	-	-	-	-	-	-	4:1340608	10	10	4	28	99	48	0.057897	0.046236	0.025597	0.125066	0.369102	0.218631	0.043244	0.2376	7.02E-07	2.52E-05	DOWN
ENSMUSC	Kcnq1	potassium	GO:00325	developm	K04926	potassium	mmu0426	Adrenergi	7:1431063	10	14	0	55	90	37	0.13449	0.150371	0	0.570669	0.779444	0.391474	0.094954	0.580529	7.61E-07	2.73E-05	DOWN
ENSMUSC	Epsti1	epithelial	-	-	-	-	-	-	14:779042	15	29	5	68	110	58	0.293447	0.453105	0.108117	1.026337	1.385786	0.892669	0.28489	1.101598	8.07E-07	2.89E-05	DOWN
ENSMUSC	Isg15	ISG15 ubi	GO:00325	developm	K12159	ubiquitin c	mmu0516	Human pa	4:1561994	10	22	9	66	69	42	0.807654	1.419046	0.803327	4.112401	3.588625	2.6686	1.010009	3.456542	8.13E-07	2.90E-05	DOWN
ENSMUSC	Acta2	actin, alph	GO:00325	developm	K12313	actin, aort	mmu0437	Apelin sig	19:342410	371	575	352	777	1471	1020	12.71903	15.7432	13.33732	20.5509	32.47481	27.51001	13.93319	26.84524	8.90E-07	3.17E-05	-
ENSMUSC	Gal3st2b	galactose	GO:00081	metabolic	-	-	-	-	1:9391831	4	0	0	14	24	16	0.068474	0	0	0.184882	0.264548	0.215464	0.022825	0.221631	8.99E-07	3.20E-05	DOWN
ENSMUSC	Szrd1	SUZ RNA	-	-	-	-	-	-	4:1411130	384	877	558	423	265	173	6.270752	11.43756	10.07091	5.329161	2.786689	2.222512	9.25974	3.446121	9.47E-07	3.36E-05	UP
ENSMUSC	Apol6	apolipopr	GO:00081	metabolic	-	-	-	-	15:770447	2	4	0	14	41	19	0.015486	0.024738	0	0.083647	0.204474	0.115759	0.013408	0.134627	9.62E-07	3.41E-05	DOWN
ENSMUSC	Spon2	spondin 2	GO:00511	localizatio	-	-	-	-	5:3319818	2	1	1	16	28	13	0.019309	0.007713	0.010673	0.1192	0.174114	0.098755	0.012565	0.13069	1.01E-06	3.58E-05	DOWN
ENSMUSC	Lgals3	lectin, gal	GO:00325	developm	-	-	-	-	14:473677	44	153	70	259	634	257	0.820816	2.279476	1.443247	3.727585	7.616254	3.771737	1.514513	5.038525	1.13E-06	3.99E-05	DOWN
ENSMUSC	Ggt6	gamma-g	GO:00081	metabolic	K00681	gamma-g	mmu0110	Metabolic	11:724355	0	5	0	15	19	17	0	0.062901	0	0.182298	0.192738	0.210682	0.020967	0.195239	1.18E-06	4.17E-05	DOWN

ENSMUS(	Spint2	serine pro	GO:00325	developm	-	-	-	-	7:2925632	190	374	192	521	986	598	2.063137	3.243338	2.304217	4.364585	6.894564	5.108432	2.536897	5.45586	1.22E-06	4.30E-05	DOWN
ENSMUS(	Sec16b	SEC16 ho	GO:00081	metabolic	-	-	-	-	1:1575067	2	12	5	29	52	31	0.017555	0.084133	0.048517	0.196414	0.293971	0.214101	0.050068	0.234829	1.30E-06	4.57E-05	DOWN
ENSMUS(	Ptprg	protein ty	GO:00325	developm	-	-	-	-	14:115535	2048	2910	1802	1885	1950	1849	8.1231	9.217871	7.899389	5.768111	4.980593	5.769519	8.413453	5.506074	1.32E-06	4.61E-05	-
ENSMUS(	C1s1	complem	GO:00081	metabolic	K01331	complem	mmu0532	Systemic l	6:1245303	32	33	26	85	151	84	0.382059	0.314661	0.343083	0.782942	1.160953	0.788996	0.346601	0.910963	1.45E-06	5.07E-05	DOWN
ENSMUS(	Btnl6	butyrophi	GO:00160	membran	-	-	-	-	17:345078	0	0	0	9	16	5	0	0	0	0.17995	0.267013	0.10194	0	0.182968	1.52E-06	5.31E-05	DOWN
ENSMUS(	Fut4	fucosyltra	GO:00081	metabolic	K07632	galactosid	mmu0110	Metabolic	9:1474832	4	3	4	18	47	25	0.059952	0.035913	0.066261	0.208131	0.453603	0.294762	0.054042	0.318832	1.54E-06	5.36E-05	DOWN
ENSMUS(	Rab19	RAB19, m	GO:00038	catalytic a	-	-	-	-	6:3938117	0	1	0	8	22	8	0	0.030965	0	0.23927	0.549221	0.243972	0.010322	0.344154	1.56E-06	5.41E-05	DOWN
ENSMUS(	Neurl3	neuralizec	GO:00081	metabolic	-	-	-	-	1:3626459	6	24	26	85	198	70	0.096304	0.307647	0.461232	1.052567	2.046554	0.883913	0.288394	1.327678	1.56E-06	5.41E-05	DOWN
ENSMUS(	Fermt1	fermitin fa	GO:00325	developm	-	-	-	-	2:1329043	30	22	5	68	188	92	0.268429	0.15721	0.049448	0.469402	1.083222	0.647594	0.158362	0.733406	1.58E-06	5.46E-05	DOWN
ENSMUS(	Cldn23	claudin 23	GO:00051	structural	K06087	claudin[-]	mmu0453	Tight junc	8:3582471	11	12	4	31	72	43	0.322915	0.28132	0.129782	0.702061	1.361025	0.993032	0.244672	1.018706	1.61E-06	5.54E-05	DOWN
ENSMUS(	Hmcn2	hemicenti	GO:00400	locomotic	-	-	-	-	2:3131441	8	15	18	53	144	52	0.027008	0.040441	0.067159	0.138033	0.313035	0.138099	0.044869	0.196389	1.72E-06	5.94E-05	DOWN
ENSMUS(	Phf11d	PHD finge	GO:00081	metabolic	-	-	-	-	14:593474	10	27	18	61	119	60	0.098212	0.211774	0.195383	0.462194	0.752599	0.463577	0.168457	0.559457	1.74E-06	5.99E-05	DOWN
ENSMUS(	Upp1	uridine ph	GO:00081	metabolic	K00757	uridine ph	mmu0110	Metabolic	11:911810	38	56	34	110	233	111	0.709539	0.83509	0.701658	1.584595	2.801598	1.630527	0.748762	2.005573	1.85E-06	6.36E-05	DOWN
ENSMUS(	Gm20503	predicted	GO:00048	signal trar	-	-	-	-	4:5900321	153	195	104	107	86	68	7.2194	7.348327	5.423622	3.895159	2.613133	2.524239	6.663783	3.010843	1.94E-06	6.65E-05	UP
ENSMUS(	Irf1	interferon	GO:00325	developm	K09444	interferon	mmu0516	Human pa	11:537700	131	174	121	295	467	319	1.386967	1.47126	1.41588	2.409604	3.183944	2.657022	1.424702	2.75019	1.97E-06	6.73E-05	-
ENSMUS(	Zfp990	zinc finge	GO:00081	metabolic	-	-	-	-	4:1455107	2	0	0	12	29	10	0.031419	0	0	0.145458	0.293421	0.123609	0.010473	0.187496	2.00E-06	6.82E-05	DOWN
ENSMUS(	AC149222	-	GO:00081	metabolic	-	-	-	-	7:1277856	4	0	0	414	0	639	0.044742	0	0	3.572408	0	5.622666	0.014914	3.065024	2.11E-06	7.19E-05	DOWN
ENSMUS(	Naip5	NLR famil	GO:00081	metabolic	K12807	baculovira	mmu0462	NOD-like	13:100211	22	35	20	62	135	90	0.258122	0.327962	0.259353	0.561217	1.019983	0.830725	0.281812	0.803975	2.17E-06	7.36E-05	DOWN
ENSMUS(	Itpr3	inositol 1,4	GO:00048	signal trar	K04960	inositol 1,4	mmu0516	Kaposi's sa	17:270573	91	155	97	272	460	242	0.618053	0.840742	0.728116	1.425222	2.011852	1.293034	0.72897	1.576703	2.18E-06	7.39E-05	DOWN
ENSMUS(	Grb7	growth fa	GO:00081	metabolic	-	-	-	-	11:984463	18	32	7	70	86	66	0.222744	0.316255	0.095745	0.668306	0.685325	0.642537	0.211582	0.665389	2.22E-06	7.50E-05	DOWN
ENSMUS(	Ikzf3	IKAROS fa	GO:00325	developm	-	-	-	-	11:984649	3	2	3	17	38	17	0.019261	0.010257	0.021289	0.084207	0.157109	0.085868	0.016936	0.109061	2.41E-06	8.15E-05	DOWN
ENSMUS(	Dtx3l	deltex 3-l	GO:00081	metabolic	K06058	deltex[2.3	mmu0433	Notch sigi	16:359265	163	193	139	343	517	358	1.373136	1.298461	1.29416	2.229206	2.804596	2.372568	1.321919	2.46879	2.65E-06	8.94E-05	-
ENSMUS(	Srgap3	SLIT-ROB	GO:00987	molecular	K07526	SLIT-ROB	mmu0436	Axon guic	6:1127179	9973	12405	3291	4394	4591	4486	38.71154	38.45539	14.11853	13.15846	11.47563	13.69887	30.42849	12.77765	3.00E-06	0.000101	UP
ENSMUS(	Parp9	poly (ADP	GO:00081	metabolic	-	-	-	-	16:359384	87	111	76	221	318	195	0.861087	0.877398	0.831361	1.687528	2.026797	1.518357	0.856615	1.744227	3.02E-06	0.000101	DOWN
ENSMUS(	Mlph	melanoph	GO:00511	localizatio	-	-	-	-	1:9091508	42	36	29	103	206	94	0.427549	0.292678	0.326273	0.808917	1.350386	0.752795	0.348833	0.9707	3.03E-06	0.000102	DOWN
ENSMUS(	Cidec	cell death	GO:00081	metabolic	-	-	-	-	6:1134246	2	0	0	9	18	14	0.037383	0	0	0.129811	0.2167	0.205901	0.012461	0.184137	3.11E-06	0.000104	DOWN
ENSMUS(	Prom1	prominin	GO:00325	developm	K06532	prominin	mmu0520	Transcript	5:4399362	188	251	186	400	665	469	0.99782	1.063932	1.091072	1.637891	2.272849	1.958297	1.050942	1.956346	3.54E-06	0.000118	-
ENSMUS(	Eif4ebp3	eukaryotic	GO:00081	metabolic	K18645	eukaryotic	mmu0301	RNA trans	18:366640	6	1	3	18	43	24	0.492392	0.065551	0.272125	1.139682	2.272442	1.549482	0.276689	1.653869	3.56E-06	0.000119	DOWN
ENSMUS(	Slc3a1	solute car	GO:00081	metabolic	K14210	solute car	mmu0497	Protein di	17:850283	12	8	9	40	79	33	0.31759	0.1691	0.263249	0.816753	1.346416	0.68711	0.249979	0.950093	3.84E-06	0.000128	DOWN
ENSMUS(	Hoxc8	homeobo	GO:00325	developm	-	-	-	-	15:102990	7	18	4	2	0	0	0.104477	0.214549	0.065986	0.023032	0	0	0.128337	0.007677	4.39E-06	0.000146	UP
ENSMUS(	Cdc42ep5	CDC42 eff	GO:00325	developm	-	-	-	-	7:4151260	12	20	14	50	102	46	0.405243	0.539413	0.522538	1.302697	2.218206	1.222118	0.489065	1.581007	4.43E-06	0.000147	DOWN
ENSMUS(	Dynlt1c	dynein lig	GO:00325	developm	-	-	-	-	17:660167	20	19	7	67	82	43	0.180807	0.137176	0.069945	0.467292	0.477363	0.30582	0.129309	0.416825	4.58E-06	0.000151	DOWN
ENSMUS(	Mxd1	MAX dime	GO:00081	metabolic	-	-	-	-	6:8664704	402	521	323	796	1363	807	3.093693	3.202098	2.747264	4.726008	6.754634	4.885799	3.014352	5.45548	4.59E-06	0.000151	-
ENSMUS(	Anxa2	annexin A	GO:00325	developm	-	-	-	-	9:6945362	140	287	174	392	854	493	2.091546	3.424263	2.872991	4.518093	8.215824	5.79424	2.796267	6.176053	4.68E-06	0.000154	DOWN
ENSMUS(	Pls1	plastin 1 (	GO:00325	developm	-	-	-	-	9:9575264	139	215	151	378	546	334	2.057971	2.54219	2.470866	4.317646	5.205612	3.890291	2.357009	4.471183	4.71E-06	0.000155	-
ENSMUS(	Hpd	4-hydroxy	GO:00081	metabolic	K00457	4-hydroxy	mmu0110	Metabolic	5:1231718	1	0	0	7	34	11	0.021415	0	0	0.115616	0.468733	0.185269	0.007138	0.256539	4.87E-06	0.00016	DOWN
ENSMUS(	Tnfrsf11a	tumor nec	GO:00325	developm	K05147	tumor nec	mmu0406	Cytokine-	1:1057807	68	86	53	157	291	162	0.663039	0.669691	0.571149	1.18103	1.827163	1.242667	0.634627	1.416954	4.93E-06	0.000161	DOWN
ENSMUS(	Tmem102	transmem	GO:00081	metabolic	-	-	-	-	11:698036	3	3	5	18	41	22	0.094759	0.075691	0.174565	0.438657	0.833976	0.546702	0.115005	0.606445	5.48E-06	0.000179	DOWN
ENSMUS(	Skp1a	S-phase k	GO:00081	metabolic	K03094	S-phase k	mmu0520	Pathways	11:522319	4665	5682	4436	4086	5420	3899	99.1769	96.47305	104.2311	67.01741	74.20154	65.21133	99.96034	68.81009	5.50E-06	0.00018	-
ENSMUS(	Pcdhb21	protocadh	GO:00325	developm	-	-	-	-	18:375136	20	0	13	73	103	119	0.259548	0	0.186453	0.730863	0.860749	1.214905	0.148667	0.935506	5.56E-06	0.000181	DOWN
ENSMUS(	Nexmif	neurite ex	GO:00325	developm	-	-	-	-	X:1040774	535	1338	1024	688	623	471	1.197877	2.392544	2.533991	1.18844	0.898257	0.82964	2.041471	0.972112	5.65E-06	0.000184	UP
ENSMUS(	Liph	lipase, me	GO:00081	metabolic	-	-	-	-	16:219538	15	6	7	35	105	44	0.241458	0.077135	0.124547	0.434666	1.088438	0.557211	0.147713	0.693438	5.89E-06	0.000191	DOWN
ENSMUS(	Cyp2c66	cytochron	GO:00325	developm	K07413	cytochron	mmu0110	Metabolic	19:391138	0	0	0	2	33	14	0	0	0	0.038601	0.531551	0.27549	0	0.28188	6.47E-06	0.000209	DOWN
ENSMUS(	Glra1	glycine re	GO:00048	signal trar	K05193	glycine re	mmu0408	Neuroacti	11:555142	6	22	2	0	0	0	0.10527	0.308276	0.038786	0	0	0	0.150777	0	6.55E-06	0.000212	UP
ENSMUS(	Ly6g6c	lymphocy	GO:00329	macromo	-	-	-	-	17:350653	0	0	0	4	19	7	0	0	0	0.086741	0.343947	0.154816	0	0.195168	6.93E-06	0.000224	DOWN
ENSMUS(	Shroom3	shroom fa	GO:00325	developm	-	-	-	-	5:9268343	70	128	74	196	332	215	0.188725	0.275607	0.220501	0.407679	0.576402	0.456018	0.228278	0.480033	7.24E-06	0.000233	DOWN
ENSMUS(	Dqx1	DEAQ RN	GO:00081	metabolic	-	-	-	-	6:8305784	6	13	7	38	53	26	0.08946	0.154802	0.115362	0.437124	0.508884	0.304982	0.119875	0.416997	8.45E-06	0.000272	DOWN
ENSMUS(	Kcnk5	potassium	GO:00511	localizatio	K04916	potassium	mmu0497	Protein di	14:201400	9	8	9	28	65	37	0.148116	0.105152	0.163697	0.355512	0.688873	0.479054	0.138988	0.507813	8.63E-06	0.000276	DOWN
ENSMUS(	Maoa	monoami	GO:00081	metabolic	K00274	monoami	mmu0110	Metabolic	X:1661969	597	821	596	1206	2135	1285	8.747709	9.607469	9.651909	13.63319	20.14524	14.81269	9.335696	16.19704	8.63E-06	0.000276	-
ENSMUS(	Cyp2e1	cytochron	GO:00081	metabolic	K07415	cytochron	mmu0110	Metabolic	7:1407637	5	4	1	22	35	15	0.089441	0.057144	0.019773	0.30364	0.403204	0.211109	0.055453	0.305985	8.60E-06	0.000276	DOWN
ENSMUS(	Ube2v1	ubiquitin-	GO:00081	metabolic	-	-	-	-	2:1676076	533	112	713	109	194	107	5.377396	0.902419	7.950253	0.848399	1.260378	0.849256	4.743356	0.986011	8.66E-06	0.000276	UP
ENSMUS(	Defa17	defensin,	GO:00508	response	-	-	-	-	8:2165578	0	0	0	6	12	6	0	0	0	0.711903	1.188518	0.725984	0	0.875468	8.84E-06	0.000282	DOWN
ENSMUS(	Pgm1	phosphog	GO:00081	metabolic	K15779	phosphog	mmu0110	Metabolic	5:6409295	125	191	102	299	405	300	2.134892	2.605231	1.925369	3.939758	4.45427	4.030881	2.221831	4.141636	9.47E-06	0.000301	-
ENSMUS(	Areg	amphireg	GO:00325	developm	K09782	amphireg	mmu0439	Hippo sig	5:9113959	1	2	2	11	37	13	0.050269	0.08028	0.11109	0.426491	1.197363	0.513932	0.080546	0.712595	9.64E-06	0.000306	DOWN
ENSMUS(	Apoa1	apolipopr	GO:00325	developm	K08757	apolipopr	mmu0332	PPAR sign	9:4622858	0	0	4	16	40	13	0	0	0.182028	0.508226	1.060503	0.421057	0.060676	0.663262	9.77E-06	0.000309	DOWN
ENSMUS(	Ugt1a8	UDP gluc	GO:00081	metabolic	-	-	-	-	1:8808781	0	0	0	6	6	48	0	0	0	0.126909	0.105928	1.035329	0	0.422722	9.98E-06	0.000315	DOWN
ENSMUS(	Gzma	granzyme	GO:00081	metabolic	K01352	granzyme	mmu0408	Neuroacti	13:113093	2	2	4	16	29	18	0.092495	0.073894	0.204506	0.570984	0.863814	0.654999	0.123632	0.696599	1.00E-05	0.000317	DOWN
ENSMUS(	Gsdmd	gasdermi	GO:00511	localizatio	K20917	gasdermi	mmu0462	NOD-like	15:758623	37	41	28	94	149	84	1.272058	1.125725	1.063907	2.493201	3.298679	2.271917	1.153897	2.687932	1.02E-05	0.00032	DOWN
ENSMUS(	St6galnac	ST6 (alpha	GO:00081	metabolic	K03479	alpha-N-	mmu0110	Metabolic	11:116765	0	0	0	6	7	11	0	0	0	0.118154	0.115055	0.220898	0	0.151369	1.04E-05	0.000328	DOWN
ENSMUS(	Fam83h	family wit	GO:00325	developm	-	-	-	-	15:760010	6	24	20	51	108	65	0.079174	0.252926	0.291695	0.519211	0.917747	0.674786	0.207932	0.703914	1.06E-05	0.000333	DOWN
ENSMUS(	Usp18	ubiquitin s	GO:00081	metabolic	-	-	-	-	6:1212459	22	28	19	58	89	72	0.389691	0.396104	0.371971	0.79261	1.015191	1.003334	0.385922	0.937045	1.12E-05	0.000351	DOWN
ENSMUS(	Guca2b	guanylate	GO:00081	metabolic	-	-	-	-	4:1196566	0	0	0	8	22	3	0	0	0	0.432663	0.993138	0.165423	0	0.530408	1.13E-05	0.000354	DOWN
ENSMUS(	Agpat2	1-acylglyc	GO:00325	developm	K13509	lysophosp	mmu0110	Metabolic	2:2659305	15	33	13	61	107	56	0.334985	0.588579	0.320871	1.05101	1.53881	0.983882	0.414812	1.191234	1.18E-05	0.000368	DOWN
ENSMUS(	Acp5	acid phos	GO:00325	developm	K14379	tartrate-re	mmu0110	Metabolic	9:2212672	6	23	11	41	108	49	0.11031	0.33772	0.223514	0.581546	1.278656	0.708724	0.223848	0.856309	1.22E-05	0.00038	DOWN
ENSMUS(	Lilrb4a	leukocyte	GO:00023	immune s	-	-	-	-	10:514806	10	48	16	2	9	3	0.205654	0.788356	0.363666	0.031736	0.119189	0.048529	0.452559	0.066485	1.23E-05	0.000383	UP
ENSMUS(	Ckm	creatine k	GO:00081	metabolic	K00933	creatine k	mmu0110	Metabolic	7:1940477	0	0	0	7	15	4	0	0	0	0.097729	0.174799	0.056944	0	0.109824	1.27E-05	0.000393	DOWN
ENSMUS(	Gbp6	guanylate	GO:00023	immune s	-	-	-	-	5:1052707	40	88	45	143	170	162	0.275132	0.483401	0.342087	0.75883	0.752976	0.876601	0.366873	0.796136	1.35E-05	0.000417	DOWN
ENSMUS(	Nkx2-3	NK2 hom	GO:00325	developm	-	-	-	-	19:436123	0	0	0	6	18	4	0	0	0	0.133819	0.335105	0.09097	0	0.186631	1.39E-05	0.000429	DOWN
ENSMUS(	Fam83c	family wit	GO:00055	protein bi	-	-	-	-	2:1558275	0	0	0	6	24	4	0	0	0	0.071154	0.237574	0.048371	0	0.119033	1.46E-05	0.000452	DOWN
ENSMUS(	Lrp3	low densit	GO:00160	membran	-	-	-	-	7:3520002	872	977	710	1603	1846	1776	7.507428	6.717613	6.755848	10.64728	10.23437	12.02897	6.993629	10.9702	1.54E-05	0.000475	-
ENSMUS(	Gramd1b	GRAM do	GO:00160	membran	-	-	-	-	9:4029323	5348	6526	2318	2711	2722	3487	15.79542	15.39334	7.566588	6.177315	5.177053	8.102194	12.91845	6.48552	1.58E-05	0.000487	-
ENSMUS(	Myh14	myosin, he	GO:00325	developm	K10352	myosin he	mmu0481	Regulatior	7:4460580	883	1038	745	1613	2361	1593	4.901304	4.601442	4.570392	6.907414	8.4392	6.956285	4.691046	7.4343	1.59E-05	0.000487	-
ENSMUS(	Slc35g1	solute car	GO:00511	localizatio	-	-	-	-	19:383959	34	46	27	84	153	89	0.5981	0.646237	0.524924	1.139988	1.733142	1.231661	0.589754	1.368264	1.59E-05	0.000489	DOWN
ENSMUS(	Ifi202b	interferon	GO:00081	metabolic	K12971|K1	interferon	mmu0462	Cytosolic l	1:1739625	0	5	1	13	35	14	0	0.143667	0.039768	0.36085	0.810923	0.396271	0.061145	0.522681	1.66E-05	0.000507	DOWN
ENSMUS(	Mab21l2	mab-21-l	GO:00325	developm	-	-	-	-	3:8654558	2	6	0	15	32	16	0.023111	0.05538	0	0.133747	0.238161	0.145478	0.026164	0.172462	1.69E-05	0.000518	DOWN
ENSMUS(	Plekhg6	pleckstrin	GO:00987	molecular	-	-	-	-	6:1253626	3	3	2	17	25	14	0.055523	0.04435	0.040914	0.242744	0.297955	0.203841	0.046929	0.24818	1.83E-05	0.00056	DOWN
ENSMUS(	Coro2a	coronin, a	GO:00329	macromo	-	-	-	-	4:4653693	449	499	329	840	1088	810	2.841823	2.522302	2.301411	4.101669	4.434399	4.033172	2.555179	4.189747	1.90E-05	0.00058	-
ENSMUS(	Usp43	ubiquitin s	GO:00081	metabolic	-	-	-	-	11:678545	11	18	12	41	65	41	0.102638	0.134126	0.123743	0.295127	0.390544	0.30095	0.120169	0.328874	1.94E-05	0.000589	DOWN
ENSMUS(	Cd68	CD68 anti	GO:00508	response	K06501	CD68 anti	mmu0414	Lysosome	11:696642	192	380	160	146	182	153	5.671588	8.964618	5.223592	3.32725	3.462007	3.555532	6.619932	3.448263	2.00E-05	0.000606	-
ENSMUS(	Golm1	golgi mer	GO:00081	metabolic	-	-	-	-	13:596346	777	1040	754	1195	3642	2545	6.504269	6.952755	6.975829	7.717484	19.63235	16.76009	6.810951	14.70331	2.00E-05	0.000606	DOWN
ENSMUS(	Pnma2	paraneop	GO:00056	cell|intrace	-	-	-	-	14:669111	237	250	200	270	1589	1282	1.467774	1.236506	1.368946	1.290042	6.337077	6.246109	1.357742	4.624409	2.02E-05	0.00061	DOWN
ENSMUS(	Kif12	kinesin fa	GO:00990	supramole	-	-	-	-	4:6316563	0	0	0	5	15	5	0	0	0	0.044817	0.112231	0.045703	0	0.067584	2.10E-05	0.000633	DOWN
ENSMUS(	Acnat1	acyl-coen	GO:00081	metabolic	-	-	-	-	4:4944710	0	3	3	15	26	11	0	0.036752	0.050857	0.17749	0.256786	0.132727	0.029203	0.189001	2.14E-05	0.000644	DOWN
ENSMUS(	2010003K	RIKEN cDI	-	-	-	-	-	-	19:449679	0	0	0	3	17	7	0	0	0	0.121319	0.573762	0.288635	0	0.327905	2.24E-05	0.000673	DOWN
ENSMUS(	Cd3e	CD3 antig	GO:00325	developm	K06451	T-cell surf	mmu0516	HTLV-I inf	9:4499874	0	0	0	5	10	7	0	0	0	0.0525	0.087646	0.074958	0	0.071701	2.29E-05	0.000687	DOWN
ENSMUS(	Mptx2	mucosal p	GO:00055	extracellul	-	-	-	-	1:1732743	2	0	0	10	19	8	0.15377	0	0	0.593292	0.940873	0.483949	0.051257	0.672705	2.34E-05	0.000702	DOWN
ENSMUS(	Pycard	PYD and (	GO:00081	metabolic	K12799	apoptosis	mmu0421	Necroptos	7:1279897	50	72	47	122	197	130	0.720711	0.828829	0.748745	1.356675	1.82856	1.474153	0.766095	1.55313	2.36E-05	0.000705	DOWN
ENSMUS(	Creb3l4	cAMP resp	GO:00325	developm	K09048	cyclic AMI	mmu0415	PI3K-Akt s	3:9023750	0	4	3	15	18	20	0	0.096028	0.099685	0.347901	0.34847	0.473012	0.065238	0.389794	2.49E-05	0.000744	DOWN
ENSMUS(	Hoxa13	homeobo	GO:00081	metabolic	-	-	-	-	6:5225769	0	0	0	6	13	4	0	0	0	0.048304	0.087361	0.032837	0	0.056167	2.55E-05	0.00076	DOWN
ENSMUS(	Mtfr1l	mitochon	GO:00081	metabolic	-	-	-	-	4:1345255	541	705	475	441	554	466	3.475635	3.617193	3.372699	2.185776	2.29193	2.355231	3.488509	2.277646	2.56E-05	0.000762	-
ENSMUS(	Ffar2	free fatty	GO:00325	developm	K04328	free fatty	mmu0402	cAMP sigr	7:3081834	0	0	0	4	13	6	0	0	0	0.026491	0.071872	0.040525	0	0.046296	2.57E-05	0.000764	DOWN
ENSMUS(	Fgfr4	fibroblast	GO:00048	signal trar	K05095	fibroblast	mmu0520	Pathways	13:551526	0	0	0	7	16	3	0	0	0	0.08229	0.156996	0.035958	0	0.091748	2.58E-05	0.000765	DOWN
ENSMUS(	Cwc22	CWC22 sp	GO:00081	metabolic	-	-	-	-	2:7788115	328	529	349	677	1064	964	1.960363	2.525015	2.305336	3.121629	4.095048	4.532629	2.263572	3.916435	2.59E-05	0.000768	-
ENSMUS(	Cbll1	Casitas B-	GO:00081	metabolic	-	-	-	-	12:314848	236	662	519	60	351	91	1.521455	3.408407	3.697957	0.298421	1.457167	0.461528	2.87594	0.739039	2.60E-05	0.00077	UP
ENSMUS(	Slc12a8	solute car	GO:00511	localizatio	-	-	-	-	16:335173	9	18	12	40	58	41	0.080209	0.128115	0.118198	0.275033	0.332867	0.287464	0.108841	0.298455	2.61E-05	0.000771	DOWN
ENSMUS(	S100a10	S100 calci	GO:00325	developm	-	-	-	-	3:9355508	140	216	142	326	527	332	5.273379	6.497695	5.911455	9.473456	12.78278	9.838045	5.894176	10.6981	2.73E-05	0.000803	-
ENSMUS(	Trim25	tripartite r	GO:00081	metabolic	K10652	tripartite r	mmu0516	Influenza	11:889993	147	224	148	365	570	321	1.542988	1.877751	1.716931	2.955752	3.852775	2.650702	1.712557	3.153076	2.77E-05	0.000816	-
ENSMUS(	Smim22	small inte	GO:00160	membran	-	-	-	-	16:500728	0	2	0	10	11	9	0	0.062848	0	0.303527	0.278667	0.278543	0.020949	0.286913	2.84E-05	0.000835	DOWN
ENSMUS(	Slc37a1	solute car	GO:00511	localizatio	-	-	-	-	17:312954	72	69	57	151	284	147	1.212409	0.927926	1.060807	1.961662	3.07956	1.947344	1.067047	2.329522	2.86E-05	0.00084	DOWN
ENSMUS(	Tep1	telomeras	GO:00081	metabolic	-	-	-	-	14:508240	77	127	83	203	342	200	0.415874	0.547801	0.495445	0.845863	1.18947	0.849794	0.486374	0.961709	2.91E-05	0.000851	-
ENSMUS(	Mfsd6l	major faci	GO:00160	membran	-	-	-	-	11:685561	0	0	0	3	12	8	0	0	0	0.06861	0.229049	0.186532	0	0.161397	2.91E-05	0.000851	DOWN
ENSMUS(	Tspan5	tetraspani	GO:00081	metabolic	-	-	-	-	3:1387421	841	3243	2280	1104	1418	1039	4.201083	12.93774	12.58772	4.254659	4.561381	4.083119	9.908847	4.29972	2.95E-05	0.00086	UP
ENSMUS(	B2m	beta-2 mi	GO:00325	developm	K08055	beta-2-m	mmu0461	Antigen p	2:1221476	1519	2016	1711	3132	5043	3069	107.8456	114.3092	134.2585	171.5525	230.5624	171.4165	118.8045	191.1771	3.06E-05	0.000891	-
ENSMUS(	Rab17	RAB17, m	GO:00325	developm	-	-	-	-	1:9095813	0	0	0	2	12	11	0	0	0	0.023562	0.11799	0.13213	0	0.091227	3.07E-05	0.000892	DOWN
ENSMUS(	Myzap	myocardia	GO:00508	response	-	-	-	-	9:7150434	5	18	6	36	52	32	0.047705	0.137165	0.063279	0.26501	0.319514	0.240212	0.082716	0.274912	3.10E-05	0.0009	DOWN
ENSMUS(	Pou2af1	POU dom	GO:00081	metabolic	-	-	-	-	9:5121370	0	3	3	12	28	13	0	0.037544	0.051953	0.145048	0.282506	0.160233	0.029832	0.195929	3.15E-05	0.000912	DOWN
ENSMUS(	Serpinb1a	serine (or	GO:00081	metabolic	K13963	serpin B[-	mmu0514	Amoebias	13:328420	55	102	77	164	352	185	1.229657	1.82125	1.902657	2.828762	5.06779	3.253901	1.651188	3.716818	3.25E-05	0.000939	DOWN
ENSMUS(	Igf2	insulin-lik	GO:00325	developm	K13769	insulin-lik	mmu0520	Pathways	7:1426507	777	1132	593	512	733	724	3.60667	4.19641	3.042196	1.833522	2.19101	2.643838	3.615092	2.22279	3.49E-05	0.001009	-
ENSMUS(	Naip2	NLR famil	GO:00081	metabolic	K12807	baculovira	mmu0462	NOD-like	13:100144	34	47	32	78	155	102	0.385373	0.425443	0.400863	0.682054	1.131315	0.909509	0.403893	0.907626	3.52E-05	0.001015	DOWN
ENSMUS(	Acpp	acid phos	GO:00081	metabolic	-	-	-	-	9:1042882	0	2	4	20	31	10	0	0.011244	0.031118	0.1086	0.140507	0.055371	0.014121	0.101492	3.53E-05	0.001017	DOWN
ENSMUS(	Ugt2b38	UDP gluc	GO:00081	metabolic	K00699	glucuronc	mmu0110	Metabolic	5:8740994	0	0	0	6	12	4	0	0	0	0.149458	0.24952	0.101601	0	0.16686	3.57E-05	0.001026	DOWN
ENSMUS(	Apoh	apolipopr	GO:00325	developm	K17305	beta-2-gl	mmu0497	Cholester	11:108343	0	0	0	13	4	6	0	0	0	0.277716	0.071329	0.13071	0	0.159918	3.59E-05	0.001029	DOWN
ENSMUS(	Rap1gap2	RAP1 GTF	GO:00325	developm	-	-	-	-	11:743833	3041	3793	2573	1908	2067	3120	11.84925	11.80329	11.08054	5.735657	5.186441	9.564007	11.57769	6.828702	3.65E-05	0.001047	-
ENSMUS(	Nek2	NIMA (ne	GO:00325	developm	-	-	-	-	1:1918214	14	28	14	54	62	57	0.156735	0.250343	0.173221	0.466396	0.446965	0.502013	0.193433	0.471791	4.43E-05	0.001266	DOWN
ENSMUS(	Zfp639	zinc finge	GO:00081	metabolic	-	-	-	-	3:3251025	194	265	149	502	500	372	1.205012	1.314564	1.022876	2.405602	1.999931	1.817792	1.180817	2.074442	4.45E-05	0.001268	-
ENSMUS(	Hoxd10	homeobo	GO:00325	developm	K09295	homrobox	mmu0520	Proteogly	2:7469192	0	0	0	4	9	7	0	0	0	0.103745	0.194863	0.185165	0	0.161258	4.45E-05	0.001268	DOWN
ENSMUS(	Elf4	E74-like fa	GO:00325	developm	-	-	-	-	X:4841104	33	67	32	99	181	107	0.184636	0.299379	0.197878	0.427333	0.652127	0.47097	0.227298	0.51681	4.49E-05	0.001277	DOWN
ENSMUS(	Perp	PERP, TP5	GO:00325	developm	K10136	TP53 apo	mmu0411	p53 signa	10:188450	77	132	60	176	337	214	2.408535	3.297488	2.074255	4.247233	6.788096	5.26608	2.593426	5.433803	4.51E-05	0.001281	DOWN
ENSMUS(	Pdlim3	PDZ and l	GO:00325	developm	-	-	-	-	8:4588546	12	33	13	53	105	54	0.11051	0.242711	0.132317	0.376563	0.622692	0.391229	0.161846	0.463494	4.71E-05	0.001337	DOWN
ENSMUS(	Lima1	LIM doma	GO:00099	cellular pr	-	-	-	-	15:997784	322	461	283	645	1048	661	2.442024	2.792159	2.372068	3.773843	5.118108	3.943716	2.535417	4.278555	4.78E-05	0.001354	-
ENSMUS(	Zc3h12a	zinc finge	GO:00325	developm	-	-	-	-	4:1251184	16	18	10	39	65	47	0.317906	0.285623	0.219591	0.597832	0.831669	0.734668	0.274373	0.72139	4.82E-05	0.001363	DOWN
ENSMUS(	Ly6g	lymphocy	GO:00160	membran	-	-	-	-	15:751552	0	2	0	8	17	6	0	0.076383	0	0.295106	0.52344	0.225698	0.025461	0.348081	5.42E-05	0.001531	DOWN
ENSMUS(	Lrrc8e	leucine ric	GO:00511	localizatio	-	-	-	-	8:4226827	0	0	0	3	15	6	0	0	0	0.033732	0.140757	0.068787	0	0.081092	5.56E-05	0.001568	DOWN
ENSMUS(	Ngp	neutrophi	GO:00325	developm	-	-	-	-	9:1104197	8	38	16	6	0	5	0.399749	1.516348	0.883571	0.231281	0	0.19654	0.933222	0.142607	5.67E-05	0.001595	UP
ENSMUS(	Myo1d	myosin ID	GO:00325	developm	-	-	-	-	11:804821	314	488	295	677	996	676	2.784235	3.455747	2.89098	4.631217	5.687092	4.715564	3.043654	5.011291	5.73E-05	0.001609	-
ENSMUS(	Psmb8	proteasor	GO:00325	developm	K02740	20S prote	mmu0305	Proteason	17:341977	37	69	59	127	225	128	0.587104	0.874392	1.034683	1.554687	2.29903	1.597824	0.832059	1.81718	5.83E-05	0.001636	DOWN
ENSMUS(	Tnni3k	TNNI3 int	GO:00048	signal trar	-	-	-	-	3:1547862	3	3	3	18	17	18	0.045884	0.036651	0.050717	0.212406	0.167443	0.216583	0.044417	0.198811	6.06E-05	0.001697	DOWN
ENSMUS(	Adamts19	a disinteg	GO:00081	metabolic	-	-	-	-	18:588367	0	1	0	7	11	6	0	0.010452	0	0.070668	0.09269	0.061769	0.003484	0.075043	6.65E-05	0.00186	DOWN
ENSMUS(	Mmp7	matrix me	GO:00325	developm	K01397	matrix me	mmu0431	Wnt signa	9:7692090	0	0	0	5	15	3	0	0	0	0.220518	0.552221	0.13491	0	0.30255	6.70E-05	0.001869	DOWN
ENSMUS(	Eps8l2	EPS8-like	GO:00987	molecular	-	-	-	-	7:1413388	72	164	67	199	406	245	0.550902	1.002143	0.566577	1.174691	2.000421	1.474745	0.706541	1.549953	7.07E-05	0.001972	DOWN
ENSMUS(	Gm4070	predicted	GO:00056	cell|intrace	-	-	-	-	7:1058951	188	352	122	437	680	524	1.160308	1.735019	0.832191	2.080785	2.70258	2.544237	1.242506	2.442534	7.09E-05	0.001973	-
ENSMUS(	Myl7	myosin, lig	GO:00452	synapse|su	K12754	myosin re	mmu0481	Regulatior	11:589663	0	0	0	6	9	4	0	0	0	0.479026	0.599782	0.32564	0	0.468149	7.47E-05	0.002074	DOWN
ENSMUS(	Tcf21	transcripti	GO:00325	developm	-	-	-	-	10:228172	0	3	1	9	21	11	0	0.047892	0.022091	0.138776	0.27027	0.172958	0.023328	0.194001	7.50E-05	0.002079	DOWN
ENSMUS(	Ifi44	interferon	GO:00056	cell|intrace	-	-	-	-	3:1517309	28	34	11	71	117	59	0.227432	0.220559	0.098748	0.444923	0.611985	0.377021	0.182246	0.477976	7.61E-05	0.002107	DOWN
ENSMUS(	Steap4	STEAP far	GO:00325	developm	-	-	-	-	5:7960457	9	15	4	31	56	28	0.174171	0.23184	0.085562	0.462848	0.697892	0.426305	0.163858	0.529015	8.39E-05	0.002319	DOWN
ENSMUS(	Nacc2	nucleus ac	GO:00081	metabolic	-	-	-	-	2:2605553	272	523	420	2184	519	2110	1.677386	2.575799	2.862601	10.39076	2.061041	10.23664	2.371929	7.562814	8.86E-05	0.002446	DOWN
ENSMUS(	Itgb6	integrin b	GO:00511	localizatio	K06589	integrin b	mmu0415	PI3K-Akt s	2:6059829	0	6	0	19	43	9	0	0.032657	0	0.099898	0.188715	0.048253	0.010886	0.112289	9.00E-05	0.002481	DOWN
ENSMUS(	Cd4	CD4 antig	GO:00325	developm	K06454	T-cell surf	mmu0451	Cell adhes	6:1248646	5	2	0	13	27	13	0.061557	0.019669	0	0.123485	0.214078	0.125918	0.027076	0.154494	9.16E-05	0.00252	DOWN
ENSMUS(	Creb3l1	cAMP resp	GO:00081	metabolic	K09048	cyclic AMI	mmu0415	PI3K-Akt s	2:9198232	98	112	79	200	351	187	2.298774	2.098132	2.048071	3.619343	5.301892	3.450822	2.148326	4.124019	0.0001	0.002752	-
ENSMUS(	Rdh16	retinol de	GO:00081	metabolic	K11154	retinol de	mmu0110	Metabolic	10:127801	0	2	1	10	22	7	0	0.011554	0.007994	0.055801	0.102467	0.039832	0.006516	0.066033	0.0001	0.002752	DOWN
ENSMUS(	Crcp	calcitonin	GO:00048	signal trar	-	-	-	-	5:1300292	444	531	351	341	442	351	6.750448	6.447485	5.89798	3.999766	4.327393	4.198248	6.365305	4.175135	0.000101	0.002757	-
ENSMUS(	Rsad2	radical S-	GO:00325	developm	K15045	radical S-	mmu0516	Influenza	12:264427	41	70	38	105	175	111	0.326639	0.445378	0.33459	0.645369	0.897802	0.695697	0.368869	0.74629	0.000104	0.002845	DOWN
ENSMUS(	BC025446	cDNA seq	-	-	-	-	-	-	15:752163	0	0	0	5	13	3	0	0	0	0.111565	0.242137	0.068254	0	0.140652	0.000104	0.00285	DOWN
ENSMUS(	4930539E	RIKEN cDI	GO:00099	cellular pr	-	-	-	-	17:288963	76	96	67	175	262	153	0.976113	0.984689	0.951049	1.734011	2.166904	1.545916	0.970617	1.81561	0.000105	0.002864	-
ENSMUS(	Clic5	chloride ir	GO:00325	developm	-	-	-	-	17:441885	147	200	155	325	469	321	1.529057	1.661429	1.781896	2.608068	3.141469	2.626769	1.657461	2.792102	0.000106	0.002888	-
ENSMUS(	Baiap2l2	BAI1-asso	GO:00508	response	-	-	-	-	15:792581	12	20	10	41	68	36	0.153024	0.203688	0.140936	0.403365	0.558407	0.361163	0.165883	0.440978	0.000108	0.002931	DOWN
ENSMUS(	Galnt3	polypepti	GO:00081	metabolic	K00710	polypepti	mmu0110	Metabolic	2:6608276	72	62	63	147	217	150	0.842829	0.579624	0.81507	1.327557	1.635764	1.381365	0.745841	1.448229	0.00011	0.002994	-
ENSMUS(	Kcnd3	potassium	GO:00511	localizatio	-	-	-	-	3:1054523	2122	550	704	515	568	548	10.82417	2.240567	3.968885	2.026686	1.865745	2.199077	5.677873	2.030503	0.00012	0.003246	UP
ENSMUS(	S100a8	S100 calci	GO:00325	developm	-	-	mmu0465	IL-17 sign	3:9066897	7	13	9	3	4	0	0.434366	0.644223	0.61719	0.143635	0.159838	0	0.56526	0.101157	0.00012	0.003246	UP
ENSMUS(	Gjb3	gap juncti	GO:00325	developm	-	-	-	-	4:1273252	3	7	3	17	26	19	0.093169	0.173623	0.102981	0.40733	0.519972	0.464213	0.123258	0.463838	0.000121	0.003258	DOWN
ENSMUS(	Gm45736	predicted	GO:00056	cell|intrace	-	-	-	-	19:873584	0	0	0	0	359	0	0	0	0	0	10.44037	0	0	3.480125	0.000123	0.003321	DOWN
ENSMUS(	S100a6	S100 calci	GO:00511	localizatio	-	-	-	-	3:9061288	38	65	45	115	165	102	1.202172	1.642269	1.573424	2.806807	3.361438	2.538613	1.472622	2.902286	0.000123	0.003322	-
ENSMUS(	Tnik	TRAF2 an	GO:00325	developm	-	-	-	-	3:2826321	5165	5759	4719	3756	6518	3805	12.49317	11.12488	12.61535	7.009035	10.15246	7.240491	12.0778	8.133996	0.000127	0.003405	-
ENSMUS(	Mfrp	membran	GO:00325	developm	-	-	-	-	9:4410172	138	342	178	48	141	148	1.009827	1.998668	1.439581	0.270979	0.664421	0.852003	1.482692	0.595801	0.00013	0.003487	UP
ENSMUS(	Irs3	insulin rec	GO:00048	signal trar	K17445	insulin rec	mmu0402	cGMP-PK	5:1376430	0	0	0	10	6	3	0	0	0	0.130203	0.065202	0.039824	0	0.07841	0.000133	0.003573	DOWN
ENSMUS(	Palld	palladin, c	GO:00325	developm	-	-	-	-	8:6151143	154	94	45	260	367	288	0.793897	0.387008	0.256392	1.034068	1.218331	1.168015	0.479099	1.140138	0.000135	0.003624	DOWN
ENSMUS(	Frrs1	ferric-che	GO:00081	metabolic	-	-	-	-	3:1168594	16	31	15	73	51	70	0.11422	0.176737	0.118349	0.402048	0.234451	0.393126	0.136435	0.343208	0.000136	0.00363	DOWN
ENSMUS(	Zfp710	zinc finge	GO:00081	metabolic	-	-	-	-	7:8002481	447	529	415	430	394	356	2.397696	2.266148	2.460264	1.779449	1.360935	1.50227	2.374702	1.547551	0.000137	0.003659	-
ENSMUS(	Fgl2	fibrinoger	GO:00081	metabolic	-	-	-	-	5:2137264	54	92	54	126	242	154	0.870411	1.184314	0.961992	1.566875	2.511905	1.952832	1.005572	2.010538	0.000141	0.003747	-
ENSMUS(	Aldh1b1	aldehyde	GO:00081	metabolic	K00128	aldehyde	mmu0110	Metabolic	4:4579902	165	155	139	310	561	288	3.672834	2.755474	3.419641	5.323651	8.041458	5.043372	3.28265	6.13616	0.000143	0.003791	-
ENSMUS(	Bdkrb2	bradykini	GO:00048	signal trar	K03916	bradykini	mmu0520	Pathways	12:105563	10	12	7	24	56	34	0.16102	0.15431	0.12458	0.298141	0.580656	0.430685	0.146637	0.436494	0.000149	0.003955	DOWN
ENSMUS(	Mpeg1	macropha	GO:00160	membran	-	-	-	-	19:124607	625	1211	582	601	757	602	8.80307	13.62211	9.05992	6.530694	6.866018	6.670552	10.49503	6.689088	0.000151	0.004016	-
ENSMUS(	Lilr4b	leukocyte	GO:00055	extracellul	-	-	-	-	10:514806	26	35	26	11	22	14	0.073107	0.078596	0.080798	0.023863	0.039835	0.030968	0.0775	0.031555	0.000152	0.004033	UP
ENSMUS(	Top2a	topoisom	GO:00325	developm	K03164	DNA topo	mmu0152	Platinum c	11:989929	40	84	47	126	285	116	0.227534	0.381597	0.29548	0.552946	1.043953	0.519098	0.301537	0.705332	0.000155	0.004107	DOWN
ENSMUS(	Mme	membran	GO:00325	developm	K01389	neprilysin[	mmu0501	Alzheimer	3:6324153	126	170	117	82	98	119	0.527626	0.568526	0.541488	0.264911	0.264264	0.392026	0.54588	0.307067	0.000156	0.004125	-

ENSMUSC	Sectm1a	secreted a	GO:00023	immune s	-	-	-	-	11:121067	0	0	0	3	14	4	0	0	0	0.048586	0.189227	0.066046	0	0.101286	0.000158	0.004168	DOWN
ENSMUSC	Rab11fip1	RAB11 far	GO:00511	localizatio	K12484	Rab11 fan	mmu0414	Endocytos	8:2713877	94	161	81	224	426	213	0.407256	0.557074	0.387859	0.748719	1.188514	0.725991	0.45073	0.887741	0.000162	0.004269	-
ENSMUSC	Vsig10	V-set and	GO:00160	membran	-	-	-	-	5:1173190	26	51	27	79	133	77	0.171327	0.268389	0.196634	0.401612	0.564361	0.399165	0.212117	0.455046	0.000163	0.004276	DOWN
ENSMUSC	Parp12	poly (ADP	GO:00038	catalytic a	-	-	-	-	6:3908641	205	258	157	412	517	380	2.952106	2.967175	2.498751	4.577252	4.794272	4.304988	2.80601	4.558837	0.000168	0.004419	-
ENSMUSC	Ccl11	chemokin	GO:00325	developm	K16597	C-C motif	mmu0406	Cytokine-	11:820578	0	5	0	11	29	9	0	0.225326	0	0.478915	1.053821	0.399537	0.075109	0.644091	0.000176	0.004601	DOWN
ENSMUSC	Tmem156	transmem	GO:00160	membran	-	-	-	-	5:6505434	0	0	0	12	3	5	0	0	0	0.121377	0.025332	0.051572	0	0.066094	0.000176	0.004601	DOWN
ENSMUSC	Cd300lb	CD300 mo	GO:00160	membran	-	-	-	-	11:114922	8	8	6	2	2	0	0.13949	0.111397	0.115626	0.026906	0.02245	0	0.122171	0.016452	0.000176	0.004601	UP
ENSMUSC	Psd4	pleckstrin	GO:00987	molecular	K12494	PH and SE	mmu0414	Endocytos	2:2436758	17	19	10	37	107	42	0.059762	0.053341	0.038851	0.100346	0.242219	0.116153	0.050651	0.152906	0.000177	0.00461	DOWN
ENSMUSC	Bicdl2	BICD fami	GO:00055	protein bi	-	-	-	-	17:236605	0	2	0	10	11	5	0	0.050988	0	0.246249	0.22608	0.125547	0.016996	0.199292	0.000177	0.00461	DOWN
ENSMUSC	Upk3b	uroplakin	GO:00160	membran	-	-	-	-	5:1360384	0	0	0	4	11	4	0	0	0	0.053371	0.122519	0.054427	0	0.076773	0.000181	0.004705	DOWN
ENSMUSC	Hoxb7	homeobo	GO:00325	developm	-	-	-	-	11:962866	2	2	0	9	17	10	0.073506	0.058724	0	0.255245	0.402428	0.289189	0.044077	0.315621	0.000184	0.004774	DOWN
ENSMUSC	Cnih4	cornichon	GO:00511	localizatio	-	-	-	-	1:1811446	659	754	244	397	347	308	9.284106	8.483435	3.799183	4.314943	3.148026	3.413626	7.188908	3.625532	0.000185	0.004786	-
ENSMUSC	Mcpt2	mast cell	GO:00081	metabolic	-	-	-	-	14:560420	0	0	0	2	7	10	0	0	0	0.088807	0.259389	0.452727	0	0.266974	0.000189	0.004887	DOWN
ENSMUSC	Arhgap26	Rho GTPa	GO:00987	molecular	-	-	-	-	18:386015	873	1131	932	911	854	842	2.583923	2.67346	3.048793	2.080243	1.627712	1.960596	2.768726	1.889517	0.00019	0.004912	-
ENSMUSC	Lrrc26	leucine ric	GO:00511	localizatio	-	-	-	-	2:2528991	0	0	1	10	14	4	0	0	0.056712	0.39586	0.462573	0.161452	0.018904	0.339962	0.000192	0.004959	DOWN
ENSMUSC	Srpx	sushi-rep	GO:00081	metabolic	-	-	-	-	X:1003797	2	2	0	10	21	8	0.029888	0.023878	0	0.115318	0.202123	0.094065	0.017922	0.137169	0.000192	0.004959	DOWN
ENSMUSC	Casz1	castor zin	GO:00325	developm	-	-	-	-	4:1488044	17	40	17	71	141	50	0.072924	0.137032	0.080596	0.234965	0.389485	0.168731	0.096851	0.264394	0.000197	0.005071	DOWN
ENSMUSC	Aspg	asparagin	GO:00081	metabolic	-	-	-	-	12:112106	24	44	24	65	103	80	0.313325	0.458745	0.34628	0.654664	0.865903	0.821627	0.372783	0.780731	0.000199	0.005107	DOWN
ENSMUSC	Mylk	myosin, lig	GO:00325	developm	K00907	myosin-lig	mmu0481	Regulation	16:347452	488	839	513	1007	1683	1158	2.885843	3.962425	3.352874	4.594235	6.409026	5.387321	3.400381	5.463528	0.000204	0.005236	-
ENSMUSC	Bak1	BCL2-anta	GO:00325	developm	K14021	Bcl-2 hom	mmu0520	Pathways	17:270198	149	217	144	289	507	348	2.069531	2.407082	2.210519	3.096806	4.534697	3.802555	2.229044	3.811353	0.000205	0.005261	-
ENSMUSC	Stra6l	STRA6-lik	GO:00511	localizatio	-	-	-	-	4:4584866	5	9	0	19	45	18	0.02623	0.037711	0	0.076902	0.152029	0.074291	0.021314	0.101074	0.000206	0.005272	DOWN
ENSMUSC	C2	compleme	GO:00081	metabolic	K01332	compleme	mmu0532	Systemic l	17:348626	11	13	6	33	41	29	0.119026	0.112337	0.071756	0.275476	0.285677	0.246852	0.10104	0.269335	0.000207	0.005296	DOWN
ENSMUSC	Gal3st2	galactose	GO:00081	metabolic	-	-	-	-	1:9386130	0	0	0	4	7	6	0	0	0	0.054185	0.079152	0.082892	0	0.072076	0.000209	0.005341	DOWN
ENSMUSC	Sgpp2	sphingosi	GO:00081	metabolic	K04717	sphingosi	mmu0407	Sphingolip	1:7831034	205	238	154	376	527	372	3.127668	2.899941	2.596779	4.425724	5.177634	4.464991	2.874796	4.68945	0.000215	0.005487	-
ENSMUSC	Mzb1	marginal	GO:00081	metabolic	-	-	-	-	18:356472	0	2	0	4	20	8	0	0.10778	0	0.208178	0.86891	0.424592	0.035927	0.50056	0.000221	0.005601	DOWN
ENSMUSC	Gsta1	glutathion	GO:00081	metabolic	K00799	glutathion	mmu0520	Pathways	9:7823065	0	0	0	4	6	7	0	0	0	0.163685	0.204953	0.292146	0	0.220261	0.00022	0.005601	DOWN
ENSMUSC	Rdh9	retinol del	GO:00081	metabolic	K11154	retinol del	mmu0110	Metabolic	10:127776	1	0	0	17	4	10	0.013809	0	0	0.181056	0.03556	0.108601	0.004603	0.108406	0.000222	0.005622	DOWN
ENSMUSC	Gm8909	predicted	GO:00023	immune s	K06751	major hist	mmu0516	Human pa	17:361644	0	0	0	6	3	9	0	0	0	0.109375	0.045654	0.167299	0	0.107443	0.000223	0.005638	DOWN
ENSMUSC	Dhrs11	dehydrog	GO:00081	metabolic	-	-	-	-	11:848208	57	90	45	120	215	147	1.656491	2.088834	1.445364	2.69048	4.023553	3.360813	1.730229	3.358282	0.000226	0.005712	-
ENSMUSC	Melk	maternal	GO:00325	developm	-	-	-	-	4:4430087	6	7	0	17	29	20	0.112031	0.104386	0	0.244897	0.348696	0.293788	0.072139	0.295793	0.000228	0.005746	DOWN
ENSMUSC	Fkbp1a	FK506 bin	GO:00325	developm	-	-	-	-	2:1515424	8367	4605	7031	5009	5914	4502	68.95306	30.30814	64.03946	31.84675	31.38483	29.18772	54.43355	30.80643	0.00023	0.005803	-
ENSMUSC	Casp8	caspase 8	GO:00325	developm	K04398	caspase 8	mmu0520	Pathways	1:5879537	36	84	40	114	236	113	0.591523	1.102278	0.726392	1.445122	2.497093	1.460686	0.806731	1.800967	0.000233	0.005871	DOWN
ENSMUSC	Crip2	cysteine ri	GO:00325	developm	-	-	-	-	12:113140	292	373	270	817	579	643	5.822657	5.940095	5.950442	12.5687	7.434834	10.08696	5.904398	10.03016	0.000238	0.005983	-
ENSMUSC	Dnah8	dynein, ax	GO:00990	supramole	K10408	dynein he	mmu0501	Huntingto	17:306243	8	10	8	31	52	21	0.033497	0.033437	0.037018	0.100136	0.140202	0.069172	0.034651	0.10317	0.000239	0.005983	DOWN
ENSMUSC	Mdfic	MyoD fan	GO:00081	metabolic	-	-	-	-	6:1572066	80	127	96	52	98	66	0.36768	0.466157	0.487638	0.184381	0.290044	0.238636	0.440492	0.237687	0.000238	0.005983	-
ENSMUSC	Plin2	perilipin 2	GO:00511	localizatio	K17284	perilipin-2	mmu0332	PPAR sign	4:8664838	126	210	121	116	130	104	1.46847	1.954608	1.558576	1.042995	0.975644	0.953543	1.660551	0.990727	0.000245	0.006142	-
ENSMUSC	Espn	espin [Sou	GO:00452	synapse|su	-	-	-	-	4:1521203	35	42	29	95	125	66	0.14984	0.143602	0.137217	0.313773	0.344609	0.222288	0.143553	0.293557	0.000247	0.006154	DOWN
ENSMUSC	Crb3	crumbs fa	GO:00325	developm	K06090	crumbs 3[	mmu0516	Human pa	17:570591	5	14	6	20	45	36	0.078494	0.175543	0.10412	0.242248	0.454954	0.444651	0.119385	0.380617	0.000247	0.006154	DOWN
ENSMUSC	Mst1r	macropha	GO:00048	signal trar	-	-	-	-	9:1079068	21	38	24	57	113	71	0.16456	0.237805	0.207848	0.344586	0.570201	0.437686	0.203404	0.450824	0.00025	0.006228	DOWN
ENSMUSC	Snrk	SNF relate	GO:00081	metabolic	-	-	-	-	9:1221172	414	590	426	1308	758	1263	2.810233	3.198455	3.195939	6.849838	3.313341	6.744603	3.068209	5.635927	0.000254	0.006316	-
ENSMUSC	E2f7	E2F transc	GO:00325	developm	-	-	-	-	10:110745	0	2	3	10	12	15	0	0.007598	0.01577	0.036694	0.036754	0.056124	0.007789	0.04319	0.00026	0.006472	DOWN
ENSMUSC	Hoxd11	homeobo	GO:00081	metabolic	-	-	-	-	2:7467955	0	0	0	3	12	4	0	0	0	0.031562	0.105369	0.042905	0	0.059945	0.000265	0.006569	DOWN
ENSMUSC	Abhd17a	abhydrola	GO:00081	metabolic	-	-	-	-	10:805836	854	1160	905	868	989	841	16.31527	17.69865	19.10874	12.79341	12.16712	12.63989	17.70756	12.53347	0.000272	0.006736	-
ENSMUSC	Itgb4	integrin b	GO:00325	developm	K06525	integrin b	mmu0415	PI3K-Akt s	11:115974	104	213	112	269	430	291	0.680897	1.113715	0.810422	1.358724	1.812889	1.498826	0.868345	1.556813	0.000272	0.00674	-
ENSMUSC	B3galt1	UDP-Gal:I	GO:00081	metabolic	K07819	beta-1,3-	mmu0110	Metabolic	2:6756587	198	204	166	824	234	934	1.336596	1.099792	1.238484	4.291332	1.017196	4.960123	1.224957	3.422884	0.000278	0.006875	DOWN
ENSMUSC	Naaladl1	N-acetyla	GO:00081	metabolic	-	-	-	-	19:610578	1	2	3	7	33	16	0.024519	0.039157	0.081277	0.132372	0.520881	0.308534	0.048318	0.320596	0.000281	0.006931	DOWN
ENSMUSC	Il1rn	interleukir	GO:00325	developm	-	-	-	-	2:2433685	3	8	4	20	32	16	0.022608	0.04815	0.033319	0.11628	0.155296	0.09486	0.034692	0.122145	0.000296	0.007303	DOWN
ENSMUSC	Ahcyl2	S-adenos	GO:00081	metabolic	K01251	adenosylh	mmu0110	Metabolic	6:2976801	1622	2343	1482	4837	2694	5026	3.006382	3.468258	3.035907	6.916714	3.215478	7.328697	3.170183	5.820296	0.000298	0.007325	-
ENSMUSC	Ccna2	cyclin A2 |	GO:00325	developm	K06627	cyclin A[-]	mmu0516	Human pa	3:3656486	41	61	41	102	147	98	0.398942	0.474031	0.440918	0.765701	0.921089	0.750182	0.437964	0.812324	0.000304	0.007468	-
ENSMUSC	Plpp2	phospholi	GO:00081	metabolic	K01080	phosphati	mmu0110	Metabolic	10:795264	17	23	21	48	108	49	0.314356	0.339664	0.429182	0.684752	1.286014	0.712802	0.361067	0.894523	0.000307	0.007521	DOWN
ENSMUSC	C3	compleme	GO:00325	developm	K03990	compleme	mmu0520	Viral carci	17:572039	56	156	66	180	459	203	0.601984	1.339258	0.784131	1.49279	3.177331	1.716726	0.908458	2.128949	0.000307	0.007523	DOWN
ENSMUSC	Bcl2l1	BCL2-like	GO:00325	developm	K04570	Bcl-2-like	mmu0520	Pathways	2:1527806	700	1626	1218	886	1029	880	3.590145	6.660094	6.904113	3.505726	3.398474	3.550648	5.718117	3.484949	0.000313	0.007645	-
ENSMUSC	Lgals12	lectin, gal	GO:00325	developm	-	-	-	-	19:759666	7	4	4	19	42	16	0.066805	0.030483	0.042192	0.139886	0.25811	0.120125	0.046493	0.172707	0.000318	0.007767	DOWN
ENSMUSC	Sgk2	serum/glu	GO:00081	metabolic	K13303	serum/glu	mmu0415	PI3K-Akt s	2:1629873	24	36	25	67	96	63	0.305296	0.365726	0.351466	0.657518	0.78637	0.630457	0.340829	0.691448	0.00032	0.007801	DOWN
ENSMUSC	Pglyrp1	peptidogl	GO:00325	developm	-	-	-	-	7:1887133	32	44	40	77	136	95	0.762033	0.836798	1.052759	1.414649	2.085546	1.779763	0.883863	1.759986	0.000324	0.007883	-
ENSMUSC	Phf3	PHD finge	GO:00081	metabolic	-	-	-	-	1:3080233	2183	2629	1775	1311	1401	2342	5.529781	5.318518	4.969349	2.562047	2.285321	4.667152	5.272549	3.171507	0.000333	0.008102	-
ENSMUSC	Chrnb2	cholinergi	GO:00325	developm	K04813	nicotinic a	mmu0408	Neuroacti	3:8974619	1157	622	510	573	517	502	8.035058	3.449785	3.914464	3.070014	2.312069	2.742648	5.133102	2.708243	0.000348	0.008441	-
ENSMUSC	Nectin2	nectin cell	GO:00325	developm	K06531	poliovirus	mmu0516	Herpes sir	7:1971664	86	80	58	157	255	146	1.261646	0.937298	0.940406	1.776937	2.408998	1.685025	1.04645	1.956987	0.000362	0.008778	-
ENSMUSC	Cd5l	CD5 antig	GO:00511	localizatio	-	-	-	-	3:8735788	6	29	10	5	5	0	0.179931	0.694569	0.331435	0.115675	0.096557	0	0.401978	0.070744	0.000363	0.008795	UP
ENSMUSC	Htr2b	5-hydroxy	GO:00325	developm	K04157	5-hydroxy	mmu0408	Neuroacti	1:8609902	5	17	8	3	3	2	0.117274	0.318459	0.207387	0.054297	0.045321	0.036914	0.214373	0.045511	0.000365	0.00881	UP
ENSMUSC	Fabp4	fatty acid	GO:00325	developm	K08753	fatty acid-	mmu0332	PPAR sign	3:1020408	6	12	6	25	51	21	0.071453	0.11413	0.078978	0.229698	0.391117	0.196749	0.088187	0.272521	0.000364	0.00881	DOWN
ENSMUSC	Slc37a2	solute car	GO:00081	metabolic	-	-	-	-	9:3722758	74	144	48	179	320	194	0.482517	0.749876	0.345917	0.900463	1.343653	0.995163	0.526104	1.07976	0.000366	0.00882	DOWN
ENSMUSC	Filip1l	filamin A i	GO:00160	membran	-	-	-	-	16:573530	127	183	92	245	365	271	0.894187	1.029012	0.715912	1.330825	1.654899	1.50108	0.879704	1.495601	0.000383	0.009225	-
ENSMUSC	Abcb1a	ATP-bind	GO:00511	localizatio	K05658	ATP-bind	mmu0522	Gastric ca	5:8660077	735	968	729	1314	1968	1413	6.403781	6.735506	7.019785	8.832323	11.04152	9.68506	6.719691	9.852968	0.000403	0.009698	-
ENSMUSC	Rbp2	retinol bin	GO:00081	metabolic	K14622	retinol bin	mmu0497	Vitamin d	9:9848611	0	0	0	10	15	0	0	0	0	0.259545	0.324943	0	0	0.194829	0.000424	0.010176	DOWN
ENSMUSC	Ifi27l2a	interferon	GO:00325	developm	-	-	-	-	12:103442	5	13	19	41	82	35	0.659316	1.369148	2.769296	4.171304	6.963527	3.631106	1.599253	4.921979	0.000428	0.010275	DOWN
ENSMUSC	Avil	advillin [S	GO:00325	developm	-	-	-	-	10:127000	0	0	3	13	13	6	0	0	0.049058	0.14838	0.123856	0.069837	0.016353	0.114024	0.000432	0.010349	DOWN
ENSMUSC	2610001J(	RIKEN cDI	GO:00160	membran	-	-	-	-	6:1386773	203	220	202	182	198	136	1.870345	1.618809	2.056951	1.293681	1.174753	0.985775	1.848701	1.151403	0.000439	0.010497	-
ENSMUSC	Dhx58	DEXH (As	GO:00023	immune s	K12649	ATP-depe	mmu0462	RIG-I-like	11:100694	26	31	28	69	82	69	0.36286	0.345516	0.431885	0.742928	0.73694	0.757579	0.380087	0.745816	0.000444	0.010602	-
ENSMUSC	Tmem237	transmem	GO:00508	response	-	-	-	-	1:5910059	381	531	322	344	395	349	5.946604	6.61889	5.554525	4.142216	3.970049	4.285295	6.040006	4.13252	0.000444	0.010602	-
ENSMUSC	Lax1	lymphocy	GO:00081	metabolic	-	-	-	-	1:1336790	0	6	0	15	14	10	0	0.106274	0	0.256661	0.19995	0.17448	0.035425	0.210364	0.000452	0.010767	DOWN
ENSMUSC	Ikbke	inhibitor c	GO:00048	signal trar	K07211	inhibitor c	mmu0516	Human pa	1:1312543	21	54	18	77	146	66	0.10828	0.222361	0.102575	0.306296	0.48476	0.267715	0.144405	0.352924	0.000459	0.010928	DOWN
ENSMUSC	Itgae	integrin al	GO:00508	response	K06524	integrin al	mmu0481	Regulation	11:730905	1	2	0	16	14	4	0.013025	0.020802	0	0.160738	0.117394	0.040974	0.011276	0.106369	0.000462	0.010967	DOWN
ENSMUSC	Cd7	CD7 antig	GO:00023	immune s	K06457	T-cell surf	mmu0464	Hematopo	11:121036	3	2	1	13	20	9	0.126499	0.067362	0.046607	0.422903	0.543069	0.298549	0.080156	0.421507	0.000478	0.011334	DOWN
ENSMUSC	Ifi208	interferon	GO:00081	metabolic	-	-	-	-	1:1736736	3	2	0	20	15	6	0.029181	0.015539	0	0.150088	0.093958	0.045916	0.014907	0.096654	0.000488	0.011571	DOWN
ENSMUSC	Tap1	transporte	GO:00511	localizatio	K05653	ATP-bind	mmu0516	Herpes sir	17:341875	77	102	75	174	246	156	0.400431	0.423629	0.431069	0.698099	0.823812	0.638226	0.418376	0.720046	0.0005	0.011827	-
ENSMUSC	Gatad2b	GATA zinc	GO:00081	metabolic	-	-	-	-	3:9029317	809	1065	747	2261	2650	1102	3.322302	3.492901	3.390455	7.163442	7.007955	3.560274	3.401886	5.910557	0.000501	0.011827	-
ENSMUSC	Cd300lf	CD300 mo	GO:00325	developm	-	-	-	-	11:115116	9	28	11	7	5	5	0.080822	0.20082	0.109176	0.048498	0.028915	0.035325	0.130273	0.037579	0.000508	0.01199	UP
ENSMUSC	Fam109b	family wit	GO:00081	metabolic	-	-	-	-	15:823389	6	11	4	1	1	2	0.057429	0.084085	0.042318	0.007386	0.006162	0.015063	0.061277	0.009537	0.000513	0.012087	UP
ENSMUSC	Hnf1b	HNF1 hor	GO:00325	developm	K08034	transcripti	-	-	11:838500	6	14	7	21	66	28	0.068642	0.127916	0.088516	0.18535	0.486234	0.252013	0.095024	0.307865	0.000524	0.012327	DOWN
ENSMUSC	F7	coagulatic	GO:00325	developm	K01320	coagulatic	mmu0461	Complem	8:1302603	3	9	7	0	0	2	0.098531	0.236086	0.25412	0	0	0.051688	0.196246	0.017229	0.000527	0.01239	UP
ENSMUSC	Ttc22	tetratricop	-	-	-	-	-	-	4:1066224	14	19	17	47	84	34	0.242919	0.263282	0.326005	0.629152	0.938554	0.464097	0.277402	0.677268	0.000554	0.01301	DOWN
ENSMUSC	Lats2	large tum	GO:00081	metabolic	-	-	-	-	14:576420	4	9	3	15	31	20	0.074307	0.133521	0.061594	0.214964	0.370823	0.292269	0.089807	0.292685	0.000558	0.013085	DOWN
ENSMUSC	Ifi47	interferon	GO:00508	response	K17072	interferon	mmu0466	TNF signa	11:490765	16	19	13	49	38	61	0.148446	0.140782	0.133302	0.350731	0.227033	0.445237	0.140844	0.341001	0.00056	0.013119	DOWN
ENSMUSC	Aldh1a7	aldehyde	GO:00081	metabolic	K07249	retinal del	mmu0110	Metabolic	19:206929	2	2	5	24	21	10	0.059097	0.047212	0.163327	0.547216	0.399648	0.232499	0.089879	0.393121	0.000565	0.013201	DOWN
ENSMUSC	Adgrg5	adhesion	GO:00048	signal trar	-	-	-	-	8:9492369	0	0	0	2	9	5	0	0	0	0.023123	0.08684	0.058938	0	0.0563	0.00057	0.013301	DOWN
ENSMUSC	Batf2	basic leuc	GO:00325	developm	-	-	-	-	19:614098	0	4	8	23	33	15	0	0.034132	0.094475	0.189611	0.227077	0.126096	0.042869	0.180928	0.000575	0.013397	DOWN
ENSMUSC	Suclg2	succinate-	GO:00081	metabolic	K01900	succinyl-C	mmu0110	Metabolic	6:9547300	185	225	178	354	492	364	1.915184	1.860232	2.036599	2.827301	3.279884	2.964495	1.937338	3.023893	0.000604	0.014051	-
ENSMUSC	Itgam	integrin al	GO:00081	metabolic	-	-	-	-	7:1280626	0	0	0	0	120	1	0	0	0	0	0.893602	0.009099	0	0.3009	0.000613	0.014253	DOWN
ENSMUSC	Cd38	CD38 anti	GO:00081	metabolic	K01242	ADP-ribos	mmu0110	Metabolic	5:4386855	168	215	165	326	561	319	2.471152	2.525655	2.68239	3.699463	5.313832	3.69141	2.559732	4.234902	0.000641	0.014872	-
ENSMUSC	Slco2a1	solute car	GO:00511	localizatio	-	-	-	-	9:1029887	102	122	80	191	282	194	0.408846	0.39054	0.354403	0.590638	0.727884	0.611744	0.384596	0.643422	0.000659	0.015275	-
ENSMUSC	Arhgef39	Rho guan	GO:00400	locomotic	-	-	-	-	4:4349614	0	3	3	12	13	12	0	0.017649	0.024422	0.068185	0.061659	0.069533	0.014024	0.066459	0.000665	0.015402	DOWN
ENSMUSC	Map2k7	mitogen-	GO:00048	signal trar	-	-	-	-	8:4238865	0	3	222	0	6	0	0	0.05082	5.203572	0	0.081939	0	1.751464	0.027313	0.000678	0.015688	UP
ENSMUSC	Slc1a5	solute car	GO:00511	localizatio	K05616	solute car	mmu0497	Protein dig	7:1678134	19	33	34	61	105	74	0.168081	0.233146	0.332428	0.416323	0.598152	0.515007	0.244552	0.509828	0.000696	0.016063	DOWN
ENSMUSC	Cxcl13	chemokin	GO:00325	developm	K10032	C-X-C mo	mmu0406	Cytokine-	5:9595695	1	0	0	4	14	6	0.05311	0	0	0.163827	0.478663	0.250622	0.017703	0.297704	0.000719	0.016591	DOWN
ENSMUSC	Trpm4	transient r	GO:00325	developm	K04979	transient r	mmu0491	Insulin sec	7:4530263	152	127	107	229	378	279	0.663722	0.442887	0.516386	0.771452	1.062893	0.958426	0.540999	0.930924	0.000727	0.016735	-
ENSMUSC	Tk1	thymidine	GO:00081	metabolic	K00857	thymidine	mmu0110	Metabolic	11:117815	0	5	2	16	22	7	0	0.033013	0.018277	0.102058	0.117132	0.045533	0.017097	0.088241	0.000734	0.016867	DOWN
ENSMUSC	Fxyd3	FXYD don	GO:00511	localizatio	-	-	-	-	7:3106817	4	4	2	9	25	18	0.031398	0.025072	0.017351	0.054501	0.12636	0.111145	0.024607	0.097335	0.000734	0.016867	DOWN
ENSMUSC	1500015C	RIKEN cDI	GO:00325	developm	-	-	-	-	1:4373060	83	234	68	38	73	88	5.618404	12.65026	5.087332	1.984506	3.182086	4.68631	7.78533	3.284301	0.000738	0.016911	UP
ENSMUSC	Gm5431	predicted	GO:00508	response	K17072	interferon	mmu0466	TNF signa	11:488874	2	11	9	26	40	21	0.016097	0.070715	0.080068	0.161467	0.207347	0.13299	0.055627	0.167268	0.000737	0.016911	DOWN
ENSMUSC	Defa24	defensin,	GO:00023	immune s	-	-	-	-	8:2173449	0	0	0	4	8	3	0	0	0	0.449645	0.750742	0.343875	0	0.514754	0.000752	0.0172	DOWN
ENSMUSC	Muc20	mucin 20	GO:00081	metabolic	-	-	-	-	16:327774	0	0	0	3	8	4	0	0	0	0.035097	0.078113	0.04771	0	0.05364	0.000753	0.017203	DOWN
ENSMUSC	Noxo1	NADPH o	GO:00081	metabolic	-	-	-	-	17:246962	20	40	24	58	104	64	0.1803	0.287984	0.239119	0.403385	0.603741	0.453896	0.235801	0.487007	0.000754	0.017217	DOWN
ENSMUSC	Mcoln2	mucolipin	GO:00400	locomotic	-	-	-	-	3:1461498	0	2	0	13	9	3	0	0.019665	0	0.12346	0.071345	0.029049	0.006555	0.074618	0.000765	0.017435	DOWN
ENSMUSC	Adat3	adenosine	GO:00081	metabolic	-	-	-	-	10:806060	81	34	428	73	39	37	0.835282	0.280011	4.877967	0.580765	0.258979	0.300167	1.997753	0.379971	0.000787	0.017913	UP
ENSMUSC	Trim6	tripartite r	GO:00325	developm	-	-	-	-	7:1042187	6	6	1	13	32	17	0.051779	0.041353	0.009539	0.086553	0.177838	0.115421	0.034224	0.126604	0.000807	0.018338	DOWN
ENSMUSC	Fabp6	fatty acid	GO:00081	metabolic	K08755	fatty acid-	mmu0332	PPAR sign	11:435960	0	0	0	2	7	6	0	0	0	0.19671	0.574554	0.601703	0	0.457656	0.000834	0.018941	DOWN
ENSMUSC	Slc43a1	solute car	GO:00511	localizatio	-	-	-	-	2:8483885	0	2	0	8	7	7	0	0.011013	0	0.042548	0.031073	0.037965	0.003671	0.037195	0.00084	0.019038	DOWN
ENSMUSC	Aldob	aldolase B	GO:00081	metabolic	K01623	fructose-b	mmu0110	Metabolic	4:4953599	22	26	24	52	105	53	0.479738	0.452797	0.578411	0.874821	1.474445	0.909232	0.503649	1.086166	0.000896	0.020279	DOWN
ENSMUSC	Nktr	natural kil	GO:00081	metabolic	-	-	-	-	9:1217191	1833	1996	1505	3133	3372	3156	3.968917	3.451565	3.601579	5.233599	4.701668	5.375979	3.67402	5.103748	0.000904	0.020441	-
ENSMUSC	Pitx1	paired-lik	GO:00325	developm	-	-	-	-	13:558250	0	0	0	3	11	2	0	0	0	0.045666	0.139739	0.031046	0	0.07215	0.00095	0.021448	DOWN
ENSMUSC	Calcrl	calcitonin	GO:00325	developm	K04577	calcitonin	mmu0408	Neuroacti	2:8433062	179	202	138	146	170	122	1.377195	1.241189	1.173461	0.866611	0.842259	0.738434	1.263948	0.815768	0.000969	0.021847	-
ENSMUSC	Fbxo34	F-box pro	-	-	-	-	-	-	14:474504	1073	148	108	142	161	141	3.699552	0.407529	0.411549	0.377719	0.357462	0.382455	1.50621	0.372545	0.000981	0.022106	UP
ENSMUSC	Xaf1	XIAP asso	GO:00508	response	-	-	-	-	11:723016	58	92	63	167	175	130	0.613755	0.777501	0.736812	1.363369	1.192506	1.082238	0.709356	1.212704	0.000987	0.022211	-
ENSMUSC	Car9	carbonic a	GO:00325	developm	K01672	carbonic a	mmu0091	Nitrogen r	4:4350696	4	4	11	26	46	18	0.065447	0.052259	0.198898	0.328169	0.484637	0.231672	0.105535	0.348159	0.000989	0.022232	DOWN
ENSMUSC	Mogat2	monoacyl	GO:00081	metabolic	K14457	2-acylglyc	mmu0056	Glycerolip	7:9921908	0	0	0	2	9	4	0	0	0	0.048197	0.181008	0.098275	0	0.10916	0.000995	0.0223	DOWN
ENSMUSC	Lrrc31	leucine ric	-	-	-	-	-	-	3:3067905	0	0	0	2	9	4	0	0	0	0.014266	0.053576	0.029088	0	0.03231	0.000995	0.0223	DOWN
ENSMUSC	Lgals3bp	lectin, gal	GO:00511	localizatio	-	-	-	-	11:118392	336	578	292	686	1520	637	4.744567	6.518249	4.557084	7.473294	13.82153	7.076328	5.2733	9.457049	0.000996	0.022301	-
ENSMUSC	Sumo3	small ubic	-	-	-	-	-	-	10:776061	4	4	0	9	17	16	0.477044	0.380922	0	0.82805	1.305532	1.501092	0.285989	1.211558	0.001006	0.02249	DOWN
ENSMUSC	Evpl	envoplaki	GO:00325	developm	-	-	-	-	11:116220	42	72	41	103	190	95	0.401698	0.549956	0.43339	0.760007	1.170196	0.714804	0.461681	0.881669	0.001018	0.022735	-
ENSMUSC	Olfr56	olfactory r	GO:00048	signal trar	K04257	olfactory r	mmu0474	Olfactory	11:489788	2	0	0	9	6	10	0.015665	0	0	0.054396	0.030267	0.06163	0.005222	0.048764	0.001026	0.022867	DOWN
ENSMUSC	Sfrp4	secreted f	GO:00325	developm	K02185	secreted f	mmu0431	Wnt signa	13:196231	44	73	45	31	40	43	0.38722	0.513072	0.437692	0.210477	0.226683	0.297709	0.445995	0.244956	0.001036	0.023072	-
ENSMUSC	Pdik1l	PDLIM1 ir	GO:00081	metabolic	-	-	-	-	4:1342750	283	336	203	226	80	191	1.563469	1.482475	1.239495	0.963253	0.284608	0.830132	1.42848	0.692665	0.001087	0.024169	UP
ENSMUSC	Gm20517	predicted	GO:00081	metabolic	-	-	-	-	17:476115	23	28	9	49	58	58	0.200334	0.194775	0.086636	0.329267	0.325317	0.397435	0.160582	0.350673	0.0011	0.02444	DOWN
ENSMUSC	Trim30c	tripartite r	GO:00081	metabolic	-	-	-	-	7:1043820	0	0	0	2	12	3	0	0	0	0.054909	0.274966	0.083965	0	0.137947	0.001107	0.024547	DOWN
ENSMUSC	Lpp	LIM doma	GO:00056	cell|cell jur	-	-	-	-	16:243933	314	393	218	517	742	576	1.198715	1.198186	0.919791	1.522675	1.824085	1.729895	1.105564	1.692218	0.001131	0.025028	-
ENSMUSC	Sapcd1	suppressc	-	-	-	-	-	-	17:350259	0	0	0	4	5	5	0	0	0	0.098331	0.102605	0.125351	0	0.108762	0.00113	0.025028	DOWN
ENSMUSC	Rhbg	Rhesus bl	GO:00511	localizatio	-	-	-	-	3:8824287	0	0	0	5	6	3	0	0	0	0.121587	0.121787	0.074385	0	0.105919	0.001184	0.026167	DOWN
ENSMUSC	H2-Q10	histocomp	GO:00023	immune s	K06751	major hist	mmu0516	Human pa	17:354700	0	0	0	4	4	5	0	0	0	0.027388	0.022864	0.034914	0	0.028389	0.00119	0.026254	DOWN
ENSMUSC	Zfp260	zinc finge	GO:00325	developm	-	-	-	-	7:3009477	820	452	170	223	265	320	4.494	1.978353	1.029707	0.942876	0.935233	1.379687	2.500687	1.085932	0.001194	0.026316	UP
ENSMUSC	Txnip	thioredox	GO:00325	developm	K20910	thioredox	mmu0462	NOD-like	3:9655795	458	398	266	622	1230	691	4.044054	2.806599	2.59585	4.237132	6.993766	4.799991	3.148834	5.34363	0.001205	0.026501	-
ENSMUSC	Sycn	syncollin [	GO:00511	localizatio	-	-	-	-	7:2854088	0	0	0	6	8	1	0	0	0	0.508319	0.565757	0.08641	0	0.386829	0.001205	0.026501	DOWN
ENSMUSC	Crip1	cysteine-r	GO:00508	response	-	-	-	-	12:113146	19	34	25	52	99	59	0.253684	0.362551	0.368913	0.535644	0.851202	0.619739	0.328382	0.668861	0.001207	0.026511	DOWN
ENSMUSC	Npc1l1	NPC1 like	GO:00081	metabolic	K14461	Niemann-	mmu0497	Fat digest	11:621101	5	4	5	12	34	18	0.068337	0.043661	0.075539	0.126539	0.299267	0.193552	0.062512	0.206453	0.001222	0.026793	DOWN
ENSMUSC	Calml4	calmoduli	GO:00431	ion bindin	K02183	calmoduli	mmu0474	Olfactory	9:6285808	38	96	44	115	211	123	0.442954	0.893702	0.566862	1.0342	1.583857	1.12796	0.634506	1.248672	0.001233	0.026998	-
ENSMUSC	Card14	caspase re	GO:00081	metabolic	K20913	caspase re	mmu0406	NF-kappa	11:119307	16	10	8	36	41	30	0.144302	0.072024	0.079738	0.250487	0.238121	0.212855	0.098688	0.233821	0.001248	0.027308	DOWN
ENSMUSC	Irgm2	immunity-	GO:00081	metabolic	-	-	-	-	11:581996	150	228	145	306	410	325	0.875847	1.063203	0.93573	1.378442	1.541613	1.4929	0.95826	1.470985	0.001255	0.027428	-
ENSMUSC	Lilrb4a	leukocyte	GO:00023	immune s	K06512	leukocyte	mmu0438	Osteoclas	10:514909	26	63	17	7	21	20	0.07938	0.15361	0.057363	0.016488	0.041286	0.048037	0.096784	0.03527	0.001261	0.027507	UP
ENSMUSC	Pla2g3	phospholi	GO:00325	developm	-	-	mmu0110	Metabolic	11:348828	29	39	27	68	110	61	0.259441	0.278644	0.266961	0.469333	0.633705	0.429319	0.268349	0.510786	0.001309	0.028538	-
ENSMUSC	Dapk2	death-ass	GO:00081	metabolic	K08803	death-ass	mmu0520	Pathways	9:6615822	0	16	11	37	76	26	0	0.193701	0.184282	0.432699	0.74186	0.310055	0.125994	0.494871	0.001321	0.028721	DOWN
ENSMUSC	Slfn2	schlafen 2	GO:00099	cellular pr	-	-	-	-	11:830651	49	69	47	118	136	106	1.79799	2.022031	1.906059	3.340429	3.213546	3.059886	1.908693	3.204621	0.001321	0.028721	-
ENSMUSC	Ahnak	AHNAK n	GO:00081	metabolic	-	-	-	-	19:898928	916	1376	896	1613	2503	1801	3.01619	3.618493	3.260754	4.097587	5.307364	4.665391	3.298479	4.690114	0.001326	0.028795	-
ENSMUSC	Senp8	SUMO/se	GO:00081	metabolic	-	-	-	-	9:597342	52	142	78	232	214	190	0.325811	0.710554	0.540138	1.121452	0.863435	0.936539	0.525501	0.973809	0.001334	0.028934	-
ENSMUSC	Trf	transferrin	GO:00325	developm	K14736	transferrin	mmu0406	HIF-1 sign	9:1032040	2060	3289	2066	2322	2710	2250	25.0059	31.8849	27.7174	21.74543	21.18358	21.48665	28.20273	21.47189	0.001337	0.028952	-
ENSMUSC	Gm28048	predicted	GO:00081	metabolic	-	-	-	-	11:228347	62	77	66	62	48	40	1.378591	1.367343	1.621947	1.063574	0.687286	0.699699	1.45596	0.816853	0.001343	0.029041	-
ENSMUSC	Ifih1	interferon	GO:00081	metabolic	K12647	interferon	mmu0516	Herpes sir	2:6259579	145	161	95	266	332	238	0.783072	0.694396	0.567031	1.108272	1.154588	1.011167	0.6815	1.091342	0.001374	0.029681	-
ENSMUSC	Sct	secretin [S	GO:00325	developm	-	-	-	-	7:1412783	0	0	0	8	10	0	0	0	0	0.216705	0.226096	0	0	0.1476	0.001376	0.0297	DOWN
ENSMUSC	Capn8	calpain 8	GO:00081	metabolic	-	-	-	-	1:1825650	0	0	0	0	13	6	0	0	0	0	0.111362	0.062792	0	0.058051	0.001396	0.030082	DOWN
ENSMUSC	Zfp180	zinc finge	GO:00971	organic cy	-	-	-	-	7:2408192	598	217	183	1050	946	989	3.006895	0.87141	1.016989	4.073212	3.063115	3.912233	1.631765	3.682854	0.001408	0.030305	DOWN
ENSMUSC	H2-Q4	histocomp	GO:00023	immune s	K06751	major hist	mmu0516	Human pa	17:353796	128	180	170	291	421	304	2.000888	2.24714	2.937011	3.509418	4.278774	3.738487	2.395013	3.828593	0.001424	0.03062	-
ENSMUSC	Styk1	serine/thre	GO:00081	metabolic	-	-	-	-	6:1312991	11	6	6	28	44	53	0.100084	0.043596	0.060337	0.168462	0.301517	0.155992	0.068006	0.20499	0.001439	0.030906	DOWN
ENSMUSC	Mpst	mercapto	GO:00325	developm	K01011	thiosulfate	mmu0110	Metabolic	15:7840667	179	272	186	356	502	401	7.24282	8.7896	8.317886	11.11309	13.08014	12.76467	8.116769	12.3193	0.001453	0.031155	-
ENSMUSC	Hmga2	high mob	GO:00325	developm	K09283	high mob	mmu0520	Transcript	10:1203611	0	0	4	11	21	7	0	0	0.037498	0.07198	0.114693	0.046709	0.012499	0.077794	0.001477	0.031635	DOWN
ENSMUSC	Lsamp	limbic sys	GO:00160	membran	-	-	-	-	16:399843	10853	2942	2460	3249	2948	2645	30.61085	6.626957	7.668447	7.069775	5.354362	5.868973	14.96875	6.097703	0.001491	0.031904	UP
ENSMUSC	Sphkap	SPHK1 int	GO:00055	extracellul	-	-	-	-	1:8325413	6067	7921	5550	5805	7245	6756	23.74765	24.76124	24.00968	17.52989	18.26164	20.80401	24.17286	18.86518	0.00151	0.0322	-
ENSMUSC	Rassf7	Ras assoc	GO:00508	response	-	-	-	-	7:1412158	7	12	13	27	47	29	0.063246	0.086586	0.129811	0.188197	0.27345	0.206124	0.093214	0.222591	0.001508	0.0322	DOWN
ENSMUSC	Ffar4	free fatty	GO:00325	developm	-	-	-	-	19:380970	0	0	0	4	8	2	0	0	0	0.135249	0.225816	0.068979	0	0.143348	0.001511	0.0322	DOWN
ENSMUSC	Iqgap3	IQ motif c	GO:00325	developm	K05767	Ras GTPas	mmu0481	Regulation	3:8808197	8	9	10	25	44	22	0.034674	0.031153	0.047899	0.083593	0.1228	0.075012	0.037909	0.093802	0.001528	0.032535	DOWN
ENSMUSC	Bcl3	B cell leuk	GO:00325	developm	K09258	B-cell CLL	mmu0466	TNF signa	7:1980846	6	15	3	25	51	19	0.106185	0.212016	0.058684	0.341351	0.581234	0.264534	0.125629	0.395706	0.001538	0.032695	DOWN
ENSMUSC	Irgm1	immunity-	GO:00081	metabolic	K14139	immunity-	mmu0514	Toxoplasr	11:4868619	176	272	188	387	479	377	1.314847	1.622844	1.552265	2.230507	2.304369	2.215718	1.496652	2.250198	0.00156	0.033133	-
ENSMUSC	Mx1	MX dynar	GO:00038	catalytic a	-	-	-	-	16:974470	8	0	6	18	39	19	0.092887	0	0.076996	0.161233	0.291579	0.173539	0.056628	0.208784	0.001576	0.033434	DOWN
ENSMUSC	Stat4	signal trar	GO:00048	signal trar	K11222	signal trar	mmu0520	Pathways	1:5198714	0	2	1	8	18	4	0	0.020679	0.014307	0.079892	0.150041	0.040731	0.011662	0.090221	0.001586	0.033507	DOWN
ENSMUSC	Barhl2	BarH-like	GO:00325	developm	-	-	-	-	5:1064525	0	0	0	2	4	7	0	0	0	0.040719	0.067969	0.145315	0	0.084668	0.001647	0.034852	DOWN
ENSMUSC	Trim14	tripartite r	GO:00325	developm	-	-	-	-	4:4649378	9	18	6	28	42	30	0.097743	0.156122	0.072024	0.234605	0.293736	0.25632	0.10863	0.261554	0.001667	0.03523	DOWN
ENSMUSC	A2m	alpha-2-r	GO:00325	developm	K03910	alpha-2-r	mmu0461	Complem	6:1216353	117	348	122	105	134	142	0.937876	2.22784	1.080854	0.649352	0.6917	0.895483	1.415524	0.745512	0.001671	0.035272	-

ENSMUSC	Slc6a17	solute car	GO:00325	developm	-	-	-	-	3:1074675	3958	4469	8061	3185	4886	4631	12.79344	11.53633	28.79699	7.94239	10.16997	11.77598	17.70892	9.962779	0.001688	0.035548	-
ENSMUSC	Gpr171	G protein-	GO:00325	developm	-	-	-	-	3:5909644	1	0	0	6	8	3	0.02745	0	0	0.127023	0.141376	0.064757	0.00915	0.111052	0.001687	0.035548	DOWN
ENSMUSC	Slc12a7	solute car	GO:00511	localizatio	K13627	solute car	mmu0496	Collecting	13:737330	72	121	57	149	244	163	0.243503	0.326815	0.213054	0.388765	0.53139	0.433678	0.261124	0.451278	0.001701	0.035777	-
ENSMUSC	Chdh	choline de	GO:00081	metabolic	K00108	choline de	mmu0110	Metabolic	14:300090	89	57	84	111	353	245	0.674553	0.345018	0.703637	0.649049	1.722871	1.460832	0.574403	1.277584	0.001705	0.035802	DOWN
ENSMUSC	Kcnn4	potassium	GO:00511	localizatio	K04945	potassium	mmu0497	Protein di	7:2437026	6	12	7	23	34	23	0.050537	0.080721	0.065169	0.14946	0.184415	0.15241	0.065475	0.162095	0.001707	0.035802	DOWN
ENSMUSC	Col6a1	collagen,	GO:00325	developm	K06238	collagen, t	mmu0415	PI3K-Akt s	10:767087	652	749	543	1131	1269	1148	8.788057	8.062561	8.08894	11.76083	11.01442	12.17301	8.313186	11.64942	0.001716	0.035965	-
ENSMUSC	Clic1	chloride ir	GO:00325	developm	-	-	-	-	17:350499	138	208	111	282	469	242	4.058764	4.885693	3.608145	6.398759	8.88267	5.599412	4.184201	6.96028	0.001739	0.036344	-
ENSMUSC	Treml4	triggering	GO:00511	localizatio	-	-	-	-	17:482642	6	4	3	0	2	0	0.103603	0.055156	0.057257	0	0.022234	0	0.072005	0.007411	0.001738	0.036344	UP
ENSMUSC	Gm28035	predicted	GO:00325	developm	-	-	-	-	2:3024056	74	87	17	170	173	150	1.066137	1.001033	0.270696	1.889564	1.605035	1.700142	0.779289	1.73158	0.001741	0.036347	DOWN
ENSMUSC	Elmo3	engulfmer	GO:00400	locomotic	K19241	engulfmer	mmu0510	Bacterial i	8:1053056	19	39	20	47	109	60	0.221097	0.362445	0.257227	0.421953	0.816796	0.549278	0.280256	0.596009	0.0018	0.037534	DOWN
ENSMUSC	H2-Q6	histocomp	GO:00023	immune s	K06751|K0	major hist	mmu0516	Human pa	17:354248	27	26	40	71	108	74	0.734665	0.564987	1.202885	1.490414	1.892343	1.584038	0.834179	1.655598	0.001805	0.037584	-
ENSMUSC	Lipi	lipase, me	GO:00081	metabolic	-	-	-	-	16:755405	0	0	0	2	9	3	0	0	0	0.033772	0.126836	0.051643	0	0.07075	0.001839	0.038255	DOWN
ENSMUSC	Ttc39a	tetratricop	-	-	-	-	-	-	4:1094066	42	51	22	73	140	81	0.437244	0.424023	0.253136	0.586311	0.938554	0.663392	0.371468	0.729419	0.001843	0.038304	-
ENSMUSC	Krt84	keratin 84	GO:00325	developm	-	-	-	-	15:101525	0	0	0	6	10	0	0	0	0	0.109971	0.152989	0	0	0.087653	0.001846	0.038319	DOWN
ENSMUSC	Zbtb41	zinc finge	GO:00081	metabolic	-	-	-	-	1:1394222	702	776	529	475	544	721	3.71042	3.275624	3.090214	1.936914	1.851569	2.998006	3.358752	2.262163	0.001856	0.038481	-
ENSMUSC	Cps1	carbamoy	GO:00325	developm	K01948	carbamoy	mmu0110	Metabolic	1:6712302	6	6	6	18	22	25	0.065948	0.052668	0.072893	0.152642	0.15572	0.216176	0.063836	0.174846	0.001914	0.039622	DOWN
ENSMUSC	Uba7	ubiquitin-	GO:00081	metabolic	K10698	ubiquitin-	mmu0501	Parkinson	9:1079755	55	71	48	109	160	111	0.272117	0.280541	0.262472	0.416053	0.509763	0.432041	0.27171	0.452619	0.001935	0.040019	-
ENSMUSC	Hsh2d	hematopc	GO:00023	immune s	-	-	-	-	8:7218963	0	0	0	1	8	5	0	0	0	0.01754	0.117111	0.089416	0	0.074689	0.001953	0.040338	DOWN
ENSMUSC	Hoxa10	homeobo	GO:00325	developm	K17443	homrobox	mmu0520	Transcript	6:5223119	0	1	0	3	14	3	0	0.007219	0	0.02092	0.081478	0.021327	0.002406	0.041241	0.001959	0.040405	DOWN
ENSMUSC	Plekho1	pleckstrin	GO:00325	developm	-	-	-	-	3:9598842	184	264	234	468	495	400	2.919615	3.345477	4.103654	5.729079	5.057871	4.993201	3.456248	5.26005	0.002035	0.041841	-
ENSMUSC	Cth	cystathion	GO:00325	developm	K01758	cystathion	mmu0110	Metabolic	3:1578942	43	37	25	83	91	76	0.737504	0.5068	0.473887	1.098255	1.005052	1.025455	0.57273	1.04292	0.002035	0.041841	-
ENSMUSC	Prr15	proline ric	GO:00325	developm	-	-	-	-	6:5432583	5	11	7	22	35	20	0.08515	0.149617	0.131774	0.289073	0.383859	0.267974	0.12218	0.313635	0.002032	0.041841	DOWN
ENSMUSC	Sfrp5	secreted f	GO:00325	developm	K02222	secreted f	mmu0431	Wnt signa	19:421979	13	42	23	7	22	8	0.417766	1.077925	0.816876	0.173546	0.455275	0.20224	0.770856	0.27702	0.002038	0.041843	UP
ENSMUSC	Igf1	insulin-like	GO:00325	developm	K05459	insulin-like	mmu0520	Pathways	10:878582	260	363	191	230	272	215	1.207785	1.346692	0.980609	0.824279	0.813652	0.785715	1.178362	0.807882	0.002043	0.041897	-
ENSMUSC	Slc30a10	solute car	GO:00325	developm	-	-	-	-	1:1854548	295	363	228	523	671	499	3.086385	3.033056	2.636396	4.221433	4.520698	4.107141	2.918612	4.283091	0.002068	0.042357	-
ENSMUSC	Kcnc3	potassium	GO:00511	localizatio	-	-	-	-	7:4459066	1400	1390	1061	1387	1209	1015	10.157	8.053755	8.507457	7.763264	5.64831	5.793145	8.906072	6.401573	0.002079	0.042527	-
ENSMUSC	Ndc80	NDC80 kir	GO:00511	localizatio	-	-	-	-	17:714961	3	3	2	20	14	8	0.083221	0.066475	0.061324	0.428033	0.250096	0.174582	0.07034	0.284237	0.002121	0.043342	DOWN
ENSMUSC	Olfr1033	olfactory r	GO:00048	signal trar	K04257	olfactory r	mmu0474	Olfactory	2:8602063	4	4	2	0	0	0	0.023922	0.019102	0.01322	0	0	0	0.018748	0	0.002134	0.043554	UP
ENSMUSC	CN725425	cDNA seq	-	-	-	-	-	-	15:911994	3	5	2	0	0	0	0.071551	0.095235	0.052724	0	0	0	0.07317	0	0.002137	0.043562	UP
ENSMUSC	Hmgn2	high mob	GO:00325	developm	-	-	-	-	4:1339647	159	354	232	704	364	722	1.144705	2.035377	1.845995	3.910205	1.687534	4.08926	1.675359	3.228999	0.002146	0.043706	-
ENSMUSC	Elmod1	ELMO/CE	GO:00511	localizatio	-	-	-	-	9:5391145	5192	2975	4443	3533	3723	3523	49.57987	22.68838	46.89147	26.02823	22.89383	26.46636	39.71991	25.12947	0.002162	0.04398	-
ENSMUSC	Lrrc18	leucine ric	GO:00508	response	-	-	-	-	14:329913	0	5	4	15	13	15	0	0.043614	0.048291	0.126402	0.09144	0.128894	0.030635	0.115579	0.002168	0.044044	DOWN
ENSMUSC	Ceacam12	carcinoem	-	-	-	-	-	-	7:1806592	0	0	0	3	4	5	0	0	0	0.042816	0.047646	0.072757	0	0.054407	0.002175	0.044081	DOWN
ENSMUSC	Lmod1	leiomodin	GO:00990	supramole	-	-	-	-	1:1353248	37	39	22	69	120	62	0.572233	0.481699	0.37605	0.82328	1.195091	0.754344	0.47666	0.924238	0.002174	0.044081	-
ENSMUSC	Fam46a	family witl	GO:00081	metabolic	-	-	-	-	9:8532043	62	249	133	290	492	338	0.654384	2.098866	1.551453	2.361395	3.343951	2.806515	1.434901	2.837287	0.002185	0.044244	-
ENSMUSC	Plin4	perilipin 4	GO:00160	membran	K20254	perilipin-4	mmu0332	PPAR sign	17:561005	58	132	108	146	321	300	0.38368	0.697367	0.789612	0.745116	1.367419	1.561257	0.623553	1.224598	0.002202	0.04453	-
ENSMUSC	Mkrn1	makorin, r	GO:00081	metabolic	-	-	-	-	6:3939780	1500	1917	1280	1498	1920	1076	13.10635	13.37699	12.36082	10.09793	10.80305	7.396288	12.94805	9.432424	0.002214	0.044722	-
ENSMUSC	Rab7	RAB7, me	GO:00081	metabolic	K07897	Ras-relate	mmu0414	Endocytos	6:8799910	2682	3623	4140	2937	3530	3202	23.02233	24.83732	39.27694	19.45023	19.51282	21.62334	29.04553	20.19546	0.002232	0.045024	-
ENSMUSC	Enpp2	ectonucle	GO:00325	developm	K01122	ectonucle	mmu0056	Ether lipid	15:548389	6619	19058	5243	3707	8138	7930	56.52358	129.9752	49.4839	24.42251	44.75171	53.27475	78.66089	40.81632	0.002257	0.045433	-
ENSMUSC	Plekhm2	pleckstrin	GO:00511	localizatio	K15348	pleckstrin	mmu0513	Salmonell	4:1416257	1521	1940	1363	1516	1614	1656	14.84485	15.12147	14.70244	11.41501	10.1439	12.71505	14.88959	11.42465	0.002257	0.045433	-
ENSMUSC	Ube2c	ubiquitin-	GO:00081	metabolic	K06688	ubiquitin-	mmu0412	Ubiquitin	2:1647698	7	8	4	22	31	14	0.153858	0.14043	0.097173	0.373047	0.438767	0.24207	0.130487	0.351295	0.002285	0.045948	DOWN
ENSMUSC	Ccdc137	coiled-co	GO:00056	cell|intrac	-	-	-	-	11:120458	142	155	187	389	277	499	1.470779	1.282149	2.140658	3.108416	1.847538	4.066035	1.631195	3.007329	0.002302	0.046235	-
ENSMUSC	P2ry4	pyrimidine	GO:00048	signal trar	K04271	purinergic	mmu0408	Neuroacti	X:1005901	0	0	0	0	8	7	0	0	0	0	0.070943	0.075837	0	0.048927	0.002315	0.046445	DOWN
ENSMUSC	Gh	growth hc	GO:00325	developm	K05438	growth hc	mmu0415	PI3K-Akt s	11:106300	0	0	0	10	6	0	0	0	0	0.54649	0.273667	0	0	0.273386	0.002357	0.047069	DOWN
ENSMUSC	Sp100	nuclear ar	GO:00325	developm	K15413	nuclear au	mmu0520	Viral carci	1:8564998	87	125	101	205	252	190	0.53382	0.612537	0.684927	0.970421	0.995707	0.917151	0.610428	0.961093	0.002356	0.047069	-
ENSMUSC	Dnajb4	DnaJ heat	GO:00987	molecular	-	-	-	-	3:1521785	1006	754	798	755	898	706	5.13025	3.070843	4.497696	2.97042	2.948975	2.832411	4.23293	2.917268	0.00236	0.047069	-
ENSMUSC	Tmem260	transmem	GO:00160	membran	-	-	-	-	14:484461	164	326	273	555	622	437	0.386906	0.614223	0.711821	1.01015	0.944946	0.811063	0.570983	0.922053	0.00236	0.047069	-
ENSMUSC	Dlgap5	discs, larg	GO:00081	metabolic	-	-	-	-	14:473877	5	14	7	24	50	19	0.030387	0.067956	0.047025	0.112537	0.195695	0.090846	0.048456	0.133026	0.002357	0.047069	DOWN
ENSMUSC	Insrr	insulin rec	GO:00325	developm	K05086	insulin rec	mmu0481	Regulatior	3:8779695	1	0	0	5	6	8	0.008098	0	0	0.031227	0.031278	0.050949	0.002699	0.037818	0.002391	0.047591	DOWN
ENSMUSC	Kng2	kininogen	GO:00081	metabolic	K03898	kininogen	mmu0461	Complem	16:229858	2	6	2	0	0	0	0.029195	0.06996	0.032275	0	0	0	0.04381	0	0.002392	0.047591	UP
ENSMUSC	Lhx5	LIM home	GO:00325	developm	K18493	LIM home	mmu0455	Signaling	5:1204316	0	0	1	7	5	7	0	0	0.025097	0.122625	0.073109	0.12505	0.008366	0.106928	0.002396	0.04762	DOWN
ENSMUSC	Gm7030	predicted	GO:00023	immune s	K06751	major hist	mmu0516	Human pa	17:361097	0	0	0	0	9	6	0	0	0	0	0.381738	0.310912	0	0.230884	0.002399	0.047633	DOWN
ENSMUSC	H2-Eb1	histocomp	GO:00023	immune s	K06752	major hist	mmu0516	HTLV-I inf	17:343058	18	56	25	70	131	67	0.928226	2.306371	1.424863	2.784986	4.350296	2.718176	1.553153	3.284486	0.002417	0.04792	DOWN
ENSMUSC	Pi4k2b	phosphati	GO:00081	metabolic	K13711	phosphati	mmu0110	Metabolic	5:5274157	26	60	36	80	132	83	0.334777	0.616992	0.512314	0.794696	1.094488	0.840759	0.488028	0.909981	0.002432	0.048168	-
ENSMUSC	Gzmk	granzyme	GO:00081	metabolic	-	-	-	-	13:113171	1	0	0	4	6	6	0.02734	0	0	0.084334	0.105596	0.129013	0.009113	0.106314	0.00247	0.048871	DOWN
ENSMUSC	Cdh3	cadherin 3	GO:00325	developm	K06796	cadherin 3	mmu0451	Cell adhes	8:1065108	21	64	10	4	21	8	0.292484	0.711863	0.153924	0.042975	0.188338	0.087649	0.38609	0.106321	0.002486	0.049124	UP
ENSMUSC	Pofut2	protein O	GO:00325	developm	K03691	peptide-C	mmu0051	Other type	10:772592	350	201	168	556	595	516	1.935719	0.887804	1.026908	2.372355	2.119075	2.245098	1.283477	2.245509	0.00249	0.049158	-
ENSMUSC	Serpinb7	serine (or	GO:00325	developm	-	-	-	-	1:1073996	0	0	0	6	4	2	0	0	0	0.139224	0.077478	0.047334	0	0.088012	0.002511	0.049513	DOWN
ENSMUSC	Nuak2	NUAK fan	GO:00081	metabolic	-	-	-	-	1:1323161	17	27	17	37	91	46	0.160359	0.203399	0.17723	0.269259	0.552755	0.341355	0.180329	0.38779	0.002541	0.049981	DOWN
ENSMUSC	Erich4	glutamate	-	-	-	-	-	-	7:2561462	0	0	0	2	10	2	0	0	0	0.103429	0.431593	0.105475	0	0.213499	0.002539	0.049981	DOWN
ENSMUSC	Pcdha4	protocadh	GO:00325	developm	-	-	-	-	18:369526	125	76	77	71	78	57	0.623651	0.302823	0.424592	0.273287	0.250599	0.223728	0.450355	0.249205	0.002552	0.050146	-
ENSMUSC	Hoxc6	homeobo	GO:00325	developm	-	-	-	-	15:102998	3	6	1	0	0	0	0.060753	0.097039	0.022384	0	0	0	0.060059	0	0.002606	0.051149	-
ENSMUSC	Lrig3	leucine-ri	GO:00325	developm	-	-	-	-	10:125966	29	52	33	75	106	78	0.141113	0.202077	0.177473	0.281554	0.332145	0.29859	0.173554	0.304096	0.002633	0.051625	-
ENSMUSC	Vsnl1	visinin-like	GO:00511	localizatio	-	-	-	-	12:113252	3269	3886	7364	11803	13140	11531	102.0445	96.87755	254.0593	284.2479	264.1341	283.1733	150.9938	277.1851	0.002641	0.051731	-
ENSMUSC	Banf1	barrier to	GO:00081	metabolic	-	-	-	-	19:536464	340	413	154	228	290	193	25.31677	24.5598	12.67355	13.09768	13.90535	11.30568	20.85004	12.76957	0.00265	0.051845	-
ENSMUSC	Plcb3	phospholi	GO:00048	signal trar	K05858	phosphati	mmu0110	Metabolic	19:695371	362	498	315	659	905	631	5.261371	5.78051	5.059977	7.389356	8.470205	7.214908	5.367286	7.69149	0.002697	0.052693	-
ENSMUSC	Stat1	signal trar	GO:00325	developm	K11220	signal trar	mmu0520	Pathways	1:5211944	546	818	526	1100	1243	1041	2.167604	2.593499	2.307912	3.36907	3.177699	3.251236	2.356339	3.266001	0.002749	0.053661	-
ENSMUSC	Trpm6	transient r	GO:00081	metabolic	K04981	transient r	mmu0497	Mineral al	19:187499	8	12	4	31	53	12	0.07506	0.089912	0.041479	0.224383	0.320203	0.088573	0.068817	0.211053	0.002818	0.054814	-
ENSMUSC	Mecom	MDS1 and	GO:00325	developm	K04462	ecotropic	mmu0520	Pathways	3:2995129	50	68	53	94	185	116	0.136737	0.148515	0.160189	0.198322	0.325791	0.249563	0.14848	0.257892	0.002815	0.054814	-
ENSMUSC	Aplp2	amyloid b	GO:00325	developm	-	-	-	-	9:3114955	9558	10444	8572	8882	10927	9763	64.48546	56.27396	63.91808	46.23127	47.47341	51.81899	61.55917	48.50789	0.002817	0.054814	-
ENSMUSC	Ttc33	tetratricop	-	-	-	-	-	-	15:518552	658	911	582	510	953	495	17.33229	19.16435	16.94338	10.36407	16.16506	10.25761	17.81334	12.26225	0.002963	0.057575	-
ENSMUSC	Cd79a	CD79A an	GO:00325	developm	K06506	CD79A an	mmu0466	B cell rece	7:2489738	0	3	0	7	7	8	0	0.100419	0	0.226313	0.188889	0.263731	0.033473	0.226311	0.002977	0.057776	-
ENSMUSC	Shb	src homol	GO:00325	developm	-	-	-	-	4:4542327	107	127	89	199	248	202	1.789923	1.696683	1.645461	2.568229	2.671507	2.658358	1.710689	2.632698	0.002985	0.057867	-
ENSMUSC	Etv5	ets varian	GO:00325	developm	K15593	ets translc	mmu0520	Transcript	16:223813	946	1160	724	1672	1821	1520	8.135347	7.966887	6.881299	11.09307	10.08439	10.28346	7.661178	10.48697	0.003004	0.058168	-
ENSMUSC	Gm4951	predicted	GO:00508	response	-	-	-	-	18:602120	8	13	7	18	51	25	0.184685	0.239666	0.178604	0.320576	0.758132	0.454011	0.200985	0.510906	0.003019	0.05833	-
ENSMUSC	Ctdsp2	CTD (carb	GO:00081	metabolic	-	-	-	-	10:126978	378	418	1011	461	393	372	2.37913	2.101112	7.032741	2.238507	1.592847	1.841964	3.837661	1.891106	0.003016	0.05833	-
ENSMUSC	Sh3rf2	SH3 doma	GO:00081	metabolic	-	-	-	-	18:420536	9	13	0	21	53	23	0.063956	0.07378	0	0.115132	0.242537	0.128587	0.045912	0.162085	0.003044	0.05875	-
ENSMUSC	Cd53	CD53 anti	GO:00325	developm	-	-	-	-	3:1067599	67	129	59	67	73	53	2.348378	3.611014	2.285567	1.811761	1.64767	1.461452	2.74832	1.640294	0.00313	0.060347	-
ENSMUSC	Ticrr	TOPBP1-i	GO:00081	metabolic	-	-	-	-	7:7966019	1	3	1	10	17	5	0.003739	0.008956	0.004131	0.028837	0.040918	0.014702	0.005609	0.028152	0.003172	0.061082	-
ENSMUSC	Slc4a5	solute car	GO:00325	developm	K13857	solute car	mmu0497	Bile secret	6:8321982	138	432	102	32	120	158	1.097706	2.744343	0.896716	0.196378	0.614672	0.988725	1.579588	0.599925	0.003192	0.0614	-
ENSMUSC	Pla2r1	phospholi	GO:00325	developm	K06560	mannose	mmu0515	Tuberculo	2:6041754	10	10	5	21	35	22	0.05391	0.043052	0.029793	0.08734	0.121502	0.093305	0.042252	0.100716	0.003258	0.062605	-
ENSMUSC	Cd3g	CD3 antig	GO:00048	signal trar	K06452	T-cell surf	mmu0516	HTLV-I inf	9:4496957	0	1	4	8	12	10	0	0.013623	0.075404	0.105265	0.131799	0.134174	0.029676	0.123746	0.003324	0.063805	-
ENSMUSC	Hk1	hexokinas	GO:00081	metabolic	K00844	hexokinas	mmu0110	Metabolic	10:622688	7342	11013	7970	8643	10350	8145	36.20486	43.37146	43.43679	32.88118	32.86605	31.59763	41.00437	32.44829	0.003338	0.063996	-
ENSMUSC	Irf8	interferon	GO:00325	developm	K10155	interferon	mmu0513	Pertussis -	8:1207363	94	144	96	203	300	185	0.661915	0.809808	0.747119	1.102812	1.360347	1.024842	0.739614	1.162667	0.003397	0.065067	-
ENSMUSC	Edn2	endothelir	GO:00325	developm	-	-	-	-	4:1201612	0	0	0	5	1	6	0	0	0	0.16109	0.026888	0.197138	0	0.128372	0.003435	0.065716	-
ENSMUSC	Col6a2	collagen,	GO:00508	response	K06238	collagen, t	mmu0415	PI3K-Akt s	10:765957	298	301	192	461	626	457	2.155843	1.739052	1.535143	2.572957	2.916284	2.600926	1.810013	2.696722	0.003494	0.066779	-
ENSMUSC	Reps2	RALBP1 as	GO:00431	ion bindin	-	-	-	-	X:1624119	5616	5272	4609	3945	6142	5297	38.26181	28.68532	34.70499	20.73556	26.94653	28.39091	33.88404	25.35767	0.003525	0.067295	-
ENSMUSC	Cwh43	cell wall b	GO:00081	metabolic	-	-	-	-	5:7340607	5	23	19	40	95	33	0.080715	0.296555	0.339023	0.498215	0.987643	0.419133	0.238764	0.634997	0.003583	0.068334	-
ENSMUSC	Igfbp1	insulin-like	GO:00325	developm	-	-	-	-	11:719778	0	0	0	5	9	0	0	0	0	0.154435	0.232047	0	0	0.128827	0.003589	0.068371	-
ENSMUSC	Mrgprf	MAS-rela	GO:00048	signal trar	-	-	-	-	7:1453008	10	12	7	20	35	32	0.15628	0.149768	0.120913	0.241131	0.352223	0.393427	0.142321	0.328927	0.003616	0.068793	-
ENSMUSC	Thbs2	thrombos	GO:00325	developm	K04659	thrombos	mmu0415	PI3K-Akt s	17:146655	133	233	114	107	141	143	1.371282	1.918568	1.299049	0.85112	0.936159	1.159902	1.529633	0.982394	0.003645	0.069284	-
ENSMUSC	Lrrtm4	leucine ric	GO:00325	developm	-	-	-	-	6:8001887	122	169	623	128	236	125	0.425904	0.471178	2.403746	0.344743	0.530543	0.343301	1.100276	0.406196	0.003673	0.069727	-
ENSMUSC	Tubb1	tubulin, be	GO:00051	structural	K07375	tubulin be	mmu0414	Phagoson	2:1744506	3	4	2	0	0	0	0.130277	0.138714	0.095998	0	0	0	0.121663	0	0.003729	0.070722	-
ENSMUSC	Ankrd6	ankyrin re	GO:00325	developm	-	-	-	-	4:3280403	326	430	519	840	962	834	2.716283	2.861359	4.779387	5.399672	5.161639	5.466823	3.452343	5.342711	0.003735	0.07075	-
ENSMUSC	Gm20537	predicted	-	-	-	-	-	-	9:8840743	17	1	0	0	0	0	0.847349	0.039812	0	0	0	0	0.29572	0	0.003761	0.071171	-
ENSMUSC	Pcdhgb1	protocadh	GO:00325	developm	-	-	-	-	18:376802	380	424	304	340	385	6065	2.775702	2.473437	2.454194	1.916012	1.81094	34.85224	2.567778	12.85973	0.003775	0.07136	-
ENSMUSC	D630039A	RIKEN cDI	-	-	-	-	-	-	4:5790848	3	18	2	26	47	18	0.064724	0.310148	0.047694	0.432765	0.652996	0.305512	0.140855	0.463758	0.003781	0.071389	-
ENSMUSC	Golt1a	golgi tran	GO:00511	localizatio	-	-	-	-	1:1333098	0	0	3	4	16	8	0	0	0.10118	0.094154	0.314384	0.192032	0.033727	0.20019	0.003795	0.07159	-
ENSMUSC	Zscan26	zinc finge	GO:00081	metabolic	-	-	-	-	13:214421	509	887	664	1090	2243	1050	6.985521	9.721886	10.07154	11.54085	19.82279	11.33656	8.926315	14.2334	0.003803	0.071623	-
ENSMUSC	Irx6	Iroquois r	GO:00325	developm	-	-	-	-	8:9267428	0	2	0	6	5	7	0	0.019777	0	0.057304	0.03986	0.068179	0.006592	0.055115	0.003805	0.071623	-
ENSMUSC	Foxa2	forkhead	GO:00325	developm	K08035	forkhead	mmu0421	Longevity	2:1480428	0	0	0	3	6	3	0	0	0	0.036999	0.061754	0.037718	0	0.04549	0.003852	0.072425	-
ENSMUSC	Il11ra2	interleukir	GO:00048	signal trar	-	-	-	-	JH584293	6	5	8	17	25	22	0.282017	0.187685	0.415572	0.616483	0.756701	0.81353	0.295091	0.728904	0.003895	0.073148	-
ENSMUSC	Ly6a	lymphocy	GO:00160	membran	-	-	-	-	15:749948	129	200	152	259	531	265	2.281716	2.825199	2.971418	3.534295	6.048138	3.687471	2.692778	4.423301	0.003909	0.07334	-
ENSMUSC	Nemp2	nuclear er	GO:00160	membran	-	-	-	-	1:5263070	66	87	54	37	76	50	0.326251	0.343457	0.295015	0.141104	0.241921	0.19444	0.321574	0.192488	0.003916	0.073387	-
ENSMUSC	Xdh	xanthine c	GO:00325	developm	K00106	xanthine c	mmu0110	Metabolic	17:738839	188	301	215	388	613	388	2.502491	3.199826	3.162999	3.984521	5.254477	4.063092	2.955105	4.43403	0.003926	0.073494	-
ENSMUSC	Kcna3	potassium	GO:00511	localizatio	-	-	-	-	3:1070361	123	125	105	109	106	90	3.948551	3.204708	3.72537	2.699533	2.19125	2.272919	3.62621	2.387901	0.003946	0.073782	-
ENSMUSC	Zc3hav1	zinc finge	GO:00081	metabolic	-	-	-	-	6:3830528	172	246	137	286	503	325	1.34417	1.535348	1.183297	1.724334	2.531326	1.998113	1.354272	2.084591	0.004024	0.075166	-
ENSMUSC	Tm4sf5	transmem	GO:00160	membran	-	-	-	-	11:705052	1	0	0	5	6	4	0.042741	0	0	0.16481	0.165081	0.134449	0.014247	0.15478	0.004051	0.075375	-
ENSMUSC	Mms22l	MMS22-li	GO:00081	metabolic	-	-	-	-	4:2449645	10	10	8	20	35	28	0.062445	0.049868	0.055208	0.096349	0.140738	0.137553	0.05584	0.12488	0.004051	0.075375	-
ENSMUSC	Uba1y	ubiquitin-	GO:00081	metabolic	K03178	ubiquitin-	mmu0501	Parkinson	Y:818649-	0	4	0	8	6	11	0	0.044518	0	0.086019	0.053846	0.120609	0.014839	0.086825	0.004047	0.075375	-
ENSMUSC	Mfsd4b3	major faci	GO:00511	localizatio	-	-	-	-	10:399469	1	0	0	5	6	4	0.034178	0	0	0.131793	0.13201	0.107514	0.011393	0.123772	0.004052	0.075375	-
ENSMUSC	Acads	acyl-Coer	GO:00081	metabolic	K00248	butyryl-Co	mmu0110	Metabolic	5:1151102	75	105	80	163	194	167	1.493109	1.669415	1.760227	2.503501	2.487059	2.615521	1.640917	2.53536	0.004072	0.075665	-
ENSMUSC	5730559C	RIKEN cDI	GO:00081	metabolic	-	-	-	-	1:1362135	43	72	51	102	176	97	0.452831	0.60554	0.593589	0.828695	1.193527	0.803613	0.550653	0.941945	0.004166	0.077326	-
ENSMUSC	Grtp1	GH regula	GO:00511	localizatio	-	-	-	-	8:1317202	36	56	39	98	116	74	0.373636	0.464175	0.447355	0.784697	0.775274	0.604212	0.428389	0.721395	0.004184	0.077569	-
ENSMUSC	Cpt2	carnitine p	GO:00081	metabolic	K08766	carnitine C	mmu0332	PPAR sign	4:1079039	70	97	76	132	193	187	0.807185	0.893292	0.968587	1.174306	1.433145	1.69641	0.889688	1.43462	0.004204	0.077855	-
ENSMUSC	Tmem127	transmem	GO:00508	response	-	-	-	-	2:1272479	266	318	255	2825	354	360	2.801701	2.674935	2.968435	22.95561	2.401034	2.983002	2.815024	9.446547	0.004218	0.077951	-
ENSMUSC	Tph1	tryptopha	GO:00325	developm	K00502	tryptopha	mmu0110	Metabolic	7:4664464	14	14	6	32	37	27	0.105158	0.083982	0.049812	0.185435	0.178964	0.159546	0.079651	0.174648	0.004213	0.077951	-
ENSMUSC	Tmem82	transmem	GO:00160	membran	-	-	-	-	4:1416133	8	4	10	18	30	28	0.07965	0.0318	0.110028	0.138256	0.192328	0.219304	0.073826	0.183296	0.004261	0.078659	-
ENSMUSC	Dpp4	dipeptidyl	GO:00081	metabolic	K01278	dipeptidyl	mmu0497	Protein di	2:6233007	32	43	28	65	124	62	0.272466	0.292397	0.263492	0.426978	0.679891	0.415305	0.276118	0.507391	0.004284	0.079004	-
ENSMUSC	Gm43518	predicted	-	-	-	-	-	-	5:1239274	3	3	7	18	13	22	0.2818	0.225093	0.726782	1.304498	0.786388	1.625808	0.411225	1.238898	0.004296	0.079142	-
ENSMUSC	Bace2	beta-site	GO:00081	metabolic	K07747	beta-site	mmu0501	Alzheimer	16:973567	67	62	37	105	160	107	1.094988	0.809239	0.668312	1.323906	1.683882	1.375721	0.857513	1.46117	0.004303	0.079186	-
ENSMUSC	Slc26a9	solute car	GO:00081	metabolic	K14706	solute car	mmu0497	Mineral al	1:1317440	0	1	0	3	7	5	0	0.008089	0	0.023443	0.045648	0.039836	0.002696	0.036309	0.004365	0.080251	-
ENSMUSC	Prkab1	protein kir	GO:00325	developm	K07199	5'-AMP-a	mmu0453	Tight junc	5:1160135	170	243	175	317	435	364	1.501722	1.714318	1.708536	2.160375	2.474479	2.529599	1.641525	2.388151	0.004377	0.080373	-
ENSMUSC	Retnla	resistin lik	GO:00508	response	-	-	-	-	16:488425	0	0	0	2	7	3	0	0	0	0.157566	0.46022	0.240943	0	0.286243	0.004388	0.080407	-
ENSMUSC	Ms4a18	membran	GO:00160	membran	-	-	-	-	19:109970	0	0	0	2	7	3	0	0	0	0.064848	0.189409	0.099163	0	0.117807	0.004386	0.080407	-
ENSMUSC	Tmem72	transmem	GO:00160	membran	-	-	-	-	6:1166790	232	679	138	61	258	219	0.802348	1.875388	0.527476	0.162756	0.574578	0.59584	1.068404	0.444392	0.004418	0.080878	-
ENSMUSC	Mamdc4	MAM dor	GO:00511	localizatio	-	-	-	-	2:2556311	9	17	7	34	27	31	0.088275	0.133164	0.075889	0.257281	0.17054	0.239209	0.099109	0.222343	0.004427	0.08096	-
ENSMUSC	Cd96	CD96 anti	GO:00023	immune s	-	-	-	-	16:460356	0	0	0	0	14	3	0	0	0	0	0.252043	0.065972	0	0.106005	0.004443	0.081171	-
ENSMUSC	Cd247	CD247 an	GO:00048	signal trar	K06453	CD3Z anti	mmu0465	Natural ki	1:1657886	0	2	0	7	11	2	0	0.011802	0	0.039896	0.052328	0.011626	0.003934	0.034617	0.00445	0.081205	-
ENSMUSC	Dao	D-amino	GO:00081	metabolic	K00273	D-amino-	mmu0110	Metabolic	5:1140037	0	6	4	0	0	0	0	0.072779	0.067151	0	0	0	0.046643	0	0.004471	0.081496	-
ENSMUSC	Col8a1	collagen,	GO:00325	developm	-	-	-	-	16:576242	151	393	109	49	181	147	1.804963	3.751719	1.440016	0.451872	1.393238	1.382355	2.332233	1.075822	0.004503	0.081993	-
ENSMUSC	Cyp2d9	cytochron	GO:00081	metabolic	K07414	cytochron	mmu0472	Serotoner	15:824523	0	0	0	0	5	9	0	0	0	0	0.119074	0.261841	0	0.126972	0.004521	0.08225	-
ENSMUSC	Hoxa3	homeobo	GO:00325	developm	-	-	-	-	6:5216906	0	3	2	6	9	14	0	0.0226	0.020849	0.043655	0.05466	0.103873	0.014483	0.067396	0.004559	0.082852	-
ENSMUSC	Amigo1	adhesion	GO:00325	developm	-	-	-	-	3:1081863	2053	530	393	500	595	590	9.482017	1.954942	2.006092	1.781607	1.769636	2.143753	4.481017	1.898332	0.004589	0.083314	-
ENSMUSC	Ada	adenosine	GO:00325	developm	K01488	adenosine	mmu0110	Metabolic	2:1637265	4	12	8	22	32	20	0.114348	0.273944	0.252729	0.485171	0.589047	0.44976	0.213674	0.507993	0.004607	0.083467	-
ENSMUSC	Smtn	smootheli	GO:00056	cell|intrac	-	-	-	-	11:351752	115	237	131	264	429	289	1.230153	2.024666	1.548735	2.178679	2.955098	2.432028	1.601184	2.521935	0.004606	0.083467	-
ENSMUSC	Atp6v1g2	ATPase, H	GO:00452	synapse|c	K02152	V-type H-	mmu0110	Metabolic	17:352336	5368	3748	4840	4455	4962	3748	52.01713	29.00543	51.83535	33.30519	30.96316	28.57225	44.28597	30.94687	0.004633	0.083844	-
ENSMUSC	Gm11437	predicted	GO:00160	membran	-	-	-	-	11:841483	0	0	0	6	7	0	0	0	0	0.228108	0.222124	0	0	0.150077	0.004674	0.084504	-
ENSMUSC	Itga10	integrin, a	GO:00508	response	K06586	integrin al	mmu0415	PI3K-Akt s	3:9664558	54	67	41	26	46	49	0.459209	0.45503	0.385343	0.170575	0.251903	0.32781	0.433194	0.250096	0.00473	0.085423	-
ENSMUSC	Dgka	diacylglyc	GO:00081	metabolic	K00901	diacylglyc	mmu0110	Metabolic	10:128720	264	290	223	407	598	473	2.831934	2.484404	2.643811	3.368242	4.1308	3.991632	2.653383	3.830225	0.004736	0.085446	-
ENSMUSC	Pdzk1ip1	PDZK1 int	GO:00055	extracellul	-	-	-	-	4:1150887	3	7	0	10	17	15	0.045531	0.084848	0	0.117095	0.166153	0.179099	0.04346	0.154116	0.004753	0.085663	-
ENSMUSC	Atp2b2	ATPase, C	GO:00325	developm	K05850	Ca2+ tran	mmu0402	cAMP sigr	6:1137438	9905	11519	8364	8571	13591	8248	27.08619	25.15673	25.27868	18.08237	23.93314	17.74406	25.84053	19.91986	0.004818	0.086746	-
ENSMUSC	Hand2	heart and	GO:00325	developm	-	-	-	-	8:5732098	0	0	0	5	8	0	0	0	0	0.100176	0.133799	0	0	0.077992	0.004897	0.088064	-
ENSMUSC	Gm44790	predicted	-	-	-	-	-	-	6:8642665	0	0	7	13	15	31	0	0	0.59199	0.767372	0.739063	1.866008	0.19733	1.124148	0.004983	0.089516	-
ENSMUSC	Slc9a2	solute car	GO:00511	localizatio	-	-	-	-	1:4068057	563	663	567	919	1368	1015	4.341462	4.083076	4.832346	5.467313	6.793111	6.157502	4.418962	6.139309	0.005002	0.089771	-
ENSMUSC	Npy2r	neuropep	GO:00325	developm	K04205	neuropep	mmu0408	Neuroacti	3:8253838	764	912	695	707	817	832	4.866811	4.639722	4.893087	3.474568	3.351411	4.169514	4.799873	3.665164	0.00503	0.09014	-
ENSMUSC	Hpse2	heparanas	GO:00038	catalytic a	K07965	heparanas	mmu0110	Metabolic	19:427889	0	0	0	1	7	4	0	0	0	0.026482	0.1547	0.107998	0	0.096393	0.005033	0.09014	-
ENSMUSC	Ptpn18	protein ty	GO:00081	metabolic	-	-	-	-	1:3445976	13	31	16	42	77	39	0.105217	0.200376	0.143123	0.262252	0.401315	0.24832	0.149572	0.303962	0.005048	0.090325	-
ENSMUSC	Heph	hephaesti	GO:00081	metabolic	K14735	hephaesti	mmu0497	Mineral al	X:9645535	158	224	182	276	508	358	1.014744	1.14893	1.29187	1.367539	2.100961	1.808812	1.151848	1.759104	0.005086	0.090907	-
ENSMUSC	Ahnak2	AHNAK nu	GO:00990	supramole	-	-	-	-	12:112772	37	50	28	27	29	30	0.132987	0.14352	0.111226	0.074867	0.06712	0.084826	0.129244	0.075604	0.005104	0.091127	-
ENSMUSC	Mmp8	matrix me	GO:00325	developm	-	-	-	-	9:7558456	0	10	5	0	0	1	0	0.201074	0.139148	0	0	0.019812	0.113407	0.006604	0.005255	0.093636	-
ENSMUSC	Myo19	myosin XI	GO:00325	developm	-	-	-	-	11:848801	119	145	106	207	286	230	0.707625	0.688607	0.696644	0.949638	1.09516	1.075959	0.697625	1.040253	0.00526	0.093636	-
ENSMUSC	Accsl	1-aminoc	GO:00081	metabolic	-	-	-	-	2:9385536	0	2	0	3	6	9	0	0.018754	0	0.027175	0.045357	0.083119	0.006251	0.051883	0.005258	0.093636	-
ENSMUSC	Map1s	microtubu	GO:00325	developm	-	-	-	-	8:7090593	936	1181	921	506	1461	470	4.294761	4.327717	4.670569	1.791198	4.31686	1.696572	4.431016	2.601544	0.005315	0.094504	-
ENSMUSC	Rtp1	receptor t	GO:00511	localizatio	-	-	-	-	16:234291	5	16	8	19	32	38	0.097841	0.250073	0.173022	0.286855	0.403271	0.585043	0.173645	0.425056	0.005407	0.096056	-
ENSMUSC	Uxs1	UDP-gluc	GO:00081	metabolic	K08678	UDP-gluc	mmu0110	Metabolic	1:4374696	703	592	647	517	674	647	6.252563	4.205039	6.359952	3.547511	3.860266	4.527077	5.605851	3.978285	0.005427	0.096316	-
ENSMUSC	Il11	interleukir	GO:00081	metabolic	K05417	interleukir	mmu0406	Cytokine-	7:4772373	10	10	11	4	6	5	0.242585	0.193725	0.29491	0.074852	0.093723	0.09542	0.24374	0.087998	0.005437	0.096382	-
ENSMUSC	Car4	carbonic a	GO:00081	metabolic	K18246	carbonic a	mmu0496	Proximal t	11:849577	509	612	492	895	1179	835	10.62152	10.1992	11.34701	14.40862	15.84301	13.70777	10.72258	14.65313	0.005519	0.097648	-
ENSMUSC	Wdr81	WD repea	GO:00081	metabolic	-	-	-	-	11:754409	528	671	475	634	465	425	2.410008	2.445979	2.396212	2.232565	1.36676	1.526104	2.4174	1.708476	0.005515	0.097648	-
ENSMUSC	Sgo1	shugoshir	GO:00329	macromo	K11580	shugoshir	mmu0411	Oocyte m	17:536747	4	1	6	19	19	10	0.057416	0.011465	0.095186	0.210388	0.175612	0.112916	0.054689	0.166305	0.005547	0.098034	-
ENSMUSC	Il18r1	interleukir	GO:00325	developm	K05173	interleukir	mmu0406	Cytokine-	1:4046555	0	0	0	0	9	4	0	0	0	0	0.041306	0.022426	0	0.021244	0.005659	0.099914	-
ENSMUSC	Steap2	six transm	GO:00081	metabolic	K14738	metallore	mmu0497	Mineral al	5:5664829	216	411	209	161	228	288	0.387966	0.589561	0.414891	0.223099	0.263712	0.406953	0.46414	0.297921	0.005761	0.101509	-
ENSMUSC	Hoxa9	homeobo	GO:00325	developm	K09294	homeobo	mmu0520	Transcript	6:5222310	2	4	3	8	26	10	0.016673	0.026633	0.027647	0.051461	0.139596	0.065594	0.023651	0.08555	0.005756	0.101509	-
ENSMUSC	Ooep	oocyte ex	GO:00325	developm	-	-	-	-	9:7837610	9	5	5	0	0	5	0.25702	0.114033	0.157827	0	0	0.112335	0.176294	0.037445	0.005816	0.102372	-
ENSMUSC	Ube2e2	ubiquitin-	GO:00081	metabolic	K20217	ubiquitin-	mmu0412	Ubiquitin	14:185735	876	1078	825	1700	1483	1828	7.36633	7.23955	7.667391	11.02875	8.030503	12.093	7.424424	10.38408	0.005848	0.102827	-

ENSMUSC	Hs3st3b1	heparan s	GO:00081	metabolic	K07809	[heparan :	mmu0053	Glycosam	11:638857	12	12	8	20	39	34	0.149192	0.11915	0.109922	0.191835	0.312241	0.332551	0.126088	0.278876	0.005862	0.102979	-
ENSMUSC	Pkmyt1	protein ki	GO:00081	metabolic	K06633	membran	mmu0411	Cell cycle	17:237263	22	37	20	69	54	59	0.658787	0.884849	0.661928	1.594071	1.041289	1.389929	0.735188	1.341763	0.005964	0.104663	-
ENSMUSC	Tyrobp	TYRO pro	GO:00325	developm	K07992	TYRO pro	mmu0438	Osteoclas	7:3041376	99	162	81	84	121	83	4.649789	6.076585	4.204694	3.043768	3.659668	3.066831	4.977023	3.256756	0.005976	0.104762	-
ENSMUSC	Acot3	acyl-CoA	GO:00081	metabolic	K01068	acyl-coen	mmu011C	Metabolic	12:840521	9	12	18	114	11	70	0.08554	0.091087	0.189087	0.835939	0.067324	0.523413	0.121905	0.475559	0.005989	0.104884	-
ENSMUSC	Myo5c	myosin V(	GO:00511	localizatio	-	-	-	-	9:7523202	25	95	27	93	212	98	0.114135	0.34638	0.136235	0.327562	0.623266	0.351981	0.198917	0.43427	0.006023	0.105265	-
ENSMUSC	Zfp750	zinc finge	GO:00325	developm	-	-	-	-	11:121510	2	5	0	8	19	8	0.037776	0.075434	0	0.1166	0.231143	0.118891	0.037737	0.155544	0.006022	0.105265	-
ENSMUSC	Nat2	N-acetylti	GO:00081	metabolic	K00622	arylamine	mmu011C	Metabolic	8:6749485	7	18	5	22	33	32	0.149499	0.307002	0.118025	0.362478	0.453835	0.537642	0.191509	0.451318	0.006049	0.105605	-
ENSMUSC	Clec12a	C-type le	GO:00160	membran	-	-	-	-	6:1293426	10	18	12	5	10	7	0.093879	0.134951	0.124504	0.036211	0.060452	0.0517	0.117778	0.049454	0.006054	0.105605	-
ENSMUSC	Wisp2	WNT1 ind	GO:00099	cellular pr	-	-	-	-	2:1638208	8	19	5	4	5	4	0.211286	0.40073	0.145949	0.081488	0.085031	0.0831	0.252655	0.083206	0.006107	0.106414	-
ENSMUSC	Glrx2	glutaredo	GO:00325	developm	-	-	-	-	1:1437163	470	569	431	1168	892	737	1.581468	1.529046	1.602827	3.032045	1.932777	1.950927	1.571114	2.30525	0.006157	0.107185	-
ENSMUSC	Fscn1	fascin acti	GO:00325	developm	-	-	-	-	5:1429603	1149	1461	1022	1549	2685	2462	12.66806	12.86431	12.45341	13.17565	19.06292	21.3545	12.66193	17.86436	0.006284	0.109277	-
ENSMUSC	Clic6	chloride ir	GO:00511	localizatio	-	-	-	-	16:924857	347	976	273	240	451	421	5.265201	11.82717	4.578193	2.809481	4.40673	5.025489	7.223522	4.080566	0.006341	0.109882	-
ENSMUSC	Golga1	golgi autc	GO:00160	membran	-	-	-	-	2:3901615	672	850	625	755	699	676	3.121183	3.15293	3.208314	2.705373	2.090653	2.470057	3.160809	2.422028	0.006326	0.109882	-
ENSMUSC	Kif22	kinesin fai	GO:00081	metabolic	K10403	kinesin far	mmu0491	Progester	7:1270277	14	22	11	34	50	33	0.122049	0.153169	0.105981	0.228666	0.280689	0.226322	0.127066	0.245226	0.006332	0.109882	-
ENSMUSC	Rhbdl2	rhomboid	GO:00081	metabolic	-	-	-	-	4:1237878	5	4	11	23	49	14	0.083451	0.053317	0.202922	0.296183	0.526681	0.183836	0.11323	0.335566	0.006344	0.109882	-
ENSMUSC	Slc2a4	solute car	GO:00325	developm	K07191	MFS trans	mmu0491	Insulin sig	11:699425	7	10	13	26	36	25	0.067726	0.077263	0.139005	0.194062	0.224289	0.19028	0.094665	0.202877	0.006387	0.110439	-
ENSMUSC	Lrat	lecithin-re	GO:00081	metabolic	K00678	phosphati	mmu0083	Retinol m	3:8289257	5	2	2	7	22	13	0.057048	0.018228	0.025224	0.061622	0.161656	0.116694	0.0335	0.113324	0.006388	0.110439	-
ENSMUSC	Vnn1	vanin 1 [S	GO:00325	developm	K08069	pantethei	mmu0077	Pantother	10:238946	1	0	1	7	6	6	0.020345	0	0.022481	0.109839	0.078581	0.096008	0.014275	0.09481	0.006444	0.111286	-
ENSMUSC	Gm45837	predicted	GO:00508	response	-	-	-	-	7:1014108	213	165	1749	300	210	341	3.142151	1.943917	28.51573	3.414279	1.9949	3.957434	11.2006	3.122204	0.006503	0.112196	-
ENSMUSC	Prlr	prolactin r	GO:00325	developm	K05081	prolactin r	mmu0415	PI3K-Akt s	15:101772	302	872	216	168	439	301	0.813713	1.876405	0.643227	0.349223	0.7617	0.63803	1.111115	0.582984	0.00656	0.11306	-
ENSMUSC	Gm14025	predicted	GO:00071	cell adhes	-	-	-	-	2:129025C	0	0	0	3	4	3	0	0	0	0.033318	0.037077	0.033966	0	0.034787	0.006677	0.114969	-
ENSMUSC	Birc3	baculovira	GO:00081	metabolic	K16060	baculovira	mmu052C	Pathways	9:7848699	28	44	25	55	108	64	0.248093	0.311355	0.244818	0.375974	0.616227	0.446123	0.268088	0.479441	0.006689	0.115065	-
ENSMUSC	Orc1	origin rec	GO:00081	metabolic	K02603	origin rec	mmu0411	Cell cycle	4:1085794	1	4	0	5	19	6	0.013859	0.044255	0	0.053441	0.169515	0.0654	0.019371	0.096118	0.006721	0.115494	-
ENSMUSC	Gbp10	guanylate	GO:00023	immune s	-	-	-	-	5:1052156	8	15	5	23	42	19	0.115456	0.17288	0.079753	0.256071	0.390306	0.215704	0.122696	0.28736	0.006797	0.116687	-
ENSMUSC	Ccl8	chemokin	GO:00325	developm	K16596	C-C motif	mmu0406	Cytokine-	11:821151	0	0	0	4	3	3	0	0	0	0.368691	0.230862	0.281964	0	0.293839	0.006805	0.116716	-
ENSMUSC	H2-T23	histocomp	GO:00325	developm	K06751	major hist	mmu0516	Human pa	17:360297	110	131	98	227	217	215	1.041139	0.990223	1.02515	1.65757	1.322608	1.600904	1.018837	1.527028	0.006904	0.118296	-
ENSMUSC	F5	coagulatic	GO:00508	response	K03902	coagulatic	mmu0461	Complem	1:1641518	326	1155	289	135	435	444	2.678993	7.580229	2.624814	0.855893	2.301965	2.870442	4.294679	2.009433	0.007017	0.119887	-
ENSMUSC	Tnc	tenascin C	GO:00325	developm	K06252	tenascin[-	mmu0415	PI3K-Akt s	4:6395978	158	268	127	301	403	348	1.216694	1.648181	1.080872	1.788222	1.99841	2.108214	1.315249	1.964949	0.007018	0.119887	-
ENSMUSC	Dera	deoxyribo	GO:00081	metabolic	K01619	deoxyribo	mmu0003	Pentose p	6:1377545	24	36	24	59	75	53	0.128455	0.153882	0.141966	0.243622	0.258494	0.223164	0.141435	0.24176	0.007011	0.119887	-
ENSMUSC	Ugt1a10	UDP glycc	GO:00081	metabolic	K00699	glucuronc	mmu011C	Metabolic	1:8805538	14	0	0	13	42	70	0.291553	0	0	0.208855	0.563229	1.146795	0.097184	0.639626	0.00704	0.120158	-
ENSMUSC	Car13	carbonic a	GO:00081	metabolic	K01672	carbonic a	mmu0091	Nitrogen r	3:1464172	22	24	11	42	82	32	0.584283	0.509043	0.322875	0.860559	1.402398	0.668603	0.472067	0.977187	0.007049	0.12018	-
ENSMUSC	Lhb	luteinizinc	GO:00508	response	K08521	luteinizing	mmu0408	Neuroacti	7:4542082	1	8	1	0	0	0	0.090083	0.575388	0.099538	0	0	0	0.255003	0	0.007056	0.12018	-
ENSMUSC	Ugdh	UDP-gluc	GO:00325	developm	K00012	UDPgluco	mmu011C	Metabolic	5:6541322	376	430	322	562	964	618	2.66456	2.433616	2.521971	3.072588	4.399154	3.445381	2.540049	3.639041	0.007083	0.12043	-
ENSMUSC	Scml4	sex comb	GO:00081	metabolic	-	-	-	-	10:428603	43	68	39	84	102	161	0.180448	0.227896	0.180879	0.271952	0.275637	0.531518	0.196408	0.359702	0.007084	0.12043	-
ENSMUSC	Ces2g	carboxyle	GO:00038	catalytic a	K03927	carboxyle:	mmu0098	Drug met:	8:1049617	0	1	0	3	10	2	0	0.01178	0	0.034139	0.094971	0.02321	0.003927	0.050773	0.007149	0.121408	-
ENSMUSC	Kcnk6	potassiun	GO:00511	localizatio	-	-	-	-	7:2922192	62	110	67	133	221	142	0.842557	1.193834	1.006296	1.394405	1.933992	1.518116	1.014229	1.615504	0.007187	0.121933	-
ENSMUSC	Slc52a3	solute car	GO:00511	localizatio	K14620	riboflavin	mmu0497	Vitamin d	2:1519965	25	23	22	50	66	49	0.223949	0.164549	0.217818	0.345551	0.380726	0.345316	0.202105	0.357198	0.007213	0.122267	-
ENSMUSC	Cul5	cullin 5 [S	GO:00081	metabolic	K10612	cullin 5[-]	mmu0412	Ubiquitin	9:5361458	1819	2114	1509	1857	2229	1607	8.617685	7.998502	7.901216	6.787345	6.800213	5.989417	8.172468	6.525658	0.007252	0.122808	-
ENSMUSC	Pmaip1	phorbol-1	GO:00081	metabolic	K10131	phorbol-1	mmu052C	Pathways	18:664586	24	20	13	51	63	33	0.553824	0.368578	0.331543	0.907932	0.936153	0.599079	0.417982	0.814388	0.007263	0.122873	-
ENSMUSC	Cast	calpastati	GO:00081	metabolic	-	-	-	-	13:746923	183	173	151	303	533	248	0.771662	0.582596	0.703719	0.985707	1.447292	0.822693	0.685992	1.085231	0.00727	0.122875	-
ENSMUSC	Smyd5	SET and N	GO:00081	metabolic	-	-	-	-	6:8543198	500	532	398	1015	1140	615	3.653114	3.104202	3.213829	5.721231	5.363554	3.534913	3.323715	4.873233	0.007281	0.122932	-
ENSMUSC	Syt17	synaptota	GO:00325	developm	-	-	-	-	7:1183807	793	999	705	1707	1253	1607	5.434238	5.467352	5.33951	9.024633	5.529314	8.663479	5.4137	7.739142	0.007313	0.123359	-
ENSMUSC	Gimap4	GTPase, IN	GO:00056	cell|organ	-	-	-	-	6:4868454	6	14	2	33	59	8	0.060432	0.112617	0.022266	0.256435	0.382676	0.063388	0.065105	0.234166	0.007347	0.123809	-
ENSMUSC	Edem1	ER degrac	GO:00081	metabolic	K10084	ER degrac	mmu0414	Protein pr	6:1088286	268	418	257	466	717	560	1.40207	1.746456	1.48599	1.880839	2.41551	2.304805	1.544839	2.200385	0.007395	0.124499	-
ENSMUSC	Csnk1g1	casein kin	GO:00325	developm	K08958	casein kin	mmu0434	Hedgehoc	9:6590892	415	609	640	580	529	403	0.958871	1.123764	1.634327	1.033879	0.787087	0.732535	1.238987	0.851167	0.007514	0.126318	-
ENSMUSC	Fam187b	family witl	GO:00160	membran	-	-	-	-	7:3097379	0	2	0	3	10	5	0	0.015092	0	0.021869	0.060836	0.037161	0.005031	0.039955	0.007524	0.126318	-
ENSMUSC	Tomt	transmem	GO:00325	developm	-	-	mmu011C	Metabolic	7:1018983	1	0	0	5	3	5	0.016606	0	0	0.064033	0.032075	0.0653	0.005535	0.053802	0.007528	0.126318	-
ENSMUSC	Gm4756	predicted	-	-	-	-	-	-	12:725760	0	0	0	3	2	5	0	0	0	0.024989	0.0139	0.042463	0	0.027117	0.007532	0.126318	-
ENSMUSC	Plk1	polo like k	GO:00081	metabolic	K06631	polo-like	mmu0411	Cell cycle	7:1221594	18	30	13	34	66	49	0.339204	0.451501	0.27076	0.494314	0.800935	0.726442	0.353822	0.673897	0.007542	0.126354	-
ENSMUSC	Postn	periostin,	GO:00325	developm	-	-	-	-	3:543611C	70	187	63	163	431	195	0.353577	0.754357	0.351705	0.635195	1.401912	0.77488	0.486546	0.937329	0.007569	0.126691	-
ENSMUSC	Krtcap3	keratinocy	GO:00160	membran	-	-	-	-	5:3125169	3	9	3	8	27	22	0.03169	0.075931	0.035028	0.065199	0.18367	0.182833	0.04755	0.143901	0.007584	0.126815	-
ENSMUSC	Kcnj13	potassiun	GO:00511	localizatio	K05006	potassiun	mmu0497	Protein di	1:8738636	115	280	70	34	139	98	4.586356	8.918106	3.0854	1.046098	3.56974	3.074719	5.529954	2.563519	0.007627	0.127414	-
ENSMUSC	Atp8a1	ATPase, a	GO:00400	locomotic	-	-	-	-	5:6761814	3327	4415	4514	4178	4931	3034	9.281747	9.836793	13.91827	8.992402	8.858632	6.658915	11.01227	8.169983	0.007657	0.127791	-
ENSMUSC	Pla2g2f	phospholi	GO:00081	metabolic	-	-	mmu011C	Metabolic	4:1387505	29	51	43	82	108	79	0.718329	1.008881	1.177174	1.567008	1.722684	1.539453	0.968128	1.609715	0.007671	0.127906	-
ENSMUSC	Trim30a	tripartite r	GO:00081	metabolic	-	-	-	-	7:104409C	70	119	66	155	225	138	0.951691	1.292091	0.991732	1.625782	1.969865	1.476012	1.078505	1.690553	0.007684	0.127936	-
ENSMUSC	Rbm47	RNA bind	GO:00325	developm	-	-	-	-	5:6601654	52	130	42	137	257	129	0.180728	0.360835	0.16133	0.367342	0.575186	0.352713	0.234298	0.431747	0.007688	0.127936	-
ENSMUSC	Kcna1	potassiun	GO:00325	developm	-	-	-	-	6:1266403	1975	2379	1763	2074	2455	1971	15.21446	14.63624	15.01026	12.32618	12.17855	11.94502	14.95365	12.14991	0.007786	0.129458	-
ENSMUSC	Cyp2s1	cytochron	GO:00081	metabolic	K07420	cytochron	mmu011C	Metabolic	7:2580247	84	89	64	134	221	138	0.737968	0.624449	0.621423	0.908226	1.250277	0.95378	0.66128	1.037428	0.007838	0.13019	-
ENSMUSC	Cnnm4	cyclin M4	GO:00325	developm	-	-	-	-	1:3647162	329	428	295	566	865	528	3.393832	3.526017	3.363286	4.504447	5.746005	4.28489	3.427712	4.845114	0.007864	0.130322	-
ENSMUSC	Tsku	tsukushi, s	GO:00325	developm	-	-	-	-	7:9835066	22	26	12	46	50	45	0.171051	0.161445	0.103117	0.275929	0.250344	0.275251	0.145204	0.267175	0.007868	0.130322	-
ENSMUSC	Cyp2d10	cytochron	GO:00081	metabolic	K07414	cytochron	mmu0472	Serotoner	15:824028	0	0	0	4	8	0	0	0	0	0.114391	0.19099	0	0	0.101794	0.007853	0.130322	-
ENSMUSC	Psme2b	protease (	GO:00081	metabolic	K06697	proteason	mmu0461	Antigen p	11:489453	71	142	77	158	229	196	5.179355	8.27282	6.208071	8.892142	10.7574	11.24826	6.553415	10.29927	0.007891	0.130578	-
ENSMUSC	Scube2	signal pep	GO:00325	developm	-	-	-	-	7:1097986	18	25	11	48	37	47	0.176379	0.195645	0.119128	0.362871	0.233477	0.362323	0.163717	0.319557	0.007911	0.130785	-
ENSMUSC	Ccl28	chemokin	GO:00400	locomotic	K05513	C-C motif	mmu0406	Cytokine-	13:119623	75	110	74	144	292	136	0.891621	1.044373	0.972293	1.320717	2.235406	1.271949	0.969429	1.609357	0.007973	0.131685	-
ENSMUSC	Best2	bestrophi	GO:00511	localizatio	K13879	bestrophi	mmu0497	Salivary se	8:850072C	0	0	0	1	4	5	0	0	0	0.019198	0.064091	0.097869	0	0.060386	0.008077	0.133273	-
ENSMUSC	Lrriq1	leucine-ri	-	-	-	-	-	-	10:103046	63	126	71	56	79	77	0.414469	0.662011	0.516241	0.284226	0.334682	0.39852	0.530907	0.339143	0.008128	0.133853	-
ENSMUSC	Gm45785	predicted	-	-	-	-	-	-	7:140818C	1	0	0	3	8	3	0.061818	0	0	0.143053	0.318382	0.145834	0.020606	0.202423	0.008124	0.133853	-
ENSMUSC	Sorcs1	sortilin-re	GO:00160	membran	-	-	-	-	19:501432	374	429	303	342	393	353	1.877169	1.719627	1.680815	1.324305	1.270219	1.393851	1.759204	1.329458	0.008164	0.134316	-
ENSMUSC	Scn8a	sodium ch	GO:00511	localizatio	-	-	-	-	15:100869	9848	7969	5636	7284	8328	7426	19.56848	12.64616	12.37733	11.1663	10.65625	11.60847	14.86399	11.14367	0.008347	0.137187	-
ENSMUSC	Pced1b	PC-estera	-	-	-	-	-	-	15:972471	68	44	71	50	52	37	0.938082	0.48476	1.08252	0.532145	0.461945	0.401557	0.835121	0.465216	0.008354	0.137187	-
ENSMUSC	Tmem138	transmem	GO:00099	cellular pr	-	-	-	-	19:105704	77	80	71	63	65	69	2.956893	2.453481	3.013355	1.866439	1.607371	2.084534	2.80791	1.852781	0.008417	0.138093	-
ENSMUSC	Serinc2	serine inc	GO:00081	metabolic	-	-	-	-	4:1302534	98	176	95	197	308	221	2.24277	3.216734	2.402868	3.478191	4.539029	3.978879	2.620791	3.998699	0.008453	0.138557	-
ENSMUSC	Khnyn	KH and N	-	-	-	-	-	-	14:558849	325	424	263	534	715	567	1.689993	1.760813	1.511484	2.142265	2.394209	2.319506	1.654097	2.285327	0.008484	0.138938	-
ENSMUSC	Snapin	SNAP-ass	GO:00325	developm	-	-	-	-	3:9048802	377	301	288	268	306	322	3.108565	1.98212	2.624562	1.704841	1.624782	2.088741	2.571749	1.806121	0.008531	0.139572	-
ENSMUSC	Cd84	CD84 anti	GO:00511	localizatio	-	-	-	-	1:1718396	95	139	76	57	108	94	0.76343	0.892082	0.675002	0.353384	0.558886	0.594271	0.776838	0.50218	0.00856	0.139921	-
ENSMUSC	Fxyd1	FXYD don	GO:00081	metabolic	K13360	FXYD don	mmu0402	cAMP sigr	7:3105167	237	294	232	242	267	233	2.991067	2.963261	3.236024	2.356258	2.169916	2.313369	3.063451	2.279848	0.008598	0.140408	-
ENSMUSC	Ptch1	patched 1	GO:00325	developm	K06225	patched 1	mmu052C	Pathways	13:635083	1004	1210	848	1228	715	768	2.374679	2.285612	2.216734	2.240784	1.08901	1.429037	2.292342	1.586277	0.008656	0.141209	-
ENSMUSC	Elf1	E74-like f	GO:00325	developm	-	-	-	-	14:794811	133	191	135	245	352	268	1.128663	1.294469	1.266175	1.604018	1.923577	1.789198	1.229769	1.772264	0.008737	0.142407	-
ENSMUSC	Rab2b	RAB2B, m	GO:00452	synapse|c	-	-	-	-	14:522617	498	544	319	461	466	360	3.046175	2.657485	2.156566	2.175492	1.835549	1.732364	2.620075	1.914469	0.008777	0.142926	-
ENSMUSC	Tmem141	transmem	GO:00099	cellular pr	-	-	-	-	2:2562006	89	98	71	180	179	149	1.981838	1.742798	1.74735	3.092286	2.566753	2.610196	1.823995	2.756412	0.008851	0.143993	-
ENSMUSC	Nostrin	nitric oxid	GO:00081	metabolic	-	-	-	-	2:691358C	56	86	49	111	184	105	1.712204	2.099953	1.655812	2.618296	3.622749	2.525616	1.822656	2.92222	0.008906	0.144746	-
ENSMUSC	Sytl1	synaptota	GO:00511	localizatio	-	-	-	-	4:133253C	9	4	7	22	25	18	0.159602	0.056646	0.137208	0.300995	0.285493	0.251118	0.117819	0.279202	0.00894	0.145029	-
ENSMUSC	Sh3bgrl2	SH3 dom:	GO:00055	extracellul	-	-	-	-	9:8354832	520	730	422	873	1153	890	2.918489	3.272071	2.61766	3.780063	4.167141	3.929664	2.936074	3.958956	0.008937	0.145029	-
ENSMUSC	Kif15	kinesin fai	GO:00990	supramole	-	-	-	-	9:122951C	8	21	8	25	45	29	0.029263	0.061345	0.032339	0.070548	0.105992	0.083447	0.040982	0.086662	0.008994	0.145781	-
ENSMUSC	Dlk1	delta-like	GO:00325	developm	-	-	-	-	12:109452	43	170	46	394	104	242	0.156383	0.493756	0.184895	1.105466	0.24356	0.692381	0.278345	0.680469	0.009243	0.149667	-
ENSMUSC	Prrg1	proline ric	GO:00055	extracellul	-	-	-	-	X:7844961	92	163	102	123	75	25	0.378861	0.536071	0.464234	0.390773	0.198887	0.080991	0.459722	0.22355	0.009277	0.150079	-
ENSMUSC	Tmem196	transmem	GO:00160	membran	-	-	-	-	12:119945	181	164	140	148	184	80	1.496075	1.082591	1.278944	0.943773	0.979373	0.520204	1.28587	0.81445	0.009365	0.151371	-
ENSMUSC	E2f8	E2F transc	GO:00325	developm	-	-	-	-	7:4886642	11	13	3	24	40	18	0.107414	0.101378	0.032378	0.180801	0.251516	0.138269	0.08039	0.190195	0.009409	0.151935	-
ENSMUSC	Gm21974	predicted	GO:00160	membran	-	-	-	-	7:1278862	0	0	0	20	1	0	0	0	0	0.462494	0.019298	0	0	0.160597	0.009458	0.152583	-
ENSMUSC	Entpd2	ectonucle	GO:00081	metabolic	K01509	adenosine	mmu0023	Purine me	2:2539587	81	148	74	179	230	181	1.486536	2.169198	1.50096	2.534396	2.718153	2.613248	1.718898	2.621932	0.009471	0.152653	-
ENSMUSC	Wdr76	WD repea	GO:00508	response	-	-	-	-	2:1215067	42	63	35	76	111	105	0.267296	0.320204	0.246186	0.373155	0.454904	0.525709	0.277895	0.451256	0.009541	0.15364	-
ENSMUSC	Pnliprp2	pancreatic	GO:00081	metabolic	K14075	pancreatic	mmu011C	Metabolic	19:587597	0	2	4	6	23	7	0	0.062247	0.17227	0.180361	0.577111	0.214588	0.078172	0.32402	0.009605	0.154531	-
ENSMUSC	Dnajc1	DnaJ heat	GO:00081	metabolic	K09521	DnaJ hom	mmu0414	Protein pr	2:1819565	656	779	534	770	559	513	2.562973	2.430654	2.305835	2.320925	1.406392	1.576769	2.433154	1.768029	0.009667	0.155372	-
ENSMUSC	Cyp4b1	cytochron	GO:00081	metabolic	-	-	-	-	4:1156247	2	2	4	10	16	9	0.042043	0.033588	0.092957	0.162216	0.216626	0.148863	0.056196	0.175902	0.009676	0.155372	-
ENSMUSC	Gramd1c	GRAM do	GO:00160	membran	-	-	-	-	16:439803	37	74	45	109	103	111	0.309858	0.49492	0.416501	0.704226	0.555455	0.731288	0.407093	0.663657	0.009706	0.15571	-
ENSMUSC	Ksr1	kinase sup	GO:00048	signal trar	K14958	kinase sup	mmu0401	Ras signal	11:790134	3185	3978	2872	4150	2971	2391	17.71291	17.66816	17.65273	17.8057	10.63991	10.46095	17.67793	12.96885	0.009733	0.156014	-
ENSMUSC	Pou4f1	POU dom	GO:00325	developm	-	-	-	-	14:104461	2	4	0	4	29	8	0.023283	0.037191	0	0.035927	0.217436	0.073275	0.020158	0.108879	0.009795	0.156851	-
ENSMUSC	Sostdc1	sclerostin	GO:00325	developm	-	-	-	-	12:363141	122	334	96	73	143	155	4.18252	9.144736	3.637435	1.930776	3.156964	4.180445	5.654897	3.089395	0.009857	0.157711	-
ENSMUSC	Ldah	lipid drop	-	-	-	-	-	-	12:820810	494	756	456	224	802	338	2.822376	3.449494	2.879381	0.98734	2.950642	1.5192	3.050417	1.81906	0.009867	0.157718	-
ENSMUSC	Hmgb3	high mob	GO:00325	developm	-	-	-	-	X:7155591	185	206	116	130	179	150	3.561083	3.166822	2.467826	1.930557	2.218801	2.271501	3.065243	2.140286	0.00988	0.157777	-
ENSMUSC	Rps27l	ribosomal	GO:00451	translatior	K02978	small subu	mmu0301	Ribosome	9:6694608	45	82	38	106	125	103	1.445349	2.103406	1.348922	2.62663	2.585381	2.602613	1.632559	2.604875	0.00989	0.157799	-
ENSMUSC	Ip6k3	inositol he	GO:00081	metabolic	K07756	inositol-h	mmu0407	Phosphati	17:271439	4	2	1	12	10	10	0.105872	0.04228	0.029253	0.245016	0.17043	0.208211	0.059135	0.207886	0.009921	0.15815	-
ENSMUSC	Foxm1	forkhead	GO:00325	developm	K09406	forkhead	mmu0421	Cellular se	6:1283629	24	21	14	46	52	41	0.160717	0.112308	0.103614	0.237648	0.224234	0.215992	0.125546	0.225958	0.01001	0.158977	-
ENSMUSC	Hmox1	heme oxy	GO:00325	developm	K00510	heme oxy	mmu011C	Metabolic	8:7509362	138	198	88	107	141	118	3.596242	4.120805	2.534547	2.151232	2.366168	2.419156	3.417198	2.312185	0.009993	0.158977	-
ENSMUSC	F13a1	coagulatic	GO:00081	metabolic	K03917	coagulatic	mmu0461	Complem	13:368671	47	122	59	47	76	51	0.411899	0.853896	0.571474	0.317782	0.428909	0.351624	0.612423	0.366105	0.010004	0.158977	-
ENSMUSC	B3gnt6	UDP-GlcN	GO:00081	metabolic	K00739	acetylgala	mmu011C	Metabolic	7:9819241	3	3	6	10	27	12	0.077254	0.061708	0.170781	0.198682	0.44775	0.243117	0.103248	0.296516	0.01001	0.158977	-
ENSMUSC	Rpl36a	ribosomal	GO:00081	metabolic	K02929	large subu	mmu0301	Ribosome	X:1345856	158	84	104	91	120	65	3.28806	1.396069	2.392013	1.461021	1.608118	1.064156	2.358714	1.377765	0.010147	0.161017	-
ENSMUSC	Zfpm1	zinc finge	GO:00325	developm	-	-	-	-	8:1222821	714	829	430	600	669	621	8.931676	8.281989	5.944974	5.790503	5.389087	6.111347	7.719546	5.763646	0.010179	0.161369	-
ENSMUSC	Rnf225	ring finge	GO:00160	membran	-	-	-	-	7:1292741	16	10	9	31	39	25	0.301613	0.150542	0.187501	0.450845	0.473423	0.37075	0.213219	0.431673	0.010214	0.161776	-
ENSMUSC	C77080	expressed	GO:00325	developm	-	-	-	-	4:1292195	204	241	147	367	635	251	2.085359	1.967499	1.660791	2.894322	4.180028	2.01853	1.90455	3.03096	0.010236	0.161923	-
ENSMUSC	Krcc1	lysine-rich	GO:00508	response	-	-	-	-	6:7127167	186	266	69	116	159	110	3.050452	3.484001	1.250674	1.467701	1.679204	1.419239	2.595042	1.522048	0.010242	0.161923	-
ENSMUSC	Ppef1	protein ph	GO:00081	metabolic	-	-	-	-	X:160623C	13	9	10	7	6	4	0.247894	0.137065	0.210744	0.102979	0.073673	0.060002	0.198568	0.078885	0.010257	0.162009	-
ENSMUSC	Gfpt1	glutamine	GO:00081	metabolic	K00820	glucosam	mmu011C	Metabolic	6:8704284	1800	2349	1613	2824	3982	2926	9.92546	10.34444	9.830131	12.0136	14.1395	12.69297	10.03334	12.94869	0.010316	0.1628	-
ENSMUSC	Urah	urate (5-h	GO:00081	metabolic	K07127	5-hydroxy	mmu011C	Metabolic	7:140835C	5	0	7	12	20	21	0.095934	0	0.148463	0.17764	0.247129	0.317012	0.081466	0.24726	0.010372	0.163541	-
ENSMUSC	Igsf9	immunog	GO:00325	developm	-	-	-	-	1:1724817	35	48	44	63	151	87	0.203178	0.222536	0.282299	0.282149	0.564472	0.39732	0.236004	0.414647	0.010398	0.163793	-
ENSMUSC	Sdr9c7	4short ch:	GO:00081	metabolic	-	-	-	-	10:127898	0	0	0	4	6	0	0	0	0	0.028187	0.035293	0	0	0.02116	0.010425	0.164061	-
ENSMUSC	Actc1	actin, alph	GO:00325	developm	K12314	actin, alph	mmu0426	Adrenergi	2:1140472	0	0	0	0	6	4	0	0	0	0	0.145798	0.118744	0	0.088181	0.010434	0.164061	-
ENSMUSC	Olfr78	olfactory r	GO:00048	signal trar	K04257	olfactory r	mmu0474	Olfactory	7:1027385	0	5	0	7	9	8	0	0.023807	0	0.032198	0.034554	0.037521	0.007936	0.034758	0.010455	0.16425	-
ENSMUSC	Metrnl	meteorin,	GO:00325	developm	-	-	-	-	11:121701	86	80	55	132	186	132	2.321384	1.724595	1.640809	2.748873	3.23309	2.803074	1.895596	2.928345	0.010523	0.165171	-
ENSMUSC	Slco1b2	solute car	GO:00325	developm	K05043	solute car	mmu0497	Bile secret	6:1416295	0	4	4	0	0	0	0	0.032702	0.045263	0	0	0	0.025988	0	0.010568	0.165731	-
ENSMUSC	Slc9a3r1	solute car	GO:00325	developm	K13365	Na(+)/H(+	mmu0516	Human pa	11:115163	759	1041	835	1361	1790	1409	24.07435	26.36993	29.27155	33.30437	36.5612	35.15884	26.57194	35.00813	0.010601	0.16595	-
ENSMUSC	Far1	fatty acyl	GO:00081	metabolic	K13356	alcohol-fo	mmu0414	Peroxisom	7:1135138	850	948	653	1644	1254	1515	4.294176	3.824853	3.646036	6.407562	4.079558	6.021217	3.921688	5.502779	0.010601	0.16595	-
ENSMUSC	Olfr1507	olfactory r	GO:00048	signal trar	K04257	olfactory r	mmu0474	Olfactory	14:524887	2	8	1	0	1	1	0.022015	0.070341	0.012169	0	0.007088	0.008663	0.034842	0.00525	0.010697	0.1673	-
ENSMUSC	Maff	v-maf mu	GO:00325	developm	-	-	-	-	15:793475	13	8	8	20	47	23	0.37459	0.184102	0.254756	0.444597	0.872099	0.521387	0.27115	0.612694	0.01072	0.167504	-
ENSMUSC	Angpt2	angiopoie	GO:00325	developm	K05466	angiopoie	mmu0415	PI3K-Akt s	8:1869026	49	61	42	44	45	39	0.784852	0.780311	0.743507	0.543717	0.464144	0.49143	0.769557	0.499764	0.010785	0.168367	-
ENSMUSC	Folr1	folate rec	GO:00325	developm	K13649	folate rec	mmu0414	Endocytos	7:1018583	75	204	60	31	68	102	0.829295	1.801452	0.733239	0.264449	0.484182	0.887273	1.121329	0.545301	0.010838	0.169038	-
ENSMUSC	Timp1	tissue inhi	GO:00325	developm	K16451	metallope	mmu0406	HIF-1 sigr	X:2087016	14	24	7	10	9	5	0.617654	0.845586	0.341335	0.340371	0.255687	0.173534	0.601525	0.256531	0.01089	0.169675	-
ENSMUSC	Akr1b8	aldo-keto	GO:00081	metabolic	K00011	aldehyde	mmu011C	Metabolic	6:3435411	8	17	14	20	53	36	0.287349	0.487616	0.555735	0.554177	1.225811	1.017204	0.443567	0.932397	0.010898	0.169675	-
ENSMUSC	Igflr1	IGF-like fa	GO:00160	membran	-	-	-	-	7:3056542	0	0	0	24	0	0	0	0	0	0.508798	0	0	0	0.169599	0.010993	0.170998	-
ENSMUSC	Hpgd	hydroxypr	GO:00325	developm	K00069	15-hydrox	mmu052C	Transcript	8:5629458	117	184	129	207	432	242	4.244685	5.33117	5.172433	5.793753	10.09247	6.90694	4.916096	7.597721	0.011032	0.171451	-
ENSMUSC	Timd4	T cell imm	GO:00160	membran	-	-	-	-	11:468108	0	0	0	4	7	0	0	0	0	0.086701	0.12665	0	0	0.071117	0.011173	0.173485	-
ENSMUSC	Iigp1	interferon	GO:00081	metabolic	-	-	-	-	18:603760	87	181	117	272	425	159	1.702033	2.827971	2.529776	4.105335	5.354187	2.447132	2.35326	3.968884	0.01119	0.173605	-
ENSMUSC	Sulf1	sulfatase 1	GO:00325	developm	-	-	-	-	1:1269227	310	884	245	236	409	399	1.171939	2.668959	1.023661	0.688313	0.995684	1.186663	1.62152	0.956887	0.011205	0.173684	-
ENSMUSC	Ndufaf1	NADH de	GO:00056	cell|intrac	-	-	-	-	2:1196554	297	379	278	274	395	277	6.1161	6.233101	6.327177	4.353111	5.238055	4.487549	6.225459	4.692905	0.011219	0.17374	-
ENSMUSC	H2-Q7	histocomp	GO:00325	developm	K06751	major hist	mmu0516	Human pa	17:354391	9	49	33	67	92	77	0.074338	0.323241	0.301263	0.42696	0.489353	0.50036	0.232947	0.472224	0.011292	0.174715	-
ENSMUSC	Ube2g2	ubiquitin-	GO:00081	metabolic	K04555	ubiquitin-	mmu0414	Protein pr	10:776222	1171	1466	908	710	1746	494	23.49624	23.49206	20.13603	10.99082	22.56006	7.797931	22.37478	13.78294	0.011445	0.176928	-
ENSMUSC	S100a9	S100 calci	GO:00325	developm	-	-	mmu0465	IL-17 sign	3:9069263	11	28	9	7	2	12	0.922614	1.875511	0.834224	0.452936	0.107995	0.791802	1.210783	0.450911	0.011585	0.178926	-
ENSMUSC	Ehhadh	enoyl-Coe	GO:00081	metabolic	K07514	enoyl-Co/	mmu011C	Metabolic	16:217612	27	51	42	64	147	77	0.546252	0.82402	0.939115	0.998923	1.91512	1.225535	0.769796	1.379859	0.011693	0.180279	-
ENSMUSC	Phf11a	PHD finge	GO:00023	immune s	-	-	-	-	14:592769	0	0	0	0	7	3	0	0	0	0	0.203258	0.106413	0	0.103224	0.011687	0.180279	-
ENSMUSC	Cntn2	contactin	GO:00325	developm	K06760	contactin	mmu0451	Cell adhes	1:1325094	1905	1674	1493	1672	2038	1567	5.886124	4.130816	5.098485	3.985668	4.055023	3.809029	5.038475	3.949907	0.011717	0.180488	-
ENSMUSC	D6Ertd527	DNA segr	-	-	-	-	-	-	6:8710474	1	0	0	3	5	4	0.007698	0	0	0.017814	0.024778	0.024216	0.002566	0.022269	0.011796	0.181543	-
ENSMUSC	Tbx3	T-box 3 [S	GO:00325	developm	K10177	T-box pro	mmu0455	Signaling	5:1196706	57	93	55	127	158	115	0.250976	0.327032	0.267651	0.431415	0.447993	0.398356	0.281886	0.425921	0.011809	0.18158	-
ENSMUSC	Il7	interleukir	GO:00325	developm	K05431	interleukir	mmu052C	Pathways	3:7569994	3	2	2	18	14	4	0.017761	0.009458	0.013088	0.082219	0.053376	0.01863	0.013436	0.051408	0.011923	0.183173	-
ENSMUSC	Asgr1	asialoglyc	GO:00511	localizatio	K10063	asialoglyc	mmu0491	Thyroid ho	11:700540	0	12	3	12	21	26	0	0.28105	0.097243	0.271494	0.396577	0.599857	0.126098	0.422643	0.011967	0.183682	-
ENSMUSC	Gdi2	guanosine	GO:00511	localizatio	-	-	-	-	13:353806	3022	2466	2644	2558	3253	2566	12.91145	8.414333	12.48501	8.431633	8.949925	8.624786	11.27027	8.668781	0.012038	0.184542	-
ENSMUSC	Dsg1c	desmogle	GO:00160	membran	K07596	desmogle	mmu0515	Staphyloc	18:202473	3	5	2	0	0	1	0.044807	0.059639	0.033017	0	0	0.011752	0.045821	0.003917	0.012044	0.184542	-
ENSMUSC	Zc4h2	zinc finge	GO:00325	developm	-	-	-	-	X:9563919	313	209	299	250	305	179	3.31216	1.766277	3.496922	2.040975	2.078364	1.490154	2.858453	1.869831	0.012104	0.185306	-
ENSMUSC	1700020L	RIKEN cDI	-	-	-	-	-	-	11:834376	3	5	2	0	2	0	0.121064	0.161138	0.089209	0	0.051963	0	0.123804	0.017321	0.012152	0.185874	-
ENSMUSC	Slc9a6	solute car	GO:00325	developm	K12041	solute car	mmu0426	Cardiac m	X:5660975	880	2311	822	990	1288	1121	4.616868	9.683018	4.766323	4.007103	4.351466	4.626809	6.355403	4.328459	0.012185	0.186209	-
ENSMUSC	Cbs	cystathior	GO:00325	developm	K01697	cystathior	mmu011C	Metabolic	17:316126	348	415	328	591	667	687	3.859107	3.675369	4.020019	5.056209	4.763079	5.993418	3.851498	5.270902	0.012223	0.186628	-
ENSMUSC	Me1	malic enz	GO:00081	metabolic	K00029	malate de	mmu011C	Metabolic	9:8658137	930	758	558	1303	1546	1296	10.1636	6.615758	6.739777	10.986	10.88	11.14245	7.839712	11.00282	0.012246	0.186822	-
ENSMUSC	Acvrl1	activin A r	GO:00325	developm	-	-	-	-	15:101128	121	185	104	264	273	223	1.182088	1.443381	1.122907	1.989744	1.717439	1.713878	1.249459	1.80702	0.012383	0.188077	-
ENSMUSC	Fasl	Fas liganc	GO:00325	developm	K04389	tumor nec	mmu052C	Pathways	1:1617806	0	0	0	3	4	2	0	0	0	0.060323	0.067128	0.041011	0	0.056154	0.012362	0.188077	-
ENSMUSC	Ms4a1	membran	GO:00023	immune s	K06466	B-lympho	mmu0464	Hematop	19:112496	0	0	0	2	4	3	0	0	0	0.011236	0.018755	0.017181	0	0.015724	0.012375	0.188077	-
ENSMUSC	Myl9	myosin, lig	GO:00508	response	K12755	myosin re	mmu0481	Regulatior	2:1567754	213	266	234	369	601	386	6.172454	6.156115	7.494475	8.249649	11.21523	8.799888	6.607682	9.421588	0.012365	0.188077	-
ENSMUSC	Amy2a3	amylase 2	GO:00081	metabolic	K01176	alpha-am	mmu011C	Metabolic	3:1133124	0	0	0	2	4	3	0	0	0	0.02535	0.042314	0.038763	0	0.035476	0.01237	0.188077	-
ENSMUSC	Wnt9a	wingless-	GO:00325	developm	K01064	wingless-	mmu052C	Pathways	11:593069	142	136	164	118	183	80	1.358759	1.039293	1.734385	0.871098	1.127616	0.602217	1.377479	0.866977	0.012441	0.188804	-
ENSMUSC	Kif23	kinesin fai	GO:00511	localizatio	K17387	kinesin far	mmu052C	MicroRNA	9:619159C	28	29	16	48	86	41	0.157844	0.130564	0.099688	0.208757	0.312196	0.181832	0.129365	0.234262	0.012468	0.188872	-
ENSMUSC	Mbnl3	musclebli	GO:00325	developm	-	-	-	-	X:5111726	1	8	2	13	31	6	0.005185	0.033117	0.011458	0.051983	0.103472	0.024467	0.016586	0.059974	0.012459	0.188872	-
ENSMUSC	Scn1a	sodium ch	GO:00511	localizatio	K04833	voltage-g	mmu0472	Dopamine	2:6627077	2366	2157	1110	1778	1473	1812	5.88446	4.284378	3.051137	3.411567	2.359116	3.545364	4.406658	3.105349	0.01248	0.188898	-
ENSMUSC	Tinag	tubulointe	GO:00081	metabolic	-	-	-	-	9:7695169	0	2	0	0	15	8	0	0.04107	0	0	0.248325	0.161792	0.01369	0.136706	0.012526	0.18943	-
ENSMUSC	Clps	colipase, p	GO:00081	metabolic	K14460	colipase[-	mmu0497	Fat digest	17:285582	0	0	0	4	3	2	0	0	0	0.205007	0.128368	0.104557	0	0.145977	0.012569	0.189917	-
ENSMUSC	Gale	galactose	GO:00081	metabolic	K01784	UDP-gluc	mmu011C	Metabolic	4:1359637	148	175	157	254	388	276	2.404625	2.270762	2.81924	3.18384	4.059505	3.527817	2.498209	3.590388	0.012581	0.189935	-
ENSMUSC	Gxylt2	glucoside	GO:00081	metabolic	K13676	UDP-xylo:	mmu0051	Other typ	6:1007047	55	78	54	44	60	58	0.378175	0.428321	0.410363	0.233407	0.265668	0.313741	0.40562	0.270938	0.012593	0.189939	-
ENSMUSC	Aldh3b3	aldehyde	GO:00081	metabolic	K00129	aldehyde	mmu011C	Metabolic	19:395864	0	0	0	4	3	2	0	0	0	0.023305	0.014593	0.011886	0	0.016595	0.012616	0.190119	-

ENSMUSC	Ankrd61	ankyrin re	GO:00971	organic cy	-	-	-	-	5:1438907	0	0	0	2	3	4	0	0	0	0.025521	0.031953	0.052039	0	0.036504	0.012631	0.190159	-
ENSMUSC	Ckap2l	cytoskelet	GO:00056	cell|intrace	-	-	-	-	2:1292682	19	22	9	38	53	30	0.369811	0.341982	0.193597	0.570617	0.66429	0.459366	0.301797	0.564758	0.01264	0.190159	-
ENSMUSC	Fhod1	formin ho	GO:00081	metabolic	-	-	-	-	8:1053291	35	66	29	79	109	81	0.340942	0.513457	0.312219	0.593704	0.683747	0.620739	0.388872	0.63273	0.012778	0.19206	-
ENSMUSC	Spata21	spermato	GO:00431	ion bindin	-	-	-	-	4:1410883	0	2	3	7	12	6	0	0.034057	0.070692	0.115132	0.164749	0.100634	0.034916	0.126838	0.012802	0.192172	-
ENSMUSC	Ttr	transthyre	GO:00081	metabolic	K20731	transthyre	-	-	18:206652	6316	23633	5257	1913	8633	8923	316.3601	945.3755	291.0211	73.92392	278.4557	351.6102	517.5856	234.6633	0.012807	0.192172	-
ENSMUSC	Hoxa7	homeobo	GO:00325	developm	-	-	-	-	6:5221449	5	12	4	16	35	15	0.054983	0.105394	0.048622	0.135753	0.247863	0.129776	0.069666	0.171131	0.012839	0.192489	-
ENSMUSC	Lnpep	leucyl/cys	GO:00081	metabolic	K01257	cystinyl ar	mmu0461	Renin-anc	17:175277	1851	2338	1536	1938	2244	1934	21.81405	22.00494	20.00635	17.62033	17.02971	17.9307	21.27511	17.52691	0.012895	0.193164	-
ENSMUSC	Ifngr1	interferon	GO:00048	signal trar	K05132	interferon	mmu0520	Pathways	10:195919	246	321	232	417	631	412	3.607178	3.759092	3.759819	4.717365	5.95822	4.752706	3.708696	5.142764	0.012981	0.193619	-
ENSMUSC	Clec2d	C-type lec	GO:00325	developm	-	-	-	-	6:1291806	49	71	65	122	137	112	2.466492	2.854205	3.616141	4.737738	4.440768	4.435174	2.978946	4.537893	0.012948	0.193619	-
ENSMUSC	Hand1	heart and	GO:00325	developm	K09071	heart-anc	mmu0455	Signaling	11:578287	0	0	0	2	5	2	0	0	0	0.023716	0.049482	0.024185	0	0.032461	0.012966	0.193619	-
ENSMUSC	Hsd3b2	hydroxy-c	GO:00081	metabolic	K00070	3beta-hyc	mmu0110	Metabolic	3:9870925	0	0	0	3	8	0	0	0	0	0.02354	0.052392	0	0	0.025311	0.01295	0.193619	-
ENSMUSC	Defa23	defensin,	GO:00508	response	-	-	-	-	8:2105504	0	0	0	2	5	2	0	0	0	0.229256	0.478323	0.233791	0	0.31379	0.012971	0.193619	-
ENSMUSC	Trpv4	transient r	GO:00325	developm	K04973	transient r	mmu0421	Cellular se	5:1146221	72	209	52	34	90	83	0.638611	1.48045	0.50974	0.232652	0.514043	0.579152	0.876267	0.441949	0.012999	0.193716	-
ENSMUSC	Csrp2	cysteine a	GO:00325	developm	-	-	-	-	10:110919	43	94	56	133	178	96	0.781869	1.365015	1.125372	1.865712	2.084195	1.37323	1.090752	1.774379	0.013022	0.193895	-
ENSMUSC	Ly75	lymphocy	GO:00048	signal trar	-	-	-	-	2:6029210	50	55	20	66	148	84	0.416782	0.36614	0.184255	0.424433	0.794422	0.550843	0.322392	0.5899	0.013049	0.194122	-
ENSMUSC	Tma7	translatior	-	-	-	-	-	-	9:1090745	185	296	214	233	261	142	6.370984	8.14089	8.145058	6.190419	5.788015	3.847093	7.552311	5.275176	0.013098	0.194688	-
ENSMUSC	Tbx15	T-box 15	GO:00325	developm	-	-	-	-	3:9924038	5	10	10	22	24	24	0.079289	0.126651	0.175273	0.269175	0.245102	0.299432	0.127071	0.271237	0.013185	0.19581	-
ENSMUSC	Herc6	hect dom	GO:00325	developm	-	-	-	-	6:5758100	118	153	103	199	275	223	0.677788	0.701856	0.653874	0.881847	1.017179	1.007689	0.677839	0.968905	0.013228	0.196283	-
ENSMUSC	Agbl1	ATP/GTP	GO:00081	metabolic	-	-	-	-	7:7622988	0	0	0	4	4	1	0	0	0	0.0354	0.029553	0.009027	0	0.02466	0.013452	0.199447	-
ENSMUSC	Ttc16	tetratricop	-	-	-	-	-	-	2:3275702	10	21	10	27	44	30	0.061427	0.103021	0.067888	0.127952	0.174045	0.144973	0.077445	0.14899	0.013483	0.199724	-
ENSMUSC	Ak2	adenylate	GO:00081	metabolic	K00939	adenylate	mmu0110	Metabolic	4:1289919	298	373	262	513	738	451	7.200362	7.197693	6.99658	9.562825	11.48284	8.572852	7.131545	9.872839	0.013598	0.201255	-
ENSMUSC	Pmp22	periphera	GO:00325	developm	-	-	-	-	11:631289	195	345	214	375	584	430	2.644091	3.735998	3.207015	3.922859	5.099279	4.586912	3.195701	4.53635	0.013718	0.202863	-
ENSMUSC	Mpzl2	myelin prc	GO:00160	membran	-	-	-	-	9:4503979	28	50	17	52	89	71	0.202272	0.288467	0.135732	0.289813	0.414028	0.403508	0.208824	0.369116	0.013736	0.202958	-
ENSMUSC	Dcun1d2	DCN1, de	GO:00081	metabolic	-	-	-	-	8:1325596	744	953	680	986	598	579	4.229721	4.326911	4.272626	4.324602	2.189247	2.589574	4.276419	3.034474	0.013773	0.203339	-
ENSMUSC	Mettl27	methyltrai	-	-	-	-	-	-	5:1349323	19	61	58	89	153	99	0.172839	0.443168	0.58313	0.624614	0.896266	0.708498	0.399713	0.743126	0.01394	0.205627	-
ENSMUSC	Lyve1	lymphatic	GO:00048	signal trar	-	-	-	-	7:1108506	11	24	8	22	53	37	0.198365	0.345626	0.159429	0.306062	0.61544	0.524894	0.234473	0.482132	0.013977	0.205998	-
ENSMUSC	Rps2	ribosomal	GO:00081	metabolic	K02981	small subu	mmu0301	Ribosome	17:247181	1381	2346	1928	1722	2248	1815	17.49767	23.73888	26.99853	16.83255	18.3416	18.09148	22.74503	17.75521	0.014062	0.207074	-
ENSMUSC	Tal2	T cell acut	GO:00325	developm	-	-	-	-	4:5377970	0	0	2	9	5	4	0	0	0.04543	0.1427	0.06617	0.064668	0.015143	0.091179	0.014124	0.207635	-
ENSMUSC	Xkr4	X-linked k	GO:00160	membran	-	-	-	-	1:3205901	988	1327	935	1063	1197	1143	5.598126	6.004855	5.855235	4.64675	4.367518	5.094987	5.819405	4.703085	0.01412	0.207635	-
ENSMUSC	Klc4	kinesin lig	GO:00990	supramole	K10407	kinesin lig	mmu0513	Salmonell	17:466306	318	448	343	588	775	597	8.361967	9.408163	9.968329	11.92857	13.12314	12.34999	9.246153	12.46723	0.014203	0.208617	-
ENSMUSC	Cdc42bpg	CDC42 bir	GO:00081	metabolic	-	-	-	-	19:630645	151	209	102	233	366	274	1.462292	1.616403	1.091704	1.740779	2.28241	2.08747	1.390133	2.036887	0.01426	0.209283	-
ENSMUSC	C1s2	complem	GO:00081	metabolic	-	-	-	-	6:1246246	5	4	4	11	22	12	0.114674	0.073265	0.101408	0.19466	0.324952	0.216541	0.096449	0.245385	0.014297	0.20965	-
ENSMUSC	Stard10	START do	GO:00511	localizatio	-	-	-	-	7:1013170	197	302	187	375	463	382	1.992122	2.438948	2.08996	2.925577	3.014992	3.038959	2.173677	2.993176	0.014317	0.209756	-
ENSMUSC	Eif4a2	eukaryotic	GO:00081	metabolic	K03257	translatior	mmu0301	RNA trans	16:231074	4127	5708	7348	4936	6282	5403	15.66108	17.29885	30.81792	14.45083	15.35112	16.12996	21.25928	15.31063	0.014337	0.209876	-
ENSMUSC	Igf2bp1	insulin-like	GO:00325	developm	K17391	insulin-like	mmu0520	MicroRNA	11:959571	2	0	0	2	14	5	0.01169	0	0	0.009022	0.052706	0.022996	0.003897	0.028241	0.014415	0.21084	-
ENSMUSC	Lbp	lipopolysa	GO:00081	metabolic	K05399	lipopolysa	mmu0515	Tuberculo	2:1583064	126	390	134	113	196	177	1.417861	3.504887	1.666539	0.98101	1.420278	1.566927	2.196429	1.322739	0.014446	0.211119	-
ENSMUSC	Igfn1	immunog	GO:00990	supramole	-	-	-	-	1:1359535	30	110	27	37	25	35	0.182701	0.534999	0.181729	0.173839	0.098041	0.167684	0.299809	0.146521	0.01446	0.211148	-
ENSMUSC	Pkhd1l1	polycystic	GO:00160	membran	-	-	-	-	15:444575	3	7	4	14	15	15	0.013031	0.024285	0.019205	0.046918	0.041959	0.05126	0.01884	0.046712	0.014514	0.211761	-
ENSMUSC	Prkg2	protein kii	GO:00081	metabolic	K19477	cGMP-de	mmu0474	Olfactory	5:9892977	69	101	69	145	172	137	0.560243	0.654929	0.619184	0.908286	0.899307	0.875096	0.611452	0.89423	0.01471	0.21444	-
ENSMUSC	4933411K	RIKEN cDI	-	-	-	-	-	-	19:420522	4	5	4	1	1	2	0.17699	0.176668	0.195614	0.034139	0.028486	0.069629	0.183091	0.044085	0.014879	0.216724	-
ENSMUSC	Gm20431	predicted	GO:00081	metabolic	-	-	-	-	2:1676078	194	1012	169	1166	1119	1003	5.004337	20.84834	4.818144	23.20459	18.58788	20.35434	10.22361	20.7156	0.014895	0.216773	-
ENSMUSC	Osgin1	oxidative	GO:00508	response	-	-	-	-	8:1194341	11	11	3	20	23	27	0.11452	0.091454	0.03452	0.160631	0.154192	0.221133	0.080165	0.178652	0.014917	0.216905	-
ENSMUSC	Hkdc1	hexokinas	GO:00081	metabolic	K00844	hexokinas	mmu0110	Metabolic	10:623831	89	137	79	175	245	162	0.893342	1.098224	0.876398	1.355168	1.583605	1.279235	0.955988	1.406002	0.014963	0.217398	-
ENSMUSC	Prex2	phosphati	GO:00325	developm	-	-	-	-	1:1099346	2423	2812	1914	2493	2709	2440	7.183829	6.658306	6.271787	5.702367	5.172094	5.691193	6.704641	5.521885	0.014999	0.217739	-
ENSMUSC	9130008F	RIKEN cDI	-	-	-	-	-	-	17:408754	1	8	0	6	24	11	0.028289	0.180692	0	0.130906	0.437074	0.244737	0.06966	0.270906	0.015201	0.220481	-
ENSMUSC	Pmm2	phosphor	GO:00081	metabolic	K17497	phosphon	mmu0110	Metabolic	16:863767	215	339	255	440	630	412	2.447337	3.081772	3.208057	3.864001	4.617957	3.689461	2.912389	4.057139	0.01526	0.220973	-
ENSMUSC	Itgb8	integrin b	GO:00325	developm	K06591	integrin b	mmu0415	PI3K-Akt s	12:119158	1372	1732	1129	1368	1671	1454	6.149288	6.19961	5.592567	4.730283	4.822828	5.126794	5.980488	4.893302	0.015256	0.220973	-
ENSMUSC	Cox16	cytochron	GO:00160	membran	-	-	-	-	12:813590	89	170	115	103	111	117	0.885337	1.35055	1.26433	0.790467	0.711037	0.915614	1.166739	0.805706	0.015279	0.221058	-
ENSMUSC	Cytip	cytohesin	GO:00056	cell|intrace	-	-	-	-	2:5812913	2	8	0	11	24	8	0.013623	0.04353	0	0.05782	0.105294	0.042876	0.019051	0.068663	0.015402	0.222467	-
ENSMUSC	Nr3c2	nuclear re	GO:00081	metabolic	K08555	mineraloc	mmu0496	Aldostero	8:7689944	2206	4138	1884	2446	3142	2495	9.649962	14.45628	9.10852	8.254808	8.850769	8.58621	11.07159	8.563929	0.015392	0.222467	-
ENSMUSC	Gsta2	glutathior	GO:00081	metabolic	K00799	glutathior	mmu0520	Pathways	9:7833101	4	0	2	9	10	9	0.165143	0	0.09126	0.286654	0.265843	0.292292	0.085468	0.281596	0.015415	0.222469	-
ENSMUSC	Hif3a	hypoxia ir	GO:00325	developm	-	-	-	-	7:1703150	99	43	104	157	254	219	0.819291	0.284193	0.951229	1.002387	1.353611	1.425801	0.684904	1.2606	0.015611	0.225121	-
ENSMUSC	Rrbp1	ribosome	GO:00325	developm	K14000	ribosome-	mmu0414	Protein pr	2:1439473	1065	1683	896	1828	2601	1799	6.880422	8.683514	6.397647	9.111125	10.82083	9.143399	7.320527	9.691785	0.015644	0.225408	-
ENSMUSC	Gsx2	GS homeo	GO:00325	developm	-	-	-	-	5:7507560	5	6	4	2	0	3	0.107223	0.102765	0.094818	0.033096	0	0.050609	0.101602	0.027902	0.015933	0.229375	-
ENSMUSC	Slc7a2	solute car	GO:00081	metabolic	-	-	-	-	8:4086239	244	483	230	275	298	299	0.802705	1.268991	0.836256	0.697957	0.631301	0.773834	0.969317	0.701031	0.015959	0.229558	-
ENSMUSC	Nfkb2	nuclear fa	GO:00325	developm	K04469	nuclear fa	mmu0520	Pathways	19:463047	44	54	41	93	136	67	0.793892	0.778133	0.817601	1.294567	1.580184	0.95104	0.796542	1.275263	0.016061	0.230655	-
ENSMUSC	Astn1	astrotactir	GO:00325	developm	-	-	-	-	1:1583622	7176	4171	3070	9457	10397	9299	26.92515	12.49861	12.73095	27.37533	25.12109	27.44877	17.38491	26.6484	0.016058	0.230655	-
ENSMUSC	Anxa8	annexin A	GO:00508	response	-	-	-	-	14:340859	13	20	13	39	31	37	0.348291	0.427933	0.384933	0.806115	0.534837	0.779855	0.387052	0.706936	0.016135	0.231527	-
ENSMUSC	Chrnb4	cholinergi	GO:00048	signal trar	K04815	nicotinic a	mmu0408	Neuroacti	9:5502815	0	6	8	16	21	14	0	0.078585	0.144998	0.202444	0.221776	0.180626	0.074528	0.201616	0.016211	0.232272	-
ENSMUSC	H2-T24	histocomp	GO:00023	immune s	K06751	major hist	mmu0516	Human pa	17:360056	57	76	37	107	115	111	1.256875	1.338372	0.901702	1.820273	1.632946	1.925541	1.16565	1.79292	0.016214	0.232272	-
ENSMUSC	Rhpn2	rhophilin,	GO:00508	response	-	-	-	-	7:3533417	88	157	79	164	286	186	0.658632	0.938436	0.653485	0.946966	1.378414	1.095176	0.750184	1.140186	0.016315	0.233532	-
ENSMUSC	Mgl2	macropha	GO:00160	membran	-	-	-	-	11:701303	7	6	1	14	14	16	0.108043	0.073957	0.017059	0.166703	0.139146	0.194277	0.066353	0.166709	0.016454	0.235326	-
ENSMUSC	Smagp	small cell	GO:00099	cellular pr	-	-	-	-	15:100621	7	32	9	34	83	27	0.342207	1.249323	0.486241	1.282287	2.61286	1.038388	0.69259	1.644512	0.016581	0.236745	-
ENSMUSC	Ccnb1ip1	cyclin B1 i	GO:00325	developm	-	-	-	-	14:507892	0	3	2	3	12	12	0	0.074916	0.069111	0.072369	0.241598	0.295152	0.048009	0.20304	0.016576	0.236745	-
ENSMUSC	Abo	ABO bloo	GO:00081	metabolic	K00709	histo-bloc	mmu0110	Metabolic	2:2684250	0	0	0	2	9	0	0	0	0	0.049075	0.184308	0	0	0.077794	0.016631	0.237274	-
ENSMUSC	Slco4a1	solute car	GO:00511	localizatio	-	-	-	-	2:1804562	16	19	12	30	38	42	0.14142	0.134118	0.117223	0.204571	0.216286	0.292047	0.13092	0.237635	0.016721	0.238357	-
ENSMUSC	Galnt7	polypeptic	GO:00081	metabolic	K00710	polypeptic	mmu0110	Metabolic	8:5752382	217	315	174	394	515	357	1.714272	1.987358	1.519202	2.401303	2.619885	2.218706	1.740277	2.413298	0.016743	0.238474	-
ENSMUSC	Fam71e1	family witl	GO:00056	cell|intrace	-	-	-	-	7:4449658	31	41	29	30	31	20	0.38107	0.402514	0.393998	0.284513	0.245399	0.193414	0.392527	0.241108	0.016768	0.238635	-
ENSMUSC	1700001C	RIKEN cDI	-	-	-	-	-	-	2:3079476	37	50	33	68	87	88	0.364795	0.39369	0.359589	0.517229	0.55235	0.682553	0.372691	0.584044	0.016807	0.238999	-
ENSMUSC	Zbtb7b	zinc finge	GO:00325	developm	-	-	-	-	3:8937764	258	396	233	436	651	490	2.224996	2.727414	2.220815	2.900861	3.61531	3.324432	2.391075	3.280201	0.016828	0.239095	-
ENSMUSC	Cgnl1	cingulin-li	GO:00508	response	-	-	mmu0453	Tight junc	9:7162650	637	1088	483	582	776	737	4.235895	5.778042	3.549766	2.98579	3.322941	3.855535	4.521234	3.388089	0.016859	0.239342	-
ENSMUSC	Hnrnpd	heteroger	GO:00325	developm	-	-	-	-	5:9995593	2850	3546	2736	2894	3870	3169	13.79216	13.70477	14.63356	10.80478	12.06016	12.06481	14.0435	11.64325	0.017017	0.241386	-
ENSMUSC	Hcfc1	host cell fa	GO:00081	metabolic	K14966	host cell fa	mmu0516	Herpes sir	X:7394279	2369	2551	1760	2590	2107	2111	9.758258	8.391963	8.012477	8.23071	5.588902	6.840791	8.720899	6.886801	0.017236	0.244104	-
ENSMUSC	Alox12b	arachidon	GO:00325	developm	K08021	arachidon	mmu0110	Metabolic	11:691569	126	157	124	241	285	222	3.164671	3.149222	3.442138	4.669873	4.609541	4.386546	3.25201	4.55532	0.017227	0.244104	-
ENSMUSC	Gsn	gelsolin [S	GO:00325	developm	K05768	gelsolin[-	mmu0520	Viral carci	2:3525638	923	1589	953	1716	2274	1846	12.79669	17.59404	14.60278	18.35455	20.30211	20.1344	14.99784	19.59702	0.017256	0.244186	-
ENSMUSC	Zbtb20	zinc finge	GO:00081	metabolic	-	-	-	-	16:428758	9832	14437	10182	12072	15202	8828	16.30383	19.11924	18.66069	15.44391	16.23316	11.51651	18.02792	14.39786	0.01736	0.245448	-
ENSMUSC	Gstt1	glutathior	GO:00081	metabolic	K00799	glutathior	mmu0520	Pathways	10:757838	66	73	55	130	128	116	2.159623	1.907659	1.98902	3.281739	2.697095	2.986063	2.018767	2.988299	0.017412	0.245988	-
ENSMUSC	Il17rc	interleukir	GO:00048	signal trar	K05166	interleukir	mmu0406	Cytokine-	6:1134714	15	39	33	63	104	46	0.269764	0.560161	0.655945	0.87412	1.204458	0.650836	0.49529	0.909805	0.017438	0.246162	-
ENSMUSC	Cenpf	centrome	GO:00325	developm	-	-	-	-	1:1896406	50	69	47	85	159	97	0.170393	0.187791	0.177021	0.223473	0.348924	0.260052	0.178402	0.277483	0.017515	0.246232	-
ENSMUSC	Itgav	integrin al	GO:00325	developm	K06487	integrin al	mmu0520	Pathways	2:8372439	1197	1286	1049	700	850	1534	5.295745	4.543803	5.129265	2.389247	2.421621	5.339111	4.989604	3.383326	0.017496	0.246232	-
ENSMUSC	Aadac	arylacetar	GO:00081	metabolic	-	-	-	-	3:6002571	0	0	0	3	6	0	0	0	0	0.072258	0.120604	0	0	0.064287	0.017528	0.246232	-
ENSMUSC	Adamts12	a disinteg	GO:00325	developm	-	-	-	-	15:110647	14	21	13	8	8	14	0.071486	0.085635	0.073362	0.031514	0.026305	0.056236	0.076828	0.038019	0.017475	0.246232	-
ENSMUSC	Apol7b	apolipopr	GO:00081	metabolic	-	-	-	-	15:774222	0	0	0	0	6	3	0	0	0	0	0.090453	0.055247	0	0.048567	0.01751	0.246232	-
ENSMUSC	1810010D	RIKEN cDI	GO:00160	membran	-	-	-	-	7:1452058	0	0	1	8	6	0	0	0	0.033082	0.18473	0.115638	0	0.011027	0.100123	0.017513	0.246232	-
ENSMUSC	Tagln2	transgelin	GO:00325	developm	-	-	-	-	1:1725000	131	244	135	249	436	282	2.666213	3.966045	3.036709	3.909771	5.714303	4.515251	3.222989	4.713108	0.017571	0.246628	-
ENSMUSC	Tmc8	transmem	GO:00511	localizatio	-	-	-	-	11:117782	3	0	6	15	15	9	0.035239	0	0.077899	0.135934	0.113461	0.083166	0.037713	0.110854	0.017634	0.24732	-
ENSMUSC	Saa2	serum am	GO:00400	locomotic	-	-	-	-	7:4675179	0	1	0	2	9	2	0	0.013703	0	0.026475	0.09943	0.026999	0.004568	0.050968	0.017663	0.247528	-
ENSMUSC	Dclre1a	DNA cros	GO:00081	metabolic	-	-	-	-	19:565291	104	118	71	172	196	183	0.582413	0.527746	0.439442	0.743116	0.706817	0.806229	0.516533	0.752054	0.017751	0.248557	-
ENSMUSC	Tnfaip1	tumor nec	GO:00325	developm	-	-	-	-	11:785228	810	1051	721	410	1349	393	12.46712	12.91903	12.26488	4.868496	13.37051	4.758665	12.55034	7.665889	0.017908	0.250351	-
ENSMUSC	Cldn1	claudin 1	GO:00325	developm	K06087	claudin[-]	mmu0453	Tight junc	16:263566	87	172	62	58	79	107	1.635986	2.583066	1.288549	0.841431	0.956631	1.582906	1.835867	1.126989	0.017899	0.250351	-
ENSMUSC	Acadsb	acyl-Coer	GO:00081	metabolic	K09478	short/brar	mmu0110	Metabolic	7:1314106	1092	762	553	795	694	736	6.149737	3.427165	3.441964	3.454071	2.516795	3.26079	4.339622	3.077218	0.017941	0.250426	-
ENSMUSC	Cldn12	claudin 12	GO:00051	structural	-	-	-	-	5:5489537	289	321	350	356	933	777	1.191801	1.057197	1.595218	1.132625	2.477662	2.520796	1.281405	2.043694	0.017957	0.250426	-
ENSMUSC	Bcl2a1a	B cell leuk	GO:00511	localizatio	K02162	hematopc	mmu0520	Transcript	9:8895690	4	7	3	0	0	3	0.298225	0.41678	0.247205	0	0	0.175926	0.320737	0.058642	0.017952	0.250426	-
ENSMUSC	Trem2	triggering	GO:00325	developm	K14378	triggering	mmu0438	Osteoclas	17:483464	147	285	147	172	171	179	1.466835	2.271192	1.621164	1.324105	1.098787	1.405162	1.786397	1.276018	0.018125	0.252569	-
ENSMUSC	Nav2	neuron na	GO:00325	developm	-	-	-	-	7:4890871	1458	1257	1201	1300	1315	1391	2.131464	1.46758	1.940486	1.466203	1.237943	1.599773	1.84651	1.43464	0.018177	0.253091	-
ENSMUSC	Zfp385c	zinc finge	GO:00508	response	-	-	-	-	11:100627	3	7	4	3	0	0	0.048177	0.08978	0.071001	0.037174	0	0	0.069653	0.012391	0.01821	0.253357	-
ENSMUSC	Ndufb10	NADH de	GO:00081	metabolic	K03966	NADH de	mmu0110	Metabolic	17:247220	1303	1732	881	1191	1305	1399	26.60827	28.24658	19.88359	18.76352	17.16079	22.47506	24.91281	19.46646	0.01836	0.255233	-
ENSMUSC	Col9a3	collagen,	GO:00325	developm	K08131	collagen, t	mmu0415	PI3K-Akt s	2:1805977	233	561	206	219	287	321	2.455815	4.722244	2.399682	1.780794	1.947947	2.661683	3.19258	2.130141	0.018448	0.256251	-
ENSMUSC	Ripk4	receptor-	GO:00325	developm	-	-	-	-	16:977419	6	17	5	16	33	27	0.092674	0.209701	0.085362	0.190665	0.328236	0.328092	0.129245	0.282331	0.01853	0.256837	-
ENSMUSC	Mrps21	mitochon	GO:00081	metabolic	K02970	small subu	mmu0301	Ribosome	3:9586263	200	205	247	194	239	194	7.670606	6.279138	10.46993	5.740278	5.902718	5.853456	8.13989	5.832151	0.018532	0.256837	-
ENSMUSC	Ube2n	ubiquitin-	GO:00081	metabolic	K10580	ubiquitin-	mmu0412	Ubiquitin	10:955151	837	2492	562	2545	3058	2305	7.581316	18.02656	5.626032	17.78431	17.83656	16.42483	10.4113	17.34857	0.018534	0.256837	-
ENSMUSC	Rnf26	ring finge	GO:00081	metabolic	-	-	-	-	9:4406632	5	0	4	4	32	19	0.10299	0	0.091075	0.063563	0.424496	0.307909	0.064689	0.265323	0.0187	0.258924	-
ENSMUSC	Rbl1	retinoblas	GO:00081	metabolic	K04681	retinoblas	mmu0516	Human pa	2:1571458	39	69	43	97	122	80	0.337769	0.477257	0.411594	0.648122	0.680408	0.545071	0.408873	0.624534	0.018849	0.260779	-
ENSMUSC	Gpatch4	G patch d	GO:00325	developm	-	-	-	-	3:8804310	290	350	202	302	207	255	3.439565	3.315268	2.647905	2.763388	1.580995	2.379333	3.134246	2.241239	0.018959	0.262089	-
ENSMUSC	Fbxo11	F-box prc	GO:00081	metabolic	-	-	-	-	17:879908	4411	5547	3791	4611	5649	4805	50.86446	51.08359	48.31455	41.02067	41.94726	43.58948	50.08753	42.18581	0.018989	0.262295	-
ENSMUSC	Bdh2	3-hydroxy	GO:00325	developm	K00019	3-hydroxy	mmu0110	Metabolic	3:1352812	39	35	28	30	34	17	0.498173	0.357053	0.395293	0.295643	0.279671	0.170838	0.41684	0.248717	0.019045	0.262868	-
ENSMUSC	Sepsecs	Sep (O-pl	GO:00081	metabolic	K03341	O-phospl	mmu0097	Aminoacy	5:5264008	275	325	256	149	135	363	1.612501	1.521938	1.659032	0.674038	0.509747	1.674498	1.597824	0.952761	0.01907	0.263009	-
ENSMUSC	Tnpo2	transporti	GO:00325	developm	-	-	-	-	8:8503691	1969	2242	1659	2089	2392	1902	12.71532	11.56281	11.84064	10.4076	9.947128	9.662806	12.03959	10.00584	0.019098	0.263181	-
ENSMUSC	Cd79b	CD79B an	GO:00325	developm	K06507	CD79B an	mmu0466	B cell rece	11:106311	4	0	0	3	13	10	0.116032	0	0	0.067143	0.242828	0.228192	0.038677	0.179387	0.01914	0.263545	-
ENSMUSC	Gpr160	G protein	GO:00048	signal trar	-	-	-	-	3:3085595	7	18	7	21	58	18	0.059363	0.121905	0.065612	0.137391	0.316732	0.120083	0.082294	0.191402	0.019259	0.264974	-
ENSMUSC	Ddx58	DEAD (As	GO:00081	metabolic	K12646	ATP-depe	mmu0516	Epstein-B	4:4020377	133	196	125	242	315	258	0.743725	0.87531	0.772536	1.044013	1.134292	1.134988	0.79719	1.104431	0.019283	0.265105	-
ENSMUSC	Spata6	spermato	GO:00325	developm	-	-	-	-	4:1117199	32	90	25	92	125	100	0.190955	0.428913	0.164879	0.423549	0.480337	0.469457	0.261582	0.457781	0.019538	0.26776	-
ENSMUSC	Nhsl1	NHS-like	GO:00325	developm	-	-	-	-	10:183189	452	801	895	1347	1593	1252	1.506375	2.131929	3.296582	3.463317	3.418728	3.282534	2.311628	3.388193	0.019514	0.26776	-
ENSMUSC	Pde9a	phosphoc	GO:00081	metabolic	K13761	high affini	mmu0023	Purine me	17:313862	274	351	259	480	626	446	3.055131	3.125597	3.191727	4.129063	4.494784	3.91224	3.124152	4.178696	0.019516	0.26776	-
ENSMUSC	Sstr5	somatosta	GO:00048	signal trar	K04221	somatosta	mmu0408	Neuroacti	17:254898	4	1	0	7	11	6	0.089369	0.017845	0	0.12065	0.158248	0.105458	0.035738	0.128119	0.019528	0.26776	-
ENSMUSC	Pcdh8	protocadh	GO:00325	developm	-	-	-	-	14:797667	932	1016	961	610	754	1250	4.517077	3.932613	5.147686	2.280876	2.353248	4.766096	4.532458	3.133406	0.019593	0.268297	-
ENSMUSC	Baz1a	bromodo	GO:00081	metabolic	-	-	-	-	12:548929	36	45	44	65	130	78	0.216348	0.215976	0.292246	0.301364	0.503091	0.36877	0.241524	0.391075	0.019643	0.268772	-
ENSMUSC	Gas2l1	growth ar	GO:00325	developm	-	-	-	-	11:505413	746	1032	688	608	1237	479	3.866013	4.271201	3.940573	2.430852	4.128086	1.952859	4.025929	2.837266	0.019712	0.269509	-
ENSMUSC	Slc8a1	solute car	GO:00325	developm	K05849	solute car	mmu0474	Olfactory	17:813886	1981	2421	1602	1960	2252	2207	41.22568	40.23683	36.84618	31.46804	30.1791	36.13238	39.43623	32.59317	0.019783	0.270268	-
ENSMUSC	Slc35f5	solute car	GO:00511	localizatio	-	-	-	-	1:1255605	280	344	217	424	600	458	5.041668	4.94675	4.318387	5.889958	6.956996	6.487719	4.768935	6.444891	0.01983	0.270695	-
ENSMUSC	Sat1	spermidin	GO:00325	developm	K00657	diamine N	mmu0110	Metabolic	X:1552131	381	354	328	590	796	554	4.752424	3.526462	4.521803	5.677706	6.393791	5.436397	4.266896	5.835965	0.019883	0.271208	-
ENSMUSC	Adam8	a disinteg	GO:00325	developm	-	-	-	-	7:1399789	128	170	85	101	154	92	1.218691	1.292636	0.894431	0.741878	0.944183	0.689097	1.135253	0.791719	0.020066	0.272584	-
ENSMUSC	Slc25a10	solute car	GO:00511	localizatio	K13577	solute car	mmu0496	Proximal t	11:120491	109	177	167	243	352	277	1.296574	1.681476	2.195514	2.230019	2.696305	2.592165	1.724521	2.506163	0.020078	0.272584	-
ENSMUSC	Slc23a4	solute car	GO:00511	localizatio	-	-	-	-	6:3494527	7	4	2	12	14	15	0.093773	0.042789	0.029613	0.124013	0.120768	0.158077	0.055391	0.134286	0.02003	0.272584	-
ENSMUSC	Il1rapl1	interleukir	GO:00511	localizatio	-	-	-	-	X:8674087	325	370	272	274	367	336	1.956414	1.778789	1.809645	1.272501	1.422647	1.59121	1.848283	1.428786	0.020047	0.272584	-
ENSMUSC	Serhl	serine hyc	GO:00038	catalytic a	-	-	-	-	15:830895	64	88	62	131	156	117	0.303417	0.33319	0.324864	0.479139	0.476256	0.436372	0.32049	0.463923	0.020065	0.272584	-
ENSMUSC	A230065H	RIKEN cDI	-	-	-	-	-	-	12:111406	0	0	1	4	3	4	0	0	0.100727	0.281196	0.176075	0.286758	0.033576	0.24801	0.02005	0.272584	-
ENSMUSC	Cpne5	copine V	GO:00325	developm	-	-	-	-	17:291565	380	232	195	561	584	479	1.680459	0.819366	0.953072	1.913979	1.663073	1.666444	1.150966	1.747832	0.020193	0.273923	-
ENSMUSC	Stat2	signal trar	GO:00325	developm	K11221	signal trar	mmu0520	Pathways	10:128270	572	705	556	999	1166	938	4.290566	4.223318	4.609357	5.781159	5.632123	5.535189	4.374413	5.64949	0.020214	0.273927	-
ENSMUSC	Cdh12	cadherin 1	GO:00160	membran	-	-	-	-	15:204492	312	442	342	355	454	303	1.907876	2.158556	2.311364	1.674766	1.787747	1.457635	2.125932	1.640049	0.020225	0.273927	-
ENSMUSC	Naa11	N(alpha)-	GO:00081	metabolic	-	-	-	-	5:9738219	5	2	0	0	0	0	0.089154	0.028487	0	0	0	0	0.039214	0	0.02024	0.273927	-
ENSMUSC	Sppl2a	signal pep	GO:00081	metabolic	-	-	-	-	2:1268903	820	1080	731	1322	1888	1256	5.476663	5.76065	5.395927	6.811832	8.12005	6.599375	5.544413	7.177086	0.02032	0.274791	-
ENSMUSC	Rbm22	RNA bind	GO:00081	metabolic	K12872	pre-mRN	mmu0304	Spliceoso	18:605607	682	911	664	743	863	777	6.263787	6.682155	6.740114	5.264671	5.104083	5.614155	6.562019	5.327636	0.020342	0.274859	-
ENSMUSC	Usp46	ubiquitin s	GO:00081	metabolic	-	-	-	-	5:7399845	774	1055	698	980	3892	1143	3.823232	4.161864	3.81059	3.734617	12.3799	4.441678	3.931895	6.852065	0.020356	0.274859	-
ENSMUSC	Gpr19	G protein	GO:00048	signal trar	-	-	-	-	6:1348690	78	83	69	120	172	153	0.527997	0.448702	0.516215	0.626685	0.749755	0.814777	0.497638	0.730405	0.020427	0.275596	-
ENSMUSC	Taf9b	TATA-box	GO:00081	metabolic	K03133	transcripti	mmu0516	Herpes sir	X:1062068	242	331	259	913	425	405	4.365153	4.768246	5.163337	12.70532	4.936608	5.747122	4.765579	7.79635	0.020461	0.275841	-
ENSMUSC	Spib	Spi-B trar	GO:00325	developm	-	-	-	-	7:4452599	5	0	0	9	10	6	0.092898	0	0	0.129021	0.119654	0.08771	0.030966	0.112128	0.020584	0.2772	-
ENSMUSC	Trim29	tripartite r	GO:00081	metabolic	-	-	-	-	9:4331084	0	1	0	3	3	5	0	0.012872	0	0.037302	0.031135	0.063387	0.004291	0.043941	0.020594	0.2772	-
ENSMUSC	Serpinb6b	serine (or	GO:00325	developm	K13963	serpin B[-	mmu0514	Amoebias	13:329652	15	29	10	34	42	47	0.234954	0.362787	0.173115	0.410871	0.42365	0.579177	0.256952	0.471233	0.020631	0.277493	-
ENSMUSC	Xpnpep3	X-prolyl a	GO:00081	metabolic	-	-	-	-	15:814001	93	330	93	132	132	108	0.573285	1.624608	0.633607	0.627759	0.523984	0.523749	0.943833	0.558497	0.020657	0.277628	-
ENSMUSC	Tnfrsf18	tumor nec	GO:00325	developm	K05154	tumor nec	mmu0406	Cytokine-	4:1560261	0	0	0	2	7	0	0	0	0	0.024455	0.071428	0	0	0.031961	0.020785	0.279132	-
ENSMUSC	Abhd3	abhydrola	GO:00081	metabolic	-	-	-	-	18:106444	713	822	652	820	875	420	9.211687	8.4814	9.309882	8.173232	7.279684	4.268837	9.000989	6.573918	0.020931	0.279976	-
ENSMUSC	Spata1	spermato	-	-	-	-	-	-	3:1464571	32	33	22	26	28	11	0.341225	0.281029	0.259281	0.213889	0.192268	0.092279	0.293845	0.166145	0.020945	0.279976	-
ENSMUSC	Ccnb2	cyclin B2 [	GO:00325	developm	K05868	cyclin B[-]	mmu0516	HTLV-I inf	9:7040769	15	13	6	27	35	24	0.598203	0.414053	0.264482	0.830724	0.898848	0.752981	0.425579	0.827518	0.02089	0.279976	-
ENSMUSC	Tmem184	transmem	GO:00325	developm	-	-	-	-	5:1398024	10	26	2	18	72	35	0.091064	0.189087	0.02013	0.126461	0.422212	0.250739	0.100094	0.266471	0.020945	0.279976	-
ENSMUSC	Begain	brain-enr	-	-	-	-	-	-	12:109032	427	2056	394	606	661	625	5.570897	21.42232	5.681202	6.099588	5.553328	6.414874	10.89147	6.022597	0.020885	0.279976	-
ENSMUSC	Swi5	SWI5 recc	GO:00081	metabolic	-	-	-	-	2:3227881	1536	1971	1120	1462	1791	1599	14.76468	15.13092	11.89865	10.84203	11.08621	12.09184	13.93142	11.34003	0.02094	0.279976	-
ENSMUSC	Trpm3	transient r	GO:00511	localizatio	-	-	-	-	19:221391	1288	2234	1142	1269	1649	1704	7.942922	11.00255	7.783544	6.037487	6.548467	8.266938	8.909672	6.950964	0.02108	0.281575	-
ENSMUSC	Snap91	synaptosc	GO:00511	localizatio	-	-	-	-	9:8676592	5947	6468	5163	6589	6626	5923	26.30298	22.84666	25.23805	22.48313	18.87179	20.60912	24.7959	20.65468	0.021109	0.281744	-
ENSMUSC	Tbl1xr1	transducir	GO:00325	developm	K04508	transducir	mmu0431	Wnt signa	3:2207665	2428	1520	2288	1390	2801	1748	6.838374	3.418956	7.122082	3.020295	5.080095	3.87308	5.793137	3.991157	0.021169	0.282206	-
ENSMUSC	Vwc2l	von Willet	GO:00325	developm	-	-	-	-	1:7072571	202	173	121	339	310	288	0.896088	0.612902	0.593242	1.160189	0.885553	1.005084	0.700744	1.016942	0.021176	0.282206	-
ENSMUSC	Il3ra	interleukir	GO:00325	developm	K04737	interleukir	mmu0520	Pathways	14:143462	17	28	21	15	20	15	0.230462	0.303146	0.314644	0.15688	0.174592	0.159972	0.282751	0.163815	0.021265	0.283173	-
ENSMUSC	Sap18	Sin3-asso	GO:00329	macromo	K14324	histone de	mmu0301	RNA trans	14:577981	21	315	15	546	77	872	0.173253	2.075434	0.13677	3.475169	0.409069	5.659528	0.795153	3.181255	0.021476	0.285759	-
ENSMUSC	Wdr72	WD repea	GO:00325	developm	-	-	-	-	9:7411035	22	25	8	4	8	17	0.141934	0.12881	0.05704	0.019907	0.033238	0.086285	0.109262	0.046477	0.021504	0.285913	-
ENSMUSC	Ect2	ect2 onco	GO:00325	developm	-	-	-	-	3:2709722	11	18	18	30	57	29	0.065869	0.086076	0.119123	0.138587	0.219786	0.136607	0.090356	0.164994	0.021545	0.286246	-
ENSMUSC	Tmem252	transmem	GO:00160	membran	-	-	-	-	19:246740	24	23	14	43	66	33	0.595215	0.455552	0.383732	0.82272	1.054033	0.643852	0.478166	0.840202	0.021587	0.286581	-
ENSMUSC	Slc38a8	solute car	GO:00511	localizatio	-	-	-	-	8:1194796	0	2	5	0	0	0	0	0.021325	0.073772	0	0	0	0.031699	0	0.021707	0.287948	-
ENSMUSC	Ttbk2	tau tubulii	GO:00325	developm	-	-	-	-	2:1207328	769	839	2070	2788	3293	2845	3.449688	3.005807	10.26291	9.648868	9.512611	10.04029	5.572802	9.733924	0.021758	0.288413	-
ENSMUSC	Mapk6	mitogen-	GO:00325	developm	K06855	mitogen-a	mmu0465	IL-17 sign	9:7536906	649	613	364	1048	1084	802	3.734143	2.816776	2.314696	4.651978	4.016337	3.630207	2.955205	4.099508	0.021799	0.288731	-
ENSMUSC	1110059G	RIKEN cDI	-	-	-	-	-	-	9:1229450	153	192	173	165	187	156	1.408186	1.411286	1.759787	1.17161	1.108315	1.129547	1.52642	1.136491	0.021868	0.289424	-
ENSMUSC	Grrp1	glycine/ar	-	-	-	-	-	-	4:1342511	26	37	30	53	75	64	0.868442	0.986984	1.10748	1.365761	1.613185	1.681749	0.987635	1.553565	0.021939	0.290151	-
ENSMUSC	Pyroxd2	pyridine n	GO:00081	metabolic	-	-	-	-	19:427258	21	22	34	55	57	67	0.411109	0.343956	0.735627	0.830662	0.71855	1.031844	0.496897	0.860352	0.021977	0.290423	-
ENSMUSC	Kif11	kinesin fa	GO:00990	supramole	-	-	-	-	19:373764	23	54	21	71	80	69	0.418122	0.546421	0.294077	0.694021	0.652727	0.687779	0.41954	0.678176	0.022129	0.291993	-
ENSMUSC	Chrna4	cholinergi	GO:00048	signal trar	K04806	nicotinic a	mmu0408	Neuroacti	2:1810183	528	564	354	579	438	197	5.338413	4.55411	3.955746	4.516349	2.851719	1.566954	4.61609	2.978341	0.022138	0.291993	-
ENSMUSC	Pafah2	platelet-a	GO:00081	metabolic	K01062	platelet-a	mmu0110	Metabolic	4:1343963	76	112	71	139	205	143	0.87539	1.030266	0.903836	1.23518	1.520525	1.295782	0.936497	1.350496	0.022179	0.292424	-

ENSMUSC	Zfp800	zinc finge	GO:00081	metabolic	-	-	-	-	6:2823992	203	298	229	147	196	298	1.397697	1.638626	1.742605	0.780845	0.869016	1.614153	1.592976	1.088005	0.022224	0.292798	-
ENSMUSC	Dclre1c	DNA cros	GO:00325	developm	K10887	DNA cros	mmu0534	Primary in	2:3424131	159	177	163	303	323	281	0.598425	0.532023	0.678027	0.879802	0.782833	0.832011	0.602825	0.831549	0.022339	0.29409	-
ENSMUSC	Bsg	basigin [S	GO:00325	developm	-	-	-	-	10:797044	4139	7337	4008	5304	5996	4861	65.96699	93.38896	70.60016	65.21764	61.53858	60.94916	76.65204	62.56846	0.022357	0.294112	-
ENSMUSC	Fam234a	family witl	GO:00055	extracellul	-	-	-	-	17:262118	167	266	204	324	465	362	1.809847	2.30226	2.443448	2.708954	3.245144	3.08636	2.185185	3.013486	0.022523	0.296019	-
ENSMUSC	Tigd4	tigger trar	GO:00056	cell|intrace	-	-	-	-	3:8459357	1	1	0	1	6	8	0.017657	0.0141	0	0.01362	0.068199	0.111089	0.010586	0.064303	0.022553	0.296019	-
ENSMUSC	Npy4r	neuropep	GO:00048	signal trar	K04206	neuropep	mmu0408	Neuroacti	14:341439	0	0	0	3	3	2	0	0	0	0.038968	0.032526	0.026492	0	0.032662	0.022537	0.296019	-
ENSMUSC	Ly9	lymphocy	GO:00325	developm	-	-	-	-	1:1715886	19	30	15	9	26	5	0.222967	0.281158	0.194549	0.081484	0.196476	0.04616	0.232891	0.10804	0.022676	0.297408	-
ENSMUSC	Apc	adenoma	GO:00081	metabolic	K02085	adenoma	mmu0520	Pathways	18:342209	3777	5808	3477	4519	5106	4412	8.502929	10.44225	8.651135	7.848638	7.402136	7.813903	9.198772	7.688226	0.022698	0.297464	-
ENSMUSC	Aim2	absent in	GO:00081	metabolic	K12966	absent in	mmu0462	NOD-like	1:1733508	71	64	71	116	176	125	0.79456	0.571996	0.878159	1.001512	1.268341	1.100503	0.748238	1.123452	0.022802	0.297935	-
ENSMUSC	Cd209c	CD209c a	GO:00360	small mol	K06563	CD209 an	mmu0515	Tuberculo	8:3940222	0	0	0	3	2	3	0	0	0	0.043636	0.024273	0.044484	0	0.037464	0.022802	0.297935	-
ENSMUSC	Klf1	Kruppel-li	GO:00325	developm	-	-	-	-	8:8490192	0	0	0	0	8	2	0	0	0	0	0.205595	0.062803	0	0.089466	0.022771	0.297935	-
ENSMUSC	Mpig6b	megakary	GO:00325	developm	-	-	-	-	17:350626	0	0	0	0	8	2	0	0	0	0	0.123454	0.037711	0	0.053722	0.022775	0.297935	-
ENSMUSC	Igsf23	immunog	GO:00160	membran	-	-	-	-	7:1993730	0	0	0	2	8	0	0	0	0	0.029199	0.097478	0	0	0.042226	0.022844	0.298264	-
ENSMUSC	Il17b	interleukir	GO:00400	locomotic	K05490	interleukir	mmu0406	Cytokine-	18:616879	0	0	0	2	4	2	0	0	0	0.136162	0.227284	0.138856	0	0.167434	0.022886	0.298364	-
ENSMUSC	Trim30d	tripartite r	GO:00081	metabolic	-	-	-	-	7:1044700	37	23	25	60	70	59	0.433373	0.215149	0.323621	0.542173	0.527971	0.543653	0.324048	0.537932	0.022886	0.298364	-
ENSMUSC	Tpd52	tumor prc	GO:00325	developm	-	-	-	-	3:8925593	727	820	506	1063	1360	1064	3.326284	2.996295	2.558718	3.75222	4.006991	3.829811	2.960432	3.863007	0.023058	0.300372	-
ENSMUSC	Nrm	nurim (nu	GO:00160	membran	-	-	-	-	17:358613	8	11	11	19	33	26	0.253894	0.278781	0.385805	0.465171	0.674384	0.649118	0.30616	0.596224	0.023158	0.301452	-
ENSMUSC	Fap	fibroblast	GO:00325	developm	-	-	-	-	2:6250094	28	83	23	20	38	32	0.244929	0.579842	0.222359	0.134971	0.214055	0.220218	0.349043	0.189748	0.023204	0.301599	-
ENSMUSC	S100b	S100 prot	GO:00325	developm	-	-	-	-	10:762538	1141	1555	1043	1343	1452	1213	46.94563	51.09587	47.42862	42.63004	38.47075	39.26279	48.49004	40.12119	0.023197	0.301599	-
ENSMUSC	Kdf1	keratinocy	GO:00325	developm	-	-	-	-	4:1335189	7	9	3	15	23	15	0.065959	0.067729	0.031244	0.109041	0.139558	0.111191	0.054977	0.11993	0.023258	0.301854	-
ENSMUSC	Ackr1	atypical cl	GO:00508	response	K06574	Duffy bloc	mmu0514	Malaria -	1:1733318	45	23	38	71	79	104	1.125147	0.459283	1.050082	1.369569	1.271983	2.045705	0.878171	1.562419	0.023245	0.301854	-
ENSMUSC	Lcp1	lymphocy	GO:00325	developm	-	-	-	-	14:751311	231	448	220	183	411	207	1.071685	1.659886	1.128038	0.654991	1.227867	0.755503	1.286536	0.879454	0.023446	0.304057	-
ENSMUSC	Gfra3	glial cell li	GO:00325	developm	-	-	-	-	18:346899	0	0	0	2	2	4	0	0	0	0.047301	0.039468	0.09645	0	0.061073	0.023557	0.305268	-
ENSMUSC	Camsap2	calmoduli	GO:00325	developm	-	-	-	-	1:1362681	4633	4560	3913	4523	5235	4577	16.2913	12.80573	15.20724	12.27017	11.854	12.66151	14.76809	12.26189	0.023574	0.305268	-
ENSMUSC	Nans	N-acetyln	GO:00081	metabolic	K05304	sialic acid	mmu0110	Metabolic	4:4648924	194	247	178	358	472	288	2.296039	2.334647	2.328331	3.268826	3.597285	2.681521	2.319673	3.182544	0.023618	0.305608	-
ENSMUSC	Ace	angiotens	GO:00325	developm	K01283	peptidyl-c	mmu0514	Chagas di	11:105967	569	1226	384	285	629	754	5.12495	8.818889	3.822573	1.980397	3.648238	5.342688	5.922138	3.657108	0.02377	0.307344	-
ENSMUSC	Vim	vimentin [	GO:00325	developm	K07606	vimentin[-	mmu0516	Epstein-B	2:1357392	742	880	484	681	832	644	5.801654	5.495112	4.182538	4.107953	4.189152	3.961366	5.159768	4.086157	0.023812	0.307659	-
ENSMUSC	Atat1	alpha tub	GO:00325	developm	-	-	-	-	17:358975	735	958	653	819	950	726	5.087598	5.295865	4.995577	4.373607	4.234514	3.953422	5.126347	4.187181	0.02389	0.308437	-
ENSMUSC	Mpz	myelin prc	GO:00325	developm	K06770	myelin prc	mmu0451	Cell adhes	1:1711507	1	7	0	0	0	0	0.017652	0.098654	0	0	0	0	0.038769	0	0.023925	0.308666	-
ENSMUSC	Zzz3	zinc finge	GO:00081	metabolic	-	-	-	-	3:1523954	747	1402	815	888	1132	980	2.666962	3.997518	3.215889	2.445907	2.602541	2.752538	3.293456	2.600329	0.023958	0.308862	-
ENSMUSC	Vipr1	vasoactive	GO:00048	signal trar	K04589	vasoactive	mmu0408	Neuroacti	9:1216427	459	537	402	726	961	705	4.366046	4.0794	4.226191	5.327741	5.886459	5.275652	4.223879	5.496617	0.024064	0.309934	-
ENSMUSC	A730017C	RIKEN cDI	GO:00160	membran	-	-	-	-	18:590622	185	269	316	288	180	204	1.548424	1.798108	2.92316	1.859691	0.970164	1.343255	2.089897	1.391037	0.02409	0.309934	-
ENSMUSC	Mia	melanom	GO:00508	response	-	-	-	-	7:2717974	6	18	6	4	7	5	0.193933	0.464647	0.214358	0.099736	0.145692	0.127142	0.290979	0.12419	0.024095	0.309934	-
ENSMUSC	Tgm7	transgluta	GO:00081	metabolic	-	-	-	-	2:1210935	0	6	2	10	13	7	0	0.136017	0.062749	0.219002	0.237635	0.156327	0.066255	0.204321	0.024187	0.310887	-
ENSMUSC	Ticam1	toll-like re	GO:00048	signal trar	K05842	toll-like re	mmu0516	Human pa	17:562693	79	110	76	143	183	163	1.61217	1.792747	1.714127	2.251382	2.404852	2.616854	1.706348	2.424363	0.02425	0.311472	-
ENSMUSC	Mcph1	microcepl	GO:00325	developm	-	-	-	-	8:1859513	190	235	160	184	213	187	1.123912	1.110177	1.046034	0.839706	0.811361	0.870228	1.093375	0.840432	0.024539	0.314956	-
ENSMUSC	Syk	spleen tyr	GO:00325	developm	K05855	spleen tyr	mmu0415	PI3K-Akt s	13:525831	100	132	80	179	246	157	0.447312	0.471553	0.395503	0.617724	0.708599	0.552484	0.438122	0.626269	0.024646	0.316023	-
ENSMUSC	Ifi203	interferon	-	-	-	-	-	-	1:1739204	87	128	87	162	207	184	0.613826	0.721246	0.67841	0.881803	0.940486	1.021303	0.67116	0.947864	0.024677	0.316023	-
ENSMUSC	Hmgb2	high mob	GO:00325	developm	-	-	-	-	8:5751190	20	49	26	52	95	58	0.329868	0.645435	0.47394	0.661669	1.008986	0.752572	0.483081	0.807742	0.024667	0.316023	-
ENSMUSC	Arhgef9	CDC42 gu	GO:00987	molecular	-	-	-	-	X:9504893	9415	12534	6187	6611	7005	11790	18.2276	19.3796	13.23842	9.874309	8.733172	17.95701	16.94854	12.18816	0.024779	0.317091	-
ENSMUSC	Palm2	paralemm	GO:00325	developm	-	-	-	-	4:5743424	217	357	292	288	291	271	1.890371	2.483713	2.811361	1.935575	1.632429	1.857236	2.395148	1.808413	0.024993	0.3196	-
ENSMUSC	H2-M3	histocomp	GO:00325	developm	K06751	major hist	mmu0516	Human pa	17:372702	18	31	15	33	65	45	0.764269	1.051206	0.70392	1.081023	1.777274	1.503161	0.839798	1.453819	0.025021	0.31972	-
ENSMUSC	Tmem56	transmem	GO:00160	membran	-	-	-	-	3:1212017	984	1145	728	1134	689	863	5.946275	5.52588	4.862151	5.286804	2.681166	4.102718	5.444768	4.023563	0.025174	0.321436	-
ENSMUSC	Serpinb6a	serine (or	GO:00325	developm	K13963	serpin B[-	mmu0514	Amoebias	13:339179	520	709	403	862	1049	826	3.635663	3.958878	3.114097	4.649619	4.722912	4.543293	3.569546	4.638608	0.025242	0.322068	-
ENSMUSC	Htra1	HtrA serin	GO:00325	developm	-	-	-	-	7:1309361	1923	2651	1888	3112	3861	3519	27.89612	30.71284	30.27006	34.82847	36.06774	40.16008	29.62634	37.01876	0.025282	0.322346	-
ENSMUSC	Cyb5rl	cytochron	GO:00081	metabolic	K00326	cytochron	mmu0052	Amino su	4:1070669	68	76	70	53	72	71	0.253276	0.226069	0.288156	0.152297	0.172691	0.208043	0.255834	0.177677	0.025397	0.323259	-
ENSMUSC	Erv3	endogenc	GO:00051	structural	-	-	-	-	2:1318536	9	15	8	7	5	7	0.162673	0.216535	0.159807	0.097613	0.058197	0.099544	0.179672	0.085118	0.025437	0.323259	-
ENSMUSC	Bend3	BEN dom	GO:00081	metabolic	-	-	-	-	10:434788	10	14	109	139	193	216	0.047332	0.052921	0.570202	0.507573	0.588256	0.804301	0.223485	0.633376	0.025383	0.323259	-
ENSMUSC	Cys1	cystin 1 [S	GO:00055	extracellul	-	-	-	-	12:246658	113	122	129	98	109	130	0.930361	0.802197	1.173841	0.622488	0.577903	0.842033	0.9688	0.680808	0.025447	0.323259	-
ENSMUSC	Xlr4b	X-linked l	GO:00325	developm	-	-	-	-	X:7321433	20	5	13	5	10	6	0.285721	0.057043	0.205256	0.055104	0.091994	0.067435	0.182673	0.071511	0.025416	0.323259	-
ENSMUSC	Hmcn1	hemicenti	GO:00099	cellular pr	-	-	-	-	1:1505625	134	179	127	119	154	152	0.361212	0.38535	0.37836	0.247476	0.267319	0.322336	0.374974	0.279044	0.025538	0.324185	-
ENSMUSC	Iqcj	IQ motif c	-	-	-	-	-	-	3:6789222	3	1	1	16	7	3	0.116004	0.030887	0.04274	0.477327	0.174295	0.09125	0.06321	0.247624	0.0257	0.326006	-
ENSMUSC	Aspm	asp (abnc	GO:00325	developm	-	-	-	-	1:1394547	18	25	16	36	83	29	0.078317	0.086871	0.076942	0.120844	0.232556	0.099265	0.08071	0.150888	0.025737	0.326229	-
ENSMUSC	Stx11	syntaxin 1	GO:00511	localizatio	K08487	syntaxin 1	mmu0413	SNARE int	10:129382	3	3	4	7	24	8	0.020475	0.016355	0.030175	0.036859	0.105482	0.042953	0.022335	0.061765	0.025798	0.326767	-
ENSMUSC	Mgp	matrix Gla	GO:00325	developm	-	-	-	-	6:1368724	166	260	144	155	204	189	7.609354	9.51829	7.295377	5.481522	6.021766	6.815713	8.141007	6.106334	0.025894	0.32775	-
ENSMUSC	Nlrc5	NLR famil	GO:00081	metabolic	-	-	-	-	8:9443435	38	58	51	104	86	105	0.17632	0.214928	0.261542	0.372293	0.256964	0.383286	0.217597	0.337514	0.025918	0.327813	-
ENSMUSC	Olfr288	olfactory r	GO:00048	signal trar	-	-	-	-	15:981860	10	31	18	2	5	19	0.229803	0.568925	0.457169	0.035463	0.07399	0.343486	0.418632	0.15098	0.026017	0.328829	-
ENSMUSC	Cdt1	chromatir	GO:00081	metabolic	-	-	-	-	8:1225680	11	12	16	28	38	29	0.130674	0.113842	0.210063	0.256605	0.290682	0.271009	0.151526	0.272765	0.02607	0.329243	-
ENSMUSC	Bcas1	breast car	GO:00055	extracellul	-	-	-	-	2:1703469	2284	3846	2462	3426	3000	2336	20.41228	27.45052	24.31814	23.62181	17.26519	16.42401	24.06031	19.10367	0.026088	0.329243	-
ENSMUSC	Cd180	CD180 an	GO:00023	immune s	-	-	-	-	13:102693	78	92	69	77	77	71	0.785897	0.740294	0.76836	0.598541	0.499591	0.56278	0.76485	0.553638	0.026195	0.330354	-
ENSMUSC	Srr	serine rac	GO:00325	developm	K12235	serine rac	mmu0110	Metabolic	11:749063	828	1465	728	1556	1791	1697	3.387796	4.787079	3.292044	4.911646	4.718864	5.462356	3.822306	5.030955	0.026255	0.330868	-
ENSMUSC	Slc25a35	solute car	GO:00511	localizatio	-	-	-	-	11:689681	110	173	76	190	277	201	1.176662	1.477923	0.898505	1.567988	1.908065	1.691479	1.184364	1.722511	0.026274	0.330868	-
ENSMUSC	Cryz	crystallin,	GO:00081	metabolic	-	-	-	-	3:1545967	222	239	153	131	299	129	1.518753	1.305808	1.156843	0.69141	1.317228	0.694282	1.327135	0.900973	0.02636	0.331715	-
ENSMUSC	1700014D	RIKEN cDI	-	-	-	-	-	-	13:597408	6	1	3	12	10	15	0.083411	0.011104	0.046098	0.1287	0.089522	0.164052	0.046871	0.127425	0.026387	0.331818	-
ENSMUSC	Rasl10b	RAS-like,	GO:00511	localizatio	-	-	-	-	11:834091	1153	1119	1487	1398	1271	1100	7.617398	5.904097	10.85763	7.125494	5.407264	5.717169	8.126375	6.083309	0.026435	0.331989	-
ENSMUSC	Prr32	proline ric	-	-	-	-	-	-	X:4509090	53	102	34	46	59	46	2.915406	4.480931	2.067045	1.952139	2.089891	1.990621	3.154461	2.010884	0.026439	0.331989	-
ENSMUSC	Med6	mediator	GO:00325	developm	-	-	-	-	12:815735	149	132	137	120	177	102	3.204529	2.267241	3.256464	1.991088	2.451348	1.725792	2.909411	2.056076	0.026527	0.332686	-
ENSMUSC	Dcst1	DC-STAM	GO:00081	metabolic	-	-	-	-	3:8935021	0	0	0	1	2	5	0	0	0	0.0068	0.011348	0.034667	0	0.017605	0.026533	0.332686	-
ENSMUSC	Abcc6	ATP-bind	GO:00511	localizatio	K05669	ATP-bind	mmu0201	ABC trans	7:4596755	14	9	16	36	30	31	0.126082	0.064732	0.159251	0.250118	0.173974	0.219627	0.116688	0.214573	0.026657	0.333996	-
ENSMUSC	Rhod	ras homol	GO:00400	locomotic	K07530	Ras homo	mmu0436	Axon guic	19:442545	7	8	7	15	26	18	0.155424	0.141859	0.171785	0.256941	0.371732	0.3144	0.156356	0.314358	0.026732	0.334692	-
ENSMUSC	Zfp189	zinc finge	GO:00081	metabolic	-	-	-	-	4:4952117	492	640	757	575	700	617	9.346789	9.710089	15.89426	8.427438	8.563482	9.221331	11.65038	8.737417	0.026812	0.335453	-
ENSMUSC	Pcdhga4	protocadl	GO:00325	developm	-	-	-	-	18:376852	93	5	7	344	365	10	1.027948	0.044135	0.085519	2.933445	2.597988	0.086956	0.385868	1.872796	0.026854	0.335748	-
ENSMUSC	Trim12c	tripartite r	GO:00048	signal trar	-	-	-	-	7:1043387	51	88	58	106	141	123	0.590546	0.813796	0.742267	0.946942	1.051381	1.120473	0.715536	1.039599	0.026987	0.33716	-
ENSMUSC	Prx	periaxin [S	GO:00325	developm	-	-	-	-	7:2749932	2	11	9	22	17	21	0.006634	0.029146	0.033001	0.056313	0.036322	0.054813	0.022927	0.049149	0.027013	0.337242	-
ENSMUSC	Irak2	interleukir	GO:00081	metabolic	K04731	interleukir	mmu0515	Tuberculo	6:1136384	234	227	239	229	246	225	1.82031	1.410267	2.054821	1.374342	1.232307	1.376962	1.761799	1.32787	0.027073	0.337755	-
ENSMUSC	Flna	filamin, al	GO:00325	developm	K04437	filamin[-]	mmu0401	MAPK sigı	X:7422346	1618	1999	1271	2221	3026	2814	6.264953	6.181555	5.439157	6.63465	7.545063	8.571842	5.961888	7.583852	0.027215	0.339285	-
ENSMUSC	Csde1	cold shoc	GO:00081	metabolic	-	-	-	-	3:1030204	3904	5424	3720	3834	4886	5185	13.42934	14.90083	14.14278	10.17484	10.82314	14.03152	14.15765	11.6765	0.027312	0.34025	-
ENSMUSC	1700028P	RIKEN cDI	GO:00160	membran	-	-	-	-	19:235587	23	34	22	23	25	15	1.617906	1.910074	1.71042	1.248199	1.132436	0.830087	1.746133	1.070241	0.027345	0.340412	-
ENSMUSC	Adora2b	adenosine	GO:00048	signal trar	K04267	adenosine	mmu0408	Neuroacti	11:622489	43	56	36	33	45	40	1.423043	1.480078	1.316745	0.842553	0.95898	1.041395	1.406622	0.947643	0.027404	0.340589	-
ENSMUSC	Cpeb1	cytoplasm	GO:00325	developm	K02602	cytoplasm	mmu0411	Oocyte m	7:8134702	615	1300	657	1248	1620	1565	5.211759	8.798294	6.153486	8.159326	8.840546	10.43361	6.72118	9.144493	0.027418	0.340589	-
ENSMUSC	Klra2	killer cell l	GO:00160	membran	-	-	-	-	6:1312192	0	4	0	6	11	3	0	0.100636	0	0.145834	0.223164	0.074347	0.033545	0.147781	0.027413	0.340589	-
ENSMUSC	Shcbp1	Shc SH2-c	GO:00508	response	-	-	-	-	8:4735976	11	18	6	18	48	26	0.077865	0.101753	0.046942	0.098299	0.218791	0.144784	0.07552	0.153958	0.027465	0.340928	-
ENSMUSC	Nrip2	nuclear re	GO:00081	metabolic	-	-	-	-	6:1283992	174	247	190	326	372	375	1.553911	1.761657	1.875331	2.246084	2.139321	2.634632	1.7303	2.340012	0.027668	0.341862	-
ENSMUSC	Sis	sucrase is	GO:00081	metabolic	K01203	sucrase-is	mmu0110	Metabolic	3:7288855	0	4	2	7	18	4	0	0.023623	0.016349	0.039939	0.085725	0.023271	0.013324	0.049645	0.027756	0.341862	-
ENSMUSC	Svep1	sushi, von	GO:00055	extracellul	-	-	-	-	4:5804244	9	25	12	21	68	33	0.028036	0.062197	0.041315	0.05047	0.136411	0.080876	0.043849	0.089252	0.027715	0.341862	-
ENSMUSC	Cyp2w1	cytochron	GO:00081	metabolic	-	-	-	-	5:1393526	0	0	0	4	4	0	0	0	0	0.105903	0.08841	0	0	0.064771	0.027695	0.341862	-
ENSMUSC	Hoxa11	homeobo	GO:00325	developm	K09296	homeobo	mmu0520	Transcript	6:5224210	0	0	0	0	4	4	0	0	0	0	0.035868	0.043815	0	0.026561	0.027738	0.341862	-
ENSMUSC	Tnfsf10	tumor nec	GO:00325	developm	K04721	tumor nec	mmu0406	Cytokine-	3:2731702	73	136	110	173	268	177	0.782516	1.164278	1.303211	1.430708	1.849965	1.492652	1.083335	1.591108	0.027589	0.341862	-
ENSMUSC	D330045A	RIKEN cDI	GO:00081	metabolic	-	-	-	-	X:1394803	9	5	9	18	20	26	0.112328	0.049837	0.124145	0.173329	0.160745	0.255295	0.095437	0.196456	0.027722	0.341862	-
ENSMUSC	Cd300c2	CD300C n	GO:00511	localizatio	-	-	-	-	11:114996	38	60	41	40	50	33	1.192288	1.503482	1.421768	0.968268	1.010251	0.814575	1.372513	0.931031	0.027729	0.341862	-
ENSMUSC	Gvin1	GTPase, v	GO:00971	organic cy	-	-	-	-	7:1061565	213	321	276	447	515	471	1.430575	1.721797	2.048741	2.316163	2.227378	2.488646	1.733705	2.344062	0.027621	0.341862	-
ENSMUSC	Zfp827	zinc finge	GO:00081	metabolic	-	-	-	-	8:7902843	952	719	863	590	900	980	3.124788	1.88477	3.130697	1.494053	1.902308	2.530583	2.713418	1.975648	0.027727	0.341862	-
ENSMUSC	Prob1	proline ric	-	-	-	-	-	-	18:356503	92	175	107	50	174	79	0.667225	1.013603	0.857659	0.279757	0.81262	0.450737	0.846162	0.514371	0.027651	0.341862	-
ENSMUSC	Cd163	CD163 an	GO:00511	localizatio	-	-	-	-	6:1243046	17	43	11	40	58	63	0.149246	0.301478	0.106727	0.27092	0.327889	0.43511	0.185817	0.34464	0.027861	0.342912	-
ENSMUSC	Fam46b	family witl	GO:00038	catalytic a	-	-	-	-	4:1334801	0	0	0	4	0	4	0	0	0	0.082379	0	0.084009	0	0.055463	0.027937	0.343599	-
ENSMUSC	Kntc1	kinetocho	GO:00990	supramole	-	-	-	-	5:1237497	13	36	14	35	54	50	0.086873	0.192129	0.103398	0.180441	0.232373	0.262856	0.127467	0.225223	0.027975	0.343831	-
ENSMUSC	Nkx2-9	NK2 hom	GO:00325	developm	-	-	-	-	12:566113	0	3	3	0	0	0	0	0.115752	0.160175	0	0	0	0.091976	0	0.028066	0.344705	-
ENSMUSC	Dsc3	desmocol	GO:00325	developm	-	-	-	-	18:199609	37	39	27	85	75	53	0.351842	0.296176	0.283758	0.623573	0.459259	0.396488	0.310592	0.493107	0.028098	0.344854	-
ENSMUSC	Slc1a4	solute car	GO:00511	localizatio	-	-	-	-	11:203021	1205	865	552	1698	1889	1365	10.5696	6.059456	5.351283	11.49052	10.66985	9.419225	7.326781	10.52653	0.028148	0.345221	-
ENSMUSC	Trank1	tetratricor	-	-	-	-	-	-	9:1113117	4428	5939	3917	9051	7048	7531	18.86445	20.20672	18.44323	29.74839	19.33558	25.24063	19.17146	24.77486	0.028324	0.347136	-
ENSMUSC	Oaz2	ornithine	GO:00081	metabolic	-	-	-	-	9:6566800	663	871	1417	863	1098	798	8.582044	9.004123	20.27189	8.618239	9.152392	8.126273	12.61935	8.632301	0.028392	0.347723	-
ENSMUSC	Acer3	alkaline ce	GO:00081	metabolic	K04711	dihydroce	mmu0060	Sphingolip	7:9820638	467	546	367	539	477	369	1.458375	1.361728	1.266674	1.298588	0.959236	0.906545	1.362259	1.054789	0.028426	0.347898	-
ENSMUSC	Ino80b	INO80 co	GO:00081	metabolic	-	-	-	-	6:8312176	119	167	150	279	323	209	3.083998	3.456457	4.296419	5.578305	5.39045	4.261128	3.612291	5.076628	0.028517	0.34876	-
ENSMUSC	Gpt	glutamic p	GO:00081	metabolic	K00814	alanine tra	mmu0110	Metabolic	15:766967	114	202	129	239	296	257	3.97978	5.631834	4.977247	6.436984	6.654252	7.058259	4.862954	6.716498	0.028599	0.349273	-
ENSMUSC	Mthfs	5, 10-met	GO:00081	metabolic	K01934	5-formylt	mmu0110	Metabolic	9:8921067	53	66	39	48	48	44	0.305696	0.304019	0.24861	0.213589	0.178281	0.199651	0.286109	0.197174	0.028597	0.349273	-
ENSMUSC	Shox2	short statu	GO:00325	developm	-	-	-	-	3:6697172	0	35	2	0	3	3	0	0.226325	0.017899	0	0.015644	0.019107	0.081408	0.011584	0.028645	0.349586	-
ENSMUSC	Sspo	SCO-spor	GO:00325	developm	-	-	-	-	6:4844822	24	30	13	56	43	44	0.081331	0.081189	0.048688	0.146404	0.093834	0.117299	0.070403	0.119179	0.028724	0.35031	-
ENSMUSC	Cenpe	centrome	GO:00081	metabolic	-	-	-	-	3:1352125	57	57	52	93	167	85	0.368987	0.294685	0.372037	0.464463	0.696162	0.43288	0.345236	0.531168	0.028825	0.351295	-
ENSMUSC	Palb2	partner ar	GO:00325	developm	K10897	partner ar	mmu0346	Fanconi a	7:1221072	17	23	20	34	46	51	0.09253	0.099979	0.120313	0.142768	0.161229	0.218378	0.104274	0.174125	0.028939	0.35244	-
ENSMUSC	Adgre1	adhesion	GO:00048	signal trar	-	-	-	-	17:573586	103	139	77	90	110	101	1.742098	1.877573	1.439378	1.174383	1.198071	1.3439	1.68635	1.238785	0.02896	0.352443	-
ENSMUSC	Ppp2r2a	protein pl	GO:00325	developm	K04354	serine/thr	mmu0415	PI3K-Akt s	14:670140	1866	2388	1664	1650	2990	1569	14.94614	15.27557	14.7305	10.19605	15.42209	9.8867	14.98407	11.83495	0.029046	0.353243	-
ENSMUSC	Lrp8	low densit	GO:00325	developm	-	-	-	-	4:1078022	1633	1085	723	1088	1155	1027	7.012793	3.721179	3.431547	3.604659	3.194049	3.469654	4.721839	3.422787	0.02908	0.35341	-
ENSMUSC	Banp	BTG3 assc	GO:00325	developm	-	-	-	-	8:1219497	325	405	300	301	491	217	1.823888	1.815163	1.860728	1.3032	1.774398	0.958044	1.83326	1.345214	0.02931	0.35596	-
ENSMUSC	Dnajb2	DnaJ heat	GO:00081	metabolic	K09508	DnaJ hom	mmu0414	Protein pr	1:7523640	908	841	756	889	1015	759	6.570369	4.860107	6.046062	4.962907	4.729601	4.320722	5.825513	4.671076	0.029405	0.356613	-
ENSMUSC	Lrrc38	leucine ric	GO:00325	developm	-	-	-	-	4:1433497	15	25	18	14	18	12	0.394083	0.524553	0.522675	0.283767	0.304536	0.248034	0.480437	0.278779	0.029402	0.356613	-
ENSMUSC	Derl3	Der1-like	GO:00081	metabolic	K13989	Derlin-2/3	mmu0414	Protein pr	10:758934	8	9	4	20	22	13	0.116641	0.104795	0.064457	0.224952	0.206548	0.149099	0.095298	0.193533	0.029558	0.358223	-
ENSMUSC	Klhl41	kelch-like	GO:00325	developm	-	-	-	-	2:6967012	0	0	0	5	3	0	0	0	0	0.093125	0.046647	0	0	0.046591	0.029673	0.359357	-
ENSMUSC	Taok1	TAO kinas	GO:00325	developm	K04429	thousand	mmu0401	MAPK sigı	11:775291	5592	7355	5043	6699	7938	4016	27.00601	28.36754	26.91714	24.95937	24.68647	15.25801	27.43023	21.63461	0.029707	0.359525	-
ENSMUSC	Ncapg	non-SMC	GO:00511	localizatio	-	-	-	-	5:4566991	6	13	5	19	21	20	0.031622	0.054719	0.029127	0.077254	0.071271	0.082925	0.038489	0.07715	0.029755	0.359615	-
ENSMUSC	Rhbdf2	rhomboid	GO:00081	metabolic	-	-	-	-	11:116598	25	31	19	34	67	62	0.211418	0.209368	0.177588	0.221825	0.364867	0.412486	0.199458	0.333059	0.029759	0.359615	-
ENSMUSC	Tro	trophinin	GO:00099	cellular pr	-	-	-	-	X:1506453	2141	2346	1689	4234	2623	4202	13.86713	12.13511	12.09055	21.15685	10.94014	21.41099	12.6976	17.83599	0.029777	0.359615	-
ENSMUSC	Mknk2	MAP kina	GO:00325	developm	K04372	MAP kina	mmu0401	MAPK sigı	10:806653	413	488	496	833	903	725	4.014172	3.788009	5.328115	6.246269	5.651817	5.543632	4.376765	5.813906	0.029819	0.359878	-
ENSMUSC	Rem1	rad and g	GO:00511	localizatio	-	-	-	-	2:1526269	3	8	2	12	15	12	0.053154	0.113208	0.039168	0.164031	0.171147	0.167276	0.06851	0.167485	0.02991	0.360728	-
ENSMUSC	Cabcoco1	ciliary ass	GO:00056	cell|intrace	-	-	-	-	10:684309	20	37	19	62	51	45	0.751029	1.109598	0.788551	1.796171	1.233246	1.329364	0.883059	1.452927	0.030125	0.362812	-
ENSMUSC	Lat	linker for	GO:00048	signal trar	K07362	linker for	mmu0401	Ras signal	7:1263638	4	7	7	11	20	20	0.047519	0.066409	0.091908	0.100814	0.152989	0.186904	0.068612	0.146902	0.030116	0.362812	-
ENSMUSC	Pdlim1	PDZ and l	GO:00081	metabolic	-	-	-	-	19:402211	79	94	87	144	270	122	0.741296	0.704429	0.902253	1.042447	1.631477	0.900601	0.782659	1.191508	0.030347	0.36524	-
ENSMUSC	Wac	WW dom	GO:00081	metabolic	-	-	-	-	18:786883	1385	2557	2288	1910	2510	1958	6.358309	9.374936	11.60899	6.764806	7.420278	7.071561	9.114079	7.085548	0.030389	0.36549	-
ENSMUSC	Mcm5	minichron	GO:00081	metabolic	K02209	DNA repli	mmu0411	Cell cycle	8:7510956	97	135	84	172	229	170	1.084533	1.205458	1.037996	1.483647	1.648772	1.49531	1.109329	1.542577	0.030445	0.365907	-
ENSMUSC	Kcnj15	potassium	GO:00511	localizatio	K05008	potassium	mmu0497	Gastric ac	16:952575	7	11	5	2	7	3	0.020251	0.025413	0.015988	0.004464	0.013039	0.006827	0.020551	0.00811	0.030477	0.366037	-
ENSMUSC	Btg1	B cell tran	GO:00325	developm	K14443	protein Tc	mmu0301	RNA degr	10:966170	381	542	368	618	874	690	4.103566	4.662105	4.380578	5.135178	6.061816	5.846515	4.382083	5.68117	0.030506	0.366133	-
ENSMUSC	Pnpla2	patatin-lik	GO:00081	metabolic	K16816	patatin-lik	mmu0110	Metabolic	7:1414551	255	299	254	445	512	441	2.228077	2.086449	2.452852	2.999722	2.880813	3.031376	2.255793	2.970637	0.03057	0.366144	-
ENSMUSC	Mga	MAX gen	GO:00081	metabolic	-	-	-	-	2:1198972	1228	2003	1082	1428	1656	1441	2.712614	3.533592	2.64158	2.433595	2.355616	2.504175	2.962595	2.431128	0.03055	0.366144	-
ENSMUSC	Lyz1	lysozyme	GO:00081	metabolic	K13915	lysozyme	mmu0497	Salivary se	10:117287	0	3	3	6	18	4	0	0.115844	0.160301	0.223773	0.560366	0.15212	0.092048	0.312087	0.030533	0.366144	-
ENSMUSC	Gm8113	predicted	GO:00160	membran	-	-	-	-	14:439253	0	0	2	3	9	4	0	0	0.049604	0.051943	0.130052	0.070609	0.016535	0.084201	0.030608	0.366352	-
ENSMUSC	Pld1	phospholi	GO:00081	metabolic	K01115	phospholi	mmu0110	Metabolic	3:2793869	136	233	110	249	326	277	0.463931	0.634771	0.414721	0.655305	0.716122	0.74337	0.504474	0.704933	0.030691	0.367091	-
ENSMUSC	Gm29094	predicted	GO:00511	localizatio	-	-	-	-	9:8919937	4	0	2	0	0	0	0.367841	0	0.203274	0	0	0	0.190372	0	0.030751	0.36755	-
ENSMUSC	Tyrp1	tyrosinase	GO:00325	developm	K00506	tyrosinase	mmu0110	Metabolic	4:8083412	1	5	1	7	16	5	0.012685	0.050634	0.014016	0.068481	0.130651	0.04988	0.025778	0.083004	0.030815	0.368067	-
ENSMUSC	Tgm2	transgluta	GO:00325	developm	K05625	transgluta	mmu0501	Huntingto	2:1581164	283	427	271	481	716	500	3.36766	4.058025	3.564167	4.415876	5.486672	4.680826	3.663284	4.861125	0.030853	0.368263	-
ENSMUSC	Acap1	ArfGAP w	GO:00048	signal trar	K12489	Arf-GAP w	mmu0414	Endocytos	11:698815	4	2	3	8	21	6	0.038187	0.01525	0.031654	0.05892	0.129092	0.045062	0.028364	0.077691	0.030878	0.368308	-
ENSMUSC	Bach2	BTB and C	GO:00081	metabolic	-	-	-	-	4:3223880	159	252	224	275	137	104	0.497221	0.62936	0.77419	0.663461	0.275885	0.255857	0.63359	0.398401	0.030993	0.369436	-
ENSMUSC	Chrdl2	chordin-li	GO:00055	extracellul	-	-	-	-	7:1000061	4	2	1	6	14	7	0.059997	0.02396	0.016577	0.069424	0.135215	0.082599	0.033511	0.095746	0.03102	0.369495	-
ENSMUSC	Hexb	hexosami	GO:00325	developm	K12373	hexosami	mmu0110	Metabolic	13:971763	947	1379	1034	1135	1411	1096	25.07461	29.16044	30.25878	23.18516	24.05835	22.82998	28.16461	23.35783	0.03128	0.37183	-
ENSMUSC	Hsd17b14	hydroxyst	GO:00081	metabolic	-	-	-	-	7:4555489	0	0	0	2	6	0	0	0	0	0.028372	0.071033	0	0	0.033135	0.031259	0.37183	-
ENSMUSC	Ces2h	carboxyle	GO:00081	metabolic	K03927	carboxyle	mmu0098	Drug met	8:1050008	0	0	0	2	6	0	0	0	0	0.038538	0.096483	0	0	0.045007	0.031252	0.37183	-
ENSMUSC	Elf2	E74-like f	GO:00325	developm	-	-	-	-	3:5125272	506	706	354	417	721	400	1.541456	1.717636	1.191875	0.980048	1.414394	0.958631	1.483656	1.117691	0.031354	0.37246	-
ENSMUSC	Kcne2	potassium	GO:00325	developm	K04896	potassium	mmu0497	Gastric ac	16:922923	108	330	66	34	120	148	3.731916	9.106815	2.520581	0.906381	2.670186	4.023267	5.119771	2.533278	0.03141	0.372871	-
ENSMUSC	Cyp1b1	cytochron	GO:00325	developm	K07410	cytochron	mmu0520	MicroRNA	17:797069	113	219	108	85	182	123	1.345468	2.082503	1.421239	0.780804	1.39547	1.152156	1.616403	1.109476	0.03155	0.374269	-
ENSMUSC	Gnb5	guanine n	GO:00048	signal trar	K04539	guanine n	mmu0520	Pathways	9:7530628	970	1421	1016	1288	1311	1044	11.45357	13.40012	13.25895	11.73316	9.968419	9.697958	12.70421	10.46651	0.031623	0.374882	-
ENSMUSC	Atp7a	ATPase, C	GO:00325	developm	K17686	Cu+-expc	mmu0152	Platinum c	X:1060272	186	298	158	175	263	190	1.150642	1.472273	1.080263	0.835206	1.047697	0.924678	1.234392	0.935861	0.03165	0.37495	-
ENSMUSC	Lrrc25	leucine ric	GO:00160	membran	-	-	-	-	8:7061615	9	11	10	10	2	4	0.311867	0.304413	0.382975	0.267352	0.04462	0.10904	0.333085	0.140337	0.03168	0.375048	-
ENSMUSC	Rnf126	ring finge	GO:00081	metabolic	-	-	-	-	10:797585	505	667	628	995	1153	947	10.99263	11.5953	15.10834	16.70951	16.16197	16.21704	12.56542	16.36284	0.031785	0.376039	-
ENSMUSC	Tmem253	transmem	GO:00160	membran	-	-	-	-	14:520156	3	0	8	12	32	10	0.07664	0	0.225869	0.236507	0.526446	0.200981	0.100837	0.321311	0.031878	0.37688	-
ENSMUSC	Syngr2	synaptogy	GO:00452	synapse|c	-	-	-	-	11:117809	66	102	50	108	155	146	0.999716	1.233896	0.837049	1.262069	1.511881	1.739785	1.023554	1.504578	0.031922	0.377146	-
ENSMUSC	Ifitm3	interferon	GO:00511	localizatio	-	-	-	-	7:1410095	75	121	78	166	213	131	6.754228	8.70254	7.763484	11.53329	12.35231	9.281058	7.740084	11.05555	0.032036	0.378224	-
ENSMUSC	Poglut1	protein O	GO:00325	developm	K13667	protein gl	mmu0051	Other typ	16:385251	640	400	294	757	998	834	5.546802	2.768648	2.816154	5.061605	5.569904	5.686427	3.710535	5.439312	0.032057	0.378224	-
ENSMUSC	Anpep	alanyl (me	GO:00325	developm	K11140	aminopep	mmu0110	Metabolic	7:7982180	111	180	109	213	312	201	1.065974	1.380517	1.156905	1.578092	1.929445	1.518553	1.201132	1.675363	0.032148	0.379037	-
ENSMUSC	Mcemp1	mast cell e	GO:00160	membran	-	-	-	-	8:3665754	0	0	0	1	1	6	0	0	0	0.031534	0.026312	0.192916	0	0.083587	0.032276	0.380297	-
ENSMUSC	Stx6	syntaxin 6	GO:00452	synapse|c	K08498	syntaxin 6	mmu0413	SNARE int	1:1551587	961	1270	1561	2000	2708	2284	4.597416	4.852217	8.25354	7.381606	8.342454	8.596025	5.901057	8.106695	0.032325	0.380616	-
ENSMUSC	Slc25a25	solute car	GO:00325	developm	-	-	-	-	2:3241448	661	874	704	709	968	737	2.783024	2.938819	3.275931	2.30299	2.624493	2.441148	2.999258	2.45621	0.032606	0.383657	-
ENSMUSC	Tk2	thymidine	GO:00081	metabolic	K00857	thymidine	mmu0110	Metabolic	8:1042266	245	633	331	274	543	261	2.112585	4.359114	3.154453	1.822761	3.015113	1.770516	3.208717	2.202797	0.032664	0.383932	-
ENSMUSC	Hrct1	histidine r	GO:00160	membran	-	-	-	-	4:4372718	13	14	14	11	9	8	0.546664	0.470169	0.650654	0.356877	0.243713	0.264639	0.555829	0.28841	0.032673	0.383932	-
ENSMUSC	Tmem140	transmem	GO:00160	membran	-	-	-	-	6:3486314	31	33	31	60	68	63	0.259569	0.220677	0.286884	0.387596	0.366655	0.415002	0.25571	0.389751	0.032747	0.384545	-
ENSMUSC	Sigirr	single imr	GO:00081	metabolic	-	-	-	-	7:1410911	8	19	12	25	40	26	0.091358	0.173273	0.151445	0.220247	0.294133	0.233571	0.138692	0.249317	0.032933	0.386462	-
ENSMUSC	Phldb3	pleckstrin	GO:00055	protein bi	-	-	-	-	7:2461076	1	2	0	4	14	1	0.015088	0.024096	0	0.046542	0.135984	0.011869	0.013061	0.064798	0.032991	0.386877	-
ENSMUSC	Mylpf	myosin lic	GO:00325	developm	K12758	fast skelet	mmu0481	Regulation	7:1272088	8	15	11	8	6	7	0.277868	0.416073	0.422235	0.214363	0.134187	0.191274	0.372059	0.179941	0.033031	0.387092	-

ENSMUS(	Rgs6	regulator	GO:00048	signal trar	-	-	-	-	12:825882	230	140	116	288	408	279	0.897741	0.436413	0.500412	0.867254	1.025506	0.85672	0.611522	0.916493	0.03308	0.387408	-
ENSMUS(	Rfk	riboflavin	GO:00081	metabolic	K00861	riboflavin	mmu011C	Metabolic	19:173940	348	1224	917	1449	2061	1498	6.109329	17.16094	17.79221	19.62513	23.29949	20.68886	13.6875	21.2045	0.033371	0.390552	-
ENSMUS(	Tardbp	TAR DNA	GO:00081	metabolic	-	-	-	-	4:1486123	825	1979	1209	1019	1893	838	1.583208	3.033023	2.564231	1.50865	2.339318	1.265141	2.393487	1.70437	0.033405	0.390681	-
ENSMUS(	Enpep	glutamyl a	GO:00325	developm	K11141	glutamyl a	mmu0461	Renin-anc	3:1292691	65	91	70	142	168	112	0.655997	0.733456	0.780785	1.105624	1.091819	0.889234	0.723413	1.028892	0.033458	0.391041	-
ENSMUS(	Tlr7	toll-like re	GO:00048	signal trar	K05404	toll-like re	mmu0516	Influenza	X:1673049	59	79	50	52	68	56	0.313038	0.334747	0.2932	0.212852	0.23233	0.233745	0.313661	0.226309	0.033485	0.391089	-
ENSMUS(	Eci3	enoyl-Coe	GO:00081	metabolic	-	-	mmu0414	Peroxisom	13:349466	0	3	1	5	8	6	0	0.09231	0.042578	0.148589	0.198461	0.18184	0.044963	0.176297	0.033635	0.391793	-
ENSMUS(	Tnfrsf4	tumor nec	GO:00023	immune s	K05142	tumor nec	mmu0406	Cytokine-	4:1560138	5	13	0	14	22	17	0.150302	0.31212	0	0.324705	0.42591	0.40207	0.154141	0.384228	0.033632	0.391793	-
ENSMUS(	Clip1	CAP-GLY	GO:00511	localizatio	K10421	CAP-Gly c	mmu0415	mTOR sig	5:1235777	3501	3946	2920	3612	4249	3661	10.6941	9.626208	9.857844	8.511976	8.357834	8.797575	10.05939	8.555795	0.033597	0.391793	-
ENSMUS(	Olfr46	olfactory r	GO:00048	signal trar	K04257	olfactory r	mmu0474	Olfactory	7:1406012	2	3	3	2	28	11	0.012405	0.014866	0.020571	0.009574	0.11186	0.053687	0.015947	0.058374	0.033615	0.391793	-
ENSMUS(	Plaur	plasminoc	GO:00048	signal trar	K03985	plasminoc	mmu052C	Proteoglyc	7:2446248	9	15	3	21	34	15	0.105736	0.140746	0.038957	0.190343	0.25723	0.138641	0.095146	0.195405	0.033978	0.395526	-
ENSMUS(	Gpx3	glutathior	GO:00081	metabolic	K00432	glutathior	mmu0059	Arachidor	11:549024	70	105	62	137	149	143	1.801873	2.158556	1.763878	2.720684	2.469837	2.895843	1.908103	2.695455	0.034039	0.395965	-
ENSMUS(	8-Mar	membran	GO:00081	metabolic	-	-	-	-	6:1163380	919	526	699	1203	1533	1442	3.29937	1.508158	2.773575	3.33205	3.544152	4.072787	2.527034	3.649663	0.034136	0.396828	-
ENSMUS(	Dnal1	dynein, ax	GO:00990	supramole	K10411	dynein lig	mmu0501	Huntingto	12:841143	350	407	361	2303	467	341	1.873934	1.740307	2.136194	9.512844	1.61012	1.436322	1.916812	4.186429	0.034244	0.397826	-
ENSMUS(	Aspn	asporin [S	GO:00325	developm	-	-	-	-	13:495444	35	36	29	43	93	80	0.538564	0.442411	0.493192	0.510468	0.921533	0.968434	0.491389	0.800145	0.034308	0.398303	-
ENSMUS(	Pik3cb	phosphati	GO:00325	developm	K00922	phosphati	mmu052C	Pathways	9:9903665	716	903	645	666	767	910	5.001442	5.037507	4.979532	3.589111	3.450105	5.000743	5.00616	4.01332	0.034364	0.398687	-
ENSMUS(	Jup	junction p	GO:00325	developm	K10056	junction p	mmu052C	Pathways	11:100368	466	591	448	801	996	729	3.044411	3.083552	3.234765	4.037204	4.190169	3.74676	3.120909	3.991378	0.034413	0.398983	-
ENSMUS(	Cdc42	cell divisic	GO:00325	developm	K04393	cell divisic	mmu052C	Pathways	4:1373196	4422	5599	4035	4496	5902	5261	89.40362	90.40505	90.16266	70.12825	76.84051	83.67883	89.99044	76.88253	0.034652	0.40069	-
ENSMUS(	Tgm1	transgluta	GO:00325	developm	-	-	-	-	14:557000	4	13	4	0	2	6	0.053492	0.138834	0.059121	0	0.017219	0.063122	0.083816	0.02678	0.034614	0.40069	-
ENSMUS(	Nr4a2	nuclear re	GO:00325	developm	K08558	nuclear re	mmu0492	Aldostero	2:5710683	1231	989	813	968	933	1144	11.81244	7.579201	8.622215	7.166171	5.765245	8.636111	9.33795	7.189176	0.034635	0.40069	-
ENSMUS(	Gm20547	predicted	GO:00081	metabolic	-	-	-	-	17:348564	7	20	3	0	2	8	0.105301	0.240271	0.049879	0	0.019369	0.094667	0.131817	0.038012	0.034642	0.40069	-
ENSMUS(	Vegfd	vascular e	GO:00325	developm	K05449	vascular e	mmu052C	Pathways	X:1643733	65	84	56	117	120	145	1.460722	1.50757	1.390871	2.028473	1.736555	2.5635	1.453054	2.109509	0.034681	0.400757	-
ENSMUS(	Atp4a	ATPase, H	GO:00511	localizatio	K01542	H+/K+-ex	mmu0019	Oxidative	7:3071220	11	13	13	3	11	10	0.131673	0.124273	0.171979	0.027708	0.084786	0.094167	0.142641	0.068887	0.034748	0.401268	-
ENSMUS(	Acaa1b	acetyl-Co	GO:00081	metabolic	K07513	acetyl-Co	mmu011C	Metabolic	9:1191480	26	30	35	48	76	71	0.699653	0.644717	1.040946	0.996497	1.316973	1.503062	0.795105	1.272177	0.034834	0.401989	-
ENSMUS(	Pja1	praja ring	GO:00081	metabolic	-	-	-	-	X:9946573	937	1206	881	3224	1505	1259	4.824307	4.958933	5.013233	12.80621	4.989839	5.099551	4.932158	7.631866	0.035039	0.40382	-
ENSMUS(	Socs3	suppressc	GO:00325	developm	K04696	suppressc	mmu0516	Herpes sir	11:117966	14	48	23	48	82	55	0.259986	0.711871	0.472039	0.687668	0.98057	0.803494	0.481298	0.823911	0.035027	0.40382	-
ENSMUS(	Ddx11	DEAD/H (	GO:00325	developm	-	-	-	-	17:661235	18	25	15	42	49	32	0.095675	0.106125	0.08812	0.172232	0.16772	0.133814	0.09664	0.157922	0.035112	0.404396	-
ENSMUS(	Apln	apelin [So	GO:00081	metabolic	-	-	-	-	X:4802514	306	398	246	354	367	227	5.933251	6.163109	5.271716	5.295466	4.582378	3.462644	5.789359	4.446829	0.035159	0.404668	-
ENSMUS(	Alkbh8	alkB homo	GO:00081	metabolic	-	-	-	-	9:3335140	724	991	740	750	1147	732	2.550548	2.788137	2.881199	2.038384	2.602033	2.028696	2.739962	2.223037	0.035272	0.4057	-
ENSMUS(	Tmem165	transmem	GO:00081	metabolic	-	-	-	-	5:7618388	320	420	302	326	419	351	5.273571	5.527768	5.500594	4.144797	4.446552	4.550651	5.433978	4.380667	0.035306	0.405826	-
ENSMUS(	Synm	synemin, i	GO:00051	structural	-	-	-	-	7:6773016	1366	1683	1113	1296	2049	878	4.647573	4.573046	4.185216	3.401816	4.489234	2.350071	4.468612	3.413707	0.035466	0.407395	-
ENSMUS(	Tbrg4	transform	GO:00081	metabolic	-	-	-	-	11:661559	244	319	309	499	597	449	2.267955	2.367996	3.17431	3.578282	3.57333	3.283233	2.60342	3.478282	0.035641	0.408471	-
ENSMUS(	Arrdc2	arrestin dc	GO:00160	membran	-	-	-	-	8:7083512	117	64	111	186	246	194	1.038948	0.45387	1.08937	1.27424	1.406687	1.35525	0.860729	1.345392	0.035649	0.408471	-
ENSMUS(	Tert	telomeras	GO:00325	developm	K11126	telomeras	mmu052C	Pathways	13:736269	22	18	22	36	51	51	0.132577	0.086628	0.14653	0.167371	0.197913	0.241785	0.121912	0.202356	0.035662	0.408471	-
ENSMUS(	Ptprc	protein ty	GO:00081	metabolic	K06478	receptor-t	mmu0451	Cell adhes	1:1380628	96	162	81	167	242	205	0.217497	0.293119	0.202824	0.291898	0.353065	0.365384	0.237813	0.336782	0.035645	0.408471	-
ENSMUS(	Ppcs	phosphop	GO:00081	metabolic	K01922	phosphop	mmu011C	Metabolic	4:1194185	39	34	42	66	111	68	1.031745	0.718347	1.22801	1.347043	1.890965	1.415236	0.992701	1.551081	0.035678	0.408471	-
ENSMUS(	Scai	suppressc	GO:00081	metabolic	-	-	-	-	2:3906621	1750	2167	1386	1798	2369	1640	7.388445	7.306674	6.46733	5.856456	6.440725	5.447156	7.05415	5.914779	0.035706	0.408526	-
ENSMUS(	Slc35d1	solute car	GO:00325	developm	-	-	-	-	4:1031706	206	157	207	345	440	314	1.024596	0.623636	1.137899	1.32384	1.409269	1.228647	0.92871	1.320585	0.035863	0.410053	-
ENSMUS(	Gpr137b	G protein-	GO:00160	membran	-	-	-	-	13:133576	192	345	219	214	269	263	1.089008	1.562768	1.37284	0.936426	0.982509	1.173536	1.341539	1.030824	0.035891	0.410107	-
ENSMUS(	Ccl21a	chemokin	GO:00325	developm	-	-	-	-	JH584294	0	3	2	4	11	7	0	0.363957	0.335756	0.46866	1.075851	0.836467	0.233238	0.793659	0.036037	0.4115	-
ENSMUS(	Fam129a	family witl	GO:00081	metabolic	-	-	-	-	1:1515711	86	122	77	92	332	200	0.525202	0.595028	0.519719	0.433462	1.305638	0.960884	0.54665	0.899995	0.036084	0.41177	-
ENSMUS(	Lmnb1	lamin B1 [	GO:00081	metabolic	K07611	lamin B[-]	mmu0421	Apoptosis	18:567078	182	246	192	364	463	279	3.467265	3.742801	4.042624	5.349921	5.680038	4.181488	3.750897	5.070483	0.036166	0.412431	-
ENSMUS(	B3gnt7	UDP-GlcN	GO:00081	metabolic	K09664	beta-1,3-	mmu0053	Glycosam	1:8630283	23	10	10	32	64	23	0.234801	0.081526	0.112824	0.252031	0.420734	0.184721	0.14305	0.285829	0.036249	0.413112	-
ENSMUS(	Mib1	mindbom	GO:00325	developm	-	-	-	-	18:107255	3439	3413	3063	3982	3703	3030	13.9595	11.0642	13.74142	12.47008	9.679348	9.675897	12.92171	10.60844	0.036489	0.415356	-
ENSMUS(	Tbr1	T-box bra	GO:00325	developm	-	-	-	-	2:6180293	1281	580	444	1624	1804	1453	11.39669	4.121004	4.365754	11.14668	10.33524	10.16965	6.627815	10.55052	0.036494	0.415356	-
ENSMUS(	Spata31d:	spermato	GO:00160	membran	-	-	-	-	13:596998	1	1	0	5	4	3	0.00536	0.00428	0	0.02067	0.013804	0.012646	0.003214	0.015706	0.036562	0.415859	-
ENSMUS(	Aasdhppt	aminoadi	GO:00081	metabolic	K06133	4'-phospł	mmu0077	Pantother	9:4294793	248	555	232	370	312	248	2.123756	3.795707	2.195776	2.444477	1.720534	1.670771	2.70508	1.945261	0.036624	0.416292	-
ENSMUS(	Ifit3	interferon	GO:00023	immune s	-	-	-	-	19:345835	214	323	182	482	401	366	7.48793	9.026017	7.038286	13.01145	9.0354	10.07489	7.850744	10.70724	0.036716	0.417065	-
ENSMUS(	Slc6a3	solute car	GO:00325	developm	K05036	solute car	mmu0503	Alcoholisr	13:735367	5	4	4	0	2	3	0.088329	0.056433	0.07811	0	0.022749	0.041691	0.07429	0.02148	0.036768	0.417109	-
ENSMUS(	Blnk	B cell linke	GO:00048	signal trar	K07371	B-cell link	mmu0438	Osteoclas	19:409289	68	97	82	126	214	134	0.664738	0.757282	0.885931	0.95026	1.34713	1.030522	0.769317	1.109304	0.036755	0.417109	-
ENSMUS(	Lig3	ligase III, [	GO:00081	metabolic	K10776	DNA ligas	mmu0341	Base excis	11:827811	300	608	492	416	497	462	1.114808	1.804383	2.020652	1.192624	1.189298	1.350617	1.646614	1.244179	0.036936	0.418475	-
ENSMUS(	Rnase4	ribonuclea	GO:00081	metabolic	-	-	-	-	14:51091C	202	247	185	303	489	332	4.141625	4.044475	4.192143	4.792826	6.456275	5.355094	4.126081	5.534732	0.036921	0.418475	-
ENSMUS(	Ctnna1	catenin (c	GO:00325	developm	K05691	catenin al	mmu052C	Pathways	18:351188	1202	1791	1112	1916	2657	2067	19.84636	23.61663	20.29216	24.40631	28.25028	26.84899	21.25172	26.50186	0.037435	0.423854	-
ENSMUS(	Slc35a3	solute car	GO:00511	localizatio	-	-	-	-	3:1166694	322	478	309	561	738	565	1.576011	1.86843	1.671506	2.118341	2.326023	2.175515	1.705316	2.206626	0.037506	0.42438	-
ENSMUS(	Fam162a	family witl	GO:00081	metabolic	-	-	-	-	16:360438	293	445	278	484	718	530	28.17331	34.17243	29.54344	35.90417	44.45791	40.09189	30.62972	40.15132	0.037766	0.427044	-
ENSMUS(	Cep55	centroson	GO:00325	developm	-	-	-	-	19:38055C	2	16	6	14	32	20	0.039926	0.255143	0.132414	0.215656	0.411447	0.314155	0.142494	0.313753	0.037819	0.427367	-
ENSMUS(	Fcrls	Fc recepto	GO:00511	localizatio	-	-	-	-	3:8725075	383	500	282	402	460	335	8.742156	9.114548	7.114029	7.079074	6.761328	6.015553	8.323578	6.618651	0.037846	0.427388	-
ENSMUS(	Vsig2	V-set and	GO:00160	membran	-	-	-	-	9:3753925	18	16	4	37	29	26	0.349117	0.247848	0.085752	0.553657	0.362209	0.396729	0.227572	0.437532	0.037967	0.428479	-
ENSMUS(	Pcdhb2	protocadł	GO:00325	developm	-	-	-	-	18:372948	95	100	81	79	113	78	1.745048	1.466995	1.644435	1.119536	1.336645	1.127169	1.618826	1.19445	0.038046	0.429096	-
ENSMUS(	Gstk1	glutathior	GO:00325	developm	K13299	glutathior	mmu052C	Chemical	6:4224593	42	73	61	130	116	94	1.127721	1.565388	1.810216	2.692931	2.00569	1.985606	1.501108	2.228076	0.038087	0.429276	-
ENSMUS(	Tgfbr1	transform	GO:00325	developm	K04674	TGF-beta	mmu052C	Pathways	4:4735322	630	858	555	635	853	707	4.213674	4.583035	4.102604	3.276606	3.673871	3.720062	4.299771	3.556846	0.03819	0.429387	-
ENSMUS(	Il13ra1	interleukir	GO:00048	signal trar	K05076	interleukir	mmu052C	Pathways	X:3611211	130	176	110	220	317	205	2.138352	2.312028	1.999754	2.791824	3.357759	2.652772	2.150045	2.934118	0.038135	0.429387	-
ENSMUS(	Alkbh4	alkB homo	GO:00081	metabolic	-	-	-	-	5:1361361	67	74	42	34	52	68	0.750206	0.661739	0.519759	0.293705	0.374944	0.599003	0.643901	0.422551	0.03815	0.429387	-
ENSMUS(	Cpa2	carboxype	GO:00081	metabolic	K01298	carboxype	mmu0497	Pancreatic	6:3054158	6	8	4	12	17	19	0.089811	0.095639	0.06618	0.138576	0.163872	0.223742	0.083877	0.175396	0.038196	0.429387	-
ENSMUS(	Insr	insulin rec	GO:00325	developm	K04527	insulin rec	mmu0415	PI3K-Akt s	8:3122061	683	957	560	922	1849	1115	2.740892	3.067108	2.48374	2.854517	4.778185	3.520117	2.763914	3.717606	0.038334	0.430661	-
ENSMUS(	Slc35a1	solute car	GO:00511	localizatio	-	-	-	-	4:3466325	215	265	208	334	478	382	3.95169	3.889875	4.225266	4.7361	5.657524	5.523551	4.022277	5.305725	0.038485	0.43208	-
ENSMUS(	Pole2	polymeras	GO:00081	metabolic	K02325	DNA poly	mmu011C	Metabolic	12:692017	5	11	5	17	20	16	0.086698	0.152336	0.095835	0.227439	0.223335	0.218279	0.111623	0.223018	0.038524	0.432234	-
ENSMUS(	Rad51b	RAD51 pa	GO:00325	developm	K10869	RAD51-lik	mmu0344	Homologc	12:792972	4	4	0	7	13	8	0.057524	0.045933	0	0.077658	0.120384	0.090498	0.034486	0.09618	0.038649	0.433088	-
ENSMUS(	Gng5	guanine n	GO:00325	developm	K04542|KC	guanine n	mmu052C	Pathways	3:1464998	27	25	26	49	55	57	0.475783	0.351821	0.506356	0.666137	0.624109	0.790181	0.444654	0.693476	0.038638	0.433088	-
ENSMUS(	Ezh1	enhancer	GO:00325	developm	K17451	histone-ly	mmu0031	Lysine deg	11:101191	1479	1336	1463	1372	1687	1547	8.0991	5.842798	8.854411	5.796327	5.948922	6.664533	7.59877	6.136594	0.038854	0.435104	-
ENSMUS(	Kcnj14	potassium	GO:00511	localizatio	K05007	potassium	mmu0492	Oxytocin s	7:4581646	22	22	26	43	57	48	0.409532	0.327073	0.534916	0.617542	0.68328	0.702951	0.42384	0.667924	0.038907	0.435415	-
ENSMUS(	Itgb7	integrin b	GO:00325	developm	K06590	integrin b	mmu0415	PI3K-Akt s	15:102215	10	18	6	22	33	23	0.21877	0.314482	0.145081	0.37131	0.464893	0.395851	0.226111	0.410684	0.038979	0.435943	-
ENSMUS(	Uhrf1	ubiquitin-	GO:00081	metabolic	-	-	-	-	17:563033	27	36	31	56	97	46	0.310703	0.330849	0.394263	0.497154	0.718787	0.416432	0.345272	0.544125	0.039056	0.436139	-
ENSMUS(	Ctsd	cathepsin	GO:00081	metabolic	K01379	cathepsin	mmu0515	Tuberculo	7:1423759	4931	7683	5356	6506	8027	4965	83.49349	103.8949	100.2317	84.98886	87.52368	66.1375	95.87336	79.55002	0.039094	0.436139	-
ENSMUS(	Esco2	establishn	GO:00325	developm	-	-	-	-	14:65819C	7	6	4	13	19	14	0.049688	0.034012	0.031382	0.071188	0.086846	0.078176	0.038361	0.078737	0.039082	0.436139	-
ENSMUS(	Gm2897	predicted	-	-	-	-	-	-	14:304928	17	28	16	37	56	35	0.524244	0.689582	0.545315	0.880259	1.112045	0.849092	0.58638	0.947132	0.039097	0.436139	-
ENSMUS(	Kl	klotho [Sc	GO:00325	developm	K14756	klotho[3.2	mmu011C	Metabolic	5:1509526	1114	2756	872	889	1616	1578	6.518325	12.87882	5.639158	4.01313	6.089013	7.263883	8.345433	5.788675	0.039138	0.436312	-
ENSMUS(	Sbspon	somatom	GO:00511	localizatio	-	-	-	-	1:1585386	3	12	2	0	0	5	0.066656	0.212935	0.049117	0	0	0.087399	0.109569	0.029133	0.039492	0.439982	-
ENSMUS(	4930404N	RIKEN cDI	-	-	-	-	-	-	10:81363C	0	2	0	5	6	2	0	0.029612	0	0.071498	0.071615	0.029171	0.009871	0.057428	0.039756	0.442641	-
ENSMUS(	Ctnnd1	catenin (c	GO:00325	developm	K05690	catenin (c	mmu0401	Rap1 sign	2:8460007	1071	1463	978	1698	2401	1642	2.891972	3.154964	2.918705	3.537304	4.17495	3.488095	2.988547	3.73345	0.039937	0.444365	-
ENSMUS(	Ptger1	prostaglaı	GO:00048	signal trar	K04258	prostaglaı	mmu052C	Pathways	8:8366667	3	5	10	28	43	4	0.020235	0.026934	0.074547	0.145708	0.186776	0.021225	0.040572	0.117903	0.039996	0.444734	-
ENSMUS(	Xcr1	chemokin	GO:00048	signal trar	K04193	XC chemc	mmu0406	Cytokine-	9:1238523	0	0	1	0	9	3	0	0	0	0.015155	0	0.079468	0.032357	0.005052	0.037275	0.040111	0.445734	-
ENSMUS(	Gbp7	guanylate	GO:00023	immune s	K20899	guanylate	mmu0462	NOD-like	3:1425303	184	299	201	372	468	336	1.717578	2.229034	2.073678	2.679001	2.813196	2.467459	2.006763	2.653219	0.04058	0.450658	-
ENSMUS(	4931406P	RIKEN cDI	-	-	-	-	-	-	7:3423670	730	954	685	778	998	810	2.655809	2.77184	2.754301	2.183654	2.338079	2.318299	2.727317	2.280011	0.040768	0.452457	-
ENSMUS(	F11r	F11 recep	GO:00325	developm	K06089	junctional	mmu0453	Tight junc	1:1714375	151	238	98	219	446	257	2.546892	3.205957	1.826863	2.849762	4.844224	3.410192	2.526571	3.701392	0.040937	0.454042	-
ENSMUS(	Mylk3	myosin lig	GO:00325	developm	K00907	myosin-lig	mmu0481	Regulatior	8:8532430	42	73	74	24	82	47	0.411826	0.571654	0.801942	0.181556	0.517763	0.362556	0.595141	0.353958	0.040974	0.454164	-
ENSMUS(	B430306N	RIKEN cDI	GO:00160	membran	-	-	-	-	17:483161	6	13	3	3	3	5	0.038212	0.066122	0.021118	0.014743	0.012305	0.025052	0.041818	0.017367	0.041248	0.456907	-
ENSMUS(	Gpam	glycerol-3	GO:00325	developm	K00629	glycerol-3	mmu011C	Metabolic	19:550697	884	1134	848	1031	1136	970	14.08543	14.43034	14.93345	12.6738	11.65604	12.15907	14.48307	12.16297	0.041466	0.458902	-
ENSMUS(	Kif28	kinesin fai	GO:00511	localizatio	-	-	-	-	1:1796952	16	4	2	5	1	2	0.224994	0.044918	0.031086	0.054241	0.009053	0.02213	0.100333	0.028475	0.041481	0.458902	-
ENSMUS(	Akap13	A kinase (	GO:00325	developm	-	-	-	-	7:7545553	1781	2059	1723	2900	3223	2865	3.763826	3.475104	4.024369	4.72818	4.386122	4.763226	3.754433	4.625843	0.041513	0.458967	-
ENSMUS(	Tenm1	teneurin t	GO:00325	developm	-	-	-	-	X:4252786	1727	2114	1415	1881	1966	1916	5.150305	5.034907	4.663842	4.327726	3.775538	4.495179	4.949685	4.199481	0.0417	0.459525	-
ENSMUS(	Shc3	src homol	GO:00325	developm	K17448	SHC-trans	mmu0401	Ras signal	13:514310	975	1301	777	1100	1125	1038	12.66631	13.49797	11.15612	11.02474	9.411363	10.60849	12.44013	10.3482	0.041655	0.459525	-
ENSMUS(	Gbp2	guanylate	GO:00023	immune s	K20897	guanylate	mmu0462	NOD-like	3:1426206	101	209	155	273	332	240	2.179109	3.601209	3.696018	4.544121	4.61264	4.073601	3.158779	4.410121	0.041662	0.459525	-
ENSMUS(	Fam109a	family witl	GO:00081	metabolic	-	-	-	-	5:1218489	15	100	64	112	174	131	0.181788	0.967903	0.857263	1.047211	1.357969	1.249019	0.668985	1.218066	0.04171	0.459525	-
ENSMUS(	Rbm33	RNA bind	GO:00971	organic cy	-	-	-	-	5:2831712	1121	1375	1018	1758	2060	1833	3.963297	3.882394	3.977824	4.795128	4.690001	5.098287	3.941172	4.861139	0.041728	0.459525	-
ENSMUS(	P2ry10	purinergic	GO:00048	signal trar	K04274	purinergic	mmu0408	Neuroacti	X:1070884	0	0	0	2	2	3	0	0	0	0.016285	0.013588	0.024902	0	0.018258	0.041749	0.459525	-
ENSMUS(	Luzp2	leucine zi	GO:00055	extracellul	-	-	-	-	7:5483549	3446	4153	3144	3877	4346	3840	34.40259	33.11186	34.69007	29.86084	27.9396	30.15908	34.06818	29.31984	0.041724	0.459525	-
ENSMUS(	Creb1	cAMP resı	GO:00325	developm	K05870	cyclic AMI	mmu0415	PI3K-Akt s	1:6453264	1323	1655	1070	1461	1708	1269	3.902976	3.899237	3.488722	3.32519	3.244729	2.945157	3.763645	3.171692	0.041785	0.459632	-
ENSMUS(	Glp2r	glucagon-	GO:00048	signal trar	K04582	glucagon-	mmu0408	Neuroacti	11:676615	62	91	62	103	156	137	0.63763	0.747416	0.704719	0.817229	1.033132	1.108426	0.696589	0.986262	0.041813	0.459649	-
ENSMUS(	2610318N	RIKEN cDI	-	-	-	-	-	-	16:171133	3	7	2	11	23	6	0.129357	0.241061	0.09532	0.365951	0.638654	0.203542	0.155246	0.402715	0.041873	0.460018	-
ENSMUS(	Mcf2	mcf.2 trar	GO:00325	developm	-	-	-	-	X:6005595	100	140	101	111	123	104	1.060772	1.186034	1.184105	0.908398	0.8402	0.867896	1.143637	0.872165	0.041915	0.460186	-
ENSMUS(	Zbtb16	zinc finge	GO:00325	developm	K10055	zinc finge	mmu052C	Pathways	9:4865429	1644	2008	1349	1733	1951	1832	17.49382	17.06445	15.86506	14.22694	13.36887	15.33625	16.80778	14.31069	0.041959	0.460383	-
ENSMUS(	Arhgap11	Rho GTPa	GO:00987	molecular	-	-	-	-	2:1138314	31	81	64	76	174	127	0.137797	0.287551	0.314421	0.260633	0.498067	0.444116	0.24659	0.400939	0.04203	0.460578	-
ENSMUS(	Acsf2	acyl-CoA	GO:00081	metabolic	-	-	-	-	11:945571	221	251	221	356	517	350	3.34669	3.035585	3.698809	4.159139	5.041595	4.169672	3.360361	4.456802	0.042004	0.460578	-
ENSMUS(	Sccpdh	saccharop	GO:00081	metabolic	-	-	-	-	1:1796682	867	1247	849	1023	1226	982	5.757815	6.6138	6.231508	5.241368	5.243051	5.130519	6.201041	5.204979	0.042187	0.462008	-
ENSMUS(	Cald1	caldesmo	GO:00160	membran	K12327	caldesmo	mmu0427	Vascular s	6:3459850	657	693	617	977	1235	1088	1.688704	1.422549	1.752751	1.937373	2.044137	2.200027	1.621335	2.060513	0.042361	0.463626	-
ENSMUS(	Wdr83	WD repea	GO:00081	metabolic	-	-	-	-	8:8507503	172	290	206	361	428	359	1.353699	1.822793	1.791874	2.191952	2.169161	2.222792	1.656122	2.194635	0.042532	0.464619	-
ENSMUS(	1700001C	RIKEN cDI	GO:00056	cell|intrac	-	-	-	-	5:3046604	8	15	9	3	10	8	0.214722	0.321519	0.266952	0.062126	0.172827	0.168903	0.267731	0.134619	0.042524	0.464619	-
ENSMUS(	Mcoln3	mucolipin	GO:00325	developm	-	-	-	-	3:1461174	7	9	4	5	5	0	0.102596	0.105349	0.064798	0.056532	0.047189	0	0.090914	0.034574	0.042513	0.464619	-
ENSMUS(	Tmem176	transmem	GO:00325	developm	-	-	-	-	6:4884091	92	142	96	175	249	169	0.971863	1.197985	1.120808	1.426209	1.693832	1.404471	1.096885	1.508171	0.042578	0.464826	-
ENSMUS(	Madcam1	mucosal v	GO:00325	developm	K06779	mucosal v	mmu0451	Cell adhes	10:796645	0	0	2	3	8	4	0	0	0.075573	0.079136	0.176126	0.107574	0.025191	0.120945	0.042645	0.465097	-
ENSMUS(	Exd1	exonuclea	GO:00081	metabolic	-	-	-	-	2:1195165	10	27	13	40	34	33	0.057391	0.123752	0.082457	0.177108	0.125653	0.148995	0.087867	0.150585	0.042656	0.465097	-
ENSMUS(	Ltbr	lymphoto:	GO:00325	developm	K03159	lymphoto:	mmu0516	HTLV-I inf	6:1253065	109	158	97	170	274	208	2.400906	2.779411	2.361385	2.88888	3.886473	3.604337	2.513901	3.459896	0.04304	0.468505	-
ENSMUS(	Six4	sine oculis	GO:00325	developm	K15615	homeobo	mmu052C	Transcript	12:730996	38	43	29	17	18	45	0.175519	0.158619	0.148043	0.06058	0.05354	0.163518	0.160727	0.092546	0.043049	0.468505	-
ENSMUS(	Car14	carbonic a	GO:00511	localizatio	K01672	carbonic a	mmu0091	Nitrogen r	3:9589776	138	257	142	139	201	179	2.38494	3.547142	2.712275	1.853294	2.236915	2.433679	2.881452	2.174629	0.043015	0.468505	-
ENSMUS(	Odf3l1	outer den	-	-	-	-	-	-	9:5684865	0	0	0	1	4	2	0	0	0	0.02431	0.081156	0.049581	0	0.051682	0.043215	0.469541	-
ENSMUS(	Dpep2	dipeptida:	GO:00081	metabolic	-	-	-	-	8:1059849	2	1	2	0	0	0	0.019718	0.007876	0.021798	0	0	0	0.016464	0	0.043226	0.469541	-
ENSMUS(	Xkr9	X-linked K	GO:00160	membran	-	-	-	-	1:1366877	0	0	0	2	4	1	0	0	0	0.048922	0.081662	0.024945	0	0.051843	0.043203	0.469541	-
ENSMUS(	Epyc	epiphycar	GO:00325	developm	-	-	-	-	10:97644C	0	0	0	10	0	0	0	0	0	0.136464	0	0	0	0.045488	0.043279	0.469824	-
ENSMUS(	Gapvd1	GTPase ac	GO:00511	localizatio	-	-	-	-	2:3467459	1677	2173	1399	1792	2043	1938	4.728663	4.893401	4.359831	3.898284	3.70961	4.299025	4.660632	3.968973	0.043306	0.469831	-
ENSMUS(	Tmem30a	transmem	GO:00325	developm	-	-	-	-	9:7976894	4523	8794	3928	5479	6851	5357	43.00978	66.78409	41.28184	40.19503	41.95168	40.07498	50.35857	40.74056	0.04336	0.470117	-
ENSMUS(	Pum2	pumilio RI	GO:00081	metabolic	-	-	-	-	12:867413	3751	4526	3287	4102	5387	3400	12.31869	11.87074	11.93064	10.39305	11.3925	8.784297	12.04002	10.18995	0.043468	0.471001	-
ENSMUS(	Eif2ak2	eukaryotic	GO:00325	developm	K16195	eukaryotic	mmu0516	Human pa	17:788525	213	284	156	343	470	318	2.217831	2.361639	1.795228	2.755335	3.151387	2.604877	2.124899	2.8372	0.043548	0.471572	-
ENSMUS(	Sox12	SRY (sex c	GO:00325	developm	-	-	-	-	2:1523936	297	421	266	114	105	451	2.103252	2.38102	2.081919	0.622834	0.478827	2.512595	2.18873	1.204752	0.043874	0.474806	-
ENSMUS(	Ndst4	N-deacet	GO:00081	metabolic	K02579	heparan s	mmu011C	Metabolic	3:125404C	397	482	324	403	492	383	1.454112	1.40994	1.311594	1.138787	1.160453	1.103613	1.391882	1.134285	0.044007	0.475949	-
ENSMUS(	Prdm1	PR domai	GO:00325	developm	-	-	-	-	10:444371	20	15	11	22	58	32	0.142344	0.085261	0.086524	0.120798	0.26582	0.179172	0.10471	0.188597	0.044079	0.476421	-
ENSMUS(	Sox5	SRY (sex c	GO:00325	developm	-	-	-	-	6:1438284	596	627	490	604	610	612	1.851469	1.555549	1.682338	1.447563	1.220267	1.495659	1.696452	1.38783	0.044105	0.476421	-
ENSMUS(	Tmem169	transmem	GO:00160	membran	-	-	-	-	1:7228436	384	465	336	282	598	322	7.29053	7.050606	7.050389	4.130542	7.311105	4.80943	7.130509	5.417026	0.044172	0.476849	-
ENSMUS(	Luzp1	leucine zi	GO:00325	developm	-	-	-	-	4:1364697	1742	2288	2700	2308	2585	2210	5.151259	5.403402	8.824212	5.2654	4.922441	5.141248	6.459624	5.109696	0.044344	0.478404	-
ENSMUS(	Chst7	carbohydı	GO:00081	metabolic	K04743	chondroit	mmu0053	Glycosam	X:2005956	32	26	40	31	25	25	0.885699	0.57472	1.223606	0.661961	0.445582	0.54436	0.894675	0.550635	0.044465	0.479415	-
ENSMUS(	Prc1	protein re	GO:00990	supramole	-	-	-	-	7:8029445	30	53	17	53	111	52	0.119348	0.168389	0.074747	0.162667	0.284359	0.162745	0.120828	0.203257	0.044716	0.481818	-
ENSMUS(	Arhgap25	Rho GTPa	GO:00511	localizatio	-	-	-	-	6:8745854	82	113	52	69	100	58	0.80147	0.882057	0.561718	0.5203	0.629399	0.445977	0.748415	0.531892	0.04488	0.483287	-
ENSMUS(	Tha1	threonine	GO:00081	metabolic	K01620	threonine	mmu011C	Metabolic	11:117867	38	56	26	70	95	65	0.622536	0.732692	0.470761	0.884739	1.002218	0.837733	0.608663	0.90823	0.045073	0.485064	-
ENSMUS(	Tcam1	testicular	GO:00160	membran	-	-	-	-	11:106276	3	3	2	12	6	10	0.034021	0.027175	0.025069	0.104987	0.043815	0.089217	0.028755	0.07934	0.045141	0.485301	-
ENSMUS(	Plagl2	pleiomorp	GO:00325	developm	-	-	-	-	2:1532277	186	211	176	296	405	297	1.063075	0.963115	1.111756	1.305186	1.490594	1.335418	1.045982	1.377066	0.045151	0.485301	-
ENSMUS(	Tubd1	tubulin, de	GO:00325	developm	-	-	-	-	11:865449	95	115	48	148	199	136	2.11467	2.044385	1.180886	2.541615	2.852498	2.3816	1.77998	2.591904	0.04525	0.48577	-
ENSMUS(	Rilp	Rab intera	GO:00081	metabolic	K13883	Rab-inter	mmu0414	Phagoson	11:75510C	18	33	10	38	40	50	0.493719	0.722899	0.303145	0.804144	0.706526	1.078939	0.506588	0.863203	0.045249	0.48577	-
ENSMUS(	Shank2	SH3 and r	GO:00325	developm	K15009	SH3 and r	mmu0472	Glutamate	7:1440019	2503	2963	1833	2439	3214	2408	9.271322	8.765126	7.503953	6.969842	7.66622	7.016945	8.513467	7.217669	0.045298	0.485983	-
ENSMUS(	Birc5	baculovira	GO:00081	metabolic	K08731	baculovira	mmu052C	Pathways	11:117849	16	18	9	30	46	22	0.234219	0.210433	0.145604	0.33881	0.433629	0.253363	0.196752	0.341934	0.045499	0.487536	-
ENSMUS(	Vasp	vasodilatc	GO:00325	developm	K06274	vasodilatc	mmu0401	Rap1 sign	7:1925692	163	221	112	270	361	240	3.004072	3.252827	2.281315	3.838975	4.284336	3.479714	2.846071	3.867675	0.045476	0.487536	-
ENSMUS(	Kif18b	kinesin fai	GO:00990	supramole	-	-	-	-	11:102905	3	9	0	6	24	13	0.049399	0.118361	0	0.07622	0.254489	0.1684	0.05592	0.16637	0.045582	0.488124	-
ENSMUS(	Ugt1a7c	UDP gluci	GO:00081	metabolic	K00699	glucuronc	mmu011C	Metabolic	1:8809506	15	67	42	51	158	91	0.18502	0.660024	0.572575	0.485331	1.255015	0.883058	0.47254	0.874468	0.045653	0.488582	-
ENSMUS(	Taz	tafazzin [S	GO:00325	developm	K13511	monolyso	mmu0056	Glyceroph	X:7428191	246	299	196	414	436	397	1.0129	0.983216	0.891939	1.31511	1.156039	1.285977	0.962685	1.252376	0.045778	0.489025	-
ENSMUS(	Agt	angiotens	GO:00325	developm	K09821	angiotens	mmu0492	Renin secı	8:1245565	332	419	314	544	644	586	10.71989	10.80469	11.20543	13.55129	13.39037	14.8854	10.91	13.94235	0.045753	0.489025	-
ENSMUS(	Msl2	male-spe	GO:00081	metabolic	-	-	-	-	9:1010741	598	1354	1169	1657	2120	1850	4.867714	8.802148	10.51685	10.40584	11.11257	11.84696	8.062237	11.12179	0.045763	0.489025	-
ENSMUS(	Ska2	spindle ar	GO:00990	supramole	-	-	-	-	11:871092	226	237	188	206	268	205	3.272259	2.740525	3.008457	2.301108	2.498791	2.335094	3.00708	2.378331	0.045817	0.489135	-
ENSMUS(	Sirt1	sirtuin 1 [S	GO:00325	developm	K11411	NAD-dep	mmu0421	Cellular se	10:633190	272	357	200	293	238	283	1.576441	1.652429	1.281105	1.310106	0.88826	1.290346	1.503325	1.162904	0.046015	0.490046	-
ENSMUS(	Antxr2	anthrax tc	GO:00511	localizatio	K20909	anthrax tc	mmu0462	NOD-like	5:9788278	56	98	62	123	153	114	0.473583	0.661877	0.57949	0.802491	0.833204	0.758442	0.57165	0.798046	0.045992	0.490046	-
ENSMUS(	Slc39a9	solute car	GO:00511	localizatio	-	-	-	-	12:806438	204	1062	214	422	333	260	0.732439	3.045169	0.849182	1.168918	0.76991	0.734387	1.542263	0.891071	0.045968	0.490046	-
ENSMUS(	Zfp455	zinc finge	GO:00081	metabolic	-	-	-	-	13:671945	41	53	35	28	51	38	0.903089	0.932335	0.852059	0.475812	0.723397	0.65849	0.895828	0.619233	0.045958	0.490046	-
ENSMUS(	C1ra	complem	GO:00081	metabolic	K01330	complem	mmu0414	Phagoson	6:1245124	35	61	41	64	132	79	0.716881	0.997835	0.928132	1.011322	1.741047	1.27298	0.880949	1.341783	0.046277	0.492531	-
ENSMUS(	Flnb	filamin, be	GO:00325	developm	K04437	filamin[-]	mmu0401	MAPK sigı	14:781795	1527	1882	1561	2422	2968	2616	10.12222	9.963272	11.43632	12.38628	12.66937	13.64223	10.50727	12.89929	0.046313	0.492615	-
ENSMUS(	Spata25	spermato	GO:00325	developm	-	-	-	-	2:1648263	0	4	4	0	2	0	0	0.154542	0.213905	0	0.062298	0	0.122816	0.020766	0.046419	0.493446	-
ENSMUS(	Sgo2a	shugoshir	GO:00511	localizatio	-	-	-	-	1:5799597	22	24	21	42	57	38	0.165386	0.144089	0.174484	0.243588	0.275937	0.22474	0.16132	0.248088	0.046455	0.493521	-
ENSMUS(	Hadh	hydroxyac	GO:00081	metabolic	K00022	3-hydroxy	mmu011C	Metabolic	3:1312334	276	414	298	441	756	521	3.114406	3.730883	3.716458	3.839145	5.493417	4.625032	3.520582	4.652531	0.046527	0.493988	-
ENSMUS(	Slc24a5	solute car	GO:00081	metabolic	-	-	-	-	2:1250681	24	65	16	18	30	26	0.551117	1.192019	0.406064	0.318888	0.443605	0.469689	0.7164	0.410727	0.046736	0.4959	-
ENSMUS(	Gm10570	predicted	-	-	-	-	-	-	4:1303081	3	3	1	9	9	6	0.357056	0.285206	0.131553	0.826436	0.689808	0.561824	0.257939	0.692689	0.046878	0.497103	-
ENSMUS(	Tsacc	TSSK6 act	GO:00081	metabolic	-	-	-	-	3:8828275	10	22	11	8	17	2	0.411169	0.722424	0.499858	0.253771	0.450123	0.064706	0.544484	0.2562	0.047053	0.498086	-
ENSMUS(	Adal	adenosine	GO:00081	metabolic	-	-	-	-	2:1211404	219	218	197	348	425	333	0.993219	0.789594	0.98745	1.217619	1.24121	1.188109	0.923421	1.215646	0.047209	0.498086	-
ENSMUS(	Cckar	cholecystc	GO:00325	developm	K04194	cholecystc	mmu0408	Neuroacti	5:5369777	0	0	0	0	4	3	0	0	0	0	0.026469	0.024248	0	0.016906	0.047129	0.498086	-
ENSMUS(	Sval1	seminal ve	GO:00055	extracellul	-	-	-	-	6:4195163	0	0	0	3	4	0	0	0	0	0.229815	0.255741	0	0	0.161852	0.047192	0.498086	-
ENSMUS(	Cd3d	CD3 antig	GO:00325	developm	K06450	T-cell surf	mmu0516	HTLV-I inf	9:4498178	0	0	0	0	4	3	0	0	0	0	0.072883	0.066767	0	0.04655	0.047149	0.498086	-
ENSMUS(	Cd160	CD160 an	GO:00048	signal trar	-	-	-	-	3:9679876	0	0	0	3	4	0	0	0	0	0.04549	0.050621	0	0	0.032037	0.047176	0.498086	-
ENSMUS(	Cradd	CASP2 an	GO:00081	metabolic	-	-	-	-	10:951747	87	103	76	94	45	86	0.885489	0.837232	0.854921	0.738111	0.294935	0.688609	0.859214	0.573885	0.047129	0.498086	-
ENSMUS(	Cxcr5	chemokin	GO:00325	developm	K04190	C-X-C ch	mmu0406	Cytokine-	9:4451178	0	0	0	3	4	0	0	0	0	0.022132	0.024629	0	0	0.015587	0.047228	0.498086	-
ENSMUS(	Cyp2c68	cytochron	GO:00081	metabolic	K07413	cytochron	mmu011C	Metabolic	19:396888	0	0	0	3	4	0	0	0	0	0.081982	0.09123	0	0	0.057737	0.047208	0.498086	-

ENSMUS(	Pfdn4	prefoldin	GO:00329	macromo	-	-	-	-	2:1704964	188	169	112	169	171	112	3.246302	2.330572	2.137442	2.251375	1.901436	1.521455	2.571439	1.891422	0.047311	0.498653	-
ENSMUS(	Ecel1	endotheli	GO:00081	metabolic	-	-	-	-	1:8714765	36	73	55	51	60	34	0.396411	0.641964	0.669344	0.433253	0.425452	0.294528	0.56924	0.384411	0.047486	0.499434	-
ENSMUS(	Zfp764	zinc finge	GO:00081	metabolic	-	-	-	-	7:1274036	56	74	60	106	119	121	0.712497	0.751919	0.843706	1.040472	0.974976	1.21112	0.769374	1.075523	0.047441	0.499434	-
ENSMUS(	Olfr317	olfactory r	GO:00048	signal trar	-	-	-	-	11:587310	0	2	0	2	3	7	0	0.022188	0	0.021434	0.026836	0.076492	0.007396	0.041587	0.047474	0.499434	-
ENSMUS(	9530053A	RIKEN cDI	-	-	-	-	-	-	7:2812946	0	0	0	3	0	3	0	0	0	0.01025	0	0.010449	0	0.0069	0.0475	0.499434	-
ENSMUS(	Cped1	cadherin-	GO:00056	cell|intrace	-	-	-	-	6:2198591	61	93	50	109	131	127	0.343243	0.417929	0.310952	0.473183	0.47468	0.562195	0.357375	0.503353	0.047623	0.500426	-
ENSMUS(	Lgals1	lectin, gal	GO:00325	developm	-	-	-	-	15:789267	136	251	130	138	199	165	10.37988	15.29935	10.96587	8.125719	9.780502	9.907145	12.21503	9.271122	0.047661	0.500524	-
ENSMUS(	Obscn	obscurin,	GO:00081	metabolic	-	-	-	-	11:589942	68	74	62	126	133	112	0.124758	0.108427	0.125719	0.178345	0.157133	0.161655	0.119635	0.165711	0.047809	0.501773	-
ENSMUS(	Ttc6	tetratricop	-	-	-	-	-	-	12:575641	0	0	3	0	14	12	0	0	0.026372	0	0.071703	0.075086	0.008791	0.048929	0.04787	0.502113	-
ENSMUS(	Mgat4e	MGAT4 fa	GO:00081	metabolic	-	-	-	-	1:1345409	0	0	0	3	0	4	0	0	0	0.031619	0	0.042982	0	0.024867	0.047937	0.502515	-
ENSMUS(	Slc17a2	solute car	GO:00511	localizatio	-	-	-	-	13:238069	0	0	0	4	3	0	0	0	0	0.01759	0.011014	0	0	0.009534	0.048	0.502782	-
ENSMUS(	Klri2	killer cell l	GO:00160	membran	-	-	-	-	6:1297290	0	0	0	4	3	0	0	0	0	0.048148	0.030148	0	0	0.026099	0.04802	0.502782	-
ENSMUS(	Cenpx	centrome	GO:00081	metabolic	K15360	centrome	mmu0346	Fanconi a	11:120710	39	137	108	173	196	212	1.20938	3.392839	3.701429	4.138807	3.913881	5.171838	2.767883	4.408175	0.048131	0.503636	-
ENSMUS(	Snx31	sorting ne	GO:00511	localizatio	-	-	-	-	15:365040	4	4	0	13	7	7	0.058432	0.046658	0	0.146498	0.06584	0.080445	0.03503	0.097594	0.048441	0.50649	-
ENSMUS(	Vamp1	vesicle-as	GO:00452	synapse|lc	K08510	vesicle-as	mmu0413	SNARE int	6:1252155	1637	1921	1415	1483	2182	1850	7.420351	6.954228	7.088902	5.186175	6.369203	6.597181	7.154494	6.050853	0.048469	0.50649	-
ENSMUS(	Vat1l	vesicle am	GO:00081	metabolic	-	-	-	-	8:1142056	592	998	497	723	770	662	4.093124	5.510739	3.797838	3.856577	3.428298	3.600827	4.467234	3.628567	0.048491	0.50649	-
ENSMUS(	Bid	BH3 intera	GO:00325	developm	K04726	BH3 intera	mmu0520	Pathways	6:1208919	144	224	179	95	139	239	1.60915	1.999062	2.210715	0.819004	1.000233	2.10107	1.939642	1.306769	0.048603	0.507355	-
ENSMUS(	Fbxo36	F-box prc	-	-	-	-	-	-	1:8483984	23	32	27	19	31	21	0.620293	0.689224	0.804776	0.395313	0.538381	0.445553	0.704764	0.459749	0.049076	0.51199	-
ENSMUS(	Dkk2	dickkopf \	GO:00325	developm	K02165	dickkopf[-	mmu0431	Wnt signa	3:1320852	8	12	7	15	27	22	0.131847	0.157934	0.127506	0.190712	0.286536	0.285229	0.139095	0.254159	0.049351	0.512713	-
ENSMUS(	Prss8	protease,	GO:00081	metabolic	-	-	-	-	7:1279257	10	8	6	23	20	16	0.2422	0.154745	0.160619	0.429765	0.311925	0.304863	0.185855	0.348851	0.04926	0.512713	-
ENSMUS(	Aff2	AF4/FMR2	GO:00081	metabolic	-	-	-	-	X:6936029	287	369	275	328	368	294	2.093126	2.149246	2.216629	1.84552	1.728292	1.686835	2.153001	1.753549	0.049277	0.512713	-
ENSMUS(	Ccl5	chemokin	GO:00081	metabolic	K12499	C-C motif	mmu0406	Cytokine-	11:835257	3	6	5	11	25	7	0.152642	0.243811	0.281195	0.431822	0.819128	0.280217	0.225883	0.510389	0.049299	0.512713	-
ENSMUS(	Enpp1	ectonucle	GO:00325	developm	K01513	ectonucle	mmu0110	Metabolic	10:246379	76	166	83	92	99	105	0.376996	0.65762	0.455034	0.35208	0.316236	0.409753	0.49655	0.359356	0.049352	0.512713	-
ENSMUS(	S100pbp	S100P bin	GO:00056	cell|intrace	-	-	-	-	4:1291480	415	609	544	485	597	568	1.637952	1.919625	2.373005	1.476811	1.517337	1.763648	1.976861	1.585932	0.049343	0.512713	-
ENSMUS(	Asgr2	asialoglyc	GO:00325	developm	K10064	asialoglyc	mmu0491	Thyroid h	11:700926	3	0	2	0	0	0	0.077878	0	0.057387	0	0	0	0.045088	0	0.049243	0.512713	-
ENSMUS(	Gstm7	glutathior	GO:00081	metabolic	K00799	glutathior	mmu0520	Pathways	3:1079263	253	413	331	298	404	333	6.707645	8.744698	9.698951	6.09532	6.897396	6.945512	8.383765	6.646076	0.049579	0.513817	-
ENSMUS(	Rarres2	retinoic ac	GO:00325	developm	-	-	-	-	6:4856969	57	76	64	129	122	108	0.964336	1.026864	1.196701	1.683752	1.329144	1.437445	1.062634	1.483447	0.049781	0.513817	-
ENSMUS(	Mybl2	myeloblas	GO:00325	developm	K09421	myb-relat	mmu0516	HTLV-I inf	2:1630546	16	21	10	35	45	23	0.175139	0.183584	0.120976	0.29557	0.317196	0.198067	0.159899	0.270278	0.049852	0.513817	-
ENSMUS(	Gpr132	G protein-	GO:00048	signal trar	-	-	-	-	12:112850	0	0	0	0	5	2	0	0	0	0	0.071618	0.035005	0	0.035541	0.049778	0.513817	-
ENSMUS(	Syt2	synaptota	GO:00325	developm	-	-	-	-	1:1346466	709	793	536	807	816	330	4.394943	3.925782	3.672138	3.859323	3.257256	1.609282	3.997621	2.90862	0.049865	0.513817	-
ENSMUS(	Galnt6	polypeptic	GO:00081	metabolic	K00710	polypeptic	mmu0110	Metabolic	15:100691	129	281	195	305	459	324	1.271221	2.211482	2.123801	2.318796	2.912725	2.511817	1.868835	2.581112	0.049522	0.513817	-
ENSMUS(	Pogk	pogo tran	GO:00081	metabolic	-	-	-	-	1:1663846	426	385	926	498	588	504	1.085501	0.783479	2.607822	0.978995	0.964833	1.010327	1.492267	0.984718	0.049872	0.513817	-
ENSMUS(	Hoxd9	homeobo	GO:00325	developm	-	-	-	-	2:7469772	0	0	0	0	5	2	0	0	0	0	0.092037	0.044985	0	0.045674	0.049788	0.513817	-
ENSMUS(	Gchfr	GTP cyclo	GO:00081	metabolic	-	-	-	-	2:1191677	0	0	0	2	0	4	0	0	0	0.146084	0	0.297872	0	0.147985	0.0496	0.513817	-
ENSMUS(	Plg	plasminog	GO:00325	developm	K01315	plasminog	mmu0408	Neuroacti	17:123786	0	0	0	4	0	2	0	0	0	0.068385	0	0.034878	0	0.034421	0.049518	0.513817	-
ENSMUS(	Bpifb6	BPI fold cc	GO:00055	extracellul	-	-	-	-	2:1539003	0	0	0	2	5	0	0	0	0	0.055622	0.116051	0	0	0.057225	0.04986	0.513817	-
ENSMUS(	Serpina1b	serine (or	GO:00325	developm	K03984	alpha-1-a	mmu0461	Complem	12:103728	0	0	0	2	5	0	0	0	0	0.033604	0.070111	0	0	0.034571	0.04985	0.513817	-
ENSMUS(	Gm14226	predicted	GO:00051	structural	K08725	agouti sig	mmu0491	Melanoge	2:1550182	2	0	3	0	0	0	0.037648	0	0.06243	0	0	0	0.033359	0	0.049809	0.513817	-
ENSMUS(	Cyp2d12	cytochron	GO:00081	metabolic	K07414	cytochron	mmu0472	Serotoner	15:825551	0	0	0	0	5	2	0	0	0	0	0.121803	0.059534	0	0.060446	0.049795	0.513817	-
ENSMUS(	Scube1	signal pep	GO:00325	developm	-	-	-	-	15:836025	728	969	754	862	859	918	3.011741	3.201518	3.447503	2.751211	2.288411	2.98772	3.220254	2.675781	0.050026	0.515095	-
ENSMUS(	Fam46c	family witl	GO:00038	catalytic a	-	-	-	-	3:1004516	65	140	72	69	106	80	0.608615	1.046896	0.745088	0.498438	0.639128	0.589288	0.8002	0.575618	0.050251	0.516808	-
ENSMUS(	Fam58b	family witl	GO:00081	metabolic	-	-	-	-	11:787505	122	161	125	235	239	234	6.08584	6.414084	6.891605	9.043984	7.677392	9.183069	6.463843	8.634815	0.050226	0.516808	-
ENSMUS(	Zbtb21	zinc finge	GO:00081	metabolic	-	-	-	-	16:979474	257	232	243	405	429	468	1.417649	1.022043	1.481456	1.723535	1.523868	2.030915	1.307049	1.75944	0.050288	0.516882	-
ENSMUS(	Nckap1l	NCK asso	GO:00325	developm	K05750	NCK-asso	mmu0481	Regulation	15:103453	202	295	197	222	258	241	2.614746	3.049614	2.818326	2.216976	2.150558	2.454173	2.827562	2.273902	0.050388	0.517101	-
ENSMUS(	Epha6	Eph recep	GO:00048	signal trar	K05107	Eph recep	mmu0436	Axon guic	16:596534	2053	2809	1959	2425	2812	2422	18.28092	19.97592	19.27926	16.65909	16.12424	16.96658	19.1787	16.5833	0.050399	0.517101	-
ENSMUS(	Gm42878	predicted	GO:00325	developm	-	-	-	-	5:1215186	361	381	291	593	700	472	4.424328	3.729152	3.941659	5.606927	5.524494	4.550849	4.031713	5.227423	0.050358	0.517101	-
ENSMUS(	C5ar1	complem	GO:00325	developm	K04010	C5a anapl	mmu0408	Neuroacti	7:1624674	27	47	14	21	29	19	0.296402	0.412057	0.169858	0.177852	0.205004	0.164085	0.292772	0.182314	0.050526	0.518103	-
ENSMUS(	Pla2g2c	phospholi	GO:00081	metabolic	-	-	mmu0110	Metabolic	4:1387247	6	6	2	1	2	3	0.078194	0.062449	0.02881	0.010056	0.016781	0.030754	0.056484	0.019197	0.050674	0.519312	-
ENSMUS(	Entpd5	ectonucle	GO:00081	metabolic	K01511	ectonucle	mmu0023	Purine me	12:843738	496	698	432	803	1032	808	2.104282	2.364957	2.025597	2.628258	2.8194	2.696774	2.164945	2.714811	0.050859	0.520905	-
ENSMUS(	Ccnt2	cyclin T2 [	GO:00325	developm	K15188	cyclin T[-]	mmu0520	Transcript	1:1277741	1390	1734	1153	1502	1656	1567	4.863376	4.845268	4.458607	4.05437	3.731109	4.313233	4.722417	4.032904	0.050984	0.521361	-
ENSMUS(	Acvr1	activin A r	GO:00325	developm	K04675	activin rec	mmu0406	Cytokine-	2:5838864	688	611	672	627	815	680	6.020926	4.270329	6.499657	4.233236	4.592899	4.681604	5.59697	4.50258	0.050994	0.521361	-
ENSMUS(	Rif1	replication	GO:00325	developm	K11138	telomere-	mmu0455	Signaling	2:5207283	677	792	520	682	826	659	2.133158	1.992991	1.81086	1.657866	1.67598	1.633544	1.979003	1.655797	0.050936	0.521361	-
ENSMUS(	Cd300ld	CD300 mc	GO:00023	immune s	-	-	-	-	11:114982	7	9	7	3	9	2	0.161594	0.165929	0.178604	0.053435	0.133789	0.036328	0.168709	0.074517	0.051216	0.523325	-
ENSMUS(	Cnksr2	connector	GO:00452	synapse|re	-	-	-	-	X:1578214	9894	12620	8880	11111	13327	11274	64.20544	65.40411	63.6883	55.62671	55.69125	57.55576	64.43262	56.29124	0.051335	0.523499	-
ENSMUS(	Homer2	homer sca	GO:00452	synapse|re	K15010	homer[-]	mmu0406	FoxO sign	7:8160048	2342	2729	1883	2425	2871	2513	9.753637	9.076724	8.66716	7.791515	7.699591	8.233475	9.16584	7.908193	0.051504	0.523499	-
ENSMUS(	Slc50a1	solute car	GO:00081	metabolic	-	-	-	-	3:8926824	202	237	176	325	421	303	3.383747	3.170588	3.258405	4.200099	4.541327	3.993	3.270913	4.244809	0.05135	0.523499	-
ENSMUS(	Rsad1	radical S-	GO:00081	metabolic	-	-	-	-	11:945397	169	176	157	236	60	68	1.901387	1.581398	1.952221	2.048452	0.434702	0.601873	1.811669	1.028342	0.051454	0.523499	-
ENSMUS(	Prdm16	PR domai	GO:00325	developm	-	-	-	-	4:1543161	361	466	282	207	502	374	1.437272	1.481708	1.240873	0.635817	1.287033	1.171422	1.386618	1.031424	0.051394	0.523499	-
ENSMUS(	Ptger4	prostaglai	GO:00325	developm	K04261	prostaglai	mmu0520	Pathways	15:520666	11	24	26	32	71	39	0.088516	0.154229	0.231224	0.198656	0.367899	0.246881	0.15799	0.271145	0.051379	0.523499	-
ENSMUS(	Mettl11b	methyltrai	GO:00081	metabolic	-	-	-	-	1:1637022	12	17	10	33	27	25	0.318695	0.360569	0.29352	0.676168	0.461772	0.52234	0.324261	0.553427	0.051302	0.523499	-
ENSMUS(	Otop2	otopetrin	GO:00160	membran	-	-	-	-	11:115307	4	2	0	5	12	6	0.074511	0.029756	0	0.071847	0.143942	0.087924	0.034756	0.101238	0.051486	0.523499	-
ENSMUS(	Dspp	dentin sia	-	-	-	-	-	-	5:1041707	2	4	2	13	7	6	0.027554	0.044015	0.030461	0.1382	0.06211	0.065045	0.03401	0.088452	0.051417	0.523499	-
ENSMUS(	Trim30b	tripartite r	GO:00081	metabolic	-	-	-	-	7:1043553	0	6	0	11	11	3	0	0.022365	0	0.039611	0.03306	0.011014	0.007455	0.027895	0.0516	0.524164	-
ENSMUS(	Ercc6l	excision re	GO:00038	catalytic a	-	-	-	-	X:1021417	3	8	6	8	20	19	0.034547	0.073578	0.076371	0.071077	0.148314	0.172132	0.061499	0.130508	0.051636	0.524223	-
ENSMUS(	Creg1	cellular re	GO:00081	metabolic	-	-	-	-	1:1657637	539	759	497	637	255	637	13.50445	15.18712	13.76229	12.31283	4.114177	12.55562	14.15129	9.660874	0.051785	0.525432	-
ENSMUS(	Ifi209	interferon	GO:00081	metabolic	-	-	-	-	1:1736309	6	9	7	21	15	19	0.097147	0.116384	0.125274	0.262321	0.156397	0.242016	0.112935	0.220245	0.05184	0.525685	-
ENSMUS(	Gsto1	glutathior	GO:00081	metabolic	K00799	glutathior	mmu0520	Pathways	19:478549	415	592	412	707	917	703	10.16819	11.58411	11.15679	13.36426	14.46837	13.55069	10.96969	13.79444	0.051906	0.525736	-
ENSMUS(	Yy2	Yy2 transc	GO:00081	metabolic	-	-	-	-	X:1575661	1	1	4	10	1	28	0.021303	0.017011	0.094157	0.164309	0.013711	0.469128	0.044157	0.215716	0.051885	0.525736	-
ENSMUS(	Nsd2	nuclear re	GO:00325	developm	K11424	histone-ly	mmu0520	Transcript	5:3382072	1131	1833	1290	1207	2134	1177	2.159777	2.795468	2.722595	1.778214	2.624191	1.76821	2.55928	2.056872	0.051949	0.52587	-
ENSMUS(	Sfxn5	sideroflex	GO:00511	localizatio	-	-	-	-	6:8521304	3273	4012	3122	3869	4350	3584	18.45274	18.06432	19.45336	16.82844	15.79279	15.89622	18.65681	16.17248	0.052262	0.528421	-
ENSMUS(	Themis2	thymocyte	GO:00023	immune s	-	-	-	-	4:1327818	23	32	15	41	55	43	0.456103	0.506789	0.328755	0.627254	0.702351	0.670837	0.430549	0.666814	0.052244	0.528421	-
ENSMUS(	Myd88	myeloid d	GO:00325	developm	K04729	myeloid d	mmu0401	MAPK sigi	9:1193359	73	116	78	138	197	141	0.964556	1.224079	1.13907	1.406746	1.676213	1.465679	1.109235	1.516213	0.052715	0.532696	-
ENSMUS(	Hmgcll1	3-hydroxy	GO:00081	metabolic	K01640	hydroxym	mmu0110	Metabolic	9:7601485	247	375	221	141	376	280	2.036096	2.468754	2.013446	0.896707	1.99592	1.815811	2.172766	1.56948	0.052833	0.532954	-
ENSMUS(	Zwint	ZW10 inte	GO:00511	localizatio	-	-	-	-	10:726548	8901	10160	8100	15405	16662	11793	97.74788	89.10624	98.31061	130.5151	117.8285	101.8835	95.05491	116.7423	0.052824	0.532954	-
ENSMUS(	Ror2	receptor t	GO:00325	developm	-	-	-	-	13:531093	10	18	8	26	29	23	0.091515	0.131552	0.080909	0.183562	0.170898	0.16559	0.101326	0.17335	0.05278	0.532954	-
ENSMUS(	Knl1	kinetocho	GO:00081	metabolic	-	-	-	-	2:1190471	7	21	2	23	40	16	0.029457	0.070574	0.009302	0.074668	0.108389	0.052968	0.036444	0.078675	0.052909	0.53341	-
ENSMUS(	Spata20	spermato	GO:00325	developm	-	-	-	-	11:944789	0	0	0	5	2	0	0	0	0	0.064383	0.021493	0	0	0.028625	0.05295	0.53352	-
ENSMUS(	Glra3	glycine re	GO:00048	signal trar	K05195	glycine re	mmu0408	Neuroacti	8:5594046	127	153	105	143	138	102	0.80157	0.771214	0.732443	0.696313	0.560881	0.506463	0.768409	0.587886	0.053021	0.53392	-
ENSMUS(	Zglp1	zinc finge	GO:00325	developm	-	-	-	-	9:2106239	11	0	1	1	1	1	0.476019	0	0.047829	0.033389	0.02786	0.03405	0.174616	0.031766	0.053097	0.534378	-
ENSMUS(	Zfp605	zinc finge	GO:00081	metabolic	-	-	-	-	5:1101100	184	248	380	219	298	260	1.889452	2.033832	4.312693	1.734971	1.970559	2.1004	2.745326	1.93531	0.053133	0.534438	-
ENSMUS(	Fcer1g	Fc recepto	GO:00325	developm	K07983	Fc recepto	mmu0515	Tuberculo	1:1712295	110	196	108	125	163	118	4.184666	5.954832	4.540881	3.668668	3.993094	3.531515	4.89346	3.731092	0.053186	0.534654	-
ENSMUS(	Flt4	FMS-like	GO:00325	developm	K05097	FMS-like	mmu0520	Pathways	11:496092	73	107	79	152	149	160	0.514749	0.602568	0.615675	0.826891	0.676574	0.887579	0.577664	0.797015	0.053507	0.536346	-
ENSMUS(	Mfsd7a	major faci	GO:00511	localizatio	-	-	-	-	5:1084410	12	12	11	24	40	18	0.292137	0.233312	0.295968	0.450777	0.627084	0.344736	0.273806	0.474199	0.053436	0.536346	-
ENSMUS(	Kap	kidney an	GO:00055	extracellul	-	-	-	-	6:1338498	0	7	0	0	0	0	0	0.308071	0	0	0	0	0.10269	0	0.053508	0.536346	-
ENSMUS(	Ahdc1	AT hook,	GO:00971	organic cy	-	-	-	-	4:1330112	343	464	253	418	397	218	1.526118	1.648763	1.244119	1.43483	1.137469	0.763067	1.473	1.111789	0.05343	0.536346	-
ENSMUS(	Cntnap2	contactin	GO:00325	developm	K07380	contactin	mmu0451	Cell adhes	6:4505935	2062	3637	1724	2366	2794	2609	7.036374	9.911726	6.501959	6.228814	6.139618	7.003984	7.816686	6.457472	0.053468	0.536346	-
ENSMUS(	Ephb4	Eph recep	GO:00325	developm	K05113	Eph recep	mmu0436	Axon guic	5:1373501	99	159	106	166	258	222	0.656465	0.842018	0.77684	0.849215	1.10167	1.15809	0.758441	1.036325	0.053666	0.537618	-
ENSMUS(	Ccr2	chemokin	GO:00325	developm	K04177	C-C chem	mmu0406	Cytokine-	9:1241019	8	12	7	17	27	19	0.0339	0.040607	0.032783	0.055573	0.073672	0.063334	0.035763	0.064193	0.053873	0.539385	-
ENSMUS(	Xbp1	X-box bin	GO:00325	developm	K09027	X box-bin	mmu0516	HTLV-I inf	11:552065	849	1118	330	1390	1591	1178	11.51451	12.10948	4.946512	14.54397	13.89514	12.56881	9.523502	13.66931	0.053966	0.540002	-
ENSMUS(	Ms4a6d	membran	GO:00160	membran	-	-	-	-	19:115866	16	40	22	23	20	19	0.420726	0.840016	0.639382	0.466596	0.338658	0.393042	0.633375	0.399432	0.054037	0.540399	-
ENSMUS(	Etv1	ets varian	GO:00325	developm	K09431	ets translc	mmu0520	Transcript	12:387793	2103	2789	984	2472	1641	913	5.849096	6.195036	3.024762	5.304302	2.939088	1.997703	5.022965	3.413698	0.05409	0.540623	-
ENSMUS(	Hlcs	holocarbc	GO:00081	metabolic	K01942	biotin--pr	mmu0110	Metabolic	16:941288	206	129	97	307	313	221	0.733579	0.366872	0.381766	0.843429	0.71776	0.619133	0.494072	0.726774	0.054228	0.541692	-
ENSMUS(	Ifitm2	interferon	GO:00325	developm	-	-	-	-	7:1409548	68	99	49	100	184	119	6.338856	7.370264	5.04833	7.191681	11.04523	8.72691	6.252483	8.987942	0.054285	0.541947	-
ENSMUS(	Dclk1	doublecor	GO:00325	developm	-	-	-	-	3:5524236	11475	13932	9742	13761	12416	12630	19.11032	18.52997	17.93124	17.68055	13.31532	16.54739	18.52384	15.84775	0.054355	0.542331	-
ENSMUS(	Zfp36	zinc finge	GO:00325	developm	K15308	tristetrapr	mmu0516	HTLV-I inf	7:2837678	53	115	60	107	209	121	0.615814	1.067126	0.770494	0.95915	1.563765	1.106026	0.817811	1.209647	0.05442	0.54267	-
ENSMUS(	Ybx2	Y box pro	GO:00325	developm	-	-	-	-	11:699357	37	70	31	83	101	73	0.634956	0.959354	0.587959	1.098872	1.116123	0.985536	0.727423	1.066844	0.054559	0.543746	-
ENSMUS(	Kif20a	kinesin fai	GO:00511	localizatio	-	-	-	-	18:346246	8	30	7	26	63	24	0.128686	0.38537	0.124449	0.322636	0.652545	0.303692	0.212835	0.426291	0.054641	0.544221	-
ENSMUS(	Cdk1	cyclin-dep	GO:00325	developm	K02087	cyclin-dep	mmu0520	Viral carci	10:693351	4	15	7	18	37	13	0.037576	0.112532	0.072678	0.13045	0.223815	0.096066	0.074262	0.15011	0.054693	0.544221	-
ENSMUS(	Sowahb	sosondow	-	-	-	-	-	-	5:9304112	84	83	49	108	181	118	1.315097	1.037768	0.847861	1.304462	1.824784	1.453354	1.066908	1.527533	0.054701	0.544221	-
ENSMUS(	Ispd	isoprenoic	GO:00325	developm	-	-	mmu0110	Metabolic	12:363814	181	197	119	147	215	138	0.935934	0.81354	0.680082	0.586428	0.715912	0.561379	0.809852	0.62124	0.054843	0.545324	-
ENSMUS(	Car8	carbonic a	GO:00081	metabolic	K01672	carbonic a	mmu0091	Nitrogen r	4:8143362	95	102	71	148	201	134	1.50546	1.290899	1.243507	1.809408	2.051135	1.670553	1.346622	1.843699	0.054924	0.545817	-
ENSMUS(	Lsm7	LSM7 hon	GO:00081	metabolic	K12626	U6 snRNA	mmu0304	Spliceosoi	10:808528	86	93	85	58	102	95	1.067053	0.921551	1.165613	0.555197	0.81498	0.927314	1.051406	0.76583	0.054963	0.545893	-
ENSMUS(	Cyp4f16	cytochron	GO:00081	metabolic	-	-	-	-	17:325365	39	70	39	88	103	80	0.435571	0.624361	0.481395	0.75824	0.740777	0.702899	0.513776	0.733972	0.055198	0.547916	-
ENSMUS(	Cdk6	cyclin-dep	GO:00325	developm	K02091	cyclin-dep	mmu0520	Pathways	5:3341485	95	131	89	156	228	171	0.338737	0.373039	0.350732	0.429134	0.523513	0.479674	0.35417	0.47744	0.055265	0.548271	-
ENSMUS(	Pawr	PRKC, apc	GO:00325	developm	-	-	-	-	10:108332	51	56	30	68	116	80	1.338181	1.173504	0.869993	1.376536	1.960008	1.651374	1.127226	1.662639	0.0553	0.548298	-
ENSMUS(	Dnm3	dynamin 3	GO:00325	developm	K01528	dynamin (	mmu0414	Endocytos	1:1619824	2806	3389	2398	3174	3336	3124	6.077869	5.862476	5.74063	5.303968	4.653122	5.323357	5.893659	5.093482	0.05537	0.548688	-
ENSMUS(	Glcci1	glucocorti	GO:00056	cell|protei	-	-	-	-	6:8509600	279	200	394	201	297	321	1.569343	0.898442	2.44938	0.872249	1.075784	1.420464	1.639055	1.122832	0.055405	0.548722	-
ENSMUS(	Chchd5	coiled-co	GO:00081	metabolic	-	-	-	-	2:1291297	60	73	52	110	115	106	1.204688	1.170566	1.153913	1.703921	1.486889	1.674334	1.176389	1.621715	0.055538	0.549519	-
ENSMUS(	Gm9040	predicted	GO:00971	organic cy	-	-	-	-	10:118324	8	5	4	3	3	2	0.202778	0.101205	0.112058	0.05867	0.048971	0.039887	0.13868	0.049176	0.055549	0.549519	-
ENSMUS(	Ppp1r18	protein pl	GO:00056	cell|intrace	-	-	-	-	17:358655	137	186	129	159	147	149	0.712155	0.772169	0.741121	0.637646	0.492068	0.609327	0.741815	0.57968	0.055652	0.550034	-
ENSMUS(	Fbxo6	F-box prc	GO:00081	metabolic	K10100	F-box prc	mmu0414	Protein pr	4:1481457	110	126	98	179	227	180	1.486256	1.35962	1.463431	1.865885	1.975067	1.913306	1.436436	1.918086	0.055664	0.550034	-
ENSMUS(	Mcm10	minichron	GO:00081	metabolic	-	-	-	-	2:4989714	10	21	11	23	43	26	0.056804	0.095267	0.069057	0.100794	0.157289	0.116188	0.073709	0.124757	0.055803	0.551093	-
ENSMUS(	F9	coagulatic	GO:00081	metabolic	K01321	coagulatic	mmu0461	Complem	X:5999946	2	2	7	2	0	2	0.044657	0.035677	0.17279	0.034464	0	0.035146	0.084375	0.023203	0.055939	0.551805	-
ENSMUS(	Hmbs	hydroxym	GO:00081	metabolic	K01749	hydroxym	mmu0110	Metabolic	9:4433633	135	193	147	245	322	249	1.219533	1.392402	1.467658	1.707488	1.873146	1.769587	1.359864	1.783407	0.055938	0.551805	-
ENSMUS(	Mlh3	mutL hom	GO:00081	metabolic	K08739	DNA misn	mmu0343	Mismatch	12:852345	538	773	556	576	842	146	3.131782	3.59364	3.577096	2.586795	3.156285	0.668612	3.434173	2.137231	0.056037	0.552152	-
ENSMUS(	Olfr538	olfactory r	GO:00048	signal trar	K04257	olfactory r	mmu0474	Olfactory	7:1405678	2	2	3	1	0	0	0.042795	0.034189	0.070964	0.016513	0	0	0.049316	0.005504	0.056038	0.552152	-
ENSMUS(	Caprin1	cell cycle a	GO:00325	developm	-	-	-	-	2:1037629	4770	4351	4576	5158	5822	4255	18.30132	13.33211	19.40425	15.26777	14.38437	12.84324	17.01256	14.16513	0.056228	0.553286	-
ENSMUS(	Slfn5	schlafen 5	GO:00325	developm	-	-	-	-	11:829513	194	318	148	362	429	329	1.563319	2.046535	1.318119	2.250531	2.226171	2.085705	1.642658	2.187469	0.056226	0.553286	-
ENSMUS(	Atp11c	ATPase, cl	GO:00325	developm	-	-	-	-	X:6022329	391	679	339	442	652	268	2.302189	3.192861	2.206028	2.007784	2.472106	1.241397	2.567026	1.907096	0.056248	0.553286	-
ENSMUS(	Gsdma	gasdermir	GO:00099	cellular pr	-	-	-	-	11:986643	1	0	0	3	2	4	0.022405	0	0	0.051848	0.028841	0.07048	0.007468	0.050389	0.056335	0.553702	-
ENSMUS(	Gimap5	GTPase, IM	GO:00160	membran	-	-	-	-	6:4874619	5	12	4	14	18	20	0.057048	0.109353	0.050448	0.123243	0.132264	0.179534	0.072283	0.145014	0.056436	0.553702	-
ENSMUS(	Ackr2	atypical cl	GO:00048	signal trar	-	-	-	-	9:1218983	5	7	4	17	6	26	0.048562	0.054302	0.042944	0.127396	0.037528	0.19868	0.048603	0.121201	0.05645	0.553702	-
ENSMUS(	Tgif1	TGFB-indi	GO:00325	developm	K19383	homeobo	mmu0435	TGF-beta	17:708442	23	42	30	49	102	48	0.11546	0.168384	0.166445	0.189769	0.329731	0.189565	0.150097	0.236355	0.056375	0.553702	-
ENSMUS(	H2-K1	histocomp	GO:00325	developm	K06751	major hist	mmu0516	Human pa	17:339960	416	584	574	735	1297	783	3.493357	3.916591	5.327308	4.76175	7.013648	5.172757	4.245752	5.649385	0.056449	0.553702	-
ENSMUS(	Slc40a1	solute car	GO:00325	developm	K14685	solute car	mmu0497	Mineral al	1:4590806	110	175	104	214	286	181	0.624142	0.793005	0.652185	0.936774	1.044987	0.807943	0.689777	0.929901	0.056528	0.554034	-
ENSMUS(	Pdf	peptide d	GO:00081	metabolic	-	-	-	-	8:1070446	234	355	223	340	173	232	6.872347	8.326505	7.23837	7.703679	3.271831	5.360287	7.479074	5.445266	0.056547	0.554034	-
ENSMUS(	Reln	reelin [Sou	GO:00325	developm	K06249	reelin [EC:	mmu0415	PI3K-Akt s	5:2188445	1700	2026	1474	1957	1941	1888	3.863573	3.67727	3.702411	3.431322	2.840669	3.375618	3.747752	3.21587	0.056634	0.55457	-
ENSMUS(	Pgd	phosphog	GO:00081	metabolic	K00033	6-phosph	mmu0110	Metabolic	4:1491499	516	657	473	820	1075	787	4.30351	4.376072	4.359944	5.27615	5.77346	5.163672	4.346509	5.404427	0.056743	0.555328	-
ENSMUS(	Tor4a	torsin fam	GO:00160	membran	-	-	-	-	2:2519296	52	71	38	77	123	96	0.723405	0.788825	0.584256	0.826421	1.101889	1.050651	0.698829	0.992987	0.056856	0.555805	-
ENSMUS(	Gm2004	predicted	GO:00081	metabolic	-	-	-	-	2:1761415	84	89	61	66	99	65	2.827385	2.392457	2.269257	1.713878	2.145835	1.721187	2.496366	1.8603	0.056835	0.555805	-
ENSMUS(	Hoxb8	homeobo	GO:00325	developm	-	-	-	-	11:962819	8	16	3	15	40	16	0.110021	0.175727	0.045599	0.159141	0.354218	0.173099	0.110449	0.228819	0.057011	0.557012	-
ENSMUS(	Cxcl5	chemokin	GO:00400	locomotic	K05506	C-X-C mc	mmu0406	Cytokine-	5:9075936	4	9	3	0	2	5	0.107787	0.193682	0.089347	0	0.034696	0.105989	0.130272	0.046895	0.057114	0.557134	-
ENSMUS(	Btla	B and T ly	GO:00160	membran	-	-	-	-	16:452243	5	7	1	15	11	11	0.050191	0.056123	0.011096	0.116177	0.07111	0.086877	0.039137	0.091388	0.05711	0.557134	-
ENSMUS(	Parp10	poly (ADP	GO:00081	metabolic	-	-	-	-	15:762311	38	57	39	64	115	77	0.31941	0.382636	0.362306	0.415026	0.622471	0.509177	0.354784	0.515558	0.05712	0.557134	-
ENSMUS(	Tbc1d8	TBC1 dom	GO:00511	localizatio	-	-	-	-	1:3937149	156	244	187	326	331	344	1.3505	1.686961	1.789193	2.177297	1.845238	2.342819	1.608884	2.121784	0.057161	0.557219	-
ENSMUS(	Snrnp25	small nucl	GO:00081	metabolic	-	-	-	-	11:322054	119	148	95	124	90	127	3.2336	3.211804	2.853072	2.59951	1.574844	2.7149	3.099492	2.296418	0.057413	0.559369	-
ENSMUS(	Sri	sorcin [So	GO:00325	developm	-	-	-	-	5:8046078	420	566	397	635	994	679	3.532771	3.802144	3.690658	4.120696	5.384033	4.493113	3.675191	4.665947	0.057472	0.559562	-
ENSMUS(	Xkr7	X-linked K	GO:00160	membran	-	-	-	-	2:1530318	22	17	17	14	10	19	0.495487	0.305772	0.423169	0.243259	0.145032	0.336644	0.408143	0.241645	0.057497	0.559562	-
ENSMUS(	Lamc2	laminin, g	GO:00325	developm	K06246	laminin, g	mmu0520	Pathways	1:1531227	62	83	45	128	126	90	0.427415	0.45696	0.34286	0.680766	0.55935	0.488099	0.409078	0.576072	0.057647	0.560702	-
ENSMUS(	Ppm1j	protein pl	GO:00081	metabolic	-	-	-	-	3:1047810	0	2	3	10	14	1	0	0.045772	0.095007	0.221058	0.258312	0.022545	0.046927	0.167305	0.057863	0.560739	-
ENSMUS(	Dusp3	dual speci	GO:00325	developm	K17614	dual speci	mmu0401	MAPK sigi	11:101971	1678	1964	1995	2039	2558	1646	18.12409	16.9415	23.81519	16.99073	17.79179	13.98638	19.62693	16.2563	0.057866	0.560739	-
ENSMUS(	Nrcam	neuronal c	GO:00325	developm	K06756	neuronal c	mmu0451	Cell adhes	12:443288	8823	10536	7518	10468	10996	8973	22.88803	21.82799	21.55467	20.95011	18.36884	18.31221	22.09023	19.21039	0.057873	0.560739	-
ENSMUS(	Adipoq	adiponect	GO:00325	developm	K07296	adiponect	mmu0493	Non-alco	16:231465	0	3	0	0	23	3	0	0.091444	0	0	0.565208	0.090052	0.030481	0.21842	0.057876	0.560739	-
ENSMUS(	4931406C	RIKEN cDI	GO:00038	catalytic a	-	-	-	-	9:1528333	423	638	412	712	975	723	1.794452	2.161515	1.931684	2.33025	2.663492	2.41291	1.96255	2.468884	0.057742	0.560739	-
ENSMUS(	2310007B	RIKEN cDI	-	-	-	-	-	-	1:9315134	2	0	0	2	10	3	0.042305	0	0	0.032649	0.136238	0.049925	0.014102	0.072937	0.057701	0.560739	-
ENSMUS(	Gm8369	predicted	GO:00160	membran	-	-	-	-	19:114859	2	3	2	12	5	7	0.017019	0.020395	0.018814	0.078792	0.027403	0.046872	0.018743	0.051022	0.057748	0.560739	-
ENSMUS(	Zfp14	zinc finge	GO:00081	metabolic	-	-	-	-	7:3003635	89	138	176	136	138	106	0.733459	0.908256	1.603035	0.864678	0.732347	0.687225	1.081584	0.761417	0.057913	0.560778	-
ENSMUS(	BC024139	cDNA seq	GO:00056	cell|intrace	-	-	-	-	15:761195	9	21	16	35	37	26	0.078625	0.14652	0.154489	0.235898	0.208155	0.178695	0.126545	0.207583	0.058042	0.561716	-
ENSMUS(	Slc38a3	solute car	GO:00325	developm	K13576	solute car	mmu0472	Glutamate	9:1076506	744	1111	861	945	1149	882	3.946417	4.706416	5.047548	3.867163	3.924685	3.680524	4.566794	3.824124	0.058139	0.562345	-
ENSMUS(	Thegl	theg sperı	-	-	-	-	-	-	5:7701602	3	10	4	10	16	18	0.070126	0.186679	0.103349	0.18035	0.240843	0.33101	0.120051	0.250734	0.058217	0.562616	-
ENSMUS(	Duox1	dual oxida	GO:00325	developm	-	-	-	-	2:1223156	0	0	0	0	6	1	0	0	0	0	0.044789	0.009122	0	0.01797	0.058232	0.562616	-
ENSMUS(	Endov	endonucle	GO:00081	metabolic	-	-	-	-	11:119491	532	467	564	393	491	669	2.061361	1.445123	2.415285	1.174804	1.22512	2.039292	1.973923	1.479739	0.058345	0.563394	-
ENSMUS(	Slc1a3	solute car	GO:00325	developm	K05614	solute car	mmu0472	Glutamate	15:863412	13604	16000	12197	14483	17504	15707	70.8914	66.58751	70.24683	58.22584	58.73797	64.39188	69.24191	60.45189	0.058639	0.565297	-
ENSMUS(	Bcar3	breast car	GO:00325	developm	-	-	-	-	3:1222941	199	254	154	312	399	309	1.25419	1.278467	1.072699	1.517031	1.619335	1.532071	1.201785	1.556145	0.05861	0.565297	-
ENSMUS(	Ppp1r36	protein pl	GO:00081	metabolic	-	-	-	-	12:764175	33	45	39	27	42	38	0.456693	0.497353	0.596507	0.288268	0.374294	0.41372	0.516851	0.358761	0.058578	0.565297	-
ENSMUS(	Mtr	5-methylt	GO:00325	developm	K00548	5-methylt	mmu0110	Metabolic	13:121827	624	771	523	653	807	650	3.881442	3.830089	3.595487	3.133665	3.232487	3.180778	3.769006	3.18231	0.058764	0.566091	-
ENSMUS(	Klk10	kallikrein r	GO:00081	metabolic	-	-	-	-	7:4378103	14	18	12	12	15	6	0.622602	0.63927	0.589786	0.41169	0.42955	0.209917	0.617219	0.350386	0.058787	0.566091	-
ENSMUS(	Trak1	trafficking	GO:00325	developm	-	-	-	-	9:1212975	3907	4534	3324	4342	3343	4675	12.2135	11.31942	11.4843	10.47169	6.729575	11.49712	11.6724	9.566129	0.058933	0.566868	-
ENSMUS(	Sirpb1c	signal-rec	GO:00055	extracellul	-	-	-	-	3:1579514	2	5	2	1	0	1	0.018341	0.036623	0.020275	0.007077	0	0.007217	0.02508	0.004765	0.058911	0.566868	-
ENSMUS(	Tlr11	toll-like re	GO:00023	immune s	K14142	toll-like re	mmu0514	Toxoplasr	14:503579	2	3	4	1	2	0	0.019692	0.023599	0.04354	0.007599	0.012681	0	0.028944	0.00676	0.059013	0.567332	-
ENSMUS(	Gm6685	predicted	-	-	-	-	-	-	11:283391	71	92	46	65	81	51	5.674241	5.871963	4.063083	4.007674	4.16859	3.206494	5.203096	3.794253	0.059055	0.567413	-
ENSMUS(	Ccl2	chemokin	GO:00325	developm	K14624	C-C motif	mmu0406	Cytokine-	11:820355	3	4	2	2	0	0	0.095951	0.102165	0.070704	0.049358	0	0	0.089606	0.016453	0.05912	0.567729	-
ENSMUS(	M1ap	meiosis 1	GO:00325	developm	-	-	-	-	6:8294690	4	6	0	2	0	0	0.084573	0.101306	0	0.032626	0	0	0.06196	0.010875	0.059193	0.568116	-
ENSMUS(	Klf6	Kruppel-li	GO:00081	metabolic	-	-	-	-	13:586148	665	893	668	1012	1492	1145	7.466652	8.007585	8.289486	8.766273	10.78767	10.11394	7.921241	9.889295	0.059249	0.568339	-
ENSMUS(	Iqub	IQ motif a	GO:00508	response	-	-	-	-	6:2444486	54	107	33	39	68	55	0.903073	1.429098	0.609952	0.503187	0.732306	0.723607	0.980707	0.653034	0.059362	0.569106	-

ENSMUSC	Myh6	myosin, h	GO:00325	developm	K17751	myosin he	mmu0426	Adrenergi	14:549419	3	6	2	1	0	3	0.021715	0.034686	0.016002	0.005585	0	0.017082	0.024134	0.007556	0.059532	0.570408	-
ENSMUSC	Rtbdn	retbindin	GO:00055	extracellul	-	-	-	-	8:8494699	6	9	7	27	12	18	0.08385	0.100454	0.108127	0.291105	0.107998	0.197894	0.097477	0.198999	0.059563	0.570408	-
ENSMUSC	Dcc	deleted in	GO:00325	developm	K06765	deleted in	mmu0520	Pathways	18:712587	481	589	482	523	589	573	2.937191	2.872421	3.252972	2.463875	2.316096	2.752656	3.020861	2.510876	0.05974	0.571791	-
ENSMUSC	Hspg2	perlecan (	GO:00055	extracellul	K06255	heparan s	mmu0520	Proteogly	4:1374687	289	424	234	449	602	516	1.055434	1.236645	0.944486	1.265055	1.415742	1.482498	1.078855	1.387765	0.059791	0.571948	-
ENSMUSC	Mettl7a2	methyltra	GO:00081	metabolic	-	-	-	-	15:100353	6	0	0	0	0	0	0.101761	0	0	0	0	0	0.03392	0	0.05985	0.571948	-
ENSMUSC	AC163623	-	-	-	-	-	-	-	9:2203638	6	0	0	0	0	0	0.503906	0	0	0	0	0	0.167969	0	0.059855	0.571948	-
ENSMUSC	Mlxipl	MLX inter	GO:00081	metabolic	K09113	MAX-like	mmu0493	Non-alco	5:1350898	39	60	43	89	88	82	0.267168	0.328259	0.325562	0.47037	0.388203	0.44192	0.306996	0.433498	0.059899	0.572046	-
ENSMUSC	Hspb7	heat shoc	GO:00056	cell|intrac	-	-	-	-	4:1414207	2	3	6	11	15	9	0.044093	0.052839	0.146234	0.187138	0.212991	0.156121	0.081055	0.185417	0.06008	0.57284	-
ENSMUSC	Cdca8	cell divisi	GO:00511	localizatio	-	-	-	-	4:1249184	16	22	18	29	56	33	0.149217	0.163861	0.185535	0.208655	0.336314	0.24212	0.166204	0.262363	0.060019	0.57284	-
ENSMUSC	5730522E	RIKEN cDI	-	-	-	-	-	-	11:256027	32	31	20	58	49	56	0.289631	0.224079	0.20007	0.404999	0.285591	0.398745	0.237927	0.363112	0.060048	0.57284	-
ENSMUSC	Cdk2ap2	CDK2-ass	GO:00990	supramole	-	-	-	-	19:409718	181	216	160	277	355	308	3.248533	3.09605	3.17377	3.835468	4.102912	4.348811	3.172784	4.09573	0.060329	0.574895	-
ENSMUSC	Rxra	retinoid X	GO:00325	developm	K08524	retinoid X	mmu0520	Pathways	2:2767644	647	787	527	993	1182	978	4.766479	4.630355	4.290924	5.643823	5.607452	5.668178	4.562586	5.639818	0.060652	0.577531	-
ENSMUSC	Col22a1	collagen,	-	-	K16630	collagen, t	mmu0497	Protein di	15:717957	22	42	25	57	56	57	0.142567	0.21737	0.179053	0.284973	0.233692	0.290594	0.179663	0.269753	0.060672	0.577531	-
ENSMUSC	Plpbp	pyridoxal	GO:00055	extracellul	-	-	-	-	8:2704255	751	603	695	721	772	702	2.222931	1.425442	2.27362	1.646462	1.471493	1.634685	1.973998	1.584213	0.060719	0.577658	-
ENSMUSC	Tusc5	tumor sup	GO:00511	localizatio	-	-	-	-	11:766798	1	8	6	10	17	13	0.018497	0.118145	0.122629	0.142663	0.202433	0.189106	0.086423	0.178067	0.060823	0.578338	-
ENSMUSC	C1galt1	core 1 syr	GO:00325	developm	K00731	glycoprote	mmu0110	Metabolic	6:7844842	303	440	269	507	655	503	3.036864	3.521937	2.979771	3.920319	4.227458	3.966087	3.179524	4.037955	0.060954	0.579266	-
ENSMUSC	Stab1	stabilin 1	GO:00325	developm	-	-	-	-	14:311390	270	416	258	298	381	318	1.028172	1.265146	1.085847	0.875485	0.934288	0.952663	1.126388	0.920812	0.061032	0.579691	-
ENSMUSC	Mgst3	microsom	GO:00081	metabolic	K00799	glutathion	mmu0520	Pathways	1:1673719	557	679	511	829	1139	874	31.00215	30.18229	31.43432	35.59768	40.82395	38.27006	30.87292	38.23056	0.061131	0.580069	-
ENSMUSC	Echdc3	enoyl Coe	GO:00081	metabolic	-	-	-	-	2:6188465	5	15	7	21	28	15	0.092058	0.220584	0.142463	0.298315	0.332004	0.217284	0.151702	0.282534	0.061139	0.580069	-
ENSMUSC	Atf3	activating	GO:00325	developm	K09032	activating	mmu0516	HTLV-I int	1:1911702	18	19	16	26	59	32	0.428802	0.361486	0.421273	0.477849	0.905103	0.599734	0.403854	0.660895	0.061611	0.584231	-
ENSMUSC	Gimap9	GTPase, II	GO:00971	organic c	-	-	-	-	6:4867612	7	5	2	9	26	8	0.208801	0.119102	0.065937	0.207114	0.499396	0.187716	0.13128	0.298075	0.0617	0.584756	-
ENSMUSC	Stat6	signal trar	GO:00325	developm	K11225	signal trar	mmu0520	Pathways	10:127642	163	262	143	266	428	284	1.72248	2.21112	1.67012	2.168592	2.912487	2.360997	1.867907	2.480692	0.061937	0.586361	-
ENSMUSC	Gabpb1	GA repeat	GO:00081	metabolic	-	-	-	-	2:1266274	316	446	298	238	515	333	1.544659	1.741111	1.609939	0.89754	1.621094	1.280563	1.631903	1.266399	0.061932	0.586361	-
ENSMUSC	Naa50	N(alpha)-	GO:00081	metabolic	-	-	-	-	16:441398	1395	1332	977	1226	1675	1189	6.442485	4.912799	4.986784	4.368173	4.981365	4.319883	5.447356	4.556474	0.062051	0.586801	-
ENSMUSC	Gm9955	predicted	-	-	-	-	-	-	18:247090	0	0	0	6	0	1	0	0	0	0.914646	0	0.155482	0	0.356709	0.062021	0.586801	-
ENSMUSC	Rps28	ribosomal	GO:00081	metabolic	K02979	small subu	mmu0301	Ribosome	17:338190	64	58	53	121	91	119	1.771398	1.282065	1.621277	2.583776	1.621933	2.591172	1.558247	2.265627	0.062106	0.587007	-
ENSMUSC	Jmjd1c	jumonji dc	GO:00325	developm	K11449	jumonji dc	mmu0520	Transcript	10:670961	2043	2645	1844	2285	2461	2540	4.400217	4.549642	4.389483	3.796839	3.413279	4.303782	4.446447	3.837967	0.062172	0.587304	-
ENSMUSC	Frmpd4	FERM anc	GO:00452	synapse|n	-	-	-	-	X:1674713	2545	2154	2068	1627	3468	2119	11.59562	7.837864	10.41366	5.719053	10.17513	7.595355	9.949049	7.829846	0.062226	0.587502	-
ENSMUSC	Txnl4a	thioredox	GO:00081	metabolic	K12859	U5 snRNP	mmu0304	Spliceosor	18:802067	164	386	151	248	210	180	3.903901	7.338201	3.972651	4.554464	3.219062	3.37085	5.071584	3.714792	0.062277	0.587658	-
ENSMUSC	Rxfp1	relaxin/ins	GO:00325	developm	K04306	relaxin far	mmu0408	Neuroacti	3:7964161	237	297	105	210	182	192	1.219413	1.220407	0.597089	0.833591	0.603014	0.777169	1.012303	0.737925	0.06238	0.588314	-
ENSMUSC	Itgax	integrin al	GO:00511	localizatio	K06462	integrin al	mmu0481	Regulatior	7:1281295	27	41	17	21	33	17	0.310761	0.376869	0.216251	0.186468	0.244583	0.153931	0.301294	0.194994	0.062554	0.589635	-
ENSMUSC	Sh3bp1	SH3-dom	GO:00081	metabolic	K07758	pyridoxal	mmu0110	Metabolic	15:788996	1526	1804	1840	2697	3181	2655	15.02333	14.18385	20.02056	20.48438	20.16647	20.56302	16.40924	20.40463	0.062837	0.591981	-
ENSMUSC	Mmp28	matrix me	GO:00081	metabolic	-	-	-	-	11:834407	35	41	36	64	79	68	0.356646	0.333659	0.40544	0.503129	0.518388	0.545121	0.365248	0.522213	0.063024	0.593426	-
ENSMUSC	Slc25a18	solute car	GO:00511	localizatio	-	-	-	-	6:1207735	650	765	562	1071	1121	990	20.76805	19.52045	19.84567	26.39991	23.06445	24.88448	20.04473	24.78295	0.063272	0.595201	-
ENSMUSC	6430548M	RIKEN cDI	-	-	-	-	-	-	8:1201141	2945	5579	2427	5674	6289	5586	13.13677	19.87493	11.9652	19.52644	18.06507	19.60267	14.9923	19.06473	0.063281	0.595201	-
ENSMUSC	Fblim1	filamin bir	GO:00325	developm	-	-	-	-	4:1415760	29	47	32	63	105	48	0.270416	0.350009	0.329787	0.453213	0.63049	0.352119	0.316737	0.478607	0.063343	0.595461	-
ENSMUSC	Khdc3	KH domai	GO:00325	developm	-	-	-	-	9:7310183	6	13	6	26	18	14	0.088467	0.153083	0.097783	0.295756	0.170911	0.162393	0.113111	0.209687	0.063567	0.597243	-
ENSMUSC	Rspo1	R-spondir	GO:00081	metabolic	-	-	-	-	4:1249864	14	15	7	17	34	29	0.374927	0.320814	0.207197	0.351233	0.586327	0.610959	0.300979	0.516173	0.063658	0.597776	-
ENSMUSC	Lrp1b	low densit	GO:00329	macromo	-	-	-	-	2:4059524	1868	2350	1774	2147	2435	2218	3.776958	3.794712	3.964292	3.349095	3.170435	3.528073	3.845321	3.349201	0.063727	0.598099	-
ENSMUSC	Kif4	kinesin fa	GO:00990	supramole	-	-	-	-	X:1006257	32	45	24	47	77	69	0.341463	0.383488	0.283039	0.386925	0.529102	0.57924	0.335997	0.498422	0.063768	0.598158	-
ENSMUSC	Shisa3	shisa fami	GO:00325	developm	-	-	-	-	5:6760788	2	9	0	9	21	6	0.022321	0.080235	0	0.077507	0.150946	0.05269	0.034185	0.093714	0.063915	0.599221	-
ENSMUSC	Ggact	gamma-c	GO:00081	metabolic	-	-	-	-	14:122816	279	301	279	146	389	306	2.453935	2.114319	2.712124	0.990698	2.203246	2.117339	2.426793	1.770428	0.064025	0.599922	-
ENSMUSC	Tnfaip3	tumor nec	GO:00325	developm	K11859	tumor nec	mmu0516	Epstein-B	10:190009	93	141	101	186	182	217	0.406819	0.492591	0.488301	0.627717	0.512679	0.746778	0.46257	0.629058	0.064066	0.599981	-
ENSMUSC	Sfi1	Sfi1 homc	GO:00081	metabolic	-	-	-	-	11:313185	241	323	193	386	411	463	1.01392	1.085261	0.897409	1.252864	1.113481	1.532421	0.998864	1.299589	0.06417	0.600637	-
ENSMUSC	Spa17	sperm aut	GO:00099	cellular pr	-	-	-	-	9:3760329	30	37	23	15	39	24	1.36089	1.340421	1.153094	0.524953	1.139239	0.856474	1.284802	0.840222	0.064325	0.601215	-
ENSMUSC	Dnase1l1	deoxyribc	GO:00081	metabolic	-	-	-	-	X:7427321	14	26	16	36	46	31	0.219526	0.325586	0.277279	0.435491	0.464475	0.382402	0.27413	0.427456	0.06437	0.601215	-
ENSMUSC	Kif3c	kinesin fa	GO:00511	localizatio	-	-	-	-	12:336513	3868	4905	3636	3746	5841	4469	17.4684	17.69096	18.14836	13.05163	16.98668	15.87771	17.76924	15.30534	0.06431	0.601215	-
ENSMUSC	Abhd14b	abhydrola	GO:00081	metabolic	-	-	-	-	9:1064474	185	192	131	238	320	322	1.668995	1.383344	1.306173	1.656497	1.859038	2.285332	1.452837	1.933622	0.064339	0.601215	-
ENSMUSC	Trh	thyrotropi	GO:00081	metabolic	-	-	-	-	6:9224206	3	6	47	13	6	6	0.132348	0.211396	2.291678	0.442459	0.170449	0.208248	0.878474	0.273719	0.06448	0.601921	-
ENSMUSC	Ppef2	protein pl	GO:00081	metabolic	-	-	-	-	5:9222667	4	7	6	18	10	16	0.063755	0.0891	0.105696	0.221332	0.102632	0.200616	0.086184	0.17486	0.06458	0.602526	-
ENSMUSC	Cd80	CD80 anti	GO:00081	metabolic	K05412	CD80 anti	mmu0451	Cell adhes	16:384589	8	12	4	3	9	3	0.28718	0.344001	0.158699	0.08309	0.208037	0.084705	0.263293	0.125278	0.064696	0.603288	-
ENSMUSC	Gm5640	predicted	-	-	-	-	-	-	X:7463911	5	10	0	9	16	17	0.106698	0.170432	0	0.148187	0.219882	0.285426	0.092377	0.217832	0.064749	0.603455	-
ENSMUSC	Gna14	guanine n	GO:00048	signal trar	K04636	guanine n	mmu0402	Calcium si	19:164356	17	21	18	37	56	26	0.306829	0.302698	0.359059	0.515197	0.650857	0.36917	0.322862	0.511741	0.064789	0.60351	-
ENSMUSC	Cdkn1a	cyclin-dep	GO:00325	developm	K06625	cyclin-dep	mmu0520	Pathways	17:290909	106	176	93	189	328	165	2.105446	2.791882	2.0416	2.896214	4.195345	2.578307	2.312976	3.223289	0.065063	0.605735	-
ENSMUSC	Gm3055	predicted	GO:00081	metabolic	-	-	-	-	10:817428	43	63	44	46	61	36	0.509018	0.595587	0.575654	0.4201	0.464994	0.335255	0.560086	0.406783	0.065146	0.606186	-
ENSMUSC	BC147527	cDNA seq	-	-	-	-	-	-	13:120300	0	0	1	2	9	0	0	0	0.032152	0.04489	0.16859	0	0.010717	0.07116	0.065285	0.607154	-
ENSMUSC	Col15a1	collagen,	GO:00051	structural	K08135	collagen, t	mmu0497	Protein di	4:4720816	89	122	79	145	207	154	0.581011	0.636062	0.569991	0.73028	0.870198	0.790904	0.595688	0.797128	0.065404	0.607932	-
ENSMUSC	Adgrl3	adhesion	GO:00325	developm	-	-	-	-	5:8102013	2727	3444	2245	3067	3370	3040	4.845204	4.886929	4.408493	4.204082	3.855774	4.249242	4.713542	4.103032	0.065479	0.608303	-
ENSMUSC	Ccdc129	coiled-co	GO:00055	protein bi	-	-	-	-	6:5583689	35	48	22	49	81	71	0.52779	0.578076	0.366669	0.570058	0.786565	0.842294	0.490845	0.732972	0.065548	0.608624	-
ENSMUSC	Cdh8	cadherin 8	GO:00452	synapse|re	-	-	-	-	8:9902447	1312	1659	1271	1586	1805	1406	7.765435	7.841954	8.314276	7.242116	6.879612	6.546786	7.973888	6.889504	0.065619	0.608956	-
ENSMUSC	Slc4a4	solute car	GO:00511	localizatio	K13575	solute car	mmu0497	Pancreatic	5:8888681	4511	5792	4306	5194	6100	5468	21.93463	22.49219	23.1408	19.48452	19.10038	20.91688	22.52254	19.83393	0.065679	0.609182	-
ENSMUSC	Igtp	interferon	GO:00508	response	K14140	interferon	mmu0514	Toxoplasr	11:581995	108	156	112	189	299	179	1.287697	1.48545	1.475883	1.73852	2.295696	1.679005	1.416343	1.904407	0.065727	0.609303	-
ENSMUSC	Angptl1	angiopoie	GO:00508	response	-	-	-	-	1:1568385	24	26	15	17	25	13	0.367918	0.318311	0.254139	0.201057	0.246787	0.156773	0.313456	0.201539	0.0659	0.610433	-
ENSMUSC	F930015N	RIKEN cDI	-	-	-	-	-	-	11:644351	5	5	1	3	1	0	0.203781	0.162752	0.045051	0.09435	0.026242	0	0.137195	0.040197	0.065919	0.610433	-
ENSMUSC	Cdca3	cell divisi	GO:00081	metabolic	-	-	-	-	6:1248295	19	27	13	30	56	35	0.233608	0.265123	0.176654	0.28457	0.443385	0.338543	0.225128	0.355499	0.065995	0.610816	-
ENSMUSC	Fscn2	fascin acti	GO:00325	developm	-	-	-	-	11:120361	0	9	11	3	2	3	0	0.163579	0.276664	0.052678	0.029303	0.053702	0.146748	0.045228	0.066086	0.611333	-
ENSMUSC	Pafah1b1	platelet-a	GO:00325	developm	K16794	platelet-a	mmu0110	Metabolic	11:746739	9590	10426	7749	3588	12773	10007	24.34	21.13321	21.73674	7.02562	20.8761	19.98098	22.40331	15.9609	0.066167	0.611751	-
ENSMUSC	Serpina3g	serine (or	GO:00055	extracellul	-	-	-	-	12:104236	22	31	18	51	50	37	0.403383	0.453944	0.364774	0.721438	0.590375	0.533721	0.407367	0.615178	0.066231	0.612021	-
ENSMUSC	Cdk2	cyclin-dep	GO:00325	developm	K02206	cyclin-dep	mmu0520	Pathways	10:128697	42	50	28	67	76	80	0.214921	0.204335	0.158356	0.264506	0.250437	0.322054	0.192537	0.278999	0.066267	0.612025	-
ENSMUSC	Tspan32	tetraspani	GO:00023	immune s	-	-	-	-	7:143005C	7	3	2	2	3	0	0.03939	0.013484	0.012439	0.008683	0.010872	0	0.021771	0.006518	0.066435	0.612441	-
ENSMUSC	Ckmt1	creatine k	GO:00081	metabolic	K00933	creatine k	mmu0110	Metabolic	2:1213577	2189	2741	2074	3192	4129	3738	22.06641	22.06687	23.10688	24.82442	26.80312	29.64395	22.41338	27.09049	0.06643	0.612441	-
ENSMUSC	Sbno2	strawberr	GO:00325	developm	-	-	-	-	10:800569	300	446	308	545	627	547	1.196122	1.420152	1.357226	1.676414	1.609818	1.715747	1.3245	1.667326	0.066347	0.612441	-
ENSMUSC	Serpina3f	serine (or	GO:00081	metabolic	-	-	-	-	12:104214	0	4	2	3	14	7	0	0.060852	0.042114	0.044099	0.171751	0.104917	0.034322	0.106922	0.066453	0.612441	-
ENSMUSC	Stk32a	serine/thr	GO:00081	metabolic	-	-	-	-	18:432076	119	171	107	108	135	151	1.929853	2.214726	1.917829	1.351235	1.409825	1.926493	2.020803	1.562518	0.066748	0.614834	-
ENSMUSC	Cyp4f18	cytochron	GO:00081	metabolic	K17726	leukotrien	mmu0110	Metabolic	8:7198848	0	4	3	0	1	0	0	0.082119	0.085247	0	0.016552	0	0.055788	0.005517	0.066788	0.614884	-
ENSMUSC	Fgf2	fibroblast	GO:00325	developm	K18497	fibroblast	mmu0520	Pathways	3:3734834	174	281	121	187	167	196	1.224538	1.579339	0.941145	1.015305	0.756823	1.085153	1.248341	0.952427	0.066961	0.616146	-
ENSMUSC	Irf9	interferon	GO:00081	metabolic	K04693	interferon	mmu0516	Human pa	14:556035	189	257	194	357	429	295	1.245817	1.352915	1.41332	1.815477	1.820976	1.529767	1.337351	1.722073	0.067296	0.618667	-
ENSMUSC	Slc2a12	solute car	GO:00511	localizatio	-	-	-	-	10:226450	133	301	106	95	195	181	1.778912	3.215247	1.566953	0.980293	1.679545	1.904551	2.187037	1.521463	0.067342	0.618667	-
ENSMUSC	Jaml	junction a	GO:00400	locomotic	-	-	-	-	9:4507918	2	0	3	7	14	3	0.026279	0	0.043577	0.070972	0.118481	0.031012	0.023286	0.073489	0.067325	0.618667	-
ENSMUSC	Als2cl	ALS2 C-te	GO:00099	cellular pr	-	-	-	-	9:1108798	148	189	153	245	330	258	0.690817	0.704546	0.789299	0.882265	0.991908	0.9474	0.728221	0.940524	0.067423	0.618759	-
ENSMUSC	Gm4737	predicted	GO:00081	metabolic	K01251	adenosylh	mmu0110	Metabolic	16:461529	204	272	193	309	493	337	5.951195	6.337079	6.222694	6.954449	9.26138	7.734182	6.170323	7.983337	0.067394	0.618759	-
ENSMUSC	Mif4gd	MIF4G do	GO:00081	metabolic	-	-	-	-	11:115607	149	210	183	136	211	204	1.909267	2.149043	2.591668	1.34447	1.741079	2.056463	2.216659	1.714004	0.067506	0.619197	-
ENSMUSC	Layn	layilin [So	GO:00160	membran	-	-	-	-	9:5105464	103	113	94	97	120	102	1.858993	1.628794	1.875071	1.350654	1.394685	1.448277	1.787619	1.397872	0.06758	0.619546	-
ENSMUSC	Rps6ka1	ribosomal	GO:00325	developm	K04373	ribosomal	mmu0401	MAPK sigr	4:1338472	193	263	229	333	486	355	1.773659	1.930256	2.325922	2.360959	2.876107	2.56657	2.009946	2.601212	0.0677	0.619666	-
ENSMUSC	Lims2	LIM and s	GO:00325	developm	-	-	-	-	18:319313	56	147	122	217	258	153	0.838262	1.757331	2.018354	2.505997	2.486928	1.801737	1.537982	2.264887	0.067653	0.619666	-
ENSMUSC	Sp110	Sp110 nuc	GO:00081	metabolic	-	-	-	-	1:8557689	23	43	23	67	55	48	0.152232	0.227293	0.168245	0.342123	0.234422	0.249938	0.18259	0.275494	0.067694	0.619666	-
ENSMUSC	Tmem168	transmem	GO:00160	membran	-	-	-	-	6:1358068	278	394	280	130	535	199	2.36047	2.671749	2.627598	0.851582	2.925247	1.329283	2.553272	1.702037	0.067856	0.620772	-
ENSMUSC	Togaram1	TOG array	GO:00990	supramole	-	-	-	-	12:649658	1103	1444	1048	1374	1553	1092	3.572602	3.735269	3.751605	3.433413	3.239181	2.782548	3.686492	3.151714	0.067943	0.621242	-
ENSMUSC	Selp	selectin, p	GO:00400	locomotic	K06496	selectin, p	mmu0451	Cell adhes	1:1641152	1	3	0	2	22	1	0.009983	0.023915	0	0.015401	0.14139	0.007853	0.011299	0.054881	0.068088	0.622235	-
ENSMUSC	Upk1a	uroplakin	GO:00325	developm	-	-	-	-	7:3060309	2	3	6	12	16	7	0.085921	0.102963	0.284955	0.397784	0.442704	0.236636	0.157946	0.359041	0.068508	0.624046	-
ENSMUSC	Ifitm1	interferon	GO:00325	developm	K19831	interferon	mmu0466	B cell rece	7:1409672	12	28	14	26	43	42	0.56144	1.046262	0.723946	0.938488	1.295553	1.545948	0.777216	1.259996	0.068527	0.624046	-
ENSMUSC	Bmp2	bone mor	GO:00325	developm	K04662	bone mor	mmu0520	Pathways	2:1335521	61	75	42	103	130	88	1.047689	1.028757	0.797257	1.364807	1.437806	1.189044	0.957901	1.330552	0.068448	0.624046	-
ENSMUSC	Snx8	sorting ne	GO:00511	localizatio	-	-	-	-	5:1403402	167	196	161	190	193	167	1.35847	1.273315	1.447462	1.192389	1.010991	1.068719	1.359749	1.0907	0.068537	0.624046	-
ENSMUSC	Ercc2	excision re	GO:00325	developm	K10844	DNA excis	mmu0342	Nucleotid	7:1938201	333	442	307	613	567	591	2.79022	2.957758	2.843017	3.962647	3.059374	3.895767	2.863665	3.639263	0.068398	0.624046	-
ENSMUSC	Ciapin1	cytokine ir	GO:00325	developm	-	-	-	-	8:9481980	765	977	710	899	1022	819	3.746648	3.821393	3.843144	3.39681	3.223199	3.15556	3.803728	3.258523	0.068371	0.624046	-
ENSMUSC	Inppl1	inositol pc	GO:00325	developm	K15909	phosphati	mmu0491	Insulin sig	7:1018226	525	723	469	784	1007	972	2.866193	3.152322	2.829865	3.302119	3.540222	4.174682	2.94946	3.672341	0.06838	0.624046	-
ENSMUSC	Hnmt	histamine	GO:00325	developm	K00546	histamine	mmu0034	Histidine r	2:2400291	341	385	338	416	418	307	6.202213	5.592409	6.794467	5.837354	4.89579	4.392795	6.196363	5.041979	0.068637	0.624306	-
ENSMUSC	Upf1	UPF1 regu	GO:00081	metabolic	K14326	regulator	mmu0301	RNA trans	8:7033152	1332	1576	1483	2203	2665	2239	15.83208	14.96014	19.48146	20.20127	20.39791	20.93624	16.7579	20.51181	0.068637	0.624306	-
ENSMUSC	Gm10093	predicted	GO:00081	metabolic	-	-	-	-	17:784915	226	349	186	386	515	365	6.979827	8.608109	6.348857	9.197179	10.24233	8.868302	7.312264	9.435938	0.06871	0.624643	-
ENSMUSC	Hagh	hydroxyac	GO:00081	metabolic	K01069	hydroxyac	mmu0062	Pyruvate r	17:248401	1063	1051	1072	1074	1425	1063	24.55723	19.39076	27.37081	19.14172	21.19902	19.31925	23.77293	19.88666	0.068883	0.625887	-
ENSMUSC	AC154682	-	-	-	-	-	-	-	14:122839	7	14	16	7	13	7	0.136817	0.218529	0.345622	0.10555	0.163621	0.107638	0.233656	0.125603	0.068927	0.62596	-
ENSMUSC	Xlr3c	X-linked l	-	-	-	-	-	-	X:7325454	13	9	9	7	4	10	0.501109	0.277073	0.383406	0.208168	0.099293	0.303247	0.387196	0.203569	0.069061	0.626849	-
ENSMUSC	Selenoh	selenopro	GO:00971	organic c	-	-	-	-	2:8466921	92	92	61	76	110	44	1.736973	1.387192	1.272861	1.107003	1.337365	0.653526	1.465675	1.032631	0.06928	0.628509	-
ENSMUSC	Raver1	ribonuclec	GO:00081	metabolic	-	-	-	-	9:2107415	356	755	379	544	360	498	6.264172	10.60979	7.370544	7.38487	4.079163	6.893726	8.081503	6.119253	0.069402	0.628961	-
ENSMUSC	Slc35d2	solute car	GO:00160	membran	-	-	-	-	13:640963	5	9	10	14	28	17	0.059205	0.085121	0.130877	0.127904	0.213523	0.158379	0.091734	0.166602	0.069388	0.628961	-
ENSMUSC	Nmbr	neuromec	GO:00048	signal trar	K04168	neuromec	mmu0408	Neuroacti	10:147055	55	62	39	53	57	39	0.707584	0.637024	0.554528	0.526045	0.472221	0.394718	0.633045	0.464328	0.069567	0.630128	-
ENSMUSC	Cept1	choline/et	GO:00081	metabolic	K13644	choline/et	mmu0110	Metabolic	3:1065022	354	222	318	527	678	482	4.1439	2.07541	4.11414	4.759343	5.110814	4.438779	3.444483	4.769645	0.069682	0.630514	-
ENSMUSC	Setd1a	SET doma	GO:00325	developm	K11422	histone-ly	mmu0031	Lysine deg	7:1277766	1013	1344	1064	1039	1741	993	7.707394	8.166643	8.947193	6.098795	8.530052	5.943723	8.273743	6.857523	0.069671	0.630514	-
ENSMUSC	Btbd6	BTB (POZ	GO:00325	developm	-	-	-	-	12:112976	123	88	339	104	919	723	1.002957	0.57307	3.05509	0.654247	4.82556	4.637955	1.543706	3.372587	0.069936	0.630735	-
ENSMUSC	Ppp1r42	protein pl	GO:00081	metabolic	-	-	-	-	1:9968624	8	8	10	9	3	4	0.228588	0.182552	0.315771	0.198391	0.055204	0.089905	0.242304	0.1145	0.069918	0.630735	-
ENSMUSC	Tulp4	tubby like	GO:00081	metabolic	-	-	-	-	17:610643	3687	4787	3343	3598	5319	4466	9.218724	9.558878	9.238061	6.940475	8.564112	8.784708	9.338554	8.096432	0.069854	0.630735	-
ENSMUSC	Arl4d	ADP-ribo	GO:00508	response	-	-	-	-	11:101665	109	144	123	105	163	118	4.815711	5.080928	6.006043	3.578952	4.637421	4.101362	5.300894	4.105912	0.070033	0.630735	-
ENSMUSC	Gm11992	predicted	-	-	-	-	-	-	11:904859	21	45	26	51	71	54	0.53427	0.9143	0.731052	1.000995	1.163175	1.080769	0.726541	1.081646	0.069982	0.630735	-
ENSMUSC	Lingo1	leucine ric	GO:00325	developm	-	-	-	-	9:5661847	2933	3810	2883	3653	4112	3226	17.26272	17.90886	18.75377	16.58735	15.58493	14.93731	17.97512	15.7032	0.070022	0.630735	-
ENSMUSC	Adnp	activity-d	GO:00325	developm	-	-	-	-	2:1681809	2573	2124	1495	2161	2082	2311	25.27796	16.66489	16.23268	16.37899	13.17157	17.8613	19.39184	15.80395	0.070023	0.630735	-
ENSMUSC	Rab7b	RAB7B, m	GO:00325	developm	K07898	Ras-relate	mmu0414	Phagosom	1:1316886	27	36	25	19	26	32	0.25711	0.273781	0.263106	0.13958	0.15943	0.239725	0.264666	0.179578	0.06977	0.630735	-
ENSMUSC	Zfx	zinc finge	GO:00325	developm	-	-	-	-	X:9407463	328	447	144	489	590	535	1.395226	1.51853	0.676985	1.604759	1.616134	1.790339	1.196914	1.670411	0.069826	0.630735	-
ENSMUSC	Itgad	integrin, a	GO:00023	immune s	K06594	integrin al	mmu0481	Regulatior	7:1281543	0	4	0	4	9	3	0	0.037528	0	0.036252	0.068091	0.027724	0.012509	0.044023	0.070498	0.634591	-
ENSMUSC	P4ha3	procollag	GO:00081	metabolic	K00472	prolyl 4-h	mmu0110	Metabolic	7:1002855	46	87	46	61	68	31	0.566838	0.856181	0.626477	0.579911	0.539586	0.30052	0.683166	0.473339	0.070642	0.635558	-
ENSMUSC	Dcn	decorin [S	GO:00325	developm	K04660	decorin[-]	mmu0520	Proteogly	10:974796	813	1018	742	1497	1402	1221	7.125052	7.125104	7.187013	10.12161	7.912255	8.418292	7.145723	8.817385	0.070742	0.636129	-
ENSMUSC	Mov10	Moloney l	GO:00081	metabolic	-	-	-	-	3:1047948	136	195	140	267	297	230	1.041111	1.192168	1.184499	1.576885	1.464097	1.385149	1.139259	1.475377	0.070883	0.636411	-
ENSMUSC	Pcolce2	procollag	GO:00081	metabolic	-	-	-	-	9:9563760	83	178	59	67	121	94	1.081708	1.852676	0.849831	0.673658	1.01549	0.963771	1.261405	0.884306	0.070855	0.636411	-
ENSMUSC	Mboat2	membran	GO:00081	metabolic	K13517	lysophosp	mmu0110	Metabolic	12:248308	1150	1472	448	1283	761	582	5.034546	5.146549	2.167643	4.333307	2.14537	2.004456	4.116246	2.827711	0.070812	0.636411	-
ENSMUSC	Gpr34	G protein-	GO:00048	signal trar	-	-	-	-	X:1363208	173	189	153	178	222	141	2.696726	2.352862	2.635901	2.140624	2.228423	1.729104	2.56183	2.032717	0.071178	0.638729	-
ENSMUSC	BC067074	cDNA seq	GO:00160	membran	-	-	-	-	13:113293	96	164	72	73	94	131	0.496321	0.677147	0.411407	0.29117	0.312951	0.532816	0.528292	0.378979	0.071235	0.638909	-
ENSMUSC	Tlr13	toll-like re	GO:00081	metabolic	-	-	-	-	X:1061432	41	113	69	58	80	63	0.599034	1.318547	1.114204	0.653775	0.75269	0.724143	1.010595	0.710203	0.071308	0.63924	-
ENSMUSC	Wnt2b	wingless-	GO:00325	developm	K00182	wingless-t	mmu0520	Pathways	3:1049452	7	8	9	18	25	15	0.092394	0.084331	0.131283	0.183295	0.212485	0.155753	0.102669	0.183844	0.071523	0.640831	-
ENSMUSC	Vps4b	vacuolar p	GO:00081	metabolic	K12196	vacuolar p	mmu0414	Endocytos	1:1067640	844	1111	571	785	1087	843	5.066667	5.326472	3.788457	3.635631	4.202075	3.981239	4.727199	3.939648	0.071745	0.642494	-
ENSMUSC	Zfp846	zinc finge	GO:00081	metabolic	-	-	-	-	9:2058129	646	296	188	671	903	752	2.198883	0.804649	0.707251	1.762066	1.979302	2.013716	1.236928	1.918361	0.071884	0.643403	-
ENSMUSC	Fbxo5	F-box prc	GO:00325	developm	K10292	F-box prc	mmu0411	Oocyte m	10:579916	21	14	9	27	40	29	0.548908	0.292246	0.259981	0.544451	0.673256	0.596313	0.367045	0.604673	0.072023	0.644316	-
ENSMUSC	Mpv17l	Mpv17 tra	GO:00081	metabolic	K13349	Mpv17-lik	mmu0414	Peroxisom	16:139031	1001	670	516	788	1934	1513	5.200303	2.779813	2.962723	3.158283	6.470022	6.183645	3.647613	5.27065	0.072375	0.647129	-
ENSMUSC	Lgals2	lectin, gal	GO:00973	carbohydr	-	-	-	-	15:788508	0	0	2	0	7	9	0	0	0.228769	0	0.46646	0.732711	0.076256	0.399724	0.072556	0.648414	-
ENSMUSC	Camp	catheliciди	GO:00325	developm	K13916	catheliciди	mmu0515	Tuberculo	9:1098473	3	0	4	0	1	0	0.241649	0	0.356131	0	0.051861	0	0.19926	0.017287	0.072638	0.648815	-
ENSMUSC	Ccl3	chemokin	GO:00325	developm	K05408	C-C motif	mmu0406	Cytokine-	11:836478	8	17	5	3	9	7	0.637719	1.082177	0.440515	0.184512	0.461973	0.438981	0.720137	0.361822	0.07279	0.648924	-
ENSMUSC	Trim21	tripartite r	GO:00081	metabolic	K10651	tripartite r	mmu0532	Systemic l	7:1025579	42	49	31	53	95	83	0.398328	0.37114	0.324939	0.387793	0.58019	0.619273	0.364803	0.529085	0.072799	0.648924	-
ENSMUSC	Hist1h1c	histone cl	GO:00081	metabolic	-	-	-	-	13:237388	190	244	164	347	418	253	7.436551	7.62701	7.094303	10.478	10.53536	7.790235	7.385954	9.601201	0.072704	0.648924	-
ENSMUSC	Pcdha8	protocadh	GO:00325	developm	-	-	-	-	18:369923	6	9	17	3	6	11	0.068629	0.082219	0.214914	0.026477	0.044193	0.098986	0.12192	0.056552	0.072758	0.648924	-
ENSMUSC	Helb	helicase (I	GO:00081	metabolic	-	-	-	-	10:120083	94	135	84	174	181	179	0.98854	1.133828	0.976317	1.411708	1.225742	1.480914	1.032895	1.372788	0.072894	0.649433	-
ENSMUSC	Epha4	Eph recep	GO:00325	developm	K05105	Eph recep	mmu0436	Axon guic	1:7736718	8343	10700	7745	6173	12647	10153	40.76893	41.75771	41.82879	23.272	39.7969	39.03124	41.45181	34.03338	0.073002	0.650068	-
ENSMUSC	Morc4	microrchic	GO:00056	cell|intrac	-	-	-	-	X:1398216	115	132	105	115	140	111	0.554025	0.507866	0.55907	0.427421	0.434324	0.42069	0.54032	0.427478	0.073242	0.651873	-
ENSMUSC	Exph5	exophilin	GO:00325	developm	-	-	-	-	9:5330167	83	117	86	129	222	158	0.461296	0.519319	0.528261	0.553128	0.794533	0.69083	0.502959	0.679497	0.073494	0.653776	-
ENSMUSC	Scn2a	sodium ch	GO:00325	developm	K04834	voltage-g	mmu0474	Taste tran	2:6562077	5836	7746	5660	6864	8256	7055	22.1175	23.44467	23.70741	20.06912	20.14861	21.03433	23.08986	20.41736	0.073549	0.653932	-
ENSMUSC	5330417C	RIKEN cDI	GO:00081	metabolic	-	-	-	-	3:1084556	621	768	563	963	1192	965	4.608297	4.551518	4.617473	5.513227	5.696134	5.633615	4.592429	5.614325	0.07362	0.654232	-
ENSMUSC	Nrg1	neureguli	GO:00325	developm	K05455	neureguli	mmu0401	ErbB signa	8:3181455	55	107	53	66	71	66	0.151968	0.236113	0.161849	0.14069	0.126329	0.143464	0.18331	0.136828	0.073756	0.655098	-
ENSMUSC	Bcl2l11	BCL2-like	GO:00325	developm	K16341	Bcl-2-like	mmu0520	Pathways	2:1281260	34	24	52	118	81	55	0.13372	0.075381	0.226026	0.358034	0.20514	0.17017	0.145042	0.244448	0.073878	0.655855	-
ENSMUSC	Usp9x	ubiquitin s	GO:00325	developm	-	-	-	-	X:1307149	5184	6656	4611	5861	6933	6112	17.8495	18.30292	17.54701	15.56909	15.37223	16.556	17.89981	15.83244	0.074031	0.656595	-
ENSMUSC	Haspin	histone H	GO:00081	metabolic	-	-	-	-	11:731354	4	5	2	10	13	8	0.08692	0.086762	0.048033	0.167642	0.181905	0.136746	0.073905	0.162097	0.074064	0.656595	-
ENSMUSC	Olfr690	olfactory r	GO:00048	signal trar	K04257	olfactory r	mmu0474	Olfactory	7:1053282	3	0	4	0	2	0	0.035588	0	0.052448	0	0.015275	0	0.029345	0.005092	0.074075	0.656595	-
ENSMUSC	Tifa	TRAF-inte	GO:00508	response	-	-	-	-	3:1277898	77	109	20	114	156	142	0.91043	1.029266	0.261361	1.039906	1.187782	1.320855	0.733686	1.182848	0.074144	0.656869	-
ENSMUSC	Haao	3-hydroxy	GO:00081	metabolic	K00452	3-hydroxy	mmu0110	Metabolic	17:838313	2	6	0	0	2	0	0.099913	0.239421	0	0	0.064338	0	0.113111	0.021446	0.074772	0.659158	-
ENSMUSC	Ifi35	interferon	GO:00056	cell|intrac	-	-	-	-	11:101448	36	48	35	66	96	59	0.730994	0.778394	0.78547	1.033912	1.255263	0.942489	0.764952	1.077222	0.07468	0.659158	-
ENSMUSC	Gpx8	glutathior	GO:00081	metabolic	K00432	glutathior	mmu0059	Arachidor	13:113042	56	122	51	64	69	74	3.358812	5.843902	3.380776	2.961445	2.66503	3.491729	4.194497	3.039401	0.074681	0.659158	-
ENSMUSC	Slc7a4	solute car	GO:00511	localizatio	-	-	-	-	16:175720	668	854	619	683	1032	666	4.339947	4.431102	4.44473	3.423408	4.3176	3.404025	4.40526	3.715011	0.074675	0.659158	-
ENSMUSC	Pim1	proviral in	GO:00325	developm	K04702	proto-onc	mmu0520	Pathways	17:294908	32	69	24	69	99	68	0.740657	1.275458	0.613931	1.232112	1.475567	1.238197	0.876682	1.315292	0.074622	0.659158	-
ENSMUSC	AU040320	expressed	GO:00055	extracellul	-	-	-	-	4:1267535	1261	1601	1209	2149	2063	2219	9.452951	9.584943	10.01672	12.42851	9.958779	13.08641	9.68487	11.82456	0.07476	0.659158	-
ENSMUSC	Cotl1	coactosin	GO:00508	response	-	-	-	-	8:1198092	731	949	1060	1013	1242	593	11.57807	12.00413	18.55545	12.37824	12.66763	7.388986	14.04589	10.81162	0.074808	0.659158	-
ENSMUSC	Zfp518b	zinc finge	GO:00971	organic c	-	-	-	-	5:3866848	339	386	296	405	432	191	1.465186	1.332377	1.413943	1.35045	1.202352	0.649439	1.403835	1.067414	0.074819	0.659158	-
ENSMUSC	Gm4924	predicted	GO:00081	metabolic	-	-	-	-	10:823542	41	64	44	86	105	76	0.315961	0.393892	0.37476	0.511306	0.521071	0.460762	0.361538	0.497713	0.074522	0.659158	-
ENSMUSC	Eef1akmt	EEF1A lysi	GO:00081	metabolic	-	-	-	-	10:127031	0	6	0	0	0	0	0	0.107603	0	0	0	0	0.035868	0	0.074715	0.659158	-
ENSMUSC	Gm20532	predicted	-	-	-	-	-	-	2:2658663	0	6	0	0	0	0	0	0.375093	0	0	0	0	0.125031	0	0.074757	0.659158	-
ENSMUSC	Atp2c2	ATPase, C	GO:00325	developm	-	-	-	-	8:1197000	37	63	38	68	94	87	0.647143	0.879988	0.734546	0.917559	1.058709	1.19708	0.753892	1.057783	0.074897	0.659506	-
ENSMUSC	Fhl1	four and a	GO:00325	developm	K14365	four and a	mmu0463	Jak-STAT	X:5673178	545	620	522	755	861	1307	3.842129	3.490702	4.067176	4.106319	3.908709	7.24873	3.800002	5.087919	0.075063	0.659969	-
ENSMUSC	Sema4g	sema don	GO:00325	developm	K06521	semaphor	mmu0436	Axon guic	19:449891	459	561	459	754	945	710	2.868835	2.800283	3.170683	3.635758	3.803466	3.491102	2.9466	3.643442	0.075055	0.659969	-
ENSMUSC	Sft2d1	SFT2 dom	GO:00511	localizatio	-	-	-	-	17:831110	135	153	138	134	164	147	1.445602	1.30844	1.633214	1.107011	1.130878	1.238349	1.462419	1.158746	0.075026	0.659969	-
ENSMUSC	Dram2	DNA-dan	GO:00081	metabolic	-	-	-	-	3:1065477	269	371	285	439	628	466	2.559949	2.819674	2.997583	3.223104	3.848519	3.488796	2.792402	3.520139	0.075146	0.66015	-

ENSMUSC(	Rnf152	ring finge	GO:00081	metabolic	K15705	E3 ubiquit	mmu0415	mTOR sig	1:1052769	184	243	103	155	185	178	0.664853	0.701229	0.411332	0.432086	0.430462	0.505985	0.592471	0.456178	0.075159	0.66015	-
ENSMUSC(	Wfikkn1	WAP, FS,	GO:00325	developm	-	-	-	-	17:258776	0	6	0	5	6	9	0	0.112441	0	0.090512	0.090661	0.166141	0.03748	0.115772	0.075335	0.661355	-
ENSMUSC(	Slc16a8	solute car	GO:00511	localizatio	-	-	-	-	15:79251C	11	16	6	4	15	2	0.371906	0.432019	0.224209	0.10432	0.326563	0.053205	0.342711	0.161362	0.075381	0.661431	-
ENSMUSC(	Pde10a	phosphoc	GO:00081	metabolic	-	-	mmu0023	Purine me	17:852537	1816	2122	1502	859	2352	2174	4.357695	4.066607	3.983434	1.590247	3.634401	4.104038	4.135912	3.109562	0.075592	0.662938	-
ENSMUSC(	C1ql1	complem	GO:00325	developm	-	-	-	-	11:102939	74	126	85	152	186	155	3.067407	4.171164	3.894082	4.860862	4.964867	5.054564	3.710884	4.960098	0.075629	0.662938	-
ENSMUSC(	Jph2	junctophil	GO:00325	developm	-	-	-	-	2:1633362	16	24	23	48	75	22	0.19708	0.236089	0.313106	0.456133	0.594896	0.213188	0.248758	0.421406	0.07588	0.664461	-
ENSMUSC(	Ccdc73	coiled-co	-	-	-	-	-	-	2:1048678	10	10	2	6	4	2	0.114321	0.091295	0.025271	0.052916	0.029448	0.017991	0.076962	0.033452	0.075864	0.664461	-
ENSMUSC(	AL731706	-	-	-	-	-	-	-	2:1368812	35	183	34	62	75	35	1.901265	7.939136	2.041299	2.598375	2.623578	1.495731	3.960567	2.239228	0.075981	0.665014	-
ENSMUSC(	Net1	neuroepit	GO:00325	developm	-	-	-	-	13:388201	645	765	562	945	1701	792	5.041926	4.775775	4.855343	5.699002	8.562404	4.870492	4.891015	6.3773	0.076303	0.667502	-
ENSMUSC(	1700066M	RIKEN cDI	GO:00055	extracellul	-	-	-	-	1:5737762	371	441	344	394	470	407	5.8188	5.523866	5.962997	4.767447	4.746913	5.021859	5.768554	4.845406	0.076726	0.670525	-
ENSMUSC(	Spag16	sperm ass	GO:00325	developm	-	-	-	-	1:6982697	37	49	43	40	40	42	0.306661	0.324338	0.393883	0.255769	0.213483	0.273851	0.341628	0.247701	0.076706	0.670525	-
ENSMUSC(	Gm10479	predicted	GO:00081	metabolic	-	-	-	-	12:204159	0	0	0	2	2	2	0	0	0	0.296303	0.247234	0.302164	0	0.2819	0.076812	0.670942	-
ENSMUSC(	Zc3h12c	zinc finge	GO:00081	metabolic	-	-	-	-	9:5211134	319	409	304	312	401	399	1.67003	1.710024	1.758948	1.260139	1.351863	1.643299	1.713001	1.418433	0.077019	0.672067	-
ENSMUSC(	Plekhj1	pleckstrin	GO:00081	metabolic	-	-	-	-	10:807960	189	259	208	231	281	186	2.020308	2.211063	2.457341	1.905013	1.934264	1.564154	2.229571	1.801144	0.076982	0.672067	-
ENSMUSC(	Diablo	diablo, IAI	GO:00081	metabolic	K10522	diablo[-]	mmu0421	Apoptosis	5:1235097	197	259	175	283	595	272	1.116846	1.172664	1.096509	1.237784	2.172199	1.21313	1.128673	1.541038	0.077101	0.672115	-
ENSMUSC(	Rps21	ribosomal	GO:00081	metabolic	K02971	small subu	mmu0301	Ribosome	2:1802573	296	484	377	378	489	377	12.30308	16.06619	17.31846	12.12115	13.08835	12.32748	15.22924	12.51233	0.077066	0.672115	-
ENSMUSC(	Spata48	spermato	-	-	-	-	-	-	11:11462C	3	11	8	2	5	6	0.08681	0.254206	0.255843	0.044656	0.093171	0.136595	0.198953	0.091474	0.07726	0.673158	-
ENSMUSC(	Parp4	poly (ADP	GO:00081	metabolic	K10798	poly [ADP	mmu0421	Necroptos	14:565756	151	221	146	219	388	285	1.27662	1.492193	1.364223	1.428432	2.112382	1.895581	1.377678	1.812132	0.077541	0.674979	-
ENSMUSC(	Zfp804b	zinc finge	GO:00971	organic cy	-	-	-	-	5:676901C	27	51	24	33	23	31	0.204059	0.307822	0.200464	0.192414	0.111936	0.184314	0.237448	0.162888	0.077546	0.674979	-
ENSMUSC(	S100a11	S100 calci	GO:00099	cellular pr	-	-	-	-	3:9352048	27	52	32	41	94	86	3.226201	4.962158	4.225922	3.779479	7.232788	8.084077	4.138094	6.365448	0.077671	0.675728	-
ENSMUSC(	Tnk2	tyrosine k	GO:00081	metabolic	-	-	-	-	16:326438	1948	2789	1342	3437	3114	3087	11.72528	13.4069	8.927573	15.9604	12.07	14.61779	11.35325	14.21606	0.077821	0.676014	-
ENSMUSC(	Klc2	kinesin lig	GO:00038	catalytic a	K10407	kinesin lig	mmu0513	Salmonell	19:510774	3530	4270	3185	5975	5434	5820	27.83253	26.88758	27.75455	36.34513	27.59004	36.10039	27.49156	33.34519	0.077756	0.676014	-
ENSMUSC(	Slc22a12	solute car	GO:00081	metabolic	-	-	-	-	19:653584	0	4	1	4	9	4	0	0.068313	0.023638	0.06599	0.123948	0.067295	0.03065	0.085744	0.077797	0.676014	-
ENSMUSC(	Igfbp2	insulin-lik	GO:00325	developm	-	-	-	-	1:7282450	429	1057	402	498	764	562	9.719463	19.12517	10.06602	8.704516	11.14635	10.01687	12.97022	9.955911	0.078155	0.67858	-
ENSMUSC(	Plekhg2	pleckstrin	GO:00987	molecular	-	-	-	-	7:2835960	88	151	88	168	208	181	0.433911	0.59462	0.479564	0.639082	0.660444	0.702114	0.502699	0.667213	0.078264	0.679134	-
ENSMUSC(	Pcdhga5	protocadh	GO:00160	membran	-	-	-	-	18:376943	248	313	227	293	295	259	3.246652	3.272469	3.284416	2.959254	2.486916	2.667449	3.267846	2.70454	0.078297	0.679134	-
ENSMUSC(	D11Wsu4	DNA segr	GO:00055	extracellul	-	-	-	-	11:113684	123	172	109	120	174	124	1.978436	2.209487	1.937724	1.48912	1.802278	1.569098	2.041883	1.620165	0.07848	0.680039	-
ENSMUSC(	Zscan18	zinc finge	GO:00081	metabolic	-	-	-	-	7:1276809	345	431	330	470	323	344	3.200406	3.193073	3.383348	3.36367	1.92949	2.510468	3.258943	2.601209	0.078476	0.680039	-
ENSMUSC(	Tmc6	transmem	GO:00511	localizatio	-	-	-	-	11:117765	31	74	38	78	110	76	0.334059	0.636862	0.452577	0.648469	0.763332	0.644303	0.474499	0.685368	0.078914	0.682382	-
ENSMUSC(	Prickle3	prickle pla	GO:00325	developm	-	-	-	-	X:765726C	9	23	10	23	38	26	0.074967	0.153011	0.09206	0.147809	0.203834	0.170382	0.106679	0.174008	0.078946	0.682382	-
ENSMUSC(	Lrrc1	leucine ric	GO:00508	response	-	-	-	-	9:7743082	102	146	105	167	264	182	0.509403	0.582315	0.579556	0.643435	0.84902	0.71506	0.557091	0.735838	0.078897	0.682382	-
ENSMUSC(	9130230L	RIKEN cDI	-	-	-	-	-	-	5:6597935	0	0	0	3	1	2	0	0	0	0.014963	0.004162	0.010172	0	0.009765	0.078946	0.682382	-
ENSMUSC(	Hist1h3g	histone cl	GO:00081	metabolic	K11253	histone H:	mmu0503	Alcoholisr	13:235354	2	1	1	0	0	0	0.230365	0.092019	0.127334	0	0	0	0.149906	0	0.078908	0.682382	-
ENSMUSC(	Wfdc2	WAP four	GO:00081	metabolic	-	-	-	-	2:1645624	13	43	8	3	22	13	0.467741	1.23558	0.318107	0.083286	0.509737	0.367959	0.673809	0.320327	0.079019	0.682672	-
ENSMUSC(	Rpp40	ribonuclea	GO:00081	metabolic	K14530	ribonuclea	mmu0301	RNA trans	13:358934	50	75	51	80	127	109	0.391502	0.468996	0.441348	0.483259	0.640353	0.671426	0.433949	0.598346	0.079088	0.682709	-
ENSMUSC(	Ly6c2	lymphocy	GO:00160	membran	-	-	-	-	15:751081	10	8	8	16	25	19	0.140011	0.089455	0.123785	0.172827	0.225396	0.209274	0.11775	0.202499	0.079102	0.682709	-
ENSMUSC(	Gjd3	gap juncti	GO:00511	localizatio	-	-	-	-	11:989821	1	5	4	6	12	12	0.072971	0.291281	0.322518	0.337665	0.56373	0.688688	0.228923	0.530027	0.079265	0.683505	-
ENSMUSC(	Irak4	interleukir	GO:00081	metabolic	K04733	interleukir	mmu0401	MAPK sig	15:945436	37	45	27	65	78	57	0.417207	0.40523	0.336475	0.565441	0.566359	0.505628	0.386304	0.545809	0.079273	0.683505	-
ENSMUSC(	Adra2a	adrenergi	GO:00048	signal trar	K04138	adrenergi	mmu0408	Neuroacti	19:540451	266	331	196	460	469	376	4.272944	4.246386	3.479758	5.700775	4.851466	4.75165	3.999696	5.101297	0.079349	0.683823	-
ENSMUSC(	Cr2	complem	GO:00325	developm	K04012	complem	mmu0516	Epstein-B	1:1951368	7	5	6	10	16	21	0.037582	0.021437	0.035604	0.04142	0.055314	0.088695	0.031541	0.06181	0.079464	0.68448	-
ENSMUSC(	P4hb	prolyl 4-h	GO:00081	metabolic	K09580	protein di	mmu0414	Protein pr	11:12056C	1612	2144	1441	2432	3224	2451	26.06613	27.68738	25.75266	30.33928	33.57075	31.17924	26.50206	31.69643	0.079597	0.685077	-
ENSMUSC(	Maml2	mastermir	GO:00081	metabolic	K06061	mastermir	mmu0516	Human pa	9:1329795	568	795	475	488	818	669	3.422244	3.825389	3.163028	2.268364	3.173735	3.171024	3.47022	2.871041	0.079612	0.685077	-
ENSMUSC(	Trnt1	tRNA nucl	GO:00081	metabolic	K00974	tRNA nucl	mmu0301	RNA trans	6:1067691	445	437	371	470	526	371	4.952767	3.88432	4.563609	4.035668	3.769884	3.248423	4.466899	3.684658	0.079699	0.685483	-
ENSMUSC(	Plin1	perilipin 1	GO:00081	metabolic	K08768	perilipin-1	mmu0437	Apelin sig	7:7972021	0	5	7	12	19	7	0	0.038322	0.074255	0.088848	0.117424	0.052854	0.037525	0.086376	0.079872	0.686634	-
ENSMUSC(	Prkd2	protein kii	GO:00325	developm	K06070	protein kii	mmu0401	Rap1 sign	7:168429C	51	75	62	100	113	120	0.437783	0.514162	0.588207	0.662245	0.624632	0.810371	0.513384	0.699083	0.080107	0.68831	-
ENSMUSC(	Pla2g7	phospholi	GO:00081	metabolic	K01062	platelet-a	mmu011C	Metabolic	17:43568C	2351	2647	2126	2722	3065	2401	37.48959	33.70992	37.46865	33.48701	31.47336	30.12042	36.22272	31.6936	0.080465	0.691048	-
ENSMUSC(	Pgf	placental	GO:00325	developm	K16859	placenta g	mmu052C	Pathways	12:851666	40	65	50	86	112	82	0.512882	0.665598	0.708556	0.850709	0.924758	0.827139	0.629012	0.867535	0.080803	0.691865	-
ENSMUSC(	Tcf3	transcripti	GO:00325	developm	K09063	transcripti	mmu0516	HTLV-I int	10:804095	341	440	319	582	654	541	2.205363	2.272603	2.280144	2.903879	2.723691	2.752541	2.252703	2.79337	0.080667	0.691865	-
ENSMUSC(	Jade1	jade famil	GO:00081	metabolic	-	-	-	-	3:4155573	604	791	523	600	984	460	1.941211	2.030288	1.857737	1.487706	2.036505	1.163064	1.943079	1.562425	0.080604	0.691865	-
ENSMUSC(	Ptbp3	polypyrim	GO:00325	developm	-	-	-	-	4:5947186	655	823	561	933	1383	972	2.118614	2.125964	2.005488	2.328212	2.880629	2.473362	2.083355	2.560734	0.080803	0.691865	-
ENSMUSC(	Dusp4	dual speci	GO:00325	developm	K04459	dual speci	mmu0401	MAPK sig	8:3480729	368	429	338	350	478	418	8.200496	7.634768	8.324462	6.017161	6.859232	7.327913	8.053242	6.734769	0.080998	0.691865	-
ENSMUSC(	Arhgap36	Rho GTPa	GO:00987	molecular	-	-	-	-	X:4946394	30	24	16	41	48	48	0.360795	0.230508	0.21267	0.380404	0.371733	0.454142	0.267991	0.402093	0.080854	0.691865	-
ENSMUSC(	Uvssa	UV stimul	GO:00081	metabolic	-	-	-	-	5:3337854	232	224	220	244	246	231	1.503445	1.159295	1.575686	1.219889	1.026574	1.177668	1.412808	1.141377	0.080954	0.691865	-
ENSMUSC(	Lrrc17	leucine ric	GO:00325	developm	-	-	-	-	5:2154355	3	0	3	4	17	5	0.048651	0	0.053774	0.05004	0.177538	0.063791	0.034142	0.097123	0.080726	0.691865	-
ENSMUSC(	Vwc2	von Willet	GO:00325	developm	-	-	-	-	11:111142	72	62	61	74	63	52	0.595449	0.409497	0.557555	0.472142	0.335509	0.338318	0.520834	0.38199	0.080737	0.691865	-
ENSMUSC(	Zfp870	zinc finge	GO:00081	metabolic	-	-	-	-	17:328792	226	142	170	95	139	246	1.286511	0.645567	1.069548	0.417214	0.509535	1.10167	1.000542	0.676139	0.080993	0.691865	-
ENSMUSC(	H2al1m	H2A histo	GO:00081	metabolic	K11251	histone H:	mmu0503	Alcoholisr	X:9283764	0	0	0	1	4	1	0	0	0	0.08806	0.293982	0.089802	0	0.157281	0.080939	0.691865	-
ENSMUSC(	Phox2b	paired-lik	GO:00325	developm	-	-	-	-	5:6709439	0	0	0	0	7	0	0	0	0	0	0.065683	0	0	0.021894	0.081771	0.693017	-
ENSMUSC(	Dcdc2c	doublecoi	GO:00508	response	-	-	-	-	12:284377	0	0	0	0	7	0	0	0	0	0	0.052915	0	0	0.017638	0.081675	0.693017	-
ENSMUSC(	1700011H	RIKEN cDI	-	-	-	-	-	-	14:492195	0	0	0	0	7	0	0	0	0	0	0.049704	0	0	0.016568	0.081719	0.693017	-
ENSMUSC(	Ribc2	RIB43A dc	-	-	-	-	-	-	15:85132C	0	5	3	12	6	7	0	0.14758	0.122555	0.342162	0.142797	0.203547	0.090045	0.229502	0.081783	0.693017	-
ENSMUSC(	Oca2	oculocuta	GO:00325	developm	-	-	-	-	7:5623976	17	60	14	8	31	22	0.178504	0.503143	0.162468	0.064806	0.209612	0.181733	0.281372	0.15205	0.081772	0.693017	-
ENSMUSC(	Trim66	tripartite r	GO:00081	metabolic	-	-	-	-	7:1094490	286	501	275	352	482	266	1.585063	2.217499	1.684453	1.505056	1.720212	1.159771	1.829005	1.461679	0.081367	0.693017	-
ENSMUSC(	Bmp5	bone mor	GO:00325	developm	K04663	bone mor	mmu0439	Hippo sig	9:7577536	26	57	29	47	92	76	0.376723	0.659579	0.464401	0.525388	0.858399	0.866308	0.500234	0.750032	0.081384	0.693017	-
ENSMUSC(	Rnf43	ring finge	GO:00081	metabolic	-	-	-	-	11:876627	23	58	26	69	75	57	0.126998	0.255764	0.158666	0.293933	0.266676	0.247601	0.180476	0.269403	0.081765	0.693017	-
ENSMUSC(	Fzd4	frizzled cl	GO:00325	developm	K02354	frizzled 4[	mmu052C	Pathways	7:8940435	319	481	232	321	396	356	2.898447	3.490317	2.329745	2.250143	2.316991	2.544693	2.906169	2.370609	0.081332	0.693017	-
ENSMUSC(	Zfp952	zinc finge	GO:00081	metabolic	-	-	-	-	17:329931	264	356	270	114	403	317	3.34635	3.603814	3.78249	1.114817	3.289473	3.161087	3.577551	2.521792	0.081219	0.693017	-
ENSMUSC(	Cyct	cytochron	GO:00081	metabolic	K08738	cytochron	mmu011C	Metabolic	2:7635394	0	0	0	0	0	6	0	0	0	0	0	0.440024	0	0.146675	0.08181	0.693017	-
ENSMUSC(	Maml3	mastermir	GO:00081	metabolic	K06061	mastermir	mmu0516	Human pa	3:516859C	249	299	264	237	347	292	1.431049	1.372375	1.676895	1.050834	1.284219	1.320228	1.493439	1.218427	0.081314	0.693017	-
ENSMUSC(	Krt79	keratin 79	GO:00051	structural	-	-	-	-	15:101929	2	4	2	11	6	7	0.058223	0.093005	0.064365	0.247108	0.112495	0.160353	0.071864	0.173319	0.081562	0.693017	-
ENSMUSC(	Aox1	aldehyde	GO:00081	metabolic	K00157	aldehyde	mmu011C	Metabolic	1:5802993	117	146	124	188	239	239	0.74867	0.74611	0.876948	0.928097	0.984817	1.203128	0.790576	1.038681	0.081542	0.693017	-
ENSMUSC(	Olfr658	olfactory r	GO:00048	signal trar	K04257	olfactory r	mmu0474	Olfactory	7:1046428	0	0	0	0	0	6	0	0	0	0	0	0.052884	0	0.017628	0.081759	0.693017	-
ENSMUSC(	Gm5127	predicted	GO:00160	membran	-	-	-	-	X:1065831	4	7	5	3	0	5	0.083162	0.116222	0.114891	0.048123	0	0.081775	0.104758	0.043299	0.081373	0.693017	-
ENSMUSC(	Tldc2	TBC/LysM	GO:00508	response	-	-	-	-	2:157087C	0	0	0	0	7	0	0	0	0	0	0.297848	0	0	0.099283	0.081626	0.693017	-
ENSMUSC(	Ptgds	prostaglaı	GO:00081	metabolic	K01830	prostaglaı	mmu011C	Metabolic	2:254667C	3223	5727	3131	5354	6197	7850	74.26048	105.383	79.73103	95.17134	91.94617	142.2912	86.45816	109.8029	0.082162	0.695414	-
ENSMUSC(	Draxin	dorsal inh	GO:00325	developm	-	-	-	-	4:1480984	17	41	22	50	48	54	0.199616	0.384478	0.285509	0.452938	0.36294	0.498816	0.289868	0.438231	0.08238	0.695414	-
ENSMUSC(	Gdf3	growth di	GO:00325	developm	-	-	-	-	6:1226054	2	3	0	0	0	0	0.047842	0.057332	0	0	0	0	0.035058	0	0.082533	0.695414	-
ENSMUSC(	Zfp719	zinc finge	GO:00081	metabolic	-	-	-	-	7:4357961	344	508	362	397	493	433	3.796818	4.477861	4.415867	3.380506	3.503989	3.759761	4.230182	3.548085	0.082405	0.695414	-
ENSMUSC(	Cbln1	cerebellin	GO:00325	developm	-	-	-	-	8:874684C	97	118	78	116	110	62	0.955109	0.927917	0.848838	0.881189	0.697474	0.480269	0.910621	0.686311	0.082531	0.695414	-
ENSMUSC(	Mif	macropha	GO:00325	developm	K07253	phenylpyr	mmu0035	Tyrosine r	10:758593	541	585	489	613	707	464	28.87451	24.93554	28.84517	25.24109	24.29916	19.48258	27.55174	23.00761	0.082508	0.695414	-
ENSMUSC(	Card11	caspase re	GO:00325	developm	K07367	caspase re	mmu0466	T cell rece	5:1408729	6	0	2	2	26	11	0.063403	0	0.02336	0.016307	0.176924	0.091448	0.028921	0.094893	0.08252	0.695414	-
ENSMUSC(	Mustn1	musculosl	GO:00325	developm	-	-	-	-	14:308792	13	34	17	22	60	57	0.642198	1.341379	0.928163	0.838451	1.908681	2.215192	0.97058	1.654108	0.082183	0.695414	-
ENSMUSC(	Mc2r	melanoco	GO:00325	developm	K04200	melanoco	mmu0408	Neuroacti	18:684069	2	3	0	0	0	0	0.078165	0.093669	0	0	0	0	0.057278	0	0.082517	0.695414	-
ENSMUSC(	Rpl7a-ps5	ribosomal	-	-	-	-	-	-	17:578384	4	4	3	6	12	13	0.30266	0.241676	0.250881	0.350217	0.584686	0.773763	0.265072	0.569556	0.082458	0.695414	-
ENSMUSC(	Foxd1	forkhead	GO:00325	developm	-	-	-	-	13:983542	9	6	8	13	17	28	0.108513	0.057775	0.106601	0.120925	0.131997	0.265598	0.090963	0.17284	0.082454	0.695414	-
ENSMUSC(	Prr13	proline ric	GO:00081	metabolic	-	-	-	-	15:102459	457	636	410	735	990	693	7.251444	8.059562	7.190163	8.997579	10.11575	8.650722	7.50039	9.254684	0.082935	0.695981	-
ENSMUSC(	Lhx4	LIM home	GO:00325	developm	-	-	-	-	1:155698C	0	3	2	0	0	0	0	0.024748	0.02283	0	0	0	0.01586	0	0.08291	0.695981	-
ENSMUSC(	Samhd1	SAM dom	GO:00081	metabolic	-	-	-	-	2:1570975	327	431	337	510	753	544	1.724775	1.81555	1.964542	2.075322	2.557611	2.257327	1.834956	2.296753	0.082919	0.695981	-
ENSMUSC(	Slc10a4	solute car	GO:00511	localizatio	-	-	-	-	5:7300688	3	10	0	9	11	16	0.083525	0.222346	0	0.193325	0.197215	0.35046	0.101957	0.247	0.082676	0.695981	-
ENSMUSC(	Epgn	epithelial	GO:00325	developm	-	-	-	-	5:9102746	0	3	2	0	0	0	0	0.066839	0.06166	0	0	0	0.042833	0	0.082934	0.695981	-
ENSMUSC(	Trim56	tripartite r	GO:00056	cell|organ	-	-	-	-	5:1371056	347	476	280	494	757	558	2.088229	2.287712	1.862316	2.293537	2.933588	2.641759	2.079419	2.622962	0.082792	0.695981	-
ENSMUSC(	Gm28047	predicted	GO:00081	metabolic	-	-	-	-	15:102523	0	0	1	13	0	0	0	0	0.041891	0.380114	0	0	0.013964	0.126705	0.082936	0.695981	-
ENSMUSC(	Gm10308	predicted	-	-	-	-	-	-	17:910884	3	4	3	2	0	1	0.24686	0.262847	0.272858	0.126987	0	0.064749	0.260855	0.063912	0.082854	0.695981	-
ENSMUSC(	Gm4881	predicted	GO:00081	metabolic	-	-	-	-	7:2492744	0	3	2	0	0	0	0	0.127006	0.117165	0	0	0	0.08139	0	0.082961	0.695981	-
ENSMUSC(	Apba3	amyloid b	GO:00325	developm	-	-	-	-	10:812669	199	250	186	160	323	179	1.82004	1.82605	1.880121	1.128954	1.902322	1.287925	1.84207	1.439734	0.083383	0.696954	-
ENSMUSC(	Fer1l4	fer-1-like	GO:00160	membran	-	-	-	-	2:1560191	0	0	0	0	3	3	0	0	0	0	0.01281	0.015646	0	0.009485	0.083247	0.696954	-
ENSMUSC(	Glrb	glycine re	GO:00325	developm	K05196	glycine re	mmu0408	Neuroacti	3:8084359	2154	2947	2283	2492	3540	2416	22.57065	24.66177	26.43936	20.14543	23.88671	19.91619	24.55726	21.31611	0.083275	0.696954	-
ENSMUSC(	Piwil4	piwi-like F	GO:00325	developm	-	-	-	-	9:1469623	0	0	0	0	3	3	0	0	0	0	0.017137	0.02093	0	0.012689	0.083249	0.696954	-
ENSMUSC(	Tlr8	toll-like re	GO:00081	metabolic	K10170	toll-like re	mmu0462	Toll-like re	X:1672426	10	15	2	3	8	5	0.07818	0.093656	0.017282	0.018097	0.040277	0.030752	0.06304	0.029708	0.083327	0.696954	-
ENSMUSC(	Actl10	actin-like	-	-	-	-	-	-	2:1545517	0	0	0	2	0	3	0	0	0	0.062774	0	0.095992	0	0.052922	0.083397	0.696954	-
ENSMUSC(	Olfr5	olfactory r	GO:00048	signal trar	K04257	olfactory r	mmu0474	Olfactory	7:6478322	0	0	0	3	0	2	0	0	0	0.035123	0	0.023879	0	0.019667	0.083318	0.696954	-
ENSMUSC(	Gm36028	predicted	-	-	-	-	-	-	16:378509	0	0	0	2	0	3	0	0	0	0.053264	0	0.081449	0	0.044904	0.083382	0.696954	-
ENSMUSC(	Tctex1d2	Tctex1 do	GO:00511	localizatio	-	-	-	-	16:324197	60	64	59	124	124	85	1.154208	0.983241	1.254398	1.840296	1.536074	1.286364	1.130616	1.554245	0.083797	0.698701	-
ENSMUSC(	Lypd2	Ly6/Plaur	GO:00099	cellular pr	-	-	-	-	15:747322	0	0	0	2	0	3	0	0	0	0.106228	0	0.162439	0	0.089556	0.083707	0.698701	-
ENSMUSC(	Rtp4	receptor t	GO:00511	localizatio	-	-	-	-	16:235202	60	58	31	115	87	77	0.848023	0.654684	0.48425	1.253967	0.79183	0.856172	0.662319	0.967323	0.083661	0.698701	-
ENSMUSC(	Col10a1	collagen,	GO:00325	developm	K19479	collagen,	mmu0497	Protein di	10:343899	4	4	2	2	2	0	0.078034	0.062311	0.043123	0.030104	0.025118	0	0.061156	0.018407	0.083807	0.698701	-
ENSMUSC(	H2-DMb1	histocomp	GO:00023	immune s	K06752	major hist	mmu0516	HTLV-I int	17:34153C	23	31	22	38	69	42	0.84701	0.911721	0.895443	1.079629	1.636309	1.216805	0.884725	1.310914	0.083772	0.698701	-
ENSMUSC(	Ifit1bl2	interferon	GO:00023	immune s	-	-	-	-	19:34617C	24	27	14	38	59	33	0.22264	0.200031	0.143535	0.271958	0.352443	0.240833	0.188735	0.288411	0.083928	0.699373	-
ENSMUSC(	Nek7	NIMA (ne	GO:00081	metabolic	K20876	NIMA (ne	mmu0462	NOD-like	1:1384828	277	753	565	689	369	364	1.205239	2.616578	2.716992	2.312824	1.033889	1.245962	2.179603	1.530892	0.084006	0.699689	-
ENSMUSC(	Gbx2	gastrulatic	GO:00325	developm	-	-	-	-	1:8992795	3	2	0	0	0	0	0.054596	0.029073	0	0	0	0	0.02789	0	0.084152	0.700564	-
ENSMUSC(	Slc2a2	solute car	GO:00325	developm	K07593	MFS trans	mmu0493	Insulin res	3:286979C	0	0	0	0	4	2	0	0	0	0	0.022065	0.01348	0	0.011849	0.084267	0.701014	-
ENSMUSC(	Als2cr12	amyotrop	GO:00990	supramole	-	-	-	-	1:586469C	0	0	0	0	4	2	0	0	0	0	0.035288	0.021559	0	0.018949	0.084286	0.701014	-
ENSMUSC(	Vipas39	VPS33B in	GO:00325	developm	-	-	-	-	12:872388	828	1213	911	832	1529	862	7.965351	9.319259	9.685899	6.174877	9.4719	6.523684	8.99017	7.390154	0.084434	0.70124	-
ENSMUSC(	Il21r	interleukir	GO:00048	signal trar	K05075	interleukir	mmu0406	Cytokine-	7:1256034	33	39	28	24	42	27	0.396016	0.373771	0.371364	0.222199	0.324565	0.254903	0.380384	0.267222	0.084408	0.70124	-
ENSMUSC(	Cited4	Cbp/p300	GO:00325	developm	-	-	-	-	4:1206665	12	13	9	23	20	31	0.586613	0.507539	0.486241	0.867444	0.629594	1.192213	0.526798	0.896417	0.084401	0.70124	-
ENSMUSC(	Cd300e	CD300E n	GO:00023	immune s	-	-	-	-	11:115051	3	5	2	2	0	1	0.087725	0.116764	0.064643	0.045127	0	0.02301	0.08971	0.022712	0.084632	0.702548	-
ENSMUSC(	Gli1	GLI-Krupp	GO:00325	developm	K16797	zinc finge	mmu052C	Pathways	10:127329	36	31	19	38	63	72	0.368994	0.253758	0.21524	0.300491	0.415823	0.580576	0.279331	0.432297	0.084795	0.703309	-
ENSMUSC(	Tmem269	transmem	GO:00081	metabolic	-	-	-	-	4:119205C	5	12	10	12	25	25	0.139199	0.266824	0.307707	0.257752	0.448223	0.547587	0.23791	0.417854	0.084845	0.703309	-
ENSMUSC(	Noc4l	NOC4 like	GO:00081	metabolic	-	-	-	-	5:1106484	247	331	279	292	353	289	3.883947	4.156712	4.848717	3.542325	3.574413	3.575074	4.296459	3.563938	0.084825	0.703309	-
ENSMUSC(	Epha7	Eph recep	GO:00325	developm	K05108	EphA7[2.7	mmu0436	Axon guic	4:2881313	2545	3444	2543	3106	3947	2731	12.82861	13.86439	14.16723	12.07879	12.8119	10.8299	13.62007	11.90686	0.084918	0.70358	-
ENSMUSC(	Sell	selectin, ly	GO:00400	locomotic	K06495	selectin, ly	mmu0451	Cell adhes	1:1640619	0	4	4	3	12	12	0	0.026018	0.036012	0.018855	0.062946	0.076899	0.020677	0.0529	0.084968	0.703652	-
ENSMUSC(	Lypla2	lysophosp	GO:00081	metabolic	K06130	lysophosp	mmu0056	Glyceroph	4:1359682	619	893	731	829	903	763	11.26527	12.97921	14.7033	11.63954	10.58263	10.92412	12.98259	11.04876	0.085008	0.703652	-
ENSMUSC(	Zfp809	zinc finge	GO:00081	metabolic	-	-	-	-	9:2222571	129	277	125	246	362	295	1.232627	2.11382	1.320082	1.813462	2.227436	2.217563	1.55551	2.086154	0.085283	0.705591	-
ENSMUSC(	Lama3	laminin, a	GO:00325	developm	K06240	laminin, a	mmu011C	Metabolic	18:123338	75	120	89	149	176	163	0.426186	0.544584	0.558951	0.653212	0.644027	0.728676	0.509907	0.675305	0.085369	0.705965	-
ENSMUSC(	B4galt1	UDP-Gal:I	GO:00325	developm	K07966	beta-1,4-	mmu011C	Metabolic	4:408046C	117	225	123	207	361	238	1.687247	2.59132	1.960404	2.302996	3.352387	2.700107	2.079657	2.785163	0.085425	0.706095	-
ENSMUSC(	Gm609	predicted	GO:00099	cellular pr	-	-	-	-	16:454117	2	8	6	14	24	7	0.026279	0.083968	0.087155	0.141945	0.203109	0.072376	0.065801	0.139143	0.085523	0.706574	-
ENSMUSC(	Trib1	tribbles ps	GO:00325	developm	-	-	-	-	15:596483	147	212	142	217	320	309	1.211114	1.394917	1.293006	1.379296	1.697743	2.002793	1.299679	1.693277	0.085584	0.706742	-
ENSMUSC(	Gm5464	predicted	-	-	-	-	-	-	14:668688	3	5	6	2	2	3	0.036415	0.04847	0.080501	0.018732	0.01563	0.028645	0.055129	0.021003	0.085659	0.707022	-
ENSMUSC(	Ap1g2	adaptor p	GO:00511	localizatio	K12391	AP-1 com	mmu0414	Lysosome	14:550985	73	104	94	129	207	150	0.652785	0.742725	0.929022	0.889962	1.191998	1.055243	0.774844	1.045734	0.085725	0.707234	-
ENSMUSC(	Nfe2l2	nuclear fa	GO:00325	developm	K05638	nuclear fa	mmu052C	Pathways	2:7567551	342	526	289	561	788	538	5.324285	6.539839	4.972557	6.737968	7.899807	6.589139	5.612227	7.075638	0.085892	0.708057	-
ENSMUSC(	Ncald	neurocalc	GO:00508	response	K19695	neurocalc	mmu0474	Olfactory	15:373661	9209	11332	8665	10346	11538	11590	35.34377	34.73384	36.75495	30.63397	28.51581	34.9941	35.61086	31.38129	0.085971	0.708057	-
ENSMUSC(	Gbx1	gastrulatic	GO:00325	developm	-	-	-	-	5:2450275	4	5	0	0	3	0	0.070287	0.070159	0	0	0.033948	0	0.046815	0.011316	0.085988	0.708057	-
ENSMUSC(	Zfp429	zinc finge	GO:00081	metabolic	-	-	-	-	13:673879	27	14	25	53	50	39	0.374084	0.154909	0.382809	0.56651	0.446097	0.425081	0.303934	0.479229	0.085944	0.708057	-
ENSMUSC(	1110065P	RIKEN cDI	-	-	-	-	-	-	4:124849C	86	171	108	186	185	277	1.156601	1.836662	1.605312	1.929884	1.602189	2.930745	1.532858	2.154273	0.086038	0.70813	-
ENSMUSC(	Lfng	LFNG O-f	GO:00325	developm	K05948	fringe[2.4.	mmu0516	Human pa	5:1406073	185	240	188	302	353	361	3.780373	3.916701	4.245871	4.761019	4.645062	5.803382	3.980982	5.069821	0.086136	0.708504	-
ENSMUSC(	Fam149b	family wit	-	-	-	-	-	-	14:203481	478	745	476	820	1058	821	2.144749	2.66963	2.360495	2.838528	3.056957	2.898031	2.391625	2.931172	0.086204	0.708504	-
ENSMUSC(	Nuggc	nuclear G	GO:00325	developm	-	-	-	-	14:655985	0	0	1	2	8	0	0	0	0.0217	0.030297	0.101145	0	0.007233	0.043814	0.086205	0.708504	-
ENSMUSC(	Kera	keratocan	GO:00325	developm	-	-	-	-	10:976068	0	0	0	4	2	0	0	0	0	0.082632	0.034483	0	0	0.039038	0.086861	0.71033	-
ENSMUSC(	Hk3	hexokinas	GO:00081	metabolic	K00844	hexokinas	mmu011C	Metabolic	13:550059	38	49	29	38	42	30	0.380048	0.391384	0.320555	0.293206	0.270497	0.236041	0.363996	0.266582	0.086743	0.71033	-
ENSMUSC(	Trim63	tripartite r	GO:00081	metabolic	-	-	-	-	4:1343151	0	0	0	0	2	4	0	0	0	0	0.015899	0.038853	0	0.018251	0.086892	0.71033	-
ENSMUSC(	Pkd1l2	polycystic	GO:00160	membran	-	-	-	-	8:1169956	0	0	0	4	2	0	0	0	0	0.020215	0.008436	0	0	0.00955	0.086833	0.71033	-
ENSMUSC(	Slc43a2	solute car	GO:00511	localizatio	-	-	-	-	11:755316	310	1041	923	1353	1668	1284	1.044015	2.799896	3.435531	3.515392	3.617398	3.401899	2.426481	3.511563	0.086862	0.71033	-
ENSMUSC(	Ankrd42	ankyrin re	GO:00081	metabolic	-	-	-	-	7:9258172	276	248	157	236	269	204	1.446528	1.03804	0.909417	0.954245	0.907872	0.84112	1.131328	0.901079	0.086877	0.71033	-
ENSMUSC(	Dmrt2	doublesex	GO:00325	developm	-	-	-	-	19:256724	23	27	18	18	21	21	0.598356	0.560972	0.517553	0.36128	0.3518	0.429796	0.55896	0.380958	0.086522	0.71033	-
ENSMUSC(	Chrna10	cholinergi	GO:00325	developm	K04811	nicotinic a	mmu0408	Neuroacti	7:1021112	0	0	0	0	2	4	0	0	0	0	0.027204	0.06648	0	0.031228	0.086917	0.71033	-
ENSMUSC(	Alg6	asparagin	GO:00081	metabolic	K03848	alpha-1,3	mmu011C	Metabolic	4:9971566	83	98	94	222	171	117	0.528061	0.497942	0.660974	1.089661	0.700581	0.585604	0.562326	0.791948	0.086701	0.71033	-
ENSMUSC(	Gm14401	predicted	GO:00081	metabolic	-	-	-	-	2:1770743	0	0	0	4	2	0	0	0	0	0.084599	0.035303	0	0	0.039967	0.086818	0.71033	-
ENSMUSC(	Gm28053	predicted	-	-	-	-	-	-	6:487186C	0	2	0	0	7	5	0	0.085188	0	0	0.240359	0.209757	0.028396	0.150039	0.08675	0.71033	-
ENSMUSC(	Hist1h3d	histone cl	GO:00081	metabolic	K11253	histone H:	mmu0503	Alcoholisr	13:235757	12	8	7	4	12	3	0.586613	0.31234	0.378229	0.150842	0.377776	0.115359	0.425728	0.214659	0.086531	0.71033	-
ENSMUSC(	Tnip1	TNFAIP3 i	GO:00081	metabolic	-	-	-	-	11:549107	467	524	400	652	946	675	3.695457	3.311529	3.498308	3.980423	4.820556	4.202097	3.501764	4.334359	0.087219	0.71246	-
ENSMUSC(	Plcd1	phospholi	GO:00325	developm	K05857	phosphati	mmu011C	Metabolic	9:1190715	138	174	111	225	257	221	2.323771	2.339976	2.065777	2.923003	2.786782	2.927647	2.243175	2.879144	0.087448	0.712566	-
ENSMUSC(	Pla2g10	phospholi	GO:00325	developm	-	-	mmu011C	Metabolic	16:13715C	6	0	0	4	18	6	0.128766	0	0	0.066222	0.248767	0.101306	0.042922	0.138765	0.087473	0.712566	-
ENSMUSC(	Zfp12	zinc finge	GO:00081	metabolic	-	-	-	-	5:1432351	184	204	561	180	238	386	0.760899	0.67373	2.564009	0.574267	0.633784	1.255765	1.332879	0.821272	0.087478	0.712566	-
ENSMUSC(	Pbx3	pre B cell	GO:00325	developm	K15610	pre-B-cel	mmu052C	Transcript	2:3417145	96	139	84	127	100	89	0.657346	0.760126	0.635695	0.670902	0.440939	0.479432	0.684389	0.530425	0.087416	0.712566	-
ENSMUSC(	Defb11	defensin b	GO:00508	response	-	-	-	-	8:2190537	1	8	2	0	0	3	0.19267	1.230641	0.425785	0	0	0.454523	0.616365	0.151508	0.087361	0.712566	-
ENSMUSC(	A730009L	RIKEN cDI	-	-	-	-	-	-	16:845151	8	19	10	8	13	7	0.180056	0.3415	0.248729	0.138905	0.188408	0.123944	0.256762	0.150419	0.087392	0.712566	-
ENSMUSC(	Deptor	DEP dom	GO:00081	metabolic	K20402	DEP dom	mmu0415	mTOR sig	15:551123	335	397	207	480	624	450	1.763166	1.668723	1.204107	1.949034	2.114888	1.863249	1.545332	1.975724	0.087688	0.713699	-
ENSMUSC(	Kel	Kell blood	GO:00325	developm	-	-	-	-	6:4168633	3	0	1	0	0	0	0.050005	0	0.018424	0	0	0	0.02281	0	0.087699	0.713699	-
ENSMUSC(	Mlf1	myeloid le	GO:00325	developm	K15622	myeloid le	mmu052C	Transcript	3:6737409	16	45	17	14	26	22	0.387362	0.870071	0.454881	0.261489	0.405339	0.419023	0.570771	0.36195	0.08774	0.713699	-
ENSMUSC(	Pcdh11x	protocadh	GO:00081	metabolic	-	-	-	-	X:1202902	85	199	112	235	242	204	0.205404	0.384051	0.299125	0.438114	0.376582	0.38782	0.296194	0.400839	0.087809	0.713923	-
ENSMUSC(	Pml	promyelo	GO:00325	developm	K10054	probable	mmu052C	Pathways	9:5821807	137	293	94	290	333	280	0.535393	0.914461	0.406001	0.874339	0.838011	0.860837	0.618618	0.857729	0.087872	0.714102	-
ENSMUSC(	Dscc1	DNA repli	GO:00081	metabolic	-	-	-	-	15:55076C	0	3	0	4	4	5	0	0.028067	0	0.036141	0.030171	0.046072	0.009356	0.037461	0.087942	0.714212	-
ENSMUSC(	Pcp4l1	Purkinje c	-	-	-	-	-	-	1:1711732	813	1392	839	1059	1287	1005	31.47764	43.04242	35.90214	31.63284	32.08817	30.61177	36.8074	31.44426	0.087967	0.714212	-
ENSMUSC(	Cxadr	coxsackie	GO:00325	developm	K06788	coxsackie	mmu0541	Viral myo	16:783014	368	435	360	571	735	590	3.398269	3.208082	3.674179	4.067965	4.370715	4.286208	3.426843	4.241629	0.088057	0.714305	-
ENSMUSC(	Rrh	retinal pig	GO:00048	signal trar	-	-	-	-	3:1298044	13	6	6	3	3	9	0.099605	0.036714	0.050812	0.017736	0.014804	0.054248	0.062377	0.028929	0.08817	0.714305	-

ENSMUSC	Smc2	structural	GO:00329	macromo	-	-	-	-	4:5243924	369	461	286	414	441	356	3.752571	3.744115	3.21452	3.24813	2.887991	2.848157	3.570402	2.994759	0.088142	0.714305	-
ENSMUSC	C1qc	complem	GO:00325	developm	K03988	compleme	mmu0532	Systemic l	4:1368898	378	559	386	503	578	336	10.73985	12.68424	12.12108	11.02568	10.57522	7.510306	11.84839	9.703735	0.088225	0.714305	-
ENSMUSC	Dxo	decapping	GO:00160	membran	-	-	-	-	17:348370	114	171	72	196	228	186	1.283303	1.537324	0.895782	1.702194	1.652772	1.647206	1.238803	1.667391	0.08819	0.714305	-
ENSMUSC	Rps12-ps	ribosomal	GO:00081	metabolic	-	-	-	-	19:593222	610	1021	764	1054	1294	1630	75.39567	100.7831	104.3654	100.5049	102.9924	158.4948	93.51472	120.664	0.088169	0.714305	-
ENSMUSC	Mtmr11	myotubul	GO:00055	extracellul	-	-	-	-	3:9616200	76	95	39	102	196	99	0.713585	0.712362	0.404704	0.738855	1.185058	0.731269	0.610217	0.88506	0.088275	0.714376	-
ENSMUSC	Zkscan17	zinc finge	GO:00081	metabolic	-	-	-	-	11:594855	125	195	134	210	307	246	1.399897	1.744082	1.658591	1.814421	2.214017	2.167371	1.600857	2.06527	0.088738	0.715505	-
ENSMUSC	Lpin3	lipin 3 [So	GO:00081	metabolic	K15728	phosphati	mmu011C	Metabolic	2:1608806	25	25	23	39	89	32	0.284411	0.22714	0.289187	0.342302	0.652015	0.286402	0.266913	0.426906	0.088587	0.715505	-
ENSMUSC	Tacr3	tachykinin	GO:00325	developm	K04224	tachykinin	mmu0408	Neuroacti	3:1348290	67	67	30	113	98	91	1.08742	0.868453	0.538137	1.414929	1.024252	1.161918	0.831337	1.200366	0.088679	0.715505	-
ENSMUSC	Tesk2	testis-spe	GO:00048	signal trar	-	-	-	-	4:1167209	86	90	72	141	146	143	0.800697	0.669204	0.740885	1.012795	0.875347	1.047414	0.736928	0.978519	0.088705	0.715505	-
ENSMUSC	Tgfbi	transform	GO:00325	developm	-	-	-	-	13:566095	72	115	68	128	198	121	0.781824	0.997288	0.816072	1.072301	1.384508	1.033641	0.865061	1.163484	0.088635	0.715505	-
ENSMUSC	Ppp1r26	protein pł	GO:00081	metabolic	-	-	-	-	2:2844680	294	481	348	577	174	123	1.498045	1.95735	1.959762	2.268214	0.570929	0.493053	1.805052	1.110732	0.088458	0.715505	-
ENSMUSC	Krt80	keratin 80	GO:00051	structural	-	-	-	-	15:101349	0	5	8	11	11	16	0	0.137431	0.3043	0.2921	0.243795	0.433235	0.147244	0.323043	0.088561	0.715505	-
ENSMUSC	Hipk2	homeodo	GO:00325	developm	K08826	homeodo	mmu0421	Cellular se	6:3869439	1754	2327	1524	2087	2216	2065	8.202814	8.691115	7.877077	7.529843	6.673562	7.597385	8.257002	7.26693	0.088744	0.715505	-
ENSMUSC	Ccl21a	chemokin	GO:00325	developm	K16062|K1	C-C motif	mmu0406	Cytokine-	4:4277286	1	0	1	6	3	3	0.070203	0	0.077571	0.324857	0.135598	0.165613	0.049258	0.208689	0.088961	0.716924	-
ENSMUSC	Fign	fidgetin [S	GO:00081	metabolic	-	-	-	-	2:6397150	31	63	26	29	45	33	0.128272	0.208189	0.118902	0.092577	0.119905	0.107425	0.151788	0.106636	0.089055	0.717345	-
ENSMUSC	Col8a2	collagen,	GO:00325	developm	-	-	-	-	4:1262867	99	265	60	65	131	141	1.29299	2.764099	0.866085	0.654944	1.101765	1.448749	1.641058	1.068486	0.089118	0.717527	-
ENSMUSC	Prkacb	protein ki	GO:00325	developm	K04345	protein ki	mmu0474	Olfactory	3:1467295	6321	6807	5262	4678	8052	7601	25.10233	21.58886	23.09542	14.33239	20.59144	23.74706	23.2622	19.55696	0.089267	0.717698	-
ENSMUSC	Swap70	SWA-70 p	GO:00325	developm	-	-	-	-	7:1102217	244	265	179	359	409	363	1.659596	1.439475	1.345589	1.883807	1.791388	1.942358	1.481553	1.872518	0.089206	0.717698	-
ENSMUSC	Col4a2	collagen,	GO:00325	developm	K06237	collagen, t	mmu052C	Pathways	8:1131280	1417	1828	1187	2237	2408	2200	12.59746	12.97881	11.66301	15.34297	13.78555	15.38674	12.4131	14.83842	0.089226	0.717698	-
ENSMUSC	Vmn1r203	vomerona	GO:00048	signal trar	-	-	-	-	13:225181	1	0	3	0	0	0	0.006623	0	0.021954	0	0	0	0.009526	0	0.089305	0.717698	-
ENSMUSC	Afmid	arylforma	GO:00081	metabolic	K01432	arylforma	mmu011C	Metabolic	11:117825	10	32	9	43	20	51	0.117466	0.300193	0.116836	0.389676	0.151282	0.471289	0.178165	0.337416	0.089377	0.717805	-
ENSMUSC	Rabep1	rabaptin,	GO:00511	localizatio	K12480	Rab GTPa	mmu0414	Endocytos	11:708447	2304	2984	1955	2845	2912	2474	12.61798	13.05125	11.83316	12.02043	10.2696	10.65904	12.5008	10.98302	0.089401	0.717805	-
ENSMUSC	Tmem8	transmem	GO:00038	catalytic a	-	-	-	-	17:261133	183	275	217	345	419	352	3.140433	3.768906	4.115697	4.567583	4.630263	4.752166	3.675012	4.650004	0.08945	0.717874	-
ENSMUSC	Lox	lysyl oxida	GO:00325	developm	-	-	-	-	18:525160	22	52	16	27	19	24	0.180159	0.340084	0.144813	0.170579	0.100195	0.154616	0.221685	0.141797	0.089657	0.719062	-
ENSMUSC	F830016B	RIKEN cDI	GO:00508	response	-	-	-	-	18:602933	6	8	3	9	16	18	0.111113	0.118324	0.061408	0.12859	0.190805	0.262238	0.096948	0.193878	0.089681	0.719062	-
ENSMUSC	Msh4	mutS hor	GO:00325	developm	-	-	-	-	3:1538571	0	1	3	0	0	0	0	0.005802	0.024085	0	0	0	0.009962	0	0.089806	0.719427	-
ENSMUSC	Arhgap19	Rho GTPa	GO:00987	molecular	-	-	-	-	19:417665	40	91	74	76	66	47	0.250548	0.455212	0.512279	0.36726	0.266212	0.2316	0.406013	0.288357	0.089809	0.719427	-
ENSMUSC	Pcnx	pecanex l	GO:00160	membran	-	-	-	-	12:818600	4167	3467	3480	3213	5859	3242	12.14807	8.072041	11.21269	7.226447	10.99922	7.435456	10.4776	8.553707	0.08986	0.719503	-
ENSMUSC	C030005K	RIKEN cDI	GO:00160	membran	-	-	-	-	10:977248	0	0	0	4	0	1	0	0	0	0.100107	0	0.025528	0	0.041878	0.09007	0.720847	-
ENSMUSC	Rab38	RAB38, m	GO:00325	developm	-	-	-	-	7:8843027	12	24	10	32	26	35	0.339833	0.542806	0.312988	0.699165	0.474149	0.779778	0.398542	0.651031	0.090341	0.722684	-
ENSMUSC	Srgn	serglycin		GO:00325	developm	-	-	-	-	10:624938	116	137	140	128	189	53	1.820753	1.717352	2.428679	1.550012	1.910339	0.65446	1.988928	1.371604	0.090454	0.722925	-
ENSMUSC	Fam181a	family witl	-	-	-	-	-	-	12:103310	5	12	9	19	19	19	0.139009	0.26646	0.276555	0.407554	0.340188	0.415593	0.227341	0.387778	0.090426	0.722925	-
ENSMUSC	Rarg	retinoic ac	GO:00325	developm	-	-	-	-	15:102234	135	152	119	217	244	222	1.462271	1.314876	1.424588	1.813369	1.701919	1.891732	1.400579	1.80234	0.090592	0.723348	-
ENSMUSC	Mapkapk5	MAP kina	GO:00325	developm	K04442	mitogen-a	mmu0401	MAPK sigi	5:1215250	143	386	261	137	217	341	1.628369	3.510348	3.284758	1.203555	1.591224	3.054796	2.807825	1.949859	0.090663	0.723348	-
ENSMUSC	Kcns2	K+ voltag	GO:00511	localizatio	-	-	-	-	15:348373	561	671	554	731	406	648	3.305694	3.157678	3.607912	3.323131	1.540568	3.003902	3.357095	2.622534	0.090673	0.723348	-
ENSMUSC	Gm15517	predicted	GO:00160	membran	-	-	-	-	7:4426144	0	0	0	1	0	4	0	0	0	0.057314	0	0.233732	0	0.097015	0.090634	0.723348	-
ENSMUSC	Otx2	orthodent	GO:00325	developm	-	-	-	-	14:486576	64	172	40	66	81	80	0.370048	0.794244	0.255613	0.29441	0.301591	0.363897	0.473302	0.319966	0.090759	0.723702	-
ENSMUSC	Dmpk	dystrophia	GO:00325	developm	-	-	-	-	7:1908384	186	227	146	280	393	260	1.051169	1.024545	0.911923	1.220807	1.430231	1.155959	0.995879	1.268999	0.09101	0.725372	-
ENSMUSC	Tyms	thymidyla	GO:00325	developm	K00560	thymidyla	mmu011C	Metabolic	5:3005820	28	53	31	86	53	71	0.163834	0.24767	0.200475	0.38822	0.199701	0.326828	0.203993	0.304916	0.091203	0.72591	-
ENSMUSC	Bub1b	BUB1B, m	GO:00081	metabolic	K06637	mitotic ch	mmu0516	HTLV-I inf	2:1185982	47	48	41	68	119	69	0.593895	0.484402	0.572591	0.662914	0.968317	0.685929	0.550296	0.772387	0.091176	0.72591	-
ENSMUSC	Lingo2	leucine ric	GO:00325	developm	-	-	-	-	4:3570664	187	248	208	399	290	412	0.768103	0.813533	0.944254	1.264392	0.767063	1.331333	0.841963	1.120929	0.091168	0.72591	-
ENSMUSC	Sdsl	serine del	GO:00038	catalytic a	K17989	L-serine/L	mmu011C	Metabolic	5:1204581	34	32	33	66	70	51	1.065164	0.800618	1.142603	1.595164	1.412166	1.256932	1.002795	1.421421	0.09128	0.725998	-
ENSMUSC	Znhit6	zinc finge	GO:00511	localizatio	-	-	-	-	3:1455762	198	234	183	209	280	175	3.249869	3.067347	3.319703	2.646535	2.959463	2.259693	3.212306	2.621897	0.091298	0.725998	-
ENSMUSC	Gm43064	predicted	GO:00511	localizatio	-	-	-	-	3:1038095	2	7	6	11	5	31	0.038834	0.10857	0.128792	0.164817	0.062529	0.473624	0.092065	0.233657	0.091493	0.727221	-
ENSMUSC	Bpifb1	BPI fold cc	GO:00023	immune s	-	-	-	-	2:1541908	0	0	0	0	5	1	0	0	0	0	0.046807	0.011439	0	0.019415	0.091914	0.729944	-
ENSMUSC	Gm10128	predicted	-	-	-	-	-	-	14:764089	2	2	2	0	2	0	0.198526	0.158602	0.21947	0	0.127838	0	0.192199	0.042613	0.091962	0.729944	-
ENSMUSC	Klra7	killer cell l	GO:00160	membran	-	-	mmu0465	Natural ki	6:1302186	0	0	0	0	5	1	0	0	0	0	0.093704	0.0229	0	0.038868	0.091951	0.729944	-
ENSMUSC	Nr2e3	nuclear re	GO:00325	developm	-	-	-	-	9:5994277	0	0	0	1	5	0	0	0	0	0.004867	0.020311	0	0	0.008393	0.09212	0.730532	-
ENSMUSC	Rbbp8nl	RBBP8 N-	GO:00055	extracellul	-	-	-	-	2:1802776	0	0	0	1	5	0	0	0	0	0.020316	0.084774	0	0	0.03503	0.092088	0.730532	-
ENSMUSC	Cd200	CD200 an	GO:00023	immune s	-	-	-	-	16:453821	313	417	228	654	570	387	2.866101	3.049508	2.307439	4.620144	3.361066	2.787854	2.741016	3.589688	0.092168	0.73058	-
ENSMUSC	Ogn	osteoglyc	GO:00325	developm	-	-	-	-	13:496080	100	172	77	166	205	226	2.379502	3.268595	2.025002	3.047378	3.141193	4.230639	2.5577	3.47307	0.092425	0.731954	-
ENSMUSC	Raly	hnRNP-as	GO:00081	metabolic	-	-	-	-	2:1547910	510	954	486	691	628	698	4.847394	7.241561	5.105295	5.066942	3.843726	5.219201	5.731417	4.709957	0.092406	0.731954	-
ENSMUSC	Ptchd1	patched c	GO:00325	developm	-	-	-	-	X:1555734	85	106	57	80	94	76	0.986121	0.982114	0.730854	0.716027	0.702252	0.693639	0.899696	0.703973	0.092687	0.733354	-
ENSMUSC	Dph6	diphtham	GO:00081	metabolic	-	-	-	-	2:1145164	316	393	319	378	440	317	1.647821	1.636671	1.838487	1.520708	1.477512	1.300449	1.70766	1.43289	0.092652	0.733354	-
ENSMUSC	Tinagl1	tubulointe	GO:00081	metabolic	-	-	-	-	4:1301644	130	205	129	219	311	241	1.190396	1.499158	1.30552	1.547111	1.833848	1.736103	1.331691	1.705687	0.092854	0.734346	-
ENSMUSC	Zfp54	zinc finge	GO:00081	metabolic	-	-	-	-	17:214232	6	15	7	43	16	12	0.066414	0.132606	0.085643	0.367211	0.114047	0.104502	0.094888	0.195253	0.092927	0.734571	-
ENSMUSC	Nags	N-acetylg	GO:00081	metabolic	K11067	N-acetylg	mmu011C	Metabolic	11:102145	0	0	3	4	8	3	0	0	0.046035	0.042838	0.071524	0.032761	0.015345	0.049041	0.092967	0.734571	-
ENSMUSC	Akap2	A kinase (	GO:00511	localizatio	-	-	-	-	4:5771765	1216	567	585	1024	1354	2419	4.611881	1.717407	2.452149	2.996226	3.306876	7.217573	2.927146	4.506892	0.093021	0.734659	-
ENSMUSC	Tnfrsf10b	tumor nec	GO:00325	developm	K04722	tumor nec	mmu0421	Necroptos	14:697674	10	9	4	15	27	13	0.095538	0.068672	0.042239	0.11056	0.166111	0.097704	0.068816	0.124792	0.093101	0.734961	-
ENSMUSC	Bhlha15	basic helix	GO:00325	developm	K08040	class B ba	mmu0495	Maturity c	5:1441902	3	3	0	4	15	4	0.052424	0.041875	0	0.053921	0.168796	0.054988	0.031433	0.092568	0.0933	0.735866	-
ENSMUSC	Ankrd44	ankyrin re	-	-	-	-	-	-	1:5464534	220	298	207	280	301	191	0.586661	0.634639	0.610073	0.57604	0.516876	0.400691	0.610458	0.497869	0.093288	0.735866	-
ENSMUSC	Pdia5	protein di	GO:00081	metabolic	-	-	-	-	16:353973	35	45	43	67	94	66	1.181981	1.213672	1.604942	1.745623	2.044223	1.753472	1.333532	1.847773	0.09351	0.736855	-
ENSMUSC	Cntn1	contactin	GO:00325	developm	K06759	contactin	mmu0451	Cell adhes	15:920511	7085	8769	6312	8203	9553	8055	34.17576	33.78113	33.65054	30.5268	29.67377	30.56713	33.86914	30.2559	0.093478	0.736855	-
ENSMUSC	Sox30	SRY (sex c	GO:00081	metabolic	-	-	-	-	11:459803	6	1	3	0	4	0	0.122236	0.016273	0.067554	0	0.052479	0	0.068688	0.017493	0.093622	0.737115	-
ENSMUSC	Osr1	odd-skipp	GO:00325	developm	-	-	-	-	12:957011	35	62	29	68	64	102	0.827021	1.170014	0.757347	1.239628	0.973843	1.8961	0.918127	1.369857	0.093628	0.737115	-
ENSMUSC	Hdac10	histone de	GO:00081	metabolic	K18671	histone de	mmu0516	Human pa	15:891233	138	179	129	233	256	249	1.28897	1.335254	1.331682	1.678998	1.539783	1.829683	1.318635	1.682821	0.093761	0.737602	-
ENSMUSC	Nat8f5	N-acetyltı	GO:00325	developm	-	-	-	-	6:8581721	14	11	19	9	17	10	0.86697	0.543991	1.300389	0.429982	0.677923	0.487164	0.903783	0.53169	0.093775	0.737602	-
ENSMUSC	Hist1h2ad	histone cl	GO:00329	macromo	K11251	histone H:	mmu0503	Alcoholisr	13:235743	3	4	7	3	3	1	0.320227	0.340965	0.825888	0.247092	0.206242	0.083993	0.495694	0.179109	0.093991	0.738966	-
ENSMUSC	Ankrd33b	ankyrin re	GO:00971	organic cy	-	-	-	-	15:312914	1672	1167	957	1235	1487	1364	4.797648	2.674291	3.034945	2.733943	2.747627	3.079056	3.502295	2.853542	0.094395	0.740228	-
ENSMUSC	Gm6377	predicted	-	-	-	-	-	-	X:1091967	2	3	3	0	3	0	0.038107	0.045665	0.06319	0	0.03682	0	0.048987	0.012273	0.094395	0.740228	-
ENSMUSC	Ticam2	toll-like re	GO:00048	signal trar	K05409	TRIF-relat	mmu0421	Necroptos	18:465591	19	29	22	22	17	22	0.287794	0.350817	0.368307	0.257089	0.165823	0.262162	0.335639	0.228358	0.094407	0.740228	-
ENSMUSC	Pla2g4a	phospholi	GO:00081	metabolic	K16342	cytosolic p	mmu011C	Metabolic	1:1498296	54	117	64	108	176	132	0.43366	0.750393	0.568051	0.669131	0.910176	0.833954	0.584035	0.80442	0.094372	0.740228	-
ENSMUSC	Tmem178	transmem	GO:00160	membran	-	-	-	-	6:3987327	3094	4370	2703	3952	4345	3220	18.63055	21.01516	17.98864	18.35915	16.84806	15.25359	19.21145	16.82027	0.094216	0.740228	-
ENSMUSC	Gm42742	predicted	GO:00325	developm	-	-	-	-	7:1270150	164	435	209	245	321	224	1.46718	3.107962	2.066494	1.690976	1.849272	1.576518	2.213879	1.705588	0.094325	0.740228	-
ENSMUSC	Zbtb49	zinc finge	GO:00081	metabolic	-	-	-	-	5:3818973	134	172	104	122	172	134	0.569248	0.58354	0.488288	0.399838	0.47052	0.447829	0.547025	0.439396	0.094501	0.74063	-
ENSMUSC	Pigx	phosphati	GO:00081	metabolic	K07541	phosphati	mmu011C	Metabolic	16:320844	110	125	97	116	126	106	1.161402	1.054012	1.131897	0.944882	0.856676	0.880451	1.11577	0.894003	0.094628	0.741299	-
ENSMUSC	Hist2h2aa	histone cl	GO:00081	metabolic	K11251	histone H:	mmu0503	Alcoholisr	3:9623974	57	71	91	73	75	67	6.591454	6.557104	11.6305	6.512713	5.585041	6.095304	8.259686	6.064353	0.094847	0.742673	-
ENSMUSC	Itgb1bp2	integrin b	GO:00990	supramole	-	-	-	-	X:1014490	3	8	6	2	2	5	0.087683	0.186746	0.193835	0.045105	0.037635	0.11497	0.156088	0.065903	0.094934	0.742686	-
ENSMUSC	Bcorl1	BCL6 co-r	GO:00081	metabolic	-	-	-	-	X:4834135	393	616	500	502	631	524	2.044288	2.559034	2.874524	2.014574	2.11365	2.144329	2.492615	2.090851	0.094909	0.742686	-
ENSMUSC	Spata33	spermato	GO:00056	cell|intrace	-	-	-	-	8:1232128	34	175	36	41	77	60	0.17444	0.717043	0.204134	0.162282	0.254392	0.24217	0.365205	0.219615	0.095229	0.744662	-
ENSMUSC	Gm13212	predicted	GO:00081	metabolic	-	-	-	-	4:1455851	18	21	14	24	48	34	0.155668	0.145047	0.133817	0.160134	0.267321	0.231326	0.144844	0.219594	0.095347	0.745162	-
ENSMUSC	Gm29609	predicted	-	-	-	-	-	-	5:3114473	7	16	9	35	17	19	0.120027	0.219103	0.170546	0.462985	0.187709	0.256288	0.169892	0.302327	0.095379	0.745162	-
ENSMUSC	Ralgapa1	Ral GTPas	GO:00081	metabolic	-	-	-	-	12:556028	3427	4323	2783	4009	4243	3781	11.03502	11.11707	9.904195	9.959226	8.798059	9.578043	10.68543	9.445109	0.095554	0.745779	-
ENSMUSC	Uts2r	urotensin	GO:00325	developm	K04241	urotensin	mmu0408	Neuroacti	11:121160	0	2	2	5	6	5	0	0.057276	0.079256	0.138293	0.138521	0.141029	0.045511	0.139281	0.095505	0.745779	-
ENSMUSC	Rps13	ribosomal	GO:00081	metabolic	K02953	small subı	mmu0301	Ribosome	7:1163315	23	30	19	59	53	30	0.167883	0.174879	0.15328	0.332246	0.249119	0.172269	0.165347	0.251211	0.095586	0.745779	-
ENSMUSC	Gfap	glial fibrill	GO:00325	developm	K05640	glial fibrill	mmu0463	Jak-STAT	11:102887	6974	11823	7594	9418	10911	9465	89.89218	121.7064	108.1826	93.65457	90.56475	95.97795	106.5937	93.39909	0.095772	0.745889	-
ENSMUSC	Rerg	RAS-like,	GO:00508	response	-	-	-	-	6:1370548	387	378	376	400	462	399	4.319838	3.369721	4.638646	3.444661	3.320873	3.503801	4.109402	3.423112	0.095737	0.745889	-
ENSMUSC	Casp3	caspase 3	GO:00325	developm	K02187	caspase 3	mmu052C	Pathways	8:4661729	156	188	126	220	352	227	1.955064	1.881659	1.745227	2.127104	2.84075	2.238067	1.86065	2.401974	0.095757	0.745889	-
ENSMUSC	Ccdc69	coiled-co	GO:00099	cellular pr	-	-	-	-	11:550497	4	3	5	6	14	15	0.11967	0.071686	0.165328	0.138474	0.269702	0.353021	0.118895	0.253732	0.095757	0.745889	-
ENSMUSC	Gbp8	guanylate	GO:00023	immune s	-	-	-	-	5:1050141	0	9	15	11	47	24	0	0.0809	0.186587	0.095518	0.340641	0.2125	0.089163	0.21622	0.095876	0.746367	-
ENSMUSC	Srrm2	serine/arg	GO:00081	metabolic	-	-	-	-	17:237906	5917	7419	5274	8118	10125	10777	20.74538	20.77357	20.43651	21.95833	22.85965	29.72547	20.65182	24.84781	0.095974	0.746459	-
ENSMUSC	D830044I	RIKEN cDI	-	-	-	-	-	-	7:1269717	0	3	0	6	3	3	0	0.026597	0	0.051377	0.021445	0.026192	0.008866	0.033005	0.095932	0.746459	-
ENSMUSC	Wdr24	WD repea	GO:00081	metabolic	K20408	WD repea	mmu0415	mTOR sig	17:258236	358	439	396	611	723	615	3.785079	3.706826	4.627359	4.983824	4.922487	5.115371	4.039755	5.007228	0.09617	0.747649	-
ENSMUSC	Pdgfd	platelet-d	GO:00325	developm	K05450	platelet de	mmu0415	PI3K-Akt s	9:6168584	114	99	66	68	85	114	1.073841	0.744758	0.687113	0.494168	0.515594	0.844794	0.835237	0.618186	0.096381	0.748952	-
ENSMUSC	Ccdc12	coiled-co	GO:00081	metabolic	K12871	coiled-co	mmu0304	Spliceosoı	9:1106565	204	267	167	240	259	180	2.412522	2.521732	2.182757	2.189686	1.972406	1.674652	2.372337	1.945581	0.096534	0.749809	-
ENSMUSC	Rgs9bp	regulator	GO:00508	response	-	-	-	-	7:3557899	7	3	2	10	8	15	0.064858	0.022202	0.020482	0.071483	0.047733	0.109335	0.035847	0.076184	0.096601	0.749991	-
ENSMUSC	Atp6ap1l	ATPase, H	GO:00511	localizatio	-	-	-	-	13:908834	10	15	15	4	21	3	0.230063	0.275605	0.3814	0.070988	0.311105	0.054289	0.295689	0.145461	0.096717	0.75056	-
ENSMUSC	Slc17a7	solute car	GO:00325	developm	K12302	MFS trans	mmu0472	Retrograd	7:4516394	15363	21190	12073	16861	20827	17539	109.8274	120.9794	95.38863	92.99257	95.87735	98.63939	108.7318	95.83644	0.096769	0.750631	-
ENSMUSC	Erap1	endoplasr	GO:00325	developm	-	-	-	-	13:746395	193	267	172	316	419	299	1.470817	1.625026	1.448695	1.857888	2.056225	1.792606	1.514846	1.90224	0.09705	0.752472	-
ENSMUSC	Hecw2	HECT, C2	GO:00081	metabolic	-	-	-	-	1:5380687	820	1029	671	907	762	1018	2.522681	2.528189	2.281488	2.152712	1.509587	2.46381	2.444119	2.042037	0.097124	0.752708	-
ENSMUSC	Pcsk4	proprotei	GO:00325	developm	-	-	-	-	10:803212	12	28	19	51	29	38	0.124711	0.232404	0.218244	0.408917	0.194082	0.310695	0.191786	0.304565	0.097265	0.752886	-
ENSMUSC	Apbb1	amyloid b	GO:00325	developm	K04529	amyloid b	mmu0501	Alzheimer	7:1055584	2867	3760	2795	5657	4857	4272	15.89085	16.64382	17.12174	24.19	17.33573	18.6278	16.55214	20.05118	0.097221	0.752886	-
ENSMUSC	Tmed9	transmem	GO:00511	localizatio	-	-	-	-	13:555737	861	1285	750	1453	1815	1269	8.802916	10.49234	8.47484	11.46092	11.94964	10.20695	9.256699	11.20584	0.097276	0.752886	-
ENSMUSC	Pcsk6	proprotei	GO:00325	developm	-	-	-	-	7:6586173	455	526	425	513	603	459	3.179021	2.935032	3.281838	2.76522	2.713023	2.522932	3.131964	2.667058	0.097464	0.753995	-
ENSMUSC	Scamp4	secretory	GO:00511	localizatio	-	-	-	-	10:806028	385	586	77	671	737	651	6.096307	7.410535	1.347551	8.197078	7.514996	8.109573	4.951464	7.940549	0.097506	0.753995	-
ENSMUSC	Zdbf2	zinc finge	-	-	-	-	-	-	1:6327326	528	855	619	867	677	575	1.286614	1.663897	1.667062	1.629911	1.062326	1.102283	1.539191	1.26484	0.097651	0.754082	-
ENSMUSC	Zcchc2	zinc finge	GO:00056	cell|organ	-	-	-	-	1:1059904	393	605	424	749	1012	580	1.666726	2.049143	1.987393	2.450664	2.763801	1.93513	1.901087	2.383198	0.097574	0.754082	-
ENSMUSC	Hook2	hook micı	GO:00511	localizatio	-	-	-	-	8:8499060	204	243	167	290	384	331	2.261	2.15091	2.045666	2.479701	2.740668	2.886091	2.152526	2.702153	0.097691	0.754082	-
ENSMUSC	Zfp131	zinc finge	GO:00081	metabolic	-	-	-	-	13:119765	622	494	708	1117	1152	958	3.63462	2.305373	4.572447	5.035612	4.334867	4.403979	3.504147	4.591486	0.097628	0.754082	-
ENSMUSC	Pdgfrb	platelet de	GO:00325	developm	K05089	platelet-d	mmu052C	Pathways	18:610451	314	484	325	672	771	432	2.494112	3.070279	2.853098	4.117997	3.943626	2.699487	2.80583	3.587037	0.097796	0.754225	-
ENSMUSC	Kcnj3	potassium	GO:00511	localizatio	K04997	potassium	mmu0492	Oxytocin s	2:5543597	936	1435	1092	1179	1470	1261	5.318788	6.512305	6.858141	5.168695	5.379091	5.637195	6.229745	5.394994	0.097796	0.754225	-
ENSMUSC	Gm12216	predicted	-	-	-	-	-	-	11:537834	21	19	11	38	47	21	0.433339	0.313109	0.250858	0.604942	0.624528	0.340903	0.332435	0.523458	0.097923	0.754868	-
ENSMUSC	Xpo6	exportin 6	GO:00511	localizatio	-	-	-	-	7:1261017	1216	1890	1287	1368	2066	1522	2.96773	3.68382	3.471492	2.575771	3.246951	2.922243	3.374348	2.914989	0.098116	0.75602	-
ENSMUSC	R3hdml	R3H dom	GO:00081	metabolic	-	-	-	-	2:1634923	0	0	0	5	1	0	0	0	0	0.215277	0.035933	0	0	0.083737	0.098189	0.756246	-
ENSMUSC	Tmem47	transmem	GO:00160	membran	-	-	-	-	X:8107069	2509	3044	2180	2820	3537	2615	25.0646	24.28569	24.06929	21.73399	22.75358	20.55146	24.47319	21.67967	0.098339	0.756713	-
ENSMUSC	Lmntd1	lamin tail	GO:00051	structural	-	-	-	-	6:1453651	13	9	7	22	21	20	0.092394	0.051086	0.054989	0.120629	0.096109	0.111825	0.066156	0.109521	0.09838	0.756713	-
ENSMUSC	Cd2ap	CD2-asso	GO:00081	metabolic	K13738	CD2-asso	mmu051C	Bacterial i	17:427929	636	917	614	928	1461	1088	7.192623	8.282189	7.674409	8.096702	10.63983	9.679874	7.716407	9.472137	0.098352	0.756713	-
ENSMUSC	Eri2	exoribonu	GO:00081	metabolic	-	-	-	-	7:1197686	169	178	152	142	162	209	0.633562	0.532927	0.629787	0.410697	0.391085	0.616394	0.598759	0.472726	0.098529	0.757524	-
ENSMUSC	Zfp652	zinc finge	GO:00081	metabolic	-	-	-	-	11:957126	1370	1525	1128	1434	1734	1454	3.932565	3.495997	3.578587	3.175667	3.20523	3.283452	3.66905	3.22145	0.098905	0.760078	-
ENSMUSC	Myom1	myomesir	GO:00325	developm	-	-	-	-	17:710195	58	82	58	102	135	104	0.541824	0.611777	0.598833	0.735125	0.812116	0.764322	0.584145	0.770521	0.09937	0.762891	-
ENSMUSC	Map3k21	mitogen-	GO:00048	signal trar	-	-	-	-	8:1259104	42	57	57	72	143	82	0.450455	0.488225	0.675652	0.595749	0.987626	0.691873	0.53811	0.758416	0.099407	0.762891	-
ENSMUSC	Arhgap10	Rho GTPa	GO:00987	molecular	K13736	Rho GTPa	mmu051C	Bacterial i	8:7725036	182	225	153	141	237	212	3.340122	3.29776	3.103343	1.996367	2.800874	3.06082	3.247075	2.619354	0.099446	0.762891	-
ENSMUSC	Gm44965	predicted	-	-	-	-	-	-	6:4859394	0	3	0	5	0	9	0	0.199878	0	0.32174	0	0.590573	0.066626	0.304104	0.099396	0.762891	-
ENSMUSC	Olfml3	olfactome	GO:00055	extracellul	-	-	-	-	3:1037222	174	625	371	542	223	152	0.984898	2.825324	2.320938	2.36686	0.812834	0.676855	2.04372	1.285517	0.099535	0.763236	-
ENSMUSC	Lrp5	low densit	GO:00325	developm	K03068	low densit	mmu052C	Pathways	19:358482	265	340	224	397	496	441	1.355141	1.388559	1.266001	1.566245	1.633336	1.774142	1.336567	1.657908	0.099641	0.763711	-
ENSMUSC	Fgd2	FYVE, Rhc	GO:00081	metabolic	-	-	-	-	17:293609	57	88	62	132	111	114	0.489286	0.603282	0.588207	0.874167	0.613573	0.769852	0.560258	0.752531	0.100081	0.766743	-
ENSMUSC	Bbx	bobby sox	GO:00325	developm	-	-	-	-	16:501918	680	1066	636	848	933	851	2.062466	2.582148	2.131974	1.984285	1.822277	2.030571	2.258863	1.945711	0.100254	0.76773	-
ENSMUSC	Uqcr11	ubiquinol-	GO:00081	metabolic	K00420	ubiquinol-	mmu011C	Metabolic	10:804029	582	834	554	876	1170	1062	32.0431	36.67109	33.71072	37.20883	41.4813	45.99886	34.14164	41.563	0.100425	0.76836	-
ENSMUSC	Ube2j2	ubiquitin-	GO:00081	metabolic	K04554	ubiquitin-	mmu0414	Protein pr	4:1559438	391	451	353	393	564	373	2.81231	2.590654	2.806133	2.18077	2.612285	2.110601	2.736366	2.301219	0.100396	0.76836	-
ENSMUSC	Ano4	anoctamir	GO:00511	localizatio	-	-	-	-	10:889489	334	446	299	339	463	388	1.763678	1.880845	1.744983	1.381029	1.574377	1.611814	1.796502	1.522407	0.100528	0.768813	-
ENSMUSC	Lrrc42	leucine ric	-	-	-	-	-	-	4:1072335	297	326	238	400	569	442	5.172344	4.534144	4.580949	5.3743	6.381141	6.055704	4.762479	5.937048	0.100622	0.769193	-
ENSMUSC	Cfap206	cilia and f	GO:00990	supramole	-	-	-	-	4:3468855	35	65	32	36	43	44	0.620506	0.920319	0.627017	0.492393	0.490911	0.613676	0.722614	0.532327	0.100897	0.769939	-
ENSMUSC	Slc25a20	solute car	GO:00511	localizatio	-	-	-	-	9:1086620	171	246	158	291	380	270	1.989505	2.285761	2.031667	2.612002	2.847001	2.471288	2.102311	2.64343	0.100871	0.769939	-
ENSMUSC	Btbd10	BTB (POZ	GO:00081	metabolic	-	-	-	-	7:1133156	1266	1177	836	1591	2075	1625	8.992502	6.676815	6.562962	8.718617	9.49117	9.080532	7.410759	9.096773	0.100883	0.769939	-
ENSMUSC	Rasl2-9	RAS-like,	GO:00511	localizatio	K07936	GTP-bind	mmu0516	HTLV-I inf	7:5124938	0	1	0	5	2	1	0	0.048144	0	0.232491	0.077612	0.047427	0.016048	0.119177	0.100857	0.769939	-
ENSMUSC	Ccnc	cyclin C [S	GO:00081	metabolic	-	-	-	-	4:2172770	567	750	556	771	385	679	5.096415	5.383796	5.523357	5.346464	2.228419	4.80134	5.334522	4.125408	0.100966	0.770115	-
ENSMUSC	P2ry12	purinergic	GO:00325	developm	K04298	purinergic	mmu0461	Platelet ac	3:5921627	357	572	559	748	951	805	2.462745	3.15132	4.261962	3.980917	4.224607	4.368754	3.292009	4.191426	0.101008	0.770115	-
ENSMUSC	Ret	ret proto-	GO:00325	developm	K05126	proto-onc	mmu052C	Pathways	6:1181517	83	133	57	82	105	82	0.72563	0.928618	0.550757	0.553075	0.591129	0.56398	0.735002	0.569395	0.101264	0.771726	-
ENSMUSC	Il1rl1	interleukir	GO:00048	signal trar	-	-	-	-	1:4042957	3	5	4	4	22	10	0.016558	0.02204	0.024403	0.017031	0.078198	0.043423	0.021	0.046217	0.101336	0.771937	-
ENSMUSC	Prrc2b	proline-ri	GO:00325	developm	-	-	-	-	2:3215108	8996	10279	8048	10566	10974	10148	31.40351	28.65665	31.05013	28.45572	24.66882	27.86892	30.3701	26.99782	0.101392	0.772023	-
ENSMUSC	Abca4	ATP-bind	GO:00511	localizatio	K05644	ATP-bind	mmu0201	ABC trans	3:1220444	100	341	65	78	134	173	0.427547	1.164352	0.307148	0.257281	0.368929	0.58189	0.633016	0.4027	0.101673	0.772813	-
ENSMUSC	Hrh2	histamine	GO:00325	developm	K04150	histamine	mmu0408	Neuroacti	13:541921	131	156	123	216	253	214	1.141358	1.085472	1.184411	1.45189	1.419462	1.466808	1.13708	1.446053	0.101627	0.772813	-
ENSMUSC	Skap1	src family	GO:00081	metabolic	K17699	src kinase	mmu0401	Rap1 sign	11:964645	25	24	12	36	56	30	0.15912	0.121994	0.084413	0.176775	0.229527	0.150217	0.121842	0.185506	0.101647	0.772813	-
ENSMUSC	Eid3	EP300 inte	GO:00081	metabolic	-	-	-	-	10:828666	3	2	1	8	8	4	0.14036	0.074744	0.051714	0.288774	0.241043	0.147224	0.088939	0.22568	0.101594	0.772813	-
ENSMUSC	Wisp3	WNT1 ind	GO:00325	developm	-	-	-	-	10:391509	0	0	1	1	2	5	0	0	0.047559	0.033201	0.055406	0.169254	0.015853	0.085954	0.101729	0.772898	-
ENSMUSC	Bcas3	breast car	GO:00325	developm	-	-	-	-	11:853531	885	1116	880	1061	1185	1039	6.237612	6.281814	6.854951	5.769267	5.378337	5.761049	6.458125	5.636218	0.101846	0.7733	-
ENSMUSC	Gm8206	predicted	-	-	-	-	-	-	14:601675	8	11	8	11	4	4	0.170861	0.187609	0.188817	0.181247	0.055012	0.067201	0.182429	0.101154	0.101871	0.7733	-
ENSMUSC	Zfp239	zinc finge	GO:00081	metabolic	-	-	-	-	6:1178621	388	375	412	347	599	333	2.727124	2.104992	3.200497	1.881626	2.711157	1.841316	2.677538	2.1447	0.102027	0.774153	-
ENSMUSC	Lzts1	leucine zip	GO:00325	developm	-	-	-	-	8:6913266	1315	1577	1154	1481	1660	1503	9.678327	9.269409	9.387011	8.4093	7.86751	8.702512	9.444916	8.326441	0.102274	0.775687	-
ENSMUSC	Cox6b2	cytochron	GO:00160	membran	K02267	cytochron	mmu011C	Metabolic	7:4751792	11	23	15	28	40	28	0.239281	0.399562	0.360611	0.469879	0.560287	0.479156	0.333151	0.503107	0.102462	0.776775	-
ENSMUSC	Dusp22	dual speci	GO:00081	metabolic	-	-	-	-	13:306599	208	177	124	273	305	289	2.090551	1.420743	1.377422	2.11685	1.974018	2.285105	1.629572	2.125324	0.102549	0.777097	-
ENSMUSC	Apip	APAF1 int	GO:00081	metabolic	K08964	methylthic	mmu011C	Metabolic	2:1030736	101	115	83	185	154	171	3.373566	3.067696	3.06401	4.767266	3.312388	4.493398	3.168424	4.191017	0.102603	0.777166	-
ENSMUSC	Itgb2	integrin b	GO:00325	developm	K06464	integrin b	mmu0516	HTLV-I inf	10:775302	118	231	118	143	178	152	1.54578	2.416696	1.708414	1.445213	1.501551	1.566454	1.890297	1.504406	0.102833	0.778141	-
ENSMUSC	Rsl24d1	ribosomal	GO:00081	metabolic	K02896	large subı	mmu0301	Ribosome	9:7311342	258	339	234	329	211	301	5.590124	5.866084	5.60357	5.499561	2.944018	5.13073	5.686593	4.52477	0.102866	0.778141	-
ENSMUSC	Atp6v0a4	ATPase, H	GO:00081	metabolic	K02154	V-type H-	mmu011C	Metabolic	6:3804848	2	8	3	12	13	8	0.020104	0.064237	0.033338	0.093076	0.084168	0.063273	0.039226	0.080172	0.102822	0.778141	-
ENSMUSC	Abi1	abl-intera	GO:00325	developm	-	-	-	-	2:2294007	2847	4509	3252	4320	4408	3190	10.63585	13.45275	13.4271	12.45085	10.60428	9.37533	12.50523	10.81015	0.103006	0.77886	-
ENSMUSC	Col6a3	collagen,	GO:00081	metabolic	K06238	collagen, t	mmu0415	PI3K-Akt s	1:9076592	74	156	81	155	252	141	0.33055	0.556514	0.39989	0.534157	0.724872	0.495494	0.428985	0.584841	0.103277	0.780569	-
ENSMUSC	Zfp955b	zinc finge	GO:00081	metabolic	-	-	-	-	17:332896	210	254	197	253	257	209	7.827956	7.561532	8.116023	7.2758	6.16903	6.128948	7.83517	6.524593	0.103453	0.781561	-
ENSMUSC	Slfn1	schlafen 1	GO:00081	metabolic	-	-	-	-	11:831168	4	3	2	6	13	8	0.131033	0.078493	0.072411	0.151623	0.274223	0.206146	0.093979	0.210664	0.103525	0.781761	-
ENSMUSC	Zfp708	zinc finge	GO:00081	metabolic	-	-	-	-	13:670693	96	121	114	81	175	22	1.194043	1.20194	1.567115	0.777259	1.401662	0.215273	1.321033	0.798064	0.103639	0.782282	-
ENSMUSC	Wars2	tryptopha	GO:00325	developm	K01867	tryptopha	mmu0097	Aminoacy	3:9914106	87	173	83	198	215	166	0.571126	0.906998	0.602194	1.002787	0.908879	0.857301	0.693439	0.922989	0.103839	0.783119	-
ENSMUSC	Emp3	epithelial	GO:00099	cellular pr	-	-	-	-	7:4591802	17	23	16	16	17	16	0.584461	0.631514	0.607952	0.42438	0.37637	0.432747	0.607976	0.411166	0.103832	0.783119	-
ENSMUSC	Tspan17	tetraspani	GO:00160	membran	-	-	-	-	13:547893	161	204	191	145	279	164	2.966306	3.001695	3.889295	2.061048	3.310167	2.377089	3.285765	2.582768	0.104006	0.784034	-
ENSMUSC	Sun3	Sad1 and	GO:00511	localizatio	-	-	-	-	11:901605	0	3	4	7	13	3	0	0.112633	0.207812	0.25384	0.393496	0.110919	0.106815	0.252752	0.104149	0.784433	-
ENSMUSC	Zfp974	zinc finge	GO:00081	metabolic	-	-	-	-	7:2790739	148	190	134	158	204	145	1.030162	1.056194	1.030853	0.848464	0.914386	0.794007	1.039069	0.852285	0.104116	0.784433	-

ENSMUSC	C1rb	complem	GO:00081	metabolic	-	-	mmu0414	Phagoson	6:1245702	9	18	9	17	40	21	0.226229	0.36135	0.250027	0.329688	0.647479	0.415287	0.279202	0.464151	0.104325	0.785415	-
ENSMUSC	Zbtb48	zinc finge	GO:00081	metabolic	-	-	-	-	4:1520197	157	143	131	149	178	132	1.418909	1.03213	1.308493	1.038893	1.035928	0.938507	1.253177	1.004443	0.104442	0.785622	-
ENSMUSC	Cchcr1	coiled-co	GO:00325	developm	-	-	-	-	17:355171	47	50	41	69	95	81	0.369569	0.313989	0.356312	0.418585	0.481039	0.501068	0.346623	0.466897	0.104438	0.785622	-
ENSMUSC	Aste1	asteroid h	-	-	-	-	-	-	9:1053953	65	71	66	73	63	68	0.334072	0.291427	0.374905	0.289454	0.208507	0.274947	0.333468	0.257636	0.1045	0.785714	-
ENSMUSC	Ptprcap	protein ty	GO:00508	response	-	-	-	-	19:415460	2	6	2	6	11	10	0.131708	0.315612	0.145603	0.304882	0.46655	0.51817	0.197641	0.429867	0.104584	0.786005	-
ENSMUSC	Lhx3	LIM home	GO:00325	developm	-	-	-	-	2:2620021	0	3	3	1	0	0	0	0.030985	0.042876	0.009977	0	0	0.02462	0.003326	0.104694	0.786494	-
ENSMUSC	Parp3	poly (ADP	GO:00081	metabolic	K10798	poly [ADP	mmu0421	Necroptos	9:1064703	71	88	56	121	152	98	0.586144	0.580198	0.510952	0.770661	0.808062	0.636479	0.559098	0.7384	0.104858	0.786709	-
ENSMUSC	Rbp1	retinol bir	GO:00325	developm	-	-	-	-	9:9842296	171	260	159	201	238	193	3.937	4.780679	4.045882	3.570234	3.528589	3.495723	4.25452	3.531515	0.104853	0.786709	-
ENSMUSC	Klhl11	kelch-like	GO:00081	metabolic	-	-	-	-	11:100462	816	1031	766	902	1083	981	20.82048	21.00899	21.60112	17.75569	17.79443	19.69158	21.14353	18.4139	0.104768	0.786709	-
ENSMUSC	Ptdss1	phosphati	GO:00081	metabolic	K08729	phosphati	mmu011C	Metabolic	13:669328	709	994	598	703	1146	700	6.291264	7.044079	5.864614	4.812576	6.548339	4.886533	6.399986	5.415816	0.104917	0.786809	-
ENSMUSC	Gm28557	predicted	GO:00081	metabolic	-	-	-	-	13:670693	17	10	5	79	12	10	0.163413	0.076764	0.053123	0.585853	0.074282	0.075621	0.097767	0.245252	0.105041	0.787397	-
ENSMUSC	Gfi1	growth fa	GO:00325	developm	-	-	-	-	5:1077166	1	0	0	3	5	0	0.012565	0	0	0.029076	0.040442	0	0.004188	0.023173	0.105206	0.78762	-
ENSMUSC	Fam3c	family witl	GO:00325	developm	-	-	-	-	6:2230652	992	1179	843	869	1370	1156	12.60817	11.9674	11.84172	8.520995	11.21284	11.5587	12.1391	10.43085	0.105182	0.78762	-
ENSMUSC	C2cd4b	C2 calciur	GO:00160	membran	-	-	-	-	9:6775943	32	50	46	68	102	66	1.472383	1.837319	2.339258	2.413865	3.022242	2.389055	1.882987	2.608388	0.105151	0.78762	-
ENSMUSC	Anxa5	annexin A	GO:00508	response	-	-	-	-	3:3644892	570	904	552	691	881	685	15.25783	19.32557	16.33068	14.27007	15.18619	14.42513	16.97136	14.62713	0.105396	0.78839	-
ENSMUSC	Jazf1	JAZF zinc	GO:00081	metabolic	-	-	-	-	6:5276879	367	485	344	500	465	325	8.836097	9.325713	9.153765	9.287401	7.209441	6.155859	9.105192	7.5509	0.1054	0.78839	-
ENSMUSC	Lrguk	leucine-ri	GO:00325	developm	-	-	-	-	6:3402944	136	171	111	174	133	118	0.647983	0.650678	0.584511	0.639592	0.408066	0.442301	0.627724	0.496653	0.105611	0.78963	-
ENSMUSC	Chrnb1	cholinergi	GO:00325	developm	K04812	nicotinic a	mmu0408	Neuroacti	11:697840	8	14	4	16	21	18	0.143253	0.200201	0.079164	0.221026	0.242133	0.253548	0.140873	0.238902	0.105768	0.790465	-
ENSMUSC	8030474K	RIKEN cDI	-	-	-	-	-	-	X:1017946	2	0	0	2	3	7	0.042305	0	0	0.032649	0.040877	0.116514	0.014102	0.063347	0.105916	0.791232	-
ENSMUSC	Gltp	glycolipid	GO:00511	localizatio	-	-	-	-	5:1146693	404	671	567	814	1053	814	7.622823	10.11121	11.82402	11.84922	12.79434	12.08288	9.852682	12.24214	0.105971	0.7913	-
ENSMUSC	St18	suppressi	GO:00081	metabolic	-	-	-	-	1:6487231	618	898	511	956	1238	980	2.853009	3.310831	2.607247	3.404891	3.680358	3.559194	2.923696	3.548148	0.106025	0.791367	-
ENSMUSC	Ccl19	chemokin	GO:00325	developm	K05512	C-C motif	mmu0406	Cytokine-	4:4275452	1	0	3	6	7	3	0.036076	0	0.119587	0.166938	0.162558	0.085106	0.051888	0.138201	0.106101	0.791592	-
ENSMUSC	Cdkl5	cyclin-dep	GO:00325	developm	-	-	-	-	X:1607719	3393	4845	3048	3718	4592	4504	14.00745	15.97404	13.90711	11.84172	12.20764	14.62799	14.62953	12.89245	0.106319	0.79254	-
ENSMUSC	Il15	interleukir	GO:00325	developm	K05433	interleukir	mmu052C	Pathways	8:8233163	6	15	6	19	16	22	0.079501	0.158736	0.087874	0.194225	0.13652	0.229335	0.108704	0.186693	0.106286	0.79254	-
ENSMUSC	Macc1	metastasi	GO:00081	metabolic	-	-	-	-	12:119314	2	2	0	0	14	6	0.010436	0.008338	0	0	0.047052	0.024636	0.006258	0.023896	0.106635	0.794552	-
ENSMUSC	AW55491	expressed	-	-	-	-	-	-	18:251689	213	252	206	179	342	202	1.074113	1.014887	1.148114	0.696394	1.110584	0.801371	1.079038	0.86945	0.107066	0.797424	-
ENSMUSC	Add2	adducin 2	GO:00325	developm	-	-	-	-	6:8602868	3637	9803	7347	4007	4659	9868	14.83621	31.93629	33.12352	12.61041	12.23846	31.66793	26.632	18.83893	0.107157	0.797762	-
ENSMUSC	Ncam2	neural cel	GO:00325	developm	K06491	neural cel	mmu0451	Cell adhes	16:812006	1825	2472	1663	2324	2559	1975	18.08359	19.56212	18.21213	17.76593	16.32851	15.39571	18.61928	16.49672	0.107294	0.797882	-
ENSMUSC	Plekha2	pleckstrin	GO:00511	localizatio	-	-	-	-	8:2503914	770	1138	818	918	1410	600	5.716771	6.747586	6.712134	5.258155	6.741153	3.504469	6.392164	5.167926	0.107311	0.797882	-
ENSMUSC	Pdzd3	PDZ dom	GO:00081	metabolic	-	-	-	-	9:4424730	7	23	21	30	44	32	0.162763	0.427102	0.539664	0.538147	0.658799	0.585346	0.37651	0.594098	0.107234	0.797882	-
ENSMUSC	Fam228a	family witl	-	-	-	-	-	-	12:471367	47	32	36	69	78	70	0.505407	0.274816	0.427861	0.572439	0.540125	0.592181	0.402695	0.568249	0.107579	0.799534	-
ENSMUSC	Nop58	NOP58 rit	GO:00081	metabolic	K14565	nucleolar	mmu030C	Ribosome	1:5968497	593	1088	519	1151	1280	1044	2.475893	3.627871	2.394923	3.707514	3.441452	3.429167	2.832895	3.526044	0.107997	0.801924	-
ENSMUSC	Tmem74b	transmem	GO:00160	membran	-	-	-	-	2:151702C	25	28	15	24	24	11	0.538615	0.481783	0.357176	0.398923	0.332972	0.186446	0.459191	0.306114	0.108039	0.801924	-
ENSMUSC	Ap5s1	adaptor-r	GO:00081	metabolic	-	-	-	-	2:131207C	87	125	61	71	64	115	1.020762	1.171282	0.791013	0.642676	0.483553	1.061491	0.994353	0.72924	0.107981	0.801924	-
ENSMUSC	Clec4d	C-type lec	GO:00325	developm	-	-	-	-	6:1232621	3	10	0	0	4	0	0.076544	0.203763	0	0	0.065725	0	0.093436	0.021908	0.108155	0.80244	-
ENSMUSC	Fgf7	fibroblast	GO:00325	developm	K04358	fibroblast	mmu052C	Pathways	2:1260346	22	17	15	27	49	33	0.243566	0.150308	0.183542	0.230614	0.349338	0.287427	0.192472	0.289127	0.108226	0.802626	-
ENSMUSC	Stxbp5l	syntaxin b	GO:00511	localizatio	-	-	-	-	16:371149	1776	2136	1550	2002	2400	1959	10.68261	10.26081	10.30417	9.290283	9.296091	9.270004	10.41586	9.285459	0.108501	0.802957	-
ENSMUSC	Tapbp	TAP bindi	GO:00023	immune s	K08058	TAP bindi	mmu0461	Antigen p	17:339158	478	652	427	786	1003	685	6.197865	6.751619	6.119124	7.862629	8.374722	6.987412	6.356203	7.741588	0.108478	0.802957	-
ENSMUSC	Srp72	signal rec	GO:00511	localizatio	K03108	signal rec	mmu0306	Protein ex	5:7697468	1220	1506	1867	1606	1931	1564	9.260433	9.129397	15.66254	9.404745	9.438612	9.339401	11.35079	9.394253	0.108469	0.802957	-
ENSMUSC	Neurod4	neurogen	GO:00325	developm	-	-	-	-	10:130268	6	5	0	9	16	6	0.109519	0.072886	0	0.126745	0.188067	0.086163	0.060801	0.133658	0.108485	0.802957	-
ENSMUSC	Cdca2	cell divisic	GO:00081	metabolic	-	-	-	-	14:676763	6	12	2	16	17	12	0.042499	0.067882	0.015658	0.087436	0.077544	0.066871	0.042013	0.077284	0.108329	0.802957	-
ENSMUSC	Sult1c1	sulfotrans	GO:00081	metabolic	-	-	-	-	17:539616	9	6	4	0	9	2	0.353155	0.188029	0.173488	0	0.227424	0.061753	0.238224	0.096392	0.108581	0.803206	-
ENSMUSC	Ccl4	chemokin	GO:00081	metabolic	K12964	C-C motif	mmu0406	Cytokine-	11:836625	0	5	0	0	0	0	0	0.369398	0	0	0	0	0.123133	0	0.108649	0.803338	-
ENSMUSC	Rex2	reduced e	GO:00081	metabolic	-	-	-	-	4:1470218	0	5	0	0	0	0	0	0.053395	0	0	0	0	0.017798	0	0.108692	0.803338	-
ENSMUSC	Anxa3	annexin A	GO:00325	developm	-	-	-	-	5:9679333	129	167	122	135	173	140	1.55416	1.606829	1.62448	1.254792	1.342174	1.326925	1.595157	1.307964	0.109177	0.806581	-
ENSMUSC	Zfp638	zinc finge	GO:00081	metabolic	-	-	-	-	6:838671C	1199	1364	1515	1494	1730	1405	3.12457	2.838776	4.363454	3.003669	2.903166	2.880437	3.442267	2.929091	0.109253	0.8068	-
ENSMUSC	Tap2	transporte	GO:00511	localizatio	K05654	ATP-bind	mmu0516	Herpes sir	17:342035	180	242	152	285	376	273	2.050454	2.201604	1.913682	2.504685	2.758168	2.44654	2.055247	2.569798	0.109471	0.808068	-
ENSMUSC	Clec2i	C-type lec	GO:00325	developm	-	-	-	-	6:1288875	0	5	3	0	2	1	0	0.04453	0.036979	0	0.01436	0.008775	0.02717	0.007712	0.109657	0.808806	-
ENSMUSC	Bst2	bone mar	GO:00081	metabolic	-	-	-	-	8:7153425	62	106	52	108	145	120	3.356038	4.582328	3.11086	4.510107	5.054266	5.11008	3.683075	4.891484	0.109664	0.808806	-
ENSMUSC	Taf4	TATA-box	GO:00325	developm	K03129	transcripti	mmu0516	Herpes sir	2:1799121	448	497	370	423	576	474	5.521608	4.892038	5.040063	4.022151	4.571564	4.595966	5.151236	4.39656	0.109824	0.809644	-
ENSMUSC	Zfp36l1	zinc finge	GO:00325	developm	K18753	butyrate r	mmu0421	Cellular se	12:801077	505	788	453	666	317	662	5.352254	6.669875	5.306281	5.445655	2.163513	5.519684	5.776137	4.376284	0.109913	0.809954	-
ENSMUSC	Cd248	CD248 an	GO:00325	developm	-	-	-	-	19:506807	43	88	56	103	103	118	1.025592	1.67623	1.476171	1.895268	1.581963	2.214095	1.392664	1.897109	0.110025	0.810441	-
ENSMUSC	Col1a1	collagen,	GO:00325	developm	K06236	collagen, t	mmu0415	PI3K-Akt s	11:949362	242	609	216	255	449	320	2.130955	4.282751	2.102129	1.732324	2.546014	2.216771	2.838612	2.165036	0.110121	0.810802	-
ENSMUSC	Rnf217	ring finge	GO:00081	metabolic	-	-	-	-	10:314931	407	506	327	432	538	410	2.256481	2.240442	2.003699	1.847784	1.920769	1.788267	2.166874	1.852273	0.110259	0.811474	-
ENSMUSC	Hdac9	histone de	GO:00325	developm	K11409	histone de	mmu0516	Human pa	12:340475	949	1341	793	1156	1246	1060	3.57371	4.032996	3.300449	3.358468	3.021523	3.140292	3.635718	3.173428	0.110453	0.811544	-
ENSMUSC	Arsg	arylsulfata	GO:00081	metabolic	K12381	arylsulfata	mmu0414	Lysosome	11:109473	212	350	291	328	304	276	2.342868	3.08905	3.55427	2.796506	2.163411	2.399554	2.995396	2.453157	0.110405	0.811544	-
ENSMUSC	Hsd17b1C	hydroxyst	GO:00081	metabolic	K08683	3-hydroxy	mmu011C	Metabolic	X:1520018	209	268	188	362	359	351	6.415874	6.57035	6.378413	8.57329	7.096725	8.476703	6.454879	8.048906	0.110455	0.811544	-
ENSMUSC	Nrsn2	neurensin	GO:00160	membran	-	-	-	-	2:1523687	819	1068	772	1413	1480	1241	34.36878	35.79294	35.80508	45.74598	39.99417	40.96974	35.32227	42.23663	0.110333	0.811544	-
ENSMUSC	Zfp457	zinc finge	GO:00081	metabolic	-	-	-	-	13:672881	4	9	1	9	14	11	0.034818	0.062564	0.00962	0.060436	0.078469	0.075323	0.035667	0.071409	0.110647	0.812613	-
ENSMUSC	Mkks	McKusick-	GO:00325	developm	-	-	-	-	2:1368737	193	203	153	361	330	235	3.049738	2.561814	2.672056	4.400922	3.357958	2.921356	2.761203	3.560079	0.110947	0.814211	-
ENSMUSC	Slx4ip	SLX4 inter	-	-	-	-	-	-	2:1368912	112	215	144	288	338	186	0.624006	0.956656	0.886709	1.237928	1.212672	0.815261	0.822457	1.08862	0.110958	0.814211	-
ENSMUSC	Pcdh7	protocadh	GO:00160	membran	-	-	-	-	5:5771796	1492	1946	664	1607	1198	1196	3.820614	3.979723	1.879227	3.174756	1.975494	2.409385	3.226521	2.519878	0.111006	0.814213	-
ENSMUSC	Best1	bestrophi	GO:00511	localizatio	-	-	-	-	19:998517	7	22	12	16	43	29	0.179511	0.450567	0.340099	0.316547	0.710078	0.585043	0.323392	0.537223	0.111115	0.814675	-
ENSMUSC	Limd2	LIM doma	GO:00056	cell|intrace	-	-	-	-	11:106156	702	976	769	1036	1401	1493	8.594901	9.543321	10.40584	9.785752	11.04579	14.38052	9.514688	11.73735	0.111537	0.817076	-
ENSMUSC	Col6a5	collagen,	-	-	K06238	collagen, t	mmu0415	PI3K-Akt s	9:1058560	46	55	36	81	91	68	0.266706	0.254674	0.23069	0.362317	0.339757	0.310167	0.25069	0.337414	0.111494	0.817076	-
ENSMUSC	Ash1l	ash1 (abs	GO:00325	developm	K06101	histone-ly	mmu0031	Lysine deg	3:8895062	4680	5714	4301	5338	6411	5520	16.14418	15.74186	16.39783	14.20623	14.24133	14.98029	16.09462	14.47595	0.111683	0.81739	-
ENSMUSC	Ociad1	OCIA don	GO:00325	developm	-	-	-	-	5:7329278	4950	6346	4742	6505	6761	5380	33.85279	34.66052	35.84243	34.32154	29.77521	28.94559	34.78525	31.01411	0.111743	0.81739	-
ENSMUSC	Paqr7	progestin	GO:00325	developm	-	-	-	-	4:1344966	487	616	447	1344	812	558	3.590788	3.627332	3.642631	7.645223	3.855415	3.236722	3.62025	4.912453	0.111635	0.81739	-
ENSMUSC	Umad1	UMAP1-M	-	-	-	-	-	-	6:8259288	439	675	396	497	685	483	2.425092	2.977919	2.417714	2.118118	2.436737	2.099042	2.606908	2.217966	0.111768	0.81739	-
ENSMUSC	Mcrip2	MAPK reg	GO:00329	macromo	-	-	-	-	17:258636	58	62	45	103	108	78	4.339907	3.705044	3.721457	5.94594	5.203942	4.591554	3.922136	5.247145	0.111887	0.817919	-
ENSMUSC	Zdhhc4	zinc finge	GO:00038	catalytic a	-	-	-	-	5:1433164	226	269	267	394	458	413	1.959823	1.862973	2.558982	2.635943	2.557582	2.817542	2.12726	2.670355	0.112033	0.818644	-
ENSMUSC	Plppr3	phospholi	GO:00160	membran	-	-	-	-	10:798604	488	476	423	545	606	383	3.555224	2.769494	3.405919	3.063192	2.842987	2.195112	3.243546	2.70043	0.112158	0.818864	-
ENSMUSC	Pclaf	PCNA clar	GO:00081	metabolic	-	-	-	-	9:6589023	9	18	11	21	44	18	0.103252	0.164923	0.139476	0.185872	0.325067	0.162458	0.135884	0.224466	0.112151	0.818864	-
ENSMUSC	Gimap3	GTPase, IM	GO:00099	cellular pr	-	-	-	-	6:4876446	2	8	4	6	22	10	0.029685	0.094849	0.065633	0.068715	0.210314	0.116787	0.063389	0.131939	0.112352	0.819942	-
ENSMUSC	C1rl	complem	GO:00081	metabolic	-	-	-	-	6:1244931	4	7	6	11	18	12	0.081145	0.113403	0.134525	0.172155	0.23513	0.191505	0.109691	0.199597	0.112645	0.82173	-
ENSMUSC	Epo	erythropo	GO:00325	developm	K05437	erythropo	mmu052C	Pathways	5:1374830	1	2	10	3	2	1	0.053064	0.084744	0.586274	0.122794	0.068306	0.041741	0.241361	0.077614	0.113264	0.825212	-
ENSMUSC	Jmjd4	jumonji d	-	-	-	-	-	-	11:594500	260	316	219	893	286	342	1.302414	1.264177	1.212456	3.451096	0.922562	1.347757	1.259682	1.907138	0.113231	0.825212	-
ENSMUSC	Gm765	predicted	-	-	-	-	-	-	6:9823570	2	0	1	6	5	3	0.032506	0	0.017968	0.075246	0.05234	0.038361	0.016825	0.055316	0.113225	0.825212	-
ENSMUSC	Rps6ka5	ribosomal	GO:00081	metabolic	K04445	ribosomal	mmu052C	Pathways	12:100548	422	554	328	669	739	645	1.487761	1.559826	1.278031	1.819603	1.677721	1.78892	1.441873	1.762081	0.113642	0.826886	-
ENSMUSC	Polr3g	polymeras	GO:00081	metabolic	K03024	DNA-dire	mmu011C	Metabolic	13:816738	104	90	93	122	81	82	0.928098	0.641426	0.917258	0.839941	0.465478	0.575685	0.828928	0.627035	0.113773	0.826886	-
ENSMUSC	Clasp1	CLIP asso	GO:00325	developm	-	-	-	-	1:1183890	1344	1865	1682	2956	3055	1957	1.820406	2.017408	2.51792	3.0889	2.664618	2.085311	2.118578	2.612943	0.113779	0.826886	-
ENSMUSC	Pnp2	purine-nu	GO:00081	metabolic	K03783	purine-nu	mmu011C	Metabolic	14:509559	5	5	3	9	13	10	0.17904	0.142993	0.118745	0.248658	0.299796	0.281726	0.146926	0.276727	0.113712	0.826886	-
ENSMUSC	Tmsb15b2	thymosin	GO:00511	localizatio	-	-	-	-	X:1369552	24	48	17	29	30	17	2.492209	3.980664	1.951038	2.323236	2.006029	1.388781	2.80797	1.906015	0.113564	0.826886	-
ENSMUSC	Ankrd31	ankyrin re	-	-	-	-	-	-	13:967482	2	4	1	6	8	7	0.018544	0.029622	0.01025	0.042926	0.047777	0.051072	0.019472	0.047258	0.113635	0.826886	-
ENSMUSC	Ttll1	tubulin tyr	GO:00325	developm	-	-	-	-	15:834837	619	568	489	587	677	618	8.716552	6.387755	7.610442	6.377084	6.138998	6.846264	7.571583	6.454115	0.113933	0.827194	-
ENSMUSC	Cox8b	cytochron	GO:00081	metabolic	K02273	cytochron	mmu011C	Metabolic	7:1408989	0	0	0	5	0	0	0	0	0	0.654204	0	0	0	0.218068	0.113997	0.827194	-
ENSMUSC	Klrb1a	killer cell l	GO:00160	membran	-	-	-	-	6:1286092	0	0	0	5	0	0	0	0	0	0.075703	0	0	0	0.025234	0.114048	0.827194	-
ENSMUSC	Star	steroidog	GO:00325	developm	K16931	steroidog	mmu0492	Aldostero	8:2580655	29	51	27	36	37	28	0.413614	0.580914	0.425604	0.396122	0.339826	0.314177	0.473377	0.350042	0.11406	0.827194	-
ENSMUSC	Krt26	keratin 26	GO:00051	structural	-	-	-	-	11:993285	0	0	0	5	0	0	0	0	0	0.04226	0	0	0	0.014087	0.113974	0.827194	-
ENSMUSC	Zc2hc1a	zinc finge	GO:00431	ion bindin	-	-	-	-	3:7503483	973	790	839	863	1229	835	13.29073	8.618041	12.66615	9.094459	10.81041	8.972913	11.52497	9.625928	0.114126	0.827328	-
ENSMUSC	Gpr65	G-protein	GO:00048	signal trar	-	-	-	-	12:982686	6	13	8	6	10	3	0.177061	0.306387	0.260913	0.1366	0.190035	0.069639	0.24812	0.132091	0.114263	0.827824	-
ENSMUSC	Isg20	interferon	GO:00081	metabolic	-	-	-	-	7:7891342	10	17	16	33	31	23	0.16136	0.219067	0.285339	0.410812	0.322118	0.291971	0.221922	0.341634	0.114289	0.827824	-
ENSMUSC	Iqcf5	IQ motif c	GO:00055	protein bi	-	-	-	-	9:1065145	0	0	0	0	0	5	0	0	0	0	0	0.435883	0	0.145294	0.114427	0.828477	-
ENSMUSC	Gm5741	predicted	GO:00048	signal trar	K04347	guanine n	mmu052C	Pathways	8:8506756	0	0	0	0	0	5	0	0	0	0	0	0.732231	0	0.244077	0.114488	0.828574	-
ENSMUSC	Gpihbp1	GPI-anch	GO:00511	localizatio	-	-	-	-	15:755966	0	0	0	0	0	5	0	0	0	0	0	0.059804	0	0.019935	0.114546	0.828651	-
ENSMUSC	Pla1a	phospholi	GO:00081	metabolic	K13618	phospholi	mmu0401	Ras signal	16:383961	19	22	21	23	17	15	0.588284	0.544016	0.71864	0.54941	0.338962	0.365373	0.61698	0.417915	0.114683	0.82878	-
ENSMUSC	Grm3	glutamate	GO:00048	signal trar	K04605	metabotr	mmu0408	Neuroacti	5:9485541	913	1178	873	1168	1126	1065	18.28338	18.83984	19.32178	18.04509	14.52042	16.77825	18.815	16.44792	0.114776	0.82878	-
ENSMUSC	Runx1t1	runt-relat	GO:00325	developm	K10053	runt-relat	mmu052C	Pathways	4:1374343	514	709	353	549	607	545	2.119957	2.335375	1.60911	1.746897	1.612158	1.768364	2.021481	1.70914	0.114803	0.82878	-
ENSMUSC	Ampd2	adenosine	GO:00325	developm	K01490	AMP dear	mmu011C	Metabolic	3:1080740	829	1077	737	917	1131	963	4.659593	4.83454	4.578338	3.97643	4.093653	4.258248	4.690824	4.109443	0.114669	0.82878	-
ENSMUSC	Zfp280b	zinc finge	GO:00971	organic cy	-	-	-	-	10:760326	435	364	261	482	732	578	4.851195	3.241952	3.216963	4.147027	5.256839	5.071055	3.770037	4.824974	0.114799	0.82878	-
ENSMUSC	Nfe2	nuclear fa	GO:00081	metabolic	-	-	-	-	15:103248	3	6	2	0	2	3	0.019927	0.031829	0.014684	0	0.008553	0.015675	0.022147	0.008076	0.114859	0.828845	-
ENSMUSC	Cpb1	carboxype	GO:00081	metabolic	K01291	carboxype	mmu0497	Pancreatic	3:2024826	0	1	0	0	1	8	0	0.01354	0	0	0.010913	0.106678	0.004513	0.039197	0.114919	0.828933	-
ENSMUSC	C130026I2	RIKEN cDI	GO:00056	cell|intrace	-	-	-	-	1:8499269	0	1	1	4	0	6	0	0.009128	0.012631	0.035261	0	0.053943	0.007253	0.029735	0.115021	0.829326	-
ENSMUSC	Apoc2	apolipopr	GO:00081	metabolic	-	-	mmu0497	Cholester	7:1967157	0	13	0	0	0	2	0	0.519177	0	0	0	0.078696	0.173059	0.026232	0.115095	0.829514	-
ENSMUSC	Rnf151	ring finge	GO:00325	developm	-	-	-	-	17:247158	0	0	4	0	0	0	0	0	0.227037	0	0	0	0.075679	0	0.115237	0.830193	-
ENSMUSC	Nrxn1	neurexin I	GO:00325	developm	K07377	neurexin[-	mmu0451	Cell adhes	17:900336	5112	5282	4320	5638	6469	5146	6.734177	5.556967	6.289616	5.729926	5.487638	5.333034	6.193587	5.516866	0.115341	0.830589	-
ENSMUSC	Ovca2	candidate	GO:00508	response	-	-	-	-	11:751759	205	294	217	414	404	340	3.665279	4.198026	4.288038	5.710622	4.651451	4.782365	4.050447	5.048146	0.115387	0.830589	-
ENSMUSC	Cdc20	cell divisic	GO:00325	developm	K03363	cell divisio	mmu0516	HTLV-I int	4:1184329	18	18	1	25	40	22	0.478042	0.381783	0.029355	0.512249	0.684092	0.459667	0.296393	0.552003	0.115589	0.831692	-
ENSMUSC	Cubn	cubilin (in	GO:00081	metabolic	K14616	cubilin[-]	mmu0497	Vitamin d	2:1327633	12	14	0	20	27	16	0.046104	0.042958	0	0.059281	0.066802	0.048361	0.029687	0.058148	0.115808	0.832693	-
ENSMUSC	Hdhd5	haloacid c	GO:00081	metabolic	-	-	-	-	6:1205094	202	305	230	382	400	409	2.202064	2.655362	2.771097	3.212707	2.807967	3.507612	2.542841	3.176095	0.115853	0.832693	-
ENSMUSC	Hist1h4m	histone cl	GO:00325	developm	K11254	histone H4	mmu052C	Viral carci	13:218117	10	6	4	6	3	4	1.518871	0.727793	0.671511	0.703048	0.293458	0.47793	0.972725	0.491479	0.115871	0.832693	-
ENSMUSC	Kptn	kaptin [Sc	GO:00452	synapse|c	-	-	-	-	7:1611989	135	187	145	256	290	222	1.468262	1.624273	1.742951	2.148032	2.031063	1.899482	1.611829	2.026192	0.116035	0.833523	-
ENSMUSC	Rpusd3	RNA pseu	GO:00081	metabolic	-	-	-	-	6:1134153	94	154	134	199	235	213	1.298227	1.698597	2.045383	2.120343	2.089999	2.314271	1.680736	2.174871	0.116127	0.833841	-
ENSMUSC	Klk1b5	kallikrein	GO:00081	metabolic	K01325	tissue kalli	mmu0496	Endocrine	7:4421594	0	0	0	0	6	0	0	0	0	0	0.169591	0	0	0.05653	0.1163	0.834742	-
ENSMUSC	Rpl10	ribosomal	GO:00325	developm	K02866	large subu	mmu0301	Ribosome	X:7427081	62	62	53	58	81	41	0.734215	0.586369	0.693665	0.529892	0.617689	0.381966	0.671416	0.509849	0.116393	0.835063	-
ENSMUSC	Gk	glycerol k	GO:00081	metabolic	K00864	glycerol k	mmu011C	Metabolic	X:8570193	520	606	496	417	722	649	3.500578	3.258024	3.690322	2.165723	3.12988	3.437098	3.482975	2.9109	0.116525	0.835651	-
ENSMUSC	Btaf1	B-TFIID T	GO:00511	localizatio	-	-	-	-	19:369260	1013	1194	904	1186	1460	902	8.585764	8.082016	8.468081	7.755049	7.96851	6.014325	8.37862	7.245961	0.116571	0.835651	-
ENSMUSC	Egf	epiderma	GO:00325	developm	K04357	epiderma	mmu052C	Pathways	3:1296775	30	36	19	45	56	53	0.124229	0.119056	0.086957	0.143761	0.149328	0.172658	0.110081	0.155249	0.116687	0.836138	-
ENSMUSC	Vps26a	VPS26 ret	GO:00511	localizatio	K18466	vacuolar p	mmu0414	Endocytos	10:624552	1195	1394	985	1189	1686	1246	11.53223	10.74371	10.50578	8.852332	10.4775	9.45964	10.92724	9.596492	0.116787	0.836246	-
ENSMUSC	Lrrc71	leucine ric	-	-	-	-	-	-	3:8773692	4	20	11	7	7	10	0.032666	0.130434	0.099276	0.0441	0.036808	0.064243	0.087459	0.048384	0.116804	0.836246	-
ENSMUSC	Lat2	linker for	GO:00511	localizatio	-	-	-	-	5:1346000	35	47	31	35	44	32	0.343628	0.368526	0.336382	0.265105	0.278181	0.247165	0.349512	0.263484	0.116883	0.836246	-
ENSMUSC	Alyref2	Aly/REF e:	GO:00971	organic cy	K12881	THO com	mmu0516	Herpes sir	1:1715034	58	50	59	85	124	95	2.781904	1.915257	3.127618	3.145297	3.829929	3.584691	2.60826	3.519972	0.116895	0.836246	-
ENSMUSC	Erbin	Erbb2 inte	GO:00081	metabolic	K12796	erbb2-int	mmu0462	NOD-like	13:103818	1607	2281	1457	2178	3222	2904	8.404315	9.527032	8.421564	8.787689	10.85092	11.94797	8.784304	10.52886	0.117044	0.836276	-
ENSMUSC	B4galnt3	beta-1,4-	GO:00038	catalytic a	K09656	beta-1,4-	mmu011C	Metabolic	6:1202030	16	20	16	23	51	32	0.178695	0.17839	0.197498	0.198178	0.366793	0.281163	0.184861	0.282045	0.116954	0.836276	-
ENSMUSC	Trpt1	tRNA pho	GO:00081	metabolic	-	-	-	-	19:699613	48	47	54	97	83	91	2.566356	2.006883	3.190914	4.001105	2.857673	3.827614	2.588051	3.562131	0.117091	0.836276	-
ENSMUSC	Npm3	nucleopla	GO:00081	metabolic	-	-	-	-	19:457477	41	68	45	52	60	41	1.693749	2.243497	2.054607	1.657307	1.596163	1.332488	1.997285	1.528653	0.116996	0.836276	-
ENSMUSC	Aurka	aurora kir	GO:00325	developm	K11481	aurora kir	mmu0411	Oocyte m	2:1723561	15	29	22	37	49	38	0.236105	0.364564	0.382739	0.44932	0.496674	0.470568	0.327802	0.472187	0.117198	0.836354	-
ENSMUSC	Synpo2	synaptopc	GO:00081	metabolic	-	-	-	-	3:1230765	162	198	108	240	296	232	0.432582	0.422245	0.318732	0.494417	0.508978	0.487363	0.391186	0.496919	0.117155	0.836354	-
ENSMUSC	Lrrc7	leucine ric	-	-	-	-	-	-	3:1580828	2535	3323	2239	2825	3398	3141	10.78021	11.28562	10.52326	9.268258	9.305237	10.5082	10.86303	9.693898	0.117588	0.83845	-
ENSMUSC	Ganc	glucosida:	GO:00081	metabolic	K12317	neutral alp	mmu011C	Metabolic	2:1204038	123	201	156	232	301	261	0.392277	0.511954	0.549868	0.57083	0.618173	0.654846	0.4847	0.614616	0.117547	0.83845	-
ENSMUSC	Teddm2	transmem	GO:00160	membran	-	-	-	-	1:1538495	33	44	26	53	63	68	0.304094	0.323808	0.264794	0.37679	0.373839	0.492962	0.297565	0.41453	0.1179	0.840324	-
ENSMUSC	Clca3a1	chloride c	GO:00081	metabolic	K05030	calcium-a	mmu0497	Pancreatic	3:1447296	4	11	0	5	24	15	0.046817	0.102813	0	0.045143	0.180879	0.138109	0.049877	0.121377	0.118032	0.840922	-
ENSMUSC	Fam83d	family witl	GO:00325	developm	-	-	-	-	2:1587680	30	28	31	26	28	31	0.445034	0.331723	0.508251	0.297553	0.267474	0.361777	0.428336	0.308935	0.118314	0.842585	-
ENSMUSC	Zfp622	zinc finge	GO:00081	metabolic	-	-	-	-	15:259843	675	904	455	991	1220	984	4.900623	5.241572	3.650946	5.550747	5.703766	5.620223	4.597714	5.624912	0.118365	0.842607	-
ENSMUSC	Cbfa2t2	core-binc	GO:00325	developm	-	-	-	-	2:1544364	469	209	260	307	316	318	2.725963	0.97015	1.670194	1.376624	1.182741	1.454069	1.788769	1.337811	0.118801	0.845367	-
ENSMUSC	Grp	gastrin rel	GO:00511	localizatio	-	-	-	-	18:658728	237	296	227	400	460	362	6.610681	6.593786	6.997953	8.607719	8.262472	7.943604	6.73414	8.271265	0.119002	0.846449	-
ENSMUSC	Slc25a1	solute car	GO:00511	localizatio	-	-	-	-	16:179252	478	569	387	714	888	664	7.706825	7.326664	6.89614	8.881297	9.219672	8.422231	7.309876	8.841067	0.11911	0.846771	-
ENSMUSC	Fam96a	family witl	GO:00099	cellular pr	-	-	-	-	9:6612661	164	184	153	262	343	230	8.075409	7.235774	8.326471	9.953002	10.87602	8.909636	7.879218	9.912886	0.119145	0.846771	-
ENSMUSC	2610028H	RIKEN cDI	-	-	-	-	-	-	10:764490	4	2	5	0	0	5	0.105552	0.042152	0.145823	0	0	0.103791	0.097842	0.034597	0.119455	0.848285	-
ENSMUSC	Glb1l2	galactosic	GO:00081	metabolic	-	-	-	-	9:2676304	20	31	14	13	28	16	0.094893	0.117463	0.073413	0.047584	0.085549	0.059722	0.095256	0.064285	0.119449	0.848285	-
ENSMUSC	G6pc3	glucose 6	GO:00081	metabolic	K01084	glucose-6	mmu011C	Metabolic	11:102189	380	551	417	525	621	297	5.038458	5.834605	6.110783	5.37036	5.302247	3.097995	5.661282	4.590201	0.11958	0.848823	-
ENSMUSC	Tst	thiosulfate	GO:00325	developm	K01011	thiosulfate	mmu011C	Metabolic	15:783995	183	213	161	274	365	271	10.19495	9.476735	9.913003	11.77644	13.09424	11.87716	9.861562	12.24928	0.11965	0.848975	-
ENSMUSC	Sesn3	sestrin 3 [	GO:00081	metabolic	K10141	sestrin 1/3	mmu0411	p53 signa	9:1427506	493	1032	751	1052	3491	513	1.757864	2.938758	2.959544	2.893906	8.015723	1.439018	2.552055	4.116216	0.120079	0.85027	-
ENSMUSC	Mb21d1	Mab-21 c	GO:00325	developm	K17834	cyclic GM	mmu0462	Cytosolic I	9:7843052	28	37	36	53	62	71	0.192047	0.202674	0.272904	0.280455	0.273843	0.38311	0.222542	0.312469	0.120118	0.85027	-
ENSMUSC	Prr5	proline ric	GO:00081	metabolic	K20411	proline-ri	-	-	15:846696	95	132	88	140	218	172	1.938683	2.151302	1.984774	2.204144	2.864791	2.761355	2.02492	2.610097	0.120066	0.85027	-
ENSMUSC	Dennd4c	DENN/MA	GO:00511	localizatio	-	-	-	-	4:8674855	387	600	356	604	781	727	2.476369	3.066203	2.517682	2.981749	3.218176	3.659734	2.686751	3.286553	0.120107	0.85027	-
ENSMUSC	Ubxn10	UBX dom	GO:00329	macromo	-	-	-	-	4:1387098	33	60	56	34	58	51	0.227856	0.330858	0.427343	0.181112	0.257884	0.277028	0.328686	0.238675	0.120106	0.85027	-
ENSMUSC	Ifit3b	interferon	GO:00023	immune s	-	-	-	-	19:346079	69	94	75	169	132	112	2.106513	2.29186	2.530591	3.980431	2.59503	2.689933	2.309655	3.088465	0.120126	0.85027	-
ENSMUSC	Krt75	keratin 75	GO:00325	developm	-	-	-	-	15:101563	5	2	3	0	3	2	0.093467	0.029865	0.06199	0	0.036121	0.029421	0.061774	0.021847	0.120267	0.85092	-
ENSMUSC	Rps11	ribosomal	GO:00325	developm	K02949	small subu	mmu0301	Ribosome	7:4512237	604	699	524	1022	948	903	24.31056	22.46884	23.30963	31.73505	24.57088	28.59277	23.36301	28.29957	0.120509	0.851939	-
ENSMUSC	Enpp6	ectonucle	GO:00081	metabolic	K08743	ectonucle	mmu0056	Ether lipid	8:4698688	105	222	146	125	181	174	0.745133	1.258175	1.145097	0.684358	0.827134	0.97141	1.049468	0.827634	0.120469	0.851939	-
ENSMUSC	Scube3	signal pep	GO:00325	developm	-	-	-	-	17:281423	55	95	46	47	55	80	0.292832	0.403949	0.270683	0.193058	0.188572	0.335084	0.322488	0.238905	0.120711	0.85302	-
ENSMUSC	Bmpr2	bone mor	GO:00325	developm	K04671	bone mor	mmu0406	Cytokine-	1:5976340	6376	7788	5517	7171	8510	7425	21.21561	20.69565	20.28883	18.40846	18.2344	19.43635	20.73336	18.69307	0.120789	0.853224	-
ENSMUSC	Ccdc85a	coiled-co	-	-	-	-	-	-	11:283856	1289	1689	1176	1400	1966	1446	4.662829	4.879463	4.701654	3.907103	4.579677	4.115058	4.747982	4.200613	0.120989	0.854291	-
ENSMUSC	C1qb	complem	GO:00325	developm	K03987	complem	mmu0532	Systemic I	4:1368801	555	909	615	721	851	757	31.14638	40.74028	38.14487	31.21619	30.75381	33.42115	36.67718	31.79705	0.121127	0.854839	-
ENSMUSC	Vgll2	vestigial li	GO:00325	developm	-	-	-	-	10:520225	0	6	1	1	0	0	0	0.12671	0.029228	0.020404	0	0	0.051979	0.006801	0.121165	0.854839	-
ENSMUSC	Chn1	chimerin	GO:00325	developm	-	-	-	-	2:7361066	19347	24345	18273	23721	31224	12062	125.1766	125.7955	130.667	118.4057	130.0926	61.39594	127.213	103.2981	0.121308	0.8555	-
ENSMUSC	Rhoc	ras homol	GO:00325	developm	-	-	-	-	3:1047883	131	185	89	200	302	182	2.673341	3.015083	2.00733	3.14878	3.968659	2.921898	2.565252	3.346446	0.121592	0.856455	-
ENSMUSC	Pafah1b3	platelet-a	GO:00325	developm	K16795	platelet-a	mmu011C	Metabolic	7:2529504	78	87	58	113	137	127	1.326242	1.181392	1.089939	1.482306	1.500042	1.698797	1.199191	1.560382	0.121706	0.856455	-
ENSMUSC	Pi16	peptidase	GO:00055	extracellul	-	-	-	-	17:293188	10	25	16	15	18	10	0.081793	0.163304	0.144638	0.094653	0.094808	0.064346	0.129912	0.084603	0.121739	0.856455	-
ENSMUSC	Rbpms2	RNA bind	GO:00325	developm	-	-	-	-	9:6562964	30	36	27	40	65	68	0.370352	0.35493	0.368381	0.380964	0.516725	0.660405	0.364554	0.519365	0.121539	0.856455	-
ENSMUSC	Gm4841	predicted	GO:00508	response	-	-	-	-	18:602683	0	0	2	2	17	0	0	0	0.047559	0.033201	0.235529	0	0.015853	0.089577	0.121569	0.856455	-
ENSMUSC	AC175538	-	-	-	-	-	-	-	13:115035	2	2	14	3	6	0	0.044592	0.035625	0.345051	0.05162	0.086158	0	0.141756	0.045926	0.121721	0.856455	-
ENSMUSC	Nadsyn1	NAD synt	GO:00081	metabolic	K01950	NAD+ syr	mmu011C	Metabolic	7:1437955	39	63	48	84	103	75	0.464547	0.599305	0.63191	0.771927	0.790056	0.702805	0.565254	0.754929	0.121886	0.857143	-
ENSMUSC	4930426L	RIKEN cDI	-	-	-	-	-	-	2:1899834	11	22	7	7	14	10	0.460358	0.735298	0.323789	0.226002	0.377287	0.329228	0.506482	0.310839	0.121996	0.857573	-

ENSMUS(	Tc2n	tandem C	GO:00081	metabolic	-	-	-	-	12:101645	10	37	14	33	47	37	0.084277	0.249028	0.1304	0.214563	0.255069	0.245313	0.154568	0.238315	0.122112	0.85803	-
ENSMUS(	4833427G	RIKEN cDI	-	-	-	-	-	-	9:5108111	2	8	4	3	2	2	0.125481	0.400939	0.277438	0.145258	0.080802	0.098754	0.267953	0.108272	0.12216	0.85803	-
ENSMUS(	Man2c1	mannosid	GO:00081	metabolic	K01191	alpha-ma	mmu0051	Other glyc	9:5713069	733	1032	749	1221	1387	1285	3.562493	4.005674	4.023264	4.578214	4.340914	4.913195	3.86381	4.610775	0.122244	0.858274	-
ENSMUS(	Bard1	BRCA1 as	GO:00081	metabolic	K10683	BRCA1-as	mmu0344	Homolog	1:7102749	11	12	6	20	21	20	0.118689	0.103401	0.071554	0.166478	0.145904	0.169763	0.097881	0.160715	0.122428	0.859222	-
ENSMUS(	6030458C	RIKEN cDI	-	-	-	-	-	-	15:128081	411	665	628	871	1118	824	2.235424	2.888588	3.775065	3.654827	3.915749	3.525791	2.966359	3.698789	0.122655	0.860462	-
ENSMUS(	Panx1	pannexin	GO:00511	localizatio	K03443	pannexin	mmu0462	NOD-like	9:1500212	255	315	186	373	462	375	2.238005	2.207889	1.804181	2.525573	2.611064	2.589187	2.083359	2.575275	0.122708	0.860491	-
ENSMUS(	Il2rg	interleukir	GO:00325	developm	K05070	interleukir	mmu0520	Pathways	X:1012643	24	32	22	42	61	40	0.403363	0.429508	0.408655	0.544571	0.660182	0.528867	0.413842	0.577873	0.122805	0.860822	-
ENSMUS(	Nlrp10	NLR famil	GO:00511	localizatio	-	-	-	-	7:1089218	4	0	0	6	7	3	0.054229	0	0	0.06275	0.061104	0.03199	0.018076	0.051948	0.122982	0.861719	-
ENSMUS(	Atrx	ATRX, chr	GO:00325	developm	-	-	-	-	X:1057976	4744	5957	4018	7098	8329	6839	7.935664	7.958148	7.428401	9.160199	8.971935	8.999991	7.774071	9.044042	0.123238	0.862467	-
ENSMUS(	Neu4	sialidase 4	GO:00081	metabolic	K12357	sialidase-:	mmu0060	Sphingolip	1:9402045	46	142	111	165	187	193	0.489233	1.206123	1.304739	1.353851	1.280711	1.614816	1.000032	1.416459	0.123146	0.862467	-
ENSMUS(	Ttn	titin [Sour	GO:00325	developm	K12567	titin[2.7.11	mmu0541	Dilated ca	2:7670398	55	117	71	67	87	87	0.024963	0.04241	0.035616	0.023461	0.025428	0.031065	0.03433	0.026651	0.123194	0.862467	-
ENSMUS(	Triobp	TRIO and	GO:00325	developm	-	-	-	-	15:789477	138	228	127	240	320	242	0.547854	0.722881	0.557232	0.735068	0.818073	0.755811	0.609322	0.769651	0.12344	0.863534	-
ENSMUS(	Klhl3	kelch-like	GO:00325	developm	-	-	-	-	13:580002	2051	2314	1103	1878	2552	1501	17.10327	15.41072	10.16567	12.08202	13.70405	9.84703	14.22655	11.8777	0.123574	0.864125	-
ENSMUS(	Map4k5	mitogen-	GO:00048	signal trar	-	-	-	-	12:698037	770	970	714	1093	1756	1090	3.161291	3.180471	3.239808	3.461979	4.642514	3.520557	3.193857	3.875017	0.123769	0.864704	-
ENSMUS(	Bank1	B cell scaf	GO:00081	metabolic	-	-	-	-	3:1360533	13	20	20	30	43	31	0.081004	0.099526	0.137736	0.144216	0.172538	0.151962	0.106089	0.156239	0.123738	0.864704	-
ENSMUS(	Gm28539	predicted	-	-	-	-	-	-	16:188365	31	50	109	47	68	0	0.634734	0.817613	2.466667	0.74245	0.896597	0	1.306338	0.546349	0.123806	0.864704	-
ENSMUS(	Isyna1	myo-inos	GO:00081	metabolic	K01858	myo-inos	mmu0110	Metabolic	8:7059437	187	286	194	352	322	410	2.334461	2.851399	2.676668	3.390145	2.588548	4.026616	2.620843	3.335103	0.12387	0.864804	-
ENSMUS(	Fst	follistatin	GO:00325	developm	K04661	follistatin[	mmu0435	TGF-beta	13:114452	22	28	31	50	60	42	0.220316	0.223942	0.343113	0.386301	0.386933	0.330894	0.262457	0.368043	0.124071	0.865856	-
ENSMUS(	Xkr6	X-linked I	GO:00160	membran	-	-	-	-	14:636065	113	105	96	102	149	81	0.67869	0.503649	0.637249	0.472632	0.576279	0.382726	0.606529	0.477212	0.124328	0.867306	-
ENSMUS(	Sncg	synuclein,	GO:00511	localizatio	-	-	-	-	14:343702	53	69	57	50	75	60	3.798241	3.949131	4.514653	2.764408	3.461152	3.3827	4.087341	3.202753	0.124538	0.868037	-
ENSMUS(	Zgpat	zinc finge	GO:00081	metabolic	-	-	-	-	2:1813649	345	591	466	820	761	679	1.7811	2.436702	2.658894	3.265976	2.529929	2.757716	2.292232	2.851207	0.124583	0.868037	-
ENSMUS(	Gm35911	predicted	GO:00511	localizatio	-	-	-	-	5:9990625	0	5	0	1	8	9	0	0.122884	0	0.023746	0.158549	0.217893	0.040961	0.133396	0.124581	0.868037	-
ENSMUS(	Gm10184	predicted	GO:00511	localizatio	-	-	-	-	17:899085	46	58	56	89	110	83	1.448522	1.458605	1.948942	2.162152	2.23055	2.056153	1.61869	2.149618	0.124637	0.86807	-
ENSMUS(	Spx	spexin ho	GO:00081	metabolic	-	-	-	-	6:1424134	16	3	2	5	4	3	0.479591	0.071827	0.066261	0.115618	0.077212	0.070733	0.205893	0.087854	0.124907	0.869598	-
ENSMUS(	Shisa5	shisa fami	GO:00048	signal trar	K10135	scotin[-]	mmu0411	p53 signa	9:1090385	515	551	458	848	921	674	3.480731	2.974137	3.421177	4.421701	4.008459	3.583721	3.292015	4.004627	0.125044	0.870205	-
ENSMUS(	Ube2a	ubiquitin-	GO:00325	developm	K10573	ubiquitin-	mmu0412	Ubiquitin	X:3687390	330	566	451	333	663	452	3.398409	4.655045	5.13316	2.645676	4.396734	3.66194	4.395538	3.568116	0.125116	0.870333	-
ENSMUS(	Dus4l	dihydrour	GO:00081	metabolic	-	-	-	-	12:316400	66	99	78	142	151	118	1.747553	2.093462	2.282585	2.900704	2.574637	2.457971	2.0412	2.644438	0.125232	0.870333	-
ENSMUS(	Irf4	interferon	GO:00325	developm	K09445	interferon	mmu0465	Th17 cell (	13:307492	8	10	0	11	23	12	0.092764	0.092595	0	0.098402	0.171731	0.109463	0.061786	0.126532	0.125313	0.870333	-
ENSMUS(	Parp16	poly (ADP	GO:00081	metabolic	-	-	-	-	9:6519535	50	62	65	90	101	125	0.420688	0.416607	0.604434	0.584198	0.547219	0.827386	0.480576	0.652934	0.12519	0.870333	-
ENSMUS(	Mansc4	MANSC d	GO:00160	membran	-	-	-	-	6:1470750	8	5	8	1	5	8	0.293037	0.146252	0.323832	0.028262	0.117931	0.230507	0.254374	0.125567	0.125284	0.870333	-
ENSMUS(	Mdk	midkine [S	GO:00325	developm	-	-	-	-	2:9192980	159	164	159	138	242	140	5.305051	4.370001	5.863212	3.552227	5.199507	3.674785	5.179421	4.142173	0.125655	0.872359	-
ENSMUS(	Ldlr	low densit	GO:00081	metabolic	K12473	low-densi	mmu0414	Endocytos	9:2172348	379	494	363	617	839	532	1.337941	1.392741	1.416285	1.680401	1.907279	1.477474	1.382322	1.688385	0.125839	0.872592	-
ENSMUS(	Zfp217	zinc finge	GO:00325	developm	-	-	-	-	2:1701086	112	121	95	136	232	196	0.782435	0.675094	0.733507	0.732997	1.043698	1.077207	0.730345	0.951301	0.125821	0.872592	-
ENSMUS(	Hnrnpa3	heteroger	GO:00081	metabolic	K12741	heteroger	mmu0304	Spliceosor	2:7565926	1463	2055	170	1513	189	446	12.06973	13.53975	1.550056	9.629909	1.004083	2.894666	9.053177	4.509553	0.125785	0.872592	-
ENSMUS(	Lnpk	lunapark,	GO:00325	developm	-	-	-	-	2:7452029	858	548	544	728	645	709	3.312332	1.689558	2.321092	2.168244	1.603471	2.153296	2.440994	1.975004	0.126307	0.872762	-
ENSMUS(	Map2k3	mitogen-	GO:00048	signal trar	K04432	mitogen-i	mmu0401	MAPK sig	11:609320	77	158	93	168	184	195	0.927859	1.520531	1.238579	1.561822	1.427793	1.848578	1.228989	1.612731	0.126356	0.872762	-
ENSMUS(	Pik3cg	phosphati	GO:00325	developm	K00922	phosphati	mmu0415	PI3K-Akt s	12:321734	64	82	54	66	75	66	0.240445	0.246036	0.22422	0.191298	0.181449	0.19507	0.2369	0.189272	0.126365	0.872762	-
ENSMUS(	Capn1	calpain 1	GO:00325	developm	K01367	calpain-1	mmu0421	Cellular se	19:598854	422	501	449	664	848	688	8.077284	7.658366	9.498308	9.805077	10.45209	10.35981	8.411319	10.20566	0.126229	0.872762	-
ENSMUS(	6430571L	RIKEN cDI	GO:00160	membran	-	-	-	-	9:1073406	46	66	46	87	101	82	0.915776	1.049347	1.012128	1.33622	1.294805	1.284263	0.992417	1.305096	0.126242	0.872762	-
ENSMUS(	Sbsn	suprabasi	-	-	-	-	-	-	7:3075147	58	89	54	105	138	96	0.612057	0.750072	0.6298	0.854841	0.937774	0.796977	0.663977	0.863197	0.12635	0.872762	-
ENSMUS(	Dmrta2	doublesex	GO:00325	developm	-	-	-	-	4:1099780	26	23	20	42	51	37	0.543296	0.383836	0.4619	0.677079	0.686262	0.608244	0.463011	0.657195	0.126285	0.872762	-
ENSMUS(	Fbxl22	F-box and	GO:00081	metabolic	-	-	-	-	9:6650845	6	9	6	10	19	17	0.093098	0.111533	0.102902	0.119714	0.189848	0.207523	0.102511	0.172362	0.126116	0.872762	-
ENSMUS(	Wfdc17	WAP four	GO:00081	metabolic	-	-	-	-	11:837039	8	10	0	0	7	0	0.916497	0.914831	0	0	0.516344	0	0.610443	0.172115	0.125919	0.872762	-
ENSMUS(	Arhgap8	Rho GTPa	GO:00987	molecular	-	-	-	-	15:847200	3	0	1	9	10	0	0.03489	0	0.012855	0.080755	0.074892	0	0.015915	0.051882	0.126278	0.872762	-
ENSMUS(	Mrpl17	mitochon	GO:00081	metabolic	K02879	large subu	mmu0301	Ribosome	7:1058032	367	473	371	348	555	449	2.248253	2.314118	2.511885	1.644707	2.189407	2.163897	2.358086	1.999337	0.12642	0.872798	-
ENSMUS(	Abhd15	abhydrola	GO:00081	metabolic	-	-	-	-	11:775151	18	16	12	10	16	14	0.297434	0.211157	0.219154	0.12749	0.170252	0.181995	0.242582	0.159912	0.127449	0.875695	-
ENSMUS(	Fam131c	family wit	-	-	-	-	-	-	4:1413682	33	26	26	33	29	18	1.406091	0.884739	1.224372	1.084795	0.795698	0.603349	1.171734	0.827947	0.127457	0.875695	-
ENSMUS(	Wt1	Wilms tun	GO:00081	metabolic	K09234	Wilms tun	mmu0520	Transcript	2:1051265	2	4	0	4	5	10	0.011242	0.017959	0	0.017348	0.018102	0.044231	0.009734	0.02656	0.127035	0.875695	-
ENSMUS(	Mttp	microsom	GO:00081	metabolic	K14463	microsom	mmu0497	Fat digest	3:1380898	215	245	193	221	298	208	1.98962	1.810686	1.973946	1.577806	1.775833	1.51428	1.924751	1.62264	0.127155	0.875695	-
ENSMUS(	Ablim3	actin bind	GO:00081	metabolic	K07520	actin-binc	mmu0436	Axon guic	18:617993	795	1004	704	493	1169	1001	10.09383	10.18048	9.878884	4.829094	9.557799	9.998468	10.05106	8.128453	0.127514	0.875695	-
ENSMUS(	Nod1	nucleotide	GO:00081	metabolic	K08727	nucleotide	mmu0462	NOD-like	6:5492394	91	116	80	154	168	153	0.922206	0.938833	0.896036	1.204029	1.09635	1.219798	0.919025	1.173392	0.127427	0.875695	-
ENSMUS(	Gm11639	predicted	GO:00431	ion bindin	-	-	-	-	11:104685	3	13	10	19	16	22	0.009493	0.032854	0.034973	0.046384	0.032603	0.054769	0.025773	0.044586	0.127409	0.875695	-
ENSMUS(	Hmga1	high mob	GO:00325	developm	-	-	-	-	17:275566	294	417	482	357	122	529	2.867125	3.247743	5.195092	2.685954	0.766152	4.058506	3.769986	2.503537	0.127236	0.875695	-
ENSMUS(	Tgoln1	trans-gol	GO:00511	localizatio	-	-	-	-	6:7260843	3320	4205	2822	4813	6530	4531	40.43745	40.90321	37.98827	45.22638	51.21692	43.41608	39.77631	46.61979	0.127445	0.875695	-
ENSMUS(	Zfp729b	zinc finge	GO:00081	metabolic	-	-	-	-	13:675894	236	349	157	251	329	197	1.542134	1.8213	1.133851	1.265361	1.384397	1.012718	1.499095	1.220825	0.127576	0.875695	-
ENSMUS(	Zfp462	zinc finge	GO:00081	metabolic	-	-	-	-	4:5494504	1338	1588	1207	1533	1733	1541	4.705705	4.46031	4.691618	4.159499	3.92484	4.263656	4.619211	4.115998	0.12704	0.875695	-
ENSMUS(	Celsr2	cadherin,	GO:00048	signal trar	-	-	-	-	3:1083908	6889	8314	6069	8168	8448	8192	29.8488	28.76912	29.06261	27.3034	23.57107	27.92361	29.22684	26.26603	0.127646	0.875695	-
ENSMUS(	Gm15319	predicted	-	-	-	-	-	-	8:2033766	10	16	12	7	9	14	0.242104	0.309368	0.321085	0.130744	0.140314	0.266642	0.290852	0.179233	0.127627	0.875695	-
ENSMUS(	Dthd1	death dor	GO:00508	response	-	-	-	-	5:6281382	4	10	2	1	2	5	0.100637	0.200908	0.055613	0.019412	0.032394	0.098958	0.119053	0.050255	0.127453	0.875695	-
ENSMUS(	1810049J	RIKEN cDI	-	-	-	-	-	-	15:123214	9	11	4	2	9	6	1.189415	1.160985	0.584302	0.203949	0.765955	0.623844	0.978234	0.531249	0.127325	0.875695	-
ENSMUS(	Pcdhgb7	protocadh	GO:00160	membran	-	-	-	-	18:377516	246	280	221	258	340	237	3.186991	2.897013	3.164353	2.57867	2.836481	2.415491	3.082786	2.610214	0.126968	0.875695	-
ENSMUS(	Usp45	ubiquitin s	GO:00081	metabolic	-	-	-	-	4:2176715	458	632	644	729	762	1663	2.295382	2.5296	3.567151	2.818687	2.459228	6.556803	2.797378	3.944906	0.127732	0.87594	-
ENSMUS(	Vps25	vacuolar p	GO:00081	metabolic	K12189	ESCRT-II (	mmu0414	Endocytos	11:101253	256	305	249	252	321	321	3.864246	3.676806	4.154041	2.934647	3.120218	3.811899	3.898364	3.288921	0.127893	0.876701	-
ENSMUS(	Gm17732	predicted	GO:00160	membran	-	-	-	-	18:626621	17	20	13	34	37	25	1.264315	1.187891	1.068529	1.950762	1.77198	1.462678	1.173578	1.728474	0.127956	0.876784	-
ENSMUS(	Stk10	serine/thr	GO:00081	metabolic	K08837	serine/thr	mmu0491	Progester	11:325333	147	218	166	259	361	251	1.640271	1.942673	2.047165	2.229603	2.593934	2.20334	1.876703	2.342292	0.128014	0.876836	-
ENSMUS(	Adamts13	a disinteg	GO:00081	metabolic	-	-	-	-	2:2697341	11	20	8	27	26	23	0.123173	0.178848	0.098997	0.233236	0.187468	0.202608	0.133673	0.207771	0.128123	0.877182	-
ENSMUS(	Tti1	TELO2 int	GO:00508	response	K20403	TELO2-int	mmu0415	mTOR sig	2:1579818	353	243	310	500	585	493	2.680118	1.473435	2.601284	2.92873	2.860154	2.944673	2.251612	2.911186	0.128266	0.877182	-
ENSMUS(	Mob1a	MOB kina	GO:00508	response	K06685	MOB kina	mmu0439	Hippo sig	6:8332601	536	797	209	797	980	838	3.02721	3.594856	1.304581	3.472691	3.564169	3.723338	2.642216	3.586733	0.128205	0.877182	-
ENSMUS(	Actn4	actinin alp	GO:00325	developm	K05699	actinin alp	mmu0520	Viral carci	7:2889324	2343	3174	2284	3513	4665	3749	13.89059	15.02799	14.9655	16.06781	17.80962	17.48536	14.62803	17.12093	0.128219	0.877182	-
ENSMUS(	Gm1141	predicted	-	-	-	-	-	-	X:7192100	9	5	0	12	11	14	0.170602	0.075692	0	0.175489	0.134273	0.208777	0.082098	0.172846	0.128372	0.877561	-
ENSMUS(	Efemp2	epiderma	GO:00099	cellular pr	-	-	-	-	19:547397	137	155	116	122	175	144	1.701928	1.537796	1.592664	1.169252	1.399951	1.407321	1.610796	1.325508	0.128571	0.878233	-
ENSMUS(	Tdrd1	tudor dor	GO:00325	developm	-	-	-	-	19:568262	9	20	12	39	25	20	0.094841	0.168322	0.139761	0.317076	0.16965	0.165807	0.134308	0.217511	0.128555	0.878233	-
ENSMUS(	Atg7	autophag	GO:00325	developm	K08337	ubiquitin-	mmu0414	Autophag	6:1146430	344	375	333	501	602	598	1.929451	1.679778	2.064265	2.167918	2.174334	2.638684	1.891165	2.326979	0.128636	0.87833	-
ENSMUS(	Cad	carbamoy	GO:00325	developm	K11540	carbamoy	mmu0110	Metabolic	5:3105478	297	360	287	396	356	286	1.61581	1.564163	1.725694	1.662109	1.247208	1.224083	1.635223	1.3778	0.129171	0.878533	-
ENSMUS(	Tom1l1	target of r	GO:00081	metabolic	-	-	-	-	11:906434	277	380	243	442	524	452	1.507543	1.65165	1.461649	1.855845	1.836435	1.935256	1.540281	1.875845	0.128875	0.878533	-
ENSMUS(	Efcab11	EF-hand (	GO:00431	ion bindin	-	-	-	-	12:996930	3	2	2	3	11	7	0.024596	0.013098	0.018125	0.018979	0.058076	0.045154	0.018606	0.040736	0.129139	0.878533	-
ENSMUS(	Slc4a10	solute car	GO:00325	developm	-	-	-	-	2:6204646	5050	4756	4298	4590	6362	5496	39.82737	29.95559	37.46308	27.92757	32.31012	34.09949	35.74868	31.44573	0.128939	0.878533	-
ENSMUS(	Nipsnap2	nipsnap h	GO:00081	metabolic	-	-	-	-	5:1297250	716	742	700	1054	1346	1065	9.543244	7.898289	10.31165	10.83812	11.55268	11.16718	9.251061	11.18599	0.128799	0.878533	-
ENSMUS(	Bmp2k	BMP2 ind	GO:00325	developm	K08854	BMP2 ind	mmu0520	Transcript	5:9699768	394	584	304	478	469	438	2.785005	3.296774	2.374925	2.606682	2.134802	2.435653	2.818901	2.392379	0.129026	0.878533	-
ENSMUS(	Mchr1	melanin-(	-	-	K04320	melanin-(	mmu0408	Neuroacti	15:812354	395	408	317	429	457	371	11.02283	9.092887	9.776936	9.235996	8.212332	8.144812	9.964217	8.531047	0.129146	0.878533	-
ENSMUS(	Arhgap20	Rho GTPa	GO:00987	molecular	-	-	-	-	9:5176533	2096	2613	1539	2080	2826	2268	8.151451	8.115754	6.61499	6.240757	7.077347	6.939013	7.627399	6.752372	0.129127	0.878533	-
ENSMUS(	Slamf8	SLAM fan	GO:00160	membran	-	-	-	-	1:1725817	0	5	2	5	8	8	0	0.077892	0.043123	0.075244	0.100499	0.122765	0.040338	0.099503	0.129069	0.878533	-
ENSMUS(	Vgll3	vestigial li	GO:00081	metabolic	-	-	-	-	16:658152	192	227	86	65	285	100	1.275922	1.204744	0.631636	0.333246	1.219612	0.5228	1.037434	0.691886	0.1288	0.878533	-
ENSMUS(	Il17rd	interleukir	GO:00048	signal trar	-	-	-	-	14:270389	119	185	118	162	180	42	0.514254	0.638483	0.563587	0.540104	0.500909	0.142789	0.572108	0.394601	0.129229	0.878584	-
ENSMUS(	Sh2b3	SH2B ada	GO:00325	developm	K12459	SH2B ada	-	-	5:1218154	302	428	262	457	609	503	1.504165	1.702463	1.442235	1.756041	1.953263	1.970911	1.549621	1.893405	0.129298	0.878709	-
ENSMUS(	Pcdh17	protocadh	GO:00325	developm	-	-	-	-	14:844435	2689	3306	2404	3227	3617	2995	12.33455	12.11104	12.18748	11.41989	10.68405	10.80788	12.21102	10.97061	0.12942	0.879197	-
ENSMUS(	Tnpo1	transporti	GO:00511	localizatio	-	-	-	-	13:988390	1387	1842	1250	979	2181	1730	7.161136	7.59523	7.132835	3.899584	7.251298	7.026878	7.2964	6.059253	0.129923	0.881825	-
ENSMUS(	Htr7	5-hydroxy	GO:00048	signal trar	K04163	5-hydroxy	mmu0408	Neuroacti	19:359587	61	104	111	104	85	86	0.383814	0.522602	0.771899	0.504843	0.344403	0.425697	0.559438	0.424981	0.129986	0.881825	-
ENSMUS(	Gpr39	G protein-	GO:00048	signal trar	-	-	-	-	1:1256769	14	17	11	22	38	24	0.223837	0.217059	0.194367	0.27136	0.39123	0.301863	0.211754	0.321484	0.13001	0.881825	-
ENSMUS(	Cyp2j8	cytochron	GO:00081	metabolic	K07418	cytochron	mmu0110	Metabolic	4:9644459	5	4	2	4	0	0	0.201894	0.12899	0.089268	0.124604	0	0	0.140051	0.041535	0.129952	0.881825	-
ENSMUS(	Kctd11	potassium	GO:00325	developm	-	-	-	-	11:698782	22	31	15	29	54	44	0.426976	0.480494	0.321753	0.434222	0.674885	0.671806	0.409741	0.593638	0.130348	0.883778	-
ENSMUS(	Hdc	histidine c	GO:00081	metabolic	K01590	histidine c	mmu0110	Metabolic	2:1265936	35	41	20	32	22	32	0.284822	0.266465	0.179884	0.200906	0.11529	0.204867	0.243724	0.173688	0.130479	0.88415	-
ENSMUS(	Zfp551	zinc fingr	GO:00081	metabolic	-	-	-	-	7:1241515	108	104	101	113	127	95	1.319387	1.014671	1.363687	1.065018	0.999094	0.913027	1.232581	0.99238	0.130505	0.88415	-
ENSMUS(	Acbd5	acyl-Coer	GO:00081	metabolic	-	-	-	-	2:2306816	1692	2223	1648	2296	2599	1288	3.730557	3.91434	4.015848	3.90549	3.690067	2.234087	3.886915	3.276548	0.130668	0.88478	-
ENSMUS(	Bmpr1b	bone mor	GO:00325	developm	K13578	bone mor	mmu0406	Cytokine-	3:1418371	464	501	382	279	682	482	2.794257	2.409526	2.542484	1.296231	2.644766	2.283526	2.582089	2.074841	0.1307	0.88478	-
ENSMUS(	Marco	macropha	GO:00511	localizatio	K13884	macropha	mmu0414	Phagoson	1:1204745	0	0	2	3	6	3	0	0	0.067051	0.070212	0.117189	0.071577	0.02235	0.086326	0.130824	0.885275	-
ENSMUS(	Cd72	CD72 anti	GO:00160	membran	K06504	CD72 anti	mmu0466	B cell rece	4:4344646	17	33	10	14	15	20	0.161557	0.250455	0.105028	0.102642	0.091793	0.149523	0.172346	0.114653	0.130939	0.885658	-
ENSMUS(	Lrrc47	leucine ric	GO:00038	catalytic a	-	-	-	-	4:1540117	955	1222	460	474	1495	586	7.835323	8.007017	4.171176	3.000285	7.898587	3.782355	6.671172	4.893742	0.130982	0.885658	-
ENSMUS(	Pacsin2	protein ki	GO:00325	developm	-	-	-	-	15:833756	740	1464	669	930	1218	930	8.387418	13.25205	8.380492	8.13223	8.88993	8.29259	10.00665	8.43825	0.131079	0.885967	-
ENSMUS(	Asb2	ankyrin re	GO:00325	developm	-	-	-	-	12:103321	34	59	34	62	84	73	0.464639	0.643914	0.513523	0.653665	0.739206	0.784811	0.540692	0.725894	0.131483	0.887942	-
ENSMUS(	Mmp2	matrix me	GO:00325	developm	K01398	matrix me	mmu0520	Pathways	8:9282729	51	83	34	76	117	97	0.665231	0.864622	0.490156	0.764804	0.982758	0.995375	0.673336	0.914312	0.131589	0.887942	-
ENSMUS(	Saa3	serum am	GO:00081	metabolic	-	-	-	-	7:4671199	9	14	7	14	25	22	0.179813	0.223392	0.154584	0.215797	0.321647	0.345803	0.18593	0.294416	0.131611	0.887942	-
ENSMUS(	Sytl5	synaptota	GO:00452	synapse|lc	-	-	-	-	X:9885622	70	100	52	74	76	75	1.857472	2.119206	1.525011	1.514917	1.298658	1.565657	1.833896	1.459744	0.131514	0.887942	-
ENSMUS(	Ecscr	endotheli	GO:00325	developm	-	-	-	-	18:357130	30	41	41	68	74	60	0.813753	0.888177	1.229125	1.423012	1.292577	1.280347	0.977019	1.331979	0.131626	0.887942	-
ENSMUS(	Gp1bb	glycoprot	GO:00508	response	K06262	platelet gl	mmu0461	Platelet ac	16:186203	64	98	70	101	251	92	1.5681	1.917637	1.895565	1.909175	3.960261	1.773346	1.793767	2.547594	0.131797	0.888747	-
ENSMUS(	Zfp59	zinc finge	GO:00081	metabolic	-	-	-	-	7:2783860	134	165	137	133	194	149	1.581637	1.555359	1.787184	1.211108	1.474543	1.38356	1.641393	1.356404	0.132024	0.889932	-
ENSMUS(	Plk4	polo like k	GO:00325	developm	K08863	polo-like	mmu0406	FoxO sign	3:4080001	64	59	48	53	73	50	0.330433	0.243278	0.273902	0.211112	0.242706	0.203088	0.282538	0.218969	0.132337	0.891698	-
ENSMUS(	Malt1	MALT1 pa	GO:00325	developm	K07369	mucosa-a	mmu0515	Tuberculo	18:654309	165	202	136	283	343	206	0.538777	0.52677	0.490808	0.712922	0.721229	0.52918	0.518785	0.654444	0.132677	0.893644	-
ENSMUS(	Nufip2	nuclear fr	GO:00329	macromo	-	-	-	-	11:776861	1123	1439	987	1344	1559	1210	9.944631	10.1769	9.659908	9.182029	8.890166	8.429572	9.927148	8.833922	0.132974	0.895042	-
ENSMUS(	Gimap7	GTPase, IN	GO:00081	metabolic	-	-	-	-	6:4871862	2	1	2	4	11	3	0.045253	0.018076	0.050027	0.06983	0.160301	0.053404	0.037786	0.094512	0.132988	0.895042	-
ENSMUS(	Gnas	GNAS (gu	GO:00325	developm	K04632	guanine n	mmu0520	Pathways	2:1742843	10241	12726	10213	17135	18425	15107	40.02151	39.71814	44.11144	51.66133	46.36746	46.44514	41.2837	48.15797	0.133113	0.895541	-
ENSMUS(	Alg12	asparagin	GO:00081	metabolic	K03847	alpha-1,6	mmu0110	Metabolic	15:888052	133	166	134	199	266	249	2.114774	2.10798	2.354854	2.441155	2.723631	3.11475	2.192536	2.759845	0.133169	0.895568	-
ENSMUS(	Podxl2	podocaly	GO:00400	locomotic	-	-	-	-	6:8884255	1033	1715	1074	1391	1628	1319	7.067004	9.370117	8.120563	7.341634	7.172067	7.098894	8.185895	7.204198	0.133586	0.897678	-
ENSMUS(	Npepl1	aminopep	GO:00081	metabolic	-	-	-	-	2:1741103	163	202	108	216	294	263	2.247626	2.224498	1.645916	2.297843	2.610584	2.853006	2.039347	2.587144	0.133557	0.897678	-
ENSMUS(	Pnpo	pyridoxine	GO:00081	metabolic	K00275	pyridoxan	mmu0110	Metabolic	11:969378	576	803	214	851	1056	802	10.09167	11.23574	4.143813	11.50272	11.91404	11.05416	8.490407	11.49031	0.133703	0.897804	-
ENSMUS(	Tnnt3	troponin T	GO:00990	supramole	-	-	-	-	7:1424988	2	7	0	0	0	2	0.040657	0.113667	0	0	0	0.031997	0.051441	0.010666	0.133708	0.897804	-
ENSMUS(	Map3k7cl	Map3k7 C	GO:00048	signal trar	-	-	-	-	16:875533	12	23	19	19	19	9	0.443511	0.678916	0.77614	0.541761	0.452211	0.261682	0.632856	0.418551	0.133852	0.898424	-
ENSMUS(	Amn	amnionles	GO:00325	developm	-	-	-	-	12:111271	42	69	23	68	80	87	1.039918	1.36442	0.629385	1.298945	1.275544	1.69465	1.011241	1.423047	0.134272	0.900038	-
ENSMUS(	Cdyl2	chromodo	GO:00081	metabolic	-	-	-	-	8:1165744	73	105	67	97	64	82	1.848699	2.12364	1.875274	1.895173	1.04372	1.633694	1.949204	1.524196	0.134276	0.900038	-
ENSMUS(	Ppp1r9a	protein pl	GO:00325	developm	-	-	-	-	6:4902917	2930	3513	2502	3061	4101	3336	6.913753	6.620186	6.524996	5.572368	6.231471	6.192745	6.686312	5.998861	0.134299	0.900038	-
ENSMUS(	Tmem64	transmem	GO:00325	developm	-	-	-	-	4:1526583	474	601	428	492	678	540	6.197336	6.275477	6.184678	4.962741	5.708353	5.554324	6.219164	5.408472	0.134205	0.900038	-
ENSMUS(	Acot8	acyl-CoA	GO:00081	metabolic	K11992	acyl-CoA	mmu0110	Metabolic	2:1647927	130	165	105	190	247	215	2.831809	2.87045	2.527882	3.193034	3.464748	3.684421	2.74338	3.447401	0.134652	0.902051	-
ENSMUS(	Rbm15	RNA bind	GO:00325	developm	-	-	-	-	3:1073254	344	420	312	411	434	367	2.037245	1.986458	2.042144	1.877831	1.65512	1.709866	2.021949	1.747606	0.134756	0.902401	-
ENSMUS(	Ifnlr1	interferon	GO:00048	signal trar	K05140	interferon	mmu0406	Cytokine-	4:1356862	3	9	4	13	20	6	0.0438	0.104946	0.06455	0.146428	0.188035	0.068918	0.071098	0.134461	0.13503	0.903889	-
ENSMUS(	Cdr2	cerebellar	GO:00056	cell|intrace	-	-	-	-	7:1209570	157	257	146	185	207	204	1.50112	1.962426	1.542815	1.364635	1.274501	1.534457	1.668787	1.391198	0.13514	0.904277	-
ENSMUS(	Map1b	microtubu	GO:00325	developm	-	-	-	-	13:994214	43282	53513	37683	51042	58190	49560	134.3663	132.6748	129.2931	122.2478	116.3285	121.039	132.1114	119.8718	0.135257	0.904713	-
ENSMUS(	Myl12a	myosin, lig	GO:00325	developm	K12757	myosin re	mmu0481	Regulation	17:709936	321	328	263	429	598	451	3.684839	3.006994	3.336688	3.799278	4.420476	4.07287	3.34284	4.097542	0.135314	0.904748	-
ENSMUS(	Lypd1	Ly6/Plaur	GO:00511	localizatio	-	-	-	-	1:1258676	1758	2512	1598	1142	2737	2377	10.48039	11.95981	10.52888	5.252365	10.50723	11.14804	10.98969	8.969211	0.135509	0.905704	-
ENSMUS(	Gabrg2	gamma-a	GO:00325	developm	K05186	gamma-a	mmu0408	Neuroacti	11:419102	2330	2954	1946	2613	3239	2608	16.68215	16.89089	15.39876	14.43331	14.93351	14.68976	16.32393	14.68553	0.135786	0.906169	-
ENSMUS(	Adgra2	adhesion	GO:00325	developm	-	-	-	-	8:2708558	202	205	146	329	311	264	1.188911	0.9636	0.949722	1.493904	1.178726	1.222398	1.034078	1.298343	0.13564	0.906169	-
ENSMUS(	Zfp365	zinc finge	GO:00325	developm	-	-	-	-	10:678861	7486	8755	6051	9160	4317	8780	54.57684	50.97538	48.7565	51.521	20.26727	50.35747	51.43624	40.71525	0.135787	0.906169	-
ENSMUS(	Ezr	ezrin [Sou	GO:00325	developm	K08007	villin 2 (ez	mmu0481	Regulation	17:673804	1370	1981	1203	2080	2830	2059	23.79789	27.482	23.09566	27.87479	31.65622	28.13748	24.79185	29.22283	0.13572	0.906169	-
ENSMUS(	Clptm1	cleft lip ar	GO:00325	developm	-	-	-	-	7:1963157	1822	2436	1895	3318	2905	3275	9.722766	10.3816	11.17629	13.65992	9.982567	13.74876	10.42689	12.46375	0.136363	0.907225	-
ENSMUS(	Gpaa1	GPI ancho	GO:00081	metabolic	K05289	glycosylph	mmu0110	Metabolic	15:763312	215	232	283	403	421	416	2.157002	1.858855	3.137942	3.119222	2.719871	3.283338	2.3846	3.04081	0.136164	0.907225	-
ENSMUS(	Mctp2	multiple C	GO:00325	developm	-	-	-	-	7:7207783	37	48	26	52	109	49	0.166336	0.172333	0.12918	0.180349	0.315545	0.173294	0.15595	0.223062	0.136245	0.907225	-
ENSMUS(	Serpinf2	serine (or	GO:00325	developm	K03983	alpha-2-a	mmu0461	Complem	11:754317	15	14	5	11	8	7	0.233814	0.174288	0.086146	0.132292	0.080307	0.085846	0.164749	0.099481	0.136074	0.907225	-
ENSMUS(	Gimap6	GTPase, IN	GO:00056	cell|organ	-	-	-	-	6:4870158	69	87	59	101	158	106	0.777742	0.78316	0.734993	0.878284	1.14682	0.939939	0.765298	0.988348	0.13629	0.907225	-
ENSMUS(	Rex1bd	required f	-	-	-	-	-	-	8:7050429	169	258	168	280	345	326	2.637738	3.215956	2.898021	3.371574	3.467516	4.002889	2.917238	3.613993	0.136073	0.907225	-
ENSMUS(	Smim20	small inte	GO:00099	cellular pr	-	-	-	-	5:5326708	174	224	168	285	136	120	2.312602	2.37764	2.467797	2.922314	1.163984	1.254717	2.386013	1.780338	0.136325	0.907225	-
ENSMUS(	Phf11c	PHD finge	GO:00160	membran	-	-	-	-	14:593808	6	3	10	11	20	15	0.105909	0.042299	0.195086	0.149808	0.227338	0.208302	0.114431	0.195149	0.136154	0.907225	-
ENSMUS(	Srp54a	signal rec	GO:00511	localizatio	K03106	signal rec	mmu0306	Protein ex	12:550802	98	126	87	76	136	115	0.919439	0.944088	0.902114	0.5501	0.821657	0.848801	0.92188	0.740186	0.13643	0.907322	-
ENSMUS(	Gpcpd1	glyceroph	GO:00325	developm	K18695	glyceroph	mmu0523	Choline m	2:1325290	937	1263	1486	2161	2581	1544	5.452865	5.869949	9.557639	9.702197	9.672256	7.068763	6.960151	8.814405	0.136674	0.908254	-
ENSMUS(	Ano1	anoctamir	GO:00325	developm	-	-	-	-	7:1445885	50	88	57	93	145	96	0.333617	0.468927	0.420335	0.478725	0.623015	0.503913	0.407626	0.535218	0.13663	0.908254	-
ENSMUS(	Steap1	six transm	GO:00081	metabolic	K14737	metallore	mmu0497	Mineral ab	5:5736317	10	49	14	4	26	19	0.343412	1.34387	0.531355	0.105962	0.574952	0.513297	0.739546	0.398071	0.136806	0.908434	-
ENSMUS(	Zbtb6	zinc finge	GO:00081	metabolic	-	-	-	-	2:3742550	346	76	54	66	154	156	2.197887	0.385555	0.379112	0.323447	0.629946	0.77959	0.987518	0.577661	0.136762	0.908434	-
ENSMUS(	Retsat	retinol sat	GO:00081	metabolic	K09516	all-trans-r	mmu0083	Retinol me	6:7259847	129	226	137	222	324	255	1.828764	2.558722	2.146529	2.428018	2.957792	2.84393	2.178005	2.743247	0.136987	0.909293	-
ENSMUS(	Traf3ip2	TRAF3 int	GO:00511	localizatio	-	-	mmu0421	Cellular se	10:396129	24	33	21	39	69	38	0.29797	0.327201	0.288157	0.373554	0.551647	0.371154	0.304443	0.432118	0.137387	0.90986	-
ENSMUS(	Slc4a8	solute car	GO:00511	localizatio	-	-	-	-	15:100761	986	2826	1596	2604	1150	1156	4.125492	9.443147	7.380376	8.405625	3.098499	3.805112	6.983005	5.103079	0.137342	0.90986	-
ENSMUS(	Polr3c	polymeras	GO:00081	metabolic	K03023	DNA-dire	mmu0110	Metabolic	3:9671149	309	376	258	423	505	583	3.217973	3.12721	2.969551	3.398556	3.386647	4.776428	3.104911	3.853877	0.137334	0.90986	-
ENSMUS(	Prrc2c	proline-ri	GO:00325	developm	-	-	-	-	1:1626707	5074	6413	3781	6126	5822	5473	11.08003	11.18401	9.125237	10.32043	8.186864	9.402146	10.4631	9.303148	0.137171	0.90986	-
ENSMUS(	Bend7	BEN dom	GO:00055	extracellul	-	-	-	-	2:4717831	22	34	21	24	29	20	0.190588	0.235236	0.201072	0.160407	0.161781	0.136306	0.208966	0.152832	0.137342	0.90986	-
ENSMUS(	Gm3095	predicted	-	-	-	-	-	-	14:396354	9	16	10	14	7	6	0.350453	0.497593	0.430358	0.420584	0.175517	0.183811	0.426135	0.259971	0.137331	0.90986	-
ENSMUS(	Acy3	aspartoac	GO:00081	metabolic	-	-	-	-	19:398666	5	16	10	13	24	29	0.202833	0.518423	0.448373	0.406886	0.627006	0.925572	0.389876	0.653155	0.13749	0.910144	-
ENSMUS(	Gem	GTP bindi	GO:00511	localizatio	-	-	-	-	4:1170445	20	26	21	22	58	54	0.500275	0.51939	0.580564	0.424549	0.934236	1.062616	0.53341	0.807134	0.137534	0.910144	-
ENSMUS(	Etv4	ets varian	GO:00325	developm	K15592	ets transl	mmu0520	Transcript	11:101769	96	147	82	199	171	165	0.962805	1.177423	0.908923	1.539756	1.104382	1.301859	1.016384	1.315332	0.137629	0.910146	-
ENSMUS(	Fam168a	family wit	GO:00081	metabolic	-	-	-	-	7:1007066	4225	5492	3785	1560	6107	5514	14.21638	14.75838	14.07586	4.04965	13.2326	14.59621	14.35021	10.62615	0.137639	0.910146	-
ENSMUS(	Zfp60	zinc finge	GO:00081	metabolic	-	-	-	-	7:2773138	749	893	661	820	1041	805	3.261252	3.105273	3.180904	2.754524	2.918824	2.757459	3.182476	2.810269	0.137784	0.91076	-
ENSMUS(	Gfra1	glial cell li	GO:00325	developm	-	-	-	-	19:582356	713	1367	1047	1580	1830	1553	3.588101	5.494011	5.823293	6.134265	5.930357	6.148336	4.968468	6.070986	0.137862	0.910928	-
ENSMUS(	Lipm	lipase, fan	GO:00081	metabolic	-	-	-	-	19:341009	6	8	6	16	12	15	0.135631	0.144433	0.149915	0.279045	0.174691	0.266759	0.143326	0.240165	0.138251	0.91315	-
ENSMUS(	Ecm2	extracellu	GO:00099	cellular pr	-	-	-	-	13:495048	191	238	169	198	282	182	1.452859	1.445816	1.420773	1.161944	1.381321	1.089114	1.439816	1.210793	0.138385	0.913344	-
ENSMUS(	Gm3739	predicted	-	-	-	-	-	-	14:729364	19	28	9	30	37	38	0.576875	0.678952	0.301997	0.702722	0.723419	0.907675	0.519274	0.777939	0.138356	0.913344	-
ENSMUS(	Fgfrl1	fibroblast	GO:00325	developm	-	-	-	-	5:1086923	183	318	97	185	219	180	2.224943	3.087733	1.303417	1.735277	1.714609	1.721671	2.205364	1.723852	0.138518	0.913533	-
ENSMUS(	Afap1l1	actin filar	GO:00329	macromo	-	-	-	-	18:617302	187	279	202	333	395	337	2.47837	2.953082	2.958848	3.404864	3.371138	3.513706	2.796767	3.429903	0.138572	0.913533	-
ENSMUS(	Tmem39b	transmem	GO:00160	membran	-	-	-	-	4:1296763	120	145	104	132	134	130	2.500674	2.413178	2.395279	2.122167	1.798185	2.131233	2.436377	2.017195	0.138539	0.913533	-
ENSMUS(	Kcnh7	potassium	GO:00511	localizatio	-	-	-	-	2:6269341	1975	2345	1523	2101	2569	2137	6.611645	6.269473	5.634938	5.426235	5.538104	5.628047	6.172018	5.530795	0.138658	0.913758	-

ENSMUS(	Zbtb3	zinc finge	GO:00081	metabolic	-	-	-	-	19:880253	55	65	61	57	73	59	1.648598	1.556003	2.020831	1.318121	1.409053	1.391294	1.741811	1.372823	0.138832	0.914555	-
ENSMUS(	Rel	reticuloen	GO:00081	metabolic	K09254	c-Rel prot	mmu0401	Ras signal	11:237368	133	141	118	121	173	133	1.087695	0.920919	1.066557	0.763436	0.911082	0.855692	1.025057	0.843403	0.139001	0.914754	-
ENSMUS(	Slc22a3	solute car	GO:00511	localizatio	K08200	MFS trans	mmu0523	Choline m	17:124199	3	6	3	11	8	11	0.052319	0.083568	0.057829	0.148011	0.089849	0.150925	0.064572	0.129595	0.138953	0.914754	-
ENSMUS(	Mrc1	mannose	GO:00048	signal trar	K06560	mannose	mmu0515	Tuberculo	2:1422939	64	108	54	80	164	155	0.525017	0.707565	0.489591	0.506308	0.866353	1.000319	0.574058	0.790993	0.139182	0.914754	-
ENSMUS(	Mier1	MEIR1 tre	GO:00048	signal trar	-	-	-	-	4:1031143	819	1078	716	1115	1640	1303	2.267358	2.383422	2.190765	2.38145	2.923713	2.837863	2.280515	2.714342	0.139262	0.914754	-
ENSMUS(	Eda2r	ectodyspl	GO:00325	developm	K05163	tumor nec	mmu0406	Cytokine-	X:9733384	10	17	11	5	17	9	0.087376	0.118624	0.106223	0.033703	0.095654	0.061863	0.104075	0.06374	0.139271	0.914754	-
ENSMUS(	Klhl13	kelch-like	GO:00081	metabolic	K10447	kelch-like	mmu0412	Ubiquitin	X:2321927	365	573	379	338	626	478	2.871191	3.59973	3.295	2.05124	3.171018	2.958071	3.255307	2.726776	0.139062	0.914754	-
ENSMUS(	Gabpb2	GA repeat	GO:00081	metabolic	-	-	-	-	3:9518176	466	603	466	751	908	740	1.033004	1.067529	1.141691	1.284359	1.296155	1.290503	1.080741	1.290339	0.139169	0.914754	-
ENSMUS(	Bcl2a1b	B cell leuk	GO:00511	localizatio	K02162	hematopc	mmu0520	Transcript	9:8919920	5	19	13	10	12	7	0.373639	1.134013	1.073761	0.57659	0.57753	0.411579	0.860471	0.521899	0.139283	0.914754	-
ENSMUS(	Skor1	SKI family	GO:00325	developm	-	-	-	-	9:6313817	3	2	2	0	2	1	0.039172	0.02086	0.028865	0	0.016814	0.010275	0.029632	0.009029	0.139635	0.916722	-
ENSMUS(	Tbxa2r	thrombox	GO:00325	developm	K04264	thrombox	mmu0408	Neuroacti	10:813287	9	12	5	16	22	17	0.286352	0.304922	0.175839	0.392756	0.450768	0.425536	0.255704	0.42302	0.140018	0.918885	-
ENSMUS(	Fbxo9	f-box pro	GO:00325	developm	-	-	-	-	9:7808149	1283	1738	905	1360	1602	1445	14.88458	16.10296	11.60394	12.17249	11.96815	13.1883	14.19716	12.44298	0.140177	0.918963	-
ENSMUS(	Kpna4	karyopher	GO:00511	localizatio	-	-	-	-	3:6906714	1103	1335	1002	1372	3318	1335	5.964144	5.765004	5.988072	5.723436	11.55323	5.678905	5.90574	7.651858	0.140557	0.918963	-
ENSMUS(	Grpr	gastrin rel	GO:00048	signal trar	K04169	gastrin-re	mmu0408	Neuroacti	X:1635139	23	35	33	22	36	30	0.556616	0.676462	0.882653	0.410751	0.561033	0.571158	0.705244	0.514314	0.140421	0.918963	-
ENSMUS(	Atp2c1	ATPase, C	GO:00325	developm	-	-	-	-	9:1054035	3683	4824	3252	4032	5588	4220	13.51587	14.13824	13.18984	11.41545	13.20545	12.18332	13.61465	12.26807	0.14026	0.918963	-
ENSMUS(	Dagla	diacylglyc	GO:00325	developm	K13806	sn1-speci	mmu0472	Retrograd	19:102452	3273	4195	3082	4254	5145	1922	20.97651	21.47163	21.83065	21.03367	21.23378	9.690618	21.42626	17.31935	0.140332	0.918963	-
ENSMUS(	Gstm2	glutathior	GO:00081	metabolic	K00799	glutathior	mmu0520	Pathways	3:1079817	43	65	22	58	102	73	0.987036	1.191571	0.558137	1.027115	1.507713	1.318247	0.912248	1.284358	0.140536	0.918963	-
ENSMUS(	Id1	inhibitor c	GO:00325	developm	K04680	DNA-binc	mmu0401	Rap1 sign	2:1527362	102	146	112	161	249	190	2.974191	3.399896	3.609358	3.621782	4.675429	4.35844	3.327815	4.218551	0.140558	0.918963	-
ENSMUS(	Agap1	ArfGAP w	GO:00511	localizatio	K12491	Arf-GAP v	mmu0414	Endocytos	1:8945480	1247	1840	1242	1620	1743	1616	2.862278	3.372943	3.150749	2.868738	2.576306	2.918084	3.128657	2.787709	0.140344	0.918963	-
ENSMUS(	Sugct	succinyl-(	GO:00081	metabolic	-	-	-	-	13:168574	46	41	58	44	65	36	0.564106	0.401544	0.786096	0.416278	0.5133	0.347308	0.583915	0.425629	0.14038	0.918963	-
ENSMUS(	Ttll9	tubulin tyr	GO:00081	metabolic	-	-	-	-	2:1529624	17	18	4	10	12	8	0.200232	0.169313	0.052073	0.090871	0.091019	0.074123	0.140539	0.085338	0.140209	0.918963	-
ENSMUS(	Esrra	estrogen i	GO:00325	developm	-	-	-	-	19:691097	578	655	438	930	873	806	8.652025	7.830273	7.246199	10.73997	8.415084	9.491514	7.909499	9.548855	0.140661	0.919293	-
ENSMUS(	Il33	interleukir	GO:00325	developm	K12967	interleukir	mmu0421	Necroptos	19:299251	503	725	220	260	256	700	5.843273	6.72623	2.824602	2.330194	1.915061	6.397305	5.131368	3.54752	0.140923	0.920339	-
ENSMUS(	Slc9a7	solute car	GO:00511	localizatio	-	-	-	-	X:2010575	371	472	350	422	520	414	4.194924	4.262239	4.373852	3.681226	3.786237	3.682649	4.277005	3.716704	0.140927	0.920339	-
ENSMUS(	Def6	differentia	GO:00160	membran	-	-	-	-	17:282077	39	57	28	42	48	31	0.906804	1.058441	0.719535	0.753399	0.718693	0.56705	0.894927	0.679714	0.141004	0.920497	-
ENSMUS(	Sgcg	sarcoglyc	GO:00325	developm	K12564	gamma-s	mmu0541	Dilated ca	14:612191	2	3	2	0	0	2	0.02858	0.034249	0.031595	0	0	0.022493	0.031475	0.007498	0.141264	0.921358	-
ENSMUS(	Atxn7l3	ataxin 7-l	GO:00081	metabolic	-	-	-	-	11:102289	1424	2197	1304	2273	2764	2402	12.1214	14.93545	12.26781	14.92699	15.15079	16.0852	13.10822	15.38766	0.141232	0.921358	-
ENSMUS(	Qk	quaking [	GO:00325	developm	-	-	-	-	17:102064	7350	9463	6234	8610	10093	8420	45.0851	46.35754	42.2629	40.74546	39.86765	40.63205	44.56851	40.41505	0.141295	0.921358	-
ENSMUS(	C1qtnf6	C1q and t	GO:00329	macromo	-	-	-	-	15:785233	27	36	17	48	58	38	0.373914	0.398159	0.260197	0.512826	0.51723	0.414002	0.34409	0.481353	0.141395	0.921618	-
ENSMUS(	Fam122a	family witl	-	-	-	-	-	-	19:244765	244	400	244	427	555	421	17.42479	22.81301	19.25808	23.52531	25.52259	23.65212	19.83196	24.23334	0.141441	0.921618	-
ENSMUS(	Fam89b	family witl	GO:00400	locomotic	-	-	-	-	19:572808	82	56	54	112	126	104	1.411952	0.770091	1.027643	1.487831	1.397112	1.408802	1.069896	1.431248	0.141612	0.922388	-
ENSMUS(	Cfap126	cilia and f	GO:00099	cellular pr	-	-	-	-	1:1711139	27	68	37	43	35	43	0.471025	0.947397	0.713375	0.578725	0.393182	0.590145	0.710599	0.520684	0.141773	0.92309	-
ENSMUS(	Apobec3	apolipopr	GO:00325	developm	-	-	-	-	15:798916	53	89	59	89	141	112	0.238563	0.319935	0.29351	0.309061	0.408694	0.396599	0.284002	0.371451	0.14196	0.923619	-
ENSMUS(	Ncapd2	non-SMC	GO:00329	macromo	-	-	-	-	6:1251680	338	492	342	587	650	593	2.315196	2.691424	2.58907	3.101978	2.867072	3.195484	2.531897	3.054845	0.141936	0.923619	-
ENSMUS(	Sptlc3	serine pal	GO:00081	metabolic	K00654	serine pal	mmu0110	Metabolic	2:1394939	6	3	2	2	2	2	0.102962	0.041122	0.037935	0.026482	0.022097	0.027006	0.060673	0.025195	0.142053	0.923876	-
ENSMUS(	Drc7	dynein re	GO:00325	developm	-	-	-	-	8:9505510	143	354	95	140	192	208	2.431445	4.807027	1.785255	1.83648	2.102253	2.782295	3.007909	2.240343	0.142153	0.924179	-
ENSMUS(	Rapgef1	Rap guan	GO:00325	developm	K06277	Rap guan	mmu0401	Rap1 sign	2:2961972	1510	2512	1543	1930	2170	2198	6.509301	8.648145	7.3514	6.418672	6.023818	7.454113	7.502949	6.632201	0.142404	0.925467	-
ENSMUS(	Tnni1	troponin I	GO:00325	developm	-	-	-	-	1:1357794	29	49	58	46	45	40	0.410641	0.554128	0.907693	0.502522	0.410324	0.445588	0.624154	0.452811	0.142578	0.926254	-
ENSMUS(	Gm14308	predicted	GO:00081	metabolic	-	-	-	-	2:1766133	118	277	163	304	351	263	1.747485	3.276088	2.667869	3.473235	3.347277	3.064058	2.563814	3.294857	0.142687	0.926612	-
ENSMUS(	Dnase1	deoxyribc	GO:00081	metabolic	-	-	-	-	16:403694	3	2	2	2	0	0	0.024335	0.012959	0.017932	0.012518	0	0	0.018409	0.004173	0.1431	0.9286	-
ENSMUS(	Apeh	acylpeptic	GO:00081	metabolic	-	-	-	-	9:1080854	246	309	209	353	469	390	1.75717	1.762718	1.649956	1.945287	2.157279	2.191564	1.723281	2.098043	0.143056	0.9286	-
ENSMUS(	Sgf29	SAGA con	GO:00081	metabolic	-	-	-	-	7:1266493	102	151	125	180	237	209	1.660788	1.963516	2.24942	2.261079	2.484935	2.677125	1.957908	2.47438	0.143325	0.92937	-
ENSMUS(	Gas2l3	growth ar	GO:00099	cellular pr	-	-	-	-	10:894088	36	54	42	80	87	62	0.15553	0.186316	0.200541	0.266642	0.242037	0.210723	0.180796	0.239801	0.1433	0.92937	-
ENSMUS(	Zbtb32	zinc finge	GO:00325	developm	-	-	-	-	7:3058968	0	0	0	2	1	2	0	0	0	0.031704	0.013227	0.032331	0	0.025754	0.143755	0.930561	-
ENSMUS(	Hmg20b	high mob	GO:00325	developm	-	-	-	-	10:813460	274	332	259	473	492	408	2.280209	2.206526	2.382159	3.036801	2.636603	2.671138	2.289631	2.781514	0.143614	0.930561	-
ENSMUS(	Fgf20	fibroblast	GO:00325	developm	K04358	fibroblast	mmu0520	Pathways	8:4027916	0	0	0	2	1	2	0	0	0	0.048098	0.020067	0.04905	0	0.039071	0.143777	0.930561	-
ENSMUS(	Klhl18	kelch-like	GO:00081	metabolic	-	-	-	-	9:1104259	243	282	497	856	913	314	1.317684	1.221236	2.978571	3.581039	3.18809	1.339509	1.839163	2.702879	0.143697	0.930561	-
ENSMUS(	Dynlt1a	dynein lig	GO:00325	developm	-	-	-	-	17:631054	12	20	10	11	16	9	0.987439	1.314365	0.909436	0.698364	0.84782	0.582614	1.070413	0.709599	0.143709	0.930561	-
ENSMUS(	4930451G	RIKEN cDI	-	-	-	-	-	-	16:496893	52	51	41	45	49	52	1.439268	1.127331	1.254198	0.960908	0.873344	1.132278	1.273599	0.988843	0.143888	0.930806	-
ENSMUS(	Mast4	microtubu	GO:00081	metabolic	-	-	-	-	13:102732	1497	2318	1663	2219	2562	1348	1.90037	2.350042	2.333218	2.173221	2.094354	1.346224	2.194543	1.871266	0.144028	0.930806	-
ENSMUS(	Abca7	ATP-bind	GO:00048	signal trar	K05645	ATP-bind	mmu0201	ABC trans	10:799964	324	444	315	505	653	543	2.627201	2.875262	2.82297	3.159151	3.409702	3.463848	2.775144	3.344234	0.143959	0.930806	-
ENSMUS(	6430550D	RIKEN cDI	GO:00081	metabolic	-	-	-	-	2:1559732	10	5	6	20	9	21	0.084265	0.033646	0.055882	0.130017	0.048837	0.139212	0.057931	0.106022	0.143996	0.930806	-
ENSMUS(	Vegfb	vascular e	GO:00325	developm	K16858	vascular e	mmu0520	Pathways	19:698247	484	724	551	874	918	948	13.71966	16.39011	17.26221	19.11347	16.75696	21.14059	15.79066	19.00367	0.144087	0.930839	-
ENSMUS(	Gsta3	glutathior	GO:00325	developm	K00799	glutathior	mmu0520	Pathways	1:2124058	11	10	5	16	20	18	0.141373	0.102632	0.071024	0.15864	0.165515	0.181982	0.10501	0.168713	0.144253	0.931564	-
ENSMUS(	Tiam1	T cell lym	GO:00325	developm	K05731	T-lympho	mmu0401	Ras signal	16:897871	2821	2514	1793	2477	3393	2318	10.61733	7.556544	7.458288	7.192313	8.223387	6.863356	8.544053	7.426352	0.144321	0.931592	-
ENSMUS(	Rsu1	Ras suppr	GO:00055	extracellul	-	-	-	-	2:1307682	306	441	240	464	577	506	1.56954	1.806486	1.360533	1.83611	1.905817	2.041796	1.578853	1.927907	0.144364	0.931592	-
ENSMUS(	Lix1	limb and (	GO:00081	metabolic	-	-	-	-	17:174026	1139	1533	1132	1398	1731	1315	20.25196	21.76863	22.24519	19.177	19.81959	18.39415	21.42193	19.13025	0.144446	0.931771	-
ENSMUS(	Arl5b	ADP-ribo	GO:00511	localizatio	-	-	-	-	2:1504939	194	181	517	294	1048	702	0.620658	0.462463	1.828052	0.725652	2.159072	1.766847	0.970391	1.550524	0.144557	0.932142	-
ENSMUS(	Rps25	ribosomal	GO:00329	macromo	K02975	small sub	mmu0301	Ribosome	9:4440713	563	805	646	737	1000	531	7.777308	8.881018	9.862801	7.854504	8.89561	5.770677	8.840375	7.506931	0.144836	0.932235	-
ENSMUS(	Spns3	spinster h	GO:00400	locomotic	-	-	-	-	11:724949	0	4	1	3	6	6	0	0.030252	0.010468	0.021923	0.036591	0.044705	0.013573	0.034406	0.144839	0.932235	-
ENSMUS(	Rida	reactive ir	GO:00325	developm	-	-	-	-	15:344840	535	699	480	623	841	469	6.781419	7.076029	6.724412	6.092357	6.86463	4.67682	6.86062	5.877936	0.144801	0.932235	-
ENSMUS(	Trim39	tripartite r	GO:00081	metabolic	-	-	-	-	17:362588	298	618	239	364	463	362	1.324644	2.193901	1.174162	1.248289	1.325315	1.265911	1.564236	1.279838	0.144751	0.932235	-
ENSMUS(	Ntf5	neurotrop	GO:00325	developm	K12457	neurotrop	mmu0415	PI3K-Akt	7:4541369	1	6	0	3	7	9	0.021597	0.103456	0	0.049977	0.097317	0.152864	0.041684	0.100053	0.144649	0.932235	-
ENSMUS(	Ccl17	chemokin	GO:00325	developm	K05514	C-C motif	mmu0406	Cytokine-	8:9481045	2	0	0	2	3	4	0.237536	0	0	0.183316	0.229514	0.373789	0.079179	0.262206	0.144961	0.932671	-
ENSMUS(	Etfa	electron t	GO:00081	metabolic	-	-	-	-	9:5545450	270	359	246	394	599	409	7.507153	7.971714	7.559488	8.451581	10.72487	8.946327	7.679452	9.374261	0.14512	0.93335	-
ENSMUS(	Gata5	GATA bin	GO:00325	developm	-	-	-	-	2:1803251	0	0	0	1	3	1	0	0	0	0.014377	0.035999	0.014661	0	0.021679	0.145309	0.934224	-
ENSMUS(	Siglecg	sialic acid	GO:00325	developm	-	-	-	-	7:4340820	0	2	0	2	5	2	0	0.008175	0	0.007897	0.016476	0.008053	0.002725	0.010809	0.145432	0.934668	-
ENSMUS(	Eif6	eukaryotic	GO:00325	developm	K03264	translatior	mmu0300	Ribosome	2:1558198	240	344	299	481	491	446	3.598711	4.119457	4.955118	5.564307	4.741023	5.261156	4.224428	5.188829	0.145505	0.934697	-
ENSMUS(	Prmt8	protein ar	GO:00081	metabolic	-	-	-	-	6:1276890	1372	1722	1252	1637	1978	1481	30.75321	30.82586	31.01611	28.30839	28.55072	26.11574	30.86506	27.65828	0.145544	0.934697	-
ENSMUS(	Dock3	dedicator	GO:00987	molecular	-	-	-	-	9:1068928	4132	5220	3527	4762	5481	4908	13.0014	13.11735	12.26541	11.55978	11.10566	12.14913	12.79472	11.60485	0.145714	0.935444	-
ENSMUS(	Mamld1	mastermi	GO:00081	metabolic	-	-	-	-	X:7105025	1154	1284	948	1271	1485	1195	12.4931	11.10134	11.34278	10.61549	10.3525	10.17754	11.64574	10.38185	0.145908	0.93634	-
ENSMUS(	Tpr	translocat	GO:00081	metabolic	K09291	nucleoprc	mmu0520	Pathways	1:1503928	3827	4684	3123	4292	5302	4129	16.17985	15.81535	14.59267	13.99929	14.43479	13.7332	15.52929	14.05576	0.146542	0.937457	-
ENSMUS(	Slc1a7	solute car	GO:00511	localizatio	K05618	solute car	mmu0472	Glutamate	4:1079683	0	0	0	1	1	2	0	0	0	0.009833	0.008205	0.020056	0	0.012698	0.146604	0.937457	-
ENSMUS(	Med1	mediator	GO:00325	developm	K15144	mediator	mmu0491	Thyroid h	11:981521	1152	1021	976	860	1795	869	4.662533	3.300203	4.365821	2.685334	4.678304	2.766942	4.109519	3.37686	0.14659	0.937457	-
ENSMUS(	Prr11	proline ric	GO:00099	cellular pr	-	-	-	-	11:870891	18	25	11	28	55	23	0.2874	0.318792	0.194113	0.344914	0.565517	0.288917	0.266769	0.399783	0.146596	0.937457	-
ENSMUS(	Fam193b	family witl	GO:00056	cell|intrac	-	-	-	-	13:555393	943	1213	841	1491	1586	1487	3.708237	3.80945	3.655094	4.523387	4.016185	4.600211	3.724261	4.379928	0.146279	0.937457	-
ENSMUS(	Sh3tc1	SH3 dom	-	-	-	-	-	-	5:3569718	29	48	24	54	70	53	0.220728	0.291776	0.201889	0.317093	0.343095	0.317358	0.238131	0.325849	0.146289	0.937457	-
ENSMUS(	Slc25a43	solute car	GO:00511	localizatio	-	-	-	-	X:3674365	0	0	0	3	1	1	0	0	0	0.063579	0.017683	0.021612	0	0.034292	0.146464	0.937457	-
ENSMUS(	Arl9	ADP-ribo	GO:00508	response	-	-	-	-	5:7700405	0	0	0	1	1	2	0	0	0	0.078915	0.065846	0.160952	0	0.101904	0.146568	0.937457	-
ENSMUS(	Fastkd5	FAST kina	GO:00081	metabolic	-	-	-	-	2:1306138	201	240	160	225	209	220	2.256422	2.151701	1.985138	1.948669	1.510863	1.942932	2.131087	1.800821	0.146512	0.937457	-
ENSMUS(	Ugt1a2	UDP gluc	GO:00081	metabolic	K00699	glucuronc	mmu0110	Metabolic	1:8820060	0	0	0	1	1	2	0	0	0	0.021308	0.017779	0.043459	0	0.027516	0.146621	0.937457	-
ENSMUS(	Bnc2	basonucli	GO:00325	developm	-	-	-	-	4:8427509	5	3	2	10	10	6	0.018169	0.008708	0.008034	0.028038	0.023402	0.017155	0.011637	0.022865	0.147086	0.937674	-
ENSMUS(	Mutyh	mutY DN	GO:00081	metabolic	K03575	A/G-spec	mmu0341	Base excis	4:1168077	26	28	26	31	21	23	0.331699	0.285285	0.366595	0.305116	0.172519	0.230844	0.32786	0.23616	0.146907	0.937674	-
ENSMUS(	Rbbp7	retinoblas	GO:00081	metabolic	-	-	-	-	X:1627604	1918	2146	1857	2141	2821	2044	26.9651	24.09512	28.85441	23.22205	25.53943	22.60712	26.63821	23.78953	0.146713	0.937674	-
ENSMUS(	Carm1	coactivatc	GO:00325	developm	K05931	type I pro	mmu0152	Endocrine	9:2154689	888	1481	1063	1629	1862	1797	4.694338	6.252624	6.210713	6.643741	6.338635	7.473435	5.719225	6.818604	0.146973	0.937674	-
ENSMUS(	P2ry2	purinergic	GO:00048	signal trar	K04269	purinergic	mmu0408	Neuroacti	7:1009965	22	19	17	28	45	37	0.120852	0.083355	0.103213	0.118664	0.159183	0.1599	0.102473	0.145916	0.146817	0.937674	-
ENSMUS(	Wdr38	WD repea	GO:00325	developm	-	-	-	-	2:3899747	12	32	19	22	12	19	0.216257	0.460568	0.378449	0.305881	0.139268	0.269375	0.351758	0.238175	0.146827	0.937674	-
ENSMUS(	Bdkrb1	bradykinir	GO:00048	signal trar	K03915	bradykinir	mmu0520	Pathways	12:105603	1	2	0	7	2	2	0.017302	0.027632	0	0.09341	0.022272	0.02722	0.014978	0.047634	0.147024	0.937674	-
ENSMUS(	Gm20479	predicted	-	-	-	-	-	-	7:2735264	4	0	0	15	7	0	0.439292	0	0	1.270841	0.494984	0	0.146431	0.588608	0.147079	0.937674	-
ENSMUS(	Plscr4	phospholi	GO:00508	response	-	-	-	-	9:9245737	71	97	76	115	148	145	0.996809	1.087602	1.179275	1.245605	1.338042	1.601524	1.087895	1.395057	0.147288	0.938494	-
ENSMUS(	Leng9	leukocyte	GO:00431	ion bindin	-	-	-	-	7:4147915	109	110	88	152	187	171	2.705411	2.180446	2.414002	2.91059	2.988845	3.338997	2.433286	3.079477	0.147338	0.938494	-
ENSMUS(	Ccser2	coiled-co	GO:00099	cellular pr	-	-	-	-	14:368749	2012	2708	1732	2324	2793	2416	9.134427	9.818561	8.690568	8.139913	8.165427	8.629015	9.214519	8.311452	0.147377	0.938494	-
ENSMUS(	Gtf2ird2	GTF2I rep	GO:00325	developm	-	-	-	-	5:1341840	207	218	210	217	190	268	1.610272	1.354351	1.805492	1.302326	0.951781	1.640118	1.590038	1.298075	0.147432	0.938505	-
ENSMUS(	Clec2g	C-type le	GO:00325	developm	-	-	-	-	6:1289343	0	0	0	0	3	2	0	0	0	0	0.019904	0.016212	0	0.012039	0.14893	0.938603	-
ENSMUS(	Gria3	glutamate	GO:00048	signal trar	K05199	glutamate	mmu0408	Neuroacti	X:4140085	3109	3734	3814	5229	6183	5381	10.34889	9.926432	14.03138	13.42833	13.25339	14.09116	11.43557	13.59096	0.148084	0.938603	-
ENSMUS(	Aven	apoptosis	GO:00099	cellular pr	-	-	-	-	2:1124929	68	97	70	141	155	100	0.808716	0.921305	0.920091	1.293711	1.187065	0.935622	0.883371	1.138799	0.147605	0.938603	-
ENSMUS(	Cyp27b1	cytochron	GO:00325	developm	K07438	calcidiol 1	mmu0110	Metabolic	10:127048	4	9	4	16	9	13	0.062772	0.112795	0.069377	0.193703	0.090946	0.16048	0.081648	0.148376	0.148158	0.938603	-
ENSMUS(	Zfp715	zinc finge	GO:00081	metabolic	-	-	-	-	7:4329619	594	684	551	719	190	707	5.467893	5.02846	5.605728	5.106141	1.126267	5.119923	5.36736	3.78411	0.148754	0.938603	-
ENSMUS(	Prss57	protease,	GO:00081	metabolic	-	-	-	-	10:797814	7	8	4	18	17	8	0.069296	0.063248	0.043766	0.137471	0.108371	0.062298	0.05877	0.102714	0.148711	0.938603	-
ENSMUS(	Drd3	dopamine	GO:00325	developm	K04146	dopamine	mmu0408	Neuroacti	16:437622	0	0	0	0	3	2	0	0	0	0	0.081079	0.06604	0	0.04904	0.149019	0.938603	-
ENSMUS(	Soat2	sterol O-a	GO:00081	metabolic	K00637	sterol O-a	mmu0497	Cholester	15:102150	0	0	0	0	3	2	0	0	0	0	0.044517	0.03626	0	0.026926	0.14896	0.938603	-
ENSMUS(	Ms4a2	membran	GO:00023	immune s	K08090	membran	mmu0407	Phospholi	19:116155	0	0	0	0	3	2	0	0	0	0	0.026303	0.021424	0	0.015909	0.14888	0.938603	-
ENSMUS(	Il6	interleukir	GO:00325	developm	K05405	interleukir	mmu0520	Pathways	5:3001311	0	2	2	0	0	0	0	0.023872	0.033033	0	0	0	0.018968	0	0.149077	0.938603	-
ENSMUS(	4933402D	RIKEN cDI	-	-	-	-	-	-	1:6375490	0	0	0	2	3	0	0	0	0	0.053597	0.067105	0	0	0.040234	0.149066	0.938603	-
ENSMUS(	Nifk	nucleolar	GO:00081	metabolic	-	-	-	-	1:1183218	650	778	558	667	952	677	17.04068	16.28918	16.16794	13.49057	16.07184	13.96282	16.49927	14.50841	0.147867	0.938603	-
ENSMUS(	Oxt	oxytocin [	GO:00325	developm	K05243	oxytocin-	mmu0492	Oxytocin	2:1305761	0	2	2	0	0	0	0	0.18164	0.251348	0	0	0	0.144329	0	0.149014	0.938603	-
ENSMUS(	Ccdc163	coiled-co	-	-	-	-	-	-	4:1167085	22	27	13	42	44	29	0.194846	0.190978	0.12725	0.286978	0.250944	0.202058	0.171025	0.24666	0.147951	0.938603	-
ENSMUS(	Slc26a5	solute car	GO:00325	developm	-	-	-	-	5:2181065	0	2	2	0	0	0	0	0.023481	0.032492	0	0	0	0.018658	0	0.149048	0.938603	-
ENSMUS(	Stab2	stabilin 2	GO:00081	metabolic	-	-	-	-	10:868411	4	6	11	19	14	15	0.024033	0.028788	0.073038	0.088063	0.054163	0.070896	0.041953	0.071041	0.147959	0.938603	-
ENSMUS(	Matr3	matrin 3 [	GO:00325	developm	-	-	-	-	18:355621	6798	8397	5905	7394	9710	7911	26.07079	25.71834	25.02878	21.87675	23.97987	23.86796	25.60597	23.24153	0.147976	0.938603	-
ENSMUS(	Nmur2	neuromec	GO:00325	developm	K05053	neuromec	mmu0408	Neuroacti	11:560249	2	2	0	0	0	0	0.036976	0.02954	0	0	0	0	0.022172	0	0.148737	0.938603	-
ENSMUS(	Rpl39l	ribosomal	GO:00081	metabolic	K02924	large sub	mmu0301	Ribosome	16:101702	0	0	0	0	3	2	0	0	0	0	0.092308	0.075186	0	0.055832	0.148898	0.938603	-
ENSMUS(	Thbs1	thrombos	GO:00325	developm	K16857	thrombos	mmu0415	PI3K-Akt	2:1181118	102	207	83	95	174	121	0.957116	1.551242	0.860767	0.687727	1.051398	0.893217	1.123042	0.877447	0.149018	0.938603	-
ENSMUS(	Tekt3	tektin 3 [S	GO:00400	locomotic	-	-	-	-	11:630616	0	2	2	0	0	0	0	0.055801	0.077216	0	0	0	0.044339	0	0.149152	0.938603	-
ENSMUS(	Erfe	erythrofer	GO:00081	metabolic	-	-	-	-	1:9136643	0	0	0	0	3	2	0	0	0	0	0.034184	0.027844	0	0.020676	0.148989	0.938603	-
ENSMUS(	4933428G	RIKEN cDI	-	-	-	-	-	-	11:974907	0	0	0	2	3	0	0	0	0	0.066122	0.082786	0	0	0.049636	0.149035	0.938603	-
ENSMUS(	Cracr2b	calcium re	GO:00511	localizatio	-	-	-	-	7:1414610	40	38	35	56	84	62	0.47702	0.36191	0.46131	0.515215	0.645073	0.58167	0.433413	0.580653	0.147833	0.938603	-
ENSMUS(	Tacstd2	tumor-as	GO:00325	developm	-	-	-	-	6:6753406	1	2	1	7	2	6	0.035203	0.056219	0.038897	0.19005	0.045314	0.166119	0.04344	0.133828	0.148735	0.938603	-
ENSMUS(	Chil1	chitinase-	GO:00325	developm	-	-	-	-	1:1341821	99	168	82	135	111	108	1.076148	1.458462	0.985138	1.13215	0.776989	0.923578	1.17325	0.944239	0.148583	0.938603	-
ENSMUS(	Spag8	sperm ass	GO:00325	developm	-	-	-	-	4:4365133	26	30	31	31	28	26	0.852584	0.785641	1.123501	0.784257	0.591259	0.670732	0.920576	0.682083	0.147946	0.938603	-
ENSMUS(	Apoc4	apolipopr	GO:00511	localizatio	-	-	-	-	7:1967808	2	2	0	0	0	0	0.046353	0.037031	0	0	0	0	0.027795	0	0.148761	0.938603	-
ENSMUS(	Colca2	COLCA2 h	GO:00056	cell|intrac	-	-	-	-	9:5127025	6	19	4	13	25	24	0.24686	0.624318	0.181905	0.412644	0.662368	0.776829	0.351028	0.61728	0.149175	0.938603	-
ENSMUS(	4933405C	RIKEN cDI	GO:00081	metabolic	K00030	isocitrate	mmu0110	Metabolic	7:5059919	0	2	2	0	0	0	0	0.072737	0.100652	0	0	0	0.057796	0	0.149118	0.938603	-
ENSMUS(	Gm17669	predicted	GO:00081	metabolic	-	-	-	-	18:675623	0	2	2	0	0	0	0	0.209764	0.290266	0	0	0	0.166677	0	0.149048	0.938603	-
ENSMUS(	Mroh6	maestro h	GO:00511	localizatio	-	-	-	-	15:758841	0	0	0	0	3	2	0	0	0	0	0.053236	0.043361	0	0.032199	0.148888	0.938603	-
ENSMUS(	Cd2	CD2 antig	GO:00511	localizatio	K06449	T-cell surf	mmu0451	Cell adhes	3:1012758	0	0	2	0	9	4	0	0	0.048552	0	0.127292	0.069111	0.016184	0.065468	0.149276	0.938902	-
ENSMUS(	Mak	male gerr	GO:00081	metabolic	-	-	-	-	13:410250	39	53	45	52	40	44	0.175106	0.190046	0.223304	0.180124	0.11565	0.155416	0.196152	0.150397	0.149713	0.939478	-
ENSMUS(	Ccl20	chemokin	GO:00081	metabolic	K14625	C-C motif	mmu0406	Cytokine-	1:8311676	0	0	0	0	2	3	0	0	0	0	0.033874	0.062078	0	0.031984	0.149915	0.939478	-
ENSMUS(	Rnf32	ring finge	GO:00056	cell|intrac	-	-	-	-	5:2919599	146	231	161	259	391	234	0.838064	1.058966	1.021402	1.146976	1.445294	1.056696	0.972811	1.216322	0.149612	0.939478	-
ENSMUS(	Rora	RAR-relat	GO:00325	developm	K08532	RAR-relat	mmu0465	Th17 cell	9:6865378	807	1157	713	973	1104	975	3.312531	3.79285	3.234617	3.081268	2.918167	3.148486	3.446666	3.049307	0.149885	0.939478	-
ENSMUS(	Mrps10	mitochon	GO:00081	metabolic	K02946	small sub	mmu0301	Ribosome	17:473688	197	253	203	343	383	317	1.00354	1.029286	1.142908	1.348013	1.256385	1.270395	1.058578	1.291598	0.149674	0.939478	-
ENSMUS(	Abhd4	abhydrola	-	-	-	-	-	-	14:542541	310	387	298	436	514	662	2.950124	2.941273	3.134313	3.201075	3.149897	4.956188	3.00857	3.769053	0.150016	0.939478	-
ENSMUS(	Kank3	KN motif	GO:00099	cellular pr	-	-	-	-	17:338105	232	234	241	362	453	352	2.99925	2.41595	3.443419	3.610475	3.771192	3.579972	2.952873	3.65388	0.149576	0.939478	-
ENSMUS(	Igfals	insulin-lik	GO:00329	macromo	-	-	-	-	17:248787	0	0	0	3	2	0	0	0	0	0.066952	0.037243	0	0	0.034732	0.1499	0.939478	-
ENSMUS(	Gm16223	predicted	-	-	-	-	-	-	5:4206796	0	0	0	3	2	0	0	0	0	0.055864	0.031075	0	0	0.02898	0.149918	0.939478	-
ENSMUS(	Gm572	predicted	GO:00325	developm	-	-	-	-	4:1486433	0	0	0	0	2	3	0	0	0	0	0.056766	0.104032	0	0.053599	0.149951	0.939478	-
ENSMUS(	Tepp	testis, pro	-	-	-	-	-	-	8:9531056	0	0	0	3	2	0	0	0	0	0.051098	0.028424	0	0	0.026507	0.149877	0.939478	-
ENSMUS(	Pvrig	poliovirus	GO:00023	immune s	-	-	-	-	5:1383419	0	0	0	0	2	3	0	0	0	0	0.072797	0.133411	0	0.068736	0.149963	0.939478	-
ENSMUS(	Mfsd8	major faci	GO:00325	developm	K12307	MFS trans	mmu0414	Lysosome	3:4081810	172	243	230	289	426	355	0.93202	1.051594	1.377433	1.208161	1.486486	1.513338	1.120349	1.402662	0.150082	0.939551	-
ENSMUS(	Mapk7	mitogen-	GO:00325	developm	K04464	mitogen-	mmu0401	MAPK sig	11:614888	376	472	388	479	548	378	1.639846	1.644007	1.870226	1.611688	1.539043	1.296933	1.718027	1.482555	0.15083	0.942497	-
ENSMUS(	Hck	hemopoie	GO:00325	developm	K08893	hemopoie	mmu0516	Kaposi's s	2:1531084	34	55	27	70	74	55	0.728423	0.941043	0.639302	1.156991	1.020909	0.926979	0.76959	1.03496	0.150768	0.942497	-
ENSMUS(	Dpys	dihydropy	GO:00081	metabolic	K01464	dihydropy	mmu0110	Metabolic	15:397684	4	5	0	0	2	2	0.05546	0.055359	0	0	0.017852	0.021818	0.03694	0.013223	0.150717	0.942497	-
ENSMUS(	Jpt2	Jupiter mi	GO:00160	membran	-	-	-	-	17:249421	90	141	69	146	228	131	1.873582	2.344214	1.587542	2.344843	3.056473	2.145434	1.935113	2.515583	0.150674	0.942497	-
ENSMUS(	Skil	SKI-like [S	GO:00325	developm	K18499	Ski-like pr	mmu0455	Signaling	3:3109505	915	1032	724	945	1189	958	2.683519	2.417181	2.346761	2.138187	2.245536	2.210345	2.482487	2.198023	0.150867	0.942497	-
ENSMUS(	4930505A	RIKEN cDI	-	-	-	-	-	-	11:304260	0	6	0	6	6	5	0	0.276795	0	0.267385	0.22318	0.22722	0.092265	0.239262	0.150878	0.942497	-
ENSMUS(	Larp1	La ribonu	GO:00451	translatior	-	-	-	-	11:580090	2966	3617	2561	3714	3108	3558	16.44555	16.01664	15.69399	15.88727	11.09719	15.52008	16.05206	14.16818	0.150933	0.942505	-
ENSMUS(	4930579G	RIKEN cDI	GO:00056	cell|intrac	-	-	-	-	3:7962907	25	42	16	30	23	21	0.77484	1.039623	0.548083	0.717348	0.459053	0.512046	0.787515	0.562815	0.15106	0.942622	-
ENSMUS(	Trim10	tripartite r	GO:00325	developm	-	-	-	-	17:368695	0	3	1	0	0	0	0	0.065172	0.030061	0	0	0	0.031744	0	0.15102	0.942622	-
ENSMUS(	Papolg	poly(A) pc	GO:00081	metabolic	K14376	poly(A) pc	mmu0301	mRNA sur	11:238626	521	611	478	588	670	596	5.21069	4.880276	5.283634	4.536961	4.315061	4.689374	5.124867	4.513798	0.151234	0.942695	-
ENSMUS(	Knstrn	kinetocho	GO:00990	supramol	-	-	-	-	2:1188140	27	38	9	52	40	46	0.218414	0.245492	0.08046	0.324523	0.208363	0.292738	0.181456	0.275208	0.151164	0.942695	-
ENSMUS(	Rpl26	ribosomal	GO:00081	metabolic	K02898	large sub	mmu0301	Ribosome	11:689015	512	739	459	513	873	537	12.62081	14.54814	12.50481	9.755857	13.85754	10.41365	13.22459	11.34235	0.151192	0.942695	-
ENSMUS(	Cacna2d2	calcium cl	GO:00325	developm	K04859	voltage-d	mmu0401	MAPK sig	9:1073996	605	743	522	755	808	571	3.238091	3.175914	3.087816	3.117534	2.784839	2.404257	3.167274	2.768877	0.151345	0.943046	-
ENSMUS(	Rasa4	RAS p21 p	GO:00987	molecular	K17630	Ras GTPa	mmu0401	Ras signal	5:1360839	50	61	43	79	94	87	0.286311	0.27896	0.272131	0.348995	0.346615	0.391917	0.279134	0.362509	0.151412	0.943126	-
ENSMUS(	Nupl1	nucleopor	-	-	-	-	-	-	14:601846	2	0	0	3	5	1	0.033896	0	0	0.039238	0.054578	0.013338	0.011299	0.035718	0.151619	0.943424	-
ENSMUS(	Cyp4v3	cytochron	GO:00081	metabolic	-	-	-	-	8:4530494	215	284	213	213	321	262	3.447351	3.636726	3.774624	2.634851	3.314412	3.304902	3.619567	3.084722	0.151587	0.943424	-
ENSMUS(	E130218I(	RIKEN cDI	GO:00160	membran	-	-	-	-	4:1342436	0	0	1	2	2	3	0	0	0.011666	0.016288	0.01359	0.024906	0.003889	0.018262	0.151623	0.943424	-
ENSMUS(	Il7r	interleukir	GO:00325	developm	K05072	interleukir	mmu0520	Pathways	15:950578	12	28	18	13	28	8	0.139878	0.260667	0.231901	0.116908	0.21018	0.073359	0.210815	0.133482	0.151895	0.944783	-
ENSMUS(	Letm2	leucine zi	GO:00099	cellular pr	-	-	-	-	8:2557849	150	214	142	273	250	287	0.759872	0.865782	0.795028	1.066943	0.815536	1.143775	0.806894	1.008752	0.151983	0.944991	-
ENSMUS(	Nsd3	nuclear re	GO:00081	metabolic	K11425	histone-ly	mmu0031	Lysine dec	8:2560160	1458	1994	1771	1852	2341	1921	3.962377	4.32782	5.319412	3.883019	4.09689	4.107112	4.536536	4.029007	0.152042	0.945022	-
ENSMUS(	Stard5	StAR-rela	GO:00511	localizatio	-	-	-	-	7:8363195	134	229	117	207	326	246	1.32456	1.807784	1.278198	1.57858	2.075096	1.912985	1.470181	1.855554	0.152138	0.945276	-
ENSMUS(	Smox	spermine	GO:00081	metabolic	K12259	spermine	mmu0033	Arginine a	2:1314914	442	676	411	748	861	742	3.035055	3.70712	3.119122	3.962561	3.807167	4.008288	3.287099	3.926005	0.152193	0.945279	-
ENSMUS(	Mfap3	microfibri	GO:00160	membran	-	-	-	-	11:575186	545	669	523	656	838	146	3.113171	3.051957	3.301833	2.890948	3.082514	0.656102	3.155654	2.209855	0.152279	0.94548	-
ENSMUS(	Prss56	protease,	GO:00325	developm	-	-	-	-	1:8718331	2	0	1	0	0	0	0.056006	0	0.030957	0	0	0	0.028988	0	0.152342	0.945531	-
ENSMUS(	Slc6a20b	solute car	GO:00511	localizatio	-	-	-	-	9:1235908	6	3	2	0	3	3	0.030511	0.012185	0.011241	0	0.009825	0.012	0.017979	0.007275	0.152504	0.9462	-
ENSMUS(	Cpt1a	carnitine p	GO:00325	developm	K08765	carnitine (	mmu0415	AMPK sig	19:332330	610	909	519	891	1328	949	8.661735	10.30824	8.144965	9.76074	12.14304	10.60112	9.038313	10.83497	0.152682	0.946968	-

ENSMUSC	Efhc2	EF-hand c	GO:00508	response	-	-	-	-	X:1713204	38	57	39	42	45	48	0.908454	1.088279	1.030457	0.774638	0.692763	0.902771	1.009063	0.790058	0.153042	0.947502	-
ENSMUSC	Cbr2	carbonyl r	GO:00081	metabolic	K00081	carbonyl r	mmu0110	Metabolic	11:120729	7	16	0	14	21	17	0.219864	0.401351	0	0.339236	0.424729	0.420063	0.207072	0.394676	0.15287	0.947502	-
ENSMUSC	Nmur1	neuromec	GO:00048	signal trar	K05052	neuromec	mmu0408	Neuroacti	1:863863C	1	0	2	0	0	0	0.018655	0	0.041226	0	0	0	0.01996	0	0.153081	0.947502	-
ENSMUSC	Gbp3	guanylate	GO:00023	immune s	K20899	guanylate	mmu0462	NOD-like	3:1425600	150	259	197	340	363	279	1.510345	2.082716	2.19229	2.641152	2.353672	2.210038	1.92845	2.401621	0.153095	0.947502	-
ENSMUSC	Catsper4	cation cha	GO:00325	developm	-	-	-	-	4:1342119	1	0	2	0	0	0	0.025186	0	0.055659	0	0	0	0.026948	0	0.153023	0.947502	-
ENSMUSC	Ugt2a1	UDP gluc	GO:00081	metabolic	K00699	glucuronc	mmu0110	Metabolic	5:8745949	1	0	2	0	0	0	0.023765	0	0.052519	0	0	0	0.025428	0	0.153053	0.947502	-
ENSMUSC	Bmx	BMX non-	GO:00325	developm	-	-	-	-	X:1641928	14	35	26	12	25	29	0.240934	0.481036	0.494511	0.159315	0.277045	0.392612	0.405493	0.276324	0.153262	0.947861	-
ENSMUSC	Cisd3	CDGSH ir	GO:00056	cell|intrac	-	-	-	-	11:976858	122	156	106	217	243	168	7.647914	7.810049	7.344087	10.49483	9.809454	8.285215	7.600683	9.529832	0.153251	0.947861	-
ENSMUSC	Cox5b	cytochron	GO:00081	metabolic	K02265	cytochron	mmu0110	Metabolic	1:3669148	1247	1779	1137	1683	1972	966	30.20208	34.41061	30.43532	31.44741	30.75616	18.40596	31.68267	26.86984	0.153325	0.947914	-
ENSMUSC	Pltp	phospholi	GO:00081	metabolic	K08761	phospholi	mmu0332	PPAR sign	2:1648395	622	947	624	827	942	739	9.51118	11.56485	10.54571	9.756182	9.275765	8.889949	10.54058	9.307298	0.153393	0.947992	-
ENSMUSC	Tymp	thymidine	GO:00325	developm	K00758	thymidine	mmu0110	Metabolic	15:893719	11	39	14	25	11	12	0.329731	0.933602	0.463795	0.578135	0.212318	0.282981	0.575709	0.357811	0.153459	0.948066	-
ENSMUSC	Ttc3	tetratricor	GO:00081	metabolic	-	-	-	-	16:943706	17438	20333	13234	21366	20600	17130	39.11865	36.42793	32.81142	36.97777	29.75836	30.2312	36.11933	32.32244	0.15354	0.948229	-
ENSMUSC	Arpc1b	actin relat	GO:00508	response	K05757	actin relat	mmu0414	Endocytos	5:1451142	212	371	203	305	562	414	1.608792	2.24845	1.702573	1.78564	2.746342	2.471577	1.853272	2.33452	0.153893	0.948601	-
ENSMUSC	Rreb1	ras respor	GO:00325	developm	-	-	-	-	13:377784	1151	1300	1131	1807	1817	1931	2.216619	1.999423	2.40727	2.684751	2.253332	2.925558	2.207771	2.621214	0.153927	0.948601	-
ENSMUSC	D1Pas1	DNA segr	GO:00325	developm	K11594	ATP-depe	mmu0520	Viral carci	1:1869674	0	0	0	3	0	1	0	0	0	0.044003	0	0.014958	0	0.019653	0.153809	0.948601	-
ENSMUSC	Olfr624	olfactory r	GO:00048	signal trar	K04257	olfactory r	mmu0474	Olfactory	7:1036654	0	0	0	3	0	1	0	0	0	0.024662	0	0.008383	0	0.011015	0.153849	0.948601	-
ENSMUSC	Gm9925	predicted	-	-	-	-	-	-	18:740651	4	2	8	8	17	11	0.505686	0.201947	1.117656	0.780227	1.383918	1.09397	0.60843	1.086038	0.153814	0.948601	-
ENSMUSC	Ifi27	interferon	GO:00081	metabolic	-	-	-	-	12:103434	275	419	230	543	440	472	6.566669	7.990481	6.069969	10.00328	6.765815	8.866769	6.875706	8.545288	0.153882	0.948601	-
ENSMUSC	Atp6v0c	ATPase, H	GO:00511	localizatio	K02155	V-type H-	mmu0110	Metabolic	17:241638	2442	2711	1797	2997	3523	4320	32.74849	29.03495	26.63425	31.00721	30.42377	45.57643	29.47257	35.66914	0.15409	0.948931	-
ENSMUSC	Ndufc1	NADH de	GO:00081	metabolic	K03967	NADH de	mmu0110	Metabolic	3:5140467	96	108	93	100	111	110	2.911851	2.616198	3.117674	2.340065	2.168081	2.624852	2.881908	2.377666	0.154059	0.948931	-
ENSMUSC	0610009B	RIKEN cDI	GO:00511	localizatio	-	-	-	-	11:516853	72	121	97	90	135	82	4.405007	5.912146	6.558877	4.24802	5.318629	3.946729	5.625343	4.504459	0.154197	0.949253	-
ENSMUSC	Nr2e1	nuclear re	GO:00325	developm	-	-	-	-	10:425619	301	365	227	325	390	285	4.45431	4.313732	3.71268	3.710461	3.716491	3.317956	4.160241	3.581636	0.15428	0.949429	-
ENSMUSC	Gusb	glucuroni	GO:00081	metabolic	K01195	beta-gluc	mmu0110	Metabolic	5:1299890	172	330	165	195	280	232	1.312258	2.010714	1.391303	1.147774	1.375635	1.392482	1.571425	1.305297	0.154549	0.950746	-
ENSMUSC	Magt1	magnesiu	GO:00160	membran	-	-	-	-	X:1059680	230	300	197	394	410	341	1.616037	1.683415	1.52981	2.135751	1.85508	1.884904	1.609754	1.958578	0.154909	0.951601	-
ENSMUSC	Smco1	single-pa	GO:00160	membran	-	-	-	-	16:322714	0	0	0	1	4	0	0	0	0	0.03346	0.111705	0	0	0.048388	0.155005	0.951601	-
ENSMUSC	Scimp	SLP adapt	GO:00081	metabolic	-	-	-	-	11:707909	0	0	0	1	4	0	0	0	0	0.031429	0.104924	0	0	0.045451	0.154987	0.951601	-
ENSMUSC	Rfx8	regulatory	GO:00081	metabolic	-	-	-	-	1:3966530	0	1	3	7	3	5	0	0.022108	0.091777	0.149473	0.053477	0.108872	0.037962	0.103941	0.154934	0.951601	-
ENSMUSC	Frem1	Fras1 rela	GO:00325	developm	-	-	-	-	4:8289792	107	249	83	116	169	139	0.340662	0.633118	0.292054	0.284923	0.346483	0.34815	0.421945	0.326519	0.155016	0.951601	-
ENSMUSC	Csprs	compone	GO:00160	membran	-	-	-	-	GL456221	0	0	0	1	4	0	0	0	0	0.017327	0.057845	0	0	0.025057	0.154947	0.951601	-
ENSMUSC	Gabrp	gamma-a	GO:00048	signal trar	K05189	gamma-a	mmu0408	Neuroacti	11:335507	0	0	0	3	1	0	0	0	0	0.038744	0.010776	0	0	0.016506	0.155601	0.95188	-
ENSMUSC	Atp12a	ATPase, H	GO:00511	localizatio	K01544	non-gastr	mmu0019	Oxidative	14:563650	0	0	0	3	1	0	0	0	0	0.033572	0.009337	0	0	0.014303	0.155562	0.95188	-
ENSMUSC	Slc39a6	solute car	GO:00511	localizatio	-	-	-	-	18:245798	1199	1351	1214	1169	1930	1292	14.62126	13.1573	16.36181	10.99792	15.15576	12.39479	14.71346	12.84949	0.155523	0.95188	-
ENSMUSC	Ttf1	transcripti	GO:00325	developm	K15225	transcripti	mmu0491	Thyroid hc	2:2906026	199	244	161	278	373	310	1.501556	1.470358	1.342641	1.618311	1.812389	1.840181	1.438185	1.75696	0.15564	0.95188	-
ENSMUSC	Txn1	thioredox	GO:00081	metabolic	K03671	thioredox	mmu0462	NOD-like	4:5794337	261	335	189	398	574	311	15.16283	15.54286	12.13525	17.83832	21.47371	14.21383	14.28032	17.84195	0.155415	0.95188	-
ENSMUSC	D3Ertd25	DNA segr	GO:00081	metabolic	-	-	-	-	3:3615101	443	764	534	756	598	537	2.055843	2.831558	2.738891	2.706692	1.787073	1.960524	2.542097	2.151429	0.155719	0.95188	-
ENSMUSC	Rsrc1	arginine/s	GO:00081	metabolic	-	-	-	-	3:6698139	534	477	498	703	514	422	1.784816	1.273259	1.839621	1.812751	1.106292	1.109624	1.632565	1.342889	0.155828	0.95188	-
ENSMUSC	Oaz1	ornithine	GO:00081	metabolic	-	-	-	-	10:808266	1141	1261	765	1128	1534	945	12.95895	11.43787	9.602676	9.883788	11.21926	8.443583	11.33316	9.848877	0.155251	0.95188	-
ENSMUSC	Slc25a28	solute car	GO:00511	localizatio	-	-	-	-	19:436638	291	369	249	484	461	479	11.73573	11.88474	11.09848	15.05888	11.97217	15.19722	11.57298	14.07609	0.155226	0.95188	-
ENSMUSC	Dtx3	deltex 3, E	GO:00081	metabolic	K06058	deltex[2.3	mmu0433	Notch sigi	10:127190	1244	1492	1088	1821	1880	2171	16.07881	15.40097	15.54208	18.15824	15.64754	22.07518	15.67395	18.62699	0.155803	0.95188	-
ENSMUSC	Erich6	glutamate	-	-	-	-	-	-	3:586163C	10	28	20	16	22	13	0.278171	0.622037	0.614884	0.343371	0.394088	0.284477	0.505031	0.340645	0.155278	0.95188	-
ENSMUSC	Aoc2	amine oxi	GO:00081	metabolic	K00276	primary-a	mmu0110	Metabolic	11:101325	70	68	37	100	96	105	0.920535	0.714169	0.537759	1.014549	0.81296	1.086292	0.724154	0.971267	0.155453	0.95188	-
ENSMUSC	Zfp977	zinc finge	GO:00081	metabolic	-	-	-	-	7:4257978	11	7	9	7	8	6	0.471342	0.239539	0.426186	0.231395	0.220745	0.202257	0.379022	0.218132	0.155416	0.95188	-
ENSMUSC	Pcdhga11	protocadh	GO:00325	developm	-	-	-	-	18:377557	531	781	482	676	735	624	6.817041	8.007529	6.839039	6.695426	6.076359	6.302274	7.221203	6.35802	0.155765	0.95188	-
ENSMUSC	Lrrk1	leucine-ri	GO:00325	developm	-	-	-	-	7:6622691	75	98	65	93	176	137	0.333312	0.347824	0.319265	0.31886	0.503681	0.478981	0.333467	0.433841	0.155937	0.952211	-
ENSMUSC	Calm1	calmoduli	GO:00511	localizatio	K02183	calmoduli	mmu0474	Olfactory	12:100199	15022	18092	13863	17993	22273	15604	106.0609	102.0141	108.1762	98.00801	101.2654	86.67114	105.4171	95.31485	0.156687	0.952762	-
ENSMUSC	Epha2	Eph recep	GO:00325	developm	K05103	Eph recep	mmu0415	PI3K-Akt	4:1413012	28	40	20	33	71	58	0.306271	0.34943	0.24179	0.278486	0.500111	0.499108	0.299164	0.425902	0.156263	0.952762	-
ENSMUSC	Cybb	cytochron	GO:00325	developm	K08008	NADPH o	mmu0421	Necroptos	X:9435252	32	65	35	24	60	42	0.310678	0.503988	0.375562	0.179766	0.37512	0.320792	0.396742	0.291893	0.156093	0.952762	-
ENSMUSC	Dstn	destrin [S	GO:00099	cellular pr	-	-	-	-	2:1439153	3508	4894	3361	5440	7353	5073	112.0836	124.8799	118.6856	134.0948	151.2871	127.5142	118.5497	137.632	0.156643	0.952762	-
ENSMUSC	Ddx4	DEAD (As	GO:00325	developm	-	-	-	-	13:112598	81	117	67	87	119	75	1.323443	1.52669	1.209871	1.096652	1.252049	0.964029	1.353335	1.104243	0.156147	0.952762	-
ENSMUSC	Dct	dopachro	GO:00325	developm	K01827	dopachro	mmu0110	Metabolic	14:118012	2	11	5	0	6	5	0.017174	0.07545	0.047466	0	0.033183	0.033784	0.046697	0.022323	0.157114	0.952762	-
ENSMUSC	Rdh5	retinol del	GO:00081	metabolic	K00061	11-cis-ret	mmu0083	Retinol m	10:128913	62	116	46	38	94	79	0.804764	1.202481	0.659906	0.380532	0.785705	0.806708	0.88905	0.657648	0.157143	0.952762	-
ENSMUSC	Rnf220	ring finge	GO:00081	metabolic	-	-	-	-	4:1172714	2593	3438	1730	3678	4447	3685	12.15071	12.86621	8.959668	13.29661	13.41902	13.58462	11.32553	13.43341	0.156782	0.952762	-
ENSMUSC	Gpat3	glycerol-3	GO:00081	metabolic	K13506	glycerol-3	mmu0110	Metabolic	5:1008457	53	67	41	94	107	71	0.548399	0.553655	0.468863	0.750369	0.712948	0.577944	0.523639	0.68042	0.156313	0.952762	-
ENSMUSC	Tmtc2	transmem	GO:00160	membran	-	-	-	-	10:105187	201	298	215	282	281	227	1.167493	1.382357	1.380201	1.263682	1.051038	1.037278	1.310017	1.117333	0.156767	0.952762	-
ENSMUSC	Dsel	dermatan	GO:00081	metabolic	-	-	-	-	1:1118587	415	555	414	457	680	444	2.472109	2.640331	2.725629	2.100225	2.608451	2.080721	2.61269	2.263132	0.156894	0.952762	-
ENSMUSC	Ikzf5	IKAROS fa	GO:00081	metabolic	-	-	-	-	7:1313886	403	542	340	517	793	719	2.54409	2.732576	2.372208	2.517955	3.223701	3.570811	2.549625	3.104155	0.15661	0.952762	-
ENSMUSC	Unc5cl	unc-5 fan	GO:00081	metabolic	-	-	-	-	17:484549	0	5	4	6	14	5	0	0.036437	0.040345	0.04224	0.082269	0.035895	0.025594	0.053468	0.157053	0.952762	-
ENSMUSC	Pcdhb8	protocadh	GO:00325	developm	-	-	-	-	18:373551	43	62	41	84	103	63	0.691653	0.796448	0.728862	1.042386	1.066867	0.797206	0.738988	0.96882	0.156705	0.952762	-
ENSMUSC	Pbp2	phosphati	GO:00508	response	-	-	-	-	6:1353091	9	0	1	1	3	0	0.42964	0	0.052765	0.036835	0.092236	0	0.160802	0.043024	0.157107	0.952762	-
ENSMUSC	Cxcr6	chemokin	GO:00048	signal trar	K04191	C-X-C ch	mmu0406	Cytokine-	9:1238064	2	1	0	4	5	4	0.032096	0.012821	0	0.049527	0.05168	0.050507	0.014972	0.050571	0.156789	0.952762	-
ENSMUSC	Spock3	sparc/oste	GO:00081	metabolic	-	-	-	-	8:6295100	1403	1857	1334	1629	2105	1679	7.469875	7.896114	7.849797	6.691246	7.217095	7.032621	7.738596	6.98032	0.157178	0.952762	-
ENSMUSC	Ncf4	neutrophi	GO:00081	metabolic	K08012	neutrophi	mmu0414	Phagosom	15:782448	9	16	10	19	24	25	0.175956	0.249832	0.216075	0.28658	0.302159	0.384521	0.213954	0.32442	0.15633	0.952762	-
ENSMUSC	2900055J	RIKEN cDI	GO:00160	membran	-	-	-	-	18:402569	61	75	57	70	71	63	5.1515	5.058412	5.320172	4.560751	3.861163	4.185606	5.176695	4.202507	0.156996	0.952762	-
ENSMUSC	Kti12	KTI12 hon	GO:00971	organic cy	-	-	-	-	4:1088477	150	185	161	234	299	272	5.622304	5.537833	6.669522	6.766559	7.216855	8.02051	5.94322	7.334641	0.156945	0.952762	-
ENSMUSC	Cd101	CD101 an	GO:00325	developm	-	-	-	-	3:1009935	1	3	0	4	5	4	0.016538	0.039618	0	0.051016	0.053233	0.052025	0.018719	0.052091	0.157138	0.952762	-
ENSMUSC	Plau	plasminog	GO:00081	metabolic	K01348	urokinase	mmu0520	Proteogly	14:208366	51	80	45	46	72	62	1.172862	1.469316	1.14377	0.816148	1.066264	1.121713	1.261983	1.001375	0.157477	0.953913	-
ENSMUSC	A530084C	RIKEN cDI	-	-	-	-	-	-	13:315561	16	18	24	11	26	17	0.305289	0.274287	0.50611	0.161933	0.319458	0.255189	0.361895	0.245526	0.157438	0.953913	-
ENSMUSC	Nes	nestin [So	GO:00325	developm	-	-	-	-	3:8797107	341	481	251	365	444	376	1.577814	1.777427	1.283576	1.302941	1.322936	1.368674	1.546272	1.331517	0.157888	0.955736	-
ENSMUSC	Sox6	SRY (sex c	GO:00325	developm	-	-	-	-	7:1154708	448	441	308	445	390	471	1.058143	0.83186	0.804014	0.81088	0.593178	0.87518	0.898005	0.759746	0.157875	0.955736	-
ENSMUSC	Scrn3	secernin 3	GO:00081	metabolic	-	-	-	-	2:7331260	757	810	610	842	1017	648	7.394171	6.318653	6.585227	6.345068	6.396901	4.979432	6.766017	5.907134	0.158059	0.955923	-
ENSMUSC	Lrrc45	leucine ric	GO:00160	membran	-	-	-	-	11:120713	653	790	576	943	1104	1061	7.488902	7.235658	7.30087	8.343479	8.153218	9.572641	7.34181	8.68978	0.15808	0.955923	-
ENSMUSC	Zfand2a	zinc finge	GO:00081	metabolic	-	-	-	-	5:1394712	744	826	335	364	1169	292	6.879783	6.099974	3.423681	2.596774	6.960995	2.124201	5.467813	3.89399	0.158084	0.955923	-
ENSMUSC	Rab43	RAB43, m	GO:00511	localizatio	-	-	-	-	6:8778885	313	283	327	467	609	463	2.318472	1.674133	2.677027	2.668735	2.904894	2.69805	2.223211	2.757227	0.158163	0.956068	-
ENSMUSC	Pcdhgb6	protocadh	GO:00160	membran	-	-	-	-	18:377420	332	518	379	418	518	450	4.327779	5.392652	5.460245	4.203709	4.348211	4.614764	5.060226	4.388895	0.158367	0.956968	-
ENSMUSC	Cep290	centroson	GO:00325	developm	-	-	-	-	10:100487	520	708	492	586	782	614	2.196033	2.38789	2.296399	1.909252	2.126653	2.039928	2.293441	2.025278	0.158457	0.957184	-
ENSMUSC	Sema3a	sema don	GO:00325	developm	K06840	semaphor	mmu0436	Axon guic	5:1312541	97	161	95	61	156	135	0.338823	0.449133	0.366754	0.164386	0.350898	0.370977	0.384903	0.295421	0.158577	0.957575	-
ENSMUSC	Asl	argininosu	GO:00325	developm	K01755	argininosu	mmu0110	Metabolic	5:1300112	148	161	150	234	288	230	1.359702	1.181288	1.523076	1.65855	1.703847	1.662345	1.354689	1.674914	0.158756	0.95832	-
ENSMUSC	Tfpi	tissue fact	GO:00081	metabolic	K03909	tissue fact	mmu0461	Complem	2:8443285	61	78	59	109	126	94	0.316604	0.323316	0.338444	0.43646	0.421129	0.383821	0.326122	0.413803	0.158857	0.958598	-
ENSMUSC	Npnt	nephrone	GO:00325	developm	K06824	nephrone	mmu0451	ECM-rece	3:1328817	716	733	637	679	1141	550	4.796226	3.921358	4.715992	3.509027	4.921835	2.898413	4.477859	3.776425	0.159114	0.959817	-
ENSMUSC	Hist1h2bc	histone cl	GO:00023	immune s	K11252	histone H	mmu0520	Viral carci	13:235713	56	66	55	62	82	40	5.532801	5.207684	6.005681	4.725847	5.217011	3.109019	5.582055	4.350626	0.159174	0.959846	-
ENSMUSC	Micu1	mitochon	GO:00511	localizatio	-	-	-	-	10:597024	1063	1264	1008	1395	1406	1080	4.897364	4.650741	5.132591	4.95831	4.171279	3.914388	4.893566	4.347992	0.159394	0.960504	-
ENSMUSC	Mgmt	O-6-metl	GO:00325	developm	-	-	-	-	7:1368946	20	20	23	20	20	19	0.421967	0.336993	0.536304	0.325536	0.271722	0.315357	0.431755	0.304205	0.159359	0.960504	-
ENSMUSC	Ski	ski sarcon	GO:00325	developm	-	-	-	-	4:1551540	3528	4470	3273	4672	5429	1684	33.71095	34.11111	34.5649	34.44099	33.40543	12.65888	34.12899	26.8351	0.159888	0.963147	-
ENSMUSC	Smyd1	SET and M	GO:00325	developm	-	-	-	-	6:7121394	28	30	29	34	24	26	0.20969	0.179426	0.240033	0.19644	0.115742	0.153183	0.209716	0.155122	0.160065	0.963883	-
ENSMUSC	Hcar1	hydrocarb	GO:00048	signal trar	K08401	G protein-	mmu0402	cAMP sigr	5:1238767	17	31	13	34	36	41	0.315982	0.460162	0.267051	0.487542	0.430894	0.599518	0.347732	0.505985	0.160259	0.964718	-
ENSMUSC	Dpf3	D4, zinc a	GO:00325	developm	-		mmu0522	Hepatoce	12:832137	170	237	176	210	252	190	1.426007	1.58769	1.631665	1.359008	1.361214	1.253823	1.548454	1.324682	0.160632	0.966212	-
ENSMUSC	Tor1b	torsin fam	GO:00038	catalytic a	-	-	-	-	2:3095295	295	397	270	370	947	383	1.446991	1.555178	1.463709	1.400154	2.991221	1.477927	1.488626	1.956434	0.160815	0.966212	-
ENSMUSC	Hgf	hepatocyt	GO:00325	developm	K05460	hepatocyt	mmu0520	Pathways	5:1655349	90	111	82	79	105	117	0.488811	0.481468	0.492219	0.331021	0.367234	0.499914	0.487499	0.39939	0.160841	0.966212	-
ENSMUSC	Nlrp3	NLR famil	GO:00325	developm	K12800	NACHT, L	mmu0421	Necroptos	11:595415	21	29	17	17	28	19	0.249029	0.274644	0.222802	0.155528	0.213813	0.177247	0.248825	0.182196	0.16078	0.966212	-
ENSMUSC	Cdc14b	CDC14 ce	GO:00081	metabolic	K06639	cell divisic	mmu0411	Cell cycle	13:641892	84	102	77	111	95	72	0.313137	0.303671	0.317245	0.319234	0.228052	0.211154	0.311351	0.252813	0.160815	0.966212	-
ENSMUSC	Kmt5b	lysine met	GO:00325	developm	K11429	histone-ly	mmu0031	Lysine deg	19:376742	943	1241	1063	1228	1369	1193	2.056203	2.161085	2.561736	2.065775	1.922261	2.046472	2.259675	2.011503	0.160714	0.966212	-
ENSMUSC	Col18a1	collagen,	GO:00325	developm	K06823	collagen,	mmu0497	Protein di	10:770521	136	297	151	177	257	179	0.939568	1.63867	1.152959	0.943393	1.143344	0.972866	1.243732	1.019868	0.161158	0.966556	-
ENSMUSC	Eln	elastin [Sc	GO:00325	developm	K14211	elastin[-]	mmu0497	Protein di	5:1347025	218	332	158	215	292	254	1.375781	1.673311	1.102035	1.046794	1.186671	1.261066	1.383709	1.164844	0.161106	0.966556	-
ENSMUSC	Psenen	presenilin	GO:00081	metabolic	K06170	presenilin	mmu0501	Alzheimer	7:3056186	39	41	32	47	38	26	0.827404	0.694679	0.750329	0.769279	0.519147	0.433948	0.757471	0.574125	0.161082	0.966556	-
ENSMUSC	Polg	polymeras	GO:00325	developm	K02332	DNA poly	mmu0110	Metabolic	7:7944623	782	1083	731	1452	1301	1216	2.623199	2.901339	2.710118	3.757691	2.810324	3.208992	2.744885	3.259002	0.161151	0.966556	-
ENSMUSC	Sft2d2	SFT2 dom	GO:00511	localizatio	-	-	-	-	1:1651743	237	345	214	355	477	417	1.548505	1.800235	1.545339	1.789463	2.006954	2.143439	1.63136	1.979952	0.161176	0.966556	-
ENSMUSC	Armc6	armadillo	GO:00325	developm	-	-	-	-	8:7022017	238	342	223	431	478	353	2.372935	2.723202	2.457311	3.315249	3.068957	2.768812	2.517816	3.051006	0.161484	0.966843	-
ENSMUSC	Amot	angiomot	GO:00325	developm	K16819	angiomot	mmu0453	Tight junc	X:1454464	632	742	525	739	846	607	3.560831	3.338752	3.269189	3.212248	3.069443	2.690506	3.389591	2.990732	0.161502	0.966843	-
ENSMUSC	Hist1h2be	histone cl	GO:00023	immune s	K11252	histone H	mmu0520	Viral carci	13:235836	117	126	67	74	144	103	1.0083	0.867202	0.638149	0.492001	0.799134	0.698316	0.837884	0.66315	0.161483	0.966843	-
ENSMUSC	Pcdhb13	protocadh	GO:00325	developm	-	-	-	-	18:374425	106	139	117	132	147	117	1.744513	1.826965	2.128148	1.675997	1.557906	1.514835	1.899875	1.582913	0.161458	0.966843	-
ENSMUSC	Ppcdc	phosphop	GO:00081	metabolic	K01598	phosphop	mmu0110	Metabolic	9:5738509	76	126	118	174	215	154	0.425573	0.563474	0.730275	0.751685	0.775264	0.678405	0.573107	0.735118	0.161404	0.966843	-
ENSMUSC	Lrp10	low-densi	GO:00325	developm	-	-	-	-	14:544641	450	606	452	707	892	736	4.679976	5.033262	5.195366	5.672583	5.9738	6.021714	4.969535	5.889366	0.16171	0.967757	-
ENSMUSC	Iqcm	IQ motif c	-	-	-	-	-	-	8:7544869	1	6	1	0	0	2	0.022537	0.10796	0.024903	0	0	0.035457	0.0518	0.011819	0.161767	0.967765	-
ENSMUSC	Cfap52	cilia and f	GO:00056	cell|cell pr	-	-	-	-	11:679248	19	65	33	30	45	32	0.317313	0.866952	0.609117	0.386536	0.483949	0.420437	0.597794	0.430307	0.161847	0.967911	-
ENSMUSC	Icam1	intercellul	GO:00325	developm	K06490	intercellul	mmu0516	HTLV-I inf	9:2101598	30	63	36	66	79	75	0.396399	0.664799	0.525729	0.672793	0.672188	0.77961	0.528976	0.708197	0.161939	0.968125	-
ENSMUSC	Kcnmb1	potassium	GO:00325	developm	K04937	potassium	mmu0402	cGMP-PK	11:339630	26	32	29	41	58	56	0.378791	0.372323	0.46695	0.460824	0.544135	0.641836	0.406021	0.548932	0.162196	0.968995	-
ENSMUSC	AC132392	-	GO:00081	metabolic	-	-	-	-	7:1123493	14	9	31	41	33	48	0.312325	0.160352	0.764326	0.705632	0.474065	0.842411	0.412334	0.674036	0.162175	0.968995	-
ENSMUSC	Gpn2	GPN-loop	GO:00511	localizatio	-	-	-	-	4:1335843	79	98	63	84	82	88	1.835469	1.818399	1.617734	1.505669	1.226831	1.608467	1.757201	1.446989	0.162341	0.969528	-
ENSMUSC	Slc1a2	solute car	GO:00325	developm	K05613	solute car	mmu0472	Glutamate	2:1026586	61204	73271	56681	70930	85311	72431	248.3882	237.4811	254.2351	222.081	222.9517	231.2526	246.7015	225.4284	0.162813	0.970682	-
ENSMUSC	Sirt2	sirtuin 2 [S	GO:00325	developm	-	-	-	-	7:2876673	1086	1538	1521	1541	1607	1589	8.003514	9.052175	12.38873	8.761606	7.626431	9.212674	9.814807	8.53357	0.162693	0.970682	-
ENSMUSC	Gjb6	gap juncti	GO:00325	developm	-	-	-	-	14:571233	585	745	572	853	1128	960	6.988645	7.10787	7.552316	7.861702	8.677632	9.022347	7.216277	8.52056	0.162709	0.970682	-
ENSMUSC	Fbxl13	F-box anc	GO:00081	metabolic	-	-	-	-	5:2148384	0	4	3	6	6	7	0	0.032834	0.034084	0.04758	0.039714	0.056609	0.022306	0.047968	0.162713	0.970682	-
ENSMUSC	Gm14326	predicted	GO:00081	metabolic	-	-	-	-	2:1779359	361	435	349	425	448	430	7.250658	6.977578	7.747143	6.585497	5.794326	6.794364	7.325127	6.391396	0.162768	0.970682	-
ENSMUSC	Trpa1	transient r	GO:00048	signal trar	K04984	transient r	mmu0475	Inflammat	1:1487264	5	2	4	11	10	7	0.071608	0.022881	0.063323	0.121554	0.092231	0.078879	0.052604	0.097555	0.162987	0.971388	-
ENSMUSC	Prps2	phosphor	GO:00325	developm	-	-	mmu0110	Metabolic	X:1673463	493	651	399	542	677	540	7.316883	7.716238	6.544837	6.20596	6.470271	6.304981	7.192652	6.327071	0.163168	0.971947	-
ENSMUSC	Ptar1	protein pr	GO:00081	metabolic	-	-	-	-	19:236874	184	184	159	209	192	179	5.443158	4.347073	5.198487	4.769918	3.657553	4.165788	4.99624	4.197753	0.163193	0.971947	-
ENSMUSC	Trp53i11	transform	GO:00160	membran	-	-	-	-	2:9318754	858	489	347	1248	638	1187	5.049917	2.298539	2.257214	5.666854	2.418091	5.496152	3.20189	4.527032	0.163318	0.972358	-
ENSMUSC	Zfp995	zinc finge	GO:00081	metabolic	-	-	-	-	17:218795	103	116	78	158	191	139	1.559378	1.402547	1.305143	1.845456	1.862098	1.655545	1.422356	1.787699	0.163506	0.973149	-
ENSMUSC	Il23a	interleukir	GO:00325	developm	K05426	interleukir	mmu0520	Pathways	10:128296	3	2	0	5	7	4	0.062366	0.033211	0	0.080188	0.093705	0.065416	0.031859	0.07977	0.163798	0.974549	-
ENSMUSC	Mapk9	mitogen-	GO:00325	developm	K04440	c-Jun N-t	mmu0520	Pathways	11:498467	1231	1481	1105	1432	1622	1430	5.061109	4.862825	5.021075	4.542145	4.294307	4.625244	4.981669	4.487232	0.1639	0.974823	-
ENSMUSC	Cideb	cell death	GO:00081	metabolic	-	-	-	-	14:557540	32	43	21	41	81	51	0.999924	1.07307	0.72526	0.98839	1.629889	1.253716	0.932751	1.290665	0.16406	0.975439	-
ENSMUSC	Zdhhc17	zinc finge	GO:00048	signal trar	-	-	-	-	10:110941	1169	2327	1089	1641	1764	1600	5.311968	8.444682	5.469097	5.75281	5.161717	5.719684	6.408582	5.544737	0.164433	0.97699	-
ENSMUSC	Setd3	SET doma	GO:00325	developm	K19199	histone-ly	mmu0031	Lysine deg	12:108106	1832	3292	2287	2552	3182	2761	11.39204	16.34865	15.7177	12.24298	12.7418	13.50683	14.48613	12.83054	0.164426	0.97699	-
ENSMUSC	Ndrg1	N-myc dc	GO:00325	developm	-	-	-	-	15:669293	1336	2222	1385	2554	2535	2353	9.644555	12.81049	11.05025	14.22417	11.78442	13.36314	11.16843	13.12391	0.16469	0.977288	-
ENSMUSC	Ubl4a	ubiquitin-	GO:00511	localizatio	-	-	-	-	X:7436571	142	773	563	923	1010	805	1.170073	5.086868	5.127203	5.867555	5.359214	5.218332	3.794715	5.481701	0.164782	0.977288	-
ENSMUSC	P2rx6	purinergic	GO:00048	signal trar	K05221	P2X purin	mmu0408	Neuroacti	16:175618	36	40	35	46	35	29	0.435785	0.3867	0.468261	0.429594	0.272826	0.276167	0.430249	0.326196	0.164912	0.977288	-
ENSMUSC	2310030G	RIKEN cDI	GO:00055	extracellul	-	-	-	-	9:5073969	9	18	17	21	39	27	0.268184	0.428364	0.559888	0.482778	0.748373	0.632966	0.418812	0.621372	0.164903	0.977288	-
ENSMUSC	Dusp26	dual speci	GO:00081	metabolic	-	-	-	-	8:3108947	396	477	348	817	600	538	5.119421	4.924814	4.972227	8.148499	4.994955	5.47165	5.005487	6.205035	0.164932	0.977288	-
ENSMUSC	Gm4779	predicted	-	-	-	-	-	-	X:1017900	3	2	2	0	16	8	0.055759	0.029693	0.041088	0	0.191502	0.116973	0.04218	0.102825	0.164716	0.977288	-
ENSMUSC	Psrc1	proline/se	GO:00081	metabolic	-	-	-	-	3:1083838	141	145	138	214	256	226	1.007038	0.827069	1.089319	1.179158	1.177396	1.269835	0.974475	1.208797	0.164664	0.977288	-
ENSMUSC	Gm21093	predicted	GO:00325	developm	-	-	-	-	4:4203301	96	134	90	123	113	107	3.427811	3.821191	3.551706	3.388297	2.59825	3.005669	3.600236	2.997405	0.164666	0.977288	-
ENSMUSC	Sh2d1a	SH2 doma	GO:00019	cell killing	K07990	SH2 doma	mmu0516	Measles -	X:4250245	0	1	0	0	3	4	0	0.006432	0	0	0.015557	0.025337	0.002144	0.013631	0.165304	0.977601	-
ENSMUSC	Gabrb2	gamma-a	GO:00325	developm	K05181	gamma-a	mmu0408	Neuroacti	11:424197	2786	3585	2488	3246	3895	3340	22.14081	22.75346	21.85293	19.90175	19.93309	20.88189	22.24907	20.23891	0.165547	0.977601	-
ENSMUSC	Apool	apolipopr	GO:00329	macromo	-	-	-	-	X:1123113	40	65	33	76	93	64	0.610129	0.791801	0.556316	0.894341	0.913476	0.767983	0.652748	0.8586	0.165373	0.977601	-
ENSMUSC	Gdpd3	glyceroph	GO:00081	metabolic	-	-	-	-	7:1267663	9	14	11	19	25	22	0.164475	0.204336	0.222176	0.26788	0.29421	0.316305	0.196995	0.292798	0.16542	0.977601	-
ENSMUSC	Zfp36l2	zinc finge	GO:00325	developm	K18753	butyrate r	mmu0421	Cellular se	17:841839	416	634	385	656	920	652	7.195523	8.757976	7.359974	8.753923	10.24734	8.872113	7.771158	9.291125	0.165529	0.977601	-
ENSMUSC	Adamts6	a disinteg	GO:00325	developm	-	-	-	-	13:104287	78	85	58	100	136	133	0.314899	0.274057	0.258792	0.311462	0.353567	0.422415	0.282583	0.362481	0.165408	0.977601	-
ENSMUSC	Clec4a1	C-type lec	GO:00160	membran	-	-	-	-	6:1229218	4	6	2	5	17	9	0.156769	0.187787	0.086633	0.151164	0.429032	0.277471	0.14373	0.285889	0.16505	0.977601	-
ENSMUSC	Trim65	tripartite r	GO:00081	metabolic	-	-	-	-	11:116121	91	118	55	151	147	136	0.735835	0.76202	0.491526	0.941993	0.765439	0.865148	0.663127	0.857527	0.165525	0.977601	-
ENSMUSC	Chrac1	chromatir	GO:00081	metabolic	-	-	-	-	15:730903	100	105	88	151	189	144	1.905681	1.598032	1.853447	2.220033	2.319357	2.158859	1.78572	2.23275	0.16551	0.977601	-
ENSMUSC	Gal3st4	galactose	GO:00081	metabolic	-	-	-	-	5:1382649	34	32	0	46	57	51	0.304981	0.229235	0	0.31833	0.329243	0.359889	0.178072	0.335821	0.16533	0.977601	-
ENSMUSC	Dhh	desert hec	GO:00325	developm	K11990	desert hec	mmu0434	Hedgehog	15:988930	1	0	0	0	3	4	0.025847	0	0	0	0.049924	0.081307	0.008616	0.043744	0.165737	0.977679	-
ENSMUSC	Rad54l	RAD54 lik	GO:00325	developm	K10875	DNA repa	mmu0344	Homologo	4:1160942	5	18	10	15	30	22	0.031325	0.090069	0.069246	0.072508	0.121041	0.108443	0.063547	0.100664	0.165777	0.977679	-
ENSMUSC	Adprhl1	ADP-ribo	GO:00081	metabolic	-	-	-	-	8:1322166	0	0	1	0	3	4	0	0	0.010082	0	0.017623	0.028701	0.003361	0.015442	0.165842	0.977679	-
ENSMUSC	Fbxl21	F-box anc	GO:00081	metabolic	-	-	-	-	13:565224	72	107	72	87	93	88	0.614166	0.728924	0.678782	0.572533	0.510847	0.590535	0.673957	0.557972	0.165838	0.977679	-
ENSMUSC	Fzd7	frizzled cl	GO:00325	developm	K02432	frizzled 1/	mmu0520	Pathways	1:5948242	177	285	135	196	243	201	2.384656	3.06651	2.010178	2.037224	2.10821	2.130398	2.487115	2.091944	0.165689	0.977679	-
ENSMUSC	Vwa5b1	von Willet	GO:00055	extracellul	-	-	-	-	4:1385653	54	116	59	78	85	69	0.36655	0.628846	0.44263	0.408474	0.371549	0.368472	0.479342	0.382832	0.166184	0.979362	-
ENSMUSC	Bicd1	bicaudal I	GO:00511	localizatio	-	-	-	-	6:1494088	1315	1493	1164	1540	1683	1453	2.365547	2.144922	2.314226	2.137257	1.949596	2.056281	2.274898	2.047711	0.166257	0.979466	-
ENSMUSC	Arfgap3	ADP-ribo	GO:00511	localizatio	K12493	ADP-ribo	mmu0414	Endocytos	15:832997	274	383	285	399	645	461	2.609154	2.912691	2.999453	2.931252	3.955161	3.453515	2.840433	3.446643	0.166643	0.981403	-
ENSMUSC	Phf6	PHD finge	GO:00081	metabolic	-	-	-	-	X:5291226	601	739	539	476	909	733	3.833676	3.764704	3.799932	2.342493	3.733871	3.678369	3.799438	3.251578	0.166742	0.981654	-
ENSMUSC	Ap3m2	adaptor-r	GO:00511	localizatio	K12398	AP-3 com	mmu0414	Lysosome	8:2278735	2506	1449	2232	3353	4114	3300	31.52929	14.55953	31.0366	32.54595	33.33128	32.66314	25.70847	32.84679	0.166842	0.981913	-
ENSMUSC	Ripk1	receptor (	GO:00325	developm	K02861	receptor-	mmu0516	Epstein-B	13:340023	105	142	110	184	243	159	0.606425	0.654971	0.702146	0.81985	0.903747	0.722427	0.654514	0.815341	0.166971	0.982006	-
ENSMUSC	Trpv3	transient r	GO:00511	localizatio	K04972	transient r	mmu0475	Inflammat	11:732673	9	28	12	31	39	23	0.094175	0.233997	0.138779	0.250263	0.262796	0.189343	0.15565	0.234134	0.16696	0.982006	-
ENSMUSC	Zfp346	zinc finge	GO:00508	response	-	-	-	-	13:551053	344	375	387	262	514	446	2.370922	2.064123	2.947927	1.393128	2.281267	2.418267	2.46099	2.030887	0.167038	0.982068	-
ENSMUSC	Brinp2	bone mor	GO:00325	developm	-	-	-	-	1:1582452	674	814	729	863	2160	885	3.955894	3.815532	4.728891	3.907741	8.163816	4.086383	4.166772	5.38598	0.167693	0.982999	-
ENSMUSC	Traf4	TNF recep	GO:00325	developm	K09848	TNF recep	mmu0520	Pathways	11:781584	126	210	130	218	278	247	1.513538	2.014596	1.725889	2.020269	2.15042	2.334163	1.751341	2.168284	0.167324	0.982999	-
ENSMUSC	Kif2c	kinesin fa	GO:00511	localizatio	-	-	-	-	4:1171596	3	13	6	10	26	13	0.029439	0.101883	0.065079	0.075711	0.164298	0.100358	0.065467	0.113456	0.167635	0.982999	-
ENSMUSC	Cmpk1	cytidine m	GO:00081	metabolic	K13800	UMP-CM	mmu0110	Metabolic	4:1149593	963	1194	762	1284	1857	1343	15.27246	15.12282	13.35624	15.71006	18.96484	16.75597	14.58384	17.14363	0.167344	0.982999	-
ENSMUSC	Myo5a	myosin V	GO:00325	developm	-	-	-	-	9:7507101	9683	11684	7912	10994	12558	11127	28.76311	27.71809	25.9752	25.19486	24.02155	26.00249	27.48547	25.07296	0.167545	0.982999	-
ENSMUSC	Cox18	cytochron	GO:00511	localizatio	-	-	-	-	5:9021472	93	146	87	126	119	101	1.10906	1.390504	1.146671	1.159243	0.913849	0.947554	1.215412	1.006882	0.167508	0.982999	-
ENSMUSC	Prlhr	prolactin r	GO:00048	signal trar	K04314	prolactin r	mmu0408	Neuroacti	19:604667	0	1	0	2	0	4	0	0.031024	0	0.059939	0	0.122219	0.010341	0.060719	0.167505	0.982999	-
ENSMUSC	Aftph	aftiphilin [	GO:00511	localizatio	-	-	-	-	11:206850	1407	1773	1282	1743	1594	1788	5.87933	5.91682	5.920638	5.619031	4.289203	5.877763	5.905596	5.261999	0.167762	0.982999	-
ENSMUSC	Ttpa	tocopherc	GO:00325	developm	-	-	-	-	4:2000793	76	89	85	102	95	69	0.756388	0.707403	0.934961	0.783174	0.608845	0.540246	0.799584	0.644088	0.16776	0.982999	-
ENSMUSC	Car15	carbonic a	GO:00038	catalytic a	K01672	carbonic a	mmu0091	Nitrogen r	16:178352	25	46	46	76	87	51	1.189739	1.748316	2.419482	2.790373	2.666176	1.909401	1.785846	2.455317	0.167693	0.982999	-
ENSMUSC	1700013F	RIKEN cDI	-	-	-	-	-	-	3:1085341	26	36	20	30	26	23	0.842182	0.931292	0.716006	0.749695	0.542315	0.586111	0.829826	0.62604	0.167917	0.98358	-

ENSMUS(	Pde3a	phosphoc	GO:00325	developm	K19021	cGMP-inh	mmu0402	cAMP sigr	6:1412492	154	246	126	256	306	268	0.694712	0.886271	0.628204	0.890953	0.888918	0.951111	0.736396	0.910327	0.168074	0.984164 -
ENSMUS(	Arhgap5	Rho GTPa	GO:00325	developm	K13709	Rho GTPa	mmu0451	Focal adh	12:525039	3291	3625	2372	3736	4063	3024	11.42171	10.04748	9.098397	10.00324	9.080398	8.256504	10.1892	9.11338	0.168232	0.984386 -
ENSMUS(	Zfp930	zinc finge	GO:00081	metabolic	-	-	-	-	8:6920903	100	143	23	167	232	105	0.893706	1.020649	0.227176	1.15144	1.335175	0.73824	0.713844	1.074952	0.168338	0.984386 -
ENSMUS(	Rpl9-ps6	ribosomal	GO:00081	metabolic	-	-	-	-	19:324658	259	297	254	327	349	236	22.88574	20.95885	24.8054	22.29173	19.85847	16.40545	22.88333	19.51855	0.168296	0.984386 -
ENSMUS(	4930518I1	RIKEN cDI	-	-	-	-	-	-	2:1568566	23	17	14	36	27	40	1.055103	0.622801	0.709804	1.27408	0.797606	1.443557	0.795903	1.171748	0.16823	0.984386 -
ENSMUS(	Cd5	CD5 antig	GO:00511	localizatio	K06455	T-cell surf	mmu0464	Hematopo	19:107181	0	1	0	0	5	2	0	0.023572	0	0	0.095017	0.046442	0.007857	0.047153	0.168577	0.984891 -
ENSMUS(	Gpr37l1	G protein-	GO:00325	developm	-	-	-	-	1:1351602	2331	2853	2278	2878	3381	2631	62.72652	61.31355	67.75003	59.7489	58.588	55.69813	63.93003	58.01168	0.168603	0.984891 -
ENSMUS(	Tgfb1i1	transform	GO:00325	developm	-	-	-	-	7:1282468	123	186	152	201	272	281	0.732554	0.884696	1.000521	0.923553	1.043176	1.316593	0.87259	1.094441	0.168651	0.984891 -
ENSMUS(	Olfr53	olfactory r	GO:00048	signal trar	K04257	olfactory r	mmu0474	Olfactory	7:1406464	0	1	0	0	5	2	0	0.020771	0	0	0.083727	0.040923	0.006924	0.04155	0.168618	0.984891 -
ENSMUS(	Gm14322	predicted	GO:00081	metabolic	-	-	-	-	2:1777592	69	101	111	83	105	107	2.579931	3.01596	4.586962	2.394235	2.528136	3.147394	3.394284	2.689922	0.168742	0.985091 -
ENSMUS(	Pcf11	PCF11 cle	GO:00081	metabolic	K14400	pre-mRN/	mmu0301	mRNA sur	7:9264354	972	1040	696	957	1225	954	6.063392	5.181181	4.798501	4.605656	4.920852	4.681753	5.347691	4.736087	0.16882	0.985216 -
ENSMUS(	Higd1a	HIG1 dom	GO:00081	metabolic	-	-	-	-	9:1218485	622	357	561	836	1149	759	6.639535	3.043417	6.618461	6.884684	7.898086	6.37382	5.433804	7.052197	0.16892	0.985473 -
ENSMUS(	Gkn3	gastrokine	GO:00099	cellular pr	-	-	-	-	6:8738325	34	29	29	19	31	39	2.425239	1.652035	2.286198	1.045547	1.423942	2.188469	2.121158	1.552653	0.16921	0.9865 -
ENSMUS(	Zfp335	zinc finge	GO:00325	developm	-	-	-	-	2:1648918	542	656	470	783	923	855	6.654637	6.432426	6.377778	7.416807	7.297622	8.258518	6.48828	7.657649	0.169189	0.9865 -
ENSMUS(	Vcan	versican [S	GO:00325	developm	K06793	versican c	mmu0451	Cell adhes	13:896553	510	682	469	565	749	605	1.556749	1.662565	1.582223	1.330536	1.472261	1.452828	1.600512	1.418542	0.16936	0.987042 -
ENSMUS(	Gtf2ird1	general tr	GO:00325	developm	K03121	transcripti	mmu0516	Herpes sir	5:1343576	233	330	225	382	457	389	1.020774	1.154606	1.089441	1.291122	1.289272	1.340708	1.088274	1.307034	0.169606	0.988148 -
ENSMUS(	Rnmt	RNA (gua	GO:00081	metabolic	K00565	mRNA (gu	mmu0301	mRNA sur	18:683003	1126	1430	1055	1312	1634	1314	5.444366	5.521925	5.637776	4.8941	5.08763	4.998215	5.534689	4.993315	0.169975	0.989215 -
ENSMUS(	Mcm7	minichron	GO:00081	metabolic	K02210	DNA repli	mmu0411	Cell cycle	5:1381645	139	173	120	221	257	211	1.251596	1.244062	1.194203	1.535225	1.490174	1.494662	1.229954	1.506687	0.169921	0.989215 -
ENSMUS(	Ackr4	atypical cl	GO:00048	signal trar	-	-	-	-	9:1040841	7	9	0	9	15	12	0.047671	0.04895	0	0.047286	0.065779	0.064291	0.032207	0.059118	0.170017	0.989215 -
ENSMUS(	C2cd4d	C2 calciur	GO:00160	membran	-	-	-	-	3:9436243	1	1	0	0	3	6	0.0308	0.024594	0	0	0.059491	0.145343	0.018465	0.068278	0.169877	0.989215 -
ENSMUS(	Plp2	proteolipi	GO:00055	extracellul	-	-	-	-	X:7667941	41	54	32	83	133	33	0.869827	0.914942	0.750329	1.358508	1.817016	0.550786	0.845033	1.242103	0.170197	0.989932 -
ENSMUS(	Esam	endotheli	GO:00055	extracellul	K06787	endotheli	mmu0451	Cell adhes	9:3752807	103	129	93	179	199	150	1.838881	1.839294	1.835052	2.465478	2.287837	2.106784	1.837742	2.2867	0.170289	0.990111 -
ENSMUS(	Ifi206	interferon	GO:00081	metabolic	-	-	-	-	1:1734684	4	7	0	7	10	10	0.070267	0.0982	0	0.094861	0.113113	0.138189	0.056155	0.115388	0.170342	0.990111 -
ENSMUS(	Kng1	kininogen	GO:00081	metabolic	K03898	kininogen	mmu0461	Complem	16:230578	0	4	0	0	0	0	0	0.038062	0	0	0	0	0.012687	0	0.170468	0.990182 -
ENSMUS(	En1	engrailed	GO:00325	developm	-	-	-	-	1:1206024	0	4	0	0	0	0	0	0.074326	0	0	0	0	0.024775	0	0.170419	0.990182 -
ENSMUS(	Slco3a1	solute car	GO:00511	localizatio	-	-	-	-	7:7427541	472	610	382	722	880	655	2.723437	2.81094	2.436047	3.213979	3.269739	2.973227	2.656808	3.152315	0.170538	0.990256 -
ENSMUS(	Mfsd3	major faci	GO:00511	localizatio	-	-	-	-	15:767015	31	42	32	59	73	50	0.872649	0.944241	0.995595	1.281345	1.323302	1.107292	0.937495	1.237313	0.170782	0.99115 -
ENSMUS(	Tprn	taperin [S	GO:00056	cell|stereo	-	-	-	-	2:2526261	243	264	202	328	416	381	4.029636	3.496305	3.702176	4.196278	4.442299	4.970449	3.742706	4.536342	0.170862	0.99115 -
ENSMUS(	Gmfb	glia matur	GO:00508	response	-	-	-	-	14:468081	3149	3697	2633	3299	4763	3164	21.15902	19.83892	19.55332	17.10155	20.60905	16.72516	20.18375	18.14525	0.170837	0.99115 -
ENSMUS(	Adgrg1	adhesion	GO:00325	developm	-	-	-	-	8:9497475	918	1066	722	1298	1551	1277	5.1532	4.779004	4.479383	5.621331	5.606613	5.639443	4.803863	5.622462	0.170925	0.991182 -
ENSMUS(	Mettl26	methyltrai	-	-	-	-	-	-	17:258754	147	160	149	225	268	248	3.444192	2.993887	3.858356	4.06708	4.043516	4.571219	3.432145	4.227271	0.171465	0.993526 -
ENSMUS(	Rapgef5	Rap guan	GO:00987	molecular	K08019	Rap guan	mmu0401	Ras signal	12:117516	2081	2757	1951	2514	3011	2528	8.802351	9.313421	9.120753	8.203936	8.201474	8.412296	9.078842	8.272569	0.17149	0.993526 -
ENSMUS(	Tlr12	toll-like re	GO:00023	immune s	-	-	-	-	4:1286154	7	8	6	14	16	15	0.118826	0.108455	0.112572	0.18334	0.174891	0.20031	0.113284	0.18618	0.171501	0.993526 -
ENSMUS(	Taf6	TATA-box	GO:00081	metabolic	K03131	transcripti	mmu0516	Herpes sir	5:1381786	544	785	519	872	1039	902	5.007629	5.770969	5.280167	6.192702	6.158904	6.532066	5.352922	6.294558	0.171708	0.994327 -
ENSMUS(	Jund	jun D prot	GO:00325	developm	-	-	-	-	8:7069894	1233	1589	1142	1962	2288	1749	45.1347	46.45334	46.20196	55.40852	53.93343	50.36723	45.93	53.2364	0.171753	0.994327 -
ENSMUS(	Ptpn22	protein ty	GO:00325	developm	-	-	-	-	3:1038597	14	7	9	21	34	12	0.09948	0.039723	0.070676	0.115118	0.155571	0.067082	0.06996	0.11259	0.171837	0.994484 -
ENSMUS(	Helz	helicase w	GO:00038	catalytic a	-	-	-	-	11:107547	1865	2269	1574	2208	2326	2175	6.551222	6.365373	6.110758	5.983744	5.261481	6.010545	6.342451	5.751923	0.172094	0.994715 -
ENSMUS(	Vash1	vasohibin	GO:00325	developm	-	-	-	-	12:866787	533	663	497	649	653	642	12.52174	12.43932	12.90446	11.76284	9.87882	11.86542	12.62184	11.16902	0.172	0.994715 -
ENSMUS(	Mat1a	methionir	GO:00081	metabolic	K00789	S-adenos	mmu011C	Metabolic	14:41105C	2	0	0	6	1	2	0.030065	0	0	0.069595	0.00968	0.023661	0.010022	0.034312	0.172106	0.994715 -
ENSMUS(	Cep295nl	CEP295 N	GO:00056	cell|intrace	-	-	-	-	11:118332	3	4	8	3	3	4	0.051655	0.055001	0.152236	0.039858	0.033269	0.054182	0.086297	0.042436	0.171958	0.994715 -
ENSMUS(	Mok	MOK prot	GO:00081	metabolic	-	-	-	-	12:110807	120	167	104	206	229	188	1.642451	1.825468	1.573227	2.175246	2.018369	2.024319	1.680382	2.072645	0.172326	0.995323 -
ENSMUS(	Unc13d	unc-13 hc	GO:00325	developm	-	-	-	-	11:116062	22	22	15	31	48	30	0.186048	0.148587	0.140199	0.202256	0.261397	0.199589	0.158278	0.221081	0.172274	0.995323 -
ENSMUS(	Tpk1	thiamine p	GO:00081	metabolic	K00949	thiamine p	mmu011C	Metabolic	6:4334500	17	41	27	42	55	55	0.123572	0.23801	0.216906	0.235527	0.257445	0.314511	0.192829	0.269161	0.172467	0.99548 -
ENSMUS(	Pomgnt2	protein O	GO:00325	developm	K18207	protein O	mmu011C	Metabolic	9:1219816	493	647	477	390	709	692	3.362562	3.524304	3.595744	2.052192	3.114042	3.713129	3.494203	2.959788	0.172445	0.99548 -
ENSMUS(	Tmem79	transmem	GO:00325	developm	-	-	-	-	3:8832865	18	11	9	17	44	21	0.180345	0.088017	0.099658	0.13141	0.283887	0.165529	0.122673	0.193608	0.17476	0.995843 -
ENSMUS(	Atp5d	ATP synth	GO:00081	metabolic	K02134	F-type H+	mmu011C	Metabolic	10:801386	1452	1612	1403	2468	2372	2040	24.64046	21.84706	26.31399	32.31154	25.92099	27.23474	24.26717	28.48909	0.173609	0.995843 -
ENSMUS(	Cyp17a1	cytochron	GO:00081	metabolic	K00512	steroid 17	mmu011C	Metabolic	19:466671	0	0	0	4	0	0	0	0	0	0.072714	0	0	0	0.024238	0.17506	0.995843 -
ENSMUS(	Hlf	hepatic le	GO:00325	developm	-	-	-	-	11:903365	1805	3344	1383	2442	4021	3770	9.508229	14.06807	8.05177	9.924266	13.6399	15.62337	10.54269	13.06251	0.174652	0.995843 -
ENSMUS(	2810408A	RIKEN cDI	GO:00081	metabolic	-	-	-	-	11:698973	1	8	0	8	12	4	0.007955	0.050809	0	0.049082	0.061454	0.025023	0.019588	0.045186	0.173354	0.995843 -
ENSMUS(	Fdxr	ferredoxir	GO:00081	metabolic	-	-	-	-	11:115268	140	152	103	223	231	185	2.418831	2.097328	1.966803	2.972435	2.570064	2.514545	2.160987	2.685681	0.174122	0.995843 -
ENSMUS(	Il20ra	interleukir	GO:00048	signal trar	K05136	interleukir	mmu0406	Cytokine-	10:197125	0	0	0	4	0	0	0	0	0	0.073479	0	0	0	0.024493	0.175327	0.995843 -
ENSMUS(	Havcr2	hepatitis A	GO:00325	developm	-	-	-	-	11:464549	83	113	70	84	118	85	1.021116	1.110258	0.951793	0.79728	0.934837	0.822672	1.027722	0.851596	0.173197	0.995843 -
ENSMUS(	Csmd3	CUB and S	GO:00325	developm	-	-	-	-	15:475806	665	740	646	754	824	807	1.682425	1.495174	1.806314	1.471688	1.342443	1.606196	1.661304	1.473442	0.174823	0.995843 -
ENSMUS(	Nox3	NADPH o	GO:00325	developm	-	-	-	-	17:363524	0	0	0	4	0	0	0	0	0	0.105135	0	0	0	0.035045	0.175171	0.995843 -
ENSMUS(	Stard4	StAR-rela	GO:00081	metabolic	-	-	-	-	18:331993	341	449	326	544	692	505	3.066848	3.225	3.240426	3.774562	4.007728	3.573058	3.177424	3.785116	0.173804	0.995843 -
ENSMUS(	Cplx4	complexir	GO:00452	synapse|c	K15295	complexir	mmu0472	Synaptic v	18:659557	0	0	0	4	0	0	0	0	0	0.107596	0	0	0	0.035865	0.174996	0.995843 -
ENSMUS(	Slc25a45	solute car	GO:00511	localizatio	-	-	-	-	19:587780	64	77	46	67	72	58	0.737999	0.709107	0.58625	0.59605	0.534642	0.526161	0.677786	0.552285	0.174984	0.995843 -
ENSMUS(	Gldc	glycine de	GO:00081	metabolic	-	-	mmu011C	Metabolic	19:300984	949	1098	827	1084	1311	983	15.43531	14.26256	14.86625	13.60218	13.73114	12.57806	14.8547	13.30379	0.17327	0.995843 -
ENSMUS(	Slc18a2	solute car	GO:00325	developm	K08155	MFS trans	mmu0503	Alcoholisr	19:592608	21	35	20	53	49	34	0.337168	0.448781	0.354896	0.656485	0.506599	0.429439	0.380282	0.530841	0.174098	0.995843 -
ENSMUS(	Fgf14	fibroblast	GO:00325	developm	K04358	fibroblast	mmu052C	Pathways	14:123977	319	460	294	884	485	419	3.055783	3.519124	3.112614	6.533016	2.991764	3.15759	3.229174	4.227457	0.173675	0.995843 -
ENSMUS(	Hao1	hydroxyac	GO:00081	metabolic	K11517	(S)-2-hyd	mmu011C	Metabolic	2:1344973	0	0	3	0	0	0	0	0	0.08318	0	0	0	0.027727	0	0.174457	0.995843 -
ENSMUS(	Dll4	delta-like	GO:00325	developm	K06051	delta[-]	mmu052C	Pathways	2:1193257	44	54	45	71	90	81	0.68569	0.672078	0.775066	0.853621	0.903184	0.993056	0.710945	0.91662	0.172854	0.995843 -
ENSMUS(	Fam209	family witl	GO:00160	membran	-	-	-	-	2:1724725	0	0	0	4	0	0	0	0	0	0.269916	0	0	0	0.089972	0.175398	0.995843 -
ENSMUS(	Arfgap1	ADP-ribos	GO:00452	synapse|c	K12492	ADP-ribos	mmu0414	Endocytos	2:1809672	804	1341	900	1031	1296	1163	3.225833	4.296951	3.990939	3.191351	3.348461	3.670933	3.837908	3.403582	0.174874	0.995843 -
ENSMUS(	Supt20	suppresso	GO:00325	developm	-	-	mmu0414	Autophag	3:5469280	446	568	396	646	885	656	2.969025	3.019767	2.913541	3.317741	3.793826	3.435531	2.967444	3.515699	0.173201	0.995843 -
ENSMUS(	Coro1a	coronin, a	GO:00325	developm	K13882	coronin-1	mmu0515	Tuberculo	7:1266997	641	836	627	775	950	735	7.560022	7.874393	8.172957	7.051755	7.215115	6.819671	7.869124	7.028847	0.172963	0.995843 -
ENSMUS(	Brs3	bombesin	GO:00048	signal trar	K04170	bombesin	mmu0408	Neuroacti	X:5704307	0	0	0	4	0	0	0	0	0	0.067966	0	0	0	0.022655	0.175516	0.995843 -
ENSMUS(	Naa10	N(alpha)-	GO:00081	metabolic	-	-	-	-	X:7391687	311	382	334	466	628	535	2.339996	2.295429	2.777459	2.705024	3.042772	3.166792	2.470961	2.97153	0.174857	0.995843 -
ENSMUS(	Itga11	integrin al	GO:00325	developm	K06587	integrin al	mmu0415	PI3K-Akt s	9:6267782	50	77	41	68	125	94	0.569152	0.699986	0.515802	0.597166	0.916258	0.841772	0.59498	0.785066	0.172697	0.995843 -
ENSMUS(	Nlrp4f	NLR famil	GO:00971	organic cy	-	-	-	-	13:651771	0	0	0	4	0	0	0	0	0	0.02547	0	0	0	0.00849	0.175622	0.995843 -
ENSMUS(	Surf6	surfeit ger	GO:00329	macromo	-	-	-	-	2:2688862	268	324	183	380	522	336	2.55202	2.464	1.925966	2.791671	3.20092	2.5171	2.313996	2.836564	0.173359	0.995843 -
ENSMUS(	Ccdc38	coiled-co	GO:00056	cell|intrace	-	-	-	-	10:935406	0	0	0	4	0	0	0	0	0	0.034941	0	0	0	0.011647	0.175232	0.995843 -
ENSMUS(	C1qa	complem	GO:00081	metabolic	K03986	complem	mmu0532	Systemic l	4:1368959	664	1052	724	877	1106	835	38.98325	49.32552	46.9781	39.7228	41.8138	38.56627	45.09562	40.03429	0.17362	0.995843 -
ENSMUS(	Poli	polymeras	GO:00081	metabolic	K03510	DNA poly	mmu0346	Fanconi a	18:705086	254	336	241	296	347	300	1.712353	1.809022	1.795657	1.539505	1.506409	1.591079	1.772344	1.545664	0.172746	0.995843 -
ENSMUS(	Gng10	guanine n	GO:00048	signal trar	K04545	guanine n	mmu052C	Pathways	4:5903508	165	175	169	218	409	241	7.66711	6.494311	8.679259	7.815088	12.23843	8.809997	7.61356	9.621171	0.17285	0.995843 -
ENSMUS(	Cpa4	carboxype	GO:00081	metabolic	-	-	-	-	6:3056836	0	0	0	4	0	0	0	0	0	0.068785	0	0	0	0.022928	0.175465	0.995843 -
ENSMUS(	Dcxr	dicarbony	GO:00081	metabolic	K03331	L-xylulose	mmu011C	Metabolic	11:120725	47	67	51	76	109	92	1.403964	1.598395	1.683772	1.751492	2.096735	2.162025	1.562043	2.003417	0.173829	0.995843 -
ENSMUS(	Dnd1	DND micr	GO:00325	developm	-	-	-	-	18:367636	4	14	4	17	14	14	0.047537	0.13287	0.052539	0.155861	0.107135	0.130882	0.077649	0.131293	0.175192	0.995843 -
ENSMUS(	Rnf208	ring finge	GO:00081	metabolic	-	-	-	-	2:2524225	428	787	430	640	749	394	7.789233	11.43856	8.649007	8.985886	8.777832	5.641019	9.292267	7.801579	0.175301	0.995843 -
ENSMUS(	Pcdhb10	protocadh	GO:00325	developm	-	-	-	-	18:374116	121	112	101	115	148	105	2.591389	1.915622	2.390637	1.900084	2.041089	1.769083	2.299216	1.903418	0.174584	0.995843 -
ENSMUS(	Cox7b2	cytochron	GO:00081	metabolic	K02271	cytochron	mmu011C	Metabolic	5:7144282	0	1	0	5	2	0	0	0.089982	0	0.434527	0.145056	0	0.029994	0.193194	0.175397	0.995843 -
ENSMUS(	Lexm	lymphocy	GO:00081	metabolic	-	-	-	-	4:1065909	0	0	0	0	0	4	0	0	0	0	0	0.082707	0	0.027569	0.175224	0.995843 -
ENSMUS(	Drc3	dynein rec	GO:00056	cell|intrace	-	-	-	-	11:603533	162	209	140	179	195	191	1.859987	1.916401	1.776525	1.585546	1.44173	1.725201	1.850971	1.584159	0.175225	0.995843 -
ENSMUS(	Armc7	armadillo	GO:00329	macromo	-	-	-	-	11:115475	99	157	105	195	237	155	1.421285	1.800084	1.666037	2.159801	2.191043	1.750617	1.629135	2.03382	0.173327	0.995843 -
ENSMUS(	Kir3dl2	killer cell i	GO:00160	membran	K07980	killer cell i	mmu0461	Antigen p	X:1364481	0	0	0	4	0	0	0	0	0	0.095587	0	0	0	0.031862	0.175266	0.995843 -
ENSMUS(	Fam133b	family witl	GO:00971	organic cy	-	-	-	-	5:3543833	258	609	434	350	411	571	1.542292	2.90744	2.867375	1.614159	1.582138	2.685311	2.439036	1.960536	0.175473	0.995843 -
ENSMUS(	4930453N	RIKEN cDI	GO:00099	cellular pr	-	-	-	-	16:647517	420	446	310	443	221	502	3.928367	3.331528	3.204584	3.196666	1.331101	3.693836	3.48816	2.740534	0.175253	0.995843 -
ENSMUS(	Ipmk	inositol pc	GO:00325	developm	K00915	inositol-p	mmu011C	Metabolic	10:713477	449	618	368	527	1506	532	2.626467	2.887088	2.379143	2.378304	5.672908	2.448207	2.6309	3.499806	0.173071	0.995843 -
ENSMUS(	Rnf183	ring finge	GO:00081	metabolic	-	-	-	-	4:6242754	0	0	3	0	0	0	0	0	0.060854	0	0	0	0.020285	0	0.17437	0.995843 -
ENSMUS(	Ccr1l1	chemokin	GO:00048	signal trar	K04176	C-C chem	mmu0406	Cytokine-	9:1239772	0	0	3	0	0	0	0	0	0.173637	0	0	0	0.057879	0	0.174313	0.995843 -
ENSMUS(	Rab44	RAB44, m	GO:00038	catalytic a	-	-	-	-	17:291350	4	0	3	0	0	2	0.054025	0	0.044782	0	0	0.021254	0.032936	0.007085	0.175022	0.995843 -
ENSMUS(	Gm10300	predicted	-	-	-	-	-	-	4:1320746	0	0	0	4	0	0	0	0	0	0.082056	0	0	0	0.027352	0.175114	0.995843 -
ENSMUS(	Tmigd3	transmem	GO:00048	signal trar	-	-	-	-	3:1058709	1	1	3	8	4	4	0.021774	0.017387	0.072178	0.134349	0.056071	0.068495	0.037113	0.086305	0.172804	0.995843 -
ENSMUS(	B3galt5	UDP-Gal:I	GO:00081	metabolic	K03877	beta-1,3-	mmu011C	Metabolic	16:962358	1429	1875	1325	2060	2797	2142	7.71391	8.083314	7.905056	8.579064	9.722757	9.096466	7.90076	9.132762	0.175614	0.995843 -
ENSMUS(	Tmem92	transmem	GO:00160	membran	-	-	-	-	11:947772	0	0	0	4	0	0	0	0	0	0.053584	0	0	0	0.017861	0.175563	0.995843 -
ENSMUS(	Raet1d	retinoic ac	GO:00019	cell killing	K07987|KC	retinoic ac	mmu0465	Natural ki	10:223605	0	0	3	0	0	0	0	0	0.1405	0	0	0	0.046833	0	0.174435	0.995843 -
ENSMUS(	Cd300ld2	CD300 mc	GO:00160	membran	-	-	-	-	11:11501C	0	0	3	0	0	0	0	0	0.160811	0	0	0	0.053604	0	0.174242	0.995843 -
ENSMUS(	A030014E	RIKEN cDI	-	-	-	-	-	-	1:8292491	0	0	3	0	0	0	0	0	0.254348	0	0	0	0.084783	0	0.174181	0.995843 -
ENSMUS(	Lcorl	ligand dep	GO:00081	metabolic	-	-	-	-	5:4569718	305	427	422	582	677	598	0.593194	0.663239	0.907101	0.873273	0.847891	0.914975	0.721178	0.878713	0.175806	0.995912 -
ENSMUS(	Tpo	thyroid pe	GO:00325	developm	K00431	thyroid pe	mmu011C	Metabolic	12:300546	2	2	0	0	10	4	0.026675	0.021311	0	0	0.085904	0.041977	0.015995	0.042627	0.175738	0.995912 -
ENSMUS(	Selenop	selenopro	GO:00325	developm	-	-	-	-	15:326854	3485	5101	3760	5760	6680	6045	41.56818	48.5914	49.56703	53.00429	51.30851	56.72382	46.57554	53.67887	0.175766	0.995912 -
ENSMUS(	Ube2cbp	ubiquitin-	GO:00081	metabolic	-	-	-	-	9:8630730	18	33	19	40	49	35	0.570919	0.835935	0.66607	0.97883	1.00083	0.873352	0.690974	0.951004	0.17593	0.99629 -
ENSMUS(	Taf1d	TATA-box	GO:00081	metabolic	-	-	-	-	9:1530621	248	307	263	383	498	416	0.876397	0.866433	1.027193	1.044188	1.133271	1.156522	0.923341	1.111327	0.176011	0.996422 -
ENSMUS(	Smim8	small inte	GO:00160	membran	-	-	-	-	4:3476866	131	140	105	82	160	156	2.727118	2.327586	2.415838	1.31697	2.144898	2.554864	2.490181	2.005577	0.176153	0.9969 -
ENSMUS(	Nmnat1	nicotinam	GO:00081	metabolic	K06210	nicotinam	mmu011C	Metabolic	4:1494675	118	149	91	127	170	87	1.495099	1.507708	1.274311	1.241427	1.387044	0.867194	1.425706	1.165222	0.176475	0.997427 -
ENSMUS(	Stx18	syntaxin 1	GO:00511	localizatio	K08492	syntaxin 1	mmu0414	Phagoson	5:3803874	299	348	288	329	421	326	2.584793	2.402585	2.751647	2.194218	2.343641	2.21709	2.579675	2.25165	0.176374	0.997427 -
ENSMUS(	Celf6	CUGBP, El	GO:00081	metabolic	-	-	-	-	9:5957791	265	284	214	386	478	347	2.430582	2.080314	2.169324	2.731384	2.823238	2.503828	2.22674	2.68615	0.176429	0.997427 -
ENSMUS(	Fam167a	family witl	GO:00056	cell|intrace	-	-	-	-	14:634363	51	75	51	58	71	59	0.677092	0.795223	0.748343	0.594069	0.607006	0.616235	0.74022	0.60577	0.176437	0.997427 -
ENSMUS(	Npepps	aminopep	GO:00081	metabolic	-	-	-	-	11:972058	2602	3652	2282	2931	3575	3438	12.55418	14.07206	12.16868	10.91007	11.10739	13.04962	12.93164	11.68902	0.177713	0.9976 -
ENSMUS(	Dlst	dihydrolip	GO:00081	metabolic	K00658	2-oxoglut	mmu011C	Metabolic	12:851108	882	2554	1587	1129	1562	2284	4.133021	9.557969	8.219068	4.081532	4.713405	8.419882	7.303352	5.738273	0.178858	0.9976 -
ENSMUS(	Fcer2a	Fc recepto	GO:00081	metabolic	K06468	low affinit	mmu0516	Epstein-B	8:3681737	0	0	0	0	5	0	0	0	0	0	0.047809	0	0	0.015936	0.177631	0.9976 -
ENSMUS(	Ube2m	ubiquitin-	GO:00081	metabolic	K10579	ubiquitin-	mmu0412	Ubiquitin	7:1303512	1203	1797	1248	1592	1808	1566	13.13536	15.67004	15.06043	13.41064	12.71245	13.45175	14.62194	13.19161	0.17849	0.9976 -
ENSMUS(	Taf7l	TATA-box	GO:00325	developm	K03132	transcripti	mmu0302	Basal tran	X:1344601	10	3	3	5	4	2	0.244626	0.058618	0.081114	0.094356	0.063013	0.038497	0.128119	0.065289	0.178021	0.9976 -
ENSMUS(	Aldh1a2	aldehyde	GO:00325	developm	K07249	retinal del	mmu011C	Metabolic	9:7121578	82	128	93	135	186	173	2.211477	2.756919	2.772031	2.808872	3.230234	3.670482	2.580142	3.236529	0.178832	0.9976 -
ENSMUS(	Galnt10	polypeptic	GO:00081	metabolic	K00710	polypeptic	mmu011C	Metabolic	11:576454	143	234	155	274	323	245	1.373496	1.794955	1.645388	2.030356	1.997782	1.851261	1.604613	1.9598	0.178364	0.9976 -
ENSMUS(	Sav1	salvador f	GO:00325	developm	K16686	scaffold p	mmu0439	Hippo sig	12:699650	464	527	405	420	752	419	4.114884	3.732464	3.969554	2.873551	4.294495	2.923236	3.938967	3.363761	0.177111	0.9976 -
ENSMUS(	Ctsb	cathepsin	GO:00325	developm	K01363	cathepsin	mmu0462	NOD-like	14:631224	8586	12663	8302	10563	12755	11494	82.59728	97.28756	88.26822	78.39574	79.01508	86.98752	89.38435	81.46611	0.177413	0.9976 -
ENSMUS(	Ebf2	early B ce	GO:00325	developm	-	-	-	-	14:672332	5	6	2	5	0	2	0.027142	0.026013	0.012001	0.02094	0	0.008543	0.021719	0.009828	0.178205	0.9976 -
ENSMUS(	Pebp4	phosphati	-	-	-	-	-	-	14:698404	0	0	0	0	5	0	0	0	0	0	0.182197	0	0	0.060732	0.177565	0.9976 -
ENSMUS(	Cyp39a1	cytochron	GO:00081	metabolic	K07439	24-hydrox	mmu0012	Primary b	17:436674	19	27	23	23	62	48	0.379986	0.431248	0.508374	0.354876	0.798477	0.755217	0.439869	0.63619	0.177423	0.9976 -
ENSMUS(	Gm16286	predicted	GO:00081	metabolic	-	-	-	-	18:802067	187	1188	193	263	1689	1332	1.554083	7.884888	1.77271	1.686238	9.03894	8.708603	3.737227	6.477927	0.177013	0.9976 -
ENSMUS(	Coro1b	coronin, a	GO:00325	developm	-	-	-	-	19:414861	1132	1573	1280	1893	2248	1927	16.35924	18.15479	20.44435	21.1056	20.92028	21.90833	18.31946	21.3114	0.178561	0.9976 -
ENSMUS(	Opn3	opsin 3 [S	GO:00048	signal trar	-	-	-	-	1:1756624	18	23	10	16	16	14	0.5745	0.586288	0.352745	0.393988	0.328846	0.351527	0.504511	0.35812	0.178688	0.9976 -
ENSMUS(	Nsun7	NOL1/NC	GO:00081	metabolic	-	-	-	-	5:6625989	166	213	181	222	367	327	1.458788	1.494891	1.757962	1.505106	2.076843	2.260694	1.570547	1.947548	0.178423	0.9976 -
ENSMUS(	Cit	citron [So	GO:00081	metabolic	-	-	-	-	5:1158452	1667	1920	1235	1954	1924	1717	5.083603	4.67609	4.162455	4.597171	3.778293	4.11924	4.640716	4.164902	0.176603	0.9976 -
ENSMUS(	Slc7a9	solute car	GO:00511	localizatio	K13868	solute car	mmu0497	Protein di	7:3544879	0	0	0	0	5	0	0	0	0	0	0.053032	0	0	0.017677	0.177668	0.9976 -
ENSMUS(	Xylt1	xylosyltrar	GO:00081	metabolic	K00771	protein xy	mmu011C	Metabolic	7:1173809	1722	2045	1374	1949	2164	1936	10.12734	9.605089	8.930914	8.843099	8.195479	8.957329	9.554449	8.665302	0.178797	0.9976 -
ENSMUS(	Qprt	quinolinat	GO:00081	metabolic	K00767	nicotinate	mmu011C	Metabolic	7:1271071	7	19	8	4	18	3	0.141999	0.307803	0.179345	0.062592	0.23513	0.047868	0.209716	0.115197	0.178133	0.9976 -
ENSMUS(	Cnga4	cyclic nucl	GO:00511	localizatio	K04951	cyclic nucl	mmu0474	Olfactory	7:1054045	25	14	18	19	22	12	0.44053	0.197023	0.350562	0.258294	0.249646	0.166358	0.329372	0.224766	0.177268	0.9976 -
ENSMUS(	Zfp275	zinc finge	GO:00081	metabolic	-	-	-	-	X:7334262	410	492	346	502	609	200	3.650306	3.498301	3.404625	3.4481	3.491549	1.400834	3.517744	2.780161	0.178321	0.9976 -
ENSMUS(	Commd4	COMM dc	GO:00081	metabolic	-	-	-	-	9:5715503	213	424	257	429	507	465	2.276052	3.618387	3.035175	3.53664	3.488717	3.909016	2.976538	3.644791	0.178771	0.9976 -
ENSMUS(	Trim69	tripartite r	GO:00081	metabolic	-	-	-	-	2:1221607	0	0	0	0	5	0	0	0	0	0	0.076109	0	0	0.02537	0.177636	0.9976 -
ENSMUS(	Agl	amylo-1,6	GO:00081	metabolic	K01196	glycogen	mmu011C	Metabolic	3:1167399	881	881	630	1004	875	835	2.708433	2.163038	2.14057	2.381257	1.732228	2.019481	2.337347	2.044322	0.176638	0.9976 -
ENSMUS(	Mitf	microphth	GO:00325	developm	K09455	microphth	mmu052C	Pathways	6:9780705	244	348	194	260	298	307	1.468816	1.673022	1.290701	1.207483	1.155176	1.453871	1.477513	1.272176	0.178837	0.9976 -
ENSMUS(	1110059E	RIKEN cDI	-	-	-	-	-	-	19:215812	183	244	194	309	358	313	2.022016	2.153119	2.369089	2.634032	2.547245	2.720743	2.181408	2.634007	0.177831	0.9976 -
ENSMUS(	Reep6	receptor a	GO:00511	localizatio	-	-	-	-	10:803299	121	260	186	156	210	228	1.485931	2.549952	2.524475	1.477976	1.660678	2.202717	2.186786	1.780457	0.177027	0.9976 -
ENSMUS(	Setd1b	SET doma	GO:00081	metabolic	K11422	histone-ly	mmu0031	Lysine deg	5:1231421	906	1268	842	1187	1155	1129	3.354067	3.748942	3.445106	3.390195	2.753471	3.288126	3.516038	3.14393	0.178333	0.9976 -
ENSMUS(	Eva1c	eva-1 hor	GO:00055	extracellul	-	-	-	-	16:908267	9	21	14	28	21	34	0.092823	0.172978	0.159586	0.222796	0.139472	0.275871	0.141795	0.212713	0.178491	0.9976 -
ENSMUS(	Mthfd1l	methylene	GO:00325	developm	K13402	monofunc	mmu011C	Metabolic	10:397311	206	259	203	335	362	355	2.230132	2.239289	2.428883	2.797949	2.523638	3.023456	2.299435	2.781681	0.17779	0.9976 -
ENSMUS(	Gpr20	G protein-	GO:00048	signal trar	-	-	-	-	15:736946	5	5	2	6	14	9	0.14238	0.113714	0.062954	0.131822	0.256745	0.201632	0.106349	0.196733	0.177354	0.9976 -
ENSMUS(	Mmp13	matrix me	GO:00325	developm	K07994	matrix me	mmu0492	Relaxin sig	9:7272514	5	32	6	7	16	6	0.114203	0.583765	0.151486	0.123359	0.235347	0.107825	0.283151	0.15551	0.176913	0.9976 -
ENSMUS(	Soga1	suppresso	GO:00081	metabolic	-	-	-	-	2:1570157	936	1106	815	1026	1208	1109	6.35852	6.000417	6.119062	5.377222	5.284476	5.926833	6.159333	5.52951	0.17789	0.9976 -
ENSMUS(	Nkain3	Na+/K+ t	GO:00511	localizatio	-	-	-	-	4:2011887	299	428	300	381	395	384	2.518818	2.879483	2.793146	2.476167	2.142775	2.544878	2.730482	2.38794	0.177431	0.9976 -
ENSMUS(	Mcpt4	mast cell p	GO:00081	metabolic	-	-	mmu0461	Renin-ang	14:560596	0	0	0	0	5	0	0	0	0	0	0.177429	0	0	0.059143	0.177496	0.9976 -
ENSMUS(	Rimbp3	RIMS binc	-	-	-	-	-	-	16:172081	83	108	76	137	165	132	0.875721	0.910041	0.88624	1.115166	1.12106	1.095655	0.890667	1.110627	0.178561	0.9976 -
ENSMUS(	Prrt4	proline-ric	GO:00160	membran	-	-	-	-	6:2916923	25	49	31	52	62	67	0.447504	0.700499	0.613298	0.718118	0.714673	0.943512	0.5871	0.792101	0.177913	0.9976 -
ENSMUS(	Usp49	ubiquitin s	GO:00081	metabolic	-	-	-	-	17:476306	177	348	125	195	469	453	1.071086	1.68181	0.836008	0.91037	1.827594	2.156559	1.196302	1.631508	0.17767	0.9976 -
ENSMUS(	Gm20441	predicted	GO:00081	metabolic	-	-	-	-	10:757714	0	0	0	0	5	0	0	0	0	0	0.260041	0	0	0.08668	0.177687	0.9976 -
ENSMUS(	Gm3776	predicted	GO:00081	metabolic	-	-	-	-	9:7825712	0	0	0	0	5	0	0	0	0	0	0.224932	0	0	0.074977	0.177757	0.9976 -
ENSMUS(	Morc2b	microrchi	GO:00056	cell|intrace	-	-	-	-	17:331355	27	66	40	45	54	30	0.217808	0.425201	0.356622	0.280058	0.280511	0.190386	0.333211	0.250318	0.179008	0.997711 -
ENSMUS(	Pirb	paired Ig-	GO:00325	developm	K06512	leukocyte	mmu0438	Osteoclas	7:3711409	15	36	21	26	25	16	0.119329	0.228728	0.184646	0.159573	0.128072	0.100138	0.177568	0.129261	0.179107	0.997711 -
ENSMUS(	Gm43720	predicted	GO:00055	extracellul	-	-	-	-	5:1382649	2	24	43	20	21	1	0.019044	0.182544	0.452618	0.146951	0.12879	0.007494	0.218069	0.094412	0.179057	0.997711 -
ENSMUS(	Raver1	ribonuclec	GO:00081	metabolic	-	-	-	-	9:2106751	93	118	118	117	395	133	0.935484	0.947943	1.311848	0.907967	2.558621	1.052487	1.065091	1.506359	0.178956	0.997711 -
ENSMUS(	Zfhx4	zinc finge	GO:00081	metabolic	-	-	-	-	3:5218526	326	558	407	526	429	458	0.768144	1.050041	1.059904	0.956185	0.650936	0.848989	0.959363	0.818703	0.17923	0.997758 -
ENSMUS(	Pdap1	PDGFA as	GO:00508	response	-	-	-	-	5:1451287	1446	1763	1025	1715	1795	1358	13.70538	13.34507	10.73725	12.54057	10.95574	10.12589	12.5959	11.2074	0.179225	0.997758 -
ENSMUS(	Abca1	ATP-bind	GO:00048	signal trar	K05641	ATP-bind	mmu0497	Cholester	4:5303078	840	1278	731	900	1132	1177	4.855517	5.899741	4.670033	4.013552	4.213638	5.352337	5.141764	4.526509	0.179421	0.998502 -
ENSMUS(	Gm3629	predicted	-	-	-	-	-	-	14:658395	9	8	5	8	5	3	0.312933	0.22216	0.192161	0.214607	0.111954	0.082052	0.242418	0.136204	0.179531	0.998794 -
ENSMUS(	Il1r2	interleukir	GO:00048	signal trar	K04387	interleukir	mmu0516	HTLV-I inf	1:4007407	0	2	3	3	9	4	0	0.041613	0.086374	0.060297	0.150969	0.081966	0.042662	0.097744	0.179591	0.998806 -
ENSMUS(	Cap2	CAP, ader	GO:00325	developm	-	-	-	-	13:465018	4830	6098	4443	5832	5951	6287	46.78152	47.16941	47.56092	43.57877	37.11691	47.90508	47.17062	42.86692	0.179985	0.999996 -
ENSMUS(	Chsy1	chondroit	GO:00325	developm	K13499	chondroit	mmu011C	Metabolic	7:6610951	330	419	280	371	443	363	4.824992	4.892639	4.524678	4.184922	4.171006	4.175418	4.747436	4.177115	0.179997	0.999996 -
ENSMUS(	Prokr2	prokinetic	GO:00048	signal trar	-	-	-	-	2:1323377	107	127	94	143	124	82	0.559495	0.53035	0.543236	0.576872	0.41753	0.337316	0.54436	0.443906	0.180092	0.999996 -
ENSMUS(	Mettl7a1	methyltrai	GO:00081	metabolic	-	-	-	-	15:100304	473	652	437	713	963	726	15.33748	16.88442	15.66105	17.83662	20.10823	18.51999	15.96099	18.82161	0.179879	0.999996 -
ENSMUS(	Gulp1	GULP, eng	GO:00511	localizatio	-	-	-	-	1:4455151	67	105	66	84	81	86	0.511615	0.640332	0.557012	0.49486	0.398299	0.516629	0.569653	0.469929	0.18008	0.999996 -
ENSMUS(	Gnai3	guanine n	GO:00048	signal trar	K04630	guanine n	mmu052C	Pathways	3:1081072	482	571	408	553	800	624	9.02207	8.53575	8.440458	7.985733	9.642822	9.188724	8.666093	8.939093	0.951904	1 -
ENSMUS(	Cdc45	cell divisic	GO:00081	metabolic	K06628	cell divisio	mmu0411	Cell cycle	16:187804	26	40	28	44	53	40	0.544228	0.668672	0.647754	0.710542	0.714391	0.658681	0.620218	0.694538	0.673794	1 -

ENSMUS(	Scml2	sex comb	GO:00081	metabolic	-	-	-	-	X:1611171	20	16	5	15	30	20	0.12409	0.079283	0.034289	0.0718	0.11986	0.097621	0.079221	0.096427	0.653975	1	-
ENSMUS(	Narf	nuclear pı	GO:00081	metabolic	-	-	-	-	11:121237	930	1214	858	1239	1427	1221	10.87817	11.34063	11.09193	11.18084	10.74859	11.23568	11.10358	11.05504	0.763103	1	-
ENSMUS(	Cav2	caveolin 2	GO:00325	developm	K12958	caveolin 2	mmu0414	Endocytos	6:1728118	232	307	201	335	288	267	3.218691	3.40155	3.08201	3.585648	2.572998	2.914165	3.234084	3.02427	0.486321	1	-
ENSMUS(	Scmh1	sex comb	GO:00325	developm	-	-	-	-	4:1204052	671	813	632	899	1077	953	3.826111	3.702298	3.982894	3.954803	3.954625	4.275028	3.837101	4.061485	0.7469	1	-
ENSMUS(	Cox5a	cytochron	GO:00081	metabolic	K02264	cytochron	mmu011C	Metabolic	9:5752127	559	685	349	687	710	613	12.72614	12.45434	8.781251	12.06623	10.40873	10.97883	11.32058	11.15126	0.74888	1	-
ENSMUS(	Tbx2	T-box 2 [S	GO:00325	developm	-	-	-	-	11:858325	29	31	42	34	60	66	0.488329	0.416887	0.781646	0.441692	0.650615	0.874318	0.562288	0.655542	0.600387	1	-
ENSMUS(	Tbx4	T-box 4 [S	GO:00325	developm	-	-	-	-	11:858864	3	0	5	6	7	3	0.032891	0	0.060592	0.05075	0.049418	0.025872	0.031161	0.042014	0.619706	1	-
ENSMUS(	Ngfr	nerve gro	GO:00325	developm	K02583	nerve gro	mmu0415	PI3K-Akt s	11:955688	30	47	28	44	51	39	0.531561	0.665078	0.548317	0.601465	0.581909	0.543624	0.581652	0.575666	0.891677	1	-
ENSMUS(	Wnt3	wingless-t	GO:00325	developm	K00312	wingless-t	mmu052C	Pathways	11:103774	33	45	41	29	56	40	0.655048	0.713368	0.899467	0.444104	0.715816	0.624634	0.755961	0.594851	0.224886	1	-
ENSMUS(	Fer	fer (fms/fp	GO:00325	developm	K08889	tyrosine-p	mmu0452	Adherens	17:63896C	469	628	472	557	746	640	7.787935	8.328279	8.662406	7.135684	7.977062	8.360655	8.25954	7.824467	0.471017	1	-
ENSMUS(	Tfe3	transcripti	GO:00325	developm	K09105	transcripti	mmu052C	Transcript	X:776256C	563	783	545	893	996	926	1.664199	1.848436	1.780494	2.036474	1.895881	2.153372	1.764376	2.028576	0.254646	1	-
ENSMUS(	Axin2	axin 2 [So	GO:00325	developm	K04385	axin 2[-]	mmu052C	Pathways	11:10892C	338	483	350	497	520	445	2.400844	2.739933	2.74765	2.723541	2.378511	2.486666	2.629476	2.529573	0.586732	1	-
ENSMUS(	Brat1	BRCA1-as	GO:00081	metabolic	-	-	-	-	5:140705C	245	307	279	333	399	380	1.365265	1.36627	1.718314	1.431615	1.431789	1.665889	1.483283	1.509765	0.967859	1	-
ENSMUS(	Gna12	guanine n	GO:00325	developm	K04346	guanine n	mmu052C	Pathways	5:1407584	1409	1925	1459	1982	2341	2040	19.09251	20.8319	21.85014	20.71983	20.42717	21.74669	20.59152	20.96457	0.949116	1	-
ENSMUS(	Igsf5	immunog	GO:00160	membran	K06786	junctional	mmu0453	Tight junc	16:963616	115	168	141	181	261	141	1.78806	2.086117	2.422964	2.171152	2.613229	1.724701	2.099047	2.169694	0.948279	1	-
ENSMUS(	Pih1d2	PIH1 dom	GO:00055	protein bi	-	-	-	-	9:5061732	10	26	22	19	24	26	0.123426	0.256283	0.30011	0.180915	0.190751	0.252457	0.226606	0.208041	0.715501	1	-
ENSMUS(	Dlat	dihydrolip	GO:00081	metabolic	K00627	pyruvate c	mmu011C	Metabolic	9:5063463	2035	2538	1907	2654	3211	2587	22.50149	22.4122	23.30475	22.6401	22.86346	22.50372	22.73948	22.66909	0.753578	1	-
ENSMUS(	Sdhd	succinate	GO:00081	metabolic	K00237	succinate	mmu011C	Metabolic	9:5059635	904	1025	721	1278	1458	1069	44.83877	40.60271	39.52458	48.90424	46.56907	41.71324	41.65536	45.72885	0.496984	1	-
ENSMUS(	Fgf23	fibroblast	GO:00325	developm	K04358	fibroblast	mmu052C	Pathways	6:1270729	11	5	0	5	6	10	0.370266	0.1344	0	0.129831	0.130045	0.264798	0.168222	0.174891	1	1	-
ENSMUS(	Ccnd2	cyclin D2	GO:00081	metabolic	K10151	cyclin D2[	mmu052C	Pathways	6:1271251	1815	2355	1500	2350	2592	2112	8.970412	9.295493	8.193581	8.960536	8.249458	8.211842	8.819829	8.473945	0.476572	1	-
ENSMUS(	Gpr107	G protein-	GO:00511	localizatio	-	-	-	-	2:3115231	822	1025	785	1291	1431	1208	3.406158	3.392056	3.59509	4.127145	3.818454	3.937958	3.464435	3.961186	0.251184	1	-
ENSMUS(	Nalcn	sodium le	GO:00511	localizatio	-	-	-	-	14:123276	2362	2804	2049	2917	3290	3008	9.00526	8.537686	8.633863	8.579919	8.077314	9.022049	8.725603	8.55976	0.616264	1	-
ENSMUS(	Btbd17	BTB (POZ	GO:00055	extracellul	-	-	-	-	11:114791	79	155	75	156	170	156	0.503509	0.788963	0.528307	0.767067	0.697721	0.782194	0.606926	0.748994	0.252919	1	-
ENSMUS(	Th	tyrosine h	GO:00325	developm	K00501	tyrosine 3	mmu011C	Metabolic	7:1428927	14	16	7	16	18	7	0.21462	0.195889	0.118606	0.189229	0.177691	0.084424	0.176372	0.150448	0.613025	1	-
ENSMUS(	Ins2	insulin II [S	GO:00325	developm	K04526	insulin[-]	mmu0415	PI3K-Akt s	7:1426786	0	3	0	0	1	0	0	0.044675	0	0	0.012003	0	0.014892	0.004001	0.657956	1	-
ENSMUS(	Scnn1g	sodium ch	GO:00511	localizatio	K04827	amiloride-	mmu0474	Taste tran	7:1217344	0	4	3	0	9	4	0	0.065206	0.06769	0	0.118312	0.064235	0.044299	0.060849	0.73962	1	-
ENSMUS(	Drp2	dystrophin	GO:00325	developm	-	-	-	-	X:1344045	834	827	663	1063	1072	897	4.286404	3.39452	3.76606	4.214931	3.54794	3.626854	3.815661	3.796575	0.786337	1	-
ENSMUS(	Lhx2	LIM home	GO:00325	developm	-	-	-	-	2:3833928	1083	1439	869	1518	1706	1326	9.900576	10.50603	8.780079	10.70616	10.04305	9.536441	9.728894	10.09522	0.895431	1	-
ENSMUS(	Gmpr	guanosine	GO:00081	metabolic	K00364	GMP redu	mmu0023	Purine me	13:455074	679	855	642	860	1053	864	15.38917	15.47597	16.08153	15.03747	15.3684	15.40531	15.64889	15.27039	0.625984	1	-
ENSMUS(	Mid2	midline 2	GO:00081	metabolic	-	-	-	-	X:1406645	569	674	510	720	898	743	3.128504	2.959583	3.099142	3.054129	3.179476	3.21384	3.06241	3.149148	0.973525	1	-
ENSMUS(	Dgke	diacylglyc	GO:00081	metabolic	K00901	diacylglyc	mmu011C	Metabolic	11:890351	1178	1500	1115	1286	1766	1492	4.993157	5.077702	5.22338	4.205343	4.820316	4.975193	5.09808	4.666951	0.241107	1	-
ENSMUS(	Scpep1	serine car	GO:00081	metabolic	-	-	-	-	11:88924C	176	296	182	299	415	299	5.124581	6.88307	5.856848	6.716564	7.781228	6.849002	5.954833	7.115598	0.233285	1	-
ENSMUS(	Mnt	max bindi	GO:00325	developm	-	-	-	-	11:748309	614	860	586	849	944	896	6.550709	7.327638	6.909774	6.988076	6.48554	7.520361	6.929373	6.997992	0.889694	1	-
ENSMUS(	Hddc2	HD doma	GO:00055	extracellul	-	-	-	-	10:313133	118	174	107	162	200	165	1.35024	1.590096	1.353191	1.430121	1.473709	1.485329	1.431176	1.463053	1	1	-
ENSMUS(	Tpd52l1	tumor prc	GO:00081	metabolic	-	-	-	-	10:313323	332	483	315	450	503	403	6.639803	7.714533	6.962647	6.943195	6.477972	6.340638	7.105661	6.587268	0.383067	1	-
ENSMUS(	Pemt	phosphati	GO:00081	metabolic	K00551	phosphati	mmu011C	Metabolic	11:599706	15	28	9	26	18	23	0.298712	0.445327	0.198081	0.399454	0.230836	0.360345	0.31404	0.330212	1	1	-
ENSMUS(	Cdh4	cadherin 4	GO:00325	developm	K06797	cadherin 4	mmu0451	Cell adhes	2:1794424	342	429	294	411	463	427	2.542536	2.547092	2.415659	2.357292	2.21655	2.497356	2.501762	2.357066	0.458748	1	-
ENSMUS(	Bcl6b	B cell CLL	GO:00325	developm	-	-	-	-	11:702241	32	33	28	51	50	33	0.463988	0.382136	0.448705	0.570503	0.46686	0.376434	0.43161	0.471266	0.789898	1	-
ENSMUS(	Clec10a	C-type lec	GO:00508	response	-	-	-	-	11:701561	7	6	7	12	10	8	0.07349	0.050305	0.081226	0.097189	0.067603	0.066069	0.06834	0.076954	0.811331	1	-
ENSMUS(	Alox12	arachidon	GO:00325	developm	K00458	arachidon	mmu011C	Metabolic	11:702414	6	7	3	3	11	11	0.081427	0.075871	0.045001	0.031415	0.096131	0.117446	0.067433	0.081664	0.798244	1	-
ENSMUS(	Arvcf	armadillo	GO:00325	developm	-	-	-	-	16:183481	591	703	477	800	924	773	3.152111	2.994437	2.811766	3.291806	3.173514	3.243429	2.986105	3.23625	0.584642	1	-
ENSMUS(	Comt	catechol-	GO:00325	developm	-	-	-	-	16:184068	718	965	792	1197	1262	1021	11.51857	12.36368	14.04256	14.81491	13.03732	12.88579	12.64161	13.57934	0.64829	1	-
ENSMUS(	Rtca	RNA 3'-te	GO:00081	metabolic	-	-	-	-	3:1164889	445	570	460	606	772	602	10.04469	10.27536	11.47575	10.55308	11.22143	10.69014	10.5986	10.82155	0.985191	1	-
ENSMUS(	Dbt	dihydrolip	GO:00081	metabolic	K09699	2-oxoisov	mmu011C	Metabolic	3:116513C	461	567	360	640	809	549	7.407317	7.27593	6.393071	7.933591	8.370725	6.939735	7.025439	7.748017	0.515076	1	-
ENSMUS(	Dazap2	DAZ asso	GO:00329	macromo	-	-	-	-	15:100615	1686	2089	1393	2163	2780	1973	56.37677	55.78622	51.48025	55.79938	59.86074	51.90158	54.54775	55.8539	1	1	-
ENSMUS(	Mcts1	malignant	GO:00081	metabolic	-	-	-	-	X:3860057	442	470	362	525	710	454	12.9936	11.03445	11.76147	11.90684	13.44064	10.49963	11.92984	11.94904	0.860208	1	-
ENSMUS(	Rnf17	ring finge	GO:00325	developm	-	-	-	-	14:564025	15	7	9	19	15	16	0.132157	0.049256	0.087636	0.129147	0.085104	0.110903	0.089683	0.108385	0.683613	1	-
ENSMUS(	Trappc10	trafficking	-	-	-	-	-	-	10:781867	1660	2330	1635	2312	2710	2484	10.19478	11.42803	11.09773	10.95438	10.7175	12.00141	10.90684	11.22443	0.959181	1	-
ENSMUS(	Ccm2	cerebral c	GO:00325	developm	-	-	-	-	11:654688	615	792	593	788	1009	796	6.501164	6.68632	6.928158	6.426471	6.868502	6.619727	6.705214	6.638233	0.738689	1	-
ENSMUS(	Ndufa9	NADH de	GO:00081	metabolic	K03953	NADH de	mmu011C	Metabolic	6:1268217	1168	1514	1020	1527	1849	1612	27.15761	28.11385	26.21173	27.39163	27.68472	29.48659	27.16107	28.18765	0.890052	1	-
ENSMUS(	Egfl6	EGF-like-	GO:00325	developm	-	-	-	-	X:166523C	54	81	39	79	123	83	1.219348	1.460727	0.973294	1.376234	1.788532	1.474438	1.21779	1.546401	0.235199	1	-
ENSMUS(	Lck	lymphocy	GO:00325	developm	K05856	lymphocy	mmu0516	HTLV-I inf	4:1295483	17	29	18	25	48	29	0.220852	0.300881	0.258446	0.250566	0.401551	0.29638	0.26006	0.316166	0.474519	1	-
ENSMUS(	Tssk3	testis-spe	GO:00325	developm	-	-	-	-	4:129489C	4	4	0	3	0	0	0.190817	0.152369	0	0.110419	0	0	0.114395	0.036806	0.233979	1	-
ENSMUS(	Cttnbp2	cortactin b	GO:00325	developm	-	-	-	-	6:1836647	3450	4090	2794	3992	4672	5004	13.01436	12.32177	11.64869	11.61782	11.3491	14.85019	12.32827	12.6057	0.977478	1	-
ENSMUS(	Galnt1	polypeptic	GO:00081	metabolic	K00710	polypeptic	mmu011C	Metabolic	18:242053	929	1140	815	1102	1416	1205	6.329275	6.202817	6.136814	5.792292	6.212357	6.458573	6.222969	6.154407	0.716056	1	-
ENSMUS(	Mkrn2	makorin, r	GO:00081	metabolic	-	-	-	-	6:1156019	517	643	506	694	856	751	4.236615	4.208098	4.582752	4.387515	4.517076	4.841497	4.342488	4.582029	0.780339	1	-
ENSMUS(	Raf1	v-raf-leuk	GO:00325	developm	K04366	RAF protc	mmu052C	Pathways	6:115618C	965	1226	881	1318	1326	1350	6.046907	6.135391	6.101388	6.371646	5.350623	6.65504	6.094562	6.12577	0.843933	1	-
ENSMUS(	1-Sep	septin 1 [S	GO:00511	localizatio	K13737	septin 1[-	mmu051C	Bacterial i	7:1272144	28	38	16	25	68	40	0.355501	0.385314	0.224521	0.244887	0.555968	0.399537	0.321779	0.400131	0.457899	1	-
ENSMUS(	Pdgfb	platelet de	GO:00325	developm	K17386	platelet-d	mmu052C	Pathways	15:799959	356	484	332	493	541	407	7.44917	8.088156	7.677898	7.958571	7.289704	6.699827	7.738408	7.316034	0.489084	1	-
ENSMUS(	Grasp	GRP1 (ger	GO:00511	localizatio	-	-	-	-	15:101224	887	1189	819	1090	1432	1050	26.90438	28.80231	27.45554	25.50682	27.97031	25.05532	27.72074	26.17748	0.407362	1	-
ENSMUS(	Acvr1b	activin A r	GO:00325	developm	K13567	activin rec	mmu0406	Cytokine-	15:101174	2164	2751	1876	2758	3343	2734	29.94102	30.39805	28.68726	29.43974	29.78519	29.75903	29.67545	29.66132	0.776999	1	-
ENSMUS(	Tom1l2	target of r	GO:00511	localizatio	-	-	-	-	11:602267	4619	5867	4436	6228	7110	6633	20.16642	20.45704	21.4052	20.97779	19.98965	22.78252	20.67622	21.24999	0.971964	1	-
ENSMUS(	Zfp385a	zinc finge	GO:00325	developm	-	-	-	-	15:103313	584	713	531	804	907	717	15.56436	15.17587	15.64082	16.53121	15.56611	15.03308	15.46035	15.71013	0.94132	1	-
ENSMUS(	Itga5	integrin al	GO:00325	developm	-	-	-	-	15:103344	30	56	37	53	80	61	0.368415	0.549224	0.502175	0.502134	0.632641	0.589321	0.473271	0.574699	0.375189	1	-
ENSMUS(	Gabra2	gamma-a	GO:00048	signal trar	K05175	gamma-a	mmu0408	Neuroacti	5:7095759	1295	1615	1115	1855	2005	1556	5.899443	5.875699	5.613876	6.51952	5.881801	5.576501	5.796339	5.992607	0.92272	1	-
ENSMUS(	Wdr77	WD repea	GO:00325	developm	-	-	-	-	3:1059593	449	543	381	620	648	612	3.03264	2.929009	2.844111	3.230702	2.818414	3.251901	2.935253	3.100339	0.788569	1	-
ENSMUS(	Adora3	adenosine	GO:00048	signal trar	K04268	adenosine	mmu0408	Neuroacti	3:1058708	7	19	20	18	24	18	0.081382	0.176407	0.256982	0.161448	0.179673	0.164623	0.17159	0.168581	0.946336	1	-
ENSMUS(	Atp5f1	ATP synth	GO:00325	developm	K02127	F-type H+	mmu011C	Metabolic	3:1059426	3260	4364	2990	4353	5415	4614	34.3722	36.74687	34.84237	35.4086	36.76571	38.27175	35.32048	36.81535	0.834573	1	-
ENSMUS(	Sox9	SRY (sex c	GO:00325	developm	K18435	transcripti	mmu0402	cAMP sigr	11:112782	610	857	623	863	998	1005	9.007367	10.10635	10.16723	9.831254	9.489716	11.67467	9.760317	10.33188	0.759371	1	-
ENSMUS(	C1d	C1D nucle	GO:00081	metabolic	K12592	exosome	mmu0301	RNA degr	11:172575	399	406	326	530	236	503	5.336735	4.336855	4.819113	5.469008	2.032683	5.29275	4.830901	4.264814	0.448825	1	-
ENSMUS(	Gm2a	GM2 gang	GO:00081	metabolic	K12383	gangliosic	mmu0414	Lysosome	11:550981	1233	1724	1223	1571	1962	1630	18.24199	20.37004	19.9978	17.93145	18.69229	18.97175	19.53661	18.53183	0.404898	1	-
ENSMUS(	Krit1	KRIT1, anl	GO:00325	developm	K17705	Krev intera	mmu0401	Rap1 sign	5:3803184	898	1180	819	1264	1442	1122	1.891153	1.984622	1.906253	2.053656	1.955555	1.858892	1.927343	1.956034	0.927673	1	-
ENSMUS(	Clcn4	chloride c	GO:00511	localizatio	-	-	-	-	7:7282309	2529	3202	2347	3416	4062	3002	10.82026	10.94098	11.09811	11.27553	11.19138	10.1044	10.95312	10.8571	0.713481	1	-
ENSMUS(	Sema4f	sema don	GO:00325	developm	K06521	semaphor	mmu0436	Axon guic	6:8291188	578	744	525	712	878	688	5.903581	6.068844	5.926431	5.610454	5.774794	5.528238	5.966285	5.637829	0.427359	1	-
ENSMUS(	Myo18a	myosin XV	GO:00400	locomotic	-	-	-	-	11:777632	4051	5011	3489	5479	6755	5802	9.377352	9.263778	8.926181	9.784747	10.06927	10.5659	9.189104	10.13997	0.370778	1	-
ENSMUS(	Sez6	seizure re	GO:00325	developm	-	-	-	-	11:779308	2827	4284	3031	4451	5191	4692	17.74376	21.4741	21.0258	21.553	20.98096	23.16801	20.08122	21.90066	0.483211	1	-
ENSMUS(	Cd52	CD52 anti	GO:00160	membran	-	-	-	-	4:1340824	13	43	27	16	38	26	0.566969	1.497699	1.301437	0.538357	1.067219	0.892073	1.122035	0.83255	0.261214	1	-
ENSMUS(	Loxl3	lysyl oxida	GO:00325	developm	-	-	-	-	6:8303417	67	84	58	127	102	114	0.739226	0.740164	0.707259	1.081033	0.724705	0.989511	0.728883	0.93175	0.183442	1	-
ENSMUS(	Btn1a1	butyrophi	GO:00081	metabolic	-	-	-	-	13:234569	1	0	1	2	0	0	0.012155	0	0.01343	0.018751	0	0	0.008528	0.00625	1	1	-
ENSMUS(	Kat2b	K(lysine) a	GO:00081	metabolic	K06062	histone ac	mmu0516	HTLV-I inf	17:535668	364	574	388	510	620	590	3.851182	4.850091	4.537025	4.16287	4.224144	4.910832	4.412766	4.432615	0.870531	1	-
ENSMUS(	Rab5b	RAB5B, m	GO:00452	synapse|c	K07888	Ras-relate	mmu0414	Endocytos	10:128677	2466	3072	2272	3353	3871	3017	34.25143	34.07632	34.87714	35.92937	34.62291	32.96643	34.40163	34.50623	0.811416	1	-
ENSMUS(	Dnmt3l	DNA (cytc	GO:00325	developm	-	-	-	-	10:780419	0	8	3	7	5	1	0	0.088141	0.045743	0.0745	0.044417	0.010855	0.044628	0.043257	1	1	-
ENSMUS(	Aire	autoimmu	GO:00325	developm	K10603	autoimmu	mmu0412	Ubiquitin	10:78030C	8	18	6	10	11	8	0.076006	0.136567	0.063003	0.073296	0.067293	0.059788	0.091859	0.066792	0.353955	1	-
ENSMUS(	Icosl	icos ligan	GO:00325	developm	K06710	inducible	mmu0451	Cell adhes	10:780693	193	194	168	264	281	245	2.062697	1.655875	1.984432	2.176772	1.933925	2.059945	1.901001	2.056881	0.68583	1	-
ENSMUS(	Spg7	SPG7, par	GO:00081	metabolic	-	-	-	-	8:1230629	640	746	543	789	1005	893	4.606533	4.288234	4.319569	4.381281	4.658162	5.056571	4.404779	4.698672	0.697659	1	-
ENSMUS(	Sult5a1	sulfotrans	GO:00038	catalytic a	-	-	-	-	8:1231401	14	29	30	25	35	32	0.149159	0.246753	0.353249	0.20549	0.240121	0.268211	0.249721	0.237941	0.839956	1	-
ENSMUS(	Rpl13	ribosomal	GO:00081	metabolic	K02873	large subu	mmu0301	Ribosome	8:1231023	121	157	95	156	208	155	1.732251	1.795019	1.503131	1.722977	1.917532	1.745691	1.6768	1.7954	0.767521	1	-
ENSMUS(	Chmp1a	charged n	GO:00081	metabolic	K12197	charged n	mmu0414	Endocytos	8:1232042	908	1162	934	1292	1483	1166	14.43019	14.74816	16.40514	15.84086	15.17686	14.57791	15.19449	15.19854	0.811105	1	-
ENSMUS(	Rpa1	replicatior	GO:00325	developm	K07466	replicatior	mmu0346	Fanconi a	11:752981	839	1094	802	1155	1250	1223	7.131804	7.426779	7.534577	7.57443	6.842302	8.178522	7.364386	7.531751	0.986113	1	-
ENSMUS(	Serpinf1	serine (or	GO:00325	developm	K19614	pigment e	mmu0431	Wnt signa	11:754097	233	277	178	295	404	276	3.572711	3.392092	3.01654	3.48975	3.989127	3.329367	3.327114	3.602748	0.657649	1	-
ENSMUS(	Tubgcp3	tubulin, ga	GO:00051	structural	-	-	-	-	8:1261427	695	998	706	960	1357	1049	3.398367	3.89728	3.81537	3.621486	4.27287	4.035262	3.703673	3.976539	0.647506	1	-
ENSMUS(	Oprm1	opioid rec	GO:00325	developm	K04215	mu-type c	mmu0408	Neuroacti	10:67585C	13	29	24	26	39	39	0.054404	0.096925	0.111004	0.083943	0.1051	0.128398	0.087445	0.105814	0.507718	1	-
ENSMUS(	Polr3d	polymeras	GO:00081	metabolic	K03026	DNA-dire	mmu011C	Metabolic	14:704387	356	463	286	489	587	400	4.485489	4.658937	3.982661	4.753345	4.762695	3.964886	4.375696	4.493642	0.985417	1	-
ENSMUS(	Tcf7	transcripti	GO:00325	developm	K02620	transcripti	mmu052C	Pathways	11:522523	55	58	60	67	96	82	1.001848	0.843746	1.207919	0.941553	1.126067	1.17507	1.017838	1.080897	0.843169	1	-
ENSMUS(	Ddx3x	DEAD/H (	GO:00081	metabolic	K11594	ATP-depe	mmu052C	Viral carci	X:1328097	5110	6307	4163	5483	7617	6433	45.79582	45.14125	41.23426	37.90998	43.95857	45.35544	44.05711	42.408	0.483401	1	-
ENSMUS(	Il12rb1	interleukir	GO:00048	signal trar	-	-	-	-	8:7080844	6	17	0	7	22	14	0.070382	0.15926	0	0.06335	0.166191	0.129197	0.076547	0.119579	0.454312	1	-
ENSMUS(	Slc5a5	solute car	GO:00081	metabolic	K14385	solute car	mmu0491	Thyroid hc	8:7088288	389	449	326	580	523	482	8.111888	7.477638	7.513405	9.331045	7.023092	7.907333	7.700977	8.087156	0.845068	1	-
ENSMUS(	Kcnn3	potassium	GO:00511	localizatio	K04944	potassium	mmu0491	Insulin sec	3:8952016	620	760	517	810	876	670	3.380608	3.3095	3.11559	3.40736	3.075826	2.874014	3.268566	3.119067	0.499234	1	-
ENSMUS(	Usp32	ubiquitin s	GO:00081	metabolic	-	-	-	-	11:849844	3436	4292	2942	4204	4475	4115	20.105	20.05656	19.02569	18.97772	16.86159	18.94225	19.72908	18.26052	0.242735	1	-
ENSMUS(	Txnrd3	thioredox	GO:00081	metabolic	K00384	thioredox	mmu052C	Pathways	6:8964398	48	112	59	80	75	86	1.03342	1.925754	1.403899	1.328791	1.03981	1.456616	1.454358	1.275072	0.418292	1	-
ENSMUS(	Zfp512b	zinc finge	GO:00971	organic cy	-	-	-	-	2:1815821	1222	1547	1191	1724	1962	1894	5.605365	5.667204	6.037973	6.100991	5.795439	6.834763	5.770181	6.243731	0.562699	1	-
ENSMUS(	Dnajc5	DnaJ heat	GO:00452	synapse|c	K09525	DnaJ hom	mmu0414	Protein pr	2:1815204	2234	2507	1720	2659	3023	2507	9.435794	8.45659	8.029165	8.664508	8.2222	8.330296	8.640516	8.405668	0.564746	1	-
ENSMUS(	Tpd52l2	tumor prc	GO:00056	cell|organ	-	-	-	-	2:1814971	241	318	244	362	392	315	1.081987	1.140191	1.21071	1.253844	1.133302	1.112567	1.144296	1.166571	0.977815	1	-
ENSMUS(	Fmr1	fragile X r	GO:00325	developm	K15516	fragile X n	mmu0301	RNA trans	X:6867854	1627	1844	1290	1924	2405	1803	7.063032	6.393084	6.189282	6.443753	6.72316	6.157582	6.548466	6.441499	0.661673	1	-
ENSMUS(	Bcl11a	B cell CLL	GO:00325	developm	-	-	-	-	11:24078C	2129	2534	1802	2733	3369	2659	7.305424	6.944199	6.833944	7.23502	7.444327	7.177928	7.027855	7.285758	0.891931	1	-
ENSMUS(	Il4	interleukir	GO:00325	developm	K05430	interleukir	mmu052C	Pathways	11:536029	0	2	0	0	0	2	0	0.068739	0	0	0	0.067715	0.022913	0.022572	1	1	-
ENSMUS(	Pxmp4	peroxisom	GO:00160	membran	K13350	peroxisom	mmu0414	Peroxisom	2:1545857	170	252	188	268	395	254	3.158827	3.739577	3.860823	3.841859	4.726364	3.712968	3.586409	4.09373	0.412362	1	-
ENSMUS(	Dlg3	discs, larg	GO:00325	developm	K12075	discs large	mmu0516	Human pa	X:1007677	5036	6547	4666	6376	8058	6216	24.25181	25.17947	24.83419	23.68847	24.9885	23.54942	24.75515	24.07546	0.532743	1	-
ENSMUS(	Gnb1l	guanine n	GO:00056	cell|behav	-	-	-	-	16:184987	51	61	50	68	70	59	0.35645	0.340493	0.386234	0.366667	0.315054	0.324412	0.361059	0.335378	0.565606	1	-
ENSMUS(	Dbh	dopamine	GO:00081	metabolic	K00503	dopamine	mmu011C	Metabolic	2:2716523	6	25	8	18	28	26	0.132876	0.442169	0.195803	0.307553	0.39932	0.452993	0.256949	0.386622	0.25291	1	-
ENSMUS(	Mmp11	matrix me	GO:00325	developm	-	-	-	-	10:759232	20	20	10	21	21	25	0.281571	0.224869	0.155592	0.228087	0.190379	0.276887	0.220677	0.231784	1	1	-
ENSMUS(	Smarcb1	SWI/SNF r	GO:00325	developm	K11648	SWI/SNF-	mmu0522	Hepatoce	10:758967	823	1046	817	1064	1277	1232	3.733068	3.789167	4.095766	3.723386	3.730022	4.396302	3.872667	3.949903	0.965769	1	-
ENSMUS(	Vpreb3	pre-B lym	GO:00511	localizatio	-	-	-	-	10:75943C	0	2	2	0	3	0	0	0.072039	0.099685	0	0.087127	0	0.057241	0.029042	0.716043	1	-
ENSMUS(	Hip1r	huntingtir	GO:00325	developm	-	-	-	-	5:1239736	1253	1583	1159	1828	2052	1840	9.005979	9.086709	9.206828	10.13646	9.497559	10.4042	9.099839	10.01274	0.427376	1	-
ENSMUS(	Nsun5	NOL1/NC	GO:00081	metabolic	-	-	-	-	5:1353699	137	169	132	178	239	203	0.979162	0.964638	1.042686	0.981485	1.09998	1.141402	0.995495	1.074289	0.70679	1	-
ENSMUS(	Top1mt	DNA topc	GO:00081	metabolic	-	-	-	-	15:75657C	354	408	305	470	514	440	11.35816	10.45467	10.8156	11.63409	10.61994	11.10626	10.87615	11.1201	0.990064	1	-
ENSMUS(	Hoxa4	homeobo	GO:00325	developm	-	-	-	-	6:5218967	0	0	0	2	0	0	0	0	0	0.059112	0	0	0	0.019704	0.527191	1	-
ENSMUS(	Gm38393	predicted	GO:00056	cell|intrace	-	-	-	-	7:5997414	2075	2441	1480	2555	2947	2351	22.0955	20.75865	17.41784	20.98973	20.20787	19.6947	20.09066	20.29743	0.883328	1	-
ENSMUS(	Mmp14	matrix me	GO:00325	developm	K07763	matrix me	mmu0466	TNF signa	14:544316	252	345	213	312	454	273	1.905465	2.083368	1.780033	1.820062	2.210607	1.623957	1.922955	1.884875	0.745439	1	-
ENSMUS(	Slc7a7	solute car	GO:00081	metabolic	K13867	solute car	mmu0497	Protein di	14:543694	25	37	15	35	35	22	0.278242	0.328875	0.184513	0.300526	0.250845	0.192631	0.263876	0.248001	0.754256	1	-
ENSMUS(	Oxa1l	oxidase as	GO:00081	metabolic	K03217	YidC/Oxa1	mmu0306	Protein ex	14:543608	566	683	519	760	876	822	5.957399	5.741261	6.037467	6.171409	5.937441	6.80649	5.912042	6.305113	0.699995	1	-
ENSMUS(	Heatr6	HEAT rep	GO:00971	organic cy	-	-	-	-	11:837536	374	471	238	491	668	517	2.80572	2.821885	1.973315	2.841736	3.227033	3.051219	2.53364	3.039996	0.218012	1	-
ENSMUS(	Wfdc18	WAP four	GO:00081	metabolic	-	-	-	-	11:83709C	6	8	0	6	18	6	0.200295	0.213293	0	0.154525	0.386956	0.157581	0.137863	0.233021	0.341318	1	-
ENSMUS(	Icam4	intercellul	GO:00055	extracellul	-	-	-	-	9:2102937	3	9	8	7	17	6	0.091631	0.219551	0.270049	0.164951	0.334383	0.14418	0.193744	0.214505	0.913122	1	-
ENSMUS(	Ilf2	interleukir	GO:00081	metabolic	-	-	-	-	3:9047612	1132	1404	1014	1418	1758	1546	6.868508	6.803442	6.799869	6.637774	6.868935	7.379658	6.82394	6.962122	0.965904	1	-
ENSMUS(	Chtop	chromatir	GO:00081	metabolic	-	-	-	-	3:9049895	872	1158	908	1106	1550	1157	4.977812	5.279302	5.728678	4.870879	5.697814	5.195966	5.328597	5.254886	0.71142	1	-
ENSMUS(	S100a4	S100 calci	GO:00508	response	-	-	-	-	3:9060377	15	18	18	22	27	20	1.098141	1.05242	1.456471	1.242596	1.272919	1.151903	1.202344	1.222472	1	1	-
ENSMUS(	S100a3	S100 calci	GO:00056	cell|intrace	-	-	-	-	3:9056057	1	6	1	1	8	5	0.008582	0.041109	0.009482	0.00662	0.044199	0.033746	0.019724	0.028188	0.728018	1	-
ENSMUS(	Icam2	intercellul	GO:00055	extracellul	K06523	intercellul	mmu0451	Cell adhes	11:106377	27	37	22	28	50	21	0.546252	0.597815	0.49193	0.437028	0.651407	0.334238	0.545332	0.474225	0.501364	1	-
ENSMUS(	Epn2	epsin 2 [S	GO:00325	developm	K12471	epsin[-]	mmu0414	Endocytos	11:615172	996	1385	1052	1451	1445	1490	6.113793	6.78964	7.136971	6.871457	5.71181	7.195291	6.680134	6.592853	0.711084	1	-
ENSMUS(	B9d1	B9 proteir	GO:00325	developm	-	-	-	-	11:615051	93	152	102	145	158	116	2.846309	3.715266	3.450222	3.423716	3.113947	2.792979	3.337266	3.110214	0.543722	1	-
ENSMUS(	Sec24b	Sec24 rela	GO:00325	developm	K14007	protein tra	mmu0414	Protein pr	3:1299827	1149	1433	1059	1457	1679	1633	7.848276	7.817114	7.994607	7.677934	7.385156	8.775087	7.886665	7.946059	0.859429	1	-
ENSMUS(	N4bp3	NEDD4 bi	GO:00325	developm	-	-	-	-	11:51643C	263	302	246	349	338	339	4.971607	4.559248	5.139515	5.089755	4.11445	5.041403	4.890124	4.748536	0.669585	1	-
ENSMUS(	Rmnd5b	required f	GO:00081	metabolic	-	-	-	-	11:516236	386	619	398	651	722	678	6.921714	8.864669	7.887804	9.006119	8.337155	9.5646	7.891396	8.969291	0.351421	1	-
ENSMUS(	Nhp2	NHP2 ribc	GO:00081	metabolic	K11129	H/ACA rib	mmu030C	Ribosome	11:516197	459	535	447	626	657	463	15.91462	14.81438	17.12926	16.74513	14.66913	12.6292	15.95276	14.68115	0.37041	1	-
ENSMUS(	Vps9d1	VPS9 dor	-	-	-	-	-	-	8:1232423	512	807	403	652	638	733	3.097677	3.89929	2.694749	3.043296	2.48566	3.488838	3.230572	3.005931	0.448478	1	-
ENSMUS(	Zfp276	zinc finge	GO:00081	metabolic	-	-	-	-	8:1232541	161	197	139	164	202	211	0.897092	0.876645	0.856002	0.704996	0.724801	0.924923	0.87658	0.784907	0.333723	1	-
ENSMUS(	Mfsd10	major faci	GO:00511	localizatio	-	-	-	-	5:3463364	121	165	112	167	163	157	0.709707	0.772898	0.726032	0.755681	0.61565	0.724439	0.736213	0.69859	0.588953	1	-
ENSMUS(	Kctd10	potassium	GO:00325	developm	-	-	-	-	5:1143635	863	1048	770	1263	1289	1161	7.053971	6.84116	6.956013	7.964462	6.784695	7.465619	6.950381	7.404925	0.695522	1	-
ENSMUS(	Poldip2	polymeras	GO:00325	developm	-	-	-	-	11:785121	1251	1634	1236	1709	2122	1758	15.01252	15.66009	16.39311	15.82227	16.39821	16.59686	15.68857	16.27245	0.889345	1	-
ENSMUS(	Sebox	SEBOX hc	GO:00325	developm	-	-	-	-	11:785034	66	91	73	111	129	107	1.618416	1.782092	1.978397	2.099894	2.036984	2.064134	1.792968	2.067004	0.427361	1	-
ENSMUS(	Ift20	intraflagel	GO:00325	developm	-	-	-	-	11:785363	354	444	319	436	586	494	6.890209	6.901728	6.862239	6.547051	7.344824	7.564266	6.884725	7.152047	0.90608	1	-
ENSMUS(	Pcbp3	poly(rC) b	GO:00081	metabolic	-	-	-	-	10:767618	673	999	668	938	1076	1001	7.837514	9.291267	8.597792	8.427459	8.069199	9.170825	8.575524	8.555828	0.796975	1	-
ENSMUS(	Araf	Araf protc	GO:00325	developm	K08845	A-Raf pro	mmu052C	Pathways	X:2079781	959	1275	987	1426	1674	1467	5.754198	6.109734	6.545309	6.601097	6.468094	6.924803	6.136414	6.664665	0.534676	1	-
ENSMUS(	Cfp	complem	GO:00081	metabolic	K15412	complem	mmu0516	Herpes sir	X:2092545	90	109	71	151	165	111	2.697701	2.609292	2.352103	3.491893	3.184868	2.617488	2.553032	3.098083	0.27796	1	-
ENSMUS(	Uxt	ubiquitou	-	-	-	-	-	-	X:2094172	18	30	18	23	30	27	0.434739	0.578664	0.480497	0.428575	0.466592	0.513033	0.497967	0.4694	0.753371	1	-
ENSMUS(	Cnnm3	cyclin M3	GO:00511	localizatio	-	-	-	-	1:3651186	421	514	401	559	702	561	3.519849	3.432028	3.705386	3.605653	3.779495	3.689911	3.552421	3.691686	0.896067	1	-
ENSMUS(	Lman2l	lectin, ma	GO:00511	localizatio	-	-	-	-	1:3641987	983	1228	898	1338	1481	1309	6.307287	6.292643	6.368131	6.623315	6.119258	6.607534	6.322687	6.450036	0.964933	1	-
ENSMUS(	Mcm3ap	minichron	GO:00511	localizatio	-	-	-	-	10:764689	1189	1388	1049	1577	1786	1588	8.810447	8.213939	8.590896	9.015256	8.522211	9.257155	8.538428	8.931541	0.826573	1	-
ENSMUS(	Pcnt	pericentri	GO:00511	localizatio	-	-	-	-	10:763512	1019	1255	903	1387	1490	1337	4.35519	4.283732	4.265475	4.573403	4.100847	4.49547	4.301465	4.389906	0.96756	1	-
ENSMUS(	Gmcl1	germ cell-	GO:00325	developm	-	-	-	-	6:8669176	475	583	370	552	716	567	4.073968	3.993364	3.507301	3.652528	3.954504	3.825768	3.858211	3.810933	0.743905	1	-
ENSMUS(	Snrnp27	small nucl	GO:00081	metabolic	K12846	U4/U6.U5	mmu0304	Spliceosoı	6:8667515	310	367	289	399	448	359	8.176239	7.730431	8.42435	8.118871	7.608963	7.448993	8.11034	7.725609	0.531406	1	-
ENSMUS(	Oas1c	2'-5' oligc	GO:00023	immune s	-	-	-	-	5:1208001	13	19	10	20	19	27	0.247199	0.288536	0.210153	0.293398	0.232654	0.403907	0.248629	0.309987	0.513795	1	-
ENSMUS(	Oas1h	2'-5' oligc	GO:00023	immune s	-	-	-	-	5:1208614	0	0	0	2	0	0	0	0	0	0.043866	0	0	0	0.014622	0.528251	1	-
ENSMUS(	Ocrl	OCRL, ino	GO:00325	developm	K01099	inositol pc	mmu011C	Metabolic	X:4791238	1426	1923	1347	1888	2343	2111	6.254947	6.736422	6.530082	6.389051	6.618065	7.284558	6.50715	6.763891	0.872303	1	-
ENSMUS(	Agpat3	1-acylglyc	GO:00081	metabolic	K13523	lysophosp	mmu011C	Metabolic	10:782691	3846	4562	3447	4607	5712	4742	19.71535	18.67653	19.52914	18.2198	18.8555	19.12351	19.30701	18.73294	0.516017	1	-
ENSMUS(	Sema6b	sema don	GO:00325	developm	K06842	semaphor	mmu0436	Axon guic	17:56123C	2337	2896	2337	3156	3861	3030	35.87951	35.50849	39.65458	37.38136	38.17177	36.59664	37.01419	37.38326	0.875438	1	-
ENSMUS(	Dpp9	dipeptidyl	GO:00081	metabolic	-	-	-	-	17:561868	1070	1422	1076	1575	1740	1538	9.928904	10.53812	11.03511	11.27531	10.39732	11.22754	10.50071	10.96672	0.837499	1	-
ENSMUS(	Ramp2	receptor (	GO:00325	developm	K08448	receptor a	mmu0427	Vascular s	11:101246	300	390	342	505	634	466	3.260485	3.385105	4.108041	4.23432	4.437166	3.984356	3.584544	4.218614	0.240399	1	-
ENSMUS(	Lsr	lipolysis st	GO:00325	developm	-	-	-	-	7:3095777	95	155	83	139	215	135	1.379767	1.797875	1.332305	1.55749	2.010819	1.542504	1.503315	1.703604	0.527141	1	-
ENSMUS(	Gramd1a	GRAM do	GO:00160	membran	-	-	-	-	7:3113012	1724	1515	1006	1650	2479	2288	14.11426	9.905572	9.102608	10.42162	13.06929	14.73628	11.04081	12.7424	0.359116	1	-
ENSMUS(	Hpn	hepsin [Sc	GO:00325	developm	K08665	hepsin[3.4	mmu052C	Viral carci	7:3109872	14	26	15	20	18	23	0.13407	0.198844	0.158757	0.147757	0.111001	0.173278	0.163891	0.144012	0.576742	1	-
ENSMUS(	Gabrg1	gamma-a	GO:00048	signal trar	K05186	gamma-a	mmu0408	Neuroacti	5:7075104	307	340	267	336	409	315	1.717817	1.519373	1.651191	1.450467	1.473726	1.386631	1.62946	1.436941	0.204446	1	-
ENSMUS(	Ckb	creatine k	GO:00325	developm	K00933	creatine k	mmu011C	Metabolic	12:111669	8390	8013	7702	11921	12559	8944	89.82598	68.51426	91.13607	98.46525	86.5864	75.33255	83.15877	86.79474	0.865414	1	-
ENSMUS(	Sp1	trans-acti	GO:00325	developm	K04684	transcripti	mmu052C	Pathways	15:102406	493	641	417	526	667	669	2.503673	2.599767	2.340527	2.060852	2.181279	2.672807	2.481322	2.304979	0.383563	1	-

ENSMUSC	Myg1	melanocy	GO:00055	extracellul	-	-	-	-	15:102331	300	365	276	391	439	359	3.124238	3.035725	3.17672	3.141452	2.944035	2.941228	3.112228	3.008905	0.61745	1	-
ENSMUSC	Pfdn5	prefoldin	GO:00325	developm	-	-	-	-	15:102326	1465	2160	1493	1997	2485	2086	27.22987	32.06325	30.67006	28.63627	29.74329	30.50234	29.98773	29.6273	0.693282	1	-
ENSMUSC	Efnb2	ephrin B2	GO:00325	developm	K05463	ephrin-B[	mmu0436	Axon guic	8:8617434	961	1118	736	1278	1448	1175	7.200493	6.69001	6.094858	7.387547	6.986544	6.926083	6.661787	7.100058	0.693558	1	-
ENSMUSC	Rrp15	ribosomal	GO:00081	metabolic	-	-	-	-	1:1867209	124	165	142	191	211	189	1.796683	1.909319	2.27396	2.13507	1.968733	2.154374	1.993321	2.086059	0.884811	1	-
ENSMUSC	Rnd2	Rho famil	GO:00325	developm	-	-	-	-	11:101464	329	1056	655	1146	387	970	8.580987	21.9964	18.88123	23.05988	6.499919	19.90327	16.48621	16.48769	0.929912	1	-
ENSMUSC	Sync	syncoilin [	GO:00452	synapse|st	-	-	-	-	4:1292876	14	23	18	17	29	32	0.265311	0.348097	0.377001	0.248545	0.35389	0.47707	0.330136	0.359835	0.853135	1	-
ENSMUSC	Fndc5	fibronecti	GO:00325	developm	-	-	-	-	4:1291369	1586	1898	1519	2134	2577	2001	35.0482	33.4969	37.09941	36.38205	36.67175	34.78729	35.21484	35.94703	0.971616	1	-
ENSMUSC	Vps50	VPS50 EA	GO:00511	localizatio	-	-	-	-	6:3498382	1389	1687	1281	1767	2058	1800	6.374272	6.182861	6.497172	6.255981	6.081748	6.498482	6.351435	6.278737	0.69039	1	-
ENSMUSC	Hars	histidyl-tR	GO:00081	metabolic	K01892	histidyl-tR	mmu0097	Aminoacy	18:367665	1382	1812	1300	1919	2209	1788	42.4672	44.4682	44.15051	45.49365	43.71151	43.22389	43.6953	44.14302	0.87884	1	-
ENSMUSC	Zmat2	zinc finge	GO:00081	metabolic	K12848	U4/U6.U5	mmu0304	Spliceoso	18:367939	2992	3744	2286	3731	4658	3300	80.94191	80.88974	68.34942	77.8695	81.14578	70.23226	76.72702	76.41585	0.769663	1	-
ENSMUSC	Smg5	Smg-5 hc	GO:00081	metabolic	K11125	protein SI	mmu0301	mRNA sur	3:8833626	1047	1561	1184	1815	2007	1919	6.754844	8.042988	8.442425	9.033905	8.33817	9.739902	7.746753	9.037326	0.185926	1	-
ENSMUSC	Cct3	chaperoni	GO:00081	metabolic	-	-	-	-	3:8829711	1505	1957	1438	2055	2594	2048	9.773716	10.14985	10.32116	10.29592	10.84795	10.46318	10.08158	10.53569	0.818131	1	-
ENSMUSC	Glmp	glycosylat	GO:00048	signal trar	-	-	-	-	3:8832502	312	428	292	464	476	447	1.409452	1.544133	1.457889	1.617124	1.384702	1.588596	1.470491	1.530141	0.899791	1	-
ENSMUSC	Mef2d	myocyte e	GO:00325	developm	K09262	MADS-bc	mmu0437	Apelin sig	3:8814237	3657	5787	3691	5184	5882	5254	24.73574	31.26074	27.59248	27.05174	25.62003	27.95767	27.86299	26.87648	0.507311	1	-
ENSMUSC	Snd1	staphyloc	GO:00325	developm	K15979	staphyloc	mmu0520	Viral carci	6:2847513	1526	1956	1493	2118	2582	2257	8.536387	8.738448	9.230523	9.140624	9.301019	9.932581	8.835119	9.458075	0.615974	1	-
ENSMUSC	Slc19a1	solute car	GO:00511	localizatio	K14609	solute car	mmu0152	Antifolate	10:770322	131	152	127	191	235	174	1.037571	0.961468	1.111718	1.167102	1.198584	1.084189	1.036919	1.149958	0.569397	1	-
ENSMUSC	Kpnb1	karyophe	GO:00511	localizatio	K14293	importin s	mmu0301	RNA trans	11:971597	4085	5174	3666	5117	6621	5363	35.58093	35.99131	35.29105	34.38516	37.13672	36.74887	35.6211	36.09025	0.89628	1	-
ENSMUSC	Tbx21	T-box 21	GO:00325	developm	K10166	T-box prc	mmu0465	Th17 cell (	11:970980	4	2	2	4	2	1	0.09817	0.039204	0.05425	0.075724	0.0316	0.01931	0.063875	0.042211	0.647529	1	-
ENSMUSC	Mrpl10	mitochon	GO:00081	metabolic	-	-	mmu0301	Ribosome	11:970415	616	808	600	912	1049	852	17.76656	18.61144	19.12583	20.29305	19.48285	19.33183	18.50127	19.70258	0.696202	1	-
ENSMUSC	Cyp51	cytochron	GO:00081	metabolic	K05917	sterol 14-	mmu0110	Metabolic	5:4081145	1221	1513	1097	1828	2038	1400	13.7804	13.63738	13.68358	15.91668	14.8117	12.4304	13.70045	14.38626	0.800028	1	-
ENSMUSC	Tcf25	transcripti	GO:00081	metabolic	-	-	-	-	8:1233737	5459	6758	5139	7706	8842	7519	31.49841	31.14151	32.77186	34.30322	32.85343	34.13081	31.80393	33.76249	0.665526	1	-
ENSMUSC	Tubb6	tubulin, be	GO:00051	structural	K07375	tubulin be	mmu0414	Phagoson	18:673907	20	47	19	32	48	28	0.695033	1.304417	0.729757	0.857939	1.074153	0.765504	0.909736	0.899199	0.927264	1	-
ENSMUSC	Def8	differentia	GO:00511	localizatio	-	-	-	-	8:1234235	610	865	570	896	1013	801	5.343682	6.051632	5.518639	6.055487	5.714455	5.520189	5.637984	5.763377	0.989807	1	-
ENSMUSC	Meox1	mesenchy	GO:00325	developm	-	-	-	-	11:101877	9	13	6	11	21	14	0.246638	0.284522	0.181742	0.232579	0.37059	0.301827	0.237634	0.301665	0.570317	1	-
ENSMUSC	Sost	sclerostin	GO:00325	developm	K16834	sclerostin[	mmu0431	Wnt signa	11:101962	0	6	0	2	0	0	0	0.141613	0	0.045607	0	0	0.047204	0.015202	0.417546	1	-
ENSMUSC	Nkx2-1	NK2 home	GO:00325	developm	-	-	-	-	12:565319	2	2	5	14	5	6	0.037823	0.030217	0.104534	0.204298	0.060902	0.089286	0.057525	0.118162	0.184972	1	-
ENSMUSC	Irx2	Iroquois r	GO:00325	developm	-	-	-	-	13:726288	0	5	0	0	9	3	0	0.04804	0	0	0.069728	0.028391	0.016013	0.032706	0.494961	1	-
ENSMUSC	Itga3	integrin al	GO:00325	developm	K06482	integrin al	mmu0520	Pathways	11:950444	310	396	263	422	575	466	2.804563	2.861178	2.6297	2.945417	3.349856	3.316656	2.765147	3.203977	0.281028	1	-
ENSMUSC	Sgca	sarcoglyca	GO:00325	developm	K12565	alpha-sar	mmu0541	Dilated ca	11:949627	4	11	3	6	16	9	0.092378	0.202865	0.076574	0.106893	0.237928	0.163502	0.123939	0.169441	0.566474	1	-
ENSMUSC	Dlx3	distal-less	GO:00325	developm	-	-	-	-	11:951201	0	3	0	0	0	0	0	0.056122	0	0	0	0	0.018707	0	0.288896	1	-
ENSMUSC	Itfg2	integrin al	GO:00325	developm	-	-	-	-	6:1284094	244	297	172	272	252	317	1.347399	1.30981	1.049737	1.158791	0.896109	1.377133	1.235649	1.144011	0.47738	1	-
ENSMUSC	Tulp3	tubby-like	GO:00325	developm	-	-	-	-	6:1283211	310	388	303	452	490	491	1.708303	1.707579	1.845408	1.921642	1.738817	2.128607	1.753763	1.929689	0.542665	1	-
ENSMUSC	Gtf2h4	general tr	GO:00081	metabolic	K03144	transcripti	mmu0520	Viral carci	17:356677	156	181	135	206	256	230	1.496477	1.386661	1.431285	1.524552	1.581392	1.735734	1.438141	1.613893	0.48998	1	-
ENSMUSC	Tubb5	tubulin, be	GO:00051	structural	K07375	tubulin be	mmu0414	Phagoson	17:358339	4724	5592	4412	6560	7504	6107	57.80331	54.64567	59.66575	61.92657	59.12761	58.78705	57.37158	59.94708	0.814307	1	-
ENSMUSC	Ell2	elongatio	GO:00081	metabolic	-	-	-	-	13:757067	529	688	459	738	842	620	2.800152	2.90844	2.685254	3.013786	2.870071	2.581833	2.797949	2.821897	0.889713	1	-
ENSMUSC	Fkbp10	FK506 bin	GO:00081	metabolic	-	-	-	-	11:100415	124	136	120	131	223	165	1.69076	1.480958	1.808372	1.378034	1.958027	1.769923	1.66003	1.701995	1	1	-
ENSMUSC	Klhl10	kelch-like	GO:00325	developm	-	-	-	-	11:100441	2	6	4	6	8	0	0.041402	0.099211	0.091539	0.095838	0.106667	0	0.077384	0.067502	0.877314	1	-
ENSMUSC	Nom1	nucleolar	GO:00325	developm	-	-	-	-	5:2943466	564	721	477	726	848	720	3.640251	3.716494	3.402653	3.615092	3.524543	3.655912	3.586466	3.598515	0.84102	1	-
ENSMUSC	Ergic1	endoplasr	GO:00511	localizatio	-	-	-	-	17:265614	1403	2822	2160	3034	3349	3071	11.54042	18.53817	19.63652	19.2535	17.73919	19.87259	16.5717	18.9551	0.357716	1	-
ENSMUSC	Tcea3	transcripti	GO:00081	metabolic	-	-	-	-	4:1362477	13	29	11	21	36	35	0.450742	0.803034	0.421515	0.561734	0.803796	0.954686	0.55843	0.773405	0.279686	1	-
ENSMUSC	Ifrd1	interferon	GO:00325	developm	-	-	-	-	12:402015	379	495	371	456	656	431	2.784385	2.904297	3.012388	2.58455	3.103475	2.491025	2.900357	2.72635	0.47109	1	-
ENSMUSC	Stk38l	serine/thr	GO:00081	metabolic	-	-	-	-	6:1467249	443	657	470	615	743	674	4.540662	5.37808	5.324273	4.863192	4.9041	5.434839	5.081005	5.067377	0.809155	1	-
ENSMUSC	Brpf1	bromodo	GO:00081	metabolic	-	-	-	-	6:1133071	747	930	672	957	1153	946	3.073476	3.055893	3.055802	3.037747	3.054874	3.062045	3.061724	3.051555	0.780386	1	-
ENSMUSC	Akr1b3	aldo-keto	GO:00325	developm	K00011	aldehyde	mmu0110	Metabolic	6:3430243	99	94	102	103	122	103	0.591229	0.448329	0.673239	0.474559	0.469178	0.483917	0.570932	0.475885	0.206232	1	-
ENSMUSC	Gstt3	glutathior	GO:00081	metabolic	K00799	glutathior	mmu0520	Pathways	10:757741	25	42	27	43	61	40	0.266069	0.356991	0.31759	0.353066	0.418065	0.334909	0.31355	0.36868	0.501358	1	-
ENSMUSC	Ddt	D-dopach	GO:00081	metabolic	-	-	-	-	10:757712	171	256	185	246	276	214	8.537141	10.2071	10.20785	9.47508	8.8732	8.40506	9.650697	8.91778	0.430857	1	-
ENSMUSC	Ddx18	DEAD (As	GO:00081	metabolic	-	-	-	-	1:1215538	203	244	242	260	326	268	4.389085	4.213221	5.782845	4.336933	4.538906	4.558528	4.79505	4.478122	0.49586	1	-
ENSMUSC	Ubl3	ubiquitin-	GO:00055	extracellul	-	-	-	-	5:1485046	1905	2584	2006	2603	3301	2515	9.100672	9.858637	10.59147	9.593629	10.15497	9.452082	9.85026	9.733561	0.693253	1	-
ENSMUSC	Gramd3	GRAM do	GO:00990	supramole	-	-	-	-	18:564321	196	280	189	311	418	271	4.594018	5.241313	4.896027	5.623756	6.309085	4.99707	4.910453	5.643304	0.358406	1	-
ENSMUSC	Eef1e1	eukaryotic	GO:00325	developm	-	-	-	-	13:386442	190	226	180	282	330	201	3.12949	2.972867	3.276729	3.583443	3.500176	2.604522	3.126362	3.22938	0.948919	1	-
ENSMUSC	Akt1	thymoma	GO:00325	developm	K04456	RAC serin	mmu0520	Pathways	12:112653	2283	2942	2231	3255	3746	2978	25.50699	26.25075	27.5486	28.05656	26.95104	26.17513	26.43545	27.06091	0.997136	1	-
ENSMUSC	Il16	interleukir	GO:00081	metabolic	-	-	-	-	7:8364282	856	1125	839	1089	1379	1157	5.152889	5.408482	5.581946	5.057493	5.345593	5.479252	5.381106	5.294113	0.678026	1	-
ENSMUSC	Tcirg1	T cell, imn	GO:00081	metabolic	K02154	V-type H-	mmu0110	Metabolic	19:389605	195	257	160	246	339	269	1.631229	1.716955	1.479266	1.587618	1.826144	1.770287	1.60915	1.728016	0.710931	1	-
ENSMUSC	Naglu	alpha-N-	GO:00325	developm	K01205	alpha-N-	mmu0110	Metabolic	11:101070	88	177	94	143	150	159	1.411751	2.26774	1.666675	1.769873	1.549607	2.0067	1.782055	1.775393	0.863646	1	-
ENSMUSC	Coasy	Coenzyme	GO:00081	metabolic	K02318	phosphop	mmu0110	Metabolic	11:101082	283	414	277	420	469	435	4.213476	4.922655	4.558058	4.824286	4.496568	5.095108	4.56473	4.805321	0.820206	1	-
ENSMUSC	Smo	smoothen	GO:00325	developm	K06226	smoothen	mmu0520	Pathways	6:2973550	278	350	261	383	452	372	2.702458	2.717243	2.804151	2.872391	2.829484	2.844912	2.741284	2.848929	0.904416	1	-
ENSMUSC	Tspan33	tetraspani	GO:00081	metabolic	-	-	-	-	6:2969422	451	745	457	681	777	650	4.046021	5.337691	4.531212	4.713342	4.488768	4.587499	4.638308	4.596536	0.770754	1	-
ENSMUSC	Crnkl1	Crn, crook	GO:00081	metabolic	K12869	crooked n	mmu0304	Spliceoso	2:1459174	428	557	386	539	698	481	5.958248	6.192642	5.938941	5.78887	6.257266	5.267813	6.029944	5.771316	0.551489	1	-
ENSMUSC	Rin2	Ras and R	GO:00511	localizatio	-	-	-	-	2:1457847	641	931	545	840	1111	868	6.29637	7.303446	5.916646	6.365636	7.027501	6.707535	6.505488	6.700224	0.958469	1	-
ENSMUSC	Folh1	folate hyd	GO:00081	metabolic	K14592	glutamate	mmu0110	Metabolic	7:8671897	109	172	114	138	208	142	1.657199	2.08845	1.91558	1.618669	2.036422	1.69843	1.887076	1.784507	0.601721	1	-
ENSMUSC	Chordc1	cysteine a	GO:00081	metabolic	-	-	-	-	9:1829212	1376	1673	1031	1907	2287	1503	9.31234	9.042372	7.711626	9.956842	9.966931	8.002221	8.688779	9.308665	0.660683	1	-
ENSMUSC	Rtcb	RNA 2',3'-	GO:00325	developm	-	-	-	-	10:859386	1508	1962	1279	2016	2517	2060	32.50111	33.77082	30.4659	33.52105	34.9329	34.92809	32.24594	34.46068	0.634474	1	-
ENSMUSC	Pwp1	PWP1 hor	GO:00325	developm	-	-	-	-	10:858294	316	379	238	408	440	364	5.289017	5.066103	4.402633	5.268407	4.742376	4.792925	4.919251	4.934569	0.861034	1	-
ENSMUSC	Fbxo7	F-box prc	GO:00325	developm	-	-	-	-	10:860219	280	374	252	374	411	389	1.388362	1.481021	1.380994	1.430691	1.312324	1.517415	1.416792	1.420143	0.853062	1	-
ENSMUSC	Capns1	calpain, sr	GO:00055	extracellul	-	-	-	-	7:3018693	1190	1681	1227	1577	1887	2586	13.47786	15.20504	15.35909	13.77958	13.7626	23.04165	14.68066	16.86127	0.382217	1	-
ENSMUSC	Clpb	ClpB case	GO:00508	response	K03695	ATP-depe	mmu0421	Longevity	7:1016636	789	1033	701	1114	1352	1091	3.506688	3.666622	3.443375	3.819753	3.869467	3.814658	3.538895	3.834626	0.562981	1	-
ENSMUSC	7-Sep	septin 7 [S	GO:00329	macromo	-	-	-	-	9:2525243	5780	7252	5057	7462	8949	7120	21.32293	21.36598	20.61858	21.23756	21.25927	20.66379	21.1025	21.05354	0.74926	1	-
ENSMUSC	Rac1	RAS-relat	GO:00325	developm	K04392	Ras-relate	mmu0520	Pathways	5:1435036	4973	6091	4256	6149	7647	5904	47.4663	46.4303	44.8968	45.27952	47.00161	44.33271	46.26447	45.53795	0.632292	1	-
ENSMUSC	Nup214	nucleopoi	GO:00081	metabolic	K14317	nuclear pc	mmu0516	Epstein-B	2:3197443	798	1038	751	1033	1192	1208	3.563287	3.701613	3.706248	3.558594	3.427511	4.243514	3.657049	3.743206	0.992109	1	-
ENSMUSC	Aif1l	allograft ii	GO:00990	supramole	-	-	-	-	2:3195013	95	170	114	179	211	195	0.937082	1.339204	1.242806	1.362187	1.340265	1.513206	1.173031	1.405219	0.295495	1	-
ENSMUSC	Cpa3	carboxype	GO:00081	metabolic	K08780	carboxype	mmu0497	Pancreatic	3:2021562	1	2	0	0	1	0	0.040261	0.064298	0	0	0.025913	0	0.034853	0.008638	0.646809	1	-
ENSMUSC	Ltbp1	latent trar	GO:00325	developm	K19559	latent trar	mmu0435	TGF-beta	17:750055	172	183	112	180	234	186	0.705163	0.59918	0.507486	0.569331	0.617776	0.599909	0.603943	0.595672	0.787317	1	-
ENSMUSC	Ugp2	UDP-gluc	GO:00081	metabolic	-	-	mmu0110	Metabolic	11:213211	1182	1265	1038	1439	1748	1228	16.88598	14.43261	16.38903	15.85988	16.0807	13.80124	15.90254	15.24727	0.512834	1	-
ENSMUSC	Kcnh6	potassium	GO:00511	localizatio	-	-	-	-	11:106008	25	26	18	23	27	24	0.311327	0.258583	0.247746	0.220974	0.216523	0.235124	0.272552	0.224207	0.338616	1	-
ENSMUSC	Trmt1	tRNA met	GO:00081	metabolic	-	-	-	-	8:8468630	536	648	506	764	749	754	2.285098	2.20628	2.38417	2.512833	2.056251	2.528842	2.291849	2.365976	0.952194	1	-
ENSMUSC	Nacc1	nucleus ac	GO:00081	metabolic	-	-	-	-	8:8467048	2262	2920	1992	3131	3697	3241	32.11941	33.11339	31.26159	34.29954	33.80485	36.20469	32.16479	34.76969	0.532709	1	-
ENSMUSC	Nfix	nuclear fa	GO:00325	developm	-	-	-	-	8:8469987	5301	7321	5011	6622	9075	6058	38.7025	42.68719	40.43453	37.29931	42.66606	34.79537	40.60807	38.25358	0.357574	1	-
ENSMUSC	Uba1	ubiquitin-	GO:00081	metabolic	K03178	ubiquitin-	mmu0501	Parkinson	X:2065832	7013	8701	6477	9463	10787	8755	79.79883	79.06926	81.45418	83.07146	79.0402	78.37178	80.10743	80.16115	0.777204	1	-
ENSMUSC	Vwf	Von Willel	GO:00325	developm	K03900	von Willet	mmu0415	PI3K-Akt s	6:1255467	418	593	467	535	734	728	1.287119	1.458287	1.5893	1.270946	1.455437	1.763537	1.444902	1.49664	0.929629	1	-
ENSMUSC	Siae	sialic acid	GO:00081	metabolic	-	-	-	-	9:3755569	290	402	276	420	497	402	1.251635	1.385641	1.316541	1.398487	1.381307	1.364948	1.317939	1.381581	0.842084	1	-
ENSMUSC	Fam50a	family witl	GO:00056	cell|intrace	-	-	-	-	X:7431303	408	509	368	554	652	549	7.98708	7.957775	7.962007	8.366968	8.219218	8.454951	7.968954	8.347046	0.84137	1	-
ENSMUSC	Emd	emerin [S	GO:00325	developm	K12569	emerin[-]	mmu0541	Dilated ca	X:7425468	495	639	439	715	797	583	6.48161	6.682277	6.353149	7.222943	6.720334	6.005618	6.505679	6.649632	0.990711	1	-
ENSMUSC	Taco1	translatior	GO:00081	metabolic	-	-	-	-	11:106066	175	278	219	282	361	231	7.72608	9.801938	10.68592	9.605078	10.2632	8.023132	9.404645	9.297137	0.816189	1	-
ENSMUSC	Grik3	glutamate	GO:00048	signal trar	K05203	glutamate	mmu0408	Neuroacti	4:1254907	1408	1614	1126	1645	1767	1628	9.580948	8.771122	8.468198	8.635786	7.74278	8.715071	8.940089	8.364546	0.336094	1	-
ENSMUSC	Npas1	neuronal	GO:00325	developm	-	-	-	-	7:1645572	156	188	154	211	243	195	2.063938	1.986444	2.251855	2.153692	2.070294	2.029631	2.100746	2.084539	0.827617	1	-
ENSMUSC	Sipa1l2	signal-inc	GO:00987	molecular	K17702	signal-ind	mmu0401	Rap1 sign	8:1254180	1729	1916	1628	2247	2167	2466	7.615195	6.739497	7.924784	7.635185	6.1461	8.544567	7.426492	7.441951	0.822268	1	-
ENSMUSC	Ap4e1	adaptor-r	GO:00511	localizatio	K12400	AP-4 com	mmu0414	Lysosome	2:1270087	214	300	225	260	378	294	1.119181	1.253009	1.300518	1.049036	1.273014	1.209609	1.224236	1.17722	0.624764	1	-
ENSMUSC	Blvra	biliverdin	GO:00081	metabolic	K00214	biliverdin	mmu0110	Metabolic	2:1270706	148	157	144	153	262	187	3.169618	2.685289	3.408433	2.527942	3.61328	3.150646	3.08778	3.097289	0.907059	1	-
ENSMUSC	Pdzd4	PDZ dom	GO:00056	cell|intrace	-	-	-	-	X:7379335	2059	2550	1888	2886	3116	2690	31.66717	31.32123	32.09236	34.2436	30.86065	32.54739	31.69359	32.55055	0.978896	1	-
ENSMUSC	Srpk3	serine/arg	GO:00325	developm	-	-	-	-	X:7377440	25	65	43	51	73	43	0.361201	0.750019	0.686639	0.568478	0.679186	0.488761	0.599286	0.578809	0.824702	1	-
ENSMUSC	Idh3g	isocitrate	GO:00081	metabolic	K00030	isocitrate	mmu0110	Metabolic	X:7377896	1396	1716	1195	1592	1999	1658	15.61119	15.32547	14.76949	13.73485	14.39522	14.58633	15.23538	14.2388	0.312377	1	-
ENSMUSC	Pnck	pregnanc	GO:00081	metabolic	-	-	-	-	X:7365599	804	905	768	1035	1176	1004	16.70889	15.02055	17.64004	16.59443	15.73814	16.41483	16.4565	16.24913	0.720665	1	-
ENSMUSC	Ssr4	signal seq	GO:00329	macromo	K04571	translocor	mmu0414	Protein pr	X:7378702	175	280	214	301	306	252	1.44141	1.841848	1.94809	1.912695	1.623028	1.632905	1.743782	1.722876	0.793186	1	-
ENSMUSC	Bcap31	B cell rece	GO:00081	metabolic	K14009	B-cell rece	mmu0516	Human pa	X:7368617	572	800	577	742	957	732	28.6507	32.00188	31.94202	28.67307	30.86784	28.84443	30.86487	29.46178	0.493678	1	-
ENSMUSC	Fam98a	family witl	GO:00081	metabolic	-	-	-	-	17:755370	567	736	515	751	920	761	9.027365	9.358406	9.062165	9.224611	9.432361	9.531771	9.149312	9.396248	0.98265	1	-
ENSMUSC	Ltbp2	latent trar	GO:00099	cellular pr	-	-	-	-	12:847832	9	9	2	3	10	5	0.040361	0.032235	0.009914	0.010381	0.028879	0.01764	0.027503	0.018967	0.440796	1	-
ENSMUSC	Kmt2a	lysine (K)-	GO:00325	developm	K09186	histone-ly	mmu0520	Transcript	9:4480335	2464	3479	2219	2836	3704	3308	7.432795	8.381304	7.398021	6.600061	7.195104	7.850327	7.737374	7.215164	0.30533	1	-
ENSMUSC	Ift46	intraflagel	GO:00400	locomotic	-	-	-	-	9:4476790	489	539	336	545	708	616	3.255278	2.865587	2.472095	2.79902	3.035058	3.226048	2.86432	3.020042	0.815164	1	-
ENSMUSC	Tmem25	transmem	GO:00055	extracellul	-	-	-	-	9:4479376	464	524	432	619	754	717	3.351192	3.022446	3.448355	3.44907	3.506768	4.073905	3.273997	3.676581	0.40733	1	-
ENSMUSC	Trappc6a	trafficking	GO:00511	localizatio	-	-	-	-	7:1950839	60	51	58	71	87	51	1.061872	0.720838	1.134484	0.969417	0.991508	0.710076	0.972398	0.890334	0.596654	1	-
ENSMUSC	Supt6	suppressc	GO:00325	developm	-	-	-	-	11:782067	6746	8276	6192	7844	9948	8443	52.04675	50.99336	52.79892	46.68907	49.42398	51.24541	51.94634	49.11949	0.335424	1	-
ENSMUSC	Spag5	sperm ass	GO:00511	localizatio	-	-	-	-	11:783015	106	152	113	162	198	161	0.786504	0.900712	0.92666	0.927345	0.946056	0.939797	0.871292	0.937733	0.7267	1	-
ENSMUSC	Foxn1	forkhead	GO:00325	developm	-	-	-	-	11:783575	0	0	3	0	2	2	0	0	0.062993	0	0.024462	0.029897	0.020998	0.01812	1	1	-
ENSMUSC	Unc119	unc-119 I	GO:00325	developm	-	-	-	-	11:783434	207	217	196	276	220	223	1.960146	1.641057	2.051265	2.016315	1.341513	1.66125	1.884156	1.673026	0.320379	1	-
ENSMUSC	Rab34	RAB34, m	GO:00038	catalytic a	-	-	-	-	11:781884	106	123	137	175	185	189	1.200101	1.112148	1.714278	1.528553	1.348774	1.683391	1.342175	1.52024	0.527054	1	-
ENSMUSC	Sdf2	stromal ce	GO:00160	membran	-	-	-	-	11:782457	338	540	410	544	663	651	7.873964	10.04652	10.55618	9.776993	9.945911	11.93076	9.492222	10.55122	0.50148	1	-
ENSMUSC	Ccne1	cyclin E1 [	GO:00081	metabolic	K06626	cyclin E[-]	mmu0520	Pathways	7:3809798	179	276	179	250	315	291	2.909081	3.582257	3.215158	3.134529	3.296604	3.720536	3.235498	3.383889	0.879302	1	-
ENSMUSC	Hsf2bp	heat shoc	GO:00056	cell|protei	-	-	-	-	17:319447	2	11	1	4	10	0	0.0342	0.150245	0.018904	0.052773	0.110135	0	0.067783	0.054303	0.711228	1	-
ENSMUSC	Bbc3	BCL2 bind	GO:00325	developm	K10132	Bcl-2-bin	mmu0520	Pathways	7:1630839	64	86	63	99	102	93	1.299536	1.394615	1.413836	1.550877	1.333727	1.485608	1.369329	1.456737	0.832269	1	-
ENSMUSC	Mybpc3	myosin bi	GO:00325	developm	K12568	myosin-b	mmu0541	Dilated ca	2:9111814	2	5	0	1	3	5	0.026049	0.052017	0	0.010052	0.02517	0.051242	0.026022	0.028821	1	1	-
ENSMUSC	Psmc3	proteasor	GO:00325	developm	K03065	26S prote	mmu0516	Epstein-B	2:9105400	2257	3075	2186	2979	4456	3132	28.27995	30.77073	30.2722	28.79704	35.95396	30.87306	29.77429	31.87469	0.633092	1	-
ENSMUSC	Acp2	acid phos	GO:00325	developm	K14410	lysosomal	mmu0110	Metabolic	2:9120288	1461	1910	1370	2087	2287	1941	11.79192	12.31158	12.22086	12.99532	11.88654	12.32454	12.10812	12.40213	1	1	-
ENSMUSC	Rapsn	receptor-	GO:00452	synapse|lc	-	-	-	-	2:9103562	4	3	4	7	0	0	0.083446	0.049987	0.092227	0.112654	0	0	0.07522	0.037551	0.306176	1	-
ENSMUSC	Slc39a13	solute car	GO:00325	developm	-	-	-	-	2:9106179	276	387	242	396	431	418	2.469888	2.765823	2.393486	2.733966	2.483705	2.942765	2.543066	2.720145	0.72419	1	-
ENSMUSC	Celf2	CUGBP, El	GO:00081	metabolic	-	-	-	-	2:6539694	5761	7075	5283	7721	9080	7259	5.789551	5.678313	5.867789	5.986195	5.876078	5.73898	5.778551	5.867084	0.914884	1	-
ENSMUSC	Nr1h3	nuclear re	GO:00048	signal trar	K08536	liver X rec	mmu0493	Non-alco	2:9118406	43	66	58	90	88	72	0.506661	0.621063	0.755301	0.818125	0.667706	0.667405	0.627675	0.717745	0.533189	1	-
ENSMUSC	Ddb2	damage s	GO:00081	metabolic	K10140	DNA dam	mmu0520	Pathways	2:9121157	101	175	110	179	168	206	1.031596	1.427488	1.241734	1.410494	1.104969	1.655258	1.233606	1.390241	0.557231	1	-
ENSMUSC	Spi1	spleen foc	GO:00325	developm	K09438	transcripti	mmu0520	Pathways	2:9108239	55	82	44	59	91	80	1.016095	1.209856	0.898403	0.840919	1.082597	1.162707	1.041451	1.028741	0.84044	1	-
ENSMUSC	Sf3a1	splicing fa	GO:00081	metabolic	K12825	splicing fa	mmu0304	Spliceoso	11:416035	1549	1864	1265	2049	2299	1894	14.02625	13.47975	12.65982	14.31404	13.40552	13.49214	13.38861	13.73724	0.989004	1	-
ENSMUSC	Clgn	calmegin	GO:00325	developm	-	-	-	-	8:8338986	237	318	191	298	368	278	3.265805	3.499571	2.908852	3.168026	3.265461	3.013682	3.224742	3.149056	0.699798	1	-
ENSMUSC	Napsa	napsin A a	GO:00081	metabolic	K08565	napsin-A	mmu0414	Lysosome	7:4457238	2	7	1	9	3	7	0.030623	0.085614	0.016927	0.106336	0.029589	0.084339	0.044388	0.073421	0.455282	1	-
ENSMUSC	Vrk3	vaccinia re	GO:00325	developm	-	-	-	-	7:4474841	263	384	261	412	493	447	1.849821	2.157003	2.028899	2.235635	2.23293	2.473387	2.011908	2.313984	0.319128	1	-
ENSMUSC	Smg9	smg-9 ho	GO:00325	developm	-	-	-	-	7:2439961	279	434	314	520	532	531	3.359341	4.173352	4.178559	4.830407	4.124923	5.029855	3.903751	4.661728	0.204698	1	-
ENSMUSC	Paxip1	PAX intera	GO:00325	developm	-	-	-	-	5:2774008	972	1193	843	1255	1391	1278	5.095602	4.994769	4.884316	5.075786	4.695818	5.270732	4.991562	5.014112	0.835007	1	-
ENSMUSC	Rmnd5a	required f	GO:00081	metabolic	-	-	-	-	6:7138863	2187	2542	1750	2689	3162	2609	9.748436	9.049145	8.621264	9.247138	9.076171	9.148947	9.139615	9.157419	0.800188	1	-
ENSMUSC	Tead3	TEA doma	GO:00325	developm	K09448	transcripti	mmu0439	Hippo sig	17:283316	34	55	37	67	90	53	0.245014	0.316532	0.294679	0.372488	0.417641	0.300466	0.285408	0.363532	0.244501	1	-
ENSMUSC	Ppard	peroxisom	GO:00325	developm	K04504	peroxisom	mmu0520	Pathways	17:282327	373	490	318	561	675	555	3.528223	3.701597	3.324455	4.093929	4.111547	4.130009	3.518092	4.111828	0.229569	1	-
ENSMUSC	Peg3	paternally	GO:00081	metabolic	-	-	-	-	7:6703892	7562	9072	6273	9344	9922	9382	28.61259	27.41381	26.23268	27.27619	24.17544	27.92717	27.41969	26.4596	0.483772	1	-
ENSMUSC	Zim1	zinc finge	GO:00081	metabolic	-	-	-	-	7:6671269	100	103	94	138	177	137	0.511588	0.420826	0.531493	0.544665	0.583107	0.55138	0.487969	0.559717	0.445396	1	-
ENSMUSC	Metrn	meteorin,	GO:00325	developm	-	-	-	-	17:257945	516	573	516	657	786	557	16.67866	14.79148	18.4335	16.38351	16.3602	14.16374	16.63455	15.63582	0.451727	1	-
ENSMUSC	Lmf1	lipase ma	GO:00081	metabolic	-	-	-	-	17:255791	184	295	223	291	315	281	0.726177	0.929806	0.972695	0.886031	0.800552	0.872452	0.876226	0.853012	0.697286	1	-
ENSMUSC	Narfl	nuclear pr	GO:00325	developm	-	-	-	-	17:257737	403	560	449	585	624	679	3.61753	4.014583	4.454514	4.051288	3.607001	4.794988	4.028875	4.151092	0.968098	1	-
ENSMUSC	Angptl4	angiopoie	GO:00325	developm	K08767	angiopoie	mmu0332	PPAR sign	17:337737	30	47	34	46	64	54	0.351383	0.439643	0.440134	0.415667	0.482718	0.497572	0.410387	0.465319	0.595982	1	-
ENSMUSC	Dbf4	DBF4 zinc	GO:00081	metabolic	K06629	activator c	mmu0411	Cell cycle	5:8396973	31	36	44	57	59	32	0.149261	0.138434	0.234147	0.211735	0.182933	0.121214	0.173947	0.171961	0.944262	1	-
ENSMUSC	Daxx	Fas death	GO:00081	metabolic	K02308	death-ass	mmu0401	MAPK sigi	17:339094	348	419	266	487	532	405	3.553218	3.416666	3.001723	3.836202	3.497907	3.253178	3.323869	3.529096	0.76663	1	-
ENSMUSC	Cd320	CD320 an	GO:00081	metabolic	-	-	-	-	17:338430	116	136	123	152	167	122	2.099831	1.966122	2.460822	2.122754	1.946693	1.737388	2.175591	1.935612	0.346368	1	-
ENSMUSC	Ipo4	importin 4	GO:00511	localizatio	-	-	-	-	14:556254	989	980	988	1417	1329	1219	7.013529	5.550247	7.743618	7.752475	6.069036	6.80072	6.769131	6.874077	0.935535	1	-
ENSMUSC	Tm9sf1	transmem	GO:00081	metabolic	-	-	-	-	14:556359	189	243	197	295	302	286	0.917329	0.941921	1.05676	1.104625	0.943897	1.092041	0.972004	1.046855	0.697955	1	-
ENSMUSC	Rec8	REC8 mei	GO:00325	developm	K13054	meiotic re	mmu0411	Oocyte m	14:556180	36	46	32	48	41	45	0.557893	0.569312	0.548083	0.573871	0.409156	0.548616	0.558429	0.510548	0.577056	1	-
ENSMUSC	Gmpr2	guanosine	GO:00325	developm	K00364	GMP redu	mmu0023	Purine me	14:556719	283	402	291	401	519	401	2.716043	3.081211	3.086662	2.969099	3.20754	3.027645	2.961305	3.068095	0.921705	1	-
ENSMUSC	Mdp1	magnesiu	GO:00081	metabolic	-	-	-	-	14:556578	547	630	490	748	936	664	8.713481	8.014772	8.626758	9.192563	9.601416	8.321156	8.45167	9.038378	0.684483	1	-
ENSMUSC	Dhrs1	dehydrog	GO:00081	metabolic	-	-	-	-	14:557390	684	875	539	773	1000	878	11.69198	11.945	10.1828	10.19393	11.00745	11.80694	11.27326	11.00277	0.642741	1	-
ENSMUSC	Ncan	neurocan	GO:00071	cell adhes	-	-	-	-	8:7009308	4904	6199	4264	6362	6928	5999	41.61626	42.01264	39.99232	41.65206	37.85952	40.04998	41.20707	39.85386	0.487253	1	-
ENSMUSC	Tmem161	transmem	GO:00081	metabolic	-	-	-	-	8:7017235	166	280	329	283	397	509	0.93927	1.265279	2.057429	1.235375	1.446529	2.265742	1.420659	1.649215	0.523929	1	-
ENSMUSC	Borcs8	BLOC-1 re	GO:00325	developm	-	-	-	-	8:7013949	221	372	251	370	425	336	5.177995	6.960783	6.499643	6.688075	6.412286	6.193264	6.212807	6.431208	0.934558	1	-
ENSMUSC	Slc25a42	solute car	GO:00511	localizatio	-	-	-	-	8:7018434	550	443	469	731	741	615	3.75427	2.414975	3.538201	3.849557	3.257134	3.302545	3.235815	3.469745	0.737638	1	-
ENSMUSC	Snx9	sorting ne	GO:00081	metabolic	-	-	-	-	17:584132	240	355	248	315	458	377	6.077955	7.179927	6.941356	6.154412	7.469072	7.511018	6.733079	7.044834	0.86472	1	-
ENSMUSC	Ranbp3	RAN bind	GO:00081	metabolic	K15304	Ran-bindi	mmu0516	HTLV-I inf	17:566732	1131	1534	1192	1618	1776	1526	27.50168	29.78982	32.03462	30.35327	27.80957	29.19188	29.77538	29.11824	0.6228	1	-
ENSMUSC	Ndufa11	NADH de	GO:00160	membran	K03956|KC	NADH de	mmu0110	Metabolic	17:567177	418	539	391	538	628	595	9.59124	9.877175	9.915657	9.523815	9.279255	10.74054	9.794691	9.847871	0.866605	1	-
ENSMUSC	Bmp8b	bone mor	GO:00325	developm	K16622	bone mor	mmu0439	Hippo sig	4:1231051	2	5	3	6	4	7	0.031467	0.062836	0.052181	0.072842	0.040536	0.086665	0.048828	0.066681	0.607011	1	-
ENSMUSC	Nr2f6	nuclear re	GO:00325	developm	-	-	-	-	8:7137412	281	232	241	290	342	351	2.754867	1.816472	2.611315	2.193429	2.159113	2.707155	2.394218	2.353232	0.778418	1	-
ENSMUSC	Use1	unconven	GO:00511	localizatio	K08507	unconven	mmu0413	SNARE int	8:7136684	456	574	483	581	776	662	5.093758	5.120724	5.963036	5.007027	5.582005	5.817582	5.392506	5.468871	0.934678	1	-
ENSMUSC	Ocel1	occludin/E	-	-	-	-	-	-	8:7137129	292	330	278	374	480	379	1.898922	1.713893	1.998092	1.8764	2.010109	1.938982	1.870302	1.941831	0.910682	1	-
ENSMUSC	Dyrk1b	dual-spec	GO:00325	developm	-	-	-	-	7:2817946	376	475	313	572	549	454	6.732508	6.792479	6.194131	7.901612	6.330181	6.395228	6.573039	6.875674	0.865963	1	-
ENSMUSC	Braf	Braf trans	GO:00325	developm	K04365	B-Raf pro	mmu0520	Pathways	6:3960323	4014	5074	3491	4731	5904	5010	15.94167	16.09358	15.32333	14.49572	15.09932	15.65327	15.78619	15.08277	0.403385	1	-
ENSMUSC	Ndufb2	NADH de	GO:00081	metabolic	K03958	NADH de	mmu0110	Metabolic	6:3959257	401	482	310	572	672	487	3.440737	3.302937	2.939787	3.786467	3.713056	3.287359	3.22782	3.595627	0.467736	1	-
ENSMUSC	Hltf	helicase-l	GO:00081	metabolic	-	-	-	-	3:2005781	469	761	457	603	763	665	3.447242	4.467142	3.712463	3.419373	3.611423	3.845315	3.875616	3.625371	0.417053	1	-
ENSMUSC	Prpf6	pre-mRN.	GO:00081	metabolic	K12855	pre-mRN.	mmu0304	Spliceoso	2:1815918	1226	1590	1130	1663	2010	1587	15.56284	16.11911	15.85343	16.28624	16.43043	15.84842	15.84513	16.18837	0.981114	1	-
ENSMUSC	Rgs19	regulator	GO:00987	molecular	-	-	-	-	2:1816884	194	208	200	260	310	252	1.832781	1.569348	2.088262	1.895017	1.885927	1.872926	1.830131	1.884624	0.973729	1	-
ENSMUSC	Rgs20	regulator	GO:00987	molecular	-	-	-	-	1:4909576	218	319	194	311	273	272	2.35796	2.755601	2.319147	2.595197	1.901504	2.31451	2.477569	2.270404	0.39644	1	-
ENSMUSC	Snrpd1	small nucl	GO:00081	metabolic	K11087	small nucl	mmu0532	Systemic l	18:106177	632	735	531	738	1127	826	46.10362	42.82045	42.81138	41.53408	52.94154	47.40331	43.91182	47.29298	0.640482	1	-
ENSMUSC	Tchp	trichoplei	GO:00081	metabolic	-	-	-	-	5:1147077	159	245	150	242	271	227	1.042104	1.282407	1.086557	1.223655	1.143767	1.170445	1.137023	1.179289	0.931074	1	-
ENSMUSC	Tsc2	tuberous s	GO:00081	metabolic	K07207	tuberous s	mmu0415	PI3K-Akt s	17:245958	2299	2960	2250	3081	3501	3213	8.623986	8.867609	9.328208	8.916431	8.45699	9.481797	8.939934	8.951739	0.794054	1	-
ENSMUSC	Rpl3l	ribosomal	GO:00081	metabolic	K02925	large subu	mmu0301	Ribosome	17:247278	0	0	3	0	8	3	0	0	0.119165	0	0.185145	0.084805	0.039722	0.089983	0.419192	1	-
ENSMUSC	Slc9a3r2	solute car	GO:00051	structural	K13358	Na(+)/H(+	mmu0496	Aldostero	17:246392	539	788	600	872	1057	879	14.58145	17.02488	17.93947	18.19947	18.41372	18.70732	16.51527	18.44017	0.401266	1	-

ENSMUS(	Puf60	poly-U bi	GO:00081	metabolic	K12838	poly(U)-b	mmu0304	Spliceoso	15:760701	1988	2686	1946	2913	3137	2669	31.26025	33.73089	33.81938	35.33839	31.76473	33.01684	32.93684	33.37332	0.903628	1	-
ENSMUS(	Golga2	golgi auto	GO:00325	developm	-	-	-	-	2:3228738	2562	3116	2328	3458	4113	3533	13.97827	13.57743	14.03797	14.55559	14.45067	15.16453	13.86456	14.7236	0.677016	1	-
ENSMUS(	Uck1	uridine-cy	GO:00081	metabolic	K00876	uridine kir	mmu0110	Metabolic	2:3225500	489	631	458	687	815	683	6.670556	6.874295	6.905031	7.230034	7.159218	7.329677	6.816627	7.239643	0.728046	1	-
ENSMUS(	Ikzf4	IKAROS fa	GO:00081	metabolic	-	-	-	-	10:128630	224	386	133	269	352	300	1.16025	1.59675	0.76138	1.074946	1.174087	1.222464	1.172793	1.157165	0.825928	1	-
ENSMUS(	Mien1	migration	GO:00400	locomotic	-	-	-	-	11:984377	347	363	311	402	513	385	28.36301	23.69601	28.09507	25.3501	27.00192	24.75679	26.71803	25.70293	0.602657	1	-
ENSMUS(	Pon1	paraoxon	GO:00081	metabolic	-	-	-	-	6:5168090	5	14	0	2	8	1	0.093783	0.209735	0	0.028948	0.096639	0.01476	0.101172	0.046782	0.26025	1	-
ENSMUS(	Axl	AXL recep	GO:00325	developm	K05115	AXL recep	-	-	7:2575727	831	1049	795	1084	1187	1107	5.936506	5.984817	6.276876	5.97433	5.460531	6.221403	6.066066	5.885421	0.580907	1	-
ENSMUS(	Tgfb1	transform	GO:00325	developmental process|metabolic process|locomotion|localization|molecular function regulator|immune system process|response to stimulus|cellular process|reproductive process|extracellular region|cell|growth|reproduction|signaling|response to																						
ion|regula	K13375	transform	mmu0520	Pathways	7:2568700	79	129	79	128	140	127	0.844914	1.101837	0.933811	1.056148	0.964201	1.068555	0.960187	1.029635	-0.10074	0.776842	1	-			
ENSMUS(	Ccdc97	coiled-co	GO:00329	macromo	-	-	-	-	7:2571110	689	919	668	954	1127	934	6.406124	6.82398	6.864356	6.843135	6.747688	6.831782	6.698153	6.807535	0.940541	1	-
ENSMUS(	Zfp40	zinc finge	GO:00081	metabolic	-	-	-	-	17:231738	375	425	333	455	561	459	2.764977	2.502624	2.713633	2.588224	2.663654	2.662468	2.660411	2.638116	0.777793	1	-
ENSMUS(	Akap8l	A kinase (	GO:00081	metabolic	K15978	A-kinase a	mmu0516	Epstein-B	17:323214	1639	2025	1380	2157	2465	2237	48.22854	47.58784	44.87981	48.96725	46.7086	51.78479	46.89873	49.15355	0.799427	1	-
ENSMUS(	Shh	sonic hed	GO:00325	developm	K11988	sonic hed	mmu0520	Pathways	5:2845681	27	39	25	31	49	41	0.579062	0.667983	0.592567	0.512921	0.676712	0.691752	0.613204	0.627128	1	1	-
ENSMUS(	Pdcd2l	programn	GO:00099	cellular pr	-	-	-	-	7:3418449	88	122	83	122	127	126	2.356633	2.609251	2.456583	2.52056	2.190118	2.654534	2.474156	2.455071	0.834548	1	-
ENSMUS(	Gtf2f1	general tr	GO:00081	metabolic	K03138	transcripti	mmu0302	Basal tran	17:570034	1262	1486	907	1525	1869	1449	44.8779	42.20245	35.64732	41.83821	42.79933	40.53707	40.90923	41.72487	0.96582	1	-
ENSMUS(	Clpp	caseinolyt	GO:00081	metabolic	-	-	-	-	17:569902	444	604	451	662	688	709	22.46048	24.40165	25.215	25.83593	22.41192	28.21585	24.02571	25.4879	0.758257	1	-
ENSMUS(	Alkbh7	alkB homo	GO:00081	metabolic	-	-	-	-	17:569973	107	102	82	143	150	150	3.18383	2.423895	2.696648	3.282717	2.874173	3.511306	2.768125	3.222732	0.419113	1	-
ENSMUS(	Pspn	persephin	GO:00508	response	-	-	-	-	17:569994	0	0	1	0	0	1	0	0	0.142377	0	0	0.101359	0.047459	0.033786	1	1	-
ENSMUS(	Dennd1c	DENN/MA	GO:00987	molecular	-	-	-	-	17:570660	19	24	24	18	35	35	0.460356	0.464401	0.642679	0.336478	0.546086	0.667144	0.522479	0.516569	0.913991	1	-
ENSMUS(	Prkd1	protein ki	GO:00325	developm	K06070	protein kir	mmu0401	Rap1 sign	12:503412	200	259	206	270	275	273	3.342898	3.45732	3.805464	3.481666	2.959927	3.589777	3.535227	3.34379	0.530113	1	-
ENSMUS(	Lcp2	lymphocy	GO:00081	metabolic	K07361	lymphocy	mmu0401	Rap1 sign	11:340469	59	107	62	76	105	92	0.683057	0.989313	0.79331	0.678811	0.782792	0.837918	0.821893	0.766507	0.58444	1	-
ENSMUS(	Cse1l	chromosc	GO:00511	localizatio	-	-	-	-	2:1669060	1143	1344	1057	1308	1352	1473	8.743348	8.210638	8.936208	7.719155	6.659826	8.864315	8.630065	7.747765	0.193927	1	-
ENSMUS(	Naa20	N(alpha)-	GO:00081	metabolic	-	-	-	-	2:1459020	281	326	266	358	484	373	3.273671	3.033144	3.424977	3.217682	3.631024	3.418601	3.243931	3.422436	0.803717	1	-
ENSMUS(	Prkra	protein ki	GO:00325	developm	-	-	-	-	2:7662989	185	243	201	248	334	239	5.361061	5.623809	6.437554	5.544471	6.232745	5.448622	5.807475	5.741946	0.798917	1	-
ENSMUS(	Fkbp7	FK506 bin	GO:00081	metabolic	-	-	-	-	2:7666304	40	54	31	61	71	59	0.948115	1.022206	0.812096	1.115474	1.083704	1.100181	0.927472	1.099786	0.452286	1	-
ENSMUS(	Plekha3	pleckstrin	GO:00160	membran	-	-	-	-	2:7667528	913	1159	777	1230	1510	1191	17.5855	17.82845	16.54062	18.27761	18.72905	18.04707	17.31819	18.35124	0.722841	1	-
ENSMUS(	Ykt6	YKT6 v-SN	GO:00511	localizatio	K08516	synaptobr	mmu0413	SNARE int	11:595569	1109	1342	1079	1611	1788	1741	21.66137	20.93403	23.29285	24.27618	22.48933	26.75248	21.96275	24.50599	0.375929	1	-
ENSMUS(	Baz1b	bromodoı	GO:00081	metabolic	-	-	-	-	5:1351872	3401	4327	3049	4431	5322	4287	12.45778	12.65804	12.34349	12.52177	12.55346	12.35373	12.48644	12.47632	0.765543	1	-
ENSMUS(	Pex6	peroxison	GO:00511	localizatio	K13339	peroxin-6	mmu0414	Peroxison	17:467114	780	1076	722	1079	1188	1045	9.404681	10.36114	9.621297	10.03694	9.224034	9.912358	9.795708	9.724445	0.752586	1	-
ENSMUS(	Mrpl2	mitochon	GO:00081	metabolic	K02886	large subu	mmu0301	Ribosome	17:466462	226	369	312	325	410	331	5.069477	6.610393	7.734938	5.624306	5.922353	5.8411	6.471603	5.79592	0.315357	1	-
ENSMUS(	Mea1	male enha	GO:00325	developm	-	-	-	-	17:466811	548	763	588	838	948	744	39.83315	44.2929	47.23763	46.99359	44.37384	42.54493	43.7879	44.63745	0.97101	1	-
ENSMUS(	Gnmt	glycine N-	GO:00081	metabolic	K00552	glycine N-	mmu0026	Glycine, se	17:467256	2	7	2	4	3	11	0.077177	0.215767	0.085318	0.119091	0.07457	0.334	0.126087	0.175887	0.664425	1	-
ENSMUS(	Grin2d	glutamate	GO:00048	signal trar	K05212	glutamate	mmu0408	Neuroacti	7:4583188	268	271	181	348	353	311	1.726842	1.394548	1.288974	1.729928	1.464695	1.576482	1.470122	1.590368	0.691973	1	-
ENSMUS(	Kdelr1	KDEL (Lys	GO:00325	developm	-	-	-	-	7:4587277	354	450	293	470	567	490	5.098982	5.176526	4.664383	5.222853	5.259179	5.552483	4.979964	5.344838	0.684451	1	-
ENSMUS(	Tmem143	transmem	GO:00160	membran	-	-	-	-	7:4589694	221	264	232	349	331	295	0.962197	0.917955	1.116364	1.17227	0.928014	1.010427	0.998838	1.036903	0.918142	1	-
ENSMUS(	Ggct	gamma-c	GO:00508	response	K00682	gamma-c	mmu0048	Glutathior	6:5498258	267	325	239	301	404	299	1.794051	1.744023	1.774876	1.56034	1.748069	1.580539	1.770983	1.629649	0.369909	1	-
ENSMUS(	Jag2	jagged 2 (	GO:00325	developm	K06052	jagged[-]	mmu0520	Pathways	12:112907	600	655	526	821	777	795	3.977296	3.467559	3.85362	4.198655	3.316746	4.145858	3.766158	3.887086	0.956805	1	-
ENSMUS(	Nudt14	nudix (nu	GO:00038	catalytic a	-	-	-	-	12:112934	38	62	59	79	70	83	0.654497	0.85284	1.123119	1.049743	0.776392	1.124649	0.876819	0.983595	0.644209	1	-
ENSMUS(	Epdr1	ependymi	GO:00055	extracellul	-	-	-	-	13:195917	1067	1159	998	1402	1608	1219	15.83978	13.74087	16.37427	16.05695	15.37182	14.23637	15.3183	15.22171	0.762688	1	-
ENSMUS(	Flii	flightless I	GO:00325	developm	-	-	-	-	11:607141	1115	1404	1001	1659	1855	1645	11.54874	11.61375	11.45883	13.25672	12.37252	13.40406	11.54044	13.0111	0.296924	1	-
ENSMUS(	Top3a	topoisom	GO:00081	metabolic	K03165	DNA topo	mmu0346	Fanconi a	11:607400	103	151	115	131	206	194	0.849746	0.994891	1.048569	0.833784	1.094394	1.259114	0.964402	1.062431	0.647065	1	-
ENSMUS(	Atg4d	autophag	GO:00081	metabolic	-	-	mmu0414	Autophag	9:2126529	247	275	228	379	387	347	1.577714	1.402842	1.609583	1.867673	1.591831	1.743696	1.530046	1.7344	0.411741	1	-
ENSMUS(	Qtrt1	queuine t	GO:00081	metabolic	-	-	-	-	9:2141183	112	113	104	125	159	158	1.523387	1.227491	1.563414	1.311698	1.392665	1.690679	1.438097	1.465014	0.986205	1	-
ENSMUS(	Hdgfl2	HDGF like	GO:00099	cellular pr	-	-	-	-	17:560796	1383	1827	1366	1926	2292	1914	8.515868	8.984442	9.296173	9.149417	9.088165	9.271709	8.932161	9.169763	0.980037	1	-
ENSMUS(	Chaf1a	chromatir	GO:00081	metabolic	-	-	-	-	17:560404	85	118	79	140	176	127	1.56891	1.739423	1.611587	1.993591	2.091919	1.84413	1.639973	1.976547	0.26681	1	-
ENSMUS(	Adprh	ADP-ribo	GO:00081	metabolic	-	-	-	-	16:384453	736	972	783	1110	1373	1071	28.38836	29.94157	33.37883	33.03055	34.10262	32.49847	30.56959	33.21055	0.555315	1	-
ENSMUS(	Tmem39a	transmem	GO:00160	membran	-	-	-	-	16:385586	94	118	101	140	188	170	0.723492	0.725326	0.859159	0.831312	0.931792	1.029357	0.769326	0.93082	0.264197	1	-
ENSMUS(	Timmdc1	translocas	GO:00160	membran	-	-	-	-	16:384983	289	361	248	424	446	345	8.463216	8.442866	8.026671	9.579303	8.410595	7.948176	8.310918	8.646024	0.902648	1	-
ENSMUS(	Mcm2	minichron	GO:00325	developm	K02540	DNA repli	mmu0411	Cell cycle	6:8888347	124	150	90	173	227	148	0.845947	0.817253	0.678594	0.910537	0.997243	0.794314	0.780598	0.900698	0.42744	1	-
ENSMUS(	Tpra1	transmem	GO:00325	developm	-	-	-	-	6:8890225	123	193	139	193	209	218	0.683486	0.856502	0.853665	0.827395	0.747869	0.953	0.797884	0.842754	0.839029	1	-
ENSMUS(	Nab1	Ngfi-A bir	GO:00325	developm	-	-	-	-	1:5245729	683	954	590	937	1094	1000	7.13476	7.958904	6.811734	7.551421	7.359205	8.218065	7.301799	7.709564	0.769063	1	-
ENSMUS(	Adgre5	adhesion	GO:00048	signal trar	-	-	-	-	8:8372325	166	174	164	172	255	205	1.640605	1.373382	1.791375	1.31146	1.622896	1.593896	1.601787	1.509417	0.570372	1	-
ENSMUS(	Il17ra	interleukir	GO:00048	signal trar	K05164	interleukir	mmu0406	Cytokine-	6:1204632	164	204	152	262	280	217	0.859528	0.853873	0.880458	1.059375	0.944996	0.894723	0.86462	0.966365	0.497682	1	-
ENSMUS(	Lamb1	laminin B1	GO:00325	developm	K05636	laminin, b	mmu0520	Pathways	12:312652	888	1099	833	1094	1441	1148	4.435141	4.383673	4.598186	4.215433	4.634609	4.510736	4.472333	4.453593	0.772992	1	-
ENSMUS(	Kcnn1	potassium	GO:00511	localizatio	-	-	-	-	8:7084204	206	313	233	406	387	310	1.447904	1.75697	1.809989	2.201557	1.751619	1.714141	1.671621	1.889105	0.447422	1	-
ENSMUS(	Ppp1r17	protein ph	GO:00081	metabolic	K08067	protein ph	mmu0473	Long-term	6:5601749	0	3	8	5	11	9	0	0.083273	0.307244	0.134043	0.246154	0.246044	0.130172	0.208747	0.403675	1	-
ENSMUS(	Map2k7	mitogen-	GO:00048	signal trar	K04431	mitogen-a	mmu0401	MAPK sigr	8:4238740	618	879	258	659	863	653	4.089065	4.644837	1.886701	3.363967	3.677064	3.399068	3.540201	3.480033	0.817359	1	-
ENSMUS(	Timm44	translocas	GO:00511	localizatio	-	-	-	-	8:4259731	608	783	498	774	1008	865	5.684169	5.846164	5.145642	5.582579	6.068467	6.361959	5.558659	6.004335	0.608073	1	-
ENSMUS(	Ap2a2	adaptor-r	GO:00325	developm	K11824	AP-2 com	mmu0414	Endocytos	7:1415621	5030	6122	4676	6514	7573	6199	49.80092	48.40704	51.16709	49.75623	48.28275	48.28386	49.79169	48.77428	0.587506	1	-
ENSMUS(	Pnkp	polynucle	GO:00081	metabolic	-	-	-	-	7:4485713	215	278	202	304	442	251	1.547691	1.598217	1.607099	1.688299	2.048905	1.421443	1.584336	1.719549	0.661511	1	-
ENSMUS(	Med25	mediator	GO:00081	metabolic	-	-	-	-	7:4487676	934	1273	973	1470	1601	1327	5.146028	5.601436	5.924952	6.248459	5.6803	5.751844	5.557472	5.893534	0.716573	1	-
ENSMUS(	Bcam	basal cell	GO:00055	extracellul	-	-	-	-	7:1975613	210	298	161	259	315	330	3.401112	3.854466	2.88187	3.236177	3.285236	4.204624	3.37915	3.575345	0.824196	1	-
ENSMUS(	Relb	avian retic	GO:00325	developm	K09253	transcripti	mmu0401	MAPK sigr	7:1960621	79	132	75	156	174	124	0.807164	1.077091	0.846918	1.229668	1.14482	0.996703	0.910391	1.123731	0.230841	1	-
ENSMUS(	Tomm40	translocas	GO:00511	localizatio	K11518	mitochon	mmu0501	Amyotrop	7:1970131	731	946	721	1054	1252	1016	7.037757	7.27368	7.671832	7.828669	7.762043	7.69523	7.327756	7.761981	0.735981	1	-
ENSMUS(	Apoe	apolipopr	GO:00325	developm	K04524	apolipopr	mmu0501	Alzheimer	7:1969610	14238	23004	14430	20951	25316	24072	133.5195	172.284	149.5577	151.5761	152.8781	177.5901	151.787	160.6814	0.719408	1	-
ENSMUS(	Hbp1	high mob	GO:00056	cell|intrac	-	-	-	-	12:319262	335	438	295	468	593	523	2.662305	2.779923	2.591083	2.869387	3.034742	3.269828	2.67777	3.057986	0.337871	1	-
ENSMUS(	Prkar2b	protein ki	GO:00081	metabolic	K04739	cAMP-de	mmu0491	Insulin sig	12:319584	580	708	513	736	896	397	5.418253	5.282147	5.296577	5.304437	5.390064	2.917649	5.332326	4.537383	0.214882	1	-
ENSMUS(	Cdkn1b	cyclin-dep	GO:00325	developm	K06624	cyclin-dep	mmu0520	Pathways	6:1349204	565	1213	709	1087	873	1066	4.551168	7.803342	6.312003	6.755136	4.528381	6.755266	6.222171	6.012928	0.690019	1	-
ENSMUS(	Ap1m1	adaptor-r	GO:00325	developm	K12393	AP-1 com	mmu0414	Lysosome	8:7224001	1134	1544	1087	1727	1950	1670	13.43942	14.61371	14.23783	15.79029	14.88184	15.57022	14.09699	15.41412	0.472701	1	-
ENSMUS(	Rab8a	RAB8A, m	GO:00325	developm	K07901	Ras-relate	mmu0414	Endocytos	8:7216120	758	949	677	974	1284	909	9.226882	9.225666	9.107962	9.146924	10.06481	8.704835	9.186837	9.305523	0.919982	1	-
ENSMUS(	Fam32a	family wit	GO:00099	cellular pr	-	-	-	-	8:7221973	1186	1478	1058	1571	1954	1624	12.16853	12.11082	11.99736	12.4354	12.91019	13.10842	12.09224	12.818	0.707675	1	-
ENSMUS(	Stard3nl	STARD3 N	GO:00511	localizatio	-	-	-	-	13:193576	214	242	185	341	342	279	1.690573	1.526795	1.615242	2.078284	1.739805	1.733946	1.61087	1.850679	0.362604	1	-
ENSMUS(	Stk11	serine/thr	GO:00325	developm	K07298	serine/thr	mmu0415	PI3K-Akt s	10:801158	674	844	756	927	1148	958	4.962996	4.963319	6.152517	5.266158	5.443529	5.549587	5.359611	5.419758	0.907826	1	-
ENSMUS(	Efna2	ephrin A2	GO:00325	developm	K05462	ephrin-A[	mmu0415	PI3K-Akt s	10:801794	134	216	161	233	208	173	3.839422	4.942639	5.098382	5.150452	3.837763	3.899564	4.626814	4.295926	0.50717	1	-
ENSMUS(	Ppp5c	protein ph	GO:00048	signal trar	K04460	serine/thr	mmu0401	MAPK sigr	7:1700464	1546	1961	1449	1952	2494	2086	16.30044	16.51251	16.88515	15.87815	16.93327	17.30274	16.56604	16.70472	0.860414	1	-
ENSMUS(	Cdk12	cyclin-dep	GO:00325	developm	-	-	-	-	11:982030	694	922	637	841	1057	938	2.514499	2.667893	2.550811	2.350813	2.466162	2.673655	2.577734	2.496877	0.577033	1	-
ENSMUS(	Lipe	lipase, hor	GO:00081	metabolic	K07188	hormone-	mmu0402	cAMP sigr	7:2537952	116	188	112	213	234	196	0.732822	0.948519	0.781996	1.038128	0.951944	0.974112	0.821113	0.988061	0.247707	1	-
ENSMUS(	Pafah1b2	platelet-a	GO:00325	developm	K16795	platelet-a	mmu0110	Metabolic	9:4596285	3778	4770	3193	4808	5845	4906	22.98501	23.17645	21.46982	22.56719	22.89928	23.48124	22.54376	22.98257	0.954602	1	-
ENSMUS(	Cnot11	CCR4-NO	GO:00081	metabolic	-	-	-	-	1:3953499	436	633	418	618	704	623	3.434121	3.981796	3.638757	3.755331	3.570731	3.860368	3.684891	3.72881	0.91173	1	-
ENSMUS(	Slc2a3	solute car	GO:00511	localizatio	-	-	-	-	6:1227278	1727	2347	1635	2278	2739	2197	9.652815	10.47661	10.10011	9.823033	9.858444	9.660565	10.07651	9.780681	0.537769	1	-
ENSMUS(	Foxj2	forkhead	GO:00325	developm	-	-	-	-	6:1228199	812	1008	711	1049	1174	922	5.84522	5.794971	5.65668	5.825738	5.442118	5.221402	5.765624	5.496419	0.475452	1	-
ENSMUS(	Dgcr2	DiGeorge	GO:00160	membran	-	-	-	-	16:178394	1727	2208	891	2373	2722	2218	6.915487	7.06115	3.943258	7.330922	7.018967	6.987195	5.973298	7.112361	0.250434	1	-
ENSMUS(	Irf3	interferon	GO:00081	metabolic	K05411	interferon	mmu0520	Viral carci	7:4499764	292	287	217	388	469	388	2.126547	1.669247	1.746618	2.179986	2.199478	2.222973	1.847471	2.200812	0.223158	1	-
ENSMUS(	Bcl2l12	BCL2-like	GO:00325	developm	-	-	-	-	7:4499122	22	23	21	27	39	29	0.377853	0.315491	0.398638	0.35776	0.431341	0.391839	0.363994	0.393647	0.859633	1	-
ENSMUS(	Zfp959	zinc finge	GO:00081	metabolic	-	-	-	-	17:558920	74	22	53	7	27	77	1.389388	0.329889	1.099796	0.101395	0.32645	1.137351	0.939691	0.521732	0.19597	1	-
ENSMUS(	Mpnd	MPN dom	GO:00081	metabolic	-	-	-	-	17:560057	640	878	667	1045	1085	961	9.346381	10.24008	10.76554	11.77361	10.20346	11.04072	10.11734	11.00593	0.554492	1	-
ENSMUS(	Sh3gl1	SH3-dom	GO:00452	synapse|c	K11247	endophilir	mmu0414	Endocytos	17:560167	544	841	646	889	1073	820	16.0307	19.79227	21.03937	20.21091	20.36141	19.00983	18.95411	19.86071	0.828261	1	-
ENSMUS(	Ebi3	Epstein-B	GO:00048	signal trar	-	-	-	-	17:559526	7	13	11	9	14	6	0.365313	0.541808	0.634435	0.362361	0.47048	0.246338	0.513852	0.359726	0.291881	1	-
ENSMUS(	Ccdc94	coiled-co	-	-	-	-	-	-	17:559591	152	193	121	171	267	179	7.079197	7.178675	6.228358	6.144231	8.007668	6.558495	6.828743	6.903464	0.935522	1	-
ENSMUS(	Ranbp2	RAN bind	GO:00081	metabolic	K12172	E3 SUMO	mmu0301	RNA trans	10:584469	4216	5245	3709	4893	5968	5258	20.56735	20.43474	19.99777	18.41549	18.74827	20.17945	20.33329	19.1144	0.304694	1	-
ENSMUS(	Edar	ectodyspl	GO:00325	developm	K05162	tumor nec	mmu0406	Cytokine-	10:586007	2	8	7	4	11	6	0.03357	0.107262	0.12989	0.051801	0.118914	0.079245	0.090241	0.08332	1	1	-
ENSMUS(	Grk5	G protein-	GO:00325	developm	K08291	G protein-	mmu0414	Endocytos	19:608897	157	222	122	197	204	190	3.028799	3.420327	2.601221	2.93201	2.534279	2.883594	3.016783	2.783294	0.450032	1	-
ENSMUS(	Dvl3	dishevelle	GO:00325	developm	K02353	segment p	mmu0520	Pathways	16:205169	737	859	634	971	1086	999	6.267389	5.833886	5.958747	6.370423	5.947074	6.68337	6.020007	6.333622	0.795451	1	-
ENSMUS(	Abcf3	ATP-bind	GO:00023	immune s	-	-	-	-	16:205485	1020	1383	1034	1380	1665	1533	13.45706	14.57197	15.07709	14.04622	14.14552	15.91121	14.3687	14.70098	0.991909	1	-
ENSMUS(	Eif2b5	eukaryotic	GO:00325	developm	K03240	translatior	mmu0301	RNA trans	16:204988	971	1266	883	1265	1635	1265	16.26094	16.93192	16.34309	16.3436	17.6319	16.66588	16.51198	16.88046	0.988589	1	-
ENSMUS(	Cyth2	cytohesin	GO:00987	molecular	K18441	cytohesin[	mmu0414	Endocytos	7:4580663	1118	1499	1150	1580	1807	1546	11.59751	12.41854	13.18462	12.64476	12.07079	12.61663	12.40023	12.44406	0.827996	1	-
ENSMUS(	Sult2b1	sulfotrans	GO:00325	developm	K01015	alcohol su	mmu0014	Steroid hc	7:4572998	120	118	147	143	211	161	1.060958	0.83319	1.436418	0.975403	1.201309	1.119836	1.110189	1.09885	0.860513	1	-
ENSMUS(	Car11	carbonic a	GO:00055	extracellul	-	-	-	-	7:4569984	1628	1908	1601	2182	2646	2207	32.88211	30.77722	35.73907	34.00088	34.41515	35.06861	33.1328	34.49488	0.858722	1	-
ENSMUS(	Dlgap1	discs, larg	GO:00452	synapse|c	K15008	discs, larg	mmu0472	Glutamate	17:699690	9765	12062	8889	12397	14771	12662	18.37841	18.13013	18.48994	18.00042	17.90195	18.74774	18.33283	18.2167	0.709359	1	-
ENSMUS(	Plag1	pleiomorp	GO:00325	developm	-	-	-	-	4:3900996	72	91	47	83	115	78	0.460817	0.465137	0.332461	0.409831	0.473965	0.392737	0.419472	0.425511	0.979702	1	-
ENSMUS(	Mrpl4	mitochon	GO:00081	metabolic	K02926	large subu	mmu0301	Ribosome	9:2100273	343	508	401	571	579	528	4.493225	5.314638	5.805714	5.770732	4.884244	5.441378	5.204526	5.365451	0.959404	1	-
ENSMUS(	Keap1	kelch-like	GO:00325	developm	K10456	kelch-like	mmu0520	Pathways	9:2122973	1115	1114	925	1542	1420	1176	7.346476	5.861855	6.735853	7.838245	6.024865	6.095683	6.648061	6.652931	0.829188	1	-
ENSMUS(	Glg1	golgi app	GO:00325	developm	K06816	golgi app	mmu0451	Cell adhes	8:1111544	6201	8042	5554	7689	9300	8364	41.9711	43.47089	41.54716	40.15031	40.53464	44.53624	42.32972	41.7404	0.646539	1	-
ENSMUS(	Atp8b3	ATPase, cl	GO:00511	localizatio	-	-	-	-	10:805195	5	6	4	5	1	7	0.053696	0.051464	0.047484	0.041427	0.006915	0.059149	0.050881	0.03583	0.536592	1	-
ENSMUS(	Btbd2	BTB (POZ	GO:00325	developm	-	-	-	-	10:806426	1009	1398	1018	1470	1739	1528	10.85215	12.00818	12.10094	12.19754	12.04422	12.92882	11.65376	12.39019	0.692663	1	-
ENSMUS(	Csnk1g2	casein kin	GO:00325	developm	K08958	casein kin	mmu0434	Hedgehog	10:806228	1840	2247	1681	2445	2890	2461	13.52599	13.19169	13.65733	13.8663	13.68056	14.23226	13.45834	13.92638	0.909218	1	-
ENSMUS(	Mob3a	MOB kina	GO:00056	cell|intrac	-	-	-	-	10:806852	165	171	101	174	226	219	2.483259	2.055323	1.679987	2.020304	2.190292	2.592941	2.072856	2.267846	0.679572	1	-
ENSMUS(	Cacnb3	calcium cl	GO:00511	localizatio	K04864	voltage-d	mmu0401	MAPK sigr	15:986322	3267	4059	3152	4625	5500	4325	63.93489	63.4386	68.17446	69.8282	69.31164	66.58643	65.18265	68.57542	0.750896	1	-
ENSMUS(	Ccdc65	coiled-co	GO:00511	localizatio	-	-	-	-	15:987082	72	138	91	154	158	167	2.32603	3.56046	3.249156	3.838241	3.286944	4.244341	3.045215	3.789842	0.228115	1	-
ENSMUS(	Fkbp11	FK506 bin	GO:00081	metabolic	-	-	-	-	15:987243	2	3	11	11	11	5	0.167022	0.200151	1.015443	0.708873	0.591644	0.328552	0.460872	0.543023	0.838372	1	-
ENSMUS(	Ddx23	DEAD (As	GO:00081	metabolic	K12858	ATP-depe	mmu0304	Spliceoso	15:986451	1216	1595	1009	1585	1975	1555	8.668614	9.080769	7.949762	8.717169	9.066461	8.720817	8.566382	8.834816	0.941759	1	-
ENSMUS(	Pld3	phospholi	GO:00081	metabolic	K16860	phospholi	mmu0110	Metabolic	7:2753200	5784	7356	5747	8086	9072	7172	76.85747	78.06302	84.40065	82.89377	77.62749	74.97371	79.77372	78.49832	0.637556	1	-
ENSMUS(	Grik5	glutamate	GO:00048	signal trar	K05205	glutamate	mmu0408	Neuroacti	7:2500984	3138	4128	3141	4562	5290	4547	27.87725	29.28752	30.83981	31.26674	30.26268	31.77846	29.33486	31.10263	0.681376	1	-
ENSMUS(	Rabac1	Rab accep	GO:00452	synapse|m	-	-	-	-	7:2496975	422	552	485	648	675	629	8.866667	9.262587	11.26254	10.50396	9.132841	10.39704	9.797263	10.01128	0.987633	1	-
ENSMUS(	Etv3	ets varian	GO:00325	developm	-	-	-	-	3:8752540	563	610	429	658	784	351	1.882981	1.629347	1.585776	1.697829	1.688528	0.923541	1.699368	1.436633	0.211234	1	-
ENSMUS(	Prkcsh	protein ki	GO:00325	developm	K08288	protein kir	mmu0414	Protein pr	9:2200280	1215	1642	1102	1735	2113	1613	14.23361	15.36236	14.26814	15.68082	15.94018	14.86566	14.62137	15.49555	0.708333	1	-
ENSMUS(	Elavl3	ELAV (em	GO:00325	developm	-	-	-	-	9:2201500	4174	5481	3704	5571	6318	5438	32.15856	33.72482	31.54001	33.11369	31.34573	32.96053	32.47446	32.47331	0.770778	1	-
ENSMUS(	Rab3b	RAB3B, m	GO:00081	metabolic	-	-	-	-	4:1088790	820	972	771	1265	1206	1190	9.44851	8.944614	9.818627	11.24528	8.94852	10.78716	9.403917	10.32699	0.508773	1	-
ENSMUS(	St8sia6	ST8 alpha	GO:00081	metabolic	-	-	-	-	2:1365102	150	227	141	173	229	179	1.174046	1.418945	1.219713	1.044649	1.154204	1.10219	1.270901	1.100348	0.211372	1	-
ENSMUS(	Fcgrt	Fc recepto	GO:00048	signal trar	-	-	-	-	7:4509299	151	169	184	302	217	184	2.195701	1.962587	2.957081	3.387931	2.031947	2.104872	2.37179	2.50825	0.860721	1	-
ENSMUS(	Nosip	nitric oxid	GO:00325	developm	-	-	-	-	7:4506242	504	671	509	836	859	669	6.643622	7.063873	7.415475	8.501812	7.291594	6.937643	7.04099	7.577017	0.648404	1	-
ENSMUS(	Pih1d1	PIH1 dom	GO:00325	developm	-	-	-	-	7:4515430	197	263	224	341	378	342	3.337524	3.558442	4.194245	4.457009	4.123873	4.558217	3.696737	4.3797	0.243176	1	-
ENSMUS(	Supt5	suppresso	GO:00081	metabolic	-	-	-	-	7:2831489	2455	3369	2272	3401	3961	3451	19.93055	21.84312	20.38557	21.30123	20.70748	22.04061	20.71974	21.34977	0.945221	1	-
ENSMUS(	Dll3	delta-like	GO:00325	developm	K06051	delta[-]	mmu0520	Pathways	7:2829355	20	22	19	16	46	35	0.318262	0.279593	0.334163	0.196429	0.471375	0.438155	0.310673	0.368653	0.613976	1	-
ENSMUS(	Paf1	Paf1, RNA	GO:00325	developm	-	-	-	-	7:2839295	1167	1369	913	1347	1623	1195	11.00971	10.31464	9.519681	9.803991	9.860022	8.86918	10.28134	9.511065	0.29604	1	-
ENSMUS(	Timm50	translocas	GO:00081	metabolic	-	-	-	-	7:2830551	346	353	277	364	449	391	5.827887	4.7485	5.156578	4.730068	4.870086	5.181113	5.244322	4.927089	0.468788	1	-
ENSMUS(	Med29	mediator	GO:00081	metabolic	-	-	-	-	7:2838614	129	192	150	251	196	246	6.1487	7.308722	7.901923	9.229927	6.015949	9.22444	7.119781	8.156772	0.476953	1	-
ENSMUS(	Ncstn	nicastrin [	GO:00081	metabolic	K06171	nicastrin[-	mmu0501	Alzheimer	1:1720660	982	1212	885	1371	1558	1316	12.3729	12.19574	12.32395	13.32685	12.64102	13.04448	12.29753	13.00412	0.731905	1	-
ENSMUS(	Pex19	peroxison	GO:00511	localizatio	K13337	peroxin-1	mmu0414	Peroxison	1:1721267	1021	1363	794	1443	1239	1349	10.31954	11.00212	8.869553	11.25204	8.064182	10.72648	10.06374	10.01423	0.789961	1	-
ENSMUS(	Phyhip	phytanoyl	GO:00055	protein bi	-	-	-	-	14:704574	10189	12821	9797	13930	16031	13796	206.6161	207.6349	219.5698	217.9285	209.3379	220.0882	211.2736	215.7849	0.970924	1	-
ENSMUS(	Crhr2	corticotro	GO:00325	developm	K04579	corticotro	mmu0408	Neuroacti	6:5509004	29	42	23	33	35	26	0.210795	0.243816	0.18477	0.185061	0.163826	0.148679	0.213127	0.165855	0.188839	1	-
ENSMUS(	Inmt	indolethyl	GO:00081	metabolic	K00562	methyltrai	mmu0038	Tryptopha	6:5517062	12	21	10	12	21	19	0.367074	0.513041	0.338077	0.283192	0.413664	0.457236	0.406064	0.384697	0.810094	1	-
ENSMUS(	Impdh1	inosine m	GO:00081	metabolic	K00088	IMP dehy	mmu0110	Metabolic	6:2920043	598	800	553	691	1004	823	4.895123	5.229963	5.003053	4.36386	5.292382	5.299972	5.042713	4.985405	0.740482	1	-
ENSMUS(	Prodh	proline de	GO:00081	metabolic	-	-	-	-	16:180603	374	439	341	478	655	528	4.955676	4.645599	4.993812	4.886398	5.588905	5.503963	4.865029	5.326422	0.555979	1	-
ENSMUS(	Dgcr14	DiGeorge	GO:00329	macromo	-	-	-	-	16:179007	429	505	381	609	689	529	9.29193	8.735462	9.120526	10.17645	9.609967	9.013943	9.049306	9.60012	0.75272	1	-
ENSMUS(	Dgcr6	DiGeorge	-	-	-	-	-	-	16:180528	383	471	332	558	649	572	3.422902	3.361728	3.279289	3.847332	3.735037	4.021635	3.35464	3.868001	0.280804	1	-
ENSMUS(	Ddr1	discoidin	GO:00325	developm	-	-	-	-	17:356815	802	1202	861	1127	1358	1083	7.248169	8.67569	8.600107	7.857924	7.903297	7.70004	8.174655	7.82042	0.496187	1	-
ENSMUS(	Ier3	immediat	GO:00508	response	-	-	-	-	17:358216	32	50	43	64	83	64	1.727544	2.155722	2.565637	2.665562	2.885467	2.718158	2.149634	2.756396	0.223279	1	-
ENSMUS(	Fosb	FBJ osteo	GO:00081	metabolic	K09029	protein Fo	mmu0503	Alcoholisr	7:1930269	161	185	34	203	180	136	0.594877	0.545908	0.138845	0.578668	0.428281	0.395324	0.426543	0.467424	0.807964	1	-
ENSMUS(	Ercc1	excision re	GO:00325	developm	K10849	DNA excis	mmu0152	Platinum c	7:1934477	217	246	192	290	315	296	2.80712	2.541457	2.745045	2.89421	2.624013	3.012336	2.697874	2.84352	0.822854	1	-
ENSMUS(	As3mt	arsenic (+	GO:00081	metabolic	-	-	-	-	19:467074	101	146	124	181	206	131	3.521918	4.065895	4.778891	4.869282	4.625707	3.593694	4.122235	4.362894	0.833646	1	-
ENSMUS(	Homer3	homer sca	GO:00452	synapse|re	K15010	homer[-]	mmu0406	FoxO sign	8:7028282	832	817	803	1164	1446	1027	8.392587	6.581735	8.952313	9.058491	9.392811	8.149932	7.975545	8.867078	0.467895	1	-
ENSMUS(	Crtc1	CREB regu	GO:00325	developm	K15309	CREB-reg	mmu0516	HTLV-I inf	8:7038235	4214	4797	3670	5338	6030	5157	20.90331	19.00361	20.12025	20.42817	19.26161	20.12466	20.00906	19.93815	0.741751	1	-
ENSMUS(	Rnf215	ring finge	GO:00160	membran	-	-	-	-	11:413520	244	315	269	330	437	381	4.84022	4.990346	5.897585	5.050316	5.582267	5.945807	5.242717	5.52613	0.817166	1	-
ENSMUS(	Sec14l2	SEC14-lik	GO:00081	metabolic	-	-	-	-	11:409703	1139	1405	1060	1377	1664	1490	14.31561	14.10288	14.72443	13.35212	13.46771	14.73272	14.38097	13.85085	0.510069	1	-
ENSMUS(	Cp	ceruloplas	GO:00081	metabolic	K13624	ceruloplas	mmu0421	Ferroptos	3:1995705	290	501	293	375	544	516	1.211555	1.671583	1.352878	1.208665	1.463519	1.69592	1.412006	1.456035	0.963875	1	-
ENSMUS(	Crot	carnitine (	GO:00081	metabolic	K05940	carnitine (	mmu0414	Peroxison	5:8966033	444	530	417	631	652	533	4.681368	4.462844	4.859291	5.132746	4.426825	4.421073	4.667834	4.660215	0.821037	1	-
ENSMUS(	Calb2	calbindin	GO:00452	synapse|c	-	-	-	-	8:1101375	557	862	565	887	1005	853	17.84332	22.05329	20.00396	21.92171	20.732	21.49713	19.96685	21.38361	0.665108	1	-
ENSMUS(	Snrnp200	small nucl	GO:00325	developm	K12854	pre-mRNA	mmu0304	Spliceoso	2:1272083	2807	3533	2682	3389	4132	3425	18.15381	18.24798	19.17039	16.90936	17.20839	17.42597	18.52406	17.18124	0.248768	1	-
ENSMUS(	Ciao1	cytosolic i	GO:00081	metabolic	-	-	-	-	2:1272409	958	1080	877	1206	1486	1211	13.8087	12.43245	13.97121	13.41112	13.79307	13.73227	13.40412	13.64549	0.95036	1	-
ENSMUS(	Has1	hyalurona	GO:00081	metabolic	-	-	-	-	17:178433	13	8	2	10	6	12	0.379788	0.186657	0.064581	0.225394	0.112871	0.275805	0.210342	0.20469	1	1	-
ENSMUS(	Taf6l	TATA-box	GO:00081	metabolic	K03131	transcripti	mmu0516	Herpes sir	19:877252	171	225	178	252	328	249	0.895986	0.94153	1.030796	1.018677	1.10671	1.0264	0.956104	1.050596	0.581033	1	-
ENSMUS(	Insig2	insulin ind	GO:00325	developm	-	-	-	-	1:1213043	624	764	492	727	939	762	5.036383	4.924628	4.388809	4.526885	4.880391	4.838385	4.783273	4.748554	0.762843	1	-
ENSMUS(	Kpna6	karyopher	GO:00081	metabolic	-	-	-	-	4:1296439	2039	2466	1802	2559	3128	2506	15.53306	15.00302	15.17195	15.03974	15.3448	15.01868	15.23601	15.13441	0.723614	1	-
ENSMUS(	Man1a	mannosid	GO:00081	metabolic	K01230	mannosyl	mmu0110	Metabolic	10:539047	1436	1818	1228	1612	2205	1666	7.24444	7.324704	6.846925	6.274018	7.163306	6.612052	7.13869	6.683126	0.330943	1	-
ENSMUS(	Itpkc	inositol 1,4	GO:00081	metabolic	K00911	1D-myo-	mmu0110	Metabolic	7:2720717	157	167	141	224	272	189	1.298233	1.102846	1.288594	1.428995	1.448354	1.229487	1.229891	1.368945	0.53729	1	-
ENSMUS(	Coq8b	coenzyme	GO:00325	developm	-	-	-	-	7:2723302	308	366	366	549	640	433	2.150721	2.041083	2.824625	2.957578	2.877848	2.378659	2.33881	2.738028	0.300865	1	-
ENSMUS(	Brd8	bromodoı	GO:00081	metabolic	-	-	-	-	18:345986	1127	1496	1071	1466	1830	1441	5.51603	5.847636	5.793472	5.535629	5.767778	5.548528	5.719046	5.617312	0.653125	1	-
ENSMUS(	Farsa	phenylala	GO:00081	metabolic	K01889	phenylala	mmu0097	Aminoacy	8:8485698	480	524	445	727	715	719	4.411847	3.846418	4.520496	5.155183	4.231944	5.198988	4.259587	4.862038	0.343646	1	-

ENSMUSC	Gcdh	glutaryl-C	GO:00081	metabolic	K00252	glutaryl-C	mmu011C	Metabolic	8:8488639	334	487	364	515	612	470	5.078041	5.913222	6.116422	6.040696	5.991777	5.621586	5.702562	5.884686	0.947328	1	-
ENSMUSC	Mast2	microtubu	GO:00081	metabolic	-	-	-	-	4:1163067	1877	2446	1629	2532	2712	2834	7.233863	7.528497	6.938633	7.528352	6.730545	8.592445	7.233664	7.617114	0.774353	1	-
ENSMUSC	Dnase2a	deoxyribo	GO:00325	developm	K01158	deoxyribo	mmu0414	Lysosome	8:8490856	40	76	51	56	88	71	0.261689	0.397081	0.368759	0.282642	0.370733	0.365418	0.34251	0.339598	0.88241	1	-
ENSMUSC	Rad23a	RAD23 hc	GO:00081	metabolic	K10839	UV excisic	mmu0414	Protein pr	8:8483401	803	1063	723	993	1272	1094	10.5011	11.10193	10.44972	10.01842	10.71177	11.25506	10.68425	10.66175	0.793369	1	-
ENSMUSC	Calr	calreticulir	GO:00325	developm	K08057	calreticulir	mmu0516	HTLV-I inf	8:8484185	7051	9550	6196	11274	13409	9053	101.3228	109.5988	98.40447	124.987	124.0817	102.3434	103.1087	117.1374	0.252346	1	-
ENSMUSC	Syce2	synaptone	GO:00099	cellular pr	-	-	-	-	8:8487211	43	78	45	72	55	72	0.882526	1.278485	1.020742	1.140034	0.726904	1.162518	1.060585	1.009819	0.706137	1	-
ENSMUSC	Nfat5	nuclear fa	GO:00081	metabolic	-	-	-	-	8:1072934	981	1497	1966	1508	1666	1799	1.504748	1.833844	3.332922	1.784541	1.645601	2.170885	2.223838	1.867009	0.222483	1	-
ENSMUSC	Nob1	NIN1/RPN	GO:00081	metabolic	K11883	RNA-binc	mmu0300	Ribosome	8:1074124	359	360	330	367	558	299	3.71334	2.973845	3.772522	2.928644	3.716718	2.433061	3.486569	3.026141	0.241633	1	-
ENSMUSC	Nqo1	NAD(P)H	GO:00325	developm	K00355	NAD(P)H	mmu052C	Pathways	8:1073882	60	86	64	71	141	91	2.358955	2.700327	2.781	2.153566	3.569821	2.81464	2.613427	2.846009	0.730449	1	-
ENSMUSC	Ppfia3	protein ty	GO:00452	synapse|c	-	-	-	-	7:4533912	3196	3665	3020	3914	4705	3749	14.47961	13.26081	15.12181	13.68048	13.72665	13.36215	14.28741	13.58976	0.398059	1	-
ENSMUSC	Gys1	glycogen	GO:00325	developm	K00693	glycogen	mmu011C	Metabolic	7:4543484	231	397	209	271	448	314	1.99723	2.741272	1.997143	1.807652	2.4943	2.135779	2.245215	2.14591	0.62971	1	-
ENSMUSC	Ruvbl2	RuvB-like	GO:00081	metabolic	-	-	-	-	7:4542176	348	538	414	491	703	521	5.183763	6.400205	6.815734	5.642568	6.743342	6.105404	6.133234	6.163771	0.886605	1	-
ENSMUSC	Lin7b	lin-7 hom	GO:00452	synapse|c	-	-	-	-	7:4536789	337	440	385	502	529	473	7.476956	7.796381	9.440665	8.592686	7.557977	8.255949	8.238	8.135537	0.76297	1	-
ENSMUSC	Bax	BCL2-assc	GO:00325	developm	K02159	apoptosis	mmu052C	Pathways	7:4546169	281	279	232	344	485	364	6.64497	5.269113	6.063472	6.275891	7.385556	6.771723	5.992518	6.811057	0.407171	1	-
ENSMUSC	Tfam	transcripti	GO:00081	metabolic	K11830	transcripti	mmu0501	Huntingto	10:712254	510	702	481	604	818	594	5.861031	6.442978	6.109351	5.355138	6.053572	5.370325	6.137787	5.593012	0.275018	1	-
ENSMUSC	Zfp81	zinc finge	GO:00081	metabolic	-	-	-	-	17:333293	697	846	674	855	1046	863	6.410229	6.213795	6.850893	6.066483	6.194798	6.243992	6.491639	6.168425	0.454614	1	-
ENSMUSC	Efnb3	ephrin B3	GO:00325	developm	K05463	ephrin-B[	mmu0436	Axon guic	11:695540	1713	2424	1691	2616	2858	2448	32.85973	37.13515	35.85065	38.7146	35.30397	36.94274	35.28184	36.98711	0.791975	1	-
ENSMUSC	Mmd	monocyte	GO:00325	developm	-	-	-	-	11:902494	7263	8660	6721	9681	11534	9378	113.3892	107.974	115.9675	116.602	115.9552	115.1799	112.4436	115.9124	0.940955	1	-
ENSMUSC	Rpl8	ribosomal	GO:00081	metabolic	K02938	large subu	mmu0301	Ribosome	15:769040	1518	2194	1634	2316	2798	2441	107.5246	124.1134	127.9189	126.5625	127.6258	136.0238	119.8523	130.0707	0.522066	1	-
ENSMUSC	Ssbp2	single-stra	GO:00081	metabolic	-	-	-	-	13:914602	1362	1643	1185	1834	1860	1720	8.715276	8.396287	8.380486	9.053858	7.664285	8.658511	8.49735	8.458885	0.75637	1	-
ENSMUSC	Fancl	Fanconi a	GO:00081	metabolic	K10606	E3 ubiquit	mmu0412	Ubiquitin	11:263861	67	86	74	91	132	80	1.000703	1.025833	1.22155	1.04859	1.269584	0.940007	1.082695	1.08606	0.942788	1	-
ENSMUSC	Gstm5	glutathion	GO:00081	metabolic	K00799	glutathion	mmu052C	Pathways	3:1078958	939	1144	976	1262	1447	1189	22.98859	22.36757	26.40849	23.83617	22.81235	22.90023	23.92155	23.18292	0.57341	1	-
ENSMUSC	Stat3	signal trar	GO:00325	developm	K04692	signal trar	mmu052C	Pathways	11:100885	765	1147	849	1089	1410	1189	2.967753	3.553656	3.64016	3.259302	3.522412	3.628767	3.387189	3.47016	0.996373	1	-
ENSMUSC	Stat5a	signal trar	GO:00325	developm	K11223	signal trar	mmu052C	Pathways	11:100859	43	80	32	52	85	73	0.263051	0.390846	0.216356	0.245416	0.334845	0.351321	0.290084	0.310527	0.864687	1	-
ENSMUSC	Cavin1	caveolae a	GO:00081	metabolic	-	-	-	-	11:100956	413	612	384	628	780	661	6.077856	7.192782	6.245655	7.130006	7.391786	7.652653	6.505431	7.391482	0.335584	1	-
ENSMUSC	Map3k11	mitogen-a	GO:00048	signal trar	K04419	mitogen-a	mmu0401	MAPK sigr	19:568913	350	459	306	497	577	471	5.622294	5.888497	5.43267	6.159316	5.96865	5.952193	5.64782	6.02672	0.71725	1	-
ENSMUSC	Akt2	thymoma	GO:00325	developm	K04456	RAC serine	mmu052C	Pathways	7:2759155	679	908	709	1074	1030	952	3.268819	3.491027	3.772371	3.988928	3.193104	3.605532	3.510739	3.595855	1	1	-
ENSMUSC	Dnaja3	DnaJ heat	GO:00325	developm	K09504	DnaJ hom	mmu052C	Viral carci	16:463998	1248	1685	1157	1697	1938	1530	16.39074	17.67379	16.79439	17.19475	16.3905	15.80833	16.95297	16.46453	0.565093	1	-
ENSMUSC	Hmox2	heme oxy	GO:00081	metabolic	K00510	heme oxy	mmu011C	Metabolic	16:472636	811	1146	737	1170	1350	1099	12.32715	13.91143	12.381	13.72012	13.21387	13.14165	12.87319	13.35855	0.895019	1	-
ENSMUSC	Cdip1	cell death	GO:00508	response	-	-	-	-	16:475034	2675	3545	2555	3725	4242	2110	15.79446	16.7164	16.67318	16.96826	16.12895	9.801083	16.39468	14.29943	0.208383	1	-
ENSMUSC	Map3k20	mitogen-a	GO:00325	developm	K04424	sterile alpl	mmu0401	MAPK sigr	2:7228563	263	355	245	323	431	322	1.467047	1.581473	1.510428	1.390018	1.548174	1.413039	1.519649	1.450411	0.555223	1	-
ENSMUSC	Cwc15	CWC15 sp	GO:00081	metabolic	K12863	protein CV	mmu0304	Spliceosor	9:1450061	812	1057	630	1041	1321	902	20.46189	21.27212	17.54596	20.23816	21.43619	17.88163	19.75999	19.85199	0.863327	1	-
ENSMUSC	Col5a3	collagen,	GO:00051	structural	K19721	collagen, t	mmu0497	Protein di	9:2077005	46	70	54	62	84	77	0.406055	0.493477	0.526821	0.422231	0.477486	0.534721	0.475451	0.478146	0.951037	1	-
ENSMUSC	Dnmt1	DNA metl	GO:00325	developm	K00558	DNA (cytc	mmu011C	Metabolic	9:2090720	1165	1415	951	1469	1722	1474	7.68255	7.45215	6.931174	7.473626	7.312519	7.646939	7.355291	7.477695	0.935078	1	-
ENSMUSC	Ppan	peter pan	GO:00508	response	-	-	-	-	9:2088817	242	328	227	342	409	297	1.817471	1.967307	1.884197	1.981571	1.978019	1.754769	1.889658	1.904786	0.903784	1	-
ENSMUSC	Angptl2	angiopoie	GO:00055	extracellul	-	-	-	-	2:3321606	82	234	80	86	142	156	0.564717	1.286999	0.608912	0.456923	0.629736	0.845186	0.820209	0.643948	0.240975	1	-
ENSMUSC	Cacna1e	calcium cl	GO:00511	localizatio	K04852	voltage-d	mmu0401	MAPK sigr	1:1543907	11334	14677	10729	14641	17927	14890	20.25145	20.94383	21.18746	20.18246	20.62696	20.93045	20.79425	20.57996	0.676428	1	-
ENSMUSC	Cacna1b	calcium cl	GO:00452	synapse|c	K04849	voltage-d	mmu0401	MAPK sigr	2:2460388	2201	2428	2022	2861	2877	2571	7.638337	6.729351	7.755445	7.659969	6.429442	7.019268	7.374378	7.036226	0.443917	1	-
ENSMUSC	Trmt10a	tRNA met	GO:00081	metabolic	-	-	-	-	3:1381434	94	125	73	147	122	130	0.479527	0.509262	0.411581	0.578539	0.400775	0.521724	0.46679	0.500346	0.794019	1	-
ENSMUSC	Kifc2	kinesin far	GO:00990	supramole	-	-	-	-	15:766606	8861	10894	8095	12393	13974	11910	172.14	169.0177	173.8051	185.7401	174.8133	182.0211	171.6543	180.8582	0.734432	1	-
ENSMUSC	Psap	prosaposi	GO:00325	developm	K12382	saposin[-]	mmu0414	Lysosome	10:602776	22469	31192	21725	30381	36589	32448	343.9244	381.3014	367.5242	358.7663	360.6488	390.7313	364.25	370.0488	0.922352	1	-
ENSMUSC	Ikbkg	inhibitor c	GO:00081	metabolic	K07210	inhibitor c	mmu052C	Pathways	X:7439329	262	661	494	739	807	546	0.713302	1.437208	1.486436	1.552198	1.414817	1.169435	1.212315	1.378816	0.514829	1	-
ENSMUSC	Pax2	paired bo	GO:00325	developm	-	-	-	-	19:447560	6	11	8	12	10	7	0.046308	0.067801	0.068238	0.071451	0.049701	0.042505	0.060782	0.054552	0.73545	1	-
ENSMUSC	Atn1	atrophin 1	GO:00325	developm	-	-	-	-	6:1247425	3345	3986	2676	3971	5193	3545	39.92169	37.99234	35.29765	36.5631	39.91038	33.28434	37.73723	36.58594	0.554527	1	-
ENSMUSC	Phb2	prohibitin	GO:00325	developm	-	-	-	-	6:1247123	952	1261	904	1433	1534	1260	11.98501	12.67834	12.57816	13.91804	12.43603	12.4791	12.41384	12.94439	0.850393	1	-
ENSMUSC	Ptpn6	protein ty	GO:00325	developm	K05697	tyrosine-p	mmu052C	Proteogly	6:1247207	83	143	80	124	180	123	1.012345	1.392943	1.078429	1.16682	1.413773	1.180234	1.161239	1.253609	0.756752	1	-
ENSMUSC	Eno2	enolase 2	GO:00081	metabolic	K01689	enolase[4	mmu011C	Metabolic	6:1247600	6625	8561	6734	9485	11529	9219	31.43046	32.43659	35.30894	34.71622	35.22177	34.40801	33.05866	34.782	0.748399	1	-
ENSMUSC	Emg1	EMG1 N1	GO:00325	developm	K14568	rRNA sma	-	-	6:1247040	233	358	248	392	470	343	1.85507	2.276322	2.182241	2.407804	2.409667	2.148371	2.104545	2.321947	0.537596	1	-
ENSMUSC	Lpcat3	lysophosp	GO:00081	metabolic	K13515	lysophosp	mmu0056	Glyceroph	6:1246630	191	243	175	228	338	292	1.903083	1.933643	1.927122	1.752627	2.16868	2.288854	1.921283	2.070054	0.695064	1	-
ENSMUSC	Atp6v1f	ATPase, H	GO:00511	localizatio	K02151	V-type H-	mmu011C	Metabolic	6:2946771	539	653	481	649	762	584	30.95987	29.95498	30.53526	28.75975	28.18512	26.38965	30.48337	27.77817	0.26496	1	-
ENSMUSC	Clcn5	chloride c	GO:00511	localizatio	-	-	-	-	X:7153810	197	253	182	257	194	264	0.918692	0.942261	0.938044	0.924629	0.582587	0.968545	0.932999	0.825253	0.323109	1	-
ENSMUSC	Clcn3	chloride c	GO:00325	developm	-	-	-	-	8:6091038	3360	4406	2892	4025	5083	4001	8.007621	8.385994	7.617445	7.40049	7.800798	7.501423	8.003687	7.56757	0.353469	1	-
ENSMUSC	Gpx6	glutathion	GO:00081	metabolic	K00432	glutathion	mmu0059	Arachidor	13:213121	0	0	0	0	2	0	0	0	0	0	0.045843	0	0	0.015281	0.52754	1	-
ENSMUSC	Pde1c	phosphoc	GO:00508	response	K13755	calcium/ca	mmu0474	Olfactory	6:5606980	180	235	159	250	261	220	0.377496	0.393599	0.36854	0.404494	0.352482	0.362973	0.379879	0.373316	0.748009	1	-
ENSMUSC	Utp20	UTP20 sm	GO:00081	metabolic	-	-	-	-	10:887466	624	735	575	782	1022	825	3.266481	3.072764	3.326676	3.158151	3.445098	3.397508	3.221974	3.333585	0.923456	1	-
ENSMUSC	Spic	Spi-C trar	GO:00325	developm	-	-	-	-	10:886747	0	0	3	1	0	0	0	0	0.040243	0.009364	0	0	0.013414	0.003121	0.439489	1	-
ENSMUSC	9330159F	RIKEN cDI	-	-	-	-	-	-	10:292116	1446	1888	902	2087	2258	1860	10.35053	10.793	7.135883	11.52516	10.40813	10.47413	9.426471	10.80248	0.320638	1	-
ENSMUSC	Cul3	cullin 3 [S	GO:00325	developm	K03869	cullin 3[-]	mmu0412	Ubiquitin	1:8026492	2915	3556	2501	3775	4662	3371	28.40932	27.67769	26.93908	28.38374	29.25831	25.84592	27.67536	27.82932	0.838632	1	-
ENSMUSC	Sst	somatosta	GO:00400	locomotic	K05237	somatosta	mmu0497	Gastric ac	16:238895	3024	3277	2709	4028	4246	3837	308.2466	266.7711	305.1914	316.7643	278.7091	307.6941	293.4031	301.0559	0.992902	1	-
ENSMUSC	Large1	LARGE xyl	GO:00325	developm	K09668	glycosyltra	mmu011C	Metabolic	8:7281459	2888	3626	2866	3789	4554	4083	18.77511	18.82604	20.59246	19.00379	19.06483	20.88216	19.39787	19.65026	0.89857	1	-
ENSMUSC	Ddx56	DEAD (As	GO:00325	developm	-	-	-	-	11:625891	385	587	467	591	701	683	7.057148	8.593153	9.460892	8.357696	8.274494	9.849184	8.370398	8.827125	0.804842	1	-
ENSMUSC	Tmed4	transmem	GO:00048	signal trar	-	-	-	-	11:627036	833	1130	832	1216	1362	1141	17.6296	19.09949	19.46112	19.85461	18.56219	18.9974	18.73007	19.13806	0.983592	1	-
ENSMUSC	Col26a1	collagen,	GO:00329	macromo	-	-	-	-	5:1367417	34	51	25	44	43	36	0.19283	0.230995	0.156701	0.192518	0.15704	0.160621	0.193508	0.17006	0.452763	1	-
ENSMUSC	Ralb	v-ral simia	GO:00081	metabolic	K07835	Ras-relate	mmu052C	Pathways	1:1194703	278	323	230	367	515	368	3.228251	2.995512	2.951866	3.287902	3.851101	3.361871	3.058543	3.500291	0.354644	1	-
ENSMUSC	Ppp1cc	protein pl	GO:00325	developm	K06269|KC	serine/thre	mmu0481	Regulatior	5:1221582	504	376	110	192	371	760	4.150696	2.473001	1.001226	1.219895	1.967519	4.923968	2.541641	2.703794	0.922994	1	-
ENSMUSC	Dnajb11	DnaJ heat	GO:00081	metabolic	K09517	DnaJ hom	mmu0414	Protein pr	16:228578	1019	1469	852	1407	1753	1374	14.34261	16.51283	13.25379	15.27841	15.88877	15.21429	14.70308	15.46049	0.787373	1	-
ENSMUSC	Tbccd1	TBCC don	GO:00325	developm	-	-	-	-	16:228132	215	244	194	330	377	320	4.858443	4.403455	4.845146	5.753099	5.485984	5.688782	4.702348	5.642622	0.18994	1	-
ENSMUSC	Clec11a	C-type lec	GO:00508	response	-	-	-	-	7:4430268	54	84	52	94	82	65	1.107158	1.375442	1.178324	1.486879	1.082644	1.048433	1.220308	1.205985	0.847751	1	-
ENSMUSC	Zfp324	zinc finge	GO:00081	metabolic	-	-	-	-	7:1296583	243	272	200	276	312	282	1.11507	0.996806	1.014312	0.977093	0.921946	1.018018	1.042063	0.972352	0.442633	1	-
ENSMUSC	Gab2	growth fa	GO:00325	developm	K08091	growth fa	mmu0401	Ras signal	7:9708158	478	614	434	614	694	673	4.447013	4.561998	4.462497	4.406967	4.157716	4.925688	4.490503	4.49679	0.830908	1	-
ENSMUSC	Coro1c	coronin, a	GO:00325	developm	-	-	-	-	5:1138424	2175	1160	2024	2710	3265	1178	11.87423	5.057663	12.21247	11.41421	11.47847	5.05944	9.714788	9.317373	0.763985	1	-
ENSMUSC	Tax1bp1	Tax1 (hun	GO:00081	metabolic	-	-	mmu0413	Mitophag	6:5271372	3461	4637	3033	4839	6132	4637	19.02603	20.35774	18.42747	20.52261	21.70718	20.0537	19.27041	20.76116	0.548629	1	-
ENSMUSC	Ctse	cathepsin	GO:00081	metabolic	K01382	cathepsin	mmu0414	Lysosome	1:1316383	2	1	0	0	3	3	0.02885	0.011524	0	0	0.027876	0.034046	0.013458	0.020641	0.752033	1	-
ENSMUSC	Ndrg2	N-myc dc	GO:00325	developm	-	-	-	-	14:519052	10645	12639	9580	13144	15165	12369	114.9564	109.0049	114.3404	109.5079	105.4592	105.0831	112.7673	106.6834	0.337041	1	-
ENSMUSC	Mettl17	methyltrai	GO:00081	metabolic	-	-	-	-	14:518848	246	304	239	349	400	366	1.618915	1.597749	1.738337	1.771924	1.695131	1.894878	1.651667	1.787311	0.64787	1	-
ENSMUSC	Arhgef40	Rho guan	GO:00987	molecular	-	-	-	-	14:519847	300	483	329	558	478	498	0.826262	1.062406	1.001476	1.185663	0.847774	1.07904	0.963381	1.037492	0.701445	1	-
ENSMUSC	Pnpla6	patatin-lik	GO:00325	developm	K14676	lysophosp	mmu0056	Glyceroph	8:3515384	666	915	652	1012	1221	1061	2.914403	3.197735	3.153336	3.416541	3.440695	3.652601	3.088492	3.503279	0.310956	1	-
ENSMUSC	Mcoln1	mucolipin	GO:00048	signal trar	K04992	mucolipin	mmu0414	Lysosome	8:3500457	715	952	661	957	1063	980	4.234387	4.502643	4.326461	4.372469	4.053895	4.565849	4.354497	4.330738	0.764326	1	-
ENSMUSC	Arhgef18	rho/rac gu	GO:00325	developm	K16529	A-kinase a	mmu0453	Tight junc	8:3393006	1069	1404	975	1571	1663	1467	5.480822	5.748848	5.524838	6.214047	5.490539	5.917104	5.584836	5.873897	0.781931	1	-
ENSMUSC	Pkn2	protein kir	GO:00081	metabolic	K06071	protein kir	mmu0415	PI3K-Akt s	3:1427909	603	734	505	716	938	818	2.269497	2.206247	2.100635	2.079005	2.273368	2.422013	2.192126	2.258129	0.962555	1	-
ENSMUSC	Cd33	CD33 anti	GO:00160	membran	K06473	CD33 anti	mmu0464	Hematopc	7:4352421	192	213	128	197	243	198	1.085881	0.962069	0.800085	0.85956	0.884995	0.880959	0.949345	0.875171	0.436687	1	-
ENSMUSC	Etfb	electron tı	GO:00081	metabolic	-	-	-	-	7:4344408	281	398	293	545	530	378	6.838307	7.735206	7.880557	10.23221	8.305645	7.236772	7.48469	8.591542	0.367036	1	-
ENSMUSC	Nkg7	natural kil	GO:00160	membran	-	-	-	-	7:4343707	0	2	2	5	6	3	0	0.060963	0.084359	0.147196	0.147438	0.090052	0.04844	0.128229	0.190566	1	-
ENSMUSC	Stxbp2	syntaxin b	GO:00511	localizatio	-	-	-	-	8:3630955	417	577	389	649	795	642	3.352365	3.704567	3.456308	4.025228	4.115641	4.060331	3.504413	4.067067	0.231744	1	-
ENSMUSC	Pcp2	Purkinje c	GO:00987	molecular	-	-	-	-	8:3623371	0	2	0	0	3	0	0	0.023767	0	0	0.028745	0	0.007922	0.009582	1	1	-
ENSMUSC	Sgce	sarcoglyca	GO:00329	macromo	-	-	-	-	6:4674350	343	399	331	403	488	412	1.561623	1.450779	1.66555	1.415524	1.430727	1.475674	1.559317	1.440642	0.368187	1	-
ENSMUSC	Chn2	chimerin 2	GO:00452	synapse|m	-	-	-	-	6:5403955	317	484	306	406	574	324	2.127436	2.594113	2.269683	2.102102	2.480641	1.710619	2.330411	2.097787	0.328408	1	-
ENSMUSC	Wwox	WW dom	GO:00325	developm	-	-	-	-	8:1144396	235	253	176	294	297	324	1.261085	1.084287	1.043847	1.21718	1.026335	1.367834	1.12974	1.203783	0.783779	1	-
ENSMUSC	Slbp	stem-loop	GO:00081	metabolic	-	-	-	-	5:3363495	202	306	187	338	364	298	1.442207	1.744791	1.475582	1.861754	1.673524	1.673797	1.554193	1.736359	0.49106	1	-
ENSMUSC	Tyr	tyrosinase	GO:00325	developm	K00505	tyrosinase	mmu011C	Metabolic	7:8742477	0	2	1	0	0	0	0	0.01144	0.007915	0	0	0	0.006452	0	0.275451	1	-
ENSMUSC	Aqp1	aquaporir	GO:00325	developm	K09864	aquaporir	mmu0492	Renin secı	6:5533643	71	253	52	52	173	105	1.650217	4.696239	1.335763	0.932432	2.589317	1.91993	2.56074	1.813893	0.236528	1	-
ENSMUSC	Arid3b	AT rich int	GO:00325	developm	-	-	-	-	9:5779035	100	138	55	123	153	131	0.441873	0.486993	0.2686	0.419307	0.435355	0.455388	0.399155	0.436684	0.717649	1	-
ENSMUSC	Cnn2	calponin 2	GO:00325	developm	-	-	-	-	10:799885	90	122	106	128	221	178	1.887094	2.042957	2.456436	2.070587	2.984006	2.936175	2.128829	2.663589	0.209574	1	-
ENSMUSC	Polr2e	polymeras	GO:00081	metabolic	K03013	DNA-dire	mmu011C	Metabolic	10:800359	873	1178	989	1533	1493	1394	11.08647	11.94731	13.88104	15.01935	12.20937	13.92684	12.30494	13.71852	0.414359	1	-
ENSMUSC	Abca13	ATP-bind	GO:00511	localizatio	K05647	ATP bindi	mmu0201	ABC trans	11:919194	3	1	2	4	4	1	0.009218	0.002454	0.006793	0.009482	0.007915	0.002418	0.006155	0.006605	1	1	-
ENSMUSC	Myo9b	myosin IX	GO:00987	molecular	-	-	-	-	8:7127271	930	1156	819	1392	1279	1245	4.525346	4.492339	4.404527	5.225625	4.00769	4.765947	4.474071	4.666421	0.867238	1	-
ENSMUSC	Cd244	CD244 na	GO:00081	metabolic	K06582	natural kil	mmu0465	Natural ki	1:1715591	0	16	2	2	6	3	0	0.147352	0.025491	0.017795	0.044552	0.027211	0.057614	0.029853	0.348316	1	-
ENSMUSC	Mtfp1	mitochon	GO:00508	response	-	-	-	-	11:409148	860	1115	845	1308	1548	1295	28.8358	29.85754	31.31389	33.83542	33.42405	34.1597	30.00241	33.80639	0.310961	1	-
ENSMUSC	Rab23	RAB23, m	GO:00325	developm	-	-	-	-	1:3371988	360	393	320	398	460	439	1.064967	0.928478	1.046237	0.908337	0.876286	1.021667	1.013227	0.93543	0.373009	1	-
ENSMUSC	Rab11a	RAB11A, r	GO:00325	developm	K07904	Ras-relate	mmu0414	Endocytos	9:6471529	1261	1604	1078	1612	1908	1644	14.23448	14.46027	13.44906	14.03854	13.86946	14.59954	14.04794	14.16918	0.865134	1	-
ENSMUSC	Eif2b2	eukaryotic	GO:00325	developm	K03754	translatior	mmu0301	RNA trans	12:852194	313	407	302	458	459	442	4.365273	4.533227	4.655025	4.927917	4.122257	4.849543	4.517841	4.633239	1	1	-
ENSMUSC	Ulk2	unc-51 lik	GO:00325	developm	K08269	serine/thre	mmu0415	mTOR sig	11:617756	2209	2712	1944	2759	3201	2891	20.12794	19.73509	19.57703	19.39482	18.78209	20.72348	19.81335	19.63346	0.699508	1	-
ENSMUSC	Ccl24	chemokin	GO:00325	developm	K05514	C-C motif	mmu0406	Cytokine-	5:1355699	0	2	0	0	3	0	0	0.061423	0	0	0.074289	0	0.020474	0.024763	1	1	-
ENSMUSC	Dgkq	diacylglyc	GO:00048	signal trar	K00901	diacylglyc	mmu011C	Metabolic	5:1086466	387	509	324	534	543	529	1.80972	1.900919	1.674525	1.926512	1.635139	1.946106	1.795055	1.835919	0.98758	1	-
ENSMUSC	Grap	GRB2-rela	GO:00325	developm	-	-	-	-	11:616532	70	87	58	75	105	90	2.325378	2.308149	2.129473	1.922164	2.246162	2.352063	2.254333	2.173463	0.705331	1	-
ENSMUSC	Chmp2b	charged n	GO:00400	locomotic	K12192	charged n	mmu0414	Endocytos	16:655391	557	643	458	745	920	670	18.50346	17.05905	16.81552	19.09345	19.68069	17.50991	17.45934	18.76135	0.653981	1	-
ENSMUSC	Plod3	procollage	GO:00325	developm	K13646	lysyl hydrc	mmu0031	Lysine deg	5:1369870	330	434	369	544	540	444	2.858849	3.002705	3.533057	3.635854	3.012492	3.02602	3.131537	3.224789	0.966745	1	-
ENSMUSC	Ap1s1	adaptor p	GO:00452	synapse|c	K12394	AP-1 com	mmu0414	Lysosome	5:1370349	2394	3190	2500	3594	4214	3295	44.38904	47.23763	51.23165	51.41147	50.31538	48.06379	47.61944	49.93021	0.789773	1	-
ENSMUSC	Srpk1	serine/arg	GO:00325	developm	K15409	serine/thre	mmu0516	Herpes sir	17:285876	1051	1287	895	1495	1767	1399	5.403056	5.283977	5.085179	5.929366	5.849623	5.658031	5.257404	5.81234	0.41318	1	-
ENSMUSC	Pax3	paired bo	GO:00325	developm	K09381	paired bo	mmu052C	Transcript	1:7810126	0	3	0	0	0	0	0	0.016967	0	0	0	0	0.005656	0	0.288798	1	-
ENSMUSC	Lbr	lamin B re	GO:00081	metabolic	-	-	-	-	1:1818153	96	160	147	165	204	159	0.753704	1.003223	1.275544	0.999416	1.031374	0.982062	1.010824	1.004284	0.867611	1	-
ENSMUSC	Crabp2	cellular re	GO:00325	developm	-	-	-	-	3:8794866	14	24	17	24	41	32	0.537969	0.736495	0.72197	0.711485	1.014522	0.96735	0.665478	0.897786	0.251075	1	-
ENSMUSC	Bcan	brevican [	GO:00325	developm	-	-	-	-	3:8798753	2715	3799	2814	4345	4234	4060	28.68041	32.05019	32.85386	35.41079	28.80189	33.74057	31.19482	32.65109	0.817453	1	-
ENSMUSC	Hapln2	hyalurona	GO:00325	developm	-	-	-	-	3:8802175	184	253	186	260	244	243	2.501599	2.747054	2.794854	2.72712	2.13621	2.59907	2.681169	2.487467	0.443543	1	-
ENSMUSC	Prcc	papillary r	GO:00099	cellular pr	K13105	proline-ric	mmu052C	Transcript	3:8785890	758	987	739	987	1264	1089	22.04957	22.92947	23.75867	22.15023	23.67733	24.92124	22.91257	23.58293	0.963667	1	-
ENSMUSC	Rrnad1	ribosomal	-	-	-	-	-	-	3:8792260	281	422	308	409	451	485	1.054991	1.265321	1.278027	1.184668	1.09037	1.432503	1.199446	1.235847	0.971002	1	-
ENSMUSC	Hdgf	hepatoma	GO:00081	metabolic	-	-	-	-	3:8790632	2214	3033	2137	3381	3974	3125	33.55241	36.70831	35.79294	39.52952	38.78186	37.25693	35.35122	38.52277	0.473074	1	-
ENSMUSC	Thop1	thimet oli	GO:00081	metabolic	K01392	thimet oli	mmu0461	Renin-anc	10:810700	790	973	754	1133	1296	1177	7.690672	7.564777	8.112515	8.509376	8.124503	9.014148	7.789321	8.549342	0.484534	1	-
ENSMUSC	Matk	megakary	GO:00081	metabolic	K08888	megakary	mmu0472	Neurotror	10:812529	1762	1947	1570	2360	2443	2177	12.16743	10.73753	11.98228	12.57289	10.86353	11.82666	11.62908	11.75436	0.8807	1	-
ENSMUSC	Pias4	protein in	GO:00325	developm	K16065	E3 SUMO	mmu0463	Jak-STAT	10:811532	270	364	316	402	476	411	3.418853	3.68098	4.422325	3.927109	3.881301	4.094198	3.840719	3.967536	0.945237	1	-
ENSMUSC	Map2k1	mitogen-a	GO:00325	developm	K04368	mitogen-a	mmu052C	Pathways	9:6418577	3393	4225	3370	4601	5633	4582	50.96437	50.68218	55.94476	53.31696	54.48504	54.1438	52.53044	53.98193	0.969849	1	-
ENSMUSC	Sgta	small glut	GO:00081	metabolic	-	-	-	-	10:810440	2242	2909	2243	3152	3706	3245	29.16324	30.21968	32.24604	31.63128	31.04276	33.20672	30.54299	31.96025	0.807605	1	-
ENSMUSC	Nmrk2	nicotinam	GO:00325	developm	K10524	nicotinam	mmu011C	Metabolic	10:811981	3	0	0	0	0	0	0.098797	0	0	0	0	0	0.032932	0	0.295238	1	-
ENSMUSC	Tmem242	transmem	GO:00160	membran	-	-	-	-	17:541087	318	455	288	467	546	430	8.78574	10.03941	8.794065	9.95402	9.714003	9.346093	9.206405	9.671372	0.828297	1	-
ENSMUSC	Dtx2	deltex 2, E	GO:00081	metabolic	K06058	deltex[2.3	mmu0433	Notch sigr	5:1359948	138	166	130	160	210	172	0.803241	0.771653	0.836291	0.718484	0.78712	0.787605	0.803728	0.764403	0.599635	1	-
ENSMUSC	Hspb1	heat shoc	GO:00325	developm	K04455	heat shoc	mmu0401	MAPK sigr	5:1358879	28	35	17	47	45	40	0.932682	0.931105	0.625865	1.207848	0.965258	1.048213	0.829884	1.073773	0.2923	1	-
ENSMUSC	Syt5	synaptota	GO:00452	synapse|c	-	-	-	-	7:4539765	2119	2701	2066	2996	3379	2703	49.93521	50.83307	53.80877	54.46883	51.27655	50.11098	51.52568	51.95212	0.859032	1	-
ENSMUSC	Hnrnpa2b	heteroger	GO:00081	metabolic	-	-	-	-	6:5146093	5720	7324	4760	7395	9556	7251	32.53096	33.26557	29.91952	32.44661	34.99707	32.44215	31.90535	33.29528	0.824024	1	-
ENSMUSC	Ccdc130	coiled-co	GO:00508	response	-	-	-	-	8:8425779	171	176	120	179	250	179	2.204121	1.811747	1.709498	1.780016	2.075081	1.815112	1.908455	1.89007	0.818915	1	-
ENSMUSC	Mri1	methylthic	GO:00081	metabolic	K08963	methylthic	mmu011C	Metabolic	8:8424990	236	271	185	303	313	304	2.837673	2.602351	2.458488	2.810759	2.423537	2.875646	2.632837	2.703314	1	1	-
ENSMUSC	Sgsh	N-sulfogl	GO:00081	metabolic	K01565	N-sulfogl	mmu011C	Metabolic	11:119343	177	188	129	205	247	206	0.832032	0.705785	0.670198	0.743449	0.747686	0.761809	0.736005	0.750981	0.978859	1	-
ENSMUSC	Chd5	chromodc	GO:00325	developm	-	-	-	-	4:1523386	6479	7798	5191	7683	8360	7271	29.61931	28.47056	26.22798	27.09743	24.61092	26.15002	28.10595	25.95279	0.22233	1	-
ENSMUSC	Cstb	cystatin B	GO:00081	metabolic	-	-	-	-	10:784256	124	195	150	172	234	175	12.55592	15.76905	16.78667	13.43648	15.25794	13.9404	15.03721	14.21161	0.580206	1	-
ENSMUSC	Sh2b2	SH2B ada	GO:00325	developm	K07193	SH2B ada	mmu0491	Insulin sig	5:1362181	84	89	50	113	110	102	0.695252	0.588304	0.457387	0.721562	0.586287	0.664162	0.580314	0.657337	0.570072	1	-
ENSMUSC	Pex5	peroxisor	GO:00325	developm	K13342	peroxin-5	mmu0414	Peroxisor	6:1243968	654	693	465	854	1034	729	5.055322	4.278093	3.972561	5.092831	5.146908	4.433121	4.435326	4.890953	0.509191	1	-
ENSMUSC	Jkamp	JNK1/MAI	GO:00081	metabolic	-	-	-	-	12:720855	385	519	379	504	587	489	6.310701	6.794068	6.865983	6.373502	6.195979	6.305753	6.656917	6.291745	0.463142	1	-
ENSMUSC	Cd44	CD44 anti	GO:00325	developm	K06256	CD44 anti	mmu0516	Epstein-B	2:1028111	93	177	65	188	225	135	0.453835	0.689819	0.350574	0.707792	0.707054	0.518277	0.498076	0.644375	0.209007	1	-
ENSMUSC	Eif2ak4	eukaryotic	GO:00325	developm	K16196	eukaryotic	mmu0516	Epstein-B	2:1183886	480	626	429	674	707	669	3.364856	3.504654	3.323758	3.645152	3.191532	3.689456	3.397756	3.508713	0.947906	1	-
ENSMUSC	Wdr1	WD repea	GO:00325	developm	-	-	-	-	5:3852681	2582	3511	2830	3812	5160	3861	13.01296	14.13177	15.76352	14.82188	16.74651	15.30843	14.30275	15.62561	0.482807	1	-
ENSMUSC	Slc2a9	solute car	GO:00081	metabolic	-	-	-	-	5:3834927	41	58	39	42	91	55	0.200944	0.227022	0.211253	0.158808	0.287204	0.212064	0.213073	0.219358	1	1	-
ENSMUSC	Wisp1	WNT1 ind	GO:00508	response	-	-	-	-	15:668913	20	31	16	24	28	31	0.167605	0.207471	0.148191	0.155167	0.151101	0.204375	0.174423	0.170214	0.856722	1	-
ENSMUSC	4930550C	RIKEN cDI	-	-	-	-	-	-	9:5340232	2	15	3	9	10	6	0.028029	0.167919	0.046479	0.097328	0.090262	0.066165	0.080809	0.084585	1	1	-
ENSMUSC	Man2b1	mannosid	GO:00081	metabolic	K12311	lysosomal	mmu0414	Lysosome	8:8508327	620	857	593	767	1010	882	3.097116	3.418945	3.273913	2.95591	3.248939	3.46613	3.263325	3.223659	0.726186	1	-
ENSMUSC	Prdx2	peroxired	GO:00325	developm	-	-	-	-	8:8496958	1091	1415	1170	1638	1800	1619	12.62592	13.07799	14.96481	14.62456	13.41423	14.73996	13.55624	14.25958	0.783578	1	-
ENSMUSC	Polr2a	polymeras	GO:00081	metabolic	K03006	DNA-dire	mmu011C	Metabolic	11:697339	2334	2623	1812	2717	3326	2494	15.3815	13.80518	13.19784	13.81395	14.1148	12.9302	14.12818	13.61965	0.506074	1	-
ENSMUSC	Senp3	SUMO/se	GO:00081	metabolic	-	-	-	-	11:696731	827	1124	827	1427	1324	1182	7.999852	8.683375	8.841556	10.64954	8.247435	8.995074	8.508261	9.297349	0.534427	1	-
ENSMUSC	Corin	corin [Sou	GO:00325	developm	-	-	-	-	5:7230002	0	6	6	5	3	2	0	0.037399	0.05176	0.030105	0.01508	0.012283	0.02972	0.019156	0.619623	1	-
ENSMUSC	Plekha8	pleckstrin	GO:00511	localizatio	-	-	-	-	6:5459511	313	465	355	501	621	493	2.634206	3.125396	3.302031	3.252924	3.36552	3.264102	3.020544	3.294182	0.583179	1	-
ENSMUSC	Spc25	SPC25, NI	GO:00329	macromo	-	-	-	-	2:6919389	13	20	4	21	23	12	0.15099	0.185516	0.05135	0.188171	0.172025	0.10965	0.129286	0.156615	0.667175	1	-
ENSMUSC	Dnah2	dynein, ax	GO:00990	supramole	K10408	dynein he	mmu0501	Huntingto	11:694208	116	175	113	155	150	159	0.443447	0.53428	0.477428	0.457137	0.369259	0.478181	0.485052	0.434859	0.359345	1	-
ENSMUSC	Ufd1	ubiquitin r	GO:00081	metabolic	K14016	ubiquitin f	mmu0414	Protein pr	16:188117	510	584	434	574	673	655	4.617387	4.222647	4.342729	4.009293	3.923697	4.665286	4.394254	4.199426	0.521747	1	-
ENSMUSC	Zfp287	zinc finge	GO:00081	metabolic	-	-	-	-	11:627003	277	402	243	319	362	363	1.294339	1.500166	1.254935	1.149976	1.089257	1.334395	1.349813	1.191209	0.219123	1	-
ENSMUSC	Letm1	leucine zip	GO:00511	localizatio	-	-	-	-	5:3373967	1939	2276	1628	2540	3126	2335	10.25836	9.61652	9.519217	10.36726	10.64985	9.718467	9.798031	10.24519	0.817692	1	-
ENSMUSC	Ubqln1	ubiquilin 1	GO:00081	metabolic	K04523	ubiquilin[-	mmu0414	Protein pr	13:581761	3287	4063	3035	4315	4948	4263	26.67438	26.33221	27.22075	27.01505	25.85705	27.21578	26.74244	26.69596	0.759072	1	-
ENSMUSC	Cadm3	cell adhes	GO:00511	localizatio	K06780	cell adhes	mmu0451	Cell adhes	1:1733332	8043	9003	7342	10695	12052	11034	49.94817	44.65135	50.39212	51.24041	48.19647	53.90703	48.33055	51.11464	0.718809	1	-
ENSMUSC	Txn2	thioredox	GO:00081	metabolic	K03671	thioredox	mmu0462	NOD-like	15:779150	837	1047	847	1194	1833	948	8.055746	8.047713	9.009706	8.865735	11.3605	7.177937	8.371055	9.134723	0.596198	1	-
ENSMUSC	Slc1a6	solute car	GO:00511	localizatio	K05617	solute car	mmu0472	Glutamate	10:787804	90	112	93	162	141	146	2.362518	2.347996	2.698142	3.280793	2.383462	3.015079	2.469552	2.893111	0.386146	1	-
ENSMUSC	Crbn	cereblon [	GO:00081	metabolic	-	-	-	-	6:1067802	902	1164	834	1155	1371	1164	7.235212	7.456645	7.393621	7.147547	7.081685	7.345279	7.361826	7.191504	0.617294	1	-
ENSMUSC	Msh6	mutS hor	GO:00325	developm	K08737	DNA misn	mmu052C	Pathways	17:879750	512	596	410	638	717	645	7.34879	6.831847	6.503937	7.064756	6.627039	7.283098	6.894858	6.991631	0.923233	1	-
ENSMUSC	Tbl2	transducir	GO:00508	response	-	-	-	-	5:1351496	673	745	622	929	1042	855	4.428027	3.91469	4.523063	4.715647	4.414869	4.425604	4.288593	4.518707	0.786783	1	-
ENSMUSC	Bud23	BUD23, rF	GO:00081	metabolic	-	-	-	-	5:1350529	267	421	318	448	495	407	2.476837	3.118984	3.260309	3.206221	2.956957	2.970236	2.952043	3.044471	0.9557	1	-
ENSMUSC	Nid1	nidogen 1	GO:00325	developm	-	-	-	-	13:134375	170	343	187	278	404	294	1.313413	2.116368	1.596758	1.657013	2.009959	1.78694	1.675513	1.817971	0.682385	1	-
ENSMUSC	Mprip	myosin pł	GO:00056	cell|cell ju	-	-	-	-	11:596613	8438	11069	7630	11393	11761	11652	23.25997	24.36822	23.24561	24.22914	20.87702	25.26859	23.6246	23.45825	0.717368	1	-
ENSMUSC	Cic	capicua tr	GO:00325	developm	-	-	-	-	7:2526770	3101	3816	2810	4068	4454	3907	11.25289	11.05902	11.26978	11.38869	10.408	11.15364	11.1939	10.98345	0.61562	1	-
ENSMUSC	Il27ra	interleukir	GO:00325	developm	K19598	interleukir	mmu0463	Jak-STAT	8:8403029	23	38	25	28	34	36	0.26477	0.349355	0.318069	0.248671	0.252041	0.326027	0.310731	0.27558	0.492835	1	-

ENSMUSC	Prkaca	protein ki	GO:00325	developm	K04345	protein ki	mmu0474	Olfactory	8:8397299	3179	4222	2843	4155	4991	4009	69.5463	73.76453	68.73962	70.12693	70.3114	68.99704	70.68349	69.81179	0.6708	1	-
ENSMUSC	Asf1b	anti-silen	GO:00081	metabolic	-	-	-	-	8:8395550	8	12	11	16	25	11	0.171221	0.205098	0.260178	0.264178	0.344534	0.185204	0.212166	0.264639	0.547813	1	-
ENSMUSC	Ddx39	DEAD (As	GO:00081	metabolic	-	-	-	-	8:8371517	116	169	152	218	262	216	1.135235	1.320869	1.644067	1.645941	1.65114	1.663003	1.366724	1.653361	0.233723	1	-
ENSMUSC	Dnajb1	DnaJ heat	GO:00081	metabolic	K09507	DnaJ hom	mmu0516	Influenza	8:8360819	578	803	507	834	937	811	11.01486	12.22115	10.67839	12.2616	11.49861	12.15857	11.3048	11.97293	0.745638	1	-
ENSMUSC	Usp40	ubiquitin	GO:00081	metabolic	-	-	-	-	1:8794511	328	450	263	358	424	426	1.217451	1.333939	1.078896	1.025159	1.013439	1.243935	1.210095	1.094178	0.303184	1	-
ENSMUSC	Kbtbd4	kelch repe	GO:00081	metabolic	-	-	-	-	2:9090474	438	465	348	489	591	481	6.768783	5.73899	5.943768	5.830096	5.881372	5.847792	6.150514	5.853087	0.516688	1	-
ENSMUSC	Celf1	CUGBP, El	GO:00325	developm	-	-	-	-	2:9094038	3501	5083	3280	4705	5912	5012	7.607276	8.820698	7.876943	7.887275	8.272298	8.567597	8.101639	8.24239	0.941501	1	-
ENSMUSC	Ndufs3	NADH de	GO:00325	developm	-	-	-	-	2:9089463	686	912	681	914	1087	1029	11.9708	12.70984	13.13389	12.30484	12.21472	14.12621	12.60485	12.88192	0.983008	1	-
ENSMUSC	Por	P450 (cyto	GO:00325	developm	-	-	-	-	5:1356700	1493	1923	1497	2114	2533	2275	17.99442	18.50987	19.94099	19.65683	19.6593	21.57101	18.81509	20.29571	0.561405	1	-
ENSMUSC	Igf1r	insulin-lik	GO:00048	signal trar	K05087	insulin-lik	mmu0520	Pathways	7:6795282	1917	2507	1677	2416	2742	2523	6.180609	6.455194	5.975703	6.009475	5.692872	6.399372	6.203836	6.033906	0.553907	1	-
ENSMUSC	Trim28	tripartite r	GO:00325	developm	-	-	-	-	7:1299911	1547	1828	1361	1904	2391	2046	12.50919	11.80486	12.16309	11.8778	12.45011	13.01534	12.15905	12.44775	0.99405	1	-
ENSMUSC	Adcy9	adenylate	GO:00081	metabolic	K08049	adenylate	mmu0520	Pathways	16:428752	1654	2169	1727	2027	2273	2244	10.07584	10.5524	11.62746	9.526417	8.91661	10.75423	10.7519	9.732419	0.1825	1	-
ENSMUSC	Mef2c	myocyte e	GO:00325	developm	K04454	MADS-bo	mmu0401	MAPK sign	13:835040	1698	2100	1588	1965	2867	3003	2.468081	2.437737	2.55105	2.203508	2.683515	3.433903	2.485622	2.773642	0.440677	1	-
ENSMUSC	Ctr9	CTR9 hom	GO:00325	developm	-	-	-	-	7:1110289	1245	1685	1139	1661	2078	1641	6.500544	7.026289	6.572807	6.690833	6.986836	6.74062	6.69988	6.806097	0.932163	1	-
ENSMUSC	Eif4g2	eukaryotic	GO:00325	developm	K03260	translation	mmu0301	RNA trans	7:1110677	9958	13805	9204	13660	16559	13296	43.63547	48.31142	44.57496	46.17943	46.72575	45.83524	45.50728	46.24681	0.9286	1	-
ENSMUSC	Mrvi1	MRV inte	GO:00508	response	K12337	inositol 1,4	mmu0402	cGMP-PK	7:1108682	66	85	52	93	145	98	0.273805	0.281617	0.238419	0.29765	0.387363	0.319839	0.264613	0.334951	0.186722	1	-
ENSMUSC	Pcyt1a	phosphate	GO:00081	metabolic	K00968	choline-p	mmu0110	Metabolic	16:324309	540	788	483	777	917	671	2.290155	2.668969	2.263941	2.542277	2.504353	2.238744	2.407688	2.428458	0.892151	1	-
ENSMUSC	Zfp592	zinc finge	GO:00081	metabolic	-	-	-	-	7:8099368	735	893	579	982	1064	1039	5.147115	4.994294	4.481282	5.305404	4.798143	5.724047	4.87423	5.275865	0.600507	1	-
ENSMUSC	Psmd4	proteasor	GO:00081	metabolic	K03029	26S prote	mmu0516	Epstein-B	3:9503269	1096	1412	1017	1450	1740	1657	9.889124	10.17484	10.1418	10.09359	10.11	11.76199	10.06859	10.65519	0.736385	1	-
ENSMUSC	Tmod4	tropomoc	GO:00325	developm	-	-	-	-	3:9512447	2	4	6	5	7	7	0.04874	0.077857	0.161645	0.094017	0.109865	0.134235	0.096081	0.112706	0.875927	1	-
ENSMUSC	Snx6	sorting ne	GO:00081	metabolic	K17920	sorting ne	mmu0414	Endocytos	12:547463	580	679	557	719	906	694	8.387897	7.842249	8.902807	8.022035	8.437386	7.895789	8.377651	8.118403	0.598402	1	-
ENSMUSC	Mthfd2	methylene	GO:00081	metabolic	K13403	methylene	mmu0110	Metabolic	6:8330569	71	138	88	156	147	133	0.874016	1.356708	1.197267	1.481552	1.165289	1.288024	1.142664	1.311622	0.495014	1	-
ENSMUSC	Kit	KIT proto-	GO:00325	developm	K05091	proto-onc	mmu0520	Pathways	5:7557491	2022	2344	1717	2607	3061	2573	9.527666	8.820817	8.941751	9.477128	9.288025	9.537971	9.096745	9.434375	0.89151	1	-
ENSMUSC	Tomm40l	translocas	GO:00511	localizatio	K11518	mitochon	mmu0501	Amyotrop	1:1712160	470	637	585	618	691	858	2.979372	3.224872	4.098541	3.022355	2.820718	4.27883	3.434262	3.373968	0.752896	1	-
ENSMUSC	Nr1i3	nuclear re	GO:00048	signal trar	-	-	-	-	1:1712139	3	4	2	4	6	6	0.021252	0.022628	0.01566	0.021859	0.02737	0.03344	0.019847	0.027556	0.583738	1	-
ENSMUSC	Apoa2	apolipopr	GO:00081	metabolic	K08758	apolipopr	mmu0332	PPAR sign	1:1712250	2	7	5	6	5	14	0.079333	0.221795	0.219256	0.183642	0.127739	0.436953	0.173461	0.249445	0.541344	1	-
ENSMUSC	Pan2	PAN2 pol	GO:00081	metabolic	K12571	PAB-depe	mmu0301	RNA degr	10:128303	383	516	412	577	672	615	1.709449	1.839301	2.032363	1.986843	1.93144	2.159449	1.860371	2.025911	0.583735	1	-
ENSMUSC	Cs	citrate syn	GO:00081	metabolic	K01647	citrate syn	mmu0110	Metabolic	10:128337	4338	5411	3974	5893	7089	5595	44.67365	44.50259	45.23104	46.81971	47.01122	45.32866	44.80243	46.38653	0.895109	1	-
ENSMUSC	Ampd3	adenosine	GO:00081	metabolic	K01490	AMP dear	mmu0110	Metabolic	7:1107682	299	431	274	465	546	423	2.306265	2.654978	2.335793	2.767074	2.711969	2.566783	2.432345	2.681942	0.518533	1	-
ENSMUSC	Bcas2	breast car	GO:00081	metabolic	K12861	pre-mRNA	mmu0304	Spliceosor	3:1031716	682	896	656	845	1136	989	4.417744	4.635212	4.696419	4.222826	4.738589	5.039916	4.583125	4.66711	0.957343	1	-
ENSMUSC	Ctcf	CCCTC-bi	GO:00081	metabolic	-	-	-	-	8:1056365	1686	2291	1416	2172	2687	2122	16.50537	17.91177	15.32067	16.4043	16.93908	16.3427	16.57927	16.56203	0.782923	1	-
ENSMUSC	Pard6a	par-6 fam	GO:00081	metabolic	K06093	partitionin	mmu0516	Human pa	8:1057011	135	119	143	177	241	250	1.340079	0.943388	1.568843	1.355505	1.540528	1.952306	1.284103	1.616113	0.217955	1	-
ENSMUSC	Agrp	agouti rel	GO:00508	response	K05231	agouti rel	mmu0492	Adipocyto	8:1055666	5	5	5	4	5	8	0.264526	0.211267	0.292404	0.163259	0.170356	0.332977	0.256066	0.222197	0.878937	1	-
ENSMUSC	Pvalb	parvalbun	GO:00452	synapse|r	-	-	-	-	15:781911	254	348	204	353	414	333	9.193113	10.05896	8.160277	9.856735	9.649013	9.481633	9.137449	9.66246	0.800105	1	-
ENSMUSC	Tfap4	transcripti	GO:00081	metabolic	K09108	transcripti	mmu0520	Proteoglyc	16:454466	71	80	51	100	126	87	2.051642	1.846207	1.628789	2.229319	2.344609	1.97776	1.842212	2.183896	0.371319	1	-
ENSMUSC	Ranbp1	RAN bind	GO:00325	developm	-	-	-	-	16:182397	712	1022	622	1079	947	939	17.60768	20.18454	17.00042	20.5861	15.08089	18.26832	18.26421	17.97844	0.717886	1	-
ENSMUSC	Rfx5	regulatory	GO:00081	metabolic	K08061	regulatory	mmu0515	Tuberculo	3:9495407	146	397	136	175	279	405	0.451618	0.980742	0.464948	0.417624	0.555747	0.985562	0.632436	0.652978	0.976549	1	-
ENSMUSC	Psmb4	proteasor	GO:00081	metabolic	K02736	20S prote	mmu0305	Proteason	3:9488409	853	965	675	1068	1422	1080	19.58728	17.69694	17.13071	18.92023	21.02709	19.51011	18.13831	19.81915	0.526442	1	-
ENSMUSC	Slc30a4	solute car	GO:00511	localizatio	-	-	-	-	2:1226812	867	1119	767	1168	1398	1099	6.59409	6.796919	6.447304	6.85345	6.846961	6.575738	6.612771	6.758716	0.985776	1	-
ENSMUSC	Bloc1s6	biogenesi	GO:00325	developm	-	-	-	-	2:1227385	145	418	306	202	280	460	1.274789	2.934901	2.973302	1.370104	1.585199	3.181555	2.394331	2.045619	0.432674	1	-
ENSMUSC	Metap1	methionyl	GO:00081	metabolic	-	-	-	-	3:1384589	807	1039	750	1116	1387	1108	5.592951	5.750809	5.744807	5.967082	6.190114	6.041127	5.696189	6.066108	0.686816	1	-
ENSMUSC	Gpr108	G protein-	GO:00160	membran	-	-	-	-	17:572349	269	338	238	398	472	359	7.83992	7.867234	7.666256	8.948961	8.858422	8.231226	7.791137	8.679536	0.470058	1	-
ENSMUSC	Tnfsf14	tumor nec	GO:00325	developm	K05477	tumor nec	mmu0406	Cytokine-	17:571894	0	0	3	1	0	0	0	0	0.108908	0.025343	0	0	0.036303	0.008448	0.439466	1	-
ENSMUSC	Rsl1d1	ribosomal	GO:00325	developm	-	-	-	-	16:111929	863	1191	808	1261	1408	1135	17.85604	19.68031	18.47703	20.12887	18.75994	18.47485	18.67112	19.12122	1	1	-
ENSMUSC	Cnga2	cyclic nuc	GO:00511	localizatio	K04949	cyclic nucl	mmu0474	Olfactory	X:7199184	1	10	2	4	0	3	0.017796	0.142076	0.039328	0.054895	0	0.041982	0.0664	0.032293	0.232018	1	-
ENSMUSC	Reep5	receptor a	GO:00055	extracellul	-	-	-	-	18:343448	7703	10062	7012	10076	11895	9743	162.5192	169.5411	163.5059	164.0073	161.6084	161.7143	165.1888	162.4433	0.618817	1	-
ENSMUSC	Ergic3	ERGIC and	GO:00511	localizatio	-	-	-	-	2:1560080	967	1223	934	1333	1685	1267	15.55816	15.71462	16.60831	16.54596	17.45767	16.03685	15.96036	16.68016	0.829685	1	-
ENSMUSC	Uqcc1	ubiquinol-	GO:00081	metabolic	-	-	-	-	2:1558468	716	1050	794	1304	1047	1128	3.345138	3.917745	4.099852	4.700118	3.149941	4.145919	3.787578	3.998659	0.80291	1	-
ENSMUSC	Spo11	SPO11 me	GO:00325	developm	-	-	-	-	2:1729777	0	4	6	4	2	10	0	0.029555	0.061361	0.02855	0.011914	0.07279	0.030305	0.037751	0.895726	1	-
ENSMUSC	Ncoa2	nuclear re	GO:00081	metabolic	K11255	nuclear re	mmu0491	Thyroid ho	1:1313910	2344	2886	2085	2741	3145	2932	6.690362	6.57861	6.577258	6.035752	5.780526	6.58365	6.61541	6.133309	0.251126	1	-
ENSMUSC	Nr2c2	nuclear re	GO:00325	developm	-	-	-	-	6:9209139	2856	3531	2356	3372	4105	3498	9.922161	9.796964	9.046281	9.037865	9.183661	9.560458	9.588469	9.260661	0.499076	1	-
ENSMUSC	Nr2c1	nuclear re	GO:00048	signal trar	-	-	-	-	10:941480	143	248	170	203	323	242	1.628977	2.256188	2.140292	1.784046	2.369384	2.168728	2.008486	2.107386	0.869966	1	-
ENSMUSC	Smpd4	sphingom	GO:00081	metabolic	K12353	sphingom	mmu0110	Metabolic	16:176193	541	789	535	723	1000	1105	2.083014	2.426152	2.276647	2.14765	2.479417	3.347096	2.261938	2.658054	0.265722	1	-
ENSMUSC	Pex1	peroxisor	GO:00511	localizatio	K13338	peroxin-1	mmu0414	Peroxison	5:3596066	345	371	289	456	463	412	1.504111	1.291755	1.392529	1.533752	1.299859	1.413086	1.396132	1.415566	0.932097	1	-
ENSMUSC	Otx1	orthodent	GO:00325	developm	K09326	homeobo	mmu0455	Signaling	11:219947	41	69	65	97	76	68	0.297771	0.400221	0.521753	0.543507	0.355443	0.388529	0.406582	0.42916	0.901162	1	-
ENSMUSC	Kctd20	potassium	GO:00081	metabolic	-	-	-	-	17:289519	1006	805	916	1363	1622	1255	6.694767	4.278376	6.737203	6.997829	6.950927	6.570403	5.903448	6.83972	0.327659	1	-
ENSMUSC	Ctns	cystinosis,	GO:00325	developm	K12386	cystinosin	mmu0414	Lysosome	11:731835	234	233	175	253	120	312	2.594445	2.063149	2.144435	2.164111	0.856768	2.721406	2.267343	1.914095	0.37503	1	-
ENSMUSC	P2rx5	purinergic	GO:00048	signal trar	K05219	P2X purin	mmu0408	Neuroacti	11:731604	32	47	42	55	74	56	0.768024	0.900888	1.114091	1.01841	1.143707	1.057364	0.927668	1.07316	0.507209	1	-
ENSMUSC	Shpk	sedohept	GO:00081	metabolic	-	-	-	-	11:731994	64	76	55	81	105	102	0.819053	0.776767	0.777931	0.799741	0.865321	1.026938	0.79125	0.897333	0.553926	1	-
ENSMUSC	Ephb3	Eph recep	GO:00325	developm	K05112	Eph recep	mmu0436	Axon guic	16:212047	311	302	303	343	493	426	1.989217	1.542674	2.141957	1.692571	2.030596	2.143593	1.891283	1.955587	0.947824	1	-
ENSMUSC	Tuft1	tuftelin 1	GO:00325	developm	-	-	-	-	3:9461275	60	71	51	74	154	86	0.56886	0.537601	0.534415	0.541277	0.940224	0.641454	0.546959	0.707652	0.207256	1	-
ENSMUSC	Rcn1	reticuloca	GO:00325	developm	-	-	-	-	2:1053862	289	435	195	405	341	477	3.931775	4.726345	2.932057	4.250849	2.987443	5.105281	3.863392	4.114524	0.819892	1	-
ENSMUSC	Trap1	TNF recep	GO:00081	metabolic	-	-	-	-	16:403997	1018	1374	1047	1430	1644	1449	19.38776	20.89837	22.03806	21.01097	20.16213	21.70996	20.77473	20.96102	0.875204	1	-
ENSMUSC	Naa60	N(alpha)-	GO:00081	metabolic	-	-	-	-	16:387237	646	775	546	792	943	814	4.263247	4.084656	3.982421	4.032398	4.007505	4.226134	4.110108	4.088679	0.768451	1	-
ENSMUSC	1700037C	RIKEN cDI	-	-	-	-	-	-	16:389517	27	49	36	49	45	39	0.348686	0.505372	0.513831	0.48819	0.374221	0.39622	0.455963	0.419544	0.616502	1	-
ENSMUSC	Ankrd13d	ankyrin re	GO:00160	membran	-	-	-	-	19:427018	553	814	586	813	1060	786	8.054655	9.468745	9.433358	9.135725	9.942206	9.006489	8.985586	9.361473	0.866012	1	-
ENSMUSC	BC003331	cDNA seq	GO:00160	membran	-	-	-	-	1:1503613	1143	623	969	1471	1915	653	8.595812	3.741751	8.053993	8.534614	9.273939	3.863358	6.797186	7.22397	0.869909	1	-
ENSMUSC	Prg4	proteogly	GO:00511	localizatio	-	-	-	-	1:1504494	53	49	24	23	38	68	0.447099	0.330119	0.223757	0.149687	0.206425	0.45128	0.333658	0.269131	0.408935	1	-
ENSMUSC	Dhx34	DEAH (As	GO:00081	metabolic	-	-	-	-	7:1619714	180	199	185	235	274	225	1.623162	1.433144	1.843775	1.634889	1.591095	1.596186	1.63336	1.60739	0.775186	1	-
ENSMUSC	Napa	N-ethylm	GO:00325	developm	K15296	alpha-solu	mmu0472	Synaptic v	7:1609845	2895	3786	3067	4135	4785	4478	34.01255	35.52361	39.82458	37.47973	36.20156	41.38907	36.45358	38.35679	0.768149	1	-
ENSMUSC	Sra1	steroid re	GO:00325	developm	-	-	-	-	18:366666	633	632	508	677	906	956	6.17409	4.923021	5.476208	5.094339	5.690518	7.335637	5.52444	6.040165	0.601316	1	-
ENSMUSC	Atp5g1	ATP synth	GO:00081	metabolic	K02128	F-type H+	mmu0110	Metabolic	11:960688	529	670	463	812	824	622	3.296903	3.334811	3.189175	3.904241	3.306985	3.049661	3.273629	3.420295	0.860221	1	-
ENSMUSC	Snf8	SNF8, ESC	GO:00081	metabolic	K12188	ESCRT-II c	mmu0414	Endocytos	11:960348	543	711	481	712	890	760	8.010275	8.376511	7.842228	8.103236	8.454593	8.820077	8.076338	8.459302	0.83086	1	-
ENSMUSC	Tbcb	tubulin fo	GO:00325	developm	-	-	-	-	7:3022413	744	891	752	978	1085	808	20.95351	20.04045	23.40716	21.24973	19.67744	17.9022	21.46704	19.60979	0.275154	1	-
ENSMUSC	Inpp5k	inositol po	GO:00325	developm	K01106	inositol-1,	mmu0110	Metabolic	11:756309	367	504	427	570	672	590	2.294291	2.516279	2.950236	2.749081	2.705243	2.901649	2.586935	2.785324	0.663946	1	-
ENSMUSC	Crkl	v-crk avia	GO:00325	developm	K04438	proto-onc	mmu0520	Pathways	16:174519	718	852	622	888	987	852	11.34861	10.75484	10.86566	10.82834	10.04593	10.59422	10.9897	10.4895	0.477556	1	-
ENSMUSC	Upk3bl	uroplakin	GO:00081	metabolic	-	-	-	-	5:1360544	0	0	0	0	2	0	0	0	0	0	0.027289	0	0	0.009096	0.528789	1	-
ENSMUSC	Eps8l1	EPS8-like	GO:00987	molecular	-	-	-	-	7:4460674	119	150	100	133	227	127	0.727026	0.731881	0.675227	0.626882	0.893068	0.610404	0.711378	0.710118	0.890091	1	-
ENSMUSC	Clint1	clathrin in	GO:00511	localizatio	-	-	-	-	11:458520	1205	1544	1180	1626	2087	1705	7.844654	8.027492	8.490155	8.166533	8.749109	8.732167	8.120767	8.54927	0.765578	1	-
ENSMUSC	Prss16	protease,	GO:00081	metabolic	-	-	-	-	13:220021	6	7	3	7	9	9	0.055683	0.051884	0.030774	0.05012	0.053788	0.065709	0.046114	0.056539	0.683445	1	-
ENSMUSC	Cdkal1	CDK5 reg	GO:00081	metabolic	-	-	-	-	13:291917	204	249	186	246	289	265	0.895653	0.873081	0.902545	0.833251	0.817076	0.915305	0.890426	0.855211	0.603259	1	-
ENSMUSC	5430419D	RIKEN cDI	GO:00511	localizatio	K13912	deleted in	mmu0497	Salivary se	7:1311744	1	1	0	2	0	4	0.006888	0.0055	0	0.010626	0	0.021668	0.004129	0.010765	0.341383	1	-
ENSMUSC	Zbtb17	zinc finge	GO:00325	developm	K10500	zinc finge	mmu0520	Pathways	4:1414446	482	601	484	619	712	641	4.23819	4.220403	4.703549	4.199083	4.031514	4.434068	4.387381	4.221555	0.558443	1	-
ENSMUSC	Clcnkb	chloride c	GO:00511	localizatio	K05018	chloride c	mmu0496	Collecting	4:1414043	2	0	0	0	0	0	0.037741	0	0	0	0	0	0.01258	0	0.525726	1	-
ENSMUSC	Epor	erythropo	GO:00325	developm	K05079	erythropo	mmu0520	Pathways	9:2195889	56	61	52	92	91	72	1.523739	1.325555	1.563748	1.93126	1.594467	1.541218	1.471014	1.688982	0.502345	1	-
ENSMUSC	Ccdc159	coiled-co	-	-	-	-	-	-	9:2192747	127	138	101	172	154	183	2.202952	1.911724	1.93628	2.301761	1.720183	2.497248	2.016986	2.173064	0.760244	1	-
ENSMUSC	Mob1b	MOB kina	GO:00081	metabolic	K06685	MOB kina	mmu0439	Hippo sig	5:8872085	766	951	704	832	1127	928	2.623431	2.60116	2.664772	2.198337	2.485532	2.500342	2.629788	2.394737	0.238805	1	-
ENSMUSC	Atp6v1b1	ATPase, H	GO:00081	metabolic	K02147	V-type H+	mmu0110	Metabolic	6:8374299	0	3	3	5	4	2	0	0.025335	0.035058	0.040782	0.027235	0.016639	0.020131	0.028218	0.827839	1	-
ENSMUSC	Vax1	ventral an	GO:00325	developm	-	-	-	-	19:591661	6	6	3	3	7	6	0.283107	0.226099	0.156461	0.109224	0.212683	0.222732	0.221889	0.181546	0.756019	1	-
ENSMUSC	Atp6v1b2	ATPase, H	GO:00081	metabolic	K02147	V-type H+	mmu0110	Metabolic	8:6908864	6840	8184	6179	9464	10275	8337	63.72253	60.89034	63.62121	68.02084	61.64153	61.10231	62.74469	63.58823	0.898273	1	-
ENSMUSC	Eps15l1	epiderma	GO:00511	localizatio	K12472	epiderma	mmu0414	Endocytos	8:7234099	1055	1322	1004	1540	1690	1425	7.875814	7.881711	8.283687	8.869396	8.124275	8.368912	8.013737	8.454194	0.75473	1	-
ENSMUSC	Ttc5	tetratricop	GO:00081	metabolic	-	-	-	-	14:507654	482	602	485	663	806	687	4.379463	4.368341	4.870376	4.647483	4.715891	4.910679	4.539393	4.758017	0.828831	1	-
ENSMUSC	Osgep	O-sialogly	GO:00081	metabolic	-	-	-	-	14:509064	326	419	336	506	564	425	2.735695	2.80809	3.11628	3.275903	3.047786	2.805758	2.886688	3.043149	0.804475	1	-
ENSMUSC	Aamp	angio-ass	GO:00329	macromo	-	-	-	-	1:7427984	1803	2273	1659	2209	2925	2354	19.05622	19.18608	19.37913	18.01221	19.90772	19.57304	19.20714	19.16432	0.767325	1	-
ENSMUSC	Tmbim1	transmem	GO:00511	localizatio	-	-	-	-	1:7428824	371	515	347	526	717	446	1.316475	1.459457	1.360861	1.439973	1.638369	1.245041	1.378931	1.441128	0.860844	1	-
ENSMUSC	Arpc2	actin relat	GO:00325	developm	K05758	actin relat	mmu0414	Endocytos	1:7423608	4605	6242	4676	5993	8679	6033	98.38089	106.5003	110.4085	98.77697	119.4003	101.3971	105.0965	106.5248	0.915116	1	-
ENSMUSC	Kmt2b	lysine (K)-	GO:00325	developm	K14959	histone-ly	mmu0031	Lysine deg	7:3056885	935	1135	974	1149	1445	1183	4.570801	4.431212	5.262434	4.333423	4.548869	4.549636	4.754816	4.477309	0.395246	1	-
ENSMUSC	Tmem147	transmem	GO:00160	membran	-	-	-	-	7:3072770	273	378	262	451	414	406	5.677411	6.278056	6.02192	7.235928	5.544239	6.642388	5.992462	6.474185	0.668044	1	-
ENSMUSC	Rps9	ribosomal	GO:00451	translatior	K02997	small subu	mmu0301	Ribosome	7:3703993	1386	1654	1308	1748	1766	1701	13.64944	13.00868	14.23659	13.28077	11.19946	13.17853	13.63157	12.55292	0.256944	1	-
ENSMUSC	Tfpt	TCF3 (E2A	GO:00081	metabolic	-	-	-	-	7:3620324	181	226	146	245	327	262	3.216388	3.207339	2.867408	3.358822	3.741904	3.662709	3.097045	3.587811	0.337103	1	-
ENSMUSC	Susd2	sushi dom	GO:00511	localizatio	-	-	-	-	10:756367	52	71	39	82	80	58	0.514341	0.560854	0.426338	0.625737	0.509557	0.451324	0.500511	0.528872	0.902835	1	-
ENSMUSC	Ggt5	gamma-g	GO:00081	metabolic	K18592	gamma-g	mmu0110	Metabolic	10:755893	33	43	42	61	63	47	0.180582	0.187917	0.25401	0.257525	0.221999	0.202333	0.207503	0.227286	0.73564	1	-
ENSMUSC	Ggt1	gamma-g	GO:00081	metabolic	K18592	gamma-g	mmu0110	Metabolic	10:755616	34	35	42	43	50	34	0.290391	0.238735	0.396454	0.283332	0.274996	0.228446	0.308527	0.262258	0.413793	1	-
ENSMUSC	Cbfa2t3	core-binc	GO:00325	developm	-	-	-	-	8:1226251	644	790	540	863	1146	781	3.355641	3.287476	3.109783	3.469213	3.845291	3.201487	3.250967	3.50533	0.621994	1	-
ENSMUSC	Fbln1	fibulin 1 [S	GO:00325	developm	-	-	-	-	15:852059	252	345	221	352	424	464	3.268188	3.573322	3.167714	3.52192	3.541008	4.734085	3.336408	3.932338	0.276925	1	-
ENSMUSC	Pgrmc1	progester	GO:00055	extracellul	-	-	-	-	X:3659820	3850	4840	3688	5384	6399	4778	126.5879	127.0933	134.0199	136.5736	135.4872	123.5915	129.2337	131.8841	0.968595	1	-
ENSMUSC	Gcat	glycine C-	GO:00081	metabolic	K00639	glycine C-	mmu0026	Glycine, se	15:790308	90	116	97	132	178	154	1.850864	1.905177	2.204702	2.094291	2.357268	2.491522	1.986914	2.31436	0.376309	1	-
ENSMUSC	Tek	TEK recep	GO:00325	developm	K05121	endothelia	mmu0415	PI3K-Akt s	4:9473928	288	371	262	377	469	368	2.326638	2.393627	2.339291	2.349677	2.439855	2.338809	2.353185	2.376114	0.907574	1	-
ENSMUSC	Mpl	myelopro	GO:00325	developm	K05082	thrombop	mmu0406	Cytokine-	4:1184424	1	5	1	4	0	2	0.011344	0.045283	0.012535	0.034993	0	0.017847	0.023054	0.017613	0.635434	1	-
ENSMUSC	Elovl1	elongatior	GO:00081	metabolic	K10247	elongatior	mmu0006	Fatty acid	4:1184280	130	235	168	149	217	219	1.494833	2.158051	2.13504	1.321801	1.606807	1.981085	1.929308	1.636564	0.231891	1	-
ENSMUSC	Med8	mediator	GO:00081	metabolic	K15129	mediator	mmu0516	Herpes sir	4:1184093	244	339	251	356	425	350	2.605035	2.890475	2.961719	2.932269	2.921912	2.939695	2.819076	2.931292	0.901767	1	-
ENSMUSC	Adamts4	a disinteg	GO:00081	metabolic	-	-	-	-	1:1712504	335	574	359	511	551	477	2.53589	3.470106	3.003494	2.984259	2.685908	2.840627	3.003164	2.836932	0.501308	1	-
ENSMUSC	Nectin4	nectin cell	GO:00099	cellular pr	K06593	poliovirus	mmu0452	Adherens	1:1713700	124	172	102	103	176	173	0.982637	1.088542	0.893341	0.629706	0.898127	1.078517	0.988173	0.868783	0.359315	1	-
ENSMUSC	Pfdn2	prefoldin	GO:00329	macromo	-	-	-	-	1:1713456	1078	1426	954	1613	1874	1291	23.874	25.22156	23.35079	27.5595	26.7258	22.49281	24.14878	25.59271	0.72832	1	-
ENSMUSC	Rnf114	ring finge	GO:00325	developm	-	-	-	-	2:1674926	739	931	622	1052	1274	1109	6.757817	6.799195	6.28636	7.421783	7.502157	7.97819	6.614457	7.634043	0.234182	1	-
ENSMUSC	C330007P	RIKEN cDI	-	-	-	-	-	-	X:3682373	139	132	625	811	121	728	0.731077	0.554457	3.633086	3.290786	0.409817	3.012247	1.63954	2.237617	0.536233	1	-
ENSMUSC	Neurl1a	neuralizec	GO:00325	developm	-	-	-	-	19:471788	2729	3724	2729	3914	4612	4042	37.77547	41.16819	41.75002	41.79819	41.11026	44.01629	40.23122	42.30825	0.76125	1	-
ENSMUSC	Srm	spermidin	GO:00081	metabolic	K00797	spermidin	mmu0110	Metabolic	4:1485915	477	528	393	516	619	720	15.00501	13.2647	13.66334	12.52269	12.53901	17.8181	13.97768	14.29327	0.982818	1	-
ENSMUSC	Rbm14	RNA bind	GO:00081	metabolic	-	-	-	-	19:480056	356	572	382	661	800	550	3.086281	3.960292	3.660123	4.420966	4.46612	3.751105	3.568899	4.21273	0.221244	1	-
ENSMUSC	Actn3	actinin alp	GO:00325	developm	K05699	actinin alp	mmu0541	Arrhythmo	19:486122	17	38	12	17	28	29	0.342688	0.611745	0.267341	0.264382	0.363461	0.459883	0.407258	0.362575	0.58779	1	-
ENSMUSC	Zdhhc24	zinc finge	GO:00038	catalytic a	-	-	-	-	19:487866	83	415	130	400	181	414	0.60045	2.397699	1.039419	2.2325	0.843206	2.356197	1.345856	1.810634	0.352937	1	-
ENSMUSC	Bbs1	Bardet-Bi	GO:00325	developm	-	-	-	-	19:488689	746	996	648	986	1140	990	8.161505	8.702347	7.835254	8.322204	8.031396	8.520739	8.233036	8.291446	0.867302	1	-
ENSMUSC	Slc34a3	solute car	GO:00511	localizatio	-	-	-	-	2:2522889	2	0	4	2	0	2	0.035942	0	0.079466	0.027737	0	0.028286	0.038469	0.018675	0.609915	1	-
ENSMUSC	Ndor1	NADPH d	GO:00081	metabolic	-	-	-	-	2:2524482	256	433	407	566	326	377	1.559034	2.105954	2.739406	2.659268	1.278463	1.80621	2.134798	1.914647	0.46321	1	-
ENSMUSC	Nsmf	NMDA re	GO:00325	developm	-	-	-	-	2:2505435	6512	8463	6212	9154	11089	9859	34.00996	35.29896	35.85667	36.88357	37.29398	40.5075	35.0552	38.22835	0.451583	1	-
ENSMUSC	Pdk1	pyruvate c	GO:00081	metabolic	K12077	pyruvate c	mmu0436	Axon guic	2:7187322	652	765	901	1337	1572	786	3.523935	3.302078	5.382101	5.574963	5.471262	3.342055	4.069372	4.796093	0.365517	1	-
ENSMUSC	Ptbp1	polypyrim	GO:00325	developm	-	-	-	-	10:798544	394	560	369	553	673	630	2.186193	2.48157	2.262903	2.367272	2.404709	2.750071	2.310222	2.507351	0.606998	1	-
ENSMUSC	Mvd	mevalona	GO:00081	metabolic	K01597	diphospho	mmu0110	Metabolic	8:1224336	288	296	217	414	482	299	8.962654	7.356676	7.463634	9.939741	9.659315	7.320263	7.927655	8.973107	0.453812	1	-
ENSMUSC	Cyba	cytochron	GO:00081	metabolic	K08009	cytochron	mmu0414	Phagoson	8:1224247	49	98	64	92	126	75	2.30853	3.687309	3.332479	3.343942	3.822654	2.779765	3.109439	3.315454	0.816429	1	-
ENSMUSC	Itih3	inter-alph	GO:00081	metabolic	-	-	-	-	14:309085	236	321	212	361	395	379	2.868173	3.115617	2.847588	3.384785	3.091328	3.623628	2.943793	3.36658	0.36186	1	-
ENSMUSC	Tmem110	transmem	GO:00511	localizatio	-	-	-	-	14:308255	169	352	171	342	360	368	1.630662	2.712475	1.823555	2.545855	2.236836	2.793413	2.055564	2.525368	0.221935	1	-
ENSMUSC	Sfmbt1	Scm-like	GO:00325	developm	-	-	-	-	14:307148	793	946	734	968	1185	1005	4.644514	4.424909	4.751273	4.373942	4.469308	4.630672	4.606899	4.491307	0.621583	1	-
ENSMUSC	Prkag3	protein ki	GO:00081	metabolic	K07200	5'-AMP-a	mmu0453	Tight junc	1:7473892	0	2	0	2	0	0	0	0.022305	0	0.021547	0	0	0.007435	0.007182	1	1	-
ENSMUSC	Cryba2	crystallin,	GO:00325	developm	-	-	-	-	1:7488993	0	0	2	3	0	0	0	0	0.089983	0.094224	0	0	0.029994	0.031408	1	1	-
ENSMUSC	Atp7b	ATPase, C	GO:00325	developm	K17686	Cu++-expo	mmu0152	Platinum c	8:2199278	51	69	42	59	51	92	0.4031	0.435555	0.366893	0.359772	0.25958	0.572062	0.401849	0.397138	0.875324	1	-
ENSMUSC	Slc4a1	solute car	GO:00511	localizatio	K06573	solute car	mmu0496	Collecting	11:102348	4	6	2	2	6	6	0.046015	0.055119	0.025428	0.017751	0.044443	0.054298	0.042187	0.038831	1	1	-
ENSMUSC	Rundc3a	RUN dom	GO:00081	metabolic	-	-	-	-	11:102393	4519	5544	4307	6660	7461	5820	51.11553	50.08176	53.84336	58.11858	54.34532	51.78981	51.68021	54.75124	0.69071	1	-
ENSMUSC	Slc4a3	solute car	GO:00511	localizatio	-	-	-	-	1:7554626	1602	2019	1369	2136	2356	1966	6.877733	6.922529	6.495803	7.074802	6.513473	6.640136	6.765355	6.742804	0.757263	1	-
ENSMUSC	Snai3	snail famil	GO:00081	metabolic	-	-	-	-	8:1224542	0	1	0	0	2	4	0	0.029957	0	0	0.048293	0.118015	0.009986	0.055436	0.264703	1	-
ENSMUSC	Aprt	adenine p	GO:00325	developm	K00759	adenine p	mmu0110	Metabolic	8:1225746	151	231	166	196	264	218	2.314194	2.827364	2.811769	2.317445	2.605452	2.628405	2.651109	2.517101	0.569283	1	-
ENSMUSC	Gtf2h1	general tr	GO:00081	metabolic	K03141	transcripti	mmu0520	Viral carci	7:4679610	843	1083	520	1102	1369	1174	12.29035	12.60988	8.378901	12.39506	12.85271	13.46529	11.09304	12.90436	0.287499	1	-
ENSMUSC	Hfe	hemochro	GO:00325	developm	-	-	-	-	13:237020	70	153	82	95	125	111	1.134903	1.981074	1.469337	1.188273	1.305048	1.415779	1.528438	1.303033	0.284201	1	-
ENSMUSC	Slc5a6	solute car	GO:00511	localizatio	K14386	solute car	-	-	5:3103603	196	253	178	220	286	249	2.017774	2.080091	2.025268	1.747302	1.895988	2.016631	2.041044	1.88664	0.411891	1	-
ENSMUSC	Tcf23	transcripti	GO:00325	developm	-	-	-	-	5:3096866	6	13	13	6	8	17	0.089004	0.154013	0.213135	0.068665	0.076424	0.198397	0.15205	0.114495	0.439552	1	-
ENSMUSC	Nphs1	nephrosis	GO:00325	developm	-	-	-	-	7:3045831	35	47	27	59	32	51	0.285622	0.306317	0.243519	0.371456	0.168164	0.327421	0.278486	0.289014	1	1	-
ENSMUSC	Aplp1	amyloid b	GO:00081	metabolic	-	-	-	-	7:3043498	13820	17032	14186	19552	22550	19853	183.7594	180.8643	208.4722	200.5686	193.0824	207.6723	191.032	200.4411	0.781904	1	-
ENSMUSC	Qrich1	glutamine	GO:00056	cell|intrace	-	-	-	-	9:1085168	1159	1497	1064	1546	1891	1727	5.084153	5.244485	5.158504	5.23209	5.341725	5.959895	5.162381	5.511237	0.657807	1	-
ENSMUSC	P4htm	prolyl 4-h	GO:00325	developm	-	-	-	-	9:1085788	795	1021	890	1181	1361	1216	8.266556	8.478689	10.22808	9.47409	9.113182	9.947218	8.99111	9.511497	0.753343	1	-
ENSMUSC	Usp19	ubiquitin	GO:00325	developm	-	-	-	-	9:1084906	1911	2193	1535	2317	2979	2560	9.098014	8.338164	8.076834	8.51025	9.132953	9.588195	8.504338	9.077133	0.654292	1	-
ENSMUSC	Pola1	polymeras	GO:00081	metabolic	K02320	DNA poly	mmu0110	Metabolic	X:9330476	130	170	117	156	230	135	0.793756	0.828967	0.789543	0.73485	0.90433	0.648469	0.804089	0.762549	0.622972	1	-
ENSMUSC	Pknox1	Pbx/knott	GO:00325	developm	-	-	-	-	17:315647	179	215	215	307	351	275	0.95088	0.912127	1.262283	1.258173	1.200698	1.149251	1.041763	1.202707	0.370285	1	-
ENSMUSC	D130043K	RIKEN cDI	GO:00325	developm	-	-	-	-	13:248451	808	914	717	1014	1118	946	6.789839	6.133949	6.659072	6.573798	6.049848	6.253887	6.52762	6.292511	0.54951	1	-
ENSMUSC	Gmnn	geminin [S	GO:00325	developm	-	-	-	-	13:247518	30	45	31	52	61	38	0.929355	1.113303	1.06137	1.24277	1.216864	0.926096	1.034676	1.128577	0.768056	1	-
ENSMUSC	Acot13	acyl-CoA	GO:00038	catalytic a	-	-	-	-	13:248179	737	867	662	912	1161	897	51.78349	48.65065	51.40771	49.43659	52.53037	49.5823	50.61395	50.51642	0.797359	1	-
ENSMUSC	Zfp184	zinc finge	GO:00081	metabolic	-	-	-	-	13:219450	14	28	24	28	23	44	0.08174	0.130558	0.154862	0.126119	0.086473	0.202094	0.122387	0.138229	0.778566	1	-
ENSMUSC	Cdk4	cyclin-dep	GO:00325	developm	K02089	cyclin-dep	mmu0520	Pathways	10:127063	404	520	398	565	628	580	3.863345	3.971288	4.206417	4.168318	3.867204	4.363362	4.013683	4.132961	0.968275	1	-
ENSMUSC	B4galnt1	beta-1,4-	GO:00081	metabolic	K00725	(N-Acetyl	mmu0110	Metabolic	10:127165	1937	2364	1720	2592	3003	2401	20.87355	20.34511	20.48528	21.54925	20.839	20.35494	20.56798	20.9144	0.933628	1	-
ENSMUSC	Mettl1	methyltrai	GO:00081	metabolic	-	-	-	-	10:127041	75	99	97	125	162	118	1.002269	1.056582	1.432646	1.288731	1.394092	1.240552	1.163832	1.307792	0.549009	1	-
ENSMUSC	Tspan31	tetraspani	GO:00508	response	-	-	-	-	10:127067	579	766	502	696	956	678	10.70967	11.31547	10.26239	9.932019	11.38703	9.865938	10.76251	10.395	0.582016	1	-
ENSMUSC	Kif5b	kinesin fai	GO:00325	developm	K10396	kinesin fai	mmu0414	Endocytos	18:620100	5203	6236	4338	6725	7694	6910	33.90813	32.45653	31.24543	33.81214	32.28918	35.42738	32.5367	33.8429	0.859839	1	-
ENSMUSC	Saal1	serum am	GO:00329	macromo	-	-	-	-	7:4668610	215	197	196	207	321	267	1.232515	0.901913	1.241814	0.91549	1.184985	1.204135	1.125414	1.101537	0.763462	1	-
ENSMUSC	Tph2	tryptopha	GO:00081	metabolic	K00502	tryptopha	mmu0110	Metabolic	10:115078	7	9	12	120	96	105	0.146426	0.150354	0.277415	2.536454	2.08579	2.283631	0.191398	2.301959	0.0001	0.007417	DOWN
ENSMUSC	Krt23	keratin 23	GO:00051	structural	-	-	-	-	11:994779	6	5	2	4	11	12	0.234083	0.155784	0.086245	0.120384	0.276353	0.368327	0.158704	0.255021	0.363742	1	-
ENSMUSC	Cnp	2',3'-cycli	GO:00325	developm	-	-	-	-	11:100574	2960	4447	3234	4336	5239	4608	32.629	39.14939	39.40024	36.87496	37.18905	39.96091	37.05954	38.00831	0.985339	1	-
ENSMUSC	Ttc25	tetratricop	GO:00056	cell|intrace	-	-	-	-	11:100545	23	36	35	24	48	40	0.184297	0.230379	0.309962	0.148366	0.247676	0.252149	0.241546	0.216063	0.573924	1	-

ENSMUSC	Sulf2	sulfatase 2	GO:00325	developm	-	-	-	-	2:1660730	2829	3481	2550	3827	4283	3758	20.66931	20.31154	20.59112	21.57157	20.15096	21.60034	20.52399	21.10762	0.966402	1	-
ENSMUSC	Sod2	superoxid	GO:00325	developm	K04564	superoxid	mmu0501	Huntingto	17:130078	2677	3263	2186	3350	3904	3449	42.74387	41.60911	38.57647	41.26678	40.14116	43.3241	40.97648	41.57735	0.91139	1	-
ENSMUSC	Tmco6	transmem	GO:00511	localizatio	-	-	-	-	18:367350	113	133	114	210	205	143	3.797225	3.569327	4.233885	5.444261	4.43605	3.780375	3.866812	4.553562	0.358856	1	-
ENSMUSC	Stambp	STAM bin	GO:00081	metabolic	K11866	STAM-bir	mmu0414	Endocytos	6:8354321	355	556	415	447	692	560	1.513765	1.893439	1.955806	1.47051	1.900165	1.878579	1.78767	1.749752	0.723248	1	-
ENSMUSC	Hap1	huntingtir	GO:00325	developm	K04647	huntingtir	mmu0501	Huntingto	11:100347	1671	1986	1613	2779	2476	2447	19.22881	18.25161	20.51433	24.67147	18.34769	22.15241	19.33158	21.72386	0.35474	1	-
ENSMUSC	P3h4	prolyl 3-h	GO:00081	metabolic	-	-	-	-	11:100408	153	207	126	207	276	210	3.8876	4.200553	3.538387	4.057797	4.51599	4.197778	3.875514	4.257188	0.602036	1	-
ENSMUSC	Ctnnb1	catenin (c	GO:00325	developm	K02105	catenin be	mmu0520	Pathways	9:1209292	4743	3597	4156	5866	5037	7275	25.01283	15.14942	24.22323	23.86614	17.10553	30.18239	21.46183	23.71802	0.62335	1	-
ENSMUSC	Eif1b	eukaryotic	GO:00081	metabolic	K03113	translatior	mmu0301	RNA trans	9:1204922	1106	1455	1096	1563	1814	1453	50.81286	53.38599	55.65137	55.39972	53.66738	52.51638	53.2834	53.86116	0.890681	1	-
ENSMUSC	Klk4	kallikrein	GO:00325	developm	-	-	-	-	7:4388116	0	0	2	0	0	0	0	0	0.108065	0	0	0	0.036022	0	0.526227	1	-
ENSMUSC	Chrd	chordin [S	GO:00325	developm	K04657	chordin[-]	mmu0435	TGF-beta	16:207331	929	1121	876	1358	1463	1450	6.582681	6.343641	6.860225	7.423647	6.675539	8.082882	6.595516	7.394023	0.359509	1	-
ENSMUSC	Psmd2	proteasor	GO:00081	metabolic	K03028	26S prote	mmu0516	Epstein-B	16:206516	2042	2673	1927	2665	3120	2886	22.06347	23.0655	23.01158	22.21496	21.70837	24.53156	22.71352	22.8183	0.823236	1	-
ENSMUSC	Syngr3	synaptogy	GO:00325	developm	-	-	-	-	17:246850	1751	2418	1799	2844	2953	2676	50.91084	56.14695	57.80987	63.79461	55.28944	61.20981	54.95589	60.09795	0.469349	1	-
ENSMUSC	Vars	valyl-tRN/	GO:00081	metabolic	K01873	valyl-tRN/	mmu0097	Aminoacy	17:350009	513	635	535	698	799	701	3.230157	3.193196	3.723118	3.390716	3.239721	3.472443	3.382157	3.367627	0.79357	1	-
ENSMUSC	Vwa7	von Willet	GO:00055	extracellul	-	-	-	-	17:350165	1	8	5	4	4	0	0.010382	0.066312	0.057358	0.032025	0.026735	0	0.044684	0.019586	0.228587	1	-
ENSMUSC	Hspa1l	heat shoc	GO:00325	developm	K03283	heat shoc	mmu0401	MAPK sigı	17:349727	29	38	21	35	28	47	0.716295	0.749586	0.573284	0.666945	0.445358	0.913282	0.679722	0.675195	0.89393	1	-
ENSMUSC	Msh5	mutS hon	GO:00081	metabolic	-	-	-	-	17:350286	5	0	5	3	5	8	0.03415	0	0.037749	0.015811	0.021992	0.042986	0.023966	0.02693	0.881958	1	-
ENSMUSC	Abhd16a	abhydrola	GO:00081	metabolic	-	-	-	-	17:350892	901	1236	940	1329	1549	1365	12.81765	14.04261	14.77945	14.58609	14.19024	15.27661	13.8799	14.68432	0.731912	1	-
ENSMUSC	Neu1	neuramini	GO:00081	metabolic	K01186	sialidase-:	mmu0414	Lysosome	17:349312	746	996	763	1146	1433	1137	7.43906	7.932027	8.409115	8.816457	9.201949	8.919706	7.926734	8.979371	0.303255	1	-
ENSMUSC	Ddah2	dimethyla	GO:00081	metabolic	-	-	-	-	17:350590	100	130	86	188	162	127	2.627282	2.727692	2.49719	3.810627	2.740794	2.624949	2.617388	3.05879	0.412671	1	-
ENSMUSC	Lsm2	LSM2 hon	GO:00081	metabolic	K12621	U6 snRNA	mmu0304	Spliceosoı	17:349818	56	95	62	108	116	84	0.527584	0.71478	0.645567	0.784972	0.70374	0.622577	0.62931	0.703763	0.605156	1	-
ENSMUSC	Pole	polymeras	GO:00325	developm	K02324	DNA poly	mmu0110	Metabolic	5:1102863	54	44	47	55	89	65	0.240385	0.156427	0.23124	0.188892	0.25513	0.227634	0.20935	0.223885	0.831337	1	-
ENSMUSC	Atp1a2	ATPase, N	GO:00081	metabolic	K01539	sodium/p	mmu0402	cAMP sigr	1:1722717	18920	23565	15822	24123	27994	22541	146.1563	145.3817	135.0842	143.7666	139.2567	136.9874	142.2074	140.0036	0.631001	1	-
ENSMUSC	Casq1	calseques	GO:00325	developm	-	-	-	-	1:1722098	6	17	7	10	9	11	0.12576	0.284568	0.162172	0.161714	0.12148	0.181389	0.190834	0.154861	0.536696	1	-
ENSMUSC	Stx1a	syntaxin 1	GO:00452	synapse|c	K04560	syntaxin 1	mmu0491	Insulin sec	5:1350234	1246	1625	1158	1793	1853	1573	16.651	17.34289	17.1032	18.48557	15.94602	16.5372	17.03236	16.98959	0.777353	1	-
ENSMUSC	Ceacam9	carcinoerr	-	-	-	-	-	-	7:1672192	0	0	2	0	0	0	0	0	0.072567	0	0	0	0.024189	0	0.526157	1	-
ENSMUSC	Zfp775	zinc finge	GO:00081	metabolic	-	-	-	-	6:4860206	99	46	102	44	152	134	1.248394	0.463257	1.42156	0.428057	1.234288	1.329335	1.044404	0.997227	0.827708	1	-
ENSMUSC	Mrpl49	mitochon	GO:00081	metabolic	-	-	-	-	19:605363	533	755	546	781	926	789	18.89895	21.37978	21.39684	21.36444	21.14344	22.00888	20.55852	21.50558	0.836801	1	-
ENSMUSC	Dennd2c	DENN/M/	GO:00987	molecular	-	-	-	-	3:1031026	5	18	12	11	14	15	0.024359	0.070038	0.064617	0.041349	0.043925	0.057494	0.053004	0.047589	0.728455	1	-
ENSMUSC	Mark3	MAP/micr	GO:00081	metabolic	-	-	-	-	12:111574	1151	1505	1117	1664	1920	1671	4.592126	4.795354	4.925363	5.121794	4.932809	5.244762	4.770948	5.099788	0.641205	1	-
ENSMUSC	Gatad1	GATA zinc	GO:00081	metabolic	-	-	-	-	5:3632932	661	839	901	903	1126	809	7.678731	7.783874	11.56804	8.092938	8.423279	7.393459	9.010214	7.969892	0.241755	1	-
ENSMUSC	Pcdha11	protocadh	GO:00325	developm	-	-	-	-	18:370052	41	65	53	50	92	50	0.467132	0.59145	0.667389	0.439499	0.674994	0.448165	0.575324	0.520886	0.550135	1	-
ENSMUSC	2310003L	RIKEN cDI	-	-	-	-	-	-	5:8796945	0	2	0	0	0	0	0	0.060849	0	0	0	0	0.020283	0	0.524671	1	-
ENSMUSC	M6pr	mannose-	GO:00511	localizatio	-	-	-	-	6:1223087	1026	1355	952	1222	1600	1339	12.3391	13.01432	12.65376	11.33803	12.39113	12.66857	12.66906	12.13258	0.476344	1	-
ENSMUSC	Lrrc8a	leucine ric	GO:00325	developm	-	-	-	-	2:3023771	800	987	863	905	1289	1195	7.716663	7.603312	9.200194	6.734701	8.006583	9.068141	8.17339	7.936475	0.633263	1	-
ENSMUSC	Mc5r	melanoco	GO:00048	signal trar	K04203	melanoco	mmu0408	Neuroacti	18:683376	5	7	4	4	8	3	0.262705	0.293755	0.232313	0.162135	0.270707	0.123996	0.262924	0.185613	0.424897	1	-
ENSMUSC	Ppp2r1a	protein pl	GO:00325	developm	K03456	serine/thr	mmu0415	PI3K-Akt	17:209453	6897	8216	6897	10136	11464	8965	83.75453	79.68092	92.56677	94.96094	89.64765	85.64643	85.33407	90.08501	0.731488	1	-
ENSMUSC	Fance	Fanconi a	GO:00329	macromo	-	-	-	-	17:283135	182	219	152	223	254	214	1.37345	1.319871	1.267746	1.298305	1.234327	1.270472	1.320356	1.267701	0.612162	1	-
ENSMUSC	Tinf2	Terf1 (TRF	GO:00081	metabolic	-	-	-	-	14:556746	96	154	101	148	172	153	0.461867	0.591718	0.53705	0.549338	0.532881	0.579094	0.530212	0.553771	0.916801	1	-
ENSMUSC	Tssk4	testis-spe	GO:00325	developm	-	-	-	-	14:556501	4	5	3	4	9	4	0.045483	0.045401	0.037702	0.035084	0.065898	0.035778	0.042862	0.045587	1	1	-
ENSMUSC	Hapln4	hyalurona	GO:00325	developm	-	-	-	-	8:7008345	2111	2807	2191	2938	3440	3300	29.90573	31.75809	34.30482	32.11058	31.3819	36.77824	31.98955	33.42357	0.830411	1	-
ENSMUSC	Dus3l	dihydrour	GO:00081	metabolic	-	-	-	-	17:567647	979	1083	821	1281	1484	1259	25.95566	22.93104	24.05687	26.20167	25.33599	26.25948	24.31453	25.93238	0.680582	1	-
ENSMUSC	Gtpbp3	GTP bindi	GO:00081	metabolic	-	-	-	-	8:7148810	221	236	200	322	321	307	2.033432	1.734181	2.033821	2.285718	1.901941	2.222214	1.933811	2.136624	0.554245	1	-
ENSMUSC	Homer1	homer sca	GO:00325	developm	K15010	homer[-]	mmu0406	FoxO sign	13:932996	2067	2435	1622	2505	2706	2163	6.343022	5.967611	5.501149	5.930535	5.347345	5.221835	5.93726	5.499905	0.272444	1	-
ENSMUSC	Rad51c	RAD51 pa	GO:00081	metabolic	K10870	RAD51-lik	mmu0346	Fanconi a	11:873766	65	60	43	81	89	63	0.650726	0.479714	0.475772	0.625605	0.57376	0.496179	0.535404	0.565181	0.884321	1	-
ENSMUSC	Cav1	caveolin 1	GO:00325	developm	K06278	caveolin 1	mmu0414	Endocytos	6:1730633	226	284	202	314	318	75	1.6371	1.642977	1.617204	1.754796	1.483368	0.427403	1.632427	1.221856	0.21268	1	-
ENSMUSC	Arpp19	cAMP-reg	GO:00081	metabolic	-	-	-	-	9:7503761	887	690	837	962	1321	812	4.093924	2.543382	4.269613	3.425483	3.926213	2.948379	3.63564	3.433359	0.566368	1	-
ENSMUSC	Khsrp	KH-type s	GO:00081	metabolic	-	-	-	-	17:570210	1755	2224	1648	2423	2885	2262	26.85638	27.1801	27.8724	28.60581	28.42961	27.23165	27.30296	28.08903	0.95945	1	-
ENSMUSC	Dio2	deiodinas	GO:00081	metabolic	K17904	type II thy	mmu0491	Thyroid hc	12:907245	1329	1755	1265	1710	2084	1635	13.95464	14.71691	14.68016	13.85224	14.09114	13.50586	14.45057	13.81641	0.445333	1	-
ENSMUSC	Ccdc124	coiled-co	GO:00099	cellular pr	-	-	-	-	8:7086822	1146	1363	935	1537	1721	1367	39.24429	37.27639	35.38756	40.60656	37.95137	36.82743	37.30275	38.46179	0.948515	1	-
ENSMUSC	Cct4	chaperoni	GO:00081	metabolic	-	-	-	-	11:229905	1574	1952	1354	2092	2611	2049	15.09194	14.94741	14.34847	15.47506	16.12137	15.45586	14.79594	15.68409	0.697363	1	-
ENSMUSC	Cpt1c	carnitine p	GO:00081	metabolic	K19524	carnitine (	mmu0415	AMPK sigı	7:4495937	1753	2164	1687	2611	2762	2328	12.37111	12.19635	13.15797	14.21556	12.55178	12.92471	12.57514	13.23068	0.766727	1	-
ENSMUSC	Twist2	twist basic	GO:00325	developm	K09069	twist[-]	mmu0520	Proteogly	1:9180146	8	8	9	13	25	11	0.12675	0.101223	0.15758	0.15889	0.255048	0.137101	0.128518	0.18368	0.321078	1	-
ENSMUSC	Zfp655	zinc finge	GO:00081	metabolic	-	-	-	-	5:1452317	700	754	380	768	1024	719	3.82981	3.294552	2.297784	3.241684	3.607728	3.094702	3.140716	3.314705	0.824435	1	-
ENSMUSC	Rhoa	ras homol	GO:00325	developm	K04513	Ras homo	mmu0520	Pathways	9:1083061	2332	2965	2015	3187	3878	3240	17.02792	17.29035	16.26129	17.95335	18.23457	18.61183	16.85985	18.26659	0.514646	1	-
ENSMUSC	Zmiz1	zinc finge	GO:00325	developm	-	-	-	-	14:254591	3012	3879	2721	3924	4485	3931	11.24607	11.56676	11.22849	11.3033	10.78358	11.54675	11.34711	11.21121	0.671702	1	-
ENSMUSC	Ankrd26	ankyrin re	-	-	-	-	-	-	6:1185013	490	633	370	572	628	596	3.830302	3.95172	3.196578	3.449558	3.161196	3.665168	3.659533	3.425307	0.424301	1	-
ENSMUSC	Aldh16a1	aldehyde	GO:00081	metabolic	-	-	-	-	7:4514068	72	103	74	117	170	118	0.431804	0.493326	0.490492	0.541338	0.656533	0.556731	0.471874	0.584868	0.209312	1	-
ENSMUSC	Hnrnpa0	heteroger	GO:00081	metabolic	-	-	-	-	13:581258	1331	1504	1111	1674	1881	1357	30.34665	27.38586	27.99586	29.44547	27.61698	24.34017	28.57612	27.13421	0.439275	1	-
ENSMUSC	Prrg2	proline-ri	GO:00081	metabolic	-	-	-	-	7:4505358	55	61	40	73	76	61	0.586789	0.519753	0.471654	0.600858	0.522141	0.511988	0.526065	0.544996	0.973717	1	-
ENSMUSC	Hnrnph1	heteroger	GO:00081	metabolic	-	-	-	-	11:503769	3768	4925	2844	4337	6218	4403	18.37595	19.18188	15.32906	16.31769	19.5274	16.89268	17.62896	17.57926	0.784314	1	-
ENSMUSC	Ift43	intraflagel	GO:00511	localizatio	-	-	-	-	12:860825	86	78	49	90	147	116	2.043974	1.480535	1.287138	1.650262	2.249838	2.168935	1.603882	2.023012	0.249895	1	-
ENSMUSC	Id3	inhibitor c	GO:00325	developm	K17694	DNA-binc	mmu0455	Signaling	4:1361434	208	259	225	295	375	317	5.538638	5.50789	6.621694	6.060264	6.430209	6.640626	5.889407	6.377033	0.658385	1	-
ENSMUSC	Tcap	titin-cap [	GO:00325	developm	-	-	-	-	11:983838	7	9	6	8	16	21	0.450861	0.462957	0.427132	0.397521	0.663589	1.064062	0.446983	0.708391	0.260229	1	-
ENSMUSC	Arid1a	AT rich int	GO:00325	developm	K11653	AT-rich in	mmu0522	Hepatoce	4:1336790	1804	3175	1507	2318	3733	3232	7.236634	10.17161	6.681289	7.173712	9.643015	10.19958	8.029844	9.005437	0.45594	1	-
ENSMUSC	Crlf1	cytokine r	GO:00325	developm	-	-	-	-	8:7049315	270	324	228	377	491	390	7.899237	7.570278	7.372307	8.509277	9.250334	8.976278	7.613941	8.911963	0.25226	1	-
ENSMUSC	Rplp1	ribosomal	GO:00081	metabolic	K02942	large subu	mmu0301	Ribosome	9:6191328	1410	1757	1246	2036	2351	1667	172.5289	171.6961	168.5031	192.1989	185.2465	160.4684	170.9094	179.3046	0.796135	1	-
ENSMUSC	Ttc9b	tetratricor	-	-	-	-	-	-	7:2765391	305	419	278	433	499	442	9.075405	9.956949	9.14234	9.939942	9.561388	10.34664	9.391565	9.949322	0.772944	1	-
ENSMUSC	Phox2a	paired-lik	GO:00325	developm	-	-	-	-	7:1018183	0	1	0	0	1	0	0	0.021939	0	0	0.017683	0	0.007313	0.005894	1	1	-
ENSMUSC	Abhd8	abhydrola	GO:00038	catalytic a	-	-	-	-	8:7145670	2736	3342	2514	3891	4418	3830	32.20648	31.41806	32.70689	35.3361	33.48941	35.46801	32.11048	34.76451	0.523535	1	-
ENSMUSC	Ift22	intraflagel	GO:00511	localizatio	-	-	-	-	5:1369081	609	841	627	913	1126	878	7.848118	8.655463	8.930223	9.077144	9.344187	8.901304	8.477935	9.107545	0.63463	1	-
ENSMUSC	Fzd3	frizzled cl	GO:00325	developm	K02329	frizzled 3[	mmu0520	Pathways	14:651924	2588	3417	2159	3055	3574	3041	12.14498	12.8063	11.19779	11.06049	10.80045	11.2269	12.04969	11.02928	0.199979	1	-
ENSMUSC	1700008C	RIKEN cDI	-	-	-	-	-	-	7:4436004	33	33	14	28	50	26	0.411378	0.328537	0.192885	0.269283	0.401378	0.254982	0.310933	0.308548	0.927434	1	-
ENSMUSC	Mid1ip1	Mid1 inter	GO:00081	metabolic	-	-	-	-	X:1071501	456	541	361	628	741	461	6.456976	6.117974	5.649619	6.860482	6.756741	5.135422	6.074856	6.250882	0.970363	1	-
ENSMUSC	Ap2s1	adaptor-r	GO:00511	localizatio	K11827	AP-2 com	mmu0414	Endocytos	7:1673841	1013	1006	979	1491	1416	1019	32.98765	26.16291	35.23479	37.45847	29.69341	26.10522	31.46178	31.0857	0.765701	1	-
ENSMUSC	4930432K	RIKEN cDI	-	-	-	-	-	-	8:8414802	39	61	41	44	58	66	0.785897	0.981698	0.913123	0.684043	0.75263	1.046296	0.893573	0.827656	0.60189	1	-
ENSMUSC	Fhl2	four and a	GO:00325	developm	K14380	four and a	mmu0438	Osteoclas	1:4312307	325	430	357	453	590	360	8.126086	8.586419	9.86536	8.738292	9.499587	7.08127	8.859289	8.439717	0.577203	1	-
ENSMUSC	Emc10	ER memb	GO:00329	macromo	-	-	-	-	7:4448993	2195	2991	2207	3277	3212	3323	26.74567	29.10591	29.72131	30.80532	25.20282	31.85372	28.5243	29.28729	0.984493	1	-
ENSMUSC	Clstn3	calsynteni	GO:00325	developm	-	-	-	-	6:1244307	6032	6752	5299	7496	8628	7348	59.48999	53.18153	57.75948	57.03513	54.79581	57.01152	56.81033	56.28082	0.688774	1	-
ENSMUSC	Fbxw9	F-box and	GO:00081	metabolic	-	-	-	-	8:8506005	170	185	165	241	244	187	1.247585	1.08427	1.338293	1.364479	1.153091	1.079626	1.223383	1.199065	0.756884	1	-
ENSMUSC	Fnbp4	formin bir	GO:00056	cell|intrace	-	-	-	-	2:9074537	897	1237	884	1405	1617	1494	6.415499	7.065676	6.987746	7.752554	7.447366	8.406196	6.822973	7.868705	0.212142	1	-
ENSMUSC	Cers4	ceramide	GO:00081	metabolic	K04710	ceramide	mmu0110	Metabolic	8:4493026	910	1199	809	1224	1431	1300	2.866873	3.016699	2.816842	2.974944	2.903097	3.221969	2.900138	3.033337	0.830618	1	-
ENSMUSC	Phax	phosphor	GO:00511	localizatio	K14291	phosphor	mmu0301	RNA trans	18:565624	755	1031	718	1064	1289	979	17.87471	19.4938	18.78724	19.43408	19.65168	18.23414	18.71858	19.10663	0.978739	1	-
ENSMUSC	Tle1	transducir	GO:00081	metabolic	-	-	-	-	4:7211714	989	1265	977	1233	1538	1396	3.820962	3.903126	4.171745	3.675105	3.826374	4.242995	3.965278	3.914825	0.701728	1	-
ENSMUSC	1700109H	RIKEN cDI	GO:00431	ion bindin	-	-	-	-	5:3571566	51	54	59	77	89	87	0.82445	0.697168	1.054132	0.960329	0.926493	1.106436	0.858583	0.997752	0.480966	1	-
ENSMUSC	Relt	RELT tum	GO:00325	developm	K05156	tumor nec	mmu0406	Cytokine-	7:1008458	161	203	156	214	276	239	2.202136	2.217484	2.358248	2.258204	2.430986	2.57174	2.259289	2.42031	0.736957	1	-
ENSMUSC	Snrpb2	U2 small r	GO:00081	metabolic	K11094	U2 small r	mmu0304	Spliceosoı	2:1430630	318	350	272	441	467	340	5.887352	5.174955	5.565563	6.298851	5.567557	4.952024	5.542623	5.606144	0.924342	1	-
ENSMUSC	Ubc	ubiquitin (	GO:00055	extracellul	K08770	ubiquitin (	mmu0332	PPAR sign	5:1253859	2275	9509	6552	10410	12344	3661	23.57556	78.69756	75.04147	83.22651	82.3742	29.84635	59.10486	65.14902	0.76682	1	-
ENSMUSC	Prpf31	pre-mRN.	GO:00081	metabolic	K12844	U4/U6 sm	mmu0304	Spliceosoı	7:3629985	635	762	478	739	909	681	4.3815	4.199042	3.645224	3.933906	4.038944	3.696637	4.075255	3.889829	0.506048	1	-
ENSMUSC	Sertad1	SERTA do	GO:00081	metabolic	-	-	-	-	7:2748691	60	71	74	97	119	112	2.291093	2.165198	3.123011	2.857574	2.926147	3.364519	2.526434	3.049414	0.322955	1	-
ENSMUSC	Carhsp1	calcium re	GO:00081	metabolic	-	-	-	-	16:865858	508	674	485	749	960	725	10.88716	11.53604	11.48787	12.38407	13.24881	12.22362	11.30369	12.61883	0.400581	1	-
ENSMUSC	Elk3	ELK3, mer	GO:00325	developm	-	-	-	-	10:932474	121	220	147	201	232	221	1.124	1.632108	1.509189	1.440478	1.387786	1.615039	1.421766	1.481101	0.919685	1	-
ENSMUSC	Herpud2	HERPUD f	GO:00508	response	-	-	-	-	9:2510813	990	1230	894	1280	1513	1251	8.973806	8.904146	8.956233	8.951209	8.831506	8.92092	8.944729	8.901212	0.756546	1	-
ENSMUSC	Rdh13	retinol del	GO:00325	developm	-	-	-	-	7:4424770	253	365	240	421	461	384	1.796245	2.069588	1.88323	2.30599	2.107658	2.1448	1.916354	2.186149	0.357876	1	-
ENSMUSC	Adam21	a disinteg	GO:00081	metabolic	-	-	-	-	12:815585	5	6	6	9	11	5	0.104865	0.100506	0.1391	0.145641	0.148572	0.082505	0.114824	0.125573	1	1	-
ENSMUSC	Nutf2	nuclear tr	GO:00511	localizatio	-	-	-	-	8:1058605	285	69	233	338	400	332	6.288962	1.215995	5.682462	5.754147	5.683926	5.763452	4.395806	5.733841	0.394038	1	-
ENSMUSC	Fut1	fucosyltra	GO:00081	metabolic	K00718	galactosid	mmu0110	Metabolic	7:4561728	3	2	0	0	1	0	0.045227	0.024084	0	0	0.009706	0	0.023104	0.003235	0.261169	1	-
ENSMUSC	Arpc5	actin relat	GO:00325	developm	K05754	actin relat	mmu0414	Endocytos	1:1527665	1812	761	1788	1748	3104	1574	44.52205	14.93302	48.55471	33.13513	49.11265	30.42515	36.00326	37.55764	0.942039	1	-
ENSMUSC	Elavl2	ELAV (em	GO:00508	response	-	-	-	-	4:9125076	955	1856	713	1019	2145	1821	3.26249	5.06372	2.692046	2.685656	4.718761	4.894032	3.672752	4.099483	0.602182	1	-
ENSMUSC	Pou2f2	POU dom	GO:00325	developm	-	-	-	-	7:2508734	129	84	45	179	167	87	0.749999	0.390027	0.289155	0.802889	0.625233	0.397925	0.476394	0.608682	0.370237	1	-
ENSMUSC	Mgst1	microsom	GO:00081	metabolic	K00799	glutathior	mmu0520	Pathways	6:1381403	75	123	73	110	214	122	0.942839	1.23488	1.014249	1.066836	1.732381	1.206549	1.063989	1.335256	0.250212	1	-
ENSMUSC	Nfib	nuclear fa	GO:00325	developm	-	-	-	-	4:8229017	4390	5434	3528	4926	6473	5111	9.216239	9.110761	8.185866	7.978366	8.750835	8.441234	8.837622	8.390145	0.376372	1	-
ENSMUSC	Htr3b	5-hydroxy	GO:00048	signal trar	K04819	5-hydroxy	mmu0472	Serotoner	9:4893500	0	0	0	2	0	0	0	0	0	0.039081	0	0	0	0.013027	0.527318	1	-
ENSMUSC	Ubqln4	ubiquilin 4	GO:00081	metabolic	K04523	ubiquilin[-	mmu0414	Protein pr	3:8855375	1993	2475	1737	2496	2967	2796	21.91801	21.73776	21.11254	21.17721	21.01194	24.19034	21.58944	22.1265	1	1	-
ENSMUSC	Rbfox1	RNA bind	GO:00325	developm	-	-	-	-	16:680922	8165	10390	7179	9908	12929	9605	116.6447	118.5414	113.3495	109.2006	118.9401	107.9486	116.1785	112.0298	0.475693	1	-
ENSMUSC	Rps18	ribosomal	GO:00081	metabolic	K02964	small subu	mmu0301	Ribosome	17:339519	236	350	239	319	373	338	3.484813	4.127449	3.900434	3.634035	3.546759	3.926405	3.837565	3.7024	0.619287	1	-
ENSMUSC	Rps15a	ribosomal	GO:00081	metabolic	K02957	small subu	mmu0301	Ribosome	7:1181043	1207	1420	1033	1517	1835	1538	6.209214	5.833959	5.873221	6.020676	6.078829	6.224386	5.972132	6.107964	0.986411	1	-
ENSMUSC	Ncaph2	non-SMC	GO:00325	developm	-	-	-	-	15:893557	1611	1746	1762	2213	2634	2678	12.94612	11.20558	15.64937	13.72005	13.63059	16.93034	13.26702	14.76032	0.450077	1	-
ENSMUSC	Hipk1	homeodo	GO:00325	developm	K08826	homeodo	mmu0421	Cellular se	3:1037398	2865	3560	2408	3499	4378	3625	12.2966	12.20271	11.42257	11.58603	12.10016	12.23995	11.97396	11.97538	0.783166	1	-
ENSMUSC	Gprc5b	G protein	GO:00325	developm	-	-	-	-	7:1189720	2698	3560	2403	3490	4424	3513	23.02694	24.26556	22.66705	22.98001	24.31444	23.58758	23.31985	23.62734	0.902757	1	-
ENSMUSC	Man1a2	mannosid	GO:00325	developm	K01230	mannosyl	mmu0110	Metabolic	3:1005622	2313	2784	1922	2764	3488	2747	14.54603	13.98247	13.35885	13.41026	14.12538	13.59059	13.96245	13.70874	0.627011	1	-
ENSMUSC	Acyp1	acylphosp	GO:00038	catalytic a	K01512	acylphosp	mmu0062	Pyruvate r	12:852723	128	136	101	219	270	123	3.426334	2.907378	2.988034	4.522638	4.654109	2.590203	3.107249	3.922317	0.234076	1	-
ENSMUSC	Hdac5	histone de	GO:00325	developm	K11406	histone de	mmu0516	Human pa	11:102194	2352	2859	2200	3011	3437	3055	8.939782	8.678599	9.241855	8.82939	8.412476	9.135069	8.953412	8.792312	0.624358	1	-
ENSMUSC	Rala	v-ral simia	GO:00325	developm	K07834	Ras-relate	mmu0520	Pathways	13:178805	1315	1733	1083	1620	2046	1561	26.87129	28.28181	24.45897	25.53925	26.92298	25.09438	26.53736	25.8522	0.596159	1	-
ENSMUSC	Vdac3	voltage-d	GO:00511	localizatio	K15041	voltage-d	mmu0516	HTLV-I inf	8:2257707	572	830	665	854	1047	1001	5.386368	6.242005	6.921	6.204237	6.348947	7.415582	6.183124	6.656255	0.648865	1	-
ENSMUSC	Vps72	vacuolar p	GO:00325	developm	-	-	-	-	3:9511102	418	490	358	630	663	503	5.601907	5.244471	5.302611	6.513745	5.721737	5.303205	5.382997	5.846229	0.611209	1	-
ENSMUSC	Gabpa	GA repeat	GO:00325	developm	-	-	-	-	16:848349	814	983	623	1012	1257	954	6.868617	6.624362	5.810049	6.588031	6.830219	6.332919	6.434343	6.583723	0.994233	1	-
ENSMUSC	Bmp7	bone mor	GO:00325	developm	K16621	bone mor	mmu0406	Cytokine-	2:1728680	103	204	102	166	210	216	1.352467	2.139273	1.480257	1.681629	1.775676	2.231285	1.657332	1.896197	0.50013	1	-
ENSMUSC	Dynll1	dynein lig	GO:00325	developm	K10418	dynein lig	mmu0496	Vasopress	5:1152971	1522	1884	1510	2260	2297	2179	14.50225	14.33665	15.90174	16.61344	14.09407	16.33387	14.91355	15.68046	0.78234	1	-
ENSMUSC	Pdcl	phosducir	GO:00081	metabolic	-	-	-	-	2:3735007	445	478	273	544	675	424	4.316949	3.703315	2.927017	4.071417	4.21672	3.235883	3.649094	3.84134	0.84247	1	-
ENSMUSC	Tmem184	transmem	GO:00511	localizatio	-	-	-	-	15:793606	941	1328	842	1216	1343	1183	6.198026	6.985662	6.129465	6.179131	5.696317	6.129984	6.437718	6.001811	0.328396	1	-
ENSMUSC	Rsph14	radial spo	-	-	-	-	-	-	10:749574	3	4	4	5	10	6	0.051065	0.054372	0.075257	0.065657	0.109613	0.08035	0.060231	0.085207	0.530596	1	-
ENSMUSC	Nf2	neurofibr	GO:00325	developm	K16684	merlin[-]	mmu0453	Tight junc	11:476584	2341	2828	2104	2841	3553	2946	15.10963	14.57731	15.00876	14.14665	14.76733	14.95877	14.89856	14.62425	0.620355	1	-
ENSMUSC	Cabp7	calcium b	GO:00160	membran	-	-	-	-	11:473689	4388	5567	4447	6133	7186	6274	101.9494	103.2964	114.191	109.9313	107.5127	114.6762	106.4789	110.7067	0.860806	1	-
ENSMUSC	Zmat5	zinc finge	GO:00081	metabolic	-	-	-	-	11:470467	109	161	107	168	187	157	4.29375	5.065057	4.65847	5.105645	4.743587	4.865427	4.672426	4.904886	0.867536	1	-
ENSMUSC	Ewsr1	Ewing sar	GO:00081	metabolic	K13209	RNA-binc	mmu0520	Transcript	11:506968	3670	4654	3306	4989	5852	5332	27.44437	27.79453	27.32353	28.78266	28.18034	31.3681	27.52081	29.4437	0.606584	1	-
ENSMUSC	Ap1b1	adaptor p	GO:00325	developm	K12392	AP-1 com	mmu0414	Lysosome	11:498682	2544	3182	2414	3465	4031	3663	21.88389	21.86016	22.95046	22.9954	22.32929	24.78878	22.2315	23.37116	0.768823	1	-
ENSMUSC	Tbx1	T-box 1 [S	GO:00325	developm	-	-	-	-	16:185817	11	13	21	17	36	26	0.377976	0.356734	0.7975	0.450654	0.796559	0.702815	0.510736	0.650009	0.481946	1	-
ENSMUSC	Gnat2	guanine n	GO:00325	developm	K04631	guanine n	mmu0474	Phototran	3:1080927	0	0	4	0	3	0	0	0	0.044326	0	0.019371	0	0.014775	0.006457	0.562641	1	-
ENSMUSC	Bcl2l13	BCL2-like	GO:00099	cellular pr	K15485	Bcl-2-like	mmu0413	Mitophag	6:1208362	901	1084	881	1220	1465	1147	5.697914	5.474774	6.157631	5.952252	5.966001	5.706443	5.776773	5.874899	0.945244	1	-
ENSMUSC	Spatc1l	spermato	GO:00056	cell|intrace	-	-	-	-	10:765622	0	0	0	2	0	0	0	0	0	0.042064	0	0	0	0.014021	0.528423	1	-
ENSMUSC	Prr29	proline ric	-	-	-	-	-	-	11:106365	2	6	7	2	7	10	0.034219	0.081999	0.132401	0.026408	0.077133	0.134625	0.082873	0.079389	1	1	-
ENSMUSC	Mymk	myomake	GO:00325	developm	-	-	-	-	2:2706163	3	5	6	4	8	3	0.082845	0.110268	0.183139	0.085211	0.142271	0.065167	0.125417	0.09755	0.750865	1	-
ENSMUSC	Fam163b	family wit	GO:00160	membran	-	-	-	-	2:2711038	4075	5495	4375	5166	6974	5491	84.08646	90.55489	99.77534	82.24	92.66912	89.13755	91.47223	88.01555	0.477799	1	-
ENSMUSC	Trpm5	transient r	GO:00511	localizatio	K04980	transient r	mmu0474	Taste tran	7:1430691	0	2	0	3	2	2	0	0.009811	0	0.014216	0.007908	0.009665	0.00327	0.010596	0.327684	1	-
ENSMUSC	Ascl2	achaete-s	GO:00325	developm	-	-	-	-	7:1429668	2	3	3	4	6	3	0.050473	0.060484	0.083696	0.077884	0.09752	0.059563	0.064884	0.078322	0.841008	1	-
ENSMUSC	Pttg1ip	pituitary t	GO:00081	metabolic	-	-	-	-	10:775817	662	936	663	917	1190	1143	6.480758	7.317945	7.173447	6.925759	7.501864	8.802882	6.990717	7.743502	0.465964	1	-
ENSMUSC	Trpm2	transient r	GO:00325	developm	K04977	transient r	mmu0462	NOD-like	10:779077	589	776	499	708	790	718	2.014069	2.119176	1.885847	1.867767	1.739567	1.931498	2.006364	1.846277	0.297091	1	-
ENSMUSC	Mpo	myeloper	GO:00325	developm	K10789	myeloper	mmu0520	Transcript	11:877935	3	7	4	0	8	3	0.038042	0.070892	0.056064	0	0.065329	0.029924	0.054999	0.031751	0.328542	1	-
ENSMUSC	Met	met protc	GO:00325	developm	K05099	proto-onc	mmu0520	Pathways	6:1746380	82	107	80	127	112	126	0.432776	0.451001	0.466645	0.517109	0.380646	0.523151	0.450141	0.473636	0.884661	1	-
ENSMUSC	Slc16a12	solute car	GO:00511	localizatio	-	-	-	-	19:346684	34	84	45	75	72	76	0.512467	1.011125	0.74962	0.872115	0.698823	0.901165	0.757737	0.824034	0.807453	1	-
ENSMUSC	Syn2	synapsin I	GO:00452	synapse|c	-	-	-	-	6:1151349	10794	12992	9957	14507	16570	13273	70.32987	67.60507	71.70224	72.92314	69.52398	68.03579	69.87906	70.16097	0.810036	1	-
ENSMUSC	Elk1	ELK1, mer	GO:00325	developm	K04375	ETS doma	mmu0520	Pathways	X:2093339	1782	2183	1561	2600	2913	2301	19.34321	18.92432	18.7271	21.77331	20.36179	19.64936	18.99821	20.59482	0.521136	1	-
ENSMUSC	Nav1	neuron na	GO:00325	developm	-	-	-	-	1:1354345	2807	3511	2961	3480	4341	3625	7.469281	7.461274	8.708062	7.144074	7.438422	7.588489	7.879539	7.390329	0.329151	1	-
ENSMUSC	Myod1	myogenic	GO:00325	developm	-	-	-	-	7:4637647	0	2	4	0	5	5	0	0.052413	0.145055	0	0.105637	0.129055	0.065823	0.078231	0.847007	1	-
ENSMUSC	Otog	otogelin [	GO:00081	metabolic	-	-	-	-	7:4624098	0	0	2	1	0	0	0	0	0.011147	0.003891	0	0	0.003716	0.001297	1	1	-
ENSMUSC	Srp14	signal rec	GO:00511	localizatio	K03104	signal rec	mmu0306	Protein ex	2:1184758	603	891	928	1336	1070	927	15.55473	18.3556	26.45695	26.58777	17.77394	18.81204	20.12243	21.05791	0.882992	1	-
ENSMUSC	6330409D	RIKEN cDI	-	-	-	-	-	-	2:3273262	16	21	21	22	14	23	0.527783	0.55323	0.765617	0.559873	0.297386	0.596877	0.615543	0.484712	0.33086	1	-
ENSMUSC	Cdk9	cyclin-dep	GO:00325	developm	K02211	cyclin-dep	mmu0520	Transcript	2:3270578	982	1299	901	1378	1294	1332	6.314139	6.670487	6.402864	6.835685	5.357863	6.737796	6.462497	6.310448	0.631159	1	-
ENSMUSC	Tor2a	torsin fam	GO:00099	cellular pr	-	-	-	-	2:3275723	177	132	130	227	257	233	1.679711	1.000419	1.36349	1.66195	1.570543	1.739521	1.347873	1.657338	0.244915	1	-
ENSMUSC	Fpgs	folylpolyg	GO:00081	metabolic	K01930	folylpolyg	mmu0110	Metabolic	2:3268260	106	166	88	147	139	157	0.912855	1.141698	0.83758	0.976662	0.770842	1.063669	0.964045	0.937058	0.73139	1	-
ENSMUSC	Mkl2	MKL/myo	GO:00325	developm	-	-	-	-	16:132564	6195	7849	5769	7746	9052	8438	30.73994	31.10441	31.638	29.65306	28.92417	32.93913	31.16078	30.50546	0.587112	1	-
ENSMUSC	Cbx5	chromobc	GO:00081	metabolic	-	-	-	-	15:103191	4784	6016	4095	5686	6670	5697	22.39359	22.48983	21.18523	20.53382	20.10542	20.97924	22.02289	20.53949	0.252376	1	-
ENSMUSC	Sardh	sarcosine	GO:00081	metabolic	K00314	sarcosine	mmu0110	Metabolic	2:2718839	152	273	194	255	269	209	1.290258	1.850727	1.820045	1.669948	1.470417	1.395693	1.653677	1.512019	0.42734	1	-
ENSMUSC	Vav2	vav 2 onc	GO:00325	developm	K05730	guanine n	mmu0481	Regulatioı	2:2726210	237	313	167	303	369	288	1.740532	1.835794	1.355493	1.716747	1.745077	1.663937	1.64394	1.708587	0.916173	1	-
ENSMUSC	Tex15	testis expr	GO:00325	developm	-	-	-	-	8:3351673	23	60	38	38	46	41	0.108251	0.225528	0.197664	0.137981	0.139418	0.151809	0.177148	0.143069	0.284014	1	-
ENSMUSC	Ppp2cb	protein pl	GO:00081	metabolic	K04382	serine/thr	mmu0516	Human pa	8:3359962	1274	1786	1311	1770	2107	2011	52.95306	59.28556	60.2242	56.75774	56.39503	65.7574	57.4876	59.63672	0.894546	1	-
ENSMUSC	G0s2	G0/G1 sw	GO:00508	response	-	-	-	-	1:1932721	72	95	75	114	105	95	5.047298	5.318588	5.810771	6.165411	4.739893	5.239163	5.392219	5.381489	0.868191	1	-
ENSMUSC	Dmap1	DNA metl	GO:00081	metabolic	-	-	-	-	4:1176746	234	308	258	347	381	361	3.01936	3.17391	3.679306	3.454287	3.165759	3.664519	3.290859	3.428188	0.896347	1	-
ENSMUSC	Pla2g12b	phospholi	GO:00081	metabolic	-	-	mmu0110	Metabolic	10:594036	0	0	0	2	0	0	0	0	0	0.054496	0	0	0	0.018165	0.527326	1	-
ENSMUSC	Mcu	mitochon	GO:00511	localizatio	K20858	calcium ui	mmu0421	Cellular se	10:594469	987	1281	880	1371	1804	1230	7.494631	7.76834	7.385206	8.03158	8.821137	7.347666	7.549392	8.066794	0.661481	1	-
ENSMUSC	Oit3	oncoprote	GO:00056	cell|intrace	-	-	-	-	10:594229	0	3	4	0	3	2	0	0.049131	0.090647	0	0.039614	0.032266	0.046593	0.02396	0.462436	1	-
ENSMUSC	Tex11	testis expr	GO:00325	developm	-	-	-	-	X:1008386	5	2	3	1	4	6	0.077498	0.024763	0.051399	0.01196	0.039929	0.07317	0.05122	0.041686	0.713385	1	-
ENSMUSC	Bcr	breakpoir	GO:00325	developm	K08878	breakpoir	mmu0520	Pathways	10:750605	3606	4504	3132	4788	5110	4755	24.08923	24.02931	23.12415	24.67639	21.98228	24.98956	23.74756	23.88274	0.826044	1	-
ENSMUSC	Fxyd5	FXYD dor	GO:00511	localizatio	-	-	-	-	7:3103272	120	148	119	159	165	128	1.037962	1.022372	1.137612	1.061027	0.91905	0.871005	1.065982	0.950361	0.335594	1	-
ENSMUSC	Kcnd1	potassium	GO:00511	localizatio	-	-	-	-	X:7822289	191	222	180	234	264	219	1.93145	1.792868	2.011732	1.825559	1.719132	1.742226	1.912017	1.762306	0.404157	1	-

ENSMUSG	Tfcp2	transcripti	GO:00081	metabolic	-	-	-	-	15:100502	435	503	379	507	614	498	8.172397	7.546993	7.869473	7.348488	7.428196	7.360379	7.862954	7.379021	0.426158	1	-
ENSMUSG	Pou6f2	POU dom	GO:00325	developm	-	-	-	-	13:181210	21	18	29	24	18	30	0.148083	0.101365	0.226006	0.130563	0.081735	0.16642	0.158485	0.126239	0.389556	1	-
ENSMUSG	Pou6f1	POU dom	GO:00081	metabolic	-	-	-	-	15:100575	1936	2339	1256	2389	2717	2586	9.406264	9.075861	6.744476	8.954846	8.500725	9.88442	8.408867	9.113331	0.596612	1	-
ENSMUSG	Ubp1	upstream	GO:00325	developm	-	-	-	-	9:1139309	1145	1644	1114	1721	2121	1907	6.674134	7.653092	7.176639	7.73928	7.961314	8.744821	7.167955	8.148472	0.263373	1	-
ENSMUSG	Ick	intestinal	GO:00325	developm	-	-	-	-	9:7810919	440	578	409	629	716	630	1.644863	1.725642	1.689846	1.814089	1.723634	1.8528	1.686784	1.796841	0.712411	1	-
ENSMUSG	Sdhb	succinate	GO:00081	metabolic	K00235	succinate	mmu0110	Metabolic	4:1409612	1163	1413	1128	1648	2220	1502	27.22798	26.41945	29.18716	29.7662	33.46904	27.66411	27.61153	30.29978	0.475735	1	-
ENSMUSG	Cox4i2	cytochron	GO:00081	metabolic	K02263	cytochron	mmu0110	Metabolic	2:1527541	10	20	7	18	12	7	0.52501	0.838571	0.406198	0.729083	0.405711	0.289131	0.589926	0.474642	0.506025	1	-
ENSMUSG	Snap47	synaptosc	GO:00452	synapse|lc	-	-	-	-	11:594071	3437	4350	4394	5703	7048	4954	50.80046	51.34796	71.7786	65.03122	67.08242	57.60432	57.97567	63.23932	0.551768	1	-
ENSMUSG	Kdsr	3-ketodih	GO:00081	metabolic	K04708	3-dehydro	mmu0110	Metabolic	1:1067204	578	738	529	797	875	709	4.107018	4.187941	4.154329	4.369051	4.003699	3.963288	4.149763	4.112013	0.746529	1	-
ENSMUSG	Nxf2	nuclear RI	GO:00081	metabolic	K14284	nuclear RI	mmu0516	Herpes sir	X:1349445	0	6	2	5	5	2	0	0.094684	0.043681	0.076218	0.063622	0.031096	0.046122	0.056979	1	1	-
ENSMUSG	Kif19a	kinesin fai	GO:00990	supramole	-	-	-	-	11:114765	68	96	63	81	123	95	0.738256	0.832363	0.75594	0.678443	0.85992	0.8114	0.77552	0.783254	0.95983	1	-
ENSMUSG	Aldh3a2	aldehyde	GO:00325	developm	K00128	aldehyde	mmu0110	Metabolic	11:612234	747	936	654	1078	1182	838	6.596815	6.601392	6.383204	7.344514	6.721806	5.821956	6.527137	6.629425	0.940676	1	-
ENSMUSG	Zmynd10	zinc finge	GO:00099	cellular pr	-	-	-	-	9:1075472	45	51	36	65	79	77	0.852765	0.771847	0.754002	0.950299	0.964054	1.14794	0.792871	1.020764	0.191231	1	-
ENSMUSG	Tmem115	transmem	GO:00081	metabolic	-	-	-	-	9:1075339	321	441	317	469	531	523	8.933298	9.801463	9.750199	10.06954	9.516032	11.45036	9.494987	10.34531	0.597769	1	-
ENSMUSG	Hyal2	hyaluronc	GO:00325	developm	K01197	hyaluronc	mmu0110	Metabolic	9:1075679	65	73	64	97	122	87	0.509864	0.457309	0.554842	0.587007	0.616249	0.536871	0.507338	0.580042	0.49947	1	-
ENSMUSG	Ifrd2	interferon	GO:00056	cell|intrace	-	-	-	-	9:1075876	84	128	100	143	157	139	1.102984	1.342293	1.45123	1.448631	1.32753	1.435874	1.298836	1.404012	0.737686	1	-
ENSMUSG	Hyal1	hyaluronc	GO:00325	developm	K01197	hyaluronc	mmu0110	Metabolic	9:1075769	59	66	37	124	94	48	0.453422	0.405078	0.314262	0.735192	0.465193	0.290205	0.390921	0.496863	0.365445	1	-
ENSMUSG	Tusc2	tumor sur	GO:00325	developm	-	-	-	-	9:1075632	1134	1606	1217	1710	1891	1603	29.12911	32.94622	34.55026	33.88758	31.27954	32.39355	32.20853	32.52022	0.882982	1	-
ENSMUSG	Nprl2	nitrogen p	GO:00081	metabolic	K20405	nitrogen p	mmu0415	mTOR sig	9:1075422	291	360	298	462	471	503	4.850641	4.792412	5.489968	5.941257	5.055704	6.596069	5.04434	5.864343	0.300596	1	-
ENSMUSG	Rassf1	Ras assoc	GO:00081	metabolic	K09850	Ras assoc	mmu0520	Pathways	9:1075515	116	161	142	187	250	181	0.802848	0.889916	1.086201	0.998502	1.114221	0.985522	0.926322	1.032748	0.548075	1	-
ENSMUSG	Epn3	epsin 3 [S	GO:00055	extracellul	K12471	epsin[-]	mmu0414	Endocytos	11:944895	54	128	47	74	107	78	0.639472	1.210563	0.615144	0.676071	0.815963	0.72667	0.821726	0.739568	0.527204	1	-
ENSMUSG	Rnf112	ring finge	GO:00325	developm	-	-	-	-	11:614484	5463	6632	5275	8142	8889	7444	56.05116	54.34302	59.81673	64.4488	58.73013	60.08563	56.73697	61.08819	0.557096	1	-
ENSMUSG	Slc3a2	solute car	GO:00081	metabolic	K06519	solute car	mmu0415	mTOR sig	19:870688	1857	2120	1533	2351	2776	2179	17.07347	15.56652	15.57754	16.67604	16.43554	15.76079	16.07251	16.29079	0.905438	1	-
ENSMUSG	Nxf1	nuclear RI	GO:00325	developm	K14284	nuclear RI	mmu0516	Herpes sir	19:875707	1210	1837	1245	1763	2047	1970	7.282438	8.829707	8.281473	8.18604	7.933482	9.32757	8.131206	8.482364	0.849407	1	-
ENSMUSG	Stx5a	syntaxin 5	GO:00081	metabolic	K08490	syntaxin 5	mmu0413	SNARE int	19:874141	479	608	444	763	769	676	1.848958	1.874309	1.894182	2.272201	1.911493	2.052812	1.872483	2.078835	0.452361	1	-
ENSMUSG	Slc47a1	solute car	GO:00511	localizatio	-	-	-	-	11:613434	29	23	16	32	42	42	0.371758	0.235475	0.22669	0.316481	0.346711	0.42357	0.277974	0.362254	0.314102	1	-
ENSMUSG	Pifo	primary ci	GO:00081	metabolic	-	-	-	-	3:1059969	5	21	17	21	22	6	0.224293	0.752409	0.842917	0.726828	0.635579	0.211768	0.60654	0.524725	0.677149	1	-
ENSMUSG	Tnfrsf13b	tumor nec	GO:00325	developm	K05150	tumor nec	mmu0406	Cytokine-	11:611267	13	40	20	27	21	43	0.16792	0.412633	0.285524	0.269058	0.174672	0.436959	0.288692	0.293563	1	1	-
ENSMUSG	Spire2	spire hom	GO:00511	localizatio	-	-	-	-	8:1233327	238	368	271	359	467	373	5.789584	7.14929	7.285952	6.737425	7.315459	7.138203	6.741609	7.063696	0.851459	1	-
ENSMUSG	Prox1	prospero	GO:00325	developm	-	-	-	-	1:1901180	2963	4537	3300	4054	5044	3823	17.82065	21.79246	21.93575	18.81074	19.53536	18.08868	20.51629	18.81159	0.232685	1	-
ENSMUSG	2610507B	RIKEN cDI	GO:00055	extracellul	-	-	-	-	11:782617	6555	8315	6028	8396	10425	8706	36.11585	36.58755	36.70669	35.68847	36.98758	37.73591	36.47003	36.80399	0.855766	1	-
ENSMUSG	AI597479	expressed	GO:00325	developm	-	-	-	-	1:4309871	520	590	436	596	724	555	9.181679	8.319868	8.508465	8.118854	8.232121	7.70943	8.670004	8.020135	0.340912	1	-
ENSMUSG	Tmem86a	transmem	GO:00160	membran	-	-	-	-	7:4705060	165	256	170	222	265	241	4.720985	5.84971	5.375804	4.900409	4.882583	5.424712	5.3155	5.069235	0.58888	1	-
ENSMUSG	Optc	opticin [S	-	-	-	-	-	-	1:1338971	10	11	10	18	28	15	0.115993	0.101895	0.128192	0.16108	0.209142	0.136876	0.11536	0.169033	0.209052	1	-
ENSMUSG	Tex14	testis expr	GO:00081	metabolic	-	-	-	-	11:874050	2	12	7	3	19	7	0.020458	0.098047	0.079157	0.023682	0.125176	0.056344	0.065887	0.068401	1	1	-
ENSMUSG	Rdm1	RAD52 m	GO:00081	metabolic	-	-	-	-	11:101627	39	55	26	72	69	57	0.894877	1.007882	0.659348	1.274559	1.019542	1.028928	0.854036	1.107676	0.232868	1	-
ENSMUSG	Nedd8	neural pre	GO:00081	metabolic	-	-	-	-	14:556622	600	717	433	781	857	625	13.27351	12.66774	10.58691	13.32955	12.20871	10.8774	12.17605	12.13855	0.812763	1	-
ENSMUSG	Gosr1	golgi SNA	GO:00511	localizatio	K08495	golgi SNA	mmu0413	SNARE int	11:767266	937	1302	873	1292	1514	1348	13.19148	14.63898	13.58361	14.03288	13.72569	14.92983	13.80469	14.22947	0.949186	1	-
ENSMUSG	Mrpl52	mitochon	GO:00081	metabolic	-	-	-	-	14:544269	94	104	80	134	159	73	2.344552	2.07162	2.205317	2.578502	2.553789	1.432409	2.207163	2.188233	0.875063	1	-
ENSMUSG	Kansl3	KAT8 regu	GO:00081	metabolic	-	-	-	-	1:3633573	1418	1766	1255	1998	2182	1927	9.741266	9.688932	9.528617	10.58925	9.652696	10.41434	9.652938	10.21876	0.715265	1	-
ENSMUSG	Eya4	EYA trans	GO:00325	developm	-	-	-	-	10:231029	112	147	98	151	145	164	0.542651	0.568811	0.524777	0.564433	0.452405	0.625113	0.545413	0.547317	0.892676	1	-
ENSMUSG	Ebf3	early B ce	GO:00325	developm	-	-	-	-	7:1371936	38	53	26	36	56	53	0.125606	0.139911	0.094983	0.091804	0.119199	0.137822	0.120167	0.116275	0.783277	1	-
ENSMUSG	Myt1	myelin tra	GO:00325	developm	-	-	-	-	2:1817633	311	416	305	419	483	416	1.398827	1.494317	1.516176	1.453944	1.39896	1.472	1.469773	1.441635	0.70361	1	-
ENSMUSG	Faf1	Fas-assoc	GO:00081	metabolic	K20703	FAS-assoc	mmu0421	Necroptos	4:1096765	513	574	429	626	729	633	5.985635	5.348725	5.532186	5.635042	5.477406	5.810418	5.622182	5.640955	0.845712	1	-
ENSMUSG	Gm266	predicted	GO:00508	response	-	-	-	-	12:111484	19	27	31	51	35	35	0.954811	1.083623	1.721777	1.977274	1.132622	1.383705	1.253404	1.497867	0.544659	1	-
ENSMUSG	Mettl16	methyltrai	GO:00081	metabolic	-	-	-	-	11:747708	877	1105	711	1160	1208	848	3.500094	3.521993	3.13615	3.571648	3.104573	2.662484	3.386079	3.112902	0.32617	1	-
ENSMUSG	Dazl	deleted in	GO:00325	developm	-	-	-	-	17:502793	61	89	64	85	114	74	1.265987	1.475159	1.468012	1.360966	1.523561	1.208219	1.403053	1.364249	0.770864	1	-
ENSMUSG	Pigyl	phosphati	GO:00081	metabolic	K11001	phosphati	mmu0110	Metabolic	9:2215684	198	188	223	258	296	309	8.818024	6.686682	10.97635	8.864532	8.488904	10.82619	8.827017	9.393207	0.804992	1	-
ENSMUSG	Rbm25	RNA bind	GO:00081	metabolic	K12822	RNA-binc	mmu0304	Spliceosor	12:836312	2806	3325	2376	3333	4472	3315	7.963237	7.535976	7.452385	7.297392	8.172567	7.401103	7.650533	7.623687	0.759513	1	-
ENSMUSG	Psen2	presenilin	GO:00081	metabolic	K04522	presenilin	mmu0501	Alzheimer	1:1802270	162	237	195	251	262	235	1.354061	1.582039	1.801379	1.618554	1.410193	1.545259	1.579159	1.524669	0.664654	1	-
ENSMUSG	Fads1	fatty acid	GO:00325	developm	K10224	acyl-CoA	mmu0121	Fatty acid	19:101828	4214	5603	3728	5518	6370	5188	74.44991	79.05623	72.79345	75.21105	72.47091	72.1075	75.4332	73.26315	0.522175	1	-
ENSMUSG	Lmbr1	limb regic	GO:00325	developm	-	-	-	-	5:2922980	514	668	287	442	562	636	2.534225	2.630297	1.56391	1.68126	1.784324	2.466893	2.242811	1.977492	0.321235	1	-
ENSMUSG	Tnfrsf22	tumor nec	GO:00325	developm	-	-	-	-	7:1436348	17	23	7	36	20	33	0.131977	0.142603	0.060065	0.215613	0.099983	0.201546	0.111548	0.172381	0.199195	1	-
ENSMUSG	Cars	cysteinyl-	GO:00081	metabolic	K01883	cysteinyl-t	mmu0097	Aminoacy	7:1435572	663	863	608	879	1080	931	4.673459	4.85827	4.736698	4.780182	4.902342	5.162806	4.756142	4.948443	0.878188	1	-
ENSMUSG	Wnt2	wingless-	GO:00325	developm	K00182	wingless-t	mmu0520	Pathways	6:1798894	53	86	75	86	108	85	1.435078	1.859693	2.244426	1.796486	1.883112	1.810607	1.846399	1.830068	0.861522	1	-
ENSMUSG	Gabra1	gamma-a	GO:00048	signal trar	K05175	gamma-a	mmu0408	Neuroacti	11:421309	6821	7617	5596	8014	9704	7191	49.23482	43.90906	44.64254	44.62765	45.10553	40.83425	45.92881	43.52248	0.370545	1	-
ENSMUSG	Grid2ip	glutamate	GO:00452	synapse|re	-	-	-	-	5:1433573	26	25	27	35	29	29	0.192169	0.147568	0.220556	0.199575	0.138026	0.168622	0.186765	0.168741	0.59487	1	-
ENSMUSG	Kdelr3	KDEL (Lys	GO:00511	localizatio	-	-	-	-	15:795164	3	13	12	16	22	9	0.12954	0.448314	0.572684	0.533026	0.611756	0.305728	0.383513	0.483504	0.576227	1	-
ENSMUSG	Pdhx	pyruvate	GO:00081	metabolic	K13997	dihydrolip	mmu0110	Metabolic	2:1030210	932	1208	764	1170	1409	1131	13.28962	13.75655	12.04028	12.87102	12.93785	12.68733	13.02882	12.83207	0.687143	1	-
ENSMUSG	Vac14	Vac14 hor	GO:00081	metabolic	K15305	vacuole m	mmu0516	HTLV-I int	8:1106185	363	493	389	522	592	576	1.923299	2.086088	2.277912	2.133736	2.019838	2.400897	2.095766	2.184824	0.883219	1	-
ENSMUSG	Akt1s1	AKT1 subs	GO:00081	metabolic	K16184	proline-ri	mmu0415	mTOR sig	7:4484899	514	761	548	727	778	736	4.205259	4.972337	4.955157	4.588756	4.098869	4.737168	4.710917	4.474931	0.479255	1	-
ENSMUSG	Tbrg1	transform	GO:00081	metabolic	-	-	-	-	9:3764876	373	433	400	570	615	537	3.705007	3.434895	4.391239	4.368029	3.93378	4.196292	3.843714	4.166034	0.631549	1	-
ENSMUSG	Adssl1	adenylosu	GO:00081	metabolic	K01939	adenylosu	mmu0110	Metabolic	12:112620	174	218	177	201	252	240	3.233138	3.235029	3.634928	2.881394	3.015301	3.508305	3.367698	3.135	0.467048	1	-
ENSMUSG	Cfap161	cilia and f	GO:00160	membran	-	-	-	-	7:8377410	35	33	29	44	43	29	0.466293	0.351118	0.42701	0.452247	0.368906	0.303946	0.414807	0.375033	0.58112	1	-
ENSMUSG	Brf1	BRF1, RN/	GO:00081	metabolic	-	-	-	-	12:112960	709	947	694	1046	1206	1088	4.443665	4.74014	4.807303	5.057747	4.8674	5.36457	4.663703	5.096572	0.518898	1	-
ENSMUSG	Vipr2	vasoactive	GO:00048	signal trar	K04590	vasoactive	mmu0408	Neuroacti	12:116077	9	24	9	22	18	18	0.10635	0.226493	0.117537	0.200566	0.136974	0.167331	0.150127	0.16829	0.88376	1	-
ENSMUSG	Odc1	ornithine	GO:00325	developm	K01581	ornithine	mmu0110	Metabolic	12:175447	920	1094	771	1015	1222	1288	8.139901	7.730264	7.539328	6.928319	6.962372	8.965158	7.803164	7.618617	0.646473	1	-
ENSMUSG	Thg1l	tRNA-hist	GO:00081	metabolic	-	-	-	-	11:459468	95	164	139	139	203	203	1.535753	2.117316	2.483472	1.733568	2.113233	2.581679	2.045514	2.142827	0.896235	1	-
ENSMUSG	Adam19	a disinteg	GO:00325	developm	-	-	-	-	11:460559	331	462	302	492	530	442	2.715336	3.026796	2.738103	3.113798	2.799794	2.852519	2.826745	2.922037	0.942444	1	-
ENSMUSG	Pabpc4	poly(A) bi	GO:00325	developm	K13126	polyadeny	mmu0301	RNA trans	4:1232623	453	504	393	626	826	584	3.141319	2.791198	3.011986	3.349026	3.688489	3.185939	2.981501	3.407818	0.322519	1	-
ENSMUSG	Exoc3l2	exocyst cc	GO:00511	localizatio	-	-	-	-	7:1946333	1	7	2	4	8	9	0.010022	0.056013	0.022149	0.030916	0.051618	0.070939	0.029395	0.051158	0.357084	1	-
ENSMUSG	Zfp296	zinc finge	GO:00081	metabolic	-	-	-	-	7:1957728	10	18	7	15	18	22	0.281506	0.404665	0.2178	0.325766	0.326299	0.487218	0.301324	0.379761	0.51361	1	-
ENSMUSG	Plin5	perilipin 5	GO:00081	metabolic	K20255	perilipin-5	mmu0332	PPAR sign	17:561116	8	10	8	10	22	4	0.15572	0.155437	0.172084	0.150167	0.275749	0.061246	0.16108	0.162387	1	1	-
ENSMUSG	Sugp1	SURP and	GO:00081	metabolic	-	-	-	-	8:7004281	1068	1354	953	1458	1798	1506	9.633629	9.754013	9.50076	10.14628	10.44391	10.68697	9.629467	10.42572	0.549631	1	-
ENSMUSG	Dhdh	dihydrodi	GO:00081	metabolic	-	-	mmu0098	Metabolis	7:4547098	244	329	211	346	359	299	1.936088	2.084857	1.850396	2.118073	1.834358	1.866453	1.957113	1.939628	0.791331	1	-
ENSMUSG	Zfp790	zinc finge	GO:00081	metabolic	-	-	-	-	7:2981611	331	439	296	396	477	429	2.933698	3.107406	2.899518	2.707773	2.722454	2.991272	2.980207	2.807166	0.458265	1	-
ENSMUSG	Slc25a41	solute car	GO:00511	localizatio	-	-	-	-	17:570327	0	0	0	0	4	0	0	0	0	0	0.09452	0	0	0.031507	0.29552	1	-
ENSMUSG	Fsd1	fibronecti	GO:00990	supramole	-	-	-	-	17:559865	500	648	482	783	733	654	17.46514	18.07685	18.60782	21.10056	16.48773	17.97177	18.04994	18.52002	0.993738	1	-
ENSMUSG	Pinlyp	phospholi	GO:00987	molecular	-	-	-	-	7:2454165	0	0	0	0	2	0	0	0	0	0	0.093263	0	0	0.031088	0.528766	1	-
ENSMUSG	Fuz	fuzzy plar	GO:00325	developm	-	-	-	-	7:4489607	161	223	162	235	229	231	0.761333	0.842171	0.846665	0.857329	0.697332	0.859355	0.816723	0.804672	0.763935	1	-
ENSMUSG	Sptbn4	spectrin b	GO:00325	developm	-	-	-	-	7:2735638	4058	5053	3481	5685	6297	5781	20.4417	20.32825	19.38009	22.09356	20.42647	22.90967	20.05001	21.8099	0.476429	1	-
ENSMUSG	Pgam1	phosphog	GO:00081	metabolic	K01834	2,3-bisph	mmu0110	Metabolic	19:419118	5745	7534	5556	8098	9274	8117	191.5777	200.6441	204.7689	208.3354	199.1484	212.9421	198.9969	206.8086	0.86097	1	-
ENSMUSG	Evi5	ecotropic	GO:00511	localizatio	-	-	-	-	5:1077447	1449	1743	1097	1733	1979	1863	8.446936	8.114728	7.067802	7.793986	7.429017	8.543866	7.876489	7.922289	0.844459	1	-
ENSMUSG	Evi5l	ecotropic	GO:00511	localizatio	-	-	-	-	8:4166567	1524	2127	1359	1954	2826	2027	5.651881	6.299721	5.570245	5.590656	6.748928	5.913883	5.840616	6.084489	0.85136	1	-
ENSMUSG	Snapc2	small nucl	GO:00081	metabolic	-	-	-	-	8:4253080	234	311	267	389	356	318	4.178886	4.435582	5.269901	5.359494	4.094009	4.467681	4.628123	4.640395	0.876182	1	-
ENSMUSG	Git1	G protein-	GO:00325	developm	K05737	G protein-	mmu0414	Endocytos	11:774935	7554	9626	7063	10199	12003	11393	76.38837	77.73954	78.93802	79.56795	78.16183	90.63569	77.68864	82.78849	0.645413	1	-
ENSMUSG	Bnip2	BCL2/ade	GO:00325	developm	-	-	-	-	9:6998946	400	423	336	480	568	630	4.192836	3.541063	3.892541	3.881673	3.833986	5.195156	3.87548	4.303605	0.528305	1	-
ENSMUSG	Ccnt1	cyclin T1 [	GO:00081	metabolic	K15188	cyclin T[-]	mmu0520	Transcript	15:985386	896	1136	724	1093	1330	1060	4.228808	4.281874	3.77655	3.979791	4.042185	3.935742	4.095744	3.985906	0.604375	1	-
ENSMUSG	Scarf2	scavenger	GO:00511	localizatio	-	-	-	-	16:177972	33	63	37	49	69	62	0.610215	0.930357	0.756152	0.69902	0.821623	0.901923	0.765574	0.807522	0.887973	1	-
ENSMUSG	Brms1l	breast car	GO:00081	metabolic	-	-	-	-	12:558363	1062	1312	966	1496	1894	1366	11.93076	11.77127	11.9941	12.96599	13.70183	12.07273	11.89871	12.91351	0.540668	1	-
ENSMUSG	Med15	mediator	GO:00081	metabolic	-	-	-	-	16:176512	921	1326	1042	1595	1308	1648	16.62268	19.11308	20.78527	22.20917	15.20211	23.39965	18.84034	20.27031	0.687397	1	-
ENSMUSG	Dhdds	dehydrod	GO:00081	metabolic	K11778	ditrans,po	mmu0090	Terpenoic	4:1339690	493	1056	518	1183	564	873	1.955796	3.345694	2.271188	3.620693	1.440822	2.724593	2.524226	2.595369	0.985317	1	-
ENSMUSG	Ubxn11	UBX dom	GO:00081	metabolic	-	-	-	-	4:1341025	145	240	129	173	261	175	1.258838	1.664027	1.237766	1.158725	1.459143	1.195231	1.386877	1.271033	0.456949	1	-
ENSMUSG	Mogat1	monoacyl	GO:00081	metabolic	K14458	2-acylglyc	mmu0056	Glycerolip	1:7851099	2	0	2	0	3	3	0.04281	0	0.047326	0	0.041364	0.05052	0.030045	0.030628	1	1	-
ENSMUSG	Tjap1	tight junct	GO:00099	cellular pr	K06105	tight junct	mmu0453	Tight junc	17:462578	311	356	170	426	446	347	1.732263	1.583614	1.046521	1.830599	1.599716	1.520524	1.454133	1.65028	0.484188	1	-
ENSMUSG	Rpl15	ribosomal	GO:00081	metabolic	K02877	large subu	mmu0301	Ribosome	14:182678	646	762	578	797	1025	676	9.792343	9.224756	9.683418	9.320571	10.00535	8.061413	9.566839	9.129111	0.50967	1	-
ENSMUSG	Tmem167	transmem	GO:00511	localizatio	-	-	-	-	13:900891	804	962	705	922	1134	961	4.396447	4.201133	4.260706	3.889618	3.993129	4.134091	4.286095	4.005613	0.358533	1	-
ENSMUSG	Mplkip	M-phase	GO:00099	cellular pr	-	-	-	-	13:176951	94	140	100	124	183	139	0.758185	0.901824	0.891442	0.771612	0.950504	0.88201	0.850484	0.868042	1	1	-
ENSMUSG	Rpa3	replicatior	GO:00081	metabolic	K10740	replicatior	mmu0346	Fanconi a	6:8255936	35	56	38	53	83	36	1.580638	2.019781	1.896672	1.846606	2.413804	1.279028	1.832363	1.846479	0.973713	1	-
ENSMUSG	Tnpo3	transporti	GO:00511	localizatio	-	-	-	-	6:2954082	1266	1406	998	1429	2167	1415	4.32445	3.835557	3.767684	3.765821	4.766623	3.802461	3.975897	4.111635	0.925858	1	-
ENSMUSG	Ttll5	tubulin tyr	GO:00325	developm	-	-	-	-	12:858246	787	1014	670	1016	1135	927	2.344378	2.41233	2.205845	2.334946	2.177227	2.17242	2.320851	2.228198	0.520251	1	-
ENSMUSG	Retn	resistin [S	GO:00508	response	-	-	-	-	8:3655770	0	7	0	4	2	0	0	0.19834	0	0.109472	0.045683	0	0.066113	0.051718	0.842759	1	-
ENSMUSG	Cdh23	cadherin 2	GO:00325	developm	-	-	-	-	10:603027	38	43	30	33	49	60	0.107846	0.097461	0.0941	0.072256	0.089551	0.133962	0.099802	0.09859	0.908691	1	-
ENSMUSG	Rps5	ribosomal	GO:00081	metabolic	K02989	small subu	mmu0301	Ribosome	7:1292229	920	1204	962	1368	1588	1321	20.6672	21.60063	23.88448	23.70885	22.97201	23.34574	22.05077	23.3422	0.729412	1	-
ENSMUSG	Podnl1	podocan-	GO:00081	metabolic	-	-	-	-	8:8412598	1	0	0	0	2	2	0.012252	0	0	0	0.015771	0.019275	0.004084	0.011682	0.646136	1	-
ENSMUSG	Adgrl1	adhesion	GO:00325	developm	-	-	-	-	8:8390010	9473	11669	8521	12429	11773	11367	39.12626	38.49109	38.89718	39.60475	31.31285	36.93495	38.83818	35.95085	0.256453	1	-
ENSMUSG	Amotl1	angiomot	GO:00400	locomotic	K06104	angiomot	mmu0453	Tight junc	9:1454196	528	705	417	595	697	609	2.85071	3.039862	2.488297	2.47837	2.423296	2.586706	2.792957	2.496124	0.206986	1	-
ENSMUSG	Gfod2	glucose-f	GO:00081	metabolic	-	-	-	-	8:1057138	263	350	236	362	503	381	3.110866	3.306277	3.085206	3.303424	3.831315	3.545369	3.16745	3.560036	0.429708	1	-
ENSMUSG	Enkd1	enkurin d	GO:00990	supramole	-	-	-	-	8:1057036	94	128	88	143	135	130	0.952924	1.036305	0.985961	1.118402	0.881287	1.036778	0.99173	1.012156	0.984573	1	-
ENSMUSG	4933405L	RIKEN cDI	-	-	-	-	-	-	8:1057076	0	7	3	3	4	4	0	0.137472	0.081539	0.056922	0.063343	0.077377	0.073004	0.065881	0.852848	1	-
ENSMUSG	Atp6v0d1	ATPase, H	GO:00325	developm	K02146	V-type H-	mmu0110	Metabolic	8:1055244	2611	3369	2542	3548	4281	3459	74.91669	77.20023	80.61097	78.53904	79.09911	78.07881	77.57596	78.57232	0.898448	1	-
ENSMUSG	Ptprs	protein ty	GO:00325	developm	-	-	-	-	17:564124	9277	11235	8697	12144	13723	12522	25.52434	24.68686	26.44619	25.77739	24.31368	27.10388	25.55246	25.73165	0.835069	1	-
ENSMUSG	Slc41a1	solute car	GO:00511	localizatio	-	-	-	-	1:1318274	1465	1919	1293	1883	2233	1595	16.75724	17.53014	16.34594	16.61672	16.44781	14.35279	16.87778	15.80578	0.335519	1	-
ENSMUSG	Iglon5	IgLON far	GO:00055	extracellul	-	-	-	-	7:4347290	451	553	413	624	699	641	9.215936	9.024715	9.327379	9.837332	9.198032	10.30461	9.189343	9.779991	0.726129	1	-
ENSMUSG	Zfp651	zinc finge	GO:00971	organic cy	-	-	-	-	9:1217593	1018	1721	1325	1932	2132	1353	6.803542	9.185734	9.787007	9.961497	9.175488	7.113708	8.592094	8.750231	0.974691	1	-
ENSMUSG	Nelfb	negative e	GO:00081	metabolic	-	-	-	-	2:2519971	541	699	533	861	927	786	5.233275	5.400068	5.698368	6.42554	5.774454	5.981496	5.443904	6.060497	0.414055	1	-
ENSMUSG	Tmem175	transmem	GO:00511	localizatio	-	-	-	-	5:1086297	333	422	316	489	508	433	2.245674	2.272799	2.355252	2.544147	2.206079	2.297216	2.291242	2.349148	1	1	-
ENSMUSG	Tango2	transport	GO:00056	cell|intrace	-	-	-	-	16:183008	610	637	448	690	943	749	3.868051	3.225876	3.139694	3.375529	3.850601	3.736412	3.411207	3.654181	0.691188	1	-
ENSMUSG	Ndufs2	NADH de	GO:00081	metabolic	K03935	NADH de	mmu0110	Metabolic	1:1712348	2266	2868	2026	3048	4032	3097	46.60077	47.10404	46.04892	48.35912	53.39596	50.10548	46.58458	50.62019	0.496477	1	-
ENSMUSG	Atraid	all-trans r	GO:00325	developm	-	-	-	-	5:3104831	282	325	359	357	420	343	4.46535	4.109943	6.282724	4.361193	4.282631	4.272785	4.952672	4.305536	0.229619	1	-
ENSMUSG	Sh3bp5l	SH3 bindi	GO:00081	metabolic	-	-	-	-	11:583307	610	491	317	810	641	779	3.233955	2.07889	1.857423	3.312983	2.188349	3.249018	2.390089	2.916783	0.273847	1	-
ENSMUSG	Atad1	ATPase fa	GO:00081	metabolic	-	-	-	-	19:326725	1659	1956	1395	1940	2459	1864	36.00974	33.90692	33.46528	32.4867	34.37058	31.82954	34.46065	32.89561	0.434585	1	-
ENSMUSG	Pten	phosphata	GO:00325	developm	K01110	phosphati	mmu0520	Pathways	19:327574	4136	5101	3528	5198	6141	4774	19.48733	19.19433	18.37161	18.89463	18.63226	17.6956	19.01776	18.4075	0.501601	1	-
ENSMUSG	Pea15a	phosphop	GO:00511	localizatio	-	-	-	-	1:1721967	7144	9495	6267	10055	9817	9369	90.4602	96.01894	87.70465	98.22647	80.04784	93.32979	91.3946	90.5347	0.70359	1	-
ENSMUSG	Timm23	translocas	GO:00511	localizatio	-	-	-	-	14:321801	174	206	234	311	386	186	2.480526	2.345356	3.686869	3.420473	3.543535	2.086025	2.837584	3.016678	0.824283	1	-
ENSMUSG	Tnfaip8l2	tumor nec	GO:00023	immune s	-	-	-	-	3:9513952	25	33	20	25	45	24	1.36046	1.434202	1.202915	1.049609	1.576932	1.027464	1.332525	1.218002	0.627883	1	-
ENSMUSG	Ly6g6e	lymphocy	GO:00048	signal trar	-	-	-	-	17:350769	25	53	23	41	45	44	0.574064	0.971957	0.583702	0.726331	0.665407	0.794848	0.709908	0.728862	1	1	-
ENSMUSG	Ehmt2	euchroma	GO:00325	developm	K11420	euchroma	mmu0421	Longevity	17:348984	2607	3060	2356	3893	4161	3309	15.22657	14.27342	15.20838	17.54186	15.64996	15.20437	14.90279	16.13206	0.541337	1	-
ENSMUSG	Elof1	ELF1 hom	GO:00081	metabolic	-	-	-	-	9:2211298	245	332	252	326	416	366	3.825886	4.140482	4.349247	3.927487	4.18326	4.496337	4.105205	4.202361	1	1	-
ENSMUSG	Med16	mediator	GO:00081	metabolic	K15159	mediator	mmu0491	Thyroid h	10:798947	831	982	747	1194	1213	1166	8.067946	7.614106	8.015469	8.943269	7.583632	8.905764	7.899174	8.477555	0.650596	1	-
ENSMUSG	St3gal1	ST3 beta-	GO:00081	metabolic	K00780	beta-gala	mmu0110	Metabolic	15:671028	408	563	397	656	728	648	4.997333	5.507222	5.37422	6.198866	5.742017	6.244018	5.292925	6.061634	0.297112	1	-
ENSMUSG	Tmem259	transmem	GO:00081	metabolic	-	-	-	-	10:799749	1397	1741	1206	1823	2198	1796	8.891711	8.849799	8.483654	8.951707	9.00889	8.993035	8.741721	8.984544	0.971906	1	-
ENSMUSG	Rnf170	ring finge	GO:00081	metabolic	-	-	-	-	8:2611936	88	826	68	118	159	736	0.493717	3.701013	0.421646	0.510748	0.574444	3.248505	1.538792	1.444566	0.869944	1	-
ENSMUSG	Clip3	CAP-GLY	GO:00325	developm	-	-	-	-	7:3029172	8294	9974	7040	10822	12829	10171	81.82517	78.58471	76.76131	82.36842	81.50239	78.94006	79.05706	80.93695	0.994501	1	-
ENSMUSG	Thap8	THAP dor	-	-	-	-	-	-	7:3028009	0	0	2	2	0	2	0	0	0.041826	0.029199	0	0.029776	0.013942	0.019658	1	1	-
ENSMUSG	Myl2	myosin, lig	GO:00325	developm	K10351	myosin re	mmu0481	Regulatior	5:1221009	0	4	4	0	3	3	0	0.063861	0.088391	0	0.038628	0.047179	0.050751	0.028602	0.505722	1	-
ENSMUSG	Dedd	death effe	GO:00325	developm	-	-	-	-	1:1713291	222	317	237	365	386	318	1.227574	1.399912	1.448408	1.55711	1.374476	1.383354	1.358631	1.438313	0.79078	1	-
ENSMUSG	Nit1	nitrilase 1	GO:00081	metabolic	-	-	-	-	1:1713380	367	468	335	525	656	507	2.362254	2.405757	2.383151	2.607054	2.719058	2.567313	2.383721	2.631141	0.495204	1	-
ENSMUSG	Pax5	paired bo	GO:00325	developm	K09383	paired bo	mmu0520	Transcript	4:4452475	10	9	5	12	8	10	0.06219	0.044702	0.034369	0.057573	0.032039	0.048925	0.047087	0.046179	1	1	-
ENSMUSG	Prdm15	PR domai	GO:00056	cell|intrace	-	-	-	-	16:977914	357	528	328	488	546	492	1.074098	1.26869	1.090678	1.132729	1.057847	1.164532	1.144489	1.118369	0.678554	1	-
ENSMUSG	Rnf168	ring finge	GO:00325	developm	-	-	-	-	16:322774	708	843	676	874	1123	839	5.423306	5.157079	5.723002	5.165026	5.539429	5.055959	5.434462	5.253471	0.573226	1	-
ENSMUSG	Chp1	calcineuri	GO:00081	metabolic	-	-	-	-	2:1195476	888	1133	694	1071	1484	1152	8.549291	8.711495	7.38454	7.95494	9.200377	8.725304	8.215108	8.626874	0.808854	1	-
ENSMUSG	Tvp23b	trans-golc	GO:00511	localizatio	-	-	-	-	11:628794	323	412	260	423	517	359	7.15853	7.292311	6.368561	7.232572	7.37848	6.259317	6.939801	6.956789	0.86281	1	-
ENSMUSG	Dnajc7	DnaJ heat	GO:00099	cellular pr	-	-	-	-	11:100582	2414	3032	2096	3197	3647	3216	24.54117	24.61688	23.55028	25.07442	23.87528	25.72083	24.23611	24.89018	0.979369	1	-
ENSMUSG	Cacybp	calcyclin b	GO:00325	developm	K04507	calcyclin b	mmu0431	Wnt signa	1:1602023	1326	1801	1155	1848	2228	1622	25.83378	28.0223	24.86986	27.77645	27.95208	24.86028	26.24198	26.86294	1	1	-
ENSMUSG	Cluap1	clusterin a	GO:00325	developm	-	-	-	-	16:390880	432	554	401	589	704	567	9.546551	9.777278	9.793859	10.04172	10.01821	9.857271	9.705896	9.972398	0.98046	1	-
ENSMUSG	Zswim7	zinc finge	GO:00081	metabolic	-	-	-	-	11:622672	34	51	48	56	59	61	2.836072	3.397394	4.425095	3.60369	3.169098	4.002875	3.552854	3.591888	0.972142	1	-
ENSMUSG	Pigl	phosphati	GO:00081	metabolic	K03434	N-acetylg	mmu0110	Metabolic	11:624584	122	168	117	170	221	188	3.033008	3.335579	3.214751	3.260576	3.538039	3.676908	3.194446	3.491841	0.642461	1	-
ENSMUSG	Ndufa2	NADH de	GO:00081	metabolic	K03946	NADH de	mmu0110	Metabolic	18:367423	233	240	183	265	366	302	24.87156	20.45997	21.58968	21.82345	25.15839	25.36091	22.30707	24.11425	0.678168	1	-
ENSMUSG	Pam16	presequer	GO:00081	metabolic	-	-	-	-	16:461646	20	20	15	24	28	18	1.061889	0.848051	0.880205	0.983086	0.957326	0.751825	0.930048	0.897412	0.890618	1	-
ENSMUSG	Glis2	GLIS famil	GO:00325	developm	-	-	-	-	16:459471	69	120	82	108	111	126	0.501431	0.696447	0.658596	0.605499	0.519442	0.720345	0.618825	0.615095	0.854919	1	-
ENSMUSG	Cox6c	cytochron	GO:00081	metabolic	K02268	cytochron	mmu0110	Metabolic	15:359258	821	875	702	1040	1023	957	9.481507	8.070272	8.9602	9.266114	7.607893	8.694748	8.837326	8.522918	0.570019	1	-
ENSMUSG	Bicc1	BicC famil	GO:00325	developm	-	-	-	-	10:709228	163	258	145	213	264	250	1.198085	1.514484	1.177914	1.207838	1.249563	1.445604	1.296828	1.301002	0.877948	1	-
ENSMUSG	Ube2z	ubiquitin-	GO:00081	metabolic	K10585	ubiquitin-	mmu0412	Ubiquitin	11:960474	1684	2235	1589	2243	2766	2198	25.72475	27.2667	26.82748	26.43436	27.20922	26.41483	26.60631	26.68613	0.812388	1	-
ENSMUSG	Tmem87b	transmem	GO:00511	localizatio	-	-	-	-	2:1288181	338	424	294	493	514	501	2.07999	2.0838	1.999577	2.340567	2.036862	2.425455	2.054456	2.267628	0.522302	1	-
ENSMUSG	Anapc1	anaphase	GO:00081	metabolic	K03348	anaphase	mmu0516	HTLV-I int	2:1286101	1663	2148	1501	2077	2476	2256	5.04595	5.20512	5.033592	4.862029	4.837888	5.385185	5.094888	5.028367	0.670481	1	-
ENSMUSG	Mertk	c-mer prc	GO:00325	developm	-	-	-	-	2:1286989	859	1189	730	1140	1458	1244	8.589735	9.495421	8.067826	8.794717	9.388557	9.786285	8.717661	9.323186	0.664056	1	-
ENSMUSG	Tsg101	tumor sus	GO:00325	developm	K12183	ESCRT-I c	mmu0414	Endocytos	7:4688894	699	993	748	1042	1232	1038	9.004135	10.21551	10.64911	10.35531	10.21952	10.51897	9.956253	10.3646	0.87271	1	-
ENSMUSG	Hps5	HPS5, bio	GO:00508	response	-	-	-	-	7:4676046	222	305	208	328	416	302	0.994853	1.091573	1.03019	1.133996	1.20048	1.064693	1.038872	1.133056	0.602598	1	-
ENSMUSG	Map3k4	mitogen-a	GO:00325	developm	K04428	mitogen-a	mmu0401	MAPK sigr	17:122276	1173	1590	1137	1560	1879	1684	13.50069	14.61507	14.46323	13.85202	13.92642	15.24793	14.193	14.34212	0.88389	1	-
ENSMUSG	Piezo1	piezo-typ	GO:00511	localizatio	-	-	-	-	8:1224816	135	232	115	177	247	177	0.609721	0.836822	0.574041	0.61674	0.718373	0.628904	0.673528	0.654672	0.727601	1	-
ENSMUSG	Blk	B lymphoi	GO:00325	developm	-	-	-	-	14:633728	0	1	0	0	2	0	0	0.016544	0	0	0.026669	0	0.005515	0.00889	1	1	-
ENSMUSG	Rnf166	ring finge	GO:00081	metabolic	-	-	-	-	8:1224661	275	448	282	504	520	463	6.644627	8.644936	7.53068	9.395052	8.090891	8.800961	7.606748	8.762301	0.326968	1	-
ENSMUSG	Ankrd28	ankyrin re	GO:00056	cell|intrace	-	-	-	-	14:316987	1040	1343	1102	1366	1570	1427	4.549067	4.691492	5.327425	4.609672	4.42225	4.910479	4.855995	4.647467	0.481609	1	-
ENSMUSG	Ankrd52	ankyrin re	-	-	-	-	-	-	10:128377	1666	2143	1449	2220	2430	2289	11.4656	11.7785	11.02141	11.78705	10.76918	12.39306	11.42184	11.64976	0.959141	1	-

ENSMUSC	Pkd2l2	polycystic	GO:00511	localizatio	-	-	-	-	18:344094	2	12	0	4	8	4	0.045796	0.219484	0	0.070668	0.11799	0.072066	0.088427	0.086908	1	1	-
ENSMUSC	Srp19	signal rec	GO:00511	localizatio	K03105	signal rec	mmu0306	Protein ex	18:343308	334	426	298	482	567	457	3.323026	3.384879	3.276805	3.699686	3.632664	3.576961	3.328237	3.636437	0.56556	1	-
ENSMUSC	Wdfy2	WD repea	GO:00325	developm	-	-	-	-	14:628376	583	763	522	778	873	754	3.714198	3.882099	3.675481	3.823901	3.581503	3.779015	3.757259	3.72814	0.752169	1	-
ENSMUSC	Rbsn	rabenosyr	GO:00511	localizatio	K12481	rabenosyr	mmu0414	Endocytos	6:9218671	940	1175	791	1301	1515	1181	5.470844	5.461477	5.088029	5.841631	5.67798	5.407384	5.340117	5.642332	0.746917	1	-
ENSMUSC	Mrps25	mitochon	GO:00081	metabolic	-	-	-	-	6:9216952	588	805	509	822	861	804	4.935688	5.396494	4.72209	5.323195	4.654018	5.3093	5.018091	5.095504	0.931953	1	-
ENSMUSC	Dguok	deoxygua	GO:00325	developm	K00904	deoxygua	mmu0110	Metabolic	6:8348021	154	182	150	232	262	186	1.502304	1.417929	1.617249	1.746053	1.645864	1.427456	1.512494	1.606458	0.793079	1	-
ENSMUSC	Camta1	calmoduli	GO:00081	metabolic	-	-	-	-	4:1509173	9054	10798	8299	10667	14393	11863	24.42756	23.26638	24.74641	22.20303	25.00608	25.17938	24.14678	24.1295	0.771697	1	-
ENSMUSC	Csf1	colony sti	GO:00325	developm	K05453	macropha	mmu0415	PI3K-Akt s	3:1077410	430	539	468	586	674	553	3.300851	3.304396	3.970545	3.470451	3.331748	3.339593	3.525264	3.380597	0.559882	1	-
ENSMUSC	Strip1	striatin int	GO:00325	developm	-	-	-	-	3:1076125	1767	2243	1773	2230	2763	2264	13.2234	13.40545	14.66432	12.87483	13.31502	13.32888	13.76439	13.17291	0.442631	1	-
ENSMUSC	Kif1a	kinesin fa	GO:00325	developm	-	-	-	-	1:9301546	23756	29817	21118	30000	31334	32134	119.1177	119.4023	117.0315	116.0525	101.1751	126.759	118.5172	114.6622	0.509712	1	-
ENSMUSC	Alx3	aristaless-	GO:00325	developm	-	-	-	-	3:1075950	2	4	3	1	8	13	0.065151	0.104073	0.108037	0.02514	0.167855	0.333206	0.09242	0.1754	0.278748	1	-
ENSMUSC	Slc25a11	solute car	GO:00511	localizatio	-	-	-	-	11:706441	753	964	741	1037	1219	1121	13.09507	13.38861	14.24222	13.91303	13.6512	15.3366	13.5753	14.30028	0.783334	1	-
ENSMUSC	Cmc2	COX asse	GO:00056	cell|intrace	-	-	-	-	8:1168886	70	94	82	113	109	74	0.990738	1.062522	1.28269	1.233881	0.993446	0.823964	1.111983	1.017097	0.5183	1	-
ENSMUSC	Chfr	checkpoir	GO:00081	metabolic	-	-	-	-	5:1101358	446	619	436	633	803	672	2.798468	3.10186	3.023561	3.064218	3.24456	3.317156	2.97463	3.208645	0.623713	1	-
ENSMUSC	Ceacam16	carcinoem	GO:00055	extracellul	-	-	-	-	7:1985209	0	0	1	0	0	4	0	0	0.031788	0	0	0.090498	0.010596	0.030166	0.44309	1	-
ENSMUSC	Hoxa2	homeobo	GO:00325	developm	-	-	-	-	6:5216241	1	2	0	0	3	0	0.034409	0.054952	0	0	0.066462	0	0.029787	0.022154	1	1	-
ENSMUSC	Adam28	a disinteg	GO:00081	metabolic	-	-	-	-	14:686060	0	0	0	1	1	0	0	0	0	0.005845	0.004877	0	0	0.003574	0.521877	1	-
ENSMUSC	Tex261	testis expr	GO:00511	localizatio	-	-	-	-	6:8376888	475	661	397	654	735	613	5.063299	5.62714	4.67711	5.378338	5.045245	5.140578	5.122516	5.188054	0.918535	1	-
ENSMUSC	Fam120b	family witl	GO:00325	developm	-	-	-	-	17:153962	1174	1597	1100	1677	1961	1738	15.86592	17.23645	16.42996	17.48479	17.06591	18.47812	16.51078	17.67628	0.628628	1	-
ENSMUSC	Tbp	TATA box	GO:00081	metabolic	K03120	transcripti	mmu0516	Human pa	17:154998	490	677	484	679	710	662	1.834365	2.024064	2.002541	1.961054	1.711601	1.949657	1.953656	1.874104	0.536199	1	-
ENSMUSC	Psmb1	proteasor	GO:00081	metabolic	K02732	20S prote	mmu0305	Proteason	17:154757	1279	1540	1249	1770	2241	1738	75.37964	72.48534	81.35659	80.47984	85.05114	80.58317	76.40719	82.03805	0.608552	1	-
ENSMUSC	Pdcd2	programn	GO:00325	developm	-	-	-	-	17:155192	98	147	89	145	197	189	1.708655	2.046874	1.715	1.950403	2.211816	2.592384	1.82351	2.251535	0.216068	1	-
ENSMUSC	Dll1	delta-like	GO:00325	developm	K06051	delta[-]	mmu0520	Pathways	17:153673	103	131	109	151	173	129	1.040532	1.056904	1.217009	1.176867	1.125436	1.025227	1.104815	1.109177	0.914699	1	-
ENSMUSC	Nol3	nucleolar	GO:00081	metabolic	-	-	-	-	8:1052761	317	387	304	396	468	374	6.094273	5.941816	6.459259	5.873378	5.793801	5.656465	6.165116	5.774548	0.435173	1	-
ENSMUSC	Plekhg4	pleckstrin	GO:00987	molecular	-	-	-	-	8:1053732	8	25	21	45	21	39	0.056467	0.140916	0.163814	0.245031	0.095443	0.216545	0.120399	0.185673	0.20591	1	-
ENSMUSC	Slc9a5	solute car	GO:00511	localizatio	-	-	-	-	8:1053488	235	342	245	353	387	333	2.154772	2.504413	2.482827	2.497118	2.285074	2.40209	2.380671	2.394761	0.885738	1	-
ENSMUSC	Stc1	stanniocal	GO:00325	developm	-	-	-	-	14:690292	94	116	63	89	139	116	1.367516	1.347741	1.012963	0.998907	1.302185	1.327614	1.24274	1.209569	0.759581	1	-
ENSMUSC	4931428F	RIKEN cDI	-	-	-	-	-	-	8:1052804	368	445	353	468	554	466	3.032302	2.928406	3.214745	2.975101	2.93961	3.020798	3.058485	2.978503	0.653358	1	-
ENSMUSC	Tppp3	tubulin pc	GO:00990	supramole	-	-	-	-	8:1054674	179	468	325	336	510	349	3.988838	8.328837	8.004293	5.77646	7.318431	6.118275	6.773989	6.404389	0.652505	1	-
ENSMUSC	Msh3	mutS hom	GO:00081	metabolic	K08736	DNA misn	mmu0520	Pathways	13:922118	443	627	425	597	729	684	1.492755	1.687324	1.582781	1.551993	1.581858	1.813227	1.58762	1.649026	0.903011	1	-
ENSMUSC	Tmem208	transmem	GO:00081	metabolic	-	-	-	-	8:1053263	135	191	135	207	221	163	2.70168	3.052672	2.985948	3.195964	2.848058	2.56625	2.913433	2.870091	0.787976	1	-
ENSMUSC	E2f4	E2F transc	GO:00325	developm	K04682	transcripti	mmu0421	Cellular se	8:1052976	537	763	531	864	1035	793	12.33102	13.99249	13.47617	15.30626	15.30453	14.32548	13.26656	14.97876	0.332542	1	-
ENSMUSC	Surf4	surfeit ger	GO:00099	cellular pr	-	-	-	-	2:2692004	904	1226	868	1310	1584	1223	14.88981	16.12712	15.8011	16.64648	16.80082	15.8474	15.60601	16.43157	0.768114	1	-
ENSMUSC	Surf2	surfeit ger	GO:00160	membran	-	-	-	-	2:2691636	244	292	226	305	387	360	1.951303	1.864933	1.997516	1.881763	1.992968	2.264893	1.937917	2.046541	0.808966	1	-
ENSMUSC	Dnajb9	DnaJ heat	GO:00081	metabolic	-	-	-	-	12:442055	825	1108	690	1098	1327	1006	17.712	18.99762	16.37229	18.18638	18.34591	16.99114	17.69397	17.84115	0.879805	1	-
ENSMUSC	Naf1	nuclear as	GO:00081	metabolic	-	-	-	-	8:6686021	280	342	216	333	413	307	5.120184	4.994576	4.365427	4.697877	4.863305	4.416476	4.826729	4.659219	0.626571	1	-
ENSMUSC	Yes1	YES proto	GO:00325	developm	K05705	tyrosine-p	mmu0452	Adherens	5:3261117	320	371	238	376	501	344	1.86064	1.722791	1.529455	1.686673	1.875881	1.573555	1.704295	1.712036	0.880329	1	-
ENSMUSC	Ppp1cb	protein pł	GO:00081	metabolic	K06269	serine/thr	mmu0481	Regulatior	5:3245884	5179	2650	1814	7059	3103	2582	25.12673	10.26791	9.726895	26.42188	9.694527	9.855016	15.04051	15.32381	0.971615	1	-
ENSMUSC	Gorasp2	golgi reas	GO:00099	cellular pr	-	-	-	-	2:7066157	1848	2283	1673	2639	3098	2468	16.82353	16.59842	16.83286	18.53467	18.16148	17.67548	16.7516	18.12387	0.527795	1	-
ENSMUSC	Tsen15	tRNA splic	GO:00081	metabolic	-	-	-	-	1:1523707	170	235	186	253	308	263	1.761396	1.944553	2.129932	2.022359	2.055002	2.143749	1.945294	2.073703	0.754107	1	-
ENSMUSC	Efr3a	EFR3 hom	GO:00511	localizatio	-	-	-	-	15:657870	3055	3692	2500	3757	4505	3883	12.44712	12.01338	11.25756	11.80944	11.81972	12.44617	11.90602	12.02511	0.869678	1	-
ENSMUSC	Trappc2l	trafficking	GO:00511	localizatio	-	-	-	-	8:1226116	205	303	250	333	361	354	5.628115	6.643499	7.585694	7.053152	6.382197	7.645794	6.619102	7.027048	0.784349	1	-
ENSMUSC	Acsf3	acyl-CoA	GO:00081	metabolic	K18660	malonyl-C	mmu0028	Valine, leu	8:1227754	202	252	247	333	368	267	2.979888	2.968893	4.027089	3.789855	3.495834	3.098632	3.32529	3.46144	0.89978	1	-
ENSMUSC	Ddx19a	DEAD (As	GO:00081	metabolic	-	-	-	-	8:1109749	355	535	369	570	616	587	6.021001	7.246703	6.916941	7.458409	6.727849	7.832313	6.728215	7.339524	0.582489	1	-
ENSMUSC	Galns	galactosai	GO:00081	metabolic	K01132	N-acetylg	mmu0110	Metabolic	8:1225782	167	202	187	188	255	252	2.598541	2.510213	3.215896	2.256855	2.55511	3.084792	2.774884	2.632252	0.590382	1	-
ENSMUSC	Gata2	GATA bin	GO:00325	developm	-	-	-	-	6:8819389	80	95	62	114	114	67	0.574257	0.544614	0.491879	0.631328	0.52696	0.378359	0.536917	0.512215	0.70667	1	-
ENSMUSC	C8g	complem	GO:00081	metabolic	K03999	complem	mmu0532	Systemic l	2:2549865	3	8	14	10	8	12	0.058149	0.123845	0.29992	0.149547	0.099861	0.182994	0.160638	0.144134	0.783425	1	-
ENSMUSC	Rabl6	RAB, men	GO:00056	cell|intrace	-	-	-	-	2:2558301	2227	2841	1887	3053	3449	2763	13.55832	13.81347	12.6971	14.33979	13.52177	13.23358	13.3563	13.69838	0.989059	1	-
ENSMUSC	Edf1	endotheli	GO:00325	developm	-	-	-	-	2:2555784	924	714	591	857	936	769	21.29774	13.14333	15.05552	15.23957	13.89288	13.94436	16.49886	14.35893	0.203622	1	-
ENSMUSC	Clic3	chloride ir	GO:00511	localizatio	-	-	-	-	2:2545683	0	7	6	0	4	6	0	0.148024	0.175595	0	0.068205	0.124985	0.107873	0.064397	0.498877	1	-
ENSMUSC	Npdc1	neural prc	GO:00160	membran	-	-	-	-	2:2539935	1899	2225	1733	2554	2738	2469	14.67713	13.73384	14.8034	15.22886	13.62713	15.01234	14.40479	14.62278	0.915951	1	-
ENSMUSC	Fbxw5	F-box anc	GO:00081	metabolic	-	-	-	-	2:2550075	1081	1281	1102	1368	1842	1458	11.25384	10.65051	12.67954	10.98732	12.34866	11.94108	11.52796	11.75902	0.969961	1	-
ENSMUSC	Slc25a13	solute car	GO:00081	metabolic	-	-	-	-	6:6041218	40	50	46	58	105	55	0.451701	0.45092	0.574107	0.505294	0.763537	0.488605	0.492242	0.585812	0.434582	1	-
ENSMUSC	Ube2i	ubiquitin-	GO:00325	developm	K10577	ubiquitin-	mmu0301	RNA trans	17:252619	856	1087	757	1216	1316	1282	5.412214	5.488786	5.289858	5.931505	5.358112	6.376762	5.396953	5.888793	0.523743	1	-
ENSMUSC	Tsr3	TSR3 20S	GO:00081	metabolic	-	-	-	-	17:252401	193	286	194	312	316	237	9.886064	11.69983	10.98287	12.3297	10.4234	9.55051	10.85625	10.76787	0.812968	1	-
ENSMUSC	Unkl	unkempt	GO:00081	metabolic	-	-	-	-	17:251883	932	1142	627	1291	807	1218	6.136756	6.005304	4.56286	6.558124	3.421773	6.309302	5.568306	5.429733	0.73829	1	-
ENSMUSC	Aldh1a3	aldehyde	GO:00325	developm	K00129	aldehyde	mmu0110	Metabolic	7:6639089	15	18	5	22	26	24	0.167707	0.160725	0.06179	0.189768	0.187194	0.2111	0.130074	0.196021	0.200949	1	-
ENSMUSC	Actn1	actinin, al	GO:00325	developm	K05699	actinin alp	mmu0520	Viral carci	12:801675	1489	1853	1378	1966	2402	1954	12.81764	12.73898	13.11021	13.05652	13.315	13.23271	12.88894	13.20141	1	1	-
ENSMUSC	Hnrnpl	heteroger	GO:00081	metabolic	-	-	-	-	7:2880854	2192	2826	2059	2883	3530	2880	12.0576	12.41476	12.51767	12.23476	12.50402	12.46304	12.33001	12.40061	0.832218	1	-
ENSMUSC	Nolc1	nucleolar	GO:00325	developm	-	-	-	-	19:460758	1101	1260	1162	1229	1581	1405	6.470783	5.914062	7.547836	5.572524	5.983513	6.496164	6.644227	6.0174	0.230673	1	-
ENSMUSC	Casd1	CAS1 don	GO:00081	metabolic	-	-	-	-	6:4600839	1066	1520	936	1290	1648	1281	12.68029	14.43981	12.30538	11.83837	12.62362	11.9876	13.14183	12.14987	0.287755	1	-
ENSMUSC	Cnksr3	Cnksr fam	GO:00081	metabolic	-	-	-	-	10:711906	53	186	96	153	129	133	0.828495	2.322043	1.658544	1.845152	1.298538	1.635586	1.603027	1.593092	0.899547	1	-
ENSMUSC	Mtmr1	myotubul	GO:00081	metabolic	K18081	myotubul	mmu0110	Metabolic	X:7136476	940	1184	916	1147	1376	1221	6.289133	6.326456	6.773376	5.920478	5.928378	6.426719	6.462989	6.091858	0.383677	1	-
ENSMUSC	Map2	microtubu	GO:00325	developm	-	-	-	-	1:6617527	26192	29634	22799	29667	36087	30986	49.35755	44.59858	47.48403	43.13094	43.79156	45.93687	47.14672	44.28645	0.296673	1	-
ENSMUSC	Cyp2j9	cytochron	GO:00081	metabolic	K07418	cytochron	mmu0110	Metabolic	4:9656842	574	684	487	752	773	660	10.07398	9.587173	9.446365	10.18209	8.736209	9.112637	9.702505	9.343646	0.560928	1	-
ENSMUSC	Nipsnap3l	nipsnap h	GO:00056	cell|intrace	-	-	-	-	4:5301188	122	129	106	152	164	124	3.120682	2.635269	2.996708	2.999602	2.70141	2.495307	2.917553	2.732106	0.552304	1	-
ENSMUSC	Lage3	L antigen	GO:00081	metabolic	-	-	-	-	X:7435127	102	127	111	174	175	131	1.777385	1.767379	2.137702	2.339148	1.963686	1.795821	1.894155	2.032885	0.764394	1	-
ENSMUSC	Gdi1	guanosine	GO:00325	developm	-	-	-	-	X:7430499	7303	9834	6618	10642	12344	9826	44.16673	47.49742	44.23516	49.65324	48.07337	46.75001	45.29977	48.15887	0.650659	1	-
ENSMUSC	Sash1	SAM and	GO:00325	developm	-	-	-	-	10:872221	2123	2570	1769	2709	3172	2684	13.42722	12.98121	12.36547	13.21829	12.91885	13.35456	12.92463	13.1639	0.946402	1	-
ENSMUSC	Gadd45b	growth ar	GO:00325	developm	K04402	growth ar	mmu0520	Pathways	10:809300	142	152	111	149	189	144	3.524488	3.01299	3.044922	2.853155	3.020821	2.811782	3.194133	2.895253	0.396463	1	-
ENSMUSC	Slamf6	SLAM fan	GO:00325	developm	-	-	-	-	1:1719175	2	3	3	6	9	3	0.010083	0.012083	0.01672	0.02334	0.029224	0.011899	0.012962	0.021488	0.36884	1	-
ENSMUSC	Slamf1	signaling	GO:00325	developm	K06536	signaling l	mmu0516	Measles -	1:1717671	3	1	3	0	4	1	0.028719	0.007647	0.031744	0	0.02466	0.007533	0.022703	0.010731	0.448961	1	-
ENSMUSC	Zdhhc12	zinc finge	GO:00081	metabolic	-	-	-	-	2:3009094	59	57	65	87	77	102	1.120162	0.864262	1.363924	1.274312	0.941394	1.523483	1.116116	1.246396	0.66858	1	-
ENSMUSC	Endog	endonucle	GO:00325	developm	K01173	endonucle	mmu0421	Apoptosis	2:3017149	79	122	86	151	166	110	4.537736	5.596512	5.45952	6.691426	6.140062	4.970674	5.197923	5.934054	0.488475	1	-
ENSMUSC	Golga7	golgi autc	GO:00081	metabolic	-	-	-	-	8:2324134	947	992	678	1030	1172	1089	6.518098	5.45291	5.157595	5.46938	5.19461	5.896707	5.709534	5.520232	0.582771	1	-
ENSMUSC	Xk	X-linked k	GO:00325	developm	-	-	-	-	X:9272756	492	558	472	614	765	559	5.91816	5.360461	6.274954	5.697975	5.925672	5.289861	5.851192	5.637836	0.58179	1	-
ENSMUSC	Cd48	CD48 anti	GO:00023	immune s	K06479	CD48 anti	mmu0465	Natural ki	1:1716820	3	14	14	11	11	9	0.101479	0.378219	0.523407	0.287084	0.239608	0.239501	0.334368	0.255397	0.529787	1	-
ENSMUSC	Clpx	caseinolyt	GO:00081	metabolic	-	-	-	-	9:6529426	470	658	483	631	729	579	5.656872	6.324846	6.424986	5.859202	5.650161	5.482362	6.135568	5.663908	0.329593	1	-
ENSMUSC	Trabd	TraB dom	-	-	-	-	-	-	15:890751	253	330	264	443	472	382	1.427699	1.487222	1.646519	1.928635	1.715194	1.695863	1.52048	1.779897	0.25781	1	-
ENSMUSC	Mov10l1	Moloney l	GO:00325	developm	-	-	-	-	15:889829	0	0	0	2	0	0	0	0	0	0.018924	0	0	0	0.006308	0.527729	1	-
ENSMUSC	Dennd6b	DENN/MA	GO:00987	molecular	-	-	-	-	15:891822	682	912	679	996	1072	1071	9.03878	9.653079	9.945846	10.18391	9.149019	11.16672	9.545901	10.16655	0.707334	1	-
ENSMUSC	Cd83	CD83 anti	GO:00325	developm	-	-	-	-	13:437847	196	264	228	326	332	296	2.762562	2.971698	3.551709	3.544892	3.013336	3.282139	3.095323	3.280122	0.79987	1	-
ENSMUSC	Tmem27	transmem	GO:00081	metabolic	-	-	-	-	X:1640888	0	9	0	3	2	2	0	0.185105	0	0.05961	0.033159	0.040526	0.061702	0.044432	0.719225	1	-
ENSMUSC	Ace2	angiotens	GO:00081	metabolic	K09708	angiotens	mmu0497	Protein di	X:1641393	18	47	39	51	75	56	0.252648	0.526864	0.605009	0.552273	0.677908	0.618375	0.461507	0.616186	0.2296	1	-
ENSMUSC	Ager	advanced	GO:00325	developm	K19722	receptor f	-	-	17:345978	3	14	5	15	4	5	0.030351	0.113121	0.055913	0.117082	0.026061	0.039796	0.066462	0.06098	0.847553	1	-
ENSMUSC	Atf6b	activating	GO:00081	metabolic	K09049	cyclic AMI	mmu0415	PI3K-Akt s	17:346471	1090	1481	1049	1530	1765	1387	17.5881	19.08504	18.70745	19.04645	18.33967	17.60676	18.4602	18.33096	0.74331	1	-
ENSMUSC	Egfl8	EGF-like c	GO:00055	extracellul	-	-	-	-	17:346133	11	15	10	25	18	10	0.188828	0.205638	0.189711	0.331083	0.198972	0.135042	0.194726	0.221699	0.755899	1	-
ENSMUSC	Notch4	notch 4 [S	GO:00325	developm	K02599	Notch[-]	mmu0520	Pathways	17:345642	84	121	99	142	172	180	0.420641	0.483911	0.547915	0.548595	0.554645	0.709114	0.484156	0.604118	0.201187	1	-
ENSMUSC	Ppt2	palmitoyl-	GO:00081	metabolic	K01074	palmitoyl-	mmu0110	Metabolic	17:346166	280	379	279	448	557	334	2.928451	3.165663	3.225003	3.614825	3.751366	2.748126	3.106372	3.371439	0.642398	1	-
ENSMUSC	Prrt1	proline-ric	GO:00452	synapse|re	-	-	-	-	17:346295	3024	4245	2876	4423	5284	4452	65.80176	73.7699	69.16579	74.25111	74.04115	76.21172	69.57915	74.83466	0.56413	1	-
ENSMUSC	Rnf5	ring finge	GO:00081	metabolic	K10666	E3 ubiquit	mmu0414	Protein pr	17:346010	704	866	733	1052	1177	1042	15.92034	15.64024	18.3202	18.35379	17.13999	18.5378	16.62693	18.01053	0.590166	1	-
ENSMUSC	Fam163a	family witl	GO:00160	membran	-	-	-	-	1:1560759	301	326	237	363	438	381	4.734282	4.094979	4.119866	4.404788	4.436252	4.714372	4.316376	4.51847	0.864294	1	-
ENSMUSC	Cacfd1	calcium cl	GO:00511	localizatio	-	-	-	-	2:2700992	463	574	402	605	669	562	3.448389	3.414232	3.30909	3.476331	3.208604	3.292933	3.390571	3.325956	0.687263	1	-
ENSMUSC	Hivep2	human im	GO:00325	developm	-	-	-	-	10:139660	4733	5146	4610	5794	6584	6117	14.79562	12.84731	15.92738	13.97351	13.25382	15.0434	14.52344	14.09024	0.544929	1	-
ENSMUSC	Arnt	aryl hydro	GO:00325	developm	K09097	aryl hydro	mmu0520	Pathways	3:9543438	401	554	424	632	688	559	1.858954	2.051068	2.172386	2.260329	2.053845	2.038671	2.027469	2.117615	0.860781	1	-
ENSMUSC	Mocs2	molybden	GO:00081	metabolic	K03635	molybdop	mmu0110	Metabolic	13:114818	429	523	492	522	715	557	1.654599	1.610952	2.097236	1.553229	1.775807	1.690057	1.787596	1.673031	0.451318	1	-
ENSMUSC	Nat9	N-acetylti	GO:00038	catalytic a	-	-	-	-	11:115182	146	149	144	221	226	187	2.023261	1.649034	2.205502	2.362769	2.016799	2.038695	1.959266	2.139421	0.659784	1	-
ENSMUSC	Lpl	lipoprotei	GO:00325	developm	K01059	lipoprotei	mmu0501	Alzheimer	8:6888049	731	1054	632	839	1215	894	3.842742	4.424971	3.671875	3.402642	4.112964	3.697197	3.979863	3.737601	0.4157	1	-
ENSMUSC	Atp6v0e	ATPase, H	GO:00511	localizatio	K02153	V-type H-	mmu0110	Metabolic	17:266763	179	238	160	204	335	285	3.071786	3.261822	3.034616	2.700829	3.702007	3.847635	3.122742	3.416824	0.635893	1	-
ENSMUSC	Zfp318	zinc finge	GO:00056	cell|intrace	-	-	-	-	17:463837	1256	1632	1151	1668	1875	1796	7.251225	7.524678	7.344192	7.429306	6.970725	8.157165	7.373365	7.519065	0.959767	1	-
ENSMUSC	Ttbk1	tau tubuli	GO:00325	developm	-	-	-	-	17:464424	3289	4011	2961	4056	4472	3747	13.91299	13.5505	13.84337	13.23686	12.18185	12.46956	13.76895	12.62942	0.196665	1	-
ENSMUSC	Srf	serum res	GO:00325	developm	K04378	serum res	mmu0401	MAPK sigr	17:465468	1208	1367	979	1573	1767	1404	18.02057	16.28606	16.14103	18.10342	16.97434	16.47706	16.81588	17.18494	0.980155	1	-
ENSMUSC	Gata3	GATA bin	GO:00325	developm	K17895	GATA-bin	mmu0465	Th17 cell c	2:9857078	0	9	0	5	5	3	0	0.082157	0	0.044087	0.036801	0.026972	0.027386	0.035953	0.878306	1	-
ENSMUSC	Lama5	laminin, a	GO:00325	developm	K06240	laminin, a	mmu0110	Metabolic	2:1801763	226	352	207	321	383	376	1.116885	1.389279	1.130623	1.223874	1.218861	1.461839	1.212262	1.301525	0.719418	1	-
ENSMUSC	Hspa8	heat shoc	GO:00081	metabolic	K03283	heat shoc	mmu0401	MAPK sigr	9:4080098	15420	16001	12630	17238	24367	16954	101.9288	84.47056	92.27044	87.90824	103.7216	88.16485	92.88992	93.26489	0.830785	1	-
ENSMUSC	Serac1	serine acti	GO:00081	metabolic	-	-	-	-	17:604219	403	467	285	470	506	542	4.379149	4.052721	3.42276	3.940145	3.540705	4.633348	3.951544	4.038066	0.978641	1	-
ENSMUSC	Pdzd11	PDZ dom	GO:00452	synapse|c	-	-	-	-	X:1006228	421	585	390	644	751	512	6.635379	7.363514	6.793532	7.830691	7.62216	6.3484	6.930808	7.267084	0.835149	1	-
ENSMUSC	Psma2	proteasor	GO:00081	metabolic	K02726	20S prote	mmu0305	Proteason	13:146132	905	1104	800	1166	1455	1144	14.0712	13.70874	13.74736	13.98657	14.56801	13.99327	13.84243	14.18262	0.99965	1	-
ENSMUSC	Mrpl32	mitochon	GO:00081	metabolic	K02911	large subu	mmu0301	Ribosome	13:146081	189	222	190	252	296	244	1.562839	1.466053	1.736408	1.607618	1.576149	1.587279	1.588433	1.590349	0.870248	1	-
ENSMUSC	Setdb1	SET doma	GO:00081	metabolic	K11421	histone-ly	mmu0455	Signaling	3:9532352	771	1062	772	1076	1359	1202	5.485411	6.034275	6.070426	5.906053	6.226286	6.727755	5.863371	6.286698	0.645781	1	-
ENSMUSC	Anxa9	annexin A	GO:00048	signal trar	-	-	-	-	3:9529609	9	11	12	22	20	15	0.204583	0.199693	0.30148	0.385825	0.292764	0.268248	0.235252	0.315612	0.40229	1	-
ENSMUSC	Arnt2	aryl hydro	GO:00325	developm	K15589	aryl hydro	mmu0520	Pathways	7:8424627	4561	5291	3981	5753	6102	5532	20.11746	18.63789	19.40672	19.57663	17.33165	19.1958	19.38736	18.70136	0.474003	1	-
ENSMUSC	Prune1	prune exc	GO:00325	developm	K01514	exopolypl	mmu0023	Purine me	3:9525367	430	547	462	568	663	614	6.87123	6.980706	8.159347	7.002368	6.822359	7.718731	7.337094	7.181153	0.686999	1	-
ENSMUSC	Cers2	ceramide	GO:00325	developm	K04710	ceramide	mmu0110	Metabolic	3:9531479	505	726	583	757	1019	761	4.76206	5.467465	6.076011	5.507183	6.187745	5.645458	5.435179	5.780129	0.723306	1	-
ENSMUSC	Capza2	capping p	GO:00329	macromo	K10364	capping p	mmu0414	Endocytos	6:1763623	4562	5680	3957	5833	7354	5027	35.47467	35.2742	34.0076	34.99334	36.82488	30.75267	34.91882	34.1903	0.610727	1	-
ENSMUSC	Vps45	vacuolar p	GO:00511	localizatio	K12479	vacuolar p	mmu0414	Endocytos	3:9599983	635	844	602	888	1032	843	8.763991	9.302858	9.182721	9.45522	9.171971	9.153069	9.08319	9.260087	0.966446	1	-
ENSMUSC	Prpf3	pre-mRN.	GO:00081	metabolic	K12843	U4/U6 sm	mmu0304	Spliceoso	3:9583012	449	584	366	575	706	615	3.244002	3.369723	2.922557	3.205039	3.284692	3.495594	3.178761	3.328442	0.847063	1	-
ENSMUSC	Anp32e	acidic (leu	GO:00081	metabolic	-	-	-	-	3:9592924	1584	1728	1313	1532	2478	1513	10.01823	8.728217	9.17799	7.475242	10.09234	7.528111	9.308147	8.365232	0.227438	1	-
ENSMUSC	Aph1a	aph1 hom	GO:00325	developm	K06172	gamma-s	mmu0501	Alzheimer	3:9589396	309	469	311	410	625	491	2.772517	3.360732	3.084058	2.838108	3.611187	3.46583	3.072436	3.305042	0.68573	1	-
ENSMUSC	Tab2	TGF-beta	GO:00325	developm	K04404	TAK1-bin	mmu0401	MAPK sigr	10:790565	1583	1994	1373	2114	2533	1051	11.75566	11.82597	11.26894	12.11159	12.11311	6.140154	11.61686	10.12162	0.27783	1	-
ENSMUSC	Ppil4	peptidylpr	GO:00081	metabolic	-	-	-	-	10:779289	962	1152	838	1224	1544	1047	8.117453	7.763244	7.815118	7.968131	8.389707	6.95028	7.898605	7.769373	0.690935	1	-
ENSMUSC	Cnih1	cornichon	GO:00511	localizatio	-	-	-	-	14:467755	687	925	572	908	1157	949	15.90103	17.09843	14.63225	16.2138	17.24474	17.28007	15.87724	16.91287	0.699643	1	-
ENSMUSC	Eps8	epiderma	GO:00325	developm	-	-	-	-	6:1374772	952	800	861	1010	1702	1368	3.645717	2.446707	3.64415	2.983992	4.197206	4.121385	3.245524	3.767527	0.352061	1	-
ENSMUSC	Med22	mediator	GO:00081	metabolic	-	-	-	-	2:2690526	339	426	339	507	574	495	4.152192	4.167102	4.58907	4.79089	4.527356	4.769739	4.302788	4.695995	0.57196	1	-
ENSMUSC	Surf1	surfeit ger	GO:00081	metabolic	-	-	-	-	2:2691338	439	578	404	675	774	662	7.087398	7.452382	7.208591	8.407293	8.04669	8.407959	7.249457	8.287314	0.291878	1	-
ENSMUSC	Med28	mediator	GO:00325	developm	-	-	-	-	5:4552022	272	437	298	431	521	462	2.420962	3.106323	2.931451	2.95956	2.98615	3.234983	2.819579	3.060231	0.624831	1	-
ENSMUSC	Qdpr	quinoid d	GO:00325	developm	K00357	dihydropt	mmu0110	Metabolic	5:4543402	1158	1746	728	1201	2030	1791	15.34737	18.48057	10.66359	12.28	17.3251	18.67375	14.83051	16.09295	0.6815	1	-
ENSMUSC	Gnrh1	gonadotr	GO:00325	developm	K05252	gonadotr	mmu0491	GnRH sigr	14:677451	0	4	2	3	2	2	0	0.172138	0.11913	0.124745	0.069391	0.084809	0.097089	0.092982	1	1	-
ENSMUSC	Tnr	tenascin R	GO:00325	developm	K06252	tenascin[-	mmu0415	PI3K-Akt s	1:1595237	6905	8992	6546	9103	11019	9142	29.55322	30.73571	30.96449	30.05772	30.36952	30.78175	30.41781	30.403	0.76571	1	-
ENSMUSC	Sqstm1	sequestos	GO:00325	developm	K14381	sequestos	mmu0421	Cellular se	11:501993	5593	7484	5891	6219	7314	8477	48.69499	52.03782	56.68594	41.77249	41.00616	58.06208	52.47292	46.94691	0.190024	1	-
ENSMUSC	Rxrg	retinoid X	GO:00048	signal trar	K08526	retinoid X	mmu0520	Pathways	1:1675983	40	67	69	80	98	99	0.599348	0.801747	1.142641	0.924773	0.945579	1.166979	0.847912	1.012444	0.428018	1	-
ENSMUSC	Adamtsl4	ADAMTS-	GO:00325	developm	-	-	-	-	3:9567620	74	104	92	115	130	109	0.794355	0.891583	1.091489	0.95238	0.898629	0.920496	0.925809	0.923835	0.88164	1	-
ENSMUSC	Prpsap1	phosphor	GO:00081	metabolic	-	-	-	-	11:116470	392	490	314	503	640	572	4.097018	4.090005	3.627093	4.055834	4.307412	4.703151	3.938039	4.355466	0.501791	1	-
ENSMUSC	Fam184b	family witl	-	-	-	-	-	-	5:4552970	119	159	135	214	213	174	0.805443	0.859472	1.00988	1.117462	0.928374	0.926506	0.891598	0.990781	0.567617	1	-
ENSMUSC	Lta4h	leukotrien	GO:00081	metabolic	K01254	leukotrien	mmu0110	Metabolic	10:934534	377	532	359	589	660	574	2.991414	3.371257	3.148302	3.605622	3.372354	3.583087	3.170324	3.520354	0.462786	1	-
ENSMUSC	Amdhd1	amidohyc	GO:00081	metabolic	K01468	imidazolo	mmu0110	Metabolic	10:935233	0	4	2	2	3	0	0	0.080559	0.055751	0.03892	0.048728	0	0.045437	0.029216	0.600236	1	-
ENSMUSC	H2afy	H2A histo	GO:00325	developm	K11251	histone H2	mmu0503	Alcoholisr	13:560736	1116	1444	1051	1562	1854	1577	7.79109	8.050944	8.109303	8.412888	8.334858	8.661168	7.983779	8.469638	0.702083	1	-
ENSMUSC	Bola1	bolA-like	GO:00056	cell|intrace	-	-	-	-	3:9619658	67	76	80	84	105	76	2.663329	2.41273	3.514724	2.576106	2.687791	2.376706	2.863594	2.546867	0.426622	1	-
ENSMUSC	Gatsl2	GATS pro	GO:00508	response	-	-	-	-	5:1340997	3003	3488	2239	4045	4572	3591	22.50061	20.87187	18.54129	23.38233	22.05972	21.16729	20.63792	22.20311	0.597923	1	-
ENSMUSC	Fcgr1	Fc recepto	GO:00048	signal trar	K06498	high affini	mmu0520	Transcript	3:9628290	74	96	56	95	113	103	1.54789	1.603704	1.294621	1.533072	1.522099	1.694953	1.482071	1.583375	0.802248	1	-
ENSMUSC	Ncf1	neutrophi	GO:00081	metabolic	K08011	neutrophi	mmu0406	Chemokin	5:1342200	135	219	147	183	222	156	1.223697	1.585373	1.47267	1.279741	1.295834	1.112443	1.427247	1.229339	0.220856	1	-
ENSMUSC	Wnt11	wingless-	GO:00325	developm	K01384	wingless-t	mmu0520	Pathways	7:9883511	7	24	4	15	14	17	0.058144	0.159202	0.036723	0.096121	0.074883	0.111087	0.084689	0.09403	0.942116	1	-
ENSMUSC	Adss	adenylosu	GO:00081	metabolic	K01939	adenylosu	mmu0110	Metabolic	1:1777629	1339	1706	1245	1773	2208	1563	13.23997	13.47198	13.60576	13.52526	14.05918	12.1584	13.43923	13.24761	0.684678	1	-
ENSMUSC	Il17rb	interleukir	GO:00048	signal trar	K05165	interleukir	mmu0406	Cytokine-	14:299961	10	24	12	23	42	18	0.072854	0.139636	0.09662	0.129273	0.197039	0.103162	0.103037	0.143158	0.303038	1	-
ENSMUSC	Cacna1d	calcium cl	GO:00511	localizatio	K04851	voltage-d	mmu0401	MAPK sigr	14:300399	1047	1401	1004	1442	1469	1268	2.982822	3.187606	3.161267	3.169395	2.69499	2.841913	3.110565	2.902099	0.329514	1	-
ENSMUSC	Actr8	ARP8 acti	GO:00081	metabolic	-	-	-	-	14:299783	541	689	557	780	890	799	3.062813	3.115218	3.485178	3.40681	3.244651	3.558616	3.22107	3.403359	0.765723	1	-
ENSMUSC	Lrrc27	leucine ric	GO:00508	response	-	-	-	-	7:1392129	50	102	59	103	97	118	0.553569	0.901874	0.721933	0.879762	0.691548	1.027757	0.725792	0.866356	0.405361	1	-
ENSMUSC	Stk32c	serine/thr	GO:00081	metabolic	-	-	-	-	7:1391036	1159	1639	1217	1682	2044	1616	30.74124	34.71865	35.67592	34.41869	34.9119	33.72022	33.71194	34.35027	0.96175	1	-
ENSMUSC	Fnta	farnesyltr	GO:00081	metabolic	K05955	protein fa	mmu0090	Terpenoic	8:2599872	553	667	495	778	800	756	7.505026	7.229346	7.424701	8.14587	6.991528	8.071609	7.386358	7.736336	0.839694	1	-
ENSMUSC	Skiv2l2	superkille	GO:00081	metabolic	K12598	ATP-depe	-	-	13:112867	1365	1729	1242	1680	2216	1788	10.46645	10.58784	10.52532	9.938163	10.94186	10.78562	10.52653	10.55521	0.815913	1	-
ENSMUSC	Celsr1	cadherin,	GO:00325	developm	-	-	-	-	15:858989	185	262	229	269	339	317	0.829594	0.938297	1.134946	0.93063	0.978924	1.118313	0.967612	1.009289	0.89998	1	-
ENSMUSC	Fli1	Friend leu	GO:00325	developm	K09436	friend leul	mmu0520	Transcript	9:3242220	129	204	101	162	183	195	2.254934	2.847872	1.951247	2.184691	2.059925	2.681576	2.351351	2.308731	0.781962	1	-
ENSMUSC	Stard13	StAR-rela	GO:00325	developm	-	-	-	-	5:1510375	371	584	373	520	596	597	1.796826	2.258867	1.996584	1.942968	1.8588	2.274662	2.017426	2.025477	0.8655	1	-
ENSMUSC	Camk1g	calcium/ca	GO:00081	metabolic	K08794	calcium/ca	mmu0492	Oxytocin s	1:1933463	421	456	420	497	586	482	8.223115	7.113201	9.066736	7.489302	7.370669	7.406485	8.134351	7.422152	0.321898	1	-
ENSMUSC	Diexf	digestive	GO:00056	cell|intrace	-	-	-	-	1:1930911	267	348	224	408	430	365	1.389224	1.446055	1.288117	1.637763	1.440733	1.494046	1.374465	1.524181	0.506617	1	-
ENSMUSC	Hsd11b1	hydroxyst	GO:00325	developm	K15680	corticoste	mmu0110	Metabolic	1:1932216	246	394	275	421	514	379	2.382282	3.047193	2.943318	3.145362	3.205351	2.887408	2.790931	3.079374	0.536319	1	-
ENSMUSC	Syt14	synaptota	GO:00511	localizatio	-	-	-	-	1:1928912	549	688	575	745	868	774	2.256238	2.258118	2.611721	2.362103	2.297135	2.502441	2.375359	2.387226	0.858264	1	-
ENSMUSC	Lonrf3	LON pept	GO:00081	metabolic	-	-	-	-	X:3632835	182	236	188	211	268	229	2.541762	2.63221	2.901799	2.273396	2.410202	2.515989	2.691924	2.399862	0.278314	1	-
ENSMUSC	Atp5e	ATP synth	GO:00081	metabolic	K02135	F-type H+	mmu0110	Metabolic	2:1744610	282	351	244	402	397	383	6.832694	6.791973	6.533993	7.514493	6.194243	7.300489	6.719553	7.003075	0.896945	1	-
ENSMUSC	Nelfcd	negative	GO:00081	metabolic	-	-	-	-	2:1744158	532	587	437	664	763	615	6.896587	6.077239	6.261095	6.640806	6.369446	6.272032	6.41164	6.427428	0.841432	1	-
ENSMUSC	Ctsz	cathepsin	GO:00325	developm	K08568	cathepsin	mmu0421	Apoptosis	2:1744274	640	1057	567	784	1045	847	27.11813	35.76846	26.55275	25.62866	28.5135	28.23408	29.81311	27.45874	0.344464	1	-
ENSMUSC	Prelid3b	PRELI dor	GO:00511	localizatio	-	-	-	-	2:1744650	676	910	653	1113	1313	983	13.02469	14.00259	13.90533	16.54423	16.29073	14.89998	13.6442	15.91164	0.19841	1	-
ENSMUSC	Sertad4	SERTA do	GO:00056	cell|intrace	-	-	-	-	1:1928444	58	537	351	103	524	477	0.522788	3.865614	3.496647	0.716251	3.041477	3.382418	2.62835	2.380049	0.76372	1	-
ENSMUSC	H2-M2	histocomp	GO:00023	immune s	K06751	major hist	mmu0516	Human pa	17:374808	0	0	0	0	3	0	0	0	0	0	0.051247	0	0	0.017082	0.290532	1	-
ENSMUSC	Slc25a5	solute car	GO:00511	localizatio	K05863	solute car	mmu0516	HTLV-I inf	X:3679565	2026	2318	1755	2769	3273	2585	41.31721	37.75294	39.55628	43.56571	42.98256	41.47277	39.54215	42.67368	0.553712	1	-

ENSMUSC	Ppdpf	pancreati	GO:00325	developm	-	-	-	-	2:1811872	240	325	296	375	460	370	4.072804	4.404653	5.551634	4.909571	5.026835	4.939635	4.676364	4.95868	0.780473	1	-
ENSMUSC	Kcnq2	potassium	GO:00511	localizatio	K04927	potassium	mmu0472	Cholinerg	2:1810755	2363	2909	2520	2976	3230	3031	3.828181	3.763729	4.512072	3.719564	3.369659	3.863008	4.034661	3.650743	0.182627	1	-
ENSMUSC	Eef1a2	eukaryoti	GO:00081	metabolic	K03231	elongatio	mmu0301	RNA trans	2:1811476	14874	18316	12874	18857	22121	17214	435.7861	428.5703	416.8751	426.2345	417.3545	396.7695	427.0772	413.4528	0.500828	1	-
ENSMUSC	Col20a1	collagen,	GO:00329	macromo	-	-	-	-	2:1809865	72	105	76	100	111	101	0.727606	0.847417	0.848836	0.779634	0.722335	0.802959	0.807953	0.768309	0.627677	1	-
ENSMUSC	Pls3	plastin 3 (	GO:00325	developm	-	-	-	-	X:7578565	3813	4804	4671	4985	8049	5134	33.59517	33.80329	45.48485	33.88485	45.66745	35.58581	37.62777	38.37937	0.976298	1	-
ENSMUSC	Mpped2	metalloph	GO:00038	catalytic a	-	-	-	-	2:1066932	623	879	645	813	922	919	9.239547	10.41111	10.57228	9.302152	8.805384	10.72232	10.07432	9.609952	0.496405	1	-
ENSMUSC	Nkap	NFKB acti	GO:00325	developm	-	-	-	-	X:3712679	247	345	220	364	451	365	3.404372	3.797562	3.351266	3.870541	4.002863	3.957705	3.517733	3.943703	0.442013	1	-
ENSMUSC	Ndufa1	NADH de	GO:00081	metabolic	K03945	NADH de	mmu0110	Metabolic	X:3718758	495	567	308	534	742	380	72.13305	65.98696	49.60514	60.03454	69.62868	43.5636	62.57505	57.74227	0.47989	1	-
ENSMUSC	E2f3	E2F transc	GO:00081	metabolic	K06620	transcripti	mmu0520	Pathways	13:299065	611	800	569	223	987	809	6.388095	6.679845	6.574902	1.79873	6.64511	6.654113	6.547614	5.032651	0.221363	1	-
ENSMUSC	Cr1l	compleme	GO:00325	developm	-	-	-	-	1:1950973	321	428	351	500	550	431	1.528834	1.627964	1.847604	1.837196	1.686835	1.614892	1.668134	1.712974	0.983637	1	-
ENSMUSC	Ppfibp1	PTPRF inte	GO:00300	cell junctic	-	-	-	-	6:1468884	546	744	437	738	854	732	2.153046	2.343035	1.904531	2.245158	2.168566	2.270819	2.133537	2.228181	0.861043	1	-
ENSMUSC	Cd46	CD46 anti	GO:00325	developm	K04007	membran	mmu0516	Measles -	1:1950368	87	125	85	102	147	137	0.635869	0.729633	0.686615	0.575147	0.691864	0.787733	0.684039	0.684915	0.90618	1	-
ENSMUSC	Cd34	CD34 anti	GO:00325	developm	K06474	CD34 anti	mmu0451	Cell adhes	1:1949388	691	740	531	707	887	848	4.290773	3.669737	3.64417	3.386935	3.54679	4.142515	3.868226	3.69208	0.511617	1	-
ENSMUSC	Plgrkt	plasminog	GO:00081	metabolic	-	-	-	-	19:293485	92	133	92	165	166	151	0.875935	1.0113	0.968095	1.211985	1.017759	1.131022	0.951776	1.120255	0.338846	1	-
ENSMUSC	Cd274	CD274 an	GO:00400	locomotic	K06745	programn	mmu0451	Cell adhes	19:293674	75	132	71	116	200	134	1.26432	1.777111	1.322814	1.508628	2.171096	1.777096	1.454749	1.81894	0.235783	1	-
ENSMUSC	Pdcd1lg2	programn	GO:00023	immune s	K06708	programn	mmu0451	Cell adhes	19:294109	2	0	0	0	0	2	0.074221	0	0	0	0	0.058412	0.02474	0.019471	1	1	-
ENSMUSC	Gtf3a	general tr	GO:00971	organic cy	-	-	-	-	5:1469486	271	312	288	400	494	400	3.858845	3.548047	4.532389	4.394193	4.529706	4.480839	3.97976	4.468246	0.445309	1	-
ENSMUSC	Mtif3	mitochon	GO:00081	metabolic	-	-	-	-	5:1469515	294	401	293	362	500	420	2.310007	2.516265	2.544372	2.194347	2.529831	2.59613	2.456882	2.440103	0.798498	1	-
ENSMUSC	Lnx2	ligand of	GO:00099	cellular pr	-	-	-	-	5:1470166	178	180	116	199	225	210	2.070949	1.672509	1.491605	1.786211	1.685721	1.922115	1.745021	1.798016	0.979251	1	-
ENSMUSC	Dyrk3	dual-spec	GO:00325	developm	-	-	-	-	1:1311274	215	264	199	266	340	292	3.533651	3.465247	3.61481	3.372846	3.598475	3.775531	3.537903	3.582284	0.931545	1	-
ENSMUSC	Mapkapk:	MAP kina:	GO:00325	developm	K04443	mitogen-a	mmu0401	MAPK sig	1:1310537	365	426	371	489	598	445	7.328572	6.830978	8.232805	7.574715	7.731836	7.029063	7.464118	7.445205	0.827801	1	-
ENSMUSC	Lamp2	lysosomal	GO:00081	metabolic	K06528	lysosomal	mmu0515	Tuberculo	X:3840135	1291	1978	1209	1666	2078	1665	7.916646	9.68695	8.193849	7.881703	8.205691	8.032301	8.599148	8.039898	0.337597	1	-
ENSMUSC	Atxn10	ataxin 10	GO:00325	developm	-	-	-	-	15:853363	3736	4513	3514	4587	5704	4593	94.06725	90.7492	97.78679	89.10267	92.48383	90.97853	94.20108	90.85501	0.473041	1	-
ENSMUSC	Foxred2	FAD-depe	GO:00081	metabolic	-	-	-	-	15:779405	406	425	303	343	541	414	1.994658	1.667544	1.645246	1.300071	1.711567	1.600121	1.769149	1.537253	0.190803	1	-
ENSMUSC	Eif3d	eukaryoti	GO:00081	metabolic	K03251	translatior	mmu0301	RNA trans	15:779589	1113	1418	1143	1535	1859	1740	35.57995	36.20195	40.38339	37.85721	38.26872	43.75927	37.38843	39.96173	0.659731	1	-
ENSMUSC	H3f3b	H3 histon	GO:00325	developm	K11253|K1	histone H:	mmu0503	Alcoholisr	11:116021	1984	2720	1816	2816	3594	2683	21.22272	23.23668	21.46951	23.23927	24.75666	22.57829	21.9763	23.52474	0.612944	1	-
ENSMUSC	Nup50	nucleopor	GO:00325	developm	K14295	nuclear pc	mmu0301	RNA trans	15:849234	816	988	717	1005	1286	1095	10.50905	10.16193	10.20561	9.9855	10.6652	11.09426	10.2922	10.58165	0.974307	1	-
ENSMUSC	Phf21b	PHD finge	GO:00081	metabolic	-	-	-	-	15:847853	110	166	122	130	242	161	0.808035	0.97385	0.990479	0.736733	1.144742	0.930412	0.924122	0.937296	0.96632	1	-
ENSMUSC	Ift27	intraflagel	GO:00325	developm	-	-	-	-	15:781594	67	86	68	114	135	88	4.042366	4.143881	4.534361	5.306396	5.245051	4.176928	4.240202	4.909459	0.432081	1	-
ENSMUSC	Ttll12	tubulin tyr	GO:00081	metabolic	-	-	-	-	15:835751	328	273	288	434	493	259	3.535229	2.349917	3.430694	3.608804	3.421724	2.196114	3.10528	3.075547	0.841725	1	-
ENSMUSC	Bik	BCL2-inte	GO:00325	developm	K18452	Bcl-2-inte	mmu0152	Endocrine	15:835268	0	2	0	0	0	0	0	0.103436	0	0	0	0	0.034479	0	0.524395	1	-
ENSMUSC	Tox4	TOX high	GO:00329	macromo	-	-	-	-	14:522791	1334	1780	1228	1921	2083	1762	8.645738	9.213238	8.796129	9.605148	8.693414	8.983863	8.885035	9.094142	0.99526	1	-
ENSMUSC	Mrps18c	mitochon	GO:00081	metabolic	K02963	small subu	mmu0301	Ribosome	5:1007986	80	92	94	119	181	135	2.26245	2.077897	2.938103	2.596381	3.296275	3.00356	2.42615	2.965405	0.268753	1	-
ENSMUSC	Srsf6	serine/arg	GO:00325	developm	K12893	splicing fa	mmu0516	Herpes sir	2:1629315	1671	2087	1557	2288	2814	2369	13.88893	13.85354	14.30302	14.67164	15.0616	15.49061	14.01516	15.07462	0.575087	1	-
ENSMUSC	Plcg1	phospholi	GO:00325	developm	K01116	phosphati	mmu0110	Metabolic	2:1607313	1483	1891	1362	2111	2373	2176	7.71945	7.86109	7.835548	8.477423	7.954224	8.91077	7.805363	8.447472	0.537283	1	-
ENSMUSC	Kctd2	potassium	GO:00099	cellular pr	-	-	-	-	11:115420	581	686	571	855	982	863	6.163096	5.811556	6.694301	6.997113	6.707932	7.201853	6.222984	6.968966	0.381451	1	-
ENSMUSC	Tmprss6	transmem	GO:00081	metabolic	-	-	-	-	15:784396	13	10	10	20	23	13	0.248748	0.152806	0.21147	0.295237	0.283401	0.195684	0.204342	0.258107	0.507754	1	-
ENSMUSC	Kctd5	potassium	GO:00099	cellular pr	-	-	-	-	17:240477	276	380	259	390	508	439	6.018589	6.617809	6.242106	6.561151	7.133514	7.531148	6.292835	7.075271	0.421433	1	-
ENSMUSC	Pom121l2	POM121	GO:00511	localizatio	K14316	nuclear pc	mmu0301	RNA trans	13:219811	1	1	0	0	0	0	0.012457	0.009947	0	0	0	0	0.007468	0	0.518819	1	-
ENSMUSC	Etaa1	Ewing tun	GO:00081	metabolic	-	-	-	-	11:179387	188	185	115	179	230	211	2.742873	2.155587	1.85435	2.0148	2.160883	2.421817	2.250937	2.199167	0.745685	1	-
ENSMUSC	Matn4	matrilin 4	GO:00431	ion bindin	-	-	-	-	2:1643893	155	163	90	208	189	185	1.707686	1.434199	1.095889	1.767954	1.340891	1.603465	1.412591	1.57077	0.624849	1	-
ENSMUSC	Slpi	secretory	GO:00081	metabolic	-	-	-	-	2:1643540	0	3	5	3	5	8	0	0.088512	0.204135	0.085503	0.11893	0.232459	0.097549	0.145631	0.50721	1	-
ENSMUSC	Rbpjl	recombin	GO:00081	metabolic	K06053	recombini	mmu0516	Human pa	2:1644031	8	16	7	13	11	11	0.066679	0.106501	0.064484	0.083588	0.059035	0.072125	0.079221	0.071583	0.687754	1	-
ENSMUSC	Sdc4	syndecan	GO:00325	developm	K16338	syndecan	mmu0520	Proteoglyc	2:1644242	1413	1880	1251	1817	2438	2080	29.90472	31.77614	29.26184	29.66763	33.22659	34.63153	30.31423	32.50858	0.617287	1	-
ENSMUSC	Nbr1	neighbor	GO:00325	developm	K17987	next to BR	mmu0413	Mitophag	11:101552	2087	2585	1954	2857	3218	2771	11.63516	11.5095	12.03986	12.28826	11.55289	12.15339	11.72817	11.99818	0.987855	1	-
ENSMUSC	Cyth1	cytohesin	GO:00987	molecular	K18441	cytohesin[	mmu0414	Endocytos	11:118132	1192	1576	1242	1650	2018	1672	11.50328	12.1464	13.24689	12.28457	12.54069	12.69383	12.29886	12.50636	0.941429	1	-
ENSMUSC	Rnd3	Rho famil	GO:00508	response	-	-	-	-	2:5113043	246	303	229	345	403	350	2.444709	2.404808	2.515206	2.645095	2.579003	2.736347	2.454908	2.653482	0.653489	1	-
ENSMUSC	Brca1	breast car	GO:00325	developm	K10605	breast car	mmu0415	PI3K-Akt	11:101488	25	43	15	31	56	52	0.130409	0.179135	0.086479	0.124758	0.188112	0.213396	0.132008	0.175422	0.313211	1	-
ENSMUSC	Cntnap1	contactin	GO:00325	developm	K07379	contactin	mmu0451	Cell adhes	11:101170	4285	5332	3989	5661	6360	6090	43.92781	43.65406	45.19595	44.77258	41.98561	49.11531	44.25927	45.29116	0.986632	1	-
ENSMUSC	Nt5c3b	5'-nucleo	GO:00081	metabolic	K01081	5'-nucleo	mmu0110	Metabolic	11:100422	356	431	382	548	533	464	3.910178	3.780681	4.63721	4.643631	3.769886	4.009367	4.109356	4.140961	0.896564	1	-
ENSMUSC	Coa3	cytochron	GO:00081	metabolic	-	-	-	-	11:101277	673	983	731	1032	1160	846	34.91256	40.72548	41.91127	41.30254	38.75071	34.52613	39.1831	38.19313	0.644425	1	-
ENSMUSC	Zpbp2	zona pellu	GO:00325	developm	-	-	-	-	11:985510	0	0	1	0	0	4	0	0	0.020026	0	0	0.057011	0.006675	0.019004	0.443008	1	-
ENSMUSC	Med24	mediator	GO:00325	developm	K15167	mediator	mmu0491	Thyroid h	11:987045	1897	2431	1845	2639	2785	2781	13.06273	13.36895	14.04138	14.01963	12.34942	15.06533	13.49102	13.81146	0.991774	1	-
ENSMUSC	Gsdma2	gasdermi	GO:00099	cellular pr	-	-	-	-	11:986467	0	0	2	2	2	1	0	0	0.027563	0.019241	0.016055	0.009811	0.009188	0.015036	1	1	-
ENSMUSC	Psmd3	proteasor	GO:00081	metabolic	K03033	26S prote	mmu0516	Epstein-B	11:986825	1704	2100	1989	2888	2888	2802	13.47184	13.25936	17.37956	17.61509	14.70311	17.42755	14.70359	16.58192	0.351966	1	-
ENSMUSC	Exosc10	exosome	GO:00081	metabolic	K12591	exosome	mmu0301	RNA degr	4:1485584	855	1062	709	1156	1320	1130	9.953227	9.873432	9.122018	10.38212	9.895246	10.34873	9.649559	10.2087	0.745177	1	-
ENSMUSC	Glod4	glyoxalase	GO:00055	extracellul	-	-	-	-	11:761209	549	811	560	807	1002	797	4.4552	5.25607	5.022609	5.05241	5.236206	5.088196	4.911293	5.125604	0.852359	1	-
ENSMUSC	Vps53	VPS53 GA	GO:00511	localizatio	-	-	-	-	11:760462	1292	1838	1240	1867	1974	1640	9.555119	10.85588	10.13543	10.65242	9.401033	9.541759	10.18214	9.865071	0.551568	1	-
ENSMUSC	Dnttip1	deoxynuc	GO:00081	metabolic	-	-	-	-	2:1647459	350	552	371	533	642	549	8.510688	10.71966	9.970514	9.998948	10.05278	10.50219	9.733622	10.18464	0.85279	1	-
ENSMUSC	Tnnc2	troponin (	GO:00990	supramole	K12042	troponin (	mmu0402	Calcium si	2:1647771	0	0	2	0	0	1	0	0	0.168717	0	0	0.060055	0.056239	0.020018	1	1	-
ENSMUSC	Cd300lg	CD300 mc	GO:00511	localizatio	-	-	-	-	11:102041	0	11	0	7	13	1	0	0.186825	0	0.114851	0.17804	0.016734	0.062275	0.103208	0.638884	1	-
ENSMUSC	Mpp2	membran	GO:00452	synapse|re	-	-	-	-	11:102057	3210	3925	3006	4471	4879	4289	26.28356	25.66639	27.20288	28.24322	25.72555	27.62779	26.38428	27.19885	0.944442	1	-
ENSMUSC	Vtn	vitronectir	GO:00325	developm	K06251	vitronectir	mmu0415	PI3K-Akt	11:784990	931	1334	791	1191	1383	1333	16.77342	19.19435	15.75052	16.55442	16.04533	18.89352	17.23943	17.16443	0.777709	1	-
ENSMUSC	Nlk	nemo like	GO:00048	signal trar	K04468	nemo like	mmu0401	MAPK sig	11:785671	1076	1359	921	1186	1636	1244	8.960519	9.038287	8.476714	7.619663	8.773216	8.149899	8.825173	8.180926	0.300466	1	-
ENSMUSC	Aldoc	aldolase C	GO:00325	developm	K01623	fructose-b	mmu0110	Metabolic	11:783229	12481	14790	10293	15043	18477	14936	137.4083	130.0402	125.2427	127.7698	130.9935	129.3626	130.897	129.3753	0.668863	1	-
ENSMUSC	Stac2	SH3 and c	GO:00508	response	-	-	-	-	11:980366	1567	1700	1332	1979	2263	1804	29.88071	25.8891	28.07199	29.11375	27.78826	27.0626	27.94726	27.9882	0.805181	1	-
ENSMUSC	Rpl19	ribosomal	GO:00081	metabolic	K02885	large subu	mmu0301	Ribosome	11:980267	450	627	479	672	780	631	23.62523	26.28918	27.7937	27.21844	26.37018	26.06176	25.9027	26.55013	0.995374	1	-
ENSMUSC	Nek8	NIMA (ne	GO:00325	developm	-	-	-	-	11:781661	53	67	42	89	74	59	0.348455	0.351795	0.305184	0.451428	0.313297	0.305165	0.335145	0.35663	0.859143	1	-
ENSMUSC	Cacnb4	calcium cl	GO:00325	developm	K04865	voltage-d	mmu0401	MAPK sig	2:5242832	1239	1447	2715	1423	3087	3561	4.659462	4.34589	11.28446	4.128568	7.475763	10.53531	6.763272	7.379879	0.7938	1	-
ENSMUSC	Plxdc1	plexin dor	GO:00325	developm	-	-	-	-	11:979232	190	219	143	311	212	279	1.70053	1.565388	1.414535	2.147447	1.221864	1.964478	1.560151	1.77793	0.516058	1	-
ENSMUSC	Zfp207	zinc finge	GO:00990	supramole	-	-	-	-	11:803832	1888	2532	1767	2671	3173	2740	12.88595	13.80143	13.32901	14.06432	13.94567	14.71214	13.33879	14.24071	0.629536	1	-
ENSMUSC	Psmd11	proteasor	GO:00325	developm	K03036	26S prote	mmu0516	Epstein-B	11:804286	933	1221	1118	1604	1882	1539	5.513134	5.762068	7.301388	7.312261	7.161289	7.154291	6.192197	7.20928	0.214173	1	-
ENSMUSC	C1qtnf1	C1q and t	GO:00081	metabolic	-	-	-	-	11:118428	49	61	38	70	73	54	0.892292	0.88713	0.764778	0.983425	0.856022	0.773589	0.848067	0.871012	1	1	-
ENSMUSC	Pipox	pipecolic	GO:00081	metabolic	K00306	sarcosine	mmu0110	Metabolic	11:778806	10	21	14	24	32	25	0.200917	0.336962	0.310875	0.372014	0.414019	0.395149	0.282918	0.393727	0.242704	1	-
ENSMUSC	Timp2	tissue inhi	GO:00325	developm	-	-	-	-	11:118301	5507	7174	4444	7682	7778	7120	82.17181	85.48993	73.28728	88.4326	74.73608	83.57929	80.31634	82.24932	0.996267	1	-
ENSMUSC	Zc3h18	zinc finge	GO:00329	macromo	-	-	-	-	8:1223766	857	1113	789	1092	1336	1066	4.283467	4.442783	4.358508	4.210826	4.30007	4.191627	4.361586	4.234174	0.579682	1	-
ENSMUSC	Top2b	topoisom	GO:00325	developm	K03164	DNA topc	mmu0152	Platinum	14:163651	2635	3382	2367	3196	4144	3294	9.841456	10.08784	9.770655	9.20907	9.966739	9.678615	9.899984	9.618142	0.542244	1	-
ENSMUSC	Rarb	retinoic ac	GO:00325	developm	K08528	retinoic ac	mmu0520	Pathways	14:164308	89	88	75	141	132	138	0.625916	0.494258	0.582951	0.765021	0.597796	0.763507	0.567708	0.708775	0.209001	1	-
ENSMUSC	Igfbp4	insulin-lik	GO:00081	metabolic	-	-	-	-	11:990412	1341	1665	1271	1968	2162	1697	13.403	13.29026	14.03995	15.17499	13.91501	13.34339	13.57774	14.14446	0.854358	1	-
ENSMUSC	Cdc6	cell divisic	GO:00081	metabolic	K02213	cell divisio	mmu0411	Cell cycle	11:989078	7	9	7	13	16	15	0.044425	0.045617	0.049102	0.063649	0.065386	0.07489	0.046381	0.067975	0.298622	1	-
ENSMUSC	Suz12	suppresso	GO:00325	developm	-	-	-	-	11:799931	770	925	620	933	1152	833	5.812898	5.576861	5.172979	5.433934	5.600275	4.947185	5.520913	5.327131	0.560323	1	-
ENSMUSC	Atad5	ATPase fa	GO:00325	developm	-	-	-	-	11:800894	114	154	125	138	191	164	0.749342	0.808429	0.908098	0.699814	0.808466	0.848065	0.821956	0.785448	0.640748	1	-
ENSMUSC	Crlf3	cytokine r	GO:00081	metabolic	-	-	-	-	11:800464	175	197	186	216	266	220	1.851528	1.664577	2.174959	1.7631	1.812293	1.831157	1.897022	1.802183	0.585618	1	-
ENSMUSC	Abr	active BCI	GO:00325	developm	-	-	-	-	11:764167	13028	16842	12256	17111	20964	17863	45.48634	46.96158	47.29321	46.09024	47.13375	49.06463	46.58037	47.42954	0.942648	1	-
ENSMUSC	Rab11fip4	RAB11 far	GO:00325	developm	K12485	Rab11 fan	mmu0414	Endocytos	11:795912	6622	8314	5615	8130	9742	8165	53.13797	53.28091	49.79805	50.33113	50.34062	51.54456	52.07231	50.73877	0.543932	1	-
ENSMUSC	Cd40	CD40 anti	GO:00325	developm	K03160	tumor nec	mmu0516	HTLV-I in	2:1650556	4	5	5	13	12	4	0.04416	0.044079	0.061008	0.110715	0.085309	0.034737	0.049749	0.07692	0.388828	1	-
ENSMUSC	Slc35c2	solute car	GO:00081	metabolic	-	-	-	-	2:1652765	535	770	679	878	1007	668	5.037177	5.789882	7.065615	6.377613	6.105451	4.947898	5.964225	5.810321	0.685846	1	-
ENSMUSC	Zfp334	zinc finge	GO:00081	metabolic	-	-	-	-	2:1653742	616	729	532	736	792	757	4.58401	4.332498	4.375455	4.225448	3.795286	4.431714	4.430654	4.150816	0.384903	1	-
ENSMUSC	Elmo2	engulfme	GO:00400	locomotic	K18985	engulfme	mmu0510	Bacterial i	2:1652880	3204	4488	3442	4483	5594	4715	19.60222	21.92863	23.27397	21.15982	22.0389	22.6937	21.60161	21.96414	0.936247	1	-
ENSMUSC	Wsb1	WD repea	GO:00081	metabolic	-	-	-	-	11:792393	1292	1575	1038	1788	2023	1833	11.41967	11.11777	10.13994	12.19239	11.51441	12.74572	10.89246	12.15084	0.363243	1	-
ENSMUSC	Ttpal	tocopherc	GO:00511	localizatio	-	-	-	-	2:1636023	244	149	151	277	290	186	1.231358	0.600516	0.842206	1.078458	0.942424	0.738445	0.89136	0.919776	0.972638	1	-
ENSMUSC	Rhot1	ras homol	GO:00511	localizatio	K07870	Ras homo	mmu0413	Mitophag	11:802090	1142	1572	1155	1481	1820	1390	4.655387	5.117854	5.203774	4.657735	4.777664	4.457747	4.992338	4.631048	0.290918	1	-
ENSMUSC	Rhbdl3	rhomboid	GO:00081	metabolic	-	-	-	-	11:803009	482	580	403	585	669	578	5.537158	5.321251	5.116727	5.184734	4.949038	5.223712	5.325045	5.119161	0.552983	1	-
ENSMUSC	Serinc3	serine inc	GO:00081	metabolic	-	-	-	-	2:1636232	3597	4466	3023	4495	6059	4699	53.02415	52.57724	49.25136	51.12028	57.51603	54.49412	51.61758	54.37681	0.743293	1	-
ENSMUSC	Pgs1	phosphati	GO:00081	metabolic	K00995	CDP-diac	mmu0110	Metabolic	11:117986	930	1120	826	1366	1520	1346	9.146914	8.79742	8.978804	10.36508	9.626978	10.41472	8.974379	10.13559	0.30202	1	-
ENSMUSC	Pigt	phosphati	GO:00325	developm	K05292	phosphati	mmu0110	Metabolic	2:1644975	1312	1683	1263	1649	2102	1984	13.47036	13.79988	14.33163	13.0616	13.89738	16.025	13.86729	14.32799	0.931145	1	-
ENSMUSC	Dbndd2	dysbindin	GO:00325	developm	-	-	-	-	2:1644861	1500	2159	1622	2225	2424	2044	12.70808	14.60788	15.18751	14.54284	13.22441	13.62325	14.16782	13.79683	0.582926	1	-
ENSMUSC	Mmp9	matrix me	GO:00325	developm	K01403	matrix me	mmu0520	Pathways	2:1649407	26	34	11	18	30	34	0.402513	0.420371	0.188207	0.214991	0.299073	0.414087	0.337031	0.309384	0.686133	1	-
ENSMUSC	Slc12a5	solute car	GO:00325	developm	K14427	solute car	mmu0472	GABAergi	2:1649608	5682	6600	4637	6739	7478	6228	27.97615	25.95233	25.2331	25.59838	23.70974	24.12382	26.38719	24.47731	0.240887	1	-
ENSMUSC	Ghdc	GH3 dom	GO:00160	membran	-	-	-	-	11:100766	71	90	55	81	106	95	1.254738	1.270228	1.074243	1.10436	1.206297	1.320785	1.199737	1.210481	0.936819	1	-
ENSMUSC	Ctsa	cathepsin	GO:00081	metabolic	K13289	cathepsin	mmu0414	Lysosome	2:1648328	1066	1503	1072	1440	1677	1444	6.827645	7.68809	7.588487	7.115519	6.916741	7.275986	7.368074	7.102749	0.51919	1	-
ENSMUSC	Zswim1	zinc finge	GO:00056	cell|intrac	-	-	-	-	2:1648226	388	540	358	550	576	540	6.662129	7.404931	6.793784	7.285762	6.368822	7.294345	6.953615	6.982977	0.859451	1	-
ENSMUSC	Slc12a4	solute car	GO:00511	localizatio	-	-	-	-	8:1059435	366	525	308	406	546	512	4.153767	4.758462	3.8633	3.554818	3.990325	4.57132	4.25851	4.038821	0.522433	1	-
ENSMUSC	Myo1c	myosin IC	GO:00400	locomotic	-	-	-	-	11:756505	316	401	329	441	630	452	2.134093	2.162802	2.45567	2.297712	2.739821	2.401465	2.250855	2.479666	0.534482	1	-
ENSMUSC	Crk	v-crk avia	GO:00325	developm	K04438	proto-onc	mmu0520	Pathways	11:756792	1439	1214	1118	1752	1480	1947	9.616135	6.478943	8.257114	9.032422	6.368777	10.23568	8.117397	8.545625	0.854324	1	-
ENSMUSC	Cox7c	cytochron	GO:00081	metabolic	K02272	cytochron	mmu0110	Metabolic	13:860448	155	193	142	176	250	140	5.374217	5.344261	5.441492	4.707893	5.581862	3.818772	5.386657	4.702842	0.28325	1	-
ENSMUSC	Pitpna	phosphati	GO:00325	developm	-	-	-	-	11:755880	3923	4606	4101	5932	7094	5447	27.14232	25.45062	31.35922	31.66359	31.60636	29.6481	27.98405	30.97268	0.390921	1	-
ENSMUSC	Mlx	MAX-like	GO:00081	metabolic	K09113	MAX-like	mmu0493	Non-alco	11:101087	201	213	227	224	378	301	3.830425	3.24173	4.781061	3.293289	4.638714	4.512608	3.951072	4.148204	0.865735	1	-
ENSMUSC	Retreg3	reticuloph	GO:00325	developm	-	-	-	-	11:101096	227	1088	811	285	1445	348	2.013392	7.706854	7.950055	1.950191	8.253251	2.428244	5.8901	4.210562	0.307172	1	-
ENSMUSC	Rab5a	RAB5A, m	GO:00081	metabolic	K07887	Ras-relate	mmu0414	Endocytos	17:534792	2094	2571	1824	2704	3297	2643	54.2451	53.19021	52.22222	54.04068	54.99933	53.86317	53.21918	54.30106	0.96494	1	-
ENSMUSC	Nkiras2	NFKB inhi	GO:00508	response	-	-	-	-	11:100619	111	179	71	180	139	118	1.142526	1.471436	0.807693	1.429374	0.92132	0.955509	1.140552	1.102068	0.761354	1	-
ENSMUSC	Ppp2r5c	protein pl	GO:00081	metabolic	K11584	serine/thr	mmu0415	PI3K-Akt	12:110447	2855	4098	2944	4135	5029	4079	17.02519	19.51658	19.40307	19.02355	19.31177	19.13597	18.64828	19.1571	0.969319	1	-
ENSMUSC	Ift52	intraflagel	GO:00325	developm	-	-	-	-	2:1630173	390	573	369	548	597	528	7.738929	9.080633	8.092618	8.389323	7.6286	8.242529	8.30406	8.086817	0.65112	1	-
ENSMUSC	Eya2	EYA trans	GO:00325	developm	-	-	-	-	2:1655950	38	76	31	69	70	84	0.494078	0.789172	0.445477	0.692145	0.586097	0.859226	0.576243	0.712489	0.382195	1	-
ENSMUSC	B4galt5	UDP-Gal:	GO:00081	metabolic	K09905	beta-1,4-	mmu0051	Mucin typ	2:1672984	820	1094	767	1202	1395	1006	10.65723	11.35516	11.01723	12.05216	11.67506	10.28582	11.00987	11.33768	0.955556	1	-
ENSMUSC	Gdap1l1	ganglioside	GO:00081	metabolic	-	-	-	-	2:1634384	1123	1307	1058	1518	1682	1402	13.46323	12.51385	14.01852	14.04015	12.98525	13.22296	13.33187	13.41612	0.852778	1	-
ENSMUSC	Ptgis	prostaglar	GO:00325	developm	K01831	prostacycl	mmu0110	Metabolic	2:1671918	13	10	7	6	15	19	0.253273	0.155585	0.150737	0.09018	0.188185	0.291207	0.186532	0.189858	1	1	-
ENSMUSC	Cadps2	Ca2+-dep	GO:00452	synapse|re	-	-	-	-	6:2326277	1842	2298	1595	2412	2665	2130	6.245159	6.222281	5.976698	6.309012	5.818427	5.681256	6.148046	5.936231	0.653998	1	-
ENSMUSC	Ddx27	DEAD (As	GO:00081	metabolic	-	-	-	-	2:1670151	556	697	524	829	914	684	5.434334	5.440642	5.660432	6.251106	5.752714	5.259433	5.511803	5.754417	0.855792	1	-
ENSMUSC	Cyth3	cytohesin	GO:00511	localizatio	K18441	cytohesin	mmu0414	Endocytos	5:1436224	962	1247	946	1278	1454	1265	5.680647	5.880781	6.173917	5.822158	5.528941	5.876675	5.911782	5.742558	0.572019	1	-
ENSMUSC	Cyth4	cytohesin	GO:00987	molecular	K18441	cytohesin	mmu0414	Endocytos	15:785970	296	347	265	351	516	320	6.475536	6.062591	6.407316	5.924073	7.269023	5.507377	6.315147	6.233557	0.781113	1	-
ENSMUSC	Rac3	RAS-relat	GO:00325	developm	K07861	Ras-relate	mmu0520	Pathways	11:120721	137	146	141	163	213	147	5.138193	4.373085	5.844583	4.716361	5.144265	4.337266	5.11862	4.732631	0.500563	1	-
ENSMUSC	Rrp7a	ribosomal	GO:00325	developm	K14545	ribosomal	mmu0301	Ribosome	15:831134	1641	2150	1594	2350	2652	2336	15.12855	15.8297	16.24141	16.71425	15.74406	16.9423	15.73322	16.46687	0.806698	1	-
ENSMUSC	Cyb5r3	cytochron	GO:00325	developm	K00326	cytochron	mmu0052	Amino su	15:831534	1044	1335	987	1345	1641	1450	14.37309	14.67834	15.01805	14.28573	14.54832	15.70467	14.68983	14.84624	0.886163	1	-
ENSMUSC	Ints2	integrator	GO:00081	metabolic	-	-	-	-	11:862106	356	567	425	443	641	473	1.510646	1.921505	1.993188	1.450263	1.751561	1.57901	1.808446	1.593611	0.22007	1	-
ENSMUSC	Med13l	mediator	GO:00081	metabolic	K15164	mediator	mmu0491	Thyroid h	5:1185606	2551	3111	2285	3144	3637	3268	7.809438	7.60598	7.731118	7.425439	7.169803	7.870499	7.715512	7.48858	0.532071	1	-
ENSMUSC	Hist1h2bc	histone cl	GO:00023	immune s	K11252	histone H:	mmu0503	Viral carci	13:236384	224	304	248	338	416	309	11.83134	12.82345	14.47717	13.77312	14.14926	12.83971	13.04399	13.58736	0.891203	1	-
ENSMUSC	Mafk	v-maf mu	GO:00325	developm	-	-	-	-	5:1397915	197	245	186	291	386	272	1.87739	1.864665	1.959059	2.407316	2.368809	2.03924	1.900371	2.182517	0.348631	1	-
ENSMUSC	Stard3	START do	GO:00081	metabolic	-	-	mmu0497	Cholester	11:983583	282	397	334	482	529	425	3.220798	3.621186	4.216064	4.247095	3.890676	3.818686	3.686016	3.985486	0.649907	1	-
ENSMUSC	Mfng	MFNG O-	GO:00325	developm	K05948	fringe[2.4.	mmu0414	Human pa	15:787558	57	68	38	54	57	71	1.105204	1.052997	0.814322	0.807788	0.711705	1.083026	0.990841	0.867506	0.41529	1	-
ENSMUSC	Vmp1	vacuole m	GO:00325	developm	-	-	mmu0414	Autophag	11:865838	914	1241	798	1286	1562	1277	9.298086	10.08242	8.972162	10.09296	10.23253	10.21996	9.450891	10.18182	0.593814	1	-
ENSMUSC	Uchl5	ubiquitin	GO:00081	metabolic	-	-	-	-	1:1437772	488	646	408	637	740	594	5.591355	5.911203	5.166593	5.630767	5.45989	5.354201	5.556384	5.48162	0.7249	1	-
ENSMUSC	Trove2	TROVE dc	GO:00325	developm	K11089	60 kDa SS	mmu0532	Systemic l	1:1437507	1213	1805	1205	1458	1983	1750	6.265418	7.445815	6.878961	5.810013	6.595784	7.111119	6.863398	6.505639	0.430794	1	-
ENSMUSC	Stk4	serine/thr	GO:00325	developm	K04411	serine/thr	mmu0401	MAPK sig	2:1640703	1349	1685	1114	1752	2022	1539	12.9917	12.95985	11.8573	13.01723	12.53978	11.66014	12.60295	12.40572	0.672169	1	-
ENSMUSC	Wfdc15b	WAP four	GO:00081	metabolic	-	-	-	-	2:1642144	0	0	0	3	0	0	0	0	0	0.126081	0	0	0	0.042027	0.292757	1	-
ENSMUSC	Gdf9	growth di	GO:00325	developm	K05498	growth di	mmu0491	Ovarian st	11:534310	10	6	6	22	12	11	0.23448	0.112355	0.1555	0.397974	0.181199	0.202914	0.167445	0.260696	0.27728	1	-
ENSMUSC	Zcchc10	zinc finge	GO:00971	organic cy	-	-	-	-	11:533246	93	105	78	101	150	128	3.555658	3.206071	3.295956	2.979125	3.693052	3.849985	3.352562	3.507387	0.901193	1	-
ENSMUSC	Psmb6	proteasor	GO:00325	developm	K02738	20S prote	mmu0305	Proteason	11:705253	999	1203	909	1251	1604	1122	19.63214	18.8805	19.74298	18.96662	20.29836	17.34627	19.41854	18.87042	0.593544	1	-
ENSMUSC	Spag7	sperm ass	GO:00056	cell|intrac	-	-	-	-	11:706637	611	848	525	874	1094	934	14.48795	16.05861	13.75853	15.98851	16.70468	17.42303	14.76836	16.70541	0.330702	1	-
ENSMUSC	Pfn1	profilin 1	GO:00325	developm	K05759	profilin[-]	mmu0481	Regulatior	11:706518	858	1152	817	1342	1672	1197	12.50307	13.40688	13.15826	15.08732	15.68991	13.72254	13.02274	14.83325	0.269969	1	-
ENSMUSC	Tomm34	translocas	GO:00511	localizatio	-	-	-	-	2:1640535	1780	2303	1124	2500	2893	2430	32.12635	33.19564	22.42098	34.81061	33.62361	34.50313	29.24766	34.31245	0.210864	1	-
ENSMUSC	Ywhab	tyrosine 3	GO:00081	metabolic	K16197	14-3-3 pr	mmu0415	PI3K-Akt	2:1639949	9623	12103	9089	12651	15177	12647	171.0516	171.8128	178.5581	173.4889	173.723	176.8541	173.8075	174.6887	0.816878	1	-
ENSMUSC	Anxa6	annexin A	GO:00511	localizatio	-	-	-	-	11:549791	1949	2574	1894	2630	3000	2506	39.13261	41.27445	42.02946	40.73925	38.78849	39.58392	40.81217	39.70389	0.553312	1	-
ENSMUSC	Il12rb2	interleukir	GO:00048	signal trar	K05064	interleukir	mmu0520	Pathways	6:6729131	2	7	0	11	4	2	0.027054	0.075635	0	0.114821	0.034851	0.021291	0.03423	0.056988	0.481792	1	-
ENSMUSC	Zkscan6	zinc finge	GO:00081	metabolic	-	-	-	-	11:658071	259	347	251	368	425	371	4.40994	4.718542	4.723388	4.834049	4.659904	4.969562	4.61729	4.821172	0.874725	1	-
ENSMUSC	Kpna2	karyopher	GO:00511	localizatio	K15043	importin s	mmu0516	Influenza	11:106988	26	26	41	38	50	15	0.400381	0.319756	0.697796	0.451456	0.495825	0.181719	0.472644	0.376334	0.442628	1	-
ENSMUSC	Smurf2	SMAD spe	GO:00325	developm	K04678	E3 ubiquit	mmu0414	Endocytos	11:106820	713	964	645	917	1108	884	5.437726	5.871521	5.436684	5.395448	5.441546	5.303846	5.581977	5.38028	0.538222	1	-
ENSMUSC	Cep95	centrosor	GO:00056	cell|intrac	-	-	-	-	11:106789	201	234	165	280	336	285	2.068894	1.923555	1.87704	2.223466	2.227082	2.307803	1.956496	2.252784	0.3447	1	-
ENSMUSC	Vezf1	vascular e	GO:00081	metabolic	-	-	-	-	11:880682	634	775	550	753	896	850	7.821948	7.636135	7.499557	7.167227	7.118501	8.250038	7.652547	7.511922	0.673525	1	-
ENSMUSC	Cuedc1	CUE dom	-	-	-	-	-	-	11:880980	426	497	356	470	674	516	3.068381	3.326187	3.479178	3.206325	3.837904	3.589555	3.291249	3.544595	0.659887	1	-
ENSMUSC	Srsf1	serine/arg	GO:00325	developm	-	-	-	-	11:880473	1433	1598	1134	1701	2128	1749	7.685225	6.844361	6.721561	7.037932	7.349141	7.379231	7.083715	7.255435	0.997531	1	-
ENSMUSC	Abi3	ABI gene	GO:00400	locomotic	-	-	-	-	11:958300	70	70	54	72	108	88	0.747603	0.597058	0.637401	0.593248	0.74277	0.739383	0.660687	0.6918	0.914258	1	-
ENSMUSC	Shroom1	shroom fa	GO:00325	developm	-	-	-	-	11:534572	75	122	81	119	118	125	0.44096	0.572854	0.526346	0.539777	0.44676	0.578174	0.513387	0.52157	0.950528	1	-
ENSMUSC	Kif3a	kinesin far	GO:00325	developm	K10394	kinesin far	mmu0434	Hedgehog	11:535673	2647	3062	2092	3094	3520	3279	33.33075	30.7923	29.11385	30.05674	28.54227	32.48206	31.07897	30.36035	0.594735	1	-
ENSMUSC	8-Sep	septin 8 [	GO:00056	cell|intrac	K16939	septin 6/8	mmu0510	Bacterial i	11:535192	5781	7083	4768	7445	8566	6969	31.85137	31.16552	29.03409	31.6461	30.39191	30.20693	30.684	30.74831	0.79182	1	-
ENSMUSC	Mtmr4	myotubul	GO:00325	developm	K18082	myotubul	mmu0456	Inositol p	11:875921	1763	2186	1449	2278	2475	2155	14.30694	14.16741	12.996	14.26194	12.93375	13.75792	13.82345	13.6512	0.678904	1	-
ENSMUSC	Mrm1	mitochon	GO:00081	metabolic	-	-	-	-	11:848130	111	139	87	133	166	122	2.11862	2.1188	1.835348	1.958444	2.040289	1.831888	2.024222	1.94354	0.652463	1	-
ENSMUSC	Mapt	microtubu	GO:00325	developm	K04380	microtubu	mmu0401	MAPK sig	11:104231	8426	10259	7850	11322	12208	10198	47.78264	46.46217	49.2	49.53385	44.5808	45.49614	47.81494	46.53693	0.542653	1	-
ENSMUSC	Kansl1	KAT8 regu	GO:00081	metabolic	-	-	-	-	11:104333	1475	1879	1042	2107	1697	2058	8.190317	8.332616	6.394742	9.026171	6.067993	8.99011	7.639225	8.028091	0.83962	1	-
ENSMUSC	Gid4	GID comp	GO:00081	metabolic	-	-	-	-	11:604171	1359	1751	1203	1771	2219	1730	14.82015	15.24983	14.49924	14.89984	15.58278	14.84191	14.8564	15.10818	0.939499	1	-
ENSMUSC	Myo1b	myosin IB	GO:00511	localizatio	-	-	-	-	1:5174976	264	381	216	361	438	424	0.968072	1.115767	0.875393	1.02127	1.034264	1.223149	0.986411	1.092894	0.535009	1	-
ENSMUSC	Dhx40	DEAH (As	GO:00081	metabolic	-	-	-	-	11:867688	590	801	566	797	1013	771	7.763855	8.417902	8.231688	8.09122	8.583998	7.981613	8.137815	8.218943	0.895163	1	-
ENSMUSC	Ypel2	yippee-lik	GO:00056	cell|intrac	-	-	-	-	11:869364	609	784	488	856	902	710	7.671629	7.887367	6.79418	8.319062	7.316972	7.036224	7.451059	7.55742	0.928094	1	-
ENSMUSC	Akap1	A kinase (	GO:00325	developm	-	-	-	-	11:888307	719	1134	772	1228	1161	1196	8.913863	11.22783	10.57795	11.74535	9.268811	11.66485	10.23988	10.893	0.731553	1	-
ENSMUSC	Nol11	nucleolar	GO:00508	response	-	-	-	-	11:107166	354	401	340	453	484	465	3.160951	2.859592	3.355367	3.120636	2.783006	3.266469	3.125304	3.056704	0.694199	1	-

ENSMUSC	Derl2	Der1-like	GO:00081	metabolic	K13989	Derlin-2/3	mmu0414	Protein pr	11:710071	354	320	257	480	417	459	2.131196	1.538566	1.710017	2.229417	1.616629	2.17392	1.793259	2.006656	0.530953	1	-
ENSMUSC	C1qbp	complem	GO:00325	developm	K15414	complem	mmu0516	Herpes sir	11:709778	604	740	561	732	983	1023	9.826571	9.614847	10.08729	9.187689	10.29847	13.09337	9.842902	10.85984	0.519635	1	-
ENSMUSC	Rpain	RPA intera	GO:00081	metabolic	-	-	-	-	11:709702	132	150	132	168	205	190	1.690718	1.534385	1.86861	1.660111	1.690854	1.914532	1.697905	1.755166	0.952273	1	-
ENSMUSC	Slc13a3	solute car	GO:00511	localizatio	-	-	-	-	2:165405C	211	291	167	253	293	295	1.686071	1.857086	1.474882	1.559712	1.507703	1.854499	1.67268	1.640638	0.738595	1	-
ENSMUSC	Kcnab3	potassium	GO:00511	localizatio	-	-	-	-	11:693262	507	512	223	385	623	524	10.07043	8.121848	4.895436	5.899711	7.968597	8.188072	7.695903	7.352127	0.672167	1	-
ENSMUSC	Chd3	chromodo	GO:00329	macromo	-	-	-	-	11:693432	16516	20364	13779	20122	23281	18921	96.49179	95.01549	88.97132	90.69576	87.58744	86.9642	93.49287	88.4158	0.336081	1	-
ENSMUSC	Kdm6b	KDM1 lysi	GO:00325	developm	-	-	-	-	11:693985	836	970	650	1090	1169	952	7.021262	6.506186	6.033498	7.062616	6.322346	6.290091	6.520315	6.558351	0.859998	1	-
ENSMUSC	1700125H	RIKEN cDI	-	-	-	-	-	-	11:851704	0	2	0	0	0	0	0	0.098031	0	0	0	0	0.032677	0	0.525077	1	-
ENSMUSC	Appbp2	amyloid b	GO:00511	localizatio	-	-	-	-	11:851872	2195	2754	1885	2730	3383	2649	20.7369	20.77873	19.68191	19.89767	20.58097	19.68802	20.39918	20.05555	0.637754	1	-
ENSMUSC	Wnt9b	wingless-t	GO:00325	developm	K01064	wingless-t	mmu0520	Pathways	11:103727	8	16	13	17	10	18	0.108121	0.172693	0.194171	0.177249	0.087026	0.191368	0.158328	0.151881	0.87061	1	-
ENSMUSC	Ncor1	nuclear re	GO:00081	metabolic	-	-	mmu0520	Transcript	11:623164	5649	7135	5018	7016	8713	7605	16.47327	16.61682	16.17282	15.7844	16.36178	17.4469	16.42097	16.53103	0.83303	1	-
ENSMUSC	Trpv2	transient r	GO:00325	developm	K04971	transient r	mmu0462	NOD-like	11:625744	209	238	167	285	310	268	2.278374	2.072052	2.012064	2.396913	2.176174	2.29839	2.12083	2.290492	0.678371	1	-
ENSMUSC	Cenpv	centrome	GO:00081	metabolic	-	-	-	-	11:625249	185	258	208	316	252	245	3.931694	4.378984	4.885602	5.181134	3.448757	4.096228	4.39876	4.24204	0.673742	1	-
ENSMUSC	Pcgf2	polycomb	GO:00325	developm	K11460	polycomb	mmu0455	Signaling	11:976888	186	318	174	265	347	311	2.158273	2.946902	2.231455	2.372294	2.592844	2.838985	2.445543	2.601374	0.785578	1	-
ENSMUSC	Cwc25	CWC25 sp	GO:00056	cell|intrace	-	-	-	-	11:977454	353	271	337	271	546	489	2.81598	1.726514	2.971197	1.667839	2.804801	3.068844	2.504563	2.513828	0.908036	1	-
ENSMUSC	1700001P	RIKEN cDI	-	-	-	-	-	-	11:977714	0	3	2	1	2	1	0	0.234472	0.216304	0.0755	0.125994	0.076994	0.150259	0.092829	0.54634	1	-
ENSMUSC	Pip4k2b	phosphati	GO:00081	metabolic	K00920	1-phosph	mmu0481	Regulation	11:977151	3578	4384	2944	4435	5215	4163	40.99567	40.11566	37.28054	39.2032	38.47748	37.5245	39.46396	38.40173	0.544543	1	-
ENSMUSC	Trim37	tripartite r	GO:00081	metabolic	K10608	tripartite r	mmu0412	Ubiquitin	11:871270	7783	9740	6493	9748	11592	9538	62.31514	62.28039	57.45645	60.2133	59.76674	60.0779	60.68399	60.01931	0.672128	1	-
ENSMUSC	Ctdnep1	CTD nucle	GO:00325	developm	-	-	-	-	11:699811	732	962	739	1026	1230	1005	13.48658	14.15506	15.04812	14.58373	14.59321	14.56692	14.22992	14.58128	0.997551	1	-
ENSMUSC	Elp5	elongator	GO:00081	metabolic	-	-	-	-	11:699682	426	469	356	582	477	508	5.152691	4.530464	4.759058	5.430967	3.71533	4.833909	4.814071	4.660069	0.649009	1	-
ENSMUSC	Gabarap	gamma-a	GO:00081	metabolic	K08341	GABA(A) r	mmu0516	Kaposi's s	11:699911	1279	1676	1215	1791	2247	1643	23.45849	24.54988	24.62934	25.34284	26.53917	23.70707	24.21257	25.19636	0.859587	1	-
ENSMUSC	Phf23	PHD finge	GO:00081	metabolic	-	-	-	-	11:699957	348	411	357	415	543	420	2.912314	2.746923	3.301975	2.679398	2.926263	2.765148	2.987071	2.79027	0.437329	1	-
ENSMUSC	Acadvl	acyl-Coer	GO:00325	developm	K09479	very long	mmu0110	Metabolic	11:700101	528	671	499	806	1009	785	6.63757	6.73664	6.93302	7.817011	8.168101	7.763466	6.769077	7.916193	0.187526	1	-
ENSMUSC	Dnah11	dynein, ax	GO:00325	developm	K10408	dynein he	mmu0501	Huntingto	12:117877	52	77	30	52	47	71	0.212632	0.251455	0.135579	0.164044	0.12376	0.228399	0.199889	0.172067	0.414267	1	-
ENSMUSC	G3bp1	GTPase ac	GO:00081	metabolic	-	-	-	-	11:554696	1249	1519	1065	1686	1996	1545	10.61844	10.31339	10.00678	11.05824	10.92731	10.33326	10.31287	10.77293	0.831757	1	-
ENSMUSC	Atox1	antioxidar	GO:00511	localizatio	K07213	copper ch	mmu0497	Mineral al	11:554466	117	111	96	132	197	146	9.019956	6.834177	8.179636	7.850947	9.780026	8.85489	8.011256	8.828621	0.635889	1	-
ENSMUSC	Glra2	glycine re	GO:00048	signal trar	K05194	glycine re	mmu0408	Neuroacti	X:1651290	115	193	124	189	207	181	2.229115	2.987696	2.656456	2.826337	2.583798	2.760087	2.624422	2.723407	0.942016	1	-
ENSMUSC	Sparc	secreted a	GO:00325	developm	-	-	-	-	11:553945	3592	5005	3147	4375	5483	4612	41.94315	46.67395	40.6133	39.41241	41.22851	42.36673	43.0768	41.00255	0.393014	1	-
ENSMUSC	Glra4	glycine re	GO:00048	signal trar	-	-	-	-	X:1367576	0	1	0	1	2	0	0	0.021919	0	0.021174	0.035335	0	0.007306	0.018836	0.648077	1	-
ENSMUSC	Mief2	mitochon	GO:00325	developm	-	-	-	-	11:607283	160	199	169	223	254	198	2.838268	2.819238	3.313349	3.051884	2.901493	2.76317	2.990285	2.905516	0.706744	1	-
ENSMUSC	Crhr1	corticotro	GO:00325	developm	K04578	corticotro	mmu0408	Neuroacti	11:104132	96	119	87	140	140	123	2.382746	2.358854	2.386568	2.680813	2.237651	2.401729	2.376056	2.440064	1	1	-
ENSMUSC	Dusp14	dual speci	GO:00081	metabolic	-	-	-	-	11:840480	302	439	272	491	506	422	4.063367	4.717258	4.044783	5.096714	4.384138	4.46686	4.275136	4.649237	0.612172	1	-
ENSMUSC	Tada2a	transcripti	GO:00081	metabolic	-	-	-	-	11:840789	439	649	426	610	747	567	6.572962	7.760449	7.049404	7.046217	7.202307	6.678676	7.127605	6.975733	0.677068	1	-
ENSMUSC	Ikzf1	IKAROS fa	GO:00325	developm	-	-	-	-	11:116850	76	38	63	58	128	109	0.427809	0.170828	0.391943	0.251878	0.463979	0.482694	0.330193	0.399517	0.508696	1	-
ENSMUSC	Coq1	compone	GO:00511	localizatio	-	-	-	-	11:113649	770	946	727	1118	1263	1026	6.336216	6.216938	6.611817	7.097604	6.692643	6.64198	6.388324	6.810742	0.687684	1	-
ENSMUSC	Cbx1	chromobo	GO:00081	metabolic	-	-	-	-	11:967891	511	759	469	567	990	653	5.184559	6.150051	5.25909	4.438175	6.468165	5.212131	5.531233	5.372823	0.674596	1	-
ENSMUSC	Cdk5rap3	CDK5 reg	GO:00081	metabolic	-	-	-	-	11:969074	328	459	305	551	561	475	3.131187	3.499402	3.217963	4.058042	3.448674	3.567301	3.282851	3.691339	0.41687	1	-
ENSMUSC	Copz2	coatomer	GO:00511	localizatio	-	-	-	-	11:968498	65	117	75	118	117	121	1.395983	2.006768	1.780226	1.955144	1.618111	2.044378	1.727659	1.872544	0.753511	1	-
ENSMUSC	Slc25a39	solute car	GO:00081	metabolic	-	-	-	-	11:102402	518	569	440	599	827	634	5.021926	4.405538	4.714547	4.480205	5.162988	4.835497	4.714004	4.82623	1	1	-
ENSMUSC	Sp2	Sp2 transc	GO:00325	developm	-	-	-	-	11:969533	281	388	179	259	359	424	3.472437	3.82918	2.444717	2.469214	2.856787	4.121969	3.248778	3.149324	0.725659	1	-
ENSMUSC	Aatf	apoptosis	GO:00325	developm	-	-	-	-	11:844228	285	429	277	474	557	401	5.387479	6.476553	5.787181	6.912731	6.780333	5.96343	5.883738	6.552165	0.476215	1	-
ENSMUSC	Lhx1	LIM home	GO:00325	developm	-	-	-	-	11:845182	2	14	6	11	6	3	0.022102	0.123585	0.073302	0.093806	0.042705	0.026083	0.072997	0.054198	0.517477	1	-
ENSMUSC	Dync1h1	dynein cy	GO:00511	localizatio	K10413	dynein he	mmu0414	Phagoson	12:110601	17059	20528	15613	21082	24228	22936	59.02365	56.72375	59.7043	56.27488	53.98147	62.43111	58.4839	57.56249	0.633865	1	-
ENSMUSC	Cpsf4l	cleavage a	GO:00081	metabolic	-	-	-	-	11:113698	0	0	0	0	0	2	0	0	0	0	0	0.03387	0	0.01129	0.526666	1	-
ENSMUSC	Pex12	peroxisom	GO:00081	metabolic	K13345	peroxin-1	mmu0414	Peroxisom	11:832946	139	172	122	184	84	163	1.12352	1.110301	1.089868	1.147399	0.437222	1.036488	1.107896	0.873703	0.209736	1	-
ENSMUSC	Ndel1	nudE neu	GO:00325	developm	-	-	-	-	11:688214	1356	1668	1139	1826	2181	1538	16.24702	15.96084	15.08289	16.87894	16.82768	14.4971	15.76358	16.06791	0.96332	1	-
ENSMUSC	Zbtb4	zinc finge	GO:00081	metabolic	-	-	-	-	11:697659	2269	2855	1918	2991	3520	3066	9.00376	9.047766	8.41172	9.15664	8.994702	9.571332	8.821082	9.240891	0.797616	1	-
ENSMUSC	Tnfsfm13	tumor nec	GO:00023	immune s	-	-	-	-	11:696825	73	86	64	102	120	105	1.376108	1.294723	1.333403	1.483413	1.45669	1.557164	1.334745	1.499089	0.544727	1	-
ENSMUSC	Mpdu1	mannose-	GO:00081	metabolic	-	-	-	-	11:696566	273	380	257	385	456	398	3.098881	3.444853	3.224204	3.371577	3.333198	3.554154	3.255979	3.419643	0.833729	1	-
ENSMUSC	Fxr2	fragile X r	GO:00055	extracellul	K15516	fragile X r	mmu0301	RNA trans	11:696329	1961	2482	1819	2717	3256	2610	23.11486	23.36475	23.69699	24.70777	24.71455	24.20277	23.3922	24.54169	0.779608	1	-
ENSMUSC	Atp5g3	ATP synth	GO:00081	metabolic	K02128	F-type H+	mmu0110	Metabolic	2:7390844	1048	1116	785	1316	1589	959	21.83177	18.56682	18.07358	21.15017	21.31601	15.71654	19.49072	19.39424	0.803792	1	-
ENSMUSC	Slc35g3	solute car	GO:00160	membran	-	-	-	-	11:697598	0	0	1	0	3	0	0	0	0.04171	0	0.072911	0	0.013903	0.024304	1	1	-
ENSMUSC	Acsl1	acyl-CoA	GO:00081	metabolic	K01897	long-chai	mmu0110	Metabolic	8:4647103	849	591	376	596	880	669	3.663745	2.036814	1.793299	1.984239	2.445429	2.271197	2.497953	2.233622	0.400731	1	-
ENSMUSC	Abca5	ATP-bind	GO:00325	developm	K05648	ATP-bind	mmu0201	ABC trans	11:110269	1088	1180	841	1200	1389	1223	4.804456	4.161435	4.104481	4.08815	3.949774	4.248668	4.35679	4.09553	0.380504	1	-
ENSMUSC	Smyd4	SET and M	GO:00081	metabolic	-	-	-	-	11:753484	141	195	196	246	263	232	1.541204	1.702244	2.367805	2.074476	1.851194	1.994994	1.870418	1.973555	0.833477	1	-
ENSMUSC	Lsp1	lymphocy	GO:00048	signal trar	K14957	lymphocy	mmu0515	Tuberculo	7:1424608	41	95	54	72	125	78	0.281277	0.520505	0.409441	0.381079	0.552226	0.420978	0.403741	0.451428	0.660614	1	-
ENSMUSC	Zfyve27	zinc finge	GO:00325	developm	K19368	protrudin[	mmu0414	Endocytos	19:421639	1029	1251	874	1385	1567	1314	5.499717	5.339842	5.162779	5.710907	5.39323	5.524988	5.334113	5.543042	0.881746	1	-
ENSMUSC	Avpi1	arginine v	GO:00081	metabolic	-	-	-	-	19:421232	145	192	137	229	241	153	5.523026	5.840594	5.767374	6.729389	5.911284	4.584706	5.710331	5.741793	0.917914	1	-
ENSMUSC	Rad51d	RAD51 pa	GO:00081	metabolic	K10871	RAD51-lik	mmu0344	Homolog	11:828764	202	249	199	265	350	277	1.841684	1.813041	2.005229	1.863971	2.054878	1.986797	1.886651	1.968549	0.879088	1	-
ENSMUSC	Unc45b	unc-45 m	GO:00325	developm	-	-	-	-	11:829105	20	26	21	31	19	25	0.248206	0.257689	0.28804	0.296806	0.15184	0.244077	0.264645	0.230908	0.517796	1	-
ENSMUSC	Pank3	pantother	GO:00081	metabolic	K09680	type II par	mmu0110	Metabolic	11:357694	1660	1959	1387	2034	2431	1905	13.74139	12.95098	12.68954	12.98984	12.95872	12.4059	13.1273	12.78482	0.570212	1	-
ENSMUSC	Rars	arginyl-tR	GO:00081	metabolic	K01887	arginyl-tR	mmu0097	Aminoacy	11:358083	782	1027	760	1031	1314	1056	12.06048	12.64953	12.95442	12.26723	13.04992	12.81247	12.55481	12.70987	0.909786	1	-
ENSMUSC	Wwc1	WW, C2 a	GO:00325	developm	K16685	protein KI	mmu0439	Hippo sig	11:358384	2028	2589	1795	2432	2939	2446	22.81242	23.25848	22.31591	21.10559	21.28912	21.64566	22.79561	21.34679	0.297497	1	-
ENSMUSC	Mrpl58	mitochon	GO:00081	metabolic	-	-	-	-	11:115403	247	230	289	420	304	279	1.856854	1.380873	2.401178	2.435902	1.471663	1.650046	1.879635	1.852537	0.826065	1	-
ENSMUSC	Otop3	otopetrin	GO:00160	membran	-	-	-	-	11:115334	0	0	0	2	0	0	0	0	0	0.023197	0	0	0	0.007732	0.527644	1	-
ENSMUSC	Sult4a1	sulfotrans	GO:00081	metabolic	-	-	-	-	15:840760	6379	7816	5967	8540	10360	8382	163.4451	159.9373	168.9749	168.8136	170.9361	168.9577	164.1191	169.5691	0.917253	1	-
ENSMUSC	Mrpl45	mitochon	GO:00329	macromo	-	-	-	-	11:973157	443	520	394	600	720	627	9.850227	9.23404	9.682456	10.29259	10.30932	10.96784	9.588908	10.52325	0.524843	1	-
ENSMUSC	Mb	myoglobi	GO:00325	developm	-	-	-	-	15:770154	0	0	2	0	3	0	0	0	0.095658	0	0.083608	0	0.031886	0.027869	1	1	-
ENSMUSC	Slc22a5	solute car	GO:00325	developm	K08202	MFS trans	mmu0523	Choline m	11:538645	125	180	106	199	257	182	1.830712	2.10538	1.715793	2.24851	2.423812	2.096982	1.883962	2.256434	0.2597	1	-
ENSMUSC	P4ha2	procollag	GO:00081	metabolic	K00472	prolyl 4-h	mmu0110	Metabolic	11:541000	225	249	175	276	321	262	1.616247	1.428463	1.389341	1.529553	1.484856	1.480596	1.478017	1.498335	0.938937	1	-
ENSMUSC	Arrb1	arrestin, b	GO:00325	developm	K04439	beta-arre	mmu0474	Olfactory	7:9953546	1214	2426	1009	1474	1676	2745	4.941635	7.886581	4.539314	4.628921	4.393199	8.790334	5.789177	5.937485	0.996303	1	-
ENSMUSC	Cxcl16	chemokin	GO:00400	locomotic	K10035	C-X-C mc	mmu0406	Cytokine-	11:704539	22	54	25	41	61	34	0.292651	0.573683	0.367546	0.420765	0.522536	0.355808	0.411293	0.433036	0.9379	1	-
ENSMUSC	Pelp1	proline, gl	GO:00081	metabolic	-	-	-	-	11:703928	527	682	507	733	861	685	8.410246	8.692181	8.942387	9.024702	8.848229	8.600032	8.681605	8.824321	0.946119	1	-
ENSMUSC	Med11	mediator	GO:00081	metabolic	-	-	-	-	11:704519	91	87	75	145	138	93	2.663611	2.033738	2.426261	3.274359	2.601132	2.141526	2.374537	2.672339	0.576475	1	-
ENSMUSC	Alox15	arachidon	GO:00325	developm	K00460	arachidon	mmu0110	Metabolic	11:703441	0	0	0	0	2	0	0	0	0	0	0.030568	0	0	0.010189	0.529142	1	-
ENSMUSC	Natd1	N-acetylti	-	-	-	-	-	-	11:609022	272	299	212	321	305	330	3.761677	3.302403	3.240381	3.424906	2.716231	3.590358	3.43482	3.243832	0.52923	1	-
ENSMUSC	Ywhah	tyrosine 3	GO:00325	developm	K16198	14-3-3 pr	mmu0415	PI3K-Akt s	5:3301881	23426	11845	21835	13511	15353	30487	268.3578	108.367	276.4497	119.4081	113.2567	274.7526	217.7248	169.1391	0.232407	1	-
ENSMUSC	Sart3	squamous	GO:00325	developm	-	-	-	-	5:1137424	785	949	709	1044	1317	1124	5.740195	5.542029	5.729938	5.889628	6.201509	6.465974	5.670721	6.185704	0.526412	1	-
ENSMUSC	E2f2	E2F transc	GO:00081	metabolic	K09389	transcripti	mmu0520	Pathways	4:1361723	10	35	22	36	59	31	0.124839	0.34895	0.303546	0.346718	0.474296	0.304452	0.259112	0.375155	0.200684	1	-
ENSMUSC	Slfn3	schlafen 3	GO:00099	cellular pr	-	-	-	-	11:831913	11	14	3	23	8	15	0.150059	0.152521	0.045232	0.242055	0.070277	0.160973	0.115938	0.157768	0.511502	1	-
ENSMUSC	Nars2	asparagin	GO:00081	metabolic	K01893	asparagin	mmu0097	Aminoacy	7:9695150	160	189	201	216	287	243	1.089718	1.028016	1.512987	1.134952	1.258723	1.301999	1.21024	1.231892	0.982509	1	-
ENSMUSC	Slc35b4	solute car	GO:00081	metabolic	-	-	-	-	6:3415338	833	1088	753	1059	1246	1035	4.120001	4.297609	4.116177	4.040906	3.968487	4.027204	4.177929	4.012199	0.506481	1	-
ENSMUSC	Dnah1	dynein, ax	GO:00400	locomotic	K10408	dynein he	mmu0501	Huntingto	14:312603	133	171	138	205	226	251	0.610306	0.626668	0.699879	0.725737	0.66782	0.906108	0.645618	0.766555	0.29606	1	-
ENSMUSC	Dalrd3	DALR anti	GO:00081	metabolic	-	-	-	-	9:1085698	639	857	728	1033	1046	1098	6.198942	6.639619	7.805397	7.731206	6.53436	8.379724	6.881319	7.54843	0.536712	1	-
ENSMUSC	Fis1	fission, mi	GO:00325	developm	K17969	mitochon	mmu0413	Mitophag	5:1369532	804	1033	863	1101	1388	1031	15.94372	16.35988	18.91433	16.84424	17.72466	16.08433	17.07264	16.88441	0.737125	1	-
ENSMUSC	Plod1	procollag	GO:00325	developm	K00473	procollag	mmu0031	Lysine deg	4:1479097	335	471	353	471	553	427	2.535261	2.846718	2.952561	2.749973	2.694992	2.542239	2.77818	2.662401	0.565404	1	-
ENSMUSC	Rab3d	RAB3D, m	GO:00081	metabolic	K07884	Ras-relate	mmu0497	Pancreatic	9:2190749	233	368	252	444	125	459	1.664311	2.099289	1.989419	2.446765	0.574966	2.579305	1.917673	1.867012	0.842829	1	-
ENSMUSC	Slc25a22	solute car	GO:00511	localizatio	-	-	-	-	7:1414297	3259	3731	2814	4469	4920	3905	11.68458	10.68316	11.15063	12.36145	11.35922	11.01439	11.17279	11.57835	0.898453	1	-
ENSMUSC	Atp6ap1	ATPase, H	GO:00325	developm	K03662	V-type H-	mmu0110	Metabolic	X:7429709	2742	3369	2489	3159	3955	3508	25.3017	24.82728	25.38364	22.48859	23.50084	25.46553	25.17087	23.81832	0.363718	1	-
ENSMUSC	Aldh3a1	aldehyde	GO:00325	developm	K00129	aldehyde	mmu0110	Metabolic	11:612075	12	5	11	24	19	17	0.325491	0.108309	0.32976	0.502243	0.331876	0.362774	0.25452	0.398964	0.195748	1	-
ENSMUSC	Ccl9	chemokin	GO:00325	developm	K05510	C-C motif	mmu0406	Cytokine-	11:835729	29	51	29	30	57	43	0.549389	0.771608	0.607194	0.438466	0.695364	0.640864	0.64273	0.591565	0.6486	1	-
ENSMUSC	Scrn1	secernin 1	GO:00081	metabolic	-	-	-	-	6:5450117	3866	4731	3309	4809	5589	4617	23.65711	23.12058	22.37914	22.70309	22.02361	22.22648	23.05228	22.31773	0.497216	1	-
ENSMUSC	BC005537	cDNA seq	GO:00056	cell|intrace	-	-	-	-	13:248016	3815	4885	3461	4766	6160	4883	38.73241	39.60867	38.83545	37.33054	40.27312	39.00116	39.05884	38.86828	0.731209	1	-
ENSMUSC	Hars2	histidyl-tR	GO:00081	metabolic	K01892	histidyl-tR	mmu0097	Aminoacy	18:367830	230	309	228	300	428	349	2.142063	2.298309	2.346859	2.155537	2.566862	2.557058	2.26241	2.426486	0.707061	1	-
ENSMUSC	Cacng2	calcium ch	GO:00081	metabolic	K04867	voltage-d	mmu0401	MAPK sign	15:779917	1006	1296	946	1347	1542	1392	11.14782	11.46945	11.5859	11.51569	11.0035	12.13506	11.40106	11.55141	0.906956	1	-
ENSMUSC	Tmem160	transmem	GO:00160	membran	-	-	-	-	7:1645277	136	220	123	221	227	215	5.484743	7.085763	5.482398	6.876064	5.895195	6.82129	6.017635	6.53085	0.695965	1	-
ENSMUSC	Rab5c	RAB5C, m	GO:00452	synapse|lc	K07889	Ras-relate	mmu0414	Endocytos	11:100715	1435	2511	2043	2909	2549	3061	22.97283	32.10371	36.14746	35.9283	26.27769	38.55111	30.408	33.5857	0.548532	1	-
ENSMUSC	Styxl1	serine/thr	GO:00081	metabolic	-	-	-	-	5:1357472	1	7	3	5	6	0	0.013976	0.078111	0.04633	0.053893	0.053982	0	0.046139	0.035958	0.729544	1	-
ENSMUSC	Mdh2	malate de	GO:00081	metabolic	K00026	malate de	mmu0110	Metabolic	5:1357784	2939	4139	3060	4476	5423	4476	34.75688	39.09151	39.99535	40.83774	41.29861	41.64302	37.94791	41.25979	0.487857	1	-
ENSMUSC	H13	histocomp	GO:00081	metabolic	-	-	-	-	2:1526694	908	1164	829	1214	1453	1250	5.263531	5.388776	5.311201	5.429257	5.4239	5.700491	5.321169	5.517883	0.899233	1	-
ENSMUSC	Rnf145	ring finge	GO:00081	metabolic	-	-	-	-	11:445189	1714	2183	1538	2179	2858	2229	11.4714	11.66823	11.37651	11.25106	12.3175	11.73618	11.50538	11.76824	0.990609	1	-
ENSMUSC	Scn1b	sodium ch	GO:00325	developm	K04845	voltage-g	mmu0426	Adrenergi	7:3111652	5219	6625	5189	7214	8483	7150	110.2638	111.7834	121.1647	117.585	115.4116	118.84	114.4039	117.2789	0.993103	1	-
ENSMUSC	Atp6v1e1	ATPase, H	GO:00511	localizatio	K02150	V-type H-	mmu0110	Metabolic	6:1207943	3060	4149	3273	4436	5113	4155	32.60695	35.3084	38.5462	36.46788	35.08482	34.83137	35.48718	35.46136	0.781916	1	-
ENSMUSC	Chtf18	CTF18, ch	GO:00081	metabolic	-	-	-	-	17:257189	23	30	11	34	33	30	0.196907	0.205113	0.104079	0.224562	0.181929	0.202051	0.1687	0.202847	0.543039	1	-
ENSMUSC	Lhx9	LIM home	GO:00325	developm	-	-	-	-	1:1388251	183	431	142	201	573	243	0.931216	1.751546	0.798606	0.789087	1.87762	0.972783	1.160456	1.213163	0.916869	1	-
ENSMUSC	Etnppl	ethanolan	GO:00038	catalytic a	K14286	ethanolan	mmu0110	Metabolic	3:1306174	279	262	341	367	461	491	1.574128	1.180545	2.126362	1.597467	1.674909	2.179354	1.627012	1.817243	0.583362	1	-
ENSMUSC	Rps6kl1	ribosomal	GO:00081	metabolic	-	-	-	-	12:851352	539	724	642	872	943	722	4.683413	5.024092	6.16531	5.845481	5.276425	4.935394	5.290938	5.352433	0.919887	1	-
ENSMUSC	Ppp1r12c	protein ph	GO:00987	molecular	K17457	protein ph	mmu0481	Regulation	7:4481520	1093	1398	1003	1629	1887	1502	7.6603	7.824893	7.769145	8.80799	8.516321	8.28145	7.751446	8.535254	0.431726	1	-
ENSMUSC	Ahr	aryl-hydro	GO:00048	signal trar	K09093	aryl hydro	mmu0465	Th17 cell	12:354979	222	238	158	201	274	259	2.071027	1.773195	1.629059	1.44664	1.646036	1.900838	1.824427	1.664505	0.398008	1	-
ENSMUSC	Tmem129	transmem	GO:00081	metabolic	-	-	-	-	5:3365321	326	429	282	381	535	417	3.293335	3.461162	3.148582	2.969434	3.480386	3.314103	3.301027	3.254641	0.747123	1	-
ENSMUSC	Nop9	NOP9 nuc	GO:00971	organic cy	-	-	-	-	14:557456	173	166	147	214	268	205	2.685755	2.058134	2.522223	2.563088	2.679228	2.50371	2.422037	2.582008	0.778513	1	-
ENSMUSC	Hsd17b1	hydroxyst	GO:00325	developm	K00044	17beta-es	mmu0110	Metabolic	11:101078	5	4	0	3	0	8	0.227979	0.145655	0	0.105554	0	0.286972	0.124545	0.130842	1	1	-
ENSMUSC	Atp6v0a1	ATPase, H	GO:00081	metabolic	K02154	V-type H-	mmu0110	Metabolic	11:101009	5156	6548	4856	6980	7989	7306	32.51556	32.97864	33.84572	33.95972	32.44333	36.24673	33.11331	34.21659	0.919502	1	-
ENSMUSC	Psmc3ip	proteasor	GO:00081	metabolic	-	-	-	-	11:101091	37	48	31	66	80	49	0.741442	0.768175	0.686564	1.02034	1.032324	0.772456	0.73206	0.941707	0.224612	1	-
ENSMUSC	Zfp687	zinc finge	GO:00081	metabolic	-	-	-	-	3:9500659	434	589	450	580	799	739	1.518493	1.645827	1.740131	1.565601	1.800216	2.034128	1.634817	1.799982	0.524289	1	-
ENSMUSC	Gdpd2	glyceroph	GO:00325	developm	-	-	-	-	X:1007298	112	187	119	201	217	169	1.01416	1.352317	1.190924	1.404161	1.265334	1.203892	1.1858	1.291129	0.674563	1	-
ENSMUSC	D8Ertd738	DNA segr	GO:00056	cell|intrace	-	-	-	-	8:8424623	428	587	382	673	753	575	42.4235	46.4672	41.84781	51.46454	48.06316	44.83749	43.5795	48.12173	0.490924	1	-
ENSMUSC	Sec14l4	SEC14-lik	GO:00511	localizatio	-	-	-	-	11:403146	10	17	15	20	16	7	0.139022	0.18874	0.230473	0.214504	0.143234	0.076562	0.186079	0.144766	0.440978	1	-
ENSMUSC	Calm3	calmoduli	GO:00511	localizatio	K02183	calmoduli	mmu0474	Olfactory	7:1691537	31586	42431	31690	43457	52200	41497	531.5825	570.3024	589.4475	564.2429	565.7196	549.419	563.7775	559.7939	0.70836	1	-
ENSMUSC	Cops3	COP9 sigr	GO:00325	developm	-	-	-	-	11:598177	974	1232	815	1252	1498	1148	12.72642	12.85594	11.76934	12.62066	12.60416	11.8005	12.45057	12.34178	0.734642	1	-
ENSMUSC	Fkbp8	FK506 bin	GO:00325	developm	-	-	-	-	8:7052772	3086	4249	3326	4792	5518	4979	21.36584	23.49398	25.45031	25.59594	24.60143	27.11921	23.43671	25.77219	0.416519	1	-
ENSMUSC	Ddx39b	DEAD (As	GO:00325	developm	K12812	ATP-depe	mmu0516	Influenza	17:352417	1907	2562	1887	3011	3122	2750	19.52672	20.95094	21.35488	23.7859	20.58572	22.15247	20.61085	22.1747	0.581734	1	-
ENSMUSC	Gipc1	GIPC PDZ	GO:00081	metabolic	-	-	-	-	8:8365267	523	689	541	750	952	676	14.63493	15.39763	16.73141	16.19123	17.15459	14.88147	15.58799	16.07576	0.945271	1	-
ENSMUSC	Tlcd1	TLC doma	GO:00160	membran	-	-	-	-	11:781767	135	162	99	159	281	175	0.938391	0.899312	0.760557	0.852663	1.2578	0.956974	0.866087	1.022479	0.362152	1	-
ENSMUSC	Plscr3	phospholi	GO:00508	response	-	-	-	-	11:698463	139	179	133	232	218	222	1.796205	1.847314	1.899503	2.31292	1.814064	2.256866	1.847674	2.12795	0.395631	1	-
ENSMUSC	Arhgef25	Rho guan	GO:00987	molecular	-	-	-	-	10:127182	3959	5565	4172	6037	6966	5595	29.6164	33.2474	34.49355	34.84161	33.55714	32.92742	32.45245	33.77539	0.846934	1	-
ENSMUSC	Xab2	XPA bindi	GO:00325	developm	K12867	pre-mRN/	mmu0304	Spliceoso	8:3608421	676	912	612	952	1101	948	6.259517	6.744273	6.263152	6.800826	6.565023	6.905798	6.422314	6.757216	0.788077	1	-
ENSMUSC	Cdc37	cell divisic	GO:00081	metabolic	K09554	cell divisio	mmu0415	PI3K-Akt s	9:2113322	2410	3362	2460	3659	4231	3579	16.60648	18.50138	18.73452	19.45151	18.77407	19.40141	17.94746	19.20899	0.61309	1	-
ENSMUSC	Rab4a	RAB4A, m	GO:00452	synapse|lc	K07879	Ras-relate	mmu0414	Endocytos	8:1238059	306	401	275	463	541	460	7.994783	8.367092	7.940788	9.33245	9.101992	9.454812	8.100887	9.296418	0.308162	1	-
ENSMUSC	Trip10	thyroid hc	GO:00511	localizatio	K07196	thyroid hc	mmu0491	Insulin sig	17:572494	125	159	101	149	250	166	1.447968	1.470935	1.293058	1.331581	1.864857	1.51276	1.403987	1.569733	0.556157	1	-
ENSMUSC	Cd70	CD70 anti	GO:00023	immune s	K05470	tumor nec	mmu0406	Cytokine-	17:571459	0	2	0	0	0	0	0	0.115844	0	0	0	0	0.038615	0	0.524574	1	-
ENSMUSC	Cops6	COP9 sigr	GO:00081	metabolic	-	-	-	-	5:1381610	1159	1603	1120	1671	2034	1527	16.43435	18.15301	17.5523	18.28	18.57269	17.0341	17.37989	17.96227	0.919513	1	-
ENSMUSC	Ubb	ubiquitin I	GO:00325	developm	K04551	ubiquitin I	mmu0414	Endocytos	11:625511	11571	15395	11457	16841	18944	17214	295.7318	314.2337	323.6271	332.0668	311.7838	346.1146	311.1975	329.9884	0.681377	1	-
ENSMUSC	Ap4m1	adaptor-r	GO:00511	localizatio	K12402	AP-4 com	mmu0414	Lysosome	5:1381720	379	494	353	561	658	583	2.905703	3.024717	2.991118	3.318221	3.248574	3.516348	2.973846	3.361048	0.36717	1	-
ENSMUSC	Gyg	glycogeni	GO:00081	metabolic	K00750	glycogeni	mmu0110	Metabolic	3:2012208	329	446	343	485	532	461	4.595765	4.975569	5.295461	5.226781	4.785514	5.066104	4.955599	5.026133	0.936699	1	-
ENSMUSC	Rcn3	reticuloca	GO:00056	cell|intrace	-	-	-	-	7:4508291	107	153	117	198	199	166	1.613539	1.842609	1.94997	2.303513	1.932428	1.969316	1.80204	2.068419	0.419729	1	-
ENSMUSC	Slc6a8	solute car	GO:00325	developm	-	-	-	-	X:7367315	1199	1526	627	913	1348	1517	7.419547	7.541515	4.288172	4.358722	5.371587	7.385073	6.416412	5.705127	0.360952	1	-
ENSMUSC	Arid3a	AT rich int	GO:00081	metabolic	-	-	-	-	10:799270	119	154	98	184	202	167	0.542434	0.560619	0.49371	0.647066	0.592936	0.598866	0.532254	0.612956	0.406696	1	-
ENSMUSC	Pdk4	pyruvate c	GO:00081	metabolic	-	-	-	-	6:5483351	137	221	148	184	265	203	2.10017	2.705653	2.507504	2.176111	2.615976	2.448155	2.437776	2.413414	0.815736	1	-
ENSMUSC	Ubxn6	UBX dom	GO:00081	metabolic	K14011	UBX dom	mmu0414	Protein pr	17:560670	925	1226	1054	1476	1595	1436	5.942632	6.290325	7.483841	7.315661	6.598609	7.257757	6.572266	7.057342	0.639309	1	-
ENSMUSC	Mydgf	myeloid d	GO:00325	developm	-	-	-	-	17:561757	341	408	304	459	551	370	8.480989	8.103966	8.356255	8.807142	8.824659	7.239432	8.313737	8.290411	0.829233	1	-
ENSMUSC	Cyb561	cytochron	GO:00081	metabolic	-	-	-	-	11:105933	422	555	320	511	620	565	4.112775	4.319775	3.44682	3.842144	3.891067	4.331932	3.95979	4.021714	0.940433	1	-
ENSMUSC	Sema6a	sema don	GO:00325	developm	K06842	semaphor	mmu0436	Axon guic	18:472355	388	569	389	517	676	505	1.08742	1.273574	1.204932	1.117858	1.220021	1.113444	1.188642	1.150441	0.619508	1	-
ENSMUSC	Fmc1	formation	GO:00081	metabolic	-	-	-	-	6:3853350	41	58	47	69	82	46	2.55706	2.888902	3.239717	3.320032	3.293272	2.256986	2.895226	2.956763	1	1	-
ENSMUSC	Akt3	thymoma	GO:00325	developm	K04456	RAC serin	mmu0520	Pathways	1:1770200	5682	7050	4797	6618	8148	6584	39.49143	39.13241	36.84836	35.48613	36.46761	35.99999	38.49073	35.98458	0.270725	1	-
ENSMUSC	Mrpl24	mitochon	GO:00081	metabolic	K02895	large subu	mmu0301	Ribosome	3:8791950	425	601	422	642	850	560	6.868636	7.757141	7.537724	8.004731	8.84616	7.120005	7.387834	7.990299	0.620697	1	-
ENSMUSC	Gle1	GLE1 RNA	GO:00081	metabolic	-	-	-	-	2:2993542	895	1107	809	1289	1349	1136	7.378749	7.288748	7.371483	8.198657	7.161869	7.367988	7.346327	7.576171	0.948353	1	-
ENSMUSC	L3hypdh	L-3-hydro	GO:00081	metabolic	K18384	trans-L-3	mmu0033	Arginine a	12:720734	31	22	23	28	42	29	0.568743	0.322355	0.466365	0.396319	0.49621	0.418566	0.452488	0.437032	0.837239	1	-
ENSMUSC	Lyst	lysosomal	GO:00511	localizatio	-	-	-	-	13:135903	2610	3440	2518	3191	4024	3605	7.604948	8.004974	8.108829	7.173198	7.550371	8.263648	7.906251	7.662406	0.530517	1	-
ENSMUSC	Slc35e1	solute car	GO:00511	localizatio	-	-	-	-	8:7248064	1299	1606	1193	1811	2099	1742	16.63475	16.42474	16.88474	17.89188	17.30909	17.54956	16.64808	17.58351	0.733011	1	-
ENSMUSC	Calr3	calreticuli	GO:00325	developm	-	-	-	-	8:7242417	12	27	11	11	24	20	0.289025	0.519369	0.292816	0.204413	0.372246	0.378969	0.36707	0.318543	0.581324	1	-
ENSMUSC	Syne4	spectrin re	GO:00325	developm	-	-	-	-	7:3031480	48	66	50	54	69	68	0.356326	0.391289	0.41023	0.309267	0.329848	0.397126	0.385948	0.345414	0.440312	1	-
ENSMUSC	Polr2i	polymera	GO:00081	metabolic	K03017	DNA-dire	mmu0110	Metabolic	7:3023194	84	103	68	128	110	129	1.674462	1.63975	1.49813	1.968511	1.412027	2.023007	1.604114	1.801181	0.591666	1	-
ENSMUSC	Krt10	keratin 10	GO:00325	developm	K07604	type I kera	-	-	11:993852	89	95	55	95	86	84	2.049868	1.74745	1.400042	1.688056	1.275516	1.522038	1.732453	1.495204	0.30672	1	-
ENSMUSC	Iyd	iodotyrosi	GO:00081	metabolic	K17231	iodotyrosi	mmu0491	Thyroid hc	10:354024	77	99	102	124	144	119	2.40607	2.470585	3.522617	2.989304	2.897576	2.925346	2.799757	2.937409	0.877316	1	-
ENSMUSC	Rmnd1	required f	GO:00081	metabolic	-	-	-	-	10:440191	203	251	149	297	387	237	1.468053	1.44966	1.190913	1.657043	1.80224	1.348361	1.369542	1.602548	0.32918	1	-
ENSMUSC	Ccdc170	coiled-co	-	-	-	-	-	-	10:448250	27	50	37	31	51	41	0.409587	0.605745	0.620326	0.362804	0.498201	0.489296	0.545219	0.4501	0.314256	1	-
ENSMUSC	Esr1	estrogen r	GO:00325	developm	K08550	estrogen r	mmu0520	Pathways	10:461159	38	59	46	53	66	53	0.12209	0.151389	0.163344	0.131373	0.136552	0.133964	0.145608	0.133963	0.556924	1	-
ENSMUSC	Vip	vasoactive	GO:00081	metabolic	-	-	-	-	10:563921	293	318	244	442	464	377	4.778326	4.141719	4.39788	5.561091	4.872819	4.836823	4.439308	5.090244	0.347893	1	-
ENSMUSC	Mtrf1l	mitochon	GO:00081	metabolic	-	-	-	-	10:581188	142	157	107	185	225	150	2.41175	2.129557	2.00852	2.424078	2.460825	2.004228	2.183276	2.296377	0.858421	1	-
ENSMUSC	Rgs17	regulator	GO:00452	synapse|m	-	-	-	-	10:582566	1229	1497	963	1761	1691	1337	7.804517	7.592094	6.758764	8.627473	6.915003	6.679384	7.385125	7.407287	0.840018	1	-
ENSMUSC	Hdac2	histone de	GO:00325	developm	K06067	histone de	mmu0520	Pathways	10:369745	1281	1564	1074	1545	2103	1825	13.7172	13.37515	12.71063	12.76365	14.50141	15.37411	13.26766	14.21306	0.642968	1	-
ENSMUSC	Rwdd1	RWD don	GO:00325	developm	-	-	-	-	10:339965	341	480	369	540	646	587	9.581606	10.77136	11.45927	11.70598	11.68882	12.97576	10.60408	12.12352	0.330571	1	-
ENSMUSC	Clvs2	clavesin 2	GO:00099	cellular pr	-	-	-	-	10:335122	757	748	728	964	1193	956	5.17882	4.086794	5.504445	5.087944	5.255697	5.14522	4.923353	5.162954	0.834599	1	-
ENSMUSC	Trdn	triadin [Sc	GO:00511	localizatio	-	-	-	-	10:330805	4	2	0	0	1	5	0.021643	0.008643	0	0	0.003483	0.021282	0.010096	0.008255	0.823992	1	-

ENSMUSC	Hey2	hairy/enha	GO:00325	developm	K09091	hairy and	mmu052C	Pathways	10:308323	75	123	94	98	139	97	0.962861	1.261105	1.333761	0.970642	1.149132	0.979679	1.185909	1.033151	0.321908	1 -
ENSMUSC	Stxbp5	syntaxin b	GO:00325	developm	-	-	-	-	10:975554	2443	3160	2216	3011	3716	3077	6.996808	7.227856	7.014442	6.653001	6.853413	6.932899	7.079702	6.813104	0.466795	1 -
ENSMUSC	Hint3	histidine t	GO:00038	catalytic a	-	-	-	-	10:306081	106	141	97	163	159	143	0.998942	1.06121	1.010304	1.185096	0.964914	1.060185	1.023485	1.070065	0.900984	1 -
ENSMUSC	Trmt11	tRNA met	GO:00081	metabolic	-	-	-	-	10:305342	78	136	94	98	132	103	0.709979	0.98863	0.945642	0.688189	0.773712	0.737562	0.881417	0.733155	0.188518	1 -
ENSMUSC	Katna1	katanin p6	GO:00990	supramole	-	-	-	-	10:772600	195	217	145	233	290	205	3.278178	2.913418	2.694088	3.02192	3.139423	2.711203	2.961895	2.957515	0.855897	1 -
ENSMUSC	Pcmt1	protein-L	GO:00081	metabolic	-	-	-	-	10:762937	1268	1364	1189	1399	1915	1445	9.861381	8.471853	10.21991	8.393954	9.590512	8.840914	9.517713	8.941793	0.385573	1 -
ENSMUSC	Lrp11	low densit	GO:00511	localizatio	-	-	-	-	10:758980	1660	2129	1523	2455	2708	2594	16.36894	16.76617	16.59813	18.67644	17.19553	20.12302	16.57775	18.665	0.289817	1 -
ENSMUSC	1700021F	RIKEN cDI	GO:00056	cell|intrace	-	-	-	-	10:435251	288	399	306	404	492	379	3.950736	4.371243	4.639313	4.275601	4.34616	4.090116	4.320431	4.237292	0.715814	1 -
ENSMUSC	Sec63	SEC63-lik	GO:00325	developm	K09540	translocat	mmu0414	Protein pr	10:427614	2061	2589	1806	2679	3297	2560	9.259806	9.289702	8.967854	9.285962	9.538884	9.048463	9.172454	9.291103	0.899593	1 -
ENSMUSC	Snx3	sorting ne	GO:00081	metabolic	K17918	sorting ne	mmu0414	Endocytos	10:425020	1834	2300	1623	2538	1804	2393	39.08571	39.14645	38.22822	41.72925	24.75768	40.12105	38.82013	35.536	0.34864	1 -
ENSMUSC	Aig1	androgen	GO:00081	metabolic	-	-	-	-	10:136470	542	769	513	790	835	760	3.043361	3.44847	3.183596	3.422248	3.019222	3.35721	3.225143	3.266226	0.913685	1 -
ENSMUSC	Adat2	adenosine	GO:00081	metabolic	-	-	-	-	10:135528	77	87	69	95	109	102	3.206999	2.893845	3.176167	3.052549	2.923415	3.342103	3.092337	3.106023	0.920064	1 -
ENSMUSC	Pex3	peroxisom	GO:00511	localizatio	K13336	peroxin-3	mmu0414	Peroxisom	10:135238	215	489	357	302	367	508	1.99658	3.626622	3.664062	2.163639	2.194663	3.711266	3.095755	2.689856	0.360909	1 -
ENSMUSC	Fuca2	fucosidase	GO:00081	metabolic	K01206	alpha-L-f	mmu0051	Other glyc	10:134995	510	646	583	597	807	630	3.750862	3.794363	4.738879	3.38739	3.821982	3.64512	4.094701	3.618164	0.192808	1 -
ENSMUSC	Cep57l1	centroson	GO:00055	protein bi	-	-	-	-	10:417188	70	131	70	111	147	113	0.436706	0.652695	0.482655	0.534253	0.590562	0.554604	0.524019	0.559807	0.807764	1 -
ENSMUSC	Ltv1	LTV1 ribo	GO:00511	localizatio	-	-	-	-	10:131781	315	423	262	414	531	462	7.901938	8.474401	7.26391	8.012232	8.577721	9.117496	7.880083	8.56915	0.610212	1 -
ENSMUSC	Plagl1	pleiomorp	GO:00325	developm	-	-	-	-	10:130605	665	804	491	985	866	77	2.512913	2.426373	2.050616	2.87159	2.10731	0.228906	2.329967	1.735935	0.391095	1 -
ENSMUSC	Cd164	CD164 an	GO:00325	developm	K06546	CD164 an	mmu0414	Lysosome	10:415194	857	1292	764	1175	1601	1187	10.23408	12.32185	10.08341	10.8252	12.31157	11.15139	10.87978	11.42939	0.799775	1 -
ENSMUSC	Utrn	utrophin [	GO:00325	developm	-	-	-	-	10:123821	1459	1831	1257	1878	2077	1896	4.104298	4.11356	3.9081	4.075769	3.762484	4.195974	4.041986	4.011409	0.72188	1 -
ENSMUSC	Smpd2	sphingom	GO:00081	metabolic	K12351	sphingom	mmu011C	Metabolic	10:414856	128	175	130	175	200	145	0.68629	0.749344	0.770345	0.723875	0.690526	0.611611	0.735326	0.675337	0.439968	1 -
ENSMUSC	Mical1	microtubu	GO:00081	metabolic	-	-	-	-	10:414763	159	218	148	224	320	281	0.961593	1.052922	0.989242	1.045136	1.246233	1.336936	1.001253	1.209435	0.212174	1 -
ENSMUSC	Zbtb24	zinc finge	GO:00325	developm	-	-	-	-	10:414503	236	259	199	255	312	271	2.124072	1.861673	1.979513	1.770628	1.808286	1.918833	1.98842	1.832582	0.394744	1 -
ENSMUSC	Grm1	glutamate	GO:00048	signal trar	K04603	metabotrc	mmu0408	Neuroacti	10:106860	1291	1598	1275	1670	2027	1754	6.572123	6.496836	7.173588	6.558827	6.64489	7.024572	6.747516	6.742763	0.788155	1 -
ENSMUSC	Wasf1	WAS prot	GO:00325	developm	K05753	WAS prot	mmu0481	Regulatior	10:408834	5775	7981	4804	7521	9648	7018	93.33261	103.0112	85.80867	93.77505	100.4092	89.22892	94.05083	94.47105	0.831717	1 -
ENSMUSC	Rab32	RAB32, m	GO:00325	developm	-	-	-	-	10:105450	48	65	30	51	62	65	0.999242	1.080661	0.69024	0.819089	0.831146	1.064518	0.923381	0.904918	0.840284	1 -
ENSMUSC	Gtf3c6	general tr	GO:00081	metabolic	-	-	-	-	10:402477	490	634	462	609	787	557	7.396412	7.64293	7.707507	7.092066	7.649872	6.614412	7.582283	7.118783	0.421803	1 -
ENSMUSC	Slc16a10	solute car	GO:00511	localizatio	K08187	MFS trans	mmu0491	Thyroid hc	10:400335	83	95	76	95	145	99	0.559175	0.511143	0.565891	0.49377	0.629065	0.524709	0.545403	0.549181	0.945862	1 -
ENSMUSC	Rev3l	REV3 like,	GO:00081	metabolic	K02350	DNA poly	mmu011C	Metabolic	10:397321	1286	1640	1151	1496	1674	1672	4.188442	4.265808	4.143179	3.759009	3.510926	4.284093	4.199143	3.851342	0.242333	1 -
ENSMUSC	Fyn	Fyn proto	GO:00325	developm	K05703	tyrosine-p	mmu0451	Focal adh	10:393688	2343	2724	2196	3012	3570	3070	16.03982	14.89293	16.61523	15.90789	15.73802	16.53395	15.84933	16.05995	0.899619	1 -
ENSMUSC	Tube1	epsilon-tu	GO:00990	supramole	-	-	-	-	10:391339	28	46	38	35	64	44	0.148095	0.194308	0.222133	0.142818	0.217984	0.183082	0.188178	0.181295	0.81351	1 -
ENSMUSC	Lama4	laminin, a	GO:00325	developm	K06241	laminin, a	mmu052C	Pathways	10:389655	117	201	103	156	232	201	0.484227	0.664364	0.471137	0.498102	0.618309	0.654441	0.539909	0.590284	0.686039	1 -
ENSMUSC	Popdc3	popeye d	GO:00160	membran	-	-	-	-	10:451780	73	75	87	106	123	105	1.356013	1.112635	1.786114	1.519082	1.471308	1.534425	1.418254	1.508272	0.828999	1 -
ENSMUSC	Prep	prolyl end	GO:00081	metabolic	K01322	prolyl olig	mmu0461	Renin-ang	10:450672	679	832	594	891	991	832	3.792045	3.710848	3.66638	3.838945	3.563956	3.655428	3.723091	3.686109	0.733331	1 -
ENSMUSC	Arfgef3	ARFGEF fa	GO:00081	metabolic	-	-	-	-	10:185818	4426	5302	3966	5309	6300	5349	17.82372	17.05188	17.65169	16.49415	16.33737	16.94612	17.5091	16.59255	0.343621	1 -
ENSMUSC	Hebp2	heme bin	GO:00511	localizatio	-	-	-	-	10:185394	21	55	27	43	47	55	0.241296	0.5047	0.34287	0.38117	0.347756	0.497156	0.362955	0.408694	0.708356	1 -
ENSMUSC	Reps1	RalBP1 as	GO:00511	localizatio	-	-	-	-	10:180558	725	890	647	937	1148	963	5.339821	5.235095	5.266715	5.324253	5.444842	5.579897	5.280543	5.449664	0.942514	1 -
ENSMUSC	Fam184a	family wit	GO:00055	extracellul	-	-	-	-	10:536331	522	677	448	608	780	621	3.858637	3.996668	3.660062	3.467348	3.712896	3.611318	3.838456	3.597187	0.3966	1 -
ENSMUSC	Asf1a	anti-silen	GO:00325	developm	-	-	-	-	10:535967	140	170	119	166	213	161	1.626669	1.577483	1.528145	1.488025	1.593694	1.471663	1.577433	1.517794	0.650581	1 -
ENSMUSC	Gopc	golgi assc	GO:00325	developm	-	-	-	-	10:523358	1367	736	1163	1713	2133	1476	4.351289	1.870998	4.091438	4.206657	4.372149	3.696126	3.437908	4.091644	0.383862	1 -
ENSMUSC	Qrsl1	glutaminy	GO:00081	metabolic	K02433	aspartyl-t	mmu011C	Metabolic	10:438741	145	231	160	228	260	208	1.75454	2.232307	2.139747	2.128438	2.02593	1.980028	2.042198	2.044799	0.869396	1 -
ENSMUSC	Rtn4ip1	reticulon 4	GO:00081	metabolic	-	-	-	-	10:439018	274	359	293	379	480	321	3.621973	3.789973	4.280649	3.865141	4.085931	3.338197	3.897532	3.76309	0.641516	1 -
ENSMUSC	Vta1	vesicle (m	GO:00511	localizatio	K12199	vacuolar p	mmu0414	Endocytos	10:146547	415	524	411	536	641	511	7.066124	7.12542	7.73431	7.040895	7.028224	6.844871	7.308618	6.97133	0.519027	1 -
ENSMUSC	Smpdl3a	sphingom	GO:00081	metabolic	-	-	-	-	10:577943	364	576	379	564	640	563	8.141088	10.28843	9.36842	9.731738	9.217546	9.90603	9.26598	9.618438	0.909559	1 -
ENSMUSC	Reep3	receptor a	GO:00990	supramole	-	-	-	-	10:670091	761	1211	768	1002	1323	1154	4.374843	5.559914	4.87962	4.444019	4.897702	5.219086	4.938126	4.853602	0.698543	1 -
ENSMUSC	Fabp7	fatty acid	GO:00325	developm	K08756	fatty acid-	mmu0332	PPAR sign	10:577848	176	233	165	310	331	237	7.112009	7.519365	7.369027	9.664311	8.613145	7.534221	7.333467	8.603892	0.290662	1 -
ENSMUSC	Pkib	protein ki	GO:00081	metabolic	-	-	-	-	10:576319	45	117	100	138	95	160	0.149489	0.310405	0.36715	0.353677	0.203224	0.418147	0.275681	0.325016	0.554407	1 -
ENSMUSC	Serinc1	serine inc	GO:00081	metabolic	-	-	-	-	10:575157	19655	25024	18413	24710	31078	25961	187.0184	190.1577	193.6345	181.3904	190.4228	194.3317	190.2702	188.715	0.692987	1 -
ENSMUSC	Hsf2	heat shoc	GO:00081	metabolic	-	-	-	-	10:574863	514	656	436	610	766	662	2.426273	2.473015	2.274625	2.221451	2.328416	2.458361	2.391304	2.336076	0.653325	1 -
ENSMUSC	Rspo3	R-spondi	GO:00325	developm	-	-	-	-	10:294524	235	288	192	300	337	364	3.812069	3.731053	3.442245	3.754437	3.520288	4.645215	3.661789	3.973313	0.667394	1 -
ENSMUSC	Echdc1	enoyl Coe	GO:00081	metabolic	K18426	ethylmalo	mmu0064	Propanoa	10:293131	355	404	294	476	543	407	3.986684	3.623359	3.649035	4.124029	3.926801	3.59575	3.753026	3.882193	0.937313	1 -
ENSMUSC	Ptprk	protein ty	GO:00325	developm	-	-	-	-	10:280748	1114	1665	906	1624	2003	1628	4.154576	4.959087	3.734365	4.672593	4.810357	4.776468	4.282676	4.753139	0.418972	1 -
ENSMUSC	Nts	neurotens	GO:00452	synapse|re	-	-	-	-	10:102481	132	131	86	156	173	142	6.458057	5.118531	4.650216	5.88822	5.45043	5.465462	5.408935	5.601371	0.96024	1 -
ENSMUSC	Dcbld1	discoidin,	GO:00160	membran	-	-	-	-	10:522336	222	312	208	277	349	272	1.387113	1.5569	1.436383	1.33527	1.404237	1.337025	1.460132	1.358844	0.429161	1 -
ENSMUSC	Ros1	Ros1 prot	GO:00325	developm	-	-	-	-	10:520457	0	0	0	1	2	0	0	0	0	0.005049	0.008426	0	0	0.004492	0.277259	1 -
ENSMUSC	Slc6a15	solute car	GO:00511	localizatio	-	-	-	-	10:103367	927	1086	797	1077	1392	1104	5.207072	4.871802	4.947878	4.667236	5.035096	4.878583	5.008917	4.860305	0.579407	1 -
ENSMUSC	Ccdc59	coiled-co	GO:00081	metabolic	-	-	-	-	10:105841	353	533	351	559	694	485	4.119554	4.96762	4.527196	5.032891	5.215422	4.452753	4.538124	4.900355	0.643449	1 -
ENSMUSC	Lama2	laminin, a	GO:00325	developm	K05637	laminin, a	mmu052C	Pathways	10:269800	311	416	363	402	487	506	1.032857	1.103364	1.332394	1.029998	1.04151	1.322031	1.156205	1.13118	0.719035	1 -
ENSMUSC	Rfx6	regulatory	GO:00325	developm	K19521	regulatory	mmu0495	Maturity c	10:516777	0	0	0	2	0	0	0	0	0	0.01643	0	0	0	0.005477	0.527939	1 -
ENSMUSC	Lin7a	lin-7 hom	GO:00325	developm	-	-	-	-	10:107271	407	529	370	498	565	442	5.302032	5.503639	5.327176	5.00505	4.739705	4.529827	5.377616	4.758194	0.184571	1 -
ENSMUSC	Ppp1r12a	protein ph	GO:00081	metabolic	K06270	protein ph	mmu0481	Regulatior	10:108162	2330	3857	2549	4118	4886	3738	14.31241	18.92136	17.30508	19.51523	19.32703	18.06372	16.84629	18.96866	0.316335	1 -
ENSMUSC	P4ha1	procollage	GO:00081	metabolic	K00472	prolyl 4-h	mmu011C	Metabolic	10:593232	1238	1700	1034	1665	1872	1373	11.5634	12.68118	10.67412	11.99801	11.25966	10.08896	11.63957	11.11554	0.477761	1 -
ENSMUSC	10-Sep	septin 10	GO:00971	organic cy	-	-	-	-	10:591416	65	157	100	109	145	123	1.20339	2.321334	2.046155	1.556856	1.728688	1.791466	1.85696	1.692337	0.52199	1 -
ENSMUSC	Lims1	LIM and s	GO:00325	developm	-	-	-	-	10:583234	148	541	375	401	694	417	1.46555	4.278414	4.104104	3.063479	4.425412	3.24853	3.28269	3.57914	0.756497	1 -
ENSMUSC	Ube2d1	ubiquitin-	GO:00081	metabolic	K06689	ubiquitin-	mmu0414	Protein pr	10:712549	724	935	704	973	1106	1052	8.814775	9.091377	9.473091	9.139354	8.671257	10.07625	9.126414	9.29562	0.955667	1 -
ENSMUSC	Slc17a8	solute car	GO:00325	developm	K12302	MFS trans	mmu0472	Retrograd	10:895740	137	166	119	155	165	185	1.558595	1.508225	1.496256	1.360422	1.20879	1.655749	1.521025	1.40832	0.484335	1 -
ENSMUSC	Atp2b1	ATPase, C	GO:00325	developm	K05850	Ca2+ tran	mmu0402	cAMP sigr	10:989144	21533	21316	19228	20502	33232	27382	67.1209	53.06459	66.24211	49.30373	66.70592	67.14742	62.14253	61.05236	0.690843	1 -
ENSMUSC	Rhobtb1	Rho-relat	GO:00508	response	K07868	Rho-relat	mmu0412	Ubiquitin	10:691514	93	48	86	130	62	120	0.532682	0.219571	0.544416	0.574458	0.228682	0.540729	0.432223	0.447956	0.989216	1 -
ENSMUSC	Arid5b	AT rich int	GO:00325	developm	-	-	-	-	10:680925	708	950	561	873	1087	726	2.845335	3.049082	2.491779	2.706727	2.813092	2.295343	2.795399	2.605054	0.389457	1 -
ENSMUSC	Actr6	ARP6 acti	GO:00099	cellular pr	-	-	-	-	10:897119	168	224	153	233	284	178	3.753294	3.996662	3.777835	4.015958	4.085797	3.128483	3.842597	3.743413	0.732048	1 -
ENSMUSC	Uhrf1bp1l	UHRF1 (IC	-	-	-	-	-	-	10:897449	1873	1784	1230	1671	2651	2284	9.495337	7.222928	6.891673	6.53551	8.654399	9.109189	7.869979	8.099699	0.976972	1 -
ENSMUSC	Poc1b	POC1 cen	GO:00099	cellular pr	-	-	-	-	10:991070	122	126	116	148	190	179	1.014998	0.837188	1.066623	0.949945	1.017922	1.171575	0.972936	1.046481	0.752846	1 -
ENSMUSC	Dusp6	dual speci	GO:00325	developm	K04459	dual speci	mmu0401	MAPK sigr	10:992632	1049	1047	685	1317	1136	1336	10.80465	8.612465	7.797813	10.46529	7.53474	10.82561	9.071641	9.608549	0.802528	1 -
ENSMUSC	Tmpo	thymopoi	GO:00081	metabolic	-	-	-	-	10:911475	442	525	467	621	821	673	1.327547	1.259311	1.550211	1.438962	1.587907	1.590206	1.379023	1.539025	0.440453	1 -
ENSMUSC	Kitl	kit ligand	GO:00325	developm	K05461	KIT ligand	mmu052C	Pathways	10:100015	383	485	362	481	621	442	2.73704	2.768025	2.85915	2.651903	2.857777	2.484937	2.788072	2.664872	0.544953	1 -
ENSMUSC	Psen1	presenilin	GO:00325	developm	K04505	presenilin	mmu0516	Human pa	12:836881	1040	1426	1005	1414	1800	1528	9.746804	10.67319	10.40979	10.22371	10.86316	11.26583	10.27659	10.78423	0.79658	1 -
ENSMUSC	Sgk1	serum/glu	GO:00325	developm	K13302	serum/glu	mmu0415	PI3K-Akt s	10:218821	825	1167	784	1002	1457	1275	4.922114	5.560508	5.169647	4.61207	5.597731	5.984379	5.217423	5.39806	0.924245	1 -
ENSMUSC	Ikbip	IKBKB inte	GO:00160	membran	-	-	-	-	10:910829	241	309	185	299	371	284	2.004499	2.052542	1.700613	1.918626	1.987085	1.858308	1.919218	1.92134	0.85927	1 -
ENSMUSC	Hbs1l	Hbs1-like	GO:00081	metabolic	K14416	elongatior	mmu0301	mRNA sur	10:212959	524	702	451	711	901	707	3.106557	3.323773	2.955094	3.251984	3.439756	3.297451	3.128475	3.32973	0.715291	1 -
ENSMUSC	Epb41l2	erythrocyt	GO:00325	developm	-	-	-	-	10:253597	1161	1433	955	1279	1429	1495	3.079029	3.035103	2.799184	2.616871	2.440442	3.11913	2.971105	2.725481	0.268407	1 -
ENSMUSC	Apaf1	apoptotic	GO:00325	developm	K02084	apoptotic	mmu052C	Pathways	10:909893	346	277	291	483	541	400	1.38659	0.886539	1.288878	1.493311	1.396125	1.261081	1.187336	1.383506	0.355911	1 -
ENSMUSC	Med23	mediator	GO:00081	metabolic	-	-	-	-	10:248699	588	737	532	747	886	758	3.775205	3.778991	3.775038	3.700103	3.663121	3.828623	3.776411	3.730616	0.718249	1 -
ENSMUSC	Ahi1	Abelson h	GO:00056	cell|cell ju	-	-	-	-	10:209525	4566	5458	4027	5584	6591	5580	28.26352	26.98172	27.54982	26.6665	26.27219	27.17286	27.59835	26.70385	0.48378	1 -
ENSMUSC	Nedd1	neural pre	GO:00511	localizatio	-	-	-	-	10:926847	51	66	48	61	84	72	0.656812	0.678833	0.683225	0.606088	0.696641	0.729486	0.672957	0.677405	0.948108	1 -
ENSMUSC	Enpp3	ectonucle	GO:00081	metabolic	K01513	ectonucle	mmu011C	Metabolic	10:247724	5	13	3	11	26	9	0.039202	0.081407	0.026	0.066545	0.131278	0.055516	0.048869	0.084446	0.186348	1 -
ENSMUSC	Pde7b	phosphoc	GO:00081	metabolic	K18436	high affini	mmu0023	Purine me	10:203980	409	527	348	507	614	525	1.605141	1.651756	1.509437	1.535068	1.551718	1.620914	1.588778	1.569233	0.741296	1 -
ENSMUSC	Mtfr2	mitochon	GO:00081	metabolic	-	-	-	-	10:203477	1	5	6	1	14	3	0.022596	0.090197	0.149804	0.01743	0.203649	0.053305	0.087532	0.091461	1	1 -
ENSMUSC	Map7	microtubu	GO:00099	cellular pr	-	-	-	-	10:201484	1350	1839	1267	1978	2360	1874	6.702043	7.291237	6.951795	7.575829	7.54467	7.319037	6.981691	7.479845	0.610746	1 -
ENSMUSC	Ctgf	connectiv	GO:00325	developm	K06827	connectiv	mmu0439	Hippo sig	10:245954	220	242	111	245	218	311	2.824385	2.481202	1.57496	2.4266	1.80224	3.14103	2.293516	2.456624	0.824063	1 -
ENSMUSC	Stx7	syntaxin 7	GO:00511	localizatio	K08488	syntaxin 7	mmu0414	Phagoson	10:241493	1730	2174	1497	2246	2727	2074	16.66885	16.72881	15.94146	16.69551	16.91996	15.72097	16.44637	16.44548	0.787107	1 -
ENSMUSC	Moxd1	monooxy	GO:00081	metabolic	-	-	-	-	10:242235	166	221	157	200	230	192	2.878631	3.060669	3.009004	2.675703	2.568388	2.61933	2.982768	2.62114	0.227675	1 -
ENSMUSC	Pex7	peroxisom	GO:00325	developm	K13341	peroxin-7	mmu0414	Peroxisom	10:198539	318	446	345	462	508	449	1.476314	1.653605	1.770182	1.654716	1.518692	1.639869	1.633367	1.604426	0.721266	1 -
ENSMUSC	Vnn3	vanin 3 [S	GO:00081	metabolic	K08069	pantethei	mmu0077	Pantother	10:238514	0	1	2	0	4	0	0	0.02693	0.07453	0	0.086847	0	0.03382	0.028949	1	1 -
ENSMUSC	Cfap54	cilia and f	GO:00325	developm	-	-	-	-	10:927756	113	163	144	160	201	209	0.430926	0.496429	0.606923	0.470733	0.4936	0.62702	0.511426	0.530451	0.945814	1 -
ENSMUSC	Cdk17	cyclin-dep	GO:00081	metabolic	-	-	-	-	10:931608	3219	3949	2897	4097	4872	4199	27.90658	27.34127	27.75754	27.40198	27.19867	28.63798	27.66846	27.74621	0.801068	1 -
ENSMUSC	Hal	histidine a	GO:00081	metabolic	K01745	histidine a	mmu011C	Metabolic	10:934887	0	5	3	0	12	2	0	0.03468	0.0288	0	0.067118	0.013668	0.02116	0.026929	0.890676	1 -
ENSMUSC	Snrpf	small nucl	GO:00081	metabolic	K11098	small nucl	mmu0304	Spliceosor	10:935830	104	170	129	170	219	161	7.375202	9.62796	10.11058	9.300783	10.00089	8.9821	9.037912	9.427924	0.905093	1 -
ENSMUSC	Ntn4	netrin 4 [S	GO:00325	developm	K06845	netrin 4[-	mmu0436	Axon guic	10:936406	116	181	84	145	179	137	1.675196	2.087535	1.340704	1.615495	1.664626	1.556463	1.701145	1.612195	0.612239	1 -
ENSMUSC	Fgd6	FYVE, Rhc	GO:00987	molecular	-	-	-	-	10:940360	459	570	404	621	755	639	3.306081	3.278854	3.216102	3.450827	3.501891	3.620872	3.267012	3.52453	0.623273	1 -
ENSMUSC	Ndufa12	NADH de	GO:00081	metabolic	K11352	NADH de	mmu011C	Metabolic	10:941989	916	954	706	1141	1485	804	27.74266	23.07529	23.63221	26.66052	28.96235	19.15667	24.81672	24.92651	0.880434	1 -
ENSMUSC	Tmcc3	transmem	GO:00160	membran	-	-	-	-	10:943119	930	1326	1038	1590	1908	1523	3.981492	4.533694	4.911422	5.251586	5.260125	5.129486	4.475536	5.213732	0.183591	1 -
ENSMUSC	Cep83	centroson	GO:00511	localizatio	-	-	-	-	10:946886	743	934	679	996	1160	918	5.129021	5.149184	5.180386	5.304386	5.156538	4.985388	5.152864	5.148771	0.802578	1 -
ENSMUSC	Socs2	suppressc	GO:00325	developm	K04695	suppresso	mmu0463	Jak-STAT	10:953853	276	275	261	322	435	350	1.230885	0.979459	1.286456	1.107883	1.249259	1.227969	1.1656	1.195037	0.997677	1 -
ENSMUSC	Nudt4	nudix (nuc	GO:00081	metabolic	-	-	-	-	10:955470	1832	2634	1692	2741	3579	2869	34.84688	40.01289	35.57013	40.2233	43.8384	42.93187	36.80996	42.33119	0.191708	1 -
ENSMUSC	Nuak1	NUAK fan	GO:00325	developm	-	-	-	-	10:843709	1743	2017	1235	2021	2242	1902	18.40295	17.00756	14.41132	16.46216	15.24335	15.79835	16.60728	15.83462	0.456012	1 -
ENSMUSC	Tcp11l2	t-complex	-	-	-	-	-	-	10:845766	300	380	304	390	484	377	4.45679	4.508483	4.991388	4.469884	4.630218	4.406099	4.65222	4.502067	0.628617	1 -
ENSMUSC	Rfx4	regulatory	GO:00325	developm	-	-	-	-	10:847560	212	255	169	254	283	265	1.699625	1.632683	1.497446	1.571018	1.461026	1.67137	1.609918	1.567804	0.681619	1 -
ENSMUSC	Cry1	cryptochr	GO:00048	signal trar	K02295	cryptochrc	mmu0471	Circadian	10:851317	217	322	203	291	371	234	3.219832	3.815706	3.329016	3.331175	3.544882	2.731489	3.454851	3.202515	0.445415	1 -
ENSMUSC	Btbd11	BTB (POZ	GO:00081	metabolic	-	-	-	-	10:853868	395	458	285	502	490	379	1.648301	1.52634	1.314413	1.61612	1.31671	1.244198	1.496351	1.392343	0.444061	1 -
ENSMUSC	Timp3	tissue inhi	GO:00081	metabolic	K16866	metallopr	mmu052C	Proteogly	10:863003	2358	3376	2001	2676	3494	3017	15.93878	18.22467	14.94876	13.95494	15.20859	16.04344	16.37074	15.06899	0.249477	1 -
ENSMUSC	Hsp90b1	heat shoc	GO:00081	metabolic	K09487	heat shoc	mmu052C	Pathways	10:866902	6525	8693	5892	9667	10433	8866	73.77854	78.49918	73.63073	84.32797	75.96499	78.86571	75.30282	79.71956	0.692227	1 -
ENSMUSC	Pah	phenylala	GO:00081	metabolic	K00500	phenylala	mmu011C	Metabolic	10:875217	0	1	0	0	2	0	0	0.013593	0	0	0.021912	0	0.004531	0.007304	1	1 -
ENSMUSC	Ascl1	achaete-s	GO:00325	developm	-	-	-	-	10:874908	161	208	159	169	264	191	3.962248	4.08815	4.324739	3.208731	4.183846	3.697952	4.125045	3.696843	0.336629	1 -
ENSMUSC	Washc3	WASH co	GO:00511	localizatio	K18463	WASH co	mmu0414	Endocytos	10:882010	119	156	115	193	190	182	1.179146	1.2345	1.259405	1.475399	1.212355	1.418747	1.22435	1.368834	0.534412	1 -
ENSMUSC	Dram1	DNA-dan	GO:00081	metabolic	-	-	-	-	10:883228	19	38	29	27	39	28	0.346297	0.553127	0.584175	0.379653	0.457736	0.40149	0.494533	0.41296	0.382476	1 -
ENSMUSC	Sycp3	synaptone	GO:00325	developm	K19528	synaptone	mmu0344	Homologo	10:884595	5	13	13	6	17	8	0.146691	0.304621	0.421558	0.135812	0.321207	0.184649	0.290957	0.21389	0.410121	1 -
ENSMUSC	Mybpc1	myosin bi	GO:00325	developm	-	-	-	-	10:885182	40	54	19	52	37	42	0.436523	0.470635	0.229166	0.437802	0.260017	0.360583	0.378775	0.352801	0.692135	1 -
ENSMUSC	Herc4	hect dom	GO:00325	developm	K10615	E3 ubiquit	mmu0412	Ubiquitin	10:632438	496	602	441	628	714	555	2.531753	2.454042	2.487854	2.473031	2.346889	2.228658	2.491216	2.349526	0.437264	1 -
ENSMUSC	Mypn	myopallac	GO:00325	developm	-	-	-	-	10:631157	22	17	21	28	19	15	0.198151	0.122282	0.209051	0.194564	0.110201	0.106286	0.176495	0.137017	0.332095	1 -
ENSMUSC	Hnrnph3	heteroger	GO:00325	developm	-	-	-	-	10:630146	773	1101	653	1116	1422	1009	9.398226	10.69054	8.774589	10.46794	11.13322	9.650923	9.621119	10.41736	0.59031	1 -
ENSMUSC	Rufy2	RUN and	GO:00056	cell|intrace	-	-	-	-	10:629802	1339	1616	1154	1648	2040	1764	6.703577	6.461198	6.385261	6.365221	6.57674	6.947611	6.516678	6.629857	0.939649	1 -
ENSMUSC	Pbld2	phenazine	GO:00081	metabolic	-	-	-	-	10:630243	3	15	8	12	18	11	0.057098	0.22801	0.168276	0.1762	0.220618	0.164709	0.151128	0.187176	0.639182	1 -
ENSMUSC	Ccar1	cell divisio	GO:00081	metabolic	-	-	-	-	10:627439	974	1174	761	1264	1605	1208	3.725293	3.586043	3.216865	3.729745	3.953042	3.634791	3.5094	3.772526	0.623024	1 -
ENSMUSC	Ddx21	DEAD (As	GO:00325	developm	-	-	-	-	10:625802	609	761	583	730	1003	810	6.628232	6.614714	7.012865	6.129621	7.029691	6.935475	6.751937	6.698262	0.758482	1 -
ENSMUSC	Ddx50	DEAD (As	GO:00081	metabolic	-	-	-	-	10:626158	1160	1531	1037	1604	1939	1483	5.447005	5.741439	5.381774	5.810781	5.863167	5.478375	5.523406	5.717441	0.908191	1 -
ENSMUSC	Supv3l1	suppressc	GO:00325	developm	-	-	-	-	10:624292	396	433	327	445	616	614	4.592407	4.010313	4.191213	3.981398	4.600239	5.601761	4.264644	4.727799	0.53175	1 -
ENSMUSC	Tacr2	tachykinin	GO:00048	signal trar	K04223	tachykinin	mmu0408	Neuroacti	10:622524	2	9	3	7	10	4	0.054071	0.194368	0.089664	0.146031	0.174128	0.085088	0.112701	0.135082	0.774414	1 -
ENSMUSC	Fam241b	family wit	GO:00160	membran	-	-	-	-	10:621076	233	300	225	355	355	277	2.02024	2.077376	2.156141	2.374686	1.982122	1.889461	2.084586	2.08209	0.851488	1 -
ENSMUSC	Aifm2	apoptosis	GO:00081	metabolic	-	-	-	-	10:617152	51	119	79	142	142	104	0.616379	1.148611	1.055251	1.324035	1.105155	0.988838	0.94008	1.139343	0.361177	1 -
ENSMUSC	H2afy2	H2A histo	GO:00325	developm	K11251	histone H2	mmu0503	Alcoholisr	10:617386	225	275	165	265	313	250	6.316368	6.165411	5.119356	5.739316	5.658262	5.521212	5.867045	5.639596	0.61794	1 -
ENSMUSC	Tysnd1	trypsin do	GO:00081	metabolic	-	-	-	-	10:616955	220	252	191	335	347	301	4.131891	3.779837	3.964664	4.854025	4.196722	4.447369	3.958797	4.499372	0.400776	1 -
ENSMUSC	Sar1a	secretion	GO:00511	localizatio	K07953	GTP-bind	mmu0414	Protein pr	10:616802	652	829	852	1056	1016	1183	7.841225	7.962259	11.32459	9.797842	7.868363	11.19262	9.042691	9.61961	0.777008	1 -
ENSMUSC	Ppa1	pyrophos	GO:00081	metabolic	K01507	inorganic	mmu0019	Oxidative	10:616485	913	1101	869	1194	1467	1289	16.24302	15.64333	17.08687	16.38818	16.80664	18.04097	16.32441	17.0786	0.829497	1 -
ENSMUSC	Npffr1	neuropep	GO:00048	signal trar	K04240	neuropep	mmu0408	Neuroacti	10:615954	43	63	30	73	80	51	0.754675	0.883023	0.581912	0.98842	0.904137	0.704157	0.73987	0.865571	0.51655	1 -
ENSMUSC	Eif4ebp2	eukaryotic	GO:00451	translatior	K18644	eukaryotic	mmu0301	RNA trans	10:614284	1135	1540	1040	1609	1882	1557	9.871933	10.69727	9.99738	10.79674	10.54097	10.65383	10.18886	10.66385	0.813715	1 -
ENSMUSC	Pald1	phosphat	GO:00056	cell|intrace	-	-	-	-	10:613196	198	281	202	278	325	330	2.460218	2.788429	2.773995	2.664911	2.60044	3.225769	2.674214	2.830374	0.800679	1 -
ENSMUSC	Tbata	thymus, b	GO:00325	developm	-	-	-	-	10:611719	120	155	122	178	190	146	1.53928	1.587871	1.729593	1.761518	1.569439	1.473335	1.618915	1.60143	0.822897	1 -
ENSMUSC	Sgpl1	sphingosi	GO:00325	developm	K01634	sphingani	mmu011C	Metabolic	10:610986	430	576	387	535	694	599	2.636306	2.820305	2.62232	2.530534	2.739943	2.889116	2.692977	2.719864	0.900483	1 -
ENSMUSC	Pcbd1	pterin 4 a	GO:00081	metabolic	K01724	4a-hydro	mmu0079	Folate bio	10:610893	17	23	20	15	35	24	0.971003	1.049176	1.262554	0.66098	1.287311	1.078405	1.094245	1.008899	0.759465	1 -
ENSMUSC	Unc5b	unc-5 net	GO:00325	developm	K07521	netrin rec	mmu0436	Axon guic	10:607625	349	565	384	597	623	509	2.106699	2.723776	2.561861	2.780238	2.421697	2.417164	2.464112	2.5397	0.958767	1 -
ENSMUSC	Slc29a3	solute car	GO:00511	localizatio	-	-	-	-	10:607120	305	536	270	537	673	421	1.785326	2.505695	1.746741	2.425056	2.536805	1.938699	2.012587	2.300187	0.411705	1 -
ENSMUSC	Vsir	V-set imn	GO:00325	developm	-	-	-	-	10:603468	543	661	431	604	682	614	4.815472	4.681519	4.224387	4.13244	3.89474	4.2837	4.573793	4.103627	0.213411	1 -
ENSMUSC	Slc16a7	solute car	GO:00511	localizatio	-	-	-	-	10:125219	353	1288	943	1082	1421	1120	1.543837	4.498715	4.558115	3.650768	4.001986	3.853503	3.533556	3.835419	0.759388	1 -
ENSMUSC	Anapc16	anaphase	GO:00081	metabolic	-	-	-	-	10:599874	535	630	528	727	843	627	4.123466	3.877887	4.497685	4.322883	4.183992	3.80178	4.166346	4.102885	0.721144	1 -
ENSMUSC	Ddit4	DNA-dan	GO:00325	developm	K08270	DNA-dan	mmu0415	PI3K-Akt s	10:599496	538	666	490	628	831	623	18.71753	18.50488	18.84123	16.85607	18.61752	17.05161	18.68788	17.5084	0.398382	1 -
ENSMUSC	Dnajb12	DnaJ heat	GO:00081	metabolic	K09518	DnaJ hom	mmu0414	Protein pr	10:598795	767	779	558	845	1008	927	5.863472	4.756012	4.714553	4.983643	4.962208	5.575072	5.111346	5.173641	0.907204	1 -
ENSMUSC	Cand1	cullin assc	GO:00325	developm	-	-	-	-	10:119199	2982	3747	2674	3571	4442	3670	19.42758	19.49578	19.25393	17.94863	18.63564	18.80997	19.39243	18.46474	0.39298	1 -
ENSMUSC	Tbk1	TANK-bin	GO:00081	metabolic	K05410	TANK-bin	mmu0516	Human pa	10:121546	933	1405	824	1377	1778	1406	9.696546	11.66158	9.464756	11.04078	11.89931	11.4956	10.2743	11.47856	0.375899	1 -
ENSMUSC	Pno1	partner of	GO:00056	cell|intrace	-	-	-	-	11:172031	314	401	325	384	508	382	9.938953	10.1368	11.3695	9.377189	10.3545	9.512305	10.48175	9.748	0.410351	1 -
ENSMUSC	Plek	pleckstrin	GO:00325	developm	-	-	-	-	11:169712	287	277	164	313	417	306	2.438243	1.879409	1.539879	2.051497	2.281321	2.04516	1.95251	2.125993	0.679601	1 -
ENSMUSC	Srgap1	SLIT-ROB	GO:00400	locomotic	K07526	SLIT-ROB	mmu0436	Axon guic	10:121780	269	783	338	780	812	370	1.617553	3.760222	2.246309	3.61852	3.144247	1.750326	2.541362	2.837697	0.659669	1 -
ENSMUSC	Egfr	epiderma	GO:00325	developm	K04361	epiderma	mmu052C	Pathways	11:167522	392	590	351	509	669	499	1.950039	2.343988	1.929794	1.953463	2.143078	1.952849	2.074607	2.016464	0.651984	1 -
ENSMUSC	Avpr1a	arginine v	GO:00325	developm	K04226	arginine v	mmu0408	Neuroacti	10:122448	0	4	5	9	6	2	0	0.073046	0.12638	0.158787	0.088352	0.035988	0.066475	0.094376	0.546666	1 -
ENSMUSC	Usp15	ubiquitin s	GO:00081	metabolic	K11835	ubiquitin c	mmu0413	Mitophag	10:123105	1926	2429	1658	2328	2675	2361	5.476039	5.515482	5.210047	5.106502	4.897659	5.281012	5.400523	5.095058	0.343066	1 -
ENSMUSC	Vps54	VPS54 GA	GO:00511	localizatio	-	-	-	-	11:212392	611	805	522	893	1106	869	3.306726	3.479356	3.122302	3.728542	3.85449	3.699883	3.302795	3.760972	0.290926	1 -
ENSMUSC	Tbc1d15	TBC1 dom	GO:00511	localizatio	K20168	TBC1 dom	mmu0413	Mitophag	10:115197	699	878	622	951	1187	946	8.423051	8.449534	8.283803	8.841037	9.210807	8.967978	8.385463	9.006607	0.628139	1 -
ENSMUSC	Rab21	RAB21, m	GO:00325	developm	-	-	-	-	10:115287	1585	2193	1493	2095	2618	2150	17.77357	19.63948	18.50343	18.12424	18.9047	18.96684	18.63883	18.66526	0.799531	1 -
ENSMUSC	2310011J(	RIKEN cDI	-	-	-	-	-	-	10:803182	255	305	196	311	410	262	9.441964	9.019212	8.020957	8.884103	9.776012	7.631939	8.827378	8.764018	0.818548	1 -
ENSMUSC	Peli1	pellino 1 [	GO:00081	metabolic	-	-	-	-	11:210912	754	994	750	976	1289	1041	5.992164	6.308774	6.587498	5.984016	6.596599	6.508396	6.296145	6.363004	0.899113	1 -
ENSMUSC	Apc2	adenoma	GO:00511	localizatio	K02085	adenoma	mmu052C	Pathways	10:802959	3178	3573	2739	4090	4290	3981	9.711358	8.719769	9.250494	9.64228	8.441858	9.57038	9.227207	9.218173	0.774838	1 -
ENSMUSC	Thap2	THAP dor	GO:00056	cell|intrace	-	-	-	-	10:115368	380	558	401	566	699	502	2.528012	2.964666	2.948402	2.904971	2.994522	2.6273	2.813693	2.842264	0.913511	1 -
ENSMUSC	Lgr5	leucine ric	GO:00325	developm	-	-	-	-	10:115450	26	56	36	26	50	44	0.119596	0.205721	0.183019	0.092265	0.148105	0.159221	0.169445	0.133197	0.247921	1 -
ENSMUSC	Dock2	dedicator	GO:00325	developm	K12367	dedicator	mmu0406	Chemokin	11:342268	107	184	125	171	178	129	0.539311	0.74066	0.696329	0.66494	0.577739	0.511513	0.658767	0.584731	0.349213	1 -
ENSMUSC	Rab1a	RAB1A, m	GO:00325	developm	K07874	Ras-relate	mmu0513	Legionellc	11:202014	3131	4140	2943	4266	5272	4058	25.36123	26.78141	26.34658	26.65866	27.49903	25.8589	26.16307	26.6722	0.960056	1 -
ENSMUSC	Gamt	guanidinc	GO:00325	developm	K00542	guanidinc	mmu011C	Metabolic	10:802581	92	150	125	147	188	140	2.056138	2.677312	3.087601	2.534604	2.705677	2.461514	2.607017	2.567265	0.823162	1 -
ENSMUSC	Ptprr	protein ty	GO:00325	developm	K04458	receptor-	mmu0401	MAPK sigr	10:116018	365	411	287	438	502	435	2.59202	2.330955	2.252554	2.399665	2.295643	2.430221	2.391843	2.375176	0.785697	1 -

ENSMUSC	Actr2	ARP2 acti	GO:00325	developm	K17260	actin-rela	mmu0453	Tight junc	11:200623	6607	8413	6009	8227	10814	8490	71.61564	72.82832	71.98676	68.79794	75.48222	72.39727	72.14358	72.22581	0.785007	1	-
ENSMUSC	Ndufs7	NADH de	GO:00081	metabolic	K03940	NADH de	mmu011C	Metabolic	10:802491	429	617	485	714	818	787	9.057383	10.40343	11.31706	11.62983	11.12124	13.07166	10.25929	11.94091	0.247438	1	-
ENSMUSC	Ptprb	protein ty	GO:00325	developm	K05694	receptor-	mmu0452	Adherens	10:116275	1105	1363	947	1195	1472	1356	4.739671	4.669041	4.489343	3.954431	4.065823	4.575688	4.632685	4.198648	0.198475	1	-
ENSMUSC	Mum1	melanom	GO:00081	metabolic	-	-	-	-	10:802264	494	647	521	748	870	659	1.571057	1.643292	1.831258	1.835257	1.781718	1.648774	1.681869	1.75525	0.859478	1	-
ENSMUSC	Meis1	Meis hom	GO:00325	developm	K15613	homeobo	mmu052C	Transcript	11:188798	27	41	26	40	67	40	0.073421	0.089039	0.078139	0.083916	0.117322	0.085569	0.0802	0.095602	0.460141	1	-
ENSMUSC	Cnot2	CCR4-NC	GO:00325	developm	K12605	CCR4-NO	mmu0301	RNA degr	10:116485	865	1065	797	1089	1550	1037	5.371772	5.281983	5.470243	5.217469	6.198517	5.066302	5.374666	5.494096	0.992832	1	-
ENSMUSC	Best3	bestrophi	GO:00511	localizatio	-	-	-	-	10:116986	19	42	38	35	49	36	0.426663	0.753239	0.943104	0.606358	0.708569	0.635986	0.707669	0.650304	0.648635	1	-
ENSMUSC	Frs2	fibroblast	GO:00325	developm	K12461	fibroblast	mmu052C	Proteogly	10:117069	1289	1490	1125	1688	1812	1632	8.51313	7.85902	8.211753	8.600805	7.706359	8.479444	8.194634	8.262203	0.861667	1	-
ENSMUSC	Yeats4	YEATS do	GO:00081	metabolic	-	-	-	-	10:117215	280	347	297	375	464	533	5.990292	5.928763	7.022518	6.189431	6.392373	8.970719	6.313858	7.184174	0.436218	1	-
ENSMUSC	Cobl	cordon-b	GO:00325	developm	-	-	-	-	11:122366	454	509	377	700	670	498	2.087536	1.869139	1.915868	2.483169	1.983843	1.801433	1.957514	2.089481	0.738198	1	-
ENSMUSC	Rab36	RAB36, m	GO:00511	localizatio	-	-	-	-	10:750370	436	562	388	567	619	642	2.572853	2.648568	2.530508	2.581322	2.352201	2.980402	2.583976	2.637975	0.973863	1	-
ENSMUSC	Grb10	growth fa	GO:00081	metabolic	K20064	growth fa	mmu0415	mTOR sig	11:119305	487	758	562	660	924	576	2.944382	3.65999	3.755331	3.078502	3.597424	2.739671	3.453234	3.138533	0.321228	1	-
ENSMUSC	Adora2a	adenosine	GO:00325	developm	K04266	adenosine	mmu0408	Neuroacti	10:753168	25	31	39	50	47	20	0.36614	0.362589	0.631275	0.56495	0.443266	0.230436	0.453335	0.412884	0.691025	1	-
ENSMUSC	Snrpd3	small nucl	GO:00081	metabolic	K11088	small nucl	mmu0532	Systemic l	10:755175	1062	1475	1061	1412	1758	1711	16.22715	17.99931	17.91761	16.64497	17.29782	20.56737	17.38136	18.17005	0.839397	1	-
ENSMUSC	Nav3	neuron na	GO:00400	locomotic	-	-	-	-	10:109681	2170	2656	1978	2695	2924	2716	7.431075	7.263834	7.486264	7.120018	6.447985	7.316992	7.393724	6.961665	0.33465	1	-
ENSMUSC	Ddc	dopa dec	GO:00325	developm	K01593	aromatic-	mmu011C	Metabolic	11:118141	90	152	76	115	190	171	1.026184	1.384119	0.957735	1.011604	1.395057	1.533881	1.122679	1.313514	0.440497	1	-
ENSMUSC	Cpm	carboxype	GO:00081	metabolic	-	-	-	-	10:117629	181	325	240	333	424	381	1.369626	1.964053	2.007161	1.944009	2.066064	2.268087	1.78028	2.09272	0.306425	1	-
ENSMUSC	Mdm2	transform	GO:00325	developm	K06643	E3 ubiquit	mmu052C	Pathways	10:117688	743	889	637	942	1177	1043	4.630148	4.424389	4.387249	4.52884	4.723206	5.113298	4.480595	4.788448	0.673773	1	-
ENSMUSC	Osbpl8	oxysterol	GO:00325	developm	-	-	-	-	10:111164	1840	2205	1515	2067	2520	2123	10.59976	10.14455	9.64579	9.186482	9.348323	9.621425	10.13004	9.38541	0.252569	1	-
ENSMUSC	Zpbp	zona pellu	GO:00099	cellular pr	-	-	-	-	11:112796	14	12	7	9	24	16	0.201232	0.137746	0.111207	0.099803	0.222138	0.180923	0.150062	0.167621	0.806823	1	-
ENSMUSC	Cabin1	calcineuri	GO:00987	molecular	-	-	-	-	10:756461	1610	2081	1569	2261	2572	2306	6.419195	6.626323	6.913923	6.954816	6.603594	7.233107	6.653147	6.930506	0.850387	1	-
ENSMUSC	Ap3d1	adaptor-r	GO:00325	developm	K12396	AP-3 com	mmu0414	Lysosome	10:807069	3371	4311	3066	4820	5245	4545	19.0193	19.42494	19.11854	20.98037	19.05619	20.17347	19.18759	20.07001	0.804046	1	-
ENSMUSC	Phlda1	pleckstrin	GO:00081	metabolic	-	-	-	-	10:111506	204	208	140	281	295	238	5.38981	4.388879	4.088084	5.727698	5.019029	4.946885	4.622258	5.231204	0.468824	1	-
ENSMUSC	Sf3a2	splicing fa	GO:00325	developm	K12826	splicing fa	mmu0304	Spliceosor	10:807981	369	467	402	494	620	545	7.517662	7.598346	9.051691	7.764505	8.13398	8.735023	8.0559	8.211169	0.972765	1	-
ENSMUSC	Mdm1	transform	GO:00325	developm	-	-	-	-	10:118141	129	143	71	149	152	161	1.183544	1.047794	0.719945	1.05466	0.898035	1.162072	0.983761	1.038256	0.889525	1	-
ENSMUSC	Jsrp1	junctional	GO:00511	localizatio	-	-	-	-	10:808084	33	33	28	47	34	43	0.737259	0.588794	0.691364	0.810094	0.489142	0.755765	0.672472	0.685	1	1	-
ENSMUSC	Wif1	Wnt inhib	GO:00325	developm	K01691	WNT inhib	mmu0431	Wnt signa	10:121033	10	18	9	25	24	19	0.126888	0.182402	0.126208	0.244734	0.196102	0.18966	0.145166	0.210165	0.216016	1	-
ENSMUSC	Timm13	translocas	GO:00511	localizatio	-	-	-	-	10:808994	344	283	364	502	599	405	8.285601	5.443741	9.689764	9.328234	9.29066	7.674165	7.806368	8.764353	0.541229	1	-
ENSMUSC	Vps13d	vacuolar p	GO:00055	extracellul	-	-	-	-	4:1449726	3094	3824	2870	3645	4451	4097	6.144736	6.065222	6.29958	5.584841	5.692397	6.401178	6.169846	5.892805	0.42419	1	-
ENSMUSC	Llph	LLP homo	GO:00325	developm	-	-	-	-	10:120227	411	459	330	492	583	417	7.307767	6.517815	6.484915	6.74898	6.67523	5.832976	6.770166	6.419062	0.508003	1	-
ENSMUSC	Tmbim4	transmem	GO:00508	response	-	-	-	-	10:120201	175	235	152	278	256	271	2.438423	2.615073	2.340789	2.988447	2.297024	2.970645	2.464762	2.752039	0.53288	1	-
ENSMUSC	Irak3	interleukir	GO:00325	developm	K04732	interleukir	mmu0472	Neurotrop	10:120141	18	41	21	28	48	33	0.156645	0.284961	0.201989	0.187992	0.268998	0.225935	0.214532	0.227642	0.922899	1	-
ENSMUSC	Slc5a4a	solute car	GO:00511	localizatio	-	-	-	-	10:761474	0	0	0	0	3	0	0	0	0	0	0.057406	0	0	0.019135	0.290432	1	-
ENSMUSC	Prmt2	protein ar	GO:00081	metabolic	-	-	-	-	10:762072	935	1155	790	1280	1465	1185	8.960807	8.840229	8.367764	9.464031	9.04124	8.934389	8.722933	9.146553	0.808666	1	-
ENSMUSC	Dip2a	disco inte	GO:00325	developm	-	-	-	-	10:762594	911	1394	988	1237	1504	1445	4.104179	5.015527	4.919395	4.299399	4.363252	5.121377	4.6797	4.594676	0.678744	1	-
ENSMUSC	Fzr1	fizzy/cell c	GO:00325	developm	K03364	cell divisio	mmu0412	Ubiquitin	10:813662	1038	1253	913	1327	1714	1297	9.295737	8.96155	9.036576	9.168272	9.884437	9.13771	9.097954	9.396806	0.930186	1	-
ENSMUSC	Ncln	nicalin [Sc	GO:00508	response	-	-	-	-	10:814862	644	774	626	1042	1096	986	5.190945	4.982503	5.576763	6.479758	5.688868	6.252425	5.25007	6.14035	0.192899	1	-
ENSMUSC	Hcfc2	host cell f	GO:00160	membran	K14966	host cell f	mmu0516	Herpes sir	10:826961	403	501	379	543	658	533	3.911372	3.883355	4.065457	4.06587	4.112485	4.069692	3.953394	4.082682	0.943327	1	-
ENSMUSC	Nfyb	nuclear tr	GO:00081	metabolic	K08065	nuclear tr	mmu0516	HTLV-I inf	10:827487	603	733	531	726	943	633	4.416222	4.287295	4.298084	4.102048	4.447336	3.647105	4.333867	4.065496	0.412653	1	-
ENSMUSC	Txnrd1	thioredox	GO:00325	developm	K00384	thioredox	mmu052C	Pathways	10:828339	845	1213	866	1339	1731	1312	4.600454	5.274127	5.210851	5.624127	6.068701	5.619387	5.028477	5.770738	0.233768	1	-
ENSMUSC	Glt8d2	glycosyltr	GO:00038	catalytic a	-	-	-	-	10:826504	271	306	239	368	439	408	2.387006	2.152546	2.326644	2.500707	2.490029	2.827198	2.288732	2.605978	0.376401	1	-
ENSMUSC	Ppm1m	protein pl	GO:00081	metabolic	-	-	-	-	9:1061949	174	282	161	319	355	262	2.932402	3.795504	2.9988	4.147595	3.852638	3.473659	3.242235	3.82463	0.281856	1	-
ENSMUSC	D10Wsu1	DNA segr	-	-	-	-	-	-	10:833602	873	1213	833	1254	1292	1080	0.432734	0.48019	0.456351	0.479551	0.412405	0.421154	0.456425	0.437703	0.506776	1	-
ENSMUSC	Aldh1l2	aldehyde	GO:00081	metabolic	K00289	formyltetr	mmu0067	One carbo	10:834874	103	149	101	129	159	159	0.566574	0.654563	0.614028	0.547447	0.563214	0.688063	0.611721	0.599575	0.765032	1	-
ENSMUSC	Wdr82	WD repea	GO:00081	metabolic	K14962	COMPASS	mmu0301	mRNA sur	9:1061709	2256	2642	1921	2998	3539	2692	18.90832	17.68449	17.79459	19.38544	19.10069	17.75006	18.12913	18.7454	0.916318	1	-
ENSMUSC	Glyctk	glycerate	GO:00081	metabolic	K11529	glycerate	mmu011C	Metabolic	9:1061528	76	75	63	124	105	77	0.50167	0.395376	0.459611	0.63147	0.446319	0.399856	0.452219	0.492548	0.737939	1	-
ENSMUSC	Slc36a1	solute car	GO:00511	localizatio	K14209	solute car	mmu0497	Protein di	11:552043	1088	492	281	1472	502	1307	9.050573	3.268577	2.583453	9.446818	2.689092	8.553304	4.967534	6.896405	0.331598	1	-
ENSMUSC	Adarb1	adenosine	GO:00325	developm	-	-	-	-	10:772907	1882	4312	2388	3442	3902	1865	4.80861	8.798822	6.743433	6.784865	6.420101	3.748778	6.783622	5.651248	0.201599	1	-
ENSMUSC	Appl2	adaptor p	GO:00329	macromo	-	-	-	-	10:836000	1125	1461	958	1565	2037	1401	6.774199	7.025893	6.37555	7.270262	7.898618	6.636733	6.725214	7.268538	0.577175	1	-
ENSMUSC	Slc36a2	solute car	GO:00511	localizatio	-	-	-	-	11:551584	4	3	2	4	3	2	0.072822	0.043623	0.040243	0.056172	0.035173	0.028649	0.052229	0.039998	0.833017	1	-
ENSMUSC	Sumo3	small ubic	GO:00081	metabolic	K12160	small ubic	mmu0301	RNA trans	10:776060	1092	1679	1156	1444	2013	1553	8.833526	10.84696	10.33514	9.011744	10.486	9.883116	10.00521	9.793621	0.652041	1	-
ENSMUSC	Hint1	histidine t	GO:00081	metabolic	-	-	-	-	11:548663	671	778	609	960	1015	790	33.69243	31.1986	33.79668	37.18873	32.81938	31.20668	32.8959	33.73827	0.99362	1	-
ENSMUSC	Lyrm7	LYR motif	GO:00081	metabolic	-	-	-	-	11:548268	197	255	87	166	351	260	2.03183	2.100424	0.991716	1.320874	2.33122	2.109625	1.70799	1.920573	0.643137	1	-
ENSMUSC	Fbxw11	F-box anc	GO:00081	metabolic	K03362	F-box anc	mmu0421	Cellular se	11:326427	2844	3411	2433	3471	4250	3317	19.19837	18.38918	18.15197	18.07672	18.47474	17.61535	18.57984	18.0556	0.544518	1	-
ENSMUSC	Pfkl	phosphof	GO:00081	metabolic	-	-	mmu011C	Metabolic	10:779869	4663	5458	4504	5737	7334	5617	36.24159	33.87827	38.68896	34.39989	36.70603	34.3445	36.26961	35.15014	0.519151	1	-
ENSMUSC	Pus10	pseudouri	GO:00081	metabolic	-	-	-	-	11:236656	142	176	105	138	229	171	1.530763	1.515227	1.250998	1.1477	1.58968	1.4502	1.432329	1.39586	0.752686	1	-
ENSMUSC	Rhbdf1	rhomboid	GO:00081	metabolic	-	-	-	-	11:322095	163	209	126	217	289	255	0.975352	0.998767	0.833276	1.00176	1.11359	1.200393	0.935798	1.105248	0.287739	1	-
ENSMUSC	Pex13	peroxison	GO:00325	developm	K13344	peroxin-1	mmu0414	Peroxisom	11:236464	458	578	449	650	682	493	5.252561	5.293935	5.691135	5.751075	5.036681	4.447983	5.412544	5.078579	0.44854	1	-
ENSMUSC	1810043G	RIKEN cDI	GO:00325	developm	-	-	-	-	10:779785	279	462	253	434	391	384	1.181688	1.562739	1.184312	1.418138	1.066426	1.2795	1.30958	1.254688	0.608379	1	-
ENSMUSC	1700093K	RIKEN cDI	GO:00160	membran	-	-	-	-	11:235162	5	5	0	3	2	4	0.123289	0.098466	0	0.057083	0.031753	0.077596	0.073918	0.055477	0.704097	1	-
ENSMUSC	Mpg	N-methyl	GO:00081	metabolic	K03652	DNA-3-m	mmu0341	Base excis	11:322265	133	160	121	225	220	186	1.517326	1.457786	1.525668	1.980346	1.616232	1.669366	1.50026	1.755315	0.333994	1	-
ENSMUSC	Ahsa2	AHA1, act	GO:00987	molecular	-	-	-	-	11:234878	352	422	331	496	592	446	2.07116	1.983032	2.152519	2.251561	2.243097	2.06451	2.068904	2.186389	0.783389	1	-
ENSMUSC	Nprl3	nitrogen p	GO:00325	developm	K20406	nitrogen p	mmu0415	mTOR sig	11:322256	293	382	292	433	501	505	1.396133	1.453676	1.537758	1.591756	1.537274	1.893046	1.462522	1.674025	0.347787	1	-
ENSMUSC	Xpo1	exportin 1	GO:00081	metabolic	K14290	exportin-1	mmu0516	HTLV-I inf	11:232560	1545	1924	1442	1962	2323	1807	8.707289	8.659753	8.981865	8.530683	8.430598	8.011678	8.782969	8.324319	0.382421	1	-
ENSMUSC	Hbq1a	hemoglob	GO:00511	localizatio	-	-	-	-	11:323000	0	3	0	3	0	2	0	0.242236	0	0.234001	0	0.159086	0.080745	0.131029	0.759949	1	-
ENSMUSC	Nsg2	neuron sp	GO:00511	localizatio	-	-	-	-	11:320004	16008	20074	13380	21469	24691	17682	225.4193	225.7532	208.2366	233.2363	223.8966	195.8828	219.803	217.6719	0.705367	1	-
ENSMUSC	4930524B	RIKEN cDI	-	-	-	-	-	-	11:319655	1	2	0	0	1	4	0.030538	0.04877	0	0	0.019655	0.096064	0.026436	0.038573	1	1	-
ENSMUSC	Cpeb4	cytoplasm	GO:00451	translation	K02602	cytoplasm	mmu0411	Oocyte m	11:318722	1830	1727	1896	2412	1734	2437	14.02489	10.57029	16.05956	14.2612	8.557607	14.69315	13.55158	12.50399	0.450091	1	-
ENSMUSC	Stc2	stanniocal	GO:00325	developm	-	-	-	-	11:313573	117	138	96	150	147	137	0.76006	0.715956	0.689251	0.751769	0.614941	0.700152	0.721756	0.688954	0.62522	1	-
ENSMUSC	Asb3	ankyrin re	GO:00081	metabolic	-	-	-	-	11:308854	162	250	188	251	278	262	1.453337	1.791171	1.864041	1.737222	1.606022	1.849114	1.70285	1.730786	0.954807	1	-
ENSMUSC	Cdc34	cell divisic	GO:00081	metabolic	K02207	ubiquitin-	mmu0516	Herpes sir	10:796821	204	325	282	412	531	360	2.188308	2.784248	3.343294	3.409618	3.667985	3.038029	2.77195	3.371878	0.198784	1	-
ENSMUSC	Tpgs1	tubulin pc	GO:00325	developm	-	-	-	-	10:796693	290	468	364	484	590	482	9.897642	12.7563	13.73032	12.74407	12.96702	12.94168	12.12809	12.88426	0.760574	1	-
ENSMUSC	Chac2	ChaC, cati	GO:00081	metabolic	-	-	-	-	11:309767	112	128	87	117	169	152	2.931197	2.675381	2.516484	2.36235	2.848202	3.12955	2.707687	2.780034	1	1	-
ENSMUSC	Erlec1	endoplasr	GO:00081	metabolic	K14008	endoplasr	mmu0414	Protein pr	11:309307	857	1186	821	1029	1382	1196	13.02634	14.397	13.79213	12.06667	13.52709	14.30156	13.73849	13.29844	0.569724	1	-
ENSMUSC	Shc2	SHC (Src l	GO:00081	metabolic	K17447	SHC-trans	mmu0401	Ras signal	10:796180	667	846	723	1043	1088	946	5.222589	5.290252	6.256699	6.300498	5.485849	5.827231	5.589846	5.871192	0.81152	1	-
ENSMUSC	Sptbn1	spectrin b	GO:00081	metabolic	-	-	-	-	11:300993	26685	33939	26105	38033	38417	38842	75.38334	76.56902	81.50396	82.88942	69.88538	86.32184	77.81877	79.69888	0.993589	1	-
ENSMUSC	Theg	testicular	GO:00325	developm	-	-	-	-	10:795763	2	2	2	9	0	0	0.051494	0.041139	0.056927	0.178811	0	0	0.049853	0.059604	1	1	-
ENSMUSC	Wdpcp	WD repea	GO:00325	developm	-	-	-	-	11:215722	137	183	99	147	180	137	1.175682	1.254194	0.938965	0.973231	0.994708	0.924909	1.122947	0.964283	0.224875	1	-
ENSMUSC	Mdh1	malate de	GO:00081	metabolic	K00025	malate de	mmu011C	Metabolic	11:215567	3993	3775	3510	5135	6135	4873	71.28802	53.8245	69.25819	70.72746	70.53205	68.4423	64.79024	69.9006	0.601088	1	-
ENSMUSC	Fstl3	follistatin-	GO:00325	developm	-	-	-	-	10:797772	24	32	32	36	50	41	0.3745	0.398774	0.551865	0.433376	0.502414	0.503301	0.441713	0.479697	0.77802	1	-
ENSMUSC	Ccng1	cyclin G1	GO:00099	cellular pr	K10145	cyclin G1[	mmu052C	MicroRNA	11:407485	1562	2096	1401	2039	2595	2111	22.09749	23.68095	21.90518	22.25407	23.64036	23.49422	22.56121	23.12955	0.991663	1	-
ENSMUSC	Fgf22	fibroblast	GO:00508	response	K04358	fibroblast	mmu052C	Pathways	10:797550	20	26	9	30	25	8	0.342353	0.355434	0.170259	0.39618	0.275569	0.107725	0.289349	0.259825	0.711698	1	-
ENSMUSC	Nudcd2	NudC dor	GO:00325	developm	-	-	-	-	11:407336	164	178	122	195	226	187	2.579471	2.235906	2.120779	2.366212	2.28903	2.313882	2.312052	2.323042	0.899077	1	-
ENSMUSC	Polrmt	polymeras	GO:00081	metabolic	-	-	-	-	10:797361	403	486	357	467	569	542	2.645	2.547429	2.589617	2.364655	2.404846	2.798536	2.594016	2.522679	0.644097	1	-
ENSMUSC	Hmmr	hyalurona	GO:00160	membran	K06267	hyalurona	mmu0451	ECM-rece	11:407013	15	26	14	26	38	31	0.153924	0.213081	0.158781	0.205836	0.25111	0.250265	0.175262	0.235737	0.264846	1	-
ENSMUSC	Hcn2	hyperpola	GO:00511	localizatio	K04955	hyperpola	mmu0402	cAMP sigr	10:797166	1467	2082	1597	1965	2430	2092	20.63876	23.39269	24.83164	21.32782	22.01479	23.15401	22.95437	22.16554	0.522522	1	-
ENSMUSC	Acsl6	acyl-CoA	GO:00325	developm	K01897	long-chai	mmu011C	Metabolic	11:543037	4865	5846	2898	6194	7344	6118	17.01402	16.32784	11.20132	16.71189	16.53908	16.83233	14.84772	16.69443	0.387777	1	-
ENSMUSC	Slc22a4	solute car	GO:00081	metabolic	K08202	MFS trans	mmu0523	Choline m	11:539831	64	104	63	95	107	100	1.279955	1.661096	1.392534	1.465784	1.378024	1.573365	1.444528	1.472391	0.980089	1	-
ENSMUSC	Zfp354b	zinc finge	GO:00081	metabolic	-	-	-	-	11:509218	76	89	70	80	127	113	1.037898	0.970682	1.05653	0.842865	1.116857	1.214026	1.021703	1.057916	0.963268	1	-
ENSMUSC	Cyfip2	cytoplasm	GO:00325	developm	K05749	cytoplasm	mmu0481	Regulation	11:461938	21883	27692	20152	28637	32607	29659	126.7319	128.0794	128.9865	127.949	121.6031	135.1284	127.9326	128.2268	0.788481	1	-
ENSMUSC	Mgat1	mannosid	GO:00325	developm	K00726	alpha-1,3	mmu011C	Metabolic	11:492441	218	219	145	319	288	151	0.914133	0.733403	0.671996	1.031985	0.777678	0.498128	0.773177	0.769264	0.877585	1	-
ENSMUSC	Ppp2ca	protein pl	GO:00325	developm	K04382	serine/thr	mmu0415	PI3K-Akt s	11:520986	6164	7884	5644	8139	10269	8186	49.08218	50.13646	49.6701	49.99915	52.65554	51.27951	49.62958	51.3114	0.908596	1	-
ENSMUSC	Sgcd	sarcoglyc	GO:00325	developm	K12563	delta-sarc	mmu0541	Dilated ca	11:468962	36	34	35	38	45	45	0.258053	0.19464	0.277284	0.210146	0.207715	0.253762	0.243326	0.223874	0.630537	1	-
ENSMUSC	Hnrnpab	heteroger	GO:00325	developm	-	-	-	-	11:516001	3288	4029	2933	4375	5518	4432	52.30824	51.18957	51.57001	53.69659	56.52944	55.46884	51.68927	55.23162	0.6137	1	-
ENSMUSC	Phykpl	5-phosph	GO:00038	catalytic a	K18202	5-phosph	mmu011C	Metabolic	11:515847	77	71	53	82	122	87	0.379977	0.279815	0.28906	0.312186	0.387689	0.337752	0.316284	0.345876	0.70754	1	-
ENSMUSC	Hspa4	heat shoc	GO:00511	localizatio	K09489	heat shoc	mmu0453	Tight junc	11:532598	6175	7822	5265	7522	8600	7075	61.39617	62.11091	57.85614	57.69895	55.06269	55.34031	60.45441	56.03398	0.226539	1	-
ENSMUSC	Cnot6	CCR4-NC	GO:00081	metabolic	K12603	CCR4-NO	mmu0301	RNA degr	11:496715	916	1198	898	1258	1425	1189	5.967692	6.23324	6.465977	6.322985	5.978341	6.094021	6.222303	6.131783	0.684855	1	-
ENSMUSC	Gfpt2	glutamine	GO:00081	metabolic	K00820	glucosam	mmu011C	Metabolic	11:497941	91	124	89	151	150	168	0.956004	1.040362	1.033367	1.223845	1.014763	1.388472	1.009911	1.209027	0.307211	1	-
ENSMUSC	Zfp354a	zinc finge	GO:00325	developm	-	-	-	-	11:510592	161	194	170	191	249	241	0.944226	0.908656	1.101908	0.8642	0.940385	1.111933	0.98493	0.972173	0.794793	1	-
ENSMUSC	Canx	calnexin [	GO:00325	developm	K08054	calnexin[-	mmu0516	HTLV-I inf	11:502939	6740	9011	6033	8840	10777	8286	54.49307	58.18355	53.90903	55.13965	56.10918	52.70323	55.52855	54.65068	0.634422	1	-
ENSMUSC	Rack1	receptor f	GO:00325	developm	K14753	guanine n	mmu0516	Measles -	11:488003	1492	2494	1877	2293	3375	2800	11.27459	15.05131	15.67628	13.368	16.4233	16.64566	14.00073	15.47898	0.470451	1	-
ENSMUSC	Rasgef1c	RasGEF dc	GO:00987	molecular	-	-	-	-	11:499018	20	36	34	28	37	30	0.238278	0.342534	0.447692	0.257356	0.283863	0.281177	0.342834	0.274132	0.278634	1	-
ENSMUSC	Rufy1	RUN and	GO:00511	localizatio	K12482	RUN and	mmu0414	Endocytos	11:503892	720	964	698	1033	1286	1044	13.31774	14.24035	14.26922	14.74105	15.31769	15.1918	13.94244	15.08351	0.581192	1	-
ENSMUSC	Rnf130	ring finge	GO:00081	metabolic	-	-	-	-	11:500253	612	818	535	773	862	727	17.29979	18.46667	16.71439	16.85774	15.69103	16.16721	17.49362	16.23866	0.328471	1	-
ENSMUSC	Ltc4s	leukotrien	GO:00081	metabolic	K00807	leukotrien	mmu011C	Metabolic	11:502364	26	73	28	47	49	36	0.579173	1.298683	0.689347	0.807729	0.702881	0.630881	0.855734	0.713831	0.374894	1	-
ENSMUSC	Rad50	RAD50 dc	GO:00081	metabolic	K10866	DNA repa	mmu0421	Cellular se	11:536495	532	660	494	715	926	637	4.872195	4.827281	5.000197	5.051835	5.461078	4.589477	4.899891	5.03413	0.975695	1	-
ENSMUSC	Mrnip	MRN com	GO:00081	metabolic	-	-	-	-	11:501744	144	185	137	209	219	252	4.061152	4.166804	4.270254	4.547395	3.977258	5.591096	4.16607	4.70525	0.496924	1	-
ENSMUSC	Clk4	CDC like k	GO:00081	metabolic	-	-	-	-	11:512617	885	1072	818	1188	1432	1356	5.432981	5.255761	5.550029	5.626543	5.660992	6.548856	5.412924	5.945464	0.480785	1	-
ENSMUSC	Sar1b	secretion	GO:00511	localizatio	K07953	GTP-bind	mmu0414	Protein pr	11:517636	367	477	355	429	560	504	16.2027	16.81843	17.32194	14.61197	15.92076	17.50502	16.78103	16.01258	0.534032	1	-
ENSMUSC	Jade2	jade famil	GO:00325	developm	-	-	-	-	11:518134	1211	1452	997	1492	1513	1469	3.814359	3.652498	3.470718	3.625573	3.068818	3.640074	3.645858	3.444822	0.403479	1	-
ENSMUSC	Pdlim4	PDZ and l	GO:00452	synapse|s	-	-	-	-	11:540549	64	98	64	110	136	110	1.193557	1.459607	1.319149	1.582659	1.633275	1.613875	1.324104	1.609936	0.257074	1	-
ENSMUSC	Cdkl3	cyclin-der	GO:00325	developm	-	-	-	-	11:520042	178	187	150	193	234	215	1.128944	0.947197	1.051456	0.944366	0.955701	1.072757	1.042532	0.990941	0.578978	1	-
ENSMUSC	Ube2b	ubiquitin-	GO:00325	developm	K10574	ubiquitin-	mmu0412	Ubiquitin	11:519854	1799	2100	1656	2433	2887	2251	38.40683	35.8049	39.0737	40.0728	39.68978	37.80626	37.76181	39.18961	0.884176	1	-
ENSMUSC	Cdkn2aipı	CDKN2A i	GO:00508	response	-	-	-	-	11:519676	657	870	611	836	985	836	16.93337	17.90784	17.4047	16.62321	16.34817	16.951	17.41531	16.6408	0.482463	1	-
ENSMUSC	Kremen1	kringle co	GO:00325	developm	-	-	-	-	11:519155	301	360	285	443	514	427	2.368361	2.262191	2.478414	2.689154	2.604348	2.643142	2.369655	2.645548	0.447222	1	-
ENSMUSC	Itk	IL2 induci	GO:00325	developm	K07363	IL2-induci	mmu0406	Chemokin	11:463251	7	7	4	9	17	8	0.069386	0.055413	0.043823	0.068825	0.108512	0.062379	0.056207	0.079905	0.415568	1	-
ENSMUSC	Nefh	neurofilan	GO:00325	developm	K04574	neurofilan	mmu0501	Amyotrop	11:493875	1353	1728	1097	1920	2052	1582	20.68392	21.0972	18.53484	22.64471	20.20074	19.02623	20.10532	20.62389	0.989106	1	-
ENSMUSC	Med7	mediator	GO:00325	developm	-	-	-	-	11:464369	283	257	195	437	185	318	2.41367	1.750531	1.838116	2.875431	1.016055	2.133678	2.000772	2.008388	0.917706	1	-
ENSMUSC	Vdac1	voltage-d	GO:00325	developm	K05862	voltage-d	mmu0516	HTLV-I inf	11:523608	5292	6175	4635	6211	8246	5708	93.87551	87.48131	90.87178	85.00106	94.19557	79.65762	90.74287	86.28475	0.400188	1	-
ENSMUSC	Slu7	SLU7 splic	GO:00081	metabolic	K12819	pre-mRN	mmu0304	Spliceosor	11:434337	1381	1707	1152	1750	2082	1773	17.70711	17.47968	16.325	17.31101	17.19053	17.88437	17.1706	17.46197	0.935962	1	-
ENSMUSC	Nipal4	NIPA-like	GO:00511	localizatio	-	-	-	-	11:461481	39	92	43	79	97	62	0.691425	1.302607	0.842548	1.080528	1.1074	0.864735	0.945527	1.017554	0.836366	1	-
ENSMUSC	Ascc2	activating	GO:00081	metabolic	-	-	-	-	11:463774	609	757	534	897	935	845	6.831597	6.781824	6.620537	7.762972	6.754157	7.45715	6.744653	7.32476	0.568622	1	-
ENSMUSC	Hus1	HUS1 che	GO:00325	developm	K10903	HUS1 che	mmu0421	Cellular se	11:899313	171	191	144	248	256	243	0.844803	0.753597	0.786264	0.945238	0.814432	0.944447	0.794888	0.901372	0.440767	1	-
ENSMUSC	Pttg1	pituitary t	GO:00081	metabolic	K06635	securin[-]	mmu0516	HTLV-I inf	11:434202	210	234	217	332	315	256	2.704533	2.406777	3.088726	3.298692	2.61239	2.593725	2.733345	2.834936	0.930619	1	-
ENSMUSC	Zfp607a	zinc finge	GO:00081	metabolic	-	-	-	-	7:2785752	31	59	31	56	57	36	0.208043	0.316224	0.229936	0.289943	0.246335	0.190069	0.251401	0.242116	0.793722	1	-
ENSMUSC	Tns3	tensin 3 [S	GO:00325	developm	-	-	-	-	11:843165	1366	1894	1254	1901	2147	1861	7.049729	7.806343	7.152638	7.568929	7.13524	7.555778	7.336237	7.419983	0.889211	1	-
ENSMUSC	Btg2	B cell tran	GO:00325	developm	K14443	protein Tc	mmu0301	RNA degr	1:1340751	177	288	183	275	372	299	3.939943	5.119827	4.502113	4.722598	5.332314	5.236003	4.520627	5.096972	0.455158	1	-
ENSMUSC	Gatsl3	GATS pro	GO:00508	response	-	-	-	-	11:421822	35	37	25	51	58	55	1.357701	1.146256	1.071816	1.526294	1.448837	1.678456	1.191924	1.551196	0.215802	1	-
ENSMUSC	Igfbp3	insulin-lik	GO:00325	developm	K10138	insulin-lik	mmu052C	Transcript	11:720608	308	443	281	499	586	475	4.971161	5.71027	5.012567	6.213517	6.090593	6.031314	5.231333	6.111808	0.246337	1	-
ENSMUSC	Gabra6	gamma-a	GO:00048	signal tran	K05175	gamma-a	mmu0408	Neuroacti	11:423064	0	0	2	0	0	2	0	0	0.029457	0	0	0.020971	0.009819	0.00699	1	1	-
ENSMUSC	Pes1	pescadillo	GO:00081	metabolic	-	-	-	-	11:396397	617	754	539	793	947	732	11.077	10.8107	10.69478	10.98349	10.94817	10.33853	10.86083	10.75673	0.74114	1	-
ENSMUSC	Adcy1	adenylate	GO:00325	developm	K08041	adenylate	mmu052C	Pathways	11:706348	23215	29263	21323	27182	34668	29400	88.00844	88.59727	89.34099	79.50005	84.63288	87.6827	88.6489	83.93854	0.338338	1	-
ENSMUSC	Tcn2	transcoba	GO:00511	localizatio	K14619	transcoba	mmu0497	Vitamin d	11:391719	371	679	341	450	629	516	8.872929	12.96905	9.013508	8.303005	9.687195	9.70853	10.28516	9.23291	0.312935	1	-
ENSMUSC	4921536K	RIKEN cDI	-	-	-	-	-	-	11:388608	6	11	11	22	14	16	0.141444	0.207095	0.286598	0.400125	0.212534	0.296741	0.211712	0.303133	0.311766	1	-
ENSMUSC	Osbp2	oxysterol	GO:00325	developm	-	-	-	-	11:370373	1646	2081	1574	2132	2698	2088	12.3801	12.50006	13.08416	12.3712	13.06745	12.3548	12.65477	12.59782	0.747856	1	-
ENSMUSC	Myo1g	myosin IG	GO:00400	locomotic	-	-	-	-	11:650654	13	24	10	25	36	15	0.0864	0.127386	0.073452	0.128188	0.154075	0.078428	0.095746	0.12023	0.487175	1	-
ENSMUSC	Arf5	ADP-ribo	GO:00511	localizatio	K07940	ADP-ribo	mmu0414	Endocytos	6:2842356	1309	1788	1548	2161	2468	2051	33.10897	36.11764	43.27368	42.16879	40.19814	40.81151	37.5001	41.05948	0.491276	1	-
ENSMUSC	2310033P	RIKEN cDI	GO:00971	organic c	-	-	-	-	11:592083	188	268	202	301	313	259	8.933016	10.16998	10.6081	11.03409	9.577213	9.681699	9.9037	10.09767	0.982984	1	-
ENSMUSC	Guk1	guanylate	GO:00081	metabolic	-	-	mmu011C	Metabolic	11:591838	760	1013	752	1040	1206	1151	18.48034	19.67216	20.20977	19.51013	18.88419	22.01825	19.45409	20.13753	0.92165	1	-
ENSMUSC	Rnf185	ring finge	GO:00081	metabolic	K10666	E3 ubiquit	mmu0414	Protein pr	11:341598	563	701	98	163	206	656	10.5967	10.53723	2.038608	2.366904	2.496798	9.713529	7.72418	4.859077	0.191433	1	-
ENSMUSC	Limk2	LIM motif	GO:00048	signal tran	K05744	LIM doma	mmu0481	Regulation	11:334425	1349	1730	1222	1912	2080	1810	11.12321	11.39227	11.13618	12.16288	11.04425	11.74107	11.21722	11.6494	0.880769	1	-
ENSMUSC	Patz1	POZ (BTB	GO:00325	developm	-	-	-	-	11:328887	518	556	321	578	535	657	2.688321	2.304476	1.841208	2.314247	1.787969	2.682425	2.278002	2.261547	0.809849	1	-
ENSMUSC	Eif4enif1	eukaryotic	GO:00325	developm	-	-	-	-	11:320239	1056	1338	1020	1379	1717	1402	5.592623	5.65918	5.970336	5.634381	5.85567	5.841314	5.740713	5.777122	0.851871	1	-
ENSMUSC	Trim11	tripartite r	GO:00325	developm	-	-	-	-	11:589780	354	511	334	504	633	524	2.971894	3.426075	3.099007	3.264306	3.422066	3.460764	3.165659	3.382379	0.706165	1	-
ENSMUSC	Ogdh	oxoglutar	GO:00325	developm	K00164	2-oxoglut	mmu011C	Metabolic	11:629163	5287	6312	4581	6835	7916	6775	39.49764	37.65951	37.82413	39.39405	38.08222	39.81824	38.32709	39.09817	0.957634	1	-
ENSMUSC	Drg1	developm	GO:00329	macromo	-	-	-	-	11:318736	664	830	674	886	981	838	9.037579	9.022104	10.13889	9.303538	8.598204	8.973028	9.399526	8.958257	0.47759	1	-
ENSMUSC	Rtn4	reticulon	GO:00325	developm	K20720	reticulon-	mmu0501	Alzheimer	11:296929	7653	10147	8309	11444	11107	9715	45.861	48.56186	55.031	52.90783	42.86109	45.8	49.81795	47.18964	0.400212	1	-
ENSMUSC	Mtif2	mitochon	GO:00081	metabolic	-	-	-	-	11:295264	528	688	430	705	874	684	4.988191	5.190911	4.489775	5.138412	5.317103	5.083657	4.889626	5.179724	0.752054	1	-
ENSMUSC	Rps27a	ribosomal	GO:00081	metabolic	K02977	small subu	mmu0301	Ribosome	11:295458	51	68	54	67	68	79	1.126609	1.19967	1.318388	1.141855	0.967312	1.37292	1.214889	1.160696	0.69173	1	-
ENSMUSC	Clhc1	clathrin he	-	-	-	-	-	-	11:295479	2	0	1	3	5	3	0.035868	0	0.019826	0.041521	0.057753	0.042328	0.018564	0.0472	0.225465	1	-
ENSMUSC	Cfap36	cilia and f	GO:00056	cell|intrac	-	-	-	-	11:292215	1513	1768	1375	2051	2351	1726	25.88426	24.15599	25.99839	27.07028	25.90026	23.22996	25.34621	25.40017	0.816269	1	-
ENSMUSC	Ppp4r3b	protein pl	GO:00081	metabolic	K17491	protein pl	mmu0492	Glucagon	11:291728	578	764	530	905	1039	852	4.796359	5.063171	4.860782	5.793784	5.552054	5.562038	4.906771	5.635959	0.248821	1	-
ENSMUSC	Pnpt1	polyribon	GO:00325	developm	K00962	polyribon	mmu0023	Purine me	11:291307	541	660	506	719	842	699	7.453166	7.261617	7.704442	7.64193	7.469827	7.575856	7.473075	7.562537	0.910144	1	-
ENSMUSC	Efemp1	epiderma	GO:00325	developm	-	-	-	-	11:288532	158	229	155	216	266	221	4.322207	5.002992	4.686259	4.558624	4.685816	4.756115	4.670486	4.666852	0.855751	1	-
ENSMUSC	Pold2	polymeras	GO:00081	metabolic	K02328	DNA poly	mmu011C	Metabolic	11:587218	169	181	179	256	300	256	3.824622	3.271348	4.477149	4.469626	4.371967	4.557765	3.857706	4.466453	0.361924	1	-
ENSMUSC	Aebp1	AE bindin	GO:00081	metabolic	-	-	-	-	11:586194	290	428	227	277	395	408	3.974608	4.684735	3.4385	2.928903	3.486158	4.399133	4.032615	3.604731	0.325756	1	-
ENSMUSC	Polm	polymeras	GO:00325	developm	K03513	DNA poly	mmu0345	Non-hom	11:582786	109	149	95	152	169	134	0.972857	1.062074	0.937122	1.046638	0.971328	0.940892	0.990684	0.986286	0.842941	1	-
ENSMUSC	Pgam2	phosphog	GO:00081	metabolic	K01834	2,3-bisph	mmu011C	Metabolic	11:580164	107	133	99	163	193	152	7.777641	7.720794	7.953253	9.140758	9.033929	8.691952	7.81723	8.955546	0.417109	1	-
ENSMUSC	Dbnl	drebrin-li	GO:00325	developm	-	-	-	-	11:578848	1305	1712	1297	2060	2243	1939	20.53629	21.51599	22.55789	25.00974	22.72979	24.00493	21.53672	23.91482	0.370097	1	-
ENSMUSC	Mrps24	mitochon	GO:00081	metabolic	-	-	-	-	11:570398	88	101	86	150	151	114	1.769221	1.621687	1.910922	2.326596	1.954927	1.80308	1.767277	2.028201	0.470649	1	-
ENSMUSC	Ccdc117	coiled-co	-	-	-	-	-	-	11:552888	246	305	228	373	372	322	4.454416	4.410641	4.562872	5.21068	4.337645	4.58693	4.475976	4.711751	0.825513	1	-
ENSMUSC	Dynll2	dynein lig	GO:00511	localizatio	K10418	dynein lig	mmu0496	Vasopress	11:879795	4547	5474	3708	5702	4439	5499	79.45942	76.39608	71.6155	76.87374	49.95285	75.59883	75.82367	67.47514	0.198006	1	-
ENSMUSC	Supt4a	suppressc	GO:00081	metabolic	-	-	-	-	11:877375	323	401	256	402	455	379	13.2717	13.15874	11.62551	12.74329	12.039	12.25108	12.68532	12.34446	0.65766	1	-
ENSMUSC	4-Sep	septin 4 [S	GO:00325	developm	K16943	septin 4[-	mmu0421	Apoptosis	11:875689	1435	2312	1696	2256	2641	2216	10.18581	13.10622	13.30505	12.35416	12.07166	12.37441	12.19903	12.26674	0.855604	1	-
ENSMUSC	Btnl10	butyrophi	GO:00160	membran	-	-	-	-	11:589179	0	0	2	0	1	0	0	0	0.018117	0	0.005277	0	0.006039	0.001759	1	1	-

ENSMUSC	2810021J	RIKEN cDI	GO:00081	metabolic	-	-	-	-	11:588672	199	265	202	253	363	260	3.059831	3.254134	3.432741	3.001199	3.594225	3.145046	3.248902	3.246823	0.856811	1	-
ENSMUSC	Smg8	smg-8 ho	GO:00081	metabolic	-	-	-	-	11:870777	254	364	265	380	470	406	3.66205	4.191189	4.222625	4.226729	4.363574	4.604971	4.025288	4.398424	0.580392	1	-
ENSMUSC	Rnf187	ring finge	GO:00081	metabolic	-	-	-	-	11:589322	4029	6400	4616	6781	8095	6420	105.0398	133.2546	133.0053	136.3894	135.9027	131.6747	123.7666	134.6556	0.501284	1	-
ENSMUSC	Mrpl22	mitochon	GO:00081	metabolic	K02890	large subu	mmu0301	Ribosome	11:581716	211	260	189	294	358	301	10.02589	9.866429	9.925409	10.77749	10.95414	11.25167	9.939242	10.99443	0.527398	1	-
ENSMUSC	Cnot8	CCR4-NC	GO:00081	metabolic	K12581	CCR4-NO	mmu0301	RNA degr	11:581041	849	1125	854	1260	1378	1243	18.42158	19.49475	20.47971	21.09211	19.2541	21.21784	19.46535	20.52135	0.76852	1	-
ENSMUSC	Rps6kb1	ribosomal	GO:00325	developm	K04688	ribosomal	mmu0520	Pathways	11:864988	1214	1499	1072	1442	1901	1544	6.845634	6.750603	6.680925	6.273224	6.902904	6.849414	6.759054	6.675181	0.694984	1	-
ENSMUSC	Sap30l	SAP30-lik	GO:00081	metabolic	-	-	-	-	11:578016	278	387	264	381	466	361	14.7473	16.39549	15.47813	15.59276	15.91873	15.0656	15.54031	15.52569	0.835512	1	-
ENSMUSC	Rnft1	ring finge	GO:00081	metabolic	-	-	-	-	11:864846	256	359	195	296	419	322	3.158391	3.537257	2.658935	2.817397	3.328849	3.125304	3.118194	3.090517	0.800279	1	-
ENSMUSC	Fam114a2	family witl	-	-	-	-	-	-	11:574829	643	830	634	884	1060	768	9.587397	9.883555	10.44782	10.16885	10.17771	9.008694	9.972922	9.785085	0.68052	1	-
ENSMUSC	Gria1	glutamate	GO:00048	signal trar	K05197	glutamate	mmu0408	Neuroacti	11:570113	24874	32586	23051	32579	39083	32176	126.7217	132.5813	129.7902	128.0482	128.2177	128.958	129.6977	128.4079	0.674277	1	-
ENSMUSC	Ppm1d	protein pł	GO:00081	metabolic	K10147	protein pł	mmu0411	p53 signa	11:853112	326	425	259	375	433	385	6.00994	6.257317	5.277143	5.333525	5.14038	5.583732	5.848133	5.352546	0.332165	1	-
ENSMUSC	Znhit3	zinc finge	GO:00056	cell|intrace	-	-	-	-	11:849109	167	240	246	340	375	315	3.167653	3.635633	5.157078	4.975442	4.58045	4.700502	3.986788	4.752131	0.285268	1	-
ENSMUSC	Prpsap2	phosphor	GO:00325	developm	-	-	-	-	11:617296	356	465	369	568	649	536	7.584326	7.911636	8.688411	9.335676	8.903619	8.983449	8.061458	9.074248	0.394847	1	-
ENSMUSC	Ggnbp2	gametoge	GO:00325	developm	-	-	-	-	11:848323	1133	1346	847	1246	1794	1397	8.823829	8.371786	7.290507	7.486444	8.99714	8.559227	8.16204	8.347604	0.989386	1	-
ENSMUSC	Acaca	acetyl-Co	GO:00081	metabolic	K11262	acetyl-Co	mmu0110	Metabolic	11:841296	1175	1364	1068	1490	1760	1628	3.539206	3.281166	3.555383	3.46246	3.413779	3.857744	3.458585	3.577994	0.923218	1	-
ENSMUSC	Shmt1	serine hyc	GO:00451	translatior	K00600	glycine hy	mmu0110	Metabolic	11:607881	54	66	81	82	121	98	0.577328	0.563535	0.957118	0.676357	0.833054	0.824268	0.699327	0.777893	0.65967	1	-
ENSMUSC	Llgl1	lethal giar	GO:00325	developm	K06094	lethal(2) g	mmu0516	Human pa	11:606997	870	1077	774	1260	1299	1212	8.401168	8.305805	8.260535	9.386872	8.07763	9.207329	8.322503	8.89061	0.672421	1	-
ENSMUSC	Drg2	developm	GO:00160	membran	-	-	-	-	11:604545	650	774	685	927	979	886	11.88256	11.30014	13.83995	13.07395	11.52483	12.74212	12.34088	12.44697	0.88516	1	-
ENSMUSC	Srebf1	sterol regı	GO:00325	developm	K07197	sterol regı	mmu0493	Non-alco	11:601990	622	813	578	810	1049	915	4.126713	4.307749	4.238271	4.145998	4.481712	4.775798	4.224244	4.467836	0.753803	1	-
ENSMUSC	Myocd	myocardiı	GO:00325	developm	-	-	-	-	11:651765	9	16	8	11	25	14	0.044683	0.063443	0.043896	0.042136	0.079928	0.054681	0.050674	0.058915	0.731139	1	-
ENSMUSC	Cox11	cytochron	GO:00081	metabolic	K02258	cytochron	mmu0110	Metabolic	11:906381	135	119	88	138	187	110	4.405572	3.101433	3.173941	3.474385	3.929749	2.824066	3.560315	3.4094	0.690976	1	-
ENSMUSC	Stxbp4	syntaxin b	GO:00511	localizatio	-	-	-	-	11:904764	502	689	455	664	762	602	2.379749	2.608511	2.383886	2.428431	2.326147	2.245096	2.457382	2.333225	0.471947	1	-
ENSMUSC	Bzw2	basic leuc	GO:00325	developm	-	-	-	-	12:360918	588	682	552	650	882	693	5.340204	4.946643	5.540723	4.554324	5.158262	4.951358	5.275857	4.887981	0.343204	1	-
ENSMUSC	1700086D	RIKEN cDI	-	-	-	-	-	-	11:651519	0	1	0	0	1	0	0	0.04374	0	0	0.035256	0	0.01458	0.011752	1	1	-
ENSMUSC	Elac2	elaC ribor	GO:00081	metabolic	K00784	ribonuclea	mmu0301	RNA trans	11:649790	576	655	495	695	830	655	5.246823	4.764983	4.9834	4.884154	4.868631	4.693822	4.998402	4.815536	0.558755	1	-
ENSMUSC	Pctp	phosphati	GO:00082	lipid bindi	-	-	-	-	11:899826	44	34	18	46	50	39	0.986607	0.608867	0.446088	0.795767	0.721979	0.687965	0.680521	0.735237	0.844039	1	-
ENSMUSC	Twistnb	twist basic	GO:00081	metabolic	K03004	DNA-dire	mmu0110	Metabolic	12:334296	214	292	193	284	348	275	2.347967	2.558628	2.340359	2.403962	2.458734	2.373674	2.415651	2.412123	0.839434	1	-
ENSMUSC	Efcab10	EF-hand (	GO:00431	ion bindin	-	-	-	-	12:333948	16	9	9	23	20	22	1.451599	0.65213	0.9024	1.609862	1.168445	1.570245	1.002043	1.449517	0.233052	1	-
ENSMUSC	Atxn7l1	ataxin 7-l	-	-	-	-	-	-	12:331476	597	709	484	673	951	762	2.442162	2.316286	2.188225	2.123955	2.505159	2.452257	2.315557	2.360457	0.966906	1	-
ENSMUSC	Atp6v1c2	ATPase, H	GO:00511	localizatio	K02148	V-type H-	mmu0110	Metabolic	12:172847	5	13	11	14	16	3	0.082104	0.170499	0.199647	0.177374	0.169199	0.038753	0.15075	0.128442	0.731686	1	-
ENSMUSC	Sypl	synaptopł	GO:00452	synapse|e	-	-	-	-	12:329538	307	350	594	436	550	780	1.696671	1.544805	3.628216	1.858988	1.957388	3.391291	2.289897	2.402556	0.917727	1	-
ENSMUSC	Pdia6	protein di	GO:00081	metabolic	K09584	protein di	mmu0414	Protein pr	12:172665	1376	1973	1210	2435	2306	1958	18.79131	21.51848	18.26295	25.65476	20.27929	21.03596	19.52424	22.32333	0.266686	1	-
ENSMUSC	Nampt	nicotinam	GO:00081	metabolic	K03462	nicotinam	mmu0110	Metabolic	12:328195	1428	1764	1329	1857	2351	1850	16.42324	16.20224	16.89283	16.47679	17.41157	16.73837	16.5061	16.87558	0.986282	1	-
ENSMUSC	Nbas	neuroblas	GO:00081	metabolic	-	-	-	-	12:132691	763	1053	723	1010	1156	1116	4.380158	4.82769	4.587226	4.473183	4.273439	5.040112	4.598358	4.595578	0.807472	1	-
ENSMUSC	Tspan13	tetraspani	GO:00511	localizatio	-	-	-	-	12:360145	2869	3472	2581	3737	4532	3668	62.20729	60.12239	61.85083	62.51211	63.27836	62.5678	61.3935	62.78609	0.985333	1	-
ENSMUSC	Rock2	Rho-asso	GO:00325	developm	K17388	Rho-asso	mmu0520	Pathways	12:168948	7781	9586	6455	9333	11491	9625	32.49389	31.97051	29.79266	30.06892	30.90144	31.62117	31.41902	30.86385	0.615165	1	-
ENSMUSC	Matn3	matrilin 3	GO:00055	extracellul	-	-	-	-	12:894792	0	2	0	0	0	0	0	0.032362	0	0	0	0	0.010787	0	0.524526	1	-
ENSMUSC	Laptm4a	lysosomal	GO:00160	membran	K12387	lysosomal	mmu0414	Lysosome	12:892166	2047	2807	1897	2649	3333	2511	70.49404	77.20099	72.20191	70.37961	73.91363	68.0287	73.29898	70.77398	0.504634	1	-
ENSMUSC	Fam49a	family witl	GO:00056	cell|intrace	-	-	-	-	12:122621	6845	8819	5929	8593	10437	8397	29.52407	30.37862	28.26383	28.5942	28.98899	28.49298	29.38884	28.69206	0.5607	1	-
ENSMUSC	Snx13	sorting ne	GO:00511	localizatio	-	-	-	-	12:350471	1875	2350	1664	2236	2735	2206	11.61802	11.62906	11.39543	10.6889	10.91297	10.75344	11.5475	10.7851	0.285663	1	-
ENSMUSC	Ntsr2	neurotens	GO:00048	signal trar	K04212	neurotens	mmu0408	Neuroacti	12:166533	943	1032	1142	1172	1498	1373	8.301297	7.255376	11.11085	7.959616	8.491819	9.508582	8.889175	8.653339	0.666628	1	-
ENSMUSC	Lpin1	lipin 1 [So	GO:00325	developm	K15728	phosphati	mmu0110	Metabolic	12:165356	402	503	329	473	575	440	1.548994	1.547881	1.401092	1.406097	1.426744	1.333788	1.499323	1.388876	0.369762	1	-
ENSMUSC	Rgs9	regulator	GO:00325	developm	K13765	regulator	mmu0503	Cocaine a	11:109225	257	305	217	334	381	291	2.121394	2.010637	1.979674	2.126987	2.025195	1.889692	2.037235	2.013958	0.774685	1	-
ENSMUSC	Trib2	tribbles ps	GO:00325	developm	-	-	-	-	12:157917	725	1083	656	999	1018	836	7.029873	8.386561	7.030086	7.473183	6.356414	6.377151	7.482173	6.735582	0.220763	1	-
ENSMUSC	Hs1bp3	HCLS1 bir	GO:00325	developm	-	-	-	-	12:831343	215	368	216	297	356	319	3.981654	5.442742	4.421038	4.243376	4.245501	4.647572	4.615145	4.378816	0.549923	1	-
ENSMUSC	Fam84a	family witl	-	-	-	-	-	-	12:141468	2019	2491	1961	2368	3227	2653	27.18331	26.78463	29.18035	24.59677	27.9782	28.10051	27.7161	26.89183	0.547562	1	-
ENSMUSC	Smc6	structural	GO:00325	developm	-	-	-	-	12:112658	1099	1416	915	1304	1732	1327	3.939349	4.053554	3.624888	3.606073	3.997874	3.742042	3.872597	3.781996	0.619708	1	-
ENSMUSC	Amz2	archaelysi	GO:00081	metabolic	-	-	-	-	11:109425	693	875	669	1067	1164	999	5.251739	5.295709	5.603291	6.238275	5.680385	5.955888	5.383579	5.958183	0.441565	1	-
ENSMUSC	Gna13	guanine n	GO:00325	developm	K04639	guanine n	mmu0520	Pathways	11:109362	911	1118	802	1119	1440	1185	8.662816	8.490405	8.428728	8.209202	8.817752	8.864817	8.527317	8.63059	0.902623	1	-
ENSMUSC	Prkar1a	protein kiı	GO:00325	developm	K04739	cAMP-de	mmu0491	Insulin sig	11:109649	12399	14839	10101	14326	19096	15714	166.0947	158.7521	149.5479	148.0554	164.7275	165.6024	158.1315	159.4618	0.854854	1	-
ENSMUSC	Fam20a	family witl	GO:00325	developm	-	-	-	-	11:109669	200	242	197	310	332	238	2.12857	2.056928	2.317247	2.545368	2.275366	1.992723	2.167582	2.271152	0.858866	1	-
ENSMUSC	Abca8b	ATP-bind	GO:00511	localizatio	K05650	ATP-bind	mmu0201	ABC trans	11:109932	741	841	644	900	1105	863	5.731447	5.195032	5.505271	5.370553	5.503806	5.251313	5.47725	5.375224	0.671671	1	-
ENSMUSC	Rdh14	retinol del	GO:00325	developm	-	-	-	-	12:103907	325	442	274	422	495	353	13.40804	14.56296	12.49333	13.4315	13.15046	11.45689	13.48811	12.67962	0.470434	1	-
ENSMUSC	Map2k6	mitogen-;	GO:00048	signal trar	K04433	mitogen-;	mmu0401	MAPK sigı	11:110399	288	409	283	380	469	437	4.761638	5.400489	5.171274	4.847061	4.993345	5.684031	5.111134	5.174812	0.924104	1	-
ENSMUSC	Klhl29	kelch-like	GO:00081	metabolic	-	-	-	-	12:507747	1122	1471	1124	1470	1729	1485	9.194365	9.626917	10.17986	9.293439	9.123857	9.573399	9.667048	9.330232	0.523047	1	-
ENSMUSC	Trappc12	trafficking	GO:00081	metabolic	-	-	-	-	12:286906	858	1272	1032	1193	1654	1181	4.337101	5.135056	5.765525	4.652464	5.383969	4.696487	5.079227	4.910973	0.594162	1	-
ENSMUSC	Adi1	acireducta	GO:00081	metabolic	K08967	1,2-dihyd	mmu0110	Metabolic	12:286752	188	270	216	307	372	357	3.925761	4.502725	4.985008	4.945772	5.002236	5.864698	4.471164	5.270902	0.269982	1	-
ENSMUSC	Rnaseh1	ribonuclea	GO:00081	metabolic	K03469	ribonuclea	mmu0303	DNA repli	12:286496	123	167	128	169	188	147	1.785154	1.935681	2.053178	1.89229	1.75705	1.678409	1.924671	1.775916	0.46219	1	-
ENSMUSC	Ubxn2a	UBX dom:	GO:00081	metabolic	-	-	-	-	12:487903	751	894	679	964	1057	943	9.723203	9.243854	9.71597	9.628901	8.812501	9.60488	9.561009	9.348761	0.636878	1	-
ENSMUSC	Fkbp1b	FK506 bin	GO:00325	developm	-	-	-	-	12:483317	422	508	381	534	641	558	24.28514	23.34734	24.23257	23.70827	23.7542	25.26229	23.95502	24.24159	0.914166	1	-
ENSMUSC	Allc	allantoica:	GO:00081	metabolic	K01477	allantoica:	mmu0110	Metabolic	12:285537	0	2	3	0	1	1	0	0.059259	0.123002	0	0.023882	0.029188	0.060754	0.01769	0.341518	1	-
ENSMUSC	Cmpk2	cytidine m	GO:00081	metabolic	K13809	UMP-CM	mmu0110	Metabolic	12:264692	307	347	264	453	516	416	3.746717	3.382109	3.56093	4.265223	4.055247	3.994083	3.563252	4.104851	0.306591	1	-
ENSMUSC	Pfn4	profilin fa	GO:00511	localizatio	K05759	profilin[-]	mmu0481	Regulatioı	12:476902	132	79	129	168	230	150	1.109992	0.530542	1.198899	1.089898	1.245461	0.992315	0.946478	1.109225	0.493629	1	-
ENSMUSC	Itsn2	intersectir	GO:00325	developm	-	-	-	-	12:459263	1962	2420	1614	2403	3033	2413	6.535161	6.437518	5.941658	6.175063	6.505554	6.323031	6.304779	6.334549	0.827369	1	-
ENSMUSC	Rnf144a	ring finge	GO:00081	metabolic	-	-	-	-	12:263009	363	188	355	520	576	505	1.789158	0.740026	1.933826	1.977316	1.828183	1.958144	1.48767	1.921215	0.235541	1	-
ENSMUSC	Id2	inhibitor c	GO:00325	developm	K17693	DNA-binc	mmu0520	Transcript	12:250937	714	999	711	989	1315	1046	10.54815	11.78662	11.60899	11.27209	12.51003	12.15683	11.31459	11.97965	0.74174	1	-
ENSMUSC	Ncoa1	nuclear re	GO:00325	developm	K09101	nuclear re	mmu0520	Pathways	12:424736	3778	4306	3236	5075	5419	4653	19.39608	17.65517	18.36146	20.10103	17.91537	18.79299	18.4709	18.93646	0.997503	1	-
ENSMUSC	Rrm2	ribonucle(	GO:00081	metabolic	K10808	ribonucle(	mmu0110	Metabolic	12:247082	19	36	32	39	64	38	0.311938	0.472031	0.580652	0.493987	0.676636	0.490813	0.454874	0.553812	0.426421	1	-
ENSMUSC	Bcap29	B cell rece	GO:00325	developm	-	-	-	-	12:315909	542	684	397	664	807	608	3.687299	3.716298	2.985012	3.485036	3.535387	3.254045	3.46287	3.424823	0.754497	1	-
ENSMUSC	Slc26a4	solute car	GO:00325	developm	K14702	solute car	mmu0491	Thyroid ho	12:315198	188	266	216	283	360	288	3.69216	4.17206	4.688376	4.287853	4.552813	4.449652	4.184199	4.430106	0.789397	1	-
ENSMUSC	Cenpo	centrome	GO:00329	macromo	-	-	-	-	12:419600	41	66	44	65	80	91	0.244469	0.314292	0.289963	0.29901	0.307177	0.426869	0.282908	0.344352	0.370198	1	-
ENSMUSC	Klf11	Kruppel-li	GO:00081	metabolic	-	-	-	-	12:246512	65	80	48	96	132	71	0.785897	0.772482	0.64142	0.895475	1.027735	0.675336	0.733266	0.866182	0.419988	1	-
ENSMUSC	Adcy3	adenylate	GO:00081	metabolic	K08043	adenylate	mmu0474	Olfactory	12:413310	1016	1248	968	1360	1367	1337	6.155488	6.038505	6.481731	6.356796	5.333251	6.372522	6.225241	6.020857	0.554079	1	-
ENSMUSC	Grhl1	grainyhea	GO:00325	developm	-	-	-	-	12:245722	694	923	679	928	1205	955	5.793593	6.153693	6.264768	5.97677	6.477837	6.27195	6.070684	6.242186	0.968465	1	-
ENSMUSC	Dnajc27	DnaJ heat	GO:00081	metabolic	-	-	-	-	12:408258	2309	2846	2057	2762	3344	3050	18.59936	18.30859	18.31282	17.16438	17.34583	19.32791	18.40692	17.94604	0.56977	1	-
ENSMUSC	Efr3b	EFR3 hor	GO:00081	metabolic	-	-	-	-	12:396255	1082	4190	3234	4465	5139	4742	4.30811	13.32353	14.23136	13.71549	13.17628	14.85361	10.621	13.91513	0.229434	1	-
ENSMUSC	Pomc	pro-opior	GO:00081	metabolic	K05228	proopiom	mmu0492	Aldostero	12:395495	12	24	21	23	21	14	0.233263	0.372585	0.45118	0.344934	0.26287	0.214095	0.352343	0.273966	0.351358	1	-
ENSMUSC	Dnmt3a	DNA metl	GO:00325	developm	K17398	DNA (cytc	mmu0110	Metabolic	12:380600	501	1588	1242	1974	2134	777	1.046996	2.650356	2.868641	3.182624	2.871817	1.277438	2.188664	2.443959	0.699023	1	-
ENSMUSC	Dld	dihydrolip	GO:00325	developm	K00382	dihydrolip	mmu0110	Metabolic	12:313312	2029	2571	1815	2588	3111	2508	29.48284	29.83563	29.14813	29.01228	29.10998	28.66987	29.48887	28.93071	0.617285	1	-
ENSMUSC	Sh3yl1	Sh3 doma	GO:00081	metabolic	-	-	-	-	12:309116	201	197	155	242	259	233	2.860765	2.239225	2.438166	2.657243	2.373783	2.608877	2.512718	2.546634	0.943022	1	-
ENSMUSC	Rab10	RAB10, m	GO:00325	developm	K07903	Ras-relate	mmu0414	Endocytos	12:324742	3887	5098	3429	4864	6208	4677	67.55847	70.76375	65.86875	65.22124	69.48188	63.95043	68.06366	66.21785	0.544809	1	-
ENSMUSC	Sntg2	syntrophir	GO:00051	structural	-	-	-	-	12:301744	25	27	34	32	32	29	0.253056	0.218269	0.380375	0.2499	0.208588	0.230933	0.2839	0.229807	0.319905	1	-
ENSMUSC	Pxdn	peroxidas	GO:00081	metabolic	-	-	-	-	12:299376	1744	1591	1502	2104	2358	2019	5.865023	4.273062	5.582643	5.458826	5.106481	5.341588	5.240242	5.302298	0.898548	1	-
ENSMUSC	Ddx52	DEAD (As	GO:00081	metabolic	-	-	-	-	11:839420	345	470	324	500	649	506	6.034109	6.565039	6.263041	6.746734	7.309588	6.962342	6.287397	7.006221	0.446951	1	-
ENSMUSC	Taf15	TATA-bo›	GO:00081	metabolic	K14651	transcripti	mmu0520	Transcript	11:834730	1074	1483	1059	1505	1926	1376	17.5104	19.30983	19.08245	18.93034	20.22099	17.64903	18.63423	18.93345	0.941215	1	-
ENSMUSC	Gas2l2	growth ar	GO:00990	supramol(	-	-	-	-	11:834219	4	8	4	11	17	4	0.087075	0.139078	0.096238	0.184737	0.2383	0.068495	0.107464	0.163844	0.357816	1	-
ENSMUSC	Cdc27	cell divisic	GO:00081	metabolic	K03350	anaphase	mmu0516	HTLV-I inf	11:104502	1460	1755	1317	1712	2304	1566	11.62253	11.1576	11.58726	10.51434	11.81096	9.807325	11.4558	10.71087	0.338482	1	-
ENSMUSC	Itgb3	integrin b	GO:00325	developm	K06493	integrin b	mmu0415	PI3K-Akt s	11:104608	62	84	46	63	96	83	0.438403	0.474356	0.35949	0.343676	0.437125	0.461712	0.424083	0.414171	0.780459	1	-
ENSMUSC	Mettl2	methyltraı	GO:00081	metabolic	-	-	-	-	11:105126	198	255	169	255	275	255	2.988754	3.074045	2.819418	2.969577	2.673081	3.028135	2.960739	2.890264	0.698761	1	-
ENSMUSC	Nle1	notchless	GO:00325	developm	-	-	-	-	11:829007	113	127	100	156	179	127	1.33894	1.201804	1.309571	1.426062	1.365814	1.18385	1.283438	1.325242	0.971784	1	-
ENSMUSC	Tlk2	tousled-li	GO:00325	developm	-	-	-	-	11:105178	1117	1355	843	1353	1565	1119	5.648657	5.472394	4.711581	5.27862	5.096372	4.451774	5.277544	4.942255	0.379539	1	-
ENSMUSC	Mrc2	mannose	GO:00325	developm	K06560	mannose	mmu0515	Tuberculo	11:105292	113	175	91	148	145	160	0.892569	1.103949	0.794425	0.901895	0.737544	0.994249	0.930314	0.877896	0.588336	1	-
ENSMUSC	Rffl	ring finge	GO:00081	metabolic	-	-	-	-	11:828024	116	212	129	167	182	179	0.748624	1.092668	0.920115	0.831481	0.756365	0.908803	0.920469	0.832216	0.398589	1	-
ENSMUSC	Cct6b	chaperoni	GO:00511	localizatio	-	-	-	-	11:827192	4	0	1	0	0	3	0.117032	0	0.032337	0	0	0.069039	0.04979	0.023013	0.591039	1	-
ENSMUSC	Map3k3	mitogen-;	GO:00325	developm	K04421	mitogen-;	mmu0401	MAPK sigı	11:106084	473	683	471	653	799	750	6.186907	7.134759	6.80895	6.589558	6.729986	7.717642	6.710205	7.012396	0.856394	1	-
ENSMUSC	Tmem132	transmem	GO:00160	membran	-	-	-	-	11:823889	127	157	151	197	192	201	0.933027	0.92116	1.226068	1.11657	0.908337	1.161709	1.026752	1.062205	0.957107	1	-
ENSMUSC	Asic2	acid-sens	GO:00325	developm	K04828	acid-sens	mmu0475	Inflammat	11:808801	1202	1586	1221	1733	1959	1339	17.21197	18.13741	19.32362	19.14498	18.06405	15.08403	18.22433	17.43102	0.498785	1	-
ENSMUSC	Ddx42	DEAD (As	GO:00081	metabolic	K12835	ATP-depe	mmu0304	Spliceosoı	11:106216	2350	2951	2117	2952	3692	3067	32.20076	32.29336	32.06016	31.20648	32.57726	33.06152	32.18476	32.28175	0.807079	1	-
ENSMUSC	Ftsj3	FtsJ RNA ı	GO:00081	metabolic	-	-	-	-	11:106249	603	822	573	842	1011	865	6.399799	6.967338	6.721254	6.89432	6.909628	7.22231	6.69613	7.008753	0.829802	1	-
ENSMUSC	Rnf135	ring finge	GO:00081	metabolic	-	-	-	-	11:801838	33	57	52	68	70	59	0.927257	1.279091	1.614841	1.474091	1.266595	1.304218	1.27373	1.348301	0.873161	1	-
ENSMUSC	Psmc5	protease (	GO:00081	metabolic	K03066	26S prote	mmu0516	Epstein-B	11:106256	2212	3050	2174	3271	3860	3244	84.20231	92.72239	91.46299	96.06161	94.61958	97.14731	89.46257	95.94283	0.593351	1	-
ENSMUSC	Adap2	ArfGAP w	GO:00325	developm	-	-	-	-	11:801541	93	128	85	131	144	116	2.726062	2.996479	2.753713	2.962476	2.718129	2.674985	2.825418	2.785197	0.807324	1	-
ENSMUSC	Ern1	endoplasr	GO:00081	metabolic	K08852	serine/thr(	mmu0501	Alzheimer	11:106394	248	359	237	339	490	420	1.423959	1.646218	1.50398	1.501677	1.811745	1.897172	1.524719	1.736865	0.387064	1	-
ENSMUSC	Nf1	neurofibrc	GO:00325	developm	K08052	neurofibrc	mmu0401	MAPK sigı	11:793396	2819	3638	2538	3419	3953	3474	8.587248	8.850493	8.544711	8.035046	7.754261	8.325296	8.660817	8.038201	0.249665	1	-
ENSMUSC	Pecam1	platelet/eı	GO:00325	developm	K06471	platelet/eı	mmu0451	Cell adhes	11:106654	387	506	340	470	565	556	2.634576	2.75103	2.558145	2.468466	2.476865	2.977729	2.647917	2.64102	0.814153	1	-
ENSMUSC	Polg2	polymera:	GO:00325	developm	K02333	DNA poly	mmu0110	Metabolic	11:106768	33	50	27	51	48	25	0.322389	0.3901	0.291522	0.384382	0.301966	0.192137	0.33467	0.292828	0.500998	1	-
ENSMUSC	Ddx5	DEAD (As	GO:00325	developm	K12823	ATP-depe	mmu0520	Proteogly(	11:106780	16507	21698	14741	21577	26508	21612	106.1045	111.3861	104.7223	107.0008	109.7229	109.2877	107.4043	108.6704	0.882645	1	-
ENSMUSC	Psmd12	proteasor	GO:00081	metabolic	K03035	26S prote	mmu0516	Epstein-B	11:107479	1083	1464	1069	1429	1850	1585	21.16708	22.85173	23.09176	21.54739	23.28406	24.37096	22.37019	23.06747	0.943842	1	-
ENSMUSC	Cacng1	calcium cl	GO:00511	localizatio	K04866	voltage-d	mmu0401	MAPK sigı	11:107703	0	0	0	0	2	0	0	0	0	0	0.062947	0	0	0.020982	0.528787	1	-
ENSMUSC	Cacng4	calcium cl	GO:00511	localizatio	K04869	voltage-d	mmu0401	MAPK sigı	11:107732	408	601	357	528	634	628	6.588655	7.750986	6.37165	6.578101	6.592965	7.978245	6.903764	7.04977	0.978659	1	-
ENSMUSC	Cep112	centroson	GO:00452	synapse|c	-	-	-	-	11:108425	80	105	65	90	116	83	0.544491	0.570739	0.488951	0.472581	0.508409	0.444419	0.534727	0.475136	0.355592	1	-
ENSMUSC	Rab37	RAB37, m	GO:00038	catalytic a	-	-	-	-	11:115091	52	91	34	60	78	50	0.562848	0.786635	0.40674	0.501037	0.543668	0.425761	0.585408	0.490155	0.321695	1	-
ENSMUSC	Grin2c	glutamate	GO:00048	signal trar	K05211	glutamate	mmu0408	Neuroacti	11:115249	632	897	629	1037	1099	953	7.079201	8.024267	7.786896	8.961412	7.927195	8.397909	7.630121	8.428839	0.453033	1	-
ENSMUSC	Nt5c	5',3'-nucle	GO:00081	metabolic	K01081	5'-nucleot	mmu0110	Metabolic	11:115490	256	305	291	349	424	388	5.552709	5.283368	6.975966	5.84011	5.922226	6.620747	5.937348	6.127695	0.955926	1	-
ENSMUSC	Jpt1	Jupiter mi	GO:00160	membran	-	-	-	-	11:115497	548	710	529	748	984	692	10.66619	11.03655	11.37971	11.2321	12.33328	10.59609	11.02748	11.38716	0.935359	1	-
ENSMUSC	Sumo2	small ubic	GO:00081	metabolic	K12160	small ubic	mmu0301	RNA trans	11:115523	297	332	65	418	157	205	7.386655	6.594413	1.786713	8.020437	2.514466	4.011034	5.255927	4.848646	0.73022	1	-
ENSMUSC	Nup85	nucleopoı	GO:00081	metabolic	K14304	nuclear pc	mmu0301	RNA trans	11:115564	299	449	302	415	525	458	3.714423	4.454634	4.146429	3.977393	4.199852	4.476064	4.105162	4.21777	0.983911	1	-
ENSMUSC	Gga3	golgi assc	GO:00081	metabolic	K12404	ADP-ribo:	mmu0414	Lysosome	11:115584	654	1189	906	936	1560	1319	3.81759	5.54293	5.845032	4.215196	5.863958	6.057135	5.068517	5.378763	0.781133	1	-
ENSMUSC	Cluh	clustered	GO:00511	localizatio	-	-	-	-	11:746494	1899	2411	1716	2543	3026	2581	13.23019	13.41479	13.21311	13.6684	13.57578	14.1462	13.28603	13.79679	0.875163	1	-
ENSMUSC	Slc25a19	solute car	GO:00511	localizatio	-	-	-	-	11:115614	428	597	334	596	742	527	3.09778	3.450859	2.671773	3.327999	3.458323	3.000737	3.073471	3.262353	0.760508	1	-
ENSMUSC	Tmem94	transmem	GO:00160	membran	-	-	-	-	11:115765	1028	1144	935	1356	1377	1378	8.287266	7.365292	8.330607	8.433502	7.148366	8.739332	7.994388	8.107067	0.914767	1	-
ENSMUSC	Recql5	RecQ pro	GO:00081	metabolic	-	-	-	-	11:115892	418	540	447	627	622	512	2.780812	2.869027	3.286623	3.218052	2.66465	2.679636	2.978821	2.854113	0.566984	1	-
ENSMUSC	Sap30bp	SAP30 bir	GO:00081	metabolic	-	-	-	-	11:115933	797	968	771	1113	1340	1116	8.730411	8.468322	9.334213	9.405931	9.452266	9.617264	8.844315	9.49182	0.635183	1	-
ENSMUSC	Galk1	galactokir	GO:00081	metabolic	K00849	galactokir	mmu0110	Metabolic	11:116008	125	119	85	109	144	129	5.428334	4.127156	4.079647	3.651858	4.026929	4.407162	4.545046	4.02865	0.359923	1	-
ENSMUSC	Unk	unkempt	GO:00325	developm	-	-	-	-	11:116030	385	492	386	513	577	503	4.014921	4.097584	4.448881	4.127286	3.874781	4.126627	4.187128	4.042898	0.594256	1	-
ENSMUSC	Trim47	tripartite r	GO:00056	cell|intrace	-	-	-	-	11:116105	36	43	27	26	37	50	0.40273	0.384166	0.333824	0.224391	0.266544	0.440036	0.373573	0.310324	0.363344	1	-
ENSMUSC	Aspa	aspartoac	GO:00325	developm	K01437	aspartoac	mmu0110	Metabolic	11:733049	123	146	122	126	237	219	1.081063	1.024814	1.185096	0.854372	1.341371	1.514259	1.096991	1.236668	0.575273	1	-
ENSMUSC	Mrpl38	mitochon	GO:00329	macromo	-	-	-	-	11:116131	481	622	507	751	809	862	7.123205	7.356417	8.298223	8.580253	7.714954	10.04264	7.592615	8.779284	0.281526	1	-
ENSMUSC	Fbf1	Fas (TNFR	GO:00990	supramol(	-	-	-	-	11:116142	1483	1775	1297	2056	2133	1908	6.550141	6.261145	6.331351	7.005887	6.066736	6.629778	6.380879	6.567467	0.963746	1	-
ENSMUSC	Acox1	acyl-Coer	GO:00081	metabolic	K00232	acyl-CoA	mmu0110	Metabolic	11:116171	3893	4748	3500	5065	6251	5138	37.94082	36.95548	37.69964	38.08308	39.23073	39.39374	37.53198	38.90252	0.887883	1	-
ENSMUSC	Ten1	TEN1 telo	GO:00081	metabolic	-	-	-	-	11:116198	134	163	138	194	205	191	2.30994	2.244023	2.629189	2.580046	2.275636	2.590237	2.394384	2.481973	0.94186	1	-
ENSMUSC	Srp68	signal rec(	GO:00511	localizatio	K03107	signal rec(	mmu0306	Protein ex	11:116245	1069	1437	1056	1588	1808	1464	21.71361	23.31078	23.70636	24.88486	23.64872	23.3941	22.91025	23.97589	0.816817	1	-
ENSMUSC	Tsen54	tRNA splic	GO:00081	metabolic	-	-	-	-	11:115814	59	53	84	97	124	91	0.786903	0.564533	1.238204	0.998091	1.064985	0.954813	0.863213	1.005963	0.518586	1	-
ENSMUSC	Ncbp3	nuclear ca	GO:00081	metabolic	-	-	-	-	11:730477	539	679	550	703	797	695	1.838361	1.849513	2.073249	1.849812	1.75047	1.864823	1.920374	1.821702	0.46506	1	-
ENSMUSC	Camkk1	calcium/ca	GO:00081	metabolic	K07359	calcium/ca	mmu0503	Alcoholisr	11:730190	1494	1784	1567	2013	2168	2596	15.48741	14.7696	17.95328	16.09912	14.47245	21.17111	16.0701	17.24756	0.679321	1	-
ENSMUSC	P2rx1	purinergic	GO:00048	signal trar	K05215	P2X purin	mmu0408	Neuroacti	11:729991	3	6	3	7	7	11	0.044599	0.071238	0.049296	0.080287	0.06701	0.128655	0.055044	0.091984	0.321121	1	-
ENSMUSC	Ankfy1	ankyrin re	GO:00511	localizatio	-	-	-	-	11:726900	708	827	591	769	984	907	2.739665	2.555732	2.527541	2.295729	2.451962	2.761101	2.607646	2.502931	0.528507	1	-
ENSMUSC	Exoc7	exocyst cc	GO:00511	localizatio	K07195	exocyst cc	mmu0491	Insulin sig	11:116288	987	1216	881	1246	1515	1185	11.2245	11.04408	11.07318	10.93196	11.09475	10.60179	11.11392	10.87617	0.62853	1	-
ENSMUSC	Galr2	galanin re	GO:00325	developm	K04231	galanin re	mmu0408	Neuroacti	11:116280	0	0	1	1	0	0	0	0	0.018893	0.013189	0	0	0.006298	0.004396	1	1	-
ENSMUSC	Ube2q1	ubiquitin-	GO:00081	metabolic	K10575	ubiquitin-	mmu0414	Protein pr	11:726072	644	790	736	1036	1301	1026	7.832949	7.673833	9.893824	9.721423	10.18994	9.817429	8.466869	9.909598	0.222026	1	-
ENSMUSC	Tekt1	tektin 1 [S	GO:00400	locomotic	-	-	-	-	11:723447	37	70	42	69	56	85	1.211354	1.830231	1.519704	1.742794	1.180616	2.189233	1.52043	1.704214	0.695844	1	-
ENSMUSC	Med31	mediator	GO:00325	developm	-	-	-	-	11:722117	98	130	87	139	171	136	3.116515	3.301644	3.05779	3.410254	3.501811	3.402463	3.15865	3.438176	0.686467	1	-
ENSMUSC	Ube2o	ubiquitin-	GO:00081	metabolic	K10581	ubiquitin-	mmu0412	Ubiquitin	11:116537	1887	3247	2539	3528	3809	3468	19.98901	27.46929	29.72548	28.83223	25.98275	28.90077	25.72793	27.90525	0.587216	1	-
ENSMUSC	Txndc17	thioredox	GO:00081	metabolic	-	-	-	-	11:722075	251	312	225	367	452	354	9.824111	9.75258	9.733041	11.08191	11.3923	10.90016	9.76991	11.12479	0.362545	1	-
ENSMUSC	Aanat	arylalkylar	GO:00081	metabolic	K00669	arylalkylar	mmu0110	Metabolic	11:116586	8	5	1	4	8	8	0.144867	0.072302	0.020014	0.055872	0.093286	0.113955	0.079061	0.087704	1	1	-
ENSMUSC	Slc13a5	solute car	GO:00511	localizatio	-	-	-	-	11:722419	120	130	85	105	171	161	0.906692	0.784452	0.709811	0.612067	0.832011	0.957009	0.800318	0.800362	0.896444	1	-
ENSMUSC	4933427D	RIKEN cDI	GO:00511	localizatio	-	-	-	-	11:721539	539	594	462	784	747	754	3.325957	2.92725	3.15077	3.732278	2.968268	3.660241	3.134659	3.453596	0.5215	1	-
ENSMUSC	Pimreg	PICALM ir	GO:00099	cellular pr	-	-	-	-	11:720420	4	8	2	10	9	0	0.067156	0.107262	0.037111	0.129523	0.097298	0	0.07051	0.075607	1	1	-
ENSMUSC	Cygb	cytoglobiı	GO:00081	metabolic	-	-	-	-	11:116645	1060	1381	980	1533	1746	1375	27.76559	28.88954	28.37097	30.97943	29.45099	28.33443	28.34203	29.58828	0.838506	1	-
ENSMUSC	Wscd1	WSC dom	GO:00038	catalytic a	-	-	-	-	11:717499	1900	2359	2032	2631	3119	2524	20.53645	20.36318	24.27405	21.93931	21.7091	21.46209	21.72456	21.7035	0.787302	1	-
ENSMUSC	1810032C	RIKEN cDI	-	-	-	-	-	-	11:116671	31	29	9	24	26	22	0.39196	0.292844	0.125764	0.234117	0.211693	0.218839	0.270189	0.22155	0.428712	1	-
ENSMUSC	Mxra7	matrix-reı	GO:00055	extracellul	-	-	-	-	11:116803	254	249	195	266	326	266	5.023901	3.933248	4.262738	4.058997	4.15221	4.139033	4.406629	4.116746	0.473897	1	-
ENSMUSC	Mfsd11	major faci	GO:00160	membran	-	-	-	-	11:116852	424	548	293	576	526	546	1.866385	1.926469	1.425443	1.956088	1.490994	1.890773	1.739432	1.779285	0.986549	1	-
ENSMUSC	Kif1c	kinesin fa	GO:00511	localizatio	-	-	-	-	11:707005	2537	3378	2436	3323	4341	3437	12.41918	13.2062	13.17943	12.54969	13.68412	13.23619	12.93494	13.15667	0.939817	1	-
ENSMUSC	Sec14l1	SEC14-lik	GO:00511	localizatio	-	-	-	-	11:117115	4636	5119	4504	5950	7405	5660	31.4972	27.77535	33.82002	31.18716	32.39727	30.25213	31.03086	31.27885	0.855708	1	-
ENSMUSC	Mink1	misshapeı	GO:00325	developm	-	-	-	-	11:705628	5834	7602	6016	8091	9194	8229	31.71412	33.0035	36.14439	33.93273	32.1844	35.19202	33.62067	33.76972	0.816733	1	-
ENSMUSC	Pld2	phospholi	GO:00081	metabolic	K01115	phospholi	mmu0110	Metabolic	11:705400	333	405	279	464	473	390	2.600043	2.525444	2.407617	2.795019	2.378223	2.395587	2.511035	2.522943	0.870747	1	-
ENSMUSC	Slc46a1	solute car	GO:00511	localizatio	K14613	MFS trans	mmu0497	Mineral ał	11:784656	120	156	110	170	180	173	1.696059	1.760877	1.718308	1.853698	1.638275	1.923604	1.725081	1.805192	0.896051	1	-
ENSMUSC	0610010K	RIKEN cDI	GO:00081	metabolic	-	-	-	-	11:702352	96	102	101	106	138	112	1.306924	1.108993	1.519667	1.113316	1.209802	1.199524	1.311861	1.174214	0.397749	1	-
ENSMUSC	Eral1	Era (G-prı	GO:00099	cellular pr	-	-	-	-	11:780733	192	245	200	338	340	284	4.32749	4.41008	4.982074	5.877344	4.934785	5.035748	4.573215	5.282626	0.340635	1	-
ENSMUSC	Dhrs13	dehydrog	GO:00081	metabolic	-	-	-	-	11:780322	60	43	39	64	68	65	0.82214	0.470552	0.590617	0.67656	0.600011	0.700681	0.62777	0.659084	0.926203	1	-
ENSMUSC	Coro6	coronin 6	GO:00099	cellular pr	-	-	-	-	11:774624	342	319	153	248	420	493	4.911064	3.658365	2.428228	2.74746	3.883778	5.569393	3.665886	4.066877	0.693078	1	-
ENSMUSC	Slc6a4	solute car	GO:00325	developm	K05037	solute car	mmu0472	Serotoner	11:769986	3	4	0	0	13	6	0.03157	0.033615	0	0	0.088099	0.049675	0.021728	0.045925	0.352417	1	-
ENSMUSC	Blmh	bleomycir	GO:00081	metabolic	-	-	-	-	11:769248	826	1063	812	1063	1375	1090	6.262758	6.436722	6.804379	6.217979	6.713407	6.501643	6.501286	6.477676	0.782193	1	-
ENSMUSC	Cpd	carboxype	GO:00081	metabolic	-	-	-	-	11:767784	2641	3564	2409	3357	4229	3283	18.49461	19.93243	18.64491	18.1367	19.0708	18.08666	19.02398	18.43139	0.524192	1	-

ENSMUSG	Timm22	translocas	GO:00511	localizatio	-	-	-	-	11:764069	227	292	203	294	310	268	3.42227	3.515745	3.382444	3.419524	3.009568	3.178588	3.440153	3.20256	0.427171	1	-
ENSMUSG	Nxn	nucleored	GO:00325	developm	-	-	-	-	11:762571	141	249	113	224	306	253	2.386794	3.366215	2.114079	2.925339	3.3356	3.369217	2.622362	3.210052	0.23177	1	-
ENSMUSG	Rflnb	refilin B [S	GO:00325	developm	-	-	-	-	11:760191	214	265	203	296	439	302	3.720512	3.679432	3.900598	3.970187	4.914829	4.130535	3.766847	4.338517	0.346214	1	-
ENSMUSG	Rph3al	rabphilin :	GO:00511	localizatio	-	-	-	-	11:758309	73	94	63	104	154	97	1.045313	1.074981	0.997046	1.148921	1.420039	1.092718	1.039113	1.220559	0.382394	1	-
ENSMUSG	Doc2b	double C2	GO:00452	synapse|lc	-	-	-	-	11:757689	2510	3003	2189	3560	3757	3209	35.14236	33.57827	33.87271	38.45365	33.87296	35.34581	34.19778	35.89081	0.787414	1	-
ENSMUSG	Ywhae	tyrosine 3	GO:00325	developm	K06630	14-3-3 pr	mmu0415	PI3K-Akt	11:757328	14253	18569	12978	18880	23686	16644	252.396	262.6095	253.9982	257.9339	270.0987	231.8701	256.3346	253.3009	0.683704	1	-
ENSMUSG	Prpf8	pre-mRN.	GO:00081	metabolic	K12856	pre-mRN.	mmu0304	Spliceosor	11:754868	5130	6248	4686	6360	7638	6791	36.70355	35.70077	37.0544	35.10574	35.19047	38.22393	36.48624	36.17338	0.691918	1	-
ENSMUSG	Nme2	NME/NM.	GO:00325	developm	K00940	nucleoside	mmu0110	Metabolic	11:939498	319	188	455	380	393	251	12.72212	5.987878	20.05516	11.69183	10.09289	7.875035	12.92172	9.886586	0.199273	1	-
ENSMUSG	Spag9	sperm ass	GO:00325	developm	-	-	-	-	11:939960	8017	9942	6939	9552	12020	10409	23.22781	23.00467	22.21979	21.35114	22.42619	23.72555	22.81742	22.50096	0.647493	1	-
ENSMUSG	Luc7l3	LUC7-like	GO:00081	metabolic	-	-	-	-	11:942878	2267	2937	2020	3062	3791	3673	10.91114	11.28934	10.74526	11.36983	11.74971	13.90754	10.98191	12.34236	0.313374	1	-
ENSMUSG	Ankrd40	ankyrin re	-	-	-	-	-	-	11:943280	1715	2398	1580	2064	2856	2190	17.06006	19.05072	17.37082	15.84004	18.29487	17.13845	17.8272	17.09112	0.470365	1	-
ENSMUSG	Cacna1g	calcium cl	GO:00511	localizatio	K04854	voltage-d	mmu0401	MAPK sigi	11:944083	1295	1446	1082	1617	1731	1540	5.102622	4.550277	4.71192	4.915459	4.392132	4.773702	4.788273	4.693764	0.638462	1	-
ENSMUSG	Xylt2	xylosyltrar	GO:00081	metabolic	K00771	protein xy	mmu0110	Metabolic	11:946638	450	564	423	655	709	633	5.046123	5.050922	5.242426	5.666526	5.119725	5.58419	5.113157	5.456814	0.701103	1	-
ENSMUSG	Lrrc59	leucine ric	GO:00160	membran	-	-	-	-	11:946297	1536	1882	1362	2108	2556	2132	26.99633	26.41672	26.45681	28.58343	28.92871	29.47892	26.62329	28.99702	0.48341	1	-
ENSMUSG	Cdc34b	cell divisic	GO:00081	metabolic	-	-	-	-	11:947417	0	3	5	2	2	4	0	0.058878	0.135789	0.037917	0.031638	0.077315	0.064889	0.048957	0.836713	1	-
ENSMUSG	Dlx4	distal-less	GO:00325	developm	-	-	-	-	11:951404	0	4	0	0	0	6	0	0.050331	0	0	0	0.074378	0.016777	0.024793	0.837394	1	-
ENSMUSG	Slc35b1	solute car	GO:00511	localizatio	-	-	-	-	11:953846	383	449	307	464	589	477	6.748995	6.318766	5.978951	6.30795	6.683591	6.612554	6.348904	6.534698	0.972689	1	-
ENSMUSG	Snx11	sorting ne	GO:00511	localizatio	-	-	-	-	11:967675	445	513	354	569	699	534	4.282925	3.943148	3.765566	4.224962	4.332238	4.043266	3.997213	4.200155	0.822824	1	-
ENSMUSG	Scrn2	secernin 2	GO:00081	metabolic	-	-	-	-	11:970299	36	51	42	65	57	43	0.642719	0.727162	0.828728	0.895281	0.65531	0.603949	0.732869	0.71818	0.829004	1	-
ENSMUSG	Lrrc46	leucine ric	GO:00511	localizatio	-	-	-	-	11:970346	9	16	2	16	11	9	0.41131	0.584002	0.101028	0.564146	0.323721	0.323577	0.365447	0.403815	0.928894	1	-
ENSMUSG	Cacnb1	calcium cl	GO:00511	localizatio	K04862	voltage-d	mmu0401	MAPK sigi	11:980015	2380	2873	2178	3176	3468	2894	23.07373	22.24447	23.33702	23.75481	21.65083	22.07243	22.88507	22.49269	0.634788	1	-
ENSMUSG	Fbxl20	F-box anc	GO:00081	metabolic	-	-	-	-	11:980825	1173	1460	1010	1365	1739	1448	5.39316	5.360977	5.132319	4.841815	5.148723	5.237508	5.295485	5.076015	0.477276	1	-
ENSMUSG	Dlg4	discs, larg	GO:00325	developm	K11828	discs large	mmu0501	Huntingto	11:700170	12069	15046	11244	16124	19564	13777	108.3851	107.9109	111.6005	111.7124	113.1386	97.33384	109.2988	107.3949	0.636756	1	-
ENSMUSG	Dvl2	dishevelle	GO:00325	developm	K02353	segment p	mmu0520	Pathways	11:700005	246	355	222	407	429	416	1.705494	1.965573	1.701043	2.176909	1.91526	2.268922	1.790703	2.120364	0.228401	1	-
ENSMUSG	Nr1d1	nuclear re	GO:00325	developm	K03728	nuclear re	mmu0471	Circadian	11:987679	1270	1542	1144	1737	2056	1927	22.14272	21.47126	22.04448	23.36455	23.08368	26.43138	21.88615	24.2932	0.388956	1	-
ENSMUSG	Gucy2e	guanylate	GO:00081	metabolic	K12321	guanylate	mmu0474	Olfactory	11:692181	106	113	73	76	143	149	0.603688	0.513964	0.459491	0.333929	0.524443	0.667583	0.525715	0.508651	0.767169	1	-
ENSMUSG	Alox8	arachidon	GO:00325	developm	K08022	arachidon	mmu0110	Metabolic	11:691839	130	168	131	179	251	200	1.744902	1.800875	1.943324	1.85358	2.169481	2.111875	1.8297	2.044979	0.516786	1	-
ENSMUSG	Aloxe3	arachidon	GO:00325	developm	-	-	-	-	11:691258	155	144	147	214	265	199	1.154568	0.856636	1.210187	1.229794	1.271127	1.166143	1.073797	1.222355	0.469485	1	-
ENSMUSG	Per1	period circ	GO:00081	metabolic	K02633	period circ	mmu0516	Herpes sir	11:690952	1200	1365	946	1374	1625	1548	8.865063	8.053393	7.723933	7.831006	7.730517	8.996683	8.21413	8.186069	0.774602	1	-
ENSMUSG	Vamp2	vesicle-as	GO:00452	synapse|lc	K13504	vesicle-as	mmu0491	Insulin sec	11:690884	17287	21341	15043	22603	26368	21648	212.8906	209.8927	204.7473	214.75	209.1071	209.7324	209.1769	211.1965	0.859773	1	-
ENSMUSG	Tmem107	transmem	GO:00325	developm	-	-	-	-	11:690708	70	88	58	71	90	99	1.029394	1.033513	0.942671	0.805513	0.852282	1.145337	1.00186	0.934377	0.56922	1	-
ENSMUSG	Aurkb	aurora kir	GO:00325	developm	-	-	-	-	11:690456	2	9	6	15	8	10	0.021014	0.075539	0.069694	0.121616	0.054141	0.082675	0.055416	0.086144	0.317286	1	-
ENSMUSG	Ctc1	CTS telom	GO:00325	developm	-	-	-	-	11:690159	564	696	480	733	880	773	3.527274	3.476285	3.317781	3.53667	3.544031	3.803213	3.440447	3.627972	0.781478	1	-
ENSMUSG	Pfas	phosphor	GO:00081	metabolic	K01952	phosphor	mmu0110	Metabolic	11:689856	327	431	449	498	733	611	1.408536	1.482667	2.137538	1.654933	2.033195	2.070486	1.676247	1.919538	0.40868	1	-
ENSMUSG	Myh10	myosin, h	GO:00325	developm	K10352	myosin he	mmu0481	Regulatior	11:686915	6931	8141	5972	8342	9475	8366	25.21111	23.64938	24.00837	23.40974	22.19371	23.94004	24.28962	23.18117	0.39301	1	-
ENSMUSG	Pik3r5	phosphoir	GO:00081	metabolic	K02649	phosphoir	mmu0415	PI3K-Akt	11:684321	53	74	39	62	76	48	0.461443	0.514539	0.375273	0.416451	0.426096	0.328772	0.450418	0.39044	0.363556	1	-
ENSMUSG	Ntn1	netrin 1 [S	GO:00325	developm	K06843	netrin 1[-]	mmu0436	Axon guic	11:682093	163	184	173	237	263	232	1.414711	1.275389	1.659478	1.586931	1.469904	1.584085	1.449859	1.546973	0.759539	1	-
ENSMUSG	Stx8	syntaxin 8	GO:00511	localizatio	K08501	syntaxin 8	mmu0413	SNARE int	11:679661	256	256	237	270	351	265	1.858385	1.484161	1.901477	1.51213	1.640807	1.513393	1.748008	1.555443	0.276458	1	-
ENSMUSG	Rcvrn	recoverin	GO:00511	localizatio	K13764	recoverin[	mmu0474	Phototran	11:676953	6	4	3	6	4	5	0.35225	0.187531	0.194674	0.271755	0.151231	0.230934	0.244818	0.217974	0.869648	1	-
ENSMUSG	Myh3	myosin, h	GO:00081	metabolic	K10352	myosin he	mmu0453	Tight junc	11:670783	23	24	11	22	30	25	0.210989	0.175825	0.111522	0.155698	0.177215	0.180417	0.166112	0.17111	1	1	-
ENSMUSG	Adprm	ADP-ribo	GO:00038	catalytic a	K01517	manganes	mmu0023	Purine me	11:670379	176	239	119	217	266	134	2.331579	2.528603	1.742331	2.217826	2.2692	1.396537	2.200838	1.961188	0.414565	1	-
ENSMUSG	Krt12	keratin 12	GO:00051	structural	-	-	-	-	11:994156	16	26	17	33	33	17	0.527498	0.684574	0.619444	0.839369	0.700602	0.44093	0.610505	0.6603	0.858143	1	-
ENSMUSG	Acly	ATP citrat	GO:00081	metabolic	K01648	ATP citrat	mmu0110	Metabolic	11:100476	2814	3515	2434	3692	4236	3358	20.97894	20.92814	20.05519	21.23496	20.33621	19.69478	20.65409	20.42198	0.684979	1	-
ENSMUSG	Kat2a	K(lysine) a	GO:00325	developm	K06062	histone ac	mmu0516	HTLV-I int	11:100704	1125	1511	1121	1643	1866	1561	15.04059	16.13328	16.56398	16.9465	16.06493	16.41822	15.91262	16.47655	0.905943	1	-
ENSMUSG	Stat5b	signal trar	GO:00325	developm	K11224	signal trar	mmu0520	Pathways	11:100780	360	416	283	453	526	489	3.749092	3.459892	3.257294	3.639589	3.527474	4.006299	3.488759	3.724454	0.730673	1	-
ENSMUSG	Tmem101	transmem	GO:00048	signal trar	-	-	-	-	11:102152	238	324	258	398	458	321	5.70772	6.205487	6.838365	7.363737	7.073029	6.056218	6.250524	6.830995	0.595091	1	-
ENSMUSG	Lsm12	LSM12 hc	-	-	-	-	-	-	11:102162	713	888	622	979	1121	819	13.61728	13.5444	13.12919	14.42492	13.78673	12.3054	13.43029	13.50568	0.861238	1	-
ENSMUSG	Ubtf	upstream	GO:00056	cell|intrace	-	-	-	-	11:102304	2225	2810	2098	3000	3574	2703	10.47531	10.56548	10.91665	10.89654	10.83543	10.01138	10.65248	10.58111	0.727565	1	-
ENSMUSG	Ccdc43	coiled-co	-	-	-	-	-	-	11:102684	344	491	361	467	589	478	3.440458	3.921799	3.990362	3.603339	3.793389	3.760941	3.784206	3.719223	0.719668	1	-
ENSMUSG	Adam11	a disinteg	GO:00081	metabolic	-	-	-	-	11:102761	4122	4851	3813	5562	6209	5501	26.33203	24.74876	26.92092	27.41181	25.54187	27.64578	26.00057	26.86649	0.922204	1	-
ENSMUSG	Higd1b	HIG1 dom	GO:00160	membran	-	-	-	-	11:102835	16	23	18	20	22	15	0.907924	1.042351	1.128901	0.875558	0.803925	0.669624	1.026392	0.783036	0.274592	1	-
ENSMUSG	Eftud2	elongatior	GO:00081	metabolic	K12852	116 kDa U	mmu0304	Spliceosor	11:102838	1706	2086	1629	2219	2717	2072	9.514538	9.291133	10.04099	9.547641	9.75782	9.09095	9.615554	9.46547	0.656107	1	-
ENSMUSG	Ccdc103	coiled-co	GO:00325	developm	-	-	-	-	11:102881	32	34	24	47	45	66	0.742345	0.629918	0.615331	0.84118	0.672233	1.204512	0.662531	0.905975	0.194947	1	-
ENSMUSG	Dcakd	dephosph	GO:00081	metabolic	-	-	-	-	11:102994	884	1078	864	1168	1305	1216	12.37683	12.05374	13.36957	12.61626	11.76582	13.39373	12.60005	12.59194	0.800394	1	-
ENSMUSG	Nmt1	N-myristc	GO:00325	developm	-	-	-	-	11:103028	3934	4764	3570	4983	6262	5194	43.29535	41.87208	43.42321	42.30855	44.3787	44.96967	42.86355	43.88564	0.994917	1	-
ENSMUSG	Plcd3	phospholi	GO:00325	developm	K05857	phosphati	mmu0110	Metabolic	11:103070	132	132	116	182	166	159	1.762829	1.407851	1.712148	1.875159	1.427578	1.670494	1.62761	1.657744	0.975529	1	-
ENSMUSG	1700023F	RIKEN cDl	GO:00431	ion bindin	-	-	-	-	11:103198	15	9	3	14	14	7	0.436327	0.209092	0.096456	0.314185	0.26225	0.1602	0.247291	0.245545	1	1	-
ENSMUSG	Map3k14	mitogen-:	GO:00048	signal trar	K04466	mitogen-:	mmu0401	MAPK sigi	11:103219	59	105	55	78	116	103	0.429783	0.610844	0.442795	0.438347	0.544135	0.590261	0.494474	0.524248	0.868516	1	-
ENSMUSG	Gosr2	golgi SNA	GO:00511	localizatio	K08496	golgi SNA	mmu0413	SNARE int	11:103676	1085	1409	1053	1523	1854	1356	14.60173	15.14368	15.6621	15.81266	16.06715	14.35639	15.13584	15.41207	0.956701	1	-
ENSMUSG	Klhl28	kelch-like	GO:00081	metabolic	-	-	-	-	12:649388	567	662	505	797	830	597	3.9963	3.72631	3.93381	4.333748	3.767105	3.310247	3.885473	3.8037	0.690499	1	-
ENSMUSG	Fkbp3	FK506 bin	GO:00081	metabolic	-	-	-	-	12:650624	1211	1617	1100	1604	1930	1447	5.874889	6.264861	5.897867	6.003305	6.029309	5.52249	6.012539	5.851701	0.583939	1	-
ENSMUSG	Foxg1	forkhead	GO:00325	developm	K09385	forkhead	mmu0406	FoxO sign	12:493826	2327	782	2085	3030	908	2895	17.39073	4.66739	17.22163	17.47006	4.369804	17.02083	13.09325	12.95356	0.895966	1	-
ENSMUSG	Scfd1	Sec1 fami	GO:00325	developm	-	-	-	-	12:513775	475	637	502	603	751	613	1.823717	1.953214	2.130173	1.786124	1.856772	1.851552	1.969035	1.831483	0.367434	1	-
ENSMUSG	Coch	cochlin [S	GO:00325	developm	-	-	-	-	12:515933	64	109	49	101	121	112	0.830896	1.130157	0.703095	1.011623	1.011603	1.143922	0.888049	1.055716	0.392004	1	-
ENSMUSG	Strn3	striatin, ca	GO:00081	metabolic	-	-	-	-	12:516096	1500	1910	1248	1844	2210	1596	11.66567	11.86308	10.72703	11.06393	11.0679	9.764771	11.4186	10.6322	0.307488	1	-
ENSMUSG	Ap4s1	adaptor-r	GO:00511	localizatio	K12403	AP-4 com	mmu0414	Lysosome	12:516910	264	300	210	383	359	247	11.29598	10.25149	9.93084	12.64295	9.891651	8.314332	10.49277	10.28298	0.757284	1	-
ENSMUSG	Dtd2	D-tyrosyl-	GO:00081	metabolic	-	-	-	-	12:519883	109	202	144	208	246	228	1.96961	2.915087	2.875834	2.899674	2.862497	3.241169	2.586843	3.001113	0.3949	1	-
ENSMUSG	Ston2	stonin 2 [S	GO:00325	developm	-	-	-	-	12:916330	686	1038	763	908	1200	920	3.116043	3.7655	3.830458	3.181967	3.510066	3.287594	3.570667	3.326542	0.37477	1	-
ENSMUSG	Gtf2a1	general tr	GO:00081	metabolic	K03122	transcripti	mmu0520	Viral carci	12:915552	764	1105	768	969	1174	948	6.128269	7.078688	6.808519	5.996513	6.064115	5.982243	6.671825	6.01429	0.205079	1	-
ENSMUSG	Tshr	thyroid sti	GO:00325	developm	K04249	thyroid sti	mmu0408	Neuroacti	12:913845	7	3	2	2	6	3	0.059068	0.02022	0.018653	0.013022	0.032601	0.019912	0.032647	0.021845	0.482027	1	-
ENSMUSG	Sel1l	sel-1 supr	GO:00081	metabolic	K14026	SEL1 prote	mmu0414	Protein pr	12:918060	4254	5250	2427	5552	4071	3522	16.50827	16.27082	10.40928	16.62203	10.17326	10.75238	14.39612	12.51589	0.250426	1	-
ENSMUSG	Dnaaf2	dynein, ax	GO:00400	locomotic	-	-	-	-	12:691890	66	128	95	133	142	127	1.188748	1.841199	1.891107	1.848101	1.646974	1.799523	1.640351	1.764866	0.770919	1	-
ENSMUSG	Klhdc2	kelch dor	GO:00160	membran	-	-	-	-	12:692966	1684	2086	1485	2114	2573	2234	12.69876	12.56259	12.37634	12.29857	12.49435	13.25297	12.5459	12.68196	0.878129	1	-
ENSMUSG	Nemf	nuclear ex	GO:00511	localizatio	-	-	-	-	12:693105	1261	1706	1104	1814	2108	1612	5.076435	5.484899	4.912013	5.633927	5.464733	5.105279	5.157782	5.401313	0.809877	1	-
ENSMUSG	Sec23a	SEC23 ho	GO:00511	localizatio	K14006	protein tra	mmu0414	Protein pr	12:589583	2705	3260	2268	3445	3894	3379	28.33456	27.27173	26.25664	27.83997	26.26636	27.84507	27.28764	27.31714	0.78894	1	-
ENSMUSG	L2hgdh	L-2-hydr	GO:00081	metabolic	K00109	2-hydroxy	-	-	12:696904	251	303	216	337	371	362	3.853561	3.715147	3.665115	3.991605	3.667887	4.372278	3.744608	4.01059	0.724647	1	-
ENSMUSG	Cdkl1	cyclin-dep	GO:00325	developm	-	-	-	-	12:697468	115	151	139	196	252	211	3.259848	3.418378	4.354723	4.286305	4.599944	4.705341	3.677649	4.53053	0.19393	1	-
ENSMUSG	Trappc6b	trafficking	GO:00511	localizatio	-	-	-	-	12:590430	883	1373	748	1251	1579	1004	8.207389	10.19202	7.684088	8.970814	9.451067	7.341569	8.6945	8.587817	0.756484	1	-
ENSMUSG	Pnn	pinin [Sou	GO:00329	macromo	K13114	pinin[-]	mmu0301	RNA trans	12:590668	1918	2290	1437	2154	2907	2382	16.80434	16.02338	13.91479	14.55958	16.40108	16.4182	15.58084	15.79295	0.910483	1	-
ENSMUSG	Ctage5	melanom.	GO:00511	localizatio	-	-	-	-	12:590957	943	1471	916	1308	1773	1333	3.233255	4.027974	3.471127	3.459922	3.914638	3.595584	3.577452	3.656714	0.992837	1	-
ENSMUSG	Galc	galactosyl	GO:00081	metabolic	K01202	galactosyl	mmu0110	Metabolic	12:982022	581	674	520	682	890	746	2.112097	1.956786	2.089238	1.912724	2.083446	2.133474	2.052707	2.043215	0.784591	1	-
ENSMUSG	Spata7	spermato	GO:00511	localizatio	-	-	-	-	12:986281	299	417	289	405	526	458	1.379298	1.536274	1.473434	1.441358	1.562525	1.662123	1.463002	1.555335	0.749496	1	-
ENSMUSG	Ptpn21	protein ty	GO:00081	metabolic	-	-	-	-	12:986767	184	250	189	188	300	280	0.974471	1.057395	1.106267	0.768141	1.023121	1.166598	1.046044	0.985954	0.565235	1	-
ENSMUSG	Npas3	neuronal	GO:00081	metabolic	-	-	-	-	12:532486	276	407	269	401	385	311	1.769244	2.083618	1.905802	1.98314	1.589255	1.568372	1.919555	1.713589	0.269056	1	-
ENSMUSG	Zc3h14	zinc finge	GO:00081	metabolic	-	-	-	-	12:987469	1267	1301	1217	1588	2016	1694	6.259963	5.133556	6.645572	6.053074	6.414167	6.584448	6.01303	6.350563	0.767287	1	-
ENSMUSG	Ttc8	tetratricor	GO:00325	developm	-	-	-	-	12:989205	373	440	362	456	533	488	3.945717	3.717193	4.232255	3.721448	3.630768	4.061138	3.965055	3.804451	0.565275	1	-
ENSMUSG	Polr2h	polymeras	GO:00081	metabolic	K03016	DNA-dire	mmu0110	Metabolic	16:207176	125	165	118	206	214	176	4.936765	5.204315	5.150658	6.276697	5.442544	5.468372	5.097246	5.729205	0.496587	1	-
ENSMUSG	Ppp2r3c	protein pl	GO:00325	developm	K11583	serine/thr	mmu0415	PI3K-Akt	12:552789	375	459	309	460	521	431	3.586587	3.50598	3.266297	3.394212	3.208802	3.242937	3.452955	3.281984	0.506961	1	-
ENSMUSG	1110008L	RIKEN cDl	GO:00081	metabolic	-	-	-	-	12:552995	99	149	116	169	154	154	0.688544	0.827616	0.891667	0.906807	0.689719	0.842616	0.802609	0.813048	0.94845	1	-
ENSMUSG	Psma6	proteasor	GO:00325	developm	K02730	20S prote	mmu0305	Proteason	12:553842	888	1137	891	1165	1493	1121	28.4468	29.08887	31.54606	28.79232	30.79888	28.25121	29.69391	29.2808	0.702278	1	-
ENSMUSG	Nfkbia	nuclear fa	GO:00325	developm	K04734	NF-kappa	mmu0520	Pathways	12:554894	270	360	233	312	495	401	8.330323	8.870443	7.94511	7.426477	9.834607	9.733138	8.381958	8.998074	0.712785	1	-
ENSMUSG	Mbip	MAP3K12	GO:00081	metabolic	-	-	-	-	12:563283	123	164	119	158	254	195	2.964921	3.157166	3.170313	2.938304	3.942732	3.697886	3.097467	3.526307	0.455844	1	-
ENSMUSG	Ngb	neuroglob	GO:00511	localizatio	-	-	-	-	12:870975	20	40	19	34	33	34	0.421821	0.673754	0.442896	0.553221	0.448192	0.564131	0.512824	0.521848	0.958732	1	-
ENSMUSG	Gstz1	glutathior	GO:00081	metabolic	K01800	maleylace	mmu0110	Metabolic	12:871471	491	642	457	663	798	641	4.613651	4.817754	4.745988	4.806261	4.828597	4.738407	4.725798	4.791089	0.926	1	-
ENSMUSG	Sptlc2	serine pal	GO:00081	metabolic	K00654	serine pal	mmu0110	Metabolic	12:873050	600	829	591	887	1132	859	3.320482	3.663953	3.614798	3.787074	4.034139	3.739847	3.533078	3.853687	0.528473	1	-
ENSMUSG	Ahsa1	AHA1, act	GO:00987	molecular	-	-	-	-	12:872664	1913	2347	1676	2624	2920	2275	18.59939	18.22396	18.00962	19.68239	18.28189	17.40107	18.27765	18.45512	0.872794	1	-
ENSMUSG	Snw1	SNW dor	GO:00325	developm	K06063	SNW dor	mmu0520	Viral carci	12:874490	1203	1519	1021	1553	2061	1562	9.42429	9.503565	8.840068	9.386092	10.39718	9.626643	9.255974	9.803306	0.718557	1	-
ENSMUSG	Slirp	SRA stem	GO:00325	developm	-	-	-	-	12:874438	97	82	65	95	120	76	1.490345	1.006184	1.103769	1.126079	1.187272	0.918626	1.2001	1.077326	0.45236	1	-
ENSMUSG	Adck1	aarF dom	GO:00081	metabolic	-	-	-	-	12:883605	445	532	505	584	684	676	3.441966	3.286271	4.317026	3.484893	3.40688	4.113425	3.681754	3.668399	0.816849	1	-
ENSMUSG	Nova1	neuro-on	GO:00081	metabolic	-	-	-	-	12:466945	792	999	659	1034	1267	1050	5.040972	5.078089	4.63576	5.077381	5.193021	5.257621	4.918274	5.176008	0.789557	1	-
ENSMUSG	Mthfd1	methylene	GO:00325	developm	-	-	mmu0110	Metabolic	12:762552	703	912	662	919	1100	916	7.965086	8.252316	8.28972	8.033064	8.025692	8.164727	8.169041	8.074494	0.717416	1	-
ENSMUSG	Ppp2r5e	protein pl	GO:00987	molecular	K11584	serine/thr	mmu0415	PI3K-Akt	12:754508	2305	2767	1937	2931	3464	2722	17.50702	16.78403	16.2599	17.17463	16.94236	16.26448	16.85032	16.79383	0.753091	1	-
ENSMUSG	Sgpp1	sphingosi	GO:00081	metabolic	K04716	sphingosi	mmu0407	Sphingolip	12:757142	826	954	720	1076	1413	988	7.731717	7.131656	7.448609	7.770312	8.51712	7.275518	7.437327	7.854317	0.764061	1	-
ENSMUSG	Esr2	estrogen	GO:00325	developm	K08551	estrogen	mmu0520	Pathways	12:761204	1	4	3	3	4	3	0.011526	0.036805	0.038207	0.026672	0.029681	0.027191	0.028846	0.027848	1	1	-
ENSMUSG	Akap5	A kinase (	GO:00081	metabolic	-	-	-	-	12:763248	1879	2456	1769	2661	3333	1851	7.177689	7.492591	7.468485	7.842114	8.198753	5.562567	7.379588	7.201145	0.66525	1	-
ENSMUSG	Sptb	spectrin b	GO:00051	structural	-	-	-	-	12:765804	7423	8694	6700	8867	10516	9449	34.55847	32.32515	34.4744	31.84799	31.52683	34.60763	33.78601	32.66082	0.488346	1	-
ENSMUSG	Rab15	RAB15, m	GO:00511	localizatio	-	-	-	-	12:767979	3859	4995	3615	5275	6481	5240	39.4811	40.81275	40.87612	41.63584	42.69837	42.17516	40.38999	42.16979	0.816421	1	-
ENSMUSG	Fut8	fucosyltra	GO:00081	metabolic	K00717	glycoprot	mmu0110	Metabolic	12:772381	1481	1803	1262	1874	2338	1924	7.769983	7.554514	7.317639	7.585162	7.898854	7.941107	7.547379	7.808375	0.91317	1	-
ENSMUSG	Atl1	atlastin G	GO:00325	developm	-	-	-	-	12:698926	2091	2487	1964	2506	3001	2564	10.32365	9.806195	10.71685	9.545319	9.541126	9.958821	10.28223	9.681755	0.332221	1	-
ENSMUSG	Nin	ninein [So	GO:00325	developm	-	-	-	-	12:700114	1147	1255	1218	1557	1736	1573	3.392939	2.964846	3.982049	3.553297	3.306868	3.6606	3.446611	3.506922	0.946681	1	-
ENSMUSG	Pygl	liver glyco	GO:00081	metabolic	K00688	glycogen	mmu0110	Metabolic	12:701908	61	87	65	86	104	95	0.467085	0.532026	0.550085	0.508036	0.512809	0.572274	0.516399	0.531039	1	1	-
ENSMUSG	Trim9	tripartite r	GO:00081	metabolic	-	-	-	-	12:702445	4008	4926	3414	5066	6001	4832	9.176919	9.007607	8.63932	8.948798	8.848048	8.703763	8.941282	8.833536	0.663953	1	-
ENSMUSG	Tmx1	thioredox	GO:00081	metabolic	-	-	-	-	12:704530	765	1015	677	970	1193	892	12.26292	12.99404	11.9941	11.99595	12.31481	11.24884	12.41702	11.8532	0.488792	1	-
ENSMUSG	Actr10	ARP10 act	GO:00329	macromo	-	-	-	-	12:709378	1729	1955	1602	2121	2380	2010	9.279199	8.379309	9.502214	8.781863	8.225213	8.486382	9.053574	8.497819	0.328298	1	-
ENSMUSG	Timm9	translocas	GO:00511	localizatio	-	-	-	-	12:711231	242	229	214	309	360	290	1.612929	1.218937	1.576377	1.588873	1.545104	1.520583	1.469414	1.55152	0.818286	1	-
ENSMUSG	Rtn1	reticulon :	GO:00160	membran	-	-	-	-	12:722117	12264	10213	7949	10946	12738	17178	114.1663	75.92851	81.78337	78.61247	76.35925	125.8025	90.62606	93.5914	0.971729	1	-
ENSMUSG	Lrrc9	leucine ric	-	-	-	-	-	-	12:724418	28	44	32	34	46	40	0.166126	0.208488	0.209838	0.155629	0.175752	0.186704	0.194818	0.172695	0.483004	1	-
ENSMUSG	Serpina3n	serine (or	GO:00055	extracellul	-	-	-	-	12:104406	1195	1799	1308	1601	2027	1664	35.35098	42.50213	42.76496	36.53888	38.61376	38.72557	40.20603	37.95941	0.396392	1	-
ENSMUSG	Dhrs7	dehydrog	GO:00081	metabolic	-	-	-	-	12:726503	358	486	384	482	533	442	5.015794	5.437983	5.946127	5.209952	4.80881	4.871798	5.466635	4.96352	0.290286	1	-
ENSMUSG	Ppm1a	protein pl	GO:00048	signal trar	K04457	protein pl	mmu0401	MAPK sigi	12:727574	2654	3275	2552	3314	4588	3082	10.2329	10.08451	10.8749	9.857815	11.39137	9.348488	10.39744	10.19922	0.648301	1	-
ENSMUSG	Clmn	calmin [Sc	GO:00325	developm	-	-	-	-	12:104763	2473	3748	2572	3607	4222	3719	11.08884	13.4217	12.74617	12.47781	12.19087	13.11895	12.4189	12.59588	0.917558	1	-
ENSMUSG	4930447C	RIKEN cDl	GO:00325	developm	-	-	-	-	12:728811	64	157	63	96	85	154	0.543038	1.063891	0.590801	0.628425	0.464436	1.027978	0.732577	0.706946	0.795758	1	-
ENSMUSG	Glrx5	glutaredo	GO:00325	developm	-	-	-	-	12:105032	308	333	199	299	440	329	4.235563	3.657219	3.024554	3.172215	3.896439	3.559321	3.639112	3.542658	0.704545	1	-
ENSMUSG	Mnat1	menage a	GO:00325	developm	K10842	CDK-activ	mmu0342	Nucleotid	12:731237	216	310	220	319	353	292	2.264903	2.595996	2.549564	2.580583	2.383556	2.408741	2.470154	2.457627	0.811484	1	-
ENSMUSG	Prkch	protein kir	GO:00325	developm	K18051	novel prot	mmu0427	Vascular s	12:735847	77	84	60	79	135	105	0.457741	0.398799	0.394211	0.362315	0.516794	0.491058	0.416917	0.456722	0.709384	1	-
ENSMUSG	Hif1a	hypoxia ir	GO:00325	developm	K08268	hypoxia-ir	mmu0520	Pathways	12:739013	1738	2183	1528	2033	2560	2125	15.26673	15.31424	14.83427	13.77729	14.48074	14.68472	15.13841	14.31425	0.357008	1	-
ENSMUSG	Papola	poly (A) p	GO:00081	metabolic	K14376	poly(A) pc	mmu0301	mRNA sur	12:105784	941	1811	788	1256	1478	1357	4.062196	6.243605	3.759624	4.183031	4.10866	4.608524	4.688475	4.300072	0.383658	1	-
ENSMUSG	Mpp5	membran	GO:00325	developm	K06091	MAGUK p	mmu0516	Human pa	12:787489	860	1162	904	1036	1378	1154	3.982858	4.297819	4.627128	3.701573	4.109605	4.204484	4.302602	4.005221	0.336936	1	-
ENSMUSG	Snapc1	small nucl	GO:00081	metabolic	-	-	-	-	12:739644	827	703	604	765	1063	741	5.400542	3.666344	4.359287	3.854103	4.470124	3.806808	4.475391	4.043678	0.301753	1	-
ENSMUSG	Atp6v1d	ATPase, H	GO:00511	localizatio	K02149	V-type H-	mmu0110	Metabolic	12:788407	3368	4415	3318	4775	5480	4756	37.68439	39.45172	41.03104	41.21858	39.48431	41.86414	39.38905	40.85568	0.881495	1	-
ENSMUSG	Vrk1	vaccinia re	GO:00325	developm	-	-	-	-	12:106010	247	344	196	315	419	337	2.389317	2.657546	2.095462	2.350806	2.610025	2.564582	2.380775	2.508471	0.828848	1	-
ENSMUSG	Eif2s1	eukaryotic	GO:00325	developm	K03237	translatior	mmu0516	Herpes sir	12:788618	655	867	649	848	1031	809	7.779239	8.223578	8.518973	7.770015	7.885137	7.558844	8.17393	7.737999	0.43125	1	-
ENSMUSG	Pigh	phosphati	GO:00081	metabolic	K03858	phosphati	mmu0110	Metabolic	12:790806	183	234	177	320	297	277	1.998151	2.04051	2.135975	2.695609	2.088275	2.379396	2.058212	2.38776	0.336308	1	-
ENSMUSG	Rdh12	retinol del	GO:00081	metabolic	K11153	retinol del	mmu0110	Metabolic	12:792089	43	49	36	46	85	48	0.575645	0.523876	0.532645	0.475088	0.732752	0.50552	0.544055	0.57112	0.889481	1	-
ENSMUSG	Vti1b	vesicle tra	GO:00511	localizatio	K08493	vesicle tra	mmu0413	SNARE int	12:791560	1411	1702	1064	1645	1863	1647	33.66667	32.43235	28.05831	30.28093	28.62467	30.9156	31.38578	29.9404	0.45389	1	-
ENSMUSG	Arg2	arginase t	GO:00325	developm	K01476	arginase[3	mmu0110	Metabolic	12:791307	23	20	19	25	46	38	0.983433	0.682954	0.897888	0.824693	1.266574	1.278245	0.854758	1.123171	0.312467	1	-
ENSMUSG	Galnt16	polypeptic	GO:00081	metabolic	K00710	polypeptic	mmu0110	Metabolic	12:805182	4187	2911	3859	5723	3369	2973	22.95917	12.74797	23.38701	24.21069	11.89622	12.82504	19.69805	16.31065	0.240674	1	-
ENSMUSG	Erh	enhancer	GO:00325	developm	-	-	-	-	12:806340	240	236	175	249	289	237	4.007096	3.146848	3.229259	3.207359	3.107212	3.112991	3.461068	3.142521	0.358796	1	-
ENSMUSG	Susd6	sushi dor	GO:00508	response	-	-	-	-	12:807905	1767	2316	1698	2291	2810	2420	12.27973	12.85395	13.04177	12.28309	12.57515	13.23057	12.72515	12.69627	0.763832	1	-
ENSMUSG	Srsf5	serine/arc	GO:00325	developm	K12893	splicing fa	mmu0516	Herpes sir	12:809455	1892	2090	1711	2503	3073	2423	10.78838	9.517594	10.78281	11.01098	11.28372	10.86925	10.36293	11.05465	0.654858	1	-
ENSMUSG	Smoc1	SPARC rel	GO:00325	developm	-	-	-	-	12:810268	235	242	205	283	344	320	2.497151	2.053706	2.407566	2.320037	2.353918	2.675094	2.319474	2.449683	0.811848	1	-
ENSMUSG	Gm20498	predicted	GO:00325	developm	-	-	-	-	12:813588	61	151	89	219	275	73	0.857003	1.694244	1.381945	2.373709	2.487946	0.806842	1.311064	1.889499	0.180488	1	-
ENSMUSG	Pacs2	phosphof	GO:00081	metabolic	-	-	-	-	12:113014	2015	1733	1278	1862	2940	1942	10.35362	7.111509	7.257629	7.381206	9.727902	7.850139	8.24092	8.319749	0.900679	1	-
ENSMUSG	Mta1	metastasis	GO:00081	metabolic	-	-	-	-	12:113098	1153	1192	1025	1491	1652	1652	11.68077	9.64416	11.47658	11.65333	10.77723	13.16628	10.93384	11.86561	0.583588	1	-
ENSMUSG	Wdr37	WD repea	GO:00081	metabolic	-	-	-	-	13:880296	706	928	1184	864	1879	1515	2.394169	2.513297	4.437607	2.260447	4.103286	4.04179	3.115024	3.468508	0.632165	1	-
ENSMUSG	Gtpbp4	GTP bindi	GO:00325	developm	K06943	nucleolar	mmu0300	Ribosome	13:896633	792	1108	690	1038	1357	1113	4.321541	4.82835	4.161108	4.369591	4.768125	4.777703	4.437	4.638473	0.839511	1	-
ENSMUSG	Zmynd11	zinc finge	GO:00081	metabolic	-	-	-	-	13:968483	4721	5776	3937	6123	7186	6045	11.39709	11.1361	10.50443	11.40394	11.17126	11.48067	11.01254	11.35196	0.941403	1	-
ENSMUSG	Esyt2	extended	GO:00511	localizatio	-	-	-	-	12:116281	671	1028	859	1136	1178	1167	3.433911	4.201509	4.858549	4.485131	3.882099	4.698382	4.164656	4.355204	0.855555	1	-
ENSMUSG	Cdca7l	cell divisic	GO:00081	metabolic	-	-	-	-	12:117804	15	36	23	34	47	43	0.250027	0.479248	0.423714	0.437231	0.504498	0.563884	0.38433	0.501871	0.292232	1	-
ENSMUSG	Tdp1	tyrosyl-DI	GO:00081	metabolic	-	-	-	-	12:998845	214	321	223	319	374	319	1.239465	1.484809	1.427487	1.425416	1.394912	1.453523	1.38392	1.424617	0.977	1	-
ENSMUSG	Psmc1	protease (	GO:00081	metabolic	K03062	26S prote	mmu0516	Human pa	12:100110	1744	1962	1495	2151	2512	1853	43.32195	38.923	41.04399	41.22231	40.18239	36.2116	41.09631	39.20543	0.436938	1	-
ENSMUSG	Nrde2	nrde-2 ne	GO:00081	metabolic	-	-	-	-	12:100125	335	434	362	469	589	521	4.311654	4.461018	5.149363	4.656948	4.88167	5.275306	4.640678	4.937975	0.742858	1	-
ENSMUSG	Ccdc88c	coiled-co	GO:00081	metabolic	-	-	-	-	12:100911	1136	1279	987	1563	1719	1585	4.8658	4.375144	4.6724	5.164941	4.741402	5.340924	4.637782	5.082422	0.485237	1	-
ENSMUSG	Dglucy	D-glutam	GO:00081	metabolic	-	-	-	-	12:100779	93	111	103	73	165	140	0.696735	0.664131	0.852844	0.421928	0.796022	0.825136	0.737903	0.681028	0.597985	1	-
ENSMUSG	Fbln5	fibulin 5 [S	GO:00081	metabolic	-	-	-	-	12:101746	58	115	69	87	114	94	0.430613	0.681876	0.566179	0.49832	0.545029	0.549035	0.559556	0.530795	0.659671	1	-
ENSMUSG	Trip11	thyroid hc	GO:00325	developm	-	-	-	-	12:101834	1071	1505	1386	1917	1650	2142	5.182127	5.815683	7.411881	7.156007	5.141111	8.153588	6.136564	6.816902	0.526204	1	-
ENSMUSG	Atxn3	ataxin 3 [S	GO:00081	metabolic	K11863	Ataxin-3 [	mmu0414	Protein pr	12:101918	597	758	506	841	990	845	5.820167	5.901682	5.452034	6.325389	6.215139	6.480802	5.724628	6.340443	0.434227	1	-
ENSMUSG	Lgmn	legumain	GO:00325	developm	K01369	legumain[	mmu0414	Lysosome	12:102394	1890	2753	1755	2370	2832	2531	35.95011	41.82061	36.89455	34.77899	34.68856	37.87401	38.22175	35.78052	0.3158	1	-
ENSMUSG	Golga5	golgi autc	GO:00511	localizatio	-	-	-	-	12:102469	435	596	351	575	728	654	6.131185	6.708836	5.467749	6.25249	6.607561	7.251765	6.10259	6.703939	0.539055	1	-
ENSMUSG	Pitrm1	pitrilysin n	GO:00081	metabolic	-	-	-	-	13:654814	681	839	626	925	1012	935	6.403909	6.300946	6.50608	6.710736	6.128206	6.917042	6.403645	6.585328	0.975303	1	-
ENSMUSG	Chga	chromogr	GO:00081	metabolic	-	-	-	-	12:102554	2451	2920	2164	3509	3676	3028	46.57748	44.31608	45.45029	51.44538	44.98449	45.26885	45.44795	47.23291	0.874229	1	-
ENSMUSG	Pfkp	phosphof	GO:00081	metabolic	-	-	mmu0110	Metabolic	13:657976	4418	5118	3742	5462	5910	5225	16.95931	15.6902	15.8757	16.17575	14.60914	15.779	16.17507	15.5213	0.437134	1	-
ENSMUSG	Unc79	unc-79 hc	GO:00325	developm	-	-	-	-	12:102948	1723	2082	1429	2151	2489	2189	6.151869	5.936746	5.638984	5.925059	5.722705	6.148632	5.909199	5.932132	0.815988	1	-
ENSMUSG	Otub2	OTU dom	GO:00081	metabolic	-	-	-	-	12:103388	648	751	553	822	987	862	4.515081	4.179036	4.258561	4.418683	4.428557	4.725074	4.317559	4.524105	0.827637	1	-
ENSMUSG	Ppp4r4	protein pl	GO:00081	metabolic	-	-	-	-	12:103532	1043	1203	926	1327	1524	1369	12.81876	11.80792	12.57823	12.58238	12.06151	13.23659	12.40163	12.62683	0.947186	1	-

ENSMUSC	Akr1c18	aldo-ketc	GO:00081	metabolic	K05295	20alpha-h	mmu0014	Steroid hc	13:413261	44	41	29	49	89	57	1.749044	1.301619	1.274079	1.50271	2.278232	1.782537	1.441581	1.854493	0.259152	1	-
ENSMUSC	Tshz3	teashirt zi	GO:00081	metabolic	-	-	-	-	7:3669811	380	324	224	476	383	383	4.196436	2.857509	2.733959	4.055407	2.723644	3.327407	3.262635	3.368819	0.974642	1	-
ENSMUSC	Dcaf4	DDB1 anc	GO:00081	metabolic	-	-	-	-	12:835204	256	347	267	381	402	371	3.237547	3.5047	3.731937	3.717328	3.273837	3.691145	3.491395	3.56077	0.97632	1	-
ENSMUSC	Papln	papilin, pr	GO:00081	metabolic	-	-	-	-	12:837636	22	22	7	18	23	25	0.197046	0.157371	0.069299	0.124384	0.132659	0.176156	0.141239	0.1444	1	1	-
ENSMUSC	Numb	numb hor	GO:00325	developm	K06057	numb[-]	mmu0433	Notch sigi	12:837940	304	375	388	430	604	539	1.849323	1.821865	2.608665	2.01808	2.366087	2.579521	2.093284	2.321229	0.553198	1	-
ENSMUSC	Acot2	acyl-CoA	GO:00081	metabolic	K01068	acyl-coen	mmu011C	Metabolic	12:839878	105	147	140	201	185	128	2.919456	3.264186	4.302168	4.311594	3.312358	2.799829	3.49527	3.474594	0.871574	1	-
ENSMUSC	Fam161b	family witl	-	-	-	-	-	-	12:843453	252	328	203	343	354	349	1.97797	2.056074	1.76101	2.077039	1.789278	2.155042	1.931685	2.00712	0.927237	1	-
ENSMUSC	Coq6	coenzyme	GO:00081	metabolic	K06126	ubiquinor	mmu011C	Metabolic	12:843616	118	151	112	180	196	159	0.975875	0.997316	1.023702	1.148456	1.043808	1.034471	0.998964	1.075578	0.730943	1	-
ENSMUSC	Aldh6a1	aldehyde	GO:00325	developm	K00140	malonate-	mmu011C	Metabolic	12:844307	771	954	1073	988	1154	1433	7.417015	7.329414	11.40831	7.332668	7.148839	10.84506	8.718246	8.442189	0.690954	1	-
ENSMUSC	Vsx2	visual syst	GO:00325	developm	-	-	-	-	12:845697	8	7	5	12	5	7	0.079702	0.055693	0.055055	0.092226	0.032076	0.054864	0.063483	0.059722	0.903801	1	-
ENSMUSC	Abcd4	ATP-bind	GO:00081	metabolic	K05678	ATP-bind	mmu0414	Peroxisor	12:846014	137	203	165	223	282	229	0.613853	0.726414	0.817098	0.770866	0.813669	0.807214	0.719122	0.797249	0.557451	1	-
ENSMUSC	Isca2	iron-sulfu	GO:00081	metabolic	-	-	-	-	12:847732	164	195	191	249	272	220	1.563145	1.484348	2.012039	1.830988	1.669473	1.649642	1.686511	1.716701	0.97233	1	-
ENSMUSC	Npc2	NPC intra	GO:00081	metabolic	K13443	Niemann-	mmu0414	Lysosome	12:847545	411	1080	406	931	1149	864	3.820197	8.017029	4.170772	6.67612	6.87731	6.317843	5.335999	6.623758	0.22384	1	-
ENSMUSC	Fcf1	FCF1 rRN	GO:00081	metabolic	K14566	U3 small r	mmu030C	Ribosome	12:849708	41	57	46	63	79	72	1.718162	1.907663	2.130539	2.036814	2.131903	2.373708	1.918788	2.180808	0.563741	1	-
ENSMUSC	Ylpm1	YLP motif	GO:00081	metabolic	-	-	-	-	12:849963	3561	4001	3054	4317	4871	4356	15.09706	13.54674	14.30989	14.11994	13.29822	14.52845	14.3179	13.9822	0.579245	1	-
ENSMUSC	Tmed10	transmem	GO:00325	developm	-	-	-	-	12:853406	1848	2554	1711	2504	2430	2537	15.66506	17.29006	16.02975	16.3755	13.2645	16.91848	16.32829	15.51949	0.427712	1	-
ENSMUSC	Fos	FBJ osteo	GO:00325	developm	K04379	proto-onc	mmu052C	Pathways	12:854738	99	151	61	130	187	150	1.594073	1.941768	1.085557	1.614906	1.938966	1.900106	1.540466	1.817993	0.417033	1	-
ENSMUSC	Erg28	ergostero	GO:00081	metabolic	-	-	-	-	12:858154	566	689	580	740	901	749	6.149265	5.978225	6.964374	6.202535	6.303568	6.401763	6.363955	6.302622	0.749221	1	-
ENSMUSC	Tgfb3	transform	GO:00325	developm	K13377	transform	mmu052C	Pathways	12:860567	222	322	197	373	360	383	4.006761	4.641333	3.929662	5.193737	4.184066	5.438141	4.192585	4.938648	0.27676	1	-
ENSMUSC	Gpatch2l	G patch d	-	-	-	-	-	-	12:862418	666	806	602	860	985	788	3.513754	3.396083	3.51027	3.50047	3.346481	3.270655	3.473369	3.372535	0.594675	1	-
ENSMUSC	Esrrb	estrogen	GO:00325	developm	K08553	estrogen-	mmu0455	Signaling	12:863611	29	65	37	51	53	57	0.163362	0.292423	0.230355	0.221643	0.192257	0.252604	0.228713	0.222168	0.805673	1	-
ENSMUSC	Angel1	angel hor	GO:00056	cell|intrace	-	-	-	-	12:867005	138	167	138	135	189	175	1.634528	1.579711	1.806515	1.233615	1.441556	1.630661	1.673585	1.435277	0.218984	1	-
ENSMUSC	Ccnk	cyclin K [S	GO:00081	metabolic	-	-	-	-	12:108179	487	574	381	568	696	607	6.118378	5.759234	5.29028	5.50536	5.630813	5.999385	5.722631	5.711853	0.812322	1	-
ENSMUSC	Cyp46a1	cytochron	GO:00081	metabolic	K07440	cholesterc	mmu0012	Primary b	12:108334	2016	2719	1897	2879	3187	2605	29.69678	31.98702	30.88395	32.7183	30.23122	30.18822	30.85592	31.04591	0.840205	1	-
ENSMUSC	Hhipl1	hedgehog	GO:00511	localizatio	-	-	-	-	12:108306	167	202	171	247	235	226	1.777353	1.716939	2.011409	2.028087	1.610578	1.892247	1.835234	1.843637	0.883315	1	-
ENSMUSC	Evl	Ena-vaso	GO:00325	developm	-	-	-	-	12:108554	1120	1488	1018	1614	1760	1522	14.82764	15.73269	14.89528	16.48496	15.00449	15.85184	15.15187	15.78043	0.85978	1	-
ENSMUSC	Yy1	YY1 trans	GO:00325	developm	-	-	-	-	12:108792	1557	1831	1349	1915	2378	2049	14.24659	13.38001	13.6421	13.51828	14.01163	14.74942	13.75623	14.09311	1	1	-
ENSMUSC	Slc25a29	solute car	GO:00511	localizatio	-	-	-	-	12:108825	209	282	173	290	326	256	4.079659	4.396136	3.732234	4.367223	4.097785	3.931218	4.069343	4.132075	0.948251	1	-
ENSMUSC	Wars	tryptopha	GO:00325	developm	K01867	tryptopha	mmu0097	Aminoacy	12:108860	1603	1278	940	1330	1521	2308	20.28941	12.91852	13.14954	12.98728	12.3971	22.98173	15.45249	16.12203	0.92955	1	-
ENSMUSC	Hsp90aa1	heat shoc	GO:00325	developm	K04079	molecular	mmu052C	Pathways	12:110690	8521	10792	7244	11288	13659	10850	79.17763	80.08656	74.39387	80.92062	81.73074	79.31449	77.88602	80.65528	0.894404	1	-
ENSMUSC	Zfp839	zinc finge	GO:00971	organic cy	-	-	-	-	12:110850	189	251	124	256	323	279	2.199352	2.332662	1.594785	2.298275	2.420412	2.554159	2.042267	2.424282	0.291213	1	-
ENSMUSC	Fdft1	farnesyl d	GO:00081	metabolic	K00801	farnesyl-c	mmu011C	Metabolic	14:631451	815	873	904	1062	1319	1191	7.524934	6.437318	9.224869	7.564837	7.842313	8.651022	7.72904	8.019391	0.914904	1	-
ENSMUSC	Tecpr2	tectonin b	-	-	-	-	-	-	12:110889	1657	2116	1543	2193	2359	2360	6.029398	6.14912	6.205318	6.156308	5.527567	6.755762	6.127945	6.146546	0.813369	1	-
ENSMUSC	Cinp	cyclin-dep	GO:00081	metabolic	-	-	-	-	12:110872	435	578	406	667	707	511	3.564661	3.782702	3.677063	4.216821	3.730807	3.294283	3.674809	3.747304	0.975908	1	-
ENSMUSC	Traf3	TNF recep	GO:00081	metabolic	K03174	TNF recep	mmu052C	Pathways	12:111166	1214	1259	923	1814	1390	1625	3.673168	3.042241	3.08653	4.234379	2.708268	3.868	3.267313	3.603549	0.552479	1	-
ENSMUSC	Cdc42bpb	CDC42 bi	GO:00081	metabolic	-	-	-	-	12:111292	3397	4163	3010	4094	4507	4630	22.16203	21.69033	21.70337	20.60593	18.9346	23.76325	21.85191	21.10126	0.510663	1	-
ENSMUSC	Exoc3l4	exocyst cc	GO:00511	localizatio	-	-	-	-	12:111417	18	30	19	27	28	34	0.108161	0.143968	0.126187	0.125169	0.108348	0.160728	0.126105	0.131415	1	1	-
ENSMUSC	Tnfaip2	tumor nec	GO:00325	developm	-	-	-	-	12:111442	8	17	7	13	24	12	0.055234	0.09373	0.053416	0.069241	0.106699	0.065178	0.06746	0.080372	0.668249	1	-
ENSMUSC	Eif5	eukaryotic	GO:00081	metabolic	K03262	translatior	mmu0301	RNA trans	12:111538	1966	4189	1511	3671	3179	3974	6.316244	10.7481	5.365212	9.098928	6.576892	10.04418	7.476519	8.573332	0.463086	1	-
ENSMUSC	Ppp1r13b	protein pl	GO:00508	response	-	-	-	-	12:111828	2319	3002	2172	3134	3779	3217	24.34974	25.17388	25.20579	25.38768	25.55201	26.57392	24.9098	25.83787	0.884313	1	-
ENSMUSC	Zfyve21	zinc finge	GO:00056	cell|cell jui	-	-	-	-	12:111814	104	118	98	114	143	151	1.726026	1.564015	1.797566	1.459656	1.528283	1.971526	1.695869	1.653155	0.756006	1	-
ENSMUSC	Xrcc3	X-ray rep	GO:00081	metabolic	K10880	DNA-repa	mmu0344	Homologo	12:111803	66	86	63	103	118	60	0.737124	0.767076	0.777649	0.887486	0.848655	0.527174	0.760616	0.754439	0.889685	1	-
ENSMUSC	Klc1	kinesin lig	GO:00511	localizatio	K10407	kinesin lig	mmu0513	Salmonell	12:111758	11393	13491	10640	15523	18610	15113	100.5398	95.08018	103.774	105.6832	105.755	104.9208	99.79799	105.453	0.712101	1	-
ENSMUSC	2010107E	RIKEN cDI	GO:00329	macromo	-	-	-	-	12:111961	210	231	215	265	292	261	7.006662	6.155311	7.928238	6.821318	6.273795	6.850832	7.03007	6.648648	0.544214	1	-
ENSMUSC	Kif26a	kinesin fai	GO:00325	developm	-	-	-	-	12:112146	127	136	123	199	157	163	1.077597	0.921585	1.153467	1.302673	0.85784	1.088053	1.050883	1.082855	0.988588	1	-
ENSMUSC	Hecw1	HECT, C2	GO:00081	metabolic	-	-	-	-	13:142264	1209	1192	1035	1021	1842	1566	3.414713	2.688748	3.230837	2.224764	3.350211	3.479609	3.111433	3.018195	0.662184	1	-
ENSMUSC	Ggps1	geranylge	GO:00081	metabolic	K00804	geranylge	mmu011C	Metabolic	13:140506	996	1330	878	1249	1500	1254	5.289545	5.641004	5.153472	5.117427	5.129853	5.239228	5.36134	5.162169	0.508152	1	-
ENSMUSC	Gng4	guanine n	GO:00048	signal trar	K04541	guanine n	mmu052C	Pathways	13:137840	742	838	515	908	944	803	12.5673	11.33517	9.640333	11.86463	10.29591	10.69953	11.18093	10.95336	0.681383	1	-
ENSMUSC	Ryr2	ryanodine	GO:00325	developm	K04962	ryanodine	mmu0402	cAMP sigr	13:115531	8006	10089	7931	10182	11975	11081	14.71984	14.81431	16.11619	14.44279	14.17809	16.02793	15.21678	14.88294	0.585946	1	-
ENSMUSC	Amph	amphiphy	GO:00452	synapse|c	K12562	amphiphy	mmu0414	Endocytos	13:189482	4601	5455	4367	6001	6988	5782	45.78363	43.35095	48.02728	46.06933	44.77809	45.26338	45.72062	45.37027	0.702382	1	-
ENSMUSC	Gli3	GLI-Krupp	GO:00325	developm	K06230	zinc finge	mmu052C	Pathways	13:154632	228	248	172	239	262	301	1.343233	1.166844	1.119929	1.086288	0.993966	1.395059	1.210002	1.158438	0.617099	1	-
ENSMUSC	Aoah	acyloxyac	GO:00081	metabolic	-	-	-	-	13:207941	4	4	0	7	6	2	0.036498	0.029144	0	0.049273	0.035251	0.014359	0.021881	0.032961	0.598698	1	-
ENSMUSC	Trim27	tripartite	GO:00081	metabolic	-	-	-	-	13:211794	377	493	360	528	604	475	1.750755	1.828421	1.847708	1.891679	1.806238	1.735356	1.808961	1.811091	0.841868	1	-
ENSMUSC	Zkscan3	zinc finge	GO:00081	metabolic	-	-	-	-	13:213870	540	753	452	774	808	369	2.217605	2.469628	2.05152	2.452234	2.136767	1.192142	2.246251	1.927048	0.264737	1	-
ENSMUSC	Scgn	secretago	GO:00452	synapse|c	-	-	-	-	13:239534	14	8	17	13	31	10	0.450861	0.205756	0.605069	0.322976	0.642877	0.253346	0.420562	0.4064	0.950201	1	-
ENSMUSC	Carmil1	capping p	GO:00325	developm	-	-	-	-	13:240123	585	770	529	691	911	743	2.850903	2.996838	2.849242	2.597976	2.858906	2.848567	2.898994	2.768483	0.492853	1	-
ENSMUSC	Mrs2	MRS2 ma	GO:00081	metabolic	-	-	-	-	13:249874	1946	2401	1527	2478	2861	2529	14.71443	14.49902	12.76105	14.45548	13.93071	15.0439	13.9915	14.4767	0.915618	1	-
ENSMUSC	Gpld1	glycosylpl	GO:00325	developm	-	-	mmu0056	Glycosylpl	13:249431	537	532	453	643	664	615	5.663878	4.481226	5.280615	5.232162	4.509854	5.103	5.141906	4.948339	0.580921	1	-
ENSMUSC	Prl	prolactin	GO:00325	developm	K05439	prolactin[-	mmu0415	PI3K-Akt	13:270575	0	0	2	2	2	0	0	0	0.050195	0.035041	0.029238	0	0.016732	0.021426	1	1	-
ENSMUSC	Exoc2	exocyst cc	GO:00511	localizatio	K17637	exocyst cc	mmu0401	Ras signal	13:308139	883	1332	888	1076	1397	1340	6.860203	8.264679	7.624931	6.449389	6.989196	8.190147	7.583271	7.209577	0.471967	1	-
ENSMUSC	Tfap2a	transcripti	GO:00325	developm	-	-	-	-	13:407153	2	6	0	0	3	2	0.010938	0.026211	0	0	0.010569	0.008609	0.012383	0.006392	0.468168	1	-
ENSMUSC	Gcnt2	glucosam	GO:00325	developm	K00742	N-acetylla	mmu011C	Metabolic	13:408597	236	369	354	466	387	561	1.017491	1.270547	1.68682	1.550012	1.074446	1.902798	1.324953	1.509085	0.517263	1	-
ENSMUSC	Tmem14c	transmem	GO:00325	developm	-	-	-	-	13:410162	250	375	261	331	507	310	16.70081	20.00663	19.2701	17.05907	21.81021	16.29184	18.65918	18.38704	0.781074	1	-
ENSMUSC	Elovl2	elongatioi	GO:00081	metabolic	K10205	elongatioi	mmu0121	Fatty acid	13:411823	291	341	259	96	472	392	2.584421	2.418635	2.54224	0.657767	2.699403	2.738848	2.515099	2.032006	0.35575	1	-
ENSMUSC	Nedd9	neural pre	GO:00099	cellular pr	-	-	-	-	13:413095	297	379	218	399	516	398	1.900465	1.936821	1.541725	1.969732	2.126221	2.003542	1.793003	2.033165	0.401202	1	-
ENSMUSC	Hivep1	human im	GO:00081	metabolic	-	-	-	-	13:420520	1399	1611	990	1683	1790	1608	7.716376	7.096383	6.035007	7.161602	6.357751	6.977384	6.949255	6.832246	0.676922	1	-
ENSMUSC	Edn1	endotheli	GO:00325	developm	K16366	endotheli	mmu0541	Fluid shea	13:423014	23	29	24	34	44	36	0.656542	0.661123	0.757153	0.74875	0.808792	0.808437	0.691606	0.78866	0.629011	1	-
ENSMUSC	Tbc1d7	TBC1 dor	GO:00081	metabolic	K20396	TBC1 dor	mmu0415	mTOR sig	13:431517	67	82	64	86	117	104	0.663457	0.648488	0.700437	0.657002	0.746073	0.810187	0.670794	0.737754	0.662311	1	-
ENSMUSC	Mcur1	mitochon	GO:00511	localizatio	-	-	-	-	13:435383	541	760	490	683	941	803	4.331554	4.859661	4.336004	4.218881	4.851668	5.057934	4.509073	4.709494	0.85655	1	-
ENSMUSC	Nup153	nucleopoi	GO:00511	localizatio	K14296	nuclear pc	mmu0301	RNA trans	13:466799	969	1184	857	1164	1396	1159	5.934929	5.791478	5.801221	5.500155	5.505946	5.584521	5.842543	5.530207	0.402573	1	-
ENSMUSC	Kif13a	kinesin fai	GO:00511	localizatio	-	-	-	-	13:467490	673	836	538	905	961	882	3.609001	3.580338	3.188612	3.74413	3.318565	3.72093	3.459317	3.594541	0.897672	1	-
ENSMUSC	Tpmt	thiopurine	GO:00081	metabolic	K00569	thiopurine	mmu0098	Drug met	13:470224	157	218	117	222	273	195	2.031394	2.25266	1.673115	2.216036	2.274633	1.984901	1.985723	2.158523	0.673852	1	-
ENSMUSC	Dek	DEK onco	GO:00081	metabolic	-	-	-	-	13:470847	1067	1428	901	1267	1640	1405	11.27731	12.05355	10.52477	10.33113	11.16193	11.6823	11.28521	11.05845	0.644732	1	-
ENSMUSC	Id4	inhibitor c	GO:00325	developm	K17695	DNA-binc	mmu0455	Signaling	13:482612	1534	1993	1439	1834	2477	1866	24.33441	25.24925	25.22917	22.44529	25.30324	23.28724	24.93761	23.67859	0.403411	1	-
ENSMUSC	Susd3	sushi dor	GO:00160	membran	-	-	-	-	13:492306	10	25	22	34	20	29	0.151098	0.301673	0.367395	0.396332	0.194596	0.344713	0.273389	0.31188	0.75606	1	-
ENSMUSC	Ippk	inositol 1,	GO:00081	metabolic	K10572	inositol-p	mmu011C	Metabolic	13:494212	589	797	621	762	953	757	4.584221	4.953981	5.341815	4.575471	4.77637	4.635081	4.960006	4.662307	0.400754	1	-
ENSMUSC	Cenpp	centrome	GO:00099	cellular pr	-	-	-	-	13:494640	12	23	18	22	23	22	0.266526	0.407992	0.441869	0.376983	0.328968	0.384422	0.372129	0.363457	0.889991	1	-
ENSMUSC	Nol8	nucleolar	GO:00081	metabolic	-	-	-	-	13:496530	707	907	594	931	1066	584	3.202381	3.281004	2.97363	3.25337	3.10932	2.081023	3.152338	2.814571	0.278918	1	-
ENSMUSC	Spin1	spindlin 1	GO:00325	developm	-	-	-	-	13:511008	3583	4163	3141	4261	5273	4567	27.24084	25.27703	26.39298	24.99284	25.81584	27.31591	26.30362	26.04153	0.685174	1	-
ENSMUSC	Nxnl2	nucleored	GO:00162	antioxidar	-	-	-	-	13:511710	3	0	0	1	2	1	0.044994	0	0	0.011573	0.019312	0.011802	0.014998	0.014229	1	1	-
ENSMUSC	Wrnip1	Werner he	GO:00081	metabolic	-	-	-	-	13:328020	949	1132	857	1339	1467	1229	9.525585	9.0744	9.507213	10.36899	9.482229	9.70484	9.369066	9.85202	0.786446	1	-
ENSMUSC	Serpinb9b	serine (or	GO:00081	metabolic	K13963	serpin B[-	mmu0514	Amoebias	13:330274	0	2	0	0	0	0	0	0.027523	0	0	0	0	0.009174	0	0.525285	1	-
ENSMUSC	Pxdc1	PX domai	GO:00431	ion bindin	-	-	-	-	13:346278	107	143	112	142	161	111	1.162288	1.240539	1.3446	1.190006	1.126187	0.948554	1.249142	1.088249	0.273574	1	-
ENSMUSC	Prpf4b	pre-mRN	GO:00081	metabolic	-	-	-	-	13:348753	1976	2605	1774	2805	3041	2582	8.537419	8.988627	8.471108	9.349812	8.460789	8.776209	8.665718	8.86227	0.986542	1	-
ENSMUSC	Fam217a	family witl	-	-	-	-	-	-	13:349099	4	19	9	12	14	10	0.039356	0.149289	0.097859	0.091081	0.088698	0.0774	0.095502	0.085727	0.787092	1	-
ENSMUSC	Eci2	enoyl-Co	GO:00081	metabolic	K13239	peroxisor	mmu0414	Peroxisor	13:349777	379	484	373	547	658	606	2.635351	2.687762	2.866522	2.934385	2.946321	3.314995	2.729879	3.065234	0.409642	1	-
ENSMUSC	Fars2	phenylala	GO:00081	metabolic	K01889	phenylala	mmu0097	Aminoacy	13:361174	186	299	207	283	334	294	1.313992	1.686927	1.616204	1.542397	1.519425	1.633945	1.539041	1.565256	0.967428	1	-
ENSMUSC	Ly86	lymphocy	GO:00023	immune s	-	-	-	-	13:373452	104	157	77	120	153	166	1.663187	2.005175	1.360963	1.480539	1.575624	2.088465	1.676442	1.714876	0.987036	1	-
ENSMUSC	Ssr1	signal seq	GO:00511	localizatio	K13249	translocor	mmu0414	Protein pr	13:379666	1490	2159	1423	1837	3710	2019	7.801112	9.027526	8.234214	7.4201	12.50832	8.316058	8.354284	9.414825	0.437885	1	-
ENSMUSC	Riok1	RIO kinas	GO:00081	metabolic	K07178	RIO kinas	mmu030C	Ribosome	13:380369	229	275	214	308	362	287	0.900282	0.86342	0.92983	0.934167	0.916446	0.887639	0.897844	0.912751	0.959396	1	-
ENSMUSC	Snrnp48	small nucl	GO:00081	metabolic	-	-	-	-	13:382049	327	412	299	391	591	472	1.501428	1.510777	1.517314	1.385046	1.747428	1.704943	1.50984	1.612472	0.726165	1	-
ENSMUSC	Slc35b3	solute car	GO:00511	localizatio	-	-	-	-	13:389321	134	118	119	121	224	166	0.869942	0.611803	0.853844	0.60604	0.936455	0.847821	0.77853	0.796772	1	1	-
ENSMUSC	Sema4d	sema don	GO:00325	developm	K06521	semaphor	mmu0436	Axon guic	13:517012	809	1021	733	1039	1144	956	4.518064	4.553815	4.524333	4.476615	4.114192	4.200228	4.532071	4.263678	0.392111	1	-
ENSMUSC	Gadd45g	growth ar	GO:00081	metabolic	K04402	growth ar	mmu052C	Pathways	13:518466	191	296	181	241	291	286	6.030039	7.463184	6.315575	5.869939	5.916079	7.103351	6.602933	6.296456	0.58045	1	-
ENSMUSC	2010111I(	RIKEN cDI	GO:00081	metabolic	-	-	-	-	13:629648	273	378	289	456	411	453	0.709525	0.78459	0.830135	0.914323	0.687863	0.92622	0.77475	0.842802	0.640147	1	-
ENSMUSC	Auh	AU RNA b	GO:00081	metabolic	K05607	methylglu	mmu011C	Metabolic	13:528351	817	1036	739	1049	1376	1123	3.950933	4.001134	3.949743	3.913663	4.284997	4.272362	3.96727	4.157007	0.817684	1	-
ENSMUSC	Fancc	Fanconi a	GO:00325	developm	K10890	fanconi ar	mmu0346	Fanconi a	13:632850	70	64	64	74	94	83	0.308885	0.225541	0.312126	0.251921	0.267107	0.288132	0.282184	0.269053	0.706919	1	-
ENSMUSC	Sptlc1	serine pal	GO:00081	metabolic	K00654	serine pal	mmu011C	Metabolic	13:533327	504	595	374	591	786	590	8.350952	7.873516	6.848961	7.554813	8.386546	7.690749	7.691143	7.877369	1	1	-
ENSMUSC	Msx2	msh hom	GO:00325	developm	K09341	homeobo	mmu0516	HTLV-I int	13:534668	10	15	13	10	12	20	0.151812	0.181864	0.218116	0.117124	0.117315	0.238858	0.18393	0.157766	0.559812	1	-
ENSMUSC	Ercc6l2	excision r	GO:00081	metabolic	-	-	-	-	13:638152	403	578	334	553	636	621	1.244824	1.425857	1.140238	1.317826	1.265069	1.509055	1.270306	1.363983	0.692488	1	-
ENSMUSC	Sfxn1	sideroflex	GO:00325	developm	-	-	-	-	13:540718	719	878	666	992	1102	964	4.293054	4.186758	4.394998	4.569616	4.237152	4.528197	4.291604	4.444988	0.914843	1	-
ENSMUSC	Habp4	hyaluronic	GO:00081	metabolic	-	-	-	-	13:641618	2280	2966	2076	3016	3436	2842	17.94438	18.64278	18.05792	18.31282	17.41411	17.59659	18.21502	17.77451	0.571678	1	-
ENSMUSC	Ctsl	cathepsin	GO:00325	developm	K01365	cathepsin	mmu052C	Proteogly	13:643593	996	1525	870	1205	1761	1330	7.093654	8.674135	6.848202	6.621067	8.076527	7.452003	7.538664	7.383199	0.674334	1	-
ENSMUSC	Drd1	dopamine	GO:00325	developm	K04144	dopamine	mmu0408	Neuroacti	13:540511	71	103	64	120	139	76	0.577477	0.66905	0.575315	0.75299	0.728023	0.486295	0.607281	0.65577	0.764376	1	-
ENSMUSC	Aaed1	AhpC/TSA	GO:00081	metabolic	-	-	-	-	13:642752	175	239	153	251	281	213	1.382656	1.508066	1.336028	1.529961	1.429673	1.323938	1.408917	1.427857	0.941475	1	-
ENSMUSC	Cdk20	cyclin-dep	GO:00325	developm	-	-	-	-	13:644323	126	118	79	151	193	113	0.530683	0.39691	0.36774	0.490652	0.523453	0.374416	0.431778	0.46284	0.78616	1	-
ENSMUSC	Lman2	lectin, ma	GO:00511	localizatio	K10082	lectin, ma	mmu0414	Protein pr	13:553438	1418	1905	1337	2090	2358	2000	9.537384	10.23279	9.938739	10.84501	10.21296	10.58264	9.902971	10.54687	0.658532	1	-
ENSMUSC	Mxd3	Max dime	GO:00081	metabolic	-	-	-	-	13:553251	15	16	8	22	16	13	0.217748	0.185503	0.128347	0.246392	0.149568	0.148461	0.1772	0.181474	1	1	-
ENSMUSC	Prelid1	PRELI dor	GO:00325	developm	-	-	-	-	13:553205	1158	1458	1109	1629	1938	1566	19.52107	19.629	20.66205	21.18587	21.03795	20.76813	19.93737	20.99732	0.76049	1	-
ENSMUSC	Nsd1	nuclear re	GO:00081	metabolic	K15588	histone-ly	mmu0031	Lysine deg	13:552097	3791	4947	3371	4764	5766	4951	9.497037	9.897422	9.3334	9.207388	9.301729	9.757493	9.575953	9.422203	0.628373	1	-
ENSMUSC	Slc34a1	solute car	GO:00325	developm	-	-	-	-	13:553981	0	0	1	2	0	2	0	0	0.008698	0.012144	0	0.012384	0.002899	0.008176	0.410988	1	-
ENSMUSC	F12	coagulatic	GO:00081	metabolic	K01328	coagulatic	mmu0461	Complem	13:554179	1	5	4	2	3	5	0.027376	0.109279	0.120998	0.042234	0.052878	0.107652	0.085885	0.067588	0.846688	1	-
ENSMUSC	Pdlim7	PDZ and l	GO:00325	developm	-	-	-	-	13:554957	515	612	493	703	803	687	3.692018	3.503916	3.906167	3.888145	3.707035	3.874576	3.700701	3.823252	0.939152	1	-
ENSMUSC	Ddx41	DEAD (As	GO:00325	developm	-	-	-	-	13:555304	607	746	599	898	997	926	9.373366	9.200074	10.22304	10.69826	9.91417	11.24938	9.598827	10.6206	0.452239	1	-
ENSMUSC	Pcbd2	pterin 4 a	GO:00081	metabolic	K01724	4a-hydrox	mmu0079	Folate bio	13:557273	42	59	31	55	65	61	3.330439	3.736371	2.71683	3.364722	3.319117	3.805331	3.261214	3.49639	0.854459	1	-
ENSMUSC	Txndc15	thioredox	GO:00099	cellular pr	-	-	-	-	13:557146	821	1174	794	1153	1343	1125	10.41095	11.88943	11.12793	11.27994	10.96674	11.22304	11.14277	11.15657	0.818234	1	-
ENSMUSC	Catsper3	cation cha	GO:00325	developm	-	-	-	-	13:557845	2	3	0	2	0	0	0.064668	0.077495	0	0.049907	0	0	0.047388	0.016636	0.498376	1	-
ENSMUSC	Ddx46	DEAD (As	GO:00081	metabolic	K12811	ATP-depe	mmu0304	Spliceosor	13:556350	1920	2387	1630	2339	2967	2270	14.62105	14.51696	13.71866	13.74162	14.54955	13.59922	14.28556	13.96346	0.595607	1	-
ENSMUSC	Caml	calcium m	GO:00081	metabolic	-	-	-	-	13:556230	534	632	490	650	776	639	13.92782	13.16451	14.12489	13.07933	13.03342	13.11155	13.73907	13.07477	0.48089	1	-
ENSMUSC	B4galt7	xylosylpro	GO:00081	metabolic	K00733	xylosylpro	mmu011C	Metabolic	13:555998	343	384	359	497	535	452	4.421136	3.952912	5.114246	4.942276	4.44067	4.583416	4.496098	4.655454	0.931853	1	-
ENSMUSC	Cxcl14	chemokin	GO:00325	developm	K10033	C-X-C mc	mmu0406	Cytokine-	13:562886	1206	1538	1148	1801	2025	1696	36.39945	37.07231	38.29447	41.93643	39.35746	40.27024	37.25541	40.52138	0.509761	1	-
ENSMUSC	Slc25a48	solute car	GO:00511	localizatio	-	-	-	-	13:564383	0	5	0	0	4	2	0	0.072259	0	0	0.046615	0.028479	0.024086	0.025031	1	1	-
ENSMUSC	Zfp729a	zinc finge	GO:00081	metabolic	-	-	-	-	13:676127	415	540	361	552	604	518	2.20878	2.295322	2.123532	2.266592	2.07012	2.168926	2.209211	2.168546	0.697151	1	-
ENSMUSC	Zfp369	zinc finge	GO:00081	metabolic	K12458	zinc finge	mmu0472	Neurotrop	13:652788	501	667	410	680	798	614	3.786839	4.026347	3.425074	3.965325	3.884158	3.651061	3.746087	3.833515	0.997336	1	-
ENSMUSC	Mterf3	mitochon	GO:00325	developm	-	-	-	-	13:669069	238	323	224	338	429	347	1.912338	2.072694	1.989217	2.095239	2.219726	2.193445	1.991416	2.16947	0.603356	1	-
ENSMUSC	Uqcrb	ubiquinol-	GO:00081	metabolic	K00417	ubiquinol-	mmu011C	Metabolic	13:669006	878	1154	781	1146	1528	1118	48.73547	51.15666	47.91246	49.07561	54.61709	48.82061	49.2682	50.83777	0.938139	1	-
ENSMUSC	Fastkd3	FAST kina	GO:00081	metabolic	-	-	-	-	13:685822	126	181	120	181	135	190	0.835322	0.958317	0.87925	0.925745	0.576329	0.990942	0.890963	0.831005	0.560198	1	-
ENSMUSC	1700001L	RIKEN cDI	-	-	-	-	-	-	13:685974	89	120	76	153	136	94	2.183286	2.350965	2.060534	2.895606	2.148389	1.814089	2.198262	2.286028	0.940692	1	-
ENSMUSC	Adcy2	adenylate	GO:00081	metabolic	K08042	adenylate	mmu052C	Pathways	13:686200	2927	3687	2696	3593	4302	3536	42.44054	42.69501	43.20408	40.19255	40.16828	40.33493	42.77988	40.23192	0.315579	1	-
ENSMUSC	Cetn3	centrin 3	GO:00099	cellular pr	-	-	-	-	13:817830	561	535	469	519	661	769	7.647601	5.824544	7.066132	5.458335	5.802539	8.247063	6.846092	6.502646	0.564656	1	-
ENSMUSC	Smad5	SMAD far	GO:00325	developm	K16790	mothers a	mmu0455	Signaling	13:567030	606	621	251	728	881	670	2.630169	2.152526	1.204014	2.437656	2.462304	2.287689	1.99557	2.395883	0.323177	1	-
ENSMUSC	Trpc7	transient	GO:00511	localizatio	-	-	-	-	13:567731	43	50	32	50	49	53	0.496974	0.461503	0.408754	0.445822	0.364679	0.481895	0.455744	0.430798	0.662092	1	-
ENSMUSC	Hnrnpk	heteroger	GO:00081	metabolic	K12886	heteroger	mmu052C	Viral carci	13:583911	2219	2706	1966	2757	3487	2688	7.490901	7.295421	7.335119	7.180323	7.580249	7.138665	7.373814	7.299745	0.695591	1	-
ENSMUSC	Ccnh	cyclin H [S	GO:00081	metabolic	K06634	cyclin H[-	mmu0411	Cell cycle	13:851894	596	880	695	989	1123	993	7.586122	8.945451	9.776993	9.711811	9.204667	9.943377	8.769522	9.619951	0.511224	1	-
ENSMUSC	Rasa1	RAS p21 p	GO:00325	developm	K04352	Ras GTPas	mmu0401	MAPK sigi	13:852147	1289	1608	1186	1560	1902	1567	8.15162	8.121254	8.289393	7.611076	7.74562	7.795984	8.187422	7.71756	0.357807	1	-
ENSMUSC	2210016F	RIKEN cDI	GO:00081	metabolic	-	-	-	-	13:583798	152	216	162	212	323	239	3.041897	3.452233	3.583133	3.273156	4.162537	3.762782	3.359088	3.732825	0.543291	1	-
ENSMUSC	Gkap1	G kinase a	GO:00508	response	-	-	-	-	13:582333	177	243	140	244	287	251	1.567178	1.718295	1.370006	1.666734	1.636377	1.748358	1.551827	1.683823	0.667171	1	-
ENSMUSC	Slc28a3	solute car	GO:00511	localizatio	-	-	-	-	13:585453	11	28	2	10	18	8	0.078585	0.159749	0.015792	0.055116	0.082806	0.044958	0.084708	0.06096	0.432787	1	-
ENSMUSC	Naa35	N(alpha)-	GO:00081	metabolic	-	-	-	-	13:595852	712	887	581	967	1021	900	3.500552	3.482782	3.157039	3.667866	3.232488	3.48105	3.380124	3.460468	0.994839	1	-
ENSMUSC	Agtpbp1	ATP/GTP	GO:00325	developm	-	-	-	-	13:594457	4980	6721	4881	6672	7294	6565	11.57301	12.47374	12.5364	11.96199	10.91535	12.00225	12.19439	11.62653	0.391609	1	-
ENSMUSC	Dapk1	death ass	GO:00081	metabolic	K08803	death-ass	mmu052C	Pathways	13:606019	3330	3632	2985	3589	5136	4100	14.8141	12.90395	14.67649	12.31785	14.71332	14.34914	14.13151	13.79343	0.605079	1	-
ENSMUSC	Slc6a19	solute car	GO:00511	localizatio	K05334	solute car	mmu0497	Protein di	13:736797	8	9	7	11	22	5	0.058265	0.052348	0.056346	0.061807	0.103175	0.028646	0.055653	0.064543	0.778464	1	-
ENSMUSC	Nkd2	naked cut	GO:00081	metabolic	K03213	naked cut	mmu0431	Wnt signa	13:738185	292	330	242	402	429	378	2.329367	2.102396	2.133624	2.474069	2.203775	2.372232	2.188462	2.350025	0.696496	1	-
ENSMUSC	Trip13	thyroid hc	GO:00325	developm	-	-	-	-	13:739113	15	13	14	18	24	16	0.198236	0.137211	0.204491	0.183533	0.204252	0.166355	0.179979	0.184713	1	1	-
ENSMUSC	Cep72	centroson	GO:00511	localizatio	-	-	-	-	13:740365	29	35	35	45	43	33	0.134265	0.129414	0.179095	0.160734	0.1282	0.120197	0.147591	0.136377	0.651774	1	-
ENSMUSC	Tppp	tubulin pc	GO:00990	supramole	-	-	-	-	13:740094	13663	17926	12397	18187	21585	18503	156.1072	163.5709	156.5454	160.3128	158.8121	166.3144	158.7412	161.8131	0.953538	1	-
ENSMUSC	Ahrr	aryl-hydrc	GO:00081	metabolic	-	-	-	-	13:742111	22	36	23	35	42	43	0.117965	0.154166	0.136301	0.144787	0.145024	0.181392	0.136144	0.157067	0.578222	1	-
ENSMUSC	Pdcd6	programn	GO:00325	developm	-	-	-	-	13:743035	435	535	461	636	670	535	5.722967	5.621231	6.703163	6.455343	5.676243	5.537281	6.015787	5.889622	0.696732	1	-
ENSMUSC	Sdha	succinate	GO:00325	developm	K00234	succinate	mmu011C	Metabolic	13:743222	7938	9439	7040	10203	12406	9923	82.07947	77.94626	80.45313	81.39199	82.60568	80.71929	80.15962	81.57232	0.935809	1	-
ENSMUSC	Ccdc127	coiled-co	GO:00160	membran	-	-	-	-	13:743503	1346	1591	1138	1961	1911	1503	5.837778	5.510847	5.454959	6.561607	5.337252	5.128288	5.601195	5.675716	0.914241	1	-
ENSMUSC	Lrrc14b	leucine ric	-	-	-	-	-	-	13:743595	5	7	0	12	2	2	0.110803	0.123899	0	0.205173	0.028537	0.034878	0.078234	0.089529	1	1	-
ENSMUSC	Pcsk1	proprotei	GO:00081	metabolic	-	-	-	-	13:750898	601	667	468	788	804	648	5.908219	5.236649	5.084804	5.97638	5.089706	5.011501	5.409891	5.359196	0.761077	1	-
ENSMUSC	Rhobtb3	Rho-relat	GO:00325	developm	-	-	-	-	13:758695	410	539	347	500	577	446	3.442965	3.614793	3.220512	3.239286	3.120182	2.946421	3.42609	3.101963	0.268619	1	-
ENSMUSC	Spata9	spermato	GO:00325	developm	-	-	-	-	13:759671	1	0	0	2	1	0	0.02854	0	0	0.04403	0.018369	0	0.009513	0.0208	0.648787	1	-
ENSMUSC	Glrx	glutaredo	GO:00081	metabolic	-	-	-	-	13:758398	318	247	225	306	414	422	2.670768	1.656733	2.088521	1.98272	2.239049	2.788255	2.138674	2.336674	0.679865	1	-
ENSMUSC	Arsk	arylsulfata	GO:00081	metabolic	-	-	-	-	13:760604	140	152	107	196	164	164	1.249364	1.083302	1.055339	1.349415	0.942453	1.15137	1.129335	1.147746	0.96493	1	-
ENSMUSC	Srd5a1	steroid 5 a	GO:00325	developm	K12343	3-oxo-5-	mmu0014	Steroid hc	13:695734	157	225	137	185	260	208	2.487325	2.846821	2.398832	2.261173	2.652527	2.592429	2.577659	2.502043	0.692399	1	-
ENSMUSC	Nsun2	NOL1/NC	GO:00325	developm	-	-	-	-	13:695337	1295	1633	1094	1671	2112	1707	9.843175	9.91283	9.190302	9.79879	10.33747	10.20728	9.648769	10.11451	0.801699	1	-
ENSMUSC	Mctp1	multiple C	GO:00452	synapse|r	-	-	-	-	13:763845	854	1096	780	1140	1356	1097	4.757193	4.875841	4.802137	4.899238	4.864156	4.807407	4.811724	4.856934	0.881405	1	-
ENSMUSC	Slf1	SMC5-SM	GO:00081	metabolic	-	-	-	-	13:770430	228	265	222	290	355	294	2.031116	1.885346	2.185743	1.993095	2.03649	2.06043	2.034069	2.030005	0.838717	1	-
ENSMUSC	Med10	mediator	GO:00325	developm	-	-	-	-	13:698023	391	499	420	538	625	553	3.441998	3.508167	4.086306	3.653817	3.542983	3.829746	3.678824	3.675515	0.829913	1	-
ENSMUSC	8030423J	RIKEN cDI	-	-	-	-	-	-	13:708829	2	0	0	0	0	0	0.140499	0	0	0	0	0	0.046833	0	0.526391	1	-
ENSMUSC	Ndufs6	NADH de	GO:00325	developm	K03939	NADH de	mmu011C	Metabolic	13:733198	475	465	306	506	629	431	14.77462	11.55107	10.5194	12.14237	12.59878	10.54657	12.2817	11.76257	0.600444	1	-
ENSMUSC	Mrpl36	mitochon	GO:00081	metabolic	K02919	large subu	mmu0301	Ribosome	13:733285	130	152	148	178	231	148	3.421363	3.194808	4.304909	3.614148	3.914916	3.064273	3.64036	3.531112	0.729266	1	-
ENSMUSC	Lpcat1	lysophosp	GO:00325	developm	K13510	lysophosp	mmu011C	Metabolic	13:734671	231	277	418	306	667	247	1.991309	1.907004	3.982443	2.035066	3.7026	1.675074	2.626919	2.470913	0.714309	1	-

ENSMUSC	Clptm1l	CLPTM1-l	GO:00099	cellular pr	-	-	-	-	13:736040	1118	1526	1141	1495	1879	1437	17.71684	19.31278	19.98375	18.27747	19.17458	17.9148	19.00445	18.45562	0.573686	1	-
ENSMUSC	Hapln1	hyalurona	GO:00325	developm	-	-	-	-	13:895397	950	1107	740	1121	1247	1045	9.387476	8.736117	8.08172	8.545963	7.934987	8.123674	8.735104	8.201542	0.381371	1	-
ENSMUSC	Xrcc4	X-ray rep	GO:00081	metabolic	K10886	DNA-repa	mmu0345	Non-hom	13:897740	177	183	144	201	269	217	2.383079	1.967715	2.142766	2.087819	2.332241	2.298461	2.16452	2.239507	0.948387	1	-
ENSMUSC	Atg10	autophag	GO:00081	metabolic	K17888	ubiquitin-	mmu0414	Autophag	13:909353	107	151	95	154	206	119	2.773013	3.125291	2.721075	3.079064	3.437866	2.426199	2.873126	2.981043	0.934975	1	-
ENSMUSC	Acot12	acyl-CoA	GO:00081	metabolic	K01067	acetyl-Co	mmu0062	Pyruvate r	13:917415	6	14	14	11	9	14	0.095625	0.1782	0.246606	0.135261	0.09237	0.175534	0.173477	0.134389	0.415102	1	-
ENSMUSC	Zcchc9	zinc finge	GO:00081	metabolic	-	-	-	-	13:917965	161	203	144	201	272	191	1.806715	1.819307	1.785966	1.740169	1.965566	1.686201	1.803996	1.797312	0.840494	1	-
ENSMUSC	Slc30a5	solute car	GO:00511	localizatio	-	-	-	-	13:100802	686	943	643	909	934	1032	4.462112	4.898622	4.622469	4.561533	3.912173	5.280889	4.661068	4.584865	0.702714	1	-
ENSMUSC	Rad17	RAD17 ch	GO:00325	developm	-	-	-	-	13:100617	291	339	246	306	452	332	1.818432	1.691805	1.69897	1.47522	1.818851	1.632123	1.736402	1.642064	0.517077	1	-
ENSMUSC	Ocln	occludin [	GO:00508	response	K06088	occludin[-	mmu0453	Tight junc	13:100496	266	386	233	362	534	369	2.148342	2.489747	2.079811	2.255592	2.777268	2.344545	2.2393	2.459135	0.570763	1	-
ENSMUSC	Gtf2h2	general tr	GO:00081	metabolic	K03142	transcripti	mmu0520	Viral carci	13:100460	311	346	263	416	564	359	3.221216	2.862081	3.010661	3.324173	3.761779	2.925262	3.031319	3.337071	0.555518	1	-
ENSMUSC	Serf1	small EDR	-	-	-	-	-	-	13:100108	80	93	42	78	101	62	2.460771	2.28461	1.427833	1.850995	2.000587	1.500327	2.057738	1.78397	0.374271	1	-
ENSMUSC	Smn1	survival m	GO:00325	developm	K13129	survival m	mmu0301	RNA trans	13:100124	206	260	233	333	372	347	3.08737	3.112018	3.859443	3.850322	3.590216	4.091307	3.352944	3.843948	0.373229	1	-
ENSMUSC	Mccc2	methylcro	GO:00081	metabolic	K01969	3-methylc	mmu0028	Metabolic	13:999485	334	479	362	502	608	502	5.032925	5.764409	6.02877	5.835901	5.899727	5.950977	5.608702	5.895535	0.818906	1	-
ENSMUSC	Cartpt	CART prep	GO:00325	developm	-	-	-	-	13:998984	10	20	9	30	16	18	0.39986	0.638676	0.397718	0.925458	0.411973	0.566207	0.478751	0.634546	0.452108	1	-
ENSMUSC	Ptcd2	pentatric	GO:00325	developm	-	-	-	-	13:993196	302	410	292	419	478	437	10.82134	11.73285	11.56387	11.58291	11.02952	12.31874	11.37269	11.64372	1	1	-
ENSMUSC	Btf3	basic tran	GO:00325	developm	-	-	-	-	13:983098	263	418	372	487	528	490	5.067306	6.431963	7.921567	7.23903	6.551027	7.427256	6.473612	7.072438	0.623193	1	-
ENSMUSC	Ankra2	ankyrin re	GO:00081	metabolic	-	-	-	-	13:982630	403	538	420	603	741	564	3.163595	3.372903	3.643947	3.651933	3.74583	3.483094	3.393481	3.626952	0.695468	1	-
ENSMUSC	Arhgef28	Rho guan	GO:00987	molecular	-	-	-	-	13:978994	1742	2178	1762	2135	2708	2395	13.02035	13.00104	14.5555	12.31127	13.034	14.08286	13.52563	13.14271	0.561	1	-
ENSMUSC	Gfm2	G elongat	GO:00081	metabolic	-	-	-	-	13:971379	469	600	393	616	774	651	2.090084	2.135443	1.935664	2.117885	2.221194	2.282353	2.05373	2.207144	0.653278	1	-
ENSMUSC	Polk	polymera	GO:00081	metabolic	K03511	DNA poly	mmu0520	Pathways	13:964806	209	259	177	272	319	230	1.750984	1.73293	1.63891	1.758061	1.720996	1.515909	1.707608	1.664989	0.703553	1	-
ENSMUSC	Col4a3bp	collagen,	GO:00325	developm	-	-	-	-	13:965426	781	1053	1029	968	1110	1410	4.169119	4.489186	6.070932	3.986566	3.81567	5.921386	4.909746	4.574541	0.482324	1	-
ENSMUSC	Hmgcr	3-hydroxy	GO:00325	developm	K00021	hydroxym	mmu0110	Metabolic	13:966489	1808	2198	1470	2429	2824	2101	10.94192	10.62353	9.832394	11.34104	11.00562	10.00304	10.46595	10.78324	0.949181	1	-
ENSMUSC	Poc5	POC5 cen	GO:00099	cellular pr	-	-	-	-	13:963882	769	948	752	944	1205	1007	6.238866	6.142329	6.742854	5.908556	6.295363	6.427166	6.374683	6.210362	0.621726	1	-
ENSMUSC	F2rl2	coagulatic	GO:00048	signal trar	K04235	coagulatic	mmu0408	Neuroacti	13:956968	3	12	4	4	3	7	0.073209	0.233871	0.107893	0.0753	0.04715	0.134396	0.138324	0.085616	0.264168	1	-
ENSMUSC	Iqgap2	IQ motif c	GO:00508	response	K05767	Ras GTPas	mmu0481	Regulatio	13:956271	3415	4304	2971	4141	5899	4620	36.13125	36.36724	34.74096	33.80087	40.19068	38.45432	35.74648	37.48196	0.789074	1	-
ENSMUSC	Crhbp	corticotro	GO:00081	metabolic	-	-	-	-	13:954313	419	562	400	574	663	572	8.995557	9.635982	9.49119	9.507269	9.166046	9.660974	9.374243	9.444763	0.881056	1	-
ENSMUSC	Aggf1	angiogen	GO:00325	developm	-	-	-	-	13:953506	1247	1763	1140	1742	2187	1638	17.64939	19.92789	17.83261	19.02136	19.93273	18.23845	18.46996	19.06418	0.930301	1	-
ENSMUSC	Pde8b	phosphoc	GO:00081	metabolic	K18437	high affini	mmu0023	Purine me	13:950244	1232	1349	991	1544	1841	1382	6.189205	5.412301	5.502305	5.98414	5.955695	5.461891	5.701271	5.800576	0.944989	1	-
ENSMUSC	Ap3b1	adaptor-r	GO:00325	developm	K12397	AP-3 com	mmu0414	Lysosome	13:943589	1354	1809	1231	1747	2391	1856	16.67796	17.79544	16.75828	16.60151	18.96526	17.98512	17.07723	17.85063	0.822187	1	-
ENSMUSC	Scamp1	secretory	GO:00452	synapse|c	-	-	-	-	13:942013	3602	4693	3054	4437	5536	4749	36.14319	37.6079	33.86871	34.34808	35.7712	37.48831	35.87326	35.8692	0.776436	1	-
ENSMUSC	Jmy	junction-r	GO:00081	metabolic	-	-	-	-	13:934301	1485	1871	1274	1850	2225	1899	8.80817	8.862962	8.351713	8.465666	8.498533	8.861249	8.674282	8.608483	0.720648	1	-
ENSMUSC	Dimt1	DIM1 dim	GO:00081	metabolic	-	-	-	-	13:106947	206	276	189	345	375	268	2.194338	2.347966	2.225079	2.835217	2.572308	2.245862	2.255794	2.551129	0.430656	1	-
ENSMUSC	Kif2a	kinesin fa	GO:00325	developm	-	-	-	-	13:106958	1910	2203	1568	2331	2734	2238	13.55743	12.48832	12.3009	12.76487	12.49675	12.49727	12.78222	12.5863	0.653489	1	-
ENSMUSC	Ercc8	excision r	GO:00081	metabolic	K10570	DNA excis	mmu0412	Ubiquitin	13:108158	95	167	142	170	201	167	0.540289	0.758515	0.892555	0.745899	0.736123	0.747187	0.730453	0.74307	0.977686	1	-
ENSMUSC	Elovl7	ELOVL far	GO:00081	metabolic	K10250	elongatior	-	-	13:108214	164	347	172	237	326	266	1.711418	2.891931	1.983753	1.90806	2.190715	2.183762	2.195701	2.094179	0.640125	1	-
ENSMUSC	Depdc1b	DEP dom	GO:00400	locomotic	-	-	-	-	13:108316	3	4	14	5	8	4	0.040142	0.042742	0.207046	0.051614	0.068937	0.042106	0.096643	0.054219	0.273477	1	-
ENSMUSC	Pde4d	phosphoc	GO:00325	developm	K13293	cAMP-spe	mmu0402	cAMP sigr	13:108449	636	815	515	697	850	793	2.23847	2.290855	2.003309	1.892593	1.926493	2.195726	2.177544	2.004937	0.305788	1	-
ENSMUSC	Rab3c	RAB3C, m	GO:00452	synapse|c	-	-	-	-	13:110054	12667	15055	9810	15341	16598	13676	54.13474	51.38412	46.33596	50.58087	45.6786	45.98035	50.61827	47.41327	0.308253	1	-
ENSMUSC	Plk2	polo like k	GO:00325	developm	K08861	polo-like	mmu0406	FoxO sign	13:110395	3669	4359	3408	4698	5764	4527	30.39238	28.83697	31.20063	30.02343	30.74649	29.50811	30.14333	30.09034	0.759814	1	-
ENSMUSC	Thbs4	thrombos	GO:00325	developm	K04659	thrombos	mmu0415	PI3K-Akt s	13:927515	270	350	241	410	447	394	4.546527	4.70684	4.485181	5.326344	4.847049	5.219421	4.579516	5.130938	0.440674	1	-
ENSMUSC	Serinc5	serine inc	GO:00325	developm	-	-	-	-	13:926110	1179	1546	1060	1596	1944	1725	9.816924	10.28057	9.754708	10.25239	10.42348	11.29956	9.950732	10.65847	0.626916	1	-
ENSMUSC	Mtx3	metaxin 3	GO:00511	localizatio	-	-	-	-	13:928447	1154	1481	1017	1431	1686	1403	8.627533	8.842636	8.403274	8.253738	8.116954	8.251813	8.624481	8.207502	0.425354	1	-
ENSMUSC	Zfyve16	zinc finge	GO:00511	localizatio	K04679	MAD, mo	mmu0414	Endocytos	13:924871	315	460	305	456	550	422	2.110536	2.461418	2.258542	2.357094	2.373008	2.224362	2.276832	2.318155	0.966243	1	-
ENSMUSC	Dhfr	dihydrofo	GO:00325	developm	K00287	dihydrofo	mmu0110	Metabolic	13:923547	79	113	88	111	154	112	0.902116	1.030523	1.110612	0.977882	1.132419	1.006147	1.014417	1.038816	1	1	-
ENSMUSC	Rasgrf2	RAS prote	GO:00987	molecular	-	-	-	-	13:918804	2169	2800	2030	2781	3468	2793	12.93437	13.3349	13.37915	12.79432	13.3174	13.10291	13.21614	13.07154	0.684303	1	-
ENSMUSC	Nln	neurolysir	GO:00325	developm	K01393	neurolysir	mmu0461	Renin-ang	13:104023	560	700	488	658	780	652	2.870668	2.865755	2.764788	2.602264	2.574807	2.629381	2.833737	2.602151	0.286807	1	-
ENSMUSC	Trappc13	trafficking	-	-	-	-	-	-	13:104142	717	913	702	1029	1151	862	4.232687	4.304409	4.580158	4.686436	4.375492	4.00327	4.372418	4.355066	0.787725	1	-
ENSMUSC	Trim23	tripartite r	GO:00081	metabolic	-	-	-	-	13:104178	1370	1639	1241	1703	1992	1691	9.683908	9.252412	9.695022	9.286991	9.067218	9.403395	9.543781	9.252535	0.538508	1	-
ENSMUSC	Ppwd1	peptidylp	GO:00081	metabolic	-	-	-	-	13:104205	141	195	157	261	313	205	1.257363	1.388745	1.54735	1.79562	1.797389	1.438161	1.397819	1.677057	0.24494	1	-
ENSMUSC	Cenpk	centrome	GO:00081	metabolic	-	-	-	-	13:104228	16	20	10	14	21	20	0.156559	0.156291	0.108141	0.105685	0.132319	0.153956	0.14033	0.130654	0.756003	1	-
ENSMUSC	Cwc27	CWC27 sp	GO:00081	metabolic	-	-	-	-	13:104631	330	412	286	433	507	354	6.269187	6.250877	6.004971	6.346223	6.202405	5.29068	6.175011	5.946436	0.603526	1	-
ENSMUSC	Srek1ip1	splicing re	GO:00081	metabolic	-	-	-	-	13:104792	201	256	202	226	302	278	1.55804	1.584776	1.730534	1.351514	1.507456	1.695268	1.62445	1.518079	0.478204	1	-
ENSMUSC	Rgs7bp	regulator	GO:00508	response	-	-	-	-	13:104945	8348	10453	7174	9906	12490	9838	72.02389	72.02461	68.40729	65.93603	69.3923	66.77469	70.8186	67.36767	0.372883	1	-
ENSMUSC	Rnf180	ring finge	GO:00081	metabolic	-	-	-	-	13:105130	292	389	229	355	327	390	2.323296	2.47182	2.013738	2.179116	1.675421	2.441164	2.269618	2.098567	0.432746	1	-
ENSMUSC	Htr1a	5-hydroxy	GO:00048	signal trar	K04153	5-hydroxy	mmu0408	Neuroacti	13:105443	746	922	568	1007	1082	870	10.1582	10.02661	8.548157	10.57883	9.487669	9.319831	9.577657	9.795444	0.988787	1	-
ENSMUSC	Parp8	poly (ADP	GO:00038	catalytic a	-	-	-	-	13:116854	574	871	537	972	936	891	3.334351	4.040762	3.447631	4.356079	3.501306	4.071816	3.607581	3.9764	0.499954	1	-
ENSMUSC	Emb	embigin [	GO:00452	synapse|c	-	-	-	-	13:117208	483	866	419	637	807	671	3.337604	4.779158	3.199992	3.395921	3.591002	3.647714	3.772251	3.544879	0.487737	1	-
ENSMUSC	Hcn1	hyperpola	GO:00325	developm	-	-	-	-	13:117602	2951	3469	2292	3481	3862	3344	9.404095	8.828725	8.072524	8.558196	7.925283	8.383493	8.768448	8.288991	0.368917	1	-
ENSMUSC	Mrps30	mitochon	GO:00081	metabolic	-	-	-	-	13:118378	363	425	305	480	557	455	3.11206	2.909889	2.889932	3.174775	3.075046	3.068762	2.970627	3.106194	0.860898	1	-
ENSMUSC	Fgf10	fibroblast	GO:00325	developm	K04358	fibroblast	mmu0520	Pathways	13:118669	275	361	239	415	542	377	3.344149	3.50595	3.212168	3.893425	4.244307	3.606665	3.354089	3.914799	0.263777	1	-
ENSMUSC	Slc4a7	solute car	GO:00452	synapse|c	-	-	-	-	14:147022	737	823	737	1006	1236	604	2.242927	2.00029	2.478916	2.361978	2.42226	1.446089	2.240711	2.076776	0.473574	1	-
ENSMUSC	Psmd6	proteasor	GO:00081	metabolic	K03037	26S prote	mmu0516	Epstein-B	14:141121	592	637	583	727	857	645	7.919891	6.805864	8.620108	7.50348	7.383007	6.788409	7.781954	7.224965	0.374064	1	-
ENSMUSC	Atxn7	ataxin 7 [	GO:00081	metabolic	-	-	-	-	14:139614	426	380	285	426	432	374	2.538132	1.808144	1.876707	1.95814	1.657458	1.753018	2.074328	1.789539	0.187865	1	-
ENSMUSC	Fezf2	Fez family	GO:00325	developm	-	-	-	-	14:123420	863	1023	753	1139	1290	1008	10.05403	9.518108	9.695518	10.23725	9.67773	9.238484	9.755885	9.717821	0.779153	1	-
ENSMUSC	4930452B	RIKEN cDI	-	-	-	-	-	-	14:843119	168	202	156	227	277	235	2.896852	2.781722	2.97295	3.019765	3.075751	3.187834	2.883841	3.09445	0.717597	1	-
ENSMUSC	Pdhb	pyruvate	GO:00081	metabolic	-	-	mmu0110	Metabolic	14:816599	1693	1907	1532	2208	2599	2142	13.9918	12.58673	13.99336	14.07816	13.83176	13.92665	13.52396	13.94552	0.944317	1	-
ENSMUSC	Fam107a	family wit	GO:00099	cellular pr	-	-	-	-	14:829627	8429	9511	6848	10785	12410	10238	90.70476	81.73838	81.44503	89.5374	85.99644	86.67225	84.62939	87.40203	0.927636	1	-
ENSMUSC	Acox2	acyl-Coer	GO:00081	metabolic	K10214	3alpha,7a	mmu0110	Metabolic	14:822551	2	11	6	6	12	8	0.025184	0.110638	0.083523	0.058297	0.097327	0.079261	0.073115	0.078295	1	1	-
ENSMUSC	Kctd6	potassium	GO:00081	metabolic	-	-	-	-	14:821414	697	834	630	982	1069	928	9.187717	8.779844	9.178294	9.986576	9.074179	9.62351	9.048619	9.561422	0.759105	1	-
ENSMUSC	Map3k1	mitogen-	GO:00081	metabolic	K04416	mitogen-a	mmu0401	MAPK sigr	13:111746	267	288	197	334	425	308	1.969619	1.696712	1.606143	1.900848	2.018898	1.787442	1.757491	1.902396	0.662897	1	-
ENSMUSC	Il6st	interleukir	GO:00325	developm	K05060	interleukir	mmu0520	Pathways	13:112464	1278	1771	923	1709	1840	1795	4.450088	4.924952	3.552106	4.59103	4.125817	4.917145	4.309048	4.544664	0.787566	1	-
ENSMUSC	Plpp1	phospholi	GO:00081	metabolic	K01080	phosphati	mmu0110	Metabolic	13:112800	142	239	157	233	260	263	3.683195	4.950864	4.500724	4.662537	4.342753	5.366657	4.378261	4.790649	0.639298	1	-
ENSMUSC	Ndufs4	NADH de	GO:00325	developm	K03937	NADH de	mmu0110	Metabolic	13:114287	446	492	378	509	688	481	9.050148	7.973198	8.477335	7.968365	8.990092	7.678507	8.500227	8.212321	0.614766	1	-
ENSMUSC	Kat6b	K(lysine) a	GO:00081	metabolic	-	-	-	-	14:214814	1333	1849	1258	1786	2156	1886	2.503706	2.773549	2.611444	2.588003	2.607693	2.786799	2.629566	2.660832	0.894173	1	-
ENSMUSC	Dusp13	dual spec	GO:00081	metabolic	-	-	-	-	14:217333	1	0	3	0	9	0	0.010572	0	0.035046	0	0.061255	0	0.015206	0.020418	0.855434	1	-
ENSMUSC	Samd8	sterile alp	GO:00081	metabolic	-	-	-	-	14:217505	1781	2150	1544	2311	2627	2102	12.90276	12.4395	12.36269	12.91663	12.25557	11.98016	12.56832	12.38412	0.658355	1	-
ENSMUSC	Vdac2	voltage-d	GO:00452	synapse|c	K15040	voltage-d	mmu0516	HTLV-I inf	14:218252	1884	2344	1694	2552	3207	2596	17.49825	17.3867	17.38897	18.28625	19.18083	18.96834	17.42464	18.8118	0.542728	1	-
ENSMUSC	Nkiras1	NFKB inhi	GO:00508	response	-	-	-	-	14:182711	1259	1476	1131	1585	1946	1582	10.48161	9.813745	10.40667	10.18033	10.43278	10.36143	10.23401	10.32485	0.869276	1	-
ENSMUSC	Comtd1	catechol-	GO:00081	metabolic	-	-	-	-	14:218458	67	135	69	115	146	119	1.115591	1.795203	1.269774	1.477267	1.565455	1.558802	1.393523	1.533841	0.681442	1	-
ENSMUSC	Ube2e1	ubiquitin-	GO:00081	metabolic	K20217	ubiquitin-	mmu0412	Ubiquitin	14:182827	1000	1284	882	1264	1574	1299	11.86054	12.1623	11.56166	11.56598	12.02165	12.12062	11.8615	11.90275	0.825485	1	-
ENSMUSC	Nr1d2	nuclear re	GO:00325	developm	-	-	-	-	14:182040	1583	2027	1432	2094	2460	2102	14.07119	14.38962	14.06824	14.36008	14.0812	14.69919	14.17635	14.38016	0.91069	1	-
ENSMUSC	Thrb	thyroid hc	GO:00325	developm	K08362	thyroid hc	mmu0408	Neuroacti	14:176602	914	1081	703	1112	1246	1191	4.79525	4.529355	4.076307	4.500906	4.20957	4.915725	4.466971	4.542067	0.937917	1	-
ENSMUSC	Dlg5	discs, larg	GO:00325	developm	-	-	-	-	14:241339	994	1309	980	1618	1577	1509	5.436867	5.71805	5.924279	6.827651	5.55455	6.493255	5.693065	6.291819	0.441094	1	-
ENSMUSC	Ngly1	N-glycan	GO:00081	metabolic	K01456	peptide-N	mmu0414	Protein pr	14:162492	321	462	345	552	616	484	1.786008	2.052892	2.121509	2.36945	2.207058	2.118531	1.986803	2.23168	0.417568	1	-
ENSMUSC	Oxsm	3-oxoacy	GO:00081	metabolic	K09458	3-oxoacyl	mmu0110	Metabolic	14:162386	167	214	128	206	266	192	1.348232	1.379779	1.142102	1.283059	1.382883	1.219445	1.290038	1.295129	0.884833	1	-
ENSMUSC	Dydc1	DPY30 do	GO:00081	metabolic	-	-	-	-	14:410729	0	0	0	0	0	2	0	0	0	0	0	0.064187	0	0.021396	0.526852	1	-
ENSMUSC	Dydc2	DPY30 do	GO:00081	metabolic	-	-	-	-	14:410491	10	8	3	15	11	8	0.746438	0.476911	0.247507	0.863799	0.528719	0.46975	0.490285	0.620756	0.669844	1	-
ENSMUSC	Fam213a	family wit	GO:00325	developm	-	-	-	-	14:409937	1789	2441	1663	2598	3050	2653	25.11103	27.36325	25.79842	28.13349	27.56822	29.29559	26.0909	28.33243	0.500003	1	-
ENSMUSC	Glud1	glutamate	GO:00325	developm	K00261	glutamate	mmu0110	Metabolic	14:343107	3261	4111	3006	4368	5492	4635	42.53589	42.82507	43.33517	43.95595	46.13069	47.56257	42.89871	45.88307	0.605385	1	-
ENSMUSC	Bmpr1a	bone mor	GO:00325	developm	K04673	bone mor	mmu0406	Cytokine-	14:344107	1215	1614	1000	1520	1897	1628	10.5934	11.23851	9.636203	10.22429	10.65077	11.16669	10.48937	10.68058	0.950481	1	-
ENSMUSC	Ldb3	LIM doma	GO:00325	developm	-	-	-	-	14:345266	90	161	57	104	222	174	0.502811	0.718349	0.351952	0.448257	0.798676	0.764756	0.524371	0.670563	0.27979	1	-
ENSMUSC	Cdhr1	cadherin-	GO:00325	developm	-	-	-	-	14:370778	120	179	142	198	225	160	1.691361	2.014902	2.212019	2.15303	2.042167	1.774133	1.972761	1.989776	0.932719	1	-
ENSMUSC	Nid2	nidogen 2	GO:00055	extracellul	-	-	-	-	14:197512	102	195	94	109	162	140	0.941205	1.437016	0.958647	0.775958	0.962612	1.0163	1.112289	0.91829	0.192926	1	-
ENSMUSC	Rtraf	RNA trans	GO:00081	metabolic	-	-	-	-	14:198113	976	1270	860	1241	1621	1307	28.90045	30.03339	28.14493	28.35025	30.90953	30.44677	29.02625	29.90218	0.95243	1	-
ENSMUSC	Nudt13	nudix (nu	GO:00038	catalytic a	-	-	-	-	14:202946	144	267	151	257	325	208	0.677641	1.003445	0.785345	0.933038	0.98486	0.770036	0.822144	0.895978	0.670153	1	-
ENSMUSC	Ecd	ecdysonel	GO:00081	metabolic	-	-	-	-	14:203198	717	933	668	1026	1234	998	6.047614	6.284813	6.227134	6.676401	6.702464	6.622261	6.186521	6.667042	0.60845	1	-
ENSMUSC	Dnajc9	DnaJ heat	GO:00055	extracellul	-	-	-	-	14:203846	364	459	331	513	607	458	5.472832	5.511494	5.500292	5.950568	5.876972	5.41735	5.494873	5.748297	0.851724	1	-
ENSMUSC	Anxa7	annexin A	GO:00325	developm	-	-	-	-	14:204552	1126	1472	1046	1557	1757	1975	6.183241	6.45552	6.34828	6.596241	6.213039	8.532095	6.329014	7.113792	0.375498	1	-
ENSMUSC	Mss51	MSS51 m	GO:00076	behavior|i	-	-	-	-	14:204828	3	13	5	5	12	9	0.100312	0.347161	0.184794	0.128978	0.258402	0.236747	0.210756	0.208042	1	1	-
ENSMUSC	Ppp3cb	protein pl	GO:00325	developm	K04348	serine/thr	mmu0401	MAPK sigr	14:204993	4489	9082	6494	5552	10429	9069	19.26954	31.135	30.80922	18.3866	28.82829	30.62611	27.07125	25.947	0.648351	1	-
ENSMUSC	Zswim8	zinc finge	GO:00056	cell|intrac	-	-	-	-	14:207075	2629	3249	2390	3396	3896	3567	9.735674	9.608839	9.781829	9.70227	9.290712	10.39177	9.708781	9.794916	0.858315	1	-
ENSMUSC	Camk2g	calcium/c	GO:00325	developm	K04515	calcium/c	mmu0474	Olfactory	14:207348	1632	2133	1557	2000	2381	2777	8.569557	8.94489	9.035946	8.102119	8.051046	11.47163	8.850131	9.208265	0.891171	1	-
ENSMUSC	Vcl	vinculin [	GO:00325	developm	K05700	vinculin[-]	mmu0481	Regulatio	14:209293	1168	1320	937	1538	1840	1361	8.552092	7.718788	7.582555	8.687926	8.675646	7.83968	7.951145	8.401084	0.75213	1	-
ENSMUSC	Ap3m1	adaptor-r	GO:00511	localizatio	K12398	AP-3 com	mmu0414	Lysosome	14:210314	849	960	741	1056	1342	986	5.900106	5.328063	5.691376	5.661694	6.00564	5.390635	5.639849	5.68599	0.87905	1	-
ENSMUSC	Txndc16	thioredox	GO:00099	cellular pr	-	-	-	-	14:451334	1447	1659	1323	1644	1948	1876	4.379669	4.010192	4.425674	3.838883	3.796789	4.467007	4.271845	4.034226	0.386437	1	-
ENSMUSC	Ero1l	ERO1-like	GO:00325	developm	K10950	ERO1-like	mmu0414	Protein pr	14:452830	655	802	584	877	1082	861	6.904881	6.752043	6.804154	7.132546	7.345081	7.1405	6.820359	7.206043	0.756179	1	-
ENSMUSC	Psmc6	proteasor	GO:00081	metabolic	K03064	26S prote	mmu0516	Epstein-B	14:453297	1328	1522	1068	1579	1984	1551	15.19302	13.90615	13.50406	13.93666	14.61647	13.95947	14.20108	14.17086	0.779398	1	-
ENSMUSC	Bmp4	bone mor	GO:00325	developm	K04662	bone mor	mmu0520	Pathways	14:463835	31	54	42	49	85	62	0.462555	0.6435	0.692632	0.564067	0.816736	0.727798	0.599562	0.702867	0.502969	1	-
ENSMUSC	Samd4	sterile alp	GO:00451	translatior	-	-	-	-	14:468828	593	759	476	664	872	677	2.465944	2.520671	2.187674	2.130232	2.335069	2.214767	2.39143	2.226689	0.366071	1	-
ENSMUSC	Mapk1ip1	mitogen-	-	-	-	-	-	-	14:472982	848	1192	584	1150	1478	1244	4.019661	4.512488	3.059519	4.205541	4.51152	4.639008	3.863889	4.452023	0.300261	1	-
ENSMUSC	Ktn1	kinectin 1	GO:00511	localizatio	-	-	-	-	14:476484	2305	2821	1946	2817	3347	2792	7.791561	7.61557	7.270145	7.346332	7.285575	7.424716	7.559092	7.352208	0.546286	1	-
ENSMUSC	Peli2	pellino 2 [	GO:00081	metabolic	-	-	-	-	14:481208	459	664	434	555	755	721	2.007856	2.31971	2.098249	1.873025	2.126775	2.481227	2.141939	2.160342	0.894038	1	-
ENSMUSC	Slc35f4	solute car	GO:00511	localizatio	-	-	-	-	14:492985	128	152	124	163	209	207	0.889031	0.84313	0.951864	0.87342	0.934772	1.131063	0.894675	0.979752	0.64787	1	-
ENSMUSC	Anxa11	annexin A	GO:00511	localizatio	-	-	-	-	14:258421	343	422	341	453	577	378	4.141373	4.069196	4.550423	4.219678	4.486225	3.590481	4.253664	4.098794	0.620084	1	-
ENSMUSC	Tmem254	transmem	GO:00160	membran	-	-	-	-	14:262033	2	4	2	4	3	3	0.034636	0.055328	0.03829	0.053447	0.033467	0.040875	0.042752	0.042596	1	1	-
ENSMUSC	Ppif	peptidylp	GO:00081	metabolic	K09565	peptidyl-p	mmu0501	Huntingto	14:256941	400	558	402	556	723	681	3.58165	3.990277	3.978284	3.840856	4.168846	4.797118	3.85007	4.26894	0.484678	1	-
ENSMUSC	Slmap	sarcolemr	GO:00511	localizatio	-	-	-	-	14:264131	2288	2781	1931	2829	3466	2844	6.969367	6.765254	6.500793	6.648147	6.798615	6.815188	6.745138	6.753983	0.791752	1	-
ENSMUSC	Arf4	ADP-ribo	GO:00325	developm	-	-	-	-	14:266380	993	1388	997	1432	1828	1381	8.116563	9.060624	9.006676	9.030173	9.621741	8.880292	8.727954	9.177402	0.778244	1	-
ENSMUSC	Dnah12	dynein, ax	GO:00038	catalytic a	K10408	dynein he	mmu0501	Huntingto	14:266932	25	47	26	41	31	40	0.124841	0.187443	0.143496	0.157955	0.099689	0.157142	0.151927	0.138262	0.591698	1	-
ENSMUSC	Rnase6	ribonucle	GO:00081	metabolic	-	-	-	-	14:511239	6	8	5	10	11	8	0.083946	0.089393	0.077322	0.107945	0.099104	0.088051	0.083554	0.098367	0.802985	1	-
ENSMUSC	Hacl1	2-hydrox	GO:00081	metabolic	K12261	2-hydrox	mmu0414	Peroxison	14:315987	85	101	78	100	140	98	0.768654	0.729423	0.779568	0.697653	0.815258	0.697185	0.759215	0.736699	0.749179	1	-
ENSMUSC	Eaf1	ELL assoc	GO:00081	metabolic	-	-	-	-	14:314943	294	379	229	405	373	296	2.635986	2.713824	2.269225	2.801441	2.153575	2.087845	2.539679	2.34762	0.4275	1	-
ENSMUSC	Mettl6	methyltrar	GO:00081	metabolic	-	-	-	-	14:314735	312	408	265	385	483	435	1.798711	1.878504	1.688492	1.71237	1.793115	1.972905	1.788569	1.82613	0.979766	1	-
ENSMUSC	Sh3bp5	SH3-dom	GO:00081	metabolic	-	-	-	-	14:313598	2616	3339	2397	3476	4120	3224	43.54635	44.38909	44.099	44.63999	44.16374	42.22017	44.01148	43.67464	0.714639	1	-
ENSMUSC	Capn7	calpain 7	GO:00081	metabolic	-	-	-	-	14:313366	961	1202	867	1236	1689	1230	7.265579	7.257672	7.244574	7.209348	8.223018	7.315823	7.255942	7.58273	0.832804	1	-
ENSMUSC	Arhgef3	Rho guan	GO:00987	molecular	-	-	-	-	14:271148	1943	2374	1717	2503	3194	2388	13.89504	13.55857	13.57077	13.80951	14.70878	13.43484	13.67479	13.98438	0.988483	1	-
ENSMUSC	Asb14	ankyrin re	GO:00081	metabolic	-	-	-	-	14:268945	0	0	0	0	0	2	0	0	0	0	0	0.025246	0	0.008415	0.52667	1	-
ENSMUSC	Btd	biotinidas	GO:00081	metabolic	K01435	biotinidas	mmu0110	Metabolic	14:316410	124	181	127	192	195	200	2.644506	3.082814	2.993444	3.159029	2.678002	3.355542	2.906922	3.064191	0.85028	1	-
ENSMUSC	Bap1	Brca1 ass	GO:00081	metabolic	-	-	-	-	14:312514	2021	2466	1898	2641	3025	2888	13.83235	13.47934	14.3573	13.94532	13.33245	15.55027	13.88967	14.27601	0.975485	1	-
ENSMUSC	Phf7	PHD finge	GO:00160	membran	-	-	-	-	14:312376	50	76	71	85	100	82	0.3363	0.408235	0.527785	0.441062	0.433119	0.433888	0.424107	0.436023	1	1	-
ENSMUSC	Galnt15	polypepti	GO:00081	metabolic	K00710	polypeptic	mmu0110	Metabolic	14:320289	75	135	95	121	175	129	0.507128	0.729015	0.70995	0.631208	0.761989	0.686209	0.648698	0.693135	0.800568	1	-
ENSMUSC	Sema3g	sema don	GO:00325	developm	K06840	semaphor	mmu0436	Axon guic	14:312178	99	123	69	109	149	132	1.331437	1.321104	1.025608	1.13095	1.290408	1.396598	1.22605	1.272652	0.952984	1	-
ENSMUSC	Dph3	diphtham	GO:00081	metabolic	-	-	-	-	14:320805	399	504	335	552	584	562	3.868243	3.90227	3.589483	4.128667	3.645928	4.286356	3.786665	4.020317	0.758981	1	-
ENSMUSC	Oxnad1	oxidoredu	GO:00081	metabolic	-	-	-	-	14:320853	118	182	121	207	138	149	0.723164	0.890778	0.81957	0.97871	0.544611	0.718374	0.811171	0.747232	0.535852	1	-
ENSMUSC	Nisch	nischarin	GO:00081	metabolic	-	-	-	-	14:311709	5598	5230	5293	6438	6689	8264	14.65081	10.93141	15.31007	12.999	11.27313	17.01492	13.63076	13.76235	0.888607	1	-
ENSMUSC	Parg	poly (ADP	GO:00081	metabolic	-	-	-	-	14:322019	702	938	656	912	1169	904	4.020521	4.290363	4.152373	4.029678	4.311364	4.073097	4.154419	4.138046	0.781774	1	-
ENSMUSC	Ogdhl	oxoglutar	GO:00081	metabolic	K00164	2-oxoglut	mmu0110	Metabolic	14:323220	1432	1777	1340	1981	2191	1869	18.67476	18.50739	19.31361	19.9309	18.39962	19.17485	18.83192	19.16846	0.943758	1	-
ENSMUSC	Glt8d1	glycosyltr	GO:00081	metabolic	-	-	-	-	14:310014	439	547	344	551	666	560	3.724404	3.706166	3.225503	3.606397	3.638489	3.737582	3.552025	3.660823	0.963588	1	-
ENSMUSC	Spcs1	signal pep	GO:00081	metabolic	K12946	signal pep	mmu0306	Protein ex	14:309998	286	548	257	481	446	577	3.740924	5.724517	3.715294	4.853869	3.75666	5.937443	4.393578	4.849324	0.629079	1	-
ENSMUSC	Nek4	NIMA (ne	GO:00325	developm	-	-	-	-	14:309513	382	560	326	507	521	546	1.658079	1.941224	1.563891	1.697774	1.456245	1.864428	1.721064	1.672815	0.657095	1	-
ENSMUSC	Chat	choline a	GO:00038	catalytic a	K00623	choline O	mmu0472	Cholinerg	14:324082	0	4	2	0	0	2	0	0.035812	0.024784	0	0	0.017644	0.020199	0.005881	0.347627	1	-
ENSMUSC	Itih4	inter alph	GO:00081	metabolic	-	-	-	-	14:308864	2	4	2	0	0	3	0.024067	0.038445	0.026606	0	0	0.028402	0.029706	0.009467	0.193761	1	-
ENSMUSC	Ebpl	emopami	GO:00081	metabolic	-	-	-	-	14:613317	179	245	163	247	311	203	0.665009	0.726919	0.669281	0.707949	0.744028	0.593307	0.687069	0.681761	0.824851	1	-
ENSMUSC	Kpna3	karyophe	GO:00511	localizatio	-	-	-	-	14:613652	1986	2438	1847	2258	2989	2294	26.0106	25.50061	26.73529	22.81525	25.20877	23.63608	26.08217	23.8867	0.205534	1	-
ENSMUSC	Spryd7	SPRY dom	GO:00160	membran	-	-	-	-	14:615319	726	1062	763	1062	1303	1117	8.562524	10.00312	9.945723	9.663178	9.896105	10.36405	9.503789	9.974443	0.807266	1	-
ENSMUSC	Rnaseh2b	ribonucle	GO:00325	developm	K10744	ribonucle	mmu0303	DNA repli	14:622925	82	98	81	122	157	109	0.739664	0.705976	0.80752	0.848999	0.911954	0.773494	0.751053	0.844816	0.53477	1	-
ENSMUSC	Gucy1b2	guanylate	GO:00081	metabolic	K12319	guanylate	mmu0023	Purine me	14:623926	3	3	8	9	10	7	0.054629	0.043636	0.160999	0.126443	0.117263	0.100286	0.086421	0.114664	0.604374	1	-
ENSMUSC	Mapk8	mitogen-	GO:00325	developm	K04440	c-Jun N-t	mmu0520	Pathways	14:333778	1983	2352	1799	2519	2891	2699	7.530677	7.133366	7.550736	7.380229	7.069913	8.063529	7.404926	7.504557	0.90053	1	-
ENSMUSC	Pspc1	paraspeck	GO:00081	metabolic	-	-	-	-	14:567207	1041	977	1038	906	1505	1204	10.1374	7.598295	11.17172	6.806662	9.43772	9.223873	9.635806	8.489418	0.23354	1	-
ENSMUSC	Ptpn20	protein ty	GO:00081	metabolic	-	-	-	-	14:335892	8	10	6	15	17	16	0.11897	0.118754	0.098617	0.172087	0.162794	0.18718	0.112114	0.17402	0.19749	1	-
ENSMUSC	Gdf10	growth di	GO:00325	developm	-	-	-	-	14:339235	203	239	197	275	307	293	2.379035	2.236916	2.55164	2.486389	2.316856	2.701371	2.389197	2.501538	0.870963	1	-
ENSMUSC	Gata4	GATA bin	GO:00325	developm	K09183	GATA-bin	mmu0421	Cellular se	14:631989	0	0	1	0	0	2	0	0	0.009724	0	0	0.013846	0.003241	0.004615	1	1	-
ENSMUSC	Zmym2	zinc finge	GO:00325	developm	-	-	-	-	14:568866	1836	2289	1754	2375	2841	2271	10.61277	10.5669	11.20553	10.59132	10.57504	10.32724	10.79507	10.49786	0.55204	1	-
ENSMUSC	Cryl1	crystallin,	GO:00081	metabolic	K13247	L-gulonat	mmu0110	Metabolic	14:572749	153	234	186	253	361	263	3.494913	4.26881	4.695731	4.458571	5.310141	4.726192	4.153152	4.831635	0.332241	1	-
ENSMUSC	Prkcd	protein ki	GO:00325	developm	K06068	novel prot	mmu0406	Chemokir	14:305953	295	556	332	496	736	554	1.762613	2.653113	2.192397	2.286373	2.83183	2.604087	2.202708	2.574097	0.303672	1	-
ENSMUSC	Eef1akmt	EEF1A alp	GO:00081	metabolic	-	-	-	-	14:575495	187	204	155	262	272	250	10.46549	9.11789	9.587287	11.3123	9.802606	11.007	9.723556	10.7073	0.581815	1	-
ENSMUSC	Xpo4	exportin 4	GO:00511	localizatio	-	-	-	-	14:575775	396	459	349	468	580	457	1.598825	1.480009	1.557318	1.457746	1.507953	1.451551	1.545384	1.472417	0.517729	1	-
ENSMUSC	Tkt	transketol	GO:00081	metabolic	K00615	transketol	mmu0110	Metabolic	14:305483	2172	2726	2126	2976	3353	3123	13.81008	13.84229	14.93986	14.59821	13.72855	15.62138	14.19741	14.64938	0.938094	1	-
ENSMUSC	Pinx1	PIN2/TERI	GO:00081	metabolic	-	-	-	-	14:638603	75	122	61	105	148	92	1.530026	1.987669	1.375353	1.65256	1.944251	1.476505	1.631016	1.691105	0.949759	1	-
ENSMUSC	Lats2	large tum	GO:00325	developm	K08791	serine/thr	mmu0439	Hippo sig	14:576898	1258	1420	914	1293	1695	1451	7.76105	6.996383	6.232076	6.154158	6.733862	7.04236	6.996505	6.64346	0.449898	1	-
ENSMUSC	Dcp1a	decapping	GO:00325	developm	K12610	mRNA-de	mmu0301	RNA degr	14:304795	288	536	369	496	368	544	1.126937	1.675013	1.595808	1.497337	0.927279	1.674623	1.465919	1.366413	0.549016	1	-
ENSMUSC	Ska3	spindle ar	GO:00990	supramole	-	-	-	-	14:578065	15	9	13	19	25	15	0.236837	0.113495	0.226859	0.231443	0.25419	0.186324	0.192397	0.223986	0.702757	1	-
ENSMUSC	Mrpl57	mitochon	GO:00081	metabolic	-	-	-	-	14:578262	234	283	198	294	313	261	4.207188	4.06357	3.934491	4.078055	3.623887	3.691703	4.068416	3.797881	0.446055	1	-
ENSMUSC	Zdhhc20	zinc finge	GO:00081	metabolic	-	-	-	-	14:578327	729	943	566	852	1162	920	4.819333	4.97871	4.135452	4.345398	4.946752	4.784738	4.644498	4.692296	0.89797	1	-
ENSMUSC	Hmbox1	homeobo	GO:00081	metabolic	-	-	-	-	14:648116	951	1321	834	1243	1426	1222	4.794112	5.31834	4.646652	4.83425	4.629155	4.846291	4.919701	4.769899	0.565777	1	-

ENSMUSC	Micu2	mitochon	GO:00511	localizatio	-	-	-	-	14:579162	289	415	278	473	597	448	4.729511	5.423902	5.028164	5.97186	6.291397	5.76776	5.060526	6.010339	0.191598	1	-
ENSMUSC	Fgf9	fibroblast	GO:00325	developm	K04358	fibroblast	mmu0520	Pathways	14:580705	249	270	217	336	368	316	2.738869	2.371819	2.63802	2.851288	2.606604	2.734455	2.582903	2.730782	0.796274	1	-
ENSMUSC	Ints9	integrator	GO:00081	metabolic	-	-	-	-	14:649500	237	341	258	328	470	395	1.999279	2.297336	2.405427	2.134664	2.553155	2.621396	2.234014	2.436405	0.610531	1	-
ENSMUSC	Extl3	exostoses	GO:00081	metabolic	K02370	alpha-1,4	mmu0110	Metabolic	14:650520	2265	2900	1824	2835	3297	2857	14.60674	14.93582	13.0004	14.10485	13.69174	14.4946	14.18098	14.09706	0.734237	1	-
ENSMUSC	Cab39l	calcium b	GO:00055	extracellul	K08272	calcium b	mmu0415	mTOR sig	14:594409	544	1019	498	702	847	741	4.381428	6.554461	4.43295	4.361987	4.392938	4.695113	5.122946	4.483346	0.198514	1	-
ENSMUSC	Cdadc1	cytidine a	GO:00038	catalytic a	-	-	-	-	14:595593	796	1051	743	1030	1301	1073	6.011408	6.338871	6.201534	6.001097	6.326965	6.374907	6.183938	6.234323	0.874003	1	-
ENSMUSC	Atp8a2	ATPase, a	GO:00325	developm	-	-	-	-	14:596385	1392	1627	1258	1694	1896	1691	5.24065	4.891922	5.234481	4.920281	4.596625	5.008418	5.122351	4.841775	0.378551	1	-
ENSMUSC	Amer2	APC mem	GO:00508	response	-	-	-	-	14:603732	2065	2548	1338	2466	2834	2446	9.498645	9.360235	6.802121	8.751146	8.394523	8.851337	8.553667	8.665669	0.913336	1	-
ENSMUSC	Mtmr6	myotubul	GO:00081	metabolic	K18083	myotubul	mmu0110	Metabolic	14:602652	3007	3805	2717	3981	4796	3955	36.07107	36.4524	36.02152	36.84241	37.04754	37.32355	36.18166	37.07117	0.998281	1	-
ENSMUSC	Spata13	spermato	GO:00987	molecular	K05769	Rho guan	mmu0481	Regulation	14:606340	356	612	424	548	690	536	1.254208	1.721935	1.650943	1.489467	1.565394	1.485579	1.542362	1.51348	0.727744	1	-
ENSMUSC	Cacna2d3	calcium cl	GO:00511	localizatio	K04860	voltage-d	mmu0401	MAPK sig	14:289049	1699	2087	1468	2137	2683	2184	10.21845	10.02444	9.758082	9.915775	10.39123	10.33369	10.00032	10.21356	0.974212	1	-
ENSMUSC	Mipep	mitochon	GO:00081	metabolic	-	-	-	-	14:607845	536	726	524	790	864	761	4.366534	4.723393	4.717907	4.965113	4.532522	4.877164	4.602612	4.7916	0.877085	1	-
ENSMUSC	Wnt5a	wingless-t	GO:00325	developm	K00444	wingless-t	mmu0520	Pathways	14:285047	167	196	71	148	188	145	0.813327	0.762344	0.382167	0.556086	0.589608	0.555558	0.652613	0.567084	0.376788	1	-
ENSMUSC	Esd	esterase D	GO:00081	metabolic	-	-	mmu0120	Carbon m	14:747322	324	322	348	417	405	419	4.054689	3.218206	4.813246	4.026052	3.263786	4.125124	4.028714	3.804987	0.549233	1	-
ENSMUSC	Lrrc63	leucine ric	-	-	-	-	-	-	14:750843	0	0	0	2	0	0	0	0	0	0.037435	0	0	0	0.012478	0.5276	1	-
ENSMUSC	Zc3h13	zinc finge	GO:00056	cell|intrace	-	-	-	-	14:752843	2430	3059	2033	3049	3807	2978	18.99759	19.09931	17.56615	18.38993	19.16593	18.31589	18.55435	18.62391	0.818022	1	-
ENSMUSC	Slc25a30	solute car	GO:00511	localizatio	-	-	-	-	14:757601	159	212	153	196	273	226	1.908817	2.032586	2.030048	1.815317	2.110498	2.134452	1.990484	2.020089	0.958423	1	-
ENSMUSC	Gpalpp1	GPALPP n	-	-	-	-	-	-	14:760862	1219	1633	1132	1629	1895	1613	13.81144	14.77635	14.17517	14.23923	13.82608	14.3774	14.25432	14.14757	0.728601	1	-
ENSMUSC	Nufip1	nuclear fr	GO:00081	metabolic	-	-	-	-	14:761108	180	250	190	270	328	258	2.765584	3.067617	3.226381	3.200434	3.245217	3.118505	3.019861	3.188052	0.803719	1	-
ENSMUSC	Tsc22d1	TSC22 do	GO:00081	metabolic	-	-	-	-	14:764149	5061	4664	3622	6419	7359	5444	14.83154	10.91577	11.73127	14.51268	13.88748	12.55102	12.49286	13.65039	0.528865	1	-
ENSMUSC	Enox1	ecto-NOX	GO:00081	metabolic	-	-	-	-	14:771567	923	1119	731	1124	1365	1179	5.856451	5.670338	5.126215	5.502114	5.577251	5.885153	5.551001	5.654839	0.954277	1	-
ENSMUSC	Dnajc15	DnaJ heat	GO:00081	metabolic	-	-	-	-	14:778206	1182	1408	1069	1554	1824	1557	10.21669	9.719439	10.21215	10.36273	10.15248	10.58747	10.04943	10.36756	0.942048	1	-
ENSMUSC	Akap11	A kinase (	GO:00081	metabolic	-	-	-	-	14:784922	4746	7287	4527	5924	8835	5947	21.92659	26.88673	23.11536	21.11488	26.28479	21.61485	23.97622	23.00484	0.505605	1	-
ENSMUSC	Rgcc	regulator	GO:00325	developm	-	-	-	-	14:792887	101	154	143	188	211	144	6.717733	8.180279	10.51193	9.646947	9.037302	7.534842	8.469982	8.739697	0.967071	1	-
ENSMUSC	Tdrd3	tudor dor	GO:00081	metabolic	-	-	-	-	14:874166	442	575	375	523	634	628	3.726555	3.871672	3.494326	3.401862	3.442143	4.165389	3.697517	3.669798	0.778285	1	-
ENSMUSC	Naa16	N(alpha)-	GO:00081	metabolic	-	-	-	-	14:793252	159	226	83	257	278	201	0.531027	0.602802	0.306367	0.66219	0.597888	0.528113	0.480065	0.596064	0.260608	1	-
ENSMUSC	Diaph3	diaphano	GO:00099	cellular pr	K05745	diaphano	mmu0481	Regulation	14:866553	33	58	28	43	75	39	0.264948	0.371892	0.248455	0.266343	0.387758	0.246329	0.295098	0.300143	1	1	-
ENSMUSC	Mtrf1	mitochon	GO:00048	signal trar	-	-	-	-	14:793977	128	185	141	151	224	159	3.049344	3.519754	3.712439	2.775258	3.436346	2.979906	3.427179	3.063837	0.342204	1	-
ENSMUSC	Wbp4	WW dom	GO:00081	metabolic	-	-	-	-	14:794599	900	893	596	934	1158	852	5.669275	4.492442	4.149335	4.53902	4.697299	4.222168	4.770351	4.486163	0.446395	1	-
ENSMUSC	Sugt1	SGT1, sup	GO:00329	macromo	K12795	suppresso	mmu0462	NOD-like	14:795876	2401	3065	2170	3025	3508	3114	17.08631	17.41937	17.06721	16.60779	16.07571	17.43354	17.19096	16.70568	0.538787	1	-
ENSMUSC	Cnmd	chondron	GO:00325	developm	-	-	-	-	14:796376	18	29	13	27	49	26	0.309061	0.397678	0.246699	0.35766	0.541788	0.35121	0.317813	0.416886	0.326139	1	-
ENSMUSC	Olfm4	olfactome	GO:00325	developm	-	-	-	-	14:799840	30	120	73	151	185	30	0.458169	1.46362	1.232168	1.779136	1.819394	0.360438	1.051319	1.319656	0.555262	1	-
ENSMUSC	Elp3	elongator	GO:00325	developm	-	-	-	-	14:655304	1657	2035	1469	2358	2596	2070	23.15179	22.70765	22.68456	25.41769	23.35723	22.75323	22.848	23.84272	0.834717	1	-
ENSMUSC	Scara5	scavenger	GO:00081	metabolic	-	-	-	-	14:656664	16	47	16	12	43	33	0.121312	0.284597	0.134077	0.070191	0.209946	0.196841	0.179995	0.158993	0.665393	1	-
ENSMUSC	Pbk	PDZ bindi	GO:00081	metabolic	-	-	-	-	14:658058	11	10	8	13	32	13	0.326525	0.237047	0.262435	0.297697	0.61167	0.303562	0.275336	0.40431	0.271743	1	-
ENSMUSC	Ccdc25	coiled-co	GO:00055	extracellul	-	-	-	-	14:658373	651	792	481	872	972	719	6.911637	6.715385	5.644059	7.142444	6.645396	6.005369	6.423694	6.597736	0.984191	1	-
ENSMUSC	Clu	clusterin [	GO:00325	developm	K17252	clusterin[-	mmu0461	Complem	14:659684	11885	16381	10890	15489	17357	15849	128.6431	141.6033	130.2753	129.3424	120.9808	134.9584	133.5072	128.4272	0.456541	1	-
ENSMUSC	Ephx2	epoxide h	GO:00081	metabolic	K08726	soluble ep	mmu0110	Metabolic	14:660843	269	319	276	352	371	296	5.726859	5.423768	6.49411	5.78145	5.086188	4.95754	5.881579	5.27506	0.282926	1	-
ENSMUSC	Chrna2	cholinergi	GO:00048	signal trar	K04804	nicotinic a	mmu0408	Neuroacti	14:661350	8	11	4	12	10	13	0.122001	0.13396	0.067419	0.141172	0.098197	0.155951	0.107793	0.131773	0.74339	1	-
ENSMUSC	Trim35	tripartite r	GO:00400	locomotic	-	-	-	-	14:662970	2696	2720	2478	2842	3479	3326	28.976	23.34712	29.43518	23.5653	24.0784	28.12236	27.25277	25.25535	0.310508	1	-
ENSMUSC	Stmn4	stathmin-	GO:00325	developm	-	-	-	-	14:663442	3006	3302	2212	3314	4037	2747	47.45105	41.62745	38.59125	40.35893	41.0365	34.11351	42.55658	38.50298	0.207552	1	-
ENSMUSC	Dpysl2	dihydropy	GO:00325	developm	K07528	dihydropy	mmu0436	Axon guic	14:668028	4216	5598	3769	5608	6674	5342	56.95155	60.39258	56.27012	58.44444	58.05582	56.7701	57.87141	57.75679	0.755398	1	-
ENSMUSC	Bnip3l	BCL2/ade	GO:00081	metabolic	K15465	BCL2/ade	mmu0413	Mitophag	14:669852	1315	1694	1194	1634	2055	1546	15.66661	16.11791	15.72174	15.01869	15.7658	14.49005	15.83542	15.09151	0.432784	1	-
ENSMUSC	Nefm	neurofilan	GO:00325	developm	K04573	neurofilan	mmu0501	Amyotrop	14:680825	4014	4896	2984	4998	6162	4003	42.38797	41.29073	34.82659	40.71851	41.90266	33.25537	39.50176	38.62551	0.640669	1	-
ENSMUSC	Nefl	neurofilan	GO:00325	developm	K04572	neurofilan	mmu0501	Amyotrop	14:680838	8903	11140	8501	12139	14504	11701	160.8285	160.7155	169.7241	169.1764	168.721	166.2879	163.756	168.0618	0.981298	1	-
ENSMUSC	Nkx3-1	NK-3 tran	GO:00325	developm	K09348	homeobo	mmu0520	Pathways	14:691906	16	16	19	22	20	21	0.305767	0.244202	0.401309	0.324358	0.246123	0.315722	0.317093	0.295401	0.726263	1	-
ENSMUSC	Pibf1	progester	GO:00081	metabolic	-	-	-	-	14:990994	221	323	263	341	380	299	1.172665	1.368767	1.542352	1.395938	1.298432	1.248141	1.361261	1.31417	0.643873	1	-
ENSMUSC	Entpd4b	ectonucle	GO:00038	catalytic a	K12305	ectonucle	mmu0023	Purine me	14:695554	1982	2428	1890	2462	3061	2548	11.79734	11.54184	12.43338	11.30574	11.73272	11.93139	11.92419	11.65662	0.593096	1	-
ENSMUSC	Bora	bora, aurc	GO:00511	localizatio	-	-	-	-	14:990462	22	34	35	39	56	43	0.186332	0.229983	0.327633	0.254839	0.305431	0.286518	0.247982	0.282263	0.634264	1	-
ENSMUSC	Rhobtb2	Rho-relat	GO:00508	response	K07868	Rho-relat	mmu0412	Ubiquitin	14:697849	1465	1829	1239	1993	2164	1608	7.167479	7.146416	6.699569	7.522572	6.817745	6.189074	7.004488	6.843131	0.618924	1	-
ENSMUSC	Klhl1	kelch-like	GO:00325	developm	-	-	-	-	14:961027	39	44	23	50	60	47	0.358355	0.322882	0.233569	0.354443	0.355023	0.339749	0.304935	0.349739	0.592619	1	-
ENSMUSC	Bin3	bridging i	GO:00325	developm	-	-	-	-	14:701001	156	239	180	243	301	260	3.317685	4.059329	4.230873	3.987003	4.122228	4.350045	3.869295	4.153092	0.72044	1	-
ENSMUSC	Pdlim2	PDZ and l	GO:00055	extracellul	-	-	-	-	14:701642	76	142	102	154	176	111	0.61528	0.918105	0.912648	0.96185	0.917537	0.706951	0.815345	0.862113	0.85707	1	-
ENSMUSC	Sorbs3	sorbin anc	GO:00081	metabolic	-	-	-	-	14:701804	415	643	380	586	671	661	6.104341	7.55349	6.177614	6.649957	6.355769	7.648966	6.611815	6.884897	0.893167	1	-
ENSMUSC	Ppp3cc	protein pl	GO:00325	developm	K04348	serine/thr	mmu0401	MAPK sig	14:702178	352	392	323	517	547	500	5.758958	5.121934	5.840512	6.525632	5.76293	6.435509	5.573801	6.241357	0.444562	1	-
ENSMUSC	Slc39a14	solute car	GO:00511	localizatio	K14720	solute car	mmu0421	Ferroptos	14:703034	658	932	655	914	1054	880	2.744841	3.104939	3.01981	2.941492	2.831303	2.887919	2.95653	2.886905	0.634482	1	-
ENSMUSC	Fam160b2	family witl	-	-	-	-	-	-	14:705832	775	918	653	1008	1259	989	11.73321	11.09951	10.92635	11.77351	12.27427	11.77938	11.25302	11.94239	0.726295	1	-
ENSMUSC	Hr	hairless [S	GO:00081	metabolic	-	-	-	-	14:705522	302	314	202	359	380	387	1.734835	1.440542	1.282474	1.591019	1.405688	1.748933	1.48595	1.58188	0.783432	1	-
ENSMUSC	Sftpc	surfactant	GO:00055	extracellul	-	-	-	-	14:705209	6	10	8	8	10	10	0.275237	0.366345	0.405581	0.283133	0.295403	0.360889	0.349054	0.313142	0.812651	1	-
ENSMUSC	Bmp1	bone mor	GO:00325	developm	-	-	-	-	14:704745	901	1050	923	1224	1316	1130	4.435846	4.128447	5.022269	4.64904	4.172173	4.376641	4.528854	4.399285	0.600717	1	-
ENSMUSC	Dmtn	dematin a	GO:00325	developm	-	-	-	-	14:706012	3950	4487	3752	5281	5329	5042	13.69716	12.42611	14.37947	14.12797	11.89964	13.75457	13.50091	13.26073	0.63669	1	-
ENSMUSC	Xpo7	exportin 7	GO:00511	localizatio	-	-	-	-	14:706542	1411	1779	1594	2050	2424	2012	3.841829	3.868412	4.796738	4.306208	4.250091	4.309726	4.168993	4.288675	0.964482	1	-
ENSMUSC	Fgf17	fibroblast	GO:00325	developm	K04358	fibroblast	mmu0520	Pathways	14:706362	3	4	0	0	2	4	0.063579	0.067696	0	0	0.027289	0.066688	0.043758	0.031326	0.643539	1	-
ENSMUSC	Dok2	docking p	GO:00048	signal trar	-	-	-	-	14:707660	3	9	15	10	16	14	0.047552	0.113937	0.262782	0.122293	0.163313	0.174577	0.141423	0.153395	0.934087	1	-
ENSMUSC	Gfra2	glial cell li	GO:00325	developm	-	-	-	-	14:708901	624	658	536	698	855	632	7.675309	6.463728	7.286575	6.623645	6.772237	6.115607	7.141871	6.50383	0.276643	1	-
ENSMUSC	Rb1	RB transcr	GO:00325	developm	K06618	retinoblas	mmu0520	Pathways	14:731836	854	1183	844	1143	1510	1120	7.672689	8.488298	8.380682	7.922577	8.736183	7.916239	8.180556	8.191666	0.824422	1	-
ENSMUSC	Rcbtb2	regulator	GO:00056	cell|intrace	-	-	-	-	14:731230	267	379	261	408	394	324	0.893535	1.012943	0.965353	1.053393	0.849083	0.853013	0.957277	0.918496	0.59625	1	-
ENSMUSC	Itm2b	integral m	GO:00325	developm	-	-	-	-	14:733622	10497	13324	10636	14417	14187	14509	184.3333	186.8612	206.4257	195.3187	160.4294	200.4412	192.5401	185.3964	0.495188	1	-
ENSMUSC	Med4	mediator	GO:00081	metabolic	K15146	mediator	mmu0491	Thyroid h	14:735100	337	461	316	509	640	505	4.482916	4.897531	4.645849	5.223706	5.482331	5.284856	4.675432	5.330298	0.3322	1	-
ENSMUSC	Sucla2	succinate-	GO:00081	metabolic	K01900	succinyl-C	mmu0110	Metabolic	14:735253	2453	3174	2216	3197	4025	3061	26.70272	27.59374	26.66088	26.84917	28.21489	26.21393	26.98578	27.09266	0.821077	1	-
ENSMUSC	Uchl3	ubiquitin c	GO:00325	developm	-	-	-	-	14:101653	274	330	230	367	351	349	2.930956	2.819156	2.719151	3.028694	2.417804	2.936944	2.823087	2.794481	0.777772	1	-
ENSMUSC	Gpc5	glypican 5	GO:00055	extracellul	-	-	-	-	14:115092	702	933	723	958	1116	949	7.153333	7.592751	8.142479	7.531253	7.323027	7.607615	7.629521	7.487298	0.668051	1	-
ENSMUSC	Spry2	sprouty h	GO:00325	developm	K17383	protein sp	mmu0520	MicroRNA	14:105891	554	783	520	693	933	770	16.20804	18.29482	16.81401	15.64172	17.57751	17.7224	17.10563	16.98054	0.766744	1	-
ENSMUSC	Rbm26	RNA bind	GO:00081	metabolic	-	-	-	-	14:105106	1508	1870	1296	1844	2196	2041	9.433014	9.341928	8.959851	8.89898	8.845777	10.04391	9.244931	9.26289	0.804393	1	-
ENSMUSC	Rnf219	ring finge	-	-	-	-	-	-	14:104477	244	356	211	334	398	307	2.839374	3.308476	2.713702	2.998537	2.982429	2.810485	2.953851	2.930483	0.797429	1	-
ENSMUSC	Ednrb	endotheli	GO:00325	developm	K04198	endotheli	mmu0520	Pathways	14:103814	316	443	586	743	887	335	2.065111	2.3121	4.232541	3.74607	3.732804	1.722316	2.869918	3.067064	0.832101	1	-
ENSMUSC	Scel	sciellin [Sc	GO:00325	developm	-	-	-	-	14:103513	28	34	27	33	51	38	0.670171	0.649919	0.714234	0.609373	0.786067	0.715537	0.678108	0.703659	1	1	-
ENSMUSC	Fbxl3	F-box anc	GO:00081	metabolic	K10269	F-box anc	mmu0471	Circadian	14:103080	1109	1518	1086	1608	1104	1532	5.950739	6.505142	6.440443	6.656648	3.814723	6.467091	6.298775	5.646154	0.304664	1	-
ENSMUSC	Cln5	ceroid-lip	GO:00325	developm	K12390	ceroid-lip	mmu0414	Lysosome	14:103070	177	280	135	211	314	221	3.939943	4.977623	3.321227	3.62351	4.500922	3.870087	4.079598	3.998173	0.763761	1	-
ENSMUSC	Acod1	aconitate	GO:00325	developm	-	-	-	-	14:103046	0	0	0	0	0	3	0	0	0	0	0	0.054931	0	0.01831	0.292136	1	-
ENSMUSC	Tgds	TDP-gluc	GO:00038	catalytic a	-	-	-	-	14:118111	50	82	50	83	112	82	0.767647	1.005425	0.848414	0.983099	1.107291	0.990404	0.873829	1.026931	0.415076	1	-
ENSMUSC	Gpr180	G protein-	GO:00508	response	-	-	-	-	14:118137	209	261	157	291	315	259	6.80596	6.787778	5.650509	7.310819	6.605515	6.635191	6.414749	6.850508	0.752386	1	-
ENSMUSC	Cldn10	claudin 1C	GO:00511	localizatio	K06087	claudin[-]	mmu0453	Tight junc	14:118787	363	412	328	489	544	401	5.97254	5.413721	5.964491	6.207143	5.763763	5.190482	5.783584	5.720463	0.771531	1	-
ENSMUSC	Dnajc3	DnaJ heat	GO:00081	metabolic	K09523	DnaJ hom	mmu0516	Influenza	14:118937	1537	2075	1205	2015	2518	1884	18.25449	19.68157	15.8172	18.46295	19.25777	17.60303	17.91775	18.44125	0.95799	1	-
ENSMUSC	Mbnl2	muscleblir	GO:00081	metabolic	-	-	-	-	14:120275	2807	4046	2550	4105	4349	3586	8.893681	10.2379	8.929478	10.0342	8.87326	8.93841	9.353685	9.281955	0.730175	1	-
ENSMUSC	Nipbl	Nipped-B	GO:00325	developm	-	-	-	-	15:829122	1684	2186	1437	2054	2474	2223	11.66442	12.09253	11.00081	10.9762	11.03508	12.11355	11.58592	11.37494	0.630336	1	-
ENSMUSC	Nup155	nucleopor	GO:00081	metabolic	K14312	nuclear pc	mmu0301	RNA trans	15:810931	436	686	470	639	807	742	4.15828	5.225135	4.954185	4.701739	4.956279	5.567276	4.7792	5.075098	0.74681	1	-
ENSMUSC	Gdnf	glial cell li	GO:00325	developm	-	-	-	-	15:781101	3	2	5	4	4	7	0.0522	0.027797	0.096163	0.053691	0.044822	0.095828	0.05872	0.06478	1	1	-
ENSMUSC	Osmr	oncostatir	GO:00048	signal trar	K05057	oncostatir	mmu0415	PI3K-Akt s	15:681357	126	208	112	175	242	228	0.536045	0.70671	0.526616	0.574379	0.662981	0.763093	0.58979	0.666817	0.508621	1	-
ENSMUSC	Fyb	FYN bindi	GO:00511	localizatio	K17698	FYN bindi	mmu0401	Rap1 sign	15:652285	241	322	245	322	370	323	1.856786	1.981278	2.086202	1.913946	1.835694	1.95775	1.974755	1.902464	0.602793	1	-
ENSMUSC	Dab2	disabled 2	GO:00325	developm	K12475	disabled h	mmu0414	Endocytos	15:629978	259	358	178	297	302	287	1.229995	1.357791	0.934266	1.088155	0.923561	1.072251	1.174017	1.027989	0.243482	1	-
ENSMUSC	Mettl3	methyltrai	GO:00081	metabolic	-	-	-	-	14:522948	338	384	304	448	486	425	2.079152	1.886454	2.066755	2.126068	1.925132	2.056689	2.010787	2.035963	0.922583	1	-
ENSMUSC	Dad1	defender	GO:00325	developm	K12668	oligosacch	mmu0110	Metabolic	14:542354	482	721	421	644	759	599	6.736094	8.047157	6.50264	6.943487	6.830581	6.585651	7.095297	6.786573	0.532214	1	-
ENSMUSC	Rem2	rad and g	GO:00511	localizatio	-	-	-	-	14:544761	226	253	229	276	378	288	4.078959	3.646769	4.567965	3.843097	4.393272	4.089261	4.097897	4.108543	0.875664	1	-
ENSMUSC	Haus4	HAUS aug	GO:00990	supramole	-	-	-	-	14:545417	27	72	46	59	53	62	0.447378	0.95276	0.842387	0.754203	0.565503	0.808182	0.747508	0.709296	0.744876	1	-
ENSMUSC	Ajuba	ajuba LIM	GO:00325	developm	K16682	LIM doma	mmu0439	Hippo sig	14:545674	19	38	24	33	52	48	0.219051	0.349882	0.305807	0.293525	0.386059	0.435362	0.29158	0.371649	0.335783	1	-
ENSMUSC	4931414P	RIKEN cDI	GO:00055	extracellul	-	-	-	-	14:545836	74	91	68	106	107	108	1.229132	1.20713	1.248306	1.358331	1.144479	1.411246	1.228189	1.304685	0.826117	1	-
ENSMUSC	Slc7a8	solute car	GO:00511	localizatio	K13781	solute car	mmu0497	Protein di	14:547222	1577	2026	1487	2006	2256	2078	16.37005	16.79591	17.05988	16.06496	15.08037	16.96973	16.74195	16.03835	0.447666	1	-
ENSMUSC	C6	complem	GO:00325	developm	K03995	complem	mmu0532	Systemic l	15:472717	0	2	0	0	11	0	0	0.022983	0	0	0.101907	0	0.007661	0.033969	0.246986	1	-
ENSMUSC	Fbxo4	F-box prc	GO:00325	developm	K10291	F-box prc	mmu0412	Ubiquitin	15:396544	64	82	49	81	118	104	1.667109	1.705877	1.41069	1.6278	1.979351	2.131233	1.594558	1.912795	0.3509	1	-
ENSMUSC	Acin1	apoptotic	GO:00325	developm	K12875	apoptotic	mmu0301	RNA trans	14:546421	1570	2067	1507	2546	2616	2447	5.335407	5.609887	5.660149	6.67508	5.724798	6.542032	5.535148	6.31397	0.231979	1	-
ENSMUSC	Oxct1	3-oxoacic	GO:00325	developm	K01027	3-oxoacic	mmu0028	Valine, leu	15:402638	4020	5314	4006	4732	6538	7306	51.871	54.76032	57.12896	47.10572	54.32481	74.16332	54.58676	58.53128	0.679753	1	-
ENSMUSC	Drosha	drosha, ril	GO:00325	developm	K03685	ribonuclea	mmu0520	Proteogly	15:128248	2358	3114	1991	3192	3282	3254	18.09186	19.08112	16.88331	18.89438	16.21559	19.64119	18.01876	18.25039	0.899183	1	-
ENSMUSC	Psmb5	proteasor	GO:00081	metabolic	K02737	20S prote	mmu0305	Proteason	14:546141	732	949	708	1042	1168	997	29.38496	30.42468	31.4119	32.27099	30.19336	31.4862	30.40718	31.31685	0.959139	1	-
ENSMUSC	Pabpn1	poly(A) bi	GO:00081	metabolic	K14396	polyadeny	mmu0516	Influenza	14:548925	1051	1459	794	1414	1484	1573	9.978556	11.06282	8.33167	10.35725	9.073051	11.7491	9.791016	10.39313	0.748448	1	-
ENSMUSC	Pdzd2	PDZ dom	GO:00056	cell|intrace	-	-	-	-	15:123597	1549	2333	1778	2094	2685	2430	4.633046	5.572831	5.877514	4.831947	5.171473	5.717845	5.36113	5.240422	0.631934	1	-
ENSMUSC	Slc22a17	solute car	GO:00048	signal trar	-	-	-	-	14:549063	5923	7639	5104	7996	6483	7609	26.01591	26.7966	24.77735	27.0957	18.33699	26.29274	25.86328	23.90848	0.322153	1	-
ENSMUSC	Golph3	golgi pho	GO:00081	metabolic	-	-	-	-	15:123214	1147	1508	1018	1559	1808	1382	10.54724	11.07446	10.34594	11.05991	10.70603	9.997564	10.65588	10.58784	0.748995	1	-
ENSMUSC	Zfr	zinc finge	GO:00325	developm	-	-	-	-	15:121178	6954	9082	6265	9050	10625	9166	52.98865	55.26823	52.76137	53.20189	52.13529	54.94636	53.67275	53.42785	0.726991	1	-
ENSMUSC	Efs	embryona	GO:00071	cell adhes	-	-	-	-	14:549165	135	197	135	198	273	152	1.611815	1.878427	1.781409	1.823802	2.098943	1.427697	1.757217	1.78348	0.969267	1	-
ENSMUSC	Ngdn	neuroguic	GO:00081	metabolic	-	-	-	-	14:550154	179	249	161	258	292	250	5.116768	5.684428	5.086447	5.689735	5.375021	5.62202	5.295881	5.562259	0.848073	1	-
ENSMUSC	Sub1	SUB1 hon	GO:00081	metabolic	-	-	-	-	15:119813	3319	3563	2591	3898	4637	3147	45.34613	38.87718	39.12435	41.08707	40.79668	33.82523	41.11589	38.56966	0.367708	1	-
ENSMUSC	Npr3	natriuretic	GO:00325	developm	-	-	-	-	15:118398	649	809	513	764	872	739	2.715834	2.703665	2.372588	2.466506	2.349792	2.43284	2.597362	2.416379	0.34546	1	-
ENSMUSC	Jph4	junctophil	GO:00511	localizatio	-	-	-	-	14:551068	8967	11344	8126	12355	14318	11068	52.6906	53.2351	52.77274	56.00916	54.17801	51.16418	52.89948	53.78378	0.930854	1	-
ENSMUSC	Dhrs4	dehydrog	GO:00081	metabolic	K11147	dehydrog	mmu0110	Metabolic	14:554787	141	182	146	186	246	215	2.011487	2.073556	2.301966	2.04712	2.259902	2.412952	2.129003	2.239991	0.847373	1	-
ENSMUSC	Carmil3	capping p	GO:00160	membran	-	-	-	-	14:554906	721	845	588	1099	1063	1080	4.106233	3.843356	3.701111	4.828764	3.898485	4.838857	3.883567	4.522035	0.228043	1	-
ENSMUSC	Cpne6	copine VI	GO:00325	developm	-	-	-	-	14:555104	7519	9577	7699	10433	12263	9841	58.80568	59.81836	66.54881	62.95043	61.76047	60.54933	61.72428	61.75341	0.782426	1	-
ENSMUSC	Dcaf11	DDB1 anc	GO:00081	metabolic	-	-	-	-	14:555600	686	983	649	1115	1089	1230	2.103125	2.406803	2.199038	2.63722	2.149928	2.966589	2.236322	2.584579	0.280599	1	-
ENSMUSC	Psme1	proteasor	GO:00081	metabolic	K06696	proteason	mmu0461	Antigen p	14:555781	236	336	206	369	388	337	2.200291	2.501804	2.122669	2.654147	2.329458	2.471773	2.274921	2.485126	0.603027	1	-
ENSMUSC	Emc9	ER memb	GO:00329	macromo	-	-	-	-	14:555815	150	177	126	225	248	189	2.700098	2.544521	2.506705	3.124649	2.874705	2.676454	2.583775	2.891936	0.513123	1	-
ENSMUSC	Adcy4	adenylate	GO:00081	metabolic	K08044	adenylate	mmu0520	Pathways	14:557690	57	65	47	59	93	91	0.468536	0.426707	0.42699	0.374157	0.492278	0.588467	0.440744	0.484968	0.713636	1	-
ENSMUSC	Sdr39u1	short chai	GO:00081	metabolic	-	-	-	-	14:558972	416	454	363	555	569	625	3.307317	2.882595	3.189592	3.404126	2.913057	3.909064	3.126501	3.408749	0.612863	1	-
ENSMUSC	Cma1	chymase	GO:00325	developm	K01329	chymase[	mmu0461	Renin-ang	14:559414	0	0	0	0	0	3	0	0	0	0	0	0.088831	0	0.02961	0.292016	1	-
ENSMUSC	Sema5a	sema don	GO:00055	extracellul	K06841	semaphor	mmu0436	Axon guic	15:322448	3177	4366	3294	3899	5349	4265	8.02738	8.810207	9.198711	7.600464	8.703298	8.477865	8.678766	8.260542	0.414101	1	-
ENSMUSC	Cct5	chaperoni	GO:00081	metabolic	-	-	-	-	15:315908	2453	3293	2445	3093	3951	3290	36.37978	39.00318	40.07635	35.38936	37.73325	38.38569	38.48643	37.16943	0.5037	1	-
ENSMUSC	Cmbl	carboxym	GO:00038	catalytic a	K01061	carboxym	mmu0110	Metabolic	15:315653	61	97	62	115	99	107	1.976929	2.510606	2.220764	2.875339	2.066107	2.728076	2.2361	2.556507	0.527341	1	-
ENSMUSC	Ropn1l	ropporin	-	-	-	-	-	-	15:314412	5	6	4	7	14	11	0.318979	0.305719	0.282077	0.344553	0.575196	0.552129	0.302258	0.490626	0.262107	1	-
ENSMUSC	Ctnnd2	catenin (c	GO:00325	developm	-	-	-	-	15:301725	5203	6074	4286	6094	7025	6143	21.60831	20.14594	19.67279	19.52539	18.78745	20.07051	20.47568	19.46112	0.36922	1	-
ENSMUSC	Tars	threonyl-t	GO:00081	metabolic	K01868	threonyl-t	mmu0097	Aminoacy	15:113823	576	703	501	772	934	718	4.545032	4.430126	4.369172	4.699612	4.745873	4.457074	4.44811	4.634186	0.869545	1	-
ENSMUSC	Amacr	alpha-me	GO:00081	metabolic	K01796	alpha-me	mmu0110	Metabolic	15:109817	123	166	132	200	262	186	2.484335	2.677685	2.946631	3.11649	3.407695	2.95549	2.702884	3.159892	0.325777	1	-
ENSMUSC	Rai14	retinoic ac	GO:00325	developm	-	-	-	-	15:105689	163	240	153	220	274	248	1.074435	1.263425	1.114631	1.118779	1.163047	1.286041	1.15083	1.189289	0.960548	1	-
ENSMUSC	Brix1	BRX1, bio	GO:00099	cellular pr	-	-	-	-	15:104747	407	531	360	532	631	495	7.665215	7.986767	7.493421	7.729873	7.652693	7.334098	7.715134	7.572221	0.699582	1	-
ENSMUSC	Rad1	RAD1 che	GO:00325	developm	K02830	cell cycle c	mmu0421	Cellular se	15:104860	67	77	54	71	95	66	0.377004	0.346026	0.335824	0.308219	0.344232	0.292164	0.352951	0.314872	0.409274	1	-
ENSMUSC	Nadk2	NAD kina	GO:00081	metabolic	K00858	NAD+ kin	mmu0110	Metabolic	15:907126	668	827	566	919	1112	980	3.770623	3.728104	3.531019	4.002051	4.041998	4.351845	3.676582	4.131964	0.355921	1	-
ENSMUSC	Mtdh	metadher	GO:00325	developm	-	-	-	-	15:340826	994	1741	815	1195	1699	1182	2.98093	4.169751	2.701283	2.7648	3.281057	2.78865	3.283988	2.944836	0.271958	1	-
ENSMUSC	Laptm4b	lysosomal	GO:00081	metabolic	K12387	lysosomal	mmu0414	Lysosome	15:342380	769	960	1015	1326	1672	1251	12.11708	12.08061	17.67602	16.11927	16.96534	15.50744	13.9579	16.19735	0.286049	1	-
ENSMUSC	Sdc2	syndecan	GO:00325	developm	K16336	syndecan	mmu0520	Proteogly	15:329207	671	936	652	863	1167	946	11.6161	12.94075	12.47477	11.52601	13.00958	12.88368	12.34387	12.47309	0.898571	1	-
ENSMUSC	Dnah5	dynein, ax	GO:00325	developm	K10408	dynein he	mmu0501	Huntingto	15:282037	197	258	167	232	284	265	0.769229	0.804555	0.720699	0.69889	0.714107	0.81404	0.764828	0.742346	0.667047	1	-
ENSMUSC	Trio	triple func	GO:00325	developm	-	-	-	-	15:277306	5196	6543	4946	7240	7145	7339	11.38513	11.44963	11.9776	12.23876	10.08151	12.65076	11.60412	11.65701	0.823007	1	-
ENSMUSC	Ank	progressiv	GO:00325	developm	-	-	-	-	15:274666	3148	4196	2989	4189	5251	4603	38.02393	40.47655	39.90202	39.03578	40.84312	43.73952	39.4675	41.20614	0.824301	1	-
ENSMUSC	11-Mar	membran	GO:00081	metabolic	-	-	-	-	15:263090	35	35	28	40	48	38	0.544326	0.434721	0.481278	0.479941	0.480715	0.464935	0.486775	0.475197	0.850156	1	-
ENSMUSC	Retreg1	reticuloph	GO:00081	metabolic	-	-	-	-	15:258431	703	855	669	833	1177	855	4.957709	4.815462	5.214335	4.532116	5.345111	4.743545	4.995835	4.873591	0.642665	1	-
ENSMUSC	Myo10	myosin X	GO:00325	developm	K12559	myosin X[	mmu0466	Fc gamma	15:256225	1074	1481	872	1419	1704	1539	4.314807	4.751797	3.871865	4.398146	4.408403	4.864146	4.312823	4.556898	0.754934	1	-
ENSMUSC	Rnf19a	ring finge	GO:00081	metabolic	-	-	-	-	15:362399	946	1245	918	1203	1498	1109	7.896236	8.299356	8.468736	7.746846	8.051846	7.282347	8.221443	7.69368	0.347612	1	-
ENSMUSC	Pabpc1	poly(A) bi	GO:00081	metabolic	K13126	polyadeny	mmu0301	RNA trans	15:365956	4033	5043	3782	5689	6425	5993	27.4707	27.43319	28.47147	29.89563	28.18184	32.11417	27.79178	30.06388	0.519422	1	-
ENSMUSC	Ywhaz	tyrosine 3	GO:00511	localizatio	K16197	14-3-3 pr	mmu0415	PI3K-Akt s	15:367707	13572	18089	13007	17883	22019	17757	102.8012	109.4246	108.8876	104.5021	107.4006	105.812	107.0378	105.9049	0.677462	1	-
ENSMUSC	Rrm2b	ribonucle	GO:00325	developm	K10808	ribonucle	mmu0110	Metabolic	15:379239	1096	1428	1072	1434	1648	1383	6.632937	6.901911	7.170289	6.695373	6.422545	6.584584	6.901712	6.567501	0.430914	1	-
ENSMUSC	Atp6v1c1	ATPase, H	GO:00511	localizatio	K02148	V-type H-	mmu0110	Metabolic	15:386619	3226	3937	3157	4285	5196	4631	25.59759	24.94859	27.68574	26.23106	26.54963	28.90816	26.07731	27.22962	0.826814	1	-
ENSMUSC	Baalc	brain and	GO:00452	synapse|n	-	-	-	-	15:389331	3996	5145	3930	5384	6174	5285	37.79833	38.86676	41.0853	39.29004	37.60698	39.32811	39.25013	38.74171	0.65458	1	-
ENSMUSC	Fzd6	frizzled cl	GO:00325	developm	-	-	-	-	15:390060	189	195	190	228	249	258	1.509286	1.243622	1.676908	1.404669	1.280454	1.62084	1.476605	1.435321	0.705361	1	-
ENSMUSC	Slc25a32	solute car	GO:00081	metabolic	-	-	-	-	15:390912	441	596	349	555	704	540	3.327151	3.591088	2.910085	3.230407	3.420278	3.205078	3.276108	3.285254	0.854212	1	-
ENSMUSC	Dcaf13	DDB1 anc	GO:00081	metabolic	-	-	-	-	15:391128	512	629	493	710	830	676	2.739135	2.687449	2.914994	2.930436	2.859409	2.845122	2.780526	2.878322	0.921459	1	-
ENSMUSC	Lrp12	low densit	GO:00325	developm	-	-	-	-	15:398705	871	662	472	1136	720	820	6.80768	4.132236	4.077274	6.849987	3.623834	5.042033	5.00573	5.171951	0.975442	1	-
ENSMUSC	Zfpm2	zinc finge	GO:00325	developm	K17442	zinc finge	mmu0520	MicroRNA	15:406550	665	784	588	747	900	879	8.16318	7.685981	7.977404	7.074383	7.114343	8.488626	7.942188	7.559117	0.478496	1	-
ENSMUSC	Oxr1	oxidation	GO:00081	metabolic	-	-	-	-	15:414474	6500	8538	5522	8654	9636	7993	55.51513	58.23714	52.12443	57.02237	52.99678	53.7055	55.29223	54.57489	0.659972	1	-
ENSMUSC	Angpt1	angiopoie	GO:00325	developm	K05465	angiopoie	mmu0415	PI3K-Akt s	15:424247	135	215	110	213	224	181	1.423631	1.810707	1.282053	1.732904	1.521132	1.501602	1.505464	1.585213	0.862531	1	-
ENSMUSC	Eif3h	eukaryotic	GO:00081	metabolic	K03247	translatior	mmu0301	RNA trans	15:517865	1890	2181	1647	2514	3130	2500	54.35697	50.09506	52.35205	55.78135	57.96855	56.56455	52.26802	56.77148	0.50643	1	-
ENSMUSC	Utp23	UTP23 sm	GO:00081	metabolic	-	-	-	-	15:518774	196	95	74	105	293	91	2.469036	0.955744	1.030265	1.020448	2.376801	0.901823	1.485015	1.433024	0.839507	1	-
ENSMUSC	Rad21	RAD21 co	GO:00081	metabolic	K06670	cohesin cc	mmu0411	Cell cycle	15:519622	2640	3240	2273	3457	4076	3239	38.08917	37.33262	36.24465	38.47932	37.86923	36.76373	37.22215	37.7041	0.897809	1	-
ENSMUSC	Cdh10	cadherin 1	GO:00160	membran	-	-	-	-	15:188189	685	876	611	858	996	845	3.594433	3.671042	3.543463	3.47342	3.365531	3.488249	3.60298	3.4424	0.479646	1	-
ENSMUSC	Matn2	matrilin 2	GO:00325	developm	-	-	-	-	15:343066	1105	1445	1021	1386	1770	1557	7.400374	7.728674	7.557254	7.161184	7.633425	8.203344	7.562101	7.665984	0.913306	1	-
ENSMUSC	Pop1	processin	GO:00081	metabolic	K01164	ribonuclea	mmu0301	RNA trans	15:344953	173	218	144	240	271	179	1.825627	1.837246	1.679475	1.953915	1.841571	1.486033	1.780782	1.760506	0.804921	1	-
ENSMUSC	Stk3	serine/thr	GO:00325	developm	K04412	serine/thr	mmu0401	MAPK sig	15:348754	518	671	424	724	520	683	3.693145	3.820628	3.34102	3.982315	2.387399	3.830877	3.618264	3.400197	0.518283	1	-
ENSMUSC	Khdrbs3	KH domai	GO:00081	metabolic	-	-	-	-	15:689284	3655	4475	3011	5023	6024	4554	118.2657	115.6406	107.6785	125.3906	125.5195	115.9245	113.8616	122.2782	0.580245	1	-
ENSMUSC	Zfat	zinc finge	GO:00325	developm	-	-	-	-	15:680837	96	154	97	201	135	158	0.5741	0.735504	0.641113	0.927352	0.519882	0.743336	0.650239	0.73019	0.59771	1	-

ENSMUSC	Eif3e	eukaryotic	GO:00081	metabolic	K03250	translation	mmu0301	RNA trans	15:432500	1175	1567	1208	1635	2100	1628	9.722626	10.35525	11.04739	10.43744	11.18973	10.59769	10.37509	10.74162	0.905884	1	-
ENSMUSC	Emc2	ER memb	GO:00329	macromo	-	-	-	-	15:434772	1020	1241	860	1346	1672	1252	15.3738	14.93821	14.32605	15.65152	16.22827	14.84555	14.87935	15.57511	0.819575	1	-
ENSMUSC	Eny2	enhancer	GO:00081	metabolic	-	-	-	-	15:444280	772	1029	724	1002	1275	1052	4.687438	4.989755	4.858515	4.693707	4.985204	5.025099	4.845236	4.901337	0.902944	1	-
ENSMUSC	Ebag9	estrogen	GO:00099	cellular pr	-	-	-	-	15:446191	496	656	466	754	893	622	6.449068	6.811851	6.696496	7.563399	7.476893	6.362336	6.652472	7.134209	0.667116	1	-
ENSMUSC	Sybu	syntabulin	GO:00325	developm	-	-	-	-	15:446718	896	1834	1306	1911	2001	1094	4.812041	7.866221	7.751949	7.917937	6.920266	4.622206	6.810071	6.486803	0.638971	1	-
ENSMUSC	Kcnv1	potassium	GO:00511	localizatio	-	-	-	-	15:451062	743	910	715	854	1131	986	7.856978	7.685174	8.356409	6.967141	7.701653	8.202653	7.966187	7.623816	0.503048	1	-
ENSMUSC	Myc	myelocytc	GO:00325	developm	K04377	Myc protc	mmu0520	Pathways	15:619853	56	113	51	80	121	118	0.411217	0.662683	0.413906	0.453211	0.572167	0.68167	0.495935	0.569016	0.573444	1	-
ENSMUSC	Washc5	WASH coi	GO:00325	developm	K18464	WASH coi	mmu0414	Endocytos	15:593319	848	1036	807	1019	1280	1034	12.11731	11.82268	12.74473	11.2335	11.7781	11.62364	12.22824	11.54508	0.40697	1	-
ENSMUSC	Sqle	squalene	GO:00081	metabolic	K00511	squalene	mmu0110	Metabolic	15:593150	785	999	679	985	1426	988	5.6033	5.694893	5.356618	5.424264	6.554632	5.548072	5.551604	5.842323	0.793338	1	-
ENSMUSC	Mtss1	metastasis	GO:00099	cellular pr	-	-	-	-	15:589412	648	879	619	1010	1057	794	2.238256	2.424764	2.363046	2.69145	2.351064	2.157575	2.342022	2.40003	0.996565	1	-
ENSMUSC	Ndufb9	NADH de	GO:00081	metabolic	K03965	NADH de	mmu0110	Metabolic	15:589337	1726	2140	1519	2373	2768	1964	90.69387	89.80424	88.21487	96.19785	93.66082	81.18759	89.57099	90.34875	0.871291	1	-
ENSMUSC	Klhl38	kelch-like	GO:00081	metabolic	-	-	-	-	15:583145	4	7	5	15	8	3	0.110419	0.154314	0.152547	0.319434	0.142207	0.065137	0.139093	0.175593	0.643776	1	-
ENSMUSC	Fbxo32	F-box prc	GO:00081	metabolic	K10305	F-box prc	mmu0406	FoxO sign	15:581758	273	474	319	433	560	495	2.389461	3.313301	3.085843	2.923848	3.156308	3.408421	2.929535	3.162859	0.671879	1	-
ENSMUSC	Wdyhv1	WDYHV n	GO:00081	metabolic	-	-	-	-	15:581413	80	91	77	106	97	115	0.91541	0.831598	0.973792	0.935762	0.714746	1.035232	0.906934	0.895247	0.823489	1	-
ENSMUSC	Atad2	ATPase fa	GO:00081	metabolic	-	-	-	-	15:580940	187	196	158	214	298	196	0.577856	0.483704	0.539612	0.510179	0.592993	0.476481	0.533724	0.526551	0.79944	1	-
ENSMUSC	Zhx1	zinc finge	GO:00081	metabolic	-	-	-	-	15:580470	1522	1802	1352	1819	2237	1805	6.756611	6.388735	6.633423	6.229848	6.394918	6.303801	6.592923	6.309522	0.444956	1	-
ENSMUSC	Gm29394	predicted	-	-	-	-	-	-	15:580282	9	30	34	24	38	33	0.258958	0.68938	1.081254	0.532775	0.704103	0.747013	0.676531	0.661297	0.901713	1	-
ENSMUSC	Tbc1d31	TBC1 don	GO:00056	cell|intrace	-	-	-	-	15:579121	126	143	111	161	207	134	0.883781	0.801042	0.860483	0.871225	0.934976	0.739419	0.848436	0.84854	0.897533	1	-
ENSMUSC	Derl1	Der1-like	GO:00081	metabolic	K11519	Derlin-1[-	mmu0414	Protein pr	15:578695	771	1021	777	998	1342	1040	13.17911	13.93809	14.6791	13.16112	14.77199	13.98545	13.9321	13.97285	0.839742	1	-
ENSMUSC	Has2	hyalurona	GO:00325	developm	-	-	-	-	15:566656	10	10	7	7	11	8	0.144108	0.115083	0.111496	0.077823	0.102075	0.09069	0.123562	0.090196	0.352797	1	-
ENSMUSC	Mtbp	Mdm2, tra	GO:00511	localizatio	-	-	-	-	15:555574	33	46	31	42	63	51	0.298685	0.332507	0.310104	0.293274	0.367189	0.363143	0.313765	0.341202	0.769128	1	-
ENSMUSC	Mrpl13	mitochon	GO:00081	metabolic	K02871	large subu	mmu0301	Ribosome	15:555340	240	340	269	357	453	337	1.744103	1.973268	2.160529	2.001519	2.119893	1.926642	1.9593	2.016018	0.968826	1	-
ENSMUSC	Col14a1	collagen,	GO:00325	developm	K08133	collagen,	mmu0497	Protein di	15:553077	33	59	32	58	86	50	0.229726	0.328013	0.246203	0.311495	0.38552	0.273825	0.267981	0.323613	0.407636	1	-
ENSMUSC	Sla	src-like ac	GO:00325	developm	-	-	-	-	15:667808	129	161	102	145	174	143	0.624672	0.622638	0.545895	0.541701	0.542584	0.544765	0.597735	0.543017	0.391623	1	-
ENSMUSC	Lrrc6	leucine ric	GO:00325	developm	-	-	-	-	15:663798	60	91	84	87	102	98	1.757897	2.129275	2.720008	1.966501	1.924432	2.258827	2.202393	2.04992	0.574644	1	-
ENSMUSC	Adcy8	adenylate	GO:00081	metabolic	K08048	adenylate	mmu0520	Pathways	15:646970	385	419	305	514	511	461	2.229665	1.937936	1.952205	2.296531	1.905701	2.100345	2.039935	2.100859	0.974825	1	-
ENSMUSC	Asap1	ArfGAP w	GO:00325	developm	K12488	Arf-GAP v	mmu0414	Endocytos	15:640868	1527	1837	1230	1812	2049	1774	7.023937	6.748334	6.253074	6.430284	6.069293	6.419573	6.675115	6.306383	0.370992	1	-
ENSMUSC	Fam49b	family witl	GO:00019	cell killing	-	-	-	-	15:639290	1731	2302	1655	2095	2672	2265	7.547782	8.016288	7.975669	7.047524	7.502628	7.769648	7.84658	7.439933	0.379743	1	-
ENSMUSC	Wnt7b	wingless-	GO:00325	developm	K00572	wingless-t	mmu0520	Pathways	15:855354	699	919	772	1006	1154	903	9.671335	10.15479	11.80521	10.73836	10.28182	9.828968	10.54378	10.28305	0.647649	1	-
ENSMUSC	Ppara	peroxisom	GO:00325	developm	K07294	peroxisom	mmu0402	cAMP sigr	15:857349	24	27	25	37	50	46	0.242058	0.217476	0.278665	0.287894	0.324735	0.36498	0.246066	0.32587	0.217756	1	-
ENSMUSC	Gtse1	G two S p	GO:00990	supramole	K10129	G-2 and S	mmu0411	p53 signa	15:858597	9	16	13	8	27	21	0.201951	0.286743	0.322404	0.138497	0.390156	0.370721	0.270366	0.299791	0.83265	1	-
ENSMUSC	Trmu	tRNA 5-m	GO:00081	metabolic	-	-	-	-	15:858793	150	184	129	237	249	186	1.461422	1.431683	1.389055	1.781404	1.562207	1.425634	1.427387	1.589748	0.544417	1	-
ENSMUSC	Brd1	bromodo	GO:00081	metabolic	-	-	-	-	15:886873	1400	1692	1192	1742	2030	1862	8.061237	7.780721	7.585717	7.738416	7.527041	8.434596	7.809225	7.900018	0.888385	1	-
ENSMUSC	Ttll8	tubulin tyr	GO:00081	metabolic	-	-	-	-	15:888906	24	29	25	34	31	27	0.267852	0.25848	0.30836	0.292739	0.222792	0.237058	0.278231	0.250863	0.574051	1	-
ENSMUSC	Tef	thyrotropl	GO:00325	developm	-	-	-	-	15:818024	4388	5395	3920	6463	7197	6184	20.09925	19.73561	19.84477	22.83903	21.2285	22.28402	19.89321	22.11719	0.319842	1	-
ENSMUSC	Zc3h7b	zinc finge	GO:00081	metabolic	-	-	-	-	15:817448	4203	5221	3683	5445	6441	5579	43.07271	42.73094	41.71491	43.0498	42.50606	44.97903	42.50619	43.51163	0.992283	1	-
ENSMUSC	Rangap1	RAN GTPa	GO:00511	localizatio	K14319	Ran GTPa	mmu0301	RNA trans	15:817042	2800	4019	2514	3681	4886	3880	42.66601	48.9089	42.33857	43.27331	47.94377	46.51217	44.63783	45.90975	0.959414	1	-
ENSMUSC	L3mbtl2	l(3)mbt-lil	GO:00325	developm	-	-	-	-	15:816638	589	675	507	712	830	648	2.773441	2.538362	2.638511	2.586512	2.516729	2.400434	2.650105	2.501225	0.435715	1	-
ENSMUSC	Rbx1	ring-box	GO:00081	metabolic	K03868|K0	RING-box	mmu0520	Pathways	15:814663	1185	1431	1070	1537	1783	1403	43.71833	42.16292	43.62905	43.74713	42.35955	40.72058	43.1701	42.27575	0.629957	1	-
ENSMUSC	St13	suppressic	GO:00329	macromo	-	-	-	-	15:813636	2908	3713	2472	3584	4429	3223	38.23364	38.98719	35.92083	36.35377	37.4983	33.33668	37.71388	35.72958	0.383337	1	-
ENSMUSC	Slc25a17	solute car	GO:00081	metabolic	K13354	solute car	mmu0414	Peroxisom	15:813189	438	527	389	554	733	588	16.04287	15.41573	15.74724	15.65481	17.28887	16.94323	15.73528	16.62897	0.775985	1	-
ENSMUSC	Adsl	adenylosu	GO:00081	metabolic	K01756	adenylosu	mmu0110	Metabolic	15:809484	233	273	253	333	348	314	1.597769	1.49509	1.917458	1.761704	1.536709	1.693943	1.670105	1.664119	0.83392	1	-
ENSMUSC	Mief1	mitochon	GO:00325	developm	-	-	-	-	15:802339	438	511	362	497	642	598	2.374453	2.212363	2.168923	2.078626	2.24119	2.550361	2.251913	2.290059	0.949071	1	-
ENSMUSC	Tab1	TGF-beta	GO:00325	developm	K04403	TAK1-bin	mmu0401	MAPK sig	15:801331	301	396	287	448	542	440	6.27038	6.588226	6.6078	7.200051	7.270783	7.210921	6.488802	7.227252	0.454102	1	-
ENSMUSC	Syngr1	synaptogy	GO:00452	synapse|c	-	-	-	-	15:800913	8474	10590	7171	11561	12989	10291	95.25155	95.06604	89.08614	100.2558	94.01864	91.00223	93.13457	95.09221	0.970304	1	-
ENSMUSC	Cacna1i	calcium cl	GO:00400	locomotic	K04856	voltage-d	mmu0401	MAPK sig	15:802872	1648	1959	1372	2324	2274	2023	8.576861	8.142382	7.89173	9.331204	7.621079	8.282816	8.203658	8.4117	0.997507	1	-
ENSMUSC	Dnal4	dynein, ax	GO:00990	supramole	K10412	dynein lig	mmu0501	Huntingto	15:797614	565	797	585	633	1037	836	9.976244	11.23886	11.41618	8.622877	11.79103	11.61276	10.8771	10.67556	0.716401	1	-
ENSMUSC	Nptxr	neuronal	-	-	-	-	-	-	15:797863	20403	26835	19660	28463	32031	26896	123.0632	129.2652	131.0583	132.4479	124.4111	127.6241	127.7955	128.1611	0.796449	1	-
ENSMUSC	Josd1	Josephin	GO:00081	metabolic	-	-	-	-	15:796742	956	1194	896	1364	1551	1245	17.19845	17.15464	17.81502	18.93111	17.96792	17.62024	17.38937	18.17309	0.831666	1	-
ENSMUSC	Tomm22	transloca	GO:00511	localizatio	-	-	-	-	15:796708	503	759	556	802	945	770	18.10863	21.82253	22.12274	22.27522	21.90805	21.80816	20.68463	21.99714	0.717512	1	-
ENSMUSC	Cby1	chibby far	GO:00325	developm	-	-	-	-	15:796592	500	653	532	690	894	741	27.28246	28.45589	32.08276	29.04644	31.41273	31.80845	29.2737	30.75587	0.81052	1	-
ENSMUSC	Dmc1	DNA meic	GO:00325	developm	-	-	-	-	15:795615	9	13	2	19	18	11	0.250689	0.289195	0.061576	0.408298	0.322875	0.241053	0.200486	0.324075	0.252193	1	-
ENSMUSC	Smc1b	structural	GO:00329	macromo	K06636	structural	mmu0411	Cell cycle	15:850646	0	3	0	2	7	2	0	0.035426	0	0.022815	0.066637	0.023266	0.011809	0.037573	0.208611	1	-
ENSMUSC	Csnk1e	casein kin	GO:00325	developm	K08960	casein kin	mmu0439	Hippo sig	15:794178	1167	1599	1122	1840	2100	1785	8.139687	8.906993	8.649211	9.901135	9.432151	9.794585	8.565297	9.70929	0.262803	1	-
ENSMUSC	Fam118a	family witl	GO:00160	membran	-	-	-	-	15:850370	404	458	338	459	601	475	9.70014	8.782307	8.969338	8.502373	9.292365	8.972261	9.150595	8.922333	0.664615	1	-
ENSMUSC	Upk3a	uroplakin	GO:00325	developm	K19520	uroplakin-	mmu0521	Bladder ca	15:850171	0	0	0	3	0	0	0	0	0	0.127791	0	0	0	0.042597	0.292887	1	-
ENSMUSC	Samm50	SAMM50	GO:00511	localizatio	-	-	-	-	15:841922	889	1187	731	1143	1442	1171	32.44512	34.59743	29.48569	32.1828	33.88966	33.62135	32.17608	33.23127	0.934721	1	-
ENSMUSC	Parvb	parvin, be	GO:00325	developm	K06275	parvin[-]	mmu0451	Focal adh	15:842320	381	599	372	586	674	558	4.872877	6.118337	5.258368	5.782147	5.551053	5.614431	5.416527	5.64921	0.874681	1	-
ENSMUSC	Parvg	parvin, ga	GO:00099	cellular pr	K06275	parvin[-]	mmu0451	Focal adh	15:843240	213	270	214	251	330	255	2.350933	2.379965	2.610482	2.137299	2.345472	2.214173	2.447127	2.232315	0.351836	1	-
ENSMUSC	Efcab6	EF-hand	GO:00081	metabolic	-	-	-	-	15:838667	136	164	126	242	236	201	1.033466	0.995283	1.058213	1.41874	1.154847	1.201613	1.028988	1.2584	0.195071	1	-
ENSMUSC	Myh9	myosin, h	GO:00325	developm	K10352	myosin he	mmu0481	Regulatior	15:777605	3305	4092	2866	4526	5953	4437	14.16816	14.00952	13.57891	14.96879	16.43359	14.96381	13.91886	15.4554	0.342079	1	-
ENSMUSC	Adamts20	a disinteg	GO:00325	developm	-	-	-	-	15:942701	316	403	290	456	534	394	2.0069	2.044043	2.035558	2.234262	2.183913	1.968548	2.028834	2.128908	0.834247	1	-
ENSMUSC	Ndufa6	NADH de	GO:00081	metabolic	K03950	NADH de	mmu0110	Metabolic	15:823501	727	872	635	1093	1290	1019	79.26655	75.93074	76.52016	91.94028	90.57318	87.40586	77.23915	89.97311	0.198528	1	-
ENSMUSC	Twf1	twinfilin a	GO:00081	metabolic	-	-	-	-	15:945779	1203	1453	1144	1433	2035	1571	18.91654	18.24683	19.88148	17.38411	20.60606	19.43403	19.01495	19.1414	0.859847	1	-
ENSMUSC	Smdt1	single-pa	GO:00511	localizatio	-	-	-	-	15:823389	486	704	619	720	957	736	15.28812	17.68626	21.52064	17.47352	19.38583	18.21406	18.16501	18.3578	0.917355	1	-
ENSMUSC	Naga	N-acetyl	GO:00081	metabolic	K01204	alpha-N-	mmu0414	Lysosome	15:823295	305	467	259	454	586	404	9.73483	11.90394	9.136381	11.17931	12.04428	10.14426	10.25838	11.12262	0.640746	1	-
ENSMUSC	Nell2	NEL-like 2	GO:00099	cellular pr	-	-	-	-	15:952194	16609	20954	16454	20923	26606	23207	280.918	283.0407	307.5774	273.0175	289.7813	308.792	290.5121	290.5303	0.775136	1	-
ENSMUSC	Wbp2nl	WBP2 N-	GO:00099	cellular pr	-	-	-	-	15:822989	0	0	2	0	0	0	0	0	0.102408	0	0	0	0.034136	0	0.526525	1	-
ENSMUSC	3-Sep	septin 3 [S	GO:00452	synapse|c	K16938	septin 3/9	mmu0510	Bacterial i	15:822749	11062	14477	10367	14568	17655	14482	107.3637	112.2143	111.2048	109.0822	110.3433	110.5766	110.2609	110.0007	0.748907	1	-
ENSMUSC	Slc38a2	solute car	GO:00325	developm	K14207	solute car	mmu0472	Glutamate	15:966873	1347	1805	1054	1732	2192	2092	17.77891	19.02658	15.37536	17.63664	18.63087	21.72253	17.39362	19.33001	0.415325	1	-
ENSMUSC	Srebf2	sterol regi	GO:00081	metabolic	-	-	-	-	15:821472	3826	4571	3634	5079	5829	4848	46.82468	44.6773	49.15431	47.95549	45.93869	46.67705	46.88543	46.85708	0.769069	1	-
ENSMUSC	Slc38a4	solute car	GO:00511	localizatio	-	-	-	-	15:969948	13	22	11	12	22	20	0.1855	0.250713	0.173472	0.132099	0.202155	0.224512	0.203228	0.186255	0.701523	1	-
ENSMUSC	Rpap3	RNA polyi	GO:00329	macromo	-	-	-	-	15:976751	420	544	373	495	625	496	11.34707	11.73762	11.13758	10.3174	10.87351	10.5421	11.40742	10.57767	0.362704	1	-
ENSMUSC	Endou	endonucle	GO:00081	metabolic	-	-	-	-	15:977110	9	25	2	14	21	35	0.202174	0.448514	0.049659	0.242633	0.30378	0.618544	0.233449	0.388319	0.266852	1	-
ENSMUSC	Rapgef3	Rap guan	GO:00325	developm	K08014	Rap guan	mmu0401	Rap1 sign	15:977447	456	566	423	591	687	658	2.08137	2.063225	2.133887	2.081146	2.019278	2.36277	2.092827	2.154398	0.971321	1	-
ENSMUSC	Xrcc6	X-ray rep	GO:00325	developm	K10884	ATP-depe	-	-	15:819878	272	400	270	398	471	379	2.536317	2.978791	2.782568	2.863176	2.828199	2.780254	2.765892	2.823876	0.985488	1	-
ENSMUSC	Desi1	desumoyl	GO:00081	metabolic	-	-	-	-	15:819925	1426	1835	1438	1638	1993	2059	14.21068	14.60419	15.83802	12.5933	12.78962	16.1422	14.8843	13.84171	0.325516	1	-
ENSMUSC	Pmm1	phosphor	GO:00081	metabolic	K17497	phosphon	mmu0110	Metabolic	15:819511	1079	1377	1021	1558	1874	1574	15.72356	16.02541	16.44379	17.51568	17.58545	18.04451	16.06425	17.71521	0.421863	1	-
ENSMUSC	Hdac7	histone de	GO:00325	developm	K11408	histone de	mmu0516	Human pa	15:977926	323	463	288	486	512	511	1.269831	1.453687	1.251361	1.474044	1.29619	1.580433	1.32496	1.450222	0.579866	1	-
ENSMUSC	Polr3h	polymeras	GO:00081	metabolic	K03022	DNA-dire	mmu0110	Metabolic	15:819150	403	486	394	574	667	503	9.787764	9.426703	10.57598	10.75524	10.43179	9.610741	9.93015	10.26592	0.935098	1	-
ENSMUSC	Aco2	aconitase	GO:00325	developm	K01681	aconitate	mmu0110	Metabolic	15:818723	9572	11657	9365	13134	15052	11761	113.9497	110.8264	123.2155	120.6252	115.3877	110.1453	115.9972	115.386	0.731467	1	-
ENSMUSC	Col2a1	collagen,	GO:00325	developm	K19719	collagen,	mmu0415	PI3K-Akt s	15:979756	10	10	12	13	13	9	0.0843	0.067321	0.111801	0.084546	0.070572	0.059685	0.087807	0.071601	0.554489	1	-
ENSMUSC	Hoxc10	homeobo	GO:00325	developm	-	-	-	-	15:102966	0	2	0	0	3	0	0	0.051095	0	0	0.061797	0	0.017032	0.020599	1	1	-
ENSMUSC	Hoxc5	homeobo	GO:00325	developm	-	-	-	-	15:102967	0	2	1	1	0	0	0	0.027569	0.019075	0.013316	0	0	0.015548	0.004439	0.646787	1	-
ENSMUSC	Pde1b	phosphoc	GO:00325	developm	K13755	calcium/ca	mmu0474	Olfactory	15:103503	1721	2255	1693	2204	2647	2323	13.91433	14.56042	15.12812	13.74748	13.7813	14.77548	14.53429	14.10142	0.536471	1	-
ENSMUSC	Ppp1r1a	protein pl	GO:00081	metabolic	K08050	protein pl	mmu0426	Adrenergi	15:103530	1032	1546	953	1333	1630	1386	35.76163	42.78512	36.49866	35.63672	36.37305	37.78431	38.34847	36.59803	0.464824	1	-
ENSMUSC	Glycam1	glycosylat	GO:00055	extracellul	K06815	glycosylat	mmu0451	Cell adhes	15:103562	3	4	7	6	6	0	0.051772	0.055125	0.133524	0.079883	0.066676	0	0.08014	0.048853	0.440496	1	-
ENSMUSC	Shisa9	shisa fami	GO:00452	synapse|c	-	-	-	-	16:119841	1329	1788	1069	1760	1963	1729	9.376731	10.07488	8.335874	9.580089	8.918696	9.596933	9.262494	9.36524	0.890733	1	-
ENSMUSC	Tnfrsf17	tumor nec	GO:00023	immune s	K05153	tumor nec	mmu0406	Cytokine-	16:113138	2	5	0	5	4	6	0.149808	0.299144	0	0.288973	0.192982	0.353639	0.149651	0.278532	0.454043	1	-
ENSMUSC	Txndc11	thioredox	GO:00508	response	-	-	-	-	16:110749	387	411	308	498	660	505	1.691447	1.434613	1.487799	1.679218	1.857571	1.736399	1.537953	1.757729	0.344158	1	-
ENSMUSC	Litaf	LPS-induc	GO:00325	developm	K19363	lipopolysa	mmu0414	Lysosome	16:109592	129	270	171	255	331	232	2.46757	4.124676	3.615117	3.763138	4.077212	3.491239	3.402455	3.777197	0.586239	1	-
ENSMUSC	Nubp1	nucleotide	GO:00081	metabolic	-	-	-	-	16:104119	142	250	164	200	294	258	1.733355	2.437167	2.212537	1.883476	2.311009	2.477597	2.127686	2.224027	0.893619	1	-
ENSMUSC	Emp2	epithelial	GO:00325	developm	-	-	-	-	16:102817	313	401	280	445	550	372	5.555571	5.684271	5.492749	6.093622	6.286403	5.194446	5.57753	5.858157	0.830411	1	-
ENSMUSC	1810013L	RIKEN cDI	-	-	-	-	-	-	16:883010	927	1117	736	1152	1400	1057	13.92053	13.396	12.2152	13.34624	13.53813	12.4871	13.17724	13.12382	0.779173	1	-
ENSMUSC	Bcl6	B cell leuk	GO:00325	developm	K15618	B-cell lym	mmu0520	Transcript	16:239650	1133	1428	983	1462	1720	1532	9.275786	9.336736	8.894492	9.234187	9.067845	9.867126	9.169005	9.389719	0.997307	1	-
ENSMUSC	Trp63	transform	GO:00325	developm	K10149	tumor prc	mmu0520	MicroRNA	16:256837	36	46	35	43	60	40	0.174799	0.178377	0.187826	0.161077	0.187605	0.152793	0.180334	0.167158	0.639098	1	-
ENSMUSC	Il1rap	interleukir	GO:00325	developm	K04723	interleukir	mmu0401	MAPK sig	16:265817	1188	1531	1137	1449	1971	1647	4.813338	4.953944	5.091402	4.529277	5.142466	5.249703	4.952895	4.973815	0.835977	1	-
ENSMUSC	Anks3	ankyrin re	GO:00055	protein bi	-	-	-	-	16:494141	400	561	437	637	717	664	9.836194	11.0173	11.87666	12.08472	11.35377	12.84534	10.91005	12.09461	0.469612	1	-
ENSMUSC	Nudt16l1	nudix (nuc	GO:00081	metabolic	K16867	NUDT16-	mmu0520	Proteoglyc	16:493911	303	383	304	453	546	439	14.57886	14.71724	16.16596	16.81553	16.91721	16.61719	15.15402	16.78331	0.487347	1	-
ENSMUSC	Mgrn1	mahogun	GO:00081	metabolic	K10604	E3 ubiquit	mmu0412	Ubiquitin	16:488610	3254	3977	3072	4040	4798	4499	58.23068	56.8376	60.75785	55.77583	55.29033	63.33755	58.60871	58.13457	0.711742	1	-
ENSMUSC	Srl	sarcalume	GO:00511	localizatio	-	-	-	-	16:448021	253	309	239	307	423	329	1.778665	1.734912	1.857029	1.665105	1.914998	1.819619	1.790202	1.799907	0.887248	1	-
ENSMUSC	Crebbp	CREB binc	GO:00081	metabolic	K04498	E1A/CREB	mmu0520	Pathways	16:408132	2487	3029	2359	3034	3307	3243	8.489967	8.258006	8.900304	7.990538	7.269742	8.709395	8.549426	7.989892	0.306565	1	-
ENSMUSC	Fgf12	fibroblast	GO:00081	metabolic	K04358	fibroblast	mmu0520	Pathways	16:281601	2143	2483	1718	2545	3026	2276	42.69088	39.50348	37.82535	39.11388	38.8183	35.66942	40.00657	37.8672	0.382248	1	-
ENSMUSC	Hrasls	HRAS-like	GO:00160	membran	-	-	-	-	16:292096	210	270	182	278	386	341	3.224086	3.310527	3.08821	3.292777	3.816183	4.118637	3.207608	3.742532	0.297189	1	-
ENSMUSC	Zfp251	zinc finge	GO:00081	metabolic	-	-	-	-	15:768521	1120	1494	1154	1433	1798	1469	19.55535	20.83263	22.26898	19.30298	20.21585	20.17811	20.88566	19.89898	0.441882	1	-
ENSMUSC	Hes1	hairy and	GO:00325	developm	K06054	hairy and	mmu0520	Pathways	16:300643	142	172	98	219	210	183	3.031553	2.932592	2.312324	3.607041	2.887026	3.073537	2.758823	3.189201	0.419332	1	-
ENSMUSC	Zfp263	zinc finge	GO:00081	metabolic	-	-	-	-	16:374409	330	436	244	496	280	548	1.42407	1.502623	1.163733	1.651314	0.778091	1.86041	1.363475	1.429938	0.913188	1	-
ENSMUSC	Atp13a3	ATPase ty	GO:00511	localizatio	-	-	-	-	16:303124	1516	2055	1309	1903	2295	1887	8.950308	9.689391	8.541324	8.66778	8.725215	8.764389	9.060341	8.719128	0.486176	1	-
ENSMUSC	Mefv	Mediterra	GO:00081	metabolic	K12803	pyrin[-]	mmu0462	NOD-like	16:370721	0	3	0	1	2	2	0	0.029588	0	0.009527	0.015899	0.019431	0.009863	0.014953	1	1	-
ENSMUSC	Glyr1	glyoxylate	GO:00081	metabolic	-	-	-	-	16:501390	4009	4855	3621	5400	6107	5311	71.13687	68.80084	71.01236	73.92355	69.78166	74.13886	70.31669	72.61469	0.925938	1	-
ENSMUSC	Tmem44	transmem	GO:00160	membran	-	-	-	-	16:305118	585	830	446	862	1055	911	2.586269	2.930502	2.179215	2.940057	3.003485	3.168456	2.565329	3.037333	0.183746	1	-
ENSMUSC	Lsg1	large 60S	GO:00511	localizatio	K14539	large subu	mmu0300	Ribosome	16:305604	210	368	225	372	394	354	1.553264	2.1738	1.839315	2.122756	1.876623	2.059872	1.85546	2.01975	0.638281	1	-
ENSMUSC	Rogdi	rogdi hon	GO:00325	developm	-	-	-	-	16:500873	2042	2300	1918	2634	2987	2614	28.33008	25.48389	29.40948	28.19279	26.6859	28.53044	27.74115	27.80304	0.805706	1	-
ENSMUSC	Eef2kmt	eukaryotic	GO:00081	metabolic	-	-	-	-	16:524415	27	62	63	72	110	78	0.38763	0.710867	0.999625	0.797461	1.016938	0.880957	0.699374	0.898452	0.292194	1	-
ENSMUSC	Ercc4	excision re	GO:00081	metabolic	K10848	DNA excis	mmu0346	Fanconi a	16:131096	476	630	437	648	707	642	2.953323	3.121697	2.996622	3.101768	2.824737	3.133647	3.023881	3.020051	0.811093	1	-
ENSMUSC	Apod	apolipopr	GO:00325	developm	-	-	-	-	16:312961	1231	2083	1170	1707	2345	1893	17.17608	23.21139	18.04259	18.37511	21.06995	20.77914	19.47669	20.07474	0.945277	1	-
ENSMUSC	Adck5	aarF dom	GO:00160	membran	-	-	-	-	15:765763	130	145	119	209	207	155	0.953461	0.849323	0.964613	1.182596	0.977653	0.894339	0.922466	1.018196	0.6071	1	-
ENSMUSC	Cyc1	cytochron	GO:00081	metabolic	K00413	ubiquinol-	mmu0110	Metabolic	15:763435	1521	1988	1714	2224	2863	2165	77.78007	81.18976	96.87165	87.74132	94.27901	87.09794	85.28049	89.70609	0.776128	1	-
ENSMUSC	Sharpin	SHANK-a	GO:00325	developm	K20894	shank-ass	mmu0421	Necroptos	15:763470	330	398	342	463	574	516	2.118735	2.04076	2.426811	2.293369	2.373172	2.606296	2.195435	2.424279	0.520947	1	-
ENSMUSC	Maf1	MAF1 hor	GO:00081	metabolic	-	-	-	-	15:763512	367	450	409	581	656	593	2.668931	2.613541	3.287311	3.259696	3.072056	3.392632	2.856594	3.241462	0.379143	1	-
ENSMUSC	Hgh1	HGH1 hor	-	-	-	-	-	-	15:763688	71	101	70	98	108	90	2.212925	2.51407	2.411305	2.356495	2.167645	2.206785	2.379433	2.243642	0.576403	1	-
ENSMUSC	Dgat1	diacylglyc	GO:00081	metabolic	K11155	diacylglyc	mmu0110	Metabolic	15:765020	187	218	154	199	242	213	2.079375	1.93595	1.892608	1.707164	1.732853	1.863302	1.969311	1.767773	0.300776	1	-
ENSMUSC	Hsf1	heat shoc	GO:00325	developm	K09414	heat shoc	mmu0513	Legionella	15:764774	420	535	424	590	719	567	1.951924	1.9857	2.177843	2.115419	2.15178	2.073043	2.038489	2.113414	0.911834	1	-
ENSMUSC	Bop1	block of p	GO:00081	metabolic	-	-	-	-	15:764529	822	1128	890	1284	1413	1298	20.27049	22.21506	24.25659	24.42801	22.43828	25.1813	22.24738	24.01586	0.59612	1	-
ENSMUSC	Mroh1	maestro h	-	-	-	-	-	-	15:763802	862	1050	754	1218	1290	1338	2.452453	2.38577	2.370891	2.673443	2.363403	2.99475	2.403038	2.677199	0.411556	1	-
ENSMUSC	Fbxl6	F-box anc	-	-	-	-	-	-	15:765357	175	238	209	238	277	294	6.241337	6.77895	8.238213	6.548582	6.361702	8.24894	7.086167	7.053075	0.843469	1	-
ENSMUSC	Slc52a2	solute car	GO:00511	localizatio	-	-	-	-	15:765389	134	200	114	147	221	178	3.030299	3.612069	2.849248	2.564643	3.218305	3.16672	3.163872	2.983223	0.575332	1	-
ENSMUSC	Oplah	5-oxopro	GO:00081	metabolic	K01469	5-oxopro	mmu0048	Glutathior	15:762965	340	377	302	411	466	413	2.55725	2.264548	2.510434	2.384878	2.257016	2.443744	2.444077	2.361879	0.613338	1	-
ENSMUSC	Grina	glutamate	GO:00508	response	-	-	-	-	15:762468	6421	8489	6420	9230	11066	9798	231.5736	244.5056	255.8988	256.8138	256.9992	277.9935	243.9927	263.9355	0.51509	1	-
ENSMUSC	Plec	plectin [Sc	GO:00051	structural	-	-	-	-	15:761709	4322	5199	3781	5634	6410	4876	7.049746	6.772584	6.816199	7.089831	6.732889	6.256959	6.879509	6.693226	0.54585	1	-
ENSMUSC	Scrib	scribbled	GO:00325	developm	K16175	protein sc	mmu0516	Human pa	15:760471	743	900	643	966	1120	978	7.016114	6.787285	6.710659	7.037445	6.810519	7.265367	6.838019	7.037777	0.967049	1	-
ENSMUSC	Tsta3	tissue spe	GO:00081	metabolic	K02377	GDP-L-fu	mmu0110	Metabolic	15:759246	245	296	240	384	436	346	11.41054	11.00976	12.35376	13.79756	13.07621	12.67734	11.59135	13.1837	0.37644	1	-
ENSMUSC	Pycrl	pyrroline-	GO:00081	metabolic	K00286	pyrroline-	mmu0110	Metabolic	15:759164	149	191	153	234	264	202	6.724063	6.883742	7.63106	8.146877	7.67193	7.171475	7.079621	7.663427	0.681796	1	-
ENSMUSC	Naprt	nicotinate	GO:00081	metabolic	K00763	nicotinate	mmu0110	Metabolic	15:758909	77	91	74	112	158	106	2.487545	2.347836	2.642166	2.791454	3.286944	2.693995	2.492516	2.924131	0.378408	1	-
ENSMUSC	Ly6h	lymphocy	GO:00987	molecular	-	-	-	-	15:755512	1664	1734	1274	2442	2162	2241	15.2852	12.72074	12.93403	17.30588	12.78875	16.19461	13.64666	15.42974	0.354078	1	-
ENSMUSC	Rhpn1	rhophilin,	GO:00987	molecular	-	-	-	-	15:757042	101	158	106	142	164	178	1.029185	1.285802	1.193782	1.116326	1.076146	1.426926	1.16959	1.206466	0.98711	1	-
ENSMUSC	Ly6i	lymphocy	GO:00160	membran	-	-	-	-	15:749795	4	0	0	0	0	2	0.147225	0	0	0	0	0.057919	0.049075	0.019306	0.550664	1	-
ENSMUSC	Ly6e	lymphocy	GO:00160	membran	-	-	-	-	15:749550	1786	1809	1882	1754	2925	2875	8.734465	7.065439	10.17235	6.617815	9.211605	11.06124	8.657417	8.963552	0.942609	1	-
ENSMUSC	Gm9747	predicted	-	-	-	-	-	-	1:8223311	3	0	0	4	1	1	0.064908	0	0	0.066762	0.01393	0.017025	0.021636	0.032572	1	1	-
ENSMUSC	Lynx1	Ly6/neurc	GO:00987	molecular	-	-	-	-	15:747478	7558	8901	6708	10033	11625	9587	92.62891	87.12128	90.86141	94.86385	91.74602	92.43432	90.20386	93.01473	0.938998	1	-
ENSMUSC	Slurp1	secreted L	GO:00400	locomotic	-	-	-	-	15:747243	0	0	2	0	0	0	0	0	0.040827	0	0	0	0.013609	0	0.526168	1	-
ENSMUSC	Zbtb11	zinc finge	GO:00056	cell|intrace	-	-	-	-	16:559738	1402	1755	1176	1792	2039	1786	9.940019	9.937158	9.214958	9.801839	9.309174	9.961657	9.697379	9.69089	0.782985	1	-
ENSMUSC	Arc	activity re	GO:00325	developm	K15867	activity-re	mmu0503	Amphetar	15:746690	2207	2546	2059	2460	2736	3031	25.92444	23.88424	26.73069	22.29321	20.69559	28.00941	25.51312	23.66607	0.315363	1	-
ENSMUSC	Cep97	centroson	GO:00329	macromo	-	-	-	-	16:558998	417	521	375	550	513	488	2.107716	2.1031	2.09486	2.144716	1.669738	1.940471	2.101892	1.918308	0.31294	1	-
ENSMUSC	Ptk2	PTK2 prot	GO:00325	developm	K05725	focal adhe	mmu0520	Pathways	15:732053	1832	2507	1657	2404	2873	2471	7.102576	7.762301	7.100023	7.190434	7.172666	7.536574	7.321633	7.299891	0.758261	1	-
ENSMUSC	Mapk12	mitogen-	GO:00325	developm	K04441	p38 MAP	mmu0401	MAPK sig	15:891305	52	54	29	62	70	72	1.680796	1.393967	1.03599	1.546095	1.45702	1.830859	1.370251	1.611325	0.493828	1	-
ENSMUSC	Lmf2	lipase mat	GO:00081	metabolic	-	-	-	-	15:893510	212	273	211	319	393	323	4.454351	4.580963	4.899792	5.170934	5.317347	5.339013	4.645035	5.275765	0.390274	1	-
ENSMUSC	Chkb	choline ki	GO:00325	developm	K14156	choline/et	mmu0110	Metabolic	15:894164	254	289	266	411	410	459	1.48951	1.353482	1.724002	1.859435	1.548275	2.117547	1.522331	1.841753	0.205088	1	-
ENSMUSC	Mapk8ip2	mitogen-	GO:00325	developm	K04435	mitogen-a	mmu0401	MAPK sig	15:894539	7174	8993	6712	9658	11121	9439	73.74257	73.82565	76.25278	76.59042	73.61315	76.32974	74.607	75.5111	0.883199	1	-
ENSMUSC	Arsa	arylsulfata	GO:00325	developm	K01134	arylsulfata	mmu0414	Lysosome	15:894724	357	442	349	489	578	513	3.226907	3.1907	3.486501	3.410024	3.364345	3.647927	3.301369	3.474098	0.813141	1	-
ENSMUSC	Rabl2	RAB, mem	GO:00325	developm	-	-	-	-	15:895825	271	355	255	396	439	407	2.737711	2.86413	2.847124	3.086342	2.85587	3.234629	2.816322	3.058947	0.617721	1	-
ENSMUSC	Acr	acrosin pr	GO:00081	metabolic	-	-	-	-	15:895683	13	15	8	11	14	20	0.527763	0.486341	0.358928	0.344539	0.365999	0.638755	0.457677	0.449764	0.932058	1	-
ENSMUSC	Shank3	SH3 and r	GO:00325	developm	K15009	SH3 and r	mmu0472	Glutamate	15:894996	4477	5431	3999	5574	6037	5418	22.51892	21.81655	22.23098	21.63006	19.55403	21.43929	22.18882	20.87446	0.306666	1	-
ENSMUSC	Kif21a	kinesin fa	GO:00990	supramole	-	-	-	-	15:909332	4654	6267	3931	6258	7488	6108	43.8864	47.19642	40.96881	45.52704	45.46991	45.31203	44.01721	45.43633	0.924905	1	-
ENSMUSC	Yaf2	YY1 assoc	GO:00081	metabolic	-	-	-	-	15:932842	1402	1798	1279	1839	2275	1926	26.20246	26.83674	26.4187	26.51589	27.3798	28.31792	26.48597	27.40454	0.911025	1	-
ENSMUSC	Zcrb1	zinc finge	GO:00081	metabolic	-	-	-	-	15:933860	583	701	569	793	967	774	7.821769	7.511046	8.43714	8.208055	8.354446	8.169362	7.923319	8.243954	0.878633	1	-
ENSMUSC	Alcam	activated	GO:00325	developm	K06547	activated	mmu0451	Cell adhes	16:522489	2225	2673	2078	2984	3464	2870	15.4802	14.85222	15.97861	16.01681	15.51956	15.70868	15.43701	15.74835	0.962298	1	-
ENSMUSC	Cblb	Casitas B-	GO:00325	developm	K04707	E3 ubiquit	mmu0520	Pathways	16:520312	878	1193	893	1105	1491	1167	3.737643	4.055921	4.201471	3.629074	4.087293	3.908275	3.998345	3.874881	0.575775	1	-
ENSMUSC	Retnlg	resistin lik	GO:00400	locomotic	-	-	-	-	16:488726	0	3	0	0	3	0	0	0.240643	0	0	0.19403	0	0.080214	0.064677	1	1	-
ENSMUSC	Morc1	microrchic	GO:00325	developm	-	-	-	-	16:484312	0	0	2	3	2	5	0	0	0.044254	0.04634	0.025777	0.078745	0.014751	0.050287	0.199979	1	-
ENSMUSC	Nectin3	nectin cell	GO:00325	developm	K06592	poliovirus	mmu0451	Cell adhes	16:463877	677	1052	687	1048	1283	1145	2.661366	3.302765	2.984828	3.178393	3.247861	3.541052	2.982986	3.322435	0.416751	1	-
ENSMUSC	Tagln3	transgelin	GO:00081	metabolic	-	-	-	-	16:457112	2624	3585	2684	4023	4165	3618	129.2068	140.9797	146.0666	152.8277	132.0661	140.1526	138.7511	141.6821	0.974281	1	-
ENSMUSC	Gcsam	germinal	GO:00400	locomotic	-	-	-	-	16:456103	0	0	0	0	2	0	0	0	0	0	0.015794	0	0	0.005265	0.527794	1	-
ENSMUSC	Atg3	autophag	GO:00081	metabolic	K08343	ubiquitin-	mmu0516	Kaposi's s	16:451587	893	1192	790	1207	1504	1079	15.85486	16.90182	15.5019	16.5329	17.19545	15.07105	16.08619	16.26647	0.902426	1	-
ENSMUSC	Slc35a5	solute car	GO:00511	localizatio	-	-	-	-	16:451395	728	1025	718	998	1241	928	3.922199	4.410301	4.275329	4.148197	4.305514	3.933301	4.20261	4.129004	0.687568	1	-

ENSMUSC	Ccdc80	coiled-co	GO:00099	cellular pr	-	-	-	-	16:450934	91	118	61	105	154	85	0.854157	0.884552	0.63281	0.760355	0.930829	0.627659	0.790507	0.772948	0.798332	1	-
ENSMUSC	Cd200r1	CD200 rec	GO:00329	macromo	-	-	mmu0516	Kaposi's sa	16:447657	3	4	3	3	9	4	0.075503	0.080393	0.083455	0.058259	0.145866	0.079195	0.079783	0.09444	0.864538	1	-
ENSMUSC	Gtpbp8	GTP-bind	GO:00056	cell|intrace	-	-	-	-	16:447367	393	507	312	565	622	574	5.026362	5.178626	4.410243	5.574929	5.122781	5.775419	4.871744	5.491043	0.401797	1	-
ENSMUSC	Mzt2	mitotic sp	GO:00329	macromo	-	-	-	-	16:158484	208	263	197	214	329	251	5.622	5.677124	5.884925	4.462428	5.72634	5.337175	5.728016	5.175314	0.333611	1	-
ENSMUSC	Prkdc	protein kii	GO:00325	developm	K06642	DNA-dep	mmu0411	Cell cycle	16:156378	746	862	669	880	1083	838	3.365302	3.105546	3.335479	3.062656	3.14607	2.973994	3.268776	3.060907	0.371957	1	-
ENSMUSC	Mcm4	minichron	GO:00081	metabolic	K02212	DNA repli	mmu0411	Cell cycle	16:156238	214	255	215	280	364	254	3.64069	3.464617	4.04254	3.675007	3.987734	3.399502	3.715949	3.687414	0.821113	1	-
ENSMUSC	Ube2v2	ubiquitin-	GO:00325	developm	-	-	-	-	16:155526	635	684	484	647	834	663	6.150364	5.290891	5.181066	4.834611	5.201731	5.051863	5.540774	5.029402	0.263473	1	-
ENSMUSC	Snai2	snail famil	GO:00325	developm	K05706	snail 2[-]	mmu0439	Hippo sig	16:147058	9	14	8	19	28	11	0.270561	0.336133	0.265794	0.440664	0.54206	0.260161	0.290829	0.414295	0.329066	1	-
ENSMUSC	Fopnl	Fgfr1op N	GO:00099	cellular pr	-	-	-	-	16:142992	224	148	290	418	328	375	5.612238	2.961397	8.030306	8.079677	5.291954	7.391455	5.534647	6.921029	0.336242	1	-
ENSMUSC	Nde1	nudE neu	GO:00325	developm	-	-	-	-	16:141632	347	453	304	491	641	495	2.908727	3.032613	2.816393	3.1753	3.460078	3.264298	2.919244	3.299892	0.376402	1	-
ENSMUSC	Pdxdc1	pyridoxal-	GO:00081	metabolic	-	-	-	-	16:138331	1433	1984	1494	2311	2717	2345	7.245469	8.011389	8.348678	9.014684	8.846358	9.327689	7.868512	9.06291	0.184656	1	-
ENSMUSC	Ntan1	N-termina	GO:00081	metabolic	-	-	-	-	16:138192	323	469	393	527	585	520	5.114562	5.93096	6.877733	6.43795	5.965084	6.477699	5.974418	6.293577	0.813908	1	-
ENSMUSC	Rrn3	RRN3 RN/	GO:00325	developm	-	-	-	-	16:137806	1272	1591	1148	1677	1886	1661	21.59185	21.56845	21.53731	21.96172	20.61576	22.18112	21.56587	21.5862	0.798754	1	-
ENSMUSC	Bfar	bifunction	GO:00081	metabolic	-	-	-	-	16:136718	556	714	495	715	900	710	4.386083	4.49828	4.315727	4.351497	4.57193	4.406276	4.40003	4.443235	0.893217	1	-
ENSMUSC	Parn	poly(A)-sp	GO:00081	metabolic	K01148	poly(A)-sp	mmu0301	RNA degr	16:135379	530	708	525	717	875	663	11.1512	11.89666	12.2082	11.63846	11.8552	10.97414	11.75202	11.48927	0.658573	1	-
ENSMUSC	B3gnt5	UDP-GlcN	GO:00325	developm	K03766	beta-1,3-	mmu0110	Metabolic	16:197602	35	27	22	5	57	31	0.138247	0.085173	0.096044	0.015236	0.144984	0.09633	0.106488	0.085517	0.559856	1	-
ENSMUSC	Boc	biregional	GO:00325	developm	K20020	brother of	mmu0436	Axon guic	16:444850	237	340	244	345	435	351	3.25113	3.72487	3.699317	3.651193	3.842642	3.787949	3.558439	3.760595	0.793299	1	-
ENSMUSC	Sidt1	SID1 trans	GO:00511	localizatio	-	-	-	-	16:442401	933	1154	835	1270	1537	1383	7.209214	7.121277	7.130819	7.570776	7.647769	8.406969	7.15377	7.875171	0.448611	1	-
ENSMUSC	Ccdc191	coiled-co	-	-	-	-	-	-	16:438898	74	86	58	95	115	101	0.851223	0.790054	0.737372	0.843074	0.851852	0.913994	0.792883	0.86964	0.661093	1	-
ENSMUSC	Hira	histone ce	GO:00325	developm	-	-	-	-	16:188770	864	1125	750	1087	1333	1229	7.00029	7.27949	6.715996	6.794577	6.954841	7.833671	6.998592	7.194363	0.976736	1	-
ENSMUSC	Qtrt2	queuine tl	GO:00081	metabolic	-	-	-	-	16:438614	223	301	224	339	386	285	1.929151	2.079569	2.141691	2.262516	2.15032	1.939627	2.050137	2.117488	0.94658	1	-
ENSMUSC	Mrpl40	mitochon	GO:00329	macromo	-	-	-	-	16:188720	272	330	243	373	469	361	9.206099	8.920035	9.089934	9.739696	10.22196	9.612254	9.072022	9.857971	0.611134	1	-
ENSMUSC	Gbe1	glucan (1,	GO:00081	metabolic	K00700	1,4-alpha	mmu0110	Metabolic	16:703139	170	215	132	219	264	180	1.072304	1.083057	0.920213	1.065717	1.072327	0.893206	1.025192	1.010416	0.776505	1	-
ENSMUSC	Usp7	ubiquitin s	GO:00081	metabolic	K11838	ubiquitin c	mmu0520	Viral carci	16:868959	3284	4121	2865	4406	4985	4398	16.65406	16.69034	16.05788	17.23819	16.27934	17.5462	16.46743	17.02124	0.918088	1	-
ENSMUSC	Tmem114	transmem	GO:00160	membran	-	-	-	-	16:840927	36	28	26	44	40	54	1.56337	0.971104	1.247884	1.474146	1.118582	1.84484	1.260786	1.47919	0.567485	1	-
ENSMUSC	Dgcr8	DGCR8, m	GO:00081	metabolic	-	-	-	-	16:182539	651	785	526	789	938	752	3.143939	3.027671	2.807537	2.939683	2.917096	2.857078	2.993049	2.904619	0.593761	1	-
ENSMUSC	Trmt2a	TRM2 tRN	GO:00081	metabolic	-	-	-	-	16:182486	452	515	432	624	692	601	2.950742	2.685007	3.116903	3.142729	2.909063	3.086582	2.917551	3.046125	0.866818	1	-
ENSMUSC	Arl6	ADP-ribo	GO:00325	developm	-	-	-	-	16:596129	244	367	279	370	437	331	4.327097	5.197778	5.468361	5.062191	4.990479	4.617913	4.997745	4.890194	0.717038	1	-
ENSMUSC	Crybg3	beta-gam	GO:00329	macromo	-	-	-	-	16:594907	90	134	85	143	165	161	0.586657	0.697581	0.612361	0.719135	0.692599	0.825621	0.632199	0.745785	0.338573	1	-
ENSMUSC	Riox2	ribosomal	GO:00081	metabolic	-	-	-	-	16:594717	219	309	204	283	354	308	1.857955	2.09361	1.912795	1.852291	1.933972	2.05567	1.954787	1.947311	0.823774	1	-
ENSMUSC	Gsc2	goosecoid	GO:00081	metabolic	-	-	-	-	16:179131	0	2	0	0	0	0	0	0.083226	0	0	0	0	0.027742	0	0.524583	1	-
ENSMUSC	Cpox	copropor	GO:00081	metabolic	K00228	copropor	mmu0110	Metabolic	16:586702	891	1176	784	1154	1434	1161	17.07557	17.9991	16.60583	17.06215	17.69708	17.50414	17.22683	17.42112	0.896596	1	-
ENSMUSC	Cldnd1	claudin dc	GO:00160	membran	-	-	-	-	16:587279	474	677	541	675	823	751	6.543423	7.463821	8.254109	7.188872	7.316125	8.156002	7.420451	7.553666	0.960474	1	-
ENSMUSC	St3gal6	ST3 beta-	GO:00081	metabolic	K03792	beta-gala	mmu0110	Metabolic	16:584681	231	257	225	326	356	301	1.459936	1.297179	1.571633	1.589532	1.448858	1.496573	1.442916	1.511655	0.860982	1	-
ENSMUSC	Cmss1	cms small	GO:00329	macromo	-	-	-	-	16:573021	94	100	69	119	125	87	6.171465	5.243326	5.006731	6.027512	5.284742	4.493556	5.473841	5.268604	0.711064	1	-
ENSMUSC	Tbc1d23	TBC1 dom	GO:00325	developm	-	-	-	-	16:571688	670	807	637	908	1064	885	3.368098	3.239885	3.539129	3.521488	3.444344	3.49997	3.382371	3.488601	0.948067	1	-
ENSMUSC	Klhl22	kelch-like	GO:00081	metabolic	-	-	-	-	16:177596	1297	1611	1114	1738	1920	1674	8.928115	8.856479	8.475231	9.229962	8.510902	9.06538	8.753275	8.935415	0.969117	1	-
ENSMUSC	Nit2	nitrilase fa	GO:00081	metabolic	K13566	omega-ai	mmu0025	Alanine, a	16:571566	78	79	65	110	138	94	3.720732	3.009581	3.426859	4.048144	4.239033	3.527553	3.385724	3.938243	0.422754	1	-
ENSMUSC	Tomm70a	translocas	GO:00055	extracellul	-	-	-	-	16:571217	4504	5711	4328	5869	7287	5941	72.79135	73.7122	77.30641	73.17721	75.83765	75.53563	74.60332	74.85016	0.8022	1	-
ENSMUSC	Tmem30c	transmem	GO:00160	membran	-	-	-	-	16:572661	6	3	0	5	8	5	0.079346	0.03169	0	0.05101	0.068131	0.052019	0.037012	0.057053	0.512024	1	-
ENSMUSC	Tmem45a	transmem	GO:00160	membran	-	-	-	-	16:568051	9	11	6	10	14	5	0.157543	0.153777	0.11609	0.135056	0.157816	0.068857	0.14247	0.120576	0.579963	1	-
ENSMUSC	Tfg	Trk-fused	GO:00048	signal trar	K09292	protein TF	mmu0520	Pathways	16:566903	940	1103	870	1246	1199	1381	16.08594	15.07439	16.45449	16.45003	13.21272	18.59187	15.87161	16.08487	0.913835	1	-
ENSMUSC	Lrrc74b	leucine ric	-	-	-	-	-	-	16:175444	9	26	16	22	22	30	0.127201	0.293479	0.249936	0.23989	0.200237	0.333572	0.223539	0.2579	0.759728	1	-
ENSMUSC	Thap7	THAP dor	GO:00081	metabolic	-	-	-	-	16:175279	183	179	111	190	318	163	2.200839	1.719235	1.475381	1.762867	2.462736	1.542176	1.798485	1.922593	0.80305	1	-
ENSMUSC	Lztr1	leucine-zi	GO:00056	cell|intrace	-	-	-	-	16:175086	2166	2725	2185	3056	3531	3263	14.12948	14.19645	15.75309	15.37982	14.83269	16.7454	14.69301	15.65263	0.662683	1	-
ENSMUSC	Aifm3	apoptosis	GO:00081	metabolic	-	-	-	-	16:174896	997	1202	856	1454	1572	1464	10.17125	9.79331	9.65161	11.4439	10.32729	11.74982	9.872056	11.17367	0.295956	1	-
ENSMUSC	Snap29	synaptosc	GO:00081	metabolic	K08509	synaptosc	mmu0414	Autophag	16:174060	579	733	536	777	942	758	10.30989	10.42379	10.54843	10.674	10.80144	10.61832	10.42737	10.69792	0.989699	1	-
ENSMUSC	Serpind1	serine (or	GO:00081	metabolic	K03912	heparin cc	mmu0461	Complem	16:173313	26	39	29	45	42	45	0.415253	0.497449	0.511898	0.554476	0.431961	0.565406	0.474867	0.517281	0.817248	1	-
ENSMUSC	Ccdc116	coiled-co	GO:00056	cell|intrace	-	-	-	-	16:171390	34	40	28	64	59	52	0.295811	0.277931	0.269236	0.429574	0.330547	0.355914	0.280993	0.372012	0.181634	1	-
ENSMUSC	Sdf2l1	stromal ce	GO:00081	metabolic	-	-	-	-	16:171301	119	151	124	239	233	166	6.55767	6.645474	7.5522	10.16091	8.268255	7.196534	6.918448	8.541899	0.207669	1	-
ENSMUSC	Dlg1	discs, larg	GO:00325	developm	K12076	disks large	mmu0516	Human pa	16:316634	1907	2342	1564	2505	2777	2284	6.279004	6.158466	5.691457	6.363236	5.888036	5.916257	6.042976	6.055843	0.804786	1	-
ENSMUSC	Ppil2	peptidylpr	GO:00081	metabolic	K10598	peptidyl-p	mmu0412	Ubiquitin	16:170865	982	1234	971	1428	1554	1360	6.985793	7.010761	7.634317	7.837235	7.118852	7.611214	7.210291	7.522434	0.849287	1	-
ENSMUSC	Senp5	SUMO/se	GO:00081	metabolic	-	-	-	-	16:319625	650	865	433	642	968	845	6.085211	6.467322	4.480202	4.636902	5.835693	6.223438	5.677578	5.565344	0.724516	1	-
ENSMUSC	Ypel1	yippee-lik	GO:00056	cell|intrace	-	-	-	-	16:170696	350	436	290	425	572	455	2.713694	2.69976	2.485061	2.542211	2.855905	2.77533	2.632838	2.724482	0.934435	1	-
ENSMUSC	Ncbp2	nuclear ca	GO:00081	metabolic	K12883	nuclear ca	mmu0301	RNA trans	16:319485	901	1107	798	1159	1449	1135	16.77748	16.46248	16.42297	16.65006	17.37501	16.6268	16.55431	16.88395	0.967518	1	-
ENSMUSC	Top3b	topoisom	GO:00081	metabolic	K03165	DNA topo	mmu0346	Fanconi a	16:168707	497	716	463	707	826	729	1.994342	2.294573	2.053388	2.188731	2.134408	2.301342	2.114101	2.20816	0.856136	1	-
ENSMUSC	Meltf	melanotra	GO:00325	developm	-	-	-	-	16:318788	10	8	5	4	25	6	0.147734	0.09439	0.081644	0.045585	0.237831	0.069735	0.107923	0.117717	0.935365	1	-
ENSMUSC	Pak2	p21 prote	GO:00325	developm	K04410	p21-activ	mmu0401	MAPK sigr	16:320162	821	1103	744	1146	1390	1025	7.342717	7.878341	7.354166	7.907305	8.005396	7.211879	7.525075	7.708193	0.998435	1	-
ENSMUSC	Spag6l	sperm ass	GO:00325	developm	-	-	-	-	16:167530	115	183	139	167	188	186	1.204824	1.531158	1.609484	1.349801	1.268344	1.533021	1.448489	1.383722	0.632889	1	-
ENSMUSC	Wdr53	WD repea	GO:00056	cell|intrace	-	-	-	-	16:322472	152	180	113	166	227	183	3.187097	3.014192	2.618641	2.685293	3.065016	3.018652	2.939977	2.922987	0.827185	1	-
ENSMUSC	Fgd4	FYVE, Rhc	GO:00081	metabolic	-	-	-	-	16:164169	1430	1669	1228	1625	2035	1543	4.037414	3.763303	3.831885	3.539573	3.699867	3.427236	3.877534	3.555559	0.229817	1	-
ENSMUSC	Dnm1l	dynamin 1	GO:00325	developm	K17065	dynamin 1	mmu0421	Necroptos	16:163122	2671	3394	2528	3362	3975	3399	38.32814	38.89569	40.09292	37.21961	36.73124	38.37125	39.10558	37.4407	0.43055	1	-
ENSMUSC	Igsf11	immunog	GO:00055	extracellul	-	-	-	-	16:388926	727	924	678	959	1041	849	8.622623	8.752307	8.887534	8.775133	7.950791	7.921793	8.754155	8.215906	0.37926	1	-
ENSMUSC	Yars2	tyrosyl-tR	GO:00081	metabolic	K01866	tyrosyl-tR	mmu0097	Aminoacy	16:163029	136	145	106	137	192	172	1.241798	1.057367	1.069709	0.965076	1.128935	1.235528	1.122958	1.109846	0.812598	1	-
ENSMUSC	B4galt4	UDP-Gal:l	GO:00081	metabolic	K07969	beta-1,4-	mmu0110	Metabolic	16:387422	523	710	537	691	904	770	5.9125	6.410231	6.709507	6.026668	6.581008	6.84811	6.344079	6.485262	0.990682	1	-
ENSMUSC	Tfrc	transferrin	GO:00325	developm	K06503	transferrin	mmu0414	Endocytos	16:326089	1735	2104	1757	2044	2717	2349	10.98578	10.63954	12.2956	9.984869	11.07836	11.70106	11.30697	10.92143	0.527797	1	-
ENSMUSC	Arhgap31	Rho GTPa	GO:00987	molecular	-	-	-	-	16:385983	807	976	639	1095	1172	941	4.093196	3.953526	3.582093	4.28483	3.827993	3.754824	3.876272	3.955882	0.972222	1	-
ENSMUSC	Fyttd1	forty-two	GO:00081	metabolic	-	-	-	-	16:328775	371	452	471	573	576	561	1.937443	1.885121	2.718455	2.308549	1.937011	2.304775	2.18034	2.183445	0.865515	1	-
ENSMUSC	Lrch3	leucine-ri	GO:00508	response	-	-	-	-	16:329141	645	852	649	933	1001	928	3.99944	4.219149	4.447653	4.463253	3.996944	4.526871	4.222081	4.329023	0.997027	1	-
ENSMUSC	Lmln	leishmanc	GO:00081	metabolic	-	-	-	-	16:330625	438	493	359	565	606	583	3.399016	3.055424	3.079078	3.382653	3.028352	3.559252	3.177839	3.323419	0.864847	1	-
ENSMUSC	Popdc2	popeye do	GO:00325	developm	-	-	-	-	16:383622	3	2	8	7	8	9	0.039872	0.021232	0.117507	0.071776	0.068472	0.094101	0.059537	0.078116	0.605569	1	-
ENSMUSC	Maats1	MYCBP-a	GO:00329	macromo	-	-	-	-	16:382977	37	72	44	56	74	52	0.446389	0.693721	0.586687	0.521221	0.574904	0.49354	0.575599	0.529889	0.568232	1	-
ENSMUSC	Osbpl11	oxysterol	GO:00325	developm	-	-	-	-	16:331850	446	530	348	540	689	557	6.024755	5.717772	5.195537	5.627676	5.993473	5.919313	5.646021	5.846821	0.928175	1	-
ENSMUSC	Snx4	sorting ne	GO:00511	localizatio	K17919	sorting ne	mmu0414	Endocytos	16:332514	645	837	582	852	1052	887	16.12714	16.71356	16.08303	16.43493	16.93823	17.44745	16.30791	16.9402	0.890549	1	-
ENSMUSC	Zfp148	zinc finge	GO:00325	developm	-	-	-	-	16:333807	2452	3272	2367	3045	3717	3228	12.03202	12.82264	12.83699	11.52752	11.74534	12.46128	12.56388	11.91138	0.372638	1	-
ENSMUSC	Gsk3b	glycogen	GO:00325	developm	K03083	glycogen	mmu0520	Pathways	16:380890	5374	6768	4589	6609	7672	6540	30.39895	30.57504	28.68969	28.84211	27.94629	29.10379	29.88789	28.63073	0.415365	1	-
ENSMUSC	Umps	uridine mo	GO:00325	developm	K13421	uridine mo	mmu0110	Metabolic	16:339547	317	323	268	411	457	377	3.33484	2.713715	3.116003	3.335706	3.095896	3.120101	3.054852	3.183901	0.891863	1	-
ENSMUSC	Fstl1	follistatin-	GO:00508	response	-	-	-	-	16:377768	740	848	676	923	1157	929	7.365996	6.741266	7.436918	7.088129	7.416304	7.274884	7.181393	7.259773	0.897732	1	-
ENSMUSC	Itgb5	integrin b	GO:00325	developm	K06588	integrin b	mmu0415	PI3K-Akt s	16:338296	541	815	518	762	955	871	7.17161	8.628257	7.589201	7.793003	8.152253	9.083406	7.796356	8.342887	0.679077	1	-
ENSMUSC	Ndufb4	NADH de	GO:00081	metabolic	K03960|KC	NADH de	mmu0110	Metabolic	16:376471	46	40	33	51	72	34	1.955909	1.358299	1.550789	1.672972	1.971401	1.137297	1.621666	1.59389	0.88812	1	-
ENSMUSC	Abcc5	ATP-bind	GO:00511	localizatio	K05668	ATP-bind	mmu0201	ABC trans	16:203313	2339	2990	2151	3586	3772	2922	8.970785	9.158351	9.117746	10.61062	9.315939	8.816401	9.082294	9.580988	0.745057	1	-
ENSMUSC	Rabl3	RAB, mem	GO:00971	organic cy	-	-	-	-	16:375398	141	181	113	169	268	179	1.03625	1.062361	0.917848	0.958216	1.268344	1.034929	1.005486	1.087163	0.709564	1	-
ENSMUSC	Gtf2e1	general tr	GO:00081	metabolic	K03136	transcripti	mmu0520	Viral carci	16:375097	344	475	358	546	574	405	3.05202	3.365643	3.510418	3.737245	3.279406	2.826796	3.30936	3.281149	0.796115	1	-
ENSMUSC	Hcls1	hematopo	GO:00325	developm	K06106	cortactin[-	mmu0520	Proteoglyc	16:369349	100	137	76	108	184	134	2.183763	2.389306	1.834288	1.819528	2.587492	2.302089	2.135786	2.23637	0.901688	1	-
ENSMUSC	Ccdc14	coiled-co	GO:00325	developm	-	-	-	-	16:346906	67	64	38	61	84	68	0.996074	0.759875	0.624373	0.69965	0.804181	0.795316	0.793441	0.766383	0.745104	1	-
ENSMUSC	Iqcb1	IQ calmod	GO:00325	developm	-	-	-	-	16:368283	358	475	378	471	593	426	2.971161	3.148342	3.467215	3.015738	3.169216	2.781397	3.195573	2.988784	0.435342	1	-
ENSMUSC	Eaf2	ELL associ	GO:00325	developm	-	-	-	-	16:367928	15	11	14	19	20	19	0.149746	0.0877	0.154472	0.146336	0.128575	0.149222	0.13064	0.141378	0.876478	1	-
ENSMUSC	Adcy5	adenylate	GO:00081	metabolic	K08045	adenylate	mmu0520	Pathways	16:351548	2427	3107	2125	3124	3394	3142	21.14857	21.62213	20.46525	21.00161	19.04486	21.53914	21.07865	20.52854	0.561917	1	-
ENSMUSC	Ap2m1	adaptor-r	GO:00081	metabolic	K11826	AP-2 com	mmu0414	Endocytos	16:205354	2112	2109	2403	2721	3500	2241	16.96996	13.53348	21.33966	16.8673	18.10963	14.16575	17.28103	16.3809	0.517285	1	-
ENSMUSC	Ece2	endotheli	GO:00081	metabolic	-	-	-	-	16:206298	403	497	398	610	622	531	3.53287	3.479563	3.856134	4.125552	3.511293	3.662081	3.622855	3.766309	0.899824	1	-
ENSMUSC	Clcn2	chloride c	GO:00325	developm	K05011	chloride c	mmu0497	Mineral ab	16:207029	715	877	769	1099	1361	1070	4.005921	3.924116	4.761785	4.750338	4.910314	4.71619	4.230607	4.792281	0.330141	1	-
ENSMUSC	Thpo	thrombop	GO:00325	developm	K06854	thrombop	mmu0406	Cytokine-	16:207251	112	133	115	151	132	170	3.722636	3.530467	4.224527	3.872066	2.825292	4.44523	3.825876	3.714196	0.731747	1	-
ENSMUSC	Dirc2	disrupted	GO:00511	localizatio	-	-	-	-	16:356940	1117	1491	1089	1517	1875	1553	10.8257	11.54054	11.6648	11.34276	11.70197	11.84091	11.34368	11.62855	0.99329	1	-
ENSMUSC	Hspbap1	Hspb asso	GO:00056	cell|intrace	-	-	-	-	16:357703	85	90	85	103	109	110	1.130709	0.956131	1.24967	1.057056	0.93371	1.15116	1.11217	1.047309	0.607134	1	-
ENSMUSC	Senp2	SUMO/se	GO:00325	developm	K03345	sentrin-sp	mmu0301	RNA trans	16:220094	1877	2445	1750	2421	3053	2469	25.47368	26.50038	26.24896	25.34851	26.68141	26.36085	26.07434	26.13026	0.802393	1	-
ENSMUSC	Tmem41a	transmem	GO:00160	membran	-	-	-	-	16:219343	104	149	109	144	196	184	4.672589	5.346317	5.41251	4.991318	5.670663	6.503575	5.143806	5.721852	0.573911	1	-
ENSMUSC	Tra2b	transform	GO:00325	developm	K12897	transform	mmu0304	Spliceosor	16:222445	863	1090	724	1062	1351	1017	7.07101	7.132517	6.556256	6.713133	7.12821	6.555447	6.919928	6.79893	0.676415	1	-
ENSMUSC	Chodl	chondrole	GO:00325	developm	-	-	-	-	16:789309	0	11	6	1	10	4	0	0.136726	0.103217	0.012009	0.100225	0.048975	0.079981	0.053736	0.515177	1	-
ENSMUSC	Dgkg	diacylglyc	GO:00325	developm	K00901	diacylglyc	mmu0110	Metabolic	16:224684	3555	4464	3242	4798	5437	4913	33.8894	33.98554	34.15734	35.287	33.3763	36.84528	34.01076	35.16953	0.912952	1	-
ENSMUSC	Btg3	B cell tran	GO:00099	cellular pr	K14443	protein Tc	mmu0301	RNA degr	16:783598	45	75	51	62	102	69	1.78532	2.376369	2.236277	1.897709	2.605923	2.153612	2.132655	2.219081	0.953263	1	-
ENSMUSC	D16Ertd47	DNA segr	GO:00325	developm	-	-	-	-	16:785440	118	174	114	154	169	189	1.859804	2.190179	1.985795	1.872554	1.715244	2.343451	2.011926	1.977083	0.772505	1	-
ENSMUSC	Usp25	ubiquitin s	GO:00081	metabolic	K11849	ubiquitin c	mmu0465	IL-17 sign	16:770140	1009	1191	890	1385	1703	1371	13.96366	13.16332	13.61272	14.78726	15.17669	14.92644	13.5799	14.96346	0.435589	1	-
ENSMUSC	Fetub	fetuin bet	GO:00081	metabolic	-	-	-	-	16:229183	0	9	0	0	4	4	0	0.18325	0	0	0.06567	0.08022	0.061083	0.04863	0.878802	1	-
ENSMUSC	Samsn1	SAM dom	GO:00081	metabolic	-	-	-	-	16:758587	28	48	28	39	49	36	0.285267	0.390561	0.315284	0.306547	0.321475	0.288544	0.330371	0.305522	0.611415	1	-
ENSMUSC	Rfc4	replication	GO:00081	metabolic	K10755	replication	mmu0342	Nucleotid	16:231139	8	22	15	22	43	22	0.20194	0.443489	0.418452	0.42841	0.698924	0.436864	0.354627	0.5214	0.188573	1	-
ENSMUSC	Robo1	roundabo	GO:00325	developm	K06753	roundabo	mmu0436	Axon guic	16:726631	1637	2105	1412	2060	2449	2045	13.21594	13.5721	12.59884	12.83059	12.73189	12.98833	13.12896	12.85027	0.599972	1	-
ENSMUSC	St6gal1	beta gala	GO:00081	metabolic	K00778	beta-gala	mmu0110	Metabolic	16:232247	388	546	317	512	542	488	2.515987	2.827581	2.271871	2.5614	2.26324	2.489474	2.53848	2.438038	0.575028	1	-
ENSMUSC	Masp1	mannan-l	GO:00081	metabolic	K03992	mannan-l	mmu0461	Complem	16:234517	127	168	127	185	191	226	2.817729	2.97681	3.114183	3.166628	2.728866	3.944702	2.969574	3.280066	0.61037	1	-
ENSMUSC	Mrpl39	mitochon	GO:00081	metabolic	-	-	-	-	16:847175	551	677	489	786	961	700	11.94285	11.71902	11.71419	13.14346	13.41327	11.93619	11.79202	12.83097	0.557699	1	-
ENSMUSC	Atp5j	ATP synth	GO:00325	developm	K02131	F-type H+	mmu0110	Metabolic	16:848278	757	905	842	1207	1431	1120	8.727524	8.332778	10.72888	10.73577	10.62404	10.15838	9.26306	10.50606	0.334347	1	-
ENSMUSC	App	amyloid b	GO:00325	developm	K04520	amyloid b	mmu0501	Alzheimer	16:849496	26357	33780	25356	36958	39235	38307	80.60649	82.50486	85.7041	87.19926	77.26848	92.16438	82.93848	85.54404	0.939499	1	-
ENSMUSC	Adamts1	a disinteg	GO:00325	developm	-	-	-	-	16:857938	370	554	333	360	529	549	3.576304	4.276495	3.55732	2.68451	3.292631	4.174607	3.803373	3.383916	0.287522	1	-
ENSMUSC	Adamts5	a disinteg	GO:00325	developm	-	-	-	-	16:858581	32	45	22	32	51	60	0.241485	0.271205	0.183493	0.186306	0.247838	0.356206	0.232061	0.26345	0.699916	1	-
ENSMUSC	Ets2	E26 avian	GO:00325	developm	K02678	C-ets[-]	mmu0516	HTLV-I inf	16:957020	1468	1692	1353	1960	2542	1921	15.10759	13.90642	15.38911	15.56164	16.84609	15.55275	14.80104	15.98683	0.561802	1	-
ENSMUSC	Dyrk1a	dual-spec	GO:00081	metabolic	-	-	-	-	16:945700	1347	2316	1182	2256	2506	2260	6.385501	8.768227	6.192868	8.250814	7.650032	8.428432	7.115532	8.10976	0.305508	1	-
ENSMUSC	Dscr3	DSCR3 ar	GO:00511	localizatio	-	-	-	-	16:944977	301	366	349	438	567	405	3.996191	3.880676	5.120964	4.486263	4.847501	4.230052	4.33261	4.521272	0.876691	1	-
ENSMUSC	Slc15a2	solute car	GO:00511	localizatio	-	-	-	-	16:367501	330	489	289	451	517	485	1.904098	2.253359	1.842976	2.007625	1.920971	2.20155	2.000144	2.043382	0.98301	1	-
ENSMUSC	Cd86	CD86 anti	GO:00023	immune s	K05413	CD86 anti	mmu0516	Kaposi's sa	16:366038	29	47	37	37	53	47	0.379486	0.491183	0.535108	0.373535	0.446609	0.483848	0.468592	0.434664	0.642424	1	-
ENSMUSC	Kpna1	karyopher	GO:00511	localizatio	K15042	importin s	mmu0516	Influenza	16:359787	3220	1132	2497	3990	4977	3606	16.62218	4.666856	14.24615	15.89042	16.54453	14.6443	11.84506	15.69308	0.246735	1	-
ENSMUSC	Arl13b	ADP-ribo	GO:00325	developm	-	-	-	-	16:627936	197	229	170	266	341	179	2.112846	1.961475	2.0151	2.20097	2.35511	1.510309	2.029807	2.02213	0.86276	1	-
ENSMUSC	Pros1	protein S	GO:00508	response	K03908	protein S[	mmu0461	Complem	16:628543	238	351	210	321	334	319	3.171509	3.735437	3.092821	3.300078	2.866083	3.344182	3.333256	3.170115	0.545953	1	-
ENSMUSC	Psmg1	proteasor	GO:00325	developm	-	-	-	-	16:959799	92	124	81	98	148	114	3.139949	3.379868	3.055396	2.580421	3.252735	3.060902	3.191738	2.964686	0.517176	1	-
ENSMUSC	Brwd1	bromodo	GO:00325	developm	-	-	-	-	16:959920	2569	2908	2141	3009	3396	2972	5.812795	5.254861	5.354075	5.252588	4.948155	5.290305	5.47391	5.163683	0.346879	1	-
ENSMUSC	Grik1	glutamate	GO:00048	signal trar	K05201	glutamate	mmu0472	Glutamate	16:878959	266	310	184	319	356	344	1.158367	1.078132	0.885585	1.071731	0.998318	1.178511	1.040694	1.082853	0.914088	1	-
ENSMUSC	Pigp	phosphati	GO:00081	metabolic	K03861	phosphati	mmu0110	Metabolic	16:943587	45	57	53	65	72	75	0.890635	0.900961	1.159331	0.9925	0.917644	1.167772	0.983642	1.025972	0.949128	1	-
ENSMUSC	Ripply3	ripply trar	GO:00325	developm	-	-	-	-	16:943284	3	0	4	2	6	4	0.119641	0	0.176321	0.061544	0.154083	0.125492	0.098654	0.113706	1	1	-
ENSMUSC	Chaf1b	chromatin	GO:00081	metabolic	-	-	-	-	16:938839	11	10	8	17	15	13	0.098354	0.071402	0.079049	0.117266	0.086363	0.091438	0.082935	0.098356	0.705397	1	-
ENSMUSC	Dopey2	dopey far	GO:00511	localizatio	-	-	-	-	16:937119	1984	2576	1806	2785	3000	2807	9.143969	9.481668	9.199362	9.902585	8.903668	10.17762	9.274999	9.661292	0.852091	1	-
ENSMUSC	Cbr3	carbonyl r	GO:00081	metabolic	-	-	mmu0110	Metabolic	16:936832	72	82	72	102	107	115	3.757433	3.417585	4.152753	4.106645	3.595818	4.721366	3.775924	4.141276	0.670717	1	-
ENSMUSC	Setd4	SET doma	-	-	-	-	-	-	16:935834	80	90	71	99	126	95	0.65636	0.589712	0.64381	0.626644	0.665703	0.613183	0.629961	0.635177	0.941397	1	-
ENSMUSC	Rcan1	regulator	GO:00325	developm	K17901	calcipressi	mmu0516	Kaposi's sa	16:923919	715	893	568	843	1109	818	17.4208	17.37639	15.29527	15.84602	17.39997	15.6793	16.69749	16.30843	0.64764	1	-
ENSMUSC	Runx1	runt relate	GO:00325	developm	K08367	runt-relat	mmu0520	Pathways	16:926014	18	49	27	28	42	31	0.060306	0.131113	0.099979	0.072374	0.090616	0.08171	0.097133	0.081567	0.414171	1	-
ENSMUSC	Atp5o	ATP synth	GO:00081	metabolic	K02137	F-type H+	mmu0110	Metabolic	16:916843	1128	1457	1183	1646	1805	1280	22.10319	22.80086	25.61991	24.88319	22.77602	19.7318	23.50799	22.46367	0.498702	1	-
ENSMUSC	Itsn1	intersectin	GO:00081	metabolic	-	-	-	-	16:917292	3572	4552	2950	4438	4752	4544	6.415448	6.529255	5.855775	6.149406	5.495999	6.42044	6.266826	6.021948	0.462808	1	-
ENSMUSC	Donson	downstrea	GO:00325	developm	-	-	-	-	16:916772	173	229	164	232	280	234	2.218667	2.345448	2.324535	2.295431	2.312372	2.360864	2.296217	2.322889	0.929942	1	-
ENSMUSC	Son	Son DNA	GO:00081	metabolic	-	-	-	-	16:916475	6759	8664	6140	9030	10298	9452	19.01367	19.4647	19.08968	19.59754	18.65482	20.91791	19.18935	19.72342	0.969959	1	-
ENSMUSC	Gart	phosphor	GO:00325	developm	K11787	phosphor	mmu0110	Metabolic	16:916211	497	674	493	698	881	676	6.431942	6.966124	7.051468	6.969007	7.342028	6.882445	6.816511	7.064493	0.909507	1	-
ENSMUSC	Tmem50b	transmem	GO:00160	membran	-	-	-	-	16:915745	1543	1919	1301	2111	2521	2109	32.1545	31.93717	29.96401	33.93863	33.8301	34.57509	31.35189	34.1146	0.493067	1	-
ENSMUSC	Ifngr2	interferon	GO:00048	signal trar	K05133	interferon	mmu0520	Pathways	16:915470	1130	1399	1174	1701	2070	1607	19.69054	19.46896	22.60966	22.86725	23.2276	22.02957	20.58972	22.70814	0.433114	1	-
ENSMUSC	Ifnar1	interferon	GO:00048	signal trar	K05130	interferon	mmu0520	Pathways	16:914852	688	912	677	1024	1039	890	4.32537	4.57905	4.70403	4.966665	4.206344	4.401854	4.53615	4.524954	0.796058	1	-
ENSMUSC	Il10rb	interleukir	GO:00160	membran	K05135	interleukir	mmu0406	Cytokine-	16:914061	148	197	95	119	257	196	1.710502	1.818337	1.213487	1.061061	1.912714	1.782092	1.580775	1.585289	0.918465	1	-
ENSMUSC	Ifnar2	interferon	GO:00048	signal trar	K05131	interferon	mmu0520	Pathways	16:913727	260	348	274	378	406	330	2.59652	2.775509	3.024229	2.912328	2.610952	2.592645	2.798752	2.705308	0.631565	1	-
ENSMUSC	1110004E	RIKEN cDI	GO:00325	developm	-	-	-	-	16:909258	250	386	248	434	445	485	5.779832	7.12703	6.336845	7.740959	6.625059	8.821205	6.414569	7.729074	0.195629	1	-
ENSMUSC	Synj1	synaptoja	GO:00325	developm	K20279	synaptoja	mmu0110	Metabolic	16:909360	9493	11359	8497	11690	13505	12619	35.03322	33.47823	34.65684	33.28292	32.09415	36.63637	34.38943	34.00448	0.671689	1	-
ENSMUSC	Paxbp1	PAX3 and	GO:00325	developm	-	-	-	-	16:910140	899	1088	793	1175	1339	1188	7.165026	6.925203	6.985182	7.224805	6.87216	7.448783	7.025137	7.181916	0.982988	1	-
ENSMUSC	Mis18a	MIS18 kin	GO:00081	metabolic	-	-	-	-	16:907193	81	127	76	102	126	87	1.147496	1.436866	1.189946	1.114797	1.149457	0.969607	1.258103	1.077954	0.244257	1	-
ENSMUSC	Sod1	superoxid	GO:00325	developm	K04565	superoxid	mmu0501	Huntingto	16:902207	1684	2127	1492	2246	2861	1949	159.1669	160.5551	155.8571	163.7763	174.1339	144.9218	158.5264	160.944	0.929583	1	-
ENSMUSC	Scaf4	SR-relatec	GO:00056	cell|intrace	-	-	-	-	16:902289	876	1042	736	1133	1224	1109	8.975822	8.526748	8.334787	8.956336	8.076179	8.939482	8.612452	8.657332	0.846165	1	-
ENSMUSC	Zfp641	zinc finge	GO:00325	developm	-	-	-	-	15:982859	189	83	202	260	291	108	1.544846	0.541807	1.824821	1.639558	1.531686	0.694476	1.303825	1.288574	0.902126	1	-
ENSMUSC	Kansl2	KAT8 reg	GO:00325	developm	-	-	-	-	15:985188	338	448	303	482	554	513	5.634087	5.963897	5.582069	6.198463	5.94663	6.727203	5.726684	6.290766	0.540063	1	-
ENSMUSC	Adcy6	adenylate	GO:00081	metabolic	K08046	adenylate	mmu0520	Pathways	15:985899	835	982	764	1187	1456	1248	3.721152	3.495009	3.762973	4.081051	4.178372	4.375388	3.659711	4.211603	0.229599	1	-
ENSMUSC	Enah	enabled h	GO:00325	developm	K05746	enabled[-	mmu0481	Regulation	1:1818963	3454	4642	3223	4497	4314	4843	7.285798	7.819982	7.513838	7.318263	5.859891	8.036745	7.539873	7.071633	0.353514	1	-
ENSMUSC	Wnt10b	wingless-t	GO:00325	developm	K01357	wingless-t	mmu0520	Pathways	15:987707	7	11	9	15	7	7	0.042875	0.053804	0.06092	0.070878	0.027607	0.033731	0.052533	0.044072	0.603542	1	-
ENSMUSC	Lmbr1l	limb regic	GO:00511	localizatio	-	-	-	-	15:989039	62	99	54	105	136	106	0.658138	0.839277	0.633523	0.859894	0.929649	0.885197	0.710313	0.89158	0.202713	1	-
ENSMUSC	Tuba1b	tubulin, al	GO:00508	response	K07374	tubulin alp	mmu0414	Phagosom	15:989314	4948	6097	5294	7137	7855	7018	133.1492	131.03	157.4489	148.1681	136.1162	148.5707	140.5427	144.285	0.820214	1	-
ENSMUSC	Prpf40b	pre-mRNA	-	-	K12821	pre-mRNA	mmu0304	Spliceosor	15:992950	966	1323	828	1549	1458	1442	6.185208	6.765235	5.859416	7.651715	6.011591	7.263621	6.269953	6.975642	0.420219	1	-
ENSMUSC	Fmnl3	formin-lik	GO:00325	developm	-	-	-	-	15:993172	182	208	154	209	263	229	1.980853	1.807968	1.852462	1.754922	1.843275	1.960767	1.880428	1.852988	0.767647	1	-
ENSMUSC	Nckap5l	NCK-assoc	GO:00990	supramole	-	-	-	-	15:994220	380	463	348	556	539	460	4.427881	4.308628	4.481647	4.998244	4.044415	4.216774	4.406052	4.419811	0.858673	1	-
ENSMUSC	Tmbim6	transmem	GO:00160	membran	-	-	-	-	15:993928	4017	5454	3674	5815	7016	6021	48.61653	52.71608	49.14373	54.29529	54.67974	57.32733	50.15878	55.43412	0.35258	1	-
ENSMUSC	Faim2	Fas apopt	GO:00325	developm	-	-	-	-	15:994970	6101	7530	5611	8529	9665	7721	82.41493	81.23548	83.77068	88.886	84.07395	82.05204	82.4737	85.004	0.940932	1	-
ENSMUSC	Racgap1	Rac GTPas	GO:00325	developm	-	-	-	-	15:996204	105	123	111	141	171	141	0.546884	0.511628	0.638958	0.566571	0.573529	0.577745	0.565824	0.572615	0.957268	1	-
ENSMUSC	Asic1	acid-sens	GO:00452	synapse|c	K04829	acid-sens	mmu0475	Inflammat	15:996703	529	650	480	815	730	682	3.687608	3.618664	3.698081	4.383055	3.276924	3.740106	3.668118	3.800028	0.92777	1	-
ENSMUSC	Smarcd1	SWI/SNF r	GO:00325	developm	K11650	SWI/SNF-	mmu0520	Hepatoce	15:997022	2007	2623	1792	2895	3115	2729	38.86573	40.56612	38.35342	43.25121	38.84473	41.57515	39.26176	41.2237	0.778362	1	-
ENSMUSC	Gpd1	glycerol-3	GO:00081	metabolic	K00006	glycerol-3	mmu0056	Glyceroph	15:997175	2554	3094	2372	3586	4330	3350	39.47909	38.19552	40.52357	42.76484	43.10115	40.73821	39.3994	42.2014	0.607022	1	-
ENSMUSC	Cox14	cytochron	GO:00099	cellular pr	-	-	-	-	15:997256	191	195	186	283	308	249	16.13015	13.15178	17.36057	18.43836	16.74983	16.54307	15.5475	17.24375	0.546756	1	-

ENSMUSC	Cers5	ceramide	GO:00081	metabolic	K04710	ceramide	mmu0110	Metabolic	15:997348	245	256	267	397	436	395	2.427262	2.02552	2.923543	3.034393	2.781585	3.078641	2.458775	2.964873	0.211187	1	-
ENSMUSC	Larp4	La ribonu	GO:00325	developm	-	-	-	-	15:999700	1312	1580	1082	1588	1952	1587	11.66568	11.21965	10.63287	10.89324	11.17663	11.10105	11.17273	11.05697	0.707133	1	-
ENSMUSC	Dip2b	disco inte	GO:00081	metabolic	-	-	-	-	15:100038	1425	1805	1224	1697	1906	1734	8.37338	8.470501	7.949032	7.693042	7.21213	8.015786	8.264304	7.640319	0.256305	1	-
ENSMUSC	Atf1	activating	GO:00325	developm	K09053	activating	mmu0516	HTLV-I inf	15:100227	174	241	144	224	283	256	2.25039	2.489276	2.058344	2.235056	2.356953	2.604713	2.266004	2.398907	0.819225	1	-
ENSMUSC	Slc11a2	solute car	GO:00325	developm	K12347	natural re:	mmu0414	Lysosome	15:100387	567	693	481	681	801	742	4.785065	4.67072	4.486383	4.433864	4.353033	4.926289	4.647389	4.571062	0.69668	1	-
ENSMUSC	Cela1	chymotryp	GO:00325	developm	-	-	-	-	15:100674	24	14	13	20	39	14	1.328576	0.618936	0.795342	0.854126	1.390223	0.609675	0.914284	0.951341	1	1	-
ENSMUSC	Nr4a1	nuclear re	GO:00325	developm	K04465	nuclear re	mmu0415	PI3K-Akt s	15:101266	843	981	675	1020	976	1012	20.80517	19.33563	18.41171	19.42112	15.5113	19.64876	19.5175	18.19372	0.378692	1	-
ENSMUSC	Pcdhgc4	protocadh	GO:00099	cellular pr	-	-	-	-	18:378150	216	266	172	263	155	271	1.593395	1.567106	1.402312	1.496773	0.736305	1.572715	1.520938	1.268598	0.244482	1	-
ENSMUSC	Csad	cysteine s	GO:00081	metabolic	K01594	sulfinoala	mmu0110	Metabolic	15:102176	218	274	204	278	330	293	5.876682	5.898917	6.077875	5.781622	5.728544	6.213749	5.951158	5.907972	0.797553	1	-
ENSMUSC	Igfbp6	insulin-lik	GO:00508	response	-	-	-	-	15:102144	108	164	129	188	196	177	5.930122	7.191638	7.828422	7.963937	6.930244	7.645816	6.983394	7.513332	0.741251	1	-
ENSMUSC	Amhr2	anti-Mulle	GO:00325	developm	K04672	anti-mulle	mmu0406	Cytokine-	15:102445	0	4	3	2	6	4	0	0.07118	0.073891	0.034388	0.086095	0.07012	0.048357	0.063534	0.839774	1	-
ENSMUSC	Map3k12	mitogen-	GO:00048	signal trar	K04423	mitogen-	mmu0401	MAPK sig	15:102497	2038	2712	1684	2800	2638	3007	9.527327	10.12517	8.700733	10.09846	7.941388	11.05888	9.451077	9.699578	0.992876	1	-
ENSMUSC	Tarbp2	TARBP2, F	GO:00325	developm	-	-	-	-	15:102518	108	156	86	175	185	154	0.63954	0.737755	0.562843	0.799485	0.705454	0.717421	0.646712	0.740787	0.456408	1	-
ENSMUSC	Npff	neuropep	GO:00325	developm	-	-	-	-	15:102523	8	13	2	19	12	16	0.318236	0.412974	0.087931	0.583054	0.307389	0.50069	0.273047	0.463711	0.184375	1	-
ENSMUSC	Calcoco1	calcium b	GO:00081	metabolic	-	-	-	-	15:102706	1891	2323	1682	2665	2886	2692	23.0231	22.58746	22.63316	25.03225	22.62681	25.78448	22.74791	24.48118	0.579093	1	-
ENSMUSC	Rttn	rotatin [Sc	GO:00325	developm	-	-	-	-	18:889717	57	72	63	51	93	82	0.478851	0.483066	0.584949	0.330543	0.503116	0.541942	0.515622	0.458534	0.456669	1	-
ENSMUSC	Nus1	NUS1 deh	GO:00325	developm	K19177	dehydrod	mmu0090	Terpenoic	10:524175	1466	1774	1177	1886	2325	1732	17.82737	17.2287	15.8189	17.69397	18.20668	16.5696	16.95832	17.49008	0.941465	1	-
ENSMUSC	Rgn	regucalcir	GO:00325	developm	-	-	mmu0110	Metabolic	X:2054978	0	7	8	0	7	8	0	0.21241	0.335923	0	0.171258	0.239113	0.182778	0.13679	0.757009	1	-
ENSMUSC	Cep89	centroson	GO:00329	macromo	-	-	-	-	7:3539703	265	327	235	360	390	356	2.158536	2.127192	2.115574	2.26228	2.045657	2.281261	2.133767	2.196399	0.977719	1	-
ENSMUSC	Mospd1	motile spe	GO:00325	developm	-	-	-	-	X:5334459	135	175	118	157	223	140	2.08468	2.158201	2.013889	1.870412	2.217513	1.700773	2.08559	1.929566	0.495801	1	-
ENSMUSC	Akirin1	akirin 1 [S	GO:00160	membran	-	-	-	-	4:1237345	1095	1267	1022	1387	1690	1292	16.64806	15.3841	17.17305	16.26883	16.54593	15.45337	16.40174	16.08938	0.651721	1	-
ENSMUSC	Noct	nocturnin	GO:00325	developm	-	-	-	-	3:5122444	256	1391	1047	1486	413	410	0.970079	4.20959	4.384907	4.344263	1.007793	1.222254	3.188192	2.191437	0.267558	1	-
ENSMUSC	Abcc1	ATP-bind	GO:00511	localizatio	K05665	ATP-bind	mmu0520	MicroRNA	16:143615	315	443	293	383	535	454	1.261201	1.416521	1.296545	1.183048	1.379374	1.430015	1.324756	1.330812	0.872625	1	-
ENSMUSC	Ndufa5	NADH de	GO:00081	metabolic	K03949	NADH de	mmu0110	Metabolic	6:2451866	638	838	555	819	1002	733	28.79162	30.20197	27.68121	28.5141	29.11842	26.02317	28.8916	27.88523	0.563083	1	-
ENSMUSC	Msrb2	methionir	GO:00081	metabolic	-	-	-	-	2:1937144	353	370	341	469	542	415	12.98404	10.86883	13.86234	13.30877	12.83775	12.00865	12.57174	12.71839	0.925445	1	-
ENSMUSC	Rfc2	replicatior	GO:00081	metabolic	K10755	replicatior	mmu0342	Nucleotid	5:1345813	347	475	338	523	582	496	2.781929	3.041276	2.994883	3.234806	3.004652	3.128301	2.939362	3.122587	0.745724	1	-
ENSMUSC	Denr	density-re	GO:00081	metabolic	-	-	-	-	5:1239071	1022	1303	859	1158	1643	1356	8.492299	8.646982	7.888861	7.423574	8.791572	8.864306	8.342714	8.359817	0.824013	1	-
ENSMUSC	Prmt5	protein ar	GO:00325	developm	K02516	type II prc	mmu0301	RNA trans	14:545071	1065	1256	1003	1346	1694	1302	11.81447	11.12757	12.29738	11.51968	12.10132	11.36284	11.74648	11.66128	0.743926	1	-
ENSMUSC	Sympk	symplekin	GO:00081	metabolic	K06100	symplekin	mmu0453	Tight junc	7:1902437	2007	2502	1743	2777	3344	2971	9.871407	9.82799	9.474926	10.53751	10.59138	11.49596	9.724774	10.87495	0.314146	1	-
ENSMUSC	Gm853	predicted	GO:00081	metabolic	K01581	ornithine (	mmu0110	Metabolic	4:1302091	2	0	2	2	0	0	0.04609	0	0.050953	0.03557	0	0	0.032348	0.011857	0.502088	1	-
ENSMUSC	Sult1c2	sulfotrans	GO:00081	metabolic	-	-	-	-	17:538296	11	31	7	9	31	28	0.363848	0.81887	0.255909	0.229665	0.66029	0.728597	0.479542	0.539517	0.840276	1	-
ENSMUSC	Nagpa	N-acetylg	GO:00081	metabolic	K01125	N-acetylg	mmu0414	Lysosome	16:519528	112	106	65	122	134	190	1.790179	1.353106	1.148267	1.504418	1.379236	2.389156	1.430517	1.757603	0.34524	1	-
ENSMUSC	Wrb	tryptopha	GO:00511	localizatio	-	-	-	-	16:961454	534	872	583	762	888	865	6.963908	9.081849	8.402857	7.666504	7.457262	8.874402	8.149538	7.999389	0.698885	1	-
ENSMUSC	Ivns1abp	influenza	GO:00508	response	K15046	influenza	mmu0516	Influenza	1:1513444	1594	2017	1469	2308	2880	2332	10.77098	10.88476	10.97073	12.03187	12.53183	12.39671	10.87549	12.32014	0.244729	1	-
ENSMUSC	Lrrc69	leucine ric	-	-	-	-	-	-	4:1462362	0	1	0	2	0	0	0	0.027476	0	0.053084	0	0	0.009159	0.017695	1	1	-
ENSMUSC	Tmem52	transmem	GO:00160	membran	-	-	-	-	4:1554691	5	10	4	7	18	10	0.111329	0.177828	0.098449	0.120254	0.258112	0.17518	0.129202	0.184515	0.370657	1	-
ENSMUSC	Rpp14	ribonuclea	GO:00081	metabolic	K14529	ribonuclea	mmu0301	RNA trans	14:808036	368	460	330	529	590	506	7.807282	7.793919	7.737734	8.658412	8.060456	8.445286	7.779645	8.388052	0.645666	1	-
ENSMUSC	Psg29	pregnanc	GO:00224	reproduct	-	-	-	-	7:1720347	0	0	1	1	0	0	0	0	0.012394	0.008652	0	0	0.004131	0.002884	1	1	-
ENSMUSC	Slc38a1	solute car	GO:00511	localizatio	K14990	solute car	mmu0472	Glutamate	15:965714	3678	1178	777	970	5359	952	26.7634	6.845748	6.248809	5.445426	25.11123	5.449763	13.28599	12.00214	0.747248	1	-
ENSMUSC	Gps2	G protein	GO:00325	developm	K15307	G protein	mmu0516	HTLV-I inf	11:699138	328	434	294	442	579	413	7.807826	8.250709	7.734817	8.117241	8.875423	7.734234	7.931117	8.2423	0.900152	1	-
ENSMUSC	Vwa5a	von Willet	-	-	-	-	-	-	9:3871826	263	392	230	359	495	406	2.156633	2.567158	2.084463	2.271144	2.613845	2.619131	2.269418	2.501373	0.548579	1	-
ENSMUSC	P3h3	prolyl 3-h	GO:00081	metabolic	-	-	-	-	6:1248410	196	390	293	457	392	388	1.582782	2.515212	2.61504	2.847151	2.038474	2.464939	2.237678	2.450188	0.655567	1	-
ENSMUSC	Grm2	glutamate	GO:00048	signal trar	K04605	metabotr	mmu0408	Neuroacti	9:1066430	785	904	661	986	1271	973	6.404413	5.890115	5.960157	6.206069	6.677436	6.245015	6.084895	6.376173	0.824893	1	-
ENSMUSC	Il15ra	interleukir	GO:00325	developm	K05074	interleukir	mmu0520	Pathways	2:1170529	52	70	39	78	91	75	0.25995	0.279465	0.215473	0.300821	0.292941	0.294955	0.251629	0.296239	0.418	1	-
ENSMUSC	Epb42	erythrocyt	GO:00325	developm	-	-	-	-	2:1210178	3	0	0	0	1	2	0.021848	0	0	0	0.004689	0.011461	0.007283	0.005383	1	1	-
ENSMUSC	Serping1	serine (or	GO:00325	developm	K04001	C1 inhibit	mmu0461	Complem	2:8476538	112	217	136	171	279	240	2.414718	3.736417	3.240683	2.84431	3.87355	4.070724	3.130606	3.596195	0.469532	1	-
ENSMUSC	Ccl25	chemokin	GO:00081	metabolic	K13072	C-C motif	mmu0406	Cytokine-	8:4325210	147	167	113	195	204	200	1.009171	0.915607	0.857374	1.03279	0.901843	1.080157	0.927384	1.00493	0.695274	1	-
ENSMUSC	Scg5	secretogr	GO:00081	metabolic	-	-	-	-	2:1137763	2152	2745	2112	2994	3530	3079	72.95783	74.32206	79.13533	78.309	77.06533	82.12025	75.47174	79.16486	0.785198	1	-
ENSMUSC	Slc26a6	solute car	GO:00511	localizatio	-	-	-	-	9:1088532	67	87	90	90	112	103	0.426133	0.441914	0.632648	0.441617	0.458718	0.515372	0.500232	0.471902	0.640333	1	-
ENSMUSC	Acy1	aminoacy	GO:00081	metabolic	K14677	aminoacy	mmu0110	Metabolic	9:1064329	73	98	87	117	137	104	0.849317	0.910585	1.118704	1.050183	1.02642	0.951908	0.959535	1.009504	0.87959	1	-
ENSMUSC	Frs3	fibroblast	GO:00508	response	-	-	-	-	17:476890	244	284	224	347	336	299	3.178621	2.954694	3.225103	3.487452	2.818659	3.064296	3.119473	3.123469	0.857681	1	-
ENSMUSC	Gabrr2	gamma-a	GO:00048	signal trar	K05190	gamma-a	mmu0408	Neuroacti	4:3306299	10	7	11	13	16	19	0.232693	0.130085	0.282884	0.23337	0.239742	0.347806	0.215221	0.273639	0.479469	1	-
ENSMUSC	Creld2	cysteine-r	GO:00055	extracellul	-	-	-	-	15:888196	205	271	188	340	384	303	9.176633	9.688224	9.301073	11.7419	11.06916	10.67044	9.388643	11.1605	0.215548	1	-
ENSMUSC	Twf2	twinfilin a	GO:00325	developm	-	-	-	-	9:1062031	351	442	378	478	475	451	3.96291	3.985425	4.716772	4.163558	3.453462	4.005843	4.221702	3.874288	0.355991	1	-
ENSMUSC	Bmp15	bone mor	GO:00325	developm	K05498	growth di	mmu0491	Ovarian st	X:6314107	2	6	0	0	4	4	0.0399	0.095612	0	0	0.051399	0.062787	0.045171	0.038062	1	1	-
ENSMUSC	5-Mar	membran	GO:00325	developm	-	-	-	-	19:372075	1043	1075	890	1403	1381	1335	15.13034	12.45428	14.26926	15.70191	12.90068	15.23549	13.95129	14.61269	0.839866	1	-
ENSMUSC	Ache	acetylchol	GO:00325	developm	K01049	acetylchol	mmu0472	Cholinerg	5:1372875	811	752	815	940	779	972	5.391784	3.992777	5.988472	4.821363	3.335058	5.083803	5.124344	4.413408	0.198829	1	-
ENSMUSC	Dtwd1	DTW dom	-	-	-	-	-	-	2:1261521	91	130	84	101	156	121	2.508484	2.861922	2.559141	2.147929	2.769167	2.624003	2.643182	2.513699	0.633322	1	-
ENSMUSC	Wfdc1	WAP four	GO:00081	metabolic	-	-	-	-	8:1196663	46	94	65	99	134	101	1.776524	2.899254	2.774434	2.949706	3.332499	3.068614	2.483404	3.11694	0.242146	1	-
ENSMUSC	Poc1a	POC1 cen	GO:00325	developm	-	-	-	-	9:1062810	99	147	103	164	187	116	1.134095	1.344869	1.304068	1.449409	1.379467	1.045402	1.261011	1.291426	1	1	-
ENSMUSC	Trip6	thyroid hc	GO:00081	metabolic	K12792	thyroid re	mmu0462	NOD-like	5:1373096	101	105	67	99	129	105	1.713974	1.423039	1.256611	1.296133	1.409697	1.401794	1.464541	1.369208	0.559678	1	-
ENSMUSC	Clec4n	C-type le	GO:00511	localizatio	-	-	-	-	6:1232298	8	17	1	9	29	10	0.185598	0.31495	0.025641	0.16108	0.43322	0.182501	0.175396	0.258934	0.461863	1	-
ENSMUSC	4921501E	RIKEN cDI	GO:00056	cell|intrac	-	-	-	-	17:330641	2	0	0	2	0	0	0.031178	0	0	0.024061	0	0	0.010393	0.00802	1	1	-
ENSMUSC	Agap3	ArfGAP w	GO:00987	molecular	K12491	Arf-GAP v	mmu0414	Endocytos	5:2445217	3649	4500	3477	5095	5854	4798	29.60811	29.16052	31.18088	31.89417	30.58752	30.62725	29.98317	31.03631	0.90119	1	-
ENSMUSC	Dlx2	distal-less	GO:00325	developm	-	-	-	-	2:7154340	66	109	60	102	108	109	1.300373	1.715111	1.306533	1.550428	1.370254	1.689507	1.440672	1.53673	0.832893	1	-
ENSMUSC	Stk31	serine thr	GO:00081	metabolic	-	-	-	-	6:4939560	0	7	0	4	0	0	0	0.084054	0	0.046393	0	0	0.028018	0.015464	0.70416	1	-
ENSMUSC	Nfatc4	nuclear fa	GO:00325	developm	K17334	nuclear fa	mmu0516	HTLV-I inf	14:558231	17	26	5	30	32	25	0.099502	0.121533	0.032346	0.135465	0.12061	0.115113	0.08446	0.123729	0.230816	1	-
ENSMUSC	Gnb3	guanine n	GO:00048	signal trar	K07825	guanine n	mmu0520	Pathways	6:1248342	0	0	2	4	4	2	0	0	0.033862	0.047266	0.039458	0.024106	0.011287	0.036943	0.192255	1	-
ENSMUSC	Pisd	phosphati	GO:00081	metabolic	K01613	phosphati	mmu0110	Metabolic	5:3273630	697	379	735	1046	1200	609	2.949443	1.280833	3.437488	3.414829	3.269964	2.027379	2.555921	2.904057	0.618403	1	-
ENSMUSC	Tpi1	triosepho:	GO:00325	developm	K01803	triosepho:	mmu0110	Metabolic	6:1248105	4826	5901	4066	5923	6391	6215	74.63689	72.88492	69.49929	70.67052	63.64873	75.6168	72.34037	69.97868	0.50156	1	-
ENSMUSC	Rab12	RAB12, m	GO:00081	metabolic	-	-	-	-	17:664945	1580	2084	1640	2147	818	2215	24.13001	25.41815	27.6816	25.29664	8.044679	26.61247	25.74326	19.9846	0.184417	1	-
ENSMUSC	Tulp2	tubby-like	GO:00511	localizatio	-	-	-	-	7:4548187	1	0	0	3	0	0	0.01094	0	0	0.025315	0	0	0.003647	0.008438	0.65847	1	-
ENSMUSC	Celsr3	cadherin,	GO:00325	developm	-	-	-	-	9:1088263	2133	2545	1319	2808	2992	2701	9.234044	8.799028	6.310922	9.378395	8.340983	9.198912	8.114665	8.972764	0.483374	1	-
ENSMUSC	Prph	peripherir	GO:00051	structural	K07607	peripherir	mmu0501	Amyotrop	15:990551	15	40	27	37	45	30	0.424201	0.903435	0.843915	0.807278	0.819508	0.667453	0.72385	0.764746	0.926581	1	-
ENSMUSC	Pcbp4	poly(rC) b	GO:00081	metabolic	-	-	-	-	9:1064532	848	1145	944	1333	1121	1003	5.364963	5.785255	6.600698	6.506259	4.567005	4.992096	5.916972	5.35512	0.282361	1	-
ENSMUSC	C1qtnf12	C1q and t	GO:00081	metabolic	-	-	-	-	4:1559623	82	111	75	109	133	92	2.677424	2.894475	2.706514	2.745729	2.796455	2.363197	2.759471	2.635127	0.662821	1	-
ENSMUSC	Ccndbp1	cyclin D-t	GO:00099	cellular pr	-	-	-	-	2:1210084	578	757	632	799	1002	756	6.719651	7.028459	8.120493	7.166314	7.501374	6.914351	7.289535	7.194013	0.729787	1	-
ENSMUSC	Ogfod2	2-oxoglut	GO:00081	metabolic	-	-	-	-	5:1241122	118	132	149	218	188	150	1.11964	1.000264	1.562529	1.595808	1.148704	1.119689	1.227478	1.288067	0.889358	1	-
ENSMUSC	Mrps23	mitochon	GO:00081	metabolic	-	-	-	-	11:882043	495	691	472	674	881	642	13.49877	15.04918	14.22584	14.18011	15.47105	13.77321	14.25793	14.47479	0.941993	1	-
ENSMUSC	Rhebl1	Ras homc	GO:00081	metabolic	-	-	-	-	15:988777	37	55	29	54	52	55	0.955658	1.134506	0.827828	1.076025	0.864877	1.11755	0.972664	1.019484	0.968966	1	-
ENSMUSC	Hes7	hairy and	GO:00325	developm	K09087	hairy and	mmu0516	Human pa	11:691204	7	9	5	20	11	8	0.236272	0.242611	0.186531	0.520785	0.239078	0.212413	0.221805	0.324092	0.371538	1	-
ENSMUSC	Tiam2	T cell lym	GO:00048	signal trar	-	-	-	-	17:332657	554	734	516	679	954	772	2.038336	2.156791	2.098276	1.927375	2.260315	2.234572	2.097801	2.140754	0.977285	1	-
ENSMUSC	Synj2	synaptoja	GO:00081	metabolic	K20279	synaptoja	mmu0110	Metabolic	17:594128	1094	1450	828	1478	1599	1268	3.970609	4.202944	3.321369	4.138517	3.737171	3.620512	3.83164	3.832066	0.815642	1	-
ENSMUSC	Rsph3b	radial spo	GO:00056	cell|intrac	-	-	-	-	17:690471	189	243	158	261	289	253	7.149939	7.341614	6.606066	7.617478	7.040319	7.529596	7.03254	7.395798	0.839431	1	-
ENSMUSC	Rps6ka2	ribosomal	GO:00325	developm	K04373	ribosomal	mmu0401	MAPK sig	17:717011	909	1198	979	1238	1406	1228	10.2705	10.81013	12.22525	10.79143	10.22982	10.91534	11.10196	10.64553	0.499929	1	-
ENSMUSC	Park2	Parkinson	GO:00325	developm	K04556	parkin[2.3	mmu0414	Protein pr	17:108403	103	124	113	162	167	149	0.495741	0.476634	0.601094	0.601539	0.517594	0.564178	0.52449	0.561104	0.784668	1	-
ENSMUSC	Agpat4	1-acylglyc	GO:00081	metabolic	K13523	lysophosp	mmu0110	Metabolic	17:121187	599	891	554	920	1251	818	10.0782	11.97236	10.30179	11.94193	13.55403	10.82731	10.78412	12.10776	0.392918	1	-
ENSMUSC	Igf2r	insulin-lik	GO:00325	developm	K06564	insulin-lik	mmu0414	Endocytos	17:126824	1200	1456	1002	1428	1779	1529	7.517159	7.284164	6.937243	6.901302	7.176338	7.535128	7.246189	7.204256	0.748661	1	-
ENSMUSC	Acat2	acetyl-Co	GO:00081	metabolic	K00626	acetyl-Co	mmu0110	Metabolic	17:129428	509	654	473	738	912	655	8.869458	9.101288	9.109347	9.921239	10.2336	8.979078	9.026698	9.711307	0.638785	1	-
ENSMUSC	Chd1	chromod	GO:00081	metabolic	K07179	RIO kinas	mmu0300	Ribosome	17:157049	1155	1517	1079	1504	1882	1563	5.197681	5.452038	5.366558	5.221624	5.453832	5.533468	5.338759	5.402975	0.897893	1	-
ENSMUSC	Mpc1	mitochon	GO:00081	metabolic	-	-	-	-	17:828290	304	404	246	379	340	363	4.528337	4.8061	4.049923	4.355473	3.261365	4.253853	4.461453	3.956897	0.251121	1	-
ENSMUSC	1700010I1	RIKEN cDI	-	-	-	-	-	-	17:898833	19	19	22	20	35	30	0.446706	0.356753	0.571677	0.362765	0.529894	0.554883	0.458379	0.482514	0.906269	1	-
ENSMUSC	Phf10	PHD finge	GO:00056	cell|intrac	-	-	mmu0522	Hepatoce	17:149450	1339	1562	1132	1674	1955	1557	14.2508	13.27656	13.31532	13.74498	13.39862	13.03641	13.61423	13.39334	0.660127	1	-
ENSMUSC	Smoc2	SPARC rel	GO:00508	response	-	-	-	-	17:142795	420	600	478	613	743	626	9.05522	10.33111	11.39004	10.19626	10.31558	10.61782	10.25879	10.37655	0.916324	1	-
ENSMUSC	Zfp51	zinc finge	GO:00081	metabolic	-	-	-	-	17:214503	78	85	57	103	110	85	1.831745	1.594168	1.479417	1.86611	1.663476	1.570354	1.63511	1.69998	0.959734	1	-
ENSMUSC	Zscan10	zinc finge	GO:00325	developm	-	-	-	-	17:236008	0	3	0	0	3	2	0	0.014733	0	0	0.011879	0.009676	0.004911	0.007185	1	1	-
ENSMUSC	Mmp25	matrix me	GO:00325	developm	-	-	-	-	17:236294	6	11	4	4	9	4	0.108609	0.15902	0.080032	0.055856	0.104913	0.05696	0.115887	0.072576	0.267288	1	-
ENSMUSC	Hcfc1r1	host cell f	GO:00056	cell|intrac	-	-	-	-	17:236735	435	600	472	645	784	588	16.0874	17.7212	19.29235	18.40291	18.67097	17.10743	17.70032	18.06044	0.982079	1	-
ENSMUSC	Tnfrsf12a	tumor nec	GO:00325	developm	K05149	tumor nec	mmu0406	Cytokine-	17:236754	19	37	19	36	32	32	1.204669	1.873527	1.331447	1.760956	1.306547	1.596177	1.469881	1.55456	0.957612	1	-
ENSMUSC	Paqr4	progestin	GO:00160	membran	-	-	-	-	17:237361	772	971	697	1125	1293	1061	18.8021	18.88661	18.76155	21.13839	20.2788	20.32898	18.81676	20.58206	0.50274	1	-
ENSMUSC	Flywch2	FLYWCH f	GO:00971	organic cy	-	-	-	-	17:237769	54	51	44	58	83	45	2.073659	1.564083	1.867435	1.718318	2.052492	1.359463	1.835059	1.710091	0.652177	1	-
ENSMUSC	Slc25a27	solute car	GO:00325	developm	-	-	-	-	17:436419	477	566	404	635	715	651	10.83913	10.27159	10.14622	11.13217	10.46252	11.63771	10.41898	11.07747	0.732715	1	-
ENSMUSC	Tnfrsf21	tumor nec	GO:00325	developm	K05157	tumor nec	mmu0406	Cytokine-	17:430165	2239	2670	2004	2939	3451	2633	37.70245	35.90647	37.29581	38.18084	37.42098	34.88007	36.96824	36.8273	0.752318	1	-
ENSMUSC	Adgrf4	adhesion	GO:00048	signal trar	-	-	-	-	17:426568	19	24	23	33	36	27	0.243666	0.245808	0.325995	0.326509	0.297308	0.272411	0.271823	0.298743	0.771741	1	-
ENSMUSC	Cenpq	centrome	GO:00511	localizatio	-	-	-	-	17:409230	50	69	52	66	97	75	1.14342	1.260172	1.314251	1.16441	1.428422	1.349279	1.239281	1.314037	0.854386	1	-
ENSMUSC	Mut	methylma	GO:00325	developm	K01847	methylma	mmu0110	Metabolic	17:409346	779	1088	689	983	1245	1059	12.94266	14.43646	12.65179	12.59998	13.32018	13.84181	13.34364	13.25399	0.76213	1	-
ENSMUSC	Tbc1d5	TBC1 don	GO:00081	metabolic	-	-	-	-	17:507331	631	823	581	1204	968	659	1.863039	1.94061	1.895903	2.742519	1.840444	1.530697	1.899851	2.037887	0.735829	1	-
ENSMUSC	Satb1	special AT	GO:00325	developm	-	-	-	-	17:517361	1883	1875	1390	1988	2271	2009	3.470028	2.759495	2.831029	2.826372	2.694973	2.91255	3.020184	2.811298	0.321322	1	-
ENSMUSC	Efhb	EF hand c	GO:00431	ion bindin	-	-	-	-	17:533988	3	9	9	8	22	18	0.068835	0.164932	0.228228	0.14162	0.325075	0.324915	0.153998	0.26387	0.189318	1	-
ENSMUSC	Cdc5l	cell divisic	GO:00325	developm	K12860	pre-mRN	mmu0304	Spliceoso	17:453918	1090	1332	950	1457	1856	1396	22.2661	21.73038	21.44806	22.96188	24.41463	22.43436	21.81485	23.27029	0.66535	1	-
ENSMUSC	Spats1	spermato	-	-	-	-	-	-	17:454489	18	11	18	10	18	17	0.947431	0.462392	1.047153	0.406098	0.610123	0.703968	0.818992	0.573397	0.218558	1	-
ENSMUSC	Aars2	alanyl-tRI	GO:00081	metabolic	K01872	alanyl-tRI	mmu0097	Aminoacy	17:455068	518	610	450	746	769	693	9.446861	8.884517	9.070198	10.49608	9.031061	9.942645	9.133859	9.823262	0.65228	1	-
ENSMUSC	Mrpl14	mitochon	GO:00081	metabolic	K02874	large subu	mmu0301	Ribosome	17:456863	81	117	92	139	148	140	3.100756	3.576954	3.892408	4.105133	3.648366	4.216211	3.523373	3.989903	0.509416	1	-
ENSMUSC	Slc29a1	solute car	GO:00325	developm	K15014	solute car	mmu0503	Alcoholisr	17:455852	183	210	162	240	292	224	1.10107	1.009085	1.07727	1.114047	1.13136	1.060281	1.062475	1.101896	0.930947	1	-
ENSMUSC	Hsp90ab1	heat shoc	GO:00325	developm	K04079	molecular	mmu0520	Pathways	17:455677	30415	35599	25673	39160	44671	37210	321.0168	300.0707	299.477	318.8695	303.613	308.9659	306.8548	310.4828	0.878532	1	-
ENSMUSC	Slc5a7	solute car	GO:00081	metabolic	K14387	solute car	mmu0472	Cholinerg	17:542735	19	17	11	23	16	21	0.22413	0.160152	0.14341	0.209319	0.121538	0.194886	0.175897	0.175248	0.943314	1	-
ENSMUSC	Nfkbie	nuclear fa	GO:00081	metabolic	K05872	NF-kappa	mmu0516	Epstein-B	17:455557	29	51	37	55	62	80	0.731083	1.026793	1.030889	1.069709	1.006503	1.586601	0.929588	1.220938	0.233412	1	-
ENSMUSC	Tcte1	t-comple:	-	-	-	-	-	-	17:455234	53	79	56	78	76	68	1.052733	1.253176	1.229342	1.195259	0.972092	1.062578	1.178417	1.076643	0.48646	1	-
ENSMUSC	Vegfa	vascular e	GO:00325	developm	K05448	vascular e	mmu0520	Pathways	17:460169	826	924	676	1027	1029	880	6.293236	5.622271	5.692295	6.036634	5.048519	5.274578	5.869267	5.453244	0.343895	1	-
ENSMUSC	Gtpbp2	GTP bindi	GO:00038	catalytic a	-	-	-	-	17:461610	901	941	791	1251	1547	1123	4.232774	3.530497	4.106987	4.534068	4.679992	4.150407	3.956753	4.454822	0.357199	1	-
ENSMUSC	Polh	polymera:	GO:00081	metabolic	K03509	DNA poly	mmu0152	Platinum (	17:461720	67	81	50	76	109	122	1.592399	1.537487	1.313407	1.393557	1.668247	2.281125	1.481098	1.780976	0.389258	1	-
ENSMUSC	Enpp5	ectonucle	GO:00081	metabolic	-	-	-	-	17:440788	3488	4132	2934	4166	5053	4189	24.97311	23.62666	23.21684	23.01154	23.29701	23.59486	23.93887	23.30114	0.548177	1	-
ENSMUSC	Enpp4	ectonucle	GO:00081	metabolic	K18424	ectonucle	mmu0023	Purine me	17:440963	154	606	215	530	660	218	1.178902	3.7049	1.819042	3.130143	3.253544	1.312884	2.234281	2.565524	0.64258	1	-
ENSMUSC	Fbxl17	F-box and	-	-	-	-	-	-	17:630574	807	1009	738	989	1165	997	5.078212	5.070774	5.132636	4.801356	4.720826	4.935639	5.093874	4.819274	0.420695	1	-
ENSMUSC	Rsph9	radial spo	GO:00400	locomotic	-	-	-	-	17:461220	109	165	117	175	192	155	3.517607	4.252575	4.17306	4.357023	3.990058	3.935186	3.981081	4.094089	0.987187	1	-
ENSMUSC	Mrps18a	mitochon	GO:00081	metabolic	K02963	small subu	mmu0301	Ribosome	17:461109	333	405	389	533	643	461	3.781353	3.672861	4.882027	4.669399	4.701854	4.11827	4.11208	4.496508	0.59233	1	-
ENSMUSC	Crip3	cysteine-r	GO:00023	immune s	-	-	-	-	17:464289	7	12	13	9	18	25	0.114374	0.156582	0.23475	0.11345	0.189388	0.321343	0.168569	0.20806	0.60238	1	-
ENSMUSC	Rrp36	ribosomal	GO:00081	metabolic	-	-	-	-	17:466674	194	269	193	281	334	292	9.912356	10.97673	10.89883	11.07675	10.98945	11.73732	10.59597	11.26784	0.761045	1	-
ENSMUSC	Ptk7	PTK7 prot	GO:00325	developm	-	-	-	-	17:465644	171	216	166	204	257	258	2.453801	2.475385	2.632688	2.258414	2.374823	2.912559	2.520625	2.515265	0.846231	1	-
ENSMUSC	Cnpy3	canopy F(	GO:00023	immune s	-	-	-	-	17:467357	774	965	680	993	1234	957	9.522258	9.481396	9.246021	9.424946	9.77617	9.262368	9.416558	9.487828	0.872508	1	-
ENSMUSC	Ubr2	ubiquitin	GO:00081	metabolic	-	-	-	-	17:469282	1740	2206	1511	2292	2601	2352	8.474205	8.580272	8.133194	8.611816	8.157269	9.011517	8.39589	8.593534	0.991488	1	-
ENSMUSC	Guca1b	guanylate	GO:00081	metabolic	K08328	guanylate	mmu0474	Phototran	17:473853	6	6	3	8	13	7	0.119174	0.095177	0.065862	0.122593	0.166282	0.109388	0.093404	0.132754	0.42629	1	-
ENSMUSC	Taf8	TATA-box	GO:00325	developm	K14649	transcripti	mmu0302	Basal tran	17:474880	269	356	254	387	434	357	5.910267	6.246698	6.167877	6.55989	6.140442	6.170701	6.108281	6.290344	0.969415	1	-
ENSMUSC	Guca1a	guanylate	GO:00081	metabolic	K08328	guanylate	mmu0474	Phototran	17:473945	0	0	0	0	2	0	0	0	0	0	0.090265	0	0	0.030088	0.528262	1	-
ENSMUSC	Pgc	progastric	GO:00081	metabolic	-	-	-	-	17:477268	0	3	0	0	0	0	0	0.096384	0	0	0	0	0.032128	0	0.288975	1	-
ENSMUSC	Bysl	bystin-like	GO:00325	developm	-	-	-	-	17:475993	455	599	382	658	751	562	7.208475	7.578863	6.6887	8.042442	7.661715	7.004532	7.158679	7.569563	0.771434	1	-
ENSMUSC	Tfeb	transcripti	GO:00325	developm	K15590	transcripti	mmu0413	Mitophag	17:477370	98	113	71	145	160	136	0.649063	0.597701	0.519713	0.740895	0.682393	0.708616	0.588826	0.710635	0.274332	1	-
ENSMUSC	Foxp4	forkhead	GO:00325	developm	-	-	-	-	17:478671	641	597	447	900	962	681	4.343384	3.230657	3.347534	4.704821	4.197591	3.630179	3.640525	4.17753	0.352654	1	-
ENSMUSC	Treml1	triggering	GO:00508	response	-	-	-	-	17:483599	0	0	2	0	3	0	0	0	0.040484	0	0.035384	0	0.013495	0.011795	1	1	-
ENSMUSC	Nfya	nuclear tr	GO:00325	developm	K08064	nuclear tr	mmu0515	Tuberculo	17:483868	392	451	296	495	566	416	1.559472	1.432894	1.301458	1.519243	1.449981	1.301954	1.431275	1.423726	0.806905	1	-
ENSMUSC	Tspo2	translocat	GO:00511	localizatio	-	-	-	-	17:484479	0	0	0	0	3	0	0	0	0	0	0.022441	0	0	0.00748	0.290463	1	-
ENSMUSC	Kif6	kinesin fa	GO:00990	supramole	-	-	-	-	17:496151	47	80	57	97	77	79	0.403899	0.549055	0.541377	0.643105	0.426112	0.534096	0.49811	0.534438	0.812853	1	-
ENSMUSC	Brd4	bromodo	GO:00325	developm	-	-	-	-	17:321962	1660	2216	1947	2219	3293	2169	9.215063	9.824391	11.94546	9.503371	11.77163	9.472413	10.3283	10.24914	0.770224	1	-
ENSMUSC	Stk38	serine/thr	GO:00081	metabolic	-	-	-	-	17:289708	652	810	583	919	1125	866	3.181483	3.156549	3.144104	3.459617	3.534996	3.324378	3.160712	3.439664	0.541889	1	-
ENSMUSC	Ppil1	peptidylpr	GO:00081	metabolic	K12733	peptidyl-p	mmu0304	Spliceosor	17:292508	132	165	153	210	279	176	2.378181	2.374114	3.046569	2.918921	3.236912	2.494572	2.599621	2.883468	0.574401	1	-
ENSMUSC	Mtch1	mitochon	GO:00081	metabolic	-	-	-	-	17:293320	3264	4479	3059	3843	5070	4084	45.15036	49.48094	46.76677	41.01208	45.16205	44.44344	47.13269	43.53919	0.233344	1	-
ENSMUSC	Ccdc167	coiled-co	GO:00160	membran	-	-	-	-	17:296893	166	167	134	207	306	192	1.327352	1.06645	1.184212	1.276963	1.57563	1.207787	1.192671	1.35346	0.472839	1	-
ENSMUSC	Cmtr1	cap meth	GO:00081	metabolic	-	-	-	-	17:296605	1867	2141	1655	2706	3019	2656	11.02791	10.09977	10.80423	12.33125	11.4833	12.34206	10.64397	12.0522	0.259346	1	-
ENSMUSC	Glo1	glyoxalase	GO:00325	developm	K01759	lactoylglut	mmu0062	Pyruvate r	17:305928	946	1427	905	1298	1617	1274	25.60327	30.84424	27.07072	27.10249	28.18181	27.12593	27.83941	27.47008	0.705041	1	-
ENSMUSC	Glp1r	glucagon-	GO:00048	signal trar	K04581	glucagon-	mmu0408	Neuroacti	17:309018	7	26	10	31	21	11	0.288795	0.856643	0.455947	0.98668	0.557888	0.357019	0.533795	0.633862	0.727868	1	-
ENSMUSC	Abcg1	ATP bindi	GO:00325	developm	K05679	ATP-bind	mmu0201	ABC trans	17:310576	1055	1434	1012	1461	1715	1348	15.33721	16.64904	16.26003	16.38606	16.0551	15.41683	16.08209	15.95266	0.735316	1	-
ENSMUSC	Rsph1	radial spo	GO:00325	developm	-	-	-	-	17:312550	67	117	79	89	110	92	3.514497	4.90141	4.580008	3.601728	3.715668	3.796546	4.331971	3.704647	0.247921	1	-
ENSMUSC	Tmprss3	transmem	GO:00081	metabolic	-	-	-	-	17:311792	0	0	0	2	0	0	0	0	0	0.03119	0	0	0	0.010397	0.527266	1	-
ENSMUSC	Wdr4	WD repea	GO:00081	metabolic	-	-	-	-	17:314943	205	357	189	371	359	260	1.169697	1.626796	1.191865	1.63314	1.319072	1.167085	1.329453	1.373099	0.960139	1	-
ENSMUSC	Ndufv3	NADH de	GO:00081	metabolic	K03944	NADH de	mmu0110	Metabolic	17:315201	461	495	534	822	689	549	8.733417	7.489176	11.18076	12.01394	8.405367	8.182126	9.134451	9.53381	0.896915	1	-
ENSMUSC	Cryaa	crystallin,	GO:00325	developm	K09541	crystallin,	mmu0414	Protein pr	17:316779	3	0	1	4	3	5	0.034573	0	0.012738	0.035561	0.022267	0.045333	0.015771	0.034387	0.270196	1	-
ENSMUSC	Sik1	salt induci	GO:00325	developm	K19008	serine/thr	mmu0492	Glucagon	17:318442	172	246	190	279	267	252	2.328087	2.659205	2.842305	2.913431	2.327212	2.683379	2.609866	2.641341	0.928977	1	-
ENSMUSC	Arhgap28	Rho GTPa	GO:00987	molecular	-	-	-	-	17:678427	7	29	12	11	32	13	0.062106	0.205484	0.117666	0.075296	0.182825	0.090733	0.128419	0.116285	0.773327	1	-
ENSMUSC	Epb41l3	erythrocyt	GO:00325	developm	-	-	-	-	17:690756	2935	3432	2417	3882	4169	3910	11.3673	10.61555	10.34601	11.59939	10.39767	11.91344	10.77629	11.3035	0.797041	1	-
ENSMUSC	Akap8	A kinase (	GO:00081	metabolic	-	-	-	-	17:323036	1320	1549	1075	1735	1995	1663	21.81237	20.44212	19.63289	22.11865	21.22885	21.61882	20.62913	21.65544	0.795176	1	-
ENSMUSC	Wiz	widely-int	GO:00081	metabolic	-	-	-	-	17:323540	674	901	715	894	1033	1031	2.415375	2.578669	2.8319	2.471682	2.383854	2.906659	2.608648	2.587399	0.758345	1	-
ENSMUSC	Lpin2	lipin 2 [So	GO:00081	metabolic	K15728	phosphati	mmu0110	Metabolic	17:711825	1102	1346	924	1446	2530	1311	4.020197	3.921539	3.725498	4.069718	5.943482	3.762525	3.889078	4.591908	0.240747	1	-
ENSMUSC	Emilin2	elastin mi	GO:00329	macromo	-	-	-	-	17:712521	53	103	74	86	118	88	0.827647	1.284541	1.27716	1.036081	1.186598	1.081087	1.129783	1.101255	0.797733	1	-
ENSMUSC	Smchd1	SMC hing	GO:00325	developm	-	-	-	-	17:713444	1006	1387	906	1339	1643	1293	2.943195	3.240733	2.929518	3.022261	3.095375	2.975981	3.037815	3.031206	0.784627	1	-
ENSMUSC	Cyp4f13	cytochron	GO:00081	metabolic	K17726	leukotrien	mmu0110	Metabolic	17:329246	93	134	113	139	179	134	1.175649	1.352844	1.578775	1.355629	1.457151	1.332638	1.369089	1.381806	0.943891	1	-

ENSMUSC	Clip4	CAP-GLY	GO:00056	cell|intrace	-	-	-	-	17:717819	958	1275	890	1214	1458	1257	14.42507	15.33234	14.81117	14.10268	14.13725	14.89013	14.85619	14.37669	0.543835		1	-
ENSMUSC	Lbh	limb-bud	GO:00325	developm	-	-	-	-	17:729183	671	808	636	858	1092	791	12.42645	11.95038	13.01752	12.25864	13.02272	11.52423	12.46478	12.26853	0.701325		1	-
ENSMUSC	Galnt14	polypeptic	GO:00081	metabolic	K00710	polypeptic	mmu0110	Metabolic	17:734932	333	420	306	456	470	432	3.116544	3.139237	3.165172	3.292489	2.832577	3.18071	3.140318	3.101926	0.749036		1	-
ENSMUSC	Ehd3	EH-doma	GO:00081	metabolic	K12476	EH domai	mmu0414	Endocytos	17:738048	3609	4612	3478	4752	5617	4591	60.24021	61.48013	64.16172	61.19365	60.37522	60.2862	61.96069	60.61836	0.580969		1	-
ENSMUSC	Dpy30	dpy-30, h	GO:00081	metabolic	-	-	-	-	17:742994	127	152	182	178	210	162	7.961387	7.609811	12.60966	8.608649	8.477303	7.989305	9.393619	8.358419	0.396603		1	-
ENSMUSC	Spast	spastin [S	GO:00325	developm	-	-	-	-	17:743389	1248	1557	1205	1616	1896	1643	10.1533	10.11642	10.83494	10.14295	9.933119	10.51577	10.36822	10.19728	0.655816		1	-
ENSMUSC	Slc30a6	solute car	GO:00511	localizatio	-	-	-	-	17:743956	182	192	134	231	255	199	4.967629	4.185281	4.042307	4.864298	4.482007	4.273077	4.398405	4.539794	0.96941		1	-
ENSMUSC	Prkd3	protein ki	GO:00081	metabolic	K06070	protein ki	mmu0401	Rap1 sign	17:789494	302	492	386	396	586	506	1.189496	1.547629	1.680312	1.203319	1.486306	1.567897	1.472479	1.419174	0.646763		1	-
ENSMUSC	Yipf4	Yip1 dom	GO:00160	membran	-	-	-	-	17:744894	511	628	431	628	791	537	14.6276	14.35681	13.63568	13.86892	14.58088	12.09311	14.2067	13.5143	0.504567		1	-
ENSMUSC	Birc6	baculovira	GO:00325	developm	K10586	baculovira	mmu0412	Ubiquitin	17:745282	3590	4808	3328	4568	5341	4934	5.177716	5.538014	5.304859	5.082767	4.960449	5.59827	5.340196	5.213829	0.569583		1	-
ENSMUSC	Crim1	cysteine ri	GO:00081	metabolic	-	-	-	-	17:782002	3108	3858	2718	3820	4423	3948	31.65448	31.38067	30.595	30.01566	29.00853	31.63314	31.21005	30.21911	0.510008		1	-
ENSMUSC	Vit	vitrin [Sou	GO:00099	cellular pr	-	-	-	-	17:785080	110	150	109	157	178	162	2.469261	2.689123	2.704265	2.718975	2.573062	2.860876	2.620883	2.717638	0.945505		1	-
ENSMUSC	Strn	striatin, ca	GO:00452	synapse|re	-	-	-	-	17:786499	2178	2830	1913	2633	3269	2740	13.15637	13.65245	12.77144	12.27041	12.71592	13.02086	13.19342	12.66906	0.45556		1	-
ENSMUSC	Ttc27	tetratricop	-	-	-	-	-	-	17:747177	231	332	258	296	371	343	5.001574	5.74088	6.173928	4.944432	5.172762	5.842503	5.638794	5.319899	0.512245		1	-
ENSMUSC	Cebpz	CCAAT/er	GO:00081	metabolic	-	-	-	-	17:789190	857	965	656	946	1260	988	12.84409	11.55036	10.86609	10.93815	12.16039	11.64904	11.75351	11.58253	0.710835		1	-
ENSMUSC	Ndufaf7	NADH de	GO:00081	metabolic	-	-	-	-	17:789371	453	546	376	625	628	622	12.01012	11.56079	11.01754	12.78379	10.72171	12.97331	11.52948	12.1596	0.805058		1	-
ENSMUSC	Pja2	praja ring	GO:00081	metabolic	-	-	-	-	17:642810	14109	16981	12289	16632	20704	17003	87.07863	83.6998	83.82599	79.19361	82.28568	82.5566	84.86814	81.3453	0.422615		1	-
ENSMUSC	Qpct	glutaminy	GO:00081	metabolic	-	-	-	-	17:790519	134	129	113	185	192	132	4.252499	3.269432	3.963348	4.529398	3.923695	3.295506	3.828426	3.9162	1		1	-
ENSMUSC	Man2a1	mannosid	GO:00325	developm	K01231	alpha-ma	mmu0110	Metabolic	17:646007	373	465	319	457	618	483	2.918708	2.905902	2.758791	2.758851	3.114043	2.973307	2.861133	2.948734	0.960303		1	-
ENSMUSC	Vapa	vesicle-as	GO:00325	developm	K06096	vesicle-as	mmu0497	Cholestero	17:655800	1488	1742	1577	2253	2635	2196	55.39907	51.79567	64.89003	64.71293	63.17348	64.31952	57.36159	64.06864	0.357364		1	-
ENSMUSC	Hnrnpll	heteroger	GO:00081	metabolic	-	-	-	-	17:800294	843	989	788	1042	1297	950	3.279098	3.072336	3.387661	3.126978	3.248789	2.907108	3.246365	3.094291	0.478189		1	-
ENSMUSC	Ralbp1	ralA bindi	GO:00511	localizatio	K08773	RalA-binc	mmu0520	Pathways	17:658484	1242	1548	1149	1681	1956	1670	17.49347	17.4129	17.88632	18.26637	17.74099	18.50468	17.59756	18.17068	0.932049		1	-
ENSMUSC	Srsf7	serine/arg	GO:00081	metabolic	K12896	splicing fa	mmu0516	Herpes sir	17:802000	1212	1369	1087	1674	1899	1488	32.47145	29.29196	32.18661	34.60069	32.76263	31.36265	31.31667	32.90865	0.789585		1	-
ENSMUSC	Twsg1	twisted ga	GO:00325	developm	-	-	-	-	17:659230	652	934	581	857	1103	945	9.839323	11.25668	9.690378	9.977659	10.71884	11.21915	10.26213	10.63855	0.909087		1	-
ENSMUSC	Ndufv2	NADH de	GO:00325	developm	-	-	mmu0110	Metabolic	17:660787	983	1438	1123	2006	1629	1656	24.42822	28.53927	30.84358	38.45912	26.06838	32.37498	27.93702	32.30083	0.284593		1	-
ENSMUSC	Washc1	WASH co	GO:00325	developm	K18461	WAS prot	mmu0414	Endocytos	17:661115	480	548	451	677	717	689	9.798696	8.934151	10.17536	10.66217	9.425417	11.06515	9.63607	10.38425	0.648362		1	-
ENSMUSC	Washc2	WASH co	GO:00511	localizatio	K18462	WASH co	mmu0414	Endocytos	6:1162080	2126	2636	1956	2642	3186	2599	11.76024	11.64513	11.95827	11.27499	11.34889	11.3102	11.78788	11.31136	0.446581		1	-
ENSMUSC	Cacna1h	calcium ch	GO:00081	metabolic	K04855	voltage-d	mmu0401	MAPK sign	17:253742	1876	3194	2525	3260	4201	2586	7.133649	9.69973	10.61175	9.563719	10.28694	7.736035	9.148378	9.195566	0.883987		1	-
ENSMUSC	Prss41	protease,	GO:00081	metabolic	-	-	-	-	17:238367	0	0	0	0	3	0	0	0	0	0	0.033658	0	0	0.011219	0.2904		1	-
ENSMUSC	Tedc2	tubulin ep	-	-	-	-	-	-	17:242150	124	98	86	158	150	146	1.440491	0.909199	1.104161	1.416043	1.122109	1.334298	1.151284	1.290817	0.603715		1	-
ENSMUSC	Lrpprc	leucine-ri	GO:00081	metabolic	-	-	-	-	17:847052	1479	1922	1284	1740	2249	1787	8.387199	8.704581	8.047491	7.612514	8.21283	7.972307	8.379757	7.932551	0.379901		1	-
ENSMUSC	Pdpk1	3-phosph	GO:00325	developm	K06276	3-phosph	mmu0415	PI3K-Akt s	17:240736	2782	3472	2354	3495	3889	3414	10.53222	10.49756	9.849542	10.20799	9.481029	10.16804	10.29311	9.952355	0.505022		1	-
ENSMUSC	Sbpl	spermine	-	-	-	-	-	-	17:239530	0	0	0	0	0	2	0	0	0	0	0	0.110446	0	0.036815	0.526986		1	-
ENSMUSC	Prepl	prolyl end	GO:00081	metabolic	-	-	-	-	17:850634	7040	9292	6922	9235	10844	8739	112.2321	118.304	121.9616	113.5826	111.324	109.6017	117.4993	111.5028	0.357656		1	-
ENSMUSC	Abca3	ATP-bind	GO:00511	localizatio	K05643	ATP-bind	mmu0201	ABC trans	17:243519	1815	2178	1662	2379	2496	2356	10.07734	9.657679	10.19875	10.19046	8.924182	10.29095	9.977921	9.801865	0.637152		1	-
ENSMUSC	Eci1	enoyl-Co	GO:00081	metabolic	K13238	3,2-trans-	mmu0007	Fatty acid	17:244266	176	281	181	310	384	261	9.849904	12.55943	11.19553	13.38476	13.83899	11.49131	11.20162	12.90502	0.362843		1	-
ENSMUSC	Six2	sine oculis	GO:00325	developm	-	-	-	-	17:856842	2	2	3	6	5	4	0.031059	0.024813	0.051504	0.071897	0.05001	0.048875	0.035792	0.056927	0.434241		1	-
ENSMUSC	Srbd1	S1 RNA b	GO:00081	metabolic	-	-	-	-	17:859846	183	256	159	298	339	236	3.165343	3.53634	3.039569	3.976635	3.775927	3.211378	3.247084	3.654647	0.463175		1	-
ENSMUSC	Dnase1l2	deoxyribo	GO:00325	developm	-	-	-	-	17:244400	38	57	36	69	45	44	0.453872	0.543714	0.475234	0.63582	0.346111	0.413439	0.49094	0.465123	0.707869		1	-
ENSMUSC	E4f1	E4F transc	GO:00325	developm	-	-	-	-	17:244437	317	335	300	446	424	417	2.24906	1.898158	2.352397	2.441224	1.937145	2.327497	2.166538	2.235288	0.964077		1	-
ENSMUSC	Epas1	endotheli	GO:00325	developm	K09095	hypoxia-i	mmu0520	Pathways	17:867539	3412	4315	3093	4577	5088	4491	39.24096	39.63303	39.31491	40.61083	37.68187	40.63353	39.3963	39.64208	0.829118		1	-
ENSMUSC	Rhoq	ras homol	GO:00325	developm	K07194	Ras homo	mmu0491	Insulin sig	17:869630	539	754	473	760	943	723	7.360846	8.223486	7.139153	8.007235	8.292874	7.767615	7.574495	8.022574	0.747108		1	-
ENSMUSC	Pigf	phosphati	GO:00081	metabolic	K05287	phosphati	mmu0110	Metabolic	17:869972	92	113	71	88	145	80	5.685596	5.577134	4.849427	4.195648	5.770458	3.889421	5.370719	4.618509	0.302007		1	-
ENSMUSC	Cript	cysteine-r	GO:00452	synapse|lc	-	-	-	-	17:870255	282	361	284	401	472	404	7.228542	7.390166	8.045761	7.930063	7.791097	8.14692	7.554823	7.956027	0.812628		1	-
ENSMUSC	Mcfd2	multiple c	GO:00081	metabolic	-	-	-	-	17:872546	795	1039	903	1151	1331	1213	9.808288	10.23735	12.31291	10.95548	10.57447	11.77328	10.78618	11.10108	0.965119		1	-
ENSMUSC	Msh2	mutS hon	GO:00325	developm	K08735	DNA misn	mmu0520	Pathways	17:876723	517	664	447	715	857	727	4.469349	4.584244	4.270795	4.768595	4.770784	4.944242	4.441463	4.827874	0.567462		1	-
ENSMUSC	Gtf2a1l	general tr	GO:00081	metabolic	K03122	transcripti	mmu0520	Viral carci	17:886686	0	0	0	0	2	0	0	0	0	0	0.036721	0	0	0.01224	0.528989		1	-
ENSMUSC	Spsb3	splA/ryan	GO:00081	metabolic	-	-	-	-	17:248866	450	599	464	517	729	617	3.47184	3.690793	3.956512	3.077288	3.621843	3.744928	3.706382	3.481353	0.43752		1	-
ENSMUSC	Mapk8ip3	mitogen-a	GO:00325	developm	K04436	mitogen-a	mmu0401	MAPK sign	17:248921	8210	8638	6608	10914	10477	10914	19.86239	16.68966	17.66873	20.37055	16.32225	20.77224	18.07359	19.15501	0.734		1	-
ENSMUSC	Tmem204	transmem	GO:00325	developm	-	-	-	-	17:250577	96	149	108	143	159	134	2.261403	2.803104	2.811773	2.598818	2.411912	2.483272	2.625426	2.498	0.614265		1	-
ENSMUSC	Ift140	intraflagel	GO:00325	developm	-	-	-	-	17:250160	631	809	536	776	990	806	3.993744	4.089269	3.74941	3.789162	4.034976	4.013257	3.944141	3.945798	0.816426		1	-
ENSMUSC	Telo2	telomere	GO:00081	metabolic	K11137	telomere	mmu0415	mTOR sig	17:250995	285	319	350	427	429	399	1.253624	1.120623	1.70152	1.449041	1.215164	1.380721	1.358589	1.348309	0.821403		1	-
ENSMUSC	St6gal2	beta galac	GO:00081	metabolic	-	-	-	-	17:554453	350	420	277	422	488	471	2.20654	2.11465	1.930056	2.052517	1.981154	2.336013	2.083749	2.123228	0.962038		1	-
ENSMUSC	Pot1b	protectior	GO:00325	developm	-	-	-	-	17:556520	106	130	82	143	152	122	1.849712	1.811705	1.581458	1.92516	1.708038	1.674824	1.747625	1.769341	0.954235		1	-
ENSMUSC	Tekt4	tektin 4 [S	-	-	-	-	-	-	17:254715	9	15	14	11	36	14	0.358688	0.477452	0.616677	0.338242	0.923946	0.43895	0.484272	0.567046	0.694585		1	-
ENSMUSC	Sox8	SRY (sex c	GO:00325	developm	-	-	-	-	17:255658	560	847	575	794	951	885	8.897354	10.74737	10.09689	9.73248	9.729898	11.06184	9.913872	10.17474	0.988149		1	-
ENSMUSC	Nme4	NME/NM	GO:00081	metabolic	K00940	nucleoside	mmu0110	Metabolic	17:260917	32	40	48	62	44	33	1.041499	1.039721	1.726636	1.556809	0.922179	0.844969	1.269285	1.107986	0.509622		1	-
ENSMUSC	Mrpl28	mitochon	GO:00051	structural	K02902	large subu	mmu0301	Ribosome	17:261235	414	527	377	619	719	491	12.88381	13.09787	12.96678	14.86159	14.40881	12.02088	12.98282	13.76376	0.761802		1	-
ENSMUSC	Axin1	axin 1 [So	GO:00048	signal trar	K02157	axin 1[-]	mmu0520	Pathways	17:261386	553	638	470	741	903	758	6.562704	6.046781	6.164566	6.784326	6.900819	7.076814	6.258017	6.920653	0.460161		1	-
ENSMUSC	Pdia2	protein di	GO:00081	metabolic	-	-	-	-	17:261959	0	0	1	0	0	1	0	0	0.024363	0	0	0.017344	0.008121	0.005781	1		1	-
ENSMUSC	Rgs11	regulator	GO:00048	signal trar	-	-	-	-	17:262029	376	468	366	597	579	552	2.466199	2.4515	2.653188	3.020959	2.445532	2.848332	2.523629	2.771608	0.547334		1	-
ENSMUSC	Luc7l	Luc7-like	GO:00325	developm	-	-	-	-	17:262529	735	828	581	821	1019	907	3.477544	3.128684	3.038145	2.996808	3.104661	3.37601	3.214791	3.15916	0.684504		1	-
ENSMUSC	Dusp1	dual speci	GO:00325	developm	K04459	dual speci	mmu0401	MAPK sign	17:265055	187	241	138	225	255	303	2.220507	2.285467	1.811083	2.061223	1.949874	2.83051	2.105686	2.280536	0.720638		1	-
ENSMUSC	Bnip1	BCL2/ade	GO:00081	metabolic	K08497	protein tra	mmu0413	SNARE int	17:267810	162	213	159	320	263	223	0.943927	0.991173	1.023922	1.438481	0.986809	1.02221	0.986341	1.149167	0.370662		1	-
ENSMUSC	Phf1	PHD finge	GO:00081	metabolic	-	-	-	-	17:269331	513	656	482	761	846	701	12.89536	13.16939	13.39089	14.75811	13.69434	13.86261	13.15188	14.10502	0.659657		1	-
ENSMUSC	Cuta	cutA dival	GO:00511	localizatio	-	-	-	-	17:269379	182	255	147	273	316	240	16.66055	18.64244	14.87242	19.28015	18.62772	17.2838	16.72514	18.39722	0.582917		1	-
ENSMUSC	Plin3	perilipin 3	GO:00511	localizatio	-	-	-	-	17:562789	257	312	217	318	452	320	7.268158	7.046792	6.782607	6.938223	8.231585	7.11954	7.032519	7.429783	0.801216		1	-
ENSMUSC	Kdm4b	lysine (K)-	GO:00081	metabolic	-	-	-	-	17:563260	1420	1822	1441	2033	2402	2231	10.09225	10.34174	11.31906	11.14724	10.99328	12.47413	10.58435	11.53822	0.497865		1	-
ENSMUSC	Rfx2	regulatory	GO:00325	developm	-	-	-	-	17:567758	56	78	49	89	114	80	1.019765	1.134355	0.986178	1.250347	1.336808	1.146063	1.046766	1.244406	0.352414		1	-
ENSMUSC	Uqcc2	ubiquinol-	GO:00325	developm	-	-	-	-	17:271226	276	273	251	319	447	307	11.26474	8.898581	11.32224	10.04461	11.74826	9.857361	10.49519	10.55008	0.897502		1	-
ENSMUSC	1700061G	RIKEN cD	GO:00081	metabolic	K15013	long-chai	mmu0110	Metabolic	17:568754	2	2	4	8	8	5	0.036875	0.02946	0.081531	0.113817	0.095005	0.072538	0.049288	0.093787	0.248028		1	-
ENSMUSC	Grm8	glutamate	GO:00048	signal trar	K04608	metabotro	mmu0408	Neuroacti	6:2727511	177	237	123	219	202	217	0.859219	0.91881	0.65991	0.820173	0.631449	0.828711	0.812647	0.760111	0.526841		1	-
ENSMUSC	Mllt1	myeloid/ly	GO:00081	metabolic	K15187	YEATS do	mmu0520	Transcript	17:568926	1164	1454	1098	1606	1944	1503	19.69835	19.65111	20.53649	20.96778	21.18496	20.00999	19.96198	20.72091	0.884569		1	-
ENSMUSC	Nudt3	nudix (nu	GO:00081	metabolic	-	-	-	-	17:275793	4321	5990	3641	5906	6926	5224	57.20558	63.33251	53.27476	60.32229	59.0461	54.40867	57.93762	57.92569	0.786208		1	-
ENSMUSC	Snrpc	U1 small r	GO:00081	metabolic	K11095	U1 small r	mmu0304	Spliceoso	17:278399	191	289	229	306	408	353	1.705733	2.061204	2.260264	2.108289	2.346348	2.480065	2.009067	2.311567	0.363377		1	-
ENSMUSC	Taf11	TATA-box	GO:00081	metabolic	K03135	transcripti	mmu0302	Basal tran	17:279011	393	486	327	480	616	550	7.074249	6.986654	6.505524	6.665904	7.140406	7.788631	6.855476	7.198314	0.833026		1	-
ENSMUSC	Anks1	ankyrin re	GO:00325	developm	-	-	-	-	17:279093	374	511	397	503	632	524	1.875781	2.046808	2.200631	1.946294	2.041181	2.067532	2.041073	2.018336	0.758723		1	-
ENSMUSC	Zfp523	zinc finge	GO:00081	metabolic	-	-	-	-	17:281772	1150	1405	1117	1586	1795	1968	10.61158	10.35391	11.39153	11.29056	10.666	14.28625	10.78567	12.08094	0.389639		1	-
ENSMUSC	Fkbp5	FK506 bin	GO:00081	metabolic	K09571	FK506-bir	mmu0491	Estrogen s	17:283990	1251	1604	1248	1836	2195	1706	6.857321	7.021781	7.560637	7.76426	7.747941	7.356768	7.14658	7.62299	0.6551		1	-
ENSMUSC	Pdzph1	PDZ and p	-	-	-	-	-	-	17:588788	7	9	4	6	3	11	0.08851	0.090885	0.055901	0.058527	0.02443	0.10942	0.078432	0.064125	0.625865		1	-
ENSMUSC	Nudt12	nudix (nu	GO:00081	metabolic	K03426	NAD+ dip	mmu0110	Metabolic	17:589996	113	231	115	195	258	191	0.993171	1.621451	1.117095	1.322245	1.460225	1.320659	1.243905	1.36771	0.658349		1	-
ENSMUSC	Cul2	cullin 2 [S	GO:00081	metabolic	K03870	cullin 2[-]	mmu0520	Pathways	18:338298	1027	1418	1103	1397	1875	1389	8.849375	9.758075	10.50424	9.286862	10.40395	9.415761	9.703895	9.702191	0.812758		1	-
ENSMUSC	Bambi	BMP and	GO:00325	developm	K10162	BMP and	mmu0431	Wnt signa	18:350795	65	108	90	100	136	128	0.787456	1.044922	1.205043	0.934633	1.06098	1.219926	1.012474	1.071846	0.85831		1	-
ENSMUSC	Mtpap	mitochon	GO:00081	metabolic	-	-	-	-	18:437559	309	370	307	398	507	385	7.116928	6.805832	7.814812	7.072076	7.519626	6.975979	7.245857	7.189227	0.793196		1	-
ENSMUSC	Map3k8	mitogen-a	GO:00048	signal trar	K04415	mitogen-a	mmu0401	MAPK sign	18:433132	19	38	23	19	37	28	0.151925	0.242663	0.203256	0.117206	0.190518	0.176138	0.199281	0.161288	0.332499		1	-
ENSMUSC	Svil	supervillin	GO:00325	developm	-	-	-	-	18:492054	269	386	199	330	478	405	1.01814	1.166779	0.832446	0.963605	1.165032	1.205927	1.005788	1.111521	0.568267		1	-
ENSMUSC	Zeb1	zinc finge	GO:00325	developm	K09299	zinc finge	mmu0520	Transcript	18:559186	1501	1847	1301	1450	2215	1997	5.509362	5.414186	5.2777	4.106	5.235388	5.766462	5.400416	5.03595	0.361662		1	-
ENSMUSC	Epc1	enhancer	GO:00325	developm	-	-	-	-	18:643595	793	1047	706	1055	1325	1085	8.305157	8.757237	8.17196	8.524277	8.936048	8.939547	8.411451	8.799957	0.829963		1	-
ENSMUSC	Sos1	son of sev	GO:00325	developm	K03099	son of sev	mmu0520	Pathways	17:803937	1889	2291	1479	2284	2479	2173	12.9318	12.52559	11.19031	12.06295	10.92844	11.70302	12.2159	11.5648	0.382692		1	-
ENSMUSC	Map4k3	mitogen-a	GO:00048	signal trar	K04406	mitogen-a	mmu0401	MAPK sign	17:805805	2151	2091	1974	2805	2491	2254	29.82875	23.15765	30.2544	30.00944	22.24452	24.59005	27.74693	25.61467	0.336611		1	-
ENSMUSC	Tmem178	transmem	GO:00325	developm	-	-	-	-	17:809446	876	1002	763	1080	1234	975	31.5371	28.80918	30.35909	29.99658	28.60798	27.61423	30.23512	28.73959	0.453369		1	-
ENSMUSC	Thumpd2	THUMP d	GO:00081	metabolic	-	-	-	-	17:810263	49	80	46	76	90	82	1.611991	2.10185	1.672519	1.928906	1.906613	2.122218	1.795453	1.985912	0.677578		1	-
ENSMUSC	Pkdcc	protein ki	GO:00325	developm	-	-	-	-	17:832152	173	119	154	147	308	223	2.866506	1.574703	2.820166	1.879115	3.286331	2.906849	2.420458	2.690765	0.670131		1	-
ENSMUSC	Cox7a2l	cytochron	GO:00081	metabolic	K02270	cytochron	mmu0501	Huntingto	17:835019	1049	1401	1013	1633	1852	1447	55.84127	59.56127	59.5986	67.06509	63.48566	60.59813	58.33371	63.71629	0.491112		1	-
ENSMUSC	Thada	thyroid ac	GO:00099	cellular pr	-	-	-	-	17:841900	289	327	234	351	435	389	2.238179	2.022505	2.002899	2.09717	2.169406	2.370047	2.087861	2.212208	0.784538		1	-
ENSMUSC	Dync2li1	dynein cy	GO:00325	developm	K10417	dynein lig	mmu0496	Vasopress	17:846264	228	309	203	310	349	260	10.51455	11.38045	10.3466	11.02929	10.3642	9.432763	10.7472	10.27542	0.578493		1	-
ENSMUSC	Abcg8	ATP bindi	GO:00511	localizatio	K05684	ATP-bind	mmu0497	Bile secret	17:846763	0	0	0	2	0	0	0	0	0	0.018886	0	0	0	0.006295	0.528456		1	-
ENSMUSC	Adcyap1	adenylate	GO:00325	developm	K05262	adenylate	mmu0491	Insulin sec	17:931994	94	123	85	134	133	116	2.748785	2.872516	2.747118	3.023072	2.504488	2.668579	2.789473	2.732046	0.760706		1	-
ENSMUSC	Polr2d	polymeras	GO:00081	metabolic	K03012	DNA-dire	mmu0110	Metabolic	18:317891	173	210	150	216	293	203	11.04924	10.7115	10.58825	10.64315	12.05058	10.19979	10.78299	10.96451	0.969519		1	-
ENSMUSC	Slc25a46	solute car	GO:00511	localizatio	-	-	-	-	18:315801	2069	2599	1894	2585	3153	2591	28.90168	28.99444	29.24081	27.85822	28.36228	28.4735	29.04564	28.23134	0.540057		1	-
ENSMUSC	Sap130	Sin3A ass	GO:00081	metabolic	-	-	-	-	18:316343	941	1159	856	1216	1342	1221	12.64987	12.44302	12.71794	12.6113	11.61725	12.91287	12.60361	12.38047	0.658662		1	-
ENSMUSC	Syt4	synaptota	GO:00325	developm	-	-	-	-	18:314378	2958	3463	2415	3690	4125	3432	46.29835	43.28782	41.77643	44.55783	41.57631	42.25962	43.78753	42.79792	0.592875		1	-
ENSMUSC	Adad2	adenosine	GO:00081	metabolic	-	-	-	-	8:1196127	0	0	4	2	0	0	0	0	0.14016	0.048922	0	0	0.04672	0.016307	0.55657		1	-
ENSMUSC	Celf4	CUGBP, El	GO:00325	developm	-	-	-	-	18:254776	6991	8005	6028	9093	10979	8774	29.63463	27.09988	28.24101	29.73704	29.96938	29.25963	28.32518	29.65535	0.796884		1	-
ENSMUSC	Tpgs2	tubulin pc	-	-	-	-	-	-	18:251272	917	1152	808	1192	1383	1117	7.989481	8.015816	7.780496	8.012278	7.759365	7.656204	7.928598	7.809282	0.682331		1	-
ENSMUSC	Elp2	elongator	GO:00081	metabolic	-	-	-	-	18:246039	1291	1576	1190	1681	2187	1783	28.26319	27.55477	28.7931	28.39179	30.83177	30.70839	28.20369	29.97732	0.684855		1	-
ENSMUSC	2700062C	RIKEN cD	-	-	-	-	-	-	18:244708	131	188	139	189	222	183	7.690998	8.814847	9.019302	8.560541	8.393014	8.452225	8.508382	8.468594	0.838154		1	-
ENSMUSC	Zfp397	zinc finge	GO:00081	metabolic	-	-	-	-	18:239546	737	815	660	953	1034	874	4.48429	3.960314	4.438299	4.473518	4.051362	4.183583	4.294301	4.236155	0.712293		1	-
ENSMUSC	Mapre2	microtubu	GO:00990	supramole	-	-	-	-	18:237523	1456	1859	1365	1992	2780	1648	5.649147	5.760322	5.853299	5.962673	6.945778	5.030248	5.754256	5.979567	0.880482		1	-
ENSMUSC	Ccny	cyclin Y [S	GO:00081	metabolic	-	-	-	-	18:931404	2145	2665	1939	2472	3178	2540	32.60384	32.35077	32.57364	28.98813	31.10643	30.37289	32.50942	30.15582	0.251485		1	-
ENSMUSC	Thoc1	THO com	GO:00325	developm	K12878	THO com	mmu0301	RNA trans	18:995818	546	709	522	668	953	712	8.647926	8.968323	9.137694	8.162554	9.720005	8.871756	8.917981	8.918105	0.827561		1	-
ENSMUSC	Rock1	Rho-asso	GO:00325	developm	K04514	Rho-asso	mmu0520	Pathways	18:100644	779	1048	700	989	1313	997	7.687779	8.259813	7.634988	7.529911	8.34416	7.740505	7.86086	7.871525	0.827597		1	-
ENSMUSC	Cyp4f14	cytochron	GO:00081	metabolic	K17726	leukotrien	mmu0110	Metabolic	17:329050	106	152	97	135	224	170	2.482602	2.843098	2.51084	2.439312	3.378349	3.132293	2.61218	2.983318	0.469455		1	-
ENSMUSC	Esco1	establishn	GO:00081	metabolic	-	-	-	-	18:105666	280	418	285	447	405	440	2.047215	2.440772	2.303016	2.521403	1.906839	2.53086	2.263668	2.319701	1		1	-
ENSMUSC	Zfp871	zinc finge	GO:00081	metabolic	-	-	-	-	17:327712	1220	1489	1202	1349	1993	1358	8.205653	7.998229	8.9352	6.999957	8.632076	7.185615	8.379694	7.605882	0.231452		1	-
ENSMUSC	Adamts10	a disinteg	GO:00081	metabolic	-	-	-	-	17:335242	312	351	295	403	458	428	1.82002	1.635213	1.90191	1.813661	1.720448	1.964157	1.785714	1.832755	0.994998		1	-
ENSMUSC	Myo1f	myosin IF	GO:00038	catalytic a	-	-	-	-	17:335557	50	80	62	64	89	67	0.480777	0.614336	0.658885	0.474764	0.551081	0.506822	0.584666	0.510889	0.361364		1	-
ENSMUSC	Kifc5b	kinesin fa	GO:00990	supramole	-	-	-	-	17:269170	39	46	28	51	66	40	0.906459	0.85386	0.719261	0.914498	0.98783	0.731395	0.826527	0.877908	0.872354		1	-
ENSMUSC	Dtna	dystrobre	GO:00452	synapse|m	-	-	-	-	18:234151	2970	3877	2752	3017	4491	4002	9.084846	9.471149	9.303711	7.119781	8.846242	9.630504	9.286569	8.532176	0.265152		1	-
ENSMUSC	Cdh2	cadherin 2	GO:00325	developm	K06736	cadherin 2	mmu0451	Cell adhes	18:165888	2353	3033	2154	2977	3517	3138	14.90814	15.34687	15.08322	14.5516	14.34923	15.64103	15.11274	14.84728	0.626688		1	-
ENSMUSC	Ccdc178	coiled coil	-	-	-	-	-	-	18:218108	0	0	0	2	0	0	0	0	0	0.024987	0	0	0	0.008329	0.528155		1	-
ENSMUSC	Pfdn6	prefoldin	GO:00329	macromo	-	-	-	-	17:339388	303	344	282	512	488	289	6.657279	6.036138	6.847797	8.678714	6.904454	4.995327	6.513738	6.859498	0.849203		1	-
ENSMUSC	Wdr46	WD repea	GO:00081	metabolic	-	-	-	-	17:339406	184	222	139	262	262	236	3.514132	3.386092	2.934024	3.860407	3.222227	3.545875	3.278083	3.542837	0.699816		1	-
ENSMUSC	Rnf138	ring finge	GO:00081	metabolic	-	-	-	-	18:210013	248	270	210	301	430	305	4.868933	4.23343	4.556691	4.559103	5.436337	4.710795	4.553018	4.902078	0.688943		1	-
ENSMUSC	Vps52	VPS52 GA	GO:00325	developm	-	-	-	-	17:339558	1343	1820	1394	1955	2111	1882	15.06265	16.30205	17.27964	16.91618	15.24643	16.60565	16.21478	16.25608	0.815707		1	-
ENSMUSC	Ring1	ring finge	GO:00325	developm	-	-	-	-	17:340207	410	573	356	634	749	456	5.050191	5.636697	4.846424	6.024818	5.941018	4.418755	5.177771	5.46153	0.808693		1	-
ENSMUSC	Slc39a7	solute car	GO:00511	localizatio	-	-	-	-	17:340282	797	1038	748	1084	1382	952	7.474015	7.773899	7.752538	7.842512	8.345618	7.023339	7.666817	7.737156	0.891521		1	-
ENSMUSC	Col11a2	collagen, t	GO:00325	developm	K19721	collagen, t	mmu0497	Protein di	17:340394	399	563	392	654	690	571	3.111789	3.506642	3.378854	3.935001	3.465302	3.503355	3.332428	3.634553	0.569193		1	-
ENSMUSC	H2-Oa	histocomp	GO:00325	developm	K06752	major hist	mmu0516	HTLV-I inf	17:340923	4	7	3	5	6	13	0.111886	0.156364	0.092744	0.107885	0.108063	0.286041	0.120331	0.16733	0.600368		1	-
ENSMUSC	Brd2	bromodo	GO:00081	metabolic	-	-	-	-	17:341120	3024	3602	2946	3893	5119	4234	15.31263	14.56658	16.48723	15.20838	16.69198	16.86669	15.45548	16.25568	0.767787		1	-
ENSMUSC	Pfdn1	prefoldin	GO:00329	macromo	-	-	-	-	18:364036	466	619	462	705	735	677	26.41882	28.02618	28.94788	30.83524	26.83302	30.19445	27.79763	29.28757	0.795533		1	-
ENSMUSC	Psd2	pleckstrin	GO:00987	molecular	K12494	PH and SE	mmu0414	Endocytos	18:359648	671	1047	565	956	1010	929	3.3527	4.177962	3.120094	3.685196	3.249736	3.65173	3.550252	3.528887	0.784397		1	-
ENSMUSC	Tmem173	transmem	GO:00081	metabolic	K12654	transmem	mmu0462	NOD-like	18:357336	52	57	50	66	80	61	1.379246	1.207413	1.465744	1.350554	1.366417	1.272852	1.350801	1.329941	0.841329		1	-
ENSMUSC	Dnajc18	DnaJ heat	GO:00160	membran	-	-	-	-	18:356711	2150	2703	1840	2762	3111	2615	25.39659	25.49933	24.02157	25.17044	23.66418	24.30073	24.9725	24.37845	0.577808		1	-
ENSMUSC	Spata24	spermato	GO:00325	developm	-	-	-	-	18:356566	25	42	33	54	59	57	1.470555	1.973081	2.145408	2.450601	2.234855	2.637743	1.863014	2.441066	0.206172		1	-
ENSMUSC	Slc23a1	solute car	GO:00325	developm	K14611	solute car	mmu0497	Vitamin d	18:356146	2	5	1	0	0	3	0.029132	0.058173	0.016103	0	0	0.034379	0.034469	0.01146	0.205002		1	-
ENSMUSC	Sil1	endoplasr	GO:00511	localizatio	K14001	nucleotide	mmu0414	Protein pr	18:352664	219	288	237	365	381	314	7.888909	8.28536	9.435587	10.14372	8.837976	8.89845	8.536619	9.293383	0.624818		1	-
ENSMUSC	Hspa9	heat shoc	GO:00325	developm	K04043	molecular	mmu0515	Tuberculo	18:349374	4518	5516	3894	5773	6714	5772	55.08398	53.70926	52.47129	54.30138	52.71267	55.36257	53.75484	54.12554	0.831803		1	-
ENSMUSC	Etf1	eukaryotic	GO:00081	metabolic	K03265	peptide ch	mmu0301	mRNA sur	18:349027	1990	2647	1978	2639	3394	2567	31.83276	33.8159	34.96989	32.56799	34.96131	32.30412	33.53952	33.27781	0.722395		1	-
ENSMUSC	Nelfe	negative e	GO:00081	metabolic	-	-	-	-	17:348503	226	337	220	266	309	337	3.404667	4.054549	3.662993	3.091568	2.997641	3.99399	3.707403	3.361066	0.34301		1	-
ENSMUSC	Cdc23	CDC23 ce	GO:00081	metabolic	K03355	anaphase	mmu0516	HTLV-I inf	18:346309	828	1047	803	1123	1252	1054	4.383222	4.426452	4.69814	4.586417	4.26798	4.389499	4.502605	4.414632	0.655527		1	-
ENSMUSC	Epb41l4a	erythrocyt	GO:00051	structural	-	-	-	-	18:337963	108	121	95	129	148	145	1.71147	1.531358	1.66386	1.577124	1.510289	1.80769	1.635563	1.631701	0.865369		1	-
ENSMUSC	Tslp	thymic str	GO:00508	response	K05436	thymic str	mmu0406	Cytokine-	18:328153	3	0	0	4	9	0	0.161668	0	0	0.166285	0.312331	0	0.053889	0.159539	0.266305		1	-
ENSMUSC	Bin1	bridging i	GO:00325	developm	K12562	amphiphy	mmu0414	Endocytos	18:323772	3831	5103	3803	5569	6241	5306	95.436	101.5246	104.7064	107.0305	100.1172	103.9868	100.5557	103.7115	0.93353		1	-
ENSMUSC	Ercc3	excision re	GO:00325	developm	K10843	DNA excis	mmu0342	Nucleotid	18:322403	604	738	558	746	916	806	10.06252	9.819094	10.27425	9.588232	9.826964	10.56368	10.05195	9.992959	0.767736		1	-
ENSMUSC	Map3k2	mitogen-a	GO:00048	signal trar	K04420	mitogen-a	mmu0401	MAPK sign	18:321630	860	1054	746	1080	1219	1032	4.866092	4.762861	4.665165	4.714502	4.441605	4.593801	4.764706	4.583303	0.518345		1	-
ENSMUSC	Iws1	IWS1, SUF	GO:00081	metabolic	-	-	-	-	18:320677	766	767	683	982	971	974	3.121573	2.496237	3.076184	3.087354	2.548112	3.122585	2.897998	2.91935	0.874692		1	-
ENSMUSC	Proc	protein C	GO:00325	developm	K01344	protein C	mmu0461	Complem	18:321231	0	0	0	0	2	0	0	0	0	0	0.050205	0	0	0.016735	0.528958		1	-
ENSMUSC	Csnk2b	casein kin	GO:00325	developm	K03115	casein kin	mmu0516	Epstein-B	17:351161	1005	1273	1035	1543	1384	1506	15.78683	15.96993	17.96867	18.69929	13.99973	18.61079	16.57514	17.10327	0.952934		1	-
ENSMUSC	Apom	apolipopr	GO:00081	metabolic	-	-	-	-	17:351289	3	0	0	0	1	0	0.180819	0	0	0	0.038806	0	0.060273	0.012935	0.66165		1	-
ENSMUSC	Bag6	BCL2-ass	GO:00325	developm	-	-	-	-	17:351351	2801	3472	2691	3683	4045	3610	15.18052	15.02793	16.11885	15.39946	14.11714	15.39188	15.44244	14.9695	0.526681		1	-
ENSMUSC	Prrc2a	proline-ri	GO:00325	developm	-	-	-	-	17:351490	9123	11476	8122	11696	13120	11659	60.17426	60.45183	59.20834	59.51696	55.72648	60.49859	59.94481	58.58068	0.566925		1	-
ENSMUSC	Aif1	allograft i	GO:00081	metabolic	-	-	-	-	17:351709	37	73	54	66	69	62	0.704891	1.110668	1.136981	0.970039	0.84649	0.92922	0.98418	0.91525	0.622242		1	-
ENSMUSC	Ltb	lymphoto	GO:00325	developm	K03157	lymphoto	mmu0406	Cytokine-	17:351944	3	0	5	5	12	4	0.052605	0	0.096908	0.067637	0.135509	0.055177	0.049838	0.086108	0.310704		1	-
ENSMUSC	Wdr33	WD repea	GO:00081	metabolic	K15542	polyadeny	mmu0301	mRNA sur	18:318040	947	1301	951	1447	1676	1419	9.01638	9.892481	10.00713	10.62872	10.27569	10.62859	9.638664	10.511	0.501554		1	-
ENSMUSC	Tnf	tumor nec	GO:00325	developm	K03156	tumor nec	mmu0516	Human pa	17:351993	0	3	0	0	8	1	0	0.048034	0	0	0.10327	0.015773	0.016011	0.039681	0.486244		1	-
ENSMUSC	Lta	lymphoto	GO:00325	developm	K05468	lymphoto	mmu0516	HTLV-I inf	17:352031	0	2	0	0	0	0	0	0.070528	0	0	0	0	0.023509	0	0.524735		1	-
ENSMUSC	Riok3	RIO kinas	GO:00081	metabolic	-	-	-	-	18:121288	853	1053	784	1148	1338	1120	14.22633	14.02549	14.45129	14.77121	14.36992	14.6951	14.23437	14.61208	0.985308		1	-
ENSMUSC	Psors1c2	psoriasis s	-	-	-	-	-	-	17:355332	0	0	0	1	1	0	0	0	0	0.055886	0.046631	0	0	0.034173	0.521851		1	-
ENSMUSC	3110002H	RIKEN cD	GO:00081	metabolic	-	-	-	-	18:121687	479	611	446	686	688	702	3.745751	3.815841	3.854654	4.138643	3.464548	4.318683	3.805415	3.973958	0.868544		1	-
ENSMUSC	Aqp4	aquaporir	GO:00511	localizatio	K09866	aquaporir	mmu0497	Bile secret	18:153893	2976	4564	2830	4104	5010	4059	35.7554	43.79254	37.57871	38.04051	38.76154	38.3653	39.04222	38.38912	0.654798		1	-
ENSMUSC	Npc1	NPC intra	GO:00081	metabolic	K12385	Niemann-	mmu0414	Lysosome	18:121896	1262	1747	1168	1714	2048	1872	4.918637	5.437808	5.031239	5.153786	5.140079	5.73987	5.129228	5.344578	0.854219		1	-
ENSMUSC	Mrpl27	mitochon	GO:00081	metabolic	K02899	large subu	mmu0301	Ribosome	11:946537	158	159	146	190	245	108	5.528462	4.443151	5.646083	5.128983	5.520383	2.972916	5.205899	4.540761	0.371811		1	-
ENSMUSC	Zfp521	zinc finge	GO:00325	developm	-	-	-	-	18:136870	178	255	189	265	273	250	1.766635	2.021214	2.073171	2.029098	1.744797	1.951989	1.953674	1.908628	0.713925		1	-
ENSMUSC	Dhx16	DEAH (As	GO:00081	metabolic	K12813	pre-mRN	mmu0304	Spliceoso	17:358798	421	540	396	632	723	600	3.95347	4.049821	4.109966	4.578714	4.372095	4.432598	4.037752	4.461136	0.478443		1	-

ENSMUSC	Impact	impact, R\	GO:00325	developm	-	-	-	-	18:129722	3732	4134	2787	4433	5045	4004	66.39535	58.73708	54.79991	60.8449	57.79789	56.04038	59.97745	58.22772	0.554587	1	-
ENSMUSC	Ttc39c	tetratricop	-	-	-	-	-	-	18:125999	418	489	410	564	645	615	4.279391	3.998162	4.639126	4.454669	4.252262	4.953264	4.30556	4.553398	0.782595	1	-
ENSMUSC	Ndfip1	Nedd4 fa	GO:00325	developm	-	-	-	-	18:384189	3481	4595	3200	4646	5561	4522	228.5412	240.9305	232.1973	235.326	235.1083	233.5618	233.8896	234.6654	0.804522	1	-
ENSMUSC	Spry4	sprouty h	GO:00325	developm	-	-	-	-	18:385862	341	369	233	338	393	355	4.539095	3.922714	3.427812	3.471054	3.368697	3.717526	3.963207	3.519092	0.247124	1	-
ENSMUSC	Gnl1	guanine n	GO:00508	response	-	-	-	-	17:359798	2588	3272	2265	3564	4038	3207	47.52432	47.98559	45.96917	50.49166	47.74992	46.32992	47.15969	48.1905	0.979614	1	-
ENSMUSC	Cabyr	calcium-b	GO:00325	developm	-	-	-	-	18:127413	20	14	8	12	16	9	0.415931	0.232522	0.183865	0.192524	0.214265	0.147241	0.277439	0.184677	0.183882	1	-
ENSMUSC	Nr3c1	nuclear re	GO:00048	signal trar	K05771	glucocorti	mmu0408	Neuroacti	18:394105	1772	1631	1498	2174	2308	2152	6.462873	4.750745	6.03838	6.117189	5.420663	6.174689	5.750666	5.90418	0.994183	1	-
ENSMUSC	Mrps18b	mitochon	GO:00081	metabolic	K16174	small subı	mmu0520	Viral carci	17:359103	172	255	208	279	318	190	2.982675	3.531528	3.986462	3.732601	3.551068	2.59204	3.500222	3.291903	0.562978	1	-
ENSMUSC	Pcdh12	protocadh	GO:00325	developm	-	-	-	-	18:382670	13	12	5	18	17	10	0.07873	0.058039	0.033469	0.084103	0.0663	0.047644	0.056746	0.066015	0.723584	1	-
ENSMUSC	0610009C	RIKEN cDI	GO:00081	metabolic	-	-	-	-	18:382502	431	603	474	671	736	682	10.94677	12.23129	13.30559	13.14806	12.03764	13.62712	12.16122	12.9376	0.730054	1	-
ENSMUSC	Rpp21	ribonuclea	GO:00081	metabolic	K03540	ribonuclea	mmu0301	RNA trans	17:362556	134	158	128	198	205	149	3.60432	3.394065	3.805162	4.108777	3.550794	3.152932	3.601183	3.604168	0.882639	1	-
ENSMUSC	Arap3	ArfGAP w	GO:00325	developm	K12490	Arf-GAP v	mmu0414	Endocytos	18:379726	204	243	139	259	299	226	2.384351	2.268255	1.795573	2.335449	2.250432	2.078067	2.149393	2.221316	0.955235	1	-
ENSMUSC	Hdac3	histone de	GO:00081	metabolic	K11404	histone de	mmu0516	Human pa	18:379358	904	1157	1101	1214	1373	1710	7.967166	8.143568	10.72431	8.254383	7.792212	11.85612	8.945016	9.300904	0.914313	1	-
ENSMUSC	Diaph1	diaphano	GO:00325	developm	K05740	diaphanoı	mmu0481	Regulatior	18:378436	1507	1995	1371	2095	2367	2031	7.323669	7.742909	7.363761	7.854702	7.407442	7.764908	7.476779	7.675684	0.981522	1	-
ENSMUSC	Trim26	tripartite r	GO:00081	metabolic	-	-	-	-	17:368371	397	510	257	663	393	617	2.155245	2.211174	1.54201	2.776837	1.373896	2.635134	1.969476	2.261956	0.511804	1	-
ENSMUSC	H2-M5	histocomp	GO:00023	immune s	K06751	major hist	mmu0516	Human pa	17:369840	18	21	13	17	17	16	0.293385	0.273366	0.234186	0.213778	0.178439	0.205168	0.266979	0.199128	0.239253	1	-
ENSMUSC	Gabbr1	gamma-a	GO:00048	signal trar	K04615	gamma-a	mmu0408	Neuroacti	17:370459	13976	17838	11317	17666	21218	16173	64.7506	66.00136	57.94808	63.14361	63.30238	58.94709	62.90001	61.79769	0.63108	1	-
ENSMUSC	Dcp2	decapping	GO:00081	metabolic	K12613	mRNA-de	mmu0301	RNA degr	18:443805	1771	2107	1460	2018	2455	1941	11.99621	11.3982	10.93013	10.54575	10.70858	10.34337	11.44152	10.53257	0.224305	1	-
ENSMUSC	Ik	IK cytokin	GO:00081	metabolic	-	-	-	-	18:367446	2594	3376	2167	3164	4128	3298	66.60419	69.22768	61.49463	62.67556	68.25365	66.61822	65.7755	65.84914	0.797906	1	-
ENSMUSC	Pggt1b	protein ge	GO:00081	metabolic	-	-	-	-	18:462399	173	214	128	211	231	191	3.860773	3.814061	3.157061	3.632781	3.319666	3.353296	3.610632	3.435248	0.577222	1	-
ENSMUSC	Mal2	mal, T cell	GO:00325	developm	-	-	-	-	15:545711	4653	5626	3866	5835	7569	5874	96.30623	92.99656	88.43619	93.17353	100.8822	95.64586	92.57966	96.56719	0.829281	1	-
ENSMUSC	Ap3s1	adaptor-r	GO:00511	localizatio	K12399	AP-3 com	mmu0414	Lysosome	18:467418	298	356	302	410	476	324	5.654231	5.394523	6.333033	6.001655	5.815921	4.836301	5.793929	5.551292	0.595181	1	-
ENSMUSC	Lvrn	laeverin [S	GO:00081	metabolic	-	-	-	-	18:468500	0	0	0	0	2	0	0	0	0	0	0.024424	0	0	0.008141	0.52864	1	-
ENSMUSC	Ankhd1	ankyrin re	GO:00023	immune s	-	-	-	-	18:365599	1497	1722	1409	2165	2391	1883	6.033266	5.54255	6.276079	6.731611	6.205332	5.970243	5.950632	6.302395	0.71995	1	-
ENSMUSC	Slc4a9	solute car	GO:00511	localizatio	-	-	-	-	18:365281	2	0	0	0	0	0	0.040388	0	0	0	0	0	0.013463	0	0.526245	1	-
ENSMUSC	Hbegf	heparin-b	GO:00325	developm	K08523	heparin-b	mmu0520	Proteoglyı	18:365049	171	271	175	238	305	251	4.399883	5.568789	4.97657	4.724472	5.053585	5.080775	4.981748	4.952944	0.8187	1	-
ENSMUSC	Yipf5	Yip1 dom	GO:00511	localizatio	-	-	-	-	18:402048	651	771	596	783	1006	720	17.06691	16.14262	17.269	15.83675	16.98347	14.84969	16.82617	15.88997	0.431547	1	-
ENSMUSC	Rbm27	RNA bind	GO:00081	metabolic	-	-	-	-	18:422753	1273	1676	1093	1605	1928	1542	12.08629	12.70822	11.46917	11.75629	11.78763	11.51755	12.08789	11.68716	0.529055	1	-
ENSMUSC	Lars	leucyl-tRN	GO:00081	metabolic	K01869	leucyl-tRN	mmu0097	Aminoacy	18:422023	693	755	582	875	1033	854	10.97336	9.547711	10.18536	10.68918	10.53322	10.63837	10.23548	10.62026	0.905237	1	-
ENSMUSC	Tcerg1	transcripti	GO:00081	metabolic	K12824	transcripti	mmu0304	Spliceosoı	18:425115	1855	2494	1616	2311	2932	2274	24.43115	26.23265	23.52274	23.48174	24.86673	23.56141	24.72885	23.96996	0.53474	1	-
ENSMUSC	Ppp2r2b	protein pl	GO:00325	developm	K04354	serine/thr	mmu0415	PI3K-Akt s	18:426374	1107	2002	1484	1956	1515	1970	11.88314	17.16302	17.60615	16.1988	10.47252	16.63646	15.55077	14.43593	0.459839	1	-
ENSMUSC	Dpysl3	dihydropy	GO:00325	developm	-	-	-	-	18:433209	1123	1430	971	1447	1604	1404	9.144869	9.299929	8.739043	9.090688	8.411178	8.994479	9.06128	8.832115	0.593176	1	-
ENSMUSC	Jakmip2	janus kina	GO:00056	cell|intrace	-	-	-	-	18:435314	1479	1717	1243	1808	2115	1886	25.72058	23.84672	23.89079	24.25723	23.68522	25.8027	24.48603	24.58172	0.820736	1	-
ENSMUSC	Dtwd2	DTW dor	GO:00511	localizatio	-	-	-	-	18:496961	60	71	48	82	100	63	0.587563	0.555277	0.519514	0.619515	0.63061	0.485356	0.554118	0.578494	0.930338	1	-
ENSMUSC	Hsd17b4	hydroxyst	GO:00325	developm	K12405	3-hydroxy	mmu0110	Metabolic	18:501282	1149	1504	1017	1417	1767	1491	17.90144	18.71379	17.512	17.0321	17.72798	18.27497	18.04241	17.67835	0.632507	1	-
ENSMUSC	Rab27b	RAB27B, r	GO:00452	synapse|lc	K07886	Ras-relate	mmu0497	Pancreatic	18:699791	975	606	880	1314	1493	1182	4.751888	2.358739	4.740137	4.940686	4.685717	4.532001	3.950255	4.719468	0.327815	1	-
ENSMUSC	Dynap	dynactin a	GO:00160	membran	-	-	-	-	18:702404	0	0	2	0	0	1	0	0	0.110003	0	0	0.039156	0.036668	0.013052	1	1	-
ENSMUSC	Mbd2	methyl-C	GO:00325	developm	-	-	-	-	18:705681	818	950	775	1030	1387	1089	11.50554	10.67144	12.04764	11.17689	12.56275	12.05015	11.40821	11.92993	0.838437	1	-
ENSMUSC	Smad4	SMAD far	GO:00325	developm	K04501	mothers a	mmu0520	Pathways	18:736390	707	901	646	737	1192	956	7.557439	7.691764	7.631929	6.07789	8.205145	8.039406	7.627044	7.440814	0.663554	1	-
ENSMUSC	Sec11c	SEC11 ho	GO:00081	metabolic	K13280	signal pep	mmu0306	Protein ex	18:658005	596	792	532	787	1018	800	7.72953	8.203097	7.625448	7.874296	8.501771	8.162207	7.852692	8.179425	0.867873	1	-
ENSMUSC	Gnal	guanine n	GO:00048	signal trar	K04633	guanine n	mmu0474	Olfactory	18:670883	2341	2864	2209	3025	3723	3082	24.81115	24.24177	25.87545	24.73439	25.40933	25.69739	24.97612	25.28037	0.8906	1	-
ENSMUSC	Impa2	inositol (m	GO:00081	metabolic	K01092	myo-inos	mmu0110	Metabolic	18:672892	58	51	37	53	72	49	2.132068	1.497226	1.503199	1.503078	1.704354	1.417022	1.710831	1.541484	0.517252	1	-
ENSMUSC	Cidea	cell death	GO:00081	metabolic	-	-	-	-	18:673435	116	152	90	138	184	143	6.084818	6.367659	5.217713	5.584687	6.215317	5.901152	5.890064	5.900385	0.88895	1	-
ENSMUSC	Afg3l2	AFG3-like	GO:00325	developm	-	-	-	-	18:674047	1267	1660	1108	1725	2167	1772	25.07638	26.23869	24.23677	26.3395	27.6186	27.5907	25.18395	27.18293	0.56402	1	-
ENSMUSC	Srfbp1	serum res	GO:00081	metabolic	-	-	-	-	18:524656	278	342	275	381	516	329	11.60228	11.3991	12.68463	12.26744	13.86767	10.80204	11.89534	12.31238	0.928916	1	-
ENSMUSC	Prelid3a	PRELI don	GO:00511	localizatio	-	-	-	-	18:674648	344	413	290	384	508	478	9.692662	9.293511	9.030852	8.3473	9.217277	10.59554	9.339009	9.386707	0.87277	1	-
ENSMUSC	Spire1	spire hom	GO:00325	developm	-	-	-	-	18:674882	3778	4631	3300	4572	5696	4557	14.17983	13.88129	13.68894	13.23871	13.76683	13.45548	13.91669	13.48701	0.505332	1	-
ENSMUSC	Sncaip	synuclein,	GO:00081	metabolic	K04558	synuclein,	mmu0501	Parkinson	18:527677	110	157	123	169	193	148	0.762966	0.869676	0.9429	0.904335	0.862036	0.807578	0.858514	0.857983	0.884608	1	-
ENSMUSC	Snx24	sorting ne	GO:00511	localizatio	-	-	-	-	18:532456	118	175	127	197	254	179	2.891204	3.424368	3.439098	3.723841	4.0076	3.450316	3.251557	3.727252	0.411212	1	-
ENSMUSC	Psmg2	proteasor	GO:00329	macromo	-	-	-	-	18:676415	92	148	109	193	208	153	5.304409	6.814847	6.945799	8.584902	7.722637	6.939834	6.355019	7.749124	0.241138	1	-
ENSMUSC	Ppic	peptidylpı	GO:00081	metabolic	-	-	-	-	18:534063	23	58	31	41	65	36	1.092024	2.19925	1.626718	1.501799	1.987341	1.34467	1.63933	1.61127	0.883155	1	-
ENSMUSC	Ptpn2	protein ty	GO:00325	developm	K18026	tyrosine-p	mmu0463	Jak-STAT	18:676655	430	594	349	578	652	494	6.485911	7.155419	5.818013	6.726068	6.33294	5.861933	6.486448	6.30698	0.652403	1	-
ENSMUSC	Cep192	centroson	GO:00081	metabolic	-	-	-	-	18:678001	237	226	175	266	334	263	0.934201	0.711454	0.762389	0.808917	0.847798	0.815566	0.802681	0.824094	1	1	-
ENSMUSC	Ldlrad4	low densi	GO:00325	developm	-	-	-	-	18:679332	425	556	341	551	664	519	10.40903	10.87531	9.23044	10.41125	10.47234	9.999985	10.17159	10.29452	0.917888	1	-
ENSMUSC	Setbp1	SET bindir	GO:00056	cell|intrace	-	-	-	-	18:787503	1462	1862	1123	1652	1945	1695	8.96885	9.12252	7.614054	7.818612	7.683578	8.180313	8.568475	7.894168	0.260784	1	-
ENSMUSC	Slc14a2	solute car	GO:00511	localizatio	-	-	-	-	18:781469	8	9	13	16	12	12	0.144696	0.130001	0.259853	0.223252	0.139764	0.170744	0.178183	0.17792	1	1	-
ENSMUSC	Galr1	galanin re	GO:00048	signal trar	K04230	galanin re	mmu0408	Neuroacti	18:823924	16	8	5	13	13	9	0.340274	0.135881	0.117532	0.213293	0.17804	0.150575	0.197895	0.180636	0.785313	1	-
ENSMUSC	Me2	malic enz	GO:00081	metabolic	-	-	mmu0120	Carbon m	18:737700	354	503	360	537	750	556	8.804312	9.990927	9.895589	10.30377	12.01179	10.87872	9.563609	11.06476	0.278035	1	-
ENSMUSC	Mapk4	mitogen-	GO:00048	signal trar	K06855	mitogen-a	mmu0465	IL-17 sign	18:739284	892	1279	811	655	1541	1428	7.289059	8.346853	7.324435	4.129314	8.108934	9.180061	7.653449	7.139436	0.559094	1	-
ENSMUSC	Cxxc1	CXXC fing	GO:00081	metabolic	-	-	-	-	18:742161	799	1072	737	1116	1190	1052	18.63461	19.96707	18.99713	20.08021	17.87209	19.30191	19.1996	19.08474	0.761199	1	-
ENSMUSC	Mbd1	methyl-C	GO:00056	cell|intrace	-	-	-	-	18:742676	864	1145	821	1216	1494	1193	6.299759	6.667482	6.616071	6.840285	7.014805	6.84324	6.527771	6.899443	0.740786	1	-
ENSMUSC	Smad2	SMAD far	GO:00325	developm	K04500	mothers a	mmu0520	Pathways	18:762415	253	287	258	332	405	299	2.942981	2.666215	3.316913	2.979443	3.033723	2.736208	2.97537	2.916458	0.73384	1	-
ENSMUSC	Sall3	sal-like 3	GO:00325	developm	-	-	-	-	18:809663	100	126	93	126	146	158	0.886698	0.892263	0.911397	0.861941	0.833651	1.102157	0.896786	0.932583	0.941516	1	-
ENSMUSC	Atp9b	ATPase, cl	GO:00511	localizatio	-	-	-	-	18:807341	988	1186	806	1281	1265	1077	3.87546	3.715324	3.494202	3.876555	3.195299	3.323478	3.694995	3.465111	0.381818	1	-
ENSMUSC	Rbfa	ribosome	GO:00081	metabolic	-	-	-	-	18:801922	391	489	348	561	753	555	17.33747	17.31666	17.05434	19.19122	21.50102	19.36035	17.23616	20.01753	0.254127	1	-
ENSMUSC	Pde6a	phosphoc	GO:00081	metabolic	K08718	rod cGMP	mmu0023	Purine me	18:612204	0	2	2	7	0	6	0	0.021575	0.029855	0.072935	0	0.06375	0.017143	0.045562	0.254821	1	-
ENSMUSC	Csnk1a1	casein kin	GO:00325	developm	K08957	casein kin	mmu0516	Human pa	18:615552	5595	6743	4878	7124	8262	6219	20.95572	20.16979	20.19254	20.58528	19.92702	18.32458	20.43935	19.61229	0.4429	1	-
ENSMUSC	Pcyox1l	prenylcyst	GO:00081	metabolic	-	-	-	-	18:616968	377	393	311	506	506	413	7.544716	6.281147	6.878733	7.812348	6.520885	6.502232	6.901532	6.945155	0.885584	1	-
ENSMUSC	Grpel2	GrpE-like	GO:00511	localizatio	-	-	-	-	18:617124	291	372	280	437	530	456	4.408908	4.501191	4.688599	5.107992	5.170934	5.435178	4.532899	5.238035	0.284797	1	-
ENSMUSC	Napg	N-ethylm	GO:00511	localizatio	-	-	-	-	18:629778	3458	4241	3046	4740	5386	4520	33.23453	32.55207	32.35498	35.14578	33.33386	34.17541	32.71386	34.21835	0.803401	1	-
ENSMUSC	Txnl1	thioredox	GO:00081	metabolic	-	-	-	-	18:636628	941	1256	940	1326	1512	1256	22.46118	23.94299	24.79804	24.41836	23.2407	23.58541	23.73407	23.74816	0.805752	1	-
ENSMUSC	Nars	asparagin	GO:00081	metabolic	K01893	asparagin	mmu0097	Aminoacy	18:644996	3999	5162	3525	5325	6540	4867	92.77028	95.63597	90.37814	95.30319	97.69895	88.82388	92.92813	93.942	0.880673	1	-
ENSMUSC	Fech	ferrochela	GO:00081	metabolic	K01772	ferrochela	mmu0110	Metabolic	18:644565	1096	1526	1137	1561	1912	1666	23.0678	25.65051	26.44861	25.34714	25.91422	27.58555	25.05564	26.2823	0.798217	1	-
ENSMUSC	Nedd4l	neural pre	GO:00325	developm	K13305	E3 ubiquit	mmu0414	Endocytos	18:648877	2810	4370	3922	4782	6158	5143	8.107229	10.06916	12.50606	10.64403	11.44091	11.6733	10.22748	11.25274	0.498247	1	-
ENSMUSC	C330018D	RIKEN cDI	GO:00081	metabolic	-	-	-	-	18:569558	124	124	113	128	190	142	2.125553	1.697525	2.140787	1.692742	2.097285	1.914906	1.987955	1.901645	0.667669	1	-
ENSMUSC	Megf10	multiple E	GO:00325	developm	-	-	-	-	18:571330	554	704	501	650	823	688	4.293753	4.357582	4.291527	3.886611	4.107542	4.194949	4.314288	4.063034	0.415374	1	-
ENSMUSC	Prrc1	proline-ri	GO:00056	cell|intrace	-	-	-	-	18:573547	333	435	282	419	558	419	4.235021	4.418212	3.963764	4.111078	4.569837	4.192143	4.205666	4.291019	0.980074	1	-
ENSMUSC	Slc12a2	solute car	GO:00325	developm	K10951	solute car	mmu0497	Pancreatic	18:578786	1207	2291	1207	1747	2567	1970	11.30325	17.13431	12.49251	12.62173	15.48019	14.51353	13.64336	14.20515	0.875418	1	-
ENSMUSC	Fbn2	fibrillin 2 [	GO:00325	developm	-	-	-	-	18:580086	142	167	119	192	210	246	0.827313	0.777043	0.76626	0.863006	0.787871	1.127529	0.790205	0.926135	0.361667	1	-
ENSMUSC	Slc27a6	solute car	GO:00081	metabolic	K08749	solute car	mmu0493	Insulin res	18:585562	0	3	4	1	6	6	0	0.058878	0.108631	0.018959	0.09493	0.115982	0.055836	0.076624	0.712743	1	-
ENSMUSC	Isoc1	isochorisn	GO:00081	metabolic	-	-	-	-	18:586594	214	237	217	309	379	269	5.098103	4.509088	5.71348	5.67915	5.814182	5.041485	5.10689	5.511606	0.679202	1	-
ENSMUSC	Dctn4	dynactin 4	GO:00511	localizatio	K10426	dynactin 4	mmu0501	Huntingto	18:605261	1645	2332	1740	2421	2818	2430	7.085254	8.021656	8.282962	8.044791	7.816015	8.233923	7.796624	8.031576	0.947999	1	-
ENSMUSC	Rps14	ribosomal	GO:00325	developm	K02955	small subı	mmu0301	Ribosome	18:607745	542	619	617	800	924	730	16.35055	14.91314	20.57144	18.61887	17.94979	17.32474	17.27838	17.96447	0.895777	1	-
ENSMUSC	Tcof1	treacle rib	GO:00325	developm	K14562	Treacher (	mmu0300	Ribosome	18:608137	473	551	391	612	764	584	4.102341	3.816528	3.747949	4.094985	4.26696	3.984687	3.88894	4.115544	0.76523	1	-
ENSMUSC	Tmx3	thioredox	GO:00081	metabolic	-	-	-	-	18:905101	1218	1493	1030	1461	1850	1408	16.12856	15.78897	15.07413	14.92549	15.77519	14.66769	15.66389	15.12279	0.527627	1	-
ENSMUSC	Camk2a	calcium/ca	GO:00325	developm	K04515	calcium/ca	mmu0474	Olfactory	18:609256	45502	49121	42255	55937	64058	53721	248.0816	213.8834	254.6179	235.2847	224.9014	230.4194	238.861	230.2018	0.481025	1	-
ENSMUSC	Csf1r	colony sti	GO:00325	developm	K05090	macropha	mmu0520	Pathways	18:611055	1231	1801	1296	1686	2103	1823	12.81981	14.979	14.91678	13.54601	14.10319	14.93554	14.23853	14.19492	0.7761	1	-
ENSMUSC	Hmgxb3	HMG box	GO:00056	cell|intrace	-	-	-	-	18:611312	902	1063	765	1091	1223	1107	10.79679	10.16172	10.12037	10.07495	9.426905	10.4243	10.35962	9.975384	0.526337	1	-
ENSMUSC	Gnaq	guanine n	GO:00325	developm	K04634	guanine n	mmu0520	Pathways	19:161328	6005	7389	5118	6919	8890	6642	55.16082	54.20622	51.95948	49.03332	52.58648	47.99848	53.77551	49.87276	0.238644	1	-
ENSMUSC	Psat1	phosphos	GO:00081	metabolic	K00831	phosphos	mmu0110	Metabolic	19:159046	1424	1986	1445	1958	2393	2142	12.49594	13.91823	14.01437	13.25568	13.52248	14.78731	13.47618	13.85516	0.967747	1	-
ENSMUSC	Tle4	transducir	GO:00081	metabolic	-	-	-	-	19:144480	932	1230	830	1209	1441	1280	8.19029	8.63246	8.061365	8.19672	8.154591	8.849209	8.294705	8.400173	0.904724	1	-
ENSMUSC	Cndp2	CNDP dip	GO:00081	metabolic	K08660	cytosolic r	mmu0110	Metabolic	18:846674	639	850	665	840	1114	878	17.66236	18.76343	20.31497	17.91254	19.82839	19.09207	18.91358	18.94433	0.833786	1	-
ENSMUSC	Timm21	translocas	GO:00511	localizatio	-	-	-	-	18:849472	92	132	80	140	185	129	4.271764	4.894827	4.105411	5.015057	5.531513	4.712144	4.424001	5.086238	0.430364	1	-
ENSMUSC	Cyb5a	cytochron	GO:00081	metabolic	-	-	-	-	18:848513	317	373	262	513	555	375	5.607013	5.268995	5.121772	7.000365	6.3215	5.218128	5.332594	6.179997	0.317229	1	-
ENSMUSC	Cbln2	cerebellin	GO:00325	developm	-	-	-	-	18:867111	133	128	53	173	142	141	1.347392	1.035617	0.593419	1.352134	0.926372	1.12376	0.992143	1.134088	0.599731	1	-
ENSMUSC	Slc22a6	solute car	GO:00511	localizatio	-	-	-	-	19:861799	69	129	36	110	116	146	1.046455	1.562441	0.603426	1.28704	1.132871	1.741946	1.070774	1.387286	0.296921	1	-
ENSMUSC	Asrgl1	asparagin	GO:00081	metabolic	-	-	-	-	19:910986	4256	5056	3533	5294	6098	4778	57.55556	54.60577	52.80511	55.23316	53.10406	50.83264	54.98881	53.05662	0.482873	1	-
ENSMUSC	Anxa1	annexin A	GO:00325	developm	-	-	-	-	19:203734	30	60	23	42	88	60	0.486652	0.777305	0.412344	0.525618	0.919249	0.765691	0.558767	0.736853	0.276598	1	-
ENSMUSC	Incenp	inner cent	GO:00990	supramol	-	-	-	-	19:987229	242	307	263	348	445	335	4.593113	4.653473	5.516893	5.095679	5.43885	5.002053	4.921159	5.17886	0.822211	1	-
ENSMUSC	Fth1	ferritin he	GO:00081	metabolic	K00522	ferritin he	mmu0421	Necroptos	19:998270	24130	33987	22826	27212	42346	33121	1701.307	1913.745	1778.697	1480.186	1922.616	1837.13	1797.917	1746.644	0.575314	1	-
ENSMUSC	Rab3il1	RAB3A int	GO:00511	localizatio	-	-	-	-	19:100016	136	183	123	176	200	198	1.071886	1.151877	1.071428	1.070169	1.015065	1.227679	1.098397	1.104304	0.893829	1	-
ENSMUSC	Fads3	fatty acid	GO:00081	metabolic	-	-	-	-	19:100415	429	608	359	556	738	592	8.012833	9.069386	7.410885	8.011868	8.876456	8.698837	8.164368	8.529054	0.86369	1	-
ENSMUSC	Fads2	fatty acid	GO:00081	metabolic	K10226	acyl-CoA	mmu0332	PPAR sign	19:100641	2283	2759	2041	2860	3325	3023	92.43746	89.21533	91.33388	89.33845	86.69387	96.2922	90.99556	90.77484	0.757581	1	-
ENSMUSC	Tmem216	transmem	GO:00329	macromo	-	-	-	-	19:105338	40	48	22	48	48	49	0.709159	0.679626	0.431081	0.656519	0.547993	0.683413	0.606622	0.629308	1	1	-
ENSMUSC	Sdhaf2	succinate	GO:00325	developm	-	-	-	-	19:105005	532	615	470	726	884	701	10.4919	9.686423	10.24441	11.04611	11.22663	10.87606	10.14091	11.0496	0.554113	1	-
ENSMUSC	Cd6	CD6 antig	GO:00511	localizatio	K06456	T-cell surf	mmu0451	Cell adhes	19:107893	6	6	6	6	17	6	0.060164	0.048049	0.0665	0.046416	0.109777	0.047334	0.058238	0.067842	0.813784	1	-
ENSMUSC	Ms4a7	membran	GO:00160	membran	-	-	-	-	19:113210	8	25	11	11	24	7	0.087874	0.219295	0.133529	0.093216	0.169751	0.060489	0.146899	0.107819	0.361953	1	-
ENSMUSC	Ms4a4c	membran	GO:00160	membran	-	-	-	-	19:114047	5	2	5	8	3	0	0.132263	0.042262	0.146202	0.16328	0.051114	0	0.106909	0.071464	0.65636	1	-
ENSMUSC	Ms4a6b	membran	GO:00160	membran	-	-	-	-	19:115165	16	28	25	19	41	27	0.11892	0.166205	0.205361	0.108946	0.196236	0.157875	0.163495	0.154352	0.829593	1	-
ENSMUSC	Mrpl16	mitochon	GO:00081	metabolic	K02878	large subı	mmu0301	Ribosome	19:117704	194	210	173	264	358	287	10.55728	9.12673	10.40502	11.08369	12.54552	12.28692	10.02967	11.97204	0.225504	1	-
ENSMUSC	Osbp	oxysterol	GO:00511	localizatio	-	-	-	-	19:119658	1360	1731	1181	1877	2028	1757	18.13871	18.43786	17.40862	19.31355	17.41768	18.4353	17.99506	18.38884	0.978824	1	-
ENSMUSC	Fam111a	family wit	GO:00081	metabolic	-	-	-	-	19:125457	55	75	43	79	106	76	0.329914	0.359292	0.285071	0.36559	0.409447	0.358642	0.324759	0.377893	0.445155	1	-
ENSMUSC	Zfp91	zinc finge	GO:00081	metabolic	-	-	-	-	19:127636	3250	4070	2709	4000	5076	4034	18.55783	18.56026	17.0962	17.62114	18.66466	18.12135	18.07143	18.13572	0.813802	1	-
ENSMUSC	Lpxn	leupaxin [	GO:00081	metabolic	-	-	-	-	19:127986	9	23	21	31	24	26	0.328066	0.669594	0.846064	0.871813	0.563369	0.745614	0.614574	0.726932	0.643643	1	-
ENSMUSC	Pcsk5	proprotei	GO:00325	developm	-	-	-	-	19:174328	279	387	270	436	529	425	1.822728	2.019175	1.949524	2.197528	2.225503	2.184324	1.930476	2.202452	0.339951	1	-
ENSMUSC	Ostf1	osteoclast	GO:00055	extracellul	-	-	-	-	19:185793	347	487	334	505	593	434	3.792905	4.251253	4.034923	4.258577	4.173993	3.732016	4.02636	4.054862	0.88995	1	-
ENSMUSC	Carnmt1	carnosine	GO:00081	metabolic	K19787	carnosine	mmu0034	Histidine r	19:186707	62	85	58	93	113	91	1.811297	1.983176	1.872714	2.096083	2.125841	2.091452	1.889062	2.104459	0.605504	1	-
ENSMUSC	Ms4a10	membran	GO:00160	membran	-	-	-	-	19:109622	0	0	0	1	0	2	0	0	0	0.035002	0	0.071388	0	0.035463	0.277549	1	-
ENSMUSC	Ccdc86	coiled-co	GO:00056	cell|intrace	-	-	-	-	19:109414	151	198	159	210	252	162	1.296365	1.357571	1.508672	1.39092	1.393178	1.094148	1.387536	1.292749	0.508173	1	-
ENSMUSC	Prpf19	pre-mRN	GO:00325	developm	K10599	pre-mRNA	mmu0412	Ubiquitin	19:108952	2140	2781	2269	3188	3466	3233	13.22782	13.72844	15.50085	15.20278	13.79614	15.72139	14.15237	14.90677	0.761736	1	-
ENSMUSC	Tmem132	transmem	GO:00055	extracellul	-	-	-	-	19:108578	2221	2692	2097	3185	3516	2820	23.30471	22.55879	24.31869	25.78308	23.75738	23.2785	23.39406	24.27299	0.885157	1	-
ENSMUSC	Slc15a3	solute car	GO:00511	localizatio	-	-	-	-	19:108397	26	55	38	38	60	37	0.218095	0.368454	0.352287	0.245916	0.324101	0.244167	0.312945	0.271395	0.462906	1	-
ENSMUSC	Ddb1	damage s	GO:00081	metabolic	K10610	DNA dam	mmu0520	Viral carci	19:106056	3765	4648	3582	4796	5646	4986	55.32702	54.54868	58.17611	54.37281	53.42787	57.64153	56.01727	55.1474	0.634138	1	-
ENSMUSC	Fen1	flap struct	GO:00081	metabolic	K04799	flap endo	mmu0303	DNA repli	19:101991	154	214	150	215	156	219	1.342124	1.489476	1.444813	1.44558	0.875493	1.501507	1.425471	1.274194	0.390295	1	-
ENSMUSC	Syt7	synaptota	GO:00452	synapse|lc	-	-	-	-	19:103890	5077	2859	4880	6654	7781	5161	23.60225	10.61467	25.07338	23.86489	23.29359	18.87518	19.76343	22.01122	0.631125	1	-
ENSMUSC	Tmc1	transmem	GO:00325	developm	-	-	-	-	19:207834	0	0	0	0	0	2	0	0	0	0	0	0.023591	0	0.007864	0.526627	1	-
ENSMUSC	Zfand5	zinc finge	GO:00325	developm	-	-	-	-	19:212722	2068	2698	2047	2630	3418	3171	14.57389	15.18493	15.94371	14.29918	15.51142	17.58053	15.23418	15.79704	0.896164	1	-
ENSMUSC	Tmem2	transmem	GO:00325	developm	-	-	-	-	19:217783	340	438	281	399	500	459	3.113807	3.203562	2.844239	2.819137	2.94875	3.30702	3.053869	3.024969	0.770553	1	-
ENSMUSC	Rtn3	reticulon 3	GO:00511	localizatio	K20723	reticulon-	mmu0501	Alzheimer	19:742590	15242	18111	13352	19704	23399	18607	143.1325	135.8265	138.5765	142.7516	141.4974	137.4623	139.1785	140.5704	0.863574	1	-
ENSMUSC	Atl3	atlastin G	GO:00038	catalytic a	-	-	-	-	19:749404	682	839	596	730	1044	774	6.217029	6.108098	6.004703	5.133946	6.128487	5.550727	6.109943	5.604387	0.287387	1	-
ENSMUSC	Naa40	N(alpha)-	GO:00081	metabolic	-	-	-	-	19:722566	396	502	377	515	575	556	7.382908	7.474491	7.768187	7.407461	6.903261	8.154886	7.541862	7.488536	0.780206	1	-
ENSMUSC	Lipo3	lipase, me	GO:00081	metabolic	-	-	-	-	19:335177	72	99	84	79	154	104	0.806938	0.886109	1.040473	0.683065	1.111425	0.916962	0.911173	0.903818	0.887508	1	-
ENSMUSC	Otub1	OTU dom	GO:00081	metabolic	-	-	-	-	19:719820	3137	3327	2928	3947	4732	4088	48.52782	41.10312	50.06037	47.10572	47.13849	49.75057	46.56377	47.99826	0.952396	1	-
ENSMUSC	Lipn	lipase, fan	GO:00081	metabolic	-	-	-	-	19:340673	0	2	0	0	0	0	0	0.044116	0	0	0	0	0.014705	0	0.52446	1	-
ENSMUSC	Ehd1	EH-doma	GO:00325	developm	-	-	-	-	19:627672	1419	1847	1374	1916	2451	1865	16.91555	17.58395	18.10245	17.62096	18.81489	17.49015	17.53398	17.97533	0.990197	1	-
ENSMUSC	Atg2a	autophag	GO:00081	metabolic	-	-	-	-	19:624166	880	1191	849	1232	1408	1497	5.661866	6.119766	6.037142	6.115305	5.83357	7.577221	5.939592	6.508699	0.514818	1	-
ENSMUSC	Ankrd22	ankyrin re	-	-	-	-	-	-	19:341225	0	2	0	2	3	0	0	0.037719	0	0.036436	0.045619	0	0.012573	0.027352	0.713627	1	-
ENSMUSC	Stambpl1	STAM bin	GO:00081	metabolic	-	-	-	-	19:341922	300	358	287	453	456	398	2.829817	2.696912	2.992038	3.296603	2.769855	2.953468	2.839589	3.006642	0.783497	1	-
ENSMUSC	Ppp2r5b	protein pl	GO:00325	developm	-	-	-	-	19:622776	1653	2002	1524	2253	2717	2295	28.05143	27.13264	28.58341	29.49672	29.69111	30.63908	27.9225	29.9423	0.600628	1	-
ENSMUSC	Fas	Fas (TNF r	GO:00325	developm	K04390	tumor nec	mmu0520	Pathways	19:342906	25	45	18	36	60	25	0.847549	1.21839	0.674457	0.941588	1.3099	0.666773	0.913465	0.972754	0.901572	1	-
ENSMUSC	Cdc37l1	cell divisic	GO:00099	cellular pr	-	-	-	-	19:289903	2373	3331	1852	3272	3809	2778	8.367942	9.380832	7.217861	8.901527	8.649411	7.70662	8.322212	8.419186	0.904576	1	-
ENSMUSC	Lipa	lysosomal	GO:00325	developm	K01052	lysosomal	mmu0414	Lysosome	19:344923	327	537	355	434	540	448	6.495119	8.518436	7.79318	6.650577	6.906976	7.000492	7.602245	6.852682	0.279502	1	-
ENSMUSC	Ak3	adenylate	GO:00081	metabolic	K00944	nucleoside	mmu0023	Purine me	19:290208	1097	1460	1031	1569	1875	1593	20.21754	21.48922	21.00039	22.30874	22.25246	23.09663	20.90238	22.55261	0.569414	1	-
ENSMUSC	Rcl1	RNA term	GO:00081	metabolic	K11108	RNA 3'-te	mmu0300	Ribosome	19:291013	188	221	181	278	343	297	6.36657	5.977046	6.774444	7.263103	7.479902	7.912517	6.372687	7.551841	0.239233	1	-
ENSMUSC	Majin	membran	GO:00511	localizatio	-	-	-	-	19:618438	0	0	0	3	0	0	0	0	0	0.057547	0	0	0	0.019182	0.292945	1	-
ENSMUSC	Snx15	sorting ne	GO:00511	localizatio	-	-	-	-	19:611939	330	435	320	467	603	422	6.340203	6.674587	6.794943	6.922084	7.460385	6.378417	6.603245	6.920296	0.840515	1	-
ENSMUSC	Jak2	Janus kina	GO:00325	developm	K04447	Janus kina	mmu0520	Pathways	19:292518	963	1233	813	1263	1533	1280	11.68966	11.9532	10.90719	11.82794	11.98318	12.22352	11.51668	12.01155	0.850919	1	-
ENSMUSC	Sac3d1	SAC3 don	-	-	-	-	-	-	19:611600	75	120	90	141	136	121	3.332872	4.258771	4.420246	4.833997	3.891806	4.230106	4.003963	4.318636	0.755259	1	-
ENSMUSC	Cdca5	cell divisic	GO:00081	metabolic	K17390	sororin[-]	mmu0520	MicroRNA	19:608498	0	3	3	2	5	4	0	0.068338	0.094564	0.044009	0.091822	0.089737	0.0543	0.07519	0.827092	1	-
ENSMUSC	Zfpl1	zinc finge	GO:00511	localizatio	-	-	-	-	19:608076	290	367	271	375	472	418	4.157521	4.201913	4.293905	4.147602	4.357453	4.714366	4.21778	4.406474	0.869537	1	-
ENSMUSC	Tnfrsf25	tumor nec	GO:00325	developm	K05160	tumor nec	mmu0406	Cytokine-	4:1521159	194	152	144	223	244	190	3.217074	2.013024	2.639175	2.852956	2.605573	2.4787	2.623091	2.645743	0.925993	1	-
ENSMUSC	Kif20b	kinesin fa	GO:00325	developm	-	-	-	-	19:349223	29	47	22	40	84	51	0.195203	0.252658	0.163669	0.207722	0.364102	0.270065	0.203843	0.28063	0.184128	1	-
ENSMUSC	Vps51	VPS51 GA	GO:00081	metabolic	-	-	-	-	19:606784	689	900	655	899	1146	934	4.589681	4.787976	4.822274	4.620121	4.915898	4.894645	4.73331	4.810221	0.940789	1	-
ENSMUSC	Tm7sf2	transmem	GO:00081	metabolic	K00222	Delta14-s	mmu0110	Metabolic	19:606282	103	159	113	158	182	158	0.881179	1.086353	1.068444	1.042836	1.002658	1.063396	1.011992	1.036297	1	1	-
ENSMUSC	Rpp30	ribonuclea	GO:00081	metabolic	K03539	ribonuclea	mmu0301	RNA trans	19:360837	105	129	98	140	206	145	5.550758	5.446222	5.725754	5.709783	7.012652	6.030328	5.574245	6.250921	0.540841	1	-
ENSMUSC	Ankrd1	ankyrin re	GO:00325	developm	-	-	-	-	19:361119	2	1	0	0	0	0	0.069175	0.027632	0	0	0	0	0.032269	0	0.277085	1	-
ENSMUSC	Pcgf5	polycomb	GO:00081	metabolic	K11489	polycomb	mmu0455	Signaling	19:363483	99	278	220	252	110	293	0.407189	0.913175	1.000075	0.799639	0.291346	0.948073	0.77348	0.679686	0.562994	1	-
ENSMUSC	Syvn1	synovial a	GO:00081	metabolic	K10601	E3 ubiquit	mmu0414	Protein pr	19:604657	769	1008	727	1174	1258	1170	4.295463	4.496656	4.488124	5.0592	4.525	5.141385	4.426748	4.908528	0.423642	1	-
ENSMUSC	Tnks2	tankyrase	GO:00325	developm	-	-	-	-	19:368342	3778	4955	3287	4463	5620	4599	19.13066	20.03815	18.39563	17.43514	18.32564	18.32071	19.18815	18.02716	0.302766	1	-
ENSMUSC	Tjp2	tight junct	GO:00325	developm	K06098	tight junct	mmu0453	Tight junc	19:240945	638	901	566	901	1076	954	8.485094	9.569889	8.319545	9.244665	9.215162	9.981485	8.791509	9.480438	0.614835	1	-
ENSMUSC	Frmd8	FERM dor	GO:00056	cell|intrace	-	-	-	-	19:584970	194	301	177	285	326	252	2.012447	2.493646	2.029286	2.280855	2.177688	2.056528	2.17846	2.17169	0.832371	1	-
ENSMUSC	Uhrf2	ubiquitin-	GO:00325	developm	-	-	-	-	19:300305	1140	1387	949	1494	1915	1329	5.980431	5.810988	5.502257	6.046565	6.469208	5.484833	5.764558	6.000202	0.867777	1	-
ENSMUSC	Rad9a	RAD9 che	GO:00081	metabolic	-	-	-	-	19:419519	121	130	88	150	162	159	3.610973	3.098317	2.902465	3.453506	3.113199	3.732893	3.203919	3.4332	0.782192	1	-
ENSMUSC	Dpf2	D4, zinc a	GO:00081	metabolic	-	-	-	-	19:589651	758	929	713	1110	1176	1027	5.314277	5.201594	5.524737	6.003831	5.309297	5.664432	5.346869	5.659187	0.749172	1	-
ENSMUSC	Mrpl21	mitochon	GO:00081	metabolic	K02888	large subı	mmu0301	Ribosome	19:328290	264	330	195	328	422	292	5.337539	5.328392	4.357309	5.116125	5.494188	4.644403	5.007746	5.084905	0.950178	1	-

ENSMUSC	Rps6kb2	ribosomal	GO:00081	metabolic	-	-	-	-	19:415671	354	407	325	415	545	451	4.261551	3.912947	4.324086	3.854275	4.224889	4.271214	4.166194	4.116793	0.7595	1	-
ENSMUSC	Ighmbp2	immunog	GO:00038	catalytic a	-	-	-	-	19:326092	172	207	158	256	307	281	1.587364	1.525685	1.611577	1.82271	1.824489	2.040162	1.574875	1.895787	0.199948	1	-
ENSMUSC	Pola2	polymera:	GO:00081	metabolic	K02321	DNA poly	mmu011C	Metabolic	19:594054	86	107	84	132	168	112	1.828332	1.816726	1.97371	2.165024	2.299967	1.873212	1.872923	2.112735	0.531596	1	-
ENSMUSC	Dmrt1	doublesex	GO:00325	developm	-	-	-	-	19:255056	0	2	1	0	0	0	0	0.024174	0.016725	0	0	0	0.013633	0	0.275415	1	-
ENSMUSC	Eif1ad	eukaryotic	GO:00081	metabolic	-	-	-	-	19:536681	331	443	303	425	482	408	8.185598	8.749271	8.281539	8.10851	7.67581	7.937683	8.40547	7.907334	0.445649	1	-
ENSMUSC	Cabp4	calcium b	GO:00325	developm	-	-	-	-	19:413542	1	0	0	0	1	0	0.042681	0	0	0	0.02747	0	0.014227	0.009157	1	1	-
ENSMUSC	Chka	choline ki	GO:00081	metabolic	K14156	choline/et	mmu011C	Metabolic	19:385177	695	885	562	946	1155	912	2.189085	2.226213	1.956414	2.298789	2.342687	2.25987	2.123904	2.300449	0.580675	1	-
ENSMUSC	Cst6	cystatin E/	GO:00325	developm	-	-	-	-	19:534470	27	28	11	31	38	36	0.417259	0.345576	0.187874	0.369599	0.378159	0.437673	0.316903	0.395144	0.435952	1	-
ENSMUSC	Aip	aryl-hydrc	GO:00081	metabolic	-	-	-	-	19:411444	990	1244	949	1405	1542	1360	20.96689	21.04094	22.21324	22.95649	21.02992	22.6594	21.40702	22.21527	0.893107	1	-
ENSMUSC	Pitpnm1	phosphati	GO:00511	localizatio	-	-	-	-	19:409999	2537	2671	2027	3328	3752	3163	15.08907	12.68709	13.32425	15.27059	14.37009	14.79968	13.70014	14.81345	0.560427	1	-
ENSMUSC	Sf3b2	splicing fa	GO:00081	metabolic	K12829	splicing fa	mmu0304	Spliceosor	19:527392	5375	6848	4770	7429	8404	6562	101.5745	103.3513	99.62575	108.3096	102.2697	97.5559	101.5172	102.7118	0.88479	1	-
ENSMUSC	Pold4	polymera:	GO:00081	metabolic	-	-	-	-	19:423189	119	144	101	155	157	147	7.984504	7.71631	7.489787	8.023502	6.783533	7.759416	7.7302	7.52215	0.712856	1	-
ENSMUSC	Pacs1	phosphof	GO:00511	localizatio	-	-	-	-	19:513368	1518	2012	1510	2066	2222	2134	21.3612	22.6114	23.4843	22.42923	20.13504	23.62431	22.48563	22.06286	0.634353	1	-
ENSMUSC	Grk2	G protein-	GO:00325	developm	K00910	beta-adre	mmu0474	Olfactory	19:428600	3724	4330	3254	4805	5955	5330	27.45809	25.4973	26.51703	27.33281	28.27464	30.9171	26.49081	28.84151	0.480942	1	-
ENSMUSC	Mbl2	mannose-	GO:00081	metabolic	K03991	mannose-	mmu0414	Phagoson	19:302329	0	0	0	2	0	0	0	0	0	0.085581	0	0	0	0.028527	0.527606	1	-
ENSMUSC	Pip5k1b	phosphati	GO:00081	metabolic	K00889	1-phosph	mmu011C	Metabolic	19:242947	1085	1308	947	1160	1570	1535	14.71852	14.17059	14.19813	12.14011	13.71477	16.3815	14.36241	14.07879	0.666446	1	-
ENSMUSC	Nudt8	nudix (nuc	GO:00038	catalytic a	-	-	-	-	19:400062	66	81	57	83	85	139	1.032238	1.011733	0.985268	1.001482	0.856066	1.710249	1.009746	1.189266	0.500925	1	-
ENSMUSC	Rab1b	RAB1B, m	GO:00081	metabolic	K07875	Ras-relate	mmu0513	Legionellc	19:509920	1910	2437	1914	2944	3280	2412	63.55369	64.76027	70.38757	75.57452	70.28065	63.13869	66.23384	69.66462	0.770979	1	-
ENSMUSC	Doc2g	double C2	GO:00160	membran	-	-	-	-	19:400332	13	16	20	21	23	26	0.148199	0.145673	0.251992	0.184692	0.168845	0.233178	0.181955	0.195572	0.841401	1	-
ENSMUSC	Cnih2	cornichon	GO:00452	synapse|c	-	-	-	-	19:509287	2792	3853	3179	4148	4861	3938	137.8126	151.8863	173.4247	157.9584	154.5091	152.9186	154.3745	155.1287	0.838707	1	-
ENSMUSC	Yif1a	Yip1 inter	GO:00511	localizatio	-	-	-	-	19:508853	114	156	103	196	159	151	4.66218	5.095102	4.655513	6.183992	4.187324	4.858168	4.804265	5.076495	0.854107	1	-
ENSMUSC	Cbwd1	COBW do	GO:00971	organic cy	-	-	-	-	19:249199	252	329	242	305	389	325	7.914933	8.252558	8.400594	7.390566	7.867763	8.030483	8.189361	7.762937	0.50957	1	-
ENSMUSC	Rin1	Ras and R	GO:00511	localizatio	K17638	Ras and R	mmu0401	Ras signal	19:505048	1541	1897	1530	2079	2595	2259	13.51681	13.28877	14.83235	14.06878	14.65763	15.5883	13.87931	14.77157	0.670457	1	-
ENSMUSC	Aldh3b1	aldehyde	GO:00081	metabolic	K00129	aldehyde	mmu011C	Metabolic	19:391349	63	96	59	80	128	98	2.051566	2.496646	2.123444	2.00984	2.684149	2.510611	2.223886	2.401533	0.767663	1	-
ENSMUSC	Asah2	N-acylspl	GO:00081	metabolic	K12349	neutral ce	mmu011C	Metabolic	19:319846	234	295	217	296	312	275	3.051604	3.072411	3.127648	2.978065	2.620122	2.821336	3.083888	2.806507	0.3235	1	-
ENSMUSC	Rce1	Ras conve	GO:00081	metabolic	K08658	prenyl prc	mmu009C	Terpenoic	19:462259	146	144	141	210	175	138	1.985851	1.564233	2.119621	2.203658	1.532804	1.476669	1.889902	1.737711	0.515307	1	-
ENSMUSC	Slc29a2	solute car	GO:00511	localizatio	-	-	-	-	19:502400	175	196	195	216	266	246	4.322479	3.866305	5.323249	4.116039	4.230883	4.780141	4.504011	4.375687	0.715959	1	-
ENSMUSC	Pcx	pyruvate c	GO:00081	metabolic	K01958	pyruvate c	mmu011C	Metabolic	19:451047	1162	1470	1008	1610	1732	1563	11.57981	11.69926	11.10204	12.37803	11.1147	12.25365	11.46037	11.91546	0.877681	1	-
ENSMUSC	Minpp1	multiple ir	GO:00081	metabolic	K03103	multiple ir	mmu011C	Metabolic	19:324857	744	937	664	964	1233	932	17.34525	17.44589	17.10893	17.33864	18.51081	17.09365	17.30002	17.6477	0.972652	1	-
ENSMUSC	Apba1	amyloid b	GO:00325	developm	-	-	-	-	19:237588	5036	6180	4208	6104	6936	6073	46.36439	45.43946	42.81751	43.35545	41.1209	43.98585	44.87379	42.82073	0.39172	1	-
ENSMUSC	Peli3	pellino 3 [	GO:00508	response	-	-	-	-	19:493065	447	525	300	518	620	470	4.568626	4.285324	3.388806	4.084495	4.080605	3.779091	4.080919	3.981397	0.685765	1	-
ENSMUSC	Mrpl11	mitochon	GO:00081	metabolic	K02867	large subu	mmu0301	Ribosome	19:496230	473	590	424	575	709	548	10.02099	9.982687	9.928	9.39826	9.672762	9.13357	9.977225	9.401531	0.435919	1	-
ENSMUSC	Lao1	L-amino a	GO:00081	metabolic	K03334	L-amino-	mmu011C	Metabolic	4:1189615	0	8	5	4	7	5	0	0.123964	0.107224	0.059867	0.087452	0.076318	0.077063	0.074546	1	1	-
ENSMUSC	Tesmin	testis expr	GO:00325	developm	-	-	-	-	19:338885	0	6	0	2	3	2	0	0.051392	0	0.016551	0.020722	0.016878	0.017131	0.01805	1	1	-
ENSMUSC	Mus81	MUS81 st	GO:00081	metabolic	K08991	crossover	mmu0346	Fanconi a	19:548234	198	268	181	298	303	314	1.641704	1.774637	1.658653	1.906236	1.617808	2.04819	1.691665	1.857411	0.593687	1	-
ENSMUSC	Gal	galanin [S	GO:00325	developm	-	-	-	-	19:340991	4	4	9	4	4	7	0.343043	0.273922	0.85297	0.264609	0.2209	0.472275	0.489978	0.319261	0.400988	1	-
ENSMUSC	Ppp6r3	protein pl	GO:00081	metabolic	-	-	-	-	19:345492	1520	1818	1302	1864	2357	1969	6.36022	6.075308	6.021247	6.017344	6.351009	6.481644	6.152258	6.283332	0.973508	1	-
ENSMUSC	Ctsw	cathepsin	GO:00081	metabolic	K08569	cathepsin	mmu0421	Apoptosis	19:546524	1	0	2	1	4	8	0.049575	0	0.109557	0.03824	0.127663	0.311896	0.053044	0.159266	0.199843	1	-
ENSMUSC	Fibp	fibroblast	GO:00508	response	-	-	-	-	19:546061	232	330	255	345	377	368	2.077351	2.359839	2.52354	2.383262	2.173798	2.592274	2.320243	2.383111	0.990467	1	-
ENSMUSC	Fosl1	fos-like ar	GO:00325	developm	K04502	fos-like ar	mmu0516	HTLV-I inf	19:544770	0	7	0	2	13	3	0	0.199967	0	0.055199	0.299445	0.084408	0.066656	0.14635	0.356202	1	-
ENSMUSC	Drap1	Dr1 assoc	GO:00081	metabolic	-	-	-	-	19:542280	1220	1679	1389	1708	2133	1807	14.31417	15.73268	18.01172	15.46053	16.11579	16.67919	16.01952	16.08517	0.840098	1	-
ENSMUSC	Smarca2	SWI/SNF r	GO:00325	developm	K11647	SWI/SNF-	mmu0522	Hepatoce	19:266050	4337	5305	3792	6051	6343	5138	10.69719	10.44989	10.33702	11.51431	10.07465	9.969776	10.4947	10.51958	0.801005	1	-
ENSMUSC	Vldlr	very low c	GO:00325	developm	-	-	-	-	19:272164	1700	2156	1508	1995	2553	2020	7.267803	7.361206	7.12529	6.580026	7.028455	6.79386	7.251433	6.80078	0.311395	1	-
ENSMUSC	Rnaseh2c	ribonuclea	GO:00081	metabolic	K10745	ribonuclea	mmu0303	DNA repli	19:560187	100	111	86	136	132	98	1.951983	1.730393	1.855329	2.048081	1.659227	1.504922	1.845902	1.73741	0.605067	1	-
ENSMUSC	Kat5	K(lysine) a	GO:00081	metabolic	K11304	histone ac	mmu0516	HTLV-I inf	19:560301	758	1020	811	1121	1303	1098	23.04883	24.76999	27.25507	26.29756	25.51401	26.26593	25.02463	26.02583	0.878561	1	-
ENSMUSC	Rela	v-rel retic	GO:00325	developm	K04735	transcripti	mmu052C	Pathways	19:563748	336	472	280	492	574	512	7.553574	8.474261	6.956942	8.533136	8.309599	9.055118	7.661592	8.632618	0.410824	1	-
ENSMUSC	Slc1a1	solute car	GO:00511	localizatio	K05612	solute car	mmu0472	Glutamate	19:288350	2254	2742	2028	2807	3316	2930	16.5216	16.05135	16.42907	15.87344	15.65192	16.89573	16.33401	16.14036	0.674281	1	-
ENSMUSC	Ehbp1l1	EH domai	GO:00160	membran	-	-	-	-	19:570737	229	332	200	338	492	349	2.157427	2.497961	2.082462	2.456677	2.984841	2.586649	2.24595	2.676056	0.222346	1	-
ENSMUSC	Ltbp3	latent trar	GO:00325	developm	-	-	-	-	19:574090	385	573	356	530	559	604	4.529357	5.383643	4.628848	4.810409	4.234894	5.59015	4.847283	4.878484	0.88009	1	-
ENSMUSC	Scyl1	SCY1-like	GO:00081	metabolic	-	-	-	-	19:575842	1029	1363	959	1308	1615	1441	23.82585	25.20432	24.54136	23.3653	24.08022	26.24873	24.52384	24.56475	0.812433	1	-
ENSMUSC	Smc5	structural	GO:00325	developm	-	-	-	-	19:232064	702	860	621	836	1041	789	5.374642	5.258441	5.254739	4.937981	5.132373	4.752261	5.295941	4.940872	0.350095	1	-
ENSMUSC	Arl2	ADP-ribo:	GO:00511	localizatio	-	-	-	-	19:613437	341	463	433	546	696	423	5.284474	5.730256	7.416213	6.527848	6.945622	5.157019	6.143648	6.210163	0.935764	1	-
ENSMUSC	Men1	multiple e	GO:00325	developm	-	-	-	-	19:633497	962	1294	816	1408	1313	1169	5.285512	5.677957	4.955063	5.968221	4.645489	5.052864	5.306177	5.222191	0.697558	1	-
ENSMUSC	Map4k2	mitogen-.	GO:00048	signal trar	-	-	-	-	19:634113	681	900	620	1038	1104	1054	2.335988	2.465539	2.350509	2.746951	2.438637	2.844297	2.384012	2.676628	0.361365	1	-
ENSMUSC	Sf1	splicing fa	GO:00325	developm	-	-	-	-	19:636369	2057	2541	1779	2331	2800	2503	9.53439	9.406089	9.113419	8.335491	8.357404	9.12705	9.351299	8.606648	0.219073	1	-
ENSMUSC	Rps6ka4	ribosomal	GO:00081	metabolic	K16510	ribosomal	mmu0401	MAPK sig	19:682908	669	832	682	908	1099	863	10.1234	10.0547	11.40594	10.60025	10.70908	10.27357	10.52801	10.52763	0.818099	1	-
ENSMUSC	Prdx5	peroxired	GO:00081	metabolic	K11187	peroxired	mmu0414	Peroxisom	19:690669	1998	2428	1696	2761	3027	2341	31.48234	30.55386	29.5355	33.56359	30.71417	29.0191	30.5239	31.09895	0.953666	1	-
ENSMUSC	Kcnk4	potassium	GO:00511	localizatio	-	-	-	-	19:692571	100	128	103	127	167	158	3.560235	3.639455	4.052875	3.488301	3.828686	4.425339	3.750855	3.914109	0.914189	1	-
ENSMUSC	Gpr137	G protein-	GO:00160	membran	-	-	-	-	19:693806	947	1175	948	1297	1504	1234	14.48811	14.35639	16.02939	15.30847	14.81713	14.8521	14.95796	14.99256	0.822218	1	-
ENSMUSC	Bad	BCL2-ass(	GO:00325	developm	K02158	Bcl-2-ant	mmu052C	Pathways	19:694186	272	370	281	423	458	370	4.640347	5.041145	5.298279	5.567403	5.031554	4.965858	4.993257	5.188272	0.905538	1	-
ENSMUSC	Dnajc4	DnaJ heat	GO:00160	membran	-	-	-	-	19:698791	151	182	129	262	248	223	6.829445	6.57394	6.4483	9.142017	7.222962	7.93462	6.617228	8.099866	0.184061	1	-
ENSMUSC	Fermt3	fermitin fa	GO:00325	developm	K17084	kindlin 3[-	mmu0461	Platelet ac	19:699895	57	86	55	60	88	69	1.345299	1.621031	1.434678	1.09252	1.337477	1.281178	1.467002	1.237059	0.242332	1	-
ENSMUSC	Stip1	stress-ind	GO:00329	macromo	K09553	stress-ind	mmu0502	Prion dise	19:702069	3830	4419	3332	5371	6204	5190	107.6174	99.16387	103.475	116.4311	112.2561	114.726	103.4187	114.4711	0.358719	1	-
ENSMUSC	Rcor2	REST core	GO:00081	metabolic	-	-	-	-	19:726732	211	295	208	319	365	336	2.742872	3.062599	2.988362	3.19922	3.055416	3.436161	2.931277	3.230265	0.553341	1	-
ENSMUSC	Mark2	MAP/micr	GO:00325	developm	-	-	-	-	19:727539	1219	1671	1210	1581	2112	1712	4.66879	5.111199	5.121919	4.671569	5.208938	5.158403	4.967303	5.01297	0.878025	1	-
ENSMUSC	AI846148	expressed	GO:00081	metabolic	-	-	-	-	19:735646	321	411	328	466	559	390	6.157605	6.296433	6.953888	6.89641	6.905152	5.885483	6.469309	6.562348	0.94603	1	-
ENSMUSC	Smc3	structural	GO:00325	developm	K06669	structural	mmu0411	Cell cycle	19:536003	1913	2440	1555	2305	2851	2194	19.41236	19.77421	17.43977	18.04532	18.6301	17.51503	18.87544	18.06348	0.448617	1	-
ENSMUSC	Pdcd4	programn	GO:00325	developm	K16865	programn	mmu052C	Proteogly	19:538922	1808	2245	1406	2287	2766	2103	39.27181	38.94437	33.75318	38.32472	38.68917	35.93625	37.32312	37.65004	0.86899	1	-
ENSMUSC	Shoc2	soc-2 (sup	GO:00987	molecular	K19613	leucine-ri	mmu0401	Ras signal	19:539443	1322	1724	1262	1830	2156	1743	19.20505	20.00171	20.26236	20.50998	20.16914	19.92014	19.82304	20.19975	0.956959	1	-
ENSMUSC	Acsl5	acyl-CoA	GO:00081	metabolic	K01897	long-chai	mmu011C	Metabolic	19:552519	1117	1454	974	1472	1832	1411	12.13341	12.61363	11.69326	12.33582	12.81475	12.05779	12.14677	12.40278	0.977324	1	-
ENSMUSC	Zdhhc6	zinc finge	GO:00081	metabolic	-	-	-	-	19:552712	290	437	330	410	417	377	1.414627	1.702435	1.779116	1.542968	1.309887	1.446758	1.632059	1.433204	0.20125	1	-
ENSMUSC	Vti1a	vesicle tra	GO:00081	metabolic	K08493	vesicle tra	mmu0413	SNARE int	19:553162	1134	1454	1033	1564	1836	1339	4.289956	4.392888	4.319037	4.564643	4.472672	3.985026	4.33396	4.34078	0.818134	1	-
ENSMUSC	Tcf7l2	transcripti	GO:00325	developm	K04491	transcripti	mmu052C	Pathways	19:557418	89	175	75	160	233	116	0.247448	0.388576	0.230462	0.343195	0.417159	0.253722	0.288829	0.338025	0.468636	1	-
ENSMUSC	Hhex	hematopo	GO:00325	developm	-	-	-	-	19:374348	42	50	55	47	76	70	0.852823	0.810815	1.23429	0.736277	0.993752	1.118192	0.965976	0.949407	0.855254	1	-
ENSMUSC	Cyp26a1	cytochron	GO:00081	metabolic	K07437	cytochron	mmu011C	Metabolic	19:376978	3	12	0	7	11	7	0.103311	0.330031	0	0.185977	0.243932	0.189656	0.144447	0.206522	0.652106	1	-
ENSMUSC	Rbp4	retinol bir	GO:00325	developm	-	-	-	-	19:381166	77	117	62	125	125	81	3.624873	4.398798	3.225843	4.539871	3.789368	2.999839	3.749838	3.776359	0.933458	1	-
ENSMUSC	Eif3a	eukaryotic	GO:00081	metabolic	K03254	translatior	mmu0301	RNA trans	19:607611	5323	6809	4325	6780	8487	6352	62.79217	64.14728	56.38739	61.7034	64.47004	58.94819	61.10895	61.70721	0.871617	1	-
ENSMUSC	Pde6c	phosphoc	GO:00325	developm	K13757	cone cGM	mmu0023	Purine me	19:381327	3	0	0	0	2	3	0.052155	0	0	0	0.022386	0.041026	0.017385	0.021137	1	1	-
ENSMUSC	Fam45a	family witl	-	-	-	-	-	-	19:608115	542	742	549	758	525	796	4.363596	4.770841	4.884998	4.708086	2.721819	5.041606	4.673145	4.15717	0.320345	1	-
ENSMUSC	Prdx3	peroxired	GO:00325	developm	-	-	-	-	19:608640	456	596	425	639	719	598	18.83794	19.66352	19.40459	20.36572	19.12725	19.43485	19.30202	19.64261	0.954499	1	-
ENSMUSC	Plce1	phospholi	GO:00325	developm	K05860	phosphati	mmu011C	Metabolic	19:384811	367	569	354	446	511	544	1.029322	1.27451	1.097324	0.965052	0.922913	1.200313	1.133719	1.029426	0.318112	1	-
ENSMUSC	Noc3l	NOC3 like	GO:00325	developm	-	-	-	-	19:387881	305	437	294	481	499	399	4.682608	5.358154	4.988636	5.697224	4.933359	4.819158	5.009799	5.149914	0.98051	1	-
ENSMUSC	Hells	helicase, l	GO:00325	developm	-	-	-	-	19:389309	71	86	64	130	141	94	0.516578	0.499715	0.514644	0.72971	0.660621	0.538044	0.510312	0.642792	0.185589	1	-
ENSMUSC	Sorbs1	sorbin anc	GO:00329	macromo	K06086	sorbin anc	mmu0491	Insulin sig	19:402947	2077	2597	1768	2486	2887	2518	7.27124	7.260889	6.840707	6.714341	6.508387	6.934874	7.124278	6.719201	0.340888	1	-
ENSMUSC	Aldh18a1	aldehyde	GO:00081	metabolic	K12657	delta-1-p	mmu011C	Metabolic	19:405502	430	479	406	572	646	582	4.771895	4.245254	4.979607	4.897218	4.616467	5.08109	4.665586	4.864925	0.88	1	-
ENSMUSC	Tctn3	tectonic fa	GO:00508	response	-	-	-	-	19:405964	298	357	267	382	454	398	4.051506	3.876273	4.011978	4.006763	3.974761	4.256913	3.979919	4.079479	1	1	-
ENSMUSC	Ccnj	cyclin J [S	GO:00056	cell|intrace	-	-	-	-	19:408312	192	236	169	218	319	225	1.666407	1.635824	1.621115	1.459705	1.782888	1.536287	1.641115	1.59296	0.690749	1	-
ENSMUSC	Tll2	tolloid-lik	GO:00325	developm	-	-	-	-	19:410839	12	8	8	10	16	21	0.212803	0.113306	0.15679	0.136821	0.182714	0.29298	0.160966	0.204172	0.548189	1	-
ENSMUSC	Dntt	deoxynuc	GO:00081	metabolic	K00977	DNA nucl	mmu0464	Hematopo	19:410292	0	0	4	0	2	0	0	0	0.126855	0	0.036946	0	0.042285	0.012315	0.553603	1	-
ENSMUSC	Tm9sf3	transmem	GO:00160	membran	-	-	-	-	19:412108	3847	5102	3302	4644	6316	4822	38.23088	40.49286	36.26736	35.6053	40.41931	37.69904	38.33037	37.90789	0.689521	1	-
ENSMUSC	Pik3ap1	phosphoi	GO:00023	immune s	K12230	phosphoi	mmu0415	PI3K-Akt s	19:412742	40	75	37	54	70	62	0.933975	1.398559	0.954804	0.972743	1.052509	1.138871	1.09578	1.054708	0.741366	1	-
ENSMUSC	Lcor	ligand der	GO:00081	metabolic	-	-	-	-	19:414826	248	318	262	330	380	334	1.614499	1.653332	1.885101	1.657413	1.593033	1.710583	1.717644	1.653676	0.611183	1	-
ENSMUSC	Slit1	slit homol	GO:00325	developm	K06838	slit 1[-]	mmu0436	Axon guic	19:416002	5433	6343	5223	6805	8082	7025	42.99231	40.08591	45.67915	41.54412	41.18363	43.73291	42.91912	42.15355	0.61866	1	-
ENSMUSC	Smndc1	survival m	GO:00081	metabolic	K12839	survival of	mmu0304	Spliceosor	19:533792	488	638	437	745	781	608	14.0218	14.64032	13.87753	16.51471	14.45074	13.74356	14.17989	14.903	0.814208	1	-
ENSMUSC	Mxi1	MAX inter	GO:00081	metabolic	-	-	-	-	19:533105	1265	1578	1065	1636	1895	1730	13.74596	13.69421	12.7903	13.71506	13.26014	14.78908	13.41016	13.92143	0.890199	1	-
ENSMUSC	Add3	adducin 3	GO:00452	synapse|st	-	-	-	-	19:531404	3352	4481	3205	4251	5035	4184	41.44731	44.24998	43.7993	40.55213	40.09096	40.70004	43.16553	40.44771	0.285216	1	-
ENSMUSC	Xpnpep1	X-prolyl a	GO:00081	metabolic	-	-	-	-	19:529434	805	1074	770	1033	1373	973	5.486917	5.846315	5.80056	5.432039	6.026395	5.217425	5.711264	5.55862	0.617399	1	-
ENSMUSC	Trim8	tripartite r	GO:00325	developm	-	-	-	-	19:465016	1457	1704	1276	1861	2338	1807	27.5423	25.72505	26.65865	27.14048	28.46033	26.87261	26.642	27.49114	0.936303	1	-
ENSMUSC	Arl3	ADP-ribo:	GO:00325	developm	-	-	-	-	19:465311	1430	1827	1151	1899	2198	1444	106.2203	108.3817	94.4917	108.8245	105.1366	84.38196	103.0312	99.4477	0.564839	1	-
ENSMUSC	Sfxn2	sideroflex	GO:00511	localizatio	-	-	-	-	19:465733	72	87	73	107	115	74	1.369532	1.321613	1.534644	1.570198	1.408607	1.107346	1.408596	1.36205	0.757176	1	-
ENSMUSC	Fundc1	FUN14 dc	GO:00081	metabolic	K17986	FUN14 dc	mmu0413	Mitophag	X:1755656	375	492	335	492	614	544	6.399322	6.705241	6.318213	6.477361	6.747243	7.303192	6.474259	6.842599	0.782812	1	-
ENSMUSC	Nt5c2	5'-nucleot	GO:00081	metabolic	K01081	5'-nucleot	mmu011C	Metabolic	19:468868	825	1251	959	1410	1507	1097	8.76508	10.61464	11.26076	11.55717	10.31027	9.168956	10.21349	10.34547	0.926457	1	-
ENSMUSC	Pdcd11	programn	GO:00081	metabolic	-	-	-	-	19:470907	594	728	493	799	1025	654	6.00373	5.876407	5.507172	6.23034	6.671338	5.20024	5.79577	6.033972	0.879713	1	-
ENSMUSC	Taf5	TATA-box	GO:00081	metabolic	K03130	transcripti	mmu0516	Herpes sir	19:470677	110	141	119	161	207	160	2.061507	2.110368	2.464826	2.327812	2.498147	2.358976	2.212234	2.394978	0.69552	1	-
ENSMUSC	Pcgf6	polycomb	GO:00081	metabolic	K11470	polycomb	mmu0455	Signaling	19:470336	137	143	116	185	212	164	3.98712	3.323676	3.731145	4.153747	3.973095	3.754848	3.680647	3.960563	0.73551	1	-
ENSMUSC	5430427C	RIKEN cDI	-	-	-	-	-	-	X:8587035	6	11	5	8	6	8	0.097587	0.142881	0.089887	0.100386	0.06284	0.102359	0.110118	0.088528	0.525171	1	-
ENSMUSC	Slk	STE20-like	GO:00081	metabolic	K08836	STE20-like	mmu0411	Oocyte m	19:475796	2831	3695	2481	3564	4399	3596	17.00497	17.72541	16.47061	16.51596	17.01549	16.99286	17.06699	16.84144	0.666749	1	-
ENSMUSC	Col17a1	collagen, t	GO:00329	macromo	K07603	collagen, t	mmu0497	Protein di	19:476463	4	25	2	0	3	13	0.038782	0.193563	0.021431	0	0.018731	0.099147	0.084592	0.039293	0.266528	1	-
ENSMUSC	Sfr1	SWI5 dep	GO:00081	metabolic	-	-	-	-	19:477317	1867	2337	1552	2355	2870	2114	72.01232	71.98913	66.16084	70.07832	71.28515	64.1473	70.0541	68.50359	0.608171	1	-
ENSMUSC	Gsto2	glutathior	GO:00081	metabolic	K00799	glutathior	mmu052C	Pathways	19:478655	8	14	5	8	19	9	0.293919	0.410762	0.203029	0.226745	0.44949	0.260108	0.30257	0.312114	1	1	-
ENSMUSC	Nhlrc2	NHL repe	GO:00099	cellular pr	-	-	-	-	19:565482	229	274	219	304	455	326	3.554216	3.396301	3.75664	3.640099	4.547525	3.980504	3.569053	4.056042	0.40412	1	-
ENSMUSC	Afap1l2	actin filar	GO:00081	metabolic	-	-	-	-	19:569123	219	370	236	345	436	360	2.038991	2.751183	2.428459	2.478112	2.614045	2.63685	2.406211	2.576336	0.723241	1	-
ENSMUSC	Ablim1	actin-binc	GO:00325	developm	K07520	actin-binc	mmu0436	Axon guic	19:570327	1784	2476	1764	2388	3471	2302	3.33	3.691016	3.639107	3.438858	4.172139	3.380382	3.553375	3.663793	0.936923	1	-
ENSMUSC	Trub1	TruB pseu	GO:00081	metabolic	-	-	-	-	19:574529	489	523	447	604	780	624	4.060023	3.467907	4.101799	3.868894	4.170318	4.075825	3.876576	4.038346	0.880893	1	-
ENSMUSC	Hspa12a	heat shoc	GO:00055	extracellul	-	-	-	-	19:587957	6605	8111	5631	8481	9558	8007	70.34516	68.98928	66.28171	69.68492	65.55157	67.08758	68.53872	67.44136	0.626045	1	-
ENSMUSC	3110040N	RIKEN cDI	-	-	-	-	-	-	7:8178218	83	102	71	110	157	94	3.099572	3.0421	2.930418	3.169189	3.775544	2.761631	3.02403	3.235455	0.797063	1	-
ENSMUSC	Btbd1	BTB (POZ	GO:00325	developm	-	-	-	-	7:8179207	2253	2895	2068	2915	3514	2930	33.93289	34.82203	34.42366	33.87103	34.0813	34.71668	34.39286	34.223	0.73451	1	-
ENSMUSC	Hdgfl3	HDGF like	GO:00325	developm	-	-	-	-	7:8188125	2385	2981	2104	2871	3524	2749	13.89008	13.86514	13.54281	12.89971	13.21621	12.59511	13.76601	12.90368	0.302758	1	-
ENSMUSC	Bnc1	basonuclii	GO:00056	cell|intrace	-	-	-	-	7:8196667	0	0	2	0	0	2	0	0	0.029052	0	0	0.020682	0.009684	0.006894	1	1	-
ENSMUSC	Gcgr	glucagon	GO:00048	signal trar	K04583	glucagon	mmu0408	Neuroacti	11:120530	0	0	2	3	0	0	0	0	0.026295	0.027535	0	0	0.008765	0.009178	1	1	-
ENSMUSC	Bhlhe22	basic helix	GO:00325	developm	-	-	-	-	3:1805417	1995	2326	1720	2372	2962	1885	36.42671	33.91819	34.70981	33.41351	34.82706	27.07692	35.01823	31.7725	0.217512	1	-
ENSMUSC	Arhgdia	Rho GDP	GO:00325	developm	K12462	Rho GDP-	mmu0472	Neurotrop	11:120578	3687	4790	3549	5243	6139	2095	62.88303	65.24417	66.89799	68.98752	67.4237	28.10963	65.00839	54.84028	0.26523	1	-
ENSMUSC	Ints4	integrator	GO:00081	metabolic	-	-	-	-	7:9748095	454	589	504	603	741	681	3.076625	3.187717	3.774818	3.152579	3.233637	3.630582	3.346387	3.338933	0.817648	1	-
ENSMUSC	Alyref	Aly/REF e:	GO:00081	metabolic	K12881	THO com	mmu0516	Herpes sir	11:120592	406	537	350	536	676	540	5.4905	5.799706	5.231185	5.59217	5.886907	5.745004	5.507131	5.74136	0.87597	1	-
ENSMUSC	Anapc11	anaphase	GO:00081	metabolic	K03358	anaphase	mmu0516	HTLV-I inf	11:120598	430	578	485	649	688	646	4.283029	4.597865	5.33914	4.987212	4.412918	5.062046	4.740011	4.820725	0.953084	1	-
ENSMUSC	Pcyt2	phosphat	GO:00081	metabolic	-	-	mmu011C	Metabolic	11:120610	823	1080	779	1243	1348	1178	6.289214	6.591222	6.579316	7.328214	6.633473	7.08195	6.486584	7.014546	0.574702	1	-
ENSMUSC	Sirt7	sirtuin 7 [S	GO:00081	metabolic	-	-	-	-	11:120618	388	394	256	422	573	459	2.040177	1.654543	1.487729	1.711903	1.940196	1.898717	1.727483	1.850272	0.729643	1	-
ENSMUSC	Tollip	toll interac	GO:00325	developm	K05402	toll-intera	mmu0462	Toll-like r	7:1418748	2679	3319	2472	3583	4258	3515	21.29601	21.07068	21.71805	21.97368	21.79646	21.98173	21.36158	21.91729	0.98598	1	-
ENSMUSC	Pycr1	pyrroline-	GO:00081	metabolic	K00286	pyrroline-	mmu011C	Metabolic	11:120635	19	38	22	40	34	22	0.246724	0.394083	0.315747	0.400733	0.28431	0.22475	0.318851	0.303265	0.803267	1	-
ENSMUSC	Myadml2	myeloid-a	GO:00160	membran	-	-	-	-	11:120646	10	14	11	10	17	13	0.435511	0.486937	0.52945	0.336001	0.476771	0.445383	0.483966	0.419385	0.636343	1	-
ENSMUSC	Aspscr1	alveolar sc	GO:00081	metabolic	K15627	tether cor	mmu052C	Transcript	11:120672	225	314	268	343	499	357	1.350049	1.504669	1.777248	1.587783	1.928063	1.685179	1.543989	1.733675	0.464124	1	-
ENSMUSC	Mob2	MOB kina	GO:00325	developm	-	-	-	-	7:1420085	256	222	142	250	500	235	1.550221	1.073623	0.950359	1.167948	1.949749	1.11952	1.191401	1.412406	0.415736	1	-
ENSMUSC	Maged1	melanoma	GO:00325	developm	K12464	melanoma	mmu0472	Neurotrop	X:9453547	8238	9704	8238	11041	12183	11469	67.39873	63.40544	74.49009	69.68971	64.18578	73.81869	68.43142	69.2314	0.882952	1	-
ENSMUSC	Fasn	fatty acid	GO:00325	developm	K00665	fatty acid	mmu011C	Metabolic	11:120805	5099	6596	5158	7184	7454	6738	22.36283	23.10299	25.00172	24.30739	21.05163	23.2479	23.48918	22.86897	0.555276	1	-
ENSMUSC	Dus1l	dihydrour	GO:00081	metabolic	-	-	-	-	11:120789	336	456	322	493	567	484	2.97197	3.221189	3.147806	3.364206	3.229561	3.367921	3.113655	3.320563	0.719979	1	-
ENSMUSC	Gps1	G protein	GO:00081	metabolic	-	-	-	-	11:120784	322	451	343	506	571	446	4.110544	4.597965	4.839319	4.983373	4.693895	4.479077	4.515942	4.718782	0.862289	1	-
ENSMUSC	Zdhhc16	zinc finge	GO:00325	developm	-	-	-	-	19:419334	380	312	238	405	494	413	3.582782	2.349293	2.48005	2.945925	2.999286	3.063359	2.804042	3.002857	0.756296	1	-
ENSMUSC	Rfng	RFNG O-f	GO:00325	developm	K05948	fringe[2.4.	mmu0516	Human pa	11:120780	816	1038	357	577	1332	691	11.46156	11.64386	5.542031	6.25259	12.04793	7.635596	9.549151	8.645373	0.52588	1	-
ENSMUSC	Mms19	MMS19 (N	GO:00081	metabolic	-	-	-	-	19:419410	497	586	472	690	752	729	1.912155	1.800569	2.007036	2.048076	1.863113	2.206504	1.906587	2.039231	0.694141	1	-
ENSMUSC	Slc16a3	solute car	GO:00511	localizatio	K08180	MFS trans	mmu0523	Central ca	11:120948	28	39	31	48	54	33	0.195384	0.217343	0.239081	0.258406	0.24265	0.181161	0.217269	0.227406	0.929369	1	-
ENSMUSC	Csnk1d	casein kin	GO:00325	developm	K08959	casein kin	mmu0439	Hippo sig	11:120961	1974	2379	1724	2660	2654	2188	19.71356	18.97397	19.02839	20.49415	17.06764	17.19001	19.23864	18.2506	0.405526	1	-
ENSMUSC	Ogfod3	2-oxoglut	GO:00081	metabolic	-	-	-	-	11:121177	97	164	104	141	179	134	4.528007	6.113992	5.365571	5.077914	5.380763	4.92098	5.335856	5.126552	0.68045	1	-
ENSMUSC	Rab40b	Rab40B, n	GO:00081	metabolic	-	-	-	-	11:121356	368	481	319	480	545	543	8.141072	8.498162	7.799581	8.1923	7.763998	9.450281	8.146272	8.46886	0.907841	1	-
ENSMUSC	Ubtd1	ubiquitin c	GO:00055	protein bi	-	-	-	-	19:419817	106	160	126	142	172	165	4.238469	5.10941	5.568268	4.3805	4.428806	5.190384	4.972049	4.666563	0.553337	1	-
ENSMUSC	Ankrd2	ankyrin re	GO:00325	developm	-	-	-	-	19:420360	1	6	0	0	9	1	0.055524	0.265975	0	0	0.321701	0.043676	0.107166	0.121793	1	1	-
ENSMUSC	Wdr45b	WD repea	GO:00081	metabolic	-	-	-	-	11:121327	678	998	703	1046	1194	1207	7.50089	8.817782	8.595772	8.927808	8.506325	10.50512	8.304815	9.313085	0.395872	1	-
ENSMUSC	Fn3k	fructosam	GO:00325	developm	-	-	-	-	11:121434	248	310	191	396	380	282	2.474246	2.470014	2.106066	3.048015	2.44135	2.213358	2.350108	2.567574	0.635694	1	-
ENSMUSC	Hoga1	4-hydroxy	GO:00081	metabolic	K18123	4-hydroxy	mmu011C	Metabolic	19:420456	12	22	21	32	26	34	0.214046	0.313409	0.41401	0.440374	0.298646	0.477113	0.313822	0.405377	0.393685	1	-
ENSMUSC	Pi4k2a	phosphati	GO:00081	metabolic	K13711	phosphati	mmu011C	Metabolic	19:420904	941	1347	1053	1429	1742	1502	15.2362	17.41808	18.84353	17.85045	18.16309	19.13232	17.16593	18.38196	0.647387	1	-
ENSMUSC	R3hcc1l	R3H dom	GO:00329	macromo	-	-	-	-	19:425187	311	358	263	464	465	379	2.090617	1.921955	1.953963	2.406369	2.012897	2.004305	1.988845	2.14119	0.682221	1	-
ENSMUSC	Loxl4	lysyl oxida	GO:00081	metabolic	-	-	-	-	19:425939	8	7	2	7	6	4	0.09112	0.063672	0.025177	0.061507	0.044003	0.035838	0.059989	0.047116	0.668571	1	-
ENSMUSC	Hps1	HPS1, bio	GO:00987	molecular	-	-	-	-	19:427551	250	291	222	364	349	366	1.633794	1.518784	1.603455	1.835224	1.468715	1.881695	1.585344	1.728545	0.630895	1	-
ENSMUSC	Cnnm1	cyclin M1	GO:00511	localizatio	-	-	-	-	19:434404	2434	3095	2200	3177	3632	3322	25.08701	25.4762	25.06097	25.26247	24.1062	26.93636	25.20806	25.43501	0.860216	1	-
ENSMUSC	Got1	glutamic-	GO:00081	metabolic	K14454	aspartate	mmu011C	Metabolic	19:434997	3005	3378	2785	3902	4602	4815	47.83098	42.94082	48.9933	47.9162	47.17002	60.2937	46.58837	51.79331	0.406067	1	-
ENSMUSC	Entpd7	ectonucle	GO:00081	metabolic	-	-	-	-	19:436896	279	390	208	359	431	386	1.729464	1.930713	1.425009	1.716846	1.720438	1.882372	1.695062	1.773219	0.877518	1	-
ENSMUSC	Cutc	cutC copp	GO:00099	cellular pr	-	-	-	-	19:437529	61	71	57	79	103	89	1.586258	1.474511	1.638196	1.584897	1.724798	1.820739	1.566322	1.710145	0.690738	1	-
ENSMUSC	Abcc2	ATP-bind	GO:00081	metabolic	K05666	ATP-bind	mmu0152	Platinum c	19:437821	4	1	1	5	3	4	0.02169	0.004331	0.005993	0.020914	0.010476	0.017061	0.010671	0.01615	0.537601	1	-
ENSMUSC	Dnmbp	dynamin l	GO:00987	molecular	-	-	-	-	19:438468	250	306	212	336	477	374	1.405574	1.373983	1.317337	1.457412	1.726978	1.65423	1.365631	1.612873	0.235033	1	-
ENSMUSC	Cpn1	carboxype	GO:00081	metabolic	-	-	-	-	19:439563	6	8	0	10	19	3	0.093933	0.100029	0	0.120788	0.191552	0.036945	0.064654	0.116428	0.303361	1	-
ENSMUSC	Erlin1	ER lipid ra	GO:00081	metabolic	-	-	-	-	19:440349	371	420	312	464	559	455	3.463169	3.131078	3.218851	3.341545	3.360205	3.341343	3.271032	3.347698	0.998445	1	-
ENSMUSC	Chuk	conservec	GO:00325	developm	K04467	inhibitor c	mmu052C	Pathways	19:440733	359	549	371	520	682	552	2.6419	3.226566	3.017471	2.952271	3.231926	3.195748	2.961979	3.126648	0.789625	1	-
ENSMUSC	Cwf19l1	CWF19-lil	-	-	-	-	-	-	19:441086	232	253	177	330	366	331	3.963474	3.45188	3.342019	4.349433	4.026468	4.44865	3.585791	4.274851	0.223811	1	-
ENSMUSC	Scd3	stearoyl-c	GO:00081	metabolic	K00507	stearoyl-C	mmu0415	AMPK sig	19:442032	36	44	34	50	53	52	0.633458	0.618314	0.661216	0.678754	0.600541	0.719829	0.637663	0.666375	0.933366	1	-
ENSMUSC	Scd2	stearoyl-C	GO:00081	metabolic	K00507	stearoyl-C	mmu0415	AMPK sig	19:442936	25120	31206	20943	30578	35022	27483	281.2726	279.0563	259.1753	264.148	252.5245	242.0934	273.1681	252.9219	0.227048	1	-
ENSMUSC	Ndufb8	NADH de	GO:00081	metabolic	K03964	NADH de	mmu011C	Metabolic	19:445485	863	1059	833	1213	1349	1233	10.63649	10.42388	11.34696	11.53396	10.70666	11.95532	10.80244	11.39865	0.76426	1	-

ENSMUSC	Mrpl43	mitochon	GO:00329	macromo	-	-	-	-	19:450050	441	644	467	626	743	653	22.19838	25.88892	25.98041	24.31012	24.08386	25.8587	24.68923	24.75089	0.84588	1	-
ENSMUSC	Twnk	twinkle m	GO:00081	metabolic	-	-	-	-	19:450065	250	326	209	389	416	298	4.3763	4.557543	4.043527	5.253472	4.689376	4.103874	4.32579	4.682241	0.668434	1	-
ENSMUSC	Sfxn3	sideroflex	GO:00511	localizatio	-	-	-	-	19:450475	1264	1536	1117	1779	2054	1606	23.85702	23.15293	23.3007	25.90451	24.96457	23.84654	23.43688	24.90521	0.69004	1	-
ENSMUSC	Kazald1	Kazal-typ	GO:00325	developm	-	-	-	-	19:450752	25	30	26	42	43	19	0.671551	0.643583	0.771898	0.870403	0.743817	0.401515	0.695677	0.671912	0.853248	1	-
ENSMUSC	Lbx1	ladybird h	GO:00325	developm	-	-	-	-	19:452326	0	3	0	1	0	3	0	0.058901	0	0.018966	0	0.058005	0.019634	0.025657	1	1	-
ENSMUSC	Btrc	beta-tran	GO:00325	developm	K03362	F-box anc	mmu0421	Cellular se	19:453637	1354	1772	1567	1876	2427	1961	4.386982	4.585185	5.611295	4.689326	5.063743	4.998453	4.861154	4.917174	0.902356	1	-
ENSMUSC	Poll	polymera:	GO:00081	metabolic	K03512	DNA poly	mmu0341	Base excis	19:455522	77	137	110	180	167	170	2.023004	2.874569	3.1941	3.648472	2.825386	3.513721	2.697224	3.329193	0.250533	1	-
ENSMUSC	Mgea5	meningioı	GO:00081	metabolic	K15719	protein O	mmu0493	Insulin res	19:457502	4108	5211	3559	5704	6706	5206	49.9257	50.57788	47.80447	53.48149	52.48219	49.77472	49.43602	51.9128	0.764077	1	-
ENSMUSC	Kcnip2	Kv channe	GO:00325	developm	-	-	-	-	19:457918	1918	2429	1696	2814	3118	2391	20.70169	20.93777	20.23158	23.43212	21.67145	20.30242	20.62368	21.802	0.723322	1	-
ENSMUSC	Ldb1	LIM doma	GO:00325	developm	K15617	LIM doma	mmu0520	Transcript	19:460325	1285	1614	1244	1784	1964	1771	9.567082	9.59679	10.23632	10.24712	9.416133	10.37304	9.800064	10.0121	0.976561	1	-
ENSMUSC	Gbf1	golgi-spe	GO:00081	metabolic	K18443	golgi-spe	mmu0414	Endocytos	19:461525	1498	1925	1335	1943	2248	1941	6.533219	6.704895	6.434921	6.537602	6.313442	6.659654	6.557678	6.503566	0.713171	1	-
ENSMUSC	Fbxl15	F-box anc	GO:00325	developm	-	-	-	-	19:463281	127	160	143	190	251	169	4.015739	4.040429	4.997387	4.634943	5.110808	4.203958	4.351185	4.649903	0.769921	1	-
ENSMUSC	Mfsd13a	major faci	GO:00160	membran	-	-	-	-	19:463411	259	337	228	396	456	331	4.861373	5.051671	4.729789	5.734353	5.511611	4.887635	4.880944	5.377866	0.539567	1	-
ENSMUSC	Actr1a	ARP1 acti	GO:00329	macromo	-	-	-	-	19:463768	2732	3792	2720	3958	4831	3958	60.83543	67.43573	66.94092	67.99579	69.27357	69.33661	65.07069	68.86866	0.701674	1	-
ENSMUSC	Sufu	suppressc	GO:00325	developm	K06229	suppresso	mmu0520	Pathways	19:463968	221	383	233	358	375	401	1.549949	2.145207	1.806039	1.93704	1.693599	2.212483	1.833732	1.947707	0.791647	1	-
ENSMUSC	Hexa	hexosami	GO:00325	developm	K12373	hexosami	mmu0110	Metabolic	9:5953954	842	1300	840	1225	1422	1027	25.75697	31.75937	28.39938	28.91007	28.01154	24.7152	28.63858	27.21227	0.470883	1	-
ENSMUSC	Arih1	ariadne R	GO:00081	metabolic	-	-	-	-	9:5938825	2032	2483	1755	2372	3004	2376	13.00798	12.69429	12.41681	11.7147	12.38342	11.96585	12.70636	12.02132	0.361066	1	-
ENSMUSC	Bbs4	Bardet-Bi	GO:00325	developm	-	-	-	-	9:5932199	852	1146	813	1179	1455	1208	8.054111	8.651837	8.494063	8.598488	8.857185	8.983714	8.400004	8.813129	0.801376	1	-
ENSMUSC	Adpgk	ADP-dep	GO:00081	metabolic	K08074	ADP-dep	mmu0110	Metabolic	9:5929155	279	327	267	382	378	358	1.117063	1.045602	1.181495	1.179959	0.974586	1.127631	1.11472	1.094059	0.724085	1	-
ENSMUSC	Parp6	poly (ADP	GO:00038	catalytic a	-	-	-	-	9:5961728	1481	1900	1427	2161	2385	2206	7.995319	8.191814	8.514349	9.000482	8.291321	9.369086	8.233828	8.886963	0.559794	1	-
ENSMUSC	Limd1	LIM doma	GO:00325	developm	K16682	LIM doma	mmu0439	Hippo sig	9:1234787	142	218	127	202	265	194	1.014894	1.244329	1.003188	1.113821	1.219646	1.0908	1.08747	1.141422	0.862637	1	-
ENSMUSC	Sacm1l	SAC1 sup	GO:00081	metabolic	-	-	mmu0110	Metabolic	9:1235297	1440	1868	1222	1915	2185	1730	19.50393	20.20608	18.29268	20.01053	19.05748	18.43386	19.33423	19.16729	0.719604	1	-
ENSMUSC	Fyco1	FYVE and	GO:00081	metabolic	-	-	-	-	9:1237895	1441	1927	1000	1405	2302	1364	5.96264	6.367987	4.573213	4.485196	6.133872	4.440175	5.634613	5.019748	0.251103	1	-
ENSMUSC	Lztfl1	leucine zip	GO:00511	localizatio	-	-	-	-	9:1236944	742	915	687	1047	1141	981	2.857826	2.814483	2.924389	3.111063	2.829908	2.972431	2.865566	2.971134	0.907758	1	-
ENSMUSC	Tbl1x	transducir	GO:00325	developm	K04508	transducir	mmu0431	Wnt signa	X:7751101	1159	1462	939	1557	1805	1552	9.282047	9.350908	8.311372	9.620095	9.308771	9.778294	8.981442	9.569053	0.678844	1	-
ENSMUSC	Ribc1	RIB43A dc	-	-	-	-	-	-	X:1520045	6	5	4	5	10	5	0.213859	0.142325	0.157588	0.137486	0.229528	0.140205	0.171257	0.169073	1	1	-
ENSMUSC	Huwe1	HECT, UB	GO:00325	developm	K10592	E3 ubiquit	mmu0412	Ubiquitin	X:1518008	9587	11964	8509	12098	13267	12370	19.2181	19.15358	18.85181	18.70991	17.12597	19.5078	19.0745	18.44789	0.490092	1	-
ENSMUSC	Fam120c	family witl	GO:00056	cell|intrace	-	-	-	-	X:1513441	1296	1576	1090	1505	1681	1545	13.14474	12.76582	12.21856	11.77643	10.97917	12.32782	12.70971	11.69447	0.24612	1	-
ENSMUSC	Tsr2	TSR2 20S	-	-	-	-	-	-	X:1510870	869	1517	927	1570	1865	1546	5.860339	8.170228	6.909225	8.16832	8.099095	8.202065	6.979931	8.156493	0.201169	1	-
ENSMUSC	Fgd1	FYVE, Rhc	GO:00325	developm	K05720	FYVE, Rhc	mmu0481	Regulatioı	X:1510461	295	449	312	406	479	439	3.34736	4.068858	3.912749	3.554158	3.50002	3.918828	3.776322	3.657668	0.634197	1	-
ENSMUSC	Gnl3l	guanine n	GO:00081	metabolic	K14538	nuclear G	mmu0300	Ribosome	X:1509831	4539	3543	2606	5566	4202	3822	42.61767	26.56725	27.04274	40.31838	25.40624	28.23132	32.07589	31.31865	0.726319	1	-
ENSMUSC	Maged2	melanom	GO:00511	localizatio	-	-	-	-	X:1508063	492	756	546	762	819	813	4.736767	5.812795	5.809738	5.659819	5.077568	6.157702	5.4531	5.631696	0.944115	1	-
ENSMUSC	Apex2	apurinic/a	GO:00081	metabolic	K10772	AP endon	mmu0341	Base excis	X:1505195	82	107	73	95	173	121	0.347404	0.362034	0.341813	0.310508	0.471977	0.403287	0.350417	0.395257	0.551929	1	-
ENSMUSC	Alas2	aminolevı	GO:00325	developm	K00643	5-aminole	mmu0110	Metabolic	X:1505473	40	71	61	48	124	29	0.764188	1.083276	1.287995	0.707463	1.5255	0.435853	1.045153	0.889605	0.545829	1	-
ENSMUSC	Pfkfb1	6-phosph	GO:00325	developm	K19028	6-phosph	mmu0415	AMPK sigı	X:1505882	12	16	5	22	15	14	0.14341	0.152716	0.066047	0.202843	0.115438	0.131625	0.120724	0.149969	0.624933	1	-
ENSMUSC	Abhd6	abhydrola	GO:00081	metabolic	K13700	abhydrola	mmu0472	Retrograd	14:800290	513	755	783	790	915	1141	7.838546	9.213207	13.22289	9.312693	9.003132	13.71559	10.09155	10.67714	0.83101	1	-
ENSMUSC	Polr3a	polymera:	GO:00081	metabolic	K03018	DNA-dire	mmu0110	Metabolic	14:244486	472	699	531	596	748	821	3.75987	4.446867	4.674902	3.662752	3.836962	5.144997	4.29388	4.214904	0.725834	1	-
ENSMUSC	Acot9	acyl-CoA	GO:00081	metabolic	-	-	-	-	X:1552624	149	180	138	204	281	223	3.070421	2.962311	3.142959	3.243183	3.728827	3.615166	3.058564	3.529059	0.36562	1	-
ENSMUSC	Prdx4	peroxired	GO:00325	developm	-	-	-	-	X:1553239	74	111	106	129	124	114	1.835957	2.199378	2.906599	2.469183	1.981105	2.225093	2.313978	2.225127	0.722303	1	-
ENSMUSC	Rps24	ribosomal	GO:00081	metabolic	K02974	small subı	mmu0301	Ribosome	14:244871	888	1231	892	1287	1651	1166	4.900985	5.425933	5.441045	5.479986	5.867756	5.062673	5.255988	5.470138	0.865347	1	-
ENSMUSC	Ptprj	protein ty	GO:00081	metabolic	K05698	receptor-	mmu0452	Adherens	2:9042975	2898	3509	2650	3335	4452	3162	19.38926	18.7496	19.59546	17.21429	19.18106	16.64316	19.24478	17.6795	0.227734	1	-
ENSMUSC	Car5a	carbonic a	GO:00081	metabolic	K01672	carbonic a	mmu0091	Nitrogen ı	8:1219161	3	5	2	4	8	1	0.12818	0.170611	0.094453	0.131841	0.220127	0.033621	0.131081	0.12853	1	1	-
ENSMUSC	Jph3	junctophil	GO:00048	signal trar	-	-	-	-	8:1217296	4112	4801	3736	5302	6244	3428	43.92424	40.95705	44.10673	43.69394	42.95055	28.80732	42.996	38.48394	0.230149	1	-
ENSMUSC	Sp4	trans-acti	GO:00081	metabolic	-	-	-	-	12:118234	577	793	519	697	885	757	5.139392	5.640985	5.109168	4.7896	5.076144	5.30448	5.296515	5.056742	0.491879	1	-
ENSMUSC	Atp10a	ATPase, cl	GO:00511	localizatio	-	-	-	-	7:5865616	269	373	275	385	489	372	2.689476	2.978315	3.038746	2.969659	3.148319	2.925969	2.902179	3.014649	0.904221	1	-
ENSMUSC	Ube3a	ubiquitin	GO:00325	developm	K10587	ubiquitin-	mmu0516	Human pa	7:5922875	868	982	709	925	1230	1074	1.824198	1.648199	1.646817	1.49977	1.664609	1.775692	1.706405	1.64669	0.563975	1	-
ENSMUSC	Padi4	peptidyl a	GO:00325	developm	-	-	-	-	4:1407458	0	11	2	5	4	0	0	0.091924	0.023132	0.040362	0.026955	0	0.038352	0.022439	0.578047	1	-
ENSMUSC	Kdm5c	lysine (K)-	GO:00081	metabolic	-	-	-	-	X:1522330	1298	1603	1119	1738	1817	1709	6.445462	6.357096	6.141244	6.658244	5.810172	6.676246	6.314601	6.381554	0.88205	1	-
ENSMUSC	Gpr143	G protein	GO:00048	signal trar	-	-	-	-	X:1527819	0	0	0	0	0	3	0	0	0	0	0	0.052778	0	0.017593	0.292205	1	-
ENSMUSC	Sbds	SBDS ribo	GO:00325	developm	K14574	ribosome	mmu0300	Ribosome	5:1302457	983	1349	732	1219	1412	1045	19.52509	21.39919	16.06931	18.67986	18.06046	16.32927	18.99786	17.68986	0.372519	1	-
ENSMUSC	Rabgef1	RAB guan	GO:00081	metabolic	-	-	-	-	5:1301717	542	594	404	657	723	463	4.110995	3.598154	3.386695	3.844529	3.531349	2.762736	3.698614	3.379538	0.352926	1	-
ENSMUSC	Itga7	integrin al	GO:00325	developm	K06583	integrin al	mmu0415	PI3K-Akt	10:128933	466	674	498	685	877	754	2.860755	3.304461	3.378865	3.24426	3.466962	3.641472	3.18136	3.450898	0.590692	1	-
ENSMUSC	Cd63	CD63 anti	GO:00325	developm	K06497	CD63 anti	mmu0520	Proteogly	10:128900	544	795	481	698	982	689	6.595632	7.697874	6.445389	6.528946	7.666969	6.571852	6.912965	6.922589	0.844614	1	-
ENSMUSC	Gdf11	growth di	GO:00325	developm	-	-	-	-	10:128882	549	721	467	661	775	606	8.252321	8.65535	7.758304	7.665401	7.501706	7.166167	8.221992	7.444425	0.239502	1	-
ENSMUSC	Ormdl2	ORM1-lik	GO:00081	metabolic	-	-	-	-	10:128817	39	51	33	58	69	54	0.669272	0.698958	0.625895	0.767882	0.762507	0.729018	0.664708	0.753136	0.587248	1	-
ENSMUSC	Dnajc14	DnaJ heat	GO:00511	localizatio	-	-	-	-	10:128804	858	1324	769	1140	1492	1117	4.99884	6.160501	4.951711	5.124098	5.597648	5.119721	5.370351	5.280489	0.697797	1	-
ENSMUSC	Mmp19	matrix me	GO:00325	developm	-	-	-	-	10:128790	21	32	20	24	30	20	0.262272	0.319166	0.276063	0.231243	0.241265	0.1965	0.285834	0.223003	0.233779	1	-
ENSMUSC	Pmel	premelan	GO:00099	cellular pr	-	-	-	-	10:128704	6	6	0	6	7	5	0.139346	0.111287	0	0.107503	0.104683	0.091355	0.083544	0.10118	0.878233	1	-
ENSMUSC	Rps26	ribosomal	GO:00081	metabolic	K02976	small subı	mmu0301	Ribosome	10:128624	376	404	349	545	625	391	11.20442	9.614546	11.49405	12.52934	11.99323	9.166196	10.77101	11.22959	0.888635	1	-
ENSMUSC	Pa2g4	proliferati	GO:00325	developm	-	-	-	-	10:128557	1359	1688	1341	1794	2340	1708	18.16506	18.0192	19.81038	18.49993	20.14132	17.9604	18.66488	18.86722	0.891914	1	-
ENSMUSC	Esyt1	extended	GO:00511	localizatio	-	-	-	-	10:128509	156	232	149	217	261	246	0.905597	1.075584	0.955968	0.971854	0.975675	1.123456	0.97905	1.023662	0.890109	1	-
ENSMUSC	Smarcc2	SWI/SNF	GO:00325	developm	K11649	SWI/SNF	mmu0522	Hepatoce	10:128459	5980	7814	5318	7737	9022	8249	45.74959	47.74251	44.96568	45.66558	44.44711	49.6476	46.1526	46.58676	0.858927	1	-
ENSMUSC	Cdh9	cadherin !	GO:00325	developm	-	-	-	-	15:167287	755	940	713	910	1127	1035	9.650172	9.595375	10.07221	8.97346	9.276129	10.40734	9.772585	9.552308	0.646971	1	-
ENSMUSC	Chmp6	charged r	GO:00081	metabolic	K12195	charged n	mmu0414	Endocytos	11:119913	225	349	237	368	468	339	3.960248	4.905803	4.610355	4.997088	5.304443	4.694071	4.492135	4.998534	0.493546	1	-
ENSMUSC	Baiap2	brain-spe	GO:00325	developm	K05627	BAI1-assc	mmu0481	Regulatioı	11:119942	3906	6287	3639	5678	6170	5237	32.99571	42.41451	33.97458	37.00419	33.56333	34.80316	36.4616	35.12356	0.511715	1	-
ENSMUSC	Rnf41	ring finge	GO:00325	developm	K11981	E3 ubiquit	mmu0414	Endocytos	10:128411	921	1223	893	1224	1421	1384	7.090472	7.519479	7.598248	7.269877	7.044727	8.382286	7.402733	7.56563	0.980681	1	-
ENSMUSC	Nabp2	nucleic ac	GO:00081	metabolic	-	-	-	-	10:128401	933	1254	921	1116	1631	1190	12.87395	13.8189	14.04548	11.88022	14.49234	12.91778	13.57944	13.09678	0.5511	1	-
ENSMUSC	Aatk	apoptosis	GO:00081	metabolic	-	-	-	-	11:120007	3790	5450	4008	5916	6831	5739	14.34479	16.47394	16.76601	17.27482	16.64923	17.08843	15.86158	17.00416	0.586626	1	-
ENSMUSC	Tepsin	TEPSIN, a	GO:00329	macromo	-	-	-	-	11:120090	219	312	273	342	358	316	2.77594	3.158402	3.824506	3.344441	2.922166	3.151123	3.252949	3.139244	0.652466	1	-
ENSMUSC	Cnpy2	canopy F	GO:00325	developm	-	-	-	-	10:128322	282	518	355	482	534	465	6.389012	9.37261	8.889142	8.42485	7.790777	8.287984	8.216921	8.16787	0.821383	1	-
ENSMUSC	Faap100	Fanconi a	GO:00081	metabolic	K10993	fanconi ar	mmu0346	Fanconi a	11:120369	156	241	158	253	308	253	0.944105	1.16482	1.056813	1.181263	1.20033	1.204556	1.055246	1.195383	0.432027	1	-
ENSMUSC	Pde6g	phosphoc	GO:00081	metabolic	K13759	retinal roc	mmu0023	Purine me	11:120447	2	3	0	3	8	3	0.031795	0.038102	0	0.036806	0.081917	0.037522	0.023299	0.052082	0.305382	1	-
ENSMUSC	Atp5b	ATP synth	GO:00325	developm	K02133	F-type H+	mmu0110	Metabolic	10:128083	15681	19989	14720	20139	25504	21298	138.4001	140.8963	143.5878	137.1295	144.9525	147.8811	140.9614	143.321	0.928505	1	-
ENSMUSC	Prim1	DNA prim	GO:00081	metabolic	K02684	DNA prim	mmu0110	Metabolic	10:128015	77	73	62	96	110	72	0.808089	0.611841	0.719134	0.777269	0.743389	0.594447	0.713021	0.705035	0.8611	1	-
ENSMUSC	Tac2	tachykinin	GO:00508	response	-	-	-	-	10:127724	21	56	31	46	55	35	0.664034	1.41416	1.083355	1.122151	1.119906	0.870638	1.05385	1.037565	0.878423	1	-
ENSMUSC	Nab2	Ngfi-A bi	GO:00325	developm	-	-	-	-	10:127660	574	811	610	887	942	748	7.812609	8.815575	9.17616	9.31404	8.256382	8.009333	8.601448	8.526585	0.760642	1	-
ENSMUSC	Shmt2	serine hyc	GO:00081	metabolic	K00600	glycine hy	mmu0110	Metabolic	10:127517	310	438	327	496	502	520	3.225629	3.639766	3.760521	3.981671	3.363657	4.256647	3.541972	3.867325	0.597311	1	-
ENSMUSC	R3hdm2	R3H dom	GO:00056	cell|intrace	-	-	-	-	10:127380	3115	3791	2372	3886	4149	3402	13.42244	13.04587	11.29627	12.91834	11.51255	11.53237	12.58819	11.98776	0.429188	1	-
ENSMUSC	Ddit3	DNA-dan	GO:00325	developm	K04452	DNA dam	mmu0401	MAPK sigı	10:127290	209	299	191	319	399	275	5.545925	6.336423	5.601532	6.530524	6.817954	5.740762	5.82796	6.36308	0.609604	1	-
ENSMUSC	Mbd6	methyl-C	GO:00056	cell|intrace	-	-	-	-	10:127281	412	583	409	714	516	546	3.643677	4.117723	3.99772	4.871592	2.938644	3.798799	3.919707	3.869679	0.781341	1	-
ENSMUSC	Dctn2	dynactin 2	GO:00511	localizatio	K10424	dynactin 2	mmu0501	Huntingto	10:127266	3095	3872	3312	4609	5480	4678	30.74762	30.72076	36.36536	35.32546	35.05791	36.56133	32.61125	35.64823	0.462555	1	-
ENSMUSC	Ttc4	tetratricop	-	-	-	-	-	-	4:1066622	465	589	611	640	1098	975	4.148459	4.196571	6.024509	4.404975	6.307982	6.843035	4.789846	5.851997	0.206422	1	-
ENSMUSC	Pip4k2c	phosphati	GO:00081	metabolic	K00920	1-phosph	mmu0481	Regulatioı	10:127197	2432	3075	2145	3180	3665	3198	25.10454	25.35009	24.47162	25.3248	24.36224	25.97036	24.97542	25.21913	0.866687	1	-
ENSMUSC	Katnal2	katanin p	GO:00990	supramole	-	-	-	-	18:769771	81	125	90	110	112	120	0.711101	0.876399	0.873245	0.745022	0.63317	0.828781	0.820248	0.735657	0.396029	1	-
ENSMUSC	Hdhd2	haloacid c	GO:00081	metabolic	-	-	-	-	18:769301	960	1280	899	1379	1583	1533	7.160493	7.62478	7.411022	7.935351	7.603389	8.995489	7.398765	8.178076	0.429716	1	-
ENSMUSC	Agap2	ArfGAP w	GO:00325	developm	K17848	Arf-GAP v	mmu0414	Endocytos	10:127075	19018	23308	19374	24346	28674	22781	193.8569	189.7437	218.2643	191.4584	188.2173	182.6839	200.6216	187.4532	0.280229	1	-
ENSMUSC	Pias2	protein in	GO:00325	developm	K16063	E3 SUMO	mmu0463	Jak-STAT	18:770652	1361	1671	1151	1714	2078	1693	15.29543	14.99774	14.29637	14.86089	15.03848	14.96827	14.86318	14.95588	0.843827	1	-
ENSMUSC	St8sia5	ST8 alpha	GO:00081	metabolic	K03369	alpha-N-	mmu0110	Metabolic	18:771858	710	789	641	834	1006	842	13.14073	11.6623	13.11192	11.90851	11.98984	12.25982	12.63831	12.05272	0.480749	1	-
ENSMUSC	Rnf165	ring finge	GO:00325	developm	-	-	-	-	18:774561	1756	2208	1580	2126	2609	2136	7.306169	7.336859	7.265561	6.824311	6.990272	6.991612	7.302863	6.935398	0.381982	1	-
ENSMUSC	Atp5a1	ATP synth	GO:00081	metabolic	K02132	F-type H+	mmu0110	Metabolic	18:777737	9392	12354	9097	13032	16440	12760	108.7949	114.2888	116.465	116.464	122.6328	116.2818	113.1829	118.4596	0.792124	1	-
ENSMUSC	Pstpip2	proline-se	GO:00400	locomotic	-	-	-	-	18:777945	134	156	123	162	222	165	5.865095	5.453038	5.950072	5.47034	6.257158	5.681517	5.756068	5.803005	0.926053	1	-
ENSMUSC	Crisp1	cysteine-r	GO:00055	extracellul	-	-	-	-	17:402937	0	0	0	0	3	0	0	0	0	0	0.083312	0	0	0.027771	0.289995	1	-
ENSMUSC	Atp23	ATP23 me	GO:00081	metabolic	-	-	-	-	10:126868	90	110	56	131	132	86	1.167456	1.139558	0.802846	1.310992	1.102626	0.877621	1.03662	1.09708	0.858721	1	-
ENSMUSC	Usp33	ubiquitin	GO:00325	developm	-	-	-	-	3:1523464	2854	3413	2447	3628	4323	3700	11.92254	11.38667	11.29784	11.69264	11.62933	12.15983	11.53569	11.82727	0.995196	1	-
ENSMUSC	Clns1a	chloride c	GO:00081	metabolic	K05019	chloride c	mmu0301	RNA trans	7:9769663	969	1230	871	1284	1585	1292	12.32611	12.49549	12.24523	12.60078	12.98332	12.92932	12.35561	12.83781	0.879382	1	-
ENSMUSC	Paip1	polyadeny	GO:00451	translatior	K14322	polyadeny	mmu0301	RNA trans	13:119428	918	1160	883	1139	1443	1238	10.23395	10.32772	10.87948	9.796135	10.35909	10.85756	10.48038	10.3376	0.694696	1	-
ENSMUSC	Nnt	nicotinam	GO:00081	metabolic	K00323	NAD(P) tr	mmu0110	Metabolic	13:119335	256	413	278	317	460	385	1.871065	2.410706	2.245641	1.787468	2.165017	2.213708	2.175804	2.055398	0.52528	1	-
ENSMUSC	Cd163l1	CD163 mo	GO:00511	localizatio	-	-	-	-	7:1402182	5	7	6	5	11	15	0.049483	0.055332	0.065638	0.038177	0.070107	0.116796	0.056818	0.075027	0.571064	1	-
ENSMUSC	Paox	polyamine	GO:00081	metabolic	K00308	N1-acetyl	mmu0414	Peroxisom	7:1401157	84	101	90	123	86	122	0.834506	0.801344	0.98819	0.942725	0.550178	0.9535	0.87468	0.815468	0.594087	1	-
ENSMUSC	Echs1	enoyl Coe	GO:00081	metabolic	K07511	enoyl-Co/	mmu0110	Metabolic	7:1401057	360	488	375	566	543	547	1.975103	2.138222	2.273871	2.395707	1.918413	2.360945	2.129065	2.225021	0.87071	1	-
ENSMUSC	Fuom	fucose mı	GO:00325	developm	-	-	-	-	7:1400967	149	163	160	199	225	187	1.500521	1.310957	1.780829	1.546105	1.459123	1.481526	1.530769	1.495585	0.748545	1	-
ENSMUSC	Caly	calcyon n	GO:00511	localizatio	K15493	neuron-sp	mmu0472	Dopamine	7:1400698	2302	2753	2136	2923	3523	2924	37.3223	35.64635	38.27463	36.56134	36.78157	37.29506	37.08109	36.87933	0.731994	1	-
ENSMUSC	Msx3	msh hom	GO:00325	developm	K09341	homeobo	mmu0516	HTLV-I int	7:1400461	0	2	0	0	0	0	0	0.022038	0	0	0	0	0.007346	0	0.525209	1	-
ENSMUSC	Zfp511	zinc finge	GO:00081	metabolic	-	-	-	-	7:1400363	171	248	152	280	327	275	2.631281	3.047673	2.585014	3.323992	3.240217	3.329005	2.754656	3.297738	0.211264	1	-
ENSMUSC	Tubgcp2	tubulin, g	GO:00051	structural	-	-	-	-	7:1399959	751	979	737	1039	1237	1112	7.624647	7.937953	8.269785	8.13815	8.087314	8.881693	7.944128	8.369052	0.780136	1	-
ENSMUSC	Adgra1	adhesion	GO:00048	signal trar	-	-	-	-	7:1398341	1325	1692	1138	1907	1858	1754	12.65676	12.90782	12.01422	14.0536	11.42897	13.18096	12.52627	12.88784	0.968893	1	-
ENSMUSC	Inpp5a	inositol pc	GO:00081	metabolic	K01106	inositol-1,	mmu0110	Metabolic	7:1393891	653	757	608	869	1116	921	5.398905	4.998438	5.555759	5.542973	5.941716	5.990506	5.317701	5.825065	0.508747	1	-
ENSMUSC	Dpysl4	dihydropy	GO:00325	developm	-	-	-	-	7:1390860	734	925	659	1021	1090	1032	4.650481	4.680466	4.614594	4.990656	4.447159	5.143894	4.648513	4.86057	0.845752	1	-
ENSMUSC	Syce1	synaptone	GO:00099	cellular pr	-	-	-	-	7:1407772	0	5	5	2	3	7	0	0.151807	0.210109	0.05867	0.073456	0.209377	0.120639	0.113834	1	1	-
ENSMUSC	Odf3	outer den	GO:00325	developm	-	-	-	-	7:1408478	3	1	1	2	0	1	0.053294	0.01419	0.019635	0.027415	0	0.013978	0.02904	0.013798	0.548359	1	-
ENSMUSC	Bet1l	Bet1 golg	GO:00511	localizatio	K08504	blocked e	mmu0413	SNARE int	7:1408533	297	323	246	331	328	338	2.621691	2.277056	2.399976	2.254154	1.864463	2.347215	2.432908	2.155278	0.240609	1	-
ENSMUSC	Ric8a	RIC8 guar	GO:00325	developm	-	-	-	-	7:1408569	575	723	514	765	931	831	2.037989	2.04653	2.013462	2.091824	2.124897	2.317104	2.03266	2.177942	0.659977	1	-
ENSMUSC	Sirt3	sirtuin 3 [S	GO:00081	metabolic	K11413	NAD-dep	mmu0523	Central ca	7:1408636	489	610	429	688	846	630	3.509333	3.496164	3.402672	3.809209	3.909679	3.556862	3.46939	3.758583	0.593734	1	-
ENSMUSC	Psmd13	proteasor	GO:00081	metabolic	K03039	26S prote	mmu0516	Epstein-B	7:1408819	664	840	671	888	1113	974	6.002751	6.064675	6.704277	6.193351	6.479361	6.927108	6.257234	6.533273	0.851696	1	-
ENSMUSC	Ptdss2	phosphati	GO:00081	metabolic	K08730	phosphati	mmu0110	Metabolic	7:1411223	656	790	592	844	921	763	4.967648	4.777706	4.954676	4.930825	4.491184	4.545508	4.90001	4.655839	0.455416	1	-
ENSMUSC	Drd4	dopamine	GO:00048	signal trar	K04147	dopamine	mmu0408	Neuroacti	7:1412920	0	5	0	0	4	0	0	0.066341	0	0	0.042797	0	0.022114	0.014266	0.7953	1	-
ENSMUSC	Hras	Harvey ra	GO:00325	developm	K02833	GTPase H	mmu0520	Pathways	7:1411891	1072	1502	1048	1497	1935	1571	7.742404	8.663572	8.365448	8.341289	8.999465	8.926231	8.257141	8.755661	0.70825	1	-
ENSMUSC	Lmntd2	lamin tail	GO:00081	metabolic	-	-	-	-	7:1412099	0	0	2	0	5	0	0	0	0.052275	0	0.076139	0	0.017425	0.02538	1	1	-
ENSMUSC	Taldo1	transaldol	GO:00081	metabolic	K00616	transaldol	mmu0110	Metabolic	7:1413921	643	897	700	930	1241	937	13.39488	14.92333	16.11657	14.94654	16.64769	15.356	14.81159	15.65008	0.757926	1	-
ENSMUSC	Tmem80	transmem	GO:00160	membran	-	-	-	-	7:1413277	104	118	145	161	127	126	0.809749	0.733743	1.247759	0.967102	0.63676	0.771785	0.930417	0.791882	0.311363	1	-
ENSMUSC	Pidd1	p53 induc	GO:00081	metabolic	K10130	leucine-ri	mmu0421	Apoptosis	7:1414381	19	36	13	30	27	32	0.09424	0.142606	0.071264	0.114799	0.08624	0.124867	0.102704	0.108635	1	1	-
ENSMUSC	Rplp2	ribosomal	GO:00081	metabolic	K02943	large subı	mmu0301	Ribosome	7:1414473	606	765	633	885	1151	839	13.11635	13.22353	15.14226	14.77794	16.04244	14.28609	13.82738	15.03549	0.561505	1	-
ENSMUSC	Cd151	CD151 an	GO:00400	locomotic	-	-	-	-	7:1414673	270	378	261	378	512	438	2.464968	2.756037	2.633503	2.662376	3.010036	3.145809	2.618169	2.939407	0.434251	1	-
ENSMUSC	Tspan4	tetraspani	GO:00508	response	-	-	-	-	7:1414752	57	83	88	68	114	72	1.077155	1.252645	1.837959	0.991396	1.387285	1.070409	1.389253	1.149697	0.274533	1	-
ENSMUSC	Chid1	chitinase	GO:00081	metabolic	-	-	-	-	7:1414931	788	984	735	1076	1155	1022	2.48496	2.478187	2.561694	2.617795	2.34547	2.535449	2.50828	2.499571	0.783241	1	-
ENSMUSC	Tktl2	transketol	GO:00081	metabolic	K00615	transketol	mmu0110	Metabolic	8:6651175	3	4	5	3	9	2	0.024403	0.025984	0.044955	0.01883	0.047145	0.012802	0.031781	0.026259	0.86924	1	-
ENSMUSC	Tmem192	transmem	GO:00055	extracellul	-	-	-	-	8:6494718	128	274	142	220	238	197	2.590477	4.428587	3.176158	3.434952	3.101696	3.136505	3.398407	3.224384	0.628651	1	-
ENSMUSC	Zfp711	zinc finge	GO:00081	metabolic	-	-	-	-	X:1126005	291	305	208	313	382	307	2.047937	1.714233	1.617835	1.699413	1.73118	1.699702	1.793335	1.710098	0.568216	1	-
ENSMUSC	Chm	choroider	GO:00081	metabolic	-	-	-	-	X:1130405	1306	1515	1040	1597	1878	1838	7.486799	6.936039	6.589203	7.062985	6.932712	8.289137	7.004014	7.428278	0.728568	1	-
ENSMUSC	Phkg1	phosphor	GO:00081	metabolic	K00871	phosphor	mmu0402	Calcium si	5:1298634	332	438	338	517	598	489	2.739738	2.886626	3.082717	3.29148	3.177796	3.174609	2.903027	3.214628	0.484237	1	-
ENSMUSC	Sumf2	sulfatase	GO:00056	cell|intrace	-	-	-	-	5:1298469	129	193	118	156	248	181	1.998601	2.388034	2.020532	1.864628	2.474245	2.206109	2.135722	2.181661	1	1	-
ENSMUSC	Tm9sf2	transmem	GO:00055	extracellul	-	-	-	-	14:122107	2004	2560	1895	2790	3823	2774	32.64687	33.30654	34.11928	35.06536	40.10539	35.55176	33.35756	36.9075	0.385711	1	-
ENSMUSC	Clybl	citrate lya	GO:00038	catalytic a	-	-	-	-	14:122181	201	252	221	284	355	250	7.114598	7.123622	8.645563	7.75539	8.091655	6.961528	7.627928	7.602858	0.846353	1	-
ENSMUSC	Farp1	FERM, Rh	GO:00325	developm	-	-	-	-	14:121035	1122	1425	1066	1427	1640	1543	11.57021	11.7357	12.14933	11.35279	10.89047	12.51772	11.81841	11.58699	0.640497	1	-
ENSMUSC	Dock9	dedicator	GO:00987	molecular	-	-	-	-	14:121542	7205	9446	6540	8835	10572	9732	22.60656	23.66978	22.67906	21.38638	21.36055	24.02224	22.98514	22.25639	0.502518	1	-
ENSMUSC	Tnrc6c	trinucleoti	GO:00081	metabolic	-	-	-	-	11:117654	2682	3219	2310	3405	3859	3524	6.366945	6.102942	6.060816	6.236197	5.899318	6.581413	6.176901	6.238976	0.86848	1	-
ENSMUSC	6030468B	RIKEN cD	GO:00055	extracellul	-	-	-	-	11:117797	0	0	4	1	0	0	0	0	0.252052	0.043989	0	0	0.084017	0.014663	0.311386	1	-
ENSMUSC	Cant1	calcium a	GO:00048	signal trar	K12304	soluble ca	mmu0023	Purine me	11:118406	680	1031	676	995	1110	858	5.745849	6.957447	6.313044	6.486325	6.039805	5.703528	6.33878	6.076553	0.524101	1	-
ENSMUSC	Rbfox3	RNA bind	GO:00325	developm	-	-	-	-	11:118489	3275	3920	2758	4091	5051	3911	22.57199	21.57698	21.00873	21.75296	22.41768	21.20593	21.71923	21.79219	0.811004	1	-
ENSMUSC	Cbx2	chromobc	GO:00325	developm	-	-	-	-	11:119022	28	43	34	48	70	59	0.383062	0.469814	0.514098	0.506621	0.616695	0.635013	0.455658	0.58611	0.234246	1	-
ENSMUSC	Cbx8	chromobc	GO:00081	metabolic	-	-	-	-	11:119036	148	198	140	220	211	236	1.886157	2.015246	1.971939	2.163066	1.731631	2.366137	1.957781	2.086945	0.786679	1	-
ENSMUSC	Gaa	glucosida	GO:00325	developm	K12316	lysosomal	mmu0110	Metabolic	11:119267	5689	6765	5172	7455	8381	7268	57.01897	54.14983	57.29131	57.64497	54.09212	57.30721	56.15337	56.3481	0.801018	1	-
ENSMUSC	Eif4a3	eukaryotic	GO:00325	developm	K13025	ATP-depe	mmu0301	RNA trans	11:119288	618	774	579	783	1066	840	11.11455	11.11707	11.50878	10.86415	12.34569	11.88486	11.2468	11.69823	0.880184	1	-
ENSMUSC	Nptx1	neuronal	GO:00325	developm	-	-	-	-	11:119538	12832	15486	12094	17330	20618	15955	150.1816	144.7463	156.4369	156.4773	155.3902	146.9028	150.455	152.9235	0.926925	1	-
ENSMUSC	Rptor	regulatory	GO:00081	metabolic	K07204	regulatory	mmu0415	PI3K-Akt	11:119602	1592	1995	1505	2192	2395	2284	4.76096	4.76475	4.974332	5.057341	4.612236	5.373516	4.833347	5.014364	0.891711	1	-
ENSMUSC	Pde8a	phosphoc	GO:00081	metabolic	K18437	high affini	mmu0023	Purine me	7:8121359	266	446	339	452	514	464	3.766573	5.043649	5.305314	4.937801	4.68686	5.168848	4.705178	4.93117	0.854678	1	-
ENSMUSC	Nat1	N-acetyl t	GO:00325	developm	K00622	arylamine	mmu0110	Metabolic	8:6748092	14	27	20	17	33	25	0.334572	0.515304	0.528247	0.313429	0.507834	0.470003	0.459374	0.430422	0.759512	1	-
ENSMUSC	Tma16	translatior	GO:00329	macromo	-	-	-	-	8:6647311	248	322	228	375	425	306	1.405978	1.457904	1.428598	1.64017	1.551571	1.36477	1.430827	1.518837	0.76929	1	-
ENSMUSC	Dach2	dachshun	GO:00325	developm	-	-	-	-	X:1132975	14	16	14	11	23	33	0.065575	0.059852	0.072472	0.03975	0.069372	0.121599	0.065966	0.076907	0.713205	1	-
ENSMUSC	Klhl4	kelch-like	GO:00081	metabolic	-	-	-	-	X:1144743	164	218	172	241	250	226	1.923825	2.042325	2.22996	2.181069	1.888503	2.085652	2.06537	2.051741	0.822393	1	-
ENSMUSC	Zfp202	zinc finge	GO:00081	metabolic	-	-	-	-	9:4019231	75	89	60	111	114	121	0.66435	0.629612	0.5874	0.758557	0.650271	0.843198	0.627121	0.750676	0.320756	1	-
ENSMUSC	Copg2	coatomer	GO:00511	localizatio	-	-	-	-	6:3074755	1181	1434	1082	1518	1732	1471	10.51314	10.19477	10.64527	10.4252	9.928531	10.30164	10.45106	10.21846	0.613653	1	-
ENSMUSC	Podxl	podocalyx	GO:00325	developm	-	-	-	-	6:3151948	674	914	720	1014	1096	1059	7.190838	7.787747	8.489826	8.346185	7.529822	8.88846	7.822804	8.254822	0.780766	1	-
ENSMUSC	Mkln1	muskelin	GO:00325	developm	-	-	-	-	6:3139873	2148	2676	1815	2855	3137	2715	4.109055	4.088271	3.837347	4.21351	3.864354	4.085907	4.011558	4.05459	0.876979	1	-
ENSMUSC	Bach1	BTB and C	GO:00081	metabolic	-	-	-	-	16:876989	246	333	232	350	445	411	2.150671	2.325036	2.241679	2.360685	2.505266	2.826779	2.239129	2.564243	0.348692	1	-
ENSMUSC	Cct8	chaperoni	GO:00081	metabolic	-	-	-	-	16:874833	2450	3080	2031	3156	3820	3037	27.33779	27.44694	25.0469	27.16844	27.44829	26.65956	26.61054	27.09209	0.945762	1	-
ENSMUSC	Usp16	ubiquitin	GO:00081	metabolic	-	-	-	-	16:874547	448	542	379	531	672	508	2.227891	2.152588	2.083058	2.037232	2.151988	1.987423	2.154512	2.058881	0.530326	1	-
ENSMUSC	Hprt	hypoxantl	GO:00325	developm	K00760	hypoxantl	mmu0110	Metabolic	X:5298813	1750	2086	1401	2071	2593	1848	82.89503	78.91336	73.34582	75.68346	79.09474	68.86574	78.38474	74.54798	0.420857	1	-
ENSMUSC	Ccdc51	coiled-co	GO:00160	membran	-	-	-	-	9:1090824	55	60	69	85	120	77	2.200652	1.917284	3.051284	2.623829	3.091887	2.423764	2.38974	2.71316	0.554285	1	-
ENSMUSC	Atrip	ATR intera	GO:00081	metabolic	K10905	ATR intera	mmu0346	Fanconi a	9:1090597	58	53	61	113	95	90	0.48759	0.355835	0.566767	0.732887	0.514287	0.595221	0.470064	0.614132	0.187324	1	-
ENSMUSC	Pfkfb4	6-phosph	GO:00081	metabolic	K19030	6-phosph	mmu0415	AMPK sigı	9:1089917	79	131	124	180	184	131	0.55913	0.740457	0.969959	0.98285	0.838603	0.729403	0.756516	0.850285	0.579134	1	-
ENSMUSC	Col7a1	collagen,	GO:00325	developm	K16628	collagen,	mmu0497	Protein di	9:1089535	70	62	47	91	79	65	0.381645	0.269963	0.283211	0.38277	0.277362	0.278796	0.311606	0.312976	0.93581	1	-
ENSMUSC	Uqcrc1	ubiquinol	GO:00081	metabolic	-	-	-	-	9:1089366	2551	3587	2888	4046	4888	3986	25.20784	28.30756	31.54054	30.84476	31.10362	30.98656	28.35198	30.97831	0.478405	1	-
ENSMUSC	Tmem89	transmem	GO:00160	membran	-	-	-	-	9:1089146	0	3	0	0	0	0	0	0.098659	0	0	0	0	0.032886	0	0.289199	1	-
ENSMUSC	Rps6ka6	ribosomal	GO:00081	metabolic	K04373	ribosomal	mmu0401	MAPK sigı	X:1113881	82	129	77	107	125	101	0.588687	0.73961	0.610953	0.592632	0.577873	0.570427	0.646417	0.580311	0.391573	1	-

ENSMUSG	Alox5	arachidon	GO:00081	metabolic	K00461	arachidon	mmu0110	Metabolic	6:1164100	7	20	11	9	29	15	0.057704	0.131667	0.100214	0.057238	0.15394	0.097275	0.096529	0.102818	0.944159	1	-
ENSMUSG	Myo3a	myosin III	GO:00081	metabolic	-	-	-	-	2:2222750	0	2	0	2	8	0	0	0.01105	0	0.010675	0.035636	0	0.003683	0.015437	0.199095	1	-
ENSMUSG	Wdr73	WD repea	GO:00099	cellular pr	-	-	-	-	7:8089072	332	443	212	400	342	460	1.830698	1.950867	1.291997	1.701643	1.214391	1.995475	1.691187	1.63717	0.701792	1	-
ENSMUSG	Nmb	neuromec	GO:00508	response	-	-	-	-	7:8090222	17	10	14	18	19	24	0.522134	0.245274	0.475226	0.426521	0.375781	0.579887	0.414211	0.46073	0.832604	1	-
ENSMUSG	Sec11a	SEC11 ho	GO:00081	metabolic	K13280	signal pep	-	-	7:8090488	309	382	259	444	570	343	2.86384	2.827475	2.65299	3.174704	3.401881	2.500889	2.781435	3.025825	0.628307	1	-
ENSMUSG	Slc28a1	solute car	GO:00511	localizatio	-	-	-	-	7:8111479	0	0	0	0	0	2	0	0	0	0	0	0.032266	0	0.010755	0.52651	1	-
ENSMUSG	A930017K	RIKEN cD	-	-	-	-	-	-	17:259463	10	18	8	18	19	20	0.240483	0.345694	0.212614	0.33396	0.294231	0.378372	0.266264	0.335521	0.561749	1	-
ENSMUSG	Pigq	phosphati	GO:00081	metabolic	K03860	phosphati	mmu0110	Metabolic	17:259264	832	1202	946	1188	1497	1517	3.231573	3.72856	4.060961	3.559898	3.744271	4.635401	3.673698	3.979857	0.602992	1	-
ENSMUSG	Rab40c	Rab40C, r	GO:00081	metabolic	-	-	-	-	17:258821	390	421	264	547	650	484	3.532515	3.045416	2.642832	3.822405	3.791289	3.448859	3.073588	3.687518	0.194205	1	-
ENSMUSG	Rhot2	ras homol	GO:00511	localizatio	K07871	Ras homo	mmu0413	Mitophag	17:258384	922	1101	771	1184	1450	1103	5.306395	5.060599	4.904223	5.257158	5.373925	4.994076	5.090406	5.208386	0.993917	1	-
ENSMUSG	Rhbdl1	rhomboid	GO:00081	metabolic	-	-	-	-	17:258344	290	434	378	499	550	554	6.112144	7.305167	8.805074	8.11382	7.464697	9.185766	7.407462	8.254761	0.507594	1	-
ENSMUSG	Jmjd8	jumonji do	GO:00055	extracellul	-	-	-	-	17:258288	234	264	171	299	314	293	2.302225	2.074346	1.859403	2.269519	1.98937	2.267828	2.078658	2.175572	0.885778	1	-
ENSMUSG	Fbxl16	F-box and	GO:00081	metabolic	-	-	-	-	17:258090	17709	21351	16735	23923	28370	24774	310.8908	299.349	324.703	324.011	320.7213	342.1534	311.6476	328.9619	0.719249	1	-
ENSMUSG	Gng13	guanine n	GO:00048	signal trar	K04547	guanine n	mmu0474	Olfactory	17:257171	90	131	111	145	164	129	5.883529	6.839322	8.019816	7.312969	6.903925	6.634336	6.914222	6.95041	0.914562	1	-
ENSMUSG	Sdc3	syndecan	GO:00400	locomotic	K16337	syndecan	mmu0451	Cell adhes	4:1307925	3962	5262	3620	5731	5912	5424	34.6927	36.79767	35.03314	38.71546	33.33595	37.36408	35.50784	36.47183	0.976195	1	-
ENSMUSG	Hadha	hydroxyac	GO:00081	metabolic	K07515	enoyl-Co/	mmu0110	Metabolic	5:3011830	1278	1733	1209	1725	2300	1728	7.298194	7.903672	7.630579	7.599827	8.457984	7.76317	7.610815	7.940327	0.837734	1	-
ENSMUSG	Hspa4l	heat shoc	GO:00508	response	K09485	heat shoc	mmu0414	Protein pr	3:4074449	4253	5154	3401	5116	5887	5035	18.64581	18.0458	16.47932	17.304	16.62013	17.36585	17.72364	17.09666	0.479499	1	-
ENSMUSG	Larp1b	La ribonu	GO:00329	macromo	-	-	-	-	3:4095035	205	276	215	281	352	307	0.805102	0.865668	0.933214	0.8514	0.890213	0.948518	0.867995	0.89671	0.950752	1	-
ENSMUSG	D3Ertd751	DNA segr	-	-	-	-	-	-	3:4174261	353	191	195	49	554	400	1.294194	0.559247	0.790145	0.138595	1.307944	1.153707	0.881195	0.866749	0.912238	1	-
ENSMUSG	Crispld1	cysteine-r	GO:00325	developm	-	-	-	-	1:1772704	163	230	168	206	267	187	1.54374	1.739639	1.758502	1.505161	1.628362	1.393282	1.680627	1.508935	0.323421	1	-
ENSMUSG	Gdap1	gangliosic	GO:00081	metabolic	-	-	-	-	1:1714536	2163	2587	2158	3140	3338	3087	12.85716	12.28091	14.17708	14.39953	12.77701	14.43563	13.10505	13.87073	0.722686	1	-
ENSMUSG	Ly96	lymphocy	GO:00048	signal trar	K05400	lymphocy	mmu0514	Toxoplasr	1:1668805	9	22	15	16	26	16	0.346913	0.677272	0.639038	0.47582	0.645369	0.485202	0.554408	0.535464	0.882921	1	-
ENSMUSG	Itih5	inter-alph	GO:00081	metabolic	-	-	-	-	2:1015357	884	1102	812	1050	1396	1233	6.832324	6.80211	6.93616	6.260888	6.947947	7.497047	6.856864	6.901961	0.860186	1	-
ENSMUSG	Atp5c1	ATP synth	GO:00081	metabolic	K02136	F-type H+	mmu0110	Metabolic	2:1005601	2340	2577	1970	2717	4000	2380	39.87608	35.0717	37.10304	35.72042	43.89461	31.90689	37.35027	37.17397	0.786208	1	-
ENSMUSG	Taf3	TATA-box	GO:00081	metabolic	K14650	transcripti	mmu0516	Herpes sir	2:9914552	377	512	314	517	563	435	1.874505	2.033108	1.725527	1.983197	1.802637	1.70155	1.877713	1.829128	0.665676	1	-
ENSMUSG	Clec3b	C-type lec	GO:00325	developm	-	-	-	-	9:1231509	22	53	29	47	92	45	1.354099	2.605275	1.972768	2.231841	3.646466	2.178977	1.97738	2.685762	0.230995	1	-
ENSMUSG	Exosc7	exosome	GO:00081	metabolic	K12589	exosome	mmu0301	RNA degr	9:1231132	132	215	159	224	285	214	1.121109	1.458338	1.492513	1.467756	1.558742	1.429877	1.35732	1.485458	0.618024	1	-
ENSMUSG	Zdhhc3	zinc finge	GO:00081	metabolic	-	-	-	-	9:1230661	701	944	685	960	1168	1128	2.252724	2.422744	2.432918	2.380079	2.417058	2.851738	2.369462	2.549625	0.638474	1	-
ENSMUSG	Tgm4	transgluta	GO:00081	metabolic	-	-	-	-	9:1230347	12	10	12	17	16	9	0.126937	0.084478	0.140294	0.138741	0.108989	0.074896	0.117236	0.107542	0.791341	1	-
ENSMUSG	St8sia2	ST8 alpha	GO:00081	metabolic	-	-	-	-	7:7393911	108	122	76	115	136	111	1.137536	1.026236	0.884711	0.934473	0.922432	0.919755	1.016161	0.925554	0.43621	1	-
ENSMUSG	Pgm2	phosphog	GO:00081	metabolic	K01835	phosphog	mmu0110	Metabolic	4:9992941	1019	1421	1043	1464	1775	1504	21.30032	23.72202	24.09588	23.60931	23.89268	24.73264	23.03941	24.07821	0.830015	1	-
ENSMUSG	Hgs	HGF-regu	GO:00081	metabolic	K12182	hepatocyt	mmu0414	Endocytos	11:120467	1221	1461	1100	1775	1819	1566	15.99827	15.2881	15.9293	17.94263	15.34775	16.1421	15.73856	16.4775	0.821072	1	-
ENSMUSG	Rpl14	ribosomal	GO:00081	metabolic	K02875	large subu	mmu0301	Ribosome	9:1205714	461	557	402	661	781	589	9.180627	8.858751	8.847984	10.15553	10.0156	9.227778	8.962454	9.799637	0.538291	1	-
ENSMUSG	Rassf3	Ras assoc	GO:00508	response	-	-	-	-	10:121410	238	396	193	363	465	368	3.719441	4.942434	3.333529	4.376602	4.679586	4.524371	3.998468	4.526853	0.447327	1	-
ENSMUSG	Ccr1	chemokin	GO:00325	developm	K04176	C-C chem	mmu0406	Cytokine-	9:1239621	5	20	4	13	39	11	0.107753	0.344249	0.095287	0.216154	0.541269	0.186512	0.18243	0.314645	0.229545	1	-
ENSMUSG	Itgb1	integrin b	GO:00325	developm	K05719	integrin b	mmu0520	Pathways	8:1286856	1375	2067	1259	1876	2814	2082	14.84614	17.82367	15.02392	15.62694	19.56544	17.68489	15.89791	17.62576	0.419098	1	-
ENSMUSG	Nrp1	neuropilin	GO:00325	developm	K06724	neuropilin	mmu0516	HTLV-I inf	8:1283586	1653	2046	1445	2063	2296	2009	8.813226	8.711911	8.514843	8.485778	7.882948	8.426609	8.679993	8.265112	0.404617	1	-
ENSMUSG	Pard3	par-3 fam	GO:00325	developm	K04237	partitionin	mmu0516	Human pa	8:1270638	441	570	377	572	855	555	1.109736	1.145518	1.0485	1.110472	1.385484	1.098714	1.101251	1.198224	0.598733	1	-
ENSMUSG	Dhtkd1	dehydrog	GO:00325	developm	-	-	-	-	2:5896115	88	124	109	147	121	153	0.654062	0.736042	0.895387	0.842914	0.579132	0.894618	0.76183	0.772221	0.960794	1	-
ENSMUSG	Sec61a2	Sec61, alp	GO:00511	localizatio	K10956	protein tra	mmu0414	Phagoson	2:5870987	784	1044	783	1035	1334	1178	6.223291	6.618361	6.869303	6.33833	6.818907	7.356316	6.570318	6.837851	0.875192	1	-
ENSMUSG	Nudt5	nudix (nu	GO:00081	metabolic	K13987	ADP-suga	mmu0023	Purine me	2:5845019	246	301	209	344	409	292	1.857797	1.815411	1.744443	2.004249	1.989028	1.73483	1.805884	1.909369	0.791694	1	-
ENSMUSG	Zfp282	zinc finge	GO:00081	metabolic	-	-	-	-	6:4787720	361	482	359	502	610	429	2.767705	2.951243	3.041962	2.969246	3.011597	2.587503	2.920303	2.856115	0.694125	1	-
ENSMUSG	Pdia4	protein di	GO:00081	metabolic	K09582	protein di	mmu0414	Protein pr	6:4779614	1035	1306	770	1433	1720	1236	23.01353	23.19165	18.92258	24.58212	24.62782	21.62081	21.70926	23.61025	0.557377	1	-
ENSMUSG	Iscu	iron-sulfu	GO:00081	metabolic	-	-	-	-	5:1137727	239	352	280	391	474	367	3.639121	4.280434	4.711984	4.593101	4.647635	4.39619	4.210513	4.545642	0.66228	1	-
ENSMUSG	Fam20c	family wit	GO:00325	developm	-	-	-	-	5:1387545	529	631	367	701	730	566	3.245554	3.091775	2.488546	3.318037	2.884101	2.731873	2.941958	2.978004	0.919942	1	-
ENSMUSG	Prkar1b	protein ki	GO:00325	developm	K04739	cAMP-de	mmu0491	Insulin sig	5:1390173	7561	9186	7171	10434	11262	9835	59.41575	57.64937	62.28004	63.25626	56.9892	60.80056	59.78172	60.34867	0.859584	1	-
ENSMUSG	Pdgfa	platelet de	GO:00325	developm	K04359	platelet-d	mmu0520	Pathways	5:1389760	299	426	294	460	507	386	3.496723	3.97874	3.800001	4.150287	3.818144	3.551305	3.758488	3.839912	0.990932	1	-
ENSMUSG	Dnaaf5	dynein, ax	GO:00099	cellular pr	-	-	-	-	5:1391502	122	144	133	153	220	191	1.701477	1.603893	2.050058	1.646223	1.975805	2.095619	1.785143	1.905882	0.782344	1	-
ENSMUSG	Get4	golgi to E	GO:00081	metabolic	-	-	-	-	5:1392523	498	663	512	792	878	758	6.704944	7.128952	7.61873	8.226625	7.612286	8.028712	7.150875	7.955874	0.423286	1	-
ENSMUSG	Xiap	X-linked i	GO:00081	metabolic	K04725	E3 ubiquit	mmu0520	Pathways	X:4205967	1113	770	1413	2452	664	491	5.576703	3.08119	7.824761	9.478355	2.142421	1.935415	5.494218	4.518731	0.503067	1	-
ENSMUSG	Stag2	stromal ar	GO:00325	developm	K06671	cohesin cc	mmu0411	Cell cycle	X:4214931	1785	2171	1480	2220	2762	2124	9.693917	9.416006	8.883207	9.301315	9.659163	9.074582	9.331043	9.34502	0.800386	1	-
ENSMUSG	Cplx2	complexir	GO:00325	developm	K15294	complexir	mmu0472	Synaptic v	13:543713	27615	35350	25199	36717	44158	35964	342.1509	349.7904	345.0665	350.9701	352.3195	350.5513	345.6693	351.2803	0.922625	1	-
ENSMUSG	Higd2a	HIG1 dom	GO:00081	metabolic	-	-	-	-	13:545902	705	985	724	1033	1162	996	66.42892	74.12257	75.39695	75.09291	70.50649	73.83096	71.98281	73.14345	0.94093	1	-
ENSMUSG	Nop16	NOP16 nu	GO:00056	cellintrace	-	-	-	-	13:545841	265	386	294	438	477	347	5.862459	6.819741	7.188335	7.475479	6.795284	6.039125	6.623512	6.769963	0.998253	1	-
ENSMUSG	Arl10	ADP-ribo	GO:00508	response	-	-	-	-	13:545750	165	261	204	292	276	311	4.180325	5.280947	5.712194	5.707407	4.502873	6.198647	5.057822	5.469642	0.704268	1	-
ENSMUSG	4833439L	RIKEN cD	GO:00081	metabolic	-	-	-	-	13:545512	1950	2617	1797	2874	2985	2565	16.31004	17.48115	16.61175	18.54546	16.07754	16.87791	16.80098	17.16697	0.979055	1	-
ENSMUSG	Thoc3	THO com	GO:00081	metabolic	K12880	THO com	mmu0301	RNA trans	13:544588	406	629	489	622	736	611	7.457765	9.227392	9.92746	8.814612	8.705919	8.829465	8.870872	8.783332	0.771299	1	-
ENSMUSG	Faf2	Fas associ	GO:00081	metabolic	-	-	-	-	13:546217	963	1311	924	1324	1583	1430	5.760087	6.262552	6.108313	6.109717	6.09731	6.728987	6.043651	6.312005	0.838928	1	-
ENSMUSG	Unc5a	unc-5 net	GO:00325	developm	K07521	netrin rec	mmu0436	Axon guic	13:549494	2728	3228	2561	3596	4091	3851	32.41224	30.62979	33.62955	32.96209	31.30033	35.99558	32.22386	33.41933	0.895778	1	-
ENSMUSG	Uimc1	ubiquitin i	GO:00081	metabolic	K20775	BRCA1-A	mmu0344	Homologo	13:550278	238	318	198	361	363	316	1.715483	1.830551	1.577326	2.007456	1.684886	1.791873	1.707787	1.828072	0.736643	1	-
ENSMUSG	Smad7	SMAD far	GO:00325	developm	K19631	mothers a	mmu0439	Hippo sig	18:753675	239	364	257	315	492	370	1.888556	2.297099	2.244466	1.920317	2.503528	2.300091	2.143374	2.241312	0.86889	1	-
ENSMUSG	Myo5b	myosin VB	GO:00038	catalytic a	-	-	-	-	18:744409	2237	2915	2242	3196	3904	3338	10.54889	10.97805	11.68485	11.62727	11.8551	12.38334	11.07059	11.95524	0.544223	1	-
ENSMUSG	Casp12	caspase 1	GO:00081	metabolic	K04741	caspase 1	mmu0501	Alzheimer	9:5345430	10	33	10	25	43	24	0.103437	0.272603	0.114315	0.199502	0.286413	0.195293	0.163451	0.227069	0.321283	1	-
ENSMUSG	Snca	synuclein,	GO:00325	developm	K04528	alpha-syn	mmu0501	Alzheimer	6:6073157	4721	5636	4215	6656	8109	5649	78.88761	75.21278	77.84293	85.80616	87.2563	74.2604	77.31444	82.44095	0.644624	1	-
ENSMUSG	Gria4	glutamate	GO:00048	signal trar	K05200	glutamate	mmu0408	Neuroacti	9:4417896	1274	1761	1127	1717	1892	1637	8.45706	9.335875	8.268388	8.793271	8.087712	8.548884	8.687108	8.476622	0.600209	1	-
ENSMUSG	Kbtbd3	kelch repe	GO:00081	metabolic	-	-	-	-	9:4309833	104	110	90	123	150	120	1.477099	1.247708	1.412751	1.347752	1.371901	1.340816	1.379186	1.35349	0.779829	1	-
ENSMUSG	Cwf19l2	CWF19-lil	-	-	-	-	-	-	9:3403592	316	375	251	349	464	343	1.640954	1.555202	1.440557	1.398189	1.55161	1.401247	1.545571	1.450349	0.462137	1	-
ENSMUSG	Rp1	retinitis pi	GO:00325	developm	-	-	-	-	1:3999557	1	2	0	0	1	4	0.004907	0.007836	0	0	0.003158	0.015434	0.004247	0.006197	1	1	-
ENSMUSG	Sox17	SRY (sex c	GO:00325	developm	K04495	transcripti	mmu0431	Wnt signa	1:4490931	58	67	65	42	112	77	0.308562	0.284667	0.382189	0.172382	0.383698	0.322267	0.325139	0.292782	0.572139	1	-
ENSMUSG	Lypla1	lysophosp	GO:00081	metabolic	K06128	lysophosp	mmu0523	Choline m	1:4807788	411	496	343	610	741	550	2.720606	2.622114	2.509373	3.115186	3.158615	2.864163	2.617365	3.045988	0.247188	1	-
ENSMUSG	Oprk1	opioid rec	GO:00048	signal trar	K04214	kappa-typ	mmu0408	Neuroacti	1:5588466	13	32	9	29	30	22	0.056701	0.111465	0.043383	0.097583	0.084259	0.075489	0.070516	0.085777	0.561476	1	-
ENSMUSG	Rb1cc1	RB1-indu	GO:00325	developm	K17589	RB1-indu	mmu0414	Autophag	1:6206197	1287	1509	1151	1592	2010	1591	3.488183	3.266299	3.447806	3.328848	3.508096	3.392358	3.400763	3.409767	0.817108	1	-
ENSMUSG	Sntg1	syntrophi	GO:00051	structural	-	-	-	-	1:8361475	453	535	410	629	717	606	1.508059	1.422394	1.508519	1.615479	1.537071	1.587098	1.479657	1.579883	0.701444	1	-
ENSMUSG	Adhfe1	alcohol de	GO:00081	metabolic	-	-	-	-	1:9547948	289	331	316	357	403	345	3.459283	3.164186	4.180445	3.296757	3.106331	3.248764	3.601305	3.217284	0.27269	1	-
ENSMUSG	Mybl1	myeloblas	GO:00081	metabolic	-	-	-	-	1:9667415	59	81	51	62	100	70	0.553796	0.607195	0.529075	0.448974	0.604436	0.516895	0.563355	0.523435	0.581688	1	-
ENSMUSG	Sgk3	serum/glu	GO:00081	metabolic	K13304	serum/glu	mmu0415	PI3K-Akt s	1:9798107	134	182	112	178	170	168	0.342938	0.371985	0.316791	0.351447	0.280165	0.338243	0.343905	0.323285	0.541471	1	-
ENSMUSG	Cops5	COP9 sigr	GO:00081	metabolic	-	-	-	-	1:1002460	528	631	508	704	797	561	5.673826	5.415231	6.033273	5.836404	5.515121	4.742595	5.707443	5.364706	0.440749	1	-
ENSMUSG	Stau2	staufen (R	GO:00511	localizatio	-	-	-	-	1:1622867	2402	2125	2251	2126	3831	2835	6.278322	4.435833	6.502685	4.287105	6.448182	5.829542	5.738947	5.52161	0.619002	1	-
ENSMUSG	Rdh10	retinol de	GO:00325	developm	K11151	retinol del	mmu0110	Metabolic	1:1610577	165	201	143	209	224	212	1.816549	1.767284	1.739985	1.775173	1.588055	1.836161	1.774606	1.73313	0.724416	1	-
ENSMUSG	Terf1	telomeric	GO:00081	metabolic	-	-	-	-	1:1580564	147	200	128	231	276	176	1.504705	1.634968	1.448067	1.824214	1.819266	1.417285	1.529247	1.686922	0.596282	1	-
ENSMUSG	Tfap2b	transcripti	GO:00325	developm	-	-	-	-	1:1920891	5	4	3	0	3	6	0.030615	0.01956	0.020305	0	0.011831	0.028905	0.023493	0.013579	0.372444	1	-
ENSMUSG	Msc	musculin	GO:00325	developm	-	-	-	-	1:1475334	18	29	19	38	28	36	0.247583	0.318572	0.288845	0.403249	0.248012	0.389558	0.285	0.34694	0.480644	1	-
ENSMUSG	Paqr8	progestin	GO:00325	developm	-	-	-	-	1:2089060	2394	950	2107	3256	3778	2845	11.86374	3.759817	11.54008	12.44836	12.0563	11.09151	9.054544	11.86539	0.245325	1	-
ENSMUSG	Eya1	EYA trans	GO:00325	developm	K15616	eyes abse	mmu0520	Transcript	1:1416895	279	338	255	412	501	444	1.197723	1.158817	1.209873	1.364519	1.384984	1.4995	1.188804	1.416334	0.187653	1	-
ENSMUSG	Tmem14a	transmem	GO:00511	localizatio	-	-	-	-	1:2121857	244	264	250	269	368	313	6.919738	5.979282	7.835849	5.88549	6.72051	6.983199	6.911623	6.529733	0.540378	1	-
ENSMUSG	Tram1	translocat	GO:00511	localizatio	K14010	translocat	mmu0414	Protein pr	1:1356469	483	610	456	716	902	626	8.02915	8.098384	8.377877	9.182602	9.655693	8.186675	8.16847	9.008323	0.487678	1	-
ENSMUSG	Lactb2	lactamase	GO:00081	metabolic	-	-	-	-	1:1362333	364	478	305	470	586	475	4.54131	4.762702	4.205577	4.52384	4.707951	4.662129	4.503196	4.631307	0.977524	1	-
ENSMUSG	Slco5a1	solute car	GO:00511	localizatio	-	-	-	-	1:1286654	73	74	51	82	85	89	0.167956	0.135973	0.129687	0.145553	0.125936	0.161093	0.144539	0.144194	0.871386	1	-
ENSMUSG	Ube2w	ubiquitin-	-	-	K10688	ubiquitin-	mmu0412	Ubiquitin	1:1654079	929	1188	786	1318	1504	1107	10.14359	10.35949	9.485183	11.10252	10.57496	9.509001	9.996088	10.39549	0.877601	1	-
ENSMUSG	Tmem70	transmem	GO:00099	cellular pr	-	-	-	-	1:1666520	289	408	310	444	535	395	4.271558	4.816082	5.064035	5.062926	5.092097	4.592995	4.717225	4.916006	0.88099	1	-
ENSMUSG	Pth2r	parathyro	GO:00048	signal trar	K04586	parathyro	mmu0408	Neuroacti	1:6528205	4	5	8	3	11	12	0.093331	0.093161	0.206277	0.054007	0.165262	0.220264	0.130923	0.146511	0.907984	1	-
ENSMUSG	Pikfyve	phosphoi	GO:00325	developm	K00921	1-phosph	mmu0481	Regulatio	1:6518668	1138	1547	1033	1480	1714	1422	3.09948	3.364982	3.109522	3.109844	3.00616	3.046891	3.191328	3.054298	0.463943	1	-
ENSMUSG	Idh1	isocitrate	GO:00325	developm	K00031	isocitrate	mmu0110	Metabolic	1:6515861	684	683	474	731	924	939	9.239768	7.368375	7.07669	7.618204	8.037696	9.978868	7.894944	8.544923	0.653103	1	-
ENSMUSG	Mettl21a	methyltra	GO:00081	metabolic	-	-	-	-	1:6460647	108	146	121	143	185	168	1.306577	1.410617	1.617867	1.334681	1.44124	1.59893	1.44502	1.458284	0.921944	1	-
ENSMUSG	Klf7	Kruppel-li	GO:00325	developm	-	-	-	-	1:6402944	1029	1335	913	1319	1524	1357	7.120214	7.377428	6.98225	7.0413	6.790748	7.387009	7.159964	7.073019	0.697036	1	-
ENSMUSG	Fastkd2	FAST kina	GO:00081	metabolic	-	-	-	-	1:6373061	236	334	202	385	430	348	1.836333	2.075542	1.73715	2.311165	2.154582	2.130245	1.883008	2.198664	0.274403	1	-
ENSMUSG	Mdh1b	malate de	GO:00081	metabolic	-	-	-	-	1:6369881	14	15	11	15	13	18	0.497285	0.425513	0.431814	0.411046	0.297354	0.502966	0.451537	0.403789	0.671225	1	-
ENSMUSG	Adam23	a disinteg	GO:00081	metabolic	-	-	-	-	1:6344589	1758	2397	1635	2327	2592	2329	5.137369	5.594169	5.280637	5.246244	4.877659	5.354294	5.337392	5.159399	0.508437	1	-
ENSMUSG	Eef1b2	eukaryoti	GO:00081	metabolic	-	-	-	-	1:6317682	862	1063	836	1043	1334	1387	9.630758	9.484889	10.323	8.99017	9.597627	12.19103	9.812884	10.25961	0.855851	1	-
ENSMUSG	Ndufs1	NADH de	GO:00081	metabolic	K03934	NADH de	mmu0110	Metabolic	1:6314359	2468	2993	2169	2941	3912	3215	13.53434	13.10825	13.14615	12.44278	13.81484	13.87024	13.26291	13.37595	0.859958	1	-
ENSMUSG	Nrp2	neuropilin	GO:00325	developm	-	-	-	-	1:6270328	2025	2798	1869	2698	3343	2544	8.898997	9.819956	9.077622	9.147205	9.460347	8.795167	9.265525	9.134239	0.667091	1	-
ENSMUSG	Maip1	matrix AA	GO:00511	localizatio	-	-	-	-	1:5740632	278	360	284	398	472	329	2.017369	2.086357	2.277752	2.2282	2.205657	1.878225	2.127159	2.104027	0.782022	1	-
ENSMUSG	Boll	bol, boule	GO:00325	developm	-	-	-	-	1:5524865	0	0	0	0	3	0	0	0	0	0	0.028879	0	0	0.009626	0.290718	1	-
ENSMUSG	Rftn2	raftlin fam	GO:00511	localizatio	-	-	-	-	1:5517015	519	829	534	903	1036	895	4.823313	6.152875	5.484868	6.47435	6.200013	6.543529	5.487019	6.405964	0.210127	1	-
ENSMUSG	Mob4	MOB fam	GO:00452	synapsellc	-	-	-	-	1:5513123	318	325	252	312	575	305	1.918057	1.565538	1.679893	1.451842	2.233348	1.447256	1.721162	1.710815	0.850848	1	-
ENSMUSG	Hspd1	heat shoc	GO:00325	developm	K04077	chaperoni	mmu0515	Tuberculo	1:5507783	2388	3184	2030	3107	4157	2996	52.50513	55.9096	49.3299	52.70345	58.85755	51.82272	52.58154	54.46124	0.89814	1	-
ENSMUSG	Coq10b	coenzyme	-	-	-	-	-	-	1:5505277	173	197	155	227	225	185	1.957938	1.780589	1.938782	1.982018	1.63979	1.647155	1.892436	1.756321	0.464245	1	-
ENSMUSG	Sf3b1	splicing fa	GO:00081	metabolic	K12828	splicing fa	mmu0304	Spliceoso	1:5498516	5484	5792	4151	5968	7362	6148	19.99777	16.8678	16.72953	16.78971	17.2876	17.63717	17.86503	17.23816	0.49845	1	-
ENSMUSG	Ccdc150	coiled-co	-	-	-	-	-	-	1:5425068	0	0	0	3	0	0	0	0	0	0.020883	0	0	0	0.006961	0.292701	1	-
ENSMUSG	Slc39a10	solute car	GO:00081	metabolic	-	-	-	-	1:4680754	2707	3449	2369	3611	4281	3536	20.98849	21.35659	20.3004	21.59984	21.37434	21.5683	20.88182	21.51416	0.946828	1	-
ENSMUSG	Wdr75	WD repea	GO:00081	metabolic	K14552	NET1-ass	mmu0300	Ribosome	1:4579516	443	499	389	552	662	479	3.652765	3.285975	3.544991	3.511458	3.515047	3.107169	3.494577	3.377891	0.612121	1	-
ENSMUSG	Ikzf2	IKAROS fa	GO:00081	metabolic	-	-	-	-	1:6953121	87	144	89	152	217	141	0.307215	0.406098	0.347344	0.414091	0.493444	0.391701	0.353552	0.433079	0.240828	1	-
ENSMUSG	Lancl1	LanC (bac	GO:00508	response	-	-	-	-	1:6700051	1345	2603	2132	2978	3444	2974	10.68755	16.51871	18.72362	18.25623	17.62276	18.59122	15.30996	18.15673	0.224018	1	-
ENSMUSG	Acadl	acyl-Coer	GO:00081	metabolic	-	-	mmu0110	Metabolic	1:6683083	614	878	551	888	1165	955	18.38632	20.99744	18.2358	20.51491	22.46502	22.49781	19.20652	21.82591	0.310034	1	-
ENSMUSG	Kansl1l	KAT8 regu	GO:00329	macromo	-	-	-	-	1:6671924	300	394	294	405	538	415	1.812528	1.901103	1.963171	1.887771	2.093155	1.972526	1.892267	1.984484	0.836768	1	-
ENSMUSG	Rpe	ribulose-5	GO:00081	metabolic	K01783	ribulose-p	mmu0110	Metabolic	1:6670083	185	709	475	674	320	323	2.84027	8.693207	8.059876	7.983221	3.163684	3.901225	6.531118	5.016043	0.268519	1	-
ENSMUSG	Icos	inducible	GO:00325	developm	K06713	inducible	mmu0451	Cell adhes	1:6097792	0	3	6	5	10	4	0	0.041625	0.115198	0.067002	0.111858	0.054659	0.052274	0.07784	0.553985	1	-
ENSMUSG	Ctla4	cytotoxic	GO:00325	developm	-	-	-	-	1:6088700	0	0	2	4	0	2	0	0	0.046704	0.065191	0	0.033248	0.015568	0.032813	0.570725	1	-
ENSMUSG	Cd28	CD28 anti	GO:00325	developm	-	-	-	-	1:6071680	0	6	4	3	6	5	0	0.043082	0.039751	0.020812	0.034737	0.035366	0.027611	0.030305	1	1	-
ENSMUSG	Raph1	Ras assoc	GO:00325	developm	-	-	-	-	1:6048229	1023	1206	797	1295	1437	1255	2.812103	2.647577	2.421368	2.74635	2.543707	2.714003	2.627016	2.66802	0.927295	1	-
ENSMUSG	Carf	calcium re	GO:00081	metabolic	-	-	-	-	1:6009824	248	313	312	337	330	336	0.898338	0.905481	1.249081	0.941778	0.769765	0.957502	1.017633	0.889681	0.244556	1	-
ENSMUSG	Ica1l	islet cell a	GO:00325	developm	-	-	-	-	1:5998249	408	550	376	865	637	545	3.040608	3.273476	3.09696	4.973326	3.056991	3.195272	3.137015	3.741863	0.269023	1	-
ENSMUSG	Wdr12	WD repea	GO:00081	metabolic	-	-	-	-	1:6006978	533	710	524	711	786	690	2.250934	2.394637	2.445754	2.316516	2.137531	2.292426	2.363775	2.248824	0.477126	1	-
ENSMUSG	Sumo1	small ubic	GO:00325	developm	K12160	small ubic	mmu0301	RNA trans	1:5958655	860	1030	715	1169	1398	941	19.80015	18.93882	18.19378	20.76418	20.72677	17.04396	18.97758	19.51164	0.970741	1	-
ENSMUSG	Cdk15	cyclin-de	GO:00081	metabolic	-	-	-	-	1:5925690	5	12	5	12	8	0	0.100383	0.192419	0.110962	0.185877	0.10344	0	0.134588	0.096439	0.543815	1	-
ENSMUSG	Als2	amyotrop	GO:00325	developm	K04575	amyotrop	mmu0501	Amyotrop	1:5916292	1434	1870	1392	1884	2341	1887	8.406846	8.75531	9.019246	8.521094	8.837718	8.702959	8.727134	8.687257	0.749927	1	-
ENSMUSG	Stradb	STE20-rel	GO:00325	developm	K17532	STE20-rel	mmu0415	mTOR sig	1:5897352	381	466	326	519	604	616	2.462226	2.405109	2.328456	2.587625	2.513592	3.131807	2.398597	2.744341	0.342665	1	-
ENSMUSG	Trak2	trafficking	GO:00325	developm	K15374	trafficking	mmu0110	Metabolic	1:5890044	4171	5234	3624	5257	6584	5339	28.17183	28.23284	27.05265	27.39323	28.63646	28.36911	27.81911	28.13293	0.877382	1	-
ENSMUSG	Cflar	CASP8 an	GO:00325	developm	K04724	CASP8 an	mmu0421	Necropto	1:5871150	497	771	442	687	953	779	1.690391	2.09426	1.661497	1.802676	2.087267	2.084388	1.815383	1.991444	0.536738	1	-
ENSMUSG	Ndufb3	NADH de	GO:00325	developm	K03959	NADH de	mmu0110	Metabolic	1:5858638	679	911	564	907	1106	805	54.33608	58.22145	49.88212	55.99592	56.99391	50.67869	54.14655	54.55617	0.878804	1	-
ENSMUSG	Clk1	CDC-like	GO:00081	metabolic	K08823	CDC-like	mmu0513	Legionello	1:5841018	1084	1377	978	1581	1851	1672	5.80282	5.886944	5.786231	6.529384	6.380742	7.041371	5.825332	6.650499	0.24172	1	-
ENSMUSG	Ppil3	peptidylp	GO:00081	metabolic	-	-	-	-	1:5843099	117	188	154	195	211	202	1.17768	1.511283	1.713206	1.514282	1.367663	1.599572	1.46739	1.493839	0.97889	1	-
ENSMUSG	Nif3l1	Ngg1 inte	GO:00325	developm	-	-	-	-	1:5844515	298	353	318	441	517	376	1.664865	1.575013	1.963524	1.900779	1.859979	1.652578	1.734467	1.804445	0.896673	1	-
ENSMUSG	Orc2	origin rec	GO:00081	metabolic	K02604	origin rec	mmu0411	Cell cycle	1:5846277	517	682	489	673	787	645	6.720676	7.080324	7.025522	6.749448	6.587978	6.596189	6.942174	6.644538	0.512596	1	-
ENSMUSG	Col5a2	collagen,	GO:00325	developm	K19721	collagen,	mmu0497	Protein di	1:4537432	194	315	176	324	294	319	1.17245	1.520369	1.17558	1.510665	1.144182	1.516683	1.289466	1.39051	0.723239	1	-
ENSMUSG	Col3a1	collagen,	GO:00325	developm	K19720	collagen,	mmu0492	Relaxin si	1:4531153	436	1117	449	507	1103	575	2.480092	5.074355	2.822769	2.224947	4.040285	2.573122	3.459072	2.946118	0.328893	1	-
ENSMUSG	Kdelc1	KDEL (Lys	GO:00081	metabolic	-	-	-	-	1:4410654	133	221	143	195	267	225	1.361853	1.807247	1.618305	1.540439	1.760532	1.812473	1.595802	1.704481	0.768547	1	-
ENSMUSG	Ercc5	excision re	GO:00081	metabolic	K10846	DNA excis	mmu0342	Nucleotid	1:4414774	331	536	431	492	638	498	2.54089	3.286007	3.656639	2.913754	3.153794	3.007447	3.161179	3.024998	0.589486	1	-
ENSMUSG	Tex30	testis expr	GO:00038	catalytic a	-	-	-	-	1:4408661	32	35	31	40	67	53	0.377775	0.329991	0.404478	0.364317	0.509347	0.492237	0.370748	0.4553	0.363271	1	-
ENSMUSG	Khdrbs2	KH domai	GO:00081	metabolic	-	-	-	-	1:3217271	131	213	118	194	248	193	0.710169	0.922179	0.706998	0.811376	0.865755	0.823107	0.779782	0.833413	0.775143	1	-
ENSMUSG	Ptp4a1	protein ty	GO:00325	developm	-	-	-	-	1:3094030	446	507	242	389	594	646	2.484662	2.255726	1.490032	1.671908	2.130952	2.831236	2.076807	2.211365	0.819306	1	-
ENSMUSG	Slc9a4	solute car	GO:00325	developm	K13961	solute car	mmu0497	Gastric ac	1:4058008	434	532	394	622	619	562	4.165888	4.078261	4.17985	4.606158	3.826164	4.243903	4.141333	4.225408	0.972498	1	-
ENSMUSG	Il18rap	interleukir	GO:00048	signal trar	K05174	interleukir	mmu0406	Cytokine-	1:4051536	1	3	5	2	8	9	0.008198	0.019639	0.045293	0.012648	0.042223	0.058027	0.024377	0.037632	0.543974	1	-
ENSMUSG	Il1r1	interleukir	GO:00048	signal trar	K04386	interleukir	mmu0401	MAPK sigi	1:4022508	307	380	271	356	503	344	1.909814	1.887913	1.863244	1.708573	2.015002	1.683536	1.88699	1.80237	0.56364	1	-
ENSMUSG	Map4k4	mitogen-	GO:00325	developm	K04407	mitogen-	mmu0401	MAPK sigi	1:3990091	2884	3912	2425	3927	4583	3860	13.54238	14.67049	12.58515	14.22627	13.85812	14.25929	13.59934	14.11456	0.880513	1	-
ENSMUSG	Npas2	neuronal	GO:00081	metabolic	K09026	neuronal	mmu0471	Circadian	1:3919373	552	621	405	741	764	728	6.824073	6.131148	5.533586	7.067293	6.082081	7.080214	6.162935	6.743196	0.562852	1	-
ENSMUSG	Pdcl3	phosducir	GO:00325	developm	-	-	-	-	1:3898560	295	347	235	379	576	363	3.513194	3.30031	3.093104	3.48217	4.417306	3.400926	3.302203	3.766801	0.389444	1	-
ENSMUSG	Chst10	carbohydr	GO:00081	metabolic	K09674	carbohydr	mmu0110	Metabolic	1:3886386	238	340	440	319	360	655	0.637167	0.726943	1.301891	0.658865	0.620629	1.379519	0.888667	0.886338	0.898635	1	-
ENSMUSG	Rev1	REV1, DN	GO:00081	metabolic	K03515	DNA repa	mmu0346	Fanconi a	1:3805278	520	615	504	663	814	714	3.401933	3.21324	3.644178	3.346307	3.429267	3.674778	3.419784	3.483451	0.961133	1	-
ENSMUSG	Eif5b	eukaryoti	GO:00081	metabolic	K03243	translatior	mmu0301	RNA trans	1:3799801	2122	2730	1782	2604	3505	2503	11.5642	11.88171	10.73309	10.94817	12.30022	10.73105	11.393	11.32648	0.746897	1	-
ENSMUSG	Mrpl30	mitochon	GO:00081	metabolic	K02907	large subu	mmu0301	Ribosome	1:3789047	273	345	247	354	472	348	3.657862	3.691727	3.657696	3.659299	4.072502	3.668209	3.669095	3.800003	0.926966	1	-
ENSMUSG	Mitd1	MIT, micr	GO:00511	localizatio	-	-	-	-	1:3787480	85	119	98	171	162	111	1.295868	1.448884	1.651247	2.011257	1.590413	1.331291	1.465333	1.644321	0.573639	1	-
ENSMUSG	2010300C	RIKEN cD	-	-	-	-	-	-	1:3761167	9510	12774	9876	12306	15210	13537	76.93953	82.53568	88.30735	76.80973	79.24154	86.15934	82.59419	80.73687	0.58647	1	-
ENSMUSG	Stk17b	serine/thr	GO:00081	metabolic	-	-	-	-	1:5375550	70	109	59	115	175	86	0.775548	0.964458	0.722457	0.982968	1.24855	0.749585	0.820821	0.993701	0.341455	1	-
ENSMUSG	Asnsd1	asparagin	GO:00081	metabolic	-	-	-	-	1:5334461	225	172	204	329	485	92	2.655218	1.621031	2.660685	2.995315	3.685641	0.854113	2.312312	2.51169	0.824539	1	-
ENSMUSG	Osgepl1	O-sialogl	GO:00081	metabolic	-	-	-	-	1:5331362	187	206	133	208	253	198	1.259136	1.107759	0.989761	1.080503	1.096999	1.048834	1.118885	1.075445	0.632057	1	-
ENSMUSG	Ormdl1	ORM1-lik	GO:00081	metabolic	-	-	-	-	1:5329709	100	128	108	145	164	120	1.24812	1.275892	1.489803	1.396221	1.318125	1.178285	1.337938	1.297543	0.728952	1	-
ENSMUSG	Pms1	PMS1 hor	GO:00081	metabolic	-	-	-	-	1:5318918	95	91	84	91	121	99	0.616423	0.471563	0.602391	0.455541	0.505586	0.50536	0.563459	0.488829	0.292151	1	-
ENSMUSG	Mstn	myostatin	GO:00325	developm	-	-	-	-	1:5306164	8	5	2	11	6	6	0.094559	0.047194	0.026127	0.100307	0.045664	0.055791	0.05596	0.067254	0.794326	1	-
ENSMUSG	Inpp1	inositol pc	GO:00081	metabolic	K01107	inositol pc	mmu0110	Metabolic	1:5278542	523	681	451	720	873	678	4.100862	4.264481	3.908374	4.355477	4.408004	4.182286	4.091239	4.315256	0.779638	1	-
ENSMUSG	Gls	glutamina	GO:00081	metabolic	K01425	glutamina	mmu0110	Metabolic	1:5216344	4961	6509	4504	7607	8110	6607	12.34198	12.9323	12.384	14.60022	12.99245	12.93096	12.55276	13.50788	0.55975	1	-
ENSMUSG	Nabp1	nucleic ac	GO:00081	metabolic	-	-	-	-	1:5146586	228	275	203	308	377	267	0.694674	0.66915	0.683578	0.72398	0.739677	0.639982	0.682467	0.701213	0.983773	1	-
ENSMUSG	Tmeff2	transmem	GO:00325	developm	-	-	-	-	1:5090064	1307	1711	1341	1799	2164	1781	11.13944	11.64618	12.63174	11.82903	11.87681	11.9416	11.80579	11.88248	0.849815	1	-
ENSMUSG	Mgat4a	mannosid	GO:00081	metabolic	K00738	alpha-1,3	mmu0110	Metabolic	1:3743934	666	827	608	832	1087	781	2.606209	2.58456	2.629576	2.511821	2.739172	2.404347	2.606782	2.55178	0.661045	1	-
ENSMUSG	Unc50	unc-50 ho	GO:00511	localizatio	-	-	-	-	1:3743013	190	241	169	296	327	219	2.862329	2.899549	2.813853	3.440241	3.172263	2.5955	2.858577	3.069335	0.717856	1	-

ENSMUSC	Coa5	cytochron	GO:00099	cellular pr	-	-	-	-	1:3741708	1354	1781	1281	1683	2289	1745	15.44706	16.22695	16.15188	14.81295	16.81619	15.6615	15.94196	15.76355	0.703879	1	-
ENSMUSC	Inpp4a	inositol pc	GO:00160	membran	K01109	inositol pc	mmu011C	Metabolic	1:3729986	1601	2338	1386	2026	3224	2132	6.17719	7.20427	5.910302	6.030724	8.010309	6.471392	6.430587	6.837475	0.709426	1	-
ENSMUSC	Cnga3	cyclic nucl	GO:00325	developm	K04950	cyclic nucl	mmu0474	Olfactory	1:3721443	5	6	0	3	2	13	0.054327	0.052068	0	0.025153	0.013992	0.111128	0.035465	0.050091	0.729614	1	-
ENSMUSC	Tmem131	transmem	GO:00160	membran	-	-	-	-	1:3679219	2113	2988	2197	2482	3304	2986	9.727497	10.98571	11.17837	8.815231	9.794816	10.8144	10.63052	9.808148	0.261437	1	-
ENSMUSC	Zap70	zeta-chai	GO:00325	developm	K07360	tyrosine-p	mmu0401	Ras signal	1:3676179	67	102	65	93	119	95	0.976109	1.186789	1.046619	1.045291	1.116418	1.088836	1.069839	1.083515	0.959816	1	-
ENSMUSC	Sema4c	sema don	GO:00325	developm	K06521	semaphor	mmu0436	Axon guic	1:3654863	144	186	128	191	235	207	1.161479	1.198137	1.141046	1.188532	1.220593	1.313498	1.166887	1.240874	0.785288	1	-
ENSMUSC	Plekhb2	pleckstrin	GO:00325	developm	-	-	-	-	1:3484997	3520	4741	3322	4961	5883	4979	25.36881	27.28809	26.46085	27.58398	27.30303	28.22997	26.37259	27.70566	0.758414	1	-
ENSMUSC	Cfc1	cripto, FRI	GO:00325	developm	-	-	-	-	1:3453564	1	0	1	1	0	3	0.058727	0	0.064891	0.0453	0	0.138542	0.041206	0.061281	1	1	-
ENSMUSC	Imp4	IMP4, U3	GO:00081	metabolic	K14561	U3 small r	mmu030C	Ribosome	1:3443985	434	569	415	587	714	539	2.595926	2.718072	2.743454	2.708761	2.750146	2.53631	2.685817	2.665072	0.77514	1	-
ENSMUSC	Dst	dystonin [	GO:00325	developm	-	-	-	-	1:3390822	11760	14939	10383	13978	16430	15407	13.27718	13.46995	12.95592	12.17514	11.94513	13.68446	13.23435	12.60158	0.386623	1	-
ENSMUSC	Prim2	DNA prim	GO:00081	metabolic	K02685	DNA prim	mmu011C	Metabolic	1:3345381	92	130	90	145	156	134	0.92088	1.039206	0.995642	1.119723	1.005525	1.055187	0.985243	1.060145	0.742657	1	-
ENSMUSC	Zfp142	zinc finge	GO:00971	organic cy	-	-	-	-	1:7456512	738	845	642	890	937	891	2.143102	1.959698	2.060479	1.993916	1.75219	2.035521	2.054427	1.927209	0.389533	1	-
ENSMUSC	Col19a1	collagen,	GO:00325	developm	-	-	-	-	1:2426189	390	428	296	510	488	512	3.037332	2.662053	2.547803	3.064276	2.447382	3.136952	2.749063	2.88287	0.861951	1	-
ENSMUSC	Rhbdd1	rhomboid	GO:00325	developm	-	-	-	-	1:8231645	170	210	166	214	295	232	1.488585	1.468549	1.606494	1.445665	1.663413	1.59817	1.52121	1.569082	0.960255	1	-
ENSMUSC	Col9a1	collagen,	GO:00329	macromo	K08131	collagen,	mmu0415	PI3K-Akt	1:2417761	0	3	8	11	6	8	0	0.027752	0.102395	0.09829	0.044746	0.072886	0.043383	0.071974	0.358932	1	-
ENSMUSC	Mff	mitochon	GO:00325	developm	-	-	-	-	1:8272489	1507	1985	1531	2174	2498	2113	5.961037	6.270683	6.693149	6.634352	6.362908	6.575352	6.30829	6.524204	0.912846	1	-
ENSMUSC	Fam135a	family witl	GO:00081	metabolic	-	-	-	-	1:2401109	713	870	593	837	811	910	2.535199	2.470515	2.330366	2.296035	1.85694	2.54551	2.44536	2.232828	0.292479	1	-
ENSMUSC	Sdhaf4	succinate	GO:00325	developm	-	-	-	-	1:2399593	57	56	42	78	99	60	3.138222	2.462348	2.555679	3.313098	3.50996	2.598792	2.718749	3.140617	0.4919	1	-
ENSMUSC	Smap1	small ArfC	GO:00325	developm	K12486	stromal m	mmu0414	Endocytos	1:2384484	1089	1564	1123	1419	1719	1707	12.74531	14.61857	14.5261	12.81254	12.95547	15.7169	13.96333	13.8283	0.733767	1	-
ENSMUSC	B3gat2	beta-1,3-	GO:00081	metabolic	K10157	galactosyl	mmu011C	Metabolic	1:2376175	341	482	313	544	538	513	2.344688	2.646813	2.378602	2.885754	2.382142	2.774971	2.456701	2.680956	0.589255	1	-
ENSMUSC	Ogfrl1	opioid grc	GO:00160	membran	-	-	-	-	1:2336642	4778	6112	4236	6028	7674	6272	31.46755	32.14741	30.83327	30.62812	32.54567	32.49629	31.48274	31.89003	0.893513	1	-
ENSMUSC	Agfg1	ArfGAP w	GO:00325	developm	K15044	Arf-GAP c	mmu0516	Influenza	1:8283948	1849	1956	1512	2250	2805	2332	8.124382	6.863857	7.342636	7.627221	7.936721	8.061075	7.443625	7.875006	0.730862	1	-
ENSMUSC	Nhej1	nonhomo	GO:00081	metabolic	-	-	-	-	1:7496713	12	23	21	24	22	20	0.655349	1.003193	1.267583	1.011225	0.773724	0.859291	0.975375	0.881413	0.691929	1	-
ENSMUSC	Wnt10a	wingless-	GO:00325	developm	K01357	wingless-t	mmu052C	Pathways	1:7479151	34	27	22	45	40	49	0.73073	0.463429	0.522578	0.746132	0.553582	0.828466	0.572246	0.709394	0.407462	1	-
ENSMUSC	Cyp27a1	cytochron	GO:00081	metabolic	K00488	cholestan	mmu011C	Metabolic	1:7471357	26	55	37	33	79	54	0.510783	0.862926	0.803351	0.500164	0.999415	0.83457	0.725686	0.77805	0.847527	1	-
ENSMUSC	Rnf25	ring finge	GO:00081	metabolic	-	-	-	-	1:7459374	465	594	405	645	750	586	7.050422	7.192733	6.786784	7.544874	7.322806	6.989889	7.00998	7.285856	0.893339	1	-
ENSMUSC	Bcs1l	BCS1-like	GO:00099	cellular pr	-	-	-	-	1:7458828	273	394	281	330	422	407	5.007173	5.771268	5.696168	4.669533	4.984213	5.872663	5.491536	5.17547	0.493902	1	-
ENSMUSC	Plcd4	phospholi	GO:00048	signal trar	K05857	phosphati	mmu011C	Metabolic	1:7454288	225	318	236	384	368	324	1.536185	1.733937	1.780816	2.022654	1.617942	1.740268	1.683646	1.793621	0.755544	1	-
ENSMUSC	Cnot9	CCR4-NC	GO:00081	metabolic	K12606	CCR4-NO	mmu0301	RNA degr	1:7450605	423	573	390	575	675	568	6.068504	6.56511	6.183774	6.364141	6.235911	6.410628	6.272463	6.336893	0.901291	1	-
ENSMUSC	Ctdsp1	CTD (carb	GO:00325	developm	-	-	-	-	1:7439150	244	474	340	414	626	440	2.643399	4.101062	4.070972	3.460215	4.367182	3.750037	3.605144	3.859145	0.744135	1	-
ENSMUSC	Slc11a1	solute car	GO:00081	metabolic	K12347	natural re:	-	-	1:7437519	31	52	30	43	52	40	0.24668	0.330466	0.263844	0.263983	0.266463	0.250407	0.28033	0.260284	0.630494	1	-
ENSMUSC	Pnkd	paroxysm	GO:00081	metabolic	-	-	-	-	1:7428493	3798	4611	3290	4983	5628	4549	17.95159	17.4056	17.18663	18.1706	17.12998	16.91511	17.51461	17.40523	0.717839	1	-
ENSMUSC	Cxcr2	chemokin	GO:00325	developm	K05050	C-X-C ch	mmu0406	Cytokine-	1:7415398	0	3	0	4	0	0	0	0.027784	0	0.035777	0	0	0.009261	0.011926	1	1	-
ENSMUSC	Ppm1f	protein pl	GO:00081	metabolic	-	-	-	-	16:168964	1095	1429	1091	1574	1746	1434	13.35036	13.91417	14.70113	14.80519	13.70812	13.75432	13.98855	14.08921	0.860373	1	-
ENSMUSC	Igfbp5	insulin-lik	GO:00325	developm	-	-	-	-	1:7285793	3751	5320	3112	5316	6349	5020	23.0481	26.10631	21.13365	25.20016	25.12164	24.26625	23.42935	24.86269	0.685646	1	-
ENSMUSC	Xrcc5	X-ray rep	GO:00325	developm	K10885	ATP-depe	mmu0345	Non-hom	1:7230742	313	366	273	383	490	372	7.479907	6.985216	7.210429	7.061247	7.540547	6.993696	7.225184	7.198497	0.81666	1	-
ENSMUSC	Pecr	peroxison	GO:00081	metabolic	K07753	peroxison	mmu0414	Peroxison	1:7225916	45	55	57	59	72	83	1.216294	1.187239	1.702738	1.230295	1.253179	1.764887	1.368757	1.41612	0.976819	1	-
ENSMUSC	Atic	5-aminoir	GO:00325	developm	K00602	phosphor	mmu011C	Metabolic	1:7155715	404	563	406	612	734	636	2.80312	3.119716	3.113386	3.275988	3.279529	3.471591	3.012074	3.342369	0.456578	1	-
ENSMUSC	Fn1	fibronecti	GO:00325	developm	K05717	fibronecti	mmu052C	Pathways	1:7158552	1562	2282	1433	2121	2698	2194	5.275918	6.155713	5.349461	5.526982	5.868317	5.829946	5.593697	5.741748	0.978439	1	-
ENSMUSC	Zfand2b	zinc finge	GO:00511	localizatio	-	-	-	-	1:7516864	186	249	209	277	336	303	2.702721	2.889562	3.356454	3.10525	3.143992	3.463704	2.982912	3.237649	0.646155	1	-
ENSMUSC	Abcb6	ATP-bind	GO:00325	developm	K05661	ATP-bind	mmu0201	ABC trans	1:7517171	277	369	240	324	458	384	1.654095	1.759754	1.583939	1.49264	1.761167	1.803939	1.66593	1.685915	0.925199	1	-
ENSMUSC	Ankzf1	ankyrin re	GO:00508	response	-	-	-	-	1:7519215	335	440	325	504	581	406	2.018004	2.116776	2.163749	2.342275	2.253761	1.924038	2.09951	2.173358	0.927781	1	-
ENSMUSC	Glb1l	galactosic	GO:00081	metabolic	-	-	-	-	1:7519823	128	157	100	161	178	144	0.787532	0.771439	0.67999	0.764209	0.705233	0.696996	0.74632	0.722146	0.686914	1	-
ENSMUSC	Stk16	serine/thr	GO:00081	metabolic	-	-	-	-	1:7521083	395	587	411	596	743	631	2.183805	2.591796	2.51134	2.542104	2.645212	2.744461	2.42898	2.643926	0.577473	1	-
ENSMUSC	Tuba4a	tubulin, al	GO:00051	structural	K07374	tubulin alp	mmu0414	Phagoson	1:7521422	2454	3252	2524	3555	2489	3803	19.48714	20.62386	22.15183	21.77928	12.72779	23.75805	20.75428	19.4217	0.48845	1	-
ENSMUSC	Ptprn	protein ty	GO:00325	developm	K07817	receptor-	mmu0494	Type I dia	1:7524702	10144	11887	8970	13963	14913	13871	124.1229	116.1611	121.3059	131.8111	117.5067	133.5247	120.53	127.6141	0.703802	1	-
ENSMUSC	Slc23a3	solute car	GO:00511	localizatio	-	-	-	-	1:7512554	0	2	2	0	10	3	0	0.039426	0.054557	0	0.158925	0.058239	0.031328	0.072388	0.352062	1	-
ENSMUSC	Speg	SPEG com	GO:00325	developm	-	-	-	-	1:7537529	2263	3274	2342	3148	3394	3376	7.091328	8.193461	8.111041	7.61041	6.84872	8.322547	7.79861	7.593892	0.584157	1	-
ENSMUSC	Dnpep	aspartyl a	GO:00081	metabolic	-	-	-	-	1:7530789	270	304	270	437	463	407	2.081529	1.871706	2.300537	2.599143	2.298551	2.468446	2.084591	2.45538	0.244918	1	-
ENSMUSC	Obsl1	obscurin-	GO:00325	developm	-	-	-	-	1:7547931	234	288	175	285	338	286	0.957679	0.941329	0.791568	0.899867	0.890788	0.920831	0.896858	0.903829	0.893	1	-
ENSMUSC	Stk11ip	serine/thr	GO:00511	localizatio	-	-	-	-	1:7552152	436	533	364	548	582	513	2.956609	2.88656	2.728071	2.866942	2.541474	2.736755	2.85708	2.715057	0.495539	1	-
ENSMUSC	Trip12	thyroid hc	GO:00325	developm	K10590	E3 ubiquit	mmu0412	Ubiquitin	1:8472118	3825	4839	3419	4876	5269	4828	10.67108	10.78148	10.542	10.49472	9.465855	10.59632	10.66485	10.18563	0.402907	1	-
ENSMUSC	Slc16a14	solute car	GO:00511	localizatio	-	-	-	-	1:8490589	154	177	155	191	228	140	2.624315	2.40888	2.919266	2.511076	2.501992	1.876879	2.65082	2.296649	0.258502	1	-
ENSMUSC	Itm2c	integral m	GO:00325	developm	-	-	-	-	1:8589428	8119	10965	8710	11195	13253	10823	129.3998	139.5679	153.425	137.653	136.0192	135.7031	140.7976	136.4584	0.521441	1	-
ENSMUSC	4933407L	RIKEN cDI	-	-	-	-	-	-	1:8592848	24	13	11	29	37	17	0.32916	0.142389	0.166732	0.306842	0.326774	0.183424	0.21276	0.272347	0.435829	1	-
ENSMUSC	Spata3	spermato	GO:00160	membran	-	-	-	-	1:8601681	3	7	4	8	4	5	0.029019	0.054078	0.042767	0.059704	0.024918	0.03805	0.041955	0.04089	1	1	-
ENSMUSC	2810459N	RIKEN cDI	GO:00055	protein bi	-	-	-	-	1:8604586	269	303	242	351	392	325	4.270575	3.84169	4.246166	4.299035	4.007515	4.05909	4.119477	4.12188	0.849719	1	-
ENSMUSC	Psmd1	proteasor	GO:00081	metabolic	K03032	26S prote	mmu0516	Epstein-B	1:8606438	2403	3091	2173	3070	3719	3318	17.25742	17.7283	17.24761	17.00948	17.199	18.74604	17.41111	17.6515	0.904564	1	-
ENSMUSC	Ncl	nucleolin	GO:00325	developm	-	-	-	-	1:8634471	6604	8353	5369	7801	10227	7486	33.26138	33.59863	29.88639	30.31195	33.16929	29.66156	32.2488	31.0476	0.477769	1	-
ENSMUSC	Ptma	prothymo	GO:00099	cellular pr	K13784	prothymo	mmu0516	Epstein-B	1:8652672	547	674	660	880	1087	883	11.34084	11.15999	15.12337	14.07574	14.5125	14.40222	12.5414	14.33015	0.332155	1	-
ENSMUSC	Pde6d	phosphoc	GO:00987	molecular	K13758	retinal roc	mmu0023	Purine me	1:8654299	223	457	392	266	627	538	3.66217	5.993716	7.11486	3.370125	6.630648	6.950674	5.590249	5.650482	0.955961	1	-
ENSMUSC	Cops7b	COP9 sigr	GO:00081	metabolic	-	-	-	-	1:8658290	227	322	263	330	445	372	1.254657	1.421348	1.606576	1.407158	1.583849	1.617534	1.427527	1.53618	0.689557	1	-
ENSMUSC	Nppc	natriuretic	GO:00325	developm	K12336	natriuretic	mmu0541	Fluid shea	1:8666629	25	31	36	31	41	34	1.46491	1.450704	2.331472	1.401427	1.547098	1.56733	1.749029	1.505285	0.47724	1	-
ENSMUSC	Farsb	phenylala	GO:00081	metabolic	K01890	phenylala	mmu0097	Aminoacy	1:7841797	612	811	608	843	1000	919	10.51422	11.12737	11.54454	11.17336	11.0632	12.42088	11.06204	11.55248	0.852084	1	-
ENSMUSC	Mrpl44	mitochon	GO:00081	metabolic	-	-	-	-	1:7977601	214	285	212	359	370	248	5.658916	6.018806	6.195878	7.323922	6.30051	5.159201	5.957867	6.261211	0.847293	1	-
ENSMUSC	Serpine2	serine (or	GO:00325	developm	-	-	-	-	1:7979419	2271	2909	2139	3001	3288	2931	15.74642	16.10847	16.39163	16.05318	14.68085	15.9879	16.08217	15.57398	0.514548	1	-
ENSMUSC	Eif4e2	eukaryotic	GO:00325	developm	K03259	translatior	mmu0415	PI3K-Akt	1:8721391	929	953	745	1120	1391	1142	6.367643	5.216774	5.643726	5.922586	6.139671	6.158008	5.742714	6.073422	0.761009	1	-
ENSMUSC	Efhd1	EF hand c	GO:00325	developm	-	-	-	-	1:8726436	301	327	290	349	467	347	5.641033	4.894239	6.006709	5.046013	5.635913	5.116018	5.513994	5.265981	0.572856	1	-
ENSMUSC	Ngef	neuronal	GO:00325	developm	K07525	neuronal	mmu0436	Axon guic	1:8747683	1068	1504	1032	1467	1511	1475	15.62291	17.57051	16.68466	16.55584	14.23345	16.97438	16.62603	15.92122	0.48857	1	-
ENSMUSC	Ndufa10	NADH de	GO:00081	metabolic	K03954	NADH de	mmu011C	Metabolic	1:9243901	1786	2063	1799	2438	2824	2516	22.36001	20.62698	24.8925	23.54803	22.76722	24.78061	22.6265	23.69862	0.813667	1	-
ENSMUSC	Rnpepl1	arginyl an	GO:00081	metabolic	-	-	-	-	1:9291078	380	482	387	605	663	525	1.339845	1.357262	1.508095	1.645722	1.505355	1.456267	1.401734	1.535781	0.54809	1	-
ENSMUSC	Capn10	calpain 1C	GO:00081	metabolic	-	-	-	-	1:9293437	278	368	298	444	469	371	2.69945	2.853804	3.198116	3.326168	2.932637	2.834105	2.917123	3.03097	0.906584	1	-
ENSMUSC	Gpr35	G protein	GO:00048	signal trar	K04276	G protein	mmu0408	Neuroacti	1:9295086	16	16	7	23	27	19	0.068202	0.05447	0.03298	0.075638	0.074114	0.063714	0.051884	0.071155	0.335645	1	-
ENSMUSC	Mterf4	mitochon	GO:00325	developm	-	-	-	-	1:9329921	205	235	189	304	344	255	1.802034	1.649763	1.836186	2.06164	1.947252	1.763433	1.762661	1.924108	0.613804	1	-
ENSMUSC	Pask	PAS dom	GO:00081	metabolic	-	-	-	-	1:9330877	46	63	62	65	114	55	0.233745	0.255662	0.348196	0.254815	0.373029	0.219864	0.279201	0.28257	1	1	-
ENSMUSC	Ppp1r7	protein pl	GO:00081	metabolic	-	-	-	-	1:9334285	1988	2435	1821	2405	2783	2423	11.15145	10.90836	11.28942	10.40783	10.0527	10.6925	11.11641	10.38434	0.285907	1	-
ENSMUSC	2-Sep	septin 2 [S	GO:00325	developm	K16942	septin 2[-	mmu051C	Bacterial i	1:9347896	974	1320	819	1310	1418	1339	5.056162	5.472451	4.698861	5.246431	4.74016	5.468315	5.075825	5.151635	0.923077	1	-
ENSMUSC	Stk25	serine/thr	GO:00325	developm	-	-	-	-	1:9361975	3116	3800	3058	4288	4972	4631	25.61005	24.94264	27.77777	27.1893	26.31472	29.94323	26.11015	27.81575	0.66022	1	-
ENSMUSC	Bok	BCL2-rela	GO:00325	developm	K02561	Bcl-2-rela	mmu0421	Apoptosis	1:9368566	569	712	633	518	1085	802	9.589302	9.582983	11.79032	6.734963	11.77496	10.63311	10.32087	9.714345	0.551481	1	-
ENSMUSC	Thap4	THAP dor	GO:00971	organic cy	-	-	-	-	1:9370193	96	100	93	127	146	142	0.627441	0.521975	0.671792	0.640381	0.614485	0.730132	0.607069	0.661666	0.702236	1	-
ENSMUSC	Atg4b	autophag	GO:00081	metabolic	-	-	mmu0414	Autophag	1:9375150	868	1082	801	1158	1299	1278	5.051318	5.028727	5.151866	5.199049	4.867981	5.850955	5.077304	5.305995	0.84579	1	-
ENSMUSC	Dtymk	deoxythyr	GO:00325	developm	K00943	dTMP kin	mmu011C	Metabolic	1:9379257	262	340	301	378	406	374	4.588989	4.755994	5.826797	5.107852	4.579278	5.153452	5.05726	4.946861	0.713763	1	-
ENSMUSC	Ing5	inhibitor c	GO:00081	metabolic	-	-	-	-	1:9380396	276	340	256	360	426	364	1.899893	1.869154	1.947633	1.911843	1.888357	1.971205	1.90556	1.923802	0.905839	1	-
ENSMUSC	Inpp5d	inositol pc	GO:00325	developm	K03084	phosphati	mmu0407	Phosphati	1:8762031	214	283	195	265	316	301	1.052387	1.11146	1.059848	1.005397	1.0007	1.164497	1.074565	1.056865	0.742075	1	-
ENSMUSC	Atg16l1	autophag	GO:00081	metabolic	K17890	autophag	mmu0462	NOD-like	1:8775587	848	1129	861	1201	1289	1242	3.169713	3.370264	3.556922	3.463358	3.102643	3.652217	3.365633	3.406073	0.902506	1	-
ENSMUSC	Iqca	IQ motif c	GO:00971	organic cy	-	-	-	-	1:9004213	19	24	32	32	25	27	0.183356	0.184968	0.341307	0.238249	0.155359	0.204986	0.236544	0.199531	0.479343	1	-
ENSMUSC	Lrrfip1	leucine ric	GO:00081	metabolic	-	-	-	-	1:9099873	1053	1609	905	1547	2114	1691	5.181254	6.322784	4.921552	5.872555	6.698325	6.545775	5.475197	6.372218	0.204354	1	-
ENSMUSC	Scly	selenocys	GO:00081	metabolic	K01763	selenocyst	mmu011C	Metabolic	1:9129833	246	308	246	319	357	344	2.481872	2.48165	2.743	2.482937	2.319353	2.730321	2.56884	2.51087	0.704117	1	-
ENSMUSC	Klhl30	kelch-like	GO:00081	metabolic	-	-	-	-	1:9135101	2	3	0	0	5	0	0.039525	0.047365	0	0	0.063642	0	0.028963	0.021214	1	1	-
ENSMUSC	Ilkap	integrin-li	GO:00081	metabolic	-	-	-	-	1:9137386	349	471	352	490	556	525	2.544389	2.742367	2.836272	2.756038	2.610284	3.011126	2.707676	2.792483	0.958587	1	-
ENSMUSC	Asb1	ankyrin re	GO:00325	developm	-	-	-	-	1:9154054	583	684	477	773	748	694	3.850808	3.608154	3.482161	3.939064	3.181557	3.606233	3.647041	3.575618	0.684172	1	-
ENSMUSC	Cdh7	cadherin	GO:00160	membran	-	-	-	-	1:1099824	79	72	85	147	97	116	0.244024	0.177615	0.29018	0.350308	0.192942	0.281884	0.237273	0.275045	0.528812	1	-
ENSMUSC	Hdac4	histone de	GO:00325	developm	K11406	histone de	mmu0516	Human pa	1:9192877	1048	1126	885	1175	1345	1191	3.461667	2.970355	3.230829	2.994278	2.860888	3.094899	3.22095	2.983355	0.302631	1	-
ENSMUSC	Serpinb8	serine (or	GO:00081	metabolic	-	-	-	-	1:1075900	75	100	62	98	109	78	0.592415	0.630827	0.541257	0.597202	0.554428	0.484697	0.588166	0.545442	0.526479	1	-
ENSMUSC	Cln8	ceroid-lip	GO:00325	developm	-	-	-	-	8:1488133	447	551	395	578	733	633	3.06078	3.013161	2.989291	3.053395	3.23209	3.409882	3.021077	3.231789	0.690522	1	-
ENSMUSC	2310035C	RIKEN cDI	-	-	-	-	-	-	1:1056638	727	797	600	982	978	763	2.6986	2.362696	2.46151	2.812196	2.337745	2.228125	2.507602	2.459356	0.688967	1	-
ENSMUSC	Htr4	5 hydroxy	GO:00048	signal trar	K04160	5-hydroxy	mmu0408	Neuroacti	18:623242	276	369	259	355	389	355	3.618649	3.863751	3.753038	3.590839	3.284288	3.661647	3.745146	3.512258	0.446141	1	-
ENSMUSC	Gin1	gypsy retr	GO:00081	metabolic	-	-	-	-	1:9777017	135	188	115	175	229	162	0.996473	1.10825	0.93816	0.996553	1.088485	0.940714	1.014294	1.008584	0.846722	1	-
ENSMUSC	Pam	peptidylgl	GO:00325	developm	-	-	-	-	1:9779511	2514	3302	2286	3220	3588	3186	8.975563	9.414979	9.020269	8.869163	8.249042	8.948559	9.136937	8.688922	0.389431	1	-
ENSMUSC	Ccdc93	coiled-co	GO:00511	localizatio	-	-	-	-	1:1214310	833	1060	760	1143	1357	1072	3.959935	4.024343	3.993036	4.191983	4.154105	4.009116	3.992438	4.118401	0.943075	1	-
ENSMUSC	Actr3	ARP3 acti	GO:00511	localizatio	K18584	actin-rela	mmu0453	Tight junc	1:1253929	2971	5069	2914	3749	7019	5377	21.19449	28.87942	22.97506	20.63317	32.2441	30.17667	24.34966	27.68465	0.378099	1	-
ENSMUSC	Tmem163	transmem	GO:00452	synapse|m	-	-	-	-	1:1274865	110	151	76	147	181	109	0.962233	1.054898	0.734766	0.992054	1.019579	0.750112	0.917299	0.920582	0.90795	1	-
ENSMUSC	Ubxn4	UBX dom	GO:00081	metabolic	-	-	-	-	1:1282439	2097	2613	1766	2701	3299	2507	24.30964	24.19163	22.6265	24.15655	24.62731	22.86363	23.70926	23.8825	0.850906	1	-
ENSMUSC	Lct	lactase [S	GO:00081	metabolic	K01229	lactase-pl	mmu011C	Metabolic	1:1282847	825	1280	891	1148	1689	1257	7.564637	9.373247	9.029403	8.120948	9.972833	9.06735	8.655762	9.05371	0.833578	1	-
ENSMUSC	Mcm6	minichron	GO:00081	metabolic	K02542	DNA repli	mmu0411	Cell cycle	1:1283315	316	419	307	393	593	375	3.317458	3.513009	3.562082	3.18304	4.008928	3.097142	3.464183	3.429703	0.794325	1	-
ENSMUSC	Dars	aspartyl-t	GO:00081	metabolic	K01876	aspartyl-t	mmu0097	Aminoacy	1:1283637	1054	1380	1027	1392	1619	1351	13.37113	13.98146	14.39941	13.62376	13.22602	13.48322	13.91733	13.44433	0.528138	1	-
ENSMUSC	Rgs18	regulator	GO:00987	molecular	-	-	-	-	1:1447526	0	0	2	3	4	2	0	0	0.066457	0.06959	0.07744	0.047311	0.022152	0.06478	0.274582	1	-
ENSMUSC	Rgs1	regulator	GO:00987	molecular	-	-	-	-	1:1442422	12	14	8	10	26	10	0.040515	0.037751	0.029851	0.026049	0.056529	0.026562	0.036039	0.03638	1	1	-
ENSMUSC	Rgs2	regulator	GO:00325	developm	K18154	regulator	mmu0474	Olfactory	1:1439993	376	582	382	584	650	484	4.894021	6.049881	5.495257	5.864367	5.44811	4.956035	5.479719	5.422837	0.773461	1	-
ENSMUSC	Cdc73	cell divisic	GO:00325	developm	-	-	-	-	1:1435988	1247	1561	1129	1531	1899	1577	3.739291	3.738279	3.741653	3.541837	3.66693	3.720193	3.739741	3.642987	0.580878	1	-
ENSMUSC	Cfh	complem	GO:00081	metabolic	K04004	complem	mmu0461	Complem	1:1400847	422	781	431	546	645	690	1.973389	2.916735	2.227533	1.969803	1.942291	2.5384	2.372552	2.150165	0.340315	1	-
ENSMUSC	Tsn	translin [S	GO:00081	metabolic	-	-	-	-	1:1182947	1103	1381	1031	1432	1707	1308	5.627736	5.627268	5.813846	5.636784	5.608493	5.250209	5.689616	5.498495	0.5361	1	-
ENSMUSC	Epb41l5	erythrocyt	GO:00325	developm	-	-	-	-	1:1195450	153	198	129	180	268	163	0.504777	0.521697	0.470374	0.458154	0.569374	0.423063	0.498949	0.48353	0.709477	1	-
ENSMUSC	Ptpn4	protein ty	GO:00081	metabolic	-	-	-	-	1:1196524	1810	2400	1668	2219	2507	2339	7.42061	7.858114	7.55795	7.018571	6.618661	7.544011	7.612225	7.060414	0.258685	1	-
ENSMUSC	Dbi	diazepam	GO:00325	developm	K08762	diazepam	mmu0332	PPAR sign	1:1201132	788	1139	829	1205	1528	1167	16.31529	18.83382	18.97012	19.24802	20.3726	19.00861	18.03974	19.54308	0.564942	1	-
ENSMUSC	Sctr	secretin re	GO:00048	signal trar	K04588	secretin re	mmu0408	Neuroacti	1:1200068	1	3	13	1	7	4	0.022397	0.053653	0.321695	0.017276	0.100921	0.070454	0.132582	0.062884	0.275862	1	-
ENSMUSC	3110009E	RIKEN cDI	-	-	-	-	-	-	1:1201211	12	18	10	18	16	20	0.267206	0.320101	0.246098	0.309235	0.229425	0.35036	0.277802	0.29634	0.934738	1	-
ENSMUSC	Steap3	STEAP far	GO:00511	localizatio	K10142	metallore	mmu0411	p53 signa	1:1201907	91	145	99	124	87	150	0.543137	0.691164	0.653053	0.570978	0.33438	0.704321	0.629118	0.536559	0.33157	1	-
ENSMUSC	Cd55	CD55 mol	GO:00081	metabolic	K04006	decay acc	mmu0464	Hematop	1:1304390	105	189	94	158	271	189	1.029734	1.48027	1.018851	1.195426	1.711425	1.458164	1.176285	1.455005	0.245606	1	-
ENSMUSC	Ddx59	DEAD (As	GO:00081	metabolic	-	-	-	-	1:1364152	140	184	145	165	221	175	2.036728	2.137803	2.33141	1.851909	2.070389	2.002873	2.168647	1.975057	0.386188	1	-
ENSMUSC	C4bp	complem	GO:00081	metabolic	K04002	complem	mmu0461	Complem	1:1306347	9	7	9	9	17	5	0.074723	0.046416	0.082583	0.057651	0.090894	0.032659	0.067907	0.060401	0.836537	1	-
ENSMUSC	Cacna1s	calcium cl	GO:00511	localizatio	K04857	voltage-d	mmu0401	MAPK sig	1:1360527	9	12	16	27	10	5	0.05609	0.059727	0.11021	0.129819	0.040133	0.024515	0.075342	0.064822	0.72049	1	-
ENSMUSC	Pfkfb2	6-phosph	GO:00081	metabolic	K19029	6-phosph	mmu0415	AMPK sig	1:1306891	550	631	508	563	713	696	2.187749	2.004517	2.233294	1.727722	1.826328	2.177988	2.141853	1.910679	0.212074	1	-
ENSMUSC	Tmem9	transmem	GO:00511	localizatio	-	-	-	-	1:1360081	612	798	418	863	984	852	8.631914	8.988855	6.515972	9.390675	8.937287	9.453795	8.04558	9.260586	0.302011	1	-
ENSMUSC	Tnnt2	troponin	GO:00325	developm	K12045	troponin T	mmu0426	Adrenergi	1:1358363	54	55	41	43	67	49	0.376943	0.306616	0.31631	0.231569	0.301171	0.269083	0.33329	0.267275	0.192418	1	-
ENSMUSC	Csrp1	cysteine a	GO:00508	response	-	-	-	-	1:1357200	3118	4712	3408	5095	5965	5660	97.33088	117.4696	117.5766	122.7013	119.9056	138.9959	110.7924	127.2009	0.208914	1	-
ENSMUSC	Srgap2	SLIT-ROB	GO:00325	developm	K07526	SLIT-ROB	mmu0436	Axon guic	1:1312852	1780	2332	1644	2308	2652	2373	7.317765	7.656544	7.469769	7.320226	7.020801	7.674799	7.481359	7.338609	0.618069	1	-
ENSMUSC	Arl8a	ADP-ribo	GO:00508	response	-	-	-	-	1:1351468	1897	2376	1550	2516	3062	2742	41.92083	41.93288	37.85655	42.89468	43.57349	47.66952	40.57009	44.71256	0.414126	1	-
ENSMUSC	Eif2d	eukaryotic	GO:00081	metabolic	-	-	-	-	1:1311531	394	555	422	614	728	594	2.535505	2.852381	3.001425	3.048369	3.016859	3.007224	2.796437	3.02415	0.61784	1	-
ENSMUSC	Ube2t	ubiquitin-	GO:00081	metabolic	K13960	ubiquitin-	mmu0346	Fanconi a	1:1349625	9	16	8	16	19	21	0.087502	0.12424	0.08596	0.120016	0.118957	0.160626	0.099234	0.1332	0.397425	1	-
ENSMUSC	Rassf5	Ras assoc	GO:00081	metabolic	K08015	Ras assoc	mmu052C	Pathways	1:1311764	355	417	334	524	468	508	4.981752	4.673436	5.180207	5.67304	4.229169	5.608268	4.945131	5.170159	0.879719	1	-
ENSMUSC	Avpr1b	arginine v	GO:00048	signal trar	K04227	arginine v	mmu0408	Neuroacti	1:1315992	10	18	22	12	29	18	0.131422	0.188919	0.319553	0.121664	0.245423	0.186095	0.213298	0.184394	0.660545	1	-
ENSMUSC	Rab29	RAB29, m	GO:00325	developm	-	-	-	-	1:1318672	64	60	35	60	97	56	1.379839	1.033113	0.834007	0.998005	1.346711	0.949835	1.08232	1.098184	1	1	-
ENSMUSC	Nucks1	nuclear ca	GO:00081	metabolic	-	-	-	-	1:1319105	3647	4922	3524	4660	5653	4521	24.97241	26.91609	26.66903	24.61732	24.92632	24.35398	26.18584	24.63254	0.307323	1	-
ENSMUSC	Slc45a3	solute car	GO:00325	developm	K15379	solute car	mmu052C	Transcript	1:1319629	6	15	11	13	36	18	0.053969	0.107757	0.109353	0.090213	0.208528	0.127372	0.09036	0.142037	0.186831	1	-
ENSMUSC	Elk4	ELK4, mer	GO:00325	developm	K04376	ETS doma	mmu0401	MAPK sig	1:1320076	530	694	479	597	858	546	3.789761	3.963161	3.785464	3.293373	3.950738	3.071426	3.846129	3.438512	0.228657	1	-
ENSMUSC	Cdk18	cyclin-dep	GO:00081	metabolic	-	-	-	-	1:1321122	413	502	304	593	634	464	4.168103	4.046109	3.390844	4.617142	4.12033	3.683979	3.868352	4.140484	0.718288	1	-
ENSMUSC	Rbbp5	retinoblas	GO:00081	metabolic	-	-	-	-	1:1324773	1114	1362	1017	1493	1714	1389	7.767354	7.584221	7.837106	8.031158	7.695796	7.619057	7.729561	7.782004	0.854971	1	-
ENSMUSC	Nfasc	neurofasc	GO:00325	developm	K06757	neurofasc	mmu0451	Cell adhes	1:1325646	5439	7466	5177	7208	8291	7359	18.26003	20.01779	19.20911	18.66927	17.92437	19.43623	19.16231	18.67662	0.548797	1	-
ENSMUSC	Lrrn2	leucine ric	GO:00325	developm	-	-	-	-	1:1328802	3562	4827	3467	5169	5837	5315	53.22785	57.60605	57.25925	59.59116	56.16805	62.48261	56.03105	59.41394	0.681509	1	-
ENSMUSC	Pik3c2b	phosphati	GO:00081	metabolic	K00923	phosphati	mmu011C	Metabolic	1:1330456	394	555	376	597	686	577	2.318064	2.607765	2.444921	2.709781	2.599014	2.670648	2.456917	2.659814	0.611	1	-
ENSMUSC	Chit1	chitinase	GO:00081	metabolic	K01183	chitinase[	mmu011C	Metabolic	1:1341112	0	0	2	2	0	0	0	0	0.049861	0.034808	0	0	0.01662	0.011603	1	1	-
ENSMUSC	Klhl12	kelch-like	GO:00325	developm	-	-	-	-	1:1344555	712	937	748	967	1149	984	3.111908	3.270637	3.613226	3.260647	3.233862	3.383399	3.331924	3.292636	0.727919	1	-
ENSMUSC	Cyb5r1	cytochron	GO:00081	metabolic	K00326	cytochron	mmu0052	Amino su	1:1344055	189	261	170	330	301	278	1.619649	1.786257	1.610101	2.181737	1.661041	1.874193	1.672002	1.905657	0.414949	1	-
ENSMUSC	Adipor1	adiponect	GO:00325	developm	K07297	adiponect	mmu0493	Non-alco	1:1344153	951	1303	975	1361	1744	1424	7.411141	8.109511	8.397612	8.18263	8.751963	8.730222	7.972755	8.554938	0.621174	1	-
ENSMUSC	Ppfia4	protein ty	GO:00452	synapse|c	-	-	-	-	1:1342967	2193	2789	2015	2905	3396	2893	9.015649	9.156991	9.15546	9.213718	8.990437	9.356593	9.109367	9.186916	0.855445	1	-
ENSMUSC	Atp2b4	ATPase, C	GO:00325	developm	K05850	Ca2+ tran	mmu0402	cAMP sig	1:1336994	2576	3377	2274	3626	3692	3121	7.933304	8.305871	7.740082	8.61522	7.321914	7.561587	7.993086	7.832907	0.622822	1	-
ENSMUSC	Tor1aip1	torsin A ir	GO:00511	localizatio	-	-	-	-	1:1560045	464	572	468	637	774	695	2.135286	2.102226	2.380292	2.261559	2.293683	2.516134	2.205935	2.357125	0.696223	1	-
ENSMUSC	Xpr1	xenotropi	GO:00511	localizatio	-	-	-	-	1:1552757	2405	4392	3026	4235	5029	4295	6.567591	9.578536	9.132858	8.922256	8.843563	9.227096	8.426328	8.997638	0.679194	1	-
ENSMUSC	Mr1	major hist	GO:00023	immune s	-	-	-	-	1:1551272	141	224	103	185	213	125	0.799291	1.014101	0.645314	0.809076	0.777538	0.557454	0.819569	0.71469	0.331346	1	-
ENSMUSC	Glul	glutamate	GO:00081	metabolic	K01915	glutamine	mmu011C	Metabolic	1:1538999	26215	32187	23137	34787	41858	33832	431.9046	423.5106	421.3003	442.1659	444.0901	438.507	425.5718	441.5877	0.873797	1	-
ENSMUSC	Rgs16	regulator	GO:00987	molecular	-	-	-	-	1:1537403	141	197	117	178	182	147	3.680703	4.107001	3.375558	3.58479	3.059414	3.018841	3.721087	3.221015	0.228215	1	-
ENSMUSC	Lamc1	laminin, g	GO:00325	developm	K05635	laminin, g	mmu052C	Pathways	1:1532189	904	1313	813	1294	1407	1198	5.405065	6.269646	5.37242	5.96894	5.417281	5.635083	5.682377	5.673768	0.796407	1	-
ENSMUSC	Ncf2	neutrophi	GO:00081	metabolic	K08010	neutrophi	mmu0414	Phagoson	1:1528001	49	86	44	60	88	62	0.281057	0.393951	0.278931	0.26551	0.32504	0.27977	0.31798	0.290107	0.530052	1	-
ENSMUSC	Rgl1	ral guanin	GO:00987	molecular	K17635	ral guanin	mmu0401	Ras signal	1:1525167	2125	2696	1889	2598	3049	2605	12.5896	12.75613	12.36893	11.8747	11.63229	12.14149	12.57155	11.88283	0.347066	1	-
ENSMUSC	Rnf2	ring finge	GO:00325	developm	-	-	-	-	1:1514580	774	965	587	836	1169	930	6.378589	6.351217	5.346487	5.315211	6.203732	6.029449	6.025431	5.849464	0.611744	1	-
ENSMUSC	Coq8a	coenzyme	GO:00081	metabolic	-	-	-	-	1:1801652	712	999	550	1053	1216	805	5.141125	5.760893	4.389228	5.86593	5.654143	4.572829	5.097082	5.364301	0.809449	1	-
ENSMUSC	Cdc42bpa	CDC42 bi	GO:00081	metabolic	-	-	-	-	1:1799604	6493	6936	5987	6784	11444	6935	13.00135	11.0917	13.24949	10.47995	14.75624	10.92448	12.44752	12.05356	0.604251	1	-
ENSMUSC	Ahctf1	AT hook c	GO:00325	developm	-	-	-	-	1:1797448	1170	1445	1208	1401	1713	1422	3.51842	3.470367	4.014907	3.25035	3.317215	3.364125	3.667898	3.310564	0.184742	1	-
ENSMUSC	Tfb2m	transcripti	GO:00081	metabolic	-	-	-	-	1:1795280	198	313	236	302	381	315	2.976979	3.758383	3.921653	3.503059	3.688838	3.725909	3.552338	3.639269	1	1	-
ENSMUSC	Kif26b	kinesin fa	GO:00325	developm	-	-	-	-	1:1785291	721	966	748	969	1139	974	2.814945	3.012017	3.227622	2.918694	2.863602	2.991603	3.018195	2.924633	0.585356	1	-
ENSMUSC	Efcab2	EF-hand	GO:00431	ion bindin	-	-	-	-	1:1784060	354	539	315	520	563	479	4.923596	5.987053	4.842133	5.579726	5.042455	5.241136	5.250927	5.287772	0.886024	1	-
ENSMUSC	Parp1	poly (ADP	GO:00325	developm	K10798	poly [ADP	mmu0421	Necropto	1:1805689	2280	2866	2025	2988	3560	2828	18.89164	18.96517	18.54414	19.10054	18.99501	18.43424	18.80032	18.84326	0.801073	1	-
ENSMUSC	Acbd3	acyl-Coer	GO:00160	membran	-	-	-	-	1:1807260	813	1025	654	1074	1288	943	7.05117	7.099708	6.26896	7.186302	7.193517	6.434178	6.806612	6.937999	0.966378	1	-
ENSMUSC	Cox20	COX20 Cc	GO:00081	metabolic	-	-	-	-	1:1783191	122	185	140	232	224	161	7.014189	8.494473	8.896008	10.29053	8.293178	7.282098	8.13489	8.621937	0.824603	1	-

ENSMUSC	Desi2	desumoyl	GO:00081	metabolic	-	-	-	-	1:1781874	397	534	374	526	625	480	1.915149	2.057309	1.994029	1.957622	1.941544	1.821651	1.988829	1.906939	0.551378	1	-
ENSMUSC	Sdccag8	serologica	GO:00325	developm	-	-	-	-	1:1768146	417	457	378	573	690	580	5.721621	5.007784	5.732202	6.065519	6.096595	6.260689	5.487202	6.140934	0.429272	1	-
ENSMUSC	Capn2	calpain 2	GO:00325	developm	K03853	calpain-2	mmu0451	Focal adh	1:1824672	1496	1879	1366	1818	2291	1853	10.98003	11.014	11.08076	10.29429	10.82809	10.69938	11.02493	10.60725	0.478731	1	-
ENSMUSC	Trp53bp2	transform	GO:00508	response	K16823	apoptosis	mmu0439	Hippo sig	1:1824091	835	784	679	1038	1269	1054	9.716696	7.286081	8.732707	9.318796	9.509302	9.649034	8.578495	9.492377	0.488684	1	-
ENSMUSC	Srp9	signal reco	GO:00081	metabolic	K03109	signal reco	mmu0306	Protein ex	1:1821247	723	960	681	909	1184	1029	6.292049	6.67223	6.550098	6.103066	6.635294	7.044986	6.504792	6.594449	0.921771	1	-
ENSMUSC	Cnih3	cornichon	GO:00452	synapse|c	-	-	-	-	1:1813526	549	722	653	621	901	825	6.776011	7.116798	8.907618	5.913212	7.161118	8.010619	7.600143	7.028316	0.392622	1	-
ENSMUSC	Nvl	nuclear V(	GO:00081	metabolic	K14571	ribosome	mmu0300	Ribosome	1:1810871	1177	1476	1041	1578	1797	1624	4.779874	4.787097	4.672371	4.943985	4.699402	5.188441	4.746447	4.943943	0.860244	1	-
ENSMUSC	Tmem63a	transmem	GO:00511	localizatio	-	-	-	-	1:1809423	260	493	323	515	623	491	2.602906	3.941642	3.57383	3.977609	4.016309	3.867023	3.372793	3.953647	0.284695	1	-
ENSMUSC	Pycr2	pyrroline-	GO:00081	metabolic	K00286	pyrroline-	mmu0110	Metabolic	1:1809042	270	311	245	412	503	307	2.849734	2.621479	2.857934	3.354805	3.418712	2.549112	2.776382	3.107543	0.486537	1	-
ENSMUSC	Fh1	fumarate	GO:00081	metabolic	K01679	fumarate	mmu0110	Metabolic	1:1756003	1046	1290	931	1481	1707	1399	13.57423	13.36963	13.35304	14.82754	14.265	14.28277	13.4323	14.45844	0.592941	1	-
ENSMUSC	Rgs7	regulator	GO:00048	signal trar	-	-	-	-	1:1750590	1302	1768	1177	1471	2174	1384	9.985887	10.82938	9.976976	8.703998	10.73717	8.350699	10.26408	9.263957	0.217152	1	-
ENSMUSC	Spta1	spectrin a	GO:00325	developm	K06114	spectrin a	mmu0421	Apoptosis	1:1741727	0	1	3	0	3	0	0	0.004728	0.019628	0	0.011437	0	0.008119	0.003812	0.517119	1	-
ENSMUSC	Ifi211	interferon	GO:00081	metabolic	-	-	-	-	1:1738963	13	19	8	9	17	16	0.444681	0.519041	0.302425	0.237514	0.374472	0.430565	0.422049	0.347517	0.494249	1	-
ENSMUSC	Dusp23	dual speci	GO:00081	metabolic	-	-	-	-	1:1726307	49	77	55	76	87	63	2.121883	2.662925	2.632277	2.539042	2.426031	2.146236	2.472361	2.370436	0.716281	1	-
ENSMUSC	Cfap45	cilia and f	GO:00056	cell|intrace	-	-	-	-	1:1725208	81	114	74	116	126	84	0.741596	0.833555	0.748792	0.819351	0.742864	0.605027	0.774648	0.722414	0.548158	1	-
ENSMUSC	Slamf9	SLAM fam	GO:00160	membran	-	-	-	-	1:1724753	4	9	7	3	5	10	0.186022	0.33426	0.359793	0.107644	0.149726	0.365837	0.293358	0.207736	0.443874	1	-
ENSMUSC	Copa	coatomer	GO:00511	localizatio	-	-	-	-	1:1720825	3483	4383	3280	4612	5412	4666	14.41018	14.48215	14.99811	14.72094	14.41877	15.18698	14.63015	14.77556	0.864037	1	-
ENSMUSC	Dcaf8	DDB1 anc	GO:00081	metabolic	-	-	-	-	1:1721480	3061	3900	2962	4060	4212	4161	11.21371	11.41028	11.99272	11.47472	9.936398	11.99208	11.5389	11.1344	0.501519	1	-
ENSMUSC	Vangl2	vang-like	GO:00325	developm	K04510	vang-like[	mmu0431	Wnt signa	1:1720009	263	352	222	314	424	365	1.883008	2.012728	1.756694	1.734425	1.954862	2.05589	1.884143	1.915059	0.952899	1	-
ENSMUSC	Uck2	uridine-cy	GO:00081	metabolic	K00876	uridine kir	mmu0110	Metabolic	1:1672228	593	716	505	684	892	630	5.171017	4.986322	4.866981	4.601585	5.00888	4.321877	5.008107	4.644114	0.348565	1	-
ENSMUSC	Tada1	transcripti	GO:00081	metabolic	-	-	-	-	1:1663790	378	488	324	552	653	521	4.699642	4.845504	4.452103	5.294716	5.22807	5.095917	4.66575	5.206234	0.435601	1	-
ENSMUSC	Dusp27	dual speci	GO:00081	metabolic	-	-	-	-	1:1660981	7	9	4	9	5	5	0.056852	0.058378	0.035907	0.056393	0.026149	0.031946	0.050379	0.038163	0.418705	1	-
ENSMUSC	Pou2f1	POU dom	GO:00325	developm	-	-	-	-	1:1658651	494	645	452	596	706	615	1.374219	1.43296	1.389676	1.279101	1.264701	1.345905	1.398951	1.296569	0.336135	1	-
ENSMUSC	Mpzl1	myelin pro	GO:00508	response	K06770	myelin pro	mmu0451	Cell adhes	1:1655922	297	212	266	330	432	383	2.796767	1.594345	2.7684	2.39742	2.619622	2.837328	2.386504	2.618123	0.665811	1	-
ENSMUSC	Adcy10	adenylate	GO:00081	metabolic	K11265	adenylate	mmu0402	cAMP sigr	1:1654851	3	2	3	7	5	2	0.01611	0.008579	0.017807	0.029001	0.01729	0.008451	0.014165	0.018247	0.85382	1	-
ENSMUSC	Mpc2	mitochon	GO:00081	metabolic	-	-	-	-	1:1654606	428	623	432	669	670	642	2.930352	3.406512	3.268938	3.533719	2.953963	3.457974	3.201934	3.315219	0.928332	1	-
ENSMUSC	Dcaf6	DDB1 anc	GO:00081	metabolic	-	-	-	-	1:1653286	1720	2155	1608	2446	2992	2455	12.9319	12.93978	13.36185	14.18798	14.48605	14.52099	13.07784	14.39834	0.40408	1	-
ENSMUSC	Nme7	NME/NM	GO:00081	metabolic	K00940	nucleoside	mmu0110	Metabolic	1:1643041	245	287	209	302	343	268	1.826968	1.709204	1.722499	1.737411	1.647077	1.572213	1.75289	1.652234	0.493473	1	-
ENSMUSC	Atp1b1	ATPase, N	GO:00081	metabolic	K01540	sodium/p	mmu0402	cAMP sigr	1:1644371	35032	44557	31927	45646	54723	43367	565.2716	574.1878	569.3733	568.2315	568.613	550.5066	569.6109	562.4503	0.652002	1	-
ENSMUSC	Blzf1	basic leuc	GO:00511	localizatio	-	-	-	-	1:1642898	441	513	449	628	674	585	3.501513	3.252969	3.94013	3.846866	3.446128	3.654127	3.564871	3.64904	0.999356	1	-
ENSMUSC	Ccdc181	coiled-co	GO:00990	supramole	-	-	-	-	1:1642755	485	605	459	687	814	585	15.5124	15.45391	16.22546	16.95214	16.7655	14.71987	15.73059	16.14584	0.984547	1	-
ENSMUSC	Sele	selectin, e	GO:00048	signal trar	K06494	selectin, e	mmu0451	Cell adhes	1:1640482	0	0	2	2	5	2	0	0	0.046351	0.032357	0.067511	0.032997	0.01545	0.044288	0.279997	1	-
ENSMUSC	Scyl3	SCY1-like	GO:00081	metabolic	-	-	-	-	1:1639291	424	491	277	390	518	452	1.915548	1.771556	1.3831	1.359321	1.506994	1.606485	1.690068	1.490933	0.233707	1	-
ENSMUSC	Kifap3	kinesin-as	GO:00511	localizatio	-	-	-	-	1:1637795	5603	7122	5191	7157	8731	6740	85.27133	86.56259	87.31327	84.03182	85.56597	80.69622	86.3824	83.43134	0.476802	1	-
ENSMUSC	Prrx1	paired rel	GO:00325	developm	-	-	-	-	1:1632451	173	223	135	176	234	183	0.661474	0.680951	0.570488	0.519168	0.57615	0.550461	0.637638	0.548593	0.192028	1	-
ENSMUSC	Ralgps2	Ral GEF w	GO:00987	molecular	-	-	-	-	1:1568041	1180	1420	949	1571	1861	1450	3.325421	3.195941	2.955819	3.415632	3.37727	3.214723	3.15906	3.335875	0.749445	1	-
ENSMUSC	Abl2	v-abl Abe	GO:00325	developm	K08887	abelson ty	mmu0401	Ras signal	1:1565587	2163	2553	1741	2493	3034	2591	10.30338	9.712245	9.165764	9.161691	9.306648	9.709602	9.72713	9.392647	0.50287	1	-
ENSMUSC	Soat1	sterol O-a	GO:00325	developm	K00637	sterol O-a	mmu0497	Cholester	1:1564245	675	963	614	828	1031	856	3.137026	3.57426	3.153763	2.968758	3.085515	3.12967	3.288349	3.061314	0.345716	1	-
ENSMUSC	Axdnd1	axonemal	-		-	-	-	-	1:1563235	0	5	8	11	10	7	0	0.031774	0.070354	0.067534	0.051242	0.043824	0.034043	0.0542	0.356419	1	-
ENSMUSC	Nphs2	nephrosis	GO:00325	developm	-	-	-	-	1:1563107	4	1	0	1	0	1	0.065254	0.01303	0	0.012587	0	0.012836	0.026095	0.008474	0.35601	1	-
ENSMUSC	Smyd2	SET and M	GO:00325	developm	-	-	-	-	1:1898804	764	896	791	870	1099	1002	12.29206	11.51291	14.06548	10.79894	11.38634	12.68266	12.62349	11.62264	0.321274	1	-
ENSMUSC	Ptpn14	protein ty	GO:00325	developm	-	-	-	-	1:1897282	363	453	317	408	516	394	1.197089	1.193063	1.155384	1.038031	1.095782	1.022178	1.181845	1.051997	0.211181	1	-
ENSMUSC	Kctd3	potassium	GO:00099	cellular pr	-	-	-	-	1:1889710	1331	1784	1238	1780	2203	1865	7.079739	7.578443	7.277911	7.304486	7.545856	7.804211	7.312031	7.551517	0.927409	1	-
ENSMUSC	Ush2a	usherin [S	GO:00325	developm	-	-	-	-	1:1882620	1	3	0	0	0	1	0.003284	0.007868	0	0	0	0.002584	0.003717	0.000861	0.412986	1	-
ENSMUSC	Esrrg	estrogen-	GO:00325	developm	-	-	-	-	1:1876087	365	482	297	469	478	400	1.567683	1.653327	1.409839	1.554063	1.322052	1.351564	1.543616	1.409227	0.324588	1	-
ENSMUSC	Spata17	spermato	GO:00056	cell|protei	-	-	-	-	1:1870446	3	5	10	9	13	13	0.038538	0.051294	0.141974	0.089199	0.107543	0.131375	0.077269	0.109372	0.457756	1	-
ENSMUSC	Eprs	glutamyl-	GO:00081	metabolic	K14163	bifunction	mmu0110	Metabolic	1:1853630	1936	2540	1702	2271	2907	2492	5.601503	5.869196	5.44259	5.069283	5.416251	5.672286	5.637763	5.38594	0.428841	1	-
ENSMUSC	Bpnt1	bisphosph	GO:00081	metabolic	K01082	3'(2'), 5'-b	mmu0110	Metabolic	1:1853321	1069	1329	982	1492	1942	1451	21.06199	20.91186	21.38355	22.67885	24.63915	22.49056	21.11913	23.26952	0.436305	1	-
ENSMUSC	Iars2	isoleucine	GO:00081	metabolic	K01870	isoleucyl-t	mmu0097	Aminoacy	1:1852847	935	1170	836	1386	1562	1402	5.054387	5.051134	4.994707	5.780303	5.437421	5.96233	5.033409	5.726684	0.261032	1	-
ENSMUSC	Mark1	MAP/micr	GO:00325	developm	-	-	-	-	1:1848967	1430	1732	1154	1763	1892	1833	18.78914	18.17458	16.75805	17.87118	16.00834	18.94715	17.90725	17.60889	0.659888	1	-
ENSMUSC	1-Mar	mitochon	GO:00081	metabolic	-	-	-	-	1:1847867	0	3	0	0	3	0	0	0.033488	0	0	0.027002	0	0.011163	0.009001	1	1	-
ENSMUSC	Lpgat1	lysophosp	GO:00081	metabolic	K13514	lysophosp	mmu0056	Glyceroph	1:1917178	3892	5007	3363	4831	5476	4451	22.83226	23.45846	21.80468	21.8647	20.68684	20.54209	22.69847	21.03121	0.23167	1	-
ENSMUSC	Ppp2r5a	protein pl	GO:00081	metabolic	K11584	serine/thr	mmu0415	PI3K-Akt s	1:1913519	1194	1403	965	1492	1869	1493	6.909621	6.484155	6.171973	6.661145	6.964879	6.797047	6.521916	6.807691	0.844308	1	-
ENSMUSC	Tmem206	transmem	GO:00160	membran	-	-	-	-	1:1913259	152	195	138	205	241	211	4.132158	4.233631	4.146299	4.299493	4.218962	4.512603	4.170696	4.343686	0.911061	1	-
ENSMUSC	Batf3	basic leuc	GO:00325	developm	-	-	-	-	1:1910978	12	0	6	8	16	6	0.229464	0	0.126815	0.118024	0.197019	0.090265	0.11876	0.135103	0.870054	1	-
ENSMUSC	Tatdn3	TatD DNa	GO:00081	metabolic	-	-	-	-	1:1910458	160	188	153	211	227	228	1.794182	1.683644	1.896203	1.825397	1.639178	2.01137	1.791343	1.825315	0.968467	1	-
ENSMUSC	Angel2	angel hor	GO:00081	metabolic	-	-	-	-	1:1909251	695	882	592	871	1173	930	5.432776	5.506186	5.114525	5.252731	5.904588	5.719138	5.351163	5.625485	0.798082	1	-
ENSMUSC	Traf5	TNF recep	GO:00081	metabolic	-	-	-	-	1:1919972	13	22	22	28	31	42	0.123006	0.166248	0.23007	0.204393	0.188888	0.312639	0.173108	0.235307	0.263764	1	-
ENSMUSC	Plxna2	plexin A2	GO:00325	developm	K06820	plexin A[-	mmu0436	Axon guic	1:1946182	3515	4386	3192	4260	5046	4551	10.63788	10.60094	10.67676	9.946487	9.834035	10.83546	10.63853	10.20533	0.431693	1	-
ENSMUSC	Usf1	upstream	GO:00081	metabolic	-	-	-	-	1:1714113	573	1069	829	1045	1189	1047	5.589756	8.328406	8.93799	7.864748	7.469215	8.035185	7.618717	7.789716	0.99791	1	-
ENSMUSC	Nmt2	N-myristo	GO:00081	metabolic	-	-	-	-	2:3284212	1799	2238	1506	2294	2620	2078	13.77521	13.68589	12.74497	13.55161	12.91883	12.51767	13.40202	12.99604	0.535524	1	-
ENSMUSC	Acbd7	acyl-Coer	GO:00480	cofactor b	-	-	-	-	2:3336168	0	4	0	0	2	0	0	0.299129	0	0	0.120584	0	0.09971	0.040195	0.733299	1	-
ENSMUSC	Suv39h2	suppresso	GO:00081	metabolic	K11419	histone-ly	mmu0031	Lysine deg	2:3455815	66	93	91	129	149	123	0.481983	0.542393	0.73447	0.726786	0.700688	0.706644	0.586282	0.711373	0.289499	1	-
ENSMUSC	Meig1	meiosis ex	GO:00325	developm	-	-	-	-	2:3409043	22	33	23	38	49	27	0.596477	0.714553	0.689194	0.79486	0.855505	0.575909	0.666741	0.742091	0.721148	1	-
ENSMUSC	Fam107b	family wit	GO:00508	neurologi	-	-	-	-	2:3570488	313	155	231	124	198	425	3.316183	1.311516	2.70491	1.013552	1.350875	3.542373	2.444203	1.968933	0.385328	1	-
ENSMUSC	Fcgr2b	Fc recepto	GO:00160	membran	K12560	low affinit	mmu0515	Tuberculo	1:1709586	53	100	61	76	96	53	0.429989	0.647926	0.546961	0.475691	0.501538	0.338274	0.541626	0.438501	0.202342	1	-
ENSMUSC	Frmd4a	FERM dor	GO:00099	cellular pr	-	-	-	-	2:4017717	1371	1797	1268	1928	2013	1819	2.378144	2.4894	2.430898	2.580109	2.248533	2.482243	2.432814	2.436962	0.804872	1	-
ENSMUSC	Dusp12	dual speci	GO:00081	metabolic	-	-	-	-	1:1708734	125	163	126	185	180	164	1.372211	1.429042	1.528721	1.566804	1.272447	1.416338	1.443325	1.418529	0.780379	1	-
ENSMUSC	Sephs1	selenopho	GO:00081	metabolic	K01008	selenide, v	mmu0110	Metabolic	2:4881564	424	470	395	481	674	508	3.206419	2.838562	3.301401	2.806274	3.28223	3.022241	3.115461	3.036915	0.673097	1	-
ENSMUSC	Atf6	activating	GO:00325	developm	K09054	cyclic AMI	mmu0501	Alzheimer	1:1707046	2141	2772	2052	2793	3464	2763	14.00979	14.48619	14.84019	14.09991	14.59647	14.22351	14.44539	14.30663	0.697473	1	-
ENSMUSC	Phyh	phytanoyl	GO:00081	metabolic	K00477	phytanoyl	mmu0414	Peroxisom	2:4919019	1093	1404	1101	1450	1823	1395	30.17022	30.95074	33.58862	30.87853	32.40408	30.29308	31.56986	31.1919	0.707215	1	-
ENSMUSC	Uhmk1	U2AF hon	GO:00325	developm	-	-	-	-	1:1701934	5705	6907	819	6791	8195	957	42.9992	41.57574	6.822369	39.48833	39.77483	5.674501	30.46577	28.31255	0.820397	1	-
ENSMUSC	Ucma	upper zor	GO:00325	developm	-	-	-	-	2:4976122	23	33	25	24	46	30	0.509373	0.583669	0.611911	0.410065	0.656027	0.522681	0.568318	0.529591	0.716331	1	-
ENSMUSC	Uap1	UDP-N-a	GO:00081	metabolic	K00972	UDP-N-a	mmu0110	Metabolic	1:1701419	829	1221	748	1282	1666	1013	7.28619	8.570526	7.265982	8.692892	9.429221	7.004328	7.707566	8.37548	0.58415	1	-
ENSMUSC	Optn	optineurir	GO:00081	metabolic	K19946	optineurir	mmu0413	Mitophag	2:5020642	361	490	341	549	600	555	3.939589	4.270565	4.112866	4.622173	4.216474	4.764827	4.107673	4.534492	0.501194	1	-
ENSMUSC	Ddr2	discoidin	GO:00325	developm	-	-	-	-	1:1699723	260	121	250	382	518	206	1.012057	0.376152	1.075518	1.147161	1.29842	0.630826	0.821242	1.025469	0.411849	1	-
ENSMUSC	Hsd17b7	hydroxyst	GO:00081	metabolic	K13373	17beta-es	mmu0110	Metabolic	1:1699495	359	394	284	432	512	365	3.966675	3.476755	3.468151	3.682528	3.642982	3.172753	3.637194	3.499421	0.601711	1	-
ENSMUSC	Ccdc3	coiled-co	GO:00325	developm	-	-	-	-	2:5137776	237	330	215	304	423	362	5.419768	6.02687	5.433961	5.363351	6.229122	6.512565	5.626866	6.035013	0.710727	1	-
ENSMUSC	Rgs5	regulator	GO:00987	molecular	-	-	-	-	1:1696555	2374	3159	1991	3278	3835	3346	31.1993	33.15581	28.91893	33.23559	32.45519	34.59402	31.09134	33.42827	0.582932	1	-
ENSMUSC	Enkur	enkurin, T	GO:00056	cell|intrace	-	-	-	-	2:2118073	28	55	34	45	64	69	0.718322	1.12688	0.964042	0.890656	1.057315	1.392609	0.936414	1.113527	0.487402	1	-
ENSMUSC	Nuf2	NUF2, ND	GO:00329	macromo	-	-	-	-	1:1694979	42	76	71	100	87	84	0.334347	0.483175	0.624672	0.614153	0.445985	0.526066	0.480731	0.528734	0.725587	1	-
ENSMUSC	Lmx1a	LIM home	GO:00325	developm	-	-	-	-	1:1676892	10	28	10	13	23	13	0.174753	0.390776	0.193132	0.175261	0.258825	0.178714	0.252887	0.204267	0.428443	1	-
ENSMUSC	Aldh9a1	aldehyde	GO:00081	metabolic	K00149	aldehyde	mmu0110	Metabolic	1:1673499	489	627	464	656	769	583	7.02362	7.192263	7.365758	7.269195	7.112677	6.587673	7.19388	6.989848	0.620518	1	-
ENSMUSC	Fmo4	flavin con	GO:00081	metabolic	K00485	dimethyla	mmu0098	Drug meta	1:1627931	0	0	0	1	1	1	0	0	0	0.008824	0.007363	0.008999	0	0.008395	0.273069	1	-
ENSMUSC	Mettl13	methyltra	GO:00081	metabolic	-	-	-	-	1:1625321	207	244	221	272	381	283	0.820503	0.772406	0.968164	0.831781	0.972499	0.882485	0.853691	0.895588	0.85018	1	-
ENSMUSC	Vamp4	vesicle-as	GO:00452	synapse|c	K08513	vesicle-as	mmu0413	SNARE int	1:1625705	1178	1268	1026	1472	1625	1363	9.316894	8.009237	8.968505	8.981819	8.27626	8.480723	8.764878	8.579601	0.637435	1	-
ENSMUSC	Myoc	myocilin [	GO:00325	developm	-	-	-	-	1:1626391	352	618	336	702	645	569	10.36279	14.5301	10.93252	15.94418	12.22782	13.17824	11.94181	13.78341	0.349581	1	-
ENSMUSC	Pigc	phosphati	GO:00081	metabolic	K03859	phosphati	mmu0110	Metabolic	1:1619691	146	203	151	195	268	239	0.820477	0.911077	0.93786	0.845434	0.969848	1.056627	0.889805	0.957303	0.718221	1	-
ENSMUSC	Prdx6	peroxired	GO:00081	metabolic	K11188	peroxired	mmu0110	Metabolic	1:1612401	1331	1475	1210	1751	2244	1644	8.896369	7.873589	8.938548	9.029242	9.658561	8.644652	8.569502	9.110818	0.696815	1	-
ENSMUSC	Klhl20	kelch-like	GO:00081	metabolic	-	-	-	-	1:1610883	331	494	305	419	465	517	1.161041	1.38386	1.182403	1.133871	1.050333	1.42666	1.242435	1.203621	0.651616	1	-
ENSMUSC	Nsun6	NOL1/NC	GO:00081	metabolic	-	-	-	-	2:1499513	111	153	96	154	153	156	0.958488	1.055116	0.916175	1.025919	0.850761	1.059739	0.976593	0.978807	0.890472	1	-
ENSMUSC	Cenpl	centrome	GO:00056	cell|intrace	-	-	-	-	1:1610707	13	15	10	23	24	18	0.053162	0.048989	0.045195	0.072563	0.0632	0.057906	0.049115	0.064557	0.383257	1	-
ENSMUSC	Dars2	aspartyl-t	GO:00081	metabolic	K01876	aspartyl-t	mmu0097	Aminoacy	1:1610406	316	393	310	469	509	423	1.966201	1.952897	2.131818	2.251357	2.039453	2.070585	2.016972	2.120465	0.822287	1	-
ENSMUSC	Serpinc1	serine (or	GO:00325	developm	K03911	antithrom	mmu0461	Complem	1:1609785	3	3	0	2	8	3	0.017036	0.013608	0	0.008763	0.029256	0.013401	0.010215	0.01714	0.551317	1	-
ENSMUSC	Stam	signal trar	GO:00511	localizatio	K04705	signal trar	mmu0414	Endocytos	2:1407409	1281	1474	1143	1623	1739	1505	6.886982	6.328825	6.791607	6.731758	6.02052	6.36542	6.669138	6.372566	0.454525	1	-
ENSMUSC	Rabgap1l	RAB GTPa	GO:00325	developm	-	-	-	-	1:1602191	4537	5670	3973	5478	6807	5415	10.70901	10.68832	10.36444	9.975458	10.34644	10.05518	10.58726	10.12569	0.401432	1	-
ENSMUSC	Trdmt1	tRNA asp	GO:00081	metabolic	-	-	-	-	2:1350901	81	123	81	124	171	123	1.216357	1.475114	1.344344	1.436572	1.653585	1.453087	1.345272	1.514415	0.524562	1	-
ENSMUSC	Tnn	tenascin N	GO:00325	developm	K06252	tenascin[-	mmu0415	PI3K-Akt s	1:1600850	2	3	1	6	0	0	0.020288	0.024312	0.011214	0.046963	0	0	0.018605	0.015654	0.819056	1	-
ENSMUSC	Pter	phosphot	GO:00325	developm	-	-	-	-	2:1292404	32	26	36	50	43	28	0.235887	0.153064	0.293299	0.284351	0.204116	0.16238	0.227417	0.216949	0.812992	1	-
ENSMUSC	Pip4k2a	phosphati	GO:00325	developm	K00920	1-phosph	mmu0481	Regulation	2:1884225	1359	1917	1340	1926	2403	1942	21.81909	24.58018	23.77763	23.85633	24.84422	24.52885	23.3923	24.4098	0.832902	1	-
ENSMUSC	Bmi1	Bmi1 poly	GO:00325	developm	K11459	polycomb	mmu0520	Transcript	2:1867701	1009	1301	517	1204	1513	730	9.551574	9.835734	5.40905	8.793069	9.223105	5.436475	8.265453	7.81755	0.636009	1	-
ENSMUSC	Mllt10	myeloid/ly	GO:00081	metabolic	-	-	-	-	2:1805523	471	597	440	628	773	911	2.717157	2.750515	2.805387	2.795011	2.871624	4.134499	2.757686	3.267045	0.243785	1	-
ENSMUSC	Plxdc2	plexin dor	GO:00055	extracellul	-	-	-	-	2:1635630	677	984	680	920	1080	902	4.5127	5.238281	5.009607	4.73114	4.635818	4.730043	4.920196	4.699	0.48373	1	-
ENSMUSC	Nek6	NIMA (ne	GO:00048	signal trar	-	-	-	-	2:3851164	539	769	455	777	1074	795	2.397664	2.731944	2.236956	2.666557	3.076511	2.782129	2.455521	2.841732	0.250847	1	-
ENSMUSC	Psmb7	proteasor	GO:00081	metabolic	K02739	20S prote	-	-	2:3858803	1338	1766	1211	1838	2379	1769	22.94824	24.18967	22.95537	24.32031	26.27498	23.86889	23.36443	24.82139	0.683885	1	-
ENSMUSC	Ppp6c	protein pl	GO:00081	metabolic	-	-	-	-	2:3919435	889	1148	908	1206	1318	1107	8.348305	8.609621	9.42386	8.737237	7.970149	8.17815	8.793929	8.295179	0.399791	1	-
ENSMUSC	Arpc5l	actin relat	GO:00400	locomotic	K05754	actin relat	mmu0414	Endocytos	2:3900534	915	1436	954	1235	1461	1365	9.978245	12.50643	11.49815	10.39035	10.25977	11.71052	11.32761	10.78688	0.467013	1	-
ENSMUSC	Orc4	origin rec	GO:00081	metabolic	K02606	origin rec	mmu0411	Cell cycle	2:4890282	619	761	573	792	1058	831	3.715589	3.648107	3.801354	3.66769	4.089566	3.924179	3.721683	3.893812	0.834366	1	-
ENSMUSC	Kif5c	kinesin fa	GO:00325	developm	K10396	kinesin fa	mmu0414	Endocytos	2:4961929	20317	24887	16699	22966	27432	24100	155.0841	151.7141	140.8786	135.2457	134.8403	144.7223	149.2256	138.2694	0.226539	1	-
ENSMUSC	Lypd6b	LY6/PLAU	GO:00987	molecular	-	-	-	-	2:4978768	93	115	83	143	156	160	2.03235	2.007068	2.004656	2.410929	2.195313	2.750732	2.014692	2.452325	0.247081	1	-
ENSMUSC	Mmadhc	methylma	GO:00081	metabolic	-	-	-	-	2:5027988	260	380	298	385	464	347	4.614861	5.386589	5.845858	5.272009	5.30344	4.845345	5.282436	5.140264	0.680646	1	-
ENSMUSC	Mindy3	MINDY ly	GO:00081	metabolic	-	-	-	-	2:1234726	685	805	362	676	1049	840	3.465476	3.252473	2.024076	2.63845	3.417449	3.343201	2.914008	3.133033	0.734173	1	-
ENSMUSC	Itga8	integrin al	GO:00325	developm	K06584	integrin al	mmu0415	PI3K-Akt s	2:1210663	376	517	322	524	504	578	2.353927	2.584888	2.227961	2.530852	2.031842	2.846717	2.388925	2.469804	0.953376	1	-
ENSMUSC	Il2ra	interleukir	GO:00325	developm	K05068	interleukir	mmu0520	Pathways	2:1164280	4	7	4	9	10	5	0.041209	0.057591	0.045545	0.071531	0.066337	0.040522	0.048115	0.059463	0.676732	1	-
ENSMUSC	Spopl	speckle-ty	GO:00081	metabolic	K10523	speckle-ty	mmu0434	Hedgehoc	2:2350622	439	557	348	562	660	497	2.170408	2.199266	1.901525	2.143597	2.101236	1.933052	2.090399	2.059295	0.731682	1	-
ENSMUSC	Pfkfb3	6-phosph	GO:00325	developm	K01103	6-phosph	mmu0415	AMPK sigr	2:1147143	768	513	669	1011	726	1012	2.482405	1.324263	2.389926	2.521116	1.511133	2.573373	2.065532	2.201874	0.832398	1	-
ENSMUSC	4931423N	RIKEN cDI	-	-	-	-	-	-	2:2320747	3	3	2	0	3	3	0.068271	0.054533	0.050307	0	0.04397	0.053702	0.057703	0.032557	0.471323	1	-
ENSMUSC	Yme1l1	YME1-like	GO:00081	metabolic	-	-	-	-	2:2315636	1906	2588	1758	2329	3113	2443	12.75921	13.83601	13.00668	12.0282	13.41945	12.86576	13.20064	12.77114	0.521141	1	-
ENSMUSC	Prkcq	protein kii	GO:00081	metabolic	K18052	novel prot	mmu0516	Measles -	2:1117210	144	245	151	194	248	182	0.962074	1.307244	1.114985	0.999948	1.066966	0.956595	1.128101	1.007836	0.322258	1	-
ENSMUSC	Mastl	microtubu	GO:00081	metabolic	-	-	-	-	2:2311560	18	28	29	27	49	38	0.178152	0.221328	0.317229	0.206166	0.312303	0.295888	0.238903	0.271452	0.667232	1	-
ENSMUSC	Abi2	abl-intera	GO:00325	developm	K05751	abl interac	mmu0481	Regulation	1:6040961	1440	3163	1173	2973	2537	2214	4.55398	7.988653	4.099899	7.253602	5.166586	5.508293	5.547511	5.97616	0.729235	1	-
ENSMUSC	Pdss1	prenyl (so	GO:00081	metabolic	K12504	decapreny	mmu0090	Terpenoic	2:2289552	117	161	116	200	236	166	1.229147	1.350798	1.346859	1.620982	1.596558	1.371948	1.308935	1.529829	0.340659	1	-
ENSMUSC	Pkn3	protein kii	GO:00081	metabolic	K06071	protein kii	mmu0415	PI3K-Akt s	2:3007768	19	30	13	35	44	27	0.182376	0.229974	0.137913	0.259187	0.271971	0.20389	0.183421	0.245016	0.265827	1	-
ENSMUSC	Apbb1ip	amyloid b	GO:00023	immune s	K17704	amyloid b	mmu0401	Rap1 sign	2:2277409	103	177	108	152	192	179	0.789378	1.083346	0.914788	0.898713	0.947557	1.079225	0.92917	0.975165	0.889497	1	-
ENSMUSC	Gad2	glutamic a	GO:00081	metabolic	K01580	glutamate	mmu0110	Metabolic	2:2262220	3980	4622	3388	4880	5460	4720	40.5695	37.62637	38.16864	38.37662	35.83966	37.85031	38.78817	37.35553	0.461522	1	-
ENSMUSC	Zbtb43	zinc finge	GO:00081	metabolic	-	-	-	-	2:3345028	279	329	237	357	379	320	2.125682	2.001868	1.995673	2.098423	1.859465	1.918029	2.041074	1.958639	0.583288	1	-
ENSMUSC	Odf2	outer den	GO:00325	developm	-	-	-	-	2:2988922	1414	1687	1277	1875	2140	1947	5.28147	5.032298	5.271605	5.403024	5.147233	5.721134	5.195124	5.423797	0.837888	1	-
ENSMUSC	Slc2a8	solute car	GO:00081	metabolic	-	-	-	-	2:3297299	110	169	135	178	204	233	1.199784	1.472116	1.62739	1.497825	1.432832	1.9993	1.433096	1.643319	0.459771	1	-
ENSMUSC	Lrsam1	leucine ric	GO:00081	metabolic	-	-	-	-	2:3292521	616	787	611	935	1000	895	4.386727	4.475901	4.808927	5.136906	4.585794	5.01411	4.557185	4.91227	0.616626	1	-
ENSMUSC	Fam129b	family wit	GO:00081	metabolic	-	-	-	-	2:3287611	401	637	375	615	728	582	4.701284	5.964279	4.859045	5.562602	5.496151	5.367925	5.174869	5.47556	0.774432	1	-
ENSMUSC	Stxbp1	syntaxin b	GO:00325	developm	K15292	syntaxin-b	mmu0472	Synaptic v	2:3278760	14593	11966	13145	19045	13949	12501	93.40822	61.16955	92.9925	94.04849	57.49608	62.95006	82.52342	71.49821	0.227174	1	-
ENSMUSC	Coq4	coenzyme	GO:00081	metabolic	-	-	-	-	2:2978749	167	256	155	202	262	208	1.563668	1.91432	1.604007	1.459189	1.579736	1.532161	1.693998	1.523696	0.320172	1	-
ENSMUSC	Med27	mediator	GO:00325	developm	K15170	mediator	mmu0491	Thyroid hc	2:2934681	309	363	306	424	421	349	4.125733	3.870745	4.515543	4.367559	3.619754	3.665881	4.170674	3.884398	0.432824	1	-
ENSMUSC	Ddx31	DEAD/H (	GO:00081	metabolic	-	-	-	-	2:2884040	109	132	77	114	166	133	1.580834	1.528907	1.234241	1.275552	1.550329	1.517482	1.447994	1.447788	0.881866	1	-
ENSMUSC	Ak8	adenylate	GO:00325	developm	K00939	adenylate	mmu0110	Metabolic	2:2870016	16	23	16	36	33	26	0.436322	0.500924	0.482235	0.757392	0.579507	0.557795	0.47316	0.631564	0.265089	1	-
ENSMUSC	Spaca9	sperm acr	GO:00990	supramole	-	-	-	-	2:2869208	0	12	13	0	11	8	0	0.476115	0.713802	0	0.351906	0.312657	0.396639	0.221521	0.426524	1	-
ENSMUSC	Dpm2	dolichol-p	GO:00081	metabolic	K09658	dolichyl-p	mmu0110	Metabolic	2:3257085	220	234	157	281	321	251	3.795642	3.224224	2.9937	3.74024	3.566341	3.406804	3.337856	3.571128	0.743672	1	-
ENSMUSC	St6galnac	ST6 (alph	GO:00081	metabolic	K03376	N-acetylg	mmu0110	Metabolic	2:3259970	1321	1633	1252	2014	2642	1154	9.185485	9.068416	9.62167	10.80411	11.83006	6.312711	9.291857	9.648961	0.911934	1	-
ENSMUSC	Tsc1	tuberous	GO:00325	developm	K07206	tuberous	mmu0415	PI3K-Akt s	2:2864122	1296	1625	1208	1804	1966	1715	4.560882	4.567127	4.698482	4.89791	4.455353	4.748089	4.60883	4.700451	0.961927	1	-
ENSMUSC	Eng	endoglin	GO:00325	developm	-	-	-	-	2:3264659	295	386	261	427	425	509	2.464042	2.574895	2.409422	2.751592	2.285967	3.344681	2.482786	2.79408	0.468835	1	-
ENSMUSC	Gtf3c5	general tr	GO:00325	developm	-	-	-	-	2:2856631	332	350	299	473	515	481	3.0094	2.5337	2.995433	3.307751	3.0061	3.430022	2.846178	3.247958	0.362015	1	-
ENSMUSC	Ak1	adenylate	GO:00081	metabolic	K00939	adenylate	mmu0110	Metabolic	2:3262175	1599	1773	1243	2101	2416	1997	15.99737	14.16624	13.74415	16.21647	15.56509	15.7177	14.63592	15.83308	0.558108	1	-
ENSMUSC	Ptges2	prostaglai	GO:00081	metabolic	K05309	microsom	mmu0110	Metabolic	2:3239589	347	449	276	527	540	493	3.657374	3.779479	3.215106	4.285298	3.665121	4.087875	3.550653	4.012765	0.403352	1	-
ENSMUSC	Ralgds	ral guanin	GO:00987	molecular	K08732	ral guanin	mmu0520	Pathways	2:2851312	1247	1595	1252	1826	1933	1775	6.803632	6.949941	7.549628	7.686093	6.791426	7.618752	7.101067	7.365424	0.896614	1	-
ENSMUSC	Lcn2	lipocalin 2	GO:00081	metabolic	-		mmu0465	IL-17 sign	2:3238463	11	70	29	20	64	62	0.138003	0.701332	0.402093	0.193569	0.517035	0.611906	0.413809	0.440837	0.926049	1	-
ENSMUSC	Dnm1	dynamin 1	GO:00081	metabolic	K01528	dynamin (	mmu0414	Endocytos	2:3230847	23252	29454	22567	31040	37015	29610	105.5792	106.8091	113.2501	108.7352	108.2307	105.7712	108.5461	107.579	0.688303	1	-
ENSMUSC	Gpd2	glycerol p	GO:00325	developm	K00111	glycerol-3	mmu0056	Glyceroph	2:5723763	3483	3874	2984	4273	4968	4318	27.03603	24.01568	25.59971	25.58896	24.8328	26.36835	25.55047	25.5967	0.794046	1	-
ENSMUSC	Gbgt1	globoside	GO:00081	metabolic	K00722	globoside	mmu0110	Metabolic	2:2849689	9	19	7	10	24	12	0.351574	0.592763	0.302245	0.301391	0.603739	0.368798	0.415527	0.424643	1	1	-
ENSMUSC	Ermn	ermin, ERI	GO:00325	developm	-	-	-	-	2:5804511	1010	1652	993	1375	1607	1325	16.96525	22.16128	18.43466	17.81854	17.38239	17.50917	19.18706	17.57003	0.266262	1	-
ENSMUSC	1700007K	RIKEN cDI	-	-	-	-	-	-	2:2846200	16	29	29	39	20	27	0.733979	1.062466	1.470312	1.380268	0.590806	0.974415	1.088919	0.98183	0.644148	1	-
ENSMUSC	Olfm1	olfactome	GO:00325	developm	-	-	-	-	2:2819299	15225	19729	14408	20488	25515	21104	174.3454	180.4281	182.3491	181.0019	188.1497	190.1205	179.0409	186.424	0.840718	1	-
ENSMUSC	Acvr1c	activin A r	GO:00325	developm	K13568	activin rec	mmu0455	Signaling	2:5826745	595	786	523	701	852	755	2.794796	2.948504	2.715078	2.540281	2.577079	2.789917	2.819459	2.635759	0.367158	1	-
ENSMUSC	Col5a1	collagen,	GO:00325	developm	K19721	collagen, t	mmu0497	Protein di	2:2788642	353	326	217	510	514	376	2.454003	1.809941	1.667272	2.735275	2.301007	2.056361	1.977072	2.364214	0.270815	1	-
ENSMUSC	Upp2	uridine ph	GO:00081	metabolic	K00757	uridine ph	mmu0110	Metabolic	2:5856729	447	503	441	510	638	557	3.163302	2.842806	3.449201	2.784414	2.907427	3.100985	3.15177	2.930942	0.395511	1	-
ENSMUSC	Lamc3	laminin ga	GO:00325	developm	K06247	laminin, g	mmu0520	Pathways	2:3188729	78	136	88	117	145	228	0.700064	0.974824	0.872915	0.810137	0.838044	1.609865	0.849268	1.086015	0.271231	1	-
ENSMUSC	Fibcd1	fibrinoger	GO:00160	membran	-	-	-	-	2:3181329	2426	2841	2209	3545	3695	3303	31.17806	29.15919	31.37623	35.14835	30.57929	33.39472	30.57116	33.04079	0.552662	1	-
ENSMUSC	Abl1	c-abl onc	GO:00325	developm	K06619	abelson ty	mmu0520	Pathways	2:3168837	1019	1461	948	1368	1868	682	3.000204	3.435363	3.084834	3.10737	3.541669	1.579689	3.173467	2.742909	0.297218	1	-
ENSMUSC	Fubp3	far upstre	GO:00081	metabolic	-	-	-	-	2:3157265	496	679	671	743	904	801	2.565858	2.805217	3.836371	2.965308	3.01144	3.259825	3.069149	3.078858	0.86883	1	-
ENSMUSC	Tor1a	torsin fam	GO:00325	developm	-	-	-	-	2:3096062	431	546	417	614	716	550	6.193468	6.266064	6.622771	6.806995	6.625585	6.217707	6.360768	6.550096	0.962072	1	-
ENSMUSC	BC005624	cDNA seq	GO:00056	cell|intrace	-	-	-	-	2:3097217	1303	1587	1068	1651	1969	1364	21.1987	20.61995	19.20362	20.7225	20.62839	17.45783	20.34076	19.6029	0.535056	1	-
ENSMUSC	Crat	carnitine a	GO:00081	metabolic	K00624	carnitine (	mmu0414	Peroxisom	2:3040047	445	760	459	791	900	629	2.160191	2.946397	2.462586	2.962365	2.813384	2.402114	2.523058	2.725954	0.644281	1	-
ENSMUSC	Usp20	ubiquitin s	GO:00081	metabolic	-	-	-	-	2:3098227	1408	1116	1434	1317	1394	1928	6.880891	4.355639	7.745293	4.965444	4.386915	7.412417	6.327274	5.588259	0.330151	1	-
ENSMUSC	Dolpp1	dolichyl p	GO:00081	metabolic	K07252	dolichyldi	mmu0051	N-Glycan	2:3039225	191	252	187	304	338	283	1.33663	1.408395	1.446324	1.641278	1.523172	1.558028	1.397116	1.574159	0.436064	1	-
ENSMUSC	Ntmt1	N-termina	GO:00081	metabolic	-	-	-	-	2:3080782	131	295	175	154	307	252	1.512888	2.72083	2.233668	1.372097	2.283113	2.289526	2.155795	1.981578	0.549331	1	-
ENSMUSC	Miga2	mitoguarc	GO:00325	developm	-	-	-	-	2:3036423	553	720	891	909	1428	1141	3.001343	3.120823	5.344604	3.806143	4.990839	4.871777	3.822257	4.556253	0.309172	1	-

ENSMUS(	Sh3glb2	SH3-dom	GO:00056	cell|intrace	K11248	endophilir	mmu0414	Endocytos	2:3034480	1684	2009	1778	2432	2744	2441	11.80097	11.2435	13.77064	13.1483	12.38267	13.45719	12.2717	12.99605	0.724653	1 -
ENSMUS(	Hspa5	heat shoc	GO:00325	developm	K09490	heat shoc	mmu0414	Protein pr	2:3477197	2205	1983	2547	2387	6031	2429	19.3661	13.9092	24.72346	16.17396	34.10968	16.78309	19.33292	22.35558	0.507176	1 -
ENSMUS(	Kynu	kynurenin	GO:00081	metabolic	K01556	kynurenin	mmu011C	Metabolic	2:4355532	0	1	0	4	0	0	0	0.007128	0	0.027536	0	0	0.002376	0.009179	0.440645	1 -
ENSMUS(	Psmd5	proteasor	GO:00329	macromo	-	-	-	-	2:3484973	594	663	511	704	834	701	3.927291	3.50079	3.733996	3.590952	3.550808	3.646159	3.720692	3.595973	0.575922	1 -
ENSMUS(	Zeb2	zinc finge	GO:00325	developm	K09299	zinc finge	mmu052C	MicroRNA	2:4498363	7015	9048	6238	8623	10488	9306	12.53028	12.90719	12.31474	11.88288	12.0637	13.07697	12.58407	12.34118	0.599399	1 -
ENSMUS(	Phf19	PHD finge	GO:00325	developm	-	-	-	-	2:3489375	14	19	12	27	42	17	0.102596	0.111196	0.097189	0.152645	0.198198	0.098008	0.10366	0.149617	0.210708	1 -
ENSMUS(	Hc	hemolytic	GO:00325	developm	K03994	complem	mmu0516	Herpes sir	2:3498333	1	12	2	5	7	4	0.008817	0.084473	0.019485	0.033999	0.03973	0.027736	0.037592	0.033822	0.892387	1 -
ENSMUS(	Traf1	TNF recep	GO:00081	metabolic	K03172	TNF recep	mmu052C	Pathways	2:3494175	13	15	20	21	46	26	0.059422	0.054758	0.101038	0.074054	0.1354	0.093495	0.071739	0.100983	0.239292	1 -
ENSMUS(	Rab14	RAB14, m	GO:00325	developm	K07881	Ras-relate	mmu0415	AMPK sigi	2:3518020	4227	5211	3630	5301	6619	5122	14.05274	13.83553	13.33774	13.59618	14.1702	13.39613	13.742	13.72083	0.759698	1 -
ENSMUS(	Stom	stomatin [	GO:00511	localizatio	-	-	-	-	2:3531398	247	337	199	354	475	353	4.101541	4.469156	3.652167	4.535062	5.079229	4.611423	4.074288	4.741905	0.295616	1 -
ENSMUS(	Dab2ip	disabled 2	GO:00325	developm	K19901	disabled h	mmu0421	Apoptosis	2:3555826	1427	1675	1217	1875	1961	1825	4.489614	4.208676	4.231771	4.551102	3.972988	4.51709	4.31002	4.34706	0.862604	1 -
ENSMUS(	Ttll11	tubulin tyr	GO:00081	metabolic	-	-	-	-	2:3575124	491	654	477	644	749	660	3.514184	3.738238	3.773192	3.555984	3.452072	3.716195	3.675205	3.57475	0.620544	1 -
ENSMUS(	Mrrf	mitochon	GO:00081	metabolic	-	-	-	-	2:3613638	348	452	321	508	589	479	3.918172	4.064325	3.994434	4.412631	4.270449	4.242778	3.99231	4.308619	0.6382	1 -
ENSMUS(	Grb14	growth fa	GO:00508	response	-	-	-	-	2:6491247	340	586	396	592	630	554	2.408324	3.314968	3.100116	3.235104	2.873633	3.087142	2.941136	3.065293	0.885572	1 -
ENSMUS(	Rbm18	RNA bind	GO:00971	organic cy	-	-	-	-	2:3611607	657	691	491	736	922	674	5.286658	4.44058	4.366612	4.56903	4.777512	4.266649	4.69795	4.53773	0.590158	1 -
ENSMUS(	Lhx6	LIM home	GO:00325	developm	-	-	-	-	2:3608195	375	472	326	562	308	518	2.689625	2.703641	2.584195	3.109764	1.422545	2.922814	2.659153	2.485041	0.580615	1 -
ENSMUS(	Gca	grancalcir	GO:00081	metabolic	-	-	-	-	2:6266428	189	245	184	254	321	214	3.207338	3.32043	3.451024	3.325423	3.50786	2.856973	3.326264	3.230085	0.689826	1 -
ENSMUS(	Morn5	MORN re	-	-	-	-	-	-	2:3604945	15	7	14	3	16	18	1.282702	0.478071	1.323178	0.19795	0.881068	1.210923	1.027984	0.763314	0.470378	1 -
ENSMUS(	Ndufa8	NADH de	GO:00081	metabolic	K03952	NADH de	mmu011C	Metabolic	2:3603632	723	978	636	1115	1277	996	51.2718	55.38918	49.84759	61.00219	58.31572	55.56616	52.16952	58.29469	0.38793	1 -
ENSMUS(	Psmd14	proteasor	GO:00081	metabolic	K03030	26S prote	mmu0516	Epstein-B	2:6171169	794	909	774	967	1237	873	12.50778	11.43587	13.47558	11.75212	12.54827	10.81894	12.47308	11.70645	0.412512	1 -
ENSMUS(	Strbp	spermatid	GO:00325	developm	-	-	-	-	2:3748322	2207	3679	2441	2925	3583	3338	6.451324	8.588595	7.886077	6.596335	6.744472	7.676154	7.641998	7.005654	0.261673	1 -
ENSMUS(	Wdr5	WD repea	GO:00325	developm	-	-	-	-	2:2751515	454	593	433	558	725	577	6.638024	6.924417	6.997097	6.294294	6.826146	6.636969	6.853179	6.585803	0.554283	1 -
ENSMUS(	Brd3	bromodo	GO:00081	metabolic	-	-	-	-	2:2744557	1040	1318	847	1222	1447	1407	5.073946	5.135392	4.567125	4.599534	4.546065	5.400294	4.925488	4.848631	0.680386	1 -
ENSMUS(	Egfl7	EGF-like c	GO:00508	response	-	-	-	-	2:2658001	247	333	222	399	403	404	1.957856	2.108017	1.944838	2.439983	2.057042	2.519273	2.00357	2.338766	0.28009	1 -
ENSMUS(	Notch1	notch 1 [S	GO:00325	developm	K02599	Notch[-]	mmu052C	Pathways	2:2645790	737	923	666	868	1120	1114	2.843054	2.843575	2.839478	2.583254	2.78221	3.380752	2.842035	2.915405	0.996581	1 -
ENSMUS(	Sec16a	SEC16 ho	GO:00511	localizatio	-	-	-	-	2:2640943	977	1327	981	1308	1565	1317	4.430615	4.806024	4.916826	4.576225	4.570231	4.698573	4.717822	4.61501	0.625589	1 -
ENSMUS(	Inpp5e	inositol pc	GO:00081	metabolic	K20278	inositol pc	mmu011C	Metabolic	2:2639624	374	343	385	357	515	647	1.917038	1.404102	2.181053	1.411749	1.69989	2.608998	1.834064	1.906879	0.940351	1 -
ENSMUS(	Pmpca	peptidase	GO:00081	metabolic	-	-	-	-	2:2638933	1751	2316	1624	2412	2838	2410	20.10772	21.24032	20.6115	21.369	20.98668	21.77232	20.65318	21.376	0.905031	1 -
ENSMUS(	Sdccag3	serologica	GO:00511	localizatio	-	-	-	-	2:2638280	901	1173	857	1319	1470	1377	6.199387	6.445663	6.517058	7.001626	6.513222	7.453646	6.387369	6.989498	0.489821	1 -
ENSMUS(	Card9	caspase re	GO:00081	metabolic	K12794	caspase re	mmu0515	Tuberculo	2:2635217	26	38	17	36	39	35	0.568797	0.663911	0.411039	0.607596	0.549415	0.602366	0.547916	0.586459	0.87757	1 -
ENSMUS(	Gpsm1	G-protein	GO:00325	developm	K15839	G-protein	mmu0503	Cocaine a	2:2631551	518	423	523	703	731	715	3.961931	2.583827	4.421057	4.148232	3.600386	4.302236	3.655605	4.016951	0.621708	1 -
ENSMUS(	1700019N	RIKEN cDI	-	-	-	-	-	-	19:587858	0	2	0	0	4	0	0	0.070274	0	0	0.113315	0	0.023425	0.037772	1	1 -
ENSMUS(	Camsap1	calmoduli	GO:00325	developm	-	-	-	-	2:2592683	2202	2891	1982	2838	3312	2865	9.596716	10.06234	9.546754	9.542188	9.295018	9.822921	9.735271	9.553376	0.616131	1 -
ENSMUS(	Fcna	ficolin A [S	GO:00048	signal trar	-	-	-	-	2:2562466	2	5	0	2	12	3	0.078215	0.156184	0	0.060361	0.302269	0.092302	0.078133	0.151644	0.325177	1 -
ENSMUS(	Traf2	TNF recep	GO:00081	metabolic	K03173	TNF recep	mmu052C	Pathways	2:2551798	157	200	126	222	233	220	1.455093	1.480356	1.290641	1.587352	1.390593	1.604069	1.408697	1.527338	0.687362	1 -
ENSMUS(	Abca2	ATP-bind	GO:00081	metabolic	K05642	ATP-bind	mmu0414	Lysosome	2:2542870	4593	6279	4608	6379	7176	7279	27.95454	30.52053	30.99668	29.95289	28.12502	34.85286	29.82392	30.97693	0.879229	1 -
ENSMUS(	Nmi	N-myc (a	GO:00081	metabolic	-	-	-	-	2:5194848	60	102	46	129	106	84	1.039574	1.411417	0.880873	1.724365	1.182678	1.144984	1.110621	1.350675	0.370402	1 -
ENSMUS(	Neb	nebulin [S	GO:00325	developm	-	-	-	-	2:5213664	114	114	76	117	129	128	0.240727	0.192252	0.177371	0.190606	0.175414	0.212638	0.20345	0.192886	0.63043	1 -
ENSMUS(	Sapcd2	suppressc	GO:00325	developm	-	-	-	-	2:2537232	16	24	22	39	37	33	0.114341	0.136972	0.173765	0.215021	0.170271	0.185529	0.141692	0.190274	0.239748	1 -
ENSMUS(	Uap1l1	UDP-N-a	GO:00081	metabolic	K00972	UDP-N-a	mmu011C	Metabolic	2:2535988	113	173	129	169	201	192	1.145535	1.400628	1.445327	1.321744	1.31214	1.531241	1.330497	1.388375	0.916941	1 -
ENSMUS(	Dpp7	dipeptidyl	GO:00081	metabolic	-	-	-	-	2:2535227	229	323	235	363	392	323	1.788016	2.014118	2.027922	2.186622	1.970959	1.984037	1.943352	2.047206	0.819444	1 -
ENSMUS(	Grin1	glutamate	GO:00325	developm	K05208	glutamate	mmu0408	Neuroacti	2:2529118	7039	8086	6513	8704	10328	8846	29.54276	27.10314	30.2112	28.18313	27.91328	29.20774	28.95237	28.43472	0.614595	1 -
ENSMUS(	Arl6ip6	ADP-ribo	GO:00160	membran	-	-	-	-	2:5319172	132	124	106	182	191	138	2.738585	2.054565	2.430561	2.91309	2.551764	2.252385	2.407903	2.572413	0.79336	1 -
ENSMUS(	Anapc2	anaphase	GO:00325	developm	K03349	anaphase	mmu0516	HTLV-I inf	2:2527247	1238	1589	1166	1878	2077	1765	15.75119	16.1459	16.396	18.43396	17.01703	17.66641	16.0977	17.7058	0.429462	1 -
ENSMUS(	Ssna1	SS nuclea	GO:00511	localizatio	-	-	-	-	2:2527103	193	250	222	275	314	284	4.713675	4.876283	5.992433	5.181634	4.938412	5.456737	5.194131	5.192261	0.86018	1 -
ENSMUS(	Fam166a	family witl	GO:00056	cell|intrace	-	-	-	-	2:2521874	2	3	0	3	0	0	0.103381	0.123887	0	0.119675	0	0	0.075756	0.039892	0.759195	1 -
ENSMUS(	Rbms1	RNA bind	GO:00081	metabolic	-	-	-	-	2:6075019	304	431	240	346	431	384	2.540952	2.877044	2.217082	2.231153	2.319824	2.525021	2.545026	2.358666	0.408499	1 -
ENSMUS(	Arrdc1	arrestin do	GO:00081	metabolic	-	-	-	-	2:2492535	65	71	68	90	116	97	0.561435	0.489765	0.64914	0.599735	0.645203	0.659124	0.56678	0.634687	0.591015	1 -
ENSMUS(	Zmynd19	zinc finge	GO:00452	synapse|n	-	-	-	-	2:2494979	451	534	337	598	668	615	6.096351	5.764747	5.034656	6.236268	5.814661	6.54002	5.631918	6.196983	0.532278	1 -
ENSMUS(	Dph7	diphtham	GO:00081	metabolic	-	-	-	-	2:2496240	205	259	285	333	381	340	2.468335	2.490552	3.792642	3.093317	2.954125	3.220622	2.917177	3.089355	0.81143	1 -
ENSMUS(	Pax8	paired bo	GO:00325	developm	K09293	paired bo	mmu052C	Pathways	2:2442056	2	3	0	1	6	5	0.016097	0.019289	0	0.006211	0.031101	0.031664	0.011795	0.022992	0.370993	1 -
ENSMUS(	7-Mar	membran	GO:00325	developm	-	-	-	-	2:6020988	752	997	692	975	1248	846	3.133964	3.318311	3.187344	3.134809	3.349232	2.773685	3.213206	3.085909	0.544456	1 -
ENSMUS(	Il1f8	interleukir	GO:00325	developm	-	-	-	-	2:2415316	0	0	0	2	2	0	0	0	0	0.077423	0.064602	0	0	0.047342	0.274801	1 -
ENSMUS(	Baz2b	bromodo	GO:00056	cell|intrace	-	-	-	-	2:5989936	821	1062	752	1090	1285	1128	2.189888	2.262293	2.216885	2.243031	2.207173	2.367001	2.223022	2.272401	0.984412	1 -
ENSMUS(	Wdsub1	WD repea	GO:00081	metabolic	-	-	-	-	2:5985236	118	126	128	146	210	135	1.095466	0.934183	1.313322	1.045678	1.255419	0.985964	1.114324	1.095687	0.819258	1 -
ENSMUS(	Dapl1	death ass	GO:00325	developm	-	-	-	-	2:5948465	3	2	0	3	2	0	0.23574	0.125535	0	0.1819	0.101185	0	0.120425	0.094362	0.782659	1 -
ENSMUS(	Pkp4	plakophili	GO:00452	synapse|c	-	-	-	-	2:5916085	4824	6712	4698	6790	8040	6931	26.01752	28.91055	28.00389	28.25261	27.92346	29.40799	27.64398	28.52802	0.926552	1 -
ENSMUS(	Nup35	nucleopor	GO:00081	metabolic	K14313	nuclear pc	mmu0301	RNA trans	2:8061723	144	120	108	163	146	140	1.881934	1.252473	1.559956	1.643458	1.228708	1.439396	1.564787	1.437187	0.504837	1 -
ENSMUS(	Dusp19	dual speci	GO:00081	metabolic	-	-	-	-	2:8061704	236	320	233	292	391	326	2.490013	2.696412	2.717016	2.376853	2.656574	2.705945	2.63448	2.579791	0.710979	1 -
ENSMUS(	Nckap1	NCK-asso	GO:00325	developm	K05750	NCK-asso	mmu0481	Regulatior	2:8050051	13918	16404	12655	15919	20606	17537	123.1783	115.9452	123.7845	108.6934	117.4372	122.1022	120.9693	116.0776	0.441581	1 -
ENSMUS(	Frzb	frizzled-re	GO:00325	developm	-	-	-	-	2:8041197	410	555	404	637	705	619	6.869867	7.426836	7.481584	8.234456	7.606921	8.159562	7.259429	8.000313	0.497355	1 -
ENSMUS(	Dnajc10	DnaJ heat	GO:00081	metabolic	K09530	DnaJ hom	mmu0414	Protein pr	2:8031546	1057	1428	1008	1444	1817	1521	11.15617	12.03688	11.75838	11.75811	12.3495	12.62932	11.65048	12.24564	0.781278	1 -
ENSMUS(	Ssfa2	sperm spe	GO:00160	membran	-	-	-	-	2:7963535	903	1283	861	1124	1475	1299	8.369073	9.496473	8.819418	8.036862	8.803115	9.471303	8.894988	8.770427	0.699504	1 -
ENSMUS(	Itga4	integrin al	GO:00325	developm	K06483	integrin al	mmu0415	PI3K-Akt s	2:7925542	993	1308	890	1219	1673	1249	3.885589	4.08753	3.848969	3.679946	4.215587	3.844862	3.940696	3.913465	0.749639	1 -
ENSMUS(	Slc25a12	solute car	GO:00081	metabolic	-	-	-	-	2:7127106	3010	3876	2675	4039	4788	4247	21.28372	21.88823	20.90507	22.03359	21.80167	23.62513	21.35901	22.4868	0.749394	1 -
ENSMUS(	Ube2e3	ubiquitin-	GO:00081	metabolic	K20217	ubiquitin-	mmu0412	Ubiquitin	2:7886812	622	749	578	688	936	670	6.574025	6.322209	6.751741	5.609961	6.370474	5.57092	6.549325	5.850452	0.190048	1 -
ENSMUS(	Dync1i2	dynein cyt	GO:00511	localizatio	K10415	dynein int	mmu0414	Phagoson	2:7121170	1478	1813	1405	2014	2401	1948	6.817549	6.678786	7.162704	7.167101	7.131825	7.06893	6.886346	7.122618	0.913757	1 -
ENSMUS(	Cybrd1	cytochron	GO:00081	metabolic	K08370	cytochron	mmu0497	Mineral al	2:7111792	107	141	72	144	210	149	1.223678	1.287803	0.910043	1.2705	1.546524	1.340543	1.140508	1.385856	0.275098	1 -
ENSMUS(	Zfp385b	zinc finge	GO:00508	response	-	-	-	-	2:7741063	748	919	442	1057	937	747	4.161787	4.083568	2.717987	4.537157	3.357166	3.269717	3.654448	3.721346	0.964702	1 -
ENSMUS(	Hat1	histone ar	GO:00081	metabolic	K11303	histone ac	mmu0503	Alcoholisr	2:7138895	276	415	236	373	420	365	4.685029	5.62596	4.427532	4.884741	4.591	4.874238	4.91284	4.783326	0.674266	1 -
ENSMUS(	Xirp2	xin actin-l	GO:00325	developm	-	-	-	-	2:6744600	3	0	0	0	0	0	0.013147	0	0	0	0	0	0.004382	0	0.295379	1 -
ENSMUS(	Stk39	serine/thr	GO:00081	metabolic	-	-	-	-	2:6821044	1285	1889	1139	1496	2098	1664	10.03577	11.78218	9.831466	9.013833	10.55135	10.22379	10.5498	9.929656	0.390293	1 -
ENSMUS(	Cers6	ceramide	GO:00081	metabolic	K04710	ceramide	mmu011C	Metabolic	2:6886144	1609	1912	1293	1955	2438	1911	8.971096	8.513799	7.967733	8.409431	8.753422	8.38226	8.484209	8.515038	0.822014	1 -
ENSMUS(	Ssrp1	structure s	GO:00081	metabolic	-	-	-	-	2:8503723	1794	2242	1512	2439	2963	2364	17.95709	17.92235	16.72677	18.83457	19.09852	18.61538	17.5354	18.84949	0.58106	1 -
ENSMUS(	Lrp2	low densit	GO:00325	developm	K06233	low densit	mmu0491	Thyroid hc	2:6942434	40	21	35	46	55	43	0.140171	0.058771	0.135555	0.124361	0.124113	0.118544	0.111499	0.122339	0.775016	1 -
ENSMUS(	P2rx3	purinergic	GO:00048	signal trar	K05217	P2X purin	mmu0408	Neuroacti	2:8499858	19	36	29	23	31	33	0.275885	0.417476	0.465395	0.257654	0.289868	0.376971	0.386252	0.308164	0.297454	1 -
ENSMUS(	Slc43a3	solute car	GO:00511	localizatio	-	-	-	-	2:8493657	23	33	22	23	48	35	0.22355	0.256156	0.236333	0.172467	0.300428	0.267622	0.23868	0.246839	1	1 -
ENSMUS(	Timm10	translocas	GO:00511	localizatio	-	-	-	-	2:8482699	163	269	200	252	313	257	8.737919	11.51641	11.84938	10.42199	10.80485	10.83836	10.70124	10.6884	0.863113	1 -
ENSMUS(	Ube2l6	ubiquitin-	GO:00081	metabolic	K04553	ubiquitin-	mmu0501	Parkinson	2:8479882	65	107	90	128	92	133	1.196858	1.573473	1.831549	1.81832	1.090861	1.926597	1.53396	1.611926	0.916467	1 -
ENSMUS(	Clp1	CLP1, clea	GO:00325	developm	K14399	polyribon	mmu0301	mRNA sur	2:8472210	72	50	61	98	57	88	0.869501	0.482224	0.814168	0.913049	0.443267	0.836047	0.721964	0.730787	0.950827	1 -
ENSMUS(	Med19	mediator	GO:00081	metabolic	-	-	-	-	2:8467840	436	567	381	552	712	581	9.383612	9.745694	9.062658	9.165438	9.867759	9.837191	9.397322	9.623463	1	1 -
ENSMUS(	Fastkd1	FAST kina	GO:00081	metabolic	-	-	-	-	2:6968681	239	241	161	267	281	299	1.653771	1.331808	1.231262	1.425344	1.252098	1.627647	1.405613	1.43503	0.976133	1 -
ENSMUS(	Phospho2	phosphata	GO:00081	metabolic	K13248	pyridoxal	mmu011C	Metabolic	2:6978962	375	480	414	420	642	470	2.960535	3.026397	3.612322	2.558107	3.263838	2.919096	3.199751	2.91368	0.338406	1 -
ENSMUS(	Zc3h15	zinc finge	GO:00508	response	-	-	-	-	2:8364443	1878	2350	1624	2314	2800	2135	27.71075	27.69278	26.48413	26.34187	26.60514	24.78345	27.29589	25.91015	0.386797	1 -
ENSMUS(	Mtx2	metaxin 2	GO:00511	localizatio	-	-	-	-	2:7482580	363	523	383	515	651	497	5.142481	5.917166	5.99669	5.628639	5.938838	5.539022	5.685446	5.702167	0.860581	1 -
ENSMUS(	Hoxd8	homeobo	GO:00325	developm	-	-	-	-	2:7470461	2	11	2	9	9	6	0.02467	0.108381	0.027273	0.085666	0.071504	0.058237	0.053441	0.071802	0.607077	1 -
ENSMUS(	Atf2	activating	GO:00325	developm	K04450	cyclic AMI	mmu0415	PI3K-Akt s	2:7381650	1744	1290	944	1850	2407	2009	6.097421	3.60193	3.647694	4.990012	5.41914	5.52574	4.449015	5.311631	0.258788	1 -
ENSMUS(	Chrna1	cholinergi	GO:00325	developm	K04803	nicotinic a	mmu0408	Neuroacti	2:7356321	32	36	29	37	44	42	0.445475	0.400247	0.446189	0.397381	0.394438	0.459978	0.430637	0.417266	0.810774	1 -
ENSMUS(	Ola1	Obg-like	GO:00081	metabolic	-	-	-	-	2:7309280	2748	2766	2574	3526	3291	3366	32.62448	26.22556	33.77397	32.29531	25.15992	31.43778	30.87467	29.631	0.525735	1 -
ENSMUS(	Sp3	trans-acti	GO:00325	developm	-	-	-	-	2:7293642	784	1027	932	1112	1512	1064	5.032014	5.264321	6.611327	5.506312	6.249313	5.372525	5.635887	5.709383	0.928932	1 -
ENSMUS(	Itga6	integrin al	GO:00325	developm	K06485	integrin al	mmu052C	Pathways	2:7174561	457	573	388	653	770	696	2.267107	2.270158	2.127329	2.499192	2.459807	2.716291	2.221532	2.55843	0.268729	1 -
ENSMUS(	Kif18a	kinesin fai	GO:00325	developm	-	-	-	-	2:1092807	17	26	23	30	33	24	0.087367	0.106711	0.130634	0.118944	0.109209	0.09703	0.108237	0.108394	1	1 -
ENSMUS(	Arl14ep	ADP-ribo	GO:00160	membran	-	-	-	-	2:1069625	686	866	640	985	1056	904	6.160589	6.211008	6.352217	6.824409	6.106846	6.386716	6.241271	6.439324	0.946251	1 -
ENSMUS(	Slc12a6	solute car	GO:00511	localizatio	-	-	-	-	2:1122658	2537	2767	2022	3037	2748	3002	10.24839	8.926684	9.02741	9.464776	7.148363	9.540185	9.400828	8.717775	0.321092	1 -
ENSMUS(	Emc4	ER memb	GO:00329	macromo	-	-	-	-	2:1123630	1011	1276	937	1349	1558	1310	40.37263	40.69419	41.35443	41.56024	40.06435	41.15457	40.80708	40.92638	0.821254	1 -
ENSMUS(	Katnbl1	katanin p8	GO:00099	cellular pr	-	-	-	-	2:1123792	187	215	137	200	245	232	2.558335	2.349091	2.071499	2.110945	2.158429	2.496983	2.326308	2.255452	0.67766	1 -
ENSMUS(	Nop10	NOP10 rib	GO:00081	metabolic	K11130	H/ACA rib	mmu030C	Ribosome	2:1122619	184	214	146	190	259	170	10.13046	9.409623	8.884053	8.070401	9.182624	7.363289	9.474712	8.205438	0.222512	1 -
ENSMUS(	Lpcat4	lysophosp	GO:00081	metabolic	K13512	lysophosp	mmu011C	Metabolic	2:1122394	857	995	801	1098	1310	1154	13.34187	12.37099	13.7821	13.18768	13.13293	14.13358	13.16499	13.48473	0.99988	1 -
ENSMUS(	Ccdc34	coiled-co	-	-	-	-	-	-	2:1100178	148	204	155	247	298	181	0.858256	0.944783	0.993421	1.105056	1.112826	0.825746	0.932153	1.014543	0.665881	1 -
ENSMUS(	Lin7c	lin-7 hom	GO:00325	developm	-	-	-	-	2:1098908	2683	3264	2221	3132	4095	2924	34.25024	33.2766	31.33562	30.84573	33.6629	29.36508	32.95415	31.29124	0.404107	1 -
ENSMUS(	Commd9	COMM dc	GO:00081	metabolic	-	-	-	-	2:1018862	271	318	271	343	459	339	8.063706	7.556821	8.912154	7.87392	8.794952	7.935541	8.17756	8.201471	0.87323	1 -
ENSMUS(	Traf6	TNF recep	GO:00325	developm	K03175	TNF recep	mmu052C	Pathways	2:1016784	362	532	387	556	669	534	2.531846	2.971572	2.991487	3.000091	3.013074	2.938199	2.831635	2.983788	0.798888	1 -
ENSMUS(	B230118H	RIKEN cDI	GO:00056	cell|cilium	-	-	-	-	2:1015607	372	448	258	418	419	382	3.129458	3.009886	2.398798	2.712893	2.269837	2.52814	2.846047	2.503623	0.214527	1 -
ENSMUS(	Dnajc24	DnaJ heat	GO:00431	ion bindin	-	-	-	-	2:1059667	51	82	69	95	87	85	1.331885	1.710254	1.991557	1.914045	1.46309	1.74633	1.677899	1.707822	0.979741	1 -
ENSMUS(	Elp4	elongator	GO:00081	metabolic	-	-	-	-	2:1057010	189	240	190	252	301	238	3.092176	3.13588	3.435594	3.180774	3.1712	3.063304	3.221217	3.138426	0.698233	1 -
ENSMUS(	Pax6	paired bo	GO:00325	developm	K08031	paired bo	mmu0455	Signaling	2:1056689	119	176	83	185	183	140	0.321003	0.379158	0.247449	0.385004	0.317885	0.2971	0.31587	0.333329	0.87111	1 -
ENSMUS(	Eif3m	eukaryotic	GO:00081	metabolic	-	-	-	-	2:1049996	517	622	525	662	860	777	9.635841	9.258397	10.81447	9.518917	10.32173	11.39283	9.902903	10.41116	0.815449	1 -
ENSMUS(	Prrg4	proline ric	GO:00055	extracellul	-	-	-	-	2:1048307	17	33	12	28	39	32	0.3044	0.471899	0.237471	0.386789	0.449682	0.450768	0.337923	0.42908	0.402092	1 -
ENSMUS(	Depdc7	DEP dom	GO:00508	response	-	-	-	-	2:1047217	39	40	25	29	39	38	1.038948	0.851011	0.736055	0.596013	0.66903	0.796394	0.875338	0.687146	0.204475	1 -
ENSMUS(	Tcp11l1	t-complex	GO:00990	supramole	-	-	-	-	2:1046572	564	846	512	821	796	708	3.440581	4.121629	3.451987	3.863903	3.126946	3.397791	3.671399	3.46288	0.448709	1 -
ENSMUS(	Cstf3	cleavage s	GO:00081	metabolic	K14408	cleavage s	mmu0301	mRNA sur	2:1045905	456	620	421	594	836	671	3.840342	4.170065	3.918626	3.859424	4.533839	4.445683	3.976344	4.279648	0.646809	1 -
ENSMUS(	Hipk3	homeodo	GO:00081	metabolic	K08826	homeodo	mmu0421	Cellular se	2:1044264	1765	2149	1454	2148	2469	2138	11.58666	11.26668	10.54933	10.87873	10.43733	11.04161	11.13422	10.78589	0.52283	1 -
ENSMUS(	Fbxo3	F-box pro	GO:00056	cell|intrace	-	-	-	-	2:1040277	1015	1377	852	1397	1542	1179	4.056421	4.394979	3.76325	4.307285	3.968403	3.706822	4.07155	3.99417	0.663167	1 -
ENSMUS(	Nat10	N-acetyltı	GO:00081	metabolic	K14521	N-acetyltı	mmu030C	Ribosome	2:1037212	544	654	479	711	784	720	3.089249	2.966045	3.006331	3.114972	2.866979	3.216604	3.020542	3.066185	0.930382	1 -
ENSMUS(	Elf5	E74-like f	GO:00325	developm	K17101	E74-like fa	mmu0491	Prolactin s	2:1034116	5	7	4	6	10	6	0.034614	0.038705	0.03061	0.032047	0.044583	0.032681	0.034643	0.036437	1	1 -
ENSMUS(	Cat	catalase [S	GO:00325	developm	K03781	catalase[1	mmu0406	FoxO sign	2:1034538	927	1276	922	1346	1653	1305	18.04875	19.84101	19.84014	20.21821	20.72502	19.98889	19.2433	20.31071	0.747072	1 -
ENSMUS(	Pamr1	peptidase	GO:00055	extracellul	-	-	-	-	2:1025500	183	217	133	250	226	233	1.653398	1.565779	1.328078	1.74259	1.314888	1.656123	1.515752	1.571201	0.952607	1 -
ENSMUS(	Trim44	tripartite r	GO:00081	metabolic	-	-	-	-	2:1023001	8176	9910	6987	10209	12025	10077	81.63714	79.02534	77.10533	78.64306	77.31908	79.15695	79.25594	78.37303	0.667354	1 -
ENSMUS(	Api5	apoptosis	GO:00329	macromo	-	-	-	-	2:9441168	1658	2298	1709	2134	2747	2068	18.90111	20.92178	21.53236	18.76841	20.16583	18.5466	20.45175	19.16028	0.324332	1 -
ENSMUS(	Ttc17	tetratricop	GO:00099	cellular pr	-	-	-	-	2:9430076	880	1210	878	1202	1556	1104	5.454935	5.99016	6.015174	5.748332	6.21114	5.383777	5.82009	5.781083	0.762572	1 -
ENSMUS(	Hsd17b12	hydroxyst	GO:00081	metabolic	K10251	17beta-es	mmu011C	Metabolic	2:9403268	444	530	804	654	1485	646	6.652712	6.342166	13.3143	7.56003	14.32837	7.614808	8.769726	9.834402	0.649175	1 -
ENSMUS(	Ext2	exostoses	GO:00325	developm	K02367	glucurony	mmu011C	Metabolic	2:9366102	532	624	472	678	873	582	3.213586	3.010294	3.151134	3.159643	3.395838	2.765745	3.125005	3.107075	0.793946	1 -
ENSMUS(	Gatm	glycine an	GO:00325	developm	-	-	-	-	2:1225944	1447	2221	1502	2133	2313	2056	26.0009	31.87237	29.82886	29.56931	26.76395	29.06391	29.23404	28.46572	0.585251	1 -
ENSMUS(	Sema6d	sema don	GO:00325	developm	K06842	semaphor	mmu0436	Axon guic	2:1240899	934	1142	845	1228	1334	1201	2.959587	2.889988	2.959292	3.002016	2.722042	2.993905	2.936289	2.905988	0.718415	1 -
ENSMUS(	Myef2	myelin ba	GO:00081	metabolic	-	-	-	-	2:1250846	1227	1426	1001	1455	1847	1459	6.940091	6.441485	6.25751	6.349127	6.727324	6.492125	6.546362	6.522859	0.76977	1 -
ENSMUS(	Slc12a1	solute car	GO:00511	localizatio	-	-	-	-	2:1251525	0	3	0	2	0	0	0	0.019999	0	0.012879	0	0	0.006666	0.004293	1	1 -
ENSMUS(	Dut	deoxyurid	GO:00081	metabolic	K01520	dUTP pyrc	mmu011C	Metabolic	2:1252471	322	474	284	463	557	432	6.698716	7.875157	6.529813	7.430983	7.461832	7.070172	7.034562	7.320996	0.895237	1 -
ENSMUS(	Fbn1	fibrillin 1 [	GO:00325	developm	-	-	-	-	2:1253005	264	438	274	400	588	394	1.097078	1.453627	1.258431	1.2824	1.573493	1.28807	1.269712	1.381321	0.627093	1 -
ENSMUS(	Cops2	COP9 sigr	GO:00325	developm	-	-	-	-	2:1258303	1593	1977	1504	2152	2593	1908	17.53796	17.38265	18.3003	18.27832	18.38318	16.52543	17.7403	17.72898	0.786109	1 -
ENSMUS(	Galk2	galactokir	GO:00081	metabolic	-	-	-	-	2:1258591	216	280	217	322	447	306	1.572872	1.628339	1.74641	1.808947	2.096053	1.75296	1.649207	1.885987	0.368784	1 -
ENSMUS(	Fam227b	family witl	-	-	-	-	-	-	2:1259834	0	0	0	2	0	0	0	0	0	0.029668	0	0	0	0.009889	0.528071	1 -
ENSMUS(	Meis2	Meis hom	GO:00325	developm	-	-	-	-	2:1158630	1114	1269	787	1398	1588	1209	3.707752	3.37313	2.894992	3.589742	3.403539	3.16565	3.325292	3.386311	0.954319	1 -
ENSMUS(	Cd82	CD82 anti	GO:00508	response	K06509	CD82 anti	mmu0411	p53 signa	2:9341911	240	387	258	349	545	416	2.890896	3.722866	3.434697	3.243225	4.227389	3.942078	3.349486	3.804231	0.4134	1 -
ENSMUS(	Tspan18	tetraspani	GO:00508	response	-	-	-	-	2:9320176	609	776	583	758	1011	841	4.340421	4.416949	4.592293	4.167868	4.640024	4.71543	4.449888	4.507774	0.917536	1 -
ENSMUS(	Slc28a2	solute car	GO:00511	localizatio	-	-	-	-	2:1224264	0	3	0	2	5	2	0	0.019224	0	0.01238	0.02583	0.012625	0.006408	0.016945	0.282613	1 -
ENSMUS(	Syt13	synaptota	GO:00452	synapse|c	-	-	-	-	2:9291509	3775	4415	3430	4741	5595	4393	61.28539	57.24224	61.54331	59.38002	58.49178	56.10636	60.02365	57.99272	0.489326	1 -
ENSMUS(	Chst1	carbohydı	GO:00081	metabolic	K01022	keratan su	mmu0053	Glycosam	2:9259970	4146	5372	4038	5758	6819	5762	94.45781	97.74407	101.6767	101.207	100.0424	103.2744	97.95954	101.5079	0.887532	1 -
ENSMUS(	Pex16	peroxisor	GO:00511	localizatio	K13335	peroxin-1	mmu0414	Peroxisor	2:9237467	205	263	196	304	328	284	5.049184	5.173305	5.335441	5.776582	5.202296	5.502962	5.185977	5.493947	0.787893	1 -
ENSMUS(	Mapk8ip1	mitogen-	GO:00081	metabolic	K04434	mitogen-	mmu0401	MAPK sigi	2:9238367	5128	6592	4681	7042	7959	6851	51.60804	52.98249	52.06607	54.67587	51.58014	54.24182	52.21887	53.49928	0.999253	1 -
ENSMUS(	Sord	sorbitol de	GO:00081	metabolic	K00008	L-iditol 2-	mmu011C	Metabolic	2:1222347	239	290	216	314	421	370	6.204453	6.012426	6.197348	6.288765	7.037898	7.556465	6.138076	6.961043	0.405443	1 -
ENSMUS(	Patl2	protein as	GO:00451	translatior	-	-	-	-	2:1221201	0	0	0	2	2	0	0	0	0	0.038712	0.032301	0	0	0.023671	0.274783	1 -
ENSMUS(	Eif3j1	eukaryotic	GO:00081	metabolic	K03245	translatior	mmu0301	RNA trans	2:1220285	848	1087	716	1050	1351	951	8.619487	8.823907	8.043504	8.233897	8.84292	7.604614	8.495633	8.227144	0.583952	1 -
ENSMUS(	Frmd5	FERM dor	GO:00400	locomotic	-	-	-	-	2:1215455	499	591	403	556	652	566	2.173491	2.055848	1.940035	1.868369	1.828773	1.939477	2.056458	1.878873	0.287949	1 -
ENSMUS(	Harbi1	harbinger	GO:00081	metabolic	-	-	-	-	2:9171085	70	70	50	96	69	43	0.500415	0.399646	0.395052	0.529464	0.317644	0.241834	0.431705	0.362981	0.358074	1 -
ENSMUS(	Atg13	autophag	GO:00081	metabolic	K08331	autophag	mmu0414	Autophag	2:9167461	1062	1450	1095	1497	1746	1461	8.941491	9.74988	10.18935	9.723836	9.466389	9.677134	9.626908	9.622453	0.797536	1 -
ENSMUS(	Hypk	huntingtir	GO:00329	macromo	-	-	-	-	2:1214532	201	273	166	261	367	267	1.654669	1.794833	1.510326	1.657626	1.945518	1.729168	1.653276	1.777437	0.70902	1 -
ENSMUS(	Ell3	elongatior	GO:00325	developm	-	-	-	-	2:1214390	0	2	0	3	1	0	0	0.030003	0	0.043475	0.012092	0	0.010001	0.018522	0.719132	1 -
ENSMUS(	Arhgap1	Rho GTPa	GO:00511	localizatio	-	-	-	-	2:9164986	821	1091	827	1056	1172	1199	6.266091	6.650036	6.975988	6.217956	5.760173	7.199189	6.630705	6.392439	0.553342	1 -
ENSMUS(	Pdia3	protein di	GO:00081	metabolic	K08056	protein di	mmu0414	Protein pr	2:1214137	4118	5354	3559	5707	7378	5365	63.65501	66.09528	60.80245	68.05883	73.44117	65.24194	63.51758	68.91398	0.49459	1 -
ENSMUS(	F2	coagulatic	GO:00325	developm	K01313	coagulatic	mmu0408	Neuroacti	2:9162532	0	0	1	0	1	0	0	0	0.01223	0	0.007124	0	0.004077	0.002375	1	1 -
ENSMUS(	Lrp4	low densit	GO:00325	developm	-	-	-	-	2:9145751	1043	1260	895	1280	1554	1299	4.687443	4.52239	4.445508	4.43805	4.497358	4.592739	4.55178	4.509382	0.717069	1 -
ENSMUS(	Map1a	microtubu	GO:00452	synapse|su	-	-	-	-	2:1212896	35634	42903	30506	42894	47634	41679	154.6921	148.7432	146.3645	143.6582	133.1605	142.3416	149.9333	139.7201	0.248233	1 -
ENSMUS(	Arfgap2	ADP-ribo	GO:00511	localizatio	K12493	ADP-ribos	mmu0414	Endocytos	2:9126497	732	899	714	981	1235	1013	9.003733	8.831148	9.70637	9.309168	9.782128	9.80239	9.180417	9.631229	0.813434	1 -
ENSMUS(	Pacsin3	protein ki	GO:00081	metabolic	-	-	-	-	2:9125595	303	358	254	399	388	314	2.450408	2.312201	2.270269	2.489429	2.020612	1.997731	2.344292	2.169257	0.406786	1 -
ENSMUS(	Tubgcp4	tubulin, g	GO:00051	structural	-	-	-	-	2:1211706	296	375	280	678	445	328	1.401568	1.418074	1.465301	2.476749	1.356865	1.221818	1.428314	1.685144	0.39989	1 -
ENSMUS(	Lamp5	lysosomal	GO:00452	synapse|n	-	-	-	-	2:1360522	533	742	527	803	863	730	6.229709	6.926122	6.807653	7.240789	6.495395	6.712341	6.654494	6.816175	1	1 -
ENSMUS(	Ubr1	ubiquitin	GO:00081	metabolic	-	-	-	-	2:1208602	1542	1875	1348	1922	2319	1880	8.330525	8.08975	8.048684	8.010725	8.067582	7.990184	8.15632	8.02283	0.646169	1 -
ENSMUS(	Snap25	synaptosc	GO:00325	developm	K18211	synaptosc	mmu0491	Insulin sec	2:1367134	41726	50563	37203	54575	66176	50192	345.2182	334.0917	340.1823	348.3462	352.5674	326.6874	339.8308	342.5337	0.846224	1 -
ENSMUS(	Jag1	jagged 1		GO:00325	developm	K06052	jagged[-]	mmu052C	Pathways	2:1370814	184	265	181	192	299	262	1.648764	1.896414	1.792533	1.327311	1.725309	1.846943	1.779237	1.633188	0.425109	1 -
ENSMUS(	Mtch2	mitochon	GO:00081	metabolic	-	-	-	-	2:9084715	986	1360	1021	935	1885	1567	4.828239	5.318589	5.525663	3.532267	5.943986	6.036589	5.224164	5.170947	0.798671	1 -
ENSMUS(	Cdan1	congenita	GO:00081	metabolic	-	-	-	-	2:1207161	400	403	246	428	470	368	1.344746	1.082008	0.914031	1.110079	1.017494	0.97328	1.113595	1.033618	0.448186	1 -
ENSMUS(	Haus2	HAUS auc	GO:00990	supramole	-	-	-	-	2:1206093	318	337	310	329	469	329	4.678671	3.95978	5.04085	3.7344	4.443476	3.808045	4.559767	3.995307	0.218559	1 -
ENSMUS(	Lrrc57	leucine ric	GO:00055	extracellul	-	-	-	-	2:1206042	461	548	423	575	667	623	7.288401	6.919225	7.391251	7.013409	6.790656	7.748724	7.199625	7.184263	0.809798	1 -
ENSMUS(	Snap23	synaptosc	GO:00452	synapse|c	K08508	synaptosc	mmu0461	Platelet ac	2:1205676	200	322	205	310	377	272	2.295846	2.951989	2.600841	2.745398	2.786826	2.456366	2.616225	2.662863	0.976897	1 -
ENSMUS(	Zfp106	zinc finge	GO:00971	organic cy	-	-	-	-	2:1205068	7384	9650	5945	8792	11166	8552	29.81635	31.11974	26.53148	27.38929	29.03455	27.16699	29.15586	27.86361	0.425664	1 -

ENSMUSC	Vps39	VPS39 HC	GO:00081	metabolic	-	-	-	-	2:1203164	1713	2140	1596	2427	2740	2226	8.376113	8.356893	8.625117	9.155568	8.627609	8.562909	8.452707	8.782029	0.871918	1	-
ENSMUSC	Ehd4	EH-doma	GO:00081	metabolic	K12477	EH domai	mmu0414	Endocytos	2:1200891	198	276	185	291	388	299	2.703984	3.010185	2.792262	3.065933	3.412127	3.212337	2.835477	3.230132	0.379217	1	-
ENSMUSC	Itpka	inositol 1,	GO:00325	developm	K00911	1D-myo-	mmu011C	Metabolic	2:1197423	2765	3678	2887	3910	4786	4099	92.86341	98.65225	107.1625	101.311	103.5087	108.3024	99.5594	104.374	0.791833	1	-
ENSMUSC	Ltk	leukocyte	GO:00325	developm	-	-	-	-	2:1197513	177	218	161	248	304	243	1.447337	1.423638	1.455022	1.56451	1.600756	1.563201	1.441999	1.576156	0.605905	1	-
ENSMUSC	Tyro3	TYRO3 pr	GO:00325	developm	-	-	-	-	2:1197977	1565	2058	1374	2044	2335	1987	10.33484	10.85377	10.0282	10.41359	9.929584	10.32282	10.4056	10.222	0.635255	1	-
ENSMUSC	Ubox5	U box dor	GO:00081	metabolic	K10600	U-box do	mmu0412	Ubiquitin	2:1305900	225	294	236	325	344	378	2.073989	2.16429	2.404262	2.311196	2.04191	2.741102	2.21418	2.364736	0.756463	1	-
ENSMUSC	Ptpra	protein ty	GO:00081	metabolic	-	-	-	-	2:1304502	5122	7018	4996	6956	8662	7385	78.57779	85.98436	84.70899	82.3285	85.57229	89.12954	83.09038	85.67678	0.934612	1	-
ENSMUSC	Rtf1	RTF1, Paf1	GO:00325	developm	-	-	-	-	2:1196750	2358	3141	2065	2932	3734	3014	22.09561	23.50585	21.38598	21.19613	22.53156	22.21858	22.32915	21.98209	0.650295	1	-
ENSMUSC	Nusap1	nucleolar	GO:00511	localizatio	-	-	-	-	2:1196182	15	30	14	23	63	24	0.150608	0.240563	0.155361	0.178168	0.407344	0.189576	0.182177	0.258363	0.260929	1	-
ENSMUSC	4930402H	RIKEN cDI	-	-	-	-	-	-	2:1307062	1997	2584	1854	2564	2751	2678	9.248563	9.557285	9.489703	9.161035	8.204299	9.75703	9.43185	9.040788	0.455783	1	-
ENSMUSC	Atrn	attractin [	GO:00325	developm	-	-	-	-	2:1309064	4771	5945	4372	5702	7023	6036	19.18395	19.09089	19.42923	17.6883	18.18469	19.09364	19.23469	18.32221	0.375597	1	-
ENSMUSC	Chac1	ChaC, cati	GO:00325	developm	-	-	-	-	2:1193512	112	116	83	141	152	146	4.177448	3.455388	3.421509	4.057368	3.650839	4.284101	3.684782	3.997436	0.72551	1	-
ENSMUSC	Gfra4	glial cell li	GO:00048	signal trar	-	-	-	-	2:1310396	287	638	512	724	412	751	3.553772	6.309204	7.006883	6.916355	3.285189	7.315757	5.623287	5.8391	0.949102	1	-
ENSMUSC	Adam33	a disinteg	GO:00081	metabolic	-	-	-	-	2:1310505	10	11	14	23	20	26	0.051868	0.045564	0.080254	0.092035	0.066799	0.106091	0.059228	0.088309	0.183058	1	-
ENSMUSC	Siglec1	sialic acid	GO:00160	membran	K06548	sialoadhes	mmu0451	Cell adhes	2:1310692	9	30	12	23	25	36	0.071635	0.1907	0.105563	0.141238	0.128139	0.225426	0.122633	0.164934	0.381667	1	-
ENSMUSC	Rad51	RAD51 re	GO:00081	metabolic	K04482	DNA repa	mmu0520	Pathways	2:1191127	19	36	15	26	29	20	0.110181	0.166729	0.096138	0.116319	0.108295	0.091242	0.124349	0.105285	0.438081	1	-
ENSMUSC	Rpusd2	RNA pseu	GO:00081	metabolic	-	-	-	-	2:1190347	86	129	92	134	143	129	1.940491	2.324607	2.294306	2.332659	2.07781	2.2899	2.186468	2.233456	1	1	-
ENSMUSC	1700037H	RIKEN cDI	-	-	-	-	-	-	2:1311463	326	413	273	479	264	342	4.961346	5.019701	4.591886	5.624037	2.587269	4.09467	4.857644	4.101992	0.239827	1	-
ENSMUSC	Spef1	sperm flag	GO:00400	locomotic	-	-	-	-	2:1311702	101	113	61	89	130	117	1.29638	1.158337	0.865342	0.881314	1.074501	1.181425	1.106686	1.045747	0.630618	1	-
ENSMUSC	Cdc25b	cell divisic	GO:00325	developm	K05866	M-phase	mmu0401	MAPK sigi	2:1311869	222	284	191	288	383	367	1.996593	2.039866	1.898531	1.998299	2.218151	2.59666	1.97833	2.271036	0.368624	1	-
ENSMUSC	Ivd	isovaleryl	GO:00081	metabolic	K00253	isovaleryl-	mmu011C	Metabolic	2:1188619	659	1254	886	1359	1711	890	5.947866	9.038968	8.838043	9.462924	9.944437	6.31941	7.941626	8.575591	0.693089	1	-
ENSMUSC	Adra1d	adrenergi	GO:00048	signal trar	K04137	adrenergi	mmu0408	Neuroacti	2:1315458	633	885	624	907	1147	882	20.35271	22.72519	22.1743	22.49862	23.74852	22.3099	21.75074	22.85235	0.798126	1	-
ENSMUSC	Rassf2	Ras assoc	GO:00325	developm	K09851	Ras assoc	mmu0439	Hippo sig	2:1319894	1070	1300	1004	1353	1638	1378	4.037836	3.917906	4.187409	3.939064	3.980462	4.090958	4.047717	4.003495	0.707152	1	-
ENSMUSC	Slc23a2	solute car	GO:00081	metabolic	-	-	-	-	2:1320524	2311	3060	2085	2758	3377	2902	15.54708	16.44054	15.5025	14.31441	14.62967	15.35879	15.83004	14.76763	0.276656	1	-
ENSMUSC	Tmem23C	transmem	GO:00452	synapse|c	-	-	-	-	2:1322394	119	264	198	134	308	271	1.340324	2.374727	2.464773	1.164393	2.233929	2.401285	2.059941	1.933202	0.669209	1	-
ENSMUSC	Pcna	proliferati	GO:00081	metabolic	K04802	proliferati	mmu0516	HTLV-I inf	2:1322491	267	364	331	402	435	426	4.644598	5.056891	6.363728	5.39502	4.87282	5.82984	5.355072	5.365893	0.868423	1	-
ENSMUSC	Fsip1	fibrous sh	GO:00056	cell|cilium	-	-	-	-	2:1182048	10	16	7	8	13	15	0.286391	0.36596	0.22158	0.176759	0.239752	0.337953	0.29131	0.251488	0.632696	1	-
ENSMUSC	Rasgrp1	RAS guan	GO:00325	developm	K04350	RAS guan	mmu0520	Pathways	2:1172799	12021	7378	11262	14024	16845	14856	79.53831	38.98704	82.35654	71.58763	71.77306	77.3301	66.96063	73.5636	0.659374	1	-
ENSMUSC	Fam98b	family witl	GO:00081	metabolic	-	-	-	-	2:1172497	733	1071	739	969	1370	1090	10.21584	11.9208	11.38312	10.41896	12.29547	11.95109	11.17326	11.55517	0.923319	1	-
ENSMUSC	Chgb	chromogr	GO:00055	extracellul	-	-	-	-	2:1327812	13945	17237	11762	18316	23436	17006	310.7501	306.7611	289.6815	314.8863	336.3032	298.1301	302.3976	316.4399	0.801226	1	-
ENSMUSC	Spred1	sprouty pi	GO:00325	developm	-	-	-	-	2:1171213	3112	3606	2408	3695	4158	3354	26.90638	24.8993	23.01012	24.6468	23.1502	22.81338	24.9386	23.53679	0.364168	1	-
ENSMUSC	Mcm8	minichron	GO:00081	metabolic	-	-	-	-	2:1328161	85	100	72	89	118	106	1.162363	1.092115	1.088179	0.93895	1.039104	1.140347	1.114219	1.039467	0.550417	1	-
ENSMUSC	Tmco5	transmem	GO:00160	membran	-	-	-	-	2:1168786	19	23	11	17	25	20	0.751357	0.726405	0.480757	0.51866	0.636628	0.622207	0.65284	0.592498	0.667802	1	-
ENSMUSC	Crls1	cardiolipir	GO:00081	metabolic	K08744	cardiolipir	mmu011C	Metabolic	2:1328466	697	432	665	378	1168	905	11.15531	5.521776	11.76298	4.667358	12.03777	11.39484	9.480022	9.366658	0.867661	1	-
ENSMUSC	Slc27a2	solute car	GO:00081	metabolic	K08746	solute car	mmu0493	Insulin res	2:1265524	112	97	75	164	170	117	1.606786	1.111368	1.189187	1.815166	1.570528	1.320498	1.302447	1.568731	0.334299	1	-
ENSMUSC	Usp8	ubiquitin s	GO:00081	metabolic	K11839	ubiquitin (	mmu0414	Endocytos	2:1267073	1736	2174	1517	2198	2707	2311	15.70091	15.70293	15.16378	15.33674	15.76588	16.44319	15.52254	15.8486	0.97038	1	-
ENSMUSC	Usp50	ubiquitin s	GO:00081	metabolic	-	-	-	-	2:1267090	42	63	36	64	54	48	0.573957	0.687564	0.543732	0.674743	0.475199	0.516042	0.601751	0.555328	0.568341	1	-
ENSMUSC	Trpm7	transient r	GO:00081	metabolic	K04982	transient r	mmu0421	Cellular se	2:1267915	1061	1366	995	1385	1739	1419	3.787575	3.894414	3.925688	3.814395	3.997605	3.985098	3.869226	3.932366	0.93594	1	-
ENSMUSC	Stard7	START do	GO:00511	localizatio	-	-	-	-	2:1272702	1232	1514	1082	929	1894	1573	9.567998	9.390357	9.287194	5.566167	9.472073	9.610588	9.415183	8.216276	0.222456	1	-
ENSMUSC	Dusp2	dual speci	GO:00325	developm	K04459	dual speci	mmu0401	MAPK sigi	2:1273361	7	11	3	17	5	2	0.180116	0.226033	0.085318	0.337468	0.082845	0.040492	0.163822	0.153602	0.927283	1	-
ENSMUSC	Fahd2a	fumarylac	GO:00081	metabolic	-	-	-	-	2:1274362	114	138	105	166	189	159	2.220298	2.146498	2.260177	2.494282	2.370405	2.436204	2.208991	2.43363	0.606583	1	-
ENSMUSC	Mrps5	mitochon	GO:00081	metabolic	K02988	small subu	mmu0301	Ribosome	2:1275872	660	872	616	953	1136	777	4.68313	4.941455	4.830809	5.216939	5.190696	4.33735	4.818464	4.914995	0.976891	1	-
ENSMUSC	Mal	myelin an	GO:00325	developm	-	-	-	-	2:1276332	3592	5740	4214	5519	7139	5744	69.89189	89.19657	90.6216	82.84784	89.45044	87.92568	83.23669	86.74132	0.844457	1	-
ENSMUSC	Prom2	prominin	GO:00081	metabolic	-	-	-	-	2:1275264	4	13	3	12	0	10	0.031548	0.08188	0.026151	0.073011	0	0.062044	0.046526	0.045018	0.929197	1	-
ENSMUSC	Nphp1	nephrono	GO:00099	cellular pr	-	-	-	-	2:1277407	310	413	244	417	464	399	4.789472	5.095911	4.166412	4.970422	4.616354	4.849644	4.683932	4.81214	0.990887	1	-
ENSMUSC	Bub1	BUB1, mit	GO:00081	metabolic	K02178	checkpoir	mmu0411	Cell cycle	2:1278011	3	13	2	8	22	10	0.035198	0.121813	0.025937	0.072415	0.166223	0.092303	0.060983	0.110314	0.19024	1	-
ENSMUSC	Ndufaf5	NADH de	GO:00081	metabolic	-	-	-	-	2:1401706	307	345	295	374	458	389	8.71042	7.817471	9.2506	8.186582	8.367998	8.682829	8.59283	8.41247	0.704359	1	-
ENSMUSC	Fbln7	fibulin 7 [	GO:00055	extracellul	-	-	-	-	2:1288629	21	31	18	27	37	20	0.254314	0.299808	0.240916	0.252249	0.288535	0.190537	0.265013	0.243774	0.677162	1	-
ENSMUSC	Zc3h8	zinc finge	GO:00325	developm	-	-	-	-	2:1289262	87	124	84	118	139	120	3.270965	3.723266	3.490453	3.422706	3.365315	3.549366	3.494895	3.445796	0.799234	1	-
ENSMUSC	Ttl	tubulin tyi	GO:00325	developm	-	-	-	-	2:1290659	1176	1409	996	1398	1709	1317	11.129	10.64893	10.41729	10.20673	10.41468	9.804959	10.73174	10.14212	0.391323	1	-
ENSMUSC	Polr1b	polymeras	GO:00325	developm	K03002	DNA-dire	mmu011C	Metabolic	2:1291009	222	274	195	293	340	304	2.032824	2.003756	1.973466	2.06988	2.004849	2.189941	2.003349	2.088224	0.890888	1	-
ENSMUSC	Slc20a1	solute car	GO:00048	signal trar	-	-	-	-	2:1291987	1358	1773	1242	1866	2212	1838	8.300822	8.655178	8.390538	8.799606	8.706852	8.838482	8.448846	8.781647	0.869505	1	-
ENSMUSC	Il1b	interleukir	GO:00325	developm	-	-	mmu0401	MAPK sigi	2:1293645	2	3	0	6	5	4	0.05191	0.062207	0	0.120164	0.083585	0.081687	0.038039	0.095145	0.219293	1	-
ENSMUSC	Il1a	interleukir	GO:00325	developm	K04383	interleukir	mmu0401	MAPK sigi	2:1292996	10	23	12	12	14	25	0.309314	0.568171	0.410221	0.286348	0.278856	0.608338	0.429235	0.391181	0.725574	1	-
ENSMUSC	Pdyn	prodynorp	GO:00508	response	K15840	proenkep	mmu0503	Alcoholisr	2:1296865	146	204	138	159	276	173	2.791884	3.115446	2.916563	2.34569	3.398661	2.602559	2.941298	2.782303	0.60845	1	-
ENSMUSC	Tgm3	transgluta	GO:00325	developm	-	-	-	-	2:1300123	11	10	6	19	17	12	0.162434	0.117921	0.097925	0.216442	0.161652	0.139403	0.126093	0.172499	0.354422	1	-
ENSMUSC	Tgm6	transgluta	GO:00081	metabolic	-	-	-	-	2:1301124	1	0	1	0	4	0	0.017953	0	0.019837	0	0.046232	0	0.012597	0.015411	1	1	-
ENSMUSC	Snrpb	small nucl	GO:00081	metabolic	K11086	small nucl	mmu0532	Systemic l	2:1301714	562	654	510	780	866	710	10.88664	10.11768	10.91877	11.65687	10.80264	10.81998	10.64103	11.09316	0.868689	1	-
ENSMUSC	Nop56	NOP56 rit	GO:00081	metabolic	K14564	nucleolar	mmu0300	Ribosome	2:1302744	802	1070	819	1096	1173	1111	3.208323	3.41848	3.621045	3.382552	3.021735	3.496459	3.415949	3.300249	0.558873	1	-
ENSMUSC	Idh3b	isocitrate	GO:00081	metabolic	K00030	isocitrate (	mmu011C	Metabolic	2:1302793	2960	4037	2992	4246	5148	4373	39.45253	42.97221	44.07491	43.66097	44.18514	45.8536	42.16655	44.56657	0.714455	1	-
ENSMUSC	Cpxm1	carboxype	GO:00038	catalytic a	-	-	-	-	2:1303907	90	123	106	113	138	133	1.869757	2.040767	2.433869	1.811147	1.846193	2.173732	2.114798	1.943691	0.493763	1	-
ENSMUSC	Vps16	VSP16 CC	GO:00081	metabolic	-	-	-	-	2:1304243	841	1062	765	1086	1365	1271	8.758288	8.832705	8.805046	8.725366	9.153997	10.4131	8.798679	9.430821	0.6521	1	-
ENSMUSC	Pcsk2	proprotei	GO:00325	developm	-	-	-	-	2:1435461	5229	6389	4836	6724	8110	7078	37.96799	37.04911	38.80897	37.66665	37.92054	40.43156	37.94202	38.67292	0.950265	1	-
ENSMUSC	Bfsp1	beaded fil	GO:00325	developm	-	-	-	-	2:1438265	12	21	13	22	36	14	0.255645	0.357302	0.306093	0.361593	0.493889	0.234638	0.306347	0.363373	0.597267	1	-
ENSMUSC	Snx5	sorting ne	GO:00511	localizatio	K17920	sorting ne	mmu0414	Endocytos	2:1442501	1026	1281	917	1306	1457	1255	11.3984	11.36558	11.25934	11.19361	10.42343	10.9686	11.34111	10.86188	0.468096	1	-
ENSMUSC	Mgme1	mitochon	GO:00081	metabolic	-	-	-	-	2:1442706	124	160	99	158	221	157	1.6349	1.684748	1.442611	1.607152	1.87636	1.628463	1.58742	1.703992	0.750271	1	-
ENSMUSC	Kat14	lysine ace	GO:00081	metabolic	-	-	-	-	2:1443689	454	425	324	599	608	476	3.278967	2.451412	2.586264	3.337631	2.827738	2.704572	2.772214	2.956647	0.756753	1	-
ENSMUSC	Polr3f	polymeras	GO:00081	metabolic	K03025	DNA-dire	mmu011C	Metabolic	2:1445277	538	738	527	699	845	681	3.399489	3.724204	3.680345	3.407522	3.438291	3.385237	3.601346	3.41035	0.446247	1	-
ENSMUSC	Rbbp9	retinoblas	GO:00038	catalytic a	-	-	-	-	2:1445422	492	739	446	681	733	660	11.28497	13.5371	11.30621	12.05072	10.82665	11.90941	12.04276	11.59559	0.568446	1	-
ENSMUSC	Sec23b	SEC23 hoi	GO:00511	localizatio	K14006	protein tra	mmu0414	Protein pr	2:1445562	584	748	601	830	933	912	4.72729	4.83556	5.376756	5.183318	4.863351	5.807714	4.979869	5.284794	0.739484	1	-
ENSMUSC	Dtd1	D-tyrosyl-	GO:00081	metabolic	-	-	-	-	2:1445998	884	1141	883	1114	1394	1154	17.76089	18.30813	19.6074	17.26745	18.03558	18.24018	18.55881	17.84774	0.514511	1	-
ENSMUSC	Xrn2	5'-3' exor	GO:00325	developm	K12619	5'-3' exor	mmu0301	RNA degr	2:1470129	1165	1486	1151	1378	1884	1551	15.65766	15.95017	17.09711	14.28828	16.30558	16.39922	16.23498	15.66436	0.531455	1	-
ENSMUSC	Nkx2-2	NK2 hom	GO:00325	developm	K08029	homeobo	mmu0495	Maturity c	2:1471775	61	81	69	109	91	117	0.957218	1.015113	1.196674	1.319587	0.919554	1.444369	1.056335	1.227836	0.483814	1	-
ENSMUSC	Cd93	CD93 anti	GO:00055	extracellul	-	-	-	-	2:1484366	179	313	148	197	283	276	1.636875	2.285876	1.49579	1.389818	1.666493	1.985553	1.80618	1.680621	0.526224	1	-
ENSMUSC	Napb	N-ethylm	GO:00452	synapse|c	-	-	-	-	2:1486939	7096	8405	6092	8266	10649	8457	57.25761	54.16305	54.32833	51.4571	55.33286	53.68424	55.24966	53.4914	0.499312	1	-
ENSMUSC	Gzf1	GDNF-inc	GO:00325	developm	-	-	-	-	2:1486810	878	1013	779	1084	1236	1072	9.621144	8.865182	9.434446	9.164141	8.721786	9.2414	9.306924	9.042443	0.58868	1	-
ENSMUSC	9230104L	RIKEN cDI	GO:00081	metabolic	-	-	-	-	2:1488467	0	0	2	0	0	0	0	0	0.122148	0	0	0	0.040716	0	0.526454	1	-
ENSMUSC	Cst3	cystatin C	GO:00325	developm	K13899	cystatin-C	mmu0497	Salivary se	2:1488717	9890	10956	8758	12124	14756	9847	302.9928	268.0612	296.5431	286.5578	291.1115	237.3291	289.199	271.6661	0.353925	1	-
ENSMUSC	Acss1	acyl-CoA	GO:00081	metabolic	K01895	acetyl-Co	mmu011C	Metabolic	2:1506181	600	665	503	730	978	632	7.40248	6.552291	6.858684	6.948307	7.769968	6.134143	6.937818	6.950806	0.849082	1	-
ENSMUSC	Gins1	GINS com	GO:00325	developm	-	-	-	-	2:1509054	9	4	7	11	22	12	0.295594	0.104912	0.25412	0.278745	0.465316	0.310076	0.218209	0.351379	0.206077	1	-
ENSMUSC	Nsfl1c	NSFL1 (p9	GO:00081	metabolic	K14012	UBX dom	mmu0414	Protein pr	2:1514941	1073	1467	1007	1556	1835	1477	9.348653	10.20764	9.696738	10.45897	10.29532	10.12374	9.751009	10.29268	0.744016	1	-
ENSMUSC	Snph	syntaphilii	GO:00325	developm	-	-	-	-	2:1515905	3095	4461	6831	4553	11519	10408	11.08227	12.75693	27.0333	12.57755	26.56058	29.3188	16.9575	22.81898	0.214912	1	-
ENSMUSC	Fam110a	family witl	GO:00056	cell|intrace	-	-	-	-	2:1519693	101	115	87	95	114	134	1.798973	1.635866	1.712648	1.305437	1.307561	1.877667	1.715829	1.496888	0.309115	1	-
ENSMUSC	Angpt4	angiopoie	GO:00325	developm	K05467	angiopoie	mmu0415	PI3K-Akt s	2:1519112	3	1	0	0	2	0	0.078445	0.020887	0	0	0.03367	0	0.033111	0.011223	0.719472	1	-
ENSMUSC	Tbc1d20	TBC1 dom	GO:00325	developm	-	-	-	-	2:1522938	918	1312	901	1280	1540	1344	10.40493	11.87616	11.28673	11.19275	11.24014	11.98413	11.18927	11.47234	0.992762	1	-
ENSMUSC	Rbck1	RanBP-tyi	GO:00081	metabolic	K10630	RanBP-tyi	mmu0421	Necroptos	2:1523163	1076	1363	1040	1606	1779	1674	10.28307	10.40285	10.98476	11.84094	10.94816	12.58568	10.55689	11.79159	0.359136	1	-
ENSMUSC	Tpx2	TPX2, mic	GO:00081	metabolic	-	-	-	-	2:1528479	56	70	55	67	130	91	0.472667	0.471852	0.513065	0.436285	0.70658	0.604249	0.485861	0.582371	0.379046	1	-
ENSMUSC	Pdrg1	p53 and D	GO:00329	macromo	-	-	-	-	2:1530088	158	242	136	222	295	223	2.35584	2.881708	2.241175	2.553718	2.832473	2.615809	2.492908	2.667333	0.750344	1	-
ENSMUSC	Ccm2l	cerebral c	GO:00325	developm	-	-	-	-	2:1530659	65	65	44	80	98	78	1.30595	1.042968	0.977039	1.24003	1.267929	1.232875	1.108652	1.246945	0.581242	1	-
ENSMUSC	Kif3b	kinesin fai	GO:00511	localizatio	-	-	-	-	2:1532914	1703	1980	1505	2081	2452	1988	18.45287	17.13406	18.02323	17.3961	17.10899	16.94637	17.87006	17.15049	0.45609	1	-
ENSMUSC	Dnmt3b	DNA metl	GO:00325	developm	K17399	DNA (cytc	mmu011C	Metabolic	2:1536494	16	17	8	20	27	18	0.10862	0.092167	0.060021	0.104748	0.118036	0.096131	0.086936	0.106305	0.547295	1	-
ENSMUSC	Mapre1	microtubu	GO:00511	localizatio	-	-	-	-	2:1537412	1495	1934	1296	2030	2312	1954	11.24577	11.6185	10.77456	11.78078	11.19929	11.56334	11.21294	11.51447	0.9797	1	-
ENSMUSC	Cdk5rap1	CDK5 reg	GO:00081	metabolic	-	-	-	-	2:1543353	97	136	118	152	196	140	1.300534	1.456243	1.748554	1.572255	1.692233	1.476692	1.501777	1.580394	0.85534	1	-
ENSMUSC	Snta1	syntrophir	GO:00081	metabolic	-	-	-	-	2:1543763	446	626	429	648	732	429	6.818201	7.642849	7.248355	7.642588	7.206108	5.159457	7.236469	6.669384	0.410497	1	-
ENSMUSC	Necab3	N-termina	GO:00081	metabolic	-	-	-	-	2:1545443	235	273	225	305	392	362	3.947353	3.66225	4.177041	3.952479	4.240138	4.783645	3.928881	4.325421	0.564753	1	-
ENSMUSC	E2f1	E2F transc	GO:00325	developm	K17454	transcripti	mmu0520	Pathways	2:1545594	107	182	102	140	159	136	1.393605	1.893093	1.468258	1.40674	1.333548	1.3935	1.584985	1.37793	0.267538	1	-
ENSMUSC	Fam210b	family witl	GO:00325	developm	-	-	-	-	2:1723455	840	1085	732	1097	1365	1084	14.17993	14.62751	13.65691	14.28669	14.83823	14.39577	14.15479	14.5069	0.997075	1	-
ENSMUSC	Cstf1	cleavage s	GO:00081	metabolic	K14406	cleavage s	mmu0301	mRNA sur	2:1723706	429	465	387	600	710	601	7.184298	6.219076	7.162831	7.751905	7.656668	7.917937	6.855402	7.775503	0.359619	1	-
ENSMUSC	Pkia	protein kii	GO:00081	metabolic	K15985	cAMP-de	mmu0503	Alcoholisr	3:7366669	2427	3062	2199	3054	3756	3000	35.22416	35.49129	35.27304	34.19559	35.10356	34.25334	35.3295	34.5175	0.582174	1	-
ENSMUSC	Stmn2	stathmin-	GO:00325	developm	-	-	-	-	3:8509360	4753	5522	4393	6117	7162	5329	45.77432	42.47138	46.75866	45.44885	44.41637	40.37483	45.00145	43.41335	0.48771	1	-
ENSMUSC	Rtfdc1	replicatior	GO:00325	developm	-	-	-	-	2:1724405	1161	1439	1096	1602	1994	1660	24.63116	24.38143	25.69863	26.22077	27.24159	27.70586	24.90374	27.05607	0.519517	1	-
ENSMUSC	Pag1	phosphop	GO:00048	signal trar	-	-	-	-	3:9687479	587	659	488	731	737	660	2.58333	2.316182	2.373605	2.481929	2.088642	2.285056	2.424372	2.285209	0.438521	1	-
ENSMUSC	Rae1	ribonuclei	GO:00511	localizatio	K14298	mRNA exp	mmu0516	Influenza	2:1730001	597	798	680	857	1036	843	10.59027	11.30527	13.33178	11.72853	11.83442	11.76443	11.74244	11.7758	0.847349	1	-
ENSMUSC	Rbm38	RNA bind	GO:00325	developm	-	-	-	-	2:1730204	33	42	32	56	67	36	0.209235	0.212675	0.224242	0.273925	0.273556	0.179569	0.215384	0.24235	0.635715	1	-
ENSMUSC	Rab22a	RAB22A, r	GO:00511	localizatio	K07891	Ras-relate	mmu0414	Endocytos	2:1736597	1014	902	955	1397	1611	271	4.414154	3.135896	4.594726	4.691766	4.51606	0.928088	4.048258	3.378638	0.465914	1	-
ENSMUSC	Stx16	syntaxin 1	GO:00511	localizatio	K08489	syntaxin 1	mmu0413	SNARE int	2:1740763	474	614	415	626	1149	553	3.84197	3.974565	3.717666	3.914532	5.997219	3.526234	3.844734	4.479328	0.348306	1	-
ENSMUSC	Edn3	endotheli	GO:00325	developm	-	-	-	-	2:1747606	113	131	100	120	157	111	1.087744	1.007083	1.063885	0.891171	0.973201	0.840584	1.052904	0.901652	0.219343	1	-
ENSMUSC	Phactr3	phosphata	GO:00987	molecular	-	-	-	-	2:1781189	3125	3925	2687	4031	4785	4016	22.01784	22.08565	20.92373	21.91128	21.71007	22.26021	21.67574	21.96052	0.89686	1	-
ENSMUSC	Impa1	inositol (n	GO:00081	metabolic	K01092	myo-inos	mmu011C	Metabolic	3:1031195	615	756	558	809	1061	867	4.819757	4.731709	4.833158	4.89135	5.354516	5.345397	4.794875	5.197088	0.574618	1	-
ENSMUSC	Fabp5	fatty acid	GO:00081	metabolic	K08754	fatty acid-	mmu0332	PPAR sign	3:1001254	943	1364	888	1204	1471	1130	13.62788	15.74261	14.18327	13.42374	13.68938	12.84712	14.51792	13.32008	0.253914	1	-
ENSMUSC	Snx16	sorting ne	GO:00511	localizatio	-	-	-	-	3:1041781	483	543	405	576	742	533	6.770218	6.078568	6.274192	6.228856	6.697524	5.87751	6.374326	6.267963	0.71732	1	-
ENSMUSC	Ptpn1	protein ty	GO:00081	metabolic	K05696	tyrosine-p	mmu0491	Insulin sig	2:1679320	932	1253	823	1213	1438	1116	9.371893	10.06256	9.146567	9.410286	9.311615	8.828497	9.527006	9.183466	0.528181	1	-
ENSMUSC	Nfatc2	nuclear fa	GO:00325	developm	K17332	nuclear fa	mmu0516	HTLV-I inf	2:1684764	201	275	202	271	309	302	0.680643	0.743706	0.755999	0.707983	0.673808	0.804528	0.726783	0.728773	0.865397	1	-
ENSMUSC	Atp9a	ATPase, cl	GO:00511	localizatio	-	-	-	-	2:1686344	5745	7290	5407	7638	9068	7984	45.08725	45.69168	46.89936	46.24596	45.8279	49.29413	45.89276	47.12266	0.978584	1	-
ENSMUSC	Sall4	sal-like 4	GO:00325	developm	-	-	-	-	2:1687483	7	11	5	2	8	12	0.052489	0.065869	0.041439	0.011571	0.03863	0.070789	0.053266	0.04033	0.501454	1	-
ENSMUSC	Lrrcc1	leucine ric	GO:00056	cell|intrace	-	-	-	-	3:1453378	387	470	327	488	555	458	2.334701	2.264455	2.180292	2.271277	2.156093	2.173684	2.259816	2.200351	0.649066	1	-
ENSMUSC	Zfp64	zinc finge	GO:00325	developm	-	-	-	-	2:1688933	226	283	250	325	369	404	2.037372	2.037484	2.490858	2.260352	2.142118	2.865195	2.188571	2.422555	0.564167	1	-
ENSMUSC	E2f5	E2F transc	GO:00325	developm	K04682	transcripti	mmu0421	Cellular se	3:1457864	103	142	103	165	189	155	2.183671	2.404288	2.413431	2.698771	2.580285	2.585199	2.333797	2.621418	0.508543	1	-
ENSMUSC	Dok5	docking p	GO:00325	developm	-	-	-	-	2:1707318	241	266	214	337	377	303	8.322979	7.336501	8.168093	8.978853	8.384123	8.232175	7.942524	8.531717	0.69858	1	-
ENSMUSC	Car2	carbonic a	GO:00325	developm	K18245	carbonic a	mmu0497	Pancreatic	3:1488627	1726	2684	1644	2607	3304	2592	34.46248	42.79906	36.27894	40.15848	42.48157	40.71476	37.84683	41.11827	0.518341	1	-
ENSMUSC	Psma7	proteasor	GO:00081	metabolic	K02731	20S prote	mmu0305	Proteason	2:1800363	813	886	845	1193	1262	933	11.59277	10.08965	13.31681	13.12403	11.58806	10.4662	11.66641	11.7261	0.869993	1	-
ENSMUSC	Ntsr1	neurotens	GO:00048	signal trar	K04211	neurotens	mmu0408	Neuroacti	2:1804999	77	70	73	100	108	89	0.757933	0.550279	0.794165	0.759399	0.684575	0.689198	0.700793	0.711057	0.980976	1	-
ENSMUSC	Mrgbp	MRG/MO	GO:00081	metabolic	-	-	-	-	2:1805813	253	306	236	313	345	343	1.851577	1.7885	1.908886	1.767243	1.625905	1.974812	1.849654	1.78932	0.633927	1	-
ENSMUSC	Gid8	GID comp	GO:00056	cell|cell jur	-	-	-	-	2:1807101	641	908	632	938	1104	943	11.06538	12.51815	12.05791	12.49227	12.27247	12.80651	11.88048	12.52375	0.772824	1	-
ENSMUSC	Nkain4	Na+/K+ t	GO:00511	localizatio	-	-	-	-	2:1809347	205	216	168	269	301	335	7.450552	6.269502	6.74825	7.542536	7.044593	9.578335	6.822768	8.055155	0.298184	1	-
ENSMUSC	Srms	src-relate	GO:00081	metabolic	-	-	-	-	2:1812055	0	6	3	3	0	4	0	0.114197	0.079024	0.055166	0	0.074991	0.064407	0.043386	0.552578	1	-
ENSMUSC	Stmn3	stathmin-	GO:00325	developm	-	-	-	-	2:1813064	5405	6947	5510	8362	9296	6935	290.5097	298.2003	327.3131	346.7412	321.748	293.2395	305.3411	320.5762	0.777563	1	-
ENSMUSC	Zbtb46	zinc finge	GO:00325	developm	-	-	-	-	2:1813877	156	163	139	183	215	138	0.895972	0.747656	0.882332	0.810868	0.795173	0.623532	0.841987	0.743191	0.31431	1	-
ENSMUSC	Oprl1	opioid rec	GO:00048	signal trar	K04216	nociceptir	mmu0408	Neuroacti	2:1817150	441	535	385	554	598	503	1.857778	1.799926	1.792515	1.800509	1.622224	1.666993	1.81674	1.696575	0.399328	1	-
ENSMUSC	Pcmtd2	protein-L	GO:00081	metabolic	-	-	-	-	2:1818378	741	1615	960	1540	1859	1503	4.316356	7.51307	6.18041	6.920702	6.973217	6.887621	6.003278	6.92718	0.326614	1	-
ENSMUSC	a	nonagout	GO:00081	metabolic	-	-	-	-	2:1547914	0	0	1	1	2	0	0	0	0.033228	0.023197	0.03871	0	0.011076	0.020636	0.648489	1	-
ENSMUSC	Ahcy	S-adenos	GO:00081	metabolic	K01251	adenosylh	mmu011C	Metabolic	2:1550593	178	209	151	238	310	222	3.454653	3.239485	3.238986	3.563628	3.874364	3.389604	3.311041	3.609198	0.629084	1	-
ENSMUSC	Itch	itchy, E3 u	GO:00325	developm	K05632	atrophin-	mmu0414	Endocytos	2:1551335	1431	1859	1326	1691	2209	1769	6.691746	6.94265	6.85315	6.100619	6.65197	6.507868	6.829182	6.420152	0.339449	1	-
ENSMUSC	Armc1	armadillo	GO:00511	localizatio	-	-	-	-	3:1913140	1106	1263	1041	1330	1655	1352	18.38559	16.76763	19.12581	17.05707	17.71638	17.68113	18.09301	17.48486	0.541267	1	-
ENSMUSC	Mtfr1	mitochon	GO:00081	metabolic	-	-	-	-	3:1918732	79	106	81	119	161	105	0.724158	0.775989	0.820613	0.841553	0.950352	0.757192	0.773587	0.849699	0.642369	1	-
ENSMUSC	Map1lc3a	microtubu	GO:00081	metabolic	K10435	microtubu	-	-	2:1552762	852	1090	750	1147	1198	1106	20.55374	21.00021	19.99674	21.3474	18.6107	20.99024	20.51689	20.31611	0.72987	1	-
ENSMUSC	Ggt7	gamma-g	GO:00081	metabolic	K00681	gamma-g	mmu011C	Metabolic	2:1554903	1083	1381	1100	1629	1703	1540	16.37186	16.67282	18.37845	18.99857	16.57825	18.31475	17.14104	17.96386	0.814329	1	-
ENSMUSC	Acss2	acyl-CoA	GO:00081	metabolic	K01895	acetyl-Co	mmu011C	Metabolic	2:1555179	846	1065	784	1255	1399	1113	4.035244	4.056906	4.132971	4.618208	4.297063	4.176431	4.075041	4.363901	0.645777	1	-
ENSMUSC	Dnajc5b	DnaJ heat	GO:00160	membran	K09525	DnaJ hom	mmu0414	Protein pr	3:1950859	0	0	0	1	0	1	0	0	0	0.021611	0	0.022039	0	0.01455	0.520585	1	-
ENSMUSC	Gss	glutathior	GO:00325	developm	K01920	glutathior	mmu011C	Metabolic	2:1555631	240	306	277	332	392	319	1.818559	1.851756	2.319756	1.940816	1.912745	1.901594	1.99669	1.918385	0.618232	1	-
ENSMUSC	Procr	protein C	GO:00508	response	K06557	protein C	mmu0461	Complem	2:1557511	17	21	9	26	15	21	0.275259	0.271554	0.161049	0.324775	0.156397	0.267497	0.235954	0.249556	1	1	-
ENSMUSC	Mmp24	matrix me	GO:00325	developm	-	-	-	-	2:1557753	776	1009	822	1127	1223	1088	9.566146	9.93372	11.19937	10.71838	9.708589	10.55151	10.23308	10.32616	0.884433	1	-
ENSMUSC	Hps3	HPS3, bio	GO:00329	macromo	-	-	-	-	3:1999594	214	249	160	264	332	254	0.766495	0.712262	0.633376	0.729507	0.765752	0.715716	0.704045	0.736992	0.867847	1	-
ENSMUSC	Nfs1	nitrogen f	GO:00081	metabolic	-	-	mmu011C	Metabolic	2:1561236	505	650	497	711	837	642	6.676989	6.863551	7.262604	7.25253	7.126393	6.677829	6.934381	7.018917	0.916558	1	-
ENSMUSC	Rbm39	RNA bind	GO:00081	metabolic	-	-	-	-	2:1561472	2716	3116	2321	3374	4038	3328	10.41735	9.544888	9.838969	9.983963	9.973522	10.04204	9.933736	9.99984	0.839441	1	-
ENSMUSC	Epb41l1	erythrocyt	GO:00051	structural	-	-	-	-	2:1564209	9574	13013	8517	12041	15294	9466	42.19506	45.80274	41.486	40.94124	43.40542	32.82053	43.16127	39.05573	0.200816	1	-
ENSMUSC	Aar2	AAR2 spli	GO:00081	metabolic	-	-	-	-	2:1565475	405	736	520	682	878	785	3.524591	5.115377	5.001544	4.57898	4.920427	5.374452	4.547171	4.957953	0.602875	1	-
ENSMUSC	Ndrg3	N-myc dc	GO:00508	response	-	-	-	-	2:1569273	8357	10422	7748	10395	12962	10307	52.10483	51.89488	53.39051	50.00143	52.04204	50.55577	52.4634	50.86641	0.504997	1	-
ENSMUSC	Dsn1	DSN1 hor	GO:00329	macromo	-	-	-	-	2:1569952	32	46	33	42	66	43	0.357194	0.410071	0.407118	0.361687	0.474411	0.377606	0.391461	0.404568	0.966742	1	-
ENSMUSC	Sla2	Src-like-a	GO:00325	developm	-	-	-	-	2:1568724	22	15	11	14	34	16	0.266892	0.145332	0.147484	0.13103	0.265612	0.152704	0.186569	0.183115	0.950495	1	-
ENSMUSC	1110008F	RIKEN cDI	GO:00056	cell|intrace	-	-	-	-	2:1568631	292	370	294	417	512	385	4.207924	4.258263	4.682512	4.636089	4.751267	4.36472	4.3829	4.584026	0.856298	1	-
ENSMUSC	Rpn2	ribophorir	GO:00325	developm	K12667	oligosacch	mmu011C	Metabolic	2:1572790	1639	2010	1448	2126	2613	2025	15.5226	15.20294	15.15658	15.53386	15.93601	15.08765	15.29404	15.51917	0.916355	1	-
ENSMUSC	Ghrh	growth hc	GO:00325	developm	-	-	-	-	2:1573294	2	0	0	0	1	0	0.141969	0	0	0	0.04571	0	0.047323	0.015237	1	1	-
ENSMUSC	Src	Rous sarc	GO:00325	developm	K05704	tyrosine-p	mmu0414	Endocytos	2:1574184	1398	1616	1353	1844	2057	1836	8.353813	7.711956	8.935564	8.500973	7.915282	8.631	8.333778	8.349085	0.808852	1	-
ENSMUSC	Ctnnbl1	catenin, b	GO:00325	developm	K12864	beta-cate	mmu0304	Spliceosoi	2:1577374	529	632	427	725	823	649	10.77736	10.28299	9.6146	11.39528	10.7972	10.40189	10.22498	10.86479	0.733126	1	-
ENSMUSC	Rprd1b	regulatior	GO:00081	metabolic	-	-	-	-	2:1580284	161	196	143	176	263	178	1.11002	1.079212	1.089652	0.936156	1.167657	0.965463	1.092961	1.023092	0.526402	1	-
ENSMUSC	Ralgapb	Ral GTPas	GO:00987	molecular	-	-	-	-	2:1584098	2299	2782	2000	2789	3426	2886	8.029551	7.759884	7.720207	7.515035	7.705384	7.929748	7.836547	7.716722	0.645271	1	-
ENSMUSC	Dhx35	DEAH (As	GO:00081	metabolic	-	-	-	-	2:1587948	265	297	238	346	437	353	2.520702	2.256203	2.502073	2.539114	2.676775	2.641564	2.426326	2.619151	0.660255	1	-
ENSMUSC	Slc2a10	solute car	GO:00511	localizatio	-	-	-	-	2:1655037	28	30	19	38	53	18	0.435901	0.372989	0.326921	0.456406	0.531331	0.220448	0.378604	0.402728	0.894667	1	-
ENSMUSC	Zmat3	zinc finge	GO:00099	cellular pr	K10137	zinc finge	mmu0411	p53 signa	3:3233479	1588	2058	1357	2086	2253	2045	5.102098	5.280675	4.818646	5.170623	4.661376	5.168952	5.06714	5.000317	0.674557	1	-
ENSMUSC	Pik3ca	phosphati	GO:00325	developm	K00922	phosphati	mmu0520	Pathways	3:3239767	1054	1199	820	1578	1946	1064	4.76635	4.330221	4.098318	5.505319	5.666865	3.785278	4.398297	4.985821	0.391788	1	-
ENSMUSC	Mfn1	mitofusin	GO:00325	developm	K06030	mitofusin	mmu0462	NOD-like	3:3252946	1058	1338	820	1328	1652	1397	8.104317	8.185258	6.942102	7.847998	8.148832	8.41856	7.743892	8.138463	0.79532	1	-
ENSMUSC	Gnb4	guanine n	GO:00325	developm	K04538	guanine n	mmu0520	Pathways	3:3258033	173	347	160	198	323	246	0.692116	1.108681	0.707454	0.611121	0.832126	0.774244	0.836084	0.739163	0.350347	1	-
ENSMUSC	Actl6a	actin-like	GO:00325	developm	K11340	actin-like	mmu0522	Hepatoce	3:3270629	132	189	109	167	225	168	1.402655	1.603927	1.280126	1.369063	1.539622	1.40442	1.428902	1.437702	0.904787	1	-
ENSMUSC	Ndufb5	NADH de	GO:00081	metabolic	K03961	NADH de	mmu011C	Metabolic	3:3273699	481	427	495	467	651	495	4.542765	3.220684	5.166857	3.402689	3.959225	3.677826	4.310102	3.679914	0.180977	1	-

ENSMUS(	Pex5l	peroxison	GO:00511	localizatio	K13342	peroxin-5	mmu0414	Peroxison	3:3294940	4329	4490	3246	5415	6098	5632	19.16894	15.87825	15.88568	18.49862	17.38812	19.61933	16.97763	18.50202	0.510305	1	-
ENSMUS(	Ccdc39	coiled-co	GO:00325	developm	-	-	-	-	3:3381236	471	562	442	583	612	633	3.328903	3.172211	3.452621	3.178914	2.785389	3.519611	3.317912	3.161304	0.516353	1	-
ENSMUS(	Ttc14	tetratricop	GO:00971	organic cy	-	-	-	-	3:3379983	1918	2467	1698	2715	2885	2800	5.275913	5.419559	5.162185	5.761681	5.110338	6.059238	5.285886	5.643752	0.648237	1	-
ENSMUS(	Ncoa3	nuclear re	GO:00325	developm	K11256	nuclear re	mmu0520	Pathways	2:1659926	821	1118	730	1253	1416	1174	4.909287	5.339036	4.824419	5.78038	5.452467	5.522736	5.024247	5.585194	0.39568	1	-
ENSMUS(	Dnajc19	DnaJ heat	GO:00325	developm	-	-	-	-	3:3405602	202	274	168	269	320	286	2.484641	2.691589	2.283856	2.552671	2.534643	2.767502	2.486695	2.618272	0.843532	1	-
ENSMUS(	Fxr1	fragile X r	GO:00325	developm	K15516	fragile X n	mmu0301	RNA trans	3:3401994	1485	1971	1309	1993	2480	1818	9.899695	10.49368	9.644543	10.25021	10.64637	9.534546	10.01264	10.14371	0.908955	1	-
ENSMUS(	Nceh1	neutral ch	GO:00081	metabolic	K14349	neutral ch	mmu0497	Bile secret	3:2718296	2406	2868	2153	3152	3754	3039	26.02867	24.77886	25.74229	26.30709	26.15202	25.86414	25.51661	26.10775	0.989547	1	-
ENSMUS(	Lrrc34	leucine ric	GO:00325	developm	-	-	-	-	3:3062426	6	12	5	15	13	7	0.193728	0.309437	0.178442	0.373658	0.270308	0.177821	0.227202	0.273929	0.749665	1	-
ENSMUS(	Lrriq4	leucine-ri	GO:00511	localizatio	-	-	-	-	3:3064450	0	0	0	0	2	0	0	0	0	0	0.026742	0	0	0.008914	0.528908	1	-
ENSMUS(	Sec62	SEC62 hoı	GO:00511	localizatio	K12275	translocat	mmu0414	Protein pr	3:3079287	2996	3748	2495	3715	4339	3441	36.18795	36.15494	33.30731	34.61875	33.74944	32.69774	35.21673	33.68864	0.432163	1	-
ENSMUS(	Dcun1d1	DCN1, de	GO:00081	metabolic	-	-	-	-	3:3589210	808	992	733	989	1330	951	3.739764	3.666826	3.749586	3.531501	3.96405	3.462774	3.718725	3.652775	0.686369	1	-
ENSMUS(	Mccc1	methylcro	GO:00081	metabolic	K01968	3-methylc	mmu0110	Metabolic	3:3595931	388	472	319	476	539	469	1.780707	1.730013	1.618073	1.685384	1.59296	1.693344	1.709598	1.657229	0.620079	1	-
ENSMUS(	Acad9	acyl-Coer	GO:00081	metabolic	-	-	-	-	3:3606597	480	650	468	646	820	603	2.566591	2.775714	2.765717	2.664885	2.823474	2.536552	2.702674	2.67497	0.753981	1	-
ENSMUS(	1810062G	RIKEN cDI	-	-	-	-	-	-	3:3647593	0	3	0	0	0	1	0	0.160605	0	0	0	0.052738	0.053535	0.017579	0.657206	1	-
ENSMUS(	Exosc9	exosome	GO:00081	metabolic	K03678	exosome	mmu0301	RNA degr	3:3655260	382	512	392	593	614	520	3.480184	3.72524	3.947038	4.167966	3.602154	3.726948	3.717487	3.832356	0.958117	1	-
ENSMUS(	Trpc3	transient r	GO:00511	localizatio	K04966	transient r	mmu0436	Axon guic	3:3662048	86	96	60	108	111	102	0.763666	0.680805	0.588852	0.739878	0.634722	0.712554	0.677774	0.695718	1	1	-
ENSMUS(	Il21	interleukir	GO:00325	developm	K05434	interleukir	mmu0406	Cytokine-	3:3722275	0	0	2	0	0	0	0	0	0.023498	0	0	0	0.007833	0	0.52657	1	-
ENSMUS(	Spata5	spermato	GO:00325	developm	K14575	AAA famil	mmu0300	Ribosome	3:3741989	222	253	235	306	345	245	2.191217	1.994345	2.563587	2.33016	2.192843	1.902435	2.249716	2.141813	0.588522	1	-
ENSMUS(	Slc7a11	solute car	GO:00325	developm	K13869	solute car	mmu0421	Ferroptos	3:4989252	819	979	619	899	1045	1066	3.185944	3.041466	2.661287	2.698014	2.617734	3.262291	2.962899	2.859346	0.578735	1	-
ENSMUS(	Rab33b	RAB33B, r	GO:00325	developm	K07920	Ras-relate	mmu0414	Autophag	3:5148392	335	444	272	352	548	398	4.24191	4.489988	3.806559	3.438656	4.468396	3.964703	4.179485	3.957252	0.526055	1	-
ENSMUS(	Cog6	compone	GO:00081	metabolic	-	-	-	-	3:5298187	657	864	594	897	806	886	5.602676	5.884232	5.598398	5.901377	4.426088	5.94395	5.695102	5.423805	0.50497	1	-
ENSMUS(	Stoml3	stomatin (	GO:00508	response	-	-	-	-	3:5348865	16	18	25	21	28	22	0.419462	0.376865	0.724362	0.424737	0.472703	0.453746	0.506896	0.450395	0.665359	1	-
ENSMUS(	Ufm1	ubiquitin-	GO:00081	metabolic	-	-	-	-	3:5385337	1126	1494	1067	1453	1747	1351	10.70561	11.34409	11.21203	10.65783	10.69598	10.10507	11.08725	10.4863	0.392984	1	-
ENSMUS(	Trpc4	transient r	GO:00325	developm	K04967	transient r	mmu0436	Axon guic	3:5415603	951	1187	805	1216	1235	1100	5.019996	5.00402	4.6964	4.952066	4.198019	4.568001	4.906805	4.572695	0.334174	1	-
ENSMUS(	Exosc8	exosome	GO:00081	metabolic	K12586	exosome	mmu0301	RNA degr	3:5472867	129	155	110	154	202	172	0.855768	0.821194	0.806507	0.788165	0.862926	0.897651	0.827823	0.849581	1	1	-
ENSMUS(	Sucnr1	succinate	GO:00048	signal trar	K10042	succinate	mmu0402	cAMP sigr	3:6008186	0	2	2	0	3	0	0	0.050356	0.069682	0	0.060904	0	0.040013	0.020301	0.716126	1	-
ENSMUS(	Mbnl1	musclebliı	GO:00325	developm	-	-	-	-	3:6047283	1238	1553	1057	1548	1869	1613	1.679179	1.682263	1.584521	1.619861	1.632452	1.721162	1.648654	1.657825	0.839231	1	-
ENSMUS(	P2ry1	purinergic	GO:00325	developm	K04270	purinergic	mmu0408	Neuroacti	3:6100279	58	58	57	74	98	65	0.448387	0.358095	0.48702	0.441355	0.487875	0.395319	0.431168	0.441517	1	1	-
ENSMUS(	Dhx36	DEAH (As	GO:00081	metabolic	K14442	ATP-depe	mmu0301	RNA degr	3:6246801	1574	1875	1388	1900	2284	1786	8.662831	8.241424	8.442897	8.067504	8.094797	7.73299	8.449051	7.965097	0.351157	1	-
ENSMUS(	Gfm1	G elongat	GO:00081	metabolic	-	-	-	-	3:6743009	1094	1341	897	1285	1695	1357	13.77269	13.48268	12.48075	12.48059	13.74124	13.43979	13.24537	13.22054	0.786448	1	-
ENSMUS(	Mfsd1	major faci	GO:00511	localizatio	-	-	-	-	3:6758274	401	509	371	509	633	474	7.944285	8.053309	8.123283	7.779627	8.075499	7.387546	8.040293	7.747557	0.584365	1	-
ENSMUS(	Il12a	interleukir	GO:00325	developm	K05406	interleukir	mmu0520	Pathways	3:6869064	8	3	5	6	18	8	0.116419	0.034869	0.080418	0.067356	0.168671	0.091577	0.077235	0.109201	0.442126	1	-
ENSMUS(	Schip1	schwannc	-	-	-	-	-	-	3:6806480	3910	5186	4106	5709	5934	4913	36.30984	38.46148	42.14183	40.90138	35.48541	35.89263	38.97105	37.42647	0.467203	1	-
ENSMUS(	Ift80	intraflagel	GO:00325	developm	-	-	-	-	3:6889249	476	745	480	804	781	849	1.728333	2.160343	1.926231	2.2522	1.826108	2.425154	1.938302	2.167821	0.444489	1	-
ENSMUS(	Ppm1l	protein pl	GO:00081	metabolic	-	-	-	-	3:6931686	3176	4047	2777	3748	4547	3763	17.32052	17.62623	16.73799	15.76922	15.96835	16.14454	17.22825	15.96071	0.239284	1	-
ENSMUS(	Nmd3	NMD3 rib	GO:00081	metabolic	K07562	nonsense-	mmu0301	RNA trans	3:6972198	468	655	480	615	754	634	2.54931	2.849468	2.889776	2.584533	2.644858	2.71692	2.762851	2.648771	0.532205	1	-
ENSMUS(	Bche	butyrylchc	GO:00325	developm	-	-	-	-	3:7363580	81	140	68	134	165	114	0.643888	0.888792	0.597419	0.821791	0.844626	0.712922	0.710033	0.793113	0.605661	1	-
ENSMUS(	Ccna1	cyclin A1 [	GO:00325	developm	K06627	cyclin A[-]	mmu0516	Human pa	3:5504546	18	14	18	35	11	14	0.322293	0.200201	0.356216	0.483486	0.12683	0.197206	0.292903	0.269174	0.798372	1	-
ENSMUS(	Smad9	SMAD far	GO:00325	developm	K16791	mothers a	mmu0455	Signaling	3:5475558	272	372	274	355	414	359	3.093853	3.379247	3.444511	3.115226	3.032393	3.212447	3.30587	3.120022	0.480461	1	-
ENSMUS(	Nbea	neurobea	GO:00452	synapse|lc	-	-	-	-	3:5562519	6365	8036	7390	7647	10752	10283	10.38048	10.46658	13.32019	9.62145	11.2918	13.19319	11.38908	11.36881	0.803155	1	-
ENSMUS(	Tm4sf1	transmem	GO:00160	membran	-	-	-	-	3:5728561	85	145	112	115	121	145	0.86456	1.177847	1.259036	0.902405	0.792532	1.160258	1.100481	0.951732	0.311276	1	-
ENSMUS(	Tm4sf4	transmem	GO:00325	developm	-	-	-	-	3:5742531	0	0	0	0	1	2	0	0	0	0	0.026277	0.06423	0	0.030169	0.278398	1	-
ENSMUS(	Wwtr1	WW dom	GO:00325	developm	K16820	WW dom	mmu0439	Hippo sig	3:5745564	374	536	321	502	546	473	2.777059	3.178512	2.634296	2.875723	2.610719	2.763027	2.863289	2.749823	0.571798	1	-
ENSMUS(	Ppid	peptidylpı	GO:00081	metabolic	K05864	peptidyl-p	mmu0421	Cellular se	3:7959134	1567	1931	1208	2021	2343	1692	15.68492	15.43625	13.36372	15.60666	15.10221	13.3237	14.82829	14.67752	0.723975	1	-
ENSMUS(	Pfn2	profilin 2 [	GO:00081	metabolic	K05759	profilin[-]	mmu0481	Regulatior	3:5784189	7361	9603	6756	9409	12252	9481	80.98176	84.37293	82.14607	79.85906	86.79846	82.05696	82.50025	82.90482	0.820895	1	-
ENSMUS(	Tsc22d2	TSC22 do	GO:00081	metabolic	-	-	-	-	3:5841471	903	1126	814	1096	1414	1111	3.059851	3.047173	3.048484	2.865192	3.085437	2.961677	3.051836	2.970769	0.597196	1	-
ENSMUS(	Serp1	stress-ass	GO:00325	developm	-	-	-	-	3:5851981	822	1081	736	1217	1594	1114	10.88479	11.43193	10.77142	12.4328	13.59224	11.60498	11.02938	12.54334	0.287077	1	-
ENSMUS(	Etfdh	electron tı	GO:00081	metabolic	-	-	-	-	3:7960378	627	845	591	896	1058	880	3.995352	4.30021	4.162186	4.404795	4.341376	4.411445	4.152583	4.385872	0.757277	1	-
ENSMUS(	Eif2a	eukaryotic	GO:00081	metabolic	-	-	-	-	3:5852582	739	925	671	920	1210	884	5.559629	5.557621	5.579187	5.33973	5.861938	5.231961	5.565479	5.477876	0.693894	1	-
ENSMUS(	Slc33a1	solute car	GO:00081	metabolic	K03372	MFS trans	mmu0110	Metabolic	3:6393350	426	504	379	559	446	472	4.287256	4.050848	4.215558	4.340215	2.890405	3.736992	4.184554	3.655871	0.20515	1	-
ENSMUS(	Gmps	guanine n	GO:00081	metabolic	K01951	GMP syntl	mmu0110	Metabolic	3:6397610	1758	2237	1530	2180	2743	2132	13.2437	13.45867	12.7388	12.67002	13.30671	12.63538	13.14706	12.8707	0.606346	1	-
ENSMUS(	Vmn2r1	vomerona	GO:00048	signal trar	-	-	-	-	3:6408164	3	10	5	7	3	8	0.066875	0.178023	0.123196	0.120386	0.043071	0.14029	0.122698	0.101249	0.687886	1	-
ENSMUS(	Kcnab1	potassium	GO:00325	developm	-	-	-	-	3:6510938	2066	2741	1962	2787	3403	2838	11.15744	11.82195	11.71063	11.61186	11.83453	12.05751	11.56334	11.83463	0.994897	1	-
ENSMUS(	Ssr3	signal seq	GO:00511	localizatio	K13251	translocor	mmu0414	Protein pr	3:6537965	2771	3589	2529	3869	4824	3527	34.21477	35.39129	34.51221	36.85582	38.3565	34.26048	34.70609	36.49094	0.761971	1	-
ENSMUS(	Ccnl1	cyclin L1 [	GO:00081	metabolic	-	-	-	-	3:6594615	460	526	481	667	818	676	2.023248	1.847662	2.338206	2.263329	2.316858	2.339099	2.069706	2.306429	0.445221	1	-
ENSMUS(	Veph1	ventricula	GO:00160	membran	-	-	-	-	3:6605355	3	4	0	0	1	5	0.02258	0.024042	0	0	0.004846	0.029607	0.015541	0.011484	0.663129	1	-
ENSMUS(	Ptx3	pentraxin	GO:00081	metabolic	-	-	-	-	3:6621991	9	21	7	9	12	12	0.178181	0.332046	0.15318	0.137471	0.152997	0.18691	0.221136	0.159126	0.266136	1	-
ENSMUS(	Serpini1	serine (or	GO:00325	developm	-	-	-	-	3:7555754	4024	4818	3224	4897	5573	4726	30.43074	29.09828	26.94613	28.57032	27.13928	28.11637	28.82505	27.94199	0.515121	1	-
ENSMUS(	Pdcd10	programn	GO:00325	developm	-	-	-	-	3:7551649	485	650	484	650	842	706	7.331806	7.847457	8.086517	7.580768	8.196651	8.39625	7.75526	8.057889	0.893373	1	-
ENSMUS(	Dclre1b	DNA cros	GO:00081	metabolic	-	-	-	-	3:1038006	55	131	135	92	254	155	0.180937	0.344177	0.490847	0.233499	0.538089	0.401152	0.338654	0.390913	0.611561	1	-
ENSMUS(	Syt6	synaptota	GO:00452	synapse|lc	-	-	-	-	3:1035752	89	65	101	45	202	122	0.496591	0.289644	0.622837	0.193709	0.725795	0.535521	0.469691	0.485008	0.981622	1	-
ENSMUS(	Nras	neuroblas	GO:00508	response	K07828	GTPase N	mmu0520	Pathways	3:1030582	709	353	246	713	314	503	4.979318	1.979907	1.909436	3.863165	1.420063	2.779089	2.95622	2.687439	0.627437	1	-
ENSMUS(	Sike1	suppressc	GO:00056	cell|intrace	K12656	suppresso	mmu0462	RIG-I-like	3:1029957	830	1049	650	1067	1338	1051	3.707802	3.742481	3.209213	3.677333	3.84901	3.693616	3.553166	3.739986	0.789916	1	-
ENSMUS(	Tspan2	tetraspani	GO:00325	developm	-	-	-	-	3:1027345	754	3308	326	3071	511	386	8.62454	30.21872	4.121253	27.10035	3.763921	3.473467	14.32151	11.44591	0.616605	1	-
ENSMUS(	Ngf	nerve gro	GO:00325	developm	K02582	nerve gro	mmu0415	PI3K-Akt s	3:1024699	91	88	64	88	107	104	1.708577	1.319541	1.328073	1.274692	1.293698	1.536166	1.452064	1.368185	0.605772	1	-
ENSMUS(	Vangl1	vang-like	GO:00325	developm	K04510	vang-like[	mmu0431	Wnt signa	3:1021535	82	122	88	101	138	130	0.45624	0.542108	0.541138	0.43354	0.494438	0.569028	0.513162	0.499002	0.744352	1	-
ENSMUS(	Casq2	calseques	GO:00325	developm	-	-	-	-	3:1020864	31	36	21	39	38	39	0.467583	0.433667	0.350088	0.453838	0.369097	0.462779	0.417113	0.428572	1	1	-
ENSMUS(	Ptgfrn	prostaglaı	GO:00099	cellular pr	-	-	-	-	3:1010402	328	411	256	459	622	416	1.826121	1.827441	1.575226	1.971509	2.229977	1.822047	1.742929	2.007844	0.324212	1	-
ENSMUS(	Gdap2	gangliosic	GO:00508	response	-	-	-	-	3:1001623	645	908	679	894	1127	880	5.784736	6.503628	6.730393	6.18574	6.508823	6.208941	6.339586	6.301168	0.772963	1	-
ENSMUS(	Spag17	sperm ass	GO:00990	supramole	-	-	-	-	3:9988540	17	21	15	31	19	22	0.109714	0.108237	0.10699	0.154348	0.078961	0.111698	0.108314	0.115002	0.948713	1	-
ENSMUS(	Notch2	notch 2 [S	GO:00325	developm	K02599	Notch[-]	mmu0520	Pathways	3:9801352	715	1103	671	915	1133	1027	3.896165	4.800128	4.041114	3.846652	3.975722	4.402636	4.245802	4.075003	0.540013	1	-
ENSMUS(	Sec22b	SEC22 hoı	GO:00081	metabolic	K08517	vesicle tra	mmu0414	Phagoson	3:9790119	772	1030	768	1057	1245	967	7.709662	8.214875	8.476684	8.143729	8.006488	7.597232	8.13374	7.915816	0.610838	1	-
ENSMUS(	Prpf38b	PRP38 pre	GO:00081	metabolic	K12850	pre-mRN	mmu0304	Spliceosor	3:1089028	1001	851	589	1150	1568	891	4.117432	2.795553	2.677654	3.649392	4.153299	2.883243	3.19688	3.561978	0.54527	1	-
ENSMUS(	Stxbp3	syntaxin b	GO:00325	developm	-	-	-	-	3:1087931	264	511	307	392	573	482	2.394796	3.701952	3.077859	2.743344	3.347134	3.439708	3.058202	3.176729	0.915473	1	-
ENSMUS(	Gpsm2	G-protein	GO:00325	developm	-	-	-	-	3:1086786	316	341	288	383	588	417	4.099943	3.533387	4.129809	3.83372	4.912735	4.256362	3.921046	4.334272	0.534994	1	-
ENSMUS(	Clcc1	chloride c	GO:00511	localizatio	-	-	-	-	3:1086539	373	442	338	508	555	452	3.556877	3.366107	3.562241	3.737263	3.408061	3.390854	3.495075	3.51206	0.868689	1	-
ENSMUS(	Sypl2	synaptopl	GO:00325	developm	-	-	-	-	3:1082114	57	82	54	84	84	76	0.716256	0.822921	0.749953	0.814344	0.679723	0.751311	0.763044	0.748459	0.79189	1	-
ENSMUS(	Gstm4	glutathior	GO:00081	metabolic	K00799	glutathion	mmu0520	Pathways	3:1080404	139	167	113	173	180	144	2.942812	2.823652	2.644065	2.825697	2.454004	2.3984	2.80351	2.559367	0.407765	1	-
ENSMUS(	Ahcyl1	S-adenos	GO:00081	metabolic	K01251	adenosylh	mmu0110	Metabolic	3:1076631	5733	7506	4944	7270	9081	7630	30.72197	32.1234	29.28144	30.05605	31.33686	32.16641	30.70894	31.18644	0.918801	1	-
ENSMUS(	Kcnc4	potassium	GO:00452	synapse|lc	-	-	-	-	3:1074383	1086	1382	950	1546	1694	1514	18.08266	18.3775	17.48248	19.85969	18.16354	19.83212	17.98088	19.28511	0.626574	1	-
ENSMUS(	Slc16a4	solute car	GO:00511	localizatio	-	-	-	-	3:1072912	39	85	41	47	60	78	0.515315	0.896957	0.598736	0.479114	0.510524	0.8108	0.670336	0.600146	0.52945	1	-
ENSMUS(	Chil6	chitinase-	GO:00081	metabolic	-	-	-	-	3:1063873	0	0	0	0	0	3	0	0	0	0	0	0.086381	0	0.028794	0.292253	1	-
ENSMUS(	Ddx20	DEAD (As	GO:00325	developm	K13131	ATP-depe	mmu0301	RNA trans	3:1056782	210	246	191	314	386	316	2.533029	2.36975	2.546251	2.922002	2.998214	2.998609	2.48301	2.972942	0.192526	1	-
ENSMUS(	Npr1	natriuretic	GO:00048	signal trar	K12323	atrial natri	mmu0402	cAMP sigr	3:9045059	69	125	63	97	124	128	0.677118	0.979645	0.683286	0.734372	0.78359	0.988175	0.780016	0.835379	0.822344	1	-
ENSMUS(	Slc27a3	solute car	GO:00081	metabolic	K08772	solute car	mmu0493	Insulin res	3:9038523	94	141	97	136	177	160	1.018357	1.21994	1.161419	1.136692	1.234808	1.363652	1.133239	1.245051	0.643844	1	-
ENSMUS(	Ints3	integrator	GO:00081	metabolic	-	-	-	-	3:9039138	1293	1632	1151	1750	1982	1797	8.728373	8.798341	8.587306	9.113877	8.615749	9.543193	8.704673	9.09094	0.835342	1	-
ENSMUS(	Rab13	RAB13, m	GO:00325	developm	K06109	Ras-relate	mmu0453	Tight junc	3:9021369	75	93	69	101	140	97	1.593377	1.577923	1.620132	1.655415	1.915317	1.621208	1.597144	1.730647	0.71968	1	-
ENSMUS(	Crtc2	CREB regu	GO:00081	metabolic	K16333	CREB-reg	mmu0415	PI3K-Akt s	3:9025416	415	513	371	549	633	529	3.692134	3.644956	3.647961	3.768186	3.626503	3.702507	3.661684	3.699065	0.901669	1	-
ENSMUS(	Jtb	jumping t	GO:00081	metabolic	-	-	-	-	3:9023159	168	215	157	245	305	223	3.415835	3.491173	3.52804	3.843127	3.993393	3.567012	3.478349	3.801177	0.617529	1	-
ENSMUS(	Nup210l	nucleopoı	GO:00325	developm	K14314	nuclear pc	mmu0301	RNA trans	3:9010413	6	13	8	7	11	9	0.055347	0.095772	0.081557	0.049817	0.065341	0.065312	0.077559	0.060157	0.491109	1	-
ENSMUS(	Tpm3	tropomyo	GO:00051	structural	K09290	tropomyo	mmu0520	Pathways	3:9007264	2185	2715	2002	2708	3467	2592	11.32914	11.24246	11.47246	10.8324	11.57588	10.57284	11.34802	10.9937	0.525932	1	-
ENSMUS(	4933434E	RIKEN cDI	GO:00160	membran	-	-	-	-	3:9005163	527	564	420	617	671	597	2.782807	2.378472	2.451147	2.513553	2.281657	2.480037	2.537476	2.425082	0.522583	1	-
ENSMUS(	Hax1	HCLS1 ass	GO:00325	developm	-	-	-	-	3:8999544	326	305	278	411	456	369	3.2429	2.42305	3.056383	3.154193	2.921028	2.88771	2.907444	2.987644	0.986771	1	-
ENSMUS(	Il6ra	interleukir	GO:00325	developm	K05055	interleukir	mmu0520	Pathways	3:8986405	153	178	129	166	242	204	0.852597	0.792168	0.794488	0.71366	0.868404	0.894318	0.813084	0.825461	0.955636	1	-
ENSMUS(	Adar	adenosine	GO:00325	developm	K12968	double-st	mmu0516	Influenza	3:8971502	2604	3233	2188	2627	4084	3502	16.04716	15.91142	14.90224	12.48958	16.20682	16.97792	15.62027	15.22477	0.620378	1	-
ENSMUS(	Pmvk	phosphor	GO:00081	metabolic	K13273	phosphon	mmu0110	Metabolic	3:8945454	571	688	456	833	938	669	11.12805	10.70822	9.821878	12.52444	11.77174	10.25699	10.55272	11.51772	0.558465	1	-
ENSMUS(	Efna1	ephrin A1	GO:00325	developm	K05462	ephrin-A[	mmu0415	PI3K-Akt s	3:8927173	89	110	72	135	106	100	1.44719	1.428468	1.293934	1.693552	1.109922	1.279223	1.389864	1.360899	0.79205	1	-
ENSMUS(	Fam198b	family witl	GO:00160	membran	-	-	-	-	3:7988453	89	116	104	113	153	131	0.314534	0.327399	0.406214	0.308095	0.348193	0.364216	0.349382	0.340168	0.760884	1	-
ENSMUS(	Tmem144	transmem	GO:00511	localizatio	-	-	-	-	3:7981256	204	318	200	325	345	352	2.571398	3.201197	2.786218	3.160479	2.800356	3.490546	2.852937	3.15046	0.563409	1	-
ENSMUS(	Sass6	SAS-6 cer	GO:00099	cellular pr	-	-	-	-	3:1165949	72	101	85	132	153	120	0.508878	0.570096	0.663964	0.719753	0.696346	0.667227	0.580979	0.694442	0.305672	1	-
ENSMUS(	Lrrc39	leucine ric	GO:00990	supramole	-	-	-	-	3:1165629	2	9	2	2	13	12	0.022146	0.079609	0.024483	0.017091	0.092718	0.104559	0.042079	0.071456	0.366411	1	-
ENSMUS(	Vcam1	vascular c	GO:00325	developm	K06527	vascular c	mmu0516	HTLV-I inf	3:1161099	519	843	526	761	907	778	3.282831	4.258477	3.677167	3.713608	3.694391	3.871433	3.739491	3.759811	0.86697	1	-
ENSMUS(	Extl2	exostoses	GO:00081	metabolic	K02369	alpha-1,4	mmu0110	Metabolic	3:1160074	976	1382	1144	1694	1976	1585	6.742021	7.624198	8.733994	9.027844	8.789857	8.613517	7.700071	8.810406	0.261741	1	-
ENSMUS(	Olfm3	olfactome	GO:00325	developm	-	-	-	-	3:1149040	331	459	318	485	573	413	2.496324	2.764597	2.650612	2.82192	2.782801	2.450384	2.637178	2.685035	0.968004	1	-
ENSMUS(	Col11a1	collagen,	GO:00325	developm	K19721	collagen,	mmu0497	Protein di	3:1140305	373	413	292	442	486	494	1.592969	1.40862	1.378249	1.4563	1.336564	1.659726	1.459946	1.484197	0.94608	1	-
ENSMUS(	Neurog2	neurogen	GO:00325	developm	-	-	-	-	3:1276331	22	26	18	21	29	27	0.603173	0.5693	0.545441	0.444191	0.512005	0.582374	0.572638	0.512856	0.566662	1	-
ENSMUS(	Larp7	La ribonu	GO:00081	metabolic	-	-	-	-	3:1275367	313	378	267	356	496	394	5.041193	4.86214	4.752781	4.423542	5.144299	4.99225	4.885371	4.853364	0.798901	1	-
ENSMUS(	Ndst3	N-deacet	GO:00081	metabolic	K02578	heparan s	mmu0110	Metabolic	3:1235261	793	951	670	1003	1169	939	2.568106	2.45961	2.398069	2.505942	2.437862	2.392303	2.475262	2.445369	0.719205	1	-
ENSMUS(	Prss12	protease,	GO:00081	metabolic	-	-	-	-	3:1234469	389	459	303	515	627	442	9.128212	8.601913	7.858232	9.32338	9.474538	8.159607	8.529452	8.985842	0.811879	1	-
ENSMUS(	Rnpc3	RNA-binc	GO:00325	developm	-	-	-	-	3:1136050	274	386	247	434	485	404	1.520347	1.710513	1.514733	1.857858	1.732961	1.763537	1.581864	1.784786	0.403944	1	-
ENSMUS(	Cyp2u1	cytochron	GO:00081	metabolic	K07422	cytochron	mmu0110	Metabolic	3:1312884	179	209	157	226	229	198	1.559785	1.454463	1.512017	1.51932	1.284993	1.357333	1.508755	1.387215	0.411102	1	-
ENSMUS(	Lef1	lymphoid	GO:00325	developm	K04492	lymphoid	mmu0520	Pathways	3:1311104	141	233	137	232	222	165	0.931123	1.22883	0.999903	1.181976	0.944055	0.857205	1.053285	0.994412	0.586306	1	-
ENSMUS(	Trim2	tripartite r	GO:00081	metabolic	-	-	-	-	3:8416043	5705	9586	7305	4680	12198	8852	8.055887	10.81038	11.40052	5.098399	11.09177	9.833541	10.08893	8.674571	0.2753	1	-
ENSMUS(	Mcub	mitochon	GO:00511	localizatio	-	-	-	-	3:1299149	61	90	61	78	84	104	1.912961	2.254052	2.114243	1.887122	1.69634	2.565799	2.093752	2.049753	0.801859	1	-
ENSMUS(	Tlr2	toll-like re	GO:00325	developm	K10159	toll-like re	mmu0415	PI3K-Akt s	3:8383627	27	41	24	35	66	34	0.546977	0.663334	0.537343	0.54701	0.860992	0.541857	0.582551	0.649953	0.732336	1	-
ENSMUS(	Sfrp2	secreted f	GO:00325	developm	K02176	secreted f	mmu0431	Wnt signa	3:8376632	13	16	12	12	30	17	0.281175	0.276382	0.286849	0.200231	0.417834	0.289268	0.281469	0.302444	0.880983	1	-
ENSMUS(	Casp6	caspase 6	GO:00325	developm	K04396	caspase 6	mmu0421	Apoptosis	3:1299014	53	70	43	53	118	67	0.531816	0.56095	0.476867	0.41029	0.762465	0.528894	0.523211	0.567216	0.772711	1	-
ENSMUS(	Plrg1	pleiotropi	GO:00081	metabolic	K12862	pleiotropi	mmu0304	Spliceosor	3:8305552	491	664	489	686	839	622	7.626432	8.236713	8.394525	8.220433	8.391835	7.600491	8.08589	8.070919	0.814256	1	-
ENSMUS(	Pla2g12a	phospholi	GO:00081	metabolic	-	-	mmu0110	Metabolic	3:1298786	232	321	241	281	389	316	2.740994	3.028804	3.146917	2.561283	2.959545	2.937105	2.972238	2.819311	0.534739	1	-
ENSMUS(	Fga	fibrinoger	GO:00081	metabolic	K03903	fibrinoger	mmu0461	Platelet ac	3:8302607	0	0	0	1	0	2	0	0	0	0.011188	0	0.022818	0	0.011335	0.27747	1	-
ENSMUS(	Gucy1b3	guanylate	GO:00081	metabolic	K12319	guanylate	mmu0023	Purine me	3:8203200	2508	3253	2264	3147	3701	3258	26.33426	27.27869	26.27343	25.49299	25.02461	26.9126	26.62879	25.81007	0.520596	1	-
ENSMUS(	Snx7	sorting ne	GO:00511	localizatio	-	-	-	-	3:1177816	236	291	212	331	449	323	1.942009	1.912398	1.92807	2.101346	2.379253	2.090993	1.927492	2.190531	0.388553	1	-
ENSMUS(	Gar1	GAR1 ribc	GO:00081	metabolic	K11128	H/ACA rib	mmu0300	Ribosome	3:1298249	264	280	200	278	293	270	4.699524	3.980647	3.934822	3.817893	3.358704	3.781143	4.204998	3.65258	0.187888	1	-
ENSMUS(	Tdo2	tryptopha	GO:00081	metabolic	K00453	tryptopha	mmu0110	Metabolic	3:8195709	99	116	78	128	194	122	1.210883	1.133107	1.054415	1.207842	1.528007	1.17392	1.132802	1.303256	0.448547	1	-
ENSMUS(	Ppa2	pyrophos	GO:00081	metabolic	K01507	inorganic	mmu0019	Oxidative	3:1333101	554	738	459	679	814	801	3.094236	3.291893	2.83337	2.925794	2.927675	3.51955	3.073167	3.12434	0.942743	1	-
ENSMUS(	Ctso	cathepsin	GO:00081	metabolic	K01374	cathepsin	mmu0421	Apoptosis	3:8193260	399	509	376	469	658	512	4.209087	4.288234	4.383793	3.816959	4.469876	4.249085	4.293705	4.17864	0.653693	1	-
ENSMUS(	Ints12	integrator	GO:00081	metabolic	-	-	-	-	3:1330918	190	223	178	220	248	225	1.302315	1.220712	1.348435	1.163364	1.094634	1.213266	1.290487	1.157088	0.293479	1	-
ENSMUS(	Gstcd	glutathior	GO:00055	extracellul	-	-	-	-	3:1329817	65	117	99	123	133	133	0.490821	0.70557	0.82621	0.716546	0.646719	0.790081	0.6742	0.717782	0.82578	1	-
ENSMUS(	Pdgfc	platelet-d	GO:00325	developm	K05450	platelet de	mmu0415	PI3K-Akt s	3:8103641	104	127	66	133	138	129	0.858811	0.837557	0.602362	0.847317	0.733833	0.83804	0.766243	0.806397	0.895402	1	-
ENSMUS(	Alpk1	alpha-kin	GO:00081	metabolic	-	-	-	-	3:1276703	153	189	109	220	205	211	1.035343	1.021408	0.815206	1.148537	0.893306	1.123274	0.957319	1.055039	0.634401	1	-
ENSMUS(	Aimp1	aminoacy	GO:00325	developm	-	-	-	-	3:1326604	505	602	502	569	831	610	4.645824	4.42297	5.104134	4.038445	4.922968	4.41481	4.724309	4.458741	0.466295	1	-
ENSMUS(	Tbck	TBC1 dom	GO:00081	metabolic	-	-	-	-	3:1326841	989	1166	815	1241	1537	1214	4.585851	4.317852	4.17665	4.439414	4.589358	4.428465	4.360118	4.485746	0.966561	1	-
ENSMUS(	Papss1	3'-phospl	GO:00081	metabolic	K13811	3'-phospl	mmu0110	Metabolic	3:1315647	1226	1573	1110	1743	2115	1958	17.40057	17.82984	17.41177	19.08537	19.33027	21.86233	17.54739	20.09266	0.227648	1	-
ENSMUS(	Kcnq5	potassium	GO:00511	localizatio	K04930	potassium	mmu0472	Cholinerg	1:2139840	1763	2388	1894	1459	3199	2654	7.536613	8.152747	8.948511	4.81182	8.806287	8.925567	8.212624	7.514558	0.417361	1	-
ENSMUS(	Fubp1	far upstre	GO:00325	developm	-	-	-	-	3:1522104	1928	2481	1631	2499	3276	2211	3.846054	3.952582	3.595912	3.845963	4.208302	3.469829	3.798183	3.841365	0.89817	1	-
ENSMUS(	Ptgfr	prostaglaı	GO:00048	signal trar	K04262	prostaglaı	mmu0408	Neuroacti	3:1517965	8	12	6	11	22	16	0.057892	0.069346	0.047988	0.061411	0.102515	0.091083	0.058409	0.085003	0.302567	1	-
ENSMUS(	Efna3	ephrin A3	GO:00325	developm	K05462	ephrin-A[	mmu0415	PI3K-Akt s	3:8931389	983	1172	782	1264	1443	1133	21.08189	20.0738	18.53572	20.91382	19.92861	19.11599	19.89714	19.98614	0.841866	1	-
ENSMUS(	Efna4	ephrin A4	GO:00325	developm	K05462	ephrin-A[	mmu0415	PI3K-Akt s	3:8933339	6	3	8	6	11	5	0.229106	0.091501	0.337604	0.176751	0.270476	0.150201	0.219404	0.199143	1	1	-
ENSMUS(	Adam15	a disinteg	GO:00325	developm	-	-	-	-	3:8933854	1233	1470	1179	1664	1721	1582	6.927825	6.596244	7.321402	7.213025	6.226861	6.992799	6.948491	6.810895	0.640342	1	-
ENSMUS(	Cks1b	CDC28 pr	GO:00081	metabolic	K02219	cyclin-der	mmu0520	Pathways	3:8941547	17	18	12	24	22	28	0.604192	0.510895	0.471348	0.658061	0.503506	0.782882	0.528812	0.64815	0.533107	1	-
ENSMUS(	Thbs3	thrombos	GO:00325	developm	K04659	thrombos	mmu0415	PI3K-Akt s	3:8921518	332	351	301	442	537	378	2.278957	1.924201	2.283554	2.340722	2.373701	2.041266	2.162237	2.251897	0.89127	1	-
ENSMUS(	Gba	glucosida	GO:00325	developm	K01201	glucosylce	mmu0110	Metabolic	3:8920292	422	627	425	672	769	646	6.310687	7.488198	7.024243	7.752889	7.405337	7.599888	6.941042	7.586038	0.54405	1	-
ENSMUS(	Scamp3	secretory	GO:00511	localizatio	-	-	-	-	3:8917747	406	529	381	549	569	613	5.09236	5.299005	5.281586	5.312461	4.595785	6.048732	5.224317	5.318993	0.955707	1	-
ENSMUS(	Hcn3	hyperpola	GO:00452	synapse|lc	-	-	-	-	3:8914607	250	253	210	315	305	272	2.757322	2.228508	2.559846	2.680326	2.166214	2.360081	2.515225	2.402207	0.591265	1	-
ENSMUS(	Rit1	Ras-like w	GO:00508	response	-	-	-	-	3:8871683	302	473	282	481	578	380	2.298051	2.874478	2.371639	2.823761	2.832272	2.274819	2.514723	2.643617	0.832063	1	-
ENSMUS(	Arhgef2	rho/rac gu	GO:00325	developm	K12791	Rho guan	mmu0453	Tight junc	3:8860596	2644	2742	2021	3225	3198	3329	8.239977	6.824611	6.961097	7.753978	6.417971	8.161862	7.341895	7.444604	0.90809	1	-
ENSMUS(	2810403A	RIKEN cDI	GO:00081	metabolic	-	-	-	-	3:8868580	732	906	610	986	1226	1105	3.761216	3.717846	3.46413	3.908628	4.056603	4.466738	3.64773	4.143989	0.313237	1	-
ENSMUS(	Lamtor2	late endo	GO:00081	metabolic	K20398	ragulator	mmu0415	mTOR sig	3:8854981	370	563	421	638	740	619	9.835225	11.95189	12.36832	13.08379	12.66685	12.94447	11.38515	12.89837	0.36581	1	-
ENSMUS(	Lmna	lamin A [S	GO:00325	developm	K12641	lamin A/C	mmu0421	Apoptosis	3:8848014	581	796	540	740	1068	737	3.626166	3.967619	3.724874	3.563145	4.292369	3.618678	3.772886	3.824731	0.932239	1	-
ENSMUS(	Sema4a	sema don	GO:00325	developm	K06521	semaphor	mmu0436	Axon guic	3:8843595	357	461	372	571	676	557	2.335057	2.408106	2.689175	2.881348	2.847283	2.866127	2.477446	2.864919	0.272508	1	-
ENSMUS(	Pmf1	polyamine	GO:00081	metabolic	-	-	-	-	3:8839414	46	64	54	81	81	95	0.495184	0.550213	0.642458	0.672701	0.561496	0.804533	0.562619	0.679577	0.366544	1	-
ENSMUS(	Naxe	NAD(P)H)	GO:00081	metabolic	-	-	-	-	3:8805652	554	693	565	715	884	646	37.25351	37.21658	41.99069	37.09315	38.27936	34.17439	38.82026	36.51563	0.425227	1	-
ENSMUS(	Sh2d2a	SH2 dom	GO:00508	response	K08273	SH2 dom	mmu0437	VEGF sign	3:8784675	0	0	2	2	6	2	0	0	0.017832	0.012449	0.031167	0.012695	0.005944	0.01877	0.201802	1	-
ENSMUS(	Ntrk1	neurotrop	GO:00325	developm	K03176	neurotrop	mmu0520	Pathways	3:8777824	2	2	0	0	4	0	0.047177	0.03769	0	0	0.060773	0	0.028289	0.020258	1	1	-
ENSMUS(	Pear1	platelet er	GO:00511	localizatio	-	-	-	-	3:8774789	83	112	55	81	108	117	0.361908	0.390019	0.265051	0.272482	0.303251	0.401345	0.338993	0.325693	0.717268	1	-
ENSMUS(	Cd1d1	CD1d1 an	GO:00160	membran	K06448	T-cell surf	mmu0453	Tight junc	3:8699583	7	11	6	15	4	8	0.105405	0.132275	0.099857	0.174251	0.038787	0.094761	0.112512	0.1026	0.83714	1	-
ENSMUS(	Dclk2	doublecoı	GO:00325	developm	-	-	-	-	3:8678615	1350	1584	1181	1873	2052	1818	4.663296	4.369787	4.508749	4.991458	4.564481	4.940422	4.513944	4.83212	0.632953	1	-
ENSMUS(	Lrba	LPS-respc	GO:00160	membran	-	-	-	-	3:8622468	1042	1391	906	1330	1590	1369	2.932184	3.126054	2.817727	2.887391	2.881214	3.030666	2.958655	2.933091	0.726876	1	-
ENSMUS(	Rps3a1	ribosomal	GO:00325	developm	K02984	small subu	mmu0301	Ribosome	3:8613794	2287	3470	2200	3306	4327	3666	142.9272	173.1905	151.9562	159.3977	174.1367	180.2404	156.0246	171.2583	0.446918	1	-
ENSMUS(	Sh3d19	SH3 dom	GO:00325	developm	-	-	-	-	3:8597110	538	905	480	738	992	809	1.963377	2.637642	1.936017	2.077821	2.331244	2.322636	2.179012	2.2439	0.96174	1	-
ENSMUS(	Gatb	glutamyl-	GO:00081	metabolic	K02434	aspartyl-t	mmu0110	Metabolic	3:8557411	292	382	306	376	494	354	3.154454	3.295719	3.6535	3.133713	3.436549	3.008536	3.367891	3.192933	0.521107	1	-
ENSMUS(	Fbxw7	F-box anc	GO:00325	developm	K10260	F-box anc	mmu0412	Ubiquitin	3:8481526	2318	2971	2146	2820	3382	3116	13.64697	13.96919	13.96366	12.80861	12.82186	14.43215	13.85994	13.35421	0.482015	1	-
ENSMUS(	Fmo5	flavin con	GO:00081	metabolic	K00485	dimethyla	mmu0098	Drug met	3:9762880	61	108	71	111	130	136	0.442186	0.625241	0.568827	0.620769	0.606838	0.775582	0.545418	0.66773	0.276501	1	-
ENSMUS(	Chd1l	chromodc	GO:00081	metabolic	-	-	-	-	3:9756074	200	268	170	267	328	310	2.565463	2.745468	2.410077	2.642276	2.709347	3.128308	2.573669	2.826644	0.584018	1	-
ENSMUS(	Acp6	acid phos	GO:00325	developm	-	-	-	-	3:9715877	209	273	181	266	361	287	1.17301	1.223667	1.122743	1.151772	1.304716	1.267205	1.17314	1.241231	0.597275	1	-

ENSMUSC	Gpr89	G protein-	GO:00325	developm	-	-	-	-	3:9686828	500	646	494	684	804	635	3.357795	3.464672	3.666554	3.543807	3.476921	3.354813	3.49634	3.458514	0.743215	1	-
ENSMUSC	Rnf115	ring finge	GO:00081	metabolic	-	-	-	-	3:9672766	1080	1389	1040	1379	1897	1353	14.48019	14.873	15.41098	14.26412	16.37843	14.27115	14.92139	14.97123	0.837978	1	-
ENSMUSC	Nudt17	nudix (nuc	GO:00038	catalytic a	-	-	-	-	3:9670606	70	118	99	126	184	163	1.044233	1.405818	1.632234	1.450116	1.767561	1.912931	1.360761	1.710203	0.212153	1	-
ENSMUSC	Pias3	protein in	GO:00081	metabolic	K16064	E3 SUMO	mmu0463	Jak-STAT	3:9669638	265	324	202	356	428	428	1.720768	1.680226	1.449688	1.783436	1.789679	2.186411	1.616894	1.919842	0.237715	1	-
ENSMUSC	Pex11b	peroxison	GO:00508	response	K13352	peroxin-1	mmu0414	Peroxisom	3:9663537	220	266	203	286	357	288	2.177464	2.102599	2.220603	2.183859	2.275365	2.242498	2.166889	2.233907	0.966217	1	-
ENSMUSC	Polr3gl	polymeras	GO:00081	metabolic	K03024	DNA-dire	mmu011C	Metabolic	3:9657787	317	442	249	410	451	360	7.287428	8.114902	6.326467	7.271583	6.676461	6.510716	7.242933	6.819587	0.484473	1	-
ENSMUSC	Rprd2	regulation	GO:00329	macromo	-	-	-	-	3:9576034	1226	990	713	1030	1893	1575	5.673584	3.658877	3.646728	3.677344	5.641207	5.73401	4.326396	5.01752	0.403163	1	-
ENSMUSC	Tars2	threonyl-t	GO:00081	metabolic	K01868	threonyl-t	mmu0097	Aminoacy	3:9573997	388	483	434	509	585	514	2.280128	2.26684	2.818803	2.307681	2.213798	2.376303	2.455257	2.299261	0.44251	1	-
ENSMUSC	Ecm1	extracellul	GO:00325	developm	-	-	-	-	3:9573414	94	139	91	125	214	146	1.195223	1.411504	1.278822	1.2262	1.752223	1.460448	1.295183	1.479624	0.481897	1	-
ENSMUSC	Ctsk	cathepsin	GO:00081	metabolic	K01371	cathepsin	mmu0421	Apoptosis	3:9549925	33	57	31	49	40	39	1.332625	1.838271	1.383571	1.526563	1.040163	1.238975	1.518155	1.268567	0.32701	1	-
ENSMUSC	Mettl14	methyltra	GO:00325	developm	-	-	-	-	3:1233682	318	413	501	419	483	625	1.427473	1.480594	2.485565	1.451058	1.396183	2.207147	1.797877	1.684796	0.593719	1	-
ENSMUSC	Bnipl	BCL2/ade	GO:00099	cellular pr	-	-	-	-	3:9524127	3	3	0	0	4	3	0.051539	0.041168	0	0	0.044255	0.040541	0.030902	0.028265	1	1	-
ENSMUSC	Gclm	glutamate	GO:00325	developm	K11205	glutamate	mmu011C	Metabolic	3:1222455	934	959	885	1265	1484	1279	6.888306	5.648459	7.213658	7.197579	7.047807	7.420734	6.583474	7.22204	0.511198	1	-
ENSMUSC	Pip5k1a	phosphati	GO:00081	metabolic	K00889	1-phosph	mmu011C	Metabolic	3:9505853	851	1503	822	1497	1194	1072	4.67186	6.589692	4.987454	6.340339	4.22104	4.62984	5.416336	5.06374	0.475756	1	-
ENSMUSC	Abcd3	ATP-bind	GO:00081	metabolic	K05677	ATP-bind	mmu0414	Peroxisom	3:1217587	1286	1591	1127	1462	2022	1611	8.612561	8.509565	8.341848	7.553863	8.720217	8.487855	8.487991	8.253978	0.579936	1	-
ENSMUSC	F3	coagulatic	GO:00325	developm	K03901	coagulatic	mmu0493	AGE-RAG	3:1217235	648	750	590	826	939	824	11.76149	10.87162	11.83549	11.56639	10.97508	11.76591	11.48953	11.43579	0.776183	1	-
ENSMUSC	Rwdd3	RWD dom	GO:00081	metabolic	-	-	-	-	3:1211553	108	105	151	126	308	153	0.752945	0.584619	1.16349	0.677703	1.38275	0.839151	0.833685	0.966535	0.558337	1	-
ENSMUSC	Ptbp2	polypyrim	GO:00325	developm	-	-	-	-	3:1197187	1578	2054	1565	2024	2559	1994	6.960174	7.235348	7.629118	6.887379	7.268388	6.919093	7.27488	7.024953	0.504923	1	-
ENSMUSC	Snx27	sorting ne	GO:00511	localizatio	-	-	-	-	3:9449754	2259	2832	1942	2882	3406	2682	11.95444	11.96883	11.35818	11.76623	11.60679	11.16561	11.76048	11.51288	0.60096	1	-
ENSMUSC	Celf3	CUGBP, El	GO:00081	metabolic	-	-	-	-	3:9447829	2466	3147	2303	3218	3568	3523	11.27691	11.49317	11.63959	11.35307	10.50695	12.6742	11.46989	11.51141	0.816664	1	-
ENSMUSC	Adh5	alcohol de	GO:00081	metabolic	K00121	S-(hydrox	mmu011C	Metabolic	3:1384430	498	654	495	729	867	684	4.167615	4.371013	4.578362	4.706697	4.672317	4.503245	4.37233	4.62742	0.752491	1	-
ENSMUSC	Riiad1	regulatory	-	-	-	-	-	-	3:9446498	20	17	15	19	26	29	1.321615	0.89713	1.095494	0.968602	1.106347	1.507561	1.104746	1.19417	0.896089	1	-
ENSMUSC	Mrpl9	mitochon	GO:00081	metabolic	K02939	large subu	mmu0301	Ribosome	3:9444331	507	646	422	691	774	591	9.880781	10.05452	9.089538	10.38941	9.713559	9.061109	9.674945	9.721361	0.86096	1	-
ENSMUSC	Oaz3	ornithine	GO:00081	metabolic	-	-	-	-	3:9443241	0	6	6	4	4	0	0	0.049255	0.068169	0.031717	0.026478	0	0.039141	0.019399	0.412544	1	-
ENSMUSC	Them4	thioestera	GO:00081	metabolic	K16339	thioestera	mmu0415	PI3K-Akt s	3:9431008	361	494	307	461	569	450	4.758636	5.200528	4.472596	4.688204	4.829939	4.66657	4.810587	4.728238	0.718231	1	-
ENSMUSC	Rap1gds1	RAP1, GTI	GO:00508	response	-	-	-	-	3:1389259	5299	6542	4737	6708	8269	6722	23.73959	23.40646	23.45468	23.18477	23.85542	23.69129	23.53358	23.57716	0.787026	1	-
ENSMUSC	Rorc	RAR-relat	GO:00325	developm	K08534	RAR-relat	mmu0465	Th17 cell	3:9437279	48	48	40	69	79	48	0.534326	0.426733	0.492119	0.592582	0.566305	0.420361	0.484393	0.526416	0.762324	1	-
ENSMUSC	Eif4e	eukaryotic	GO:00325	developm	K03259	translatior	mmu0415	PI3K-Akt s	3:1385261	1445	1844	1114	1614	2219	1511	12.64931	12.89157	10.77782	10.90016	12.50867	10.40578	12.10624	11.27154	0.33944	1	-
ENSMUSC	Dapp1	dual adap	GO:00160	membran	K12229	dual adap	mmu0466	B cell rece	3:1379310	42	121	95	142	64	102	0.322004	0.740874	0.804979	0.839908	0.315973	0.615209	0.622619	0.590363	0.775221	1	-
ENSMUSC	Ppp3ca	protein pl	GO:00325	developm	K04348	serine/thre	mmu0401	MAPK sign	3:1366701	37440	45571	34346	44214	60618	47755	199.3908	193.8223	202.1586	181.66	207.8862	200.0779	198.4572	196.5414	0.689917	1	-
ENSMUSC	Nfkb1	nuclear fa	GO:00325	developm	K02580	nuclear fa	mmu052C	Pathways	3:1355846	345	420	342	477	627	538	1.29815	1.262118	1.422258	1.384693	1.519243	1.592568	1.327508	1.498835	0.3923	1	-
ENSMUSC	Manba	mannosid	GO:00081	metabolic	K01192	beta-man	mmu0414	Lysosome	3:1354856	188	254	212	223	297	240	1.206784	1.302122	1.504025	1.104351	1.227674	1.211973	1.337644	1.181333	0.251509	1	-
ENSMUSC	Cisd2	CDGSH irc	GO:00325	developm	-	-	-	-	3:1354064	903	1121	808	1139	1342	1029	6.252603	6.199042	6.183456	6.084535	5.983846	5.60531	6.2117	5.89123	0.428623	1	-
ENSMUSC	Wls	wntless hc	GO:00325	developm	-	-	-	-	3:1598396	333	503	363	505	673	325	1.762054	2.125637	2.122893	2.061566	2.293216	1.352913	2.003528	1.902565	0.61325	1	-
ENSMUSC	Rpe65	retinal pig	GO:00325	developm	K11158	retinoid is	mmu0083	Retinol me	3:1595991	0	1	0	0	0	2	0	0.022351	0	0	0	0.044037	0.00745	0.014679	1	1	-
ENSMUSC	Depdc1a	DEP dom	GO:00081	metabolic	-	-	-	-	3:1594954	1	5	1	3	9	4	0.013333	0.05322	0.014732	0.030853	0.077248	0.04194	0.027095	0.050013	0.363953	1	-
ENSMUSC	Zranb2	zinc finge	GO:00081	metabolic	-	-	-	-	3:1575341	1264	1448	1056	1574	1824	1398	6.500249	5.946992	6.001965	6.244792	6.040354	5.65589	6.149735	5.980346	0.582513	1	-
ENSMUSC	Lrriq3	leucine-ri	-	-	-	-	-	-	3:1550934	12	8	10	19	20	20	0.13319	0.070917	0.122669	0.162696	0.142949	0.174639	0.108925	0.160094	0.240647	1	-
ENSMUSC	Adgrl2	adhesion	GO:00325	developm	-	-	-	-	3:1488155	1071	1313	826	1279	1356	1291	2.131323	2.086749	1.816714	1.963634	1.737697	2.021142	2.011595	1.907491	0.432037	1	-
ENSMUSC	Uox	urate oxic	GO:00081	metabolic	K00365	urate oxid	mmu011C	Metabolic	3:1465704	6	12	6	9	15	9	0.056377	0.09005	0.062315	0.065245	0.090762	0.066528	0.069581	0.074178	1	1	-
ENSMUSC	Rpf1	ribosome	GO:00081	metabolic	-	-	-	-	3:1465059	192	233	199	254	335	252	2.261851	2.19212	2.590974	2.308484	2.541336	2.335467	2.348315	2.395095	0.991164	1	-
ENSMUSC	Ctbs	chitobiase	GO:00081	metabolic	-	-	-	-	3:1464497	149	199	161	185	215	187	1.06755	1.138678	1.274895	1.022596	0.99196	1.054036	1.160374	1.022864	0.264247	1	-
ENSMUSC	Bcl10	B cell leuk	GO:00325	developm	K07368	B-cell CLL	mmu0515	Tuberculo	3:1459228	147	207	139	213	259	189	1.803408	2.028115	1.884684	2.015976	2.046118	1.824097	1.905403	1.962064	0.978311	1	-
ENSMUSC	Ddah1	dimethyla	GO:00325	developm	-	-	-	-	3:1457586	1877	2340	1760	2457	2852	2229	19.25505	19.17089	19.95446	19.44535	18.84016	17.98877	19.46013	18.75809	0.488065	1	-
ENSMUSC	Cyr61	cysteine ri	GO:00325	developm	-	-	-	-	3:1456469	55	114	55	73	111	83	1.663296	2.753333	1.838292	1.703176	2.161638	1.97468	2.084973	1.946498	0.623014	1	-
ENSMUSC	Col24a1	collagen,	GO:00325	developm	K19721	collagen,	mmu0497	Protein di	3:1452924	21	20	6	14	21	36	0.133013	0.101168	0.042004	0.06841	0.085651	0.179382	0.092062	0.111148	0.67646	1	-
ENSMUSC	Asph	aspartate-	GO:00325	developm	-	-	-	-	4:9448069	1978	2634	1963	2442	3170	2259	6.196027	6.58944	6.796011	5.901506	6.394416	5.566907	6.527159	5.954277	0.20236	1	-
ENSMUSC	Trp53inp1	transform	GO:00325	developm	K15310	tumor prc	mmu0516	HTLV-I inf	4:1115643	313	350	265	361	585	295	2.656911	2.372724	2.486142	2.364126	3.197743	1.970003	2.505259	2.510624	0.892221	1	-
ENSMUSC	Ccne2	cyclin E2 [	GO:00081	metabolic	K06626	cyclin E[-]	mmu052C	Pathways	4:1119135	71	84	48	72	112	78	0.556927	0.526216	0.416132	0.435714	0.565737	0.481335	0.499758	0.494262	0.853534	1	-
ENSMUSC	Fam92a	family witl	GO:00099	cellular pr	-	-	-	-	4:1215340	520	690	495	675	946	780	4.724036	5.006159	4.970054	4.730901	5.534207	5.574615	4.900083	5.279908	0.630368	1	-
ENSMUSC	Tmem55a	transmem	GO:00081	metabolic	K13084	phosphati	mmu0407	Phosphati	4:1486407	615	779	483	813	979	697	16.05418	16.24039	13.935	16.37322	16.457	14.31385	15.40985	15.71469	0.971942	1	-
ENSMUSC	Calb1	calbindin	GO:00325	developm	K14757	calbindin	mmu0496	Endocrine	4:1588126	1862	2506	1909	2486	3092	2375	20.96444	22.53361	23.75508	21.5941	22.41805	21.03673	22.41771	21.68296	0.521856	1	-
ENSMUSC	Decr1	2,4-dieno	GO:00081	metabolic	-	-	-	-	4:1591724	301	329	246	380	508	366	4.566083	3.985832	4.124381	4.447254	4.962445	4.36787	4.225432	4.592523	0.621237	1	-
ENSMUSC	Nbn	nibrin [So	GO:00325	developm	K10867	nijmegen	mmu0421	Cellular se	4:1595792	338	428	286	415	546	407	7.05561	7.135212	6.598283	6.683379	7.339469	6.683793	6.929701	6.902214	0.811542	1	-
ENSMUSC	Mmp16	matrix me	GO:00325	developm	K07996	matrix me	mmu052C	MicroRNA	4:1785289	525	683	527	656	759	718	2.742835	2.849752	3.042969	2.644079	2.553505	2.951043	2.878519	2.716209	0.43826	1	-
ENSMUSC	Cpne3	copine III	GO:00081	metabolic	-	-	-	-	4:1951925	336	455	366	473	636	426	2.750068	2.974142	3.310794	2.986731	3.352094	2.742994	3.011668	3.027273	0.887968	1	-
ENSMUSC	Rmdn1	regulator	GO:00990	supramole	-	-	-	-	4:1957516	81	114	81	105	162	136	2.37318	2.667456	2.62289	2.373374	3.056433	3.134707	2.554509	2.854838	0.57597	1	-
ENSMUSC	Tmem68	transmem	GO:00081	metabolic	-	-	-	-	4:3549041	470	634	562	618	978	716	5.868571	6.32222	7.755641	5.953236	7.863717	7.033292	6.648811	6.950081	0.861373	1	-
ENSMUSC	Tgs1	trimethylg	GO:00081	metabolic	K14292	trimethylg	mmu0301	RNA trans	4:3574875	685	799	671	880	1098	871	6.472428	6.029329	7.007225	6.414894	6.680878	6.474492	6.502994	6.523421	0.842265	1	-
ENSMUSC	Rps20	ribosomal	GO:00081	metabolic	K02969	small subu	mmu0301	Ribosome	4:3831334	637	674	480	740	897	856	8.22979	6.954332	6.853892	7.375845	7.462711	8.700297	7.346005	7.846284	0.711186	1	-
ENSMUSC	Ubxn2b	UBX dom	GO:00081	metabolic	-	-	-	-	4:6191098	1102	1421	989	1438	1739	1398	10.13039	10.4324	10.04818	10.19844	10.29435	10.11027	10.20366	10.20102	0.795042	1	-
ENSMUSC	Nsmaf	neutral sp	GO:00987	molecular	K18953	factor ass	mmu0407	Sphingolip	4:6396207	536	744	547	822	987	842	2.81524	3.120822	3.175299	3.330832	3.338275	3.479151	3.03712	3.382753	0.405247	1	-
ENSMUSC	Faxc	failed axo	GO:00160	membran	-	-	-	-	4:2193132	1168	1611	1171	1544	1713	1527	5.685713	6.263012	6.300076	5.798552	5.369747	5.847792	6.082934	5.67203	0.310576	1	-
ENSMUSC	Coq3	coenzyme	GO:00081	metabolic	K00591	polypreny	mmu011C	Metabolic	4:2187967	261	342	258	356	434	343	4.860061	5.085968	5.309691	5.114257	5.204113	5.024659	5.08524	5.114343	0.883578	1	-
ENSMUSC	Pnisr	PNN inter	GO:00056	cell|intrace	-	-	-	-	4:2184758	2537	2691	2112	2799	3418	3389	8.508894	7.207958	7.828768	7.242457	7.38209	8.942011	7.84854	7.855519	0.802475	1	-
ENSMUSC	Sdcbp	syndecan	GO:00325	developm	-	-	-	-	4:6365650	2074	2557	1865	2602	3445	2613	14.49239	14.26947	14.40314	14.02714	15.50155	14.36421	14.38833	14.63097	0.936891	1	-
ENSMUSC	Tstd3	thiosulfate	GO:00038	catalytic a	-	-	-	-	4:2175738	297	425	284	429	575	394	11.41955	13.05052	12.06864	12.72567	14.23689	11.9179	12.17957	12.96015	0.743311	1	-
ENSMUSC	Odf2l	outer den	GO:00056	cell|intrace	-	-	-	-	3:1451185	236	253	169	275	349	255	0.795984	0.68149	0.629981	0.715577	0.758007	0.676618	0.702485	0.716734	0.983415	1	-
ENSMUSC	Fhl5	four and a	GO:00081	metabolic	-	-	-	-	4:2519990	1	3	0	0	0	1	0.052158	0.124945	0	0	0	0.041028	0.059034	0.013676	0.412941	1	-
ENSMUSC	Ndufaf4	NADH de	GO:00099	cellular pr	-	-	-	-	4:2489808	661	847	113	844	207	914	6.153287	6.29702	1.162598	6.06148	1.240885	6.693646	4.537635	4.665337	1	1	-
ENSMUSC	Spaca1	sperm acr	GO:00325	developm	-	-	-	-	4:3402487	0	0	0	0	0	2	0	0	0	0	0	0.041293	0	0.013764	0.526255	1	-
ENSMUSC	Lmo4	LIM doma	GO:00325	developm	-	-	-	-	3:1441885	1450	1607	1317	1857	2049	2446	13.33749	11.80505	13.38871	13.17796	12.13676	17.70001	12.84375	14.33824	0.440024	1	-
ENSMUSC	Gtf2b	general tr	GO:00081	metabolic	K03124	transcripti	mmu052C	Viral carci	3:1427650	614	729	537	780	913	731	8.053643	7.636551	7.784757	7.893114	7.711685	7.543139	7.824984	7.715979	0.707548	1	-
ENSMUSC	Pdlim5	PDZ and L	GO:00325	developm	-	-	-	-	3:1422395	522	706	523	743	854	707	2.51637	2.718031	2.78646	2.763267	2.65104	2.681229	2.67362	2.698512	0.890856	1	-
ENSMUSC	Rngtt	RNA guar	GO:00081	metabolic	K13917	mRNA-ca	mmu0301	mRNA sur	4:3331031	590	784	471	752	914	703	4.313769	4.577903	3.806033	4.241827	4.303341	4.043626	4.232568	4.196265	0.758544	1	-
ENSMUSC	Ube2j1	ubiquitin-	GO:00325	developm	K10578	ubiquitin-	mmu0414	Protein pr	4:3303141	1831	2361	1690	2604	3011	2524	30.42934	31.33618	31.04114	33.38685	32.22327	32.99928	30.93555	32.8698	0.670522	1	-
ENSMUSC	Rragd	Ras-relate	GO:00508	response	K16186	Ras-relate	mmu0415	mTOR sig	4:3298303	1780	525	1787	549	2769	546	17.86675	4.208536	19.82426	4.251366	17.89794	4.311509	13.96652	8.820272	0.191594	1	-
ENSMUSC	Gabrr1	gamma-a	GO:00048	signal trar	K05190	gamma-a	mmu0408	Neuroacti	4:3313252	2	2	0	2	0	0	0.053409	0.042669	0	0.041218	0	0	0.032026	0.013739	0.714055	1	-
ENSMUSC	Casp8ap2	caspase 8	GO:00081	metabolic	-	-	-	-	4:3261545	444	104	224	412	295	300	3.794234	0.709773	2.115613	2.716242	1.62337	2.016851	2.20654	2.118821	0.808129	1	-
ENSMUSC	Map3k7	mitogen-	GO:00325	developm	K04427	mitogen-a	mmu0401	MAPK sign	4:3196409	1181	1507	1138	1499	687	1662	9.085253	9.258617	9.67556	8.896495	3.403286	10.0584	9.33981	7.452728	0.190561	1	-
ENSMUSC	Akirin2	akirin 2 [S	GO:00325	developm	-	-	-	-	4:3455093	892	1163	771	1115	1336	1047	25.00636	26.03821	23.88839	24.11519	24.1183	23.09101	24.97765	23.77483	0.450345	1	-
ENSMUSC	Rars2	arginyl-tR	GO:00081	metabolic	K01887	arginyl-tR	mmu0097	Aminoacy	4:3461495	318	445	340	467	587	376	5.279088	5.899807	6.238173	5.981072	6.275156	4.910555	5.80569	5.722261	0.76568	1	-
ENSMUSC	3110043C	RIKEN cDI	GO:00325	developm	-	-	-	-	4:3519128	313	415	194	218	488	391	2.474257	2.619961	1.694923	1.329496	2.484137	2.431582	2.263047	2.081738	0.535221	1	-
ENSMUSC	Rnf20	ring finge	GO:00081	metabolic	-	-	-	-	4:4963200	1433	1789	1224	1844	2187	1775	8.044156	8.020295	7.593857	7.985928	7.905653	7.838692	7.886103	7.910091	0.814608	1	-
ENSMUSC	Ppp3r2	protein pl	GO:00081	metabolic	K06268	serine/thre	mmu0401	MAPK sign	4:4967874	7	5	6	10	7	7	0.141248	0.080569	0.133814	0.155675	0.090949	0.111124	0.118544	0.119249	1	1	-
ENSMUSC	Polr1e	polymeras	GO:00081	metabolic	K03005	DNA-dire	mmu011C	Metabolic	4:4501858	105	97	78	123	139	121	1.111692	0.820181	0.912718	1.00468	0.94768	1.007832	0.948197	0.986731	0.93589	1	-
ENSMUSC	Exosc3	exosome	GO:00325	developm	K03681	exosome	mmu0301	RNA degr	4:4531661	111	118	76	103	169	141	3.100396	2.63221	2.346143	2.219528	3.039733	3.098308	2.692916	2.785856	0.971705	1	-
ENSMUSC	Tmod1	tropomoc	GO:00325	developm	-	-	-	-	4:4603893	585	821	595	803	942	801	8.382766	9.395523	9.423144	8.877232	8.692356	9.029738	9.067144	8.866442	0.648021	1	-
ENSMUSC	Xpa	xeroderm	GO:00325	developm	K10847	DNA-repa	mmu0152	Platinum c	4:4615534	139	158	102	133	194	167	3.230708	2.932824	2.620173	2.384873	2.90362	3.053599	2.927902	2.780697	0.606199	1	-
ENSMUSC	Ncbp1	nuclear ca	GO:00081	metabolic	K12882	nuclear ca	mmu0301	RNA trans	4:4613861	1281	1587	1140	1707	2165	1660	21.08233	20.85894	20.73582	21.67371	22.94468	21.49257	20.89236	22.03699	0.747212	1	-
ENSMUSC	Trmo	tRNA met	GO:00081	metabolic	-	-	-	-	4:4637650	42	34	39	56	46	58	0.779939	0.504241	0.800422	0.802281	0.550081	0.84733	0.694867	0.733231	0.916334	1	-
ENSMUSC	Hemgn	hemogen	GO:00325	developm	-	-	-	-	4:4639398	0	3	0	0	0	0	0	0.055358	0	0	0	0	0.018453	0	0.289033	1	-
ENSMUSC	Anp32b	acidic (leu	GO:00325	developm	-	-	-	-	4:4645090	905	1119	783	1147	1490	1124	32.2014	31.79817	30.79177	31.48618	34.14026	31.46322	31.59711	32.36322	1	1	-
ENSMUSC	Nr4a3	nuclear re	GO:00325	developm	K08559	nuclear re	mmu052C	Transcript	4:4804515	728	1834	660	846	2234	1847	4.030675	8.109455	4.038659	3.613658	7.964974	8.044971	5.39293	6.541201	0.403016	1	-
ENSMUSC	Erp44	endoplasr	GO:00081	metabolic	-	-	-	-	4:4819332	797	1129	717	1068	1378	1122	12.63982	14.29955	12.56748	13.06725	14.073	13.99866	13.16895	13.71297	0.867947	1	-
ENSMUSC	Invs	inversin [S	GO:00508	response	K19626	inversin[-]	mmu0431	Wnt signa	4:4827976	275	328	212	335	438	342	1.329031	1.265965	1.132364	1.249045	1.36311	1.300286	1.242453	1.304147	0.845839	1	-
ENSMUSC	Tex10	testis expr	GO:00329	macromo	-	-	-	-	4:4843085	212	289	252	285	324	258	1.993278	2.170073	2.618657	2.06731	1.961689	1.908368	2.260669	1.979122	0.219831	1	-
ENSMUSC	Tmeff1	transmem	GO:00325	developm	-	-	-	-	4:4858517	1751	2179	1454	2246	2707	2234	29.50132	29.31961	27.07486	29.19411	29.3696	29.61073	28.63193	29.39148	0.982815	1	-
ENSMUSC	Cavin4	caveolae a	GO:00325	developm	-	-	-	-	4:4866351	14	15	8	17	14	16	0.416179	0.356113	0.262818	0.389879	0.267995	0.374177	0.345037	0.344017	1	1	-
ENSMUSC	Brinp1	bone mor	GO:00325	developm	-	-	-	-	4:6876151	4712	5844	4153	5653	6964	5952	43.61161	43.19693	42.48206	40.36511	41.50595	43.33818	43.09687	41.73641	0.493306	1	-
ENSMUSC	Fmn2	formin 2 [	GO:00325	developm	-	-	-	-	1:1745018	2126	2459	1786	2530	2813	2436	15.13284	13.97854	14.0503	13.89339	12.89383	13.64098	14.38723	13.47606	0.308074	1	-
ENSMUSC	Zfp618	zinc finge	GO:00081	metabolic	-	-	-	-	4:6296557	77	122	63	89	107	114	0.405788	0.513472	0.366943	0.361852	0.36312	0.472635	0.428735	0.399202	0.553211	1	-
ENSMUSC	Slc44a5	solute car	GO:00160	membran	K15377	solute car	mmu0523	Choline m	3:1539734	875	1013	763	1219	1292	1053	5.955395	5.506275	5.739495	6.400834	5.662651	5.638216	5.733722	5.900567	0.967409	1	-
ENSMUSC	Tnfsf8	tumor nec	GO:00325	developm	K05471	tumor nec	mmu0406	Cytokine-	4:6383130	0	3	3	0	3	0	0	0.038432	0.053181	0	0.030988	0	0.030538	0.010329	0.244412	1	-
ENSMUSC	Pappa	pregnanc	GO:00081	metabolic	-	-	-	-	4:6512417	23	53	40	48	65	49	0.127355	0.234373	0.244788	0.205047	0.231769	0.213446	0.202172	0.216754	0.828682	1	-
ENSMUSC	Astn2	astrotactir	GO:00325	developm	-	-	-	-	4:6538080	680	851	496	888	889	869	7.467537	7.463523	6.020003	7.523362	6.286735	7.507568	6.983688	7.105889	0.94694	1	-
ENSMUSC	Ptgr1	prostaglar	GO:00081	metabolic	-	-	-	-	4:5896543	34	35	26	47	67	37	0.904183	0.743343	0.764166	0.96428	1.147361	0.774081	0.803897	0.961907	0.447666	1	-
ENSMUSC	Ugcg	UDP-gluc	GO:00081	metabolic	K00720	ceramide	mmu011C	Metabolic	4:5918925	1937	2549	1611	2268	2912	2342	20.17563	21.20378	18.54556	18.22514	19.53186	19.1909	19.97499	18.98263	0.400946	1	-
ENSMUSC	Hsdl2	hydroxyst	GO:00081	metabolic	-	-	-	-	4:5958156	571	775	491	755	872	771	8.302975	9.000056	7.890891	8.469834	8.165234	8.819882	8.397974	8.484983	0.893869	1	-
ENSMUSC	Snx30	sorting ne	GO:00511	localizatio	-	-	-	-	4:5980584	1208	1563	1033	1492	1650	1469	7.380985	7.626972	6.975809	7.033097	6.492112	7.061226	7.327922	6.862145	0.334593	1	-
ENSMUSC	Zfp37	zinc finge	GO:00325	developm	-	-	-	-	4:6218954	252	376	234	292	415	337	1.329413	1.58414	1.364339	1.188427	1.409818	1.398621	1.425964	1.332289	0.457664	1	-
ENSMUSC	Wdr31	WD repea	-	-	-	-	-	-	4:6244865	74	117	80	122	124	155	0.741798	0.936667	0.886327	0.943503	0.800443	1.222356	0.854931	0.988767	0.475539	1	-
ENSMUSC	Alad	aminolevu	GO:00081	metabolic	K01698	porphobil	mmu011C	Metabolic	4:6250916	179	233	173	247	300	279	5.27227	5.48083	5.631658	5.612704	5.690095	6.464867	5.461586	5.922555	0.64773	1	-
ENSMUSC	Pole3	polymeras	GO:00081	metabolic	K02326	DNA poly	mmu011C	Metabolic	4:6252264	314	375	327	403	517	436	7.915871	7.549984	9.110957	7.83798	8.392944	8.64702	8.192271	8.292648	0.928173	1	-
ENSMUSC	Kdm4c	lysine (K)-	GO:00325	developm	-	-	-	-	4:7424249	711	938	653	929	1151	959	6.905096	7.275261	7.009064	6.960587	7.1983	7.327051	7.06314	7.161979	0.920715	1	-
ENSMUSC	Dmac1	distal mer	GO:00329	macromo	-	-	-	-	4:7527735	124	160	87	160	190	145	10.21757	10.52911	7.923019	10.17126	10.08169	9.399499	9.556566	9.884147	0.9611	1	-
ENSMUSC	Ptprd	protein ty	GO:00325	developm	-	-	-	-	4:7594123	3937	5524	3473	5154	5989	5332	7.37538	8.264537	7.190692	7.448928	7.224836	7.858145	7.610203	7.510636	0.666071	1	-
ENSMUSC	Mpdz	multiple P	GO:00452	synapse|m	K06095	multiple P	mmu0453	Tight junc	4:8127850	1840	2313	1562	2310	2821	2375	6.663518	6.68971	6.251923	6.453987	6.578752	6.766441	6.53505	6.599727	0.870797	1	-
ENSMUSC	Zdhhc21	zinc finge	GO:00325	developm	-	-	-	-	4:8279873	1054	1235	929	1296	1518	1324	3.982627	3.726848	3.87964	3.778018	3.693646	3.935751	3.863038	3.802472	0.669354	1	-
ENSMUSC	Aco1	aconitase	GO:00325	developm	K01681	aconitate	mmu011C	Metabolic	4:4014308	497	646	435	689	809	686	6.109503	6.342017	5.909976	6.534298	6.404018	6.634138	6.120499	6.524151	0.704089	1	-
ENSMUSC	Smim27	small inte	-	-	-	-	-	-	4:4026957	7	9	11	14	14	9	0.319206	0.32777	0.554361	0.492513	0.411099	0.322852	0.400445	0.408822	1	1	-
ENSMUSC	Smu1	smu-1 su	GO:00081	metabolic	-	-	-	-	4:4073654	1154	1437	1053	1654	1814	1470	19.04353	18.93844	19.20512	21.05753	19.27674	19.08402	19.06236	19.8061	0.879861	1	-
ENSMUSC	Dnaja1	DnaJ heat	GO:00081	metabolic	K09502	DnaJ hom	mmu0414	Protein pr	4:4072215	1865	2109	1410	2179	2732	1939	8.248105	7.449	6.891937	7.434693	7.780561	6.746279	7.529681	7.320511	0.582535	1	-
ENSMUSC	Aptx	aprataxin	GO:00081	metabolic	-	-	-	-	4:4068238	503	577	401	568	603	546	3.580767	3.280423	3.155	3.119511	2.764265	3.057818	3.33873	2.980532	0.211015	1	-
ENSMUSC	Slc44a1	solute car	GO:00511	localizatio	K06515	solute car	mmu0523	Choline m	4:5344041	1151	1738	1210	1667	2187	1808	8.865636	10.69129	10.3007	9.906046	10.84771	10.95579	9.952542	10.56985	0.698141	1	-
ENSMUSC	Fktn	fukutin [S	GO:00325	developm	K19872	fukutin [E	mmu011C	Metabolic	4:5371399	735	915	636	856	1072	871	3.595969	3.57516	3.439005	3.230969	3.377366	3.352417	3.536711	3.320251	0.37808	1	-
ENSMUSC	Bag1	BCL2-ass	GO:00325	developm	K09555	BCL2-ass	mmu0414	Protein pr	4:4093639	1237	1836	1232	1839	2213	1599	21.23984	25.17678	23.3797	24.36092	24.46908	21.59937	23.26544	23.47646	0.883902	1	-
ENSMUSC	Chmp5	charged r	GO:00081	metabolic	K12198	charged n	mmu0414	Endocytos	4:4094840	989	1243	897	1336	1685	1151	39.7541	39.90273	39.84967	41.43071	43.61544	36.39752	39.8355	40.48122	0.942501	1	-
ENSMUSC	Tmem38b	transmem	GO:00511	localizatio	-	-	-	-	4:5382604	210	230	160	225	272	243	2.971539	2.599177	2.50224	2.456271	2.47848	2.705081	2.690985	2.546611	0.538862	1	-
ENSMUSC	Nfx1	nuclear tr	GO:00081	metabolic	K12236	transcripti	mmu0516	Human pa	4:4097090	1710	2149	1462	2238	2644	2272	10.10935	10.14634	9.552594	10.20746	10.06568	10.56687	9.936094	10.28	0.911701	1	-
ENSMUSC	Rad23b	RAD23 hc	GO:00325	developm	K10839	UV excisic	mmu0414	Protein pr	4:5535004	4314	5369	3815	5447	6623	5361	38.6791	38.44461	37.80398	37.67766	38.23893	37.81403	38.30923	37.91021	0.674275	1	-
ENSMUSC	Aqp7	aquaporir	GO:00511	localizatio	K08771	aquaporir	mmu0332	PPAR sign	4:4103307	0	2	0	0	0	0	0	0.032054	0	0	0	0	0.010685	0	0.524636	1	-
ENSMUSC	Nol6	nucleolar	GO:00081	metabolic	K14544	U3 small r	mmu030C	Ribosome	4:4111442	2444	3020	2280	3492	3564	3139	25.28826	24.95575	26.0735	27.87548	23.74707	25.55171	25.43917	25.72475	0.883772	1	-
ENSMUSC	Ikbkap	inhibitor c	GO:00081	metabolic	-	-	-	-	4:5674968	1035	1386	1033	1405	1776	1437	6.58832	7.046009	7.267438	6.89987	7.280008	7.196175	6.967256	7.125351	0.991651	1	-
ENSMUSC	Ubap2	ubiquitin-	GO:00081	metabolic	-	-	-	-	4:4119431	1084	1361	917	1415	1625	1315	9.726225	9.752573	9.093508	9.794942	9.389085	9.28222	9.524102	9.488749	0.767468	1	-
ENSMUSC	Epb41l4b	erythrocyt	GO:00081	metabolic	-	-	mmu0453	Tight junc	4:5699197	809	983	803	1049	1377	1175	5.257689	5.102063	5.767786	5.259596	5.762823	6.00752	5.375846	5.676646	0.764291	1	-
ENSMUSC	Aqp3	aquaporir	GO:00325	developm	K09876	aquaporir	mmu0496	Vasopress	4:4109272	0	0	0	0	0	2	0	0	0	0	0	0.04036	0	0.013453	0.52907	1	-
ENSMUSC	Dcaf12	DDB1 anc	GO:00081	metabolic	-	-	-	-	4:4129130	689	903	593	928	1112	866	12.22226	12.79281	11.6261	12.7002	12.70258	12.0854	12.21372	12.49606	0.99509	1	-
ENSMUSC	Ubap1	ubiquitin-	GO:00081	metabolic	-	-	-	-	4:4134899	679	1034	653	1096	986	829	7.716067	9.384101	8.201375	9.608765	7.215361	7.411261	8.433848	8.078462	0.554306	1	-
ENSMUSC	Kif24	kinesin fa	GO:00990	supramole	-	-	-	-	4:4139074	17	16	18	30	28	18	0.099141	0.07452	0.116017	0.134974	0.10515	0.082579	0.096559	0.107568	0.796603	1	-
ENSMUSC	Fam219a	family witl	-	-	-	-	-	-	4:4151769	2705	3234	2400	3525	4030	3271	21.88738	20.89837	21.46271	22.00473	20.9984	20.82178	21.41615	21.27497	0.722549	1	-
ENSMUSC	1110017D	RIKEN cDI	GO:00325	developm	-	-	-	-	4:4150500	11	23	19	24	23	22	0.227991	0.380709	0.435229	0.383757	0.30697	0.358715	0.347976	0.349814	1	1	-
ENSMUSC	Nudt2	nudix (nuc	GO:00038	catalytic a	K01518	bis(5'-nuc	mmu0023	Purine me	4:4146515	177	251	184	269	300	227	12.78966	14.48459	14.69442	14.99581	13.95925	12.90398	13.98956	13.95301	0.844756	1	-
ENSMUSC	Cntfr	ciliary neu	GO:00325	developm	K05059	ciliary neu	mmu0406	Cytokine-	4:4165749	1054	918	626	1356	1251	1180	19.55493	13.60204	12.83621	19.4091	14.94609	17.22298	15.33106	17.19272	0.488885	1	-
ENSMUSC	Enho	energy hc	GO:00508	response	-	-	-	-	4:4163814	372	524	408	576	658	483	17.89882	20.13535	21.69643	21.38132	20.38743	18.28268	19.9102	20.01714	0.881419	1	-
ENSMUSC	Dctn3	dynactin 3	GO:00990	supramole	-	-	-	-	4:4171479	606	695	511	777	960	613	20.53342	18.80699	19.13621	20.31141	20.94665	16.3403	19.49221	19.19946	0.732141	1	-
ENSMUSC	1700022I1	RIKEN cDI	-	-	-	-	-	-	4:4296960	3	2	0	0	0	2	0.028951	0.015417	0	0	0	0.015187	0.014789	0.005062	0.501691	1	-
ENSMUSC	Vcp	valosin co	GO:00081	metabolic	K13525	transitiona	mmu0414	Protein pr	4:4297996	1905	2240	1734	2475	2861	2220	20.27819	19.04268	20.39999	20.32543	19.61132	18.5908	19.90695	19.50918	0.619135	1	-
ENSMUSC	Fancg	Fanconi a	GO:00325	developm	K10894	fanconi ar	mmu0346	Fanconi a	4:4300234	102	136	141	169	146	146	0.943487	1.004655	1.441445	1.206008	0.869644	1.062426	1.129862	1.046026	0.552476	1	-
ENSMUSC	Pigo	phosphati	GO:00081	metabolic	K05288	phosphati	mmu011C	Metabolic	4:4301763	217	252	217	280	402	312	1.97903	1.835437	2.187252	1.970067	2.360879	2.238504	2.000573	2.189817	0.594374	1	-
ENSMUSC	Stoml2	stomatin (	GO:00081	metabolic	-	-	-	-	4:4302769	364	348	344	430	571	379	3.717205	2.838177	3.88258	3.387769	3.754959	3.044841	3.479321	3.395856	0.717306	1	-
ENSMUSC	Unc13b	unc-13 hc	GO:00325	developm	K15293	protein ur	mmu0472	Synaptic v	4:4305895	1079	1178	925	1267	1370	1525	2.286924	1.993983	2.166799	2.071749	1.869844	2.542792	2.149235	2.161462	0.854309	1	-
ENSMUSC	Atp8b5	ATPase, cl	GO:00511	localizatio	-	-	-	-	4:4326715	2	0	3	0	3	7	0.015482	0	0.025673	0	0.014959	0.04264	0.013719	0.0192	0.698116	1	-
ENSMUSC	Tesk1	testis spec	GO:00048	signal trar	-	-	-	-	4:4344193	1279	1590	1130	1615	1835	1634	11.26237	11.18156	10.99726	10.97141	10.40519	11.31938	11.14707	10.89866	0.606513	1	-
ENSMUSC	Sit1	suppressic	GO:00023	immune s	-	-	-	-	4:4348208	0	0	0	0	4	0	0	0	0	0	0.079314	0	0	0.026438	0.29548	1	-
ENSMUSC	Ccdc107	coiled-co	GO:00160	membran	-	-	-	-	4:4349290	148	210	193	238	275	260	2.579669	2.923263	3.717984	3.200449	3.086672	3.565224	3.073639	3.284115	0.764099	1	-
ENSMUSC	Tln1	talin 1 [So	GO:00511	localizatio	K06271	talin[-]	mmu0516	HTLV-I inf	4:4353151	918	1251	830	1325	1604	1478	4.919375	5.353902	4.915777	5.477892	5.535107	6.230923	5.063018	5.747974	0.27733	1	-
ENSMUSC	Creb3	cAMP res	GO:00081	metabolic	K09048	cyclic AMI	mmu0415	PI3K-Akt s	4:4356233	423	550	429	630	755	627	6.238539	6.478161	6.992737	7.168266	7.170418	7.274807	6.569812	7.204497	0.518294	1	-
ENSMUSC	Gba2	glucosida	GO:00325	developm	K17108	non-lysos	mmu011C	Metabolic	4:4356692	1073	1299	974	1277	1488	1420	7.267372	7.026394	7.290938	6.672657	6.489858	7.566185	7.194901	6.909567	0.497041	1	-
ENSMUSC	Rgp1	RAB6A GE	GO:00081	metabolic	-	-	-	-	4:4357871	722	957	734	1116	1353	715	5.523611	5.847144	6.206243	6.586893	6.665591	4.303314	5.858999	5.851933	0.854048	1	-
ENSMUSC	Npr2	natriuretic	GO:00325	developm	K12324	atrial natri	mmu0023	Purine me	4:4363193	844	1013	756	1031	1271	1152	7.036192	6.744511	6.965684	6.631074	6.823311	7.555417	6.915462	7.003267	0.907433	1	-
ENSMUSC	Hint2	histidine t	GO:00081	metabolic	-	-	-	-	4:4365422	77	125	104	126	136	122	1.500147	1.944912	2.239364	1.893849	1.706235	1.869882	1.894808	1.823322	0.706561	1	-
ENSMUSC	Spaar	small regu	GO:00325	developm	-	-	-	-	4:4373003	8	16	6	1	27	5	0.09243	0.14763	0.076617	0.008914	0.200873	0.045444	0.105559	0.085077	0.708701	1	-
ENSMUSC	Reck	reversion-	GO:00325	developm	K17461	reversion-	mmu052C	MicroRNA	4:4387553	148	266	186	242	306	283	1.392385	1.998603	1.934005	1.756483	1.853853	2.094576	1.774998	1.901637	0.753804	1	-
ENSMUSC	Clta	clathrin, li	GO:00452	synapse|c	K04644	clathrin lic	mmu0414	Endocytos	4:4400445	2731	3608	2779	4105	4644	3932	45.93655	48.46733	51.66211	53.26969	50.30181	52.03088	48.68866	51.86746	0.652581	1	-

ENSMUSC	Gne	glucosam	GO:00081	metabolic	K12409	bifunction	mmu0110	Metabolic	4:4403407	731	834	599	927	1164	1079	3.667197	3.341405	3.321165	3.587789	3.760321	4.258429	3.443256	3.868846	0.372098	1	-
ENSMUSC	Glipr2	GLI patho	GO:00325	developm	-	-	-	-	4:4395740	32	65	43	86	49	52	0.813427	1.319558	1.20805	1.686543	0.802081	1.039886	1.113678	1.17617	0.934318	1	-
ENSMUSC	Snapc3	small nucl	GO:00081	metabolic	-	-	-	-	4:8341772	395	524	352	583	736	499	2.266298	2.401029	2.232078	2.58059	2.71927	2.252328	2.299802	2.517396	0.556019	1	-
ENSMUSC	Psip1	PC4 and S	GO:00081	metabolic	-	-	-	-	4:8345568	4598	4229	2998	4457	5393	4949	38.5586	28.32278	27.7863	28.83535	29.12309	32.64981	31.5559	30.20275	0.507937	1	-
ENSMUSC	Sh3gl2	SH3-dom	GO:00325	developm	K11247	endophilir	mmu0414	Endocytos	4:8520512	4162	4397	3584	5527	6102	5118	34.99847	29.52903	33.30901	35.8564	33.04257	33.85775	32.61217	34.25224	0.785308	1	-
ENSMUSC	Saxo1	stabilizer	GO:00508	response	-	-	-	-	4:8644464	0	0	0	0	2	0	0	0	0	0	0.029238	0	0	0.009746	0.528435	1	-
ENSMUSC	Rps6	ribosomal	GO:00325	developm	K02991|KC	small subu	mmu0415	PI3K-Akt s	4:8685466	481	568	375	514	669	454	11.98245	11.30041	10.32473	9.878549	10.732	8.897482	11.20253	9.836011	0.182992	1	-
ENSMUSC	Mllt3	myeloid/ly	GO:00325	developm	K15187	YEATS do	mmu0520	Transcript	4:8776992	934	1147	790	1071	1269	1109	3.726608	3.654903	3.483697	3.296754	3.260492	3.481041	3.621736	3.346096	0.284349	1	-
ENSMUSC	Hacd4	3-hydroxy	GO:00081	metabolic	K10703	very-long	mmu0121	Fatty acid	4:8839614	54	95	57	63	94	67	0.394864	0.554789	0.460657	0.355406	0.442629	0.385428	0.470104	0.394488	0.250569	1	-
ENSMUSC	Usp24	ubiquitin s	GO:00081	metabolic	-	-	-	-	4:1063162	2227	2714	2019	2807	2555	2896	8.154019	7.9361	8.170235	7.929106	6.024167	8.341821	8.086785	7.431698	0.273859	1	-
ENSMUSC	Plpp3	phospholi	GO:00325	developm	K01080	phosphati	mmu0110	Metabolic	4:1051573	5578	6460	4765	6775	7280	6100	107.8133	99.71766	101.7896	101.026	90.61076	92.75439	103.1068	94.79706	0.203352	1	-
ENSMUSC	Prkaa2	protein kir	GO:00081	metabolic	K07198	5'-AMP-a	mmu0415	PI3K-Akt s	4:1050298	680	940	606	899	1229	934	5.210127	5.751927	5.131673	5.314096	6.063815	5.629855	5.364576	5.669255	0.756006	1	-
ENSMUSC	Dab1	disabled 1	GO:00325	developm	-	-	-	-	4:1036193	2486	3236	2359	3127	3629	3127	17.28821	17.97228	18.13108	16.77672	16.25139	17.10755	17.79719	16.71188	0.311178	1	-
ENSMUSC	Tctex1d1	Tctex1 do	-	-	-	-	-	-	4:1029786	5	3	3	5	4	6	0.055746	0.026719	0.036972	0.043008	0.028722	0.052633	0.039812	0.041454	1	1	-
ENSMUSC	Sgip1	SH3-dom	GO:00511	localizatio	-	-	-	-	4:1027412	3001	4891	3530	4803	5604	3738	10.0304	13.05557	13.03989	12.38498	12.06161	9.828847	12.04195	11.42514	0.462601	1	-
ENSMUSC	Pde4b	phosphoc	GO:00081	metabolic	K13293	cAMP-spe	mmu0402	cAMP sigr	4:1020875	1413	1684	1328	1918	2094	1900	5.17392	4.924537	5.374306	5.418217	4.93752	5.473208	5.157588	5.276315	0.986727	1	-
ENSMUSC	Ak4	adenylate	GO:00325	developm	K00939	adenylate	mmu0110	Metabolic	4:1014192	677	734	306	930	1000	684	6.997856	6.059243	3.495781	7.41634	6.656272	5.56216	5.517627	6.544924	0.34628	1	-
ENSMUSC	Dnajc6	DnaJ heat	GO:00081	metabolic	K09526	DnaJ hom	mmu0414	Endocytos	4:1014966	7141	8248	5904	8318	10041	8261	37.48095	34.57372	34.24876	33.68223	33.93777	34.11105	35.43448	33.91035	0.415062	1	-
ENSMUSC	Jak1	Janus kina	GO:00325	developm	K11217	Janus kina	mmu0520	Pathways	4:1011523	4338	5309	3792	5475	6429	5316	28.90017	28.24681	27.92068	28.14008	27.58094	27.86165	28.35589	27.86089	0.611428	1	-
ENSMUSC	Cachd1	cache dor	GO:00511	localizatio	-	-	-	-	4:1007766	1076	1479	1029	1435	1742	1569	9.817473	10.77709	10.37647	10.10113	10.23506	11.26215	10.32368	10.53278	0.96801	1	-
ENSMUSC	St3gal3	ST3 beta-	GO:00081	metabolic	K00781	N-acetylla	mmu0110	Metabolic	4:1179321	704	1192	469	1076	1167	892	8.453279	11.43075	6.224033	9.967698	9.023558	8.426126	8.702686	9.139127	0.85176	1	-
ENSMUSC	Artn	artemin [S	GO:00325	developm	-	-	-	-	4:1179261	1	9	4	9	5	6	0.014988	0.107701	0.066245	0.10404	0.048243	0.070728	0.062978	0.074337	0.887925	1	-
ENSMUSC	Dph2	DPH2 hor	GO:00081	metabolic	-	-	-	-	4:1178886	141	155	112	161	221	159	1.286678	1.129617	1.129576	1.133465	1.298674	1.141459	1.181957	1.191199	0.914043	1	-
ENSMUSC	B4galt2	UDP-Gal:l	GO:00325	developm	K07967	beta-1,4-	mmu0110	Metabolic	4:1178692	463	734	455	642	741	675	3.089269	3.911261	3.355312	3.304761	3.183812	3.543151	3.451947	3.343908	0.611636	1	-
ENSMUSC	Slc6a9	solute car	GO:00452	synapse|lc	-	-	-	-	4:1178345	627	1033	556	978	751	973	3.917268	5.154206	3.839165	4.713949	3.021411	4.782328	4.303547	4.172562	0.679227	1	-
ENSMUSC	Bend5	BEN dom	GO:00081	metabolic	-	-	-	-	4:1114150	85	132	67	130	162	120	1.038198	1.287597	0.904438	1.22499	1.274176	1.153065	1.076744	1.21741	0.548868	1	-
ENSMUSC	Elavl4	ELAV (em	GO:00325	developm	-	-	-	-	4:1102037	1626	1030	616	2128	1303	2078	8.263732	4.180601	3.460056	8.343681	4.264369	8.308302	5.301463	6.972117	0.235779	1	-
ENSMUSC	Itgb3bp	integrin b	GO:00081	metabolic	-	-	-	-	4:9976540	72	66	61	89	115	81	0.662777	0.485203	0.620599	0.632054	0.681687	0.586581	0.589526	0.63344	0.777116	1	-
ENSMUSC	Atg4c	autophag	GO:00081	metabolic	-	-	mmu0414	Autophag	4:9919393	560	739	488	755	912	726	6.434424	6.781285	6.1971	6.692667	6.747938	6.562503	6.470936	6.667703	0.956369	1	-
ENSMUSC	Cdkn2c	cyclin-dep	GO:00325	developm	K06622	cyclin-dep	mmu0516	HTLV-I inf	4:1096608	28	53	27	49	76	42	0.604956	0.91452	0.644732	0.81676	1.0574	0.713893	0.721403	0.862684	0.479045	1	-
ENSMUSC	Eps15	epiderma	GO:00081	metabolic	K12472	epiderma	mmu0414	Endocytos	4:1092802	4227	5186	3567	5173	6172	5098	23.6804	23.20251	22.08547	22.35782	22.26575	22.46816	22.98946	22.36391	0.535351	1	-
ENSMUSC	Angptl3	angiopoie	GO:00325	developm	-	-	mmu0497	Cholester	4:9903095	0	6	0	2	3	0	0	0.073382	0	0.023633	0.029589	0	0.024461	0.017741	0.809228	1	-
ENSMUSC	Dock7	dedicator	GO:00325	developm	-	-	-	-	4:9893667	655	770	574	723	955	829	1.897925	1.781861	1.838215	1.616241	1.781951	1.889745	1.839334	1.762646	0.513671	1	-
ENSMUSC	Rnf11	ring finge	GO:00081	metabolic	-	-	-	-	4:1094510	3685	4780	3211	4883	5747	4528	36.36644	37.67358	35.02275	37.17751	36.52238	35.15447	36.35426	36.28479	0.758954	1	-
ENSMUSC	Calr4	calreticulir	GO:00099	cellular pr	-	-	-	-	4:1092344	13	22	9	18	18	13	0.192612	0.260325	0.147371	0.205757	0.171741	0.151523	0.200103	0.17634	0.626913	1	-
ENSMUSC	Osbpl9	oxysterol	GO:00511	localizatio	-	-	-	-	4:1090611	571	652	494	718	843	724	3.005016	2.740335	2.873324	2.91518	2.856885	2.997505	2.872892	2.92319	0.949612	1	-
ENSMUSC	Usp1	ubiquitin s	GO:00325	developm	K11832	ubiquitin c	mmu0346	Fanconi a	4:9892381	471	564	451	570	803	634	6.018918	5.756022	6.369717	5.619563	6.607957	6.373788	6.048219	6.200436	0.995013	1	-
ENSMUSC	Tm2d1	TM2 dom	GO:00048	signal trar	-	-	-	-	4:9835537	263	320	242	370	442	269	9.827596	9.549656	9.994342	10.66654	10.63575	7.907789	9.790531	9.736691	0.832355	1	-
ENSMUSC	Nfia	nuclear fa	GO:00325	developm	-	-	-	-	4:9777273	1389	2301	1538	1278	2763	1976	4.316674	5.710967	5.282632	3.064138	5.529459	4.831087	5.103424	4.474895	0.254698	1	-
ENSMUSC	Txndc12	thioredox	GO:00325	developm	K05360	protein-d	mmu0048	Glutathior	4:1088346	392	526	360	475	711	507	9.15638	9.81226	9.293677	8.559768	10.69454	9.316576	9.420772	9.523627	0.91919	1	-
ENSMUSC	Btf3l4	basic tran	-	-	-	-	-	-	4:1088142	806	1088	749	1030	1370	1057	9.111798	9.822992	9.358315	8.983313	9.973426	9.400586	9.431035	9.452442	0.831064	1	-
ENSMUSC	Hook1	hook micr	GO:00325	developm	-	-	-	-	4:9596724	1331	1137	1172	1268	1581	1727	6.062086	4.135714	5.899548	4.455471	4.636933	6.187959	5.365783	5.093454	0.537475	1	-
ENSMUSC	Fggy	FGGY carl	GO:00081	metabolic	-	-	-	-	4:9555750	130	147	107	157	176	172	0.921044	0.831765	0.837854	0.858159	0.802978	0.958688	0.863555	0.873275	0.934884	1	-
ENSMUSC	Eqtn	equatorin	GO:00325	developm	-	-	-	-	4:9490726	4	7	6	3	7	9	0.145993	0.20403	0.242033	0.084481	0.164502	0.258398	0.197352	0.169127	0.776963	1	-
ENSMUSC	Ift74	intraflagel	GO:00325	developm	-	-	-	-	4:9461449	565	740	468	669	787	687	12.40038	12.97071	11.35216	11.32772	11.12284	11.86188	12.24108	11.43748	0.377659	1	-
ENSMUSC	Plaa	phospholi	GO:00325	developm	K14018	phospholi	mmu0414	Protein pr	4:9456751	1077	1352	1003	1387	1798	1327	7.830389	7.850366	8.059632	7.779904	8.418055	7.590133	7.913462	7.929364	0.820658	1	-
ENSMUSC	Caap1	caspase a	GO:00099	cellular pr	-	-	-	-	4:9450008	166	165	140	218	281	205	2.423639	1.923929	2.259101	2.45553	2.641921	2.354636	2.202223	2.484029	0.476985	1	-
ENSMUSC	Pum1	pumilio RI	GO:00325	developm	-	-	-	-	4:1306633	2504	2463	1832	2642	2924	2706	16.0497	12.60791	12.97792	13.06459	12.06882	13.64493	13.87851	12.92612	0.326151	1	-
ENSMUSC	Laptm5	lysosomal	GO:00511	localizatio	K12387	lysosomal	mmu0414	Lysosome	4:1309131	491	758	523	661	818	716	5.426154	6.689998	6.387927	5.635631	5.82129	6.22494	6.168026	5.893954	0.523246	1	-
ENSMUSC	Cc2d1b	coiled-co	GO:00081	metabolic	-	-	-	-	4:1086199	419	523	369	597	657	588	3.354755	3.344215	3.265275	3.687662	3.387403	3.703694	3.321415	3.59292	0.620011	1	-
ENSMUSC	Pdpn	podoplan	GO:00325	developm	-	-	-	-	4:1432674	206	340	256	286	361	294	5.788286	7.629727	7.950071	6.199827	6.531987	6.498935	7.122694	6.410249	0.319496	1	-
ENSMUSC	Gpx7	glutathior	GO:00081	metabolic	K00432	glutathior	mmu0059	Arachidor	4:1084003	48	58	56	70	68	64	1.809122	1.745824	2.332715	2.035446	1.650402	1.897677	1.962554	1.861175	0.678017	1	-
ENSMUSC	Tnfrsf1b	tumor nec	GO:00325	developm	K05141	tumor nec	mmu0406	Cytokine-	4:1452134	63	125	80	120	124	109	0.982049	1.556128	1.378253	1.443119	1.244702	1.336682	1.305477	1.341501	1	1	-
ENSMUSC	Podn	podocan	GO:00081	metabolic	-	-	-	-	4:1080147	68	67	27	58	74	70	0.448326	0.352783	0.19674	0.295014	0.314177	0.363071	0.332616	0.324087	0.830817	1	-
ENSMUSC	Echdc2	enoyl Coe	GO:00081	metabolic	-	-	-	-	4:1081654	167	241	173	250	387	291	1.73886	2.004071	1.990864	2.008258	2.594869	2.383706	1.911265	2.328944	0.181905	1	-
ENSMUSC	Tnfrsf8	tumor nec	GO:00325	developm	K05145	tumor nec	mmu0406	Cytokine-	4:1452671	21	20	17	30	35	37	0.225669	0.17164	0.201903	0.248711	0.242192	0.312793	0.199737	0.267899	0.26415	1	-
ENSMUSC	Scp2	sterol carr	GO:00081	metabolic	K08764	sterol carr	mmu0110	Metabolic	4:1080438	1061	1289	963	1409	1726	1294	11.02684	10.69878	11.06136	11.29735	11.5513	10.57988	10.92899	11.14284	0.96496	1	-
ENSMUSC	0610037L	RIKEN cDI	GO:00055	extracellul	-	-	-	-	4:1078898	630	707	545	794	943	770	3.522263	3.156794	3.367629	3.424771	3.395059	3.386745	3.348895	3.402192	0.937309	1	-
ENSMUSC	Magoh	mago hor	GO:00081	metabolic	K12877	protein m	mmu0301	RNA trans	4:1078797	155	188	167	173	303	176	8.666664	8.395092	10.32012	7.462738	10.90983	7.741853	9.127293	8.704808	0.658648	1	-
ENSMUSC	Dmrtb1	DMRT-lik	GO:00081	metabolic	-	-	-	-	4:1076762	3	0	4	2	4	4	0.056586	0	0.083394	0.029109	0.048588	0.059354	0.04666	0.045683	1	1	-
ENSMUSC	Ndc1	NDC1 trar	GO:00511	localizatio	K14315	nucleopor	mmu0301	RNA trans	4:1073677	406	556	357	517	631	523	2.947635	3.2238	2.864591	2.895798	2.950063	2.987169	3.012009	2.944343	0.672025	1	-
ENSMUSC	Tmem59	transmem	GO:00081	metabolic	-	-	-	-	4:1071783	710	818	647	843	1095	881	9.922468	9.129781	9.993378	9.089067	9.854397	9.686086	9.681876	9.543183	0.706567	1	-
ENSMUSC	Tceanc2	transcripti	GO:00081	metabolic	-	-	-	-	4:1071341	346	463	332	489	570	397	2.267235	2.422967	2.40439	2.472066	2.405193	2.04655	2.364864	2.307936	0.685705	1	-
ENSMUSC	Mrpl37	mitochon	GO:00081	metabolic	-	-	-	-	4:1070558	459	563	457	662	696	660	4.674837	4.5794	5.144192	5.201663	4.564762	5.288217	4.799476	5.018214	0.855752	1	-
ENSMUSC	Col9a2	collagen,	GO:00329	macromo	K08131	collagen, t	mmu0415	PI3K-Akt s	4:1210393	62	119	34	92	103	121	1.037722	1.590675	0.628953	1.187979	1.11015	1.593247	1.085783	1.297125	0.498405	1	-
ENSMUSC	Exo5	exonuclea	GO:00081	metabolic	-	-	-	-	4:1209211	128	141	110	191	163	171	1.536361	1.351598	1.459227	1.768663	1.259862	1.614691	1.449062	1.547738	0.796647	1	-
ENSMUSC	Dyrk2	dual-spec	GO:00081	metabolic	-	-	-	-	10:118855	1084	1455	1007	1615	2232	1133	9.527416	10.21304	9.781883	10.95088	12.63266	7.834063	9.840779	10.47253	0.740714	1	-
ENSMUSC	Kcnq4	potassium	GO:00325	developm	K04929	potassium	mmu0472	Cholinerg	4:1206961	86	131	86	152	135	120	1.304913	1.587457	1.442214	1.779337	1.319083	1.432448	1.444861	1.510289	0.910265	1	-
ENSMUSC	Ctps	cytidine 5'	GO:00081	metabolic	K01937	CTP synth	mmu0110	Metabolic	4:1205398	516	679	516	692	880	714	8.014754	8.422786	8.858023	8.29233	8.801928	8.724678	8.431854	8.606312	0.979554	1	-
ENSMUSC	Hivep3	human im	GO:00325	developm	-	-	-	-	4:1197337	1194	1545	1030	1496	1726	1537	2.949525	3.048049	2.812105	2.85108	2.745636	2.986981	2.93656	2.861232	0.587481	1	-
ENSMUSC	Ccdc30	coiled-co	-	-	-	-	-	-	4:1193228	238	334	215	327	374	326	1.494739	1.675249	1.492355	1.584405	1.512565	1.610708	1.554115	1.569226	0.908853	1	-
ENSMUSC	Ybx1	Y box pro	GO:00325	developm	-	-	-	-	4:1192779	1922	2368	1605	2539	3637	2690	23.82816	23.44577	21.99171	24.28455	29.03588	26.23617	23.08855	26.51887	0.212821	1	-
ENSMUSC	Tfap2c	transcripti	GO:00325	developm	-	-	-	-	2:1725495	2	2	0	4	0	8	0.015141	0.012096	0	0.023363	0	0.047651	0.009079	0.023671	0.221449	1	-
ENSMUSC	P3h1	prolyl 3-h	GO:00325	developm	-	-	-	-	4:1192329	126	168	121	148	169	157	0.684824	0.729231	0.726845	0.620585	0.591495	0.671302	0.713633	0.627794	0.272353	1	-
ENSMUSC	Svbp	small vasc	GO:00081	metabolic	-	-	-	-	4:1191953	72	52	81	109	124	129	3.394747	1.958041	4.220929	3.964875	3.764865	4.784919	3.191239	4.171553	0.215955	1	-
ENSMUSC	Ermap	erythrobla	GO:00160	membran	-	-	-	-	4:1191754	10	17	4	13	11	17	0.052442	0.071197	0.023185	0.052595	0.037146	0.070137	0.048942	0.053293	0.930642	1	-
ENSMUSC	Slc2a1	solute car	GO:00325	developm	K07299	MFS trans	mmu0520	Pathways	4:1191087	1274	1758	1404	1966	2495	2236	14.15873	15.60341	17.24522	16.85655	17.85583	19.54958	15.66912	18.08732	0.206831	1	-
ENSMUSC	Rragc	Ras-relate	GO:00081	metabolic	K16186	Ras-relate	mmu0415	mTOR sig	4:1239174	1084	1380	987	1362	1651	1406	11.64239	11.83689	11.71593	11.28549	11.41864	11.8798	11.73174	11.52798	0.652672	1	-
ENSMUSC	Mycbp	MYC bind	GO:00081	metabolic	-	-	-	-	4:1239048	66	75	48	72	91	77	1.132937	1.028179	0.910646	0.9535	1.005899	1.039827	1.023921	0.999742	0.790276	1	-
ENSMUSC	Ndufs5	NADH de	GO:00081	metabolic	K03938	NADH de	mmu0110	Metabolic	4:1237127	167	326	216	316	367	305	7.941337	12.38062	11.35215	11.59299	11.23825	11.4101	10.55803	11.41378	0.689712	1	-
ENSMUSC	Macf1	microtubu	GO:00325	developm	-	-	-	-	4:1233496	9479	11713	8470	11630	14258	12817	13.03536	12.86395	12.87332	12.33873	12.62623	13.86621	12.92421	12.94372	0.78512	1	-
ENSMUSC	Ppie	peptidylpr	GO:00081	metabolic	K09564	peptidyl-p	mmu0304	Spliceosor	4:1231271	127	167	144	187	198	189	3.558693	3.737224	4.459588	4.042582	3.572782	4.166375	3.918502	3.927246	0.89671	1	-
ENSMUSC	Trit1	tRNA isop	GO:00081	metabolic	-	-	mmu0110	Metabolic	4:1230165	201	205	186	282	310	280	5.382755	4.384381	5.505127	5.826238	5.345942	5.898997	5.090754	5.690393	0.502305	1	-
ENSMUSC	Mycl	v-myc avi	GO:00325	developm	-	-	-	-	4:1229956	470	505	540	762	435	696	4.785278	4.106264	6.076445	5.985421	2.852026	5.574806	4.989329	4.804084	0.709574	1	-
ENSMUSC	Mfsd2a	major faci	GO:00325	developm	-	-	-	-	4:1229468	267	350	245	382	473	392	6.062677	6.34697	6.148437	6.691846	6.916216	7.002447	6.186028	6.87017	0.481819	1	-
ENSMUSC	Cap1	CAP, ader	GO:00325	developm	-	-	-	-	4:1228590	4622	6348	4721	6500	8135	6227	58.1398	63.77146	65.63332	63.07936	65.89551	61.62165	62.51486	63.53217	0.931425	1	-
ENSMUSC	Ppt1	palmitoyl-	GO:00325	developm	K01074	palmitoyl-	mmu0110	Metabolic	4:1228362	1496	2008	1405	1933	2336	1958	15.9245	17.0704	16.52941	15.87436	16.0126	16.39675	16.5081	16.09457	0.575659	1	-
ENSMUSC	Epha8	Eph recep	GO:00325	developm	K05109	Eph recep	mmu0436	Axon guic	4:1369294	71	69	77	100	106	101	0.919822	0.71391	1.102515	0.99948	0.884311	1.029382	0.912083	0.971058	0.834364	1	-
ENSMUSC	Ephb2	Eph recep	GO:00325	developm	K05111	Eph recep	mmu0436	Axon guic	4:1366475	662	858	585	791	1002	917	2.354134	2.436723	2.299193	2.170101	2.294538	2.565391	2.36335	2.343343	0.7495	1	-
ENSMUSC	Eloa	elongin A	GO:00081	metabolic	-	-	-	-	4:1360033	768	927	681	939	1115	1055	9.960201	9.601357	9.761149	9.395132	9.311858	10.76392	9.774236	9.823636	0.851005	1	-
ENSMUSC	Pithd1	PITH (C-te	GO:00508	response	-	-	-	-	4:1359754	747	892	650	966	1126	1083	12.05667	11.49788	11.59491	12.02858	11.70308	13.75138	11.71648	12.49435	0.702048	1	-
ENSMUSC	Hmgcl	3-hydroxy	GO:00325	developm	K01640	hydroxym	mmu0110	Metabolic	4:1359464	176	255	186	290	341	272	3.878111	4.487373	4.529661	4.929871	4.838558	4.715052	4.298382	4.827827	0.453091	1	-
ENSMUSC	Fuca1	fucosidase	GO:00081	metabolic	K01206	alpha-L-f	mmu0414	Lysosome	4:1359207	436	690	424	684	948	745	6.38707	8.072552	6.864802	7.730398	8.942905	8.58584	7.108142	8.419714	0.187373	1	-
ENSMUSC	Pnrc2	proline-ric	GO:00081	metabolic	-	-	-	-	4:1358709	826	1002	737	1034	1196	992	20.05328	19.42758	19.77514	19.36673	18.69782	18.94645	19.752	19.00367	0.525795	1	-
ENSMUSC	Srsf10	serine/arg	GO:00081	metabolic	K12900	FUS-inter	mmu0304	Spliceosor	4:1358557	764	992	550	1043	1212	1035	4.481974	4.647653	3.566037	4.720531	4.578608	4.776695	4.231888	4.691944	0.466951	1	-
ENSMUSC	Plk3	polo like k	GO:00081	metabolic	K08862	polo-like	mmu0515	Tuberculo	4:1171286	129	169	131	174	177	150	2.057598	2.152796	2.309347	2.141164	1.818019	1.882236	2.173247	1.94714	0.336341	1	-
ENSMUSC	Ptch2	patched 2	GO:00325	developm	K11101	patched 2	mmu0520	Pathways	4:1170960	35	21	25	18	36	45	0.263472	0.126252	0.207994	0.10454	0.174514	0.266496	0.199239	0.18185	0.69185	1	-
ENSMUSC	Eif2b3	eukaryotic	GO:00325	developm	K03241	translatior	mmu0301	RNA trans	4:1170194	206	246	206	226	340	304	1.651733	1.575269	1.825523	1.398014	1.755526	1.917601	1.684175	1.69038	0.888173	1	-
ENSMUSC	Urod	uroporphy	GO:00081	metabolic	K01599	uroporphy	mmu0110	Metabolic	4:1169899	434	407	327	539	561	473	5.277677	3.952692	4.394889	5.056759	4.393093	4.525069	4.541753	4.658307	1	1	-
ENSMUSC	Toe1	target of E	GO:00081	metabolic	-	-	-	-	4:1167943	129	147	94	136	206	151	1.344798	1.223861	1.08304	1.093802	1.382894	1.238389	1.217233	1.238362	0.974268	1	-
ENSMUSC	Mmachc	methylma	GO:00081	metabolic	K14618	methylma	mmu0497	Vitamin d	4:1167022	272	399	274	415	496	408	6.427168	7.529567	7.155624	7.565358	7.547228	7.584419	7.037453	7.565668	0.674883	1	-
ENSMUSC	Prdx1	peroxired	GO:00081	metabolic	K13279	peroxired	mmu0414	Peroxisom	4:1166855	762	1064	854	1247	1507	1019	11.3784	12.6886	14.09389	14.36557	14.49086	11.97048	12.7203	13.60897	0.675938	1	-
ENSMUSC	Akr1a1	aldo-ketc	GO:00081	metabolic	-	-	mmu0110	Metabolic	4:1166365	2433	3111	2261	3166	4084	3125	43.71818	44.64428	44.90218	43.88956	47.25637	44.17544	44.42154	45.10712	0.924046	1	-
ENSMUSC	Nasp	nuclear au	GO:00325	developm	-	-	-	-	4:1166010	352	467	336	552	631	492	2.320245	2.458409	2.447809	2.807119	2.678401	2.551335	2.408821	2.678952	0.457558	1	-
ENSMUSC	Ipp	IAP prom	GO:00081	metabolic	-	-	-	-	4:1165075	164	214	136	185	288	234	3.343408	3.484231	3.064312	2.909703	3.78089	3.752947	3.297317	3.48118	0.832705	1	-
ENSMUSC	Pik3r3	phosphoir	GO:00081	metabolic	K02649	phosphoir	mmu0520	Pathways	4:1162216	987	1212	775	1172	1351	1111	8.887233	8.715612	7.712548	8.141557	7.833568	7.869998	8.438464	7.948374	0.390787	1	-
ENSMUSC	Pomgnt1	protein O	GO:00081	metabolic	K09666	beta-1,2-	mmu0110	Metabolic	4:1161238	759	1101	803	1214	1302	1043	5.130424	5.943529	5.998932	6.330828	5.667311	5.546341	5.690961	5.84816	0.97312	1	-
ENSMUSC	Lurap1	leucine ric	GO:00400	locomotic	-	-	-	-	4:1161324	404	500	372	472	568	479	3.97798	3.931852	4.04829	3.585534	3.601511	3.710464	3.986041	3.632503	0.2859	1	-
ENSMUSC	Lrrc41	leucine ric	GO:00081	metabolic	-	-	-	-	4:1160752	536	665	546	714	840	730	6.905923	6.842643	7.774923	7.097167	6.969321	7.399297	7.174497	7.155261	0.801308	1	-
ENSMUSC	Nsun4	NOL1/NC	GO:00081	metabolic	-	-	-	-	4:1160328	116	114	105	164	128	180	0.857889	0.673326	0.858243	0.935725	0.609591	1.047266	0.796486	0.864194	0.748258	1	-
ENSMUSC	Dmbx1	diencepha	GO:00325	developm	-	-	-	-	4:1159151	1	3	6	4	4	6	0.006438	0.015422	0.042682	0.019859	0.016579	0.03038	0.021514	0.022272	1	1	-
ENSMUSC	Mknk1	MAP kina	GO:00081	metabolic	K04372	MAP kinas	mmu0401	MAPK sigr	4:1158391	357	516	324	457	573	478	3.286754	3.793975	3.296785	3.245976	3.397099	3.462084	3.459171	3.368386	0.656031	1	-
ENSMUSC	Mob3c	MOB kina	GO:00081	metabolic	-	-	-	-	4:1158280	63	100	75	105	118	89	0.984815	1.248411	1.295745	1.266286	1.187813	1.094495	1.176324	1.182864	0.923799	1	-
ENSMUSC	Atpaf1	ATP synth	GO:00099	cellular pr	-	-	-	-	4:1157848	275	417	313	361	391	421	2.472897	2.994716	3.110749	2.504442	2.264152	2.978287	2.859454	2.582294	0.315656	1	-
ENSMUSC	Tal1	T cell acut	GO:00325	developm	-	-	-	-	4:1150564	22	45	22	47	34	33	0.132826	0.21698	0.146806	0.218925	0.132189	0.156745	0.165537	0.169286	1	1	-
ENSMUSC	Stil	Scl/Tal1 ir	GO:00511	localizatio	-	-	-	-	4:1150001	9	14	6	12	19	21	0.081967	0.101833	0.0604	0.084315	0.111434	0.150467	0.0814	0.115405	0.334904	1	-
ENSMUSC	Ebna1bp2	EBNA1 bir	GO:00081	metabolic	-	-	-	-	4:1186207	717	946	671	1069	1166	1021	8.838821	9.313482	9.142073	10.16677	9.256104	9.90174	9.098125	9.774871	0.635213	1	-
ENSMUSC	Cfap57	cilia and f	-	-	-	-	-	-	4:1185545	20	24	11	18	46	19	0.278045	0.26646	0.169009	0.193061	0.411809	0.207797	0.237838	0.270889	0.70378	1	-
ENSMUSC	Pax7	paired bo	GO:00325	developm	K09381	paired bo	mmu0520	Transcript	4:1397370	0	0	0	1	1	0	0	0	0	0.007365	0.006145	0	0	0.004503	0.521876	1	-
ENSMUSC	Aldh4a1	aldehyde	GO:00081	metabolic	K00294	1-pyrrolin	mmu0110	Metabolic	4:1396228	301	477	338	483	601	471	2.68495	3.398086	3.332217	3.323898	3.452226	3.305228	3.138418	3.360451	0.702445	1	-
ENSMUSC	Mrto4	mRNA tur	GO:00081	metabolic	-	-	-	-	4:1393474	159	259	157	287	328	206	2.506	3.260091	2.734823	3.489762	3.328986	2.554242	2.833638	3.12433	0.595599	1	-
ENSMUSC	Akr7a5	aldo-ketc	GO:00081	metabolic	K15303	aflatoxin E	mmu0098	Metabolis	4:1393107	177	247	198	325	373	268	5.430785	6.052485	6.714338	7.693146	7.369773	6.468986	6.06587	7.177302	0.256573	1	-
ENSMUSC	Pqlc2	PQ loop r	GO:00511	localizatio	-	-	-	-	4:1392940	119	162	122	173	203	191	1.307757	1.421807	1.481797	1.466759	1.436588	1.651299	1.403787	1.518215	0.698763	1	-
ENSMUSC	Capzb	capping p	GO:00325	developm	K10365	capping p	mmu0414	Endocytos	4:1391928	4151	5514	3519	5503	6239	6041	35.23595	37.38057	33.01409	36.03821	34.10382	40.34159	35.2102	36.82787	0.813938	1	-
ENSMUSC	Htr6	5-hydroxy	GO:00325	developm	K04162	5-hydroxy	mmu0408	Neuroacti	4:1390611	53	70	54	67	70	65	0.913121	0.963144	1.028223	0.890544	0.776611	0.880991	0.968163	0.849382	0.362333	1	-
ENSMUSC	Pla2g2e	phospholi	GO:00081	metabolic	-	-	mmu0110	Metabolic	4:1388779	0	0	0	0	2	0	0	0	0	0	0.069823	0	0	0.023274	0.528358	1	-
ENSMUSC	Cda	cytidine d	GO:00081	metabolic	K01489	cytidine d	mmu0110	Metabolic	4:1383384	4	10	5	10	16	7	0.155968	0.31137	0.215475	0.300814	0.401713	0.214725	0.227604	0.305751	0.565409	1	-
ENSMUSC	Pink1	PTEN indu	GO:00325	developm	K05688	PTEN indu	mmu0501	Parkinson	4:1383134	4462	4887	4038	5988	6051	5735	64.6208	56.52374	64.63323	66.90443	56.43189	65.3412	61.92592	62.8925	0.916674	1	-
ENSMUSC	Ddost	dolichyl-c	GO:00081	metabolic	K12670	oligosacch	mmu0110	Metabolic	4:1383047	1254	1694	1271	1816	2103	1782	36.11645	38.9643	40.45758	40.35094	39.00331	40.37626	38.51277	39.91017	0.897863	1	-
ENSMUSC	Kif17	kinesin fai	GO:00325	developm	-	-	-	-	4:1382504	358	489	369	490	570	542	3.722553	4.060804	4.240631	3.930828	3.816688	4.433716	4.007996	4.060411	0.925609	1	-
ENSMUSC	Hp1bp3	heterochr	GO:00081	metabolic	-	-	-	-	4:1382162	3787	4987	3580	5308	6157	5298	15.74901	16.56317	16.45462	17.03018	16.48853	17.33329	16.2556	16.95067	0.829491	1	-
ENSMUSC	Eif4g3	eukaryotic	GO:00971	organic cy	K03260	translatior	mmu0301	RNA trans	4:1379930	5430	6781	4699	7130	8196	6993	22.71171	22.65111	21.72212	23.00752	22.0753	23.01041	22.36165	22.69774	0.910681	1	-
ENSMUSC	Alpl	alkaline pl	GO:00325	developm	K01077	alkaline pl	mmu0110	Metabolic	4:1377417	136	147	106	154	174	162	1.815457	1.567152	1.56387	1.58598	1.495725	1.701264	1.648826	1.594323	0.681945	1	-
ENSMUSC	Ptpn12	protein ty	GO:00325	developm	-	-	-	-	5:2098664	600	667	487	700	795	736	5.960767	5.292032	5.347202	5.365115	5.085957	5.752275	5.533334	5.401116	0.645348	1	-
ENSMUSC	Zcchc17	zinc finge	GO:00329	macromo	-	-	-	-	4:1303153	777	949	763	1190	1395	1192	8.69222	8.478553	9.433677	10.2704	10.04936	10.49052	8.86815	10.27009	0.210707	1	-
ENSMUSC	Fabp3	fatty acid	GO:00081	metabolic	K08752	fatty acid-	mmu0332	PPAR sign	4:1303085	565	654	469	645	867	561	41.9172	38.74959	38.4559	36.91763	41.42069	32.74292	39.70756	37.02708	0.404727	1	-
ENSMUSC	Gnat3	guanine n	GO:00048	signal trar	K19729	guanine n	mmu0474	Taste tran	5:1796254	0	0	0	1	3	0	0	0	0	0.034616	0.086679	0	0	0.040432	0.277722	1	-
ENSMUSC	Hcrtr1	hypocretir	GO:00048	signal trar	K04238	hypocretir	mmu0408	Neuroacti	4:1301302	32	43	38	39	52	33	0.587801	0.6308	0.77145	0.552688	0.615093	0.476884	0.663351	0.548222	0.290756	1	-
ENSMUSC	Pef1	penta-EF	GO:00325	developm	-	-	-	-	4:1301025	631	767	590	907	1016	879	13.32215	12.93261	13.76714	14.77347	13.81317	14.59973	13.34064	14.39546	0.605336	1	-
ENSMUSC	Sema3c	sema don	GO:00325	developm	K06840	semaphor	mmu0436	Axon guic	5:1757428	519	762	464	685	1042	794	2.848721	3.340284	2.8148	2.900702	3.683026	3.428574	3.001268	3.337434	0.455691	1	-
ENSMUSC	Adgrb2	adhesion	GO:00325	developm	-	-	-	-	4:1299848	7122	8600	6736	9364	11321	8982	39.36416	37.96149	41.14792	39.92921	40.29382	39.05557	39.49119	39.75953	0.836014	1	-
ENSMUSC	Spocd1	SPOC dor	GO:00081	metabolic	-	-	-	-	4:1299292	3	3	0	6	7	1	0.055188	0.044083	0	0.085154	0.08292	0.014475	0.03309	0.06085	0.449438	1	-
ENSMUSC	Hpca	hippocalc	GO:00325	developm	-	-	-	-	4:1291115	18850	22275	18807	23324	31573	23021	201.9911	190.627	222.7344	192.821	217.867	194.0689	205.1175	201.5856	0.641314	1	-
ENSMUSC	Ptp4a2	protein ty	GO:00081	metabolic	-	-	-	-	4:1298112	2442	2631	2289	3378	3309	3616	17.42682	14.99475	18.05366	18.59784	15.20631	20.30074	16.82508	18.03496	0.664866	1	-
ENSMUSC	Azin2	antizyme	GO:00081	metabolic	K01583	arginine d	mmu0110	Metabolic	4:1289302	258	321	254	373	434	334	3.109548	3.089787	3.383445	3.468301	3.368395	3.166909	3.19426	3.334535	0.873955	1	-
ENSMUSC	Khdrbs1	KH domai	GO:00081	metabolic	-	-	-	-	4:1297031	2247	2726	2022	2779	3328	2340	17.00305	16.47386	16.91031	16.22343	16.21667	13.92998	16.79574	15.45669	0.240764	1	-
ENSMUSC	Rnf19b	ring finge	GO:00081	metabolic	-	-	-	-	4:1290582	653	876	654	830	1127	766	8.41869	9.019478	9.318724	8.255441	9.356433	7.769112	8.918964	8.460329	0.469476	1	-
ENSMUSC	A3galt2	alpha 1,3-	GO:00081	metabolic	K20736	alpha-1,3	mmu0060	Glycosphi	4:1287553	10	2	2	8	12	10	0.117601	0.018787	0.025996	0.072583	0.090879	0.092516	0.054128	0.085326	0.372556	1	-
ENSMUSC	Ccdc28b	coiled coi	GO:00099	cellular pr	-	-	-	-	4:1296192	190	275	199	253	332	242	2.723244	3.147833	3.152352	2.797596	3.064266	2.728728	3.00781	2.86353	0.568298	1	-
ENSMUSC	Phc2	polyhome	GO:00325	developm	-	-	-	-	4:1286547	1058	1210	1108	1534	1960	1448	6.36323	5.811965	7.365095	7.117826	7.591063	6.851271	6.51343	7.18672	0.461456	1	-
ENSMUSC	Tmem234	transmem	GO:00160	membran	-	-	-	-	4:1296006	498	751	450	689	795	646	2.869931	3.456432	2.866165	3.063318	2.950287	2.928772	3.064176	2.980793	0.634377	1	-
ENSMUSC	Eif3i	eukaryotic	GO:00081	metabolic	K03246	translatior	mmu0301	RNA trans	4:1295919	660	735	654	899	1032	841	9.470818	8.4232	10.37214	9.952534	9.536264	9.494028	9.422054	9.660942	0.995242	1	-
ENSMUSC	Zfp362	zinc finge	GO:00971	organic cy	-	-	-	-	4:1287730	498	587	364	668	719	609	6.265592	5.898165	5.061519	6.483957	5.82528	6.027829	5.741759	6.112355	0.736892	1	-
ENSMUSC	Hdac1	histone de	GO:00325	developm	K06067	histone de	mmu0520	Pathways	4:1295161	322	397	350	496	590	488	3.346508	3.295125	4.020222	3.976927	3.948594	3.989938	3.553952	3.97182	0.452289	1	-
ENSMUSC	Stpg1	sperm tail	GO:00511	localizatio	-	-	-	-	4:1354947	57	84	72	89	106	85	1.017628	1.19768	1.420679	1.225851	1.218642	1.193836	1.211996	1.212777	0.896854	1	-
ENSMUSC	Nipal3	NIPA-like	GO:00511	localizatio	-	-	-	-	4:1354454	1545	2106	1411	2308	2524	2171	9.533579	10.37843	9.622796	10.98737	10.02932	10.53898	9.844935	10.51855	0.631018	1	-
ENSMUSC	Csmd2	CUB and S	GO:00160	membran	-	-	-	-	4:1279878	1427	1631	1225	1817	1974	1653	4.835717	4.414043	4.587961	4.750311	4.307633	4.406771	4.612573	4.488238	0.57546	1	-
ENSMUSC	Zbtb8a	zinc finge	GO:00081	metabolic	-	-	-	-	4:1293536	65	101	84	102	155	136	1.709944	2.121957	2.442265	2.070131	2.625762	2.814618	2.091389	2.503504	0.340989	1	-
ENSMUSC	Srrm1	serine/arg	GO:00081	metabolic	K13171	serine/arg	mmu0301	RNA trans	4:1353204	2091	2547	1811	2563	3062	2521	10.30282	10.02251	9.862049	9.742735	9.715425	9.772049	10.06246	9.743403	0.514297	1	-
ENSMUSC	Yars	tyrosyl-tR	GO:00081	metabolic	K01866	tyrosyl-tR	mmu0097	Aminoacy	4:1291897	1475	1875	1449	2051	2445	2015	11.48294	11.65757	12.46741	12.31848	12.2573	12.34091	11.86931	12.30556	0.892003	1	-
ENSMUSC	CK137956	cDNA seq	-	-	-	-	-	-	4:1279275	12	24	22	22	28	25	0.315946	0.504653	0.640209	0.446884	0.474741	0.517835	0.486936	0.47982	0.900023	1	-
ENSMUSC	Sfpq	splicing fa	GO:00081	metabolic	-	-	-	-	4:1270213	3496	4110	2836	4795	5405	3941	35.94193	33.74564	32.22427	38.03199	35.7833	31.87479	33.97062	35.23003	0.891206	1	-
ENSMUSC	Syf2	SYF2 hom	GO:00325	developm	K12868	pre-mRN	mmu0304	Spliceosor	4:1349308	741	922	643	963	1159	884	16.64608	16.54133	15.96436	16.68978	16.7661	15.62274	16.38392	16.35954	0.799469	1	-
ENSMUSC	Tmem50a	transmem	GO:00160	membran	-	-	-	-	4:1348978	812	1198	845	1214	1407	1168	31.22113	36.78711	35.90844	36.01157	34.83707	35.33025	34.63889	35.39296	0.985914	1	-
ENSMUSC	Tmem57	transmem	GO:00325	developm	-	-	-	-	4:1348027	1040	1312	1016	1361	1649	1395	6.47369	6.522261	6.989709	6.535927	6.609886	6.831308	6.661887	6.65904	0.794776	1	-

ENSMUSG	Stmn1	stathmin 1	GO:00325	developm	K04381	stathmin[-	mmu0401	MAPK sigı	4:1344683	1955	2360	1425	2559	2877	2213	40.61539	39.1563	32.7194	41.01515	38.48915	36.16896	37.49703	38.55775	0.969859	1	-
ENSMUSG	Ncdn	neurochoı	GO:00325	developm	-	-	-	-	4:1267437	36960	45110	36258	50290	61314	50694	500.4896	487.8449	542.6424	525.3819	534.6597	540.0458	510.3257	533.3624	0.809909	1	-
ENSMUSG	Slc30a2	solute car	GO:00511	localizatio	-	-	-	-	4:1343431	0	5	0	0	4	0	0	0.034348	0	0	0.022158	0	0.011449	0.007386	0.795256	1	-
ENSMUSG	Psmb2	proteasor	GO:00081	metabolic	K02734	20S prote	mmu0305	Proteason	4:1266776	578	746	561	762	951	801	21.59829	22.2626	23.16866	21.9673	22.88372	23.54695	22.34319	22.79932	0.974375	1	-
ENSMUSG	Extl1	exostoses	GO:00081	metabolic	K02368	alpha-1,4	mmu0110	Metabolic	4:1343563	1041	1152	831	1209	1397	1288	14.38694	12.71499	12.69304	12.89063	12.4328	14.00375	13.26499	13.10906	0.710375	1	-
ENSMUSG	Zfp593	zinc finge	GO:00081	metabolic	-	-	-	-	4:1342432	110	101	94	132	129	100	1.673241	1.226971	1.58031	1.549066	1.2636	1.196682	1.493507	1.336449	0.418176	1	-
ENSMUSG	Ago3	argonaute	GO:00081	metabolic	-	-	-	-	4:1263317	1433	1839	1252	1798	2038	1781	5.03344	5.158771	4.860374	4.87235	4.609751	4.921453	5.017528	4.801185	0.443684	1	-
ENSMUSG	Sh3bgrl3	SH3 domi	GO:00081	metabolic	-	-	-	-	4:1341274	1263	1859	1396	1849	2168	1789	47.3397	55.64775	57.83016	53.46747	52.32826	52.75256	53.60587	52.84943	0.687607	1	-
ENSMUSG	Tekt2	tektin 2 [S	GO:00400	locomotic	-	-	-	-	4:1263221	24	22	13	14	28	18	0.498611	0.365022	0.29849	0.224388	0.374591	0.294181	0.387374	0.29772	0.321467	1	-
ENSMUSG	Trappc3	trafficking	GO:00511	localizatio	-	-	-	-	4:1262623	654	831	609	870	966	781	20.21873	20.51743	20.80849	20.75037	19.23128	18.99495	20.51488	19.65886	0.507789	1	-
ENSMUSG	Map7d1	MAP7 dor	GO:00051	structural	-	-	-	-	4:1262321	3797	4833	2775	5202	5932	3831	45.59245	46.34629	36.82665	48.18957	45.86783	36.18891	42.9218	43.41544	0.90895	1	-
ENSMUSG	Gpatch3	G patch d	GO:00081	metabolic	-	-	-	-	4:1335747	70	92	83	121	132	142	1.866395	1.95903	2.445856	2.489	2.266402	2.978557	2.090427	2.577987	0.243698	1	-
ENSMUSG	Nudc	nudC nuc	GO:00325	developm	-	-	-	-	4:1335325	1367	1655	1157	1739	2131	1441	46.89125	45.33849	43.86354	46.02069	47.07188	38.88646	45.36443	43.99301	0.577373	1	-
ENSMUSG	Slc9a1	solute car	GO:00325	developm	K05742	solute car	mmu0481	Regulatioı	4:1333697	1220	1449	1052	1652	1857	1545	10.7786	10.2239	10.27224	11.26011	10.56499	10.73845	10.42492	10.85452	0.862808	1	-
ENSMUSG	Tmem222	transmem	GO:00160	membran	-	-	-	-	4:1332660	758	1017	804	1075	1200	1077	15.45826	16.56374	18.1215	16.91337	15.75895	17.27898	16.7145	16.65043	0.784218	1	-
ENSMUSG	Csf3r	colony stiı	GO:00325	developm	K05061	granulocy	mmu0520	Pathways	4:1260245	93	141	108	128	127	155	1.02888	1.245802	1.32055	1.092507	0.904778	1.349044	1.19841	1.115443	0.552337	1	-
ENSMUSG	Mrps15	mitochon	GO:00081	metabolic	K02956	small subı	mmu0301	Ribosome	4:1260469	347	458	303	445	556	484	10.3352	10.89434	9.974205	10.22539	10.66396	11.34086	10.40125	10.74341	0.948422	1	-
ENSMUSG	Map3k6	mitogen-.	GO:00048	signal trar	K04425	mitogen-ı	mmu0401	MAPK sigı	4:1332408	61	68	53	88	111	89	0.648648	0.57748	0.622873	0.721926	0.760074	0.744531	0.616333	0.742177	0.309179	1	-
ENSMUSG	Meaf6	MYST/Esa	GO:00081	metabolic	-	-	-	-	4:1250851	1435	1862	1370	1835	2194	1768	12.48837	12.94134	13.17714	12.32027	12.29547	12.1045	12.86895	12.24008	0.403096	1	-
ENSMUSG	Cd164l2	CD164 sia	GO:00160	membran	-	-	-	-	4:1332121	20	30	17	42	22	23	0.473323	0.567001	0.444655	0.766832	0.33528	0.428224	0.494993	0.510112	1	1	-
ENSMUSG	Wasf2	WAS prot	GO:00325	developm	K05748	WAS prot	mmu0481	Regulatioı	4:1331305	460	560	359	624	878	589	3.511719	3.414253	3.02903	3.675162	4.316294	3.53743	3.318334	3.842962	0.27634	1	-
ENSMUSG	Gnl2	guanine n	GO:00038	catalytic a	K14537	nuclear G	mmu0300	Ribosome	4:1250165	825	1027	872	1123	1367	1261	10.31386	10.25378	12.04845	10.83123	11.00502	12.40206	10.87203	11.41277	0.811677	1	-
ENSMUSG	Fgr	FGR protc	GO:00325	developm	K08891	tyrosine-ţ	mmu0516	Epstein-B	4:1329740	0	12	2	10	10	3	0	0.123762	0.028548	0.099635	0.08316	0.030475	0.05077	0.07109	0.599469	1	-
ENSMUSG	Epha10	Eph recep	GO:00048	signal trar	-	-	-	-	4:1248808	655	782	661	914	982	965	5.056653	4.821416	5.639881	5.44375	4.881888	5.86084	5.17265	5.395493	0.870125	1	-
ENSMUSG	Fam76a	family witl	GO:00056	cell|intrace	-	-	-	-	4:1328992	818	1142	820	1148	1400	1146	11.43442	12.7489	12.66838	12.38036	12.60211	12.60249	12.2839	12.52832	0.969842	1	-
ENSMUSG	Stx12	syntaxin 1	GO:00081	metabolic	K13813	syntaxin 1	mmu0414	Phagoson	4:1328535	2331	2784	2094	2502	3812	2822	42.19581	40.24779	41.89387	34.94175	44.43596	40.18787	41.44582	39.85519	0.505749	1	-
ENSMUSG	Ppp1r8	protein pł	GO:00325	developm	-	-	-	-	4:1328269	548	686	540	729	874	771	8.588257	8.586067	9.353296	8.814184	8.820441	9.505823	8.84254	9.046816	0.994218	1	-
ENSMUSG	Rpa2	replicatior	GO:00081	metabolic	K10739	replicatior	mmu0346	Fanconi a	4:1327683	118	133	76	130	187	153	1.749182	1.574525	1.245125	1.486702	1.785035	1.78424	1.522944	1.685326	0.623022	1	-
ENSMUSG	Smpdl3b	sphingom	GO:00081	metabolic	-	-	-	-	4:1327329	24	38	23	40	56	29	0.730518	0.923717	0.773711	0.939306	1.097631	0.69441	0.809315	0.910449	0.707452	1	-
ENSMUSG	Eya3	EYA trans	GO:00325	developm	-	-	-	-	4:1326389	435	569	400	385	783	591	1.684763	1.759979	1.712207	1.150378	1.952836	1.800727	1.718983	1.634647	0.597604	1	-
ENSMUSG	Yrdc	yrdC dom	GO:00081	metabolic	-	-	-	-	4:1248506	162	208	156	254	357	213	4.014372	4.116355	4.27241	4.855874	5.696723	4.152351	4.134379	4.901649	0.276276	1	-
ENSMUSG	Mtf1	metal resţ	GO:00081	metabolic	-	-	-	-	4:1248021	669	839	524	852	788	699	2.737979	2.742278	2.37018	2.690133	2.076751	2.250566	2.616812	2.33915	0.215144	1	-
ENSMUSG	Sesn2	sestrin 2 [	GO:00081	metabolic	K20394	sestrin 2[-	mmu0415	mTOR sig	4:1324920	340	381	288	404	479	415	5.974029	5.34637	5.592772	5.47646	5.419743	5.736504	5.637724	5.544235	0.722454	1	-
ENSMUSG	Inpp5b	inositol pc	GO:00325	developm	K01099	inositol pc	mmu0110	Metabolic	4:1247418	690	843	641	895	994	973	5.850589	5.708523	6.006973	5.854691	5.427398	6.490444	5.855362	5.924178	0.901711	1	-
ENSMUSG	Rcc1	regulator	GO:00987	molecular	-	-	-	-	4:1323319	87	137	95	157	202	110	0.920792	1.158	1.111259	1.281957	1.376733	0.9159	1.06335	1.19153	0.566029	1	-
ENSMUSG	Trnau1ap	tRNA sele	GO:00081	metabolic	-	-	-	-	4:1323117	111	184	169	164	252	207	1.303359	1.725454	2.193182	1.485644	1.905438	1.91215	1.740665	1.767744	0.981201	1	-
ENSMUSG	Taf12	TATA-box	GO:00081	metabolic	K03126	transcripti	mmu0302	Basal tran	4:1322743	309	379	272	420	494	440	4.417461	4.327123	4.297642	4.632264	4.547735	4.948539	4.347409	4.709513	0.626313	1	-
ENSMUSG	Gmeb1	glucocorti	GO:00081	metabolic	-	-	-	-	4:1322210	339	331	289	478	467	370	1.461049	1.139303	1.376605	1.589364	1.296097	1.254521	1.325653	1.379994	0.906471	1	-
ENSMUSG	Sf3a3	splicing fa	GO:00081	metabolic	K12827	splicing fa	mmu0304	Spliceosoı	4:1247147	591	732	506	787	940	791	11.25206	11.13015	10.64736	11.55977	11.52462	11.84764	11.00986	11.64401	0.753839	1	-
ENSMUSG	Epb41	erythrocyt	GO:00325	developm	-	-	-	-	4:1319234	624	793	564	756	1070	819	1.597695	1.621544	1.59601	1.493351	1.764201	1.649699	1.605083	1.63575	0.966961	1	-
ENSMUSG	Utp11	UTP11 sm	GO:00325	developm	-	-	-	-	4:1246781	311	436	304	448	559	400	3.486792	3.903894	3.766913	3.875018	4.035824	3.528064	3.7192	3.812969	0.994028	1	-
ENSMUSG	Ptpru	protein ty	GO:00325	developm	-	-	-	-	4:1317684	1261	1343	1093	1791	1955	1678	8.903132	7.57268	8.528932	9.755589	8.888515	9.320311	8.334915	9.321471	0.362379	1	-
ENSMUSG	Mecr	mitochon	GO:00081	metabolic	K07512	mitochon	mmu0110	Metabolic	4:1318434	180	186	162	225	271	211	2.224784	1.836012	2.212984	2.145508	2.15695	2.051681	2.09126	2.118046	0.949989	1	-
ENSMUSG	Srsf4	serine/arg	GO:00081	metabolic	K12893	splicing fa	mmu0516	Herpes sir	4:1318736	1161	1534	1075	1717	2097	1620	15.40384	16.25429	15.76347	17.57508	17.91637	16.90919	15.8072	17.46688	0.402329	1	-
ENSMUSG	Casp9	caspase 9	GO:00325	developm	K04399	caspase 9	mmu0520	Pathways	4:1417936	386	461	379	542	390	503	3.108472	2.964873	3.373224	3.36736	2.022453	3.186679	3.148857	2.858831	0.38359	1	-
ENSMUSG	Arhgef19	Rho guan	GO:00987	molecular	-	-	-	-	4:1412394	258	339	242	410	483	366	2.508435	2.632265	2.600439	3.07537	3.024026	2.799471	2.580379	2.966289	0.318903	1	-
ENSMUSG	Fbxo42	F-box prc	-	-	-	-	-	-	4:1411479	515	777	539	734	837	756	5.052205	6.08752	5.843983	5.555209	5.287545	5.834539	5.661236	5.559098	0.690781	1	-
ENSMUSG	Necap2	NECAP er	GO:00511	localizatio	-	-	-	-	4:1410665	285	357	260	408	466	428	3.258719	3.25999	3.285652	3.599091	3.431171	3.849967	3.26812	3.626743	0.486516	1	-
ENSMUSG	Cdk14	cyclin-deţ	GO:00081	metabolic	K08821	cyclin-deţ	mmu0520	Transcript	5:4803391	5159	6494	5206	7023	8804	7586	14.30577	14.38149	15.955	15.02446	15.72101	16.54891	14.88075	15.76479	0.693072	1	-
ENSMUSG	Padi2	peptidyl a	GO:00325	developm	-	-	-	-	4:1409063	560	911	544	869	996	944	6.625175	8.607433	7.113038	7.931575	7.587932	8.786021	7.448549	8.101843	0.580274	1	-
ENSMUSG	Kcnab2	potassium	GO:00325	developm	-	-	-	-	4:1523907	11475	13760	12146	16846	19503	17078	59.11591	56.61298	69.1563	66.95435	64.70053	69.21491	61.6284	66.95659	0.513568	1	-
ENSMUSG	Psmc2	proteasor	GO:00325	developm	K03061	26S prote	mmu0516	Epstein-B	5:2178528	1370	1795	1300	1698	2172	1670	15.93022	16.66908	16.70673	15.23244	16.26357	15.27667	16.43534	15.59089	0.395786	1	-
ENSMUSG	Xrcc2	X-ray rep.	GO:00325	developm	K10879	DNA-repa	mmu0344	Homologı	5:2568981	106	117	88	137	175	125	1.034054	0.911526	0.948785	1.031069	1.099338	0.959313	0.964788	1.029907	0.790731	1	-
ENSMUSG	Rpl22	ribosomal	GO:00325	developm	K02891	large subı	mmu0301	Ribosome	4:1523257	966	1188	851	1265	1601	1374	9.285601	9.120005	9.040846	9.381101	9.91012	10.39036	9.148817	9.893861	0.566416	1	-
ENSMUSG	Acot7	acyl-CoA	GO:00081	metabolic	K17360	acyl-coen	mmu0104	Biosynthe:	4:1521781	3682	4640	3650	5284	5837	4986	67.12928	67.56036	73.5475	74.32267	68.52871	71.51406	69.41238	71.45515	0.954631	1	-
ENSMUSG	Prkag2	protein kiı	GO:00081	metabolic	K07200	5'-AMP-a	mmu0453	Tight junc	5:2486274	1948	2347	1898	2633	3154	2637	13.48081	12.97138	14.51677	14.0575	14.05541	14.35649	13.65632	14.15647	0.894205	1	-
ENSMUSG	Rheb	Ras homc	GO:00325	developm	K07208	Ras homo	mmu0415	PI3K-Akt :	5:2480282	1227	1573	1004	1636	1934	1590	8.69525	8.902506	7.863542	8.944399	8.825692	8.864328	8.487099	8.87814	0.822713	1	-
ENSMUSG	Nol9	nucleolar	GO:00081	metabolic	-	-	-	-	4:1520393	375	504	358	478	590	527	2.590426	2.780461	2.733193	2.547406	2.624504	2.863923	2.70136	2.678611	0.771865	1	-
ENSMUSG	Smarcd3	SWI/SNF ı	GO:00325	developm	K11650	SWI/SNF-	mmu0522	Hepatoce	5:2459081	613	857	646	986	1125	1023	5.194093	5.799301	6.049623	6.445493	6.138413	6.819235	5.681005	6.467714	0.295139	1	-
ENSMUSG	Tas1r1	taste rece	GO:00048	signal trar	K04624	taste rece	mmu0474	Taste tran	4:1520279	1	0	2	0	3	5	0.021333	0	0.047144	0	0.041205	0.083888	0.022826	0.041698	0.610519	1	-
ENSMUSG	Abcf2	ATP-bind	GO:00038	catalytic a	-	-	-	-	5:2456534	1701	2148	1663	2261	2615	2323	40.42812	40.77175	43.68358	41.45817	40.02263	43.43495	41.62782	41.63859	0.786642	1	-
ENSMUSG	Nub1	negative ı	GO:00081	metabolic	-	-	-	-	5:2468553	1913	2365	1737	2524	2871	2476	18.86068	18.6217	18.92731	19.19826	18.22763	19.20453	18.80323	18.87681	0.814965	1	-
ENSMUSG	Vamp3	vesicle-as	GO:00325	developm	K13505	vesicle-as	mmu0414	Phagoson	4:1510473	305	454	276	423	554	412	5.672473	6.743322	5.673198	6.069368	6.63494	6.028105	6.029664	6.244137	0.927413	1	-
ENSMUSG	Per3	period cirı	GO:00081	metabolic	K02633	period cirı	mmu0516	Herpes sir	4:1510036	595	723	574	866	1050	974	2.858794	2.774282	3.048069	3.21007	3.248705	3.681593	2.893715	3.380123	0.201225	1	-
ENSMUSG	Tmub1	transmem	GO:00081	metabolic	-	-	-	-	5:2444545	237	312	255	330	391	386	4.086637	4.296533	4.859649	4.389967	4.341596	5.236194	4.414273	4.655919	0.819248	1	-
ENSMUSG	Fastk	Fas-activa	GO:00081	metabolic	-	-	-	-	5:2443844	506	707	495	748	865	771	2.644476	2.950898	2.859174	3.015929	2.911119	3.169964	2.818183	3.032337	0.634448	1	-
ENSMUSG	Ube4b	ubiquitina	GO:00325	developm	K10597	ubiquitin (	mmu0414	Protein pr	4:1493284	2264	2767	1953	2910	3171	2782	12.71131	12.40707	12.1189	12.60484	11.46476	12.28803	12.41243	12.11921	0.579267	1	-
ENSMUSG	Slc4a2	solute car	GO:00325	developm	K13855	solute car	mmu0497	Pancreatic	5:2442383	510	886	475	571	926	745	2.618759	3.633328	2.695663	2.261995	3.061896	3.009485	2.982583	2.777792	0.455449	1	-
ENSMUSG	Park7	Parkinson	GO:00081	metabolic	K05687	protein D.	mmu0501	Parkinson	4:1508971	1426	1740	1067	2031	2047	1800	21.68589	21.1326	17.93367	23.82857	20.04612	21.53482	20.25072	21.80317	0.614795	1	-
ENSMUSG	Tnfrsf9	tumor nec	GO:00325	developm	K05146	tumor nec	mmu0406	Cytokine-	4:1509145	6	14	9	7	8	10	0.085673	0.159655	0.142029	0.077114	0.073564	0.112335	0.129119	0.087671	0.217404	1	-
ENSMUSG	Errfi1	ERBB rece	GO:00325	developm	-	-	-	-	4:1508539	796	962	725	941	1191	1017	7.897665	7.622661	7.950066	7.202877	7.609431	7.93812	7.823464	7.583476	0.582352	1	-
ENSMUSG	Cdk5	cyclin-deţ	GO:00325	developm	K02090	cyclin-deţ	mmu0501	Alzheimer	5:2441824	1204	1466	1119	1682	1893	1655	29.28842	28.48062	30.08475	31.56646	29.65342	31.67222	29.2846	30.96404	0.731108	1	-
[truncated: 2,043,272 more chars]
