# Supplementary material for: Brain Short-Chain Fatty Acids Induce ACSS2 to Ameliorate Depressive-Like Behavior via PPARγ–TPH2 Axis
Source: Research (Wash D C). 2024 Jun 27;7:0400. doi: 10.34133/research.0400 (PMC11210491; doi:10.34133/research.0400)

Supplemental Material – Original Blots

Relevant areas for cropped blots in the main and Extended Data figures are shown with a solid box.

Supplemental Material to Fig 1B (original blots)

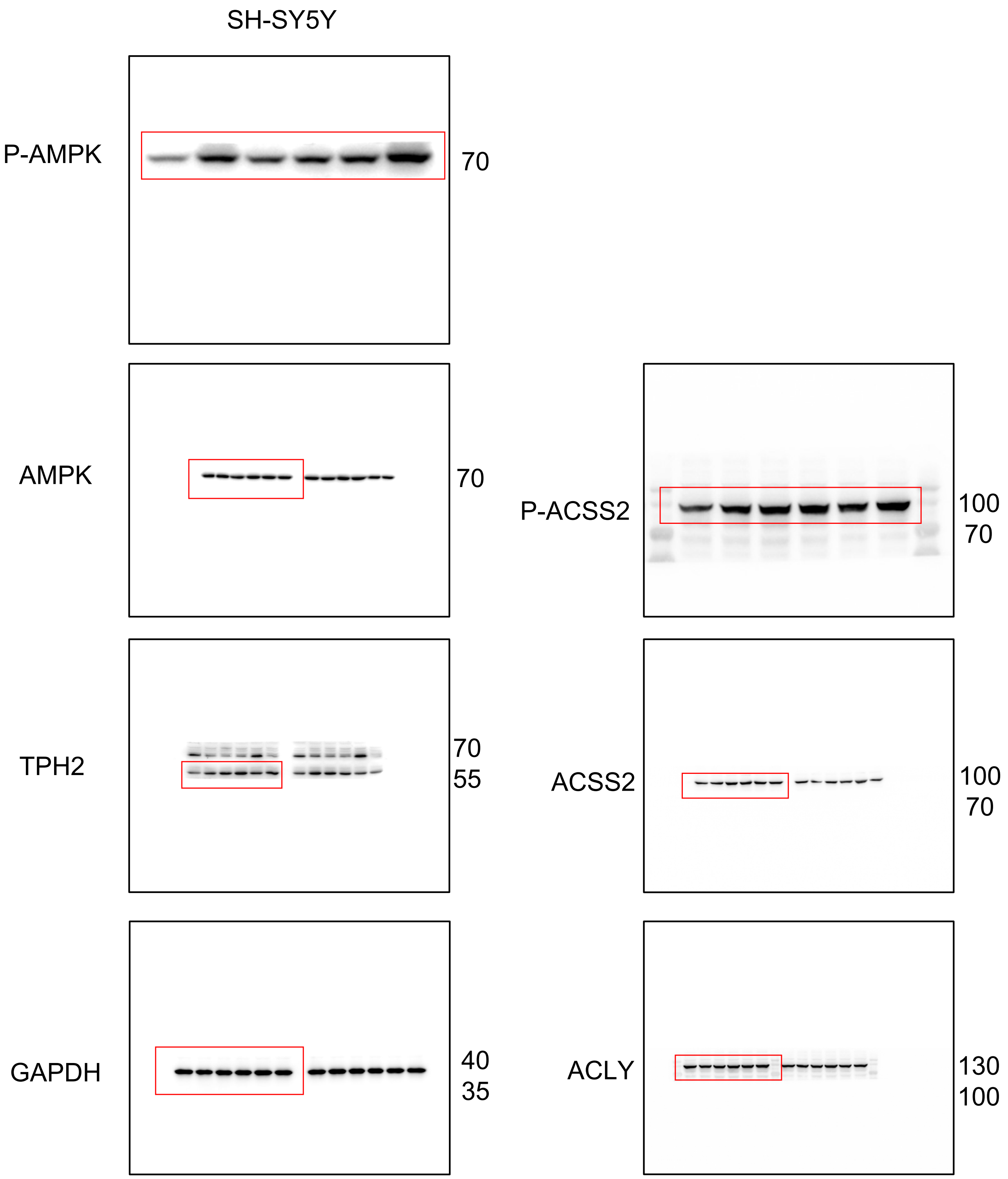

Supplemental Material to Fig 1C and 1H (original blots)

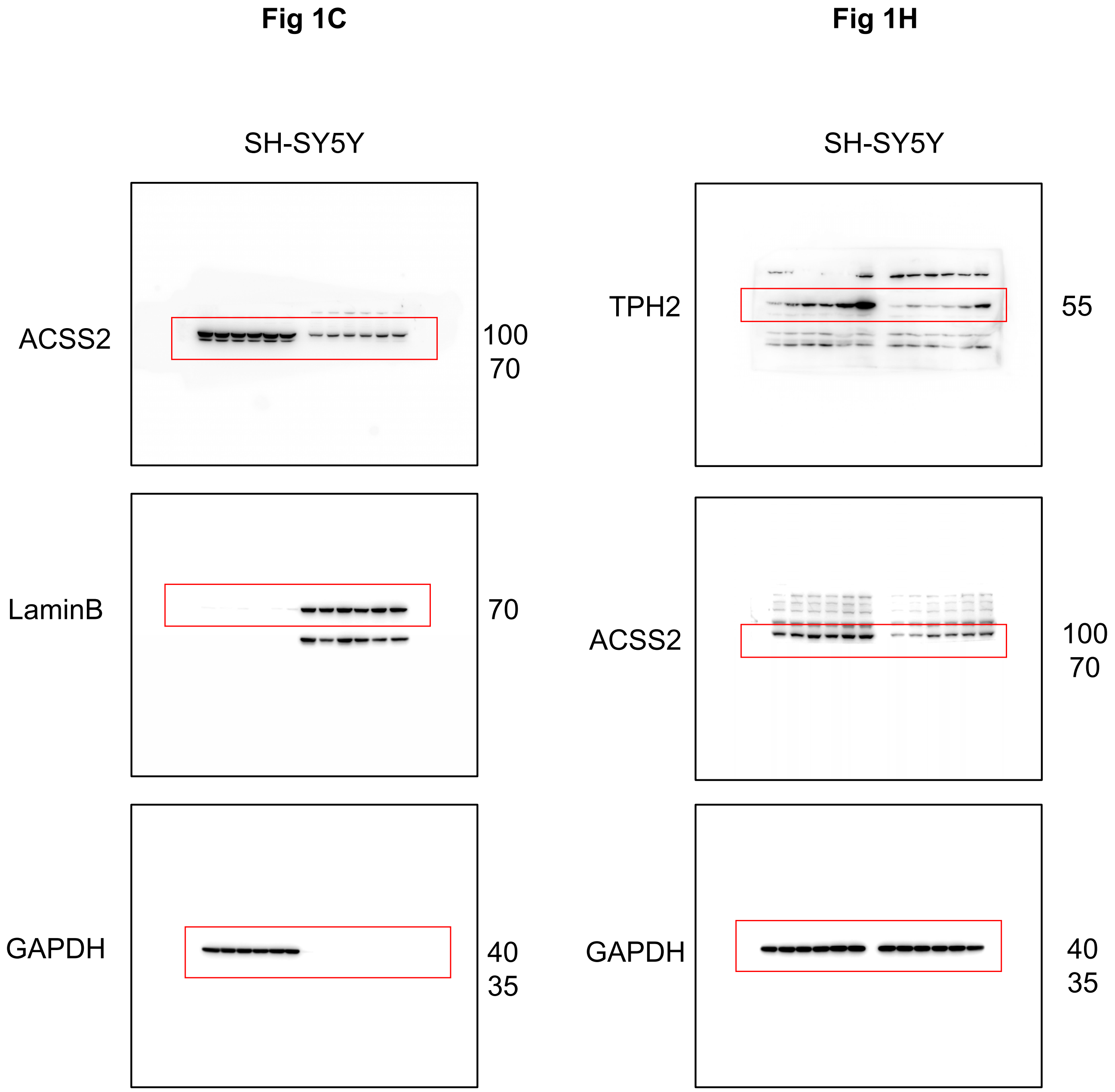

Supplemental Material to Fig 1I and 2I (original blots)

Fig 1I

SH-SY5Y

TPH2

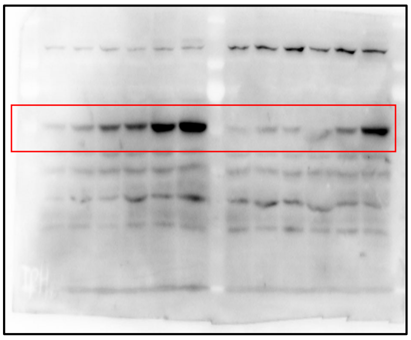

55

ACSS2

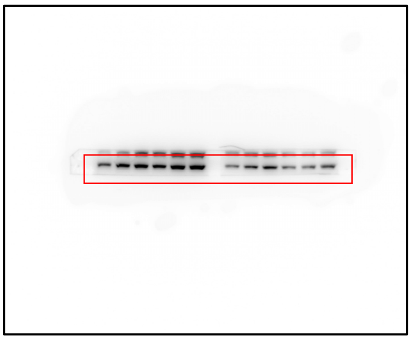

100
70

GAPDH

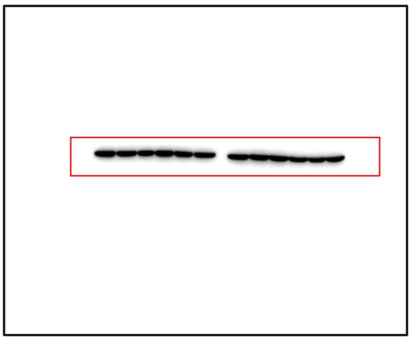

40
35

Fig 2I

Hippocampus

TPH2

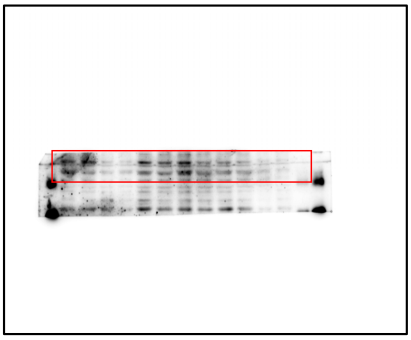

55

ACSS2

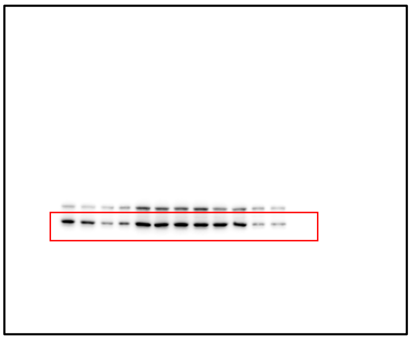

100
70

ACLY

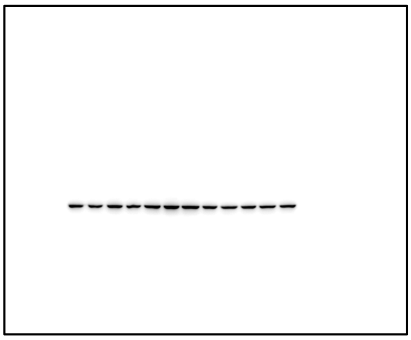

130

GAPDH

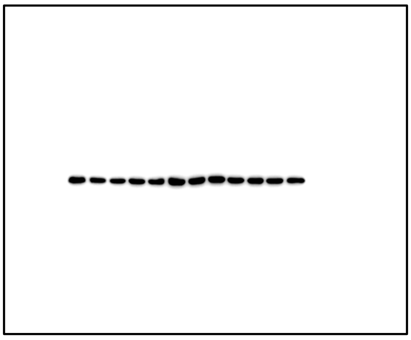

40
35

Supplemental Material to Fig 3A-B (original blots)

Fig 3A

SH-SY5Y

TPH2

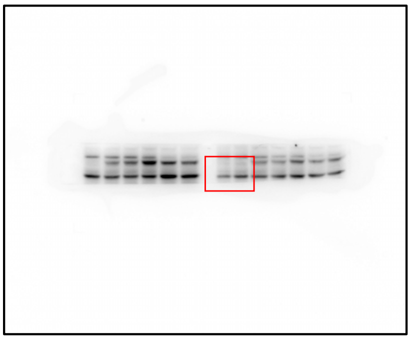

55

PPARγ

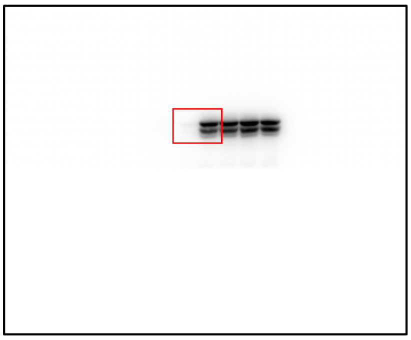

55

P-AMPK

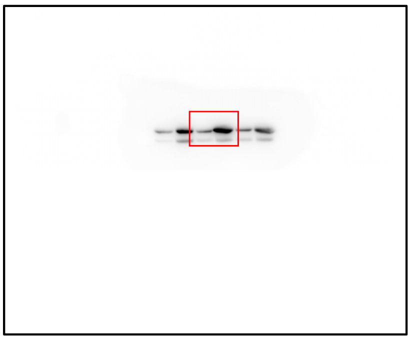

70

AMPK

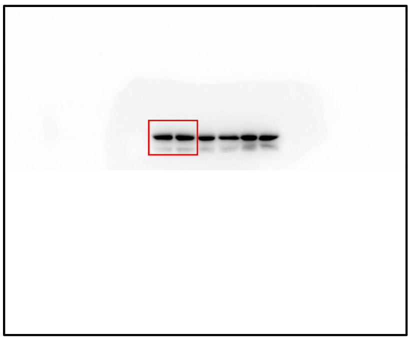

70

GAPDH

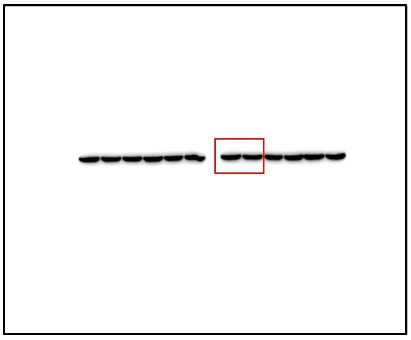

40
35

Fig 3B

SH-SY5Y

TPH2

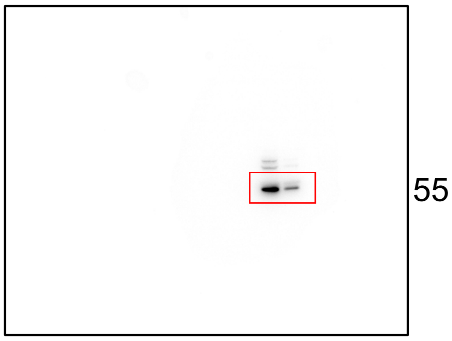

55

PPARγ

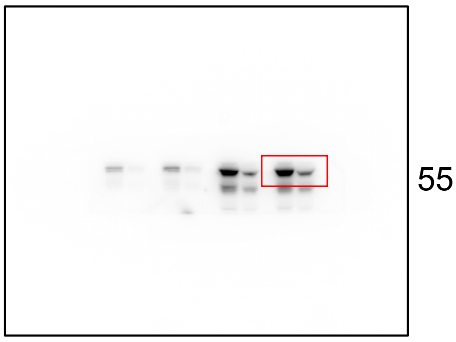

55

GAPDH

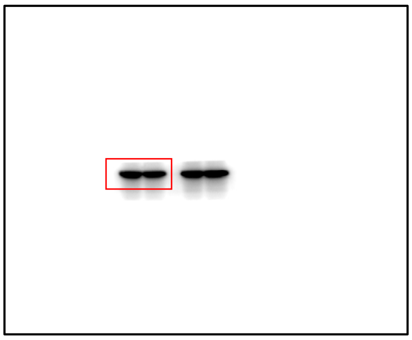

40
35

Supplemental Material to Fig 3C-D and 3H (original blots)

Fig 3C

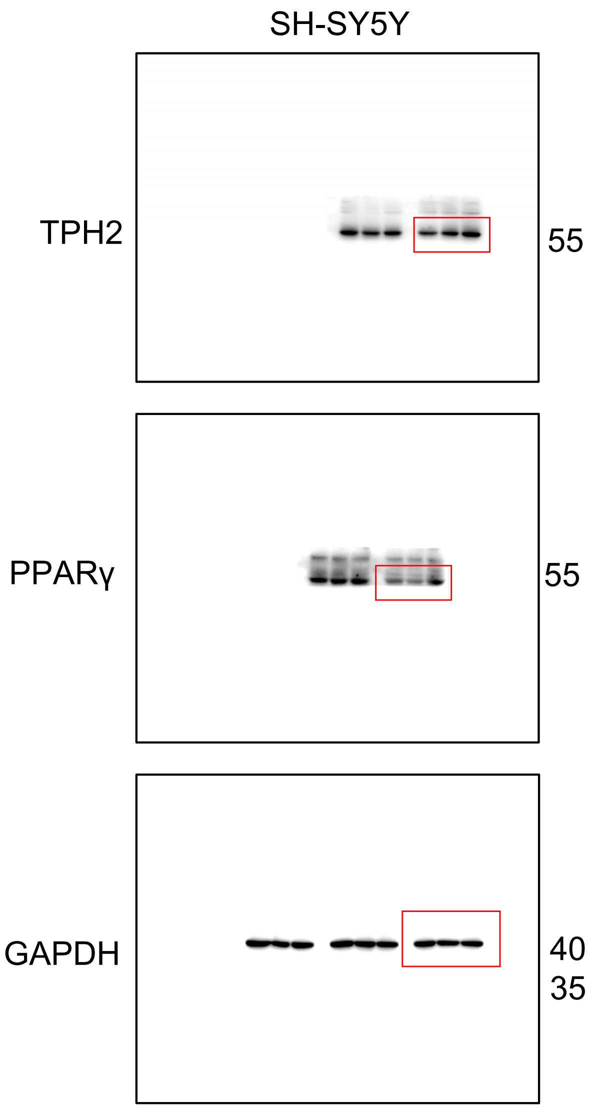

Fig 3D

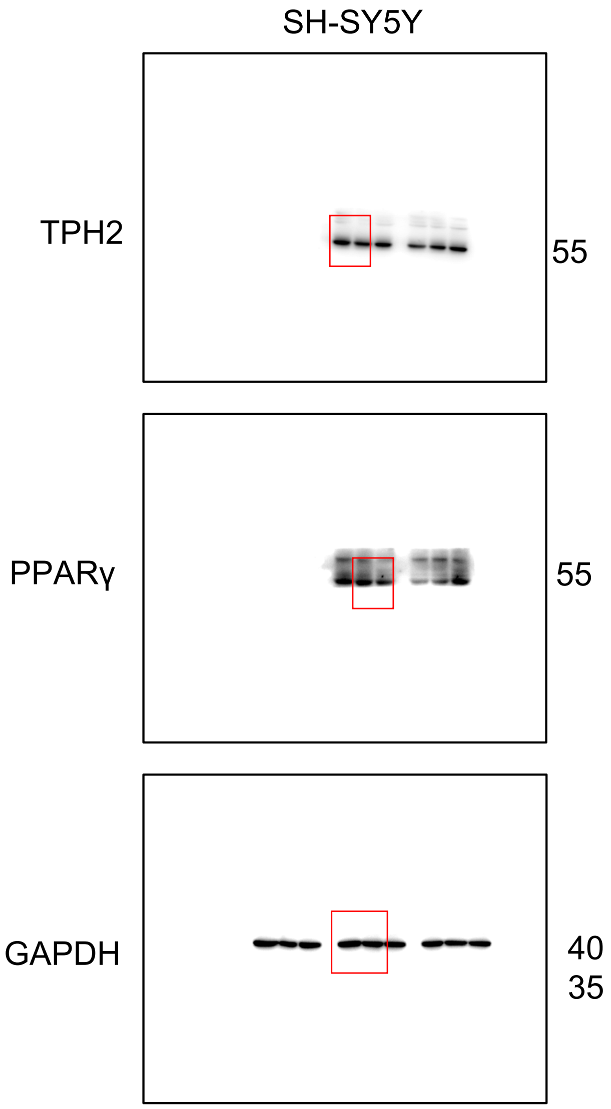

Fig 3H

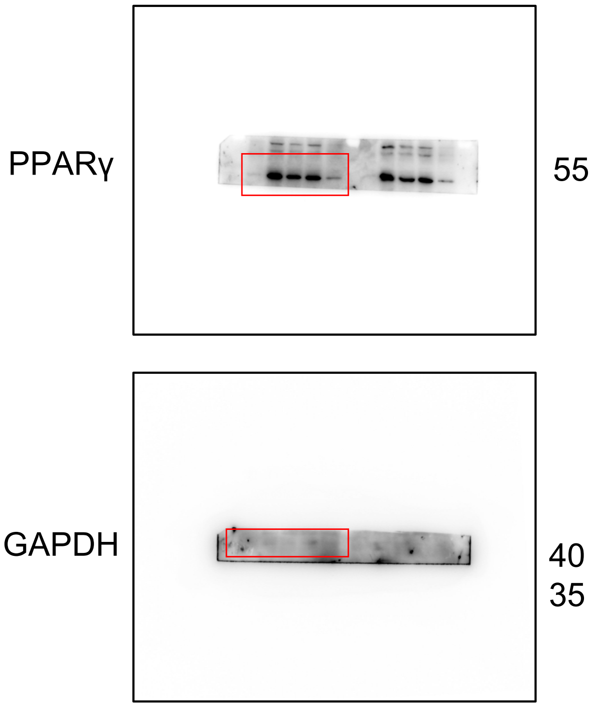

Supplemental Material to Fig 4B (original blots)

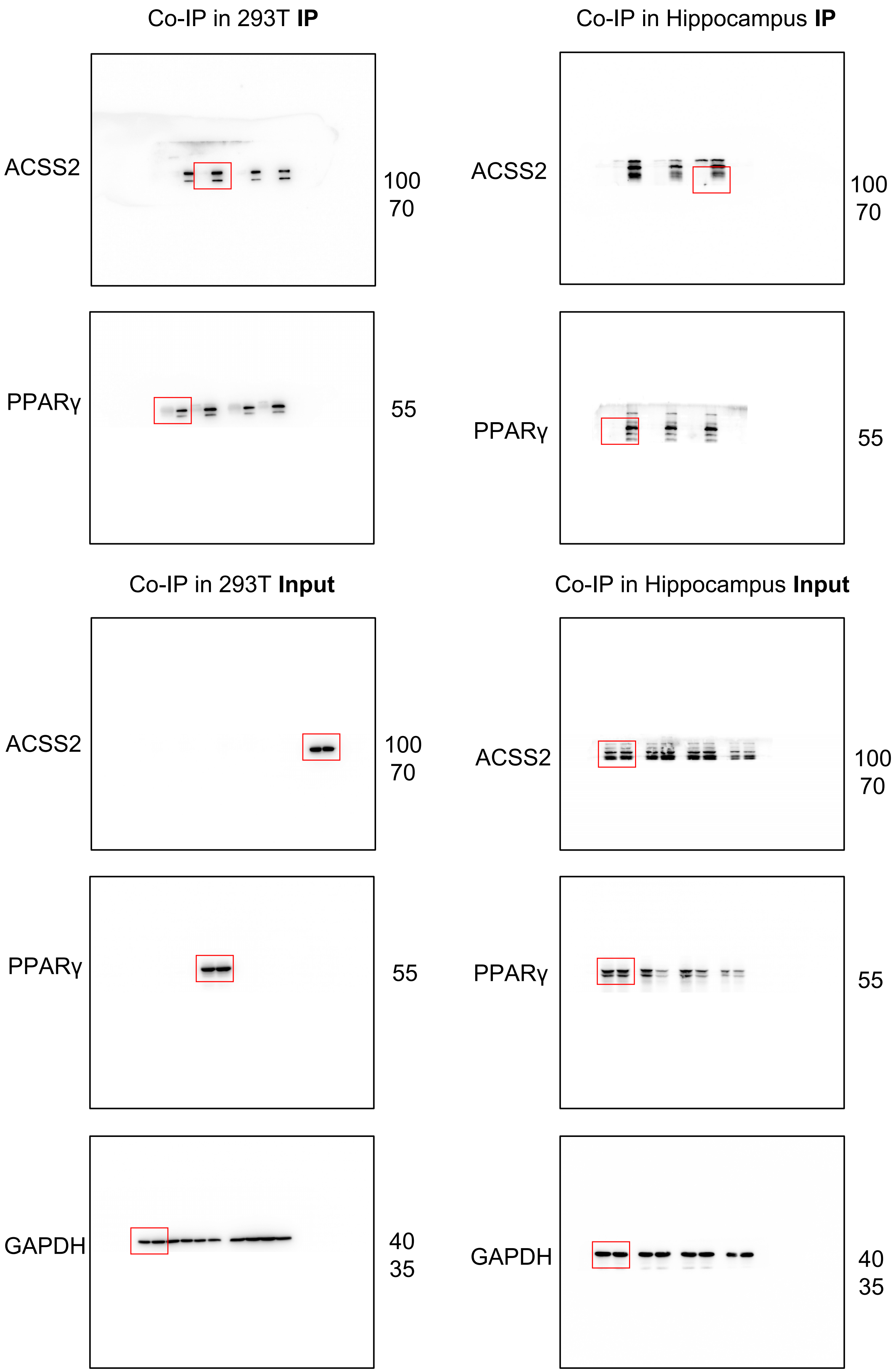

Supplemental Material to Fig 4C (original blots)

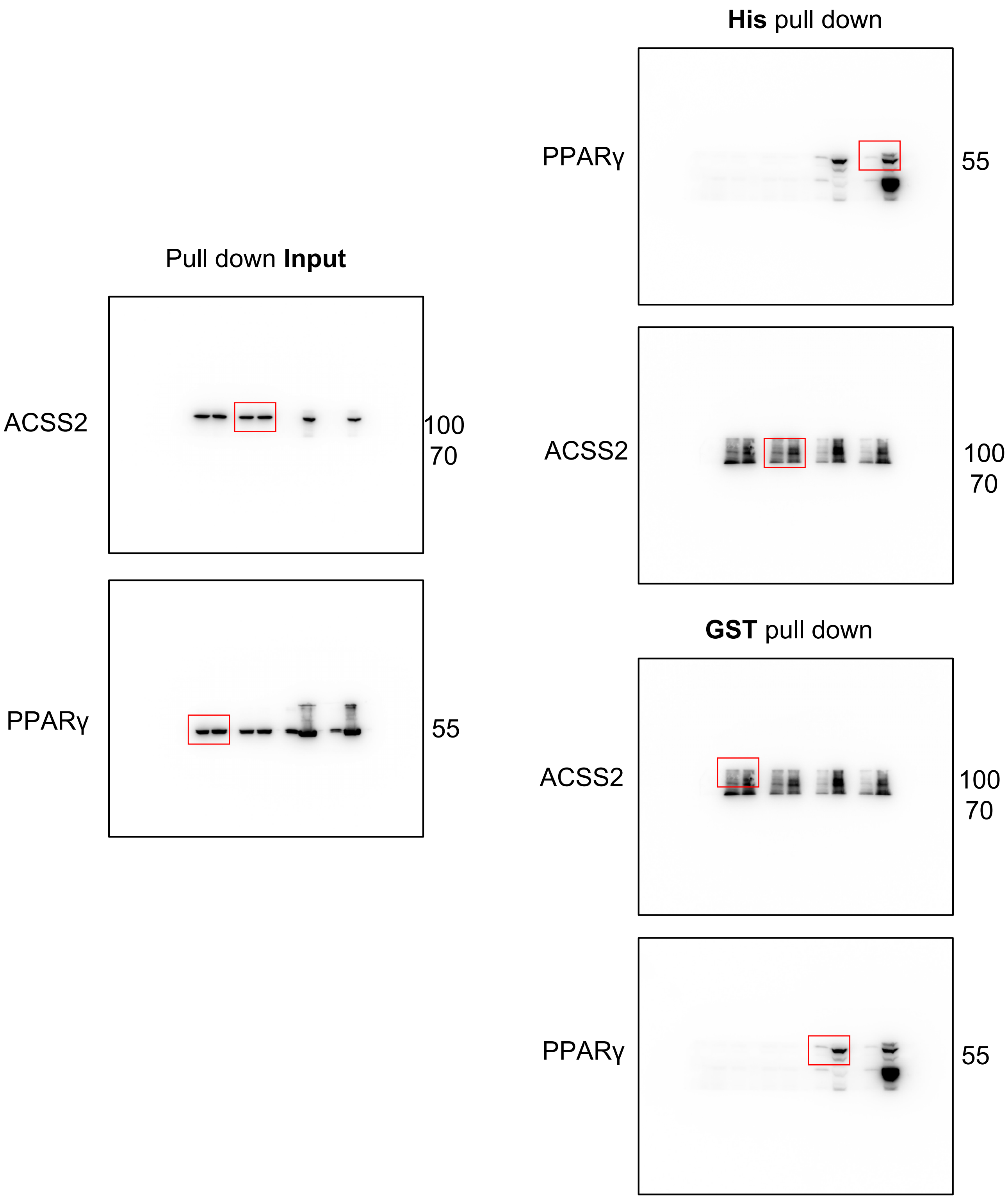

Supplemental Material to Fig 4D and 5B (original blots)

Fig 5B

SH-SY5Y

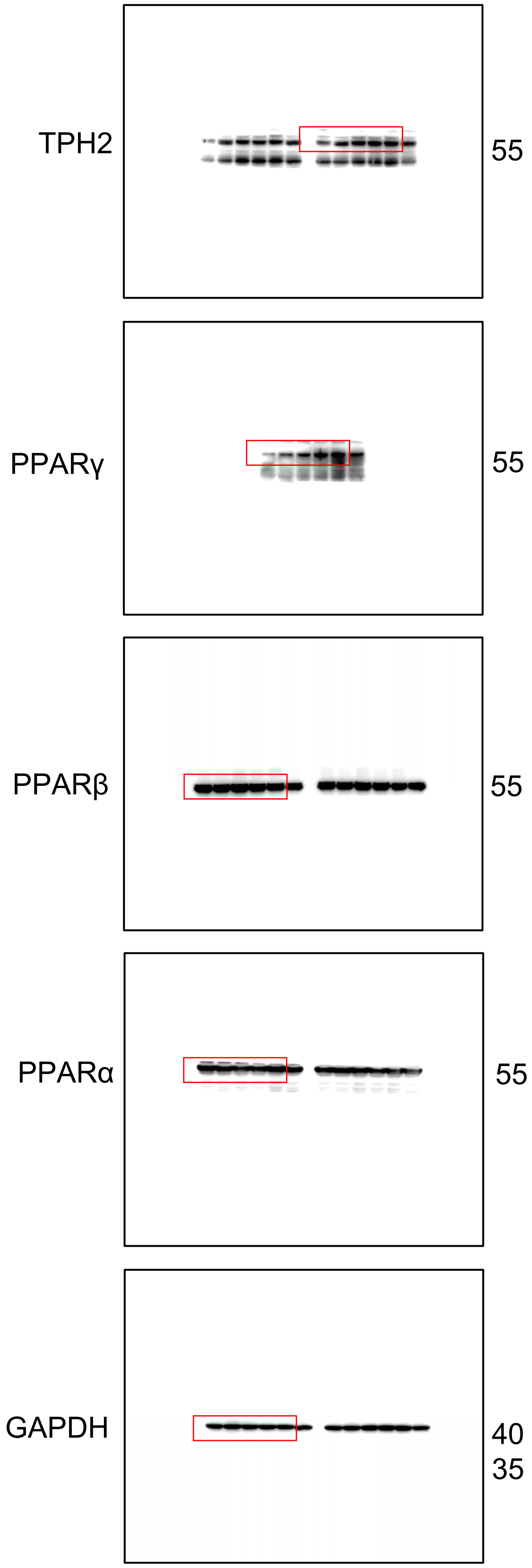

Fig 4D

DNA probe

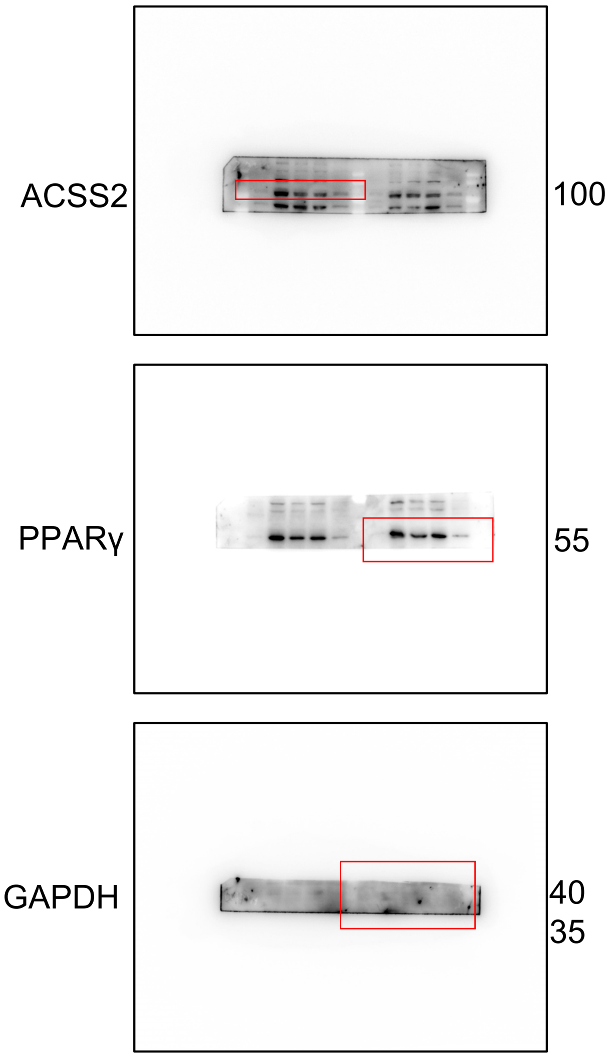

Supplemental Material to Fig 5M, 7J and 7L (original blots)

Fig 5M

Hippocampus

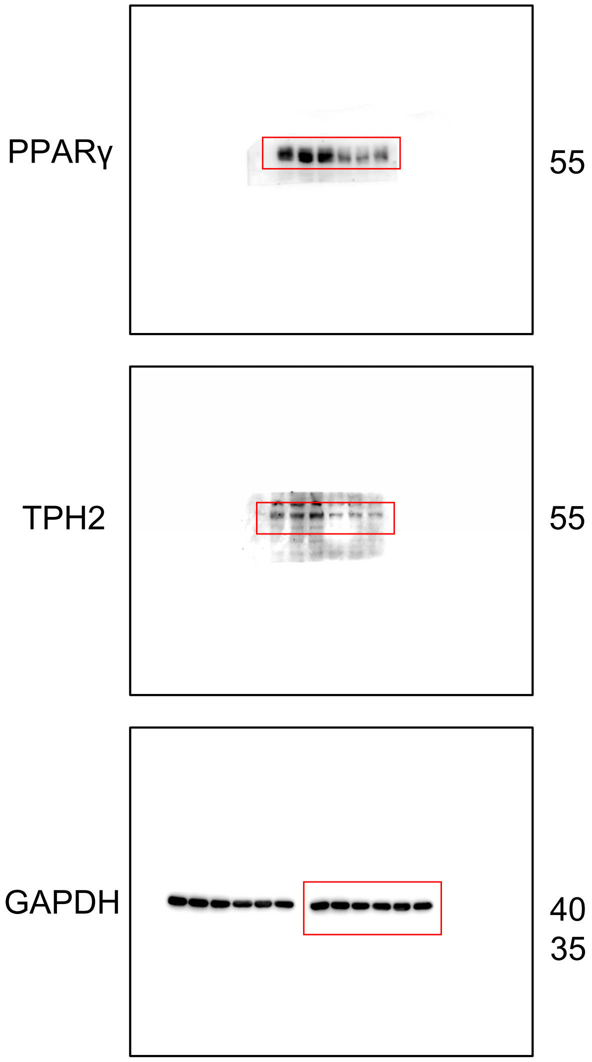

Fig 7L

HEK-293T

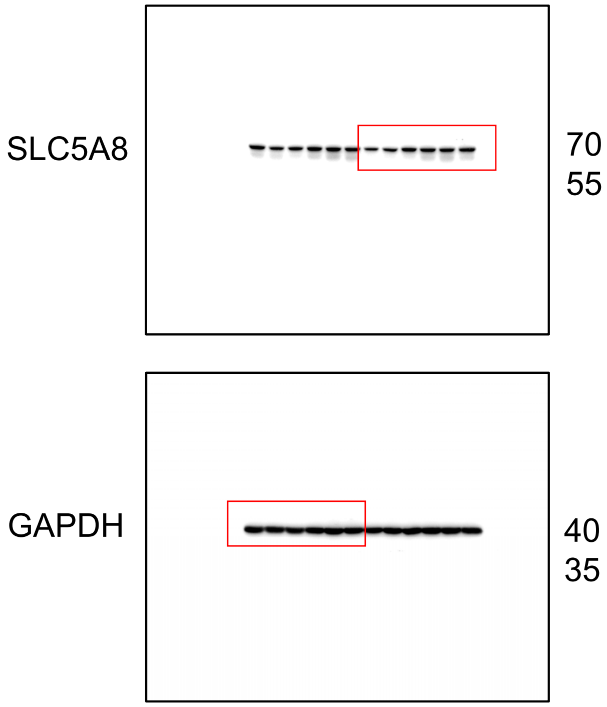

Fig 7J

Hippocampus

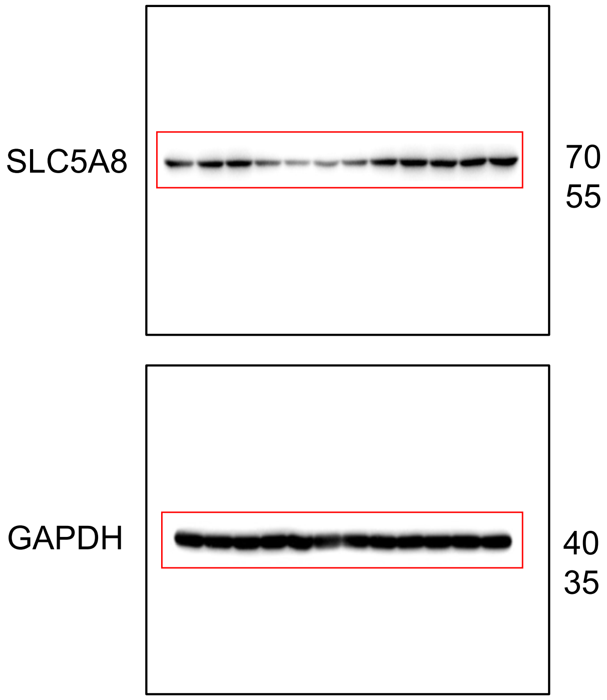

Supplemental Material to Fig 8B-C (original blots)

Fig 8B

Hippocampus

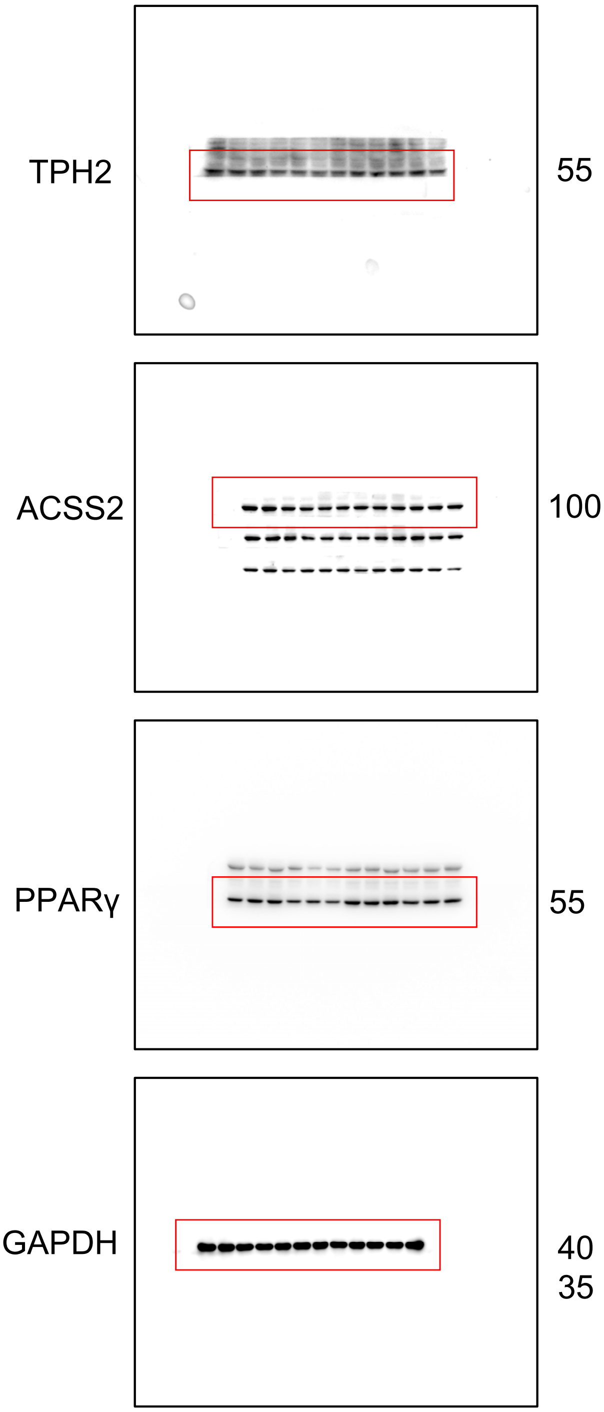

Fig 8C

Hippocampus

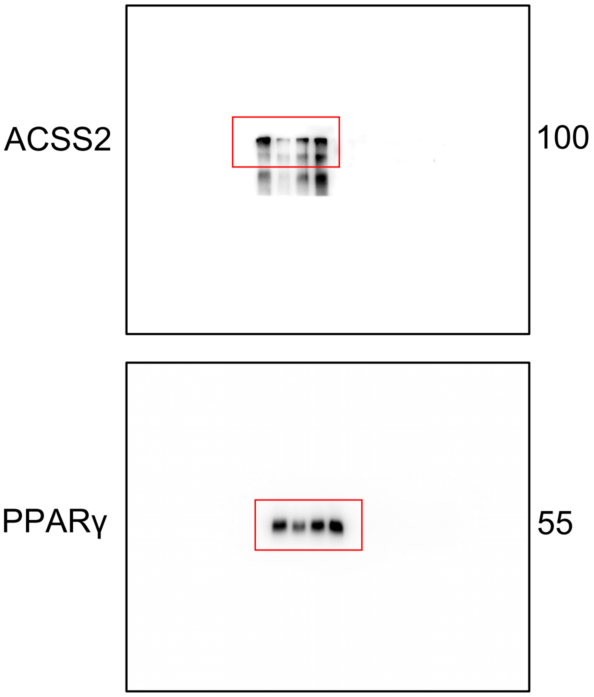

Supplemental Material to Fig S1G (original blots)

Fig S1G

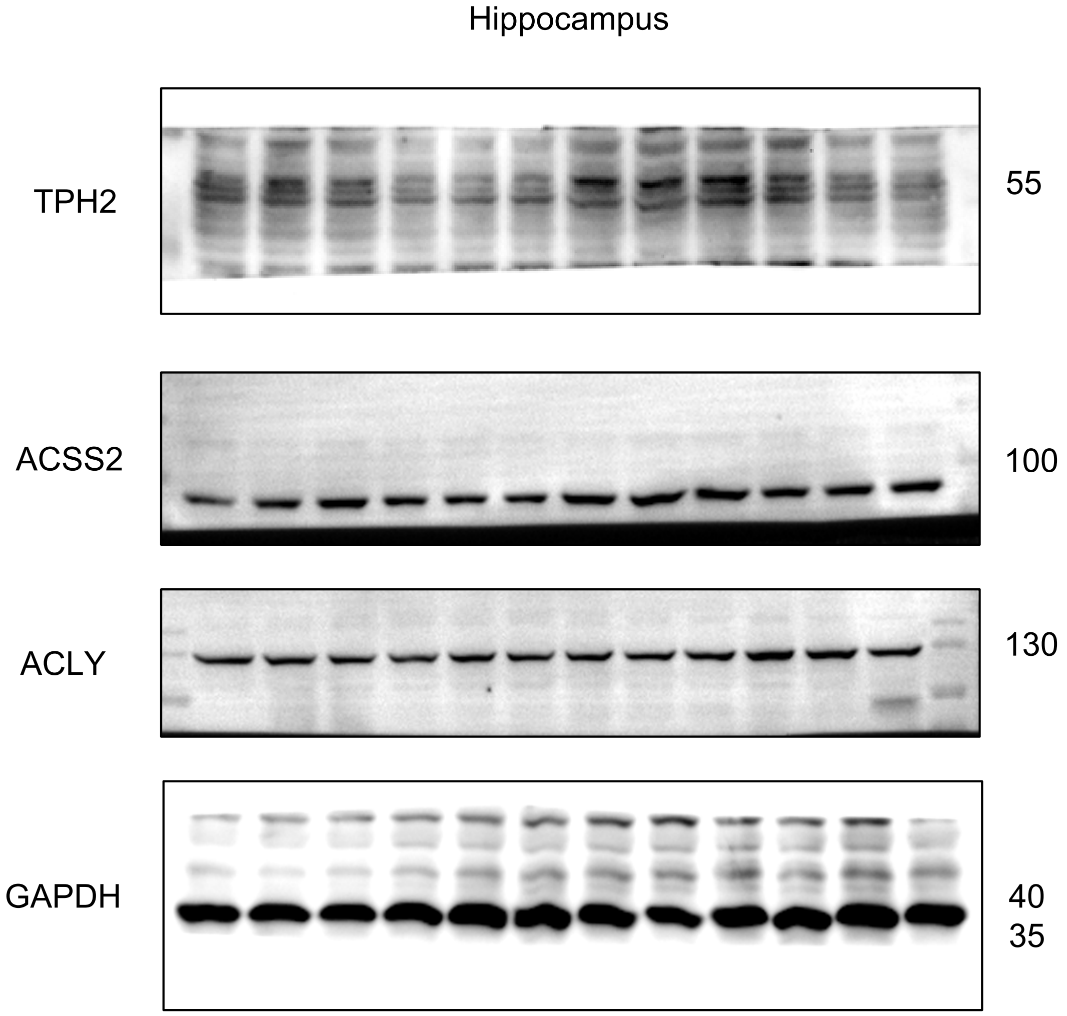

Supplemental Material to Fig S1H-I (original blots)

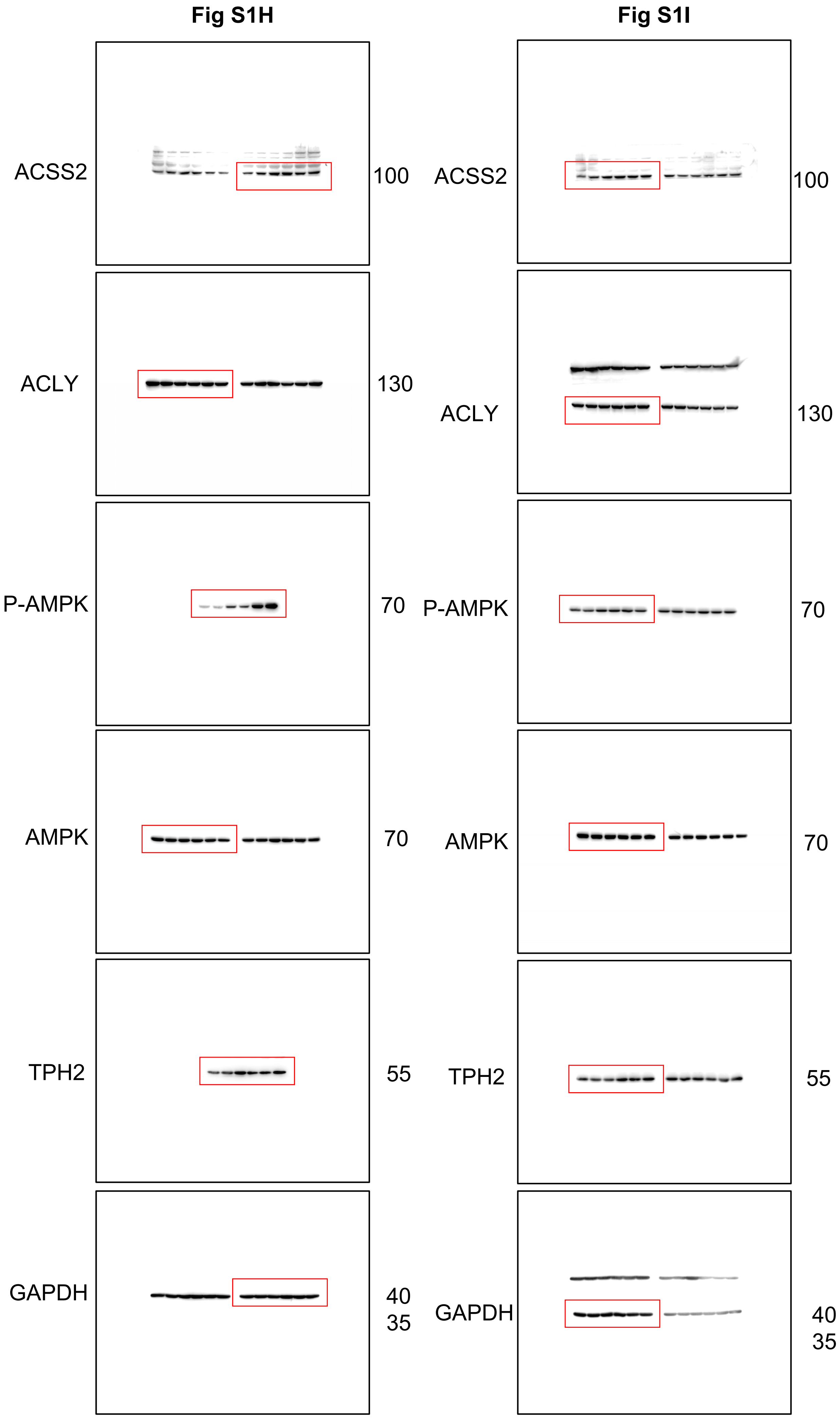

Supplemental Material to Fig S1J (original blots)

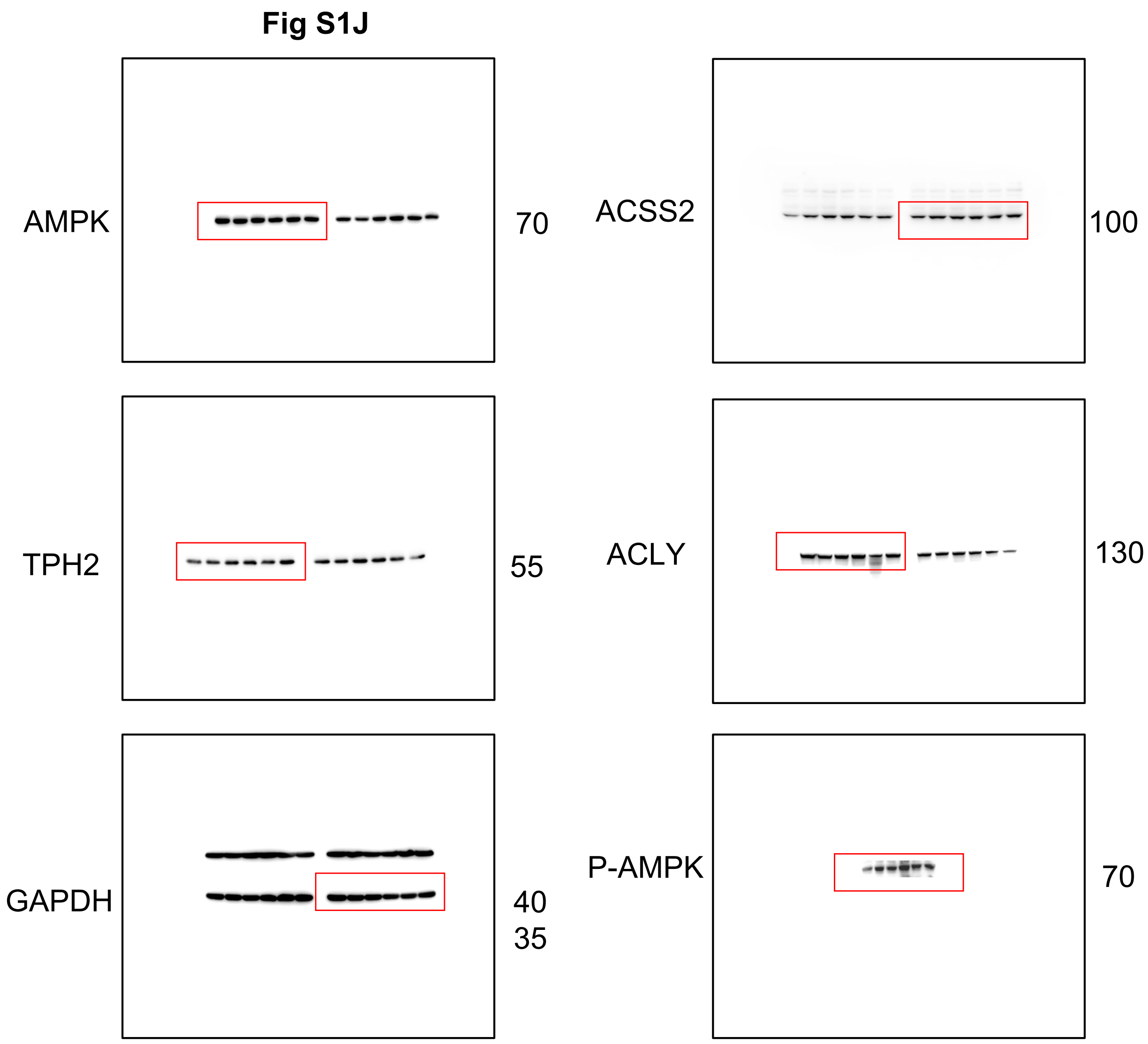

Supplemental Material to Fig S1L-M (original blots)

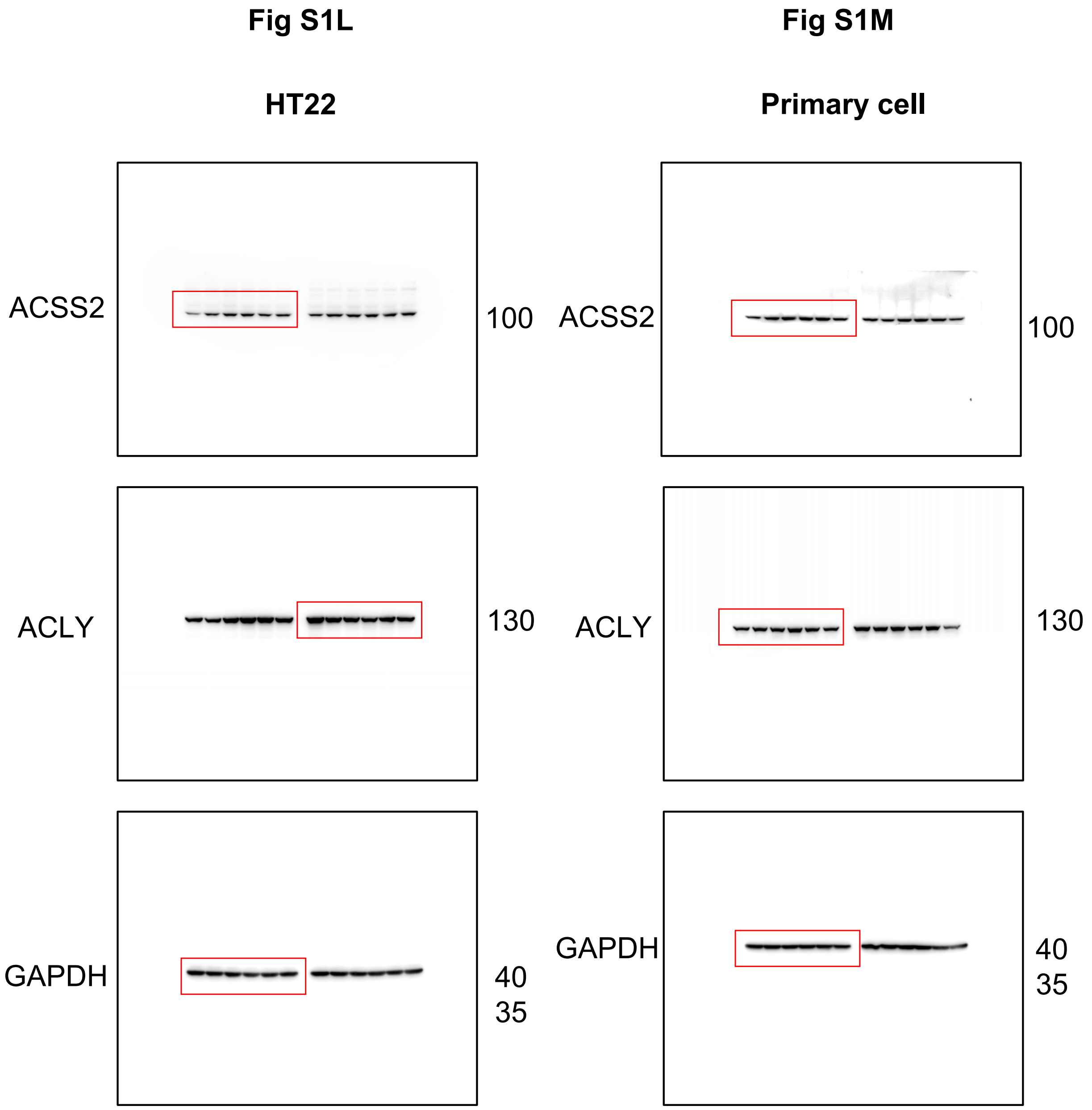

Supplemental Material to Fig S3A (original blots)

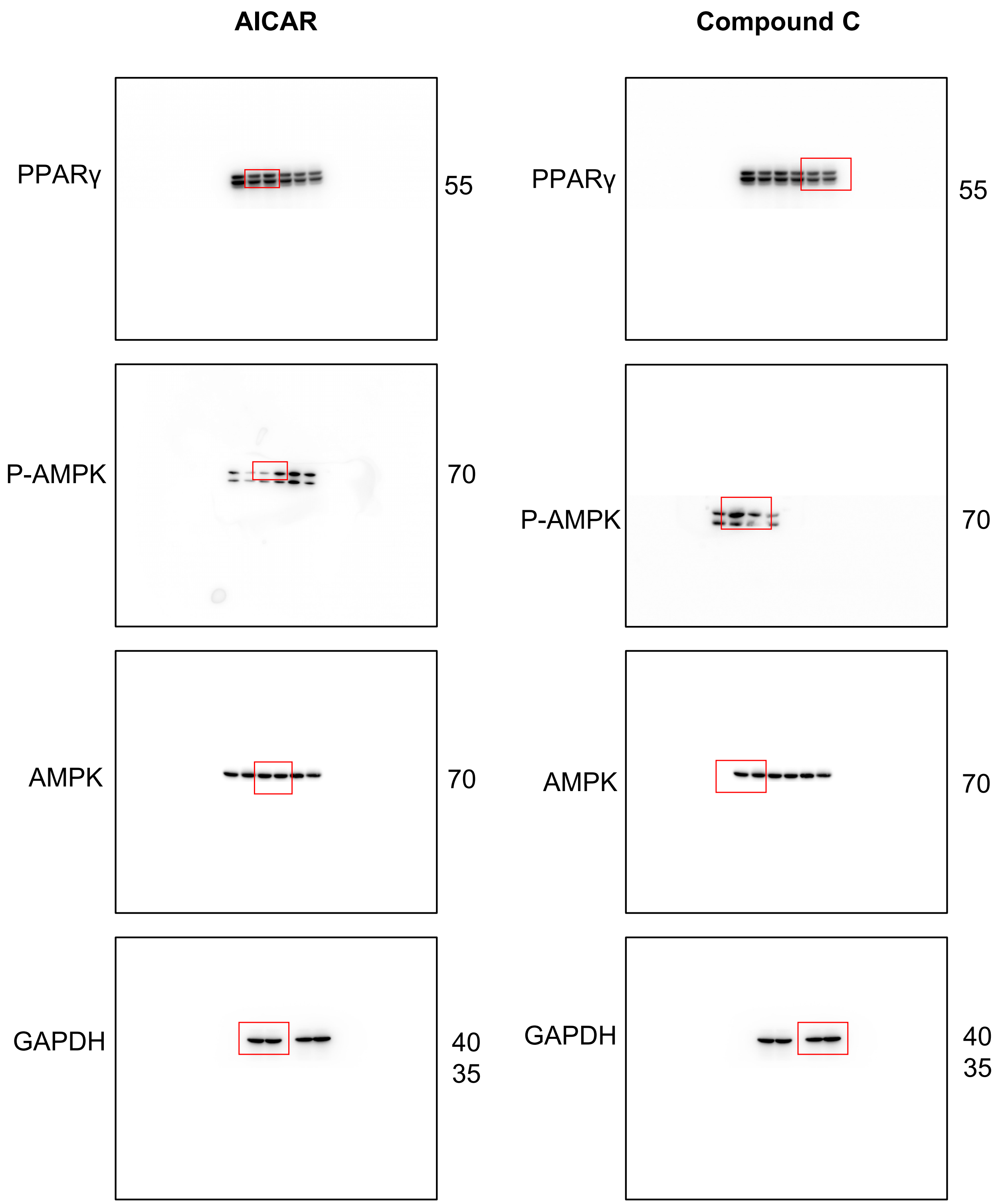

Supplemental Material to Fig S5 B (original blots)

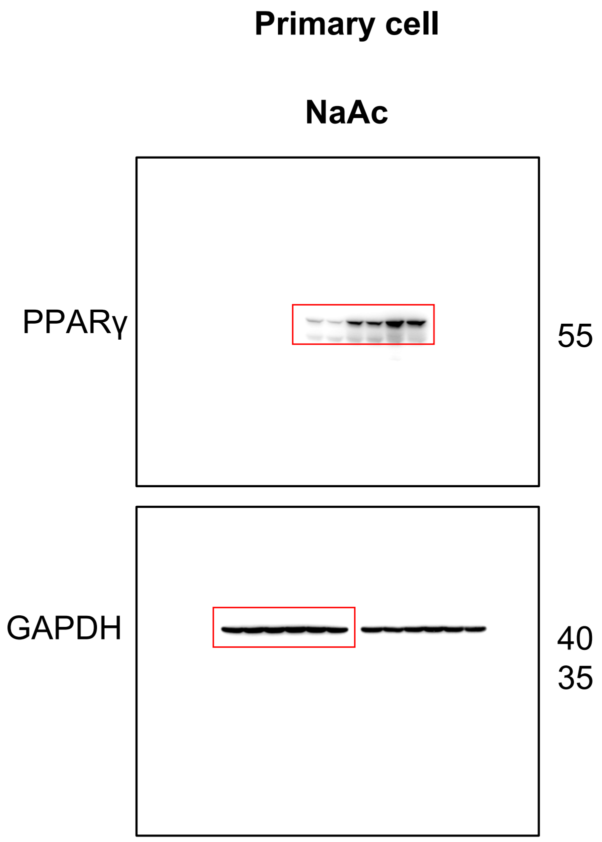

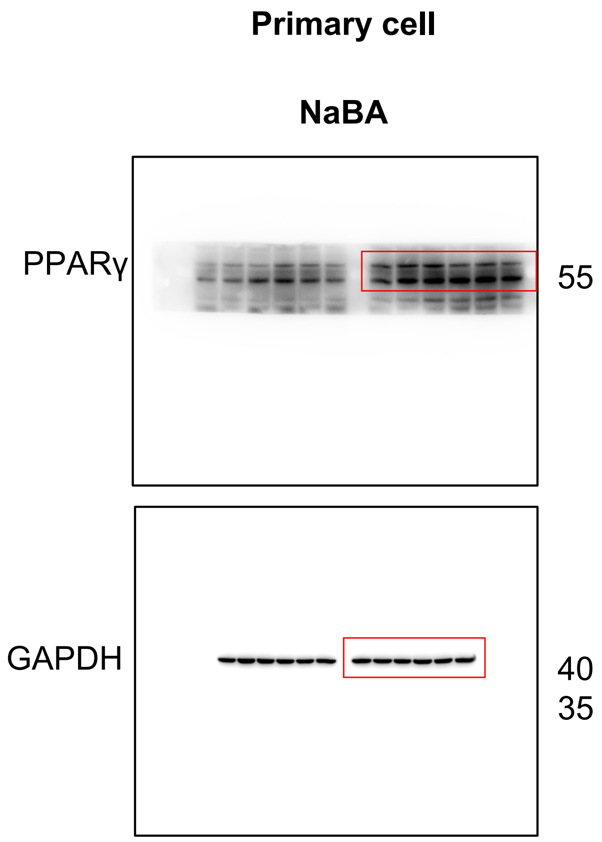

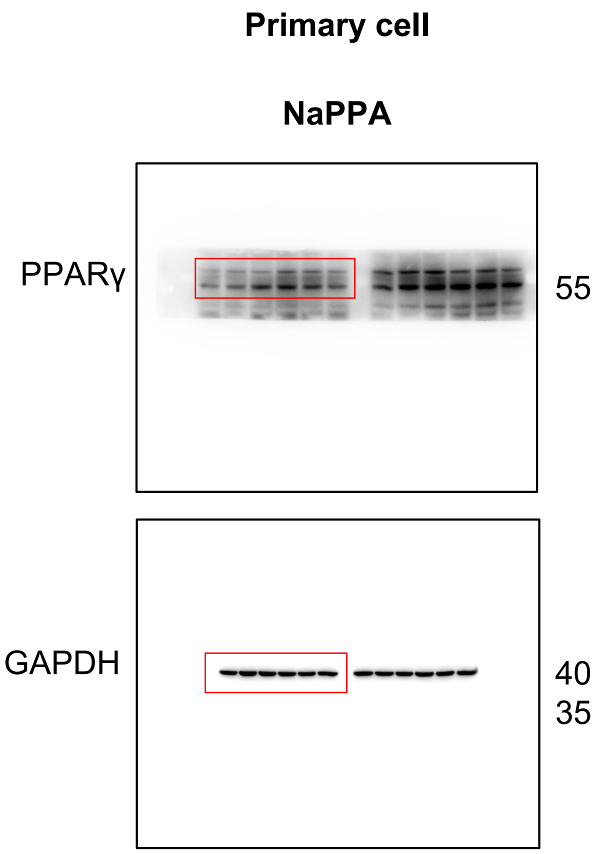

Supplemental Material to Fig S5 C (original blots)

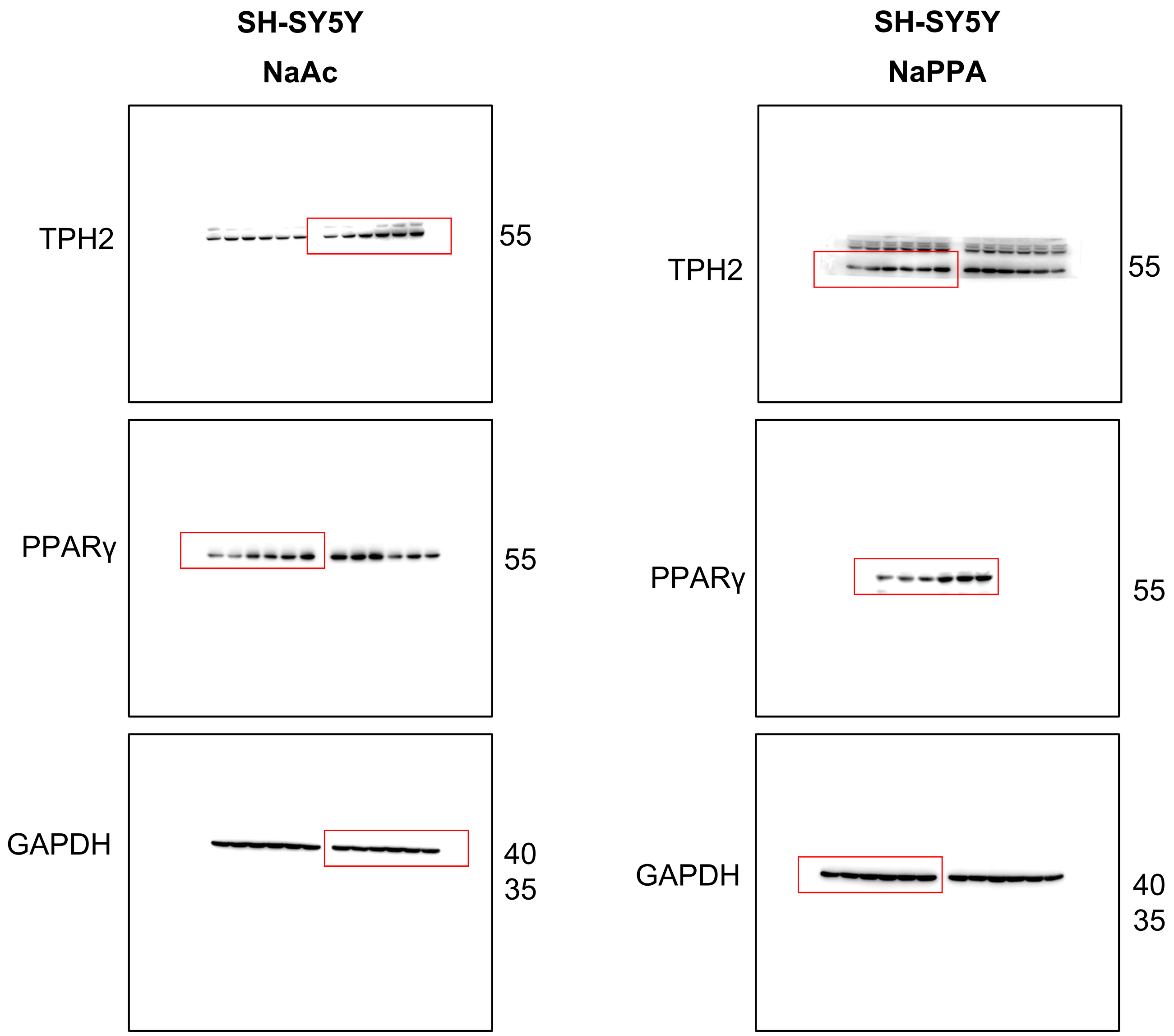

Supplemental Material to Fig S5 C (original blots)

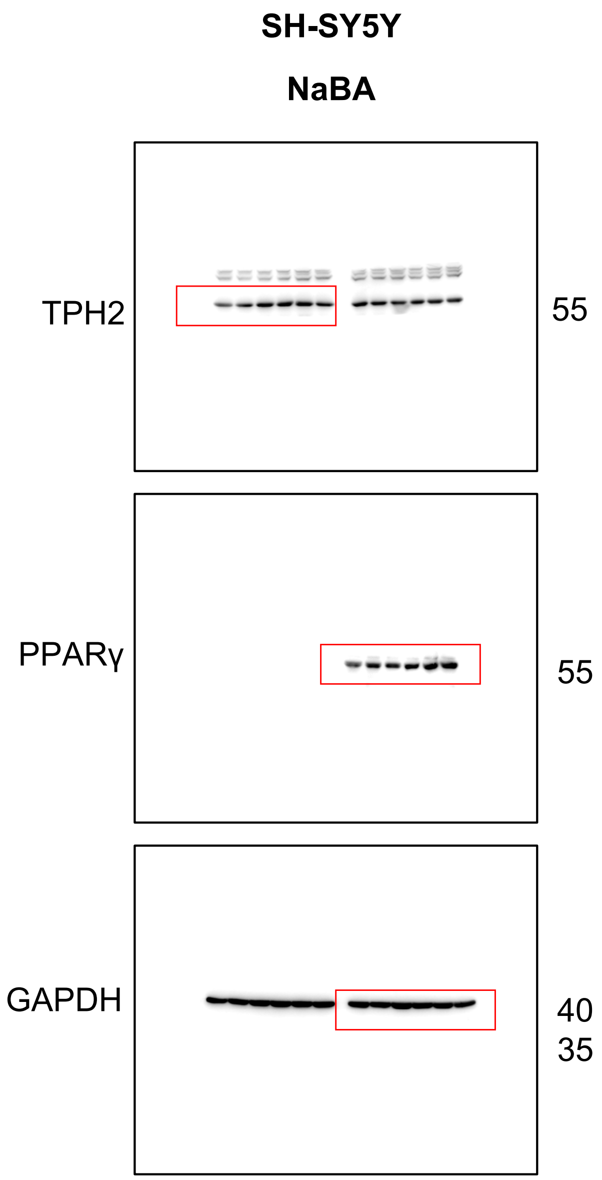

Supplement: Supplementary 1 — Figs. S1 to S6 Tables S1 to S3 Control VS Mannose CRS-Control VS CRS-Mannose RNA sequencing for mannose-treated MG1655 [file research.0400.f1.zip › renamed_4b8e0.pdf]
