# Supplementary material for: Brain Short-Chain Fatty Acids Induce ACSS2 to Ameliorate Depressive-Like Behavior via PPARγ–TPH2 Axis
Source: Research (Wash D C). 2024 Jun 27;7:0400. doi: 10.34133/research.0400 (PMC11210491; doi:10.34133/research.0400)
Supplement: Supplementary 1 — Figs. S1 to S6 Tables S1 to S3 Control VS Mannose CRS-Control VS CRS-Mannose RNA sequencing for mannose-treated MG1655 [file research.0400.f1.zip › Table S1-TPH2 putative binding site in different species.docx]

# Homo sapiens chromosome 12, GRCh38.p13 Primary Assembly

NCBI Reference Sequence: NC_000012.12

1 actttgttat aagttttgat ttgtctaact gcttctcaca ctttgaacca ctctttttca

61 gtctcatcta ccttcaattc cagaggtatt tgagccttta gggtattctg tcatgtaaag

121 tgcgttgctt ctaagtcttc ttcattgata gcttaaggtt cagctttccc atgactgcaa

181 agcctttgat cagttttttc ttctttcaag ctgctaattt tttgttgcca ttgttttcca

241 acttatgcga ctcttgtgta ccttgaaaag gaatttttta tcttgtttta atagggtttc

301 cagggtgagc aaaagtagtt aaatctttaa tattctaaat atatattata aataaattac

361 ttttaatatc tttttttccc tcatttccac atccaagcag ggacactatc tagtggattt

421 tatcccttca catctctggt tctttcatta catctcagta acattagcta ctattattgt

481 cattagttca tttgccttat ttaaacttgc ttctcctatt agctgattag agctgaccct

541 tacagaaaat gtaagccaaa taatattttc agaattagat caattaataa ttatgtctct

601 tcaagactcc agaaacaatt aaacttcagt caatattagt tcatcccgac ttgttttgaa

661 attttttttc tatatagaca agcagtgcca gcaccagatt ttgtttagat tttcagtcaa

721 acaagcacat ttggtcaaga gtttttcatt cttcaggaga ttggtaacat tttcctgtgg

781 cctggtgaat tttatgactt tctaaggttt gaatagtaag aatattgatt aatctatgca

841 tgtcaagttg ctggatttga tctagttgac attgatattt gacagtatgt ttagtcatta

901 aaagctcaaa tgtcatagta ctcttaacct ctgctttctc ctcaccacat aacgcacaga

961 tctcgtatat ttaagtagca ctttttatat gaaagggtca tgtcaaagat taaatttcca

1021 tgatttccag tagagagaaa aaccacaaga gtattaaaat gaatggctga tatatgggaa

1081 tctagtttcc ttttatttct catttagtaa ggaagattcc aggattagta tctgagctat

1141 atgtaatcta catgtggcta aattgaaccc ttacctttcc tttgcaatac attttcctcc

1201 atataactct gcatagaggc atcacaggat taagaagaag cccttttatg aaagccatta

1261 cacatatata cactcacaca tttgcatgca caaaattaga atatgtcaag tcagaaaaag

1321 cttattaaca taaaatggag ttggtcaatg agtaaaaaaa atatgctgat gggagggata

1381 agatctagtg ttcgggagca caataattta ttttcttttg tattttaaaa taactggaag

1441 agtggaattg gaatgtttct aacacaaaaa gaaatgataa atgcttgagg caatggatat

1501 cttgattacc ttatttgatc attacacatt gtacgcttgt gtcaaaatat cacatgtgcc

1561 ttataaatgt gtacaactat tagttatcca taaaaattaa aaattaaaaa atccgtaaaa

1621 tggtttaagc attcagcagt gctgatcttt cttaaattat ttttctaatt ttggaaagaa

1681 agcacaaaat ctttgaattc acaattgctt aaagactgag gttaacttgc cagtggcagg

1741 cttgagagat gagagaacta acgtcagagg atagatggtt tcttgtacaa ataacacccc

1801 cttatgtatt gttctccacc acccccgccc aaaaagctac tcgacctatg aaacaaatca

1861 cactatgagc acagataacc ccaggcttca ggtctgtaat ctgactgtgg ccatcggcaa

1921 ccagaaatga gtttctttct aatcagtctt gcatcagtct ccagtcattc atataaagga

1981 gcccggggat gggaggattc

| **Matrix ID** | **Name** | **Score** | **Relative score** | **Sequence ID** | **Start** | **End** | **Strand** |
| --- | --- | --- | --- | --- | --- | --- | --- |
| [**MA0065.1**](http://jaspar.genereg.net/matrix/MA0065.1) | PPARG::RXRA | 14.449 | 0.833150753439 | NC_000012.12:71936845-71938844 | 899 | 918 | + |
| [**MA0065.2**](http://jaspar.genereg.net/matrix/MA0065.2) | Pparg::Rxra | 9.12914 | 0.819384107715 | NC_000012.12:71936845-71938844 | 1171 | 1185 | - |
| [**MA0065.2**](http://jaspar.genereg.net/matrix/MA0065.2) | Pparg::Rxra | 8.63358 | 0.81131433842 | NC_000012.12:71936845-71938844 | 925 | 939 | - |

# Macaca mulatta isolate AG07107 chromosome 11, Mmul_10, whole genome shotgun sequence

NCBI Reference Sequence: NC_041764.1

1 ttaaggttca gctttcccat gactgcaaag cctttgatca tttttttctt ctttcaagct

61 gctaattttt tgttgctgtt gttttccaat ttatgcattt cttatgtacc ttgaaaagga

121 atttttatgt tgttttaata gggtttctag ggtgagaaaa ggtagttaaa tctttaatat

181 tgtaaataca tattttaaat aaattacttt taatatcttt tttccctcgc tttcacatcc

241 aagcagggac actgtctagt ggattttatc ccttcacatc tctggttttt tcattacatc

301 tcagtaacat tagctattat tgtcattact tcattcatct tatttaaact tgcttctcct

361 attagctgat tagagctgac ctttacagaa tatgtaaacc aaataatatt ttcagaatta

421 gatcaattaa taattatatc ccttcaagat tccagaaaca attaaacttc agtcaatatt

481 agttcatccc aacttgtttt gaaacccctt ttttatatat atatatatag gcaagcagtg

541 ccagcaccag attttgttta gatttttagt gaaacaagca catttggcca agagtttttc

601 attcttcagg agattggtaa cattttcctg tggccttgtg aattttatga ctttctaagg

661 tttgaatagt aagaattgat taatctatgc atgtcaagtt gctggatttg atctagttga

721 cactgatgtt tgacagtata tgtagtcatt aaaagctcag atgtcttagt actcttaacc

781 tctgcctcct cctcaccaca taacacacag atctcatata tttaagtagc agtttttata

841 tgaaagggtt atgtcaatga ttaaatttcc atgattttca gtggagagaa aaaattcagt

901 aaccacaaga gtgttaaaat gaatggctga tatatgggaa tctagtctcc ttttatttct

961 catttagtaa ggaagattcc agggttagta tctgagctat acgtaaactc cctgtggcta

1021 aatcaaaccc ttacattttc tttgcaatac attttcctcc atataactct acatagagga

1081 atcacaggat taagaagaag tccttctatg aaagccatta cacatatgta cactcacatg

1141 tttgcatgca caaaattaca gtatgtcagg tcagaaaaag cttattaaca taaaatggag

1201 ttggtcaatg agtaaaaaaa taggctgata ggaggaataa gatctagtat tcaggagcac

1261 aataatttat tttcttttgt atgttaaaat aactgaaaga gtggaattgg aatgtttcta

1321 tcacaaagaa atgataaatg cttgaggcaa tggatatctt gattacctta tttgatcatt

1381 acatattgca cacttttatc aaaatatcac atgtacctta taagtatgca caactattcg

1441 ttatccataa aaattaaaaa ttaaaaaatc cataaaatgg tttaaggatt cagcagtgct

1501 gatctttcgt aaattatttt tctaattttg gaaagaaagc acaaaatctt tgaattcaca

1561 gttgcttaaa gactgaggtt aacttgctag cggcaggctt gagagatgag agagctaacg

1621 tcaggggata gatggtttct tgtacaaata acaccccctt atgtattgtt ctccaccacc

1681 cccgcccaga aagctactcg acctatgaaa caaatcacat catgagcaca gataacccca

1741 ggcttcaggt ctgtaatctg actgtggcca tcggcaacca gaaatgagtt tctttctaat

1801 cagccttcca tcagtctcca gtcattcata taaaggagcc cggggatggg aggattctca

1861 ttgctcttca gcaccagtgt tctggacagc gccccaagca ggcagctgat cgcacgcccc

1921 ttcctctcaa tctccgccag cgctgctact gcccctctag tcccccctgc tgcagagaaa

1981 gaatattaca ctgggatcca

| **Matrix ID** | **Name** | **Score** | **Relative score** | **Sequence ID** | **Start** | **End** | **Strand** |
| --- | --- | --- | --- | --- | --- | --- | --- |
| [**MA0065.2**](http://jaspar.genereg.net/matrix/MA0065.2) | Pparg::Rxra | 10.7705 | 0.846111811648 | NC_041764.1:71508031-71510030 | 777 | 791 | - |

# Sus scrofa isolate TJ Tabasco breed Duroc chromosome 5, Sscrofa11.1, whole genome shotgun sequence

NCBI Reference Sequence: NC_010447.5

1 attatgttct ggcttttgaa aggtgaaata taaatagatt tcataattta tccaaaaagg

61 aagtaatatt cagttccatc aactcctgtt gattcttttt tttttttctt tttcttttta

121 gggccatacc tacagcatgt ggaagttcct tggctagggg tctaattgga gctttagttg

181 ccggcctata ccacagccac agctatgctg agacctacac cacagctcat ggcaatgcca

241 gatccttaac ccactgaaca aggccaggga tcgaacctgc agcctcatgg atactagtca

301 gctgagacaa gatggaaact ccccaactcc ttgttgattc ttatgttgac attccttttt

361 ctgtttataa ctgccaccac tgtagttcaa actttttgtt aattttcttc cctctaacag

421 gttgtcctgc ctagactaga tcctactgag acattttcat taggctatag tctcaataca

481 aaataataaa tgcttccttt ttaaatacag attttttatc ctggcattca aatttccaaa

541 taatcagtcc ttcattttat ttttcaagtt taattattgt ctttgaaaaa cttaccttca

601 cttggctttc ctttacctac ctatctgaat ttttttcttt cttattttcc ttttcctttt

661 tgtttttgtt tttcctttta ttttaaactg tttaagtttg gtgttcctca gtgttctgtt

721 ctgtttgttt gtttgttttt gtttttgttc catagcatat ggtcttccta ggatctaagc

781 tgcattttga gacctatgct gcagctgtgg tgatgctgaa tgctcaaccc tctgggctgg

841 gccagggatc aaacctacgt cccagtcctc cagagatgct gctgatcctg tcgcaccaga

901 gtgggaactc ccccagggtt ccatttttga ctgtcttctc aaacaccata ttctttgtga

961 atgaagctat attcacatag attcagttat ttgatgattc ctaaatttct atatccagct

1021 taggtctcat ttcaggttta aaatctatat actcaaatcc ttattggaga gcttcacttg

1081 acattcacat gcaatatgta taagatctaa cttttaagag tttttaaaaa ttgctaatat

1141 ttatttatgt gtggtctagt tcaatgaatg acgctaattt acccagtccc ctaagtgaga

1201 aacttgggag ttaattctgg caccttttta tctgcttcca catccaagta gagatgttgt

1261 tctatggatt tcacccctta acatcttttg ttctttttat tacctgcctg atcttttgcc

1321 ataatccaac tcctcacctc ctggtgagac tatttcaagt cctaagtggt ttatgccttc

1381 agtcttaaag tccccctcca agctgctacc tttctaaaat accaatatgt ttatgctatc

1441 gccttgcata aagcccttcc aattgatctc tttcctctac aggataaagt gataaaggga

1501 aaagcccact gaatgccatt caaggtttat caagatttgg accttgcttt tgctttcagc

1561 cttatctttc actggtgccc ttaccgcaaa ttcatactcc atccttgttg agcaacaaca

1621 gttctctata tatttccttt tttttttctt ttttggctgc acctgcaata tgtggaggtt

1681 cccgggccag ggatcaaacc catgccacag ttgtggcctg taccacagct gcagcaatac

1741 catatcctta tgctgtgcca caagggagct tcctctgtat attttcttat attgttcttt

1801 ctcccaggaa ttatctccca ttccattaca attaaaccca aattcacttt ttttttcttt

1861 tttgttttgt tttgaagggc cgcacccaca gcatatggaa gttcccaggc tacgggtcga

1921 atcagggctg cagctgctgg cctatgccat agccacagca acacccagtc cttaacccag

1981 tgagcaaggc cagggattga

| **Matrix ID** | **Name** | **Score** | **Relative score** | **Sequence ID** | **Start** | **End** | **Strand** |
| --- | --- | --- | --- | --- | --- | --- | --- |
| [**MA0065.2**](http://jaspar.genereg.net/matrix/MA0065.2) | Pparg::Rxra | 11.4554 | 0.857265107765 | NC_010447.5:35915159-35917158 | 837 | 851 | + |
| [**MA0065.2**](http://jaspar.genereg.net/matrix/MA0065.2) | Pparg::Rxra | 11.043 | 0.850549773219 | NC_010447.5:35915159-35917158 | 1938 | 1952 | - |
| [**MA0065.2**](http://jaspar.genereg.net/matrix/MA0065.2) | Pparg::Rxra | 10.0122 | 0.833764224462 | NC_010447.5:35915159-35917158 | 1682 | 1696 | + |
| [**MA0065.2**](http://jaspar.genereg.net/matrix/MA0065.2) | Pparg::Rxra | 9.24331 | 0.821243231114 | NC_010447.5:35915159-35917158 | 1793 | 1807 | - |
| [**MA0065.2**](http://jaspar.genereg.net/matrix/MA0065.2) | Pparg::Rxra | 8.90983 | 0.815812804237 | NC_010447.5:35915159-35917158 | 1714 | 1728 | - |

**Mus musculus strain C57BL/6J chromosome 10, GRCm39**

NCBI Reference Sequence: NC_000076.7

1 atcttgaagt cctcttccaa ggtactagtg ttctaaaatg ctactatgtt tacatgactg

61 cactgtagaa agcctttcca atcaactttc agttacatga agaggtgata aagtgccagg

121 ctgtgcagca tcatggaaat cttgtcatac gctagatggt gcttctcttt taaatttgtc

181 atcctttccc tacatctaga ttacaccatt ttcgaacaat aatggtttcc tacacatttt

241 gttatgttgt tccctatatt ttagaccatc cccttgctgt tttccattac ctatgacact

301 gccaaaatca cttttaaaaa attcagttca aataactccc tcaagagtct taggactcca

361 tagcatatgt agtactgacc actctgtgtc ttgtgctttt tgtgtcttct gcagagaggt

421 tgacactctt tgttctactt ggttattgca cagcttgatt ctctcagatg gactgtgatc

481 tctgtgggtc agtgctgtgt tggacacttc tttgcatcct caggattcag ttcccaatgc

541 ttaatataaa ctcagagctc aacaaaaagt ttttgagtta tacatctgtt cttatgtata

601 gctctatagt ctgtgatccg ttgagtccac ttttcttcac acccttctcc ttatttggag

661 tacttgggga gattgtacag tgtaatggta gagaaagcca aaattgcctg aattaaaatc

721 ctactttgaa cattctttat ttgtgtgagc acgtgaacct ttgttaattt acctacagct

781 tttgtgcctt agtatacata cacacatata tacacacaca tgcatacaca cacacacaca

841 cacacacaca cacacacaca cacacacaca caatcagtaa ctctttgtga gtgttgttat

901 gatgactgtt ctgacataca aacatgcata gaaagtgtcc agaattagca ttgtcaacca

961 tcatttttca taattacttc ggttcattta ttaacacttg cttttcctat tagctgctca

1021 gtgctggttt tcagaggaca agtaagctgg atgattattt ccagagttgg atcaaacaaa

1081 aatcctaaac cctcaagtct ccagaaacaa ttaaaatcca accaatgtta acatatacag

1141 tcttgctttg aagaaccttc attctgcttt ctacataatc aaactgtata ggtcccagat

1201 tttggttagt tcttcagtta aacatgtgct cttgcttaag aattttccat tcttcaaagg

1261 attgtcaagc tttcctgtgg ctttctaaag ttggaaaagt acaaatataa tcttgtctat

1321 gcctgtcaaa ttgctgggtc tgatcaggtc atagatggag agcaataaaa ttgtatcaga

1381 agagtatcaa aggaatgatg ggcctatggg catttcattt ccatatattt cttacttaat

1441 gaagaacatt ctagaaggtt agcatctgag ctaagttcaa gcctcgaatt tcaaaagcct

1501 gaatatcctt ctacatggaa acaccatggg ttttggaagc agacttctta tgaaagccgt

1561 cacacacaca cacacacaca cacacacaca cacacacaca cacacacagg taaaatttgc

1621 ataatgtaac gtcatgccag cagaaaagcc tttcttaagt tattttttta cttttataaa

1681 atacaaagca cttaaattca tagttactta aagacggagg gtaaccttcc aatagcagac

1741 ttgagattca aaattagacg acggctgttt tgttgcaaga aaaaaaaata ataaccctga

1801 tgtattgttc ccctccatct cttcccaaag agctactcga cctacgaaac aaacaaatct

1861 catcaggagc acagataacc ccaagcttca gacgtgtaat ctgactgtgg ccatcagcaa

1921 ccagaaatga gttttttcta atcagctttc catcagtcct cagtcactca tataaaggaa

1981 cacggggagg ggaggaagcg

| **Matrix ID** | **Name** | **Score** | **Relative score** | **Sequence ID** | **Start** | **End** | **Strand** |
| --- | --- | --- | --- | --- | --- | --- | --- |
| [**MA0065.2**](http://jaspar.genereg.net/matrix/MA0065.2) | Pparg::Rxra | 9.32797 | 0.82262189241 | NC_000076.7:c115022927-115020928 | 179 | 193 | - |

# Canis lupus familiaris isolate SID07034 breed Labrador retriever chromosome 10, ROS_Cfam_1.0, whole genome shotgun sequence

NCBI Reference Sequence: NC_051814.1

1 cccacgcaag gagcccaatg tgggactcga tcctggggct ccaggattgc gccctgggcc

61 aaaggcaggc actaaaccgc tgagccaccc agggatcccc aaataaatat ttttaaaaac

121 aaaaaaaccc ccaaaccccc ccccccccaa agttttgttg gattgcacac catcccaaac

181 cctattctgg ctgaaattat ctttgtgtgc tctcttgagc ctacttatgt ttccttgttt

241 ggaataccta aggggctgta taatgtgata gttgaggaca cagagtttga agccagacta

301 cctgaattta aatcctgcct cagccactct ttaattggac tttttggcaa gttacttaaa

361 ttctttgtgc ctcagttttc tctttatata aaatggaaat accagttact gccttgcaag

421 tgttgttgtg atgatcaatc agatttttat ctaaaagttt tcttagaata acgattggca

481 agcactcagt aatattagtt attcttatta tgatttcaat caaggcattt tttatttaaa

541 aattttaata gaaagaagta gcatgatttt attgaaatgc tctagtacag ggttggacaa

601 tctcaaaatc ctatatgcta aacttcaatc aatgtattta agcttcacct tactgaaggc

661 acctgcatgg ctcagtccgt agagcatgtg actcttgatc ttggggatgt aagttccagc

721 cccgagttgg atatagagat tacttaaaaa taaaatctta aaaagaaaaa aaaaaaaccc

781 ttcgctttcc tattagttta tcagagctga tttttagagg aaattcattt caaataatgt

841 ttccaaaatg aggtcaaata ataatcatgt cccctcaaga ttccagaaac tattaatatc

901 cagttaatat gaagtcatcc atttcagttt tgaacgtttt tcatcttgtt ttctgtgcag

961 agaagcagtg ccagccccag gttttgctta gttttgtagt cgggcaagca cattcggtcg

1021 agtcttacag ccttaggggg ttggtagtat tttcccgtga catggtgaat tttctcactt

1081 ggtaaagttg gaacagtact tacctcaagt gatctatgca tttcagatgg ttggatttga

1141 tctaggtaca agcggagagg gaaaaattca gcaaatatca atagcgccaa gagattgatt

1201 gatgtacagg catcaatcaa tgatgagaat gattgattct catcaatcat caaaaagttt

1261 ccttttcctt cccacacagt gaggaagatg ctagggttag tgtctcccct ttatctgtct

1321 gtagccaaat cagagcctta cattgccttc acgctacagc tgcctgcgcg tcaccgcata

1381 gcggcatcac aggggtggga gcagtgcctt ttaggaaagc catcacccgc atccagtcat

1441 gacgcgtcag atcagaaaac acttattaac acagaatgga atgaattaag gattcagcgg

1501 tgctgatctt tctaattttt ctcctttggg ggaagaaagc acagaacctt tgaactcaca

1561 gtcccctaaa ggctgaggtt agcttgccgg gagcgggttt gagagatgag agctggctac

1621 caggggacag agctaatatg ggtttctttc acatacagca ccccattgtg tcttgttctc

1681 ccccacgcat gcccacacga ctattcgacc tatgaaacaa actgcgccgg aggcacagat

1741 aaccccaggc gtcgggtctg taatctgatc gtggccatcg gcagccagaa atgagtttct

1801 ttctaatcag cctcccatca gcccccgtca ctcatataaa ggcttctggg gggaggacgg

1861 tcctcatcgc tctcccttca gcaccagggc tctggacaga gcctgggcag aggctcctgc

1921 tggccgtcct ggtggtggcc gccagcccag gacgtcgctc cggtccccct gctgcacaga

1981 cagaaagttg cagcggatcc

| **Matrix ID** | **Name** | **Score** | **Relative score** | **Sequence ID** | **Start** | **End** | **Strand** |
| --- | --- | --- | --- | --- | --- | --- | --- |
| [**MA0065.2**](http://jaspar.genereg.net/matrix/MA0065.2) | Pparg::Rxra | 9.52153 | 0.825773874754 | NC_051814.1:13576664-13578663 | 54 | 68 | + |
| [**MA0065.2**](http://jaspar.genereg.net/matrix/MA0065.2) | Pparg::Rxra | 9.42627 | 0.824222521668 | NC_051814.1:13576664-13578663 | 689 | 703 | - |
| [**MA0065.1**](http://jaspar.genereg.net/matrix/MA0065.1) | PPARG::RXRA | 13.1074 | 0.814146946764 | NC_051814.1:13576664-13578663 | 1541 | 1560 | - |
| [**MA0065.2**](http://jaspar.genereg.net/matrix/MA0065.2) | Pparg::Rxra | 8.32017 | 0.806210659317 | NC_051814.1:13576664-13578663 | 1156 | 1170 | + |

# Protobothrops mucrosquamatus isolate PMUCROS unplaced genomic scaffold, P.Mucros_1.0 scaffold49, whole genome shotgun sequence

NCBI Reference Sequence: NW_015386431.1

1 aatgtgaccc tcatgacatt tgtcaggatc tagaaatccc cttttcataa tgacacccta

61 atccatgttt tgtcaataga aaagctggct tgctttttcc aatataatta aaggtaggtg

121 ggtagctgta ggaaaaagaa aaggaaatat taagtcattc ccaattattt agcaaataca

181 atctatctcc acaaaggatt caatttcttg tatatacatt ctgaaaaaga acttaacaga

241 gagtgaagaa atatgcccat gcttttagtt gctcaaaaga taaagcaaat tgttgtgaga

301 gaactgatga aatggcaagt tattaacaca gatatcggaa ggtgagatct ctgtagttct

361 tggctttaat tcatttggga tacttatctt ttatcagtta ataacaaaat accccatttg

421 gcaaatggca agaaagcaag acccagcatc tctaggcttt catttccttc ttacaggaaa

481 taatttttgg attatcttgc cccttttgtt ctctcataga actttggctt ttacattatc

541 atgttaatac aattgcggga tatatatttt ttctgtgctg tctgttggca tgattccaaa

601 atgaacacaa agctaacatg gaataattct cccctccttc tcatacacac acacacacac

661 aattcaacat ggcagaaatc tggaaagcat aattaggata tttatgcaca tagtgggact

721 tctgattttt tcttttttac ttatcttaca tgacaacttc attatcaaaa caatgttgaa

781 tgtcgtcata aaaatagtca aactgatttt gtgttgaaaa caaccgaata attgtagcac

841 aatagcccca gtgataatac ttatttaaaa aatatatgac atgcgtaaag cctgtctacc

901 ttaagatagc cagatagaat cagaagagac tctttaagga aggtattccc atcatttaca

961 gtagatgcaa ccacatgcat cctgctgcaa ccaaattagc tcttcaatag atataggagg

1021 gggaatttat aagaactgat cattatagta catgtcctgt attgaattgc tactgagttc

1081 tagaaaccta catttaagtc tactgaaatc atgtgccaaa gttcttggga atttaacctc

1141 acctgtattt ggctaaagaa cacatccatg actctaagca gaattgtgta attggattct

1201 tatttcctgg gatacagtag tttaacatag tattggataa tacaactatg atatttaaat

1261 atttgcagag tctctacact agaatttatg cactaggtaa aatttatttt tatatatgat

1321 acttcaccaa tacgtaacag aaggatgcct ttggtctaat gtcctcattt ctatattcaa

1381 agtaggtaaa tgaggaatgg taaatcaatc aaacaacttt ataatacttt taatcctgta

1441 atcacacact catttgatta tagtgtgaag ggttctgaca atattaaaac acccctggga

1501 aaagtaccaa tttctcttca ttcttttaaa aaatgtaatc tttactcaag tgacacgtaa

1561 aatgcatttc ttgttaactg aaatatcaag ctgcccatct tactttgagt aaccatcttc

1621 gtaatggaat gccatgcaag tgttcaaatg gctctctgta aagaactgct aaacgttttc

1681 tgaagtctac tgataaaagt atagtacttt taaaaatagt acaactcaaa ggaaacaaac

1741 aaagtgctag ttgttgtacg ggaagacaag aggagtcaag aaagaagggt gtttctctgc

1801 tactttcatg tgacgatctt catcagtctt tcatcagttc ttcaagaggc gtatataaac

1861 tcttgctggg ggaggtgaga ggcacttgaa gcgtttcttg ttcagcctca gagtgctgaa

1921 cagctggaca gctccctgcg ggcagcattg caagcctgtt tccttctgct ctgaacaggg

1981 aaaacagtac agttcccacc

| **Matrix ID** | **Name** | **Score** | **Relative score** | **Sequence ID** | **Start** | **End** | **Strand** |
| --- | --- | --- | --- | --- | --- | --- | --- |
| [**MA0065.2**](http://jaspar.genereg.net/matrix/MA0065.2) | Pparg::Rxra | 8.62868 | 0.811234468467 | NW_015386431.1:c582009-580010 | 1111 | 1125 | + |

# Gallus gallus isolate bGalGal1 chromosome 1, bGalGal1.mat.broiler.GRCg7b, whole genome shotgun sequence

NCBI Reference Sequence: NC_052532.1

1 taatgagtaa acatgaagga gattatctct tggtaacttt gggtatgaga cagaactaac

61 acctgcagac attcattttt tcctcaactg gaaaaatgaa aaaaaaaaac tttgtgggtt

121 atcaagacaa aagtgattca tttgcattac aggagcaggt aagggaacca aatattatgg

181 caattttatt gtcaagcaaa aggaagaacc attattacat ggagcagctg tgatcctaac

241 tcagtcctga acatcaggca tacgtttcca ttttatgaaa gggctcatct ttctgacaag

301 ggctggttta gacttgaagc ttttgctggt ttgttagttg tggtaaatgg tgagatctca

361 ctgtacaaaa cagtagaaaa cttcctgttg tccttttgag ctggacccta tgaccaaaag

421 ggattcctcg gtgatgcagg tatgtaaata ttagctaaaa gatactgagg ctagcaagga

481 tagagacact gactttacgt aaagttagca agctatagcc gagccttagc tgtagcatga

541 aacatagcac catgagaact aagcctttct gctactctcc ttgcgtagct ccctgctcag

601 tctttcagtt tcagagagtg tgattttaac tctttattct tgatccatgt ggtctttgta

661 tgcacgtagg agtaaaggcc atgtgatgcc agctagggct gctgcaggga aggcaccact

721 ggagatttcc aaggggaagc agcgaccatt ttacctgtct ggtgtttcag aacagagaaa

781 agtgagcacg tatttgaaaa aacttactga cataacattc ccagtccagc tgtggtgttc

841 agttctatgc tgtataactt actcaaagta gccaatggaa aagttttgga aggtgagaga

901 tgtggagagg gaaaaaatag gaagaaaaag tgacaacagt aagtgacaaa atgagtatta

961 gttttcagta tgtgggtatg agagaaataa aagatcttaa aactatttca caagtagatt

1021 tgagtgccaa gttattgtct gagcaattac tttctgctca tttacagtct gtgaggttct

1081 acaggcctga tattaactag aaagcaaatt cttcagaaaa attttttgcc cttgagatat

1141 aagcaactca gagacaggtg aagcggtcca atacgaaaac agacccacag gatccataca

1201 ggacggtttc acctccacag attattaatt gctttattga tgttgtggtt agaagagagt

1261 cttatgatca gagcccggcc attatatagc cgaggtagaa aacacagctg ctgctgcctc

1321 ggttaaaaag ctttctccaa ttaagcatcc tttttcagta gaagtactct ttccatgtca

1381 tcaatcatgc tttaatgcag aaggtttgct aagtctttca ttataagaca gatctcctac

1441 cccttaatgc ttcctctctg aacctgccta catttttcac tgaagaactt cataaaaaaa

1501 tgaggaaaga tgagtttcta catcaaagaa ttcctcacat caaagctgac gtggaaataa

1561 ctgcacaact tttcactcct tttatttatt tatcgttggc atgcactgag gcctgctgtc

1621 cccgtgtcca cacatagtcc ccagggccat ctgcatcctg gctgcccaaa gccagctggt

1681 ggggctggag ctcctgacac ggggtggttt gggactctgg ttatgttcaa ggcgagccat

1741 cacggtgggc actgaggcgt gaggagctgc agccgctgcc ctgaggccac cccgcagcca

1801 ggccgcgatg gggtccggtg cagctctccc ggggctcccg cagcgctgct gctccggggg

1861 ctgctgaggg gcgagggctg agggctgccc agcggcgtat gaggtaagcg aggccgaggg

1921 cgcccgcccg cagccggctc cgtcaccggc gcccggtccc ccctcggcgg ctttccgtca

1981 gcccccgcgg ggccgtataa

| **Matrix ID** | **Name** | **Score** | **Relative score** | **Sequence ID** | **Start** | **End** | **Strand** |
| --- | --- | --- | --- | --- | --- | --- | --- |
| [**MA0065.2**](http://jaspar.genereg.net/matrix/MA0065.2) | Pparg::Rxra | 9.39106 | 0.823649284318 | NC_052532.1:36619578-36621577 | 667 | 681 | + |
| [**MA0065.2**](http://jaspar.genereg.net/matrix/MA0065.2) | Pparg::Rxra | 8.41153 | 0.80769837113 | NC_052532.1:36619578-36621577 | 122 | 136 | + |

# Chelonia mydas isolate rCheMyd1 chromosome 1, rCheMyd1.pri, whole genome shotgun sequence

NCBI Reference Sequence: NC_051241.1

1 tgggagctgt ttgactcatg gaatttacac atgttcttga gtgtgcctgt acagtttgta

61 aactcactta atgtagttag tggtctgtag actttaggag agtcatgggg ataatagcaa

121 taaacacagc ctacaagttc tgcataatca ttccacaaac tttgaaagtg atcctacctg

181 tggaccttga aagctctgta gtgctaaatg tggtccttgt agtgagcaag gttaggccta

241 gtgtgtgggt tgggctgtag ctatggatct tctaggacag gggttggcaa cctttgagaa

301 gtggcatgcc aagtcttaat taaggtttcg cgtgccaata ataaatttta cggggccccc

361 aacggaaccc caaactggca gtgggctgag cgggtccggt ggccaggacc ccggctggaa

421 ccccagacca gtagcgaggt gagtggggcc agcggctggt atcccaggcc agcagcaggc

481 tgagcagggc ccatggctgg gatcctggct ggcaaggggc cagcggctgg aaccccagac

541 tgtcagtagg ctgagcgggg tggcagacag aaccccagac cggcagcggc tcacctgccg

601 ccggtctggg gttccgtccg tcggctcctg ccagccgggg tcctgtccgc cggccccact

661 cagcctgctg ccggcctggg gtcccattca tccaggcagg cagtgggctg agtggggcca

721 gcggctggga ccccggctgg cagcagcgtg ccagtaaaaa tcggtttgcg tgctgcaggt

781 tgccgaccct gttctagggt ctgtagagaa cacctgagga cctggaatgt tcaataacag

841 atctgtggtc cctggaagct ccatagtagg gaatgattat gcactggctg gacgatgaat

901 taggatgaga cctttaaatc aagcttggac ggctgtctgg aagatttgat aggtctttcc

961 cctctctaac agctgtgatt ttatgcaaat aggaagtaag ttgagtacca ttcctctcca

1021 gctctccttt aaaggccact gctggcatta actgagggaa acctgggaaa tcataagtgc

1081 agggctgcag gccacaactc aggctgttgt gctgtgtttt ttaagtcacc tttcccccca

1141 tgccggtctg agtgaaggag gaactacctc cccgacttgg agctgtgttg tgctggcttc

1201 tgttcatttt gcctcaggac gatcataatg ttaactaaca ttgagttcaa taatgttcta

1261 ggtgcgtagc ccagtgacag ggacaaaaca gtgtgctaca gaaggaatta gggggtggag

1321 gaggaaggga ggaagtgaca ggccagaagt attatgttgc tggaccccac aggtgtaagg

1381 cagcccgggc tgtggccgga tcgctgggcc ccacagatgt aaggcagttt ggcgggggac

1441 tgacccctat gggtgtaagg cagccaggga ggtggccaga gggctgggcc ctacatgtgt

1501 aaggtagccc agcggggggc tgggccccac aggtgtcagg cagccctgga ggtggctgag

1561 tcgctgcccc accccccaca ggtgtaaggt agcctggagg ggggctgggt cgctgggctc

1621 cttccacagc ttcccctggg gcgggcatca gcccagatgt gagcagctgc ggaacgtttc

1681 cacaccgcaa gcagcctcat tagctcccgg gctggggcgg ggggttcccg gcaccagcca

1741 cagcagccac gccggctcga gcctggctgg ggcccctttg cccgcaggtg aagccgcccc

1801 cctccaggat cggtgtaaaa acctaacccg gccgctcctc cgggctgcga cagcgcagcc

1861 agcgtgtgaa cgaaccagct cccgccaagg caacctgcgc tggcatccgc ccgccaccgc

1921 cagccacacg cgagcctccc tatcagcctc ccatcagctg ccggccgggc gcataaaagc

1981 cgccgccgcc gccaggggag

| **Matrix ID** | **Name** | **Score** | **Relative score** | **Sequence ID** | **Start** | **End** | **Strand** |
| --- | --- | --- | --- | --- | --- | --- | --- |
| [**MA0065.2**](http://jaspar.genereg.net/matrix/MA0065.2) | Pparg::Rxra | 13.5654 | 0.891624484286 | NC_051241.1:c281595471-281593472 | 1126 | 1140 | - |
| [**MA0065.2**](http://jaspar.genereg.net/matrix/MA0065.2) | Pparg::Rxra | 9.91501 | 0.832181268158 | NC_051241.1:c281595471-281593472 | 1471 | 1485 | + |
| [**MA0065.2**](http://jaspar.genereg.net/matrix/MA0065.2) | Pparg::Rxra | 9.62879 | 0.827520515542 | NC_051241.1:c281595471-281593472 | 272 | 286 | + |

# Danio rerio strain Tuebingen chromosome 18, GRCz11 Primary Assembly

NCBI Reference Sequence: NC_007129.7

1 agcattcata aaatgtgaaa atgaaagtga aagtgccaaa ctgcagttca agttgacata

61 ctgaaaataa aacacctgaa attaaattta ctctggagga aatgtggatg gtgtcgtaaa

121 gcaatggcgt taatcgaatg atgtgctgta acatgtaaaa cagaaccatg aaaggagcat

181 tcaaaaatac actcatgtaa acaacttatt tatattattg gactattcag attaaggcaa

241 atatttgaat actgatgtcc atgtaaactt attcaatgtg cacttgtttt gtctcaataa

301 catgaatttt gattctcttt aaggacttgc cccattcagt gagtgagatt cttggtctga

361 acacagtcag tgaaccctct gcagcattag aaaccagtga atatggaata cttgagtcac

421 ctcaaacctc accgagatcc tcacacagac aagatcacgt cgtctccaac ggccacagcc

481 acttaaaaga aacaacacaa aacggctgca aagtagcctt catttcctag tgcacagcac

541 tacatgatgt atagtacaga ttttatagcc tctgtattcc agcaaaaaga acagtattgg

601 gtgtcatgta gatatttgct accaaatgcc acattgtaaa atatcccaga gaaaatatat

661 gaatgtaaaa ctaggaccca aaaaggatcc taaagcatta tttttgtaat atcttgtacc

721 atattgtctt tcattgaaat aatcattcct tctcactgta gagagagaga gaaagttttc

781 ataatgtttc aggggtgggg gattcattgt tgaatattat atattttttg ttcttatcag

841 tttgcccttt tcctaaataa ttcagattca aattaaaatc tgtcttcctg ccattagtct

901 gagttatatt ttggaaagct gatgaagatc ggtaagattg ccttttttat tacataattt

961 gtaaatgaaa tgcatcattg aggctactgt tatgcacgtt tttttgtcca gtgaagccat

1021 tttgtataat gtaataatac ttaagttaat ttgttacgta attctcagat ttaattagga

1081 attttcagat ttaattagga aagaggactt tataattaat tgatcaccta agataaaaat

1141 ttgctaagag cgacaaagtt aaataattta attgatttga ggagagtaaa ggagaaaatt

1201 tgcttggctg tagaaaccaa aatcagttcc aactggcatg agatcatcag tccgcagact

1261 tggttcatgt cactatgaca tttccagatg agacgtggaa aatacagatt gaggatgaaa

1321 tgtcttttat ccactcgtaa ccatggccaa caacaaagac acaccgaatc aacttccatc

1381 tctttcattt cctgcttgac aggaaattgc ctcagaggaa gctgatagct cactgctcac

1441 aacagtaatg ttatctctcc tccagctccc tttcattaaa ggaagccagc attttgtgca

1501 ctgacaaaca catacccgtt cgatgaaaac ataaacaata tgcatctatc cttaaaggac

1561 gattcaagaa cttctataaa tgtttagctg atgtcgatgt taacatgtga accaagatat

1621 cagtatttct aattaaatca catccctctt acttgctttt aatattgtac atgtctaaaa

1681 tcttcctgtc aagtatgcac ttcacaaatt tcttatattt tacaaatatg gttgctaatg

1741 tttgtcgatt tctccctctc tccatcttcc aatggctgca tttatgaaaa tctttaccag

1801 ttattttcct ctagatttgt ttcttctaga gagcgaagtg agcttatcca cttcaatcag

1861 gacttggacg tctcgacatt tcctagaaag agccatcccc tcaaaagcta atatgagtga

1921 tggcctatcc tgcgatagat cggcccttct ccgcgtcatt cgtcacagta tataatccca

1981 gctctgatgc tgacagggga

| **Matrix ID** | **Name** | **Score** | **Relative score** | **Sequence ID** | **Start** | **End** | **Strand** |
| --- | --- | --- | --- | --- | --- | --- | --- |
| [**MA0065.2**](http://jaspar.genereg.net/matrix/MA0065.2) | Pparg::Rxra | 10.3926 | 0.839959200753 | NC_007129.7:c17003056-17001057 | 843 | 857 | - |
| [**MA0065.2**](http://jaspar.genereg.net/matrix/MA0065.2) | Pparg::Rxra | 8.34735 | 0.806653182028 | NC_007129.7:c17003056-17001057 | 1451 | 1465 | - |

# Rana temporaria chromosome 3, aRanTem1.1, whole genome shotgun sequence

NCBI Reference Sequence: NC_053491.1

1 tttccctaca gacgatcgtt tttttctatc ggttttttta accatcagat cattttaaaa

61 caggttctaa gttttttcac cgatgggaaa aacaccgatg gggcccacac acgatcggtt

121 tgtctgatga aaacggtcca tcagatcgtt ttcatcagac gaaccgatcg tgtgtacgcg

181 acataagagt ctcctctctt cttttttata ctcagttatg acatgacact actcttatat

241 caaggcatcg cttgtatatc aaggcaaaat gtattaaaac attttgcttg tcttgcaaaa

301 cgctctcaaa ccaagttgct ctcaaaccaa ggttttactg tatttaataa atcgaattat

361 taattgtaca gggattgtaa tgtgtatagt gacccacaga aatattttcc gataagatgt

421 aaagtttatt gcttcctttt tttttttttt ttttctattt ttagttcttt tattctgtgt

481 gacagagggg attatttacg aaaaggcaaa tccacgttgc actataaatg caaagcgcac

541 ttggaattgc actgaaagtg ctcttggaag tgcagtcgct gtaaatctga ggggaagatc

601 tgaaatgagg ggaatctctg ctgattgtat catgtgcaag ctaaaatgct gttttttatt

661 ttccttgcat gtccccctcg gatctacagc gactgcactt ccaagtgcac taagggggtt

721 atttactaaa ggcaaattca ctttgcacta caagtgcact gcaagtgcat tgaaagtgta

781 gatctgaggg ggacatgcaa ggaaaataaa aaacagcatt ttagcttgca catgattgga

841 tgataaaatc agcagagctt cccctcattt cagatctacc cctcatattt gcagtgcact

901 tgtagtgcaa cgtggatttg cttttcgtaa ataacaccct ttcagtgcaa tttcaagtgc

961 actttgcagt tgtatagcca gattcacgta ggcaggcgta gcgtatggca tatacgctac

1021 gccactgtaa gttagagagg caagtgctgt attcacaaag cacttgcctc ctaagttacg

1081 gcggcgtagc gtaaatgggc cggcctaagc acgcctaatt caaatgtgga acaggggggc

1141 gtgttttatg taaatgactg gtgacccgac gtgattgact ttttcaacga acgacgcatg

1201 cgccgtccgt ggacatatcc cagtgtgcat tgctccaaag tacgccgttt cgacgtgaac

1261 gtaaattacg tcaagcccga ttcgcgtaca acaaacaacg taaaaagata ggcctgttcc

1321 gacgtccata ccttgcatgg gatgcgccac ctagggagca gctttatctt tacgccggcg

1381 tatctctaac gtaaacggcg taactaattg cgacgggcgc acgtacgttc gtgaatcggc

1441 gtatctagtc atttgcatat tctacgccga actcaacgga agcgccacct attggccagc

1501 gtaaatatgc accctaagat acgacagcgt aggagactta cgccgctcgt atcttagccg

1561 aatttaagcg tatctggttt ccagaataca cttaaatttg cgacggcgta gattcagagt

1621 tacgacagcg tatctactga tacgccaccg taactctctg tgaatctacc tagtagtttg

1681 cacttgtagt gcaaagtgga tttgcctttc gtaaataacc ccccagtgtg tctctgaggg

1741 ttgtgtactt gtaaataaaa tttgataata tatatatata tatatatata tatatatata

1801 tatatatata tatatatata tatatatata tatatatata tatatatata taataatgta

1861 ttgcttagta tgaaacagga gaaatctata gatcatgatg gcagatccta ataataatgt

1921 cttgaataaa catgtatcta gtctacattg gcatattcct ccaatcagga cccacagcct

1981 gtaaatacag gcaaacaaca

| **Matrix ID** | **Name** | **Score** | **Relative score** | **Sequence ID** | **Start** | **End** | **Strand** |
| --- | --- | --- | --- | --- | --- | --- | --- |
| [**MA0065.2**](http://jaspar.genereg.net/matrix/MA0065.2) | Pparg::Rxra | 12.2178 | 0.869681041764 | NC_053491.1:c190339719-190337720 | 477 | 491 | + |
| [**MA0065.2**](http://jaspar.genereg.net/matrix/MA0065.2) | Pparg::Rxra | 10.9776 | 0.849484441918 | NC_053491.1:c190339719-190337720 | 1686 | 1700 | + |
| [**MA0065.2**](http://jaspar.genereg.net/matrix/MA0065.2) | Pparg::Rxra | 9.67467 | 0.828267578359 | NC_053491.1:c190339719-190337720 | 737 | 751 | - |

# Latimeria chalumnae isolate SAIAB 97564 unplaced genomic scaffold, LatCha1 scaffold01670, whole genome shotgun sequence

NCBI Reference Sequence: NW_005820680.1

1 gtttataatg gaaggactct gctccaactt tactttgctg agggtggcat gatatatcat

61 gattaaatat cactaagata tcatggtttt ttttcttgcc ccatatttta aaatcggttt

121 ttcaggaagc attcactgac tccagtttca gatagaagtc ttatcaggcc agagtgacac

181 tttcaaaagg tgtatttatt tattttttaa ttttttcccc tgtactccct ctctctctgc

241 tttcaggttg cggcaatcct ttctgatggt gcatggaact gtggagacca gctgtcctct

301 gatgttcttt cctagtggct gttcttcatt agagcgcctc atcagccagc gttgatgagc

361 ctagtgatcc tgggagacag caggatcgaa ctcagtctgt cccagcctgg ctgcctctta

421 aggcgcccac tgccaggggt tggaacttga agtaggaacc ctggccggtt tttccttccc

481 taaccctggg gggggcgctg tgggctaatt tggtgcctcc ctccactggc tggcagagga

541 aaggtgcagt taaagccagt tatatgtcaa tacactgtac attttttccc acttatagtt

601 tcattggggt aagagcccaa cctttctttt tagcaatctt taagtgttct gaaataaagt

661 cagatgtcta ataagattat agttcatttg gtgatcaacc tttaatgaga gaagagacac

721 tttcagactg agtattatga aaagagctac aaagtgataa attcataaaa acacaatttt

781 atcaaaatat aaaattcagt gaaatccatg atcactatca taatgttcat accacactac

841 ctattattac cacattatta ttaagtaata atatgcagta gtaatatgta acatggtatt

901 aacatttatg atattgttta tggatttcat tgaattttac atttgagtat tttttttttt

961 ttaatgaatt tgtattaaac atatacctgt caacatttgg aaagtcaaag ggtgagggcc

1021 tgaacatact agggaagtcc aggggcatgc cccctggaaa agtttgaatt ttagatgctg

1081 tgaaatgcca tttcctacat tctgagggca ggaaaaaaac aactaatatt gcttgaaaat

1141 gcacaatttt ttaatcaaaa caatctaaat ttgagaaaac tttacatcac aatcactcct

1201 tgttgatgcc attgtaaatt ttatatgtac tgcacatgtg cagaataatg tagcataagt

1261 cttaattatg tattagtatc agcttaaagt aggcaacatt ttttaagcaa atttacaaaa

1321 aagcatggtt aattttagct tattttaaga ctaataaaga ttatgttggg gggatgaaat

1381 cttcaaagga ccacattgca ggccctaaga agccacagcc tgaggaccac tgttgtagat

1441 gaagcatttg acagtaacag cccagttagg tatagctctt acctagaaag cagggcttcg

1501 attttctgat cactttcaaa gcaaagtgca aattctgaca aaactattaa taaagtgtac

1561 tggaacatgt ggacttcatc ctgcaacaat caatgttatg tacattatat agttaatcct

1621 tttatgaaac agttccaccc tcccacaaaa aaaaagaatc atttaaaaat attttatgat

1681 tttagatgaa ctatataaaa tgtttcatac aagtcactct ggcattttag gaactgtgta

1741 tgtggctcaa aactaaagtc tttattttat acacagcaca tgcacaattt agaaagtata

1801 tgtttcatag atattgtgtg tgtgtggtat gcaattgtta aaatgaatgg ccttaaatga

1861 tctatgaacc tagggctaga cgaccaatga gaaatgattg tgtttttatc ttgtaattaa

1921 ttgctggact cccacatata aaaaccacag caagaagagc ccagtgtgcc agagaaaaag

1981 gagagacctg aagtgctgca

| **Matrix ID** | **Name** | **Score** | **Relative score** | **Sequence ID** | **Start** | **End** | **Strand** |
| --- | --- | --- | --- | --- | --- | --- | --- |
| [**MA0065.2**](http://jaspar.genereg.net/matrix/MA0065.2) | Pparg::Rxra | 9.7053 | 0.82876635014 | NW_005820680.1:406077-408076 | 533 | 547 | + |
| [**MA0065.2**](http://jaspar.genereg.net/matrix/MA0065.2) | Pparg::Rxra | 9.29629 | 0.822105944629 | NW_005820680.1:406077-408076 | 1516 | 1530 | + |
| [**MA0065.2**](http://jaspar.genereg.net/matrix/MA0065.2) | Pparg::Rxra | 8.08699 | 0.802413505417 | NW_005820680.1:406077-408076 | 13 | 27 | - |

# Amblyraja radiata isolate CabotCenter1 chromosome 19, sAmbRad1.1.pri, whole genome shotgun sequence

NCBI Reference Sequence: NC_045974.1

1 tccagttaga tttatttact tttatcgtcc atgtttgaaa cgttctcgac tattgctcct

61 taattttaaa ggattattat ttgaaacaaa catcactcct atatatttca aagtgcatgg

121 cagccaattg atttatcttt cagattcatt caagccacat gtcaatggtt ttaagatcaa

181 agaaataaat aacatctata ttgccggtta tccaataatt ccggattgaa ggaagttaat

241 ttttgaaact cctgattatg cagtttggcc tgaatttaag atacatcaac aatggcgccg

301 aacaaactcc catctccatt accatttgaa gtcatctttt tttctcttgg ggtttgaact

361 cattagtgct gtgattaata actcacatcc tccttttctg aatttcccgt ctccccaaat

421 catctaaact gcttaccgtg gctgacacaa atcccattaa actggttgga caaggaactg

481 cagatatgtg tttacaaaac aagacacaaa gtgctggagt aactcagcgg gtcatgtagc

541 atctctggag cacatggata ggtgatgttt cgggtcggga cccttcttca gactgaatca

601 tttccatgtt cttcagaggt gttgcctgac ccgcttcaga ggtgctgcct gacccgcttc

661 agaggtgttg cctgacccgt taccccagca ctttgtgtct ttttctgtta aactggttat

721 ctggcacaaa gtcaaaggga gaatgtgcga gaatatttgt agttgtataa aacttgcaga

781 actctccaaa ttgcatcgac gtgtgttcat tatcttacaa acaattggta ctgaaactat

841 tgagtcgaaa gatcgaatga atttcagtat gttttgtcaa tgcaaacaaa ttaggtttaa

901 aattatctcc atcgtggtaa tttagtttat aaagggaaag aaagacgccc ttgcagtcaa

961 agtctagaga aaagatgcag aacgagcgaa aactatagag atgggttaga taagccgaac

1021 acaatgaaac cggagagcga tacaataacc gaacaatact gatcacgaac taaaagagca

1081 atgaagaaaa tagtaagata gaattaccgt gagctcgctg tcagaggtgg caccgtataa

1141 gtctttgata ggttttacct tttactttaa atcttcagag cgctatttca ttcaaacaac

1201 aattgatgat caaacggagg gacttggcga ccgattaatg ggggcaggat caatgagtgg

1261 acagaagtga aagataacgt ggccgaagtc atcccacggc gcggagtctg gtatcagcta

1321 agaagtagac atataattct atctctaaat cattgctact ttgcttccat ttattctctg

1381 ggcaaaaaaa aaaagtgtca ttaatcttgg cacaaaaaaa cattgttctg cttttataaa

1441 aaaatatagt tgacgttttt agaaagtatg cctctttgta ctacacagtt tgcaaggggg

1501 gaaaaaaccc tccaaatatc aaatagtatt ctgcagtgtg acgtaaaacc agtcgaaaat

1561 cttgcattag ttcagcttgc aaagtggaaa taacatctca aggcaaacaa ctgtgcccgg

1621 gacccgatgc acgtgtaaag ttttggcgct tacacaacag cccatgtttg agaaagtatc

1681 ttccagcacc agtctcaata cattattttt cttccagcac caaacagccc aaataaaatc

1741 tgcttcccag gcctgtgttg agaggtgggt cactgaattt gacctgaagt ctttaaaggt

1801 ttcattgcaa tctgatctcc agagtgcacc atacagggtt aaagcgacct tcatatttgc

1861 ttcattaatg caccctttct gcgcaattga ctggaggcga gggtggctgt gccgtgattg

1921 tgtttccgtg atgcttcgcg cagaacagca catcccttat caccccatca gcgctgaagc

1981 ctatttaagg tcccagaact

| **Matrix ID** | **Name** | **Score** | **Relative score** | **Sequence ID** | **Start** | **End** | **Strand** | **Predicted sequence** |
| --- | --- | --- | --- | --- | --- | --- | --- | --- |
| No data available in table | | | | | | | | |

# Oryctolagus cuniculus breed Thorbecke inbred unplaced genomic scaffold, OryCun2.0 chrUn0035, whole genome shotgun sequence

NCBI Reference Sequence: NW_003159359.1

1 attgtagctt aacaggttaa gccactgcct gcaacgccag catccaacat gggcatcagt

61 tcaaatccca gctgctccac ttctgttaaa gctgtgcagc agccacagtg ccagctccac

121 cactagattc tttaaaaggg actaaagaaa atttaatcac actggcaaag actcaagttg

181 agagagcaaa ggagaaatcg aacattttaa gattaaatat atccatgata tacaagtgtt

241 aaatcccatt attaaaagtc agaaattttt ctgttaaaac acattcctat aacgttccag

301 aaaaggcaaa accatgggga caagtttgaa atccgaccag tgtttgccag ggtctgggtg

361 tgggggagag ggctttgtta agggaacttt tctggagtga tggaaatgtt ccatatgttg

421 actgcttggt ggttacagac tataggttgt caaaactcag agaattgtac acctcaaaag

481 ggtgacaatc actgtatgta aattgaacat caataaacct gacttcaaaa actggagaaa

541 agtggctggc gccgtagctc acttggctaa tcctccacct gcggcactgg cacaccaggt

601 tctagtcctg gttggggcgc cggattctgt cctggttgct tctattccag tccagctctg

661 tgctgtggcc cgggagtgcg gtggaggatg gcccaagtgc ttgggacctg tacccgcatg

721 ggagaccggg aggaagcacc tggctcctgt ctttggactg gtgcagcgct ggcggtggtg

781 gccattaggg gagttgaacc aacagaagga agacctttct ctgtgtctct cactgtctat

841 aactctacct gtcaaaaaaa aaaaaaaaaa aatggagaaa agaatgtcct gcaatagagt

901 gcgtgacaca tgaggtccac tctctacatg catgggctcc acatgtgggg attcagccaa

961 tcaggaattg aaaatactcg agaaaaaaaa aaattgtgcc tgtgctgaac atggactctt

1021 gttgttgtca ttatgtccta gacaatacag tccaatagct attcccatag cattttcgtt

1081 gtatgataag tgataaccta gatcatctct aggtaataga taatagtctc tagataatct

1141 agagatgatt taaaagcata taggaggatg tgcatgggtt cgatgtaagt accgggccat

1201 cttttataga ggtcttcagc atccttggat tttggtatcc agagggctct gggaacccct

1261 cagccccacc cactgtggat actgaggagg gctggttagc ccttttggag gccatcttaa

1321 tggtccaaac acttaaccca taaataacct attccaagtc tgtgccatgt tgtcacagag

1381 cctgtaactt gattttctgc taatagagca cgcccatctg cttgcaactt aagcattttt

1441 aaattcaaaa ggcactgcac acacaccttc taaaagctta gcgattgctc tgcctcaagg

1501 aaccaagcgc aggatgcggc aagactgggc tttgagatgg ggattgggga tggggcgggg

1561 gttggatagg acattcctga tttggtcccc aacacgggag aagcagtgca aaccctcagc

1621 aggtaaatca tagatgtcac ccaggttgag agcgaatttc caactctgga ttgtttgttt

1681 agtattagaa gtctgagatc atggatggac taacattagc ttaattaaaa caccagtcag

1741 atacacttga aagctaaggc gcaatcgggc gggcgccgtc agcaccccgc ggaggtcagc

1801 tggcggctaa cgctgccccg cccgcaggag aaccctcaac gctgtgcctg cggcttctcc

1861 cttctcattg gtcacttccc ggccgcctcg gttgcctatt ggcccgcccc gacgcccgct

1921 tctataagag gcgggccccg cgggaccgcg aacagactcg gtttcccgct agtggcgccc

1981 aggtgagtgg catggccagt

| **Matrix ID** | **Name** | **Score** | **Relative score** | **Sequence ID** | **Start** | **End** | **Strand** |
| --- | --- | --- | --- | --- | --- | --- | --- |
| [**MA0065.2**](http://jaspar.genereg.net/matrix/MA0065.2) | Pparg::Rxra | 13.8344 | 0.896005218407 | NW_003159359.1:c2085690-2083691 | 360 | 374 | + |
| [**MA0065.2**](http://jaspar.genereg.net/matrix/MA0065.2) | Pparg::Rxra | 8.55433 | 0.81002376236 | NW_003159359.1:c2085690-2083691 | 181 | 195 | + |

# Aplysia californica isolate F4 #8 unplaced genomic scaffold, AplCal3.0 scaffold00183, whole genome shotgun sequence

NCBI Reference Sequence: NW_004797453.1

1 ttctgtttct gcttgattgt ctgtgtttta cgctctcacc gatattgttt agattattaa

61 gaggcgagct gtttaaactt tgcttttcaa aggaccaata ccggctcaat gaggtacaag

121 atcccaccaa gaaggcggac acactcgata aaatagactt accctcatgg caataaaagc

181 cacgtgtcat acgaacaaca gagaaaaact ctattcaata gagcattgac ttcagtgatg

241 cctttcaaat aaatgctgac ccacaaatat ctcctacagg gagatgaaaa gccgttctgt

301 gtcggatgcg acgcagatct taccattaga cacatcttaa ccgaatgtct ggactttgga

361 gagatccgaa ggaaatatta taaatgcaag aaatggaaga catttttacc gtctttggtc

421 ccgacagaat tttgaatttt atgaaggcca ttggccttta tggcaaatta taaagagaaa

481 caatattgct ttaaaaaatg tattgttatt tataagagtt gttttaaatt gtaaacatat

541 ttacaccgaa ttttagtatt ttagcatctt aaagtctaat tgtcgaccaa ccgtacagtg

601 aactgttgcg aatgctacca acttgtgttt cgcgcgctca tatgacctta tgcagttgcg

661 agcgccgtaa aaccctgtac taaacaaaac aaacagcatg gaactgtttc ctacagaagc

721 ggctcttggt agcaaaccta cgtcataacg tatcggcgtc tggggtatat cactttgacg

781 cttcgctgtc acacgctgga gacccgagta cgattcatgg gtggggactt aggccttctc

841 agaactgaga aggcctaggt ggggacacat tttatctagt ctttctgcta ggatggcgta

901 tatccctctc agcccgggct ggccctgtca gacaggatcg aacgcctgcc tccaaaatga

961 cctaatctat atcatcgatt atagtatagt cgaagctttc taaggggaaa attgacgttt

1021 tagacagatg gatgtctcac aaaatgtcaa tataaagttg ctgtttttta accaaaaata

1081 aaaacaacca aaaaatggca agggtagaaa tgggaagcgg ccatttgtac aggtgactgt

1141 catatacagg tgtacgttag agtaggttcg actgtatgat tttatcaatt tttttctgtt

1201 caacgtaatc aaaatgggaa atgaagccca aaaatatttt catccatttc tcagaatcaa

1261 acctcggcgt tgcagtgacc atctcatccc tccaacggca gcgccatctt gtgtgtccca

1321 gcaccagagg atatataaga agcatcgaag cagaaaacag aaaccagcga ctcgaaaacg

1381 atagagggag ctctacgtgc aagaactaag agggcgagcg gttcggacga tagagcagga

1441 gttgttgtgt ccagaaactt ctgctgcaac ccagaaaatc aacatcaaca gccatcaata

1501 atacaaaaat agtagcgata caaacgatta gaataataac agtggagacg aaataatcag

1561 taacagtgca gacgatagaa attgtaacaa cagtaaagag gaaaaaaacc agctacggtg

1621 gtagtgtaag cgttataaat agtaagaaca gtgtatgcca ttagaataac aaacaagagt

1681 acagacaata gaaatagtat cagtgcagcc actaaaaata gtaacaacag cgcagacgct

1741 aaaaatagta acaaaagcac agacgcttca attagtaacg acagtccaaa cacttactta

1801 aagttgtaac gaaattactg acgcccaaaa atagcaacaa aagtgcagac gctaaaatta

1861 gtaacaaaat tacagacgcg taacataata acaacagtcg aaacacttaa acttgtaaca

1921 aaattactaa cgccgaaaat agtaacaaag tgctgacgct aaaattagta acaaaaatac

1981 taacgccgaa aaaaaccacc

| **Matrix ID** | **Name** | **Score** | **Relative score** | **Sequence ID** | **Start** | **End** | **Strand** |
| --- | --- | --- | --- | --- | --- | --- | --- |
| [**MA0065.2**](http://jaspar.genereg.net/matrix/MA0065.2) | Pparg::Rxra | 8.48802 | 0.808943895132 | NW_004797453.1:c454490-452491 | 74 | 88 | - |

# Solenopsis invicta isolate M01_SB chromosome 9, UNIL_Sinv_3.0, whole genome shotgun sequence

NCBI Reference Sequence: NC_052672.1

1 taaaatgtca ataataataa aaagcacatt aaaaattttc agcataaaag tataatgtga

61 aaatataaca aattgtgtaa taaaaagtgc acgcgaaaca tttgcgaaat aatgatttga

121 acgcgaatac aactgtatgt ctttttaata aataatatct ctatttgcag tatgtaacac

181 gatcgttcga ttgtgaatat taaaaagcaa aaatcaatgt attaaacata tagatataat

241 ttgaaactgt ggtaattaaa catgtcttta ttacggagac tagactgagt ttcatttgtg

301 aaatctcgag gaaaaattca ggcaatacga agaaaagaaa tcgacaatat cgactgcata

361 tgacctagta gcctttgcag cgaggataga agacagaggc taaattcgtg ctacccagca

421 tatctcattt aaaactccct cgtttgattt cataaccgac ctcttgccga cgacatatcg

481 tcgctgataa ttgcgctatt cctttgcaac ttcatcacca tattgcagcg aaggatgtct

541 cgtgtcaccg acatacgaaa tgatggatcc agcaatgcgg gtaacgtaag attgcgttac

601 gctcgcggtt tcacgaagag gatctccgaa tctctctatt ctcaaacgat tttgtagcgt

661 cgtcatcaaa atcgaaaaat tttacggcac gctcgacaac gaggttaacg aaatcggtgg

721 tgaattaaat tgattaaata ataaatagtt accagtgtcc cgagaacgct cgtgagaggc

781 atccccttgg aactggctgt gcgaacgcct cctctgcgaa cgcgttccga taagggagta

841 tctccgtcga ggatcgaacg atactctcaa tctcgcttaa tgccggtgaa acacttggta

901 taatttactg agggaactgc gttgcggcag ttgatagtgg taagtgatag accactcgta

961 atgtgctgca tctactttcc tcgagagtgg gagtaccctt cgggcgttcg agcttttatt

1021 aaacagtctc tttgtttcgg agcagaatcc ggagaacgaa gaatctcctt gaagaaactt

1081 ccttccctag atagaaaatt gcctctccaa ctgacccctt ctttgacgtt ttgatatttg

1141 caaacttttg cggcctctat aattttacac agcgtgaact aagcatcttg tattacagaa

1201 atgttgtttt acgatactat tgtaataatt tatataaaat taaaagaaga aaatctctct

1261 cttgtcgatg tattcatatt acgagctcta taattataat attatactta ataaatattt

1321 ctgaaatgtg cagactttta tctgattttt atagcaagtg tgattaagca tataaaaata

1381 aaagaataat ttacaaatag atgtacaaat aaacagtttt ggtatcaata tataattgaa

1441 attatgttat caatttaata gtgacaatgt aatttaaaat aagtaaaatc ctgtaattat

1501 attttatata aatattaata gttaataatt ggtaataagt attataatta tatatataat

1561 ttgtgttaat taattaattt tttattataa ttaagaagta tatttttcaa tattatagct

1621 tttttctctg tcaatttaga atatatatcc tctttttttt actcgtttaa acaaataaaa

1681 agaaatactg cgctttattt cagaaattta ttgcaaaaat aaaagtgtac agtgtcgcac

1741 ctcatttatt ggaaactttg caattcttgg gtgcgcttct cgttaccggt gctcgcatca

1801 acccccgtgg gctccaatga cgtcactgag gaatacattc gaagaccagc gccacttgcc

1861 gctaaacggg ggtgagccaa cacttgtctt caaccccatg agaagattta tccgaccaat

1921 gggaatcgct gatgttgccg tgcgccgccc agccacggcg agatcgtcat tgaaaaggac

1981 aagcgcaaac ggggagaccg

NO

# Apis florea unplaced genomic scaffold, Aflo_1.1 scaffold01062, whole genome shotgun sequence

NCBI Reference Sequence: NW_003790165.1

1 gattaaattt atttctatat attaataaac gacattgtaa taattaattt tcaatctttt

61 acagatcgaa cagctggtca tcttcagtag aatagtctct tgatcgatcg ttcgaaatta

121 tttattccct atttatcaat acacaaatat cttgcataaa tataattgcg aaaagcgatt

181 tagggactcg gtgataaaac atcctctcga agctgatcga acgatactct caattctctt

241 aatgccagag gaactcggct cggtataatt cagcaagggg tggtagctga tagcggtaag

301 tgatggacat tggtaatgtg ctgcatctgc tttgccacga ggcgaggcgt tcgagctttt

361 attaaacacg ctctttgttc cgtggaaaaa gaaacacgaa agcgagaacg attgtcttcc

421 taaaagaaag gagggggcag aaaaattaaa taatcccacc cttcgaaaag cactttctcg

481 ttcaaggata tgatttatac ttgtcaatat attgttcctt caactttgaa ttattagatt

541 attggatgta tatataaata ttaatatgtt ataatacatt gcgcaacatt ttgtaaatta

601 cacgtgttgc gtatatttaa gacaggaggt aaattattaa taaaatctat ggatctattg

661 atctataatc taataattaa aaaatttgaa ttttacagtt ttgtaataaa cattttattc

721 ggttcatcaa ttggcatatt tataacactt gtgaaattga agagggagaa aaggtgtata

781 atagtggaaa aagacaggag gtaaattatt aataaaatat atctattgat ccattgatct

841 aatccaataa ttaaaaaact tgaattttac agttttgtat ccggataaac attttattca

901 taacacttgc aaaattgaag agggagaaaa ggtgtataat agtggaaaaa gacaggaggt

961 aaattattaa taaaatatat tgatctattg atctaatcca ataattaaaa aattttatag

1021 ttttgtatcc ggataaacat tttattcata atacttgcaa aattgaagag ggaaaaaagg

1081 tgtataatag tggaaaaaga caggaggtaa attattaata aaatatattg atccattgat

1141 ctaatccaat aattaaaaaa tttgaatttt acagttttgt atccggataa acattttatt

1201 cataacactt gtgaacctga agaggaagaa aaggtatgca ataatggaaa aataagatat

1261 aatacaatta catataaagt aataataata ataataataa atatcacagt gataattctg

1321 gatgacgcta cggtaacgat cttcgttaca attgaatgaa aatcgtagcg aatattcacg

1381 agccatatca aggaatcgaa gtgtcggatt cgtgtctttt cctcggcaga ctaatttcac

1441 ttggcccaga aatccaaagg agaaactgtt tttcgtcgga tcaaagaaca ggatgaattc

1501 attggtgaac gaaggaggat aaaaaaagaa aaaaaaaaaa aggaccttgc gatttcttct

1561 ccttgcattc cacccacagt tttgtggacc ccagtgacgt caccgggaat agattgtgga

1621 accggcacgg cagttaaatg gggcgagcca acaacgctcc tctcttccca accccatgaa

1681 aagtgttatc cgaccaatag ggaaacttcg taccactgtg cgtcgaccaa tcgcgagcag

1741 atcgtcattg aggaagacgc gtactcggag gggaccgagg tcttaagagg agcggaggat

1801 accggtaccg ggacactgtt gctcgcggcc cttcaacagg cgaatcgttc aactttgccc

1861 ggtgatttct tcttttcaat ctcgtcgaga aatcaacgtg tcaacgtgtt gtcccgcaac

1921 ttggaatata taactagacg tacggtttga agagattgga aggatttaaa aattgtgtga

1981 aagtattctg tttcttcttt

| **Matrix ID** | **Name** | **Score** | **Relative score** | **Sequence ID** | **Start** | **End** | **Strand** |
| --- | --- | --- | --- | --- | --- | --- | --- |
| [**MA0065.2**](http://jaspar.genereg.net/matrix/MA0065.2) | Pparg::Rxra | 9.02168 | 0.817634081423 | NW_003790165.1:c6873296-6871297 | 1849 | 1863 | - |

# Drosophila melanogaster chromosome 3L

NCBI Reference Sequence: NT_037436.4

1 gtccaagtgg gcgtgcattg cagagcacga gagctcctac cgcaccggag tggtggggcc

61 tcccaacacc gatggatcca acgactatgg cattttccag atcaacgaca tgtactggtg

121 ccagccgtcc agtggaaagt tctcccacaa tggctgcgat gtgagttgca acgctctctt

181 gaccgatgac atcaaaagtt ccgtaagatg tgccctaaag gtcctgggtc aacagggctg

241 gtcagcctgg tccacctggc actactgcag tggatacctg cccccgatcg atgattgctt

301 tgtttaattc aagtctctta accatggaca tttcaaaatg taattgtttt gcaatctaat

361 aaacactttt tatacgttta tactttttga tacgttgatt cacgatatat acataaataa

421 aagtaaatat ctggtacgac atttgtaatt aaacaaagaa atgtgcggac aaataaatca

481 aagataagcg acagcgaaca aaattagtca ttgcaatttt tcaacgaatt cataatatat

541 agaaaggtaa ttactatgta ctgtatacta tatatactat actatatact ataaatatat

601 aaaaaaaaac aaatagttca gaaacactct aaaatcttaa aaagtacgtt cttaaagcag

661 acacatgtaa atgttgtttc ttaagtgttg atcaaattac cctttccaat tcccgaatta

721 aaaccgagtc acagtttaga cccccattca aattagatgt ctaagttaat tattccgaaa

781 tcaagagctg ccattcatcg tgacctcgac tccgttccgg gcttcatctc cgtctggctg

841 ccttcacatc ggcggagtgg gggacatcct gcatcccttg gggcaaccat aaatccttta

901 gctcgtgggc aaatagaaag tggatggctc ctgggagcca gtcgtttgtc ggtcaatcag

961 ttagccagca ccattaagtt aatcgcctca gctgatgagt tggggtgaag cgctgcccac

1021 gcatgtgttt agtgggcctt tcgcatattt taatagtcaa gtggcagtga ctctggctcc

1081 cagcctgacc acccggccca cccaacgctt ttcccatctt ttccgtctcc cctggcgcta

1141 tttgcttttc tcgaagctca aacaaaggcc caaagaagcg ggcggaaaga aattgccatt

1201 tatctactat tgactctgtg gaagcatcaa gtggttggct ggaaagcgct tttcctttat

1261 agctcgactt gacttggcca actacctagg aactcattta tttgaccact ctcaatgtcg

1321 cacgctctgc acttctttta acttcatatt gattttatcc aacgggagtt tcccgattcc

1381 aacaagggtt tcactaggtt ttccttacgg tacactagtt actgctgtac cagcataaat

1441 taaacgcctg aatttgaaag tcaagcgcaa tgtctggctg tctgtccacc aaccctagtc

1501 ccgtgacttt tcctcattcg gttggaagct gttgctgctg attagaaagt gaaattacga

1561 ttatgagccg agatctgtat actctcatat atgttcctca tttcaatata ttacaattca

1621 ttcattcttg taattacata actaatatag aatacataat atattataac taatacagaa

1681 tgtaaaaata tatttacatt tgctgactaa aacaaactac tttaaatata agaaggactt

1741 tctttgtaag ggcacggtga cgtcatgcgt tagcgatttg tcctgactcc aacatgattt

1801 ggccaatcag gacgtgccac cctaaaagga ggtgggtcct ctgaatagaa cagaaaaact

1861 cacttgtagc ttttatatcc ttgaacgaat ttatctcgcc acgcacactt aataaataca

1921 attgaatatg tatctcctgt gtgcccgcgt gtgtgcgagt cctgcgggct gcctataaaa

1981 ggactttcgc atccgaagga

| **Matrix ID** | **Name** | **Score** | **Relative score** | **Sequence ID** | **Start** | **End** | **Strand** |
| --- | --- | --- | --- | --- | --- | --- | --- |
| [**MA0066.1**](http://jaspar.genereg.net/matrix/MA0066.1) | PPARG | 13.0936 | 0.800581828836 | NT_037436.4:c1195173-1193174 | 1747 | 1766 | + |

# Tribolium castaneum strain Georgia GA2 linkage group LG3, Tcas5.2, whole genome shotgun sequence

NCBI Reference Sequence: NC_007418.3

1 acgattttgg tgtctgttaa cccggcgcct ccaccatttt ttgattacgt tacaaagatt

61 tttcagtcgt ttaattgatc cacaacagtt tttttacttg tttagaattt ttttatgctt

121 tattgcaagt tactgtacaa catttttaag ttcttgtttc taggtaaagt gttttacagc

181 cagatattct actttgaaat agtattccaa accctttagt agcttttttt cttttcacaa

241 actgtgcaca cttaatccat aaaattaaaa atttaaaaca cgattcaatt tcgctttatg

301 gaatcaacaa gatttcataa tacgtacctt ttttttagaa aacagtgaag aataataaat

361 taataatttg ttattttatt cgtttgaata atgaaaaaaa taataatttt tgtattacat

421 gctaccattt ccttactttt aagcatgtag aacacacgaa tattttgttt aatttttacc

481 ataacaattt ttgcttactt gtttatattt ttttatgctt tattgcaagt ttctttccaa

541 aatctttaag tagctgtttc tgaatacagc tatttttaaa gtgttttaca gtcggatgtt

601 ctacttttct ttagaaaaga gtagagcata ataaatttaa aaatttttac tccatttgtt

661 taaataatga gaaaagtaat aatttttgca ttacatacca ccacttcctt actttcaaac

721 ttgtagaaca cacggaattt ttttctcatt cggcctttaa atctttggct ttttaaaatt

781 ttactattaa tgcattttgt aattaggagg taaaaacaaa taaatcaaag ttacacacgt

841 tgtgacctca gaataaagaa taaacaattt ttgtctgtag acccggcgca tccaccattt

901 tttaaatacg ttacattgag ccgtttaatt tttaccataa taatttttac ttacttgttt

961 attctttttt atgctttatt gcaagtttct ttccaaaatc tttaagtagc tgtttctgaa

1021 tacagctatt cttaaagtgt tttacagccg gatattctac ttttctttag aaaacagtaa

1081 agcataataa atttaaaaat ttttactcca tttgtttaaa taatgaaaaa aataataatt

1141 tttgcattac ataccaccac ttccttactt tcaaactagt agaacacacg gaattttttt

1201 ctcattcggc ctttaaatct ttggcttttt aaaattttac tattaatgca ttttgtaatt

1261 agaaggtaaa aacaaataaa tcaaagttac acacgttgtg acctcaggat aaagaatata

1321 cactttttgt tgacccggcg caaaatggcg catccaccat ttttaacagg cgtttaaata

1381 aacgtaaacg tacatggtta taaatttatt tttctactag tacaatgtta gttgttttca

1441 tgatgaaaca ttaaaagaat ttccacttgt tcccatcgag ttaccatact aaaacgtgcc

1501 tttcacgaga gactgcggat agaagagcaa gtttatggtt tattcagcaa agaggtatag

1561 aatcgtttca attactctac ttcagattca ggatacctcc cactctacgt tttatcaatt

1621 tctctccatt tgatagaacg ggggaagcta atacatattg aaagcgctat atttcttttt

1681 aattcctctc gtgttgcgca aagaaaacat aaataacgca acacatcgga aaggcttcaa

1741 gataatttaa tccagcgtca agtgcaaagg tgcatttgag acagaaattc aattttcgtg

1801 aatatggcgc aaataaaccg atgacctagt gacatttcca ccgaatattt gacggttttt

1861 tggtacaagt gtcttggaag aatctaaaat tgtgtcaaag aatcgccata aatcgtgatt

1921 ctgccaatca aagttaagga ttctgcgcca gtcctgttga gtttataaaa gctgatgcgg

1981 gaatcttaat cgttacaaac

| **Matrix ID** | **Name** | **Score** | **Relative score** | **Sequence ID** | **Start** | **End** | **Strand** |
| --- | --- | --- | --- | --- | --- | --- | --- |
| [**MA0065.2**](http://jaspar.genereg.net/matrix/MA0065.2) | Pparg::Rxra | 8.76307 | 0.813422855474 | NC_007418.3:26735850-26737849 | 1759 | 1773 | + |

PIO75699.1 putative tryptophan 5-monooxygenase partial Teladorsagia circumcincta No available (18265-18991, 726bp, TELCIR_02257)

aagcct tctcatagaa gcaggcaagc gatttttgat

18301 caccaaccgg tcagccaagt taagctcaca tttaagcgta gaatgggggc attttaagga

18361 ctgcggtcgg aacagataaa ggaccaatag t

| **Matrix ID** | **Name** | **Score** | **Relative score** | **Sequence ID** | **Start** | **End** | **Strand** | **Predicted sequence** |
| --- | --- | --- | --- | --- | --- | --- | --- | --- |

# Arabidopsis thaliana chromosome 2 sequence

NCBI Reference Sequence: NC_003071.7

1 atttaaaaaa tttagagtaa caattgttta tttacgtttt aatctacagt agcgtttgag

61 tgttttaaat aattactaaa atactataac atacatacgt ttgaaacaat cattatctag

121 aacgaaaaaa gaatcatatt taagacgtaa gagattaaat tttaaaacct tggatgttat

181 ctcatttact ctgtttttgt cctacaataa ttataaactg aacattgatt ttctagcatt

241 tgttcgtttg ttcgtaaatt aactagtgtg tttcaatatg catctttgta cgtgtagact

301 tctgctacat agaagagata aacggggcgt cacgtgtcat gtgcgaccag aacaacaggc

361 aatatccatg tgcaccggcc aagagctacc atggccgtgg tccgctccta ctatcatgga

421 acttcaacta tggagcatgc ggccagagtc tcggcttaga cctcctacgc cagcccgagc

481 tggtgagtag taacccagtc gtggctttca ggacggctct gtggttttgg atgaagagcg

541 tgaggcctgt attgaaccag ggatttggag ccaccataag agccatcagc ggtttcgact

601 gtgacggtag gaacttaggt ggggtgaacg caaggattgg ctactataga gactattgtg

661 ggcagcttgg tttggaccct ggcgccaaca taacttgcta aaacgctctt gggacataca

721 tggcacgaaa gtattaaagg tgaaataaga agtgttgttg taattgtagt actttttttt

781 tttttttttt tgataaaaag ctagagtaat cttgtgaagt gaaatcacat tattaaaaga

841 ttaataaatc gacttataca aataaaatgc ttgattcttg atatttatac cgcacccaaa

901 gaacaacaga acataacttt gttgcataag tttttgactc ctatatgtaa aacttcattc

961 ctttcttatc tagtattgtt ggtttcttat ctcaattgag cgatcggatt agaattggtc

1021 tgacaactag tttattatgt gaaactttta aaatgaagag ttttgttgtg aatcagaggg

1081 tttgaaagaa tttgatttca gtttaaaagt aaaccgaatt tgttcaagtc gttgtaggcc

1141 tatgttttgt ctgtttctat acatgattga tcagtctctt gaaagtatga tatatagatg

1201 atagagcttg ttttctctat gatatacatg ttcctgcaat atatcttgtt agattactaa

1261 ctggactaag tctggcacgt aagacaacga tcttccacaa gtaattctag tttacaagtc

1321 attctaataa ttttaattaa tttcaaaatg gttggaataa aattacctag ttttactatt

1381 taagagaaga ccaacatcaa agattattct tatctaatat tcaaattatg gatattaatg

1441 cattcatata cgactcggga ttagtatttc ttacaagact tgttcaataa aatttcttgc

1501 caacaattat gacttttcct gttgtttaca atttttcaca agtctattgt cgattttaaa

1561 tttgtgttat cacacacaac atgagactag taaaaccaat tgacattgac tacaggcttc

1621 taccgattaa acttatcttg tataacaaac tcaaaccata ttttaatttt tgttttccaa

1681 ttggcgacta ctttcagtaa aagtcactta gtttcttgct ttttagaacc acaaaaacca

1741 aatatacttt tttcacctat ctcattttat ctctgataat ttttctttac actaaatgtt

1801 tgcatcacag gaacaaaaaa atatttggtt taaccaattt cgtagattga aacgaaaatg

1861 ttggtgtttg tttgaaaatt tcccaacgtg ttacgtgtaa cgtttcgaat ggtctaagaa

1921 ctaataagca tgcgtggcat tggcgttgac gttgactcag aaaactacaa gtatatttta

1981 tttacaacaa aacgtatgct

Filter:

| **Matrix ID** | **Name** | **Score** | **Relative score** | **Sequence ID** | **Start** | **End** | **Strand** | **Predicted sequence** |
| --- | --- | --- | --- | --- | --- | --- | --- | --- |
| No data available in table | | | | | | | | |

BAD01049.1 tryptophan 5-hydroxylase Hemicentrotus pulcherrimus. NO

AFH41116.1 plastid 3-phosphoglycerate kinase partial Triticum sphaerococcum. NO

<https://blast.ncbi.nlm.nih.gov/Blast.cgi>

NP_001035035.1

XP_020947662.1

NP_775567.2

NP_001184049.1

XP_015667296.1

NP_001001301.1

XP_007054860.1

NP_001296997.1

XP_040199920.1

XP_006008042.1

XP_032894074.1

NP_001075741.1

NP_001191619.1

XP_011157665.1

XP_012341006.1

NP_612080.1

XP_967413.1

PIO75699.1

BAD01049.1

AFH41116.1

# Homo sapiens chromosome 3, GRCh38.p13 Primary Assembly

NCBI Reference Sequence: NC_000003.12

1 gaaccctgac acattgctgg tgggattgta aaatggtgtg cccactttgg aaaacagact

61 ggcagttcct caaaaacacc gagttacctt atgatcctgc agttctgtcc ctaggtatat

121 actcaagaga aataaaaata tatgtccaca agtaaccttg tacatgaatg ctcacagcag

181 cattattcat aatagcccat aaaagtagaa acaacctaaa tattcatcaa ttcatgggat

241 gaataaacaa aatgtggtat atgtgtataa tggaatattg accataaaaa ggaatgaaat

301 attaatataa gctataacat ggatgagcct cacaaatact atgctaagtg aaagaagaaa

361 gtcacaaagg acttcatatt ctatgattct atttatatga attgtccaga ataggtaaat

421 ctatagagaa agaatatctc tatctagagt tggtggaatg actgttaatg gagagggggt

481 tcctttttgg agtgatgaaa atgttctaag ggtagatttg gtgatgatgg cacaactctg

541 tcaataaact aaaactcatt gaactgtaca ttttatttat ttatttttga gatggagtct

601 tgctctgggg ctgaagtgca gtggcgcaat ctcggcttgt aacctctgcc tcccagggtc

661 aagcgattct actgcctcag ccccccgagt agctgagatt acaggcacgt gccaccacgc

721 ccagctaatt tttgtatttc ttagtagaga tggagtttca ccatgttggc caggctggtc

781 ttgaactccc ggcctcaagt gatccacctg cctcggcctc ccaaagtgct gggattacag

841 gcgtgagctg ccatacccgg cctgaattgt acattttact tctatggtat ttacatttta

901 gattatatta attattcctc aataaagctg tgattttaaa aagcaggcta ggcgcagtgg

961 ctggtgccta taatcccagc actttggaaa gctgaggcag gaggatcact tgagcccagg

1021 agtttcagac tagtctaggc aacatgtcaa gacacagtct ctactaaaca attaaaatta

1081 aaaaaaaaaa ttagccaggc atggtggtgt gcacctgtag tcccagctac ttgggagcct

1141 ggggtgggag gattccttga gcccgggaag tcgaggctga agtgagccgt gattgcgcca

1201 cagcactcca gcctgggcga cacagcaaca ccctgtctca tggaagaaag aaagaaaaga

1261 aaggaagaaa gaaaaaaaaa aagcagattg gaactctgga attaacaaga agtaggacgc

1321 acggagcact tccgcctgag tggagactgt ggatccgggt caacctgact acctaaatca

1381 caggccaata aatggtcttt cagtggtcag tccctgtaag atccgtggct ctcagcttct

1441 tatcttaggg gctgtggagg aaggacatga ttatgttgat ttaagcgctg aatattttcc

1501 cttgtgatac ccatcctcgc aaaactttgc ttcaaccaca aacgaggacc ttctgtacca

1561 gaggggcaat aaccacaatg aagctaggaa gaaatgcaga gcaccccagc atacagtcca

1621 taagcttcct gaagtggggg gcctcaggca tcgctgcctc cccaaagagg atcaggccca

1681 gaacagtatg ctccagaaat aagactggaa aaagggaaag aggggcctca agtccaggag

1741 accagcggct ttctgaacgc gcacctgcca acccactttg gacaggtcac gatggacagc

1801 gtggcaggaa aagaaaaggt cactgtctac ccaacacatg agaaactgtt tctcgtgcct

1861 cacgtcccca ctccgtcccc acccatgttg tctgagtccc tcggtgtcag aaacactgct

1921 aagaaattta agaaattctg ttaatgagtt taagaaatgt ttttaatgat taaaagtcag

1981 tgacttgtga ataaccatgt

| **Matrix ID** | **Name** | **Score** | **Relative score** | **Sequence ID** | **Start** | **End** | **Strand** |
| --- | --- | --- | --- | --- | --- | --- | --- |
| [**MA0065.2**](http://jaspar.genereg.net/matrix/MA0065.2) | Pparg::Rxra | 12.4621 | 0.873658155418 | NC_000003.12:12285368-12287367 | 640 | 654 | - |
| [**MA0065.2**](http://jaspar.genereg.net/matrix/MA0065.2) | Pparg::Rxra | 10.3975 | 0.840038915407 | NC_000003.12:12285368-12287367 | 1808 | 1822 | + |
| [**MA0065.2**](http://jaspar.genereg.net/matrix/MA0065.2) | Pparg::Rxra | 8.87135 | 0.815186097658 | NC_000003.12:12285368-12287367 | 1373 | 1387 | + |
