# Supplementary figures and images for: Brain Short-Chain Fatty Acids Induce ACSS2 to Ameliorate Depressive-Like Behavior via PPARγ–TPH2 Axis
Source: Research (Wash D C). 2024 Jun 27;7:0400. doi: 10.34133/research.0400 (PMC11210491; doi:10.34133/research.0400)

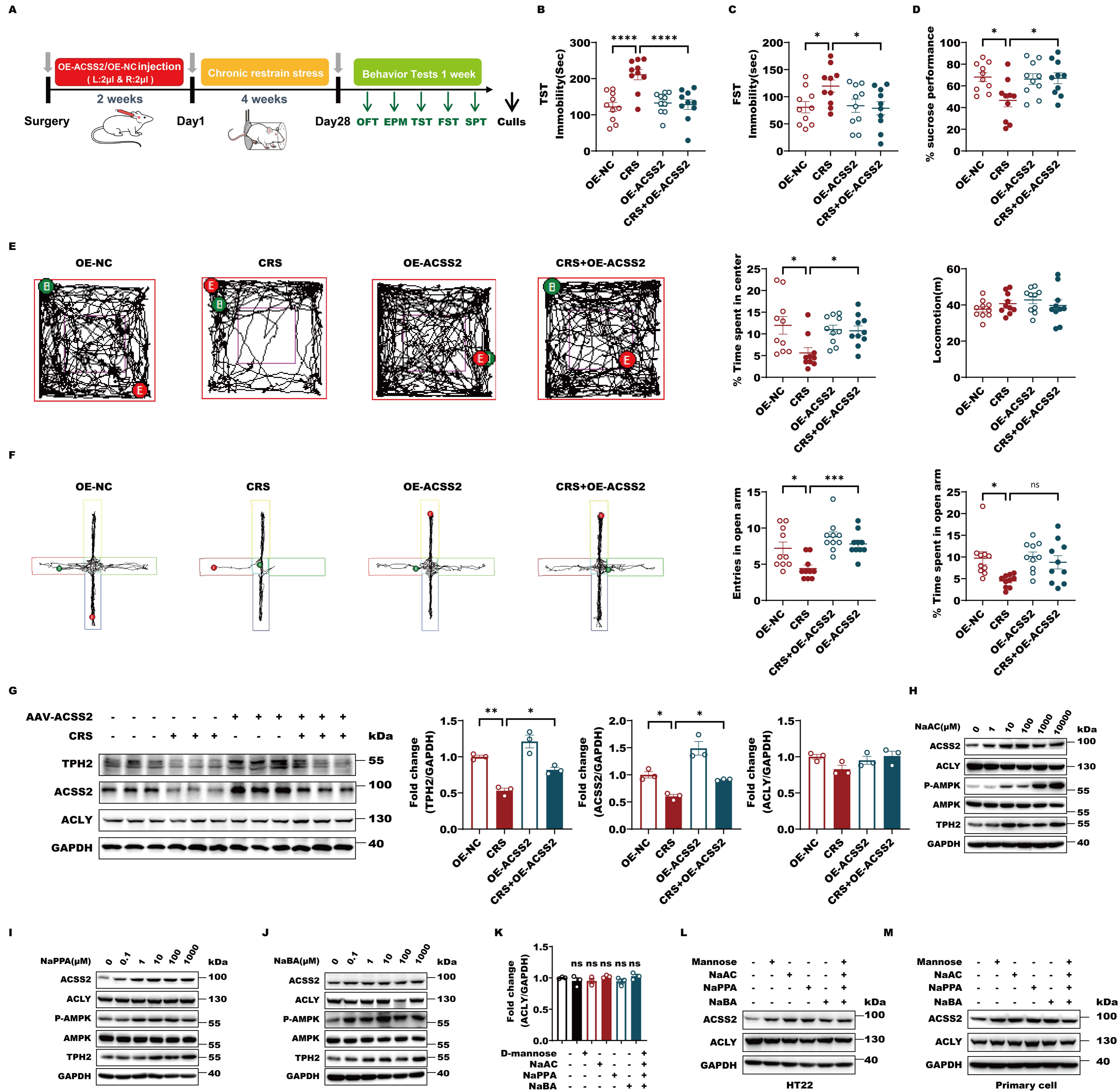

Supplement: Supplementary 1 — Figs. S1 to S6 Tables S1 to S3 Control VS Mannose CRS-Control VS CRS-Mannose RNA sequencing for mannose-treated MG1655 [file research.0400.f1.zip › FS1.pdf]

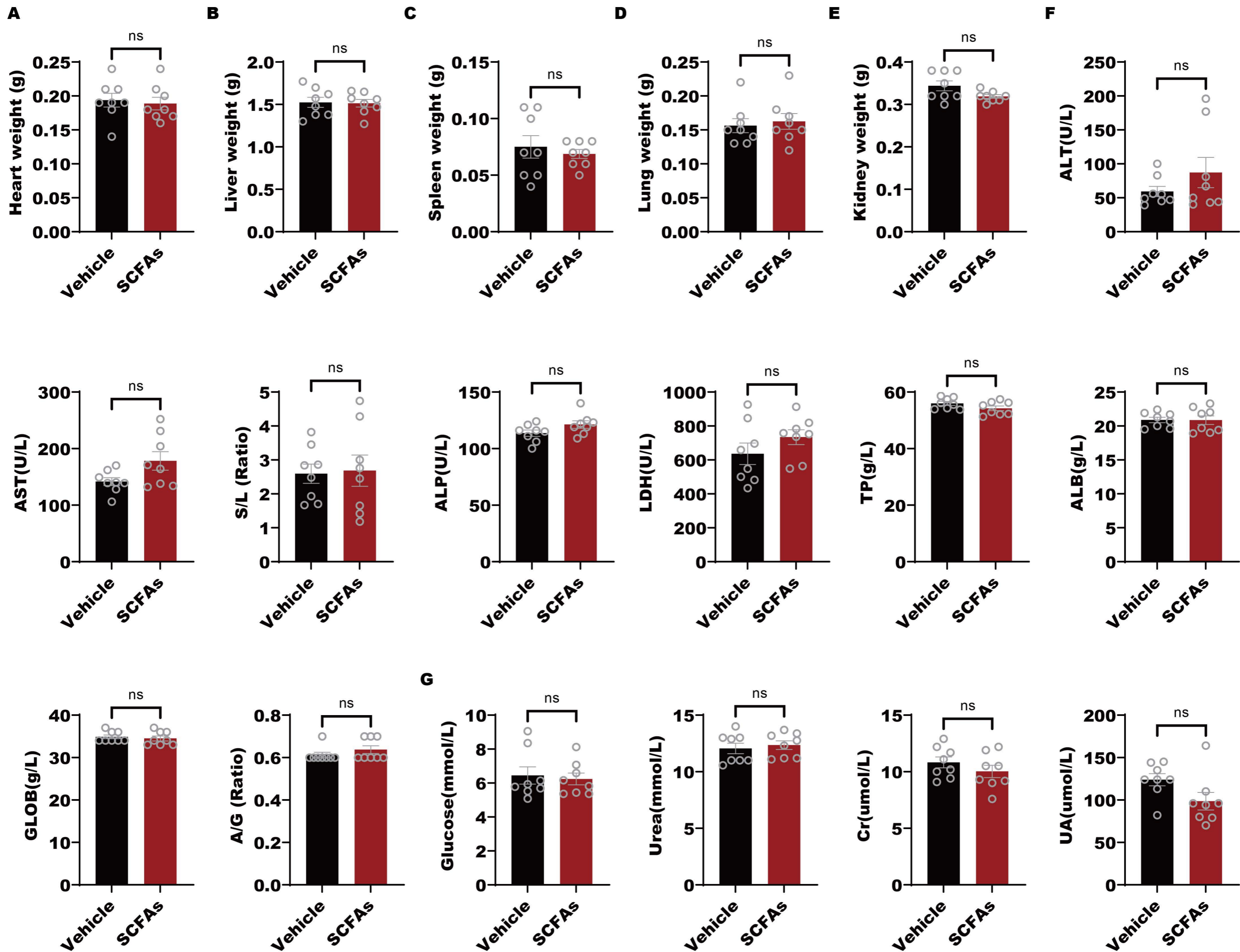

Supplement: Supplementary 1 — Figs. S1 to S6 Tables S1 to S3 Control VS Mannose CRS-Control VS CRS-Mannose RNA sequencing for mannose-treated MG1655 [file research.0400.f1.zip › FS2.pdf]

**A**

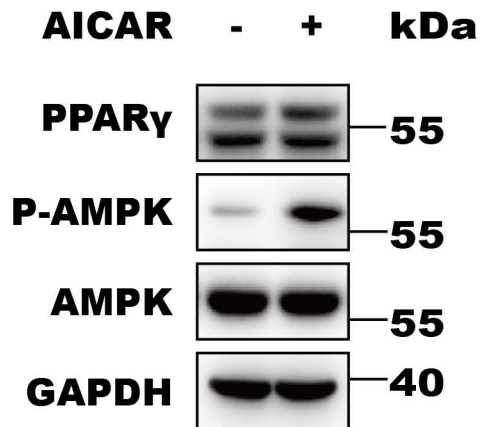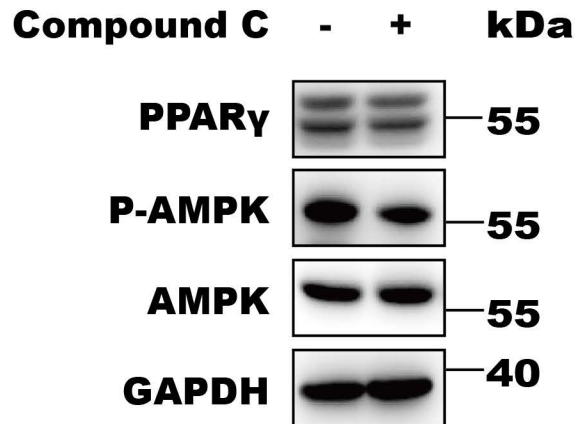

Supplement: Supplementary 1 — Figs. S1 to S6 Tables S1 to S3 Control VS Mannose CRS-Control VS CRS-Mannose RNA sequencing for mannose-treated MG1655 [file research.0400.f1.zip › FS3.pdf]

**A**

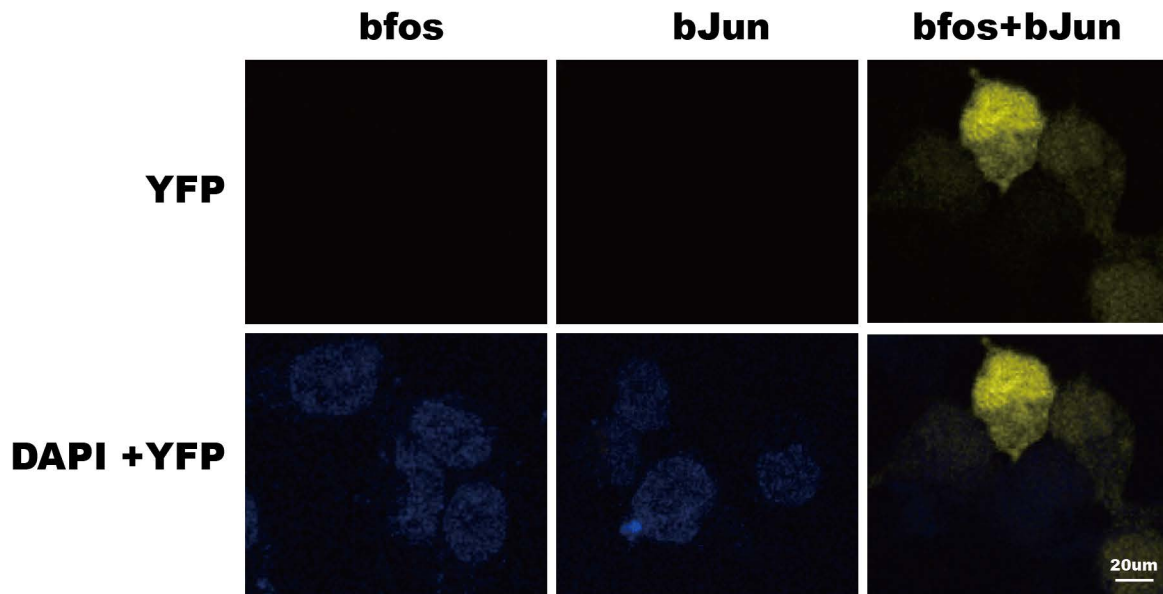

Supplement: Supplementary 1 — Figs. S1 to S6 Tables S1 to S3 Control VS Mannose CRS-Control VS CRS-Mannose RNA sequencing for mannose-treated MG1655 [file research.0400.f1.zip › FS4.pdf]

**A**

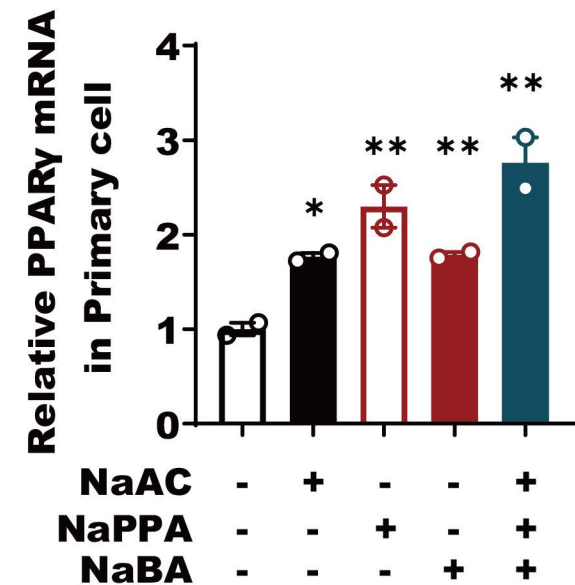

**B**

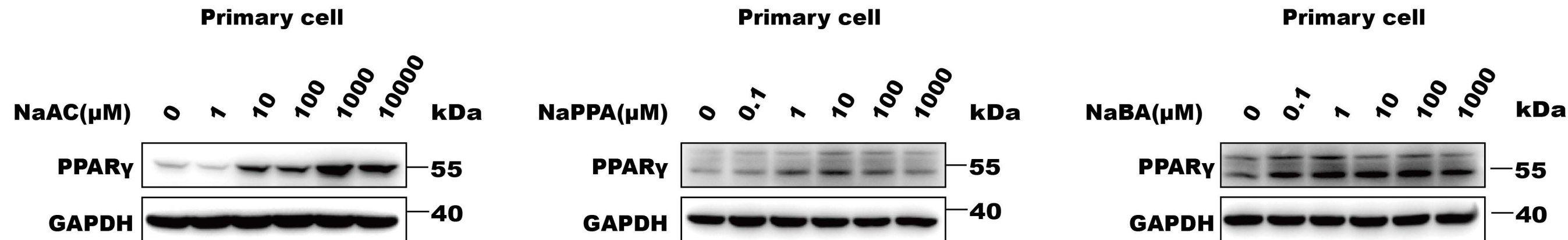

**C**

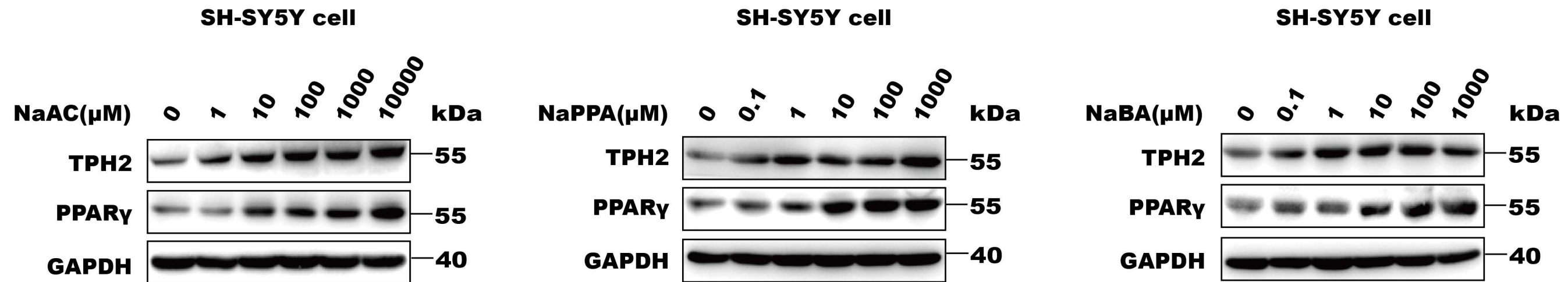

Supplement: Supplementary 1 — Figs. S1 to S6 Tables S1 to S3 Control VS Mannose CRS-Control VS CRS-Mannose RNA sequencing for mannose-treated MG1655 [file research.0400.f1.zip › FS5.pdf]

**A**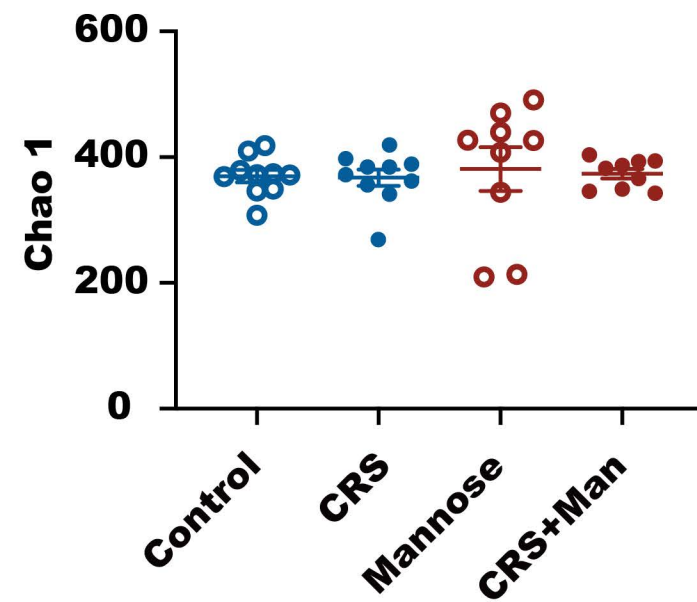**B**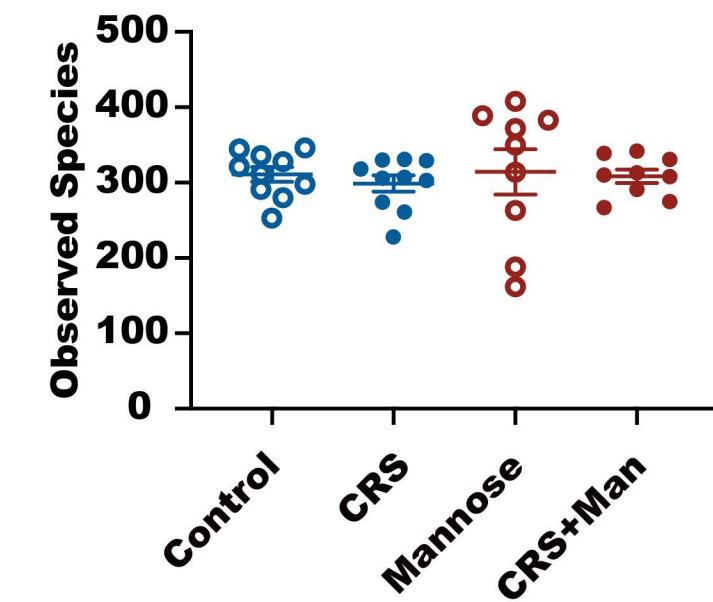**C**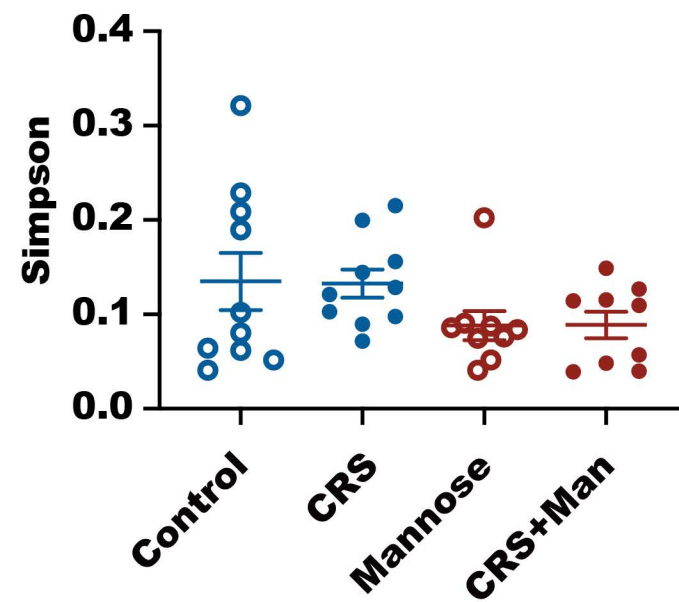**D**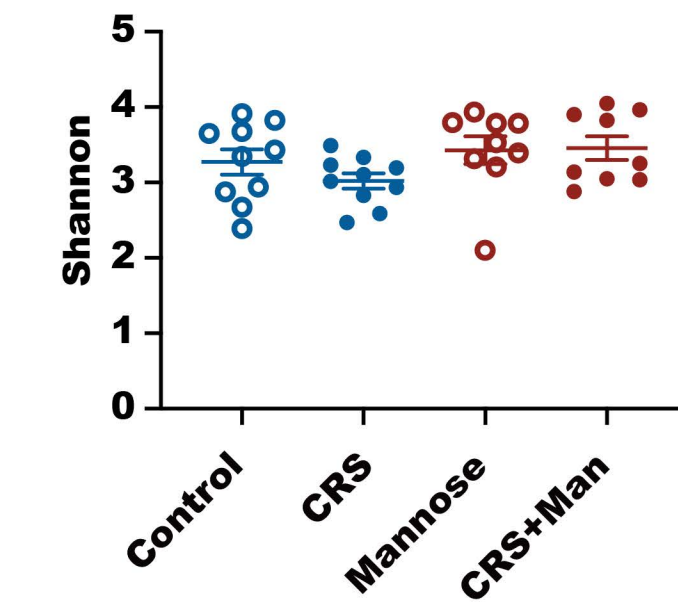**E**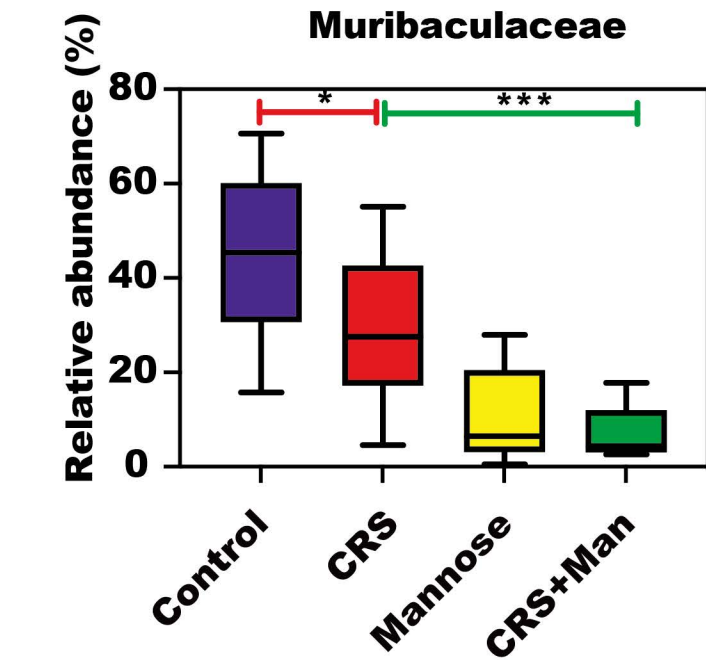

Supplement: Supplementary 1 — Figs. S1 to S6 Tables S1 to S3 Control VS Mannose CRS-Control VS CRS-Mannose RNA sequencing for mannose-treated MG1655 [file research.0400.f1.zip › FS6.pdf]
